# A High-Density Genetic Linkage Map for Cucumber (*Cucumis sativus* L.): Based on Specific Length Amplified Fragment (SLAF) Sequencing and QTL Analysis of Fruit Traits in Cucumber

**DOI:** 10.3389/fpls.2016.00437

**Published:** 2016-04-19

**Authors:** Wen-Ying Zhu, Long Huang, Long Chen, Jian-Tao Yang, Jia-Ni Wu, Mei-Ling Qu, Dan-Qing Yao, Chun-Li Guo, Hong-Li Lian, Huan-Le He, Jun-Song Pan, Run Cai

**Affiliations:** ^1^School of Agriculture and Biology, Shanghai Jiaotong UniversityShanghai, China; ^2^Biomarker Technologies CorporationBeijing, China; ^3^Shanghai Seed Management StationShanghai, China

**Keywords:** *Cucumis sativus* L., SLAF-seq, high-density genetic map, SNPs, QTL analysis

## Abstract

High-density genetic linkage map plays an important role in genome assembly and quantitative trait loci (QTL) fine mapping. Since the coming of next-generation sequencing, makes the structure of high-density linkage maps much more convenient and practical, which simplifies SNP discovery and high-throughput genotyping. In this research, a high-density linkage map of cucumber was structured using specific length amplified fragment sequencing, using 153 F_2_ populations of S1000 × S1002. The high-density genetic map composed 3,057 SLAFs, including 4,475 SNP markers on seven chromosomes, and spanned 1061.19 cM. The average genetic distance is 0.35 cM. Based on this high-density genome map, QTL analysis was performed on two cucumber fruit traits, fruit length and fruit diameter. There are 15 QTLs for the two fruit traits were detected.

## Introduction

Cucumber (*Cucumis sativus* L.), a diploid species (2*n* = 14), which belongs to the family of Cucurbits. The cultivated area of cucumber is second only to tomato in the world. Cucumber has seven chromosomes and 100s of known functional genes (Xie and Wehner, 2001), and the genome size is only 367 Mb, which is smaller than other Cucurbits commercial crops (Ren et al., 2009). Because of its economic importance and nutritional value, the cucumber has been the research model plant of cucurbitaceae plants, and the genetics and molecular biology study of cucumber has progressed rapidly in the latest 20 years. Recent years, the whole genome of cucumber varieties 9930 (‘Chinese long’ inbred line; Huang S. et al., 2009), ‘Gy14’ (North American pickling type; Cavangnaro et al., 2010), and ‘B10’ (European inbred line; Wóycicki et al., 2011) have been sequenced. Cucumber has become model plant for Cucurbits’ genetic research.

Currently, genetic linkage maps commonly include restriction fragment length polymorphisms (RFLP), random amplified polymorphic DNA (RAPD), amplified fragment length polymorphism (AFLP), sequence characterized amplified region (SCAR), simple sequence repeat (SSR), and single-nucleotide polymorphism (SNPs) markers. A genetic map, especially a high-density genetic map provides an important foundation for quantitative trait loci (QTL) mapping (Esteras et al., 2012; Petroli et al., 2012; Song et al., 2012) and anchoring sequence scaffolds (Wang et al., 2011; Ren et al., 2012), and the utility of genetic linkage maps depends on the types and numbers of markers used. Since 1994, several kinds of markers have been used to assess the genetic diversity of cucumber accessions including RAPD, AFLP, SCAR, SRAP, SSR (Dijkhuizen et al., 1994; Kennard and Havey, 1995; Serquen et al., 1997; Park et al., 2000; Bradeen et al., 2001; Fazio et al., 2003; Li et al., 2005; Wang et al., 2005; Sun et al., 2006; Yuan et al., 2008a,b; Ren et al., 2009; Zhang et al., 2012), but no SNP markers were available for cucumber. With the rapid development of the next-generation sequencing (NGS), makes it possible to obtain thousands of SNPs throughout the cucumber genome and construct the high-density SNP genetic maps. There are several methods have been used to discover SNPs markers, for example RAD-seq (restriction site-associated sequencing; Miller et al., 2007), double digest RAD-seq (Peterson et al., 2012) and GBS (two-enzyme genotyping-by-sequencing; Poland et al., 2012). Sun et al. (2013) developed a new technique SLAF-seq (specific length amplified fragment sequencing), and discovered large-scale *de novo* SNP and genotyping by this method. The efficiency of this method was tested on rice and soybean, in addition, SLAF-seq was used to create a high-density genetic map for common carp (*Cyprinus carpio* L.). Through the method of SLAF-seq, SNPs marker has been wildly applied for high-density genetic map construction in several crop and animal species, such as sesame (Zhang et al., 2013), soybean (Qi et al., 2014), common carp (Sun et al., 2013). And it is possible to construct a high-density genetic map based on SNPs markers of cucumber. SNPs markers may provide a more saturated genetic linkage map of cucumber. Wei et al. (2014) constructed the first SNP genetic map of cucumber by SLAF-seq, which contained 1,800 SNPs, the average distance between adjacent markers was 0.50 cM. And Xu et al. (2015b) constructed a SNP map of cucumber by SLAF-seq, which contained 1,892 SLAFs with the total length is 845.87 cM and average distance is 0.45 cM. After then, Xu et al. (2015a) constructed anther SNP map using 949 F_2_ populations, on this basis, QTL analysis on fruit flesh thickness, and find the candidate genes.

There are a lot of important QTLs and horticultural traits associations have been researched in cucumber. Which include sexual expression, lateral branch, disease resistance, parthenocarpy, fruit shape, formation of bisexual flowers, fruit warty and fruit flesh thickness (Kennard and Havey, 1995; Serquen et al., 1997; Dijkuizen and Staub, 2002; Fazio et al., 2003; Pan et al., 2005; Sakata et al., 2005; Sun et al., 2006; Li et al., 2009; Yang et al., 2014; Xu et al., 2015a). And some of these research results have been successfully used in cucumber marker-assisted selection breeding.

In our study, genotype data was generated and SNPs markers were discovered by SLAF-seq for cucumber. Using these SNP markers, we constructed a high-density genetic map of cucumber. This high-density genetic linkage map might have utility in locating QTL associated with economically important traits of cucumber. And we used the new genetic map to define QTLs for several fruit traits of cucumber.

## Materials and Methods

### Ethical Standards

The authors declare that this study complies with the current laws of the countries in which the experiments were performed.

### Plant Materials

Cucumber gynoecious line S1000 was crossed with the monoecious line S1002. The S1000 plants have little leaves and normally produce short fruit (10–15 cm), the diameter size is about 55–60 mm. In contrast, the plants of S1002 have large leaves and longer fruit (40–45 cm), the diameter size is about 30–35 mm. Parent materials provided by Huang Sanwen. The two parental lines S1000 and S1002 were planted in greenhouse in the spring of 2012, the F_1_ of S1000 × S1002 were grown in August of 2012, and the F_2_ population were planted in the spring of 2013 and self-pollinated to obtain 150 F_2:3_ families.

### DNA Extraction

Young healthy leaves from the two parents and the 153 F_2_ plants were compiled and DNA was extracted by the method of CTAB (Doyle and Doyle, 1987). DNA was quantified with an ND-2000 spectrophotometer (NanoDrop, Wilmington, DE, USA) and by electrophoresis in 0.8% agarose gels with a lambda DNA standard.

### Phenotypic Data Collection and Genotyping

Phenotypic data were collected in three environments over 3 years (2013 autumn, 2014 spring, and 2015 spring) with F_3_ families, S1000, S1002 and their F_1_ were included in all experiments. F_3_ Families were arranged in a randomized complete block design two replications respectively in 2013 autumn, 2014 spring and 2015 spring with 10 plants in each replication. We collected the data of fruit length (fl, cm, length from the apex of fruit to the pedicel attachment), fruit diameter (fd, mm, at the maximum fruit width). The fruit-related traits were measured according to the standards published by Yuan et al. (2008a). We measured the numbers of fl and fd on individual plants, and averaged within each F_3_ family. Considering that there might be some diseases and other factors, 12 plants were randomly selected from the F_3_ families and planted, and three fruits were measured of each plant. Phenotypic data from 10 healthy plants were used to represent each F_2_ individual.

### SLAF Library Construction and High-throughput Sequencing

The method of SLAF-seq was used to genotype 153 F_2_ plants, and the two parents, as above described (Sun et al., 2013). Genome DNA was digested to completion with RsaI+Hpy166II. A single-nucleotide overhang were added to the digested fragments with Klenow Fragment (3′→5′ exo–; NEB) and dATP at 37°C, and then the Duplex Tag-labeled Sequencing adapters (PAGE purified, Life Technologies) were ligated to the A-tailed DNA with T4 DNA ligase. The PCR reaction was performed using diluted restriction-ligation samples, dNTP, Q5^®^ High-Fidelity DNA Polymerase and PCR primers: AATGATACGGCGACCACCGA and CAAGCAGAAGACGGCATACG (PAGE purified, Life Technologies). The PCR productions were purified using Agencourt AMPure XP beads (Beckman Coulter, High Wycombe, UK) and pooled. The pooled sample was separated by electrophoresis in a 2% agarose gel. Fragments with 244~344 bp (with indexes and adaptors) in size were excised, purified using QIAquick Gel Extraction Kit (QIAGEN). The gel-purified product was sequenced on the Illumina HiSeq 2500 system (Illumina, Inc., San Diego, CA, USA) according to the manufacturer’s recommendations.

### SLAF-seq Data Analysis and Genotyping

SLAF-seq data was operated using the software developed by Sun et al. (2013), and the genotyping methods with reference to Sun et al. (2013) and Wei et al. (2014). According to sequence similarity, the generated pair-end reads from SLAF-seq were clustered, and the reads could be inferred from one to one alignment by BLAT (-tileSize = 10 -stepSize = 5). Identical reads were merged, and the reads with over 90% similarity sequences were grouped into one SLAF locus (Sun et al., 2013). In each SLAF locus, minor allele frequency (MAF) evaluation was used to define alleles.

In order to ensure the quality of genetic map, according to the following rules to filter SLAFs: (1) SLAFs with parents sequence depth of less than 10×; (2) SLAFs with complete degree below 30%; (3) SLAFs with serious partial separation (*p*-value < 0.05); (4) Heterozygous SLAFs in two parents. One SLAF locus can contain no more than four SLAF tags in the mapping populations of cucumber, according to this principle, the groups which contain over four tags were considered as repetitive SLAFs and excluded. Polymorphic SLAFs which contained 2–4 tags were considered as potential markers. Those polymorphic SLAF markers were then assorted into eight segregation patterns as following: ab × cd, ef × eg, hk × hk, lm × ll, nn × np, aa × bb, ab × cc, and cc × ab. Since the F_2_ mapping populations were derived from two homozygous cucumber inbred lines with a genotype of aa or bb, therefore only the SLAF markers which had segregation patterns of aa × bb were used in map construction.

### Linkage Map Construct and QTL Analysis

**FIGURE 1:**
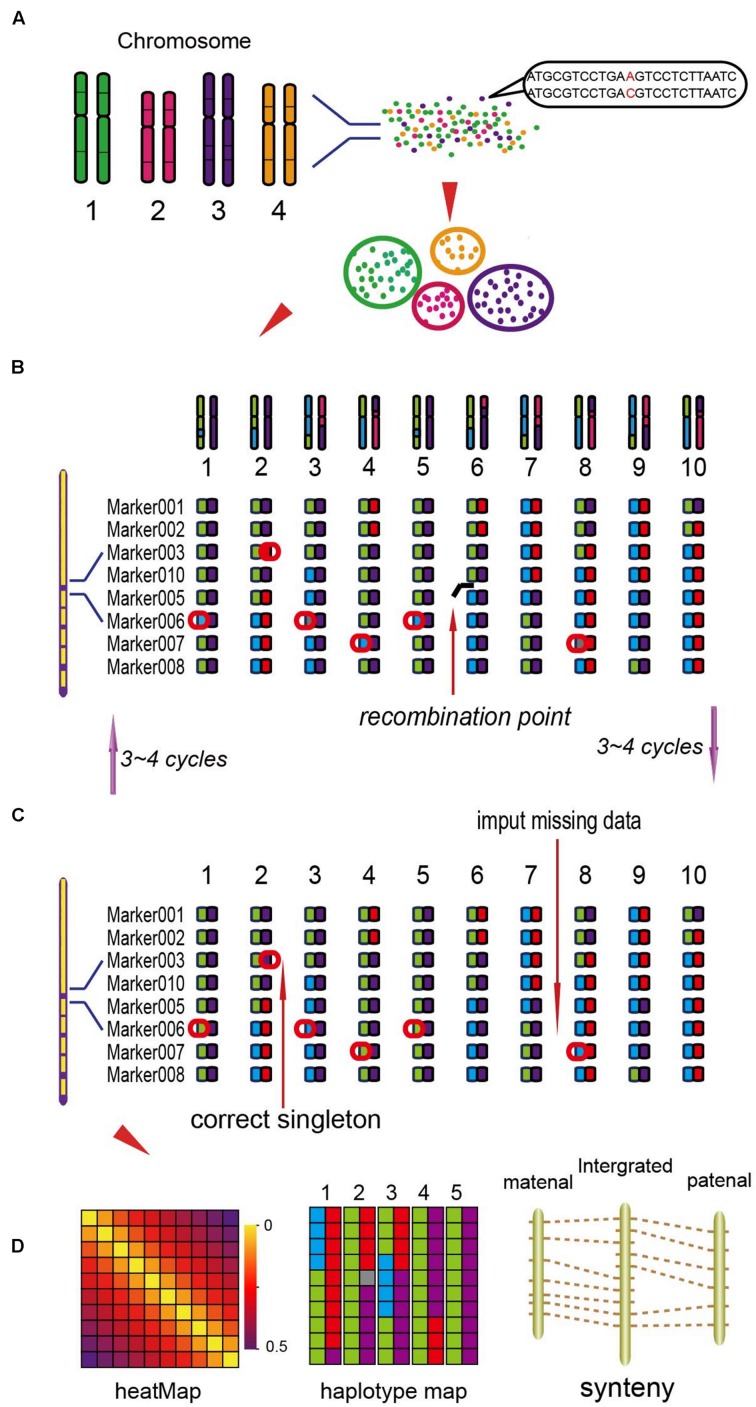
**The process of the genetic map construction (Liu et al., 2014). (A)** Calculate the mLOD value of the high quality molecular tags, using this value to analysis seven linkage groups (LGs). **(B)** Using the software of HighMap to construct the genetic map for each LG. **(C)** According to the result of mapping to correct the tags genotyping. **(D)** Get the high-density genetic map and evaluate the quality of the map.

Based on the locations of SLAF markers on chromosomes, they were assigned into seven linkage groups (LGs). The NGS data may exist some genotyping errors and deletion, which can reduce the quality of the genetic map. These errors were corrected by the High Map Strategy (Liu et al., 2014). All the genotype data from the F_2_ mapping population was used to perform linkage analysis using JoinMap 4.0 software (Van Ooijen, 2006). The SLAFs can be divided into seven LGs with the position of the reference genome. By computing the MLOD value between two adjacent markers (Vision et al., 2000), to filter out the SLAFs with the MLOD values are less than 5. Using Marker HighMap (Liu et al., 2014) software to analysis the linear array of markers in each LG, and estimate the genetic distances of between two adjacent markers. The method of Internal Mapping and the software of R/qtl were used in QTLs analysis, the confidence intervals of QTLs were calculated according 95% Bayes credible interval method (Sen and Churchill, 2001) with R/qtl. The process of the genetic map construction was shown in **Figure 1** (Liu et al., 2014).

## Results

### Analysis of SLAF-sqe Data and SLAF Markers

After DNA high-throughput sequencing, generated a total of 22.98 Gb of raw data, containing 143.63 M reads of ~80 bp in length left over after preprocessing. The Q30 (it is a quality score of 30 which indicating a 0.1% chance of an error and 99.9% confidence) ratio was 81.35% and guanine–cytosine (GC) content was 39.04%. There were 12,417,012 reads from female parent and 8,916,362 reads from male parent, the average number of reads from F_2_ population is 799,335.

The numbers of SLAFs in the male and female parent were 96,617 and 107,488, respectively. The average sequencing depths of male and female parent were 29.05- and 39.95-fold, respectively. In F_2_ population, the average number of SLAFs was 80,403, and the coverage ranged with an average of 3.45-fold (**Table 1**).

**Table 1:** Summary of marker depth.

Sample ID	SLAF number	Total depth	Average depth
S1002	96,617	2,806,591	29.05
S1000	107,488	4,294,668	39.95
Average of F_2_	80,403	277,265	3.45

**FIGURE 2:**
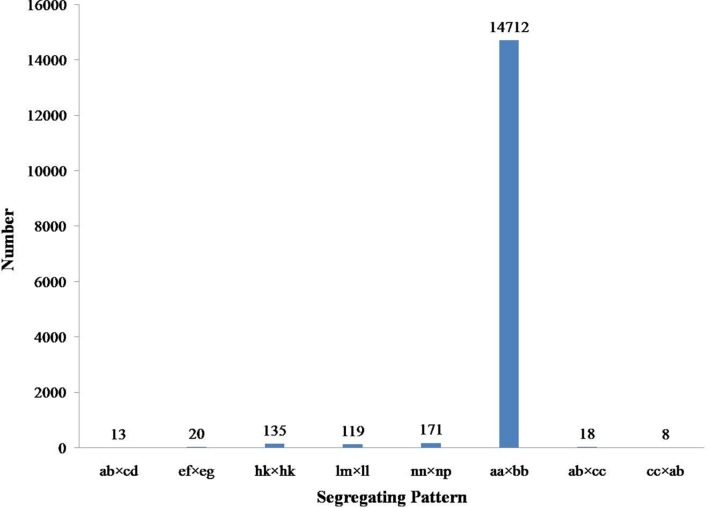
**Gnotype distribution of SLAF markers.** The Y axis is the number of SLAFs, the X axis is the type of SLAFs.

Among the 115,789 high-quality SLAFs detected, 15,946 were polymorphic with a rate of 13.77% (**Table 2**). Of the 15,946 polymorphic SLAFs, 15,196 were classified into eight segregation patterns (**Figure 2**). F_2_ population was obtained by selfing the F_1_ of a cross between two parents with homozygous genotype of aa or bb. So, only the F_2_ plants with aa × bb segregation pattern were used to construct the high-density genetic linkage map, and there are totally 14,712 markers fell into this type. Among these 14,712 markers, 3,077 markers were used for the high-density genetic map construction, which are homozygous in the two parents, with sequence depth more than 10-fold, and over 70% integrity of SLAF tags.

**Table 2:** Specific length amplified fragment (SLAF) markers number on each chromosome.

Chr ID	SLAF number	Polymorphic SLAF
Chr 1	17,193	2,449
Chr 2	13,617	1,343
Chr 3	23,612	3,545
Chr 4	13,785	2,128
Chr 5	16,776	2,153
Chr 6	17,442	2,262
Chr 7	11,694	1,841
Scaffold	1,670	225
Total	115,789	15,946

### Basic Characteristics of the Genetic Map

After linkage analysis, 3,057 of the 3,077 SLAF markers were mapped on the genetic map, while the other 20 SLAFs were not link to any group. These markers with 52.60-fold sequence depth in S1002 (the male parent), and 76.58-flod in S1000 (the female parent), and 4.97-fold in each F_2_ population on average.

The final genetic map included 3,057 markers on the seven LGs (**Table 3** and **Supplementary Figure S1**), and was 1,061.19 cM in length with an inter-marker distance of 0.35 cM (**Table 3**). The largest LG was LG3 with 649 markers, a length of 212.14 cM, and an average distance of only 0.33 cM between adjacent markers. The smallest LG is LG7 which only has 336 markers, with the length of 100.69 cM and an average distance of 0.30 cM. The largest gap on this map was 2.36 cM located in LG5. And this genetic map included 4,475 SNPs (**Table 3**, **Supplementary Tables S1**–**S3**).

**Table 3:** Description on basic characteristics of seven linkage groups.

Linkage group ID	Total marker	Total distance (cM)	Average distance (cM)	Largest gap (cM)	SNP number
Chr 1	404	136.93	0.34	1.31	954
Chr 2	326	139.54	0.43	1.77	751
Chr 3	649	212.14	0.33	2.29	604
Chr 4	503	165.68	0.33	1.96	593
Chr 5	399	151.07	0.38	2.36	625
Chr 6	440	155.15	0.35	1.96	461
Chr 7	336	100.69	0.30	1.44	487
Total	3,057	1,061.19	0.35	2.36	4,475

Until now, this map might be the most density SNP genetic linkage map to data for cucumber.

### QTL Analysis Using the High-Density Genetic Map

**FIGURE 3:**
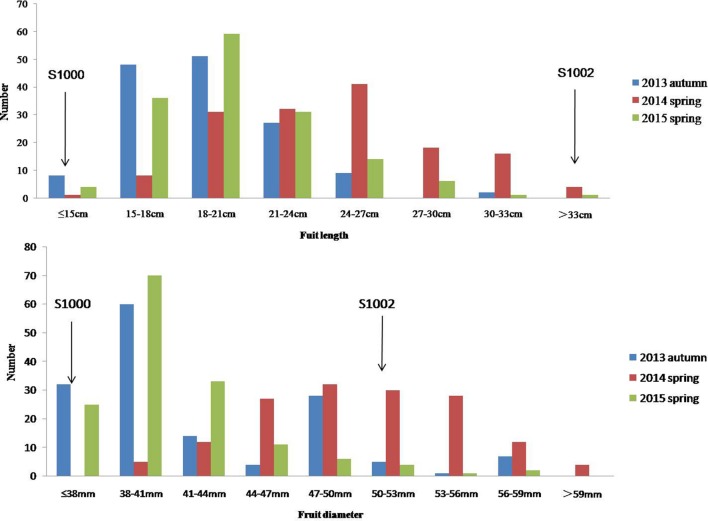
**Phenotypic evaluation of fruit length and fruit diameter for S1000, S1002, and F_3_.** The Y axis is the number of plant individuals. The X axis is continuous, for the figure fruit length, it means the fruit length (fl) ≤ 15 cm, 15 cm < fl ≤ 18 cm, 18 cm < fl ≤ 21 cm, 21 cm < fl ≤ 24 cm, 24 cm < fl ≤ 27 cm, 27 cm < fl ≤ 30 cm, 30 cm < fl ≤ 33 cm, fl > 33 cm; for the fruit diameter, it means the fruit diameter (fd) ≤ 38 mm, 38 mm < fd ≤ 41 mm, 41 mm < fd ≤ 44 mm, 44 mm < fd ≤ 47 mm, 47 mm < fd ≤ 50 mm, 50 mm < fd ≤ 53 mm, 53 mm < fd ≤ 56 mm, 56 mm < fd ≤ 59 mm, fd > 59.

**FIGURE 4:**
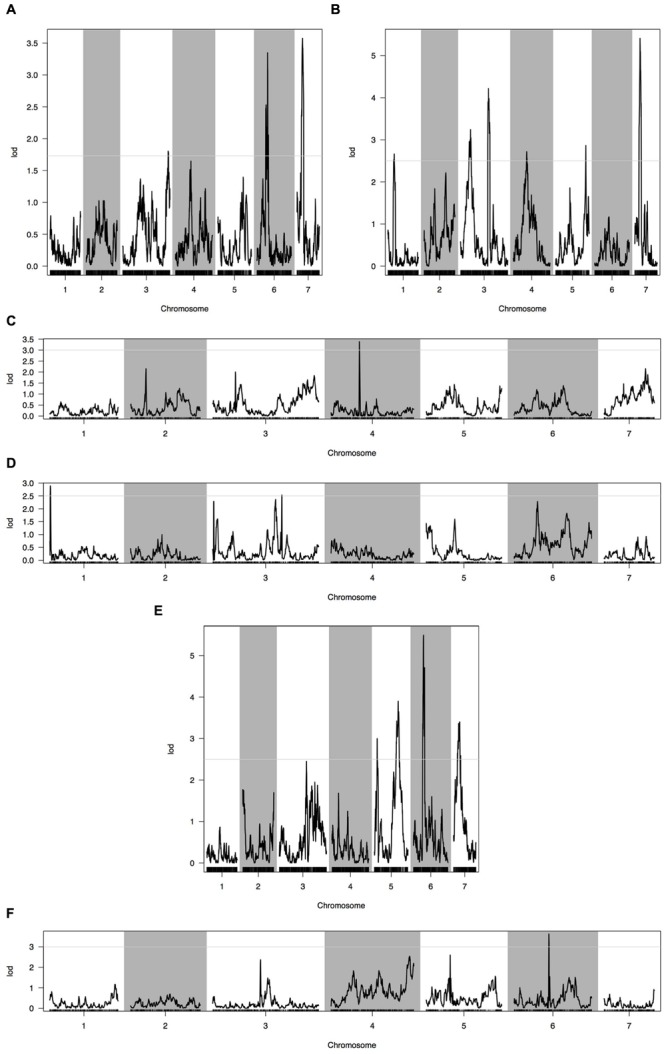
**Quantitative trait loci (QTL) analysis with two fruit traits using the high-density genetic map. (A)** QTL analysis of fruit length in 2013 autumn; **(B)** QTL analysis of fruit length in 2014 spring; **(C)** QTL analysis of fruit length in 2015 spring; **(D)** QTL analysis of fruit diameter in 2013 autumn; **(E)** QTL analysis of fruit diameter in 2014 spring; **(F)** QTL analysis of fruit diameter in 2015 spring. Horizontal line on the chart represents LOD threshold.

**FIGURE 5:**
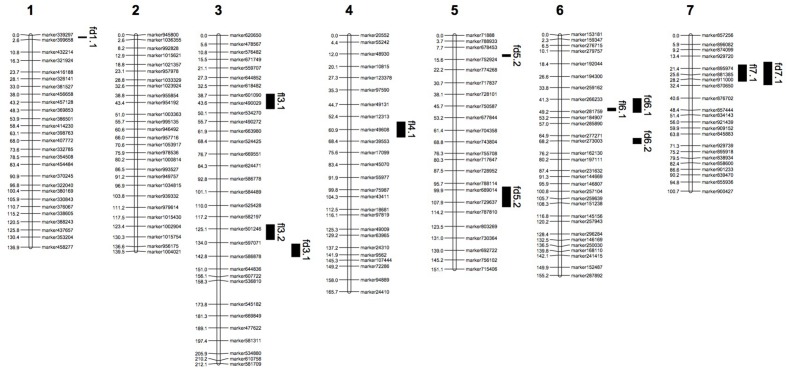
**Genetic linkage map with QTL locations on seven LGs.** fd: QTLs of fruit length; fl: QTLs of fruit diameter.

Phenotypic data of two parents, F_1_, F_2_, and F_2:3_ families are presented in **Figure 3**. QTLs for two fruit traits were detected in F_3_ families (**Table 4**; **Figures 4** and **5**), and mapped to unique positions. QTLs were detected for all fruit traits. A total of 15 QTLs were detected with seven and eight QTLs indentified per trait. The proportion of phenotypic variance explained by a single QTL (*r^2^*) ranged from 5.7 to 13.6% and LOD scores from 2.05 to 5.49. Seven QTLs were detected for fruit length, which were localized on chromosomes 3, 4, 6 and 7, accounting for 7.6–13.6% of the phenotypic variation. There were eight QTLs were detected for fruit diameter, which were localized on chromosome 1, 3, 5, 6 and 7, and the phenotypic variation was from 5.7 to 13.3%. (**Table 4**; **Figures 4** and **5**.

**Table 4:** Quantitative trait loci (QTL) analysis of cucumber fruit length and diameter in F_3_ families.

Fruit trait	Season	QTL	Chr	Marker interval	Interval (cM)	LOD	Additive effect	*r^2^*(%)	The donor parent
Fruit length	2013 autumn	*fl6.1*	6	Marker148176-248082	47.137–48.771	3.35	1.27	9.4	S1002
		*fl7.1*	7	Marker853155-903830	20.072–29.683	3.57	1.19	10.9	S1002
	2014 spring	*fl3.1*	3	Marker478460-625238	38.046–47.524	3.24	1.68	8.3	S1002
		*fl3.2*	3	Marker472756-604311	122.380–131.992	3.99	0.35	9.6	S1002
		*fl4.1*	4	Marker73031-85989	59.893–61.854	2.72	1.37	7.6	S1002
		*fl7.1*	7	Marker929060-911000	19.091–28.242	5.41	1.88	13.6	S1002
	2015 spring	*fl4.1*	4	Marker73728-82290	55.970–65.776	3.38	11.14	8.5	S1002
Fruit diameter	2013 autumn	*fd1.1*	1	Marker341596-393565	0.983–1.636	2.87	-0.45	7.2	S1000
		*fd3.1*	3	Marker559541-471326	134.933–143.104	2.55	-0.40	5.7	S1000
		*fd6.1*	6	Marker194932-264617	44.196–48.117	2.05	1.66	6.1	S1002
	2014 spring	*fd5.1*	5	Marker725009-785644	12.358–13.665	3.00	-0.68	8.1	S1000
		*fd5.2*	5	Marker810508-803274	97.956–110.903	3.65	-2.21	13.3	S1000
		*fd6.1*	6	Marker187297-251021	40.927–49.884	5.49	1.54	11.2	S1002
		*fd7.1*	7	Marker934821-888279	17.390–31.971	3.35	-1.21	10.8	S1000
	2015 spring	*fd6.2*	6	Marker265447-297311	66.552–70.147	2.60	-0.66	7.6	S1000

## Discussion

SLAF-seq is not a new but also a highly automated technique, it was developed based on the high-throughput sequencing. Because of the SLAF-seq was measured by sequencing the paired-ends of the sequence-specific restriction fragment length, which makes the repeatability of it is better than other techniques (such as RAD-seq, double digest RAD-seq and GBS). The SLAF-seq method provided significant advantages making it is very suitable for analysis of cucumber which with low polymorphism, such as the development of large numbers of markers having high accuracy with less sequencing. Usually, those conventional methods of developing markers were inefficient, expensive, and time-consuming, (Kennedy et al., 2003; Huang X. et al., 2009; Xie et al., 2010), while SLAF-seq has higher density, uniformity, and efficiency. SLAF-seq has been used in several studies since it was developed, such as Zhang et al. (2013) using this method constructed the first sesame high-density SNP genetic linkage map, Huang et al. (2013) obtained the draft of kiwifruit *Actinidia chinensi* genome, Chen et al. (2013) researched the development of 7E chromosome-specific molecular markers for *Thinopyrum elongatum*, Wei et al. (2014) and Xu et al. (2015a,b) constructed the high-density SNP genetic map for cucumber, respectively.

In this research, we developed thousands SNP markers for cucumber. We obtained 22.98 Gbp raw data based on high-throughput sequencing, consisting of 143.63 M reads, including 115,789 high-quality SLAF markers with a polymorphism rate of 12.71%. The obtained markers covered seven cucumber chromosomes, which had from 1,343 to 3,545 polymorphic SLAF markers on each chromosome, and Aa total of 15,946 polymorphic SLAF markers were used to construct the high-density SNP map. The integrity and accuracy of markers were much higher and the quality and quantity of markers met the requirements for construction of a high-density genetic linkage map. Therefore, the technique of SLAF-seq is suitable for discovering plant chromosome-specific molecular markers with higher success rates, specificity, and stability at low cost.

Compared with the other two SNP maps of cucumber (Wei et al., 2014; Xu et al., 2015a), this genetic map reported in this paper is the highest density map and had the smallest average distance (0.35 cM) between adjacent markers for cucumber. The map spans 1,061.19 cM with an average number of 436.71 markers per LG with an average distance of 0.35 cM between adjacent markers. This map had the most number of markers (3,057) and the minimum average distance (0.35 cM) in all of the cucumber genetic maps (**Table 5**). These markers could be used for different populations. More important, the SNP markers on this map are the most abundant type of genetic variation between different individuals. SNPs are very suitable and favorable for the studies of comparative genomic (Luo et al., 2009) and association mapping (Cogan et al., 2006; Chan et al., 2010).

**Table 5:** Comparison of three high-density genetic SNP maps.

Author	Number of F_2_	Raw	Total SLAF	Polymorphic	aa × bb	Total	Average	SNP
	population	data (Gb)	number	SLAF number	SLAF	distance (cM)	distance (cM)	number
Wei Qing-zhen	148	10.76	52,684	5,044	4,227	890.79	0.50	1,800
Xu Xue-wen	102	18.69	79,092	16,784	1,892	845.87	0.45	–
Our group	153	22.98	115,789	15,946	3,057	1,046.97	0.35	4,475

This map not only provides large scale SNP markers for cucumber, but also provides useful data for cucumber QTL analysis, gene fine mapping, map-based gene clone and molecular breeding. For this genetic SNP map, all the seven LGs were structured based on the level of whole genome molecular markers, so, it could be served as a reference data for positioning sequence scaffolds on the physical map of cucumber.

Fruit is the most important commodity character of cucumber (Marcelis, 1992). So high fruit yield and quality always are the most important objective for cucumber breeding. Using F_3_ family plants and BC_1_ population Kennard and Havey (1995) obtained the QTLs for several important cucumber fruit quality traits, which including fruit length, fruit diameter, seed-cavity diameter, fruit skin color, the ratio of fruit length/diameter, and seed-cavity/fruit diameter ratio. Subsequently, Serquen et al. (1997, using F_3_ families); Dijkuizen and Staub (2002) using S_3_ and BC families obtained the QTLs for fruit number, fruit weight, fruit length, fruit diameter, and fruit length/diameter ratio, and Fazio et al. (2003) identified additional QTLs for the same cucumber fruit traits. In the previous studies in our group, Yuan et al. (2008b) identified 38 QTLs for eight yield and quality components (fruit length, weight, pedicel length, flesh thickness, seed-cavity diameter, stalk length, the ratio of fruit length/diameter and fruit length/stalk) using F_2_ and F_3_ populations. Weng et al. (2015) identified three QTLs for fruit length which were localized on chromosomes 3, 4, and 6; and three QTLs for fruit diameter localized on chromosomes 2, 5, and 6. Zhou et al. (2015) detected two QTLs for fruit length which were localized on chromosomes 5 and 7.

We identified 15 QTLs for fruit length and fruit diameter in this study. There were seven QTLs for fruit length. In these seven QTLs, there are two couples of repeat QTLs, *fl7.1* in 2013 autumn and *fl7.1* in 2014 spring, *fl4.1* in 2014 spring and *fl4.1* in 2015 spring. We search the genes in the repeat regions on the web of http://www.icugi.org/cgi-bin/ICuGI/index.cgi, and we found several genes which were related to auxin response and multi cellular development. Eight QTLs were detected for fruit diameter, including two repeat QTLs *fd6.1* in 2013 autumn and *fd6.1* in 2014 spring. In this region there are several genes which were related to plant hormone response, many cells, and cell mitosis. So we suspect that the genes associated with fruit length and diameter may be existed in these areas.

In our study, the LOD value varies among most of the QTLs identified for fruit length and diameter of cucumber between 2.0 and 3.5. The LOD value is low to ascertain the accuracy of QTLs identified. In our opinion, there are several reasons for this phenomenon. First of all, the character data might exist a certain error, which is one of the important factors which affecting LOD values. The other reason of the low LOD value might be the different analysis software. In our study, we used the R/qtl to analysis the QTL, the reference coefficient is different from the software MapQTL, which might cause low LOD value of the QTL analysis results.

In this study, none of the QTLs for fruit length and fruit diameter are common across 2013 autumn, 2014 spring and 2015 spring seasons. We speculated that one of the most important reasons may be the fruit length and diameter are easily influenced by environment. Statistical characters results showed that, the results are relatively similar in 2014 spring and 2015 spring, which are different from 2013 autumn. In the process of cucumber growth, the temperature and humidity of 2013 autumn were much higher than that of 2014 spring and 2015 spring. Under the environment of high temperature and high humidity, plant diseases and insect pests such as powdery mildew and root rot occurred frequently. Which leads the QTL analysis also appeared similar phenomenon: in 2014 spring and 2015 spring, there are common QTLs (both fruit length and fruit diameter). The deviation when we collected phenotypic data might be another reason which caused there no common QTL in three seasons. In addition, the detected QTLs are less in each season, this is probably one of the reasons for no common QTL in three seasons.

In our study, some of the 15 QTLs for fruit length and fruit diameter are consistent with the previous reported, such as *fl4.1* is the same with the main effect QTL in Yuan’s result, and the *fd5.1* are the same with the QTL in Yuan’s report (Yuan et al., 2008a). But some are different from the QTLs for the two fruit traits in previous reports. We suspected that there are two reasons for this result. First of all, the gene of the same trait may be different in different materials; Second, under the influence of environment, climate and other conditions, the fruit characters are different in different season, which caused the different positioning results.

## Conclusion

We obtained a high-density SNP genetic linkage map for cucumber. We constructed this map by a F_2_ family plants, and polymorphic markers developed by using the method of SLAF-seq. We generated a total of 22.98 Gb of raw data, containing 143.63 M reads of ~80 bp in length left over after preprocessing. The final genetic map included 3,057 markers on the seven LGs, and was 1,061.19 cM in length with an inter-marker distance of 0.35 cM. Based on this high-density genetic map, QTL analysis on fruit length and fruit diameter, seven QTLs for fruit length and eight QTLs for fruit diameter were detected. The results of this study will not only provide a platform for gene/QTL fine mapping, map-based gene isolation, and molecular breeding for cucumber, but also provide a reference to help position sequence scaffolds on the physical map and assist in the process of assembling the cucumber genome sequence.

## Author Contributions

W-YZ constructed the mapping populations, surveyed fruit traits, performed genetic analysis, marker development, and wrote the paper. LH performed genome sequencing, constructed the map and mapping analysis. J-TY constructed the mapping populations. LC, M-LQ, and D-QY performed some of the field work and assisted with extracted DNA. H-LL and H-LH provided valuable research ideas. J-SP and RC designed and supervised the study.

## Conflict of Interest Statement

The authors declare that the research was conducted in the absence of any commercial or financial relationships that could be construed as a potential conflict of interest.

## Supplementary Material

FIGURE S1 **High-density cucumber genetic map composing of SNPs**.

TABLE S1 **Sequence information of total SLAFs**.

TABLE S2 **Sequence information of mapped SNPs**.

TABLE S3 **Physical position of mapped markers**.
