# Supplementary figures and images for: A High-Density Genetic Linkage Map for Cucumber (Cucumis sativus L.): Based on Specific Length Amplified Fragment (SLAF) Sequencing and QTL Analysis of Fruit Traits in Cucumber
Source: Front Plant Sci. 2016 Apr 19;7:437. doi: 10.3389/fpls.2016.00437 (PMC4835494; doi:10.3389/fpls.2016.00437)

Chr1

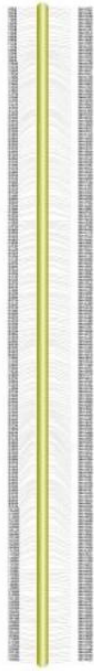

Chr2

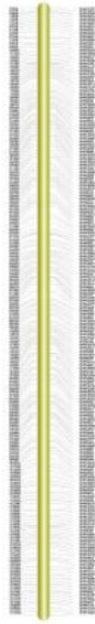

Chr3

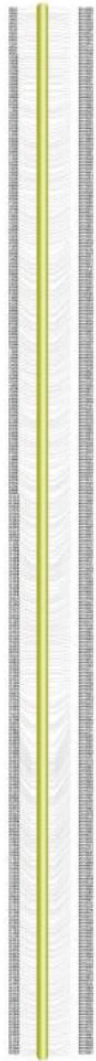

Chr4

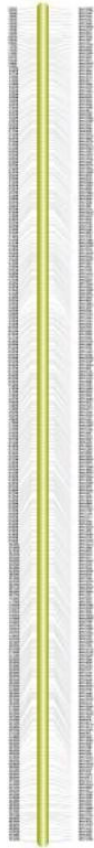

Chr5

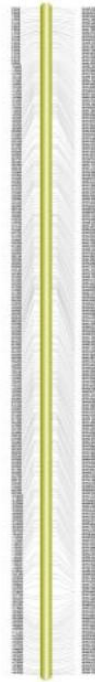

Chr6

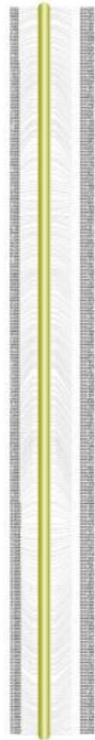

Chr7

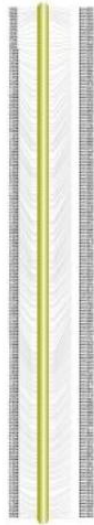

Supplement: FIGURE S1 — High-density cucumber genetic map composing of SNPs. [file Image_1.PDF]
