# Supplementary material for: A High-Density Genetic Linkage Map for Cucumber (Cucumis sativus L.): Based on Specific Length Amplified Fragment (SLAF) Sequencing and QTL Analysis of Fruit Traits in Cucumber
Source: Front Plant Sci. 2016 Apr 19;7:437. doi: 10.3389/fpls.2016.00437 (PMC4835494; doi:10.3389/fpls.2016.00437)
Supplement: TABLE S1 — Sequence information of total SLAFs. [file Table_1.PDF]

>Marker3285
ACTCTCTTGTCTTGTGATGTATGCTTATATTCATGTTGTCTTTGTAGGTTCAACTAAAGCCAACAACTTCATGAG
TTTTTXXXXXXXXXXATTCTGATGGTATTTACACTAGTTTTGATTAAGGGCTGAAATTCCATATTTGTTAATTGT
TCAAACTTGATTGGAAATGT
ACTCTCTTGTCTTGTGATGTATGCTTATATTCATGTTGTCTTTGTAGGTTCAACTAAAGCCAACAACTTCATGAG
TTTTTXXXXXXXXXXATTCTGATGGTATTTACACTAGTTTTGATTAAGGGCTAAAATTCCATATTTGTTAATTGT
TCAAACTTGATTGGAAATGT
>Marker3624
TACAAATCTGAGCTTTCAGGTTAGTTTATGTTTCTTGAAAGTGGGTATCAGTATCAAAATTTGAACAGAAAGCCT
TCAGAXXXXXXXXXXTGTCCATTTCCTGAAGTTTTCTGTTAGTGAAGAGTTGCAAGGCACTCGACCCTCCAACAG
AATTGAAGAAAAAGTCAGTT
TACAAATCTGAGCTTTCAGGTTAGTTTATGTTTCTTGAAAGTGGGTATCAGTATCAAAATTTGAACAGAAAGCCT
TCAAAXXXXXXXXXXTGTCCATTTCCTGAAGTTTTCTGTTAGTGAAGAGTTGCAAGGCACTCGACCCTCCAACAG
AATTGAAGAAAAAGTCAGTT
>Marker4017
AACTCCATCCACTGTGTCCCATCGGTGCCATAAAATTCAGGTCAAATCTTGTAGTCTGTAGGGATTGTATAAACA
ATCCTXXXXXXXXXXAGCCAAGCAAGGTTACTTATGTCAGTTGCAATTGCAGCTAGTTCAAAACTGGGGAAGGAT
GGTGGAGGCTCTGAGAAGTC
AACTCCATCCACTGTGTCCCATTGGTGCCATAAAATTCAGGTCAAATCTTGTAGTCTGTAGGGATTGTATAAACA
ATCCTXXXXXXXXXXAGCCAAGCAAGGTTACTTATGTCAGTTGCAATTGCAGCTAGTTCAAAACTGGGGAAGGAT
GGTGGAGGCTCTGAGAAGTC
>Marker4282
GACCTAACCATATTTTTCTTTTACTTATTATAAACTTCATTTTGATATTAAATATGGATCTTACCTTCTTATTCT
TCTTAXXXXXXXXXXTTTATATATATAACAATTAAGTGAAAGAATTGGAGATGAGTGTGGTGCAGATATTTTTTG
AAATTTTTTAATAGAAATGT
GACCTAACCATATTTTTCTTTTACTTATTATAAACTTCATTTTGATATTAAATATGGATCTTACCTTCTTATTCT
TCTTAXXXXXXXXXXTTTATATATATAACAATTTAGTGAAAGAATTGGAGATGAGTGTGGTGCAGATATTTTTTG
AAATTTTTTAATAGAAATGT
>Marker5195
TACATCATCAGCCTCTAAAGTTGCATTTAATTTTCATTTTTTCGAGAGAGTGAAAAGTGAGACAATGAGAGAGCG
AGAGGXXXXXXXXXXATGATAGCGAGAAAAGCTAATTCACATAAGTGAGTTAATTTTGCACAAATATGATGTAAG
AAAAGGGTTAAAAAGACGTC
TACATCATCAGCCTCTAAAGTTGCATTTAATTTTCATTTTTTGGAGAGAGTGAAAAGTGAGACAATGAGAGAGCG
AGAGGXXXXXXXXXXATGATAGCGAGAAAAGCTAATTCACGTAAGTGAGTTAATTTTGCACAAATATGATGTAAG
AAAAGGGTTAAAAAGACGTC
>Marker5704
ACTTAGATTATATAACCCTATTCTTATATAATAAATTGACGAATCAATCTGTATGATCTATCTCAAGAATCTTTG
AATTGXXXXXXXXXXCCTTAGAGTATTATTAAACACGAAGCTTCTTGTGTGGTTAAATCGGACTTCGCTACTCTT
ACTATCAAGAAGTTGTGGTT
ACTTAGATTATATAACCCTATTCTTATATAATAAATTGACGAATCAATCTGTATGATCTATCTCAAGAATCTTTC
AATTGXXXXXXXXXXCCTTAGAGTATTATTAAACACGAAGCTTCTTGTGTGGTTAAATCGGACTTCGCTACTCTT
ACTATCAAGAAGTTGTGGTT
>Marker5942
ACTGCTCAAACTCTTGATCAATGGTTGGTTTGATAGGCTTGGATTCTGTCTCTGCATATTTGAATGATTCAGTAT
TAGTTXXXXXXXXXXGCTGTATTGGTCTGGTTTAAATCTTCGTTCCATCACTAAATTTTGCCACTTATTTCATTC
AAATTTTCATATAATATGTG

Create PDF with GO2PDF for free, if you wish to

ACTGCTCAAACTCTTGATCAATGGTTGGTTTGATAGGCTTGGATTCTGTCTCTGCATATTTGAATGATTCAGTAT
TAGTTXXXXXXXXXXGCTGTATTGGTCTGGTTTAAATCTTCGTTCCATCATTAAATTTTGCCACTTATTTCATTC
AAATTTTCATATAATATGTG
>Marker6029
CACAATTACATAACAATGAACTCATGGTTTTGGTTAGTTGGCGTTTTTGTAATGGTGGTATTGGAACAAAACTTT
AGGGTXXXXXXXXXXTTTCTAGGTCACAGGGTGAGTATCCAAATCCTGTTTTCAATAGATTCAATTAATTTGAAA
ACTAGAAATTTAACTTCGGT
CACAATTACATAACAATGAACTCATGGTTTTGGTTAGTTGGCGTTTTTGTAATGGTGGTATTGGAACAAAACTTT
AGGGTXXXXXXXXXXTTTCTAGGTCACAGGGTGAGTATCCAAATCCTGTTTTCTATAGATTCAATTAATTTGAAA
ACTAGAAATTTAACTTTGGT
>Marker6085
AACCAAAACCAACCCCTTGAATGTTTCTATCCCTAAACCCACACAGAATCCCAACCTACTAACTAATCAAATATA
AGCAAXXXXXXXXXXTCTCTAGAAGCTAACTCTTACTCACCCAACTTTATGGATGTGTATTAGCATTGAGTATGA
TCTCCAAAGCATACAAAGTA
AACCAAAACCAACCCCTTGAATGTTTCTATCCCTAAACCCACACAGAATCCCAACCTACTAACTAATCAAATATA
AGCAAXXXXXXXXXXTCTCTGGAAGCTAACTCTTACTCACCCAACTTTATGGATGTGTATTAGCATTGAGTATGA
TCTCCAAAGCATACAAAGTA
>Marker6235
TACAACACGAGATGATAGTAAGCATCGTAACCACGGTGGAGGATCCTCTCCAGATGCTGAGAGATGAGGAAAGCG
CCTCTXXXXXXXXXXAATGGTTCCAGGTTTTGTTAATAATTATATATGGTGAGGCTGTATCTTTGATCATTAGCA
ATGTAAATGGTTAATAGGTA
TACAACACGAGATGATAGTAAGCATCGTAACCACGGTGGAGGATCCTCTCCAGATGCTGAGAGATGAGGAAAGCG
CCTCCXXXXXXXXXXAATGGTTCCAGGTTTTGTTAATAATTATATATGGTGAGGCTGTATCTTTGATCATTAGCA
ATGTAAATGGTTAATAGGTA
>Marker6481
GACTGAATTGAATAGATTCCTCCATTGGCTTTGTAATTTCATAGTTCTGCAGATTTATCATTTATGTCCTTGACT
TTCTTXXXXXXXXXXAACTACCTAAACAATTCATAATTCGAAAAGGTAACTTTTCATTTATTCTTCATTTTCTTG
TTCCACAGATGCCCCGGGTG
GACTGAATTGAATAGATTCCTCCATTGGCTTTGTAATTTCATAGTTCTGCAGATTTATCATTTATGGCCTTGACT
TTCTTXXXXXXXXXXAACTACCTAAACAATTCATAATTCGAAAAGGTAACTTTTCATTTATTCTTCATTTTCTTG
TTCCACAGATGCCCCCGGTG
>Marker6518
AACAAATGATGGATCATTAAATTTCTTTGTTTGCACTTGTTATGAATTACCAAGTATATAGTTTGAAAATTTGAG
GAAGTXXXXXXXXXXTTTAAAAGCATATATATGCATCAACTAATTCAATGCTAACGCATTTGTTTTTCATTTTCA
TTTTCTTTCGGTTTTAAGTT
AACAAATGATGGATCATTAAATTTCTTTGTTTGCACTTGTTATGAATTACCAAGTATATAGTTTGAAAATTTGAC
GAAGTXXXXXXXXXXTTTAAAAGCATATATATGCATCAACTAATTCAATGCTAACGCATTTGTTTTTCATTTTCA
TTTTCTTTCGGTTTTAAGTT
>Marker6611
ACTTGTCGGGCATTCAGGTTAAGTATTCTAAATCTAGTTTTGATGATTTACATCATGGTTCATTTAAGATTTTAA
TTTTAXXXXXXXXXXATATGCTCTTTTTGTTCTCCAATTATTTTGTTTGTTTGCCTCAAGTATTTTTTTTCCTTG
TATAATTGGATTTGTATGTA
ACTTGTCGGGCATTCAGGTTAAGTATTCTAAATCTAGTTTTGATGATTTACATCATGGTTCATTTAAGATTTTAA
TTTTAXXXXXXXXXXATATGCTCTTTTTGCTCTCCAATTATTTTGTTTGTTTGCCTCAAGTATTTTTTTTCCTTG
TATAATTGGATTTGTATGTA
>Marker7005

Create PDF with GO2PDF for free, if you wish to

CACAAGTAAAGAAAAAAACAACTTTATAAAAAAAAAATTAATGATACAAATCAAATTATGCACGTCTAGGGTTAT
AGATTXXXXXXXXXXTATATGTATATTTCCAAGTTAATAAGTCTAACTCCTTAAGATGTTTGGGACAACGGTTGA
GGTTATGAAAATTGAAAAGT
CACAAGTAAAGAAAAAAACAACTTTATAAAAGAAAAATTAATGATACAAATCAAATTATGCACGTCTAGGGTTAA
AGATTXXXXXXXXXXTATATGTATATTTCCAAGTTAATAAGTCTAACTCCTTAAGATGTTTGGGACAACGGTTGA
GGTTATGAAAATTGAAAAGT
>Marker7294
AACAAGTGTTGGCATATGTGTATTGTGGTGTGTATGAGATGAGATGGACCGTTTGGGAATAATAACTCAAACCTT
TAGGCXXXXXXXXXXCAGAGTGAAGAACAGAACAGAATGACGAGCAACCACCATAGCCTATCAATCATTTTTTCA
TTCCTACATCGTGGGTCGTT
AACAAGTGTTGGCATATGTGTATTGTGGTGTGTATGAGATGAGATGGACCGTTTGGGAATAATAAATCAAACCTT
AGGCTXXXXXXXXXXCAGAGTGAAGAACAGAACAGAATGACGAGCAACCACCATAGCCTATCAATCATTTTTTCA
TTCCTACATCGTGGGTCGTT
>Marker7439
CACGAAAGGAACTCATGATTACTAAAATTGAAGATTCCTTTATCAGAAAAAAGGACAAAATGATTCCAGTGCCTC
TCTCTXXXXXXXXXXATTAAATTAGGCCCAATTTAGGCGTTTGAATTTAACTAATTTTATAATTTTAACCTTCAA
AAAATCCTATTCATTCAAGT
CACGAAAGGAACTCATGATTACTAAAATTGAAGATTCCTTTATCAGAAAAAAGGACAAAATGATTCCAGTGCTTC
TCTCTXXXXXXXXXXATTAAATTAGGCTCAATTTAGGCGTTTGAATTTAACTAATTTTATAATTTTAACCTTAAA
AAAATCCTATTCATTCAAGT
>Marker7446
ACTGATGGTAAGTTCCTGAATTTTGTCCATTTAGTTTATATAGTGTCAATGAGTATTCTACAGGCTTGAGCTCTC
TGCCAXXXXXXXXXXTAACCGTGCAAAAACAACAGTCAAGCAGGGTTATTCCACATATTTTGGTTAGGTGTGCCG
ATTATCTCGTCTTGTCAGGT
ACTGATGGTAAGTTCCTGAATTTTGTCCATTTAGTTTATATAATGTCAATGAGTATTCTACAGGCTTGAGCTCTC
TGCCAXXXXXXXXXXTAACCGTGCAAAGACAACAGTCAAGCAGGGTTATTCCACATATTTTGGTTAGGTGTGCCG
ATTATCTCGTCTTGTCAGGT
>Marker7782
GACAAGCTCATTATCGAACATATTTCTTCTAAGGCCCAAGCTCCACGAATGAGTAACAATACACCAACATGCAGC
CACATXXXXXXXXXXAAAGCTGAGAATTTAAAGGTCTCCTTGTGCTTCAAAATACCAGCCCATATCTATAAGATT
TTCCCCTATTCGGTTTAGTA
GACAAGCTCATTATCGAACATATTTCTTCTAAGGCCCAAGCTCCACGAATGAGTAACAATACACCAACATGCAGC
CACATXXXXXXXXXXAAAGCTGAGAATTTAAAGGTCTCCTTGTGCTTCAAAATACCAGCCCATATCTATAAGATT
TTCCCCTGTTCGGTTTAGTA
>Marker7905
ACAGGAATTGACAGATGATATTGGAGAACTTGAGAACCAAAAGGATCGGCTGGAGGCTGAATTGAAAAAGGTTGG
ATTTCXXXXXXXXXXGATTGGTTGTGCTTTTATTTATTTTCCATATTTTTCCAATGGGAAGTTCGTATATGCTTC
ATGGGTTACTTTAGTGTGTT
ACAGGAATTGACAGATGATATTGGAGAACTTGAGAACCAAAAGGATCGACTGGAGGCTGAATTGAAAAAGGTTGG
GTTTCXXXXXXXXXXGATTGGTTGTGCTTTTATTTATTTTCCATATTTTTCCAATGGGAAGTTCGTATACGCTTC
ATGGGTTACTTTAGTGTGTT
>Marker8022
TACAACTCAACCATATTTCCAAAACTCCATCCCCTAAACACAACTAACCTTTCAGCTTATGACAAATTGCTTGGC
GGAGTXXXXXXXXXXAAAACTTGGACATTGAACACTAAAAATTTTAGTATAAGATAATACCTCAGTAATTGGGGC
AGGTGTATCAAAATCAGGTT

Create PDF with GO2PDF for free, if you wish to

TACAACTCAACCATATTTCCAAAACTCCATCCCCTAAACACAACTAACCTTTCAGCTTATGACAAATTGCTTGGC
GGAGTXXXXXXXXXXAAAACTTGGACATTGAACACTAAAAATTTTAGCATAAGATAATACCTCAGTAATTGGGGC
AGGTGTATCAAAATCAGGTT
>Marker8083
AACCTGGAGACACCTTAACCAAAACATAACCAACAAAAATTTATACAACCACGGTTCCAAAAGCTTACACAAACT
GTAGTXXXXXXXXXXGTGGGTAAAACATTTTGGAAGGGTAAGATATAAAGCTCAGTGAGTGACTCGGTTTCAAAT
AGAATTTTAAAGGTAAGAGT
AACCTGGAGACACCTTAACCAAAACATAACCAACAAAAATTTATACAACCACGGTTCCAAAAGCTTACACAAACT
GTAGAXXXXXXXXXXGTGGGTAAAACATTTTGGAAGGGTAAGCTATAAAGCTCAGTGAGTGACTCGGTTTCAAAT
AGAATTTTAAAGGTAAGAGT
>Marker8123
CACTGTCGCATTATTCTTATTGTTGAAAGAATTTTGGGAAGGATACTTACCTCTAGCATACCCCGTAAAGTTAGG
CTCATXXXXXXXXXXGATCGGATAAAGGTGCCATCAATTTATCGAATTGGTTACGATATTCCTCCACCATATCCT
TGAGATTCGACCAATTGGTA
CACTGTCGCATTATTCTTATTGTTGAAAGAATTTTGGGAAGGATACTTACCTCTAGCATACCCCGTAAAGTTAGG
CTCATXXXXXXXXXXGATCGGATAAAGGTGCCATCAATTTATCGAATTGGTTACGATATTCCTCCACCATATCCT
TGAGATTCGACCAATCGGTA
>Marker8165
TACCTAATACAGTTCATCCGAGATGAGGGCTAAATTATGAGCAAATGGAATTTGAAAATTTTCATTTGCATGAGG
AGTAGXXXXXXXXXXAATAGCAAAGATGAGCATATAAACCAATTGATGAAATCATTTACCTCAAAATTTATAGAT
GAAAATATATGTTTATAAGT
TACCTCATACAGTTCATCCGAGATGAGGGCTAAATTATGAGCAAATGGAATTTGAAAATTTTCATTTGCATGAGG
AGTAGXXXXXXXXXXAATAGCAAAGATGAGCATATAAACCAATTGATGAAATCATTTACCTCAAAATTTATAGAT
GAAAATATATGTTTATAAGT
>Marker9562
TACGGTTTCATGAATACTTATTTTTATGGTAGGTATTTTTGAGCATCCATAATTACTTTAATTGAAGTGTGGAGA
TAGAAXXXXXXXXXXTATTGGAAGTAGCAGCAATTATATTCTTTTATTTGGGAAATGAAGAAAATAAACCAACTT
GGTGACTCTCCACCATTGTT
TACGGTTTCATGAATACTTATTTTTATGGTAGGTATTTTTGAGCATCCATAATTACTTTAATTGAAGTGTGGAGA
TAGAAXXXXXXXXXXTATTGGAAGTAGCAGCAATTATATTCTTTTATTTGGGAAATGAAGAAAATACACCAACTT
GGTGACTCTCCACCATTGTT
>Marker9754
TACCTATATATTTATCTTTTTATTTCTTTTATATAGTTGCATTGATGTTTGTGCCTCAAGACGTCTCAGCTGCCC
AAAGAXXXXXXXXXXATGCTTGTTATTGTTTCTTTGATTGCGGTGGTCAAAAGAATCGATCTGCATACTCGCATC
TTCCTTATTTAATGTAATGT
TACCTATATATTTATCTTTTTATTTCTTTTATATAGTTGCATTGATGTTTGTGCCTCAAGACGTCTCAGCTGCCC
AAAGAXXXXXXXXXXATGCTTGTTATTGTTTCTTTGATTGCAGTGGTCAAAAGAATCGATCTGCATACTCGCATC
TTCCTGATTTAATGTAATGT
>Marker10414
CACACGAGGTTTCGGAAAAATGTAATTCGTGGAATTGAAATTTGTATATTGATGAATATTGTGAATACAATTTCT
AGTATXXXXXXXXXXCCTTCGATCTTATGGAATCCAAGGAAAGTTCAATCTTCCATGTCTTCAATTTTTCAGAAG
TTGTTGATCTCGAGGTTGGT
CACACGAGGTTTCGGAAAAATGTAATTCGTGGAATTGAAATTTGTATATTGATGAATATTGTGAATACAATTTCT
AGTATXXXXXXXXXXCCTTCGATCTTCTGGAATCCAAGGAAAGTTCAATCTTCCATGTCTTCAATTTTTCAGAAG
TTGTTGATCTCGAGGTTGGT
>Marker10491

Create PDF with GO2PDF for free, if you wish to

ACAAGAAGTTTGAGTCAGTTATGGGGGAGTAAGGCTACACAAAAACTATTTCTGGTCACTGTGTTTTGTCCATAA
TTATTXXXXXXXXXXTAAAGAAAGTTCTTGGAATTTGAATAGTTCGAGATAAAACATCCAAGAAGTTATACATGT
CACATGACCAAAGAGAAAGT
ACAAGAAGTTTGAGTCAGTTATGGGGGAGTAAGGCTACACAAAAACTATTTCTGGTCACTGTGTTTTGTCCATAA
TTATTXXXXXXXXXXTAAAGAAAGTTCTTGGAATTTGAATAGTTCGAGATAAAACATCCAAGAAGTTATACATGT
CACATGACCAAAAAGAAAGT
>Marker10815
GACATTGCTGGGTTAGTGAAAGGAGCCAGTCAAGGAGAGGTAAAGTTTATTCTCACCTCAGTATTATCATCATCA
TCATCXXXXXXXXXXTTATCATAATTTATTTTTTTCCATCATTTCAATTAGAATGCATACTTAAGTAGTAGCGGA
TTTAAAGAGTAATATTAGTT
GACATTGCTGGGTTAGTGAAAGGAGCCAGTCAAGGAGAGGTAAAGTTTATTCTCACCTCAGTATTATCATCATCA
TCATCXXXXXXXXXXTTATCGTAATTTATTTTTTTCCATCATTTCAATTAGAATGCATACTTAAGTAGTAGCGGA
TTTAAAGAGTAATATTAGTT
>Marker10958
GACGTTTTTTTCGTGGTTAGAAATATCTTTTATGACCACGATTTTAATTTGCAAGGTCATTTCTGCCGACACAAC
ATAAAXXXXXXXXXXTGCCCATGTTTTCCTGCATCGGTAAAGACAAAAATCTTTGTAGTGAAAACTCCCGACTAA
TTACTAATGGCTTCAAGAGT
GACGTTTTTTTTGTGGTTAGAAATATCTTTTATGACCACGATTTTAATTTGCAAGGTCATTTCTGCCGACACAAC
ATAAAXXXXXXXXXXTGCCCATGTTTTCCTGCATCGGTAAAGACAAAAATCTTTGTAGTGAAAACTCCCGACTAA
TTACTAATGGCTTCAAGAGT
>Marker11011
ACATGGACTTTCGATTCCAAGACTATTTGATAGAGCATGAAATCCAATTACAATTCTCTGCACCTAATACACCTT
AGCAAXXXXXXXXXXTGTCTATATTTTGAACAATGTTCCCACTAAAAGTGTTTTAGAAACAACTTATGAGCTTTA
AAAAGGGCGTAAAGGAAGTT
ACATGGACTTTCGATTCCAAGACTATTTGATAGAACATGAAATCCAGTTACAATTCTCTGCACCTAATACACCTT
AGCAAXXXXXXXXXXTGTCTATATTTTGAACAATGTTCCCACTAAAAGTGTTTTAGAAACAACTTATGAGCTTTA
AAAAGGGCGTAAAGGAAGTT
>Marker11303
TACTAGTCGTGTGCTCACGTATGAACTTAACTAGTGTTATATAGATTTGAGTATTATCCTCCTTAAGAAAATACT
TTTTGXXXXXXXXXXAGGAGATCTAAGTTCGAAAAGATTTTTCATGAGAAAAATTAATTTCGGCAAAAGTGTTGA
TAAACACTCATGAATGTGTA
TACTAGTCGTGTGCTCACGTATGAACTTAACTAGTGTTATATAGATTTGAGTATTATCCTCCTTAAGAAAATACT
TTTTGXXXXXXXXXXAGGATATCTAAGTTCGAAAAGATTTTTCATGAGAAAAATTAATTTCGGCAAAAGTGTTGA
TAAACACTCATGAATGTGTA
>Marker11362
CACTTCGCAAATCTTCCAATCAAGCTTCTTATGCCTACCTCTTTTACCAACATCACTGAAATTGCTCTTAAAACC
ATTCCXXXXXXXXXXAGGGATCTTGATCGCAAAGCCTGATTACAAGAAGGCCTATGTTACTCTCAGAAATCCCTT
GTCAATATCTCCGGACATGT
CACTTCGCAAATCTTCCAATCAAGCTTCTTATGCCTACCTCTTTTACCAACATCACTGAAATTGCTCTTAAAACC
ATTCCXXXXXXXXXXAGGGATTTTGATCGCAAAGCCTGATTACAAGAAGGCCTATGTTACTCTCAGAAATCCCTT
GTCAATATCTCCGGACATGT
>Marker11510
AACCACAGTAACATAACAAACAAAGTAAATAAATATACAGGAGTTTAAAAAATAATATTAATAATAAAAAACCTT
CTGAAXXXXXXXXXXGGAATTTTATTAGTTGGAAATGAATTTGACCTGGACTAAATTAAAATCCTGAATTTTAGT
AGAAAACATGGTAGAGGGTC

Create PDF with GO2PDF for free, if you wish to

AACCACAGTAACATAACAAACAAAGTAAATAAATATACAGGAGTTTAAAAAATAATATTAATAATAAAAAACCTT
CTGAAXXXXXXXXXXXGGAATTTTATTAGTTGGAAATGAATTTGACCTGGACTCAATTAAAATCCTGAATTTTAGT
AGAAAACATGGTAGAGGGTC
>Marker11576
ACCAATATTTCAATGAAAAGCATCCATTCATCAAATTTCCGATACTTTAAGTTTCTTCTCAAGTTTTCGATTTAA
GCAGCXXXXXXXXXXXGCACCTACGAATCAGAGCTCATCAGGTTATTTTGCTTTGAGTCTCTTTTGCATTTGTCCT
CACATAGTCAAGGGTTTTGT
ACCAATATTTCAATGAAAAGCATCCATTCATCAAATTTCCGATACTTTAAGTTTCTTCTCAAGTTTTCGATTTAA
GCAGCXXXXXXXXXXXGCACCTACGAATCAGAGCTCATCAAGTTATTTTGCTTTGAGTCTCTTTTGCATTTGTCCT
CACATAGTCAAGGGTTTTGT
>Marker12313
ACCTGTCTTGATCTGTGATCAACGTTTGCTTGGCTTTCTGCATCTTGGGGCTTGAAACATTCAAAGCTGATGAGT
TTTGAXXXXXXXXXXXTTCCATTATGGTGAGCTTTCCTTACAGGAGAACTAGAGGAAGACAACATACCAATACCAC
CACCATGATTAACAGTTGGT
ACCTTTCTTGATCTGTGATCAACGTTTGCTTGGCTTTCTGCATCTTGGGGCTTGAAACATTCAAAGCTGATGAAT
TTTGAXXXXXXXXXXXTTCCATTATGGTGAGCTTTCCTTACAGGAGAACTAGAGGAAGACAACATACCAATACCAC
CACCATGATTAACAGTTGGT
>Marker12900
ACTAAACAATGTCCGATAGAACTGTATTAGGGAATAGATCTTAAACTAATATATTTCGTTGATGGCTAGGAAGAA
AGAATXXXXXXXXXXXGGCGATTCGTTATAAGGATTGCTGCAACACAATAAACAGGAAGCTCATCATCACCTTGCT
TAGTTGATGATATTGCAGTG
ACTAAACAATGTCCGATAGAACTGTATTAGGGAATAGAGCTTAAACTAATATATTTCGTTGATGGCTAGGAAGAA
AGAATXXXXXXXXXXXGGCGATTCGTTATAAGGATTGCTGCAACACAATAAACAGGAAGCTCATCATCACCTTGCT
TAGTTGATGATATTGCAGTG
>Marker13242
TACCAACTTAAGTCGAGATGAGTGGGTTAAAACCTACTCATTTCGCAACTCTTCAATAACTTTATTCCACTAATT
TTATAXXXXXXXXXXXAGTTACTCGATAAATGTGTAAACTTCAATAGTAAATACTATTGTATTGAAGTGAAGTTGT
TACCAACTTAAAAGTGGGTG
TACCAACTTAAGTCGAGATGAGTGGGTTAAAACCTACTCATTTCGCAACTCTTAAATAACTTTATTCCACTAATT
TTATAXXXXXXXXXXXAATTACTCGATAAATGTGTAAACTTCAATAGTAAATACTATTGTATTGAAGTGAAGTTGT
TACCAACTTAAAAGTGGGTG
>Marker13326
ACAAATATATTGTTGTGTTTTGAACACATTGGAAAAGATTAGGCTTTGTAGAAATCAATCTATGAGTTATGACGA
CATACXXXXXXXXXXXTAATAAAATGTAGTTTATTAATACGCGTGATTAAGAGGAATGATCGATCAGTTAAACAAA
TAATTATTTAAAGAAAAGTG
ACAAATATATTGTTGTGTTTTGAACACATTGGAAAAGATTAGGCTTTGTAGAAATCAATCTATGAGTTATGACGA
CATACXXXXXXXXXXXTAATAAAATGTAGTTTATTAATACGCGTGATTAAGAGGAATGATCGATCAGTTAAATAAA
TAATTATTTAAAGAAAAGTG
>Marker13330
CACTAAGTGATGGCATTTAAATTATATTGAATTACACGAATAACATTAGCCAACTTCCCATGCATAAGGTGAAGT
TCGAGXXXXXXXXXXXGATTTGATTTAATTTCTATTGATTGTTGTAAAATTTACAAAAATGATGCATTTACTGTTC
AAGTTATGAAATAGATTTGT
CACTAAGTGATGGCATTTAAATTATATTGAATTACACGAATAACATTAGCCAACTTCCCATGCATAAGGTGAAGT
TCGAGXXXXXXXXXXXGATTTGATTTAATTTCTATTGATTGTTGTAAAATTTACAAAATTGATGCATTTACTGTTC
AAGTTATGAAATAGATTTGT
>Marker13417

Create PDF with GO2PDF for free, if you wish to

TACGTAAAATTGATTAACATTAATTTTCATTGATATACAAATTCTAACTTTTTGACGCCACAATGATATAGATTT
AATAAXXXXXXXXXXXGTTTGAAGTGAGAGTATGAGATTTAATTGCATCTAATCATATTACTGTAAAAATAACATG
TGAAGATATCTAATTAAGTA
TACGTAAAATTGATTAACATTAATTTCCATTGATATACAAATTCTAACTTTTTGACGCCACAATGATATAGATTT
AATAAXXXXXXXXXXXGTTTGAAGTGAGAGTATGAGATTTAATTGCATCTAATCATATTACTGTAAAAATAACATG
TGAAGATATCTAATTAAGTA
>Marker13650
AACACACAAAATTTCTCAAACCTAATTCCAAGCGTCTTTAAAGAAAATAAATCTTTGTTTAAAATACCAATTTGA
GTATTXXXXXXXXXXXACATTGAGCTAATGGATGATACAGCTTCAAATGCTTTTGTAGTCTATATGGGTATCGAAA
CAAACTAAATGCATAACGTT
AACACACAAAATTTCTCAAACCTAATTCCAAGCGTCTTTAAAGAAAATAAATCTTTGTTTAAAATACCATTTTGA
GTATTXXXXXXXXXXXACATTGAGCTAATGGATGATACAGCTTCAAATGCTTTTGTAGTCTATATGGGTATCGAAA
CAAACTAAATGCATAACGTT
>Marker13764
CACTCTCTGGTTTTCCACGGTTGAGGAATTGAGACTCTTCGTCGTCAGATTGAGCTCCAATTTCATCGGAGTAAG
CATATXXXXXXXXXXXAACGCAAGAATGTTCCAAAAGGCAGTTATGATCCAAGCACTCTGCCACCTTTCATTGAAA
GGATCAGTCGCCTTGAAGTA
CACTCTCTGGTTTTCCACGGTTGAGGATTTGAGACTCTTCGTCGTCAGATTGAGCTCCAATTTCATCGGAGTAAG
CATATXXXXXXXXXXXAACGCAAGAATGTTCCAAAAGGCAGTTATGATCCAAGCACTCTGCCACCTTTCATTGAAA
GGATCAGTCGCCTTGAAGTA
>Marker13842
GACTGTTCTAGGAATAACCTTAGATGCTCAGAATACAAGCCAGGTGCAATCAAGTCAGACTAACATGGTGGATTC
AAGTAXXXXXXXXXXXTTCTCACACAGACAAGATTTTTCTCCCAACAATCTTTTCTTCTAATGTCAGAAGGGAGCT
TTCATTTTCAGCTGAGAGTA
GACTGTTCTAGGAATAACCTTAGATGCTCAGAATACAAGCCAGGTGCAATCAAGTCAGACTAACATGGTGGATTC
AAGTAXXXXXXXXXXXTCCTCACACAGACAAGATTTTTCTCCCAACAATCTTTTCTTCTAATGTCAGAAGGGAGCT
TTCATTTTCAGCTGAGAGTA
>Marker13979
GACTAAGAGTAGGGAACAAAAAACAACGTATAACATTATACTAATACCCGATTCCTTAAAGCTGCAACCAAAAGT
TCAGCXXXXXXXXXXXTAATGCTATTCCATTGTGAAATAAAAGAAAGTGAGATAGCAATAAAATATGGAACATGAA
TATTCACTATGAGTGAAGTA
GACTAAGAGTAGGGAACAAAAAACAATGTATAACATTATACTAATACCCGATTCCTTAAAGCTGCAACCAAAAGT
TCAGCXXXXXXXXXXXTAATGCTATTCCATTGTGAAATAAAAGAAAGTGAGATAGCAATAAAATATGGAACATGAA
TATTCACTATGAGTGAAGTA
>Marker14084
AACAAATGACCTTCCTAATCCTGATCACCACATATGTGCAAACGACCTTTTCTGAAGGATATTAAGAATTTATCA
GCCCAXXXXXXXXXXXTATTTTCACAGTGGGCCTGCTTTTCAGAACACTACTTTTTTTCTCAGAACACTTACAATC
TTTCTTCTTCCAAAACAGTG
AACAAATGACCTTCCTAATCCTGATCACCACATATGTGCAAACGACCTTTTCTGAAGGATATTAAGAATTTATCA
GCCCAXXXXXXXXXXXTATTTTCACAGTGGGCCTGCTTTTCAGAACACTACTTTTTTTCTCAGAACACTTATAATC
TTTCTTCTTCCAAAACAGTG
>Marker14377
ACATTGACAAGTAAGAATATTTGGAAAACCTAAGGAGACCCTTACAAATAAGGGAGGAAAGTAAGCAAAAAACCA
GTCTAXXXXXXXXXXXGACTAATCTTATGAGACAACCCTGCCTAACACTACAATATTTGGGTATCATGAAAACTCC
TAAGATATTAAATCCTAGGT

Create PDF with GO2PDF for free, if you wish to

ACATTGACAAGTAAGAATATTTGGAAAACCTAAGGAGACCCTTACAAATAAGGGAGGAAAGTAAGCAAAAAACCA
GTCTAXXXXXXXXXXGACTAATCTTATGAGACAACCCCGCCTAACACTACAATATTTGGGTATCATGAAAACTCC
TAAGATATTAAATCCTAGGT
>Marker14837
AACCCTATAAAGGTTTTAGAGTAAAGTAGAATATAAATAGGGTGTGGCACAAAAGTTTAGGATCCTTTTGTGACC
CAAATXXXXXXXXXXAAAAATGTGAATGAATATTGTAGTTGAAGCTTGAGGCTTTAATTTCTTCGAGATAGTTCT
CATTCTCTTTAATGATTAGT
AACCCTATAAAGGTTTTAGAGTAAAGTAGAATATAAATAGGGTGTGGCACAAAAGTTTAGGATCCTTTTGTGACC
CAAATXXXXXXXXXXAAAAATGCGAATGAATATTGTAGTTGAAGCTTGAGGCTTTAATTTCTTCGAGATAGTTCT
CATTCTCTTTAATGATTAGT
>Marker15147
CACCAAAGGATTGATCTGACAAATCAATACTATCATATGGAAAATAAAACTCAGAAGGGAAAGAGAAAAGAAAAA
AAAAAXXXXXXXXXXTCAGTGTGTAGAGAAAAGAATCTAAGGGGACCATTAATTGGTAAATCATCATCCTCTTAC
CTTTTACATATCAGTTAGTC
CACCAAAGGATTGATCTGACAAATCAATACTATCATATGGAAAATAAAACTCAGAAGGGAAAGAGAAAAGAAAAA
AAAAGXXXXXXXXXXTCAGTGTGTAGAGAAAAGAATCTAAGGGGACCATTAATTGGTAAATCATCATCCTCTTAC
CTTTTACATATCAGTTAGTC
>Marker15171
ACTGTCTTTTTGATTATTTGACACATTATAAATGATTTGTTTTTAATAGTTGATTAGTGGGAAGTGCTTAAATTT
AAGATXXXXXXXXXXCTCGGCCTCTTTCTGGCTACCCAATCACCAATCATCATGGAATTGGAAGCAAACGGACAA
TGAATGAATCAAAGTGTGTA
ACTGTCCTTTTGATTATTTGACACATTATAAATGATTTGTTTTTAATAGTTGATTAGTGGGAAGTGCTTAAATTT
AAGATXXXXXXXXXXCTCGGCCTCTTTCTGGCTACCCAATCACCAATCATCATGGAATTGGAAGCAAACGGACAA
TGAATGAATCAAAGTGTGTA
>Marker15346
CACATTAAATAATACTAAATAAATCTAAAGTCTTTTTATTTAAAATATAAAACCATTCATCTTCATCTTTAATCA
CATTGXXXXXXXXXXATATCTTCTATTACAATATATAGCAAAATATCTTTATTTTATAAAAAAATTACTTGAAAC
TCCATTATTTACAAATTGTA
CACATTAAATAATACTAAATAAATCTAAAATCTTTTTATTTAAAATATAAAACGATTCATCTTCATCTTTAATCA
CATTGXXXXXXXXXXATATCTTCTATTACAATATATAGCAAAATATCTTTATTTTATAAAAAAATTACTTGAAAC
TCCATTATTTACAAATTGTA
>Marker15874
ACCTCCATTCAATCAGTCTTAGATAATATCAGGTCAGTATCTCCACGTCCCACTTCTGTTCATTTTAATTACTCA
AGCTTXXXXXXXXXXCTTATAGTGTCAGCAGTGAAGATTCTCTAGGATTAAGGCAAATTCAAGAAGACAGAGCAA
ATGTTGAAATTGAAGTAGTG
ACCTCCATTCAATCAGTCTTAGATAATATCAGGTCAGTATCTCCACGTCCCACTACTGTTCATTTTAATTACTCA
AGCTTXXXXXXXXXXCTTATAGTGTCAGCAGTGAAGATTCTCTAGGATTAACGCAAATTCAAGAAGACAGAGCAA
ATGTTGAAATTGAAGTAGTG
>Marker16151
AACCTTCAAGAATTTGTTACATAAACATCTAAGTTATTTTCTTTGTGGTGGTTTATTTTAAATTGGAAATAGTAA
CAAGAXXXXXXXXXXGAGAATTGGAAATAACAATTTAAGTTTGAAGATCTAATTTTCAAATTTGAAATTCAAATA
AAGAAGCTATCAATAAAGTC
AACCTTCAAGAATTTGTTACATAAACATCTAAGTTATTTTCTTTGTGGTGGTTTATTTTAAATTGGAAATAGTAA
CAAGAXXXXXXXXXXGAGAATTGGAAATAACAATATAAGTTTGAAGATCTAATTTTCAAATTTGAAATTCAAATA
AAGAAGCTATCAATAAAGTC
>Marker16158

Create PDF with GO2PDF for free, if you wish to

AACAATATCAGAAGACGCAAACGGTTGCTTTGATTGCAACATTTGCTTAGACTCAGCAGCTGACCCTGTTGTCAC
CCTCTXXXXXXXXXXTCCTCGTTGGTTCCGCTCTATGGGCGTGGCACGTCGAACTCAGATTCTGAATCCAAGAAA
TCTCATTTGGGTATGGCTGT
AACAATATCAGAAGACGCAAACGGTTGCTTTGATTGCAACATTTGCTTAGACTCAGCAGCTGACCCTGTTGTCAC
CCTCTXXXXXXXXXXTCCTCGTTGGTTCCGCTCTATGGGCGTGGCACGTCGAACTCAGATTCCGAATCCAAGAAA
TCTCATTTGGGTATGGCTGT
>Marker16548
ACTACATATCAAAATATTACGTTAAAAGGAGAAAGCATGGTGAAAGTGGAAATATGAAAAAAAAGACCTCAAACA
CAAGAXXXXXXXXXXXAGCGCCTAATGCACGCCTTATTTTGTGAGCCTGGTCTTTCCAAGGTGAGGCACTAGACCT
GTGCCTTGAGCCTAAACGTG
ACTACATATCAAAATATTACGTTAAAAGGAGAAAGCATGGTGAAAGTGGAAATATGAAAAAAAAGACCTCAAACA
CAAGAXXXXXXXXXXXAGCGCCTAATGAACGCCTTATTTTGTGAGCCTGGTCTTTCCAAGGTGAGGCACTAGACCT
GTGCCTTGAGCCTAAACGTG
>Marker17092
GACCCAATTAGCTCATGTTATCAAAACTAAATGACGATTCTTGATATGATAATTGTTGCTATGCAGGAAGGTTTT
TTTTTXXXXXXXXXXCTTCCTTAAACTTCAATGGATCGTATTTGCTGTTTATAACTTCTAAACTTTTTTCAAGAC
TGCTTCCTGATACCGCTTGT
GACCCAATTAGCTCATGTTATCAAAACTAAATGACGATTCTTGATATGATAATTGTTGCTATGCAGGAAGTTTTT
TTTTTXXXXXXXXXXCTTCCTTAAACTTCAATGGATCGTGTTTGCTGTTTATAACTTCTAAACTTTTTTCAAGAC
TGCTTCCTGATACCGCTTGT
>Marker17099
ACTTATAAACCTGTAGATAGAGAAATTCAGCCCTCTTTTACAAATTTGAAGATGAATATAAAAAAAATTAACAAT
TTGAAXXXXXXXXXXXCTTGCTAACACAACCGTAGGTGGTCAATACAGATATCAAAATGCAGTGTAACACCGTTTA
AATTGTCATTACAAATTGTT
ACTTATAAACCTGTAGATAGAGAAATTCAGCCCTCTTTTACAAATTTGAAGATGAATATAAAAAAAATTAACAAT
TTGAAXXXXXXXXXXXTCTTGTAACACAACCGTAGGTGGTCAATACAGATATCAAAATGCAGTGTAACACCGTTTA
AATTGTCATTACAAATTGTT
>Marker17423
CACACTAATCCTAGATTATAAATATTTCAATGTCTTTAAATTAATTGTGATAAAATATCGAACATAAATGTGTAG
AATGAXXXXXXXXXXXCTCAAAATTCATAAAATGAACAACTTAAAATATTGTTTGAAAATGTTACTCAAAACTCGA
CCATTTTTTTCTTATTTTGT
CACACTAATCCTAGATTATAAATATTTCAATGTCTTTAAATTAATTGTGATAAAATATCGAACATAAATGTGTAG
AATGAXXXXXXXXXXXCTCAAAATTCATAAAATGAACAACTTAAAATATTGTTTGAAAATGTTACTCAAAACTCAA
CCATTTTTTTCTTATTTTGT
>Marker17503
TACTTATATTTTGGTATTTTTATTCTGAATCTCGGCTTCTTTTGGTGATTCTGTAGTTTAAACATATTGTATATG
AAGAAXXXXXXXXXXXTTGAGTGTTATGTTTTCTTTTAAAAAAGAAAAAGAAAAAGAAAAACAGTTAAGAGAGAGT
TTTGGTGGGTGTGGAGTGTG
TACTTATATTTTGGTATTTTTATTCTCAATCTCGGTTTCTTTTGGTGATTCTGTAGTTTAAACATATTGTATATG
AAGAAXXXXXXXXXXXTTGAGTGTTATGTTTTCTTTTAAAAAAGAAAAAGAAAAAGAAAAACAGTTAAGAGAGAGT
TTTGGTGGGTGTGGAGTGTG
>Marker17601
TACTCTTACTCCCTTCAATGGAGTCAAATAAATGATAATTCTGTAGGTGTATAAAGGTTCCTTAAAATAAACAGA
GTAATXXXXXXXXXXCAACATCCATCATAGTCAAAGATAATGACATAAATATAAAATCCAAACATTTGAAATTAT
CTAATATTCAAAAGAACGTC

Create PDF with GO2PDF for free, if you wish to

TACTCTTACTCCCTTCAATGGAGTCAAATACATGATAATTCTGTAGGTGTATAAAGGTTCCTTAAAATAAACAGA
GTAATXXXXXXXXXXCAACATCCATCATAGTCAAAGATAATGACATAAATATAAAATCCAAACATTTGAAATTAT
CTAATATTCAAAAGAACGTC
>Marker18306
ACCCTCATCACATCCAAGAAACAAAAATAAACCCTTTAAACTAGGATCCTATTCCCAATACAAGATCAAAACAAA
GTAAAXXXXXXXXXXCGAAGACTTCAAGGCTATAAAGGCGAGCTAATTTAACAAAATTTCCTGCGAAGAGTAATC
TTGAAAATATTTGGATGGTG
ACCCTCATCACATCCAAGAAACAAAAATAAACCCTTTAAACTAGGATCCTATTCCCAATACAAGATCAAAACAAA
GTAAAXXXXXXXXXXTGAAGACTTCGAGGCTATAAAGGCGAGCTAATTTAACAAAATTTCCTGCGAAGAGTAATC
TTGAAAATATTTGGATGGTG
>Marker18424
CACTTCCCTATTGTTCAACGGCTTTGAAACGTATGGTTTATCTCTTATTGCTAATGCTTTTCTGCTTGCAGGGTG
CAGTCXXXXXXXXXXGTGTATTAGTCCATCCGTTACCTTGAATTTTTTTGTGTCTAATTCAAGTTTCCTGTAAGG
TGTGCAGGTATATTCTGGTG
CACTTCCCTATTGTTCAACGGCTTTGAAACATATGGTTTATCTCTTATTGCTAATGCTTTTCTGCTTGCAGGGTG
CAGTCXXXXXXXXXXGTGTATTAGTCCATCCGTTACCTTGAATTTTTTTGTGTCTAATTCAAGTTTCCTGTAAGG
TGTGCAGGTATATTCTGGTG
>Marker18645
AACTTTTTTAGGGTGAGAAAAAGTATAAAGTGATTAGGAAATCAGGAGATTATATACAAATAAAGGAAGCATATT
AAAAAXXXXXXXXXXCATGTAATTAGACCCTCTCAACAAAGATAGTATATAAAGACAAACATAAAGGCAAAAGGG
GACTTAAGAAGTGAGTAGTA
AACATTTTTACGGTGAGAAAAAGTATAAAGTGATTAGGAAATCAGGAGATTATATACAAATAAAGGAAGCATATT
AAAAAXXXXXXXXXXCATGTAATTAGACCCTCTCAACAAAGATAGTATATAAAGACAAACATAAAGGCAAAAGGG
GACTTAAGAAGTGAGTAGTA
>Marker18681
ACAGAGACAAAACATAATTGTCTAAACAACCCCTCTCAAACGAGGCAAGGGAGGGAATCGAAGTCGGAGGTTTGT
CATGAXXXXXXXXXXCAAGCATGATGAATCAGATTGCAAGTAATGGCTATAGAAAAAATGTTATAATACTATGTA
GTTGAGGGAGAACAGAGGTT
ACAGAGACAAAACATAATTGTCTAAACAACCCCTCTCAAACGAGGCAAGGGAGGGAATCGAAGTCGGAGGTTTGT
CATGAXXXXXXXXXXCAAGCATGATGAATCAGATTGCGAGTAATGGCTATAGAAAAAATGTTATAATACTATGTA
GTTGAGGGAGAACAGAGGTT
>Marker18683
AACGAACCCACGACTATTAAAATTAACCTTGTGTGATTGATTTATATACCTTGTAAAAATAACGCATAGTGAAAT
TTCATXXXXXXXXXXCTTAACTGAAATAGTTCTTGTAACTACTTATTGTAACCTTTCACTAATTGAATGCTTTAT
TTCTTTAAAAAAATGTAGGT
AACGAACCCACGACTATTAAAATTAACCTTGTGTGATTGATTTATATATCTTGTAAAAATAACGCATGGTGAAAT
TTCATXXXXXXXXXXCTTAACTGAAATAGTTCTTGTAACTACTTATTGTAACCTTTCACTAATTGAATGTTTTAT
TTCTTTAAAAAAATGTAGGT
>Marker19259
ACTTGCTTTTCTTTGTTTCTTTGGAACCATTGTAGTATTGAATTAAACAAACAACAACTTAGATGTTTTCAAGTT
TCTGCXXXXXXXXXXTGTTTAAAGAGGGCAGTAAAAAAAACAGAACAATGATATAATGGTGTTTAAAGAGGATAG
TAAAAAGGAAGAAAGATGGT
ACTTGCTTTTCTTTGTTTCTTTGGAACCATTGTAGTATTGAATTAAACAAACAACAACTTAGATGTTTTCAAGTT
TCTGCXXXXXXXXXXTGTTTAAAGAGGGCAGTAAAAAAAACAGAACAACGATATAATGGTGTTTAAAGAGGATAG
TAAAAATGAAGAAAGATGGT
>Marker19633

Create PDF with GO2PDF for free, if you wish to

ACTGCTCCTTGGTAAAACTATAGCTACTGAATCAGAATATTCCTTTCAGAACTGTTGGAGAGTTCTTATATGTTC
TATATXXXXXXXXXXTATGTATTATATCTATCTTCTTTTCCATTAGCTACTTACCTCCTAGTCATCTTACTTCTA
ATAATAAGATATTTCAGAGT
ACTGCTCCTTGGTAAAACTATAGCTACTGAATCAGAATATTCCTTTCAGAACTGTTGGAGAGTTCTTATATGGTC
TATATXXXXXXXXXXTATGTATTATATCTATCTTCTTTTCCATTAGCTACTTACCTCCTAGTCATCTTACTTCTA
ATAATAAGATATTTCAGAGT
>Marker19658
ACATTTGACTTTGTTTTCAGTGTTCTTCAATGTTTAATTCTTGTTTCATATTTAAATCAGGATTTCATATTGAGG
GGAGGXXXXXXXXXXCTAGATGGGATGACACTATTAATTAATCCATAGATTGATTGATCTTTCGTTTCGTCGTAG
GAATTAAAAAACATAAAGTT
ACATTTGACTTTGTTTTCAGTGTTCTTCAATGTTTAATTCTTGTTTCATATTTAAATCAGGATTTCATATTGAGG
GGAGGXXXXXXXXXXCTAGATGGGATGACACTATTAATTAATCCATAGATTGATTGATCTTTCGTTTCGTTGTAG
GAATTAAAAAACATAAAGTT
>Marker19684
TACATATAGAATCGTAAAATACTCTATTACCCTTAATTTTATATCCTTTCACATGTTTCATCATTTACTTGAACC
ACAAAXXXXXXXXXXGGTAAATGTTGTTATAGATTTGGCTGCACTCGTAACAGATCAGATGCTTATACACAGATG
CTCATATTTTGTGATTTGTG
TACATATAGAATCGTAAAATACTCTATTACCCTTAATTTTATATCCTTTCACATGTTTCATCAGTTACTTGAACC
ACAAAXXXXXXXXXXGGTAAATGTTGTTATAGATTTGGCTGCACTCGTAACAGATCAAATGCTTATACACAGATG
CTCATATTTTGTGATTTGTG
>Marker20138
ACCTAGAGACAAATCTAGCATTCAATCAGCCTAACATAACAAACGAGTCAGTTAAGATTCCAAAAAAACCTAACA
TACAAXXXXXXXXXXAGCCTCATCGAATCCTATATGCCTGGAAGGCGTGGATGCATTGTATGGTTTTCCAATTAA
ACTTGATTGTCGATAAAGTA
ACCTAGAGACAAATCTAGCATGCAATCAGCCTAACATAACAAACGAGTCAGTTAAGATTCCAAAAAAACCTAACA
TACAAXXXXXXXXXXAGCCTCATCGAATCCTATATGCCTGGAAGGCGTGGATGCATTGTATGGTTTTCCAATTAA
ACTTGATTGTCGATAAAGTA
>Marker20552
CACTGATACTAACAGAATTACTGGAAGCCAACAATTAAGTCTTTTTAAATAATCATATGGGTTGAACTTCTAGGG
ATTTGXXXXXXXXXXAGCTCCAGGAGAAGTGAAATCTTCACAAACATGGATATGTTCAACAAGATCATCGATGCA
TATGTGCTCGTCTTCCATGT
CACTGATACTAACAGAATTACTGGAAGCCAACAATTAAATCTTTTTAAATAATCATATGGGTTGAACTTGTAGGG
ATTTGXXXXXXXXXXAGCTCCAGGAGAAGTGAAATCTTCACAAACATGGATATGTTCAACAAGATCATCGATGCA
TATGTGCTCGTCTTCCATGT
>Marker20562
CACTCTATCCTCCCATTCCATACATTGTCATTTTTGGTTACAGCCCAAACTTAGTCTAGATAATATGTTTTCACA
TGTTTXXXXXXXXXXAACTACATGGCCACATCATCACAAAATTACATTATCAACATAGAAGTGTTAAATTCAATA
GAGTGCCAAACCTTACAAGT
CACTCTATCCTCCCATTCCATACATTGTCATTTTTGGTTACAGCCCAAACTTAGTCTAGATAATATGTTTTTACA
TGTTTXXXXXXXXXXAACTACATGGCCACATCATCACAAAATTACATTATCAACGTAGAAGTGTTAAATTCAATA
GAGTGCCAAACCTTACAAGT
>Marker20640
CACCAAGTTCAACATGCTTAAGTAGCATGGCAATGGATAGATGTTTCCTGGTTCTAATCATCAACGAAATACAAG
CCAAGXXXXXXXXXXTATGCCGCTGGCATTCGTTCCATCACTTTAGAGAAAGAGCGAGTGATAAAGTAAATGGCA
AGGGCCCCCATGTTGAGGTC

Create PDF with GO2PDF for free, if you wish to

CACCAAGTTCAACATGCTCAAGTAGCATGGCAATGGATAGATGTTTCCTGGTTCTAATCATCAACGAAATACAAG
CCAAGXXXXXXXXXXXTATGCCGCTGGCATTCATTCCATCACTTTAGAGAAAGAGCGAGTGATAAAGTAAATGGCA
AGGGCCCCCATGTTGAGGTC
>Marker20644
CACCTCCAAAGGAACAATGAAGCTGAGAGGAAATGTGAGAGGGAAAGAGGTAGTGGTCCTTATTGATAGTAGAGC
CACTAXXXXXXXXXXXATTTTCTAGCGGTGGAATTGGGAAGGTATTGACCTAGTTTTGAGGATGCAGTGGCTGGAT
ACCACCGGAACAATGAAGGT
CACCTCCAAAGGAACAATGAAGCTGAGAGGAAATGTGAGAGGGAAAGAGGTAGTGGTCCTTATTGATAGTAGAGC
CACTAXXXXXXXXXXXATTTTCTAGCAGTGGAATTGGGAAGGTATTGACCTAGTTCTGAGGATGCAGTGGCTGGAT
ACCACCGGAACAATGAAGGT
>Marker20723
CACATGAAATTGTCCTTAAATCTACATAGGTGAGGATAGCTCATTAGCACTAGCCAAATAAGCTTTCCATTTTAG
GGGTAXXXXXXXXXXXGAATAATGGGCCCTACCCTCTCATTGTCTCGAGAGAAATTCGATTTATAGGTTAGATCTT
AAACCAATTGTTCAACAGTG
CACATGAAATTGTCCTTAAATCTACGTAGGTGAGGATAGCTCATTAGCACTAGCCAAATAAGCTTTCCATTTTAG
GGGTAXXXXXXXXXXXGAATAATGGGTCCTACCCTCTCATTGTCCCGAGAGAAATTCGATTTATAGGTTAGATCTT
AAACCAATTGTTCAACAGTG
>Marker21074
GACACCTCAGTGCCCTCATCATATCCAAGAACGCACCAAACATGGAATAAGAGTGCCTAGACACTCCTAACAATA
CTGAGXXXXXXXXXXXCAGCAACTTCTTCATGGAGCTTGAGGATCTTGGCCTGCTAAATTAAGATGGAGATCAGAA
ATGCACCGAAAAAGCCAAGT
GACACCTCAGTGCCCTCATCATATCCAAGAACGCACAAAACATGGAATAAGAGTGCCTAGACACTCCTAACAATA
CTGAGXXXXXXXXXXXCAGCAACTTCTTCATGGAGCTTGAGGATCTTGGCCTGCTAAATTAAGACGGAGATCAGAA
ATGCACCGAAAAAGCCAAGT
>Marker22068
ACTTCGAGCATTAGTCTCATTAATTTTTAATAAAGAGACCTGTCATCGTTTCAAAAAAAAGAAAATTTTGTTAAG
GTATTXXXXXXXXXXXACAACACCTTGTTGGTGGTTGTGGAAATGCCATTGCTTGTAGAAAATAGCCTGATCACGT
GAAGGAAGAAATTAAAGAGT
ACTTCGAGCATTAGTCTCATTAATTTTTAATAAAGAGACCTGTCATCGTTTCAAAAAAAAAAAATTTTTGTTAAG
GTATTXXXXXXXXXXXACAACACCTTGTTGGTGGTTGTGGAAATGCCATTGCTTGTAGAAAATGGCCTGATCACGT
GAAGGAAGAAATTAAAGAGT
>Marker22455
TACCTTGGCTAGGTGGGATTGTAAAATGGTGAGAAGGCCCTGACATTGCTTAGCATTCATGTTTGAAAGAGGGTC
AGTGTXXXXXXXXXXXTGAGCGAGATTGTGGTGGATTTTGTGAAACATTTTTCACTAACATGGCTGAGTTTTCATA
ATTTATAGGAAGGTTAGGTG
TACCTTGGCTAGGTGGGATTGTAAAATGGTGAGAAGGCCCTGGCATTGCTTAGCATTCATGTTTGAAAGAGGGTC
AGTGTXXXXXXXXXXXTGAGCGAGATTGTGGTGGATTTTATGAAACATTTTTCACTAACATGGCTGAGTTTTCATA
ATTTATAGGAAGGTTAGGTG
>Marker22480
CACTGCGCAGGGCTATCAATTTCATAAATGTTTTGCATTTTAACTTATCAAGCTCTGATAGTATCTGTATTGAAC
TAAAGXXXXXXXXXXXTTTTTCTCCAACTAAAAACCTGAGTTATATAGAGGGAAGAAGACATTCCGATTGGCAGAG
GGAATAGGGCTTCGAAGGTT
CACTGCGCAGGGCTATCAATTTCATAAATGTTTTGCATTTTAACTTATCAAGCTCTGATAGTATCTGTATTGAAC
TAAAGXXXXXXXXXXXTTTTTCTCCAACTAAAAACCTGAGTTATATAGAAGGAAGAAGACATTCCGATTGGCAGAG
GGAATAGGGCTTCGAAGGTT
>Marker22683

Create PDF with GO2PDF for free, if you wish to

CACTTTCGTAATCATTTTGATCTTAATTACTATTTTACTTTATTTTGTTTCAATTCTGTATTATAATTTTCAAAA
CCCTAXXXXXXXXXXGAAAAAAACCTAAATGAGACATTCAAACTTGAAAATAATTTATCAACTAGAATTATTTTA
AACCCATATAATAATAAGTT
CACTTTGGTAATCATTTTGATCTTAATTACTATTTTACTTTCTTTTGTTTCAATTCTGTATTATAATTTTCAAAA
CCCTAXXXXXXXXXXGAAAAAAACCTAAATGAGACATTCAAACTTGAAAATAATTTATCAACTAGAATTATTTTA
AACCCATATAATAATAAGTT
>Marker22790
CACACGATGGAACTTAGTTTGTTCATAACTTTGACATCCCTTAAAGAGTTGGATCTTAGTTATGATATTTTCAAT
GTAGAXXXXXXXXXXATTTTGGTAACTAGATAGTAGATCTAACTTGACGAAATAATCATACTACGTCTAAAAATG
TCAAAAAGGGTTGTGGAGTG
CACACGATGGAACTTAGTTTATTCATAACTTTGACATCCCTTAAAGAGTGGGATCTTAGTTATGATATTTTCAAT
GTAGAXXXXXXXXXXATTTTGGTAACTAGATAGTAGATCTAACTTGACGAAATAATCATACTACGTCTAAAAATG
TCAAAAAGGGTTGTGGAGTG
>Marker23361
ACAACAAAATGAAGAATAATTGTATGAGATTTTATCATTTTCAAACGAATTGAGTAAAATAATGTTGATGTAATA
TATATXXXXXXXXXXTTTCCATAGAGAATAAATACATGAATAATAATATATTTATTTGTGGTTATAGACATATAT
TTGATATATATTAATATGTT
ACAACAAAATGAAGAATAATTGTATGAGATTTTATCATTTTCAAACGAATTGAGTAAAATAATGTTGATGTAATA
TATATXXXXXXXXXXTTTCCATAGAGAATAAATACATGAATAATAATATATTTATTTGTTGTTATAGACATATAT
TTGATATATATTAATATGTT
>Marker24214
ACAAGCCTTCCTTGGCTTGGCATCCTTTTACAGAAGATTCATTCGAAACTTCAGCTGAATAGTGGCCCCCTTAAC
CGACTXXXXXXXXXXATCACCATTTGAAGTAGCTGTTGATGCTTGCAGAACTGGGATTGGAGCTGTATTATCACA
GCAAGGCCACCCTATTGAGT
ACAAGCCTTCCTTGGCTTGGCATCCTTTTACAGAAGATTCATTCGAAACTTCAGCTCAATAGTGGCCCCCTTAAC
CGACTXXXXXXXXXXATCACCATTTGAAGTAGCTGTTGATGCTTGCAGAACTGGGATTGGAGCTGTATTATCACA
GCAAGGCCACCCTATTGAGT
>Marker24279
ACTCTATAAATACATCCCATTTAATTTCTAATTTTCTATTAGGTGTCAAACCCTAATAAATGAATTTAGGAAAAT
AAAAAXXXXXXXXXXTAGTGATATCATATGAGTGTCAAACGATAATCAAAGGCTATTAGACAATAATCATAAGGT
TATCGGCTTTTAAATTTGTT
ACTCTATAAATACATCCCATTTAATTTCTAATTTTCTATTAGGTGTCAAACCCTAATAAATGAATTTAGGAAAAT
AAAAAXXXXXXXXXXTAGTGATATCATATGGGTATCAAACGATAATCAAAGGCTATTAGACAATAATCATAAGAT
TATCGGCTTTTAAATTTGTT
>Marker24310
CACCTCATTTGAAATTTTTAAATACGTAGATCAATTCTAATGGGATGAGTTCTAATGGGATGATAGACTGAAGCC
AAGATXXXXXXXXXXTCAATCCTACTAAAAGAGATCAAAATTTTGCATCCATTTTTGTTGTTCTATGATAATATA
AGAATTTTGTTGTTTATTGT
CACCTCATTTGAAATTTTTATATACGTAGATCAATTCTAATGGGATGAGTTCTAATGGGATGATAGACTGAAGCC
AAGATXXXXXXXXXXTCAATCCTACTAAAAGAGATCAAAATTTTGCATCCATTTTTGTTGTTCTATGATAATATA
AGAATTTTGTTGTTTATTGT
>Marker24410
ACAGTAATATTATATCATGAGGGGTGAAAAAAAATCACGAATCTTTAGTGACTAAATGCATATTTTTACTGAGGT
TTGGTXXXXXXXXXXTCTTGATCGTCTCTTCATCAGCTTCTTCTATAGGATCGAGAAATCCACTACCTTTTACGT
TATTTTCGTTTGAGCGTGTA

Create PDF with GO2PDF for free, if you wish to

ACAGTAATATTATATCATGAGGGGTGAAAAAAAATCACGAATCTTTAGTGACTAAATGCATACTTTTACTGAGGT
TTGGTXXXXXXXXXXTCTTGATCGTCTCTTCATCAGCTTCTTCTATAGGATCGAGAAATCCACTACCTTTTACGT
TATTTTCGTTTGAGCGTGTA
>Marker24415
ACTCTCGTTTTCTATATAGTTAATATATCTGTAATAAACGTGTCAATTTTTAATTTCTTCCATTTTAGAATATTA
TATTTXXXXXXXXXXTATTATCAATGGCTTCTAACAGATGTTTATTTAAGAAACAAAATCAAATTTTGTTTTAAA
TCTTTTAGAAATGGAAAGTA
ACTCTCGTTTTCTATATAATTAATATATCTGTAATAAACGTGTCAATTTTTAATTTCTTCCATTTTAAAATATTA
TATTTXXXXXXXXXXTATTATCAATGGCTTCTAACAGATGTTTATTTAAGAAACAAAATCAAATTTTGTTTTAAA
TCTTTTAGAAATGGAAAGTA
>Marker24535
AACAATATCTTTGATGTGCAGATTAACTAATAACATCAATTTGGAATTTCTATAGATTATGAATTGTAGTTAGAC
CATCAXXXXXXXXXXCTCTATCTCAATCAATGTCATGAGATTGGCTTGAACCATTCCCCAATAACTTTATGAAAT
TATTCCACAAATTTTCTTGT
AACAATATCTTTGATGTGCAAATTAACTAATAACATCAATTTGGAATTTCTATAGATTATGAATTGTAGTTAGAC
CATGAXXXXXXXXXXCTCTACCTCAATCAATGTCATGAGATTGGCTTGAACCATTCCCCAATAACTTTATGAAAT
TATTCCACAAATTTTCTTGT
>Marker24691
ACATACACATACATATTGGTTGGTCCAGTATTCAATACACACCCTTTCAAATTTAAATACAAAGCTTGAATTCGT
TATTTXXXXXXXXXXTTAGGTCCAAACGCGCGTGAATTGAACTCATTTGTGGTAGGGCACGTAATGATGTAAAAT
AATTTTTTTCTATTTTGGTG
ACATACACATACATACTGGTTGGTCCAGTATTCAATACACACCCTTTCAAATTTAAATACAAAGCTTGAATTCGT
TATTTXXXXXXXXXXTTAGGTCCAAACGCGCGTGAATTGAACTCATTTGTGGTAGGGCACGTAATGATGTAAAAT
AATTTTTTTCTATTTTGGTG
>Marker24695
TACCATTTTTTCTCTCTCTAGACACTTCTCTCGTTTAAATGGCCATTGGCCAGTGACCTTAAGGTTGAAAAGGAA
ATGGGXXXXXXXXXXGAGCCATATTGTTTTTCAATCTCTCTTTCTTCTCTTACTAAAGGTGGCGCCTCCTGAGAT
GAAAGGGACTTACTGGAAGT
TACCATTTTTTCTCTCTCTAGACACTTCTCTCGTTTAAATGGCCATTGGCCAGTGACCTTAAGGTTGAAAAGGAA
ATGGGXXXXXXXXXXGAGCCACATTGTTTTTCAATCTCTCTTTCTTCTCTTACTAAAGGTGGCGCCTCCTGAGAT
GAAAGGGACTTACTGGAAGT
>Marker24745
AACCTGCACCCGTGGTGACAATTGTGCCACCAGCCAAGCCAACAGATAATGACATAAAATCCAGTGGGGGATCAA
ATCAGXXXXXXXXXXTCAGGAAACACTTCTATAATTGGAGTCTATACTTCCTTACATTGTTCGGACAATATATTA
CATTACAGGCAGTTTCATGT
AACCTGCACCCGTGGTGACAATTGTGCCACCAGCCAAGCCAACAGATAATGACATAAAATCCAGTGGGGGATCAA
ATCAGXXXXXXXXXXTCAGGAAACACTTCTATAATTGGAGTCTATACTTCCTTACATTGTTCGGAGAATATATTA
CATTACAGGCAGTTTCATGT
>Marker25820
TACTACTTCAGTCCAACAACAACATTCAAATATAGGTAGAAAAGTTAGGAGAACATGTCCTTTTAAACTGATGAA
CACTAXXXXXXXXXXAATATTGCATCAAAATCTCCAAACCATATCTATTAATTGACAATGGGGGTTTCTGAACTC
CATAATCAAAAATGCTTTGT
TACTACTTCAGTCCAACAACAACATTCAAATATAGGTAGAAAAGTTAGGAGAACATGTCCTTTTAAACTGATGAA
CACTAXXXXXXXXXXAATATTGCATCAAAATCTCCAAACCATATCTATTAATTGACAATGGGGGTTTCTAAACTC
CATAATCAAAAATGCTTTGT
>Marker25864

Create PDF with GO2PDF for free, if you wish to

TACCCTTTTCTTCTTCTTTTTTGTTAAATTATACAAAACCAAAATCAATTTTAGGGTATAATATTCCAACCAAAA
GTTCTXXXXXXXXXXTCATATAATTGGTTACAAATTTGGGTAATATATGGTTTTTCATAAGTATCAAACTTTAAC
TTTAACTTTATATCACAAGT
TACCCTTTTCTTCTTCTTTTTTGTTAAATTATACAAAACCAAAATGAATTTTAGGGTATAATATTCCAACCAAAA
GTTCTXXXXXXXXXXTCATATAATTGGTTACAAATTTGGGTAATATATGGTTTTTCATAAGTATCAAACTTTAAC
TTTAACTTTATATCACAAGT
>Marker26265
ACTTAAATTGTTGCAAGGTATTTATCGTTTCTTGTTTTTGCTTCTGGTTTTGATTCAAAGCCACTATGTTCTTCA
TTATTXXXXXXXXXXCTTCTTTCTTTTACTTGCTATGTAATAATGATAAGAGGGAGAATTTCTAAATTTTAATTA
GACGAGAATCTGTTTTAGTG
ACTTAAATTGTTGCAAGGTATTTATCGTTTCTTGTTTTTGCTTCTGGTTTTGATTCAAAGCCACTATGTTCTTCA
TTATTXXXXXXXXXXCTTCTTTCTTTTACTTGCTATGTAATAATGATAAGAGGGAGAATTTCTAAATTTTAATTA
GACGAGGATCTGTTTTAGTG
>Marker26363
TACTGTAAAAGCTCGATCTGCAGAACGAGAAAAATGATGTTGGTATCTAACTGCTTCACCCATGATATTTTGTTT
CCGAAXXXXXXXXXXCCATAATGCATGGCAGTGTCAATGTCACAGTTGGAGAACAAGAAGAACGGATGATGATCA
CAGGATTGCACACCGTTGTT
TACTGTAAAAACTCGATCTGCAGAACGAGAAAAATGATGTTGGTATCTAACTGCTTCACCCATGATATTTTGTTT
CCAAAXXXXXXXXXXCCATAATGCATGGCAGTGTCAATGTCACAGTTGGAGAACAAGAAGAACGGATGATGATCA
CAGGATTGCACACCGTTGTT
>Marker26389
ACAATCGAAAAAAATGGACTCCGATTTTGGGTTCGATCGGTGAGAAAACTGAGAATTGGGATTTAAATCATTTTC
TTTCTXXXXXXXXXXCGTGTCTTCTCAATGAGGTGGTAAGTTCTTATTGGATGCAATGAAGTCTTCTTACCGGTC
TTAAGGTCTGATGTGACGTG
ACAATCGAAAAAAATGGACTCCGATTTTTGGTTCGATCGGTGAGAAAACTGAGAATTGGGATTTAAATCATTTTC
TTTCTXXXXXXXXXXCGTGTCTTCTCAATGAGGTGGTAAGTTCTTATTGGATGCAATGAAGTCTTCTTACCGGTC
TTAAGGTCTGATGTGACGTG
>Marker26415
TACTTCAGATTATAAAACTAACTGCTCCCTTAATAAAAATTATATATACTCTACTTTTTCCTTCATATCAGTTTT
CATTTXXXXXXXXXXTTGGATATCTGGATCATCCCCAGCTTCTTTATCCTAAAGTTTAGAAAACAATAATATTAA
TAGTAATAATAACAAAAGTT
TACTTCAGATTATAAAACTAACTGCTCCCTTGATAAAAATTATATATACTCTACTTTTTCCTTCATATCAGTTTT
CATTTXXXXXXXXXXTTGGATATCTGGATCATCCCCAGCTTCTTTATCCTAAAGTTTAGAAAATAATAATATTAA
TAGTAATAATAACAAAAGTT
>Marker26880
AACTCCATCTCATAGTATGTTATTCATGTAGTTAAGTTTTATAAATCAAAGTAATATATGTTCAAAAGATGTTAA
TTCAAXXXXXXXXXXGTGTTATGAAGTATAACCGACAAAGAGCTGAGTTGAAGCCTTGATAGTATTTAACATTAT
TTAACAAGGAAAGATGAGGT
AACTCCATCTCATAGTATGTTATTCATGTAGTTAAGTTTTATAAATCAAAGTAATATATGTTCAAAAGATGTTAA
TTCAAXXXXXXXXXXGTGTTATGAAGTATAACTGACAAAGAGCCGAGTTGAAGCCTTGATAGTATTTAACATTAT
TTAACAAGGAAAGATGAGGT
>Marker27176
AACAGTGAAATGGTGTTGTTTAACTATGCTTGCTTCATGAAGATGGTGGGATTAATCCCTTGCCCGCAACAACAC
TCAAGXXXXXXXXXXAAATTTTGGAATCTCTACATTATTTCCTTTTCAATATTCATTTTCCAAAAGAAATTGAAA
GTAGTGGATGATTGTGTGTG

Create PDF with GO2PDF for free, if you wish to

AACAGTGAAATGGTGTTGTTTAACTATGCTTGCTTCATGAAGATGGTGGGATTAATCCCTTGCCCGCAACAACAC
CCAAGXXXXXXXXXXAAATTTTGGAATCTCTACATTATTTCCTTTTCAATATTCATTTTCCAAAAGAAATTGAAA
GTAGTGGAAGATTGTGTGTG
>Marker27245
TACAACCTAATAGATCCTAATAGATCTTCTCTCTCTCTCTTTTGGCCTAGTAGCTAGAGGGCCTGGAAGGTGCAA
CGTAAXXXXXXXXXXGTTGTAACAGGATAATGGGGAGGTTAAATGTAAATGTCCTCCAGGATTTAAAGGTGATGG
TGTCAAAACATGTGAAGGTA
TACAACCTAATAGATCCTAATAGATCTTCTCTCTCTCTCTTTTGGCCTAGTAGCTAGAGGGCCTGGAAGGTGCAA
CGTAAXXXXXXXXXXGTTGTAACAGGATAATGGGGAGGTTAAATGTAAATGTCCTCCAGGATTTAAAGGTGATGG
TGTCAAAACTTGTGAAGGTA
>Marker27377
AACTAAAACAATAGATATCACACATCTTCTCATTATAACAAAGTTTGTTTTCATGATTGTGTTCGTTTTGGCCTT
GAACAXXXXXXXXXXTTAAAAGCATAATGCTTACCCTAAAACCACACTAGCACTACTTCGTCATTAGTAGAATGG
GTGAAAGATTTCAATGTAGT
AACTAAAACAATAAATATCACACATCTTCTCATTATAACAAAGTTTGTTTTCATGATTGTGTTCGTTTTGGCCTT
GAACAXXXXXXXXXXTTAAAAGCATAATGCTTACCCTAAAACCACACTAGCACTACTTCGTCATTAGTAGAATGG
GTGAAAGATTTCAATGTAGT
>Marker27416
ACTCCATTAACTAGTTAGGTTTCTATTTTACTAGGATGACCTAAGTAACTTAGTTTTGCTTATAATTCTTATTGG
ATTAAXXXXXXXXXXAGGAGAACAAGTGTAGGCGTTAGGTGCCAAGAGGTTACTATCCTTAAACACGAAGCTCTA
TAAGCCTTATAAGTGAAGTA
ACTCCATTAACTAGTTAGGTTTCTATTTTACTAGGATAACCTAAGTAACTTAGTTTTGCTTATAATTCTTATTGG
ATTAAXXXXXXXXXXAGGAGAACAAGTGTGGGCGTTAGGTGCCAAGAGGTTACTATCCTTAAACACGAAGCTCTA
TAAGCCTTATAAGTGAAGTA
>Marker27562
ACATTTGGTAGTGTCTTGCCATTGCGTCTAGCACACATAAGTTATAAGTTATTTGTGAGAGAGTTCAATTCAAGG
TTGGGXXXXXXXXXXTTTGCTATATAAATTTTCTAGGATTATGAAATTCTATAATATTTTCTTTAATAAAATTGT
CGGTTTTCCTCTATCTCGTA
ACATTTGGTAGTTTCTTGCCATTGCGTCTAGCACACATAAGTTATAAGTTATTTGTGAGAGAGTTCAATTCAAGG
TTGGGXXXXXXXXXXTTTGCTATATAAATTTTCTAGGATTATGAAATTCTATAATATTTTCTTTAATAAAATTGT
CGGTTTTCCTCTATCTCGTA
>Marker27820
CACTCTGAGGACCAGTATTTTTGCTCTTTTCCTATCATTTTCATTTTTCTGTTTTAGGTAACATTATCATTCAAC
TGTTCXXXXXXXXXXTTACGATAACCCATTAAGTTGATGACCATCCTTATCCCTGCAGTTTTGTTCATAATGACC
TTAATGAATCTACTGTGCGT
CACTCTGAGGACCAGTATTTTTGCTCTTTTCCTATCATTTTCATTTTTCTGTTTTAGGTAACATAATCATTCAAC
TGTTCXXXXXXXXXXTTACGATAACCCATTAAGTTGATGACCATCCTTATCCCTGCAGTTTTGTTCATAATGACC
TTAATGAATCTACTGTGCGT
>Marker27982
AACGTCAAGCCGTTAAGCCTTTGAAACAAGCACATTCACACATGATTATCAAAGATGAAACATGAAGGTCACCTG
TATAAXXXXXXXXXXTCAACTTTAACTCCATCGACACCAGCAGATGCAAGATACGAGTGTTGTTCATTGTAAAAG
TTGAAAACTTTTTCAGGGTT
AACGTCAAACCGTTAAGCCTTTGAAACAAGCACATCCACACATGATTATCAAAGATGAAACATGAAGGTCACCTG
TATAAXXXXXXXXXXTCAACTTTAACTCCATCGACACCAGCAGATGCAAGATACGAGTGTTGTTCATTGTAAAAG
TTGAAAACTTTTTCAGGGTT
>Marker28424

Create PDF with GO2PDF for free, if you wish to

AACCTTCAAAGCCTTACCTCTCTATAACTATATGTTAGACCTCCAATATTATATCACTACAGGAGGAAGGGGAAT
AAGTTXXXXXXXXXXXAGGGAGGGAGGGATATGATTATTTTTTCAGTATTTGAATCTTTCTGTTATTTCTTGGGAG
AGAGGGAAACAGAGGATTGT
AACCTTCAAAGCCTTACCTCTCTATAACTATATGTTAGACCTCCAATATTATATCACTACAGGAGGAAGGGGAAA
AAGTTXXXXXXXXXXXAGGGAGGGAGGGATATGATTATTTTTTCAGTATTTGAATCTTTCTGTTATTTCTTGGAAG
AGAGGGAAACAGAGGATTGT
>Marker28855
AACTATTTCATTAAGAAGATGATAAAGTTCGAGCAATGAAAGAAAAACTAAACCTTGGGATCTAATTAAACATGG
AAAGAXXXXXXXXXXXAAGAATGGTTTAATTTGGATAAATAAACTCAAACCAGTCATCCAACGACCCTTGACCAAG
GATAAGGTAGAGGTTGAGTA
AACTATTTCATTAAGAAGATGATAAAGTTCGAGCAATGAAAGAAAAACTAAACCTTAGGATCTAATTAAACATGG
AAAGAXXXXXXXXXXXAAGAATGGTTTAATTTGGATAAATAAACTCAAACCAGTCATCCAACGACCCTTGACCAAG
GATAAGGTAGAGGTTGAGTA
>Marker28924
ACAAAGGAGGACTTTGAGTCAAAGGCCAGATGCAGTGTTTGTGATTGTGAGCGTGATTATTTTTAGTAAAAAGGA
TTTCTXXXXXXXXXXXATGAAATGATCATAACTTGCATTAGAAATGAAGGCAAAATAATTTAAAGGATGCATGTGA
ATAAGAAAATGGTGCTTAGT
ACAAAGGAGGACTTTGAGTCAAAGGCCAGATGCAGTGTTTGTGATTGTGAGCGTGATTATTTTTAGTAAAAAGGA
TTTCTXXXXXXXXXXXTTGAAATGATCGTAACTTGCATTAGAAATGAAGGCAAAATAATTTAAAGGATGCATGCGA
ATAAGAAAATGGTGCTTAGT
>Marker29108
ACATGAGGGACATACAAAGAGCAACCGGGGAGGGGGAGGATCAGCAATTGAATTGAAACATTTAATTGATTAATG
AAATAXXXXXXXXXXXTTTGACTTTTAGGTATATAATTTCCCCGTATATGTTAGGTTCTATTTCTTTCTCTGACAA
ACTGTCTTGTCCTTTTCGTT
ACATGAGGGGCATACAAAGAGCAACCGGGGAGGGGGAGGATCAGCAATTGAATTGAAACATTTAATTGATTAATG
AAATAXXXXXXXXXXXTTTGACTTTTAGGTATATAATTTCCCCGTATATGTTAGGTTCTATTTCTTTCTCTGACAA
ACTGTCTTGTCCTTTTCGTT
>Marker29116
AACCCTATTGCACTCCTAGTCCTATTTGTAAAAGAACATTACATACCACAATGAGTCGTAACAATCAAATCATGG
TGGGTXXXXXXXXXXXTATTCTACCTCAAGCCAATTTAAAACCAACTTTTAGTTAATCTGATCATGCCGTCTGCCT
GATCACTAGTCAGGGTAAGT
AACCCTATTGCACTCCTAGTCCTATTTGTAAAAGAACATTACATACCACAATGAGTCGTAACAATCAAATCATGG
TGGGCXXXXXXXXXXXTATTCTACCTCAAGCCAATTTAAAACCAACTTTTAGTTAATCTGATCATGCCGTCTGCCT
GATCACTAGTCAGGGTAAGT
>Marker29547
TACCATCTGTCCCAAATTACAAGTAGCCATTACTTTATAAACCTACAACATTTGCAGAGACCACTGCTTAAACCA
CTTGCXXXXXXXXXXXCAAGCTTAGATTTTTCAACAAATATGTTTTAGTCATGGACTTCAAGTTCTATTTAATCTT
GTTTCTAAATTTTAAATGTG
TACCATCTGTCCCAAATTACAAGTAGCCATTACTTTACAAACCTACAACATTTGCAGAGACCACTGCTTAAACCA
CTTGCXXXXXXXXXXXCAAGCTTAGATTTTTCAACAAATATGTTTTAGTCATGGACTTCAAGTTCTATTTAATCTT
GTTTCTAAATTTTAAATGTG
>Marker30762
CACAAAGAAGAATATCACGTTATCGATATTGAGTATGTTCCATGACAACAATGCAACCGTAAGTGTCGAGCATAC
CCCACXXXXXXXXXXXGCCCTTCCACAACGTAATAAAGTTAAGCCAGTGATTCTAAGTCACGAGTTACTTTGCTAG
GGATACTTGCCTAGAGTAGT

Create PDF with GO2PDF for free, if you wish to

CACAAAGAAGAATATCACGTTGTCGATATTGAGTATGTTCCATGACAACAATGCAACCGTAAGTGTCGAGCATAC
CCCACXXXXXXXXXXXGCCCTTCCACAACGTAATAAAGTTAAGCCAGTGATTCTAAGTCATGAGTTACTTTGCTAG
GGATACTTGCCTAGAGTAGT
>Marker30833
ACCACATTGGTCATCCCTCAATGTGGGACAAAAGCTTCCCGTAACTACTTTGGTTCCTAACAATACCCCATCCCT
AGAAAXXXXXXXXXXXXTTTGACATTTTGGAGTGGAACTGAGAGGGTTTTGATTGGGAGCGGCTGATAAGTATTGTC
AAAGTAGCTGCTGGGACGTT
ACCACATTGGTGATCCCTCAATGTGGGACAAAAGCTTCCCGTAACTACTTTGGTTCCTAACAATACCCCATCCCT
AGAAAXXXXXXXXXXXXTTTGACATTTTGGAGTCGAACTGAGAGGGTTTTGATTGGGAGCGGCTGATAAGTATTGTC
AAAGTAGCTGCTGGGACGTT
>Marker30896
TACAAGGGAATAAAAAAGGAAAATATTTAGGAAATAAGGAAAATATTCCCATAATCTATCCATAAATATTCTAGG
ATTCTXXXXXXXXXXXCCATGCAAATCAACAATTTTGAGCATAATTGCACTCTACAAGGATGTGCTAAATGTCTTC
CTCTTCTCCTTTGCACGGTG
TACAAGGGAATAAAAAAGGAAAATATTTAGGAAATAAGGAAAATATTCCCATAATCTATCCATAAATATTCTAGG
ATTCTXXXXXXXXXXXCCATGCAAATCAACAATTTTGAGCATAATTGCACTCTACAAGGATGTGCTAAATGTCTTG
CTCTTCTCCTTTGCACGGTG
>Marker31602
ACATCACAATAAAAAATATTTTAATATTTCAAAATATTCTCCATCAACATACTTTAGAAAGATCTATTTGATGAA
AATTTXXXXXXXXXXXATTTCTTTCCATAAAAGATTTAAGAATAGTGTGTATAGTCTAACTTGGGTGTGGGAGTTG
GGGTTTCCATATTAGTGGTG
ACATCACAATAAAAAATATTTTAATATTTCAAAATATTCTCCATCAACATACTTTACAAAGATCTATTTGATGAA
AATTTXXXXXXXXXXXATTTCTTTCCATAAAAGATTTAAGAATAGTGTGTATAGTCTAACTTGGGTGTGGGAGTTG
GGGTTTCCATATGAGTGGTG
>Marker31740
TACAACTGTCATAATAGGATATTTTTGTTGCAGTATTCACTAGCCATTTACCCGCCTAGACCTAAGATTCACTTC
CACCAXXXXXXXXXXXXAAATTTTTTACATACAACACATCGCCTCACATTAGTAACATTCTCAAATGTTAATAATCA
AACATAAGATTACAAAGAGT
TACAACTGTCATAATAGGACATTTTTGTTGCAGTATTCACTAGCCATTTACCCGCATAGACCTAAGATTCACTTC
CACCAXXXXXXXXXXXXAAATTTTTTACATACAACACATCGCCTCACATTAGTAACATTCTCAAATGTTAATAATCA
AACATAAGATTACAAAGAGT
>Marker31765
ACAAACAAGAAAATTAACAGAATGAAAGAATAAATAAGATTAAAACTTTGCTACGCGATAACGATTTGAACTGAG
ATCTTXXXXXXXXXXXTCAATTCATTACGTTAAGATATGAAAAAACCAAAAGTTATATGCTTACCAATGCCATATC
CAAAGAGATTAATAACGGTC
ACAAACAAGAAAATTAACAGAATGAAAGAATAAATAAGATTAAAACTTTGCTACGCAATAACGATTTGAACTGAG
ATCTTXXXXXXXXXXXTCAATTCATTACGTTAAGATATGAAAAAACCAAAAGTTATATGCTTACCAATGCCATATC
CAAAGAGATTAATAACGGTC
>Marker31803
ACTTTTAATCAATGTTTTTTTAGCATATTTTTACAGAGCTCGAAGTCTTGAAGTATAATCACCCATTCACTCTAC
CATTTXXXXXXXXXXXTCAGAATAACCAAAAAACAATGAACAAAACAAAAATTCATCCAAGATTGAATTAACCTCT
TTATTGCATTAAAACAAGTA
ACTTTTAATCAATGTTTTTTTAGCATATTTTTACAGAGCTCGAAGTCTTGAAGTATAATCACCCATTCACTCTAC
CATTTXXXXXXXXXXXTCAGAATAACCAAAAAACAATGAACAAAACAAAAACTCATCCAAGATTGAATTAACCTCT
TTATTGCATTAAAACAAGTA
>Marker32525

Create PDF with GO2PDF for free, if you wish to

AACGTAATTGTTTAATTTATAGCTTGATGTATATTCTAGGGCTTAAATTCATAATATCATACAATGTATTGATAT
GTAAAXXXXXXXXXXXTTAAACTTACAAGTGTATGCATATTTTGGAATGAAAACTTAGATTGTTTAAATTTGAGAT
TTTGACTGATTAGGCAAGTA
AACGTAATTGTTTAATTTATAGCTTGATGTATATTCTAGGGCTTAAATTCATAATATCATACAGTGTATTGATAT
GTAAAXXXXXXXXXXXTTAAACTTACAAGTGTATGCATATTTTAGAATGAAAACTTAGATTGTTTAAATTTGAGAT
TTTGACTGATTAGGCAAGTA
>Marker32628
AACAATTTGTTTAGATTTGACTATCTAATATTTTTACGATAGACATGAAAAGAAGTAATATTGTTGAAGATGGTA
AAGAAXXXXXXXXXXXTTTTCTTAAATTGTTGTATGGACGGTAAATATTTTACCATTTTGTTATATTTATGAAAAT
TAATCTAATTTTTATTAGTA
AACAATTTGTTTAGATTTGACTATCCAATATTTTTACGATAGACATGAAAAGAAGTAATATTGTTGAAGATGGTA
AAGAAXXXXXXXXXXXTTTTCTTAAATTGTTGTATGGACGGTAAATATTTTACCATTTTGTTATATTTATGAAAAT
TAATCTAATTTTTATTAGTA
>Marker33207
AACCATAAGATTCATAACTCTAACCCAAGCTCCAAACAACTTTAATGTATAACTATGAGTTGGATATGTTTTGAG
CCAGGXXXXXXXXXXXCACAAGTATTCCCCTTTCTTCTAGTTCTCTCTCTTTATTTCCATGCTAAGGATGGAACAG
GTCCTCCCTTCAGATTCGTT
AACCATAGGATTCATAACTCTAACCCAAGCTCCAAACAACTTTAATGTATAACTATGAGTTGGATATGTTTTGAG
CCAGGXXXXXXXXXXXCACAAGTATTCCCCTTTCTTCTAGTTCTCTCTCTTTATTTCCATGCTAAGGATGGAACAG
GTCCTCCCTTCAGATTCGTT
>Marker33376
AACCCATGTCAAATCAAATTTGACATCTTCTCGCCAAACCTTTCTTCTCGCTTAACTCCATTTCGTGAATAGCTT
TAACAXXXXXXXXXXXTAACTTCTTAATCGCAAAACTTATACTTAATCATATAAATTTTTATCACTCAATCATTTT
CACTAATTGATTCAAACGTC
AACCCATGTCAAATCAAATTTGACATCTTCTCGCCAAACCTTTCTTCTCGCTTAACTCCATTTCGTGAATAGCTT
TAACAXXXXXXXXXXXTAACTTCTTAATAGCAAAATTTATACTTAATCATATAAATTTTTATCACTCAATCCTTTT
CACTAATTGATTCAAACGTC
>Marker33791
GACTAATTAAAGAAAGATAAGTGTAGCATCACAGCCAACATTTAAATATGTATTTTAGAATAGTTAAATGGATTG
GATGGXXXXXXXXXXXCCTCTCAAATCATGCTATCCTTTTGACACGTCTGATTCATACTTAACATAAAACATCTTA
AATATTATCTTCATTATGTT
GACTAATTAAAGAAAGATAAGTGTAGCATCACAGCCAACATTTAAATATGTATTTTAGAATAGTTAAATGGATTG
GATGGXXXXXXXXXXXCCTCTTAAATCATGCTATCCTTTTGACACGTCTGATCCATACTTAACATAAAACATCTTA
AATATTATCTTCATTATGTT
>Marker33896
CACTTGGAGAGATAAGTATGAAAAGCATGGGGATGCCCATCACGTGACCCAATGGCAAATATAAGATATAACCTG
AAGGCXXXXXXXXXXXACAACTTTACAATATTGACTTCTGAAGTTTGACCATGCTTTCCTTTTACTCTTTTCAGAG
AACTTCTTTCTTAACATTGT
CACTTGGAGAGATAAGTATGAAAAGCATGGGGATGCCCATCACGTGACCCAATGGCAAATATAAGATATAACCTG
AAGGCXXXXXXXXXXXACAACTTTACAATATTGACTTCTGAAGTTTGACCATGCTTTCCTTTTCCTCTTTTCAGAG
AACTTCTTTCTTAACATTGT
>Marker34185
GACCTTGTTGATGAGGAGAAGGATGAATTACTAAAACTGGCATTTGTGCGAATTGTTGAGGCATATAAGCAAATA
GCAGTXXXXXXXXXXXTGGTCTGGTTAAATGATCCATATTTTGTTCAACGCTCTCTGTTCATATCATACACTTAAC
TGTTACTAATGCAGCATGTT

Create PDF with GO2PDF for free, if you wish to

GACCTTGTTGATGAAGAGAAGGATGAATTACTAAAACTGGCATTTGTGCGAATTGTTGAGGCATATAAGCAAATA
GCAGTXXXXXXXXXXTGGTCTGGTTAAATGATCCATATTTTGTTCAACGCTCTCTGTTCATATCATACACTTAAC
TGTTACTAATGCAGCATGTT
>Marker34548
ACATAAATTGAATAAAGTAATTATGTAAAGAGAGAAAAAGAAAAGAAAGAAAAATGAGAAATGAAGAGAGAAATG
ATTGGXXXXXXXXXXACTTACTAAAACCTTCATACACTAACTCTTTCTTTCAAAATACTTACCTTTTTATTGCTA
TTATATTATTAAATGGAGTG
ACATAAATTGAATAAAGTAATTATGTAAAGAGAGAAAAAGAAAAGAAAGAAAAATGAGAAATGAAGAGAGAAATG
ATTGGXXXXXXXXXXACTTACTAAAACCTTCATACACTAACTCTTTCTTTCAAAATACTTACCTTTTTATTACTA
TTATATTATTAAATGGAGTG
>Marker34851
AACAATAAAGAACATGTTGATATTAATTATTTAAGATAATCAGACCAAAATTATTCGGTATAAACATTATGCTTT
GTTTCXXXXXXXXXXTTTCACATAGAAATATGCAAGAACTCTAATGAAGATATGAAGTTCAAATAGAAAACACTG
ATAAACATCAAACCTTTGTT
AACAATAAAGAACATGTTGATATTAATTATTTAAGATAATCAGACCAAAATTATTCGGTATAAACATTATGCTTC
GTTTCXXXXXXXXXXTTTCACATAGAAATATGCAAGAACTCTAATGAAGATATGAAGTTCAAATAGAAAACACTG
ATAAACATCAAACCTTTGTT
>Marker35761
ACCCTCACTGCTAGATATGAGTGTGGTTGAGAAGGAGATTTAGGCCGATGAAAAGTTGAAAGGGATTTATGATCA
AGATGXXXXXXXXXXTGGGACATTTGCCCACTTACAAGCGTATTGCGGCGGAGTTATTTTGGGAAGGGATGAGCA
ATGACATTAAAAAGTATGTG
ACCCTCACTGATAGATATGAGTGTGGTTGAGAAGGATATTTAGGCCGATGAAAAGTTGAAAGGGATTTATGATCA
AGATGXXXXXXXXXXTGGGACATTTGCCCACTTACAAGCGTATTGCAGCGGAGTTATTTTGGGAAGGGATGAGCA
ATGACATTAAAAAGTATGTG
>Marker35867
AACTTTTAATAAGTTAAACTGATTCATTACGTTTGATAGACCAATATATTTGACATTCATAAAAAAAAATACAAT
AAAATXXXXXXXXXXCAAAGCAGTTGTTGGTCACCTATAAAGCACCAACCCTCTTGGAATGACAAAGTGCAGGCC
TCGTCTAACACTGACACGTT
AACTTTTAATAAGTTAAACTGATTCATTACGTTTGATAGACCAATATATTTGACATTCATAGAAAAAAATACAAT
AAAATXXXXXXXXXXCAAAGCAGTTGTTGGTCACCTATAAAGCACCAACCCTCTTGGAATGGCAAAGTGCAGGCC
TCGTCTAACACTGACACGTT
>Marker35963
TACAATCACTCTGAATCGTCTTCTCATTGTTTTTATTATTATTTTCCGTCCATTTCTGTGTTGCCTGACTTCGGC
TAATTXXXXXXXXXXTCTGTTATGGTGTTTGATTGTTGTTATTGAAGTTCGTTCTGTTATATGAAATTTTGTAGG
GATTTTTGAAGATTTAAGTG
TACAATCACTCTGAATCGTCTTCTCATTGTTTTTATTCTTATTTTCCGTCCATTTCTGTGTTGCCTGACTTCGGC
TAATTXXXXXXXXXXTCTGTTATGGTGTTTGATTGTTGTTATTGAAGTTCGTTCTGTTATATGAAATTTTGTAGG
GATTTTTGAAGATTTAAGTG
>Marker36295
AACGAGGCTCAAGATCGCCATTGGAGCAGCAAAGGGGCTGGACTTCCTTCATGGGGCAGAGAAACCTGTCATTTA
TAGGGXXXXXXXXXXCAGCAAAGTTGTCTGACTTTGGATTGGCAAAGATGGGACCTGAGGGATCAGACACTCATG
TAACCACCAGAGTAATGGGT
AACGAGGCTCAAGATCGCCATTGGAGCAGCAAAGGGGCTCGACTTCCTTCATGGGGCAGAGAAACCTGTCATTTA
TAGGGXXXXXXXXXXCAGCAAAGTTGTCTGACTTTGGATTGGCAAAGATGGGACCTGAGGGATCAGACACTCATG
TAACCACCAGAGTAATGGGT
>Marker37014

Create PDF with GO2PDF for free, if you wish to

CACTTATCACTATTGGATGTCTATATTATATGAAAAAGATTAATATATTTATAAGTTTCTTCATATTCCTTGCAT
TATGAXXXXXXXXXXTCTTGAAAAGTATGTCCAAAATCCAAGGCATATTGAGTTTCAGGTAATTATTCCAAAATC
TCAGGAAATTATTTTTTAGT
CACTTATCACTATTGGATGTCTATATTATATGAAAAAGATTAATATATTTGTAAGTTTCTTCATATTCCTTGCAT
TATGAXXXXXXXXXXTCTTGAAAAGTATGTCCAAAATCCAAGGCATATCGAGTTTCAGGTAATTATTCCAAAATC
TCAGGAAATTATTTTTTAGT
>Marker37640
ACATAACAGCGAATGTGGTAGATAGAAACAATGGACGAGTCGTTGCAACTGCATCCACAGTGGAACACTCCATAA
AAAACXXXXXXXXXXGAAGAGGGATTCACGCAGACATAAAAAAAGAAATTGAGAAGAAAGGCTTCAAAAACCACA
CAAAAATCTGGGCTATAGTG
ACATAACAGCGAATGTGGTAGATAGAAACAATGGACGAGTCGTTGCAACTGCATCCACAGTGGAGCACTCCATAA
AAAACXXXXXXXXXXGAAGAGGGATTCACGCAGACATAAAAAAAGAAATTGAGAAGAAAGGCTTCAAAAACCACA
CAAAAATCTGGGCTATAGTG
>Marker38062
TACTAGAATACTATCAACCAACTTATAATATGGTTGTGTGATGATTAACTAATTAATAACTTATTTTAGTTAATT
AGTCCXXXXXXXXXXGCTTAGCCTTCATACTTAGCTGAACAAAAATCTTCTCTTATTCCTAATTAGTCCTTGACA
CAATAGTTTCTCTCTTAGTC
TACTAGAATACTATCAACCAACTTATAATATGGTTGTGTGATGATTAACTAATTAATAACTTATTTTAGTTAATG
AGTCCXXXXXXXXXXGCTTAGCCTTCATACTTAGCTGAACAAAAATCTTCTCTTATTCCTAATTAGTCCTTGACA
CAATAGTTTCTCTCTTAGTC
>Marker38619
AACTCACACGTGTGATATAGAACTCACACACGTGCAACCGACGCCTTTTCGCTTCCCCTTCCTTGAATCCAACAC
GGAATXXXXXXXXXXGTAAATGGGAATCAGCTCATGCAGCTGGAAACAGATCCAAAATGAGACTCGGCTAAAAGG
TAAAGTAGCTTGGAAAAGTG
AACTCACACGTGTGATATAGAACTCACACACGTGCAACCGACGCCTTTTCGCTTCCCCTTCCTTGAATCCAACAC
GGAATXXXXXXXXXXGTAAATGAGAATCAGCTCATGCAGCTGGAAACAGATCCAAAATGAGACTCGGCTAAATGG
TAAAGTAGCTTGGAAAAGTG
>Marker38717
ACTTTTAACTATTGTTTTTGGTATTTTGCAATATCGCGAACATCTCGTAGCTGTCAAACTTTAAATGCCCAAGGC
TGAATXXXXXXXXXXAATCAAATTTTGCAGGAATTTAAAAAGTTAAAGGTGGTTTGAGGATGTCACATGTTCGAA
TTTTAGTGTGTTACGGTGGT
ACTTTTAACTATTGTTTTTGGTATTTTGCAATATCGCGAACATCTCGTAATTGTCAAACTTTAAATGCCCAAGGC
TGAATXXXXXXXXXXAATCAAATTTTGCAGGAATTTAAAAAGTTAAAGGTGGTTTGAGGATGTCACATGTTCGAA
TTTTAGTGTGTTACGGTGGT
>Marker38800
ACTCAAAACCTTTGTTTTCTCTCTAGAGCTAGCCTTGGTGTGGGGAAGGCCTCTTGGCAACTAGCAGGGTTGGTT
CTATAXXXXXXXXXXGTCTGTAAATGCATTTTTAAGGTTACATGCTCACTTGTGGGTCATTTGTGTTTTTTGAAT
ATTGAAATATATTTGAACGT
ACTCAAAACCTTTGTTTTCTCTCTAGAGCTAGCCTTGGTGTGGGGAAGGCCTCTTGGCAATTAGCAGGGTTGGTT
CTATAXXXXXXXXXXGTCTGTAAATGCATTTTTAAGGTTACATGCTCACTTGTGGGTCATTTGTGTTTTTTGAAT
ATTGAAATATTTTTGAACGT
>Marker39046
CACCTATTCAATTGAAAAAATAGAATTACTTAAAAGTATATTTTAAATTAAGATACAAAATTTTAGCTATATAGG
GCTATXXXXXXXXXXTAAAGATAACATCTTTTATAAGGAGAGACGGATCGAGACCATATAAAAACTCAGGAAAAT
AAATTGAGACAAAAAAAGTC

Create PDF with GO2PDF for free, if you wish to

CACCTATTCAATCGAAAAAATAGAATTAGTTAAAAGTATATTTTAAATTAAGATACAAAATTTTAGCTATATATG
GCTATXXXXXXXXXXTAAAGATAACATCTTTTATAAGGAGAGACGGATCGAGACCATATAAAAACTCAGGAAAAT
AAATTGAGACAAAAAAAGTC
>Marker39553
ACTTTAGATTCTTTGGATTTTGTTGATGGGGGCTTGTTGCTTTTGTCTGATTTTTCTGTTTAGCTTTAGTTTGGG
GTTACXXXXXXXXXXCCATTTGAAGACTATAATTTCTTCTGTTCATTTGTTTCTTTGCTTTGATGAATCATTTTC
ACAATTTATTTGAAGGGGTA
ACTTTAGATTCTTTGGATTTTGTTGATGGGGGCTTGTTGCTTTTGTTTGATTTTTCTGTTTAGCTTTAGTTTGGG
GTTACXXXXXXXXXXCCATTTGCAGACTATAATTTCTTCTGTTCATTTGTTTCTTTGCTTTGATGAATCATTTTC
ACAATTTATTTGAAGGGGTA
>Marker39728
GACCCATTAGAGGAAAAGCACGAAGAAGGCAAAATCAATGGGAAAGAAGAAGACCCAGATGGAAAAAAGAAGACC
CAAGAXXXXXXXXXXTTTTGAGTAGTGTGAGAGGTTGTGTTATGTAAGGAGGAATGAAATGCAGAGAGAATTAAA
GAGAGTGAAAATGAAAGGTC
GACCCATTAGAGGAAAAGCACGAAGAAGGCAAAATCAATGGGAAAGAAGAAGAGCCAGATGGAAAAAAGAAGACC
CAAGAXXXXXXXXXXTTTTGAGTAGTGTGAGAGGTTGTGTTATGTAAGGAGGAATGAAATGCAGAGAGAATTAAA
GAGAGTGAAAATGAAAGGTC
>Marker39931
AACAACAGGGTAAGAAGAAGCTCACCCTTTAACACAACATGTTAAGCAAGAACTTCAACATAACACAGACTAAAA
TACAGXXXXXXXXXXGTGAAAATCCACCCTTAAACACAACAAGTTAAGCAAAAACTCCAACATAACACTTAGGAC
TAAAAAATAAGTAAAAAAGT
AACAACAGGGTAAGAAGAAGCTCACCCTTTAACACAACATGTTAAGCAAGAACTTCAACATAACACATACTAAAA
TACAGXXXXXXXXXXGTGAAAATCCACCCTTAAACACAACAAGTTAAGCAAAAACTCCAACATAACACTTAGGGC
TAAAAAATAAGTAAAGAAGT
>Marker40151
ACTTATAAATTTTTTCAAGAAGTCACACCAAACTTTAGAGTTTCCACGACGAACGAACCACAAAGAAAAGAAGGT
GTATCXXXXXXXXXXCATGTCTCTCCTAAACTTACATTTCCAAGTTGAGAATATTTTCAAAATATTCTTTGGATG
AGTGGAAGGTATAAGAAGTA
ACTTATAAATTTTTTCAAGAAGTCACACCAAACTTTAGAGTTTCCACGACGAACGAACCACAAAGAAAAGAAGGT
GTATCXXXXXXXXXXCATGTATCTCTTAAACTTACATTTCCAAGTTGAGAATATTTTCAAAATATTCTTTGGATG
AGTGGAAGGTATAAGAAGTA
>Marker40288
ACAGCATTAAGGTTCATTTTATCATTTTTTTTAAAAAAGTGTAAGTTGAGAACAAAAGAGAGTGACTGTTTTAGA
TAACAXXXXXXXXXXAATGGCTTATGTTCCCTTACCTTTTATCTTCTTTTTGTTATATTAGCTTTAAGTATAGCT
CAATTGATATAAAGTTTGGT
ACAGCATTAAGGTTCATTTTATCATTTTTTTTAAAAAAGTGTAAGTTGAGAACAAAAGAGAGTGACTGTTTTAGA
TAACAXXXXXXXXXXAATGGCTTATGTTCCCTTACCTTTTATCTTCTTTTTGTTATATTAGCTTTAAGCATAGCT
CAATTGATATAAAGTTTGGT
>Marker40343
ACCTTTTATTTATAGGCATAAAATCGTAGGAGCCCAAACCAAAGATTTCTCTAAAATTCTAACTATTCTAGATAT
ATATTXXXXXXXXXXCTTCAAAATTATGATGGCATTTCTTTTTTGATTCAAGTGGCTTTCATGATAATGCTTCAT
CATGTGTAATTTTAAAGGTA
ACCTTTTATTTATAGGCATAAAATCGTAGGAGCCCAAACCAAAGATTTCTCTAAAATTCTAACTATTCTAGATAT
ATATTXXXXXXXXXXCTTCAAAATTATGATGGCATTTCTTTTTTGATTCAAGTGGCTTTCATGATAATGCTTCAT
CATGTGTAATTTTAAATGTA
>Marker41649

Create PDF with GO2PDF for free, if you wish to

ACATTTTAAAGCTTATGTTTATTTGATCTAACATTTAAAAGAGTCTTAAATATAAATTTATCCATTATAACGGAA
AACTTXXXXXXXXXXXAATAAAAAACGTTAATTGTTAGAGATTTTTTAAACGACTTTAGAGTTCAAAATTACTACC
AAAGTTGATGGATTGAAGTT
ACATTTTAAAGCTTATGTTTATTTGATCTAACATTTAAAAGAGTCTTAAATATAAATTTATCCATTATAACGAAA
AACTTXXXXXXXXXXXAATAAAAAACGTTAATTGTTAGAGATTTCTTAAACGACTTTAGAGTTCAAAATTACTACC
AAAGTTGATGGATTGATGTT
>Marker41805
ACTAAATAATGAGAATGGCCAAGTTAGTTGGTATATTTTATGTTATTGGGAAATCAATGATTAAGTTATAAATAA
GTAGGXXXXXXXXXXXAATCCCCTTATTACTCTACTGTAAGCCTTTTCAACTGATAGGTGGAGTGCCTCTTTGTGG
TTGGACCTATACTATGTTGT
ACTAAATAATGAGAATGGCCAAGTTAGTTAGTATATTTTATGTTATTGGGAAATCAATGATTAAGTTATAAATAA
GTAGGXXXXXXXXXXXAATCCCCTTATTATTCTACTGTAAGCCTTTTCAACTGATAGGTGGAGTGCCTCTTTGTGG
TTGGACCTATACTATGTTGT
>Marker42259
GACTAATCAGGCTCCTCCTTGCCTACCTTGTCACCTGATAATTGGTTAAGTGTTTCCTGTCACCTTCCCTTCTTG
TGTTAXXXXXXXXXXXTATCCGAACTTCAAAAATTAATTTACAAGAAACCTCTTTATTAGGATCCAAGTTTCACAC
ATGGGTAGCCAATTGTTGTT
GACTAATCAGGCTCCTCCTTGCCTACCTTGTCACCTGATAATTGGTTAAGTGTTTCCTGTCACCTTCCCTTCTTG
TGTTAXXXXXXXXXXXTATCCGAACTTCAAAAATTAATTTACAAGAAACCTCTTTATTAGGATCCAGGTTTCACAC
ATGGGTAGCCAATTGTTGTT
>Marker43411
AACGTGTTAGATTAAGAAAATGTCTATTCCCTCTCTACGAGAACAAAAGGAAGAGGAGAATTTTTTGTTAAAGTG
TCGTGXXXXXXXXXXXAGTTTGTGTATTCCAACATTATAAAAATCATGTTAAAGCTTGATTTAATGATTCCTTAAG
ATGATGCTAACTTTAATGTG
AACGTGTTAGATTAAGAAAATGTCTATTCCCTCTCTACGAGAACAAAAGGAAGAGGAGAATTTTTTGTTAAAGTG
TCGTGXXXXXXXXXXXAGTTTGTGTATTCCAGCATTATAAAAATCATGTTAAAGCTTGATTTAATGATTCCTTAAG
ATGATGCTAACTTTAATGTG
>Marker43570
ACCGAAGCATTGCTTCTCTGATGCACATATATCTATATAGAATATCATGAAATATTGGTTCTCATCTCTATATGC
TTGTTXXXXXXXXXXXATGATTTATGTATATATAATCTGATTATATTGGAACAAACAGAAAATTCCTTACTTCAAC
GTGCAAAGTCATTGCGCGTG
ACCGAAGCATTGCTTCTCTGATGCACATATATCTATATAGAATATCATGAAATATTGGTTCTCATCTCTACATGC
TTGTTXXXXXXXXXXXATGATTTATGTATATATAATCTGATTATATTGGAACAAACAGAAAATTCCTTACTTCAAC
GTGCAAAGTCATTGCGCGTG
>Marker43843
AACAGAGAACCAATTTTCTGGAAATACAAGGTCATGGACAAGGAGAAAGATCCATCAACATTGAAACATCTGCAG
TCACTXXXXXXXXXXXATTAGGTTATGATTGAACATTCTTCAAACTATTTCATTTGACCACAGCTCCAAGTTGTGT
ATTGTGTTTAGAATGAAGTG
AACAGAGAACCAATTTTCTGGAAATACAAGGTCATGGACAAGGAGAAAGATCCATCAACATTGAAACATCTGCAG
TCACTXXXXXXXXXXXATTAGGTTATGATTGAACATTCTTCAAACTATTTCATTTGGCCACAGCTCCAAGTTGTGT
ATTGTGTTTAGAATGAAGTG
>Marker44177
AACATTTAGCTATCATGTAACTAAAATATTAATTTTTTACTTATTCTCCATAATCTTTTGATTAATTTTTTTTAA
ATTTTXXXXXXXXXXXTAGACTATAACTTGTCAATATTTGGTAGAGCTTAAATCTGAAATTAATTTCCATATATTG
AATTAATCGTGGTTATAAGT

Create PDF with GO2PDF for free, if you wish to

AACATTTAGCTATCATGTAACTAAAATATTAATTTTTTACTTATTCTCCATAATCTTTTGATTAATTTTTTTAAA
ATTTTXXXXXXXXXXTAGACTATAACTTGTCAATCTTTGGTAGAGCTTAAATCTGAAATTAATTTCCATATATTG
AATTAATCGTGGTTATAAGT
>Marker44211
AACCAGATCCCCCTTTAGATTGATCATGCCTGTAATTGCAAAGTAGAATATGTTCCAAACCATACTGTCATCAGA
GCATTXXXXXXXXXXXAGCCGACCCTGGTATTATGTCTAAATTTGATAATCGGGTAACAGCCCCCAACAATAATCA
GGGTTTATCATCAAAGGGTT
AACCAGATCCCCCTTTAGATTGATCATGCCTGTAATTGCAAAGTGGAATATGTTCCAAACCATACTGTCATCAGA
GCATTXXXXXXXXXXXAGCCGACCCTGGTATTATGTCTAAATTTGATAATCGGGTAACAGCCCCCAACAATAATCA
GGGTTTATCATCAAAGGGTT
>Marker44411
CACCGGATGGCCCTCCCAATCCTTGGCTCTCAGCTCTAGCAACCCCTTCACTTCCCTTACTGTGGTCATTGTCGT
CCCTTXXXXXXXXXXTGAAGTTCATATACACACAATTAGATAAGTAACTTATAGTTTAAAGATCATGCTTACCTC
ATATTCGGTTGTCTTATGTG
CACCGGATGGCCCTCCCAATCCTTGACTCTCACCTCTAGCAACCCCTTCACTTCCCTTACTGTGGTCATTGTCGT
CCCTTXXXXXXXXXXTGAAGTTCATATACACACAATTAGATAAGTAACTTATAGTTTAAAGATCATGCTTACCTC
ATATTCGGTTGTCTTATGTG
>Marker45070
ACAAGAGAATAAAATTAAAATTGAAAAAAAAAAGAAAAAAAGTTAGTTGTGAAGTTATGAAAGAATATCCTTATC
ATTTAXXXXXXXXXXXTTTACGCCCTTCCTCTTCTCCCTTCTCATTCCTCCATTTCTGTTCTATACTGTGTTTGAT
CTTCCATGGCCGACCGTGTT
ACAAGAGAATAAAATTAAAATTGAAAAAAAAAAGAAAAAACGTTAGTTGTGAAGTTATGAAAGAATATCCTTATC
ATTTAXXXXXXXXXXXTTTACGCCCTTCCTCTTCTCCCTTCTCATTCCTCCATTTCTGTTCTATACTGTGTTTGAT
CTTCCATGGCCGACCGTGTT
>Marker45074
ACTAATCGAAGCAGTATCTTTACTTTCAAAGTTAATAGAATAATATTAACTATATGATTTTTATATTAACTGCGT
CAGCAXXXXXXXXXXCCATTTATAGCTTCCCTTTTTACACGAATCCGATAAAAAAGTTATTTCATGATCTATCTC
TTTAACATAAATTAGTAGTG
ACTAATCGAAGCAGTATCTTTACTTTCAAAGTTAATAGAATAATATTAACTATATGATTTTTATATTAACTGCGT
CAGCAXXXXXXXXXXCCATTTATAGCTTCCCTTTTTACACGAATCCGATAAAAAAGTTAATTCATGATCTACCTC
TTTAACATAAATTAGTAGTG
>Marker45093
ACAAAACGTGGCTGTGTATTTATGCTCTGTTTCGGCATCCAAATGAATGTGGGTTATAGTATTGGTTGATTATGA
ATGCAXXXXXXXXXXATCATCAAAAGAACTGTTGGAGGCTACTTCGTTGTTCAAGGTAGGGCTGATGACACCATG
AACCTTGGCGGCATCAAGGT
ACAAAACTTGGCTGTGTATTTATGCTCTGTTTCGGCATCCAAATGAATGTGGGTTATAGTATTGGTTGATTATGA
ATGCAXXXXXXXXXXATCATCAAAAGAACTGTTGGAGGCTACTTCGTTGTTCAAGGTAGGGCTGATGACACCATG
AACCTTGGCGGCATCAAGGT
>Marker45133
TACTTGCTTTCTAGGTGGCACCTATAAACCTTTTACACAACCATTTGGCTTATATGTTTTAGTGTTTTGAGATTT
CACTTXXXXXXXXXXAACTAGGAAAAATCTTAAGCAATAAAATAAAAGTTAAATAATGCATTTGATTTCAAATTT
TTGTTTAGTTCTTAAGAAGT
TACTTGCTTTCTAGGTGGCACCTATAAACCTTTTACACAACCATTTGGCTTATATGTTTTAGTGTTTTGAGATTT
CACTTXXXXXXXXXXAAGTAGGAAAAATCTTAAGCAATAAAATAAAAGTTAAATAATGCATTTGATTTCAAATTT
TTGTTTAGTTTTTAAAAAGT
>Marker45220

Create PDF with GO2PDF for free, if you wish to

GACTTATCCCATAGTCGATGTTGAAAGTGTTGAGTATTCCAAGCCTGAAATGGATTGGACTGATGCTAAAGATGA
AACTGXXXXXXXXXXAGTTAAGATGTCTCGCCTTTATATCATAACCTCTCAGTTTGAGGCTCTTAAAATGTTGGA
AGGTGAATCCATAGTTGAGT
GACTTATCCCATAGTCGATGTTGAAAGTGTTGAGTATTCCAAGCCTGAAATGGATTGGACTGATGCTAAAGATGA
AACTGXXXXXXXXXXAGTTAAGATGTCTCGTCTTTATATCATAACCTCTCAGTTTGAGGCTCTTAAGATGTTGGA
AGGTGAATCCATAGTTGAGT
>Marker45298
AACTAGTTTGTTACAAATCAGATTATTGTTGAATAAAATTTGCATGTATTTATTGGTTATGATGGAATTAAATAT
TCTTGXXXXXXXXXXATACTTGGATTACTCTCATTGCCTATTAGTTTTTAGATGAACCCTTGTTTATCTAATGAT
TCTACCATGATATCAAAGTA
AACTAGTTTGTTACAAATCAGATTATTGTTGAATAAAATTTGCATGTATTTATTGGTTATGATGGAATTAAATAT
TCTTGXXXXXXXXXXATACTTGGATTACTTTCATTGCCTATTAGTTTTTAGATGAACCCTTGTTTATCTAATGAT
TCTACCATGATATCAAAGTA
>Marker45484
AACACAAGAACAGAGGGATGCAGGACAAAAACTACAATAAACGGGATTTCAAGCTCAAGAACATCCCATAAATGA
ACCACXXXXXXXXXXGTAGAGAAGGAAATGGAGAGGCTCCCAGTAATTGAAAACTTCGTCGCAATCGTGAATGAT
GTTGGATGTGGCACTCATGT
AACACAAGAACAGGGGGATGCAGGACAAAAACTACAATAAACGGGATTTCAAGCTCAAGAACATCCCATAAATGA
ACCACXXXXXXXXXXGTAGAGAAGGAAATGGAGAGGCTCCCAGTAATTGAAAACTTCGTCGCAATCGTGAATGAT
GTTGGATGTGGCACTCATGT
>Marker46113
AACTTCTTTCTATGAAGGCAGTCATTTGGTTTGCATGGGTGAGAGTGACCTATGTTGCTGACTTAATATGCCAAC
TATTTXXXXXXXXXXACCACACACTCTATCTTGGCTCGAGAGGAGTTCAATTATAGTGAAACTATAACTTATTGT
TCATTAGATGAATTAGTGGT
AACTTCTTTCTATGAAGGCAGTCATTTGGTTTGCATGGGTGAGAGTGACCTATGTTGCTGACTCAATATGCCAAC
TATTTXXXXXXXXXXACCACACACTCTCTCTTGGCTCGAGAGGAGTTCAATTATAGTGAAACTATAACTTATTGT
TCATTAGATGAATTAGTGGT
>Marker46197
AACTCTAAAAAGCAAAGCAAAGATCGGATACGTTAGTTTATGTGTAAGGGTTGAATAGCTCACCTCCATACTTTC
TAGTTXXXXXXXXXXTGGGATGGGTTATCTATATCGACAATTCATATTGACTAACAATATCGACTTAGTTAATGA
AATCAGAACAAAAGAGAGTG
AACTCTAAAAAGCAAAGCAAAGATCGGATACGTTAGTTTATGTGTAAGGGTTGAATAGCTCACCTCCATACTTTC
TAGTTXXXXXXXXXXTGGGATGGGTTATCTATATCGACAATTCATATGGACTAACAATATCGACTTAGTTAATGA
AATCAGAACAAAAGAGAGTG
>Marker46302
ACAACATATCCATTTTAGACTTCGTATTTCTTCACCTTTTCCATTTTGATTTCTGTTCTGTCCATTTTTGCTTTG
CATACXXXXXXXXXXGTCAAAATCGCCATATTTTAGAAAGTGTAGTTCTAAAACAAACATTTTAAAAGTAAATGT
AATAAAATTAATAGAAGGTT
ACAACATATCCATTTTAGACTTCGTATTTCTTCACCTTTTCCATTTTGATTTCTGTTCTGTCCATTTTTGCTTTG
CATACXXXXXXXXXXGTTAAAATCGCCATATTTTAGAAAGTGTAGTTCTAAAACAAACATTTTAAAAGTAAATGT
AATAAAATTAATAGAAGGTT
>Marker47870
ACTACATCTGTATGTTTAAAAATGGGGCACTTGCTTTACTGTAAAATGCCTTCTCTGATGTGAGTTACTTCATGT
TTTCTXXXXXXXXXXGATAAACATTTTTTTTTTAAATACTGCATGTTACTGTTGAAACCGGTGTAAATATTTTAT
GTTGGGTGGCTTCTTTTGTT

Create PDF with GO2PDF for free, if you wish to

ACTACATCTGTATGTTTAAAAATGGGGCACTTGCTTTACTGTAAAATGCCTTCTCTGATGTGAGTTACTTCATGT
TTTCTXXXXXXXXXXXGATAAACATTTTTTTTTTAAATACTGCATGTTACTGTTGAAACCGGTGTAAATATTTTAT
GTTGGGTGGCTTCCTTTGTT
>Marker48024
ACCAACCTCTAGATCCAAGGGCACCTTCTTCTCCTCCGTCTCTAAAACGCTTGGGAAATTAAATCCCTTCCGTCA
CGGATXXXXXXXXXXXTCTGCTCGTTTTGTATAAAGTTGCTTTCTTTTACTCCTTTTAAAGTTGGTGTTTCAATGT
TTTGTTGAAGTTGTGTTGTG
ACCAACCTCTAGATCCAAGGGCACCTTCTTCTCCTCCGTCTCTAAAACGCTTGGGAAATTAAATCCCTTCCGTCA
CGGATXXXXXXXXXXXTCTGTTCGTTTTGTATAAAGTTGCTTTGTTTTACTCCTTTTAAAGTTGGTGTTTCAATGT
TTTGTTGAAGTTGTGTTGTG
>Marker48054
ACCCTTGAAGACAAAAGAGTGTAGGGTCTGAGTGTCCCACACTAACACATTTTTTCTCAAAACAGTCCCCACTCC
GAATAXXXXXXXXXXXGTTTCTCTTTGTTTAGTCTCTTTTGTTTTTCAAACTAACATCTCTTAAACATCTTTACAT
TGGTGTGATTTCGATAAGTT
ACCCTTGAAGACAAAAGAGTGTAGGGTCTGAGTGTCCCACACTAACACATTTTTTCTCAAAACAGTCCCCACTCC
GAATAXXXXXXXXXXXGTTTGTCTTTGTTTAGTCTCTTTTGTTTTTCAAACTAACATCTCTTAAACATCTTTACAT
TGGTGTGATTTCGATAAGTT
>Marker48194
ACCATCAATTTTCAAGCACAAAAACTATTAAGTATCCTATCATTATCTATCTCAAATAGACTCTAATAGTATTTT
ATCTTXXXXXXXXXXXGCATATCTCTATCTAGCTATCTAGGATAGAAAATGATACATCACTATGATTGTCTAAGTC
TCGTTGAGTCGCACAGGGTA
ACCATCAATTTTCAAACACAAAAACTATTAAGTATCCTATCATTATCTATCTCAAATAGACTCTAATAGTATTTT
ATCTTXXXXXXXXXXXGCATATCTCTATCTAGCTATCTAGGATAGAAAATGATACATCACTATGATTGTCTAAGTC
TCGTTGAGTCGCACAGGGTA
>Marker48653
AACAGAAGCATTAGAGATTGACGGAAGTTTGAAAGCAGGGGTTAAGTTATTCCATTTTAAAATTACAGGAACCAA
ATCGTXXXXXXXXXXXAATTTACTAAACTTCGTGTGTTTAGTTAAGCACATTTTGTTTTTTAAGTTAAATTTACAT
GATTTTATCTATAAAGAAGT
AACAGAAGCATTAGAGATTGACGGAAGTTTGAAAGCAGGGGTTAAGTTATTCCATTTTAAAATTACAGAAACCAA
ATCGTXXXXXXXXXXXAATTTACTAAACTTCGTGTGTTTAGTTAAGCACATTTTGTTTTTTAAGTTAAATTTACAT
GATTTTATCTATAAAGAAGT
>Marker48805
ACCAACATAACACTTCCCCAAACACTTATCATAATACTATCATAACACTACCCACATAACTCTTTCCCTAAACAC
ATCTTXXXXXXXXXXXCCAAATATAAAGTCGTTTTATGATTTAGACAAAAGAATTATTGGTTTTGGTTTTTGGTTG
TAAGTGTAAATATTTTATGT
ACCAACATAACACTTCCCCAAACACTTATCATAATACTATCATAACACTACTCACATAACCCTTTCCCTAAACAC
ATCTTXXXXXXXXXXXCCAAATATAAAGTCGTTTTATGATTTGGACAAAAGAATTATTGGTTTTGGTTTTTGGTTG
TAAGTGTAAATATTTTATGT
>Marker48929
ACCATTTACCAATAGTAGATGAGAATTTTAAGAGATTAGGATCAAATGGAGAATAACCTAAAAGATAAGTAACCA
ACCTCXXXXXXXXXXXGAAGAACATTCAGCACCTACAGAACATGGATAATGTTGCATTTTTCAAAACTAAGTTATA
AAAGTCTGTTTTCAGTAGTT
ACCATTTACCAAGAGTAGATGAGAATTTTAAGAGATTAGGATCAAATGGAGAATAACCTAAAAGATAAGTAACCA
ACCTCXXXXXXXXXXXGAAGAACATTCAGCACCTACAGAACATGGATAATGTTGCATTTTTCAAAACTAAGTTATA
AAAGTCTGTTTTCAGTAGTT
>Marker48930

Create PDF with GO2PDF for free, if you wish to

AACATATGCATCAATTTGTTTGCTGAAAAATCTCAAATGGCATCAAACAAAGGGGTCATAAAAATTTAATGGTAT
ATCTAXXXXXXXXXXXTTTGATTACTCACTTGACTTCAAAGAGGATGAATATGATACTGAAAACAAAGAGGAATGG
AGAAGAAATGGAGAGGCGTT
AACATATGCATCAATTTGTTTGCTGAAAAATCTCAAATGACATCAAACAAAGGGGTCATAAAAATTTAATGGTAT
ATCTAXXXXXXXXXXXTTTGATTACTCACTTGACTTCAAAGAGGATGAATATGATACTGAAAACAAAGAGGAATGG
AGAAGAAATGGAGAGGCGTT
>Marker49009
TACCTTGCAGAGTCATATATATCTCACTTGCTCCACACGACGATAATTCACAAATAAAATCAAAAGAATATAAAT
GTTTCXXXXXXXXXXXCAATATATAAGAATATAAATTTCATTCTATTTTTAAATCTTTCATCTGGCAAACAAAATT
GTTGAATTAATTATTTTAGT
TACCTTGCAGAGTCATATATATCTCACTTGCTCCACACGACGATAATTCACAAATAAAATCAAAAGAATATAAAT
GTTTCXXXXXXXXXXXCAATATATAAGAATATAAATTTTATTCTATTTTTAAATCTTTCATCTTGCAAACAAAATT
GTTGAATTAATTATTTTAGT
>Marker49019
CACTAAGAAACTCTCATCATCTACATCAGAAACATTGCAAATAAAATCCCTCAGACTGTCTTCAAGAACTTCTTA
CAAAAXXXXXXXXXXXTTACCCTTGACCTTTAAAATTTGTTTTAAAAAATAACCTTGTAATTTGAAAAATACACTT
CCTATTAAAGCAAATAGGTT
CACTAAGAAACTCTCATTATCTACATCAGAAACATTGCAAATAAAATCCCTCAGACTGTCTTCAAGAACTTCTTA
CAAAAXXXXXXXXXXXTTACCCTTGACCTTTAAAATTTGTTTTAAAAAATAACCTTGTAATTTGAAAAATACACTT
CCTATTAAAGCAAATAGGTT
>Marker49131
ACTCTATTTTGGCTCCTCTGATTTCATTCATACTTGATGAAGCATCTAGAATTATAATATTGTTTTTACTTGCAG
GTTGGXXXXXXXXXXXTCGATGAGCTCTTTGTTGATACTCAGGTAATTGAGAATTTTGTAAATCTAGTATCCTGTG
ACATATTATAATATGCTGTG
ACTCTATTTTGGCTCCTCTGATTTCATTCATACTTGATGAAGCATCTAGAATTATAATATTGTTTATACTTGCAG
GTTGGXXXXXXXXXXXTCGATGAGCTCTTTGTTGATACTCAGGTAATTGAGAATTTTGTAAATCTAGTATCCTGTG
ACATATTATAATATGCTGTG
>Marker49202
AACAAATCAATGGTGTATTTTCTCTGAGATATAGAGATGCCTTCTTTAGATTTGGCCACCACCATGCCAAGGAAA
TATTCXXXXXXXXXXXCCAACATACACTATCAGAACTGCAATCTTTCCATCTTGGAAACCTTTGTAAATAAATATG
ATCAGAATGCCCCTGACTGT
AACAAATCAATGGTGTATTTTCTCTGAGATATAGAGATGCCTTCTTTAGATTTGGCCACCACCATGCCAAGGAAA
TATTCXXXXXXXXXXXCCAACATACACTATCAGAACTGCAATCTTTCCATCTTGGAAACCTTTGTAAATAAATATG
ATCAAAATGCCCCTGACTGT
>Marker49608
ACCTTCCCAATTCAATACATCTATGTCCTTGGATCCAAGACGAGTTTTGAACCAACCATTCTGCAAATGCAAAAG
TCCCCXXXXXXXXXXXAAAAAACAAAACCAACTCCTAAACATAAACAAAACATCAAGAACTGGCAAATAGCATTAC
AATTTGAACCATTCTAAGTA
ACCTTCCCAATTCAATACATCTATGTCCTTGGATCCAAGACGAGTTTTGAACCAACCATTCTGCAAATGCAAAAG
TCCCCXXXXXXXXXXXAAAAAACAAAACCAACTCCTAAACATAAACAAAACATCAAGAACTGGCATATAGCATTAC
AATTTGAACCATTCTAAGTA
>Marker49726
GACGGTGAATTTTAAAAAGTAAAAATAAGTAACAATTTTGTAAGTTTAGAAATTTAAGAATCATATAGGCATGAA
TACAAXXXXXXXXXXXATGATGAACACTAGTAGATTTTTATTAGTGTAATGTTATTAATAGAAATCGATTGAAATC
TATATTTTGTAGGTTTGGTA

Create PDF with GO2PDF for free, if you wish to

GACGGTGAATTTTAAAAAGTAAAAATAAGTAACAATTTTGTAAGTTTAGAAATTTAAGAATCATATAGGCATGAA
TACAAXXXXXXXXXXATGATGAACACTAGTAGATTTTTATTAGTGTAATGTTATTAATAGAAATCGATTGAAGTC
TATATTTTGTAGGTTTGGTA
>Marker49820
TACTGCATGAGGGAAATTGAAAAAGATGTGCTGAAGGTCTTCCTGATTGTTACAGCATAGATGGCACATATGGGG
TGAGAXXXXXXXXXXGTGAGATGATTAACCAAAGATTTAACAGTGAAGGAACCTTCTGGTTCTAGGGACCAAACT
CTCTTATTTGTGGAGAAGTT
TACTGCATGAGGGAAATTGAAAAAGATGTGCTGAAGGTCTTCCTGATTATTACAGCATAGATGGCACATATGGGG
TGAGAXXXXXXXXXXGTGAGATGATTAACCAAAGATTTAACAGTGAAGGAACCTTCTGGTTCTAGGGACCAAACT
CTCTTATTTGTGGAGAAGTT
>Marker50323
CACTATAAAAAGTGGAACACTTTGGATTCCATTGTCAATAAGACAAAAAAAGAGACGATGTTAAATGTGTTAAAA
ATAAAXXXXXXXXXXGATAAAAACTTATATAAGTTTATTGATAAAACTAATAGAATTTTATCAGTGATGGGAATC
AATAGAAACTAGCATAAGTC
CACTATAAAAAGTGGAACACTTTGGATTCCATTGTCAATAAGACAAAAAAAGAGACGATGTTAAATGTGTTAAAA
ATAAAXXXXXXXXXXGATAAAAACTTATATAAGTTTATTGATAAAACTAATAGAATTTTATCAGTGATGGAAATC
AATAGAAACTAGCATAAGTC
>Marker50847
CACATCCAGGCTTTATAGTTTTCTTCCCTATCTTGCATTGTGATAAAATCTTTAACTTAACTTTTGATTAATGGA
GAAGCXXXXXXXXXXAACCAGAGGGAGCATTTGCTTCTACTCCTTGCTAACATGCAAGCTCGACTAACAAATAAG
CAGAAGTCTGGCTCAAAGGT
CACATCCAGGCTTTGTAGTTTTCTTCCCTATCTTGCATTGTGATAAAATCTTTAACTTAACTTTTGATTAATGGA
GAAGCXXXXXXXXXXAACCAGAGGGAGCATTTGCTTCTACTCCTTGCTAACATGCAAGCTCGACTAACAAATAAG
CAGAAGTCTGGCTCAAAGGT
>Marker51406
ACCAATGGTGATGAAGTATTAGTGAATTGCTACCCTTGAATCTAGTGTCACAACAATGGGATTTTATTTGCCATG
AGACAXXXXXXXXXXTGCTGCAATCAGAGAATTGTTATATATTTGAAAGAGCATTCCAGCTTCCTAGCTTAGCTC
ATATTTTTGCCGTGTATGTA
ACCAATGGTGATGAAGTATTAGTGAATTGCTACCCTTGAATCTAGTGTCACAACAATGGGATTTTATTTGCCATG
AGACAXXXXXXXXXXTGCTGCAATCAGAGAATTGTTATATATTTGAAAGAGCATTCCAGCTTCCAAGCTTAGCTC
ATATTTTTGCCGTGTATGTA
>Marker51550
TACTAGTTCATAACATTGGTTGATGTCTTCTCATGCAGCAAGACCAATTTGGTCGGCTACTTCCAACCTTCATTC
AGGTCXXXXXXXXXXTAGCATCAATACGAAGGTTTTGTTCTAATTACTAGACAAAATTACCTGTTTGAAAAACCT
TTTTAATAAATCTACAAAGT
TACTAGTTCATAACATTGGTTGATGTCTTCTCATGCAGCAAGACCAATTTGGTCGGCTACTTCCAACCTTCATTC
AGGTCXXXXXXXXXXTAGCATCAATACGAAGGTTTTGTTCTAATTACTAAACAAAATTACCTGTTTGAAAAACCT
TTTTAATAAATCTACAAAGT
>Marker52338
ACCACACACTGAAAACACACTTTTTATTAAGTCTAACATCATGTTAAAGGACTTGTTACTCCAACCATTTAAAAC
CTTTAXXXXXXXXXXAATATTGTAGGGTATCTCATTCTCCAAACCTTCCTCTATTTCCCGTTCATGTTCAATCAG
AGCTTGTAAATCATTAAGTA
ACCACACATTGAAAACACACTTTTTATTAAGTCTAACATCATGTTAAAGGACTTGTTACTCCAACCATTTAAAAC
CTTTAXXXXXXXXXXAATATTGTAGGGTATCTCATTCTCCAAACCTTCCTCTATTTCCCGTTCGTGTTCAATCAG
AGCTTGTAAATCGTTAAGTA
>Marker52515

Create PDF with GO2PDF for free, if you wish to

AACTTTTCCTTGCTTCACTAATAAAGCTGTAGATGAATAATGCCGTGACCTCTTTAACTATGACAAGAGTCTCTA
TTTAAXXXXXXXXXXXTAGTATTCTACTACTTAATGGAACTCCTTTTTTCTTTTTCATTCTTCCTATAATTTAGGT
TGCTTAGTTTAGTTGATGTC
AACTTTTCCTTGCTTCACTAATAAAGCTGTAGATGAATAATGCCGTGACCTCATTAACTATGACAAGAGTCTCTA
TTTAAXXXXXXXXXXXTAGTATTCTACTACTTAATGGAACTCCTTTTTTCTTTTTCATTCTTCCTATAATTTAGGT
TGCTTAGTTTAGTTGATGTC
>Marker52675
TACCTCAATCCCTACCTTGCTCGGAACCACCAAAGCCACAACTGAGGTAGTATTCGTCACTCATCAATAAACACT
ACTACXXXXXXXXXXXACAAAATTGAAAAAATGCATAATTTTTTTTAAAAAAATTCAATACTTGACACATTAAAAT
CGTCAAGTCTAGTTTAATGT
TACCTCAATCCCTACCTTGCTCGGAACCACCAAAGCCACAACTGAGGTAGTATTCGTCACTCATCAATAAACACT
ACTACXXXXXXXXXXXACAAAATTGAAAAAATGCATAATTTTTTTAAAAAAAATTCAATACTTAACACATTAAAAT
CGTCAAGTCTAGTTTAATGT
>Marker53505
AACTAAAACTCAGTGAATGGCAAGTTTTTAAAAGTAAAAGCTCATAAAACAATACTTTCAAACTATTTAAATATG
TCATCXXXXXXXXXXXGACGCCTAAATATACGACCAAGAATAGCTCGCACATGATTAAAGAATCATGCGACCACAA
CTAGCTCACACATATTTAGT
AACTAAAACTCAGTGAATGACAAGTTTTTAAAAGTAAAAGCTCATAAAACAATACTTTCAAACTATTTAAATGTG
TCATCXXXXXXXXXXXGACGCCTAAATATACGACCAAGAATAGCTCGCACATGATTAAAGAATTATGCGACCACAA
CTAGCTCACACATATTTAGT
>Marker53941
CACACATAATGTTCGGCTCAAACTTAATTTCCTGCAATAAAGCAAGTCTTAGGAATATCTACAAGTATCATTAAC
AAGGCXXXXXXXXXXXTCAACCAACTAACAGTGCTACAAAGGCATTTAATCATACTGACCATAAGAACATCAAAAC
CTGATTCTTTAAGTTAAAGT
CACACATAATGTTCGGCTCAAACTTAATTTCCTGCAATAAAGCAAGTCTTAAGAATATCTACAAGTATCATTAAC
AAGGCXXXXXXXXXXXTCAACCAACTAACAGTGCTACAAAGGCATTTAATCATACTGACCATAAGAACATCAAAAG
CTGATTCTTAAAGTTAAAGT
>Marker53990
TACCCATTTAACAAAATGCCAATGATTTAGCCTAATGATAATAAACACAAAATACTAATATATCGAGAACATTTT
AACTCXXXXXXXXXXXGTATCGTATTATTTAAAATATGTAATGTATAAAATTCAAATCCACTAATTGGCTTAAAAG
GGAATATGCTCTGTTTGGTA
TACCCATTTAACAAAATGCCAATGATTTAACCTAATGATAATAAACACAAAATACTAATATATTGAGAACATTTT
AACTCXXXXXXXXXXXGTATCATATTATTTAAAATATGTAATGTATAAAATTCAAATCCACTAATTGGCTTAAAAG
GGAATATGCTCTGTTTGGTA
>Marker54098
ACATGGATTTGAGATTTCAGAACTATATGATAGAACATGAAATCTAATTCCAACTCTCGGCTCCTGATGCACCTC
AACAAXXXXXXXXXXXAGAGACTGCAATTCATATCTTGAACAATGTTCCCTCGAAAAGTGTTTCTGAAACACCTTT
TGAGTTATGGAGATGACGTA
ACATGGATTTGAGATTTCAGGACTATATGATAGAACATGAAATCTAATTCCAACTCTCGGCTCCTGATGCACCTC
AACAAXXXXXXXXXXXAGAGACTGCAATTCATATCTTGAACAATGTTCCCTCGAAAAGTGTTTCTGAAACACCTTT
TGAGTTATGGAGATGACGTA
>Marker54247
AACAATCTTTAGTGATAATACAAAAACTTCTCTACGTCATACTTTATGCTGTTCTGAATATTGCACATCTTAACA
AACTTXXXXXXXXXXXAAAATGAAGAAGGCTGGAAAACTCATAAAGACTACCTCAAATATCTTTTTTGCAATTTCA
GGATAACATCTTGTGCTGTC

Create PDF with GO2PDF for free, if you wish to

AACAATCTTTAGTGATAATACAAAAACTTCTCTACGTCATACTTTATGCTGTTCTGAATATTGCACATCTTAACA
AACTTXXXXXXXXXXXAAAATGAAGAAGGCTGGAAAACTCATAAAGACTACCTCAAATATCTTTTTTGCAATTTCA
GGATAACATCTTGTTCTGTC
>Marker54630
AACCTTAAAATTTTCCTATTGATTTTGTTAATTTTAATCCATTAGTTTTCTACACCTAATCCTACCTGCTTGTAA
AAAATXXXXXXXXXXXTACCATTTAAGCTCAGACTTGACTAAGTTGTGTTACAATGGAAATGGGATTTGAAAAAAT
TAAAGGGAAAGTGATGAAGT
AACCTTAAAATTTTCCTATTGGTTTTGTTAATTTTAATCCATTAGTTTTCTACACCTAATCCTACCTGCTTGTAA
AAAATXXXXXXXXXXXTACCATTTAAGCTCAGACTTGACTAAGTTGTGTTACAATGGAAATGGGATTTGAAAAAAT
TAAAGGGAAAGTGATGAAGT
>Marker54662
GACCTATCATTTCTATTGATGGCAAGTTTTTGAAGTTTAAGTTTGGTAACCTCCTATTAACAGCCTAATCACAGG
ATGGTXXXXXXXXXXXTTAGTCATTGTTTCTTATAGACATCTTAGCATCTCTAAAGGAGTTTTATGAGTGTTTCCT
AATGTTGAGTATTGTGTGTG
GACCTATCATTTCTATTGATGGCAAGTTTTTGAAGTTTAAGTTTGGTAACCTCCTATTAATAGCCTAATCACAGG
ATGGTXXXXXXXXXXXTTAGTCATTGTTTCTTATAGACATTTTAGCATCTCTAAAGGAGTTTTATGAGTGTTTCCT
AATGTTGAGTATTGTGTGTG
>Marker54753
ACCTCATAAAAACCCTAAAATTGAAAATCTTGAACTAACCTAACACAACTAATTAATTAAAAAAACAACTTAAAA
TTTATXXXXXXXXXXXATATCTAAATGCTTTGGTGATCATTGAAAGGATTTGATTCGATGGCAACTTATTGTGTTA
ATGACAATTTATTAAAAGTA
ACCTCATAAAAACCCTAAAATTGAAAATCTTGAACTAACCTAACACAACTAATTAATTAAAAAAACAACTTAAAA
TTTATXXXXXXXXXXXATATCTAAATGCTTTGGTGATCATTGAAAGGATTTGATTCGATGGCAACTTATTGTGTTA
AGGACAATTTATTAAAAGTA
>Marker54856
TACCAAATTAACCACTCAGCAGAAACATAAAAACCTTCACTGGCAAGTAAAAGTAGGATTTCGTGTCTATATGCA
GCATGXXXXXXXXXXXTTCATTTCCCTAGTTTCCAGTTCCTTTTCCCATAATTGGCACCTGAAAAAAATAAGCAAA
GTGATGCTTGAAAATAAGTA
TACCAAATTAACCACTCGGCAGAAACATAAAAACCTTCACTGGCAAGTAAAAGTAGGATTTCGTGTCTATATGCA
GCATGXXXXXXXXXXXTTCATTTCCCTAGTTTCCAGTTCCTTTTCCCATAATTGGCACCTGAAAAAAATAAGCAAA
GTGATGCTTGAAAATAAGTA
>Marker55043
AACAATTATTTCTACAAAAGGTCGGTAGTGAAAGTTGTAAGATCGAGTGTTGATCCACGCGGAATCTATGCAAAG
TTTCTXXXXXXXXXXXTATAGATAAAATAAGTTCTACAGTTTGAACCTTGTCACAACATATCAGAAAATGCAAGTG
CGAGTGTGTAGCATTTGGGT
AACAATTATTTCTACAAAAGGCCGGTAGTGAAAGTTGTAAGATCGAGTGTTGATCCACGCGGAATCTATGCAAAG
TTTCTXXXXXXXXXXXTATAGATAAAATAAGTTCTACAGTTTGAACCTTGTCACAACATATCAGAAAATGCGAGTG
CGAGTGTGTAGCATTTGGGT
>Marker55063
AACCTAGTTGAGATATCCGAGTGTGCTCGTTGATCTTTCAACCTCTAGTTCGATCTCCTTTCAAAAAAGAAAATA
AATCAXXXXXXXXXXXTTGGCTCAACGATAATTGATATGATCTTTCATTTTAAAGGTGGAAGGTTTGAATCCCTCA
ACCTCAAATGTTGTGTCGTA
AACCTAGTTGAGATATCCGAGTGTGCTCGTTGATCTTTCAACCTCTAGTTCGATCTCTTTTCAAAAAAGAAAATA
AATCAXXXXXXXXXXXTTGGCTCAACGATAATTGACATGATCTTTCATTTTAAAGGTGGAAGGTTTGAATCCCTCA
ACCTCAAATGTTGTGTCGTA
>Marker55242

Create PDF with GO2PDF for free, if you wish to

TACCTGCAAAGAACAATCCAAGTGATTTTCTTTCAAGAGAAAATAAACTTTGAAAGCTCAATGGTAGCCAAACAC
TAAAAXXXXXXXXXXGCAAAATCTCAATGTTGCAATTGATAGTAAATTTCAGGATTGCTGCCAATCGCAGTAAAT
CTTTCATCCGAAACTCAGTA
TACCTGCAAAGAACAATCCAAGTGATTTTCTTTCAAGAGAAAATAAACTTTGAAAGCTCAATGGTAGCCAAACAC
TAAAAXXXXXXXXXXGCAAAATCTCAATGTTGCAATTGATAGTAAATTTCAGGATTGCTGCCAATCGCAGTAAAT
CTTTCACCCGAAACTCAGTA
>Marker55523
AACCTTTGTAATTTAGTTTAAAAGATAAAATTTGGTAGGTTTTTTTTATATATTTTTGGGTGACCAATCAGTTAG
TGGAGXXXXXXXXXXCTTTTATTACTTAAATTCTTAAAAGTTTTCAGATAAATTCCATTGATATCTAAAGCCTAA
TCTTGTCTCTTCTAAACGTG
AACCTTTGTAATTTAGTTTAAAAGATAAAATTTGGTAGGTTTTTTTTATATATTTTTGGGTGACCAATCGGTTAG
TGGAGXXXXXXXXXXCTTTTATTACTTAAATTCTTAAAAGTTTTCAGATAAATTCCATTGATATCTAAAGCCTAA
TCTTGTCTCTTCTAAACGTG
>Marker55977
CACTAAACAATGAAGTTACATTTGATATTAAAAGACTTTGAACCACAAATATTGACAACTAAGAAGGAAAATTAA
AAGAAXXXXXXXXXXCAGAGAGAATCCTAAAAAGTTTTCATTGGCAGGGGCACAATGGCAGAAAGATTAACCATT
TGGTAAAATGGTTAATTGTT
CACTAAACAATGAAGTTATATTTGATATCAAAAGACTTTGAACCACAAATATTGACAACTAAGAAGGAAAATTAA
AAGAAXXXXXXXXXXCAGAGAGAATCCTAAAAAGTTTTCATTGGCAGGGGCACAATGGCAGAAAGATTAACCATT
TGGTAAAATGGTTAATTGTT
>Marker56001
TACTTGTAAAATAACATATTTATCTTTAGTCTTTAAAATATGTGGATAATTTTTTGCCATGTTTTCGAGTGGATT
ACTTTXXXXXXXXXXTCAATTTAAGCTTTGGTTTGATAGCTATTTTGTTTTTAAGTTTTTAAAAATTAACCTAAC
GATTGACATCTTTCTAAAGT
TACTTGTAAAATAACATATTTATCTTTAGTCTTTAAAATGTGTGGATAATTTTTTGCCATGTTTTCGAGTGGATT
ACTTTXXXXXXXXXXTCAATTTAAGCTTTGGTTTGATAACTATTTTGTTTTTAAGTTTTTAAAAATTAATCTAAC
GATTGACATCTTTCTAAAGT
>Marker56294
TACACATTTTATTACATTTGTTTATACTTAACAAATTTCAACAATATTTACATTGTATTAACAAAATCAAAGTAT
TAGTCXXXXXXXXXXTAGGACAAATTTAGGTCGAAATGACAAAAATACCCTTATTTAATTTTACTTCACATTTAC
TCTTCTCTTCAAGGTAGAGT
TACACATTTTATTACATTTGTTTATACTTAACAAATTTCAACAATATTTACATTGTATTAACAAAATCAAAGTAT
TAGTCXXXXXXXXXXTAGGACAAATTTAGGTCGAAATGACAAAAATACCCTTATTTAATTTTACTTCACATTTAC
TCTTCTCTTCAATGTAGAGT
>Marker56699
TACCATCAATACTAAATGCAGATTCAAAAGTTGGGAACAACTAGAACATCTTTTGCAATTTCATACAAAACAAGA
TACTAXXXXXXXXXXCCTGCTTTCCTCTTTTGCCCAAATGATGTTCCCTTATCAAACCTCTTTGACTGAACCTCA
TCCTTCTGAATACTCATGTC
TACCATCAATACTAAATGTAGATTCAAAAGTTGGGAACAAGTATAACATCTTTTGCAATTTCATACAAAACAAGA
TACTAXXXXXXXXXXCCTGCTTTCCTCTTTTGCCCAAATGATGTTCCCTTATCAAACCTCTTTGACTGAACCTCA
TCCTTCTGAATACTCATGTC
>Marker56865
ACCCATGCAATAAGACGCTTTACACTTGTTGTGTGCTTTAGTGTGGGATGTCTTATCTTTGTTGTTTGGTTTGGT
ATAAGXXXXXXXXXXCGCAACTCAACAAAAGAGTGTGGTCAATGCTTGAGATGAGGAAAGACCACAAAGATATAA
TTAAACATGCATAATGAGTC

Create PDF with GO2PDF for free, if you wish to

ACCCATGCAATAAGACGCCTTACACTTGTTGTGTGCTTTAGTGTGGGATGTCTTATCTTTGTTGTTTGGTTTGGT
ATAAGXXXXXXXXXXCGCAACTCAACAAAAGAGTGTGGTCAATGCTTGAGATGAGGAAAGACCACAAAGATATAA
TTAAACATGCATAATGAGTC
>Marker56917
ACCTTAAAGTGAGCATTTTGTTAAATAAAAATATCCCTTGTAACGTTTAAAATGATTATGTATATGAAATGTTTG
ATATGXXXXXXXXXXTAAAACATAATAAAAAATATTCTTTGAGTTAATTTCATCATTTGAACTTCTAATCATTTT
AAAATGATACTTCACATGTT
ACCTTAAAGTGAGCATTTTGTTAAATAAAAATATCCCTTGTAACGTTTAAAATGATTATGTATATGAAATGTTTG
ATATGXXXXXXXXXXTAAAACATAATAAAAAATATTCTTTGAGTTAATTTGATCATTTGAACTTCTAATCATTTT
AAAATGATACTTCACATGTT
>Marker56919
AACCCAACAACCCAAACCATTTTTCCCTCAATATTTCTTTAACCCAACCAAATCCAAAACATAATTTAACCCAAT
CCAATXXXXXXXXXXTACACAGTCCTAAAAAGCTTCATCTTCGATGCACAATGAGATAAAACAACCATGGAAGAA
ACTTCCATGTTTCAGATGTC
AACCCAACAACCCAAACCATTTTTCCCTCAATATTTCTTTAACCCAACCAAATCCAAAACATAATTTAACCCAAT
CTAATXXXXXXXXXXTACACAGTCCTAAAAAGCTTCATCTTCGATGCACAATGAGATAAGACAACCATGGAAGAA
ACTTCCATGTTTCAGATGTC
>Marker56931
AACCAGGTATTTAGGGACAAGGATGGGACTCTCTGTGGCTCCCATTCCCATCCTCTCCTTACTTTACACACAAAA
GATATXXXXXXXXXXTCGATCTCTCTTTTTCTTTTACCCGAATATTACACATTTATCCACTCTTTGCGTCCAATC
CATCAACATTTATTTATGTG
AACCAGGTATTTAGGAACAAGGATGGGACTCTCTGTGGCTCCCATTCCCATCCTCTCCTTACTTTACACACAAAA
GATATXXXXXXXXXXTCGATCTCTCTTTTTCTTTTACCCGAATATTACACATTTATCCACTCTTTGCGTCCAATC
CATCAACATTTATTTATGTG
>Marker56960
AACAGAAAATATGAAAATGTAAAAGAAATAACAAACTTTCTCGTTTTTTGTTTATCCTATCTCTCAAGGATGAAA
ACAGAXXXXXXXXXXCTTCTTCCCTTTTCTACTATATTGTAAAATAATAGGTGGTCTAACGTATTGATAGGCTTC
CAACTTGTTATACGAAAGGT
AACAGAAAATATGAAAATGTAAAAGAAATAACAAACTTTCTCGTTTTTTGTTTATCCTATCTCTCAAGGATGAAA
ACAGAXXXXXXXXXXCTTCTTCCCTTTTCTACTATATTGTAAAATAATAGGTGGTCTAACATATTGATAGGCTTC
CAACTTGTTATACAAAAGGT
>Marker57114
AACCTCTGCAAATCTTGGTTCTTGCCTTCATAAACATGCTCATATTTCCCGTGAATGCTTGTTGGTAGTATTATA
AATTTXXXXXXXXXXTCATCTTTAGCTTCCCCCGACGATCTGTTTAACTTCTTCAGTGAGATGCGAGGTTAGTTC
TGCAAATTTGTATTGTTGTT
AACCTCTGCAAATCTTGGTTCTTGCCTTCATAAACATGCTCATATTTCCAGTGAATGCTTGTTGGTAGTATTATA
AATTTXXXXXXXXXXTCATCTTTAGCTTCCCCCGACGATCTGTTTAACTTCTTCAGTGAGATGCGAGGTTAGTTC
TGCAAATTTGTATTGTTGTT
>Marker57282
ACTCCAAAATCGCATTATTTGAAGCCTCCTACTTTTCTCCTAAATTTGAACATATTTCTAATCAGAATTGGCTAA
TGAGTXXXXXXXXXXATTTTTCTACTTCAAAAATCATTCACTTAACCTCAGAATTTGCTCAAGCTTTATTCACGA
AACTTGTTAGAAGTTGAGTT
ACTCCAAAATCGCATTATTTGAAGCCTCCTACTTTTCTCCTAAATTTGAACATATTTCTAATCAGAATTGGCTAA
TGAGTXXXXXXXXXXATTTTTCTACTTCAAAAATCATTCACTTAACCTCAGAATTTACTCAAGCTTTATTCACGA
AACTTGTTAGAAGTTGAGTT
>Marker57308

Create PDF with GO2PDF for free, if you wish to

CACCTCGAAGGCCATGAATAAAAACAACATCTACCAAAGGAGAATCTGGCCTAGAATCATTCTGAGACACATGAG
ATAAAXXXXXXXXXXXGAACCTTCCAGTGAGGCAGTTCAGGATTTATTAAAAAAACCATATCATCATAACGGGGAC
ATTTCTTTTTCCTGTTTGTA
CACCTCGGAGGCCATGAATAAAAACAACATCTACCAAAGGAGAATCTGGCCTAGAATCATTCTGAGACACATGAG
ATAAAXXXXXXXXXXXGAACCTTCCAGTGAGGCAGTTCAGGATTTATTAAAAAAACCATATCATCATAACGGGGAC
AATTCTTTTTCCTGTTTGTA
>Marker57386
ACTAATGTTTTAAATCAGTCAAGTTAAGTTTTATGTGTTTATTTGAGAGGTAGAAAACACAATTGAGAACTTGAT
AAGTAXXXXXXXXXXXTAATCTAATTGAAAGATTAACACGAACGCGATAATCAAGACGTCTCACTAGAGATTTTGG
ACTTCCTTTACCAAAGTTGT
ACTAATGTTTTAAATCAGTCAAGTTAAGTTTTATGTATTTATTTGAGAGGTAGAAAACACAATTGAGAACTTGAT
AAGTAXXXXXXXXXXXTAATCTAATTGAAAGATTAACACGAACGCGATAATCAAGACGTCTCACTAGAGATTTTGG
ACTTCCTTTACCAAAGTTGT
>Marker57561
TACACTAATTGAGTAATTATGTTGATGTGGTTATTCAAATGCTTGAATCATTCCAAATATGTCCAAAATCCTAAT
AAACTXXXXXXXXXXXTGCTTCCAATTTTTGAAGGAAAAATTAAGATACGTAAGCATTTACTATTTACTATTTTAT
GATTGACATTTGATTGAAGT
TACACTAATTGAGTAATTATGTTGATGTGGTTATTCAAATGTTTGAATCATTCCAAATATGTCCAAAATCCTAAT
AAACTXXXXXXXXXXXTGCTTCCAATTTTTGAAGGAAAAATTAAGATACGTAAGCATTTACTATTTACTATTTTAT
GATTGACATTTGATTGAAGT
>Marker58122
CACGATATGGACAAAGCTACTATATATCATTTTGTTTGGCAAAATAGGGAGATGAGGAACAATTTGTTGTTTGAG
GGACTXXXXXXXXXXXTGTGGAAGTTGAATTTTGATGCCAATTGGAATTCAAAATAAACAATAGAATATTGGTTGG
TAGGGTGAGTTTGTTAGGTT
CACGATATGGACAAAGCTACTATATATCATTTTGTTTGGCAAAATAGGGAGATGAGGAACAGTTTGTTGTTTGAG
GGACTXXXXXXXXXXXTGTGGAAGTTGAATTTTGATGCCAATTGGAATTCAAAATAAACAATAGAATATTGGTTGG
TAGGGTGAGTTTGTTAGGTT
>Marker58493
AACGTAGCTCTGTTGTTTTTAGAAACCTTCAAATTCTGCACCTCTTGTTTTCTTTTAATTTTGCCTCTTTTACTT
AGATTXXXXXXXXXXXCGAATTTGTGACTGTGGCTCTATCGACTTGTCCATGTGTTCTTTTCCAAAATTGGTTACT
TGATTATTGCAGTTACCAGT
AACGTAGCTCTGTTGTTTTTAGAAACCTTCAAATTCTGCACCTCTTGTTTTCTTTTAATTTTGCCTCTTTTACTT
AGATTXXXXXXXXXXXCGAATTTGTGACTGTGGCTCTACCGACCTGTCCATGTGTTCTTTTCCAAAATTGGTTACT
TGATTATTGCAGTTACCAGT
>Marker58638
ACTCTACTGGCTCTCTGGCCTTACGTGTTCGGATGAAAATTTTATAATCAAAACGGGGGCACAACGCACTGCTTC
CAGTGXXXXXXXXXXXTAATAATTCTTTTAAGTTCTAATATATTATAAATTAACAAATTTTGCATTCTAAGATATG
TGTTTTGATGATATCTAGTT
ACTCTACTGGCTCTCTGGCCTTACGTGTTCGGATGAAAATTTTATAATCAAAGCGGGGGCACAACGCACTGCTTC
CAGTGXXXXXXXXXXXTAATAATTCTTTTAAGTTCTAATATATTATAAATTAACAAATTTTGCATTCTAAGATATG
TGTTTTGATGATATCTAGTT
>Marker59598
ACTTAGGTGTTTCACGCACATGAACTATTGAACTCTACCAATTTTGGTTGTAGTTGCTCGTTATAATTTAGAAAT
ACTCGXXXXXXXXXXXTGATTGGTTATCTCTCAATGGTATTAAAATTTATATCTTGATGCCATATTAATGGTCTTA
TGCATCAATTTTGGTAGGTG

Create PDF with GO2PDF for free, if you wish to

ACTTAGGTGTTTCACGCACATGAACTATTGAACTCTACAAATTTTGGTTGTAGTTGCTCGTTATAATTTAGAAAT
ACTCGXXXXXXXXXXXTGATTGGTTATCTCTCAATGGTATTAAAATTTATATCTTGATGCCATATTAATGGTCTTA
TGCATCAATTTTGGTAGGTG
>Marker60345
CACATGAAATTTTTGTTCCATGTATAATGGTGGGATTTTCTCTCTCTTCTTCATGTTCTTTCTTCTTTCTTTTC
TTCCTXXXXXXXXXXXAAGCAAAACAATACCCAACGAGTAATTAGAAAAAGAAAACCTTTGCAACGGATATCCAAA
TGGAACCTAGCAAGGGAGTA
CACATGAAATTTTTGTTCCATGTATAATGGTGGGATTTTCTCTCTCTTCTTCATGTTCTTTCTTCTTTCTTTTC
TTCCTXXXXXXXXXXXAAGCAAAACAATACCCAATGAGTAATTAGAAAAAAAAAACCTTTGCAACGGATATCCAAA
TGGAACCTAGCAAGGGAGTA
>Marker60423
AACCCAGTTCCCCTAGACCGCGGGTTCAACAAGGTCCGCCTCTCGAATAGCCATGTCAAACTCCTCCATGTCCCC
CATGCXXXXXXXXXXXACAAAACCTCTACCTTCTCCCCAGAGATCAAATCTGTTACCACACCATAAATAAACTGGT
CGGCAACCTCATAAGTCGTG
AACCCAGTTCCCCTAGACCGCGGGTTCAACAAGGTCCGCCTCTCGAATAGCCATGTCAAACTCCTCCATGTCCCC
CATGTXXXXXXXXXXXACAAAACCTCTACCTTCTCCCCAGAGATCAAATCTGTTACCACACCATAAATAAACTGGT
CGGCAACCTCATAAGTCGTG
>Marker60513
GACAACGGTATATCCCTTCTCAACACAGTTCTCTACAGTCACAAGGGGCCTGCTCTGGTAGCTTTCGCTGCAACT
CGTGTXXXXXXXXXXXACATAGAGGAGAAGCTGTTTAATCTTAGCCAACCAATCGGGGTCATCGATCGGCATTCCA
CTCGTATCATCGGTGATGTA
GACAACGGTATATCCCTTCTCAACACAGTTCTCTACAGTCACAAGGGGCCTGCTCTGGTAGCTTTCGCTGCAACT
CGTGTXXXXXXXXXXXACATAGAGGAGAAGCTGTTTAATCTTAGCCAACCAATCGGGGTCATCGATCGGCATTCCA
CTCGTGTCATCGGTGATGTA
>Marker60722
ACCAATATAGGCAAGAAAGGAGAATATATTATCTAAGTAGGGCTGGTGAAAGTTGAGGAATAAACAATACTGTGG
TCATGXXXXXXXXXXXCACAGTGAAGTAATAAAAAAGTAAGCTAATTTTCTCTTCCAATTTATTTGTGGGAAAAAA
AGGTAAAGTATATATGTGTC
ACCAATATAGGCAAGAAAGGAGAATATATTATCTAAGTAGGGCTGGTGAAAGTTGAGGAATAAACAATACTGTGG
TCATGXXXXXXXXXXXCACAGTGAAGTAATAAAAAAGTAAGCTAATTTTCTCTTCCAATTTATTTGTGGGAAAAAA
AGGTAAAGTATATACGTGTC
>Marker60817
TACAAATTTAATTCTAAAAAACGAAATGTTTTTCAAAATAGGCCTAACACATCCTAAGACTTAATAGCTATCCTC
AAGCTXXXXXXXXXXXTTTTCCAATAGAATTTGCCTCGTCTTCCAATGATTTGAAAAGTGTTATCAGGTTGAAATT
GTGTTTTTCTTTTGACTTGT
TACAAATTTAATTCTCAAAAACGAAATGTTTTTCAAAATAGGCCTAACACATCCTAAGACTTAATAGCTATCCTC
AAGCTXXXXXXXXXXXTTTTCCAATAGAATTTGCCTCGTCTTCCAATGATTTGAAAAGTGTTATCAGGTTGAAATT
GTGTTTTTCTTTTGACTTGT
>Marker60883
AACTTTCTTTTAAAAATAGCATGAAGAGGTTTGTTATCCATTAAAGAGTTGTTTATGATGAGACAAGAATATATA
GTGAGXXXXXXXXXXXCTTCTCAACACAATCCCACCATATATATCTTTTCTCCCTTTTCTATCATTATTTCCATCC
ATTAAATTTGCAACATATGT
AACTTTCTTTTAAAAATAGCATGAAGAGGTTTGTTATCCATTAAAGAGTTGTTTAAGATGAGACAAGAATATATA
GTGAGXXXXXXXXXXXCTTCTCAACACAGTCCCACCATATATATCTTTTCTCCCTTTTCTATCATTATTTCCATCC
ATTAAATTTGCAACATATGT
>Marker60959

Create PDF with GO2PDF for free, if you wish to

GACCCTAAATTTTGAAAAAGGGGTGGATTCCATTCTTTAAAATGGTCCATTTAATCCGTAAGTTAATAAGTTTAA
AATGTXXXXXXXXXXATGTGGACCTACGAATTTGTGGATATTTTGTTAATCCTGATTATGTTCGTGATTATAGG
TTAATTCGTAGATTCAAAGT
GACCCTAAATTTTGAAAAAGGGGTGGATTCCATTCTTTAAAATGGTCCATTTAATCCGTAAGTTAATAAGTTTAA
AATGTXXXXXXXXXXATGTGGACCTACGAATTTGTGGATATTTTGTTAATCCTGACTATGTTCGTGATTATAGG
TTAATTCGTAGATTCAAAGT
>Marker60989
AACTTCCTCCCTGATTTAAAACACAATATACTAGCAGTTTGGGCTGATTACCAAGTAAAGACACCTATTCTATGA
AGTTTXXXXXXXXXXGTTTGGTTACCGGCTTTTGACATTCAATATGGGCTGAAGAGATGAAAGATCCAATTTCAG
AATAGAAGCATGGTTTGAGT
AACTTCCTCCCTGATTTAAAACACAATATACTAGCAGTTTGGGCTGATTACCAAGTAAAGACACCTATTCTATGA
AGTTTXXXXXXXXXXGTTTGGTTACCGGCTTTTGACATTCAATATGGGCTGAAGAGATGAAAGATCCAATTTTAG
AATAGAAGCATGGTTTGAGT
>Marker61010
TACCAGAGAGGCTAATCGGCAATGAAGTTGAAGGGAAGGAGGAGAAGGAGAGAAAGGGAGAGAGAGAGAACTTCC
CTAGCXXXXXXXXXXAGGTAGATGAATAAGAACTCACAGTTCCATTAAAATAAGTGAGAAAGGATAGCAAGATCC
CATTTTCAAGGGTTGATTGT
TACCAGAGAGGCTAATCGGCAATGAAGTTGAAGGGAAGGAGGAGAAGGAGAGAAAGGGAGAGAGAGAGAACTTCC
CTAGCXXXXXXXXXXAGGTAAATGAATAAGAACTCACAGTTCCATTAAAATAAGTGAGAAAGGATAGCAAGATCC
CATTTTCAAGGGTTGATTGT
>Marker61156
ACTTAGTTTGAGCAATTTTGGGGTGGTCTTTTGAGAATTTTTCTAGTAGTATATAGGTGGAAACAAATCATGTGA
AAAAAXXXXXXXXXXXXAAATTTCATGGTTGAAAAGTTACAATACTAATCATTTGCCTATTCAATTGGAGAAAATTT
TAAAATCTCATTTGGTTAGT
ACTTAGTTTGAGCAATTTTGGGGTGGCCTTTTGAGAATTTTTCTAGTAGTATATAGGTGGAAACAAATCATGTGA
AAAAAXXXXXXXXXXXXAAATTTCATGGTTGAAAAGTTACAATACTAATCATTTGCCTATTCAATTGGAGAAAATTT
TAAAATCTCATTTGGTTAGT
>Marker61303
ACATGATTTTGGGTAAAACAGAGCAAGTTTGTTTTGAGGTTGATTCTGATTGGAAGAAGCAATTTTAGAGTTCCA
AAACCXXXXXXXXXXAAAATGATAGGTTTTACGCATCGAAAGTTTGGTTGATTCAAGGAGTTCAAGAGTGTTTCA
AATGTCCAAATTTTGAGGTA
ACATGATTTTGGGTAAAACAGAGCAAGTTTGTTTTGAGGTTGATTCTGATTGGAAGAAGCAATTTTAGAGTTCCA
AAACCXXXXXXXXXXAAAATGATAGGTTTTACGCATCGAAAGTTTGGTTGATTCAAGGAGTTCGAGAGTGTTTCA
AATGTCCAAATTTTGAGGTA
>Marker61467
CACATCTTGTCATATAGTGCATAAGTTGCAATTATATACCTCTGGTGGAGTCACAGATATATAATTAATTCCTAG
TTTGAXXXXXXXXXXTAAATGTATTAAGGGTAACTAAATAGATTATAATGGATGATGTATTTACTATAAATAGCT
ACAATTCATCACATGAGGTA
CACATCTTGTCATATAGTGCATAAGTTGCAATTATATACCTTTGGTGGAGTCACAGATATATAATTAATTCCTAG
TTTGAXXXXXXXXXXTAAATGTATTAAGGGTAACTAAATAGATTATAATGGATGATGTATTTACTATAAATAGCT
ATAATTCATCACATGAGGTA
>Marker61597
GACCCAACTCAACTTTAATGTTTATATTATTTTTATTTTATTTTTTCATCCATTAAAATTATTCCTTTCTCTTTT
TCTTAXXXXXXXXXXCTTTTAACTTTTAAAACTGATAAGAAAATTGAAAAAAAATATAACTTACACAAATATTTT
TAATCTTGTATTTGTTTTGT

Create PDF with GO2PDF for free, if you wish to

GACCCAACTCAACTTTAATGTTTATATTATTTTTATTTTATTTTTTCATCCATTAAAATTATTCCTTTCTCTTTT
TCTTAXXXXXXXXXXCTTTTAACTTTTAAAACTGATAAGAAAATTGAAAAAAAATATAACTTACACAAATATTTT
TAATATTGTATTTGTTTTGT
>Marker61746
CACACTACCTTGAGGTGACGCTCGCTTAGGGAAATTTGAGATAGGAAGGCGTAATGTTTAGATCGAGCTTGCATT
TTTCAXXXXXXXXXXTAGGGAATTCATTTCGTTTATTAATCCCTTGTGTTTTTCTTTTGTTTTTATTTTATTCGA
GTTTGAGCTCTGTTTAAGTC
CACACTACCTTGAGGTGACGCTCGCTTAGGGAAATTTGAGATAGGAAGGCGTAATGTTTAGATCGAGCTTGCATT
TTTCAXXXXXXXXXXGAAGGAATTCATTTCGTTTATTAATCCCTTGTGTTTTTCTTTTGTTTTTATTTTATTCGA
GTTTGAGCTCTGTTTAAGTC
>Marker61945
ACAGTTGGATTAGGGAGATACGAACCAAGTCACTAATACATACAAATGTCAGTTGAACTATGTTCTCTATGCTCT
CTTTGXXXXXXXXXXCAAAAGTTCCATGAAAATGAAAAAGACTACAAACTCAATGTATTTGAGTAGATTTAAAAG
CTTTAAAAGTTATCAGTAGT
ACAGTTGGATTAGGGAGATACGAACCAGGTCACTAATACATGCAAATATCAGTTGAACTATGTTCTCTATGCTCT
CTTTGXXXXXXXXXXCAAAAGTTCCATGAAAATGAAAAAGACTACAAACTCAATGTATTTGAGTAGATTTAAAAG
CTTTAAAAGTTATCAGTAGT
>Marker62496
ACTGTTACACCTAATTATTTATGTTATTGTATTGTTACTACATGTAGAAGAAAGAAGATAAAAAAATTAGAACCA
TAGTGXXXXXXXXXXCCAAGGACGTAGAACTCAAAAAGTTACATGGAATCAACTAACTAGTAGTCTTAGTAGCAA
GTTGAACCACAAATATTGGT
ACTGTTACACCTAATTATTTATGTTATTGTATTGTTACTGCATGTAGAAGAAAGAAGATAAAAAAATTAGAACCA
TAGTGXXXXXXXXXXCCAAGGACGTAGAACTCAAAAAGTTACATGGAATCAACTAACTAGTAGTCTTAGTAGCAA
GTTGAACCACAAATATTGGT
>Marker63448
AACACAACACAACAAACTGTAATATAAACTTGGTGCAATTATGGATGCAAACCAATGAACTCAATATATATATAT
AACTTXXXXXXXXXXTAAAATGTTTAATTAAATCTAAGTATCAATATCTCTTTCGGAATGAACTGAGAAAAAATG
TAACAAATCGTTTAGAAGTG
AACACAACACAACAAACTCTAATATAAACTTGGTGCAATTATGGATGCAAACCAATGAACTCAATATATATATAT
AACTTXXXXXXXXXXTAAAATGTTTAATTAAATCTAAGTATCAATATCTCTTTCGGAATGAACTGAGAAAAAATG
TAACAAATCGTTTAGAAGTG
>Marker63740
TACTCTTACCTTTCGAACCAAAAAAGTTGTTCAATTGAAAGCAACATAAATTGGCTTTTACAACAATAGACTCAA
ATCGTXXXXXXXXXXAAAAAAAAAAAAAGGAAAGTTTGTAAGGTCCTCTTCTTCATCTCTAAATAAATGCACTTA
CCTCTTAAACCAGCATTGTT
TACTCTTACCTTTCGAACCAAAAAAGTTGTTCAATTCAAAGCAACATAAATTGGCTTTTACAACAATAGACTCAA
ATCGTXXXXXXXXXXAAAAAAAAAGAAAGGAAAGTTTGTAAGGTCCTCTTCTTCATCTCTAAATAAATGCACTTA
CCTCTTAAACCAGCATTGTT
>Marker63965
GACTTCCTTACTTATCATTTTCTTTAAATTGTGGGAAGGAGTTAGATAATTGACATAGTTTATGGTAGTAAGAGT
TTGAAXXXXXXXXXXATGCTTTGTAAGATGAAGAATTTAGGGAATAAAGCCTTCTAAGATGGACCTATATGGCTT
TACCCAGATGAAGCTGGGTG
GACTTCCTTACTTATCATTTTCTTTAAGTTGTGGGAAGGAGTTAGATAATTGAAATAGTTTATGGTAGTAAGAGT
TTGAAXXXXXXXXXXATGCTTTGTAAGATGAAGAATTTAGGGAATAAAGCCTTCTAAGATGGACCTATATGGCTT
TACCCAGATGAAGCTGGGTG
>Marker64118

Create PDF with GO2PDF for free, if you wish to

TACCTCAGCCTGTGAGTATGATCTTCGTGTATCAATCTATAAACATGCAAAATAAATTAAATGAATTAACTCACA
ATCCAXXXXXXXXXXCTCGGTGCTCTCAACTTGTAGAGAACGCTATATAAAAACAACAAATTAGAATAACGAAGT
AATCCTAATTGCTAGTAGTC
TACCTCAGCCAGTGAGTATGATCTTCGTGTATTAATCTATAAACATACAAAATAAATTAAATGAATTAACTCACA
ATCCAXXXXXXXXXXCTCGGTGCTCTCAACTTGTAGAGAACGCTATATAAAAACAACAAATTAGAATAACGAAGT
AATCCTAATTGCTAGTAGTC
>Marker64188
ACAAGTTCATGGATTAGCATTTTCCCAATAAGACTAGGTCACAAAAACTCAATTCAACTATACACACACAGCAAA
ACCCAXXXXXXXXXXTCTTCATAGTCATGCCTTCCTTGGTGGAATACTGCAACAACTCCTTCACAATAGCCAAAT
GCCCCTTCTCGGCTGCGGTA
ACAAGTTCATGGATTAGCATTTTCCAAATAAGACTAGGTCACAAAAACTCCATTCAACTATACACACACAGCAAA
ACCCAXXXXXXXXXXTCTTCATAGTCATGCCTTCCTTGGTGGAATACTGCAACAACTCCTTCACAATAGCCAAAT
GCCCCTTCTCGGCTGCGGTA
>Marker64666
ACCCTTGGTTTTCAATTGATGAAGTCTAGATCAAGTTTATAGGGTGATATGTGCTGTCTAATTCCTCATAACTAA
CAAACXXXXXXXXXXATGTCTAAAATAAATTTTGTCTAGGAAAAGGGTTACTGCTTCATCTACAGTTTTTTGTTC
TTTCTATATATTCTAATGTT
ACCCTTGGTTTTCAATTGATGAAGTCTAGATCAAGTTTATAGGGTGATATGTGCTGTCTAATTCCTCATAACTAA
CAAACXXXXXXXXXXATGTCTAAAATAAATTTTGTCTAGGAAAAGGGTTACTGCTTCATCTACAGTTTTTTGTTC
TTCCTATATATTCTAATGTT
>Marker64707
AACAAAATTCACAAATTATAACAAACATATATTAGCTCTAATGTCATTTTTTTCTAAAGTTTTATTACCAATTAA
TTCCAXXXXXXXXXXATTTACTTAGTTAAAGTCTATATTGTGACACTTCTCTATTTAATCTTGCAATGCGAATTT
AGTGAAATCATATTTACGTT
AACAAAATTCACAAATTATAACAAACATATATTAGCTCTAATGTCATTTTTTTCTAAAATTTTATTACCAATTAA
TTCCAXXXXXXXXXXATTTACTTAGTTAAAGTCTATATTGTGACACTTCTCTATTTAATCTTGCAATGCGAATTT
AGTGAAATCAAATTTACGTT
>Marker65056
ACTAAATGAAGACTTAGAAGCAACTATGGATATCAAAATTGCTAAAATATCTCTTGCAATTCGACTAAAAACCTT
ATATCXXXXXXXXXXTCCATATTTTCAAAATGGATACGAAAAACGTCTGAAGCATAAGAAGAAGTTCTAGATGTA
GTGGAAGTAGTTATAGATGT
ACTAAATGAAGACTTAGAAGCAACTATGGATATCAGAATTGCTAAAATATCTCTTGCAATTCGACTAAAAACCTT
ATATCXXXXXXXXXXTCCATATTTTCAAAATGGATACGAAAAACGTCTGAAGCATAAGAAGAAGTTCTAGATGTA
GCGGAAGTAGTTATAGATGT
>Marker65404
ACTCTCGGAGTCCCTCTCCCCTTGGTCTTGTCCCGAGGCCTTGCCTGAACATTTTCCCCCCGAAAAAAAATTCAA
AATATXXXXXXXXXXCTCTCCGTTTTCTTATCGCCTCTGCCACAATATACTGAAAAAAAAGTAAAGACAGAACAG
AAATTAAGCATTGAAAAGTG
ACTCTCGGAGTCCCTCTCCCCTTGGTCTTGTCCCGAGGCCTTGCCTGAACATTTTCCCCCCGAAAAAAAATTCAA
AATATXXXXXXXXXXCTCTCCGTTTTCTTATCGCCTCTGCCACAATATACTGAAAAAAAAGTAAAGACAGAACAG
AAATTAAGCAATGAAAAGTG
>Marker66302
GACTATAAAAATTTGAACCCAATCCAACCCATATCATTTTTGTTTGGATTAAATTTAATTTTGGGTAGAAGAATT
GGGCTXXXXXXXXXXTTGCTTTTTAATTATAAATACAGCACTTGTCTTCTTAGATTTGCAACTTTTAAACATTAA
TAAAATTTTCTGATTTGGTA

Create PDF with GO2PDF for free, if you wish to

GACTATCAAAATTTGAACCCAATCCAACCCATATCATTTTTGTTTGGATTAAATTTAATTTTGGGTAGGAGAATT
GGGCTXXXXXXXXXXXTTGCTTTTTAATTATAAATACAGCACTTGTCTTCTTAGATTTGCAACTTTTAAACATTAA
TAAAATTTTCTGATTTGGTA
>Marker66303
AACTATTAAATGTTGTTACTCTAGAAAGAGAAGTATTTGGATCGGTTGTTTGAATGTTTTCTTCAAAAGCATTTC
ATATGXXXXXXXXXXXATGTTTTATATGATTCAAATGATTGTGAATGATGTTTAAACTATTAGTCTTCTTCTTATC
ATAGGACTAACTGTTGTGTG
AACTATTAAATGTTGTTACTCTAGAAAGAGAAGTATTTGGATCGGTTGTTTGAATGTTTTCTTCAAAAGCATTTC
ATATGXXXXXXXXXXXATGTTTTATATGATTCAAATGATTGTGAATGATGTTTAAACTATTAGTCTTCTTCTTATC
ACAGGACTAACTGTTGTGTG
>Marker67015
TACACCATATTCTCAATCAACATGTAATTTGTGTATATCTACTGGTAAAAAAGCATCTTCAAATCTTAAGCTACA
ATATTXXXXXXXXXXXGATTTAAGTGGGTGTAATTTTGTTTATATAACAGTTGCAATGAAATTGGAAAAGGTAATT
GGATTGTTTTCTAAAATGTG
TACACCATATTCTCAATCAACATGTAATTTGTGTATATCTATTGGTAAAAAAGCATCTTCAAATCTTAAGCTACA
ATATTXXXXXXXXXXXGATTTAAGTGGGTGTAATTTTGTTTATATAACAGTTGCAATGAAATTGGAAAAGGTAATT
GGATTGTTTTCTAAAATGTG
>Marker67607
ACTTCATTGGAACATAGCCTTCATATAGTTTTCTGAATTTCTTTAACTGGCGTGGGAGGAAACGAAGAAATTATG
CAATAXXXXXXXXXXXATGAAGCGCAATACTGGTCTGAATGGGCAAGAACAATGTTCAATAAAATTTACTGCACGA
ATTTGGTATAAAGTAACAGT
ACTTCATTGGAACATAGCCTTCATATAGTTTTCTGAATTTCTTTAACTGGCGTGGGAGGAAACGAAGAAATTATG
CAATAXXXXXXXXXXXATGAAGCACAATACTGGTCTGAATGGGCAAGAACAATGTTCAATAAAATTTACTGCACGA
ATTTGGTATAAAGTAACAGT
>Marker67953
ACTGATCCCTGCATAATTCAGGTCAAACGGAAATTAGTTAGTTCCCCATGGAAACGAAACCACTACTCAACAAGC
AGAGAXXXXXXXXXXXAAAGATTTTAAAACAATTGTGTTTTCACTGCTCAGCTTGGGATCAAGCAAAGATGGAAAT
CTAAATCTTTTATCCCTGTC
ACTGATCCCTGCATAATTCAGGTCAAACGGAAATTAGTTAGTTCCCCATGGAAACGAAACCACTACTCAACAGGC
AGAGAXXXXXXXXXXXAAAGATTTTAAAACAATTGTGTTTTCACTGCTCAGCTTGGGATCAAGCAAAGATGGAAAT
CTAAATCTTTTATCCCTGTC
>Marker68063
ACCCGATTGTTGGACAAAAAGGTGACTGAACTCAGAATTCAAACAAAAACTAATTTGCACTTTTCTACAATCGAC
ATTTCXXXXXXXXXXXGTAGAGCTTCAAGCCCATCGCTTTCTTAACCCTTTCAGGTGTGAGATTACTCTCTTGTAA
TATTTTGCCAAAAAAATAGT
ACCCGATTGTTGGACAAAAAGGTGACTGAAATCAGAATTCAAACAAAAACTAATTTGCACTTTTCTACAATCGAC
ATTTCXXXXXXXXXXXGTAGAGCTTCAAGCCCATCGCTTTCTTAACCCTTTCAGGTGTGAGATTACTCTCTTGTAA
TATTTTGCCAAAAAAATAGT
>Marker68142
CACCTTTTCTGATTTTGATGAACATACAGACCTCTCTAGTCCTACGTCAATGCAATATTTGAAGAACAACAAAGG
GTTTGXXXXXXXXXXXACTTATTGAAGTTAATGATAATCAAAGCCATGCCGGAGTCCATGGTGGAATTCGCTATTA
TAGCTCAACGTTTCCTTGGT
CACCTTTTCTGATTTTGATGAACAAACAGACCTCTCTAGTCCTACGTCAATGCAATATTTGAAGAACAACAAAGG
GTTTGXXXXXXXXXXXACTTATTGAAGTTAATGATAATCAAAGCCATGCCCGAGTCCATGGTGGAATTTGCTATTA
TAGCTCAACGTTTCCTTGGT
>Marker68666

Create PDF with GO2PDF for free, if you wish to

ACTATTACAGCAGGTGTTTAATATCACCATTGAAGTATGTTTTTGAGTAGTCATCCAAAATACGTTGAAGTATGT
GTGCTXXXXXXXXXXCTCATGCTCATGGGAATTACCCAAGTGTGAGTCTGTTTGAAAGGACCTCAAACATGGGTG
CGATCATACCAGTCCTAGTG
ACTATTACAGCAGGTGTTTAATATCACCATTGAAGTATGTTTTTGAGTAGTCATCCAAAATACGTTGAAGTATGT
GTGCTXXXXXXXXXXCTCATGCTCATGGGAATTACCCAAGTGTGAGTCTGTTTGAAAGGACCTCAAACATGGGTG
CGATCATACCAGCCCTAGTG
>Marker68731
CACACAAATACGTTTTCAATCTTTTATATTTGATAGAATAAAATCACTATGTTTTTCTATATAACCTGGAGCTTG
TCATTXXXXXXXXXXATTAATGAAGCTTGTAAGTTTTTCTTTTGATCTTTCATTAATGATTGAAAAATTATAAAA
GACTTCAAGAATTTAAGGTC
CACACAAATACGTTTTCAATCTTTTATATTTGATAGAATAAAATCACTATGTTTTTCTATATAACCTGGAGCTTG
TCATTXXXXXXXXXXATTAATGAAGCTTGTAAGTTTTTCTTTTGATCTTTCATCAATGATTGAAAAATTATAAAA
GACTTCAAGAATTTAAGGTC
>Marker68878
CACATGGTTGTTTTTGGAGGGAATTAAACTTTCACTTTAAAACTAATTTACTTGATTTAAGTTCATGCTTTCCAA
TCTCAXXXXXXXXXXGTATGACAATTTTTTTTAAATGGTCCATAAATGTTTAATTATAATGTAATTAGCTAATGC
AGAAGTAGAGAAATTATGTC
CACATGGTTGTTTTTGGACGGAATTAAACTTTCACTTTAAAACTAATTTACTTGATTTAAGTTCATGCTTTCCAA
TCTCAXXXXXXXXXXGTATGACAATTTTTTTTAAATGGTCCATAAATGTTTAATTATAATGTAATTAGCTAATGC
AGAAGTAGAGAAATTATGTC
>Marker69017
ACCGACGCTGAATTCGGTATTTGACCATTGCCAACAAAGAAAAAGGGCCTACAAAAAGATACAGACGTCATTATA
ATAGAXXXXXXXXXXTACCCATTGATTCACACACGAACAAGATGAAGTTTCAACACTATGGGGCGTTTTGGTTTA
TCCAGCAAGAAGAAGAAAGT
ACCGACGCTGAATTCGGTATTTGACCATTGCCAACAAAGAAAAAGGGCCTACAAAAAAATACAGATGTCATTATA
ATAGAXXXXXXXXXXTACCCATTGATTCACACACGAACAAGATGAAGTTTCAACACTATGGGGCGTTTTGGTTTA
TCCAGCAAGAAGAAGAAAGT
>Marker69461
ACTCTATAAATCTGAATTGAATAAAAATAAGATTACTTGACTATAATATGAACATTGAACTTTATTTAGTAACAT
AAAAGXXXXXXXXXXTTAATATAAACGAATAAATACAAGAAGCAAGAAAGAAAACAAACTAAGGACCGGAGGAAA
GCCGAGGAGAGACCCAAAGT
ACTCTATAAATCTGAATTGAATAAAAATAAGATTACTTGACTATAATATGAACATTGAACTTTATTTAGTAACAT
AAAAGXXXXXXXXXXTTAATATAAACGAATAAATACAAGAAGCAAGAAAGAAAACAAACTAAGGACCGGAGGAAA
GCCGAAGAGAGACCCAAAGT
>Marker70097
ACCTTCGTCGGCCGGCAGCCGAGCAGCGGCGTAGGCGAGAGAGAAGAAAAGAAAAACGGCGAGGAATCGAGCCAT
GAAAAXXXXXXXXXXGTTTTATTAACTTCCTTTTTTTAGTTACAAAACAAAACCAAATTATTTTTAAATTAAGTT
AAGAATTGTATATTTTGGTA
ACCTTCGTCGGCCGGCAGCCGAGCAGCGGCGTAGGCGAGAGAGAAGAAAAGAAAAACGGCGAGGAATCGAGCCAT
GAAAAXXXXXXXXXXGTTTTATTAACTTCCTTTTTTTAGTTACAAAACAAAACCAAATTATTTTAAAATTAAGTT
AAGAATTGTATATTTTGGTA
>Marker70216
TACAAAATAAAATGACTTTAAATGTTTGTTTCTTGCTTTTTTTAATGTTCTTTTGTGATTGAAACATCGGATCAA
TAAACXXXXXXXXXXCATTTGTGACACCCTCCAAACTTCTAATCCAACCCAAAAATGAAATCAAACCAATTATTA
GGCAAAAACTTTAATGGGTA

Create PDF with GO2PDF for free, if you wish to

TACAAAATAAAATGACTTTAAATGTTTGTTTCTTGCTTTTTTTAATGTTCTTTTGTGATTGAAACATCGGATCAA
TAAACXXXXXXXXXXCATTTGTGACACCCTCCAAACTTCTAATCCAACCCAAAAATGAATTCAAACCAATTATTA
GGCAAAAACTTTAATGGGTA
>Marker70541
ACAGCCTGAACGATGCTCGATGCCAGGTATATATGCTTTTTAACTGGTATTTAATCTATATGCACAAACTAGACA
AAACAXXXXXXXXXXAACTTATCAAGAATTCATACAAAGTTATCAGGTGCGGTCCAACCCCAAACCCCACATCCC
TCTTTTCCTTTTCCATGGTT
ACAGCCTGAACGATGCTCGATGCCAGGTATATATGCTTTTTAACTGGTATGTAATCTATATGCACAAACTAGACA
AAACAXXXXXXXXXXAACTTATCAAGAATTCATACAAAGTTATCAGGTGCGGTCCAACCCCAAACCCCACATCCC
TCTTTTCCTTTTCCATGGTT
>Marker70626
TACTAAGAGCACAAAGGACTAAGAGAACAAATACAACATAGTCCAAGACAAGAGAGAGCATAAAGAAATTATAAT
GAGCAXXXXXXXXXXCACCATGAATAGGCAACCAGCCATGGAATCCTGTGCGCTTGAAGACTTTCTGAAATCTTT
CATATCATTAATAAAAAGTG
TACTAAGAGCACAAAGGACTAACAGAACAAATACAACATAGTCCAAGACAAGAGAGAGCATAAAGAAATTATAAT
GAGCAXXXXXXXXXXCACCATGAATAGGCAACCAGCCATGGAATCCTGTGCGCTTGAAGACTTTCTGAAATCTTT
CATATCATTAATAAAAAGTG
>Marker70639
TACAAAAATACGTGACAAAAAACAAGTCTAATCAAAAATTGAAATTTGCATACACATGTGGTTCCGACTTCAACA
CATCTXXXXXXXXXXATGTAACTCTAGGCTTCGCTAGTAAGTTCGGCTTAATTATAATCTTTTAACAAAATCATA
TAGTCTAGCTCAACTAGGTT
TACAAAAATACGTGACAAAAAACAAGTCTAATCAAAAATTGAAATTTGCATCCACATGTGGTTCCGACTTGAACA
CATCTXXXXXXXXXXATGTAACTCTAGGCTTCGCTAGTAAGTTCGGCTTAATTATAATCTTTTAACAAAATCATA
TAGTCTAGCTCAACTAGGTT
>Marker71093
CACAAACTCAGGTTTCTTTAATTAATCTGCGTTTCATTAGAGCTTTTATTTGTTATTTACTTTTTAATTTTAAAT
TTACCXXXXXXXXXXATTTTGTTTCTTTATATATGTTTCACAGGATCTTGTTTGTAAGACATTCTATGAGTTCTG
GTTTGAGGAGCCTTCCAGTT
CACAAACTCAGGTTTCTTTAATTAATCTGCGTTTCATTAGAGCTTTTATTTGTTATTTAATTTTTAATTTTAAAT
TTACCXXXXXXXXXXATTTTGTTTCTTTATATATGTTTCACAGGATCTTGTTTGTAAGACATTCTATGAGTTCTG
GTTTGAGGAGCCTTCCAGTT
>Marker71094
ACCACAACAGCAGCCTTTCAACCTTTTTAGATTCCAACTCAAAAACATACAACCAACCACATAACCACACTCCAC
CTTGCXXXXXXXXXXTGGTAAGATCTGCTATAACCTCGTTCTAATCTTGAATGGAAACACTCAGAGACCGAGACT
CATAAGATAGGAGTTGTGTA
ACCACAACAGCAGCCTTTCAACCTTTTTAGATTCCAACTCAAAAACATACAACCAACCACATAATCACACTCCAC
CTTGCXXXXXXXXXXTGGTAAGATCTGCTATAACCTCGTTCTAATCTTGAATGGAAATACTCAGAGACCGAGACT
CATAAGATAGGAGTTGTGTA
>Marker71244
CACTATAGCCACCCTACCTAATGAGCTCCAGCGATTTCAGAATCTTACCATGGGTAAAGTGAAGGAAGTAGAAGC
AAATGXXXXXXXXXXTAGTAAGAGAAAGAATATTGCGAAAGGTAAATGTTACCACAGCAACAACAATAGGCATTG
GTTGAGAAATTACCCGTAGT
CACTATAGCCACCCTACCTAATGAGCTCCAGCGATTTCAGAATCTTACCATGGGTAAAGTGAAGGAAGTAGAAGC
AAATGXXXXXXXXXXTAGTAAGAGAAAGAATATTGCGAAAGGTAAATGTTACCATTGCAACAACAATAGGCATTG
GTTGAGAAATTACCCGTAGT
>Marker71378

Create PDF with GO2PDF for free, if you wish to

ACTTACAACAGCACGAAAGTTCTTAAACATGAAACATTCGAGTTGTTGAAGGGTCGATGGGCAATCCTACGAGGA
AAGCCXXXXXXXXXXXTCTCCTTATTATGATGACCTGTCATATGTTTTTGAAAAAGATTAGGCTACGGGAGCACGA
TTAGAGACCTTTGCTGATGT
ACTTACAACAGCACGAAAGTTCTTAAACATGAAACATTCGAGTTGTTGAAGGGTCGATGGGCAATCCTACGAGGA
AAGCCXXXXXXXXXXXTCTCCTTATTATGATGACCTGTCATATGTTTTTGAAAAAGATTAGGCTACGGGAGCACGA
TTAGAGACCTTCGCTGATGT
>Marker71461
ACCAAAATTGCCAGCAGGGAAGCAGAACTGTTGCTGATTACATAGAAGAATTCCACCGATTGGGAGCAAGAACTA
ATTTGXXXXXXXXXXXCATCACTCATGCAGAATCTGTAGAAGAGATTAATGAATTGAATTCAAGAAAAAAATACAA
GAAGAGGTCCGTGGAATGTC
ACCAAAATTGCCAGCAGGGAAGCAGAACTGTTGCTGATTACATAGAAGAATTCCACCGATTGGGAGCAAGAACTA
ATTTGXXXXXXXXXXXCATCACTCATGCAGAATCTGTAGAAGAGATTAATGAATTGAATTCAAGAAAAAAATACAA
GAAGATGTCCGTGGAATGTC
>Marker72078
ACTCTGATATAGTGATATTTCCCACTTGTAGTAAATCTATTGGCCATATTATTACACACAGGGTGGAGACTTGAA
CCTATXXXXXXXXXXXAGTTATGCCATATTATTTCCATATATTTTTGTGATTACATCCTTTTCCTTTGATTCTTGC
CAGCTGAGATACATATTTGT
ACTCTGATATAGTGATATTTCCCACTTATAGTAAATCTATTGGCCATATTATTACACACAGGGTGGAGACTTGAA
CCTATXXXXXXXXXXXAGTTATGCCATATTATTTCCATATATTTTTGTGATTACATCCTTTTCCTTTGATTCTTGC
CAGCTGAGATACATATTTGT
>Marker72110
ACAAGCACTTACGAAATGACGCGCATAATCATATGTGTGAAAACTGCAAAATTATAGTCATTTCGTAAAGGTCGC
ACAAAXXXXXXXXXXXTTATTCTTGTAAAAGGTTGGAGGAAACAAAACTTTCATACTATGCTCCTATATATAAAGG
TTCAGGAAGGCAAATTCAGT
ACAAGCACTTACGAAATGACGCGCATAATCATATGTGTGAAAACTGCAAAATTATAGTCATTTTGTAAAGGTCGC
ACAAAXXXXXXXXXXXTTATTCTTGTAAAAGGTTGGAGGAAACAAAACTTTCATACTATGCTCCTATATATAAAGG
TTCAGGAAGGCAAATTCAGT
>Marker72286
CACTTCAATATGGATGGATACATATAGGTGCATTTGTGTTTATATAGGTCAATTTAAGTGTTACGGTGATCTCTG
CTTTAXXXXXXXXXXXAGCACTACCTAGATTTTAGATAGAAAAGCTTCTGTGCAAGGCTGCAAATTTCATATATTT
CACAAAAATGATGTGAGAGT
CACTTCAATATGGATGGATACATATAGGTGCATTCGTGTTTATATAGGTCAATTTAAGTGTTACGGTGATCTCTG
CTTTAXXXXXXXXXXXAGCACTACCTAGATTTTAGATAGAAAAGCTTCTGTGCAAGGCTGCAAATTTCATATATTT
CACAAAAATGATGTGAGAGT
>Marker72295
ACAGATTATAGTAAGAAAACTTCATACATTTTTCCTAAGTAGCTAAGCTTTAAAATAGAGATCTAATAAAATTAG
TAAATXXXXXXXXXXXCAAGACTTACTAATGACAGGCAGGTCCATCTCCAGCCCAACAAGCTCTAGCAGCTTAAAT
CCATCCATGTCAGGCATGTG
ACAGATTATAGTAAGAAAACTTCATACATTTTTCCTAAGTAGCCAAGCTTTAAAATAGAGATCTAATAAAATTAG
TAAATXXXXXXXXXXXCAAGACTTACTAATGACAGGCAGGTCCATCTCCAGCCCAACAAGCTCTAGCAGCTTAAAT
CCATCCATGTCAGGCATGTG
>Marker72525
ACAGTAAAATTTAAAAATTTTAAATAGAAAGGATGATAAACCACCAAAGCTGAACTCACAGATATATCACTCTCT
TGGTTXXXXXXXXXXXTGCTCTGAAGCTCAAAAGTGCCTCACCTACAATAAACAAGAGACACCAAGCACTGAGAAA
GAGCGTCATAATATGAAGGT

Create PDF with GO2PDF for free, if you wish to

ACAGTAAAATTTAAAAATTTTAAATAGAAAGGATGATAAACCACCAAAGCTGAACTCACAAATATATCACTCTCT
TGGTTXXXXXXXXXXTGCTCTGAAGCTCAAAAGTGCCTCACCTACAATAAACAAGAGACACCAAGCACTGAGAAA
GAGCGTCATAATATGAAGGT
>Marker72609
CACTAATTTGGTTCTCCTTGGGCTGCCATCTTGCAGCCAAATGCCAAGATTATTCCAATTACACACAGCAAGAAC
TTCAGXXXXXXXXXXCAGCAACTGAAAGAATCTTTTGAAAGATATCCCATTCATGATAAGTTGCTCCATTCGGTT
TTAACTTTGGCAGAACCGGT
CACTAATTTGGTTCTCCTTGGGCTGCCATCTTGCAGCCAAATGCCAAGATTATTCCAATTACACACAGCAAGAAC
TTCAGXXXXXXXXXXCAGCAACTGAAAGAATCTTTTGAAAGATATCCCATTCATGATAAGTTGCTCCATTCGGTT
TTAACTTTGGCAGAACTGGT
>Marker72798
TACTACTTCAAAGCGACATTTTTCTGTTGAACGATTCTTTGTATTGTTGAAAGCAAACTCAGCTTGAGAAACTAC
TAGATXXXXXXXXXXTTGTGTCAAAATTATTCCATAGGGTTTGCCAGAAATGGCTTAGAAACTTAACATCTTTGT
CGGACACAATAGTTTTAGGT
TACTACTTCAAAGCGACATTTTTCTGTTGAACGATTCTTTGTATTGTTGAAAGCAAACTCAGCTTGAGAATCTAC
TAGATXXXXXXXXXXTTGTGTCAAAATTATTCCATAGGGTTTGCCAGAAATGGCTTAGAAACTTAACATCTTTGT
CGGACACAATAGTTTTAGGT
>Marker73031
ACAATGAACTTCCAATAAATCTGGATTAGTTTCTAAAGATTTCTTGAAACTGCTTGACTGTAATGAGAGATTTGA
ATTTGXXXXXXXXXXGACCAATGGCTATAGAAGAAAACTAGAGATTATAAAAAGCGTTCCAGATAATCAATAGGT
TGTTTAGACTGCAATTTGTG
ACAATGAACTTCCAATAAATCTGGATTAGTTTCTAAAGATTTCTTGAACCTGCTTGACTGTAATGAGAGATTTGA
ATTTGXXXXXXXXXXGACCAATGGCTATAGAAGAAAACTAGAGATTATAAAAAGCGTTCCAGATAATCAATAGGT
TGTTTAGACTGCAATTTGTG
>Marker73145
AACGCCTAGCTCACCAACAGTTAGCCAAAATTTCACTTAACTTCCTCTCTTTCCTTCTCTTGGCTGCCAATCCTA
AGTCCXXXXXXXXXXAATAAGTTTAGGGTTTATAATATTTAATTGATTCAATTTCCTTTGCCAGCGAGTGCATGT
TTGAGATTAGAGGTTGTGTA
AACGCCTAGCTCACCAACACTTAGCCAAAATTTCACTTAACTTCCTCTCTTTCCTTCTCTTGGCTGCCAATCCTA
AGTCCXXXXXXXXXXAATAAGTTTAGGATTTATAATATTTAATTGATTCAATTTCCTTTGCCAGCGAGTGCATGT
TTGAGATTAGAGGTTGTGTA
>Marker73273
AACACGTTCGAAGCCTTAGTAAGGAGGTTCGTATTGCTAAAGAATCCACGGATAGAGCTAAGAGAGAGGTTGAGT
GAAATXXXXXXXXXXGCATTTTTCCATCTATCTTTGGGTTCTCATTTGAGTTGTAATTCTTTGTGTTCTATGATT
AATTCAAAATGTTTTAGGTT
AACACGTTCGAAGCCTTAGTAAGGAGCTTCGTATTGCTAAAGAATCCACGGATAGAGCTAAGAGAGAGGTTGAGC
GAAATXXXXXXXXXXGCATTTTTCCATCTATCTTTGGGTTCTCATTTGAGTTGTAATTCTTTGTGTTCTATGGTT
AATTCAAAATGTTTTAGGTT
>Marker73671
AACAATCTCCTTGGAGGCTAGAAATTCACTAAGGTTATGGTTTTGGAATTCCAGACCATTATCAATTCTAAAAAT
AGCAAXXXXXXXXXXCAATGTAAGTTACAAATCACCGTTTCTCAGATGAGGTGGTGACCTTGGAGGGACCCCAAA
TGTCACTATGGATAAGGGTA
AACAATCTCCTTGGAGGCTAGAAATTCACTAAGGTTATGGTTTTGGAATTCCAGACCATTATCAATTCTAAAAAC
AGCAAXXXXXXXXXXCAATGAAAGTTACAAATCACCGTTTCTCAGATGAGGTGGTGACCTTGGAGGGACCCCAAA
TGTCACTATGGATAAGGGTA
>Marker73728

Create PDF with GO2PDF for free, if you wish to

AACACCAATAAAACAAACACGCACGTATATCAACAGGACTTGAATCTATAGATTATTTATTTTAAAAAAAAAAAA
AAAAAXXXXXXXXXXXTATTCTGCAAACTTCTTAAGGTATTAAGGTATGTCTTTCAAAATGATTAAAAATGTTAAG
AGTGTTAAACCAATAAAAGT
AACACCAATGAAACAAACACGCACGTATATCAACAGGACTTGAATCTATAGATTATTTATTTTAAAAAAAAAAAA
AAAAAXXXXXXXXXXXTATTCTGCAAACTTCTTAAGGTATTAAGGTATGTCTTTCAAAATGATTAAAAATGTTAAG
AGTGTTAAACCAATAAAAGT
>Marker73855
TACCCAATAAGTGCAATCCTACTGATACTCATCTCATTCTTCTACCTAATATCCAAGTGATCATTTTTCCTTTCC
CCCTTXXXXXXXXXXXACTAAGTAAAAGATAAGCAACATGTTTATCTTTCCTTTAAATAATGTTGTTTAATATTGC
TGGTCATACAAAGAGCCTGT
TACCCAATAAGTGCAATCCTACCGATACTCATCTCATTCTTCTACCTAATATCCAAGTGATCATTTTTCCTTTCC
CCCTTXXXXXXXXXXXACTAAGTAAAAGATAAGCAACATGTTTATCTTTCCTTTAAATAATGTTGTTTAATATTGC
TGGTCATACAAAGAGCCTGT
>Marker74018
CACTGGAGTTCTGTGTAAATCAGTTATTATTGACTGAGTCCATCTGAGTTGGCTAACGGAAATAGAAATGCTTCT
GCAGAXXXXXXXXXXXTACGAGAGAAAGAGAGAGGAGAGAGAAGGGAAAGAACTTACCATTCAACCAAGTAGTAGG
AGGAATTGGTTTTAATCAGT
CACTGGAGTTCTGTGTAAATCAGTTATTATTGACTGAGTCCATCTGAGTTGGCTAACGGAAATAGAAATGCTTCT
GCAGAXXXXXXXXXXXTACAAGAGAAAGAGAGAGGAGAGAGAAGGGAAAGAACTTACCATTCAACCAAGTAGTAGG
AGGAATTGGTTTTAATCAGT
>Marker74354
CACCATGATAGAACTAACCATGTTGAGATTGATCGACAATTCATCAAAGAAAGACTTAACCATCAGAGCATATGC
ATTTTXXXXXXXXXXXAAGGGATTTGCCCTAAATATTTTTTTTCTTTTTTTTCCTCACTATATTTTTTCTATTTAT
TCTCCTCTTGTAACAATTGT
CACCATGATAGAACTAACCATGTTGAGATTGATCGACAATTCATCAAAGAAAGACTTAACCATCAGAGCATATGC
ATTCTXXXXXXXXXXXAAGGGATTTGCCCTAAATATTTTTTTTCTTTTTTTTCCTCACTATATTTTTTCTATTTAT
TCTCCTCTTGTAACAATTGT
>Marker74393
TACAGCAAGCTAAGTAAGAAATAAATTTTGAACTAAATAAAATAAATACAGGTGGGCTGATAAATCCAATGGAAA
AACATXXXXXXXXXXXATAGGAATGTAATTAAATCAAGATAAGGTTTGGGTTTTTTATTCTTTCCTCAATTATATT
TTTCCTATAAAAAGATAGGT
TACAGCAAGCTAAGTAAGAAATAAATTTTGAACTAAATAAAATAAATACAGGTAGGCTGATAAATCCAATGGAAA
AACATXXXXXXXXXXXATAGGAATGTAATTAAATCAAGATAAGGTTTGGGTTTTTTATTCTTTCCTCAATTATATT
TTTCCTATAAAAAGATAGGT
>Marker74771
AACTAAACACTTAATTATTAATTAAACAGAAAACTAAAAACGGAATTGTTATAAAAACAGAACACTTCCGTTTGT
TATAAXXXXXXXXXXXCTATGATGAGAGAAGAATTAAGTGTGTGTGTGTATATATATATACCTGACATTTTGCA
GCTTTTGATGAAGTGCTTGT
AACTAAACACTTAATTATTAATTAAACAGAAAACTAAAAACGGAATTGTTATAAAAACAGAACACTTCCGTTTGT
TAAAAXXXXXXXXXXXCTATGATGAGAGAAGAATTAAGTGTGTGTGTGTATATATATATACCTGACATTTTGCA
GCTTTTGATGAAGTGCTTGT
>Marker75301
GACCATAAACAAAACAACCAAAGTGAAGGATCGAAGATGGTGAGTCAGAGAGATAAAAATCAAAACCAAATTTCA
TTATGXXXXXXXXXXXTATGAATGAATAGATAAGATGTTGAAAAGTGGAATGAGAGTGATGTCCAATCAAATCGAT
GGCAGAAAGAAACCAAAGGT

Create PDF with GO2PDF for free, if you wish to

GACCATAAACAAAACAACCAAAGTGAAGGATCGAAGATGGTGAGTCAGAGAGATAAAAATCAAAACCAAACTTCA
TTATGXXXXXXXXXXTATGAATGAATAGATAAGATGTTGAAAAGTGGAATGAGAGTGATGTCCAATCAAATCGAT
GGCAGAAAGAAACCAAAGGT
>Marker75987
CACGTTCAACCAAAGCCTTCTCATTTTCCTTTACCTAAAATTTTCAAATCATAAGAACATATTACATTCTTAGAG
GCTATXXXXXXXXXXACACATAATGGAAGAAAGAAATCATAGTCCTATATGCATAAATAATGATACCAAAACATT
AATATTAAAACTAAGTTGTT
CACGTTCAACCAAAGCCTTCTCATTTTCCTTTACCTAAAATTTTCAAATCATAAGAACATATTACATTCTTAGAG
GCTATXXXXXXXXXXACACATAATGGAAGAAAGAAATTATAGTCCTATATGCATAAATAATGATACCAAAACATT
TATATTAAAACTAAGTTGTT
>Marker76099
ACAAATTGGTGTGTGGATACTAATAATAGGGTTACGAATTTGATGTAATTGGAGTTGGTATGGAGCAGATTAAAG
AAGAGXXXXXXXXXXTTGAATGGATTGAATTACCCTATCTCTCCTTTGCCTTCTTTTGGAAGCTTTTGAATTTTG
TTTTTTACTCAATTGGCGTA
ACAAATTGGTGTGTGGATACTAATAATAGGGTTACGAATTTGATGTAATTGGAGTTGGTATGAAGCAGATTAAAG
AAGAGXXXXXXXXXXTTGAATGGATTGAATTACCCTATCTCTCCTTTGCCTTCTTTTGGAAGCTTTTGAATTTTG
TTTCTTACTCAATTGGCGTA
>Marker76149
ACTTTTGATGATCTAATGTCTAGAACTGAGGTGGTGATGATGACTTGCTCGTGTATTTGCCTCAATGGAATCTGA
TTAATXXXXXXXXXXAAGTTAGCGTGTTGCAAAGTGTAGCCAATAAGCAAGTCGATAGAATCTTCTCCTTTGACA
AGGTAAGTTTCTGAAACGTT
ACTTTTGATGATCTAATCTCTAGAACTGAGGTGGTGATGATGACTTGCTCGTGTATTTGCCTCAATGGAATCTGA
TTAATXXXXXXXXXXAAGTTAGCGTGTTGCAAAGTGTAGCCAATAAGCAAGTCGATAGAATCTTCTCCTTTGACA
AGGTAAGTTTCTGAAACGTT
>Marker76165
ACGTATATTGTGCTCACAAATTGATTTCACAAATAAGTTATAAATTTTGTGTTCAAAAGATGGCAAAAGAGCAGG
AAAAGXXXXXXXXXXTTCATCGAGATCAAACAACTAAACTTCGTGATGTTGTAAATGATTCACATGTCTTTAATA
TATGCACTGATAATGAAGGT
ACGTATATTGTGCTCACAAATTGATTTCACAAATAAATTATAAATTTTGTGTTCGAAAGATGGCAAAAGAGCAGG
AAAAGXXXXXXXXXXTTCATCGAGATCAAACAACTAAACTTCGTGATGTTGTAAATGATTCACATGTCTTTAATA
TATGCACTGATAATGAAGGT
>Marker76253
ACAAAACTCTTTGGAAGAGATTGTAAAATAAAGCTCACTTGATGTGCCTCATCAATGGGATCGCCATTTACTTCA
ACGATXXXXXXXXXXAATGAATCCATAATCTCTTTAGTCGTGGCTAAGGGTTCATGTTTCTTTGTGAAAACAACA
GACATGCTGGCAAGAATGTA
ACAAAACTCTTTGGAAGAGATTGTAAAATAAAGCTCACTTGATGTGCCTCATCAATGGGATCGCCATTTACTTCA
ACGATXXXXXXXXXXAATGAATCCATAATCTCTTTAGTCGTGGCTAAGGGTTCATGTTTCTTTGTGAAAACAACA
GACAGGCTGGCAAGAATGTA
>Marker76385
AACACAAACCAAGAGGCCGGTTGCCTATTTCAGCATAACACTGTGTATGAGGGATAGGGCACGACCTGTCTATGA
GAGAGXXXXXXXXXXTTTTGGGCTATTCTTTTGAGGTGGTGACCAGTTTGGAAAACAAGGCAGCTGATGCTTTGT
CTAGAATGCCTCCTACAGTG
AACACAAACCAAGAGGCCGGTTGCCTATTTCAGCATAACACTGTGTATGAGGGATAGGGCACGACCTGTCTATGA
GAGAGXXXXXXXXXXTTTTGGGCTATTCTTTTGAGGTGGTGACCAGTTTGGAAAACAAGGCAGCCGATGCTTTGT
CTAGAATGCCTCCTACAGTG
>Marker76443

Create PDF with GO2PDF for free, if you wish to

AACTGTTGCTTCTTTCTAGATCCTCCTTCCCCATATAACTCATTCCATTGCCTCAATTCACTCATCATCGCTCCT
TCTGCXXXXXXXXXXGGGCAACTTGTGCAACTAGAATCATGTCGTTGGTTTGTTTTCTTTTTGCTGTCTTTTGTT
TGAACAAATATTGGAACAGT
AACTGTTGCTTCTTTCTAGATCCTCCTTCCCCATATAACTCATTCCATTGCCTCAATTCACTCATCATTGCTCCT
TCTGCXXXXXXXXXXGGGCAACTTGTGCAACTAGAATCATGTCGTTGGTTTGTTTTCTTTTTGCTGTCTTTTGTT
TGAACAAATATTGGAACAGT
>Marker77051
GACGTTCCTGACAATAGAAAATATGGGAAAAGTTATATATGGAATAAAATAGAAAGTATGCAAAAACTTATATAT
CAAACXXXXXXXXXXTTAAGGATCCTTTCCAAATCTGCCATTTCTTTCAGTTCTCCTAAATATTACTTGCTGAAA
ATTGAGTTTATCATTAGGTT
GACGTTCCTGACAATAGAAAATATGGGAAAAGTTATATATGGAATAAAATAGAAAGTATGCAAAAAGTTATATAT
CAAACXXXXXXXXXXTTAAGGATCCTTTCCAAATCTGCCATTTCTTTCAGTTCTCCTAAATATTACTTGCTGGAA
ATTGAGTATATCATTAGGTT
>Marker77061
ACAGGAAGTGCGATGAGATCGAAAACAGTATCTGCACAATCAGAAATGAGAGAATCCGCAACAATCTTGATCTGA
TGAGCXXXXXXXXXXCGTTCGCAACCGGAACCAAAACCTAATCTCAACCAACGACGAGGTTGCAAACACAAACAA
AATGTCATAAATCAAACAGT
ACAGGAAGTGCGATGAGATCGAAAACAGTATCTGCACAATCAGAAATGAGAGAATCCGCAACAATCTTGATCTGA
TGAGCXXXXXXXXXXCGTTCGCAACCGGAACCAAAACCTAATCTCAACCAACGATGAGGTTGCAAACACAAACAA
AATGTCAAAAATCTAACAGT
>Marker77152
TACCTAAGTCCTTCCCTTTAGGATTTGGAGTTGCAGAGGAAAAGAGAAACTATTCCCACCAAGGAAAATATATAG
ATAGAXXXXXXXXXXTGCATTAAAGAGAAGTTCTAGGTGTAGGAGCGAAGCAATACGACTGGAGAACTCCACCAG
AGGATAGTCTTCATGAGAGT
TACCTAAGTCCTTCCCTTTAGGATTTGGAGTTGCAGCGGAAAAGAGAAACTATTCCCACCAAGGAAAATATATAG
ATAGAXXXXXXXXXXTGCATTAAAGAGAAGTTCCAGGTGTAGGAGCGAAGCAATACGACTGGAGAACTCCACCAG
AAGATAGTCTTCATGAGAGT
>Marker77657
ACTTTGAGTTTCTATTATTAATAAAGAAGTTTGTCTCTGTTTAAAAAAAAGACCAAAAAGCATACTTTAAAGTTC
TTGAGXXXXXXXXXXAGTTCCACTGATTGGGCTGACGATCGTTCTGTCCTCTCCGACCAAAGCATTCAAAATACT
ACTTTTACCAACATTAGGTC
ACTTTGAGTTTCTATTATTAATAAAGAAGTTTGTCTCTGTTTAAAAAAAAAACCAAAAAGCATACTTTTAAGTTC
TTGAGXXXXXXXXXXAGTTCCACTGATTGGGCTGACGATCGTTCTGTCCTCTCCGACCAAAGCATTCAAAATACT
ACTTTTACCAACATTAGGTC
>Marker77899
AACTGATTTCATTTTGATGAACCAGTTAGTGATGAACTACCCCCAATTCAATATCTCTGGTCCAATTTACTGAAT
TTGATXXXXXXXXXXTCTTCCTAGTTCAATCATTAGTGGTCATCTACTGAGAATTAATTTTCTATACAGTAGAGT
AAGGTTGATTTGTCCAGGTG
AACTGATTTCATTTTGATGAATCAGTTAGTGATGAACTACCCCCAATTCAATATCTCTGGTCCAATTTACTGAAT
TTGATXXXXXXXXXXTCTTCCTAGTTCAATCATTAGTGGTCATCTACTGAGAATTAATTTTCTATACAGTAGAGT
AAGGTTGATTTGTCCAGGTG
>Marker78315
ACATCCCTTAACCAGAAAGATAAAGCAATCGTCCAAGGAAGCTATGGCGGTAAAGAGAAGGGCGTTGACGAGCCT
TCAACXXXXXXXXXXCCACGGTCCAATTGGGTTTGGTGATCACGAACATGGAACGCTTGGGCCAAATCAGGAGCA
AAATGTGACCTGTGAACAGT

Create PDF with GO2PDF for free, if you wish to

ACATCCCTTAACCAGAAAGATGAAGCAATCGTCCAAGGAAGCTATGGCGGTAAAGAGAAGGGCGTTGACGAGCCT
TCAACXXXXXXXXXXXCCACGGTCCAATTGGGTTTGGTGATCACGAACATGGAACGCTTGGGCCAAATCAGGAGCA
AAATGTGACCTGTGAACAGT
>Marker78609
CACCCTTAAATGGGAAACCTTTAACAATATGGTAGTCATCAACACCTGTAATTGGAGCATTTGCATCAAATATTC
TCAAAXXXXXXXXXXXXTTTGATTACGAAATCAAATCTTTGAAGGAAGAAAATCCAGCGTGCGTGCATTCTGCTGAT
ATTTTTTTTAGATGGGAGGT
CACCCTTAAATGGGAAACCTTTAACAATATGGTAGTCATCAACACCTCTAATTGGAGCATTTGCACCAAATATTC
TCAAAXXXXXXXXXXXXTTTGATTACGAAATCAAATCTTTGAAGGAAGAAAATCCAGCGTGCGTGCATTCTGCTGAT
ATTTTTTTTAGATGGGAGGT
>Marker78656
CACCTCTTCATCTAGCTGCTAGAAGTGGTTCTTTAGAATGTGTTCGAGAATTACTTGCGTGGGGAGCAGAAAGAT
TACAAXXXXXXXXXXXXACTGGTTATGCTTATACCATTTCAATCGTTGGCGCTGCGAATTACGTGAGCTACATAAGC
ACATAAGTTCTGAATACGTA
CACCTCTTCATCTAGCTGCTAGAAGTGGTTCTTTAGAATGTGTTCGAGAATTACTTGCGTGGGGAGCAGAAAGAT
TACAAXXXXXXXXXXXXACTGGTTATGCTTATACCATTTCAATCGTTGGCACTGCGAATTACGTGAGCTACATAAGC
ACATAAGTTCTGAATACGTA
>Marker78702
ACTGCAGCAACCTGATATATTTACCATTAGAGCAAGTTGTAAGCTTAAAATGATTGAAATTGCTAAAAGAAATGA
AATTGXXXXXXXXXXXATAAACCAAACCCTCTGGCTTTGTGACAGTTGTTCATCCTTTCTAATTCCTAGAATTTAA
GCCCTTTATTTATTATTGTG
ACTGCAGCAACCTGATATATTTACCATTAGAGCAAGTTGTAAGCTTAAAATGATTGAAATTGCTATAAGAAATGA
AATTGXXXXXXXXXXXATAAACCAAACCCTCTGGCTTTGTGACAGTTGTTCATCCTTTCTAATTCCTAGAATTTAA
GCCCTTTATTTATTATTGTG
>Marker78758
CACTAGCCTTATCCTTTTCTTGATCTTTAGTGGATTGTGCAGCAGAAGGCTTAGAGCTAGTGTTGATATTCCACG
GTCCCXXXXXXXXXXXTAAATTGATTCTTGCACCCAACCGCTGGAATTCTTCTATATAATCAGCAACTATTCTTGT
TCCTTGCCGGCAATTATGGT
CACTAGCCTTATCCTTTTCTTGATCTTTAGTGGATTGTGCAGCAGAAGGCTTAGAGCTAGTGTTGATATTCCACG
GTCCCXXXXXXXXXXXTAAATTGATTCTTGCACCCAACCGCTGGAATTCTTCTATATAATCAGCAATTATTCTTGT
TCCTTGCCGGCAATTATGGT
>Marker78771
ACTTATATATGATAAATAAACTAAATAGTATTCACAAATATAATTCATGCATTCACGAAACGGGACGAATAACTT
AGTTTXXXXXXXXXXXAGAGTTGAGTTTTATAAAGGGGGTATCAATATGGATTAAGATTCTAAAATAAAGACCCAA
AAACAAGACAAATTTATTGT
ACTTATATATGATAAATAAACTAAATAGTATTCGCAAATATAATTCATGCATTCACGAAACGGGACGAATAACTT
AGTTTXXXXXXXXXXXAGAGTTGAGTTTTATAAAGGGGGTATCAATATGGATTAAGATTCTAAAATAAAGACCCAA
AAACAAGACAAATTTATTGT
>Marker79028
ACAAGAGGATCTAAATATTAAAGACGAGTTAATTTGACTATAACGACAAATTTAAACTATTTATTAGTTGAAATG
ATTGTXXXXXXXXXXXAAGCACCGTTTGATTAAATCTATGGCATTGCCTAGTATTTAAAAATCAACCTTAAATTTT
TGACTATTGATTAATTGGTC
ACAGGAGGATCTAAATATTAAAGACGAGTTAATTTGACTATAACGACAAATTTAAACTATTTATTAGTTGAAATG
ATTGTXXXXXXXXXXXAAGCACCGTTTGACTAAATCTATGGCATTGCCTAGTATTTAAAAATCAACCTTAAATTTT
TGACTATTGATTAATTGGTC
>Marker79121

Create PDF with GO2PDF for free, if you wish to

ACTTTATATCGAACTCTTAAATCTGGGACCCTCTATAACTTACTTGAAGAAATGAAAGTGAGATTCCCACTGGAT
TTTTCXXXXXXXXXXGTAACGAGTGCTTCATGAAAGTTATAGGACATTGAATAAGATTTCCAACCATATTCAATA
GGGCTTTTAGTTCCATCTGT
ACTTTATATCGAACTCTTAAATCTGGGACCCTCTATAACTTACTTGAAGAAATGAAAGTGAGATTCCCACTGGAT
TTTTCXXXXXXXXXXGTAACGAGTGCTTCATGAAAGTTATAGGACATTGAATAAGATTTCCAACCATATCCAGTA
GGGCTTTTAGTTCCATCTGT
>Marker79285
TACACCAGACCTCTGTCCCAACAGGAAATGCTGCTTTCCACAAATCTTCCTGCATTCAGTAATTGCTATTCGTAA
ATCAAXXXXXXXXXXXTTAAAAAAACAAACAAACTTCATTTCCAAGTTCAAGTAAATCATTAACAAAGCAAAATAC
AAAAGTTTATTGAAGCTCGT
TACACCAGACCTCTGTCCCAACAGGAAATGCTGCTTTCCACAAATCTTCCTGCATTCAGTAATTGCTATTCGTAA
ATCAAXXXXXXXXXXXAAAAAAAAACAAACAAACTTCATTTCCAAGTTCAAGTAAATCATTAACAAAGCAAAATAC
AAAAGTTTATTGAAGCTCGT
>Marker79371
ACTTGTCTTTAAAGATTTGTAGATGTAGAATGTGAGATATATTGCAAATACTTTGTGTCCATACATGTGCTGTTT
TGACCXXXXXXXXXXCGATAATCGACAATAGCAAATACGTAATGATGTTTTGTCCATTGTCATCCTCCCACTGAA
TGTAAAACGTTCAGTTGGTT
ACTTGTCTTTAAAGATTTGTAGATGCAGAATGTGAGATATATTGCAAATACTTTGTGTCCATACATGTGCTGTTT
TGACCXXXXXXXXXXCGATCATCGACAATAGCAAATACGTAATGATGTTTTGTCCATTGTCATCCTCCCACTGAA
TGTAAAACGTTCAGTTGGTT
>Marker79388
ACTATAGAGGAAGTTGGCAGTCCTGATGTTCAAGGAATTCAAGACTCAAAAATTGAATTAGTTGTAAGTGCAAGT
AGTGAXXXXXXXXXXAAATCAGAGACGAAATCCCCAACACTAGCACGACATCATTGAAGCTTGTGGCTTATCTTT
CTTTTGAGTTCTATAATGTT
ACTATAGAGGAAGTTGGCAGTCCTGATGTTCAAGGAATTCAAGACTCAAAAATTGAATCAGTTGTAAGTGCAAGT
AGTGAXXXXXXXXXXAAATCAGAGACGAAATCCCCAACACTAGCACGACATCATTGAAGCTTGTGGCTTATCTTT
CTTTTGAGTTCTATAATGTT
>Marker79463
ACTAGTAATAGATAAGCTTAAGATCATTGCACAACGCATGAAAACAAGCATTAGCACGAAAGCAAGATGTAATGT
GAGCCXXXXXXXXXXATAAGACAAACTATAATCTGGTATGTGCAAGTCATTAATAGTCTGTAGTGAAAGACTGAA
AGCAATAACAAATTTTAGTT
ACTAGTAATAGATAAGCTTAAGATCATTGCACAACGCATGAAAACAAGCATTAGCACGAAAGCAAGATGTAATGT
GAGCCXXXXXXXXXXATAAGACAAACTATAATCTGGTATGTGCAAGTCATTAATAGTCTGTAGTGAAAGACTGAA
AGCAATAACTAATTTTAGTT
>Marker79552
TACTTTGTTCCCTCTTGAAAGCTCCACCCCTTTCTCATACTTCAATTGTCTTTGTTCCAGAGCTGCTATTGGAAT
GCTTGXXXXXXXXXXTGTTCCACAACCAGCAGCAGCAGAAGGCGCTAAAATAATATTGAAAGACCTAAGCGTTCT
TAGCAAGGATAGCCAGGAGT
TACTTTGTTCCCTCTTGAAAGCTCCACCCCTTTCTCATACTTCAATTGTCTTTGTTCCAGTGCTGCTATTGGAAC
GCTTGXXXXXXXXXXTGTTCCACAACCAGCAGCAGCAGAAGGCGGTAAAATAATATTGAAAGACCTAAGCGTTCT
TAGCAAGGATAGCCAGGAGT
>Marker79564
GACAACTAGGCTACTCAAAAGGTATTGGTTGGGGTCCATGGAAAAAGTTTCGAAACATTGCAGACAATTCTTCAT
CTACAXXXXXXXXXXGAAAATGCCGACAGATGAAAGAAATGACGATGATTGAAGATTTTACTCGGGCACAGAGAG
GACCTTGATTCTATTGTGGT

Create PDF with GO2PDF for free, if you wish to

GACAACTAGGCTACTCAAAAGGTATTGGTTGGGGTCCATGGAAAAAGTCTCGAAACATTGCAGACAATTCTTCAT
CTACAXXXXXXXXXXGAAAATGCCGACAGATGAAAGAAATGACGATGATTGAAGATTTTACTCGGGCACAGAGAG
GACCTTGATTCTATTGTGGT
>Marker79703
ACTGAATTTTCCCTGGAGTATGTGATTGATCTTTTTCCCCCCTTCCCTTTCAAGATCCCAAACTAGAAGGCCTGA
ACAGAXXXXXXXXXXCTTCAATCAGCCAAAATGTCAGTCTAAGCAATACAGATAGGACTCGGCACTGTCATTTGC
TAATTTTAAAAAAACCAGTA
ACTGAATTTTCCCTGGAGTATGTGATTGATCTTTTTCCCCCCTTCCCTTTCAAGATCCCAAACTAGAAGGCCTGA
ACAGAXXXXXXXXXXCTTCAATCAGCCAAAATGTCAGTCTAAGCAATACAGATAGGACTCGACACTGTCATTTGC
TAATTTTAGAAAAACCAGTA
>Marker79842
ACGATAGACGTGTGTCCGGTAATTGAATTGTCTCTAAACTCGGTGGTAGGACTCACTACACCAGGAGCTTTCAAG
GTCAAXXXXXXXXXXCAATGATGACCATCGTGGAAGCTTCCTACCCTTGGAGCTCCACAACTTGGATATGGTTTT
GGGTATGCAGTGGCTAGGTA
ACGATAGACGTGTGTCCGGTAGTTGAATTGTCTCTAAACTCGGTGGTAGGACTCACTACACCAGGAGCTTTCAAG
GTCAAXXXXXXXXXXCAATGATGACCATCGTGGAAGCTTCCTACCATTGGAGCTCCACAACTTGGATATGGTTTT
GGGTATGCAGTGGCTAGGTA
>Marker79982
ACTTCACCCCAGTAACTTGAAATAAATAATAAGAGAAACCTTTTTTTAACCCAAAATAGAAAAATGCACACTGTG
CTGTCXXXXXXXXXXCCACCTACAATTTTAGCCGGTTAAAAAGCTTTCTTACCCAAGTTCATTAGAGGAAATGGG
CTTTGAGAAAAAAATGGGTT
ACTTCACCCCAGTAACTTGAAATAAATAATAAGAGAAACCTTTTTTTAACCCAAAATAGAAAAATGCACACTGTG
CTGTCXXXXXXXXXXCCACCTACAATTTTAGCCGATTAAAAAGCTTTCTTACCCAAGTTCATTAGAGGAAATGGG
CTTTGAGAAAAAAATGGGTT
>Marker80529
AACAAACCCAGCACCATTTTCATATATATGTTTTTCAATCCTCCATTACTGATATTAATTTAGTGCGTCCCAGGA
ATTCTXXXXXXXXXXAGTGTCACCAAGTCAGAATCTCTATATCCAATCTCCAACAGTTTCTCTCCTCTCGAGCAC
ACAACAACGTTCCTTGGGTG
AACAAACCCAACACCATTTTCATATATATGTTTTTCAATCCTCCATTACTGATATTAATTTAGTGCGTCCCAGGA
ATTCTXXXXXXXXXXAGTGTCACCAAGTCAGAATCTCTATATCCAATCTCCAACAGTTTCTCTCCTCTCGAGCAC
TCAACAACGTTCCTTGGGTG
>Marker80836
TACAAATCAAAAACTCCAATTGACATATTTGACATCAATCAAGTTTAACATCAGGATCAACAAATTGACTAGAGA
GCTTTXXXXXXXXXXCAGATCATTGGACATTTTTCTGTATTATATTTCAGGAAAGTAGAACATGAAATGGATGAT
ACCTTAAGATCTCTGTGGTA
TACAAATCAAAAACTCCAATTGACATATTTGACATCAATCAAGTTTAACATAAGGATCAACAAATTGACTAGAGA
GCTTTXXXXXXXXXXCAGATCATTGGACATTTTTCTGTATTATATTTCAGGAAAGTAGAACATGAAATGGATGAT
ACCTTAAGATCTCTGTGGTA
>Marker81733
CACTAACACACATAATGACAATAAGGTGGGTGAGAATGACGAGTCTGAGAATAACGAATCCAAGACAGTTTTTCT
TGAAGXXXXXXXXXXACAAAACCAAGTAGGACCATTTAGAAAACATTAGTAAATATGATCTTTACCTTGATCTTC
CTATTGCCCTGAGGAAAGGT
CACTAACACACATAATGACAATAAGGTGGGTGAGAATGATGAGTCTGAGAATAACGAATCCAAGACAGTTTTTCT
TGAAGXXXXXXXXXXACAAAACCAAGTAGGACCATTTAGAAAACATTAGTAAATATGATCTTTACCTTGATCTTC
CTATTGCCCTGAGGAAAGGT
>Marker81869

Create PDF with GO2PDF for free, if you wish to

ACATTCAGCTTTTTGTCAAGGCACTTGGAGGAAACGTCAGTGGATCAGATAAAAGAATTCTATTCCTTTGTGGGG
TAAGCXXXXXXXXXXCACAACTGTTCAAGAATTTTCTCACTTCAAAACAACAAACATTTTTTTGTAACGCCTGTG
AGGGTATGAGTATCTAGGTC
ACATTCAGCTTTTTGTCAAAGCACTTGGAGGAAACGTCAGTGGATCAGATAAAAGAATTCTATTCCTTTGTGGGG
TAAGCXXXXXXXXXXCACAACTGTTCAAGAATTTTCTCACTTCAAAACAACAAACATTTTTTTGTAACGCCTGTG
AGGGTATGAGTATCTAGGTC
>Marker82290
CACATATGGGATTCGAACAAAATTCATATTATGTAGGTTAAAAATTCAACTGTCTGAAGACACAATCAGTTCAAA
AGTCAXXXXXXXXXXCACTCTATCTTCTTACACATTATCTTCAGCTTCCTCTTCGTCTTCATCGTAATACTCGTC
TTCGGCTGTGGCGTCTTGGT
CACATATGGGATTCGAACAAAATTCATATTATGTAGGTTAAAAATTCAACTGTCTGAAGACACAATCAGTTCAAA
AGTCAXXXXXXXXXXCACTCTATCTTCTTACACATTATCTTCACCTTCCTCTTCGTCTTCATCGTAATACTCGTC
TTCGGCTGTGGCGTCTTGGT
>Marker82292
ACTAATTTAGGTATCAAAGTGTGATAGGTTTGACATCACATCGATTCGAGAATCCATTCTTATTTCTATTAATTT
AGAGAXXXXXXXXXXTTGATGAATTATTTTATCTATGATTGCTTCAATGCATGTTGAATTTTGACAAAACAAATT
AGAACTATAGCTTAGAAGTT
ACTAATTTAGGTATCAAAGTGTGATAGGTTTGACATCACATCGATTCGAGAATCCATTCTTATTTCTATTAATTT
AGAGAXXXXXXXXXXTTGATGAATTATTTTATCTATGACTGCTTCAATGCATGTTGAATTTTGACAAAACAAATT
AGAACTATAGCTTAGAAGTT
>Marker83400
GACAAGTTACTAAGAGCCTAAAGATGACTAAGCTATTTTGAAAAGTAATATAATTAGGAGAAGAGGTCAAGAAAG
GAGGGXXXXXXXXXXTGAGTAATTTTTCAAAATTAAGATTGATTTAAACTTCCTTCCAACGTTTCTAGTATTTTG
TGATTTGTGTGTTTCATGTT
GACAAGTTACTAAGAGCTTAAAGATGACTAAGCTATTTTGAAAAGTAATATAATTAGGAGAAGAGGTCAAGAAAG
GAGGGXXXXXXXXXXTGAGTAATTTTTCAAAATTAAGATTGATTTAAACTTCCTTCCAACGTTTCTAGTATTTTG
TGATTTGTGTGTTTCATGTT
>Marker83525
ACCTCCCTGTGAGATGACACACGAACGCCTAGTAAGCGGAACAACTCTTTTTCTTACCTTTTTACAACGCATTTC
TTAAGXXXXXXXXXXTGATAAGCACAAAAGAAAAACACAGCAGAAATAACAAATTAAAATTCCTCTCTTTATTAA
ATGAACACTTTTACAAAGTT
ACCTCCCCGTGAGATGACACACGAACGCCTAGTAAGCGGAACAACTCTTTTTCTTACCTTTTTACAACGCATTTC
TTAAGXXXXXXXXXXTGATAAGCACAAAAGAAAAACACAGCAGAAATAACAAATTAAAATTCCTCTCTTTATTAA
ATGAACACTTTTACAAAGTT
>Marker83654
TACCACGACTACAAGATGAAGATCGATCTTCCTACATATAGTGGCAAGAGTAGTATAGAGTCCTTTTTGTTGGGA
TTTATXXXXXXXXXXTTGAACAGTATGTAGAGACATATATGGATCGATGTTCAAAATCAGCTTAAAGGGTCTATA
GTATAGGAATAAGGTTGGGT
TACCACGACTACAAGATGAAGATCGATCTTCCTACATATAGTGGCAAGAGTAGTATAGAGTCCTTTTTGTTGGGA
TTTATXXXXXXXXXXTTGAACAGTATGTAGAGACATATATGGATCGATGTTCAAAATCAGCTTAAATGGTCTATA
GTATAGGAATAAGGTTGGGT
>Marker83705
ACCACACGGCGCACAATGTTGGATACGTCGGGGCATGATTCGTCGTAAAACGTGGGGCAGAGCTGAGCTTGAGAA
GCTCTXXXXXXXXXXTTTTTTTTTGTTACTAAGAAAAAAAATAATTGGGTTATGAATATGACAGGGTGGTGGTGG
CTAAGTCACCGGCGAGAGTT

Create PDF with GO2PDF for free, if you wish to

ACCACACGGCGCACAATGTTGGATACGTCGGGGCATGATTCGTCGTAAAACGTGGGGCAGAGCTGAGCTTGAGAA
GCTCTXXXXXXXXXXXTTTTTTTTTGTTACTAAGAAAAAGAATAATTGGGTTATGAATATGACAAGGTGGTGGTGG
CTAAGTCACCGGCGAGAGTT
>Marker83770
AACTTAAGATAAGGAAGTGTTAGTTTTGTGTGTCATGGTAACCGGAAAAGGGAAGACTAAAATGTTAAGGTTTAA
AAGTTXXXXXXXXXXXTAGGACGCATGCTAGGTAGCAAGATAGGAGAAGATAGAGGTTATTTTGGCGTTTGATCGG
TGGTTGAGAAGAACAAAGTG
AACTTAAGATAAGGAAGTGTTAGTTTTGTGTGTCATGGTAACTGGAAAAGGGAAGACTAAAATGTTAAGGTTTAA
AAGTTXXXXXXXXXXXTAGGACGCATGCTAGGTAGCAAGATAGGAGAAGATAGAGGTTATTTTGGCGTTTGATCGG
TGGTTGAGAAGAACAAAGTG
>Marker83816
ACCAAAGCTCATCTCATTACCATATTTCTTCATTTTCAATTCAAACTCAAATACATTTTAAGAATTTAAGATCAT
AAGAGXXXXXXXXXXXACTTTCCTGTAGTTCCTTGTCAAATTTGACCTTCTGATGGATCTAAATCTTCGAAACTCT
TTCACTTCTTTCCTTAAGTA
ACCAAAGATCATCTCATTACCATATTTCTTCATTTTCAATTCAAACTCAAATACATTTTAAGAATTTAAGATCAT
AAGAGXXXXXXXXXXXACTTTCCTGTAGTTCCTTGTCAAATTTGACCTTATGATGGATCTAAATCTTCGAAACTCT
TTCACTTCTTTCCTTAAGTA
>Marker83863
ACTATATTTCTTAGCTATACTACTCAACCTTATCTTTTCCAAAACAAAAGCAATATATTAATACTAATTTTACTT
TGTATXXXXXXXXXXXAATTGAAAAAATATATGGAGCATGTTTATTTTTTAATAGCCATATTGAGGAGATATTTCA
TTTAATTTACACATCCATGT
ACTATATTTCTTAGCTATACTACTCAACCTTATCTTTTCCAAAACAAAAGCAATATATTAATACTAATTTTACTT
TGTATXXXXXXXXXXXAATTGAAAAAAAATATGGAGCATGTTTATTTTTTAATAGCCATATTGAGGAGATATTTCA
TTTAATTTACACATCCATGT
>Marker84320
ACATTAACTGTGGATTTTTTTTTCTTTCTAAAAGACGTGTTTCCTGTGATATAAACGAAGAATGCATAATAGCGA
GAAGGXXXXXXXXXXXCTTATGGGCAGTTCCATTTTCGTTTTTTGAACTATTTTCTTTGGAATTATACTGGGCATT
GGATATACGCCGTATTTGTC
ACATTAACTGTGGATTTTTTTTTCTTTCTAAAAGACGTGTTTCCTGTGATATAAACGAAGAATGCATAAGAGCGA
GAAGGXXXXXXXXXXXCTTATGGGCAGTTCCATTTTCGTTTTTTGAACTATTTTCTTTGGAATTATACTGGGCATT
GGATATACGCTGTATTTGTC
>Marker84567
ACCTTTCCAACTCCAAGTTTCAAGACTGTTGACGTCTCGTGGAGTCGGAGTGGGTTGAGACACTTATGATGTAGA
AATCAXXXXXXXXXXXCCTGCGTTGTTTAACTCGTTGCCGAAATTGAGAAAGAGAGCGTATGTGTCATCGAAAGTA
TTTGGGAATAAATACATAGT
ACCTTTCCAACTCCAAGTTTCAAGACTGTTGACGTCTCGTGGAGTCGGAGTGGGTTGAGACACTTATGATGTAGA
AATCAXXXXXXXXXXXCCTGCGTTGTTTAACTCGTTGCCGAAATTGAGAAAGAGAGCATATGTGTCATCGAAAGTA
TTTGGGAATAAATACATAGT
>Marker84888
ACATGATTTTTATGAATGGTTTCAAGCTTAGATTTTATAATGAAATTTATATATGAGAACGTTATTATCGACTTT
TTAAAXXXXXXXXXXXCAAACATGTTTTAACGTTATTATCGCCTAGTATTACTACATGACTAGGAAGTTGAGTCCG
AGGCTTTATGATACCATGTG
ACATGATTTTTATGAATGGTTTCAAGCTTAGATTTTATAATGAAATTTATATATGAGAACGTTATTATCGACTTT
TTAAAXXXXXXXXXXXCAAACATGTTTTAACGTTATTATCGCCTAGTATTACTACATGACTAAGAAGTTGAGTCCG
AGGTTTTATGATACCATGTG
>Marker85020

Create PDF with GO2PDF for free, if you wish to

TACAAATACACTATATGTGATATTTGACATCATTATTTGCATGCAAATACACTGCAAAAAAACACGTATTATCCT
ACATAXXXXXXXXXXTTTTACTGACCCAGCAGGTGCATAATCAACAACCACAACAAAATCATTGTCAGAAAATTT
CGACATAATCAACATAAGTC
TACAAATACACTATATGTGATATTTGACATCATTATTTGCATGCAAATACACTGCAAAAAAACACGTATTATCCT
ACATAXXXXXXXXXXTTTTACCGACCCAGCAGGTGCATAATCAACAACCACAACAAAATCATTGTCAGAAAATTT
CGACATAATCAACATAAGTC
>Marker85194
AACATCAAGATTGACCCGTATGCTGGTGCAGAAAGTGGAAGGCTGCAACACAAGAACTGAAGTTAAGTGCTTACT
TCTGTXXXXXXXXXXTATAAATTATGTAAATCATGGAGTTTGAATAAAATTAATGTAACTTGTAAAGCCTGCAGT
TGGATAAACGATTTAACAGT
AACATCAAGATTGACCCGTATGCTGGTGCAGAAAGTGGAAGGCTGCAACACAAGAACTGAAGTTAAGTCCTTACT
TCTGTXXXXXXXXXXTATAAATTATGTAAATCATGGAGTTTGAATAAAATTAATGTAACTTGTAAAGCCTGCAGT
TGGATAAACGATTTAACAGT
>Marker85206
GACTTAGCTCTAATCCTTGTGCTTGTGATAATTTCTTGTCAAGATGAATACTCCATGATCTATTTTGTTCTCTTA
TATGCXXXXXXXXXXACCTCAAGTGGTCTTACCCAAGCAGTTGAAAGGGTTGTTCCGCGACCATTACAATTTTTA
GTGAGGTTTCTTGCGTGGTT
GACTTAGCTCTAATCCCTGTGCTTGTGATAATTTCTTGTCAAGATGAATACTCCATGATCTATTTTGTTCTCTTA
CATGCXXXXXXXXXXACCTCAAGTGGTCTTACCCAAGCAGTTGAAAGGGTTGTTCCGCGACCATTACAATTTTTA
GTGAGGTTTCTTGCGTGGTT
>Marker85295
CACCCTTTTATTTCTCTTTTATTCCTATGGTTCTGTTTATTTCTTCTTTAGAATCCCGGAAACTAAACATTTGAA
AAACCXXXXXXXXXXCCATTGAGAAAATCACAAATCCATCTATGATCTATCTAATATACAGAGTTTCTGTTTCTT
ACCAAAAGACCATAAATGTA
CACCCTTTTATTTCTCTTTTATTCCTGTGGTTCTGTTTATTTCTTCTTTAGAATCCCGGAAACTAAACATTTGAA
AAACCXXXXXXXXXXCCATTGAGAAAATCACAAATCCATCTATGATCTATCTAATATACAGAGTTTCTGTTTCTT
ACCAAAAGACCATAAATGTA
>Marker85526
GACCAAAAGTGCTCCAGCACCCAGGGATTGTGCAGCACCAGCAAACTACAAAAAGCTTGAAAATTAGTAAAGAAA
AGAACXXXXXXXXXXTATTGAATTTTTGTAAGATAATTTAAAGGAGATAGTATTCTATGGTCAGAACGTCATATA
CAGACATACATAAATAGGTA
GACCAAAAGTGCTCCAGCACCCAGGGATTGTGCAGCACCAGCAAACTACAAAAAGCTTGAAAATTAGTAAAGAAA
AGAACXXXXXXXXXXTATTGAATTTTTGTAAGATAATTTAAAGGAGATAGTATTCTATGGTCAGAACGTCATATA
CATACATACATAAATAGGTA
>Marker85695
AACCCAACCCAACCCAAACTAAAAAATCCGGGTTGGACAGGGTTAGGTTGGCGGGTTGGTCTTGTTTTTTTTCTT
CATTAXXXXXXXXXXAGACATCCATATTGAAAATTTTAAAAAAAAGATTGCTATAAGAGCCAACATGCAATAGAT
TCATAATGGATTTTTTGGTG
AACCCAACCCAACCCAAACTAAAAAATCCGGGTTGGACAGGGTTAGGTTGGCGGGTTGGTCTTGTTTTTTTTCTT
CATTAXXXXXXXXXXAGACATCCATATTGAAAATTTAAAAAAAAAAATTGCTATAAGAGCCAACATGCAATAGAT
TCATATTGGATTTTTTGGTG
>Marker85749
AACGACAGAGGAGATGAGATGAGGAATGTGAATGGCATAGAAGATGGGGAATCTAGGGTTGTAGAAGATGAAGAA
GTTGGXXXXXXXXXXCTCCTTACATTTCTCGTGTTATTTCATCTCCATTTTATTTAGACGGGGAAACGAGAAAAG
AAAAAAGAAGAAAGAAGGTA

Create PDF with GO2PDF for free, if you wish to

AACGACAGAGGAGATGAGATGAGGAATGTGAATGGCATAGAAGATGGGGAATCTAGGGTTGTAGAAGATGAAGAA
GTTGGXXXXXXXXXXXCTCCTTAGATTTCTCGTGTTATTTCGTCTCCATTTTATTTAGACGGGGAAACGAGAAAAG
AAAAAAGAAGAAAGAAGGTA
>Marker85899
AACCAGTTGTCAAGGCCATAATAAATGCAATGGTTGAATGTAATTTGCCGCCTGAAATGGTTACCAATCAGCAGA
TGTGCXXXXXXXXXXXGAATGCATTTTGTTGTTGGAAGACCAGATACACATTGTTCAAGATGGTCTTAAATCAAAT
TGTTTTCCTGGATTGAGGGT
AACCAGTTGTCAAGGCCATAATAAATGCAATGGTTGAATGTAATTTGCCGCCTGAAATGGTTACCAATCAGCAGA
TATGCXXXXXXXXXXXGAATGCATTTTGTTGTTGGAAGACCAGATACACATTGTTCAAGATGGTCTTAAATCAAAT
TGTTTTCCTGGATTGAGGGT
>Marker85989
TACAGGAATTTTGTAGTGACGGATCATAAATATCTCATTTCTTTTCAGAAAGGTTGGGAAGCGAAAGGCTGTCGT
TAAAGXXXXXXXXXXXTATTTGTTTGATTTTCTTAAGGAGAAAAATTCTCTTTTTTGTTTAAATTTTATATAACAG
CGAGGAGTTCAGAAAGGAGT
TACAGGAATTTTGTAGTGACGGATCATAAATATCTCATTTCTTTTCAGAAAGGTTGGGAAGCGAAAGGCTGTCGT
TAAAGXXXXXXXXXXXTATTTGTTTGATTTTCTTAAGGGGAAAAATTCTCTTTTTTGTTTAAATTTTATATAACAG
CGAGGAGTTCAGAAAGGAGT
>Marker86166
ACATCATTAATTTATGTTCATCAAAATACGTGACAAAGTTGTATTTTATGGAAAAAATTATGCTGAACTTGCTTG
GGACTXXXXXXXXXXXGGTTAAATTGTAAATTTGGTTTTATAGTTTGAAAAAAAAGCTAGAATTTAGTTTTTATGA
TTTATAAGTTGAATTTAGTC
ACATCATTAATTTATGTTCATCAAAATACGTGACAAAGTTGTATTTTATGGAAAAAATTATGCTGAACTTGCTTG
GGACTXXXXXXXXXXXGGTTAAATTGCAAATTTAGTTTTATAGTTTGAAAAAAAAGCTAGAATTTAGTTTCTATGA
TTTATAAGTTGAATTTAGTC
>Marker86388
TACAAGTTAGAATTCCAATCTACTACGTGGCGTCAAACTCATTTATGTAGCCATCTATTCAATAGGTAATAAGAA
GTTTCXXXXXXXXXXXAACCATGAAATAAGAAAATCCCAATAAAAATAGAGAAACAGAAACACTACTCTGTTATAA
TGATAAATTCCAAATGAGTT
TACAAGTTAGAATTCCAATCTACTACGTGGCGTCAAACTCATTTATGTAGCCATCTATTCAATAGGTAATAAGAA
GTTTCXXXXXXXXXXXAACCATGAAATAAGAAAATCCCAACAAAAATAGAGAAACAGAAACACTATTCTGTTATAA
TGATAAATTCCAAATGAGTT
>Marker86669
GACAAACAATTGATTATCCCTCAAAACCGCAAAACCATTCCCAAATGTTTCATATGCTCATCTATATCAGCACTA
TAGACXXXXXXXXXXXTGACTAAGAACTCATAGTGGCCTTCGTGTGTGCAAAACACTGTCTTCTCCACATCATCCT
CCTTCATCCTTATTTGTGGT
GACAAACAATTGATTATCCCTCAAAACCGCAAAACCATTCCCAAATGTTTCATATGCTCATCTATATCAGCACTA
TAGACXXXXXXXXXXXTGACTAAGAACTCATAGTGGCCTTCGTGTGTGCGAAACACTGTCTTCTCCACATCATCCT
CCTTCATCCTTATTTGTGGT
>Marker87684
CACCTTCACCGGAAGTGGTGGCATCTGCTTCATTACTTCCGGCATTTCATTTAAGCTACAAGCATGTAGATTGAA
GATAAXXXXXXXXXXXTCGGGTTTTAATGCTAAAAATCCTGACTGCTACTACGGGGAAGAATTCCTGCAACTAGGT
CGATTTTACCAAGAGCTAGT
CACCTTCACCGGAAGAGGTGGCATCTGCTTCATTACTTCCGGCATTTCATTTAAGCTACAAGCATGAAGATTGAA
GATAAXXXXXXXXXXXTCGGGTTTTAATGCTAAAAATCCTGACTGCTACTACGGGGAAGAACTCCTGCAACTAGGT
CGATTTTACCAAGAGCTAGT
>Marker87739

Create PDF with GO2PDF for free, if you wish to

TACCCATCAAGCTTTTTGATGTTATACATTTGGGAATGTTATGATGAATATCTGTTGATTCTATTTTGGCAGGTT
GCAGAXXXXXXXXXXXAATATAGCTTTGTTGGTTCATTCGACGACCTTGGACCTTACGGGAGAGGACGATATAGGG
ATGCCATGGCACCAGAGGTA
TACCCATCAAGCTTTTTGATGTTATACATTTAGGAATGTTATGATGAATATCTGTTGATTCTATTTTGGCAGGTT
GCAGAXXXXXXXXXXXAATATAGCTTTGTTGGTTCATTCGACGACCTTGGACCTTACGGGAGAGGACGATATAGGG
ATGCCATGGCACCAGAGGTA
>Marker87798
ACTAACTTTATATTTGATTGAAAACAAATAGCGATGTGACAAAAAGTAGTAGATGTGTTCAAATAACATGACATC
CCAAAXXXXXXXXXXXGATAGTTGAGGTATTTCTTTCTATTACCTAAATATGGTATTAGAACTCATATGGTTATTG
GGTCCAATCATGTTTGAGTA
ACTAACTTTATATTTGATTGAAAACAAATAGCGATGTGACAAAAAGTAGTAGATGTGTTCAAATAACATGACATC
CCAAAXXXXXXXXXXXGATAGTTGAGGTATTTCTTTCTATTACCTAAATATGGTATTAGAACTCATATGGTTATTG
GGTCCAATCATGTTTTAGTA
>Marker87842
ACTATCTTGTGCAAATTCAATGTCGAACAATTCTAGCATGTTTCCTACTACACAATCTCATCATTCGCGAGATGA
CAAATXXXXXXXXXXXGGGATGATTTGGCCGGAGAGATGTTCAATGAATGGCAGTTGCGTAACGAATAGGCTTTCG
TGTCGATTTTGTATTCCTGT
ACTATCCTGTGCAAATTCAATGTCGAACAATTCTAGCATGTTGCCTACTACACAATCTCATCATTCGCGAGATGA
CAAATXXXXXXXXXXXGGGATGATTTGGCCGGAGAGATGTTCAATGAATGGCAGTTGCGTAACGAATAGGCTTTCG
TGTCGATTTTGTATTCATGT
>Marker87930
AACCCAATCTAATTATTGAATGAAACATATCGTTTGGACCGGGATGTTGGCACTCTTCTAAAAAAAGGAGAGGTG
GAGACXXXXXXXXXXXTCAAGATAAGAGCGGTGATTTTAAAAGAAGAATATTGAAAAAGAGAGAAGGGAGAGGTAG
AAGATGGCTAGGTTGATGTT
AACCCAATCTAATTATTGAATGAAACATATCGTTTGGACCGGGATGTTGGCATTCTTCTAAAAAAAGGAGAGGTG
GAGACXXXXXXXXXXXTCAAGATAAGAGCGGTGATTTTAAAAGAAGAATATTGAAAAAGAGAGAAGGGAGAGGTAG
AAGATGGCTAGGTTGATGTT
>Marker88103
AACTTTTGACTTTGAAAAGTCAAGCTTTAATTGACTTTATATTCAAATGTGATTTGAATTTCAGAAAAATAAATG
TAAATXXXXXXXXXXXCTTAATTGGTCAAATGACCATGGACTAAAGTTAGTGGGTTTTGTTGGATAATTTCACTAA
CACTTAGTGGGACAAGTGTT
AACTTTTGACTTTGAAAAGTCAAGCTTTAACTGACTTTATATTCAAATGTGATTTGAATTTCAGAAAAATAAATG
TAAATXXXXXXXXXXXCTTAATTGGTCAAATGACCATGGACTAAAGTTAGTGGGTTTTGTTGGATAATTTCACTAA
CACTTAGTGGGACAAGTGTT
>Marker88855
TACATATAACCATTAACTTTAGAGTCTGGTGACCCTTTTCTAGAAAAAATGGATATTTTTATCAGCAAAATTAAA
CAGTGXXXXXXXXXXXTATAGGTAATGTAATCTGCATTAAAAGCATAAAACGGTGATTGGCATACATATCATTGTA
GCTATGGACAACCTTCTGTT
TACATATAACCATTAACTTTAGAGTCTGGTGACCCTTTTCTAGAAAAAATGGATACTTTTATCAGCAAAATTAAA
CAGTGXXXXXXXXXXXTATAGGTAATGTAATCTGCATTAAAAGCATAAAACGGTGATTGGCATACATATCATTGTA
GCTATGGACAACCTTCTGTT
>Marker88985
GACATTAAACAAGAGATTTAGAGGTTGAAAAATGTTTATAGGCTTAATTTTAAGAAACCAAAACAAACAACGAAA
TGGTTXXXXXXXXXXXATAATTAAGTGTATCAGCGATAGAAATCTGATAGGGCTCTTATGGCCTATCAATGATAGA
TTAATATTTACAATATGGTA

Create PDF with GO2PDF for free, if you wish to

GACATTAAACAAGAGATTTAGAGGTTGAAAAATGTTTATAGGCTTAATTTTAAGAAACCAAAACAAACAACGAGA
TGGTTXXXXXXXXXXXATAATTAAGTGTATCAACGATAGAAATCTGATAGGGCTCTTATGGCCTATCAATGATAGA
TTAATATTTACAATATGGTA
>Marker89444
TACATCCGGATAGACACCCTCTCCAAAAACGTTCTTACAAAGATCCAGATGATATCTGTCATTAGGCAAGCTATT
ATGAAXXXXXXXXXXXCTGGAAGAATACTTTGCATCCACTTTAGATGAACGCATGCTATCATTTGCTGGTGCAACC
TGGAAATATGCAACTTCAGT
TACATCCGGATAGACACCCTCTCCAAAAACGTTCTTACAAAGATCCAGATGATATCTGTCATTAGGCAAGCTATT
ATGAAXXXXXXXXXXXCCGGAAGAATACTTTGCATCCACTTTAGATGAACGCATGCTATCATTTGCTGGTGCAACC
TGGAAATATGCAACTTCAGT
>Marker89445
TACCCCCGCCGTTGTGCAGACAGACCTTCGACAACCCAAAGGACTGAGAATGGTCTCGGCCATGCAACTGAGAGA
GGGCCXXXXXXXXXXXCAGGATTCACTAGACCTGTAAGAGATCCGTATCAAGCACAAGACTTCTTACGGAAAGAAG
AGAATGGACGATTGTGTGTC
TACCCCCACCGTTGTGCAGACAGACCTTCGACAACCCAAAGGACTGAGAATGGTCTCGGCCATGCAACTGAGAGA
GGGCCXXXXXXXXXXXCAGGATTCACTAGACCTGTAAGAGATCCGTATCAAGCACAAGACTTCTTACGGAAAGAAG
AGAATGGACGGTTGTGTGTC
>Marker89573
TACAAATTCTAGATAAGTAGTGTAAATTAAAATCCTTACTAAGTAATCTAAACCCAAGGAAAAGAGGAACTCACA
GCTCGXXXXXXXXXXXGTCCATTTCAATGCAAATGATGAACACAGAGAAGCCACCATTTGAGCTCCAATATACAAT
GGCACCTGCAAACATATGTA
TACAAATTCTAGATAAGTAGTGTAAATTAAAATCCTTACCAAGTAATCTAAACCCAAGGAAAAGAGGAACTCACA
GCTCGXXXXXXXXXXXATCCATTTCAATGCAAATGATGAACACAGAGAAGCCACCATTTGAGCTCCAATATACAAT
GGCACCTGCAAACATATGTA
>Marker89858
CACAACGGCAAGCTCCTCCCCAAAAATCCAAAAAGACCAAACCACAAAGTCTAAATTGCCTGCTTCCTCCTACCA
AATCCXXXXXXXXXXXTCTAACTCTTGTCTCCAAATCAACCAACCAAACCAAACCATTGAGCCATGAGGGATCCAA
TAAGAAGATTTTCCAAGAGT
CACGACGGCAAGCTCCTCCCCAAAAATCCAAAAAGACCAAACCACAAAGTCTAAATTGCCTGCTTCCTCCTACCA
AATCCXXXXXXXXXXXTCTAACTCTTGTCTCCAAATCAACCAACCAAACCAAACCATTGAGCCATGAGGGATCCAA
TAAGAAGATTTTCCAAGAGT
>Marker89860
ACTTTACTACTACTAAGATTGTAAAAATAAAGTATATTGTTTGAGTTTGAGTTTCATAGATTCTATCTACATGGT
CATGAXXXXXXXXXXXGAATCAGCTTCAATTGTTAGAAGTTTAGCACTCTGTGTTATGAAAAACAATCAAAGAAGT
TGGGTGTTCAAAGTAAGAGT
ACTTTACTACTACTAAGATTGTAAAAATAAAGTATATTGTTTGAGTTTGAGTTTCATAGATTCTATCTACATGGT
CATGAXXXXXXXXXXXTAATCAGCTTCAATTGTTAGAAGTTTAGCACTCTGTGTTATGAAAAACAATCAAAGAAGT
TGGGTGTTCAAAGTAAGAGT
>Marker89886
AACACTTTGACTATTTCCATCAATTTCGAGCTTTTGAATATGAATCCATATTCATATTTTTAATATTTAAACCAC
ATTTAXXXXXXXXXXXATGGAGCTATAGATCATGAGCTTCAATGATTCAAAATTAACTGACTAAACTTTTTTAGAC
TAAGCAAATCAACATTCGTT
AACATTTTGACTATTTCCATCAATTTCGAGCTTTTGAATATGAATCCATATTCATATTTTTAATATTTAAACCAC
ATTTAXXXXXXXXXXXATGGAGCTATAGATCATGAGCTTCAATAATTCAAAATTAACTGACTAAACTTTTTTAAAC
TAAGCAAATCAACATTCGTT
>Marker90479

Create PDF with GO2PDF for free, if you wish to

ACCCGCCACGATAGTAACCTCCATCCAATCCGACCATGATAGCACACTAAATACACGACTCGATATATCTCACAC
ATGATXXXXXXXXXXCACAAGGAAACACATGGCCAAACGAAGCTCACGCATATTTAGCACCTACCATAGTAACAA
CATCCACCCACTCTTATGTG
ACCCGCCACGATAGTAACCTCCATCCAATCCGACCATGATAGCACACTAAATACACGACTCGATATATCTCACAC
ATGATXXXXXXXXXXCACAAGGAAACACATGGCCAAACGAAGCTCACGCATATTTAGCACCTACCATAGTAACAA
CATCCACCCACTCCTATGTG
>Marker90588
AACCCCACAGCTCTAGTTGAAGGATCAATAAATAATATCCGAGCATTCATCTATACAAGAAAAATATATATGTAT
ATACAXXXXXXXXXXAAATGTGCTTCAAACAGCAGCACATAAGAAAAACCATTAATTAAGTGGCAGAGGCAACAA
TTTAATCATCAAAGAAAGTA
AACCCCACAGCTCTAGTTGAAGGATCAATAAATAATATCCGAGCATTCATCTATACATGAAAAATATATATGTAT
ATACAXXXXXXXXXXAAATGTGCTTCAAACAGCAGCACATAAGAAAAACCATTAATTAAGTGGCAGAGGCAACAA
TTTAATCATCAAAGAAAGTA
>Marker90681
ACCCAAGGGACCAAATATAAAAATGACTAAAACCAATGTCTTCAACTTCACTTCTTCATAATTTGATTAGACAGG
TCGAAXXXXXXXXXXCAACATTCCATTTGACCTCAACCTTATCTCATACATGAACATTACAATAAAATCCTCTCC
AACAAAATTATAAATTCTGT
ACCCAAGGGACCAAATATAAAAATGACTAAAACCAATGTCTTCAACTTCACTTCTTCATAATTTGATTAGACAGG
TCGAAXXXXXXXXXXCAACATTCCATTTGACCTCAACCTTATCTCATACATGAACATTACAACAAAATCCTCTCC
AACAAAATTATAAATTCTGT
>Marker91594
GACTCTGTGAGACCCATTTCTAATTTGGAGGAAGTAAATTTAGCAAAGTATTAAGAAGTTGGCGTTGAATAAATC
CGCAAXXXXXXXXXXGGCATTTTGGGTAGTCTTCACAAGAAGAACCAAGTGTTTCATGGAGAAGAAGTCTGTGAG
GAAGCATTGGAGTTTAAGTT
GACTCTGTGAGACCCATTTCTAATTTGGAGGAAGTAAATTTAGCAAAGTATTAAGAAGTTGGGGTTGAATAAATC
CGCAGXXXXXXXXXXGGCATTTTGGGTAGTCTTCACAAGAAGAACCAAGTGTTTCATGGAGAAGAAGTCTGTGAG
GAAGCATTGGAGTTTAAGTT
>Marker91867
ACCAGTCCTTCTGTTCCATATTTGAATTCAATGGCTAAATCACAAGCCAAAGAACATGTTCATAACACAATCTAA
TAGTCXXXXXXXXXXAGAGAACTTTGGCATATAATACATTCATGGAATTATGCAAAAGAAGTATAAGTGGGTCCT
TGGATATTGTAAGCTATGTT
ACCAGTCCTTCTGTTCCATATTTGAATTCAATGGCTAAATCACAAGCCAAAGAACATGTTCATAACACAATCTAA
TAGTCXXXXXXXXXXAGAGAACTTTGGCATATCATACATTCATGGAATTATGCAAAAGAAGTATAAGTGGGTCCT
TGGATATTGTAAGCTATGTT
>Marker92600
AACCTTTCATCAATTACATCCATGATCCGACAATCTATGTTGTTTGTTTTGATAATTGATGAGAAACGAAAACAT
AAAAGXXXXXXXXXXAGGTAAGCTCAAAGGGAAGGGGCGACTTCTCCTCTCTTCGTCCATAAATCTCACAAGCTT
GTTAAGAAATGGGCGTTTGT
AACCTTTCATCAATTACATCCATGATCCAACAATCTATGTTGTTTGTTTTGATAATTGATGAGAAACGAAAACAT
AAAAGXXXXXXXXXXAGGTAAGCTCAAAGGGAAGGGGCGACTTCTCCTCTCTTCGTCCATAAATCTCACAAGCTT
GTTAAGAAATGGGCGTTTGT
>Marker92779
CACCATATAACAATCTAAATCTAGTGACAAATATGTATTCTAAAAACATCCTCTTTCATGACGTTGTTTTTCCTA
TGTTCXXXXXXXXXXAAGGAAGGAAGGGAAAAATTGACCATAGTAAGGAATAAATTGAAATAAAATAACCCACTG
TGCCTATATATAAATTTGTG

Create PDF with GO2PDF for free, if you wish to

CACCATATAACAATCTAAAACTAGTGACAAATATGTATTCTAAAAACATCCTCTTTCATGACGTTGTTTTTCCTA
TGTTCXXXXXXXXXXXAAGGAAGGAAGGGAAAAATTGACCATAGTAAGGAATAAATTGAAATAAAATAACCCACTG
TGCCTATATATATAAATTTGTG
>Marker93533
TACTTGTGTTAGTTATTATTACTTGTTTTTGCTATTCTTGTAGTTGTCATTTTACATCATGTCATATTTTTTTAA
TTATTXXXXXXXXXXXATTTGTAGGATTTATTGTCCATAGATAGGGTCTTCTCTTTTCAAACTTTCTTTTCATCCG
TAACAATTAAATGCTGAGTA
TACTTGTGTTAGTTATTATTACTTGTTTTTGCTATTCTTGTAGTTGTCATTTTACATTATGTTATATTTTTTTAA
TTATTXXXXXXXXXXXATTTGTAGGATTTATTGTCCATAGATAGGGTCTTCTCTTTTCAAACTTTCTTTTCATCCG
TAACAATTAAATGCTAAGTA
>Marker93674
AACTCTTTTCTTTCTTTTATTTATTTCTCGATATTAAATTTTCAATTTTACAAATGTATCAATAGAATATCAATA
TTGATXXXXXXXXXXXATTAAGACTTTATTTAAAGTATCCTAATTGAATTGCATTTTGAGAAACTTATTATTGGTG
TGTATCTATCTTGGTATGTT
AACTCTTTTCTTTCTTTTATTTATTTCTCGATATTAAATTTTCAATTTTACAAATGTATCAATAGAATATCAATA
TTGATXXXXXXXXXXXATTAAGACTTTATTTAAAGTATCCTAATTGAATTGCATTTTGAGAAACTTATTATTGGTG
TGTATCTATCTTGCTATGTT
>Marker93740
AACAGATTTACAAATAACTAAATATCCAAATCTCACTGTTTTTCGATTATACTATTTAATTATATCAATACTCCA
AACTCXXXXXXXXXXXCTTTTGCTTGATACTATGTTAAATCCACCCATCAACCCAAAAGCTTAAGTTTATAAGTTA
TAGTTAAGTTAATTATCAGT
AACAGATTTACAAATAACTAAATATCCAAATCTCACTGTTTTTCGATTATACTATTTAATTATATCAATACTCCA
AACTCXXXXXXXXXXXCTTTTGCTTGATACTATGTTAAATCCACCCATCAACCCAAAAGCTTAAGTTTATAAGTTA
TAGTTAAATTAATTATCAGT
>Marker93858
ACAATTACAGCATTGATATGTCGTATTTTGTCAATATTATCTAGCAGAAAGGAATCAGAAGGGAAAAACCCCTTA
TTTACXXXXXXXXXXXAACAAAAACAATACTGTCATTTCTTAGACCCATATACTATCTTATCTTTCTGGACGTGGG
TCAGAAGTTGCAAGTTTGTT
ACAATTACAGCATTGATACGTCGTATTTTGTCAATATTATCTAGCAGAAAGGAATCAGAAGGGAAAAACCCCTTA
TTTACXXXXXXXXXXXAACAAAAACAATACTGTCATTTCTTAGACCCATATACTATCTTATCTTTCTGGACGTGGG
TCAGAAGTTGCAAGTTTGTT
>Marker93885
ACATTCAGATCTATGTAGTCTTATGAAGGTTAAAGTAAGAGGAGAGTTTGAATATTTCATCACTTTTACTAATGC
TTACTXXXXXXXXXXXAGGTGGAGAGTATATGGATTTGAAATTTCAAGACTATTTGATGGAATGTGAAATTGTATC
TCAACTCTCAGCACCTGGTA
ACATTCAGATCTATGTAGTCTTATGAAGGTTAAAGTAAGAGGAGGGTTTGAATATTTCATCACTTTTACTAATGC
TTACTXXXXXXXXXXXAGGTGGAGAGTATATGAATTTGAAATTTCAAGACTATTTGATGGAATGTGAAATTGTATC
TCAACTCTCAGCACCTGGTA
>Marker93909
CACCATCAAATAGAATTTTTTCCTCCAAAAACATCTTTATGGTTCAGTAGGTTTGATGTGTATTAGATTTAATAGG
TAATCXXXXXXXXXXXAGTTTCTAACATGTATATTGGATTTGAAGAACTTTTTCTTGCATAATATATTGATGCATC
TGTTATAATGCTTACAGGTA
CACCATCAAATAGAATTTTTTCCTCCAAAAACATCCTTATGGTTCAGTAGGTTTGATGTGTATTAGATTTAATAGG
TAATCXXXXXXXXXXXAGTTTCTAACATGTATATTGGATTTGAAGAACTTTTTCTTGCATAATATATTGATGCATC
TGTTATAATGCTTACAGGTA
>Marker94212

Create PDF with GO2PDF for free, if you wish to

TACTAGCCTTACCCATCCTCATCCACGCTCTTTTTAGCGGGTAGTAGATATTGCCCTTCCACATTTTTGTATTTC
GAGCGXXXXXXXXXXTCAAGTTAAACTATGTTCGATTTAGTTCTAATATAATATTTCAATCTACAAATTTCACAT
AATTTAACAAAAAAAGAAGT
TACTAGCCTTACCCATCCTCATCCACACTCTTTTTAGCGGGTAGTAGATATTGCCCTTCCACATTTTTGTATTTC
GAGCGXXXXXXXXXXTCAAGTTAAACTATGTTCGATTTAGTTCTAATATAATATTTCAATCTACAAATTTCACAT
AATTTAACAAAAAAAGAAGT
>Marker94312
GACATCACCAAAAGATCCTCTTCCAATAAGCTCCAAAGAACTAAATCTTGCTGCCACTGCCTCTGCTATACTCGC
CACATXXXXXXXXXXCTCATCTCCAGATTTCCCTCATTTTTTGTAAGAAACAATCCAACCAGCAACCTCAAATCC
ACGACCAAAGTATTTATGTT
GACATCACCAAAAGATCCTCTTCCAATAAGCTCCAAAGAACTAAATCTTGCTGCCACTGCCTCTGCTATACTCGC
CACGTXXXXXXXXXXCTCATCTCCAGATTTCCCTCATTTTTTGTAAGAAACAATCCAACCAGCAACCTCAAATCC
ACGACCAAAGTATTTATGTT
>Marker94889
TACCTAATATTTCCTTGCCAACCCTAGATCTAGATATAAACAGAGTTCTCAAGTAGGAAGTCATTTCCAACAAGA
GGCTCXXXXXXXXXXTGTAGGAGTTGAAATTAATTGTTAGTCATTAGTTATTTGACAGTTAGTTAGTTTTACCTA
CTGGTTGGTTTCTTTTTGTG
TACCTAACATTTCCTTGCCAACCCTAGATCTAGATATAAACAGAGTTCTCAAGTAGGAAATCATTTCCAACAAGA
GGCTCXXXXXXXXXXTGTAGGAGTTGAAATTAATTGTTAGTCATTAGTTATTTGACAGTTAGTTAGTTTTACCTA
CTGGTTGGTTTCTTTTTGTG
>Marker94949
CACCTTTTATAATACAAATGATGAAAATGCGCTACGAGAACATCAAATTTCCTCTCTAATTAAGGAGAACAACCT
TTTGGXXXXXXXXXXTCTCTCGAAGAGAATCAAGTTTTGCAATTAAGCTCAATCTAAACACCATGAGTCTTATAA
CTAAAGAACCCTTATCAAGT
CACCTTTTAGGATACAAATGATGAAAATGCGCTACGAGAACATCAAATTTCCTCTCTAATTAAGGAGAACAACCT
TTTGGXXXXXXXXXXTCTCTCGAAGAGAATCAAGTTTTGCAATTAAGCTCAATCTAAACACCATGAGTCTTATAA
CTAAAGAACCCTTGTCAAGT
>Marker95464
GACACCATGGATTGAACCATGACCTCTTAATTAGTTATTGAGACTTTCTCCTATTTTTACTACTAGGCCAATCCA
TGATGXXXXXXXXXXTGATTGTGATGGTAATTTAGCCAGACGTCGTTTAGGAACAAATTGTCTATGGCATGGAAA
ATTGGAAATGAAGTGAAGTG
GACACCATGGATTGAACCATGACCTCTTAATTAGTTATTGAGACTTTCTCCTATTTTTACTACTAGGTCAATCAA
TGATGXXXXXXXXXXTGATTGTGATGGTAATTTAGCCAGACGTCGTTTAGGAACAAATTGTCTATGGCATGGAAA
ATTGGAAATGAAGTGAAGTG
>Marker95535
AACTTAAGAATGTCGAAAGCAAGAATATAAAGCATAAATCAACCCTATTGCTGATAGTAGTGATAGTAATGAGAA
TGAAGXXXXXXXXXXTGACGGGTTCTGATTACACAATGTGTTGCATGTGCCACGAATTTCATATAATCTTTTGTC
GATTAGCAAGATTACTCGTG
AACTTAAGAATGTCGAAAGCAAGAATATAAAGCATAAATCAATCCTATTGCTGATAGTAGTGATAGTAATGAGAA
TGAAGXXXXXXXXXXTGACGGGTTCTCATTACACAATGTGTTGCATGTGCCACGAATTTCATATAATCTTTTGTC
GATTAGCAAGATTACTCGTG
>Marker95620
GACCAAGTTTAAATTTGTTAGGATAAAAATTATAAATCTAATCTTACTTGCATACTTTTTAAAGTTAATAACATT
CATTAXXXXXXXXXXACTAACATAGTAATAAGTAGTGAATTGTGTTTAGATTTGAGGATTGATTAAACATATAAT
AGGGGTTTAATTAATAAAGT

Create PDF with GO2PDF for free, if you wish to

GACCAAGTTTAAATTTGTTAGGATAAAAATTATAAATCTAATCTTACTTGCATACTTTTTAAAGTTAATAACATT
CATTCXXXXXXXXXXACTAACATAGTAATAAGTAGTGAATTGTGTTTAGATTTGAGGATTGATTAAACATATAAT
AGGGGTTTAATTAATAAAGT
>Marker95765
ACTTCAAAAGTTATCATCTTTTGTATTATATTTTCTTTGAATGTCTTCAATTTTTTACGCTCATCTTTCTATATT
TCCATXXXXXXXXXXTAAAAATTGGTGTATTCCAAAAAAAAATGACTTCTAATAGGACTAATTATAAACACTACA
CAATTGTTTTCTATCAGGTT
ACTTCAAAAGTTATCATCTTTTGTATTATATTTTCTTTGAATCTCTTCAATTTTTTACGCTCATCTTTCTATATT
TCCATXXXXXXXXXXTAAAAATTGGTGTATTCCCAAAAAAAATGACTTCTAATAGGACTAATTATAAACACTATA
CAATTGTTTTCTATCAGGTT
>Marker96054
GACTCCATTGTCAATGTCGAATCATACCTTTTCCATAGTGTTTCAGCTTTCGTTACCACTCTCCATAACTTGGAG
CGTAAXXXXXXXXXXXTTCATGGAAAGCTGGTTTTCGGTGGAGTTTTGGGATTTAATTAAGTCACAGTTTGGTCA
GCTACCAAAAAAGACATGTG
GACTCCATTGTCAATGTCGAATCATACATTTTCCATAGTGTTTCAGCTTTCGTTACCACTCTCCATTACTTGGAG
CGTAAXXXXXXXXXXXTTCATGGAAAGATGGTTTTCGGTGGAGTTTTGGGATTTAATTAAGTCACAGTTTGGTCA
GCTACCAAAAAAGACATGTG
>Marker96287
AACTCCTGTGCGAGAATCGAGGCCAGGGGGAGTGACACGTGTCCCGAGAAGCCTCTCAACGACGCCGTTTTTGTA
GAGTCXXXXXXXXXXTGAAGAGGTTTGATTTGAAATTATCATATAATTGGTGAAGAGATATATGTGAAACTCTAT
CCTTTATAATTTCAAATGTT
AACTCCTGTGCGAGAATCGAGGCCAGGGGGAGTGACACGTGTCCCGAGAAGCCTCTCAACGACGCCGTTTTTGTA
GAGTCXXXXXXXXXXTGAAGAGGTTTGATTTGAAATTATCATATAATTGGTGAAGAGATATATGTCAAACTCTAT
CCTTTATAATTTCAAATGTT
>Marker96295
CACAACAGTTTATGCGCTGAAGTCAAAAGGATAGGAATGTGAAGCAGTTAAAATAATGGAAGTTAATAGCTGCAG
GAATCXXXXXXXXXXCCATGAGGTAGATAATATTTTACCAAATCTTATGATACAACCAATTCCATGAAAAAACTA
GGGCTGGTTTAGGCATTGTT
CACAACAGTTCATGCGCTGAAGTCAAAAGGATAGGAATATGAAGCAGTTAAAATAATGGAAGTTAATAGCTGCAG
GAATCXXXXXXXXXXCCATGAGGTAGATAATATTTTACCAAATCTTATGATACAACCAATTCCATGCAAAAACTA
GGGCTGGTTTAGGCATTGTT
>Marker96625
TACAAAATGGTTAGAGCATGTGTATAGATTATAGAAAGTTAAACGAGGTAACTGCTTGTTTTGGGTTTGTATGCA
CATGCXXXXXXXXXXCCAAATGCAAGCGAAGAGAGGTTTCCTGATAAATTCAGGGTTGAACACAAGAAATTAATT
TTCCTAATGATCTTATTGTG
TACAAAATGGTTAGAGCATGTGTATAGATTATAGAAAGTTAAACGAGGTAACTGCTTGTTTTGGGTTTGTATGCA
CATGCXXXXXXXXXXCCAAATGCAAGCAAAGAGAGGTTTCCTGATAAATTCAGGGTTGAACACAAGGAATTAATT
TTCCTAATGATCTTATCGTG
>Marker97106
ACTTGAGTTGATCTTTAGTCGAGAATATCCTTCAGAGTCTCATTCATCTGAAAAATCCAAATTAAATTCTTAATT
AATCTXXXXXXXXXXGATGTAGTGCTGACTAGACATAAATGATATGTGTGAAAGAATGTATTGGTAAGGTCGTCA
AAATCAAGCAATAATATGTT
ACTTGAGTTGATCTTTAGTCGAGAATATCCTTCAGAGTCTCATTCATCTGAAAAATCCAAATTAAATTCTTAATT
AATCTXXXXXXXXXXGATGTAGTGCTGACTAGACATAAATGATACGTGTGAAAGAATGTATTGGTAAGGTCGTCA
AAATCAAGCAATAATATGTT
>Marker97211

Create PDF with GO2PDF for free, if you wish to

ACTTTCTTCTATTTTTGCCCTTTTATTATTTTATATTTTTCCTCAAATGTGTTTGTCAATCTTTGAATTCAATAT
TATTGXXXXXXXXXXGATTTCTTTCCCTCAATGTTTGAATTCAAATGTGCCTTGTGTTGGGTATAATAACCTTGC
AAGGGAAGGTGAAAACTAGT
ACTTTATTCTATTTTTGCCTTTTTATTATTTTATATTTTTCCTCAAATGTGTTTGTCAATCTTTGAATTCAATAT
TATTGXXXXXXXXXXGATTTCTTTCCCTCAATGTTTGAATTCAAATGTGCCTTGTTTTGGGTATAATAACCTTGC
AAGGGAAGGTGAAAACTAGT
>Marker97342
ACTTGTTGGAGAGCTATGATTGACGTCGATCACTAATATAATAGAGTAATTGATAAATATCTGGAAGTCTATACA
AAACAXXXXXXXXXXGGTATGAAACTTTTTTTCTTTTGTTTTGGAGAAATAGAGAAAAGAAAAGACAGAAAGAAG
AGAAAGGGCCAGCGTTGGTT
ACTTGTTGGAGAGCTATGATTGACGTCGATCACTAATATAATAGAGTAATTGATAAATATCTGGAAGTCTATACA
AAACAXXXXXXXXXXGGTATGAAACTTTTTTTCTTTTGTTTTGGAGAAATAGAGAAAAGAAAAGATAGAAAGAAG
AGAAAGGGCCAGCGTTGGTT
>Marker97590
AACTTGTGAAAAAAATTTCAAGTTATCAAATTTATGAACACATCTATATCATAATAAAAAGATGTTTTCATAAAT
TAAAAXXXXXXXXXXAAGATATGTTCATTAATTATTTATAACATGTCATACATTTTACTTTAAGAAAAGAACAAT
AACATGGTGCATCTAGAGTG
AACTTGTGAAGAAAATATCAAGTTATCAAATTTATGAACACATCTATATCATAATAAAAAGATGTTTTCATAAAT
TAAAAXXXXXXXXXXAAGATATGTTCATTAATTATTTATAACATGTCATACATTTTACTTTAAGAAAAGAACAAT
AACATGGTGCATCTAGAGTG
>Marker97615
AACAAACTGTTAATCCAACTAGATCCGTGAATAATGATATAGAAATCATGAAAGCATGCATCTCTGAGATAGAAA
ATAAGXXXXXXXXXXTGATATTACTTTAGGAAGTGGAATAATTTTCAATGATAATGATGATCCAAAGTCTTCTAG
TGATCATGTTTCCTTGTGTG
AACAAACTGTTAATCCAATTAGATCCGTGAATAATGATATAGAAATCATGAAAGCATCCATCTCTGAGATAGAAA
ATAAGXXXXXXXXXXTGATATTACTTTAGGAAGTGGAATAATTTTCAATGATAATGATGATCCAAAGTCTTATAG
TGATCATGTTTCCTTGTGTG
>Marker97662
AACCAAATCATTTCAACTGTCAGTTGAGTTACAATTTATTAAAGAGTGGAAAAAAAATAAAAATAAAAAAGTGGT
CTAATXXXXXXXXXXGACCATATCCCCTTTTTTACTTTTCCTACTAATTTCAAAGAGGCCAAAAAGAGACTATGA
ATTGACTCACCACACCTGTA
AACCAAATCATTTCAACTGTCAGTTGAGTTACAATTTATTAAAGAGTGGAAAGAAAATAAAAATAAAAAAGTGGT
CTAATXXXXXXXXXXGACCATATCCCCTTTTTTACTTTTCCTACTAATTTCAAAGAGGCCAAAAAGAGACTATGA
ATTGACTCACCACACCTGTA
>Marker97712
ACTACGCCATGCAGTGCATCTCAGAATCCACCCACTTTTGGTTCTGAATTTGGAACTTTCAACCCTTCCACACTC
AACTTXXXXXXXXXXAGAAAAAAAAATTGTCGATTTTGCAGCTTTTTACTTCCATCTTTGTGAGTTAAGAATGGT
TGCTAGGTAGATGTCAAAGT
ACTACGCCATGCAGTGCATCTCAGAATCCACCCACTTTTGGTTCTGAATTTGGAACTTTCAACCCTTCCACACTC
AACTTXXXXXXXXXXAGAAAAAAAAATTGTCGATTTTGCAGCTTTTTACTTCCATCTTTGTGAGTTAAGAATGGT
TGCTAGGGAGATGCCAAAGT
>Marker97794
AACTTTTGATCACATTTATAATACATTTAGTCTGTTAATTGGATGTGTTTAAACCTATCATTTCTTGGGGTCGGA
TTATGXXXXXXXXXXTGATCACAGGTTGCTGGTATATGAGTTCATGCAAAAGGGCAGTGCAGAAAATCATCTTTT
CAGAAGTGAGTTTTCGAGTA

Create PDF with GO2PDF for free, if you wish to

AACTTTTGATCACATTTATAATACATTTAGTCTGTTAATTGGATGTGTTTGAACCTATCATTTCTTGGGGTCGGA
TTATGXXXXXXXXXXXTGATCACAGGTTGCTGGTATATGAGTTCATGCAAAAGGGCAGTGCAGAAAATCATCTTTT
CAGAAGTGAGTTTTCGAGTA
>Marker97819
ACAACCTATAGACCAGATCTTATTCAAGTAAGGTTGTGAGAAACAGAAACTGAAATTTAAACTAAAACAGTGTAA
TAAAAXXXXXXXXXXXCAATACATCTTCATCTTGAAGCTGAAGTTTGCAAATCATGTGAAGGACTTTACATCTTCT
AGAATCTCCTTTGTCGAGTT
ACAACCTATAGACCAGATCTTATTCAAGTAAGGTTGTGAGAAACAGAAACTGAAATTTAAACTAAAACAGTGTAA
TAAAAXXXXXXXXXXXCAATACATCTTCATCTTGAAGCTGAAGTTTGCAAATCATGTGAAGGACTCTACATCTTCT
AGAATCTCCTTTGTCGAGTT
>Marker97834
ACTGTTGATGCTGATACATTGTTGTTATCAGTAATCTTTAAAGACTGCACGTGATACTTTCAAAGCTTTTCAGAA
ATGACXXXXXXXXXXXTCAGCAGAACTGTCCTGTTAATTATGATGTTCATCATATAGCTAAAGCTCAGTTAGGAGA
TGAGAGTAGTTGAAAAGGTT
ACTGTTGATGCTGATACATTGTTGTTATCAGTAATCTTTAAAGACTGCACGTGATACTTTCAAAGCTTTTCAGAA
ATGACXXXXXXXXXXXTCAGCAGAACTTTCCTGTTAATTACGATGTTCATCATATAGCTAAAGCTCAGTTAGGAGA
TGAGAGTAGTTGAAAAGGTT
>Marker98294
ACTTAAAAAAACCTATGTATGATATTATGGATAGCAGGGAATGTAAATGTCCTTGTAGAACTTGCCTATGAGAAT
CTCTTXXXXXXXXXXXTTTTAAATTCTATCAAGATAAACGTTGATAGAAGCCTGTCAATATCTATCAACTATAAAC
TTTTGCAATTTTTTAGGAGT
ACTTAAAAAAACCTATGTATGATATTATGGATAGCAGGGAATGTAAATGTCCTTGTAGAACTTGCCTATGAGAAT
CTCTTXXXXXXXXXXXTTTTAAATTCTATCAAGATAAACGTTGATAGAAGCCTGTCAATATCTATCAACTATAAAC
TTTTGCAAGTTTTTAGGAGT
>Marker98438
ACAAATAGCGAAAAGATTTATGAAAATAAAATTTGTAAAATGCAATTTTTAAAGTGATGATCATTTAATAACTTA
CGATGXXXXXXXXXXXAAAATCGAATAACCTGTGACAGCTATTTAATAACCCTCTAATTGTTGTTTAATATATCTA
ATTGTCAAATTTATTTAGTG
ACAAATAGCGAAAAGATTTATGAAAATAAAATTTGTAAAATGCAATTTTTAAAGTGATGATCATTTAATAATTTA
CGATGXXXXXXXXXXXAAAATCGAATAACCTGTGACAGCTATTTAATAACCCTCTAATTGTTGTTTAATATATCTA
ATTGTCAAATTTATTTAGTG
>Marker99245
CACAGGATAAAAGAACAAACGCTTACATCAGATTTATTTTGCAGGGGAACGAGTGCTGCAACAAGTTCTTTAATG
CTTGGXXXXXXXXXXXGTCATAAAATGTTAATAAAATATTTGCTAGTGAAAGCATTTGGCTAGTTGCTAGTGACAC
TCATAAAATATTTGTAAAGT
CACAGGATAAAAGAACAAACGCTTACGTCAGATTTATTTTGCAGGGGAACGAGTGCTGCAACAAGTTCTTTAATG
CTTGGXXXXXXXXXXXGTCATAAAATGTTAATAAAATATTTGCTAGTGAAAGCATTTGGCTAGTTGCTAGTGACAC
TCATAAAATATTTGTAAAGT
>Marker99342
AACTTTTATTCAGTCCTAGCATTCAACCAATGGCCATTGTAAAAGGTATCTTTATTTGCTTTCTTCTCAAACTAA
TGCCGXXXXXXXXXXXCACCAAGAAATCCGACAAACAACATCTTCCCATGAGAAATTTAATGATGTTGGAAAAGAA
GTGCCGAGCTAAAAGTAGTG
AACTTTTATTCAGTCCTAGCATTCAACCAATGGCCATTGTAAAAGGCATCTTTATTTGCTTTCTTCTCAAACTAA
TGCCGXXXXXXXXXXXCACCAAGAAATCCGACAAACAACATCTTCCCATGAGAAATTTAATGATGTTGGAAAAGAA
GTGCCGAGCTAAAAGTAGTG
>Marker99802

Create PDF with GO2PDF for free, if you wish to

CACCTGCAGCCTCTAAAAGCACAGTAAGAACCATCAAAAGGTTGTTTTAAAGAAAATGTTCTTACCTAACTATGT
ATGATXXXXXXXXXXGATACTGTTTCACATCTGAAAAGAGAAATGAAAGGGCTCCTTATCGTTATCAAGTTAAGA
GATCCTTTTTTCGTTTTAGT
CACCTGCAGCCTCTAAAAGCACAGTAAGAACCATCAAAAGGTTTTTTTAAAGAAAATGTTCTTACCTAACTATGT
ATGATXXXXXXXXXXGATACTGTTTCACATCTGAAAAGAGAAATGAAAGGGCTCCTTATCGTTATCAAGTTAAGA
GATCCTTTTTTCGTTTTAGT
>Marker100438
ACTTAATTAGCTGACCAAGAAAACAGAAAAGAAAAGTGAATTGTCGCATATAAGGAAAAAAAGAAAATCGTCCAT
ATCCGXXXXXXXXXXAAAGCTACGGTTTCTGGGAAAGAGGATAGGTAGAGAGGTAAAATTTTTGCCGTCCAATAT
ACCGTTTCCAAAAGTAATGT
ACTTAATTAGCTGACCAAGAAAACAGAAAAGAAGAGTGAATTGTCGCATATAAGGAAAAAAAGAAAATCGTCCAT
ATCCGXXXXXXXXXXAAAGCTACGGTTTCTGGGAAAGAGGATAGGTAGAGAGGTAAAATTTTGGCCGTCGAATAT
ACCGTTTCCAAAAGTAATGT
>Marker100685
TACTGAAATCTTCTTTTTGATGCAACTTGTTGGTAGGGTGATGATGGGGTTAAAACCACAATTGAGCTTTTCATT
AAAAGXXXXXXXXXXGGTGGAGGGAAATATTGGGAAAAAAACCATGAAAAAGCCTTTTCCCTTCTTGTTCTTAAT
TGTGATGGACATTTTAAGTT
TACTGAAATCTTCTTTTTGATGCAACTTGTTGGTAGGGTGATGATGGGGTTAAAACCACAATTGAGCTTTTCATT
AAAAGXXXXXXXXXXGGTGGAGGGAAATATTGGAAAAAAAACCATGAAAAAGCCTTTTCCCTTCTTGTTCTTAAT
TGTGATGGACATTTTAAGTT
>Marker100836
CACCCTAATAAGCATACAAAATTACCAAAAAAAAAGAAGAAAAAAACTTAAAGAACCCCTATTGTGACAATATGC
AGAAGXXXXXXXXXXAGAATCCTCAAGAGATTCCTACAAACGCCTCCTTTATCATTCTTCATTAACTGTAGATGC
GAAATTTGCTAACTCTCTGT
CACCCTAATAAGCATACAAAATTACCAAAAAAAAAGAAGAAAAAAACTTAAAGAACCCCTATTGTGACAATATGC
GGAAGXXXXXXXXXXAGAATCCTCAAGAGATTCCTACAAACGCCTCCTTTATCATTCTTCATTAACTGTAGATGC
GAAATTTGCTAACTCTCTGT
>Marker101271
GACTAAGAATTAAAAATGATGTCAAGTTCATTCTTTGTGTTGATGTCAAATTTATGAGAATATAATTATACTAAT
AGAAAXXXXXXXXXXCAAGTATTAGATACTGTTTTTTTTAATTGTTAATTATTAAGAGTCAGTATGGAGACCTTG
ATCTCTCAAACTAAGTTGTG
GACTAAGAATTAAAAATGATGTCAAGTTCATTCTTTGTGTTGATGTCAAATTTATGAGAATATAATTATACTAAT
AGAAAXXXXXXXXXXCAAGTATTAGATACTGTTTTTTTTAATTGTTAATTATTAAGAGTCAGTGTGGAGACCTTG
ATCTCTCAAACTAAGTTGTG
>Marker101320
ACGAAGAAAGGTCAAGCAGTTGTCAGGAAAATCGCAACACCTTCATACGGCGATCATGTATGAAGAAACTTCGAT
GCGAAXXXXXXXXXXCTCTTGAAGCTAATGTATAAATCCATATATGCTAAAGAAACAAAAAATAATAATAATAAT
TAAAGGGGAACAACTTTGTG
ACGAAGAAAGGTCAAGCAGTAGTCAGGAAAATCGCAACACCTTCATACGGCGATCAAGTATGAAGAAACTTCGAT
GCGAAXXXXXXXXXXCTCTTGAAGCTAATGTATAAATCCATATATGCTAAAGAAACAAAAAATAATAATAATAAT
TAAAGGGGAACAACTTTGTG
>Marker101419
AACCTTTTTTCTGGGTCAAAGATTGCTATATTATTGGTGAATGTCTTAAGAAATATATAATGATTTTGGGGGATT
TTTTTXXXXXXXXXXTACTATTGCATCAGGAGAGATGGAGAATGCATGCATTGATGATGGTGATAGGATTGGAGA
ATTTTGATGGAGAGATTTGT

Create PDF with GO2PDF for free, if you wish to

AACCTTTTTTCTGGGTCAAAGATTGCTATATTATTGGTGAATGTCTTAAGAAATATATAATGATTTTGGGGGATT
TTTTCXXXXXXXXXXTACTATTGCATCAGGAGAGATGGAGAATGCATGCATTGATGATGGTGATAGGATTGGAGG
ATTTTGATGGAGATATTTGT
>Marker101500
AACATCGTTAGAATGATGCTCTAGCCCTCTAAAGATCTTATTGTTCCTCTTTTCTTAAAGCTCCTCGACACAGTA
GCACGXXXXXXXXXXCTATAATCCTTCTTGATTCTAGGAGATGAGATACCTATCCTGATTCCTCTGGATTCTATT
TCGGTGTGCAGTCTTCTGTG
AACATCGTTAGGATGATGCTCTAGCCCTCTAAAGATCTTATTGTTCCTCTTTTCTTAAAGCTCCTCGACACAGTA
GCACGXXXXXXXXXXCTATAATCCTTCTTGATTCTAGGAGATGAGATACCTATCCTGATTCCTCTGGATTCTATT
TCGGTGTGCAGTCTTCTGTG
>Marker101999
ACCGATCATCGATCGTAGCAGAAGAATCGTCTTAAAATTATTGTTGTTGCACTTTGGTGGAGCTAAGCGTCATAG
TAGGAXXXXXXXXXXCCCGTCATCCATAAAACAACCTCAGTTCTTCGTTCGGAGCTGCAGTGGAACCAAATGGAA
TACCAACAACTTTAAAGGGT
ACCGATCATCGATCGTAGCAGAAGAATCATCTTAAAATTATTGCTGTTGCACTTTGGTGGAGCTAAGCGTCATAG
TAGGAXXXXXXXXXXCCCGTCATCCATAAAACAACCTCAGTTCTTCGTTCGGAGCTGCAGTGGAACCAAATGGAA
TACCAACAACTTTAAAGGGT
>Marker102009
ACATCATTATCAATGATAATCATCCTTTCTTATTTACCAATCTTTATTAGATTCTTTGTTCATTGTGTTTTTTCC
CTTCCXXXXXXXXXXATTAAGCATCTCGTCCTATCTTCACATAAAAAAAAACTAGATCGAACAAACGTTCTAAGA
GTAGCTCATGCAAGAGAGTA
ACATCATTATCAATCATAATCATCCTTTCTTATTTACCAATCTTTATTAGATTCTTTGTTCATTGTGTTTTTTCC
CTTCCXXXXXXXXXXATTAAGCATCTCGTCCTATCTTCACATAAAAAAAAACTAGATCGAACAAACGTTCTAAGA
GTAGCTCATGCAAGAGAGTA
>Marker102614
ACTCCTGGTTTGTTCGGGGAACTCTGTCTTTTTCTTTTGGGTTCTGTTGTCATTCATCCAAATGGGAAACAACAG
TGGTGXXXXXXXXXXTGACTTTTGATGTGGTTTGGAGGATTAAGATATCTAAGAAAATTAGGTTCTTCACTTGGC
AAGTTTTTCTCGTTCAGGTG
ACTCCTGGTTTGATCGGGGAACTCTGTCTTTTTCTTTTGGGTTCTGTTGTCATTCATCCAAATGGGAAACAAAAG
AGGTGXXXXXXXXXXTGACTTTTGATGTGGTTTGGAGGATTAAGATATCTAAGAAAATTAGGTTCTTCACTTGGC
AAGTTTTTCTCGTTCAGGTG
>Marker103074
ACTCTTTCTTATCTCTTTAGTTTTATGAGTAGTGTATGAAATAATAGAAGATGTTAAAATACTACTTTGGTTCGT
ATATTXXXXXXXXXXACTACTTTACACCGCAAATACAACTTACAAATTGATAGGGTTTAATTTAGTGAGAGAAAG
AAAGTGAAAATGGATTGGGT
ACTCTTTTTTATCTCTTTAGTTTTATGAGTAGTGTATGAAATAATAGAAGATGTTAAAATACTACTTTGGTTCGT
ATATTXXXXXXXXXXACTACTTTACACCGCAAATACAACTTACAAATTGATAGGGTTTAATTTAGTGAGAGAAAG
AAAGTGAAAATGGATTGGGT
>Marker103169
ACCTAACAAAAAGCTTTTGATCATTTAGGACAAAAACATTTCAATGGCAGTTGATAATGGACTTAACATTAACAA
CTGAAXXXXXXXXXXTGTTCTTGTGAAGACTTTAGATTGTTTCTGGAGGGATCATCTCATAAACATGAATAGACT
CAGTTCTGCGGTATATAAGT
ACCTAACAAAAAGCTTTTGTTCATTTAGGACAAAAACATTTCAATGGCAGTTGATAATGGACTTAACATTAACAA
CTGAAXXXXXXXXXXTGTTCTTGTGAAGACTTTAGATTGTTTCTGGAGGGATCATCTCATAAACATGAATAGACT
CAGTTCTGCGGTATATAAGT
>Marker103351

Create PDF with GO2PDF for free, if you wish to

ACAAGAATATATTTACAATTTTTTTTGAATGAAAAGCTAAAGGAACACTTTCATGTTTAATTTATCATTAAACAT
ATGCTXXXXXXXXXXACCATTAAGTTAGTAGTATGAACACAAACACTACAGCACAGAAATTTCTCTACAATTTTT
TCTCAAACTTTCTCTTAGTT
ACAAGAATATATTTACAAATTTTTTTGAATGAAAAGCTAAAGGAACACTTTCATGTTTAATTTATCATTAAACAT
ATGCTXXXXXXXXXXACCATTAAGTTAGTAGTATGAACACAAACACTACAGCACAGAAATTTCTCTACAATTTTT
TCTCAAACTTTCTCTTAGTT
>Marker103747
ACTTCCTTGAAGCTGCAATTTTTCTTCTCTTTTGGTATTATGGTGCTTGGGATGAGTCTCATGAATGAAACACAT
ATACAXXXXXXXXXXXAATGGGAAGTTGAGCAAAACTGTAGAATAAAAGATTGTTGTTATTTCCAAAACACTCAAG
AAAAAGGTATGGCCAACGTT
ACTTCCTTGAAGCTGCAATTTTTCTTCTCTTTTGGTATTATGGTGCTTGGGATGAGTCTCATGAATGAAACACAT
ACACTXXXXXXXXXXXAATGGGAAGTTGAGCAAAACTGTAGAATAAAATATTGTTGTTATTTCCAAAACACTCAAG
AAAAAGGTATGGCCAACGTT
>Marker104898
ACCCCATCATGTCTATGATTTTGTCAGCAATTTAGAGGCTCTCTACTTTGACATAAACAGAAAATTGACAAGAAT
TTCCAXXXXXXXXXXXGAATGAAAAGATAATGAAGAGTCTGTCAAGATTAGTATTGAGGTTCTAACTATCAAATCA
TTAAATAGTGAATAATTGGT
ACCCCATCATGTCTATGATTTTGTCAGCAATTTAGAGGCTCTCTACTTTGACATAAACAGAAAATTCACAAGAAT
TTCCAXXXXXXXXXXXGAATGAAAAGATAATGAAGAGTCTGTCAAGATTAGTATTGAGGTTCTTACTATCAAATCA
TTAAATAGTGAATAATTGGT
>Marker104999
AACTAAATCGATGATCTGAATGGTTTCTTTCTTTTCTGGCGTGACATGATCTCCTAGAAGATCTAAGCGACCGAA
ACGAAXXXXXXXXXXXCCCCTCTGGGCAAGATAAATTAAATACATTGCAAGAATCTTCCAAAACATCTGTAGTTTC
TTGAACAGTATGTGAAGGTG
AACTAAATCGATGATCTGAATGGTTTCTTTCTTTTCTGGCGTGACATGATCTCCTAGAAGATCTAAGCGACCGAA
ACGAAXXXXXXXXXXXCCCCTCTGGGCGAGATAAATTAAATACATTGCAAGAATCTTCCAAAACATCTGTAGTTTC
TTGAACAGTATGTGAAGGTG
>Marker105396
ACATTAATTAATTTGTATAAAAAATATTATTATGTAGCATGGGAATGTGCAACTCGGAACCACTAAATATTCTCA
TAGATXXXXXXXXXXACGACCCACCTTGAAGTGAGTATGGAAGAACCTTTTATCCTTCATTTGACAAAACACAAT
TAAGTTAATGATCCAATGTA
ACATTAATTAATTTGTATAAAAAATATTATTATGTAGCATGGGAATGTGCAACTCGGAACCACTAAATATTCTCA
TAGATXXXXXXXXXXACGACCCACCTTGAAGTGAGTATGGAAGAACCTTTTATCCTTCATTTTACAAAACACAAT
TAAGTTAATGATCCAATGTA
>Marker105524
AACCGTAGGCTGAGCGAAGCGGTTCCCGACCTGCACCAAGCGAATTAGAGACCGCCGGTGTTATAACCGAAAAGA
AACAAXXXXXXXXXXXAAGAAGATGAGAACACGTGGCATGGTGACCAAGATAGGCTCATCGATCCAACGGGTGTTA
TAGGATGAAAATTAGGGGTC
AACCGTAGGCTGAGCGAAGCGGTTCCCGACCTGCACCAAGCGAATTAGAGACCGCCGGTGTTATAACCGAAAAGA
AACAAXXXXXXXXXXXAAGAAGATGACAACACGTGGCATGGTGACCAAGATAGGCTCATCGATCCAACGGGTGTTA
TAGGATGAAAATTAGGGGTC
>Marker105743
AACCAACTTTCTATTGTTTCACTTCACCCTTTGTCTCGTTTTATGTATCGTTCCTGGCATAGTTAAATCACGTAC
TGATTXXXXXXXXXXGACTCTACTCCTTTGTGCAAACAGAATTATTGTCTCGTCGTCCCCATAATAAAATAATTA
TCAAGTATATGAATCAAGTT

Create PDF with GO2PDF for free, if you wish to

AACCAACTTTCTATTGTTTCACTTCACCCTTTGTCTCGTTTTATGTATCGTTCCTGGCATAGTTAAATCACGTAC
TGATTXXXXXXXXXXGACTCTACTCCTTTGTGCAAACAGAATTATTGTCTCGTCGTCCCCATAATAAAATAATTA
TCAAGTATATGAATCGAGTT
>Marker105793
CACATTATATAGTGTATGTATTGGAAAAAAGTATGAGATTTTATGTATAATTTGAAATTGAAATTTTATGTGTAT
TGTATXXXXXXXXXXXTGAATTTGAATATGAATTTCTATTTAATTTCAAATCGAATTTGAATTTTTGAAAATGTTA
GATATTTAATTAAAAAGGTT
CACATTATATAGTCTATGTATTGGAAAAAAGTATGAGATTTTATGTATAATTTGAAATTGAAATTTTATGTGTAT
TGTATXXXXXXXXXXXTGAATTTGAATATGAATTTCTATTTAATTTCAAATTGAATTTGAATTTTGGAAAATGTTA
GATATTTAATTAAAAAGGTT
>Marker105850
AACCTCGGCACTAAGATCAACGTTAGGAGGTAATTTAAGAAGGTCAAATGTAAGATAAGTAGAATAAGTATAGAT
AACCTXXXXXXXXXXXACTGAGTGAAGAGAGGTTGCCTAATGTTCTATTAGTCACTTGTATTTGACCATCTGTTTG
AGGATGGCTGGCCGAATAGT
AACCTCGGCACTAAGATCAACGTTAGGAGGTAATTTAAGAAGGTCAAATGTAAGATAAGTAGAATAAGTATAGAT
AACCTXXXXXXXXXXXACTGAGTGAAGAGAGGTTGCCTAATGTTCTATTAATCACTTGTATTTGACCATCTGTTTG
AGGATGGCTGGTCGAATAGT
>Marker106267
TACATTCCTTATATATATATTACACGTTTATACCTTTTCAATCCATCATGTCTCTACTTTCCATCAGTTAATGTT
TTATTXXXXXXXXXXXACGCGTGCAAGGAAGAATTTTCTCTTCTTTTGCAAGATAACTTCCATCATTTGTATCCTG
AAAGGTGAGCAAGTGAGGGT
TACATTCCTTATATATATATTACATGTTTATACCTTTTCAATCCATCATGTCTCTACTTTCCATCAGTTAATGTT
TTATTXXXXXXXXXXXACGCGTGCAAGGAAGAATTTTCTCTTCTTTTGCAAGATAACTTCCATCATTTGTATCCTG
AAAGGTGAGCAAGTGAGGGT
>Marker106706
TACTCGCCCACAAACATCATAATCATCACATAGAAATCAATAGTTTTCTCTTTTCAAATAACATCAATATCAATG
CAACAXXXXXXXXXXXATAAAGGTCACTCATGAAAATACTTGATTCCCTTCTTCTCATAACGCCTAATTTCTTCAA
CAAGCAGCTCAACTCTTAGT
TACTCGCCCACAAACATCACAATCATCACATAGAAATCAATAGTTTTCTCTTTTCAAATAACATCAATATCAATG
CAACAXXXXXXXXXXXATAAAGGTCACTCATGAAAATACTTGATTCCCTTCTTCTCATAACGCCTAATTTCTTCAA
CAAGCAGCTCAACTCTTAGT
>Marker106789
AACAAATCAACTTAAAATTCTAAAGTCAATTACATGGGATTGTGGAAGGTCACACTATCCATTGCTTCGAAGCAG
AAGTAXXXXXXXXXXXACCTTCAAGGTGGCTGCTTATGTATGGGTCGGTGGTTGAGATGGTTGCTTGTCTTAGGCC
AATACAGACATACAGGTGTT
AACAAATCAACTTAAAGTTCTAAAGTCAATTACATGGGATTGTGGAAGGTCACACTATCCATTGCTTCGAAGCAG
AAGTAXXXXXXXXXXXACCTTCAAGGTGGCTGCTTATGTATGGGTCGGTGGTTGAGATGGTTGCTTGTCTTAGGCC
AATACAGACATACAGGTGTT
>Marker106971
CACACAAGAGTGAGATAATTGATTCAAGTGAAAAGTGTTTATCCCAACAAATTGTTCATGTAATCTCTATCATTT
AATTTXXXXXXXXXXAAAATATATGAATGCTACAAATACATTTTCTTTTTAAATGAAAATGTATGACATATTTCA
ATATGAAATTACTATATGTT
CACACAAGAGTGAGATAATTGATTCAAGTGAAAAGTGTTTATCCCAAAAAATTGTTCATGTAATCTCTATCATTT
AATTTXXXXXXXXXXAAAATATATGAATGCTACAAATACATTTTCTTTTTAAATGAAAATGTATGACATATTTCA
ATATGAAATTACCATATGTT
>Marker107254

Create PDF with GO2PDF for free, if you wish to

TACCTTTCTGAGACCTAGGCCTTTGATCCCTCATATCTGGTAGGCCAAGCATGGCTTCCAACTTGTTCATTAAAT
GGGGAXXXXXXXXXXCCATTCTGATTTGGTAACTGCTGAGGCAATAAATTGTTTAGGTGACTAGAATATTCTCCT
GGAAGCATGCCAGTTTGGTT
TACCTTTCTGAGACCTAGGCCTTTGATCCCTCATATCTGGTAGGCCAAGCATGGCTTCCAACTTGTTCATTAAAT
GGGGAXXXXXXXXXXCCATTCTGATTTGATAACTGCTGAGGCAATAAATTGTTTAGGTGACTAGAATATTCTCCT
GGAAGCATGCCAGTTTGGTT
>Marker107444
ACAATAAGATTTCTAACATTGATGAACAATAGAACAATTGAGACGCTCATACACACAAGAACTACATTTTATTTT
AAGTTXXXXXXXXXXGTAAAAAAGGAGTATTAATAATGATAACATATCTTTCCTTCCAGAAACTCCAAAAATTTC
CATTTTCAAAACACACAGTG
ACAATAAGATTTCTAACATTGATGAACAATAGAACAATTGAGACGCTCATACACACAAGAACTACATTTTATTTT
AAGTTXXXXXXXXXXGTAAAAAAGGAGTATTAATAATGATAACATATCTTTCCTTCCAGAAACTCCAAAAATTTT
CATTTTCAAAACACACAGTG
>Marker108079
CACTTTTAAGTAGAAGATCTTGGCACACTTAAGAAAAAAAGATTTTCATCTAAAACTTATGGAAAGCTAAATGAT
AATCAXXXXXXXXXXATATTACGCCCCAAATAATTTCAAGCTTGCTAACCCAATTTGAGGACGAGCCTAGTTTTA
GGGAAGTGGATTATGGTGTA
CACTTTTAAGTAGAAGATCTTGGCACACTTAAGAAAAAAAGATTTTCATCTAAAACTTATGGAAAGCTGAATGAT
AATCAXXXXXXXXXXATATTACGCCCCAAATAATTTCAAGCTTGCTAACCCAATTTGAGGACGAGCCTAGTTTTA
GGGAAGTGGATTATGGTGTA
>Marker108160
AACCCTCCGTGTGGGTGCCCTCTTTTTATAAAGATTAAACCCATCTAAACTCATAAACATTTATGATTATACTAA
ACCTTXXXXXXXXXXTGGTAACATTTTAAGACTGTTTGTTGATTATTGCTTTAGAGGAAAAAAATTGTTAATTTC
AAATCAAACATGAAATATGT
AACCCTCCTTGTGGGTGCCCTCTTTTTATAAAGATTAAACCCATCTAAACTCATAAACATTTATGATTATACTAA
ACCTTXXXXXXXXXXTGGTAACATTTTAAGACTGTTTGTTGATTATTGCTTTAGAGGAAAAAAATTGTTAATTTC
AAATCAAACATGAAATATGT
>Marker108661
CACCTTTCAAGACACAAATGATGGAAGTTATCTCGCAAATGTGGAGTTTGTCTCCAAACTCCTCCTTGCACGCAT
TAGTCXXXXXXXXXXATGAAAAGTCAAGAAATGATGGGTTGAAAAGGTATAAACATGTAATATATATATAAAGAA
AGTAGGCTTTAGGCCGTTGT
CACCTTTCAAGACACAAATGATGGAAGTTATCTCGCAAATGTGGAGTTTCTCTCCAAACTCCTCCTTGCACGCAT
TAGTCXXXXXXXXXXATGAAAAGTCAAGAAATGATGGGTTGAAAAGGTATAAACATGTAATATATATATAAAGAA
AGTAGGCTTTAGGCCGTTGT
>Marker108916
ACTGTTTAGTTGAAATTGATGATCTCCTGAAGAACAACATAATCTCTGGTAAAGAAAATTCTAATGTAGGCTTAA
ACAATXXXXXXXXXXGTCTAGATAGTGCTAAAAAGATTTGTCTCACAACATACTTGATGAACTTTTGCAATGAAC
AAAACGTATGTCAATTAGTG
ACTGTTTAGTTGAAATTGATGATCTCCTGAAGAACAACATAATCTCTGGTAAAGAAAATTCTAATATAGGCTTAA
ACAATXXXXXXXXXXGTCTAGATAGTGCTAAAAAGATTTGTCTCACAACATACTTGATGAAGTTTTGCAATGAAC
AAAACGTATGTCAATTAGTG
>Marker109212
TACGTTCATACGCCAAAGTGAGGATTGGATGTTCTAAAAACACAAAATCAATTGGAACCATCTGATAAGACTTTA
CAAGTXXXXXXXXXXTGAACACTCACATTACGCCTACTTCAGTCCTATGGCTTGAAGCCTCTTTAGTTCTTTTAT
GATGTAAGATTCTTCTTCGT

Create PDF with GO2PDF for free, if you wish to

TACGTTCATACGCCAAAGTGAGGATTGGATGTTCTAAAAACACAAAATCAATTGGAACGATCTAATAAGACTTTA
CAAGTXXXXXXXXXXCGAACACTCACATTACGCCTACTTCAGTCCTATGGCTTGAAGCCTCTTTAGTTCTTTTAT
GATGTAAGATTCTTCTTCGT
>Marker109282
TACAGTTATCACTCAGTTTCTGGTCTTTTTGGGCATATTTATTAATGTTTGGATCCTATTGAAATGGATATCATA
AGAATXXXXXXXXXXTGTGATTGTGGAAAGCAGAGAATTATTAGTTTATTACATTGAAGATGTCGCCTGCTGATT
CTTCACGGGAGGAAAATGTT
TACAGTTATCACTCAGTTTCTGGTCTTTTTGGGCATATTTATTAATGTTTGGATCCTATTGAAATGGATATCATA
AGAATXXXXXXXXXXTGTGAATGTGGAAAGCAGAGAATTATTAGTTTATTACATTGAAGATGTCGCCTGCTGATT
CTTCACGGGAGGAAAATGTT
>Marker109592
CACCAGAATTTAGTTAAGCAATTAGGTGGTGGGCGGAGATTCCTATCAAAACCTCTCTAGTTTTCATCGCCACCA
TTTCTXXXXXXXXXXTCGCGCTCCCTCTCTCACTGTCAGTTTCTTTTCCACTCTGATCTGCATTTCCTTTTGCTT
TATTCTCCGCATTTTTTGTT
CACCAGAATTTAGTTAAGCAATTAGGTGGTGGGCGGAGATTCCTATCAAAGCCTCTCTAGTTTTCATCGCCACCA
TTTCTXXXXXXXXXXTCGCGCTCCCTCTCTCACTGTCAGTTTCTTTTCCACTCTGATCTGCATTTCCTTTTGCTT
TATTCTCCGCATTTTTTGTT
>Marker110677
CACTCAAACATTGATAATTAGTATTAAGTCAATTAACATTTGCCTTTGCAATTCTTTTGAATAGATGAGTTGGTG
GAAATXXXXXXXXXXCACCAAAAACCTTGTTTAACTTTATGGCTTAAACAATTAATTGCATTAAAATGACAAATT
TGATTGAGTGATTTTGTGTG
CACTCAAACATTGATAATTAGTATTAAGTCAATTAACATTTGCCTTTGGAATTCTTTTGAATAGATGAGTTGGTG
GAAATXXXXXXXXXXCACCAAAAACCTTGTTTAACTTTACGGCTTAAACAATTAATTACATTAAAATGACAAATT
TGATTGAGTGATTTTGTGTG
>Marker110834
CACTTAAAGCTAGGGATCAAGGCTAAGATTGTAAAACATGATACAATGAATAGATTATGAAATCCCACATTGTAA
GATAAXXXXXXXXXXTGACACACTAAAATCCTTGGTTCTGAATATTAAATACGTCATTAGCTCCACTAGCCAGTT
GAATGAGTTACCAGTTCAGT
CACTTAAAGCTAGGGATCAAGGCTAAGATTGTAAAACATGATACAATGAATAGATTATGAAATCCCACATTGTAA
GATAAXXXXXXXXXXTGACACACTAAAATCCTTGGTTCTGAATATTAAATGCGTCATTAGCTCCACTAGCCAGTT
GAATGAGTTACCAGTTCAGT
>Marker110882
AACTGTAGATTCATTATCAGAAGTCAGAAGTATGGAGTTTTATGTGCCACGAGATGAAGCATTCTCCATATCAAA
ACAGGXXXXXXXXXXTTGTCCTAAACTTGTCCAAATCCTTACTGATAAGGCCATGGATATCTTACTCTTGCATTC
TTCGCAGACATCCTATGGTA
AACTGTAGATTCATTATCAGAAGTCAGAAGTATGGAGTTTTATGTGCCACGAGATGAAGCGTTCTCCATATCAAA
ACAGGXXXXXXXXXXTTGTCCTAAACTTGTTCAAATCCTTACTGATAAGGCCATGGATATCTTACTCTTGCATTC
TTCGCAGACATCCTATGGTA
>Marker111659
ACAAGATACTTAAGTAGTTCCCCGGCCTGGCTCTTTAGTGACATGATCGCTTGTTTCAATCTTGGGTTACAGCAT
GCCACXXXXXXXXXXACCAACTAATGAGGAAAACTAAACATAATTGGCAGAGTTAAGACAAGAGCATCATTCTCA
TTCACCACTCTATAAAGGTC
ACAAGATACTTAAGTAGTTCCCCGGCCTGGCTCTTTAGTGACATGATCGCTTGTTTCAATCTTGGGTTACAGCAT
GCCACXXXXXXXXXXACCAACTAATGAGGAAAACTAGACATAATTGGCAGAGTTAAGACAAGAGCATCATTCTCA
TTCACCACTCTATAAAGGTC
>Marker111776

Create PDF with GO2PDF for free, if you wish to

AACCAAACTCTAATATAATTCAACAAAATATAGTTTAAGTTTTGGAGTATGTGTGAAGGCTTACTACCATTTGTA
GGTATXXXXXXXXXXTTAGACGATAACTTCCAAATTTAATCCACGATCGAGCCTTTAATGTTTGTGTCAAATAAT
GTCACTCAATACAAATTGTC
AACCAAACTCTAATATAATTCAATAAAATATAGTTTAAGTTTTGGAGTATGTGTGAAGGCTTACTACGATTTGTA
GGTATXXXXXXXXXXTTAGACGATAACTTCCAAATTTAACCCACGATCGAGCCTTTAATGTTTGTGTCAAATAAT
GTCACTCAATACAAATTGTC
>Marker111975
CACAAAAATAATCTCAATTCTTATCCAATTATATTGACATAATTAAATTATCCATAAGTAGATAAATTGATCACA
TATAAXXXXXXXXXXTTTTGTTACCATAATATAATTATAAGACCGATGTTTGATACTAAAAAAACAAAGTTTTAA
TTAATATAATTTCCAAAGTG
CACAAAAATAATCTCAATTCTTATCCAATTATATTGACATAATTAAATTATCCATAAGTAGATAAATTGATCACA
TATAAXXXXXXXXXXTTTTGTTACCATAATATAATTATAAGACCGATGTTTGATACTAAAAAAAGAAAGTTTTAA
TTAATATAATTTCCAAAGTG
>Marker112112
CACTTCAATCAAGAAAACATTGACCTTCAAATCACCAAACGGTCATTCGTCTCTTGTATTAGTATGTGGCACATG
AAATTXXXXXXXXXXAAAGGACTCAGGTCAGCAATATTGAAAGTAGAACTTACCGAATATTCTCATGGAAAGTCA
ATTTTGTATTCATTGTTGTT
CACTTCAATCAAGAAAACATTGACCTTCAAATCACCAAACAGTCATTCGTCTCTTGTATTAGTATGTGGCACATG
AAATTXXXXXXXXXXAAAGGACTCAGGTCAGCAATATTGAAAGTAGAACTTACCGAATATTCTCATGGAAAGTCA
ATTTTGTATTCATTGTTGTT
>Marker112853
TACCTAGCTAATAATGTTATAACTGTCAAGTAAGATTTTCATGATATAGATGTTGATGCCATTGAAATTGAAAAA
GAAATXXXXXXXXXXGGAAATGATTTGCCAATTGCAAAAACTAGAAATTAATTCTATATTGAAAATTACATAGTT
TAAACAACAAAGATGACAGT
TACCTAGCTAATAATGTTACAACTGTCAAGTAAGATTTTCATGATATAGATGTTGATGCCATTGAAATTGAAAAA
GAAATXXXXXXXXXXGGAAATGATTTGCCAATTGCAAAAACTAGAAATTAATTCTATATTGAAAATTACATAGTT
TAAACAACAAAGATGACAGT
>Marker112948
AACTACTTAAACAAATTGGCAGTGTGAAATGAAACATAGAATTGCAGTGTTTTTCCATTTTCTTCAAATAAATTT
ACTAAXXXXXXXXXXAATGGAATGGAGATTGAAAAGAAAAAGAAAGGAAAAATCGAATTTGAACTGAAGGGGCCA
ATAAAAGAAGGAAAAAGGTT
AACTACTTAAACAAATTGGCAGTGTGAAATGAAACATAGAATTGCAGTATTTTTCCATTTTCTTCAAATAAATTT
ACTAAXXXXXXXXXXAATGGAATGGGGATTGAAAAGAAAAAGAAAGGAAAAATCGAATTTGAACTGAAGGGGCCA
ATAAAAGAAGGAAAAAGGTT
>Marker113318
GACTTAATTGTTTATAATAGCAATAATTGTATGAATATGAAGGTTCAATTATTATTTGTTAATAGTTAAAATCTA
TCTAAXXXXXXXXXXGAGAGTCCAATTAGCGTGAGTTAATAGGTCAAATGTATAAAGTTAATAGGTCAAGTGTAT
AGTTATGTTGTTGGAAAGTT
GACTTAATTGTTTATAATAGCAATAATTGTATGAATACGAAGGTTTAATTATTATTTGTTAATAGTTAAAATCTA
TCTAAXXXXXXXXXXGAGAGTCCAATTAGCGTGAGTTAATAGGTCAAATGTATAAAGTTAATAGGTCAAGTGTAT
AGTTATGTTGTTGGAAAGTT
>Marker113401
ACCTTGGGCTTAGATTGGGCCCAAATCTTTGATTTTGGGAATTTATTCTGGGCTCTCACGATCTGCACTAACAAG
TCGAAXXXXXXXXXXTTAAATCATCATTTCCTTCTCATTCCATGAGACTTGATCAACTTTTTCTCCATGCTTTCA
AATGTCCAAATGTGTATGTA

Create PDF with GO2PDF for free, if you wish to

ACCTTGGGCTTAGATTGGGCCCAAATCTTTGATTTAGGGAATTTATTCTGGGCTCTCACGATCTGCACTAACAAG
TCGAAXXXXXXXXXXTTAAATCATCATTTCCTTCTCATTCCATGAGACTTGATCAACTTTTTCTCCATGCTTTCA
AATGTCCAAATTTGTATGTA
>Marker113531
TACATATTTTCTCTTCACATTCTCATAATCACTTTGTTGTTTGGGATTCAATTCCCACTTATCTTGATTATTCCA
TGGAAXXXXXXXXXXCCTTGAACCAACATTCCTTGTGATGGGTATCTCCCCCCGATCGATGTTAGGTTTTTAAAT
ACCCTTATTGGAACTTGGTC
TACATATTTTCTCTTCACATTCTCATAATCACTTTGTTGTTTGGGATTCAATTCCCACTTATCTTGATTATTCCA
TGGAAXXXXXXXXXXCCTTGAACCAACATTCCTTGTGATGGATATCTCCCCCCGATCGATGTTAGGTTTTTAAAT
ACCCTTATTGGAACTTGGTC
>Marker113622
ACTTTGAATATATTTTCAAGTTTGAGATTAAGAATCCAATGTGATAAGTGGATACTATTTGATAGATTTGAAAGT
TTGTTXXXXXXXXXXACCATTTGTTCTTCTATGAAGACAGTCATATTTTAATTAGTTACATATGTTGTTGGCATA
AATATAGTTCAACCGTGGTA
ACTTTGAATATATTTTCAAGTTTGAGATTAAGAATCCAACGTGATAAGTGGATACTATTTGATAGATTTGAAAGT
TTGTTXXXXXXXXXXACCATTTGTTCTTCTATGAAGACAGTCATATTTTAATTAGTTACATATGTTGTTGGCATA
AATATAGTTCAACCGTGGTA
>Marker114255
ACTACCGTCTGAGCAAGACGTGTGGAACGAGAAACCGCAGCCACGGAAGGCGGCGAAGGGCACTTCACGGCGGAA
TCAACXXXXXXXXXXTTACAAATGAAGGGTTTATTAAATGCGACTGTTGCTCTAATCTGTTCCCCATCAGTAAAT
TTGAGGCTCATACAGGTAGT
ACTACCGTCTGAGCAAGACGTGAGGAACGAGAAACCGCAGCCACGGAAGTCGGCGAAGGGCACTTCACGGCGGAA
TCAACXXXXXXXXXXTTACAAATGAAGGGTTTATTAAATGCGACTGTTGCTCTAATCTGTTCCCCATCAGTAAAT
TTGAGGCTCATACAGGTAGT
>Marker114460
CACTTATGTATCTTGCTAACAATACTAGACCAGATATTGCATTTTTCAATAAATCTATTTGCTAGATGTAGTTCA
TCTCCXXXXXXXXXXTGATCCACACAAAAGTAGATCTCAGACAGGTTATCTATTAACATGTGGAGAAACCGCTAT
ATCTTGGCATCAATAAAGTA
CACTTATGTATCTTGCTAACAATACTAGACCCGATATTGCATTTTTCAATAAATCTATTTGCTAGATGTAGTTCA
TCTCCXXXXXXXXXXTGATCCACACAAAAGTAGATCTCAGACAGATTATCTATTAACATGTGGAGAAACCGCTAT
ATCTTGGCATCAATAAAGTA
>Marker115048
CACAAAGATCAAATCGTGAGTGCTACATTGTAATATATTCCACATGAATCCTATGTCAATCTTGATCATGTTTGC
CTCTTXXXXXXXXXXAGCAATACCATTATATCGGGTGTGTAAGGCGTCCAACTAATTTGTTAAAAAAACCATAAG
TAGGCAATAAAACGTAAGTA
CACAAAGATCAAATCGTGAGTGCTACATTGTAATATATTCCATATGAATCCTATGTCAATCTTGATCATGTTTGC
CTCTTXXXXXXXXXXAGCAATACCATTATATCGGGTGTGTAAGGCGTCCAACTAATTTGTTAAAAAAACCATAAG
TAGGCAATAAAACGTAAGTA
>Marker115526
AACAAGTTGGATTTGGACAGAATATTTACATTTCCCTTAGACCAAAGTGTGGAAATTGTAGTGTTAGTGACCTAT
GCTCAXXXXXXXXXXAGTCCTACATTTTCCCCAAGTTTTTGGTTTTCTATCTTTTTTATTTTGTTTTTGTTTTCT
TTTTTGGCACTATAATGGTT
AACAAGTTGGATTTGGACACAATATTTGCATTTCCCTTAGACCAAAGTGTGGAAATTGTAGTGTTAGTGACCTAT
GCTCAXXXXXXXXXXAGTCCTACATTTTCCCCAAGTTTTTGGTTTTCTATCTTTTTTATTTTGTTTTTGTTTTCT
TTTTTGGCACTATAATGGTT
>Marker115981

Create PDF with GO2PDF for free, if you wish to

AACAAGCAAGGGATTCTTGAGAATATGCTCGCCTTTGGATTTATGGAATGAAATCCCTCCTTGAAATGGTGTTGC
ATTCTXXXXXXXXXXTTATTTAATATAATTTGACACTATTTTCGTAAAACTGATCATCACGAACTTAATGTCATA
AATAATCTATGGTTAGGGTA
AACAAGCAAGGGATTCTTGAGAATATGCTCGCCTTTGGATTTATGGAATGAAATCCCTCCTTGAAATGGTGTTGC
ATTCTXXXXXXXXXXTTATTTAATAAAATTTGACACTATTTTCATAAAACTGATCATCACGAACTTAATGTCATA
AATAATCTATGGTTAGGGTA
>Marker116003
AACTATCTTACCTCAAAAGTTTAGCTTGAAATTCGAAGAATTGAGTTTTGTGGCCTCCAGAAACTCAACACTGCT
TAACAXXXXXXXXXXXTGAATCACTCAATTTTCACCAGAAAAATGAGAGAAATTTAATTCTAAAGTGAGCCTATTT
ATAGAGTTTTCTGCAAAGTG
AACTATCTTACCTCAAAAGTTTAGCTTGAAATTCGAAGAATTGAGTTTTGTGGCCTCCAGAAACTCAACACTGCT
TAACAXXXXXXXXXXXTGAATCACTCAATTTTCACAAGAAAAATGAGAGAAATTTAATTCTAAAGTGAGCCTATTT
ATAGAGTTTTCTGCAAAGTG
>Marker116100
TACCTTTACTGTTTTGAATTATTTGATTTGTTATTCCTATATCATATCTTCAATGACTCCAAGGTGTTTTCCTTT
CCTTTXXXXXXXXXXCACCTAGCTACAACTACGACTCCTACTTCTCGTCGCTCTGACTCTCTTAAACTTTCTAAA
GGAAGGCATGAACAAACGTC
TACCTTTACTGTTTTGAATTATTTGATTTGTTATTCCTATATCATATCTTCAATGACTCCAAGGTGTTTTCCTTT
CCTTTXXXXXXXXXXCACCTAGCTACAACTACGACTCCTACTTCTCGTCGCTCTAACTCTCTTAAACTTTCTAAA
GGAAGGCATGAACAAACGTC
>Marker116274
TACGGTCTGAACTATCGTTTGAATTACCGTTATACTTATTCCCAGGCATACGCTTACTTTAGAGCCTACCCCCTT
CAAGTXXXXXXXXXXGGGAAGATTTTAAGACGGCTCCCAACAAAATTAAAAATTGAGAGAATATTCATATAATTT
TTTTAAAGAAAAATGGGGTC
TACGGTCTGAACTATCGTTTGAATTACCGTTATACTTATTCCCAGGCATACGCTTACTTTAGAGCCTACCCCCTT
CAAGTXXXXXXXXXXGGGAAGATTTTAAGACGGCTCCCAACAAAATTAAAAATTGAGAGAATATTCATATAATTT
TTTTTAAGAAAAATGGGGTC
>Marker116475
AACATCGATTCAGAAGAAAGTCAATAATCCAATCTTACTACCAAAACTTATCTAGATCTGCTATCGTTCTTACCT
AATATXXXXXXXXXXCAGTAGCCTAACTAGATAACTTCCATTACATCCTGCACATTTCTTCATCAACACCTGATT
GATCTCTATTATATAATGTA
AACATCGATTCAGAAGAAAGTCAATAATCCAATCTTACTACCAAAACTTATCTAGATCTGCCATCGTTCTTACCT
AATATXXXXXXXXXXCAGTAGCCTAACTAGATAACTTCCATTACATCCTGCACATTTCTTCATCAACACCTGATT
GATCTCTATTATATAATGTA
>Marker116562
GACCCCAGTTCAATCTCAGCTGTTGATCCTAAACTCTATTCCAAGCGGTTCAGAGATTTTATTGGGAGAATATTT
ATTGAXXXXXXXXXXXGGAAGTAAGCTGCTGCACGGATGGGTGTAGCAAATGCTTCAATGTGGTCCAAAACTTCCA
TATCGCCATCCAGAGACGGT
GACCCCAGTTCAATCTCAGCTGTTGATCCTAAACTCTATTCCAAGCGGTTCAGAGATTTTATTGGGAGAATATTT
ATTGAXXXXXXXXXXXGGAAGTAAGCTGCTGCACGGATGGGTGTAGCAAATGCTTCAATGTGGTCCAAAACTTCCA
TATCGTCATCCAGAGACGGT
>Marker116923
ACCACCAATTCCTCTAATGAACAATAGTTATAGTCCCACTATCACTGAACTCTTCTCGGGCCAAGAGAAGGTGTG
GCACCXXXXXXXXXXGAATGGTAGGCCTATTGAGTCAGTGACAAAGGCCACTCTCACTAATGCAAATCAAAGGAC
AACCTTCATAGGCAGGAGTT

Create PDF with GO2PDF for free, if you wish to

ACCACCAATTCCTCTAATGAACAATAGTTATAGTCCCACTATAACTGAACTCTTCTCGGGCCAAGAGAAGGTGTG
GCACCXXXXXXXXXXGAATGGTAGGCCTATTGAGTCAGTGACAAAGGCCACTCTCACTAATGCAAATCAAAGGAC
AACCTTCATAGGCAGGAGTT
>Marker117010
ACTAAAATAATGAATTAAATTAACTTACAAACTTCGGCAAGGTAGAAAGAAGTCTTGTCCTTGTAAAATTCTGGA
GAGAAXXXXXXXXXXCACTTCTAGAACTACAATTTTTTCCATTGCTAAATTCAGATTAAAAAAAAGGATAAAGAT
AAAGATGGAGAGAGCAAGTA
ACTAAAATAATTAATTAAATTAACTTACAAACTTCGGCAAGGTAGAAAGAAGTCTTGTCCTTGTAAAATTCTGGA
GAGAAXXXXXXXXXXCACTTCTAGAACTACAATTTTTTCCATTGCTAAATTCAGATTAAAAAAAAGGATAAAGAT
AAAGATGGAGAGAGCAAGTA
>Marker117183
CACATAAAGAGAAAGGCAACTATATATAAAATGACTATATGGTGGAATAAACTCAGCTAGTGCATAAAAAATGTG
TATTGXXXXXXXXXXATGTGAAATGTATGTCTATGTGATATGTGTGTTTATGTAAAAGACGTGTTTAAGTGTTTT
TGATATGAACAAATGATGTT
CACATAAAGAGAAAGACAACTATATATAAAATGACTATATGGTGGAATAAACTCAGCTAGTGCATAAAAAATGTG
TATTGXXXXXXXXXXATGTGAAATGCATGTCTATGTGATATGTGTGTTTATGTAAAAGACGTGTTTAAGTGTTTT
TTATATGAACAAATGATGTT
>Marker118052
ACTCAAGCAAAAACAAGACTGACAATGACTAGAGAATCGGGAGGGACTAGAGCACCACAGAGTTCAAAATCAACG
ACGAAXXXXXXXXXXAATACAGCTGAAAATTAATATAAATAGAACGGATTATAAGCATAAATTTCACTCAAGTAA
TGAAATTTGGATGAATGGTT
ACTCGAGCAAAAACAAGACTGACAATGACTAGAGAATCGGGAGGGACTAGAGCACCACATAGTTCAAAATCAACG
ACGAAXXXXXXXXXXACTACAGCTGAAAATTAATATAAATAGAACGGATTATAAGCATAAATTTCACTCAAGTAA
TGAAATTTGGATGAATGGTT
>Marker118103
TACAAAGCTATTGTAATTAGAACAACAGGTTGGAGAATCAAAGATCTAACGTCGAGGTTGATAGTCTAAGCTATA
TAAAAXXXXXXXXXXGCATTCCAATGCTTGATTTTGTTTTAATAAAGTCGAAAGTCATACACATACATCCACACC
ATATCATTCATTTTAACGTC
TACAAAGCTATTGTAATTAGAACAACAGGTTGGAGAATCAAAGATCTAACGTCGAGGTTGATAGTCTAAGCTATA
TAAAAXXXXXXXXXXGCATTCCAATGCTAGATTTTGTTTTAATAAAGTCGAAAGTCATACACATACATCCACACC
ATATCATTCATTTTAACGTC
>Marker118476
ACCGTTTTTTCAACTCCGTTTTCTTTGCAAAATATGTCAAATAAAATAATAAGAAAAGTCAAGAGAGTGAATTAC
AATATXXXXXXXXXXGAAATGAAGGTTTTGGTCGTGCAGATTATTTCCTTGTGGGAAAGGACTTCCCCAGTTATA
TTGAATGTCAAGAGAAGGTC
ACCGTTTTTTCAACTCCGTTTTCTGTGCAAAATATGTCAAATAAAATAATAAGAAAAGTCAAGAGAGTGAATTAC
AATATXXXXXXXXXXGAAATGAAGGTTTTGGTCGTGCAGATTATTTCCTTGTGGGAAAGGACTTCCCCAGTTATA
TTGAATGTCAAGAGAAGGTC
>Marker118567
ACAAATATCGCTTTGCAGTTCAACTCATGAGTAAGCTTATACACATTAGATTATAGAAAATTTGGGGCACATACA
ACACAXXXXXXXXXXTGATTAGTGGCACCAGAGTCTAGAATCCAGAATTTATAGATTTTTTCCATCAACACCAAA
AAGACTGAAGGACTGAGGTA
ACAAATATCGCTTTGCAGTTCAACTCACGAGTAAGCTTATACACATTAGATTATAGAAAATTTGGGGCACATACA
ACACAXXXXXXXXXXTGATTAGTGGCACCAGAGTCTAGAATCCAGAATTTATAGATTTTTTCCATCAACACCAAA
AAGACTGAAGGACTGAGGTA
>Marker118751

Create PDF with GO2PDF for free, if you wish to

ACTCTCAGTTCTTAATAATAAAGAAGTTTTGTCTCCGTTTAAGAAAAAAAACAAAACCCATGATTGGGTAGATCG
ATATGXXXXXXXXXXATCCTTAGTGATGTTTGTCCTTTGTGAAGTTTATCCTTAGTCAAGTTTTGTTCTTTATGC
ATTTTTGTTCTGGATCCTGT
ACTCTCAGTTCTTAATAATAAAGAAATTTTGTCTCCGTTTAAGAAAAAAAACAAAACCCATGATTGGGTAGATCG
ATATGXXXXXXXXXXATCCTTAGTGATGTTTGTCCTTTGTGAAGTTTATCCTTAGTCAAGTTTTGTTCTTTATGC
ATTTTTGTTCTGGATCCTGT
>Marker119299
TACATTAATTTGAACACATGAGTAAGTATCATATTTGAAGATAAAGAACCATACATCAACAGTTCAATTAAGGGC
CACAAXXXXXXXXXXXACACACAAAAGATCAATAGCAGAAAACACCTTTACCTTTTCCAGAGCTAGAGCAAGCTTT
GAGAGATTGGGATAGATGGT
TACATTAATTTGAACACATGAGTAAGTATCATATTTGAAGATAAAGAACCATACATCAACAGTTCAATGAAGGGC
CACAAXXXXXXXXXXXACACACAAAAGATCAATAGCAGAAAACACCTTTACCTTTTCCAGAGCTAGAGCAAGCTTT
GAGAGATTGGGATAGATGGT
>Marker119551
CACAAAACTGGTCAAGCTTGTTGAACATGATAATCGAACTGGTTGGAAGATAAATTTGTAGATTTACCTCTGTCA
CATGAXXXXXXXXXXAAATGTGATCTCAATGCAAGCTTTCCTAATAAGAGAAATGAGAATGAGGTCCTAAAGTCC
TAAGAGAACTGACAATAAGT
CACAAAACTGGTCAAGCTTGTTGAACATGATAATCGGACTGGTTGGAAGATAAATTTGTAGATTTACCTCTGTCA
CATGAXXXXXXXXXXAAATGTGATCTCAATGCAAGCTTTCCTAATAAGAGAAATGAGAATGAGGTCCTAAAGTCC
TAAGAGAACTGACAATAAGT
>Marker119618
ACCTTTGAGAATGCATCACTCATGATTCCATGGCCTTGATTTTCTTTTTCCTTTTTTCTTTTTGATGAAAAGCAT
GACTTXXXXXXXXXXCATAAAACCCCTTAATTTTAGAAGCTTAAAAAAGAAAGCGGATTCAATGTCAAAAATGGC
CCTAAAATTCTTTCCGTGTG
ACCTTTGAGAATGTATCACTCATGATTCCATGGCCTTGATTTTCTTTTTCCTTTTTTCTTTTTGATGAAAAGCAT
GACTTXXXXXXXXXXCATAAAACCCCTTAATTTTAGAAGCTTAAAAAAGAAAGCGGATTCAATGTCGAAAATGGC
CCTAAAGTTCTTTCCGTGTG
>Marker119820
CACAACATAGCCAAAATCCAGCCAAGCCATCAATGCATTTCATCCAACTTTGTAGAAAATGATTCATTAAAAAGA
TATTAXXXXXXXXXXCTGTTAGCCATGTGCAAAAAAATATTAGATCTCAAATTGTAATCAGTAAAAGTTTGTTAA
ATAATTTGAACAATAGTGTT
CACAACATAGCCAAAATCCAGCCAAGCCATCAATGCATTTCATCCAACTTTGTAGGAAATGATTCATTAAAAAGA
TATTAXXXXXXXXXXCTGTTAGCCATGTGCAAAAAAATATTAGATCTCAAATTGTAATCAGTAAAAGTTTGTTAA
ATAATTTGAACAATAGTGTT
>Marker120304
CACCCACCTCTCTATATAGACATATCCCAAGAAGCTATATCTATAGTTTTTCTAAAATAAAGGGTTAATGAGCAT
CTAAGXXXXXXXXXXAAGGGGTTACCGAGCATTGTTTTTGTGCCTTAACTAAAAAACTGTCATAAAACAAAACTG
TAATGTGTTTTCAAAGGGTG
CACCCACCTCTCTATATAGACATATCCCAAGAAGCTATATCTATAGTTTTTCCAAAATAAAGGGTTAATGAGCAT
CTAAGXXXXXXXXXXAAGGGGTTACCGAGCATTGTTTTTGTGCCTTAACTAAAAAACTGTCATAAAACAAAACTG
TAATGTGTTTTCAAAGGGTG
>Marker120354
GACTTTGGGACAAACGTCATCCATGAGTTCTTTGATTAATGGAAAATGAGATTGGACATGACATCTTAAAGAACC
CAAGAXXXXXXXXXXTCTGGCTAGTGTTTGTGAAGAAAAAGATCATGCCTACTCGCCATGACAACAACATCTCCA
TGGAAAGGGTAATGTTGGTA

Create PDF with GO2PDF for free, if you wish to

GACTTTGGGACAAACGTCATCCATGAGTTCTTTGGTTAAAGGAAAATGAGATTGGACATGACATCTTAAAGAACC
CAAGAXXXXXXXXXXTCTGGCTAGTGTTTGTGAAGAAAAAGATCATGCCTACTCGCCATGACAGCAACATCTCCA
TGGAAAGGGTAATGTTGGTA
>Marker120517
ACTATAGGCCTTTTATTAAGGACACTTTCATCGGTTATATCTCATTCTTACCCAATTTTTGGTGTAATTGTTCCT
TTTCAXXXXXXXXXXCCAGTAATCAGCATATTGTAAATATCATGCCAGAGCTAAGGAGCAACAAGTGGCAATAAA
CAATGGGAACTACTAGAGTA
ACTATAGGCCTTTTATTAAGGACACTTTCATCGGTTATATCTCATTCTTACCCAATTTTTGGTGTAATTGTTCCT
TTTCAXXXXXXXXXXCCAGTAATCAGCATATTGTAAATATCTTGCCAGAGCTAAGGAGCAACAAGTGGCAATAAA
CAATGGGAACTACTAGAGTA
>Marker120713
ACCTTTATGAATAATCTCCAACAACTTAGTGTTACTTTTTTTAAATAGAAACAAGACCTTTTCATTGGATTAACG
AAATGXXXXXXXXXXAATCCTTACAACAAATGAAAATAACTAGTTAAACATTAAACACTAAGGGGTTGTTTGGCC
CGACTTCATTCGTCGATGTT
ACCTTTATGAATAATCTCCAACAACTTAGTGTTACTTTTTTTAAATAGAAACAATACCTTTTCATTGGATTAACG
AAATGXXXXXXXXXXAATCCTTACAACAAATGAAAATAACTAGTTAAACATTAAACACTAAGGGGTTGTTTGGCC
CGACTTCATTCGTCGATGTT
>Marker120766
CACAGTAAATGGTTTCCTAGTCACCATCACCCTTATATATTTATATCTACATTTTCTGCAGGACCAGAAACTGAA
TAATTXXXXXXXXXXTTGGGGGTCAATCTTCGTCCGATGAAGCACAGAAATCCTGAGGTAAGGTATTTTATTTTT
GGATATATGCTATCTTGGTG
CACAGTAAATAGTTTCCTAGTCACCATCACCCTTATATATTTATATCTACATTTTCTGCAGGACCAGAAACTGAA
TAATTXXXXXXXXXXTTGGGGGTCAATCTTCGTCCGATGAAGCACAGAAATCCTGAGGTAAGGTATTTTATTTTT
GGATATATGCTATCTTGGTG
>Marker120883
ACGAATAATGTGTGTGTGTGTGTATAACTATATATATAGTTTGTTGATGTGATGCTATTGGGCAGATTCATCAGC
GCTCAXXXXXXXXXXTTTTAATTTCCTCATGCTAGGTGATCTAAGATTATTATTGCATTATGAAGATGAATTTTT
CTCAAATTCCAATATTTGTA
ACGAATAATGTGTGTGTGTGTGTATAACTATATATATAGTTTGCTGATGTGATGCTATTGGGCAGATTCATCAGC
GCTCAXXXXXXXXXXTTTTAATTTCCTCATGCTAGGTGATCTAAGATTATTATTGCATTATGAAGATGAATTTTT
CTCAAATTCCAATATTTGTA
>Marker121640
ACAAAAGCTGAAAAGATTGTTTGGAAGGAAGAAAAGAAAAACCCTCCCCAAAAAAAGCGAAAATAATTAAAAAAG
GAGAAXXXXXXXXXXTTAGATGATTAAGAAACAATGCAACAGCTTTGATTTGGACGAGTGATTAAGAAGAGCATG
AGATGGATGAATTAATTGTA
ACAAAAGCTGAAAAGATTGTTTGGAAGGAAGAAAAGAAAAACCCTCCCCAAAAAAAGCGAAAATAATTAAAAAGG
AGAAAXXXXXXXXXXTTAGATGATTAAGAAACAATGCAACAGCTTTGATTTGGACGAGTGATTAAGAAGAGCATG
AGATGGATGAATTAATTGTA
>Marker121698
AACAAGAGCCTATCCAATCAAAGGGAGCGTTCCATCCAATTGAACAGTGAAGGCCTTGAGGTCAATTTCCTTATG
ATTTTXXXXXXXXXXAATGAAATTATTATTATATTCCACCATGAATATTGAAAGACTGGCTGGACATGCAAAATG
AAAATCACAAATAATGGGTC
AACAAGAGCCTATCCAATCAAAGGGAGCGTTCCATGCAATTGAACAGTGAAGGCCTTGAGGTCAATTTCCTTATG
ATTTTXXXXXXXXXXAATGAAATTATTATTATATTCCACCATGAATATTGAAAGACTGGCTGGACATGCAAAATG
AAAATCACAAATAATGGGTC
>Marker121748

Create PDF with GO2PDF for free, if you wish to

AACCCCAAATCTGAATTATTGCTTCAAAAGTTATATTTCAGCATGTCATTCTTGCTCAATTATACCCAGCATGAC
TTTTAXXXXXXXXXXXAGCTTATCGGGAACCATTCCAATGTCATTAACTAATGTTAAATCCTTGCAAGTCCTGTGA
GTGTTGTATTTATAAACGTT
AACCCCAAATCTGAATTATTGCTTCAAAAGTTATATTTCAGCATGTGATTCTTGCTCAATTATACCCAGCATGAC
TTTTAXXXXXXXXXXXAGCTTATCGGGAACCATTCCAATGTCATTAACTAATGTTAAATCCTTGCAAGTCCTGTGA
GTGTTGTATTTATAAACGTT
>Marker121801
ACTTTCCCTAACACTTGCAAGTTCTGCTGAATCATTCGAGCTTTCCTTTTCACATGTGTATGATTTATCTATACA
ATTATXXXXXXXXXXXGTATCATCCGACACTCCTCGATCAGGAACTTGTAACGTTCCCTGCATGAAAAAACTTAAC
AGTCATTTCATTTGATAGTG
ACTTTCCCTAACACTCGCAAGTTCTGCTGAATCATTCGAGCTTTCCTTTTCACATGTGTATGATTTATCTATACA
ATTATXXXXXXXXXXXGTATCATCCGACACTCCTCGATCAGGAACTTGTAACGTTCCCTGCATGAAAAAATTTAAC
AGTCATTTCATTTGATAGTG
>Marker122022
ACCAAGCAAGAACGTCCGCAGGGCGGGGTATATGATGATAAATGTTTTTCCCATTTTCTTTAATAGATAAAACAA
CTATAXXXXXXXXXXXAATATTAAGCAAAATATCTGCAATTTGTAATTTATTGAGTATAATGAAAATGAAGAATGC
TATCATAATTAATTTGAGTT
ACCAAGCAAGAACGTCCGCAGGGCGGGGTATATGATAATAAATGTTTTTCCTATTTTCTTTAATAGATAAAACAA
CTATAXXXXXXXXXXXAATATTAAGCAAAATATCTGCAATTTGTAATTTATTGAGTATAATGAAAATGAAGAATGC
TATCATAATTAATTTGAGTT
>Marker122945
ACATTTCTTCTACTTCTACCTCTCCCATCTCTCTTCCAAAATTCTTTCAATATTCATTTTCAATCCATTTAATTA
GTTTAXXXXXXXXXXXATCGCCTAACAAGTGAAAGCACGTATTTAACTAAACAAACGACGAAGAAATTGTAGCCTA
TATATATTTCTCCCTTTTGT
ACATTTCTTCTACTTCTACCTCTCCCATCTCTCTTCCAAAATTCTTTCAATATTCATTTTCAATCCATTTAATTA
GTTTAXXXXXXXXXXXATCGCCTAACAAATGAAAGCACGTATTTAACTAAACAAACGACGAAGAAATTGTAGCCTA
TATATATTTCTCCCTTTTGT
>Marker123090
CACATATCTCTCTACAGAAAACACAAAAGTGAAAACTTTAGCTTTGCTTATTATGGAGGGTAACGCGTGGAAGTG
AGTCAXXXXXXXXXXXAAAAAAAAAACCCTTTCCTCGAATATCCTAAACCACCCATTAGTTCCTATTTTCTTGTTC
TATCATCATTCATATTAGTC
CACATATCTCTCTACAGAAAACACAAAAGTGAAAACTTTAGCTTTGCTTATTATGGAGGGTAACGCGTGGAAGTG
AGTCAXXXXXXXXXXXAAAATAAAAACCCTTTCCTCGAATATCCTAAACCACCCATTAGTTCCTATTTTCTTGTTC
TATCATCATTCATATTAGTC
>Marker123378
ACTTGTAAATTGTTTCAAACTTGATTGGAAATGTTATAATTAAATCTATGGTTGTTCTTCTCTTCTGTTGTGTGT
CATTTXXXXXXXXXXXTGAGCAGTGAAGCCAAGATGGAGCAATGGATTGATATAGGACTTTTTGTATGGTTGGTAG
AAAAATCTTGATTCCAATGT
ACTTGTAAATTGTTTCAAACTTGATTGGAAATGTTATAATTAAATCTATGGTTGTTCTTCTCTTCTGTTGTGTGT
CATTTXXXXXXXXXXXTGAGCAGTGAAGCCAACATGGAGCAATGGATTGATATAGGACTTTTTGTATGGTTGGTAG
AAAAATCTTGATTCCAATGT
>Marker123715
TACTCCTCCTAATTCCAACGGTAAGAAATTAGCCGTTAATTTCCAGCCGCCCAGCTTCACTTCTACTGTTTCACA
GACTCXXXXXXXXXXXGAGATGAAATTGTGGGTAGCTCCACAATCGATCAGAATTATTACTTCCTTTTCTTTGATG
CTTCCTTTGACGTTCATAGT

Create PDF with GO2PDF for free, if you wish to

TACTCCTCCTAATTCTAACGGTAAGAAATTAGCCGTTAATTTCCAGCCGCCCAGCTTCACTTCTACTGTTTCACA
GACTCXXXXXXXXXXGAGATGAAATTGTGGGTAGCTCCACAATCGATCAAAATTATTACTTCCTTTTCTTTGATG
CTTCCTTTGACGTTCATAGT
>Marker123749
TACTATCTATGGATGCTTAGTTTAAATACCTATTCTTATGTGGTTGAGTTTTATCGTAGAGAAACTATGTCAGCA
ACATAXXXXXXXXXXCATCTATAGGTGAATTTAAAAGTTTATCAAGATGTTCTAATTGTAACCGTAAGGGTCACA
ATAGTAGGTCTTGCAGAGTT
TACTATCTATGGATGCTTAGTTTAAATACCTATTCTTATGTGGTTGAGTTTTATCGTAGAGAAACTATGTCAGCA
ACATAXXXXXXXXXXCATCTATAGGTGAATTTAAAAGTTTATCAAGATGTTCTAATTGTAACCGTAAGGGTCACA
ATAGTAGGACTTGCAGAGTT
>Marker123923
CACAACCTAGTTTAGCATGCACAAATTTCTATCATTAACTCTTTTAGCACAGAACATGAAAGGTCCAGTCAAGAG
TGGCAXXXXXXXXXXCCAAAGGTCTTGGGTTCAAGACTCATACAATTATTGTGCCCCCTACATATGATTTCTGGA
TACACCACTAAAGAGTGGTA
CACAACCTAGGTTAGCATGCACAAATTTATATCATTAACTCTTTTAGCACAGAACATGAAAGGTCCAGTCAAGAG
TGGCAXXXXXXXXXXCCAAAGGTCTTGGGTTCAAGACTCATACAATTATTGTGCCCCCTACATATGATTTCTGGA
TACACCACTAAAGAGTGGTA
>Marker123929
CACGATTCAGTTTGGTTCTATTTCAATCTAAAACCGCACCGAACCGCAAAATGTAAAAAGATCTTATACTAAACC
GAATCXXXXXXXXXXTTCGAACAATCTCGGACTAGCAAAGCACTTAGTTCTATAAAATTTCCCTTTTTATTTTAG
ATATCAGTGGATCTCGGGTC
CACGATTCAGTTTGGTTCTATTACAATCTAAAACCGCACCGAACCGCAAAATGTAAAAAGATCTTATACTAAACC
GAATCXXXXXXXXXXTTCGAACAATCTCGGACTAGCAAAGCACTTAGTTCTATAAAATTTCCCTTTTTATTTTAG
ATATCAGTGGATCTCGGGTC
>Marker124132
AACTGTTAAGAAAGAACTTTTCAACAGTTAAGCCGGGCTGTCAGCCTTGTTATATATGTGTTTAGGGTATTATTA
TTTGTXXXXXXXXXXTTTGGAAGTTAGTGGATAAAGTGAGGGGGGCTTCCTTTTTTGCTATATACCTGACCGGCC
CCACAATTTGATTGCACGTG
AACTGTTAAGAAAGAACTTTTCAACAGTTAAGCCGGGCTGTCAGCCTTGTTATATATGTGTTTAGGGTATTATTA
TTTGTXXXXXXXXXXTTTGGAAGTTAGTGGATAAAGTGAGGGGGGCTTCCTCTTTTGCTATATACCTGACCGGCC
CCACAATTTGATTGCACGTG
>Marker124221
ACATTCGGCAAGTTCCTTTATGACAACCTATTACGGCACCCTGCACATACATATAAGGATCCATTGATTTTTTTT
TTTTAXXXXXXXXXXTATCTGGAATGTTCCATATCCTCACCTTTGCATCTAATGCTCCACTTATGAAATAATCAT
CATCCATTGGGTTGAACTGT
ACATTCGGCAAGTTCCTTTATGACAACCTATTACGGCACCCTGCACATACATATAAGGATCCATTGATTTTTTTT
TTTTTXXXXXXXXXXTATCTGGAATGTTCCATATCCTCACCTTTGCATCTAATGCTCCACTTATGAAATAATCAT
CATCCATTGGGTTGAACTGT
>Marker124553
AACGCGCCATTGGATTTGAACTCAAATCAAATGCTCATTATGGCTACAAATCATTCCAAATCAAGGTTTGGATTC
TCTAAXXXXXXXXXXTGTAAGCAACTGTTAAGAGTAGAATACCCCAAATGCTATGAAATAGGATCAGTGCTGTTG
ATGAGGTTTAGAAAATAAGT
AACGCGCCATTGGATTTGAACTCAAATCAAATGCTCATTATGGCTACAAATCATTCCAAATCAAGGTTTGGATTC
TCTAAXXXXXXXXXXTGTAAGCAACTGTTAAGAGTAGAATACCCCAAATGCTATGAAATAGGATCAGTGCTATTG
ATGAGGTTTAGAAAATAAGT
>Marker124645

Create PDF with GO2PDF for free, if you wish to

CACTCAAACTGTTGAAGGAAGCTCAGAGTAGAAGTATCTGGGCTTAGTGGAATGTCAAGGGGTTTGGATCAGAA
GAAAAXXXXXXXXXXXAGTTATAGGAACATCAGATGGCTTACTATTCGTTCCGTTGGTATTGCAAGGTGAATTGTT
TCTTTCTGGAACGGGAAGTT
CACTCAAACTGTTGAAGGAAGCTCAGAGTAGAAGTATCTGGGCTTAGTGGAATGTCAAGGGGTTTGGATCAGAA
GAAAAXXXXXXXXXXXAGTTATAGGAACATCAGATGGCTTACTATTCGTTCTGTTGGTATTGCAAGGTGAATTGTT
TCTTTCTGGAACGGGAAGTT
>Marker124955
ACGAGACTATTCATGCTTGTAACTATGACTGTGTATTGTTCTAGAAGAAGTTTGAGGAGTTGCAACAATGTCCTA
CTTGTXXXXXXXXXXXATACATGTGATGACATAAGAATAAACGAGTGGAAACAGAGCATGTGTTGAGACATCCAAT
CGATGTAGAGGGATGAAAGT
ACGAGACTATTCATGCTTGTAACTATGACTGTGTATTGTTCTGGAAGAAGTTTGAGGAGTTGCAACAATGTCCTA
CTTGTXXXXXXXXXXXATACATGTGATGACATAAGAATAAACGAGTGGAAACAGAGCATGTGTTGAGACATCCAAT
CGATGTAGAGGGATGAAAGT
>Marker125079
TACAGCGCCGCGATTTTCGCCCTCTTCTATCACCACATCCTTTCCATTCTTCGAAGTTCCTCCATTTCATCTTCC
TTAATXXXXXXXXXXXCAGAGGATTCCGATTTCCCGGCGGTTGATGTGTTTATTTGCACCACGGATCCTGAAAAGG
AGCCACCTATGAGCGTGGTG
TACAGCGCCGCGATTTTAGCCCTCTTCTATCACCACATCCTTTCCATTCTTCGAACTTCCTCCATTTCATCTTCC
TTAATXXXXXXXXXXXCAGAGGATTCCGATTTCCCGGCGGTTGATGTGTTTATTTGCACCACGGATCCTGAAAAGG
AGCCACCCATGAGCGTGGTG
>Marker125099
GACACCAAAATAGAACAAAAATTTGATTTGAAGAGTAACAATTTAAAATTTTGATATGGAAAGACATTAATAAAA
TTTTAXXXXXXXXXXXTTTTAGTGGGCCTTTAGAATGAAAAGGAGAAGCCCATGATTCATGGGCACTACCTGGACC
AAAACTACCCAATTGTGGTA
GACACCAAAATAGAACAAAAATTTGATTTGAAGAGTAACAATTTAAAATTTTGATATGGAAAGACATTAATAAAA
TTTTAXXXXXXXXXXXTTTGAGTGGGCCTTTAGAATGAAAAGGAGAAGCCCATGATTCATGGGCACTACCTGGACC
AAAGCTACCCAATTGTGGTA
>Marker125459
CACTAAACAAAAATTCAGACTATTTCTTTTCCCAATTCATGTAAATTATAGTTAAACACCATTCTATCTTTAGAA
ATCAAXXXXXXXXXXXTCAATAGAGAGAGAAAGAGCCATCATCTACTAACAAAAGGATATTTATCAATTCATGAAT
AATGTAACTCAGAACACGTT
CACTAAACAAAAATTCAGACTATTTCTTTTCCCAATTCATGTAAATTATAGTTAAACACCATTCTATCTTTAGAA
ATCAAXXXXXXXXXXXTCAATAGAGAGAGAAAGAGCCATCATCTACTAACAAAAGGATATTTATCTATTCATGAAT
AATGTAACTCAGAACACGTT
>Marker126428
AACACTTACAAAACATTTAAAAACAACGAAAGTCTTTTATTAAAAATAAACATTTAAAATATACTTTGAAATCCG
AAGTGXXXXXXXXXXXAACAAGACAATGTCCTTGCATAATGAAAACTAAGTTTGCCTAGTAGTAGTAAGTTCAACA
TACGAAGATCCACAATCTGT
AACACTTACAAAACATTTAAAAACAACGAAAGTCTTTTATTAAAAATAAACATTTAAAATATACTTTGAAATCCG
AAGTGXXXXXXXXXXXAACAAGACAATGTCTTTGCATAATGAAAACTAAGTTTGCCTAGTAGTAGTAAGTTCAACA
TACGGAGATCCACAATCTGT
>Marker126685
ACCAAATTACTATTTTTATTTGATGTGCTTGCATTGACATTTACACATGTATTTTGGGTATAACATATAAGACTA
CTTTAXXXXXXXXXXXAGATGAGTTTTATCATATTTAATATTACCATTGTGGTTTAGGTTATGTTATATGGTTATA
CAAATCATAACAGAAACGGT

Create PDF with GO2PDF for free, if you wish to

ACCAAATTACTATTTTTATTTGATGTGCTTGCATTGACATTTACACATGTATTTTGGGTATAACATATAAGACTA
CTTTAXXXXXXXXXXXAGATGAGTTTTATCAAATTTAATATTACCATTGTGGTTTAGGTTATGTTATATGGTTATA
CAAATCATAACAGAAACGGT
>Marker126837
ACTTTTCCTCTTGGATTTGGTAAGAACGTTTCGTCAAGACCATCGGTTGTGAATATTGGAGCTATTTTATCTCAT
AATTCXXXXXXXXXXXGTGATCCTTTCCTTCTTTTCTTCTTTATTATTTTACCCCACTTGATTGCTTTTATTTTCT
GATGTTCTTTTGCGTGTGTT
ACTTTTCCTCTTGGATTTGGTAAGAACATTTCGTCAAGACCATCGGTTGTGAATATTGGAGCTATTTTATCTCAT
AATTCXXXXXXXXXXXGTGATCCTTTCCTTCTTTTCTTCTTTATTATTTTACCCCACTTGATTGCTTTTATTTTCT
GATGTTCTTTTGCGTGTGTT
>Marker126855
GACTACAAATATTTTGAACATGGAGCTTTTGTTTTGTGTATAAACTCTTGGCTCTTAATACAACTGTATTAGTTT
CTCTTXXXXXXXXXXXTGTTAAAATCAGTAACTTTTAGATTTATTGCAACACATGCTTGAATTTTATAACGTTTTC
TAATACATGAAATTGAGGTG
GACAACAAATATTTTGAACATGGAGCTTTTGTATTGTGTATAAACTCTTGGCTCTTAATACAACTGTATTAGTTT
CTCTTXXXXXXXXXXXTGTTAAAATCAGTAACTTTTAGATTTATTGCAACACATGCTTGAATTTTATAACGTTTTC
TAATACATGAAATTGAGGTG
>Marker126857
AACTTCATTATCTCAATTTAGTGTAATTTGGAAGTAATGTTCTTTCCGGCCAATCACTTAGAATTTTGTCACCAC
ACTCGXXXXXXXXXXXAGAGCTCCATAAAAGGGAAACACATCCCAATGGATCAAATTTGAGAATAATATAAATATG
GAAAGATATATTATGCAGTA
AACTTCATTATCTCAATTTAGTGTAATTTGGAAGTAATGTTCTTTCCGGCCAACCACTTAGAATTTTGTCACCAC
ACTCGXXXXXXXXXXXAGAGCTCCATAAAAGGGAAACACATCCCAATGGATCAAATTTGAGAATAATATAAATATG
GAAAGATATATTATGCAGTA
>Marker126948
TACTGCCACTAATGCTGATTATTCTTATTTTCTTACCATAATTGATGATGCCACTCGTTTCACATGTGTTTTTAT
GCTTAXXXXXXXXXXXCAATGCGTTCTGATAGTGCCCCAAAGTTAAAATTCATTGATTTCTTTAAGGAAAAAGGAG
TTCTACTTCAGTTTTCATGT
TACTGCCACTAATGCTGATTATTCTTATTTTCTTACCATAATTGATGATGCCACTCGTTTCACATGTGTTTTTAT
GCTTAXXXXXXXXXXXAAATGCGTTCTGATAGTGCCCCAAAGTTAAAATTCATTGATTTCTTTAAGGAAAAAGGAG
TTTTACTTCAGTTTTCATGT
>Marker127087
ACTAATCAAATTCAATGCTGGACAAACCCCTTGATTTTTTAAAAATTTATGGATGAATATGCATACTTTATATAA
ACACAXXXXXXXXXXXTATACGAAATTTGATGAAGCTATGAAGGTGAAAGAATAATAATAGATAGGAATAAGTAAT
TAGAGTGGCGGGCGGGTGTG
ACTAATCAAATTTAATTCTGGACAAACCCCTTGATTTTTTAAAAATTTATGGATGAATTTGCATACTTTATATAA
ACACAXXXXXXXXXXXTATACGAAATTTGATGAAGCTATGAAGGTGAAAGAATAATAATAGATAGGAATAAGTAAT
TAGAGTGGCGGGCGGGTGTG
>Marker127300
AACACAGGCAAGAAAATTGGTAATTAGGGAAATATCTGTCATCGACATTACAAGCATTTTTGCATACTAGCACAA
AGATGXXXXXXXXXXXGCAAAAGCCTAAGGAAAAAATGTCAAAAGAAATTTCACTCAGCTTCTCATACCTCCTAGG
CTTTCAGCACCAATTCTGTC
AACACAGGCAAGAAAATTGGTAATTAGGGAAATATCTGTCATCGACATTACAAGCATTTTTGCATACTAGCACAA
AGATGXXXXXXXXXXXGCAAAAGCCTACGGAAAAAATGTCAAAAGAAATTTCACTCAGCTTCTCATACCTCCGAGG
CTTTCAGCACCAATTCTGTC
>Marker127316

Create PDF with GO2PDF for free, if you wish to

ACTGAATTATTAAGATATAAGGAAGAGAATGGTTACAAAATTCCCATACCATTTGATACTATGAGACTCGGTGCC
TATATXXXXXXXXXXTTTGCAAATCCTCAGTTTTCCCATAGAATTAAATAATTTAAGCCTCTTATCATAGTGATT
GATTTGTATTTGTCAAAAGT
ACTGAATTATTAAGATATAAGGAAGAGAATGGTTACAAAATTCCCATACCATTTGATACTCTAAGACTCGGTGCC
TATATXXXXXXXXXXTTTGCAAATCCTCAGTTTTCCCATAGAATTAAATAATTTAAGCCTCTTATCATAGTGATT
GATTTGTATTTGTCAAAAGT
>Marker127404
ACATCAGTGCTATAACATAATTGATTCGGAATGAAATAGCCCACTTTTAGATGAAAAAAAGGGAAAAATTTCGTC
AGGAAXXXXXXXXXXTAAAGTTAGATCTAACATTCACTTTTTTTTTTAGTTATGAAGTTTATGTTTTTAGTGTTG
CTTTTAATTTGTAAGTTAGT
ACATCAGTGCTATAACATAATTGATTCGGAATGAAATAGCCCACTTTTAGATGAAAAAAAGGGAAAAATTTCGTC
AGGAAXXXXXXXXXXTAAAGTTAGATCTAACATTCACTTTTTTTCTTAGTTATGAAGTTTATGTTTTTAGTGTTG
CTTTTAATTTGTAAGTTAGT
>Marker127422
ACTCCTTTCAAGGCTAATTGTTTTGATTTATTTAAGATACCTCTAGTGTTCCACGATATGACTTTCATGTTTCGC
TTATTXXXXXXXXXXTCCAGATTATTTGCCTCGTATTCAGCCAACACGTCGTCTCTCTCCTCTAAATTAACAGGT
TCTAATTCTTCACTGCTGGT
ACTCCTTTCAAGGCTAATTGTTTTGATTTATTTAAGATACCTCTAGTGTTCCACGATATGACTTTCATGTTTCGC
TTATTXXXXXXXXXXTCCAGATTATTTGCCTCGAATTCAGCCAACACGTCGTCTCTCTCCTCTAAATTAACAGGT
TCTAATTCTTCACTGCTGGT
>Marker127794
ACTGCACACCATCATTAAGGTAGTTCTAAACAATTTCTCTACAAAGATCTATTTCCTACCAAATGAGAGAAAGTT
TGTTAXXXXXXXXXXATACAGCAATGGCTTGCCAATTTGTGGCTCTATATTGTTTATTAACATGACTTGAATTAG
AGAATCATCCCCTTTCTAGT
ACTTCACACCATCATTAAGGTAGTTCTAAACAATTTCTCTACAAAGATCTATTTCCTACCAAATGAGAGAAAGTT
TGTTAXXXXXXXXXXATACAGCAAGGGCTTGCCAATTTGTGGCTCTATATTGTTTATTAACATGACTTGAATTAG
AGAATCATCCCCTTTCTAGT
>Marker128013
ACCATGCCCAACACCAACCACACATAAAATCCAATCAAGAAAATGGAAGCTCAGTAGAAATTTTTTCACACTGAT
TTGTCXXXXXXXXXXTGCCAAAACCACTTTGCCAATAACTCCCTATTACAAGCCCTTTAAGTTCTGTAATTTCAA
CCCTCCTAGGTAAATGGGTT
ACCATGCCCAACACCAACCACACATAAAATCCAATCAAGAAAATGGAAGCTCAGTAGAAATTTTTTCACACTGAT
TTGTCXXXXXXXXXXTGCCAAAACCACTTCGCCAATAACTCCCTATTACAAGCCCTTTAAGTTCCGTAATTTCAA
CCTTCCTAGGTAAATGGGTT
>Marker128890
TACATTTCTACATAAAGTTCAAGTTTTACTCAAGATAGCCTCGAGACTTTAGTTTATTGGATTCAATATTATAGT
ATTCAXXXXXXXXXXTTTCGATTCCACGCAATATTCTTTGCACTGCACCGTGGGAGTGGAGTCAAGGACCTTTCA
ATATTTCATTCATATTTGTA
TACATTTCTACATAAAGTTCAAGTTTTACTCAAGATAGCCTCAAGACTTTAGTTTATTGGATTCAATATTATAGT
ATTCAXXXXXXXXXXTTTCGATTCCACGCAATATTCTTTGCACTGCACCGTGGGAGTGGAGTCAAGGACCTTTCA
ATATTTCATTCATATTTGTA
>Marker128991
CACGAAACTCTATGATAGAACCGAAATGTCATTCTTCGTTCTCATTCTTACTCCCCATTTCATTCTCCATGCTTG
TAAAAXXXXXXXXXXTACTAGAAACTAATGAAGTTTTGAATATTCTAAGTATGTAATGTGTATAAATGAAGGGGC
GCCGGTGGGGAGCTGGAGTT

Create PDF with GO2PDF for free, if you wish to

CACGAAACTCTATGATAGAATCGAAATGTCATTCTTCGTTCTCATTCTTACTCCCCATTTCGTTCTCCATGCTTG
TAAAAXXXXXXXXXXTACTAGAAACTAATGAAGTTTTGAATATTCTAAGTATGTAATGTGTATAAATGAAGGGGC
GCCGGTGGGGAGCTGGAGTT
>Marker129475
ACTTCAAGAAGCAAGCCGAAACCTCTATCAACCCTAACAACTTTTGCAGAATCATAGATATCACCAATTCTCAAG
TGCTAXXXXXXXXXXGACATATATAAAGAAATATGGAAACGTTTTGCATGGTTCAAGTTATCAACCACCCAATCT
TATACACCCAGCGCAAAGTG
ACTTCAAGAAGCAAGCCGAAACCTCTATCAACCCTAACAACTTTTGCAGAATCATAGATATCACCAATTCTCACG
TGCTAXXXXXXXXXXGACATATATAAAGAAATATGGAAACGTTTTGCATGGTTCAAGTTATCAACCACCCAATCT
TATACACCCAGCGCAAAGTG
>Marker129505
ACTCTTATTTGCGATGATTTATTCATCTTCTCTTCCCAATTTTCTTTAAGATCAATTGTTTAAATTTATTATTTC
ATCTCXXXXXXXXXXACTTACCATGACCAACGCGATCGTTTAAAGTATATTATTTTACTACATGATCATTTTATT
CGGACATAAACGATCGTGTA
ACTCTTATTTGCGATGATTTATTCATCTTCTCTTCCCAATTTTCTTAAAGATCAATTGTTTAAATTTATTATTTC
ATCTCXXXXXXXXXXACTTACCATGACCAACGCGATCGTTTAAAGTATATTATTTTACTACATGATCATTTTATT
CGGACATAAACGATCGTGTA
>Marker142079
AACGAGTGAAAAGGCTAAGAGAATAAGAAAGGAAACGAAAACTTGGCAATTTACAGAACTTCAGAAACATTAAAG
AGAGAXXXXXXXXXXGAATGATTGGAAGATGTCATTAGCAGTTGCATCTTTTTGCACAATAACACTGATAGTTTC
CTTCCTCTCCACTAGAAGGT
AACAAGTGAAAAGGCTAAGAGAATAAGAAAGGAAACGAAAACTTGGCAATTTACAGAACTTCAGAAACATTAAAG
AGAGAXXXXXXXXXXGAATGATTGGAAGATGTCATTAGCAGTTGCATCTTTTTGCACAATAACACTGATAGTTTC
CTTCCTCTCCACTAGAAGGT
>Marker142319
AACAATAAAGATGGCTGGGAGATAAATATAAACACGAGCCTACACAGACCTACATCAATTAGTTTAAATTTTACA
TGGTTXXXXXXXXXXCAGCTCTGCCCTTTCCCCTCCCACCCTCCATTATAAAAAATAAAAGGAAAAAGAATTTCC
ACTCTAGCAGTTGAGAAGTG
AACAATAAAGATGGCTGGGAGATAAATATAAACACGAGCCTACACAGACCTACATCAATTAGTTTAAATTTTACA
TGGTTXXXXXXXXXXCAGTTCTGCCCTTTCCCCTCCCACCCTCCATTATAAAAAATAAAAGGAAAAAGAATTTCC
ACTCTAGCAGTTGAGAAGTG
>Marker142747
TACCTTTCTAACCAATTAAAAATGTTCTAGGACTAACTTTCTAATACTATGTTAATTTGAGCCTAGTTGAATGAT
TGCCAXXXXXXXXXXCCACTTGAGTGCATGACCTGCAATGCATGCACACTATAAAAATGCTCATTCATATTTTCG
ACAGAGCCTTTATAAGTGTT
TACCTTTCTAACCAATTAAAAATGTTTTAGGACTAACTTTCTAATACTATGTTAATTTGAGCCTAGTTGAATGAT
TGCCAXXXXXXXXXXCCACTTGAGTGCATGACCTGCAATGCATGCACACTATAAAAATGCTCATTCATATTTTCG
ACAGAGCCTTTATAAGTGTT
>Marker143500
ACAAAGAAACTAAGTGTAGGAAATCTATCGAAATTTTCCAAAGGGACAAATACAAGCAAAATATAAAATAAACGT
TGGTAXXXXXXXXXXATCACTACAATAGATCAATCAGATAATAGCTTCAAACACACAAGGTCAGAGTCTGTAAAA
ATAAAAATATAAAATTAAGT
ACAAAGAAACTAAGTGTAGGAAATCTATCGAAATTTTCCAAAGGGACAAATACAAGCAAAATATAAAATAAACGT
TGGTAXXXXXXXXXXATCACTACAATAGATCGATCAGATAATAGCTTCAAACACACAAGGTCAGAGTCTGTAAAA
ATAAAAATATAAAATTAAGT
>Marker144100

Create PDF with GO2PDF for free, if you wish to

TACTACCTGTCAGCATATTGTAACTCAAGTCAAGATGCACTAATGACACCAACTCAAAAACCTCGTTCGGAATTG
CACCGXXXXXXXXXXTTGAGTGAAGCAATTGAAGAAGAAGAGAGAATGAAGGTGAGGGAGATCAAAGATGAATGA
AGGGAAAATAGCAGTAGGTT
TACTACCTGTCAGCATATTGTAACTCAAGTCAAGATGCACTAACGACACCAACTCAAAAACCTCGTTCGGAATTG
CACCGXXXXXXXXXXTTGAGTGAAGCAATTGAAGAAGAAGAGAGAATGAAGGTGAGGGAGATCAAAGATGAATGA
AGGGAAAATAGCAGTAGGTT
>Marker144284
CACATTTTATGGTGGCTGCTATGTTATCTTGTAACCATGTGTTTTTCATTTCTGACGTTGTTGCTTATTCTAGAC
TACAAXXXXXXXXXXXTTTGGCTTCAACAACATTATAAACTTCCAACGAGCATCGTGATAGGCTATAATTTAAACG
AAACAGTATTGTGGTTTGTA
CACATTTTATGGTGGCTGCTATGTTATCTTGTAACCATGTGTTTTTCATTTCTGACGTTGTTGCTTATTCTAGAC
TACAAXXXXXXXXXXXTTTGGCTTCAACAACATTATAAACTTCCAACAAGCATCGTGATAGGCTATAATTTAAACG
AAACAGTATTGTGGTTTGTA
>Marker144669
AACTTAATTGTGTTTAAGATTAAATTCTAAGGTATGCTTAAACTAATTTTAAGACTAAACAGTCACTTTTTTTTT
CCTTTXXXXXXXXXXATTGTTTAAAATTGAGATATGTCAATAATCAGCAGAGATTCTAAAGAAACAACATATTTT
TATCATTCATCCTTAATTGT
AACGTAATTGTGTTTAAGATTAAATTCTAAGGTATGCTTAAACTAATTTTAAGACTAAACAGTCACTTTTTTTTT
CCTTTXXXXXXXXXXATTGTTTAAAATTGAGATATGTCAATAATCAGCAGAGATTCTAAAGAAACAACATATTTT
TATCATTCATCCTTAATTGT
>Marker145011
CACAAATGGAGCTGAAGTTTCTTATAAATTTCCTATAGAAGGAAGCCAGCCCTAGGAAGACCTGTATTTCTTTGA
CAGTGXXXXXXXXXXTTGATGTAAAGCTCCTTTTCTGTCAATACTTGGAACAACTTTCTAAGGTGGAGCAAGTGT
TCATTGTTTCCACAGCTGTA
CACAAATGGAGCTGAAGTTTCTTATAAATTTCCTATAGAAGGAAGCCAGCCCTAGGAAGACCTGTATTTCTTTGA
CAGTGXXXXXXXXXXTTGATGTAAAGCTCCTTTTCTGTCAATACTTGGAACAGCTTTCTAAGGTGGAGCAAGTGT
TCATCGTTTCCACAGCTGTA
>Marker145082
ACCGACTCAGCTTTATCAGAATTAATTATAGTTCATGTTGTTCTGTCACATCCCAATTTGCCTATAAATAAAGGC
TTCTTXXXXXXXXXXTTAGATAAGAGTTTGAAAGAAAGAGAGAGGTCCTCAACCATTTTCTCAACGTAAAAGGCA
AAAACCAGAGCCAAACGAGT
ACCGACTCAGCTTTATCAGAATTAATTATAGTTCATGTTGTCCTGTCACATCCCAATTTGCCTATAAATAAAGGC
TTCTTXXXXXXXXXXTTAGATAAGAGTTTGAAAGAAAGAGAGTGGTCCTCAACCATTTTCTCAACGTAAAAGGCA
AAAACCAGAGCCAAACGAGT
>Marker145136
ACCATCCAAGTAAGTCTAATGTAGTAGCTGATGCTTTTAGCAGAAAAGTAACTCATCAACAGCTTTTATTATCAA
ATAGAXXXXXXXXXXXAGAAGCAGGACAAGTTGATGATTTCTCTATATTATTATTTAAGGATGGTCTCCCGTTTAA
GAGACGTTTGTATGCTAGTA
ACCATCCAAGTAAGTCTAATGTAGTAGCTGATGCTTTTAGCAGAAAAGTAACTCATCAGTAGCTTTTATTATCAA
ATAGAXXXXXXXXXXXAGAAGCATGACAAGTTGATGATTTCTCTATATTATTATTTAAGGATGGTCTCCCGTTTAA
GAGACGTTTGTATGCTAGTA
>Marker145156
TACATTTTCTTTGACATTTGAGGTTTTTGGTGGGTGAAATCCATGATCCAGCAACCTTCTTGGTAAAAGACACCG
CATTTXXXXXXXXXXCTAGTCAGACCTACAGTCAGTTAAATATAATATTCATTGAACAGAATCTAAACTATGTAA
TGCAAAATGTAAAAATAGTT

Create PDF with GO2PDF for free, if you wish to

TACATTTTCTTTGACATTTGAGGTTTTTGGTGGGTGAAATCCATGATCCAGCAACCTTCTTGGTAAAAGACACCG
CATTTXXXXXXXXXXCTAGTCAGACCTACGGTCAGTTAAATATAATATTCATTGAACAGAATCTAAACTATGAAA
TGCAAAATTTAAAAATAGTT
>Marker146044
ACCTCCTTATACCTTTTCAAATGGTGTTGGAACGGTTGCTGGAGAACATCCTTATGGAGAGCATCCTTCTAGAAC
GTTGTXXXXXXXXXXACGCAACCTGATCAGCAAATTCATACTTGAATGTTTCTTAAAATGCCACAATCTAGAGAT
TTATTTGCGTATGCAAGGTC
ACCTCCTTATACCTTTTCAAATGGTGTTGGAACGGTTGCTGGAGAACATCCTTATGGAGAGCATCCTTCTAGAAC
GTTGTXXXXXXXXXXACACAACCTGATCAGCAAATTCATACTTGAATGTTTCTTAAAATGCCACAATCTAGAGAT
TTATTTGTGTATGCAAGGTC
>Marker146191
ACATGAGAGCTGTGGCTGAGCCAATTCTTATAAGTCATTTTGGAGAAGCAATCATCGATGAATTGTTCATCAGAT
ATGGGXXXXXXXXXXTATGTGGTAATTATTGTGTGTCTTAGTATAATATAGTATATTTACTATATGTTAAGCTTA
GTTTAGTTGATAAAAATGTC
ACATGAGAGCTGTGGCTGAGCCAATTCTTATAAGTCATTTTGGAGAAGCAATCATCGATGAATTGTTCATCAGAT
ATGGGXXXXXXXXXXTATGTGGTAATTATTGTGTGTCTTAGTATAATATAGTATATTTACTATATGTTAAGCTTA
GTTTAGTTCATAAAAATGTC
>Marker146275
TACTTTCAATAATTATAAGTGTTTGTTTTGTTACTTGCTCGTATAAGAGTTATGGACAATTTTTATGGTAAGTTT
AATAAXXXXXXXXXXCTTAGAAATTAGAAGTGTATGTTTCATTGGATAATGGATACAAAAGTCTATCTTTTAACT
TGGAAGTGGATGGTTTAAGT
TACTTTCAATAATTATAAGTGTTTGTTTTGTTACTTGCTCATATAAGAGTTATGGACAATTTTTATGGTAAGTTT
AATAAXXXXXXXXXXCTTAGAAATTAGAAGTGTATGTTTCATTGGATAATGGATACAAAAGTCTATCTTTTAACT
TGGAAGTGGATGGTTTAAGT
>Marker146416
ACTCTTTATGCTGCTTGCTCTTTGTGGCGTAACATGAACATATAACAGCTCCTTAAAAGTTTTGCCTTCTTTCAC
CAATCXXXXXXXXXXCCATATTATTTTGGCATCTCTTTTGATTGAATCTCAGGATAGTTTTGAACATATGATGTA
GTTTTTCATCTTTCCCAGTT
ACTCTTTATGCTGCTTGCTCTTTGTGGCGTAACATGAACATATAACAGCTCCTTAAAAGTTTTGCCTTCTTTCAC
CAATCXXXXXXXXXXCCATATTATTTTGGCATCTCTTTCGATTGAATCTCAGGATAGTTTTGAACATATGATGTA
GTTTTTCATCTTTCCCAGTT
>Marker146512
TACGACAAGGCCATAGCACAACCAAGTAGGGTGGCATGCCCTCAGGCTACACGCGTGACTTTCCTAGAAGTGGTT
AGCGTXXXXXXXXXXCTTGAACAACCGAGACTTAGTGTTAGTAAATGGTGTATGAAAATTATATTCAGTCTAGGC
GACATATCACTAATCATGTT
TACGACAAGGCCATAGCACAACCAAGTAGGGTGGCATGCCCTCAGGCTACATGCGTGACTTTCCTAGAAGTGGTT
AGCGTXXXXXXXXXXCTTGAACAACCGAGACTTAGTGTTAGTAAATGGTGTATGAAAATTATATTCAGTCTAGGC
GACATATCACTAATCATGTT
>Marker146689
ACATTACTTCTCTCTATCTAAAGTGTTCTATAATGGTTTTAAGGGTATTTAGCTACTTTAGGATTTTATTCATGC
TAACTXXXXXXXXXXACACATGTTCATAAAGCTAGGTTAAATTAGGCTCAAGAACTAATTACGATCTTCAGGTAG
AAGGTGTTCCGTAAATCGTC
ACATTACTTCTCTCTATCTAAAGTGTTCTATAATGGTTTTAAGAGTATTTAGCTACTTTAGGATTTTATTCATGC
TAACTXXXXXXXXXXACACATGTTCATAAAGCTAGGTTAAATTAGGCTCAAGAACTAATTACGATCTTCAGGTAG
AAGGTGTTCCGTAAATCGTC
>Marker146779

Create PDF with GO2PDF for free, if you wish to

AACATCGATTCTCTGCAATTCTTCAGTGGTCGGCGCCGGAGAAAGCGCCCCCGATCTGCCGCCAGTGGCTCCGCC
GTCGCXXXXXXXXXXAGAAAATGAAGGGTTTCAAACGAAGATTGAGGCGAGGTTGGAAGATAAGGAGTGAAGAAG
GCTTCGCAGCCATGGTGGTC
AACATCGATTCTCTGCAATTCTTCAGTGGTCGGCGCCGGAGAAAGCGCCCCCGATCTGCCGCCAGTGGTTCCGCC
GTCGCXXXXXXXXXXAGAAAATGAAGGGTTTCAAACGAAGATTGAGGCTAGGTTGGAAGATAAGGAGTGAAGAAG
GCTTCGCAGCCATGGTGGTC
>Marker146807
GACACCAATGGGGCAGGTATATGGCCTTCTTCTCTCTACTTTTAATTGGTAAATGCATGGAGATACTGTGTTACT
TTTTTXXXXXXXXXXAACGTTTATTGGAATATTTTTGCATCCCCCATTTATCATATCATTTGTAGAGTTTAGACC
ATGAGGGCACACGTAGTTGT
GACACCAATGGGGCAGGTATATGGCCTTCTTCTCTCTACTTTTAATTGGTAAATGCATGGAGATACTGTGTTAGT
TTTTTXXXXXXXXXXAACGTTTATTGGAATATTTTTGCATCCCCCATTTATCATATCATTTGTAGAGTTTAGACC
ATGAGGGCACACGTAGTTGT
>Marker146957
GACAAGTCTATAGCTCATAGGCCATTAGGAAGGGTATATATTTATGCCTTAAAGAAGGAGAATTACAATGAAAGT
TGTTTXXXXXXXXXXACTGTTTCTTGATAAATGATATTCAAGCTTCAGTTTTATGTTAAGCTATTTACAAAAACT
TGTTTTATAAAATTGTTGTT
GACAAGTCTATAGCTCATAGGCCATTAGGAAGGGTATATATTTATGCCTTAAAGAAGGAGAATTACAATGAAAGT
TGTTTXXXXXXXXXXACTGTTTATTGATAAATGATATTCAAGCTTCAGTTTTATGTTAAGCTATTTACAAAAACT
TGTTTTATAAAATTGTTGTT
>Marker147191
ACTTCAGGTTGTTGGACGCCATTATCATCATCAAGAATTGCTTTGGCCTTTCTGGCCAAAGCACCCCAGAATCCA
TGTTTXXXXXXXXXXGACGAGTGCAAAACAAAAACTGATAAGTAGCACTGTAGAAAAGTGAAAGCGCCACTGCAG
AAGTAAAATGCAGAAACGGT
ACTTCAGGTTGTTGGACGCCATTATCATCATCAAGAATTGCTTTGGCCTTTCTGGCCAAAGCACCCCAGAATCCA
TGTTTXXXXXXXXXXGACAAGTGCAAAACAAAAACTGATAAGTAGCACTGTAGAAAAGTGAAAGCGCCACTGCAG
AAGTAAAATGCAGAAACGGT
>Marker147821
ACTAACACCAAAGAGGCAGGGTGCAAGAAGTATGCAATAAGTTGATATTTGTGATAACAAATTACTAACCATAGA
TTTGTXXXXXXXXXXAGGAATTCTCACTAGAGTGTCTGATCACCTAGTTGAGGATTTAGAAAGAAACTTGTAAGC
GTTACCAAAAGTAGGAGGTG
ACTAACACCAAAGAGGCAGGGTGCAAGAAGTATGCAATAAGTTGATATTTGTGATAACAAATTACTAACCATAGA
TTTGTXXXXXXXXXXAGGAATTCTCACTAGAGTGTCTGATCACCCAGTTGAGGATTTAGAAAGAAACTTGTAAGC
GTTACCAAAAGTAGGAGGTG
>Marker147869
AACTCAGAAACAAATACTAGGGAAAAGAACTGCTGTAGTCAAGTAATCGAGCAAAAAGATTGGACTTTGAAAGTA
AAAAAXXXXXXXXXXGTCTCAAGATTGTTAAATAATCAAACGAAACACCCAATGAAGCATATTGAATAAACTATA
ATGTTAAAAATAAAGATGTA
AACTCAGAAACAAATACTAGGGAAAAGAACTGCTGTAGTCAAGTAATCGAGCAAAAAGATTGGACTTTGAAAGTA
AAAAAXXXXXXXXXXGTCTCAAGATTGTTAAATAATCAAACGAAATACCCAATGAAGCATATTGAATAAACTATA
ATGTTAAAAATAAAGATGTA
>Marker148176
CACGAGGAAGTGATGGGCAAACCGAGATGGGAAATTGAGCAGCCACAGCCACAGCAACCGAAAAACCAAGTGGGG
TTCGAXXXXXXXXXXGGTGATTTTGGGTCGTTGTCGTGATCATTATCATCATGTTACCTAATTGTTGTATAATTG
GATTGGATGTGAAAAATGTT

Create PDF with GO2PDF for free, if you wish to

CACGAGGAAGTGATGGGCAAACCGAGATGGGAAATTGAGCAGCCACAGCCACAGCAACCGAAAAACCAAGTGGGG
TTCGAXXXXXXXXXXGGTGATTTTGGGTCGTTGTCGTGATCATAATCATCATGTTACCTAATTGTTGTATAATTG
GATTGGATGTGAAAAATGTT
>Marker148257
TACTCATGGACATCTTCTGCAAGGATACGCCGACTCGTCAATAAAGATCTTATCTCATTCTAATAAAGATCGTA
TTTTAXXXXXXXXXXTTGCTTGCCTCCTTTAGTATTAGGATTATGCGTGATTTCTTTGACCTCTTTCAGTAGGAA
TACCTGTAACTTCAAGGGTG
TACTCATGGACATCTTCTGCAAGGATACGCCGACTCGTCAATAAGGATCTTATCTCATTCTAATAAAGATCGTA
TTTTAXXXXXXXXXXTTGCTTGCCTCCTTTAGTATTAGGATTATGCGTGATTTCTTTGACCTCTTTCAGTAGGAA
TACCTGCAACTTCAAGGGTG
>Marker148272
AACACCCCACCAGGAACTCCAATTAGAATCATGAAGAATTTGAGAGTTTGTGCAGACTGTCATCTGGCAATCAAA
CTCATXXXXXXXXXXAATGAGTTTGCAACCTTTTAACGAGAGGTGACTTTCCATTGTATTATTGAGCTATGAATA
AAAGGTTGTGTTAGGAGGTT
AACACCCCACCAGGAACTCCAATTAGAATCATGAAGAATTTGAGAGTTTGTGCAGACTGTCATCTGGCAATCAAA
CTCATXXXXXXXXXXAATGAGTTTGCAACCTTTTAACGAGAGGTGACTTTCCATTGTATTATTGAACTATGAATA
AAAGGTTGTGTTAGGAGGTT
>Marker148455
ACTTTGAATGTTGGAGCTAGTTCTAAGTGTTGTTGTTTCTTGTTTGTATCATCATATACTATGTATTTCTCACCT
AATTTXXXXXXXXXXTGTAGCTCAAATCTCATCTAAAGGCAGATTACTGTAATGTAATAAGGATTACTAACAATA
GGAACACTATGTTTGAAGTG
ACTTTGAATGTTGGAGCTAGTTCTAAGTGTTGTTGTTTCTTGTTTGTATCATCATATACTATGTATTTCTCATCT
AATTTXXXXXXXXXXTGTAGCTCAAATCTCATCTAAAGGCAGATTACTGTAATGTAATAAGGATTACTAACAATA
GGAATACTATGTTTGAAGTG
>Marker148861
AACACCCACTGTCCCCTTTCTTCTTCCTCTTCTTCTTCTTCCTCTTCCACTCTCCCAACCCCCACTCTTCTCCAA
TATCAXXXXXXXXXXGGCATAAATCTAACTGGGGCTTCTTTTTTGGTTTAGAGGGAACTTAATCTAGGTAGTTTT
ATCACTGTCCGTGGTTGGTT
AACACCCACTGTCCCCTTTCTTCTTCCTCTTCTTCTTCTTCCTCTTCCACTCTCCCAACCCCCACTCTTCTCCAA
TATCAXXXXXXXXXXGGCATAAATCTAACTGGGGCTTCTTTTTTGGTTTAGAGGGAACTTAATCTTGGTAGTTTT
ATCACTGTCCATGGTTGGTT
>Marker149091
CACAAAGCAAGCAGCAATCTCCAATAATTACATTATGACCGATCTAAATAGCCAGGAAGAGTTTGAGAGTCACTA
TGATGXXXXXXXXXXCAGAGCACTGTATTAGAAATTTAGAATGTCAGCTCATCCTATTCCCAAAAGCAATGACAT
TCAAGAAAATATAACTAGTC
CACAAAGCAAGCAGCAATCTCCGATAATTACATTATGACCGATCTAAATAGCCAGGAAGAGTTTGAGAGTCACTA
TGATGXXXXXXXXXXCAGAGCACTGTATTAGAAATTTAGAATGTCAGCTCATCCTATTCCCAAAAGCAATGACAT
TCAAGAAAATATAACTAGTC
>Marker149392
AACTTAAATATAAGTATATAGATTTAGGTATATATCCTCCATCCAGAAGCCAGTCATAATGCTCAAATATGAGTG
TAGGAXXXXXXXXXXATTAAGTTAAAGAGTAAATATTTTTTAACCAAAATCTTGTTATTTTCTTGACTTTGTTGC
TTCTATTCTTATCTTATGTC
AACTTAAATATAAGTATATAGATTTAGGTATATATCCTCCATCCAGAAGCCATTCATAATGCTCAAATATGAGTG
TAGGAXXXXXXXXXXATTAAGTTAAAGAGTAAATTTTTTTTAACCAAAATCTTGTTATTTTCTTGACTTTGTTGC
TTCTATTCTTATCTTATGTC
>Marker150693

Create PDF with GO2PDF for free, if you wish to

ACTCCAATAATTCCAAACCACATTACCCATTGCTTGTAGCAAATGTGTGGATTCATACTTGTTTGAACACAACTT
AACAAXXXXXXXXXXAATGCATTGGGCATTGGAAAAGTTCTAAATAATGCTGTAAGTAATTCGATTAGACTTTTA
TCCAAGGATTCAACTTGGTA
ACTCCAATAATTCCAAACCACATTACCCATTGCTTGTAGCGAATGTGTGGATTCATACTTGTTTGAACACAACTT
AACAAXXXXXXXXXXAATGCGTTGGGCATTGGAAAAGTTCTAAATAATGCTGTAAGTAGTTCGATTAGACTTTTA
TCCAAGGATTCAACTTGGTA
>Marker151238
ACAAGAACATTTGAAATTGCAATGATTAAAATGGAAGAAAGAAATTACAAAAAATTAAAAAGAAAAAAGGGGAAG
TCCAAXXXXXXXXXXGATGTGGCTCCCATAACCCATCATAATGATAACCAATTGGTATATGACATGATTTGATGT
GTATTTGTTACCATTATGGT
ACAAGAACATTTGAAATTGCAATGATTAAAATGGAAGAAAGAAATTTCAAAAAATTAAAAAGAAAAAAGGGGAAG
TCCAAXXXXXXXXXXGATGTGGCTCCCATAACCCATCATAATGATAACCAATTGGTATATGACATGATTTGATGT
GTATTTGTTACCATTATGGT
>Marker151432
ACATGTAAGAACTTTCAAACACTTTTATTTTTTCTTGTAAGTGGTTAGTTAATTGATCCAAATAAATCCTTAATC
GAAAAXXXXXXXXXXATGATGTATTTACTATTTTGTTCAACTTGAAGAAAGTCTCTCAGTATTTCTTTTTTTACA
TCAATGCATGTATTTCCAGT
ACATGTAAGAACTTTCAAACACTTTTATTTTTTCTTGTAAGTGGTTAGTTAATTGATCCAAATAAATCCTTAATC
GAAAAXXXXXXXXXXATGATGTATTTACTATTTTGTTCAACTTGAAGAAAGTCTCTCAGTATTTCTTTTTTTACA
TCAATGCATGTATTTCGAGT
>Marker151881
ACCACAAATCCTCTAATGAACAACTAGTTTAAGGTCTAACCTATAAACCAAATCCCTCTCGAGTTAATAAAAGGG
TGTGGXXXXXXXXXXTTCACCTTAAATGGGAGGCATATTGGGCCAACACTGATGAGTTACTCTCATCTAAGCAGA
TCTAAGGATAATCTTGTGTG
ACCACAAATCCTCTAATGAACAACTAGTTTAAGGTCTAACCTATAAACCAAATCCCTCTCGAGTTAATAAAAGGG
TGTGGXXXXXXXXXXTTCACCTTAAATGGGAGGCATATTGGGCCAACACTGATGAGTTGCTCTCACCTAAGCAGA
TCTAAGGATAATCTTGTGTG
>Marker151896
CACAAATTCAAAGCTCAAACTGCAAAATCTTCAAATAATAATAATGAAACTACAAAATTGGGCTAAAAGATTCTG
TTTTGXXXXXXXXXXTTCTCACCCTTTCAATTTTACCTTGTTTCCATTATTATTTTAAGTTATTTACTTTCAAAT
ATCTAATTTTAGTTTGAGTA
CACAAATTCAAAGCTCAAACTGCAAAATCTTCAAATAATAATAATGAAACTACAAAATTGGGCTAAAAGATTCTG
TTTTGXXXXXXXXXXTTCTCACCCTTTTAATTTTACCTTGTTTCCATTATTATTTTAAGTTATTTACTTTCAAAT
ATCTAATTTTAGTTTTAGTA
>Marker152468
ACTAAGTTACCAACTTTACTTCTTTATCTATTCCAAAAAACAATAGGGTGAATCTCACTGCTAAATCTATCAAAT
TTACAXXXXXXXXXXATATAATAAGTGTTTGAATTAAAAAAGAAAGAGTATATTTAATAGATGGATACAAAATTA
GTTTTATGTTAAAGTTTGTG
ACTAAGTTACCAACTTTACTTCTTTATCTATTCCAAAAAACAATAGGGTGAATCTCACTGCTAAATCTATCAAAT
TTACAXXXXXXXXXXATATAATAAGTGTTTGAATTAAAAAAGAGAGAGTATATTTAATAGATGGATACAAAATTA
GTTTTATGTTAAAGTTTGTG
>Marker152487
ACCCACAAGTGACTAGGTGGGTTCGACACCAAGGATTTCGTGAAATCCCCTACCGCTCCGGCTAACATCATGTGC
TCTTTXXXXXXXXXXCAGAGGGAGCAGAGATTTGAACAGGAACACGCCTGACCGTAACCCATGGAGAATGATTA
GGAAGGTGATTGCTGCTGTG

Create PDF with GO2PDF for free, if you wish to

ACCCACAAGTGACTAGGTGGGTTCGACACCAAGGATTTCGTGAAATCCCCAACCGCTCCGGCTAACATCATGTGC
TCTTTXXXXXXXXXXCAGAGGGAGCAGAGATTTGAACAGGAACACGCCTGACCGTAACCCATGGGAGAATGATTA
GGAAGGTGATTGCTGCTGTG
>Marker152887
TACTTGAAAGAATTTTTTTTCCTGCAGCATGAAGTTCTTATACCCGGACTAGATTCTATTCTCACATCTGATGTT
GCTGAXXXXXXXXXXGTAATTCTTTTGTCACTTTCTAAATTTTTCTGTGTCTATACCCTAGAAGTTAATTTAAAA
AAAACTTAATCCCATGTGTG
TACTTGAAAGAATTTTTTTTCCTGCAGCATGAAGTTCTTATACCCGGACTAGATTCTATTCTCACATCTGATGTT
GCTGAXXXXXXXXXXGTAATTCTTTTGTCACTTTCTAAATTCTTCTGTGTCTATACCCTAGAAGTTAATTTTAAA
AAAACTTAATCCCATGTGTG
>Marker152976
ACCTTTTGCCCGCAGACAGGAACTCACCCGAAAATCACTACTTTGAGGAATATTAATCGCTTTCGGTCTCAGGTG
CAGTTXXXXXXXXXXTTATTATTTTTTATGATCTAAACTCCAAGCTGAAGCACATAAGAATGGCTCTATCTATAG
CATATTTGGAATGAGTTGTG
ACCTTTTGCCCGCAGACAGGAACTCACCCGAAAATCACTACTTTGAGGAATATTAATCGCTTTCGGTCTCAGGTG
CAGTTXXXXXXXXXXTTTTTTTTTTTTATGATCTAAACTCCAAGCTGAAGCACATAAGAATGGCTCTATCTATAG
CATATTTGGAATGAGTTGTG
>Marker153181
AACTTTCTTAGTGTTTGCCTATTTAGGTATTGTGAATGTTGGGGGTGGGTATTTTTTTTTGCTAAATGATTTTGG
TATAGXXXXXXXXXXCCTGTATTAAGTTTGGAGTCAAAGAAAATAAGAAAAACATAATTTTCCTCCCAGAAGCCA
CACAGCCCGAAGACAATAGT
AACTTTCTTAGTGTTTGCCTATTTAGGTATTGTGAATGTTGGGGGTGGGTATTTTTTTTTGCTAAATGATTTTGG
TATAGXXXXXXXXXXCCTGGATTAAGTTTGGAGTCAAAGAAAATAAGAAAAACATAATTTTCCTCCCAGAAGCCA
CACAGCCCGAAGACAATAGT
>Marker153569
CACTTGACTCTAAACTCATTTGTAATTACCACATTTCAACTATTCCTTTTACATTGGAATTATTTAGATATGGCG
AGAAAXXXXXXXXXXGCATAATTATTTTCAAAATTAAAGCAAATATATAGCTATCAAATAGAGCCTAAACGACCA
ATAATATGTTTTGGTTACGT
CACTTGACTCTAAACTCATTTGTAATTACCACATTTCAACTATTCCTTTTACATTGGAATTATTTAGATATGGCG
AGAAAXXXXXXXXXXGCATAATTATTTTCAAAATTAAAGCAAATATATAGCTATCAAATAGAGCCTAAACGACCA
ATAATATGTCTTGGTTACGT
>Marker153579
ACTGCACTATAACAGTCTCAAACCAGATTTTGACAATTTAACTTATACCAAGTTTTTAGTGTTATAATTCGTTTG
TTTTTXXXXXXXXXXAAAATGTAAAATAGAAAAGGGGGAAATAAGTAGAGAGAACCGAAATAATATCTCCTTTTT
AAAGCTCTTTCTGGGAGGTT
ACTACACTATAACAGTCTCAAACCAGATTTTGACAATTTAACTTATACCAAGTTTTTAGTGTTATAATTCGTTTG
TTTTTXXXXXXXXXXAAAATGTAAAATAGAAAAGGGGGAAATAAGTAGAGAGAACCGAAATAATATCTCCTTTTT
AAAGCTCTTTCTGGGAGGTT
>Marker153818
CACTTGTGCTTTTAGAGGACGATCCTTTTGAAAAGCTCCTTTTAGTGGTAGCAACATTTGCTTCCACCTTTTCCT
TACCCXXXXXXXXXXTTCAGAAGAGATTCTAAGATAAAGCTAACTTGATTAGCCTCTTCAATAGAACCACCATTT
AGTTCGACGATGTTGAAGTG
CACTTGTGCTTTTAGAGGACGATCCTTTTGAAAAGCTCCTTTTAGTGGTAGCAACATTTGCTTCCACCTTTTCCT
TACCCXXXXXXXXXXTTCAGAAGAGATTCTAAGATAAAACTAACTTGATTAGCCTCTTCAATAGAACCACCATTT
AGTTCGACGATGTTGAAGTG
>Marker153948

Create PDF with GO2PDF for free, if you wish to

ACAAAACCTGAGATATAAATGATACAGCCATTTGCAGAGACCAACACAAAACATGTTAAAGCAAAAGGAACATCT
CATGAXXXXXXXXXXXTCCAAACAAAGATAATGTTGAACGCTAAAACCATGTGAGGCTAGATAAAGTGGTGAAAAT
TCCATATGACAAGAGATGGT
ACAAAACCTGAGATATAAATGATACAGCCATTTGCAGAGACCAACACAAAACATGTTAAAGCAAAAGGAACATCT
CATAAXXXXXXXXXXXTCCAAACAAAGATAATGTTGAACGCTAAAACCATGTGAGGCTAGATAAAGTGGTGAAAAT
TCCATATGACAAGAGATGGT
>Marker154034
AACTGTGGCCTATAGTATAGGATAAGGAAAAAACACTAAAAGCTTATCTAACTTCTTCATACCATTACATTACCC
TCTAGXXXXXXXXXXXAGTCATGAGTCCCATGACCAAACTTGTAAAGAGATGAACAATATGTCATAATCAGCACAT
GCTATGGGATAGAAACAGTA
AACTGTGGCCTATAGTATAGGATAAGGATAAAACACTAAAAGCTTATCTAACTTCTTCATACCATTACATTACCC
TCTAGXXXXXXXXXXXAGTCATGAGTCCCATGACCAAACTTGTAAAGAGATGAACAATATGTCATAATCAGCACAT
GCTATGGGATAGAAACAGTA
>Marker154098
TACAGCTTCGGTGTAGTTCTACTTGAGCTTTTGACCGGTAGGAAACCTGTCGATCACACGATGCCTCGCGGACAG
CAAAGXXXXXXXXXXXCCTTTGTGAGGCAATTCCTCGGTTCCAGCTCCATAAAGGCTGTTAAATATTCCTGCGTCT
TGTGCTGATAGAACATTGTC
TACAGCTTCGGTGTAGTTCTACTTGAGCTTTTGACCGGTAGGAAACCTGTCGATCACACGATGCCTCGCGGACAG
CAAAGXXXXXXXXXXXCCTTTGTGAGGCAATTCCTCGGTTTCAGCTCCATAAAGGCTGTTAAATATTCCTGCGTCT
TGTGCTGATAGAACATTGTC
>Marker154708
AACAGTGAAATTTTCCAAAATACATGAACTGTAGAGCTGGGTCTGAAATTCCTTGGACATATAAGATTAGCTTCA
GATTGXXXXXXXXXXXTCAACAGAGAAAGGTCTGTCATGAGTTCTATTTTCTACACTAAGTTAATGCAATTATCTG
GTTCTTTTTATTACTCCGTA
AACAGTGAAATTTTCCAAAATACATGAACTGTAGAGCTGGGTCTGAAATTCCTTGGACATATAAGATTAGCTTCA
GATTGXXXXXXXXXXXTCAACAGAGAAAGGTCTGTCATGAGTTCTATTTTCTACACTAAGTTAATGCAATTATGTG
GTTCTTTTTATTACTCCGTA
>Marker155031
ACCAGTTGAGCTAAGCTTGTGTTGGCTTCACATAATTTATTTCATCGATGTGTTATTGAATCGCAAAGAAAAATT
AGAATXXXXXXXXXXXTTACATAAGATATAATTAAAATTTAGAGAAATGTTACAAAATTCTAAAAAGATCTTAATT
CGGCTTGTGGGGATGGTGTA
ACCAGTTGAGCTAAGCTTGTGTTGGCTTCACATAATTTATTTCATCGATGTGTTATTGAATCACAAAGAAAAATT
AGAATXXXXXXXXXXXTTACATAAGATATAATTAAAATTTAGAGAAATGTTACAAAATTCTAAAAAGATCTTAATT
CGGCTTGTGGGGATGGTGTA
>Marker156079
AACTTCCATTGATCCAATATTTCTTTCTTACAGAATACAAATACATCCAAAGAAGATATAAGCGCACTAACGACT
ACAGAXXXXXXXXXXXATTCAATGCCTGCAATTAGACATTTCAATTAGCAATCAATTCTTCAGAAAATTGATATGT
TAATAAATGTAATGAAACGT
AACTTCCATTGATCCAATATTTCTTTCTTACAGAATACAAATACATCCAAAGAAGATATAAGCGCACTAACGACT
ACAGAXXXXXXXXXXXATTCAATGCCTGCAATTAGACATTTCAATTAGCAATCAATTCTTCAGAAAATTAATATGT
TAATAAATGTAATGAAACGT
>Marker156199
ACAATTATTGTTGCAAATGAGATTATATATATATTGCTTACCAAAGTCATTCAATTTTTGCTGCCTTCCTAACTT
TTACCXXXXXXXXXXXTAGATAAGTCAAATTATTAACCTTTATATGAACATAACATGCATTCAAAAAATATTTTTA
CTTAATTATATAATTATGTA

Create PDF with GO2PDF for free, if you wish to

ACAATTATTGTTGCAAATGAGATTATATATATATTGCTTACCAAAGTCATTCAATTTTTGCTGCCTTCCTAACTT
TTACCXXXXXXXXXXXTAGATAAGTCAAATTATTAACCTTTATATGAACATAACATGCATTCAAAAAATATTTTTA
CTTAATTATATAATTAAGTA
>Marker156835
ACTTTGTGACTCCGATGGCGAATAGATCCAATGTAGATCCAAATTTCTATTCACTCGTCAGATCGAATGACTCTA
CGTAAXXXXXXXXXXXGAGTCCAGTCCGCTCGAGCAGTTTAAGGTGCAAAAAACTTTAATATTACTCCAAATGAGA
CCACTCGGTAGATAGATGTG
ACTTTGTGACTCCGATGGCGAATAGATCCAATGTAGATCCAACTTTCTATTCACTCGTCAGATCGAATGACTCTA
CGTAAXXXXXXXXXXXGAGTCCAGTCCGCTCGAGCAGTTTAAGGTGCAAAAAACTTTAATATTACTCAAAATGAGA
CCACTCGGTAGATAGATGTG
>Marker158579
AACAGAGAATAACAAAACTAACAAATTTTCCCTTGTAAGCAAAGATAATACATATAAATCCACACACTGTAAGAA
AAATAXXXXXXXXXXXATGTAACATACTTCCATGGTCAGATAGACATAAATAAGGTTATTACAGAAATGACTGTGA
ATAGAAATTAGCATTTGAGT
AACAGAGAATAACAAAACTAACAAATTTTCCCTTGTAAGCAAAGATAATACATATAAATCCACACACTGTAAGAA
AAATAXXXXXXXXXXXATGTAACAGACTTCCATGGTCAGATAGACATAAATAAGGTTATTACAGAAATGACTGTGA
ATAGAAATTAGCATTTGAGT
>Marker159347
TACAGGATTACAAACTCGAGAATCGCCCTCTTACCAAGTGCAAGAATCTCCTAATCCTGCTGCATTGGGATGTCA
TGTCAXXXXXXXXXXXCCGGATGCATATAAGAGAGACACAGCAGAGGTCGATTTGATTTCTCTGGGTTCATTTTTC
TCATTTACACTTCAGTTGTA
TACAGGATTACAAACTCGAGAATCGCCCTCTTACCAAGTGCAAGAATCTCCTAATCCTGCTGCATTGGGATGTCA
TGTCAXXXXXXXXXXXCCGGATGCATATAAGAGAGACACAGCAGAGGTCGATTTGATTTCTCTGGGTTCATTTTTC
TCATTTACAATTCAGTTGTA
>Marker159460
CACTCACCTCACCACCTTTAAATTCGTTCATCTGTTTTGTTTTATTTCTCAGACATTAGAAGAGCAAATAAATTG
AAGAAXXXXXXXXXXXATCAAAACGCTATTGAGTTTCGAGATAGAGTATATATATGTTCAATAAACTATAAAAGAA
AAACGAAATCAAGAGATGTA
CACTCACCTCACCACCTTTAAATTCATTCATCTGTTTTGTTTTATTTCTCAGACATTAGAAGAGCAAATAAATTG
AAGAAXXXXXXXXXXXATCAAAACGCTATTGAGTTTAGAGATAGAGTATATATATGTTCAATAAACTATAAAAGAA
AAACGAAATCAAGAGATGTA
>Marker159685
AACGAAAGTGATAAGTGCATCTACTATAAGTTTGATAATGGCTTATGCACTATTGTATGCTTATATGTAGATGAC
ATGTTXXXXXXXXXXXTCGGTCTGAAATAGGAATTTCTTTCGATCAATCCTCTTACATAGAGAAAATTGTGAAGAA
ATATAACTACCTTGAAAGTA
AACGAAAGTGATAAGTGCATCTACTATAAGTTTGATAATGGCTTATGCACTATTGTATGCTTATATGTAGATGAC
ATGTTXXXXXXXXXXXTCGGTCTGAAATAGGAATTTCTTTGGATCAATCCTCTTACATAGAGAAAATTTTGAAGAA
ATATAACTACCTTGAAAGTA
>Marker160266
ACTTGAAAAGTTAGAATAAAAGACCCAAAATTCCATGGACCTTTTGGGTGTTTTTAAAGAGAAAAAAAGAACGAT
AATTGXXXXXXXXXXXTATATGAAGGGGGAAGGGGCAGTGAGGGCAATTTTGTGGAAGAAAGGGGAATTTTTGCAT
GGATTCACACCGGAGATGTG
ACTTGAAAAGTTAGAGTAAAAGACCCAAAATTCCATGGACCTTTTGGGTGTTTTTAAAGAGAAAAAAAGAACGAT
AATTGXXXXXXXXXXXTATATGAAGGGGGAAGGGGCAGTGAGGGCAATTTTGTGGAAGAAAGAGGAATTTTTGCAT
GGATTCACACCGGAGATGTG
>Marker160664

Create PDF with GO2PDF for free, if you wish to

ACAATTTACAAACGATGATTTGAATCAAAGATGGAAGAAGATGGAGAAAGAAGAAGAAGATGATGATGATGAAGA
AAGATXXXXXXXXXXTACCATAACATTCCTCTATTATGAGGAATGATGAGACAAACCTACTGACTAACATCGTGT
TTGAATGAAACGATACGGTT
ACAATTTACAAACGATGATTTGAATCAAAGATGGAAGAAGATGGAGAAAGAAGAAGAAGATGATGATGAAGAAGA
AAGATXXXXXXXXXXTACCATAACATTCCTCTATTATGAGGAATGATGAGACAAACCTACTGACTAACATCGTGT
TTGAATGAAACGATACGGTT
>Marker160945
CACCAATTGATTTAGGTTTCCATTTCTCTGAGATTTTAATTGGAATCGTGAATGGATCATCAACAAACTCCTCAG
TCAATXXXXXXXXXXAATCTAGAGAATGAGAAGGGGAGTTATAAAATATGTTGGAATGTCCTAAATCTAATATCT
TGTGCTAACAACAACCAAGT
CACCAATTGATTTAGGTTTCCATTTCTCTGAGATTTTAATTGGAACCGTGAATGGATCATCAACAAACTCCTCAG
TCAATXXXXXXXXXXAATCTAGAGAATGAGAAGGGGAGTTATAAAATATGTTGGAATGTCCTAAATCTAATATCT
TGTGCTAACAACAACCAAGT
>Marker161457
GACTCATTTCTCACAATTTTCACTCCAAGGGCAAGGTAGAGAGAAGAGGGAGAGAGAATTTGAGTGGTTGGTGAT
TAAGTXXXXXXXXXXGCTAATTTGACAAGCGTCACACCTATGTGTAATTCTTGTCTATTTTGCAGACAACATACT
TTAACTAAAAATTTAGGGTC
GACTCATTTCTCACAATTTTCACTCCAAGGGCAAGGTAGAGAGAAGAGGGAGAGAGAATTTGAGTGGTTGGTGAT
TAAGTXXXXXXXXXXGCTAATTTGACAAGCGTCACACCTATGTGTAATTCTTGTCTATTTTGCAGACAACATACT
TTAACTAAAAATTTAAGGTC
>Marker162062
ACTATCACGTAGGTCTCACCGTCCAAGTTCTCATCATCTGTGGTTGGTTAGGTCAAAACGCCGATTACTGAGCAC
TGTCCXXXXXXXXXXTCTTCACAAGTTACCACATATAGTCTTTATCACGTAGCTTAAGAGGCGATTAGGGTCTCA
CAGAAACGGACCAAAAAGTT
ACTATCACGTAGGTCTTACCGTCCAAGTTCTCATCATCTGTGGTTGTTTAGGTCAAAACGCCGATTACTGAGCAC
TGCCCXXXXXXXXXXTCTTCACAAGTTACCACATATAGTCTTTATCACGTAGCTTAAGAGGCGATTAGGGTCTCA
CAGAAACGGACCAAAAAGTT
>Marker162130
CACAAAAATGGGGGAAAACCCTTTTGATATTACAACAAAAAAAAAAAGGAAAGAAAACTCTGAAGAAGATGAAAA
CTTAGXXXXXXXXXXAAGAGAGTGACAGATATAGAAAATATAAAGTTCCAGAAATAACCCTTTCCACAAATGAGA
AAAAGCTTGAAGTTCTTAGT
CACAAAAATGGGGGAAAACCCTTTCGATATTACAACAAAAAAAAAAAGGAAAGAAAACTCTGAAGAAGATGAAAA
CTTAGXXXXXXXXXXAAGAGAGTGACAGATATAGAAAATATAAAGTTCCAGAAATAACCCTTTCCACAAATGAGA
AAAAGCTTGAAGTTCTTAGT
>Marker162159
AACTCCGGCAATATACGAACATAACGTGTTCGCTCCATATCTAGTAAAGGATTGGATCTATAATCCCCCGCCACA
TAAAAXXXXXXXXXXXGAATATGAGACCACCCACAACGGAGAACATCCCTAAAAAGGAAATCCATCAACATCGTT
AGGAATTTTTTTACGACGTT
AACTCCGGCAATATACGAACATAACGTGTTCGCTCCATATCTAGTAAAGGACTGGATCTATAATCCCCCGCCACA
TAAAAXXXXXXXXXXXGAATATGAGACCACCCACAACGGAGAACATCCCTAAAAAGGAAATCCATCAACATCGTT
AGGAATTTTTTTACGACGTT
>Marker162526
GACAAATCTAGAGTTGCTATCATAAATAGTTAAATTTATCTAAATCCATCAACAAGGATATAACTTGGATGTTTG
AAGACXXXXXXXXXXAGAAGTTTTTTAGATTTTGAGTTTATGCATACAACATCAAATCATATATCTTGTCCATGC
AAGAGGAGTAGTAATGCAGT

Create PDF with GO2PDF for free, if you wish to

GACAAATCTAGAGTTGCTATCATAAATAGTTAAATTTATCTAAATCCATCAACAAGGATATAACTTGGATGTTTG
AAGACXXXXXXXXXXXAGAAGTTTTTTAGATTTTGAGTTTATGCATACAACATCAAATCATATATCTTGTCCATGC
AAGAGGTGTAGTAATGCAGT
>Marker163591
AACAATAACGGCTTGGCCATGTGTTTCTTTTTCAATCATTTTCTTTATTCTGTTGTTCTTGATTAGTTCTACTTT
CCCTAXXXXXXXXXXXGGTAGAGTCTTTTAGTTCTAGAGAGTATTACTGAGGTGGCAGATATGTCTTTTAATTCTA
GAGAGTTTTACCCCTTAAGT
AACAATAACGGCTTGGCCATGTGTTTCTTTTTCAATCATTTTCTTTATTCTGTTGTTCTTGATTAGTTCTACTTT
CCCTAXXXXXXXXXXXGGTAGATTCTTTTAGTTCTAGAGAGTATTACTGAGGTGGCAGATATGTCTTTTAATTCTA
GAGAGTTTTACCCCTTAAGT
>Marker163969
CACCAATATAAAAAGGAACGCAAATACATTATGGAAAAGCTTTGGTCTGGACACAACCAAATGAACCACAACAAA
GTCACXXXXXXXXXXXACAAGAAGATTCAGAAACTGTATGTATAATGAACGACTGAATGCAATAAAAAATTGAATA
TTATATTGGCCTAGCTAGTA
CACCAATATAAAAAGGATCGCAAATACATTATGGAAAAACTTTGGTCTGGACACAACCAAATGAACCACAACAAA
GTCACXXXXXXXXXXXACAAGAAGATTCAGAAACTGTATGTATAATGAACGACTGAATGCAATAAAAAATTGAATA
TTATGTTGGCCTAGCTAGTA
>Marker164070
AACAACTTGAAAAGAAGTTATAAATGAAGGAATTTCCACCTTAACCTCTTGACTTTTTACTTTACGTGGTTATAA
ACTCCXXXXXXXXXXXGTTGTTCTTGAATCTAGTTTCCTTTCGAGTAAATTATATATAAATTCTTACTTATGCTTG
ACAACCTCGAACATGTAGGT
AACAACTTGAAAAGAAGTTATAAATGAAGGAATTTCCACCTTAACCTCTTGACTTTTTACTTTACATGGTTATAA
ACTCCXXXXXXXXXXXGTTGTTCTTGAATCTAGTTTCCTTTCGAGTAAATTATATATAAATTCTTACTTATGCTTG
ACAACCTCGAACATGTAGGT
>Marker164287
TACATGGATATAAATATAAAATTGGTAGTAAGGTCCCATCTCCTTCTCCATATTGCTCTTTCCTTTGTTTGTTTT
CTTCTXXXXXXXXXXXAAACATTCGTTACTACCGATTTCATAAATATAACAAACCATAACAAAATTAAAAAGTTCT
AGAAATTGGCTAATATTTGT
TACATGGATATAAATATAAAATTGGTAGTAAGGTCCCATCTCCTTCTCCATATGGCTCTTTCCTTTGTTCGTTTT
CTTCTXXXXXXXXXXXAAACATTCGTTACTACCGATTTCATAAATATAACAAACCATAACAAAATTAAAAAGTTCT
AGAAATTGGCTAATATTTGT
>Marker164395
ACAGAAGTTCAACACAAATCATCAAAATCAAGTAGGCAAGATATAAAAGCAAACATGAGAAAATCAACAGACGGC
GTTCAXXXXXXXXXXXTTTTAGTTAGTCTGACGAATTTACATCACCACATATTTTAGCCTCATCTGTGACAATTTG
AAAAGTGCAACTTCTCAAGT
ACAGAAGTTCAACACAAATCATCAAAATCAAGTAGGCAAGATATAAAAGCAAACATGAGAAAATCAACAGACGGC
ATTCAXXXXXXXXXXXTTTTAGTTAGTCTGACGAATTTACATCACCACATATTTTAGCCTCATCTGTGACAATTTG
AAAAGTGCAACTTCTCAAGT
>Marker164857
ACCAAATTTAGAAAAACATTAACTAAGCTTCTCAATACATAGAAAGAAAAAAGATAAATACAAGTTTAATCATTA
ACAACXXXXXXXXXXXGAAAGAACTATTCAAGTCATTCTTCTTCTAGCCCTAGTTTCAGTGTTTTCATCTTTTTCA
GCTTATTCCTTACCCTTGTC
ACCAAATTTAGAAAAACATTAACTAAGCTTCTCAATACATAGAAAGAAAAAAGATAAATACAAGTTTTATCATTA
ACAACXXXXXXXXXXXGAAAGAACTATTCAAGTCATTCTTCTTCTAGCCCTAGTTTCAGTGTTTTCATCTTTTTCA
GCTTATTCCTTACCCTTGTC
>Marker164858

Create PDF with GO2PDF for free, if you wish to

AACTCATTAGACACCTATGCTTCCCTTTGACGTAAGCCTTCTTCTCTTCAAAACTTAAGCTATCCGTAAGTGTGA
ATAGAXXXXXXXXXXXGGTAGAATTGGGTGTTAATATTGATACGAAACTTAAATAGGAAAAAACGAAATCAAGATG
CTTCAACCGAAAGTCAAAGT
AACTCATTAGACACCTATGCTTCCCTTTGACGTAAGACTTCTTCTCTTCAAAACTTAAGCTATCCGTAAGTGTGA
ATAGAXXXXXXXXXXXGGTAGAATTGGGTGTTAATATTGATACGAAACTTAAATAGGAAAAAACGAAATCAAGATG
CTTCAACCGAAAGTCAAAGT
>Marker165285
ACACAACTTGTCCGGCAATATTCAAAACCAACAAAGGCATATATGAAATTTATGAAAAAAAAATAATCGGGCTCC
ATAGGXXXXXXXXXXXTTAAAGTTAAATTCTATTAAATAAAGTAGTTATTAAAGACTTATTAGTGAAAATTGATTA
TTATAATCTTGAATCCAGTA
ACACAACTTGTCCGGCAATATTCAAAACCAACAAAGGCATATATGAAATTTATGAAAAAAAAATAATCGGGCTCC
ATAGGXXXXXXXXXXXTTAAAGTTAAATTCTATTAAATAAAGTGGTTATTAAAGACTTATTAGTGAAAATTGATTA
TTATAATCTTGAATCCAGTA
>Marker165302
CACCATTAATGATGTTGGTGATGACACCATAGCCAGGGGATCTTCCTGCAGCATTGTCAGCACTCGAAGGTTGCC
AATTGXXXXXXXXXXXCGGCGCTCCTATTGCATTCCCTGCTGGTCCGTAGTTGTAGTTGCTAAAAAAAGTCCAATC
ATTTTAATACGATGCTAGTT
CACCATTAATGATGTTGGTGATGACACCATAGCCAGGGGATCTTCCTGCAGCATTGTCAGCGCTCGAAGGTTGCC
AATTGXXXXXXXXXXXCGGCGCTCCTATTGCATTCCCTGCTGGTCCGTAGTTGTAGTTGCTAAAAAAAGTCCAATC
ATTTTAATACGATGCTAGTT
>Marker165671
GACGTAACCACATTATTTCACACACTGATTATGCCATTGCTTTGTATTCTGACTCAACACTTGATTGTGAAACTA
CATTTXXXXXXXXXXXATTAAATATTCATGTGACCGTGATCTTTATATAGGATCCCACGTTCAGGTGCAACCTTAA
GGTAACATAAAATTTGTTGT
GACGTAACCACATTATTTCACACACTGATTATGCCATTGCTTTGTATTCTTACTCAACACTTGATTGTGAAACTA
CATTTXXXXXXXXXXXATTAAATATACATGTGACCGTGATCTTTATATAGGATCCCACGTTCAGGTGCAACCTTAA
GGTAACATAAAATTTGTTGT
>Marker165778
TACCTTTTTGCTAATCAAGAGTTATTCTCTGCTTTACTGGCATGTGGTTTGAAACAAAGTTTAGTGAAGGAGAAG
TGTAGXXXXXXXXXXXTCTCTACAAGAAACTTGGGTTCCAAGTTGATAGCTTGATTAAAGGATACTATTCTGCGGA
TCGAGATGCCTACAGAATGT
TACCTTTTTGCTAATCAAGAGTTATTCTCTGCTTTACTGGCATGTGGTTTGAAACAAAGTTTAGTGAAGGAGAAG
TGTAGXXXXXXXXXXXTCTCTACAAGAAACTTGGGTTCCAAGTTGATAGCTTGATTGAAGGATACTATTCTGCGGA
TCGAGATGCCTACAGAATGT
>Marker167669
ACATAAATCAGTTTAACAAATTGAGAGCATAACATCTACTTCATATAATATACAATTTATTGTTTCATATATACT
ATTCAXXXXXXXXXXXTATATAATAGATAACAACTAATGTTTTAATGACATATCTTACATATACTCATCATATATT
TTTTTATTCATGTTTATGTA
ACATAAATCAGTTTAACAAATTGAGAGCATAACATCTACTTCATATAATATACAATTTATTGTTTCATATATACT
ATTCAXXXXXXXXXXXTATATAATAGATAACAACTAATGTTTTAATGACATATCTTACATATACTCATCATATTTT
TTTTTATTAATGTTTATGTA
>Marker167750
GACAGCTTTCTAAGTTTTTATTTTTCTTTAAAAGGAAAAAAGTGTAGAGATTAGGAACTAAAAAGAAAAAGAAAG
GAGTAXXXXXXXXXXXCCCAAATGAAAGCCTTATCCCTTTTAAGTAACTGATGGAAACACATTAATGCATTATATT
TCATTTAAACTCATATCAGT

Create PDF with GO2PDF for free, if you wish to

CACAGCTTTCTAAGTTTTTATTTTTCTTTAAAAGGAAAAAAGTGTAGAGATTAGGAACTAAAAAGAAAAAGAAAG
GAGTAXXXXXXXXXXXCCCAAATGAAAGCCTTATCCCTTTTAAGTAACTGATGGAAACACATTAATGCATTATATT
TCATTTAAACTCATATCAGT
>Marker167996
ACTGTCTTTCTCAGCAACCCAAATACAAGAGTCTCGTAATTGCATTGTCTATCAACCAGCAAATTATAAATGCCG
CTCTGXXXXXXXXXXXAATTAAAATCTTCAACCCAAACCGAAAATGCAAACAAGGTTATTTATTTGTTTTCTTTTT
TCTTTAACTATACTTTTGTT
ACTGTCTTTCTCAGCAACCCAAATACAAGAGTCTCATAATTGCATTGTCTATCAACCAGCAAATTATAAATGTCG
CTCTGXXXXXXXXXXXAATTAAAATCTTCAACCCAAACCGAAAATGCAAACAAGGTTATTTATTTGTTTTCTTTTT
TCTTTAACTATACTTTTGTT
>Marker168110
ACTTTATTCTACCAAATTTATAGGTTGGGTAATTTTCAGCCATCACCATTGTTTCATAAACATTAAAAAGAATAA
TAATAXXXXXXXXXXXTTTTAATTAATTTAGTGACACACTTTGATTGAAATCGCATCCTTAGGTCCAATTTTTTCA
TTTGGTAGGTCTTAAGTGGT
ACTTTATTCCACCAAATTTATAGGTTGGGTAATTTTCAGCCATCACCATTGTTTCATAAACATTAAAAAGAATAA
TAATAXXXXXXXXXXXTTTTAATTAATTTAGTGACACACTTTGATTGAAATCGCATCCTTAGGTCCAATTTTTTCA
TTTGGTAGGTCTTAAGTGGT
>Marker168182
TACTCGGCGACGATAGAAGATTAGGAGAGATATGTTCAAAGAGAGTGTATAGGATACTTATGGCTCAAAATGGAA
ACATGXXXXXXXXXXXATTGCGTTTAACTGTCACAGACAGGTCCAAGGAGTATCATCGTTGGGCCATTACCTAATC
ACAAATGCCACTTGGTAGTC
TACTCGGCGACGATAGAAGATTAGGAGAGATATGTTCAAAGAGAGTGTATAGGATACTTATGGCTCAAAGTGGAA
ACATGXXXXXXXXXXXATTGCGTTTAACTGTCACAGACAGGTCCAAGGAGTATCTTCGTTGGGCCATTACCTAATC
ACAAATGCCACTTGGTAGTC
>Marker168372
AACCTACCTCTCTATCTATCTATGAGATGGCTGGATTTTTTTGAACTTCACGGACAAGCAAAAGAGAAATAATAT
TTCCAXXXXXXXXXXXTAGAATTTAATTCTCGATGTAGTTGCTACATAGTTGGTTCTTATCCCTTAGAGACCACAA
GTGTGTTAGGAGTATCTGTC
AACCTACCTCTCTATCTATCTATGAGATGTCTGGATTTTTTTGAACTTCATGGACAAGCAAAAGAGAAATAATAT
TTCCAXXXXXXXXXXXTAGAATTTAATTCTCGATGTAGTTGCTACATAGTTGGTTCTTATCCCTTAGAGACCACAA
GTGTGTTAGGAGTATCTGTC
>Marker168390
TACCTCTTTGTTCTTTCTAGGGATAATCTTTTATGATATTGTTTGAAACGGAATATTTTTAGAATGCCTTCACAG
TTTTCXXXXXXXXXXXTTATTAGCAAGTCTTTAGATTTAAATATTGGTTGCCTATTGTGATCATCGTTGATTTTAT
TGACCCGTGGGGATTTCTGT
TACCTCTTTGTTCTTTCTAGGGATAATCTTTTATGTTATTGTTTGAAACGGAATATTTTTAGAATGCCTTCACAG
TTTTCXXXXXXXXXXXTTATTAGCAAGTCTTTAGATTTAAATATTGGTTGCCTATTGTGATCATCGTTGATTTTAT
TGACCCGTGGGGATTTATGT
>Marker168925
CACTTCTCAATTTTTATGGTATCTTTTTAACTACAGTTGTGCTTGCTGTTTCTATTTTTCCGATCTTTCTCTCTC
CGTGTXXXXXXXXXXXGAAGTTCCAAAATCTGCAAGAACTCTTCTTGATATATATTACAAATCCGCTGGAAGAAAC
TGTCTCTTGTTGCTAAATGT
CACTTCTCAATTTTTATGGTATCTTTTTAACTACAGTTGTGCTTGCTGTTTCTATTTTTCCAATCTTTCTCTATC
TGTGTXXXXXXXXXXXGAAGTTCCAAAATCTGCAAGAACTCTTCTTGATATATATTACAAATCCGCTGGAAGAAAC
TGTCTCTTGTTGCTAAATGT
>Marker169083

Create PDF with GO2PDF for free, if you wish to

TACACCCAACAGAAAAACTAAAAACCATTCATTTTTATAACATAGTATTTATTTTCAATTTATTCTAGAAGGCTC
TGATAXXXXXXXXXXXTGTAATAAAGGCATGAGATATGATATCTTAGTCATTAATTCTTTCCTTTTCAATATAAAA
ACTTCACACTCATTATAAGT
TACACCCAACAGAAAAACTAAAAACCATTCATTTTTATAATATAGTATTTATTTTCAATTTATTCTAGAAGGCTC
TGATAXXXXXXXXXXXTGTAATAAAGGCATGAGATATGATATCTTAGTCATTAATTCTTTCCTTTTCAATATAAAA
ACTTCACACTCATTATAAGT
>Marker169181
ACTAGAATTACTGTTAGAAGTTGATTTAAAGACTCAAGTGTCCAAATGGAGAAAAAATCCACTTGGTATAAGATA
TTTTCXXXXXXXXXXXGATAATGATTTTTCTTTTCAAATATGGAGGTAAAATTACGTATGTATGTATGCATACATT
ATTAATGTTAAGACGAGGTT
ACTAAAATTACTGTTAGAAGTTGATTTAAAGACTCAAGTGTCCAAATGGAGAAAAAATCCACTTGGTATAAGATA
TTTTCXXXXXXXXXXXGATAATGATTTTTCTTTTCAAATATGGAGGTAAAATTACGTATGTATGTATGCATACATT
ATTAATGTTAAGACGAGGTT
>Marker169379
TACTTTTCATATTAGTTGGCAAATATATGGGTGATTCCAATGCTCACTGAGGAAAATAATTGCTATTTCTGCAAG
GCTATXXXXXXXXXXXCATAGTCGTCCCCAGATAGAACAATGTCATCAACATAAACAATCAATACTGCAACCTTCC
CAGCCTCAGAGATCTTTGTA
TACTTTTCATATTAGTTGGCAAATATATGGGTGATTTCAATGCTCACTGAGGAAAATAATTGCTATTTCTGCAAG
GCTATXXXXXXXXXXXCATAGTCGTCCCCAGATAGAACAATGTCATCAACATAAACAATCAATACTGCAACCTTCC
CAGCCTCAGAGATCTTTGTA
>Marker169482
ACCCTGCTTGAAGAAGAGAACAAAAGAGCAGATGATATTTACAAGGTCTGCAGATATCTTACTTCAGGATGCATT
CTATAXXXXXXXXXXXTCTTAGCTGTTGTAGAAATTACAGTGGCTGGAAGTTGCTCCGGATCACCAACCTATGACA
AAGAAAACCAAATAAATGTG
ACCCTGCTTGAAGAAGAGAACAAAAGAGCAGATGATATTTACAAGGTCTGCAGATATCTTACTTCAGGATGCATT
CTATAXXXXXXXXXXXTCTTAGCTGTTGTAGAAATTACAGTGGCTGGAAGTTGCTCTGGATCACCAACCTATGACA
AAGAAAACCAAATAAATGTG
>Marker170100
AACCTTATCCATAGTGACGTTTGGGGTCCCTCAAAGGGTCATGACGTTCGGTGATTTGTGACCTTTATTGATGAT
CATACXXXXXXXXXXXATTGCAACTCTTCGAAGTGATAATGGTCGTGAGTTTCAAAACATACCCTTAGTGAGTTTC
TGTCCTCCAAAGGGATTGTT
AACCTTATCCATAGTGACGTTTGGGGTCCCTCAAAGGGTCGTGACGTTCGGTGATTTGTGACCTTTATTGATGAT
CATACXXXXXXXXXXXATTGCAACTCTTCGAAGTGTTAATGGTCGTGAGTTTCAAAACATACCCTTAGTGAGTTTT
TGTCCTCCAAAGGGATTGTT
>Marker170554
ACAGAAGCACATGATGATTTGTGTTTAAAATAGGGTCCCTCTATCTGACGCACCAATCCACTACGATGACTCATC
AGCTAXXXXXXXXXXXTTTCTTATAATAGTGTGAAACTGAAACCCTTCACAAAGAGAGCATCTTGATAAAGAGAAA
AGAAAAGGAGTTTCAGAGTA
ACAGAAGCACATGATGATTTGTGTTTAAAATAGGGTCCCTCTATCTGACGCACCAATCCACTACGATGACTCATC
AGCTAXXXXXXXXXXXTTTCTTATAATAGTGTGAAACTGAAACCCTTCACAGAGAGAGCATCTTGATAAAGAGAAA
AGAAAAGGAGTTTCAGAGTA
>Marker170613
TACCAGTGAAAGATAAGAAGGCCTTATTTTCTCAAATTTCCCCTCAACAAGCAATTTACAAATTGAGATAATCAT
AATTTXXXXXXXXXXXCAAAATCGTTATAATTTCAAGGCAACAATCAGAAAGTAACTTAAATACGCAATTACTAAC
TCAATCACTGTCTTACAGTA

Create PDF with GO2PDF for free, if you wish to

TACCAGTGAAAGATAAGAAGGCCTTATTTTCTCAAATTTCCCCTCAACAAGCAATTTACAAATTGAGATAATCAC
AATTTXXXXXXXXXXCAAAATCGTTATAATTTCAAGGCAACAATCAGAAAGTAACTTAAATACGCAATTACTAAC
TCAATCACTGTCTTACAGTG
>Marker171317
AACCACTTCAACGGTAGCGGTGGCTCTGGCGGTAGCGGCGATGTCATGGTCCGTCGCCCTAGGGGCCGCCCGGCC
GGCTCXXXXXXXXXXCGTGGGATCTGTGTGTTGAGTGGAAGTGGGATTGTTAATAATGTTAGCTTACGGCAACCC
GCAGCTGCTGGATCGGTGTT
AACCATTTCAACGGTAGCGGTGGCTCTGGCGGTAGCGGCGATGTCATGGTCCGTCGCCCTAGGGGCCGCCCGGCC
GGCTCXXXXXXXXXXCGTGGGATCTGTGTGTTGAGTGGAAGTGGGATTGTTAATAATGTTAGCTTACGGCAACCC
GCAGCTGCTGGATCGGTGTT
>Marker172483
ACCATCTGTTTTGTATTTCAGAGAGAACACTCATTTGCATCCCACAATTTTGTGTCCCTTGGGGAGAGCACAAAT
TTTCCXXXXXXXXXXGAGTCAAGACTTGCTGTAAAAACTCTGAACTGTGGAGAGAGATTATCATAGGAAACATAG
TTGCAAATTGGATGTTTAGT
ACCATCTGTTTTGTATTTAAGAGAGAACACTCATTTGCATCCCACAATTTTGTGTCCCTTGGGTAGAGCACAAAT
TTTCCXXXXXXXXXXGAGTCAAGACTTGCTGTAAAAACTCTGAATTGTGGAGAGAGATTATCATAGGAAACATAG
TTGCAAATTGGATGTTTAGT
>Marker172611
GACTAAGCATTCCAACAGTAAATTTACTTACCTTGAATTGATGTTTAAACAACTAGCACAAACCCTCTTAACTTG
TAACAXXXXXXXXXXACCTCTAATTAATTACTTACCCCCTTAAGTCTTGATTCAAGGTCAATTTTAGGGTTTTTC
TAATGGATTCGAACCTAAGT
GACTAAGCATTCCTACAGTAAATTTACTTACCTTGAATTGATGTTTAAACAACTAGCACAAACCCTCTTAACTTG
TAACAXXXXXXXXXXACCTCTAATTAATTACTTACCCCCTTAAGTCTTGATTCAAGGTCAATTTTAGGGTTTTTC
TAATGGATTTGAACCTAAGT
>Marker173283
ACTAAATGAGAAGAGGAAGGAACTAGGCGTTATAAGGAAAATCCCAAGCATTTTCGTGATTTATCTTTTGTTTTA
TGAGTXXXXXXXXXXTGATGTTATTGATGTTTTTGTTGGTGTGTTATTTGAAAAGAGAATATTATTGACTTATAT
GTGATGCTTGTGTATGTGTA
ACTAAATGAGAAGAGGAAGGAACTAGGCGTTATAAGGAAAATCCCAAGTATTTTCGTGATTTATCTTTTGTTTTA
TGAGTXXXXXXXXXXTGATGTTATTGATGTTTTTGTTGGTGTGTTATTTGAAAAGAGAATATTATTGACTTATAT
GTGATGCTTGTGTATGTGTA
>Marker174765
ACCCATCATTATAGTTTTGCAACGATATTCCAAAGGTTTAGAATAATCTCCACCGAAGGGCAGTCAATTATTCCT
TCTTTXXXXXXXXXXAACTTTATAATTTTGTAAGTGTAACCCTCCATTTTCGAATCTCAGAGATTAGAATCATTA
TACTCCTGAATGAGAAAGTC
ACCCATCATTATAGTTTTGCAACGATATTCCAAAGGTTTAGAATAATCTCCACCGAAGGGCAGTCAATTATTCCT
TCTTTXXXXXXXXXXAACTTTATAATTTTGTAAGTGTAACCCTCCATTTTCGAATCTCAGAGATTAGAATCGTTA
TACTCCTGAATGAGAAAGTC
>Marker174885
CACAATGATGAACTTTGGGTTCCCACGGGAGAAGGTCTTACATCTGGATGAAACAATTGTTTTTTCTTATCTTTG
CTCGTXXXXXXXXXXTAATAATTCAATCCTGCCAGTGATGCTTATAGTTCAATCTAAACTCTTAACTCAGTTATA
TATGTAATCCATTTTGCAGT
CACAATGATGAACTTTGGGTTCCCACGGGAGAGGGTCTTACATCTGGATGAAACAATTGTTTTTTCTTATCTTTG
CTCGTXXXXXXXXXXTAATAATTCAATCCTGCCAGTGATGCTTTTAGTTCAATCTAAACTCTTAACTCAGTTATA
TATGTAATCCATTTTGCAGT
>Marker175174

Create PDF with GO2PDF for free, if you wish to

ACTAAAAACTTCAGTTTAAATAATGTAAGAAAGTAGATGAATATAAATCATCTTACACTTGAGTTATCTGCCTGC
TCTCAXXXXXXXXXXGCAAACAATACAAGTGCATTGCACATGAGAGATAATTCAACATATCAACTACCTAGCACA
TCATTCAATAGAAGACGGTG
ACTAAAAACTTCAGTTTAAATAATGTAAGAAAGTAGATGAATATAAATCATCTTACACTTGAGTTATCTGCCTGC
TCTCAXXXXXXXXXXGCAAACAATACAAGTGCATTGCACATGAGAGATAATTCAACATATCAACTACCTAGCACA
TCATTCAATAGAAGACAGTG
>Marker176045
ACTCAAATCTTTCCTCTCCTCGTCCCTCTTCAAGATCCTATCAAAATTTGCTTCTACTATTGAGAAACTCTATAG
AAGCTXXXXXXXXXXTCTTTGGCAGTTTGTTCATCCATAACTCAAGAGAAGCATTCCCAGGTTCATCAAGCTTAC
CAATCTTTCAAGTCTTTGTT
ACTCAAATCTTTCCTCTCCTCGTCCCTCTTCAAGATCCTATCAAAATTTGCTTCTACTATTGAGAAACTCTATAG
AAGCTXXXXXXXXXXTCTTTGGCAGTTTGTTCATCCAAAACTCAAGAGAAGCATTCCCAGGTTCATCAAGCTTAC
CAATCTTTCAAGTCTTTGTT
>Marker176244
ACTCTAAGAAATGTTCCTGTGGGTGGTGGATGTAGGAAAAACAAAAGGCCATCACCTTCTACTTCTTCTTCTTCA
TCTCCXXXXXXXXXXTCCAACATCAATCAAATGGGCAAATGGGTCTTTTTGATACAACCCATAACAGCTTTCAAA
ATTTGTATTCTGGGTTTGGT
ACTCTAAGAAATGTTCCTGTGGGTGGTGGATGTAGGAAAAACAAAAGGCCATCACCTTCTACTTCTTCTTCTTCA
TCTCCXXXXXXXXXXTCCAACATCAATCAAATGGGCAGATGGGTCTTTTTGATACAACCCATAACAGCTTTCAAA
ATTTGTATTCTGGGTTTGGT
>Marker176511
ACCAAAGAGACCAAACTTGATTATATATGAATATCCCTAAACCAGTGTTCAAATACAAAAAATACCCAAGCTTTC
AAAAAXXXXXXXXXXGAAGCACCATCCCAGTCAAAATGATAGTAAAAAATATATGCTTGATCATACTCTGTTGTC
TTTCAACTATCTTTTTTGTT
ACCAAAGAGACCAAACTTGATTATATATGAATATCCCTAAACCAGTGTTCAAATACAAAAAATACCTAAGCTTTC
AAAAAXXXXXXXXXXGAAGCACCATCCCAGTCAAAATGATAGTAAAAAATATATGCTTGATCATACTCTGTTGTC
TTTCAACTATCTTTTTTGTT
>Marker177307
TACTCTTTAATTATTTATTTTATCTCTCACGACGGAAAATATAATAAAAAAAAGTTTATACTTATTTTCCTTTTT
TTTAAXXXXXXXXXXCATAATCTTTTTTCGTGTTTATTGTCTTTAAAAAATTAAAATTGATTCTTAAATTTTGGA
GTTGGATTTCAAAAATGGGT
TACTCTTTAATTATTTATTTTATCTCTCACGACGGAAAATATAATAAAAAAAAGTTTATACTTATTTTCCTTTTT
TTAAAXXXXXXXXXXCATAATCTTTTTTCGTGTTTATTGTCTTTAAAAAATTAAAATTGATTCTTAAATTTTGGA
GTTGGATTTCAAAAATGGGT
>Marker178228
ACTGTCGAATAGAGTTTTAATCTCTTAAATATATCTAAATTAGTTGAATTACGTTAAGATTGATCGACTTGTGTT
GGTTCXXXXXXXXXXTGTGCTTCTATTCGAATATTGCTTAATTGTAGAAGAGTTAAATCTCACTTCTACAAATAA
TGGTTGGACTCAAAAAAGTA
ACTGTTGAATAGAGTTTTAATCTCTTAAATATATCTAAATTAGTTGAATTACGTTAAGATTGATCGACTTGTGTT
GGTTCXXXXXXXXXXTGTGCTTCTATTCGAATATTGCTTAATTATAGAAGAGTTAAATCTCACTTCTACAAATAA
TGGTTTGACTCAAAAAAGTA
>Marker178479
ACTCTGTGCTAGGAAAGTATTGATTCCTCACACATTGCAGTTCGCCATTCAAGATACTTGAAGGCTTCAGTGTAT
GATGTXXXXXXXXXXTTGCAGCAACATCTATCTCATTTCCTATATCGACTGTAGATAAAACATGTAAAGACTGAC
AAGTGGCAACATCTAGTGTG

Create PDF with GO2PDF for free, if you wish to

ACCCTGTGCTAGGAAAGTATTGATTCCTCACACATTGCAGTTCGCCATTCAAGATACTTGAAGGCTTCAGTGTAT
GATGTXXXXXXXXXXXTTGCAGCAACATCTATCTCATTTCCTATATCGACTGTAGATAAAACATGTAAAGACTGAC
AAGTGGTAACATCTAGTGTG
>Marker178517
TACAATAGATACCCTTAGAACCTTAGCTTATTGGATTAAAGATTATAGTATTCTATTTTCAGTAATAAGTTCTCA
ATAACXXXXXXXXXXXTCAATTGACTTACGCTTTAAGTTTTCCCTCCCTAGGTTGTATGAGGTAAGTGATCGTCTA
GAAAAGTAAGTGATTGGGTA
TACAATAGATACCCTTAGAACCTTAGCTTATTGGATTCAAGATTATAGTATTCTATTTTCAGTAATAAGTTCTCA
ATAACXXXXXXXXXXXTCATTTGACTTACGCTTTAAGTTTTCCCTCCCTAGGTTGTATGAGGTAAGTGATCGTCTA
GAAAAGTAAGTGATTGGGTA
>Marker179016
ACCTAGAGCACGCCGAATATGATACTGGTAACTTGAATGAGGATCTGAAGACACAGTCCTTGATGAAACAAATGC
TTTTTXXXXXXXXXXXTTAGGCCATTCCTTTTTTCCTTTGATCCTTTGATAAAATATTATGCAAGTTGTTACTCTC
TTCTTTTATGTCATGCAGTG
ACCTAGAGCACGCCGAATATGATACTGGTAACTTGAATGAGGATCTGAAGACACAGTCCTTGATGAAACAAATGC
TTTTTXXXXXXXXXXXTTAGGCCATTCCCTTTTTCCTTTGATCCTTTGATAAAATATTATGCAAGTTGTTACTCTC
TTCTTTTATGTCATGCAGTG
>Marker179098
TACTCGAAATATACCTGAGCGTCAAGAAGTGAGCAAGTAAGTTCCAGTGCCAGCTCCAGTGCCCTAAATTCAAAG
GGCAAXXXXXXXXXXXTGAAATACTATAATCAAACTATCCATTAAAGGATATGCTTGATAACCAGATTGCAATGCA
TCGCTGTGTTGAATAATTGT
TACTCGAAATATACCTGAGCGTCAAGAAGTGAGCAAGTAAGTTCCAGTGCCAGCTCCAGTGCCCTAAATTCAAAG
GGCAAXXXXXXXXXXXTGAAATACTATGATCAAACTATCCATTAAATGATATGCTTGATAACCAGATTGCAATGCA
TCGCTGTGTTGAATAATTGT
>Marker179368
CACAATATACAGCATCCAGATCTTCATCCTTCACTTGCTTCTCTAAGCTCACTGGCATCACCATATGGCCAGCCT
GAGTCXXXXXXXXXXXCACCATGATCACATGTCTTCGATTCACCTTCTCCATCTTCAGTCTTAACGTTTAAGGAAA
CAAGTCGTGGCCTCATTGTG
CACAATATACAGCATCCAGATCTTCATCCTTCACTTGCTTCTCTAAGCTCACTGGCATCACCATATGGCCAGCCT
GAGTCXXXXXXXXXXXCACCATGATCACCGGTCTTCGATTCACCTTCTCCATCTTCAGTCTTCACGTTTAAGGAAA
CAAGTCGTGGCCTCATTGTG
>Marker179482
AACTTGGTTTTGTGATTTATTGAGGTTCCATTTTTTTTTCTATATGGAGTGTATTTATTTGTTTCTTCCCTCTTT
GATTTXXXXXXXXXXXTTAGGTAAGATTGTAACACATAGTTATTCATTGTATAAAGTATGATTGAAATGAGGGTTA
AGAAGAAGCACATGGTTGTG
AACTTGGTTTTGTGATTTATTGAGGTTCCATTTTTTTTTCTATATGGAGTGTATTTATTTGTTTCTACCCTCTTT
GATTTXXXXXXXXXXXTTAGGTAAGATTGTAACACATAGTTATTCATTGTATAAAGTATGATTGGAATGAGGGTTA
AGAAGAAGCACATGGTTGTG
>Marker179503
TACGTATATGAATGTAAGGATTTCTCTTTATTCCTACACGTTAAGAAAGAAAAGAAAAATAAGGAAAGAAAGGAA
AAATGXXXXXXXXXXXACTACAAGATTTACGTGGTTCGGTCAAAGAAAGCTTACATCCACGAAAATGATCTCTTTC
CTTGATATTTGAAGATAAGT
TACGTATATGAATGTAAGGATTTCTCTTTATTCCTACACGTTAAGAAAGAAAAGAAAAATAAGGAAAGAAAGGAA
AAATGXXXXXXXXXXXACTACAAGATTTGCGTGGTTCGGTCAAAGAAAGCTTACATCCACGAAAATGATCTCTTTC
CTTGATATTTGAAGATAAGT
>Marker179536

Create PDF with GO2PDF for free, if you wish to

ACTGTGAAAGCAAACCAAATAGGCATCTTGAGATTGAGGAAGGAATGATGAAGCCAAAATTTCTAAAAATATGCA
TTAAAXXXXXXXXXXTTTTGAACCACGTTGGTAGGACAAACCATAAACACGGTGCAAGGAGTTTCATGAATACGA
TACCATAAAAACTTTTATGT
ACTGTGAAAGCAAACCAAATAGGCATCTTGAGATTGAGGAAGGAATGATGAAGCCAAAATTTCTAAAAATATGCA
TTAAAXXXXXXXXXXTTTTGAACCACGTTCGTAGGACAAACCATACACACAGTGCAAGGAGTTTCATGAATACGA
TACCATAAAAACTTTTATGT
>Marker179581
CACTGTTGCAAATATTCACTTTGCCTACTTCACTCTGACCTCTCCAGAAAACATAGTCCATGAATTGGCGATTTT
CCCTTXXXXXXXXXXTTAAGATTTCTGTCAATCAAGTATTTTCAACTCTTTTTCTATAAGCCTCTTTTCAGATCT
CGAGAAAGTTTGTGTAAGGT
CACTGTTGCAAATATTCACTTTGCCTACTTCACTCTGACCTCTCCAGAAAACATAGTCCATGAATTCGCGATTTT
CCCTTXXXXXXXXXXTTAAGATTTCTGTCAATCAAGTATTTTCAACTCTTTTTCTATAAGCCTCTTTTCAGCTCT
CGAGAAAGTTTGTGTAAGGT
>Marker180347
AACTCCAAAGGATGGAATATGAAGAATGTGTGTTGATAGCAGGGTCATTAATTGAATAACAGTCAAATACCGCTT
CCCTAXXXXXXXXXXGAAGGGTTGTTTGAATGACTTGTGATGCATTTTGGCCTTTCGAATGCCCCAAGCACATTT
ATGTGCCTAATGAACCAAGT
AACTCCAAAGGATGGAATATGAAGAATGTGTGTTGATAGCAGGGTCATTAACTGAATAACAGTCAAATACCGCTT
CCCTAXXXXXXXXXXGAAGGGTTGTTTGAATGACTTGTGATGCATTTTGGCCTTTCCAATGCCCCAAGCACATTT
ATGTGCCTAATGAACCAAGT
>Marker180350
AACTAAACAAGCGGTAGGAAAGTGATCACAATTCTATCTCATTGTCGTATTCATCCTTTGCATTCGTTACAGAGC
TGTTAXXXXXXXXXXCGTTGCTTGTCTTAATTTTGAAGTTAAGAAATGCTTTGTGTCGTCTTGAGTTATTAAACC
GCTACTCATTTCAATAAGTT
AACTAAACAAGCGGTAGGAAAGTGATCACAATTCTATCTCATTGTCGTATTCATCCTTTGCATTCGTTACAGAGC
TATTAXXXXXXXXXXCGTTGCTTGTCTTAATTTTGAAGTTAAGAAATGCTTTGTGTCGTCTTGAGTTATTAAACC
GCTACTCATTTCAATAAGTT
>Marker180451
AACATTCTGACAGGTATTAATTTTTTGACAATGATGAATACCTTTTCCTTTTATAGGAGGAAGATATTGGTTAGG
CTCAAXXXXXXXXXXTAAGATTTCTAGTAGTGGTCATCTTCCTGTTAAGGTTACGAATTTAGGATGTAATTAGTG
ATTAGCTTTGACACTTTGTA
AACATTCTGACAGGTATTAATTTTTTGACAATGATGAATACCTTTTCCTTTTATAGGAGGAAGATATTGGTTAGG
CTCAAXXXXXXXXXXTAAGATTTCTAGTAGTGGTCATCTTCCTGTAAAGGTTACGAATTTAGGATGTAATTAGTG
ATTAGCTTTGACACTTTGTA
>Marker181101
CACTATTAAATAATTGATTTTAGGTTTAACCTATAAACCAAATCCCACTCGAGCCAATAAGAGGGTAGGACGACT
CATTGXXXXXXXXXXAACAAAAGTGTTTGGTAACGTTCCATGTTTCACGTTCTAAAAAAAGTAGAAAGTTTTCCA
TTTCATAAGAAATTATTGTG
CACTATTAAATAATTGATTTTAGGTTTAACCTATAAACCAAATCCCTCTCGAGCCAATAAGAGGGTAGGACGACC
CATTGXXXXXXXXXXAACAAAAGTGTTTGGTAACGTTCCATGTTTCACGTTCTAAAAAAAGTAGAAAGTTTTCCA
TTTCATAAGAAATTATTGTG
>Marker181337
ACAACCAAATATTTAGGCTCAATAGAAAACAATGCTTTTGTTGGAAAGAGAACATGTTAAGGAATCTGACCTTGA
GGAACXXXXXXXXXXATTCTTTCGTTCCACAATTCCATATTGGGATTGAGTATCAGCACAAGATGACCGGTGAAT
GATTGCATGTGTATAAAGGT

Create PDF with GO2PDF for free, if you wish to

ACAACCAAATATTTAGGCTCAATAGAAAACAATGCTTTTGTTGGAAAGAGAACATGTTAAGGAATCTGACCTTGA
GGAACXXXXXXXXXXXATTCTTTCGTTCCACAATTCCATATTGGGATTGAGTATCAGCACAAGATGACTGGTGAAT
GATTGCATGTGTATAAAGGT
>Marker181480
AACCAAAAATCCTATTACGATACTTCCAAATTTCAACGTATGCTATACGTTAAAAGCTTGAATGATAACTAAAAA
AAATCXXXXXXXXXXXTCTCGTGGATTTATTTGTTTAATTTAATTTAGATAATGATAGATATATTTGGTAACCGTT
ATGTTTGTGTTGTGGGTGTT
AACCAAAAATCCTATTACGATACTTCCAAATTTCAACGTATGCTATACGTTAAAAGCTTGAATGATAACTAAAAA
AAATCXXXXXXXXXXXTCTTGTGGATTTATTTGTTTAATTTAATTTAGATAATGATAGATATATTTGGTAACCGTT
ATGTTTGTGTTGTGGGTGTT
>Marker181798
ACCTTTGTGTGGGGTAACCTAATTACTTCGAGAATTAAGAAGCAAAGTGTTGTTGCTAGAAACAATGCTGAAACT
AAACAXXXXXXXXXXXCCTATACAACATGATAGAATGAAACATATGGAGATTGACAGACACATTATAAAACCAAAA
CTGGACAATGACATTATGTA
ACCTTTGTGTGGGGTAACCTAATTACTTCGAGAATTAAGAAGCAAAGTGTTGTTGCTAGAAACAATGCTGAAACT
AAACAXXXXXXXXXXXCCTATACAACATGATAGAATGAAACATATGGAGATTGACAGACACATTGTAAAACCAAAA
CTGGACAATGACATTATGTA
>Marker181937
CACAGGAAACTCATGCAGATATCTCACCATCATCACCTCAGTCACAGAACATCAATGGGGTAAGTTGGAGAACAG
AGCAGXXXXXXXXXXXTCTAGTGGATATATGGATGTTGCATCTTCCAGGACTGAGGAAGATGCCTATGGACTAGGA
TTAAATTCTCCTCTTCTGTT
CACAGGAAACTCATGCAGATATCTCACCATCATCACCTCAGTCACAGAACATCAACGGGGTAAGTTGGAGAACAG
AGCAGXXXXXXXXXXXTCTAGTGGATATATGGATGTTGCATCTTCCAGGACTGAGGAAGATGCCTATGGACTAGGA
TTAAATTCTCCTCTTCTGTT
>Marker181955
AACCTGAGAGACATATTGAAATAGATTGATATAACTCCTCACATGCATATATATATTATTATGATAATCAACACG
ACGCAXXXXXXXXXXXAACTTATTAAAGCAAATACTCCTGACAAAAAAAATCAACATTGTAAATATATTTAGCTAA
GAATACATTATACCCACGTG
AACCTGAGAGACATATTGAAATAGATTGATATAACTCCTCACATGCATATATATATTATTATGATAATCAACACG
ACGCAXXXXXXXXXXXAACTTATTAAAGCAAATACTCCTGACAAAAAAAATCAACATTATAAATATATTTAGCTAA
GAATACATTATACCCACGTG
>Marker182935
GACTGGTTTAATTCACAGATCAAATGAGATTAATAATGGAGAAAATCTCTCTTTTTGGAAAGGCATCTGGAGTAG
TCTTAXXXXXXXXXXXAACAATGGAGGGAGATTAAAAGTGTGCTGACGAACCCTATTGAAAATAGAGGTCAAGATC
TTCCATTATGGAATCTGAGT
GACTGGTTTAATTCACAGATCAAATGAGATTAATAATGGAGAAAATCTCTCTTTTTGGAAAGGCATCTGGAGTAG
TCTTAXXXXXXXXXXXAACAATGGAGGGAGATTAAAAGTGTGCTGACGACCCCTATTGAAAATAGAGGTCAAGATC
TTCCATTATGGAATCTGAGT
>Marker183327
ACCATCGTTACTCTATATTTTTCTCCCATAGTAAGCCCAACCTCATTCTCCAATCTAGCCCAACTACAAGCTTCA
ATTTTXXXXXXXXXXXAAAATCCCATTTATTTTTTATACAGAAGGCTTTCCCATCACATATTTTGGTGAGTTTTGG
TATCCGCTATGTAACAAGTG
ACCATCATTACTCTATATTTTTCTCCCATAGTAAGCCCAACCTCATTCTCCAATCTAGCCCAACTACAAGCTTCA
ATTTTXXXXXXXXXXXAAAATCCCATTTATTTTTTATACAGAAGGCTTTCCCATCACATATTTTGGTGAGTTTTGG
TATCCGCTATGTAACAAGTG
>Marker183718

Create PDF with GO2PDF for free, if you wish to

TACTATTGTCTCCACTTGCATCAACATGTCCTATGTCCTATGTCCTTTACCCTTGAATTTTGAATTTTTTAAAAT
GTGTTXXXXXXXXXXTTCTGTTTTGTAATTTCTTCCTTGGATGATAATCATGAATATGAAAGAGATCTTAATTAT
AACTCCCCCCCTTCTTTGTT
TACTATTGTCTCCACTTGCATCAACATGTCCTATGTCCTATGTCCTTTACCCTTGAATTTTGAATTTTTAAAAAT
GTGTTXXXXXXXXXXTTCTGTTTTGTAATTTCTTCCTTGGATGATAATCATGAATATGAAAGAGATCTTAATTAT
AACTCCCCCCCTTCTTTGTT
>Marker184419
ACAGATGTTACATTTTGTCGCAGTTCATCTTTTCTCCTCTTTCCTTTGTAACTGTGGCCCTTGATAGTTGTTGTA
TGTGAXXXXXXXXXXTGAAAATGAATCTATCACTTGGCCAAAATCAGTTTTTAACTTCCAATTAGATTGGATATG
GGAATGATAGCAAAAAAGTG
ACAGATGTTACATTTTGTCGCAGTTCATCTTTTCTCCTCTTTCCTTTGTAACTGTGGCCCTTGATAGTTGTTGTA
TGTGAXXXXXXXXXXTGAAAATGAATCTATCACTTGGCCAAAATCAGATTTTAACTTCCAATTAGATTGGATATG
GGAATGATAGCAAAAAAGTG
>Marker184629
ACTTTTTTTTTCTTTATTCTTTTACTTGATTAAACCTTCTCAAATTTTGTCACAATCTAAGAGCGAAATTTTTTT
GAGATXXXXXXXXXXTTTGTTCATATAGAAAATAATAACCATTGTAACAAAGATGGAAAGAGATAAGCGAGAGCC
TCAAATATGGAGCGGTCGGT
ACTTTTTTTTTCTTTATTCTTTTACTTGATTAAACCTTCTCAAATTTTGTCACAATCTAAGAGCGAAATTTTTTT
TGAGAXXXXXXXXXXTTTGTTCATATAGAAAATAATAACCATTGTAACAAAGATGGAAAGAGATAAGCGAGAGCC
TCAAATATGGAGCGGTCGGT
>Marker184837
CACATCCACGTAGGAATTACAAAGGCTTGGGAGATTCTAAGTCACTTAAAGTTGATTTAGAACAACCATTTCAAA
CATGGXXXXXXXXXXAATTTTGAAGAGATGAAACAAGACACGTCTCTTATGTAGTTAAGATATCAACTAAATATT
ATTAAATTGGTATATTTGTC
CACATCCAGGTAGGAATTACAAAGGCTTGGGAGATTCTAAGTCACTTAAAGTTGATTTAGAACAACCATTTCAAA
CATGGXXXXXXXXXXAATTTTGAAGAGATGAAACAAGACACGTCTCTTATGTAGTTAAGATATCAACTAAATATT
ATTAAATTGGTATATTTGTC
>Marker184907
TACGACCGAGCTGCTATAGAACTCCAGGGTCCAAACGCCGCAACGAACTTCTCCGGCGACGGAGCAGTTAAATCG
GCGGTXXXXXXXXXXCAGCTTTTTGACTCCGATTGAGGAGATGGGGTATTGTGGAGAGGTGGATGAGTTGGGATT
GGAGATTGGAGCTGCAAGTT
TACGACCGAGCTGCTATAGAACTCCAGGGTCCAAACGCCGCAACGAACTTCTCCGGCGACGGAGCAGTTAAAACG
GCGGTXXXXXXXXXXCAGCTTTTTGACTCCGATTGAGGAGATGGGGTATTGTGGAGAGGTGGATGAGTTGGGATT
GGAGATTGGAGCTGCAAGTT
>Marker185278
CACCTCAAAAGGATAAAAAAATGAATATTATGTATATAGAGAGGTAAGTCATTATATCATTAAATTAATAAATGG
TTATAXXXXXXXXXXTATGAATAAAGAAAAGAAAGATTGAATGAATCATTAAAATTAATTAATAAAGTTAGTGGT
GACGTAAGAGTGAAATTGTG
CACCTCAAAAGAATAAAAAAATGAATATTATGTATATAGAGAGGTAAGTCATTATATCATTAAATTAATAAATGG
TTATAXXXXXXXXXXTATGAATAAAGAAAAGAAAGACTGAATGAATCATTAAAATTAATTAATAAAGTTAGTGGT
GACGTAAGAGTGAAATTGTG
>Marker185477
CACAAATTTTCAATAAAAAACTTGACACAACAAAGATTTACATATCCATTTGTGTTTAGTGGAAGAAGCCCTAAT
TCTTTXXXXXXXXXXGAGAAACAGCTTGAAAGTTCTCTTTAAAATTTTCGATTTTTCTGTGAGTAACACTTTAAC
CTTCTAACTTTACCATTTGT

Create PDF with GO2PDF for free, if you wish to

CACAAATTTTCAATAAAAAACTTGACACAATAAAGATTTACATATCCATTTGTGTTTAGTGGAAGAAGCCCTAAT
TCTTTXXXXXXXXXXXGAGAAACAGCTTGAAAGTTCTCTTTAAAATTTTCGATTTTTCTATGAGTAACACTTTAAC
CTTCTAACTTTACCATTTGT
>Marker185507
ACTTGTATTTACAACCCTTTAGGAATCAAACCAATTCCTCCCGTGTCATCTGTAACCAAAATATAAGTAAGCTCC
ACTATXXXXXXXXXXXGAAAGCACTGAACAAGATATGAAAGTCATGGAAATGACAAGAATGATTTGGTTTGACTTT
GGTTGCCTCTTCAATGAAGT
ACTTGTATTTACAACCCTTTAGGAATCAAACCAATTCCTCCCGTGTCATCTGTAACCAAAATATAAGTAAGCTCC
ATTATXXXXXXXXXXXGAAAGCACTGAACAAGATATGAAAGTCATGGAAATGACAAGAATGATTTGGTTTGACTTT
GGTTGCCTCTTCAATGAAGT
>Marker185556
GACCCAATCTTTTAGTTTGCTCAATTTCCCCCATAAATGTGTTTAGATTGGACCCAGTTAATTTAAATTACTTCG
CAATCXXXXXXXXXXXATAGTTCTTATCTTTTGTTGTGATAATATCACAATTTTGATTTTTATTAGACACAATTTC
TTCTCCAAGTTGTTGGAGTG
GACCCAATCTTTTAGTTTGCTCAATTTCCCCCATAAATGTGTTTAGATTGGACCCAATTAATTTAAATTACTTCG
CAATCXXXXXXXXXXXATTGTTCTTATCTCTTGTTGTGATAATATCACAATTTTGATTTTTATTAGACACAATTTC
TTCTCCAAGTTGTTGGAGTG
>Marker185660
TACTTTTGTTTCTATAAACCTGAAACTTATCTTACCCTTTTTTACCTTCTCAAATCTTATCCATTCACACTTTTA
CATTAXXXXXXXXXXXTGAGTAACTAATAAAGGAGAATATCGTTACTAGAAAATTCTGAAGTTTTCAACAATTAAA
AAACAAACCCAGTTCTTGTT
TACTTTTGTTTCTATAAACCTGAAACTTATCTTACCCTTTTTTACCTTCTCAAATCTTATCCATTCACACTTTTA
CATTAXXXXXXXXXXXTGAGTAACTAATAAAGGAGAATATCGTTACTAGAAAATTCTGAAGTTTTCAACAATGAAA
AAACAAACCCAGTTCTTGTT
>Marker186080
ACTTTATGACAAATTTTTCCTGTCAAATATATGTCAATCGTGACAACTTTTTTTATAAAATCAAATGTCATTGTT
CTGCTXXXXXXXXXXXTGTCTTCTTGTTAAATATGCATAGCTAGCTGAGTTAGGAATGTCAAGGAATGAAATGCAT
CTTTACCCTCTTTTTAAGTG
ACTTTATGACAAATTTTTCCTGTCAAATATATGTCAATCGTGACAACTTTTTTTATAAAATCAAATGTCATTGTT
TCGCTXXXXXXXXXXXTGTCTTCTTGTTAAATATGCATAGCTAGCTGAGTTATGAATGTCAAGGAATGAAATGCAT
CTTTACCCTCTTTTTAAGTG
>Marker186131
ACTAACGTAGCAAGAACCTTTGAAGTAAGAAACTAACACCTCCTTCTACTAATTTAGATCAATCAATAGAAATTA
AAGTTXXXXXXXXXXXCATTCTATCACAAGAATTGCAAATTAAGGAAAAACTCTAAAAAATGAGATTCAAATTGCA
TTCATCAAAAGTCTTGTGTT
ACTAACGTAGCAAGAACCTTTGAAGTAAGAAACTAACACCTCCTTCTACTAATTTAGATCAATCAATAGAAATTA
AAGTTXXXXXXXXXXXCATTCTATCACAAGAATTACAAATTAAGGAAAAACTCTAAAAAATGAGATTCAAATTGCA
TTCATCAAAAGTCTTGTGTT
>Marker186537
GACATGGATCGCCTCGAAAGTTCAGATTTGAGATTGGAATCGAGCATTTGGCCCTCCCTAGACATGCCTGAAACA
CCACAXXXXXXXXXXXCTCACATGTTCTACAATGGCTCGGGAGTTTGGCGAGTGACACAGTCCAATAGCATAGCTT
TGACAATCACCAGATGGGGT
GACATGGATCGCCTCGAAAGTTCAGATTTGAGATTGGAATCGAGCATTTGGCCCTCCCTAGACATGCCTGAAACA
TCACAXXXXXXXXXXXCTCACATGTTCTACAATGGCTCTGGAGTTTGGCGAGTGACACAGTCCAATAGCATAGCTT
TGACAATCACCAGATGGGGT
>Marker187297

Create PDF with GO2PDF for free, if you wish to

GACTTAATTATATACTAGAGTCAAGAGTTTGTGCAGCAAGTATCTGCAAACTCTCTTCTTTTACCCAAACTAACT
TCTTTXXXXXXXXXXXAAACATAGAAAATAGTGGATTAATTTGATTGAACAAGTGTGTGTGTGTGTGTGAAATA
ATGGGACCGTGAGGTAGAGT
GACTTAATTATATACTAGAGTCAAGAGTTTGTGCAGCAAGTATCTGCAAACTCTCTTCTTTTACCCAAACTAACT
TCTTTXXXXXXXXXXXAAACATAGAAAGTAGTGGATTAATTTGATTGAACAAGTGTGTGTGTGTGTGTGAAATA
ATGGGACCGTGAGGTAGAGT
>Marker187399
GACTTTAAAAAAGAGATATATAATGTAAATTGAAATATTCCTTAATGCATTCATAAAGAGAGGCCAGTTTTACAT
ATTTAXXXXXXXXXXXAGTGGCAAAAGAATTTAACCACAGTGATCTTAATAACATAAGCATCACCAACCCTGACG
TATGGCTTCAACTTAAGGGT
GACTTTAAAAAAGAGAGATATAATGTAAATTGAAATATTCCTTAATGCATTGATAAAGAGAGGCCAGTTTTACAT
ATTTAXXXXXXXXXXXAGTGGCAAAAGAATTTAACCACAGTGATCTTAATAACATAAGCATCACCAACCCTGACG
TATGGCTTCAACTTGAGGGT
>Marker187904
AACTTCTACCTCTTATATGGTTCTTAATACGGTGCAGGGTTTGACGTGGCTTCTTCCTGAACGGTTTTCTGATTC
AGAAAXXXXXXXXXXXTAAAAGTTATAAATTTACAGAAGCCTATATCAAAGATTCAAAACCCATTTTATTTTGATT
TAAACAAAAACGAAGAGGTC
AACTTCTACCTCTTATATGGTTCTTAATACGGTGCAGGGTTTGACGTGGCTTCTTCCTGAACGGTTTTCTGATTC
AGAAAXXXXXXXXXXXTAAAAGTTATAAATTTACAGAAGCCTATATCGAAGATTCAAAACCCATTTTATTTTGATT
TAAACAAAAACGAAGAGGTC
>Marker188348
AACTCATGTTGTGACTAAGTAGATGAAAAGACTGACGTCTATAGAATTTTTCAATTCTTCTTCTTTTTTCTACTC
GATCAXXXXXXXXXXXCTATAATTTAGTATTTTGGCATGTTTTAGATTAATATATTATAGTCTTCTATCACTGATA
GTCAGTAATAGTCAATGGTA
AACTCATGTTGTGACTAAGTAGATGAAAAGACTGACGTCTATAGAATTTTTCAATTCTTCTTCTTTTTTCTACTC
GATCAXXXXXXXXXXXCTATAATTTGGTATTTTGGCATGTTTTAGATTAATATATTATAGTCTTCTATCACTGATA
GTCAGTAATAGTCAATGGTA
>Marker188659
ACCCTGCACAGTCAAAGTCTTGTGATCTCGCAGGATACACATAATCAGTCTAACGCCCCGAAGGACGCATAAATC
AGTCTXXXXXXXXXXXTTGGCATATAACTTATTCACTCGAAGGGTCTCCAACACTTGATGCAAATGCTCCTCATGC
TCAGCCTCTGTCTTGGAGTA
ACCCTGCACAGTCAAAGTCTTGTGATCTCGTAGGATACACATAATCAGTCTAACGCCCCGAAGGACGCATAAATC
AGTCTXXXXXXXXXXXTTGGCATATAACTTATTCACTCGAAGGGTCTCCAACACTTGATGCAAATGCTCCTCATGC
TCAGCCTCTGTCTTGGAGTA
>Marker188663
TACTTTATTTGATTATAGTATTGCATTATATCAAATCATGAAATCGATTGGGGTAAGTGTTATGTTTGAACATAT
TTGTGXXXXXXXXXXXATATGAAAATACAATGTAATTGTGAGGGGAAATATGGAACATACGGTTTCTCATCACTTG
CATTTCTATGGTTAAAGAGT
TACTTTATTTGATTATAGTGTTACATTATATCAAATCATGAAATCGATTGGGGTAAGTGTCATGTTTGAACATAT
TTGTGXXXXXXXXXXXATATGAAAATACAATGTAATTGTGAGGGGAAATATGGAACATACGGTTTCTCATCACTTG
CATTTCTATGGTTAAAGAGT
>Marker189074
AACTAAATTTGTTGCAACAATTGTAGTCTTCATGTTCGTTTTACAATTCATGTTCGTTTTTGTTTTCAATCGCTA
TTTTTXXXXXXXXXXXATCTGCTAGTTTTCTCTTATTTTGATAATTGAATTTAGATTAATGTTTAGAATATGATTG
ATTGATGACCATGTGTGGTT

Create PDF with GO2PDF for free, if you wish to

AACTAAATTTGTTGCAACAATTGTAGTCTTCATGTTCGTTTTACAATTCATGTTCGTTTTTGTTTTCAATCGCTA
TTTTTXXXXXXXXXXATCTGCCAGTTTTCTCTTATTTTGATAATTGAATTTAGATTAATGTTTAGAATATGATTG
ATTGATGACCATGTGTGGTT
>Marker189162
ACTCCCGTTATTGAAGCTTGGAACCCTGGCTTTGAAAACTCTGAGCAAGCCACTCGGTAATAGACTCAAGAAACA
AGCTGXXXXXXXXXXTGATTGTTCTGTTCTGTTCTGATCTATACAAGAGCACATGTTGTTGAGCTGTTGTTGTTT
TATAACAAATGGTCATGGTC
ACTCCCGTTATTGAAGCTTGGAACCCTGGCTTTGAAAACTCTGAGCAAGCCACTCGGTAATAGACTCAAGAAACA
AGCTGXXXXXXXXXXTGATTGTTCTGTTCTGTTCTGATCTATACAAGAGCACGTTTTGTTGAGCTGTTGTTGTTT
TATAACAAATGGTCATGGTC
>Marker189293
GACTTCCATTCTCGGTTGATGATGGCTCTCTTGCAGATTGGGCATCGGATTTTCTCAAGGGAGAACAGTTGTTAA
GAGATXXXXXXXXXXAAAGAAATCACCGCATTGGAGCCTGCTGGAGCAACACCAAAACTATCTCCTCTGTGGTGG
GCAGAACTAGAAATCTTGTC
GACTTCCATTCTCGGTTGATGATGGCTCTCTTGCAGATTGGGCATCGGATTTTCTCAAGGGAGAACAGTTGTTAA
AAGATXXXXXXXXXXAAAGAAATCACAGCATTGGAGCCTGCTGGAGCAACACCAAAACTATCTCCTCTGTGGTGG
GCAGAACTAGAAATCTTGTC
>Marker189493
ACTCTTCTTGGTATCTAGGTGGGTAGTTAGTCATCAGTTTTCAGTGTAAAATGGAAATAATCACCAGTGCTTCCA
ATGATXXXXXXXXXXTCTTATTATCTGACTCTCGATCAACTTATAGATTCTTAACTTTCTGAGAAACAGGTCTCA
AAGAACCAATGTGTTTTGTT
ACTCTTCTTGGTATCTAGGTGGGTAGTTAGTCATCAGTTTTCACTGTAAAATGGAAATAATCACCAGTGCTTCCA
ATGATXXXXXXXXXXTCTTATTATCTGACTCTCGATCAACTTATAGATTCTTAACTTTCTGAGAAACAGGTCTCA
AAGAACCAATGTGTTTTGTT
>Marker189506
TACCCAAATATGACCCGGTTTGCTAATCCCCAAAACATTCCTTATATACCTCATCAAGGGTGAGTTCCCCCCCCA
AGAACXXXXXXXXXXATGTTTGACTATATTGTAGATGGCTGATGTTTGAATATAAAGGGCTAGTTATATCATTAG
GGTGTCTTCTTTCCATGAGT
TACCCAAATATGACCCGGTTTGCTAATCCCCAAAACATTCCTTATATACCTCATCAAGGGTGAGTTCCCCCCCAA
GAACCXXXXXXXXXXATGTTTGACTATATTGTAGATGGCTGATGTTTGAATATAAAGGGCTAGTTATATCATTAG
GGTGTCTTCTTTCCATGAGT
>Marker189848
AACAAATTCAAAGTTTTTAAATGAAAAAGAAGTAATCAGACAACTAATTAAAACTATCTTTTAAACATCAAACAA
CAACAXXXXXXXXXXTATGCACCCCACTTGAGTAAAAATAAAAACTTTGGTATCCAACATTTCAAAATGAACCTT
ATTTACTTGCAAGTTGGTGT
AACAAATTCAAAGTTTTTATATGAAAAAGAAGTAATCAGACAACTAATTAAAACTATCTTTTAAACATCAAACAA
CAACAXXXXXXXXXXTATGCACCCCACTTGAGTAAAAATAAAAACTTTGGTATCCAACATTTCAAAATGAACCTT
ATTTACTTGCAAGTTGGTGT
>Marker189938
AACAATCATCAAATCAAGATCAAACATTTTCTCGTGTTGCATCAAAATACCTCTTCCTTGCATTATCAAACCAAA
ACGCAXXXXXXXXXXATTAACAATGATTGGATGTATGCATGGTTTCACTTTTTGATAATAGAGGAGGAAAAGAAA
AAAGAAAAACTTTGAAGGTA
AACAATCATCAAATCAAGATCAAACATTTTCTCGCGTTGCATCAAAACACCTCTTCCTTGCATTATCAAACCAAA
ACGCAXXXXXXXXXXATTAACAATGATTGGATGTATGCATGGTTTCACTTTTTGATAATAGAGGAGGAAAAGAAA
AAAGAAAAACTTTGAAGGTA
>Marker190056

CACCCTTGATAGAGTGCTTGGTTGAGTTGGTGTTCGCTGGTGGGTGGAAATTGGATAACGAGACATTTGGACCCG
GTTATXXXXXXXXXXTTAGAAATGTGGGGTCCATCATGCAATGGGTTTGGTTGGAACGATGAGGTGAAGTGCATG
ATCGCTAAGAAGGAATTGTT
CACCCTTGATAGAGTGCTTGGTTGAGTTGGTGTTTGCTGGTGGGTGGAAATTGGATAACGAGACATTTGGACCCG
GTTATXXXXXXXXXXTTAGAAATGTAGGGTCCATCATGCAATGGGTTTGGTTGGAACGATGAGGTGAAGTGCATG
ATCGCTAAGAAGGAATTGTT
>Marker190212
CACTTCCAGCACAATATCGGACACGGACCCGCAGTTGCTCTCGCCGCTCTTAGTAGCCAGAACCCTGGTGTTATT
ACCATXXXXXXXXXXATAATTATTACTTTTTTATGTTTTGAGTCTGTGGTGTTGTGTATGTGATGAAATCTTTGA
TTCTTTATTTGTTTGGATGT
CACTTCCAGCACAATATCGGACACGGACCCGCAGTTGCTCTCGCCGCTCTTAGTAGCCAGAACCCTGGTGTTATT
ACCATXXXXXXXXXXATAACTATTACTTTTTTATGTTTTGAGTCTGTGGTGTTGTGTATGTGATGAAATCTTTGA
TTCTTTATTTGTTTGGATGT
>Marker190287
ACTAGTTTAATTATGTGAGAAGAACAACAACGTTGTGAGAGAAGTATTCTATGTAATTGTAACAAATTTTTACCT
ATGGAXXXXXXXXXXAGTTCGTATATGAATGTGTTTTATACCATCAAATACAAATGTTATCTTACATTTTTTCTT
TATATTTGTATTAAATTGTG
ACTAGTTTAATTATGTGAGAACAACAACAACGTTGTGAGAGAAGTATTCTATGTAATTGTAACAAATTTTTACCT
ATGGAXXXXXXXXXXAGTTTGTATATGAATGTGTTTTATACCATCAAATACAAATGTTATCTTACATTTTTTCTT
TATATTTGTATTAAATTGTG
>Marker190420
AACGATTGAACCAAATAAAATCACAACCAAACAAAATGACAGAATTCTCCCCTGAAGAAACTGACTAAGACTTAA
CCTTGXXXXXXXXXXCAAGACAAAACTTATTAGAATCCAGAAACAAAACAAAGGGAATTGAGATTGGCCCTGAGA
TAGTGAGTATAATAAAGGGT
AACGATTGAACCAAATAAAATCACAACCAAACAAAATGACAGAATTCTCCCCTGAAGAAACTGACTAAGACTTAA
CCTTGXXXXXXXXXXCAAGACAAAACTTATTAGAATCCAGAAACAAAACAAAGGGAATTGAGATTGACCCTGAGA
TAGTGAGTATAATAAAGGGT
>Marker190477
AACCTATACAGCATCTGATAAACCTTATAATTTGAAAAAATCGCAGAAACGTCATGGAGAGAAAGAAAAGCGCGG
ATATAXXXXXXXXXXGCGATTTGTTTCAACCAGAAATTTATTTCCATATTTGGAAAGAGGATAAGAACACACCAT
TTCCCATCCCAATAATTAGT
AACCTATACAGCATCTGATAAACCTTATAATTTGAAAAAATCGCAGAAACGTCATGGAGAGAAAGAAAAGCGCGG
ATATAXXXXXXXXXXGCGATTTGTTTCAACCAGAAATTTATTTCCATATTTTGAAAGAGGATAAGAACACACCAT
TTCCCATCCCAATAATTAGT
>Marker190733
AACCCGATTTAACCCAAACTCAATTCTTTTTTCATTTTTAGAAAAATAGATTTTTTAACTAAGGTAATTAGTTTG
GTAATXXXXXXXXXXAGAGAAAAAGAAAGAGTTATTAAATTTATGTATAAAATGGGAAAGCTTGGTCCTCAAGTA
ATTGACATCATTTTCTTTGT
AACCCGATTTAACCCAAACTCAATTCTTTTTTCATTTTTAGAAAAATAGATTTTTTAACTAAGGTAATTAGTTTG
GTAATXXXXXXXXXXAGAGAAAAAGAAAGAGTTATTAAATTTATGTATAAAATGGGAAAGCTTGGTCCTCAAGTA
ATTGACATCAGTTTCTTTGT
>Marker191036
ACCCGAAAACATGAAGAAAATGCTCGACTAATGGAAGAAATGAAGAAAATGATTGAGGAATTGAGTTGGATATAG
AGAAGXXXXXXXXXXGGAACTTGGCGTTTGCGACTATTAGGCAAATTAGTTTTTAGTTTTTATTTTTCATATAAT
TTTTGAACCTACAAATATGT

Create PDF with GO2PDF for free, if you wish to

ACCCGAAAACATGAAGAAAATGCTCGACTAATGGAAGAAATGAAGAAAATGATTGAGGAATTGAGTCGGATATAG
AGAAGXXXXXXXXXXXGGAACTTGGCGTTTGCGACTATTAGGCGAATTAGTTTTTAGTTTTTATTTTTCATATAAT
TTTTGAACCTACAAATATGT
>Marker191269
AACCGCAGAAACAGAGCTGACTTTGTATAGACTACATAACCTCCATTGTCTAATCAACTTAAAATTTGTAACTCT
GTCATXXXXXXXXXXXTATTTCATCAAACAGAACCTGATCATAAACTAAAACCCCACCAAAATTCTTCAACTGAAA
CGGAGACCTCTCCTCGGGGT
AACCGCAGAAACAGAGCTGACTTTGTATAGACTACATAACCTCCATTGTTTAATCAACTTAAAATTTGTAACTCT
GTCATXXXXXXXXXXXTATTTCATCAAACAGAACCTGATCATAAACTAAAACCCCACCAAAATTCTTCAACTGAAA
CGGAGACCTCTCCTCGGGGT
>Marker191350
TACCAAGCTTAATAAATTCTTTTAAATAGATTTTCTTTTTCTAGAAAAAAATCATCCAACCACCCAAATGATATC
ACAATXXXXXXXXXXXAAAGTAAAGAGGGAAGGATCCATAGGTAATTTTATGTTTCGTTTCTTCGATTCAAAACAA
TAACGTTATATAACGATGTC
TACCAAGCTTAATAAATTCTTTTAAATAGATTTTCTTTTTCTAGAAAAAAATCATCCAACCACCCAAATGATATC
ACAATXXXXXXXXXXXAAAGTAAAGAGGGAAGGATCCATAGGTAATTTTATGTTTCGTTTCTTCGTTTTAAAACAA
TAACGTTATATAACGATGTC
>Marker191847
GACAGAACAGACTGACAAACCCCTTGAAACTAAGTAATTGAAACAAAATAGAGATTGAATGTGAAATACTTTGTT
GTCTTXXXXXXXXXXXATGTTGAGAAGCCAAATCTTGTTGCCAAATTGATTACACAATTTGTTCATGAAGATTCCA
GAAAAGAGGTTCAGTTTGTT
GACAGAACAGACTGACAAACCCCTTGAAACTAAGTAATTGAAACAAAATAGAGATTGAATGTGAATGACTTTGTT
GTCTTXXXXXXXXXXXATGTTGAGAAGCCAAATCTTGTTGCCAAATTGATTACACAATTTGTTCATGAAGATTCCA
GAAAAGAGGTTCAGTTTGTT
>Marker192044
ACATCTATTTACAACCATGGTTATAATCAGTGAAGAACAAAATAAAGACAAGAACTAAATGTTTTTTTTTTTAGT
TTTAAXXXXXXXXXXXCTATCTAATCTAATAGATTCCAATTTAAGTTTGGACTGAATTTTGTATCTAATGGCAATC
CTACCTTTCAATTTTGTGTA
ACATCTATTTACAACCATGGTTATAATCAGTGAAGAACAAAATAAAGACAAGAACTAAATGTTTTTTTCTTTTAG
TTTTAXXXXXXXXXXXCTATCTAATCTAATAGATTCCAATTTAAGTTTGGACTGAATTTTGTATCTAATGGCAATC
CTACCTTTCAATTTTGTGTA
>Marker192382
ACAGTAAATACATGGTAAATGTGTATTTAATTAAATATTAGCATCTAACGTAAATAGATAAGTCTAACATGGCTC
ATGCTXXXXXXXXXXXCCATTCCCAGGTTACAGAAAAACAACAATCAACCGAAATGTGGAATATATCAGATCACAG
GGTTAGTCAGTATTGGAAGT
ACAGTAAATACATGGTAAATGTGTATTTAATTAAATATTGGCATCTAACGTAAATAGATAAGTCTAACATGGCTC
ATGCTXXXXXXXXXXXCCATTCCCAGGTTACAGAAAAACAACAATCAACCGAAATGTGGAATATATCAGATCACAG
AGTTAGTCAGTATTGGAAGT
>Marker192686
AACTAAGCAATCACTCTTCTAATTCTATAAATATATTAGTCCTCTCTCTATTTAATTTGTTCTATAACAAACAAT
AATAAXXXXXXXXXXXTCTACACATCAAAATTTGTTCAACGCCATGAGTTATATTATGTAAATGAATGATAATGTT
ATCGTTTTAAATTTAAGGTC
AACTAAGCAATCACTCTTTTAATTCTATAAATATATTAGTCCTCTCTCTATTTAATTTGTTCTATAACAAACAAT
AATAAXXXXXXXXXXXTCTACACATCAAAATTTGTTCAACGCCATGAGTTATATTATGTAAATGAATGATAATGTT
ATAGTTTTAAATTTAAGGTC
>Marker192942

Create PDF with GO2PDF for free, if you wish to

AACTGCCAACAGAATGAGTTGTGTGCATTGGAGCTGTTGAACTGATTTTCTGCAGTTGGTCCCTTCACAGCCCCA
TGGTGXXXXXXXXXXTTGCTACTGGCTCTTTGTTCAGTGTTGTCCTCTTCTTCCTCTAGGTTTCGTTGGGGATAT
CTTCATTTTAATTTGGGGTG
AACTGCCAACAGGATGAGTTGTGTGCATTGGAGCTGTTGAACTGATTTTCTGCAGTTGGTCCCTTCACAGCCCCG
TGGTGXXXXXXXXXXTTGCTACTGGCTCTTTGTTCAGTGTTGTCCTCTTCTTCCTCTAGGTTTCGTTGGGGATAT
CTTCATTTTAATTTGGGGTG
>Marker193059
ACTTCTTTTCCTATTGTCACTCTAAACACACCATATGATTGTCTCCTTCTCTTTACATAAAACTACAAGTATATC
TAAAAXXXXXXXXXXCCAAGAACTAAATATACCAATGCAAATGATGCTAATGGGAATTGACAATAATTCAAGTTG
TTAGATATTAGCGTTATGTC
ACTTCTTTTCCTATTGTCACTCTAAACACACCATATGATTGTCTCCTTCTCTTTACATAAAACTACAAGTATATA
TAAAAXXXXXXXXXXCCAAGAACTAAATATACCAATGCAAATGATGCTAATGGGAATTGACAATAATTCAAGTTG
TTAGATATTAGCGTTATGTC
>Marker193227
ACTTATAACAGTCGATGTCGCAACAGCATGTAGTCATAGCATTAATGATTTTTTCCCCCTTTTTTATTTGAATTA
CCAAAXXXXXXXXXXAAACAGCACAAGATTCAAACTGCAAACTTTGTGGCAAAGTTTATCATAAAACAAAAGAAA
CAAATAATTTTACCCTGAGT
ACTTATAACAGTCGATGTCGCAACAGCATGTAGTCATAGCATTAATGATTTTTTCCCCCTTTTTGATTTGAATTA
CCAAAXXXXXXXXXXAAACAGCACAAGATTCAAACTGCAAACTTTGTGGCAAAGTTTATCATAAAACAAAAGAAA
CAAATAATTTTACCCTGAGT
>Marker193495
ACTATGTTTTATCTTGGAAAACTACTGCTCCTAAAATTAGTCACTGGACCGAAAAAAATTGTCATAAGAAGACAG
ATAGTXXXXXXXXXXCTGTCGAGCTCCTTATAAACGTCGGACTCCAAAATATCGGAAGTGAGTTTTCGCCATGAA
ATGTTCTTCTTCTCCGCTGT
ACTATGTTTTATCTTGGAAAACTACTGCTCCTGAAATTAGTCACTGGACCGAAAAAAATTGTCATAAGAAGACAG
ATAGTXXXXXXXXXXCTGTCGAGCTCCTTATAAACGTCGGACTCCAAAATATCGGAAGTGAGTTTTCGCCATGAA
ATGTTCTTCTTCTCCGCTGT
>Marker193566
ACTTTTCTATTATAATAATTAATGATATAAAGTGATGAAACCTTCTACCACCAGAGAAGAATATTATATTATATG
TCACTXXXXXXXXXXAGTTTATTTACTTTTCATTAGTTTCTTACTTTCTTCATCTGTAATTTTTCTTTCATTAAC
CCAAAGATAATAATAATAGT
ACTTTTCTATTATAATAATTAATGATATAAAGTGATGAAACCTTCCACCACCAGAGAAGAATATTATATTATATG
TCACTXXXXXXXXXXAGTTTATTTACTTTTCATTAATTTCTTACTTTCTTCATCTGTAATTTTTCTTTCATTAAC
CTAAAGATAATAATAATAGT
>Marker193617
GACATGCCTAGTGGAAATGTTTGTGTAGAATGATGCAAAATCACATTTTTTCTTATTTAGGTGGTTGCTTTTTTG
TGTTTXXXXXXXXXXTACATATCTCTCTCTGATTGAACAAAAATATTGTAGTTTATTTTAAACCTAAGTGTCTTC
TAGAAGAGTTGTTTAAAGTT
GACATGCCTAGTGGAAGTGTTTGTGTAGAATGATGCAAAATCACATTTTTTCTTATTTAGGTGGTTGCTTTTTTG
TGTTTXXXXXXXXXXTACATATCTCTCTCTGATTGAACAAAAATATTGTAGTTTATTTTAAACCTAAGTGTCTTC
TAGAAGAGTTGTTTAAAGTT
>Marker193623
AACCCTTTAAGTTTGATTCTATGAAAAATGCTTACATGGTTTCCTGAACAGTTATCATATTATCATTTGGACTAA
AATATXXXXXXXXXXTTAAGGACTATATATTTTATAGGCATGGTATAAAAGAGTTTTGCTATCTCATTTTGTGTT
TATCCTTTTCACTTTTGGTT

Create PDF with GO2PDF for free, if you wish to

AACCCTTTAAGTTTGATTCTATGAAAAATGCTTACATGGTTTCCTGAACAGTTATCATATTATCATTCGGACTAA
AATATXXXXXXXXXXTTAAGGACTATATATTTTATAGGCATGGTATAAAAGAGTTTTGCTATCTCATTTTGTGTT
TATCCTTTTCACTTTTGGTT
>Marker194300
ACAAACTTTCATAAACCGATCATTCTCACAATCCGAATGAAAAAAAGCTAAACTATAAATAACGAAACTAGAAAG
CCAAAXXXXXXXXXXTCTAGGTTTTGATCTTGATGGGTCTCTGTCAAATGGGAATTCGAAGGCTCAGAGCCATTA
ATGGGTTTGTTGTTGATGTT
ACAAACTTTCATAAACCGATCATTCTCACAATCCGAATGAAAAAAAGCTAAACTATAAATAAAGAAACTAGAAAG
CCAAAXXXXXXXXXXTCTAGGTTTTGATCTTGATGGGTCTCTGTCAAATGGGAATTCGAAGGCTCGGAGCCATTA
ATGGGTTTGTTGTTGATGTT
>Marker194505
AACCCACATAACACCTAAATATCACAGATTCAAATACGAGAATGAAAAACGATAAGACACGACACAGGCATGGCT
ACAAGXXXXXXXXXXAATGAGCTTGTGCATTTACATCATTAAAATTCATGATGCGTTTCGCTCTCATAATTGATT
ATTTTCGTCCCATATGTGTC
AACCCACATAACACCTAAATATCACAGATTCAAATACGAGAATGAAAAACGATATGACACGACACAGGCATGGCT
ACAAGXXXXXXXXXXAATGAGCTTGTGCATTTACATCATTAAAATTCATGATGTGTTTCGCTCTCATAATTGATT
ATTTTTGTCCCATATGTGTC
>Marker194651
CACTTGGACTTTGTTATTGTATGATGGGAGTTTGTTTAACTTGCCATACTACTTAGTTCATGATTTTGTTTTGTT
TTGTTXXXXXXXXXXATGAGGGTTGCCGTTGGTATTCACAAAGATGATATTGATTCTGCTATTAAAACTTATCAC
ACAATGTCACAACGTTGGTT
CACTTGGACTTTGTTATTGTATGATGGGAGTTTGTTTAACTTGCCATACTACTTAGTTCATGATTTTGTTTTGTT
TTTCTXXXXXXXXXXATGAGGGTTGCCGTTGGTATTCACAAAGATGATATTGATTCTGCTATTAAAACTTATCAC
ACAATGTCACAACGTTGGTT
>Marker194920
TACTTTCAAATTGAAGAAGAAAAACCTGAAAATATAGCTGTCATTCTACACCAAACAACAAATTATTTCCATTTA
AAAAAXXXXXXXXXXTATAACATCTGCCTCCATTACAGCGTAGCTCTATTATATGACATGATCTGAATGTTTCCA
GGGTCCATTGATATATTAGT
TACTTTCAAATTGAAGAAGAAAAACCTGAAAATATAGCTGTCATTCTACACCAAACAACAAATTATTTCCATTTA
AAAAAXXXXXXXXXXTATAACATCTGCCTCCATTACAGCGTAGCTCTGTTATATGACATGATCTGAATGTTTCCA
GGGTCCATTGATATATTAGT
>Marker194932
GACAGCTTATGGCACAAAGAGAGGTTCAGTCTGAGGCTCTAGGCAATAATCACCAACAAGTTCAAAACTCTGATG
TGCGTXXXXXXXXXXAATGGGATAGTCATCCTACAGATGATGTGAAGGAATTAACCGATTCTAAAAGGGAACTAA
CGCATCATTAGCCACTTGTC
GACAGCTTATGGCACAAAGAGAGGTTCAGTCTGAGGCTCTAGGCAATAATCACCAACAAGTTCAAAACTCTGATG
TGCGTXXXXXXXXXXAATGGGATAGTCATCCTACAGATGATGTGAAGGAATTAACCGATTCTAAAAGGGAACTAA
CGCGTCATTAGCCACTTGTC
>Marker195333
ACAAAGGATAATAAGTAGGGAGAAGAAGTTATCTCATTGTATATGACTTTTGGTTCTACTTTACTGGTGATAAGA
AGGAAXXXXXXXXXXTTTCCTTGTGAATGATGAGGCTCAAGTTCTCTTTTTTTAATGTCATGATCAAATCTAACA
TTTCAAACGAACGATCAAGT
ACAAAGGATAATAAGTAGGGAGAAGAAGTTATCTCATTGTATATGACTTTTGGTTCTACTTTACTGGTGATAAGA
AGGAAXXXXXXXXXXTTTCCTTGTGAATGATGAGGCTCAAGTTCTCTTTTTTTAATGTCATGATCAAATCTAACG
TTTCAAACGAACCATCAAGT
>Marker195832

Create PDF with GO2PDF for free, if you wish to

TACACAATGTTCAAAAAAGAAAAAAAAAGAAAAAGAAAAAGAGGAAACTCACAAACCATAGCGAATGTCACTAAAT
TTAGTXXXXXXXXXXXTGCTTTCGCAGTCGGACCGTTGAAATTGTAAGAGTTAGATCGAGTTTGGAAACCGAACTC
ACCCGAGGTTGAGGAGGAGT
TACACAATGTTCAAAAAAGAAAAAAAAAAAAAAAGAAAAAGAGGAAACTCACAAACCATAGCGAATGTCACTAAAT
TTAGTXXXXXXXXXXXTGCTTTCGCAGTCGGACCGTTGAAATTGTAAGAGTTAGATCGAGTTTGGAAACCGAACTC
ACCCGAGGTTGAGGAGGAGT
>Marker195883
AACTCCTGCCTATGAAGGTGATCATTTGATTTGCATGGGTGAGAGTGGCCTATGTCGCAGACTCAATATTTCTAC
CATTTXXXXXXXXXXXGTGCCACACCCTCTCTTGACCCTAGAGGAGTTCAGTTACAACGGAACTATAACTTATTGT
TCATTAGAGGAACCAACAGT
AACTCCTGCCTATGAAGGTGATCATTTGATTTGCATGGGTGAGAGTGGCCTATGTCGCAGACTCAATATTTCTAC
CATTTXXXXXXXXXXXGCGCCACACCCTCTCTTGACCCTAGAGGAGTTCAGTTGCAACGGAACTATAACTTATTGT
TCATTAGAGGAACCAACAGT
>Marker196412
GACCTACACTCGTATTATTTATACGTATTTTGATTAAATTGGGTCGAATTTAAGTGAAGAACTTCCTCCAACTTA
CTACTXXXXXXXXXXXATTTTACAATATATCTCTAGCGATAATATCGATAAATACACACAACAACAATGATGATGT
TGGTATAAATAAGTTGTGTG
GACCTACACTCCTATTATTTATACGTATTTTGATTAAATTGGGTCGAATTTAAGTGAAGAACTTCCTCCAACTTA
CTACTXXXXXXXXXXXATTTTACAATATATCTCTAGCGATAATATCGATAAATACACACAACAACAATGATGATGT
TGGTATAAATAAGTTGTGTG
>Marker196480
ACCTTTTAATAAGAATGCAGTAAATGAGCTGCAAACCCAAAAACTAATGAACAGGGATATAACATCGTTAAAAAA
TGAGAXXXXXXXXXXXATTTTTCAACTTTTTTTAGAGTCGATTGGAGTAATTTCTTAATTAACATGACCTTCTGTA
TATATGCTTGGCAAAACAGT
ACCTTTTAATAAGAATGCAGTAAATGAGCTGCAAAGCCAAAAACTAATGAACAGGGATATAACATCGTTAAAAAA
TGAAAXXXXXXXXXXXATTTTTCAACTTTTTTTAGAGTCGATTGGAGTAATTTCTTAATTAACATGACCTTTTGTA
TATATGCTTGGCAAAACAGT
>Marker196495
AACGCTTATTTTCTCCATTAAAATGTCATTTCCATTGCTCTATCTCGGGGTTATTTAAATTTCAAATGCACTTCT
AATTAXXXXXXXXXXXATTAAAGCAGGGAATGAAAATAATATTTTAATCTTACTAGCTACTTATTCATATTTTATT
TTTATCATATAATCAATGTC
AACGCTTATTTTCTCCATTAAAATGTCATTTCCATTGCTCTATCTCGGGGTTATTTAAATTTCAAATGCACTTCT
AATTAXXXXXXXXXXXATTAAAGCAGGGAATGAAAATAATATTTTAATCTTACTAGCTACTTATTCATATTTTATT
TTGATCATATAATCAATGTC
>Marker196811
TACGACTAAATGAAGAAGAACCAGTATTTTAAGCTTGCAGACCTGCACCATTATCAACTTTGCTGTTATTGCCTC
CATTGXXXXXXXXXXXATTTCAAGAAGTAAAGTTATTTCCATTCTGATACCCCATTATAAGGATAGTAACACCAAA
TAGCTGTGTGACCAAATGTC
TACGACTAAATGAAGAAGAACCAGTATGTTAAGCTTGCAGACCTGCACCATTATCAACTTTGCTGTTATTGCCTC
CATTGXXXXXXXXXXXATTTCAAGAAGTAAAGTTATTTCCATTCTGATACCCCATTATAAGGATAGTAACACCAAA
TAGCTGTGTGACCAAATGTC
>Marker196896
ACCCTTACTGTTTTAGTGCAATTGAAGAGAGATATTTGAATTACAAATTTTATGATTATACATATAGTTATATAC
TCATTXXXXXXXXXXXTCAAAGTATTCCCAAGTATGTGTTTTACTTCTAAAACTACTGTCAATCAAAATCAACGTG
CGCATGCATCAACCATAGTC

Create PDF with GO2PDF for free, if you wish to

ACCCTTACTGTTTTAGTGCAATTGAAGAGAGATATTTGAATTACAAATTTTATGATTATACATATAGTTATATAC
TCATTXXXXXXXXXXXTCAAAGTATTCCCAAGTATGTGTTTTACTTCTAAAATTACTGTCAATCAAAATCAACGTG
CGCATGCATCAACCATAGTC
>Marker197111
ACTTGAAAGAGTGTAGCAATATAAGAAAGCACCAAAATTATATTTAGCCAATTTTGCCAGTGAGTCCTAGCACTA
AAATTXXXXXXXXXXXAAGTTAAAATAACTTTCAAAAGAATAAATTTATCTGTAACGATAGAGTTTATTTCATGTT
GTATTAAAAATTAGAATGGT
ACTTGAAAGAGTGTAGCAATAGAAGAAAGCACCAAAATTATATTTAGCCAATTTTGCCAGTGAGTCCTAGCAATA
AAATTXXXXXXXXXXXAAGTTAAAATAACTTTCAAAAGAATAAATTTATCTGTAACGATAGAGTTTATTTCATGTT
GTATTAAAAATTAGAATGGT
>Marker198038
CACTTTCTGTAATCTCATATAGCTTTCACAATGGTGGGCAGCCCTCACTTTTCTCTATTGTTTGTATCTTTTATG
GACAGXXXXXXXXXXXCCTATGGATGTCAAATGAATATTAATGATATGGAGGTAGTTCTATCTATCATGAAAAAAG
CTGGATACAGTGAAACTGTT
CACTTTCTGTAATCTCATATAGCTTTCACAATGGTGGGCAGCCCTCACTTTTCTCTATTGTTTGTATCTTTTATG
GAGAGXXXXXXXXXXXCCTATGGATGTCAAATGAATATTAATGATATGGAGGTAGTTCTATCTATCATGAAAAAAG
CTGGATACAGTGAAACTGTT
>Marker198455
CACTCATAGTTAATTCATTAATGATAGTTATTATCATTGATAAGACATGCATTAAAATTTTCAAACTTTAATAGA
TAATAXXXXXXXXXXXTTTTTGAAATGGTTTTAAGGGTATATGTTGTTTAATAAACAAAACTTTAATGTAACAATT
AAAAAAGAACATAGTTAGTA
CACTCATAGTTAATTCATTAATGATAGTTATTATCATTGATAAGACATGTATTAAAATTTTCAAACTTTAATAGA
TAATAXXXXXXXXXXXTTTTTGAAATGGTTTTAAGGCTATATGTTGTTTAATAAACAAAACTTTAATGTAACAATT
AAAAAAGAACATAGTTAGTA
>Marker198515
AACAACAGTATCCAAATTCAGCTTCTTTTTTTTCTTAATTCAATAAGCATGGAGGAAAGATAAAAAAAAAACCTT
TTAATXXXXXXXXXXXCAGATACAAAGCACACATCATTTTAATTATACTCCCCCATTCTTCATATATTTGCTTAGA
GAAGTGTTGAACTGAAAGTA
AACAACAGTATCCAAATTCAGCTTCTTTTTTTTCTTAATTCAATAAGCATGGAGGAAAGAGAAAAAAAAAACCTT
TTAATXXXXXXXXXXXCAGATACAAAGCACATATCATTTTAATTATACTCCCCCATTCTTCATATATTTGCTTAGA
GAAGTGTTGAATTGAAAGTA
>Marker199175
ACACTGAGGGGCACTTTTATCACACTTTATTCACAAAATTATCTAAGTCATGAAAAATTACAGTTTTGTAGTCTA
TGTTGXXXXXXXXXXXAAATACTTCCTTGGGATGGAAATTGTCATATTTAGAGAGGGTGTCTCCGTATCCCAAAGA
AAATACATAATTGGTTTGTT
ACACTGAGGGGCACTTTTATCACACTTTATTCACAAAATTATCTAAGTCATGAAAAATTACCGTTTTGTAGTCTA
TGATGXXXXXXXXXXXAAATACTTCCTTGGGATGGAAATTGTCATATTTAGAGAGGGTGTCTCCGTATCCCAAAGA
AAATACATAATTGGTTTGTT
>Marker199389
ACCTTGTTCAAGACTTGGATTCAGTCATTAAGGGAACAACCTATCTACTAACCCGAAAGCGGTTAGGAGTGAATT
CCGTCXXXXXXXXXXXGTGAATAGGAGTCCATAGTTGGCTCAAGGTTAAGATTAAGTTAAAAAGGATATCTATAAA
ATAGTAAGTTTTATTAAGTT
ACCTTGTTCAAGACTTGGATTCAGTCATTAAGGGAACAACCTATCTACTAACCCGAAAGCGGTTAGGAGTGAATT
TCGTCXXXXXXXXXXXGTGAATAGGAGTCCATAGTTGGCTCAAGGTTAAGATTAAGTTAAAAAGGATATCTATAAA
ATAGTAAGTTTTATTAAGTT
>Marker199401

Create PDF with GO2PDF for free, if you wish to

AACCCAATTCCAACATAAATGGTAGCTGCCGCTTCCTGGACTCGTGCTTGGGCTGGGTTAAGTCTTCAATTTCAA
AGGTAXXXXXXXXXXATTTATTTTCTTTTAATTGTCCAAAATTTCATTCATGAAACGTTAATCCTAGATTTTGTT
CTTCTTCTTACTTTATTGTT
AACCCAATTCCAACATAAATGGTAGCTGCCGCTTCCTGGACTCGTGCTTGGGCTGGGTTAAGTCTTCAATTTTAA
AGGTAXXXXXXXXXXATTTATTTTCTTTTAATTGTCCAAAATTTCATTCATGAAACGTTAATCCTAGATTTTGTT
CTTCTTCTTACTTTATTGTT
>Marker200248
ACTGAAATTTTAATTTCTTGTTCAAAGCTTTAGATTATATTTGTAAGCTAGAAGTATTTTCTCTCTTAAAGAGAG
AGAGGXXXXXXXXXXACAAAGAAAAGGAGAACGACCAATCTTGGATGTAAAAAATAGATGAACAATGTGAAACTA
ATATTTAAGGATCTCTGGTC
ACTGAAATTTTAATTTCTTGTTCAAAGCTTTAGATTATATTTGTAAGCTAGAAGTATTTTCTCTCTTAAAGAGAG
AGAGGXXXXXXXXXXACAAAGAAAAGGAGAACGACAAATCTTGGATGTAAAAAATAGATGAACAATGTGAAACTA
ATATTTAAGGATCTCTGGTC
>Marker200270
ACAGATTGTGTATCTCCACGTGTTGAACTTACTGATGTCAGGCCAACTTAGTTTTGTTCATAAAAGGGAATTGAT
TTATTXXXXXXXXXXATCTCTATGTTTAAAAGACTTCTGTTGTATAAAGTTTCATGCATTCATGTCATGTGTCAG
TTGAGGTCATTACATAGGTG
ACAGATTGTGTATCTCCACGTGTTGAACTTACTGATGCCAAGCCAACTTAGTTTTGTTCATAAAAGGGAATTGAT
TTATTXXXXXXXXXXATCTCTATGTTTAAAAGACTTCTGTTGTATAAAGTTTCATGCATTCATGTCATGTGTCAG
TTGAGGTCATTACATAGGTG
>Marker200273
TACATATATATACACACACATAATATTAGATTACAAGAATTTGTTGGATTTTGTTCTGGTTGAATCTTGGTATCT
CTTTTXXXXXXXXXXTTAGACTAATTTTATTGTTTTAAATTTAGAGTATAAGATAGAAAGCATAGTTGAAAAGAT
TGATGCTGTGATGCTTAAGT
TACATATATATACACACACATGATATTAGATTACAAGAATTTGTTGGATTTTGTTCTGGTTGAATCTTGGTATCT
CTTTTXXXXXXXXXXTTAGACTAATTTTATTGTTTTAAATTTAGAGTATAAGATAGAAAGCATAGTTGAAAAGAT
TGATGCTGTGATGCTTAAGT
>Marker200653
ACTCCTTTCAGAATGTTGCTACATTATGTCAATATACCTAGTGTATTTTGTCTAACTTTTGTTTAATCTTTGCAG
GTTTCXXXXXXXXXXATGTGATTGAGTTGCTTTCCTAGTAATGTATGTGTGTGTTTATATAGAAGCTCTCTTAAC
TCATTCCACTATACATTAGT
ACTCCTTTCAGAATGTTGCTACATTATGTCAATATACCTAGTGTATTTTGTCTAACTTTTGTTTAATCTTTGCAG
GTTTCXXXXXXXXXXATGTGATTGAGTTGCTTTCCTAGTGATGTATGTGTGTGTTTATATAGAAGCTCTCTTAAC
TCATTCCACTATACATTAGT
>Marker200695
CACCAAGCTATTTTCACTGATCCGGATGATCAAAGTGGTTGGTTTTATCATCTTTGGCTTCTGGACCAAACGGTG
AAAGCXXXXXXXXXXCCTCTCCATTCTGTAGTTTCTATTCGGATTCAGGAACAATACCCCTCATTCTTTACTTTG
ATCAACCTGTTCAAGGCGTT
CACCAAGCTATTTTCACTGATCCGGATGATCAAAGTGGTTGGTTTTATCATCTTTGGCTTCTGGACCAAACGGTG
AAAACXXXXXXXXXXCCTCTCCATTCTGTAGTTTCTATTCGGATTCAGGAACAATACCCCTCATTCTTTACTTTG
ATCAACCTGTTCAAGGCGTT
>Marker201297
ACAACATAATGTAGAAGTTCATAATGATTTATTTATTTTTATATGTTGAGGCTCGATTCAGTTTCGACTATGTTA
TGCAAXXXXXXXXXXGGTATTTCCATTAATTCGTGCTCGTAGGATGTATGCTTTGCTGACCCCTCTTCTTTTGTA
TTTGTTGGGTTAATAATAGT

Create PDF with GO2PDF for free, if you wish to

ACGACATAATGTAGAAGTTCATAATGATTTATTTATTTTTATATGTTGAGGCTCGATTCAGTTTCGACTATGTTA
TGCAAXXXXXXXXXXXGGTATTTCCATTAATTCGTGCTCGTAGGATGTATGCTTTGCTGACCCCTCTTCTTTTGTA
TTTGTTGGGTTAATAATAGT
>Marker201458
CACCCATGTAGTGTTCAATAGATGCAACCTTAAGTTCTGGCTTAAGACATACCCCATTCCTATTTATTCTAATAT
TTACTXXXXXXXXXXXTAAGGGTGAGTGGCTTGCATTTGGTTGAACAGTCTATATTTAATCCTCTAGTCTTGGCGG
CCTTGGGATAGAGTCCTGTG
CACCCATGTAGTGTTCAATAGATGCAACCTTAAGTTCTGGCTTAAGACATACCCCATTCCTATTTATTCTAATAT
TTACTXXXXXXXXXXXTAAGGGTGAGTGGTTTGCATTTGGTTGAACAGTCTATATTTAATCCTCTAGTCTTGGCGG
CCTTGGGATAGAGTCCTGTG
>Marker201469
ACTCCCGTTATTTTCGTCAGGGTCTTTGTCTTCTTTTTCTCTCTTCTCAATGATACTGGTATTCGGGTCCTTGTT
ATTTTXXXXXXXXXXXTGAAAATGATTTTCCATGGAGGTAACTTAGTTCTGGCCTTGGATTTGTGAATAACTGCTT
ACATAAGATGAAATTTTAGT
ACTCCTGTTATTTTCGTCAGGGTCTTTGTCTTCTTTTTCTCTCTTCTCAATGATACTGGTATTCGGGTCCTTGTT
ATTTTXXXXXXXXXXXTGAAAATGATTTTCCATGGAGGTAACTTAGTTCTGGCCTTGGATTTGTGAATAACTGCTT
ACATAAGATGAAATTTTAGT
>Marker201941
ACCAGGCACAGGACTACAATATTTGAACATTCCAGTATCAGACAGAAAGTAAATGACAATAAACAAATACACCAA
CATATXXXXXXXXXXXGCATGAGATTAGCGGGTTGAAAGTTAGAAACAAAGAAGATTTGAACCTCCGTCTGCGCTC
ACAATTTAGGCTATTGGGTA
ACCAGGCACAGGACTACAATATTTGAACATTCCAGTATCAGACAGAAAGTAAATGACAATAAACAAATACACCAA
CATATXXXXXXXXXXXGCATGAGATTAGCGGGTTGAAAGTTAGAAACAAAGAAGATTTGAACCTCCGTCTGCACTC
ACAATTTAGGCTATTGGGTA
>Marker201963
GACCATGCCCTCGAGACGGAACTGCACGACGTCCGCCATGGTCTAGCTGGGTAAGAGGGGAGTCGGAGGGAATGG
AGCTGXXXXXXXXXXXAGTTATTTACAAAAATGTTAGAGCCCACTCTCTCTTTTTTTAAAGATACAAATTACTTTC
TGAAAATCTAGCTATTGGTT
GACCATGCCCTCGAGACGGAACTGCACGACGTCCGCCATGGTCTAGCTGGGTAAGAGGGGAGTCGGAGGGAATGG
AGCTGXXXXXXXXXXXAGTTATTTACAAAAATGTTAGAGCCCACTCTCTCTTTTTTCAAAGATACAAATTACTTTC
TGAAAATCTAGCTATTGGTT
>Marker202612
TACTTGGCAATGATAAATAATGTTAAAGAGATCTGAGGGCATCTTGAAAATGGTTAATTTTTCATGAAACTGCAA
CTGTTXXXXXXXXXXXGTTGTATTTTAAAATGGTTCTTTTTATTGTATATGTAAGCTAGTTTTGACACTTACAGAT
GTCAAAAAGAATAAAGTGGT
TACCTGGCAATGATAAATAATGTTAAAGAGATCTGAGGGCATCTTGAAAATGGTTAATTTTTCATGAAACTGCAA
CTGTTXXXXXXXXXXXGTTGTATTTTAAAATGGTTCTTTTTATTGTGTATGTAAGCTAATTTTGACACTTACAGAT
GTCAAAAAGAATAAAGTGGT
>Marker205080
ACATTGTGATTCTTTGTTTGCTTTGCAACCTATCTTTTACATTTTTGTATCAATTAAGGACTTATTTGATTGATA
TGAAGXXXXXXXXXXXCTTCACAGACAAACCATGCATGAATGATTCCTAAACAAAATCATAACTAGAAACCTTAAA
TTGCTTTACTTATTAAAGTG
ACATTGTGATTCTTTGTTTGCTTTGCAACATATCTTTTACATTTTTGTATCAATTAAGGACTTATTTGATTGATA
TGAAGXXXXXXXXXXXCTTCACAGACAAACCATGCATGAATGATTCCTAAACAAAATCATAACTAGAAACCTTAAA
TTGCTTTACTTATTAAAGTG
>Marker205093

Create PDF with GO2PDF for free, if you wish to

CACAATGCTCATCAGCGTCAGGAAAATACTCCAACTTGAATTACATCAGCAGCGACGAGTATGAGACCAGCATGA
GTTCAXXXXXXXXXXXCAAGGAGTTGCCTCTGGTCGCAAAGACAAGAAAGGAACAGATGGTCGTAAATTAACTAGG
AACAGATCACTAGTAGATGT
CACAATGCTCATCAGCGTCGGGAAAATACTCCAACTTGAATTACATCAGCAGCGACGAGTATGAGACCAGCATGA
GTTCAXXXXXXXXXXXCAAGGAGTTGCCTCTGGTCGCAAAGACAAGAAAGGAACAGATGGTCGTAAATTAACTAGG
AACAGATCACTAGTAGATGT
>Marker205268
AACCTTAGGTCTTGAAAACTAAAAAGTATTGGTTCTATTTAATAACTATTGACTTACCTTTCACAATTTTTTATT
TATAAXXXXXXXXXXXAGATCGGAATTAGCCTTTGGGCTCTTTCATATGAACACACGATTAAGGGTAGATCGTATT
CAATTTTTTATAAAAATGTA
AACCTTAGGTTTTGAAAACTTAAAAGTATTGGTTCTATTTAATAACTATTGACTTACCTTTCACAATTTTTTATT
TATAAXXXXXXXXXXXAGATCGGAATTAGCCTTTGGGCTCTTTCATATGAACACACGATTAAGGGTAGATCGTATT
CAATTTTTTATAAAAATGTA
>Marker205962
AACAATTGCAAATTCTTTTAGTAATCACCTATAGGTGCTTATTTCATTTTATCAATGAGATGTTTCTTAACTAAA
CAAAAXXXXXXXXXXXTTATGAAAATGGAGGATTTGGAATCTCTCTAAAGTCGGTGAGTTTTTTTCTTTTCTGAAA
ACTTTGAGCTCTTTTTTGTG
AACAATTGCAAATTCTTTTAGTAATCACCTATAGGTGCTTATTTCATTTTATCAATGAGATGTTTCTTAACTAAA
CAAAAXXXXXXXXXXXTTATGAAAATGGAGGATTTGGAATCTCTCTAAACTCGGTGAGTTTTTTTCTTTTCTGAAA
ACTTTGAGCTCTTTTTTGTG
>Marker206194
TACAGCAGTTGTCAAAATGCATACTAACTTATGTTCAACTTTGACAATGAAGCAATGCCTGTTTGATCATAAGAG
GCCTCXXXXXXXXXXXGTTTTGCAGTGTGAGAACCAAATGTGTAGGTAGAAACAAAGTTGCTAGAATCTCCTAAAA
GCTTGAACCGTTCCAAAGGT
TACAGCAGTTGTCAAAATGCATACTAACTTATGTTCAACTTTGACAATGAAGCAATGCCTGTTTGATCACAAGAG
GCCTCXXXXXXXXXXXGTTTTGCAGTGTGAGAACCAAATGTGTAGGTAGAAACAAAGTTGCTAGAATCTCCTAAAA
GCTTGAACCGTTCCAAAGGT
>Marker206975
TACCAATGAGGCATAACAAACAAGTTTATTAGCAAGACATCAATTGTCTTAAACTTATTAGTGAAGCATTTAAGT
GTATCXXXXXXXXXXXAGGAGTATCAGGTATATCAGCTGTATCAAATGTGAATTAATAAAGAGGGCATTTGTGACA
TTTTACATATTGTATATGTG
TACCAATGAGGCATAACAAAGAAGTTTATTAGCAAGACATCAATTGTCTTAAACTTATTAGTGAAGCATTTAAGT
GTATCXXXXXXXXXXXAGGAGTATCAGGTATATCAGCTGTATCAAATGTGAATTAATAAAGAGGGCATTTGTGACA
TTTTACATATTGTATATGTG
>Marker207309
TACATGGTTTTTTTATTTTCTAGAATTTAAAAATATATATCTTCACTTAAAATAAGGATACGAACTGAGGGGAGG
GAATAXXXXXXXXXXXATCTCCGGATATTCTCTCACAGTCTCACTTTCCCTTAAATCCCTTCAATTTCTCTACTCT
TTCCAGTTTCCACATCAAGT
TACATGGTTTTTTTATTTTCTAGAATTTAAAAATATATATCTTCACTTAAAATAAGGATACGAACTGAGGGGAGG
GAATAXXXXXXXXXXXATCTCCGGATATTCTCTCACAGTCTCACTTTCCCTTAAATCCCTTCAATTTCTCTACCCT
TTCCAGTTTCCACATCAAGT
>Marker207411
AACCATGATTAATATTTGCATTTATTAGATATCTTATGTAAATATTGTAAGTGGTTAATTCTATGGTAATATTGT
AATAAXXXXXXXXXXXCCTAATTATAGTATATATTGAATAATACGATGTATATTTTATTAATGAAAAATTATTATA
TCATTTGTTTATAGAAGGTG

Create PDF with GO2PDF for free, if you wish to

AACCATGATTAATATTTGCATTTATTAGATATCTTATGTAAATATCGTAAGTGGTTAATTCTATGGTAATATTGT
AATAAXXXXXXXXXXXCCTAATTATAGTATATATTGAATAATACGATGTATATTTTATTAATGAAAAATTATTATA
TCATTTGTTTATAGAAGGTG
>Marker207863
ACATTTATTACTCATCTTCTCAACTTTTTTGCTTTGTGGTTCATTTCATGGAGAATTTGCTTAATTTGAAACTTA
TTTTCXXXXXXXXXXXTATTGTTATTTTAGCCACCGCATTTTAATTCTCTCATAGATCACTCGAGCTCCACTGTGA
ATCCTATGAGCATCTAGGTT
ACATTTCTTACTCATCTTCTCAACTTTTTTGCTTTGTGGTTCATTTCATGGAGAATTTGCTTAATTTGAAACTTA
TTTTCXXXXXXXXXXXTATTGTTATTTTAGCCACCGCATTTTAATTCTCTCATAGATCACTCGAGCTCCACTGTGA
ATCCTATGAGCATCTAGGTT
>Marker208350
TACTGAACTTGTCATGTTCGAAGTAGTAAAATTTATCATTTCATTACATGAAAATAAGTTTTGGAGAGAAATTTC
TCAAAXXXXXXXXXXXTTATCCTTTCTTTTTTTCCTTGGAAACGTGGCCTAGTTGTTTTATTTCCTGAATTCTATT
TATCATGCCAAGAATCTGTT
TACTGAACTTGTCATGTTCGAAGTAGTAAAATTTATCATTTCATTACATGAAAATAAGTTTTGGAGAGAAATTTC
TCAAAXXXXXXXXXXXTTATCCTTCCTTTTTTTCCTTGGAAACGTGGCCTAGTTGTTTTATTTCCTGAATTCTATT
TATCATGCCAAGAATCTGTT
>Marker208544
ACTTGTGTTTGATATTTGAGATGTAACGTAACCCAACTAATGGCATTATTATTTTTTTGAGAGAACTAATTGCAT
TAATTXXXXXXXXXXXTAATTGCAGGTTGGAATGGCTGCTACTCTTGCTGGGGTTTGTCAGGTGCCTCTTACTGCT
GTTTTGTTGCTTTTTGAGTT
ACTTGTGTTTGATATTTGAGATGTAACGTAACCCAACCAATGGCATTATTATTTTTTTGAGAGAACTAATTGCAT
TAATTXXXXXXXXXXXTAATTGCAGGTTGGAATGGCTGCTACTCTTGCTGGGGTTTGTCAGGTGCCTCTTACTGCT
GTTTTGTTGCTTTTTGAGTT
>Marker209058
CACTATTTCAAAGCTTTCTTAAAAATTGGTTCTATCTGCAAAGCAATTTGATGGGATGGAAATGGATGTCTATCT
ATATGXXXXXXXXXXXTCACACCTATTTTAGACACCAATTAACCTCTAGATTCCATTCCCACTAACATCAATATAA
TAAAGTATAAAGAGGCATGT
CACTATTTCAAAGCTTTCTTAAAAATTGGTTCTATCTGCAAAGCAATTTGATGGGATGGAAATGGATGTCTATCT
ATATGXXXXXXXXXXXTCACACCTATTTTAGACACCAATTAACCTCTAGATTCCCTTCCCACTAACATCAATATAA
TAAAGTGTAAAGAGGCATGT
>Marker209614
ACAAGCAATGGGAAACAATTTGAATATATGAATTTGATTATTCTATCTTCTATGAATGGGAGAGATGAAAAACAA
AAAAGXXXXXXXXXXXATTTTGAATCTCTACCATAATTTTATTTCTTTTTTAGAATTTAAAATTAGGGTTTTATGC
ATTTAATTGGGACAGTGGTT
ACAAGCAATGGGAAACAATTTGAATATATGAATTTGATTATTCTATCTTCTATGAATGGGAGAGATGAAAAACAA
AAAAGXXXXXXXXXXXATTTTGAACCTCTACCATAATTTTATTTCTTTTTTAGAATTTAAAATTAGGGTTTTATGC
ATTTAATTGGGACAGTGGTT
>Marker209644
GACGCAAACACTTGAAAATATTAAAAAGAACAAAATAAAATAAAACATATGTTCAAATACTGGTATCCTCCACTG
CATGAXXXXXXXXXXXAAAATATTAATTATATGATATAATACATAATTGAAATACCATTCCTTGCTTTTAAAAATT
ATATAGCACACGAAATAAGT
GACGCAAACACTTGAAAATATTAAAAAGAACAAAATAAAATAAAACATATGTTCAAATACTGGTATCCTCCACTG
CATGAXXXXXXXXXXXAAAATATTAATTATATGATATAATACATAATTGAAATACCATTCTTTGCTTTTAAAAATT
ATATAGCACACGAAATAAGT
>Marker210840

Create PDF with GO2PDF for free, if you wish to

AACTCTTGTTAGATAATGTAATTACATAATATATTATAATAGTTGAAAAAACCAATTGTGTTATAATGAAAGCAT
CAAAGXXXXXXXXXXTATGAATATGAAATTGATTATATAGCATCTAAGGACAATTTTGTATCTACCATGTATGTA
AATATGTTATATACATAGTA
AACTCTTGTTAGATAATGTAATTACATAATATATTATAATAGTTGAAAAAACCAATTGTGTTATAATGAAAGCAT
CAAAGXXXXXXXXXXTATGAATATGAAATTGATTATATAGCATCTAACGACAATTTTGTATCTACCATGTATGTA
AATATGTTATATACATAGTA
>Marker211004
TACATTCATGACCATGTGATACTGAATAAAACCATATTCAAAGTCAAGGCATACCCACCTTGCTCAATCTTTAAC
TCTAGXXXXXXXXXXAAAATAACAAATAAGAGGCTGATGAAGTCAGTAGAAAGATAAGAGTAAAATAGGAAATAA
AACAAACATTTAAAACCAGT
TACATTCATGACCATGTGATACTGAATAAAACCATATTCAAAGTCAAGGCATACCCACCTTGCTCAATCTTTAAC
TCTAGXXXXXXXXXXAAAATAACAAATAAGAGGCTGATGAAGTCAGTAGAAAGATAAGAGTAAAATAGGAAATAA
AACAAACATTTAAAACTAGT
>Marker211978
ACTTTGTATAACTATTAACTTCAACAAATAGCTCACTTTTATTTGCAAGTTAGGCATGTATTTTCCGTCGTTAGG
TCAAAXXXXXXXXXXXTAATGGAATTGAACTTTTAATTTTTTAGATGGATAGTATGATTTTTATGTTGTTTGAATT
ATGACATGCTTTTCCTGGTT
ACTTTGTATAACTATTAACTTCAACAAATAGCTCACTTTTATTTGCAAGTTAGGCATGTATTTTCCGTCGTTAGG
TCAAAXXXXXXXXXXXTAATGGAATTGAACTCTTAATTTTTTAGATGGATAGTATGATTTTTATGTTGTTTGAATT
ATGACATGCTTTTCCTGGTT
>Marker212876
ACCCCTATGTTACTTATAGGCCTTAAATAACAATGCTCCTCTTACGAACAACCTGTTTATGGTTCAACTAATAAA
CAAAAXXXXXXXXXXXATGTTATCAGATCCCTTTTCGATCTTGTCTCTCCAATATGATAAACTTATCGAGTCTGTA
ATCTGATCACTCTCACATGT
ACCCCTATGTTACTTATAGGCCTTAAATAACAATGCTCCTCTTACGAACAACCTGTTTATGGTTCAACTAATAAA
CAAAAXXXXXXXXXXXATGTTATCAGATCCCTTTTCGATCTTGTCTCTCCAAAATGATAAACTTATCGAGTCTGTA
ATCTGATCACTCTCACCTGT
>Marker214163
ACTTTAACACATAAATTTGTATATTAATAACGTGGCTATGTTATGAAAATGTCCTTAAACTTTGTTATTTGTTTT
TAAAAXXXXXXXXXXXCTGCTTAATAAAATTCAATTATAATGTTAGATGAAAAAGAAAGAATTGAAATATATTGTA
ATTTTTATGTTGCATTTGGT
ACTTTAACACATAAATTTGTATATTAATAACGTGGCTATGTTATGAAAATGTCCTTAAACTTTGTTATTTGTTTT
AAAAAXXXXXXXXXXXCTGCTTAATAAAATTCAATTATAATGTTAGATGAAAAACAAAGATTTGAAATATATTGTA
ATTTTTATGTTGCATTTGGT
>Marker214294
AACAAATATAATGTGTATTGATTAAATTAATGTTACATATGCAACAAAGATCTGTCACATCAAACCTAAGTCAGT
GACTAXXXXXXXXXXTTTCGTCTAGAGCCTGAAATGTGAAAACATGCTTGAGTTTTTAAACTTGCAGTGTGATCA
GATTTTGGGTAAAAGTTGGT
AACAAATATAATGTGTATTGATTAAATTAATGTTACATATGCAACAAAGATCTGTCACATCAAACCTAAGTTAGT
GACTAXXXXXXXXXXTTTCGTCTAGAGCCTGAAATGTGAAAACATGCTTGAGTTTTTAAACTTGCAGTGTGATCA
GATTTTGGGTAAAAGTTGGT
>Marker214382
CACAAGGATCTTCATGTTATGAGACAACATCTAGTAGTGACCATATGCTTGAAGATCACAATATAATATTATCAA
TTTATXXXXXXXXXXAGATCCTAAGTGTGTGGTTTCAAATCGAACATTCTTTGTGTATCAAACAAAGGTTTAAAT
AACCAACCAGGTGCAAATGT

Create PDF with GO2PDF for free, if you wish to

CACAAGGATCTTCATGTTATGAGACAACATCTAGTAGTGACCATATGCTTGAAGATCACAATATAATATTATCAA
TTTATXXXXXXXXXXAGATCCTAAGTGTGTGGTTTCAAATCGAACATTCTTTATGTATCAAACAAAGGTTTAAAT
AACCAACCAGGTGCAAATGT
>Marker214524
ACCATTCAGGCATTCCACAACCCAAGCCAAGCACCCACGCACGCTCCAAAACAAAATTCTACTTCTTTACTCTTA
TTCCTXXXXXXXXXXACTAACTTTTCTTCATATTTGGGACGACATTAGTAAATGGTTCCTACTTCGACGGCACTT
CATAACGTATATACATCTGT
ACCATTCAGGCATTCCACAACCCAAGCCAAACACCCACGCACGCTCCAAAACAAAATTCTACTTCTTTACTCTTA
TTCCTXXXXXXXXXXACTAACTTTTCTTCATATTTGGGACGACATTAGTAAATGGTTCCTACTTCGACGGCACTT
CATAACGTATATACATCTGT
>Marker214693
ACTCCTCTTTGTGCATGTTAGCCTTATCTTTGCTACTTGCCCTCTTGAGTAATTGACGATATACATTGGCCAAGC
AATAAXXXXXXXXXXGAGAGAGAAGTCAAGAAATGATGGATTAAAAAGGTATAAACATGTAATATATATAAAGAA
TGTAGGCCTTAGGCCATTGT
ACTCCTCTTTGTGCATGTTAGCCTTATTTTTGCTACTTGCCCTCTTGAGTAATTGACGATATACATTGGCCAAGC
AATAAXXXXXXXXXXGAGAGAGAAGTCAAGAAATGATGGATTAAAAAGGTATAAACATGTAATATATATAAAGAA
TGTAGGCCTTAGGCCATTGT
>Marker214874
ACGGAGAATGATTGACATTATGATAGAATTGCTATTAATTCATCCATGTGCTATATGACACATGCTCCAAAGTAT
TTCTCXXXXXXXXXXATTCTCTCTTCTTAATATTTTTTTTTTGGAATATGTTGGTTATCTTTGGTATTTGGTATT
AGTTCTTACATGTTTCTTGT
ACGGAGAATGATTGACATTATGATAGAATTGCTATTAATTCATCCATGTGCTATATGACACATGCTCCAAAGTAT
TTCTCXXXXXXXXXXATTCTCTCTTCTTAATATTTTTTTTTTGGAATATGTTGGTTATCTTTGGTATTTGGTATT
AGTTCTTACAGGTTTCTTGT
>Marker214942
AACTTTCTCATATCCAAAATGTTCAACAACCTTCTTCAATAAAATAGACTCAAATTATGAACTTTCAAGACCAAT
TTTAGXXXXXXXXXXTGAATTAATTAAACGTTAGCTAAAATGTTTGAATTAATTAAACGTTAGCTAAAACGCCCA
ACCTCTACGTTCCTTCTGTA
AACTTTCTCATATCCAAAATGTTCAACAACCTTCTTCAATAAAATAGACTCAAATTATGAACTTTCAAGACCAAT
TTTAGXXXXXXXXXXTGAATTAATTAAACGTTAGCTAAAACGTTTGAATTAATTAAACGTTAGCTAAAACGCCCA
ACCTCTACGTTCCTTCTGTA
>Marker215173
GACCACCTTTTAGCCTAATCTAGAAGATGATCATCGGCTACAAACCTCAACAACAAGGCTAACACACCAGCTGTT
ACTGCXXXXXXXXXXGTTAAGTTTCAATAACCGTGCATATTCAAACACAATTAACAACATCGATATCAGCGCCGT
CCGAGAAATGATTTAATGTA
GACCACCTTTTAGCCTAATCTAGAAGATGATCATCGGCTACAAACCTCAACAACAAGGCTAACACACCAGCTCTT
ACTGCXXXXXXXXXXGTTAAGTTTCAATAACCGTGCATATTCAAACACAATTAACAATATCGATATCAGCGCCGT
CCGATAAATGATTTAATGTA
>Marker215305
GACAACCTGAGAGAAAGACCTGATTAAAGAACCTCAAGGGGAGAGTGAGGGAATTGACCCCAACATTAAAAAATG
GATAAXXXXXXXXXXGTTTATAGGCAAACATTTCGGGAATAAATTCGGAGAAACTCACTTATTTTGAATGGAAGA
AGCTCAATGAGTTTCAATGT
GACAACCTGAGAGAAAGACCTGATTAAAGAACCTCAAGGGGAGAGTGAGGGAATTGACCCCAACATTAAAAAATG
GATAAXXXXXXXXXXGTTTATAGGCAAACATTTCGGGAATAAATTCGGAGAAACTCACTTATTTTGTATGGAAGA
AGCTCAATGAGTTTCAATGT
>Marker216324

Create PDF with GO2PDF for free, if you wish to

ACCCTTTCTACAGCTCAGCCTCGCCGCCGCTTCTCTTCCGTTCAGTTCTACCGCCGCCTCTCTTTCGCCTAAACC
TCCATXXXXXXXXXXTCTCTCTTGTCTTTGCTGAAAACTGTCTTGTTTCCTCGACAATTAAACGTCCCAGCATTC
CCTTTCTTCTTTATTTATGT
ACCCTTTCTACAGCTCAGCCTCGCCGCCGCTTCTCTTCCGTTCAGTTCTACCGCCGCCTCTCTTTAGCCTAAACC
TCCATXXXXXXXXXXTCTCTCTGGTCTTTGCTGAAAACTGTCTTGTTTCCTCGACAATTAAACGTCCCAGCATTC
CCTTTCTTCTTTATTTATGT
>Marker216436
AACTTTTTGTCAAATTTGAATAATTAACCTAATTTCTTTTTCACATTTTTAAAATTGGCGTAGGCACTAAACTTA
AAATTXXXXXXXXXXTAAAAGAATTGAAAGTAATTGAATAATTGATTAGACAAAATTTTAAAAGTATTTTGAAAA
TAGAATAGACAGAAAATAGT
AACTTTTTGTCAAATTTGAATAATTAACCTAATTTATTTTTCACATTTTTAAAATTGGTGTAGGCACTAAACTTA
AAATTXXXXXXXXXXTAAAAGAATTGAAAGTAATTGAATAATTGATTAGACAAAATTTTAAAAGTATTTTGAAAA
TAGAATAGACAGAAAATAGT
>Marker216706
ACAAAAAACTAGCAATAATTTACGTGAATTGGCAACACCAAAATATATTGAAATGAAAAATATTGTGTTTCATCA
TATTTXXXXXXXXXXTACTAATAAAGTAATGTTTGATGCTTCACCTCTTAATTCATTTGGAGTGCTTTTATGCAT
GTTTAGGAAGCCTTCTGAGT
ACAAAAAACTAGCAATAATTTACGTGAATTGGCAACACCAAAATATATTGAAATGAAAAATATTGTGTTTAATCA
TATTTXXXXXXXXXXTACTAATAAAGTAATGTTTGATGCTTCACCTCTTAATTCATTTGGAGTGCTTTTATGCAT
GTTTAGGAAGCCTTCTGAGT
>Marker217283
TACAAATTGAATAGAAAAATATAGTAGCGACTCTCGATATCTCGAACAGTTTCTGTGGGGGGAATAATAAAGTAA
TTATAXXXXXXXXXXTCAATGGCTAAGAATAATTTCGTTTATGATATATATATAAAGAAAAAGGTGGAAATCGTT
GAAGTATCGTCAGCACCGTT
TACATATTGAATAGAAAAATATAGTAGCGACTCTCGATATCTCGAACAGTTTCTGTGGGGGGAATAATAAAGTAA
TTATAXXXXXXXXXXTCAATGGCTAAGAATAATTTCGTTTATGATATATATATAAAGAAAAAGGTGGAAATCGTT
GAAGTATCGTCAGCACCGTT
>Marker218302
TACATCAACACACACTTGACATATTTGTTTTTAAGTTGATGTTTTTTAAAAAAAATAAAAATAAGTAACTCTTTC
GATCAXXXXXXXXXXTATCTTAAAAAATTTATGGAAAATAAAGTTCTTCATTTATTGTTTTCGATGTAGCTTTTG
TTAATTGCAAGCAATAATGT
TACATCAACACACACTTGACATATTTGTTTTTAAGTTGATTTTTTTTTAAAAAAATAAAAATAAGTAACTCTTTC
GATCAXXXXXXXXXXTATCTTAAAAAATTTATGAAAAATAAAGTTCTTCATTTATTGTTTTCGATGTAGCTTTTG
TTAATTGCAAGCAATAATGT
>Marker218367
CACAGTTTCAAAGAATCGGGGTTTTCATTCGCCGTCCGTCTGCAAACCATCGCCGTCAGTTGAGAAATGGTCCTC
CGATAXXXXXXXXXXGTTCGAGAGATTTCTTTACGAAGGTGTATTTTCGCTTCAACCTAAGGTTATAATCCGGAT
CGGTCGGTTCTCAGACGGTT
CACAGTTTCAAAGAATCGGGGTTTTCATTCGCCGTCCGTCTGCAAACCATCGCCGTCAGTTGAGAAATGGTCCTC
CGATAXXXXXXXXXXGTTCGAGAGATTTCTTTACGAAGGTGTATTTTCGCTTCAACCTAAGGTTACAATCCGGAT
CGGTCGGTTCTCAGACGGTT
>Marker218451
CACTTCTTCTATCATTCTTCCATTTCTCTATTGTTATTCATATTCAATCTGTTTAATTAGTTCATGAGCAATGGA
ATGTGXXXXXXXXXXTAACTAAGCAAGTGACGAGGAGATTGTAGTGTTACAATATTATCATCGAATTCATCATTC
GCATAAGTTGCAAAGATGTT

Create PDF with GO2PDF for free, if you wish to

CACTTCTTCTATCATTCTTCCATTTCTCTATTGTTATTCATATTCAATCTGTTTAATTAGTTCATGAGCAATGGA
ATGTGXXXXXXXXXXTAACTAAGCAAGTGACGAGGAGATTGTAGTGATACAATATTATCATCGAATTCATCATTC
GCATAAGTTGCAAAGATGTT
>Marker218807
CACAAATTTCTTAATTTCATATCTACTCGGACCACTGGGTGAAATTAGAATTTGACATAGTATTAATCTTTTTTC
TGTTTXXXXXXXXXXGGCTATATTTGGTGGAAGAGAAAAAGTGGGAATGCTTGAGATTCCAAAATTTCTATCCAT
TCATTTTGTGAAATCTGGTT
CACAAATTTCTTAATTTCATATCTACTTGGACCACTGGGTGAAATTAGAATTTGACATAGTATTAATCTTTTTTC
TGTTTXXXXXXXXXXGGCTATATTTGGTGGAAGAGAAAAAGTGGGAATGCTTGAGATTCCAAAATTTCTATCCAT
TCATTTTGTGAAATCTGGTT
>Marker218952
ACTATCTTCTTTGGCTGTAATTTACTGTATTTTCCAGCTGGAAGTCTTGTTTTTCAAAGATATATCATGACTAGA
TCTCCXXXXXXXXXXATGTTTTCGGCTTCCATACTACGATCAACCTTAGAAGGAATGTTAGCAAGATCAACAATT
AGTCTAGGTAATTTAGTGTA
ACTATCTTCTTTGGCTGTAATTTACTGTATTTTCCAGCTGGAAGTCTTGTTTTTCAAAGATATATCATGACTAGA
TCTCCXXXXXXXXXXATGTTTTCGGATTCCATACTAAGATCAACCTTAGAAGGAATGTTAGCAAGATCAACAATT
AGTCTAGGTAATTTAGTGTA
>Marker219360
GACTTCCTAACTATCTTGGTGCTGCTTGTTGTATTTGATCCCAGATCTAGTGTTTTCAAGGCTTCCTCCACCAAA
CCAGCXXXXXXXXXXAATCGAATTGAATTGATCGGTGGCTTTAATTCCTTTTTTGGTTTTCATCTCTAGCCAGAG
CTCTTCAGCGCGGCTTAGTA
GACTTCCTAACTATCTTGGTGCTGCTTGTTGTATTTGATCCCAGATCTAGTGTTTTCAAGGCTTCCTCCACCAAA
CCAGCXXXXXXXXXXAATCGAATTGAATTGATCGGTGGCTTTAATTCCTCTTTTGGTTTTCATCTCTAGCCAGAG
CTCTTCAGCGCGGCTTAGTA
>Marker219574
CACCAATCAACTGCAGCTCCATGACCCTCTCCTTTAATGATCTCAGGAGCCAAATATTCGTGAGTGCCAACAAAG
GAATTXXXXXXXXXXGCAGGTAGAAACCTGGGGCTGAAGCATGGGACTTGACATGATGGTTCAATGCAAAATGGT
TCGGCACAACTAGAATCTGT
CACCAATCAACTGCAGCTCCATGACCCTCTCCTTTAATGATCTCAGGAGCCAAATATTCGTGAGTGCCAACAAAG
GAATTXXXXXXXXXXGCAGGTAGAAACCTGGGGCTGAAGCATGGGACTTGACATGATGGTTCAATGCAAAATGGT
TCAGCACAACTAGAATCTGT
>Marker219688
TACCACACTTTTCTTGCTAGGTTGCAGTTGTGCGAGTGATCTCAATGCAACAACAATTCATCATCAATTCTATCA
CGCATXXXXXXXXXXAGAAATAGGGTCTTCCCAAACGCCCCCACATCGATAAAAACCCCAAACATGTTGGGAAAG
GTTACCTCGACGTCTTAGTT
TACCACACTTTTCTTGCTAGGTTGCAGTTGTGCGAGTGATCTCAATGCAACAACAATTCATCATCAATTCTATCA
CGCATXXXXXXXXXXAGAAATAGGGTCTTCCCAAACGCCCCCACATCGATAAAAACCCCAAACATGTTGGGAAAG
GTTACCCCGACGTCTTAGTT
>Marker221483
GACATACAATGTTAGATAATGTTGGGTGTTTTATTCTAGTGACGGATAATTGTTACGAGAATTGTAAAATACTAC
GAACGXXXXXXXXXXTATAACACTGTTAATTGAAGAGATTAACATTTCAATAGAATGAGCAAATGTGATTCTAAC
CTAATCATGATTGAGTTGTA
GACATACAATGTTAGATAATGTTGGGTGTTTTATTCTAGTGACGGATAATTGTTACGAGAATTGTAAAATACTAC
GAACGXXXXXXXXXXTATAATACTGTTAATTGAAGAGATTAACATTTCAATAGAATGAGCAAATGTGATTCTAAC
ATAATCATGATTGAGTTGTA
>Marker222357

Create PDF with GO2PDF for free, if you wish to

AACTCACCTCTTCTAGAGGGGATTGAATATTCGGAGGTGGTGTTGAGTGCTTTTCAGAATATACAGCTGCTTGTC
CTAATXXXXXXXXXXTAATGAAAAAAAAAGGCGGCGTTATTAATTCTAAGAAAGCTGAGACATAACCTGCAGCAA
TCTAATCAGATATAAAGGTT
AACTCACCTCTTCTAGAGGGGATTGAATATTCGGAGGTGGTGTTGAGTGCTTTTCAGAATATACAGCTGCTTGTC
CTAATXXXXXXXXXXTTAATGAAAAAAAAGGCGACGTTATTAATTCTAAGAAAGCTGAGACATAACCTGCAGCAA
TCTAATCAGATATAAAGGTT
>Marker222398
AACGTTGAAGAAAAATAACTTCACATGTTATCACTTCCATTCTTTGATACTTTGCTTCTGCAGTTGAGTGGGATA
CAGTTXXXXXXXXXXATCTGCATCAGCAAAACCACAAATGGAGAAATTTTGCACTTTTGACGAGAACATTTCTTT
TCCTGGGGAGCCTTTGATGT
AACGTTGAAGAAAAATAACTTCACATGTTATCACTTCCATTCTTTGATACTTTGCTTCTGCAGTTGAGTGGGATA
CAGTTXXXXXXXXXXATCTGCATCAGCAAAACCACAAATGGAGAAATTTTGCACTTTTGACGAGAACATTTCTTT
TCCAGGGGAGCCTTTGATGT
>Marker223472
ACCACTGATTCTTCTAATGAACAATAATTATAGTCCCACTATAACTGAACTCATCTCAGGCCAAGAGAGGGTATG
GCGCCXXXXXXXXXXAAACGGTAGGCATATTGAGTCGGCGACATAGACCACTCTCTCCCATGAAAATTAAAGGAT
CACCTTCTTAGGCAGAAGTT
ACCACTGATTCTTCTAATGAACAATAATTATAGTCCCACTATAACTGAATTCATCTCAGGCCAAGAGAGGGTATG
GCGCCXXXXXXXXXXAAACGGTAGGCATATTGAGTCGGCGACATAGACCACTCTCTCCCATGAAAATTAAAGGAT
CACCTTCTTAGGCAGAAGTT
>Marker224105
ACTAAAGAAATCTATCATCTTCCTAGTTCTCAACTAAAAGTAGTCTTTTTTAATTAAAAAGTTTCTCACTTTTTA
TGACCXXXXXXXXXXATTAAGTTTATTTTATCGTTGTGACATTTAATTTCTTTGAAACATCTCTTGTTCTAAAAA
AAATTGAAAAATGAAGGAGT
ACTAAAGAAATCTATCATCTTCCTAGTTCTCAACTAAAAGTAGTCTTTTTTAATTAAAATGTTTCTCACTTTTTA
TGACCXXXXXXXXXXATTAAGTTTATTTTATCGTTGTGACATTTAATTTCTTTAAAACATCTCTTGTTCTAAAAA
AAATTGAAAAATGAAGGAGT
>Marker224789
TACACATTACCATGGTTGCATCACTATTTTAAACTTGGAAGCCTCGATTGAGACTTAAGTGTGACTGGTAACTAT
GAATTXXXXXXXXXXGTGAATTCTTGGGCTTTTCCTGTCTTGGTTGATATCAGTAACTTCGATTTTGCCCAATGA
TGGAAAATCACTATGAAGTG
TACACATTACCATGGTTGCATCACTATTTTAAACTTGGAAGCCTCGATTGAGACTTAAGTGTGACTGGTAACTAT
GAATTXXXXXXXXXXGTGAATTCTTGGGCTTTTCCTGTCTTGGTTGATATCAGTAACTTTGATTTTGCCCAATGA
TGGAAAATCACTATGAAGTG
>Marker224949
ACCCAAATATATATGTTTCAAAAAAAGGGAAAAAAGAAAAGACCTGAAGTAAGGAGTTTTTGGCATAGCCTTTGT
CTCCAXXXXXXXXXXGACCCTTTTATCTCCAACTCTATCTCTTACGATCCTTCCCCCCATGCACATTTAATTTAA
TTTATATGCCTTTCCAGGTG
ACCCAAATATATATGTTTCAAAAAAAGGGAAAAAAGAAAAGACCTGAAGTAAGGAGTTTTTGGCATAGCCTTTGT
CTCCAXXXXXXXXXXGACCCTTTTATCTCCAACTCTATCTCTTATGATCCTTCCCCCCATGCACATTTAATTTAA
TTTATATGCCTTTCCAGGTG
>Marker225276
ACTATCATCATCTATCGTTGATAAACAACTAAAAAATTGCTATAATGGTAAATATTTTCAACAATTTTACTATTT
AAAACXXXXXXXXXXATATTGAGATGTTTGATAGTAACAGTGCCTAGCCAATGTCGAGATATAATTATTATAGGA
GGAGAATAATTGGGAGAGTT

Create PDF with GO2PDF for free, if you wish to

ACTATCATCATCTATCGTTGATAAACAACTAAAAAATTGCTATAATGGTAAATATTTTCAACAATTTTACTATTT
AAAACXXXXXXXXXXXATATTAAGATGTTTGATAGTAACAGTGCCTAGCCAATGTCGAGATATAATTATTATAGGA
GGAGAATAATTGGGAGAGTT
>Marker225421
ACAACAGTTAGTTGATGACAATTTATTACCTCTACAAGCAGACTGCAAACGTATAGCCGACCTCCGAAGCAATAC
AAAACXXXXXXXXXXXTCTAAGAAACACCTTTGTCTTACCAATCTGCCAGAATATGTTACATTTGTAACATAACAC
GCTATAGTTTATACTTTGTA
ACAACAGTTAGTTGATGACAATTTATTACCTCTACAAGCAGACTGCAGACGTATAGCCGACCTCCGAAGCAATAC
AAAACXXXXXXXXXXXTCTAAGAAACACCTTTGTCTTACCAATCTGCCAGAATATGTTACATTTGTAACATAACAC
GCTATAGTTTATACTTTGTA
>Marker225895
GACACCTTGTGATAACATGATAAGAACATCAAAATCACGAAGTAATGGCAAAGTGATATATCACAAATTGAACCA
AAAGAXXXXXXXXXXXTTTTGAACGTAGGACCTTCTGATATGATGCCATTGTAAATCACCAATCAACTTAGAAACT
TAGATCATTGGGTTGTGGTA
GACACCTTGTGACAACATGATAAGAACATCAAAATCACGAAGTAATGGCAAAGTGATATATCACAAATTGAACCA
AAAGAXXXXXXXXXXXTTTTGAACGTAGGACCTTCTGATATGATGCCATTGTAAATCACCAATCAACTTAGAAACT
TAGATCATTGGGTTGTGGTA
>Marker226152
TACAGTTGAATTCAATAGGAGTATTAGCAAGATGATTTCCGAATATACTTGGCTTGGTTAGCAAATCAAGGGTGT
ATTTTXXXXXXXXXXXAACAAGCAACTTCTAAAAGGTGATGATTTTTTGTTCGGCAACCCCATTTTGTTGGGGGTG
TAAGCATAGAACCTTTGGTG
TACAGTTGAATTCAATAGGAGTATTAGCAAGATGATTTCCGAATATACTTGGCTTGGTTAGCAAATCAAGGGCGT
ATTTTXXXXXXXXXXXAACAAGCAACTTCTAAAAGGTGATGATTTTTTGTTCGGCAACCCCATTTTGTTGGGGGTG
TAAGCATAGAACCTTTGGTG
>Marker226221
TACAAATGTTAGGGACAATTCTAATAAACTCAAATTTAATCGGAAAAGATTTTAGAATAACGTTATCGTCTAAAT
CTTGAXXXXXXXXXXXTAACCATTTCAAAACTGTCATTGAAGCTAGCATCAAGAAAGTTGAACTTCTTGACAGTTA
ATAAAACGTCAAGAATAGTG
TACAAATGTTAGGGACAATTCTAATAAACTCAAATTTAATCGGAAAAGATTTTAGAATAACATTATCGTCTAAAT
CTTGAXXXXXXXXXXXTAACCATTTCAAAACTGTCATTGAAGCTAGCGTCAAGAAAGTTGAACTTCTTGACAGTTA
ATAAAACGTCAAGAATAGTG
>Marker226758
TACTTAGGGAGTCTTTTTTGTTTTAGTTATTACGAAATGGAGTTGTCTTCTAAGCATTAGTCCATGCCAAGGAAT
TTATTXXXXXXXXXXXATTTCGTTTATACGTGGATGCACTCTTAAAGTAGAACTCATTTGTCCTGTTCAAAGTGCG
ATTTTTTATTCAAAGTTGTT
TACTTAGGGAGACTTTTTTGTTTTAGTTATTACGAAATGGAGTTGTCTTCTAAGCATTAGTCCATGCCAAGGAAT
TTATTXXXXXXXXXXXATTTCGTTTATACGTGGATGCACTCTTAAAGTAGAACTCATTTGTCCTGTTCAAAGTGCG
ATTTTTTATTCAAAGTTGTT
>Marker227459
ACTAAAAAGCACATATACGGCTTGTTTCAGTAAAATCTTCCAATTGTTAAATCTACAAAACATCACCCGAGAATT
TCATTXXXXXXXXXXXTGTGATTATAAAAAGCTCTATAACGGGACGGCTTAATTTTAAAAACAAATTATTAGAATG
ATTCATCTGTCAAAATTGTG
ACTAAAAAGCACATATACGGCTAGTTTCAGTAAAATCTTCCAATTGTTAAATCTACAAAACATCACCCGAGAATT
TCATTXXXXXXXXXXXTGTGATTATAAAAAGCTCTATAACGGGACGGCTTAATTTTAAAAACAAATTATTAGAATG
ATTCATCTGTCAAAATTGTG
>Marker227774

Create PDF with GO2PDF for free, if you wish to

TACTTATTTCTTTTTCTTTATTAATATTAACAAGATACATTAATGAGGAAGGTTAGTAGGTGGCATGGGAAAAAT
CTCAAXXXXXXXXXXXGCACTAAATTGAAATAACATTCAAAATATAAATTAGCTATTATAACGTATTTAACCTAGT
TTGTTTGATGTATATGTGTG
TACTTATTTCTTTTTCTTTATTAATATTAACAAGATACATTAATGAGGAAGGTTAGTAGGTGGCATGGGAAAAAT
CTCAAXXXXXXXXXXXGGACTAAATTGAAATAACATTCAAAATATAAATTAGCTATTATAACGTATTTAACCTAGT
TTGTTTGATGTATATGTGTG
>Marker228219
CACACTTGATTGACATAAAAATTTAAGGTTTTATTAACTCCATATGAAGCTAAAGAGTTGTTCCTATCTCAGACT
TCAGAXXXXXXXXXXXACTTAGGCCGAGAAAGCGGGCCTGGAGATGAGATGAAGACCCGCACCAGTAGAAACACAA
CCCACAGAATCAATCTCGGT
CACACTTGATTGACATAAAAATTTAAGGTTTTATTAACTCCATATGAAGCTAAAGAGTTGTTCCCATCTCAGACT
TCAGAXXXXXXXXXXXACTTAGGCCGAGAAAGCGGGCCTGGAGATGAGATGAAGACCCGCACCAGTAGAAACACAA
CCCACAGAATCAATCTCGGT
>Marker228352
ACTACAGATCGTAATGCGTAGAATCTATTGTTTAGTTTCTCCCTTCTCTGTCGTTCTGCTTCTACGTGATTCATT
GCATTXXXXXXXXXXTCCGCCACTTCGCTTATAAACCCAATATCCTCGAAGTTGAAGTCTTGATCCAAAAACGGA
AGTGGTGCATCGGTGGTGGT
ACTACAGATCGTAATGCGTAGAATCTATTGTTTAGTTTCTCCCTTCTCTGTCGTTCTGCTTCTACGTGATTCATT
GCATTXXXXXXXXXXTCCGCCACTTCGCTTATAAACCCAATATCCTCGAAGTTGAAGTCTTGATCCAAAAACGGA
AGTGGTGTATCGGTGGTGGT
>Marker228838
TACATGTCCATTTTGAAAGTAACCTCAGGTGTATATCTTTGTCTGTTCTCAGATTTGTGGCAGGGCTAATGAACA
TACCCXXXXXXXXXXTCTTTTTTTTTTCTCTGTCGAAGATTTAGGAATTTGAGATTGTCTTGTAAGTGTAAATTC
AATAATGTGACTTTTATAGT
TACATGTCCATTTTGAAAGTAACCTCGGGTGTATATCTTTGTCTGTTCTCAGATTTGTGGCAGGGCTAATGAACA
TACCCXXXXXXXXXXTCTTTTTTTTTTCTCTGTCGAAGATTTAGGAATTTGAAATTGTCTTGTAAGTGTAAATTC
AATAATGTGACTTTTATAGT
>Marker229660
ACATCAAGAGAGTCTGTAACTTTCCTCTGCCAACATATTTTCATTTCAGCCATTATATATGACCTACTTTTTCTC
ACTAAXXXXXXXXXXXTCAATTATATTAGTTTCTATGTTTCATTTAATATCTTGATGATTTTTGTTTGAAATATTT
AAACATATGCAACTAAGGTG
ACATCAAGAGAGTCTGTAACTTTCCTCTGCCAACATATTTTCATTTCAGCCATTATATATGACCTACTTTTTCTC
ACTAAXXXXXXXXXXXTCAATTATATTAGGTTCTATGTTTCATTGAATATTTTGATGATTTTTGTTTGAAATATTT
AAACATATGCAACTAAGGTG
>Marker229963
CACTTCATATTAGACATAATGTTAAGATGATTTAGTAGAGCAAAATGCTATATTTACGAACATAATGTTCGTATT
TGAAAXXXXXXXXXXXGTAATTAATGTAATTTAATGTAAGTTATATGTAACTATGTATCTATCTAATATCTATAAA
CAAAAGGAGGTAGCTTCGTG
CACTTCATATTAGACATAATGTTAAGATGATTTAGTAGAGCAAAATGCTATATTTACGAACATAATGTTTGTATT
TGAAAXXXXXXXXXXXGTAATTAATGTAATTTAATGTAAGTTATATGTAACTATGTATCTATCTAATATCTATAAA
CAAAAGGAGGTAGCTTCGTG
>Marker231632
TACGGCGACGTAAAATATCGCAATCGTGGCGTAAGAAAAGGAAATGAGAGGAATCAACTCGTGAGATTCCTCATA
CCGTTXXXXXXXXXXTTCAATTCGAATCTCTTGCATTATAGTTCTTCTACAGGTAGTTCTTCTCATAGATTAATC
TTCGTTAATCGTAATTGAGT

Create PDF with GO2PDF for free, if you wish to

TACGGCGACGTAAATTATCGCAATCGTGGCGTAAGAAAAGGAAATGAGAGGAATCAACTCGTGAGATTCCTCATA
CCGTTXXXXXXXXXXXTTCAATTCGAATCTCTTGCATTATAGTTCTTCGACAGGTAGTTCTTCTCATAGATTAATC
TTCGCTAATCGTAATTGAGT
>Marker231683
CACCGTTAAACAAGAGGGATACAAAAAGTAGAAGATTCCATTATATGAAAACTAGACACAATTGAAATGAATAAC
CACACXXXXXXXXXXXTGAGTTTCAAAAATAAGGGACCGCCCATTCCAAACATCGATCAGCGAAAGGACGTGCAAC
AAATTGGACGAATTGCAGTG
CACCGTGAAACAAGAGGGATACAAAAAGTAGAAGATTCCATTATATGAAAACTAGACACAATTGAAATGAATAAC
CACACXXXXXXXXXXXTGAGTTTAAAAAATAAGGGACCGCCCATTCCAAACATCGATCAGCGAAAGGACGTGCAAC
AAATTGGACGAATTGCAGTG
>Marker231826
TACCCCACACTGATCCCATCTCCAGCCCGATGTATCCAGCTCCAATTACAACAAGTTTCTTAGGGACCTCAGACA
AAGCTXXXXXXXXXXXACCACTGTATTACCACCATCAATGGTATCCACAGAAACTTCAGAAGGGGAAATCAATTTC
CCATAGCCTTTAACATAGTT
TACCCCACACTGATCCCATCTCCAGCCCGATGTATCCAGCTCCAATTACAACAAGTTTCTTAGGGATCTCAGACA
AAGCTXXXXXXXXXXXACCACTGTATTACCACCATCAATGGTATCCACAGAAACTTCAGAAGGGGAAATCAATTTC
CCATAGCCTTTAACATAGTT
>Marker232023
ACAGCTGACAACTCTTTTGTTATTTGAAGCAAAGATTAATTCCATAAACAAACTGTATCCCATCATCAACTATTA
CTTTTXXXXXXXXXXXTCTTACTACCTTATTTAACTAAATTAAGCATATATTCTATTAATAATACAAAATAGATAT
GCTTGTAATGGAAGGAAAGT
ACAGCTGACAACTCTTTTGTTATTTGAAGCAAAGATTAATTCCATAAACAAACTGTATCCCATCATCAACTATTA
CTTTTXXXXXXXXXXXGCTTACTACCTTATTTAACTAAATTAAGCATATATTCTATTAATAATACAAAATAGATAT
GCTTGTAATGGAAGGAAAGT
>Marker232049
CACTGCATACATTGCCTTCTTCATAACTTGTATTTTCACTTTTAGTAAATCTCTTAAATCTCAAAAATAAATATT
AATTTXXXXXXXXXXXATCATTGGATTGAATAAGCTAAAATGGTTCGATTTGGAATTAATACTTGAAATCATGAGC
CTCAAACACGCTCTAATGTT
CACTGCATACATTGCCTTCTTCATAACTTGTATTTTCACTTTTAGTGAATCTCTTAAGTCTCAAAAATAAATATT
AATTTXXXXXXXXXXXATCATTGGATTGAATAAGCTAAAATGGTTCGATTTGGAATTAATACTTGAAATCATGAGC
CTCAAACACGCTTTAATGTT
>Marker232068
CACCAGTTTTTGACAAACTTGAAGCTGAGTTTGCTAAAGCTGTTTTGTCATTACCAGCATCGAAGGGCTTTGAAA
TTGGCXXXXXXXXXXXTTTGTGGTTGCTCGTTGCCTTAGTTTTGTTTTTCTTTTTTATTCAGCACTCACATGTTCA
AAACTAAACCCCCTGCAGGT
CACCAGTTTTTGACAAACTTGAAGCTGAGTTTGCTAAAGCTGTTTTGTCATTACCAGCATCGAAGGGCTTTGAAA
TTGGCXXXXXXXXXXXTTTGTGGTTGCTCGTTGCCTTGGTTTTGTTTTTCTTTTTTATTCAGCACTCACATGTTCA
AAACTAAACCCCCTGCAGGT
>Marker232072
AACCAAGACCCTGTCGTTTATCATGAAGGGAAACAACATTACCAAGCGGACAGCTCCTCTCAGGACTCTAATTCT
CAGAGXXXXXXXXXXXTTAATGGAGAAATCCCTTGAGGAAAGCAATGGTCAGGTTCAACATGCTTCAAAAAGTTCA
TCAATTTCCTCAAACAATGT
AACCAAGACCCTGTCGTTTATCATGAAGGGAAACAACATTACCAAGCGGACAGCTCCTCTCAGGACTCTAATTCT
CAGAGXXXXXXXXXXXTTAGTGGAGAAATCCCTTGAGGAAAGCAATGGTCAGGTTCAACATGCTTCAAAAAGTTCA
TCAATTTCCTCAAACAATGT
>Marker232308

Create PDF with GO2PDF for free, if you wish to

AACCACTTTATTTGGAAATTAATAGAAACATTAAAGAAAATGAAAAGTTAGATATTAGTCTTTTTCTTTTCTTAT
AATTGXXXXXXXXXXTCTCTAGACTTCCGGTGGCTAACCCAATCCCCCAATTAAAATATGTCTCATTTTGTAGGA
TTCTTTCATACATTTACGTT
AACCACTTTATTTGGAAATTAATAGAAACATTAAAGAAAATGAAAAGTTAGATATTAGTCTTTTTCTTTTCTTAT
AATTGXXXXXXXXXXTCTCTAGACTTCCGGTGGCTAACCCAACCCCCCAATTAAAATATGTCTCATTTTGTAGGA
TTCTTTCATACATTTACGTT
>Marker232934
ACTGTGATTGATTATAGCTAAAATCACATATTATTCAATGATCATCCCATTGGCACATGAAATAATTGACTGCTT
ACTTTXXXXXXXXXXAATTTCTGGTGTTTTTGTCGTTCTATTGTATAGCAACATAAGCTCTTGCTCTCACGGAGT
TTAGTTGTTTTTACACAAGT
ACTGTGATTGATTATAGCTAAAATCACATATTATTCAATGATCACCCCATTGGCACATGAAATAATTGACTGCTT
ACTTTXXXXXXXXXXAATTTCTGGTGTTTTTGTCGTTCTATTGTATAGCAACATAAGCTCTTGCTCTCATGGAGT
TTAGTTGTTTTTACACAAGT
>Marker233002
CACCCTTAATCACCTAACCTCGCCTCTCACCAAGATTAAAAATAAATTTTCATAATTTGAATATACTGCATAGAT
GTAAAXXXXXXXXXXTTTATAAACTTGGTTTTGTATGCAGCTTTTTATTGGTCCGAAAATAGGCACATTTTTAAA
CACTTCAGTTTTTCTTAGTT
CACCCTTAATCACCTAACCTCGCCTCTCACCAAGATTAAAAATAAATTTTCATAATTTGAATATACTGCATAGAT
GTAAAXXXXXXXXXXTTTATAGACTTGGTTTTGTATGCAGCTTTTTATTGGTCCGAAAATAGGCACATTTTTAAA
CACTTCAGTTTTTCTTAGTT
>Marker233549
ACCCTATAGCAATGAGTCATGCCTTATATTAAGCGCTACCATTTTGATTTCTCTTAATACGATGCACCTATTTTT
GTCATXXXXXXXXXXAGAAGCCTTTTTACTAGGGGAATGTTCCTCTTGAATAGAATCAACAGTAGCAAGAAAGAT
CTTCTTTGAAATACCAAGTT
ACCCTATAGCAATGAGTCATGCCTTATATTAAGCGCTACCATTTTGATTTCTCTTAATACGATGCACCTATTTTT
GTCATXXXXXXXXXXAGAAGCCTTTTTACAAGGGGAATGTTCCTCTTGAATAGAATCAACAGTAGCAAGAAAGAT
CTTCTTTGAAATACCAAGTT
>Marker233972
TACTGTTTTAATTTATCATACTTTTTGTTCAACCTTCTTAGAATAGCAAGTCCGGATTGGATAGAGGTTTTTATT
GAATAXXXXXXXXXXAGGCTCTTAATTCATGGAGCAACGGACGTTGAGTTTTACCAAATTTTTGTAAAGCTGAGA
TTGTTGAAGCAGTTGAAGGT
TACTGTTTTAATTTATCATACTTTTTGTTCAACCTTCTTAGAATAGCAAGTCCGGATTGGATAGAGGTTTTTATT
GAATAXXXXXXXXXXAGGCTCTTAATTCATGGAGCAACGGACGTTGAGTTTTGCCAAATTTTTGTAAAGCTGAGA
TTGTTGAAGCAGTTGAAGGT
>Marker234992
CACTCCGCCTTTAGTGAAACTCCATCTCTTTTTATGCTTCAAGTTTCTTTCATTTTCATTTCCATGGCTTCATTT
TGAATXXXXXXXXXXTCTCTAACTATTTCACCTAAAACCTTGATGGGTGTGCCTTAAATTCTTTTTTGGTCCATT
GAATGCCCTAGTTTCTTAGT
CACTCCGCCTTTAGTGAAACTCCATCTCTTTTTATGCTTCAAGTTTCTTTCATTTTCATTTCTATGGCTTCATTT
TGAATXXXXXXXXXXTCTCTAACTATTTCACCTAAAACCTTGATGGGTGTGCCTTAAATTCTTTTTTGGTCCATT
GAATGCCCTAGTTTCTTAGT
>Marker235121
AACCACTAAAATGGAGAATAAGGGATGGCATCCTATCACATTTACCTATGCTAATGCTGGACCTCGGCAGGTCAA
GGAAAXXXXXXXXXXTACTCTTTTTCACATTGTTTGTTTTGACTTGTTGTCTTATAATGACGCTATAAGGATCGG
CGTCTATTTTATATGCCAGT

Create PDF with GO2PDF for free, if you wish to

AACCACTAAAATGGAGAATAAGGGATGGCATCCTATCACATTTACCTATGCTAATGCTGGACCTCGGCAGGTCAA
GGAAAXXXXXXXXXXXTACTCTTTTTCACATTGTTTGTTTTGACTTGTTGTCTTATAATGACGCTATAAGGATCGG
CGGCTATTTTATATGCCAGT
>Marker235217
AACCTCAAGTTTGTGAGTGACTCATGCATTTAATGGGTTCTAAAGTATATTTCAAGCTTGATTTATTAATCACAA
TTTCAXXXXXXXXXXXTGTTATAAAAGATGAAGTTTGAAAGCTTAAATGTTTTTCATTAGGCTATACTAGAACTAT
AACTACTACCTTTTAATGTG
AACCTCAAGTTTGTGAGTGACTCATGCATTTAATGGGTTCTAAAGTATATTTCAAGCTTGATTTATTAATCACGA
TTTCAXXXXXXXXXXXTGTTATAAAAGATGAAGTTTGAAAGCTTAAATGTTTTTCATTAGGCTATACTAGAACTAT
AACTACTACCTCTTAATGTG
>Marker235643
GACAACAATGATAGTGATGAAGCTGTTTTTATTCTTTGTCCAATAATGCATGGTTATTAGTGACAATAAATAGAG
TCTTTXXXXXXXXXXXAAGAGTTAAAAAGTAGTTGAAATTGGAACTAAAATTGAAACAAGAATAGGAAAGTGAGCA
AAGACTAAACCACGAAAGTG
GACAACAATGATAGTGATGAAGCTGTTTTTATTCTTTGTCCAATAATGCATGGTTATTAGTGACAATAAATAGAG
TCTTTXXXXXXXXXXXAAGAGTTAAAAAGTAGTTGAAATTGGAACTAAAATTGAAACAAGAATAGGAAAGTGAGCA
AAGACTAAACCATGAAAGTG
>Marker237194
GACTTCTGTTGCAATATGCCGAGGAGGAGGATGCGATGCAATATGATCCGATTCGATTGAATTCGGCCGATTTTA
CCATTXXXXXXXXXXXTATGTATGAATGTGGTTGGTTTAGGTGATTTGTTAAATTTCTTCAAGTGTTTTTCATGGC
AGCTGGCAATGGAGAATGTA
GACTTCTGTTGCAATATGCCGAGGAGGAGGATGCGATGCGATATGATCCGATTCGATTGAATTCGGCCGATTTTA
CCATTXXXXXXXXXXXTATGTATGAATGTGGTTGGTTTAGGTGATTTGTTAAATTTCTTCAAGTGTTTTTCATGGC
AGCTGGCAATGGAGAATGTA
>Marker237659
ACTGTAATATGATAGGGGGTCTATCATATGCCAAATCCTTTCTAGGGATATACAAGCAAGGATTTTGCGAGGCAG
CGGTAXXXXXXXXXXXATTGGAAACCATACCAAATATGTTTCCATAAACTGCCCACCATTGATCATTTAACCATAT
TATGCCAATTCATAGAGGTC
ACTGTAATATGATAGGGGGTCTATCATACGCCAAATCCTTTCTAGGGATATACAAGCAAGGATTTTGCAAGGCAG
CGGTAXXXXXXXXXXXATTGGAAACCATACCAAATATGTTTCCATAAACTGCCCACCATTGATCATTTAACCATAT
TATGCCAATTCATAGAGGTC
>Marker237800
AACTGTGTGCTCGTGCAGGTTTTAATGGCAATCACAGCATCTTCTTTTTTTTCTTTTCAATAATTAATAGCAATT
CAAGAXXXXXXXXXXXGAGGCAAAACAATGATTTATGGCCGTATTTTATTAGTCTTGGTGGCTGTGGGATCTGAAA
TCAGATTCTCAGGTTCTGTT
AACTGTGTGCTCGTGCAGGTTTTAATGGCAATCACAGCATCTTCTTTTTTTTATTTTCAATAATTAATAGCAATT
CAAGAXXXXXXXXXXXGAGACAAAACAATGATTTATGGCCGTATTTTATTAGTCTTGGTGGCTGTGGGATCTGAAA
TCAGATTCTCAGGTTCTGTT
>Marker238174
ACAAGTGGAAAGATCGGGCTGATCGATCTACCATAAATAAAAAGATTAGTTTCTCAAAATTAATTATTTTACTTA
AAGTGXXXXXXXXXXXTGATCATCCAATTATCAATGTATAATTTTTAACTTCATACCTATATCTAGCCCAATTGAT
TACAAACACTAACCAATGGT
ACAAGTGGAAAGATCGGGCTGATCGATCTACCATAAATACAAAGATTAGTTTCTCAAAATTAATTATTTTACTTA
AAGTGXXXXXXXXXXXTGATCATCCAATTATTAATGTATAATTTTTAACTTCATACCTATATCTAGCCCAATTGAT
TACAAAGACTAACCAATGGT
>Marker238349

Create PDF with GO2PDF for free, if you wish to

AACAAGTGGTTTCATGAATGGTTGATAATCTATGTTAATAAAAGATGAATCTATAGCACATCACAGTTTTTAAGC
ACTACXXXXXXXXXXXATTCAAATTAACCATAACAGAATCTGTTTTGTTCGCAAAGAAAGTTATGCTTTATCATGG
CTCAACAAAAAGGTTCCGTG
AACAAGTGGTTTCATGAATGGTTGATAATCTATGTTAATAAAAGATGAATCTATAGCACATCACAGTTTTTAAGC
ACTACXXXXXXXXXXXATTCAAATTAACCATAACAGAATCTGTTTTGTTCGCAAAGAAAGTTATGCTTTATCATGG
CTCAACAAAAAAGTTCCGTG
>Marker239111
AACTTTAACTGCTCAACTGAGATTACAACTATATTCAACTTCTAATATAATTACAAAGTTTGTATTCCTCTAAAC
CTCCAXXXXXXXXXXXGTTAAAATCAGTGTAAAGCATATCGATCTTTTAAACGATTACATTTTATATAGAATATCT
AAATCTAGTAAGTCGAGGTG
AACTTTAACTGCTCAACTGAGATTACAACTATATTCAACTTCTAATATAATTACAAAGTTTGTATTCCTCTAAAC
CTCCAXXXXXXXXXXXGTTAAAATCAGTGTAAAGCATATCGATCTTTTAAACGATTACATTTTATATAGAATATCT
AAATCTAGTAAGTCGGGGTG
>Marker239176
ACCTCACTCAAGTCACAATTGCCTGACCCAACAACATTTAGGTGCTAAAGAAACTCATGGAATATTGAATTTTAG
TAGGTXXXXXXXXXXXTGCACAATTTTGTTAGCTGATAAATAATTTACCTACATAAGAGAATAGGAAAAGTCAATG
AATTCTATCAATGTTTGGTA
ACCTCACTCAAGTCACAATTGCCTGACCCAACAACATTTAGGTGCTAAAGAAACTCATGAAATATTGAATTTTAG
TAGGTXXXXXXXXXXXTGCACAATTTTGTTAGCTGATAAATAATTTACCTACATAAGAGAATAGGAAAAGTCAATG
AATTCTATCAATGTTTGGTA
>Marker239287
CACTAAACCACTCACAAGCGATCACATTCACCACCTCACAAGCAGACTCATGCTTCATCAACATTCATTTGAGGG
GCATAXXXXXXXXXXXAAATTAGTAGAAAACAACTCTCATTCTTGAGAATTTCTTCCATTATGTATTGTTTCAGTG
ATGACTTAAGAATTCATGTT
CACTAAACCACTCACAAGCGATCACATTCACCACCTCACAAGCATACTCATGCTTCATCAACATTCATTTGAGGG
GCATAXXXXXXXXXXXAAATTAGTAGAAAACAACTCTCATTCTTGAGAATTTCTTCCATTATGTATTGTTTCAGTG
ATGACTTAAGAATTCATGTT
>Marker239314
GACCAAAAATAAATAAATACCACAGAGATAAATTACACCAATAAATTTAAGTTTTGGTTATTGAAAATTGAAAAG
GATTGXXXXXXXXXXXCCATGTCTTCCTACCACGACTCACTGTCACTCAGAATTAAACATAAGATGCTCTTATTCT
CAGGCACTCAACTCGTTGTG
GACCAAAAATAAATAAATACCACAGAGATAAATTACACCAATAAATTTAAGTTTTGGTTATTGAAAATTGAAAAG
GATTGXXXXXXXXXXXCCATGTCTTCCTACCACGACTCACTGTCACTCAGAATTAAACATAAGATGCTCTTATTCG
CAGGCACTCAACTCGTTGTG
>Marker239498
ACATTGATGCAATGTTGTCACAAGTGCTCTAATGTGCTTTGAGCCAAATAATGAAAAGATCCTATGTAAGAAGTT
AGAAAXXXXXXXXXXXTAATTGTATCAATGATTAAGTTGGCAACTAAATTTATTTGAAATGAAATCGACTATATCT
TATATGTATATAGATGTGTG
ACATTGATGCAATGTTGTCACAAGTGCTCTAATGTGCTTTGAGCCAAATAATGAAAAGATCATATGTAAGAAGTT
AGAAAXXXXXXXXXXXTAATTGTATCAATGATTAAGTTGGCAACTAAATTTATTTGAAATGAAATCGACTATATCT
TATATGTATATAGATGTGTG
>Marker239624
GACTTCTTTCCAGTGCCAGATTGCTGTTTGATTCGTGTTCTGTTTTGGATTCTGTTTCTTGTAACATTATGATGT
CTGGGXXXXXXXXXXXAATTGAGGTTTTTAAGGATATGAGGTCTGCAGGTGTAGCCCCAAATGAAGTGACAATGGC
AAGTGTTATGTCAGCATGTT

Create PDF with GO2PDF for free, if you wish to

GACTTCTTTCCAGTGCCAGATTGCTGTTTGATTCGTGTTCTGTCTTGGATTCTGTTTCTTGTAACATTATGATGT
CTGGGXXXXXXXXXXAATTGAGGTTTTTAAGGATATGAGGTCTGCAGGTGTAGCCCCAAATGAAGTGACAATGGC
AAGTGTTATGTCAGCATGTT
>Marker239805
ACTAGAGATTAAGATTCAAAATTTAATCAAAATTGTTGAATATCCCATGAGTTTCTTTGACATCCAAATGTTGCA
AAGTCXXXXXXXXXXAATCAAGATATCACAAAAAATCTCATCCATCACAGCAAAACAAAGCACATTGAGATAAAT
CGTTAATCCACAAGCATGTG
ACTAGAGATTAAGATTCAAAATTTAATCATAATTGTTGAATATCCCATGAGTTTCTTTGACATCCAAATGTTGCA
AAGTCXXXXXXXXXXAATCAAGATATCACAAAAAATCTCATCCATCACGGCAAAACAAAGCACATTGAGATAAAT
CGTTAATCCACAAGCATGTG
>Marker239971
AACGGGAACAAAAACTTCCTCTACATCACAATATCATCAAACAACAATTATTAACCTTCTAAAGGATGCAAAGTT
GATAAXXXXXXXXXXTTATTCTGATTGCTAAACCAATAAAACTTGAACCACTTCTACAGATGAGATAAAAGAATG
TAAAGATAAGAACTTCTGTA
AACGGGAACAAAAACTTCCTCTACATCACAATATCATCAAACAACGATTATTAACCTTCTAAAGGATGCAAAGTT
GATAAXXXXXXXXXXTTATTCTGATTGCTAAACCAATAAAACTTGAACCACTTCTACAGATGAGATAAAAGAATG
TAAAGATAAGAACTTCTGTA
>Marker240285
AACTATAGATAAACTTAGTTATATTGAATGACATTTCACATTCTATGCCCATAGGGGCTTTATAAAAATCTAATA
CAGGAXXXXXXXXXXTGACATGCAATGCTTTCACTTTCACAAATAGAAAGCATCTTCAAAATATGTTCAATTTGC
CTAGGGTGTCATATGCTGTT
AACTATAGATAAACTTAGTTATATTGAATGACATTTCACATTCTATGCCCATAGGGGCTTTATAAAAATCTAATA
CAGCAXXXXXXXXXXTGACATGCAATGCTTTCACTTTCACAAATAGAAAGCATCTTCAAAATATGTTCGATTTGC
CTAGGGTGTCATATGCTGTT
>Marker240420
TACTTGAAATACTACGTTGGAATTTTTAGGATTTCAAGCTATTTTGGGATTCGTTAAACATTTTAAGTAGAGGAG
ATTTGXXXXXXXXXXTTTTTCCATGGCTTGAAGTATTGATATTATATTATGCTTTAATGTCAGGAACAGATGGTG
TTTCGGAGCCCCATACAGTC
TACTTGAAATACTACGTTGGAATTTTTAGGATTTCAAGCTATTTTGGGAATCGTTAAACATTTTAAGTAGAGGAG
ATTTGXXXXXXXXXXTTTTTCCATGGCTTGAAGTATTGATATTATATTATGCTTTAATGTCAGGAACAGATGGTG
TTTCGGAGCCCCATACAGTC
>Marker240739
ACTTTTGATGTATAACCTGTAATCAAGAGCATTAACAAGTAAGAAAACAATATGCTAAATATAACTATATGCATT
TGTTGXXXXXXXXXXTTAGACGTCTGTGCTAAAATTGCACCAAATAAAAGACTAATCTCAAAATTTTACTTCTTT
ATCGAAAAGGGAAATTAAGT
ACTTTTGATGTATAACCTGTAATCAAGAGCATTAACAAGTAAGAAAACAATATGCTAAATATAACTATATGCATT
TGTTGXXXXXXXXXXATAGACGTCTGTGCTAAAATTGCACCAAATAAAAGACTAATCTCAAAATTTTACTTCTTT
ATCGAAAAGGGAAATTAAGT
>Marker240752
ACAACAATTTGTTCCACGAGAAAGGAAAGAATAGAAGAATTTTTTATTGAAGTGTGGTGAGTGGTTTTCTTTTTA
AAAGTXXXXXXXXXXTTAAAACTTGATTTGATGATTACGATTTCAATGCTATGAAATGGTTTTCAAATCATTAGT
TGATTCTTTAAGATGATGTT
ACAACAATTTGTTCCACGAGAAAGGAAAGAAGAGAAGAATTTTTTATTGAAGTGTGGTGAGTGGTTTTCTTTTTA
AAAGTXXXXXXXXXXTTAAAACTTGATTTGATGATTACGATTTCAATGCTATGAAATGGTTTTCAAATCATTAGT
TGATTCTTTAAGATGATGTT
>Marker241415

Create PDF with GO2PDF for free, if you wish to

ACTTTATCCGCAGACTGAAGTTGGAGAATAGCACGAGCCAAGCAGAGGAGGAAGGGATCATCTGCTGCAGTTTAG
CAATCXXXXXXXXXXXTCCCATTATGAAAAATGTCTCTGCAACTTGTTTGGACTGTGTTTGGTCGTTAAAGGATAG
TGGTAGCAGTGTATGTAGTT
ACTTTATCCGCAGACTGAAGTTGGAGAATAGCACGAGCCAAGCAGAGGAGGAAGGGATCATCTGCTGCAGTTTAG
CAATCXXXXXXXXXXXTCCCATTATGAAAAATGTCTCTGCAACTTGTTTGGACTGTGTTTGGTCGTTTAAGGATAG
TGGTAGCAGTGTATGTAGTT
>Marker242800
GACGAATGCTCCATCCACCTTTCAATCTTTGATGAATACTATTTTTAAACCATACTTGAGGTGGTTCGTATTGGT
GTTTCXXXXXXXXXXXGAGTATTTGGGCATATTTTATATCTGAAAAAGGAGTAGAGGTGGATCCCAAAAAAGTCAG
AGCAATTAAGGAATGTCAGT
GACGAATGCTCCATCCACCTTTCAATCTTTGATGAATACTATTTTTAAACCATACTTGAGGAGGTTCGTATTGGT
GTTTCXXXXXXXXXXXGAGTATTTGGGCATATTTTATATCTGAAAAAGGAGTAGAGGTGGATCCCAAAAAAGTCAG
AGCAATTAAGGAATGGCAGT
>Marker243412
ACATATGTCTAACACGTGTTGAACAAACAAGTGCCCTTTACGTGCCTAACAAGTGTCGGAGTGTCTGACACTGAC
ATGCTXXXXXXXXXXXTCAAAATGACCCAAGCAAATGAGAGAAACTAACTAGTTTCATACAAATGTCATAGAAGGA
TATAATATACTTTAGGTGTG
ACATATGTCTAACACATGTTGAACAAACAAGTGCCCTTTACGTGCCTAACAAGTGTCGGAGTGCCTGACACTGAC
ATGCTXXXXXXXXXXXTCAAAATGACCCAAGCAAATGAGAGAAACTAACTAGTTTCATACAAATGTCATAGAAGGA
TTTAATATACTTTAGGTGTG
>Marker243579
TACCTATTACATGGTCTAGCGACTGGAAATGATATTTCCAAGTATCTCGTTCGAATAATGATCAACAACTTTAGT
TTCAAXXXXXXXXXXXTATTAAAATTTTACTGAAATCCTTGTTTGGAGGTGATTTTTTATAGTTGTGAAGGTGACT
TTTTGTGATTTCAAGAAGTT
TACCTATTACATGGTCTAGCGACTGGAAATGATATTTCCAAGTATCTCGTTCGAATAATGATCAACAACTTTAGT
TTCAAXXXXXXXXXXXTATTAAAATTTTACTGAAATCCTTGTTTGGAGGTGATTTTTTATAGTTGTGAAGGTGACT
TTTTGTAATTTCAAGAAGTT
>Marker244111
TACGACGACAAATATTTTTTGTCATAAATAAAATTCGTGATAATTTTTTATAGTCATAGGATCGCCTGTAAAGGA
AAGACXXXXXXXXXXXTTAACTCATATTATTAGAGTAATTATGTCATCGTTTGCACTTACCACATTGGTGCAACTT
CCACTATCAATTACTAAGGT
TACGGCGACAAATATTTTTTGTCATAAATAAAATTCGTGATAATTTTTTATAGTCATAGGATCGCCTGTAAAGGA
AAGACXXXXXXXXXXXTTAACTCATATTATTAGAGTAATTATGTCATCGTTTGCACTTACCACATTGGTGCAACTT
CCACTATCAATTACTAAGGT
>Marker245068
AACCTAGTAATAATCTAAAGCAACACTATCATAAACCGCTTGAAGATATACACAAGGAAAATGTAATGAAAAAAT
TAATGXXXXXXXXXXXAAGTATTATTTCAGATAAACAGTCATAAATAGTTGAAGTTGATCTACAAAAGGTTACGCC
AAAGAGATAAAGCTGAAGTT
AACCTAGTAATAATCTAAAGCAACACTATCATAAACCGCTTGAAGATATACACAAGGAAAATGTAATGAGAAAAT
TAATGXXXXXXXXXXXAAGTATTATTTCAGATAAACAGTCGTAAATAGTTGAAGTTGATCTACAAAAGGTTACGCC
AAAGAGATAAAGCTGAAGTT
>Marker245400
AACTAATAAAACTATATAAATAAATATTTATTGTGGGCAAATGATAGAAATCTTAACTTGAAAACCCATGTGAGC
GACGGXXXXXXXXXXXTACGGAAAAGGGTTTTTTCTTCGTTTTCTTCTATAATTATTTTAATTGGATCCTTGGAAT
GCTAATAAATATTAATGGTC

Create PDF with GO2PDF for free, if you wish to

AACTAATAAAACTGTATAAATAAATATTTATTGTGGGCAAATGATAGAAATCTTAACTTGAAAACCCATGTGAGC
GACGGXXXXXXXXXXXTACGGAAAAGGGTTTTTTCTTCGTTTTCTTCTATAATTATTTTAATTGGATCCTTGGAAT
GCTAATAAATATTAATGGTC
>Marker245488
GACAAACGTGAAAATGCATGGATATCTTTATCTCTTTGCAGTTTAAAAATTGCAGTCTGAAAGTGTTTTTTAATA
GAAATXXXXXXXXXXXTGAGTGTATAGATGTGAATTACAAGAATAATTTTTACTGTTATGGGAATATATTTGTTGC
AGGATCTAATTCGTGAAGTA
GACAAACGTGAAAAGGCATGGATATCTTTATCTCTTTGCAATTTAAAAATTGCAGTCTGAAAGTGTTTTTTAATA
GAAATXXXXXXXXXXXTGAGTGTATAGATGTGAATTACAAGAATAATTTTTACTGTTATGGGAATATATTTGTTGC
AGGATCTAATTCGTGAAGTG
>Marker245730
AACATTAATAACATCTCACCTTAAGCCCCCTTTCACCTACAACCGTAGAATACTTTTCAGGAAAGTTCATGATAG
TATCGXXXXXXXXXXXCTCCTCCTCGGTAGCTGAAAGACGACCATAATGAATGTTATGGAAAATTGTATCATTGAA
GAGAACCTGTTATTCACAGT
AACATTAATAACATCTCACCTTAAGCCCCCTTTCACCTACAACCGTAGAATACTTTTCAGGAAAGTTCATGATAG
TATCGXXXXXXXXXXXCTCCTCCTCGGTAGCTGAAAGACGACCATAATGAATGTTATGGAAAATTGTATCATTGAA
GAGAACCTGTTATTAACAGT
>Marker246176
AACTCATCGGTGGATCTTTTGTCAAGCATACACTCGATACTTATAGTTCATGATTGAATGACATTTTGTTTCAAG
GAGATXXXXXXXXXXXACCATCTGATATTCATTAAGCTCATCATTTGTAAAGGTTTGCAAGTCTATTCATTATTCT
GAATGGTATGCTTGTAGGTG
AACTCATCGGTGGATCTTTTGTCAAGCATACACACGATACTTATAGTTCATGATTGAATGACATTTTGTTTCAAG
GAGATXXXXXXXXXXXACCATCTGATATTCATTAAGCTCATCATTTGTAAAGGTTTGCAAGTCTATTCATTATTCT
GAATGGTATGCTTGTAGGTG
>Marker247042
TACTACTTTTTTTAGAAGAGAAGAAAAGCAGTGCTGGGATTGTTAGAACTTGTTTCTTACAAATTTAGTCTTTGA
GTATTXXXXXXXXXXXTCCGTTTGCAGGGAAGACCATGACAACACTGACATACCATAAGAAATCTGTTCGGGCAAT
GGCATTGCATCCTAAAGAGT
TACTATTTTTTTTAGAAGAGAAGAAAAGCAGTGCTGGGATTGTTAGAACTTGTTTCTTACAAATTTAGTCTTTGA
GTATTXXXXXXXXXXXTCCGTTTGCAGGGAAGACCATGACAACACTGACATACCATAAGAAATCTGTTCGGGCAAT
GGCATTGCATCCTAAAGAGT
>Marker247319
TACCGACCAATTTCGATTCCACATACTGCTATGCATTGGGTTATGGTGCTGGAGCTCTCCTGCAGAGTGGAAAAA
CTGGAXXXXXXXXXXXCTTAGCTGCTCCTGTTGAAGAATGGACTGTTGGTGGGACTGCATTGACTTCATTAATGGA
CGTCGAGAGAAGACATGGTA
TACCGACCAATTTCGATTCCACATACTGCTATGCATTGGGTTATGGTGCTGGAGCTCTCCTGCAGAGTGGAAAAA
CTGGAXXXXXXXXXXXCTTAGCTGCTCCTGTTGAAGAATGGACTGTTGGTGGAACTGCATTGACTTCATTAATGGA
CGTCGAGAGAAGACATGGTA
>Marker247350
CACCAAGTAGTTGTGACAACACAATATTATCCACGTAAAAAAAAAGTATATTATCACGAGATGCTAATGCTAATG
CCACTXXXXXXXXXXXTGTAACTAAATAGGTAATTTTTACCCATTACCCTTTTTAATTTACTGGTATGATTAAAAG
GCATGGTTTTTGTTTGGAGT
CACCAAGTAGTTGTGACAACACAATATTATCCACGTAAAAAAAAAGTATATTATCACGACATGCTAATGCTAATG
CCACTXXXXXXXXXXXTGTAACTAAATAGGTAATTTTTACCCATTACCCTTTTTAATTTACTGGTATGATTAAAAG
GCATGGTTTTTGTTTGGAGT
>Marker247911

Create PDF with GO2PDF for free, if you wish to

TACCAACCTTGAAAGTATATAGTTGATAGTAGAGTCTTTATTTAATTTTATTAATGTTTTTTTAAAATTTATTTT
GCTACXXXXXXXXXXXTTTTAAATCCACGAGTATCCGAGCAATAGTCCTAGTGTTAATTTCATATATAAAAATAAT
CAAATAAGTTTTAACGAGTT
TACCAACCTTGAAAGTATATAGTTGATAGTAGAGTCTTTATTTAATTTTATTAATGTTTTTTTTAAATTTATTTT
GCTACXXXXXXXXXXXTTTAAAATCCACGAGTATCCGAGCAGTAGTCCTAGTGTTAATTTCATATATAAAAATAAT
CAAATAAGTTTTAACGAGTT
>Marker248082
ACTTGATATTAAATTATCAACAGACTACAATAATGATAGAGATAAGTTGGAGGCATACCTTCAATTCTAGCCGCG
TTTAAXXXXXXXXXXXTGATGACAGCAGCCCTACTGGATTGCGTAAAGTGCCTCTCTTCCTGTCATCACCATTTGC
CTACGAATTTTTTCTGAAGT
ACTTGATATTAAATTATCAACAGACTACAATAATGATAGAGATAAGTTGGAGGCATACCTTCAATTCTAGCCGCA
TTTAAXXXXXXXXXXXTGATGACAGCAGCCCTACTGGATTGCGTAAAGTGCCTCTCTTCCTGTCATCACCATTTGC
CTACGAATTTTTTCTGAAGT
>Marker248234
ACTTGAGACCAAAAGTTTAACTGTTACGTTTTTGTTAAGAAAATCTTCTCAAATCACCCTTATGATTCTTGTTTT
CTAGGXXXXXXXXXXXTCAATCTCATAAAACCTAATAAATTTAAATAAAATTTTCAAAACATTTTTAATTTAGAGT
TATCTTTTCGGTGAGATGTT
ACTTGAGACCAAAAGTTTAACTGTTAGGTTTTTGTTAAGAAAATCTTCTCAAATCACCCTTATGATTCTTGTTTT
CTAGGXXXXXXXXXXXTCAATCTCATAAAACCTAATAAAGTTAAATAAAATTTTCAAAACATTTTTAATTTAGAGT
TATCTTTTCGGTGAGATGTT
>Marker248786
CACATCTATGATGTTGAGAGACACAAATGGATAACAGGATAAGCCTTTACCAATGAATTTTCAAAAAGTTTGTTT
GCTAAXXXXXXXXXXXGAAAGTTACATATAACATGATTGATAGAGTTTGATATATTTACCATCAAATTGTAACCTA
TTATTATAATTCGTTTGGTT
CACATCTATGATGTTGAGAGACACAAATGGATAACAGGATAAGCCTTTACCAATGAATTTTCAAAAAGTTTGTTT
GCTAAXXXXXXXXXXXGAAAGTTATATATAACATGATTGATAGAGTTTGATATATTTACCATAAAATTGTAACCTA
TTATTACAATTCGTTTGGTT
>Marker249080
ACATTTAATAGACCTTCAGAAAGAGTGTCTTCCCCCCCTTCTCCAAAAACAAAATAAATGCTTAATTTCATAAAT
GATGGXXXXXXXXXXXCCCATTTAGGTGAGATTAAGAATTCAAGTATGCTTACTTATTTCTAATAATGTCCATTTA
AATGTGTGTGTGATTTAAGT
ACATTTAATAGACCTTCAGAAAGAGTGTCTTCCCCCCCTTCTCCAAAAACAAAATAAATGCTTAATTTCATAAAT
GATGGXXXXXXXXXXXCCCATTTAGGTGAGATTAAGAATTCAAGTATGCTTACTTATTTCTAATAATGTCCCTTTA
AATGTGTGTGTGATTTAAGT
>Marker249221
ACTAAAGAAGTTAGAGTAACTAAAGAAAATTAGCCCTATAGATTAGATTAACCCCAATGCAGTAGAGGGAGAGGC
TGAAAXXXXXXXXXXXAAAAAACCAGTTTAATCTTTTAAGACTCACATTTAAAGTTTAATCAGATTTTGAATTTTG
TTTGCCATTTGAAGATCGTA
ACTAAAGAAGTTAGAGTAACTAAAGAAAATTAGCCCTATAGATTAGATTAACCCCAATGCAGTAGAGGGAGAGGC
TGAAAXXXXXXXXXXXAAAAAACCAGTTTAATCTTTTAAGACTCACATTTAAAGTTTAATCAGATTTTGAATTTTG
CTTGCCATTTGAAGATCGTA
>Marker249274
AACCTATTTGATTTTCATTCAAATTTTAATATAATACATTCCTTTTAATATTCTATGTATCGAATATAATAAGAT
ATAATXXXXXXXXXXXAAATATTTCCTCATTTATTTTGTAGCCCTAATTCCTCTCTGTCCAAACTCAAATTTGAAG
ACGGTTTTTTAGTTCGAGTT

Create PDF with GO2PDF for free, if you wish to

AACCTATTTGATTTTCATTCAAATTTTAATATAATGCATTCCTTTTAATATTCTATGTATCGAATATAATAAGAT
ATAATXXXXXXXXXXXAAATATTTCCTCATTTATTTTGTAGCCCTAATTCCTCTCTGTCCAAACTCAAATTTGAAG
ACGGTTTTTTAGTTCGAGTT
>Marker249671
ACCTAAAAATCATCTCTAAGTAAAGTTCCATCATTCTCATACACCAACAAGGACGTTACTTGGTTAGGAACATAT
CCGTAXXXXXXXXXXXTACAAGCATCAATTAATCTTTTGTAGAAAGATTTCCAATCCATCCATTCTTATTTGTTCC
CCTTTGTTATAGAGCAAGTT
ACCTAAAAATCATCTCTAAGTAAAGTTCCATCATTCTCATACACCAACAAGGACGTTACTTGGTTAGGAACATAT
CTGTAXXXXXXXXXXXTACAAGCATCAATTAATCTTTTGTAGAAAGATTTCCAATCCATCCATTCTTATTTGTTCC
CCTTTGTTATAGAGCAAGTT
>Marker249705
AACATGAATCTATTACGTTTCCACAAACCCACATATGGCCAATTCCATTGCTTTAAAGTAAGTAGATGAGTCTTC
CCTTAXXXXXXXXXXXGGGTAAAACGATAAATTGACCCAACTGGTGTTACGAACACTCGTGAAGGACTAACTTACT
GTTATTGGTGTATATCCGTG
AACATGAATCTATTACGTTTCCACAAACCCACATATGGCCAATTCCACTGCTTTAAAGTAAGTAGATGAGTCTTC
CCTTAXXXXXXXXXXXGGGTAAAACGATAAATTGACCCAACTGGTGTTACGAACACTCGTGAAGGACTAACTTACT
GTTATTGGTCTATATCCGTG
>Marker250030
ACTTATATTATGATTTAAGCTTTTAGTGATATATAATAATGGCTTTAAGAAAAAAGTTAATATGAACCCTCACCA
TATATXXXXXXXXXXXTCATTTCTAAAAAATACACTTTCTTTATACATATTTAATATAAAGTTGAGTGTAAAATCT
AGAATACTAAGTTTAGAGTC
ACTTATATTATGATTTAAGCTTTTAGTGATGTATAATAATGGCTTTAAGAAAAAAGTTAATATGAACCCTCACCA
TATATXXXXXXXXXXXTCATTTCTAAAAAATACACTTTCTTTATACATATTTAATATAAAGTTGAGTGTAAAATCT
AGAATACTAAGTTTAGAGTC
>Marker250064
CACTTGAACTGAAACTACTTTAACGAATTAGAAGTCATGGTCCTCGAATTACTCTGTTTATCTATTATCTTCTTT
TTTGTXXXXXXXXXXXGTTTTATCTGAAATGACAACCAGTGTAGTTATGGTATCCAATTTATTATCTGATAGCTGA
TAGAATATGAATGCATTGTA
CACTTGAACTGAAACTACTTTAACGAATTAGAAGTCATGGTCCTCGAATTACTCTGTTTATCTATTATCTTCTTT
TTTGTXXXXXXXXXXXGCTTTATCTGAAATGACAACCAGTGTAGTTATGGTATCCAATTTATTATCTGATAGCTGA
TAGAATATGAATGCATTGTA
>Marker250468
ACATTAGCGGCCACAGAACCTTGAAATATTGATTACATTTTAGATGGAGGTTGCATAGGAACAATAAGGGATGCA
GCAGTXXXXXXXXXXXGGCTCCACTGGAGAAACAAAAGATTGACCTGCAATAAAGTCAGACAATTAAACATAAAAG
AATGTTGAAAGGACCAAGTG
ACATTAGCGGCCACAGAACCTTGAAATATTGATTACATTTTAGATGGAGGTTGCATAGGAACAATAAGGGATGCA
GCAGTXXXXXXXXXXXGGCTCCACTGGAGAAACAAAAGATTGACCTGCAATAAAGTCAGACAATTAAACATAAACG
AATGTTGAAAGGACCAAGTG
>Marker250506
TACAAATCAATGTCGTTCAACTTCCATTTCCCTGCTTACAAGGCGGCCTGCCGGAAGGCTGTGAGAATTTGGACT
TACTTXXXXXXXXXXXTTGCTCACAAATTTCACATCCCTAGGATTGTTTTTTACAGCCTTTGTTGTTTCTCTCTCC
TGTGTCAGCCCACTTTAGTA
TACAAATCAATGTCGTCCAACTTCCATTTCCCTGCTTACAAGGCGGCCTGCCGGAAGGCTGTGAGAATTTGGACT
TACTTXXXXXXXXXXXTTGCTCATAAATTTCACATCCCTAGGATTGTTTTTTACAGCCTTTGTTGTTTCTCTCTCC
TGTGTCAGCCCACTTTAGTA
>Marker250529

Create PDF with GO2PDF for free, if you wish to

GACAGTGATCTTCCTCATTTTTGTTGATAGTTGGTGCATTAAGCGCATACACGCACTTAGACCATTGGAAGGGCC
AAAAGXXXXXXXXXXAGAAGCAATATTTGACTGTTATTCGGTTAATGGCCCTACTATCAACATACATTCTCTAGC
ATCCATCCTTCTTTGGAGTT
GACAGTGATCTTCCTCATTTTTGTTGATAGTTGGTGCATTAAGCGCATACACGCACTTAGACCATTGGAAGGGCC
AAAAGXXXXXXXXXXAGAAGCAATATTTGACTGTTATTCGGTTAATGGCCCTGCTATCAACATACATTCTCTAGC
ATCCATCCTTCTTTGGAGTT
>Marker250724
TACAATGACAAGAACATTATTTAAGATGTAAATATATACTAATCAAAATGCATGGTATGAAAATCATTTCAAGAT
GGACTXXXXXXXXXXTTTATCACCACGTCATGCGAATCAACAATTTTAAATGAGTAAAGATCTAAATGCAAACAA
CATGAGCATGTATGTAAAGT
TACAATGACAAGAACATTATTTAAGATGTAAATATATACTAATCAAAATGCATGGTATGAAAATCATTTCAAGAT
GGACTXXXXXXXXXXTTTATCACCACGTCATGCAAATCAACAATTTTAAATGAGTAAAGATCTAAATGCAAACAA
CATGAGCATGTATGTAAAGT
>Marker250947
ACCAAAATAAAATTACTTCCCTACATCTACTTATATAAACTTGTTCCATTAGTGTTATAAGTTGTCTTTCTCTTT
TTGCTXXXXXXXXXXTTTTCTCTAACCAATACTTGATAAAAGAGCAGTCTAAACTATTATTTATAATTGGAAAAG
AAAAAGTCACTCATACTGTT
ACCAAAATAAAATTACTTCCCTACATCTACTTATATAAACTTGTTCCATTAGTGTTATAAGTTGTCTTTCTCTTT
TTGCTXXXXXXXXXXTTTTCTCTAACCAATACTTGATAAAAGAGCAGTCTAAACTATTATTTATAATTGGAAAAG
AAAAAGTCAGTCATACTGTT
>Marker251021
GACCTATTAGACACAATTGGAAATTCAAAGACTATAAAGTTCAATAACCGATTAGATAAAAAATTTGGTAGTTCAA
GGTTGXXXXXXXXXXGCTATAATAGATGCACTTGAGAATAAAAACAAAGGCCAATTACCTCCGAAAATGCTAATC
TATCAGCAACATCATTTGTC
GACCTATTAGACACAATTGGAAATTCAAAGACTATAAAGTTCAATAACCGATTAGATAAAAAATTTGGTAGTTCAA
GGTTGXXXXXXXXXXGCTATAATGGATGCACTTGAGAATAAAAACAAAGGCTAATTACCTCCGAAAATGCTAATC
TATCAGCAACATCATTTGTC
>Marker251666
AACTTGTAGAGATTACTCAACTTGTTACCTCCTTCCCAAAATATCTTCAAAATCCAAACCATCAGAAGCTATGAA
TAATAXXXXXXXXXXTTTAACAGACAGTTCAAGAAATTCAGTCTAATAGTTTTCTCAAGTATAGATTATGTAGGG
AGCTAGCTAGCTAGGAAGTA
AACTTGTAGAGATTACTCAACTTGTTACCTCCTTCCCAAAATATCTTCAAAATCCAAACCATCAGAAGCTATGAA
TAATAXXXXXXXXXXTTTAACAGACAGTTCAAGAAATTCAGTCTAATAGTTTTCTCAAGTATAGCTTATGTAGGG
AGCTAGCTAGCTAGGAAGTA
>Marker251722
GACTTTCATTATTTTTGTATGTATATACTCAAGTTTTGAACTGCATCATGAGACGGTTAGGAGTAGCGAGGGTTA
AGATAXXXXXXXXXXGTGAGACATACTCTGATATTATTTGGAGTATGAAGAATATTATATAGAAGTAAAAAAATT
CCCTCTTTTTAGGTTTGGTT
GACTTTCATTATTTTTGTATGTATATACTCAAGTTTTGAACTGCATCATGAGACGGTTAGGAGTAGCGAGGGTTA
AGATAXXXXXXXXXXGTGAGACATACTCTAATATTATTTGGAGTATGAAGAATATTATCTAGAAGTAAAAAAATT
CCCTCTTTTTAGGTTTAGTT
>Marker252770
TACCATGACTTGTTAATTATATAAAAAAAAAACTCGAAAACAACGTATATTGTTTTGGTTTATATATTTATACAA
TTGTCXXXXXXXXXXTTGATAGAGATGTTTAATTATTGGTATGATCTTCCACTTAATTCTTATATATAATTTAAC
TGATATAAAGAAGATTATGT

Create PDF with GO2PDF for free, if you wish to

TACCATGACTTGTTAATTATATAAAAAAAAAACTCGAAAACAAAGTATATTGTTTTGGTTTATATATTTATACAA
TTGTCXXXXXXXXXXTTGATAGAGATGTTTAATTATTGGTATGATCTTCCACTTAATTCTTATATATAATTTAAC
TGATATAAAGAAGATTATGT
>Marker253564
ACTTCAGCATAAGCATATTTTATGTAGTCACCTCTTCAACATTCTGGGCAGTTTTGGCAGATGCCTCCATGAATA
TTAAAXXXXXXXXXXXTGTTTGCATTTGCATGCTGCCTTGCATCTTCTAACCAGCTAATCAAGTGATTGAAAGTCT
CCCTTCTGAATAACGTAGTG
ACTTCAGCATAAGCATATTTTATGTAGTCACCTCTTCAACATTCTGGGCAGTTTTGGCAGATGCCTCCATGAATA
TTAAAXXXXXXXXXXXTGTTTGCGTTTGCATGCTGCCTTGCATCTTCTAACCAGCTAATCAAGTGATTGAAAGTCT
CCCTTCTGAATAACGCAGTG
>Marker253786
ACCTTAACTGTTCCCATTTTCTATTTTTCTCTATCGATCTTTGTTGTGGGTTGGACCACGAAGTGTCCAAAAACA
CACATXXXXXXXXXXTCTCAGCTTTTAAGGGCAATCATGAATATTGCACCCTTAGCAATATCCACAACAGGCCGT
GGTTCCTCTGGCGTTGGGTT
ACCTTAACTGTTCTCATTTTCTATTTTTCTTTATCGATCTTTGTTGTGGGTTGGACCACGAAGTGTCCAAAAACA
CACATXXXXXXXXXXTCTCAGCTTTTAAGGGCAATCATGAATATTGCACCCTTAGCAATATCCACAACAGGCCGT
GGTTCCTCTGGCGTTGGGTT
>Marker254817
TACCTAGCTTCTAAACCTTGGTAAAAAGACTTGGAGTGTATCCTAACTTTAAACTAAATTGGAATATTTGTTCCC
TGGGGXXXXXXXXXXTCATTTTTTTTTCTAATTTATTTTAGGACACTCTTGCACACATGTTATGCATATGACTAC
TAAATGGAAAGATACGGGGT
TACCTAGCTTCTAAACCTTGGTAAAAAGACTTGGAGTGTATCCTAACTTTAAACTAATTTGGAATATTTGTTCCC
TGGGGXXXXXXXXXXTCATTTTTTTTTTAAATTTATTTTAGGACACTCTTGCACACATGTTATGCATATGACTAC
TAAATGGAAAGATACGGGGT
>Marker254911
ACAAAATCTTTAATTCCAAATACAAGCATAAATTTTGATGCTGATTCTAATGAAAGAGAGAATGAACCCGGAAAA
AAGAAXXXXXXXXXXXACAGTCTTCCCTGTCTTTGCCATAAACTTATCTTCTTGAGATTTTTTTGCCTTTCATTAT
GAAGACTCAAATATAGTGTT
ACAAAATCTTTAATTCCAAATACAAGCATAAATTTTGATGCTGATTCTAATGAAAGAGAGAATGAACCCAGAAAA
AAGAAXXXXXXXXXXXACAGTCTTCCCTGTCTTTGCCATAAACTTATCTTCTTGAGATTTTTTTGCCTTTCATTAT
GAAGACTCAAATATAGTGTT
>Marker255321
CACACTATAATGCGTCTTTTGGAATAAAAATTTATGATGATGTTAAGAGTCGTTGCTTATTATTTTGACACTCTT
TATTGXXXXXXXXXXCATCCACAATTTACTCTTCATTGTTGTATATTGATGTGATTTTCACTCGCAGATTGTTTA
ATAAAAGTTTCATTGCTGTA
CACACTATAATGCGTCTTTTGGAATAAAAATTTATGATGATGTTAAGAGTCGTTGCTTATTATTTTGACACTCTT
TATTGXXXXXXXXXXCATCCACAATTTACTCTTCATTGTTGTATATTGATGTGATTTTCAGTCGCAGATTGTTTA
ATAAAAGTTTCATTGCTGTA
>Marker255727
ACGGCTTCGAGGGCGGAAACAGTGCCTTTCAGTTTGTCAAGTTCATCATGGAAACCCCACAACAATCCAAGGTCT
TGAAGXXXXXXXXXXTGCTTGGAGAGAATTGGAACTTGAACCTTTATATTTCGACTCTTTTTGTAATTTCTAATC
TAATCTTTAATTTGTTTGTT
ACGGCTTCGAGGGCGGAAACAGTGCCTTTCAGTTTGTCAAGTTCATCATGGAAACCCCACAACAATCCAAGGTCT
TGAAGXXXXXXXXXXTGCTTGGAGAGAATTGGAAGTTGAACCTTTATATTTCGACTCTTTTTGTAATTTCTAATC
TAATCTTTAATTTGTTTGTT
>Marker255854

Create PDF with GO2PDF for free, if you wish to

GACTCCTTTTCTTCCGCCATACCCTTCTTAAAATCAGTTAAAACCTACTTGAATCTCCGACGAATCAGACCCCAA
ACATCXXXXXXXXXXTGGCAAGCAGTTGCACTATGAAAATGACATTAGCACCAAAATGGGGTTAGTTGAAACCGC
AAGCAAATGCCAGGCGAGTA
GACTCCTTTTCTTCCGCCATACCCTTCTTAAAATCAGTTAAAACCTACTTGAATCTGCGACGAATCAGACCCCAA
ACATCXXXXXXXXXXTGGCAAGCAGTTGCACTATGAAAATGACATTAGCACCAAAATGGGGTTAGTTGAAACCGC
AAGCAAATGCCAGGCGAGTA
>Marker256042
GACAACTGCATACATTTCATTATCAACTTTTTGAAGGTTTAAAGACTAGACAATTGTAATTCTATCGGTTGAAGA
GTGATXXXXXXXXXXTTACCGTTGATGAATTTGGCATGAAACAACATCTTTATAATATTTCATGAATTTGCTAGT
TCTCATTGGATTTAAGTGTT
GACAACTGCATACATTTCATTATCAACTTTTTGAAGGTTTAAAGACTAGACAATTGTAATTCTATCGGTTGAAAA
GTGATXXXXXXXXXXTTACCGTTAATGAATTTGGCATGAAACAACATCTTTATAATATTTCATGAATTTGCTAGT
TCTCATTGGATTTAAGTGTT
>Marker256207
AACTAGTAGAAAAAGCTTTGAGATGACCAACTAATGAATCACTTATTTTATCAAAGTTTAACAGTAAATTGAATT
TTATGXXXXXXXXXXAGATATCAATCTACCAAAATGTTTAACTAATTGTGACCGAGACCCAGGTAACGAAAGGTG
TTGTCTAAATTAAAATCAGT
AACTAGTAGAAAAGGCTTTGAGATGACCAACTAATGAATCACTTATTTTATCAAAGTTTAACAGTAAATTGAATT
TTATGXXXXXXXXXXAGATATCAATCTACCAAAATGTTTAACTAATTGTGACCGAGACCCAGGTAACGAAAGGTG
TTGTCTAAATTAAAATCAGT
>Marker256255
ACCGAAGATTTGAAGATTCAAACTTCTACAAGCTCTCAAGACGTGCAAGTTCGTATCAACCTCGAGATCAACAAC
TTCTGXXXXXXXXXXTACTAGAGATTGTATTCACAATATTTATCAATATATCAAAGTTTATTTCCACGAATTACA
TTTCTTCGAAATCTCGTGTA
ACCGATGATTTGAAGATTCAAACTTCTACAAGCTCTCAAGACGTGCAAGTTCGTATCAACCTCGAGATCAACAAC
TTCTGXXXXXXXXXXTACTAGAGATTGTATTCACAATATTTATCAATATATCAAAGTTTATTTCCACGAATTACA
TTTCTTCGAAATCTCGTGTA
>Marker256666
TACCCTTGAACAAAATATACACCGACTATTACTTTAACTATTTTATTATTTTGGATGGGTGTGGATCAAATTATT
ACTTAXXXXXXXXXXAAAGAAGATCGAATTGAGTATCACCTCTATCTCTAGTATGAATAATAAGATTGAATCTCA
AACTTTGTGAGATTGACAGT
TACCCTTGAACAAAATATACACCGACTATTACTTTAACTATTTTATTATTTTGGATGGGTGTGGATCAAATTATT
ACTTAXXXXXXXXXXAAAGAAGATCGAATTGAGTATCACCTCTATCTCTAGTATGAATAATAAGATTGAATCTCA
AACTTTGTGAGATCGACAGT
>Marker256934
AACTGACACTACGATATCATACTTGAAAATGTATCTTAAAGTTTTGCCAGCAATTGATTTTGTTTGCTAGTTTAG
ATAAAXXXXXXXXXXGAAACAATTACTAAGATTCTCTTTATAACAAAATCCTACATGCTACACCAGTTGTAGTTT
AAAATGTTATCACTTTAGTG
AACTGACACTACAATATCATACTTGAAAATGTATCTTAAAGTTTTGCCAGCAATTGATTTTGTTTGCTAGTTTAG
ATAAAXXXXXXXXXXGAAACAATTACTAAGATTCTCTTTATAACAAAATCCTACATGCTACACCAGTTGTAGTTT
AAAATGTTATCACTTTAGTG
>Marker257104
AACATATTTAATGAGTCATGAGGTGTTGCTGCAATTGATGATGAAAACAACAAAATGATGAGGTAATGGAAAAT
TCAATXXXXXXXXXXCAATATCATGAGCTTAATTTCTCTAGGGATATATTTAACACTATGATACTCTTAAGGTTT
TTATATAACTTGAGTTTGTG

Create PDF with GO2PDF for free, if you wish to

AACATATTTAATGAGTCATGAGGTGTTGCTGCAATTGATGATGAAAACAACAAAATGATGAGGTAATGGGAAAAT
TCAATXXXXXXXXXXCAATATCATGAGCTTAATTTCTCTAGGGATATATTTAACACTACGATACTCTTAAGGTTT
TTATATAACTTGAGTTTGTG
>Marker257197
ACCAATTCCTTCCATTCATTTAATTACATATCACAAAATGATTTACTATTCATTTATTTTATAGACCTCAAATAA
TCTTTXXXXXXXXXXCCCTAAAATGTGTTACACTTTATGACATTCTGAAGAAGACTAACAATGCATGCTCCATTT
CTCTACACTACGCCTATGTC
ACCAATTCCTTCCATTCATTTAATTACATATCACTAAATGATTTACTATTCATTTATTTTATAGACCTCAAGTAA
TCTTTXXXXXXXXXXCCCTAAAATGTGTTACACTTTATGACATACTGAAGAAGACTAACAATGCATGCTCCATTT
CTCTACACTACGCCTATGTC
>Marker257943
ACAATAATTTTACACAGGTAATTGTATGCTGAAAATGCCCCAACTCCAAGTGCAACGATAGATATTTATGTAGTT
CCTTTXXXXXXXXXXCAGCACTGTCATTGGATGACCTCATTGTTCCACAGGTTACTTCATATCTCCACCTCGTCA
TCCTCCTTAGTAATAGGAGT
ACAGTAATTTTACACAGGTAATTGTATGCTGAAAATGCCCCAACTCCAAGTGCAACGATAGTTATTTATGTAGTT
CCTTTXXXXXXXXXXCAGCACTGTCATTGGATGACCTCATTGTTCCACAGGTTACTTCATATCTCCACCTCGTCA
TCCTCCTTAGTAATAGGAGT
>Marker258632
CACTACAATCCGAGGAGTGTTTTGTGTAACAGAGTATATGAAATTAGTTAGAAGTCGTTTTAACATGCTTAGAAC
TCAATXXXXXXXXXXAAATCAACACAAACTCTCACCATTATTGTGTTGTAACTAAATTCTTAGACGATAATTTGA
TTAAAAAAGTCAATAATGTA
CACTACAATCCGAGGAGTGTTTTGTGTAACAGAGTATATGAAATTAGTTAGAAATCGTTTTAACATGCTTAGAAC
TCAATXXXXXXXXXXAAATCAACACAAACTCTCACCATTATTGTGTTGTAACTAAATTCTTAGACGATAATTTGA
TTCAAAAAGTCAATAATGTA
>Marker258651
TACTTTGGTGCAATCCTTCATCTTCAATGAGGCATTGTATGCAACATATATCTCTACCAATATTAAATGCCTAAT
TTTAGXXXXXXXXXXAGCACTCCGCTCATGTGAGAGAGATTTGTTTAGCTTATAGTTCTAATTTGATGATGAGAG
AACTCGTGCATTATTGGGTA
TACTTTGGTGCAATCCTTCATCTTCAATGAGGCATTGTATGCAACATATATCTCTACCAATATTAAATGCCTAAT
TTTAGXXXXXXXXXXAGCACTCCGCTCATGTGAGAGAGATTTGTTTAGCTTATAGTTCTAATTTGATGATGAGAG
AACTCGTGCATTCTTGGGTA
>Marker258945
AACTGAGAATAATTGTTGAGATAATAGATTTGATGATGATTTAATTTGGTTAGATATTTGAAAGATTTGAAATTT
ATGATXXXXXXXXXXTTCATTTCTTTGTTCTTCTGCGGGCACACGAATTTTCATTTCTTTGAGTCGTTGGTTTAT
TGGACGTTTTTGTGTTGAGT
AACTGAGAATAATTGTTGAGATAATAGATTTGATGATGATTTAATTTGGTTAGATATTTGAAAGATTTGAAATTT
ATGATXXXXXXXXXXTTCATTTCTTTGTTCTTCTGCGGGCACACGAATTTTCATTTCTTTGAGTCCTTGGTTTAT
TGGACGTTTTTGTGTTGAGT
>Marker259162
AACAAATTCATCAAAAGTTTTCCTTGTTGGAAAACCAGCACAACTTATCCTTATTGCCTCCATGACTCCCTAATG
AAAAGXXXXXXXXXXAATGCCAATTTCTCTCCCATTACAGTGTGCCCAGCAGTGGTAATCACAACCAAAGATAAC
TTAAAGTTTGCTTCAGGGTA
AACAAATTCATCAAAAGTTTTCCTTGTTGGAAAACCAGCACAACTTATCCTTATTGCCTCCATGACTCCCTAATG
AAAAGXXXXXXXXXXAATGCCAATTTCTCTCCCATTACAGTGTGTCTAGCAGTGGTAATCACAACCAAAGATAAC
TTAAAGTTTGCTTCAGGGTA
>Marker259639

Create PDF with GO2PDF for free, if you wish to

ACTGTTATGAAAAAATTGAATATTCAAACCTCTAAAGTTCGTATCAACCTCGAGATCAACATCTTCTAAAAAATTG
AAGACXXXXXXXXXXXATATTAGAGATTGTATCCGCTATATCAATCAATACACAATGCTCAATTCCACGAACTACA
TTTCTTCGAAATCGCGTGTG
ACTGTTATGAAAAAATTGAATATTCAAACCTCTAAAGTTCGTATCAACCTCGAGATCAACATCTTCTAAAAAATTG
AAGACXXXXXXXXXXXATATTAGAGATTGTATCCGCTATATCAATCAATACACAATGCTCAATTCCACGAACTACA
TTTCTTCGAAATCACGTGTG
>Marker260236
ACTACCCGTTCGTAAATTCATACATCTAATTGCTAGTGGAACTATAATGAACAAGGAAGCTCTTAATACATACTC
ATATGXXXXXXXXXXXCCTCAAATAATTACCTATTTAAGGTTAAATTGCGTTGTTTTTATTTTGAAAAATTGTCCC
CCTGTTGCTTGTAGAAAGTA
ACTACCCGTTCGTGAATTCATACATCTAATTGCTAGTGGAACTATAATGAACAAGGAAGCTCTTAATACATACTC
ATATGXXXXXXXXXXXCCTCAAATAATTACCTATTTAAGGTTAAATTGCGTTGTTTTTATTTTGAAAAATTGTCCC
CCTGTTGCTCGTAGAAAGTA
>Marker261597
CACTTTAGCAAGCCTTAGTTTTGCATGATGATATGGCCTCTTTTGATTTTTATCATCGTGCCATAACTATGCTCT
TGAAAXXXXXXXXXXXATGGTCCCCATTGCTGCTCAACTGCTTCTTTAGTGATAATGACCATTCTTTGACATGCAT
ATTGATTTTTAGTGGTAGTT
CACTTTAGCAAGCATTAGTTTTGCATGATGATATGGCCTCTTTTGATTTTTATCATCGTGCCATAACTATGCTCT
TGAAAXXXXXXXXXXXATGGTCCCCATTGCTGCTCAACTGCTTCTTTAGTGATAATGACCATTCTTTGACATGCAT
ATTGATTTTTAGTGGTAGTT
>Marker262609
CACCTCTTCAGCATCTCTAATGCAGCTTTGGGCTGATCCATGGGGACCATGTGCCCCGCATCATGAACCTGTTGT
GTTGAXXXXXXXXXXXTTGAATTATAGAGGAAGAACTACAAATCAAACTAAGTATAAACCTCCCTCTTGAGTATGC
TAACACTAGGCCCAATTGTC
CACCTCTTCAGCATCTCTAATGCAACTTTGGGCTGATCCATGGGGACCATGTGCCCCGCATCATGAACCTGTTGT
GTTGAXXXXXXXXXXXTTGAATTATAGAGGAAGAACTACAAATCAAACTAAGTATAAACCTCCCTCTTGAGTATGC
TAACACTAGGCCCAATTGTC
>Marker262939
CACTTCAAGGCTCAACCAAAATTTAATAATTTTGATTTCAATTTATATCCTCTTGGAGAGATTGGGAAGGACAAA
AGGAGXXXXXXXXXXXCTCATAGTTCAAGGATTGTTTTTGCATCTTTTTCTGTTGTTTTTGTTCTTATTGAGGCCA
TGTTTAAAAGTGCAGGGGTA
CACTTCAAGGCTCAACCAAAATTTAATAATTTTGATTTCAATTTATATCCTCCTGGAGAGATTGGGAAGGACAAA
AGGAGXXXXXXXXXXXCTCATAGTTCAAGGATTGTTTTTGCATCTTTTTCTGTTGTTTTTGTTCTTATTGAGGCCA
TGTTTAAAAGTGCAGGGGTA
>Marker263600
TACCTGATCACTTATCTTTCTAAACGATCACTTACCTCTTACAATCTGAGGAGGGAAAACTTTCAAATATAAGTC
AAAAGXXXXXXXXXXXTTTCACCAAGACATGAACCATTCTCAACATGGACTACTGTGATGGTCTTTCATCTATAGT
ATCATTTAGAATTTTTAGTT
TACCTGATCACTTATCTTTCTAAACGATCACTTACCTCTTACAATCTGAGGAGGGAAAACTTTCAAATATAAGTC
AAAAGXXXXXXXXXXXTTTCACCAAGACATGAAACATTCTCAACATGGACTACTGTGATGGTCTTTCATCTATAGT
ATCATTTAGAATTTTTAGTT
>Marker263814
CACCTCAGGGCCCAGCAATTCCTCATCATTGATTTCTCCATCTACCATATGAATATACAATGGATACACAGAGAG
CATCAXXXXXXXXXXXTGCAAATATGTGACATTGGGTAGTCTATTCTTAGATGCATATAAACCATCTTTCTCCCAT
TTTCCACTCTTTTGTTATGT

Create PDF with GO2PDF for free, if you wish to

CACCTCAGGGCCCAGCAATTCCTCATCATTGATTTCTCCATCTACCATATGAATATACAATGGATACACAGAGAG
CATCAXXXXXXXXXXTGCAAATATGTGACATTGGATAGTCTATTCTTAGATGCATATAAACCATCTTTCTCCCAT
TTTCCACTCTTTTGTTATGT
>Marker264223
GACCATAGAAGGCGTTTTGACCAGACGAGATACTTACACTTTACGCGAGATACAAGGCAAGGAAACACACATTTT
GTCTAXXXXXXXXXXTCCTCTCACCTCGAGCGCCCTTTTCGCTTTGAATGAGCACATTTCAAAAGATGGAAGCAA
AAGATGTTTTTCCTCTTGTT
GACCATAGAAGGCGTTTTGACCAGACGAGATACTTACACTTTACGCGAGATACAAGGCAAGGAAACACACATTTT
GTCTAXXXXXXXXXXTCCTCTGACCTCGAGCGCCCTTTTCGCTTTGAATGAGCACATTTCAAAAGATGGAAGCAA
AAGATGTTTTTCCTCTTGTT
>Marker264617
AACTTTCATTGGAAACAAATTAAAGAATACAATCGTCATACAAAATAACAAAACCCACAACCACCCTAAACAAAA
GGAAAXXXXXXXXXXTCATTGCATTATTAATGCAAAAGCTACATCAGAAACGAGGAATATTATACTTTGTTGAGA
CACGATCTTTGGACTTTGGT
AACTTTCATTGGAAACAAATTAAAGAATACAATCGTCATACAAAATAACAAAACCCACAACCACCCTAAACAAAA
GGAAAXXXXXXXXXXTCATTGCATTTTTAATGCAAAAGCTACATCAGAAACGAGGAATATTATACTTTGTTGAGA
CACGATCTTTGGACTTTGGT
>Marker265447
ACTATGAAACCCTAAACGTTATGTTTCATCAATTTCTCTCTTTATACCTCAATAAATTAGTTTAAATGCTGTTTT
CTAAGXXXXXXXXXXTTCAAAGTGAATTAAAATATCACTTAAATAAGTGCATTTTCTTTTACTAAAAAACTAAGT
AACAAAAAACAAAATTAGTA
ACTATGAAACCCTAAACATTATGTTTCATCAATTTCTCTCTTTATACCTCAATAAATTAGTTTAAATGCTGTTTT
CTAAGXXXXXXXXXXTTCAAAGTGAATTAAAATATCACTTAAATAAGTGCATTTTCTTTTACTAAAAAACTAAGT
AACAAAAAACAAAATTAGTA
>Marker265707
TACACCAATCGTGAAAATGGAACTACTTCCTCATACAAATATGCATTATATGAATCTGGTAATATTCATGATGTA
ACCATXXXXXXXXXXTCAAATTTGTTGTCATTGGTATGTATAGAAGGAAACTTCTGCTACACAAGAAAAACTTGT
TAATGGCTTCAAGTTTTTGT
TACACCAATCGTGAAAATGGAACTACTTCCTCATACAAATATGCATTATATGAATCTGGTAATATTCATGATGTA
ACCATXXXXXXXXXXTCAAATTTGTTGTCATTGGTATGTATAGAAAGAAACTTCTGCTACACAAGAAAAACTTGT
TAATGGCTTCAAGTTTTTGT
>Marker266233
ACTGAATTGACTAGCATAAGGTAGCTTTTACATGGCTAATGTGGGAGTTTGAGGCTTAATAGAAGTGGGAGTCCA
AGATTXXXXXXXXXXTTTGGAATTGGATCTCTTTCTAAGCAGTAGATGACTACATTTGAGGGATAAAAAGGTTTA
TGAATTTTGGCCACTTTGTG
ACTGAATTGACTAGCATAAGGTAGCTTTTACATGGCTAATGTGGGAGTTTGAGGCTTAATGGAAGTGGGAGTCCA
AGATTXXXXXXXXXXTTTGGAATTGGATCTCTTTCTAAGCAGTAGATGACTACATTTGAGGGATAAAAAGGTTTA
TGAATTTTGGCCACTTTGTG
>Marker266525
ACAAACGAAGGGGGCTCTTTGGTTGGTGAAGCCTAAAGAGAGAGCCTTAGACCTAGCAAGGATGAATTGATTTTT
ATATGXXXXXXXXXXTAGTGATGCTATCCACACTCCATGGATGTCTCATTTAACTTGTATCAGTATCCATGTCTG
TTTCTTATTTATTTGTTAGT
ACAAACGAAGGGGGCTCTTTGGTTGGTGAAGCCTAAAGAGAGAGCCTTAGACCTAGCAAGGATGAATTGATTTTT
ATATGXXXXXXXXXXTAGTGATGCTATCCACACTCCATGGATGTCTCATTTAATTTGTATCAGTATCCATGTCTG
TTTCTTATTTATTTGTTAGT
>Marker267025

Create PDF with GO2PDF for free, if you wish to

AACCGGACAGGTCAGAGATTCAGAAATTATCACAAACCTCTCCCTCATGAGCACCTTCTAAATAGGTCTTCTGCT
GTAGGXXXXXXXXXXXACATTTAACATACTGATCTGATTAGAAACATAGAAACTCTTTCATATCCAACGAACATAA
CAAAAATACTAGAGTAGGGT
AACCGGACAGGTCAGAGATTCAGAAATTATCACAAACCTCTCCCTCATGAGCACCTTCTAAATAGGTCTTCTGCT
GTAGGXXXXXXXXXXXACATTTAACATACTGATCTGATTAGAAACATAGAAACTCTTTCATATCCAACGAACATAA
CAAAAATACTACAGTAGGGT
>Marker267262
ACTCAAACCATAGATATGCGGCCTGAAAAGTAACAAAACGGCTTGAACTAGAGGAAGGTAAAACCAGGACAAGAC
TGCGCXXXXXXXXXXXTAAAGGAAAATGGACGATAAGATGGCTTATGTTGCCACGAAAGAACTAGAATTTTCAGGT
ATTAAAAACATGAAGATAGT
ACTCAAACCATAGATATGCGGCCTGAAAAGTAACAAAACGGCTTGAACTAGAGGAAGGTAAAACCAGGACAAGAC
TGCGCXXXXXXXXXXXTAAAGGAAAATGGACGATAAGATGGCTTATGTTGCCACGAAAGAACTAGAATTTTCAGGT
ATTAAAAACACGAAGATAGT
>Marker267530
TACATTCTTTATATATATTACATGTTTATACCTTTTCAATCCATTATTTCTTGACTTCTTACTTGTCAATGTGTT
ATTAAXXXXXXXXXXXGGCTAATGCGTGCAAGGAAGAATTTGACAATAAACTCCAACTTAGCGAGATAACTTCCAA
CATTTGTATCTCGAAAGGTG
TACATTCTTTATATATATTACATGTTTATACCTTTTCAATCCATCATTTCTTGTCTTCTTACTTGTCAATGTGTT
ATTAAXXXXXXXXXXXGGCTAATGCGTGCAAGGAAGAATTTGACAATAAACTTCAACTTAGCGAGATAACTTCCAA
CATTTGTATCTCGAAAGGTG
>Marker269979
AACTAGAGCTGATATCCGTATTTCTAAGGGCGACAAACTTAAGTGTGCTTCTTCCAGTGATGAACTTGTTGAGGT
TTGCTXXXXXXXXXXXGATTGTGTTGAGGCTTCGTGATGATGCTTTGAAAGAGAGGGATGTTGGTCATAACTCTGC
TATCGTTACTGATTCAATGT
AACTAGAGCTGATATCCGTATTTCTAAGGGCGACAAACTTAAGTGTGCTTCTTCCAGTGATGAACTTGTTGAGGT
TTGCTXXXXXXXXXXXGATTGTGTTGAGGCTTCGTGATGATGCTTTGAAAGAGAGGGATGTTGGTCATAACTCTGC
TATTGTTACTGATTCAATGT
>Marker270085
CACGTGTCCGATGGTGGCATAATGACACGTGATCCACGGCACTTTTCCACCATTCTTAGGGGGCCGACCATGTCT
GACTCXXXXXXXXXXXAAAACGACGTGTCTAAAATTATCGTAGAAAATGTAATTATAAGATGGTAACAAAGTTAAT
AACTCATCCAGAACTTGGTT
CACGTGTCCGATGGTGGCATAATGACACGTGATCCACGGCACTTTTCCACCATTCTTAGGGGGCCGACCATGTCT
GACTCXXXXXXXXXXXAAAACGACGTGTCTAAAATTATCGTAGAAGATGTAATTATAAGATGGTAACAAAGTTAAT
AACTCATCCAAAACTTGGTT
>Marker270206
ACTTCAGAATGATCCTGAACCGTGAGAATGTTGCTAATTCTGTTGTAATGATCCAACCGTCATTGATTTCTTACA
CATTTXXXXXXXXXXXCTGGGTATCACAATCAACCGGAACATCAGGTATTTCTTTTCATTTTCCTTTTTAGTAACT
AATTGTTACTGCAGAAGAGT
ACTTCAGAATGATCCTGAACCGTGAGAATGTTGCTAATTCTGTTGTAATGATCCAACCGTCATTGATTTCTTACA
CATTTXXXXXXXXXXXCTGGGTATCACAATCAACCGGAACATCAGGTATTTCTTTTCATTTTCCTTTTTAGTAACT
AAATGTTACTGCAGAAGAGT
>Marker270228
CACTATAACTCAATATATCCCGAAGGAGGTGAGTTCCCATTATTCATTAGTTTAATAATTGACTGTTTCATTTAC
TTCTTXXXXXXXXXXXTTTGTCTTCCCTAATTCTATGTTATTTGTTCAGCTAGATTTTGTTTTTTGCACTGATAGC
ATCTTAAAATAGGTTTCAGT

Create PDF with GO2PDF for free, if you wish to

CACTATAACTCAATATATCCCGAAGGAGGTGAGTTCCCATTATTCATTAGTTTAATAATTGACTGTTTCATTTAC
TTCTTXXXXXXXXXXTTTGTCTTCCCTAATTGTATGTTATTTGTTCAGCTAGATTTTGTTTTTTGCTCTGATAGC
ATCTTAAAATAGGTTTCAGT
>Marker270264
ACCATATTTAACATTCCTTAGACACGTATTAAGGCTTTTTTGAGTATATATATTGAATTATGATAGCAAAAACAA
CTAGTXXXXXXXXXXATAGCAGAAATAAATTCAAATGGAGATCTATGGGTGTTATATGAAAGTTTTTAACTGAGT
GGATCCTTGAAAATTAAGTA
ACCATATTTAACATTCCTTAGACACGTATTAAGGCTTTTTTGAGTATATATATTGAATTATGATAGCAAAAACAA
CTAGTXXXXXXXXXXATAGCAGAAAGAAATTCAAATGGAGATCTATGGGTGTTATATGAAAGTTTTTAACTGAGT
GGATCCTTGAAAATTAAGTC
>Marker270861
AACACATAGGTATTTTCATGTTTTGTAAAGGAATTTTGTAGGACGATTTGTTTTTCTCTTGGTTCAATTTATGGC
AATTCXXXXXXXXXXGGTAAAGAGAAAAGTTCTAAAGTTGTGGGATCATAGAGTAAGAATATTATAGAACATAAA
AGAGTAGAAAGAGTAGAGTT
AACACATAGGTATTTTCATGTTTTGTAAAGGAATTTTGTAGGACGATTTGTTTTTCTCTTGGTTCAATTTATGGC
AATTCXXXXXXXXXXGGTAAAGAGAAAAGTTCTAAAGTTGTGGGATCATAGAGTAAGAATATTATAGAACATAAA
ATAGTAGAAAGAGTAGAGTT
>Marker271836
ACCTCAAGGTTTTGGATCTATAAAGTTGGTAAGTCTTTCTAAAGCTATTTCTACATGGTTCTCCGTGCTTAACTG
ATCATXXXXXXXXXXAATTCTTGTATGATTATGTTTGAATGACTCAAGTGTTCTTAAATTCTTGTATGATTAGTT
CGTTAGCACGTTAGTTAGTT
ACCTCAAGGTTTTGGATCTATAAAGTTGGTAAGTCTTTCTAAAGCTATTTCTACATGGTTCTCCGTGCTTAACTG
ATCATXXXXXXXXXXAATTCTTGTATGATTATGTTTGAATGACTTAAGTGTTCTTAAATTCTTGTATGATTAGTT
CGTTAGCACGTTAGTTAGTT
>Marker272239
ACTATTTTGATTCTTTGGTAGGTGAGAACAAGCATTGTTGATGCCACAAAAGCGAAAACAAGTGCTAAAAAATGG
CTTTTXXXXXXXXXXAAATAACTGCAAAATACAACCAAGCACAATTAAAATGCTTATTCAGCTGCAATCCAGAAG
GCGAAGAGGAAGGTTATGTT
ACTATTTTGATTCTTTGGTAGGTGAGAACAAGCATTGTTGATGCCACAAAAGCGAAAACAAGTGCTAAAAAATGG
CTTTTXXXXXXXXXXAAATAACTGCAAAATACAACCAAGCACAATTAAAATGCTTATTCAACTGCAATCCAGAAG
GCGAAGAGGAAGGTTATGTT
>Marker272585
ACTTGAAAGTATAATTTGTTTTAGAATGTTTTAACTCAAGCACACCTGCCCTTTTTCTTCTGACAGGGTAGCAGG
TTAGGXXXXXXXXXXGTAGCTGTTGCTGAAGCTAAGGTGAAAAGAAAAGCAAAAAAGTTGGCAGGCAATCCTCAT
TCACATCTGAAGGATGAAGT
ACTTGAAAGTATAATTTGTTTTAGAATGTTTTAAATCAAGCACACCTGCCCTTTTTCTTCTGACAGGGTAGCAGG
TTAGGXXXXXXXXXXGTAGCTGTTGCTGAAGCTAAGGTGAAAAGAAAAGCAAAAAAGTTGGCAGGCAATCCTCAT
TCACATCTGAAGGATGAAGT
>Marker272619
ACTGCCACATTATTTATTTTCCTTCTTTTTAGTTTTGGTGGGGAGGAGGAAACAGAGCCACTACAAATGGAGTAA
AAACAXXXXXXXXXXTAGAAGTTGAAAATGATGAAGAACTCATTATAGCTATCCAACTTATATACAATCCTATGA
GATAACAACTGAGCGTCGTT
ACTGCCACATTATTTATTTTCCTTCTTTTTAGTTTTGGTGGGGAGGAGGAAACAGAGGCACTACAAATGGAGTAA
AAACAXXXXXXXXXXTAGAAGTTGAAAATGATGAAGAACTCATTATAGCTATCCAACTTATATACAATCCTATGA
GATAACAACTGAGCGTCGTT
>Marker273003

Create PDF with GO2PDF for free, if you wish to

AACCATTGAAGAAGACTTGAATTTAAGAGAGTGTTAGTATGTGCTGTTACTACTGTTATTTATTTTTTTTATGGT
TGAAAXXXXXXXXXXXTCCAGCGTCAGTCTTTATTCACGGGATAGGCTCTCAATTTAGTTCAGCAGTTCGAACAAT
TTCCTTTTGCTAGAGAGGTG
AACCATTGAAGAAGACTTGAATTTAAGAGAGTGTTAGTATGTGCTATTACTACTGTTATTTATTTTTTTTATGGT
TGAAAXXXXXXXXXXXTCCAGCGTCAGTCTTTATTCACGGGATAGGCTCTCAATTTAGTTCAGCAGTTCGAACAAT
TTCCTTTTGCTAGAGAGGTG
>Marker273170
GACCTTATCCGACGCAGTTACACATTGAAAAAACTTCAATTTGTGTCATGCAAGGTATTTCCGACGTTTGAATCA
ACGTCXXXXXXXXXXXCGGGGAAGGCTTTTTCCGACGTGATGCGAATAAAGCCTTCCCCGACGTCGTGCAATATAC
GTCAGGAAGGCTTTCGTAGT
GACCTTATCCGACGCAGTTACACGTTGAAAAAACTTCAATTTGTGTCATGCAAGGTATTTCCGATGTTTGAATCA
ACGTTXXXXXXXXXXXCGGGGAAGGCTTTTTCCGACGTGATGCGAATAAAGCCTTCCCCGACGTCGTGCAATATAC
GTCAGGAAGGCTTTCGTAGT
>Marker273390
AACCCTAATCCTCATTTAACTGAACTTTGCTGTCTTAATAGTAAGAATGCACTTTCTCTGGGAAAAATTTCTGCT
TGGCAXXXXXXXXXXXCACAACCGGATGTGACCTTAGTATTGAATGAGTATATGACTAAAAGAATGATGAGAAATT
ATCAATGGCCATCCTTCGTT
AACCCTAATCCTCACTTAACTGAACTTTGCTGTCTTAATAGTAAGAATGCACTTTCTCTGGGAAAAAATTCTGCT
TGGCAXXXXXXXXXXXCACAACCGGATGTGACCTTAGTATTGAATGAGTATATGACTAAAAGAATGATGAGAAATT
ATCAATGGCCATCCTTCGTT
>Marker273529
ACCCTTAGTGTAGCATAATTATTCTCTAGCATCTTATTCATGTTGCTTCCGTTTCCTTGCAAGAACATTGGGATT
AGAGGXXXXXXXXXXXTGTGAAAATAAATGTTTATAGTTATCAATGTTTAAATCAATTAACAATCTTTTGCATAAG
AACTATTGCAAACCAAGGGT
ACCCTTAGTGTAGCATAATTATTCTCTAGCATCTTATTCATGTTGCTTCCGTTTCCTTGCAAGAACATTGGGATT
AGAGGXXXXXXXXXXXTGTGAAAATAAATGTTTATAGTTATCAATGTTTAAATCAATTAACAATCTTTGGCATAAG
AACTATTGCAAACCAAGGGT
>Marker273620
ACAGACAATGGGTTGTGAAGATGTCCTTAAAGTAACTAGTTAAGTTACGTGTAAGGTAAGACTAGTTAGTAAATG
CTACAXXXXXXXXXXXAAACTATGATTTCTGCATTATGCAAATAAAATCCCAAGTCATATATTGCTATATGAATAA
GAAAAGTGGATATAGCTGTT
ACAGACAATGGGTTGTGAAGATGTCCTTAAAGTAACTAGTTAAGTTACGTGTAAGGTAAGACTAGTTAGTAAATG
CTACAXXXXXXXXXXXAAACTATGATTTCTGCATCATGCAAATAAAATCCCAAGTCATATATTGCTATATGAATAA
GAAAAGTGGATATAGCTGTT
>Marker273652
ACCCATGAACTTACGAACAATTCAAATATCGCTTTTTTTAACACAGAAGCACATAAATACCATGGATAGGACGAA
TCATTXXXXXXXXXXXTTAGTTCTGAACATTACTCCCATCTTAAAAGGTAATCGAGCATGTAGTGAATCAAACGCA
TCAACGTCAATAATCTCTGT
ACCCATGAACTTATGAACAATTCAAATATCGCTTTTTTTAACACAGAAGCACATAAATACCATGGATAGGACGAA
TCATTXXXXXXXXXXXTTAGTTCTGAACATTACTCCCATCTTAAAAGGTAATCGAGCATGTAGTGAATCAAACGCA
TCAACGTCAATAATCTCTGT
>Marker274882
AACATTAATTCCTTTCAACAAAATGGCACATGCCAAGAAAAGTCCCCGATCATACAAAAATACACATGACAGGAA
CTAGCXXXXXXXXXXXATCAATAATTGGAGTAGGGTCTGCACAAGCTCTTCTAGCAGACTCTGGATCCCGGAAAGT
CACCTAAAACCCTCCATGTA

Create PDF with GO2PDF for free, if you wish to

AACATTAATTCCTTTCAACAAAATGGCACATGCCAAGAAAAATCCCCGATCATACAAAAATACACATGACAGGAA
CTAGCXXXXXXXXXXXATCAATAATTGGAGTAGGGTCTGCACAAGCTCTTCTAGCAGACTCTGGATCCCGGAAAGT
CACCTAAAACCCTCCATGTA
>Marker274933
CACCATAATCATAATATCCCCCTGATTGGCGACCATAACCACGTCCCCTTCCACGAAATGCACGACCTCTTCCAC
GTCCTXXXXXXXXXXXTTAATAATCAAGTGAAACAACATTTTAAAAAATTTACAAAAATATCAAAATCTCAATATA
GACCAATTTTGCTATATGTG
CACCATAATCATAATATCCCCCTGATTGGCGACCATAACCACGTCCCCTTCCACGAAATGCACGACCTCTTCCAC
GTCCTXXXXXXXXXXXTTAATAATCAAGTGAAACAACATTTTAAAAAATTTACAAATATATCAAAATCTCAATATA
GACCAATTTTGCTATATGTG
>Marker275699
TACCAAAGTTTGAATTGTCTAAAGATGCATCTAATCTATTAATGTCTAGCCCAACATGATCAATCCTTGATAAGA
AGTCTXXXXXXXXXXXAGAACTTTCATGTATCTTTTGAAGGGATACATCCATTTTAGATATATGGGCCAAAAAAGT
TTGACTTTTCTAACAATGTG
TACCAAAGTTTGAATTGTCTAAAGATGCATCTAATCTATTAATGCCTAGCCCAACATGATCAATCCTAGATAAGA
AGTCTXXXXXXXXXXXATAACTTTCATGTATCTTTTGAAGGGATACATCCATTTTAGATATATGGGCCAAAAAAGT
TTGACTTTTCTAACAATGTG
>Marker275952
TACATATATATATCACCATTCAAAAATTGAAGAAGAATTAAAATTTCATCTGTAAATGTGAGAAAATAGAATGAT
GTTTGXXXXXXXXXXXTATTTTAGTAAAATTATTAGAGTTTAGGGAAATTTACAACTTTTGTGAGATTTTAAGTTA
AAATTAGTTTAGACCACGTT
TACATATATATATCACCATTCAAAAATTGAAGAAGAATTAAAATTTCATCTGTAAATGTGAGAAAATAGAATGAT
GTTTGXXXXXXXXXXXTATTTTACTAAAATTATTTGAGTTTAGGGAAATTTACAACTTTGGTGAGATTTTAAGTTA
AAATTAGTTTAGACCACGTT
>Marker276124
ACCAGCATGACAGAATTACTTGATAGATAATTTTTACCAGAGCACTCAAGGTTTTAATTTCTCTTTTACACAACG
GGATGXXXXXXXXXXXTTTGTGCTGTTACACCCATTATTACTTGTTAGAGATAGATATATATGTGGCATCTGATAT
AATATTCAATTGGAAGAGTT
ACCAGCATGACAGAATTACTTGATAGATAATTTTTACCAGAGCACTCAAGGTTTTAATTTCTCTTTTACACAACG
GGATGXXXXXXXXXXXTTTGTGCTGTTACACCCATTATTACTTGTTAGAGATAGAGATATATGTGGCATCTGATAT
AATATTCAATTGGAAGAGTT
>Marker276235
ACCCTTTATTTTATTTGAAACATTGGCTTCGAGCCATCCAACAATTTGTATCATGTTATTTAACTTGCTTAATGT
ATGTGXXXXXXXXXXXAGTCCTTGGATGATCTGAAATCAATGGCTGTTGAAACTGTGTTATTGCTGCAAGAGAAGA
TTAATGATGACGAAGGTGGT
ACCCTTTATTTTATTTGAAACATTGGCTTCGAGCCATCCAACAATTTGTATCATGTTATTTAACTTGCTTAATGT
ATGTGXXXXXXXXXXXAGTCCTTGGATGATCTGAAATCAATGGCTGTTGAAACTGTGTTATTGCTGCAAGAGAAGA
TTAATGATGATGAAGGTGGT
>Marker276715
ACTGTGAGTAACACATTGATTTGCAAGCTACTAATTAAACATGGTTTCCACCTACACAAGCAATAACTTGTGAAA
TGATTXXXXXXXXXXXGCAAAGAAAATATTTCAATGTTTTCAATACATTTTCTCCTAGCCCGAATATTCTATGAGA
CCTATTGTATATATGTGGTT
ACTGTGAGTAACACATTGATTTGCAAGCTACTAATTAAACATGGTTTCAACCTACACAAGCAATAACTTGTGAAA
TGATTXXXXXXXXXXXGCAAAGAAAATATTTCAATGTTTTCAATACATTTTCTCCTAGCTCGAATATTCTATGAGA
CCTATTGTATATATGTGGTT
>Marker276855

Create PDF with GO2PDF for free, if you wish to

CACCATTTGGTTCATCAATAATTAAAGATGTTTTATCATTCGTTATTCATATCAACACATATTTTTCACTGAAAC
ACTCAXXXXXXXXXXAATCACATTTTAAAATATCATAATGTATTTGTGATAAATTAAGATAGTTTCTAATATTAT
TAACATATTATGAATAAAGT
CACCATTTGGTTCATCAACAATTAAAGATGTTTTATCATTGGTTATTCATATCAACACATATTTTTCACTGAAAC
ACTCAXXXXXXXXXXAATCACATTTTAAAATATCATAATGTATTTGTGATAAATTAAGATAGTTTCTAATATTAT
TAACATATTATGAATAAAGT
>Marker276974
GACTAATTAGAGATCCTTTTTGTAACTCCACATTCTCTATTGTAATGTTATCTTGTTTCTTAATAAACCATGAAA
AGAAAXXXXXXXXXXAATTACGCACCCACTAGCTAAACTCTCCGTATTTTTTGGTTGCTGCCTATATAATAGTAA
CAATTAGCTCAATGTTGGTG
GACTAATTAGAGATCCTTTTTGTAACTCCACATTCTCTATTGTAATGTTATCTTGTTTCTTAAGAAACCATGAAA
AGAAAXXXXXXXXXXAATTACGCACCCACTAGCTAAACTCTCCGTATTTTTTGGTTGCTGCCTATATAATAGTAA
CAATTAGCTCAATGTTGGTG
>Marker277158
GACTTTACACCCTTGAAACTAAGTATCTTTGTCTGGCATGAGAGGATCAATTAAAACTTAGTCCCATGTTATACT
TGATTXXXXXXXXXXGAACAGAGGAAACTAAGAAAGAGAGCAGATTCTTTCATGCTGAATAAGTCCAAAGCCTGT
GTGTCGATCCAGGATGGGTA
GACTTTACACCCTTGAAACTAAGTATCTTTGTCTGGCATGAGAGGATCAATTAAAACTTAGTCCCATGTTATACT
TCATTXXXXXXXXXXGAACAGAGGAAACTAAGAAAGAGAGCAGATTCTTTCATGCTGAATAAGTCCAAAGCCTGT
GTGTCGATCCAGGATGGGTA
>Marker277271
ACTAAAGATTTCAAGTGAATTTATACATGTAATATTTTTCTTTGTTTCCAAAACTATACAGATTATGTTAAATCT
TTTATXXXXXXXXXXTATATAAACCTAATTAACACTCTAGTCGAGATAATTACAACATTAAAAGTATGATTAAGG
AATTACAGAGAACAAGTGTA
ACTAAAGATTTCAAGTGAATTTATACATGTAATATTTTTCTTTGTTTCCAAAACTATACAGATTATGTTAAATCT
TCTATXXXXXXXXXXTATATAAACCTAATTAACACTCTGGTCGAGATAATTACAACATTAAAAGTATGATTAAGG
AATTACAGAGAACAAGTGTA
>Marker277404
ACTTAAGTCATCAAAATCAATCCTTAATATCCTTAGTAAAAAAAATCATGAACAAATATAAAACACAGCAAAGCT
GAAATXXXXXXXXXXAGGGAAGAAATCTGAAACTGACAAAAATAGCAAAAATTTTACCTCTCGACGAACATTAAG
TTCTCTTTGGATAAGTGGTT
ACTTAAGTCATCAAAATCAATCCTTAATATCCTTAGTAAAAAAAATCATGAATAAATATAAAACACAGCAAAGCT
GAAATXXXXXXXXXXAGGGAAGAAATCTGAAACTGACAAAAATAGCAAAAATTTTACCTCTCGACGAACATTAAG
TTCTCTCTGGATAAGTGGTT
>Marker278549
TACTTTTTAGCACCCCAAACGATTTCCTAGTGAATTTCAAATATTTCCTTCTCGAAGATTAAGATTACAGAGAGG
AACAAXXXXXXXXXXCAATGATTTTCCACCCAATAAATTTGTCACACATCAACACTCTCAACCATATCCTATCAA
TTTTTTAGATTATCAAAGTA
TACTTTTTAGCACCCCAAACGATTTCCAAGTGAAATTCAAATATTTCCTTCTCGAAGATTAAGATTACAGAGAGG
AACAAXXXXXXXXXXCAATGATTTTCCACCCAATAAATTTGTCACACATCAACACTCTCAACCATATCCTATCAA
TTTTTTAGATTATCAAAGTA
>Marker279757
AACCAGCAAATAGAACAACCTAAATGAAGACAAAGAATCAAAAAGAGAGGGAGGAAGAAACACAAGCCACAAGAT
AATAAXXXXXXXXXXAAGCAACATCCAACACTATTGGGAATGTATGAAAATTTTGTTGATTCTAAAATATATTGT
TGAAGTAGTGAATGAATGTA

Create PDF with GO2PDF for free, if you wish to

AACCAGCAAATAGAACAACCTAAATGAAGACAAAGAATCAAAAAGAGAGGGAGGAAGAAACACAAGCCACAAGAT
AATAAXXXXXXXXXXAAGCAACATCCAACACTATTGGGAATGTATGAAAATTTTCTTGATTCTAAAATATATTGT
TGAAGTAGTGAATGAATGTA
>Marker279846
ACAAAAGTGAGAAATTATAATAACTTTATGAAATCAGTGGGCATTTGAATTCTAATCTCTGACCAAACAGCTTCT
CTTTTXXXXXXXXXXTTTTACAATTGGAAATAAACAAAACAAACATTTAATTGACTGATCAATTTGTTGTATTTT
CATCATTTAAATCCCAAGTC
ACAAAAGTGAGAAATTATAATAACTTTATGAAATCAGTGGGCGTTTGAATTCTAATCTCTGACCAAACAGCTTCT
CTTTTXXXXXXXXXXTTTTACAATTGGAAATAAACAAAACAAACATTTAATTGACTGATCAATTTGTTGTATTTT
CATCATTTAAATCCCAAGTC
>Marker280361
GACTTAATGAATAGAGTGTTTCTCGTTACTTGGATCGATTTATCATTGTCTTTATTGATGACATGCATTTTGGTT
TATTCXXXXXXXXXXACGAAAAATTGAGGCAATTGAAAGTTGGGAACATCCTAACACAGTTTCTGAAGTTCGGAG
CTTCCTTGGTTTGGCATGGT
GACTTAATGAATAGAGTGTTTCTCGTTACTTGGATCGATTTATCATTGTCTTTATTGATGACATGCATTTTGGTT
TATTCXXXXXXXXXXACAAAAAATTGAGGCAATTGAAAGTTGGGAACATCCTAACACAGTTTCTGAAGTTCGGAG
CTTCCTTGGTTTGGCATGGT
>Marker280799
ACCCCAACTTCACTTCTCTACTCATCAGAAATTGACTCTCCATCATATACACAAACAACACACAAGAGACCATCC
TGAAGXXXXXXXXXXCATTTGCAGGTGCAACAGACAACAATATACCTTCATCGGTTATTCGATACCGAGAATGTT
TGAAAAACCACGCAGCAAGT
ACCCCAACTTCACTTCTCTACTCATCAGAAATTGACTCTCCATCATATACATAAACAACACACAAGAGACCATCC
TGAAGXXXXXXXXXXCATTTGCAGGTGCAACAGACAACAATATACCTTCATCGGTTATTCGATACCGAGAATGTT
TGAAAAACCACGCAGCAAGT
>Marker280893
AACACGGACACACTAGCAAGTGAGCTACAAACGTGGTTGTCAATACTTTTGCCACTAACCCTTTTTTGTGATAAT
ATTTAXXXXXXXXXXTTTTCTTTGCACATCACTTTCCTCGCAAACTAACACCCTTATTTCCCATCATTTTCTTTA
TAGACTTTTGGTTCAATTGT
AACACCGGCACACTAGCAAGTGAGCTACAAACGTGGTTGTCAATACTTTTGCCACTAACCCTTTTTTGTGATAAT
ATTTAXXXXXXXXXXTTTTCTTTGCACATCACTTTCCTCGCAAACTAACACCCTTATTTCCCATCATTTTCTTTA
TAGACTTTTGGTTCAATTGT
>Marker281759
ACTTTCTCTGTTTTGATTTGTTGGGATTTTTCTGCTTTTCAGGTTTGGGGTCCTAACAAATTGGTATCAAAGCCT
CAACAXXXXXXXXXXAGTGTGGAACAGCTTGCGAAAGACATGAAAGAGAGTGCCTCCAGTCAAGGAGGAATATCA
AGGACGTCTGATGCCTTGGT
ACTTTCTCTGTTTTGATTTGTTGGGATTTTTCTGCTTTTCAGGTTTGGGGTCCTAACAAATTGGTATCAAAGCCT
CAACAXXXXXXXXXXAGTGTGGAACAGCTTGCGGAAGACATGAAAGAGAGTGCCTCCAGTCAAGGAGGAATATCA
AGGACGTCTGATGCCTTGGT
>Marker281931
GACTCTTCAACAATATGTTGCTGGAGATGTGCCTCTATGCATCAAAAAGTTGTAGAAAAAGCATACTCATTCAAA
ATGAAXXXXXXXXXXACAAAGTATGCTTCAAGCAGCATCTCCAATTGTTCGATGTTATCTCCGCTGGTCGAGGAA
CTCATCAAGCTGCTTGAGTT
GACTTTTCAACAATATGTTGCTGGAGATGTGCCTCTATGCATCAAAAAGTTGTAGAAAAAGCATACTCATTCAAA
ATGAAXXXXXXXXXXACAAAGTATGCTTCAAGCAGCATCTCCAATTGTTCGATGTTATCTCCGCTGGTCGAGGAA
CTCATCAAGCTGCTTGAGTT
>Marker282253

Create PDF with GO2PDF for free, if you wish to

ACCAACATTTATTGTTAGATATGAATAATAGAGGTGATATGTTGATATTTTTTTAAAAAATAATTAGATTTATTT
GGTATXXXXXXXXXXAGCATGAGAATGTGGTGTAATAGGTTTTAGGCGCAACATCATTATCAATTCCAACTTACA
AGAAGTCATAATAATGAGTG
ACCAACATTTATTGTTAGATATGAATAATAGAGGTGATATGTTGATATTTTTTTTAAAAATAATTAGATTTATTT
GGTATXXXXXXXXXXAGCATGAGAATGTGGTGTAATAGGTTTTAGGCGCAACATCATTATCAATTCCAACTTACA
AGAAGTCATAATAATGAGTG
>Marker282266
CACATCATATTTTATGTGCCTCATCTTGAGGTGCCAGACCTGCACTTAATGCCTAGGCATGTTCCCGAGATCGTT
TTTTAXXXXXXXXXXTTATGGAAGCCGTGCATACACGTTGGCCTAAAGCTATTGTTCAGGTTTATTGGTTAAGAT
ATCAAATTTTTAACTGTGTT
CACATCATATTTTATGCGCCTCATCTTGAGGTGCCAGACCTGCACTTAATGCCTAGGCATGTTCCTGAGATCGTT
TTTTAXXXXXXXXXXTTATGGAAGCCGTGCATACACGTTGGCCTAAAGCTATTGTTCAGGTTTATTGGTTAAGAT
ATCAAATTTTTAACTGTGTT
>Marker282299
TACAAGTGAATAAAAAAGAAAAAGATTTAGGAAATAAGGAAAATATTCTCATAATCTTTCCATAAATATTATAGG
ATTCTXXXXXXXXXXAATGAACAAAGAGAACTCTCCCCCACTTACTGATGCTCCCAAAAGCACGTGTTTATACCA
TCCCCCACAAACAACGAGTA
TACAAGTGAATAAAAAAGAAAAAGATTTAGGAAATAAGGAAAATATTCTCATAATCTTTCCATAAATATTATAGG
ATTCTXXXXXXXXXXAATGAACAAAGAGAACTCTCCCCCACTTACTGATCCTCCCAAAAGCACGTGTTTATACCA
TCCCCCACAAACAACGAGTA
>Marker283567
ACGTAAAAACATAGAATATTTATGCTTAATTATTTTTGTTATTTCTTTCACTGTCTTATATTCATGCAAATGTGT
TCTTCXXXXXXXXXXGGTGAAAACTGGGTTCCCGGAGTAGGGAAGTAAAAGATTTCATTTTCTTGCGATGCTTCC
GATCTGGTGCTTACATTGTT
ACGTAAAAACATAGAATATTTATGCTTAATTACTTTTGTTATTTCTTTCACTGTCTTATATTCATGCAAATGTGT
TCTCTXXXXXXXXXXGGTGAAAACTGGGTTCCCGGAGTAGGGAAGTAAAAGATTTCATTTTCTTGCGATGCTTCC
GATCTGGTGCTTACATTGTT
>Marker284407
ACTGACCGCTGAAGGAATGCTGGAAACTGTTCGGCGAAATGCTTGAATGAGCCTATACCCAGAGAAAAAGCTCCC
CGTTGXXXXXXXXXXGAACCAAATTAGCAAAAATTCTCTCTTCAAATTTTACATTTTCTAACCTTTATTATTTAC
CAAATACTCATTTCCATGTT
ACTGACCGCTGAAGGAGTGCTGGAAACTGTTCGGCGAAATGCTTGAATGAGCCTATACCCAGAGAAAAAGCTCCC
CGTTGXXXXXXXXXXGAACCAAATTAGCAAAAATTCTCTCTTCAAATTTTACATTTTCTAACCTTTATTATTTAC
CAAATACTCATTTCCATGTT
>Marker284491
TACTTGTAAAAGTGAGTGTATGTAAAATTTGATTAATTTTTTAATACACCTTTATATTTATAGGTGTTTTACAAA
GACAAXXXXXXXXXXCAGTTATACTATATGTTTATACGGAGTCACTAGCAAGACTAGGGGTGTTTCGAAGGAATC
ACTCGCGCAGTTAGAGTTGT
TACTTGTAAAAGTGAGTGTATGTAAAATTTGATTAATTTTCTAATACACGTTTATATTTATAGGTGTTTTACAGA
GACAAXXXXXXXXXXCAGTTATACTATATGTTTATACGGAGTCACTAGCAAGACTAGGGGTGTTTCGAAGGAATC
ACTCGCGCAGTTAGAGTTGT
>Marker285192
AACGACGACTTCACCATAATTCTCCACCTTCGACCTTCCACGTAATGGATGGTTTGATTTTATATGTGCTGAGTA
GCAAAXXXXXXXXXXGCCGGACGGGATGACCCATTTTGCTCTTCTGGACGACAATCAATCAAACTAACCATTTTA
ATGGGCCGGGCTTTCATGTA

Create PDF with GO2PDF for free, if you wish to

AACGACGACTTCACCATAATTCTCCACCTTCGACCTTCCACGTAATGGATGGTTTGATTTTATATGTGCTGAGTA
GCAAAXXXXXXXXXXXGCCGGACGGGATGACCCATTTTGCTCTTCTGGACGACAATCAATCAAACTAACCATTTTA
ATGGGCCGGGCTTTGATGTA
>Marker285317
AACAGTGACAATTAGTCTGCCAATTGCAACACAAGGTCACTTCAAGACCTAACTTTGATCTCTCATCAAACAATT
ACCTGXXXXXXXXXXXATACTCTGCAGTCTGAAGAAAGCTAAGAACCAATTATACCTCCCATCCAAAACTTCTGGA
GCAAGGAGTCCAAAACGGTC
AACAGTGACAAATAGTCTGCCAATTGCAACACAAGGTCACTTCAAGACCTAACTTTGATCTCTCATCAAACAATT
ACCTGXXXXXXXXXXXATACTCTGCAGTCTGAAGAAAGCTAAGAACCAATTATACCTCCCATCCAAAACTTCTGGA
GCAAGGAGTCCAAAACGGTC
>Marker285351
TACAAATGTCACAAAGATTATTGATTTTTAATTTTATGCCGGTGAGCCGTTTGTGTAGAATTAGCAAATATATTA
AACAAXXXXXXXXXXXACAAAAAGAAAAAAAAAACAAAGAGAGAAATTAACCGAACACAAAATTAAAAATTTAGAA
ATGTATTAATTAACACATGT
TACAAATGTCACAAATATTATTGATTTTTAATTTTATGCCGGTGAGCCGTTTGTATAGAATTAGCAAATATATTA
AACAAXXXXXXXXXXXACAAAAAGAAAAAAAAAACAAAGAGAGAAATTAACCGAACACAAAATTAAAAATTTAGAA
ATGTATTAATTAACACATGT
>Marker285890
ACCCATTGATTTTCGTGAATCAATCTTTCTTGATCACGTTTTATAACCAAAATAGTAATTTTGAGTCTTAAACGT
TCTTAXXXXXXXXXXXACCCTTTTTCGATGTTGCTATTTTGTCCCGAACTACCTTAGAAACATCATCGAAATCACT
AGAATCAAATTTCCAATGTG
ACCCATTGATTTTCGTGAATCAATCTTTCTTGATCACGTTTTATAACCAAAATAGTAATTTTGAGTCTTAAACAT
TCTTAXXXXXXXXXXXACCCTTTTTCGATGTTGCTATTTTGTCCCGAACTACCTTAGAAACATCATCGAAATCACT
AGAATCAAATTTCCAATGTG
>Marker285969
GACTCGGTCATTAGATTGTTGGATATTGTTCTCATAGTTATGTTTCCTTTGTGGGCTCTCCAGATTGTAGCCATG
ATTTGXXXXXXXXXXXTGTGGTGTAAAACAGAAGTGTGTCAATTCTATGCGAAGTTTGTATTATTTTTTGATTGTG
ATAGTTTTGTGGATTTGGTG
GACTCGGTCATTAGATTGTTGGATATTGTTCTCATAGTTATGTTTCCTTTTTGGGCTCTCCAGATTGTAGCCATG
ATTTGXXXXXXXXXXXTGTGGTGTAAAACAGAAGTGTGTCAATTCTATGCGAAGTTTGTATTATTTTTTGATTGTG
ATAGTTTTGTGGATTTGGTG
>Marker287115
CACGGGCTTGGTTTGGAAAATTTATCGAGGTGATAGAAAGTTTTGGAATGCAAAAGAGCACGTCAGATCATTCAA
ACTTTXXXXXXXXXXXGTTGAAATACCTCCTAGGAATTTGAGGTAAAAACAGGCAAGAAAGCAATACTATTATCAC
AGAAAAAAATATGGACTTGTT
CACGGGCTTGGTTTTGAAAATTTATCGAGGTGATAGAAAGTTTTGGAATGCAAAAGAGCACGTCAGATCATTCAA
ACTTTXXXXXXXXXXXGTTGAAATACCTCCTAGGAATTTGAGGTAAAAACAGGCAAGAAAGCAATACTATTATCAC
AGAAAAAAATATGGACTTGTT
>Marker287666
ACTATTTTCAGAAGATAGTATGTGTAGTCTCTTTTGGTTAGGTCTTTTATTTTGGCAATTTATACTTTCTGTTGA
AATTGXXXXXXXXXXXTTCCTTGTGGGCACTTGTATGGCATTTCTTGTATCAATAGATGGTTGCAACAACGTAAAA
ATGCAGGGAAGGTATTTGTC
ACTATTTTCAGAAGATAGTATGTGTAGTCTCTTTTGGTTAGGTCTTTTATTTTGGCAATTTATACTTTCTGTTGA
AATTGXXXXXXXXXXXTTCCTTGTGGGCACTTGTATGGCATGTCTTGTATCAATAGATGGTTGCAACAACGTAAAA
ATGCAGGGAAGGTATTTGTC
>Marker287892

Create PDF with GO2PDF for free, if you wish to

AACTATGTTATGCTATGTTTCAAGGCTGTGGGGATGGATAAACAATGGATTTGGGTTTTAAGGTAGCAAATTTTC
TTGGTXXXXXXXXXXXATAAATTCAGAGGAAATTGCTTAGTTATGTTTGTCCTTCCATAGGTTGGCCATCTTGGAG
AAGCATATCAAGAGTGGGTT
AACTATGTTATGCTATGTTTCAAGGCTGTGGGGATGGATAAACAATGGATTTGGGTTTTAAGGTAGCAAATTTTC
TTGGTXXXXXXXXXXXATAAATTCAGAGGAAATTGCTTAGTTATGTTTGTTCTTCCATAGGTTGGCCATCTTGGAG
AAGCATATCAAGAGTGGGTT
>Marker288011
ACCCTTCGAGTCATCCAAGAGGGAGTTCGAGGATAGCAAAAAGCCTTTAGGTTTTGGATTTTCTTTAAAGACCTT
ATAACXXXXXXXXXXXACAACATATTTGAGATTGCCTATGATGCTTGCATACTCGAATTGGTTTTTGAACAACTTT
ACACTAGGGTCATAAGGAGT
ACCCTTCGAGTCATCCAAGAGGGAGTTCGAGGATAGCAAAAAGCCTTTAGGTTTTGGATTTTCTTTAAATACCTT
ATAACXXXXXXXXXXXACAACATATTTGAGATTGCCTATGATGCTTGCATACTCGAATTGGTTTTTGAACAACTTT
ACACTAGGGTCATAAGGAGT
>Marker289051
GACTCAACAATCTTTTGCAACTGCCTTCACTAAAACATCCAATTTTATACACATTTTCACCCCACAAAGAGGCAA
AAGAGXXXXXXXXXXXAAGAAAAAAAAAAAAAGGAAAAAAGACTAATGGTGTCTTGTATTTGAGCTACGTAGGGTGA
CACCATGTAAAAACTTTGTA
GACTCAACAATCTTTTGCAACTGCCTTCACTAAAACATCCAATTTTATACACATTTTCACCCCACAAAGAGGCAA
AAGAGXXXXXXXXXXXAAGAAGAAAAAAAAAAGGAAAAAAGACTAATGGTGTCTTGTATTTGAGCTACGTAGGGTGA
CACCATGTAAAAACTTTGTA
>Marker289818
ACATTAGCGTAAATGGGTTATGTTTCTCTATTTTATCAATTAGGAGAATTACAAATTCACCTTTAGGAAGAGTGA
AGCCAXXXXXXXXXXXAACTGCTTGAAAAACCTGCATATCAAGAGCCAATATATTACTACATAATTCCAACGGAGA
GAGGGAGGAAAAAGAAGGGT
ACATTAGCGTAAATGGGTTATGTTTCTCTATTTTATCAATTAGGAGAATTACAAATTCACCTTTAGGAAGAGTGA
AGCCAXXXXXXXXXXXAACTGCTTGAAAAACCTGCATATCAAGAGCCAATATATTACTACATAATACCAACGGAGA
GAGGGAGGAAAAAGAAGGGT
>Marker289826
ACAAGTAAAAACGAAGACATATTGAGTTGGTTCCTGTTAAAGAGTTGGTTCCTATTAAAGAGGCAGTTTCGGTAT
GTGAGXXXXXXXXXXXGGGGGATGATCTCTATACGATAAAAAGGGAAAAGAGCGGAAGTGATTATGACAATCTCCT
TTGGCAGCCTCATGGAGGTG
ACAAGTAAAAACGAAGACATATTGAGTTGGTTCCTGTTAAAGAGTTGGTTCCTATTAAAGAGGCAGTTTCAGTAT
GTGAGXXXXXXXXXXXGGGGGATGATCTCTATACGATAAAAAGGGAAAAGAGCGGAAGTGATTATGACAATCTCCT
TTGGCAGCCTCATGGAGGTG
>Marker290224
ACAAATGAGTGTGTATCAGCCACACTGCTTTTCTTTGATTTCTTGGCTGGAGGCTCCTCATCATTTCTTCCTGTG
GCCACXXXXXXXXXXXGAAGAAAAAAGAAACAGATAAAGTAGATTGGATACTTTGAGAAGTTTTGTTGGGCTTTTT
CTTCAACAAGAAATCTCGTT
ACAAATGAGTGTCTATCAGCCACACTGCTTTTCTTTGATTTCTTGGCTGGAGGCTCCTCATCATTTCTTCCTGTG
GCCACXXXXXXXXXXXGAAGAAAAAAGAAACAGATAAAGTAGATTGGATACTTTGAGAAGTTTTGTTGGGCTTTTT
CTTCAACAAGAAATCTCGTT
>Marker291339
AACAATAGGCTTCTCCATCAGAATTTCCATGCCATTTGACACCTTTTTTGTAGTCCCATCCCATTGCGCTGAACT
TATTGXXXXXXXXXXXATTATATATATTATACTGCAAAAGTAATATGAACAGTTACTACTAGTAGTAGCGTTGTGT
ATAAACTATTTAACTATAGT

Create PDF with GO2PDF for free, if you wish to

AACAATAGGCTTCTCCATCAGAATTTCCCTGCCATTTGACACCTTTTTTGTAGTCCCATCCCATTGCGCTGAACT
TATTGXXXXXXXXXXXATTATATATATTATACTGCAAAAGTAATATGAACAGATACTACTAGTAGTAGCGTTGTGT
ATAAACTATTTAACTATAGT
>Marker291532
CACCTGATGATAGCCATTTTGAGGAGCCTTTTGCTGGGGTGAGTCGCCATCAGCTGTCATTGACAGTAGAAACCA
TTCTTXXXXXXXXXXXTGCTCCCCAGGCTGATTCAACCTTACCCATTCATCAACAATCTCCTTCCATTTTCTGACG
AAAAACAAATTGATATCGTT
CACCTGATGATAGCCATTTTGAGGAGCTTTTTGCTGGGGTGAGTCGCCATCAGCTGTCATTGACAGTAGAAACCA
TTCTTXXXXXXXXXXXTGCTCCCCAGGCTGATTCAACCTGACCCATTCATCAACAATCTCCTTCCATTTTCTGTCG
AAAAACAAATTGATATCGTT
>Marker291778
AACCTCGCGAATAGCCATGTCAAACTCCTCCATATCCCCAGGATTTGGAGCTCCACCAAAAGCTTTAGAATGAAG
TGTAAXXXXXXXXXXXAAAGAACCTCTAACCTATCCCCCGAGTGCAAATCTTTTACCAAACCAGAGATAATTTGAT
ATGACACCTCACTAGGCGTA
AACCTCGCGAATAGCCATGTCAAACTCCTCCATATCCCCAGGATTTGGAGCTCCACCAAAAGCTTTAGAATGAAG
TGTAAXXXXXXXXXXXAAAGAACCTCTAACCTATCCCCCGAGTGCAAATCTTTTACCAAACCAGAGATAAATTGAT
ATGACACCTCACTAGGCGTA
>Marker292153
TACATGTCTTAAGGAAAAGTCAAATCGCCATTGATGGTTAGATTATGTATCGAGTTCATATTGGTGGCATAGCGC
GACGTXXXXXXXXXXXAATGCTGAACAAACGGTTCCTTCAACTGCTAGATAAAAAGAGAAAGTCGAAAGTCGAGAA
GGAAGGAATTGAGGCTAAGT
TACATGTCTTAAGAAAAAGTCAAATCGCCATTGATGGTTAGATTATGTATCGAGTTCATATTGGTGGCATAGCGC
GACGTXXXXXXXXXXXAATGCTGAACAAACGGTTCCTTCAACTGCTAGATAAAAAGAGAAAGTCGAAAGTCGAGAA
GGAAGGAATTGAGGCTAAGT
>Marker292404
GACCAAAAAAGCTGATGGCCTAACCATATCAAGATAGGCATATATGAATTTGATTGGCATTTTCCACTACCATAC
CCAACXXXXXXXXXXXTCGAAATTACATTTGAACGAAAGAAATCATGAGTTTATGAAAGTATTTTTAAGACAAACT
TTAAATAATTGAAAGTTAGT
GACCAAAAAAGCTGATGGCCTAACCATATCAAGATAGGCATATATGAATTTGATTGGCATTTTCCACTACCATAC
CCAACXXXXXXXXXXXTCGAAATTAAATTTGAACGAGAGAAATCATGAGTTTATGAAAGTATTTTTAAGACAAACT
TTAAATAATTGAAAGTTAGT
>Marker293319
ACTCATAGACTAGAAGTTTTTCTCCTCCTTCAACACAACATCCCAAGAGCCTGACCAGATTCTTATGTTGAAGCT
TCCAAXXXXXXXXXXXTCATATGGTTGTTCTAACACACCTTACAAATAATAGTAATAAAAACTTATATCTCAAACT
TCTGATTCACTAACCTTGTA
ACTCATAGACTAGAAGTTTTTCTTCTCCTTCAACACAACATCCCAAGAGCCTGACCAGATTCTTATGTTGAAGCT
TCCAAXXXXXXXXXXXTCATATGGTTGTTCTAACACACCTTACAAATAATAGTAATAAAAACTTATATCTCAAACT
TCTGATTCACTAACCTTGTA
>Marker293539
TACTTGATTGAAACCATTTTTTTATTGGGCTTCCTTTTGTTAATATTTTATTTCTATATGTCCCATGTAATCTTT
CATTTXXXXXXXXXXXATTTGATACAAAAAGGTGTTGGTATGAAATTGACTATTGAGATAGCCATGTATTGATTAT
GCTGCCAAATTCCCCATGTT
TACTTGATTGAAACCATTTTTTTATTGGGCTTCCTTTTGTTAATATTTTATTTCTATATGTCCCATGTAATCTTT
CATTTXXXXXXXXXXXATTTGATACAAAAAGGTGTTGGTATGAAATTGACTATTGAGATAGCCATGTATTGATTAT
GCTGCCAAATACCCCATGTT
>Marker293969

Create PDF with GO2PDF for free, if you wish to

AACTAGGTTGGGTTGCCTCTTTGAGTAACTTCAAATTATCGACTTTAATCTCATTCTATTTTATGAACTTTCAGC
CAACTXXXXXXXXXXTCATCGTATATGTCGAGACTAATTACCATTATACATAATATAACGGGCCCTTCTAATAGT
TATCTGGCTATCACTATGTA
AACTAGGTTGGGTTGCCTCTTTGAGTAACTTCAAATTATCGACTTTAATCTCATTCTATTTTATGAACTTTCAGC
CAACTXXXXXXXXXXTCATCGTATATGTCGAGACTAATTACCATTATACATAATATAACGGGTCCTTCTAATAGT
TATCTGGCTATCACTATGTA
>Marker294808
ACTAATTTGTGTATTTGGTCTCTAGTCCTCAGGAAGATATCACACTTTGATGTCAGACATTAAGCATGGGCAGAT
CTTGTXXXXXXXXXXGAATTTGATCGTTGGAAGTTGATGTCTATAAATACTAACTTGGAAATGCATCAAGCAAAC
CCAACCACACCGAAAATGGT
ACTAATTTTTGTATTTGGTCTCTAGTCCTCAGGAAGATATCACACTTTGATGTCAGACATTAAGCATGGGCAGAT
CTTGTXXXXXXXXXXGAATTTGATCGTTGGAAGTTGATGTCTATAAATACTAACTTGGAAATGCATCAAGCAAAC
CCAACCACACCGAAAATGGT
>Marker294940
CACCTTTGAAGAAATGTAAAAAGCTCAAACATTGAGCTGATAGATCAAACAATGGTGGCCTATACAGCAAAGTTA
AAAAGXXXXXXXXXXTGCCTAAAAATATAATCATAATTATTAATTTCAGAGGTCCATAGTTTATTTTGATGATCT
TTTGTGAAATAGGTCACGTT
CACCTTTGAAGAAATGTAAAAAGCTCAAACATTGAGCTGATAGATCAAACAATGGTGGCCTATACAGCAAAGTTA
AAAAGXXXXXXXXXXTGCCTAAAAATATAATCATCATTATTAATTTCAGAGGTCCATAGTTTATTTTGATGATCT
TTTGTGAAATAGGTCACGTT
>Marker295115
ACTTGGATCATCATGCTTCATACGAACGTTACCTCGAATAATGACCCCTTGTTTGATCTCATCAGAAGGAACCTT
AAACCXXXXXXXXXXTTAAATCTTACTCCTCATCTCCTAGATGAGTTGATAAAGTAGTCTCTTGAATATCATACT
CCTCAACATGCTAAGAGGTT
ACTTGGATCATCATGCTTCATACGAACGTTACCTCGAATAATGACCCCTTGTTTGATCTCATCAGAAGGAACCTT
AAACCXXXXXXXXXXTTAAATCTTACTCCTCATCTCCTAGGTGAGTTGATAAAGTAGTCTCTTGAATATCATACT
CCTCAACATGCTAAGAGGTT
>Marker295485
ACTGAATATGAAATTGTTCGAATACTGAACAAATTTGTGTCACAAAGGAAACGTTGAGAGCATTCATAGGTCTAT
TATAAXXXXXXXXXXTTAGCGAATGTATTCCTAAATAATATGTTTGAATATTCTACTATTTTCTTTAATATCTAG
TTACTCAGAAACACAATGTT
ACTGAATATGAAATTGTTCGAATACTGAACAAATTAGTGTCACAAAGGAAACGTTGAGAGCATTCATAGGTCTAT
TATAAXXXXXXXXXXTTAGCGAATGTATTCCTAAATAATATGTTTGAATATTCTACTATTTTCTTTAATATCTAG
TTACTCAGAAACACAATGTT
>Marker295589
CACATTTAATCTTCACAATGACAAGTCTCACATAACACTCATTCAAAACAAACCCAATAGATGAACAACTGATAT
ATTCAXXXXXXXXXXTTATAGATATAATCGAACATTTCTCACATTATTTCGAAGGATATGAAGTAGAAGCAAAAG
AAATTAACATTGGGCCAGTG
CACATTTAATCTTCACAATGACAAGTCTCACATGACACTCATTCAAAACAAACCCAATAGATGAACAACTGATAT
ATTCAXXXXXXXXXXTTATAGATATAATCGAACATTTCTCACATTATTTCGAAGGATATGAAGTAGAAGCAAAAG
AAATTAACATTGGGCCAGTG
>Marker295987
GACAAATGACAGCTCCTCCGACTCTGGCTCTCCTAGATCACTCCCCTCCGATTTCAAAGGCGTAAAGAAGGAAGA
AGCACXXXXXXXXXXAACAGCTGGACCTTAGCATCTCTTATAAACCCTTCCATGCAGAAAAGGTTGTGGTATCGA
TCGAAGATAAAAATTTGGTG

Create PDF with GO2PDF for free, if you wish to

GACAAATGACAGCTCCTCCGACTCTGGCTCTCATAGATAACTCCCCTCCGATTTCAAAGGCGTAAAGAAGGAAGA
AGCACXXXXXXXXXXXAACAGCTGGACCTTAGCATCTCTTATAAACCCTTCCATGCAGAAAAGGTTGTGGTATCGA
TCGAAGATAAAAATTTGGTG
>Marker296234
GACATGTTCTCAGAATTCAGGTTCGGTGTGTATGGCCGCAGAAGAAGCTGAGGTTCATTTTCTGCAAACTGAAGC
AGACGXXXXXXXXXXXGAAGTTAGTGCTCAAAAGCCCATCCACATTAAAAGCCAACCTAGGATAATTATTAGAGAT
ATTTCTCAAGCCCTTGTGTA
GACATGTTCTCAGAATTCAGGTTCGGTGTGTATGGCCGCAGAAGAAGCTGAGATTCATTTTCTGCAAACTGAAGC
AGACGXXXXXXXXXXXGAAGTTAGTGCTCAAAAGCCCATCCACATTAAAAGCCAACCTAGGATAATTATTAGAGAT
ATTTCTCAAGCCCTTGTGTA
>Marker296240
ACCTTCAATTTTTTAGTAGATAGATTAAATTTTTTTGGTATCGAAATATAAAATTAGAGAAGCATTTACTAGACA
TGATTXXXXXXXXXXXATAGATAAGAGTATTTCGGTTCTTTATTTTTCCTCATTTCTGTTTTATAGAAAAAAATTG
CTTTTTTGAATGCATGAGTC
ACCTTCAATTTTTTAGTAGATAGATTAAATTTTTTTGGTATCGAAATATAAAATTAGAGAAGGATTTACTAGACA
TGATTXXXXXXXXXXXATAGATAAGAGTATTTCGGTTCTTTATTTTTCCTCATTTCTGTTTTATAGAAAAAAATTG
ATTTTTTGAATGCATGAGTC
>Marker296284
CACATTTGAGGGATACATTTGTGATATGGAATTGAAAAAAATGATAAAATAGGTAGGGATAAGTAGTATATATAT
GGAACXXXXXXXXXXXCTGTAATTAAAGGTTATAAAAACAATACCACAAACATTATTCATGATCAATTAATATATA
TGAATATATTATATTGAGTC
CACATTTGAGGGATACATTTGTGATATGGAATTGAAAAAAATGATAAAATAGGTAGGGATAAGTAGTATATATAT
GGAACXXXXXXXXXXXCTGTAATTAAAGGTTATAAAAATAATACCACAAACATTATTCATGATCAATTAATATATA
TGAATATATTATATTGAGTC
>Marker297311
ACTACAAATTTCTTTCAAATACTTTAAGCATAAACTAATTAATTTCCAATGCCACAACCTAGTAAATTAATGTGA
GAAATXXXXXXXXXXXTAATCACAAAGGAAAAAAGAGAAATTATTACCTTTTACAAAGGATTTGGAGACAAATGGA
GGTTTGTCGGTTAATGTGTT
ACTACAAATTTCTTTCAAATACTTTAAGCATAAACTAATTAATTTCCAATGCCACAACCTAGTAAATTAATGTGA
GAAATXXXXXXXXXXXTAATCACAAAGGAAAAAAGAGAAATTATTACCTTTTACAAAGGATTTGGAGACAAATGGA
GGTTTGACGGTTAATGTGTT
>Marker297473
CACAATTTCACTACTTTTGCGTTGAACAACTTCTTATATAGAATCACATTTCTTCTATGAAACAATGCCTTTTAT
CATTCXXXXXXXXXXXTATGAGAACACTCGAATGATTGGCATGAAAATACGACATTGTTACACGATGGGGTGTTGT
TGGTGTGATAAAACATTGTT
CACAATTTCACTACTTTTGCGTTGAACAACTTCTTATATAGAATCACATTTCTTCTACGAAACAATGCCTTTTAT
CATTCXXXXXXXXXXXTATGAGAACACTCGAATGATTGGCATGAAAATACAACATTGTTACACGATGGGGTATTGT
TGGTGTGATAAAACATTGTT
>Marker297511
ACCCTCTTACGCTACACAAGTTCAACTGAAATCACATCCAAAATAGGCTAAGTTGGTTTCCAAAGTCGAAATAAA
GAATAXXXXXXXXXXXGTAACATAGTGACAGATCGCACATTTGACAGATGGAGCTCCATAAGGAAAGACAAGTGTT
GTTCGGCAGTTTCCACAGTT
ACCCTCTCACGCTACACAAGTTCAACTGAAATCACATCCAAAATAGTCTAAGTTGGTTTCCAAAGTCGAAATAAA
GAATAXXXXXXXXXXXGTAACATAGTGACAGATCGCACATTTGACAGATGGAGCTCCATAAGGAAAGACAAGTGTT
GTTCGGCAGTTTCCACAGTT
>Marker297846

Create PDF with GO2PDF for free, if you wish to

ACATTCATAAATCAACATATAATGACTTGTTACTAGTTACATGTAGCCCTATCACATTATGCCATCTGTGTCATC
GGTTTXXXXXXXXXXAGATGAGATGAAAGGTGTATGTGATGAGAGAGCTTATTTATTTGAGTGAATGGAAAAAGG
AGTAAGAGTGAGAGGCAGTG
ACATTCATAAATCAACATATAATGACTTGTTACTAGTTACATGTAGCCCTATCACATTATGCCATCTGTGTCATC
TGTTTXXXXXXXXXXAGATGAGATGAAAGGTGTATGTGATGAGAGAGGTTATTTATTTGAGTGAATGGAAAAAGG
AGTAAGAGTGAGAGGCAGTG
>Marker297909
ACATCCATCCCATCTATGAGCCATTACAGACAATATGAAGCCAACTAATTAGCCCACCAAATCACAGATTCAATC
CAACCXXXXXXXXXXTAAACAGAAGCTCTGAATGTGTTCTTTGGTTTTGAATAAATGGGTTGCAACAAAAGTCTC
ATAAAAAGATCGCTGTCGGT
ACATCCATCCCATCTATGAGCCATTACAGACAATATGAAGCCAACTAATTAGCCCACCAAATCACAGATTCAATC
CAACCXXXXXXXXXXTAAACAGAAGCTCTGAGTGTGTTCTTTGGTTTTGAATAAATGGGTTGCAACAAAAGTCTC
ATAAAAAGATCGCTGTCGGT
>Marker298441
CACTCTCTTATGAAACTATGAAAGAGGAAAAGGGAATTGACACTCCATTAATTCCAACTTTTCTTCTTTCATGTC
AAAATXXXXXXXXXXTATTGAACATTTAATTATGAAAAACATGCCTATTAAAGAAACATTTTTTTTTAAAAAAAA
TCAACTAAAACAAATGAGTT
CACTCTCTTATGAAACTATGAAAGAGGAAAAGGGAATTGACACTCCATTAATTCCAACTTTTCTTCTTTCATGTC
AAAATXXXXXXXXXXTATTGAACATTTAATTATGAAAAACATGCCTATTAAAGAAACATTTTTTTTAAAAAAAAA
TCAACTAAAACAAATGAGTT
>Marker298632
ACTGTTTTTCTTCTACTAATGGAGAAACAAACTTTAGGATCCCAGATTTTTTTTTAAAAAAATGCAATAAATTTC
ATTTTXXXXXXXXXXTTGTATCTGCTATCGTTATCTGTTTTTGGATTCTTATTAAAAGAATGTTTGATGCTTACT
GCTTTAGGTAATGCATTTGT
ACTGTTTTTCTTCTACTAATGGAGAAACAAACTTTAGGATCCCAGATTTTTTTTTATAAAAATGCAATAAATTTC
ATTTTXXXXXXXXXXTTGTATCTGCTATCGTTATCTGTTTTTGGATTCTTATTAAAAGAATGTTTGATGCTTACT
GCTTTAGGTAAGGCATTTGT
>Marker298968
ACTCATCATCAAGAAGTTTGGAAAAACCACCTCCCAAACCACCTCTCATCCCGACAAAACCATCGACTCCTTCGT
CAATAXXXXXXXXXXCACCTGCTGTTCCTGAAAAATCCAATTCCTCTGCTAAATTCCGCCGGAGTTTGGATTCCA
ATTCACGGACAAAATCCGTA
ACTCATCATCAAGAAGTTTGGAAAAACCACCTCCCAAACCACCTCTCATCCCGACAAAACCATCGACTCCTTCGT
CAATAXXXXXXXXXXCACCTGCTGTTCCTGAAAAATCCAATTCCTCTGCTAAATTCCGCCGGAGTTTGGATTCAA
ATTCACGGACAAAATCCGTA
>Marker299092
ACAAGCTGGGCCACTGATTAGAGTCAAATTCAATAATTAATTGAAACCACATAAGCTTTAAACTTTGAAGTCTTC
CTTTTXXXXXXXXXXCCCCCTCACTTTCAATTTGGGGTATTTTTAATTAAATAAAAACAGCGTTGTTTTTTTGGA
GGTTAGTTGAAATTGTAGGT
ACAAGCTGGGCCACTGATTAGAGTCAAATTCAATAATTAATTGAAACCACATAAGCTTTAAACTTTGAAGTCTTC
CTTTTXXXXXXXXXXCTCCCTCACTTTCAATTTGGGGTATTTTTAATTAAATAAAAACAGCGTTGTTTTTTTGGA
GGTTAGTTGAAATTGTAGGT
>Marker299383
AACTTTTTGGGCGTGGTCGAACATCCAAATCTTGTCAAGTTACTAGGCTACTGTGCCGAGGACGATGAAAGGGGG
ATCCAXXXXXXXXXXATGAAGGAATGGATTTCCAGGTAACTTGTCATTTTGCAAGATTTTTATAGCATGAGATAT
GTTTTGAGCCAAAACTAGTG

Create PDF with GO2PDF for free, if you wish to

AACTTTTTGGGCGTGGTCGAACATCCAAATCTTGTCAAGTTACTAGGCTACTGTGCCGAGGACGATGAAAGGGGG
ATCCAXXXXXXXXXXXATGAAGGAATGGATTTCCAGGTAACTTGTCATTTTGCAAGATTTTTATAGCATGAGATAT
TTTTTGAGCCAAAACTAGTG
>Marker304726
AACCAAAAAATCGTTAAGATTTGGTTACTCAAATTTAAACGATTAAATATAAACAACAAACAACGTGTAAGAAAG
AATAGXXXXXXXXXXXTATTTTTTAAAAGACCCCTATTATTGTTATTATTGTATGACATTTAATTTACCCTACATA
ACCTAGTGGAAAAAAAAGTG
AACCAAAAAATCGTTGATATTTGGTTACTCAAATTTAAACGATTAAATATAAACAACAAACAACGTGTAAGAAAG
AATAGXXXXXXXXXXXTATTTTTTAAAAGACCCCTATTATTGTTATTATTGTATGACATTTAATTTACCCTACATA
ACCTAGTGGAAAAAAAAGTG
>Marker304912
ACTATTCTTTAAGTGATTATATCTTCTTTACAACTTTTTTGGAACAGTAACTATATGATCCATATGGATATATGT
CAGTTXXXXXXXXXXXGTGATTAACTTTGGTATTTCTCATTTCACAGACCTTGTAAATGTTTTTCGAGAGTTTCTT
CCAAAAGTCGTGCAAGAGTG
ACTATTCTTTAAGTGATTATATCTTCTTTACAATTTTTTTGGAACAGTAACTATATGATCCATATGGATATTTGT
CAGTTXXXXXXXXXXXGTGATTAACTTTGGTATTTCTCATTTCACAGACCTTGTAAATGTTTTTCGAGAGTTTCTT
CCAAAAGTCGTGCAAGAGTG
>Marker305536
TACTTCTTCTCCCTCTTTTGCATTATTGATTGTTGCAGGTAAAAATTTCTTGCTTTGGAATCTCTCGACTCTAAT
TTATTXXXXXXXXXXXTCACAATTCCTGAATTTCCATTCATTTCAATCTTAACACCTGTCGCTGCAATATTTCAAA
ATAGGTTTTTGCTCGATGGT
TACTTCTTCTCCCTCTTTTGCATTATTGATTGATGCAGGTAAAAATTTCTTGCTTTGGAATCTCTCGGCTCTAAT
TTATTXXXXXXXXXXXTCACAATTCCTGAATTTCCATTCATTTCAATCTTAACACCTATCGCTGCAATATTTCAAA
ATAGGTTTTTGCTCGATGGT
>Marker306063
AACTTCTTATGCACTACTTTTTCCAAAAATGCCCAATCCACTCTGTCGAAAGCCTTTTCAAGGTCTAATTTAATG
ATCGAXXXXXXXXXXXTATTTGTATTAAAAGAGAGTTACTTGTTTTTTAAATGAGGAAAGTTATAAAGTTCATTAG
AAGGGTAGTTTGGACCTTGT
AACTTCTTATGCACTACTTTTTCTAAAAATGCCCAATCCACTCTGTCGAAAGCCTTTTCAAGGTCTAATTTAATG
ATCGAXXXXXXXXXXXTATTTGTATTAAAAGAGAGTTACTTGTTTTTTAAATGAGGAAAGTTAGAAAGTTCATTAG
AAGGGTAGTTTGGACCTTGT
>Marker306066
TACCCACCATTTCTTGGAATTGCCCACCATGTTGCATTCTGAAATGACTCTACAAAGGTCAATATCTGCAACACC
ATCTGXXXXXXXXXXXTTTGAACCTTCACCAGAAAACTTTTTTTTCTTGTTACTCTATAGTTTATTTTCTAGAATG
TTGGCCTTGGGATCTATTGT
TACCCACCATTTCTTGGAATTGCCCACCATGTTGCATTCTGAAATGACTCTACAAAGGTCAATATCTGCAACACC
ATCTGXXXXXXXXXXXTTTGAACCTTCACCAGAAAACTTTTTTTTCTTGTTACTCTATAGTCTATTTTCTAGAATG
TTGGCCTTGGGATCTATTGT
>Marker306703
ACAAGCTCTCAAGAAGTGCAAGTTTGTATCAATCTCAAGATTAACATCTTATGAAATATCGGAGACTTTGAAGAT
TAAAAXXXXXXXXXXXXATACTAGAGACTATATTCACAATATTTATCAATATACAAAGTTCAATTCCACGAAATACA
TTTTCTTGAAATCTCGTGTG
ACAAGCTCTCAAGAAGTGCAAGTTTGTATCAATCTCAAGATTAACATCTTATGAAATATCGGAGACTTTGAAGAT
TAAAAXXXXXXXXXXXXATACTAGAGACTGTATTCACAATATTTATCAATATATAAAGTTCAATTCCACGAAATACA
TTTTCTTGAAATCTCGTGTG
>Marker307032

Create PDF with GO2PDF for free, if you wish to

AACAGATCATTTGCAAGTTGGAGCTTCAAAATTTGGGAACACTATAAAAGCAATGGACAATGGAGCTATAACTAT
AGGAGXXXXXXXXXXGATATGGACAAATCTGATTCCTTGTTTCCAACCCAACTTACTGTTTTCTTAATCCCTTGC
ATGAAAGAGCTCCCTTTGTG
AACAGATCATTTGCAAGTTGGAGCTTCAAAATTTGGGAACACTATAAAAGCAATGGACAATGGAGCTATAACTAT
AGGAGXXXXXXXXXXGATATGGACAAATCTGATGCCTTGTTTCCAACCCAACTTACTGTTTTCTTAATCCCTTGC
ATGAAAGAGCTCCCTTTGTG
>Marker307653
AACATGATCATGACCTTGGTCAAACATAATCTAATGCAACCATGCTATAATTAGATTTCACTATTTATCAAAGTA
TTCCTXXXXXXXXXXAATATTTATTTAGTAACACACGATAAATTATATTCATTGGATTAAAACAATTAAACAAAA
TAAAAGTAACTAGCTAGAGT
AACATGATCATGACTTTGGTCAAACATAATCTAATGCAACCATGCTATAATTAGATTTCACTATTTATCAAAGTA
TTCCTXXXXXXXXXXAATATTTATTTAGTAACACACGATAAATTATATTCATCGGATTAAAACAATTAAACAAAA
TAAAAGTAACTAGCTAGAGT
>Marker308380
AACAGATTATATACTTATAACATAAACATTTAATTTTCATCTTTAAACTGTAAATGAGTAACAGTATTGATTTCA
ACCCTXXXXXXXXXXAAAAAATGTTATAATTGGAGATTGATTTGTAATCCTTGTTTGTAAAACGGTTAGCTTGGT
TTTAGGTTTTGAATTGAGTC
AACAGATTATATACTTATAATATAAACATTTAATTTTCATCTTTAAACTGTAAATGAGTAACAGTATTGATTTCA
ACCCTXXXXXXXXXXAAAAAATGTTATAATTGGAGATTGATTTGTAATCCTTGTTTGTAAAACCGTTAGCTTGGT
TTTAGGTTTTGAATTGAGTC
>Marker309055
ACATTTGTTTAATAAGCCATTTAATTTATCAAATTAAAACATTTTAATTATCAAACAAAATGATATTTTATTTGC
AGCCTXXXXXXXXXXTAAAGGTCCTTGTAAAAGTCCTATTGAACTACAAATTGAAGGAACACTACAAGCTTCGCC
TGACCCAAAAGGAGATGGTT
ACATTTGTTTAATAAGCCATTTAATTTATCAAATTAAAACATTTTAATTATCAAACAAAATTATATTTTATTTGC
AGCCTXXXXXXXXXXTAAAGGTCCTTGTAAAAGTCCTATTGAACTACAAATTGAAGGAACACTACAAGCTTCGCC
TGACCCAAAAGGAGATGGTT
>Marker309411
AACCATAGGAAGCAACTGACTTTTAACGATTTAACACCAAGGTCACATTGATTTTCCTTTTAAACACAATAGTTT
CCACAXXXXXXXXXXTCCTTATACGATATATGGCAACCTCGATATTCACATGATCTTACTTGTTCTAAATATCTT
GGCATTTTCACAATAGTGTA
AACCATAGGAAGCAACTGACTTTTAACGATTTAACACCAAGGTCACATTGATTTTCCTTTTAAACACAGTAGTTT
CCACAXXXXXXXXXXTCCTTATACGATATATGGCAAGCTCGATATTCACATGATCTTACTTGTTCTAAATATCTT
GGCATTTTCACAATAGTGTA
>Marker309520
AACTACGTGACTTATATTGACTGAACCAAAATCTCAAATAGAATATAAAGACAGACTCAAAAAATGCCAAACGAT
TCAGGXXXXXXXXXXTGAGGAGATTGATGAATGTCTATAGACTATATGAAACTAATAGTTACAACACTCTTCACT
TACAGAGCTCTGAGCTGAGT
AACTACGTGACTTATATTGACTGAACCAAAATCTCAAATAGAATATAAAGACAGATTCAAAAAATGCCAAACGAT
TCAGGXXXXXXXXXXTGAGGAGATTGATGAATGTCTATAGACTATATGAAACTAATAGTTACAACACTCTTCACT
TACAGAGCTCTGAGCTGAGT
>Marker309762
CACCTCCTTGCATGTAAGTTTCCCCATTCTCTCTGTCTCTCTCTTTTTTTTTGTTTTGACAAGAAATAGTTCTTTG
ATGTAXXXXXXXXXXGATCGATAAGATAAAATGTTCAAATTTCCACCGACATGTTGAAGTTAAAATAAACTTCTC
TGTCCCTCATTTATACTTGT

Create PDF with GO2PDF for free, if you wish to

CACCTCCTTGCATGTAAGTTTCCCCATTCTCTCTGTCTCTCTCTTTTTTTTGTTTTGACAAGAAATAGTTCTTTG
ATGTAXXXXXXXXXXXGATCGATAAGATAAGATGTTCAAATTTCCACCGACATGTTGAAGTTAAAATAAACTTCTC
TGTCCCTCATTTATACTTGT
>Marker309769
AACTTAAGGTGGTTGAAAAATTCAAGTTCTTACTATCCACTTTTAAACAACAAACAGGTCACCAGCTGAGAGTCG
AAGATXXXXXXXXXXXCATATCTTCTCAACAAACAAAAAGGTGTATTAAATTTATAGGAAAGGATGCTTGTAACTA
CCTAGGATTATGGTGTAGTA
AACTTAAGGTGGTTGAAAAATTCAAGTTCTTACTATCCACTTTTAAACAACAAACAGGTCACCAGCTCAGAGTGG
AAGATXXXXXXXXXXXCATATCTTCTCAACAAACAAAAAGGTGTATTAAATTTATAGGAAAGGATGCTTGTAACTA
CCTAGGATTATGGTGTAGTA
>Marker309837
CACCGGTTGCATCTTTTTGCTAGTCTCTAGATCCGAATGATTTCCTTTTGAGAAAGTTATTTTGTTATGCTTTGT
ATTTAXXXXXXXXXXXCTTTTATTGACCGAGGTCGTCTTTGGGCAGTTTGTTTGGTTGTTTGTCTTTATTTTTTGC
TCTTTGTATAATTCTCTTGT
CACCGGTTGCATCTTTTTGCTAGTCTCTAGATCCGAATGGTTTCCTTCTGAGAAAGTTATTTTGTTATGCTTTGT
ATTTAXXXXXXXXXXXCTTGTATTGACCGAGGTCGTCTTTGGGCAGTTTGTTTGGTTGTTTGTCTTTATTTTTTGC
TCTTTGTATAATTCTCTTGT
>Marker310501
AACTAACTTGCATGCAAGAGAAACGTGAAGGAAGGTCTATGCATTATTAGGCAACTAAGCAGTGTGAGAACTCTG
ATGCGXXXXXXXXXXXAGGTATATCTAGAAGACTTGACTTATAGAACTTCTCACTTAAGGACAACAACCTCTTAGT
GCCTAATGTATGCACTTGTT
AACTAACTTGCATGCAAGAGAAACGTGAAGGAAGGTCTATGCATTATTAGGCAACTAAGCAGTGTGAGAACTCTG
ATGCGXXXXXXXXXXXAGGTATATCTAGAAGACTTGACTTATAGAACTTCTCACTTAAGGACAACAACCGCTTAGT
GCCTAATGTATGCACTTGTT
>Marker311295
TACGGATATATTTCCTTTCTAGTATATATATTGTGACAAATATCAAATTATAATCCTAAACAAAGAATTGTATCT
ATTAAXXXXXXXXXXXTTAGATCGTAACTACAAGAAAATTATTTATGCATTAACTCTTAATATTTTACAAAATGAA
CCATTAGAATTAATGGCGTG
TACGGATATATTTCCTTTCTAGTATATATATTGTGACAAATATCAAATTATAATCCTAAACAAAGAATTGTATCT
ATTAAXXXXXXXXXXXATACATCGTAACTACAAGAAAATTATTTATGCATTAACTCTTAATATTTTACAAAATGAA
CCATTAGAATTAATGGTGTG
>Marker311335
AACTTCATCCCTAGCTGTCCCCACCCTTTTTTTTCTCTCTTCTTATTCCTAATTTAAAATACCAAAAGTATATCA
TTACCXXXXXXXXXXXTTATGAATGAAACTTAATGTTAGTTGTTGGGTTTAGAAAGATTTGAAACTTAAAAGCAAG
GAATAGCGTTGTAGAAGGTT
AACTTCATCCCTAGCTGTCCCCACCCTTTTTTTTCTCTCTTCTTATTCCTAATTTAAAATACCAAAAGTATATCA
TTACTXXXXXXXXXXXTTGGGAATGAAACTTAATGTTAGTTGTTGGGTTTAGAAAGATTTGAAACTTAAAAGCAAG
GAATAGCGTTGTAGAAGGTT
>Marker311736
ACTTCTACGACTAGTGTTTCCCGTCTTGTTGCTTTCAAATGGTCATCGCCGTTCTCCCTTCGTCTATTGTGTTAA
AAAATXXXXXXXXXXXGGACTCAAGCGTGGATCGCCTCCAGGATGCAACGTTTGATCGACCTCATCTTTGGTATCC
TACTTCTGAAGTAATTAGTG
ACTTCTACGACTAGTGTTTCCTGTCTTGTTGCTTTCAAATGGTCATCGCCGTTCTCCCTTCGTCTATTGTGTTAA
AAAATXXXXXXXXXXXGGACTCAAGCGTGGATCGCCTCCAGGATGCAACGTTTGATCGACCTCATCTTTGGTATCC
TACTTCTGAAGTAATTAGTG
>Marker312290

Create PDF with GO2PDF for free, if you wish to

AACCGATATCGCTCCCTTTTTTAACAACACCAGCTGATCCTTAACCTTTCACCTTTAACTAAAACGCAGGCGGAA
GCTAAXXXXXXXXXXTCTAACTTCAACCCTCTTAAATCCAAGTCCGTTACAATTAAAAGACCAACTAAATGTAAC
TTCTAAACAGCAAGACATGT
AACCGATATCGCTCCCTTTTTTAACAACACCAGCTGATCCTTAACCTTTCACCTTTAACTAAAACGCAGACGGAA
GCTAAXXXXXXXXXXTCTAACTTCAACCCTCTTAAATCCAAGTCCGTTACAATTAAAAGACCAACTAAATGTAAC
TTCTAAACAGCAAGACATGT
>Marker312526
CACAGATAAATGCATTCAAGCGTTCGATCAAATTTTTTCCTCTGTGAAATACAAAATACAATGATATTTTGGCAA
GGGTTXXXXXXXXXXATTTAAAATTACAGATTGGTTAAAATAATAAGCAAAGCTACCCATTTAGTAATCATTACA
TCTCTACTACACGTTTTGGT
CACAGATAAATGCATTCAAGAGTTCGATCAAATTTTTTCCTCTGTGAAATACAAAATACAATGATATTTTGGCAA
GGGTTXXXXXXXXXXATTTAAAATTACAGATTGGTTAAAATAATAAGCAAAGCTACCCATTTAGTAATCATTACA
TCTCTACTACACGTTTTGGT
>Marker312708
ACTTGAAAATTCCTGGTCTTTGATTTGGGAATCTTCTGCTAACTTTTTCTTCGCGAAAGTCATTTCTGTCTCATC
ATTGCXXXXXXXXXXGCTTTTCGAAGTGTTTTTTTTTAACTCAGGACCTCATGTCCGCAGATACATACAGGTGTC
ATTTGAGCTAAGCTCATGTT
ACTTGAAAATTCCTGGTCTTTGATTTGGAAATCTTCTGCTAACTTTTTCTTCACGAAAGTCATTTCTGTCTCATC
ATTGCXXXXXXXXXXGCTTTTCGAAGTGTTTTTTTTTAACTCGGGACCTCATGTCCGCAGATACATACAGGTGTC
ATTTGAGCTAAGCTCATGTT
>Marker313097
TACAACAAGAGACCTGAAATGATTGTGTATTCCAACTCTCTTATCTTCAAAAATGCAATGATTAAGGAGTCCGAA
GAAACXXXXXXXXXXCCAAAATGATTGTGCAAAGTCACAGAAAACAAAATTATCCTCCAAAACTATTGTGCAAAG
TCACAGAAAACCAAGATGTT
TACAACAAGAGACCTGAAATGATTGTGTATTCCAACTCTCTTATCTTCAAAAATGCAATGATTAAGGAGTCCGAA
GAAACXXXXXXXXXXCCAAAATGATTGTGCAAAGTCACAGAAAACAAAATTATCCTCCAAAATTATTGTGCAAAG
TCACAGAAAACCAAGATGTT
>Marker313525
ACTGCTTTCATAGGAAAACTAATCTATTGTATCCTGTGAGACAATTATTGGCAACACAATTATTCCCAACTCAGC
ACTAAXXXXXXXXXXTCAGTTTCAAAACCATCCCCACAATCACAATCAAACAAAAGAAACAGAAATGAAGACTGA
GATTAAAAAGTTTTGGGGTA
ACTGCTTTCATAGGAAAGCTAATCTATTGTATCCTGTGAGACAATTATTGGCAACACAATTATTCCCAACTCAGC
ACTAAXXXXXXXXXXTCAGTTTCAAAACCATCCCCACAATCACAATCAAACAAAAGAAACAGAAATGAAGACTGA
GATTAAAAAGTTTTGGGGTA
>Marker313528
ACTGTTAAGAACACCGACAAACAAACAGCTTCTTCAGGTATAAAATTCGAATTCCTTAGAACTCACCCAATGATA
ATAATXXXXXXXXXXCACTTTAAATTTAATGTTATACCTTGTGTCTTGCATGGAAATTAATTCTCATTCTCACAT
GCATCCATTATAAATATAGT
ACTGTTAAAAACACCGACAAACAAACAGCTTCTTCAGGTATAAAATTCGAATTCCTTAGAACTCACCCAATGATA
ATAATXXXXXXXXXXCACTTTAAATTTAATGTTATACCTTGTGTCTTGCATGGAAATTAATTCTCATTCTCACAT
GCATCCATTATAAATATAGT
>Marker313585
AACCCAAGACTTTGAATTTCTTTTTTCAATTGAACATGGGTTATAGGTCTATATGTTTCTTTTGATCATTAAATT
ATGTCXXXXXXXXXXTAATCTCTGATCAGGCATTATTAACATCCATAATTGCAGTCCCATCAAGATTTCTCAGTG
TTCAAAACTTCTGATTAGTT

Create PDF with GO2PDF for free, if you wish to

AACCCAAGACTTTGAATTTCTTTTTTCAATTGAACATGGGTTATAGGACTATATGTTTCTTTTGATCATTAAATT
ATGTCXXXXXXXXXXTAATCTCTGATCAGGCATTATTAACATCCATAATTGCAGTCCCATCAAGATTTCTCAGTG
TTCAAAACTTCTGATTAGTT
>Marker313855
CACATCAATGAGAAGGTGTTGGAGGAAGATTTCAGTAGCTCGTGGGTAGTCTCAAGACTATTTGTGTATAATGAG
AGGGAXXXXXXXXXXTGGGTGGATTTCCATCAAAAATCTACCACTTGATTACTGGAAAAATGACGTCTTTATTAC
ATTTGGAAATTACTTCGGTG
CACATCAATGAGAAGGTGTTGGAGGAAGATTTCAGTAGCTCGTGGGTAGTCTCAAGACTATTTGTGTATAATGAG
AGGGAXXXXXXXXXXTGGGTGGATTTCCATCAAAAATCTACCACTTGATTACTGGAAAAATGGCGTCTTTATTAC
ATTTGGAAATTACTTCGGTG
>Marker314131
CACATGAAATTTCTGGAGAAATTTGATTCGTGGAGTTGAACTTGAGTTGATGTTTTATTTTTGTTGATTTGATGC
GATGTXXXXXXXXXXAAGTCTTGTAAAGCTTGTAGAAATTGGAGTCTTGGAAGAAAGCTTGTATCCTAAAGGAGC
TTCAATCTTCAAGGATGGTT
CACATGAAATTTCTGGAGAAATTTGATTCGTGGAGTTGAACTTGAGTTGATGTTTTATTTTTGTTGATTTGATGC
GATGTXXXXXXXXXXAAGTCTTGTAAAGCTTGTAGAAATTGGAGTCTTGGAAGAAAGCTTGTATCTTAAAGGAGC
TTCAATCTTCAAGGATGGTT
>Marker314609
GACCTTGAGGCAAGCTGTCACAAATCTTTTAGTGCTTTGGTCAAGGAATTCCTCTTCTATCCGCCGTTTTGGGGT
AAGAGXXXXXXXXXXTTGAATTTTTTTATTTTCTCAATTAAAGTTGGTATATTGTCTAAAAAGCTACAAATGTAG
TCATATTCTAGGTTGGTGTA
GACCTTGAGGCAAGCTGTCACAAATCTTTTAGTGCTTTGGTCGAGGAATTCCTCTTCTATCCGCCGTTTTGGGGT
AAGAGXXXXXXXXXXTTGAATTTTTTTATTTTCTCAATTAAAGTTGGTATATTGTCTAAAAAGCTACAAATGTAG
TCATATTCTAGGTTGGTGTA
>Marker315017
CACAAAGATGGATGGATGCAATTTGAAATGCTCCCAACACCAATCCATTATTATGTATGTTCTCAAAGCTCTAGG
ATCCAXXXXXXXXXXATCCTAAGCTTTTCAGAAAGAATCAATTTAACTTAATTTGTTAGTAATTTGTAACTTTGT
TTGATTCTGTCCAATCAGGT
CACAAAGATGGATGGATGCAATTTGAAATGCTCCCAACACCAATCCATTATTATGTATGTTCTCAAAGCTCTAGG
ATCCAXXXXXXXXXXATCCTAAGCTTTTCAGAAAGAATCAATTTAACTTATTTTGTTAGTAATTTGTAACTTTGT
TTGATTCTGTCCAATCAGGT
>Marker315368
TACCCAAATCTTTCATGGTCAAAGTGATGGATTGCTGGGTAGCTCCCTCAGCTCGAAGGTCAGATAGTTTGTGAT
CCACAXXXXXXXXXXACTGACATAAGCTAACCAAACAAAATCAACAATTAAATTTCAAATATTCATTCAGTATAG
TCAATATTGAACAAATTAGT
TACCCAAATCTTTCATGGTCAAAGTGATGGATTGCTGGGTAGCTCCCTCAGCTCGAAGGTCAGATAGTTTGTGAT
CCACAXXXXXXXXXXACTAACATAAGCTAACCAAACAAAATCAACAATTAAATTTCAAATATTCATTCAGTATAG
TCAATATTGAACAAATTAGT
>Marker316167
AACATGATAACCTCACCAAGTTTTTCAAATTTATCTATTTCGTTGAACAAGCAGAATGAGAAATTTGAATTTTTG
ACCTCXXXXXXXXXXTTAATTTCATAATTTTAGACCATAGGAATTGAATTCCCCATCAAATTGGTTTCTTTTTTG
AAATTTTAAAGCTCCTAGTA
AACATGATAACCTCACCAAGTTTTTCAAATTTATTTATTTCGTTGAACAAGCGGAATGAGAAATTTGAATTTTTG
ACCTCXXXXXXXXXXTTAATTTCATAATTTTAGACCATAGGAATTGAATTCCCCATCAAATTGGTTTCTTTTTTG
AAATTTTAAAGCTCCTAGTA
>Marker316802

Create PDF with GO2PDF for free, if you wish to

TACTTTTGTTGGAAGGAGATCAATTATTAAGGAACGGTTCTCAAAGAAATATAGACATCCATTACTGGATTCCAA
GCTTAXXXXXXXXXXCTGGAACTTCATGTTAGGAAGCAAGGTGCATGACCAAAGCTAGGCGACTCGGAGTAGCCA
CACCAGTGTTGTATGCTGTT
TACTTTTGTTGGAAGGAGATCAATTATTAAGGAACGGTTCTCAAAGAAATATAGACATCCATTACTGGATTCCAA
GCTTAXXXXXXXXXXCTGGAACTTCATGTTAGGAAGCAAGGTGCATGACCAAAGCTAGGCGACTCGGAGTAGCTA
CACCAGTGTTGTATGCTGTT
>Marker317240
TACACCGCCGGATGCCTCACCTGCGACGGCAGCGAGAAGGAGAAGCTCTTTGAGCGGTGGTGATAAGAAAGCAAG
TTTGTXXXXXXXXXXGAAAACAAAGATTTCGAAACCAGAAACAAAACGCGCACTCTTCAACAAGATTTCTAACGA
TAAGACTATTAAATTCGGTG
TACACCGCCGGATGCCTCACCTGCGACGGCAGCGAGAAGGAGAAGCTCTTTGAGCGGTGGTGATAAGAAAGCAAG
TTTGTXXXXXXXXXXGAAAACAAAGATTTCGAAACCAGAAACAAAACGCGCACTCTTCAACAAGATTTCTGACGA
TAAGACTATTAAATTCGGTG
>Marker318357
CACCTTGTAGATTTTCAATGCCTACAAACAATTGATTACGTATTAAGATATTAGTGTGTTTTGATTTTCAATGCC
TACAAXXXXXXXXXXTGAACCAATTGCATTTCTAATAAAAAATAATCAAAAGTAGAAACTTGAAAGAAAATTGTT
TTGAGTTTTTTGAAAAATGT
CACCTTGTAGATTTTCAATGCCTACAAACAATTGATTACGTATTAAGATATTAGTGTGTTTTGATTTTGAATGCC
TACAAXXXXXXXXXXTGAACCAATTGCATTTCTAATAAAAATAATCAAAAAGTAGAAACTTGAAAGAAAATTGTT
TTGAGTTTTTTGAAAAATGT
>Marker318361
CACTCCAATTGATCAAATATATCAACTAAACAAATTATATAGAGATATTTGGTCCATCAACTTCATTAGTATTAA
ATAACXXXXXXXXXXAAAATCTAACCTATTAACTCAATTGGATTTAGATATTTTGATTGTAACCTATCAACTCGT
GACTTTAACTCAATTTAGTA
CACTCCAATTGATCAAATATATCAACTAAACAAATTATATAGAGATATTTGGTCCATCAACTTCATTAGTATTAA
ATAACXXXXXXXXXXAAAATCTAACCTATTAACTCAATTGGATTTAGATATTTTGATTGTAACCTATCAACTCGC
GACTTTAACTCAATTTAGTA
>Marker319000
CACAAATAAATGTGATTAACACTTTAGGATTCGTTATGAAATGTAATTGTATTGCAATGTTATAAAATAGAAAAC
TCAAAXXXXXXXXXXGTGATATATTTGTTGTTTCCACGGTAAAATATTGTTGTTCTTATCATGATTGCATTATAT
TATTTTTCTTCAAAACGGTC
CACAAATAAATGTGATTAACACTTTAGGATTCGTTACGAAATGTAATTGTATTGCAATGTTATAAAATAGAAAAC
TCAAAXXXXXXXXXXGTGATATATTTGTTGTTTCCACCGTAAAATATTGTTGTTCTTATCATGATTGCATTATAT
TATTTTTCTTCAAAACGGTC
>Marker319607
ACTGAAGTTATTGCCACAGTTAAGGTATGAAATGTTGAGCTTCTTTCTCTTTGAACTATGCTATTCACTACATTT
TTACAXXXXXXXXXXTCCAATGCAACCTGACAAAGGGAAGGTCTCGATCTCTGTTGATTGTAGTCCGACTGCAGA
GCCGGCATTTGAGGTGGGGT
ACTGAAGTTATTGCCACAGTTAAGGTATGAAATGTTGAGCTTCTTTCTCTTTGAACTATGCTTTTCACTACATTT
TTACAXXXXXXXXXXTCCAATGCAACCTGACAAAGGGAAGGTCTCGATCTCTGTTGATTGTAGTCCGACTGCAGA
GCCGGCATTTGAGGTGGGGT
>Marker319654
GACAGTGATAGAAAGAAGTATCAACAATACAGATAACTGTGACATTGAAATATTTAAAACTAGGTAGCAGCTGTC
AGTGCXXXXXXXXXXATATGCCAAGAACAACCAAAAGTTAAAAACTTTCATTTGATTATGAATTCAGCTTACGAG
AAATTGAGAAAGAGGAAGTA

Create PDF with GO2PDF for free, if you wish to

GACAGTGATAGAAAGAAGTATCAACAATACAGATAACTGTGACATTGAAATATTTAAAACTAGGTAGCAGCTGTC
AGTGCXXXXXXXXXXATATCCCAAGAACAACCAAAAGTTAAAAACTTTCATTTGATTATGAATTCAGCTTACGAG
AAATTGAGAAAGAGGAAGTA
>Marker320100
ACTAAATAATGCTTCTTAAAACATAAAAATCACCTGCCTTCTCCTTAAACATGAAGTTAGTAAAAGGTTAGAATT
AATACXXXXXXXXXXAATCTTTATTGAGGACTGTAGAAGTTTGGCTTATGAACAGGAGATTGAAGGAGTTTATTC
CCCCCTCCCCCCAAGATGTA
ACTAAATAATGCTTTTTAAAACATAAAAATCACCTGCCTTCTCCTTAAACATGAAGTTAGTAAAAGGTTAGAATT
AATACXXXXXXXXXXAATCTTTATTGAGGACTGTAGAAGTTTGGCTTATGAACAGGAGATTGAAGGAGTTTATTC
CCCCCTCCCCCCAAGATGTA
>Marker320187
CACTATGATATTTGGGGGCCTTGGGGCCTTTTCAAACACCCACACATGTTGGTTGTTCCTATTTTGTTACTTTGG
TTGATXXXXXXXXXXAGTTTTCAAAAGTTATTAAAACATTTAGGACTTATAATGCCCATGAGCTTGGTTTCAAAG
AATTTCTTGCTTCTATTGGT
CACTATGATATTTGGGGGCCTTGGGGCCTTTTCAAACACCCACACATGCTGGTTGTTCCTATTTTGTTACTTTGG
TTGATXXXXXXXXXXAGTTTTCAAAAGTTATTAAAACCTTTAGGACTTATAATGCCCATGAGCTTGGTTTCAAAG
AATTTCTTGCTTCTATTGGT
>Marker320278
CACTTAAAATCAACCCGGCCAACATGAAATTCACAACTTACCCAAGTTGAATGAAGCATAAACTTCCAATGAAGC
TTGATXXXXXXXXXXAATAAACTTACAACAAGACAGCGCAGCGCGGATAGATGGAATCGGTGTGTGCAAAATAGA
TATGAACGAAAATGGATGTG
CACTTAAAATCAACCCGGCCAACGTGAAATTCACAACTTACCCAAGTTGAATGAAGCATAAACTTCCAATGAAGC
TTGATXXXXXXXXXXAATAAACTTACAACAAGACAGCGCAGCGCGGATAGATGGAATCGGTGTGTGCAAAATAGA
TATGAACGAAAATGGATGTG
>Marker320417
GACTTAATTATTAACCCTACTAATACTACAATTTTAGTTAGGTTTCAAGTTTATAATTTATTTGGTGATTAGAAA
GTATTXXXXXXXXXXTCTCCGGCTATATTTAAAGCAATTAACTATAGAAATAACTCAAGCACACAAAAGTAATAA
ATAACACATAAGACTTTGTT
GACTTAATTATTAACCCTACTAATACTACAATTTTAGTTAGGTTTCAAGTTTATAATTTATTTGGTGATTAGAAA
GTATTXXXXXXXXXXTCTCCAGCTATATTTAAAGCAATTAACTATAGAAATAACTCAAGCACACAAAAGTAATAA
ATAACACATAAGACTTTGTT
>Marker320680
ACTTTTAATTTTTTAGTAAAATTACTTCTCAATTTATTTCGCAAATTTGGAACCTTTCTCACTTTTAATCACACT
TTAATXXXXXXXXXXTCCATTAAATAAACCTTTCATCTTTAATTTTAACTTTCGTATTAGAACTTGGATTGTATT
TCATTTGATTAAACAAAGTG
ACTTTTAATTTTTTAGTAAAATTACTTCTCAATTTATTTCTGAAATTTGGAACCTTTCTCACTTTTAATCACACT
TTAATXXXXXXXXXXTCCATTAAATAAACCTTTCATCTTTAATTTTAACTTTCGTATTAGAACTTGGATTGTATT
TCATTTGATTAAACAAAGTG
>Marker321070
ACCAATATTCGTTATGTTCTCCACAACCACGCTTGTGACCATGCACGTAAGAAGATGATGGATCGCTCCCTAACA
CATAGXXXXXXXXXXAGCATCATCATCATTAGACTAACGTAACCAGGCCTACTTTGTGTTTGTGTGATGGGAGGC
AATTATTTCACGTGTTGGTC
ACCAATATTCGTTATATTCTCCACAACCACGCTTGTGACCATGCACGTAAGAAGATGATGGATCGCTCCCTAAAA
CATAGXXXXXXXXXXAGCATCATCATCATTATACTAACGTAACCAGGCCTACTTTGTGTTTGTGTGATGGGAGGC
AATTATTTCACGTGTTGGTC
>Marker321528

Create PDF with GO2PDF for free, if you wish to

ACTCCTCGCCGGATAGCCATAGGTCCCAAGAGTGTAATGATTTGATACCTATTTGGTATAAACTACCAAAGTGAT
TTTTGXXXXXXXXXXACAGTAATTAACAATCAACCTTTACCTTTAAATATAGTATATAACAATACAGCTACTATA
GCTTTATTTACAGCTTTGTT
ACTCCTCGCCGGATAGCCATAGGTCCCAAGAGTGTAATGCTTTGATACCTATTTGGTATAAACTACCAAAGTGAT
TTTTGXXXXXXXXXXACAGTAATTAACAATCAACCTTTACCTTTAACTATAGTATATAACAATACAGCTACTATA
GCTTTATTTACAGCTTTGTT
>Marker321924
CACCTCATCCTTTTCCACAGCTGAAACCATCTTTGTCAATTTCCACATTCCCTCCAACTCCAACTCCTTGCCCAG
TGTCCXXXXXXXXXXTTTGGACTGATCCCATTTCCTAATTGTAACAGCTTTAATACCAAGATAACAGAATTTGAT
GCTACTAAGAACAAAAGGGT
CACCTCATCCTTTTCCACAGCTGAAACCATCTTTGTCAATTTCCACATTCCCTCCAACTCCAACTCCTTGCCCAG
TGTCCXXXXXXXXXXTTTGGACTGATCCCATTTCCTAATTGTAACAGCTTTAATACCAAGATAACAGAATTTGAT
GTTACTAAGAACAAAAGGGT
>Marker321979
AACAGTAATTTCTGTGGTAAGAAATTTGAATCACACACTTTTTCATCAATAATACCTACTCTACATATGTGGAAT
TTGAAXXXXXXXXXXTATTTACAAAGGTGGGATATGGATTATGGGGTAAATGCATATTCATATCATAAACAAAA
CAAAACTATGCATCATTGGT
AACAGTAATTTCTGTGGTAAGAAATTTGAATCACACACTTTTTCATCAATAATACCTACTCTACATATGTGGAAT
TTGAAXXXXXXXXXXTATTTACAAAGGTGGGATATGGATTATGAGGGTAAATGCATATTCATATCATAAACAAAA
CAAAACTATGCATCATTGGT
>Marker322036
TACTTTTGTCGAACAATATGATTGTTGATGTCAAATTTTGGTTAAAAGGATAAATTTACATGACTCTTACGTGAC
ATCAAXXXXXXXXXXGATTTAATATATGAGTTTTCAAATCTTAGATAAGATAGGTTATCTAACAAGCTGAGTTGG
GCCTTCTAATGGAGAAGGTG
TACTTTTGTCGAACAATATGATTGTTGATGTCAAATTTTGGTTAAAAAGATAAATTTACATGACTCTTACGTGAC
ACCAAXXXXXXXXXXGATTTAATATATGAGTTTTCAAATCTTAGATAAGATAGGTTATCTAACAAGCTGAGTTGG
GCCTTCTAATGGAGAAGGTG
>Marker322040
AACAATTACTTCTAATTTGAGAAACTTGTAAATAAAAACTTGTAATATTGATAAACAGAAAGTTATCAACTGATA
GCAACXXXXXXXXXXAGCTTTTAATTTGAGAAGTTTAAAAAGAAACGTTTAAAATGACTACACATGAGTTTTTTA
GTCTTTTATTAATTTTTGTA
AACAATTACTTCTAATTTGAGAAACTTGCAAATAAAAACTTGTAATATTGATAAACAGAAAGTTATCAACTGATA
GCAACXXXXXXXXXXAGCTTTTAATTTGAGAAGTTTAAAAAGAAACGTTTAAAATGACTACACATGAGTTTTTTA
GTCTTTTATTAATTTTTGTA
>Marker322318
ACCTATTTGTTTCAATCATTCAATTTCAGCACGAGCTTACTTGCCCCGTGAAGACGGATTCTTCTTTCTGGACCT
TAGGTXXXXXXXXXXAGAGGATGATATAATGCACATTACACTGCAGAAGAGGGACAAGGGTCAGACATGGGCTTC
CCCAATACAGGGTCAGGGTC
ACCAATTTGTTTCAATCATTCAATTTCAGCACGAGCTTACTTGCCCTGTGAAGACGGATTCTTCTTTCTGGACCT
TAGGTXXXXXXXXXXAGAGGATGATATAATGCACATTACACTGCAGAAGAGGGACAAGGGTCAGACATGGGCTTC
CCCAATACAGGGTCAGGGTC
>Marker322333
TACCTTGTGATAAGATCTTCAAACTTAGTTCCACTATCTTTTAGCCATTTGGAAAAGTTGCGTGATGCTGGACTT
TGATGXXXXXXXXXXATGTCTTTCGAGTGCGCGTCGATCTTTGATAGATAGTTTGTTCGCAGTGCATAGTTTCTT
ACTGCTTGAAGCGAGATGTT

Create PDF with GO2PDF for free, if you wish to

TACCTTGTGATAAGATCTTCAAACTTAGTTCCACTATCTTTTAGCCATTTGGAAAAGTTGCGTGATGCTGGACTT
TGATGXXXXXXXXXXXATGTCTTTTGAGTGCGCGTCGATCTTTGATAGATAGTTTGTTCGCAGTGCATAGTTTCTT
ACTGCTTGAAGCGAGATGTT
>Marker322422
AACCTAGTTCGATGAATTTGACCTATGTCTAGGGAGTTTCTCCGCTCAAATAAGAAATATTATTAAGATTCAAAC
GAGTAXXXXXXXXXXXCAACTCTTATAAATTCATCAACTGCAATTTAACTATTTCAGTATTCTTTCATATTTTTTA
TCAACAAGTGCAGCGTAAGT
AACCTAGTTCGATGAATTTGACCTATGTCTAGGGAGTTTCTCCGCTCAAATAAGAAATATTATTAAGATTCAAAC
GAGTAXXXXXXXXXXXCAACTCTTATAAATTCATCAATTGCAATTTAACTATTTCAGTCTTCTTTCATATTTTTTA
TCAACAAGTGCAGCGTAAGT
>Marker322442
AACCACTCTTTCCTTCCTCTTTCTAGTTATATGGATCGGCTCTGGCGACTTGTATCCAAGTCCTTTTCTCAACAC
AGGTAXXXXXXXXXXXTCATTCACCTTTGGGGCAAGTTTGCTTCCATCATATCTATCTTTACCTTCTATTTCGTTA
TCTTAGTTAGTAATGTAGTG
AACCACTTTTTCCTTCCTCTTTCTAGTTATATGGATCGGCTCTGGCGACTTGTATCCAAGTCCTTTTCTCAACAC
GGGTAXXXXXXXXXXXTCATTCACCTTTGGGGCAAGTTTGCTTCCATCATATCTATCTTTACCTTCCATTTCGTTA
TCTTAGTTAGTAATGTAGTG
>Marker322788
TACATACAATGCCATCATTCGAGGTATTTGCAAGGAAGGAATGGAGGATCGAGCCTTGGATTTTGTTCGACATTT
ATCAGXXXXXXXXXXXATTTTAATTAGTTCGTTTTGTCGCGAAGGGAGAGTAAGGGAAGCCGTGAATGTGTTGGAG
GTGATGAAGGAGAAAGGGTT
TACATACAATGCTATCATTCGAGGTATTTGCAAGGAAGGAATGGAGGATCGAGCCTTGGATTTTGTTCGACATTT
ATCAGXXXXXXXXXXXATTTTAATTAGTTCGTTTTGTCGCGAAGGGAGAGTAAGGGAAGCCGTGAATGTGTTGGAG
ATGATGAAGGAGAAAGGGTT
>Marker322919
ACATGTGAAAAAGAGAAGGAATCTTTAAATATTTTTGAATTAGAACATTGTAAGAATATTAAAGTTGTGTTCTCT
CCACAXXXXXXXXXXXTCAAGAGTATAGGGTTAGATGGTTAATTTATATCTACCAAGCTAATTGTTAAACATATTT
AAATAATACATGAAAGTGTG
ACATGTGAAAAAGAGAAGGAATCTTTAAATATTTTTGAATTAGAACATTGTAAGAATATTAAAGTTGTGTTCTCT
CCACAXXXXXXXXXXXACAAGAGTATAGGGTTAGATGGTTAATTTATATCTACCAAGCTAATTGTTAAACATATTT
AAATAATACATGAAAGTGTG
>Marker322944
AACCTGCAGTGCTAGAAGAGATCTCTCATCATGGATATGCCATGAGACATCTCCCCCTTTGGCCTTGAAGGCTTT
CATATXXXXXXXXXXXTCCAGGAGCAAGCCCAGCAACATCAGCAACAACAAGTTTCTCGAGGAAAGCGATAGAATC
CTTCCCCTTAAGGCTCAGTC
AACCTGCAGTGCTAGAAGAGATCTCTCATCGTGGATATGCCACGAGACATCTCCCCCTTTGGCCTTGAAGGCTTT
CATATXXXXXXXXXXXTCCAGGAGCAAGCCCAGCAACATCAGCAACAACAAGTTTCTCGAGGAAAGCGATAGAATC
CTTCCCCTTAAGGCTCAGTC
>Marker323131
TACAAAATGGTCATTATGTAAGAAAATTGTTCTAATTGTGTATGTAGATAATATTCTCTTGTCAGGAGATGATAC
GACTTXXXXXXXXXXXAAAATCAAGGGAAAGGATCTTTGTTACACAGTAGAAATACACCCTTGGCCTGTTAAAAGG
AACAAGTATGATTGGATGTA
TACAAAATGGTCATTATGTAAGAAAATTGTTCTAATTGTGTATGTAGATAATATTCTCTTGTCAGGTGATGATAC
GACTTXXXXXXXXXXXAAAATCAAGGGAAAGGATCTTTGTTACACAGCAGAAATACACCCTTGGCCTGTTAAAAGG
AACAAGTATGATTGGATGTA
>Marker323148

Create PDF with GO2PDF for free, if you wish to

TACATACCTTGTATTTGCACTAATGTGATAAGAATAACTATAACTATAGATAAGACCCACAAATATGTTATAATG
TGAAAXXXXXXXXXXXTAGTGAAAAATACCTTAACTATCTATTTTGTTGGTGCATCATCAATTGTCAAATTAAGAG
GTAGGCACATCACTCTTGTA
TACATACCTTGTATTTGCACTAATGTGATAAGAATAACTATAGCTATAGATAAGACCCACAAATATGTTATGATG
TGAAAXXXXXXXXXXXTAGTGAAAAATACCTTAACTATCTATTTTGTTGGTGCATCATCAATTGTCAAATTAAGAG
GTAGGCACATCGCTCTTGTA
>Marker323950
CACAATCAAACAAGATGATGGTGCATTGCCTCAAAACTTGAACAAAGCTGATATTGTTCTTGCTGGGGTATCTCG
AACAGXXXXXXXXXXXTGACTATAAATCCAATTGTGTTGCAAACAATAAGAAGAGCAAGAGCAAAGAGTTTGGGAT
TTAGTGAAGAAATGAGAAGT
CACAATCAAACAAGATGATGGTGCATTGCCTCAAAACTTGAACAAAGCTGATATTGTTCTTGCTGGGGTATCTCG
AACAGXXXXXXXXXXXTGACTATAAATCCAATTGTGTTGCAAACAATCAGAAGAGCAAGAGCAAAGAGTTTGGGAT
TTAGTGAAGAAATGAGAAGT
>Marker323973
AACCTAGAGAATCTGTAAAGATAGTTTAATGAGATGGTTGAGGCAGTAAAAGCATGAATGAATTAAGTTGCATTA
AATAAXXXXXXXXXXXAATCATTAAACGCCACACTGGATCTCCTTCTGGGATCCAAATGACTAAACCTAACTAAAC
AAGGTATATTTAGAAGTGTT
AACCTAGAGAATCTGTAAAGATAGTTTAATGAGATGGTTGAGGCAGTAAAAGCATGAATGAATTAAGTTGCATTA
AATAAXXXXXXXXXXXAATCATTAAACGCCACACTGGATCTCCTTCTGGGATCCAAATGACTAAATCTAACTAAAC
AAGGTATATTTAGAAGTGTT
>Marker324914
AACTCCCGTGATTAATTATTTAAAATGAAATCAAGCTTAATTCAACAAATTAAGAGAGGAATAGAAAGCCACGAA
ATTACXXXXXXXXXXXTTTTTTCCTTAGTCAAATTGATGAATGATGAGATTTTTTTTGGAGAATAATCAGTAAATG
ATCGAATTAACGGATCATGT
AACTCCGGTGATTAATTATTTAAAATGAAATCAAGCTTAATTCAACAAATTAAGAGAGGAATAGAAAGCCACGAA
ATTACXXXXXXXXXXXTTTTTTCCTTAGTCAAATTGATGAATGATGAGATTTTTTTTGGAGAATAATCGGTAAATG
ATGGAATTAACGGATCATGT
>Marker324932
CACAAAAGGGCCCTATAAAAGGGAATACAAAAGATGTTTCCAAATTGGAAATGAGAAAAAAGTAACTGTGGCTCT
TGATAXXXXXXXXXXXGAATAATCTTGCATTTTTATCGTTCCAACCCTTCCACAAAATCACTGAAACAATATTTTT
TCCCAAATACGTTCTTGGTT
CACAAAAGGGCCCTATAAAAGGGAATACAAAAGATGTTTCCAAATTGGAAATGAGAAAAAAGTAACTGTGGCTCT
TGATAXXXXXXXXXXXGAATAATCTTGCATTTTTATCGTTCCAACCCTTCCACAAAATCATTGAAACAATATTTTT
TCCCAAATACGTTCTTGGTT
>Marker324977
ACTGCAGTTAAAATATATATTAAAGGAAAAACACTGGAAGATGTGATGGGAAAGCACCTTTATACGCTCGTATTC
TCGCAXXXXXXXXXXXCTTGTATGCAATGAGGACAAGTCAGACAATAGAGTTTGCTCAGTCAGTAAAGCACTTGAG
CGTTCTGAGAATGCACCGTG
ACTGCAGTTAAAATATATATTAAAGGAAAAACACTGGTAGATGTGATGGGAAAGCACCTTTATACGCTCGTATTC
TCGCAXXXXXXXXXXXCTTGTATGCAATGAGGACAAGTCAGACAATAGAGTTTGCTCGGTCAGTAAAGCACTTGAG
CGTTCTGAGAATGCACCGTG
>Marker326141
AACATTTTCCCTTTTCAATTCTTTTCTTTTTATATTCTACTTTGTGTCATCAATGAATCCTACCTTCACATTTTA
AATTAXXXXXXXXXXXAACGATCTAATTTATAATCATCCATCTATAAGAAATATAACTAATATTGTGAGTCGGCAG
TGAAATTACAGGTTTGGGTT

Create PDF with GO2PDF for free, if you wish to

AACATTTTCCCTTTTCAATTCTTTTCTTTTTATATTCTACTTTGTGTCATGAATGAATCCTACCTTCACATTTTA
AATTAXXXXXXXXXXXAACGATCTAATTTATAATCATCCATCTATAAGAAATATAACTAATATTGTGAGTCGGCAG
TGAAATTACAGGTTTGGGTT
>Marker326633
TACTCTTGCAGTGTCTCCTCTCAAGCAAACCAATATCTTCCCTTTCACTTTTGAATGATCCAATGTTTTTGGTTT
GCAAAXXXXXXXXXXXXAGAGAGTGAAAGCTGAGACGAACCATATAACGGTGACCATTTTGGAGCTCAACAGGAGCC
TGAAACTCACGATCCATAGT
TACTCTTGCAGTGTCTCCTCTCAAGCAAACCAATATCTTCCCTTTCACTTTTGAATGATCCAATGTTTTTGGTTT
GCAAAXXXXXXXXXXXXAGAGAGTGAAAGCTGAGACGAACCATATAACGGTGACCATTTTGGAGCTCAACAGGAGCC
TGGAACTCACGATCCATAGT
>Marker326838
AACAATAACCAACCATTTTTTAAGCAAAATGCATAGTGATATAAAAATGATGAAATTTTGAAATAGTAGTGTCAC
CACGTXXXXXXXXXXXCAAAAAATAAAGTTGAATTCGGAAGTAATTTGTGTATAGGGAAGGTATTAGCACCCACAA
CACTCATTTAAAAAAACGGT
AACAATAACCAACCATTTTTTAAGCAAAATGCATAGTGATATAAAAATGATGAAACTTTGAAATAGTAGTGTCAC
CACATXXXXXXXXXXXCAAAAAATAAAGTTGAATTCGGAAGTAATTTGTGTATAGGGAAGGTATTAGCACCCACAA
CGCTCATTTAAAAAAACGGT
>Marker328363
TACAAGGGGTCCTCATTGCTTTTTGAGGGAATTTTGTCATCAATTTGCTAGATATAGGAAATAAGACTTGTTCAG
TTGTGXXXXXXXXXXXCATTGTATAATTCTCTTGTCCATTGAGCTCTTATTATTATTAATAAAGAAGTTTATCTCC
ATTTCAAAAAAATTGCATGT
TACAAGGGGTCCTCATTGCTTTTTGAGGGAATTTTGTCATCAATTTGCTAGATATAGGAAATAAGACTTGTTCAG
TTGTGXXXXXXXXXXXCATTGTATAATTCTCTTGTCCATTGAGCTCTTATTATTATTAATAAAGAAGTTTGTCTCC
ATTTCAAAAAAATTGCATGT
>Marker328495
CACCTTACCCAAGTTATTACTAGTATGGATGAAGATCTCCCAAATGGCTGGATGACTAATGAATTCAGAAAGGAC
TGTGGXXXXXXXXXXXACATACTCTATTTATAGTTGGTCGTAAGGCTGTCTTCCATAAAAAGGCTTACAAATATTT
TGAAGCTTTGACCAACAAGT
CACCTTACCCAAGTTATTACTAGTATGGATGAAGATCTCCCAAATGGCTGGATGACTAATGAATTCAGAAAGGAC
TGTGGXXXXXXXXXXXACATACTCTATTTATAGTTGGTCGTAAGGCTGTCTTTCATAAAAAGGCTTACAAATATTT
TGAAGCTTTGACCAACAAGT
>Marker328659
CACTGCGTTGGGAAATCCTTGTGTCTCCAAACAAAGACCTGCATGCTTTCCATAAGCAGCCCCTCCTTTGCCAAC
AACACXXXXXXXXXXXTGCTTCAGACCTGATTTTTCGTCACCACAGTCGAGAACATAATTGTGGTCGTATCCCATG
CCAACCTCATGAATAGAGGT
CACTGCGTTGGGAAATCCTTGTGTCTCCAAACAAAGACCTGCATGCTTTCCATAAGCAGCCCCTCCCTTGCCAAC
AACACXXXXXXXXXXXTGCTTCAGACCTGATTTTTCGTCTCCACAGTCGAGAACATAATTGTGGTCGTATCCCATG
CCAACCTCATGAATAGAGGT
>Marker329746
AACAAGTGTATTAGCAATGCATCAAGTGTGTCAAACTTATCAGTGAAGCATTTATGTGTATCAAGGCATAAGGGG
TATCAXXXXXXXXXXXATGTATCTATCAAATGTATAAAGTGTATCAAAAAGCATCAAATATATCAATGAGTATTAA
GTGTATAAATATTAAGTGTA
AACAAGTGTATTAGCAATGCATCAAGTGTGTCAAACTTATCAGCGAAGCATTTATGTGTATCAAGGCATAAGGGG
TATCAXXXXXXXXXXXATGTATCTATCAAATGTATAAAGTGTATCAAAAAGCATCAAATATATCAATGAGTATTAA
GTGTATCAGTATTAAGTGTA
>Marker329937

Create PDF with GO2PDF for free, if you wish to

AACCTCTTTAAGCTAGAGGTATAATCAATGCACATACGTCATCCGTTTTTAATTCGAGTCGGTGTCATTGCTCCT
TTCTCXXXXXXXXXXXAGAGTGATAAAGTGCATGCAAAGAGAAGCCTATATTTCTGTTTAATGTAGTCTAAGATTC
ATCTAATTGATGGTTTTGTT
AACCTCTTTAAGCTAGAGGTATAATCAATGCACATACGTCATCCGTTTTTAATTCGAGCCGGTGTCATTGCTCCT
TTCTCXXXXXXXXXXXAGAGTGATAAAGTGCATGCAAAGAGAAGCCTATATTTCTGTTTAATGTAGTCTAAGATTC
ATCTAATTGATGGTTTTGTT
>Marker330324
GACTAAGTAGAACAATTTTCGCACAACAGCTCGTCCGTGTTTGAAAGATTTCGATTGTGACATCGTGCGCCTTGT
AGCCCXXXXXXXXXXXCTTAGAAATGTCTTTATTATGTTACCTCAAATGTTCAATCCTTAATTCTAAGTTATGTGT
CCTGGAGCCTCTAGAAAGTG
GACTAAGTAGAACAATTTTCGCAAAACAGCTCGTCCGTGTTTGAAAGATTTCGATTGTGACATCGTGCGCCTTGT
AGCCCXXXXXXXXXXXCTTAGAAATGTCTTTATTATGTTACCTCAAATGTTCAACCCTTAATTCTAAGTTATGTGT
CCTGGAGCCTCTAGAAAGTG
>Marker330843
ACAAAGAGCAATTTCCTGTTTGCAACTAAAACTGCTAATATACACTAAACCAAACGCTTTTTGACAATCTCAAAT
GCCTAXXXXXXXXXXXTATGATGCGAAAATGGTTTTACCAATTAGTATAACCAAGAAATGGAAAAAGATCACAAGC
TATTGCGTTTGCTTGTTGGT
ACAAAGAGCAATTTCCTGTTTGCAACTAAAACTGCTAATAAACACTAAACCAAACGCTTTTTGACAATCTCAAAT
GCCTAXXXXXXXXXXXTATGATGCGAAAATGGTTTTACCAATTAGTATAACCAAGAAATGGAAAAAGATCACAAGC
TATTGCGTTTGCTTGTTGGT
>Marker331025
ACAGGATATTACAAGGGGACAATAACAAAACAAAAACAAAGGAACAGGAGATGCACCCGAGCATATGAACTAAGG
TGACAXXXXXXXXXXXTTTAGACCGGAAATATAACAGCATGCCAATTTACATTTAAATCTTGTATAGAAAAGTCTT
CAAAACTCTTAGATACGGTG
ACACGATATTACAAGGGGACAATAACAAAACAAAAACAAAGGAACAGGAGATGCACCCGAGCATATGAACTAAGG
TGACAXXXXXXXXXXXTTTAGACCGGAAATATAACAGCATGCCAATTTACATTTAAATCTTGTATAGAAAAGTCTT
CAAAACTCTTAGATACGGTG
>Marker332030
GACCTTTACTCGAACATTACAACCGCAATACTAGAAAATGAAAATGAAAATGGGAAATATTGAAGAAAATGAACG
TGGAAXXXXXXXXXXXTGATAACAATAATTAAAATAATTATTGACCTTAATAAATACCAAAATTTAGACTACCAAC
TTTTACAATTATGGTATAGT
GACCTTTACTCGAACATTACAACCGCAATACTAGAAAATGAAAATGAAAATGGGAAATATTGAAGAAAATGAACA
TGGAAXXXXXXXXXXXTGATAACAATAATTAAAATAATTATTGACCTTAATAAATACCAAAATTTAGACTACCAAC
TTTTACAATTATGGTATAGT
>Marker332198
CACTTCAGACTTGAAAGCAACAACATTTTTTAATGAACTACGGTTGTGGCAGGGGAAAAAAGATGTTAAAAAATA
TTCAGXXXXXXXXXXXGTAATTAACCGCCCGATCGGTTTAGAGGTCTTTGTAAGTAACTTGGTGTTCCCATTGTTA
TTATTGTTGAAATCAAGGTT
CACTTCAGACTTGAAAGCAACAACATTTTTTAATGAACTACGGTTGTGGCAGGGGAAAAAAGATGTTAAAAAATA
TTCAGXXXXXXXXXXXGTAATTAACCTCCCGATCGGTTTAGAGGTCTTTGTAAGTAACTTGGTGTTCCCATTGTTA
TTATTGTTGAAATCAAGGTT
>Marker332201
TACTACCCTTTCAAAAGGGACTAAGTGGTGGATAGGGAATGTATGGATGCTTTTTTAAAATTTTGTTCCTAAAGA
ACATTXXXXXXXXXXXTAAGCAGCGTGAAACATTCTCCAGAACTCTTCTTTCCCAAAAAGAACATGCGCCTTCAAT
AGATACTCATAGAATGAGTC

Create PDF with GO2PDF for free, if you wish to

TACTACCCTTTCAAAAGGGACTAAGTGGAGGATAGGGAATGTATGGATGCTTTTTTAAAATTTTGTTCCTAAAGA
ACATTXXXXXXXXXXXTAAGCAGCGTGAAACATTCTCCAGAACTCTTCTTTCCCAAAAAGAACATGCGCCTTCAAT
AGATACTCATAGAATGAGTC
>Marker332785
AACAGAATATGCATATCTCCCCTTCCAACTACGACAATCGCAATGAACTTTAATGGGCCTTTCACAACGTCCCCC
ACTCCXXXXXXXXXXXAAAATTTGTTGTTTCCTTCTTATTAGGAATTGGTGGATTTTCAAAATGTCACTAATTTAA
AATTATCAATTGGAAATGTA
AACAGAATATGCATATCTCCCCTTCCAACTACGACAATCGCAATGAACTTTAATGGGCCTTTCACAACGTCCCCC
ACTCCXXXXXXXXXXXATAATTTGTTGTTTCCTTCTTATTAGGAATTGGTGGATTTTCAAAATGTCACTAATTTAA
AATTATCAATTGGAAATGTA
>Marker332882
ACTAACATTTTAAAGAAAATGAGAATGAATAAATAAGAGGTCTTTAAATAAAATGTCTAAAATTTAAAATACTAA
AAAAAXXXXXXXXXXXAATATTAAACATAGGAAATAGTGAGTATATAAAATATATCTAGTAAGGGATAGACTACTA
GTCCCGCTAGGTGATCTGTT
ACTAACATTTTAAAGAAAATGAGAATGAATAAATAAGAGGTCTTTAAATAAAATGTCTAATATTTAAAATACTAA
AAAAAXXXXXXXXXXXAATATTAAACATAGGAAATAGTGAGTATATAAAATATATCTAGTAAGGGATAGACTACTA
GTCCCGCTAGGTGATCTGTT
>Marker333003
GACCAGTTGTAAATTAGTTTTATCGATCTCCTCTAAACCTAGGTTTAAGCCCTACTCTATACAGTGTAATGCCAC
ACACTXXXXXXXXXXXAAAATATATATGTGGGAAAAAAAAAATTTCCAATGCATGGTATGTTTCGTGGGAAGAATT
GCATTTTTTACCCACGTGTT
GACCAGTTGTAAATTAGTTTTATCGATCTCCTCTAAACCTAGGTTTAAGCCCTACTCTATACAGTGTAATGCCAC
ACACTXXXXXXXXXXXAAAATATATATGTGGGAAAAAAAAAGATTCCAATGCATGGTATGTTTCGTGGGAAGAATT
GCATTTTTTACCCACGTGTT
>Marker333213
TACTCCTCCAGCAATTTTTTGAGTAAGGGCAGGTAAAAAGGAGATGCAGCAGGTTCTCACTGTCCTTCATACATA
GTGGGXXXXXXXXXXXTAGGTCTTTCCTCTTTTTTGCATTAAGGAATCTACGAAAAAAGCCAGAATTTTCATCTCC
TACAGCAAGCCAATTTAGTT
TACTCCTCCAGCAATTTTTTGAGTAAGGGCAAGTAAAAAGGAGATGCAGCAGGTTCTCACTGTCCTTCATACATA
GTGGGXXXXXXXXXXXTAGGTCTTTCCTCTTTTTTGCATTAAGGAATCTATGAAAAAAGCCAAAATTTTCATCTCC
TACAGCAAGCCAATTTAGTT
>Marker334549
TACACATATATATGCCAAATGAGTAAAAAAACAGTTAAACACATTGAAAATTATAATAATAAAAATAATCCAAAT
GAAACXXXXXXXXXXXTATTCATGTATAATACATAGAATATTTATCAATAAAATATCCCCCACGTGGTATCAGAGC
TTTAAACCTACATCTATTGT
TACACATATATATGCCAAATGAGTAAAAAAACAGTTAAACACATTGAAAATTATAATAATAAAAATAATCCAAAT
GAAACXXXXXXXXXXXTATTCATGTATAATACATAGAATATTTATCAATAAAATATCCCCCACGTGGTATCAGAGC
TTTAAACCTAGATCTATTGT
>Marker335524
AACCTTCAGCTTCAGCGATAGAAATAGAGGAGAGCCGGAACCTGTCCCTCAAGAGTTCGGAAGCGGAGTCGTCTT
CTTCCXXXXXXXXXXXTTCTTTTTCTCTTTTTTGTCCTAATAAAAATTAGAATAAAATAAATAAACAAAAAATATT
AGTATAAAGCATGTAGAGTC
AACCTTCAGCTTCAGCGATAGAAATAGAGGAGAGCCGGAACCTGTCCCTCAAGAGTTCGGAAGCGGAGTCGTCTT
CTTCCXXXXXXXXXXXTTTTTTTTCTCTTTTTTGTCCTAATAAAAATTAGAATAAAATAAATAAACAAAAAATATT
AGTATAAAGCATGTAGAGTC
>Marker336156

Create PDF with GO2PDF for free, if you wish to

AACATTCATGCAGATATTGCAATTACTTGAATCAATTTTGATATTGCAATAACATGATCTAAACCTTGTCTTCGG
GATTCXXXXXXXXXXGAAGGTATGATGCTATTGCTATTGCTGGACCTCTAGCTGCCCAATCTCTTTCACAAGTTT
TAGATTATATGGGCCATAGT
AACATTCATGCAGATATTGCAATTACTTGAAGCAATTTTGATATTGCAATAACATGATCTAAACCTTGTCTTCGG
GATTCXXXXXXXXXXGAAGGTATGATGCTATTGCTATTGCTGGACCTCTAGCTGCCCAATCTCTTTCACAAGTTT
TAGATTATATGGGCCATAGT
>Marker336282
GACTCCATTGCACACATGGGGTCTGTTGTTGTCATCCTAGGTTTGGGTAGAGTAATTTGGGGTAGAGTGTGACAC
TTCTTXXXXXXXXXXTGGCATTCATGAAAGTAGTGTCTATTCAAGCTCAACTTGATTTTTCTCCGATCAATGTAT
ATCCATCGACTTGTTTGGTC
GACTCCATTGCACACATGGGGTCTGTTGTTGTCATCCTAGGTTTGGGTAGAGTAATTTGGGGTAGAGTGTGACAC
TTCTTXXXXXXXXXXTGGCATTCATGAAAGTAGTGTCTATTCAAGCTCAACTTGATTTTTCTTCGATCAATGTAT
ATCCATCGACTTGTTTGGTC
>Marker336478
ACTCCACTCTCCTTAATTACATCTTTTATAGTAATGCCACTGATACAAGCATTGCACATTAATATGCAGACAATA
ATGAGXXXXXXXXXXCAATTACCTGCATTATTTTGTAGTTGATTATAACGAAATTTGATAACATAGAAATGATGT
CATTATCAGAACTAATAGTG
ACTCCACTCTCCTTAATTACATCTTTTATAGTAATGCCACTGATACAAGCATTGCACGTTAATATGCAGACAATA
ATGAGXXXXXXXXXXCAATTACCTGCATTATTTTGTAGTTGATTATAACGAAATTTGACAACATAGAAATGATGT
CATTATCAGAACTAATAGTG
>Marker337374
TACAAGATCTGATATATTTCTGTTTAAAACGAATGTCATGGTTTTATGAAATGATTTATGAAAATGTATTACGAA
AATGAXXXXXXXXXXTTTTAAGTTTTAGTTTAGGTTGTATTGTATAATATGTATGAAACGCATGTTGATAATAAA
GTTTAAGTATTTGTTATGTT
TACAAGATCTGATATATTTCTGTTTAAAACGAATGTCATGGTTTTATGAAATGATTTATGAAAATGTATTACGAA
AAGGAXXXXXXXXXXTTTTAAGTTTTAGTTTAGGTTGTATTGTATAATATGTATGAAACGCATGTTGATAATAAA
GTTTAAGTATTTGTTATGTT
>Marker337742
GACGACGACAATAAAACCATATTTCATGTAGCAGCTGAAAATCGACAAGAAGATGTGTTTAGTCTTATACATGAG
ATCGGXXXXXXXXXXGTGGTTTAAGATAAGTTCTTTGATGCATACATTGCATGTTTTTTCAAACAATAATTGAGA
GCGTTATTTTGAAATCTTGT
GACGACGACAATAAAACCATATTTCATGTAGCAGCTGAAAATCGACAAGAAGATGTGTTTAGTCTTATACATGAG
ATCGGXXXXXXXXXXGTGGTTTAAGATAAGTTCTTTGATGCATACATTGCATGTTTTTTTAAACAATAATTGAGA
GCGTTATTTTGAAATGTTGT
>Marker337853
ACTTTTATTTATTTCCTCTTAGTGGTATAATCTACTAATCACAATCTTAATACATTTCAAAAAAATAAATAAATA
AAACAXXXXXXXXXXTCATATAAACCTTATCATATTTTTAATAAATGAACTCAAATCTCAAATTGTTAAATTCAA
TTATGATGAGTGGATGAGTT
ACTTTTATTTCTTTCCTCTTAGTGGTATAATCTACTAATCACAATCTTAATACATTTCAAAAAAATAAATAAATA
AAACAXXXXXXXXXXTCATATAAACCTTATCATATTTTTAATAAATGAACTCAAATCTCAAATTGTTAAATTCAA
TTATGATGAGTGGATGAGTT
>Marker337939
TACACTTATTGGAAGGAAAAGGTATGAATAAACCCCTAGTATTATTGGGGTTTTGTTTAGTATTTTGACATATTG
CTTTTXXXXXXXXXXAGTTTTGCTTGTAATTTTTATGGCGGAACACAGAAAGGAAGATGAGACATTCTCTAACAC
GAAGAATGCTTGCTTAAGTG

Create PDF with GO2PDF for free, if you wish to

TACACTTATTGGAAGGAAAAGGTATGAATAAACCCCTAGTATTATTGGGGTTTTGTTTAGTATTTTGACATATTG
CTTTTXXXXXXXXXXAGTTTTGCTTGTAATTTTTATGGCGGAACACAGAAAGGAAGATGAGACATTCTCTAACAC
AAAGAATGCTTGCTTAAGTG
>Marker337990
GACCTCGCTGCACTGTTTGTTACTGGGATACTATACCACACTTGTTTTATTAGCGGTTGCTGGTCTGTTGGAGGT
TGGAGXXXXXXXXXXGAAAATTGTGTATTTACACCCGTTAATATGTCCTGCCTTCAACAAAACTAGAAAGAAATG
ATATTTTTATGTGCTTTAGT
GACCTCGCTGCACTGTTTGTTACTGGGATACTATACCACACTTGTTTTATTAGCGGTTGCTGGTCTGTTGGAGGT
TGGAGXXXXXXXXXXGAAAATTGTGTATTTACACCCGTTAAAATGTCCTGCCTTCAACAAAACTAGAAAGAAATG
ATATTTTTATGTGCTTTAGT
>Marker338400
AACAAGGTTGAATTTAATTTATTAAAAACTCTAGCTTACGCTCTTGAAATACTTTCCCTCGTTTTGGATACCTCC
ACTCAXXXXXXXXXXTAGATTATTGCTTGAACGAAATCTACTACGTCTATACATAGTTACCAGGATAACAATTAT
TTTTTCAATCAATCATTGTT
AACAAGGTTGAATTTAATTTATTAAAAACTCTAGCTTACGCTCTTGAAATACTTTCCCTTGTTTTGGATACCTCC
ACTCAXXXXXXXXXXTAGATTATTGCTTGAACGAAATCTACTACGTCTATACATAGTTACCAGGATAACAATTAT
TTTTTCAATCAATCATTGTT
>Marker338605
AACGTCTTCGTCACACACCAATTTTAGTTTTTCCCGTTTTGAGTTTTTCCTTTTAAATGTGGTTTTACGATTTTG
CTTTTXXXXXXXXXXTATGAACAAATGAGTTTTTTCGATAAAAGTGTAAAGTTTAATAATGTTTTCTATTGAAAT
CGAGTTGTGTGAAGTTGGTG
AACGTCTTCGTCACACACCAATTTTAGTTTTTCCCGTTTTGAGTTTTTCCTTTTAAATGTGGTTTTACGATTTTG
CTTTTXXXXXXXXXXTATGAACAAATGAGTTTTTTCGATAAAAGTGTAAAGTTTAATAATGTTTTCTATTGAAAT
TGAGTTGTGTGAAGTTGGTG
>Marker338748
CACTCACAATATCACTAAGTATGAAGTCTGCATTATGGGACTTCAAGTAGAATGCGACGTGAGTATTAAAAAATT
GAAGGXXXXXXXXXXACCGGTTTATAGGGAGGACAATCGAATAGCAGATGCATTAGCCACCCTAACAATGATGTT
TGATCTTAATCTTGAATGTG
CACTCACAATATCACTAAGTATGAAGTCTGCATTATGGGACTTCAAGTAAAATGCGACGTGAGTATTAAAAAATT
GAAGGXXXXXXXXXXACCGGTTTATAGGGAGGACAATCGAATAGCAGATGCATTAGCCACCCTAACAATGATGTT
TGATCTTAATCTTGAATGTG
>Marker338812
AACTGCCAAAATTCCCACCATGGTAGATTTGGGGTAATAATAAGTGAGAAACGACAAACTCGATGCATGCTATGA
GATTTXXXXXXXXXXTGAAAATTCCCAATTCCTTACCATGGAGACTATTTGGAGGTTTATTAATAGGCAAAGAGT
ATTGTTCCCCATCGAGTGTG
AACTGCCAAAATTCCCACCATGGTAGATTTGGGGTAATAATAAGTGAGAAACGACAAACTCGATGCATGCTATGA
GATTTXXXXXXXXXXTGAAAATTCCCAATTCCTTACCATGGAGACTGTTTGGAGGTTTATTAATAGGCAAAGAGT
ATTGTTCCCCATCGAGTGTG
>Marker339038
AACTAATGTTGTGAGCTAATATTATTTTGATCAAATAGTCACTACTTACATCAAACAACATTACAAAAAAATTTT
CAACAXXXXXXXXXXTTCTACTTTGCATTAGGTTTGATTGAGTTAGGATAAATTTTATTTTGAATTTTAGGGTTC
ATAATTCTTTTCGTCATGTT
AACTAATGTTGTGAGCTAAGATTATTTTGATCAAATAGTCACTACTTACATCAAACAACATTACAAAAAAGTTTT
CAACAXXXXXXXXXXTTCTACTTTGCATTAGGTTGGATTGAGTTAGGATAAATTTTATTTTGAATTTTAGGGTTC
ATAATTCTTTTCGTCATGTT
>Marker339217

Create PDF with GO2PDF for free, if you wish to

AACCATTTCAAAGATCTTTACAATGGCAGTGGTAAGGAGTCCTTTCTCATAGAAAATCTGGAATAGAACAAAATT
GTAGAXXXXXXXXXXATAAGATTTTTAGAGTCATCTCAAGAAGAACATCATGGAAGTCTTTAAAGATTAGTAAAG
GCATCATCAAGAAAAATGTA
AACCATTTCAAAGATCTTTACAATGGCAGTGGTAAGGAGTCCTTTCTCATAGAAAATCTGGAATAGAACAAAATT
GTAGAXXXXXXXXXXATAAGATTTTTAGAGTCATCTCAAGAAGAACATCATGAAAGTCTTTAAAGATTAGTAAAG
ACATCATCAAGAAAAATGTA
>Marker339234
ACTTGATTGTTATTCTGTTTTACAGACTGTCTTAATGAATTTTGATTTGGAAGTTCTTTGTGTTATCTGGAAAAA
ATCATXXXXXXXXXXACAGATACCGACAACTTGCAAAATCAGGAAATGATGATTTGCGACAAGATGCTGTAAGAT
TTCAGTGATGAAATGATGGT
ACTTGATTGTTATTCTGTTTTACAGACTTTCTTAATGAATTTTGATTTGGAAGTTCTTTGTGTTATCTGGAAAAA
ATCATXXXXXXXXXXACAGATACCGACAACTTGCAAAATCAGGAAATGATGATTTGCGACAAGATGCTGTAAGAT
TTCAGTGATGAAATGATGGT
>Marker339297
AACCAATTCCATGCATTTTAGGAATTTGAGAAATATAATAATAATAATAATCATTGAACTTAAGAAATTCAAAGA
GAATAXXXXXXXXXXTGTAATTTCTTGAAAATGGTAGCTCACCAAAAATCTTTGCAACTCCTTCAGCTTTTCTTA
AGTAAATACAAGGTAAGAGT
AACCAATTCCATACATTTTAGGAATTTGAGAAATATAATAATAATAATAATCATTGAACTTAAGAAATTCAAAGA
GAATAXXXXXXXXXXTGTAATTTCTTGAAAATGGTAGCTCACCAAAAATCTTTGCAACTCCTTCAGCTTTTCTTA
AGTAAATACAAGGTAAGAGT
>Marker340443
TACCACACTTTTTTGCTGAGTTACGGCTGTGCGGAGTAATCTCCACACATACAGTAAATTTTTCATCAATTCCAT
TATGCXXXXXXXXXXAACAAGTATGAGACATCATATTTTAGAACACGCAACAAGAATAAGGCATCCTACAACATA
TCACAAAGCAAGTGTGAGGT
TACCACACTTTTTTGCTGAGTTACGGCTGTGCGGAGTAATCTCCACAGATACAGTAAATTTTTCATCAATTCCAT
TATGCXXXXXXXXXXAACAAGTATGAGACATCATATTTTAGAACACGCAACAAGAATAAGGCATCCTACAACATA
TCACAAAGCAAGTGTGAGGT
>Marker340733
ACTTTGATTAATATATCTCAATTTAGTCTGTTTGACTGTTTAAATTGAAAGCTTGTAAAATAACCTTCTTCATCA
TTGACXXXXXXXXXXTTATTGTTTTACTTGGATTTACTTTCTTAAACATCGTTCTAACTTATCTCAAACCTTAAA
TTGATTTTGCAAATATGGTT
ACTTTGATTAATATATCTCAATTTAGTCTGTTTGACTGTTTAAATTGAAAGCTTGTAAAATAACCTTCTTTATCA
TTGACXXXXXXXXXXTTATTGTTTTACTTGGATTTACTTTCTTAAACATCGTTCTAACTTATCTCAAACCTTAAA
TTGATTTTGCAAATATGGTT
>Marker340770
TACTAGCACATAAATTTTTTGATAGATTCATTCTTAATTTTAATTTTATAAATCTATAATTGGATCATTTGTTGA
AAAGAXXXXXXXXXXAGTTTTTCTTTTCAAATACCTTTTCTCTCTGTTTTCTTTTTCTTTTTCCCACAATTGATT
GTTCAAATAGAAATTTAGTT
TACTAGCACATAAATTTTTTGATAGATTCATTCTTAATTTTAATTTTATAAATCTATAATTGGATCATTTGTTGA
AAAGAXXXXXXXXXXAGTTTTTCTTTTCAAACACCTTTTCTCTCTGTTTTCTTTTGCTTTTTCCCACAATTGATT
GTTCAAATAGAAATTTAGTT
>Marker340833
ACCCCAAACTAATCAACAATCCAACCTAGTCTTCACAAATTTTTTGTATTTTCTGACTTTGTCAATAATCTTAGT
TCTTAXXXXXXXXXXGATTCTTAGTCGGCACCCAAACTTTCCTACAACTATATATTGAACTCAAATTCTCTACTT
CCCTTAACCAACTCTTCAGT

Create PDF with GO2PDF for free, if you wish to

ACCCCAAACTAATTAACAATCCAACCTAGTCTTCACAAATTTTTTGTATTTTCTGACTTTGTCAATAATCTTAGT
TCTTAXXXXXXXXXXXGATTCTTAGTCGGCACCCAAACTTTCCTACAACTATATATTGAACTCAAATTCTCTACTT
CCCTTAACCAACTCTTCAGT
>Marker341596
AACACATAGACAAATATATATGTTTAGGAAAACCCTATTTGGTCATTTATTGGATGTTAAGTTGGTGTTTATTAG
ACCCAXXXXXXXXXXXACAGGAGAAAACTCTTAGATTGAAACTGTTATAATTGGACAATAAGACAAATATGAATGA
GTTTGAGTTGGATAAAGAGT
AACACATAGACAAATATATATGTTTAGGAAAACCCTATTTGGTCATTTATTGGATGTTAAGTTGGTGTTTATTAG
ACCTAXXXXXXXXXXXACAGGAGAAAACTCTTAGATTGAGACTGTTATAATTGGACAATAAGACAAATATGAATGG
GTTTGAGTTGGATAAAGAGT
>Marker342411
CACCCTTTCTTTTTTTATTACTCCTAATAGGATGAGTTTGTAACCTAAATGTATGTTTGATTTTAGTGTTATTTT
TTACTXXXXXXXXXXXGCAAATGTGTATAATATAATATTACTATGGCATAGACTCAAATCAATTGAATTTTTCAGA
TCTCAATCATCGCCTATGTA
CACCATTTCTTTTTTTATTACTCCTAATAGGATGAGTTTGTAACCTAAATGTATGTTTGATTTTAGTGTTATTTT
TTACTXXXXXXXXXXXGCAAATGTGTATAATATAATATTACTATGGCATAGACTCAAATCAATTGAATTTTTCAGA
TCTCAATCATCGCCTATGTA
>Marker343313
CACAATAATGTAATTCATAATTCATGATTAGATAGAACGTTTTTGAGCTCGAATTGTAATTCTAAACTCATTATG
TTTCTXXXXXXXXXXXCTACTATAACAATTGATTCTTCAACCATTCAAATGTTTTAAGAAAATAAAATTAAAAGAG
AAAGAAAAGTTGGGTGTGTG
CACAATAATGTGATTCATAATTCATGATTAGATAGAGCGTTTTTGAGCTCGAATTGTAATTCTAAACTCATTATG
TTTCTXXXXXXXXXXXCTACTATAACAATTGATTCTTCAACCATTCAAATGTTTTAAGAAAATAAAATTAAAAGAG
AAAGAAAAGTTGGGTGTGTG
>Marker343315
CACAAACTTTTGGTTTCCTTAGCATTACAAGACTAGCTCCACCTTTCAGCTACTGGCACTTGTAAAAGACATGTC
ATCATXXXXXXXXXXXCAATTTTAACAAATTGGAATCTAAATGTAGAGCTAGGTTAAACTACCACACTTTCCTGAA
AAATTAAAAAATAGATCGTA
CACAAACTTTTGGTTTCCTTAGCATTACAAGACTGGCTCCACCTTTCAGCTACTGGCACTTGTAAAAGACATGTC
ATCATXXXXXXXXXXXCAATTTTAACAAATTGGAATCTAAATGTAGAGCTAGGTTAAACTACCACACTTTCCTGAA
AAATTAAAAAATAGATCGTA
>Marker343318
ACAAAGGAAGAATACCTTGAAAAAGCAAATAATATATAAGAAAATAAAAGACCAACAAGGACAATGCCAAGAAAC
CAAGAXXXXXXXXXXXAGGTCATCTAAGTAATTAGCTAAAGAGTTTGTTAGGGTTTTTAGTTATAAAAAGAGGGAG
TGGGTTGGGAGCAAGGGGTG
ACAAGGGAAGAATACCTTGAAAAAGCAAATAATATATAAGAAAATAAAAGACCAACAAGGACAATGCCAAGAAAC
CAAGAXXXXXXXXXXXAGGTCATCTAAGTAATTAGCTAAAGAGTTTGTTAGGGTTTTTAGTTATAAAAAGAGGGAG
TGGGTTGGGAGCAAGGGGTG
>Marker343885
TACTTCTATATTGTTCTGTATTTTGAGTTCAATTAATAAAGAAGTTTTTGTCTCCATTTTAAAAAACAAAAAAAC
AAAACXXXXXXXXXXXAAGAAATGGAAACTTAAACCATAAGTCAGATACTTAGTGAGAGATGTTTTGGAAAATCTT
CCTAGGAAAACAATAGAGTC
TACTTCTATATTGTTCTGTATTTTGAGTTCAATTAATAAAGAAGTTTTTGTCTCCATTTCAAAAAACAAAAAAAC
AAAACXXXXXXXXXXXAAGAAATGGAAACTTAAACCATAAGTCAGATACTTAGTGAGAGATGTTTTGGAAAATCTT
CCTAGGAAAACAATAGAGTC
>Marker344372

Create PDF with GO2PDF for free, if you wish to

CACGAGTTTTCAGACTTGACTCGAACTCCTTTCTTCTCAGAAACAATCTGTTCTTTTTTTCACAACCACACACCC
ACAAAXXXXXXXXXXXAGTAGAACGACCCATGCCACAAGCTCCATTCACGCGATTCTTGTGTATGTTGTTTGAGTC
GTTCGCTTGCTTCAAATGTT
CACGAGTTTTCAGACTTGACTCGAACTCCTTTCTTCTCAGAAACAATCTGTTCTTTTTTTCACAACCACACACCC
ACAAAXXXXXXXXXXXAGTAGAACGACCCATGCCACAAGCTCCATTCACACGATTCTTGTGTATGTTGTTTGAGTC
GTTCGCTTGCTTCAAATGTT
>Marker345442
CACAATTTATTCTGTCTGGCCACAGTAAGATTTCACTTTAATCTACCAATGATGGTTTGATGAAGTTTGTGCATT
GATGAXXXXXXXXXXXTTGCTAACTGGGGGATACTAAACTCTGGATGATACTACATGAATACTTATTTTTTATAC
ATTGACTTCACAAACGGAGT
CACAACTTATTCTGTCTGGCCACAGTAAGATTTCACTTTAATCTACCAATGATGGTTTGATGAAGTTTGTGCATT
GATGAXXXXXXXXXXXTTGCTAACTGGGGGATACTAAACTCTGGATGATACTACATGAATACTTATTTTTTATAT
ATTGACTTCACAAACGGAGT
>Marker345789
TACTAAAAAAACAAACACATCGACTTATGTGATATACTAATTAATATCCACACATTAGTCATTTGAATTCTCATT
ATCCGXXXXXXXXXXXGAAGAAGAATTTGGTGTCAAAATTTCAATTGACATTAGTAGGCAACTTCGTTGTGTTTTG
GACCTGATGGTGTCAAGGTC
TACTAAAAAAGCAAACACATCGACTTATGTGATATACTAATTAATATCCACACATTAGTTATTTGAATTCTCATT
ATCCGXXXXXXXXXXXGAAGAAGAATTTGGTGTCAAAATTTCAATTGACATTAGTAGGCAACTTCGTTGTGTTTTG
GACCTGATGGTGTCAAGGTC
>Marker345832
GACGTATAGTTGGATTTGAAAGATGTGAAAAAAAGAAGAGATTTGATGATATATGAAGATGAATAATATAAATGG
GGAAAXXXXXXXXXXXCTATAAACATATCTAATGGAACAAACACACAACACTCGACTCATAAAACCTCACTATTAT
CTTTGTTTTCATCATCTGTT
GACGTATAGTTGGATTTGAAAGATGTGAAAAAAAGAAGAGATTTGATGATATATGAAGATGAATAATATAAATGG
GGAAAXXXXXXXXXXXCTATAAACATATCTAATGGAAAAAACACACAACACTCGACTCATAAAACCTCACTATTAT
CTTTGTTTTCATCATCTGTT
>Marker345840
ACTTGAACACTGTAACCACACCAGCCAAAATTTAACTGACTACAAACTATTATGAATAAACGTGGTTTTGAAATG
GTTTAXXXXXXXXXXXCGGATAACATTCACTATTAAAGAAGGATTGTATTTGTATTTACAAATGTAGCATCAAAGA
GACTTAGAAATGGGACAGTT
ACTTGAACACTGTAACCACACCAGCCAAAATTTAACTGACTACAAACTATTATGAATAAACGTGGTTTTGAAATG
GTTTAXXXXXXXXXXXCGGATAACATTCACTATTAAAGAAGGATTGTATTTGTTTTTACATATGTAGCATCAAAGA
GACTTAGAAATGGGACAGTT
>Marker346373
ACCATAAATTTACTTTTACTCAATTTCTTTGTTTTGTTATATCAGTTTCTAGAGTCCTAATTACAAAAGCAAAAA
TGAAAXXXXXXXXXXXATATACTTCTAACATAATAACACAAATTTGTATATCAAATACAAATTTTTCAACTTACAA
ATATATTAACTTAATACGTA
ACCATAAATTTACTTTTGCTCAATTTCTTTGTTTTGTTATATCAGTTTCTAGAGTCCTAATTACAAAAGCAAAAA
TGAAAXXXXXXXXXXXATATACTTCTAACATAATAACACAAATTTGTATATCAAATACAAATTTTTCAACTTACAA
ATATATTAACTTAATACGTA
>Marker346744
ACAAGTGCTGAGTGTTTCGACAACTCCTACCAATGACAGTGACAATAACAAGCACAACATAAAACCAATTCCTGT
CTGTGXXXXXXXXXXXACCTTCTCAACCACCTGATCCACATGAAATACATACCAATCCCGGTCCCGCTACTTTAGG
TGTCATTGTCCAACCAGGTA

Create PDF with GO2PDF for free, if you wish to

ACAAGTGCTGAGTGTTTCGACAACTCCTACCAATGACAGTGACAATAACAAGCACAACATAAAACCAATTCCTGT
CTGTGXXXXXXXXXXACCTTCTCAACCACCTGATCCACATGAAATACATACCAATCCCAGTCCCGTTACTTTAGG
TGTCATTGTCCAACCAGGTA
>Marker346856
ACAATGGTCGAACGCTTATATTCTTTAATATATTACATGGCTATACTTTTTCAATCCATCATTTCTCTACCTGCC
ATCAAXXXXXXXXXXCTGAGAGAATAAGTAACAAGAACGTGGCTAATGCCCACAATTTTGCAAGATAACTTCCAT
CATTTAAATCCCGAAAGGTG
ACAATGGTCGAACGCTTATATTCTTTAATATATTACATGGCTATACTTTTTCAATCCATCATTTCTCTACCTTCC
ATCAAXXXXXXXXXXCCGAGAGAATAAGTAACAAGAACGTGGCTAATGCCCACAATTTTGCAAGATAACTTCCAT
CATTTATATCCCGAAAGGTG
>Marker347385
TACGGCGACAACTTGCATATTTCACGTAGCAGTTGAAAATCGACAAAAAAATGTATGCAGTATATATCAGATAGC
ATTTGXXXXXXXXXXCTTCTTTCTCCTTGCTTCCTATACTTATCTATGTTTTATAGTTAATGAAGCGGAAATGAC
GAGGATGCTAAGAGGATGTC
TACGGCGACAACTTGCATATTTCACGTAGCAGTTGAAAATCGACAAAAAAATGTATGCAGTATATATCAGATAGC
ATTTGXXXXXXXXXXATTCTTTCTCCTTGCTTCCTATACTTATCTATGTTTTATAGTTAATGAAGCGGAAATGAC
GAGGATGCTAAGAGGATGTC
>Marker347603
AACCTAATAATTATCATTGTGAATGGACTTGCTGCTTTTGAGAACCATCAAACACAAATATCTGAGAACAGCCAA
AAGTCXXXXXXXXXXGACACAAAACAGAAACTCAAGGACTGTGAGGTGCTAATCGGAGAGAGAATTTGCAGTAAT
AAATATGGATTAAAAGATGT
AACCTAATAATTATCATTGTGAATGGAATTGCTGCTTTTGAGAACCATCAAACACAAATATCTGAGAACAGCCAA
AAGTCXXXXXXXXXXGACACAAAACAGAAACTCAAGGACTGTGAGGTGCTAATCGGAGAGAGAATTTGCACTAAT
AAATATGGATTAAAAGATGT
>Marker347961
CACATATATTGAATTTAATTGTGTGTGATGGTCTGAAATATGTGAATGATACATTGATTCAAATTCGAAATGTCA
TTAGGXXXXXXXXXXCACATACTTTAAACTATCATAGTCATGAATCATCTTCAAGCTCGATTGCATCATCACAAC
TTCAAGTGTTTAAGGATTGT
CACATATATTGAATTTAATTGTGTGTGATGGTCTGAAATATGTGAATGATACATTGATTCAAATTCGAAATGTCG
TTAGGXXXXXXXXXXCACATACTTTAAACTATCATAGTCATGAATCATCTTCAAGCTCGATTGCATCATCACAAC
TTCAAGTGTTTAAGGATTGT
>Marker348286
ACATGTTTTGAATCTCACACGATGTGTTCCTTTGGGATCAAAAGTTCCAAACAGAATATGGTCAGGTAAGGATAT
ATCTTXXXXXXXXXXAACTAAGAAAAAGCTTATAAGAAGTCGAGATGTTATATTTGTTGAAGACCAAACAATAGC
AAACATTGAGAAAATAGGTG
ACATGTTTTGAATCTCACACCATGTGTTCCTTTGGGATCAAAAGTTCCAAACAGAATATGGTTAGGTAAGGATAT
ATCTTXXXXXXXXXXAACTAAGAAAAAGCTTATAAGAAGTCGAGATGTTGTATTTGTTGAAGACCAAACAATAGC
AAACATTGAGAAAATAGGTG
>Marker349000
GACCCTTCAAGTCTAGATTCCTACGAGGAAGATTCATAATCTTTCTAGGTTGAAGGGTTGAGTTTTCCCATAAGC
TTCTTXXXXXXXXXXCTTTGAGGTTTCAGACAAGTGATTTGACGTTTCAAACGAGGTTGAGTTTTCCAGTAAAAG
TGAAGAACACACACAAAGTG
GACCCTTCAAGTCTAGATTCCTACGAGGAAGATTCATAATCTTTCTAGGTTGAAGGGTTGAGTTTTCCCATAAGC
TTCTTXXXXXXXXXXCTTTAAGGTTTCAGACAAGTGATTTGACGTTTCAAACGAGGTTGAGTTTTCCAGTGAAAG
TGAAGAACACACACAAAGTG
>Marker349127

Create PDF with GO2PDF for free, if you wish to

CACCTCTTACCTCGACCTCCCATCAATGGGGTTACGTTGGACATATATATTATACTTCTTAGCTTTAGTGGATCG
TGCTTXXXXXXXXXXTTGAACCTCAAACTTAGTAAATGTTATGATCAATACAGACGTATGGATTTACAATTAAAA
AAGCAAATCATGAAGAGAGT
CACCTCTTACCTCGACCTCCCATCAATGGGGTTACGTTGGACATATATATTATACTTCTTAGCTTTAGTGGATCG
TGCTTXXXXXXXXXXTTGAACCTCAAACTTAGTAAATGTTACGATGAATACACACGTATGGATTTACAATTAAAA
AAGCAAATCATGAAGAGAGT
>Marker349789
AACAATTTTTAGACACCCACATTAGAGAGAGAAATAATCACCCATCTTACATAAAAACTTCCATTATCCAATAAA
ACAATXXXXXXXXXXACTCAGGAGTCGAGACACACTCGGGGACCGTTTTACTATTTCTATTATAGTTGTTGTTTC
AACTGTTTAGTTAGACAGTC
AACAATTTTTAGACACCCACATTAGAGATAGAAATAATCACCCATCTTACATAAAAACTTCGATTATCCAATAAA
ACAATXXXXXXXXXXACTCAGGAGTCGAGACACACTCGGGGACCGTTTTACTATTTCTATTATAGTTGTTGTTTC
AACTGTTTAGTTAGACAGTC
>Marker350326
ACTTCTCTTTTACTCCTTTAGGAACTGTAGCTTGGGTTTATTAAGTCCTGGGTTTTAAGCGGGTGCTTAGATTGT
GGAAAXXXXXXXXXXXATAATTGTCTCATGTAATTCATGAATTGAACTTTCAGGCGTTTATCTTTGTTCTTGTTCG
ACCCATTTCAAAGTGTTTGT
ACTTCTCTTTTACTCCTTTAGGAACTGTAGCTTGGGTTTATTAAGTCCGGGGTTTTAAGCGGGTGCTTAGATTGT
GGAAAXXXXXXXXXXXATAATTGTTTCATGTAATTCATGAATTGAACTTTCAGGCGTTTATCTTTGTTCTTGTTCG
ACCCATTTCAAAGTGTTTGT
>Marker350824
CACATCCAACCTCACTCTTTCCTCATAGCTAAGCAAGAAGAAGAAGAAGAAGAATTGGAGGAGGAGGAGGAAGAA
AGCCAXXXXXXXXXXCTCTCTTTCCTCACCTCGTCCTCGTGAGCCTCCTCCTCCTCCTCGCTCTCTCTCTCTCTA
TTTCTCTCTCATTGCCGGTT
CACATCCAACCTCACTCTTTCCTCATAGCTAAGCAAGAAGAAGAAGAAGAAGAATTGGAGGAGGAGGAGGAAGAA
AGCCAXXXXXXXXXXCTCTCTTTCCTCAGCTCGTCCTCGTGAGCCTCCTCCTCCTCCTCGCTCTCTCTCTCTCTA
TTTCTCTCTCATTGCCGGTT
>Marker351292
ACTCTTTAATCAAGCTTTTTCACAAGTGAAGTTAATCCTTTTAATTTTAGGATTTAAATTTCGTTTTATTGTAAA
TTGAAXXXXXXXXXXCGAATATTTATAGTTCTTATAAGGGGTATATTATAAAATATAATTCTATATGTTATTTGG
ATATTTTTTGTCATGTAGTC
ACTCTTTAATCAAGCTTTTTCATAAGTGAAGTTAATCCTTTTAATTTTAGGATTTAAATTTCGTTTTATTGTAAA
TTGAAXXXXXXXXXXCGAATATTTATAGTCCTTATAAGGGGCATATTATAAAATATAATTCTATATGTTATTTGG
ATATTTTTTGTCATGTAGTC
>Marker351762
ACTTGAGGTTAGTCTTTTATACTACCGCTCTATTACTTGCACCTTCATTGATGAGGAATAACCAACGGATAACCA
GTAATXXXXXXXXXXCTACATTTAAATTATGCTTCATTCCCACTTTGATTAACAACATCTAAATATTGTAGAGTA
TTTTGTCCATTTTGTTTGTT
ACTTGAGGTTAGTCTTTTATACTACCGCTCTATTACTTGCACCTTCATTGATGAGGAATAACCAATGGATAACCA
GTAATXXXXXXXXXXCTACATTTAAATTATGCTTCATTCCCACTTTGATTAACAACATCTAAATATTGTAGAGTA
TTTTGTCCATTTTGTTTGTT
>Marker351845
ACCCAACACATGGTTTGCAGTAAAATCACTTACAATCTTAGCCCAAAGAAATGAATCTACACATAAAACAAGAGT
ATAAAXXXXXXXXXXCTTTAACACTCTCCAAATCTTCAATCTAACGTCCAAAACATCATGGGTAACATCAAATCA
ATGTCCAATAATAATGGGTA

Create PDF with GO2PDF for free, if you wish to

ACCCAACACATGGTTTGCAGTAAAATCACTTACAATCTTAGCCCAAAGAAATGAATCTACACATAAAACAAGAGT
ATAAAXXXXXXXXXXXCTTTAACACTATTCAAATCTTCAATCTAACGTCCAAAACATCATGGGTAACATCAAATCA
ATGTCCAATAATAATGGGTA
>Marker352813
AACTGATTTGAACCAATCGAAAGCTTACCCCCAAATTTAAAAAACGCTGCTGCAATGGACCCAGCCTCAAGCGGA
GAAGAXXXXXXXXXXXGTGAGGTCGCCGGCGAAGAGATCTGAGCTGACGGTAAGTGATCGGCGGGTGGAAGTGGAG
GGGAGGAGATGATGAAGGGT
AACTGATTTGAACCAATCGAAAGCTTACCCCCAAATTTAAAAAACGCTGCTGCAATGGACCCAGTCTCAAGCGGA
GAAGAXXXXXXXXXXXGTGAGGTCGCCGGCGAAGAGATCTGAGCTGACGGTAAGTGATCGGCGGGTGGAAGTGGAG
GGGAGGAGATGATGAAGGGT
>Marker352948
GACCTATGATAGACATGGATAGATTGCTATTTGTGTCTATTTTATATATATCGAGATTGAAATCTATTTGTGTCT
GTCTCXXXXXXXXXXXTGGAAATCAACAATTCATTGTGAAGTTAAATTTAGGATCTTGTAACTGTCGAGTGTGGAA
TTTTGAGAAAATTCTATGTG
GACCTATGATAGACATAGATAGATTGCTATTTGTGTCTATTTTATATATATCGAGATTGAAATCTATTTGTGTCT
GTCTCXXXXXXXXXXXTGGAAATCAACAATTTATTGTGAAGTTAAATTTAGGATCTTGTAACTGTCGAGTGTGGAA
TTTTGAGAAAATTCTATGTG
>Marker353204
AACTTGAATGAGCTTAACAAGTTCAACATCTCATACAATCCTCTCATAACTGGGGAAGTAATTCCAAGTGGGCAA
TTCTCXXXXXXXXXXXCAGGATCATCAAAAAGGAATTCAAGGCTAGTTGGAATGTTGGCTTCTTTATCATTGATCC
TTGCTTTTTTTGGTATTTGGT
AACTTGAATGAGCTTAACAAGTTCAACATCTCATACAATCCTCTCATAACTGGGGAAGTAATTCCAAGTGGGCAA
TTCTCXXXXXXXXXXXCAGGATCATCAAAAAGGAATTCAAGGCTAGTTGGAATGTTGGCTTCTTTATCCTTGATCC
TTGCTTTTTTTGGTATTTGGT
>Marker354216
CACCAACAAAGAGCAAATGAACTATTTTCTCCCATTTATCATCGGCCGATCTCTTTCTTGGCGTAATTATAAGAT
TCCCTXXXXXXXXXXXTTTGCCCAAGAACAAAAAAGAAAGAAAAGGTGAAAAGGAAAATATAATAACGATAATATT
TACCCCTTCTTTTTCATTGT
CACCAACAAAGAGCAAATGAACTATTTTCTCCCATTTATCATCGGCCTATCTCTTTCTTGGCGTAATTATAAGAT
TCCCTXXXXXXXXXXXTTTGCCCAAGAACAAAAAAGAAAGAGAAGGTGAAAAGGAAAATATACTAACGATAATATT
TACCCCTTCTTTTTCATTGT
>Marker354508
ACTCCTAGACTACTAGCTAAGGAGAGTTCAAATGACCCATGAAGGAAGACCTCTGAGCCATTGAATGTGAAATAA
TTTGGXXXXXXXXXXXTTCAAGAGGCACAATTATAATATAAGCATCGGATGCACATCACCATCTTATATAAATAAC
CAAGGCCACTGGTCGAAAGT
ACTCCTAGACTACTAGCTAAGGAGAGTTCAAATGACCCATGAAGGAAGACCTTTGAACCATTGAATGTGAAATAA
TTTGGXXXXXXXXXXXTTCAAGAGGCACAATTATAATATAAGCATCGGATGCACACCACCATCTTATATAAATAAC
CAAGGCCACTGGTCGAAAGT
>Marker355094
AACGGAACAAAGTAATCCAATAGGTTAATGGTTTTTTCCTGAAAAAGCAAGGCACCACAATCATGTCAGCAGAAG
AAATAXXXXXXXXXXXGGATACAAATTGATTTAATAAGAAGAAATAGATGGATGTAGATCAGAACAGTTAATGGCA
CGATCGAAAACTAAAAGGGT
AACGGAACAAAGTAATCCAATAGGTTAATGGTTTTTTCCTGAAAAAGCAAGGCACCACAATCATGTCAGCAGAAG
AAACAXXXXXXXXXXXGGATACAAATTGATCTTATAAGAAGAAATAGATGGATGTAGATCAGAACAGTTAATGGCA
CGATCGAAAACTAAAAGGGT
>Marker355366

Create PDF with GO2PDF for free, if you wish to

TACCTTTCCGGTAGAACTCCATACTTCTCCCATACATAAAAGAACTCACGATGTGATGAATTAGCTGCCGGAATA
TCCCCXXXXXXXXXXGTCCTCATATCGGCCTCATGATACCTACATTACCAAATAATTTCAAACAGCCCCCCAACT
TAAGATAATATATTTTTGTA
TACCTTTCCGGTAGAACTCCATACTTCTCCCATACATAAAAGAACTCACGATGTGATGAATTAGCTGCTGGAATA
TCCCCXXXXXXXXXXGTCCTCATATCGGCCTCATGATACCTACATTACCAAATAATTTCAAACAGCCCCCCAACT
TAAGATAATATATTTTTGTA
>Marker355607
ACCTCCATTCTCAACTTCTTCCACAGGAGACGGATTTGGGGGATTCGACGGATTCGACGGATTCAATGAAATGCT
TCGTTXXXXXXXXXXAGCTCCAACACCACTGCATACCCCTGATACGAATCAAAACAAGAAAACTTTTAATCAATC
TTCAAAGAGGCAAACATGTG
ACCTCCATTCTCAACTTCTTCCACAGAAGACGGATTTGGGGGATTCGACGGATTCGACGGATTCAATGAAATGCT
TCGTTXXXXXXXXXXAGCTCCAACACCACTGCATACCCCTGATACGAATCAAAACAAGAAAACTTTTAATCAATC
TTCAAAGAGGCAAACGTGTG
>Marker356416
AACATCACATAATTTTACAACTAAAACAAACAAACACTTCTCCTCCTCCACTTTTTAATTCCAAAAGTATCCATT
AAAAAXXXXXXXXXXXTTATGCTAGATGGGCTAGGATGTGATCGAGAATGTGTAGTTTCTTACAAATATCATTGAT
TATTTGAACATGCTTAGGTG
AACATCACATAATTTTACAACTAAAACAAACAAACACTTCTCCTCCTCCACTTCTTAATTCCAAAAGTATCCATT
AAAAAXXXXXXXXXXXTTATGCTAGATGGGCTAGGATGTGATCGAGAATGTGTAGTTTCTTACAAATATCATTGAT
TATTTGAACATGCTTAGGTG
>Marker357085
CACAGGCAATAATTTGTGGAAGGGTTGGGCAAGCCCCTGTGCAGAAGATCTTGAGAAATGGGCGGACGATAACAA
TCTTTXXXXXXXXXXGTTCAAAAACTTTGTAGAAAGTATGATTTGAACCTCTCATAATAACTGATTTGACTGTAT
AATTTCACTGACTCTCTGTG
CACAGGCAATAATTTGTGGAAGGGTTGGGCAAGCCCCTGTGCAGAAGATCTTGAGAAATGGGCGGACGATAACAA
TCTTTXXXXXXXXXXGTTCAAAAACTTTGTAGAAAGTATGATTTGAACCTCTCATAATAACTGATTTGACTGTAT
AATTTCACTGAATCTCTGTG
>Marker357159
AACCAAACGGTGAGTTATCTTTTTTACCCACTCAATGGAACTCTTCTAAATTAACAAGTTGTCGATACTCCTAAA
TCATAXXXXXXXXXXAAAAAAAAAAAAAGATCCCAAGTAAAGTAAAGTAAGGGAAATTTGGGATGTGATGTGCAT
GCTAGTGATAGTGAATGGGT
AACCAAAGGGTGAGTTATCTTTTTTACCCACTCAATGGAACTCTTCTAAATTAACAAGTTGTCGATACTCCTAAA
TCATAXXXXXXXXXXAAAAAAAAAAAAAGATCCCAAGTAAAGTAAAGTAAGGGAAATTTGGGATGTGATGTGCAT
GCTAGTGATAGTGAATGGGT
>Marker357839
TACAAATTTGTAGAGTTTTAAACCAAGTATTGTGCTCGTTTTAATTTTTTGGTTTATTTTACCGTCGTAAAGGTT
AAACAXXXXXXXXXXTAAGTTTGAAAGAAAAATAATTAAATTTGTTTTGAATTTAGATTTATTTAACCCAAGTGT
CTTAATTATATTTATAGGTT
TACAAATTCGTAGAGTTTTAAACCAAGTATTGTGCTCGTTTTAATTTTTTGGTTTATTTTACCGTCATAAAGGTT
AAACAXXXXXXXXXXTAAGTTTGAAAGAAAAATAATTAAATTTGTTTTGAATTTAGATTTATTTAACCCAAGTGT
CTTAATTATATTTATAGGTT
>Marker358029
GACCTAGAGCCTGACTCACCGCTGCCTATTTGCTTGAGAGTTTTAGTTTCCATTCCAGTATGTCTTATATATCTA
AAACAXXXXXXXXXXCGTGGTAGAACTTAGTTGTTCATGGTATGGGCCACACTACATTTATATTAAGTTTGGTTT
CATCTTCAAGCATGAGCGTG

Create PDF with GO2PDF for free, if you wish to

GACCTAGAGCCTAACTCACCGCTGCCTATTTGCTTGAGAGTTTTAGTTTCCATTCCAGTATGTCTTATATATCTA
AAACAXXXXXXXXXXXCGTGGTAGAACTTAGTTGTTCATGGTATGGGCCACACTACATTTATATTAAGTTTGGTTT
CATCTTCAAGCATGAGCGTG
>Marker358824
CACCTCCAAATCCACCACCTGCTCCTCCGCCAGCTCCACCACCTAAAATGCCACCACCACCGCCGCCGCCAAACC
CTCCGXXXXXXXXXXXGAAAGGCAATGTCTTTTCACTCTCAATCTCTCCCCCATTTGTAATTACAAGTTTTCTGGG
CTCTGAGGCCCAAATTAGTG
CACCTCCAAATCCACCACCTGCTCCTCCGCCAGCTCCGCCACCTAAAATGCCACCACCACCGCCGCCGCCAAACC
CTCCGXXXXXXXXXXXGAAAGGCAATGTCTTTTCACTCTCAATCTCTCCCCCATTTGTAATTACAAGTTTTCTGGG
CTCTGAGGCCCAAATTAGTG
>Marker359007
ACCTCTTGTTTCAAAAAAGCAGAGAACCCAGAAGATGAAATTATGGAAGTTGAATCAGTTATCAGTGTGAGTAGT
GCAGAXXXXXXXXXXXAAGACAAAAGACTCCTTCCCCCTCAAGAGTTAATCCCATTCTTGGCAGCCTTTGAAATTA
AATTGATCTAAGGCAGGGGT
ACCTCTTGTTTCAAAAAAGCAGAGAACCCAGAAGATGAAATTATGGAAGTTGAATCAGTTATCAGTGTGAGTAGT
GCAGAXXXXXXXXXXXAAGACAAAAGACTCCTTCCCCCTCAAGAGTTAATCCCATTCTTGGCAGCCTTTTAAATTA
AATTGATCTAAGGCAGGGGT
>Marker359019
AACCATTATCTCCAATTTTATAACTTTAAAAAGTGACTCTTAAGTTGTGAGAGTGGTGCAGTAATGATGTGAAAT
AAAAAXXXXXXXXXXXXTGAATTTACACAATTTGAACAAATTCAAACTTTGTTTATACCTAAGGATAATATATATTA
AATTGTTCATTCTTGAAAGT
AACCATTATCTCCAATTTTATAACTTTAAAAAGTGACTCTTAAGTTGTGAGAGTGGTGCAGTAATGATGTGAAAT
AAAAAXXXXXXXXXXXXTGAATTTACACAATTTGAACAAATTCAAACTTCGTTTATACCAAAGGATAATATATATTA
AATTGTTCATTCTTGAAAGT
>Marker359692
ACTGTTACAGATGCCCCACCTTTTGACCCTACGAATATTGTGTCACTGCAGGCAAGATTGCTTTTCGTTTAGTTT
CTTCTXXXXXXXXXXXCCAACTTTTGTTGAAGGTCCATTTCAACTTCCATTATCAAGTATAAGGGCTTATATAAAA
TATCCTCTAACTCCCAGGTT
ACTGTTACAGATGCCCCACCTTTTGATCCTACGAATATTGTGTCACTGCAGGCAAGATTGCTTTTCTTTTAGTTT
CTTCTXXXXXXXXXXXCCAACTTTTGTTGAAGGTCCATTTCAACTTCCACTATCAAGTATAAGGGCTTATATAAAA
TATCCTCTAACTCCCAGGTT
>Marker359713
CACTGCGCTGTTCTTTTTTGCTCTGGTTCAGTTATTTTTTTGCTACATTCCCATATCGTTTATTTATTCGTGTGT
TTCTTXXXXXXXXXXXCTTATTTGGAATTTAAAAGAATGCCCTGGAGGAATTTAACCCTCAACCTTTTTCACTAGG
AAATGGTAAAGTTTAGTAGT
CACTTCGCTGTTCTTTTTTGCTCTGGTTCAGTTATTTTTTTGCTACATTCCCATATCGTTTATTTATTTGTGTGT
TTCTTXXXXXXXXXXXCTTATTTGGAATTTAAAAGAATGCCCTGGAGGAAATTAACCCTCAACCTTTTTCACTAGG
AAATGGTAAAGTTTAGTAGT
>Marker359924
AACTTTAATTTCTCTCGATATTGGAAAGATTTTGAACAACAAATGCTTTAATCCAAGAACAAGACTGTTGCTCCA
TCTTCXXXXXXXXXXXTCAAACCGAAATATGGGAATAATATTTGGACGATGGTGTTGGTAATCAACTCTTTCCCAT
TAAAGAGCTCCATCGAAGTA
AACTTTAATTTCTCTCGATATTGGAAAGATTTTGAATAACAAATGCTTTAATCCAAGAACAAGACTGTTGCTCCA
TCTTCXXXXXXXXXXXTCAAACCGAAATATGGGAATAATATTTGGACGATGGTGTTGGTAATCAACTCTTTCTCAT
TAAAGAGCTCCATCGAAGTA
>Marker359941

Create PDF with GO2PDF for free, if you wish to

AACCGACAATGTCACTAAGGATGGTGTTTGGAGTTGGTCGATGAATTTTCTAGATTGAATTAGAAAAAAAAATTA
ATCCAXXXXXXXXXXGGGCATCACTATGATCGCTTGTTAAGAGGCATGAGATTGATAATTAACGATAACGATTGG
TTGTTGTCGATCAAGATGTT
AACCGACAATGTCACTAAGGATGGTGTTTGGAGTTGGTTGATGAATTTTCTAGATTGAATTAGAAAAAAAAAATA
ATCCAXXXXXXXXXXGGGCATCACTATGATCGCTTGTTAAGAGGCATGGGATTGATAATTAACGATAACGATTGG
TTGTTGTCGATCAAGATGTT
>Marker360007
AACAACTCTGGTGACCCATTAACATTTAAATGTCAAAAAAACCTGTAAAATACTAAATCCTATATAGATTGCCCC
TATAGXXXXXXXXXXAGGAATCAAATGAGTTTGTTTAGAAAATCTACTACCAATCTACCTCCTGTTTCTAAGATC
TTTCCTTCATGTTGAATGTG
AACAACTCTGGTGACCCATTAACATTTAAATGTCAAAAAAACCTGTAAAATACTAAATCCTATACAGATTGCCTC
TATAGXXXXXXXXXXAGGAATCAAATGAGTTTGTTTAGAAAATCTACTACCAATCTACCTCCTGTTTCTAAGATC
TTTCCTTCATGTTGAATGTG
>Marker360132
TACTTGAAATGTATATACATTTTCAAGGTTGTTCTATTTTTGATAGAATATTATCAATTTTCTTATTTAAAACAA
TTAAAXXXXXXXXXXCAATTCACAAGTATTCTAACAAAGTCTTTAGTTACATCATGTTTTTTAAGATACAAGAAC
ATATTTTTTTAAGAAAAGTT
TACTTGAAATGTATATACATTTTCAAGGTTGTTCTATTTTTGATAGAATATTATCAATTTTCTTATTTAAAACAG
TTAAAXXXXXXXXXXCAATTCATAAGTATTCTAACAAAGTCTTTAGTTACATCATGTTTTTTAAGATACAAGAAC
ATATTTTTTTAAGAAAAGTT
>Marker360566
CACCTAGTTGAGATGTTTGATGCGCTCACTGATTCCTCCACCACCAATTCCCATCTCAAAAAATACAGAAATATG
ACTAAXXXXXXXXXXATCTTAACTAGATTGACACCTCTATAGCTCCATCATCCGTGCCAAATTCAAAAGCCATGT
CATTAATTGATGGAAAGGTA
CACCTAGTTGAGATGTTTGATGCGCTCACTGATTCCTCCACCACCAATTCCCATCTCAAAAAATACAGAAATATG
ACTAAXXXXXXXXXXATCTTAACTAGATTGACACCTCTATAGCTCCCTCATCCGTGCCAAATTCAAAAGCCATGT
CATTAATTGATGGAAAGGTA
>Marker360845
TACTAAAAATGTTTCTTTATACATTGGTCTATGAACAAGCATATGAATGCTAAGTTTATGAAATTATTTAAGTTA
AATGTXXXXXXXXXXGATTATTTGAGCATGTTTTAGTATATACGTGAGAGATGTCCAGAGTTTTGTGGCAACTCT
ATAGGGGAGTTTTTCTTGTG
TACTAAAAAGGTTTCTTTATACATTGGTCTATGAACAAGCATATGAATGCTAAGTTTATGAAATTATTTAAGTTA
AATGTXXXXXXXXXXGATTATTTGAGCATGTTTTAGTATATACGTGAGAGATGTCCAGAGTTTTGTGGCAACTCT
ATAGGGGAGTTTTTCTTGTG
>Marker360930
ACTAATATGAAGAAGATGTATAAGCATTCAATGTGAAATTCGAAACCAACCAAAAAATGACAATCAAATATGTGT
CATTTXXXXXXXXXXAATGCTAGCTAACAGTAAATATAGAGACTTTAAAACAGAAAATGGTGATGAATTCAAAGA
GATGAACTTCGATGGTTGTG
ACTAATATGAAGAAGATGTATAAGCATTCAATGTGAAATTCGAAACCAACCAAAAAATGACAAGCAAATATGTGT
CATTTXXXXXXXXXXAATGCTAGCTAACAGTAAATATAGAGACTTTAAAACAGAAAATGGTGATGAATTCAAAGA
GATGAACTTCGATGGTTGTG
>Marker361157
TACCATCAATATCTTGATATGAAATTCATTTACTTGTTTCTTGTTATCTATTTCAGTTCAGGCTTCTATTGCGGC
AAACAXXXXXXXXXXAATTTTTTCTTGCAAATTTTGGTCATACGTTTTAAAAAGAACAAATAGTCCCTAAATGAA
AATGACACTACAGAGATGTC

Create PDF with GO2PDF for free, if you wish to

TACCATCAATATCTTGATATGAAATTCATTTAGTTGTTTCTTGTTATCTATTTCAGTTCAGGCTTCTATTGCGGC
AAACAXXXXXXXXXXXAATTTTTTCTTGCAAATTTTGGTCATACGTTTTAAAAAGAACAAATAGTCCCTAAATGAA
AATGACACTACAGAGATGTC
>Marker361920
ACTCGTAGTTTCTTTAAATATAATTGCAGCATCTGAATGCAGATATTTTGTTTTCGTGCATTACTAACTAGGTGT
ATATAXXXXXXXXXXXCTCGGAGTTATATCATCACAGCCCTTGAAGAATCAATACAGCGAGTCAATTCAGCTATTC
ATCTTTTACTGATGGAGCGT
ACTCGTAGTTTCTTTAAATATAATTGCAGCATCTGAATGCAGATATTTTGTTTTCGTGCATTACTAACTAGGTGT
ATATAXXXXXXXXXXXCTCGGAGTTATATCATCACAGCCCTTGAAGAATCAATACAACGAGTCAATTCAGCTATTC
ATCTTTTACTGATGGAGCGT
>Marker362183
ACTCTAGAGTTAGGCAATGGACCGTTGGTTTATCAGGAGGTGGAGACCAAATAGCTGTGATCAGAGCTGGAGGAA
GCATTXXXXXXXXXXXTTTAAATATCGTTGAATGGTGTATTGGCACGGTGCCGTCTTTTATTTATCTCATTACGAT
CATCGAGCTCATACCTTGTG
ACTCTAGAGTTAGGCAATGGACCGTTGGTTTATCAGGAGGTGGAGACCAAATAGCTGTGATCAGAGCTGGAGGAA
GCATTXXXXXXXXXXXTTTAAATATCGTTGAATGGTGTATTGGCACGGTGCCATCTTTTATTTATCTCATTACGAT
CATCGAGCTCATACCTTGTG
>Marker363661
AACGCACCCCATGGATTGAAATCCACTATCTTAACACTTTCATTTCTCGTCACATACACATCCAACGTGTAATTC
TCCAAXXXXXXXXXXXAACCTCACGCTGAGAAATGCCAATCAGGTTCCGATTCTTAACAAAGCAACGAAATTCCAT
CTCCGGCCGAAGGGATGGGT
AACGCACCCCATGGATTGAAATCCACTATCTTAACACTTTCATTTCTTGTCACATACACATCCAACGTGTAATTC
TCCAAXXXXXXXXXXXAACCTCACGCTGAGAAATGCCAATCAGGTTCCGATTCTTAACAAAGCAACGAAATTCCAT
CTCCGGCCGAAGGGATGGGT
>Marker363991
TACATCTAATTTAAAATTTTCACCAGAAATATAAATATAAATCGATATCTTAGTAAAGAAAAAGATTTGAAATTG
GAAGAXXXXXXXXXXXACTCTCTTCTTCCAAAATGCCATATTCTACTTTTATTTTGTTTTCTACTTCAATTCCTAC
ACTCAACTAAACCATTTGTT
TACATCTAATTTAAAATTTTCACCAGAAATATAAATATAAATCCATATCTTAGTAAAGAAAAAGATTTGAAATTG
GAAGAXXXXXXXXXXXACTCTCTTCTTCCAAAATGCCATATTCTACTTTTATTTTGTTTTCTACTTCAATTCCTAC
ACTCAACTAAACCATTTGTT
>Marker364209
CACATTTTTATTTCATTTTGTCTCAGCCGTTGGATTGCAAAGCACCATTCCCAAGTAGAGATAAACATTACATCA
GAAAAXXXXXXXXXXXGAGGCTACCGATCACTCATTGGATAGGTAATAACACAACCTGTATAATTTTGGTTTGCTT
CTTATATCTTCAAGTAAGTA
CACATTTTTATTTCATTCTGTCTCAGCCGTTGGATTGCAAAGCACCATTCCCAAGTAGAGATAAACATTACATCA
GAAAAXXXXXXXXXXXGAGGCTACCGATCACTCATTGGATAGGTAATAACACAACCTGTATAATTTTGGTTTGCTT
CTTATATCTTCAAGTAAGTA
>Marker365332
TACATGATTCTAATTCTTTTGTTAATTGCTTGGTCATGTCCTCATCGTTCAGATTGTTTGCTGGGCGGTCTCTGA
AGATTXXXXXXXXXXXTCAAATTTTTCCTCTTAGTCCGAGTTCTAGGGTGAATAATTTTTTATTTATGTTTTGAAG
ATGCCTGTGAAAATGATTGT
TACATGATTCTAATTCTTTTGTTAATTGCTTGGTCATGTCCTCATCGTTCAGATTGTTTGCTGGGCGGTCTCTGA
AGATTXXXXXXXXXXXTCAAATTTTTCCTCTTAGTTCGAGTTCTAGGGTGAATAATTTTTTATTTATGTTTTGAAG
ATGCCTGTGAAAATGATTGT
>Marker365520

Create PDF with GO2PDF for free, if you wish to

CACATAGCACTCACAAAAGGTAGGAAAAATATGCTCCAGGCTTCCAGCTTCCTAACATAAGTGAAGGACCCCTGC
AGGGCXXXXXXXXXXTCCTTAGAGAAAAGCAATATGCACGCATCTATGATTGACTAAGAAATTCAACTTCAGACA
GCAACTTAAATGACAATGTA
CACATAGCACTCACAAAAGGTAGGAAAAATATGCTCCAGGCTTCCAGCTTCCTAACATAAGTGAAGGACCCCTGC
AGGGCXXXXXXXXXXTCCTTAGAGAAAAGCAATATGCACGCATCTATGATTGACTAAGAAATCCAACTTCAGACA
GCAACTTAAATGACAATGTA
>Marker365870
CACATCCATCTAAAGAAAAGAGGAGTTTCAATGCTATTTGGAAAAAGATTTTTTTCTTTTGATGGTATGTCAAAG
CCACTXXXXXXXXXXTATAAGAGCATGTCACATCTTTGAAGAATAATCAACACATGGTCTAGCTCCTCCCACGAA
CAAACCATAGTAAAGGTGTG
CACATCCATCTAAAGAAAAGAGGAGTTTCAATGCTATTTGGAAAAAGATTTTTTTCTCTTGATGGTATGTCAAAG
CCACTXXXXXXXXXXTATAAGAGCATGTCACATCTTTGAAGAATAACCAACACATGGTCTAGCTCCTCCCACGAA
CAAACCATAGTAAAGGTGTG
>Marker366028
CACGACGACTTCTGTAAATTGAGATCTATTTAAAATTGGCTACTGCTTTGATTCAATTTTGCAAAATACAGACAA
TAACTXXXXXXXXXXAGCCAACGTATAAGGACCCTATGGCTTCAAAAATTAGTTCATCAAAAGAATGACTTGAAT
GCTATACTTAGATGTTGGTT
CACGACGACTTCTGTAAATTGAGATCTATTTAAAATTGGCTACTGCTTTGATTCAATTATGCAAAATACAGACAA
TAACTXXXXXXXXXXAGCCAACGTATAAGGACCCTATGGCTTCAAAAATTAGTTCATCAAAAGAATGACTTGAAT
GCTATACTTAGATGTTGGTT
>Marker366572
ACCATGGTTGGCTTGGGAAATTAACAGCGCAGGCTGTGCTTTCATGCAATGATGAACTAATTCCATCTGAACTAA
CCTCTXXXXXXXXXXAATCATATTTTACCTTCACATGGTGCATTATGTGTGAATCTAGTGCTAAATATTTAGGTT
CTTTATTTGACCGTCAGGTT
ACCATGGTTGGCTTGGGAAATTAACAGCGCAGGCTGTGCTTTCATGCAATGATGAACTAATTCCATCTGAACTAA
CCTCTXXXXXXXXXXAATCATATTTTACCCTCACATGGTGCATTATGTGTGAATCTAGTGCTAAATATTTAGGTT
CTTTATTTGACCGTCAGGTT
>Marker367053
TACACCATCGACAGGGATATTGACCCTCAAAACTCCCTTTGTCAATGTCTCAAAAGAGGCATCGATACATGACAG
GATCCXXXXXXXXXXTTGCTGGCAGCAAGGAAAAGGGGAAAAAGAAGAAAGAAATATGGAAAATATCATTAATAT
GAAATCACAAAAGAATTGTA
TACACCATCGACAGGGATATTGACCCTCAAAACTCCCTTTGTCAATGTCTCAAAAGAGGCATCGATACATGACAG
GATCCXXXXXXXXXXTTGCTGGCAGCAAGGAAAAGGGGAAAAAGAAGAAAGAAATATGGAAAATATCATTAATAT
GAAATCACAGAAGAATTGTA
>Marker367544
CACCACCTCGTAAGAATCAAATTAAACAACAGTGACATGGGAGAAGGAAGAAACAATAGAATTGAGATGTTAAGA
CCCTCXXXXXXXXXXCCCACTCCACCCACCATGACACTTTTCAAGGTTTTGCAAAGCATCAATATTCACTTTCCA
TTCATTCATATAAAGGGAGT
CACCACCTCGTAAGAATCAAATTAAACAACAATGACATGGGAGAAGGAAGAAACAATAGAATTGAGATGTTAAGA
CCCTCXXXXXXXXXXCCCACTTCACCCACCATGACACTTTTCAAGGTTTTGCAAAGCATCAATATTCACTTTCCA
TTCATTCATATAAAGGGAGT
>Marker367708
AACAAGAAAAAAAATAATCTCCACTCTCTCCATTTCCCGTTTGGAATGGGTTCCATATGGGTTAAATAAATATCT
CTATTXXXXXXXXXXAAAGGATAATCCAAAAATCTAACAATTTTTCTTGGATTGTGTCGAATTGATTGAAGTTTT
AGAATTTGATATTAGAGGTT

Create PDF with GO2PDF for free, if you wish to

AACAAGAAAAAAAATAATCTCCACTCTCTCCATTTCCCGTTTGGAATGGGTTCCATATGGGTTAAATAAATATCT
CTATTXXXXXXXXXXXAAAGGATAATCCAAAAATCTAACAATTTTTCTTGGATTGTGTCGAATTGATTGAAGTTTT
AGAATTTGATATTAGAAGTT
>Marker367764
AACTGGAAGAGAAATTAGGATCCTACTGTTCTATTTGTAGTGTTTTTCTTTCTTTGTCAATTGTTTTTGAGTTTG
AACTCXXXXXXXXXXXTCAGCAGCAGTGATCGTTGGAGGACAAATTGGACTCCGGCAATGGAGCAATATTTTATTG
ATCTTATGTTGAATCAAGTG
AACTGGAAGAGAAATTAGGATCCTACTGTTCTATTTGTAGTGTTTTTCTTTCTTTGCCAATTGTTTTTGAGTTTG
AACTCXXXXXXXXXXXTCAGCAGCAGTGATCGTTGGAGGACAAATTGGACTCCGGCAATGGAGCAATATTTTATTG
ATCTTATGTTGAATCAAGTG
>Marker368497
TACTAAACATGCTTTATGTGGGTTCAAATGAAAATAATGTTTTACTAGCTATTAAACATGATTTCTGAGTCATAA
TAATCXXXXXXXXXXXTTTAGCAACTCTAACTTTGAAGACCGTTACTATGCTTGCTTCTAAAACAGGCGTGCCTAA
ATATCTGAAACCATCAAGTG
TACTAAACATGCTTTATGTGGGTTCAAATGAAAATAATGTTTTACTAGCTATTAAACACGATTTCTGAGTCATAA
TAATCXXXXXXXXXXXTTTAGCAACTCTAACTTTGAAGACCGTTACTATGCTTGCTTCTAAAACAGGCGTGCCTAA
ATATCTGAAACCATCATGTG
>Marker369382
ACTTCAGACATAACTCCATCAATTCGGTTCTTCAAAGGGTCAATCTAAAATGCAACACTCTTTGTAGGATTTATA
ACAGCXXXXXXXXXXXATGCACTTACCCGGAGTTGTAAGGGAACAATACTAGTTGGTCATACTCGGCTTCGAGCAA
TCAAGCATTCAACAACTGTG
ACTTCAGACATAACTCCATCAATTCGGTTCTTCAAAGGGTCAATCTAAAATGCAACACTCTTTGTAGGATTTATA
ACGGCXXXXXXXXXXXATGCACTTACTCGGAGTTGTAAGGGAACAATACTAGTTGGTCATACTCGGCTCCGAGCAA
TCAAGCATTCAACAACTGTG
>Marker369636
CACACGTTATTGTAATTCTTATTGGCTCAACATCGAGCTGTAATGCGTTAAATAAAAAACAAATTATAAATAAAA
TCTTAXXXXXXXXXXXATATTTAAAAAGGTAGGTTGTAGATTCTGTTGTCTTAAATATCTTATTTAGTTCAAATCA
ATGGCTGATTTTGAAATGGT
CACACGTTATTGTAATTCTTATTGGCTCAACATTGAGCTGTAATGCGTTAAATAAAAAACAAATTATAAATAAAA
TCTTAXXXXXXXXXXXTATATTTAAAAGGTAGGTTGTAGATTCTGTTGTCTTAAATATCTTATTTAGTTCAAATTA
ATGGCTGATTTTGAAATGGT
>Marker369850
AACAGGATGATGTTAATGAACAACAGTTATATTAAATTTGTGTTGTTAATGTTTAATGAAAACAGTTTGCTGAAT
TTTGAXXXXXXXXXXXGAGAAATTAAAATAGAAAGAAGATATTACAGAAGTCTAACCAATTGAGAGAGGAAACTTC
ATGCAGGTGGTGTTAGTGGT
AACAGGATGATGTTAATGAACAACAGTTATATTCAATTTGTGTTGTTAATGTTTAATGAAAACAGTTTGCTGAAT
TTTGAXXXXXXXXXXXGATAAATTAAAATAGAAAGAAGATATTACAGAAGTCTAACCAATTGAGAGAGGAAACTTC
ATGCAGGTGGTGTTAGTGGT
>Marker369853
TACTCTATGGCCTTACTCCTCACGGGTGACCTTTTCCTCTTTTCCCACCATGTAGTGGGATTTTGATCTCAATGA
GTCTCXXXXXXXXXXXACAACTCGCAACCCAAATTAATATGCTTAAAACTAAAGTCATGTTAGATGGTTTGATAGA
TCAATAAGGGTTGGAGAGGT
TACTCTATGGCCTTACTCCTCACCGGTGACCTTTTCCTCTTTTCCCACCATGTAGTGGGATTTTGATCTCAATGA
GTCTCXXXXXXXXXXXACAACTCGCAACCCAAATTAATATGCTTAAAACTAAAGTCATGTTAGATGGTTTGATAGA
TTAATAAGGGTTGGAGAGGT
>Marker370245

Create PDF with GO2PDF for free, if you wish to

TACGTTGTATCTCTTTATATCAACCATGAATTGGTGCAACAAATATTTCTTTCTAATCTGTCCATGATATAACAA
CTCAGXXXXXXXXXXCATAAACGAGCTATTTTGGAAAAGTAAAAGAAAATGATATGTTAGAACTTTTTTGGTGTG
TATTGAGAGAATGGTAAGTG
TACGTTGTATCTCTTTATATCAACCATGAATTGGTGCAACAAATATTTCTTTCTAATCTGTCCATGATATAACAA
CTCAGXXXXXXXXXXCATAAACGTGCTATTTTGGAAAAGTAAAAGAAAATGATATGTTAGAACTTTTTTGGTGTG
TATTGAGAGAATGGTAAGTG
>Marker370370
ACCATTGCTAGAATAACCTTGAGCAAAATCACTGCTATAGGTTCCAATGTCATTCCATAATTTCTTGAGCTTTTT
GAAGTXXXXXXXXXXGAGCCCATTAAATCATCTATTGAGTTTCCTTTGAGGATGTCGTCAAGGTCTTTACCTACA
CACATGTTATTTTTTTCGTC
ACCATTGCTAGAATAACCTTGAGCAAAATCACTGCTATAGGTTCCAATATCATTCCATAATTTCTTGAGCTTTTT
GAAGTXXXXXXXXXXGAGCCCATTAAATCATCTATTGAGTTTCCTTTGAGGATGTCGTCAAGGTCTTTACCTACA
CACATGTTATTTTTTTCGTC
>Marker372328
GACTTGAAATAAGATAAAGGTCCAACAAGTAGAAATCAATATTTTGTTTTTGAAACGAGGACAAAAGTAGAAATC
AATATXXXXXXXXXXTTGACCCAAAAAGCTTAAGTTAGTGGGTAACTATTGAAAGTAGAGACAAAAAACACACAA
ATTCACGTGAAAACCTGAGT
GACTTGAAATAAGATAAAGGTCCAACAAGTAGAAATCAATATTTTGTTTTTGAAACGAGGACAAAAGTAGAAATC
AATATXXXXXXXXXXTTGACCCAAAAAGCTTAAGTTAGTGGGTAACTATTGAAAGTAGAGACAAAAAACACACAA
ATTCACGTGAAAACCCGAGT
>Marker373811
ACTTTAATTCATACCTTTTGTTAGTATGGTTATGGAAGGAAATACCAGAGTTTCCAGCACTCCAATTGCAAATAT
TATGGXXXXXXXXXXTTTTCATAAAACCCCCAGTTGTATTTTTTCCTATAGCCGAGTATGTGCTTGCCCAGATCG
TCATCTTTCGACTTACAGTG
ACTTCAATTCATACCTTTTGTTGGTATGGTTATGGAAGGAAATACCAGAGTTTCCAGCACTCCAATTGCAAATAT
TATGGXXXXXXXXXXTTTTCATAAAACCCCCAGTTGTATTTTTTCCTATAGTCGAGTATGTGCTTGCCCAGATCG
TCATCTTTCGACTTACAGTG
>Marker374325
TACAAAATGGTTGAAAATGTAAGGGCCAAGGGTGGTGAGAATATGATCATTAGAGAACCTTTTGAGCTGAAAGGC
AGCCCXXXXXXXXXXAACGCTCTATTAACTTCTACCAAAAGGTAAAGTCACAAATTTTCAAAAAGTTTCGAACAA
TTTAGCCCTCAAACTTTAGT
TACAAAATGGTTGAAAATGTAAGGGCCAAGGGTGGTGAGAATATGATCATTAGAGAACCTTTTGAGCTGAAAGGC
AGCCCXXXXXXXXXXAACGCTCTATTGACTTCTACCAAAAGGTAAAATCACAAATTTTCAAAAAGTTTCGAACAA
TTTAGCCCTCAAACTTTGGT
>Marker375483
CACAAAGCGCTACTTCCTTTTCCCTTTCATCTGCATTTAGCAGAGCATACGCTGTTTTTGGAGAGTAACCTTCTT
CTTTCXXXXXXXXXXAACCGCGTTGTCCGATTTTCTTCCTTAATTTTTGAACTTCTTCCCATTTCTCTGCCTCCG
CATAAATGTTTGCCAAGAGT
CACAAAGCGCTACTTCCTTTTCCCTTTCATCTGCATTTAGCAGAGCATACGCTGTTTTTGGAGAGTAACCTTCTT
CTTTCXXXXXXXXXXAACCGCGTTGTCCGATTTTCTTCCTTAGTTTTTGAACTTCTTCCCATTTCTCTGCCTCCG
CATAAATGTTTGCCAAGAGT
>Marker375691
TACAACGTTGGACAACGAAAAGAAATCAACTTTGAAAATTTTAGATTGGTTAAAGAACATTGTTTGAAGAATCAA
TTCAAXXXXXXXXXXGAAAATAAAAAGTAATTACAAAGTAGATCGAACTCATCTTTTGAAGTCTAGCATAGCATA
CAAATCTCTCGAGAAGTGTG

Create PDF with GO2PDF for free, if you wish to

TACAATGTTGGACAACGAAAAGAAATCAACTTTGAAAGTTTTAGATTGGTTAAAGAACATTGTTTGAAGAATCAA
TTCAAXXXXXXXXXXXGAAAATAAAAAGTAATTACAAAGTAGATCGAACTCATCTTTTGAAGTCTAGCATAGCATA
CAAATCTCTCGAGAAGTGTG
>Marker375837
AACAAGCATAGTTGTCAGCTCCTATACAAAGCAGACGGTATTTATGAGTGGCATATAGATTTTCATTTTCCTATC
GAGATXXXXXXXXXXXTTTTCATCATGATTCATCTGTTGCTGCAATTGCTTTAACCACACAGCTATGCTATTTGAA
ATTTTTTCTAGAAGAATGTC
AACAAGCATAGTTGTCAGCTCCTATACAAAGCAGACGGTATTTATGAGTGGCATATAGATTTTCATTTTCCTATC
GAGATXXXXXXXXXXXTTTTCATCATGATTCATCTGTTGCTGCAGTTGCTTTAACCACACAGCTATGCTATTTGAA
ATTTTTTCTAGAAGAATGTC
>Marker376067
TACTCTTTTCCACACTAACTAGGGTAGCTAGGTTAATGTTTATATGCTTTTATATATTTAATTTGTTAATATATT
ACGGTXXXXXXXXXXXACTTTTTAAATTAATGATGAATCCTTAATAATATTTTTCGTTAATTATCAATCTTAATTG
CGTAGTTATGTCTTGATGTA
TACTCTTTTCCACACTAACTAGGGTAGCTAGGTTAATGTTTATATGCTTTTATATATTTAATTTGTTAATATATT
ACGGTXXXXXXXXXXXACTTTTTAAATTAATGATGAATCCTTGATAATATTTTTCGTTAATTATCAATCTTAATTG
CGTAGTTATGTCTTGATGTA
>Marker376344
AACCCTTGGCTTTGCAAATTTCTTTGGGTTTTATAGATCCAGCATCTTCTGTTTTCTTTTCTGTTGTTTGAGATT
TGTAGXXXXXXXXXXXATAGTTTAAAATATAAACTTATAAATCAAGTGTAGCTGAAAAAACTAAAACTTAATCGAA
CAAAACTTTGATTTGTTGTC
AACCCTTGGCTTTGCAAATTTCTTTGGGTTTTATAGATCCAGCATCTTCTGTTTTCTTTTCTGTTGTTTGAGATT
TGTAGXXXXXXXXXXXATAGTTTAAGATATAAACTTATAAATCAAGTGTAGCTGAAAAAACTAAAACTTAATCGAA
CAAAACTTTGATTTGTTGTC
>Marker377265
CACACACGGCTTATAAATTCTACAATTATTAGTCAATCGGTGAGGTTAGCTTCTTGAGACTTTTAAATTAATATC
TTATAXXXXXXXXXXXTTGTGGGAGTTGAATGACAAGATATCTACTCCAAAAGAAGCTTTTGATGCCTTATGCCTT
TGTCTCATCATGTTCAAGGT
CACACACGGCTTATAAATTCTACAATTATTAGTCAATCAGTGAGGTTAGCTTCTTGAGACTTTTAAATTAATATC
TTATAXXXXXXXXXXXTTGTGGGAGTTGAATGACAAGATATCTACTCCAAAAGAAGCTTTTGATGCCTTATGCCTT
TGTCTCATCATGTTCAAGGT
>Marker378110
ACAATAGATATATACTAAAAATAAAAATTAAGAGACCTAAAGATTAGGAGACACACCCAAACATCTCAACTAGGT
TGCCAXXXXXXXXXXXTCCAGATATCTTAGCATGTAAAAGCCGACCAATTTGAACACAAATCTTGAATAGATGTGC
TAAACTCCTTGAATAAAGTG
ACAATAGATATATACTAAAAATAAAAATTAAGAGACCTAAAGATTAGGAGACACACCCAAACATCTCAACTAGGT
TGCCAXXXXXXXXXXXTCCAGATATCTTAGCATGTAAAAGCCAACCAATTTGAACACAAATCTTGAATAGATGTGC
TAAACTCCTTGAATAAAGTG
>Marker378872
ACTTCTCCATTTTTGGTTAAACAAAATGAAACATTCATGGCTTTGAATTGTAAAATTTTAAACGACATTGTTGTG
GTCACXXXXXXXXXXXTTACGTGCAAGAAGGACAACACGATAAAGGATCTGATTTTGTTGCTCGATTCTAAGAAGA
TCAACCGTGTGTATGTGGTG
ACTTCTCCATTTTTGGTTAAACAAAATGAAACATTCATGGCTTTGAATTGTAAAATTTTAATCGACATTGTTGTG
GTCACXXXXXXXXXXXTTACGTGCAAGAAGGACAACACGATAAAGGATCTGATTTTGTTGCTCGATTCTAAGAAGA
TCAACCGTGTGTATGTGGTG
>Marker379207

Create PDF with GO2PDF for free, if you wish to

CACCTTGCGCCTAGGCTTCAGAGGGCCATTGTGCCTTAGGCATTTAAAAACACTGAGTATTCAGCTTTTTCGTGG
TTCCAXXXXXXXXXXXTTAAAAATTCACAATCTGCAAATCCCCCGAATAGAGCCAATTCTCTTACTCTTTCAACAA
GTTCCCTGCAGAAAATGGTA
CACCTTGCGCCTAGGCTTCAGAGGGCGATTGTGCCTTAGGCATTTAAAAACATTGAGTATTCAGCTTTTTCGTGG
TTCCAXXXXXXXXXXXTTAAAAATTCACAATCTGCAAATCCCCCGAATAGAGCCAATTCTCTTACTCTTTCAACAA
GTTCCCTGCAGAAAATGGTA
>Marker379621
CACATCGCCGGCATCCCCCAACAGTCCAACATCGTGTCGCTGGAAGGCCCACACTTTCAGCCACCACCGTTCTGA
CACTCXXXXXXXXXXXTGGCAACAGTTGGATTTCGACTCAAGTGGGTTTCAGCTTGGAACCGAAGGGAATTGTAAC
ATGAGGTGAATACAGAAGTG
CACATCGCCGGCATCCCCCAACAGTCCAACATCGTGTCGCTGGAAGGCCCACACTTTCAGCCACCACCGTTCTGA
CACTCXXXXXXXXXXXTCGCAACAGTTGGATTTCGACTCAAGTGGGTTTCAGCTTGGAACCGAAGGGAATTGTAAC
ATGAGGTGAATACAGAAGTG
>Marker380169
ACAAAAAAAGAACGTCAAATTACTATAAAAGAAGACGATTAAGACAAAGACTAGTCGTCACAAAAATTGATGGTT
GGAGGXXXXXXXXXXXTGAGAGTTACTTTTCATTCATATATATACATTTCATCGTCTAGTAAAATTATTCAAAACA
AACTATAAGACCTTGAGGTT
ACAAAAAAAGAACGTCGAATTACTATAAAAGAAGACGATTAAGACGATGACTAGTCGTCACAAAAATTGATGGTT
GGAGGXXXXXXXXXXXTGAGAGTTACTTTTCATTCATATATATACATTTCATCGTCTAGTAAAATTATTCAAAACA
AACTATAAGACCTTGAGGTT
>Marker380306
CACCTCCATGGCGTGAGTGTTGCTTTTTGCTTATTTTTTCTTCCTCCTTCCAAATTGAGTTCCATTTTCAGAACA
TTAGTXXXXXXXXXXXATTTTATTTCCATTCTCCCGGTTTTCCTCTTTATTTTGTTAGAATCTGCTGTTTGGAAAG
TAAATTTGTATTACTTTGTA
CACCTCCATGGCGTGAGTGTTGCTTTTTGCTTATTTTTTCTTCCTCCTTCCAAATTGAGTTCCATTTTCAGAACA
TTAGTXXXXXXXXXXXATTTTATTTCCATTCTCCCGGTTTTCCTCTTTATTTTGTTAGAATCTGCTGTTTGGAAAG
TTAATTTGTATTACTTTGTG
>Marker380691
AACCAATACGAAACAATTTTTTTTACTTAAAAAGATGTTTAAAAATAATAATAAAGAAGCAGTTGCCTGTAAAAA
AAATGXXXXXXXXXXXAAAATAGGAATGAAAAAAAGCCTAAAACCCTCCTTCTACCCTGATCGAATCGTCGCTTCC
ATTTCTGCTCTTTCACAGTC
AACCAATACGAAACAATTTTTTTTACTTAAAAAGATGTTTAAAAATAATAATAAAGAAGCAGTTGCCTGTAAAAA
AAATGXXXXXXXXXXXAAAATAGGAATAAAAAAAAGCCTAAAACCCTCCTTCTACCCTGATCGAATCGTCGCTTCC
ATTTCTGCTCTTTCACAGTC
>Marker381527
ACTTTAGTGGGCAGACTAGTGCTGCCATGTAGGCATTTCACACTGCCATTCTTTATAAACCATGCACCTCCTTAA
GTTGAXXXXXXXXXXXGTTTGTTAAGTTAAACATTTGATTGTTTTGTCAGAGTCGGGATGACAGGAGAATTTTTAT
TTAGAGTAAAAAATGCTGTT
ACTTTAGTGGGCAGACTAGTGCTGCCATGTAGGCATTTCACACTGTCATTCTTTATAAACCATGCACCTCCTTAA
GTTGAXXXXXXXXXXXGTTTGTTAAGTTAAACATTTGATTGTTTTGTCAGAGTCGGGATGACAGGAGAATTTTTAT
TTAGAGTAAAAAATGCTGTT
>Marker382136
TACCACATCTCTATATCTTCTAAGGCAAGCATGATGGCAGAAGCATTTTGATCATCGTTCCCTAGATCAATTTCA
AATCTXXXXXXXXXXXCAAACCCATGATTTTCTGTTAAATTATTCAAATACAAAGCAAGCTCCAAAGCTGCACCAT
ATATTGATGGAGAGAAATGT

Create PDF with GO2PDF for free, if you wish to

TACCACATTTCTATATCTTCTAAGGCAAGCATGATGGCAGAAGCATTTTGATCATCGTTCCCTAGATCAATTTCA
AATCTXXXXXXXXXXXCAAACCCATGATTTTCTGTTAAATTATTCAAATACAAAGCAAGCTCCAAAGCTGCACCAT
ATATTGATGGAGAGAAATGT
>Marker382154
ACTCCAGGGGTAATGTAGAAAAGAAGGAGGCACTTGAGAACTTGGACCTCATTTTACTGTGTCTCGATGAAATTA
TCGATXXXXXXXXXXXTCTGTATCTTGATTGTGCCACTCTTTTTACTCACCCTCTCTAAGTTGATGCCTGTGTCCA
TTCTTGCTAGACAAATTGTG
ACTCCAGGGGTAATGTAGAAAAGAAGGAGGCACTTGAGAACTTGGACCTCATTTTACTGTGTCTCGATGAAATTA
TCGACXXXXXXXXXXXTCTGTATCTTGATTGTGCCACTCTTTTTACTCACCCTCTCTAAGTTGATGCCTGTGTCCA
TTCTTGCTAGACAAATTGTG
>Marker382229
TACAATAGGTCTCATGGAAAATTCGAGCATGCATAAAAACAGTTAAAAACATGAAAACATTTTCTTGAACTTGAT
CATGAXXXXXXXXXXXTTACAAACTATTACTTGTAAAGCTTGAAATCATGTTTCACAATATCATTTAGAAATCAAT
GTGTTACTCACAATATTTGT
TACAATAGGTCTCATGGAATATTCGAGCATGCATAAAAACAGTTAAAAACATGAAAACATTTTCTTGAACTTGAT
CATGAXXXXXXXXXXXTTACAAACTATTACTTGTAAAGCTTGAAATCATGTTTCACAATATCATTTAGAAATCAAT
GTGTTACTCACAATATTTGT
>Marker382262
ACATGCTTCCAAGATTGGATAAGATGATGAAAGAATGAAAAATAGAAATGCAATTAAGAGATCAAACATGGACAT
GTGTTXXXXXXXXXXXTCAAATAATATACCATAAAATATACACTATTATTGGCCACATCCAATATATACCATATGC
TTTATTTTGATATTTGTGTA
ACATGGTTCCAAGATTGGATAAGATGATGAAAGAATGAAAAATAGAAATGCAATTAAGAGATCAAACATGGACAT
GTGTTXXXXXXXXXXXTCAAATAATATACCATAAAATATACACTATTATTGGCCACATCCAATATATACCATATGC
TTTATTTTGATATTTGTGTA
>Marker382626
ACCTTTATGATGACTTATAAAACCTACCTTGTCCTTTTTAGTGCTATAGCACCATGCTATAACTGTTCTAGCCTG
GTTACXXXXXXXXXXXATTGTGCAGGATCCTTTGGAACCAGTTTTGTTGTTTGCAAAACCTCTGATTACAGAGAAG
TTGGCAGCTGCAACTATAGT
ACCTTTATGATGACTTATAAAACCTACCTTGTCCTTTTTAGTGCTATAGCACCATGCTATAACTGTTCTAGCCTG
GTTACXXXXXXXXXXXATTGTGCAGGATCCTTTGGAACCAGTTTTGTTGTTTGCAAAACCTCTGATTACAGAGAAG
TTGGCAGCTGCAGCTATAGT
>Marker382913
ACAATAATTTTAATAATATAAGTTTGTAATTATATGTTTTCTATTACATGGGCACACAAAAAAAATAAATTAATG
AAAACXXXXXXXXXXXAAAGATTAGTGCAAAAGAAAGATTGAAGCTTAATTTATGATATAGACGTAGTAATTAAAA
AACTTTTTTCATTCTGATGT
ACAATAATTTTAATAATATAAGTTTGTAATTATATGTTTTCTATTACATGGGCACACAAAAAAAATAAATTAATG
AAAACXXXXXXXXXXXAAAGATTAGTGCAAAAGGAAGATTGAAGCTTAATTTATGATATAGACGTAGTAATTAAAA
AACTTTTTTCATTCTGATGT
>Marker383257
CACAAAATCACTAGAAAGAAACATTTAACTTTCCTTTCAAATAAGGTATTATTTACTTATTATTATTATAGGAAA
TGGAGXXXXXXXXXXXAGAAAAGGGTGAGGAGTAAGAAGGTGGTGCAGTCTAGAGGAAGCAAATGCTTGGGGGAAT
GGAAAAAGACCACAATGAGT
CACAAAATCACTAGAAAGAAACATTTAACTTTCCTTTCAAATAAGGTGTTATTTACTTATTATTATTATAGGAAA
TGGAGXXXXXXXXXXXAGAAAAGGGTGAGGAGTAAGAAGGTGGTGCAGTCTAGAGGAAGCAAATGCTTGGGGGAAT
GGAAAAAGACCACAATGAGT
>Marker383305

Create PDF with GO2PDF for free, if you wish to

ACATAAAATTGTATTTTAAAATAGTGTCGATATAGTAATGATATATTTGAACGAATGCATTTTTTAAAAAAGTGT
AGATGXXXXXXXXXXATTAATTTTGATTGATGCAATAATAATGTCATATTTTACATAAAATTTGAATATATACTA
GTGCTAACAAAATAATTGGT
ACATAAAATTGTATTTTAAAATAGTGTCCATATAGTAATGATATATTTGAACGAATGCATTTTTTAAAAAAGTGT
AGATGXXXXXXXXXXATTAATTTTGATTGATGCAATAATAATGCCATATTTTACATAAAATTTGAATATATACTA
GTGCTAACAAAATAATTGGT
>Marker384086
CACATGATACCTTTTGTCCCCTAGATATATCCAAAACATCTACGACATGACAAAACATCTAATCACCGACCCTTC
TTCTCXXXXXXXXXXAACAACTTCCTTTCAACTTTCATTAAACTTAGCTCTCTCTTAGAACATAGAAATTCTCAA
GTAAGGTTAGATTTAGGGTT
CACATGATACCTTTTGTCCCCTAGATATATCCAAAACATCTACGACATGACAAAACATCTAATCACCGACCCTTC
TTCTCXXXXXXXXXXAACAACTTCCTTTCAACTTTCATTAAACTTAGCTCTCTTTTAGAACATAGAAATTCTCAA
GTAAGGTTAGATTTAGGGTT
>Marker384336
ACTCTTCTGTCAATGAAGGATGACGGTGAAGATGAGAATGAGAAGCTCAATGATACTGAGATTAAGGCTTTGTTG
TTGGTXXXXXXXXXXAAACAATTTCATTACATAATTAGTAATTACAATGTTAATCAATTTCTCTATGATCGTTAT
TATTTCATACAGAACATGTT
ACTCTTCTGTCAATGAAGGATGACGGTGAAGATGAGAATGAGAAGCTCAATGATACTGAGATTAAGGCTTTGTTG
TTGGTXXXXXXXXXXAAACAATTTCATTACATAATTAGTAATTACAATGTTAATCAATTTCTGTATGATCGTTAT
TATTTCATACAGAACATGTT
>Marker384917
AACTCCCATTGTGATTTGAACTTTGAACTGCGCACACCTACATGCTTGCATGCATTTTCAACAGCAATTGATTAT
AAATCXXXXXXXXXXCTCAGACTTTCTTTGAAGATGTCCTTGATGTTCATCATTCTTGTTGATGCTACTACAAGG
ATTATAAAGAACTAAAAGGT
AACTCCCATTGTGATTTGAACTTTGAAGTGCGCACACCTACATGCTTGCATGCATTTTCAACAGCAATTGATTAT
AAATCXXXXXXXXXXCTCAGCCTTTCTTTGAAGATGTCCTTGATGTTCATCATTCTTGTTGATGCTACTACAAGG
ATTATAAAGAACTAAAAGGT
>Marker385878
CACATTGGTTGGAGAGAGAAAGGAAAGATAAGTGGAAAAGAACAGGTGATAGAAAAGTTTTTACTATTTGGTTAC
TTTATXXXXXXXXXXTTGGAAGATTCACAGACTCTGGGTTGCTTGTGTTTGTGGGGTTGCTTTTCCAGTTTCTTC
TGTAGCGGTTTGGGTTGGTG
CACATTGGTTGGAGAGAGAAAGGAAAGATAAGTGGAATAGAACAGGTGATAGAAAAGTTTTTACTATTTGGTTAC
TTTATXXXXXXXXXXTTGGAAGATTCACAGACTCTGGGTTGCTTGTGTTTGTGGGGTTGCTTTTCCAGTTTCTTC
TGTAGCGGTTTGGGTTGGTG
>Marker386072
ACATAATCGTATGATATAAAGAAATTTAATTTCTATTTGAAAAATGTTAGAAACATACGTAACAAATAATATCAA
ATGAAXXXXXXXXXXTTGAAAGAAGTATATTATTAAGTTTCACTTTTTTTTCAACCACGATGAATTGACCTAGTG
GTGAATACTTGGGATGTGTT
ACATAATCGTATGATATAAAGAAATTTAATTTCTATTTGAAAAATGTTAGAAACGTACGTAACAAATAATATCAA
ATGAAXXXXXXXXXXTTGAAAGAAGTATATTATTAAGTTTCACTTTTTTTTCAACCACGATGAATTGACCTAGTG
GTGAATACTTGGGATGTGTT
>Marker386167
TACTATACGTTGGGATATTGTGGCTTCTTTTGTTTTGGACTTAATTTTTAATTTCTTACTATTTGGGGAGTTTG
GTTTTXXXXXXXXXXTAGGGTTTTGGTTCTGTTTTGAAGCTGACTGAAGATTCTTGGAATTGCAGTGGGTTACTG
TCAGATTGTTCAGATCAGGT

Create PDF with GO2PDF for free, if you wish to

TACTATACGTTGGGATATTGTGGGCTTGTTTTGTTTTGGACTTGATTTTTAATTTCTTACTATTTGGGGAGTTTG
GTTTTXXXXXXXXXXXTAGGGTTTTGGTTCTGTTTTGAAGCTGACTGAAGATTCTTGGAATTGCAGTGGGTTACTG
TCAGATTGTTCAGATCAGGT
>Marker386227
CACTGACATATGGTTGTAGTATTTTCCATGACAACATCATAGGTCCAATCCTTTCATGTTTAGGTTAAATTGCAA
ATTTGXXXXXXXXXXXAAGTTTTATCAAACCATAATTATTAAATTCTAACTTTTAAAATCATAGGGACTCAATTCC
AATATTCTTCAAACCATGTG
CACTGACATATGGTTGTAGTATTTTCCATGACAACATCATAGGTCCAATCCTTTCATGTTTAGGTTAAATTGCAA
ATTTGXXXXXXXXXXXAAGTTTTATCAAACCATAATTATTAAATTCTAACTTTTAAAATCATAGGGATTCAATTCC
AATATTCTTCAAACCATGTG
>Marker387434
CACCTTTCGAGATACAAATTTTAGAAGATGCCTCGCTAAGGAAAAGTTTCTTACCAAACTCCTCTTTACATGCAT
TAGTCXXXXXXXXXXXTGAAAAGTTAAGAAATGGTGGATTGCAATGGGACAAACATTCAGTTATGTATAAAAAGAA
TATAGGTCTTCAGCCACTGT
CACCTTTCGAGATACAAATTTTAGAAGATGCCTCGCTAAGGAAAAGTTTCTTACCAAACTCCTTTTTACATGCAT
TAGTCXXXXXXXXXXXTGAAAAGTTAAGAAATGGTGGATTGCAAAGGGACAAACATTCAGTTATGTATAAAAAGAA
TATAGGTCTTCAGCCACTGT
>Marker387747
CACTCTTTAGCCAAACTTTTTTGTTTCTTTGCAAGGATTGACTGATGCTTACTCTGTTTAACTTTCTCAGGTTGT
GGGGCXXXXXXXXXXXTTAGATCACTTAAAATTAGTTATTCGAAAATGACTCTCAAATATATCTTAAATTGGTGTT
GAATAGAAATCTAAAGGGTT
CACTCTTTAGCCAAACTTTTTTGTTTCTTTGCAAGGATTGATTGATGCTTACCCTGTTTAACTTTCTCAGGTTGT
GGGGCXXXXXXXXXXXTTAGATCACTTAAAATTAGTTATTCGAAAATGACTCTCAAATATATCTTAAATTGGTGTT
GAATAGAAATCTAAAGGGTT
>Marker388130
CACTGTGATCTAAAACCTAACAATATACTGTTAGATGGAGATATGGTTGCACACTTGACAGATTTTGGGATTTCA
AAACTXXXXXXXXXXXCATACCGTATAACATGCTTCTTTGTTGTGCTGAGTGCAGAATTGGGATTGGATGGAATTG
TTTCTAGAAAGTGTGATGTT
CACTGTGATCTAAAACCTAACAATATACTGTTAGATGGAGATATGGTTGCACACTTGACAGATTTTGGGATTTCA
AAACTXXXXXXXXXXXCATACCGTATAACATGCTTCTTTGTTGTGCTGAATGCAGAATTGGGATTGGATGGAATTG
TTTCTAGAAAGTGTGATGTT
>Marker388243
CACTATAGAATCCCTTGAGGGCGTTCTTGATAAAAGTATCTGATCGAATGAGGACGCTTGCGACGGAGCTGTTTT
GTTGTXXXXXXXXXXXAAGATTCATAATTTAAGATCCAGGAAGAACTCAAAATTTCCATACACAACTTAAATGCTT
AACATAATTACAATGTTGTA
CACTATAGAATCCCTTGAGGGCGTTCTTGATAAAAGTATCTGATCGAATGAGGACGCTTGCGACGGAGCTGTTTT
GTTGTXXXXXXXXXXXAAGATTCATAGTTTAAGATCCAGGAAGAACTCAAAATTTCCATACACAACTTAAATGCTT
AACATAATTACAATGTTGTA
>Marker388496
ACTTAACAATGGATGATCTTGCTGATCAATTTGGAATTGGAACACTTCGTCTAACCACCAGGCAAACATTTCAGC
TACATXXXXXXXXXXXGTTCTTGCCCCAGCTGCTCCACTTGTGAGAAAAGATTACCTTTTTGCACAGCAAACTGCA
GAAAACATTGCTGCTTTGTT
ACTTAACAATGGATGATCTTGCTGATCAATTTGGAATTGGAACACTTCGTCTAACCACCAGGCAAACATTTCAGC
TACATXXXXXXXXXXXGTTCTTGCTCCAGCTGCTCCACTTGTGAGAAAAGATTACCTTTTTGCACAGCAAACTGCA
GAAAACATTGCTGCTTTGTT
>Marker388601

Create PDF with GO2PDF for free, if you wish to

TACATTCAAGACAATGTCAGCTGGGGTTGGGGCTTTGGCATTTGCCTCGCCACTAACATCATTTCTCTTGCTATT
TTCTTXXXXXXXXXXTCAAGCACGGAGAGGGATAATGGTTGCTACTATCAAGGTAAGGACCATCATTTGAGGAAT
CAAGCTGGAGATGGAGTGTT
TACATTCAAGACAGTGTCAGCTGGGGTTGGGGCTTTGGCATTTGCCTCGCCACTAACATCATTTCTCTTGCTATT
TTCTTXXXXXXXXXXTCAAGCACAGAGAGGGATAATGGTTGCTACTATCAAGGTAAGGACCATCATTTGAGGAAT
CAAGCTGGAGATAGAGTGTT
>Marker388606
TACAGTATGAGCATTGGCATCAAGAAGAGTCTCGAGTGATCTCGCCTTTCGGCTCCGAACTCTACCAGCTTCTTT
CAACAXXXXXXXXXXAGAGTCATGAGAGGAGAACTAATCAATTGCATCATTCGCTGCTCAAAGAAAACTTTCAGC
GACTTCCTACTCTAAACGTA
TACAGTATGAGCATTGGCATCAAGAAGAGTCTCAAGTGATCTCGCCTTTCGGCTCCGAACTCTACCAGCTTCTTT
CAACAXXXXXXXXXXAGAGTCATGAGAGGAGAACTAATCAATTGCATCATTCGTTGCTCAAAGAAAACTTTCAGC
GACTTCCTACTCTAAACGTA
>Marker389209
AACAAGATTCACTAGCCAAACATATTCATTCTAAATCAGGTTTAAAAAAGAGACTTCAAACCGCATTCCTTGAAC
AGAAAXXXXXXXXXXGATTCCAAATTGAGAAACGCCGTAGCACCACGGCACCGCTCACCGGCAGCTTGAACTGCT
TGAAGCTCAGCAGATTCAGT
AACAAGATTCACTAGCCAAACATATTAATTCTAAATCAGGTTTAAAAAAGAGACTTCAAACCGCATTCCTTGAAC
AGAAAXXXXXXXXXXGATTCCAAATTGAGAAACGCCGTAGCACCACGGCACCGCTCACCGGCAGCTTGAACTGCT
TGAAGCTCAGCAGATTCAGT
>Marker389841
ACAGTCAGGGGCATTTTGATCATACTTTATTTACAAAGGTTTCCAAGATATGGAAGGTTGTTGTTCAGATAGAGC
ATGTTXXXXXXXXXXAAAAAGGCATCTCCATATCTCAAAGAAAATACACCCTTGATTTCCTGGCCGAGACTAGTA
TGCTGAGATGTCGTCTTGTT
ACAGTCAGGGGCATTTTGATCATACTTTATTTACAAAGGTTTCCAAGATATGGAAGGTTGTTTTTCAGATAGAGC
ATGTTXXXXXXXXXXAAAAAGGCATCTCCATATCTCAAAGAAAATACACCCTTGATTTCCTGGCCGAGACTAGTA
TGCTGAGATGTCGTCTTGTT
>Marker390130
AACAAAAGACGAACTATTTATGGTTTTCATTGTTCAACTGATTATGCAATTGAAGAATGTCACACTCACACGTGC
CTTTTXXXXXXXXXXTTGGTTGTTACAACAATCTTTTTCGTTTAATGAAAAAGGTGAAGACTGTTTGCCTCTTTG
ATAAACAAATATAACATGTG
AACAAAAGACGAACTATTTATGGTTTTCATTGTTCAACTGATTATGCAATTGAAGAATGTCACACTCACACGTGC
CTTTTXXXXXXXXXXTTGGTTGTTACAACAATCTTTTTCGTTTAATGAAAAAGGTGAAGACTGTTTGCCTTTTTG
ATAAACAAATATAACATGTG
>Marker390710
TACCCATTTAAAATCATAAATATTGTGAGTTTAAGTAATTTATTAGTTTGTGATCCTCATCTTATCTTTACAAAT
TTCTGXXXXXXXXXXTCCAAAGAAGATGAAATCAAGGATTAGAATTAGAAATATGAGAGATATTTATCTTTAGGT
TCATAAAATCATACTGTTGT
CACCCATTTAAAATCATAAATACTGTGAGTTTAAGTAATTTATTAGTTTGTGATCCTCATCTTATCTTTACAAAT
TTCTGXXXXXXXXXXTCCAAAGAAGATGAAATCAAGGATTAGAATTAGAAATATGAGAGATATTTATCTTTAGGT
TCATAAAATCATACTGTTGT
>Marker390747
AACGTGAAAGAACGTTTGAAGTTTTGATACTATGTAATGTTATTTGAAATAACTAGTTTGATATTTACGATAGTA
AATGGXXXXXXXXXXTAAACCATAACATTGTTTGAACATAGTAAATGAAGAGGATGGGTATTTTTTATTTTATGG
GTATGGTGTTTAGATATGTT

Create PDF with GO2PDF for free, if you wish to

AACATGAAAGAACGTTTGAAGTTTTGATACTATGTAATGTTATTTGAAATAACTAGGTTGATATTTACGATAGTA
AATGGXXXXXXXXXXXTAAACCATAACATTGTTTGAACATAGTAAATGAAGAGGATGGGTATTTTTTATTTTATGG
GTATGGTGTTTAGATATGTT
>Marker391279
ACTTTTACTCTATCTGATTTTAGTGATTTTACATTTAAACTCAGTTAGACTCTTTTAAATTCACTTACAAATCAA
TTTCAXXXXXXXXXXXAATCAATTCAAAGTATGATTTCAAACACATTACTTTTGAAGGTAGATGATATTTCTTCGG
ACCGAATGACGAATTTTTGT
ACTTTTACTCTATCTGATTTTAGTAATTTTACATTTAAACTCAGTTAGACTCTTTTAAATTCACTTACAAATCAA
TTTCAXXXXXXXXXXXAATCAATTCAAAGTATGATTTCAAACACATTACTTTTGAAGGTAGATGATATTTCTTCGG
ACCGAATGACGAATTTTTGT
>Marker392028
CACCAAAAGAAAGTAATAATACCATTCATTTTTGTATGTTGTGGAATTATTCCTTTCGGTGAAATATGGGGAAGT
TTTGGXXXXXXXXXXXTCTATTGGAGATGACCTTTGCCGAAGTGCCAAAAATTCTCCCAAAAGAACTCTTTTCAAC
GTTCCTCCCACTCCATCGTC
CACCAAAAGAAAGTAATAATACCATTCATTTTTGTATGTTGTGGAATTATTCCTTTTGGTGAAATATGGGGAAGT
TTTGGXXXXXXXXXXXTCTATTGGAGATGACCTTTGCCGAAGTGCCAAAAATTCTCCCAAAAGAACTCTTTTCAAC
GTTCCTCCCACTCCATCGTC
>Marker392382
ACTTCAAGAAAGGCTTTCAAGAATTCAAAGACAAGTCCAAGAAAATCCAATAAAGCAACAAAATATTCAAGAAAT
GCTCCXXXXXXXXXXXCTCCGGATCATAAGAAGGTAAAGTTAATAGCTCTCAAGTTGAAAGGGGGAGCCTCGACAT
GGTGGGTGCAATTAGAGGTA
ACTTCAAGAAAGGCTTTCAAGAATTCAAAGACAAGTCCAAGAAAATCCAAGAAAGCAACAAAATATTCAAGAAAT
GCTCCXXXXXXXXXXXCTCCGGATCATAAGAAGGTAAAGTTGATAGCTCTCAAGTTGAAAGGGGGAGCCTCGACAT
GGTGGGTGCAATTAGAGGTA
>Marker393565
ACATAGAACTGGTGAAGAATGTGTGTAGTTATCGGCCATTTCTTTTAGACATTCTTCCAATGGGTTTGGTCACCC
CATTTXXXXXXXXXXXAAGTTTGTCACATATAAAACGATGTTCCAAAAGTTTGGATCTTTTTTCCCCTCTTGCAAA
TGTCACTGTGGATTTATGTT
ACATAGAACTGGTGAAGAATGTGTGTAGTTATCGGCCATTTCTTTTAGACATTCTTCCAATGGGTTTGGTCACCC
CAGTTXXXXXXXXXXXAAGTTTGTCACATATAAAACGATGTTCCAAAAGTTTGGATCTTTTTTCCCCTCTTGCAAA
TGTCACTGTGGATTTATGTT
>Marker394231
AACAGAGCCCTAAGACACATACCAATTCTTAATCGAGGGAAATCACGACTGCCTTTGAAATGATCAATAATACTC
CTCATXXXXXXXXXXXGATCCTTGAACTGAGTTTTCTTGGATCAGGCGCACACATGACACCGTTTAATCTTTTGGC
AACTTTCACATTTACTTGTC
AACAGAGCCCTAAGACACATACCAATTCTTAATCGAGGGAAATCACGACTGCCTTTGAAATGATCAATAATACTC
CTCATXXXXXXXXXXXGATCCTTGAACTGAGTTTTCTTGGATCAGGCGCACACATGACACCGTTAAATCTTTTGGC
AACTTTCACATTTACTTGTC
>Marker395182
AACAATTTAAACTAATTCGTATAAATATTTGATATTTTTTTAAAAAATTTGACCATTTCTCATAATATAATTCAT
TAAGAXXXXXXXXXXXGATTTTGATAGATTAATAATAATTTAGTTTGTATGATTTCCCATAATCACTTTCTAAACG
AGGCTGACGCCGACGCCGTA
AACAATTTAAACTAATTCGTATAAATATTTGATATTTTTTTAAAAAAAATTGACCATTTCTCATAATATAATTCAT
TAAGAXXXXXXXXXXXGATTTTGATAGATTAATAATAATTTAGTTTGTATGATTTCCCATAATCACTTTCTAAACG
AGGCTGACGCCGACGCCGTA
>Marker395557

Create PDF with GO2PDF for free, if you wish to

AACGAAGGTGACAAAATTTGAGAATTAAATAGCAAAAGGAAGAAACCTTTGCAAGAAAAAAAAATGAAGAAGCTGT
TCCACXXXXXXXXXXGAGGCACGCCTACGAAGAAACGTTCGATGCAAAAGCGTTATCCAACCGAGTCGAAGAAGT
TCTAGATCAGCTAGAAAGTT
AACGAAGGTGACAAAATTTGAGAATTAAATAGCAAAAGGAAGAAACCTTTGCAAGAAAAAAAAATGAAGAAGCTGT
TCCACXXXXXXXXXXGAGGCACGCCTACGAAGAAACGTTTGATGCAAAAGCGTTATCCAACCGAGTCGAAGAAGT
TCTAGATCAGCTAGAAAGTT
>Marker395618
ACATTTCTTTCTTCTTTTTTCCATTCTCTGTTTTGCTTTTGCCCAAAACTAGTAAGGATTCCTCCCCTTGTCTGA
TTTAGXXXXXXXXXXCAATGAAAGCATGTCTGTGGTTCTTGAAAGATTTGGTGCCATGGGTATTGACACCTCTGG
AGTGGTTGCTTTACTAGGTA
ACATTTCTTTCTTCTTTTTTCCATTCTCTGTTTTGCTTTTGCCCAAAACTAGTAAGGATTCCTCCCCTTGTCTGA
TTTTGXXXXXXXXXXCAATGAAAGCATGTCTGTGGTTCTTGAAAGATTTGGTGCCATGGGTATTGACACCTCTGG
AGTGGTTGCTTTACTAGGTA
>Marker395727
CACAGCATCAAACTAAATTGCTTCACACAAATCCTTTTGTAATTACATCAAGCAAGGAAACAACTAGGATAATGA
TCTTGXXXXXXXXXXAAGTCGAGCTGTTCATTTAGGGAAAGGTGCAGTAATTGTTTGTTCTCGTCTTCACTCAAG
CCTATTAGTAAAAGGAAGGT
CACAGCATCAGACTAAATTGCTTCACACAAATCCTTTTGTAATTACATCAAGCAAGGAAACAACTAGGATAATGA
TCTTGXXXXXXXXXXAAGTCGAGCTGTTCATTTAGGGAAAGGTGCAGTAATTGTTTGTTCTCGTTTTCACTCAAG
CCTATTAGTAAAAGGAAGGT
>Marker395787
GACGCTCTGTATGACCAAACAGAGGGTCTAGTGGCACCTCTAGATTGCGTTATTTGATATTTATCTCTAATAATA
AATTGXXXXXXXXXXGGGTGCTTTAGACGATGTGGTAATGGGTGGAGTAAGTGAAAGTTCATTTCAAATCGACAT
GAACGGTGGTGAAAATGGTG
GACGCTCTGTATGACCAAACAGAGGGTCTAGTGGCACCTCTAGATTGCGTTATTTGATATTTATCTCTAATAATA
AATTGXXXXXXXXXXGGGTGTTTTAGACGATGTGGTAATGGGTGGAGTAAGTGAAAGTTCATTTCAAATCGACAT
GAACGGTGGTGAAAATGGTG
>Marker397147
GACTCACGAGGCTTTTCAAAGCCACCAGTGAGATACACATACAATGCAAAAACCAACAGGTTGATGCCAAACGCA
ACTGAXXXXXXXXXXGATTTTAATGATTTTCCAGTTTCTTGAGCGTTATCTTCTCCCATCCTTTGCTAATTCATT
TGCCTATGAATTAATTAGTG
GACTCACGAGGCTTTTCAAAGCCACCAGTATGATACACATACAATGCAAAAACCAACAGGTTGATGCCAAACGCA
ACTGAXXXXXXXXXXGATTTTAATGATTTTCCAGTTTCTTGAGCGTTATCTTCTCCCATCCTTTGCTAATTCATT
TGCCTATGAATTAATTAGTG
>Marker397435
TACATTGTATCTTGGAGTATTAGTCTCTTTTCATTTTCTTCATGAAAAGTGTGTAATATCCTTTGGAAAAAAATG
GAAAGXXXXXXXXXXAAACCAGAATCGTGATGCTTGTTAAGTTCTAAATTTTCATCACGGTAGCATACTTTAAAC
TACGGTTTTCTATATTATGT
TACATTGTATCTTGGAGTATTAGTCTCTTTTCATTTTCTTCATGAAAAGTGTGTAATATCCTTTGGAAAAAAATG
GAAAGXXXXXXXXXXAAACCAGAATCACGATGCTTGTTAAGTTCTAAATTTTCATCACGGTAGCATACTTTAAAC
TACGGTTTTCTATATTATGT
>Marker397523
GACCTAGCAAGTAGAAATAAATATTAATTGGAGAGAATAACACTGCAAAGGTTTGGTCACAACACCTCCTAGACC
ACCTGXXXXXXXXXXCAATTTCACTTTCAAATCAATTGGAAGACTTCTCTTCCTCTTTTTTACATTACATTCTTG
ATTCATCGAAAAAAAAGGTT

Create PDF with GO2PDF for free, if you wish to

GACCTAGCAAGTAGAAATAAATATTAATTGGAGAGAATAACACTGCAAAGGTTTGATCACAACACCTCCTAGACC
ACCTGXXXXXXXXXXXCAATTTCACTTTCAAATCAATTGGAAGACTTCTATTCCTCTTTTTTACATTACATTCTTG
ATTCATCGAAAAAAAAGGTT
>Marker397643
CACACAAGATTTCTAGAGAAATGTCTAATTGGCTGAAATTTCTTACTCAATAGATATGATGCATAAGTATGTCGA
GTGGTXXXXXXXXXXXGAACCTTCTCATGTGCGCGTTTGGTGTGACCTTAGAAAATTTTCTCGGTTTCATTGTAAG
GTATTGGGGGATTGAAGGTA
CACACAAGATTTCTAGAGAAATGTGTAATTGGCTGAAATTTCTTACTCAATAGATATGATGCATAAGTATGTCGA
GTGGTXXXXXXXXXXXGAACCTTCTCATGTGCGCGTTTGGTGTGACCTTAGAAAATTTTCTCGGTTTCATTGTAAG
GTATTGGGGGATTGAAGGTA
>Marker397653
TACTTTGGCTAGAAGAGAGGCTGGTGAAAGAACTTCGCCATTGACAGAAACAGGGAGACCTCCCACGATTCCTAT
TTCCAXXXXXXXXXXXATGAATTGAGAAAGTATTATCTTAATATTACAACTCTAATATTGAATTATTAAAAGTGCC
TCTATTTTTCAAACTATGTA
TACTTTGGCTAGAAGAGAGGCTGGTGAAAGAACTTCGCCATTGACAGAAACAGGGAGACCTCCCACAATTCCTAT
TTCCAXXXXXXXXXXXATGAATTGAGAAAGTATTATCTTAATATTACAACTCTAATATTGAATTATTAAAAGTGCC
TCTCTTTTTCAAACTATGTA
>Marker398034
AACGAAAGAGAGGTTGTCGGTCATCCAAAGGAAAAGGAAAGCAAATTGTTTATTTTGATCAGTGATCCCATTTTT
ACCAAXXXXXXXXXXXGATTTTTGGGTTAATTTGTAGCAGTTTAGTATTTAGAAGATCATGAAGTGTAGCAAACAA
ATTAGAAGAGTTAAAAAGTG
AACGAAAGAGAGGTTGTCGGTCATCCAAAGGAAAAGGAAAGCAAATTGTTTATTTTGATCAGTGATCTCATTTTT
ACCAAXXXXXXXXXXXGATTTTTGGGTTAATTTGTAGCAGTCTAGTATTTAGAAGATCTTGAAGTGTAGCAAACAA
ATTAGAAGAGTTAAAAAGTG
>Marker398547
ACAAATATAGATCGAAAAGAACTTTGATTGTTTCCCTATATATATAAACACATAATTCAACGCAAATGAATAAAC
CAAGCXXXXXXXXXXXGATTTTGTCAGGTTCAATGCATTATCCACGCAGCACTGAAGCAATGTGGCCTGATCTTAT
TCAAAAAGCTAAAGATGGTG
ACAAATATAGATCGAAAAGAACTTTGATTGTTTCCCTATATATATAAACACATAATTCAACGCAAATGAATAAAC
CAAGCXXXXXXXXXXXGATTCTGTCAGGTTCAATGCATTATCCACGCAGCACTGAAGCAATGTGGCCTGATCTTAT
TCAAAAAGCTAAAGATGGTG
>Marker398718
ACATGTGTTCAGTTGTGTTGTATATGTTGGTTGTTGATATATACTAATCGAATACTATGTAACTAGTCTCTGTAA
GGCTTXXXXXXXXXXXTGATATTCACTATTCATCCATCACGCTCTCCTTCAAAACAATGTGGATCATTCACAAGAG
GGTGTGACACTCAGGTGGTC
ACATGTGTTCAGTTGTGTTGTATATGTTGGTTGTTCATATATACTAATTGAATACTATGTAACTAGTCTCTGTAA
GGCTTXXXXXXXXXXXTGATATTCACTATTCATCCATCACGCTCTCCTTCAAAACAATGTGGATCATTCACAAGAG
GGTGTGACACTCAAGTGGTC
>Marker398763
CACCGTAAAATAAATTTTAGAAGATGGTCTGAGAAATAATTCAAATACAGCACCAACAACACGTCTAAGTTTTTA
AATTCXXXXXXXXXXXCTCCCCAAATCAGCCAATTTCTGGTTTAACTCAAACCGGTCACAGTTTTGAAGTAAAGCT
AAGGTCATTGGATTAATGTT
CACCGTAAAATAAATTTTAGAAGATAGTCTGAGAAATAATTCAAATACAGCACCAACAACACGTCTAAGTTTTTA
AATTCXXXXXXXXXXXCTCCCCAAATCAGCCAATTTCTGGTTTAACTCAAACCAGTCACAGTTTTGAAGTAAAGCT
AAGGTCATTGGATTAATGTT
>Marker398880

Create PDF with GO2PDF for free, if you wish to

ACCTTATTAGGTTCATCAATCTTAGATCGAAATACAAAAATCGAAATGATTATCATACGAAACCTTAATAACTAG
GGTGGXXXXXXXXXXCATGTAACTATGACTTTGTTTTATACTAATAAGTTAATTTAGAAGTGCCATAAATTAGGA
AGTTCATTAATAACAAGGTC
ACCTTCTTAGGTTCATCAATCTTAGATCGAAATACAAAAATCGAAATGATTATCATACGAAACCTTAATAACTAG
GGTGGXXXXXXXXXXCATGTAACTATGACTTTGTTTTATACTAATAAGTTAATTTAGAAGTGCCATAAATTAGGA
AGTTCATTAATAACAAGGTC
>Marker399152
CACGACCTGGAAAAATTAGCTTCCAATGAGATGATTTAAAATTAAATCAAATACATATAATTTTAAGACTAAAAA
TTTATXXXXXXXXXXCTCATAATTGAAAAATAATATAATTCAATAAGTTGAAATAAACAGCATTTAGGAATTAAT
TAAATTGTATCAATACAAGT
CACGACCTGGAAAAATTAGCTTCCAATGAGATGATTTAAAATTAAATCAAATACATATAATTTTAAGACTAAAAA
TTGATXXXXXXXXXXCTCATAATTGAAAAATAATATAATTCAATAAGTTGAAATAAACAGCATTTAGGAATTAAT
TAAATTGTATCAATACAAGT
>Marker399422
TACACCCCACGCTGTATCTATCACAAAAGGGATTGAGGATATCGGAGCAGTTTTCTAGTCCAAAAAGTCCTTAAG
AAATGXXXXXXXXXXCATGAGTTCTCGTTATCTTCGACTTGTTTTTTGTCTCAGTCTTTTGAAAGCCATAAAGGT
TACAGCAGCTGAGAGAGAGT
TACACCCCACGCTGTATCTATCACAAAAGGGATTGAGGATATCGGAGCAGTTTTCTAGTCCAAAAAGTCCTTGAG
AAATGXXXXXXXXXXCATGAGTTCTCGTTATCTTCGACTTGTTTTTTGTCTCAGTCTTTTGAAAGCCATAAAGGT
TACAGCAGCTGAGAGAGAGT
>Marker399658
GACATGCCTGAAGACATTATATTATTTGCCTGATACACATAAAAGGGAAGTTAGTGTCTAGATCACGAGCCCCTG
ACTCAXXXXXXXXXXATGTTAGCATACCAAGGAAAGAGTTTCATCAGATATTAAAATTTTATATTTGGAAGATAC
TACCTGATGAAGTTGCGGTA
GACATGCCTGAAGACATTATATTATTTGCCTGATACACATAAAAGGGAAGTTAGTGTCTAGATCACGAGCCCCTG
ACTCAXXXXXXXXXXATGTTAGCATACCAAGGAAAGAGTTTCATAAGATATTAAAATTTTATATTTGGAAGATAC
TACCTGATGAAGTTGCGGTA
>Marker399909
ACCTCTTTATCGCAAGTCATTGTTGAATCCAACTTTTCATACTGCTTGTCCAAGTGCAAGCGAAACAATAGATTT
ACCTGXXXXXXXXXXTAGAAAAACTAAAGTTATTTACACTGGCCTAGAGACTCATGCATAGAGAGATAAATTATG
ATAAAATGGATGATTAAGTG
ACCTCTTTATCGCAAGTCATTGTTGAATCCAACTTTTCATACTGCTTGTCCAAGTGCAAGCGAAACAATAGATTT
ACCTGXXXXXXXXXXTAGAAAAACTAAAGTTATTTACACTGGCCTAGAGACTCATGCATAGAAAGATAAATTATG
ATAAAATGGATGATTAAGTG
>Marker400277
ACATTGCTACGTTGGACAGCAAGTCTTGCTTGTATTGGATGACCTTGAGTTATTTCTCCTCTTTGCTTCAGCCAG
TTTATXXXXXXXXXXTTGACTTCTGTAGTCGCCACCTTCTTTAAAGTTACAGTCTAACTTTGTTTGGTATTGTGT
CTGGCTGTCTGTTTCGAAGT
ACATTGCTACGTTGGACAGCAAGTCTTGCTTGTATTGGATGACCTTGAGTTATTTCTCCTCTTTGCTTCAGCCAG
TATATXXXXXXXXXXTTGACTTCTGTAGTCGCCACCTTCTTTAAAGTTACAGTCTAACTTTGTTTGGTATTGTGT
CTGGCTGTCTGTTTCGAAGT
>Marker400734
TACCACTCTTTTGCTCAGTTGCGACTAAACTCAACGCATTTAGCAAGTTTCATCAATTCCACTATGAATTATGCA
GTCTTXXXXXXXXXXTATCCAAAATCACACAACAAGGATAAGGCATCCTATACCATATCACATGACAAGTGTGAG
ACATTCTCATTGCATAGGGT

Create PDF with GO2PDF for free, if you wish to

TACCACTCTTTTGCTCAGTTGCGACTAAACTCAACGCATTTAGCAAGTTTCATCAATTCCACTATGAATTATGCA
GTCTTXXXXXXXXXXTATCCCAAATCACACAACAAGGATAAGGCATCCTATACCATATCACATGACAAGTGTGAG
ACATTCTCATTGCATAGGGT
>Marker401062
AACATTATTTCAAAACATCTAATTTACTACAACTTTTACTACGTTACGTTCTTCTATAAGAAAGCAAAATAACTG
ATGTTXXXXXXXXXXAATGTATGAAAGAAACATAAGAAATAGTGAGGAGACAGATTGGACAGATAATAGATCCCC
ACATGGCCAAAAACAACAGT
AACATTATTTCAAAACATCTAATTTACTACAACTTTTACTACGTTACGTTCTTCTATAAGAAAGCAAAATAAGTC
ATGTCXXXXXXXXXXAATGTATGAAAGAAACATAAGAAATAGTGAGGAGACAGATTGGACAGATAATAGATCCCC
ACATGGCCAAAAACAACAGT
>Marker401174
AACAAACACAAAAAAATGCCCGTCTATGATCTATAATATCAATGATTTAATCTTTTTAAAAAAATTATCAATCAA
ATTAGXXXXXXXXXXACTGAAACTAGCATATATTAACTAGTTTATGTCATGAACGATTCATAATTAATTATCAAA
GTTAGTAACCCATAAAAGTT
AACAAACACAAAAAAATGCCCGTCTATGATCTATAATATCAATGATTTAATCTTTTTAAAAAAATTATCAATCAA
ATTAGXXXXXXXXXXACTGAAACTAGCATATATTAACTAGTTTATGTCATGAACGATTCATAATTAATGATCAAA
GTTAGTAACCCATAAAAGTT
>Marker401300
ACTTTCGATTCTTCATTTGATGGATTTAAACATGATGACCTAATTATCATGTTTAATTCCAAAGCTACGAATAAG
AGAAAXXXXXXXXXXTTCTTGTCTCTATTATTAAAAGATTTGTTGTTGTTGTTATAATTACTAAATCTTTGTTAG
ATCATTTCAATTGTTATTGT
ACTTTCGATTCTTCATTTGATGGATTTAAACATGATGACCTAATTATCATGTTTAATTCCAAAGCTACGAATAAG
AGAAAXXXXXXXXXXTTCTTGTCTCTATTATTAAAAGATTTGTTGTTGTTGTTATAATTACTAAATCTTTGTTAG
ATTATTTCAATTGTTATTGT
>Marker401428
AACTTTAATTCTGGTTTTCAAATTTTAAATATAGATTTCTTGTGATGGCATTCTTTCCTTTTTTAAATACAGGGT
AAGGTXXXXXXXXXXCTTTATTTTCTTGTGTGATAAAATGATTTAGTTACCTTTTCCCCTCCATCCATGATCTAT
CTAGTATCCCATTCATTGTT
AACTTTAATTCTGGTTTTCAAATTTTAAATATAGATTTCTTGTGATGGCATTCTTTCCTTTTTTAAATACAGGGT
AAGGTXXXXXXXXXXCTTTATTTTCTTGTGTGATAAAATGATTTAGTTACCTTTTCCCCTCCATCCATGATCTAT
CTAGTATCTCATTCATTGTT
>Marker402275
TACACCAGGAAATTGTGATATTAATAACCTGATCAACAAAATTATTAAAGTCATTCAATTTGTTGTTGAAAATAT
GGTCAXXXXXXXXXXAGATGAAACCTCATACTATCAAGTTTAACATGGTATCAGAGCCCAGAAAACGCAAACGGA
TATTTGGTCCAAGATAGGTG
TACACCAGGAAATTGTGATATTAATAACCTGATCAACAAAATTATTAAAGTCATTCAATTTGTTGTTGAAAATAT
GGTCAXXXXXXXXXXAGATGAAACCTCATACTATCAAGTTTAACATGGTATCAGAGCCCAGAAAACGCAAACAGA
TATTTGGTCCAAGATAGGTG
>Marker403330
CACATAATGAGTCAAGGCAACCATGGGGTGAAAGGAGCTACATGGTGAAGAGAAGCTAGCTAATAGGTTGAAAGA
AGCATXXXXXXXXXXTCCTTGACAAAAGGAATGCTTGGTAACTAATGTGTGTTTATGCAAGATGAAACATTTATG
CAAAATGATGTATTTGTGTG
CACATAAAGAGCCAAGGCAACCATGGGGTGAAAGGAGCTACATGGTGAAGAGAAGCTAGCTAATAGGTTGAAAGA
AGCATXXXXXXXXXXTCCTTGACAAAAGGAATGCTTGGTAACTAATGTGTGTTTATGCAAGATGAAACATTTATG
CAAAATGATTTATTTGTGTG
>Marker403491

Create PDF with GO2PDF for free, if you wish to

ACTAAATAATTGATATTAATTTATAAACATGGACACGAATGTTTGTGAGTGAATAAATAATGAAAATTAAGGTTA
AAGATXXXXXXXXXXXTATATATCATTTTAAAATATAAGAACTAAATAGAAAAGGCAATGACCGAAACTTATTTTT
CTCAATGTTTAAAACTGGTT
ACTAAATAATTGATATTAATTTATAAACATGGACACGAATGTTTGTGAGTGAATAAATAATGAAAATTAAGGTTA
AAGATXXXXXXXXXXXTATATATCATTTTAAAATATAAGAACTAAATAGAAAAGGCAATGACGGAAACTTATTTTT
CTCAATGTTTAAAACTGGTT
>Marker404055
ACTTGCTATCTAGTTTATTAATACTTGACTGAACTGAAAATCATCGAATTAAGTCAAGATATAAACAAAAACCAC
CACCAXXXXXXXXXXXTATATGGAGACTGCCTATTGTATTACCATCAGTTAGACTTCTTGTTTAGTGAAATATGGG
AAGGAGACAATTTAGTGGTG
ACTTGCTATCTAGTTTATTAATACTTGACTGAACTGAAAATCATCGAATTAAGTCAAGATATAAACAAAAACCAC
CACCAXXXXXXXXXXXCATATGGAGACTGCCTATTGTATTACCATCAGTTAGACTTCTTGTTTAGTGAAATATGGG
AAGGAGACAATTTAGTGGTG
>Marker404292
ACTCATACTAGAATAAAAAGAACCCTAACAAGACAATTAGGGAGAATTGAAGTGTTAAAATGATTGGATATGCAA
ATATTXXXXXXXXXXXGGGTTGAAAGAGAAAAGGTTGGGTGAAAGGGGAGAGTGGGCCAAAAGAGGCACGCGGGTT
TTTGAACAATAATAATAGTA
ACTCATACTAGAATAAAAAGAACCCTAACAAGACAATTAGCGAGAATTGAAGTGTTAAAATGATTGGATATGCAA
ATATTXXXXXXXXXXXGGGTTGAAAGAGAAAAGGTTGGGTGAAAGGGGAGAGTGGGCCAAAAGAGGCACGCGGGTT
TTTGAACAATAATAATAGTA
>Marker404396
ACTGTTCCTTTTGAAGTTGATGATGATAAATTTGGGGAAAGAATGCAAGTTGTGTAGAATTGTCTCCTGAGTCTA
TATCAXXXXXXXXXXXTCTAGAAGTGTCTATTTTATGCCAATTAGTTGATCCATGATGGTATTATTTTCAGATTTA
TGAGGTTGCTCCATCACTGT
ACTGTTCCTTTTGAAGTTGATGGTGATAAATTTGGGGAAAGAATGCAAGTTGTGTAGAATTGTCTCCTGAGTCTA
TATCAXXXXXXXXXXXTCTAGAAGTGTCTATTTTATGCCAATTAGTTGATCCATGATGGTATTATTTTCAGATTTA
TGAGGTTGCTCCATCACTGT
>Marker404849
TACATACTTGTGTATCTTGTTTAATATAAGTTAATGTATTTGTCTTCCATCAACATTTAGCCACTTGAAAATTTG
TTAAGXXXXXXXXXXXTTAAAAATGCATATAAATCACATAGTTGTGTATGTTAGCTATAGTTCGAATCTCCTACTA
ATCTCGAACTAAGATTATGT
TACATACTTGTGTATCTTGTTTAATATAAGTTAATGTATTTGTCTTCCATCAACGTTTAGCCACTTGAAAATTTG
TTAAGXXXXXXXXXXXTTAAAAATGCATATAAATCACATAGTTGTGTATGTTAGCTATAGTTCGAATCTCCTACTA
ATCTCGAACTAAGATTATGT
>Marker405296
TACGCATCTGAATTCTGGTCATTTTGTGTCGATAGCATACAGTGTTTGTCATGATCTTGGAGATGCCTTTATTTA
CACTAXXXXXXXXXXXACATGGGTTCAATGTGACGCTCCTTGCAGCAGTTGTGGCAAGGTAATCTTCCAATGTTAT
TGAGGAATTTATGCTCTTGT
TACGCATCTGAATTCTGGTCATTTTGTGTCGATAGCATACAGTGTTTGTCATGATCGTGGAGATGCCTCTATTTA
CACTAXXXXXXXXXXXACATGGGTTCAATGTGACGCTCCTTGCAGCAGTTGTGGCAAGGTAATCTTCCAATGTTAT
TGAGGAATTTATGCTCTTGT
>Marker405828
AACCACAATGCAAATATGTTTCTAAGTTCTCATTTTCCTCTTAAAATAAACAACAATCATTTACACATGCATGAA
TGTTTXXXXXXXXXXXCTAAAACTTATATCAGTCCAACCACACAAAACTTTAAGTTTATATAAGGTCACCATAGCT
GACATCTTTATGAATTTTGT

Create PDF with GO2PDF for free, if you wish to

AACCACAATGCAAATATGTTTCTAAGTTCTCATTTTCCTCTTAAAATAAACAACAATCATTTACACATGCATGAA
TGTTTXXXXXXXXXXXCTAAAACTTTTATCAGTCCAACCACACAAAACTTTAAGTTTATATAAGGTCACCGTAGCT
GACATCTTTATGAATTTTGT
>Marker405968
AACAACGTTAGTGTTAATGGTAGAGATTTCACGAGTTTTGACACTTCAAAGTTTGGAAAGTTTCTACAGTATGAT
ATAACXXXXXXXXXXXAAATGACAATTCAGAAGAATATAATTTTTCTCAATGGATCTATTTTATATTGATATTACT
GTAATCAAACTTTTTTTGTT
AACAACGTTAGTGTTAATGGTAGAGATTTCATGAGTTTTGACACTTCAAAGTTTGGAAAGTTTCTACAGTATGAT
ATAACXXXXXXXXXXXAAATGACAATTCAGAAGAATATAATTTTTCTCAATGGATCTATTTTATATTGATATTACT
GTAATCAAACTTTTTTTGTT
>Marker406537
TACAACTACAGATTCCTTTGTGGTAATTGGTATTGTTATTTGGATTTTGCAACATTTTCACTTAACAATCCTTGT
GTGTTXXXXXXXXXXXTAAACTCTTCGTCTTTGTCTCTTTTTGTCTCCACTCTACTACAAAAGCTGGCTTACTTGA
CGTCAATACAGTATCAAGTA
TACAACTACAGATTCCTTTGTGGTAATTGGTATTGTTATTTGGATTTTGCAACATTTTCACTTAACAATCCTTGT
GTGTTXXXXXXXXXXXTAAACTCTTCGTCTTTGTCTCTTTTTGTCTCCACTCTACTACAAAAGCTGGCTTACTTGA
CGCCAATACAGTATCAAGTA
>Marker406971
CACTTGCAGTTAAGTTCCATCTGATTGCAGAAGAGATTGAAACCATATTTTAACAAGCTATGACACAAAATCTTG
CAAACXXXXXXXXXXXCAGATTAAGGTAACTTAAATCGTCAATAATATTTCAAACAATACTAGACTAAAAACCAAT
TGAAATGCTAATAAGGAGTT
CACTTGCAGTTAAGTTCCATCTGATTGCAGAAGAGATTGAAACCATATTTTAACAAGCTATGACACAAAATCTTG
CAAACXXXXXXXXXXXCAGATGAAGGTAACTTAAATCGTCAATAATATTTCAAACAATACTAGACTAAAAACCAAT
TGAAATGCTAATAAGGAGTT
>Marker407091
CACCTTTCAGGACACAAATGATGGAAGTTATCTCACAAGGTGGTGTTTGTCTCAAAACTCCTCTTTGCGCATGTT
AGTCTXXXXXXXXXXXGAATGGAAAGTTAAGAAATGATGGATTGAAAAGGTATAACCATGTAATATATATAAAGAA
TGCAGGCCTTAGGCCATTGT
CACCTTTCAGGACACAAATGATTGAAGTTATCTCACAAGGTGGTGTTTGTCTCAAAACTCCTCTTTGCGCATGTT
AGTCTXXXXXXXXXXXGAATGGAAAGTTAAGAAATGATGGATTGAAAAGGTATAACCATGTAATATATATAAAGAA
TGCAGGCCTTAGGCCATTGT
>Marker407772
AACCAATTTTTGTAGTAGTGTTTTTTGAAAAATACATTTATATCTAGACCACATTTTGTTTATATAAGATGCTTG
AGACAXXXXXXXXXXXCCTATGTCATTTCCAATTAGTATAATATCATCTACATACAAAACTAAGAATGCTATAATG
GAATTGACGACCTTTTTGTA
AACCAATTTTTGTAGTAGTGTTTTTTGAAAAATACATTTATATCTAGACCACATTTTGTTTATATAAGATGCTTG
AGACAXXXXXXXXXXXCCTATGTCATTTCCAATGAGTATAATATCATCTACATACAAAACTAAGAATGCTATAATG
GAATTGACGACCTTTTTGTA
>Marker408295
AACTGCAACATTCTTTCCCTGAAGACGATTTCAAGCTGGGAGAGAACATATTTTGGGGGAGTGGATCGGCATGGA
GGCCAXXXXXXXXXXXGGGTTGCGCTCGTGTCGTTTGTGACAATGGGGATATCTTCATGACTTGTAACTATGATCC
GCCTGGTAACTATCTGGGTG
AACTGCAACATTCTTTCCCTGAAGACGATTTCAAGCTGGGAGAGAACATATTTTGGGGGAGTGGATCGGCATGGA
GGCCAXXXXXXXXXXXGGGTTGCGCTCGTGTCGTTTGTGACAATGGGGATATCTTCATGACTTGTAACTATGATCC
GCCTGGTAACTATGTGGGTG
>Marker408458

Create PDF with GO2PDF for free, if you wish to

ACATTCTTCAAATCATTGTTTGATTGTATCACGATATTAGTTTGAGTTTCGTTGCTATTAGTTCTCTTTTTTCTC
CCTACXXXXXXXXXXCCATGCACATACAAAATAACAAAAACAAACTTTTTAAAAAGAAAATGTTATACAGCAATT
TTATGATTGAACACTTTGTG
ACATTCTTCAAATCATTGTTTGATTGTATCACGATATTAGTTTGAGTTTCGTTGCTATTAGTTCTCTTTTTTCTC
CCTACXXXXXXXXXXCCAGGCACATACAAAATAACAAAAACAAACTTTTTAAAAAGAAAATGTTATACAGCAATT
TTATGATTGAACACTTTGTG
>Marker409297
TACATCATAGCCAGAATATGGTGTTGGGGAGGTATTTGAAAGGAAGAGTTATAGCCTGATAAAACTTCTCCGGAT
GGAGAXXXXXXXXXXAACATTGGATATAGAACTGATGCTAGGTATCAAGAAACTGGTCAAGTAAAGCCAAATTCT
GCTTTAATTTTATCTGGGTA
TACATCATAGCCAGAATATGGTGTTGGGGAGGTATTTGAAAGGAAGAGTTATAGCCTGATAAAACTTCTCCGGGT
GGAGAXXXXXXXXXXAACATTGGATATAGAACTGATGCTAGGTATCAAGAAACTGGTCGAGTAAAGCCAAATTCT
GCTTTAATTTTATCTGGGTA
>Marker410571
GACTACACTCACCAAGAAGAAGACCCCGGGCTTTTCTCTCTCTAAACTTACAGAAAATTCGCTCTCTCAGTTTGT
GTTATXXXXXXXXXXAATGGTATTAGAGCTATGTTGCTGATGTTCTAGAGTGGAAACGACAGAGGCTTCTGACCA
GGTGAGGCCTTGAGTAAAGT
GACTACACTCACCAAGAAGAAGACCCCGGGCTTTTCTCTCTCTAAACTTATAGAAAATTCGCTCTCTCAGTTTGT
GTTATXXXXXXXXXXAATGGTATTAGAGCTATGTTGCTGATGTTCTAGAGTGGAAACGACAGAGACTTCTGACCA
GGTGAGGCCTTGAGTAAAGT
>Marker410782
CACGTTATATTAGTCCTTATGAGATTCTAGCAAGCGTAGGACCAATGACTTATAAGCTAAGATTGCCTGCAAAAC
TATCTXXXXXXXXXXCCCCATGGTCAAAATTTTGCGAGACAACCATGGCGTTGAAGAATCTACTTGAGAAAGTGG
GGAGCTAATAAAAACCAAGT
CACGTTATATTAGTCCTTATGAGATTCTAGCAAGCGTAGGACCAATGACTTATAAGCTAAGATTGCCTGCAAAAC
TATCTXXXXXXXXXXCCCCATGGTCAAAATTTTGCGAGACAACCATGGCGTTGAAGAAGCTACTTGAGAAAGTGG
GGAGCTAATAAAAACCAAGT
>Marker411001
GACTTAGTAAAGTGATGATTTCCATTTACTAAAAGAGTTGAGTTGTATTTTTTATATTTATTGAAGAAATTAAAA
ACCTAXXXXXXXXXXGTCTCTAATATAATCATTTTTGTTATTCATATCCACATTTTAGACTGATACTGCCTCAGT
TCTACATGAAGCAATTGAGT
GACTTAGTAAAGTGATGATTTCCATTTACTAAAAGAGTTGAGTTGTATTTTTTATATTTATTGAAGAAATTAAAA
ACCTAXXXXXXXXXXGTCTCTATTATAATCATTTTTGTTATTCATATCCACATTTTAGACTGATACTGCCTCAGT
TCTACATGAAGCAATTGAGT
>Marker411361
AACTAAAAATAGGAAAAGAAATCACTGGAGATAGAAGGTTTTCCTTAAAAGAATAAAGGAAAGGGGAAAAGGAAA
AGGAGXXXXXXXXXXACTTCTTGTCGAGGGTAGAGTAGATATATGTTAATTAACACCAAATATTCACTAAATTAA
AACCTTAGAAGTTTGGAGTT
AACTAAAAATAGGAAAAGAAATCACTGGAGATAGAAGGTTTTCCTTAAAAGAATAAAGGAAAGGGGAAAAGGAAA
AGGAGXXXXXXXXXXACTTCTTGTCGAGGGTAGAGTAGATATATGTTAATTAACACCAAAAATTCACTAAATTAA
AACCTTAGAAGTTTGGAGTT
>Marker411449
TACTTTCTGTTGTTTTTGTGCTATTTATTTTATTGTGTGGAGTCCCATCTATAGTTTTTCCACCTAGTATTCTTG
TGTGGXXXXXXXXXXATTACTGCATTTTCATCATTTGGGGTTTTTGTTTTGGATGATTTAGGAGATGGGTTGTGC
TTAGATTTGAGTTTTGTTGT

Create PDF with GO2PDF for free, if you wish to

TACTTTCTGTTGTTTTTGTGCTATTTATTTTATTGTGTGGAGTCCCATCTATAGTTTTTCCACCTAGTATTCTTG
TGTGGXXXXXXXXXXXATTACCGCATTTTCATCATCTGGGGTTTTTGTTTTGGATGATTTAGGAGATGGGTTGTGC
TTAGATTTGAGTTTTGTTGT
>Marker411882
ACGAAGATCCCCTCAAATAGAAATCTTGGAATTCTAAGTTTGGTAAGGGGTAGCATATTACATCTTGCTGCAAGT
TGCCTXXXXXXXXXXXTTTGACTTTCCATGTATCTCCATTCCTTTCAGGTCTTGAGCAAAGACAATCCTCCCCGCA
AAACTTTTGTGTATAGGGTA
ACGAAGATCCCCTCAAATAGAAATCTTGGAATTCAAAGTTAGGTAAGGGGTAGCATATTACATCTTGCTGCAAGT
TGCCTXXXXXXXXXXXTTTGACTTTCCATGTATCTCCATTCCTTTCAGGTCTTGAGCAAAGACAATCCTCCCCGCA
AAACTTTTGTGTATAGGGTA
>Marker412858
CACTTTAACCATGCTTTCTCATGAATGTAGTATGTTTATATATAATGCGCCTCATTAAAGAACTATTGCGTTGAG
CCCTAXXXXXXXXXXXTTCGTATGAAAGGAAGTTTATATATTAATGCTGTTATGAACCTGTTTGTGATGTTACTTG
TTACAGTCTCCAGACGAGTA
CACTTTAACCATGCTTTCTCATGAATGTAGTATGTTTATATATAATGCGCCTCATTGAAGAACTATTGCGTTGAG
CCCTAXXXXXXXXXXXTTCGTATGAAAGGAAGTTTATATATAAATGCTGTTATGAACCTGTTTGTGATGTTACTTG
TTACAGTCTCCAGACGAGTA
>Marker412990
CACTTATTTTGGGACACTATTTTTATCCTTCACAATTTTTATTAAAATTTTCCCTGTTTGTAGATCCTTGGTAGT
GGTATXXXXXXXXXXXGGACTGATAACAGGGGAGCAATACTTTCCTGCTGATTCTCTGTCTGATAGCCAGAAGGTA
AATAAACCCATGTTTGTTGT
CACTTATTTTGGGACACTATTTTTATCCTTCACAATTTTTATTAAAATTTGCCCTGTTTGTAGATCCTTGGTAGT
GGTATXXXXXXXXXXXGGACTGATAACAGGGGAGCAATACTTTCCTGCTGATTCTCTGTCTGATAGCCAGAAGGTA
AATAAACCCATGTTTGTTGT
>Marker413009
CACTTTAATCGTCCAAGTCTTCAATCTTTCGGAAGTTGTTGATCTCGAGGTTGATACGAACAGAGCTTCAATTCT
TTCTTXXXXXXXXXXXTCTCCTCACAAACCAACACAAGTCTTTTCAGGATGCTTTGTCCTCATTCACATGTATCGT
AGAAAAATTTCTAGAAGGTT
CACTTTAATCGTCCAAGTCTTCAATCTTTCGGAAGTTGTTGATCTCGAGGTTGATACGAACAGAGCTTCAATTCT
TTCTTXXXXXXXXXXXTCTCCTCACAAACCAACACAAGTCTTTTCAAGATGCTTTGTCCTCATTCACATGTATCAT
AGAAAAATTTCTAGAAGGTT
>Marker413445
ACTCCCAAATTATGGAATCCCAATGACCTTTATTTTAGACTTGAAATTTCCACTGCTAAATTGAAAATTGGTAGC
AAGTTXXXXXXXXXXXTAAGAATTAAAAAGTTGAACTATGAATTCATATACGTTTTTCCCAGTTTGAAAGATCTAT
TCATCCTCCAGCTTTTTTGT
ACTCCCAAATTATGGAATCCCAATGACCTTTATTTTAGACTTGAAATTTCCACTGCTAAATTGAAAATTGGTAGC
AAGTTXXXXXXXXXXXTAAGAATTAAAAAGTTGAACTATGAATTCATATACGTCTTTCCCAGTTTGAAAGATCTAT
TCATCCTCCAGCTTTTTTGT
>Marker414230
AACAATAGCTCAGACTTGATCATTGCTACACCTTGTGTGAGGAGATATCCCTTTCTCCCGAAGTCAGCAGAATGC
TATTTXXXXXXXXXXXTAGTTTATATACTTTAAAATGCTTGAAATTTGGTCAATTTTAGTTCTTATATTTTCAAAT
ATACAATTTTAGTCGTTGTA
AACAATAGCTCAGACTTGATCATTGCTACACCTTGTGTGAGGAGATATCCCTTTCTCCCGAAGTCAGTAGAATGC
TATTTXXXXXXXXXXXTAGTTTATATACTTTAAAATGCTTGAAATTTGGTCAATTTTAGTTCTTATATTTTCAAAT
ATACAATTTTGGTGGTTGTA
>Marker414421

Create PDF with GO2PDF for free, if you wish to

AACCACAAGACAATTTGTCGCATATTGAACTATTACAGCATATGACCTGGTAAATGTCACTCTATGTTATGAAAT
CATATXXXXXXXXXXTATATTCATTTGTATATAATATTTTGTTACCTAAGTGTCATTAGTCGGTTAAATATCTGT
TGATATGTTACCAACATGTT
AACCACAAGACAATTTGTCGCATATTGAACTATTACAGCATATGACCTGGTAAATGTCACTCTATGTTATGAAAT
CATATXXXXXXXXXXTATAGTCATTTGTATATAATATTTTGTTACCTAAGTGTCATTAGTCGGTTAAATATCTGT
TGATATGTTACCAACATGTT
>Marker414452
ACTCTCAAAATTGGCTCACTCCCTCAATTTCTCCTTCATTTCATAACATTGCACGTTATTGTCAGATTCTTCTTT
TAACCXXXXXXXXXXAATTCTTCTTGGTAAATTTGCGTTTTCTTCTTTGGGTGATCGATCATGCTTTGGTTTGCA
TGTTTGTTCCATTGAGGGTT
ACTCTCAAAATTGGCTCACTCCCTCAATTTCTCCTTCATTTCATAACATTGCACGTTATTGTCAGATTCTTCTTT
TAACCXXXXXXXXXXAATTCTTCTTGGTAAATTTGCGTTTTCTTCTTTGGGTGATCGATTATGCTTTGGTTTGCA
TGTTTGTTCCATTGAGGGTT
>Marker414867
ACAAGATATTTATGTCAAACATCAATAATTATAACTTTTTCAACTAACATGTCTATTTCTTTCAACTTTCTTTGA
AGTTCXXXXXXXXXXTTCAGTAAAGACAGCAAATCCACCCCTTTCACTTGAATTATTTAAATCTCACCCATTTTC
CATCAACAGTTTAAAATGTT
ACAAGATATTTATGTCAAACATCAATAATTATAACTTTTTCAACTAACATGTCTATTTCTTTCAACTTTCTTTGA
AGTTCXXXXXXXXXXTTCAGTAAAGACAGCAAATCCACCCCTTTCACTTGAATTATTTAAATCTCACCCATTTTC
CATCAACAGTTTTAAATGTT
>Marker415046
ACCCTGTTTGGAGCTGGGAATAAGGGAATCCTAGCTCTAACAATGATACCAAATTGGCCTAAACCACCAAGGACT
GAGTTXXXXXXXXXXATATAATTATTATTTTGTCTTCTGAAAAATACGATCTCTTTTTAATTTTAAATATAAATA
ACTTTGCTACGTGTGATTGT
ACCCTGTTTGGAGCTGGGAATAAGGGAATCCTAGCTCTAACAATGATACCAAATTGGCCTAAACCACCCAGGACT
GAGTTXXXXXXXXXXATATAATTATTATTTTGTCTTCTGAAAAATACGATCTCTTTTTAATTTTAAATATAAATA
ACTTTGCTACGTGTGATTGT
>Marker415086
GACGTTTATGGGATACCCACAGGCCACAGCAGATTGTAAGTCACTCCATGTGAGATGCTCCACAAAACAAATACG
AGGTTXXXXXXXXXXATCATACACAATACAAAAAGCAGTCATAGCCCTTAGTAATCAATCCAGTAGGAAAGCACT
AAAATCTAAGACTAGCAGTT
GACGTTTATGGGATACCCACAGGCCACAGCAGATTGTAAGTCACTCCATGTGAGATGCTCCACAAAACAAATACG
AGGTTXXXXXXXXXXATCATACACAATACAAAAAGCAGTCATGGTCCTTAGTAATCAATCCAGTAGGAAAGCACT
AAAATCTAAGACTAGCAGTT
>Marker415440
TACAAGTTAGCACGCATCTCCATTTCGCATCCATCTAGTCCCACTTTACAGATTATCTAGGCATACATAAGTAGT
GATTCXXXXXXXXXXCTATTGTTTCACTTATACATGGGCAAGCAATAGGAAAACTTGTATTCGACAAGGATTTAG
TGATTACACGGTAAAGAGGT
TACAAGTTAGCACGCATCTCCATTTCGCATCCATCTAGTCCCACTTTACAGATTATCTAGGCATACATAAGTAGT
GATTCXXXXXXXXXXCTATTGTTTCGCTTATACATGGGCAAGCAATAGGAAAACTTGTATTCGACAAGGATTTAG
TGATTACACGGTAAAGAGGT
>Marker415659
ACTTCGAGGACTAGTTTTTTATCAACGCACTTTACTACTTTTGAAAAAGATTGTATGTTGTGGTATTTCCAGCTG
GGTCAXXXXXXXXXXAGTCTCCTTTCCCTCACAATCATATAAACGAACCAACCTTTCACCTCTATCCATAGTGAT
GTTTAGGGGCCATCCAAGTT

Create PDF with GO2PDF for free, if you wish to

ACTTCGAGGACTAGTTTTTTATCAACGCACTTTACTACTTTTGAAAAAGATTGTATGTTGTGGTATTTCCAGCTG
GGTCAXXXXXXXXXXXAGTCTCCTTTTCCTCACAATCATATAAACGAACCAACCTTTCACCTCTATCCATAGTGAT
GTTTAGGGGCCATCCAAGTT
>Marker416016
TACAAATCTACCTACCAGACAGCATGCTTTGACGCGGTAATATACTTTTCAACAAAATAAAGTCATTGTCCAGAA
GTGACXXXXXXXXXXXTGATGCTAGTATTTCAAAACAAAAAACACCATACAAGAGCACTTAAACTGCTAATAACAA
CCTGGCTGCAAGCTAGGGTC
TACAAATCTACCTACCAGACAGCATGCTTTGACGCGGTAATATACTTTTCAACAAAATAAAGTCATTGTCCAGAA
GTGACXXXXXXXXXXXTGATGCTAGTGTTTCAAAACAAAAAACACCATACAAGAGCACTTAAACTGCTAATAACAA
CCTGGCTGCAAGCTAGGGTC
>Marker416188
CACGTCCTTTTGGAGATAAAATGAGTGAAAAAAAGTCAATAGGCATCAACCGGGGTGAAGCATGAAGAAAAGAAG
CCAAAXXXXXXXXXXXCTTTTCCAATTGAATTTGGGTTAGGTGAAGCCAACCCTTGAACCTAGTAATAAGAAATGC
ACATGAATGAATGCATTGTA
CACGTCCTTTTGGAGATAAAATGAGTGAAAAAAAGTCAATAGGCATCAACCGGGGTGAAGCATGAAGAAAAGAAG
CCAAAXXXXXXXXXXXCTTTTCCAATTGAATTTGGGTTAGATGAAGCCAACCCTTGAACCTAGTAATAAGAAACGC
ACATGAATGAATGCATTGTA
>Marker417014
CACGCTTTCAAAACTTCCGTATTATACCGGTATAAGTTGGGTTAATAATCATGTCAGGCTTATGTTTATTTTAGT
AACGAXXXXXXXXXXXAAGCTGTTCATGGGTTTGTTCATTTTAAATTTAACGATTTTGTTTAATCATGATAAAGCA
AATAAAATTAAAAATTTGTC
CACGCTTTCAAAACTTCCGTATTATACCGATATAAGTTGGGTTAATAATCATGTCAGGCTTATGTTTATTTTAGT
AACGAXXXXXXXXXXXAAGCTGTTCATGGGTTTGTTCATTTTAAATTTAACGATTTTGTTTAATCATGATAAAGCA
AATAAAATTAAAAATTTGTC
>Marker417104
TACTAGAATTGATAAGGTATAGAGAACAAAAGGTTCCCATTGCAACTCAAACAATGAGATGATAGATTTGATTCA
AAAAAXXXXXXXXXXXAAAGAAAAGGATGCATGCATGCTCAAAAACTGCTAACACGTAAGAAGAATTAGGTCATAC
CTGAAATTGGCAAATACAGT
TACTAGAATTGATAAGGTATAGAGAACAAAAGGTTCCAATTGCAACTCAAACAATGAGATGATAGATTTGATTCA
AAAAAXXXXXXXXXXXAAAGAAAAGGATGCATGCATGCTCAAAAACTGCTAACACGTAAGAAGAATTAGGTCATAC
CTGAAATTGGCAAATACAGT
>Marker417138
TACGTTTGTTCCATTTTTCAAATTCAAGATGAAATGTTTAAGTTTGTTCCATTTTTCAAATTTGAGATGAAATGA
ACCCAXXXXXXXXXXXAGCGATTTCCTGGTCATTCGCCAATCATCAAATGCAAATAGTTAGGTAACTATCTAAAGA
CTTTCAAAGTTGGTTGTGTC
TACGTTTGTTCCATTTTTCAAATTCAAGATGAAATGTTTAAGTTTGTTCCATTTTTCAAATTTGAGATGAAATGA
ACCCAXXXXXXXXXXXAGCGATTTCCTGGTCATTCGCCAATCATCAAATGCAAATAGTTAGGTAACTATCTAAAGA
CTTTCCAAGTTGGTTGTGTC
>Marker417839
AACACTTAGCCCAATAAGTTATCTAATGGTTAGTGGATTTTTAGGTGTTGAGATTTTATAAACTAATATTTTGCA
TGTAAXXXXXXXXXXXCTTCAATTTCCATCTCTCTCTCAATTTTCTTCTCTTATCGGGTCTCATAATTTGGTTCTA
AGTTTGAAGAATAAAGGGTC
AACACTTAGCCCAATAAGTTATCTAATGGTTAGTGGATTTTTAGGTGTTGAGTTTTTATAAACTAATATTTTGCA
TGTAAXXXXXXXXXXXCTTCAATTTCCATCTCTCTCTCAATTTTCTTCTCTTATCGGGTCTCATAATTTGGTTCTA
AGTTTGAAGAATAAAGGGTC
>Marker417930

Create PDF with GO2PDF for free, if you wish to

AACCAACTTCCAATCGTCCTATATCTTATTCTGATCGTCCGACTTTGGTTTGGTCAGTTATGGTCGGTCGTTTCA
ATTTTXXXXXXXXXXTATATTTCATCAACCACAAATTAAATGTGTGCTAACTAAGCAAACAAAATTTTGTATTGT
CCAATAGATCAAGTAGTAGT
AACCAACTTCCAATCGTCCTATATCTTATTCTGATCGTCCGACTTTGGTTTGGTCAGTTATGGTTGGTCGTTTCA
ATTTTXXXXXXXXXXTATATTTCATCAACCACAAATTAAATGTGTGCTAACTAAGCAAACAAAATTTTGTATTGT
CCAATAGATCAAGTAGTAGT
>Marker418013
TACTTGTTTAAACTCCAAGCCAGTTTTGAAATCTAAAAAGTAGGTTTTCAATGAACTTGTTGTGTTTCAAACTTG
GCTGAXXXXXXXXXXXAGGTCATTTGATGTTTCTTTAGGAAAGATGAAAATCATTGTAATGAATTGCGAGAAAACA
AACGCAATTTTCAAAATGTA
TACTTGTTTAAACTCCAAGCCAGTTTTGAAATCTAAAAAGTAGGTTTTCAATGAACTTGTTGTGTTTCAAACTTG
GCTGAXXXXXXXXXXXAGGTCATTTGATGTTTCTTTAGGAAAGATGAAAATCATTGTAATGAATTGCAAGAAAACA
AACGCAATTTTCAAAATGTA
>Marker418227
TACCTACTAGATAGAAAATATTTTGTAATCATTAGATAAAAAATGTGTTATGTTAGCAAAATCCTATACGTAGAA
TTATGXXXXXXXXXXXATGTGACAGACAAGATACATGTGATAAGCCAATCGATCATTATATATGTGTATATAATGC
TCAATTATCAAAACGTCAGT
TACCTACTAGATAGAAAATATTTTGTAATCATTAGATAAAAAATGTGTTATGTTAGCAAAATCCTATACGTAGAA
TTATGXXXXXXXXXXXATGTGACAGCCAAGATACATGTGATAAGCCAATCGATCATTATATATGTGTATATAATGC
TCAATTATCAAAACGTCAGT
>Marker418577
AACAACACAACTGCAAATGAAAACTAATGGGTGTTGAAAAGCAGAAACATATAATAGAAGCATAAACATTGAAAG
AAAGAXXXXXXXXXXXCTCCAATTCATTAAGAAACTGCAGAACATCGAACAGACGAGATTGATTTCAAGCTTACAA
CTGGCTGATCAACGAGAGTA
AACAACACAACTGCAAATGAAAACTAATGGGCGTTGAAAAGCAGAAACATATAATAGAAGCATAAACATTGAAAG
AAAGAXXXXXXXXXXXCTCCAATTCATTAAGAAACTGCAGAACATCGAACAGACGAGATTGATTTCAAGCTTACAA
CTGGCTGATCAACGAGAGTA
>Marker418615
ACCTGAGATCTTAAGACGCATAACAAACTCCATTTCTGGCAAAAAAAATTGAAAAGCACATGGGAGCTTTTCGCC
GAGAAXXXXXXXXXXXAAACCATGATTATGTGAATTTCCTTATGCTTCCCAATTCCTAAAATGCTCGTGATTTATG
AATGAATTGTATTTATTGTG
ACCTGAGATCTTAAGACGCATAACAAACTCCATTTCTGGCAAAAAAAATTGAAAAGCACATGGGATCTTTTCGCC
GAGAAXXXXXXXXXXXAAACCATGATTATGTGAATTTCCTTATGCTTCCCAATTCCTAAAATGCTCGTGATTTATG
AATGAATTGTATTTATTGTG
>Marker418696
GACATTATTTACCTGAAGAAGTAACGTAGTGCAGGTGAAATCGAATGCTTCAGGAGCTAACTTATTGAAATAATG
ACGAAXXXXXXXXXXXGTGGATGTGCAAGAGAGGAGGAGATATATTGAGAATGATACCCTCTTTTTATGGTGATTG
CGGAAATATATTTTCTGGTA
GACATTATTTACCTGAAGAAGTAACGTAGTGCAGGTGAAATCGAATGCTTCAGGAGCTAACTTATTGAAATAATG
ACGAAXXXXXXXXXXXGTGGATGTGCAAGAGAGGAGGAGATATATTGAGTATGATACCCTCTTTTTATGGTGATTG
CGGAAATATATTTTCTGGTA
>Marker419492
GACATCCTTTTTCTCATTTTAAGCAGCCTCAACGAGGAAGTAAAGCAAGTAAAGAACTCTACAATTGCCCGGCAT
CTGCAXXXXXXXXXXXCTTATCATTAAAAAACCATTTCAACTTTTTAAGTAAATTATCTAACAAAAAACTAACATA
AACGTTATGAAATGCTTGTG

Create PDF with GO2PDF for free, if you wish to

GACATCCTCTTTCTCATTTTAAGCAGCCTCAACGAGGAAGTAAAGCAAGTAAAGAACTCTACAATTGCCCGGCAT
CTGCAXXXXXXXXXXXCTTATCATTAAAAAACCATTTCAACTTTTTAAGTAAATTATCTAACAAAAAACTAACATA
AACGTTATGAAATGCTTGTG
>Marker419531
ACTAAAATTTCTGCTTGAATATGATCTTTTTGGTTCTGATTGTCCCAGCTTAATTTTCATATGTATTCGTTTTTG
TCTAAXXXXXXXXXXXAGAGTCTTGTTCTTGCATCTCTTATTACTTGTGTGGGGAGTAGTTCTTCAATGAATTGTT
CCATTTTATTGTTATTGGTA
ACTAAAATTTCTGCTTGAATATGATCTTTTTGGTTCTGATTGTCCCAGCTTAATTTTCATATGTATTCTTTTTTG
TCTAAXXXXXXXXXXXAGAGTCTTGTTCTTGCATCTCTTATTACTTGTGTGGGGAGTAGTTCTTCAATGAATTGTT
CCATTTTATTGTTATTGGTA
>Marker420414
ACTTGCAAATTGCTATTGTTTCTGCTAATCTATAAATTATGGGGAAATTTTAGGTCTTGACATCTGGATTATCAA
TTGCTXXXXXXXXXXXAAACTGAAAGCATGGAAGAGATCAAGGGTGGAATCAAGAAAGATGTCACTGAAGTAATTT
TCTCTTTCTATGAGTTGGTT
ACTTGCAAATTGCTATTGTTTCTGCTAATTTATAAATTATGGGGAAATTTTAGGTCTTGACATCTGGATTATCAA
TTGCTXXXXXXXXXXXAAACTGAAAGCATGGAAGAGATCAAGGGTGGAATCAAGAAAGATGTCACTGAAGTAATTT
TCTCTTTCTATGAGTTGGTT
>Marker420443
TACTCTTTTGTTAATAAATTAATAATTGGAAAGAAGCACACAAAGGAATAGATTGGGCCAATACATTTACGGCCC
AAAAAXXXXXXXXXXXTCCCACTTTTCATGATCGGAGAAATTTGTCACACTGATTTTCAGATTTGGGATTTAAAAT
GAATTTTATTACCTTACGTT
TACTCTTTTGTTAATAAATTAATAATTGGAAAGAAGCACACAAAGGAATAGATTGGGCCAATACATTTACGGCCC
AAAAAXXXXXXXXXXXTCCCACTTTTCATGATCGGAGAAATTTGTCACACTGATTTTCAGATTTGGGATTTAAAAT
GAATTTCATTACCTTACGTT
>Marker420651
ACATCAAACCAATTGTGGAACTATCCAATGGATTTCAGTGAGTTCTGTCGAAGTTCATTCATTTATCAATTTCGT
TTTCTXXXXXXXXXXXGAGCAGCTCATCTATTCTAATTATATGTTTTACTCCTTATGACTCTGCAACATTGTCACC
CAACAATTCTGCTTTATGTT
ACATCAACCCAATTGTGGAACTATCCAATGGATTTCAGTGAGTTCTGTCGAAGTTCATTCATTTATCAATTTCGT
TTTCTXXXXXXXXXXXGAGCAGCTCATCTATTCTAATTATATGTTTTACTCCTTATGACTCTGCAACATTGTCACC
CAACAATTCTGCTTTATGTT
>Marker421111
TACCTCTCTCTCACAGCGATCTGCAGAAATCTCCAACGGACAGCCTCTCCGTCGTCGTTGGCCGCCGTTCGACCA
CTCCCXXXXXXXXXXXGTGGTCTTCTCCGGAATTTCGTTTGGGTGAGTTTTTGTGTTAGTTGGAAAGATGTTTTTG
ACGCATCTAGAATGCCTGTT
TACCTCTCTCTCACAGCCATCTGCAGAAATCTCCAACGGACAGCCTCTCCGTCGTCGTTGGCCGCCGTTCGACCA
CTCCCXXXXXXXXXXXGTGGTCTTCTCCGGAATTTCGTTTGGGTGAGTTTTTGTGTTAGTTGGAAAGATGTTTTTG
ACGCATCTAGAATGCCTGTT
>Marker422019
ACTTGTAGCAAGACGACAGAATCTACTTGAAGAAGTTGCAGATATAGCTCGGTATTATGGATCACCAGGTGTAAT
CACCAXXXXXXXXXXXAAATTTCAATTTAGACAACCAAAAACAGAGTAGTTTTTTGCCAATGATTTTGTAATGCAA
ATGCAGTGGATCATTTGGTG
ACTTGTAGCAAGACGACAGAATCTACTTGAAGAAGTTGCAGATATAGCTCGCTATTATGGATCACCAGGTGTAAT
CACCAXXXXXXXXXXXAAATTTCAATTTAGACAACCAAAAACAGAGTAGTTTTTTGCCAATGATTTTGTAATGCAA
ATGCAGTGGATCATTTGGTG
>Marker422656

Create PDF with GO2PDF for free, if you wish to

ACATAACTTTCTCCATCAAATTTATTACGAAACTTCGCACTTATTTAAAGTTCAGTGTTTGGTTCTTAAAATTAA
TGAAAXXXXXXXXXXXTCACCTTATCCTTGCAGGTTGCACCTAGTGAAATGGTGGGAAAGTGGATCTTCACAATTG
ATCTGAAGAAAGCTAGGAGT
ACATAACTTTCTCCATCAAATTTATTACGAAACATCGCACTTATTTAAAGTTCAGTGTTTGGTTCTTAAAATTAA
TGAAAXXXXXXXXXXXTCACCTTATCCTTGCAGGTTGCACCTAGTGAAATGGTGGGAAAGTGGATCTTCACAATTG
ATCTGAAGAAAGCTAGGAGT
>Marker422733
GACCTTGTGTTCTTCCATTGTCAAGCTATAGATTATTATGTCATATAGGTAGATCACGATAAACTTGTCAAGGTA
TTCATXXXXXXXXXXXTTGGGCTTATCTCCCTAAACAATCATTGCTTGATAATGTCCTGATCGCAAGTCTAGTTTC
GGGAAATATTTCGCTCAAGT
GACCTTGTGTTCTTCCATTGTCAAGCTATAGATTATTATGTCATATAGGTAGATCACAATAAACTTGTCAAGATA
TTCATXXXXXXXXXXXTTGGGCTTATCTCCCTAAACAATCATTGCTTGATAATGTCCTGATCGCAAGTCTAGTTTC
GGGAAATATTTCACTCAAGT
>Marker422747
AACCTTGTTAAATGGTTTTCGCAATTACCCTTTTGTTTCCTAAGTTTGAAGGAGCAATGGTTTTATGGAGAATGT
CCTCGXXXXXXXXXXXTCCCTAGTGATATGGTAAGAAGCATGGCCACATACACATGCTACAAATACGACAGATGGC
ACAAAGATATTGGGCATGTA
AACCTTGTTAAATGGTTTTCGCAATTACCCTTTTGTTTCCTAAGTTTGAAGGAGCAATGGTTTTATGGAGAATGT
CCTCGXXXXXXXXXXXTCCCTAGTGATATGGTAAGAAGCATGGCCACATACACATGCTACAAATACGACAGATGGC
ACAAAGATATTGGGGATGTA
>Marker423145
CACCTTGCACTACCAAGCACAATAGTGTAAATATCTAGGATCAATATTATGTTGTTAATATCTTTACTATATAGT
GTATTXXXXXXXXXXXTTCTCATGATAGCAACGCATGAATATAAATGCACCTTTAATTTTATTGCATAACCCTTTT
TTAGTTGCATAATAGTCGTA
CACCTTGCACTGCCAAGCACAATAGTGTAAATATCTAGGATCAATATTATGTTGTTAATATCTTTACTATATAGT
GTATTXXXXXXXXXXXTTCTCATGATAGCAACGCATGAATATAAATGCACCTTTAATTTTATTGCATAACCCTTTT
TTAGTTGCATAATAGTCGTA
>Marker423446
ACCTCAACATTACATGAGCGTGTGAGGCGTTCAAGAATTTGTCTTCTTTTTTGCTTAAGGCTAAGATCATTTTTA
TTGCCXXXXXXXXXXXATACCACGAAGGTAGCATTATGTAAGATCCAAATGGAGTTAAGATTTCACAATAACAAAA
CAGAAAATGACCAGAAGGTC
ACCTCAACATTACATAAGCGTGTGAGGCGTTCAAGAATTTGTCTTCTTTTTTGCTTAAGGCTAAGATCATTTTTA
TTGCCXXXXXXXXXXXATACCAAGAAGGTAGCATTATGTAAGATCCAAATGGAGTTAAGATTTCACAATAACAAAA
CAGAAAATGACCAGAAGGTC
>Marker424356
CACAATGAAGTGAATTTCAATTCCACAGAAAAAAGGGAAAAAAAAGATTCGTAAAAAGCATTATCACTAATCTAT
AGTAAXXXXXXXXXXXCCAATTCACCAACTGACTCTGCAATCTTCTCAATACAAGAAATGGAATCAATGAGCAAAG
AAGCCACAGTCGCCATCGGT
CACAATGAAGTGAATTTCAATTCCACAGAGAAAAGGGAAAAAAAAGATTCGTCAAAAGCATTATCACTAATCTAT
AGTAAXXXXXXXXXXXCCAATTCACCAACTGACTCTGCAATCTTCTCAATACAAGAAATGGAATCAATGAGCAAAG
AAGCCACAGTCGCCATCGGT
>Marker425076
AACAATAGACTGAATTACACAAACAATGAAAAGGAACATGATCAAATTGATCGAGATATCAATTTGAGAAACTAT
GAAAAXXXXXXXXXXXCCCCCTTTTACATGTGCTATTCCTTTCTTTCCCTTTCTAATGTAATGCATAATAATAGGT
GGTCTAACGTAAGCCAAAGT

Create PDF with GO2PDF for free, if you wish to

AACAATAGACTGAATTACACAAACAATGAAAAGGAACATGATCAAATTGATCGAGATATCAATTTGAGAAACTAT
GAAAAXXXXXXXXXXXCCCCCTTTTACACGTGCTATTCCTTTCTTTCCCTTTCTAATGTATTGCATAATAATAGGT
GGTCTAACGTAAGCCAAAGT
>Marker425363
CACAAGGACGGATTTATCAAGAGGCGTGTCCCCCTTCACATTATTGATTTTTTTTTAATATTAATTTTGAAGTGT
CAAACXXXXXXXXXXXCGCCTATTACAACTAAAAAATAGTTTTTATTTCACCAAATTTATTGTTTTGCTCAATCTA
TTTATAGTTTAGAATTAGTT
CACAAGGACGGATTTATCAAGAGGCGTGTCCCCCTTCACATTATCGATTTTTTTTTAATATTAATTTTGAAGTGT
CAAACXXXXXXXXXXXCGCCTATTACAACTAAAAAATAGTTTTTATTTCACCAAATTTATTGTTTTGCTCAATCTA
TTTATAGTTTAGAATTAGTT
>Marker425488
ACTCATGGTGAAGAAGGAAGATGGTTATCATCAAATTAGGATGTGTAGCCAAGACATAGAAAAAACAGCCTTTAG
AACAGXXXXXXXXXXXTTGATTTACAGTAGGGGATTGGAGGAGCACTGCCAACATATAGAATTGGTATTGGAGGTG
TTGAGGAGTCATAAACTGTT
ACTCATGGTGAAGAAGGAAGATGGTTATCATCAAATTAGGATGTGTAGCCAAGACATAGAAAAAACAGCCTTTAG
AACAGXXXXXXXXXXXTTGATTTACAGTAGGGGATTGGAGTAGCACTGCCAACATATAGAATTGGTATTGGAGGTG
TTGAGGAGTCATAAACTGTT
>Marker425579
ACCCATATGTGGCTACGTCCATGTTCCATTGTAGCTAGGTCATAAATAGTTGATTTGATTTGAAATTACTAATTG
ATATTXXXXXXXXXXXGGTTGAAGTTATTTGCGGAAGGATATTAGGCGAGAAGAAAGGTTTGGCAAGAAGATGTCA
AATCTGACTTGACAAGGGTT
ACCCATATGTGGCTACGTCCATGTTCCATTGTAGCTAGGTCATAAATAGTTGATTTGATTTGAAATTACTAATTG
ATATTXXXXXXXXXXXGGTTGAAGTTATTTGCGGAAGGATATTAGGCGAGAAGAAAGGTTTGGCGAGAAGATGTCA
AATCTGACTTGACAAGGGTT
>Marker426713
AACTTGTGTGTTATTTTTGTTGTTACTTTCAATATTTACCAAACTTGTAAAAACACAAGAATAAAACTAAAAGTA
ATATCXXXXXXXXXXXGGTTCAAGCTCGGTTGCTGAAAGCAACTTCTCTTTTGTAACCAGATTAGGATACAGACTA
GTTAGAGCAGTCAGCCTTGT
AACTTGTGTGTTATTTTTGTTGTTACTTTCAATATTTACCAAACTTGTAAAAACACAAGAATAAAACTAAAAGTA
ATATCXXXXXXXXXXXGGTTCAAGCTCGGTTGTTGAAAGCAACTTCTCTTTTGTAACCAGATTAGGATACAGACTA
GTTAGAGCAGTCAGCCTTGT
>Marker426791
TACAAGAGTCACTAGAGAGGGGGTCTATCAAATAAGTCATTTCGTTGTAATTGTTAAACTCTTTATATAGTGATA
TTTCAXXXXXXXXXXXGCTGTTTTTTTATTTCTATCAAGTATATTGACTACCTTGTTCATTGAATCTTCAATATAG
TTGATTAGGGAAGGTGGGTT
TACAAGAGTCACTAGAGAGGGAGTCTATCAAATAAGTCATTTCGTTGTAATTGTTAAACTCTTTATATAGTGATA
TTTCAXXXXXXXXXXXCTGTTTTTTTTATTTCTATCAAGTATATTGACTACCTTGTTCATTGAATCTTCAATAAAG
TCGATTAGGGAAGGTGGGTT
>Marker426825
TACTGCTAAGGACATATTTGGAAGTGATTTTGAAATGGTTAAAATCAACTTGACGTGTTTAAAATCACTCGGAAA
CACACXXXXXXXXXXXCAAACATTAATTAAATGAAATAAAAGATAATACAAGTCCAAGTAGTTGTAGAGTTATGCA
CATAAACATTACACTCTTGT
TACTGCTAAGGACATATTTGGAAATGATTTTGAAATGGTTAAAATCAACTTGACGTGTTTAAAATCACTCGGAAA
CACACXXXXXXXXXXXCAAACATTAATTAAATGAAATAAAAGATAATACAAGTCCAAGTAGTTGTAGAGTTATGCA
CATAAACATTACACTCTTGT
>Marker427323

Create PDF with GO2PDF for free, if you wish to

ACCCAACCTTTTCCCAAAACTAGTCTTCTTGAAAAAGTAGGTTCCCTTTTTTCCAACTTTTGCCATTATGAAAAC
CTCAAXXXXXXXXXXCTTCTAGCCAACTCACTTGCAATAGTTGCACAACCGACTTCGGGTCCTTCCTATGTAATG
TTATAAGGATGAACTTGGTA
ACCCAACCTTTTCCCAAAACTAGTCTTCTTGAAAAAGTAGGTTCCCTTTTTTCCAACTTTTGCCATTATGAAAAC
CTCAAXXXXXXXXXXCTTCTAGCCAACTCACTTGCAATAGTTGCACAGCCGACTTCGGGTCCTTCCTATGTAATG
TTATAAGGATGAACTTGGTA
>Marker427665
TACTTTCTGTCAATCGAGCAATATGTGGCCATTATTGTGTTACGGGCAGTTATTTGAAGATTGGGCAGCAACTTT
ACATCXXXXXXXXXXTGTGTTTAATTTCTGTCAATCGAGCAATATGTGGCCATTATTGTGTAACAGGAAGGTATT
TGAAGGTTGAGCAGCAGGTT
TACTTTCTGTCAATCGAGCAATATGTGGCCATTATTGTGTTACGGGCAGTTATTTGAAGATTGGGCAGCAACTTT
ACATCXXXXXXXXXXTGTGTTTAATTTCTGTCAATCGAGCAATATGTGGCCATTATTGTGTAACAGGAAGGTATT
TGAAGATTGAGCAGCAGGTT
>Marker428001
ACTGAAAGCCTTGCATCAGTGCGAAGCTTGGTATTTTTCCATCATATGCAAAGGCTTGGCATGAGTCCTTTAGTT
TGTTTXXXXXXXXXXATTTTTTATTTTTAAAAAAGGATACTCAGCATCGTCAATGATTATATTCAAAATAGAGCT
TGGAGGAGATTGATTAAGTT
ACTGAAAGCCTTGCATCAGTGCGAAGCTTGGTATTTTTCCATGATATGCAAAGGCTTGGCATGAGTCCTTTAGTT
TGTTTXXXXXXXXXXATTTTTTATTTTTAAAAAAGGATACTCAGCATCGTCAATGATTATATTCAAAATAGAGCT
TGGAGGAGATTGATTAAGTT
>Marker428067
TACTCTGTTGAGAAAACATCTTGTTTTCCCTCTATGTTATATTTAAGATTATCATTTTTCTCTGATAAATCAGTT
CATTAXXXXXXXXXXAGGAAGAAGAAAAGCATGGGAAGATTATGGGTAATCACGTGGTTGATTACGCAGATGCTG
TTGTAATGCCTGGCTTGGTT
TACTCTGTTGAGAAAACATCTTGTTTTCCCTCTATGTTATATTCAAGATTATCATTTTTCTCTGATAAATCAGTT
CATTAXXXXXXXXXXAGGAAGAAGAAAAGCATGGGAAGATTATGGGTAATCACGTGGTTGATTACGCAGATGCTG
TTGTAATGCCTGGCTTGGTT
>Marker428201
ACATTTTTTAAAGTTATACTATTCAACACTTTTTGATTACTAACACATTCAAACATTAAATTTATCGTCAAACAT
GCGATXXXXXXXXXXCCAACAAAGAAAGAGGCAAGCCAATCAAACACAACCATATAAGGCCCTTCCTCCAAAGGC
CAAACAATTAATACTTTTGT
ACATTTTTTAAAGTTATACTATTCAACACTTTGTGATTACTAACACATTCAAACATTAAATTTATCGTCAAACAT
GCGATXXXXXXXXXXCCAACAAAGAAAGAGGCAAGCCAATCAAACACAACCATATAAGGCCCTTCCTCCAAAGGC
CAAACAATTAATACTTTTGT
>Marker428573
GACATCATTAAAAAAAGGACAATAACGCAACAATTTATTAAAAATAAAATTGTAAAAACGGACCCAAAAACAAGG
CTTTAXXXXXXXXXXTTGTGCATCATTGAATGAAATTTTGATGGAAGATAATAAGGGTGCTAAAAGGGTGGCCAT
CTAATAGGGATGCTTGGGTG
GACATCATTAAAAAAAGGACAATAACGCAACAATTTATTAAAAATAAAATTGTAAAAACGGACCCAAAAACAAGG
CTTTAXXXXXXXXXXTTGTGCATCATTGAATGAAATTTTGATGGAAGATAATAAGGGTGCTAAAAGGGTGGCCAT
CTAGTAGGGATGCTTGGGTG
>Marker429225
CACTTCATTGTTCCATAGCTGTAATAATGAGCAATTGTTTAAAGCAAACTTTTACAATCGTTGCAGAGGAGGAGA
AAACCXXXXXXXXXXTTATCTGCAGCATTCCTTTCTCAGACCATGAATCTGGCGAGAGAGACGACCGCTCAGAAA
ACGATGCTTCCGTCGCAGTG

Create PDF with GO2PDF for free, if you wish to

CACTTCATTGTTCCATGGCTGTAATAATGAGCAATTGTTTAAAGCAAACTTTTACAATCGTTGCAGAGGAGGAAA
AAACCXXXXXXXXXXTTATCTGCAGCATTCCTTTCTCAGACCATGAATCTGGCGAGAGAGACGACCGCTCAGAAA
ACGATGCGTCCGTCGCAGTG
>Marker430872
ACATTTCTTGATCTTCTTATGAATGTTGTTGATGGATCAATACCATTTCGATTTAATTAATTATAGAAGAGACCT
CCTCCXXXXXXXXXXAGCTAGAATCTAAATTTGAGAGGACAATTTTTTTGAAAATAGACATGATTTTAATTAGTG
ATTCTGTAGGGTATCAGGGT
ACATTTCTTGATCTTCTTATGAATGTTGTTGATGGATCAATACCATTTGGATTTAATTAATTATAGAAGAGACCT
CCTCCXXXXXXXXXXAGCTAGAATCTAAATTTGAGAGGACAAATTTTTTGAAAATAGACATGATTTTAATTAGTG
ATTCTGTAGGGTATCAGGGT
>Marker431451
ACCAATGTTTCCTGTGATCATACTTTCCTAATGTCTTCACTAAATTATCTACAAATATTATTGTATCGTCATCTG
TCATTXXXXXXXXXXAAAACATAGAAAGCTGATCAATTCATGAGTTATAACTTTTAGATGTTTATACATATTTAG
TCTCTCACAACTTTGAAGGT
ACCAATGTTTCTTGTGATCATACTTTCCTAATGTCTTCACTAAATTATCTACAAATATTATTGTATCGTCATCTG
TCATTXXXXXXXXXXAAAACATAGAAAGCTGATCAATTCATGAGTTATAACTTTTAGATGTTTATACATATTTAG
TCTCTCACAACTTTGAAGGT
>Marker431768
ACCTCAGCGAACAAAAAATGGGATGTGTTTATTGATTTGTAGCCTAGTTCCTTGTGCATCCTAGGTTTTTTGTTT
TTGTTXXXXXXXXXXCTATTTTGTGTTGTGCGTCGGAAATCACGCTCTAACGAGTGAAATCGTACGCCACCAATA
ATGCATCCACTGTAAGAGTG
ACCTCCGCGAACAAAAAATGGGATGTGTTTATTGATTTGTAGCCTAGTTCCTTGTGCATCCTAGGTTTTTTGTTT
TTGTTXXXXXXXXXXCTATTTTGTGTTGTGCGTCGGAAATCACGCTCTAACAAGTGAAATCGTACGCCACCAATA
ATGCATCCACTGTAAGAGTG
>Marker431956
TACTAACCGACAACAAATTGCAAGCTAATTTTTGAACATGAAGGGATGACCTTCAAATTTTGGAGCAGTTAGTAT
CTTTAXXXXXXXXXXGCCAACTTCAGAAAATAGGTTATTAATAGATGGAAGAGAGGTTTTACCAAGTATTGGACC
TCTCTAACCTCATAAAAGTC
TACTAACCAACAACAAATTGCAAGCTAATTTTTGAACATGAAGGGATGACCTTCAAATTTTGGAGCAGTTAGTAT
CTTTAXXXXXXXXXXGCCAACTTCAGAAAATAGGTTATTAATAGATGGAAGAGAGGTTTTACCAAGTATTGGACC
TCTCTAACCTCATAAAAGTC
>Marker432214
CACTAAGTGTTAGTAGAATGTGAGGTGTTAGATTTTGACGAACTAATTAATGTATTTATGCATGTAAATAGATTA
TTTAAXXXXXXXXXXTCAAATTTAAAAACTTTTTGCACTCTAATTATTTTTCTTTCAAAAAATAAGAAAGAAAAA
TCTTACCAAAGATTTTGGTG
CACTAAGTGTTAGTAGAATGTGAGGTGTTAGATTTTGACGAACTAATTAATGTATTTATGCATGTAAATAGATTA
TTTAAXXXXXXXXXXTCAAATTTAAAAACTTTTTGCACTTTAATTATTTTTCTTTTAAAAAATAAGAAAGAAAAA
TCTTACCAAAGATTTTGGTG
>Marker432892
ACCATCCAAGAGCGCTCCACATCTAAAGGATTACTGATAAGTAATGAATAATGTGTTAGAAATATAAAATTTTCA
ACAATXXXXXXXXXXATTATAGTTTGATTAGAATAGTTTGTTTTTTGGTGTAGATTATTTTAGTTTGGGTTATAA
TAGTATGTGTCTGGTGTGTA
ACCATCCAAGAGCGCTCCACATCTAAAGGATTACTGATAAGTAATGAATAATGTGTTAGAAATATAAAATTTTCA
ACAATXXXXXXXXXXATTATAGTTTGATTAGAATAGTTTGTTTTTGGGTGTAGATTATTTTAGTTTGGGTTATAA
TAGTATGTATCTGGTGTGTA
>Marker433210

Create PDF with GO2PDF for free, if you wish to

AACCCAAAAATAATGCTATAGAAACTAATGTTGAACATTGATGGGAAGGGTATAGAATATAGATACCGCATTTTC
ATCATXXXXXXXXXXXTTGAGAACAGAACAACAGCTAGAAACTGTTCATCAAAGAGAACGTGTCAGGAAAAAAACC
AGAAAGAAAAATAATTTGTT
AACCCAAAAATAATGCTATAGAAACTAATGTTGAACATTGATGGGAAGGGTATAGAATATAGATACCGCATTTTC
ATCATXXXXXXXXXXXTTGAGACCAGAACAACAGCTAGAAACTGTTCATCAAAGAGAACGTGTCAGGAAGAAAACC
AGAAAGAAAAATAATTTGTT
>Marker433546
ACTTTTATTTTATCTTTATTGTGCAGTTTAAAAAAATGAAAACCTTGCGTCTTATCTTTCTTTCCCCCCACTTTT
TGCTGXXXXXXXXXXXAGAAAAGAAAATAATCAAGCATAGACACACACACACGCACACAACCACAAAATAGTAGTT
TCTACACCCATAAATATGTT
ACTTTTATTTTATCTTTATTGTGCAGTTTAAAAAAATGAAAACCTTTCGTCCTATCTTTCTTTCCCCCCACTTTT
TGCTGXXXXXXXXXXXAGAAAAGAAAATAATCAAGCATAGACACACACACACGCACACAACCACAAAATAGTAGTT
TCTACACCCATAAATATGTT
>Marker434603
AACCTTTGCATTGCAGCTTCAAGCCTGTCACGGAAATTTTTTTAAGACTGACTGCATTTATGATAAAACTAACAA
CTTATXXXXXXXXXXXATAAAAATTTCCAGACAACATCCTAGCAACAGATTACATGGACAAAATAATCCGTTGCAA
AACCACTTCAGGTTAATGTT
AACCTTTGCATTGCAGCTTCAAGCCTGTCAGAGAAATTTTTTTAAGACTGACCGCATTTATGATAAAACTAACAA
CTTATXXXXXXXXXXXATAAAAATTTCCAGACAACATCCTAGCAACAGATTACATGGACAAAATAATCCGTTGCAA
AACCACTTCAGGTTAATGTT
>Marker434739
AACAGCCTGCATATAATACATCAATTGGTTGAAAGACCTCAGATACGGATAAACAGAAGGAAAAAGAATCAGACA
CTCTAXXXXXXXXXXXCTTTGCCCATGTATCCTCCTCAAAATTAGCAGGGAGTGTCGGTTTTGCTTCATAGAGAAA
ACGGAAAATGAGATATTGTA
AACAGCCTGCATATAATACATCAATTGGTTGAAAGACCTCAGATAGGGATAAACAGAAGGAAAAAGAATCAGAAA
CTCTAXXXXXXXXXXXCTTTGCCCATGTATCCTCCTCAAAATTAGCAGGGAGTGTCGGTTTTGCTTCATAGAGAAA
ACGGAAAATGAGATATTGTA
>Marker434832
TACTGATACAATAGATTTTTATAAGGAGAAAAGAAGATACGAAGTATATATGTGTTGTTAAGAATAATGATCGTT
AAAATXXXXXXXXXXXTAAAAATTGTTTCAGTAAATCGATGTATTGATATTTTAAGTTGAAACTTTACAATAAAAA
TTAATATGACGCGATAAGTT
TACTGATACAATAGATTTTTATAAGGAGAAAAGAAGATACGAAGTATATATGTGTTGTTAAGAATAATGATCGTT
AAAGTXXXXXXXXXXXTAAAAATTGTTTAAGTAAATCGATGTATTGATATTTTAAGTTGAAACTTTACAATAAAAA
TTAATATGACGCGATAAGTT
>Marker435658
AACAGTATCCACAGTTCTGTATCTGTTCTTGTAGATGATACAATACCAAATGCTTTTTAATCAATAGATGGTTTA
ATATGXXXXXXXXXXXTGGAGTAATTCAAGGGACCTTATAATATTTATTCCATTGATGGAGTAACCACCTATTGTC
AATTGTCAGTTGTTTTCGTG
AACAGTATCCACAGTTCTGTATCTGTTCTTGTAGATGATACAATACCAAATGCTTTTTAATCAATAGATGGTTTA
TTATGXXXXXXXXXXXTGGAGTAATTCAAGGGACCTTATAATATTTATTCCATTGATGGAGTAACCACCTATTGTC
AATTGTCAGTTGTTTTCGTG
>Marker436008
ACTTTTAGGGAATAATCCCAACTCCAGTTATAGTGTTTTCAGTTCATCTTGTTTTAATTTGAATCTCTATTAGTT
ACATAXXXXXXXXXXXGTTTTGTTACCTATGGTTAATTTGCAGGTGCAAGAACACATATTTGTAGAATCAAACAAG
TTGATCAAGTCAATTCAAGT

Create PDF with GO2PDF for free, if you wish to

ACTTTTAGGGAATAATCCCAACTCCAGTTATAGTCTTTTCAGTTCATCTTGTTTTAATTTGAATCTCTATTAGTT
ACATAXXXXXXXXXXXGTTTTGTTACCTATGGTTAATTTGCAGGTGCAAGAACACATATTTGTAGAATCAAACAAG
TTGATCAAGTCAATTCAAGT
>Marker436670
ACTTGTTCCAAATTGTTAGGAAATTCACAGGCTTTATTGTTGGCTAACTAGCTGAGAGATATTAAAAAAGATATT
AAAAGXXXXXXXXXXXTACTCTTAACCCTTGCCCTGTTCATCTCAAGAATGTAGAACAAGATTTCCATCATCCTAT
ACGCATGCAGTGCAACCGTG
ACTTGTTCCAAATTGTTAGGAAATTCACAGGCTTTATTGTTGGCTAACTAGCTGAGAGACATTAAAAAAGATATT
AAAAGXXXXXXXXXXXTACTCTTAACCCTTGCCCTGTTCATCTCAAGAATGTAGAACAAGATTTCCATCATCCTAT
ATGCATGCAGTGGAACCGTG
>Marker436867
AACCCAACACAAGAAATCCTCAAGTCATCTAAGGAACTCAGGAGGAGTTGAGGTTAGTTGGAGAAAGATGATTCA
AGATTXXXXXXXXXXXAAGAAGGATTGTTTTGGTGTGACCAAGAGGTGTCAAGATAACCTCTATAGCACAAGGCTA
GGTGGCCAAAGACATTGGTT
AACCCAACACAAGAAATCCTCAAGTCATCTAAGGAACTCAGGAGGAGTTGAGGTTAGTTGGAGAAAGATGATTCA
AGATTXXXXXXXXXXXAAGAAGGATTGTTTTGGTGTGACCAAGAAGTGTCAAGATAACCTCTATAGCACAAGGCTA
GGTGGCCAAAGACATTGGTT
>Marker436936
ACTCACACAACAGTAGTCATACCTTAACAACATTGATTAACATTGATTGTGATAATCTAAACTAAAGAAAATAGT
GAAGCXXXXXXXXXXXAATAGAGAGGTGTTTGGAAGCAAAAGATCTGACGGTCCAGATTGAATAAAAAAACCCGGT
TTTGTGATTCCCAAAACAGT
ACTCACACAACAGTAGTCATACCTTAAAAACATTGATTAACATTGATTGCCATAATCTAAACTAAAGAAAATAGT
GAAGCXXXXXXXXXXXAATAGAGAGGTGTTTGGAAGCAAAAGATCTGACGGTCCAGATTGAATAAAAAAACCCGGT
TTTGTGATTCCCAAAACAGT
>Marker437180
TACATGAGCCCCCAACTGGATTTGAAACCCAATTTGGTAAGCAGGTATGGAATCTCCAGAAATCCCTAATGGTCT
GAAACXXXXXXXXXXXCAAAACAGGGAAGATTGCAGTTCTTATAGTTTATGTGGATGACATCGTTTTGTCTTGAGA
TGAACAGACAGAAATCAGTC
TACATGAGCCCCCAACTAGATTTGAAACCCAATTTGGTAAGCAGGTATGGAATCTCCAGAAATCCCTAATGGTCT
GAAACXXXXXXXXXXXCAAAACAGGGAAGATTGCAGTTCTTATAGTTTATATGGATGACATCGTTTTGTCTTGAGA
TGAACAGACAGAAATCAGTC
>Marker437639
ACTAAAATCCTTTTGTGCTTCCATTAAGCTGTTTCTTCCTTCATCTTTCCACCTTGATTAATGATTCAGTTTGGG
CCACGXXXXXXXXXXXATTGTTGTTTGGTTCTCTATTTTGTTTGGATGTGATGAGGGTGTTAAAGGAGATGTCAAT
CTAGTTGAGATATTTGGGTG
ACTAAAATCCTTTTGTGCTTCCATTAATCTGTTTCTTCCTTCATCTTTCCACCTTGATTAATGATTCAGTTTGGG
CCACGXXXXXXXXXXXATTGTTGTTTGGTTCTCTATTTTGTTTGGATGTGATGAGGGTGTTAAAGGAGATGTCAAT
CTAGTTGAGATATTTGGGTG
>Marker437657
GACATCATTTCTTTCTTTGGAACTGTATTTTGTATTATATCAAAACAAGGATAAGTTTCTATCCAAAATTTCACA
AACTTXXXXXXXXXXXCAATGGGCCATCAAGCATGTGGAGCTCTCTCTCTCTGGAACTATCCATCATGGATGAGAA
AACTTATTGCCCACGATGTT
GACATCATTTCTTTCTTTGGAACTGTATTTTGTATTATATCAAAACAAGGATAAGTTTCTATCCAAATTTTCACA
AACTTXXXXXXXXXXXCAATGGGCCATCAAGCATGTGGAGCTCTCTCTCTCTGGAACTATCCATCATGGATGAGAA
AACTTATTGCCCACGATGTT
>Marker438324

Create PDF with GO2PDF for free, if you wish to

GACGGCACCCTTCTGGGGCAGTGTTTCAAAAGCTGAGACAGTGTTCTCAAATTTAATACGATGAAGAGGAGCTCC
ACTCTXXXXXXXXXXATATAGAAGAACGGGTTCATGAAGCAGAAAACTGAAACAAAGTCATGAGGAAAAACATGG
CAAATACAATCGTCCTAGTG
GACGGCACCCTTCTGGGGCAGTGTTTCAAAAGCTGAGACAGTGTTCTCAAATTTAATACGATGAAGAGGAGCTCC
ACTCTXXXXXXXXXXATATAGAAGAACGGGTTCATGAAGCAGAAAACTGAAACAAAGTCATGAGGAAAAACATGG
CAAATACAATCGTCATAGTG
>Marker438395
TACAACGTTATTATCCATGATAATATTATCACCGCCACTCCCGGTCCCCTGTGTGACAAACACACAATAGATTAA
TTTAGXXXXXXXXXXACCCTTAATAACTCGAGCGCCCGATTGTAATGTTTATGGCTCAAATTTCATCATTATGGG
TATAAGTGCTTTTTACCGTT
TACAACGTTATTATCCATGATAATATTATCACCGCCACTCCCGGTCCCCTGTGTGACAAACACACAATAGATTAA
TTTAGXXXXXXXXXXACCCTTAATAACTCGAGCGCCCGATTGTAATATTTAGGGCTCAAATTTCATCATTATGGG
TATAAGTGCTTTTTACCGTT
>Marker438701
ACTTTTCTTTGAGAATGCTAGTTCTACCACTAATTGCTTTCTAATTTCAATCTTTAGTTGAAATTTCAATTTTGC
TTTTAXXXXXXXXXXAAAATGGAAGTAAATTCTAGCATGAAGATTAATTCAATCAACAACTAATTAGTTCAAGAA
AATTTATCAACGTATTTGTT
ACTTTTCTTTGAGAATGCTAGTTCTACCACTAATTGCTTTCTAATTTCAATCTTTAGTTGAAATTTCAATTTTGA
TTTTAXXXXXXXXXXAAAATGGAAGTAAATTCTAGCATGAAGATTAATTCAATCAACAACTAATTAGTTCAAGAA
AATTTATCAACGTGTTTGTT
>Marker438950
CACCTTTGATTTTGAAGTAAAGTAATGAAAATCAATTAATTATAAATTTTAGTAGAAATTAGCAATGAAATTAAG
AAGTGXXXXXXXXXXATTCAAAATCTACTAAAAGAATAAATTTTCCGAAGAGAAAAGTGAATAGCTAAGAAAATA
AATAAATTGGTGATGTAGTC
CACCTTTGATTTTCAAGTAAAGTAATGAAAATCAATTAATTATAAATTTTAGTAGAAATTAGCAATGAAATTAAG
AAGTGXXXXXXXXXXATTCAAAATCTACTAAAAGAATAAATTTTCCGAAGAGAAAAGTGAATAGCTAAGAAAATA
AATAAATTGGTGATGTAGTC
>Marker439021
GACAACACTTTTCACCGATAGATATAGTCAAAAGTCATAAAATATTAGTGAAGGAAACATAAAACACTCTGAAGG
AAAAAXXXXXXXXXXAACCAGTATCAGATAATGAATTGATCTCGCCTCCTGTTGATGGAATATGTTGTCTTGAAT
TTAATCCCTTGGAGGTTGTG
GACAACACTTTTCACCGATAGATATAGTCAAAAGTCATAAAATATTAGTGAAGGAAACATAAAACACTCTGAAGT
AAAAAXXXXXXXXXXAACCAGTATCAGATAATGAATTGATCTCGCCTCCTGTTGATGGAATATGTTGTCTTGAAT
TTAATCCCTTGGAGGTTGTG
>Marker439130
ACCCTTTTAGTGATTAGTTTGAATTATATTTTGGCATAACCACTTTATTGGCGAAAATGAGATTTAATCACTTAT
TGTTGXXXXXXXXXXGGTAGTTTATAAATGGTTAATATTCATGAATTAGTGTCTGATGAGCATTTACTACAAAGC
TAAAGCTAAAGACTCAGGTG
ACCCTTTTAGTGATTAGTTTGAATTATATTTTGGCATAACCACTTTATTGGTGAAAATGAGATTTAATCACTTAT
TGTTGXXXXXXXXXXGGTAGTTTATAAATGGTTAATATTCATGAATTAGTGTCTGATGAGCATTTACTACAAAGC
TAAAGCTAAAGACTCAGGTG
>Marker439186
TACCAATTCTTCAATTTTTTCAAAGGTCATCTAAAGATCTATCAAAACTTTAAAATCTTGAAAGTCGTGTAAATG
AGTGTXXXXXXXXXXGTGATTATTTTTGTATCATTTAAATATATACACATATACATACATTATTTTAGTCTCCAT
ATGCTAAGACTACTTATGTT

Create PDF with GO2PDF for free, if you wish to

TACCAATTCTTCAATTTTTTCAAAGGTCATCTAAAGATCTATCAAAACTTTAAAATCTTGAAAGTCGTGTAAATG
AGTGTXXXXXXXXXXGTGATGATTTTTGTATAATTTAAATATATACACATATACATACATTATTTTAGTCTCCAT
ATGCTAAGACTACTTATGTT
>Marker439505
TACATCTGGGCCCCGCCTTCCACTAATTGATTATGGCTGCTTGGAAACTAAAAATGCCTCCACGAATCTTTTAAA
CCAATXXXXXXXXXXAATCATGTTTCTTCACGCTTGAATTTCGGAGGCATTTTGTAACTATTTTATGGCAGTAT
TTCGCATAGTTGGAATCGTG
TACATCTGGGCCCCGCCTTCCACTAATTGATTATGGCTGCTTGGAAACTAAAAATGCCTCCACGAATCTTTAAAA
CCAATXXXXXXXXXXAATCATGTTTCTTCACGCTTGAATTTCGGAGGCATTTTGTAACTATTTTATGGACAGTAT
TTCGCATAGTTGGAATCGTG
>Marker439635
ACCCTTTTAACTTCTCTTCTTCTGATCTTCTGAACTTCTTTTTCCCTTTAATCATTTGTTTCTTATACAGTTTTA
GTCTAXXXXXXXXXXTACTGTTACATGCAAATGGGTTGCTTCATATTGGAATTCCATATACACTTCTCAAATATA
TATTTTTTTATACAAAAGTA
ACCCTTTTAACTTCTCTTCTTCTGATCTTCTGAACTTCTTTTTCCCTTTAATCATTTGTTTCTTATACAGTTTTA
GTCTAXXXXXXXXXXTACTGTTACATGCAAATGGGTTGCTTCATATTGGAATTCCATATACACTTCTCAAATATA
TATATTTTTATACAAAAGTA
>Marker439885
ACATCAAAACGACGTCGGATTATATCCACAATACCAAAACAATTCAATGGTGATTCAAAGCGGTGGGATTGTGAA
GAAAAXXXXXXXXXXCTGGGGATTTACAAGCCAGCCCGCAGTCGCCACCGCCACCGTCGATGGTGCCCACTTCGG
CCTCTGATGAAATCTCTGTT
ACATCAAAACGACGTCGGATTATATCCACAATACCAAAACAATTCAATGGTGATTCAAAGCGGTGGGATTGAGAC
GAAAAXXXXXXXXXXCTGGGGATTTACAAGCCAGCCCGCAGTCGCCACCGCCACCGTCGATGGTGCCCACTTCGG
CCTCTGATGAAATCTCTGTT
>Marker440466
TACTAAAGTGGGAATTTTCAGATTTTTTCTCATTCGCTTTAGATATAGAACTAGCTGCCTTGGAAAATCGCCTCA
CAGACXXXXXXXXXXAGAAAGAAGAGAGTAAATAAAAGTCACGAACACCGCCAATTTTCGACCGGAAAATAAAGC
CAAACTGAATAAACCCTTGT
TACTAAAGTGGGAATTTTCAGATTTTTTCTCATTCGCTTTAGATATAGAACTAGCTGCCTTGGAAAATCGCCTCA
CAGACXXXXXXXXXXAGAAAGAAGAGAGTAAATAAAAGTCACGAACACCGCCAATTTTCGACCGGAAAATAAAGC
CAATCTGAATAAACCCTTGT
>Marker441336
ACTCCGACACAATAGGTAAAACAAAAAAAAATGCAATCGAGTCTAAAATCATAGTCTTCATGCAATTGAGTCTAA
AACCAXXXXXXXXXXAAAATTAATTTCCTACTGGTTTCCTTGACACCCAAATGTTGTAGGATTAGATGGGTTGAT
CCATGAAATTAGTCGATGTG
ACTCCGACACAATAGGTAAAACAAAAAAAAATGCAATCGAGTCTAAAATCATAGTCTTCATGCAATTGAGTCTAA
AACCAXXXXXXXXXXAAAATTAATTTCCTACTGGTTTCCTTGACACCCAAATGTTGTAGGATTAGATGGATTGAT
CCATGAAATTAGTCGATGTG
>Marker443082
TACTCATTATTGGAAACTCCAAAGAATCTAGTTCCAATATTTCGTGGTCATTCATTACTACTGAAGATGATTTAA
GCTCTXXXXXXXXXXCTATCCCTCATGCACATGTTTGATATCATTTTGCACTCCGACCGGATAAATCACCATATG
CTAGAAAGTCATTAATTGTC
TACTCATTATTGGAAACTCCAAAGAATCTAGTTCCAATATTTCGTGGTCATTCATTACTACTGAAGATGATTTAA
GCTCTXXXXXXXXXXCTATCCCTCATGCACATGTTTGATATCATTTTGCACTCCGACCGGATAAATCACCATATG
CTGGAAAGTCATTAATTGTC
>Marker443322

Create PDF with GO2PDF for free, if you wish to

CACAACTCACTCAGAATTAATGTCAAGTCTCCTATGGTCATCTTAGTGAAATATTTGTTCCTTCGAATAACAATG
TTACAXXXXXXXXXXXATTTACATCTCATGTAACAATTAAAGAGTGAGTTGCATCCACAATGTTATCAGAATAAGG
CACTCGACCTCTATTCAAGT
CACAACTCACTCAGAATTAACGTCAAGTCTCCTATGGTCATCTTAGTGAAATATTTGTTCCTTCGAATAACAATG
TTACAXXXXXXXXXXXATTTACATCTCATGTAACAATTAAAGAGTGAGTTGCATCCACAATGTTATCAGAATAAGG
CACTCGGCCTCTATTCAAGT
>Marker443736
TACCATAATAAGTGCATTCTAATTACAGCTACAAATATTAAAGAGGACAAATCAACATAAAGAAATCATGTTACC
GTAACXXXXXXXXXXXTTTTATTACTACTTTTGGAGGGATATTATTCTTACAGGAATCGTTGAGGTTCGTATGATT
TCGTAGATGAATAGGAGGTG
TACCATAATAAGTGCATTCTAATTACAGCTACAAATATTAAAGAGGACAAATCAACATAAAGAAATCATGTTACC
GTAACXXXXXXXXXXXTTTTATTACTACTTTTGGAGGGATATTATTCTTACAGGAATCGTTGAGGTTCGTATGATT
TAGTAGATGAATAGGAGGTG
>Marker445371
TACAAAATTACGAATCACGAGTTTAGGGCATAAACCCCGACAAGAGTGACACCAAATTCTCGATTTAATCATGTT
AGACAXXXXXXXXXXXTCCTCCAGCTATCTCGAAAGTAATTAACTGAGAGAATAGCTAAAGAACTAGAAAAAACAA
ATAACACATAAGGCTTTGTT
TACAAAATTACGAATCACGAGTTTAGGGCATAAACCCCGACAAGAGTGACACCAAATTCCTGATTTAATCATGTT
AGACAXXXXXXXXXXXTCCTCCAGCTATATCGAAAGTAATTAACTGAGAGAATAGCTAAAGAACTAGAAAAAACAA
ATAACACATAAGGCTTTGTT
>Marker445616
AACTTTTTCCATTCAACTCTAAATTTCTCCATCTCTCTTCATTTATGCCCAAATGAATCTCTATTCAATCACAAC
ATTTTXXXXXXXXXXXCCCTCTTAATAGGTGAGACTCAAAGGATAAATTTAAAATTACTACACACAAGGAAGATTT
TGAGATAAATTTGTTAAAGT
AACTTTTTCCATTCAACTCTAAATTTCTCCATCTCTCTTCATTTATGCCCAAATGAATCTCTATTCAATCACAAC
ATTTTXXXXXXXXXXXCCCTCTTAATAGGTGAGACTCAAATGATAAATTTAAAATTACAACACACAAGGAAGATTT
TGAGATAAATTTGTTAAAGT
>Marker446762
AACAAAATCACCACAGCATAATTAGAATAAAAGCTTTATAGTCCAATTCAAAAACATATAGAAACAATTATACCT
TAAGAXXXXXXXXXXXTTAAAATCTGCATCATACATTTATACATGACATTATATGATATCATCTCATATCATCCTG
GAGATATCTAGAAATTAGTT
AACAAAATCACCACAGCATAATTAGAATAAAAGCTTTATAGTCCAATTCAAAAACATATAGAAACAATTATACCT
TAAGAXXXXXXXXXXXTTAAAATCTGCATCATACATTTATACATGACATTATATGATATCATCTCATATCATCCTG
GAGATATGTAGAAATTAGTT
>Marker447064
GACTAGGTTTCAGACCCTGATGATAGAAAATCCACTTTTGGCTTTTGTGTCTATCTTGGTGGTAACTTAATCTCG
TAGGGXXXXXXXXXXXTCTTAGTATTATCTTACGTCACCCCCTATTTTATGGTGTGACAACTTAAGTGTAGTTCAT
CTAAGTGTTAATAATCATGT
GACTAGGCTTCAGACCCTGATGATAGAAAATCCACTTTTGGCTTTTGTGTCTATCTTGGTGGTAACTTAATCTCG
TAGGGXXXXXXXXXXXTCTTAGTATTATCTTACGTCACCCCCTATTTTATGGTGTGACAAATTAAGTGTAGTTCAT
CTAAGTGTTAATAATCCTGT
>Marker447104
ACTCCTTTGGATAAAAAGCCATCGGCCAAGCCCCAATAGAACTCTATAAAACCAGACTAGCTTAGAAATTACCTT
CTGAAXXXXXXXXXXXTCCTGAGAATGGCACCGAAACCCGTAAATCATAATAAGGTTGGAGTAGAACAGCACAATC
AATAAAGGGGACAGAAAGTT

Create PDF with GO2PDF for free, if you wish to

ACTCCTTTGGAAAAAAAGCCATCGGCCAAGCCCCAATAGAACTCTATAAAACCAGACTAGCTTAGAAATTACCTT
CTGAAXXXXXXXXXXXTCCTGAGAATGGCACCGAAACCCGTAAATCATAATAAGGTTGGAGTAGAACAGCACAATC
AATAAAGGGGACAGAAAGTT
>Marker447791
AACTTTCGGTGTCTAATAAATCTTTTTAGTTTTAGTATCACCAAAGAAAATGCTAGGCAAAATGATTAAAAGTAT
GCTTAXXXXXXXXXXXTTTTCCTTTCAGCAAGAACGTTTGTGTATATTTAAACAAACCCAAGATATGGTCTAGATC
CTCAGGGTGGAAATGATAGT
AACTTTCTGTGTCTAATAAATCTTTTTAGTTTTAGTATCACCAAAGAAAATGCTAGGCAAAATGATTAAAAGTAT
GCTTAXXXXXXXXXXXTTTTCCTTTCAGCAAGAACGTTTGTGTATATTTAAACAAACCCAAGATATGGTCTAGATC
CTCAGGGTGGAAATGATAGT
>Marker447888
ACTCCAAGCTTTAAACTCGTTCATTACATCAATAAATAGTCTTGTTTCCGTTTAAAATAAATAAATAAAGGCTTG
TAACTXXXXXXXXXXXGTTTCAACAAAAGGTGGAACATTTTGTGTTAGTTGTCACACTAAACCACTAGTCCAACTT
TTTTGCTCAATTTCAAAGTC
ACTCCAAGCTTTAAACTCGTTCATTACATCAATAAATAGTCTTGTTTCCGTTTAAAATAAATAAATAAAGGCTTG
TAACTXXXXXXXXXXXGTTTCAACAAAAGGTGGAACATTTTGTGTTAGTTGTCACACTAAACAACTAGTCCAACTT
TTTTGCTCAATTTCAAAGTC
>Marker448346
CACACAACAAGGTCATCCAAATAGATTAGTGAATTTAAAGAACTCGCACTAGATCCTGGCTTCTCATCCTCCCTG
ACAATXXXXXXXXXXXAGTTAAAATCTTTTTTTAAAAAAATATTTTCCACAGTGACGAACGGCTCAAATTTCCTGT
GAAACATTTTTTTTCTTCGTT
CACACAACAAGGTCATCCAAATAGATTAGTGAATTCAAAGAACTCGCACTAGATCCTGGCTTCTCAACCTCCCTG
ACAATXXXXXXXXXXXAGTTAAAATCTTTTTTTAAAGAAATATTTTCCACAGTGACGAACGGCTCAAATTTCCTGT
GAAACATTTTTTTTCTTCGTT
>Marker448560
ACTTGTTCCTTTCTGACCAAGTTGTTGAACTTGATGAAGAGGGGGCATGTCCATGATCATCATCAAAAGGATTGG
AACTAXXXXXXXXXXXTGTTTTCCTAACCTAAAATGAACCTTCCTTGAACCAAACATTTTCCCGATAGCTAGAATG
TCAAAATTGGCTTTAATGTA
ACTTGTTCCTTTCTGACCAAGTTGTTGAACTTGAAGAAGAGGGGGCATGTCCATGATCATCATCAAAAGGATTGG
AACTAXXXXXXXXXXXTGTTTTCCTAACCTAAAATGAACCTTCCTTGAACCAAACATTTTCCCGATAGCTAGAATG
TCAAAATTGGCTTTAATGTA
>Marker449218
TACCTTTCTAATGGCATCCAGCTCATGCAACAAAACTTGGTAGAACTGTCCTTCGCTAACCCCATCCCTACAAGA
AAAAGXXXXXXXXXXXTCCAGTTGCTCGACGGAAGGAGATGAGGAGTTCCCTGCATCACCGGGAACAAGGAAAGAC
AAAAGATAAGGACACAAGTT
TACCTTTCTAATGGCATCGAGCTCATGCAACAAAACTTGGTAGAACTGTCCTTCGCTAACCCCATCCCTACAAGA
AAAAGXXXXXXXXXXXTCCAGTTGCTCGACGGAAGGAGATGAGGAGTTCCCTGCATCACCGGGAACAAGGAAAGAC
AAAAGATAAGGACACAAGTT
>Marker449445
ACTAAGCAGTGGATTAATGTTAGGTTTCAATGCAATTTTTATTAGTATAAACGTTATGTTATTCTTCACTTGTAA
TGGAAXXXXXXXXXXXGAGATGACTTGTTGAATGAATTAAAAAGGTATGATGGAAGATGAGAATTTTTGAATAAAA
AACTATATGGCTTTTGAGTA
ACTAAGCAGTGGATTAATGTTAGGTTTCAATGCAATTTTTATTAGTATAAACGTTATGTTATTCTTCACTTGTAA
TGGAAXXXXXXXXXXXGAGATGACTTGTTGAATGAATTTAAAAGGTATGATGGAAGATGAGAATTTTTGAATAAAA
AACTATATGGCTTTTGAGTA
>Marker450697

Create PDF with GO2PDF for free, if you wish to

AACCTCAAAGTAAATGTAGATTGACATAACACCTAAAAGTTGCAGAAAACGTTAGCGAATGAGGCTGAAAAAAGT
GTTGAXXXXXXXXXXXTAAGTGGAAGAGGATGAGGTTGAAAAAAAAACATTGGGGGAAAATAGGTAAGAAAACCCA
GAATCTGTAACAAAATTGTA
AACCTCAAAGTAAATGTAGATTGACATAACACCTAAAAGTTGCAGAAAACGTTAGCGAATGAGGCTGAAAAAAGT
GTTGAXXXXXXXXXXXTAAGTGGAAGAGGATGGGGTTGAAAAAAAAACATTGGGGGAAAATAGGTAAGAAAACCCA
GAATCTGTAACAAAATTGTA
>Marker451353
ACCTTCGTTATGATCTCAAGACAATCATTTCTGCTAAGCCTAAGGAAGAGAAGCAGGCGCTTAAGGACCTCACCG
GAAAAXXXXXXXXXXXXGGGAAATTTTTGCAGTTGGATTATGCAGCAAAGATTAAGAGCAGCTCAGAAGCAGAGAAG
TATTATGCTCAGACTGTGTC
ACCTTCGTTATGATCTCAAGACAATCATTTCTGCTAAGCCTAAGGAAGAGAAGCAGGCCCTTAAGGACCTCACCG
GAAAAXXXXXXXXXXXXGGGAAATTTTTGCAGTTGGATTATGCAGCAAAGATTAAGAGCAGCTCAGAAGCAGAGAAG
TATTATGCTCAGACTGTGTC
>Marker451366
AACATAGTCTATATAGGAGACCACCAATTTATAAGCAGACTCAAGGGATTTTTCTTTTGGCCTAGTGATAATTAT
ATGAAXXXXXXXXXXXAGCCATAGATTGGGAGCATGCTAATATTGAGAATGCATGTAGGCAAAAACATCACAAACT
ATGTATTCCATGGTTTTGTG
AACATAGTCTATATAGGAGACCACCAATTTATAAGCTGACTCAAGGGATTTTTCTTTTGGCCTAGTGATAATTAT
ATGAAXXXXXXXXXXXAGCCATAGATTGGGAGCATGCTAATATTGAGAATGCATGTAGGCAAAAACATCACAAACT
ATGTATTCCATGGTTTTGTG
>Marker452204
TACAACAAGGAAAGTAATACCATTATCATATGTCCTTTTCTCTTACCAAACCATGCACGAGGACTTTTGTTTTAG
TCCATXXXXXXXXXXGAAAAATATTTTTAATGTTGAGTTAATGCAATGACTAATTGTGAATAGCAACCATAGAAA
TAAATAGTTGAATGGAAGTT
TACAACAAGGAAAGTAATACCATTATCATATGTCCTTTTCTCTTACCAAACCATGCACGAGGACTTTTGTTTTAG
TCCATXXXXXXXXXXGAAAAATATTTTTAATGTTGAGTTAATGCAATGACTAATTGTGAGTAGCAACCATAGAAA
TAAATAGTTGAATGGAAGTT
>Marker452248
TACACTTGCACGTCTAATCATATTCCACATTCACTAATTTAAATCAACAAACTTAACATCCTCTAAACTAAAGGT
CTCAAXXXXXXXXXXXTTAATTGTATGTTATGATGATTGAAAACATCATCTATGGTAACTATAGCACTAAATATGG
AGTGTGATATAATAATAGTC
TACACTTGCACGTCTAATCATATTCCACATTCACTAATTTAAATCAACAAACTTAACATCCTCTAAACTAAAGGT
CTCAAXXXXXXXXXXXTTAATTGTATGATATGATGATTGAAAACATCAACTATGGTAACTATAGCACTAAATATGG
AGTGTGATATAATAATAGTC
>Marker452468
AACATGCATGCATCCAATTAACATCATGAGTCAAAAGTCATAATGTCGTTGTGTGCTTATATTGACTCCGAAACT
TTTATXXXXXXXXXXAAAAATAATAGTGAATAAGAATACTGGCATGTGCATTAAATGAACACAAAAACATATATG
TTATAGGGTATTGTGATTGT
AACATGCATGCATCCAATTAACATCATGAGTCAAAAGTCATAATGTTGTTGTGTGCTTATATTGACTCCAAAACT
TTTATXXXXXXXXXXAAAAATAATAGTGAATAAGAATAGTGGCATGTGCATTAAATGAACACAAAAACATATATG
TTATAGGGTATTGTGATTGT
>Marker452637
AACCAAACTGCGGAAAGAGCGCAGTAGAAAACATACCGACCAAAAGGGGTTTCTAATTGTCAAACAAAAACGGAG
AGTGAXXXXXXXXXXXCAGGTGGCAAGTTGTTTCCCAGTGCCGCCGTAACTGACCGACCGTCCATGCTTCCATTGA
CTGACACGTGTCGCCTAGTG

Create PDF with GO2PDF for free, if you wish to

AACCAAACTGCGGAAAGAGCGCAGTAGAAAACATACCGACCAAAAGGGGTTTCTAATTGTCAAACAAAAACGGAG
AGTGAXXXXXXXXXXCAGGTGGCAAATTGTTTCCCAGTGCCGCCGTAACTGACCGACCGTCCATGCTTCCATTGC
CTGACACGTGTCGCCTAGTG
>Marker452900
ACTCTGTATCATAACAGTCTTGTGATCTCGTAGAATGCACATAATCAGTCGAGTGCTCCGAAGGATGCACATATC
AATCTXXXXXXXXXXACTCAAAATACAATCCAAGCTACCATCATTATATCATCATTTCAGTCCCACACATAAACA
GTCAAATTACCCATACTAGT
ACTCTGTATCATAACAGTCTCGTGATCTCGTAGAATGCACATAATCAGTCGAGTGCTCCGAAGGATGCACATATC
AATCTXXXXXXXXXXACTCAAAATACAATCCAAGCTACCATCATTATATCATCATTTCAGTCCCACACATAAACA
GTCAAATTACCCATACTAGT
>Marker452938
ACTCTAATGACTAATTAATCCATACTCTCCATAGAAAGAGTATCCATACTCTTTAATTTATTTTAGTATTAATAG
ATTTTXXXXXXXXXXGCTTATCAACTTCTTCATTAACCATCTAAACTATATATGGTTAAGTTGTCAAAAATATAT
GTATGATTAAATTTTAAGTA
ACTCTAATGACTAATTAATCCATACTCTCCATAGAAAGAGTATCCATACTCTTTAATTTATTTTAGTATTAATAG
ATTTTXXXXXXXXXXGCTTATCAACTTCTTCATTAACTATCTAAACTATATATGGTTAAGTTGTCAAAAATATAT
GTATGATTAAATTTTAAGTA
>Marker453414
TACTGCTACGGACATGTGTTGGGAGCGATTTTGAAATGGTTAAAACCAATTTTTCGTATTTAAAATCACTCTGAA
ACACAXXXXXXXXXXGATTTTAATCATTTCAGAATCACTCTCAAACATGTCATGAAATAAAAGATAATACAAATA
CAAGTAGTCGTAGAGTTGTG
TACTGCTACGGACATGTGTTGGGAGTGATTTTGAAATGGTTAAAACCAATTTTTCGTATTTAAAATCACTCTGAA
ACACAXXXXXXXXXXGATTTTAATCATTTCAGAATCACTCTCAAACATGTCATGAAATAAAAGATAATACAAATA
CAAGTAGTTGTAGAGTTGTG
>Marker453692
AACCAAGTGAGGTTGAGAAGATTTTGGCTGAGCAATGGAAATTAGAGTCTAAAAGAGATTCTAGAGATGCAATTA
CTTGGXXXXXXXXXXTTTTCTGTTCGTAGTATTTAAATATTAGCCTTGTGTTTATTGTAAAAGTAGCAATAAAGA
CATATGTTGTCAGTCTTTGT
AACCAAGTGAGGTTGAGAAGATTTTGGCTGAGCAATGGAAATTAGAGTCTAAAAGAGATTCTAGAGATGCAATTA
CTTGGXXXXXXXXXXTTTTCTGTTCGTAGTATTTAAATATTAGCCTTGTGTTTATTGTAAGAGTAGCAATAAAGA
CATATGTTGTCAGTCTTTGT
>Marker453787
TACAACCACAGCAGCGAGGCATCTCTAGGTAGATAGAGCACTCGGTGCAAGAGCAGCAGCATAGCCATGACAAAT
TAAAGXXXXXXXXXXTTCACCCTGCAACTGCTTCAAGAAAAGACGCATGAGCTTGGATCATAATCAATGCAGTTT
TAAATTTATGCAGATTAGTT
TACAACCACAGCAGCGAGGCATCTCTAGGTAGATAGCGCACTCGGTGCAAGAGCAGCAGCATAGCCATGACAAAT
TAAAGXXXXXXXXXXTTCACCCTGCAACTGCTTCAAGAAAAGACGCATGAGCTTGGATCATAATCAATGCAGTTT
TAAATTTATGCAGATTAGTT
>Marker454089
CACCGGCTAAGATTAAGTTGAATCAAAATTCAGGTATGAACAAGACATCTTTCAAAATCAGACTAGATAGTAAGT
GAATAXXXXXXXXXXAAGCATATGTGGGTTGTGGCACCAAAATCTATAATCTAGCAGCTCATTTCAAAAATGGAC
AATGGCAAAAAATGACAAGT
CACCGGCTAAGATGAAGTTGAATCAAAATTCAGGTATGAACAAGACATCTTTCAAAATCAGACTAGATAGTAAGT
GAATAXXXXXXXXXXAAGCATATGTGGGTTGTGGCACCAAAATCTATAATCTAGCAGCTCATTTCAAAAATGGAC
AATGGCAAAAAATGACAAGT
>Marker454349

Create PDF with GO2PDF for free, if you wish to

CACCCCTAACCTCAAATGGACAGTCAAACCTAACAAAGCTTTGCTCAAGGTTGCCCTTTTCTTAAATTAAACATG
TAGCGXXXXXXXXXXAAAACAAGTTGGGTAAGTCAATCCAAAATTGGGTTGATAAAAACTCAAAAGAAGACAGAT
GTCTCAAGGCAAAGCTAAGT
CACCCCTAACCTCAAATGGACAGTCGAACCTAACAAAGCTTTGCTCAAGGTTGCCCTTTTCTTAAATTAAACATG
TAGCGXXXXXXXXXXAAAACAAGTTGGGTAAGTCAATCCAAAATTGGGTTGATAAAAACTCAAAAGAAGACAGAT
GTCTCAAGGCAAAGCTAAGT
>Marker454484
ACTTAACACTTCTTGCGAATAACCTTATCGCATCGTTATATTTTTACTACAAGAAACCTTTCTCATAAACTAACC
AACTTXXXXXXXXXXTTGTCTAACACTTAGTCAAAACTTCACTTCTTAATTTAGACGAAGACTTTTTCTCTTCTT
ATCGGATAACTTATTTGGTG
ACTTAACACTTGTTGCGAATAACCTTATCGCATCGTTATATTTTTACTACAAGAAACCTTTCTCATAAACTAACC
AACTTXXXXXXXXXXTTGTCTAACACTTAGTCAAAACTTCACTTCTTAATTTAGACGAAGACTTTTTCTCTTCTT
ATCGGATAACTTATTTGGTG
>Marker454627
GACAACGACTCAATCGGATGTCGAATTATTTTCATCATCCGACAATAAGAAGATGAAAAACTGTGATGAAGAACA
AGATCXXXXXXXXXXTTTATTTTTAAGCAACAAGGTTCTTGGAAATGTCAAATACCATACAAATTTTATTGTGAT
GGTAATATATGCAATGTGTA
GACAACGACTCAATCGGATGTCGAATTATTTTCATCATCCGACAATAAGAAGATGAAAAACTGTGATGAAGAACA
AGATCXXXXXXXXXXTTTATTTTTAAGCAACAAGGTTCTTGGAAAGGTCAAATACCATAAAAAATTTATTGTGAT
GGTAATATATGCAATGTGTA
>Marker454949
CACCTTTCTAGATACAAATGTTGGGAGTTACCTCGCTAAGTTGAAACTTGTCTCCAAATTTCTTCTTGTGCGCGC
TAGCCXXXXXXXXXXATGAGATGTCAAGAAATGGTAGATTGAAAAGGCATAAATATGCGGTCTATGTATAAAGAA
TATAGGTCTTTGGCCACTGT
CACCTTTCTAGATACAAATGTTGGGAGTTACCTCGCTAAGTTGAAACTTGTCTCCAAATTTCTTCTTGTGCGCGC
TAGCCXXXXXXXXXXATGAGATGTCAAGAAATGGTAGATTGAAAAGGCATAAATATGCAGTCTATGTATAAAGAA
TATAGGTCTTTGGCCACTGT
>Marker455594
AACAAAGCAATTAAGTAAGGTTATGAAATGTTTGAATAAATATCCTAGTAATGAAAACTCCCCACATATCAGAGA
CATCAXXXXXXXXXXCTTGAGATATAGAGCATTAGAAGGGTTTTATCATTACAAAATTAAATGTCCAAACTTCAT
GAGAAGACAAAGTAAAGGGT
AACAAAGCAATTAAGTAAGGTAATGAAATGTTTGAATAAATATCCTAGTAATGAAAACTCCCCACATATCAGAGA
CATCAXXXXXXXXXXCTTGAGATATAGAGCATGAGAAGGGTTTTATCATTACAAAATTAAATGTCCAAACTTCAT
GAGAAGACAAAGTAAAGGGT
>Marker456289
ACATGAATTGCTAACCGGTTGTGACGGCCTATTAAAGAAAATAAATAATCCATATTCAAAAAATCAATTCCCAAA
GTCTCXXXXXXXXXXAAATATTTTCAGATTTTTCATGTTCCATCAGGTAGAAATCCTCATATAGTTTATATATGT
TTTAATTCTTCTCTATTGTT
ACATGAATTGCTAACCGGTTGTGACGGCCTATTAAAGAAAATAAATAATCCATATTCAAAAAATCAATTCCCAAA
GTCTCXXXXXXXXXXAAATATTTTCAGATTTTTCATGTTCCCTCAGGTAGAAATCCTCATATAGTTTATATATGT
TTTAATTCTTCTCTATTGTT
>Marker456658
AACAAGGTAGAAATTGTGAGGGTAAAAATGCTAGAACTATCAAGCATCAAAGTTTTAATTCAGTTATATGTAAGA
CACTCXXXXXXXXXXCCTCTCGTAACTTTTCTTATGAAATTATAGTATCTTTTCATACATTTCCATACTTGAGTG
TTCTTGAGTTTTAGGAGGTA

Create PDF with GO2PDF for free, if you wish to

AACAAGGTAGAAATTGTGAGGGTAAAAATGCTAGAACTATCAAGCATCAAAGTTTTAATTCAGTTATATGTAAGA
CACCCXXXXXXXXXXXCCTCTCGTAACTTTTCTTATGAAATTATAGTATCTTTTCATACATTTCCATACTTGAGTG
TTCTTGAGTTTTAGGAGGTA
>Marker457128
TACTTGTGTGTTTTCATTTTTGTTGAGTTCTTTTATATCCGGGATAATTGCTTTGATCTTACTTCAATGATCTAT
ATTTTXXXXXXXXXXXCCCACCACGAATACGAACTTCTGGATCTTTGGTGGAGGCAAACGCAATTATGCTCCCTAC
TGAAAGCGTGAAGCAAGAGT
TACTGGTGTGTTTTCATTTTTGTTGAGTTCTTTTATATCCGGGATAATTGCTTTGATCTTACTTCAATGATCTAT
ATTTTXXXXXXXXXXXCCCACCACGAATACGAACTTCTGGATCTTTGGTGGAGGCAAACGCAATTATGCTCCCTAC
TGAAAGCGTGAAGCAAGAGT
>Marker457517
CACAAGGTAAAACTGTGTTTTTCCAGACAAATTTTGAGAATTCCAAGGTTGCTGACCAAAAGGTCTCACAATGGG
ATGAAXXXXXXXXXXXCTACTATTACACAGTATATATACTATTTTGATCAAATTTAGGAAGAGGATGTGTAATATG
ATGCTCATTTGGTTTGAAGT
CACAAGGTAAAACTGTGTTTTTCCAGACAAATTTTGAGAATTCCAAGGTTGCTGACCAAAAGGTCTCACAATGGG
ATGAAXXXXXXXXXXXCTACTATTACACAGTATATATACTATTTTGATCAAATTTAGGAAGAGGATGTGTAATATG
ATGTTCATTTGGTTTGAAGT
>Marker457608
CACAGCAATCCAAGGTCTTGGAGTGCTAAAGAACCCAATTTGGTTGCAATATTTGCTGCAAGGCTGAACAGAATT
GATTCXXXXXXXXXXXAAGTGATAAGATTTCGTGTGGTATCATCGTTGCAAGCTGTCACTATAATGCCATAAATTT
CCAACCAACCCATTTTTGTT
CACAGCAATCCAAGGTCTTGGAGTGCTAAAGAACCCAATTTGGTTGCAATATTTGCTGCAAGGCTGAACAGAATT
GATTCXXXXXXXXXXXAAGTGATAAGATTTCGTGTGGTATCATCGTTGCAAGCTGTCACTATAATGCCATAAATTT
CCAACCAACCCATTTTCGTT
>Marker457847
ACCATTGCCATTTAATAAGTTAGCCCTGTGATTCCAAACATAAGCATGACCCTACACCAAGATATGAACTTAAGG
TCTAAXXXXXXXXXXXGAAAGAATAGAATATTCATTGGTAACAGAAAACCGACTTTTTTTAAAAAAAGAAGCTTCTT
TATTAAATAGAACTCGAAGT
ACCATTGCCATTTAATAAGTTAGCCCTGTGATTCCAAACATAAGCATGACCCTACACCAAGATATGAACTTAAGG
TCTAAXXXXXXXXXXXGAAAGAATAGAATATTCATTGGTAACAGAAAACCGACTTTTTTTAAAAAAGAAGCTTCTT
TATTAAATAGAACTCGAAGT
>Marker458117
GACTAGGAAAGCGTGTCAAGACTAGGGCATGCCATCAAAAAGTAGGTGATAGATGTCAATCTTGAGTCATGCAAC
CTTTCXXXXXXXXXXXTATTGAGGAGCAAAAAAAGGTTTGAGTTTGAGTTGAGAAAAGGGAGAAAGGTTAGTTTTC
AGGGCAATATGACCAAGGTG
GACTAGGAAAGCGTGTCAAAACTAGGGCATGCCATCAAAAAGTAGGTGATAGATGTCAATCTTGAGTCATGCAAC
CTTTCXXXXXXXXXXXTATTGAGGAGCAAAAAAAGGTTTGAGTTTGAGTTGAGAAAAGGGAGAAAGGTTAGTTTTC
AGGGCAATATGACCAAGGTG
>Marker458277
ACCTTTGCGGGTAATAATGGTATTCATTCTTCTTCACTTCCACCATTTTCGGGCCTTCTTCTGCTCCTGCTTGGC
CGGCGXXXXXXXXXXXACGACTTCTGATTCATTATTCTCCGGCGAAACTTCCGGCTCTTGCTTCACAATCGCGGCG
TGTTTTCCTGTCCGGCGGTG
ACCTTTGCGGGTAATAATGGTATTCATTCTTCTTCACTTCCACCATTTTCGGGCCTTCTTCTGCTGCTGCTTGGC
CGGCGXXXXXXXXXXXACGACTTCTGATTCATTATTCTCCGGCGTAACTTCCGGCTCTTGCTTCACAATCGCGGCG
TGTTTTCCCGTCCGGCGGTG
>Marker458436

Create PDF with GO2PDF for free, if you wish to

AACTTGCAATATTAATTGTGGATCTTGGCGAAGGAGTATTAGCAGGAACTCGATATTTCCAATTTCTGCTGCATC
GCGCAXXXXXXXXXXXTGATGGGCGAATGTCTGCATTAGAGCTTTATTGTAAATTCCATTGAACCCTATAGCAAAT
ACACAAAAGATTTAGTTGTA
AACTTGCAATATTAATTGTGGATCTTGGCGAAGGAGTATTAGCAGGAACTCGATATTTCCAACTTCTGCTGCATC
GCGCAXXXXXXXXXXXTGATGGGCTAATGTCTGCATTAGAGCTTTATTGTAAATTCCATTGAACCCTATAGCAAAT
ACACAAAAGATTTAGTTGTA
>Marker459111
AACTTTATCTGAATGTTTTTGTTTGATTCTTTTGAAATTCTTAGTTTAAACAAATACTCCAAAAGTCCAAAAGCA
AAGCAXXXXXXXXXXXTAAAAAGGATAAGGGTTTTTCATCAAGATTTAGTATGGAAGAACCTGTAAAGCTCAGGAG
GGATAGCGCCAATTAATAGT
AACTTTATGTGAATGTTTTTGTTTGATTCTTTTGAAATTCTTAGTTTAAACAAATACTCCAAAAGTCCAAAAGCA
AAGCAXXXXXXXXXXXTAAAAAGGATAAGGGTTTTTCATCAAGATTTAGTATGGAAGAACCTGTAAAGCTCAGGAG
GGATAGCGCCAATTAATAGT
>Marker459336
ACATGTGTTTTACTTTTATCTTTTTGAAAGGTTAGAAACTAAATGCGGGTAGTGTAATTTATAAACAGAAGTGCA
GAACGXXXXXXXXXXXCATTTTGGAAATACAGAATTTGGTCAGAAGAGAAGGGTTTCAGATCAGAAAAATCTTGAC
AGGACATGACAACACAAGTA
ACATGTGTTTTACTTTTATCTTTTTGAAAGGTTAGAAACTAAATGCGGGTAGTGTAATTTATAAACAGAAGTGCA
GAATGXXXXXXXXXXXCATTTTGGAAATACAGAATTTGGTCAGAAGAGAAGGGTTTCAGATCAGAAAAATCTTGAC
AGGACATGACAACATAAGTA
>Marker459435
GACCAATATCATCACCTTTTGATTCTCCCCCACCCTTACAACTTTGAATGCTAACAGTTTCAAATAGCTACATCA
CAAGTXXXXXXXXXXXCATCTAAATAGTTGTCACAAAAAACCTGCAAAAAACCAATGAAACGTATTAGCAAAACCC
ACAAGAATTGTGGTAACAGT
GACCATAATCATCACCTTTTGATTCTCCCCCACCCTTACAACTTTGAATGCTAACAGTTTCAAATAGCTACATCA
CAAGTXXXXXXXXXXXCATCTAAATAGTTGTCACAAAAAACCTGCAAAAAACCAATGAAACGTATTAGCAAAACCC
ACAAGAATTGTGGTAACAGT
>Marker460711
TACAAAAAGGTCGTCAATTCCATTGTAGCATTCTTAGTTTTGTATGTAGATGATATTCTATTGGAAACGACGTAG
GTTATXXXXXXXXXXXTGTCACAAGCATCTTATATAGACAAAATGTTGTCTATGTGTTAAATGTAGATTCTAACAA
GGGTTTGTTGTCATACAGGT
TACAAAAAGGTCGTCAATTCCATTGTAGCATTCTTAGTTTTGTATGTAGATGATATTCTATTGGAAACGATGTAG
GTTATXXXXXXXXXXXTGTCACAAGCATCTTATATAGACAAAATGTTGTCTATGTGTTAAATGTAGATTCTAACAA
GGGTTTGTTGTCATACAGGT
>Marker460809
AACCTTGCTCATAAATTAACTGAGAAATAACATTCTTGCCTACTCTTGATCAAAATACAAATTTCCTTGAATCTA
AAATGXXXXXXXXXXXGAGAAATCAACTGCTAAAATTGACCTATTTATAGAGTCCCTTGCATGCAATTAAGATGAC
ACTTAATCATTGATTAGGTT
AACCTTGCTCATAAATTAACTGAGAAATAACATTCTTGCCTACTCTTGATCAAAATACAAATTTCCTTGAATCTA
AAATGXXXXXXXXXXXGGGAAATCAACTGCTAAAATTGACCTATTTATAGAGTCCCTTGCATGCAATTAAGATGAC
ACGTAATCATTGATTAGGTT
>Marker461092
ACCAAGCACCTAACCCATGTATCTCATCCTCAAACTTGATATTCATCCTAGATAATTGATTTAAAATACCTTGGA
AAGTAXXXXXXXXXXXAGAGCTTATTCTACATAATTCGTGCATGAGTTTCTTCACAAATATAGTTTAGAAAATTAT
CTTCTGCCCATAGCATCGTA

Create PDF with GO2PDF for free, if you wish to

ACCAAGCACCTAACCCATGTATCTCATCCTCAAACTTGATATTCATCCTAGATAATTGATTTAAAATACCTTGGA
AAGTAXXXXXXXXXXAGAGCTTATTCTACATAATTCGTGCATGAATTTCTTCACAAATATAGTTTAGAAAATTAT
CTTCTGCCCATAGCATCGTA
>Marker461184
AACCGTGACGGTCAGGATTAATGTAAATATTTAATCACAAAATGCAACTTATCGATGAAAAACTTCGTGTAAGAA
CAAAAXXXXXXXXXXTTACCATGATTCAATGTTGAAAATTGGTTTCATCATATTCTAACAAATCCAAGTTGGATC
TAATCAAATCAAAGTCTAGT
AACCGTGACAGTCAGGATTAATGTAAATATTTAATCACAAAATGCAAATTATCGATGAAAAACTTCGTGTAAGAA
CAAAAXXXXXXXXXXTTACCATGATTCAATGTTGAAAATTGGTTTCATCATATTCTAAGAAATCCAAGTTGGATC
TAATCAAATCAAAGTCTAGT
>Marker461861
AACTCTTTACATAGGATGTCCCACTTTCATGTCTCTACATAAACGATTCAAGATTACATCGTTTGTATTAACTAC
AAAGCXXXXXXXXXXGGACTTACAGTTCCCTTTAACATTTTCTTCTTCCTCTCATTTTTTCTACTTTAAAATCAT
ATAAGTAGATTGTCGTTGTT
AACTCTTTACATAGGATGTCCCACTTTCATGTCTCTACATAAACGATTCAAGATTACATCGTTTGTATTAACTAC
AAAGCXXXXXXXXXXGGACTCACAGTTCCCTTCAACGTTTTCTTCTTCCTCTCATTTTTTCTACTTTAAAATCAT
ATAAGTAGATTGTCGTTGTT
>Marker462042
ACTTTTGATATCAAAAGAAATGAGTTGATACATTTATTATCATCATCATATACGTGTCTTTTTAACATACTGAAC
ATTTCXXXXXXXXXXTTTTCATTTTTTTTGGGTGTGATTCACAAGACATATCCAAATTTGATTAAACCAATCATC
AAGGGCTTGAAGAACCAAGT
ACTTTTTATATCAAAAGAAATGAGTTGATACATTTATTATCATCATCATATACGTGTCTTTTTAACATACTGAAC
ATTTCXXXXXXXXXXTTTCATTTTTTTTTGGGTGTGATTCACAAGACATATCCAAATTTGATTAAACCAATCATC
AAGGGCTTGAAGAACCAAGT
>Marker462243
ACTATGTTTTTCTTAGAATTAGAAGGAATGCTATATGGCTTTCATGCTTTATCTACCAAGACCTCTTTTTTGGTT
TATCCXXXXXXXXXXAAATTATGATATAAAGAGAATGCTATTACTATCAATTGGGATTAAATAAATCCTTTATTT
TTGGCTAATTTATTTAAAGT
ACTATGTTTTTCTTAGAATTAGAAGGAATGCTATATGGCTTTCATGCTTTATCTACCAAGACCTCTTTTTTGGTT
TATGCXXXXXXXXXXAAATTATGATATAAAGAGAATGCTATTACTATCAATTGGGATTAAATAAATCCTTTATTT
TTGGCTAATTTATTTAAAGT
>Marker462725
AACTCAGATGGATACTTATGAGTATCTATCACTTAAACACAATAAGATTTTACTATATTTATAGATATTTACAAC
AGTATXXXXXXXXXXCAAAATGCAAAGAGGAGAGAAGAAGTAGTTTCCTTGCTTCTTCCTTCCTTAACAATCAAA
CATTAGTATAAAACTATAGT
AACTCAGATGGATACTTATGAGTATCTATCACTTAAACACAATAAAATTTTACTATATTTATAGATATTTACAAC
CGTATXXXXXXXXXXCAGAATGCAAAGAGGAGAGAAGAAGTAGTTTCCTTGCTTCTTCCTTCCTTAACAATCAAA
CATTAGTATAAAACTATAGT
>Marker462748
GACGTTATAAACAAAAGATGTGTCGAATATATGTCGGGTTCTTATTAGATAATTGATAAAAACTTAGACAATGAC
TAAAAXXXXXXXXXXTTGTTTTTATTAAATACCTCTCAAAGGCAAAATAAAGTTTTCATTACTTTTAACATTTAG
CTTTATTAAAAAAGATATGT
GACGTTATAAACAAAAGATGTGTCGAATATATGTCGGGTTCTTATTAGATAATTGATAAAAACTTAGACAATGAC
TAAAAXXXXXXXXXXTTGTTTTTATTAAATACCTCTCAAAGGCAAAATAAAGTTTTCATTACTTTTAACATTTAG
TTTTATTAAAAAAGATATGT
>Marker462897

Create PDF with GO2PDF for free, if you wish to

AACTCAGGCTTTCTACAAAAGGAAAAAGATGACAGATTGATTTCACAAGAACTTACAGAAATTACAAAACCAAAA
GCATAXXXXXXXXXXTTATAGAAGGTAACATTCAAATGTCTATTCTCAAACTTCAGATTTAAAAATCTCTTCCTT
CACTTAGTCGAAGGGTCGTG
AACTCAGGCTTTCTACAAAAGGAAAAAGATGACAGATTGATTTCACAAGAACTTACAGAAATTACAAAACCAAAA
GCATAXXXXXXXXXXTTATAGAAGGTAACATTCAAATCTCTATTCTCAAACTTCAGATTTAAAAATCTCTTCCTT
CACTTAGTCGAAGGGTCGTG
>Marker463038
TACCCTTACAATGAACAATCTTCTCTTATAGGTCTTTGAAATGTATAAAATCTCACACAAAAGAAATGTTTTGGC
TGCAGXXXXXXXXXXTTTTCTTGTAATACTTTTAAAGATTTTAAATTTTCTGTAAGTTGGGGGGCGTATGCTCTC
TCTCAGTTGTAATTTTATGT
TACCCTTATAATGAACAATCTTCTCTTATAGGTCTTTGAAATGTATAAAATCTCACACAAAAGAAATGTTTTGCC
TGCAGXXXXXXXXXXTTTTCTTGTAATACTTTTAAAGATTTTAAATTTTCTGTAAGTTGGGGGTCGTATGCTCTC
TCTCAGTTGTAATTTTATGT
>Marker463106
GACTGGTTTTGCTTCGATTCCAAACTCCTTCTTCATGGACTTGAAGTATTGAACCCCAAGATCAACGAATCCAGC
ATGGTXXXXXXXXXXGGCATTCCAGGAAATCACATCCTTTCGTGGCATCTCGAGAAACAACTTCCATGCACTGTC
GAGATCTCCACACTTGCAGT
GACTGGTTTTGCTTCGATTCCAAACTCCTTCTTCATGGACTTGAAGTATTGAACCCCAAGATCGACGAATCCAGC
ATGGTXXXXXXXXXXGGCATTCCAGGTAATCACATCCTTTCGTGGCATCTCGAGAAACAACTTCCATGCACTGTC
GAGATCTCCACACTTGCAGT
>Marker463148
ACCAAAATAATATATGAAATAACATAACAGGTTAGGAATATACGTCCCTTGAAAGTTGAAAGCAGTCAGACTCTT
GAGCTXXXXXXXXXXTAGTATGCGGCCCACCAACAAGAAATATTGAGCATCATCATTTGGATTCATATATTCAAT
ACAGCTACAGATTTTTGGTG
ACCAAAATAATATATGAAATAACATAACAGGTTAGGAATATACGTCCCTTGAAAGTTGAAAGCAGTCAGACTCTT
GAGCTXXXXXXXXXXTAGTATGCGGCCCACCAACAAGAAATATTGAGCATCATCATTTGGATTCATATATTCAAT
ACAGCTACAGATTTTTAGTG
>Marker463807
AACGTGGTCATCAAAGTTGGCTTTTTTTGGAGTTTCCTATTGGAGATAGTTACCATAGCCCAAAATTAGTTGTTG
GAGGTXXXXXXXXXXATCTGAGTGTGGTAATGGTAATTGATCGTTGAATCTATTAGAAAAACTGGAGAGAGCTTC
ATCAACCTATAAAGTTAGTG
AACGTGGTCATCAAAGTTGGCTTTTTTTGGAGTTTCCTATTGGAGATAGTTACCATAGCCCAAAATTAGTTGTTG
GAGGTXXXXXXXXXXCTCTGAGTGTGGTAATGGTAATTGATCGTTGAATCTATTAGAAAAACTGGAGAGAGCTTC
ATCAACCTATAAAGTTAGTG
>Marker463935
CACGTTGGTTGATTTTTTTTTAATCATGGAGTGCTTTTTATTTTTTAAGGCTTTATTGAAAAACCAATATATTTT
ATTTTXXXXXXXXXXTAAATATCATTCACATCAATAAAAAAAAAAATTTAATTTTAATTGAATTATGCTGGAAAT
AGTTAATTAAAATAGTGGTA
CACGTTGGTTGATTTTTTTTTAATCATGGAGTGCTTTTTATTTTTTAAGGCTTTATTGAAAAACCAATATATTTT
ATTTTXXXXXXXXXXTAAATATCATTCACATCAATAAAAAAATATATTTAATTTTAATTGAATTATGCTGGAAAT
AGTTAATTAAAATAGTGGTA
>Marker465079
ACTCAACATTAAGTGGATGATGTTCATGAGAAATGGAGAGAAAGCGCTCATGAATTAGGGTGATACATAGGTAGC
TGCCTXXXXXXXXXXAGACTTATGAATTAGGAGAAGGGCTTTGTTATTTGAATCATCCGATTATGTCATTTTTAA
ATCACGGTTTAAGATATAGT

Create PDF with GO2PDF for free, if you wish to

ACTCAACATTAAGTGGATGATGTTCATGAGAAATGGAGAGAGAGCGCTCATGAATTAGGGTGATACATAGGTAGC
TGCCTXXXXXXXXXXAGACTTATGAATTAGGAGAAGGGCTTTGTTATTTGAATCATCCGATTATGTCATTTTTAA
ATCACGGTTTAAGATATAGT
>Marker465315
GACCTCAAATGCCGGACGAAGATTTGATTGAGCTTAAGTTCCGACTTTATGATGGATTAGATATTGGTCCATTTT
GTTCTXXXXXXXXXXGCGATCTTCCTAGAGGAGTTTTTACTATGCATGTCGTGGTGCAGCCAACTATAGCAAAAG
CAAAAGCAGAAAATAAGGTC
GACCTCAAATGCCAGACGAAGATTTGATTGAGCTTAAGTTCCGACTTTATGATGGATTAGATATTGGTCCATTTT
GTTATXXXXXXXXXXGCGATCTTCCTAGAGGAGTTTTTACTATGCATGTCGTGGTGCAGCCAACTATAGCAAAAG
CAAAAGCAGAAAATAAGGTT
>Marker465562
TACACCTCAAACACATATTATTATAGTTTAAACTAAAATAATATACAACTCAAATACAAACAATTATGATTTAAC
TACAAXXXXXXXXXXTTTCATTGTCAGAGACAAGCTTGGACAAGCCAAAATCTGCAACTTTTGCATTTAAATGTT
CATCCAATAGAATATTGGTG
TACACCTCAAACACATATTATTATAGTTTAAACTAAAATAATATACAACTCAAATACATACAATTATGATTTAAC
TACAAXXXXXXXXXXTTTCATTGTCAGAGACAAGCTTGGACAAGCCAAAATCTGCAACTTTTGCATTTAAATGTT
CATCTAATAGAATATTGGTG
>Marker466288
CACGAAAAATTATTATGACGTATAACTGTAAAGCAATTATTGTGATTTTTATGTGATTCTATGATTTTTTAATGC
ATCATXXXXXXXXXXAGTTTATTGTCATCACATCTCATTGTAAGTTCAATACAGTAGTTTGGGGTGGGGAATGAG
TTGAATTTTTTTCTCCAGTA
CACGAAAAATTATTATGACGTATAACTGTAAAGCAATTATTGTGATTTTTATGTGATTCTATGATTTTTTAATGC
ATCATXXXXXXXXXXAGTTTATTGTCATCACATCTCATTCTAAGTTCAATACAGTAGTTTGGGGTGGGGAATGAG
TTGAATTTTTTTCTCCAGTA
>Marker466763
AACCAAGCTCCTTGAACATGCTTCAATGTTGAAACTGAATCAACAAGTAAATTACACCCTACATTTTGTTTTAGA
AGTCTXXXXXXXXXXACAAACCAGAACCTGAAGGCAAAATGAAGTTGAAATCACTCAAACTGATGTCATTTTAAA
AATGAAGCAAACTACAAGTG
AACCAAGCTCCTTGAACATGCTTCAATGTTGAAACTGAATCAACAAGTAAATTACACCCTACATTTTGTTTTAGA
AGTCTXXXXXXXXXXACAAACCAGAACCTGAAGGCAAAATGAAGTTGAAATCACTCAAACTGATGCCATTTTAAA
AATGAAGCAAACTACAAGTG
>Marker467020
GACACTACCTCAAATGACTAATAATCTATAATGTTAATTCTTCTCTCTATTTTATTGTTTAGGTTTTTGCTGCTT
ATTGTXXXXXXXXXXTTATCTTGTTCGGACTAACTTAGATTTATTTTAAAAAGTAGTCATAATTTAATTAATCTC
GTTCCTATAAAAAAATTGTT
GACACTACCTCGAATGACTAATAATCTATAATGTTAATTCTTCTCTCTATTTTTTTGTTTAGGTTTTTGCTGCTT
ATTGTXXXXXXXXXXTTATCTTGTTCGAACTAACTTAGATTTATTTTAAAAAGTAGTCATAATTTAATTAATCTC
GTTCCTATAAAAAAATTGTT
>Marker467252
ACCTTTGTATTTATGTAACCAAATATGTCGTATATCCTTCACTGAAAAAATAAAGAAAAGAATAATGCTATTGCT
AGCAAXXXXXXXXXXTTTCTCACTCCTAATTAATAAATATCATTTTTAATAGTCATTATAAACCTGTATTATGAA
CACCTGTTGCTTAATTTGTT
ACCTTTGTATTTATGTAACCAAATATGTCGTATATCCTTCATTGAAAAAATAAAGAAAAGAATAATGCTATTGCT
AGCAAXXXXXXXXXXTTTCTCACTCCAAATTAATAAATATCATTTTTAATAGTCATTATAAACCTGTATTATGAA
CACGTGTTGCTTAATTTGTT
>Marker467328

Create PDF with GO2PDF for free, if you wish to

GACCATTTTGGTTTAGAAATGTAATGGATGACCTTTGTTTTTCCATGAATAGGCAATCCTTTATATTCAATATCT
CATGCXXXXXXXXXXCACACTCATCTCTGATCTTCAAATCAGTTCCACAGCTTTGTTGGACTTCACATGTTTAAG
ATATTTGCGTATGTTTTTGT
GACCATTTTGGTTTAGAAATTTAATGGATGACCTTTGTTTTTCCATGAATAGGCAATCCTTTATATTCAATATCT
CATGCXXXXXXXXXXCACACTCATCTCTGATCTTCAAATCAGTTCCACAGCTTTGTTGGACTTCACATGTTTAAG
ATATTTGCGTATGTTTTTGT
>Marker467394
CACTAAATAATAATTAATAGATGGGTGTATATCTCATTAACAGATGAGAGATGAGATAGAATCTAATGTATTAAA
TCGTTXXXXXXXXXXTTAGTATCATTAATCATATTCATTCTCACTCCTTAATTCACCTAAATGGATACAATGATC
TTTTTTTTTCTTCACTAGTG
CACTAAATAATAATTAATAGATGGGTGTATATCTCATTAACAGATGAGAGATGAGATAGAATCTAATGTATTAAA
TCGTTXXXXXXXXXXTTAGTATCATTAATCCTATTCATTCTCACTCCTTAATTCACCTAAATGGATACAATGATC
TTTTTTTTTCTTCACTAGTG
>Marker467397
ACTCATATCGTTATTGATTCGAGCAATAGCTCAATTGGCATAAATGTGTGGTATCATCCGCAAAGAAAAAGGCAT
CGATAXXXXXXXXXXACAACTTATACAATTGGTTCGGCTTTTAAGTATAAAACAGACTAACAGAGAATCTGTGAC
AAAATAAGTCCTTGAACTGT
ACTCTTATCGTTATTGATTCGAGCAATAGCTCAATTGGCATAAATGTGTGGTATCATCCGCAAAGAAAAAGGCAT
CGATAXXXXXXXXXXACAACTTATACAATTGGTTCGGTTTTTAAGTATAAAACAGACTAACAGAGAATCTGTGAC
AAAATAAGTCCTTGAACTGT
>Marker467835
CACAATAACCACTTACTCGAGCAAAAGTTGATTGTTTCTTTGGATAACCAGAGATGTGTATGCTTCCTTCAATAT
AACCTXXXXXXXXXXATTTGGAAGATATAGATGTGTAGTGATCAAACAGTTTCCATTGTTGATAACATCTTTGCA
AAAATTTACCAACTTCAGTT
CACAATAACCACTTACTCGAGCAAAAGTTGATTGTTTCTTTGGATAACCAGAGATGTGTATGCTTCCTTCAATAT
AACCTXXXXXXXXXXATTTGGAAGATATAGATGTGTAGTGATCAAACAGTTTCCATTGTTGATAACATCTTTGCA
AAAATTTACAAACTTCAGTT
>Marker467875
AACGCTAACCTCTCGATATCGGCGTAACACTCCTGCTTCTTTTGCCGGGTTTCAGCATCCTGAATAATTGAAATA
CAATTXXXXXXXXXXGTGAGCAATGAGAAGATTAACCCTAGAAGGATCAAGGGTTTGAGTGTGTTGAGAAGCTTT
GAATTTCTCGAAGAATTGTT
AACGCTAACCTCTCGATATCGGCGTAACACTCCTGCTTCTTTTGCCGGGTTTCAGCATCCTGAATAATTGAAATG
CAATTXXXXXXXXXXGTGAGCAATGAGAAGATTAACCCTAGAAGGATCAAGGGTTTGAGTGTGTTGAGAAGCTTT
GAATTTCTCGAAGAATTGTT
>Marker468180
AACCAATGACGACATCCAAATTAGAATCAAAAGGTCAAGGGTTTATCAGTATAAGTAGGTAGGCAAAAAGATGCC
ACTTAXXXXXXXXXXAAAGATATTTGAGTGGCTAGAAATATTATTAGGGAATTAAAGACAATAATACTTAGCCAA
GGAGTTTGTTAGTGGGAGTT
AACCAATGACGACATCCAAATTAGAATCAAAAGGTCAAGGGTTTATCAGTATAAGTAGGTAGGCAAAAAGATGCC
ACTTAXXXXXXXXXXAAAGATATTTGGGTGGCTAGAAATATTATTAGGGAATTAAAGACAATAATACTTAGCCAA
GGAGTTTGTTAGTGGGAGTT
>Marker468272
CACAATGTTGTATTATAAAAAACAGAGAGCTATTTGCATCTGCATATTTCTTATTCGAGATGCATATTGTAGCAT
TCAGAXXXXXXXXXXTTGCTGAAAGAGATTACAGATTCATAGCTCCCATGATAGCTAGAAAAGCCAGTCCAACAA
GGAAGAAAATCATCATAAGT

Create PDF with GO2PDF for free, if you wish to

CACAATGTTGTATCATGAAAAACAGAGAGCTATTTGCATCTGCATATTTCTTATTCGAGATGCATATTGTAGCAT
TCAGAXXXXXXXXXXTTGCTGAAAGAGATTACAGATTCATAGCTCCCATGATAGCTAGAAAAGCCAGTCCAACAA
GGAAGAAAATCATCATAAGT
>Marker468306
TACCTTTTCCAGCGTCAATCCATGTAAATGATATCTAAGTTGCCGGCTTCATATTTTGAATTGTGCATGGTGCTG
AAGAAXXXXXXXXXXCTAATAGGATTTCCTACCTCACCAGCCAAGAGGGTATTAGATCATCAGCCCAAAGAAATT
GAAGTCGCTGATCTGTCAGT
TACCTTTTCCAGCGTCAATCCATGTAAATGATATCTAAGTTGCCAGCTTCATATTTTGAATTGTGCATGGTGCTG
AAGAAXXXXXXXXXXCTAATAGGATTTCCTACCTCACCAGCCAAGAGGGTATTAGATCATCAGCCCAAAGAAATT
GAAGTCGCTGATCTGTCAGT
>Marker468478
AACTTCGAGAACTACCTTCCTGAGGACCAGAGTCACCTGAAGGGCACGATGAGCGTTGTCGTCTAGATCCCTTCT
CCGAGXXXXXXXXXXCTATTACCAGTTCATCAAAATGCAATAAATCAAACAACATTTTACATCACCAATAAGGTA
GAATAGAGAGTAGTTTTGGT
AACTTCGAGAACTACCTTCCTGAGGACCAGAGTCACCTGAAGGGCACGATGAGCGTTGTCGTCTAGATCCCTTCT
CCGAGXXXXXXXXXXCTATTACCAGTTCATCAAAATGCAATAAATCAAACAACATTTTACATCACCAATAAGGTA
AAATAGAGAGTAGTTTTGGT
>Marker469102
TACATCTATCTGGGTTGGATCTTGAATTGGCAATTATGTTAGTCTCTGTATGGGTTTTTCGTTTTTTGTATTTCT
TGTAAXXXXXXXXXXTTCTTTAAAAAAATGTCAATTATGCTAGAGGTGGATTGAGAAAAAGTTTCAGGAGTCTGT
TGATTATCTTTGTCTTTGTG
TACATCTATCTGGGTTGGATCTTGAATTGGCAATTATGTTAGTCTCTTTATGGGTTTTTCGTTTTTTGTATTTCT
TGTAAXXXXXXXXXXTTCTTTAAAAAAATGTCAATTATGCTAGAGGTGGATTGAGAAAAAGTTTCAGGAGTCTGT
TGATTATCTTTGTCTTTGTG
>Marker469128
TACCCTCCAGAGAAAGCCCTAGACATAGTCAAGGAAAGAAACCAACTTCACACCCAAAGGCATCTGCCCGTCTCC
TTATCXXXXXXXXXXACTGGAGGTGTGAAATGGCTAACTCTTTTGAACAAAATAAAATTTTATGATACTTCCTTC
CACCGCTTTCATAATATGTC
TACCCTCCAGAGAAAGCCCTAGACATAGTCAAGGAAAGAAACCAGCTTCACACCCAAAGGCATCTGCCCGTCTCC
TTATCXXXXXXXXXXACTGGAGGTGTGAAATGGCTAACTCTTTTGAAGAAAATAAAATTTTATGATACTTCCTTC
CACCGCTTTCATAATAAGTC
>Marker469434
CACAATAATTTTGATTTAACGAAGAAAAAAACCGTTAAAATAAGCATAAGTCAATTACATAATAGTGCAAGTTTT
ATACAXXXXXXXXXXAAAATCAAAACTCTTCAATATTTATAGGAGAAAAGAAAAACTTCAAACTCAGCTTGGTCA
AAATTTTTGAAGTCAAAGTG
CACAATAATTTTGATTTAACGAAGAAAAAAACCGTTAAAATAAGCATAAGTCAATTACATAATAGTGCAAGTTTT
ATACAXXXXXXXXXXAAAATCAAAACTCTTCAATATTTATAGGAGAAAAGAAAATCTTCAAATTCAGCTTGGTCA
AAATTTTTGAAGTCAAAGTG
>Marker469720
ACATTTCTTCTCAAACTTCAATGGATATCCCTTTTCACAAGTTGGATTGTTTTGGAACGCCATTCCAAAATCCTA
AATTTXXXXXXXXXXTGAGAAGTTAGGAGATATTCTGAAGGGGAAAACAGCTTCAAAAGCTACCAAGAAACCAAT
ATTCAGAATTGGTGATAGGT
ACATTTCTTCTCAAACTTCAATGGATATCCCTTTTCACAAGTTGGATTGTTTTGTAACGCCATTCCAAAATCCTA
AATTTXXXXXXXXXXTGAGAAGTTAGGAGATATTCTGAAGGGGAAAACAGCTTCAAAAGCTACCAAGAAACCAAT
ATTCAGAATTGGTGATAGGT
>Marker469731

Create PDF with GO2PDF for free, if you wish to

ACAAAACTTGGCAATTTTAAAAATTCAAATTTAGTTTCAGGAATTCATTGTGTGTTTCATTTGTAGCCTCATTGC
ATGGGXXXXXXXXXXAATATCCTTTAGACGATAAAGAACTTAATGCGGAAAGATAAAACAACTTTAAACTTTTAT
TCCTTAACAAGGCGTTTAGT
ACAAAACTTGGCAATTTTAAAAATTCAAATTTAGTTTCAGGAATTCATTGTGTGTTTCATTTATAGCCTCATTGC
ATGGGXXXXXXXXXXAATATCCTTTAGATGATAAAGAACTTAATGCGGAAAGATAAAACAACTTTAAACTTTTAT
TCCTTAACAAGGCGTTTAGT
>Marker469831
CACTTCTGAAACTGATTCAAACGGTTCATTACTTACTTCTTGCTTGCATATTGTGTTGGAATATAAATTACGTTG
TTTAAXXXXXXXXXXTGATATATCTGATACATGTGCAAGGACTCGCCGAACATGTTCATTCAACTTTAAATTCCA
AATTCACACTTCTCCTTGTG
CACTTCTGAAACTGATTCAAACGGTTCATTACTTACTTCTTGCTTGCATATTGTGTTGGATTATAAATTACGTTG
TTTAAXXXXXXXXXXTGATATATCTGATACATGTGCAAGGACTCGCCGAACATGTTCATTCAACTTTAAATTCCA
AATTCACACTTCTCCTTGTG
>Marker469899
GACCTAACTAATGATTAATGAGGGTCCATGCATAAATAATAATAGATTTTATTGAGAATGAAAGTTAAGTTACCA
ACATCXXXXXXXXXXAAGTGTGTATTTAATTACCTATTAAATTTGAAACGTTTTTTTAAAAATTTTTTAATTTAG
ATTGAAAAAACGTTTTCGTT
GACCTAACTAATGATTAATGAGGGTCCATGCATAAATAATAATAGATTTTATTGAGAATGAAAGTGAAGTTACCA
ACATCXXXXXXXXXXAAGTGTGTATTTAATTACCTATTAAATTTGAAACGTTTTTTTAAAAATTTTTTAATTTAG
ATTGAAAAAACGTTTTCGTT
>Marker469939
ACCAACGATCCGCCTTTACGTGACCAAACTTCTTGCAATGGTAACACTGAATACTAGCTTTGCTGCTTGATTGCT
CTCCTXXXXXXXXXXCTTCGTTCCTTTTCGTCGAACTGTTAATTCTCGACTCATGTGCTTGAAGAGATCCCATCA
ATTCATTAAATGAAAAAGTG
ACCAACAATCCGCCTTTACGTGACCAAACTTCTTGCAATGGTAACACTGAATACTAGCTTTGCTGCTTGATTGCT
CTCCTXXXXXXXXXXCTTCGTTCCTTTTCGTCGAACTGTTAATTCTCGACTCATGTGCTTGAAGAGATCCCATCA
ATTCATTAAATGAAAAAGTG
>Marker470406
AACATTAACGATTATTTTTTGAAAAGTTAATGAACTTAAACAAAATATCACTTTGTGTGGCAAAATTCATGAAAT
AAATGXXXXXXXXXXAACACAACTAATGATTATAATCATATTTTAAATAAATTATAAACAACTAATAGTTTTAAA
CAAAATACTTTAAATTGAGT
AACATTAACGATTATTTTTTGAAAAGTTAATGAACTTAAACAAAAGATCACTTTGTGTGGCAAAATTCATGAAAT
AAATGXXXXXXXXXXAACACAACTAATGATTATAATCATATTTTAAATAAATTATAAACAACTAATAGTTTTAAA
CAAAATACTTTAAATTGAGT
>Marker471326
ACTAAAGTTCTAATTTAGGTTAGATAAAAATTTAAATGAAATTAGAGTATGGGAAGAAAAATAAGTAGGAGACTG
AAAGTXXXXXXXXXXAGTCCTACATATTTGTATGAATGTTTATATATTGTTTCCTCCACTAAACTCAAATCTTAA
ATTCTAAACCATGATGGGTA
ACTAAAGTTCTAATTTAGGTTAGATAAAAATTTAAATGAAATTAGAGTATGGGAAGAAAAATAAGTAGGAGACTG
AAAGTXXXXXXXXXXAGTCCTACATATTTGTATGAATGTTTATATATTGTTTCATCCACTAAACTCAAATCTTAA
ATTCTAAACCATGATGGGTA
>Marker471809
ACCCTGACTTAGTAATTATCCTTGATGTTCTTCGGCTTTGATTTCGAAAATATTTTGAAGTTATCTTCTAGACTT
GATTTXXXXXXXXXXGCCCGAATATCCTAATAACACCCTAATATCATTCCAATTACCTTCTTTATTTAGGCAAAA
ATTTAAAAAAAAATCCATGTA

Create PDF with GO2PDF for free, if you wish to

ACCCTGACTTAGTAATTATCCTTGATGTTCTTCGGCTTTGATTTCGAAAATATTTTGAAGTTATCTTCTAGACTT
GATTTXXXXXXXXXXXGCCCGAATATCCTAATAACACCCTAATATCATTCCAATTACCTTCTTTATTTAGGCAAAA
ATTAAAAAAAAATCCATGTA
>Marker471870
TACATATTACCTCTCCGTATTTGGTATAATAGACAAAACATTTGTATTTGGCTTGCAGACCAATTGTGAAAATTA
TACTGXXXXXXXXXXXTTATACCGTGTTGTTTCTTTTAGTTTTATAAATTCATTTTGTTTTTTCTTTTCTTCTATC
TAAACTAGGGTCCGCATGTA
TACATATTACCCCTCCGTATTTGGTATAATAGACAAAACATTTGTATTTGGCTTGCAGACCAATTGTGAAAATTA
TACTGXXXXXXXXXXXTTATACCGTGTTGTTTCTTTTAGTTTTATAAATTCATTTTGTTTTTTCTTTTCTTCTATC
TAAACTAGGGTCCGCATGTA
>Marker471936
AACCCTACCTGAAGCACCCACAAAGAAAGAAAAAGAGCATTCTTGTCGTAAGCCAAAGCATGAGTGAAATATTAC
AAATCXXXXXXXXXXXTACAGCTTTCCTAAGGAAAGAAAGCCTTGCACCTTGCCCCATCTCGTGAGATAAACTCCC
CAACCCTCATCAAATAAAGT
AACCCTACCTGAAGCACCCACAAAGAAAGAAAAAGAGCATTCTTGTCGTAAGCCAAAGCATGAGTGAAATATTAC
AAATCXXXXXXXXXXXTACAGCTTTCCTAAGGAAAGAAAGCCTTGCACCTTGCCCCATCTCGTGAGATAAATTCCC
CAACCCTCATCAAATAAAGT
>Marker472427
TACTGGACAAGATGAAGTATCCGCCAGGCCTTAAAATTCTATTCATTTCTAGGAGAAGCTTCCCATCTAGAGAAA
GAGAGXXXXXXXXXXXCTAGGAAAAGCAAGTCTTCTATTCCCAAAAGGGCTAACCAGAGTTGGAAACCCTCGCTCA
AGTGCAACTTGAGCCAAGTC
TACTGGACAAGATGAAGTATCCGCCAGGCCTTAAAATTCTATTCATTTCTAGGAGAAGCTTCCCATCTAGAGAAA
GAGAGXXXXXXXXXXXCTAGGAAAAGCAAGTCTTCTATTCCCAAAAGGGCTAACCAGAGTTGGAAACCCCCGCTCA
AGTGCAACTTGAGCCAAGTC
>Marker472756
CACTGAAAATTTATAATGAAACCAAAAGACACATAAAATCACATTAAGTTAAAGTTAAACAAAATATCTTGAAAC
AGTAAXXXXXXXXXXXATGTAAGGTAGATGGGCTGGACGTGTTAAATTCATCGTCTTGTCTCCTTGCTTAAACGAC
ACTTCAGGTTCCTTTGTTGT
CACTGAAAATTTATAATGAAACCAAAAGACATATAAAATCACATTAAGTTAAAGTTAAACAAAATATCTTGAAAC
AGTAAXXXXXXXXXXXATGTAAGGTAGATGGGCTGGACGTGTTAAATTCATCGTCTTGTCTCCTTGCTCAAACGAC
ACTTCAGGTTCCTTTGTTGT
>Marker473697
CACTTCCTTGTATCACTCTTCTTAAATAACCACTTCTTGGTTCAAATGGCATGTGTGCATTAATGTGGAAAGTAA
CAAAAXXXXXXXXXXXAAGTTTTGATTTTGAATCATCTTTCACATAATTTCAAGTCCTAATTTTATAGAAGCTCTA
CGAAAATTTAATAAAAAGTG
CACTTCCTTGTATCACTCTTCTTAAATAACCACTTCTTGGTTCAAATAGCATGTGTGCATTAATGTGGAAAGTAA
CAAAAXXXXXXXXXXXAAGTTTTGATTTTGAATCATCTTTCACATAATTTCAAGTCCTAATTTTATAGAAGCTCTA
CGAAAATTTAATAAAAAGTG
>Marker473709
CACCTCCCTTAGCGTGCCATCCGTGCGTGCGACCACCCTGTGTGGCATGCGCCCTTTTTGCGCATCATCCTTGGT
TCATGXXXXXXXXXXXCCCCAAGATCGAGGCACTTGTTGCCTATCTCGCCTAGCTATCCGCCATTAGGTGGGGTTG
TGGCATGTCTTGACTTGGTC
CACCTCCCTTAGCGTGCCATCCGTGCGTGCGACCACCCTGTGTGGCATGCGCCCTTTTTGCGCATCATCCTTGGT
GCATGXXXXXXXXXXXCCCCAAGATCGAGGCACTTGTTGCCTGTCTCGCCTAGCTATCCGCCATCAGGTGGGGTTG
TGGCATGTCTTGACTTGGTC
>Marker474374

Create PDF with GO2PDF for free, if you wish to

TACTCGGGACTGGGATTGGTCTGGTTTCCATATGGATCAAATGGTTGAGAAGATCTAGCAGACTCACTCACATAA
GCCCGXXXXXXXXXXXTGCTTGTCACTGCCACTGGTAGAGGACCTCGCATTAAAAGTAGCAGAGTCAATAGCATGA
GTCGCCGAAATATTCATAGT
TACTCGGGACTGGGATTGGTCTAGTTTCCATATGGATCAAATGGTTGAGAAGATCTAGCAGACTCACTCACATAA
GCCCGXXXXXXXXXXXTGCTTGTCACTGCCACTGGTAGAGGACCTCACATTAAAAGTAGCAGAGTCAATAGCATGA
GTCGCCGAAATATTCATAGT
>Marker474803
TACGTATACGTGCATACATATAAATATAAGCTATACAATACATATATGTATAGACATATACACATATAAATATGT
CACACXXXXXXXXXXXGCAGCAGCCACGATGCAGATAGAACTCGTGGATGCATCGACAAAAATAAAGGCAATTTAA
TGGTCAAGATTGAGAGGGTT
TACGTATACGTGCATACATATAAATATAAGCTATACAATACATATATGTATAGACATATACACATATATATATGT
CACACXXXXXXXXXXXGCAGCAGCCACGATGCAGATAGAACTCGTGGATGCATCGACAAAAATAAAGGCAATTTAA
TGGTCAAGATTGAGAGGGTT
>Marker474904
CACACCCTAGCAAGGCATAAGAAGCACCCTTTCCAAAACAAAGACAAATTGAGGAGGAACTCCCTGATCGAGTTT
ATAGCXXXXXXXXXXXTTCTTCCGCTTTCAACAAAGAATACAAAAGAAAGGTCCCAGCAACTAAAGTGACATACTC
GTGAGCCGATCCAAGGTGTT
CACACCCTAGCAAGGCATAAGAAGCACCCTTTCCAAAACAAAGACAAATTGAGGAGGAACTCCCTGATTGAGTTT
ATAGCXXXXXXXXXXXTTCTTCCGCTTTCAACAAAGAATACAAAAGAAAGGTCCCAGCAACTAAAGTGACATACTC
GTGAGCCGATCCAAGGTGTT
>Marker475386
AACTTTTGTATCCATCTGCTATCTCTATGACTTTAGGCCTCAGTAGTTTTTGTCTAAGTGACTCTTAATCAACCA
GGAGCXXXXXXXXXXXGTCACATGTGATATGAACATCGAAGCTGCTGTATAATTTTGTATATTTCTCTGAACTCTA
TGTATTAGTTCATGACGGTT
AACTTTTGTATCCATATGCTATCTCTATGACTTTAGGCCTCAGTAGTTTTTGTCTAAGTGACTCTTAATCAACCA
GGAGCXXXXXXXXXXXGTCACATGTGATATGAACATCGAAGCTGCTGTATAATTTTGTATATTTCTCTGAACTCTA
TGTATTAGTTCATGACGGTT
>Marker475426
GACATTCGGTGCATTTGCAGTTTGTTCAACTACAAAGGTTACCTTTGCAGGCATGCGTTAAATGTTTTTAATTAT
AACGGXXXXXXXXXXXAAGTAATACAAATGTGTATAGTTCGACATACCAATATAGTCATATTCTTAATTTTGCACT
TCCAGTTGTAGAAGAAGGTG
GACATTCGGTGCATTTGCAGTTTGTTCAACTACAAAGGTTACCTTTGCAGGCATGCATTAAATGTTTTTAATTAT
AACGGXXXXXXXXXXXAAGTAATACAAATGTGTATAGTTCGACATACCAATATAGTCATATTCTTAATTTTGCACT
TCCAGTTGTAGAAGAAGGTG
>Marker475476
TACAATACACTATTACACAAAGATACTATTAGTATTATTTATTCAGATACTATGAATTATAGGCATCTCATTTTA
TACAAXXXXXXXXXXXCTAATACCAAATAGGAACTAAACTCAAAATTTAAGGCTATAAGGATTTAATGGTTACTAA
ATTCAAAACTTGGGTAATGT
TACAATACACTATTACACAAAGATACTATTAGTGTTATTTATTCAGATACTATGAATTATAGGCATCTCATTTTA
TACAAXXXXXXXXXXXCTAATACCAAATAGGAACGAAACTCAAAATTTAAGGCTATAAGGATTTAATGGTTACTAA
ATTCAAAACTTGGGTAATGT
>Marker475627
TACCCATCTTCCGACGTTACACTTTTCACGTCAGAAGAAGGGGGTTATTTCGACACATTTTTTGGGACGTTGGAG
GAACCXXXXXXXXXXXTACAGTGACGTCGCGGACAATGTCGATAGGCACATTGAGGAAAGCACGTCGGGGATACTT
TTCTTGATGCGGCTCATGTT

Create PDF with GO2PDF for free, if you wish to

TACCCATCTTCCGACGTTACACTTTTCACGTCAGAAGAAGGGGGTTATTTCGACACATTTTTTGGGACGTTGGAG
GAACCXXXXXXXXXXXTACAGTGACGTCGCAGACAATGTCGATAGGCACATTGAGGAAAGCACGTCGGGGATACTT
TTCTCGATGCGGCTCATGTT
>Marker475640
CACAATCTCAAGAACGTTATAAGCTTGCATTGCAAGAATTGGAGGAGTTATTACATAAATTAAATCTTGTAGAGG
ATGATXXXXXXXXXXXCTTCCCTCCCTCAATCATCATTTCCTAGATCCATATGTATCTGTTGAGTCCCTCTATAAA
ACTGATTCATAATGCAGGTA
CACAATCTCAAGAACGTTATAAGCTTGCATTGCAAGAATTGGAGGAGTTATTACATAAATTAAATCTTGTAGAGG
ATGATXXXXXXXXXXXCTTCCCTCCCTCAATCATCATTTCCTAGATCCATATGTATCTGTTGAGTCCCTCTATAAA
ACTGATACATAATGCAGGTA
>Marker475834
AACCTAAAGGTAAAATTTGGGCTTCAAGAAGTTTCAGACAGGGACATCTTCTTTCCCCGTTTCTTTTCATTATCG
TGGTGXXXXXXXXXXXTTTGCTTGAACCACATTATCATCTTCTTTGAAGAGATGTTGGGTTTGAAAATTAATAGAG
GTAAGAGTTGTGTTATGGGT
CACCTAAAGGTAAAATTTGGGCTTCAAGAAGTTTCAGACAGGGACATCTTCTTTCCCCGTTTCTTTTCATTATCG
TGGTGXXXXXXXXXXXTTTGCTTGAACCACATTATCATCTTCTTTGAAGAGATGTTGGGTTTGAAAATTAATAGAG
GTAAAAGTTGTGTTATGGGT
>Marker476305
ACCCTCCCATTCATCAGGTTTGTCTTTGTTGCCCTTAGTTACTTTCAGAATGTAGAGGGGCTGATTTGATTTTCA
ATTTAXXXXXXXXXXXCGATCATTCGATGTCGGCTGTAAGTTTGGGGATATGAACTGTTTTGGATGGATGTGTCAA
ATGGGAACTTGTTATTGAGT
ACCCTCCCATTCATCAGGTTTGTCTTTGTTGCCCTTAGTTACTTTCAGAATGTAGAGGGGCTGATTTGATTTTCA
ATTTAXXXXXXXXXXXCCATCATTCGATGTCGGCTGTAAGTTTGGGGATATGAACTGTTTTGGATGGATGTGTCAA
ATGGGAACTTGTTATTGAGT
>Marker477183
AACTCACCTAGAAGTTCAAATTTTGTCTCCATTTCTTTTCATTTCAAATCTCATTTCCTTCCAATAATTTGCATA
AACTAXXXXXXXXXXXGAATTCCAAAACGATTACCAATCGGGACCTTAGTTTTTTTTCCAGGTAAAGACAACAAAA
ATGAATCAAGGTGGGAAAGT
AACTCACCTAGAAGTTCAAATTTTGTCTCCATTTCTTTTCATTTCAAACCTCATTTCCTTCCAATAATTTGCATA
AACTAXXXXXXXXXXXGAATTCCAAAACTATTACCAATCGGGACCTTAGTTTTTTTTCCAGGTAAAGACAACAAAA
ATGAATCAAGGTGGGAAAGT
>Marker477622
GACTCCTAAAAAGGAGCTGAGCACCAAACTGAGGCTGTAAGGTTATGAACAGCATGGGGGAATGAGTATAGGTTG
GAGAAXXXXXXXXXXXGCTAAATGTTTCCGAGGCTCGCAAAATCTCAAAGGATTGAAGCAGTTTGCATCCTCTCCC
CAAAACTCTTTGTCATGGTG
GACTCCTAAAAAGGAGCTGAGCACCAAACTGAGGCTGTAAGGTTATGAACAGCATGGGGGAATGAGTATAGGTTG
GAGAAXXXXXXXXXXXGCTAAATGTTTCCGAGGCTCGCCAAATCTCAAAGGATTGAAGCAGTTTGCATCCTCTCCC
CAAAACTTTTTGTCGTGGTG
>Marker478460
ACCAGATAGCTTGCACTGGTGGGAAAAAACTAACAGAATAATGAATTATAGAGGAATGAGTGAAGTTCAGGCAAC
AATCAXXXXXXXXXXXAAATTAAGAACTGCATCAAAGTATAGACACATAAAAATATTGATAAATCAACGGGTTAAG
TTCAATTTTATAGAAATGTA
ACCAGACAGCTTGCACTGGTGGGAAAAAACTAACAGAATAATGAATTATAGAGGAATGAGTGAAGTTCAGGCAAC
AATCAXXXXXXXXXXXAAATTAAGAACTGCATCAAAGTATAGACACATAAAAATATTGATAAATCAACGGGTTAAG
TTCAATTTTATAGAAATGTA
>Marker478488

Create PDF with GO2PDF for free, if you wish to

ACTAATGCACCAATCATCTCCTCACACACACATACACACAGCAATCATTTAAACTGCTGCCATGGAAAGGCCAGA
AGCAAXXXXXXXXXXGATTGCTCAGGAAAAGTATAAAAAAGATGAACTGATATTGATTAAAGACTAACCTCCTCG
AGGCTAGGACTTATTCGGTG
ACTAATGCACCAATCATCTCCTCACACACACATACACAAAGCAATCATTTAAACTGCTGCAATGGAAAGGCCAGA
AGCAAXXXXXXXXXXGATTGCTCAGGAAAAGTATAAAAAAGATGAACTGATATTAATTAAAGACTAACCTCCTCG
AGGCTAGGACTTATTCGGTG
>Marker478618
AACATTGTAGATGCGATAAAAATTTAAACTTTTGACATTGTGATCAAAAGTATAATAATGTCCTAATCAATTAAG
CTTCAXXXXXXXXXXCATGAAAAAATCATACTATATCAAAATAATTTTACATTTTCTCTCTCACCTAATTAAGCA
TTCAACAATGTAAAATTGTC
AACATCGTAGATGCGATAAAAATTTAAACTCTTGACATTGTGATCAAAAGTATAATAATGTCCTAATCAATTAAG
CTTCAXXXXXXXXXXCATGAAAAAATCATACTATATCAAAATAATTTTACATTGTCTCTCTCACCTAATTAAGCA
TTCAACAATGTAAAATTGTC
>Marker478667
GACCCTATTGCTTTTGTGTATATATGGTTTGAAAGGGGATAAAATCAAATGAAATCATGTATATAAAGTGCTTGC
TTTGCXXXXXXXXXXTTTAAATTATAGCATCATGGTTTAAGTAACATGACAACAACAACTCCCACACTAATTATG
TTATTTTATTTAAGAATAGT
GACCCTATTGCTTTTGTATATATATGGTTTGAAAGGGGATAAAATCAAATGAAATCATATATATAAAGTGCTTGC
TTTGCXXXXXXXXXXTTTAAATTATAGCATCATGGTTTAAGTAACATGACAACAACAACTCCCACACTAATTATG
TTATTTTATTTAAGAATAGT
>Marker479285
AACCTTATTCTCAAGTAATGGGGTAGTTGCTCCTTGTTTTTATGCTATCATGTTATGTTTCTCTCTCTTCACACT
GTAACXXXXXXXXXXGAAGTTTTGAGATCTTTACTGTCATAAATAGAATTGAGAAGTCTTTCATTTTGCTAAAAT
GTTGAGGATCCTGAGGAGTG
AACCTTATTCTCAAGTAATGGGGTAGTTGCTCCTTGTTTTTATGCTATCATGTTATGTTTCTCACTCTTCACACT
GTAACXXXXXXXXXXGAAGTTTTGAGATCTTTACTGTCATAAATAGAATTGAGAAGTCTTTCATTTTGCTAAAAT
GTTGAGGATCCTGAGGAGTG
>Marker480039
ACTTTAAGCGTTGTATTTATTATGTATCAAAGTTGGATAAATTTAAGAGATCGAGCTTCTAGTGAATATTCTAAT
GATGTXXXXXXXXXXAACACATTGATATTGTTATGTAATTATGGTGTAGGATGAAATGATGAAATTAAAAACCAC
TTCATCACAAGAAGGGAGTG
ACTTTAAGCGTTGTATTTATTATGTATCAAAGTTGGGTAAATTTAAGAGGTCGAGCTTCTAGTGAATATTCTAAT
GATGTXXXXXXXXXXAACACATTGATATTGTTATGTAATTATGGTGTAGGATGAAATGATGAAATTAAAAACCAC
TTCATCACAAGAAGGGAGTG
>Marker480875
TACTCGAGTAAGCACATAAAGAATATTGCTTAATCTCATCAAGTTGTTGGTGTGGTCATTTACAAAGTCAATCAT
CTAACXXXXXXXXXXTGGTCGATTTGAAACTAAAGGACTTGAACCCTTTTATCAAGCTTTTCCCAAGTCTTCTAG
GTTTGAACTAGATTGATAGT
TACTCGAGTAAGCACATAAAGAATATTGCTTAATCTCATCAAGTTGTTGGTGTGGTCATTTACAAAGTCAATCAT
CTAACXXXXXXXXXXTGGTCGATTTGAAACTAAAGGACTTGAACCCTTTTATCAAGCTTTTCCCAAGTCTTCTAG
GTTTGAGCTAGATTGATAGT
>Marker481167
ACTGGTTTTGTGCATCAAACACTGGTATTGTGTATCTTAAGTGCCATGTTATATAAGCAATTTTCGGTGTAAGAT
TTGTAXXXXXXXXXXTATTAAGGATGCTCACTCTGTTGATTAGGTGAAGAAACGTAGAGAGGCAATCAACAACTT
TTTTCTAGGAATTAGAGGTA

Create PDF with GO2PDF for free, if you wish to

ACTGGTTTTGTGCATCAAACACTGGTATTGTGTATCTTAAGTGCCATGTTATATAAGCAATTTTCGGTGTAAGAT
TTGTAXXXXXXXXXXXTATAAAGGATGCTCACTCTGTTGATTAGGTGAAGAAACGTAGAGAGGCAATCAACAACTT
TTTTCTAGGAATTAGAGGTA
>Marker481176
GACCTTTTAACCATTTCCTGCTTCAATTGACTTATTCAGATGCCAAAACTTCACATAACAACAGGAAACCTATTC
TGGCCXXXXXXXXXXXTAATAATTAATAAATCAATAATTAACAAAACAAAAACTAGCATCAACGACTTCCCAACTC
AAACTTCCTCAGGGGAAGTT
GACCTTTTAACCATTTCCTGCTTCTAATGACTTATTCAGATGCCAAAACTTCACATAACAACAGGAAACCTATTC
TGGCCXXXXXXXXXXXTAATAATTAATAAATCAATAATTAACAAAACAAAAACTAGCATCAACGACTTCCCAACTC
AAACTTCCTCAGGGGAAGTT
>Marker481511
AACATCAATCTTAAGACCAAAATGGATGGGATCAAATGACCATCTTATGTAATTCTTTTTTACACGTTTTTTTTA
TATCAXXXXXXXXXXXGTGAATTCGAAAAAAAGAAAAAAGATAAATTAACTTGTCAAATTTAACCGTATGTTAAAA
TCAAGCTAATATAATTTGTC
AACATCAATCTTAAGACCAAAATGGATGGGATCAAATGACCATCTTATGTAATTCTTTTTTACACATTTTTTTTA
TATCAXXXXXXXXXXXGTGAATTCGAAAAAAAGAAAAAAGATAAATTGACTTGTCAAATTTAACCGTATGTTAAAA
TCAAGCTAATATAATTTGTC
>Marker481579
AACTTTTTTTAGGAGTGTGCAATCCAAACAAGCAGCTAAAATAATTTTTGTTCATTTAAAGAAAAACTAAAACAA
ATTTTXXXXXXXXXXXTACAAATGTATTATACTCTCGATTGTATTTTCGACTAAATAGCACGTTAGACTTATACAT
TTTTATCCCATTAATTCGTT
AACTTTTTTTGGGAGTGTGCAATCCAAACAAGCAGCTAAAATAATTTTTGTTAATTTAAAGAAAAACTAAAACAA
ATTTTXXXXXXXXXXXTACAAATGTATTATACTCTCGATTGTATTTTCGACTAAATAGCACGTTAGACTTATACAT
TTTTATCCCATTAATTCGTT
>Marker481853
AACACATTTTTTAAACATAAATGAACGTTATCATAAAAATGTTAGGTCATGTTAGCTTTCCAAGTAATATCATAA
AATAGXXXXXXXXXXXCTTGAAACAACGATTAAGGTCCCTTAGTTATCTTCTCACGTCAAGATATTACAATTATTT
CCAAAATAAGTTATAAGGTT
AACACATTTTTTAAACATAAATGAACGTTATCATAAAAATGTTAGGTCATGTTAGCTTTCCAAGTAATATCATAA
AATAGXXXXXXXXXXXCCTAAAACAACGATTAAGGTCCCTTAGTTATCTTCTCATGTCAAGATATTACAATTATTT
CCAAAATAAGTTATAAGGTT
>Marker481912
CACAAAATATCAAATTAAAATTATAGGAAACTTTCAAAAATAAAAAAATTGACAAGATATTTAAATTTAAAAAAA
GTGTAXXXXXXXXXXXTTGTTATAGGAGATTCTAGGAAATTAGGTTCTTGTGAGAATTATTTCCCATTTTTAGGAA
GTAAATTTTTTATTAAGGTT
CACAAAATATCAAATTAAAATTATAGGAAACTCTCAAAAATAAAAAGATTGACAAGAGATTTAAATTTAAAAAAA
GTGTAXXXXXXXXXXXTTGTTATAGGAGATTCTAGGAAATTAGGTTCTTGTGAGAATTATTTCCCATTTTTAGGAA
GTAAATTTTTTATTAAGGTT
>Marker482420
GACTAATGTATAAACTGAGAATTCATTGATTAAAAATGAAAGTAAAGAGAACTAATAAGCTCAAAATAACTTGCC
ACTCCXXXXXXXXXXXCTGAAAATGTTATAGATATAAGAAGTTTAGAAGAGTGATAAAAACAACAGACTATTCAAC
TGAAATCCAAGAAATGAGTT
GACTAATGTATAAACTGAGAATTCATTGATTAAAAATGAAAGTAAAGAGAACTAATAAGCTCAAAATAACTTGCC
ACTCCXXXXXXXXXXXCTGAAAATGTTATAGATATAATAAGTTTAGAAGAGTGATAAAAACAACAGACTATTCAAC
TGAAATCCAAGAAATGAGTT
>Marker482547

Create PDF with GO2PDF for free, if you wish to

AACCTTTTCACTCATGAAAACTTTAACTAATCCACCTATGAAATTCATTTCAATCTAATTTAGAATACAAGAAAT
TCATTXXXXXXXXXXXTTACGAAATAACATAGGATACCAGGAGACAGGATATAGAAAATAACAGCTTCTCTAAACA
CTAAGTCTTATATATTAGTG
AACCTTTTCACTCATGAAAACTTTAACTAATCCACCTATGAAATTCATTTCAATCTAATTTAGAATACAAGAAAT
TCATTXXXXXXXXXXXTTACGAAATAACACAGGATACCAAGAGACAGGATATAGAAAATAACAGCTTCTCTAAACA
CTAAGTTTTATATATTAGTG
>Marker482665
CACAACATTGAAATGCGGCGACTGAGAGATCTCAAGTGATGTTACCATGAGGGGAGTAAATTTTACAACATTCTT
CTTATXXXXXXXXXXXGATTTTGACTTTTTTTAAGATACTCTTGATAAAAAGTTTTAAAAGAACATTAGGTGAAAG
TCATATTTGCAGGTAATGTT
CACAACATTGAAATGCGACGACGGAGAGATCTCAAGTGATGTTACCATGAGGGAAGTAAATTTTACAACATTCTT
CTTATXXXXXXXXXXXGATTTTGACTTTTTTTAAGATACTCTTGATAAAAAGTTTTAAAAGAACATTAGGTGAAAG
TCATATTTGCAGGTAATGTT
>Marker482814
AACCTCATATCTAATAAAAATAATACATTCTATTCAAAGTTCCTCGATCTATAAAAACTATTAATCATATCTATA
GAAAGXXXXXXXXXXXCCTTTTGAGAATGAAAGCAACACATTGTTAGCTTACCATACATCAAAACCTTTCATGAAT
AAAAATTTCTTACTAATGTC
AACCTCATATCTAATAAAAATAATACATTCTATTCAAAGTTTCTCGATCTATAAAAACTATTAATCATATCCATA
GAAAGXXXXXXXXXXXCCTTTTGAGAATGAAAGCAACACATTGTTAGCTTACCATACATCAAAACCTTTCATGAAT
AAAAATTTCTTACTAATGTC
>Marker482838
AACATCCTGTAATATGCATTTACCCGTGTCCTTCCTGATTGTAACCTTTGTTCATATTAAGTATTTAGAGAAGGA
GGGGCXXXXXXXXXXXATAAAATTTTCTGCATTTATTCTTCTGGGAGCTCTGTAGTGGTCTGTCATTCAGAGGGGG
GAAATTTCTGTGAAACTGTG
AACATCCTGTAATATGCATTTACCCGTGTCCTTCCTGATTGTAACCTTTGTTCATATTAAGTATTTAGAGAAGGA
GGGGCXXXXXXXXXXXATAAAATTTTCTGCATTTATTCTTCTGGGAGCTTTGTAGTGGTCTGTCATTCAGAGGGGG
GAAATTTCTGTGAAACTGTG
>Marker483092
CACATTCATCATTTATATCCATTAACTATACCCATTCAAAATGATGAGTTTCTTTCTTCTCCACTTTTTCTCTTT
TAATAXXXXXXXXXXXGCCACCGTTAAAAAAAATGAATTAATTATATTATATTAGTTTTCTAGACTAAATTTTATA
AACTTTTAGAAAATAGAGTT
CACATTCATCATTTATATCCATTAACTATACCCATTCAAAATGATGAGTTTCTTTCTTTTCCACTTTTTCTCTTT
TAATAXXXXXXXXXXXGCCACCGTTAAAAAAAATGAATTAATTATATTATATTAGTTTTCTAGACTAAATTTTATA
AACTTTTAGAAAATAGAGTT
>Marker483785
ACAACAATACCCACAACTCATTTTTGCACTCCATAGCAAAAAATAATATATTATGTTAAGGACGACCCCTTACGT
AAATTXXXXXXXXXXXTAGGATTCATTTCTTGTAGCAAATATTCACTTGTAGCATACGATATCTAAAGGGACTGGC
GTAAGTTTTCTACTGCGGGT
ACAACAATACCCACAACTCATTTTTGCACTCCATAGCAAAAAATAATATATTATGTTAAGGACAACCCCTTAGGT
AAATTXXXXXXXXXXXTAGGATTCATTTCTTGTAGCAAATATTCACTTGTAGCATACGATATCTAAAGGGACTGGC
GTAAGTTTTCTACTGCGGGT
>Marker484086
CACCGAATTGGTAGAACAGGGAGAGCTGGAGCAAAAGGAACTGCATACACTTTCTTTACTGCTGCTAATGCCAGA
TTTGCXXXXXXXXXXXCAACACCAGGGATAAAAGGAAAAGTAATCAAGAAGCTTGGTCCATTACTTACATTTCTCT
GGTGCATGCTCGGTGGAGTT

Create PDF with GO2PDF for free, if you wish to

CACCGAATTGGTAGAACAGGGAGAGCTGGAACAAAAGGAACTGCATACACTTTCTTTACTGCTGCTAATGCCAGA
TTTGCXXXXXXXXXXXCAACACCAGGGATAAAAGGAAAAGTAATCAAGAAGCTTGGTCCATTACTTACATTTCTCT
GGTGCATGCTCGGTGGAGTT
>Marker484381
CACAGCTAAACTAAGACAACACTTTCAATAACACTTTGAAGTAATTAGAATCATAAATAATCTCTAACTCTCAAC
TTTAAXXXXXXXXXXXATAAAACTGATATTAGTGTTACAAAATCACCCAACAAAAGAATATAAAAATTTATTTAGT
TCATTACATTAGCTATGGTT
CACAGCTAAACTAAGACAACACTTTCAATAACACTTTGAAGTAATTAGAATCATAAATAATCTCTAACTCTCAAC
TTTAAXXXXXXXXXXXATAAAACTGATATTAGTGTTACAAAATCACCCAACAAAAGAATATAAAAATTTATTTAGT
TCATTATATTAGCTATGGTT
>Marker484392
CACCTTGAATTGAATTGAATTTGATGTGACTTTTGAAAGAGTTGAATGTGTCTTTCCTCCATCAATGACGGTTTT
AACAAXXXXXXXXXXXTCATACTAATTGTTTATCAATTTTGAAAAATACTTATTTTAAGTAACATTTATACTACTT
TAAAATCACTTTAAGCTGTT
CACCTTGAATTGAATTGAATTTGATGTGACTTTTGAAAGAGTTGAATGTGTCTTTCCTCCATCAATGACGGTTTT
AACAAXXXXXXXXXXXTCATACTAATTGTTTATCAATTTTGAAAAATACTTATTTTAAGTAACATTTATACTACTT
TAAAACCACTTTAAGCTGTT
>Marker484437
ACCCTAGGGAAATGGCTGATCTCAAATGACACAAACTTATCCTTGAGCTCCTTAGCCACTTTACAAAATTTAGCC
ATATTXXXXXXXXXXXTGTGATTGTGCGTAGTTTAAGTAGAATTTGACAGAAGTCATGTTTAACAGCTTCCTTGAT
CTTATCTATCTATATGTGTA
ACCCTAGGGAAATGGCTGATCTCAAATGACACAAACTTATCCTTGAGCTCCTTAGCCACTTTACAAAATTTAGCC
ATATTXXXXXXXXXXXTGTGATTGTGCGTAGTTTAAGTAAAATTTGACAGAAGTCATGTTTAACAGCTTCCTTGAT
CTTATCTATCTATATGTGTA
>Marker484510
CACCTAAGTATTATTTTTCCTTTTTGCCGGGTTAGAATTTAGTGTATGAATTTATGTATTATCTAAATATATGAG
AAATAXXXXXXXXXXXACATATATATATTTCAAGCTATATCACTTATGTAAGAAATACGTCACTCCAATTTGTTGT
TTTTCTAAAATAATATTGTA
CACCTAAGTATTATTTTTCCTTTTTGCCGGGTTAGAATTTAGTGTATGAATTTATGTATTATCTAAATATATGAG
AAATAXXXXXXXXXXXACATATGTATATTTCAAGCTATATCACTTATGTAAGAAATACGTCACTCCAATTTGTTGT
TTTTCTAAAATAATATTGTA
>Marker484859
AACCAGAAGTTACAGGGTTGAAAAGGGTATGTCTTATTTTCATGGATTGTGTTATTTAATTATTGGGTAAGTCAA
GATTTXXXXXXXXXXXTCTATCATTAAATTGCCCTCTCTATGTGTAAATGCATGTATTATGAAAAGTAATATGCTG
CTGTCTCTATATGCAATGGT
AACCAGAAGTTACAGGGTTGAAAAGGGTATGTCTTATTTTCATGGATTGTGTTGTTTAATTATTGGGTAAGTCAA
GATTTXXXXXXXXXXXTCTATCATTAAATTGCCCTCTCTATGTGTAAATGCATGTATTATGAAAAGTAATATGCTG
CTGTCTCTATATGCAATGGT
>Marker485276
ACATATTCATGAATATTAAATATATATGATGGTAAGCGAAATTATTAGCAAAATTTGACTTGTTTGTTTCAACTA
GATCAXXXXXXXXXXXACTTAAATCCATCGTTCTCAAGACGGATGGTCTTCGGCATCGGAACACCCGGCGGCTTAA
TTCCGAATTGCGTTCCTGTG
ACATATTCATGAATATTAAATATATATGATGGTAAGCGAAATTATTAGCAAAATTTGACTTGTTTGTTTCAACTA
GATCAXXXXXXXXXXXACTTAAATCCATCGTTCTCAAGACGGATGGTCTTCGGCATCGGAACAGCCGGCGGCTTAA
TTCCGAATTGCGTTCCTGTG
>Marker486515

Create PDF with GO2PDF for free, if you wish to

AACAACATAAAGAAAAAGGGAGTTGAGATATCAACTTCCTTAAGCTTTGTTGAGCCTCGAAGCCAACAATAATAC
CTCACXXXXXXXXXXTTAATTTTCATTCATCAAAACTAACTTTTAAGTGGCATTCAACCTTTGACTTTGGAAGGT
AACTGAAATGAAGGCAGAGT
AACAACATAAAGAAAAAGGGAGTTGAGATATCAACTTCCTTAAGCTTTGTTGAGCCTTGAAGCCAACAATAATAC
CTCACXXXXXXXXXXTTAATTTTCATTCATCAAAACTAACTTTTAAGTGGCATTCAACCTTTGACTTTGGAAGGT
AACTGAAATGAAGGCAGAGT
>Marker486679
TACACAAAAGCAAAGTTATTTGTTATCCAAACCCATCCTAATAGTAATTATAATTAACTTTAAAACAAGTTTTTA
AATATXXXXXXXXXXTTTTAGTTTAGGCATCAATTTAGTTAAATTGAATCTTGCATTCATCGGAAACATAATTGT
TGAATAAAGCAAACTTTGTT
AACACAAAAGCAAAGTTATTTGTTATCCAAACCTATCCTAATAGTAATTATAATTAACTTTAAAACAAGTTTTTA
AATATXXXXXXXXXXTTTTAGTTTAGGCATCAATTTAGTTAAATTGAATCTTGCATTCATCGGAAACATAATTGT
TGAATAAAGCAAACTTTGTT
>Marker486720
TACAAAAGGGACCTGATCACTTTATTAATTTATATAATTTAGAAGACCCAAAATTTCAAAGAAATTAAGCAAAAC
AGCTTXXXXXXXXXXTGATCCAACTGATAGGAATGGGCAGACATCTCTCTCCATCTAAAAAACACTTCTTCTAAT
GACATTAAATCAGAATTGTT
TACAAAAGGGACCTGATCACTTTATTAATTTATATAATTTAGAAGACCCAAAATTTCAAAGAAATTAAGCAAAAC
AGCTTXXXXXXXXXXTGATCCAACTCATAGGAATGGGCAGACATCTCTCTCCATCTAAAAAACACTTCTTCTAAT
GACATTAAATCAGAATTGTT
>Marker487192
ACCATTTCTTTTAAAAGTTGTAATACATGATCTTTTATTGGTATTTTAAAAAAAAACCCTTCAAAATTTTGAAGA
GAGCTXXXXXXXXXXTTTAGACTAAATTATTAAAAATGTGGTTCAACTTTGTGTTTTATGTAAAAAATATCTTAA
GCTTTCAATCATTTCTGGTG
ACCATTTCTTTTAAAAGTTGTAATACATGATCTTTTATTGGTATTTAAAAAAAAAACCCTTCAAAATTTTGAAGA
GAGCTXXXXXXXXXXTTTAGACTAAATTATTAAAAATGTGGTTCAACTTTGTGTTTTATGTAAAAAATATCTTAA
GCTTTCAATCATTTCTGGTG
>Marker487258
AACTTATCACTGAAGCTCTATTAGTTGGGTCATACTTTTAGAAGCAAACATGTGGTTTTTATTTATTATGTGTGT
TATAAXXXXXXXXXXGTATTGGTGTAATTTGTGAGATTGTTAATGGATGGTTACTATAACTAAGCTTAATTATGA
GCTATTTGGTATATTTGGTG
AACTTATCACTGAAGCTCTATTAGTTGGGTCATACTTTTAGAAGCAAACATGTGGTTTTTATTTATTATGTGTGT
TATAAXXXXXXXXXXGTATTGGTGTAATTTATAAGATTGTTAATGGATGGTTACTATAACTAAGCTTAATTATGA
GCTATTTGGTATATTTGGTG
>Marker487508
ACAAGGCTGAAAATTCTGCGACTCAAATGCTATTAGATCTAGTGTTGAAGAAAGGATTCGCTTTAAGAAAACTGA
CAAACXXXXXXXXXXAACTCCATTCTTAGACTAGGGTTGGGAGCATTTCTATGTGGATCACACTAATCAGTGAAA
ATTTTCTTTTAATGCAGAGT
ACAAGGCTGAAAATTCTGCGACTCAAATGCTATTAGATCTAGAGTTGAAGAAAGGATTCGCTTTAAGAAAACTGA
CAAACXXXXXXXXXXAACTCCATTCTTAGACTAGGGTTGGGAGCATTTCTATGTGGATCACACTAATCAGTGAAA
ATTTTCTTTTAATGCAGAGT
>Marker487609
CACTAGCAAGAGACTCTTAAAAACATTTTATTTTTGGTGTAATACATTTACATTTATATTTCAAAAGTTAAAAAA
GGAATXXXXXXXXXXCAAAAATGTTCCCTTAAATAGTAAGAAATATCTAACTATGTTTCATTTTAGGTTAAAGTA
TCGGTTTAAGCACTAAGGTG

Create PDF with GO2PDF for free, if you wish to

CACTAGCAAGAGACTCTTAAAAACATTTTATTTTTGGTGTAATACATTTACATTTATATTTCAAAAGTTCAAAAA
GGAATXXXXXXXXXXXCAAAAATGTTCCCTTAAATAGTAAGAAATATCTAACTATGTTTCATTTTAGGTTAAAGTA
TCGGTTTAAGCACTAAGGTG
>Marker487940
ACGTAAGCCTATTTGTTTATATGAATAGTTTCTCCGGGAGTGTTATCATAATGGATACTTCTTGCAATGAGAGAT
TAGATXXXXXXXXXXXAAAGTTTTAAAGTTGATGAGTTTTGGGAGTGGCTTGTTTTTGTTTTATAGACTATTGTGG
TCTTCGAAGATGCTTTGGTT
ACGTAAGCCTATTTGTTTATATGAATAGTTTCTCCGGGAGTGTTATCATAATGGATACTTCTTGCAATGAGAGAT
TAGATXXXXXXXXXXXAAAGTTTTAAAGTTGATGAGTTTTGGGAGTGGCTTGTTTTTGTTTTATAGACTACTGTGG
TCTTCGAAGATGCTTTGGTT
>Marker487999
CACATCAATAGACTTCGTTATTTTACATAATGCAATTCTAGTCTATCCTGCATTCTAGCATTTCTTCTCTTCTTC
ACTACXXXXXXXXXXXCCTTTCTTTTTCTTGCAACACTTTTATTAGCTAAGTTTATTTATTTACATACATTTGGCG
TTGAGTGGAAAATCATAGGT
CACATCGATAGACTTCGTTATTTTACATAATGCAATTCTAGTCTATCCTGCATTCTAGCATTTCTTCTCTTCTTC
ACTACXXXXXXXXXXXCCTTTCTTTTTCTTGCAACGCTTTTATTAGCTAAGTTTATTTATTTACATACATTTGGCG
TTGAGTGAAAAATCATAGGT
>Marker488542
AACGCGTTAAATTTAATTCTGGACTTTCTGTGACGTCTTATTCCTCTACGTCGCAGAAAGCCCCTTTCTGCAACG
TAATAXXXXXXXXXXXATCTTTATAAAGTGATATTTTCAATCGAGCTAAAGGCCCTCATATATACATAATGTGTCA
TCAAATTGGGTTACCAAAGT
AACGCGTTAAATTTAATTCTGGACTTTCTGTGACGTCTTATTCCTCTACGTCGCAGAAAGCCCCTTTCTGCAACG
TAATAXXXXXXXXXXXATCTTTATAAAATGATATTTTCAATCAAGCTAAAGGCCCTCATATATACATAATATGTCA
TCAAATTGGGTTACCAAAGT
>Marker488627
GACCAAGTGGAAGGCTTTGTTCTCACATAATGTGGATGATATTATGTTGTTAAATATATAACAGATCCAAGCTTT
TCCATXXXXXXXXXXXTCAGGCTTTTGCCTAATTCATCTCATTAATCAGATCATTGATGTCATCTTCTCTATTACC
ACTATCTCCACCTTCCTTGT
GACCAAGAGGAAGGCTTTGTTCTCACATAATGTGGATGATATTATGTTGTTAAATATATAACAGATCCAAGCTTT
TCCATXXXXXXXXXXXTCAGGCTTTTGCCTAATTCATCTCATTAATCAGATCATTGATGTCATCTTCTCTATTACC
ACTATCTCCACCTTCCTTGT
>Marker488638
AACTATCTTCAAATACGTCTCAATATGGACAATGTAACTGCTTCAAATAGTGTCACTAGTTGGTCAGGAACTTGC
TACCTXXXXXXXXXXXACAATTTTTTTTTAATTAGCGGCTGCATTGCGTAATGATATTATCTTTTGATGTATGGTC
TTGCAGGGCATGACGTTGTT
AACTATCTTCAAATACGTCTCAATATGGACAATGTAACTGCTTCAAATAGTGTCACTAGTTGGTCAGGAACTTGC
TACCTXXXXXXXXXXXACAATTTTTCTTTAATTAGCGGCTGCATTGCGTAATGATATTATCTTTTGATGTATGGTC
TTGCAGGGCATGACGTTGTT
>Marker488718
AACTATAAGTTCGCATGAAAGGCCTCAAAGCTACAGAGGTAATGATAATATTAGTATTTACAAACCTGTGCATTT
GGGACXXXXXXXXXXXCCAAACCCTGCTGGGCTGCCGGCAGGAAGAGGCTTGAACTGCTGGATTCCATACTTCAAG
TTATTTGCATTCATGTGAGT
AACTATAAGTTCGCATGAAAGGCCTCAAAGCTACAGAGGTAATGATAATATTAGTATTTACAAACCTGTGCATTT
GGGACXXXXXXXXXXXCCAAACCCTGCCGGGCTGCCGGCAGGAAGAGGCTTGAACTGCTGGATTCCATACTTCAAG
TTATTTGCATTCATGTGAGT
>Marker489458

Create PDF with GO2PDF for free, if you wish to

ACAAGGTAGATTATCCAGAGATTATCAAATAATATTGTTTATTGAAAAAAGAATCATATTTGACTGCTGTCATTT
GTGTTXXXXXXXXXXGTTGGAAAACTCTTTATTTTCTGTTTTTACTGTATCCAGTGTCCCTATTTGGTTAGATTG
ATGCTGTAGGGATTTTGGTA
ACAAGGTAGATTATCCAGAGATTATCTAATAATATTGTTTATTGAAAAAAGAATCATATTTGACTGCTGTCATTT
GTGTTXXXXXXXXXXGTTGGAAAACTCTTTATTTTCTGTTTTTACTGTATCCAGTGTCCCTATTTGGTTAGATTG
ATGCTGTAGGGATTTTGGTA
>Marker489772
ACATTTTTTTTTGAAAAAAAAATCAATATTGCACTTACAACATCAATTTTAAATAAATATATATAATTTCTTTAG
CACGTXXXXXXXXXXAAATTTTGGCAAATTAAAAATATGAATATTTGTTTATTTGACTTACAATACGATTTTTTT
TAAAAGAAAAATACAAGAGT
ACGTTTTTTTTTGAAAAAAAAATCAATATTGCACTTACAACATCAATTTTAAATAAATATATATAATTTCTTTAG
CACGTXXXXXXXXXXAAATTTTGGCAAATTAAAAATATGAATATTTGTTTATTTGACTTACAATACGATTTTTTT
TAAAAGAAAAATACAAGAGT
>Marker489878
AACCGATATCGCCCCCTTCAAGAACAACACCAACTAACCTTTTAACCTAAACCTTTAAACTGAAAAACGTTAACG
GAGACXXXXXXXXXXTATTTACATAAAACAAAACCTAACAGCAACCTTCCCAAAAACCTAAGTCTAACATTACAA
AAGACTAACTGATCGCAAGT
AACCGATATCACCCCCTTCAAGAACAACACCAACTAACCTTTTAACCTAAACCGTTAAACTGAAAAACGTTAACG
GAGACXXXXXXXXXXTATTTACATAAAACAAAACCTAACAGCAACCTTCCCAAAAACCTAAGTCTAACATTACAA
AAGACTAACTGATCGCAAGT
>Marker489943
AACGAGAAGCAACAAATTTCTTCACACACCATAAACGATAAGACTAAAAGAAATGAAAGTAAATAATTGAAAGGA
CAGTAXXXXXXXXXXCACAAGACAAAAGATAGCGTTGATCAGCAATCGCAGGTTATAAGATTATGATATATAAAC
ACTTATAATCGAGTTAATGT
AACGAGAAGCAACAAATTTCTTCACACACCATAAACGATAAGACTAAAAGAAATGAAAGTAAATAATTGAAACGA
CAGTAXXXXXXXXXXCACAAGACAAAAGATAGCGTTGATCAGCAATCGCAGGTTATAAGATTATGATATATAAAC
ACTTATAATCGAGTTAATGT
>Marker489996
ACTGTTAATGACTTAGTAAGCTGAACCCCACGAAATACTTGATGAATGGAAGAATGGCAATAATAAAGCATGCAG
ACAAAXXXXXXXXXXCTCAATAGGTAACTGAACATTACAGAGCTTTCTATTGGAAGTAATGATCAATCGTAGCCA
GCAATAGCATCTTAAAGGTA
ACTGTTAATGACTTAGTAAGCTGAACCCCACGAAATACTTGATGAATGGAAGACTGGCAATAATAAAGCATGCAG
ACAAAXXXXXXXXXXCTCAATAGGTAACTGAACATTACAGAGCTTTCTATTGGAAGTAATGATCAATCGTAGCCA
GCAATAGCATCTTAAAGGTA
>Marker490029
GACATGCTTCTACTAGTTGATAAGTTTTTAATCCCGCACTAAGTCTGTTATTAAGTTTCTTGGCTGAATTTTCCT
TTCCTXXXXXXXXXXGATCTCTAATAAGCAATTTCTTGTATTGATATTTGGTATGGATGAATTAAGGTCAAACCT
GGATCTGTTTCAATGTTGTT
GACATGCTTCTACTAGTTGATAAGATTTTAATCCCGCACTAAGTCTGTTATTAAGTTTCTTGGCTGAATTTTCCT
TTCCTXXXXXXXXXXAATCTCTAATAAGCAATTTCTTGTATTGATATTTGGTATGGGTGAATTAAGGTCAAACCT
GGATCTGTTTCAATGTTGTT
>Marker490165
AACCAAAAGTGACCTATTTATACTAAACCTTGCATGAATATTGATTGATACCTCATACTTGCCAAAATCTAAATT
AAGCAXXXXXXXXXXATTATAGTAAATGCAGTTGCAATACATCACCGATTCAGATCATTATGCATAATGCGCTCA
TTAAAATTCTTTGTATTGTA

Create PDF with GO2PDF for free, if you wish to

AACCAAAAGTGACCTATTTATACTAAACCTTGCATGAATATTGATTGATACCTCATACTTGCCAAAATCTAAATT
AAGCAXXXXXXXXXXXATTATAGTAAATGCAGTTGCAATACATCACCGATTCAGATCATTACGCATAATGCGCTCA
TTAAAATTCTTTGTATTGTA
>Marker490272
ACTTTCTGATCAAAATTTATAATCCTTGATTGCTGATATACTCCAGCAATTTCTGTGTAATGATCTATTTTTCTC
GTTTGXXXXXXXXXXXTATGAATGTATTTTAGGCTACCTTCTTATTACCAGCACACTTGATATTTTCATCTGTTCA
GTTACTTAAATGAATTTGTT
ACTTTCTGATCAAAATTTATAATCCTTGATTGCTGATATACTCTAGCAATTTCTGTGTAATGATCTATTTTTCTC
GTTTGXXXXXXXXXXXTATGAATGTATTTTAGGCTACCTTCTTATTACCAGCACACTTGATATTTTCATCTGTTCA
GTTACTTAAATGAATTTGTT
>Marker491693
AACACCAAAATAGGATTTAAAGTGTTCTTTTTTTCTTTCTAAAAGACTTTTGAATCATTTTGAAAAGAGAGATTC
CATTTXXXXXXXXXXXTCTTTGATTGAATAAGAACACAACGAAATCATTCACAACAATAACACTGAAGAATTCCCC
TATAATCTTTCACAGCTGTT
AACACCAAAATAGGATTTAAAGTGTTCTTTTTTTCTTTCTAAAAGACTTTTGAATCATTTTGAAAAGAGAGATTC
CATTTXXXXXXXXXXXTCTTTGATTGAACAAGAACACAACGAAATCATTCACAACAATAACACTGAAGAATTCCCC
TATAATCTTTCACAGCTGTT
>Marker492316
AACAATAGCCTTCTTCAACTAAGACATCACCATATAGAAAATGTGTAATTTTAGTTAATTTTTCTTATTATTCTA
CGTAAXXXXXXXXXXXTAATAATGCCAAAAGTATAGGGACTTTCTTTTTGTAGGCAGTAACAACAAATTAAATAGG
TCCAACTTTGTCTCTATTGT
AACAATAGCCTTCTTCAACTAAGACATCACCATATAGAAAATGTGTAATTTTAGTTAATTTTCCTTATTATTCTA
CGTAAXXXXXXXXXXXTAATAATGCCAAAAGTATAGGGACTTTCTTTTTGTAGGCAGTAACAACAAATTAAATAGG
TCCAACTTTGTCTCTATTGT
>Marker492820
TACTCTCTTCATTTTTTATCCATCTGGTTCAAATCGTAGATAATACTTTGTTGAAAGTTCATACTTTGTTTGTCA
TATCCXXXXXXXXXXXTTCTTGTCGAATTTTGATATCTAGATCTTTTGTAACTATTCCATAGGCATTATTTTACTT
GGTTCAAATTCCCTTTGGTG
TACTCTCTTCATTTTTTATCCATCTGGTTCAAATCGTAGATAATACTTTGTTGAAAGTTCATACTTTGTTTGTCA
TATCCXXXXXXXXXXXTTCTTGTCGAATTTTGATTTCTAGATCTTTTGTAACTATTCCATAGGCATTATTTTACTT
GGTTCAAATTCCCTTTGGTG
>Marker493115
ACTAACCTCAACCTGGCGGCTCATGAAGGATGGCATGTTTCCTCTAACATCTCTATTTGGGGCTGCTGGAGGATT
CCCCGXXXXXXXXXXXCCAAATGGAAAGAGACTAATACTAAAAAATACAAAACATTCAATTAAAAAGAAAAATAAA
GACACAAAATTAATGAAGTG
ACTAACCTCAACCTGGCGGCTCATGAAGGATGGCATGTTTCCTCTAACATCTCTATTTGGGGCTGCTGGAGGATT
CCCCGXXXXXXXXXXXCCAAATGGAAAGAGACTAATACTAAAAAATACAAAACATTCAATTAAAAAGACAAATAAA
GACACAAAATTAATGAAGTG
>Marker494048
ACAATCAATAAATTTTTATCCTCGCATCTACCATCACCATTTCTTTTTATTTATGGTGACTTTCCGAGTGCTTTA
CGATTXXXXXXXXXXXATTCAGAAACTAATGTCATATTTGATCTTTGATATTGTTTTCCAAAATTAGAATGACTCA
ACTAATATACAATTTATGTT
ACAGTCAATAAATTTTTATCCTCGCATCTACCATCACCATTTCTTTTTATTTATGGTGACTTTCCGAGTGCTTTA
CGATTXXXXXXXXXXXATTCAGAAACTAATGTCATATTTGATCTTTGATATTGTTTTCCAAAATTAGAATAACTCA
ACTAATATACAATTTATGTT
>Marker494292

Create PDF with GO2PDF for free, if you wish to

AACTTATCATTGGACAATATGGCCCGTGTTACTATATACTCTTTCGTAAAGTATCATAAGAATATTTATTTTTTG
TTTCAXXXXXXXXXXAGCCTATTATTGATGATTTTGATAATAATCTAGTCAAATTTTGAGTTAAGTGTCCTTGGG
ATAAAAGAATGAATAGAGTA
AACTTATCATTGGACAATATGGTCCGTGTTACTATATACTCTTTCGTCAAGTATCATAAGAATATTTATTTTTTG
TTTCAXXXXXXXXXXAGCCTATTATTGATGATTTTGATAATAATCTAGTCAAATTTTGAGTTAAGTGTCCTTGGG
ATAAAAGAATGAATAGAGTA
>Marker494320
ACATTCAATTTTATCATCAATTGGGGCATTTGGATGGAATACAGATAATGCACGAGCAAACTCTTCGAAGCCCAA
TATTCXXXXXXXXXXATGTAGTCCATCTAAGAAAGAGCAATTTTAATATCCTGCAGATATACATTATTAAATACA
TAATACTTAGAAAAAATAGT
ACATTCAATTTTATCATCAATTGGGGCATTTGGATGGAATACAGATAATGCACGAGCAAACTCTTCGAAGCCCAA
TATTCXXXXXXXXXXATGTAGTCCATCTAAGAAAGAGCAATTTTAATATCCTGCAGATATACATTATTAAATAAA
TAATACTTAGAAAAAATAGT
>Marker495686
TACCTTAATACTTGTATCATCATGATATTCAGGTGCTACTTTGCATGTGAAGTCTTAAATGAAAAGCTCTATACC
ATTGGXXXXXXXXXXAAATTGAAGATTCTATTGTAATGGATGGAAAGATATATATCCGTCTTCGATCTGCAGATT
CTCAAGTGTATGCTCTTGTT
TACCTTAATACTTGTATCATCATGATATTCAGGTGCTACTTTGCATGTGAAGTCTTAAATGAAAAGCTCTATACC
ATTGGXXXXXXXXXXAAATTGAAGATTCTATTGTAATGGATGGAAAGATATATATCCGTCTTCGATCTGCGGATT
CTCAAGTGTATGCTCTTGTT
>Marker495734
TACTTGGCATTCAATGCTCACGAGTATTTTGTCAATGTTTCGTTGCCCTCTCCTCCAACAAAGCGAAAATGGTTT
CGTGTXXXXXXXXXXAACAGTTCAGTTTATGAACTTTATTTGACAATGATACAGTTTAGAAAAACTATTTTAAAT
AACAAAATTCTGTAAATGTT
TACTTGGCATTCAATGCTCACGAGTATTTTGTCAATGTTTCGTTGCCCTCTCCTCCAACAAAGCGAAAATGGTTT
CGTGTXXXXXXXXXXAACAGTTTAGTTTATGAACTTTATTTGACAATGATACAGTTTAGAAAAATTATTTTAAAT
AACAAAATTCTGTAAATGTT
>Marker495856
CACTGGTTTCAAATTGTTGTAAGCATTTATCCGCGATGGCGGATGAAGAATCATTCTACATATGAAGAATCATCG
TCAGAXXXXXXXXXXGTCAAAACACTAGCCACCAATAATCAATTTGAACCGAGCTTAACTTGGCTAAAAGAACGA
ACTAAAACCCTAGGAAAGTT
CACTGGTTTCAAATAGTTGTAAGCATTTATCCGCGATGGCGGATGAAGAATCATTCTACATATGAAGAATCATCG
TCAGAXXXXXXXXXXGTCAAAACACTAGCCACCAATACTCAATTTGAACCGAGCTTAACTTGGCTAAAAGAACGA
ACTAAAACCCTAGGAAAGTT
>Marker495863
ACTACATCTAGACAGCTTCCCAACATCAGAGTCCCCACCATTTACAAAACTAGAAGTGCAGATCCAGCATATACA
AATAAXXXXXXXXXXCATTATCCAGAAGTTATTGAATTTCCATAAATAATCATTACACTGAAGATGCAAGAAATC
GTCGGGAAGGCCTACCTGTA
ACTACATCTAGACAGCTTCCCAACATCAGAGTCCCCACCATTTGCAAAACTAGAAGTCCAGATCCAGCATATACA
AATAAXXXXXXXXXXCATTATCCAGAAGTTATTGAATTTCCATAAATAATCATTACACTGAAGATGCAAGAAATC
GTCGGGAAGGCCTACCTGTA
>Marker495966
ACTATTTTTCTTCCTACCCACTTTATTTTACTAATCTTTCTTTCTACCCCTTTATTCATTATCTGATTATACACT
TACTTXXXXXXXXXXAACTTCTGACTTCGAAGTTTGGAGGTAAGTTTTTCACCCACCAATGGAATCACCTTGTTG
GAGTTGCCACCAATGTTGTT

Create PDF with GO2PDF for free, if you wish to

ACTATTTTTCTTCCTACCCACTTTATTTTACTAATCTTTCTTTCTTCCCCTTTATTCATTATCTGATTACACACT
TACTTXXXXXXXXXXXAACTTCTGACTTCGAAGTTTGGAGGTAAGTTTTTCACCCACCAATAGAATCACCTTGTTG
GAGTTGCCACCAATGTTGTT
>Marker496362
CACTCTTTAGGTGTGATCATGTTGAGATGCTGACGATACTAACTAGGCTACTGCAATCACATGTTCGATTTTTCT
TGCAGXXXXXXXXXXXTCCTATGCTTGTTAAAATGTCTGCTATTTTTCCGTATCTTTTGAAAATTATACTTGTTTC
GGTGCAAAGATAAGTAAGTG
CACTCTTTAGGTGTGATCATGTTGAGATGCTGACGATACTAACTAGGCTACTGCAATCACATGTTCGATTTTTCT
TGCAGXXXXXXXXXXXTCCTATGCTTGTTAAAATGTCTGCTATTTTTCCGTATCTTTTGAAAATTATACTTGTTTT
GGTGCAAAGATAAGTAAGTG
>Marker496412
ACTTTTATTGGATCCATCATGGGGCAAACTCATGTGGATTTGAGAAATACAGTGAGTATTTTATATAAACCATGT
TTCGAXXXXXXXXXXXTATTTCACCAATCTTAATTAAATTTTGCTATTTATCCAAAATAAACCGTTTTGCTATTTT
TCTGGTCAGCCCTTACTGGT
ACTTTTATTGGATCCATCATGGGGCAAACTCATGTGGATTTGAGAAATACAGTGAGTATTTTATATAAACCATGT
TTCGAXXXXXXXXXXXTATTTCACCAATCTTAATTAAATTTTGCTATTTATCCAAAATAAACCGTTTTGCTATTTT
TCTGGTCAGCCCTTACCGGT
>Marker496483
TACAAGTGGTAGTTGCTTGTGCTCTCTTGGTTTCTAACTGAACAATTTCACATATTGAGTTGTGAATTTTAACCT
CTTGTXXXXXXXXXXXTAGACATCTCACCAAGAATACCAGTGGAAAGCTGGATATGGCCCCACACACCTGCCTTGA
TCATGGTGGGAAATCTTTGT
TACAAGTGGTAGTTGCTTGTGCTCTCTTGGTTTCTAACTGAACAATTTCACATATTGAGTTGTGAATTTTAACCT
CTTGTXXXXXXXXXXXTAGACATCTCACCAAGAATACCAGTGGAAAGCTGGATATGATCCCACACACCTGCCTTGA
TCATGGTGGGAAATCTTTGT
>Marker496610
ACCTTCTTACCTTGCCACGCAGAAGCTAGGAGATGGAATAAATAACATCACTAACACATAGCATAGTGACTTGTC
TAAATXXXXXXXXXXXTATAGTGCCACACTTGAGTTCTAAGACCTTTTAGGCATTGTTAAGTGACTAAATCTTGAA
CAGCTGTGCATAGGTTGAGT
ACCTTCTTACCTTGCCACGCAGAAGCTAGGAGATGGAATAAATAACATCACTAACACATAGCATAGTGACTTGTC
TAAATXXXXXXXXXXXTATAGTGCCACACTTGAGTTCTAAGACCTTTTAGGCATTGTTAAGTGACTAAATCTTGAA
CAGCTGTGCATAGGATGAGT
>Marker496735
ACCAAAATATTATATAAATCAGAGCTGAACAATTTTTTACTCCCCATAACCAAAAGTAATGAACTCCTAGAGAAT
GTCACXXXXXXXXXXXGACTTACCTTATGAACCCATTCATATTCCCCGCCTTTTGAATCGAAAGTTCCATGCTGCA
AACTGATCAATTTCATTGTG
ACCAAAATGTTATATAAATCAGAGCTGAACAATTTTTTACTCCCCATAACCAAAAGTAATGAACTCCTAGAGAAT
GTCACXXXXXXXXXXXGACTTACCTTATGAACCCATTCATATTCCCCGCCTTTTGAATCGAAAGTTCCATGCTGCA
AACTGATCAATTTCATTGTG
>Marker496895
AACATATCGACAAATATTTGACTAACTAACACACACTCAGGTATCAATTGAGTAATATATGAATGAATATTACTA
CTTATXXXXXXXXXXXGTTGCGACAATTTGGTATGTTGCATATCATACTGTCATCATACTTTGCATACTCAACGCT
ACATATTGATTTAAGAGGTA
AACATATCGACAAATATTTGACTAACTAACACACACTCAGGTATCAATTGAGTAATATATGAATGAATATTACTA
CTTATXXXXXXXXXXXGTTGCGACAATTTGGTATGTTGCAGATCATACTATCATCATACTTTGCATACTCAACGCT
ACATATTGATTTAAGAGGTA
>Marker496898

Create PDF with GO2PDF for free, if you wish to

GACAACATGAGATCTCTTCAGACTATTAATAAGACTGCTCTTACCAACATTAGGCAAGCCAATGACGCCCACGGT
AATTGXXXXXXXXXXTAAAGAATACAATAATATGCTAGGAAACTAATCCTATGGAGGCAAAAAGAAAATACAACT
TCTTCATGCATATATATGTC
GACAACATGAGATCTCTTCAGACTATTAATAAGACTGCTCTTACCAACATTAGGCAAGCCAATGACGCCCACGGT
AATTGXXXXXXXXXXTAAATAATACAATAATATGCTAGGAAACTAATCCTACGGAGGCAAAAAGAAAATACAACC
TCTTCATGCATATATATGTC
>Marker497563
AACTGTGACCGCAAACGCCCCTTGGTAGTCGGTTTCTCTCTCACTCTGCTAATCCTTCTCCTCTCAATCCATTTC
TCCCTXXXXXXXXXXTTTTATGCTGAGCAGTTGAATAAAGCAGTTCTTAATGGATTTGAGGGATATAAGGTATGT
GGGTTTCTTTGATTCTTGTT
AACTGTGACCGCAAACGCCCCTTGGTAGTCGGTTTCTCTCTCACTCTGCTAATCCTTCCCCTCTCAATCCATTTC
TCCCTXXXXXXXXXXTTTTATGCTGAGCGGTTGAATAAAGCAGTTCTTAATGGATTTGAGGGATATAAGGTATGT
GGGTTTCTTTGATTCTTGTT
>Marker498763
CACATCGTTATAGATTGCTAATTGCTATTAGTGATGGTATTTCTTATTTTTCATCTTGAACCTTGTATAAGAGGA
AGCCAXXXXXXXXXXTTTTTATACCAAGCTTCTGATTGACATGTTCTATTAGTATTTCTCCTTCCTGATTGTGAC
AGCTTTGGAAATGTAGATGT
CACATCATTATAGATTGCTAATTGCTATTAGTGATGGTATTTCTTATTTTTCATCTTGAACCTTGTATAAGAGGA
AGCCAXXXXXXXXXXTTTTTATACCAAGCTTCTGATTGACATGTTCTATTAGTATTTCTCCTTCCTGATTGTGAC
AGCTTTGGAAATGTAGATGT
>Marker499631
CACTACTATAAACTTATCCCCTTCCTTCTACATCTCTGGAATTTGGTTCATATTCGAGAATCCAATGTGTTCATG
TTATTXXXXXXXXXXGACGATAAGACATGGCCATGCGCTAAAAAATGCTAACTATGCCTATGTGTTGCTACTTCT
AATGATATTCTACGTTGAGT
CACTACTATAAACTTATCCCCTTCCTTCTACATCTCTGGAATTTGGTTCATATTCGAGAATCCAATGTGTTCATG
TTATTXXXXXXXXXXGACGATAAGACATGGCTATGCGCTAAAAAATGCTAACTATGCCTATGTGTTGCTACTTCT
AATGATATTCTACGTTGAGT
>Marker499767
ACTATTTTCGTCCAACTATGTTTAACTGTGAAATTTTGGTGGAATCTGATGCATGTATTAGTGTTTTGATGTCGT
CACACXXXXXXXXXXTCTAAACCAATTCAAGTTAATGCTTTCAACCAAATGAAAACTTTATTGATTGTTTAGAAA
GATTTAATCAAAATTCAGGT
ACTATTTTCGTCCAGCTATGTTTAACTGTGAAATTTTGGTGGAATATGATGCATGTATTAGTGTTTTGATGTCGT
CACACXXXXXXXXXXTCTAAACCAATTCAAGTTAATGCTTTCAACCAAATGAAAACTTTATTGATTGTTTAGAAA
GATTTAATCAAAATTCAGGT
>Marker500044
AACTCTGTTAAAGGTTTGGATTATGATAAGAACTTGAACTCAGAGGTAGAAATCTTCAAATCTCTGGCTATCCAG
AAAGGXXXXXXXXXXTGTTCAGGTGTAGAAGAAGTTATTGTCACTTTGGTTAATACCTTAGTTAGACGTGGTAGA
GTCAGAGAAGCACTTCGGGT
AACTCTGTTAAAGGTTTGGATTATGATAAGAACTTGAACTCAGAGGTAGAAATCTTCAAATCTCTGGCTATCCAG
AAAGGXXXXXXXXXXTGTTCAGGTGTAGAAGAAGTTATTGTCACTTTGGTTAATACCTTAGTTAGACGTGGTAGA
GTTAGAGAAGCACTTCGGGT
>Marker500175
CACCTAATATTTGTTGTTAGGTAATGCCCCAAATTTACTCCTCCAATTTCATTCTCAACTTTCTTTAAGCTTAGT
TTAAGXXXXXXXXXXACATGTTCAAATCACTTTCATATAATCGAGGAGTTATAAATATCTTAGTTGCATATTATT
TTAGTTTGAATTAAACAGTA

Create PDF with GO2PDF for free, if you wish to

CACCTAATATTTGTTGTTAGGTAATGCCCCAAATTTACTCCTCCAATTTCATTCTCAACTTTCTTTAAGCTTAGT
TTAAGXXXXXXXXXXACATGTTCAAATCACTTCCATATAATCGACGAGTTATAAATATCTTAGTTGCATATTATT
TTAGTTTGAATTAAACAGTA
>Marker500976
ACAATTTGTCTCTACTTTTGATGGTTCCATCGGTTAGCTTTCACATGGATCGTTTCTTGCGTTCTTTCTTTTTGA
GATGGXXXXXXXXXXAATGAATGAAGCCCATATGGAACGTTATATCTTGAAATTAATTTGTCAAGTTGATGCTAA
AATACACAAAGGTTTTGGTG
ACAATTTGTCTCTACTTTTGATGGTTCCATCGGTTAGCTTTCACATGGATAGTTTCTTGCGTTCTTTCTTTTTGA
GATGGXXXXXXXXXXAATGAATGAAGCCCATATGGAACGTTATATCTTGAAATTAATTTGTCAAGTTGATGCTAA
AATACACAAAGGTTTTGGTG
>Marker500988
ACAACACTATAGTTTAATAAGGTCACCATTTGGGTCACATCAAAGTAATGTATGGAAGCTGTTGGAGGGGGAGAG
AGAGGXXXXXXXXXXCTTTCTCTCTTTTTCAATCAATCCCTACGGCGGAGATTTTTTTATAAAGATTTTAATATT
TCCTTTTATGAATTGTTGTG
ACAACACTATAGTTTAAAAAGGTCACCATTTGGGTCACATCAAAGTAATGTATGGAAGCTGTTGGAGGGGGAGAG
AGAGGXXXXXXXXXXCTTTCTCTCTTTTTCAATCAATCCCTACGGCGGAGATTTTTTTATAAAGATTTTAATATT
TCCTTTTATGAATTGTTGTG
>Marker501246
CACTTGTCATATTAAAAAAAAAACCTATAATAAACATGGCTAAATAGGGGTAACAAACAGCCTATACAACATAAG
ATAACXXXXXXXXXXCTCCCCCTCTCTCTCTTAATGCACCAACCAACGAACCTATCAACAAGCATAAACCCAA
TTTTGTTTCCTAGACTAGTC
CACTTGTCATATTAAAAAAAAAACCTATAATAAACATGGCTAAATAGGGGTAACAAACAGCCTATACAACATAAG
ATAACXXXXXXXXXXTCCTCCCCCTCTCTCTCTTAATGCACCAACCAACGAACCTATCAACAAGCATAAACCCAA
TTTTGTTTCCTAGACTAGTC
>Marker501347
TACTGTCGTTTTACACCATCCTGGGTTCAGTTGGGTAATTTGGGGCGGGATGTGACAACAATAATCCGATAACAT
GTATGXXXXXXXXXXGGCCTCAGACACCCCTTTCAATCTGAATGCTGATGATTTGGACCAGAGAATCAAACAAAG
AATCACAGGGGAAAACAAGT
TACTGTCGTTTTACACCATCCTGGGTTCAGTTGGGTAATTTGGGGCGGGGTGTGACAACAATAATCCGATAACAT
GTATGXXXXXXXXXXGGCCTCAGACACCCCTTTCAATCTGAATGCTGATGATTTGGACCAGAGAATCAAACAAAG
AATCACAGGGGAAAACAAGT
>Marker501399
ACTCCAAATAGTAGGCACCAAAACAGGATTGTGTTGCACCGCTATGACTTTTATTATAATTTTCTTTATTAGTAA
ATAGGXXXXXXXXXXTTATGTGATATATCTACGGCCTCCGTCGATCGATAGCCGATTTTTTGACAATGATGAAGT
CTGGAGCTTTCAAAGGAGTG
ACTCCAAATAGTAGGCACCAAAACAGGATTGTGTTGCACCGCTGTGACTTTTATTATAATTTTCTTTATTAGTAA
ATAGGXXXXXXXXXXTTATGTGATATATCTACGGCCTCCGTCGATCGATAGCCGATTTTTTGACAATGATGAAGT
CTGGAGCTTTCAAAGGAGTG
>Marker501742
AACCACAATTTTCAACCTTGTTCAGAGAAAAGGGGATAGGAATATGATAGATAGCCTTAATTATATTTGACAACA
GCATTXXXXXXXXXXCACCACCTTGGGCCCCACCTAAACACGTAAAAATTTACCCTTTAACTCTGTTTGCACACC
TTCCTCCCTTCCAACGTGTA
AACCACAATTTTCAACCTTGTTCAGAGAAAAGGGGATAGGAATATGATAGATAGCCTCAATTATATTTGACAACA
GCATTXXXXXXXXXXCACCACCTTGGGCCCCACCTAAACACGTAAAAATTTACCCTTTAACTCTGTTTGCACACC
TTCCTCCCTTCCAACGTGTA
>Marker501782

Create PDF with GO2PDF for free, if you wish to

AACATGTATTTCTTCCTGTTCTAGTGAGATTGTTTTGAGAAATATCAAAGACTTGAAGGCAGAGGTTTTTTGGGC
AAAAGXXXXXXXXXXAGTTGTATTAAGTATTGAAAGCGACGTTATACTTGTCGAATCATTCCAAGAGCTCACAAT
TTCAAGTGGGTCATTGAGTA
AACATGTATTTCTTCCTGTTCTAGTGAGATTGTTTTGAGAAATATCAAAGACTTGAAGGCAGAGGTTTTTTGGGC
AAAAGXXXXXXXXXXAGTTGTATCAAGTATTGAAAGCGACGTTATACTTGTCGAATCATTCCAAGAGCTCACAAT
TTCAAGTGGGTCATTGAGTA
>Marker501793
AACCACTTAAAAGGGTCGAGAGCTCTCTTCAACATTCTAGTGAGTCATATTCAAAGTGAAAATACATGGTGTATT
TTTCTXXXXXXXXXXTCGCGGATTTCTTTCAATCTATATCGATGTAGTAGAACTTGTTGTTGCAAAAGACTAAGA
GTAGTCCCGAGTTTGGGGTG
AACCACTTAAAAGGGTCGAGAGCTCTCTTCAACATTCTAGTGAGTCATATTCAAAGTGAAAATACATGGTGTATT
TTTCTXXXXXXXXXXTCGAGGATTTCTTTCAATCTATATCGATGTAGTAGAACTTGTTGTTGCAAAAGACTAAGA
GTAGTCCCGAGTTTGGGGTG
>Marker501939
AACATTGCCTATTATCCTTGCAGGCGATCTCTGTCATGGCCAAGTCTGGGTTTGCAGAGCTGATTGACTTCAACC
CTATTXXXXXXXXXXATTTTCATCTATTGTTAATATAGGACACTAGCTGTCTTTTTATTTTGAGTTGTATAATTT
GTTTGATTCTTTACAATGTG
AACATTGCCTATTATCCTTGCAGGCGATCTCTGTCATGGCCAAGTCTGGGTTTGCAGAGCTGATTGACTTCAACC
CTATTXXXXXXXXXXATTTTCATCTACTGTTAATATTGGACACTAGCTGTCTTTTTATTTTGAGTTGTATAATTT
GTTTGATTCTTTACAATGTG
>Marker502910
AACACTTGAATGCGGATACCCATTCGAGTCTCTGACTAATTTCTCTTCCACATTAATGCAGTTGGAGGCTTTGAT
TCATCXXXXXXXXXXACTACTGGTCCATCAGCTGTGATATATATGCCTAACTTTAGTTTATTTCAATTGTGTTGC
CAACCAGAATTGAAGGGAGT
AACACTTGAATGCGGATACCCATTCGAGTCTCTGACTAATTTCTCTTCCACATTAATGCAGTTGGAGGCTTTGAT
TCATCXXXXXXXXXXACTACTGGTCCATCAGCTGTGATATATATGCCTAACTCTAGTTTATTTCAATTGTGTTGC
CAACCAGAATTGAAGGGAGT
>Marker503065
ACTCTTGGGGAAATACCACTTGGCTTTGAAAGGTCTTTAGAGGTTTTCTCACACTCGGTTGCTGTAGGGACTTAA
AAAAGXXXXXXXXXXCTCTTTTAACATGATATACACAAGTATGGTTCTATATGGTTTGTAGCATTGTTTTCTCTA
ATATGATATTGTGATATGTT
ACTCTTGGGGAAATACCACTTGGCTTTGAAAGGTCTTTAGAGGTTTTCTCACACTCGGTTGCTGTAGGGACTTGA
AAAAAXXXXXXXXXXCTCTTTTAACATGATATACACAAGTATGGTTCTATATGATTTGTAGCATTGTTTTCTCTA
ATATGATATTGTGATATGTT
>Marker503126
ACCCTCTTCTGACCAATTAGCTTACTACCTCACAAAAACCCTCACGCATTCTCAATTTCAATATCTTCGTTCCAA
ACGCGXXXXXXXXXXTGTATCGATTTTTTATCAATAAGAAAAGTGAGAAAGATTTTCTCCCAAGATTTCAGTATT
CTTCAGACCCTACTTCTGTG
ACCCTCTTCTGACCAATTAGCTTACTACCTCACAAAAACCCTCACGCATTCTCAATTTCAATATCTTCGTTCCAA
AGGCGXXXXXXXXXXTGTATCGATTTTTTATCAATAAGAAAAGTGAGAAAGATTTTCTCCCAAGATTTCAGTATT
CTTCAAACCCTACTTCTGTG
>Marker503189
ACTCTTTTGAGTTTTTTAGATGAGGTTGATACAAGATTACGTGAAAGCAGAAGTTATAATATAAAGACAAATATA
AGTAAXXXXXXXXXXAACGGGTCCGGAATTTCTTGAAACCCTAAACCCTCGCAATATAAACTCTGCCGCCTACTT
CCTCCTTCTCTTTGGACGGT

Create PDF with GO2PDF for free, if you wish to

ACTCTTTTGAGTTTTTTAGATGAGGTTGATACAAGATTACGTGAAAGCAGAAGTTATAATATAAAGACAAATATA
AGTAAXXXXXXXXXXXAACGGGTCCGGAATTTCTTGGAACCCTAAACCCTCGCAATATAAACTCTGCCGCCTACTT
CCTCCTTCTCTTTGGACGGT
>Marker503234
GACCCAAAAATTTCAACGAGAAAAATTGATTGTGACTTTATATAAATATTGAATTCTTTCCACTTTTTTTTTCTT
AAAAAXXXXXXXXXXXAAAATCAGAAATTAAGCTTTTAAACTTATCCACTGAACTATAAATTGATTAACTTTAAAA
AAGAATTATCAAATGAAGTG
GACCCAAAAATTTCAACGAGAAAAATTGATTGTGACTTTATATAAATATTGAATTCTTTCCACTTTTTTTTCTTA
AAAATXXXXXXXXXXXAAAATTAGAAATTAAGCTTTTAAACTTATTCACTGAACTATAAATTGATTAACTTTAAAA
AAGAATTATCAAATGAAGTG
>Marker503967
GACAAAAAACAGTATAAAATATAGTTTAACCTAAAAAAAAGAAGATAAGTTGTTTAAAAGTTTGTTTAGAAGAAT
GCCTTXXXXXXXXXXXTCTAAAAAAAAAAAAGATTAATAGTTTTTAAAAACTGTTCGTCATATTTCTAAGTTCTAA
TGTTTCTGTTGGAATAAGTT
GACAAAAAACAGTATAAAATATAGTTTAACCTAAAAAAAAGAAGATAAGTTGTTTAAAAGTTTGTTTAGAAGAAT
GCCTTXXXXXXXXXXXTTCTAAAAAAAAAAAGATTAATAGTTTTTAAAAACTGTTCGTCATATTTCTAAGTTCTAA
TGTTTCTGTTGGAATAAGTT
>Marker504217
ACTAAAGTTTAGTCATACCTCTTTGCTATCTTAACATTCAGCTACACAAATCCAACTTGTGTGCTAATCAAGTCC
TAAAGXXXXXXXXXXXTTAACCATATTAGGAGTCTTAGAGGTAATCTCCCTCCCTTTGATAAGATGATATCAACAT
CAAATACTGAAACGGACTGT
ACTAAAGTTTAGTCATACCTCTTTGCTATCTTAACATTCAGCTACACAAATACAACTTGTGTGCTAATCAAGTCC
TAAAGXXXXXXXXXXXTTAACCATATTAGGAGTCTTAGAGGTAATCTCCCTCCCTTTGATAAGATGATATCAACAT
CAAATACTGAAACTGACTGT
>Marker504251
ACCAATTTTATTGGCAAAGCCTCAAACTTACATGAATTTCTGTGGAGAATCGGTTTGTCCTCTTGTCTCTCTTTT
CTTCTXXXXXXXXXXXATATTCATAGGGATAGATTGTTAGTTTCTTTCCATTGAAGCAATGACATAGCATTAATAT
CGCATTTTGATAAGTATGTG
ACCAATTTTATTGGCAAAGCCTCAAACTTACATGAATTTCTGTGGAGAATCGGTTTGTCCTCTTGTCTCTCTTTT
CTTCTXXXXXXXXXXXATATTCATAGGGATAGATTGTTAGTTTTTTTCCATTGAAACAATGACATAGCATTAATGT
CGCATTTTGATAAGTATGTG
>Marker504376
CACTGATTGCCTAAGGCATCATCTCGGGTAATAATATATGGTCAATGTATTAAGTCGTTAGAAATTTTAAAGCTC
AAGCTXXXXXXXXXXXGATTCATCCCAACTTAGTATCACTGAATTTTATATTCAGATCAAAGAAAATGCAAGGATA
TGGAATGAATATTGACAAGT
CACTGATTGCCTAAGGCATCATCTCGGGTAATAATATATGGTCAATGTATTAAGTCGTTAGAAATTTTAAAGCTC
AAGCTXXXXXXXXXXXGATTCATCCCAACTTAGTATCACTGAATTTTATATTCAGATAAAAGAAAATGCAAGGATA
TGGAATGAATATTGACAAGT
>Marker505000
ACAGGTGATTCTCGTTTAATAGTTATTTTCCAACAGAATGTGTTTTAGGTTTCAAATGACATTTATCAATGGGGG
ACTGTXXXXXXXXXXXCTATAGGTAGAACATCAACGCCTGTAGCTAATTGTTGATATGTTATGCCATTCAGAATGA
AGATGGAACAAAAATTGAGT
ACAGGTGATTCTCGTTTAATAGTTATTTTCCAACAGAATGTGTTTTAGGTTTCAAATGACATTTATCAATGGGGG
ACTGTXXXXXXXXXXXCTATAGGTAGAACATCAACGCCTGTAGCTAATTATTGATATGTTATGCCATTCAGAATGA
AGATGGAACAAAAATTGAGT
>Marker505432

Create PDF with GO2PDF for free, if you wish to

CACTTTATGTCAATACCTAACCATAGAAATCAAATTAATTCCATAAACCAACATCGTAAATTAATTTTTAACAAT
CCTTAXXXXXXXXXXXAAAACACTTTCAAATTCAAATACTTATGCCTCTTTAAGTAACCATCTGGCTTTGGCTTTT
TGGTTTTTGAAAATGTAGTC
CACTTTATGTCAATACCTAACCATAGAAATCAAATTAATTCCATAAACCAACATCGTAAATTAATTTTTAACAAT
CCTTAXXXXXXXXXXXAAAACACTTTCAAATTCAAATACTTATGCCCCTTTAAGTAACCATCTGGCTTTGGCTTTT
TGGTTTTTGAAAATGTAGTC
>Marker505510
ACTCTAATCAAATGCATCCCTGACAGAATTCTAATTTCTGTTCAGCATCTTCAAAACTACATAGTTCATGTCACA
CAAAGXXXXXXXXXXXCTTTGTAGCATTTGTTTAATTCTGAACTCTCATAAGACACAATAACGCCTCCTTTATGCT
CTTATAACTTGGAATTTGTT
ACTCTAATCAAATGCATCCCTGACAGAATTCTAATTTCTGTTCAGCATCTTCAAAACTACATAGTTCATGTCACA
CAAAGXXXXXXXXXXXCTTTGTAGCATTTGTTTAATTCTGAACTCTCATAAGACATAATAACGCCTCCTTTATGCT
CTTATAACTTGGAATTTGTT
>Marker506791
CACACGACTCACTTGACACAACCTAGCTAGCCATAATAAAGACTTATGACAACTAGTTAAGAATGAGCCAAACAA
GCGTCXXXXXXXXXXXGCTTGTAGAAATACAAGTTATTGTAGCCTCTTTTATAGAATAATGAGGCCAAAATATTAA
AGTAGTGTATCAAAACATGT
CACACGATTCACTTGACACAACCTAGCTAGCCATAATAAAGACTTATGACAACTAGTTAAGAATGAGCCAAACAA
GCGTCXXXXXXXXXXXGCTTGTAGAAATACAAGTTATTGTAGCCTCTTTTATAGAATAATGAGGCCAAAATATAAA
AGTAGTGTATCAAAACATGT
>Marker507292
ACTAAAAATTATGAAATCGTTAAGGTAATAGGGGAAAAATAAGAACGAACCTTTCCTGTTTGACTTCGCAATGTC
AATGAXXXXXXXXXXXGAGAGGGCAGGAGCATTCAAAGAGAAAGAAATCACATCCCCGGAAAACTCAAGCCCTGAT
AAACAGGGAGCGTCAATGTC
ACTAAAAATTATGAAATCGTTAAGGTAATAGGGGAAAAATAAGAACGAACCTTTCCTGTTTGACTTTGCAATGTC
AATGAXXXXXXXXXXXGAGAGGGCAGGAGCATTCAAAGAGAAAGAAATCACATCCCCGGAAAACTCAAGCCCTGAT
AAACAGGGAGCGTCAATGTC
>Marker507325
AACACCCCTATCCCTTCTTCCATCTCTGTCTTTGCCGAGAACCCGTTACGTATGATCCTACAAACGCAGCATTTC
TCCGAXXXXXXXXXXXTTCTTTGGGAGCTTGCTGGCTTTGATCTTAACTGTCTCGCGATACTCTTCGAAACGAGCT
AGGGTTCGTTGCATATTGTG
AACACCCCTATCCCTTCTTCCATCTCTGTCTTTGCTGAGAACCCGTTACGTATGATCCTACAAACGCAGCATTTC
TCCGAXXXXXXXXXXXTTCTTTGGGAGCTTGCTGGCTTTGATCTTAACTGTCTCGCGATACTCTTCGAAACGAGCT
AGGGTTCGTTGCATATTGTG
>Marker507486
CACGTTGAAATAAAATTTTAAGTGGGTGCTCAACCTTGTTATCTTCCCAAAGTAGTTCTTGCACCATTTCTAACC
TCCATXXXXXXXXXXXCATTCTAGTGATTTTTTCTTATTTTATTTATTTATCTTATTGGAAATATTTCATTAATGA
TATGAAATATTTCCACCTGT
CACATTGAAATAAAATTTTAAGTGGGTGCTCAACCTTGTTATCTTCCCAAAGTAGTTCTTGCACCATTTCTAACC
TCCATXXXXXXXXXXXCATTCTAGTGATTTTTTCTTATTTTATTTATTTATCTTATTGGAAATATTTCATTAATGA
TATGAAATATTTCCACCTGT
>Marker508178
ACAGTTTTGTTTAAGATCAATTTATCACTCAGCCCCATCTGCTCTTCCGCCATACTTTATTCGTTTCTCCTGAAT
CTTCAXXXXXXXXXXXATTCAGAAAGTATTATAGTCCAAACTGAAACAGATGACTAGAGAATTACAAACTTTTGAC
CTGGTTGATTGCAATGTGTT

Create PDF with GO2PDF for free, if you wish to

ACAGTTTTGTTTAAGATCAATTTATCACTCAGCCCCATCCGCTCTTCCGCCATACTTTATTCGTTTCTCCTGAAT
CTTAAXXXXXXXXXXXATTCAGAAAGTATTATAGTCCAAACTGAAACAGATGACTAGAGAATTACAAACTTTTGAC
CTGGTTGATTGCAATGTGTT
>Marker508338
CACCATATAGACATCTCTTAATACTTCATCATCTTGTTTCATTAATATATTTTGATCAATATTTTATTTCTTCGT
CGTGAXXXXXXXXXXXGTGACTTTTTCACCTGCAGGATCCATCCTAAGATCTAAGGATAAGTAAGTAGAGCTATAA
GAAACTCTTTTTGATTAAGT
AACCATATAGACATCTCTTAATACTTCATCATCTTGTTTCATTAATATATTTTGATCAATATTTTATTTCTTCGT
CGTGAXXXXXXXXXXXGTGACTTTTTCACCTGCAGGATCCATCCTAAGATCTAAGGATAAGTAAGTAGATCTATAA
GAAACTCTTTTTGATTAAGT
>Marker508390
AACTTTAATATTATTTATGATTGAAATTGTCCATATTAGAAAACTGTTGATATATGAACATGAAATTTGAAAAAA
AAGTTXXXXXXXXXXXAAAGGGTTGAGAAGTAGGAGTTTGGGAATAAAGTGTATGAATCGCATATTTCACTCTCTA
TTTGGTCTAAAGATTTTGTG
AACTTTAATATTATTTATGATTGAAATTGTCCATATTAGAAAACTGTTGATATATGAACATGAAATTTGAAAAAA
AAGCTXXXXXXXXXXXAAAGGGTTGAGAAGTAGGAGTTTGGGAATAAAGTGTATGAATCGCACATTTCACTCCCTA
TTTGGTCTAAAGATTTTGTG
>Marker509256
TACTTGTTTGTATGGAGGAGTTTCCAAAGGCAGCCAAATATCTTGTCTTAAATCTGGTGTTGTATGATGTTTCTG
CTCCCXXXXXXXXXXXCATACTCATACCTCTCTCATGTTTTTTACTTCCTTTTGGTTAATTCAAGTCTATGTTTCC
CCTTTTAGTGATAATAGGTT
TACTTGTTTGTATGGAGGAGTTTCCAAAGGCAGCCAAATATCTTGTCTTAAATCTGGTGTTGTATGATGTTTCTG
CTCCCXXXXXXXXXXXACTACTCATACCTCTCTCATGTTTTTTACTTCCTTTTGGTTAATTCAAGTCTATGTTTCC
CCTTTTAGTGATAATAGGTT
>Marker509450
ACCCTTTCTATTGACATAAACAACAAAATAACAGTTAGGGGTTTGCAGTTATCATAGCTTTGATGCAAATTCTAA
CATTAXXXXXXXXXXXTTAAAACTTGTGGAATGCAAAGATACCGTTACCCGGTCTTCTTCTGTTTAAGGACTAGAT
AACTCTAATAAAATATTAGT
ACCCTTTCTATTGACATAAACAACAAAATAACAGTTAGGGGTTTGCAGTTATCATAGCTTTGATGCAAATTCTAA
CATTAXXXXXXXXXXXTTAAAACTTGTGGAATGCAAAGATACCGTTACCCGGTCCTCTTCTGTTTAAGGACTAGAT
AACTCTAATTAAATATTAGT
>Marker509818
CACTCATCTCACTATAGATATATTTGTGTCCATCTAATATGACCAGTATCAGTAAGTTAATCCTTCACAGGTTGT
TCGTAXXXXXXXXXXXTGGAGGGGCCCCTTGTTCAATGCTTGGATTCAGTCCTTATGGAAATCACCTATCTACTAA
CCCTAAAGTGGGTAGGAGTG
CACTCATCTCACTATAGATATATTTGTGTGCATCTAATATGACCAGTATCAGTAAGTTAATCCTTCACAGGTTGT
TCGTAXXXXXXXXXXXTGGAGGGGCCCCTTGTTCAATGCTTGGATTCAGTCCTTATGGAAACCACCTATCTACTAA
CCCTAAAGTGGGTAGGAGTG
>Marker509971
ACTCGACGACGACGGCGATCTAGAATCGAGCTTCGAGCTAAAATTCATGACAAAGATTCGACGGCTAGCTGATTT
CACGGXXXXXXXXXXXTAGTTCATCGTCTTTAATTTCTTTTTTCTAATTTTATTTTCCGTTTTGTAGAGATTTAAG
CGCTTAGATAATGACACGTG
ACTCGACGACGACGGCGATCTAGAATCGAGCTTCGAGCTAGAATTCATGACAAAGATTCGACGGCTAGCTGATTT
CACGGXXXXXXXXXXXTAGTTCATCGTCTTTAATTTCTTTTTTCTAATTTTATTTTCCGTTTTGTAGAGATTTAAG
CGCTTAGATAATGACACGTG
>Marker510562

Create PDF with GO2PDF for free, if you wish to

ACTCCATCAAGCATATATAAATATATGTTGTAGTTTGAATGACTTTCCAATTTATGTAACATTTTCCGTCACTTT
CTCCAXXXXXXXXXXXCCAAAATTTACCCAATCCCCAGATAGCTCTGTATATATAATTGTTCTAATTATGGGTCAG
ATTCTGGTTCAGCATAGGGT
ACTCCATCAAGCATATATAAATATATGTTGTAGTTTGAATGACTTCCCAATTTATGTAACATTTTCCGTCATTTT
CTCCAXXXXXXXXXXXCCAAAATTTACCCAATCCCCAGATAGCTCTGTATATATAATTGTTCTAATTATGGGTCAG
ATTCTGGTTCAGCATAGGGT
>Marker510748
ACAAATCAATCCAATTATCAAAAAAAGTTATGATAAAAACAACAAACCATTTCTATATTTGTTTTCATCTAATTG
AAAAAXXXXXXXXXXXXATCAGAAGTTTAAATTTAAATTTTCGTCCCAATCCATGGAAATTATAAAAGTCTTATACA
TTTCAATTGCTTTTTGCGTT
ACAAATCAATCAAATTATCAAAAAAAGTTATGATAAAAACAACAAACCATTTCTATATTTGTTTTCATCTAATTG
AAAAAXXXXXXXXXXXXATCAGAAGTTTAAATTTAAATTTTCGTCCCAATCCATGGAAATTATAAAAGTCTTATACA
TTTCAATTGCTTTTTGCGTT
>Marker510802
CACTATATGGAGAGACAACAACAACCTTTCTAGGATTCTTGCATTTCTCAGAGATTCTACAGATTTCTCGAGACC
CAGCCXXXXXXXXXXXCTCCTCTGTTAAACTATCACCTTATGCTTTAGATAAACTCCTTTTAGACTTTTACTACGA
AGGTTTCAATATTATAAGTA
CACTATGTGGAGAGACAACAACAACCTTTCTAGGATTCTTGCATTTCTCAGAGATTCTACAGATTTCTCGAGACC
CAGCCXXXXXXXXXXXCTCCTCTGTTAAACTATCACCTTATGGTTTAGATAAACTTCTTTTAGACTTTTACTACGA
AGGTTTCAATATTATAAGTA
>Marker510939
ACCTAAATAAGATCAACTGTAAATTAAGGATCTCTAAAAATAGTAGAATGATTACTATGACAGCAATAAAAAAGG
TTACCXXXXXXXXXXXTAGAACTTCAGTGAGTCCCCTTTGAGGTCCAGTCACTTGGTTTCATCTAAACTTCCAAAA
TTTCAGAGTTCTAACCTGTA
ACCTAAATAAGATCAACTGTAAATTAAGGATCTCTAAAAATAGTAGAATGATTACTATGAAAGCAATAAAAAAGG
TTACCXXXXXXXXXXXTAGAACTTCAGTGAGTCCCCTTTGAGGTCCAGCCACTTGGTTTCATCTAAACTTCCAAAA
TTTCAGAGTTCTAACCTGTA
>Marker510951
AACACCTCAACTACGAAGGTTAGGACCCCAGAACAAAGGCTCCTTCTCTCCATAGTATAGAAAGATACCCAAATA
CAAACXXXXXXXXXXXATTCAATAATTTCAGCTCCTGTGAGATGCCATCTCAAAATAAGATCCCAAGAAGCATTCT
ATTTCCAACAATTGGCCGTG
AACACCTCAACTACGAAGGTTAGGACCCCAGAACAAAGGCCCCTTCTCTCCATAGTATAGAAAGATACCCAAATA
CAAACXXXXXXXXXXXATTCAATAATTTCAGCTCCTGTGAGATGCCATCTCAAAATAAGATCCCAAGAAGCATTCT
ATTTCCAACAATTGGCCGTG
>Marker511002
CACCACCGCCGACTAGTTTGTGTGCCTATGTTGAAGCAAATAACAAATCACATCACTAAACCATCAAGAACTGAA
TTCGAXXXXXXXXXXXCAAAGATATAACTATTACCCAAACAATCCTCAAAACTCTTTTTTGCTTTCTGAGTTCCCA
AAAAGAGACTTGAATAAAGT
CACCACCGCCGACTAGTTTGTGTGCCTATGTTGAAGCAAATAACAAATCACATCACTAAACCATCAAGAACTGAA
TTCGAXXXXXXXXXXXCAAAGATATAACTATTACCGAAACAATCCTCAAAACTCTTTTTTGCTTTCTGAGTTCCCA
AAAAGAGACTTGAATAAAGT
>Marker511188
AACTTCACTCCTGTTTTGGAGAAAATAGTTTCAGAAAATGGGGTTAGAGAAAAAGGTTTCATAAACGTTTGAAAG
CATTTXXXXXXXXXXXAATACATATACCTATCACCGATCAACAAGAAAGTCTGCAGTCATATTATTTCTCTCATTT
GCATTTTACAGGCATGCTGT

Create PDF with GO2PDF for free, if you wish to

AACTTCTCTCCTGTTTTGGAGAAAATAGTTTCAGAAAATGGGGTTAGAGAAAAAGGTTTCATAAACGTTTGAAAG
CATTTXXXXXXXXXXAATACATATACCTATCACCGATCAACAAGAAAGTCTGCAGTCATATTATTTCTCTCATTT
GCATTTTACTGGCATGCTGT
>Marker511940
ACATTGAGCTTGGGCCTCTCCATGATCTTTAGGAAAAGGAAATTGAAAATTTAAAGTTGCAGCCTTTTATGAGAG
ATCTTXXXXXXXXXXGTCTTTCAAACATAAGCTGAAGATAACTTTAGCTTCATTATTGTTATTGAGATTTACAAC
AAGAAATAAGGAACTCTTGT
ACATTGAGCTTGGGCCTCTCCATGATCTTTAGGGAAAGGAAATTGAAAATTTAAAGTTGCAGCCTTTTATGAGAG
ATCTTXXXXXXXXXXGTCTTTCAAACATAAGCTGAAGATAACTTTAGCTTCATTATTGTTATTGAGATTTACAAC
AAGAAATAAGGAACTCTTGT
>Marker512118
AACTTGGACTTCAATCCAAAAGCCTGACAGCTAAAATAAAATTGGCGTCTTTATACAAATGATAAGAGAAACATG
CTCAAXXXXXXXXXXAAAAGGCATAGGTTATATACCCCCCATGGAGATGCTCCTTGAACACCAGGTCAAATGCAC
GGCAAAGTTCTAGGATGGTA
AACTTGGACTTCAATCCAAAAGCCTGACAGCTAAAAAAAAATTGGCGTCTTTATACAAATGATAAGAGAAACATG
CTCAAXXXXXXXXXXAAAAGGCATAGGTTATATACCCCCCATGGAGATGCTCCTTGAACACCAGGTCAAATGCAC
GGCAAAGTTCTAGGATGGTA
>Marker512622
CACAACATTTTGTCTGCCTCCAACAGAACAAATGGTTCTCCTTTAACTTTGTTTTGGCAGGAGGGTCATGTCACA
GGTCCXXXXXXXXXXTGCAGACTCGACAAGAAATGATTTTGAGCTCCACTTCAGTGTTGCATGTATCTTTCCCTC
TTGTGCGAATCAATGTTGGT
CACAACATTTTGTCTGCCTCCAACAGAACAAATGGTTCTCCTTTAACTTTGTTTTGGCAGGAGGGTCATGTCACA
GGTCCXXXXXXXXXXTGCAGACTCGACAAGAAATGATTTTGAGCTCCACTTCAGTGTTGCATGTATCTTTCCCTC
TTGTGAGAATCAATGTTGGT
>Marker513707
AACCTACTGTTTGTTGTCATCGATAGTTTATTTAATATATATAAAAGAAGAGAGATGACCCACTGTCTAAAACTT
TTTGTXXXXXXXXXXTCAAGGTCCTTCCCCTCTCCACAAGCATATTGTTTGCTTTCCTGCGTCTGTTAAATTATG
TATAATGTTCAATTGTTGTA
AACCTACTGTTTGTTGTCATCGATAGTTTATTTAATATATATAAAAGAAGAGAGATGACCCACTGTCTAAAACTT
TTTGTXXXXXXXXXXTCAAGGCCCTTCCCCTCTCCACAAGCATATTGTTTGCTTTCCTGCGTCTGTTAAATTATG
TATAATGTTCAATTGTTGTA
>Marker514226
AACATGCCTATTTTGCTATAACATGTGATTTTAGGAGAGAGTTTAACCATAATCAGGGTGCGATTTGATCAGTTC
ATCTGXXXXXXXXXXTCAAGCCTCGAGCTAAACGCTTAGAATGACTAAAATGCTTGATCAATACCTCCAGCTATT
GCTGCTATGTCACATCTGTG
AACATGCCTATTTTGCTATAACATGTGATTTTAGGAGAGAGTTTAACCATAATCAGGGTGCGATTTGATCAGTTC
ATCTGXXXXXXXXXXTCAAGCCTCGAGCTAAACGCTTAGAATCACTAAAATGCTTGATCAATACCTCCAGCTATT
GCTGCTATGTCACATCTGTG
>Marker514917
ACAGAGGGCTACTACAAAAACAAAAAATCAGCCTAACCAACAGAGGGAGGACCTACAAAAAACATGTTAGCCATA
TGCCCXXXXXXXXXXTGCTAATTGTCAAAGTCTCATATAACCGATAGATGATTCAAAACCCACCACCAAACCATA
CAATAAAGAAGTTTCATGTA
ACAGAGGGCTACTACAAAAACAAAAAATCAGCGTAACCAACAGAGGGAGGACCTACAAAAAACATGTTAGCCATA
TGCCCXXXXXXXXXXTGCTAATTGTCAAAGTCTCATATAACCGATAGATGATTCAAAACCCACCACCAAACCATA
CAATAAAGTAGTTTCATGTA
>Marker515722

Create PDF with GO2PDF for free, if you wish to

GACAACAAAACGAAGAAATCAATTGGTAGATGTAGAGTTGTTTATAATTTATAAGCTTAATTTTCAAAACTCAAA
AAACCXXXXXXXXXXCAATAAACGGTCAAGAAAAATGATCCAAGAAATAAAAGTCACGAAAGGCATTCAATTGAT
CAGAGCATTGACAATGAAGT
GACAACAAAACGAAGAAATCAATTGGTAGATGTAGAGTTCTTTATAATTTATAAGCTTAATTTTCAATACTCAAA
AAACCXXXXXXXXXXCAATAAACGGTCAAGAAAAATGATCCAAGAAATAAAAGTCACGAAAGGCATTCAATTGAT
CAGAGCATTGACAATGAAGT
>Marker515764
TACACACACAATTTATCTAATAAATTGACAAAAACTACCATGTGCAATTGAGTAAGATTAAGCATGCTCTGATAC
CATATXXXXXXXXXXTAATAGAAAAACTCACATTTGATGCTTTTCTATTGTCATAATCTCGTCATGTTCTTGAAG
TTGAAACTACCACTAGCGTT
TACACACACAATTTATCTAATAAATTGACAAAAACTACCATGTGCAATTGAGTAAGATTAAGCATGCTCTGATAC
CATATXXXXXXXXXXTAATAGAAAAACTCACATTTGATGCTTTTCTATTGTCATAATCTCGTCATGTTCTTGAAG
TCGAAACTACCACTAGCGTT
>Marker515791
AACAAAAGTTTTAAACAAGAAAAGTGGTAGGTGCTCTACTATTTAACAGTTTTCTTCCTAGTCTTGAAATATTGA
AAGATXXXXXXXXXXTTGCATATTTTGTTCTGTTTGCAATACCAATTATGACGGCAGTGTTTAGTGGAACAATGT
CGGTGGGAGCTTATGTTGTT
AACAAAAGTTTTAAACAAGAAAAGTGGTAGGTGCTCTACTCTTTAACAGTTTTCTTCCTAGTCTTGAAATATTGA
AAGATXXXXXXXXXXTTGCATATTTTGTTCTGTTTGCAATACCAATTATGACGGCAGTGTTTAGTGGAACAATGT
CGGTGGGAGCTTATGTTGTT
>Marker515978
AACAGTAATTGATCATTCATGAACTAGGATGTGATCATTTTTTAATTAACAAAAGAATAAGAAATCAGCTTCTTT
AGGGAXXXXXXXXXXTTCATGCAAAAAAGGCCTTCTGCAGATAGTAGCTAGAAATTAGAAAAAAGGTCATTGGAA
AAGGAATTATAGAATTAAGT
AACAGTAATTGATCATTCATGAACTAGGATGTGATCGTTTTTTAATTAACAAAAGAATAAGAAATCAGCTTCTTT
AGGGAXXXXXXXXXXTTCATGCAAAAAAGGCCTTCTGCAGATAGTAGCTAGAAATTAGAAAAAAGGTCATTGGAA
AAGGAATTATAGAATTAAGT
>Marker516527
TACTCCTGGAAAGGGTGTTGGAGATTTAGACGGCGGTTCTGATGTTCCCGAGCGGCCAAGCCCTGTTTCAGTTCT
TGAGCXXXXXXXXXXACATTCCCCTGGCTAATATCAGTTTATTCTATCAGCAGGCTTACCAATACAACCAGTTCA
TATTGAATTTGATGATCGTG
TACTCATGGAAAGGGTGTTGGAGATTTAGACGGCGGTTCTGATGTTCCCGAGCGGCCAAGCCCTGTTTCAGTTCT
TGAGCXXXXXXXXXXACATTCCCCTGGCTAATATCAGTTTATTCTATCAGCAGGCTTACCAATACAACCAGTTCA
TATTGAATTTGATGATCGTG
>Marker516546
GACGTCGAAGAGAACCTCTTATCTTTGAACATCTGGTTAAATCGTGAATCAATCACAACCTTGGCTTTGTGCTTG
GGGGCXXXXXXXXXXAGTTGCTCAAGTTTTTGGAGCCCATGACGACGGCCTACAATCAGAGTGCAACTGGCGAAA
CGCAGTGATAATCGGTCGTA
GACGTCGAAGAGAACCTCTTATCTTTGAACATCTGGTTAAATCGTGAATCAATCACAACCTTGGCTTTGTGCTTG
GGGGCXXXXXXXXXXAGTTGCTCAAGTTTTTGGAGCCCATGATGACGGCCTACAATCAGACTGCAACTGGCGAAA
CGCAGTGATAATCGGTCGTA
>Marker516646
GACGTCTCAAAGCAAATGCCTAACACAGACGTGTGCTCAAACATACATAACAATTATTATATGATTGGAAAAACC
ATTAAXXXXXXXXXXGACTTTTTCTTGTGGCTCAACACTTAGTTAATCTTCCGGAATCAGGAATGACCTTGACGA
GGCCAGGTCCAGGAATAAGT

Create PDF with GO2PDF for free, if you wish to

GACATCTCAAAGCAAATGCCTAACACAGACGTGTGCTCAAACATACATAACAATTATTATATGATTGGAAAAACC
ATTAAXXXXXXXXXXGACTTTTTCTTGTGGCTCAACACTTAGTTAATCTTCCGGAATCAGGAATGACCTTGACGA
GGCCAGGTCCAGGAATAAGT
>Marker517095
CACTACATTCAGGTTATATAAACAACTACTTTTTTTTCTTCTCTTTTGTGTATATGTATTGAAACAAAAATTCAG
GTTTTXXXXXXXXXXTGGTTAAGAGCAACCGTAGAAATTGGCTTCCGTAGAAGTTTCTTGTATTCACTCACGAGC
TTTTTTCAAGATACATGGTT
CACTACATTCAGGTTATATAAACAACTACTTTTTTTTCTTCTCTTTTGTGTATATGTATTGAAACAAAAATTCAG
GTTTTXXXXXXXXXXTGGTTAAGAGCAACTGTAGAAATTGCCTTCCGTAGAAGTTTCTTGTATTCACTCACGAGC
TTTTTTCAAGATACATGGTT
>Marker517322
ACAAACTCTCTCATAACCCTGCAAACAAAACATCTTCAAATTCAGACTTGACTTGTTTAGCCATTTTTGAGCCTG
TGCTTXXXXXXXXXXTTTATGAATGAATATGAAGAGAGAATTGGCAGCATAACATTAATTTGATTGATATTATGA
AGGAATAATAATGGTTGGTA
ACAAACTCTCTCATAACCCTGCAAACAAAACATTTTCAAATTCAGACTTGACTTGTTTAGCCATTTTTGAGCCTG
TGCTTXXXXXXXXXXTTTATGAATGAATATGAAGAGAGAATTGGCAGCATAACATTAATTTGATTGATATTATGA
AGGAATAATAATGGTTGGTA
>Marker518023
TACTGCTTTGTCCAATGACATTTCGGACATCTTCAACTCTCTGTGGGCCACCTATTATTGGTTGCAAGAAGGTTA
TTTGAXXXXXXXXXXAAAATTTGGCACCTTTTTAAGAACTTTAGTTTATCGGTCTTAGTTGCCAATTGGCGCTCT
TTTTGTAATCACCTTTAGGT
TACTGCTTTGTCCAATGACATTTCGGAAATCTTCAACTCTCTGTGGGCCACCTATTATTGGTTGCAAGAAGGTTA
TTTGAXXXXXXXXXXAAAATTTGGCACCTTTTTAAGAACTTTAGTTTATTGGTCTTAGATGCCAATTGGCGCTCT
TTTTGTAATCACCTTTAGGT
>Marker518033
AACTTAAGGATGAAGAAATTGGATTGTTAAGACGAAAAGATACAACAACCATCACTCAACCAAGCACATTTCTTT
AGGATXXXXXXXXXXGAACTCGACACGAGGCCGAGTAGGAGAAGTCTTCTATCATGTTAAGTTTTTGTTGATGTG
ATAGTATTAAGACATATGTT
AACTTAAGGATGAAGAAATTGGATTGTTAAGACGAAAAGTTACAACAACCATCACTCAACCAAGCACATTTCTTT
AGGATXXXXXXXXXXGAACTCGACACGAGGCCGAGTAGGAGAAGTCTTCTATCATGTTAAGTTTTTGTTGATGTG
ATAGTATTAAGACATATGTT
>Marker518134
GACAAGATTGCACAGTTCATGCAGATCTACAGAAGTAGAGAAGTAGGACAATCGTATTTCACTTCCGTTTGGACA
ACTTTXXXXXXXXXXTCCTTTTAGAGAACCTATTGTGGTGTGTGTTTTCTTTGACCAGTTTTGTTTTGTTGCAGA
TTCTATGCAATGGCCCTGGT
GACAAGATTGCACAGTTCATGCAGATCTACAGAAGTAGAGAAGTAGGACAATCGTATTTCACTTCTGTTTGGACA
ACTTTXXXXXXXXXXTCCTTTTAGAGAACCTATTGTGGTGTGTGTTTTCTTTGACCAGTTTTGTTTTGTTGCAGA
TTCTATGCAATGGCCCTGGT
>Marker518171
CACATTTTCTTTTTTGTTCCACATTGGATGTTTTTAGTTTCGAGTAACTTTTGAGATCTACGTTTTGAAGGCAAA
CGAAGXXXXXXXXXXACACAACAATTCAATATTTACATAAAAAGTGTTTGAATCAAACCTGGAGCGCCATCACTC
CTATATATGCAGAAAAAAGT
CACATTTTCTTTTTTGTTCCACATTGGGTGTTTTTAGTTTCGAGTAACTTTTGAGATCTACGTTTTGAAGGCAAA
CGAAGXXXXXXXXXXACACAACAATTCAATATTTACATAAAAAGTGTCTGAATCAAACCTGGAGCGCCATCACTC
CTATATATGTAGAAAAAAGT
>Marker518212

Create PDF with GO2PDF for free, if you wish to

ACTAAATGCCCACCTTTCGATGATAATTAATGGAATGAAATAATGAAATTAAACTGCTGATCATTTCACTTTTTA
AATATXXXXXXXXXXCTGTATAGCTTTTGATAGAATAAGGATATTCTTGAAACAAGGGACAGTTCATAGTTTATC
TTTTTTAGCCAATTTGTGTA
ACTAAATGCCCACCTTTCGATGATAATTAATGGAATGAAATAATGAGATTAAACTGCTGATCATTTCACTTTTTA
AATATXXXXXXXXXXCTGTATAGCTTTTGATAGAATAAGGACATTCTTGAAACAAGGGACAGTTCATAGTTTATC
TTTTTTAGCCAATTTGTGTA
>Marker518645
AACTTACTCTATCTAAAGTTCTTTACAAGGCTAGTGTAGGAGATTGGATGTCAGCCACTGTAGTTCTCTTGTTGG
TGGTCXXXXXXXXXXAATTCTGTAGTTAGGCTGAGGTAATTCTGTGTGATGTTTTGAACAAGTAATCAATTTTCG
ACGTTTGATGAATTATTGGT
AACTTACTCTATCTAAAGTTCTTTACAAGGCTAGTGTAGGAGATTGGATGTCAGCCACTGTAGTTCTCTTGTTGG
TGGTCXXXXXXXXXXAATTCTGTAGTTAGGCTGAGGTAATTCTGTGTGATGTTTTGAACAACTAATCAATTTTCG
ACGTTTGATGAATTATTGGT
>Marker519569
AACCTAGTGTTATTTTTTTCTACTGTTTAACATATGCACACACATGTATATATGCACATATACTGATATACACAT
GTATGXXXXXXXXXXGAGAAGGTTACATATAATATTTTTTTGTAAAAGGAAAGTTACATATAATCTGCTGTAACT
TGCTAATGGAAAGAGCCGTT
AACCTAGTGTTATTTTTTTCTACTGTTTAACATATGCACACACATGTATATATGCACATATACTGATATACACAT
GTATTXXXXXXXXXXGAGAAGGTTACATATAATATTTTTTTGTAAAAGGAAAGTTACATATAATCTGCTGTAACT
TGCTAATGGAAAGAGCCGTT
>Marker519662
GACCCCCAACTAAAAACTTCAATTTTCTGCCTTATTAAGGCCTTACACTATGGACCCAAGAGTTCATAACTCTCA
AGATTXXXXXXXXXXGAAGAAATGGAATTCAATTTGAGATGCTTTTTCAATTTTAGCATGATATGCAATTAGGAC
CCATGCAAACAAGATCTGTT
GACCCCCAACTAAAAACTTCAATTTTCTGCCTTATTAAGGCCTTACACTATGGACCCAAGAGTTCATAACTCTCA
AGATTXXXXXXXXXXGAAGAAATGGAATTCAATTTGAGATGCTTTTTCAATTTTAGCATGATATGCAATTAGGGC
CCATGCAAACAAGATCTGTT
>Marker519948
CACCAATCTTTGTGAATGGAATGTCGATTATTCCGACCTTTGCTGTCGCTGGTCCATTGAAGGGGAAGAAAATCC
ATGTTXXXXXXXXXXAAACCATCCGGGTTGATCGGAACTGGATTTGACTCGTTGGATTTATCATACAACAGAGCC
CCGTTGGAGAAAAACCAGTC
CACCAATCTTTGTGAATGGAATGTCGATTATTCCGACCTTTGCTGTCGCTGGTCCATTGAAGGGGAAGAAAATCC
ATGTTXXXXXXXXXXAAACCGTCCGGGTTGATCGGAACTGGATTTGACTCGTTGGATTTATCATACAACAGAGCC
CCGTTGGAGAAAAACCAGTC
>Marker520039
ACAGGCCTTTTGCTGTGCATAGATCGTATCGAGGGAAATTTAAGAAATATGGTGATAAGTGATACGGTTGTGTTA
AAGCAXXXXXXXXXXATGTTCAAAATCCATATTATTTGATTATTTTGCCAGAGAGAATCCTAAGGTAGTTTTGAA
TGTGATTGTATCAAAAGGTT
ACAGGCCTTTTGCTGTGCATAGATTGTATCGAGGGAAATTTAAGAAATATGGTGATAAGTGATACGGTTGTGTTA
AAGCAXXXXXXXXXXATGTTCAAAATCCATATTATTTGGTTATTTTGCCAGAGAGAATCCTAAGGTAGTTTTGAA
TGTGATTGTATCAAAAGGTT
>Marker520519
ACAGTTTAAGTATGCTTAGTTTAGAACAATTCTTTATCGGATGTTCTTCTGAAAGATTTTATAGAAAGCATACTA
TGATTXXXXXXXXXXGCATTACACGTCTTTTTTCTTTTTAAACTATAGAAAAAAGTCTATTAGGTTCCTTGACAC
AAATTTTCTCCGGAATAGGT

Create PDF with GO2PDF for free, if you wish to

ACAGTTTAAGTATGCTTAGTTTAGAACAATTCTTTATTGGATGTTCTTCTGAAAGTTTTTATAGAAAGCATACTA
TGATTXXXXXXXXXXXGCATTACACGTCTTTTTTCTTTTTAAACTATAGAAAAAAGTCTATTAGGTTCCTTGACAC
AAATTTTCTCCGGAATAGGT
>Marker521131
TACATGGCAAGAAGAATTAGAATGCCTTGTTCGTGGGGGGTTGCCAAAAGATCTGAGGGGAGAGGTAAAGTTGAA
ATTTTXXXXXXXXXXXATTGAGAAATATTACCAAGATTTGTTGGATCAAGAAACTAATTGTAGTGCAGATAATGAG
AACAATATTCCATCTGGTGT
TACATGGCAAGAAGAATTAGAATGCCTTGTTCGTGGGGGGTTGCCAAAAGATCTGAGGGGAGAGGTAAAGTTGAA
AATTTXXXXXXXXXXXATTGAGAAATATTACCAAGATTTGTTGGATCAAGAAACTAATTGTAGTGCAGATAATGAG
AACAATATTCCATCTGGTGT
>Marker522251
CACAGCAATAGAAGAGATTCAATGAACTTTGATTATTTGAATTTGAGCTCTAAAGTTAGTATTGGCAACATTTGT
GATTCXXXXXXXXXXXGAATCAAAATTTCAATCAAGCATGTGTGATCATACTTTCTTATTTTAAGATGTCACAACC
CTCCATATTGCATTAATGTC
CACAGCAATAGAAGAGATTCAATGAACTTTGGTTATTTGAATTTGAGCTCTAAAGTTAGTATTGGCAACATTTGT
GATTCXXXXXXXXXXXGAATCAAAATTTCAATCAAGCATGTGTGATCATACTTTCTTATTTTAAGATGTCACAACC
CTCCATATTGCATTAATGTC
>Marker523145
AACATATAAGATCAATCAATAACAATATTTATAACAAAACGATAATTATAATCACGGAAAATTGAGCATAAATGG
AGTCTXXXXXXXXXXXAAAATGAAAATGCTCTAAAAGAAGAAGAGTAAGCATTTAGTAATTGTTTAGAATAATGGA
GAAATAATGGAATTAACGTC
AACATATAAGATCAATCAATAACAATATTTATAACAAAACCATAATTATAATCACAGAAAATTGAGCATAAATGG
AGTCTXXXXXXXXXXXAAAATGAAAATGCCCTAAAAGAAGAAGAGTAAGCATTTAGTAATTGTTTAGAATAATGGA
GAAATAATGGAATTAACGTC
>Marker523560
ACTGCATATGGTCAGCTAGGCAACCCAAGTTCAGATGGAGTTATTCCTTGCTTAGTGCAGGATAGATTAGTCGGT
GAATTXXXXXXXXXXXCCCAACACTCATAGAAGCTTTGAAGGATAGGCATGTTAAAAGTATATCTTGTGGGTCAAA
TTTTACTGCAAGTATATGTA
ACTGCATATGGTCAGCTAGGCAACCCAAGTTCAGATGGAGTTATTCCTTGCTTAGTGCAAGATAGATTAGTCGGT
GAATTXXXXXXXXXXXCCCAACACTCATAGAAGCTTTGAAGGATAGGCATGTTAAAAGTATATCTTGTGGGTCAAA
TTTTACTGCAAGTATATGTA
>Marker523763
GACCATTGGGACCCAAGACATTATGCAATGCATGTGGAGTAAGATACAAAAAGTCAGGAAGATTATTACCAGAGT
ATAGAXXXXXXXXXXXCTACCTTTCCCTCCTCTTCGTAAAACCGACTACCGACGCACCAAACCCGCAGTTGCAAGA
CAATTTGGTTCTCCAATGTA
GACCATTGGGACCCAAGACATTATGCAATGCATGTGGAGTAAGATACAAAAAGTCAGGAAGATTATTACCAGAGT
ATAGAXXXXXXXXXXXCTACCTTTCCCTCCTCTTCGTAAAACCGACTACCGACGCACCAAACCCACAGTTGCAAGA
CAATTTGGTTCTCCAATGTA
>Marker524093
ACTCCTATAATATCAGAAAATGTTAAAATACATTTTCTCATATAATGAACCTGTCTTAGAGTTTGTGAAGTCAAT
TCTATXXXXXXXXXXXGCACTCCATATCATACATTACTTGTTGAAGGTTTTGTAGATGGGCCTGTTGATATTTGTA
AGTGTTACTGATAAATAGTT
ACTCCTATAATATCAGAAAATGTTAAAATACATTTTCTCGTATAATGAACCTGTCTTAGAGTTTGTGAAGTCAAT
TCTATXXXXXXXXXXXGCACTCCATATCATACATTACTTGTTGAAGGTTTTGTAGATGGGCCTGTTGATATTTGTA
AGTGTTACTGATAAATAGTT
>Marker524214

Create PDF with GO2PDF for free, if you wish to

ACCTATACTCTATTATCCTACTACAATATTTGATGAAACAACTTGAATTATAATTGCAATTACAAAGGGGTTGTT
CGAAAXXXXXXXXXXXAGCCTAAAGCAGTGATTGCATGGAACTTGGGACGAATGCTCTTCTTAATCCTTAAAATAA
ACACCTCATAAAATCAAGTT
ACCTATACTCTATTATCCTACTACAATATTTGATGAAACAACTTGAATTATAATTGCAATTACAAAGGGGTTGTT
CGAAAXXXXXXXXXXXAGCCTAAAGCAGTGATTGCATGGAACTTGGGACGAATGCTCTTCTTAATCCTTAAAATAA
ACACCTGATAAAATCAAGTT
>Marker524307
GACCAACTTTTTTTGTAATGTAATCATATTATTTGGGATTTTGAAATATTAAAACGTGGTGGGCATGCTACCTTG
AATTTXXXXXXXXXXTTTCTTAAAATAAACAAAAATTTTTTTTGATGGGTTTGCATATATAATATATAGAAACTC
ATTTGATCAGTTAGTGAAGT
GACCAACTTTTTTTGTAATGTAATCATATTATTTGGGATTTTGAAATATTAAAACGTGGTGGGCATGCTACCTTG
AATTTXXXXXXXXXXTTTCTTAAAATAAACAAAAAATTTTTTTGATGGGTTTGCATATATAATATATAGAAACTC
ATTTGATCAGTTAGTGAAGT
>Marker524425
CACCTAAATGTAGCCTCGTTGGCACTCCTATAGTAATCTAATATTAACAACTAGACTTTAACAAGATTTCTGCCA
TATTTXXXXXXXXXXGAAAATGTTTATCTAAAACATAACTCAACGAAGTAACGTTTGTAAAGTAATTTGAAAGTA
ATTTTGAAATAGCTAAAAGT
TACCTAAATGTAGCCTCGTTGGCACTCCTATAGTAATCTAATATTAACAACTAGACTTTAACAAGATTTCTGCCA
TATTTXXXXXXXXXXGAAAATGTTTATCTAAAACATAACTCAACGAAGTAACGTTTGTAAAGTAATTTGAAAGTA
ATTTTGAAATAGCTAAAAGT
>Marker524719
AACCCTCCAAAGCAGACCTTGATATAAGTATAACTACATAGATATTAATGTGTCTTTCTACTTCCAACAATTAGC
TTCAAXXXXXXXXXXXCGAATGAAGATTATTAAAGCTGATAATTATCTATACGACCTAACTTAGAATAATAATCTA
AACCCTTTTAAAAAGTAGGT
AACCTTCCAAAGCAGACCTTGATATAAGTATAACTACATAGATATTAATGTGTCTTTCTACTTCCAACAATTAGC
TTCAAXXXXXXXXXXXCGAATGAAGATTATTAAAGCTGATAATTATCTATACGACCTAACTTAGAATAATAATCTA
AACCCTTTTAAAAAGTAGGT
>Marker524868
TACAGTTTTGGTGTTGTGTTCTTGGAGATTATAACTGGAAGAAGGGTTATAGACAATGCAAGACCAACAGCAGAA
CAAAAXXXXXXXXXXXXTAGATGTTGTTATCTTGTGTTCCAAACTATCTAAGCTTGAACACTGAATAATTTGTTGAG
ACCAAAACTTATTAATTGTC
TACAGTTTTGGTGTTGTGTTCTTGGAGATTATAACTGGAAGAAGGGTTATAGACAATGCAAGACCAACAGCAGAA
CAGAAXXXXXXXXXXXTAGATGTTGTTATCTTGTGTTCCAAACTATCTAAGCTTGAACACTGAATAATTTGTTGAG
ACCAAAACTTATTAATTGTC
>Marker524981
ACATTAATAGACTTACTGTTAAAAAAGAATATAAATGATCCAATCTTAGAGAAAGTGTCATTGAAGAAGAGGAAG
AGAAAXXXXXXXXXXXXTTTTTTTTACCAAGTCAATCTGTTTTTTCATGAGAGTGAAGGGCACCTCAAAAGTGTATA
ATATATAGGTAATAAATGGT
ACATTAATAGACTTACTGTTAAAAAAGAATATAAATGATCCAATCTTAGAGAAAGTGTCATTGAAGAAGAGGAAG
AGAAGXXXXXXXXXXTTTTTTTTACCAAGTCAATCTGTTTTTTCATGAGAGTGAAGGGCACCTCAAAAGTGTATA
ATATATAGGTAATAAATGGT
>Marker525127
AACAAAAATCCTGAAAGCACAACCAAAATAAAACTCTTAATAAGATGCGATAAGCACTGTTCCAGAACCTAGAAA
CAAAAXXXXXXXXXXXXTTTCCTTTACTTCCCATTTCCCTGTGAGTTGATTGGGAAAAGGAATATATTATAGATAAT
GAAATTCAGGAAACGAAGGT

Create PDF with GO2PDF for free, if you wish to

AACGAAAATCCTGAAAGCACAACCAAAATAGAACTCTTAATAAGATGCGATAAGCACTGTTCCAGAACCTAGAAA
CAAAAXXXXXXXXXXXTTTCCTTTACTTCCCATTTCCCTGTGAGTTGATTGGGAAAAGGAATATATTTTAGATAAT
GAAATTCAGGAAACGAAGGT
>Marker525237
CACGAACTTGTTCAGTTCTCCTTATTATCTTCCCTCAGTATGGATTTGGTTTGTGCGTTGGCTATTGTGTGTTGT
GTGACXXXXXXXXXXXGGCTAGAAAAGTAGTTTTTATTTTATTTTATTGTGTTATTTATTAATCTTTGCCATTTTT
TCAATTAAGAATTTTTTGTG
CACGAACTTGTTCAGTTCTCCTTATTATCTTCCCTCAGTATGGATTTGGTTTATGCGTTGGCTATTGTGTGTTGT
GTGACXXXXXXXXXXXGGCTAGAAAAGTAGTTTTTATTTTATTTTATTGTGTTATTTATTAATCTTTGCCATTTTT
TCAATTAAGAATTTTTTGTG
>Marker525428
ACTTTAGCAGTCCTTGTTGCCACGTATAATTCTTCACTTCTTCACCTTTCTGCAGTCAGTTGATGATTTCCATCA
AAAATXXXXXXXXXXXCATCGGCTGCCTTTTTTTCTAGACGGGTTTATACACTACCTCAAATGAATAGCCGAGAAA
CTTGGCAATCCATCTTTGGT
ACTTTAGCAGTCCTTGTTGCCACGTATAATTCTTCACTTCTTCACCTTTCTACAGTCTGTTGATGATTTCCATCA
AAAATXXXXXXXXXXXCATCGGCTGCCTTTTTTTCTAGACGGGTTTATACACTACCTCAAATGAATAGCCGAGAAA
CTTGGCAATCCATCTTTGGT
>Marker526334
AACACAAACCGTCACGCTCAACCCAGCAGATCGTGTCTCCAATGGAAATGTTTCACTAGGAACTAACCATGCAAG
GGGGCXXXXXXXXXXXAGTATAATGGCGATCATAAGTTGGGAGAGGAACATTTGAACTCCGGCTTCTAACAAAAGC
ATTCGTCGCCCGATCCTGTC
AACACAAACCGTCACGCTCAACCCAGCAGATCGTGTCTCCAATGGAAATGTTTCACTAGGAACTAACCATGCAAG
GGGACXXXXXXXXXXXAGTATAATGGCGATCATAAGTTGGGAGAGGAACATTTGAACTCCGGCTTCTAACAAAAGC
ATTCGTCGCCCGATCCTGTC
>Marker526476
ACCCCGAAACACATACACCTTAGACATATCAGAGAATCCCAATTCATGAACCTGAACCTGAGTCCCAAACGAAAC
AAATCXXXXXXXXXXXGGGAGGAATTCGCCTGTTGGGCTGAATCCAGAGCAGGATTAGGATGAGTGGATTGAGGAG
GAGCATATTGAACAGTGGTA
ACCCCGAAACACATACACCTTAGACATATCAGAGAATCCCAATTCATGAACCTGAACCTGAGTCCCAAACGAAAC
AAATCXXXXXXXXXXXGGGAGGGATTCGCCTGTTGGGCTGAATCCAGAGCAGGATTAGGATGAGTGGATTGAGGAG
GAGCATATTGAACAGTGGTA
>Marker527100
ACTTTTACGAAACTATTTTAAAAAATGCTACCATTCAAGATTTATTATTGTAATAACAAAGTTTTTACACATCAT
TAATTXXXXXXXXXXXTTCAATTGATACACGTCTTTATATGCTCCAACCTTAAAAGAATGCATGGCAAGAAAACGT
GGAGGCAGAATATAAATGTA
ACTTTTACGAAACTATTTAAAAAAATGCTACCATTAAAGATTTATTATTGTAATAACAAAGTTTTTACACATCAT
TAATTXXXXXXXXXXXTTCAATTGATACACGTCTTTATATGCTCCAACCTTAAAAGAATGCATGGCAAGAAAACGT
GGAGGCAGAATATAAATGTA
>Marker527481
AACCAGTTGTTTCCTTACCAAAAATGTGCAAGTATAGAAGAAAGCAAAATAAAGGTGGTGATTTTATTTATATAT
ATCTTXXXXXXXXXXXATATTTGGTATTGAAATATAGAAATATACGAAGGTTGGTCTCAAAATTAAAATTAAAGTT
TCTGCTCAAACATGCATGTC
AACCAGTTGTTTCCTTACCAAAAATGTGCAAGTATAGAACAAAGCAAAATAAAGGTGGTGATTTTATTTATATAT
ATCTTXXXXXXXXXXXATATTTGGTATTGAAATATGGAAATATACGAAGGTTGGTCTCAAAATTAAAATTAAAGTT
TCTGCTCAAACATGCATGTC
>Marker527832

Create PDF with GO2PDF for free, if you wish to

GACAATATAATATATCAGAAATAACATTAAATAAACATTCAATTTAAGTTATGGACAATATAAATTCAGTCGTCG
AAGACXXXXXXXXXXXGCGCTTCAAATGATGTATAATAAATGATTGAAATACATTTTTCAGATGAAATACGCCATG
GGAGAAGGAGAAAAAAAGTA
GACAATATAATATATCAGAAATAACATTAAATAAACATTCAATTTAAGTTATGGACAATATAAATTCAGTCGTCG
AAGACXXXXXXXXXXXGCGCTTCAAATGATGTATAATAAATTATTGAAATACATTTTTCAGATGAAATACGCCATG
GGAGAAGGAGAAAAAAAGTA
>Marker527959
ACAGATTGTGGTTCTTCGCATGCTGAACATACTGTTGCCAAGCCAACTTAGTTATCAATTATGAAATAAAGAGGT
TTGTTXXXXXXXXXXXAGTTTAAAGACTAACACTGGCAAATGTTTTAATGTTTCACATTTTCAAGTCAAGTGTCAG
TTGAGGTTGTTTCAAAGGTG
ACAAATTGTGGTTCTTCGCATGCTGAACATACTGTTGCCAAGCCAACTTAGTTATCAATTATGAAATAAAGAGGT
TTGTTXXXXXXXXXXXAGTTTAAAGACTTCCACTGGCAAATGTTTTAATGTTTCACATTTTCAAGTCAAGTGTCAG
TTGAGGTTGTTTCAAAGGTG
>Marker528224
CACAAAATATCAAAATTTTCCCATAGGAACTATGAACACACAACCATAGATGTGATGCTAAAAAAGCATTCAATT
ATGAAXXXXXXXXXXXCACCAAAAAAAGAAAAAAAATGGTATGCGTAAATTCATGAGATTTAAGTTCGGGAGTCCG
TTGTGTGTAGGGAAGGTGTT
CACAAAATATCAAAATTTTCCCATAGGAACTATGAACACACAATCATAGATGTGATGCTAAAAAAGCATTCAATT
ATGAAXXXXXXXXXXXCACCAAAAAAAGAAAAAAAATGGTATGCTTAAATTCATGAGATTTAAGTTCGGGAGTCCG
TTGTGTGTAGGGAAGGTGTT
>Marker529413
GACCTTTTCTAACATAGTTTATAACTTAACTCACGCATTTTTAAATTTGTTATAATTTAGGTCTCTTTTGATAAT
TTTTTXXXXXXXXXXXGTTAATTATAAGTTTTGAGAACTTCTTTCATTAGTTTTAAAATTTGGAAGGTTCGAATTG
TAAAATACTTCTAAAATGTA
GACCTTTTCTAACATAGTTTATAACTTAACTCACGCATTTTTAAATTTGTTATAATTTAGGTCTCTTTTGATAAT
TTTTTXXXXXXXXXXXGTTAATTATAAGTTTTGAGAACTTCTTTCATTAGTTTTAAAATTTTGAAGGTTCGAATTG
TAAAATACTTCTAAAATGTA
>Marker529842
GACTTTTTTACAGGGTTGTTATCAATTATGATTTCCCATCCGGAGTGGAGGATTATGTCCATAGGATTGGTCGAA
CTGGAXXXXXXXXXXXTAGACGTTGGGGTTCTGGCTCTGATGGTCGTGATGGTGGTCGAGGTGGGCGCAATGATTC
AAACTCTGGTGGGAGGGGTG
TACTTTTTTACAGGGTTGTTATCAATTATGATTTCCCATCCGGAGTGGAGGATTATGTCCATAGGATTGGTCGAA
CTGGAXXXXXXXXXXXTAGACGTTGGGGTTCTGGCTCTGATGGTCGTGATGGTGGTCGAGGTGGGCGCAATGATTC
AAACTCTGGTGGGAGGGGTG
>Marker529918
ACTAAAACTAAAATATCTTCTTGTCCAAAACGCCAAGTTACATACTAAGTCAAAATTTTATCTTATAACTTTTAA
TTTAGXXXXXXXXXXXCTAATCTATAATTTTTTTAAGCATGCTTAACCTTAGTTTAAATTATAAGAAAATTTGTTT
ATGTAATTGTTTGCCAATGT
ACTAAAACTAAAATATCTTCTTATCCAAAACGCCAAGTTACATACTAAGTCAAAATTTTATCTTATAACTTTTAA
TTTAGXXXXXXXXXXXCTAATCTATAATTTTTTTAAGCATGCTTAACCCTAGTTTAAATTATAAGAAAATTTGTTT
ATGTAATTGTTTGCCAATGT
>Marker530311
CACACACATATAATTTATTATTGAAAATTGTGAAATATTAGAGATGAAAAGTATATACAGATGAATTAATAATAA
TAAGAXXXXXXXXXXXCAATAGACTTTTAATCCTATATCTAATTAAGTTCCATATTAGATCATTTTCAAATTTTTT
GAAGTTTGTGAATCTTATGT

Create PDF with GO2PDF for free, if you wish to

CACACACATATAATTTATTATTGAAAATTGTGAAATATTAGAGATGAAAAGTATATACAGATGAAATAATAATAA
GAAGAXXXXXXXXXXXCAATAGACTTTTAATCCTATATCTAATTAAGTTCCATATTAGATCATTTTCAAATTTTTT
GAAGTTTGTGAATCTTATGT
>Marker530667
AACAATTATGGACCCTTTCACCATTAGCTTCATCCCAAACCTCTCTTCTATAATTATGTTCCCTAAAATTTACT
TTTTGXXXXXXXXXXXTTATGTATTTTTTAGTCATCTTATATCGTGTTTATTTTCATTTATCAATGTAACTTGAAA
AGTGAATTACATTTGTTGTT
AACAATTATGGACCCTTTCACCATTAGCTTCATCCCAAACCTCTCTTCTATAATTATGTTCCCTAAAATTTACT
TTTTGXXXXXXXXXXXTTATGTATTTTTTAGTCATCTTATATCGTGTTTATTTTCATTTATCAATGTAATTTGAAA
AGTGAATTACATTTGTTGTT
>Marker530745
AACATTGTCTTCAATAATTGGTGGTGTTATGGTAATATGTGTTTGGAGGAAGAAATATGAATGGTAGTGTTATGA
TAATAXXXXXXXXXXXAGTAGAAAAATTTGTAGGGTTACGAGAAAATGAAGTGGAGAAATTTGTATGGTTATTTAA
CCATAATCCCCAAAAATGTA
AACATTGTCTTCAATAATTGATGGTGTTATGGTAATATGTGTTTGGAGGAAGAAATATGAATGGTAGTGTTATGA
TAATAXXXXXXXXXXXAGTAGAAAAATTTGTAGGGTTACGAGAAAATGAAGTGGAGAAATTTGTATGGTTATTTAA
CCATAATCCCCAAAAATGTA
>Marker531029
CACATTTGTGTTCAACCACTGAATGAATGATACATTGTGAAATTTTGAATCTGTTGCCAAACTTGTCGATGGCAT
GTATTXXXXXXXXXXXAGGGAGGTCTCATTTAACTACATGTTGATATTTTAGGCTCTTGAAAAGCTGAAACGTCAA
TTGGCTGAAGCCGAGGCTGT
CACATTTGTGTTCAACCAATGAATGAATGATACATTGTGAAATTTTGAATCTGTTGCCAAACTTGTTGATGGCAT
GTATTXXXXXXXXXXXAGGGAGGTCTCATTTAACTACATGTTGATATTTTAGGCTCTTGAAAAGCTGAAACGTCAA
TTGGCTGAAGCCGAGGCTGT
>Marker531800
TACCGCAGCCCACTTGAGTGATAATATTATTATAATCACTTTCATGTGCTTATTATTGGCTTTTAGGGAGCATTT
AGTGTXXXXXXXXXXXAATAAATTGAACCCATTTTCAAAACAATTATTGTCTTTTTTTTCAATCCTATGTTTTTGA
AGAAATGAATTAAATAGGTT
TACCGCAGCCCACTTGAGTGATAATATTATTATAATCACTTTCATGTGCTTATTATTGGCTTTTAGGGAGCATTT
AGTGTXXXXXXXXXXXAATAAATTGAACCCATTTTCAAAACAATTATTGTCTTTTTTTTCAATCATATGTTTTTGA
AGAAATGAATTAAATAGGTT
>Marker532061
CACCTTAACTAATCTTACAGAACAACCTGTCTGACACTATAACATTTAGGTGTCAAGGAAACCTGTAGGAAATTA
ATTTCXXXXXXXXXXXCCCAGTGACTTGAAACAAATAAGTTGGAATGTCAAAGACATGAAAGCACAGAAAGTAGTC
CTCAAAAGACCGGCCAGGTA
CACCTTAACTAATCTTACAGAACAACCTGTATGACACTATAACATTTAGGTGTCAAGGAAACTTGTAGGAAATTA
ATTTCXXXXXXXXXXXCCCAATGACTTGAAACAAATAAGTTGGAATGTCAAAGACATGAAAGCACAGAAAGTAGTC
CTCAAAAGACCGGCCAGGTA
>Marker532147
CACCTCTAAGCACATTCGAATGTTGGAGTGACAACCTGACAAAGTCAAAAAATCACAACATAGCATCCATCCAAC
TTTGGXXXXXXXXXXXACTGACAACCTTCTTTGCGCTTCCACATCTTCTACTTGGAATTTTCCATCTTCAAACCAT
ATACAGTAGTGTGCTTGGTG
CACCTCTAAGCACATTCGAATGTTGGAGTGACAACCTGACAAAGTCAAAAAATCACAACATAGCATCCATCCAAC
TTTGGXXXXXXXXXXXACTGAAAACCTTCTTTACGCTTCCACATCTTCTACTTGGAATTTTCCATCTTCAAACCAT
ATACAGTAGTGTGCTTGGTG
>Marker532201

Create PDF with GO2PDF for free, if you wish to

ACCTTAGACAGAGATGGAGAAAATCGACGAGGATCCGCCGGAGACAATGCAGGAACAGAGAGAGAGAAGAACCTG
GAACAXXXXXXXXXXXAGAGAAATACGATTGTAACTACCTTTTTCCTTTTTCTTGTTTATTATTTATTAGAGAAGT
TGGGCTGGGCTGAACACGTA
ACCTTAGACAGAGATGGAGAAAATCGACGAGGATCCGCCGGAGACAATGCAGGAACAGAGAGAGAGAAGAACCTG
GAACAXXXXXXXXXXXAGAGAAATACGATTGTAACTACCTCTTTCCTTTTTCTTGTTTATTATTTATTAGAGAAGT
TGGGCTGGGCTGAACACGTA
>Marker533186
AACACTTAGGCTTCATGTCAATATCTGAAGTTGTAATTGTTAGAATTATGATATTTTCACATTTTAATTTTCTAT
GCTCGXXXXXXXXXXXTGTTCCTTAGCCTTGTAAGGCCGATACCAAATAATGCAATATTCCCCTTTATAACTTGTG
TGTTCATTTACATATTGGTT
AACACTTAGGCTTCATGTCAATATCTGAAGTTGTAATTGTTAGAATTATGATATTTTCACATTTTAATTTTCTAT
GCTCGXXXXXXXXXXXCGTTCCTTAGCCTTGTAAGGCCGATACCAAATAATGCAATATTCCCCTTTATAACTTGTG
TGTTCATTTACATATTGGTT
>Marker533223
CACAAAAAACCAACTTACCTGTGCTAAAATCATTATCTCCTACACCATACCGGCTGCAACTCTGTAAAAAGAAGG
GAAAGXXXXXXXXXXXATTCTTCACTTCTATATATCGAAAAAATATTTAGCCTAGAAAGTTGAAAATTTTATCATT
TCTAAACCCAATTGCAAGTT
CACCAAAAACCAACTTACCTGTGCTAAAATCATTATCTCCTACACCATACCGGCTGCAACTCTGTAAAAAGAAGG
GAAAGXXXXXXXXXXXATTCTTCACTTCTATATATCGAAAAAATATTTAGCCTAGAAAGTTGAAAATTTTATCATT
TCTAAACCCAATTGCAAGTT
>Marker533275
AACATATCCGTTATTATAACCTCTTATATAGAACAATGAGGCCAAAATGCATGAAGAATGTCAAAATGCATGGCT
TGTATXXXXXXXXXXXCTAGTGAATCACAGAGGTGCTCACAAGAAGACCAGGTGAAAAGACCAAGTATTTACGTGA
GAAGAACTCATCTGACAAGT
AACATATCCGTTATTATAACCTCTTATATAGAACAATGAGGCCAAAATGCATGAAGAATGTCAAAATGCATGGCT
TGTATXXXXXXXXXXXCGAGTGAATCACAGAGGTGCTCGCAAGAAGACCAGGTGAAAAGACCAAGTATTTACGTGA
GAAGAACTCATCTGACAAGT
>Marker533698
ACATATTATTGTGGGTAGATTTTGGACCAACGATAAGATAGTTAAAACATAACAAATGAAAGGGAGAAGATCAAG
ATCGAXXXXXXXXXXXAGTTTTATATTGTTTAAGATGAATGTGGAATTGTATTCAAATCATTAGTATTTTCCTATC
AAAGTCCTAACTGTTATGTG
ACATATTATTGTGGGTAGATTTTGGACCAACGATAAGATAGTTAAAACATAACAAATGAAAGGGAGAAGATCAAG
ATCGAXXXXXXXXXXXAGTTTTATATTGTTTAAGATGAATGTGAAATTGTATTCAAATCATTAGTATTTTCCTATC
AAAGTCCTAACTGTTATGTG
>Marker534270
CACTATGCCAACCCATGGGTGTTGTAGTCGATATGATCTTCCTTCCAAAACCTTTTACAAAAATGAACATATATG
TTTCAXXXXXXXXXXXAGCAAAACCTCAAAACTAATACAGGATAACAACTTACATCCAAAGCATTAGTTCCTTTGT
CCATCAAGTCAAGCTTCGTC
CACTATGCCAACCCATGGGTGTTGTAGTCGATATGATCTTCCTTCCAAAACCTTTTACAAAAATGAACATATATG
TTTCAXXXXXXXXXXXAGCAAGACCTCAAAACTAATACAGGATAACAACTTACATCCAAAGCATTAGTTCCTTTGT
CCATCAAGTCAAGCTTCGTC
>Marker534880
AACACCACTATGGCTTCTAATCCCACCACCTTCCTTAAACAAATATTAAACAATCAAACAAAGTTTGACTATAAT
ATTGGXXXXXXXXXXXCCAAGAAGAAGAAAAAAGATTGTTTTATGAAAACAATCAAGATCTACATATTGAGGAAAT
TCAAGGAATTAATTAACGTA

Create PDF with GO2PDF for free, if you wish to

AACACCACTATGGCTTCTTATCCCACCACCTTCCTTAAACAAATATTAAACAATCAAACAAAGTTTGACTATAAT
ATTGGXXXXXXXXXXXCCAAGAAGAAGAAAAAAGATTGTTTTATGAAAACAATCAAGATCTACATATTGAGGAAAT
TCAAGGAATTAATTAACGTA
>Marker535635
CACTACAATTACTAAGGATATTTAGGACATAGCCCATAAACTATATTTTAAACGTTAGGACGCCTCTCACCTATA
AACACXXXXXXXXXXXCTCTAGGATTTAAGAGATTGACATGATTTATGACTTTATTGTTGGTTTTATTCCTAGGTT
TGATGTAATTTGAGGGCGTA
CACTACAATTACTAAGGATATTTAGGACATAGCCCATAAACTATATTTTAAACATTAGGACGCCTCTCACCTATA
AACACXXXXXXXXXXXCTCTAGGATTTAAGAGATTGACATGATTTATGACTTTATTGTTGGTTTTATTCCTAGGTT
TGATGTAATTTGAGGGCGTA
>Marker536247
ACTAAAAAGTGAAATCACTACTTCAACTATATATCATTGAATATCTCAAGAAAATGCACGATATCAATAACACAA
TTCCGXXXXXXXXXXXATTACCTCATAATGGCTCTTTTCTTTTAATTAGCCACAAGGGGTATAACCAATATGCATT
TAGAAGCATATATATAAGTG
ACCAAAAAGTGAAATCACTACTTCAACTATATATCATTGAATATATCAAGAAAATGCACAATATCAATAACACAA
TCTCCXXXXXXXXXXXATTACCTCATAATGGCTCTTTTCTTTTAATTAGCCACAAGGGGTATAACCAATATGCATT
TAGAAGCATATATATAAGTG
>Marker536779
CACAATTAATATAATCCAAGACAGTGTTATGAAATAGATGGTGTATATCAAACACCCTATAATGTGAATAAAAGC
TAATGXXXXXXXXXXXTTTTATTTCCTAACACCTAACTCTTCTAATCAATACAGAGTTTATGTAATAATAAGGTTG
GTCATCATAAATAAAACGTT
CACAATTAATATAATCCAAGACAGTGTTATGAAATAGATGGTGTATATCAAACACCCTATAATGTGAATAAAAGC
TATTGXXXXXXXXXXXTTTTATTTCCTAACACCTAACTCTTCTAATCAATACACAGTTTATGTAATAATAAGGTTG
ATCATCATAAATAAAACGTT
>Marker536810
ACTCACTTTAGTGAGTTTCTCAAGAGCTCTCTAAGAATTACTAAATCAAATAACTATTTTAGACGTATGTGATAT
ATTATXXXXXXXXXXXTTTTCATTCACTTACTTTGGTGTGTTAATTTTAGATAATGACAACATGGAAAAGAATAGG
GAAAAATTACAAGATATTGT
ACTCACTTTAGTGAGTTTCTCAAGAGCTCTCTAAAAATTACTAAATCAAATAACTATTTTAGACGTATGTGATAT
ATTATXXXXXXXXXXXTTTTCATTCACTTACTTTGGTGTGTTAATTTTAGATAATGACAACATGGAAAAGAATAGG
GAAAAATTACAAGATATTGT
>Marker536941
GACTAATACTAATATTAGTTGGTGAAAAGGTTTTATGACATGATAGTTTGCAGGCAAAATTTTCTAGCAACGATG
TGAGCXXXXXXXXXXXTAAAAAAAATTCAATGTGTGAAAAGAAATGAATGCATGAGAATAGATGGCATTAAGAAGT
AAAACATAAACACAAAAGTC
GACTAATACTAATATTAGTTGGTGAAAAGGTTTTATGACATGATAGTTTGCAGGCAAAATTTTCTAGCAACGATG
TGAGCXXXXXXXXXXXTAAAAAAAATTAAATGTGTGAAAAGAAAGGAATGCATGAGAATAGATGGCATTAAGAAGT
AAAACATAAACACAAAAGTC
>Marker537256
AACCTTTCCGATATTTCCTTCTCCGGCGCCCCCAGAAGAGGCCACCGGATTTCCCCCGACGTCTCCCTCATTGCC
CTCCCXXXXXXXXXXXATATACACACAATTTTCTTTTGGGTTACGGGAACACTTGGCGCGTATGGCCCACCATTGT
CCATGAGGATGTTGGGTGTT
AACCTTTCCGATATTTCCTTCTCCGGCGCCCCCAGAAGAGGCCACCGGATTTCCCCCGACGTCTCCCTCATTGCC
CTCCCXXXXXXXXXXXATATACACACAATTTTCTTTTGGGTTACGGGAACACTTGGCGCGTATGGCCCACCATTGT
CCATAAGGATGTTGGGTGTT
>Marker537365

Create PDF with GO2PDF for free, if you wish to

TACATATATGCAAAAGCTTGAAAGAAATGATTCGATTGTAGAGGGATGGTTGGATTATTGGTGAAAGATATGGAT
GGAAAXXXXXXXXXXXTGCTCAATTTTGGTGTTTCTTTTCATGCCACGTGGAAATTGAGTATAGTGGATATAATTC
ATGGACGGCTGTTAAATGTT
TACATATATGCAAAAGCTTGAAAGAAATGATTCGGTTGTAGAGGGATGGTTGGATTATTGGTGAAAGATATGGAT
GGAAAXXXXXXXXXXXTGCTCAATTTTGGTGTTTCTTTTCATGCCACGTGGAAATTGAGTATAGTGGATATAATTT
ATGGACGGCTGTGAAATGTT
>Marker538060
ACCCAGTTGAATTATAGGGACAAAACCTGGAAAAAATATTAAGAAAAAATGAATTGATTTTTTTTCAAAAAGAAA
AAGAAXXXXXXXXXXXTTAAATTCAAATAGTCGGAAATGCAACCAGTAAAAGAATTACACGTGGAACAAACAATAA
TGTCGTTGAAGAAAAAGGTA
ACCAAGTTGAATTATAGGGACAAAACCTGGAAAAAATATTAAGAAAAAATGAATTGATTTTTTTTCAAAAAGAAA
AAGAAXXXXXXXXXXXTTAAATTCAAATAGTCGGAAATGCAACCAGTAAAAGAATTACACGTGGAACAAACAATAA
TGTCGTTGAAGAAAAAGGTA
>Marker538133
ACCTACAAAAGTTCATTCATAAAAAGTATCGAATCATATTTTAAATTTTTAGAAATTATATCAATTTAAACCTTA
CAAAGXXXXXXXXXXXTGAATCAATTTAAACTCTTAAAAATATTTTTTTAGAATTGTTAAATAAACTATTTTATTA
GTCTGATAACAAAATAAGTT
ACCTACAAAAGTTCATTCATAAAAAGTATCGAATCGTATTTTAAATTTTTAGAAATTATATCAATTTAAACCTTA
CAAAGXXXXXXXXXXXTGAATCAATTTAAACTCTTAAAAATATTTTTTTAGAATTGTTAAATAAACTATTTTATTA
GTCTGATAACAAAATAAGTT
>Marker538988
ACTTTTTTATCTATTTCTTTTTCTATGGTGCTTAATGGAAGTTCTTGTCTCATATTATGTTACAAACGAATTCTA
TGCTTXXXXXXXXXXXATAAGTTCTTGAAACATACAAATTTTGATTTAAATGGGTTTCTCTTCCTAGTTCATGGTA
TTATGAAAATACAATGATGT
ACTTTTTTGTCTATTTCTTTTTCTATGGTGCTTAATGGAAGTTCTTGTCTCATATTATGTTACAAACGAATTCTA
TGCTTXXXXXXXXXXXATAAGTTCTTGAAACATACAAATTTTGATTTAAATGGGTTTCTCTTCCTAGTTCATGGTA
TTATGAAAATACAATGATGT
>Marker539333
ACCAACAAAACAACTTTGGGCCTATCCAAAGAAATGTTGTGTTCTGCAAATATTAAATCAAAGATCAGATATCAA
TTTACXXXXXXXXXXXCCAAACTGAGGCATGTATTTCTATGAAGGGTTATTATTTTGGACGAGCTTACTTGAACAA
CGACTGTTCTATACAGTTGT
ACCAACAAAACAACTTTGGGCCTATCCAAAGAAATGTTGTGTTCTGCAAATATTAAATCAAAGATCAGATATCAA
TTTACXXXXXXXXXXXCCAAACTGAGGCATGTATTTCTATGAAGGGTTATTATTTTGGACGAGCTTACTTCAACAA
CGACTGTTCTATACAGTTGT
>Marker539358
TACTTTATATCTGAACAGATTTTTTTTAGAGTAAATAAAATGTTCCAATATTGGCAGCACAAATAAAAATCATTA
AGAATXXXXXXXXXXXTCCGTTCCGTGACTGCATGCACCGAGCATATAAACTTACAAAGGACTTCGTGGGCTGTTA
GTCTTTTCTTGGGATCTCGT
TACTTTATATCTGAACAGATTTTTTTTAGAGTAAATAAAATGTTCCAATATTGGCAGCACAAATAAAAATCATTA
AGAATXXXXXXXXXXXTCCATTCCGTGACTGCATGCACCGAGCATATAAACTTACAAAGGACTTCGTGGGCTGTTA
GTCTTTTCTTGGGATCTCGT
>Marker539519
TACATCACAATCCAAATAATGAAAACAACACATTAAAAATGTATCACTTTATTTTATGGAATGTGAGAAATCTGA
AAATGXXXXXXXXXXXGAAGGATAACAAAACTAGAGTTATTCAATATTCGTCAAATTAATAGAACAACTAAAAATG
TAATAATTTCCCGAACTGTA

Create PDF with GO2PDF for free, if you wish to

TACATCACAATCCAAATAATGAAAACAACACATTAAAAATGTATCACTTTATTTTATTGAATGTGAGAAATCTGA
AAATGXXXXXXXXXXXGAAGGATAACAAAACTAGAGTTATTCAATATTCGTCAAATTAATAGAACAACTAAAAATG
TAATAATTTCCCGAACTGTA
>Marker539528
ACTTTTATAATGGTAGAGGGATGGGTTGGTTGTCAATGTCAATGTAAATACAAAGTGAAGGCACAGTAAGCAATG
ATTGGXXXXXXXXXXXTCTCTTTTCTTTTAGCAGGGAAGAAGCAAACATAAATAAAATGATGGTGACATATGAGCA
TGTTAGTAATATTGTTGGTT
ACTTTTATAATGGTAGAGGGATGGGTTGGTTGTCAATGTCAATGTAAATACAAAGTGAAGGCACAGTAAGCAATG
ATTGGXXXXXXXXXXXTCTCTTTTCTTTTAGCAGGGAAGAAGCAAACATAAATAAAATGATGATGACATATGAGCA
TGTTAGTAATATTGTTGGTT
>Marker539563
GACCATTGTCAATCACTTTTTTGTTACCGTTTTTCAAGGAGCATTGCGGCCAACTACAAACAACTGTTTTTTGTGA
CTACCXXXXXXXXXXXTTCTTTTATTATAATTTGAAATTATAAAACATTTTTAGCATATAACAATTCAAACAATTT
TTTTTACAAAAAAGTTGGTA
GACCATTGTCAATCACTTTTTTGTTACCGTTTTTCAATGAGCATTGCGGCCAACTACAAACAACTGTTTTTTGTGA
CTACCXXXXXXXXXXXTTCTTTTATTATAATTTGAAATTATAAAACATTTTTAGCATATAACAATTCAAACAATTT
TTTTTACAAAAAAGTTGGTA
>Marker539995
CACAACGTTATTTAAATATTGAATGCTAAGAAAATTGAAATTAGAGACAAGATATTTAATTTAAGCTTGGGAAGC
TATCTXXXXXXXXXXXGTTTCTATTTAATTAATCATTAGTCTTAGGAAGTGTCAATTACAAATTTAAATTTATAAT
TATCCATCCATTAACGAGTT
CACAACCTTATTTAAATATTGAATGCTAAGAAAATTGAAATTAGAGACAAGATATTTAATTTAAGCTTGGGAAGC
TATCTXXXXXXXXXXXGTTTCTATTTAATTAATCATTAGTCTTAGGAAGTGTCAATTACAAATTTAAATTTATAAT
TATCCATCCATTAACGAGTT
>Marker540057
GACACAAAGAATGAGTAGATAAAACTAAGTCACTTTTAAAACTTTGGATAACAAATTTAACTCGAAGATGTATCT
ATATTXXXXXXXXXXXATTATTTTGTTCAAATGCCAAATGGTTACAAATAGTGTTAAATATTTTAAACATTTCTAA
AAACATTATTCATGATTGTT
GACACAAAGAATGAGTAGATAAAACTAAGTCACTTTTAAAACTTTGAATAACAAATTTAACTCGAAGATGTATCT
ATATTXXXXXXXXXXXATTATTTTGTTCAAATGCCAAATGGTTACAAATAGTGTTAAATATTTTAAACATTTCTAA
AAACATTATTCATGATTGTT
>Marker540256
AACTTGCTTTGTGACGTATTGCAATTGTATTACACGCATACAAAAGTCTAAGTTCTCCAATATAAACACCCATCA
TTCCAXXXXXXXXXXXCTTACTAAACATTCCACATCATACACCCTGAATTTTTTTCTCCATCTCCAATATCCAATC
CCCTCCTCCTTCTCGTTGTT
AACTTGCTTTATGACGTATTGCAATTGTATTACACGCATACAAAAGTCTAAGTTCTCCAATATAAACACCCATCA
TTCCAXXXXXXXXXXXCTTACTAAACATTCCACATCATACACCCTGAATTTTTTTCTCCATCTCCAATATCCAATC
CCCTCCTTCTTCTCGTTGTT
>Marker540786
ACTAAACTCAAAGAAGACGTTTTCATTTTAGGGTTTCTATGAATTCTTTAGAGTTTGCATTAACCTTCTAGGTTT
AAGTGXXXXXXXXXXXAAAAAATGTTTAATTAACATCAAACTAAGAATTTTCCAATCAATCTTGCTTCAAGAATCA
TGCTTAGCATCATTAAAGTA
ACTAAACTCAAAGAAGACGTTTTCATTTTAGGGTTTCTATGAATTCTTTAGAGTTTGCATTAACCTTCTAGGTTT
AAGTGXXXXXXXXXXXAAAAAATGTTTAATTAACATCAAACTAAGAATTTTCCAAGCAATCTTGCTTCAAGAATCA
TGCTTCCCATCATTAAAGTA
>Marker540931

Create PDF with GO2PDF for free, if you wish to

ACATTGAGTGGTGTGGATAATTGGTAGTTCATGCCATAAATCATGAGTGTTCATCCTTCAGTCTATTCAAAATGT
AGGCGXXXXXXXXXXAAATCGTAATAAGCAAAAGACTTGGTGATAACAACCATAATAAAATATTACTTAAACTTG
ACATTAATTCTATTTCACGT
ACATTGAGTGGTGTGGATGATTGGTAGTTCATGCCATAAATCATGAGTGTTCATCCTTCAGTCTATTCAAAATGT
AGGCGXXXXXXXXXXAAATCGTAATAAGCAAAAGACTTGGTGATAACAACCATAATAAAATATTACTTAAACTTG
ACATTAATTCTATTTCACGT
>Marker541042
AACTAAGTGGCATCTAAGTTCATACCATAATATTTTTTTATAAGAAAGTTCGTGCCGTAACATAAGTTTGGAAAA
TGAAAXXXXXXXXXXAAAGGTGTTGATCTAAAGATTTCTTTTGGAAGGAGGTATGGTCTCTTTTTGTCTTAAGAG
ATATTTGATACCAAAGTGTT
AACTAAGTGGCATCTAAGTTCATACCATAATATTTTTTTATAAGAAAGTTCGTGCCGTAACATAAGTTCGGAAAA
TGAAAXXXXXXXXXXAAAGGTGTTGATCTAAAGATTTCTTTTGGAAGGAGGTATGGTCTCTTTTTGTCTTAAGAG
ATATTTGATACCGAAGTGTT
>Marker541267
TACTTGGTTGGAAATATATAAAAAGTTAGTTACGGGATAGATGGATAGACCGCAGGATGATATGTTGAGATTGAT
TTACTXXXXXXXXXXTCTATTTATCTTGGAAACCTTCAACTTTAGCCTCATTGTTTTCCCACATGGACTTGGGTT
CCACATTTCAGCTTTCAGTG
TACTTGGTTGGAAATATATAAAAAGTTAGTTACGGGATAGATGGATAGACCGCAGGATGATATGTTGAGATTGAT
TTACTXXXXXXXXXXTCTATTTATCTTGGAAACCTTCAACGTTAGCCTCATTGTTTTCCCACATGGACTTGGGTT
CCACATTTCAGCTTTCAGTG
>Marker541489
ACTTCTAAAATTAGGTTACTAGACTTAGGTGATATCATTCTATGACATTGTATCAAGTAAATAGACATAGACGTG
GTAGAXXXXXXXXXXGAGAAGATTTTTTTCTAAAACCATTTCTAAAGTCAAACTAGACATAAAAACCAGTTTTGG
ACTATTAAGGGAATTTTGGT
ACTTCTAAAATTAGGTTACTAGACTTAGGTGATATCATTCTATGACATTGTATCAAGTAAATAGACATAGACGTG
GTAGAXXXXXXXXXXGAGAAGATTTTTTTCTAAAACCATTTCTAAAGTCAAACTTGACATAAAAACCAGTTTTGG
ACTATTAAGGGAATTTTGGT
>Marker541694
CACATATAATCCTGCTTTATTGGTTGATGACCCCCTGTTTATGTGGTTTGCAGTATAATCCCCCCTTTACTCTTC
CATTTXXXXXXXXXXGGTGTATAGCCACATATAATGAACTGTATATATGTCACTTTCACATTTAAATACAGCTCC
ATCAGGTATTATTGATGGTG
CACATATAATCCTGCTTTATTGGTTGATGACCCCCTGTTTATGTGGTTTGCAGTATAATCCCCCCTTTACTCTTC
CATTTXXXXXXXXXXGGTGTATAGCCACATATAATGAACTGTATATATGTCACTTTCACATTTAAATACAGCTCC
ATCACGTATTATTGATGGTG
>Marker541750
AACCGTTGCTATCATTCTATCAAAGTTATATTGGTTTGAAATGAATCCATTTTAAAAAGATCAGGTTTGGTCCTA
AAACTXXXXXXXXXXATCTTAGAAAATGAAGTAAAGATTTTGAATCACTTTATGAAGTGATATATCACAATCAAA
GATGACATTGTTTGGTTGGT
AACCGTTGCTATCATTCTATCAAAGTTATATTGGTTTGAAATGAATCCATTTTAAAAAGATCAGGTTTGGTCCTA
AAACTXXXXXXXXXXATCTTAGAAAATGAAGTAAAGATTTTGAATCACTTTATGAAGTGATATCTCACAATCAAA
GATGACATTGTTTGGTTGGT
>Marker542398
ACTTTCAACTTTCCAGCCACCTAATGAGACAAATATGTGGAGCAACAGAAGGTGATTCTCAACTCATTTGTAATC
TTTTTXXXXXXXXXXTTCAATGATTTGCAGTTACTCAAGTGTTGTATTTGTAGAAAAATTCCCTAAACAAAAACT
TCTTTTTTCCTTCAAAAGTT

Create PDF with GO2PDF for free, if you wish to

ACTTTCAACTTTCCAGCCACCTAATGAGACAAATATGTGGAGCAACAGAAGGTGATTCTCAACTCATTTGTAATC
TTTTTXXXXXXXXXXXTTCAATGATATGCAGTTACTCAAGTGTTGTATTTGTAGAAAAATTCCCTAAACACAAACT
TCTTTTTTCCTTCAAAAGTT
>Marker542722
CACAACCTACCACTATTACAAAACTTTTCTTCGATTTTTTTAAATTGAAAGCTACAGGTAGTAAGGATATCTACT
ATCACXXXXXXXXXXXTAATTTTTCAATGATGATAAAATTTTATTAATGGACTGTTTTTAATTGAGTGTTTAAGAT
ATCAATTAGAATGTTTGAGT
CACAACCTACCACTATTACAAAACTTTTCTTCGATTTTTTTAAATTGAAAGCTACAGGTAGTAAGGATATCTACT
ATCACXXXXXXXXXXXTAATTTTTCAATGATGATAAAATTTTATTAATGGACTGTTTTTAATTGAGTGTTTAAGAT
ATCAATTAGAATGTTTGGGT
>Marker542894
TACTTGAATACAACATTTAAAGAGGTAAAAACTGGTCTTCAAAGTTCAACATATGATTTCTTTATCCCACTTTCT
TGCAAXXXXXXXXXXXCACATCGGGTGCTTGTGCAAAGTTAGAGGAGGATGCATATTCAGCTATGAAATTCCCAGC
AACCTGCAACAAGAATAGTT
TACTTGAATACAACATTTAAAGAGGTAAAAACTGGTCATCAAAGTTCAACATATGATTTCTTTATCCCACTTTCT
TGCAAXXXXXXXXXXXCACATCGGGTGCTTGTGCAAAGTTAGAGGAGGATGCATATTCAGCTATGAAATTCCCAGC
AACCTGCAACAAGAATAGTT
>Marker543451
GACATCCATGCATGTCATTACCAGCAAAGAAAGCTAACGACAGGGAAACAGTCTATTACGGCCACTTTTTTAACA
TAAACXXXXXXXXXXXAAAAATAAATAAATAAAAATTGGTATTGGTATTATCACATTAGTTCCACCCATGTCTTCT
AATTGTTCATGGTATGGAGT
GACATCCATGCATGTCATTACCAGCAAAGAAAGCTAATGACAGGGAAACAGTCTATTACGGCCACTTTTTTAACA
TAAACXXXXXXXXXXXAAAAATAAATAAATAAAAATTGGTATTGGTATTATCACATTAGTTCCACCCATGTCTTCT
AATTGTTCATGGTATGGAGT
>Marker543731
AACATTTCCCCCCACTCATCTCTATCACTTTAATTTCAGTGCATATTAAGTCTAAGTCTTCCTCAAAAGAGGCTC
AAGTCXXXXXXXXXXXCTGTTATCAAAACATAATATAATCGTATAGGACAATTCATTTAATTTTTATTTATTCTGA
TATTGAAAAACATTATATGT
AACATTTCCCCCCACTCATCTCTATCACTTTAATTTCAGTGCATATTAAGTCTAAGTCTTCCTCAAAAGAGGCTC
AAGTCXXXXXXXXXXXCTGTTATCAAAACATAATATAATCGTATAGGACAATTCATTTAATTTTTATTTATTCTGA
TATTGAAAATCATTATATGT
>Marker544133
TACAGTGATTGATTGATTGATTTGGAGTTTAATTAAATAAAGTTGGAGAGATAATGCACTTTTTCATGTTCATCT
CCATAXXXXXXXXXXXTGAGTATAGTTCATATTTACTCAAAATTTCTATGCAGCAAAATTTGAGATTAGAAATTTG
ATTCTTTTGTATGCATATGT
TACAGTGATTGATTGATTGATTTGGAGTTTAATTAAATAAAGTTGGAGAGATAATGCACTTTTTCATGTTCATCT
CCATAXXXXXXXXXXXTGAGTATAGTTCATATTTACTCAAAATTTCTAGGCAGCAAAATTTGAGATTAGAAATTTG
ATTCTTTTGTATGGATATGT
>Marker545182
ACCTCAAAATGTTATTGCATTATATTATTGCGCCATCTTAGATTACATCATTTGCTTATTATAGGAAGTGATCAA
TAATTXXXXXXXXXXXTAGAAACAATTGAGGTTTAATATTAGTAATTTGGTGACTGTTATATATATACTTACAAGC
TAGTATATATTTCATTTAGT
ACCTCAAAATGTTATTGCATTATATTATTGCGCCATCTTAGATTACATCATTTGCTTATTATAGGAAGTGATCAA
TAATTXXXXXXXXXXXTAGAAACAATTGAGGTTTAATATTAGTAATTTGGTGACTGTTATATAAATACTTACAAGC
TAGTATATATTTCATTTAGT
>Marker545801

Create PDF with GO2PDF for free, if you wish to

ACATATTTGATGCTCCCACTTCGTTTTAAACACATCCAATTGCTATCAAACGTAATCACTTCCTCCATTCTATTA
TATATXXXXXXXXXXAAAACAAATTTTTGCTTGATTTTTTAAACATGTTTCTACATTGATGTAGGCAACAAATAA
AAAATCATTAGAACGACGTA
ACATATTTGATGCTCCCACTTTGTTTTAAACACATCCAATTGCTATCAAACGTAATCACTTCCTCCATTCTATTA
TATATXXXXXXXXXXAAAACAAATTTTTGCTTGATTTTTTAAACATGTTTCTACATTGATGTAGGCAACAAATAA
AAAATCATTAGAACGACGTA
>Marker545827
AACAAGCCATTGGACATGGAGTTTCAAAAGAACATCCAATCATTTTTGCTTTCTTCTCTCTAAATATGTAGTTGG
TTTCCXXXXXXXXXXATAATTTCATTCATATTCCCTCCAAATTTACTCTAAAGTTAAGTCTCGAATAAGAATTTC
GAATATTCGATCAATTGGTA
AACAAGCCATTGGACATGGAGTTTCAAAAGAACATCCAATCATTTTTGCTTTCTTCTCTCTAAATATGTAGTTGG
TTTCCXXXXXXXXXXACAATTTCATTCATATTCCCTCCAAATTTACTCTAAAGTTAAGTCTCGAGTAAGAATTTC
GAATATTCGATCAATTGGTA
>Marker546457
AACTTCCTCTTCACGTCCTCAACAACACCACACCAATTTTTCTTCCCATTAACCTTTTGCAAAACCCTCTTCATT
TCATTXXXXXXXXXXTAATTTGTGCCTCCAAATAATTAGAAAGGATCAAAAGAAAATATTAGTAACATTACCCGT
GTAAATCAATGCATAAAAGT
AACTTCCTCTTCACGTCCTCAACAACACCACACCAATTTTTCTTCCCATTAACCTTTTGCAAAACCCTCTTCATT
TCATTXXXXXXXXXXTAATTTGTGCCTCCAAATAATTAGAAAGGATCAAATGAAAATATTAGTAACGTTACCCGT
GTAAATCAATGCATAAAAGT
>Marker546960
AACTCAAAGACATTTAAATTAATTCTGCTACATGTAAAATTATCAACTAGAAATAGACTTCATTAAATCGTTTCC
TTGACXXXXXXXXXXACTCTAAGGATGAGATTATCGTAACCATATCCATTAAGCTTGTTAATTGATCTTTGTGCA
TCCTCACGATTCACAAAGTT
AACTCAAAGACATTTAAATTAATTCTGCTACATGTAAAATTATCAAGTAGAAATAGACTTCATTAAATCGTTTCC
TTGACXXXXXXXXXXACTCTAAGGATGAGATTATCGTAACCATATCCATTAAGCTTGTTAATTGATCTTTGTGCA
TCCTCACGATTCACAAAGTT
>Marker547027
AACTATAAAATTATTGTGTTTGTAAAGCAAGGGAATAAAAGTTTTTTGATAAGGAGAAGTAGATAAACATAGTAA
GATAAXXXXXXXXXXTCAAACTTCAAGTTACAAGATTGAAAGTTTAATAATTTAAAACCTTTATTGCACTTACTA
TCAAGTTCTAAAATTTAAGT
AACTATAAAATTATTGTGTTTGTAACGCAAGGGAATAAAAGTTTTTTGATAAGGAGAAGTAGATAAACATAATAA
GATAAXXXXXXXXXXTCAAACTTCAAGTTACAAGATTGAAAGTTTAATAATTTAAAACCTTTATTGCACTTACTA
TCAAGTTCTAAAATTTAAGT
>Marker547319
CACCGGACTCGGTATGGCCACGCCGAAGAAACCCAAAAGACTTTACCAAGTTTGGAGGGGAAGTAATGTGAGTTC
TTAGCXXXXXXXXXXACTCTGTTTATGAGCTGAAAATGTTGATGGGATGTCTAAGTTGTATGTGAGAGTGTTTCT
GCCTTCTATGATGTCATGTA
CACCGGACTCGGTATGGCCACGCCGAAGAAACCCAAAAGACTTTACCAAGTTTGGAGGGGAAGTAATGTGAGTTC
TTAGCXXXXXXXXXXACTCTGTTTATGAGCTGAGAATGTTGATGGAATGTCTAAGTTGTATGTGAGAGTGTTTCT
GCCTTCTATGATGTCATGTA
>Marker547393
TACTTTTGTAACCACAAGTGTTGTAATTTATTCAAGAAAACAAATAGCTACAAACAACTTTTAGATTGATGCTAT
ATTATXXXXXXXXXXTATATTTGGATATGCATCAGCTTGAACTTTACTCATATGTCTTTTTCATCTCTTTTCAAT
CATACAACACTAGTGTATGT

Create PDF with GO2PDF for free, if you wish to

TACTTTTGTAATGACAAGTGTTGTAATTTATTCAAGAAAACAAATAGCTACAAACAACTTTTAGATTGATGCTAT
ATTATXXXXXXXXXXTATATTTGGATATGCATCAACTTGAACTTTACTCATATGTCTTTTTCATCTCTTTTCAAT
CATACAACACTAGTGTATGT
>Marker547611
TACCACATTCGGACTTGGAATATGCAAAGTATATCCATATGATATGGATAAGGATTGTAATATAAGCCTCCGATC
AGTAGXXXXXXXXXXAAGATATATTGGTTAGGAAGTTAATAAGTTAAGTAATATGCTATTAACTCTCAACAGGTT
CTAAATAATGCACGGAAAGT
TACCACATTCGGACTTGGAATATGCAAAGTATATCCATATGATATGGATAAGGATTGTAATATAAGCCTCCGATC
AGTAGXXXXXXXXXXAAGATATATTGGTTAGGAATTTAATAAGTTAAGTAATATGCTATTAACTCTCAACAGGTT
CTAAATAATGCACGGAAAGT
>Marker547729
TACTTTTTATGGTCCATCTTTTACGACTAAATTGCAATCAACGACTCTAAATTAGTCATTTCTTTTTAAAAACGA
CTACTXXXXXXXXXXAATGTATTGGACGGTGAAATTGAGACTTGGACACAACCACATCGTTTTAAGATTTTGCCA
CAATATTCTTATCGAGTTGT
TACTTTTTATGGTCCATCTTTTACGACTAAATTGCAATCAACGACTCTAAATTAGTCATTTCTTTTTAAAAACGG
CTACTXXXXXXXXXXAATGTATTGGACGGTGAAATTGAGACTTGGACACAACCACATCGTTTTAAGATTTTGCCA
CAATATTCTTATCGAGTTGT
>Marker548207
AACATAGGATGACAATTACGATAATCGTATAGCAATGATAGATATATGAAGGTGAGTATGTGTCAGCCAAATGTT
TGAGTXXXXXXXXXXTATTATCAATACGGTGATTCATATCTCATTAAATAAATGAATTAGACTAAATTAAATTGA
TCAAACATTGACTTTGAGTC
AACATAGGATGACAATTACGATAATCGTATAGCAATGATAGATATATGAAGGTGAGTATGTGTCAGCCAAATGTT
TGAGTXXXXXXXXXXTATTATCAATACGGTGATTCATATTTCATTAAATAAATGAATTAGACTAAATTAAATTGA
TCAAACATTGACTTTGAGTC
>Marker548945
TACTCTGTCTGGAGTAGTCTTTTCAAGTTTTTGTTTTGTTTGTCTCTAAATTAGTTAAAAACTTAAAATGCTATG
CATTCXXXXXXXXXXTTCTGCTTCAGATTAACACATTTAATGGATTTTACTGGGAGAACTTTGAACCAAACAAGG
CTTATAAACCCAAAATTGTG
TACTCTTTCTGGAGTAGTCTTTTCAAGTTTTTGTTTTGTTTGTCTCTAAATTAGTTAAAAACTTAAAATGCTATC
CATTCXXXXXXXXXXTTCTGCTTCAGATTAACACATTTAATGGATTTTACTGGGAGAACTTTGAACCAAACAAGG
CTTATAAACCCAAAATTGTG
>Marker549515
AACAACTTTTTCGGAATATTATGACAATGACAAGCCTTGCTATGTTTCTTGTAGTTCTACCTTTTCTTTCAAGTC
TGTGAXXXXXXXXXXCAAATGATAGGTCGGGAAAATCCGATAAATTTTAGGCGTAAAGGATTAAAATCAGAAAC
TAATGTGCCCAGTGAAAGTT
AACAACTTTTTCGGAATATTATGACAATGACAAGCCTTGCTATGTTTCTTGTAGTTCTACCTTTTCTTTCAAGTC
TGTGAXXXXXXXXXXCAAATGATAGGTCGGGAAAATCCGATAAATTTTAGGCGTAAAGGATTAAAATCAGAAAC
TAATGTGCCCAGTGGAAGTT
>Marker549828
CACCACCTGGAAGATACCCCATTCCTCGCAAGCTTCAACAATGCTGTGGCGAATCATGGCCCTGCGTTCGCCGTT
GTCGTXXXXXXXXXXAGAAAAAGTCATTGTTGGTTAAGATTTTGTTAGTTTGAGGAGTGGGAAAATGAATATGAT
ATTTATTAGTGTTTTTGGTA
CACCACCTGGAAGATACCCCATTCCTCGCAAGCTTCAACGATGCTGTGGCGAATCATGGCCCTGCGTTCGCCGTT
GTCGTXXXXXXXXXXAGAAAAAGTCATTGTTGTTTAAGATTTTGTTAGTTTGAGGAGTGGGAAAATGAATATGAT
ATTTATTAGTGTTTTTGGTA
>Marker549995

Create PDF with GO2PDF for free, if you wish to

GACCTTAAAACCAAAAAACCCAAATTCAGCATTTCGAAAAAAACTCATTTGGGTTTGTTTAAAAAATAAAAAAA
TTAAAXXXXXXXXXXXATCACAAACAGAGGCAGTAACTTTGAAGCCTTTTTGTTGCCATTCCTGAAGCCTTTGGTC
AATCTCTGTTTGGTTACGTG
GACCTTAAAACCAAAAAACCCAAATTCAGAATTTCGAAAAAAACTCATTTGGGTTTGTTTAAAAAATAAAAAAA
TTAAAXXXXXXXXXXXATCACAAACAGAGGCAGTAACTTTGAAGCCTTTTTGTTGCCATTCCTGAAGCCTTTGGTC
AATCTCTGTTTGGTTACGTG
>Marker550134
ACCATTTATCGTTTGATACAGTTCAAATATAGTCTAATAAAATACGATTATGTGATATTTTTAAGTTTTTGAAAT
TCTTTXXXXXXXXXXXATTAGTCACCGATAAATTTGGAGTAGAAAACTTATGTAAATGAGTTTTACTACATCACCT
CATTTGATGTTGGGGATGGT
ACCATTTATCGTTTGATACAGTTCAAATATAGTCTAATAAAATACGATTACGTGATATTTTTAAGTTTTTGAAAT
TCTTTXXXXXXXXXXXATTAGTCACCGATAAATTTGGAGTAGAAAACTTATGTAAATGAGTTTTACTACATCACCT
CATTTGATGTTGGGGATGGT
>Marker553007
TACTATTTTTTATTATAGTTCGTTTTACCTACTCATTAACTCAAAATAAACTATTATAACCAATTTAATATATGT
TCGAGXXXXXXXXXXXCTAGTGCTCACATTTACGGATTAATTTTATCCTATTTCTTTATCTTCCTCCAATATTTCT
AATGACTCTAATTGCTCGTT
TACTATTTTTTATTATAGTTCGTTTTACCTACTCATTAACTCAAAATAAACTATTATAACCAATTTAATATATGT
TCGAGXXXXXXXXXXXCTAGTGCTCACATTTACGGATTAATTTTATCCTATTTCTTTATCTTCCTCCAATATTTCT
AATGACTCTAACTGCTCGTT
>Marker553301
GACAACATGATTCCTCCTTTGCCAAGTAAATTCAGTGATGTATTCGATTGGTCAAAAGAACTACCCCTAAAAAGG
GATATXXXXXXXXXXXTCATTAGACCAAGCACTAGTCCCTATTCCAGGTCGGTCCTATTAGTTAAAAAGAAGGATG
GAAGGTGGAGATTTTGCGTG
GACAACATGATTCCTCCTTTGCCAAGTAAATTCAGTGATGTATTCGATTGGTCAGAAGAACTACCCCTAAAAAGG
GATATXXXXXXXXXXXTCATTAGACCAAGCACTAGTCCCTATTCCAGGTCGGTCCTATTAGTTAAAAAGAAGGATG
GAAGGTGGAGATTTTGCGTG
>Marker553857
AACAACCCATTTCTTCACAGAACTACTCTGATCGTTAATCCAGTGCAATGCTATAAAAAATTTAGAAAAAAAAAT
TGCAGXXXXXXXXXXXCCCAAGTTCTCTTGAGAGGCAAACTTACAAAAGTGGCTTATGCCCTTTCTTATTCTCTGT
GCCAAAGAGGCATTCAGGTC
AACAACTCATTTCTTCACAGAACTACTCTGATCGTTAATCCAGTGCAATGCTATAAAAAATTTAGAAAAAAAAAC
TGCAGXXXXXXXXXXXCCCAAGTTCTCTTGAGAGGCAAACTTACAAAAGTGGCTTATGCCCTTTCTTATTCTCTGT
GCCAAAGAGGCATTCAGGTC
>Marker553874
AACTTTGACAGTGGAGTAGATGACCTTCATAATCCAAGTCAATATTTAGTTTCAAATGTTAATATGAAAAGCCAC
ATTTTXXXXXXXXXXXATGCCACATGTATCTTAAGGAAAAATTGATACTGAACAGGATGCATGAATGCAATGTTTC
TTTTTATTATTACTATTTGT
AACTTTGACAGTGGAGTAGATGACCTTCATAATCCAAGTCAATATTTAGTTTCAAATGTTAATGTGAAAAGCCAC
ATTTTXXXXXXXXXXXATGCCACATGTATCTTAAGGAAAAATTGATACTGAACAGGATGCATGAATGCAATGTTTC
TTTTTATTATTACTATTTGT
>Marker553910
ACAAGAGAATTATAAAAATAGCTAAATCTCTAAGGAAAAAGAATCCAGAATCCAAAAATGCGGGCAATAAATGAA
GATCTXXXXXXXXXXXTATTAGGTTAAATCTCCGTCACTACTATTTAATTGTTTTTTTTTAAAGGAGACAAACTTC
TTTATTTAGAACTCAAAAGT

Create PDF with GO2PDF for free, if you wish to

ACAAGAGAATTATAAAAATAGCTAAATCTCTAAGGAAAAAGAATCCAGAATCCAAAAATGCGGGCAATAAATGAA
GATCTXXXXXXXXXXTATTAGGTTAAATCTCCGTCACTACTATTTAATTGTTTTTTTTTAAAAGAGACAAACTTC
TTTATTTAGAACTCAAAAGT
>Marker553914
AACCAATGAAAAACCCTACCACGAAAACCTTGACAACTGGCCCATGCACAAGGTATTACTGATTCTAAAAACAAT
AATTTXXXXXXXXXXTCAAATGACTCGGATAAACCAAGGAGTTACGATCCCTCTCTTGACATACCCATTGCCTTG
AGAAAGGGCACGAGGTTGTG
AACCAATGAAAAACCCTACCACGAAAACCTTGACAACTGGCCCATGCACAAGGTATTACTGATTCTAAAAACAAT
AATTTXXXXXXXXXXTCAAATGACTCGGATAAACCAAGGAGTTACGATCCCTCTCTTGACATACCCATTGCCTTG
AGAAAGGGCGCGAGGTTGTG
>Marker554070
AACTTCCCTTAATTTCTCTCTTGTTCTTACATTATTGTTCTTTAGGTGACTCTGAATGGTTACAACCACAAATTA
AGGATXXXXXXXXXXTGAAACCTAAATTTTATCTATTAATTAAATTTGGAACCTGAATAATATCTATCAACTGTT
GATATATTTAAAATTTCAGT
AACTTCCCTTAATTTCTCTCTTGTTCCTACATTATTGTTCTTTAGGTGACTCTGAATGGTTACAACCACAAATTA
AGGATXXXXXXXXXXTGAAACCTAAATTTTATCTATTAATTAAATTTGGAACCTGAATAATATCTATCAACTGTT
GATATATTTAAAATTTCAGT
>Marker554170
ACCTAAAAGAAATGAAATGCAATCAAGATTATTCAAAGTTAGGAAAATTGTTGGCATGCATATCTAATTAAGGGC
CGAACXXXXXXXXXXATTACTTTACAATGTATTTTATTTTCAATACAAGTTCTTAAAACCCTATAGTTTGCTCCC
TTTTATCTTCCTTATTTTGT
ACCTAAAAGAAATGAAATGCAATCAAGATTATTCAAAGTTAGGAAAATTGTTGGCATGCATATCTAATTAAGGGC
CGAACXXXXXXXXXXATTACTTTACAATGTATTTTATTCTCAATACAAGTTCTTAAAACCCTATAGTTTGCTCCC
TTTTATCTTCCTTATTTTGT
>Marker554229
AACTTCGTTTGAGAAAGATATAAACAAACTTAACATAACATAGCATCAATGGCAGAAAGAAACTAACCACGACAA
AGAGTXXXXXXXXXXTTTAGAAGCTAAACTAAAATTTGAAAACTAAAAAAGGTAGCTTGTTGAAATGTTGTTTTT
GTTTTTGCTAGAGCCTTTGT
AACTTCGTTTGAGAAAGATATAAACAAACTTAACATACCATAGCATCAATGGCAGAAAGAAACTAACCACGACAA
AGAGTXXXXXXXXXXTTTAGAAGCTAAACTAAAATTTGAAAACTAAAAAAGGTAGCTTGTTGAAATGTTGTTTTT
GTTTTTGCTAGAGCCTTTGT
>Marker554667
AACCTCTTATTTTGTTATTCTTATTGTAAGACCAGGAATAATTAACATGCTTTTTAAGAGTCATTTTGAAAAATG
ACAAAXXXXXXXXXXXGCATTATGGACCTTTAATTATTTTCGTGACGTGTAAAGATATAACAACCCATATCATAAC
TTCTTTCTAATATTTTCAGT
AACCTCTTATTTTGTTGTTCTTGTTGTAAGACCAGGAATAATTAACATGCTTTTTAAGAGTCATTTTGAAAAATG
ACAAAXXXXXXXXXXXGCATTATGGACCTTTAATTATTTTCGTGACGTGTAAAGATATAACAACCCATATCATAAC
TTCTTTCTAATATTTTCAGT
>Marker554972
ACTACATATCACGTTTTCAGAGATCCAAGTGCCACTTTTTCCCTTACGAAGTGATAGTCAAGTTCTATGTATTTG
GATCTXXXXXXXXXXATCCATGTTATCTCAATAGCTGCATTAGCCATAACTTCAACTTTTGAACTTGAACGAGGC
ACAGTTGGTTGCTTATTAGT
ACTACATATCACGTTTTCAGAGATCCAAGTGCCACTTTTTCCCTTACGAAGTGATAGTCAAGTTCTATGTATTTG
GATCTXXXXXXXXXXATCCATGTTATCTCAATAGCTGCATTAGCCATAACTTCAACTTTTGAACTTGAACAAGGC
ACAGTTGGTTGCTTATTAGT
>Marker555227

Create PDF with GO2PDF for free, if you wish to

AACAGCCACATCTATTCAAATTTACTGCGTGATATCTACAGAATACTCAAATACCTGCTCAATCAAATCAAGAGC
AAGTGXXXXXXXXXXAGCTGAGTTGCCAATTCTTGAAGCTGTATAAGACAAAATACAAAATAATTCAATGTTAAA
AAGCTCCAAATAAACAAGTT
AACAGCCACATCTATTCAAATTTACTGCGTGATATCTACAGAATACTCAAATACCTGCTCAATCAAATCAAGAGC
AAGAGXXXXXXXXXXAGCTGAGTTGCCAATTCTTGAAGCTGTATAAGACAAAATACAAAATAATTCAATGTTAAA
AAGCTCCAAATAAACAAGTT
>Marker555296
ACAATAATATTAAAGAAAAAGAGAAATAAAAGTATTATAAAAATTCAAAGGTAAAGAAGTGTTCATTTAAGTTTT
ATAATXXXXXXXXXXTTTCTAGTAATTCTAAAAAGTGATAAAATGGATAATATTATTTTCATTTTATATTCCAAC
TCTAAGAGATAAAAATGGTC
ACAATAATATTAAAGAAAAAGAGAAATAAAAGTATTATAAAAATTCAAAGGTAAAGAAGTGTTCATTTAAGTTTT
ATAATXXXXXXXXXXTTTCTAGTAATTCTAAAAAGTGATAAAATGGATAATATTGTTTTCATTTTATATTCCAAC
TCTAAGAGATCAAAATGGTC
>Marker556320
TACACTCTGGCAGAGCTTCAATCCGCTACTAACAACTTTAGTCAAGAGAATCTTCTAGGAGAGGGATCTCTTGGT
GCTGTXXXXXXXXXXTTTCTATGAACTTTTTTCTGACTTCTAATTTCTAACCAGGTTTTGGCTGTGAAAAATATC
CACATGGGAACGCTCTTGTT
TACACTCTGGCAGAGCTTCAATCCGCTACCAACAACTTTAGTCAAGAGAATCTTCTAGGAGAGGGATCTCTTGGT
GCTGTXXXXXXXXXXTTTCTATGAACTTTTTTCTGACTTCTAATTTCTAACCAGGTTTTGGCTGTGAAAAATATC
CACATGGGAACGCTCTTGTT
>Marker557049
AACGTCAAAGTATAAAAGAATTAAAAAAGTTGAAGTTAGTGCAAAATCTAAAATTTGTATTTTTTTGGAAAAGAC
AATGAXXXXXXXXXXTAACGATCGTTATCTAAAATTTCTTCATAATTCAAATATATGTGTGATTGTATGTATAAG
AAAAAAAAACACTTTAGGGT
AACGTCAAAGTATAAAAGAATTAAAAAAGTTGAACTTAGTGCAAAATCTAAAATTTGTATTTTTTTGGAAAAGAC
AATGAXXXXXXXXXXTAACGATCGTTATCTAAAATTTCTTCATAATTCAAATATATGTGTGATTGTATGTATAAG
AAAAAAAAACACTTTAGGGT
>Marker557614
TACCTTGAAACCATCAATTTCAAAATTCAAAATTTTGGTAATTTGTTTAAACAATCAATTTGGATTTCAAAATTC
CAAAAXXXXXXXXXXAAACACATGATTCTTATTGTTAAACAATAATTAAATACTAAACAACCAGTAAAGATTTTT
ATAGTATAAAAATTTATGGT
TACCTTGAAACCATCAATTTCAAAATTCTAAATTTTGGTAATTTGTTTAAACAATCAATTTGGATTTCAAAATTC
CAAAAXXXXXXXXXXAAACACATGATTCTTATTGTTAAACAATAATTAAATACTAAACAACCATTAAAGATTTTT
ATAGTATAAAAATTTATGGT
>Marker557775
AACATCATTAGCAGCAGTAAGAGAAATCTTGGTAAATACTCGTGCAATAAACCAAAAATGTCAAAACAAAAAGAA
TCCACXXXXXXXXXXTTCTTTTAATTATACACGAGCTTCACAAATTTGTTATGCATTGCACTTCACAATTAAGCT
CAATTGCTCAAGTGTCAGTG
AACATCATTAGCAGCAGTAAGAGAAATCTTGGTAAATACTCGTGCAATAAACCAAAAATGTCAAAACAAAAAGAA
TCCACXXXXXXXXXXTTCTTTTAATTATACATGAGCTTCACAAATTTGTTATGCATTGCACTCCACAATTAAGCT
CAATTGCTCAAGTGTCAGTG
>Marker557893
CACCAAAACTCGTGTGCCTACACTCCTCAACAAAATGGGGTGGCTGAGCGAAAAAATCATCTTCTGGAAGTAGCA
CATTCXXXXXXXXXXAGACTCCCTTAGATTGTCTTAAGGAGTCCTACCTCTCTACTCGTCTAATTGCTGAGGTTC
CTCTTTGCGTGTTTGGGTGT

Create PDF with GO2PDF for free, if you wish to

CACCAAAACTCGTGTGCCTACACTCCTCAACAAAATGGGGTGGCTGAGCGAAAAAATCATCTTCTGGAAGTAGCA
CATTCXXXXXXXXXXAGACTCCCTTAGATTGTCTTAAGGAGTCCTACCTCTCTACTCGTCTAATTTCTGAGGTTC
CTCTTTACGTGTTTGGGTGT
>Marker558233
CACTTAGGGTTTCAAGCTTAGGGATTATTGTGTGAGCCCCAACATTCATTTTTGTAATAAAACCTTTTTGTTTTT
TTCTTXXXXXXXXXXCACTACAAACAACAAATTAGCCTATAGGAAGAATAAACACTAATCTTATATACATAGAAT
GACCCAGACAAAATGGAGTG
CACTTAGGGTTTCAAGCTTAGGGATTATTGTGTGAGCCCCAACATTCATTTTTGTAATAAAACCTTTTTGTTTTT
TTCTTXXXXXXXXXXCACTACAAACAACAAGTTAGCCTATAGGAAGAATAAACACTAATCTTATATAGATAGAAT
GACCCAGACAAAATGGAGTG
>Marker558401
ACCATCCCTCCCACTCTCGCAAAGAACCAAAACACCACAAAGCTCATAACTACCTTGATCATATGCTTATGACAT
ATATAXXXXXXXXXXAACACTGAAATTCCCCTTTTACCCTTTCAATAATATTGGAATGGAGTTGGGGGCCTAACA
GATTCTTAGAGAGGAAAGTC
ACCATCCCTCCCACTCTCGCAAAGAACCAAAACACCACAAAGCTCATAACTACCTTGATCATATGCTTATGACAT
ATATAXXXXXXXXXXAACACCGAAATTCCCCTTTTACCCTTTCGATAATATTGGAATGGAGTTGGGGGCCTAACA
GATTCTTAGAGAGGAAAGTC
>Marker558477
CACATCAAGATAGAGATTTGAACAAAAGGATAAAACAGTGTTGATTATGTTTATACTCAAAATTCTTTCTACCGA
AGATCXXXXXXXXXXTTAAATAGGAAGAACACGTATATGATAGTCTAAGATTTAAAATGGGGACGTGTTATTAAT
CATGACCATGAAAATGGGTG
CACATCAAGATAGAGATTTGAACAAAAGGATAAAACAGTGTTTATTATGCTTATACTCAAAATTCTTTCTACAGA
AGATCXXXXXXXXXXTTAAATAGGAAGAACACGTATATGATAGTCTAAGATTTAAAATGGGGACGTGTTATTAAT
CATGACCATGAAAATGGGTG
>Marker558518
TACAACATAGAGGTTCTTTTGACACATCCTTAGCCTTATAGCTCACACTTGCATACAAAGCAAACACTTAGTCCA
ATCCTXXXXXXXXXXACTTAGTCACTTGAATAAAATAACCTCACACTAAGGAAATTTCATCCTTCTATAATCAAC
TACATAGAAAGTCTAGGGTT
TACAACATAGAGGTTCTTTTGACACATCCTTAGCCTTATAGCTCACACTTGCATACAAAGCAAACACTTAGTCCA
ATCCTXXXXXXXXXXACTTAGTCACTTGAATAAAATAACCTCACACTAAGGAAATTTCATCATTCTATAATCAAC
TACATAGAAAGTCTAGGGTT
>Marker559418
AACAAGTATCACGTTTGAATTAAAGGAATTTAATGGACATGAAAGTTAAAATGATTACTAGCTATAATCTAATAC
CAACGXXXXXXXXXXGACATTTTGGTGGGTGGAAAAGTGTTTAGTCGTATAGAGAGAAGACTGTAAAAAGTAAAA
AGAAATGAAAGGACATTGTG
AACAAGTATCACGTTTGAATTAAAGGAATTTAATGGACATGAAAGTTAAAATGATTACTAGCTATAATCTACTAC
CAACGXXXXXXXXXXGACATTTTGGTGGGTGGAAAAGTGTTCAGTCGTATAGAGAGAAGACTGTAAAAAGTAAAA
AGAAATGAGAGGACATTGTG
>Marker559494
ACTCATGAAATTTTAATTTACACAGTTGTAACTAAATTTTTATTACCGAACTTATACGCTAAGAACTAAAAATAT
ATAAAXXXXXXXXXXAAATGAAAGAAAAGAAAGGAGGCTTTTCATTCGGATTCTATCTGAGGAAGAAAGTGATGG
GAGGATCTAATCAGTAGGTA
ACTCATGAAATTTTAATTAACACAGTTGTAACTAAATTTTTATTACCGAACTTATACGCTAAGAACTAAAAATAT
ATAAAXXXXXXXXXXAAATGAAAGAAAAGAAAGGAGGCTTTTCATTCGGATTCTATCTGAGGAAGAAAGTGATGG
GAGGATCTAATCAGTAGGTA
>Marker559541

Create PDF with GO2PDF for free, if you wish to

ACCTTCATACAGGCTGTATTCCAGCAATTATTCACAGGGACTTGAAAAGCAGTAATATTCTTCTTGACAGGCACA
TGAAAXXXXXXXXXXXGATCCGGAGTAAGCTTTTCCCCTTTCTTGAGAATTTAAATATATCCTGAAGTAAAGTTTG
ATGGCTATAATGTATCTGTA
ACCTTCATACAGGCTGTATTCCAGCAATTATTCACAGGGACTTGAAAAGCAGTAATATTCTTCTTGACAGGCACA
TGAAAXXXXXXXXXXXGATCCGGAGTAAGCTTTTCCTCTTTCTTGAGAATTTAAATATATCCTGAAGTAAAGTTTG
ATGGCTATAATGTATCTGTA
>Marker559641
TACCTACCCCGACAATTTGCGCTCGTGTTTTCTGGATGGTGCTAGAACGTATTTGTTTGTTGGGGTTGTTTGAAT
TTGATXXXXXXXXXXXGACAGTATTTGGCATTTGCAATGGCAATGGAGCCATCTATGCGTATGGACTAATTGAACC
GTTTATGTTTTCCTGGTTGT
TACCTACCCCGACAATTTGCGCTCGTGTTTTCTGGATGGTGCTAGAACGTATTTGTTTGTTGGGGTTGTTTGAAT
TTGATXXXXXXXXXXXGACAGCATTTGGCATTTGCAATGGCAATGGAGCCATCTATGCGTATGGACTAATTGAACC
GTTTATGTTTTCCTGGTTGT
>Marker559707
GACTGTCTGGTAAAGCTGGTAAGACAAAGTTTGCCATCGAAAATGCCACCAAGACGAGGATAGTCATTGCAGATA
CTAAGXXXXXXXXXXXAGAAAGGTTTTGATCTGTAATTTAATGTTTGTTTGTCTTGATAAATGAATTTTGCTTACA
CTAGTTGTTTGAGGAAGAGT
GACTGTCTGGTAAAGCTGGTAAGACAAAGTTTGCCATCGAAAATGCCACCAAGACGAGGATAGTTATTGCAGATA
CTAAGXXXXXXXXXXXAGAAAGGTTTTGATCTGTAATTTAATGTTTGTTTGTCTTGATAAATGAATTTTGCATACA
CTAGTTGTTTGAGGAAGAGT
>Marker559740
ACTCTTACTGATATATATATTCCTAGATATGTGGATGTGCATCATCCATTACATATTCATGATCGAAATAGAAGCAA
TTTGGXXXXXXXXXXXTCTAGAATCTGTTATTTCAGAGCAGAGTTCTCAAACTTTTGCATCTTGTGATAAATGAAA
CATGCATATGATGTGAAAGT
ACTCTTACTGATATATATATTCCTAGATATGTGGATGCGCATCATCCATTACATATTCATGATCGAAATAGAAGCAA
TTTGGXXXXXXXXXXXTCTAGAATCTGTTATTTCAGAGCATAGTTCTCAAACTTTTGCATCTTGTGATAAATGAAA
CATGCATATGATGTGAAAGT
>Marker560135
CACCACATGAACCAGTAATGACGATTCTGATATCTCCTCTAATGTTGCTCTGAATGCAGCAACCTGTAGGACTTA
ACCAGXXXXXXXXXXXGGGTAGTTGAGATTTACTTTGCCCTAATTTTTTATTATTTTAATTAGGCCCATGTTGTCA
ATAAATATTTTTTCCCTTTGT
CACCACATGAACCAGTAATGACGATTCTGATATCTCCTCTAATGTTGCTCTGAATGCAGCAACCTGTAGGACTTA
ACCAGXXXXXXXXXXXGGGTAGTTGAGATTTACTTTGCCCTAATTTTTTATTATTCTAATTAGGCCCATGTTGTCT
ATAAATATTTTTTCCCTTTGT
>Marker560488
CACACTTGATATGCTTCCATTTGATATTCTTTGGTGATGGCAAACCGAAATTTAGTGTGGAACGTTGTGAAGAAA
TCTTTXXXXXXXXXXXTTAATCCTATTGCGACTTCTCTGACAGGACCAATGACTGGTAATTTTCTAAACCTAGCAT
ATTTATTATGGCGAGATGGT
CACACTTGATATGCGTCCATTTGATATTCTTTGGTGATGGCAAACCGAAATTTAGTGTGGAACGTTGTGAAGAAA
TCTTTXXXXXXXXXXXTTAATCCTATTGCGACTTCTCTGACAGGACCAATGACTGGTAATTTTCTAAACCTAGCAT
ATTTATTATGGCGAGATGGT
>Marker560554
CACCAAGCGTCGGAGTGTCCAGGTGTCCAACATGTCAGGCATGGACATGCTAGCGAAACGAGAGTGTTTGTGCTT
CTTAAXXXXXXXXXXXCTTCATTTTGCCTAAATCCTGCTTATGCGGTAATGGTTGTGCCTCTGCACAATCCTTTGG
TTATGAGTTTAAATTTTGTC

Create PDF with GO2PDF for free, if you wish to

CACCAAGCGTCGGAGTGTCCAGGTGTCCAACATGTCAGGCATGGACATGCTAGCCAAACGAGAGTGTTTGTGCTT
CTTAAXXXXXXXXXXCTTCATTTTGCCTAAATCCTGCTTATGCGGTAATGGTTGTGCCTATGCACAATACTTTGG
TTATGAGTTTAAATTTTGTC
>Marker560910
AACCCCATGCCTGATAGGTAAAATCTTTCTTAGAGTTCTGCATCGAATATCAAACCAACATTTTGTCTATATAGG
TTGCCXXXXXXXXXXATTGGGTTGCTAGCCAATTTTTAACGTCAGTTAGATATCCTATATCATTCCTAATGAGAA
GGATATCGTCCATATAAAGT
AACCCCATGCCTGATAGGTAAAATCTTTCTTAGAGTTCTGGATCGAATATCGAACTAACATTTTGTCTATATAGG
TTGCCXXXXXXXXXXATTGGGTTGCTAGCCAATTTTTAACGTCAGTTAGATATCCTATATCATTCCTAATGAGAA
GGATATCGTCCATATAAAGT
>Marker561011
CACTAACCATTGACATTGAAATATGGATATGCAACCAAAAAAAGCTCATCTTCAGGTTTCACACGGTCAGCCATT
TTCACXXXXXXXXXXTAAAAGAGTTTTTATATGGTCAGAAAAGAGAAAAAAGAACAGCAGACAGTAAGCATATTA
CAAACAAAAGCATGATTGTT
CACTAACCATTGACATTGAAATATGGATATGCAACCAAAAAAAGCTCATCTTCAGGTTTCACACGGTCAGCCATT
TTCACXXXXXXXXXXAAAAAGAGCTTTTATATGGTCAGAAAAGAGAAAAAAGAACAGCAGACAGTAAGCATATTA
CAAACAAAAGCATGATTGTT
>Marker561048
ACTATATTTCATCTATTATTGCCCAATATTGTTTCAACTTTTTAAAATTAAAAAGAAAAAGTGGAAAAATAATTT
ACAAAXXXXXXXXXXATTTAGCTTCACCACAAAATGCTTGTTGGTGTTGAGAATTAGACATTATGATTATAAATA
AAAATTAATAGTCAAAAGTC
ACTATATATCATCTATTATTGCCCAATATTGTTTCAACTTTTTAAAATTAAAAAGAAAAAGTGGAAAAATAATTT
ACAAAXXXXXXXXXXATTTAGCTTCACCACAAAATGCTTGTTGGTGTTGAGAATTAGACATTATGATTATAAATA
AAAATTAATAGTCAAAAGTC
>Marker561793
TACCAGATTGGTGACCTTTTATAGAAAACCAAAATTACATACACCATAGGTTGGTCTGGGCTCATTTGTTTAGGA
CCCAAXXXXXXXXXXTTTTTTTTTAAGCTCTAGTTGCCTTTTTTTTCACTTGCACTTAATCAGGCTCAAAAAATC
ATCGTAGATTTTTACATGTG
TACCAGATTGGTGACCTTTTATAGAAAACCAAAATTACATACACCATAGGTTGGTCTGGGCTCATTTGTTTAGGA
CCCAAXXXXXXXXXXTATTTTTTTAAGCTCTAGTTGCCTTTTTTTTCACTTGCACTTAATCAGGCTCAAAAAATC
ATCGTAGATTTTTACATGTG
>Marker561935
CACTTTGCTCTTCATTTCTTTATGGCACCTTCAAAGTTCTATAGATGTTTTGTCCGAGTTTTATGTATGGCTTGC
TGCATXXXXXXXXXXACTAGGAGAGGCCCCCATCTGCCATGCCCCATCTTCTGAACTTTCTCATCGTATAGATAT
ATCCGGTCTTTGATAAAGTT
CACTTTGCTCTTCATTTCTTTATGGCACCTTCAAAGTTCTATAGATCTTTTGTCCGAGTTTTATGTATGGCTTGC
TGCATXXXXXXXXXXACTAGGAGAGGCCCCCATCTGCCATGCCCCATCTTCTGAACTTTCTCATCCTATAGATAT
ATCCGGTCTTTGATAAAGTT
>Marker562045
ACTGTGAATAGGACTACTTACAGGGAGTGGCAAAATCTTTTCTCCCAAACCTCTCTTCACAAAATTCTGAATTGC
TTCTTXXXXXXXXXXCCATGTATCTTCACTTGGACCATAGCCTTTCCAACGCACCTGATGAAATAGAATATTTTA
TTATGAATGTAAAGAAAAGT
ACTGTGAATAGGACTACTTACAGGGAGTGGCAAAATCTTTTCTCCCAAACTTCTCTTCACAAAATTCTGAATTGC
TTCTTXXXXXXXXXXCCATGTATCTTCACTTGGACCATAGCCTTTCCAACGCACCTGATGAAATAGAATATTTTA
TTATGAATGTAAAGAAAAGT
>Marker562210

Create PDF with GO2PDF for free, if you wish to

ACTAATTCAAGAATCTAGAAGAGAATACTTCGATAAACATTCATAAAAAGCATCTAGAGCTCAAAAGATGTTGGC
TGGAGXXXXXXXXXXGAGTATAGTCTTTGTTTAAGTTTTAGCTTGTCGCCTTGTATTTCCCTAAGTTTTTTTTGC
TCTTAGTATAATACTCTTGT
ACTAATTCAAGAATCTAGAAGAGAATACTTCGATAAACATTCATAAAAAGCATCTAGAGCTCAAAAGATGTTGGC
TGGAGXXXXXXXXXXGAGTATAGTCTTTGTTTAAGTTTTAGCTTGTCGCCTTGTATTTCCCTAAGTTCTTTTTGC
TCTTAGTATAATACTCTTGT
>Marker562355
AACCGACTCAACATCTTTGTTAGACAGACTATGTTAAGACGTAGGCTATGTTGGGCCTTTGACCCTCAAACTAAG
ATAAGXXXXXXXXXXCTAGAATCAACGGGTAAGACGGACAATAAACTAGTTTTGAAGTGTCAATTTAAGTTTAAT
AAAAATTTAAATTCAAAGGT
AACCGACTCAACATCTTTGTTAGACACACTATGTTAAGACGTAGGCTATGTTGGGCCTTTGACCCTCAAACTAAG
ATAAGXXXXXXXXXXCTAGAATCAACGGGTAAGACGGACAATAAACTAGTTTTGAAGTGTCAATTTAAGTTTAAT
AAAAATTTAAATTCAAAGGT
>Marker562720
CACCTTTGATTGTCCTCCCCTGTCTTAAAGAGAATAGGCTATTCAGTTGAAATTAATTTTTCTTTTTTTCAAAAA
AGGATXXXXXXXXXXGAAATGTAACTAAAAAGAAATGCTTCTATACACTATGAAGATTTGAATATGATCTGTAAT
TGAATTGCATAGGGCAAGTA
CACCTTTGATTGTCCTCCCCTGTCTTAAAGAGAATAGGCTATTCAGTTGAAATTAATTGTTCTTTTTTTCAAAAA
AGGATXXXXXXXXXXGAAATGTAACTAAAAAGAAATGCTTCTATACACTATGAAGATTTGAATATGATCTGTAAT
TGAATTGCATAGGGCAAGTA
>Marker562727
TACATATGCAACCCAAAATTACTTTTAAATTGATACCTCTTGTTGGAACACTAGGTGTATTCATTGATTTGTTAG
GTTGTXXXXXXXXXXTTGAAATATCTAATTTGATTCTCGACATTACATTTAGATGCTCGTATATGACTTTTATGG
AGATACCTAAATTTATAGTG
TACATATGCAACCCAAAATTACTTTTAAATTGATACCTCTTGTTGGAACACTAGGTGTATTCATTGATTTGTTAG
GTTGTXXXXXXXXXXTTGAAATATCTAATTTGATTCTCGACATTACATTTAGATGCTCGTATATGACTTTTATGG
AGATACCTAAATTTATGGTG
>Marker562951
AACTAAACAAGAAACAATTAGACATCAGAAAACAAAATCAGAATGAGCATACCTCACTTCTAGATGAGGTGTCCA
TCATAXXXXXXXXXXATCCATCAAACTTCGACCTCATGAAGACATACATGCAAAAAAATAATTACAAAAAGTAAT
GTTGATCGGGATTGAATGTT
AACTAAACAAGAAACAATTAGACATCAGAAAACAAAATCAGAATGAGCATACCTCACTTCTAGATGAGGTGTCCA
TCATAXXXXXXXXXXATCCGTCAAACTTCGACCTCATGAAGACATACATGCAAAAAAATAATTACAAAAAGTAAT
GTTGATCGGGATTGAATGTT
>Marker562962
GACTTTAGCACAAATAATAATATTTAATTGAATCAAAATAGTTGATTTAATTCAATGATCACGATAAAAACACTT
TGTATXXXXXXXXXXTGCTAGAATGCCTCTAAGTGAGGTCAAGGAGTTTTCGACGTTAATAAGGATTATGGTTGG
ATATTCTCCCCATCTTGGTT
GACTTTAGCACAAATAATAATATTTAATTGAATCAAAATAGTTGATTTAATTCAATGATCACGATAAAAACACTT
TGTATXXXXXXXXXXTGCTAGAATGCCTCTAAGTGAGGTCAAGGGGTTTTCAACGTTAATAAGGATTATGGTTGG
ATATTCTCCCCATCTTGGTT
>Marker563008
ACTACAAATCGTGATCGGATTTTATCTTCGACCTCAGAAATTGGAGAGTGGCTGAAGCTCTGTTGGTGTTGTTTT
CATGTXXXXXXXXXXCTTATTTCACATTTTTTTTACTAGAAACATATTTTTTGGGGGTTAAATTTGCTTTATTTG
TCTTCTGTCGTCTCGATGTA

Create PDF with GO2PDF for free, if you wish to

ACTACAAATCGTGATCGGCTTTTATCTTCGACCTCAGAAATTGGAGAGTGGGTGAAGCTCTGTTGGTGTTGTTTT
CATGTXXXXXXXXXXXCTTATTTCACATTTTTTTTACTAGAAACATATTTTTTGGGGGTTAAATTTGCTTTATTTG
TCTTCTGTCGTCTCGATGTA
>Marker563087
ACTATTTTCTCTTTTGTCTTGATATATCTCAGGGATTACAAGCTTTTGGCATTAATGGTTCATTACTCAGGAAAC
ATTGAXXXXXXXXXXXTTGTGAAGCTTGAGAGGGGCATTGGTTATTTCTATTCAGCAGAGGGTCTCAATTCAAATG
ACGAGAATTCCGCTCTTGTT
ACTATTTTCTCTTTCGTCTTGATATATCTCAGGGATTACAAGCTTTTGGCATTAATGGTTCATTACTCAGGAAAC
ATTGAXXXXXXXXXXXTTGTGAAACTTGAGAGGGGCATTGGTTATTTCTATCCAGCAGAGGGTCTCAATTCAAATG
ACGAGAATTCCGCTCTTGTT
>Marker563110
CACACAATTAATCTCATGGAACAACCTGTTTGACTTTGCTAGACTTGTGGCATATTAAATCCTAAGAAGGTAACT
ATAATXXXXXXXXXXXATGCGCTTTCCTTCTTCAAAGTTACTTCCAAATGCAGTTCTCGAAATAACGTCTGCTGAG
AGACGACGAACTTCTTTGTG
CACACGATTAATCTCATGGAACAACCTGTTTGACTCTGCTAGACTTGTGGCATATTAAATCCTAAGAAGGTAACT
ATAATXXXXXXXXXXXATGCGCTTTCCTTCTTCAAAGTTACTTCCAAATGCAGTTCTTGAAATAACGTCTGCTGAG
AGACGACGAACTTCTTTGTG
>Marker563120
ACTGTTATTTATGTGGTGCGGATTATATGAGTGAAATGCATCAAATGTAGATAACTGTTTTATTGGTGTTGCGAT
CTTCCXXXXXXXXXXXGTGAGGGATGATTATTCTGCCCACTGGCTTGATAACTCAGATAACCTTTTGACAGATGCA
ACACGCTTACAGGTATGGTC
ACTGTTATTTATGTGGTGCGGATTATATGGGTGAAATGCATCAAATGTAGATAACTGTTTTATTGGTGTTGCGAT
CTTCCXXXXXXXXXXXGTGAGGGATGATTATTCTGCCCACTGGCTTGATAACTCAGATAACCTTTTGACAGATGCA
ACACGCTTACAGGTATGGTC
>Marker563164
ACTGGCAACACTTGTCCGGGCAAGTGCAGCATTTGAAAGATACTGCAAAAGGAGAATGTATTAGGAACTGCGATT
GAGAGXXXXXXXXXXXTTCCAGATTAACCTGATCCCATGTTAATTTGAGATTAACTACATCATCCACCAACTTTCC
TCAAACTACAACTAGTGAGT
ACTGGCCACACTTGTCCGGGCAAGTGCAGCATTTGAAAGATACTGCAAAAGGAGAATGTATTAGGAACTGCGATT
GAGAGXXXXXXXXXXXTTCCAGATTAACCTGATCCCATGTTAATTTGAGATTAACTACATCATCCACCAACTTTCC
TCAAACTACAACTAGTGAGT
>Marker563668
TACATGTAAATATTAGATATTGAAAAAAATTTAAGAATAACTTAGAAGATACATTCTTTATTTTCTTAGCATAGT
TATTAXXXXXXXXXXXAATTTAGGAAATTTTTTGAAAAGTAAGGTAGTTATAATGTTGCATTCATTTGGATGATAG
AAAGTTATATATATATGAGT
TACATGTAAATATTAGATATTGAAAAAAATTTAAGAATAACTTAGAAGATACATTCTTTATTTTCTTAGCATAGT
TATTAXXXXXXXXXXXAATTTAGGAAATTTTTTGAAAAGTAAGGTAGTTATAATGTTGCATTCATTTGAATGATAG
AAAGTTATATATATATGAGT
>Marker564514
AACTCTACTTCACCAGAATTAGGCTTGAGTTTGGAGTGTGTGGTAGAGATATCATGCTCACTGCCAAAAGTATCC
ATTTTXXXXXXXXXXXAGAAGCCTTGGCTTTCTCAATAAATTCATCATACTCCCCACATCGAAATGCCTTCACTCT
TTCAGACCTTTCAAGATTGT
AACTCTACTTCACCAGAATTAGGCTTGAGTTTGGAGTGTGTGGTAGAGATATCATGCTCACTGCCAAAAGTATCC
ATTTTXXXXXXXXXXXAGAAGCCTTGGCTTTCTCAATAAATTCATCATACTCCCCACATCGAAATGCCTTCACTCT
TTTAGACCTTTCAAGATTGT
>Marker565646

Create PDF with GO2PDF for free, if you wish to

AACGACCAAATAAAATTTTGGTCTTCTTTCACATCATGGCGTGCTTGAAGAATGGAAAGAGAGACCCTTTTAAAT
GTCTCXXXXXXXXXXCTCGGTTCATAGAAATGGGCATATACGATATGAAAAGAAAAGGGCTAAAATAATTTCACA
CGTTGAATTTTAATCTATGT
AACGACCAAATAAAATTTTGGTCTTCTTTCACATCATGGCGTGCTTGAAGAATGGAAAGAGAGACTCTTTTAAAT
GTCTCXXXXXXXXXXCTCGGTTCATAGAAATGGGCATATACGATATGAAAAGAAAAGGGCTAAAATAATTTCACA
CGTTGAATTTTAATCTATGT
>Marker566171
AACCTTCTTTTTACTAGTATTTACATCTGGTTTAGTGGCCGGATTTTTTTGGTGGAATGTCATCTTTCTTGACAA
GCTGTXXXXXXXXXXTCTTGCTCTTTTGACTGACTTCAATCTTAGATGCAAAGGAAGTAGCATTTGAAAGAAAAG
TGAGAAGATACATGTAAGTT
AACCTTCTTTTTACTAGTATTTACATCTGGTTTAGTGGCCAGATTTTTTTGGTGGAATGTCATCTTTCTTGACAA
GCTGTXXXXXXXXXXTCTTGCTCTTTTGAGTGACTTCAATCTTAGATGCAAAGGAAGTAGCATTTGAAAGAAAAG
TGAGAAGATACATGTAAGTT
>Marker566653
AACTAAAATGAAGAGATTTGTTAAGAATGAAGTTATTGTGTTAAGTTAGAAATTGATAGGAAGAGTAGTAGAAAG
CAATGXXXXXXXXXXGGTGGAATTTAGGACCCACCACACGCACACAACTTTAATTTAATGTTGTTTTATTTGCCT
TTGAGCAAATGGTTGAAGTG
AACTAAAATGAAGAGATTTGTTAAGAATGAAGTTATTGTGTTAAGTTATAAATTGATAGGAAGAGTAGTAGAAAG
CAATGXXXXXXXXXXGGTGGAATTTAGGACCCACCACACGCACACAACTTTAATTTAATGTTGTTTTATTTGCCT
TTGAGCAAATGGTTGAAGTG
>Marker566778
CACTCTTAATTAATCTCCCAACTGCATTAAGACTTATGTGATCAAGTCTTAAGTGCCATAAATAGGCGTTAGAAG
AAACCXXXXXXXXXXATAATTTCAAAATATATTCAATACATTTGTTCTAAAATACAAGAAATAGATATTAAATTT
CTCAAGATTTTTGAGAAGTA
CACTCTTAATTAATCTTCCAACCGCATTAAGACTTATGTGATCAAGTCTTAAGTGCCATAAATAGGCGTTAGAAG
AAACCXXXXXXXXXXATAATTTCAAAATATATTCAATACATTTGTTCTAAAATACAAGAAATAGATATTAAATTT
CTCAAGATTTTTGAGAAGTA
>Marker566930
AACCCTCAGCTGCCACCGTCTCTTCATTCCGACGAAGTCACCGCCATTCTCTTCTTCTTTCACTCTCTCGCCTCT
CTGATXXXXXXXXXXGAAGAAAATGAAGAACTGTTTGTTCTCTTCATTGGATTTCTTCTTCAAATTAGTGTTAAT
GAAAATATAAAAATAATGTA
AACCCTCAGCTGCCACCGTCTCTTCATTCCGACGAAGTCACCGCCATTCTCTTCTTCTTTCACTCTCTCGCCTCT
CTGATXXXXXXXXXXGAAGAAAATGAAGAACTGTTTGTTCTCTTCATTGGATTTCTTCTTCAAATTAGTGTTAAT
GAAAATATAAAAGCAATGTA
>Marker566944
ACTCATAGAAAACATAATGCATATTTTTTATATATATCCTCTTTACCTGAAATTGAGAAACTTGTGAAGTTGTTC
CATTGXXXXXXXXXXGTTGTTAATGATGAAGGATTTGAGGAGCTTCCTGATTCCATCTCCAAACATAGCTGCATC
CCCAAATCAAATATAGTGTT
ACTCATAGAAAACATAATGCATATTTTTTATATATATCCTCTTTACCTGAAATTGAGAAACTTGTGAAGTTGTTC
CATTGXXXXXXXXXXGTTGTTAATGATGAAGGATTTGAGGAGCTTCCTGATTCCATCTCCAAACATAGCTGCATC
TCCAAATCAAATATAGTGTT
>Marker567290
GACATGGTTGGTATTCGTATTTCAATTACTTTCCTTTATCATTGTCCTATCGCATTGATAAACATTTTCCAGTGT
CAGTTXXXXXXXXXXGAGAATATTGAGAGTCAAATTTGTTATGGGTTTGTTTGAGAACCCATTAGCTGATTTAAG
CTTGATTAATGAGCTTGGTA

Create PDF with GO2PDF for free, if you wish to

GACATGGTTGGTATTCGTATTTCAATTACTTTCCCTTATCATTGTCCTATCGCATTGATAAACATTTTCCAGTCT
CAGTTXXXXXXXXXXGAGAATATTGAGAGTCAAATTTGTTATGGGTTTGTTTGAGAACCCATTAGCTGATTTAAG
CTTGATTAATGAGCTTGGTA
>Marker567482
AACCACTCAAAATTTTGCAACATTTTGAGTCTTACTTATTCGGCAATATGAGAGTCTTATATATCATTATCGCTT
TTCAAXXXXXXXXXXAAAACCAATATTCTAAGCAAAAAAAACAATTCCACTACACCTCATCACAACGCCAAGA
TGACGTTCTTACAAACAGTA
AACCACTCAAATTTTTGCAACATTTTGAGTCTTACTTATTGGGCAATATGAGAGTCTTATATATCATTATCGCTT
TTCAAXXXXXXXXXXAAAACCAATATTCTAAGCAAAAAAAACAATTCCACTACACCTCATCACAACGCCAAGA
TGACGTTCTTACAAACAGTA
>Marker567527
AACCATTATTTATGTAATGACTACATATCCTAGCGGGAATAAGCGGAGTATGCATTGACAATTGTCTTGAACATA
CAATAXXXXXXXXXXTAGAGGAAATCCTGGAATTGAATCTACACATTACTGTTTATCCAGAAAAAGAAAAGGCAC
ATATATTTATGGAAACAGTC
AACCATTATTTATGTAATGACTACATATCCTAGCGGGAATAAGCGGAGTATGCATTGACAATTGTCTTGAACATA
CAGTAXXXXXXXXXXTAGAGGAAATCCTGGAATTGAATCTACACATTACTGTTTATCCAGAAAAAGAAAAGGCAC
ATATATTTATGGAAACAGTC
>Marker567739
TACTTGAATCTTTGGTTTAACACATCCTCACAATTTTTCCCCATTGCTCAGTTGATATGTCCATCAAAGAGGGAG
GTGATXXXXXXXXXXTCAACATGGGGCTCTGAGGGTGGCTGCTGTTGTGCAGCTTGTTTGAGCAATGGTATGTAT
GTATTTATTTTTGTGATGGT
TACTTGAATCTTTGGTTTAACACATCCTCACAATTTGTCCCCATTGCTCAGTTGATATGTCCATCAAAGAGGGAG
GTGATXXXXXXXXXXTCAACATGGGGCTCTGAGGGTGGCTGCTGTTGTGCAGCTTGTTTGAGCAATGGTATGTAT
GTATTTATTTTTGTGATGGT
>Marker567944
CACGCTTACACCCACTCATACACATCCTTTTATCTACATTTTTAATCACGAAAGCTTTGCTTTACAATTGAACCT
AGAACXXXXXXXXXXCAATCGGGTTGGGATTTTCAGATTTCTATTTCTGTGTGAGTATTTGAGTGGTTCTATTCG
TGATTGTGAAGACATGTGGT
CACGCTTACACCCACTCATACACATCCTTTTATCTACATTCTTAATCACGAAAGCTTTGCTTTACAATTGAACCT
AGAACXXXXXXXXXXCAATTGGGTTGGGATTTTCAGATTTCTATTTCTGTGTGAGTATTTGAGTGGTTCTATTCG
TGATTGTGAAGACATGTGGT
>Marker568090
AACAAAAGTTTTTCTTCTTGAACTAACACATCAATTTTAGTTGCTAAATGTTGTAAATTAATCTTTTTCGAAATT
TATAAXXXXXXXXXXGTCTTACACTCTCATAGTTTGTAATATTTTGATACATTCTTTAGTTTCTTATGTTTTGTA
TTAAAGCACTCTTGTTTGTG
AACAAGAGTTTTTCTTTTTGAACTAACACATCAATTTTAGTTGCTAAATGTTGTAAATTAATCTTTTTCAAAATT
TATAAXXXXXXXXXXGTCTTACACTCTCATAGTTTGTAATATTTTGATACATTCTTTAGTTTCTTATGTTTTGTA
TTAAAGCACTCTTGTTTGTG
>Marker568273
CACTTGGCTGTGATTCTTTGTTCCTTTTTTCTGACTTTTTCATCTTTTGAATAAGAATTTCTAGTTTATTTTTTT
AAAGAXXXXXXXXXXTTGATGGAAGAGAACTTCTTTGGAGGAACTCATTTGGGCTACAATCATAAATCATCTTAG
ACCAGGAATTCTGCTTGGTC
CACTTGGCTGTGATTCGTTGTTCCTTTTTTCTGACTTTTTCATCTTTTGAATAAGAATTTCTAGTTTATTTTTTT
AAAGAXXXXXXXXXXTTGATGGAAGAGAACTTCTTTGGAGGAACTCATTTGGGCTACAATCATAAATCATCTTAG
ACCAGGAATTCTGCTTGGTC
>Marker568490

Create PDF with GO2PDF for free, if you wish to

CACTGCGATTCTTGCCCTTTAAGGTGGAAAAAATGTTTAGGAGGGAACATTAAGGAAGAAGTATCTTATTTTAAT
GAACAXXXXXXXXXXXCCCAACACCAAAAAGTGCATTACAAGAAAATCTTCAATTTAAAAGAAGAGAGGAAAGATG
GTAATTGGTAAAGGGGTGTA
CACTGCGATTCTTGCCCTTTAAGGTGGAAAAAATGTTTAGGAGGGAACATTAAGGAAGAAGTATCTTATTTTAAT
GAACAXXXXXXXXXXXCCCAACACCAAAAAGTGCATTACAAGAAAATCTTCAATTTAAAAGAAGAGAGGAAAGATG
GTAATTGGTAAAAGGGTGTA
>Marker568991
AACATCAGCTTCCAAAGGACATTGAGATGTGATGAATGTTTCCTTCAATATAGCCTTCTCTCTCTCCATGTTTGC
ATCCAXXXXXXXXXXXTTAAAGCAACCGTAAATGGTATTCTCCTGCAAATGGCCAAGCTCATCCCTTGAGAAAACT
TTATTAGGAGGTTTAATGTT
AACATCAGCTTCCAAAGGACATTGAGATGTGACGAATGTTTCCTTCAATATAGCCTTCTCTCTCTCCATGTTTGC
ATCCAXXXXXXXXXXXTTAAAGCAACCGTAAATGGTATTCTCCTGCAAATGGCCAAGCTCATCCCTTGAGAAAACT
TTATTAGGAGGTTTAATGTT
>Marker569378
TACCACAATTTTCATTGTTCTTGTGGATTTACTCCTCTACTTGAGGACTGTTTTCTTTTTCTCTCCATATAAATC
TACCCXXXXXXXXXXXTTTAGAGTGAAAATGTGAATATAATTACACTTGCACATTTCTATTAAACATATATTAAGA
ACGTAACCATTTTTATTTGT
TACCACAATTTTCATTGTTCTTGTGGATTTACTCCTCTACTTGAGGACTGTTTTCTTTTTCTCTCCATATAAATC
TACCCXXXXXXXXXXXTTTAGAGTGAAAATGTGAATATAATTACACTTGCACATTTCTATTTAACATATATTAAGA
ACGTAACCATTTTTATTTGT
>Marker570073
TACGCATATCTCGACTAATCTCACAGGACAACCTGCTTAACTCTACAACATTAGGGTGTCAAGGTAACTCATAGA
AAATTXXXXXXXXXXXTGAAAATAGGCCAATGCTCCAAGATAGGAGAGGAAAATCAATTACACAAAACTATTAATC
CATTTGGTGATGAAAATGTT
TACGCATATCTCGACTAATCTCACAGGACAACCTGCTTAACCCTACAACATTAGGGTGTCAAGGTAACTCATAGG
AAATTXXXXXXXXXXXTGAAAATAGGCCAATGCTCCAAGATAGGAGAGGAAAATCAATTACACAAAACTATTAATC
CATTTGGTGATGAAAATGTT
>Marker570172
ACTTTTGTTTGAAAGCCTTGAGAATCTCACCCACAAAATTGTTTCTACAACCATAGCTTTGATATTAATTGATTT
GGATAXXXXXXXXXXXTGATCTTCTGCTACGAACTTAAATCTTTAACATCTTAACCTCTATCTAGGAGAAAGCATC
GATTGGTCAGAGTCGAAGTC
ACTTTTGTTTGAAAGCCTTGAGAATCTCACCCACAAAATTGTTTCTACAACCATAGCTTTGATATTAATTGATTT
GGATAXXXXXXXXXXXTGATCTTCTGCTACGAACTTAAATCTTTAACATCTTAACCTCTATCTAGGAGCAAGCATC
GATTGGTCAGAGTCGAAGTC
>Marker570325
AACTTGATTTGATTCCTTTATTGTTTTGTTCTGATTTGATTCCTTTATTGTTTTGTTCTCTTTTCGAAATCACAG
TTTAAXXXXXXXXXXXTGACAAAGTTTTCATCAATTATCAGCTTTTTTATTTTATGATTGTCTGAGGGTATATCCA
ATTACAATGCCTTTTCTGTT
AACTTGATTTGATTCCTTTATTGTTTTGTTCTGATTTTATTCCTTTATTGTTTTGTTCTCTTTTCGAAATCACAG
TTTAAXXXXXXXXXXXTGACAAAGTTTTCGTCAATTATCAGCTTTTTTATTTTATGATTGTCTGAGGGTATATCCA
TTTACAATGCCTTTTCTGTT
>Marker570797
CACTTTAATTTTGGGCCAGACTCTCAATTTATGTTTCTGATTTTGACGCTCATGCTCTTATCAAGGATCTGCCTT
TGTAAXXXXXXXXXXXATGTTTCTTTCTACATCACTAGGGTTGTGATCAGCCATTGATTGTTCGTCTGGCAGACCC
TAAGAAACCCAGGATTGGTG

Create PDF with GO2PDF for free, if you wish to

CACTTTAATTTTGGGCCAGACTCTCAATTTATGTTTCTGATTTTGACGCTCATGTTCTTATCAAGGATCTGCCTT
TGTAAXXXXXXXXXXXATGTTTCTTTCTACATCACTAGGGTTGTGATCAGCCATTGATTGTTCGTCTGGCAGACCC
TAAGAAACCCAGGATTGGTG
>Marker570971
AACTCATGTTTCTTTATAATCCAAATAATCTTAAATAGGTCTCATTACTTTGCATCATTACTCCAAATCTAGCTA
AACATXXXXXXXXXXXAGATAAACATCGTTTGATTTTTATCTAAAATAAGAAATTATTTAGTCGTATAATAAATCA
ATTCTATCTAATTAAGTGTA
AACTCATGTTTCTTTAAAATCCAAATAATCTTAAATAGGTCTCATTACTTTGCATCATTACTCCAAATCTAGCTA
AACATXXXXXXXXXXXAGATAAACATCGTTTGATTTTTATCTAAAATAAGAAATTATTTAGTCGTATAATAAATCA
ATTCTATCTAATTAAGTGTA
>Marker571673
ACTATAGATTCTCAATGGTGCTTTGATTTTACTATGTTGTTTATATGCCCTTATAATATTGCACAAAATGGGAAG
AGTGTXXXXXXXXXXXCAAAATGCAGATTGGGAGACATGTGTAGCTGTGACCGATTTTGACTGGAAACAGATTGCA
GAACCCGTTATGCAATTGTA
ACTATAGATTCTCAATGGTGCTTTGATTTTACTATGTTGTTTATATGCCCTTATAATATTGCACAAAGTGGGAAG
AGTGTXXXXXXXXXXXCAAAATGCAGATTGGGAGACATGTGTAGCTGTGACCGATTTTGACTGGAAACAGATTGCA
GAACCCGTTATGCAATTGTA
>Marker572333
GACCAATACTTCGAAGACCTAATGTTCAACAATCCCATCTACAAGAAAATTGAAAATGGATAATGTTTTGAGGGG
CCATGXXXXXXXXXXXGGTAGATGGCTCGTCTCCCTTCCTTCAATTCGTGCATTTTGGCTCCTTTTGATTACCAAT
GAATCCTTGTTTGTTCAGTA
GACCAATACTTCGAAGACCTAATGTTCAACAATCCCATCTACAAGAAAATTGAAAATGGATAATGTTTTGAGGGG
CCATGXXXXXXXXXXXGGTAGATGGCTCGTCTCCCTTCCTTCAATTCGTGCATTTTGGCTCCTTTTGATTACTAAT
GAATCCTTGTTTGTTCAGTA
>Marker572516
AACTCTTCTAGATTTCCATATGCCCCTAATGAACTGAGAATCAAATTTGCCCCTTTAAGTCTCTGTTGGGATCAC
GTGTGXXXXXXXXXXXAATACAAGATTATCTAATTTTGGTGACTCCCTTCCTAATCTCACTCTTTGTTGAGTTTTA
TTTTCCTTTTCTGAAACGTA
AACTCTTCTAGATTTCCATATGCCCCTAATGAACTGAGAATCAAATTTGCCCCTTTAAGTCTCTGTTGGGATCAC
GTGTGXXXXXXXXXXXAATACAAGATTATCTAATTTTGGTGACTCCCTTCCTAATTTCACTCTTTGTTGAGTTTTA
TTTTCCTTTTCTGAAACGTA
>Marker572606
TACCCAGCAATGTATATAGAGAATGTTACCGAGCTTGCTTTTGATGATATTACACCCCAAGACCCTGATTTTGCA
TCTATXXXXXXXXXXXTCTTCTTCATTGGACGAAGACCAGGGTCCCTTATATTTCTCTCCTGAAAGGTTATTTCCT
CTTTATCCTTTATTCTGGTT
TACCCAGCAATGTATATAGAGAATGTTACCGAGCTTGCTTTTGATGATATTACACCCCAAGACCCTGATTTTGCA
TCTATXXXXXXXXXXXTCTTCTTCATTGGACGAAGACCAGGGTCCCTTATATTTCTCTCCTGAAAGGTTATTTCCA
TTTTATCCTTTATTCTGGTT
>Marker572638
ACCGAAGCTCTAAAGAATTGAATATTCAAACTTCTACAAGCTTTCTAGACGTGCAAGTTCGTTTCAACCTCGAGA
TCAACXXXXXXXXXXXTACTAGAGACTGTATTCACAATATTCATCAATATAACAAAGTTCAATTTCACGAAATACG
TTTCTTTGAAATCTCGTGTG
ACCGAAGCTCTAAAGAATTGAAGATTCAAACTTCTACAAGCTTTCTAGACGTGCAACTTCGTATCAACCTCGAGA
TCAACXXXXXXXXXXXTACTAGAGACTGTATTCACAATATTCATCAATATAACAAAGTTCAATTTCACGAAATACG
TTTCTTTGAAATCTCGTGTG
>Marker573381

Create PDF with GO2PDF for free, if you wish to

ACTTCTTCATCACATATTCCGAGAGCTTAACCTGAGCTGTGGTTATTTCAGATGTGGAATTCAAAACACTCCACA
TCCGTXXXXXXXXXXGAGTATTGTTAGCAGCCCAATCAAAATAGCTAGCATCACCTGTATTTTAATGTAATAAAT
CATCAAAGCCAGTATTAGTG
ACTTCTTCATCACATATTCTGAGAGCTTAACCTGAGCTGTGGTTATTTCAGATGTGGAATTCAAAACACTCCACA
TCCGTXXXXXXXXXXGAGTATTGTTAGCAGCCCAATCAAAATAGCTAGCATCACCTGTATTTTAATGTAATAAAT
CATCAAAGCCAGTATTAGTG
>Marker573624
GACTATTTGGAATGCCTTGTGGAGGAATTTTTTTCAAAAAGCAATTCCCATTTGACTCCACCAAACAAAGACCGG
TCTCCXXXXXXXXXXCTGTTGCTGTGGCATCAGGCTGAAGGGCTTCTGCTTCCATCCCTCATTGTTGATTGGGTG
CTGGGACAACTTGAGGTGTG
GACTATTTGGAATGCCTTGTGGAGGAATTTTTTTCAAAAAGCAATTCCCATTTGACTGCACCAAACAAAGACCGG
TCTCCXXXXXXXXXXCTGTTGCTGTGGCATCAGGCTGAAGGGCTTCTGCTTCCATCCCTCATTGTTGATTGGGTG
CTGGGACAACTTGAGGTGTG
>Marker573723
ACAAAAGTTCAAGGTATATTTCTATCGTTTATTCCAAATACCAAAACTTAAAAAAAATTGTATTTATGTGATTAC
TTAATXXXXXXXXXXTAAAAGGGACATTTAAGTTCAAAGATTAACCTTGTAGTTTAATCTGTATTTTAAAGTATC
TTTGCTACTTTACATAGGTA
ACAAAAGTTCAAGGTATATTTCTATCGTTTATTCCAAATACCAAAACTTAAAAAAAATTGTATTTATGTGATTAC
TTAATXXXXXXXXXXTAAAACGAACATTTAAGTTCAAAGATTAACCTTGTAGTTTAATCTGTAATTTAAAGTATC
TTTGCTACTTTACATAGGTA
>Marker574117
AACCGATGAATTTTATCGTAATTATGATACTGATCTTTTGAGTATCGGTTTCTAAATAAAATATATAATATTGAT
GCTTTXXXXXXXXXXCTTAAATTTTAGTAGGGATCAGGCCAAATGGAGTAATTAACACGAGAATTTCTGTATGGA
TGATCAAGTTTAGAGTTGTT
AACCGATGAATTTTATCGTAATTATGATACTGATCTTTTGAGTATCGGTTTCTAAATAAAATATATAATATTGAT
GCTTTXXXXXXXXXXCTTAAATTTTAGTAGGGATCAGGCCAAATGGAGTAATTAACATGAGAATTTCTGTATGGA
TGATCAAGTTTAGAGTTGTT
>Marker574220
ACTAAAAAAGATTCTACCCCACGTATTGAAGTTAGTTTGTAGCAATCTAAAAGTAATGTGACAAAAAAAATGTAT
CAAACXXXXXXXXXXTCTTGATGGTTAAATTTGTCACCCTTAACAAAAAAACTTTGTTATGTTAGTGTTAATTAA
CTTTTGTATATTAGGTTGTA
ACTAAAAAAGATTCTACCCCACGTATTGAAGTTAGTTTGTAGCAATCTAAAAGTAATGTGATAAAAAAAATGTAT
CAAACXXXXXXXXXXTCTTGATGGTTAAATTTGTCACCCTTAACAAAAAAACTTTGTTATGTTAGTGTTAATTAA
CTTTTGTATATTAGGTTGTA
>Marker575257
AACTTTATAAATAAACAAACATCTTGTAAAAGGAAAAAGAAAAAAGAAAAGCTACTTGATGATATAAGCTAAGTG
AGAACXXXXXXXXXXTCAACGAAGTTTCTGTTCTTACTTACATACAAAAGGATCTTCCCTATATTTAACTAATCT
GGAAACCATGTGCAGTTGTA
AACTTTATAAATAAACAAACATCTTGTAAAAGGAAAAAGAAAAAAGAAAAGCTACTTGATGATATAAGCTAAGTG
AGAACXXXXXXXXXXTCAACGAAGTTTCTGTTCTTACTTACATACAAAAGGATCTTCCCTACATTTAACTAATCT
GGAAACCATGTGCAGTTGTA
>Marker575607
AACTTTTAATTTTGTTCTATTCTTTACTTATCTCCTTCCCATTGCTCTGATATGTAGCATACCTTAGGCAACATT
TACCGXXXXXXXXXXTTTGTCAGGGATTGAACCAAATCTTTGAAATTGTTGTAGTGAAATTTTATTACTAATGAA
ACCATATCAGTAACGATGTC

Create PDF with GO2PDF for free, if you wish to

AACTTTTAATTTTTTTCTATTCTTTACTTATCTCCTTCCCATTGCTCTGATATGTAGCATACCTTAGGCAACATT
TACCGXXXXXXXXXXXTTTGTCAGGGATTGAACCAAATCTTTGAAATTGTTGTAGTGAAATTTTATTACTAATGAA
ACTATATCAGTAACGATGTT
>Marker575770
AACAAGTATGAAAAAACATACATAAAATCATACCATAACACACACCTAAAGAGTTATATTTTGATTTCCTTAGTT
GTTGGXXXXXXXXXXXATACTGGGATAGACGTTTATGATTGTTTGGGCATTATTGAGTGTTCATTTGGCACGAGCA
GCACGTGGCAAATTGGTTGT
AACAAGTATGAAAAAACATACATAAAATCATACCATAACACACACCCAAAGAGTTATATTTTGATTTCCTTAGTT
GTTGGXXXXXXXXXXXATACTGGGATAGACGTTTATGATTGTTTGGGCATTATTGAGTGTTCATTTGGCACGAGCA
GCACGTGGCAAATTGGTTGT
>Marker575863
ACTACCCTTCTGAAAAGAGTCATTTTCATATTAACAAAATTACTTTATCATTTTCAAAACCATTCCAAACATATT
CACACXXXXXXXXXXXATATTGAGTAGAAAGGTAATAATTTGAAAAGAAACATAAGAGTTTGGGTATTAGTGATGA
AGAATTGTTCAAAAGTGGTG
ACTACCCTTCTGAAAAGAGTCATTTTCATATTAACAAAATTACTTTATCATTTTCAAAACCATTCCAAACATATT
CACACXXXXXXXXXXXATATTGAATAGAAAGGTAATAATTTGAAAAGAAACATAAGAGTTTGGGTATTAGTGATGA
AGAATTGTTCAAAAGTGGTG
>Marker576004
ACATCAAAATGTGAATATTTACAAACTTATCGTCTTATAATTAAAAGTATAATTAGAAACGGTTGAAATTTTGTA
TATCAXXXXXXXXXXXTTGTAAATTTATCCTGAAGTTAGTTTAGTGGTATTAGACATGCTTCTAGTTGTTGGCTCA
ATTTTTCATTCCATGGTGGT
ACATCAAAATGTGAATATTTACAAACTTATCGTCTTATAATTAAAAGTATAATTAGAAACGGTTGATATTTTGTA
TATCAXXXXXXXXXXXTTGTAAATTTATCCTGAAGTTAGTTTAGTGGTATTAGACATGCTTCTAGTTGTTGGCTCA
ATTTTTCATTCCATGGTGGT
>Marker576482
CACATTTAATTTTTTTTCTTATACAAATTTGATTTTTTAATACATGTGTTGGTGAAACAAATTTATTACCTAAAA
GATATXXXXXXXXXXXACTTAGGTTTTTGAGACATGAAATTCAACCGATTTACTAGTAAAATATATTTTTACGTGT
TTGAATGCCAAAAACATGTT
CACATTTAATTTTTTTTCTTATACAAATTTGATTTTTTAATACATGTGTTGGTGAAACAAATTTATTACCTAAAA
GATATXXXXXXXXXXXACTTAGGTTTTTGAGACATGAAATTCAACCGATTTACTAGCAAAATATATTTTTACGTGT
TTGAATGCCAAAAACATGTT
>Marker576684
AACATTGGATTTTCGTGATTTATAGCCTGTTGCTGTAGTCTGAGAAGTATTTAGTATTCCTCTTCAATGGCGCCA
TATATXXXXXXXXXXXTTGTCTTACCAAAACTTAGCACCTCCAAGATACCATGTGGTTTTCTGTTGCTATTTTTGG
ATTGGAATACAAACAATGTG
AACATTGGATTTTCGTGATTTATAGCCTGTTGCTGTAGTCTGAGAAGTATTTAGTATTCCTCTTCAATGGCGCCA
TATATXXXXXXXXXXXTTGTCTTACCGAAACTTAGCACCTCCAAGATACCATGTGGTTTTCTGTTGCTATTTTTGG
ATTGGAATACAAACAATGTG
>Marker576880
AACGCGTCAAGAGAAGTTAAATATATTAAAAAAGTTATAATCTTCTTTCAACACCATACATAACAACATCCCGAG
AAGTTXXXXXXXXXXXTCGTATGTCAAAAAATACCTATTTTCTTATAGTGGTGTTGGGTGAGAAAATTGAAACCTT
GGTTGTTTAAGTTTGATAGT
AACGCGTCAAGAGAAGTTAAATATATTAAAAAAGTTATAATCTTCTTTCAACGCCATACATAACAACATCCCGAG
AAGTTXXXXXXXXXXXTCGTATGTCGAAAAATACCTATTTTCTTATAGTGGTGTTGGGAGAGAAAATTGAAACCTT
GGTTGTTTAAGTTTGATAGT
>Marker577667

Create PDF with GO2PDF for free, if you wish to

GACCTAAAATGTGAAGCGCTGGAGCAAAATCGACATCACCAGATATGAGCATTATGGATGATGGGGGAGGGTTAT
CTAGGXXXXXXXXXXCCTTCTCTCAGTCGTCTAGGAAAAGCATTGAAATCCCCATATGCAGAAAACATCATAACT
GCTCCTTGTATTACAGGGTG
GACCTAAAATGTGAAGCGCTGGAGCAAAATCGACATCTCCAGATATGAGCATTATGGATGATGGGGGAGGGTTAT
CTAGGXXXXXXXXXXCCTTCTCTCAGTCGTCTAGGAAAAGCATTGAAATCCCCATATGCAGAAAACATCATAACT
GCTCCTTTTATTACAGGGTG
>Marker577769
ACCTCAGGACAATACTCTCGTATCCCATCGTTATTGTGCTCCCTTGGAACAGAACTGTTCAAGCTTTGAGTTGCA
ATGAAXXXXXXXXXXAACAATCCAGTGACCCATCAACAACGAAAGAAGTCCAAAGAGCATCAGCTCTGCTCAAGA
AAACAAAACCCATAAACGTA
ACCTCAGGACAATACTCTCGTATCCCATCGTTATTGTGCTCCCTTGGAACAGAACTGTTCAAGCTTTGAGTTGCA
ATGAAXXXXXXXXXXAACAATCCAGTGACCCATCAACAACGAAAGAAGTCCAAAGAGCATCAGCTCTGCTCAAGA
AAACAAAACCCAGAAACGTA
>Marker577846
TACACGTGCAATTGTTGCACGTAATTTGTCTATTTAAAGGGTGAATTTTCAATCGTAGGTAACTTCTTTACTCAC
TATTTXXXXXXXXXXTCATGCTAGGGTGGAGGTTTGAGTGATTATGGTCCTACGAATGTGAGTTATCCCTAACTC
TAATTGTAATCTAATTAAGT
TACATGTGCAATTGTTGCACGTAATTTGTCTATTTAAAGGGTGAATTTTCAATCGTAGGTAACTTCTTTACTCAT
TATTTXXXXXXXXXXTCATGCTAGGGTGGAGGTTTGAGTGATTATAGTCCTACGAATGTGAGTTATCCCTAACTC
TAATTGTAATCTAATTAAGT
>Marker577879
CACAATTTATGTATAGGAGTTCTCCAGAGTTGAAAAAATGAACAATATTCACCTTCCCCATTAATTTAGGCTGGA
AGCTTXXXXXXXXXXCCTTTCTTAACCATAATAATAATAATAACCAATAAATAAAAATTATGGTTCCAAATAAAT
TGATAAATCATGGAATTGTT
CACAATTTATGTATAGGAGTTCTCCAGAGTTGAAAAAATGAACAATATTCACCTTCCCCATTAATTTAGGCTGGA
AGCTTXXXXXXXXXXCCTTTCTTAACCATAATAATAATAATAACCAATAAATAAAAATTATGGTTCCAAATACAT
TGATAAATCATGGAATTGTT
>Marker578350
ACTTAATTTCAGTTTTTCTCCTTACATTTTAAACATTTTTATTATAATAGAAATTGCTTAAAGAGACCCTTTCTT
GTGTAXXXXXXXXXXAATATATCTTCTCATGACCATTCATCAAAATGTTACAAAGTCTATTGAAAGAAAATGAAA
AATTACATTTTATTTACTGT
ACTTAATTTCAGTTTTTCTCCTTACATTTTAAACATTTTTATTACAATAGAAATTGCTTAAAGAGACCCTTTCTT
GTGTAXXXXXXXXXXAATATATCTTCTCATGACCATTCATCAAAATGTTACAAAGTCTATTGAAAGAAAATGAAA
AATTACATTTTATTTACTGT
>Marker579205
TACTTTTACTTCTCAATTATATTACACATATTCGTGTAATTGATATATATGCAACAATGAACCTGAAGTTCATTG
ATCCAXXXXXXXXXXTTAAACTCTCACTAGCGAAAATTGAAATTAAAACTTTCATGATTTTGGAAGATCAGATTC
ATGGTTTATTAATTCTTGTT
TACTTTTACTTCTCAATTATATTACACATATTCGTGTAATTGATATATATGCAACAATGAACCTGGAGTTCATTG
ATCCAXXXXXXXXXXTTAAACTCTCACTAGCGAAAATTGAAATTAAAACTTTCATGATTTTGGAAGATCAGATTC
ATGGTTTATTAATTCTTGTT
>Marker579342
TACTATGAAATCTATGATTGATGACTTGACAGGTGCTCTCGAGCCGTCTGAAAGGGATTTTTATTTGATCATTTG
GATGGXXXXXXXXXXTGAATCCTCTGCTCATCTATTTATTCAATGGCCTTTTGCTCGACAATTTTGGGATCTCAT
TTTGGGGCTTTTTGTTGGTC

Create PDF with GO2PDF for free, if you wish to

TACTATGAAATCTATGATTGATGACTTGATAGGTGCTCTCGAGCCTTCTGAAAGGGATTTTTATTTGATCATTTG
GATGGXXXXXXXXXXTGAATCCTCTGCTCATCTATTTATTCAATGGCCTTTTGCTCGACAATTTTGGGATCTCAT
TTTGGGGCTTTTTGTTGGTC
>Marker579512
CACCCTTGTCCCTATTTGATTTACCAGTAGAAAATTAGAAAAAAATAGATTTTGCCAAGTGGATCACCGTAACAT
TTTTCXXXXXXXXXXACTTTTTCCACTGTTTCTATCACACAACGATATCCACAGTTAATTTTGATAATTCTAGAA
AATTCCTTAACTCAAAGGTT
CACCCTTGTCCCTATTTGATTTACCAGTAGAAAATTAGAAAAAAATAGATTTTGCCAAGTGGATCACCGTAACAT
TTTTCXXXXXXXXXXACTTTTTCCACTGTTCCTATCACACAACGATATCCACAGTTAATTTTGATAATTCTAGAA
AATTCCTTAACTCAAAGGTT
>Marker579731
AACTTCTTGAGTTAGGGTTTGTGTTAATTTCTATGGTTTATCAATTGATACGGGTTTATGAATTGATGGTTCGTG
AAATTXXXXXXXXXXAGTGGATTTATTTTTTCCGAGCTCAATCCTCTGAAATCTAGTTTTCATGGATTTCCATTA
TGTAGAGTGTTTTCCTTTGT
AACTTCTTGAGTTAGGGTTTGTGTTAATTTCTATGGTTTATCGATTGATACGGGTTTATGAATTGATGGTTCGTG
AAATTXXXXXXXXXXAGTGGATTTCTTTTTTCCGAGCTCAATCCTCTGAAATCTAGTTTTCATGGATTTCCATTA
TGTAGAGTGTTTTCCTTTGT
>Marker579929
AACCAATTGAAAGAGAGATTTACCAATTTTGAGACAAAGGTTGAGCAAAGCCGGTCAAATTAGGAGTCATCCTCT
TCAAAXXXXXXXXXXAACTCAAACTTCAAAGCATTCATTCATTTAAAATACTAAATGAGTTGATTATTACAGTCT
TAGATAATTTTTTAGTCGTA
AACCAATTGAAAGAGAGATCTACCAATTTTGAGACAAAGGTTGAGCAAAGCCGGTCAAATTAGGAGTCATCCTCT
TCAAAXXXXXXXXXXAACTCAAACTTCAAAGCATTCATTCATTTAAAATACTAAATGAGTTGATTATTACAGTCT
TAGATAATTTTTTAGTCGTA
>Marker580275
CACAAAATCAAACTTTCAGTTAGACATGGTGGATATGAGCAATGTAATTATGGATTACTTGGGTAATTGAATAAA
TCTGAXXXXXXXXXXGGGTTTTGTGTTGGAGAAAGGGAGTGGGAAGAAATGTTATATGATAGGAGCAAGAGATCT
GGAGATTGTTTGGGGGAGTT
CACAAAATCAAACTTTCAGTTACACATGGTGGATATGAGCAATGTAATTATGGATTACTTGGGTAATTGAATAAA
TCTGAXXXXXXXXXXGGGTTTTGTGTTGGAGAAAGGGAGTGGGAAGAAATGTTATATGATAGGAGCAAGAGATCT
GGAGATTGTTTGGGGGAGTT
>Marker580667
GACAATTTTATATCTTGAATGCATGCATATCATACATGCAGCTCCATCATGCTATGAAGATTATAAAAAGGAATA
TGAGGXXXXXXXXXXATCCTTTGTTCGAATTGTCAGAGACGCTGTAAAATCTGCTTTCTGCAGAATGATGGTGAT
TGAGTGTGCTAAAGAATGTC
GACAATTTTATATCTTGAATGCATGCATATCATACATGCAGCTCCATCATGCTATAAAGATTATAAAAAGGAATA
TGAGGXXXXXXXXXXATCCTTTGTTCGAATTGTCAGAGACGCTGTAAAATCTGCTTTCTGCAGAATGATGGTGAT
TGAGTGTGCTAAAGAATGTC
>Marker580760
AACTCTACCCTACAGACTGTTCTTCCCTTTGAAAAGATATATATTTGATACGATATTAAGCATTACTTCCTTTTA
ACTCTXXXXXXXXXXTTCTTTCAAGTTAGTCTTGGTGAGAGTAGCTCTAGAGAGAGAGAAAGAGAGAAAAATC
TTGGTGAGAATAGATTAAGT
AACTCTACCCTACAGACTGTTCTTACCTTTGAAAAGATATATATTTGATACGATATTAAGCATTACTTCCTTTTA
ACTCTXXXXXXXXXXTTCTTTCAAGTTAGTCTTGGTGAGAGTAGCTCTAGAGAGAGAGAAAGAGAGAAAAATC
TTGGTGAGAATAGATTAAGT
>Marker581311

Create PDF with GO2PDF for free, if you wish to

ACACGTTGGCTGGCACTGTGTGATTAGATTTATGATGGATTTCATGATACCATATATAACCACAAAATTTAATTA
TTGAAXXXXXXXXXXTGGAGGAAGTTTGTCTTAATAGTGAGCCTATCTTTGATGAATGTGACGAATATGAGGTGG
ATGGTGATGATTCCTTTGTG
ACACGTTGGCTGGCACTGTATGATTAGATTTATGATGGATTTCATGATACCATATATAACCACAAAATTTAATTA
TTGAAXXXXXXXXXXTGGAGGAAGTTTGTCTTAATAGTGAGCCTATCTTTGATGAATGTGACGAATATGAGGTGG
ATGGTGATGATTCCTTTGTG
>Marker581709
AACAGATATGGGGTAGAACTTCGTTGGTATTTTGTCATGATAATGGTTTAAGATTTTTTTTTAATTAAAATTTGA
AACTAXXXXXXXXXXGGCTGCAGTGTGATAATTTATTTAGAGGTCAATAAACCTTAGAGTAATAGCAAGCAAATT
CAATCTTCAATAATTATGTG
AACAGATATGGGGTAGAACTTCGTTGGTATTTTGTCATGATAATGGTTTAAGATTTTTTTTTAATTAAAATTTGA
AACTAXXXXXXXXXXGGCTGCAGTGTGATAATTTATTTAGAGGTCAATAAACCTTAGAGTAATAACAAGCAAATT
CAATCTTCAATAATTATGTG
>Marker582056
ACCATCTAAGTTTTAAAAAGATTAGGGTAAATTTTAGAACTATTCCTAAAGTATGTTAATAGTTACAATTACACT
CTCAAXXXXXXXXXXAATTGAGGGTTCAATTTTTACATTTATACACCTTTTAAAATTTGATTCCTAGAAACTGTC
AAATGATTTTGCATTTTGTT
ACCATCTAAGTTTTAAAAAGATTAGGGTAAATTTTAGAACTATTCCTAAAGTATGTTAATAGTTACAATTACACT
CTCAAXXXXXXXXXXAATTGAGGGTTCAATTTTTACCTTTATACACCTTTTAAAATTTGATTCCTAGAAACTGTC
AAATGATTTTGCATTTTGTT
>Marker582197
ACTTACTGGACTCAATTTTATCAAGAAAGATGCACAATTCAATTGGTTATCACTGCTCACTTAGCAAGACTCGAT
TTGCTXXXXXXXXXXAAGTATCATTGTTGATGCATGTAGTATAATTATTATGTTTTAGAATGAGTTGAGGAAGTA
AGAATTTCGTGGGGGGAGTG
ACTTACTGGACTCAATTTTATCAAGAAAGATGCACAATTCAATTGGTTATCACTGCTCACTTAGCAAGACTCGAT
TTGCTXXXXXXXXXXAAGTATCATTGTTCATGCATGTAGTATAATTATTATGTTTTAGAATGAGTTGAGGAAGTA
AGAATTTCGTGGGGGGAGTG
>Marker582904
ACTTTTTATCCTTTACTGATGATTTTTCTAGTAAAAGTTGGATTTATTTTCTAATAACAAAAGACCAAGTTTTTG
AAAATXXXXXXXXXXCAAAACTGTGAGATACACACCTCAACAAAATGTGGTGGCAGAAAGACTTAATAGAACTAT
AATGGAAAGGGTAAGATGTC
ACTTCTTATCCTTTACTGATGATTTTTCTAGTAAAAGTTGGATTTATTTTCTAAAAACAAAAGACCAAGTTTTTG
AAAATXXXXXXXXXXCAAAACTGTGAGATACACACCTCAACAAAATGTGGTGGCAGAAAGACTTAATAAAACTAT
AATGGAAAGGGTAAGATGTC
>Marker583204
GACGGAAGACTGGGCACATCCGAGAACGATGGCATTGCCGTAATAGCCGTGAGGGATTTTGGGGGAAAGTTTGTT
ACGGAXXXXXXXXXXTGGAGGAGGGGATCAACAAGGGATGAGATTGGAGGGATTTAGTGATGTCGAGGTGCCAGT
TGGGGCGTGGGGTTTTGGTA
GACGGAAGATTGGGCACATCCGAGAACGATGGCATTGCCGTAATAGCCGTGAGGGATTTTGGGGGAAAGTTTGTT
ACGGAXXXXXXXXXXTGGAGGAGGGGATCAACAAGGGATGAGATTGGAGGGATTTAGTGATGTCGAGGTGCCAGT
TGGGGCGTGGGGTTTTGGTA
>Marker583236
CACCTTCATTACAAATAAAACAACTGGCGAATTTAGTTCCCCCTGCAGATCCAAGATGGAAAACAATACACATAA
TTCAGXXXXXXXXXXAATATTCTACATATTATGCAAAGCACTGATAAATATGAGTTTGAATTTGTAATAAAATTT
ACCATCTTGAAGGGGTTGTG

Create PDF with GO2PDF for free, if you wish to

CACTTTCATTACAAATAAAACAACTGGCGAATTTAGTTCCCCCTGCAGATCCAAGATGGAAAACAATACACATAA
TTCAGXXXXXXXXXXXAATATTCTACATATTATGCAAAGCACTGATAAATATGAGTTTGAATTTGTAATAAAATTT
ACCATCTTGAAGGGGTTGTG
>Marker583828
ACTGAGGAAGAATGAACTGTATGCAAATAAGAAGAAATGCACCTTTGCTCAGTCTTGAGTAGATTACTTGAGACA
TATTAXXXXXXXXXXXTACTATCGCAAGTTTGTCCAGAATTATGGGACAATTGCGGCTCCTTTGACGCAATTATTG
AAGATAGGAGGGTTTAAGTG
ACTGAGGAAGAATGAACTGTATGCAAATAAGAAGAAATGCACCTTTGCTCAGTCTCGAGTAGATTACTTGAGACA
TATTAXXXXXXXXXXXTACTATCGCAAGTTTGTCCAGAATTATGGGACAATTGCGGCTCCTTTGACGCAATTATTG
AAGATAGGAGGGTTTAAGTG
>Marker583852
TACAACCAATAAACTATTTTTCCCTTGAACATTTTTACATTAAAACAGTTCAACTTTTCAACTAGCGTCAATAAT
TAGCAXXXXXXXXXXXTGTTATTTTCAAGTAGAAAGTTTTAAAATTAATTCTCCTCTTTGAATATATATATTGAAA
AATATATGGAACACTTTTGT
TACAACCAATAAACTATTTTTCCCTTGAACATTTTTACATTAAAACAGTTCAACTTTTCAACTAGCGCCAATAAT
TAGCAXXXXXXXXXXXTGTTATTTTCAAGTAGAAAGTTTTAAAATTAATTCTCCTCTTTGAATATATATATTGAAA
AATATATGGGACACTTTTGT
>Marker583938
ACCATCTCGATGCATTATATGTTCGATGTGGAATTAATTAAAGGTATGATTATTTCATCAGGAGCCATTGTATGC
TTGAGXXXXXXXXXXXAAAAAAAACTATGGTAATTAGTAAAATGGAAATTTTGTTAAGAAAAGCTACAAATACAAT
AAAATGACACAGTCTACGTA
ACCATCTCGATGCATTATATGTTCGATGTGGAATTAATTAAAGGTATGATTATTTCATCAGGAGCCATTGTATGC
TTGAGXXXXXXXXXXXAAAAAAAACTATGGTAATTAGTAAAATGGAAATTTTGTTAAGAAAAGCTACAAATACAAT
AAAATGCCACAGTCTACGTA
>Marker584206
TACGGAGTTCAATTAATCAAGTTTCAATTGTTTCTTTTTCTCCTTTCACGTTCCTGTTTCCCTGCCTCTCATTCT
CCTGGXXXXXXXXXXXATTTCACTCAGAAAACAAATGGAACGTCCAACTTGCTTCTGCAAAAATATTGTAGGAGGC
GATATTTACAAAATATCGTA
TACGGAGTTCAATTAATCAAGTTTCAATTGTTTCTTTTTCTCCTTTCACGTTCCTGTTTCCCTGCCTCTCATTCT
CCTGGXXXXXXXXXXXATTTCACTCAGAAAACAAATGGAACGTCCGACTTGCTTCTGCAAAAATATTGTAGGAGGC
GATATTTACAAAATATCGTA
>Marker584428
ACATCTTTTTCCCAGTGCCAGCGAGGAAAAGTTTTGAAATGGTGATCCAAGCCTAGACTGAGGATTTGACGAATG
CCATTXXXXXXXXXXXTAACATAACAATTCTTTTTAATACCTACTGGTGCGGGAGCAGGAACAACTACAGTTTGAG
GCACATTTCTCACAGGGGTT
ACACCTTTTTCCCAGTGCCAGCGAGGAAAAGTTTTAAAATGGTGATCCAAGCCTAGACTGAGGATTTGACGAATG
CCATTXXXXXXXXXXXTAACATAACAATTCTTTTTAATACCTACTGGTGCGGGAGCAGGAACAACTACAGTTTGAG
GCACATTTCTCACAGGGGTT
>Marker584592
GACCATCAATACCAATTCACCAACACCCACAACTTCTTCACAATGAATTGATTGAGACCTCCCAAAATAATTTCA
AATTAXXXXXXXXXXXTAAACCTTCAAATTAAGAATGTTTCAGTCAAGAAATTTTCAAACAAAAAATATGAAAAAA
CATAAAGAAAAAAATTAGTC
GACCATCAATACCAATTCACCAACACCCACAACTTCTTCACAATGAATTGATTGAGACCTCCCAAAATAATTTCA
AATTAXXXXXXXXXXXTAAACCTTCAAATTAAGAATGTTTCAGTCAAGAAATTTTCAAACAAAAAATATGAAAAAA
CATAAAGAAAAAAAGTAGTC
>Marker584642

Create PDF with GO2PDF for free, if you wish to

GACATTTTTGACAAAAAAGAAGCAATAAATGGATATATATATATATGTAGATAGTTATGACTTGTGAGTAGGCTA
ACATGXXXXXXXXXXTTGAGTGGGAAATGGATTCAGCATCATTGGCTGCTCTCGCATCTCTTCACCCCCAGCAGA
ATCTTCACACAGTCTTCGTC
GACATTTTTGACAAAAAAGAAGCAATAAATGGATATATATATATATGTAGATAGTTATGACTTGTGAGTAGGCTA
ACATGXXXXXXXXXXTTGAGTGGGAAATGGATTCAGCATCATTGGCTGCTCTCGCATCTCTTCACTCCCAGCAGA
ATCTTCACACAGTCTTCGTC
>Marker584877
AACTTAACTTGGGCCATTGCATTAATTCGCTGCAAAAATAGATGCAAGTGCAACGAATACAATTTACAAATCATG
AGATTXXXXXXXXXXAATGAAGAAATTTCGCTTTCATTTTTCCAGTGCTGTTCTCAATGAATTCTAACCAGCCCC
ATTTGATCAAATTAAATGTC
AACTTAACTTGGGCCATTGCATTAATTCGCTGCAAAAATAGATGCAAGTGCAACGAATACAATTTACAAATCATG
AGATTXXXXXXXXXXAATGAAGAAATTTCGCTTTCATTTTTCCAGTGCTGTTCTCATTGAATTCTAACCAGCCCC
ATTTGATCAAATTAAATGTC
>Marker585484
ACTTGGGCCTTGGCCTTTTTGTCAATAATTGCTTTCTGTTTCTCCATTTCTTGTAGTTGTTTCAGTCTTGGGTCG
TTTGGXXXXXXXXXXTTCTAAATTAAAAATTTATTCGGTTCTTCTTAACGTGACGTGAAAGATTTGAACCTTAAA
TTTAGTTTTAGTGAAGAGTT
ACTTGGGCCTTGGCCTTTTTGTCAATAATTGCTTTCTGTTTCTCCATTTCTTGTAGTTGTTTCAGTCTTGGGTCG
TTTGGXXXXXXXXXXTTCCAAATTAAAAATTTATTCGGTTCTTCTTGACGTGACGTGAAAGATTTGAACCTTAAA
TTTAGTTTTAGTGAGGAGTT
>Marker585795
TACATATTGCAGGCACCGATTTAGACTAATTGGAAATATCTTCTAAGAAATTTCAAAGTCATTTTGCTTCTTCTT
CAGCCXXXXXXXXXXATATATTACACACTAATGATTCCCATTTAATTTTTTGTCAAATCCACCACTATAATCATT
ATATAATGTGTTTTCTAGTC
TACATATTGCAGGCACCGATTTAGACTAATTGGAAACATCTTCTAAGAAATTTCAAAGTCATTTTGCTTCTTCTT
TAGCCXXXXXXXXXXATATATTACACACTAATGATTCCCATTTAATTTTTTGTCAAATCCACCACTATAATCATT
ATATAATGTGTTTTCTAGTC
>Marker586437
TACCCATCATCCCCATCGAGATTTTCGTTTCTAATAGCCAGACAACACTAGTAAATAGGTTTGTCTTATGGGTAT
TTCCGXXXXXXXXXXTTTTTGGACTCTTCTCCTTGCATATGGGAATTTTCTAGCCGTCGGAGATGGGTTTTCTTT
TATGCTATGTAGTATTGGTT
TACCCATCATCCCCATCGAGATTTTCGTTTCTGATAGCCAGACAACACTAGTAAATAGGTTTGTCTTATGGGTAT
TTCCGXXXXXXXXXXTTTTTGGACTCTTCTCCTTGCATATGGGAATTTTCTAGCCGTCGGAGATGGGTTTTCTTT
TATGCTATGTAGTATTGGTT
>Marker586703
TACACCCTAGTGAAATAATCAGCGGCTACACAAAAGGAATCAATAAGGTTTCTCACTTTCTACAATGATGATGGA
ATATAXXXXXXXXXXGTCAAGAAGATTTTATTTGTTCTCTAGTTGCTGATGTATATTTTTGCCTACTCCTGTTAT
AATTGAGTAATTGTCCTGTA
TACACCCTAGTGAAATAATCAGCGGCTACACAAAAGGAATCAATAAGGTTTCTCACTTTCTACAATGATAATGGA
ATATAXXXXXXXXXXGTCAAGAAGATTTTATTTGTTCTCTAGTTGCTGATGTATATTTTTGCCTACTCCTGTTAT
AATTGAGTAATTGTCCTGTA
>Marker586874
AACTCTTGCCAAGTAAAACACCTCTCTTTGGCCATTTGTGACTAAAGATTCTTTTGTAACATTGTTTAACCTTTA
TTATTXXXXXXXXXXAGGGTGTTTATATTTCATTTTGCATGTGACTGAATGCACGTTATGTATTGTTTTGAGAAC
ACCTTTTTGCATTGAATGTA

Create PDF with GO2PDF for free, if you wish to

AACTCTTGCCAAGTAAAACACCTCTCTTTGGCCATTTGTGACTAAAGATTCTTTTGTAACATTGTTTAACCCTTA
TTATTXXXXXXXXXXXAGGGTGTTTATATTTCATTTTGCATGTGACTGAATGCACGTTATGTATTGTTTTGAGAAC
ACCTTTTTGCATTGAATGTA
>Marker586878
ACAAGAGAATGACATCTGGTGTTCTAAACTCCACTCCTTGACCTTGGAACTTCTTGTGCTCTTCCAATATTCAAA
CTGTTXXXXXXXXXXXGCCAAACTGAGGCTATGTAACATATTATACATACCCTTGACCTAGGATAGCAATCCCTGG
TCCCAAATAAAATATCGGTA
ACAAGAGAATGACATCTGGTGTTCTAAACTCCACTCCTTGACCTTGGAACTTCTTGTGCTCTTCCAATATTCAAA
CTGTTXXXXXXXXXXXGCCAAACTAAGGCTATGTAACATATTATACATACCCTTGACCTAGGATAGCAATCCCTGG
TCCCAAATAAAATATCGGTA
>Marker586926
TACTATTTTAACAAGGTGCTTGGCTTGGTCTTCTTTACTGCATGCAGGAAGCGGAATCTGCAACTCTTGAAGCCA
TCTCAXXXXXXXXXXXTTGGCTTGAAGAGAACTTTCATTTTATGCACATTTTTTGGATATGCTGCTTGGTCAGTTT
CCTGTAGCAGGTGCTTTGTC
TACTATTTTAACAAGGTGCTTGGCTTGGTCTTCTTTACTGCATGCAGGAAGCAGAATCTGCAACTCTTGAAGCCA
TCTCAXXXXXXXXXXXTTGGCTTGAAGAGAACTTTCATTTTATGCACATTTTTTGGATATGCTGCTTGGTCAGTTT
CCTGTAGCAGGTGCTTTGTC
>Marker587075
CACGTTTTCACAGGCGAAGATTAGCAGGGCGGCAAGCATTTCTAAGAATGCTAAAAAGGTCTCTCATGAAACAAA
ACTACXXXXXXXXXXXCTTTCGACGATTGGAACGAAATAGCTGAACTTTTAGAAAACTTTTTTCAAGTTAAAATTA
TCATCAATCCTTTGTTTGTA
CACGTTTTCACAGGCGAAGATTAGCAGGGCGGCAAGCATTTCTAAGAATGCTAAAAAGGTCTCTCATGAAACAAA
ACTACXXXXXXXXXXXCTTTTGACGATTGGAACGAAATAGCTGAACTTTTGGAAAACTTTTTTCAAGTTAAAATTA
TCATCAATCCTTTGTTTGTA
>Marker587087
AACAAATCCCACTATTTAATGTCAAAACTTGAGCCATTCCTAAAGTTGAAAGTGTATGTTATTAATATATATATA
TACACXXXXXXXXXXXTTTTTACTTTTTGTTTCATGGAATTATTCGTATTTTAATTGATCCTTTTTCTTTTTCTTT
TCTATTGAAATTGCAGAGTG
AACAAATCCCACTATTTAATGTCAAAACTTGAGCCATTCCTAAAGTTGAAAGTGTATGTTATTAATGTATATATA
TATACXXXXXXXXXXXTTTTTACTTTTTGTTTCATGGAATTATTCGTATTTTAATTGATCCTTTTTCTTTTTCTTT
TCTATTGAAATTGCAGAGTG
>Marker587385
AACATAGATTGAACAGCCACACCAGATGCCTCGGCCAAAGCCATCAATCCAATAGCAAATAGGGCAACAGATGAA
ACTTTXXXXXXXXXXXGCTACTGCAGTTGGTGCAACTATCACGCCACTGCAAGTCAAAGTTTGAACAAATAATGTT
AGGGGAGATAAAGTTAAAGT
AACATAGATTGAACAGCCACACCAGATGCCTCGGCCAAAGCCATCAATCCAATAGCAAATAGGGCAACAGATGAA
ACTTTXXXXXXXXXXXGCTACTGCAGTTGGTGCAACTATCACGCCACTGCAAGTCAAAGTTTGAACAAATAATGTT
AGGGAAGATAAAGTTAAAGT
>Marker587816
AACTTGTTTATCATTCTTTCTTTTTTATGTAAAACTATTGTTCTTCATTGCAGCTTGATGAGTTCGTCATTCCAC
TACTGXXXXXXXXXXXTATTTCTCTAATATTGTCTTTTACTTTATCTCACTTTAGAGTCATACTGATTGTCCCATG
AGATTAGTTGAGTGCATGTA
AACTTGTTTATTATTCTTTCTTTTTTATGTAAAACTATTGTTCTTCATTGCAGCTTGATGAGTTCGTCATTCCAC
TACTGXXXXXXXXXXXTATTTCTCTAATATTGTCTTTTACTTTATCTCACTTTAGAGTCATACTAATTGTCCCATG
AGATTAGTTGAGTGCATGTA
>Marker587880

Create PDF with GO2PDF for free, if you wish to

TACTGTGAAATCTATGATTGATGACTTGACAAGTGCTCTCAATCCTTCTGAAAGGAGTTTTTATTCGATCATTTG
GATGGXXXXXXXXXXGAATCCCATGCTCATCTTTTTATTCATTGTCCTTTTGCTTGACAATTTTGGAATCTCATT
TTGGAGGTTTTTGTTGGTC
TACTGTGAAATCTATGATTGATGACTTGACAAGTGCTCTCAATCCTTCTGAAAGGAGTTTTTATTCGATCATTTG
GATGGXXXXXXXXXXGAATCCCATGCTCATCTTTTAATTCATTGTCCTTTTGCTTGACAATTTTGGAATCTCATT
TTGGAGGTTTTTGTTGGTC
>Marker588078
CACATCAAAGCCTCAAAATTTCCCTATTTATTAATATGGTTGCATGACTAAGATTAACCAAAAACCAACTCCTCC
TACTTXXXXXXXXXXTTCTTTTTTTTTTAACCAAACTCTTTTAACAAACTTTAAAACATACCAAGGCTTAAATAA
ATAAGAAAAGATCAAGGGTT
CACATCAAAGCCTCAAAATTTCCCTATTTATTAATATGGTTGCATGACTAAGATTAACCAAAAACCAACACCTCC
TACTTXXXXXXXXXXTTCTTTTTTTTTTAACCAAACTCTTTTAACAAACTTTAAAACATACCAAGGCTTAAATAA
ATAAGAAAAGATCAAGGGTT
>Marker588496
CACCAATTCCACATCCAACATCCAACACCTGGAATCTAGACAAAGTTATTCTCCATAGTTTGAAATAAATGCAGA
AAATTXXXXXXXXXXAAAATATGCACGTTTAGATTTGAGATTGAAAAAAACAGAGACACATTCTGGTCTGTCGAA
TGCAGGAGGAGGAAGATAGT
CACCAATTCCACATCCAACATCCAACACCTGGAATCTAGACAAAGTTATTCTCCATAGTTTGAAAAAAATGCAGA
AAATTXXXXXXXXXXAAAATATGCACGTTTAGATTTGAGATTGAAAAAAACAGAGACACATTCTGGTCTGTCGAA
TGCAGGAGGAGGAAGATAGT
>Marker590648
CACATATTTCAACCCTTTCGCTATATATTTCGACCATTTACTTTTTTGGCTCAAGAGATGCTTCATTGACAAACA
CTTCCXXXXXXXXXXAGACCCAAAATAACCCAAAGAACAACCCATGCGAAGTGAAAATTCGATGTTTGCCCCTCT
AAGAAGTGGCCTATAGAGTT
CACACAATTCAACCCTTTCGCTATATATTTCGACCATTTACTTTTTTGGCTCAAGAGATGCTTCATTGACAAACA
CTTCCXXXXXXXXXXAGACCCAAAATAACCCAAAGAACAACCCATGCGAAGTGAAAATTCGATGTTTGCCCCTCT
AAGAAGTGGCCTATAGAGTT
>Marker591057
AACGGTAATACCAAAGTAACCGATTCACTGTCGTCATGTTTCAGAAAAAGAAAGGAGCTTTCCACCACTCAAGTT
TCTTGXXXXXXXXXXGGGGTTGGTAGTATTGGCTTAACTAGACTTTTTCGTTCTCTGATGGCTTTGATATCTTCA
GGCCATCTCAGCATATAGTG
AACGGTAATACCAAAGTAACCGATTCACTGTCGTCATGTTTCAGAAAAAGAAAGGAGCTTTCCACCACTCAAGTT
TCTTGXXXXXXXXXXGGGGTTGGTAGTATTGGCTTAACTAGACCTTTTCGTTCTCTGATGGCTTTGATATCTTCA
GGCCATCTCAGCATATAGTG
>Marker591355
CACTTTCTTTTACTTTTTCCATCTTTTAATCTCAGATAAGAAGAATCAACACAATTGTCTAGGAAGATAGAAACC
ACAGAXXXXXXXXXXTTGAATTTTGGTTGTTTCCAGAATCATCATGTGAAGTGGAACAAGCAAAAACTGCACTTA
TATGACAGCATTTTTCATGT
CACTTTCTTTTACTTTTTCCATCTTTTAATCTCAGATAAGAAGAATCAACACAATTATCTAGGAAGATAGAAACC
ACAGAXXXXXXXXXXTTGAATTTTGGTTGTTTCCAGAATCATCATGTGAAGTGGAATAAGCAAAAACTGCACTTA
TATGACAGCATTTTTCATGT
>Marker591491
AACTACCTCCACCGAAACAACTCTCACTGCTGTTGTGCGACGAGATGATCGAGCTGCATTTGTTCCAGCCATGCT
CCTAAXXXXXXXXXXTTTCACAAGTGATTGAACCCATGATAAATCTGGCTCTTCTCCATTGTTCCCGAGCTCAAA
AGACGACGATCGCCGAAGTT

Create PDF with GO2PDF for free, if you wish to

AACTACCTCCACCGAAACAACTTTCACTGCTGTTGTGCGACGAGATGATCGAGCTGCATTTGTTCCAGCCATGCT
CCTAAXXXXXXXXXXTTTCACAAGTGATTGAACCCATGATAAATCTGGCTCTTCTCCATTGTTCCCGAGCTCAAA
AGACGACGATCGCCGAAGTT
>Marker591557
CACTTCCTTCAATTTATCGTAGTTTTGGGACTGGTTCTCCTCATCAAAGACATAGTGTTTTCCTAGTGAAATGGC
TTTAGXXXXXXXXXXTCTTCGGTATGTCTAGAAAGAAACAAATAGTGGTTTATGAAGATATGTGTTCCTTTTTTT
GCTCCTCTTAACTTTCGGTG
CACTTCCTTCAATTTATCGTAGTTTTGGGACTGGTTCTCCTCATCAAAGACATAGTGTTTTCCTAGTGAAATGGC
TTTAGXXXXXXXXXXTCTTCGGTATGTCTAGAAAGAAACAAATAGTGGTTTATGAAGATATGTGTTCCTTTTTTT
GCTCCTCTTAACTTTCAGTG
>Marker591571
AACAAATTTGAAGACTTGTTTCAGTATAGAATTACAGATGGAGATGAAGATGAAGAGTTTCAACTCACCTCTTCT
ATCTAXXXXXXXXXXTCGGCTTGGTTTTAGATGAAGTCACTCCTCCTCTATGGGCTCCCATTAAGTAGGGTGGTT
ACCCTTAGCCCAATGAGGTT
AACAAATTTGAAGACTTGTTTCAGTATAGAATTACAGATGGAGATGAAGATGAAGAGTTTCAACTCATCTCTTCT
ATCTAXXXXXXXXXXTCGGCTTGGTTTTAGATGAAGTCACTCCTCCTCTATGGGCTCCCATTAAGTAGGGTGGTT
ACCCTTAGCCCAATGAGGTT
>Marker591591
AACAGCCAGCTTTAACTGCGAATGGTCATTCTGAAGCTACGTCCAAAGAACATCTACAAGATAAATCCATAGCAG
CGATTXXXXXXXXXXTGCAAATTAGAAACACATGAATTGGAATATATCGTTTATGATTTTGTGCTATAGTGGATT
TTGTATGTCTATATATCGTT
AACAACCAGCTTTAACTGCGAATGGTCATTCTGAAGCTACGTCCAAAGAACATCTACAAGATAAATCCATAGCAG
CGATTXXXXXXXXXXTGCAAATTAGAAACACATGAATTGGAATATATCGTTTATGATTTTGTGCTATAGTGGATT
TTGTATGTATATATATCGTT
>Marker592359
GACTAATTCTCTAATGTTATTATCGTCGATGTTGTAAATTTACTTACTTTTCTTCTGTAACTCGAGTGTCCTGTT
TGGTTXXXXXXXXXXCTACAGTTTTAGGCGTTTCTGATTTGGGATGCTCTTGGTTTGAAGATTATATAGATGCTG
AAGTTTTAGTCTATTTTTGT
GACTAATTCTCTAATGTTATTATCGTCGATGTTGTAAATTTACTTACTTTGCTTCTGTAACTCGAGTGTCCTGTT
TGGTTXXXXXXXXXXCTACAGTTTTAGGCGTTTCTGATTTGGGATGCTCTTGGTTTGAAGATTATATAGATGCTG
AAGTTTTAGTCTATTTTTGT
>Marker592420
AACGATTTTTCTTATTTTTCAAAGTTGATTGGATGTTGAAACATGGAATAGAAGTTTTTGGAAAAGAGGTTTCCA
AGCAAXXXXXXXXXXGAGAAATACTCTGAGTCGCTTCAAAATGCTTTGAAACTGGGGAAGAGGAGTGGTTTCGCA
AAAGGCTTTGGCGTGGGGTT
AACGATTTTTCTTATTTTTCAAAGTTGATTGGATGTTGAAACATGGAATAGAAGTTTTTGGAAAAGAGGTTTCCA
AGCAAXXXXXXXXXXGAGAAATACTCTGAATCGCTTCAAAATGCTTTGAAACTGGGGAAGAGGAGTGGTTTCGCA
AAAGGCTTTGGCGTGGGGTT
>Marker592492
ACCTATCCATTCTTTTTCTAATGAATGGAGGAATATCAGAGGAGGTATTGGGAGCAAAGAACTGCAGGGTTTCAC
CAAGTXXXXXXXXXXGCTCCAATCAAAAGAAGCCACATTTTGGTTATAAATAGGAACAGAGAAGTTCCAAAGAAG
GAATATATATATGATTGGTG
ACCTATCCATTCTTTTTCTAATGAAAGGAGGAATATCAGAGGAGGTATTGGGAGCAAAGAACTGCAGGGTTTCAC
CAAGTXXXXXXXXXXGCTCCAATCAAAAGAAGCCACATTTTGGTTATAAATAGGAACAGAGAAGTTCCAAAGAAG
GAATATATATATGATTGGTG
>Marker592553

AACTACTCGAGAGAGTAAGTAGCAAGGACCTCACTAAACACCTGCATAATGAATTCTGGCATTGTGCCTCCAATC
ATTTAXXXXXXXXXXXTTGTGTCCCGAAAAGAGAACAAGTGGGGTGTTTAGGCACCGAGAGGTTGCTCGCCTTAAA
TAGAAGATGGTTAAATGGTC
AACTACTCGAGAGAGTAAGTAGCAAGGACCTCACTAAACATCTGCATAATGAATTCTGGCATTGTGCCTCCAATC
ATTTAXXXXXXXXXXXTTGTGTCCAGAAAAGAGAACAAGTGGGGTGTTTAGGCACCGAGAGGTTGCTCGCCTTAAA
TAGAAGATGGTTAAATGGTC
>Marker593205
GACTGTTTTGAAACATTGATGAACATCCCATGTAAATATGAACCAAAAAAGAAAGATCCAAATTCTGACGTGATT
TATACXXXXXXXXXXXTAATAGTAATATGTTGTAATTTGCGACCACATCCTCAAATTAACATCTCCACGTAATAGT
TAAACTAATCAAATTCTTGT
GACTGTTTTGAAACATTGATGAACATACCATGTAAATATGAACCAAAAAAGAAAGATCCAAATTCTGACGTGATT
TATACXXXXXXXXXXXTAATAGTAATATGTTGTAATTTGCGACCACATCCTCAAATTAACATCTCCACGTAATAGT
TAAACTAATCAAATTCTTGT
>Marker593866
ACAAAAACCCAGTAGAGAACCATGGAGATGCAATTCTTCATTCATACTACAGTAAAATATCAAATGAACCTTCTT
AACAAXXXXXXXXXXXCTAAAATATGAGCTCAAAAGCTGCCACCAAACAATTGGGTCCGGTCGGCTAGCCCAGAAA
TGAGCTCAAAAGTTGAAGTG
ACAAAAACCAAGTAGAGAACCATGGAGATGCAATTCTTCATTCATACTACAGTAAAATATCAAATGAACCTTCTT
AACAAXXXXXXXXXXXCTAAAATATGAGCTCAAAAGCTGCCACCAAACAATTGGGTCCGGTCGGCTAGCCCAGAAA
TGAGCTCAAAAGTTGAAGTG
>Marker594418
ACCCATAAAAGAAAAAATATTGTAAATAAAAAAACTGCTGAAAATATTTAGAATCTATAGCAAAATTTATTTTGG
TTGTGXXXXXXXXXXXGGAGTGATGGTCCAATTAAGCATGATTTATATTACCTAATATTTGGAGAGGGAAAGGATT
AGGTTAAGAGAGAAAATGTT
ACCCATAAAAGAAAAAATATTGTAAATAAAAAAACTGCTGAAAATATTTAGAATCTATAGCAAAATTTATTTTGG
TTGTGXXXXXXXXXXXGGTGTGATGGTCCAATTAAGCATGATTTATATTACCTAATATTTGGAGAGGGAAAGGATT
AGGTTAAGAGAGGAAATGTT
>Marker594469
CACTAAGCAATATGACAATCTCATACGTCAAGACCTACCAATTTAATTAATGTAACATAATAAACAGAACAACCC
TTATTXXXXXXXXXXXATCCATTGATTGACTCTATATATAAACTACTTAATGTCTTCAAAAAAATCATATTGCCCA
AATCGTAGCTCCACAAAGTT
CACTAAGCAATATGACAATCTCATACGTAAAGACCTACCAATTTAATTAATGTAACATAATAAACAGAACAACCC
TTATTXXXXXXXXXXXATCCATTGATTGACTCTATATATAAACTACTTAATGTCTTCAAAAAAATCATATTGCCCA
AATCGTAGCTCCATAAAGTT
>Marker594509
CACTCCGATCTGCATAAACATATTAAAGAGATGGAAGAATCTCTGTAGTTTTGTATTTTTTTGTTCATCACATAAC
TGCCAXXXXXXXXXXXAACTGATGGATCTTGAGCTGTGATAACTGAAAGATGTGGAAACTTACAAGCACTTGCCAA
CTGATTCTTTATCTGGTAGT
CACTCCGATCTGCATAAACATATTAAAGAGATGGAAGAATCTCTGTAGTTTTGTATTTTTTTGTTCATCACATAAC
TGCCAXXXXXXXXXXXAACTGATGGATCTTGAGCTGTGACAACTGAAAGATGTGGAAACTTACAAGCACTTGCCAA
CTGATTCTTTATCTGGTAGT
>Marker594644
ACTTCAAAAGGTAGTTGGCTCAAGAGTCTTTCCAAACATCGTTAAGACTATTTTAGTTTCGTCTCATTTTAATTT
AGTGAXXXXXXXXXXXATCATTAACCATAACAACCGAAGATAAAAGCACATGAAAATGATTGAATTTTTCCTCACC
TTAGGTTTCAAAACCAAGTC

Create PDF with GO2PDF for free, if you wish to

ACTTCAAAAGGTAGTTGGCTCAAGAGTCTTTCCAAACATCGTTAAGACTATTTTAGTTTCGTCTCATTTTAATTT
AGTGAXXXXXXXXXXXATCATTAACTATAACAACCGAAGATAAAAGCACATGAAAATGATTGAATTTTTCCTCACC
TTAGGTTTCAAAACCAAGTC
>Marker595412
ACATATGTTAGTTACTAAGATGCATTCTAAACTTTATGAATAGATTGTCTATATTCGGTTATTTTCTTTTCCTTA
TACTGXXXXXXXXXXXATTTGTAAATACTACATGACAATATTATTCTTTTGAAATTAGTTCAACAGTGTTTGACAG
GTTGTATAGCATATGTTGTT
ACATATGTTAGTTACTAAGATGCATTCTAAACTTTATGAATAGATTGTCTATATTCGGTTATTTTCTTTTCCTTA
TACTGXXXXXXXXXXXATTTGTAAATACTACATGACAATATTATTCTTTTGAAATTAGTTCAACGGTGTTTGACAG
GTTGTATAGCATATGTTGTT
>Marker595939
ACAGAACATGCAAATGTTCTGTGAATGATTACTTATTTTTTTCCGAGTATCGAAGATCTTTAAGGATTTCTTTTA
TTAGTXXXXXXXXXXXCGTTGAATTTAAAAAGTATTTATTTAAATTCTTTACCTTCTAGGTGTTGAGTATTCCAGT
TGTTTTGGTCGAGAACAGTG
ACAGAACATGGAAATGTTCTGTGATTGATTACTTATTTTTTTCCGAGTATCGAAGATCTTTAAGGATTTCTTTTA
TTAGTXXXXXXXXXXXCGTTGAATTTAAAAAGTATTTATTTAAATTCTTTACCTTCTAGGTGTTGAGTATTCCAGT
TGTTTTGGTCGAGAACAGTG
>Marker596193
ACATAAGTAAGCTTTCACATCCAACTTTCTTTCTGGGATCGTTGTGCTTCTTTACGTATTGGTCTTTTTTATTAT
ATAAAXXXXXXXXXXXXTGGAGGCTTCTTCTGATGGGGGATAGCATAGGTCACCAGTGTTGAATCAGAGTCACCAGC
TAAATTTGATATAGGAATGT
ACATAAGTAAGCTTTCACATCCAACTTTCTTTCTGGGATCGTTGTGCTTCTTTACGTATTGGTCTTTTTTATTAT
ATAAAXXXXXXXXXXXXTGGAGGCTTCTTATGATGGGGGATAGCATAGGTCACCAGTGTTGAATCAGAGTCACCAGC
TAAATTTGATATAGGAATGT
>Marker596591
AACTTTGCAATTTAACTCATGAGTAAAATCTTACTTATAGCTAGCAGATTATAAAAAATTTTGGGCACATGCAAA
ACATTXXXXXXXXXXXATCAAATGATCTATGGCACCAGAGTCTAGAATCCAAGGGTTCTTTCCCATCAACACTAAT
AAGACCGAAGGACTGAGGTA
AACTTTGCAATTTAACTCATGAGTAAAATCTTACTTATAGCTAGCAAATTATAAGAAATTTTGGGCACATGCAAA
ACATTXXXXXXXXXXXATCAAATGATCTATGGCACCAGAGTCTAGAATCCAAGGGTTCTTTCCCATCAACACTAAT
AAGACCGAAGGACTGAGGTA
>Marker596602
AACGATTTACCGTCTTTGATATTTTACGAAGAGTTCTTGATATATCTTCATTATTGGTTCAAGTGTATTTGAGAG
AAGAGXXXXXXXXXXXTATTTTTAGTTTCTATTCATAACTAAAAAGAGTTCTTATACTAGAAGGAATCTCTTCTGG
GTTATTAAAAATTGTTTAGT
AACGATTTACCGTCTTTGATATTTTACGAAGAGTTCTTGATATATTTTCATTATTGGTTCAAGTGTATTTGAGAG
AAGAGXXXXXXXXXXXTATTTTTAGTTTCTATTCATAACTAAAAAGAGTTCTTATACTAGAAGGAATCTCTTCTGG
GTTATTAAAAATTGTTTAGT
>Marker597071
GACGACGGTGAGAGAATGGTTGGCGATGGAGAAGAAGAGTTCATCGTTGAGTGCAGCGTTGATTAGTCGGAGTAA
GTATGXXXXXXXXXXXTAGGTATCAGCTTAAACTAAAATTAGTTTAGTGAGCTTTTAAAAAATGGAGTCAAACAAA
GTAAGATTAATAGTGAGAGT
GACGACGGTGAGAGAATGGTTGGCGATGGAGAAGAAGAGTTCATCGTTGAGTGCAGCGTTGATTAGTCGGAGTAA
GTATGXXXXXXXXXXXTAGGTATTAACTTAAACTAAAATTAGTTTAGTGAGCTTTTAAAAAATGGAGTCAAACAAA
GTAAGATTAATAGTGAGAGT
>Marker597166

Create PDF with GO2PDF for free, if you wish to

ACCATGATTTCTTTATGATTTTCTTCCATATTGGATTTTCTTTAATTACATAACTGATCCCTCTCAGATCTCTAG
CCTAGXXXXXXXXXXTCAATAATTATTCATTTTAAAGTTGAATAATAGGATATTATTATAAGGAGCTGAGTGTCT
CCTTCTTATGAACGGGTGTT
ACCATGATTTCTTTATGATTTTCTTCCATATTGGATTTTCTTTAATTACATAACTGATCCCTCTCAGATCTCTAG
CCTAGXXXXXXXXXXTCATTAATTATTCATTTTAAAGTTGAATAATAGGATATTATTATAAGGAGCTGAGTGTCT
CCTTCTTATGAACGGGTGTT
>Marker597456
TACAGGACCTTACGATTTCACACTTTTACCTCTCTTTCTTTAATTTCTTCCTGCCCCCGTTCATAATTTGTTGTT
GTTTGXXXXXXXXXXAGGGTAAGTAATATTAATCTATGTTGTTAGTTGTGAAAAGTAGGTAGATCTAACATAGAA
CACAATATCAACTACGATGT
TACAGGACCTTACGATTTCACACTTTTACCTCTCTTTCTTTAATTTCTTCCTGCCCCCGTTCATAATTTGTTGTT
GTTTGXXXXXXXXXXAGGGTAAGTAATATTAATCTATGTTGTTAGTTGTGAAAAGTAGGTAGATCTAACATAGAA
CACAATATCATCTAGGATGT
>Marker598057
AACTCGGTTGTATATATTTGCATAGAGACACATAATTTAATTATTCTACAATGAAGAAGAGGTTGAGTTTATGGA
AGGATXXXXXXXXXXTTTTCTATATACTTTTTTTTCATCCAGTTTGCTAGTAATTATTGTCTTTTCTTTTTTCTT
TTTAAGAAAAACAATAAGTG
AACTCGGTTGTATATATTTGCATAGAGACACATAATTTAATTATTCTACAATGAAGAAGAGGTTGAGTTTATGGA
AGGATXXXXXXXXXXTTTTCTATATACTTTTTTTTCATCCAGTTTGCTAGTAATTATTGTCATTTCTTTTTTCTT
TTTAAGAAAAACAATAAGTG
>Marker598064
TACTGGCAGTTTGAAATGAAGAAAGTTGTAGGGAATGAAATGAAAAACAAAAGGTTCAAGGAATTATTACATATA
CACATXXXXXXXXXXAGAATATTAAGTTAATGTAATATGTGATTTATGGTAACCTATTGACTTATGAGCCACAAG
GGAAATGGGAGAGATGGGTG
TACTGGCAGTTTGAAATGAAGAAAGTTGTAGGGAATGAAATGAAAAACAAAAGGTTTAAGGAATTATTACATATA
CACATXXXXXXXXXXAGAATATTAAGTTAATGTAATATGTGATTTATGGTAACCTATTGACTTATGAGCCACAAG
GGAAATGGGAGAGATGGGTG
>Marker598416
AACAAATACCAACTTTCAAGAATTTTGGATGTTACTGAATTGATTAGTAGCATGCAATCCACTAGAAGTGTATCC
GTGATXXXXXXXXXXAATGCAATAAATGTAACCAAGAGCAACAAGAGAAAAACAGTAGTTTATAAAGCCGATAAG
TTGGAATAGATAAACAAGGT
AACAAATACCAACTTTCAAGAATTTTGGATGTTACTGAATTGATTAGTAGCATGCAATCCACTAGAAGTGTATCC
GTGATXXXXXXXXXXAATGCAATAAATGTAACCAAGAGCAACAAGAGAAGAACAGTAGTTTATAAAGCCGATAAG
TTGGAATAGATAAACAAGGT
>Marker599312
TACATTTTCTATGCAAAAACAACACTTTTTTAGACTAGCATAAAGCTTTTCCCTTCTAAATGCATCTAGAACACA
CCTAAXXXXXXXXXXCAGTGGAATGACCTTGTTTAGAAACTAGGGTGTGTTTGAATTTTGGTTTGCATTTTAAAG
TCTAGAAACTTAGATTAGTT
TACATTTTCTATGCAAAAACAACACTTTTTTAGATTAGCATAAAGCTTTTCCCTTCTAAATGCATCTAGAACACA
CCTAAXXXXXXXXXXCAGTGGAATGACCTTGTTTAGAAACTAGGGTGTGTTTGAATTTTGGTTTGCATTTTAAAG
TCTAGAAACTTAGATTAGTT
>Marker599688
ACTCACATAATTATATAATAGCAAAAAACTTTTTGAGGAATTCTACCAATTTCTAAATTATAACGCTGTAAGTGG
GTTAGXXXXXXXXXXAACTAGTGTAAGGACTCAGATGTTAGTTATTTTTGTAAAAGCTGTAAAGTTTATTGCGAA
CAGAGAATCTTGAAAACGTC

Create PDF with GO2PDF for free, if you wish to

ACTCACATAATTATATAATAGCAAAAAACTTTTTGAGGAATTCTACCAATTTCTAAATTATAACGCTGTAAGTGG
GTTAGXXXXXXXXXXXAACTAGTGTAAGGACTCAAATGTTAGTTATTTTTGTAAAAGCTGTAAAGTTTATTGCGAA
CAGAGAATCTTGAAAACGTC
>Marker599751
ACAAGTATTTATTACTACTTGGAAGTTTGGTCCTTACTATGTGTTGCTTAGGCTGTTGGTCATGCAACTTAATGT
TAGCAXXXXXXXXXXXCATGCACTAGTATCAGTGTTTTGACTTACAACCCTTGAGTTCTCCACCGGCTTCTCCTTT
TCACATAGAAGAAGAATGGT
ACAAGTATTTATTACTACTTGGAAGTTTGGTCCTTACAATGTGTTGCTTAGGCTGTTGGTCATGCAACTCAATGT
TAGCAXXXXXXXXXXXCATGCACTAGTATCAGTGTTTTGACTTACAACCCTTGAGTTCTCCACCGGCTTCTCCTTT
TCACATAGAAGAAGAATGGT
>Marker600016
TACTGAAGTTTCAACTTTAACTCTTCCAATATGTTATTTTGCATGCTGATGCATGGCATAAAGTAGTATTTATTT
TGTTAXXXXXXXXXXXTTTCTTTTGACGTTTGGCGTTAATTTGTGGGTTCTTTTATGAAATCTAATAAAATCTAAT
ACTCTGCTTATATTAAAGTC
TACTGAAGTTTCAACTTTAACTCTTCCAATATGTTATTTTGCATGCTGATGCATGGCATAAAGTAGTATTTATTT
TGTTAXXXXXXXXXXXTTTCTTTTGACGTTTGGCGTTAATTTGTGGGTTCTTTTATGAAATTTAATAAAATCTAAT
ACTCTGCTTATATTAAAGTC
>Marker600097
AACAATATTTAAAATTTTATGGCAGATTTAATTCTTCCTTAATGAAAAAAAAAAACCTTAGAGGAGATCTCTTAC
TGAATXXXXXXXXXXXGTTTAGATCATGAATACAATGAGAACAAGTCAATAAAATTGTCGTCACTGATAGATAGCA
ACCTTAACTCACTTTATAGT
AACAATATTTAAAATTTTATGGCAGATTTAATTCTTCCTTAATGAAAAAAAAACACCTTAGAGGAGATCTCTTAC
TGAATXXXXXXXXXXXGTTTAGATCATGAATACAATGAGAACAAGTCAATAAAATTGTCGTCACTGATAGATAGCA
ACCTTAACTCACTTTATAGT
>Marker600518
GACTTTGAATACCGTAACAGCTTTAAAAAGTCATGTCAGGTTTTGCATTTCATCTGTTGTTACTTGTTAGTTGCT
TTAAAXXXXXXXXXXXAACTACAATCTAGATCAGTTAAAGAGTGCTAATGGAGAAATAGTGAGAGAATTATTAAGA
ATGTAGTTATGTGAGTTGTT
GACTTTGAATACCGTAACAGCTTTAAAAAGTCATGTCAGGTTTTGCATTTCATCTGTTGTTACTTGTTAGTTGCT
TTAAAXXXXXXXXXXXCAATAAAATCTAGATCAGTTAAAGAGTGCTAATGGAGAAATAGTGAGAGAATTATTAAGA
ATGTAGTTATGTGAGTTGTT
>Marker601090
ACTTTGTAGTTTCCAGTTATTAACATCTTGTTTTCTTCAGCTCCCTCTCTAACATTCTTTGCTTCATAGAACAAG
AACGCXXXXXXXXXXXTCTTTGCCACTCACCTGAAACCCTTTTGAAATTATGTCATCGTGTCTTGATATTTGATTC
CTGCAATTATGGCAACTGTA
ACTTTGTAGTTTCCAGTTATTAACATCTTGTTTTCTTCAGCACCCTCTCTAACATTCTTTGCTTCATAGAACAAG
AACGCXXXXXXXXXXXTCTTTGCCACTCACCTGAAACCCTTTTGAAATTATGTCATCGTGTCTTGATATTTGATTC
CTGCAATTATGGCAACTGTA
>Marker601502
CACTTATGAAGTTTAGCATGTAACAATTTATTTCGATAGTTTAAGATTTGTAATAACCTAGTTTTTGAATTTTAG
CTTTCXXXXXXXXXXXAAAACTAAATCATTACAAAATTTAAAAACTGCATATTCCGTCTAGATTTTGACTTCGGGT
TTTTTCCAGGTTCTTGGGTG
CACTTATGAAGTTTAGCATGTAACAATTTATTTCGATAGTTTAAGATTTGTAATAACCTAGTTTTTGAATTTTAG
CTTTCXXXXXXXXXXXAAAACTAAATCATTACAAAATTTAAAAACTGCATATTCCGTCTAGATTTTGACTTTGGGT
TTTTTCCAGGTTCTTGGGTG
>Marker601688

Create PDF with GO2PDF for free, if you wish to

AACTCAAATGAATGTGGATTGAAAGTGTTCGTCCAAATTTCAATTTCTATAAATTTCCCTAAATTTTTTGTTTTG
ACTTTXXXXXXXXXXTTCAAATTTGTTACAATTTAACCTCTACAATGAAGAATTTCATTAAAATTGTTTGCCAAT
TTTTCTAATTCAGTGATGTA
AACTCAAATGAATGTGGATTGAAAGTGTTCGTCCAAATTTCAATTTCTATAAATTTCCCTAAATTTTTTGTTTTG
ACTTTXXXXXXXXXXTTCAAATTCGTTACAATTTAACCTCTACAATGAAGAATTTCATTAAAATTGTTTGCCAAT
TTTTCTAATTCAGTGATGTA
>Marker602924
TACAATAATTTTAAATATGGATGGTTGAATTTTCTACCTGTAAGGAGAATTGATTTTGAAAATTGAAAAAATATG
GAGACXXXXXXXXXXGCTTAATAATAGTGTTTATTTACATCCGCCATCCGGAAACAAATTTAGTCCGGTGAAATA
TCCTTAAATAGAGCGCAGTT
GACAATAATTTTAAATATGGATGGTTGAATTTTCTACCTCTAAGGAGAATTGATTTTGAAAATTGAAAAAATATG
GAGACXXXXXXXXXXGCTTAATAATAGTGTTTATTTACATCCGCCATCCGGAAACAAATTTAGTCCGGTGAAATA
TCCTTAAATAGAGCGCAGTT
>Marker603112
ACAAAATTGTAAATTCCATGAAATTTTAAACCAATATCTGAATTCAATAAATATTAGGGTAAGAGTAATATCTAG
TTTTCXXXXXXXXXXCCAAGATAGATCTTGAAAATCATTTCCTCTCCGCTGTGAATTATAGTTGCTTTTTAGCTC
TCAGAAACAGGCTTATTGTG
ACAAAATTGTAAATTCCATGAAATTTTAAACCAATATCTGAATTCAATAAATATTAGGGTAAGAGTAATATCTCG
TTTTCXXXXXXXXXXCCAAGATAGATCTTGAAAATCATTTCCTCTCCGCTGTGAATTATAGTTGCTTTTTAGCTC
TCAGAAACAGGCTTATTGTG
>Marker603540
CACACTTCACTTTCGTAAGATTTCAGACAATACATCTTGTATTCCCTTTTAATTTTTCATTCATCCAAGTGTTCT
CCATTXXXXXXXXXXACTTTACCCTCTATGTAACATAACAATCTTTTCCAAATAATGGATAGTGTATTTTTAAAC
TAATTGCTTCAGAGGACGTC
CACACTTCACTTTCGTAAGATTTCAGACAATACATCTTGTAATCCATTTTAATTTTTCATTCATCCAAGTGTTCT
CCATTXXXXXXXXXXACTTTACCCTCTATGTAACATAACAATCTTTTCCAAATAATGGAAAGTGTATTTTTAAAC
TAATTGCTTCAGAGGACGTC
>Marker603611
AACCGTCTAATAGAATGGAACCGTCAGATTCTATGTTTGGATGACTGGATGTCAAATAAGATAGTTTGTGGTTTT
AATTTXXXXXXXXXXTCACAAGTTTGGAATTTTTTGTGATTTTGTTTTGAATTTTGGTTTATTTAATTGAATTAT
ATTGATTATGTTTCTAGGTT
AACCGTCTAATAGAATGGAACCGTCAGATTCTATGTTTGGATGATTGGATGTCAAATAAGATAGTTTGTGGTTTT
AATTTXXXXXXXXXXTCACAAGTTTGGAATATTTTGTGATTTTGTTTTGAATTTTGGTTTATTTAATTGAATTAT
ATTGATTATGTTTGTAGGTT
>Marker603662
CACAGATCAAGGGAGGTAACTTGTTTGGTTTTTATAAGTCATTGGACTTTGAACCTACAGAAACTTGCATTATGC
AACTGXXXXXXXXXXAACTTAAAGTAGGAGGCAATATATGGTGGTAGATTCCGGATCCCTTATGTTTTAGTTCAA
AATCTGGAATCAATTCAGTG
CACAGATCAAGGGAGGTAACTTGTTTGGTTTTTATAAGTCATTGGACTTTGAACCTACAGAAACTTGCATTATGC
AACTGXXXXXXXXXXAACTTAAAGTAGGAGGCAATATGTGGTGGTAGATTCCGGATCCCTTATGTTTTAGTTCAA
AATCTGGAATCAATTCAGTG
>Marker604284
AACAACATGAGAAAAGATTGTCATTCGAAGACAATAGATTTAAGATTTAAATATCACGATAACACATGAACAATA
AAATCXXXXXXXXXXAAAGGAGGAGGAGAAGAAGAAGAAACCCAGAGGTGAAGTGCCGAAGAAGATGGAATCGTT
AATCAACTCGCATGTGAAGT

Create PDF with GO2PDF for free, if you wish to

AACAACATGAGAAAAGATTGTCATTCAAAGACAATAGATTTAAGATTTAAATATCACGATAACACATGAACAATA
AAATCXXXXXXXXXXXAAAGGAGGAGGAGAAGAAGAAGAAACCCAGAGGTGAAGTGCCGAAGAAGATGGAATCGTT
AATCAATTCGCATGTGAAGT
>Marker604311
TACAAGGGTGTAACGAGAGACAGACCTTCAATTGCCATTAAAGTAATAGACTTGGAACAAATGGATTTCTTATCG
AATGTXXXXXXXXXXXGCAGATCAGAACAGAACCATGGCTGACTCTGAAATGAAGAATAGACATAGCTTTAGATGT
AGCAGCAGCAATAATGTAGT
TACAAAGGTGTAACGAGAGACAGACCTTCAATTGCCATTAAAGTAATAGACTTGGAACAAATGGATTTCTTATCG
AATGTXXXXXXXXXXXGCAGATCAGAACAGAACCATGGCTGACTCTGAAATGAAGAATAGACATAGCTTTAGATGT
AGCAGCAGCAATAATGGAGT
>Marker605112
AACAGTATCGGAACTTCGTGACCGAATTCCAAGATTGCTTGTTTGAGAATGTTTATGGGCTTCAAGCGACTGATG
AGAACXXXXXXXXXXXTGATAGAGGAGTTCGAGGAGGCTGCTAATGGAGGAGTGCAGAGCTTGACATTAGGGGCGC
TGGATAACAGCTTCTTGGTG
AACAGTATCGGAACTTCGTGACCGAATTCCAGGATTGCTTGTTTGAGAATGTTTATGGGCTTCAAGCGACTGATG
AGAACXXXXXXXXXXXTGATAGAGGAGTTCGAGGAGGCTGCTAATGGAGGAGTGCAGAGCTTGACATTAGGGGCGC
TGGATAACAGCTTCTTGGTG
>Marker605191
AACCGCATTCAACATTGATAAACACTAGACATCCATGTCTATCATGAAATATAGATTCTAGTGTTCTTTATTTCC
CACCTXXXXXXXXXXXCAATTTAGCTAGGTTGAGATAACCTGACAACTTCTGATTAGTGAAATTTTCTATTTAATA
TTTTATCAAAATGGGTCGTT
AACCGCATTCAACATTGATAAACACTAGACATCCATGTCTATCATGAAATATAGATTCTAGTGTTCTTTATTTCC
CACCTXXXXXXXXXXXCAATTTAGCTAGGTTGAGATAACTTGACAACTTCTGATTAGTGAAATTTTCTATTTAATA
TTTTATCAAAATGGGTCGTT
>Marker605371
AACATATTTACAGATATTGTCCGAAATCGGTTTGAAAAACTTTTCATATCTAACTTCTGGATGCTATATCACAGC
ACAACXXXXXXXXXXXCCAATCACATGAACAACAATTAACAATTTTCAAACTAACACAATGATGGTATATATAGAC
TTATAGTCTATGCTCCTGTT
AACATATTTACAGATATTGTCCGAAATCGGTTTGAAAAACTTTTCATATCTAACTTCTGGATGCTATATCACAGC
ACAACXXXXXXXXXXXCCAATCACATGAACAACAATTAACAATTTTCGAACTAACACAATGATGGTATATATAGAC
TTATAGTCTATGCTCCTGTT
>Marker605521
AACTGCCTACTTGTTTGAATCTCATTTCAAACCAATGTGAAATCTTCATCTCTATGATTAATTAATTAGAATGTG
AGTAAXXXXXXXXXXXTCGGTTGTCATGACCTTTCTCTTTTTCCTGTTGAACAGTTTGTGCGTGTGGGTAATGATA
GTATTTTCAATTCATAAGTG
AACTGCCTACTTGTTTGAATCTCATTTCAAACCAATGTGAAATCTTCATCTCTATGATTAATTAATTAGAATGTG
AGTAAXXXXXXXXXXXTCGGTTGTCATGTCCTTTCTCTTTTTCCTGTTGAACAGTTTTTGCGTGTGGGTAATGATA
GTATTTTCAATTCATAAGTG
>Marker606557
CACACATGACACAAGGCTCAAACTTTATTTCCTGAAGATCAACAAATGAACTTATGATTAAGAATGATTTATAGA
TGAATXXXXXXXXXXXGAACTCTTCACAAAAAGCCTACCTACGTATCTGGCATGAGAAAAGGCATGTGAAAATGTC
ATTATCCAGAATCCGAAGTT
CACACATGACACAAGGCTCAAACTTTATTTCCTGAAGATCAACAAATGAACTTATGATTAAGAATGATTTATAGA
TGAGTXXXXXXXXXXXGAACTCTTCACAAAAAGCCTACCTACGTATCTGGCATGAAAAAAGGCATGTGAAAATGTC
ATTATCCAGAATCCGAAGTT
>Marker606636

Create PDF with GO2PDF for free, if you wish to

ACTTAGTATCAATTTTTTTGTGTCCATGGGCAAAATTTTTGAAGCAGACAACCACCCCTATCATTTCTTTTCCCC
TTCAAXXXXXXXXXXXTGATCACCATCACCACACCAGCCTTCATTGTTTGTATTGCTTAATGTCACACTTCTATAC
CTTCTTTTCTTCGTCTGGTG
ACTTAGTATCAATTTTTTTGTGTCCATGGGCAAAATTTTTGAAGCAGACAACCACCCCTATCATTTCTTTTCCCC
TTCAAXXXXXXXXXXXTGATCACCATCACCACACCAGCCTTCATTGTTTGTATTGCTTAATGTCACACTTCGATAC
CTTCTTTTCTTCGTCTGGTG
>Marker607262
ACTTATTTATTATGTTATGTTACACATTTTAGATTTGACACACAAATGGTCTTCTATACTAGATTTTGATGGTTT
TTGGTXXXXXXXXXXXTGTAACTTCATGGTTATAACTTTGGAACTAACTTTAGACATTCATTTATTTGATGTAACT
TTATACTTATAACTTAGGTT
ACTTATTTATTATGTTATGTTACACATTTTAGATTTGACACACAAATGGTCTTCTATACTAGATTTTGATGGTTT
TTGGTXXXXXXXXXXXTGTAACTTCATGGTTATAACTTTGGAACTAACTTTGGACATTCATTTATTTGATGTAACT
TTATACTTATAACTTAGGTT
>Marker607357
CACAATGCAAGTGGCAATGGTGTATCATACTTTCATTCTTGCAATTTGGAGAACCACAGTTATTATGATGGATTG
AACTCXXXXXXXXXXXATTTTGGATTATTATTAAAGAAAAATGGCTCTTGCTTATTGTGAATAGTCACCTATTTTA
TTCTCTGTTTCTTTATTGTT
CACAATGCAAGTGGCAATGGTGTATCATACTTTCATTCTTGCAATTTGGAGAACCACAGTTATTATGATGGATTG
AAGTCXXXXXXXXXXXATTTTGGATTATTATTAAAGAAAAATGGCTCTTGCTTATTGTGAATAGTCACCTATTTTA
TTCTCTGTTTCTTTATTGTT
>Marker607363
CACACTCCTTATAAGTAGTAAAAGTTGGAAAAATTTCACACATTGTCTCGAGCTCAGTGCAATTTTCTTTACCGC
TTGTAXXXXXXXXXXXGCTCAACTTCAATTTATGGATGAAATAGATTCTAACACTACTCGAATAACCCAATGAACG
CTCGACCCAATTTGGGTTGT
CACACTCCTTATAAGTAGTAAAAGTTGGAAAAATTTCACACATTGTCTCGAGCTCAGTGCAATTTTCTTTACCGC
TTGTAXXXXXXXXXXXGCTCAACTTCAGTTTATGGATGAAATAGATTCTAACACTACTCGAATAACCCAATGAACG
CTCGACCCAATTTGGGTTGT
>Marker607722
ACTTTTATTATATTTTTTCTTTTTATGTTTAAGTGATTTATTTGACATTTTTTTAAATTTTGAATTAATGGGTGT
TTTTCXXXXXXXXXXXTTTAAAATAATAATAAAATAAACGTGTGAAATTAAATGAAAAAATATTGTTTAAAATGAA
TTTGATTTAGTGGTATTTGT
ACTTTTATTATATTTTTTCTTTTTATGTTTAAGTGATTTGTTTGACATTTTTTTAAATTTTGAATTAATGGGTGT
TTTTCXXXXXXXXXXXTTTAAAATAATAAAAAAATAAACGTGTGAAATTAAATGAAAAAATATTGTTTAAAATGAA
TTTGATTTAGTGGTATTTGT
>Marker608040
ACTAATACAACCCCTTGTTAATACATTTTACAATATTTTAATGATTCGATTGCATATATATCTTAGATGAAGGTG
AGAGTXXXXXXXXXXXATGAATGAAAGGAGAGTTCTAAAATTGGAAGAATACTTATTGAAATTTTAGAATGTTGAG
AGATGCTTTTGAAACAAAGT
ACTAATACAACCCCTTGTTAATACATTTTACAATATTTTAATGATTCGGTTGCATATATATCTTAGATGAAGGTG
AGAGTXXXXXXXXXXXATGAATGAAAGGAGAGTTCTAAAATTGGAAGAATACTTATTGAAATTTTAGAATGTTGAG
AGATGCTTTTGAAACAAAGT
>Marker608258
ACAAATATATGATCCATTTTTACGGTAGGTAAGAAAACTCTTGACACTCAAGCTTATTTCTCCTCCAACAAGATA
TACATXXXXXXXXXXXTTTGTTTGAAAATATTTGATTGTAACGAGCATTTTTATCTCTTTAAGAGTTGTTTGTCTC
TTGCTTCTTTCAATATTGTT

Create PDF with GO2PDF for free, if you wish to

ACAAATATATGATCCATTTTTATGGTAGGTAAGAAAACTCTTGACACTCGAGCTTATTTCTCCTCCAACAAGATA
TACATXXXXXXXXXXTTTGTTTGAAAATATTTGATTGTAACGATCATTTTTATCTCTTTAAGAGTTGTTTGTCTC
TTGCTTCTTTCAATATTGTT
>Marker608260
AACATACAACCACCTTTCAAGGAATAAAACTACATTGAGACCAAAATTTAAGGACTTGACACAACATAAAACTCA
AGGTTXXXXXXXXXXCTATCTGTGACTGTGTTGCCTACATATACTTGCTACTAACCAAGAGCTAGCTCTCTAAGT
TCATTACTAGTTGCTAGGTA
AACATACAACCACCTTTCAAGGAATAAAACTACATTGAGACCAAAATTTAAGGACTTGACACAACATAAAACTCA
AGGTTXXXXXXXXXXCTATCTGTGACTGTGTTGCCTACATATACTTGCTACTAACCAAGAGCTAACTCTCTAAGT
TCATTACTAGTTGCTAGGTA
>Marker608481
TACTCTCTTCATCTTTGTTCCATCTGGTTCAAATAGTAGATAATACTTTGTAGAAAGTTCATTATTTGTTTGTCA
TATCCXXXXXXXXXXTCTTGTCAAATTTTGATTTCAAAGATATTTTGAAACTATTCCATGGGCATTATTTTACTT
GGTTCAAATTCCCTTTTGTG
TACTCTCTTCATCTTTGTTCCATCTGGTTCAAATAGTAGATAATACTTTGTAGAAAGTTCATTATTTGTTTGTCA
TATCCXXXXXXXXXXTCTTGTCGAATTTTGATTTCAAAGATATTTTGAAACTATTCCATGGGCATTATTTTACTT
GGTTCAAATTCCCTTTTGTG
>Marker608501
ACCCCACAGCCATTTTACCTGAAATCGTAGGGGAGATTTTTGTTAAATGATCGACGTCTTTTTTTATATATACTT
ATTATXXXXXXXXXXTATTAATTAACTCATAACATTTTGTTAAGCTATTTTATTGAATTTTAGATCGAAACTGAA
AACGAAATAGCACCGAGGTA
ACCCCACAGCCATTTTACCTGAAATCGTAGGGGAGATTTTTGTTAAATGATCGACGTCTTTTTTTATATATACTT
ATTATXXXXXXXXXXTATTAATTAACTCATAACATTTTGTTCAGCTATTTTATTGAATTTTAGATCGAAACTGAA
AACGAAATAGCACCGAGGTA
>Marker608768
ACATTGCATTAACAAATGGATAATGATACGTGTCTGACAATGAAATTAACCACGCAAAAAAATTAAACTTTTGTG
AATTGXXXXXXXXXXTGAACTTACTCTAACTCCGTTTGCAGTTAGATAAAAGACTAGTGATCAATTTCGTATCTC
TTACATGCAGAACTATCGTG
ACATTGCATTAACAAATGGATAATGATACGTGTCTGACAATGAAATTAACCACGCAAAAAAATTAAACTTTTGTG
AATTGXXXXXXXXXXTCAACTTACTCTAACTCCGTTTGCAGTTAGATAAAAGACTAGTGATCAATTTCGTATCTC
TTACATGCAGAACTATCGTG
>Marker608952
AACACTTGAAAACTTTTATGAAACCAAGATTTTAGTCTCTAAAGAATTTAGGGTTTAAGTTTTTTTTTTTTCTTT
GGGTTXXXXXXXXXXTGTTAAAGTTGTAAGGGCATAAACAACTTTGTTTTTCTCTCACTAAGGATGTGGCAAAAT
TGAGAAATATTAACGAAGTT
AACACTTGAAAACTTTTATGAAACCAAGATTTTAGTCTCTAAAGAATTTAGGGTTTAAGTTTTTTTTTTTCTTTG
GGTTTXXXXXXXXXXTGTTAAAGTTGTAAGGGCATAAACAACTTTGTTTTTCTCTCACTAAGGATGTGGCAAAAT
TGAGAAATATTAACGAAGTT
>Marker609529
ACTTCAAACAGTAGAGTAAATTGTGAAACATCAGAACTAGTGCTAGAGATCAAAATATTCTCAAGAACCATAGAA
AAATTXXXXXXXXXXTTTAGGCACCAATGGCTTTCTTATTTGGAGCCCCCAAAAGAGTCCATTCTCCTATTTCAA
GGTGAAACAGTGTAGCTGTA
ACTTCAAACAGTAGAGTAAATTGTGAAACATCAGAACTAGTGCTAGAGATCAAAATATTCTCAAGAACCATAGAA
AAATTXXXXXXXXXXTTTTAGGCACAATGGCTTTCTTATTTGGAGCCCCCAAAAGAGTCCATTCTCCTATTTCAA
GGTGAAACAGTGTAGCTGTA
>Marker609817

Create PDF with GO2PDF for free, if you wish to

GACTTAGTTAATATCTACTAGATCCCTAGTTGCATCTTCAATTAACCACAACCATATCATCCATTCTGTAGTTTT
AAACCXXXXXXXXXXXATGATGTCTTATACATGTGTTTGACATTCGAATATTTTTTAAGAAATTTGAAAAAAAATT
AAAATTTGTATTTGTTTTGT
GACTTAGTTAATATCTAGTAGATCCCTAGTTGCATCTTCAATTAACCACAACCATATCATCCATTCTGTAGTTTT
AAACCXXXXXXXXXXXATGATGTCTTATACATGTGTTTGACATTCGAATATTTTTTAAGAAATTTGAAAAAAAATT
AAAATTTGTATTTGTTTTGT
>Marker610420
CACACCATGGAATTGAAAATTAGGGGTTTTGGTCATGGTTTCTCTTTTCTTGCACTTATAAGTTGTCTTCGTATC
CAATCXXXXXXXXXXXATTGAAACATATATTATTGTATTAAAAGGTTTCAGGTTTTAAGTTTTAATTTTCGTATGT
TGTTTAGTTTAAAAGAAGTG
CACACCATGGAATTGAAAATTAGGGGTTTTGGTCATGGTTTCTCTTTTCTTGCACTTATAAGTTGTCTTCGTATC
CAATCXXXXXXXXXXXATTGAAACATACATTATTGTATTAAAAGGTTTCAGGTTTTAAGTTTTAATTTTCGTATGT
TGTTTAGTTTAAAAGAAGTG
>Marker610758
CACTATAAAAACATTCCAATGTGTAATCCCTCTTCATCTGTGTAAGCATATCTTTCTTGTTTGAAAAATTACCCT
ACACTXXXXXXXXXXXACATGTCCTCGATGTTCTTAATCCGCATAGAAACATCTTGAATATCACTCGGGTTAGCTA
TGACGAAAAGGATCAGCGTG
CACTATAAAAACATTCCAATGTGTAATCCCTCTTCATCTGTGTAAGCATATCTTTCTTGTTTGAAAAATTACCCT
ACACTXXXXXXXXXXXACATGTCCTCGATGTTCTTAATCCGCATAGAAACATCTTGAATATCACTCGCGTTAGCTA
TGACGAAAAGGATCAGCGTG
>Marker611143
CACTAGAGTGTCTATGATTCCATCAGTGTGATTCGTGATTAGTAATGAAAAATACAAAAATATTTTTTAATGATA
TGTTTXXXXXXXXXXXTGTAAGCCTGACCGTTGTCTCCAAGTCTATTGTGTAAAGTTATAGATAGTTTTACTACTT
TATTGGTTAATACTTAGGTT
CACTAGAGTGTCTATGATTCCATCAGTGTGATTCGTGATTAGTAATGAAAAATACAAAAATATTTTTTAATGATA
TGTTTXXXXXXXXXXXTGTAAGCCTGACCGTTGTCTCCAAGTCTATTGTGTAAAGTTATAGATAGTTTTACTACTC
TATTGGTTAATACTTAGGTT
>Marker611176
AACCATTTGGTCAATAATACACTCTATATATTTGTCAAATTATATTTATTTTGGCATACTTTCTCTACTAATTTT
TATTTXXXXXXXXXXXTATTTTAACAATATTTTGAATCCTCAAATATAACAATGATTTATGTTTCTTTTCATTTCG
AAGACATTTTATTTTTATGT
AACCATTTGGTCAATAATACACTCTATATATTTGTCAAATTATATTTATTTTGGCATACTTTCTCTACTATTTTT
TATTTXXXXXXXXXXXTATTTTAACAATATTTTGAATCCTCAAATATAACAATGATTTATGTTTCTTTTCATTTCG
AAGACATTTTATTTTTATGT
>Marker611413
AACAACTAGGTTTTGATAAATATACGTAACTCCCCGGAAGAAGCCGATTGATAAAACAAGTGTAATTATTAGACT
ATATGXXXXXXXXXXXGTGGTCAGTATCAAAGAATCTTAAAATTAAATAATTATTTTACATGGAGGACAAATTCTA
GGCCAGAATTCGAACTTGTT
AACAACTAGGTTTTGATAAATATACGTAACTCCCCGGAAGAAGCCGATTGATAAAACAAGTGTAATTATTAGACT
ATATGXXXXXXXXXXXGTGGTCTGTATCAAAGAATCTTAAAATTAAATAATTATTTTACATGGAGGACAAATTCTA
GGCGAGAATTCGAACTTGTT
>Marker611432
ACATAAGATGAAACTCTACTTAAATTTTTTTCTTTTGGGTTAGAAAAATAAATTGATTCATTGACACGATGAAATC
ATACAXXXXXXXXXXXGCAAATTCAGCATCCCAGCCTGTTGAACACAGATAGAGCATTAATCATTTTTAAAAGAAA
CCAAAAGTAATCACAAATGT

Create PDF with GO2PDF for free, if you wish to

ACATAAGATGAAACTCTACTTAAATTTTTTCTTTTGGGTTAGAAAAATAAATTGATTCATTGACACGATGAAATC
ATACAXXXXXXXXXXXGCAAATTCAGCATCCAGCCTGTTGAACACAGATAGAGCATTAATCATTTTTAAAAGAAA
CCAAAAGTAATCACAGATGT
>Marker611822
ACTCAATTTGAATTGATTTTTATAGGTCCTGTGGTATCCCTGGAACTGCATTAATTGGCCATTATAAGCCTCTAA
TTGGTXXXXXXXXXXXCTCCGGGTTCAATCTCTCTCTCCCTCTCTCATTTTTTTTATTCCGAGGCTTTGGTTGAAT
TCTAAGAATTTGCAATGGTC
ACTCAATTTGAATTGATTTTCATAGGTCCTGTGGTTTCCCTGAAACTGCATTAATTGGCCATTATAAGCCTCTAA
TTGGTXXXXXXXXXXXCTCCGGGTTCAATCTCTCTCTCCCTCTCTCATTTTTTTTATTCCGAGGCTTTGGTTGAAT
TCTAAGAATTTGCAATGGTC
>Marker612162
GACGTTGGCGTTGTAGGTTGAACCAAACCTCCAACTAGATGGAGCAACGTTCCAAGCTATGATGGTCTCTTTGGT
TGTGTXXXXXXXXXXXCCCACATTCCCGACACGTCGCCGCCGCCACCGACATTCATCACGTACGCCAAAAGCCAGT
AGCCATTTCCTTGTAATGTG
GACGTTGGCGTTGTAGGTCGAACCAAACCTCCAACTAGATGGAGCAACGTTCCAGGCTATGATGGTCTCTTTGGT
TGTGTXXXXXXXXXXXCCCACATCCCCGACACGTCGCCGCCGCCACCGACATTCATCACGTACGCCAAAAGCCAGT
AGCCATTTCCTTGTAATGTG
>Marker612325
TACGTTTCTTATTAATCAATAATTGAATGCTTCATTAATCATAACCAAACACCACACCCCATCAACCAATTCCTA
TTTTGXXXXXXXXXXXTAAACTCTTTCATTTCTACTATTAAATAGGAAGAAGAAGTAAGACAAATTAAAAGGTTGA
TAATGTAATATAATATATGT
TACGTTTCTTATTAATCAATAATTGAATGCTTCATTAATCATAACCAAACACCACACCCTATCAACCAATTCCTA
TTTTGXXXXXXXXXXXTAAACTCTTTCATTTCTACTATTAAATAGGAAGAAGAAGTAAGACAAATTAAAAGGTTGA
TAATGTAATATAATATATGT
>Marker612644
TACAATGTTGCATAGAGGATTGCTCGACCTTCTTCGACGCAACCCTATTTTATGTCAGCTTATCTCCATGAATCC
CCTACXXXXXXXXXXXAACTTTTGGATTGAGGATTATGATTGCCTTATCATTTATATACAAAATCACCACCTACCG
CAACCATTAAAAAATTGGTG
TACAATGTTGCATAGAGGATTCCTCGACCTTCTCCGACGCAACCCTATTTTATGTCAGCTTATCTCCATGAATCC
CCTACXXXXXXXXXXXAACCTTTGGATTGAGGATTATGATTGCCTTATCATTTATATACAAAATCACCACCTACCG
CAACCATTAAAAAATTGGTG
>Marker612827
GACTTGGGATTCGGAGAATTTGTAGAGTGGTTGGGAGTGTTTGAAGAAGTAATCGGAGAGAAATTGTTGGAATCG
GGCTCXXXXXXXXXXXGCAACATCGTTCTGCGTTGAATTCATCCAATGATATGAAGAACGTTCTTGGGTTTTTGGA
GCTCTTTCGATGTTTTTGTG
GACTTGGCATTCGGAGAATTTGTAGAGTGGTTGGGAGTGTTTGAAGAAGTAATCGGAGAGAAATTGTTGGAATCG
GGCTCXXXXXXXXXXXGCAACATCGTTCTGCGTTGAATTCATCCAATGATATGAAGAACGTTCTTGGGTTTTTGGA
GCTCTTTCGATGTTTTTGTG
>Marker613118
ACAAAGGAGAATAAATAGAGAATACAAGGGAATAAAAAAGGAAAATATTTAGGAAATAAGGAAAATATTCCCATA
ATCTTXXXXXXXXXXXTCAATGAGGCCCAACTTGCTAACACAAAAATGGAAGTTTTGGTCTGAGAAGCCCCTTGGT
AAGAACATCAGCAACTTGTT
ACAAAGGAGAATAAATAGAGAATACAAGGGAATAAAAAAGGAAAATATTTAGGAAATAAGGAAAATATTCCCATA
ATCTTXXXXXXXXXXXTCAATGAGACCTAACTTGCTAACACAAAAATGGAAGTTTTGGTCTGAGAAGCCCCTTGGT
AAGAACATCAGCAACTTGTT
>Marker613716

Create PDF with GO2PDF for free, if you wish to

TACACATTTGGTCTTGTTTTAACTATACCATTTTCATCTTCTCTCTCAATTATTTAAGGTTTTATTTGGAGTATG
TGTTGXXXXXXXXXXCTCTCAACTTCATTCATCTCAAGATAGAGTAAGCTTTGCCAATTTGGCAAATAGCTAGGG
AGATAATAGAGGTGTGTGTA
TACACATTTGGTCTTGTTTTAACTATACCATTTTCATTTTCTCTCTCAATTATTTAAGGTTTTATTTGGAGTATG
TGTTGXXXXXXXXXXCTCTCAACTTCATTCATCTCAAGATAGAGTAAGCTTTGCCAATTTGGCAAATAGCTAGGG
AGATAATAGAGGTGTGTGTA
>Marker614301
ACTAAAAAAATTGTTGCTAATTTTTTTTCTAACATAACCGTCTGTTCAATCTTGAAGTATTTTCACAATAACATA
TCAATXXXXXXXXXXTATACTTTAATTGATGGGACAATAATGTTATATGAATAATATAAAAAGAAAATTAATTAG
AAAATATACGTCGTTGTGTA
ACTAAAAAAATTGTTGCTAATTTTTTTTCTAACATAACCGTCCGTTCAATCTTGAAGTATTTTCACAATAATATA
TCAATXXXXXXXXXXTATACTTTAATTGATGGGACAATAATGTTATATGAATAATATAAAAAGAAAATTAATTAG
GAAATATACGTCGTTGTGTA
>Marker615555
CACTTAAAGAAGAGACATGAAACATAAAAGCATATTTATAATTGTATTGTATAACTGCTTCATCTAATTTTTTTC
TTATAXXXXXXXXXXTTGGTAAAGTTATGTTTTTTTTTCATAAATATTTGATGATTGACCTTACTTAAATGTCAT
CAATGATACCAGATTGTTGT
CACTTAAAGAAGAGACATGAAACATAAAAGCATATTTGTAATTGTATTGTATAACTGCTTCATATAATTTTTTTC
TTATAXXXXXXXXXXTTGGTAAAGTTATGTTTTTTTTTCATAAATATTTGATGATTGGCCTTACTTAAATGTCAT
CAATGATACCAGATTGTTGT
>Marker615712
CACCTTTTATAAACAAATTAATATAAGAACATTTCATTCAAAAAATATACACTGCATTATTCAATATATATACGATA
ATTAGXXXXXXXXXXTTATAATATCACCATAATAGAATTAATCACTTACCATATTTACAAAGAATATTTAATAAA
TGCAAATATCAATCATGGTC
CACCTTTTATAAACAAATTAATATAAGAACATTTCATTCAAAAAATATACACTGCATTATTCAATATATATACGATA
ATTAGXXXXXXXXXXTTATAATATTACCATAATAGAATTAATCACTTACCATATTTACAAAGAATATTTAATAAA
TGCAAATATCAATCATGGTC
>Marker616129
ACATTTTCAAGTATAGAGAACTTCTAATTGGAATTTGGGAGGCAAAATTATATAGCTTTATCGGTGGTTGTTCTG
GGAGTXXXXXXXXXXCTGTGAGATTAATGATAATGTAAGATAAATTGCACAAAAAGAAGGTGCATTCCCATAAAC
AGTATAAAGTCATCTCTGTA
ACATTTTCAAGTATAGAGAACTTCTAATTGGAATTTGGGAGGCAAAATTATATAGCTTTATCAGTGGTTGTTCTG
GGAGTXXXXXXXXXXCTGTGAGATTAATGATAATGTAAGATAAATTGCACAAAAAGAAGGTGCATTCCCATAAAC
AGTATAAAGTCATCTCTGTA
>Marker616200
AACTAGAAGCATTACCACGATGAGCACCCACCATACAACATGACAAATTATTGCTTGATGTGAAGAAAGAAAGCC
AACCAXXXXXXXXXXCCCTCTCTTCTACATTTATTTTACTGGAGAAAGAAGGCACAAAACTCAAAGAAAAATAAA
TCAATAATACAGTAAATGTC
AACTAGAAGCATTACCACGATGAGCACCCACCATACAACATGACAAATTATTGCTTGATGTGAAGAAAGAAAGCC
AACCAXXXXXXXXXXCCCTCTCTTCTACATTTATTTTACTGGAGAAAAAAGGCACAAAACTCAAAGAAAAATAAA
TCAATAATACAGTAAATGTC
>Marker616226
ACTTTATAAATACCAATCTTCTTTTAAACCTTTACAAATAACAATATTAGCAACGCTTATAAAATCTATTATTAA
AATACXXXXXXXXXXTGAAGTTTTCAAACGGCGAACATAAGGAGTTTGTTGTGCTGTATGGTTGGTGTATGTGAA
TTATCAGAGGCAAAGTAAGT

Create PDF with GO2PDF for free, if you wish to

ACTTTATAAATACCAATCTTCTTTTAAACCTTCACAAATAACAATATTAGCAACGCTTATAAAATCTATTATTAA
AATACXXXXXXXXXXXTGAAGTTTTCAAACGGCGAACATAAGGAGTTTGTTGTGCTGTATGGTTGGTGTATGTGAA
TTATCAGAGGCAAAGTAAGT
>Marker616317
CACTAGAAGTAAGCTTTATATATAACTTTCTTCAGCTCATTTCTGCTGTCTAGTTTTCGGTTAGATGATTCATTT
CATTCXXXXXXXXXXXACTTTCATTTCTGCGCTACCAATCAAAATATTGAGCTGTTCTATTGAGTCTAAATTTCGT
ATTTTGGCACATCTTTTGTG
CACTAGAAGTAAGCTTTATATATAACTTTCTTCAGCTCATTTCTGCTGTCTAGTTTTCGGTTAGATGATTCATTT
CATTCXXXXXXXXXXXACTTTCATTTCTGCGCTACCAATCAAAATATTGAGCTGTTCTATTGAGTCTAAATTTCCT
ATTTTGGCACATCTTTTGTG
>Marker616617
TACATTGGTGGGTTGTTTTTTTTATGGGATTAAGAAAATGGTTTGATTGGCAATAACACATGCTTTGCTTTGGAG
TCTTTXXXXXXXXXXXTCACCCATAGGCGTTGGGGTTTTCCCTACTTCATTTGTCAATGAAATATTTCTTATTCCA
GAAAAGTTCCAAGAGGATGT
TACATTGGTGGGTTGTTTTTTTGATGGGATTAAGAAAATGGTTTGATTGGCAATAACACATGCTTTGCTTTGGAG
TCTTTXXXXXXXXXXXTCACCCATAGGCGTTGGGGTTTTCCCTACTTCATTTGTTAATGAAATATTTCTTATTCCA
GAAAAGTTCCGAGAGGATGT
>Marker616638
TACTCGCATGATGAAGAAAATAATGGAGAGCATTTTGCATGCAGTGATCCATATTTAATAACACTTCATGAGGAG
TTAAAXXXXXXXXXXXXATCAATAATTAGGTTGTAGGCGTAGTTGAGTGGGTTTCAGTGGTGCAAACCCTCAAACCA
ACCTTCAATCAAGCTAAGTA
TACTCGCATGATGAAGAGAATAATGGAGAGCATTTTGCATGCAGTGATCCATATTTAATAACACTTCATGAGGAG
TTAAAXXXXXXXXXXXXATCAATAATTAGGTTGTAGGCGTAGTTGAGTGGGTTTCAGTGGTGCAAACCCTCAAACCA
ACCTTCAATCAAGCTAAGTA
>Marker617589
ACTACTTTTAACAATCAAAATTGATTTTATATAACCCTAATTTAAGGAAAATCAATTTCTACTCCTACATGTGTG
GTTTGXXXXXXXXXXXGTTATATATGTGTGTTTAATTTATTATTGAATATTGGAAGCGAATGAAAAGAGATACGAA
AAGAATGCTATGGATTTGTT
ACTACTTTTAACAATCAAAATTGATTTTATATAACCCTAATTTAAGGAAAATCAATTTCTACTCCTACATGTGTG
GTTTGXXXXXXXXXXXGTTACATATGTGTGTTTAATTTATTATTGAATATTGGAAGCGAATGAAAAGAGATACGAA
AAGAATGCTATGGATTTGTT
>Marker617610
AACTTTCTTTGGGTTCATAATTATAAGATGAAAGAAAAGATGTAATACACATTCTGTAATCAACTTGGGTGTTAG
AGAATXXXXXXXXXXXCCTTCTTTGCATAGGATTCATGTTTGGCCAATGAAGAAATTAAGAATTGTTCAACCCATC
AACTTAAGATCTACATGGTC
AACTTTCTTTGGGTTCATAATTATAAGATGAAAGAAAAGATGTAATACACATTCTATAATCAACTTGGGTGTTAG
AGAATXXXXXXXXXXXCCTTCTTTGCATAGGATTCATGTTTGGCCAATGAAGAAATTAAGAATTGTTCAACCCATC
AACTTAAGATCTACATGGTC
>Marker618214
CACTAAGTTCCACTAATTATTAGTGGATTATTAGATGTTGAGATTTGACCAACTAATTACATGCATGAATTAGGT
TATAAXXXXXXXXXXXXCTTCACCAAATTGGATCCCACAACTCGATTCTAAGTCCGAAGATTAGCGGGTCAATTCTA
GTGGTTGTCTCGATCTGAGT
CACTAAGTTCCACTAATTATTAGTGGATTATTAGATGTTGAGATTTGACCAACTAATTACATGCAGGAATTAGGT
TATAAXXXXXXXXXXXXCTTCACCAAATTGGATCCCACAACTCGGTTCTAAGTCTGAAGATTAGCGGGTCAATTCTA
GTGGTTGTCTCGATCTGAGT
>Marker618323

Create PDF with GO2PDF for free, if you wish to

TACTGTCAAATTCAACTCCAAAAACTTGATTCTTTGAGGAATCAAAAGCCAAATCCGAGCTAAATAATCCAAGAA
ATCCCXXXXXXXXXXXACCTGTGATGCAGAGGATTTTGTGATTAGGCCAAATTCATGATTGAATTTAAAGAGAAAA
CTATTTATGTTATTGATTGT
TACTGTCAAATTCAACTCCAAAAACTTGATTCTTTGAGGGATCAAAAGCCAAATCCGAGCTAAATAATCCAAGAA
ATCCCXXXXXXXXXXXACCTGTGATGCAGAGGATTTTGTGATTAGGCCAAATTCATGATTGAATTTAAAGAGAAAA
CTATTTATGTTATTGATTGT
>Marker618462
GACATACGTTTATCAACCACCAACACTTTCCCTTCCTTTCCCTCACTCATATCCCTATAGCTATTTTAATATTCT
CTATGXXXXXXXXXXXATTATAGACCACGTTTGAAAATAACTTTATTTGTTTTTTCATTATTTCGTATAAATCTTA
TAATTTCGTTTGAATTGGTA
GACATACGTTTATCAACCACCAACACTTTCCCTTCCTTTCCCTCACTCATATCCCTATAGCTATTTTAATATTCT
CTATGXXXXXXXXXXXATTATAGACCACGTTTGAAAATAACTTTATTTGTTTTTTCATTATTTCGTATAAATCTTA
TAATTTCGTTTGAATTGGTG
>Marker618738
CACTTGAATTGGCACCTAGATCAATTGCAAAAAGTGATACGATTCAGATACCACAGCACATAATCACGCCCAAAT
TTTGAXXXXXXXXXXXATATTTATGCACTTGCAGGCAATTCAATGCCACTAACTGAAATATTTCAACAGTTATTTT
GGCCACTTAAGGAAGTAGTT
CACTTGAATTGGCACCTAGATCAATTGCAAGAAGTGATACGATTCAGATACCACAGCACATAATCACGCCCAAAT
TTTGAXXXXXXXXXXXATATTTATGCACTTGCAGGCAATTCAATGCCACTAACTGAAATATTTCAACAGTTCTTTT
GGCCACTTAAGGAAGTAGTT
>Marker618984
CACGACTTTTGGTCCATTATAATATGTGATTTGATGATGGAATCAATTAATTCTTCTGCTCTTACACTTGACCCT
GTGAGXXXXXXXXXXXGCTTATAGCGATTACATTGGACAAGAATTGGTGCCGAGGCTATTGTGACTTAAGATTTAG
TAGCTAAAGTAATAGTTGTT
CACAACTTTTGGTCCATTATAATATGTGATTTGATGATGGAATCAATTAATTCTTCTGCTCTTACACTTGACCCT
GTGAGXXXXXXXXXXXGCTTATAGCGATTACATTGGACAAGAATTGGTGCCGAGGCTATTGTGACTTAAGATTTAG
TAGCTAAAGTAATAGTTGTT
>Marker619176
CACGAAGGTAGATGTAATTCCTCCAAACCATCACATAGATTGTTGTTTCCAAGGATGGAAAACATGGTTGAATTG
GAAAAXXXXXXXXXXXTGAGAGATTTAGTTCTTCTAGACCTTTCAAAGCTTTCAAAGACTCGGGAATTGTTCCTTC
AAATTGGTTGCCTCCTAAGT
CACGAAGGTAGATGTAATTCCTCCAAACCATCACATAGATTGTTGTTTCCAAGGATGGAAAACATGGTTGAATTG
GAAAAXXXXXXXXXXXTGAGAGATTTAGTTCTTCTAGACCTTTCAAATCTTTCAAAGACTCGGGAATTGTTCCTTC
AAATTGGTTGCCTCCTAAGT
>Marker619259
CACTTAAAAGGAACCAATTGCTGTGCAAAGAAAAGATAATAGATTGATAAAACAAATGCAACAGAAACTTCTCGT
GAAAGXXXXXXXXXXXATTCTTTGGAATGCCAGAAAGCTGCTTAAATAAGGGTTACTAACCTTCAATAAATACTCG
TAGAAGGAATCAATGCTCGT
CACTTAAAAGGAACCAATTGCTGTGCAAAGAAAAGATAATAGATTGATAAAACAAACGCAACAGAAACTTCTCGT
GAAAGXXXXXXXXXXXATTCTTTGGAATGCCAGAAAGCTGCTTAAATAAGGGTTACTAACCTTCAATAAATACTCG
TAGAAGGAATCAATGCTCGT
>Marker620644
TACCCAAATTTTCCCTTTCTCACACCCGAAGATCTTTGGTGCCATCACTTCGAGACCGCATCGACCCCTCTCTTT
CTACCXXXXXXXXXXXCTCCATCTCCACCTCCACCTCCCTGTTTTTCTTAGCTAATTTTATCGATTCCTCGCGTCT
ATCCAGGTAAGGATCATGTT

Create PDF with GO2PDF for free, if you wish to

TACCCAAATTTTCCCTTTCTCACACCCGAAGATCTTTGGTGCCATCACTTCGAGACCGCATCGACCCCTCTCTTT
CTACCXXXXXXXXXXXCCCCATCTCCATCTCCACCTCCCTGTTTTTCTTAGCTAATTTTATCGATTCCTCGCGTCT
ATCCAGGTAAGGATCATGTT
>Marker620650
GACAAGGATATGTATTCAAGGAAAAATCTGTGTTAGTAATTGCACAATGATTCAGTTTTAGTTCCATTATTCTAC
TGATCXXXXXXXXXXXATTGTTGTTGGAAAGTTTGTTTGATTCTTCTACCTTGTTTTAAGCTTTGTCCATTGGTGA
TTGAAATGATAGTGTTGGTT
GACAAGGATATGTATTCAAGGAAAAATCTGTGTTAGTAATTGCACAATGATTCAGTTTTAGTTCCATTATTCTAC
TGATCXXXXXXXXXXXATTGTTGTTGGAGAGTTTGTTTGATTCTTCTACCTTGTTTTAAGCTTTGTCCATTGGTGA
TTGAAATGATAGTGTTGGTT
>Marker621508
AACAACGACATCCTGCCGAATATCCTGCACAAAATGAAGAGACACATCAAATTGGTGATAAAAAATTATCATAAA
AGTAAXXXXXXXXXXXXGCATTAGAAAGTTGATTATTCACAAATTACATTATGATATATATTTTTTATAACCGAGAG
TTGGAGCTTAGCTCAGATGT
AACAACGACATCCTGCCGAATATCCTGCACAAAATGAAGAGACACATCAAATTGGTGATAAAAAATTATCATAAA
AATAAXXXXXXXXXXXXGCATTAGAAAGTTGATTATTCACAAATTACATAATGATATATATTTTTTATAACCGAGAG
TTGGAGCTTAGCTCAGATGT
>Marker621666
ACATTGACAAAAACCCCGGAAAAAGGAAAGAACAGTTAATGTTTTGATGTGTTTTTCTGAAGAGACTATCAAAAT
GGATCXXXXXXXXXXXTGTCTGGATGGTAATTTATATGAACAATGACAGGTTTCAACTGTTTCAATTTTGGATCCT
TTCGAACCGTCTTGAAGAGT
ACATTGACAAAAACCCCGGAAAAAGGAAAGAACAGTTAATGTTTTGATGTGTTTTTCTGAAGAGACTATCAAAAT
GGATCXXXXXXXXXXXTGTCTGGATGGTAATTTATATGAACAATGACGGGTTTCAACTGTTTCAATTTTGGATCCT
TTCGAACCGTCTTGAAGAGT
>Marker621798
CACTGCTTTTTTATATGTAATTTTTGGCAGATAGGAGAGCTTGGAGGCGTAGCTGTGGGGTCAGCTGTATGTATC
TCGTCXXXXXXXXXXXTTGAATTCCAAATTTGGACCCTTTAGTTTGGAGAAAATTTGTTTATTTATGAGATTTTAT
CAAACTATAAAGCTGAGGTT
CACTGCTTTTTTATATGTAATTTTTGGCAGATAGGAGAGCTTGGAGGCGTAGCTGTGGGGTCAGCTGTATGTATC
TCGTCXXXXXXXXXXXTTGAATTCCAAATTTGGACCCTATAGTTTGGAGAAAATTTGTTTATTTATGAGATTTTAT
CAAACTATAAAGCTGGGGTT
>Marker621930
ACATTACACTTGCCTTATATGCTAGGAGACTAGGAGTTATATCGTTATTTGAAACAGAAGTGAGATTCACAAATT
ACAATXXXXXXXXXXXCTAGGTCTTTGTAGTTTGGTATGGTGTGCATATTTTATTGGAGGACAACCCTAAGGAGAG
TGTTTTACGACAGGTTAGTG
ACATTACACTTGCCTTATATGCTAGGAGACTAGGAGTTATATCGTTATTTGAAACAGAAGTGAGATTCACAAATT
ACAGTXXXXXXXXXXXCTAGGTCTTTGTAGTTTGGTATGGTGTGCATATTTTATTGGAGGACAACCCTAAGGAGAG
TGTTTTACGACAGGTTAGTG
>Marker622285
CACGTCGTTCCGACTAGTTGTATAACATGAGTCGGGGTGGAAATAGATTTAATTTTTAAAAACTAAAAAAAAAAA
CAAAAXXXXXXXXXXXXXAGCAAAAAAAAAGTTGAATAAAGAAATAATATTATGTATATTTTGTTTGTTTTAGAGAAG
AAAATAAATAAAAAGTGAGT
CACGTCGTTCCGACTAGTTGTATAACATGAGTCGGGGTGGAAATAGATTTAATTTTTAAAAACTAAAAAAAAAAC
AAAAAXXXXXXXXXXXXXAGCAAAAAAAAAGTTGAATAAAGAAATAATATTATGTATATTTTGTTTGTTTTAGAGAAG
AAAATAAATAAAAAGTGAGT
>Marker622452

Create PDF with GO2PDF for free, if you wish to

AACAAAGACTTGTCTCCCAGCTCGTTTGAGAAGGTTGGATTCTTCTCCCAAAAAGCCCTCGGCAGCTTTTCGCTA
AATATXXXXXXXXXXCCCAACATGAGCTCCCCTGATTTTTTCCTTTTATATGTATAAATAATTGGGGGCCTAACA
ATACCGCTTGCCTTGAAGTG
AACAAAGACTTGTCTCCCAGCTCGTTTGAGAAGGTTGGATTCTTCTCCCAAAAAGCCCTCGGCAGCTTTTCGCAA
AATATXXXXXXXXXXCCCAACATGAGCTCCCCTGATTTTTTCCTTTTATATGTATAAATAATTGGGGGCCTAACA
ATACCGCTTGCCTTGAAGTG
>Marker622564
ACTCCAATAAATTGCCTGAGAAGTAAGTCGTTTTGTTTATCTTGATCTTGATGTTTTGCTTATTAATTGATGGTT
GGCTGXXXXXXXXXXAATGCGGTTGTTACGATGTAAAGGTGTAGCCTCCAACTTCTCCAACGTTTGCTCTAAAAT
TGAAGTCCTCACTGATAGGT
ACTCCAATAAATTGCCTGAGAAGTAAGTCGTTTTGTTTATCTTGATCTTGATGTTTTGCTTATTAATTGATGGTT
GGCTGXXXXXXXXXXAATGCGGTTGTTACGATGTAAAGGTGTAGCCTCCAACTTCTCCCACGTTTGCTCTAAGAT
TGAAGTCCTCACTGATAGGT
>Marker622723
ACTTAGAGTCATGCTTTCATAAGTGATATTAGACCTGTAAAGGTAGTGTGGCTCTTGATAGAGACTCTTAGATTT
TTGTAXXXXXXXXXXTTCAATCTTATCGTTTTTTTCTTAATATAAACAGTTTTTTTTTTTAATATGAACAGTTTT
CATTTAATAGACTTTAGGTA
ACTTAGAGTCATGCTTTCATAAGTGATATTAGACCTGTAAAGGTAGTGTGGCTCTTGATAGAGACTCTTAGATTT
TTGTAXXXXXXXXXXTTCAATCTTATCGTTTTTTTCTTAATATAAACAGTTTTTTTTTTTAATATAAACAGTTTT
CATTTAATAGACTTTAGGTA
>Marker623146
CACAAGAAGAACATTGTAAAAATGGAACAACAAGCTCATGTCATGTTATGAAATAAAAGACAAAAATTAAGTGAT
GCTTTXXXXXXXXXXTGCTAGAGCCTTTGCCTTAGAAGAACTGATTTAGAAGGCTCCCTCTTCCAAAAATATTTA
ACATGACCTTTAAGATAGTG
CACAAGAAGAACATTGTAAAAATGGAACAACAAGCTCATGTCATGTTATGAAATAAAAGACAAAAATTAAGTGAT
GCTTTXXXXXXXXXXAGCTAGAGCCTTTGCCTTAGAAGAACTGATTTAGAAGGCTCCCTCTTCCAAAAATATTTA
ACATGACCTTTAAGATAGTG
>Marker624332
AACACAAAAAGATTTAGAGTTGAAAATTAGAACAATTGTGTGCCTTTTAGACAAAAATAATAGTTAGATTTGAAT
GGAGGXXXXXXXXXXTATAATGACCTGTTGAATTTTTATTGAACACTTTATAAAGTTGAGAGTGGAGACTTTGAA
CACAGATTCGAAAATTTGGT
AACACAAAAAGATTTAGACTTGAAAATTAGAACAATTGTGTGCCTTTTAGACAAAAATAATAGTTAGATGTGAAT
GGAGGXXXXXXXXXXTATAATGACCTGTTGAATTTTTATTGAACACTTTATAAAGTTGAGAGTGGAGACTTTGAA
CACAGATTCGAAAATTTGGT
>Marker624471
CACGAGCACACATATGAATTTCTTCTTCTCCTTGCTTTATTGCTTCCTGATGCGCCAATTTGGCATTCTTCACAG
AACCAXXXXXXXXXXCTAGTTTAGGTGCAAGACAAGATACAATGATATGGTGCAATGCTTACAGAAATCTTCAAG
AAAACTGACTTGGTTGTGTT
CACGAGCACACATATGAATTTCTTCTTCTCCTTGCTTTATTGCTTCCTGATGCGCCAATTTGGCATTCTTCACAG
AACCAXXXXXXXXXXCTAGTTTAGGTGCAAGACAAGATACAATGATATGGTGCAATGCCTACAGAAATCTTCAAG
AAAACTGACTTGGTTGTGTT
>Marker625238
ACTGTTGTATCCATAAAATGACACTCCTTTATATTTTCTGCACGTAGCTCACTATGTTATTCAAGACAAGAGTGC
AGAATXXXXXXXXXXAGAGTCTTTTCCACCACATATAGACAGGCACACAAGGCCAATAAAATGGAGGAAAGGAA
TGCATAATTTGATTGCAGTT

Create PDF with GO2PDF for free, if you wish to

ACTGTTGTATCCATAAAATGACACTCCTTTATATTTTCTGCACGTAGCTCACTATGTTATTCAAGACAAAAGTGC
AGAATXXXXXXXXXXAGAGTCTTTTGCACCACATATAGACAGGCACACAAGGCCAATAAAATGGGAGGAAAGGAA
TGCATAATTTGATTGCAGTT
>Marker625365
CACCATTAACGAAAACCTATTTCACACAACAATACAAATAACAAACACACAGAGAAAAAAGAAAAAAAAAAGGAC
CGTTTXXXXXXXXXXCTCATCTTCTATGTATATATTTATATGAGAAATAAGCCTTAAACTAAACCGAATGATTAA
GAGATACATAGTTTAGTGTA
CACCATTAACGAAAACCTATTTCACACAACAATACAAATAACAAACACACAGAGAAAAAAGAAAAAAAAAAGGGA
CCGTTXXXXXXXXXXCTCATCTTCTATGTATATATTTATATGAGAAATAAGCCTTAAACTAAACCGAATGATTAA
GAGATACATAGTTTAGTGTA
>Marker625516
CACAGGCCAGTAGGGACGAGAGTCAGTTTTGATGATGATGGCAACCCACTTGCCCCTCTTGCTAAGCTTGCTGAC
ATCAAXXXXXXXXXXCCTTTGCTTTTTGATAAGCGCTGCTTTCTTTGATATGGTTTCTTCTTTCTTGTCTATTCA
ATCTTCCCACGTAAAGCTGT
CACAGGCCAGTAGGGACGAGAGTCAGTTTTGATGATGATGGCAACCCACTTGCCCCTCTTGCTAAGCTTGCTGAC
ATCAAXXXXXXXXXXCCTTTGCTTTTTGATAGGCGCTGCTTTCTTTGATATGGTTTCTTCTTTCTTGTCTATTCA
ATCTTCCCACGTAAAGCTGT
>Marker625603
ACCCTACACATAAAGTAAAATTGGTATAACATTTTATTTCGCATCTACTCAAACGTTGTCCTTTCTTTTGTCCAT
TGAAGXXXXXXXXXXGAAAATGAGAAAAATAAGTTTTATTTTCCTTCTAATGAATGTTTTAACTACGAAATACCT
TGCCATATTTGTTGTTTGTA
ACCCTACACATAAAGTAAAATTGGTATAACATTTTATTTCGCATCTACTCAAACGTTGTCCTTTCTTTTGTCCAT
TGAAGXXXXXXXXXXGAAAATGAGAAAAATAAGTTTTATTTTCCTTCTAATTAATGTTTTAACTACGAATTACCT
TGCCATATTTGTTGTTTGTA
>Marker625673
AACCAAAATGGTCCTCAAAATGAGATGTATTATTTGGATTATGTATCGACGGATAATAGCCTTGAGTGGGCATTT
CAACTXXXXXXXXXXATAGGCCAAGCACCAGAGTGATAAAATGGTTTAGTAGGTTTCGCCCCACTATTGACCGGA
GCCATTTGACTGGGATTGTG
AACCAAAATGGTCCTCAAAATGAGATGTATTATTTGGATTATGTATCGACGGATAATAGCCTTGAGTGGGCATTT
CAACTXXXXXXXXXXATAGGCCAAGCACCAGAGTGATAAAATGGTTTAGTAGGTTTCGCCCCACTATTGACTGGA
GCCATTTGACTGGGATTGTG
>Marker625755
CACTTCCACATTTGATCACACACAAAAACCATTCACATTTATGCAAAAATAATTACAGCACCAATATTATTACTG
CCTACXXXXXXXXXXTCTCACTATATTTAATTTTCACCATAATTTATGTCACCTCCTCATTGAAAACAGATAAAG
AACTTATTGTTCTGGTGGTT
CACTTCCACATTTGATCACACACAAAAACCATTCACATTTATGCAAAAAGAATTACAGCACCAATAATATTACTG
CCTACXXXXXXXXXXTCTCACTATATTTAATTTTCACCATAATTTATGTCACCTCCTCATTGAAAACAGATAAAG
AACTTATTGTTCTGGTGGTT
>Marker626989
GACACAGTTGTAGATAAGTCATGGATATATAAATCTGTGGTGTCATAATTTTACTTACCAAACAGATATATAAAT
CATAAXXXXXXXXXXACTAATGTTTTATACAAAACTCTATACATATGGATTTACTAACCACAAATTAACATAAAA
TTACATATTGAAAGAGAGTT
GACACAGTTGTAGATAAGTCATGGATATATAAATCTGTGGTGTCATAGTTTTACTTACCAAACAGATATATAAAT
CATAAXXXXXXXXXXACTAATGTTTTATACAAAACTCTATACATATGGATTTACTAACCACAAATTAACATAAAA
TTACATATTGAAAGAGAGTT
>Marker627082

Create PDF with GO2PDF for free, if you wish to

ACCAGCACATTCACTTGCACTGCCGCCAATTACCTCATCACCTTTTGTCCCCTTTCAGATAGGTGAGTCACTTCT
GTTCTXXXXXXXXXXAGAGATGGGTAGGGACTTGTAATTGTATTTTGATCGAAATTTGTTCTTTATGCAGAAACG
CACAATCCCCAAAGGGAGTG
ACCAGCACATTCACTTGCACTGCCGCCAATTACCTCATCACCTTTTGTCCCCTTTCAGATAGGTGAGTCACTTCT
ATTCTXXXXXXXXXXAGAGATGGGTAGGGACTTGTAATTTTATTTTGATCGAAATTTGTTCTTTATGCAGAAACG
CACAATCCCCAAAGGGAGTG
>Marker627366
TACTTAATTTAGTGATGCTGCTACTTTTTTGCTGTAATTTCCATAATTGAGTATGAAGGCTTTTTAGAAATGTTG
TTTTCXXXXXXXXXXTAGGATGCTGTGAGCGAGAAATCAGTTGGGGCTAAGCTGGAAAATGGAGATGGAGGGTTG
TCTGTCTCGACTGAAGAGTT
TACTTAATTTAGTGATGCTGCTACTTTTTTGCTGTAATTTCCATAATTGAGTATGAAGGCTTTTTAGAAATGTTG
TTTTCXXXXXXXXXXTAGGATGCTGTGAGCGAGAAATCAGGTGGGGCTAAGCTGGAAAATGGAGATGGAGGGTTG
TCTGTCTCGACTGAAGAGTT
>Marker627448
ACCATATTGTTCTTGTAATCTGAGTTTCATTCCATATTGTTCTTGTGTTTTCAAATCTTCAAAATCAATCTTTGC
ACTTTXXXXXXXXXXAATTTTGATAAGAATTAACTTCAAGTTATTAAAGTTAAGATTATAAAAAAGATGAAGAGT
AAATTTGGGCATTTGAAAGT
ACCATATTGTTCTTGTAATTTGAGTTTCATTCCATATTGTTCTTGTGTTTTCAAATCTTCAAAATCAATCTTTGC
ACTTTXXXXXXXXXXAATTTTGATAAGAATTAACTTCAAGTTATTAAAGTTAAGATTATAAAAAAGATGAAGAGT
AAATTTGGGCATTTGAAAGT
>Marker627676
CACGCCACAATGAGTCTCAACCTTGCACTTAAAAGGATGGCAGAGAAGGAAAAGATGGGAGATAAAAAGAAACAG
AAAAAXXXXXXXXXXTGCACATCCGAGTATTATGCATACCTCACCTCTTAGAACAAAACCTGCCAACAATTTTTT
TCATATGAAATACCAGCAGT
CACGCCACAATGAGTCTCAACCTTGCACTCAAAAGGATGGCAGAGAAGGAAAAGATGGGAGATAAAAAGAAACAG
AAAAAXXXXXXXXXXTGCACATCCGAGTATTATGCATACCTCACCTCTTAGAACAAAACCTGCCAACAATTTTTT
TCATATGAAATACCAGCAGT
>Marker628294
GACAGAGTATCAACTATAGTGCCATTAGATTTTTTTCTCTTTCCTTGATTGAGAATTACTTATTTGTCTATTCAG
TTTCCXXXXXXXXXXTGAAAGCAAGAGGAAACATTGAAAGAACAAATAAGAAATGAGATCACAATAGTGAAAAGT
AAAAGTGACAAATGTTTGTT
GACAGGGTATCAACTATAGTGCCATTAGATTTTTTTCTCTTTCCTTGATTGAGAATTACTTATTTGTCTATTCAG
TTTCCXXXXXXXXXXTGAAAGCAAGAGGAAACATTGAAAGAACAAATAAGAAATGAGATCACAATAGTGAAAAGT
AAAAGTGACAAATGTTTGTT
>Marker628577
GACAAATTTTAAATAATTTTGTATTTTTATAGATTAAATTTATAGTTTTGAAGGTTGAAGAATTAAATAGGATAC
ATCCAXXXXXXXXXXAATGACAAAATTTCTAAAAATATTTATTAAGGTAAAGATAGTAAATATCTCAATTTTCTT
TTATAGACAGTTATTTATGT
GACAAATTTTAAATAATTTTGTATTTTTATAGATTAAATTTATAGTTTTGAAGGTTGAAGAATTAAATAGGATAC
ATCCAXXXXXXXXXXAATGACAAAATTTCTAAAAATATTTATTAAGGTAAAGATAGTAAATATCTCAATTTTCTT
TTATAAACAGTTATTTATGT
>Marker628988
ACGTAGTTTCCGGTATTCTCAGGTTCCATTTCAAATAGATGTTTAGCAGCAATCTCCCCCACCTCCAAATTACCA
TGGATXXXXXXXXXXTAATGCCAAAACCCTTCCTCATAAGTTCAAAATACTTGCATCCTTGTTCAATCAACCCTG
CATGGGCACATGCATTTAGT

Create PDF with GO2PDF for free, if you wish to

ACGTAGTTTCCGGTATTCTCAGGTTCCATTTCAAATAGATGTTTAGCAGCAATCTCCCCCACCTCCAAATTACCA
TGGATXXXXXXXXXXCAATGCCAAAACCCTTCCTCATAAGTTCAAAATACTTGCATCCTTGTTCAATCAACCCTG
CATGGGCACATGCATTTAGT
>Marker628994
TACTTGGGACATTATCTTGTTATATTCGTCCTTTTTAGATTGTTCAATTAAGAATTGTCCCTTTTAATTTTCATA
AAAATXXXXXXXXXXCGGTTTCCAATTGTTTAGGCTTTGATGATGATGTATAGAAGGCCACTAGTTTCCTTATTG
TGCCACATCTTTTTACTTGT
TACTTGGGACATTATCTTGTTATATTCGTCCTTTTTAGATTGTTCAATTAAAAATTGTCCCTTTTAATTTTCATA
AAAATXXXXXXXXXXCGGTTTCCAATTGTTTAGGCTTTGATGATGATGTATAGAAGGCCACTAGTTTCCTTATTG
TGCCGCATCTTTTTACTTGT
>Marker629012
ACCTAATGATATTCGTTTGATATGGTTTATTGGGACTTTCTCAATGACTTATTGATTGCAAAGGGTTTGGCCCAA
AATGGXXXXXXXXXXGTGGGTCTATCTATTTTCCTCTATACAATTGTGGTTAAGCTTAGTAAGATGTTATTGCAA
GAACTAAAAGAAGATCCGTA
ACCCAATGATATTCGTTTGATATGGTTTATTGGGACTTTCTCAATGACTTATTGATTGCAAAGGGTTTGGCCCAA
AATGGXXXXXXXXXXGTGGGTCTATCTATTTTCCTCTATACAATTGTGGTTAAGCTTAGTAAGATGTTATTGCAA
GAACTAAAAGAAGATCCGTA
>Marker629392
ACAAGGGAAACAAAACAGAGGAAAATGAACCAAGAGACTCGAATTTTGTAAAGTGACAACTGACCAGGATAGGAA
AAAGAXXXXXXXXXXGAAAAAGTCCAAAAAAAAAAAGAAGAAAAATCTAGAAGCCGAATAAGTTGATAAGGATGT
CGCTATGACAACAATAGGTT
ACAAGGGAAACAAAACAGAGGAAAATGAACCAAGAGACTCGAATTTTGTAAAGTGACAACTGACCAGGATAGGAA
AAAGAXXXXXXXXXXGAAAAGTCCAAAAAAAAAAAAGAAGAAAAATCTAGAAGCCGAATAAGTTGATAAGGATGT
CGCTATGACAACAATAGGTT
>Marker629770
GACTTAGTGATTTTTCAATCCTTTTATTAGAATACATTTTTATTTACTCCTCACTAGTGAAGTGTTCGTTTGAAT
TCACTXXXXXXXXXXGGAAAGTAAATTTTAAAATGTAATATTAGTTTTTTTTCAACCAAAAATAACAGCACAATA
CACCGAGGATCAAAAGGGTT
GACTTAGTGATTTTTCAATCCTTTTATTAGAATACATTTTTATTTACTCCTCACTAGTGAAGTGTTCGTTTGAAT
TCACTXXXXXXXXXXGGAAAGTAAATTTTAAAATGTAATATTAGTTTTTTTTCAACCAAAAATAACAGCACAATA
CACTGAGGATCAAAAGGGTT
>Marker629903
AACACATCAACCCATTTAGTGGAACTTTCTTTTAATTAAGAATGACCTTATTGTCCAAAATGCATAATTTAATCA
ATGTGXXXXXXXXXXGACACTTTACTTATATAATTTAGGGTTAGAGAAGTCCATGGGTTTGGAACAAACACACTA
CTATTTCAATCTCCATTGTC
AACACATCAACCCATTTAGTGGAACTTTCTTTTAATTAAGAATGACGTTATTGTCCAAAATGCATAATTTAATCA
ATGTGXXXXXXXXXXGACACTTTACTTATATAATTTAGGGTTAGAGAAGTCCATGGGTTTGGGACAAACACACTA
CTATTTCAATCTCCATTGTC
>Marker629937
AACTTTTCTTTCAAGGATATAATCCTAACATTTTTCAACTCATGTCATTGTTATCATTATTTGGGATAAAAAAAG
AAATTXXXXXXXXXXAAGGTTGAAATTATTTCAACCTCTAACTTTTATTATATAGTTAAAAAGGAAATTTTGGTA
TGTTAAAATAGTATATGCGT
AACTTTTCTTTGAAGGATATAATCCTAACATTTTTCAACTCATGTCATTGTTATCATTATTTGGGATAAAAAAAG
AAATTXXXXXXXXXXAAGGTTGAAATTATTTCAACCTCTAACTTTTATTATATAGTTAAAAAGGAAATTTTGGTA
TGTTAAAATAGTATATGCGT
>Marker630326

Create PDF with GO2PDF for free, if you wish to

TACTATTGTCACTTTCATTGGCCTCAATGGATGTATGAATTTGGATACTCCAAAACAAATGTCAATGTTAATAGA
GAAAGXXXXXXXXXXATATGAACCATTAATATATAATCCATATGGAGTTTTTAGATGGAAAGATTTAACCACACA
TGCATTTTTTAACAGGTGTT
TACTATTGTCACTTTCATTGGCCTCAATGGATGTATGAATTTGGATGCTCCAAAACAAATGTCAATGCTAATAGA
GAAAGXXXXXXXXXXATATGAACCATTAATATATAATCCATATGGAGTTTTTAGATGGAAAGATTTAACCACACA
TGCATTTTTTAACAGGTGTT
>Marker631189
GACATGGTGATGCTAAGATCAGGGAATAAGAATACTGTAAATGCCATGTTCTTCCCTCATGGTCAAGGACCCTCT
TAAGTXXXXXXXXXXTTGCCGATAAATTAAGGTGGCGTTTTCATGTTATCATCATTTCTTAAGTTTATATTTCTT
TCTCGGTTCTCGCATCCGTA
GACATGGTGATGCTAAGATCAGGGAATAAGAATACTGTAAATGCCATGTTCTTCCCTCATGGTCAAGGACCCTCT
TAAGTXXXXXXXXXXTTGCCGATAAATTAAGGTGGCGTTTTCATGTTATCATCAATTCTTAAGTTTATATTTCTT
TCTCGGTTCTCGCATCCGTA
>Marker631603
TACCTTTTAATTCCTTACAGAGACGAGAGCTTCTAGTATAGCTAAAACAAAACCAATCAAATCTTAGTAAAGTTA
TTTCTXXXXXXXXXXTTACCATAACTCATAAGGTAATTAAGTAAGGTTTTATTGATGACATAAACAATTCAATCT
TCAATTCTTTTGATCGAGTA
TACCTTTTAATTCCTTACAGAGACGAGAGCTTCTGGTATAGCTAAAACAAAACCAATCAAATCTTAGTAAAGTTA
TTTCTXXXXXXXXXXTTACCATAACTCATAAGGTAATTAAGTAAGGTTTTATTGATGACATAAACAATTCAATCT
TCAATTCTTTTGATCGAGTA
>Marker632582
GACCATCCTTATGCAGGAAAAAATTCACAAGCACACAACTTTATGAAAGAATGAAAACAAACTACAGATATCACT
CAAGCXXXXXXXXXXTTTTGTTGTCACTGAACTAGAGTGTGGTGTTTCGGCATTTTCATATGCAAGTATATCAAA
GCTAAAAATGTTTAAAATGT
GACCATCCTTATGCAGGAAAAAATTCACAAGCACACAACTTTATGAAAGAATGAAAGCAAACTACAGATATCACT
CAAGCXXXXXXXXXXTTTTGTTGTCACTGAACTGGAGTGTGGTGTTTCGGCATTTTGATATGCAAGTATATCAAA
GCTAAAAATGTTTAAAATGT
>Marker632698
CACAACAAACTTCAAAAGTTCGATAAAAGGGAGACTTCTTATACTTCTTTTCCAGGTAAGTCACATTATTAAGAA
AAGAGXXXXXXXXXXTAGTGATTTGAGTTTGAGCTTTGAACCAACTCTTCTTAAGGAAAACAAATTATATGATGG
GTTGTCAATCCAATTAGGTT
CACAACAAACTTCAAAAGTTCGATAAAAGGGAGACTTCTTATACTTCTTTTCCAGGTAAGTCACATTATTAAGAA
AAGAGXXXXXXXXXXTAGTGATTTGAGTTTGAGCTTTGAACCATCTCTTCTTAAGGAAAACAAATTATATGATGG
GTTGTCAATCCAATTAGGTT
>Marker633407
TACCAGAAGGAAGGATAGTGATTCCTGCTTTCGTAACTGATGTTATACATCTATTAAAAGGAAGGATACTGTTTC
CTGCCXXXXXXXXXXAGCTAACCTTTTAATCTCACACCCAACTATAGGAAGTCCCTGAACCACTTCAAGAAAGCG
TTACTTCATGTCATGGTGGT
TACTAGAAGGAAGGATAGTGATTCCTGCTTTCGTAACTGATGTTATACATCTATTAAAAGGAAGGATACTGTTTC
CTGCCXXXXXXXXXXAGCTAACCTTTTAATCTCACACCCAACTATAGGAAGTCCCTGAACCACTTCAAGAAAGCG
TTACTTCATGTCGTGGTGGT
>Marker633918
ACATATGATGCATTAGATTTCCATTAGTAGTGAGAGAGCAAAGCTTCTAAATCAATTTAGATGATAGAAAGCATT
CACTGXXXXXXXXXXTCAGTATGATCTATTGTTGAAAAGGCATTGAGATAGTAACAAAGGTCATTTGAATCATGT
GAATATCTTCATGGAAGGTT

Create PDF with GO2PDF for free, if you wish to

ACATATGATGCATTAGATTTCCATTAGTAGTGAGAGAGCAAAGCTTCTAAATCAATTTAGATGATAGAAAGCATT
CACTAXXXXXXXXXXXTCAGTATGATCTATTGTTGAAAAGGCATTGAGATAGTAACAAAGGTCATTTGAATCATGT
GAATATCTTCATGGAAGGTT
>Marker635075
AACAAAATCAAATAATTTTCATCACGTGACAAATAAATAACGTCCAATAAATTAAATCTAATTAAATAAAACTAA
CATAAXXXXXXXXXXXGTTCTACTCAATAGGAGTTTCAACTATCCTCTAACCAAGTGAATATGTTTCTTTCAATAA
ATCAAGAATCTTCTTTAGTT
AACAAAATCAAATAATTTTCATCATGTGACAAATAAATAACGTCCAATAAATTAAATCTAATTAAATAAAACTAA
GATAAXXXXXXXXXXXGTTCTACTCAATAGGAGTTTCAACTATCCTCTAACCAAGTAAATATGTTTCTTTCAATAA
ATCAAGAATCTTCTTTAGTT
>Marker635205
TACACTAGTAATAATGAAAAGGAACCAATTTATTTATTAATCTAATTACAAAGTTGATTTTTCTTTAGTGCAAAT
GTTGAXXXXXXXXXXXGTGGTGAGACAGAGCCCCACTGGCACCAAGCCTCCAAAAAGGAGAAATGTTTTAACATAT
TTTGAAAATATTTTTTTAGT
TACACTAGTAATAATGAAAAGGAACCAATTTATTTATTAATCTAATTACAAAGTTGATTTTTCTTTAGTGCAAAT
GTTGAXXXXXXXXXXXGTGGTGAGACAGAGCCCCACTGGCACCAAGCCTCCAAAAACGAGAAATGTTTTAACATAT
TTTGAAAATATTTTTTTAGT
>Marker635432
GACTTCCCCAGAGGTTTTTATATTACGAAGATGGGGATTGGGTAGATTACCCACAAATAATTGTCAAGTTAATTA
GGGAAXXXXXXXXXXXGCAGCACATTGCTTGGATTGATGACAATGATCAATGTTTCTTTCCAGAATTTTATTCTGG
TGACAGTATGATGCATCAGT
GACTTCCCCAGAGGTTTTTATATTACGAAGATGGGGATTGGGTAGATTACCCACAAATAATTGTCAAGTTAATTA
GGGAAXXXXXXXXXXXGCAGCACATTGCTTGGATTGATGACAATGATCAATGTTTCTTTCCAGAATTTTATTCTGG
TGACAGAATGATGCATCAGT
>Marker635888
TACCTTTACGATTTGATTTCGAAGACTTATTGTCATTACTATTTAGGCAACATTATATTTTCTCAATCGCATTTC
TTTTAXXXXXXXXXXXTTTGAAGCAAATAACTACTCAAAACTATCCCTACAAAACTTGTCCCAACCAATAAAAGAA
TCTCTCAGCCTAAATGCAGT
TACCTTTACGATTTGATTTCGAAGACTTATTGTCATTACTATTTAGGCAACATTATATTTTCTCAATCGCATTTC
TTTTAXXXXXXXXXXXTTTGAAGCAAATAACTTCTCAAAACTATCCCTACAAAACTTGTCCCAACCAATAAAAGAA
TCTCTCAGCCTAAATGCAGT
>Marker637283
ACATCAGTAGATGAAGTTGAATCAAGCATAGCTACTGCAGGGTGTGCTTTTTGTGAATATATTGGCCAAGAAGTG
TTTAAXXXXXXXXXXXTGAAGAGGTAAAATGTAGTGTATAATTTATTGTTAAGCACTTAGTAAATTATTTCAAACA
AACTCTTGAAAAGCTATGTC
ACATCAGTAGATGAAGTTGAATCAAGCATAGCTACTGCAGGGTGTGCTTTTTGTGAATATATTGGCCAAGAAGTG
TTTAAXXXXXXXXXXXTGAAGAGGTAAAATGTAGTGCATAATTTATTATTAAGCACTTAGTAAATTATTTCAAACA
AACTCTTGAAAAGCTATGTC
>Marker637432
TACTGAAAATCAGGAATTTTAATTTGATGTTAAAAGTCTTAGGTTAAATTGTAAGGTTTATTACGTGAAAACAAT
TATTAXXXXXXXXXXXTATAAGATAGCAATTTGATAACAAACAATGTCAATATAAGATGATAGTTAGATAAGAATC
ACACATTTTTAACACTTGTG
TACTGAAAATCAGGAATTTTAATTTGATGTTAAAAGTCTTAGGTTAAATTGTAAGTTTTATTACGTGAAAACAAT
TATTAXXXXXXXXXXXTATAAGATAGCAATTTGATAACAAACAATGTCAATATAAGATGATAGTTAGATAAGAATC
ACACATTTTTAACACTTGTG
>Marker637509

Create PDF with GO2PDF for free, if you wish to

ACTTCTTACACCTTCTCAAAATCTCAACACTCGGCCCCATCTTTCTTTCTAGTCTTTTGGAAATGGGGATTGTGC
TTTGTXXXXXXXXXXXTGTAACATTCTAAAGAACTTCCATCATCTTCCTCTCTTCTTATTTTAAACGGGAACAAAA
CTTTTCATTGATAAAACGTC
ACTTCTTACGCCTTCTCAAAATCTCAACACTCGGCCCCATCTTTCTTTCTAGTCTTTTGGAAATGGGGATTGTGC
TTTGTXXXXXXXXXXXTGTAACATTCTAAAGAACTTCCATCATCTTCCTCTCTTCTTATTTTAAACGGGAACAAAA
CTTTTCATTGATAAAACGTC
>Marker637791
ACTCACCGTCTTGTTTCAATTGCAGTCTATGTAATTTTATATGTTAGAATGTTGAATTTTAATCCCTTTTAATAA
ATCTTXXXXXXXXXXXGATTTATTGAATATGAACTTACGTTACACATTTAAATAGACTCGAGTGAAATGAAATCAA
AATATAATATAAAATTAGTA
ACTCACCGTTTTGTTTCAATTGCAGTCTATGTAATTTTATATGTTAGAATGTTGAATTTTAATCCCTTTTAATAA
ATCTTXXXXXXXXXXXGATTTATTGAATATGAACTTACGTTACACATTTAAATAGACTCGAGTGAAATGAAATCAA
AATATAATATAAAATTAGTA
>Marker638811
ACCAAAAAAAACACAAAATTACACAGGACAAAAACCACTATATCTTGAGACTTGAAAGAGATTGCAATCATTGAG
TTTGTXXXXXXXXXXXCCAACAAAATCACATTCATCCATGACTTCCAACTCAATATCAAATCAAATGTTTATAAGG
AAATTTGGATTACGAAAAGT
ACCAAAAAAAACACAAAATTACACAGGACAAAAACCACTATATCTTGAGACTTGAAAGAGATTGCAATCATTGAG
TTTGTXXXXXXXXXXXCCAACAAAGTCACATTCATCCATGACTTCCAACTCAATATCAAATCAAATGTTTATAAGG
AAATTTGGATTACGAAAAGT
>Marker639000
AACGACAAGCTTAGCAAAATGGCCAATGTGAATGAGTGCCTCAGCTTGAGAAAATAAAGGGTTTCTATAACAACT
ATTTTXXXXXXXXXXXTACTATATTTGAAAGTATTTTCAAAAGTTTTGTCATCTAAAAGAACTTCCCTTATCATAA
TATCACCCAACCCAAAAGTT
AACGACAAGCTTAGCAAAATGGCCAATGTGAATGAGTGCCTCAGCTTGAGAAAATAAAGGGTTACTATAACAACT
ATTTTXXXXXXXXXXXTACTATATTTGAAAGTATTTTCAAAAGTTTTGTCATCTAAAAGAACTTCCCTTATCATAA
TATCACCCAACCCAAAAGTT
>Marker639661
AACATATAACAACCGAGCTTCTCAAACCTTTTTGTTATTTGAAACTATTATCATGTTACTCTTACCGTCCTGCAT
AAGTTXXXXXXXXXXXGGCCTACTCTATGGTCTTATTTCCTTTGATTCATCACCCCATAGAGATAAAGACAGCAAG
TGGCATATTAGTGAGGAGGT
AACATATAACAACCGAGCTTCTCAAACCTTTTTGTTATTTGAAACTATTATCATGTTACTCTTACCGTCCTCCAT
AAGTTXXXXXXXXXXXGGCCTACTCTATGGTCTTATTTCCTTTGATTCATCACCCCATAGAGATAAAGACAGCAAG
TGGCATATTAGTGAGGAGGT
>Marker639704
TACCTTGAAGAAATGGATCCTGGAAAACATGCACAAGAAGAGAGAAAAGATTAGCGACAACAAATTTACCACAAC
TACGGXXXXXXXXXXXGTCCTCCTCTTTTAGCTGCACTCCACCATTTTTCTTAACAATGATATCACAAACAAGCTA
AATTCAGACTCTACTTTGTT
TACCTTGAAGAAATGGATCCTGGAAAACATGCACAAGAAGAGAGAAAAGATTAGCGACAACAAATTTACCACAAC
TACTGXXXXXXXXXXXGTCCTCCTCTTTTAGCTGCACTCCACCATTTTTCTTAACAATGATATCACAAACAAGCTA
AATTCAGACTCTACTTTGTT
>Marker639761
ACTATAGGTAAAAAGAACACTAAAAAAATCTGGAAAGAAAGCTTACACGTCTAAGATTGAGATATTTCAAAGGGA
TATTGXXXXXXXXXXXACCTAAATTGATCACAATGGATCCTATGCCATAGCATTAACTAAAGTTTGCTACCCCTAT
TCAATTTCCCCTCAACAGTC

Create PDF with GO2PDF for free, if you wish to

ACTGTAGGTAAAAAGAACACTAAAAAAATCTGGAAAGAAAGCTTACACGTCTAAGATTGAGATATTTCAAAGGGA
TATTGXXXXXXXXXXXACCTAAATTGATCACAATGGATCCTATGCCATAGCATTAACTAAAGTTTGCTACCCCTAT
TCAATTTCCCCTCAACAGTC
>Marker639956
CACAAAAGACTTTGATGAACCGATACCGGCAAATCAACTCTGGGATCCTGATTCCTTGATAAGGATTGCACCTGA
TGATGXXXXXXXXXXXGACACTTCAAAGATATGCAACATTGAGATCCAAAGAAGCTATTTCTTTGATCGAAAAGCA
GACTCAATCATTGTTTGGTA
CACAAAAGACTTTGATGAACCGATACCAGCAAATCAACTCTGGGATCCTGATTCCTTGATAAGGATTGCACCTGA
TGATGXXXXXXXXXXXGACACTTCAAAGATATGCAACATTGAGATCCAAAGAAGCTATTTCTTTGATCGAAAAGCA
GACTCAATCATTGTTTGGTA
>Marker640351
CACCAAAATTCTTGTGCTTACACCCTCCAACAAAATGGGGTTGCCGAACGAAAAAACCGTTACATTTTGGAAGTT
GCATCXXXXXXXXXXXAGACATCGTTAGATTGTGTTAAAGAATCCTATCCCTCCACTCGTCACATCCCTGATGTTC
CCCTTCGGGTGTTCGGATGT
CACCAAAATTCTTGTGCTTACACCCTCCAACAAAATGGGGTTGCCGAACGAAAAAACTGTTACATTTTGGAAGTT
GCATCXXXXXXXXXXXAGACATCGTTAGATTGTGTTAAAGAATCCTATCCCTCCACTCGTCACATCCCTGATGTTC
CCCTTCGGGTGTTTGGATGT
>Marker640656
AACATCTGAATTACTTTTAAAATCTGACTACTTTTTCCCACGTCCCATTCCAGCATAATTTATGATCTCTTCCAT
TGTATXXXXXXXXXXXTTATGGCACTAGGCTTGGGGCATTGCCCATATGCTTTTGGAATGTCCGTATGCTTAATTG
CTAACAATCATGGCGAAGTG
AACATCTGAATTACTTTTAAAATCTGACTACTTTTTCCCACGTCCCATTCCAGCATAATTTATGATCTCTTCCAT
TGTATXXXXXXXXXXXTTATGGCACTAGGCTTGGGGCATTGCCCATATGCTTTTGGAATGTTCGTATGCTTAATTG
CTAACAATCATGGCGAAGTG
>Marker640996
TACTTTGATTAATTTCTTTGTAGTCAGATATTTTGGTTTTGGTCCTTTTGCTATCCCTCTCCCTTCATGTCAAAT
TTGCAXXXXXXXXXXXTAAAGCAATGAGATTAATCTGGGTTTGAATATTGGTTTGGCTTTCTGATATTTTCATCAT
CATCATTATTTATTCCTGTT
TACTTTGATTAATTTCTTTGTAGTCAGATATTTTGGTTTTGGTCCTTTTGCTATCCCTCTCCCTTCATGTCAAAT
TTGCAXXXXXXXXXXXTAAAGCAATGAGATTAATCTGGGTTTGAATATTGGTTTGGGTTTCTGATATTTTCATCAT
CATCATTATTTATTCCTGTT
>Marker641490
TACAGTGCCATTCCATGAGTATCAATGAAGCACGACGTAGCACATTTTACTTGTAAGACAAATTAAATTGGCATG
CATATXXXXXXXXXXXTCTTCTCTAGCAAGTCCAAAGTCTGCAAGCTTCACAGACCTTTGATTTGCAGTAAGCAAC
AAATTATCTACATTGCAAGT
TACAGTGCCATTCCATGAGTATCAATGAAGCACGACGTAGCACATTTTACTTGTAAGACAAATTAAATCGGCATG
CATATXXXXXXXXXXXTCTTCTCTAGCAAGTCCAAAGTCTGCAAGCTTCACAGACCTTTGATTTGCAGTAAGCAAC
AAATTATCTACATTGCAAGT
>Marker641519
TACCTTTAATTTTTTCATTTAGACATTACATCGATCAACATTATGCCAACTTCTCGTCTTTTAAGTAAAGGGCAA
AGGTTXXXXXXXXXXXGAATATATTTTATCTTACATTTATTCTCATGGCAAGTGCCGGCAATATTTATTGATGCCT
ATCAATTGACATAACTTGGT
TACCTTTAATTTTTTCATTTATACATTACATCGATCAACATTATGCCAACTTCTTGTCTTTTAAGTAAAGGGCAA
AGGTTXXXXXXXXXXXGAATATATTTTATCTTACATTTATTCTCATGGCAAGTGCCGGCTATATTTATTGATGCCT
ATCAATTGACATAACTTGGT
>Marker641536

Create PDF with GO2PDF for free, if you wish to

TACTTGGCCAAAGAACGATTTCCTCCCTAATGGAAGTCTATTCTGAAATACACCTCAAGGAGGATCACACAAGTG
CTCTGXXXXXXXXXXAAGATCAGTGTTGAAAGTTACATGGTCGTCCCCCAAGAGGTAAAAAACGCTCTCCCAACG
ACAAATACTGAGAGGCTGTA
TACTAGGCCAAAGAACGATTTCCTCCCTAATGGAAGTCTATTCTGAAATACACCTCAAGGAGGATCACACAAGTG
CTCTGXXXXXXXXXXAAGATCAGTGTTGAAAGTTACATGGTCGTCCCCCAAGAGGTAAAAAACGCTTTCCCAACG
ACAAATACTGAGAGGCTGTA
>Marker642193
ACTTCAAGTCTAGTTGTTGAAAAGAATCTTCACCAACATGTAATCTGATATTTTGATGCATTTCTTCATGACTTG
CTGATXXXXXXXXXXGGAAACGTTAGAGAGAGAGAAAGATTGGATAGAAAAATGTGCAAAGTTCTTCTGTGACCA
CTTTTGAAGTCGGTAGAGTT
ACTTCAAGTCTAGTTGTTGAAAAGAATCTTCACCAACATGTAATCTGATATTTTGATGCATTTCTTCATGACTTG
CTGATXXXXXXXXXXGGAAACGTTAGAGAGAGAGAAAGATTGGATAGAAAAATGTGCAAAGTTCTTCTGTGACCA
CTTTTGAAGTCGATAGAGTT
>Marker642234
AACTCAGAAGAACCAAAAGTTTCGTGGTTGTATGAATCATTAGCATTCCCTCGTTGATGGGCTGCAGCCTGATTA
ATAAAXXXXXXXXXXATAACCTAGGGATAGAAATTTAATTTAAATTATGTTTGCAAGTGAGGAGATTGGAAAACA
CAATGTAATTGTGAAATGTG
AACTCAGAAGAACCAAAAGTTTCGTGGTTGTATGAATCATTAGCATTCCCTCGTTGATGGGCTGCAGCCTGATTA
ATAAAXXXXXXXXXXATAACCTAGGGATAGAAATTTAATTTAAATTATGTTTGCAAGTGAGGAGATTGGGAAACA
TAATGTAATTGTGAAATGTG
>Marker642491
TACCTTTAAAAAAAATCCAAACAAGCAGCAAGGTTATCAAATGTCATTATTATTTGGTTACTCAATAGCAGAATA
GATCTXXXXXXXXXXAAATCCCATATTTTGAGTTTGTGTTTAAAAAAATGCAAAACAAATGGTTGATGAATTAAA
ACTAAAAAAAGTTTGCGGTG
TACCTTTAAAACAAATCCAAACAAGCAGCAAGGTTATCAAATGTCATTATTATTTGGTTACTCAATAGCAGAATA
GATCTXXXXXXXXXXAAATCCCATATTTTGAGTTTGTGTTTAAAAAAATGCAAAACAAATGGTTGATGAATTAAA
ACTAAAAAAAGTTTGCGGTG
>Marker643000
CACCCACTTTGATATCACTGCCTACATACTCAAGGAGATAAAAACTCTAATTATACACTCAATGAAAGCATGAAC
ATAATXXXXXXXXXXCATTATTCAAAGAGAAACTTCCATTCTGATCTGCTCTTGTCCAAAATTGGTAGCCCTGAG
TTGCCAAGCGTATTGAAGTC
CACCCACTTTGATATCACTGCCTACATACTCAAGGAGATAAAAACTCTAATTATACACTCAATGAAAGCATGAAC
ATAATXXXXXXXXXXCATTATTCAAAGAGAAACTTCCATTCTGATCTGCTCTTGTCCAAAATTGGTAGCCCTGAG
TTGCCAAACGTATTGAAGTC
>Marker643752
TACACTACCACAACCGAGAAAGAGAGTCGAAAGAAGAACTAAAGAGAACAAATCGAAACTAACTCTAAGCTTATA
TGCTTXXXXXXXXXXTATGAGAAAATGAGAAGTGAATTGTGAAAGAGGTAACTGAATTTGTGGTAATCAAATAGT
GGAACTTCGATTTTGGTGTC
TACACTACCACAACCGAGAAAGAGAGTCGAAAGAAGAACTAAAGAGAACAAATCGAAACTAACTCTAAGCTTATA
TGCTTXXXXXXXXXXTATGAGAAAATGAGAAGTGAATTGTGAAAGAGGTAACTGAATTTGTGGTAATCAAATAGT
GGAACTTCGATTCTGGTGTC
>Marker643781
AACAAAATCAAGATTTGTTAATTATACATCAACCTTTTATAATGGAAAAAAAAAATCATAAATATAAGGGTTTAG
GGTATXXXXXXXXXXAACAAAAATTTTCAATTTCACCATTTTAAGAGGAAATTTTAGGGAGCTTGAACCCAGAGC
AAGGATTACCAGTCACAAGT

Create PDF with GO2PDF for free, if you wish to

AACAAAATCAAGATTTGTTAATTATACATCAACCTTTTATAATGGAAAAAATAAATCATAAATATAAGGGTTTAG
GGTATXXXXXXXXXXXAACAAAAATTTTCAATTTCACCATTTTAAGAGGAAATTTTAGGGAGCTTGAACCCAGAGC
AAGGATTACCAGTCACAAGT
>Marker643861
ACAATAATGTAGCCAAACTTGACCCATACAACACTCTATCTCAATCTATCTTTAAATGAATTCTCTTTTTTACCA
ATCCCXXXXXXXXXXXGATATAGAATTAGAAAGAAATAGTAAAAGAGAAGCATTAATGTGAGGTTAATTAAGGTAG
GACCAATGGAGACAATGGGT
ACAATAATGTAGCCAAACTTGACCCATAGAACACTCTATCTCAATCTATCTTTAAATGAATTCTCTTTTTTACCA
ATCCCXXXXXXXXXXXGATATAGAATTAGAAAGAAATAGTAAAAGAGAAGCATTAATGTGAGGTTAATTAAGGTAG
GACCAATGGAGACAATGGGT
>Marker644003
GACAACTTCCGCAAAACTTCTTATATCTAACAAAGAAAACTGCAACTACATTTTACATCATACTTACGAATCTTT
TCAAAXXXXXXXXXXXXACCGCGGCGCCCCAAGGTGAGGTCCTCTCTGCCTCCTGAGCCGACCTGCTCTTAGTTCTC
ATGGAAGCAGAACCAACTGT
GACAACTTCCGCAAAACTTCTTATATCTAACAAAGAAAACTGCAACTACATTTTACATCATACTTACGAATCTTT
TCAAAXXXXXXXXXXXXACCGCGGCGCCCCAAGGTGAGGTCCTCTCGGCCTCCTGAGCCGACCTGCTCTTAGTTCTC
ATGGAAGCAGAACCAACTGT
>Marker644563
CACCTGTTAGGGAACTTTGATAAAAGGGTTATAACCATATTGAAGTGATTTAATTAATGTGTATATAATATCAAA
AAGATXXXXXXXXXXXTATTATAGCATAGTAAAGAAAGATGAAATTAAAAAAGTAAAAAAGGAGTCAAGTGGATTA
ATAATTCTTGTTTAATTGTT
CACCTGTTAGGGAACTTTGATAAAAGGGTTATAACCATATTGAAGTGATTTAATTAATGTGTATATAATATTAAA
AAGATXXXXXXXXXXXTATTATAGCATAGTAAAGAAAGATGAAATTAAAAAAGTAAAAAAGGAGTCAAGTGGATTA
ATAATTCTTGTTTAATTGTT
>Marker644836
AACCTGAAATAACACAATACCATTTGATTGCCGAAGTTAGTTTATTAACGGAAAGTAGTTTGCGACAAGAAAAAT
TGTTTXXXXXXXXXXXACGCACATTACTCCTTTGTGTGAAAAGCTATCTCAAACAATGTCATAATGTTAATCCATC
TTCAAAGCATTGAGCAAAGT
AACCTGAAATAACACAATACCATTTGATTGCCGAAGTTAGTTTATTAACGGAAAGTAGTTTGCGACAAGAAAAAT
TGTTTXXXXXXXXXXXACGCACATTACTCCTTTGTGTGAAAAGCTATCTCAAACAATGTCATAATGTTAATCCATC
TTCAAAACATTGAGCAAAGT
>Marker644852
ACTCTTATTTAGAGTTGTTTTTATTAGATTTAACGCGTTTGCTAGTTTATTTTTGAAGAATTATTAAAATAAATG
AGTATXXXXXXXXXXXCATTGTGGGCACTGCAACATTATACATGTTGTGCTATATATCGAAGCTGAGGCATCCGGT
CATGCTGTGCGTGAGAGGTT
ACTCTTATTTAGAGTTGTTTTTATTAGATTTAACGTGTTTGCTAGTTTATTTTTGAAGAATTATTAAAATAAATG
AGTATXXXXXXXXXXXCATTGTGGGCACTGCAACATTATACATGTTGTGCTATATATCGAAGCTGAGGCATCCGGT
CATGCTGTGCGTGAGAGGTT
>Marker644887
ACTCAAGCACAATTACTCTGGATGTTGTTTGAATGTCAAATTTTAATGTTCTTGACGTTTTTTCAAAACATTTTT
TAAACXXXXXXXXXXXAGTTTATATTGCTACAAAATAGGATTTCCTAACCAAATATTTAGATATATTTAGTGTATA
TCATTATTAATATAGTTGTA
ACTCAAGCACAATTACTCTGGATGTTGTTTGAATGTCAAATTTTAATGTTCTTGACGTTTTTTCAATACATTTTT
TAAACXXXXXXXXXXXAGTTTATATTGCTACAAAATAGGATTTCCTAACCAAATATTTAGATATATTTAGTTTATA
TCATTATTAATATAGTTGTA
>Marker645135

Create PDF with GO2PDF for free, if you wish to

AACAAGAAACTCCTTTGCTGAAACACCAACCGTAGCAGTAGAGTTGATAGAAAACCAGGGAATTCCACTAAAAAG
AAAAAXXXXXXXXXXTGTAATACTTTCTTCTAATCTTTTTTCCATTCTCTGTTTTCCAAATTTCAGCGGAAGACA
TATAGGCGAAGGTGCTTGTT
AACAAGAAACTCCTTTGCTGAAACACCAACCGTAGCAGTAGAGTTGATAGAAAACCAGGGAATTCCACTAAAAAG
AAAAAXXXXXXXXXXTGTAATACTTTCTTCTAATCTTTTTTCCATTCTCTGTTTTCCAAATTTCAGTGGAAGACA
TATAGGCGAAGGTGCTTGTT
>Marker645205
ACAAACTTCTTATTGTCTCCGTCTTTTTAATTGTTGTTTCATTTGTATTATCCAGGACTCTACTTTTGAATTTGA
GAGGTXXXXXXXXXXGTTAAAAATTCCTTCCATTCCCATTTGTTATATGCCTATATTTTTTGCAAATGCTATTTC
AGTTGTAACTCAGGATCGTT
ACAAACTTCTTATTGTCTCCGTCTTTTTAATTGTTGTTTCATTTGTATTATCCAGGACTCTACTTTTGAATTTGA
GAGGTXXXXXXXXXXGTTGAAAATTCCTTCCATTCCCATTTGTTATATGCCTATATTTTTTGCAAATGCTATTTC
AGTTGTAACTCAGGATCGTT
>Marker645938
TACTTTTAAAATTCATACACCAAAAGCATATATTCATCTAAACTTCAAGACTAAAAATGTAGTTTTTTTTTTAAT
TTTTGXXXXXXXXXXTAAAAACATTTTAGATTTTACCTCTATTTCTTTTCTTTTTTGTTTCTTCTAAAAAAATCA
ATGACATAGCGGAGTATGTA
TACTTTTAAAATTCATACACCAAAAGCATATATTCATCTAAACTTCAAGACTAAAAATGTAGTTTTTTTTTTAAT
TTTTGXXXXXXXXXXTAAAAACATTTTAGATTTTACCTCTATTTCTTTTCTTTTTTGTTTCTTCTAAAAAAATCA
TTGACATAGCGGAGTATGTA
>Marker645957
ACTCCTTACTTGGGTTATATGTATCAATCTCTGAATCTTACCGGGGTTTGTAGATTGGGAGATATTTCAATGAAA
CCATCXXXXXXXXXXTAACAAAGATACGAATATATTGATTCAATACCTTCACTTCTTAGGTTGCTTTTAATTAAG
AAAACATACATTCTTAGGTT
ACTCCTTACTTGGGTTATATGTATCAATCTCTGAATCTTACCGGGGTTTGTAGATTGGGAGATATTTCAATGAAA
CCGTCXXXXXXXXXXTAACAAAGATACGAATATATTGATTCAATACCTTCACTTCTTAGGTTGCTTTTAATTAAG
AAAACATACATTCTTAGGTT
>Marker646096
CACCCTCAAACCTTAACCATATAAGGATTTAGATTGTGAAACGAATGGTCTTTAACTTCATCTCTCTTCTCCAAA
GAAATXXXXXXXXXXTCATTTGGATTTTGGTTAAAAATTCTAAAGCTTCCTTTAGAAAGATGAAAACTATGGTAA
CAAATTGTAATAAAACCAGT
CACCCTCAAACCTTAACCATATAAGGATTTAGATTGTGAAACGAATGGTCTTTAACTTCATCTCACTTCTCCAAA
GAAATXXXXXXXXXXTCATTTGGATTTTGGTTAAAAATTCTAAAGCTTCCTTTAGAAAGATGAAAACTATGGTAA
CAAATTGTAATAAAACCAGT
>Marker646395
AACAAAAGAAAAAAATAATAGATTTTGAGCAAATTTTCTTTTTGCTAATTACCATCTCTTTCTCCACCAATGCTG
AAAGCXXXXXXXXXXCAAATTTTCCTCTTCAATTCAACTCCTGCCATTTAAAATTACCTGAAAGTAGCATACTAC
GTATAACATACCAAGTGGTT
AACAAAGGAAAAAAATAATAGATTTTGAGCAAATTTTCTTTTTGCTAATTACCATCTCTTTCTCCACCAATGCTG
AAAGCXXXXXXXXXXCAAATTTTCCTCTTCAATTCAACTCCTGCCATTTAAAATTACCTGAAAGTAGCATACTAC
GTATAACATACCAAGTGGTT
>Marker646407
ACATGACACCGTGAATCCTGTCTGGAATCAAACGTTCGACTTTCTGGTGGAGGATGCATTACATGATATGCTGAT
TGTAGXXXXXXXXXXTGGTTTGAATATTACTTCGATTAATAAAACCCAACATATAAATTATCGAAGACACCGACT
GAGCAACTAAAAAACATGTA

Create PDF with GO2PDF for free, if you wish to

ACATGACACCGTGAATCCTGTCTGGAATCAAACGTTCGACTTTCTGGTGGAGGATGCATTACATGATATGCTGAT
TGTAGXXXXXXXXXXTGGTTTGAATATTACTTTGATTAATAAAACCCAACATATAAATTATCGAAGACACTGACT
CAGCAACTAAAAAACATGTA
>Marker646410
CACATCTTCACCTTTTTTCGGCAATGTTTCAGATTGGTGACCATGTTTCTTGAATACCACAGCAATGTGACTATG
GCCCTXXXXXXXXXXGAATAGAAGCAATGAACATGCTCTTACCGTGGAATTTTTCTAATAATCATTTTTTTAAGG
GAAACTCTATCTTCTGGGTC
CACATCTTCACCTTTTTTGGGCAATGTTTCAGATTGGTGACCATGTTTCTTGAATACCACAGCAATGTGACTATG
GCCCTXXXXXXXXXXGAATAGAAGCAATGAACATGCTCTTACCGTGGAATTTTTCTAATAATCATTTTTTTAAGG
GAAACTCTATCTTCTGGGTC
>Marker646428
CACATAAAAGTTAGCATCATCTCAAGGAATCAACTAATGGTTTGAAAACCATTTCATAGCATTGAAATCATAATC
ATCAAXXXXXXXXXXAACTTTTAAGAAGAAAATCATTCACCACACTTTAACAAAAATCCTCCTATTCCTTCTCTT
CCCGCACAACAAATCGTCGT
CACATAAAAGTTAGCATCATCTCAAGGAATCAACTAATGGTTTGAAAACCATTTCATAGCATTGAAATCATAATC
ATCAAXXXXXXXXXXAACTTTTAAGAAGAAAATCATTCACCACACTTTAACAAAAATCCTCCTATTCCTTCTCTT
CTCGCACAACAAATCGTCGT
>Marker646840
CACCATTCCTGCCAGAAATGTAAAGTTATCCTCAGCAGAGACCTATGCAGAAGGACTTTAATCTTCAGACCACAA
GAGGAXXXXXXXXXXATTAAAACAAACATTTTTAATTTGGAGACTAAATGAGACAAGATTTGAGAAAAATGGTGC
TTTGCAACAATACAAGCAGT
CACCATTCCTGCCAGAAATGTAAAGTTATCCTCAGCAGAGACCTATGCAGAAGGACTTTAATCTTCAGACCACAA
GAGGAXXXXXXXXXXATTAAAACAAACATTTTTAATTTGGAGACTAAATGAGACAATATTTGAGAAAAATGGTGC
TTTGCAACAATACAAGCAGT
>Marker647047
ACTTTAAGAAGATGATGTGTGACATTTAAGTGATCATTAGTTGGTTTTTATAGAAATTGGCTAAGGCGATGTATA
GCAAAXXXXXXXXXXCAGTTTAATGTTCGAATCCATGGGCAACGAAATAGGCTTGATATCTAGATAACTAGTGTC
TTCCAGGATCTATAGTGAGT
ACTTTAAGTAGATGATGTGTGACATTTAAGTGATCATTAGTTGGTTTTTATAGAAATTGGCTAAGGCGATGTATA
GCAAAXXXXXXXXXXCAGTTTAATGTTCGAATCTATGGGCAACGAAATAGGCTTGATATCTAGATAACTAGTGTC
TTCGAGGATCTATAGTGAGT
>Marker647268
ACCATTATTCAAATGATACATAGCATAGGAAACTTTTGTGTTGTTTGACAAACTGGGTGACTTGGAAAAGATGGA
TTGGAXXXXXXXXXXATCAAGACTCGTAGGACAAGACAATAATTCACAAAAGCAAGAGCAACCCGATAAAAGATC
TGATAATTCACTAATAAAGT
ACCATTATTCAAATGATACATAGCATAGGAAACTTTTGTGTTGTTTGACAAACTGGGTGACTTGGAAAAGATGGA
TTGGAXXXXXXXXXXATTTAGACTCGTAGGATAAGACAATAATTCACAAAAGCAAGAGCAACCCGATAAAAGATC
TGATAATTCACTAATAAAGT
>Marker647283
ACATAAAAATGAAATAGATAGTATAAAATGAATCCACAAAATAGTTAATTTAAATTTTGTTGTATTGCGTGTTCA
AGAAAXXXXXXXXXXAAGAAAATTCTTTTGGAATGGACATTCTAGAGGTAAATTTATTAATGGGAAGGTGGTGTC
TTGTTTGCAAGAGGATGGTG
ACATAAAAATGAAATAGATAGTATAAAATGAATCCACAAAATAGTTAATTTAAATTTTGTTGTATTGCGTGTTCA
AGAAAXXXXXXXXXXAAGAAAATTCTTTTGGAAAGGACATTCTAGAGGTAAATTTATTAATGGGAAGGTGGTGTC
TTGTTTGCAAGAGGATGGTG
>Marker647434

Create PDF with GO2PDF for free, if you wish to

ACCATCAAGCCTCCTTCAAACAGTCATTTACCCTCCTAAATCTATGCTAACTTTCTTTCTCGGAGAAAACCGGCG
ATCCAXXXXXXXXXXATTAAACTAAGAACGTAGCTTTCATATCACTTGTTAGAGCATGAACCCTTTAAATATTTC
TGCAATAGTATGATATTGTT
ACCATCAAGCCTCCTTCAAACAGTCATTTACCCTCCTAAATCTATGCTAACTTTCTTTCTCGGAGAAAACCGGCG
ATCCAXXXXXXXXXXATTAAACTAAGAACGTAGCTTTCATATCACTTGTTAGGGCATGAACCCTTTAAATATTTC
TGCAATAGTATGATATTGTT
>Marker648037
GACATTTTTGCATCTGTAAAATCAAATCAACAAAAATTATTCACCATAGTTGAGTTTCCAAAACAGGGTTTTCAG
TTTCTXXXXXXXXXXGCTCTAAACTTTGTGATTATGTATTATAAGGCAAGGATGTTTTTCAATTGGGTTTCAACA
TTTTAGTGTTAGGTGTGGGT
GACATTTTTGCATCTGTAAAATCAAATCAACAAAAATTATTCACCATAGTTGAGTTTCCAAAACAGGGTTTTCAG
TTTCTXXXXXXXXXXGCTCTAAACTTTGTAATTATGTATTATAAGGCAAGGATGTTTTTCAATTGGGTTTCAACA
TTTTAGTGTTAGGTGTGGGT
>Marker648233
TACTGCTACGATACTTCCATAAATAGTTGGAGTCCAGTTGCTCAATTGTCATCGGCTAGGTAATGGTTATTCTTA
TACACXXXXXXXXXXTTGGTTTATATTTCTTTTTATAAGATATTGACCATTGGTTTATTAGTAACATATTTGCCT
ATGCACAATTTTCTTTGGTT
TACTGCTACGATACTTCCATAAATAGTTGGACTCCAGTTGCTCAATTGTCATCGGCTAGGTAATGGTTATTCTTA
TACACXXXXXXXXXXTTGGTTTATATTTTTTTTTATAAGATATTGACCATTGGTTTATTAGTAACATATTTGCCT
ATGCACAATTTTCTTTGGTT
>Marker648548
ACAAGCACCAAGAAATTGAACAACATTTTTGTGCCGAACTTTCCTGCAAAAATAGGAACCAGACTTTAGTATAGT
AGTAAXXXXXXXXXXTTTTTGGGATTTTTTTTTTCCTGAAGCCATTCATCTTCAATATCAATATGGGAATTTTTA
AGCAAAATTATATGGAGAGT
ACAAGCACCAAGAAATTGAACAACATTTTTGTGCCGAACTTTCCTGCAAAAATAGGAACCAGACTTTAGTATAGT
AGTAAXXXXXXXXXXCTTTTTGGGATTTTTTTTTTCCTGAAGCCATTCATCTTCAATATCAATATGGGAATTTTTA
AGCAAAATTATATGGAGAGT
>Marker648578
ACCTCAACTCTTCTATCACCATCAATACTTTAAACATTATTTAGTGTGTTTTTTAAATTTAGGTTGAGTGAAAAA
TATGAXXXXXXXXXXATTTAATAGTTCCAAGTTTTGCAAAACATATATTTATATTGCCTGCCTTATCTAACCTAG
TTCTTTTAGTATATTGAAGT
ACCTCAACTCTTCTATCACCATCAATACTTTAAACATTATTTAGTGTGTTTTTTAAATTTAGGTTGAATGAAAAA
TATGAXXXXXXXXXXATTTAATAGTTCCAAGTTTTGCAAAACATATATTTATATTGCCTGCCTTATCTAACCTAG
TTCTTTTAGTATATTGAAGT
>Marker648596
TACAGTAGCATATCATCTGTCATATAGTCTGGCTTCAATATACAGAATCCATGTCTAACTTAGGATGCTATCTTG
TTAAAXXXXXXXXXXACAATTGTTTGATCATAAAGAAGCGGCAATAACTTGGGAAGAAGCCCCTTTACAAGATTA
GGCTTATTGTGGGCAAATGT
TACAGTAGCATATCATCTGTCATATAGTCTGGCTTCAATATACAGAATCTATGTCTAACTTAGGATGCTATCTTG
TTAAAXXXXXXXXXXACAATTGTTTGATCATAAAGAAGCGGCAATAACTTGGGAAGAAGCCCCTTTACAAGATTA
GGCTTATTGTGGGCAAATGT
>Marker648667
CACACAAATCATTTAGCCCGAGATGGGCTTTCCAATGAAACAAAGAAATACCGAACTGTAACAATACCGTCACGT
TTTTAXXXXXXXXXXCCTTGACGTTTCGGTTGAGAATAAAAATTAAATGTTTGCTTAGCTATATGAATAGATACC
TGGACAATTATTCTCTATGT

Create PDF with GO2PDF for free, if you wish to

CACACAAATCATTTAGCCCGAGATGGGCTTTCCAATGAAACAAAGAAATACCGAACTGTAACAATACCGTCACGT
TTTCAXXXXXXXXXXCCTTGACGTTTTGGTTGAGAATAAAAGTTAAATGTTTGCTTAGCTATATGAATAGATACC
TGGACAATTATTCTCTATGT
>Marker648932
ACTTTCCTTGAATTAACTTAATCTGTAATTCTCTAATCGACTAGGGCCCACAAACAAATCTATAATCAACAAAAC
ACAATXXXXXXXXXXGTTTCCGTTAGTTACCATTATAGATATCAAGAAGATAATGATACTAGACAAAGTAAAGTA
AGCATTATGTATAAGGAAGT
ACTTTCCTTGAATTAACTTAATCTGTAATTCTCTAATCGACTAGGGCCCACAAACAAATCTATAATCAACAAAAC
ACAATXXXXXXXXXXGCTTCCGTTAGTTACCATTATAGATATCAAGAAGATAATGATACTAGACAAAGTAAAGTA
AGCATTATGTATAAGGAAGT
>Marker648999
GACTAGTGTTTTCCTTATTCCTTTCAAATAAAAAAGGGGATAGCAGAAAAGCATTTCCCGAGGGAAGGAGATAAC
AGAAAXXXXXXXXXXAGTTTCTAAAAAAGTGAAGCTGAAAAATCTTCTCATATTTTTGAAGCAATGCTCCATCAT
TTTAATGAAAAAACTGGGTT
GACTAGTGTTTTCCTTATTCCTTTCAAATAAAAAAGGGGATAGCAGAAAAGCATTTCCCGAGGGAAGGAGATAAC
AGAAAXXXXXXXXXXAGTTTCTAAAAAAGTGAAGCTGAAAAATCTTCTCATATTTTTGAAGCAATGTTCCATCAT
TTTAATGAAAAAACTGGGTT
>Marker649099
CACTCTTTTGATTCCAAAAGCCATTAAACAAAAACAAACCCTACTGTTCATTCCCTCTTTCTTTTTCTAATTCTC
TTAATXXXXXXXXXXTGCCTCCTGATATTTTTCACTATCTTAACCCTTCCTTTTTTTACCTGTGGTTTTTGTATT
GTTTGTGTTAGATTTTTGTT
CACTCTTTTGATTCCAAAAGCCATTAAACAAAAACAAACCCTACTGTTCATTCCCTCTTTCTTTTTCTAATTCTC
TTAATXXXXXXXXXXTGCCTCCTGATATTTTTCACTATCTTAACCCTTCCTTTTTCTACCTGTGGTTTTTGTATT
GTTTGTGTTAGATTTTTGTT
>Marker649324
ACTTACCTGATCAAACAATTAAATCTCTCCGTTATTACTTAATTCAATTGAAGATTGATCAGATACTTACAAAAA
TTTTAXXXXXXXXXXTAGTTTTAAACTCTATTGGGAAGCTATCTAATCTTATTCCATGTTTTAGAGTAGAAAACG
ACGTGAAGATAATAGTGGTT
ACTTACCTGATCAAACAATTAAATCTCTCCGTTATTACTTAATTCAATTGAAGATTGATCAGATACTTACAAAAA
TTTTAXXXXXXXXXXTAATTTTAAACTCTATTGGGAAGCTATCTAATCTTATTCCATGTTTTAGAGTAGAAAACG
ACGTGAAGATAATAGTGGTT
>Marker649430
GACTTAATTTTTTTAAAAAAAATTGATAATGAAATAGTTCCCCATAAGTCTTCGTTTTTCAATTTTAGTAAGCAA
TGGTTXXXXXXXXXXAAATTTTATCTCTGATTAAGTCATCAATCTTTATGCAGACTGTAAAATAAATGGTGATAG
CATAGTAAATTGTTCATAGT
GACTTAATTTTTTTAAAACAAAATTGATAATGAAATAGTTCCCCATAAGTCTTCGTTTTTCAATTTTAGTAAGCAA
TGGTTXXXXXXXXXXAAATTTTATCTCTGATTAAGTCATCAATCTTTATGCAGACTGTAAAATAAATGGTGATAG
CATAGTAAATTGTTCATAGT
>Marker649971
CACAGATTTATTATTATCTTTATATTTTACAGAATTCTCGTTTTGTGTAATAGTAAATCCCATATGACTTTTCCA
TTTGGXXXXXXXXXXCTTGTTCCTTAACTAATTCCTTCTGTTTTCCAAATCAGGATGATTCGTATCTGGATAGCT
ACATTAGCACTATTGGAGTT
CACAGATTTATTATTATCTTTATATTTTACAGAATTATCGTTTTGTGTAATAGTAAATCCCATATGACTTTTCCA
TTTGGXXXXXXXXXXCTTGTTCCTTAACTAATTCCTTCTGTTTTCCAAATCAGGATGATTCGTATCTGGATAGCT
ACATTAGCACTATTGGAGTT
>Marker650408

Create PDF with GO2PDF for free, if you wish to

ACTGATGACAAAAATGAACAAAATAAATGTTTCATATCAAGAAAAATGAATTCATAATTAAGAAAGATAAATGTC
TGTTCXXXXXXXXXXGGGTAGTCATTAAAAGCCCAAGAAAGTTCTTCACTTCACAAATTTCAAATTTCCAGACTG
CAAATGTGATGCTGAAAGTG
ACTGATGACAAAAATGAACAAAATAAATGTTTCATATCAAGAAAAATGAATTCATAATTAAGAAAGATAAATGTC
TATTCXXXXXXXXXXGGGTAGTCATTAAAAGCCCAAGAAAGTTCTTCACTTCACAAATTTCAAATTTCCAGACTG
CAAATGTGATGCTGAAAGTG
>Marker650951
ACTTATTGTTCAATACCCGAAATCAACTCTTAGTGGATAAATTCATTTACTTATCCTAAATGGAAAGAAGTAGAT
TTCATXXXXXXXXXXTTAGAATGAAGTTCATATAACTCATTGAGGTTTGAAGACCAGTTATATATGGTCATCCTA
TGAAATGTTAATATTTAGTC
ACTTATTGTTCAATACCCGAAATCAACTCTTAGTGGAGAAATTCATTTACTTACCCTAAATGGAAAGAAGTAGAT
TTCATXXXXXXXXXXTTAGAATGAAGCTCATATAACTCATTGAGGTTTGAAGACCAGTTATATATGGTCATCCTA
TGAAATGTTAATATTTAGTC
>Marker651103
TACAAACACCATTTTTACTTTTATGGATTATCGGCAATTGAACAAAGTGACAATCAGGAACAAATATCTTTTGCC
GCAAAXXXXXXXXXXCAACCTTTAGAATGCATTATGGGCATTGTGAATTCTTGGTAATGTCTTTTGGATTGACGA
ATGCCCCAACTGCTTTCGTG
TACAAACACCATTTTTACTTTTATGGATTATCGGCAATTGAACAAAGTGACAATCAGGAACAAATATCTTTTGCC
GCAAAXXXXXXXXXXCAACCTTTAGAATGCATTATGGGCATTATGAATTCTTGGTAATGTCTTTTGGATTGACGA
ATGCCCCAACTGCTTTTGTG
>Marker651738
ACCCCTTTAGTCCAGTGCCGGAGCTAGAACTGTCAGGTCGTCTAATTGCGGCTCGGACGCCGCTGTTGGTATTAT
CGGGGXXXXXXXXXXGCAAGTTCCTGTATATTGAAAGAATTTGTAGCAACATAGCGTGAAGAGGTGGTTGCTTGG
TTGTTTGTGGGGGATTTGTA
ACCCCTTTAGTCCAGTGCCGGAGCTAGAACTGTCAGGTCGTCTAATTGCGGCTCGGACGCCGCTGTTGGTATTAT
CGGGGXXXXXXXXXXGCAAATTCCTGTATATTGAAAGAATTTGTAGCAACATAGCGTGAAGAGGTGGTTGCTTGG
TTGTTTGTGGGGGATTTGTA
>Marker652039
TACAAGTATTTGACTTTTGACAGCTATATAATGTATAGTATGAAATTCATATGATGAAATGCGGTATATACACAT
TGCCAXXXXXXXXXXGAAACTTTATTCTAGATGACAAAGCTGCCATGTCATGTCAGTAAGTTTGGTGGAGTTGGA
ATGGGAGAAATATGCAGGTT
TACAAGTATTTGACTTTTGACAGCTATATAATGTATAGTATGAAATTCATATGATGAAATGCGGTATATACACAT
TGCCAXXXXXXXXXXGAAATTTTATTCTAGATGACAAAGCTGCCATGTCATGTCAGTAAGTTTGGTGGAGTTGGA
ATGGGAGAAATATGCAGGTT
>Marker652066
CACCTCTAGTTTCAAGGTCCCTAAACTGAAGAACGAAATCAATTTGTAGATTTGCTAGTGTGTTACTTATAGCTG
TGTGTXXXXXXXXXXTGTCTGTTTTAGGAAACTTTTAACATAAAATAGTAGTATTGTGGGCATATTGATAATATG
CCGTCAGCCTCTATAGAGTA
CACCTCTAGTTTCAAGGTCCCTAAACTGAAGAACGAAATCAATTTGTAGATTTGCTAGTGTGTTACTTATAGCTG
TGTGTXXXXXXXXXXTGTCTGTTTTAGGAAACTTTTAACATAAAATAGTAGTGTTGTGGGCATATTGATAATATG
CCGTCAGCCTCTATAGAGTA
>Marker652562
ACTTGGGTTACGGTTGTCTTCAGCTAGTCCAAAAATGTATCAAAAGTAGTTTTGTAATTATTTTCAAAATTAACC
GTTATXXXXXXXXXXTTACCAAAATCTTGTTAAAAAAAATAAATTGGTTACGAATTTAAGTTTTGTTTTAATTTG
AGTTTTTTAAAAATAAATGT

Create PDF with GO2PDF for free, if you wish to

ACTTGGGTTACGGTGGTCTTCAGCTAGTCCAAAAATGTATCAAAAGTAGTTTTGTAATTATTTTCAAAATTAACC
GATATXXXXXXXXXXTTACCAAAATCTTGTTAAAAAAAATAAATTGGTTAAGAATTTAAGTTTTGTTTTAATTTG
AGTTTTTTAAAAATAAATGT
>Marker653248
TACAAGATTCAATTTCCCCAATAAAAAACTTAGCAACAAAAAAGAAGTATATATACACACATACATATACATATA
CATATXXXXXXXXXXATAGATTAAAAACAAAATTGTTACCATACAAGTCTTTAAGTTTCAAAACTGTGAAATAAA
ATGTAAATTATGGAAATGTT
TACAAGATTCAATTTCCCCAATAAAAAACTTAGCAACAAACAAGAAGTATATATATACACATACATATACATATA
CATATXXXXXXXXXXATAGATTAAAAACAAAATTGTTACCATACAAGCCTTTAAGTTTCAAAACTGTGAAATAAA
ATGTAAATTATGGAAATGTT
>Marker653522
AACTGACTTCCCAGCAGATGCATGGAGATCTAAACTTTCATAAGCACCTTTGAAAGCTACAGCAGGCTAATGGTG
GCAAAXXXXXXXXXXTGTCAAGAGGGATGTAATGCAATAAATTGACTACGAAACAAGGGTAAAGAAAAAACCAGC
TCATACTTTAAAGGGTAGTC
AACTGACTTCCCAGCAGATGCATGGAGATCTAAACTTTCATAAGCACCTTTGAAAGCTACAGCAGGCTAATGGTG
GCAAAXXXXXXXXXXCGTCAAGAGGGATGTAATGCAATAAATTGACTACAAAACAAGGGTAAAGAAAAAACCAGC
TCATACTTTAAAGGGTAGTC
>Marker653752
CACTTTCCTGTCATCTAATTGGACAAGCCTATTGTAATTCCATATAGTTTATCCCTTTTTATAAATTCTATAAAT
CAACAXXXXXXXXXXAGGTTATTGTGAATCAAATTGCTTTATATTGCTGAAGCAGTTACATTGAGCTAGATTAAT
TATTAATGCATCTAAATGGT
CACTTTCCTGTCATCTAATTGGACAAGCCTATTGTAATTCCATATAGTTTATCCCTTTTTATAAATTCTATACAT
CAACAXXXXXXXXXXAGGTTATTGTGAATCAAATTGCTTTATATTGCTGAAGCAGTTACATTGAGCTAGATTAAT
TATTAATGCATCTAAATGGT
>Marker653766
TACCGGGGACAAACATAAACTAACAGAGAACTACAAGTGATGGAGGAACATGTATCATCCCAAAAGGGAGAGACA
TGAATXXXXXXXXXXATCCACTTTGTTCCGAGCAAGGAATCATTTATTGTTTGACAAGGGGCAGGATTGAACTGG
GACAGAGCGAGGAACGGGTA
TACCGGGGACAAACATAAACTAACAGAGAACTACAAGTGATGGAGGAACATGTATCATCTCAAAAGGGAGAGACA
TGAATXXXXXXXXXXATCCACTTTGTTCCGAGCAAGGAATCATTTATTGTTTGACAAGGGGCAGGATTGAACTGG
GACAGAGCGAGGAACGGGTA
>Marker653778
ACAGACCTCAAGTTAAAGGCAAAAGAAAAAGAAAAAAAAAACCACAAACAACGACAAAAACTTGGATCTTTTTGT
GTCCAXXXXXXXXXXTTACAAATCCATATCAAAGAAAAAATATAAAAATAACATGACTACTAAAAAAAGAAAAGA
AGCTATATGCAACAAATGTA
ACAGACCTCAAGTTAAAGGCAAAAGAAAAAGAAAAAAAAAACCACAAACAACGACAAAAACTTGGATCTTTTTGT
GTCCAXXXXXXXXXXTTACAAATCCATATCAAAGAAAAAATATAAAAATAACATGACTACTAAAAAATGAAAAGA
AGCTATATGCAACAAATGTA
>Marker654184
ACTTAAACACACGAAGAAGAACCTCAGAAATAAAACGAGTAGAGGGGTAGGATTCCTTAAGACAATCTAAGGGAG
TTTGAXXXXXXXXXXCGGGCTACTTCCAGAAGGTGACAGTTTTTTCGCTTGGCCACTTCATTTTGTTAAGGAGTG
TAGGTGCATGAGTTTTGGTG
ACTTAAACACACAAAGAGGAACCTCAGAAATAAAACGAGTAGAGGGGTAGGATTCCTTAAGAAAATCTAAGGGAG
TTTGAXXXXXXXXXXCGGGCTACTTCCAGAAGGTGACAGTTTTTTCGCTTGGCCACTTCATTTTGTTAAGGAGTG
TAGGTGCATGAGTTTTGGTG
>Marker655093

Create PDF with GO2PDF for free, if you wish to

CACTTTATTTTCTTTCATATTTTTAGGAACAAACATTTGCCTTTCACGAAAAGCATGTATATAAACTCATTTTAT
TTTTAXXXXXXXXXXXATGGAGGGGTTCTGCCATATCCTTTCCCGTCGGCGCAATACATGCAATGCAAGGACACT
ATTTTCCTTCACTGCTAAGT
CACTTTATTTTCTTTCATATTTTTAGGAACAAACATTTGCCTTTCACGAAAAGCATGTATATAAACTCATTTTAT
TTTTAXXXXXXXXXXXATGGAGGGGTTCTGCCATATCCTTTCCCGTCGGCGCAATACATGCAGTGCAAGGACACT
ATTTTCCTTCATTGCTAAGT
>Marker656252
TACTTACTACTTTTCCAGAAGCATTTCTTCAAATTAAGCAGTTCTGATAATACAAAAATTTAACGTAAAACAGTC
ATTTCXXXXXXXXXXXTCATTTCCCAGCCAAAAACATATGAACCAAGCCATACCTTTAGTTTATGGGTTATCATAG
TGGGATTTTGGAAAAGAGTT
TACTTACTACTTTTCCAGAAGCATTTCTTCAAATTAAGCAGTTCTGATAAAACAAAAATTTAACGTAAAACAGTC
ATTTCXXXXXXXXXXXTCATTTCCCAGCCAAAAACATATGAACCAAGCCATACCTTTAGTTTATGGGTTATCATAG
TGGGATTTTGGAAAAGAGTT
>Marker657231
ACTATGAAACCTTCAGGTTGTTCCGTATAAATCTCTTCTTCTAATTCACCATTTAGAAAAGCAGTTTTGACTTCC
ATTTGXXXXXXXXXXXTTATCTACTGGTCCATCAGGTTTGAGTTTGTTCCTTAAAACCCATTTACATTCTATTGCT
TTGCATTCAGGGGGTAAGTT
ACTATGAAACCTTCAGGTTGTTCCGTATAAATCTCTTCTTCTAATTCACCATTTAGAAAAGCAGTTTTGACTTCC
ATTTGXXXXXXXXXXXTTATCTACTGATCCATCAGGTTTGAGTTTGTTCCTTAAAACCCATTTACATTCTATTGCT
TTGCATTCAGGGGGTAAGTT
>Marker657322
GACTCCGATTCCTCTCAGAATTATCAAACACAACGGAGACAGTTGCTTTGGTAATACCCGCTTGCCCCTGCTTAT
ACACTXXXXXXXXXXXCAGTCCTCGTGGCGTAGGATTTGAACCCCTCCAGACAGATTTCCTTGATGTGCATCGTTC
CCAAAAATGCTTGGAAAGGT
GACTCCTATTCCTCTCAGAATTATCAAACACAACGGAGACAGTTGCTTTGGTAATACCCGCTTGCCCCTGCTTAT
ACACTXXXXXXXXXXXCAGTCCTCGTGGCGTAGGATTTGAACCCCTCCAGACAGATTTCCTTGATGTGCATCGTTC
CCAAAAATGCTTGGAAAGGT
>Marker657555
ACTCACCTTCCTTCTAGATCTTGAATTATTTCATGTAAGTGAAGCAGTGGCATCTAGATAATTCGTCACGAACCA
TTTGTXXXXXXXXXXXCATCTTCCACGATTTGTTCGATGAAGTCGCTTGCCTAAAAGAACAGTTCCATTAGGTGGT
AGTTTTTAAGGTGCTAAGGT
ACTCACCTTCCTTCCAGATCTTGAATTATTTCATGTAAGTGAAGCAGTGGCATCTAGATAATTCGTCACGAACCA
TTTGTXXXXXXXXXXXCATCTTCCACGATTTGTTCGATGAAGTCGCTTGCCTAAAAGAACAGTTCCATTAGGTGGT
AGTTTTTAAGGTGCTAAGGT
>Marker657659
TACCATGTAAATTTTACAACATATGAAAAAAAAATTAGGTGTTCAACTTATGTTTCAATATATGCATTATTTTAT
GTTATXXXXXXXXXXXAATTGCCCAAATCAATAATCTTCCTCATGATTCTTCTTTGGTTGATTTATAACATTATTC
ACTATTAAATGCAAATAGTT
TACCATGTAAATTTTACAACATATGAAAAAAAAATTAGGTGTTCAGCTTATGTTTCAATATATGCCTTATTTTAT
GTTATXXXXXXXXXXXAATTGCCCAAATCAATAATCTTCCTCATGATTCTTCTTAGGTTGATTTATAACATTATTC
ACTATTAAATGCAAATAGTT
>Marker659576
AACTATTTTAGCTAAGCTCACTCTAACAACATAGTTAATTAATAATTAAGTATTTGTTTATATAATTTCAAAAAA
ACATTXXXXXXXXXXXAACAAAATTATAAATACTAAAAAAGTATTAGACTTATATAGTTGATAGATTTATACCGAT
AGATTGCCAACGACATAGTA

Create PDF with GO2PDF for free, if you wish to

AACTATTTTGGCTAAGCTCACTCTAACAACATAGTTAATTAATAATTAAGTATTTGTTTATATAATTTCAAAAAA
ACATTXXXXXXXXXXXAACAAAATTATAAATACTAAAAAAGTATTAGACTTATATAGTTGATAGATTTATACTGAT
AGATTGCCAACGACATAGTA
>Marker659904
TACAACAATCTTCATTTTTTTTTACCGTTTATTGTGTTTTTCCTATTTTCTCACAGTTTGTTATGTTTTCATGAA
TATTTXXXXXXXXXXXAAAATGTGGTAGTGCATTATGATGTAAGCAGAAGATTATATTTTGTATGCTTGTGGGGAC
GAGTCATTAATCATAAATGT
TACAACAATCTTCATTTTTTTTTACCGTTTATTGTGTTTTTCCTATTTTCTTACAGTTTGTTATGTTTTCATGAA
TATTTXXXXXXXXXXXAAAATGTGGTAGTGCATTCTGATATAAGCAGAAGATTATATTTTGTATGCTTGTGGGGAC
GAGTCATTAATCATAAATGT
>Marker660916
CACCTAGAATAAGGGGAATTCTTAAACGTGATTGAGGTAGTTTCGTTTAGCGCTTCTACCGCTGTTGAACCAAAC
TAGCCXXXXXXXXXXXAATTGATTGGGACTTTCTGGATGCAGTTCTTCAAGCTAAGGATTTTGGTATTCTCCGGCA
TTAGAAGTTGTATGTCTGGT
CACCTAGAATAAGGGGAATTCTTAAACGTGATTGAGGTAGTTTCGTTTAGCGCTTCTACCACTGTTGAACCAAAC
TAGCCXXXXXXXXXXXAATTGATTGGGACTTTCTGGATGCAGTTCTTCAAGCTAAGGATTTTGGTATTCTCCGGCA
TTAGAAGTTGTATGTCTGGT
>Marker661197
TACTCGTAGATGCGAAGTGGGCTCAATATGCACTGTTTGAATAAAAAATTATGAAGAATTTTGTTCATAGGCTTG
CTATCXXXXXXXXXXXATTTTTTTTGGAAATTTTCCTCACTACAATTTTGATTGCTCAATAAAAGTCTGGAGGTTT
TTTGGCTTAACATTTTCAGT
TACTTGTAGATGCGAAGTGGGCTCAATATGCACTGTTTGAATAAAAAATTATGAAGAATTTTGTTCATAGGCTTG
CTATCXXXXXXXXXXXATTTTTTTTGGAAATTTTCCTCACTACAATTTTGATTGCTCAATAAAAGTCTGGAGGTTT
TTTGGCTTAACATTTTCAGT
>Marker661381
AACAAGTTGAGAACTAGATCTTACGATATATTCTTTCTTCTTTCAGGCTTATGCATCTAGGGGATATGTAGCTAT
TGCCAXXXXXXXXXXXACCTGATGTCATAATCTGGTGCAGGCTCTTATTTCTGCATGGAAAAAAGGTGATACCATG
CCATTCATATTTGACACGGT
AACAAGTTGAGAACTAGATCTTACGATATATTCTTTCTTCTTTCAGGCTTATGCATCGAGGGGATATGTAGCTAT
TGCCAXXXXXXXXXXXACCTGATGTCATAATCTGGTGCAGGCTCTTATTTCTGCATGGAAAAAAGGTGATACCATG
CCATTCATATTTGACACGGT
>Marker661591
AACCTCCTTGGTTTGAGTGAAATTTCCTGTGAAAGGGCATGAAAGTTGAAACCATAAATTTTGAAGAAAAATGGT
CACTGXXXXXXXXXXXTTTCCACAAAAGTTCAAAATCTAGCAGTTATTTGTTGTGTCCCAAATTCCAGCTATGGAA
GCATTCTTTCTTTCAGCAGT
AACCTCCTTGGTTTGAGTGAAATTTCCTGTGAAAGGGCATGAAAGTTGAAACCATAAATTTTGAAGAAAAATGGT
CACTAXXXXXXXXXXXTTTCCACAAAAGTTCAAAATCTAGCAGTTATTTGTTGTGTCCCAAATTCCAGCTATGGAA
GCATTCTTTCTTTCAGCAGT
>Marker662447
GACCCGGACTACTCGCCCGACGACGTCTGAAGCTAGTGGAGTAGTTTTTCCATGGCGACGGCGGTGGTGGCAGCT
CTCTCXXXXXXXXXXXACTAAGTTACGTGAAAATCCGAGAACCGAGAGAAAAACCATGATCTGACTTTCGTATTAT
TTTCTCACATTAACAAAGGT
GACCCGGACTACTCGCCCGACGACGTCTGAAGCTAGTGGAGTAGTTTTTCCATGGCGACGGCGGTGGTGGCAGCT
CTCTCXXXXXXXXXXXATTGAGTTACGTGAAAATCCGAGGACCGAGAGAAAAACCATGATCTGACTTTCGTATTAT
TTTCTCACATTAACAAAGGT
>Marker663910

Create PDF with GO2PDF for free, if you wish to

TACACAAAATGAAATCTTAATATTGTGTTTGTATATACATTCTACAATAATACATGAAACAAGATCCTAATACAG
GATTTXXXXXXXXXXTTAAATCCAGAGAATCTACAAAATTATGTTGATAAAGGCCAACATTTTCTTTATCCACAG
TAGCATCACAATGATTATGT
TACACAAAATGAAATCTTAATATTGTGTTTGTATATACATTCTACGATAATACATGAAACAAGATCCTAATACAG
GATTTXXXXXXXXXXTTAAATCCAGAGAATCTACAAAATTATGTTGATAAAGGCCAACATTTTCTCTATCCACAA
TAGCATCACAATGATTATGT
>Marker663980
ACTGGAGGCTCCATACGATCACACAAATACTGAAGAATTAATCAAAGATTTGTTAAGTCCGAGAAAATATAAGCC
ACTCAXXXXXXXXXXXTAGCTTTAATAGAACGAAGAGACTTCTCACAGATATCGGTAAAAAGAATATCCTCATAAT
TATCCACCGTCAATTTGGTG
ACTGGAGGCTCCATACGATCACACAAATACTGAAGAATTAATCAAAGATTTGTTAAGTCCGAGAAAATATAAGCC
ACACAXXXXXXXXXXXTAGCTTTAATAGAACGAAGAGACTTCTCACAGATATCGGTAAAAAGAATATCCTCATAAT
TATCTACTGTCAATTTGGTG
>Marker664198
TACAGCTCAAAACCTCATAACTCATCAATGTTCGAAGAAATTTCACTTCCATGTCATGCTCATAGAAAATAAATT
TATAAXXXXXXXXXXXTTATAACGGTTTTCATCCTGTATAAGTTGAAAAGTAATGAAAACTAAGAACAGAAATGTA
TTTAGCAATCGGATGGTGTG
TACAGCTCAAAACCTCATAACTCATCAATGTTCGAAGAAATTTCACTTCCATGTCATGCTCATAGAAAATAAATT
TATAAXXXXXXXXXXXTTATAACGGTTTTCATCCTGTATAAGTTGAAAAGTAACGAAAACTAAGAACAGAAATGTA
TTTAGCAATCGGATGGTGTG
>Marker665023
GACTTTAACGACGACAACGTTCTCCCAGCATTATTCACCTAACTCAAATAATCGATCAAAATCCAAAATCCAAAA
TCCAAXXXXXXXXXXXGAGCCACTCACTTTGAAGCCCATTGCTTCCCATTCTTTCAAACATTTGTCTAAATCTTCT
TGGCTTCGACAGCATGTGTG
GACTTCAACGACGACAACGTTCTCCCAGCATTATTCACCTAACTCAAATAATCGATCAAAATCCAAAATCCAAAA
TCCAAXXXXXXXXXXXGAGCCACTCACTTTGAAGCCCATTGCTTCCCATTCTTTCAAACATTTGTCTAAATCTTCT
TGGCTTCGACAGCATGTGTG
>Marker665331
CACATGACCGAGAGAGAAAGTGTGATGACCTAGACTCTCAATGTTTATCTCATTGAAAAATAACACGTTGGACAT
CTAATXXXXXXXXXXTTATACCACTTAAACAATTTCTGCTTAAGATATCATAACATATTATGCATTTGGAAGTTG
GACAACATTAGGAGGAAAGT
CACATGATCGAGAGAGAAAGTGTGATGACCTAGACTCTCAATGTTTATCTCATTGAAAAATAACACGTTGGACAT
CTAATXXXXXXXXXXTTATACCACTTAAACAATTTCTGCTTAAGATATCATAACATATTATGCATTTGGAAGTTG
GACAACATTAGGAGGAAAGT
>Marker666034
GACCATTGTCAAGTGGAGTTACTAACATACCTGAATGAGAGCTTTTACATCAAGCTCATCGATATGTTTGGGAGA
ATACTXXXXXXXXXXACCGAAGTAGATCAAAAAACCAAAAATGGATTCAAGATGAACACAACAAAACCTTCATAG
CTTGGTTACGAGAAGAGGTA
GACCATTGTCAAGTGGAGTTACTAACATACCTGAATGAGAGCTTTGACATCAAGCTCATCGATATGTTTGGGAGA
ATACTXXXXXXXXXXACCGAAGTAGATCAAAAAACCAAAAATGGATTCAAGATGAACACAACAAAACCTTCATAG
CTTGGTTACGAGAAGAGGTA
>Marker666166
CACTTTTAAGGCTGTGTGTGATAAGCTGGAAAAACATGCGTTTCACTTTCTTTACCTCACTAAGGAGGGATTCAG
GAATAXXXXXXXXXXTTTACTCATAACTTTAGATCATTATCCTTAAAATAATAACAGTATGTTCCTACACTGTCA
ATCTCTTCTAAGTTAATAGT

Create PDF with GO2PDF for free, if you wish to

CACTTTTAAGGCTGTGTGTGATAAGCTGGAAAAACATGCGTTTCACTTTCTTTACCTCACTAAGGAGGGATTCAG
GAATAXXXXXXXXXXXTTTACTCATAACTTTAGATCATCATCCTTAAAATAATAACAGTATGTTCCTACACTGTCA
ATCTCTTCTAAGTTAATAGT
>Marker666780
AACAAATGAACCCAATACCGACGCAGCATATCCTGCATCAGTGTAGGTCGTATTCCCCAACGTGGGAATCCACTG
CGTCAXXXXXXXXXXXTCTAACGTTTGAGAGAAAGACGTCGGGGAAGGTCTGACACATTGATGCGACCGTTGGATA
TACCCCGATGCCATAGCAGT
AACAAATGAACCCAATACCGACGCAGCATATCCTGCATCAATGTAGGTCGTATTCCCCAACGTGGGAATCCACTG
CGTCAXXXXXXXXXXXTCTAACGTTTGAGAGAAAGACGTCGGGGAAGGTCTGACACATTGATGCGACCGTTGGATA
TACCCTGATGCCATAGCAGT
>Marker667073
TACAATCTCCTGCTCTAAAGATGATGTATTAGGAAATCTAAACATAGTGAAGTGTTTAATTGGTTTAATCAATTT
TCATCXXXXXXXXXXXAAGCTGCAGTTTTGAAAAACAACAGATCGGTCACTACCAATGAACTATACTCCTTTTACA
GTGCTAGGAGTGACTGCTGT
TACAATCTCCTGCTCTAAAGATGATGTATTAGGAAATCTAAACATAGTGAAGTATTTAATTGGTTTAATCAATTT
TCATCXXXXXXXXXXXAAGCTGAAGTTTTGAAAAACAACAGATCGGTCACTACCAATGAACTATACTCCTTTTACA
GTGCTAGGAGTGACTGCTGT
>Marker667459
AACCATTCCAGATTATTGGTGGTGACCTGCATTATTTCAGAACTCTTCCTGAGGTATTTATTATGCACTTATTTC
CTTTCXXXXXXXXXXXTTTATAGTGTCATTCTTCATCTTTCGGTTCAGTTCAGACTAGTCAGAAATGGATTTTGAT
ACAATACTTTGAAGCAGGTC
AACCATTCCAGATTATTGGTGGTGACCTGCATTATTTCAGAACTCTTCCTGAGGTATTTATTATGCACTTATTTC
CTTTCXXXXXXXXXXXTTTATAGTGTCATTCTTCATCTTTCGGTTCAGTTCAGACTAGTCGGAAATGGATTTTGAT
ACAATACTTTGAAGCAGGTC
>Marker667496
ACTTAAAAAAGAGTTTTTAAATTTTGAAAAATAAGTTAAATCTAAGCCCTTGTTTTGTAAATCATTTTCTTTTTG
TTCTTXXXXXXXXXXXTGTTTCTTCAAAGATAGTGAAACCAGGTAATAATATTGATTGATAAAACAAGTTTAAATT
TAAAAAATCAAGTCAGGGTT
ACTTAAAAAAGAGTTTTTAAATTTTGAAAAATAAGTTAAATCTAAGCCCTTGTTTTGTAAATCATTTTCTTTTTG
TTCTTXXXXXXXXXXXTATTTCTTCAAAGATAGTGAAACCAGGTAATAATATTGATTGATAAAACAAGTTTAAATT
TAAAAAATCAAGTCAGGGTT
>Marker667506
ACCCTATTGATTCGATATAACAGCAACAAACATAATTATGAATGATCATGAAAACCATTGACAATAGCACTTATA
ATTGAXXXXXXXXXXXTTAAATTGTCGTAATTGCTCTTTAATTGTTCAATTTAATGTTTGTCATTTTAAGTTTTCT
TTTGAGTTATATAAGTTTGT
ACCCTATTGATTTGATATAACAGCAACAAACATAATTATGAATGATCATGAAAACCATTGATAATAGCACTTATA
ATTGAXXXXXXXXXXXTTAAATTGTCGTAATTGCTCTTTAATTGTTCAATTTAATGTTTGTCATTTTAAGTTTTCT
TTTGAGTTATATAAGTTTGT
>Marker668750
TACAAACAATTGGTTAAAATCAGAAGCTCATGGATATCTTTTAATAGGAGATGATTGAAAATCTAAATAGGAATC
AAATAXXXXXXXXXXXCATCGATCAACTCTAGCTACTACCATTGGCTGCCACCTACATCCAATTCAGCCAACTTTG
ATGACCTCTACCTAGAAGTC
TACAAACAATTGGTTGAAATCAGAAGCTCATGGATATCTTTTAATAGGAGATGATTGAAAATCTAAATAGGAATC
AAATAXXXXXXXXXXXCATCGATCAACTCTAGCTACTACCGTTGGCTGCCACCTACATCCAATTCAGCCAACTTTG
ATGACCTCTACCTAGAAGTC
>Marker668985

Create PDF with GO2PDF for free, if you wish to

ACAAATGTAAATCAACATTGTTGTTTTTATTTAATTCTAGTTCATATTAGATATAAAGTAAAGATTGTATAGTTT
TTATGXXXXXXXXXXXAAGGCTACAAAATTATCATTGATTTTCTTTTTCTTGCTTTCCTCTCTTATTCTCTATTTT
GTATTAATCCATTTGTGGTT
ACAAATGTAAATCAACATTGTTGTTTTTATTTAATTCTAGTTCATATTAGATATAAAGTAATGATTGTATAGTTT
TTATGXXXXXXXXXXXAAGGCTACAAAATTATCATTGATTTTCTTTTTCTTGCTTTCCTCTCTTATTCTCTATTTT
GTATTAATCCATTTGTGGTT
>Marker669000
GACTTGGTGATGATTATTAAATGAAGTTTTAGAAAAAAATATGTGCAAAATACCGAATCAAACCAAATTCGTTTA
TGTTTXXXXXXXXXXXAATGGGCGGTTATATTTGTCAGGCAATGGCCATTGGGAAAGCAGCAAGCAAGTTGATTGA
AGAGGAGCTGAAAATGGAGT
GACTGGGTGATGATTATTAAATGAAGTTTTAGAAAAAAATATGTGCAAAATACCGAATCAAACCAAATTCGTTTA
TGTTTXXXXXXXXXXXAATGGGCGGTTATATTTGTCAGGCAATGGCCATTGGGAAAGCAGCAAGCAAGTTGATTGA
AGAGGAGCTGAAAATGGAGT
>Marker669551
CACGTTTTTCATGAATTCCGTTTTCCCACTAGCGAAAAAGAGAGGTTTTATCACCAATTATGTGTAGGAAGAAAT
AGGCCXXXXXXXXXXXAAGCACTGAACAGAGTATATGGAATATGGATGGAACCAACATTTCTTTTCACTCTTCAAT
GTCTATTTCAATGCAAAGTG
CACGTTTTTCATGAATTCCGTTTTCCCACTAGCGGAAAAGAGAGGTTTTATCACCAATTATGTGTAGGAAGAAAT
AGGCCXXXXXXXXXXXAAGCACTGAACAGAGTATATGGAATATGGATGGAACCAACATTTCTTTTCACTCTTCAAT
GTCTATTTCAATGCAAAGTG
>Marker669588
ACTATAACGTGGCCCGTTCGGAAGATTGTGCCCATCCTAGCAGGAGATTACAGGTATGTTAAAAAAGCAGAACAT
GAAGAXXXXXXXXXXXGAAGTGAAGCTTGTGCAGGCAAAGGAATTAACCAATAAAGACATTATAGGAAAGTCGGAT
CCTTATGCTGTGCTGTTTGT
ACTATAACGTGGCCAGTCCGGAAGATTGTGCCCATCCTAGCAGGAGATTACAGGTATGTTAAAAAAGCAGAACAT
GAAGAXXXXXXXXXXXGAAGTGAAGCTTGTGCAGGCAAAGGAATTAACCAATAAAGACATTATAGGAAAGTCGGAT
CCTTATGCTGTGCTGTTTGT
>Marker669849
TACCCAACAGTTTCAAGATCTACTTTTTTCTTAATACTTGGTATAACTGATTATAGGTTGAAAGATGGTGGGGA
TTATTXXXXXXXXXXXTTGTCAAGAACTTTTTAGGCTTCCTACAATACTGGAAACATTCTGTATATTTTTAGGGTC
CTTTGGGTAGTCTCTTGGTC
TACCCAACAGTTTCAAGATCTACTTTTTTCTTAATACATGGTATAACTGATTATAGGTTGAAAGATGGTGGGGA
TTATTXXXXXXXXXXXTTGTCAAGAACTTTTTAGGCTTCCTACAATACTGGAAACATTCTGTATTTTTTTAGGGTC
CTTTAGGTAGTCTCTTGGTC
>Marker670015
TACCTGCAAATGACTTGGTCGATGTTATTGCTCCATCTTGCTACAGGTATTTTTTCTCATTGTTTCCCATTTCAT
TTGGTXXXXXXXXXXXAAATTAAATGTAGTCATTAAGGAATCATTCTTAAACACTTAAACCAAGATGCAATCATGA
CAGGAAAAAGATAAAAAGTG
TACCTGCAAATGACTTGGTCGATGTTATTGCTCCATCTTGCTACAGGTATTTTTTCTCATTGTTTCCCATTTCAT
TTGGTXXXXXXXXXXXAAATCAAATGTAGTCATTAAGGAATCATTCTTAAACACTTAAACCAAGATGCAATCATGA
CAGGAAAAAGATAAAAAGTG
>Marker671749
AACTAAACATATTCCGTGTATTATCTAATTCTTTTACCTCTTTTTGTGAAGAATCTAAAACTTTGAGTAATTGTC
ATGAAXXXXXXXXXXXTAAGATAAAAGAAGTGAATTGTATTGGTATGTTTTTATTCTACAAGAGACATACTATTTT
GATACACAGAAAAATACGTG

Create PDF with GO2PDF for free, if you wish to

AACTAAACATATTCCGTGTATTATCTAATTCTTTTACCTCTTTTTGTGATGAATCTAAAACTTTGAGTAATTGTC
ATGAAXXXXXXXXXXTAAGATAAAAGAAGTGAATTGTATTGGTATGTTTTTATTCTACAAGAGACATACTATTTT
GATACACAGAAAAATACGTG
>Marker677182
CACATAAGAGTATAGGCAGCCATGTAGAAAATGCATGCATGGTGGAGTAAAGGCAATCAATGCATAAAAGATGTG
CATTGXXXXXXXXXXGGAATGCAACTAATGTGTGTTATGTATATTCATTGTTTCTTGAAAGTTTAAGAACTTTAA
GGCTTAACTAGACGATTCGT
CACATAAGAGTATAGGCAGCCATGTAGAAAATGCATGCATGGTGGAGTAAAGGCAATCAATGCATAAAAGATGTG
CATTGXXXXXXXXXXGGAATGCAACTAATGTGTGTTATGTATATTCATTGTTTCTTGAAAGTTTAAGAACTTTAA
GGCTTAACCAGACGATTCGT
>Marker677609
CACCAAACATTAGAATCTTTGTCCGACCATAAACTGAAAGAGAATGTCCCAGTCTAGATGGGGGTGTCCCAGATG
TGGGAXXXXXXXXXXAGCTATGCCAGGATCTAGGCAATGGAGGAGCTCCACCAGAGATTTCCTTCCATGTTGGTT
GTTGGGCATCCAAATCGAGT
CACCAAACATTAGAATCTTTGTCCGACCATAAACTGAAAGAGAATGTCCCAGTCTAGATGGGGGTGTCCCAGATG
TGGGAXXXXXXXXXXAGCTGTGCCAGGATCTAGGCAATGGAGGAGCTCCACCAGAGATTTCCTTCCATGTTGGTT
GTTGGGCATCCAAATCGAGT
>Marker677698
ACCTTATTAACCATAATACTGAAAAAATCATGAAGTCAATTGCTAATGGATGAAAATATGAAAATCAAATAACTA
AATCTXXXXXXXXXXGCTTTCAATCTTGACTAAATCATACCAGTAAAAGATGAGCATTTTTTCAAGTAGATATCA
TGTAAAAAAACAACAAAAGT
ACCTTATTAACCATAATACTGAAAAAATCTTGAAGTCAATTGGTAATGGATGAAAATATGAAAATCAAATAACTA
AATCTXXXXXXXXXXGCTTTCAATCTTGACTAAATCATACCAGTAAAAGATGAGCATTTTTTCAAGTAGATATCA
TGTAAAAAAACAACAAAAGT
>Marker677844
AACGTGAAATTACTAATGCCAACCAGAGCTTGACTCAAATGTATGTTATATTGAGGATTACAAGATCCATAGTTT
GAATCXXXXXXXXXXATTAAAATTTAAGTCATGAAAATTAACATCAAGTAATGTTAGTTCATCCAATCGAATTGT
GTCATGTGTCAATCAAAGTC
AACGTGAAGTTACTAATGCCAACCAGAGCTTGACTCAAATGTATGTTATGTTGAGGATTACAAGATCCATAGTTT
GAATCXXXXXXXXXXATTAAAATTTAAGTCATGAAAATTAACATCAAGTAATGTTAGTTCATCCAATCGAATTGT
TTCATGTGTCAATCAAAGTC
>Marker677903
GACCAAAATATTTACAAATTATAACAAAGTTTCAGTTTCTATTCGATGATAGAATCTGAAATTTTGCTATATTTT
GTAAAXXXXXXXXXXAGTGCCCATAATGTTGATAAAATTTCTTAGAATTTTTTCTTTTGAAATCATACACTTGTT
ATCCGTAGAGCTTAGCAGTA
GACCAAAATATTTACAAATTATAACAAAGTTTCAGTTTCTATTCGATGATAGAATCTGAAATTTTGCTATATTTT
ATAAAXXXXXXXXXXGTGTCCCATAATGTTGATAAAATTTCTTAGAATTTTTTCTTTTGAAATCATACACTTGTT
ATCCGCAGAGCTTAGCAGTA
>Marker678056
ACTCGTTGAAGTGCTTTATGAATGAATTCTCTTCCAACTTATAAGAAAACATGAATAGTCACAACAATAGTGTGC
TCACAXXXXXXXXXXTTTTGATTGCACATATTATAGTTTTGAATTTAATAATAATTGTATAGGGTATTAGAAATG
ATCGTAAAAATCAAAATGTT
ACTCGTTGAAGTGCTTTATGAATGAGTTCTCTTCCAACTTATAAGAAAACATGAATAGTCACAACAATAGTGTGC
TAACAXXXXXXXXXXTTTTGATTGCACATATTATAGTTTTGAATTTAATAATAATTGTATAGGGTATTAGAAATG
ATTGTAAAAATCAAAATGTT
>Marker678115

Create PDF with GO2PDF for free, if you wish to

ACCAATTATTCTACCAACAATTTTATCCTGTTCATCCAGAGAAAAATATGTATGGAACAACGGCAACAATTCTGA
TTCCTXXXXXXXXXXXATGAAACTTCATGCAACTTGAATTATTACACTTTTTATTACCTTTCAAGTCTCTGCGAAG
GCCTGCCAGCTTAGATAGTG
ACCAATTATTCTACCAACAATTTTATCCTGTTCATCCAGAGAAAAATATGTATGGAACAACGGCAACAATTCTGA
TTCCTXXXXXXXXXXXATGAAACTTCATGCAACTTGAATTATTACACTTTTTATTACCTTTCAAGTCTCTGCGAAG
GTCTGCCAGCTTAGATAGTG
>Marker678436
TACCAAAGGAAGGGTAAGAAGGGAAATTCAACCTAGACAACATGGTTGTTAGAATGGCCAGTGGTCCCAGCAACA
AGGTAXXXXXXXXXXXAAAAACTATCGTAGAACTCTTCTGGAATTCTTGTTGAATATACCTATATCTCATCTCTTC
TCATATTGGTGTTATGTGTG
TACCAAAGGAAGGGTAAGAAGGGAAATTCAACCTATGCAACATGGTTGTTAGAATGGCCAGTGGTCCCAGCAACA
AGGTAXXXXXXXXXXXAAAAACTATCGTAGAACTCTTTTGGAATTCTTGTTGAATATACCTATATCTCATCTCTTC
TCATATTGGTGTTATGTGTG
>Marker678453
CACAGTTCTTTACAAAGGAAAAATAGGAATACTGAAAACTGATTCAGAAGATAAAGAAAATTATTATTATAAACA
CTTCAXXXXXXXXXXXTGTAGAAAGATTCATGGTTCATATCTAAATCATTATTTTAAAACTATTTAAACCACGTGG
AACACATGCAGAAACCAAGT
CACAGTTCTTTACGAAGGAAAAATAGGAATACTGAAAACTGATTCAGAAGATAAAGAAAATTATTATTATAAACA
CTTCAXXXXXXXXXXXTGTAGAAAGATTCATGGTTCATATCTAAATCATTATTTTAAAACTATTTAAACCACGTGG
AACACATGCAGAAACCAAGT
>Marker678914
ACAATATTTGGGTTATTGGATCTCTAGTAAAGGAGTGGCAGCAGATGGGGAATAGGTCAAAGGTATGGTCCAACG
GCCTCXXXXXXXXXXXCACTAAACTCTTATAAAAAATGCATTTAGATGGAGTGAGGAAGCTACAGAGTCCTTTGAA
CAATTGAAGCAAGCCATGGT
ACAATATTTGGGTTATTGCATCTCTAGTAAAGGAGTGGCAGCAGATGGGGAATAGGTCAAAGGTATGGTCCAACG
GCCTCXXXXXXXXXXXCACTAAACTCTTATCAAAAATGCATTTAGATGGAGTGAGGAAGCTACAGAGTCCTTTGAA
CAATTGAAGCAAGCCATGGT
>Marker679435
ACCGTGTTTCGCCATCTTGGGCCATCTTATATGCGTATAATGTTTTACTTTAGGAAACCTTGTAGGCAATCAACA
TATCCXXXXXXXXXXXCACGAAAGAATTCAAATGCAACGTATCATAAATCAAGCAATATACCCTTTCTAAAGACAT
CAAAAGTTATAACTAATGTG
ACCGTGTTTCGCCATCTTGGGCCATCTTATATGCGTATAATGTTCTACTTTAGGAAACCTTGTAGGCAATCAACA
TATCCXXXXXXXXXXXCACGAAAGAATTCAAATGCAACGTATCATAAATCAAGCAATATACCCTTTCTAAAGACAT
CAAAAGTTATAACTAATGTG
>Marker680014
AACCGCTCATTCTTTTGCAAATATTTTCAAAAACCACCCTATTATCACACTAAATCCCTAGACTTGATGTATAAA
CGTAGXXXXXXXXXXXTTCTAAGGGGTCTTTCCTTTTAGAACCTTCATAATGACTCAATAATGTGTGATGTTCATG
ACATGAATAGCACTTGATGT
AACCGCTCATTCTTTTGCAAATATTTTCAAAAACCACCCTATTATCACACTAAATCCCTAGACTTGATGTATAAA
CGTAGXXXXXXXXXXXTTCTAAGGGGTCTTTCCTTTTAGAACCTTCATAATGACTCAATAATGTGTGATGTTCATG
ACATGAATAGCACCTGATGT
>Marker681840
TACTAAAGAAAGGGTAAGAAGGAAAATTCAACCTAGGCAACATGGTTGTTAGGATGGCCAGTGGTCCCAACAACG
TGGCAXXXXXXXXXXXAAAAACTATCATAAAACTCTTGTGGAATTCTTATTAAATATACCTATATCTCATATCTTC
TCATATTGGTGTTGTGTGTG

Create PDF with GO2PDF for free, if you wish to

TACTAAAGAAAGGGTAAGAAGGAAAATTCAACCTAGGCAACATGGTTGTTAGGATGGCCAGTGGTCCCAACAACG
TGGCAXXXXXXXXXXXAAAAACTATCATAGAACTCTTTTGGAATTCTTATTAAATATACCTATATCTCATCTCTTC
TCATATTGGTGTTGTGTGTG
>Marker681975
CACCAGCCAATCAAGAGGAAAAATCCAGGTGAGGAGAAGAAAATGAGTCAGGAAGAAATGCTTCTTGAAGCAGCT
CAAACXXXXXXXXXXXTCATGAACTTGAGGAACTTGGAGCGTGTTTTGGCAAGGGAAGAAGAAGTCAAAAAGAGAG
CAATTGTGCATAAAGCTGTG
CACCAGCCAATCAAGAGGAAAAATCCAGGTGAGGAGAAGAAAATGAGTCAGGAAGAAATGCTTCTTGAAGCTGCT
CAAACXXXXXXXXXXXTCATGAACTTGAGGAACTTGGAGCGTGTTTTGGCAAGGGAAGAAGAAGTCAAAAAGAGAG
CAATTGTGCATAAAGCTGTG
>Marker682242
TACCTCCACAGTTTTAACCAAATCAATTTCATTCTCTAAAAGCATCAAGGAAATGAAGGAAGAAATGCAACATGG
AAAAGXXXXXXXXXXXAATCACAACCCCTCCATTCCCTTTCGGAAACCCATCTCTTAATTTCTCCCCTACTTTCTC
ACAAGAATTCAAATCCCAGT
TACCTCCACAGTTTTAACCAAATCAATTTCATTCTCTAAAAGCATCAAGGAAATGAAGGAAGAAAGGCAACAAGG
AAAAGXXXXXXXXXXXAATCACAACCCCTCCATTCCCTTTAGGAAACCCATCTCTTAATTTCTCCCCTACTTTCTC
ACAAGAATTCAAATCCCAGT
>Marker682555
CACTAGAGTCGGTGGCATTATCCAACAAAATGTTGGATTTTTCCACTAACAAAGTCAAATGGCAAAATGGTCATT
TGGTTXXXXXXXXXXXTTTGACTTTTCAAAGTAAAAAAAATCAATATTTTAACTTTTTACAACTTTGACTATTTCC
ATCATTTTTTAGCTTCGAGT
CACTAGAGTCGGTGGCATTATCCAACAAAATGTTGGATTTTTCCACTAACAAAGTCAAATGGCAAAATGGTCATT
TGGTTXXXXXXXXXXXTTTGACTTTTCAAAGTAAAAAAAGTCAATATTTTAACTTTTTACAACTTTGACTATTTCC
ATCATTTTTTAGCTTCGAGT
>Marker684046
TACCTAGAACACAAAGGGGTCATAATTCTGTTTTTGTAGTAGTAGATCGCTTTAGTAAAATGGTCCATTTCATCC
CTTGTXXXXXXXXXXXTGTCAAATCACGATGTAAAGTATTTGAGTCATTTTTGGAGATCCCTATGGAAGAAATTCG
ATACCAACCTTCTATTTGGT
TACCTAGAACACAAAGGGGTCATAATTCTGTTTTTGTAGTAGTAGATCGCTTTAGTAAAATGGCCCATTTCATCC
CTTGTXXXXXXXXXXXTGTCAAATCACGATGTAAAGTATTTGAGTCATTTTTGGAGATCCCTATGGAAGAAATTCG
ATACCAACCTTCTATTTGGT
>Marker684273
ACTTAGTGTAATGAGTTAAGTGAATAAAGCGAAAAAGTTAAGCATATTCAGCTACATCATTGTTATTATTTGCCA
TATCCXXXXXXXXXXXAAAATAGAAGCTTTGCGAAGAGATAAGTTCAAGAAGTTGCTCTGCTTACTAGGCATTTAG
CGTGTTATCTCGCGATTGGT
ACTTAGTGTAATGAGTTAAGTGAATAAAGCGAAAAAGTTAAGCATATTCAGCTACATCATTGTTATTATTTGCCA
TATCCXXXXXXXXXXXAAAGTAGAAGCTTTGCGAAGAGATAAGTTCAAGAAGTTGCTCTGCTTACTAGGCATTTAG
CGTGTTATCTCGCGATGGGT
>Marker684358
ACATCAATATTATGTTAATACATGAAATAACAAAAAATTAAAATAATATCAAACTCAAACTTCAAAAGTAGATTA
TCAAGXXXXXXXXXXXCGTGTGTTTGAGGTGTAAATTATGTTGATTTGAGAATTAAGGAAGGAAGAATAAAAAAAA
AGATGAACCAAGTGAAAGTA
ACATCAATATTATGTTAATACATGAAATAACAAAAAATTAAAATAATATCAAACTCAAACTTCAAAAGTAGATTA
TCAAGXXXXXXXXXXXTGTGTGTTTGAGGTGTAAATTATGTTGATTTGAGAATTAAGGAAGGAAGAATAAAAAAAA
AGATGAACCAAGTGAAAGTA
>Marker684533

Create PDF with GO2PDF for free, if you wish to

ACTCGCCGTTTTCATTTACCCCTTTCAGCTTGCGAACTGATGAAAGAGCAGCAAGAAGAAAGGAGGCAAGCTTTT
GTTGCXXXXXXXXXXCACAAACGAGTCACAAAAAAAGGTGCAACTTCAAACAAAACTAAAGGTAACAAATAAAAA
ATATTACTTGGTAATCTGTA
ACTCGCCGTTTTCATTTACCCCTTTCAGCTTGCGAACTGATGAAAGAGCAGCAAGAAGAAAGGAGGCGAGCTTTT
GTTGCXXXXXXXXXXCACAAACGAGTCACAAAAAAAGGTGCAACTTCAAACAAAACTAAAGGTAACAAATAAAAA
ATATTACTTGGTAATCTGTA
>Marker684690
AACATGTTAGTTTTTATTGTTTTCATAATCTAGATGATTTGAAATTAGCAAAATCAAATTTAGATCATCTGTCAA
AATAAXXXXXXXXXXXCAGGTTTCAATTAAGCAATGTTTCTGATAATAGTGAGTTTGAAAAGGTTTTCTCAGGGCA
CCAAGAGCTTTTGGATAGTG
AACATGTTAGTTTTTATTGTTTTCATAATCTAGATGATTTGAAATTAGCAAAATCAAATTTAGATCATCTGTCAA
AATAAXXXXXXXXXXXCAGGTTTCAGTTAAGCAATGTTTCTGATAATAGTGAGTTTGAAAAGGTTTTCTCAGGGCA
CCAAGAGCTTTTGGATAGTG
>Marker685403
TACACTAGATAGCCCCATGACTTTAGTTTATTGGATTCAAAATTATAATATTCAATTTTCAGAAATAAGTCGTAA
TTAATXXXXXXXXXXAAATTTACAATAATATATCTTCTATCCATCCCTACCAAATATGATAATTCCAAAATTACA
AGACCAATCCACACATGGTA
TACACTAGATAGCCCCATGACTTTAGTTTATTGAATTCAAAATTATAATATTCAATTTTCAGAAATAAGTCGTAA
TTAATXXXXXXXXXXAAATTTACAATAATATATCTTCTATCCATCCCTACCAAATATGATAATTCCAAAATTACA
AGACCAATCCACACATGGTA
>Marker687102
ACTCATAATTTATAAAATTGGGAGAAGACTCATTGTAAAGAAACATGATGTGGTTATGACTTGTTGGGGAGTATA
TTCTCXXXXXXXXXXGTCTAAACTATCGATAATCATTTGTTTTTTCATTTTCTAAAATTCCATCCATATATCTAA
ACTATTAGTGATAAATCAGT
ACTCATAATTTATAAAATTGGGAGAAGACTAATTGTAAAGAAACATGATGTGGTTATGACTTGTTGGGGAGTATA
TTCTCXXXXXXXXXXGTCTAAACTATCGATAATCATTTATTTTTTCATTTTCTAAAATTCCATCCATATGTCTAA
ACTATTAGTGATAAATCAGT
>Marker687344
CACTTGTCTAAGGAACAGTGTCCTAAGACACCTCAAGTTGTGGGCAGCTTAATATATGTTATGCTCTACATTAGG
CCAGAXXXXXXXXXXXGATTTGATCCTTATGGGATACACTGACTCTAATTTTCAAACCAATCAGGATTCTAGAAAA
TTCATGTCGGGATTAGTGTT
CACTTGTCTAAGGAACAGTGTCCTAAGACACCTCAAGTTGTGGGCAACTTAATATATGTTATGCTCTACATTAGG
CCAGAXXXXXXXXXXXGATTTGATCCTTATGGGATACACTGACTCTAATTTTCAAACCAATCAGGATTCTAGAAAA
TTCATGTCGGGATTAGTGTT
>Marker687426
ACCACTTTGAATCATAATCTTTCTTGAACCATTTAGGTTATCTGTTGTTGGGCCATTGTGACTATGAGATGCACT
GAGTTXXXXXXXXXXCTTGGGCTAAAGTTGTCCTGAAATGTATGGAAGAAAAAATCAAGCAATTATGTAGCATTG
TGTAGTATGATCTAGGTTGT
ACCACTTTGAATCATAATCTTTCTTGAACCATTTAGGTTATCTGTTGTTGGGCCATTGTGACTAAGAGATGCACT
GAGTTXXXXXXXXXXCTTCGGCTAAAGTTGTCCTGAAATGTATGGAAGAAAAAATCAAGCAATTATGTAGCATTG
TGTAGTATGATCTAGGTTGT
>Marker687707
ACATAGAACTAAAAGCGAAAATTCATAGGACCTTATGGAAGTTCAAGATAATGTCTAAACTATAAAATATGTGAT
AGTTTXXXXXXXXXXTGTAGTAGAACCTTAAAAATAGTCATGGTATTATCCATGGTTGTGCTTTATTGGTTAAGC
AGAATGCATTAGAGAGGAGT

Create PDF with GO2PDF for free, if you wish to

ACATAGAACTAAAAGCGAAAATTCATAGGACCTTATGGAAGTTCAAGATAATGTCTAAACTATAAAATATGTGAT
AGTTTXXXXXXXXXXXTGCAGTAGAACCTTAAAAATAGTCATGGTATTATCCATGGTTGTGCTTTATTGGTTAAGC
AGAATGCATTAGAGAGGAGT
>Marker687846
AACAGGAACCAGTAAATTTTTCTCAAGAACCATTTCTCAAATGCCGCTCCAAGGCCTTGTGCAATGGATGATCAT
CGATGXXXXXXXXXXXTCATGAATTCGAGAATCACCTGTTTTTCAATATTCATTCGATTCGATCTCAGAGCTGATT
ATGAATGTATGAGTGCTTGT
AACAGGAACCAGTAAATTTTTCTCAAGAACCATTCCTCAAATGCCGCTCCAAGGCCTTGTGCAATGGATGATCAT
CGATGXXXXXXXXXXXTCATGAATTCGAGAATCACCTGTTTTTCAATATTCATTCGATTCGATCTCAGAGCTGATT
ATGAATGTATGAGTGCTTGT
>Marker688861
AACCGCCCTTCCCCAAAACGGTCCTACAATGTTAATGTTTTAGTTGGACGAAGAGCAAGAGTGGGAGGGATGAAA
AAAAAXXXXXXXXXXXTATCCCCGCCACCCCTTGTCGTGAAAAAAGCTAGAAGGTATGGATTGAAACCCTAATTTG
AAACCCATAAAATTTGAAGT
AACCGCCCTTCCCCAAAACGGTCCTACAATGTTAATGTTTTAGTTGGACGAAGAGCAAGAGTGGGAGGGATGGAA
AAAAAXXXXXXXXXXXTATCCCCGCCACCCCTTGTCGTGAAAAAAGCTAGAAGGTATGGATTGAAACCCTAATTTG
AAACCCATAAAATTTGAAGT
>Marker689014
ACCTTGTTTCATGTCGTCTGGACTCTTTCTGCTTCCCATTGTAAATAAGCATTTCTGTGCTGGCACAACCTCCTC
CTATTXXXXXXXXXXXAATATTGGAAACGATTACTGATATACAAAAGAAAACGAATAAAAGAAAAATTCATGGCAA
GTGGAACCAAGAAGGACGTA
ACCTTGTTTCATGTCGTCTGGACTCTTTCTGCTTCCCATTGTAAATAAGCATCTCTGTGCTGGCACAACCTCCTC
CTATTXXXXXXXXXXXAATATTGGAAACGATTACTGATATACAAAAGAAAACGAATAAAAGAAAAATTCATGGCAA
GTGGAACCAAGAAGGACGTA
>Marker689843
TACCAACACAACTGTTAGAAAACAGTAGCATAGCCTGAGAAGAATATTTCAAAATTCCAGTTTCTACCACGTGTT
GCATGXXXXXXXXXXXATCAAGGGTGGAAATAATCAAGAGTTACTGTTTGTCTCATTAAGAACAGTTTGCTTCTTG
CTAAACACGAGATAAAAGGT
TACCAACACAACTGTTAGAAAACAGTAGCATAGCCTGAGAAGAATATTTCAAAATTCCAGTTTCTACCACGTGTT
GCATGXXXXXXXXXXXATCAAGGGTGGAAATAATCAAGAGTTACTGTTTGTCTCTTTAAGAACAGTTTGCTTCTTG
CTAAACACGAGATAAAAGGT
>Marker689933
AACAGAAACATTGATTAGTTGGATGATAACCTTATTGTTTGGACTAAGTCTTGCTCTTTCAACATGGGGAAGTAT
TATAGXXXXXXXXXXXATCTCACTAATTATCACATAATTTATTTTCCCAAGTCCTAATTTTAAATCAAGCATAAAT
GATTGATTTTCAATGTTGTT
AACAGAAACATTGATTAGTTGGATGATAACCTTATTGTTTGGACTAAGTCTTGCTCTTTCAACATGGGGAAGTAT
TATAGXXXXXXXXXXXATCTCACTAATTATCACATAATTTATTTTCCCAAGTCCTAATTTTAAATCAAGCATAAAT
GATTGATTTTTAATGTTGTT
>Marker690064
GACAAAATGGGATTGCTGAAAGAAAAAAATAGAAATTTACTTGAAATGGCACACGCCGTAATGTTTTCCATGCAT
GTTTCXXXXXXXXXXXTCAAAAATGTGAAAAATTAATATGATCAAAGTAACATTTAGCTATATCAAAAGATATTAG
CTGTAAAACTAGCTAAAGTA
GACAAAATGGGATTGCTGAAAGAAAAAAATAGAAATTTACTTGAAATGGCACACGCCCTAATGTTTTCCATGCAT
GTTTCXXXXXXXXXXXTCAAAAATGTGAAAAATTAATTTGATCAAAGTAACATTTAGCTATATCAAAAGATATTAG
CGGTAAAACTAGCTAAAGTA
>Marker690094

Create PDF with GO2PDF for free, if you wish to

GACCTAATTAACGAAAGAAAATGGTTCATTATTGTATGTGCCTAGCTTTATTAGTTTAGTATGGATTGTAAGTGT
TGGAGXXXXXXXXXXATTGATAAGAGCAATTATATATTTAACTGTTCCAATTTTGAAATTGGGCACTAGGGTTTT
GACGTTAGACTTTTTTCGTT
GACCTAATTAACGAAAGAAAGTGGTTCATTATTGTATGTGCCTAGCTTTATTAGTTTAGTATGGATTGTAAGTGT
TGGAGXXXXXXXXXXATTGATAAGAGCAATTATATATTTAACTGTTCCAATTTTGAAATTGGGCACTAGGGTTTT
GACGTTAGACTTTTTTCGTT
>Marker690249
TACATCAACTTGCATTTAATCGTTCAATTTTAAGTTCCAGCATGAAAATTCTTTAATATTTAGGAGTTAGATTAA
AAATTXXXXXXXXXXACCCAACAAAGGATAGAGAAAAATAGGGAGGACGCTCCCTGTTTCGATGTTATATTCAAA
AATGAATTCCACCACTAGTT
TACATCAACTTGCATTTAATCGTTCAATTTTAAGTTCCAGCATGAAAATTCTTTAATATTTAGGAGTTAGATTAA
AAATTXXXXXXXXXXACCGAACAAAGGATAGAGAAAAATAGGGAGGACGCTCCCTGTTTCGATGTTATATTCAAA
AATGAATTCCACCACTAGTT
>Marker690717
ACTTAAAATTGACCTTCTCACCTTCTCTTGGTCCAGACGCCAAACTTCTCGATTCCAATTTACACAATAGCCTAG
GTCTAXXXXXXXXXXGCTTGCATGATTTAGTCTTAACACCACCTACTTGACACCTTATGCTAACATTTTCACACT
TTAAACCAAACTTCCAATGT
ACTTAAAATTGACCTTCTCACCTTCTCTTGGTCCAGACGCCAAACTTCTCGATTCCAATTTACACAATAGCCTAG
GTCTAXXXXXXXXXXGCTTGCATGATTTAGTCTTAACACCACCTACTTGACACCTTATGCTAACATTTTCACACT
TTAAACCAAACATCCAATGT
>Marker691632
CACATATGTTGCCTCCTCTCCCTCCCCTTCATTGCTAACTAGATGTAATAGAATAACTAGCTAGGGATATCTTAA
TTGCAXXXXXXXXXXTATCTTGTTCAAACTCAATACGACATCTCGACCTATACCTCAACCATATAATAATGTTAA
AATATTCTCGTCCCAAAGTA
CACATATGTTGCCTCCTCTCCCTCCCCTTCATTGCTAACTAGATGTAATAGAATAACTAGCTAGGGATATCTTAA
TTGCAXXXXXXXXXXTATCTTGTTCAAACTCAATACGACATCTCGACCTATACCTCAACCATATAATAATGTTAA
AATATTCCCGTCCCAAAGTA
>Marker691798
AACAAGATGCGAGTCCTGCAGCAGAAAGAAAAGACCAATCCCCACAGTTCATTTGAAGGAGAAGTTAAAGCAATT
CCGAAXXXXXXXXXXCACCACAACTTGTTTAACAATGCCAACCAACATTCATTTTTGACAATTTTCTAGCTTCTC
AAAGAATCATCAATTCAAGT
AACAAGATGCGAGTCCTGCAGCAGAAACAAAAGACCAATCCCCACAGTTCATTTGAAGGAGAAGTTAAAGCAATT
CCGAAXXXXXXXXXXCACCACAACTTGTTTAACAATGCCAACCAACATTCATTTTTGACAATTTTCTAGCTTCTC
AAAGAATCATCAATTCAAGT
>Marker692010
ACTGAACGTTATATTTTCAAGTGTTGAGAGTCATGTTTGGAAGACTTATTATGCAAATTGTTGATATAAATATTA
TATTTXXXXXXXXXXAAACAACCCATCTCTCAGTCAATTACCATATACATATACACTGAATTCATCTCTTTCTCA
AGTAAATTATATTATAGGTT
ACTGAACGTTATATTTTCAAGTGTTGGGAGTCATGTTTGGAAGACTTATTATGAAAATTGTTGATATAAATATTA
TATTTXXXXXXXXXXAAACAACCCATCTCTCAGTCAATTACCATATACATATACACTGAATTCATCTCTTTCTCA
AGTAAATTATATTATAGGTT
>Marker692722
AACTCATGGATTGCCTCACGTGAGGATGATGCCCTTAATGAAATGAAAATGGAGGCTAACCCCTGAACTTGGGGG
AAGGCXXXXXXXXXXGATCAACCCCACGTAGCTGTTTTGGCTGGAACACGTTCGTCGCCGGACAGCCTCTATTCG
CTCTCTCCCTGGTGTGGGTC

Create PDF with GO2PDF for free, if you wish to

AACTCATGGATTGCCTCACGTGAGGATGATGCCCTTAATGAAATGAAAAGGGAGGCTAACCCCTGAACTTGGGGG
AAGGCXXXXXXXXXXGATCAACCCCACGTAGCTGTTTTGGCTGGAACACGTTCGTCGCCGGACAGCCTCTATTCG
CTCTCTCCCTGGTGTGGGTC
>Marker693409
AACCTTCTCCAAAATTCAGCAAGATTAATCCTTTTATTACCCAACTAATGTATGCATTATATAGTTAATATACAT
ACAAAXXXXXXXXXXAATGGATTAATTCCACTAACTATGGAATTATCTACTAACCATGGTCAAAGTCATCATTTG
ACTTTCAAGAGTCAGAAGTC
AACCTTCTCCAAAATTCAGCAAGATTAATCCTTTTATTACCCAACTAATGTATGCATTATATAGTTAATATACAT
ACAAAXXXXXXXXXXAATGGACTAATTCCACTAACTATGGAATTATCTACTAACCATGGTCAAAGTCATCATTTG
ACTTTCAAGAGTCAGAAGTC
>Marker693655
AACTAGGCTAACATACCAAAATTTTCTAAAATAAAAGAAAGAATGTGCAATTGGACCCAAACCTTAAAATCAAAC
CAACAXXXXXXXXXXTTTGCTATCTATTTTTACCATCATAGTTTATAAAACCAAACAATACTTTGAAAAAAAAAA
CTAGCTTTTAAAATTTTGTT
AACTAGGCTAACATACCAAAATTTTCTAAAATAAAAGAAAGAATGTGCAATTGGACCCGAACCTTAAAATCAAAC
CAACAXXXXXXXXXXTTTGCTATCTATTTTTACCATCATACTTTATAAAACCAAACAATACTTTGAAAAAAAAAA
CTAGCTTTTAAAATTTTGTT
>Marker694909
AACCTTTGCAGATAAGCGAAGATGAAATTACTTTCTTGACTGGTGGAGATGATCCCTATGATGACAATGTGGTGT
TGAAAXXXXXXXXXXCTCTCTTTTCCACTCTTTGTTAAATCACCAATAGACCCAAATACTTAACCTGTAGCATCC
AACACTTCTATTTTCTTGTG
AACCTTTGCAGATAAGCGAAGATGAAATTACTTTCTTGACTGGTGGAGATGATCCCTATGATGATAATGTGGTGT
TGAAAXXXXXXXXXXCTCTCTTTTCCACTCTTTGTTAAATCACCAATAGACCCAAATACTTAACCTGTAGCATCC
AACACTTCTATTTTCTTGTG
>Marker695531
AACGGATGGTTTCTTTTACCATTTTTTTCTCTTGTTTCTGTGTAGGAAATGTTGAACATTAGAAAATGAAAAGTT
TGCTGXXXXXXXXXXCTCCTGTCAGTTTGTGTGCCTACCTTGTTTAATACTGTTGCATCCATTCTATTTAAACTT
TTTACACAAGTATTTGAGTG
AACGGATGGTTTCTTTTACCATTTTTTTCTCTTGTTTCTGTGTAGGAAATGTTGAACATTAGAAAATGAAAAGTT
TGCTGXXXXXXXXXXCTCCTGTCAGTTTGTGTGCCTACCTTGTTTAATACTGTTGCATCCATTCTGTTTAAACTT
TTTACACAAGTATTTGAGTG
>Marker696234
AACCAATTTGTGATACTTTTGACACCCTGCACACTGAGCACAGAATAATAATTGGAAGCATTAAGAACACATTAA
ACAGCXXXXXXXXXXAATAACACACACTATATGTTTTTAATAAAAAAAACTTTAATCTGCTAGACGTTGCAAACA
TGAAATTTAGTCTATTTAGT
AACCAATTTGTGATACTTTTGACACCCTGCACACTGAGCACACAATAATAATTGGAAGCATTAAGAACACATTAA
ACAGCXXXXXXXXXXAATAACACACACTATATGTTTTTAATAAAAAAAACTTTAATCTGCTAGACGTTGCAAACA
TGAAATTTAGTCTATTTAGT
>Marker698195
TACGTATATATATCATCTACCAAGAGGTTAAAGGTTCAAATTTCATCCCCAATATGTTGCTGAACCTTTTTCAGA
ATAGAXXXXXXXXXXGAATAATGTTGGCTGGAATTCACCGTGGGTTGGTCTGGCAGTCATTACAACTAAAGAATA
GAGATTGGTTCAGTATAGTA
TACGTATATATATCATCTACCAAGAGGTTAAAGGTTCAAATTTCATCCCCAATATGTTGCTGAACCTTTTTCAGA
ATAGAXXXXXXXXXXGAATAATGTTGGCCGGAATTCACCGTGGGTTGGTCTGGCAGTCATTACAACTAAAGAATA
GAGATTGGTTCAGTATAGTA
>Marker698374

Create PDF with GO2PDF for free, if you wish to

CACAACGAGGAGTTTGGTAACAAACTTCACCTTAGTGAGGTAACTTCATTGGTCTATTTTCTTTCATTAAATACA
CTAGAXXXXXXXXXXXTTCTTGCAAATACTTGTCGAATTCAAGCTCGTGATGGATCTTTGAAAATCTTTGAAATTC
TTTCAATTCTTTCTTAAGTG
CACAACGAGGAGTTTGGTAACAAACTTCACCTTAGTGAGGTAACTTCATTGGTCTATTTTCTTTCATTAAATACA
CTATAXXXXXXXXXXXTTCTTGCAAATACTTGTCGAATTCAAGCTCGTGATGGATCTTTGAAAATCTTTGAAATTC
TTTCAATTCTTTCTTAAGTG
>Marker698681
CACGCTAAAGCTTTTGAAAAATGTTTGAAAATCCTGTGGCATCTTTTGAAAAACAAGGCATTTTAACAAAAGTTA
AAACTXXXXXXXXXXXCTTCAAAAGTAAAAACCAGAGAGTTAAGTAATAAGAGGCGATGAGAGATACAATTGAGGA
GAGAAACATATCCATAGAGT
CACGCTAAAGCTTTTGAAATATGTTTGAAAATCCTGTGGCATCGTTTGAAAAACAAGGCATTTTAACAAAAGTTA
AAACTXXXXXXXXXXXCTTCAAAAGTAAAAACCAGAGAGTTAAGTAATAAGAGGCGATGAGAGATACAATTGAGGA
GAGAAACATATCCATAGAGT
>Marker698721
ACCCACATAACACCATCCAATCTTCTGCACTCCCCCCACATCCCACTTCCCACTGACTAATTGTCAATTTGTTAA
ATAACXXXXXXXXXXXTTTTGTTTGAAGGGTTGTGAATTTTTAATCGCATTTATATAATTATGTTTCTTTCTTCTA
TCTGCTGTGTTTCATGCGGT
ACCCACATAACACCATCCAATCTTCTGCACTCCCCCCACATCCCACTTCCCACTGACTAATTGTCAATTTGTTAA
ATAACXXXXXXXXXXXTTTTGTTTGAAGGGTTGTGAATTTTTAATCGCATTTATATAATTATGTTTCTTTCTTCTA
TGTGCTGTGTTTCATGCGGT
>Marker698853
ACAATGTTTTAAGACACCTCAAGAAGTTGAGAATATAAGACATGTTTCCTATGCATCAACTGATTGGAGTCTGAT
ATATGXXXXXXXXXXXTATGGAGGTGGAAATTTGATACACTGATTTCGATTTCTAGTTAGTAAATATTTTAGAAAA
TCAACACCAGGATCAGTGTT
ACAATGTTTTGAGACACCTCAAGAAGTTGAGAATATAAGACATGTTTCCTATGCATCAACTGATTGGAGTTTGAT
ATATGXXXXXXXXXXXTATGGAGGTGGAAATTTGATACACTGATTTCGATTTCTAGTTAGTAAATATTTTAGAAAA
TCAACACCAGGATCAGTGTT
>Marker699409
CACTTCGGTTCCAGTATAGGTCTCGATTGATAAAAATGCTGGAACTAGCATATGTATCCCGGTATGTGACTAAGA
TAGTTXXXXXXXXXXXACGACCACTATCTTGTTTCATCAGTTTTTTCTTAAGATTAGCAATCGCCTCCTAATGAGA
ACTTAAATCCTCTGGTTGGT
CACTTCGGTTCCAGTATAGGTCTCGATTGATAAAAATGCTGGAACTAGCATATGTATCCCGGTATGTGACTAAGA
TAGTTXXXXXXXXXXXACGACCACTATCTTGTTTCATCAGTTTCTTCTTAAGATTAGCAATCGCCTCCTAATGAGA
ACTTAAATCCTCTGGTTGGT
>Marker700067
AACTAGATTTGTATGCCTAATGGCCAACTAGTTTTGGTTTAAAATTTGCATGCATAGCAGTAATGACCCTGGTTA
GGGACXXXXXXXXXXXCTTCGAATTTTGTAGTTTGTCTTTCTTTTTTTCTATCTACACTTGATTATATCTTCTAGT
GGGCTCATTGTTGAAGAGTA
AACTAGATTTGTATGCCTAATGGCCAACTAGTTTTGGTTTAAAATTTGCATGCATAGCAGTAATGACCCTAGTTA
GGGACXXXXXXXXXXXCTTCGAATTTTGTAGTTTGTCTTTCTTTTTTTCTATCTACACTTGATTATATCTTCTAGT
GGGCTCATTGTTGAAGAGTA
>Marker700069
CACAATTCATAAATGCCTAAATCCAATATCTACATAGATGTTAAAGCAATACTGATAACTAATAATGGCCTCAAG
TCCAAXXXXXXXXXXXTCATATAGTTGTATATTTATTATGCTTAACAAACGTTAAGTAAGCGACTAGCTAATTGAA
CTCATAGACTTTGCACAGTA

Create PDF with GO2PDF for free, if you wish to

CACAATTCATAAATGCCTAAATCCAATATCTACATAGATGTTAAAGCAATACTGATAACTAATAATGGCCCCAAG
TCCAAXXXXXXXXXXXTCATATAGTTGTATATTTATTATGCTTAGCAAACGTTAAGTAAGCGACTAGCTAATTGAA
CTCATAGACTTTGCACAGTA
>Marker700742
ACAATTTTTCGGAAGAGACTGTCATGTAGTTTGAGGATATTAAAGCTTCTTGCTGACCTCTCGAAGAAATTTCCA
TATATXXXXXXXXXXXTTGAGAACACCATTCTGTTACTCTTGGAAGAATTTTTGGAGCTAGTTCAGATTGTATTTC
GCAACAGCTACGTTTGTGTG
ACAATTTTTCGGAAGAGACTGTCATGTAGTTTGAGGATATTAAAGCTTCTTGCTGACCTCTCGAAGAAATTTCCA
TATATXXXXXXXXXXXTTGAGAACACCATTCTGTTACTCTTGGAAGAATTTTTGGAGCTAGTTCAGATTGTATTTC
GCAACATCTACGTTTGTGTG
>Marker700797
ACTGTGTTTTTCAAAGTCGTCGATCCATCCGGCAAATTCGAATCATAATAACCAGAAAGCACTCCCCAACTGTTT
TCAGCXXXXXXXXXXXAAACTATGTTGAGTTCTTTTCCAATGAAGATGTCAGTCCCTAAAGAATTCAAAGTAGTGG
TCTCCGAAACTATCCACAGT
ACTGTGTTTTTCAAAGTCGTCGATCCATCCGGCAAATTCGAATCATAATAACCAGAAAGCACTCCCCAACTGTTT
TCAGCXXXXXXXXXXXAAACTATGTTGAGTTCTTTTCCAATGAAGATGTCAATCCCTAAAGAATTCAAAGTAGTGG
TCTCCGATACTATCCACAGT
>Marker700806
ACTCTTAAAGAGTTCTATGAATTAAGCATCAATGTAAAAGTTCTTTTCCTGCATTAACCTTTAATAAACTTCGGC
AATCTXXXXXXXXXXXCCAAGAAAAAGAGTAAAAAGAAGAATTTAACCACAAAGAAAGGTCACCTTATAGGAGGGT
CAAAACAGTTCGTGCATAGT
ACTCTTAAAGAGTTCTATGAATTAAGCATCAATGTAAAAGTTCTTTTCCTGCATTAACCTTTAATAAGCTTCGGC
AATCTXXXXXXXXXXXCCAAGAAAAAGAGTAAAAAGAAGAATTTAACCACAAAGAAAGGTCACCTTATAGGAGGGT
CAAAACAGTTCGTGCATAGT
>Marker701302
AACAAACAAAAACAGAGCTATCATTGAATAATGATATCCAAAGAAGCCATTGAGTTCAAGTAATTCTAGTGCAAC
TTGATXXXXXXXXXXXACTAATTAAAATATGATTGGATGATGGAGTTGGAGTGAGTCAAAGACGTGGGGAAAGATT
TATTATATCCTACAAATGTG
AACAAACAAAAACAGAGCTATCATTGAATAATGATATCCAAAGAAGCCATTGAGTTCAAGTAATTCTAGTGCAAC
TTGATXXXXXXXXXXXACTAATTAAAATATGATTGGATGGTGGAGTTGGAGTGAGTCAAAGACGTGGGGAAAGATT
TATTATATCCTACAAATGTG
>Marker702073
ACTATTTTTGGTATCATCATCTAATCATTTTCATAACGCTCTGGATTTCAACTTGCATATTAAGCATAGCAATAC
TGTGCXXXXXXXXXXXTACATTACATGTCTTTATAAAATTGCTTTTCTTTTGTTTTTGTCCCAAATTTCGTGGAGG
TTTTCTTGGTGTTCTGGGTA
ACTATTTTTGGTATCATCATCTAATCATTTTCATAACGCTCTGGATTTCAACTTGCATATTAAGCATAGCAATAC
TGTGCXXXXXXXXXXXTACATTACATGTCTTTATAAAAATGCTTTTCTTTTGTTTTTGTCCCAAATTTCGTGGAGG
TTTTCTTGGTGTTCTGGGTA
>Marker702308
AACTTAAAACTTAAACTTGGAATTTATATGCATTGAAATCCATTGTTAATTTATATTCATTATTAAACCTAAACA
ATTAGXXXXXXXXXXXACAATAAGCATTGAGAGAAAGTAAAACAATTAGCATAACAAATTTCTTGCATTCGTGTAA
TACATAATACTATAATGGTG
AACTTAAAACTTAAACTTGGAATTTATATGCATTGAAATCCATTGTTAATTTATATTCATTATTAAACCTAAACA
ATTAGXXXXXXXXXXXACAATAAGCATTGAGAGAAAGTAAAACAATTAGCATAACAAATTTCTTGCATTCGTGTAA
TACATAATACTGTAATGGTG
>Marker702976

Create PDF with GO2PDF for free, if you wish to

CACAAAGGACTTCCATTATCAACTTATGGGAATTGGCATTTCTCTTGGTTCAAGAGATAATTTTGGTTGCGTTAT
CTACTXXXXXXXXXXGGATGTGAATAATAGAATGGAGACTATGAGATTTTGTTATTTACTTGATGATGAATTGGC
AGCTTTCATTGAAATAAGGT
CACAAAGGACTTCCATTATCAACTTATGAGAATTGGCATTTCTCTTGGTTCAAGAGATAATTTTGGTTGCGTTAT
CTACTXXXXXXXXXXGGATGTGAATAATAGAATGGAGACTATGAGATTTTGTTATTTACTTGATGATGAATTGGC
AGCTTTCATTGAAATAAGGT
>Marker703429
ACTTGACTTCGCAAATAAATCCATCTTCAATGAGCTTATTTACCTCTTCCTCGATTTGAGAAATGAGTTTTGATT
GAAATXXXXXXXXXXTTTACTAAGCTCACATACTTGTTTTCGTTACCATCAGAAAGTTGAGCGCTTATGAAGGTT
GGACGAGGATCTTTTATCGT
ACTTGACTTCGCAAATAAATCCATCTTCAATGAGCTTATTTACCTCTTCCTCGATTTGAGAAATGAGTTTTGGTT
GAAATXXXXXXXXXXTTTACTAAGCTCACATACTTGTTTTCGTTACCATCAGAAAGTTGAGCGCTTATGAAGGTT
GGACGAGGATCTTCTATCGT
>Marker704358
AACTCAACAATATATTCTCATGAACAAACTTTTATGAGTTCTGTCCAATATAATGATAGATGAGATGAAAACAAT
TGTTAXXXXXXXXXXTTGTCGAATACAACAAAGATGTGTTGTAGTTGTTGTCCTTTCTTTCTAATTCCTCATTTG
ATGATGTTTCTGTTGTTGTC
AACTCAACAATATATTCTCATGAACAAACTTTTATGAGTTCTGTCCAATATAATGATAGATGAGATGAAAACAAT
TGTTAXXXXXXXXXXTTGTCGAATACAACAAAGATGTGTTGTAGTTGTTGTCCTTTCTTTCTAATTCCTCCTTTG
ATGATGTTTCTGTTGTTGTC
>Marker704781
ACTCCTTTTATTCCTTTCAGTATGCTTCCAACAGGAAATGGAAATCTTGATATTGGCAATGTTTGATTGAGAAAC
TCGTTXXXXXXXXXXATGCCATACTTGCCTACACTAAATCATATGTAAGAAGCTTGTTGTGGATGTTTAATCCTT
AAAGAGGGGGATGGTAAGGT
ACTCCTTTTATTCCTTTCAGTATGCTTCCAACAGGAAATGGAAATCTTGATATTGGCACTGTTTGATTGAGAAAC
TCGTTXXXXXXXXXXATGCCATACTTGCCTACACTAAATCATATGTAAGAAGCTTGTTGTGGATGTTTAATCCTT
AAAGAGGGGGATGGTAAGGT
>Marker704856
ACCTAAGCAAAGAGTTACAAGGGAATTATGTTCCGGCCATACAAGCTTCCTTTCTTGATGTTGAGTAGGGCCATA
CACGTXXXXXXXXXXTCAAACTCCATAGAACCTTAATTAAGGAGTTGTCAATAGTCTCTTGTTTTGTCTCCAAAA
TTAGAACATCTGGATTGGTA
ACCTAAGCAAAGAGTTGCAAGGGGATTATGTTCCGGCCATACAAGCTTCCTTTCTTGATGTTGAGTAGGGCCATA
CACGTXXXXXXXXXXTCAAACTCCATAGAACCTTAATTAAGGAGTTGTCAATAGTCTCTTGTTTTGTCTCCAAAA
TTAGAACATCTGGATTGGTA
>Marker704897
ACTCAAAATGGAAGATTAACACCCTCAGTAATACGATATCTGCAACAAAAGAACAAGAGATTATATAAGATATAG
GAGAAXXXXXXXXXXTCATAAAACAAAAAACGGATAACTTATTAGATGAAACCAAAACGGGGGAATCATTAACTC
TGTATTATAGTAATTATTGT
ACTCAAAATGGAAGATTAACACCCTCAGTAATACGATATCTGCAACAAAAGAACAAGAGATTATATAAGATATAG
GAGAAXXXXXXXXXXTCATAAAAAAAAAAACGGATAACTTATTAGATGAAACCAAAACGGGGGAATCATTAACTC
TGTATTATAGTAATTATTGT
>Marker705185
GACCGAATTTGTGCTTCTCTATAAGAATAATATGAAAAGGTGCATTGTATTAACAATATATGTTTATCGATATG
AAAAGXXXXXXXXXXGAGGGATTTAATATATGAATGGTATTGAGTAGTGGGGAGTGTATGTTAAAAGGAGAAGGG
AATAGATGCCAAGTTTGGTT

Create PDF with GO2PDF for free, if you wish to

GACCGAATTTGTGCTTCTCTATAAGAATAATATGAAAAAGGTGCATTGTATTAACAATATATGTTTATTGATATG
AAAAGXXXXXXXXXXXGAGGGATTTAATATATGAATGGTATTGAGTAGTGGGAGTGTATGTTAAAAGGAGAAGGG
AATAGATGCCAAGTTTGGTT
>Marker705235
ACCATCTAAAGACACTGTGAAATTGAAACTAAAGCAATCAGAGAACTCAACAATAACAACAACTAACAAATACAC
TGTAAXXXXXXXXXXXGGGACTAGGAAGTCCTTGTATTTATAGAGAATGGATGCTTCATTAAAAATTCATTCGGTT
TAATACTAATTAATGTAGTA
ACCATCTAAAGACACTGTGAAATTGAAAATAAAGCAATCAGAGAACTCAACAATAACAACAACTAACAAATACAC
TATAAXXXXXXXXXXXGGGACTAGGAAGTCCTTGTATTTATAGAGAATGGATGCTTCATTAAAAATTCATTCGGTT
TAATACTAATTAATGTAGTA
>Marker705259
CACGCTCCCGCACTTCGCGAGATATTTTCTAATATTATGTTAAAATTATTGATTGATTTCAGAATTTAGTTGATG
GATTTXXXXXXXXXXXAAGATTATAAAACTGCACTACATATTTATGTATTTGAGGTGAATGCGATATATATGATGA
ACAAACAAATTTGGCCTGTG
CACGCTCCCGCACTTCGCGAGATATTTTCTAATATTATGTTAAAATTATTGATTGATTTCAGAATTTAGTTGATG
GATTTXXXXXXXXXXXAAGATTATAAAACTGCACTACATATTTATGTATTTGAGGTGAATGCAATATATATGATGA
ACAAACAAATTTGGCCTGTG
>Marker705418
AACCAAGATTTCGTCAATGACAACTAACACAAAAGTGTCTAAGAAATCCTTAAATATCATGTTCATTAAGCCCAT
GAATAXXXXXXXXXXXCCACTATTATATCCCTTTTTATTAGAATAATGGGATTAAAACATATAAGGAGATGCTCAG
AACACAAAACTTATGTTGTA
AACCAAGATTTCGTCAATGACAACTAACACAAAAGTGTCTAAGAAATCCTTAAATATCATGTTCATTAAGCCCAT
GAATAXXXXXXXXXXXCCACTATTATATCCCTTTTGATTAGAATAATGGGATTAAAACGTATAAGGAGATGCTCAG
AACGCAAAACTTATGTTGTA
>Marker705808
GACCAATTGTAGAAGGCACATTAAGGTTGATATCCCAAGCACGTTAAACATGAACTTGACATAAGGGAAGCATTT
TAAGAXXXXXXXXXXXGCATAATCTGTCAGCATAATATGAGCAAGAAAATTAACAGAGGTGATACTTGAAGAGATA
CAATAAATATGAAAGAAGTT
GACCAATTGTAGAAGGCACGTTAAGGTTGATATCCCAAGCACGTTAAACATGAACTTGACATAAGGGAAGCATTT
TAAGAXXXXXXXXXXXGCATAATCTGTCAGCATAATATGAGCAAGAAAATTAACAGAGGTGATACTTGAAGAGATA
CAATAAATATGAAAGAAGTT
>Marker705844
AACAGGGTAGATTGCCACAGAAAATCAAAACCCAAGTCACTTAGTTTATAGCTACCGTTTCGAATTGAAACGCAA
CACAAXXXXXXXXXXXTGGTTCCGGCTTCTTATCAAGCAAGTTCCGCAGCTCATCTTCTTGAATACACTCTTCCCC
AACACTTCTAACAATTTGGT
AACAGGGTAGATTGCCACAGAAAATCAAAACCCAATTCACTTAGTCTATAGCTACCGTTTCGAATTAAAACGCAA
CACAAXXXXXXXXXXXTGGTTCCGGCTTCTTATCAAGCAAGTTCCGCAGCTCATCTTCTTGAATACACTCTTCCCC
AACACTTCTAACAATTTGGT
>Marker706001
CACAATTAACAACAACAGGACAAATTGATTACATTTCTTTCTTGAATGACCGGCGGATGAGATTGCTTTTCTCCA
AGGTGXXXXXXXXXXXAAGCAATATGCAGTAACTGTAGGCTGAGTCACAGCCACAGGTGTATTGTAGTCCATCCAA
GGCCATAATGGCTGAGCTGT
CACAATTAACAACAACAGGACAAATTGATTACATTTCTTTCTTGAATGACCGGCGGATGAGATTGCTTTTCTCCA
AGGTGXXXXXXXXXXXAAGCAATATGCAGTAACTGTAGGCTGAGTCACAGCCACAGGTGTATTGTATTCCATCCAA
GGCCATAATGGCTGAGCTGT
>Marker706072

Create PDF with GO2PDF for free, if you wish to

AACTTACGAACCATCCATGCCCCACCTTATGCACGTAAATACCAACCACAAGACAAATCCTTACAATGAATATCA
AATAAXXXXXXXXXXXATTGTGAGGATTCAAAAGCTAAAAAATCAATTGTTTTGGAATTTAGATTTGACTGTTAGA
CACAATGCACAATTCTTGTT
AACTTACGAACCATCCATGCCCCACCTTATGCACGTAAATACCAACCACAAGACAAATCCTTACAATGAATATCA
AATAAXXXXXXXXXXXATTGTGAGGATTCAAAAGCTAAAAATTCAATTGTTTTGGAATTTAGATTTGACTGTTAGA
CACAATGCACAATTCTTGTT
>Marker706405
TACAAGCACAGTAAAACCATAACCAAAGAACACCAACATAGAAAAGAAGAAATAGGAAAATAGAAAGTGATGACT
AGAATXXXXXXXXXXXCTTCTGTCTATTTCCTAACATACCTTCCCCCGTCTTCCTCCTTCTGTAGGTGTTAATTAT
TGGGGGCCTAACATGTTGTT
TACAAGCACAGTAAAACCATAACCAAAGAACACCAACATAGAAAAGAAGAAATAGGAAAATAGAAAGTGATGACT
AGAATXXXXXXXXXXXCTTCTGTCTATTTCCTAACATACCTTCCCCCGTCTTCCTCCTTCTGTAGGTGTTAATAAT
TGGGGGCCTAACATGTTGTT
>Marker706661
ACAATGGCCGAAAGGCCTACAACTTTCAATACATTGCATCTTAATTCTCTGTTAATCTATCATTTCTTTACTATT
CATCTXXXXXXXXXXXCATGGCCAACGTTTATCGCCAACTACTCGAGAGGGTAAATAGCAAGGACATGGCTAAACG
CCCACAAGAAGAAGTTTGTA
ACAATGGCCGAAAGGCCTACAACTTTCAATACATTGCATCTTAATTCTCTGTTAATCTATCATTTTTTTACTATT
CATCTXXXXXXXXXXXCATGGCCAACGTTTATCGCCAACTACTCGAGAGGGTAAATAGCAAGGACATGGCTAAACG
CCCACAAGAAGAAGTTTGTA
>Marker708861
ACTTAAAATTGATACCTGATCCATGTTATTACAATTAAAAGGAAAAGCAATAGTGTGGTAGAGCATCAGAACAAG
TATGCXXXXXXXXXXXTCCTAAATTAGTAATGAAACTTCTATTTATGAGAATTGATCATCCCTTGGTTTGCATGGG
TGAGAATGACTCAAGTTGTC
ACTTAAAATTGATACCTGATCCATGTTATTACAATTAAAAGGAAAAGCAATAGTGTGGTAGAGCATCAGAACAAG
TATGCXXXXXXXXXXXTCCTAAATTAGTAATGAAACTCCTATTTATGAGAATTGATCATCCCTTGGTTTGCATGGG
TGAGAATGACTCAAGTTGTC
>Marker709393
TACATGGACTATTTTCATAAATTACATGCTTATTAGTTCTGTATATATAGTTATTTGCTGAAAATATTATATAAA
TATTTXXXXXXXXXXXCAATTGAAAATACCAAAAATGCTCCGATCAATACGCCATTAGCCGGTCACACGTGTGTGT
AGTTCTTCTAAATGATCGTG
TACATGGACTATTTTCATAAATTACATGCTTATTAGTTCTGTATATATAGTTATTTGCTGAAAATATTATATAAA
TATTTXXXXXXXXXXXCAATTGAAAATACCAAAAATGCCCCGATCAATACGCCATTAGCCGGTCACACGTGTGTGT
AGTTCTTCTAAATGATCGTG
>Marker709994
CACTGTTTCTGCCTAATAATATTCTCAGATATTATATTATCATCAATAGTGATTAATAAGTTTATCGAACAAAAG
GTTATXXXXXXXXXXXACTGTATTGCCTAGGTAGTTGTGTCAGTTATTATTTTATTTTATTATTTGTTTTTTGAGT
TTAGGGTTAGGGATTCCAGT
CACTGTTTCTGCCTAATAATATTCTCAGATATTATATTATCATCAACAGTGATTAATAAGTTTATTGAACAAAAG
GTTATXXXXXXXXXXXACTGTATTGCCTAGGTAGTTGTGTCGGTTATTATTTTATTTTATTATTTGTTTTTTGAGT
TTAGGGTTAGGGATTCCAGT
>Marker710266
TACACAAGGTTCATCAACATTTTGGTTAAAACCGTAAGATTTGATCGCAGTATCAAATCTAATCTTCTAAGATCT
AGATGXXXXXXXXXXXTAATCATAAAAAGTGGCTATGGACAAGAGATTCCTTATAAACTTTAACATAGCAACAGGG
GAAAAAGTTTCCTCATAGTA

Create PDF with GO2PDF for free, if you wish to

TACACAAGGTTCATCAACATTTTGGTTAAAACCGTAAGATTTGATCGCAATATCAAATCTAATCTTCTAAGATCT
AGATGXXXXXXXXXXTAATCATAAAAAGTGGCTATGGACAAGAGATTCCTTATAAACTTTAACATAGCAACAGGG
GAAAAAGTTTCCTCATAGTA
>Marker710347
GACTTCTCTTTTTTTTACCTAATTCAAACTTTTTGAGCTAATCCAACACATTGTTCTAAGTTAATTCAATATGAG
ATAGTXXXXXXXXXXCACTAAAATCTCGTATTTGTGCTCCCTTCAGTATAGATATATTTGTGTCCATCTGATATA
ACTATGATTAGTAAGTTAGT
GACTTCTCTTTTTTTTACCTAATTCAAACTTTTTGAGCTAATCCAACATATTGTTCTAAGTTAATTCAATATGAG
ATAGTXXXXXXXXXXCACTAAAATCTCGTATTTGTGCTCCCTTCAGTATAGATATATTTGTGTCCATCTGATATA
ACTATGATTAGTAAGTTAGT
>Marker710770
ACTCTTCTTCGCTGATCTCCCCAACTCTTAATTCATCTGGTCTCTTTCTTTCACCCACCATCTCAAGAACCAACA
TTCCAXXXXXXXXXXACAGAAGTCATCGTCCAAAAGAATGTTGTGTGGTTTGATGTCAAAATGAAAAATCCTCGT
GTTGCAGCCTCGATGCAGGT
ACTCTTCTTCACTGATCTCCCCAACTCTTAATTCATCTGGTCTCTTTCTTTCACCCACCATCTCAAGAACCAACA
TTCCAXXXXXXXXXXACAGAAGTCATCGTCCAAAAGAATGTTGTGTGGTTTGATGTCAAAATGAAAAATCCTCGT
GTTGCAGCCTCGATGCAGGT
>Marker710926
AACGACAAAGTGTATCATTACATCGAAGAACGCAGGTGGGAATATTTTTTCCAATTTACAAAGCATGATTAGGAT
ATCTTXXXXXXXXXXTAAACACCAATAGAAACAAATCCATTGGGAAATTTAAATGATTTCAGATACTTGCAAACA
TAACTTTCTCGCTGCTAGTC
AACGACAAAGTGTATCATTACATCGAAGAACGCAGGTGGGAATATTTTTTCCAATTTACAAAGCATGATTAGGAT
ATCTTXXXXXXXXXXTAAACACCAATAGAAACAAATCCATTGGGAAATTTAACTGATTTCAGATACTTGCAAACA
TAACTTTCTCGCTGCTAGTC
>Marker711008
ACCTTAACTGTTGAATGAAAAATTCTTTATGTGAGAAGACCAAGCTATGCGATATAGCTATGTTTTAAATGCTAA
TTATTXXXXXXXXXXAATATTGATCGTGACTATGCGATAACATGAGTTATGCGAGATAGCTATGTGAAAGGTTAG
AATATCTCATCGCATATGTT
ACCTTAACTGTTGAATGAAAAATTCTTTATGTGAGAAGACCAAGCTATGCGATATAGCTATGTTTTAAACGCTAA
TTATTXXXXXXXXXXAATATTGATCGTGACTATGCGATAACATGAGCTATGCGAGATAGCTATGTGAAAGGTTAG
AATATCTCATCGCATATGTT
>Marker711270
ACTAAGGGGAAATAAATAATCGCATCCTTATTAATGTATTATTGAGGAAGGTAAGGAAATGGAGTGAATAGGAGT
CGATAXXXXXXXXXXTTGAAAGTTCCACTTTACTTGGGGAGATGCAAAATGAATGAAGCCACAACATTAACACGT
TTTATGAACTAATTTTAAGT
ACTAAGGGGAAATAAATAATCGCATCCTTATTAATGTATTATTGAGGAAGGTAAGGAAATGGAGTGAATAGGAGT
CGATAXXXXXXXXXXTTGCAAGTTCCACTTTACTTGGGGAGATGCAAAATGAATGAAGCCACAACATTAACACGT
TTTATGAACTAATTTTAAGT
>Marker711829
GACAAGTCTGGCCATATATTAAACAGTAATTGAACATTAGGGTAATGTTTAACTATCAATGTAATGAGAGATACA
CCAATXXXXXXXXXXAATTTTGTCACTTCCTAGACATTATTTACCTAAAAATCTTATTAACTTTACCAATAAAAC
CAAAAGATAACATGAAGGTG
GACAAGTCTGGCCATATATTAAACAGTAATTGAACATTAGGGTAATGTTTAACTATCAATGTAATGAGATATACA
CCAATXXXXXXXXXXAATTTTGTCACTTCCTAGACATTATTTACCTAAAAATCTTATTAACTTTACCAATAAAAC
CAAAAGATAACATGAAGGTG
>Marker712279

Create PDF with GO2PDF for free, if you wish to

AACTTAGTGTATTTGAATTCAGAGAGTAGAGCAAATATGGTAGAATGTTGAGAAGCATCTCGCCTTAATTTGGAG
TTTAAXXXXXXXXXXXTTCCATTATTCATCTGTTGTCATCTCGCCTTGTCATCTTGCGCCGCCACACACACCACAT
GTATATAATAGATCAGAGTA
AACTTAGTGTATTTGAATTCAGAGAGTAGAGCAAATATGGTAGAATGTTGAGAAGCATCTCGCCTTAATTTGAAG
TTTAAXXXXXXXXXXXTTCCATTATTCATCTGTTGTCATCTCGCCTTGTCATCTTGCGCCGCCACACACACCACAT
GTATATAATAGATCAGAGTA
>Marker712437
ACTCATCGTCCGAGAATTGCTCCGCCGCGTAGTGATACGAACATCGCCAGACTCGTGAAACAGAATAGGAACGAG
ACTGCXXXXXXXXXXXGTTTGATATTATGCATTCTAACAAAGAAGACCTAATCATGGCGGCTGATATGGTGTCCCT
TCATCACACTGATCATTTGT
ACTCATCGTCCGAGAATTGCTCCGCCGCGTAGTGATACGAACATCGCCAGACTCGTGAAACAGAATAGGAACGAG
ACTGCXXXXXXXXXXXGTTTGATATTATGCATTCTAACAAAGAAGACTTAATCATGGCGGCTGATATGGTGTCCCT
TCATCACACTGATCATTTGT
>Marker712609
TACCAAAACTTCTCAGCTTTAATTCCTTTCTCGAGATACCACCTACTTGCAGCTAAAAGTTAAAATTTTCTTCCC
AAGACXXXXXXXXXXXCCATGGCAGAAGGCTGAGGTTTATATTAAGACGAATCTAATAATTTATGTCATTGGATTC
AAAGCAGTGTCGCAAGGGTT
TACCAAAACTTCTCAGCTTTAATTCCTTTCTCGAGATACCACCTACTTGCAGCTAAAAGTTAAAATTTTCTTCCC
AAGACXXXXXXXXXXXCCATGGCAGAAGCCTGAGGTTTATATTAAGACGAATCTAATAATTTATGTCATTGGATTC
AAAGCAGTGTCGCAAGGGTT
>Marker712691
TACATTTTATTGACTCAATACTTTATTTACCTATTTTTTTTACTATTGGTTCAATTTCCATTCCTGTATAAACCA
TTTCCXXXXXXXXXXXACTAAAGCAGAGAGTTCCAATATATGATAGTTTCCGATGCGAAAGTCAGAGGTTTGCTCA
CATAATTGTTGAATCGTGTG
TACATTTTATTGACTCAATACTTTATTTACCTATTTTTTTTACTGTTGGTTCAATTTCCATTCCTGTATAAACCA
TTTCCXXXXXXXXXXXACTAAAGCAGAGAGTTCCAATATATGATAGTTTCCGATGCGAAAGTCAGAGGTTTGCTCA
CATAATTGTTGAATCGTGTG
>Marker712860
AACTTGTCACCAAAGAGCTAACAATTCTTACAAAATGTGTTTGATGCTCTATTAAGTGGATAAGTGCTTGCAAGA
ACAACXXXXXXXXXXXAACAGATTCATATTCTGGGCGTAATCCCATAACACTACAGGAAAAAAGGTCTATGTTAGC
TCATTAATAACACTATCGTT
AACTTGTGACCAAAGAGCTAACAATTCTTACAAAATGTGTTTGATGCTCTATTAAGTGGATAAGTGCTTGCAAGA
ACAAAXXXXXXXXXXXAACAGATTCATATTCTGGGCGTAATCCCATAACACTACAGGAAAAAAGGTCTATGTTAGC
TCATTAATAACACTATCGTT
>Marker713920
TACATAGTGATAGCCAGGCAACTATTGGAAAGACATAGAATATTATGTATAATAATGTTAAGTCTCGACATATAT
GACGAXXXXXXXXXXXAGGTGGTTGAAAGTTCATAAAAAAGAATGAGATTAAAGCCTACAATATGAAGTTACTCAA
AGAGACAATCCAACCTAGTT
TACGTAGTGATAGCCAGGCAACTATTGGAAAGACATAGAATATTATGTATAATAATGTTAAGTCTCGACATATAT
GACGAXXXXXXXXXXXAGGTGGTTGAAAGTTCATAAAAAAGAATGAGATTAAAGCCTACAATATGAAGTTACTCAA
AGAGACAATCCAACCTAGTT
>Marker714792
ACTCATATTACAGGAACTAATTAATTAATTTTATAATTGTCATAAAATTTGAGGATTAGAAACCGTCACCCTATA
TTAAAXXXXXXXXXXXTTTAATGAAACTAAGTAGGAAAAATAGGATTTAAGGTTATAAGGAAACTCCATCAGTAAA
GTTCTGTCTAAGGCTGGAGT

Create PDF with GO2PDF for free, if you wish to

ACTCATATTACAGGAACTAATTAATTAATTTTATTATTGTCATAAAATTTGAGGATTAGAAACCGTCACTCTATA
TTAAAXXXXXXXXXXXTTTAATGAAACTAAGTAGGAAAAATAGGATTTAAGGTTATAAGGAAACTCCATCAGTAAA
GTTCTGTCTAAGGCTGGAGT
>Marker714810
TACCAAGGCTTGTTATTAATTACAAGCCCCTCAACAAAGTTCTCAAATGGATTCGGTATCCAAGTCCTAATCGTC
AAGACXXXXXXXXXXXTCGGATTGAAGAATGCTCCGTCCGAATTTCATAAGACAATGAACGATATCTTCAACCCGT
ATCAAGATTTCATAATAGTA
TACCAAGGCTTGTTATTAATTACAAGCCCCTCAACAAAGTTCTCAAATGGATTTGGTATCCAAGTCCTAATCGTC
AAGACXXXXXXXXXXXTCGGATTGAAGAATGCTCCGTCCGAATTTCATAAGACAATGAACGATATCTTCAACCCGT
ATCAAGATTTCATAATAGTA
>Marker715122
CACAGAAGGTCAAGTGATGATAAACAAATTGAAAATTATCATGAATGTGGATAAAAAATGAATAAACCCAAAAAT
CAAAAXXXXXXXXXXXXATAGGTTACAGAAAGTTTAAATAATGGGATTATATAAGGTAAGGTCCGGTAGCTTTACAA
ATGAACCAGATTATACTGTA
CACAGAAGGTCAAGTGATGATAAACAAATTGAAAATTATCATGAATGTGGATAAAAAATGAATAAACCCAAAAAT
CAAAAXXXXXXXXXXXXATAGGTTACCGAAAGTTTAAATAATGGGATTATATAAGGTAAGGTCCGGTAGCTTTACAA
ATGAACCAGATTATACTGTA
>Marker715172
AACTTCTTTTAATGAATTATGGGTAGCTTTGTTGAGAGCTGGAGACTTGAGTTGTTTTCTTAACATCTGCAAGTT
TGTTTXXXXXXXXXXXTGAAGCCAGAGCAGGCGAACAAGTTATCGAAACTAGAAACTTGGCGCTCCGAACTAATGC
TTCTAGAAGAACAAAGGGTA
AACTTCTTTTAATGAATTATGGGTAGCTTTGTTGAGAGCTGGAGACTTGAGTTGTTTTCTTAACAACTGCAAGTT
TGTTTXXXXXXXXXXXTGAAGCCAGAGCAGGCGAACAAGTTATCGAAACTAGAAACTTGGCGCTCCGAACTAATGC
TTCTAGAAGAACAAAGGGTA
>Marker715406
TACAAATTTCAACTTATATGGCTATACAGACTCTTATGCATAACTCCACACACATACAAAAAATTTGTCGGGAAA
CTTCTXXXXXXXXXXXTTATATATGTTAGCAAGTGATAAAGTTTTCAAACATCTAGATAAATCTGTAAAGCAAAGC
AACCTCGTGATTTTAATGTA
TACAAATTTCAACTTATATGGCTATACAGACTCTTATGCATAACTCTACACACATACAAAAAATTTGTCGGGAAA
CTTCTXXXXXXXXXXXTTATATATGTTAGCAAGTGATAAACTTTTCAAACATCTAGATAAATCTGTAAAGCAAAGC
AACCTCGTGATTTTAATGTA
>Marker715740
GACGAGGAGCTTATCTCCCAATTTCATAGTTTGCAGTTCTTATGTTACTGAATGAAAGGGTTTAAATTCTTTTCC
TATTTXXXXXXXXXXXGAAATATGGCCTTGTGTGGTTGAAATGTCGGTTCTTCTTGCAACTAGCATTCACAATATG
AATCCTCGTAGCTCATGGTA
GACGAGGAGCTTATCTCCCAATTTCATAGTTTGCAGTTCTTATGTTACTGAATGAAAGGGTTTAAATTCTTTTCC
TATTTXXXXXXXXXXXGAAATATGGCCTTATGTGGTTGAAATGTCGGTTCTTCTTGCAACTAGCATTCACAATATG
AATCCTCGTAGCTCATGGTA
>Marker716247
AACAAGCATGCAAGAAAGCATGTGCTAAGAGGATAATTGTTGTGAGTAGCCATTCAAATTTATAAGTTGAAAAAG
GTTCTXXXXXXXXXXXGCTTATAGTGCAAATTTATGTGGATGATATTATATTTGGTTCTACTAATTCATTTTTGTG
TGAAGAATTTTCCAAGTGTA
AACAAGCATGCAAGAAAGCATTGGCTAAGAGGATAATTGTTGTGAGTAGCCATTCAAATTTATAAGTTGAAAAAA
GGTTCXXXXXXXXXXXGCTTATAGTGCAAATTTATGTGGATGATATTATATTTGGTTCTACTAATTCATTTTTGTG
TGAAGAATTTTCCAAGTGTA
>Marker716345

Create PDF with GO2PDF for free, if you wish to

GACTGTATCGTAGTCTTCTAACCTCTCAAATGCCTTTTCAAACTTTCAGTTGCATCAAGCATCAAGTATGTGGAA
TTTCAXXXXXXXXXXCATAACTGCATTTCGAATTCGAATTAATGTATCATTTAGATTTTTCAGACCATCGCACAC
AATTAAATTAAATATATGTG
GACTGTATCGTAGTCTTCTAACCTCTCAAATGCCTTTTCAAACTTTCAGCTGCATCAAGCATCAAGTATGTGGAA
TTTCAXXXXXXXXXXCATAACTGCATTTCGAATTCGAATTAATATATCATTTAGATTTTTCAGACCATCGCACAC
AATTAAATTAAATATATGTG
>Marker716783
AACGAAAATGTCTCGAATTAAATATGCTGAGAATAAAACATTTAAATAAGAGAAATTGCATCAAATAACAAAAAT
ATTTTXXXXXXXXXXACTATCAGTAACCATAGGGCTATCGACTTTTAAATTTGCTACTTTTACAATTTAGAAAAT
GTAGAGATATGGATCCTGTT
AACGAAAATGTCTCGAATTAAATATGCTGAGAATAAAACATTTAAATAAGAGAAATTGCATCAAATAACAAAAAT
ATTTTXXXXXXXXXXACTATCAGTAACCATAGGGCTATCGACTTTTAAATTTGCTACTTTTACAATTTAGAAAAT
GTAGAGATATGGATCATGTT
>Marker716860
ACCTGAAGAAATTCCAGATTCCTCGTCTAATGATTTCCAACACAGCGACAATGGCAATCAAGGCTTGTCTATGAA
TAAATXXXXXXXXXXCTAAGTGACATCATATAGTTTCCTCTTCCAAGCTAAACGAAATGAAAAAGGTTGATTATC
CTTACAATTGCTACAAAGTA
ACCTGAAGAAATTCCAGATTCCTCGTCGAATGATTTCCAACACAGCGACAATGGCAATCAAGGCTTGTCTATGAA
TAAATXXXXXXXXXXCTAAGTGACACCATATAGTTTCCTCTTCCAAGCTAAACGAAATGAAAAAGGTTGATTATC
CTTACAATTGCTACAAAGTA
>Marker717560
ACCATTGCTCATCCACAAATGGATGGACAAACTGAGGTAACTAACCGGTCCTTGGAAAATTTAATTTGTTGCCTT
AGTGGXXXXXXXXXXCTTCACACAGAAGTCATTGACCATGTCACTAAGACTGTCGAGTCTTACAAAGAAGAGAAT
AATAAGAAGAGAAGGGAAGT
ACCATTGCTCATCCACAAATGGATGGACAAACTGAGGTAACTAACCGGTCCTTGGAAAATTTAATTTGTTGCCTT
AGTGGXXXXXXXXXXCTTCACACAGAAGTCATTGACCATATCACTAAGACTGTCGAGTCTTACAAAGAAGAGAAT
AATAAGAAGAGAAGGGAAGT
>Marker717647
CACCTATAAGACTTATAGAGCTTCTCTTTTAAGGGTGACAACCTTTCGGCACCAAATATCCACACTTGTTCTCCT
TTCATXXXXXXXXXXAACACTCGGTCTTAGAACCCTTAAGGTTTTGCTTAATCGCCTAGTAACTTTCCTTTGTCT
TAACTTACAACTTAACAAGT
CACCTATAAGACTTATAGAGCTTCTCTTTTAAGGGTGACAACCTTTCGGCACCAAATATCCACACTTGTTCTCCT
TTCATXXXXXXXXXXAACACTCGGTCTTAGATCCCTTAAGGTTTTGCTTAATCGCCTAGTAACTTTCCTTTGTCT
TAACTTACAACTTAACAAGT
>Marker717837
AACTATAAGCATAAGAAATAAAATTATGATTTAATTTTTTGCAGTTGTGACCCCAATGATGAAAAATGTCAATTT
CTTTTXXXXXXXXXXCCACTATGGTGTTTATTTGGATTCGGATCAAGTAAAGCCCAATGAGAAACATTGATTGCC
TTTTGGAATAATAGCCGAGT
AACTATAAGCATAAGAAATAAAATTATGATTTAATTTTTTGCAGTTGTGACCCCAATGATGAAAAATGTCAATTT
CTTTTXXXXXXXXXXCCACTATGGTGTTTATTTGGATTTGGATCAAGTAAAGCCCAATGAGAAACATTGATTGCC
TTTTGGAATAATAGCCGAGT
>Marker718339
GACAATCCCAAAGTAACGTAGTTCTACGTCATTAAAGGGTATTTACTTGCTTTTCAGGTAGGGGAAGATTATGAA
GAGCTXXXXXXXXXXAGTGGTGGTGCTTATTATTGCCTATCACAATTGTTTTCAATGGAATATAATTTGAACCTA
CCTTATTTACTTGCCTTGTG

Create PDF with GO2PDF for free, if you wish to

GACAATCCCAAAGTAACGTAGTTCTACGTCATTAAAGGGTATTTACTTGCTTTTCAGGTAGGGAAGATTATGAA
AAGCTXXXXXXXXXXXAGTGGTGGTGCTTATTATTGCCTATCACAATTGTTTTCAAAGGAATATAATTTGAACCTA
CCTTATTTACTTGCCTTGTG
>Marker718491
ACTTTGGACAATTTCTTAACCAATGTCCGTCTTGGTTGCAGTGGTAACATTTACCTTTAGCAACTTTTTTTCCCT
TACTAXXXXXXXXXXXACATTTTCTTTCACTTCCTTTCCCTTACCTTGATAAGGTTTTGGAATCGCTGGAGCTTAT
TTAGAAGAGTGGTCAGAGTG
ACTTTGGACAATTTCTTAACCAATGTCCGTCTTGGTTGCAGTGGTAACATTTACCTTTAGCAACTCTTTTTCCCT
TACTAXXXXXXXXXXXACATTTTCTTTCGCTTCCTTTCCCTTACCTTGATAAGGTTTTGGAATCGCTGGAGCTTAT
TTAGAAGAGTGGTCAAAGTG
>Marker718697
CACAGTATGAACTTAAGCTTTTTTTTAACCTTTGATGTTCCATTTTTTCACTGAGGTCATTTGGATTGTAAATCC
GTCGCXXXXXXXXXXXAGATTATCATAAAAAGTAACTATAGGAAACTGATTATAGGATATTCATAGTCAAGTTCCT
TTAAACATCAGCTCGTTGGT
CACAGTATGAACTTAAGCTTTTTTTTAACCTTTGATGTTCCATTTTTTCACTGAGGTCATTTGGATTGTAAATCC
ATCGCXXXXXXXXXXXAGATTATCATAAAAAGTAACTATAGGAAACTGATTATAGGATATTCATAGTCAAGTTCCT
TTAAGCATCAGCTCGTTGGT
>Marker718888
CACCTACCTAAGTCACAATTTGACACTTTAAATTGGTTGGAATTTTAGATTGTGATATCAATGATTCAATATTTT
GTGTAXXXXXXXXXXXACTCTAATTCACTAACCTCACGTATTATCGTTTAGAATCCTAAGGTTATACTTTAGCTTC
TCGAACACTCAAGACTTGTT
CACCTACCTAAGTCACAATTTGACACTTTAAATTGGTTGGAATTTTAGATTGTGATATCAATGATTCAATATTTC
GTGTAXXXXXXXXXXXACTCTAATTCACTAACCTCACGTATTATCGTTTAGAATCCTAAGGTTATACTTTAGCTTC
TCGAACACTCAAGACTTGTT
>Marker718978
AACGACCGAGTTACTCAAAAGGTCTTGGTTGGGGCCCAAAACCCAAGTCTAAGACTACCGCTTCATCTTCTTCCT
CTAATXXXXXXXXXXXCATGCACGACAAATTGAGGAGATGAAGAAGATGCTCGAAGAGATCAGTCAGGCACAAAGA
GGCCCGTGATCACTTGGAGT
AACGACCGGGTTACTCAAAAGGTCTTGGTTGGGGCCCAAAACCCAAGTCTAAGACTACCGCTTCATCTTCTTCCT
CTAATXXXXXXXXXXXCATGCACGACAAATTGAGGAGATGAAGAAGATGCTCGAGGAGATCAGTCAGGCACAAAGA
GGCCCGTGATCACTTGGAGT
>Marker719020
AACCCCAAAAGTTATCAATTCTGATTAAGAGTCAGCTCAGTTCTTGAGTAGAGGCAAGATAAAACTCTAAGGGGA
CGTTTXXXXXXXXXXXCTATTTTTGTTTGGGTTGTAAATAGCAAACAGTTTAGCTAAGAAGAAAAAAGAGGGTGGA
TGATAAATGGTAAATAATGT
AACCCCAAAAATTATCAATTCTGATTAAGAGTCAGTTCAGTTCTTGAGTAGAGGCAAGATAAAACTCTAAGGGGA
CGTTTXXXXXXXXXXXCTATTTTTGTTTGGGTTGTAAATAGCAAACAGTTTAGCTAAGAAGAAAAAAGAGGGTGGA
TGATAAATGGTAAATAATGT
>Marker719378
CACAAAGTTGCTTTCACAATGCTACAAATAAATTTTGGATGTTCATGAAATGTTTTCTCTTCTTTTCTTTCTTGA
AGAAGXXXXXXXXXXXGATTTCATGAAATCAGCCCTATGTTTGAATTCTATTTCGTGACTTTGAACTTCAAGCCGT
ATTAATTCAAATAACGATGT
CACAAAGTTGCTTTCACAATGCTACAAATAAATTTTGGATGTTCATGAAATGTTTTCTCTTCTTTTCTTTCTTGG
AGAAGXXXXXXXXXXXGATTTCATGAAATCAGCCCTATGTTTGAATTCTATTTCGTGACTTTGAACTTCAAGCCGT
ATTAATTCAAATAACGATGT
>Marker720050

Create PDF with GO2PDF for free, if you wish to

TACCACCAAGCGGGAAACTCAAGTAGTGGAAGAGCTTGGAAAGTTGAAATATTTCCTTGGAATCGAAGTTGCCTA
TTCTAXXXXXXXXXXXTATGTGCAGCCGATGAGAGTCCTTCAGTAAATAAGGAAACATAACAAAGGTTAGTTGGCA
AACTAATATATCTCTTTCGT
TACCACCAAGCGGGAAACCCAAGTAGTGGAAGAGCTTGGAAAGTTGAAATATTTCCTTGGAATCGAAGTTGCCTA
TTCTAXXXXXXXXXXXTATGTGCAGCCGATGAGAGTCCTTCAGTAAATAAGGAAACATAACAAAGGTTAGTTGGCA
AACTAATATATCTCTTTCGT
>Marker720409
AACTACGCGTCAAGGCCTTTTGTTGAGGTCTTGTGAAGGAATATGAGGTGCTAGGCCTTTTGAATGACCCTTAGA
AATTCXXXXXXXXXXXCATAAAACTCTATAAATAGACCATTTTGAAGGCTGCTTTCAATTTCCTTGTGCATTTATG
TAATTCTTGTCTCAAAAGTT
AACTAGGCGTCAAGGCCTTTTGTTGAGGTCTTGTGAAGGAAGATGAGGTGCTAGGCCTTTTGAATGACCCTTAGA
AATTCXXXXXXXXXXXCATAAAACTCTATAAATAGACCATTTTGAAGGCTGCTTTCAATTTCCTTGTGCATTTATG
TAATTCTTGTCTCAAAAGTT
>Marker721308
ACCACTAAACCTTGCCTTTACTTCAATAAAACCTCAAATCTTCTAAATGTTGCAGTATTACCATTTACATACATA
CTTCAXXXXXXXXXXXCACGAGACATTTATGGATCAAGAGTAATACTACAACTTCTGAAAAATATGAGTATTTTTA
AGCAAATGGCAAAGTGAGTT
ACCACTAAACCTTGCCTTTACTTCAATAAAACCTCAAATCTTCTAAAAGTTGCAGTATTACCATTTACATACATA
TTTCAXXXXXXXXXXXCACGAGACATTTATGGATCAAGAGTAATACTACAACTTCTGAAAAATATGAGTATTTTTA
AGCAAATGGCAAAGTGAGTT
>Marker721331
TACAGTAAATGAAGAAATTCAACCATTTTTTTCTTAAGAATATAACTTCATTCTTTCAACACAATCAAACTAAAA
ATCATXXXXXXXXXXXACTTTACATTCTCAACAACAAAATCCAATAACGATAATTTTGCAACCATAAAAATAACAA
TTTAAAATAGTATGGAAAGT
TACAGTAAATGAAGAAATTCAACCATTTTTTTCATAAGAATATAACTTCATTCTTTCAACACAATCAAACTAAAA
ATCATXXXXXXXXXXXACTTTACATTCTCAACAACAAAATCCAATAACGATAATTTTGCAACCATAAAAATAACAA
TTTAAAATAGTATGGAAAGT
>Marker721588
ACTTACGCCATGTATGCCACCGATGTGAAAGTATTGACTTCTCGTATGTTACAGAAATCAATAATATCTTCGCGG
AGAACXXXXXXXXXXXACTCACAAGTAACTTCTTTTTAATTAGGAACCTAAGAAAGTCATAAACCCGCATTCAGCT
CATGGTCTCACAAGAATGTA
ACTTACGCCATGTATGCCACCGATGTGAAAGTATTGACTTCTCGTATGTTACAGAAATCAATAATATCTTCGCGG
AGAACXXXXXXXXXXXACTCACAAGTATCTTCTTTTTAATTAGGAACCTAAGAAAGTCATAAACCCGCATTCAGCT
CATGGTCTCACAAGAATGTA
>Marker721680
GACTCTTTCTTAGATCTGCATCATCAGTATTTCGAAATGGCAAAATCTGGAGCAAACAAATGCCAACCTCTAACA
GATTTXXXXXXXXXXXAACATTGAGGAAAGGAGTGTTCATTGCTGGGGCAGTGTTTGTGGTTGCTACCATGATTCT
TAATGTGTATTATTACATGT
GACCCTTTCTTAGATCTGCATCATCAGTATTTCGAAATGGCAAAATCTGGAGCAAACAAATGCCAACCTCTAACA
GATTTXXXXXXXXXXXGACATTGAGGAAAGGAGTGTTCATTGCTGGGGCAGTGTTTGTGGTTGCTACCATGATTCT
TAATGTGTATTATTACATGT
>Marker721705
AACTCTTTGACTTTTTACAACTTTGGTCATTTCCATTCGAGCTTACGAATATGAATATGCATTCATATTTTTAAT
ATTTAXXXXXXXXXXXATGAACCTATAGATCATGGGCTTGAACGATTCAAGATTAACTAACTAAACTCTTTTACAC
TGAGCTAATCAACATTTGTT

Create PDF with GO2PDF for free, if you wish to

AACTCTTTGACTTTTTACAACTTTGGTCATTTCCATTCGAGCTTACGAATATGAATATGCATTCATATTTTTAAT
ATTTAXXXXXXXXXXXATGGACCTATAGATCATGGGCTCGAACGATTCAAGATTAACTAACTAAACTCTTTTAGAC
TGAGCTAATCAACATTTGTT
>Marker721921
ACAGTTATTATGTGATCAATATGGAAATGTTGCTGCTTTGCACAGTCGTGATTGCAGTGTTCAGAGACGCCACCA
AAAGGXXXXXXXXXXXAGGAGGGCCCAATTACTGTTGCGCAACTGGAGACAGTAAAAAAGCTAGAACAGGCAGCTA
GAAGACTGGCTAAATGTGTG
ACAGTTATTATGTGATCAATATGGAAATGTTGCTGCTTTGCACAGTCGTGATTGCAGTGTTCAGAGACGCCACCA
AAAGGXXXXXXXXXXXAGGAGGGCCCAATTACTGTTGCGCCACTGGAGACAGTAAAAAAGCTAGAACAGGCAGCTA
GAAGACTGGCTAAATGTGTG
>Marker722017
TACTTAAAATAGCTTTGAGATGGATTTAAGATTAGAGTATTCAAGTTCTCAATAACGACTTTATTGAATAGAATA
TGATTXXXXXXXXXXXAATTTTATTTTTGAATTTTGAATTTTTAAAAAATAGTCAATATGTTTTCATTCTGTCAAC
TGGGCTAATTTCCTGTCGTG
TACTTAAAATAGCTTTGAGACGGATTTAAGATTAGAGTATTCAAGTTCTCAATAACGACTTTATTGAATAGAATA
TGATTXXXXXXXXXXXAATTTTATTTTTGAATTTTGAATTTTTAAAAAATAGTCAATATGTTTTCATTCTGTCAAC
TGAGCTAATTTACTGTCGTG
>Marker722265
GACCAAATGTTATGAATTTAAGATAGTGACATGCAATGAATCATAAACTTTTATGTAGTCATTGGTATCACCTTC
GCCTAXXXXXXXXXXXTCAATAACAAAGGTCCTACCCATCAACTACTCAAACAACATCCATAAGAAATTTAAATCA
ACAAGATCATGTGCCTTAGT
GACCAAATGTTATGAATTTAAGATAGTGACATGCAATGAATCATAAACTTTTATGTAGTCATTGGTATCACCTTC
GCCTAXXXXXXXXXXXTCAATAACAAAGGTCCTACCCATCAACTACTCAAACAACATCCATAAGGAATTTAAATCA
ACAAGATCATGTGCCTTAGT
>Marker722284
ACAGAGATAATGCTTTCAAAATCGATTTACCACCACACATCCACATACACCCAGTCTTCAATGTTGCTGATCTAA
AGCCAXXXXXXXXXXXAGTTAAAACCCTTAATTAGTTAAAGAACAGCTATAATCAATAACTAACTAAAACAGTTAG
ACACTCAACTGTTTCAAGTA
ACAGAGATAATGCTTTCAAAATCGATTTACCACCACACATCCACATACACCCAGTCTTCAATGTTGTTGATCTAA
AGCCAXXXXXXXXXXXAGTTAAAACCCTTAATTAGTTAAAGAACAGCTATAATCAATAACTAACTAAAACAGTTAG
ACACTCAACTGTTTCAAGTA
>Marker722787
ACAAGGGCTGTAGATTCCATCTGGCAGCCTTAAAAGCTCAATTTCTCATTCAACTTATTAGCCGACAAAGGAAAA
GAAGAXXXXXXXXXXXTTTTATGAAAGTCGGTAAAGGTATGGTTAGTATCAAGGGATGGATAACTTTCTGAGAGTG
AGACTTTCCATTTTCTTGTT
ACAAGGGCTGTAGATTCCATCTGGCAGCCTTGAAAGCTCAATTTCTCATTCAACTTATTAGCCTACAAAGGAAAA
GAAGAXXXXXXXXXXXTTTTATGAAAGTCGGTAAAGGTATGGTTAGTATCAAGGGATGGATAACTTTCTGAGAGTG
AGACTTTCCATTTTCTTGTT
>Marker724768
CACTACTCTCTAACATGAACTACCTCAATAGATTTTGCAACTGATACGGTATTGCAGAAGAAACTGAAAAAGTGT
TGCAAXXXXXXXXXXXCTATATTTACTATTATAGTTGCCTTTAGGGGTCAAACGGTGATAGCAACATTAGATGTGT
TAAATTCTTAAATTGAAGTA
CACTACTCTCTAACATGAACTACCTCAGTAGCTTTTGCAACTGATACGGTATTGCAGAAGAAACTGAAAAAGTGT
TGCAAXXXXXXXXXXXCTATATTTACTATTATAGTTGCCTTTAGGGGTCAAACGGTGATAGCAACATTAGATGTGT
TAAATTCTTAAATCGAAGTA
>Marker725009

Create PDF with GO2PDF for free, if you wish to

ACCTTAAGTTTGAGTCGTCAGCTAAAAAGAAAAGAAAGAGATGTTAAACAAAAAGGAATTCTGAGTCGGTTAGAG
AGAGAXXXXXXXXXXTTTTTTAACAAGGAAGAAAAATAATATACTTTGAAAATTAAGGCAAAACTTACAAAAGTG
TAATAACCAAAGTTGATGTT
ACCTTAAGTTTGAGTCGTCAGCTAAAAAGAAAAGAAAGAGATGTTAAACAAAAAGGAATTCTGAGTCGGTTAGAG
AGAGAXXXXXXXXXXTTTTTTAACAATGAAGAAAAATAATATACTTTGAAAATTAAGGCAAAACTTACAAAAGTG
TAATAACCAAAGTTGATGTT
>Marker725043
CACATATTTAATCATTTATTCATATTCTTTAACCAGATTCAAGCAAGATCCCAGAAAAATTTGTTTAGAGTTACC
AACGAXXXXXXXXXXTGATGTCAAGATATTAAAGCAAGAAAGAATTATTGAGAGTCATTCATGCAGTGAAGAAGA
GGTAGTTAAAGTGTTTGGTG
CACATATTTACTCATTTATTCATATTCTTTAACCAGATTCAAGCAAGATCCCAGAAAAATTTGTTTAGAGTTACC
AACGAXXXXXXXXXXCGATGTCAAGATATTAAAGCAAGAAAGAATTATTGAGAGTCATTCATGCAGTGAAGAAGA
GGTAGTTAAAGTGTTTGGTG
>Marker725050
AACTGCTCTTGACGCTAGTGGAATTGATGTGGGAAATTTATTCCTCTAGATGGCTGTTTGAATAGCTACGAGCTT
TCTTTXXXXXXXXXXTAAGGCTCCTTGTAGGGTTGATTCCTTATGTTTGGCAAAGGGGATAGATCTCACATGCTT
CAATGAAACGTTTTCTTGTT
AACTGCTCTTGACGCTAGTGGAATTGATGTGGGAAATTTATTCCTCTAGATGGCTGTTTGAATAGCTACGAGCTT
TCTTTXXXXXXXXXXTAAGGCTCCTTGTAGGGTTGATTCCTTATGTTTGGCAAAGGGGATAGATCTCACATGCTT
CAATGAAATGTTTTCTTGTT
>Marker725429
AACTAAACAAATACAATATCTAATAATGATGATCAAAGATCAAGAAGAAAAAACAACAGAACTTTGTTCAATTTC
TTTAGXXXXXXXXXXCTCAATTTGAACTATGTGAATTAAATAAAATGACACAACTTTTGATCATCTTTCGACTGC
AACACATAAAATAAATAGGT
AACTAAACAAATACAATATCTAATAATGATGATCAAAGATCAAGAAGAAAAAACAACACAACTTTGTTCAATTTC
TTTAGXXXXXXXXXXCTCAATTTGAACCATGTGAATTAAATAAAATGACACAACTTTTGATCATCTTTCGACTGC
AACACATAAAATAAATAGGT
>Marker725567
ACATGAAATCACACAAATATGAAAGGGGAAAGGAGGGTGGAATTTCATCTTAGGTTTAGAACTTTGCCAAGGAAT
GGATGXXXXXXXXXXTTCTTCAATGCTAATGCATCTAGGAGGAGGGTAGAAAACTGGTGGATTGCAATAGAGATT
ATTATAAGCTTGATATTGTT
ACATGAAATCACACAAATATGAAAGGGGAAAGGAGGGTGGAATTTCATCTTAGGTTTAGAACTTTGCCAAGGAAT
GGATGXXXXXXXXXXTTCTTCAATGCTAATGCATCTAGGAGGAGGGTAGAAAACTGGTGGATTGCAATAGAGATA
ATTATAAGCTTGATATTGTT
>Marker725579
ACTCTGATTGAACCACTTGTAAAAATTTAGTCTTGATGTAAGTAAAAAACAGAAAATTTCAATGTAACTACTTTC
TTTTTXXXXXXXXXXTTGCAATTGGAACCTCAGAGAGGAATAGCAAAAAAGCGCTCCATCTTAATCAAAACAAGA
AACCTTTAGTTAGGAGAGTG
ACTCTGATTGAACCACTTGTAAAAATTTAGTCTTGATGTAAGTAAAAAACAGAAAATTTCAATGTAACTACTTTC
TTTTTXXXXXXXXXXTTGCAATTGGAACCTCAGAGAGGAATAGCAAAAAAGCGCTCCATCTTAATCAAAACAAGA
AACGTTTAGTTAGGAGAGTG
>Marker725592
CACCTAGTCGTGTGATATCGTGTTGTATAACGAGCATGCTTGTCCATGTCATTGTTTGTCTATGGTTATGTGCCC
AAATAXXXXXXXXXXCAAGCCTACCAGAGCTAAAAAAGTCCAAAATATATTATAAGGGAGGCATAATAGTTGAAT
CCCCGCCCAAGCATTGTTGT

Create PDF with GO2PDF for free, if you wish to

CACCTAGTCGTGTGATATCGTGTTGTATAACGAGCATGCTTGTCCATGTCATTGTTTGTCTATGGTTATGTGCCC
AAATAXXXXXXXXXXXCAAGCCTACCAAAGCTAAAAAAGTCCAAAATATATTATAAGGGAGGCATAATAGTTGAAT
CCCCGCCCAAGCATTGTTGT
>Marker725820
ACCATAAGACCCTTCCACCCAAAAAAATAATAATAACAACAACAAAATGTAGATTTGCTAGGATTCACTATATGC
CTAGGXXXXXXXXXXXGAAGCTCTTCTAACACAATAGTTGTGGCAGTTTTTCATGGAGCACAATGCTTTGTAGCAC
AGAGTTATTGTGAGAAGGTT
ACCATAAGACCCTTCCACCCAAAAAAATAATAATAATAACAACAAAATGTAGATTTGCTAGGATTCACTATATGC
CTAGGXXXXXXXXXXXGAAGCTCTTCTAACACAATAGTTGTGGCAGTTTTTCATGGAGCACAATGCTTTGTAGCAC
AGAGTTATTGTGAGAAGGTT
>Marker725906
TACTCTACCAAAGGTAAAACCCAATTTTTTAATTATACTTCATTCCTTTAGTTTCTCTACATGCAACACTCTTAA
TTACTXXXXXXXXXXXTATGGCTTTCAATTCCTGGCATGGATATATGATCTTTTAGTTAAAGTTTGTGTTTGTATT
TCTGTTTGTTCAATAATAGT
TACTCTACCAAAGGTAAAACCCAATTTTTTAATTATACTTCATTCCTTTAGTTTCACTACATGCAACACTCTTAA
TTACTXXXXXXXXXXXTATGGCTTTCAATTCCTGGCATGGATATGTGATCTTTTAGTTAAAGTTTGTGTTTGTATT
TCTGTTTGTTCAATAATAGT
>Marker727206
ACCCTCTTGGATGTATTGTGAGAATTTCACATCGAAAATGGACTTCACTCTTCATGAGATAGATAGACTACTCTT
CATTCXXXXXXXXXXXGTCTAATGCACATAAATTTCTTCCATTTTTGCTGATGTTGTGTATGTGGGTGTTCCTCCT
GGAGGGAGAGGCGTTTAGTC
ACCCTCTTGGATATATTGTGAGAATTTCACATCGAAAATGGACTTCACTCTTCATGAGATAGATAGACTACTCTT
CATTCXXXXXXXXXXXGTCTAATGCACATAAATTTCTTCCATTTTTGCTGATGTTGTGTATGTGGGTGTTCCTCCT
GGAGGGAGAGGCGTTTAGTC
>Marker727462
GACTAGAAATAGCAAAAGATATGCCATAATGTTTTGTCGATGATTGTTCTAATTTTACTTTTATTTACTTGCTTA
GAAATXXXXXXXXXXXGATAAGGGAACTAAATATGATTCAGCGTTGCCTTTAATGAGTTCTACAACTCAAAAGGAA
TAATTCGTGAGAAAATTGTG
GACTAGAAATAGCAAAAGATATGCCATAATGTTTTGTCGATGATTGTTCTAATTTTACTTTTATTTACTTGCTTA
GAAATXXXXXXXXXXXGATAAGGGAACTAAATATGATTCAGCGTTTCCTTTAATGAGTTCTACAACTCAAAAGGAA
TAATTCGTGAGAAAATTGTG
>Marker728101
TACATCTTGTAGTCCCATCGCGGTTGAATTTTCGTCATTCGATTCTCGCCATATGCATAATCAGCTCGAGCTCGT
TTCGTXXXXXXXXXXXGAATCGCAGTCGTAGCACAGTATGTTGGGTGCCCCGTTCCATCGGTAGCAATCAGGATCT
TGCACGTTCTCGCTGCATGT
TACATCTTGTAGTCCCATCGTGGTTGAATTTTCGTCATTCGATTCTCGCCATATGCATAATCAGCTCGAGCTCGT
TTCGTXXXXXXXXXXXGAATCGCAGTCGTAGCACAGTATGTTGGGTGCCCCGTTCCATCGGTAGCAATCAGGATCT
TGCACGTTCTCGCTGCATGT
>Marker728297
ACCTTATGTGATGTCTTACACTTGTTGTGTGTTCTGGTGTGGGATGCCTTATCCTTGCTATGTGATCTGGGATAA
TAAGAXXXXXXXXXXXATGCTCGATGAAATTGATGGAAAATTGTTGTGCCGTTGAGATTACTCTGCATAATCGCAA
CTCAACAAAAGCGTATGGTA
ACCTTATGTGATGTCTTACACTTGTTGTGTGTTCTGGTGTGGGATGCCTTATCATTGCTATGTGATCTGGGATAA
TAAGAXXXXXXXXXXXATGCTTGATGAAATTGATGGAAAATTGTTGTGGCGTTGAGATTACTCTGCATAATCGCAA
CTCAACAAAAGCGTATGGTA
>Marker728330

Create PDF with GO2PDF for free, if you wish to

AACAAGATAAAATATATTTTCACGATTACAGTTATCTTGTGATCATCAAGCTATGCTAACTCTGACACCAAAATA
TGGAAXXXXXXXXXXGTTTATCAATGATGAGAGTAGAAGATTTGAATTTACTGGGAAAATTAACACCTTTTTTGT
TTTATATTTCAAAAGTAGTA
AACAAGATAAAATATATTTTCACGATTACAGTTATCTTGTGATCATCAAGCTATGCTAACTCTGACATCAAAATA
TGGAAXXXXXXXXXXGTTTATCAATGATGAGAGTAGAAGATTTGAATTTACTGGGAAAATTAACACTTTTTTTGT
TTTATATTTCAAAAGTAGTA
>Marker728332
ACTCTTTTATCTTTGATGGACGATTGATATGAAATTGTTTTATTCAAATAAATCAAACTACGATCTAACTGGTTA
TGCAAXXXXXXXXXXAGCTAAAAGAGCATGTGTGTGGTTGGGGTCAATGACTCATCATATTCGAGAAAGATATGG
TTTGTCTTTTAGTGAAAGTT
ACTCTTTTATCTTTGATGGACGATTGATATGAAATTGTTTTATTCAAATAAATCAAACTACGATCTAATTGGTTA
TGCAAXXXXXXXXXXAGCTAAAAGAGCATGTGTGTGGTTGGGGTCAATGACTCATCATATTCGAGAAAGATATGG
TTTGTCTTTTAGTGAAAGTT
>Marker728444
ACTTTCAATTTTGGTAATTTGAGTGTAAATTTTACATATTTGTTATAATTAGATATCACCCTTACTTCAATTTCA
ACTATXXXXXXXXXXCTAGGAAGAAACTATCAAATTTCTATATAGATGTTGCACTAATTATGTATGTTATGTCTA
ATAACAATATGGTATAAGTT
ACTTTCAATTTTGGTAATTTGAGTGTAAATTTTACATATTTGTTATAATTATATATCACCCTTACTTCAATTTCA
ACTATXXXXXXXXXXCTAGGAAGAAACTATCAAATTTCTATATAGATGTTGCACTAATTATGTATGTTATGTCTA
ATAACAATATGGTATAAGTT
>Marker728952
TACAATGAGAATAGCTCTGCAAAAACAAACTCTGCCTACGCCCACCCTGTCAAAAGCATAGAATTGAAGAGCTCA
TCAGCXXXXXXXXXXACATACTTGCAGGAAGTCGGCAGCAGTTATATCTCCAGCAGTAGCCATTTAGCTAACCTC
CAAAACGAACTCCGCCTGTA
TACAATGAGAATAGCTCTGCAGAAACAAACTCTGCCTACGCCCACCCTGTCAAAAGCATAGAATTGAAGAGCTCA
TCAGCXXXXXXXXXXACATACTTGCAGGAAGTCGGCAGCAGTTATATCTCCAGCAGTAGCCATTTAGCTAACCTC
CAAAACGAACTCCGCCTGTA
>Marker729262
TACATTTGTATATCTTATTACCCCACTATAATTATAGGTGTCCCTTCGAACCCTAACCCATTACTGAACGTATGA
ATTTGXXXXXXXXXXTGAAAAAAAATGTGAAATTCCTCCCATGGAAAATCACTAACTCTTTGTCTTAATACCGTG
GTAGGTTACAGAAGCGATGT
TACATTTGTATATCTTATTACCCCACTATAATTATAGGTGTCCCTTCGAACCCTAACCCATTACTCAACGTATGA
ATTTGXXXXXXXXXXTGAAAAAAAATGTGAAATTCCTCCCATGGAAAATCACTAACTCTTTGTCTTAATACCGTG
GTAGGTTACAGAAGCGATGT
>Marker729432
AACATACCTATTGTGCTACATTGTGAAAAAATGTGAGCGTCTATGCGAACAATATACATTATGGTTATCAATCAT
TCAACXXXXXXXXXXAATTCATATTTAAAGTGGCCTCGTGATATTATGCAATTAAAGTATCATCCATATGGAAAA
TTTAGAGAGTGACCAATGTA
AACATACCTATTGTGCTACATTGTGAAAAAATGTGAGCGTCTATGCGAACAATATACATTATGGTTATCAATCAT
TCAACXXXXXXXXXXAATTCATATTTAAAGTGGCCTCGTGATATTATGCAATTGAAGTATCATCCATATGGAAAA
TTTAGAGAGTGACCAATGTA
>Marker729637
TACATTGTCATAGATTTTAATGGATAAGATACTAATACTTAAATAAAAAATGATATAATAAATAGATGAAATATT
TAATTXXXXXXXXXXTATACTTTGTGGGTAGGGGTTCATTTGCACTCTTCACTAGGAACTTTATTTTGCCTCTTC
TTGTGTTCTCAATTTGAGTT

Create PDF with GO2PDF for free, if you wish to

TACATTGTCATAGATTTTAGTGGATAAGATACTAATACTTAAATAAAAAATGATATAATAAATAGATGAAATATT
TGATTXXXXXXXXXXTATACTTTGTGGGTAGGGGTTCATTTGCACTCTTCACTAGGAACTTTATTTTGCCTCTTC
TTGTGTTCTCAATTTGAGTT
>Marker729669
AACCCTATATATCATGCCCTAATAAATCATTTTAGCCCAAAAATATGGCAAATATAAGATACTTGGCATTGCCTT
TGACCXXXXXXXXXXCATACAACCCACCAAACAAGGGATGTTGTGCTGTAGGTCTAACCCAAGAAAGGTGGATAA
GATAAAAAGATAGGTGTGTA
AACCCTATATATCATGCCCTAATAAATCATTTTAGCCCAAAAATATGGCAAATATAAGATACTTGGCATTGCCTT
TGACCXXXXXXXXXXCATAGAACCCACCAAACAAGGGATGTTGTGCTGTAGGTCTAACCCAAGAAAGGTGGATAA
GATAAAAAGATAGGTGTGTA
>Marker730364
ACCAACCTACCAACGAAGGAAACATCCCAATGATTAAGAAAATGATGAGAGAAAAGAGAGATAAAGAAAGAAGAA
TGAGAXXXXXXXXXXATAAAGATTCTTCTTTCTTTTTGCATGGCAATCATATGGCAAGCAAATTTGTGAGGCCAT
AAATACTCTCCCCCTTTGTA
ACCAACCTACCAACGAAGGAAACATCCCAATGATTAAGAAAATGATAAGAGAAAAGAGAGATAAAGAAAGAAGAA
TGAGAXXXXXXXXXXATAAAGATTCTTCTTTCTTTTTGCATGGCAATCATATGGCAAGCAAATTTGTGAGGCCAT
AAATACTCTCCCCCTTTGTA
>Marker730753
TACATCAACAAGAAATCTCAGGTAAATAATTCAAAACTCAAGCAAAGATGATTCGTGAAGAACAAAACTCAAGCA
ACGATXXXXXXXXXXTCATCCTGTTTATCACCTAGAAAACGACTTGTCCTAACCGTTCTGTGAAACCGTGGTTCT
GTTGTGTTCTAGGTGTGGTT
TACATCAACAAGAAATCTCAGGTAAATAATTCAAAACTCAAGCAAAGATGATTCGTGAAGAACAAAACTCAAGCA
ACGATXXXXXXXXXXTCATCCTGTTTATCTCCTAGAAAACGACTTGTCCTAACCGTTCTGTGAAACCGTGGTTCT
GTTGTGTTCTAGGTGTGGTT
>Marker730915
CACAAGATTATCAAGCATTTTCATCATCAAACTTGCAGAAAATTAAGGTGTCATCAGCGAATTGGAGAATAGAGA
CATACXXXXXXXXXXCCTTGACTATATTGAACAGTAATATTTGACATTGAAGATAATATGGGGCCAACCAAGATT
TGTTGTAAATCTTCACAGTT
CACAAGATTATCAAGCATTTTCATCATCAAACTTGCAGAAAATTAAGGTATCATCAGCGAATTGGAGAATAGAGA
CATACXXXXXXXXXXCCTTGACTATATTGAACAGTAATATTTGACATTGAAGATAATATGGGGCCAACCAAGATT
TGTTGTAAATCTTCACAGTT
>Marker730936
AACGTATTAATTAAATTAGGGTTTAAAACGTTTTTTTTAATTGATTATCGAGATTTGAGGTAAGAATTTTAAGTT
TAAAAXXXXXXXXXXGTAATATAGTGAAATCAACACGTTTATTAAAGTGGAAAGTTGGTGTATAAAAAGGTCTAT
CTAGTTTAGTAGATATGGTA
AACGTATGAATTAAATTAGGGTTTAAAACGTTTTTTTTAATTGATTATCGAGATTTGAGGTAAGAATTTTAAGTT
TAAAAXXXXXXXXXXGTAATATAGTGAAATCAACACGTTTATTAAAGTGGAAAGTTGGTGTATAAAAAGGTCTAT
CTAGTTTAGTAGATATGGTA
>Marker731989
AACTTTGGTGCAACTCATAACTTCATGACAAAGACGGAAGCCAGACGGTTGAACCTCCATCAAGAAAGTGTATCA
TGGTTXXXXXXXXXXTTCTTTGGAGACTAGAGAGCACTATTTTCTTAACCGGCAATCAAAATAGACATCATCAAG
ACTAGCTCTATATCAAATGT
AACTTTGGTGCAACTCATAACTTCATGACAAAGACGGAAGCCAGACAGTTGAACCTCCATCGAGAAAGTGTATCA
TGGTTXXXXXXXXXXTTCTTTGGAGACTAGAGAGCACTATTTTCTTAACCGGCAATCAAAATAGACATCATCAAG
ACTAGCTCTATATCAAATGT
>Marker732418

Create PDF with GO2PDF for free, if you wish to

AACTAATATTTTATAAATATTTTCTCAAATTGATCCCTTCTGAGTCTGAGTTGTGCATAACTCGACCAGTATCTC
TATTGXXXXXXXXXXTGTGCATAATGTGAATGGCAACGATCAATGCAACAAAAAAATGAATGAATGGAAACTTCC
CAAATGCTATGGATTGAGTG
AACTAATATTTTATAAACATTTTCTCAAATTGATCCCTTCTGAGTCTAAGTTGTGCATAACTCGACCAGTATCTC
TATTGXXXXXXXXXXTGTGCATAATGTGAATGACAACGATCAATGCAACAAAAAAATGAATGAATGGAAACTTCC
CAAATGCTATGGATTGAGTG
>Marker732574
TACATGGCTGATGCAACAAAATAAAACCTTACCCAATCAAGTAGATTCTCCGCATCCATAAATGAAGTAAATGAC
GAATTXXXXXXXXXXAGTGAATCGCTTCAGTTAGGATTCAGAAATTTGACAAGTTTTCTCAATGAGCAAAGCAAA
GATTCAGAAACTTACTTTGT
TACATGGCTGATGCAACAAAATAAAACCTTACCCAATCAAGTAGATTCTCCGCATCCATAAATGAAGTAAATGAC
GAATTXXXXXXXXXXAGTGAATTGCTTCAGTTAGGATTCAGAAATTTGACAAGTTTTCTCAATGAGCAAAGCAAA
GATTCAGAAACTTACTTTGT
>Marker733093
AACATTTAAGAATATTATTGATAAATATAGTATTTTTTGAGCCCATTAACAAGGATTCGAATTTCGTGTTTCTTG
AAAAAXXXXXXXXXXTTTTTCTTGATGGAATTTGCCCATGATTTATTTTTGGTCTTTATCCATCTAGAAACTCTA
GACCTATTAAGAAAATTGTA
AACATTTAAGAATATTATTGATAAATATAGTATTTTTTGAGCCCATTAACAAGGGTTCGAATTTCGTGTTTCTTG
AAAAAXXXXXXXXXXTTTTTCTTGATGGAATTTGCCCATGATTTATTTTTGGTCTTTATCCATCTAGAAACTCTA
GACCTATTAAGAAAATTGTA
>Marker733414
ACCCATCATTATAGTTTTGCAATGATATTCCAAAGGTTTAGAATAACCCCCACCGAAAGGCAGTCGATTATTCCT
CCTTTXXXXXXXXXXAGCTTAGTAATTTTGTAAGTATAACCCTCCATTTTCGAATCTCATAGGTTAGAATCATTA
TGCTCCCAAAGGAAAAAGTC
ACCCATCATTATAGTTTTGCAATGATATTCCAAAGGTTTAGAATAACCCCCATCGAAAGGCAGTCGATTATTCCT
CCTTTXXXXXXXXXXAGCTTAGTAATTTTGTAAGTATAACCCTCCATTTTCGAATCTCATAGGTTAGAATCATTA
TGCTCCCAAAGGAAAAAGTC
>Marker733437
CACTTACTTAATTGACTAATTATCTATCTTATAATAATTAGAATTTTGTGAGATGTTCTAAACTATGTGATAATA
GAGAGXXXXXXXXXXAATTGATGTCATTCTTTATACATGAACGTCAGTTATGACCCAATCACTTACTAATAATCA
TAATTTGAATGGTAAGAGGT
CACTTACTTAATTGACTAATTATCTATCTTATAATAATTAGAATTTTGTGAGATGTTCTAAACTATGTGATAATA
GAGAGXXXXXXXXXXAATTGATGTCATTCTTTATACATGAAGGTCAGTTATGACCCAATCACTTACTAATAATCA
TAATTTGAATGGTAAGAGGT
>Marker733522
AACCACACGTGATTGGGAGAATAGAAGGGTTTTATGTGGAAAACAGTGAAGAGAATAGTGGTGGGCATATGATGG
GTATGXXXXXXXXXXCTAATGGTTATGCAACTTTGAAGAAATCATTTAGAACAAAGTTTGGTGGCAAATATAATA
AGTTCATAGAATTCAATGTT
AACCTCACGTGATTGGGAGAATAGAAGGGTTTTATGTGGAAAATAGTGAAGAGAATAGTGGTGGGCATATGATGG
GTATGXXXXXXXXXXCTAATGGTTATGCAACTTTGAAGAAATCATTTAGAACAAAGTTTGGTGGCAAATATAATA
AGTTCATAGAATTCAACGTT
>Marker733718
GACCCTCATGACTGCAAAATGTTCGTATTCACACCTGTAGCCACGTTCCGTGCTGAAATGTAAATTATCCATCCT
CAACTXXXXXXXXXXGCATTGTAGCTATTTTGTGCAAGTTGTATGGTTTCTGATGGTTCAACTTTGTAAAAGCAC
TAAACAATGTTTGTGAAAGT

Create PDF with GO2PDF for free, if you wish to

GACCCTCATGACTGCAAAATGTTCGTATTCACACCTGTAGCCACATTCCGTGCTGAAATGTAAATTATCCATCCT
CAACTXXXXXXXXXXXGCATTGTAGCTATTTTGTGCAAGTTGTATGGTTTCTGATGGTTCAACTTTGTAAAAGCAC
TAAACAACGTTTGTGAAAGT
>Marker733958
GACTCTTGATTTCTCTGACTCAAGCTCCAACACCAATTGCTTGAGACGTCCAACTTCTGCCTTCATTTTCTCAAA
CTCTAXXXXXXXXXXXGATCCAGATCCAACTTAAGCTTCATTGATTCCTCAACTTTTTCATGTAATGACTCAAGTA
AAGTTTTTTTCTCCTTTGTT
GACTCTTGATTTCTCTGACTCAAGCTCCAACACCAATTGCTTGAGACGTCCAACTTCTGCCTTCATTTTCTCAAA
CTCTAXXXXXXXXXXXGATCCAGATCCAACTTAAGCTTCATTGATTCCTCAACTTTTTCTTGTAATGACTCAAGTA
AAGTTTTTTTCTCCTTTGTT
>Marker734081
ACCAAACATCAGATTGTCACACTGGTTTAGGTCATTGCTACATCTTTGAAGTTCCAAGTTCCAATTTTTGTTAAA
TAATTXXXXXXXXXXXCAAAGGTTAAGAATATATAATATTGCACCTTTTAAGATAAGCATTGCACTTTCTCAAAAA
TAAGTTGGATTATAAAAGTG
ACCAAACATCAGATTGTCACACTGGTTTAGGTCATTGCTACATCTTTGAAGTTCCAAGTTCCAATTTTTGTTAAA
TAATTXXXXXXXXXXXCAAAGGTTAAGAATATATAATATTGCACCTTTTAAGATAAGCATTGCACTTTCTCAAAAA
CAAGTTGGATTATAAAAGTG
>Marker734308
ACTTTTTTTATTCTTTCTCCGAAATCTTTCATATATAAAAGTATTGTTAAGATTAATTTTCATAAATATAACAAA
GAGAAXXXXXXXXXXXTTTGGTTGTTCCAAATATAAATAATAATTAGATATTGAATAGTCAAATCTGAACGACCCT
TTTTCCAAATATAAACGAGT
ACTTTTTTTATTCTTTCTCCGAAATCTTTCATATATAAAAGTATTGTTAAGATTAATTTTCATAAATATAACAAA
GAGAAXXXXXXXXXXXTTTGGTTGTTCCAAATATAAATAATAATTAGATATTGGATAGTCAAATCTGAACGACCCT
TTTTCCAAATATAAACGAGT
>Marker734324
CACGGAATGAAGAATCGAGTCGTCAAAAGGGATTTGGTGCAGGTCCGATGAAACAATTTCCCGATATTTCAACCT
CGTATXXXXXXXXXXXAGTCCTACTACTGCGATCTACAATTTTTTTAACTCTGCAGAATTACCGTAGGTTATTGGA
CAGGACTTACATAGGATGTC
CACGGAATGAAGAATCGAGTCGTCAAAAGGGATTTGGTGCAGGTCCGATGAAACAATTTCCCGATATTTCAACCT
CGTATXXXXXXXXXXXAGTCCTACTACTGCAATCTACAATTTTTTTAACTCTGCAGAATTACCGTAGGTTATTGGA
CAGGACTTACATAAGATGTC
>Marker735039
TACTAATAACCTAATTTAATATCTTATAAGATGTTGAAAAACAACAAAATAGAGTATGAGTTAGAAAATGAGGTT
TCACAXXXXXXXXXXXAATTTAGAGACAATCATTATGCTTCAAGGCTAAATGACAAGCTAGAGCTAAAAGACTTCG
TGATTGATTAAATACTTGTT
TACTAATAACCTAATTTAATATCTTATAAGATGTTGAAAAACAACAAAATAGAGTATGAGTTAGAAAATGAGGTT
TCACAXXXXXXXXXXXAATTTAGAGACAATCATTATGCTTCAAGGCTAAATGACAAGCTAGAGCTAAAAGACTTCA
TGATTGATTAAATACTTGTT
>Marker735101
AACTAATGTGTGTTAATGCAAGATGTTGCATTTACGCGAAAGGAAATGTTTATGTGAAATTGTTATGTTATAAAC
GTGTTXXXXXXXXXXXTCAACAAACTTTACAAGCTACATAACACTTCCAACAAACAAAACTACTAACAAATTAAAA
ACTAAGTTTAAAATAAAGTC
AACTAATGTGTGTTAATGCAAGATGTTGCATTTACGCGAAAGGATATGTTTATGTGAAATTGTTATGTTATAAAC
GTGTTXXXXXXXXXXXTCAACAAACATTACAAGCTACATAACACTTCCAACAAACAAAACTACTAACAAATGAAAA
ACTAAGTTTAAAATAAAGTC
>Marker735127

Create PDF with GO2PDF for free, if you wish to

AACAATTTACAAAGCGTGGAAGAAGCCTCCATCGAAATTCAAAATCAATAATACCCGAAGGATAGTAAAACCCTT
AATCAXXXXXXXXXXXTAAACCCTAGCAGGATCCAGAATCAAACTTAAAATTGGAAACATCAAAATACGAATCAAA
GAAGAAGAGAGCACCTGGTA
AACAATTTACAAAGCGTGGAAGAAGCCTCCATCGAAATTCAAAATCAATGATACCCGAAGGATAGTAAAACCCTT
AATCAXXXXXXXXXXXTAAACCCTAGCAGGATCCAGAATCAAACTTAAAATTGGAAACATCAAAATACGAATCAAA
GAAGAAGAGAGCACCTGGTA
>Marker735397
AACCCCTCCTATTTGATTGCCACCTTTCCTTTACCTCGGGTTTCGGGTGACAGATCTAAAGGATGTTTCATTACT
AGAAAXXXXXXXXXXXCATGTCCTTAATGAGTCAGCTAATGTTCAGGATGAAAATAGAAATTTTGGTGACAAAAAG
AAATAATCTATGAATATTGT
AACCCCTCCTATTTGATTGCCACCTTTCCTTTACCTCGGGTTTCGGGTGACAGATCTAAAGGATGTTTCATTACT
AGAAAXXXXXXXXXXXCATGTCCTTAAAGAGTCAGCTAATGTTCAGGATGAAAATAGAAATTTTGGTGACAAAAAG
AAATAATCTATGAATATTGT
>Marker735652
TACTAGCCCAATGTGCAGCCACCTAGTGCCAAAAACCATTAATTTTGAAAAATGGATTCCAAAAGAACACTTACC
ACATTXXXXXXXXXXXAAATTCATGAGCACGGCAGGTCTCTATAATCTCTGAGATCACCTAAGAAACAACAAACCA
TTTTAATCAAACAAAAGGTC
TACTAGCCCAATGTGCAGCCACCTAGTGCCAAAAACCATTAATTTTGAAAAATGAATTCCAAAAGAACACTTACC
ACATTXXXXXXXXXXXAAATTCATGAGCACGACAGGTCTCTATAATCTCTGAGATCACCTAAGAAACAACAAACCA
TTTTAATCAAACAAAAGGTC
>Marker735777
ACTCTTTTTTAATGGATCTTAGAATCATCGTGACTTAGCGAAGAATTCATTTGTCACACCCCGCTCGCAGACCTT
CTTGTXXXXXXXXXXXTGCTTTAGGCGATAAAGCTTAATGCAATCAACATAACAAAAACTTAAACTTTATTAACAA
CAGACATTTTATACATAGTA
ACTCTTTTTTAATGGATCTTAGAATCATCGTGACTTAGCGAAGAATTCATTTGTCACACCCCGCCCGCAGACCTT
CTTGTXXXXXXXXXXXTGCTTTAGGCGATAAAGCTTAATGCAATCAACATAACAAAAACTTAAACTTTACTAACAA
CAGACATTTTATACATAGTA
>Marker735986
ACTAGATCTACGATTCATTTAAAGTATAAAAATAAAATTTGATCATTCAAATATAACAATAACAAAAATAATATG
AAGTTXXXXXXXXXXXGTTAGATTCACTTTGGTCGTTGTTATCTTAAATATTAATTGCTTTCTATTTAAGAGTTGT
ATTTTAAGGAAGAAAATGTG
ACTAGATCTACGACTCATTTAAAGTATAAAAATAAAATTTGATCATTCAAATATAACAATAACAAAAATAATATG
AAGTTXXXXXXXXXXXGTTAGATTCACTTTGGTCGTTGTTATCTTAAATATTAATTGCTTTCTATTTAAGAGTTGT
ATTTTAAGGAAGAAAATGTG
>Marker736094
CACCTTGAGAACAAAATGAGGGCTATCAGAGGCTCCACCTTGTGTTTAATTTGGTATAGCAAGTAAATTGATTTA
ATTGTXXXXXXXXXXXCATTGTCTCCAAGTCCGCTGTGTAAAAATTGTGAATAGTTTAGTTACTAAGGGTCAGTTT
GTGTTTAAACCTTATCATGT
CACCTTGAGAACAAAATGAGGGCTATCAGAGGCTCCACCTTGTGTTTAATTTGGTATAGCAAGTAAATTGATTTA
ATTGTXXXXXXXXXXXCATTGTCTCCAAGTCCGTTGTGTAAAAATTGTGAATAGTTTAGTTACTAAGGGTCAGTTT
GTGTTTAAACCTTATCATGT
>Marker736587
ACCATAGTTTAGGTTACTCAAAACTTGTTTTGTTTTTCTGCTGAAAAAAAGATAATGGAAAAGAAAAGAAACCCT
CTCCTXXXXXXXXXXXTAGCATTCCGCCGCCATCATGGTTTAACTTGGTCTTTGCATAGATGTGATTCGAACCAGC
AAATATCTCAACCACTTGTG

Create PDF with GO2PDF for free, if you wish to

ACCATAGTTTAGGTTACTCAAAACTTGTTTTGTTTTTCTGCTGAAAAAAAGATAATGGAAAAGAAAAGAAACCCT
CTCCTXXXXXXXXXXXTAGCATTCCGCCGCCATCATAGTTTAACTTGGTCTTTGCATAGATGTGATTCCAACCAGC
AAATATCTCAACCACTTGTG
>Marker736933
GACTTCATGATCAATCAACTTCACAGTTGATGACCGATCGCCGATCGTTTGGACCATGACGATCAACCACCAATC
GTCTTXXXXXXXXXXXAAGTGATGACCAAACACCATGACCTTGAGGACCAAAAACTATGGAATGAACTTCTTATGA
AATTTAATTTCAAAGCTGTT
GACTTCATGATCAATCAACTTCACAGTTGATGACCGATCGCCGATCGTTTGGACCATGACGATCAACCACCAATC
GTCTTXXXXXXXXXXXAAGTGATGACCAAACACCATGACCTTGAGGACCAAAAACTATGGAATGAACTTGTTATGA
AATTTAATTTCAAAGCTGTT
>Marker737383
CACGCTGCTTTATTTCTTTATCTCATGTGCATATTTCCCTTCAGGTAGGCTCAAGGGTGTCTGTGCTACACAAGC
TCACGXXXXXXXXXXXAAAATGACAGAGAACGACGAACTCCAACTTATTCGATTACCAATCAGACTACATTTGATG
CTGTATGGCTTCTTTCGGTA
CACGCTGCTTTATTTCTTGATCTCATGTGCATATTTCCCTTCAGGTAGGCTCAAGGGTGTCGGTGCTACACAAGC
TCACGXXXXXXXXXXXAAAATGACAGAGAACGACGAACTCGAACTTATTCGATTACCAATCAGACTACATTTGATG
CTGTATGGCTTCTTTCGGTA
>Marker737981
TACTGGTTCAGACTTTTTATTCTTGAGTAGCTTTCAAAGTAATTTGCTGAGGGAAGGTTTGAAGAACCAGATTGG
CTGGGXXXXXXXXXXXTCCCATTCCTCGAGACGTTAAGAGAATGGAGAATTATGGAGTTCATTTAACGAGCTTTGG
GACTGTTGATGAAGATAGTC
TACTGGTTCAGACTTTTTATTCTTGAGTAGCTTTCAAAGTAATTTGCTGAGGGAAGGTTTGAAGAACCAGATTGG
CTGGGXXXXXXXXXXXTCCCATTCCTCGAGACATTAAGAGAATGGAGAATTATGGAGTTCATTTAACGAGCTTTGG
GACTGTTGATGAAGATAGTC
>Marker738061
AACGATGTATTTCTGTTACGTGAATGTAAATTGTGTGAGGTGTGATACATTTTATGGGCCCATAGGAATTTTGTA
AGTTGXXXXXXXXXXXCCTTGGCAAGTAAATTCACAATTCGAGAACTATCATTCTACAAGCTCGAACAGTTCTTTT
CTTTTCAATTTTCTATTGTT
AACGATGTATTTCTGTTACGTGAATGTAAATTGTGTGAGGTGTGATACATTTTATGGGCCCATAGGAATTTTGTA
AGTTGXXXXXXXXXXXCCTTGGCAAGTAAATTCACAATTCGAGAACTATCATTCTACAAGCTCGAACAGTTCTTTT
CTTTTCAATTTTCTCTTGTT
>Marker738568
AACGTCGCACATAAATTCCTAACGATGTTGATGGACTTCCTTTTTAAGGCCGTTCTCCGTTGTGGGTGGTCTCAT
ATTCCXXXXXXXXXXXGTTTATGTGATGGGGGATTATAGATCCAATCCTTTACTAGATATGGAGCGAACGCGTTAT
GTTCGTATATTACCGGAGTT
AACGTCGCACATAAATTCCTAACGATGTTGATGGACTTCCTTTTTAAGGACGTTCTCCGTTGTGGGTGGTCTCAT
ATTCCXXXXXXXXXXXGTTTATGTGATGGGGGATTATAGATCCAATCCTTTACTAGATATGGAGCGAACGCGTTAT
GTTCGTATATTACCGGAGTT
>Marker738900
CACTCAATGACAATTACGTAATGAATAGATTCTATTTGTCTATTTAGTATCGATGTATTCTAACTAAGTTATATA
TGGTTXXXXXXXXXXXTAATTTTTCTTTTTTACATATTTAACTTACTGATTTATGTGCCTTCATTTCAATTTTTAG
TATGGCAACTTCGTCATGTG
CACTCAATGACAATTACGTAATGAGTAGATTCTATTTGTCTATTTAGTATCGATGTATTCTAACTAAGTTATATA
TGGTTXXXXXXXXXXXTAATTTTTCTTTTTTACATATTTAACTTACTGATTTATGTGCCTTCATTTCAATTTTTAG
TATGGCAACTTCGTCATGTG
>Marker739775

Create PDF with GO2PDF for free, if you wish to

GACATTTTTTGATGTGCTCATCAAAGAAGTTCAAATTTGTTGTAGTGATTAGGTTAAAGTGTAAAGGATAATTAA
TTGCAXXXXXXXXXXXTTCCCATCAAATTTGAATTGGTTTGGAACTGAATTTTTGGTAAGTTAAAATTAGGAAAAA
CTAGATACGAGCTATTGGTA
AACATTTTTTGATGTGCTCATCAAAGAAGTTCAAATTTGTTGTAGTGATTAGGTTAAAGTGTAAAGGATAATTAA
TTGCAXXXXXXXXXXXTTCCCATCAAATTTGAATTGGTTTGGAACTGAATTTTTGGTAAGTTAAAATTAGGAAAAA
CTAGATACGAGCTATTGGTA
>Marker739850
GACTGTTTGCCCCGCCAAAGGCAGTCCCTATTGTTCTCTGCAACCATTCCAAGGGAGGTAAGTCCATTTTCTCTT
CTTTAXXXXXXXXXXXTATCTCAGCTAGTTTTGAAAAGGGAACATGTTTTTGTTAATAATGTGGGAATTGGTTGTG
TAGAAACGCCTGCTCAGGTA
GACTGTTTGCCCCGCCAAAGGCAGTCCCTATTGTTCTCTGCAACCATTCCAAGGGAGGTAAGTCCATTTTCTCTT
CTTTAXXXXXXXXXXXTATCTCAGCTAGTTTTGAAAAGGGAACATGTTTTTGTTAATAATGTGGGAATTGGTTGTG
TAGAAACGCCTGTTCAGGTA
>Marker739889
TACCCTATCCTTGCGCTCCTTGTAAGTCGCTTCACTTTGCACCTCTACACTTTCTCATTTGTTTTAGATGGTTCA
TGGTGXXXXXXXXXXXGTTTGTCTATGATTTGGTTCTTCTCTTCCTCTTCATATGTTTTATCCCAAAGAAAGGAGT
TGAGGTCTAAGCACTCTTGT
TACCCTATCCTTGCGCTCCTTGTAAGTCGCTTCACTTTGCACCTCTACACTTTCTGATTTGTTTTAGATGGTTCA
TGGTGXXXXXXXXXXXGTTTGTCTATGATTTGGTTCTTCTCTTCCTCTTCATATGTTTTATCCCAAAGAAAGGAGT
TGAGGTCTAAGCACTCTTGT
>Marker739933
CACCTACTATTTCGTAATTTCAAGTTTTGATTTTAAATTCAAATCAAATAAAATCGTAACTTACTCTAGTCTTAA
AATTTXXXXXXXXXXXTTCTGATTTTTGGAAGAATTCTTTCTAAACTTCTCTAACGAGCCTTGTGAGCTTGGACTT
GTTTGGCCTATGGACTTAGT
CACCTACTATTTCTTAATTTCAAGTTTTGATTTTAAATTCAAATCAAATAAAATCGTAACTTACTCTAGTCTTAA
AATTTXXXXXXXXXXXTTCTGATTTTTGGAAGAATTCTTTCTAAACTTCTCTAACGAGCCTTGTGAGCTTGGACTT
GTTTGGCCTATGGACTTAGT
>Marker740314
ACCGTTCTGTGTCCTCTTTTCAGATTTGTTAGAATTGAACTAGAACATTGCTGTAAAATTCCATGGCCACAGCAT
GGAATXXXXXXXXXXXCAAAACTTTGTATGATAGATAATAGTCAATGGGATCATATTTGGTTAAAACTCTCTTAAT
ATCAAGTTGAAGTGGGTGTG
ACCGTTCTGTGTCCTCTTTTCAGATTTGTTAGAATTGAACTAAAACATTGCTGTAAAATTCCATGGCCACAGCAT
GGAATXXXXXXXXXXXCAAAACTTTGTATGATAGAGAATAGTCAATGGGATCATATTTGGTTAAAACTCTCTTAAT
ATCAAGTTGAAGTGGGTGTG
>Marker740874
ACTCAAATGAATGTCAAATAATTATAGCAAGATGCATGCTGATTAAGGAATGCAAATAATTCGAATGAGTAATTG
ACCCAXXXXXXXXXXXTAATTAATTAATCATGGATAATTATTAACGAACTATGTGATATGAATACAAGTAATTAAC
ACATAATGTAAATACATGGT
ACTCAAATGAATGTCAAATAATTATAGCAAGATGCATGCTGATTAATGAATGCAAATAATTCGAATGAGTAATTG
ACTCAXXXXXXXXXXXTAATTAATTAATCATGGATAATTATTAACGAACTATGTGATATGAATACAAGTAATTAAC
ACATAATGTAAATACATGGT
>Marker740883
TACTCATCTAGTAGTATTTTAAAATTCAAAATTATATCTTTTCGAGATTTAATATTAATTATATAAGATTTTATT
CCAATXXXXXXXXXXXATTATTATATAAAATTAAATAACAAGAAGAGTTTAAAAGTAATCTGGACTGTAAAAATAG
TTAATTAGATTATTAATGTG

Create PDF with GO2PDF for free, if you wish to

TACTCATCTAGTAGTATTTTAAAATTCAAAATTATATCTTTTCGAGATTTAATATTAATTATATAAGATTTTTTT
CCAATXXXXXXXXXXXATTATTATATAAAATTAAATAACAAGAAGAGTTTAAAAGTAATCTGGACTGTAAAAATAG
TTAATTAGATTATTAATGTG
>Marker741148
ACTCGTCTAATCTCATTTTCTAAATATTCATTTAATTCGAATTTTATCATTTTAGCTGAGAAATAGTTGTTTGGT
ATGTTXXXXXXXXXXXCTCATTTACTTTCCGGAATATATTACTTTCGAAAAAAATAACAATAACTTTAAAAAACTT
CCATTATCTTCAATAATGTT
ACTCGTCTAATCTCATTTTCTAAATACTCATTTAATTCGAATTTTATCATTTTAGCTGAGAAATAGTTGTTTGGT
ATGTTXXXXXXXXXXXCTCATTTACTTTCCGGAATATATTACTTTCGAAAAAAATAACAATAACTTTAAAAAACTT
CCTTTATCTTCAATAATGTT
>Marker741454
TACTTCACAAGCAAAATTTTACAAATATCTTAAACTTTAATCATATTTCACAACTTAATTCACATGGTTTTTTT
TTTTTXXXXXXXXXXXGATAAAGATATGATGTGTTGATGTAATTTTATTGTGTGAGCAAATATATGATTTAGTATG
TTCTTATTCATTTTAAATGT
TACTTCACAAGCAAAATTTTACAAATATCTTAAACTTTAATCATATTTCACAACTTAATTCACATGGTTTTTTT
TTGGAXXXXXXXXXXXGATAAAGATATGATGTGTTGATGTAATTTTATTGTGTGAGCAAATATATGATTTAGTATG
TTCTTATTCATTTTAAATGT
>Marker741497
TACCATTATGTATCAACATTTATTAATGATATTTTGTATAATTAAAAAACAACCTTAAAATATTGGATGGAAGAA
GCATAXXXXXXXXXXXACACTTTCATCAACCTTACCAAACATATAAATTATTGTCATCTACCCCTAGTTTAATTTA
CCTGATTCCATGTCCGGGTC
TACCATTATGTATCAACATTTATTAATGATATTTTGTATAATTAAAAAGCAACCTTAAAATATTGGATGGAAGAA
GCATAXXXXXXXXXXXACACTTTCATCAACCTTACTAAACATATAAATTATTGTCATCTACCCCTAGTTTAATTTA
CCTGATTCCATGTCCGGGTC
>Marker741797
ACTGTATATGGTCGATTTACAAATTTTCAAACGAAATTGTAGTATATTGACGCTATTTTAAGATCCATGACACTT
ATCCTXXXXXXXXXXXGATAAAAGGAGGCTTATTGGAAAATTTGACGCGATGAACTGATAGACATTGGGCTCATTT
CACCCAAAAATGGCAACGTA
ACTGTATATGGTCGATTTACAAATTTTCAAACGAAATTGTAGTATATTGACGCTATTTTAAGATCCATGACACTT
ATCCTXXXXXXXXXXXGATAAAAGGAGGCTTATTGGAAAATTTGACGCGATGAACTGATAGACATTGGGCTCATTT
CACCCAAAAATGGCAGCGTA
>Marker742081
TACTTTGATTGTTGTTTATGAGTCTCATGTAATCAACAATAAATTAGCAATATGTGTAGTTAATTATCTAAATTT
TTTTTXXXXXXXXXXXAATACAGAAATTAATTAAAAGAGAGCATGCCACACACAACTAATTTCAATCCACTCTTCA
AATGAGTGTTTAAATAAGTT
TACTTTGATTGTTGTTTATGAGTCTCATGTAATCAACAATAAATTAGCAATATGTGTAGTTAATTATCTTAAAAT
TTTTTXXXXXXXXXXXAATACAGAAATTAATTAAAAGAGAGCATGCCACACACAACTAATTTCAATCCACTCTTCA
AATGAGTGTTTAAATAAGTT
>Marker742447
CACCCCGCTGCTCCGCAACCCCCGACGGTCCTCGTTGTATTACGTGAATTTAATTTCCATCAGAGTCGGCCGCAA
AATCGXXXXXXXXXXXGAAAAACAATAGCCAAAATTTGAATGTATAAAAATGGTTGTTGAGTTGTTGAGTTGTTGT
TGTTGTTCAGGGACGACGTT
CACCCCGCTGCTCCGCAACCCCCGACGGTCCTCGTTGTATTATGTGAATTTAATTTCCATCAGAGTCGGCCGCAA
AATCGXXXXXXXXXXXGAAAAACAATAGCCAAAATTTGAATGTATAAAAATGGTTGTTGAGTTGTTGAGTTGTTGT
TGTTGTTCAGGGACGACGTT
>Marker742682

Create PDF with GO2PDF for free, if you wish to

ACTGAAATTAATCAAGTGAAGTATGGGAACAGTTTGGAGTTCATTGTGATAGGACTAAGCTTGGCATGGGGGACA
AGAACXXXXXXXXXXCTTATGCTTCAGAACCGAACAATAATGAAAATTGGAAATATAGAAACTATTCTTAATATA
TCACATTTCCAGGGTTGGTT
ACTGAAATTAATCAAGTGAAGTATGGGAACAGTTTGGAGTTCATTGTGATAGGACTAAGCTTGGCATGGGGGACA
AGAACXXXXXXXXXXCTTATGCTTCAGAACCGAACAATAATGAAAATTGGAAATATAGAAAGTATTCTTAATATA
TCACATTTCCAGGGTTGGTT
>Marker742828
AACAAAATCTATTAAAGTTACACTTAGGTCATCGTAGGTCACAACAAAATAGAATGGAACACTAACCAAAAACTA
AAACTXXXXXXXXXXTACATATTAAAATACTATCAATCCCACTTTAGATTCAAAACTTTGTTTCATTACTATAGA
TCTATAAATCTTATCATGGT
AACAAAATCTATTAAAGTTACACTTAGGTCATCGTAGATCACAACAAAATAGAATGGAACACTAACCAAAAACTA
AAACTXXXXXXXXXXTACATATTAAAATACTATCAATCCCACTTTAGATTCAAAACTTTGTTTCATTACTATAGA
TCTATAAATCTTATCATGGT
>Marker742930
AACAGAAAGCTCCATTCATTCATACCATTATACCTCTGATAATTTCGAACCCCCTCCTACTCACACTATTTCATC
CCAATXXXXXXXXXXTGTAAAATGAAAAGAATTTTCAGCCTATGCTTTTTCTAAATTTGAAAACCATTTATGGGA
CATATCATTTCAATAATGTG
AACAGAAAGCTCCATTCATTCATACCATTATACCTCTGATAATTTCGAACCCCCTCCTACTCACACTATTACATC
CCAATXXXXXXXXXXTGTAAAATGAAAAGAATTTTCAGCCTATGCTTTTTCTAAATTTGAAAACCATTTATGGGA
CATATCATTTCAATAATGTG
>Marker743423
ACATCATCAATAGGGAAGGACTGATTGCTGCTCTCTTTTGTAGTGGATGATAGGAATTTGATGGTCGAGTCATTC
TTGTTXXXXXXXXXXGTCAATGTCTCGTCACGACGGCACCGATTTTCATATCGTGAAGGCATCCGATTTGACAAA
ATCAATGATCAATCAGGGTC
ACATCATCAATAGAGAAGGACTGATTGCTGCTCTCTTTTGTAGTGGATGATAGGAATTTGATGGTCGAGTCATTC
TTGTTXXXXXXXXXXGTCAATGTCTCGTCACGACGGCACCAATTTTCGTATCGTGAAGGCATCCGATTTGACAAA
ATCAATGATCAATCAGGGTC
>Marker743513
TACAACAAATTCTGCCTATCTACTACTTTTGGAGTTTTTCCCTGGATTTTCCTCCTAGGTTATGATTTGCCTTAC
TCTAGXXXXXXXXXXGCTATAGACAAAAACCGACCATCTGGAGAACACAACCTTGTGGAATGGGCAAAACCTTAC
CTCACTAGCAAACGAAGAGT
TACAACAAATTCTGCTTATCTACTACTTTTGGAGTTTTTCCCTGGATTTTCCTCCTAGGTTATGATTTGCCTTAC
TCTAGXXXXXXXXXXGCTATAGACAAAAACCGACCATCTGGAGAACACAACCTTGTGGAATGGGCAAAACCTTAC
CTCACTAGCAAACGAAGAGT
>Marker743804
TACGTAACCAGCATGTAAATCATTTTGCCTCGACTTTCTTTTTCTTGGCTTCCGATTTCTTCATCACGTCTGACA
GCTAGXXXXXXXXXXTGATGGTCATGAATTTTGTGCTTTACACATGTTGTCTTAGCATGCACCGCGTTAAGCTAC
CAATCCAAACTTGACATGTT
TACGTAACCAGCATGTAAATCATTTTGCCTCGACTTTCTTTTTCTTGGCTTCCGATTTCTTCATCACGTCTAACA
GCTAGXXXXXXXXXXTGATGGTCATGAATTTTGTGCTTTACACATGTTGTCTTAGCATGCACCGCGTTAAGCTAC
CAATCCAAACTTGACATGTT
>Marker743899
ACCCAAAGCTATCGTGTGTCCGATATGGAACACACTCAATCTAGTGATCCCCAAGGATCACTATCATTTATCTGT
GAATCXXXXXXXXXXATAATATGACAGACTAGTGATCGATGCACACAGTCATAAATCTGTGAAAGGTGGTGATCC
AGAGGAACACCCATGTAGGT

Create PDF with GO2PDF for free, if you wish to

ACCCAAAGCTATCGTGTGTCCGATATGGAACACACTCAATCTAGTGATCCCCAAGGATCACTATCATTTATCTGT
GAATCXXXXXXXXXXXATAATATGACAGACTAGTGATCGATGCACACAGTCATAAATCTGTGAAAGGTGGTGATCA
AGAGGAACACCCATGTAGGT
>Marker744016
GACCACGTCGCCAAAGGTTCAACCTTTAAATAATTATTGCAACATTTTTGCAATGACAAAAAGTGACACGTTAGG
ATTTTXXXXXXXXXXXGTAGTCACTTATAGCATTGCGATTGGGGAGTTCTCACGATGTTTTCGAGTTGCGTCGGGA
GAACCTTTGTCATTAAAGTT
GACCACGTCGCCAAAGGTTCAACCTTTAAATAATTATTGCAACATTTTTGCAATGACAAAAAGTGACACGTTAGG
ATTTTXXXXXXXXXXXGTAGTCACTTATAGCATTGCGATTGGGGAGTTCTCACGATGTTTTCGAGTTGCGTCGGAA
GAACCTTAGTCATTAAAGTT
>Marker744336
GACACTTACCTTTCCTCGAATTGCAGCATGGACTTGCAGAATATATAGCTGAGGTGAGAATACCAGCATCTGTTT
TTCACXXXXXXXXXXXATTTTAAACTGTAGTTGGACATTTTCACAATTTTATGAATATTTTCTTCTTTCAGGAGGA
AATGGATCGGAAAAATGGGT
GACACTTACCTTTCCTCGAATTGCAGCATGGACTTGCAGAATATATAGCTGAGGTGAGAATACCAGCATCTGTTT
TTCACXXXXXXXXXXXATTTTAAACTGTAATTGGACATTTTCACAATTTTATGAATATTTTCTTCTTTCAGGAGGA
AATGGATCGGAAAAATGGGT
>Marker744951
ACCATTTTTAGAAAATTGTAGAGATGCAGATTTCACTCAAACATTAAAATCTAAACTAATTCTTCCAAACTTAAA
CCAATXXXXXXXXXXXTACTGTATAATATTTGAAAAGGATTATGTTGATCTCGGTTCATCTTGTATAAATTTGTAT
CCAGAAAAACCAAAATTAGT
ACCATTTTTAGAAAATTGTAGAGATGCAGATTTCACTAAAATATTAAAATCTAAACTAATTCTTCCAAACTTAAA
CCAATXXXXXXXXXXXTACTGTATAATATTTGAAAAGGATTATGTTGATCTCGGTTCATCTTGTATAAATTTGTAT
CCAGAAAAACCAAAATTAGT
>Marker745914
TACCCTGAGGCCCCAGCACAAGCCAAAAGAACCTCATATGCTGTTTCTCGAAGATCGTCATCTGTGATTCCTACA
ACAAAXXXXXXXXXXXXAATTGGAATATAGTCTTTTTGCTTAACAACGGAAACAAGACCTTTCATTGCATTAATGAA
ATGTGTCTAATGCTCGAAGT
TACCCTGAGGCCCCAGCACAAGCCAAAAGAACCTCATATGCTGTTTCTCGAAGATCGTCATCTGTGATTCCTACA
ACAAAXXXXXXXXXXXXAATTGGAATATAGTCTTTTTTCTTAACAACGGAAACAAGACCTTTCATTGCATTAATGAA
ATGTGTCTAATGCTCGAAGT
>Marker745959
AACCCAGTTGGCACGTGAATCACTCTAACAGCACTATTTGTGGTATTTGCATGCTGACCTCCTGAACCGCCTGAT
CTGAAXXXXXXXXXXXTTCATTTTCTAGGAAGAAAATGGCTTGGGTTGGACCTCGTCTGCTTGAGGGAGAATGGCT
ACAGAAATAGCACTCGTGTG
AACCCAGTTGGAACGTGAATCACTCTAACAGCACTATTTGTGGTATTTGCATGCTGACCTCCTGAACCGCCTGAT
CTGAAXXXXXXXXXXXTTCATTTTCTAGGAAGAAAATGGCTTGGGTTGGACCTCGTCTGCTTGAGGGAGAATGGCT
ACAGAAATAGCACTCGTGTG
>Marker746815
TACCACACAAGCCAGCTACCACCCAAATGTGTTCTCTCCACTGTAAGTAACATACAGAAATCCATCTTCATCCTT
CTTCTXXXXXXXXXXXAGTTTTTGAAATTTTGAACATGACCTTTCATTAAATTTTCTGCTATCTTTGATATAATTG
TTATTTGAGGTGCCATGTGT
TACCACATAAGCCAGCTACCACCCAAATGTGTTCTCTCCACTGTAAGTAACATACAGAAATCCATCTTCATCCTT
CTTCTXXXXXXXXXXXAGTTTTTGAAATTTTGAACATGACCTTTCATTAAATTTTCTGTTATCTTTGATATAATTG
TTATTTGAGGTGCCATGTGT
>Marker747621

Create PDF with GO2PDF for free, if you wish to

ACTCTTGTTCTTCTAAACATCTTTGCTGATTTTTAAACTTTCTTTGTAATTTTTTTTCCTTATCGTCTTTGAATT
TATTGXXXXXXXXXXGTTTTAGGCCTTCCCATCAATCATTCTTCTCTACTGGAGATTTTTAATCTTCTGTAATTT
GCCTCAAGTCATTCTATGTT
ACTCTTGTTCTTCTAAACATCTTTGCTGATTTTTAAACTTTCTTTGTAATTTTTTTTCCTTATCGTCTTTGAATT
TATTGXXXXXXXXXXGTTTTAGGCCTTCCCATCAATCATTCTTCTCTACTGGAGATTTTTAATCTTCTGTAATTT
GCCTCAAGTCATTCTCTGTT
>Marker748292
AACGTGTCTGTAACGGATAGACCAAGGTATCGAACAAGAAAGGGAAAAGTGGCATTGAATGTCCTCGACGTATGC
GATACXXXXXXXXXXGAAGGTTCCAAAGGGTGAGATATATGTTCCAACTAACCCTCGTTAAAACAATTTGGAATT
TGTTCATTCTTTCTTGGGTT
AACGTGTCTGTAACGGATCGACCAAGGTATCGAACAAGAAAGGGAAAAGTGGCATCGAATGTCCTCGACGTATGC
GATACXXXXXXXXXXGAAGGTTCCAAAGGGTGAGATATATGTTCCAACTAACCCTCGTTAAAACAATTTGGAATT
CGTTCATTCTTTCTTGGGTT
>Marker748554
AACAACTTTGAGTCACTTACTTGAATTTAGATAGATACAACATGCAAATTTTTATTTACAAATTATTTGAAGCAA
AGGCTXXXXXXXXXXTCCTACCTAGGGGAATAAGAAGAAGGAAAAAGGTTTGGCTTTAAATGGTCCTTTAATTTA
CCAACATGACATGTTGAGTT
AACAACTTTGAGTCACTTCCTTGAATTTAGATAGATACAACATGCAAATTTTTATTTACAAATTATTTGAAGCAA
AGGCTXXXXXXXXXXTCCTACCTAGGGGAATAAGAAGAAGGAAAAAGGTTTGGCTTTAAATGGTCCTTTAATTTA
CCAACATGACATGTTGAGTT
>Marker748718
TACAACGTTGCCACCATTGGATGTGCTCCTGAGCAGCTAATTGTTCAATTTGTTCCCTCAATAAACGACTTGTGG
ATAATXXXXXXXXXXATGAACGAAAGGAACATTTTAATGCTTTGAGCTTCATCATCATTCCATGTCTAGGCCAAC
CAGCAACCCTATTGTTTGTC
TACAACGTTGCCACCATTGGATGTGCTCCTGAGCAGCTAATTGTTCAATTTGTTCCCTCAATGAACGACTTGTGG
ATAATXXXXXXXXXXATGAACGAAAGGAACATTTTAATGCTTTGAGCTTCATCATCATTCCATGTCTAGGCCAAC
CAGCAACCCTATTGTTTGTC
>Marker749101
AACATAATAATGAAGAAAGGGTTGCAAACAATTTGAAAATTGAAGATTGTTCATATCATCAAAAAAACTGTTTCT
TATGAXXXXXXXXXXTTTAGATAAGCTTGTATTAGTAAATACCTTTTGTTTGCCCTTGACTCACTTCTGTGCCCC
CATTTTATCCAAAGACAAGT
AACATAATAATGAAGAAAGGGTTGCAAACAATTTGAAAATTGAAGATTGTTCATATCATAAAAAAAACTGTTTCT
TATGAXXXXXXXXXXTTTAGATAAGCTTGTATTAGTAAATACCTTTTGTTTGCCCTTGACTCACTTCTGTGCCCC
CATTTTATCCAAAGACAAGT
>Marker750084
ACCTTTCTATGAGACGACCTTGTAACTTGGAGGAGTAAGAAGCAAAGTGTTGTGACTAGAAATAATGCTAAGGTC
GAATAXXXXXXXXXXTCAATGTGATAGAATTAAACATGTTGAGATTGATTGACATTTCATCAAAGAAAGACTTGA
CAGTGGGAGCATATGATAGT
ACCTTTCTATGAGACGATCTTGTAACTTGGAGGAGTAAGAAGCAAAGTGTTGTGACGAGAAATAATGCTAAGGTC
GAATAXXXXXXXXXXTCAATGTGATAGAATTAAACATGTTGAGATTGATTGGCATTTCATCAAAGAAAGACTTGA
CAGTGGGAGCATATGATAGT
>Marker750190
CACGACCAACACCTTGCCGCCCACTCCTTGCGCGCCAATCATGCCATTTTGGTATGTATGAGCTATCTTTGATAT
TTTGAXXXXXXXXXXAAAATGAGTTTGAAGTTGATTTCTAAATCTGATTATTTATCAAATGTTCTAAAGAAAATT
TGACGTTTTAGTGTCGAGTT

Create PDF with GO2PDF for free, if you wish to

CACGACCAACGCCTTGCCGCCCACTCCTTGCGCGCCAATCATGCCATTTTGGTATGTATGAGCTATCTTTGATAT
TTTGAXXXXXXXXXXXAAAATGAGTTTGAAGTTGATTTCTAAATCTGATTATTTATCAAATGTTTTAAAGAAAATT
TGACGTTTTAGTGTTGAGTT
>Marker750359
CACACGAGATTTCAAATAAATGTATTTCGTGAAATTGAACTTTGTTATATTGATTAATATTGTGAATACAATCTC
TAGTAXXXXXXXXXXXAGAAGATGATGATCTCGAAGTTGATAAGAACTTGCACGTCTTGAGAGCTTTATAAAAGTT
TGAATCTTCAATTCTTCAGT
CACACGAGATTTCAAATAAATGTATTTCGTGAAATTGAACTTTGTTATATCGATTAATATTGTGAATACAATCTC
TAGTAXXXXXXXXXXXAGAACATGATGATCTCGAAGTTGATAAGAACTTGCACGTCTTGAGAGCTTTATAAAAGTT
TGAATCTTCAGTTCTTCAGT
>Marker750587
ACTGCAAATATTGACCTCTGAAAATATGCCTTAGAGCAGAATTCTTATCCTTCCTCTATAGTCTCTTTTCGCACT
TTACTXXXXXXXXXXXCAAATAAGGCTTCTAAGGTGCTTTTGATCATTACCTGTTGGAACTTTGTGGTCGGGTTAC
CACATTCAGAATACGGAGTG
ACTGCAAATATTGACCTCTGAAAATATGCCTTAGAGCAGAATTCTTATCCTTCCTCTATGGTCTCTTTTCTCACT
TTACTXXXXXXXXXXXCAAATAAGGCTTCTAAGGTGCTTTTGATCATTACCTGTTGGAACTTTGTGGTCGGGTTAC
CACATTCAGAATACGGAGTG
>Marker750633
CACTACTTAAAGTTTGAATAGTCTAAGATTTTTTAGTCTTTTTTTTCAATCACCCCTAAAGCTCTCAATCAATCA
TTGCAXXXXXXXXXXXAATAGCCCAAAATCTCTGTATGTATTATTGATTAAATAATCAAATATTATCTAAATAAAT
TAAATATTTCCCTGACGGTT
CACTACTTAAAGTTTGAATAGTCTAAGATTTTTTAGTCTTTTTTTTCAATCACCCCTAAAGCTCTCAATCAATCA
TTGCAXXXXXXXXXXXAATAGCCCAAAATCTCTGTATGTATTATTGATTAAATAATCAAATATTATCTAAATAAAT
TAAATATTTCCATGACGGTT
>Marker751265
TACATTCTTGTGAGACCATGAGGTGAATGTGGGTTTATCACTTTCTTAGGTTTCTAATGAAAAAGAAGATACTTG
TGAGTXXXXXXXXXXXATTCTCTGTGAAGATATTATTGATTTCTGTAACATGCTAAAAGTCAATACTTTCACATTG
GTGGCATACATGGCGTAAGT
TACATTCTTGTGAGACCATGAGGTGAATGTGGGTTTATCACTTTCTTAGGTTTCTTATGAAAAAGAAGATACTTG
TGAGTXXXXXXXXXXXATTCTCTGTGAAGATATTATTGATTTCTGTAACATGCTAAAAGTCAATACTTTCACATTG
GTGGCATACATGGCGTAAGT
>Marker751620
CACAGACGCATGATTGAGGAAGCTAGTAAAGCTCATCAAAATCGATAAGAAAAAAAGAAAGAAACCAATTCAAGA
TTCCAXXXXXXXXXXXATCCGAGTATCCACATGTATTTATCGTAACTTCTAAGAACAAAAATAAAAGAAAAAAAAG
GGATACAGACAAAAAGAGTC
CACAGACGCATGATTGAGGAAGCTAGTAAAGCTCATCAAAATAGATAAGAAAAAAAGAAAGAAACTAATTCAAGA
TTCCAXXXXXXXXXXXATCCGAGTATCCACATGTATTTATCGTAACTTCTAAGAACAAAAATAAAAGAAAAAAAAG
GGATACAGACAAAAAGAGTC
>Marker752023
CACCTTATTCAGATTGTTCCAAAAACTGACATTTCTCCTTTTTCTCGACTCAAAATCAAACCTTCTTTGCTCCAA
GTTGTXXXXXXXXXXXAGACAGGTTGCATGGCTTGAGATTGACATGTATCACTTACATTTTGTTGGCATGCCTTAG
TCTTACACCCCTTCCTAGTC
CACCTTATTCAGATTGTTCCAAAAACTGACATTTCTCCTTTTTCTCGACTCAAAATCAAACCTTCTTTGCTCCAA
GTTGTXXXXXXXXXXXAGACAGGTTGCATGGCTCGAGATTGACATGTATCACTTACATTTTGTTGGCATGCCTTAG
TCTTACACCCCTTCCTAGTC
>Marker752179

GACCAACTTTCTCTGACCATTTTTCGGTGATTGTTGCCGGCCTACTTTCATTGACAATTTTCTTGTGGTAGTCAC
TAATCXXXXXXXXXXGGATAATATAACAGATTATGAATGACACTATTTAGCATTTGATAAGCTATAATGTGATGA
GATTTAAAGTATGATGTGTA
GACCAACTTTCTCTGACCATTTTTCAGTGATTGTTGCCAGCCTACTTTCATTGACAATTTTCTTGTGGTAGTCAC
TAATCXXXXXXXXXXGGATAATATAACAAATTATGAATGACACTATTTAGCATTTGATAAGCTATAATGTGATGA
GATTTAAAGTATGATGTGTA
>Marker752222
CACTAAAACTAGCCAATGGGAAAAACCTAACAAGTATTTTAGCAAATCATAGGCCAACTTGAATCCATTTTCATA
AAAATXXXXXXXXXXTGCACGTTAAGCTCACTATTTTTGTAGTCCCAATTGCAATTTGATTTTTATCCATGACTT
ATATAATAGGGTAGGATAGT
CACTAAAACTAGCCAATGGGAAAAACCTAACAAGTATTTTAGCAAATCATAGGCCAACTTGAATCCATTTTCATA
AAAATXXXXXXXXXXTGCACGTTAAGCTCACTATTTTTGTAGTCCCAATTGCAATTTGATTTTTATCCATGACAT
ATATAATAGGGTAGGATAGT
>Marker752402
ACTATATTTGCTAGCTGCTAAAATTTTCCTTCTGTTATAAGTTTTTGGTTGGCTTTTTTATTATATATGCTATGG
TATATXXXXXXXXXXGAAGCCAAATTTTGAAAGGTAGAAAAAAGTAATTTTCAAAAACTTGTTATTGTTTTTAGA
AATTGACTAGGATTTCAAGT
ACTATATTTGCTAGCTGCTAAAATTTTCCTTCTGTTGTAAGTTTTTGGTTGGCTTTTTTATTATATATGCTCTGG
TATATXXXXXXXXXXGAAGCCAAATTTTGAAAGGTAGAAAAAAGTAATTTTCAAAAACTTGTTATTGTTTTTAGA
AATTGACTAGGATTTCAAGT
>Marker752924
TACCCACATATATCAACGAGTTATGAGGCAAGAGATACATCAGATTGCACAAAGAATCTTTTAGTTGCTTAAAGT
TACATXXXXXXXXXXAGAAAGCTAGAGATTTTCTCAGAAGCTACTTTAATGGCAGGGCCCTCTTGTGGGGGGTAA
AGACAAGCTCCAAGCTCGTC
TACCCACATATATCAACGAGTTATGAGGGAAGAGATACATCAGATTGCACAAAGAATCTTTTAGTTGCTTAAAGT
TACATXXXXXXXXXXAGAAAGCTAGAGATTTTCTCAGAAGCTACTTTAATGGCAGGGCCCTCTTGTGGGGGGTAA
AGACAAGCTCCAAGCTCGTC
>Marker753095
AACCATGTTCTTCAACAAATCTCTGGAATGTGTTGTAAATGTGCTGAAGAAACAGCTGCAGACTTGGTTGCTTAC
TGGAAXXXXXXXXXXTTTGGTTACTAGTTTGTGGTTTTGTTTATACTAGTTGGTGCATTACAATATAATCGGTTC
AGCATGCAAGATAATTTTGT
AACCATGTTCTTCAACAAATCTCTGGAATGTGTTGTGAATGTGCTGAAGAAACAGCTGCAGACTTGGTTGCTTAC
TGGAAXXXXXXXXXXTTTGGTTACTAGTTCGTGGTTTTGTTTATACTAGTTGGTGCATTACAATATAATCGGTTC
AGCATGCAAGATAATTTTGT
>Marker754959
AACAAATAAAACAATGCAAATGAAATTCTTGATTGAAATCAAGTAAATTCACAAAAATGTTGAGCAAAAAGCTAG
AAAATXXXXXXXXXXAGTTTTAGCAAATATAATGAAAGGAAATTGCTGCCCTCTAGGATTGGATGTCAGAATTTA
CTATAGTATTTCTGGTTGTT
AACAAATAAAACAATGCAAATGAAATTCTTGATTGAAATCAAGTAAATTCACAAAAATGTTGAGCAAAAAGCTAG
AAAATXXXXXXXXXXAGTTTTAGCAAATATAATGAAAGAAAATTGCTGCCCTCTAGGATTGGATGTCAGAATTTA
CTATAGTATTTCTGGTTGTT
>Marker755656
ACCATTTTATCAGCTCAAGCGTTTATCACACACTCTTTTACAAAGTTGTAGCTATGCGGAGTAGTCTCAACACAT
TTAGAXXXXXXXXXXTATTCAAGATCACATAGCATGAATAGGGCATCTTATCCCATATCATATAGCAAGTATGAA
TTATTCTCATCGTATAGAGT

Create PDF with GO2PDF for free, if you wish to

ACCATTTTATCAGCTCAAGCGTTTATCACACACTCTTTTACAGAGTTGTAGCTATGCGGAGTAGTCTCAACACAT
TTAGAXXXXXXXXXXXTATTCAAGATCACATAGCATGAATAGGGCATCTTATCCCATATCATATAGCAAGTATGAA
TTATTCTCATCGTATAGAGT
>Marker755708
AACATTTCTTTCATACTTGGTGCTTGCATTATGGATAGCCCTTGAGCCTTCTCGGGTTTGTGATTTGTGGAATTT
TATTAXXXXXXXXXXXAGGACTCACGATTTTACGACCGGTTCAAAAGAAAATCTCCTCTTTCTCAAAGTTGAAGAG
TATTTGGTATAATCTTAGTA
AACATTTCTTTCATACTTGGTGCTTGCATTATGGATAGCCCTTGAGCCTTCTCGGGTTTGTGATTTGTGGAATTT
TATTAXXXXXXXXXXXAGGACTCACAATTTTACGACGGGTTCAAAAGAAAATCTCCTCTTTCTCAAAGTTGAAGAG
TATTTGGTATAATCTTAGTA
>Marker755888
AACATATTTTTTCAACTTCTTTTAATAATTTGCATACTTCTTGGGTAGGAAAGTTAAATTTTAAACCAAATTAAA
AAGAGXXXXXXXXXXXCTAGGGAACATGAGAGAACATGGAAGAATTGGAGTTTGTGGAATGATATCACAATACAAT
CTTGAGAAGCCAGAAGGAGT
AACATATTTTTTCAACTTCTTTTAATAATTTGCATTCTTCTTGGGTAGGAAAGTTAAATTTTAAACCAAATTAAA
AAGAGXXXXXXXXXXXCTAGGGAACATGAGAGAACATGGAAGAATTGGAGTTTGTGGAATGATATCACAATACAAT
CTTGAGAAGCCAGAAGGAGT
>Marker756024
CACAAAGTCATTGGCTGAGATTGTAAATTCTACATCTTTTTAAATAAGGGATTGGAAACAAAGAAAGATTCTGCA
TACCAXXXXXXXXXXXCACCAGGAAAAATGTCATAAAACAATTAAATGCTTAGAGGTGCAACAGCTAGTTCTGTGT
CTGACCTCATTTAGAAAGTA
CACAAAGTCATTGGCTGAGATTGTAAATTCTACATCTTTTTAAATAAGGGATTGGAAACAAAGAAAGCTTCTGCA
TACCAXXXXXXXXXXXCACCAGGAAAAATGTCATAAAACAATTAAATGCTTAGAGGTGCAACAGCTAGTTCTGTGT
CTGACCTCATTTAGAAAGTA
>Marker756102
AACAACCACTAAAACAATGATGTTCGAAAGAAGGAAATTTAATAGATGACACCCAAAAATTTAGTCTATAGTTGC
ATTGTXXXXXXXXXXXCATGAATTCTACGTTTTCTTCAATGTGTCATAAAATGCGTTGATGTTGCCTTTGGATTTC
ACTGACTTACTCCAATCGGT
AACAACCACTAGAACAATGATGTTCGAAAGAAGGAAATTTAATAGATGACACCCAAAAATTTAGTTTATAGTTGC
ATTGTXXXXXXXXXXXCATGAATTCTACGTTTTCTTCAATGTGTCATAAAATGCGTTGATGTTGCCTTTGGATTTC
ACTGACTTACTCCAATCGGT
>Marker756318
ACAGGAGTTGATAGACTTCTTGCTTGCCATCAGTTTCAAATCTAGGAGCTCTAAGACCAATTTGGTGTAACCAGA
TGCTCXXXXXXXXXXXACTTAATTTGCACAGTGACAACACTCAACTCGTGGCAAAGCTAAACATAGAAGGTCTCAT
CAAATTTAATCTAATCAAGT
ACAGGAGTTGATAGACTTCTTGCTTGCCATCAGTTTCAAATCCAGGAGCTCTAAGACCAATTTGGTGTAACCAGA
TGCTCXXXXXXXXXXXACTTAATTTGCACAGTGACAACACTCAACTCGTGGCAAAGCTAAACATAGAAGGTCTCAT
CAAATTTAATCTAATCAAGT
>Marker756335
AACTGTTGTGTTGTTAATAATGATTGTTCTTTTTTTTCTTTCTATATGAACGTAGATAAAGTGAGGGTTTGTGAAT
CAATTXXXXXXXXXXXCTCATTCTCACTTTCACTCTTTCACATTAATATTTAATTAATCTTTTCATAATTTCAATT
ATTAGTTATGGATATGAGTG
AACTGTTGTGTTGTTAATAATGATTGTTCTTTTTTTTCTTTCTATATGAACGTAGATAAAGTGAGGGTTTGTGAAT
CAATTXXXXXXXXXXXCTCATTCTCACTTTCACTCTTTCACATTAATATTTAATTAATCTTTTCATAATTTCAATT
ATTAGTTATGGATATAAGTG
>Marker756701

Create PDF with GO2PDF for free, if you wish to

GACCGGTTCTGAGCTCCAAGCACGGCGAGAAAAGGGACTTTGCTATCGGTGTGACGAACCGTTCAGCAAGGGGCA
TCGCTXXXXXXXXXXXTAATGATGGGGAAAAACCTTCATCATTCAAGACTTCCCTTATACGCAACCTGTCAATTGA
ATGGCTGAAATTTCCTTGTA
GACCGATTCTGAGCTCCAAGCACGGCGAGAAAAGGGACTTTGCTATCGGTGTGACGAACCGTTCAGCAAGGGGCA
TCGCTXXXXXXXXXXXTAATGATGGGGAAAAACCTTCATCATTCAAGACTTCCCTTATACGCAACCTGTCAATTGA
ATGGCTGAAATTTCCTTGTA
>Marker757606
ACTCTATTTATAATAGAAAGGCAAACTAGTCCTAATCCTAACTAATAAAAGAAACTAATTCTAATCCTAATTAAT
AAAAGXXXXXXXXXXXTAATACCCTATTCCTACTACATCATTCTATCCCTAAAAGAAAAAAAACTCGTCCTCAAGT
TTTAAAACGAAAATGAAGGT
ACTCTATTTATAATAGAAAGGCAAACTAGTCCTAATCCTAACTAATAAAAGAAACTAATTCTAATCCTAATTAAT
AAAAGXXXXXXXXXXXTAATACCCTATTCCTACTACATCATTCTATCCCTAAAAGAAAAAAAACTCATCCTCGAGT
TTTAAAACGAAAATGAAGGT
>Marker757710
CACTGCTATCTCTTTTACGTTCATTCCTGGCCACTGCAGAATAAAATTCACAAGAGTTCCCATCTGAGCATACAA
CACCAXXXXXXXXXXXCGATCTATTAGTTCCTCCACCAATTTATAATTGTCTGTTGTAGCCATAGGTATGACCTGA
CAATAAAATAATGATATGTT
CACTGCTATCTCTTTTACGTTCATTCCTGGCCACTGCAGAATAAAATTCACAAGAGTTCCCATCTGAGCATACAA
CACCAXXXXXXXXXXXCGATCTATTAGTTCCTCCACCAATTTATAATTGTCTGTTGCAGCCATAGGTATGACCTGA
CAATAAAATAATGATATGTT
>Marker758125
ACCATCAACAAAATAACGGTAAAGTATCGAGATCTTATTCTTAGATTAGATGACACGTTGGATGAATTACATGGT
GCAAAXXXXXXXXXXXCTTTTACGAGGGTAATGAATTATGTTTTGAGAGAATACATTGGAAAGTTTGTTATTGTAT
ATTTTGATGATATTCTTGTT
ACCATCAACAAAATAACGGTAAAGTATCGAGATCTTATTCCTAGATTAGATGACACGTTGGATGAATTACATGGT
GCAAAXXXXXXXXXXXCTTTTACGAGGGTAATGAATTATGTTTTGAGAGAATACATTGGAAAGTTTGTTATTGTAT
ATTTTGATGATATTCTTGTT
>Marker758636
AACTTACAAGCGCAAATAATTAGAGCAATTAATGAACAAATAATCAAATCCTCATATGAAGCGAATTCGATGAGT
TTCTAXXXXXXXXXXXTTCCATTGCGTAAAATGAAGATAGTGGAGAGGAATTGACGGTGACTTCTATATTGGGTTG
CTGCCATTGTTGTTCTGGTG
AACTTACAAGCGCAAATAATTACAGCAATTAATGAACAAATAATCAAATCCTCATATGAAGCGAATTCGCTGAGT
TTCTAXXXXXXXXXXXTTCCATTGCGTAAAATGAAGATAGTGGAGAGGAATTGACGGTGACTTCTATATTGGGTTG
CTGCCATTGTTGTTCTGGTG
>Marker758701
TACATAGATTATCACTTTAATGATTCTCCAATACCTCAATATGTTCTCCTTTTATTCGACCTAATCTCTACTTTT
GTTTTXXXXXXXXXXXTAAACACAAAATCAAAACTATATACTAAATCATATTATGTGATGGAAACTTTATATTAAA
GACATGAATCATATTATGTT
TACATAGATTATCACTTTAATGATTCTCCAATACCCCAATATGTTCTCCTTTTATTCGACCTAATCTCTACTTTT
GTTTTXXXXXXXXXXXTAAACACAAAATCAAAACTATATACTAAATCATATTATGTGATGGAAACTTTATATTAAA
GACATGAATCATATTATGTT
>Marker758825
AACATGAACACTTCAAATATAATAGATTAATCTAAGATCACTCATCGTAAATTTACAAGTTTTCCTCTCTTTTGT
CTTTTXXXXXXXXXXXGACACGACGATGAAGTATAACGTAATATGAACTCAGCAATTAGGTGTGTTTATATAGGAA
TATTTAACCAATTAAATGTT

Create PDF with GO2PDF for free, if you wish to

AACATGAACACTTCAAATATAATAGATTAATCTAAGATCACTCATCGTAAATTTATAAGTTTTCCTCTCTTTTGT
CTTTTXXXXXXXXXXXGACACGACGATGAAGTATAACGTAATATGAACTCAGCAATTAGGTGTGTTTATATAGGAA
TATTTAACCAATTAAATGTT
>Marker758848
CACTTAGTTATTAGTTTTGGTTTAATCTTGATTCTCCACTTCAACTCTGTTGTTTTTTCTTCACAACATATATCC
CCTATXXXXXXXXXXXTAAAGCCTTCATTTTCGTTCATTTCAGATATCAAAAAACATTCTTGGTGTTGTTGTAATG
CCCATGCCCAGGAGTCGAGT
CACTTAGTTATTAGTTTTGGTTTAATCTTGATTCTCCACTTCAACTCTGATGTTTTTTCTTCACAACACATATCC
CCTATXXXXXXXXXXXTAAAGCCTTCATTTTCGTTCATTTCAGATATCAAAAAACATTCTTGGTGTTGTTGTAATG
CCCATGCCCAGGAGTCGAGT
>Marker760218
TACATTTTTATGAGGCATTAAGAGAGCAAATTAGAGTGACATGACATGTCACCCTCTCTATTTCCTACCATCATA
TTTAGXXXXXXXXXXXTTTTTCCTAATACTTATAAAATAAACAAACTTGAGTCGTTTTTATAACCCTAATCAAATA
GGATCTATGAGAGGGCCGTG
TACATTTTTACGAGGCATTAAGAGAGCAAATTAGAGTGACATGACATGTCACCCTCTCTATTTCCTACCATCATA
TTTAGXXXXXXXXXXXTTTTTCCTAATACTTATAAAATAAACAAACTTGAGTCGTTTTTATAACCCTAATCAAATA
GGATCTATGAGAGGGCCGTG
>Marker760363
AACGTGTTGTGGGAGTCTCAACCATAGTTATTGAAAGTTAAAAAGGTCACTTACTTTCTTTAGTTAGATTATTGC
CTCACXXXXXXXXXXXATGATAGTCATTCCCAATAATCGCCCACGAATGACTACCATTGCATTCATGTGGAATTGT
TAGATAATGATTATCATTGT
AACGTGTTGTGGGAGTCTCAACCATAGTTATTGAAAGTTAAAAAGGTCACTTACTTTCTTTAGTTAGATTATTGC
CTCACXXXXXXXXXXXATGATAGTCATTCCCAATAATCGCCCACTAATGACTACCATTGCATTAATGTGGAATTGT
TAGATAATGATTATCATTGT
>Marker760538
ACTTCTGCTTTTATACTTTACTTCTCTATTACTTTTCTTGTATCAGCAACAAGATGACTCATGTTTTTATATATT
TTCTTXXXXXXXXXXXATGAGCAACAAGTGTTTGCATGGATTGTCATTGTGTTTAAGAATCTAAAGTCTCTTTACT
AACCATACCAAACATTTGTT
ACTTCTGCTTTTATACTCTACTTCTCTATTACTTTTCTTGTATCAGCAACAAGATGACTCATGTTTTTATATATT
TTCTTXXXXXXXXXXXATGAGCAACAAGTGTTTGCATGGATTGTCATTGTGTTTAAGAATGTAAAGTCTCTTTACT
AACCATACCAAACATTTGTT
>Marker761041
CACATCATATGTGCTAGTTTCTAGTGGAGAACAAATATTTATGGGTAACTGCTCTACTTCAAAGGTTGAGGGACA
ATGCAXXXXXXXXXXXATTTGATAAGTTTGTATTTTCCAAGAATGAGATTACATTGGAAATTGTTATTTGAGTGAT
GATCTCTTTAAATTAAATGT
CACATCATATGTGCTAGTTTCTAGTGGAGAACAAATATTTATGGGTAACTGCTCTACTTCAAAGGTTGAGGGACA
ATGCAXXXXXXXXXXXATTTGATAAGTTTGTATTTTCCAAGAATGAGATTACATTGGAAATGGTTATTTGAGTGAT
GATCTCTTTAAATTAAATGT
>Marker761075
CACAAGCTTGATCTATTTTGTTCAATAAGGATGCCTCCTGGTAGTTAATTAAAGGCAAGAAAAATCCTATTTTCA
ATATCXXXXXXXXXXXGAAACCAAAGAGATGAAATGTGTTGCTGAGTAAAATTTAGATCATAACGTTCAGTGAGTG
ACTTAGTTATATCATGGAGT
CACAAGCTTGATCTGTTTTGTTCAATAAGGATGCCTCCTGGTAGTTAATTAAAGGCAAGAAAAATCCTATTTTCA
ATATCXXXXXXXXXXXGAAACCAAAGAGATGAAATGTGTTGCTGAGTAAAATTTAGATCATAACGTTCAGTGAGTG
ACTTAGTTATATCATGGAGT
>Marker761377

Create PDF with GO2PDF for free, if you wish to

AACACATTGCAACTTTGATGTAGGGATCAATAGAAGAAAATCCTTTACCTTAACTTTACTTAAACTTCTTTACCA
AACCGXXXXXXXXXXCTTACCCACAAAGAAGGAGAATCGAACCCAAACCAAACCTATTGAAAAATTCAAGGATTT
GATCGTTATCCTTCAAAAGT
AACACATTGCAACTTTGATGTAGGGATCAATAGAAGAAAATCATTTACCTTAACTTTACTTAAACTTCTTTACCA
AATCGXXXXXXXXXXCTTACCCACAAAGAAGGAGAATCGAACCCAAACCAAACCTATTGAAAAATTCAAGGATTT
GATCGTTATCCTTAAAAAGT
>Marker762427
ACTGTTATAATATATGAGTGCAACGTAAGATGTTATCAAGTATTACAATAATTAAAACACTGTCATAATTAATAT
ATGATXXXXXXXXXXTATGTAAAAAAGTAATACTACGAATAATTATATAGGAATTGCCAGAGGGTCAGAGAGCGG
ACGCTGCAAGTAGTTTGGTA
ACTGTTATAATATATGAGTGCAACGTAAGATGTTATCAAGTATTACAATAATTAAAACACTGTCATAATTAATAT
ACGAGXXXXXXXXXXTATGTAAAAAAGTAATACTACGAATAATTATATAGGAATTGCCAGAGAGTCAGAGAGCGG
ACGCTGCAAGTAGTTTGGTA
>Marker762692
ACAAATGTTATGATGCAATGCCTTGTTATCACATGAGGTTTGATTTAGCCAAGATTTAAAGTCTGTCCATAAGTC
TAAATXXXXXXXXXXTAATTTTATTCAATAACCCTAAGTGATCGAGAGAAGAAAACTCAAAAGAGAACTCTAACT
TAAATCTCTATGGTTTGGTT
ACAAATGTTATGATGCAATGCCTTGTTATCACATGAGGTTTGATTTAGCCAAGATTTAAAGTCTGTCCATAAGTC
TAAATXXXXXXXXXXTAATTTTATTCAATAACTCTAAGTGATCGAGAGAAGAAAACTCAAAAGAGAACTCTAACT
TAAATCTCTATGGTTTGGTA
>Marker764144
AACAAAATGATAGAACAAGGGCTTTTTTTTCAACTAATAAACCAGCATGTAGAAAATAACATTCACTACTTGGCT
AATACXXXXXXXXXXTGTATCAACTCTTTGCTCCAATATGTTTTGAATTCCGTATTGCGAAAACAACATTCTTTT
GAACAAGTGGTTTTGAAGTC
AACAAAATGATAGAACAAGGGCTTTTTTTTCAACTAATAAACCAGCATGTAGAAAATAACATTCACTACTTGGCT
AATACXXXXXXXXXXTGTATCAACTCTTTGCTCCAATATGTTTTGAATTCCATATTGCGAAAACAACATTCTTTT
GAACAAGTGGTTTTGAAGTC
>Marker764496
AACAAGCTGCTCGCCTCGTCCAAGTAGATATTCAAAGAAGAGAAAGAATCAAGGAGTTAATAATAAATGGAAGTG
ATCTAXXXXXXXXXXAGCTGGTGGAGAAGATGGAACCAGCATGAACAGAGATGCCCTCACTGGGAGCAAGATCTA
CTTGACATACAACACAAGTG
AACAAGCTGCTCGCCTAGTCCAAGTAGATATTCAAAGAAGAGAAAGAATCAAGGAGTTAATAATAAATGGAAGTG
ATCTAXXXXXXXXXXAGCTGGTGGAGAAGATGGAACCAGCATGAACAGAGATGCCCTCACTGGGAGCGAGATCTA
CTTGACATACAACACAAGTG
>Marker765199
GACTTTTTCACAATTTCACGACCATTTCTAATGTTTGGTGTAGAATGAATTTGTTGAGCAAATGGTCATTTTCAT
ATTTGXXXXXXXXXXGTAACTAATAGCCTTTTCTTAAATATCTTCTTCTCGCATAATACTCTTTCGATGGATAAA
TTTTCTTGAATAACAATGTA
GACTTTTTCACAATTTCACGACCATTTATAATGTTTGGTGTAGAATGAATTTGTTGAGCAAATGGTCATTTTCAT
ATTTGXXXXXXXXXXGTAACTAATAGCCTTTTCTTAAATATCTTCTTCTCGCATAATACTCTTTCGATGGATAAA
TTTTCTTGAATAACAATGTA
>Marker765311
TACTTCCAACACAGACAAAGCTCAGACACGTGAAAACTGAAGCCAATCTTATAAAATTTTTGGAAAACAAATGGC
ACAAGXXXXXXXXXXACGTACGTAAGGTCTTTAGTTAAAAGTTAAGCTGCAATTCAATCAGAAAATAAATATCCA
CAAACCCTTTAATTGATGTG

Create PDF with GO2PDF for free, if you wish to

TACTTCCGACACAGACAAAGCTCAGACACGTGAAAACTGAAGCCAATCTTATAAAATTTTTGGAAAACAAATGGC
ACAAGXXXXXXXXXXACGTACGTAAGGTCTTTAGTTAAAAGTTAAGCTGCAATTCAATCAGAAAATAAATATCCA
CAAACCCTTTAGTTGATGTG
>Marker765463
CACAAATAACACCGTCACAGCGCCTACGTGTCAGTAACAGATTTTTTTCATTATAATTAGATCAAACCATGCCAC
CAAAAXXXXXXXXXXATTTCATTTTTAATTAAAAATGATATGCAATACACAAGATAAATTAATATATCATTTGAT
GATATGGCCAAAATTTTAGT
CACAAATAACACCGTCACAGCGCCTACGTGTCAGTAACAGATTTTTTTCATTATAATTAGATCAAACCATGCCAC
CAAAAXXXXXXXXXXATTTCATTTTTAATTAAAAATGATATGCAATACACAAGACAAATTAATATATCATTTGGT
GATATGGCCAAAATTTTAGT
>Marker766095
ACAAAGAAATGAACACAAAATCCCAGAAAGGAACCTGAAAATCGTAGAACTTAGCAAACCGAGGCTTGCCCTCAG
TATTCXXXXXXXXXXTGCATAAAACATACCAAATACAAACTATAACAACTGCACGGTTCAGTGGAAAAAAATTCT
TACAGATGATTTTTAAGGTA
ACAAAGAAATGAACACAAAATCCCAGAAAGGAACCTGAAAATCGTAGAACTTAGCAAACCGAGGCTTGCCCTCAG
TATTCXXXXXXXXXXTGCATAAAACATACCAAATACAAACTATAACAACTGCACGGTTCAGTGGAAAAAAATTCT
TACAAATGATTTTTAAGGTA
>Marker766665
GACCTTTAAGCCTGGATTTTTATTGGACGTTTGTTTTTTTTCTTCAGTTTCCTTCAAAGAATATTGGTTTTGCAG
AAAGAXXXXXXXXXXGCTTTTGAGGTCTTTGCTTGTTTTGGTTCTATTATATGTGTGTCACTCTATTATGGGTTC
TAGCAAATTTGTTTCTTTGT
GACCTTTAAGCCTGGATTTTTATTGGACGTTTGTTTTTTTTCTTCAGTTTCCTTCAAAGAATATTGGTTTTGCAG
AAAGAXXXXXXXXXXGCTTTTGACGTCTTTGCTTGTTTTGGTTCTATTATATGTGTGTCACTCTATTATGGGTTC
TAGCAAATTTGTTTCTTTGT
>Marker766681
TACTTTGCTTCCATTGTCTCTATTTGAAGTCCAATGTGATCAAATGAAATTAGAAAAGAAAAGAATTTGAAGAAC
AAGAGXXXXXXXXXXGAAAAAAGAAAGAATGTGAGTTTGGTTATAAGAAAGGGGAAATTGAAAGACTTGTTTTCA
AATGAAGCACCAAACTTTGT
TACTTTGCTTCCATTGTCTCTATTTGAAGTCCAATGTGATCAAATGAAATTAGAAAAGAAAGGAATTTGAAGAAC
AAGAGXXXXXXXXXXGAAAAAAGAAAGAATGTGAGTTTGGTTATAAGAAAGGGGAAATTGAAAGACTTATTTTCA
AATGAAGCACCAAACTTTGT
>Marker766791
CACAGTGAAAAATAGTCCTTTTATTGATTTGACATTTTGATTTCAAACGACAAAAATTGAATAGGCTGGAACATA
GGAGCXXXXXXXXXXCATCTAGTAGGTTCTCTCTGTGGATGTCAAGGGGTGAAACCTGTTGAATAGCACCAGGTA
ATTAAGTAATTTAGTAATGT
CACAGTGAAAAATAGTCCTTTTATTGATTTGACATTTTGATTTCAAACGACAAAAATTGAATAGGCTGGAACATA
GGAGCXXXXXXXXXXCATCTAGTAGGTTCTCTCTATGGATGTCAAGGGGTGAAACCTGTTGAATAGCACCAGGTA
ATTAAGTAATTTAGTAATGT
>Marker766822
ACTCGTTATGTGAAGCATGGTGTTCCATCTTTTAAATATTAGGTCAGGAAATATTTTTCTTTTTTCTTTATTGAG
GTTGTXXXXXXXXXXTTATTTATGTGTTCTACCTCAGTGATTCGATAGGAATGTAAATTACCACTACTTAACTAA
CATGCAACATACATGTATGT
ACTCGTTATGTGAAGCATGGTGTTCCATATTTTAAATATTAGGTCAGGAAATATTTTTCTTTTTTCTTTATTGAG
GTTGTXXXXXXXXXXTTATTTATGTGTTCTACCTCAGTGATTCGATAGGAATGTAAATTACCACTACTTAACTAA
CATGCAACATACATGTATGT
>Marker766863

Create PDF with GO2PDF for free, if you wish to

CACTCAATACCTTCAACTTCTCATTTTTCCCCTTTCTAACAGCATTAAGATCATGGGAAGCCAAAGATATTGGAA
TGGCTXXXXXXXXXXTACAATTTGTTTGGTATCTCTATTAAATGATTGTTCTTGACAACCGTTGAGTGTATTGTC
AAGATATATTTGTTTTTGTT
CACTCAATACCTTCAACTTCTCATTTTTCCCCTTTCTAACAGCATTAAGATCATGGGAAGCCAAAGATATTGGAA
TGGCTXXXXXXXXXXTACAATTTGTTTGGTATCTCTATTAAATGATTGTTCTTGACAACTGTTGAGTGTATTGTC
AAGATATATTTGTTTTTGTT
>Marker767366
CACGCAAAGCTTTTTCCATTGTCATTCATTATAATTCACAAACATATCATCGTTACCGTTTTTTGTCCTATTTTC
CAGTTXXXXXXXXXXGACGAGTGAAACCCGACTCTTCATCTTCTGATGAAGCTATGTCCCCTACATCTGTTCCCA
ACATTGTTGGGACAATTGGT
CACGCAAAGCTTTTTCCATTGTCATTCATTATAATTCACAAACATATCATCGTTACCGTTTTTTGTCCTATTTTC
CAGTTXXXXXXXXXXGACGAGTGAAACCCGAGTCTTCATCTTCTGATGAAGCTATGTCCCCTACATCTGTTCCCA
ACATTGTTGGGACAATTGGT
>Marker767975
ACTATGTCATGGAAGTCAGAAACTGCAGCATTTTTCTCATAGGAGCTGGAAACTCACGTTTAGGAATCGATGAGT
AGAATXXXXXXXXXXATTCAAATTGTTGAAAACAAATGTGTTCTGCTTCAAACCAAAATTCGACACGACGTGGGT
GCTAAATTGCTTCCAAGGTT
ACTATGTCATGGAAGTCAGAAACTGCAGCATTTTTCTCATAGGAGCTGGAAACTCACGTTTAGGAATCGATGAGT
AGAATXXXXXXXXXXATTCAAATTGTTGAAAACAAATGTGTTCTGCTTCAAAGCAAAATTCGACAGGACGTGGGT
GCTAAATTGCTTCCAAGGTT
>Marker768027
TACCGAGTAATGAGTTTTCTTACTTCGCATCTCTATTTCTCCACCACATTTATTTTCTCATGGTTGATTCAAGTA
TTAGGXXXXXXXXXXTATCTCTAGACATAACACAGCTAACTTGCATGCCTAATTGTGTTTCAACGTGCCAATGAT
GGTTCCCTTTAGAAACAGTC
TACCGAGTAATGAGTTTTCTTACTTCGCATCTCTATTTCTCCACCACATTTATTTTCTCATGGTTGATTCAAGCA
TTAGGXXXXXXXXXXTATCTCTAGACATAACACAGCTAACTTGCATGCCTAATTGTGTTTCAACGTGCCAATGAT
GGTTCCCTTTAGAAACAGTC
>Marker770162
AACGAATATTGATTAATCAGTTAATCTCGGATCGTTGAAGTCCAAGGTCTATAGATCTAGTAGGTTCCCTTGTTA
GCTCAXXXXXXXXXXTATTTAGGATCTCAAAATTGATGGAAATAATGGTCAAAGTTATAAAAAGCCAAAATAGTG
ATTTCCGACTTTGAAAAGTC
AACGAATATTGATTAATCAGTTAATCTCGGATCGTTGAAGTCCATGGTTTATAGATCTAGTAGGTTCCCTTGTTA
GCTCAXXXXXXXXXXTATTTAGGATCTCGAAATTGATGGAAATAATGGTCAAAGTTATAAAAAGCCAAAATAGTG
ATTTCCGACTTTGAAAAGTC
>Marker770437
TACTTTTTGAGAAAAAATTGAAAAACAAGCCAATTTCCAGGGTGTTATATTTATGAAAATTTCGTGAAAATAATA
AGATTXXXXXXXXXXAACACAAAAGCATAAGCTTTACCTACAACTTTCATGTCTCTCATCCCATTTCAACCTCAT
CGTTGGTTTCAAAATTAGTC
TACTTTTTGAGAAAAAATTGAAAAACAAGCCAATTTCCAGGGTGTTATATTTATGAAAATTTCGTGAAAATAATA
AGATTXXXXXXXXXXAACACAAAAGCATAAGCTTTACCTACAACTTTCATGTCTCTCATCCCATTTCAACCTCAT
CCTTGGTTTCAAAATTAGTC
>Marker770577
AACAAAACACGAGAAGGAATTTATAATTGGACAACGGAATAGCAGAGAAAAGACAAAACATTAAATGAAAATGAA
AAGAAXXXXXXXXXXTCTCGGTTCCTCTTATTTTCTTCCATTGTAAATTATGAAGGGAAAATCTCTCTTGTCATG
CAAGACAAAAGTTCTTAGTG

Create PDF with GO2PDF for free, if you wish to

AACAAAACACGAGAAGGAATTTATAATTGGACAACGGAATAGCAGAGAAAAGACAAAACATTAAATGAAAATGAA
AAGAAXXXXXXXXXXXTCTCGGTTCCTCTTATTTTTTTCCATTGTAAATTATGAAGGGAAAATCTCTCTTGTCATG
CAAGACAAAAGTTCTTAGTG
>Marker772374
CACCTTTATGTATGTGTCGTCCAATGTCCCAAGATAATTCTGTATAATTGGAAAAACTGCAGCTAAGTGCTATGA
CCTTGXXXXXXXXXXXAGCACGAGGTTGAAATGACAAGAAACAGTCTCCTCAAACTGCACAAAGTCTCTTTGGATG
ACTCTACTCTTCACATCGTG
CACCTTTATGTATGTGTCGTCCAATGTCCCAAGATAATTCTGTATAATTGGAAAAACTGCAGCTAAGTGCTATGA
CCTCGXXXXXXXXXXXAGCACGAGGTTGAAATGACAAGAAACAGTCTCCTCAGACTGCACAAAATCTCTTTGGATG
ACTCTACTCTTCACATCGTG
>Marker772527
AACAGATTGATTTTTATATTTAAGTGATGATTGTGTGTGCCACTGCCAATGATAGGCTCAGCTTGCTAGTTAATT
TACATXXXXXXXXXXXTAAAATAAAGGGAATGTTAAATTTTAAAAAACAATCCTGCTTTCTAAAAGAATTGAAAGC
TCAAAAGACATTCTAAGGTT
AACAGATTAATTTTTATATTTAAGTGATGATTGTGTGTGCCACTGCCAATGATAGGCTCAGCTTGCTAGTTAATT
TACATXXXXXXXXXXXTAAAATAAAGGGAATGTTAAATTTTAAAAAACAATCCTGCTTTCTAAAAGAATTGAAAGC
TCAAAAGACATTCTAAGGTT
>Marker772580
CACGTCTTTTCAGACTTCTACAATCATTGTGTCAAAAACAAAAATTGCCTTTCTAATTCAACTAAAATCTATAAA
TAATCXXXXXXXXXXXTAAACACCAAGGAAGTCACGTTTCTTGGTTGTTTTCATATATGGTCGAATCACCCATTCA
TTCTGAGCTTCCGTTTGGTC
CACGTCTCTTCAGACTTCTACAATCATTGTGTCAAAAACAAAAATTGCCTTTCTAATTCAACTAAAATCTATAAA
TAATCXXXXXXXXXXXTAAACACCAAGGAAGTCACGTTTCTTGGTTGTTTTCATATATGGTCGAATCACCCATTCA
TTCTGAGCTTCCGTTTGGTC
>Marker772618
ACTTTCACTTATAAGTCTGAATTTATGGCATCTCTGTATGGTTGCATGATGCTTTCTTATTTGCTTATGCTACTT
TTCATXXXXXXXXXXXATATTTAAGGACTTCACATCTTATAAGCTTCGAGTTCCTGTCACTGGGGGAATTATTTTG
GATGAAACCTTTGAGAGGGT
ACTTTCACTTATAAGTCTGAATTTATGGCATCTCTGTATGGTTGCATGATGCTTTCTTATTTGCTTATGCTACTT
TTCATXXXXXXXXXXXATATTTAAGGACTTCACGTCTTATAAGCTTCGAGTTCCTGTCACTGGGGGAATTATTTTG
GATGAAACCTTTGAGAGGGT
>Marker773329
ACATTTATATCATTTAGAAATTCAAATTTCAAGTTATCATTGATAAATTCATGAAAAACATATTGACATATTAGA
CACAAXXXXXXXXXXXAAAATTTAGCATATGTCTAGTAAAAAATTGTCATTCTTGCAAATATTGTTCGTTGTGCTA
AATTATATTTAATCAATGTT
ACATTTATATCATTTAGAAATTCAAATTTCAAGTTATCATTGATAAATTCATGAAAAACATATTGACATATTAGA
CACAAXXXXXXXXXXXAAAAATTAGCATATGTCTAGTAAAAAATTGTCATTCTTGCAAATATTGTTCGTTGTGCTA
AATTATATTTAATCAATGTT
>Marker773566
CACAAAGCTACCATTAGCTCAATTAACAACCTCTAACAATTAAATTTGTTGGAACAACTTCTTCTAGTATAGGAA
ATGAAXXXXXXXXXXXGTGTCTTAATTTTAACAAGGGAGGAAATGTTTTTTTTTATAAAGCTTGAACCACCTCACT
ATGTCACATTTGAATGGGTG
CACAAAGCTACCATTAGCTCAATTAACAACCTCTAACAATTAAATTTGTTGGAACAACTTCTTCTAGTATAGGAA
ATGAAXXXXXXXXXXXGTGTCTTAATTTTAACAAGGGAGGAAATGTTTTTTTTTATAAAGCTTGAACCACCTCACT
ACGTCACATTTGAATGGGTG
>Marker774268

Create PDF with GO2PDF for free, if you wish to

AACAATAAACAAATCACTCTCCCAGGCTTAAAATTACTTAATTAGGATTTTTTCATCATTTAGTTTCTAATCCTA
TCGTAXXXXXXXXXXXAATTAAATGCTTTGAACTTAACTTCAAAGGTGCTAATTGACATGTAGCTCATATCTTCTT
ACTTTATTTTACAAAAAGTT
AACAATAAACAAATCACTCTCCCAGGCTTAAAATTACTTAATTAGGATTTTTTCATCATTTAATTTCTAATCCTA
TCGTAXXXXXXXXXXXAATTAAATGCTTTGAACTTAACTTCAAAGGTGCTAATTGACATGTAGCTCATATCTTCTT
ACTTTATTTTACAAAAAGTT
>Marker774340
AACCTTCATCCTCATCTCGTAGTGGTTCTAATAGCGTCGAATACTGATTACGAGCATCCGCGAGACTTTGTTTCA
AAATCXXXXXXXXXXXGCTGGATTGCAGTTTTGGAAAAATTCAATTCCACTACTTGTTTGTATAGTTTTAAGTTCG
ATATGTTTTTACATTGTGTG
AACCTTCGTCCTCATCTCGTAGTGGTTCTAATAGCGTCGAATACTGATTACGAGCATCCGCGAGACTTTGTTTCA
AAATCXXXXXXXXXXXGCTGGATTGCAGTTTTGGAAAAATTCAATTCCACTACTTGTTTGTATAGTTTTAAGTTCG
ATATGTTTTTACATTGTGTG
>Marker776452
TACTAAAATTGTGCGTATAGTTATAGATTGAAACAGTGACATAGAAAGTAGGAATGTTTAACTTCATTTGTTGCT
TATTGXXXXXXXXXXXTCTTAACTACAATGGAGAAATTCAAGTAGGAGTGACCTTCACTCAGAAGGTAATTGACCA
TCTTTTAGTTATTGAATGTG
TACTAAAATTGTGCGTATAGTTATAGATTGAAACAGTGACATAGAAAGTAGGAATGTTTAACTTCATTTGTTGCT
TATTGXXXXXXXXXXXTCTTAACTACAATGGAGAAATTCAAGTAGGAGTGACCTTCACTCAGAAGGTAATTGACCA
TCTTTTAGTTCTTGAATGTG
>Marker776626
CACTTGGCTTGAATGCAACAATTGCCTCTTCAACAACAAAGAAACACGTAGGTATCTTCCTTGTTCATGTTCGTA
ACACTXXXXXXXXXXXCCCTCTCTTTGTAATTTCATCCTTTAGTGAAATTGTCTCTTAACAAAAAAGAAAAGTCTC
TTAACAATAGTAATACCGTG
CACTTGGCTTGAATGCAACAATTGCCTCTTCCACAACAAAGAAACACGTAGATATCTTCCTTGTTCATGTTCGTA
ACACTXXXXXXXXXXXCCCTCTCTTTGTAATTTCATCCTTTAGTGAAATTGTCTCTTAACAAAAAAGAAAAGTCTC
TTAACAATAGTAATACTGTG
>Marker777859
GACCAATGCACCTTATTGTTTATTGACTTCAATACGCTATGGAAACCTTTTATTTTCATTTACAATGCAGAGAAG
AAAGTXXXXXXXXXXXGGGGAAGATGCAAGCATCTGTAGCACCTTGATTTCTTGGAAGTTGAAGGGTGCAAAGAAT
TTATAGGGATAAGGTTGGGT
GACCGATGCACCTTATTGTTTATTGACTTCAATACGCTATGGAAACCTTTTATTTTCATTTACAATGCAGAGAAG
AAAGTXXXXXXXXXXXGGGGAAGATGCAAGCATCTGTAGCACCTTGATTTCTTGGAAGTTGAAGGGTGCAAAGAAT
TTATAAGGATAAGGTTGGGT
>Marker778231
AACAAATTAAAGGAATTATTACATTTCTCTTCCATCCAAAACTAATTCAAACGTTCTTTTAAAAAAGTGAAACAA
TAAACXXXXXXXXXXXCCATGCCAAGAATTAAGTTTTGCTGACTGAGAAACTCGAGCTCGGCAGAAACTTCAGAAC
CATTTGCTTGTAAGGCTTGT
AACAAATTAAAGGAATTATTACATTTCTCTTCCATCCAAAACTAATTCAAATGTTCTTTTAAAAAAGTGAAACAA
TAAACXXXXXXXXXXXCCATGCCAAGAATTAAGTTTTGCTGACTGAGAAACTCGAGCTCGGCAGAAACTTCAGAAC
CATTTGCTTGTAAGGCTTGT
>Marker778263
TACATCATTTGATGTTTATATATAATTACTACTACTACTACTATTATTGAATCATATAGTTTAAAATGAAGAAAT
GAAAAXXXXXXXXXXXTTTCTTAGTGGCAATCTCACGAGTCTCAATGCTTTGCCAAAAATTCAATTTTCTTTTTTG
TTTTGTTTCTTTAAATTAGT

Create PDF with GO2PDF for free, if you wish to

TACATCATTTGATGTTTATATATAATTACTACTACTACTACTATTATTGAATCATATAGTTTAAAATGAAGAAAT
GAAAAXXXXXXXXXXXTTTCTTAGTGGCAATCTCACGAGTCTCGATGCTTTGCCAAAAATTCAATTTTCTTTTTTG
TTTTGTTTCTTTAAATTAGT
>Marker778270
ACCAAAAAGAAGCTTTAACCGAGCAATTTTACAACGTTCAGATCAATTGGTTTAGGTTTGATTCCTTTCAAAACA
AATTTXXXXXXXXXXXACAACTTGCAACAAGATGCCATGGAACAACAACAATCACAGAAAAGATGTTGAGAATCTT
CATTGGCCGAGAAAAAAGTG
ACCAAAAAGAAGCTTTAACCGAGCAATTTTACAAGGTTCAGATCAATTGGTTTAGGTTTGATTCCTTTCAAAACA
AATTTXXXXXXXXXXXACAACTTGCAACAAGATGCCATGGAACAACAACAATCACAGAAAAGATGTTGAGAATCTT
CATTGGCCGAGAAAAAAGTG
>Marker778795
ACCAAGATTGCATTCAATCATTGTTGTATTTCCCTGAAGCATTTGAAAATTTTGCAGCATAGTCTAATCAAATAT
CATTCXXXXXXXXXXXCTGCTAACTGCTACAATAATGATCAGGTATGAACAAAAACCAACTAAATATCTTATATTC
ATAATTCAATAATTTTGGTT
ACCAAGATTGCATTCAATCATTGTTGTATTTCCCTGAAGCATTTGAAACTTTTGCAGCATAGTCTAATCAAATAT
CATTCXXXXXXXXXXXCTGCTAACTGCTACAATAATGATCAGGTATGAACAAAAACCAACTAAATATCTTATATTC
ATAATTCAATAATTTTGGTT
>Marker778816
AACTTAAAATTTTACACAAGGGAGTTATGCATACAGAAATATGCCAGGCATGAAGAAAAACATTCGCTCTTAAGA
AAATAXXXXXXXXXXXCCAATCATTGTCACGAGTAGTGTCTGGGTGAGCATAAGTAAGAAATTAGATGATAAAAAA
TTTCAATCACAATTAAGGTT
AACTTAAAATTTTACACAAGGGAGTTATGCATACAGAAATATGCCAGGCATGAAGAAAAACATTCACTCTTAAGA
AAATAXXXXXXXXXXXCCAATCATTGTCACGAGTAGTGTCTGGGTGAGCACAAGTAAGAAATTAGATGATAAAAAA
TTTCAATCACAATTAAGGTT
>Marker778935
ACATAAAGAAGTAAAAGCATAGTTGAAGGGGAAATATGAGAAAAAGACCCCTAAACTCAAGGCATTTTGCTCTTA
GACTTXXXXXXXXXXXCAAGGCCCAAGGTTTTGAGCCTTGGAGATTCACAAAAAAAATGCGCCTTATTTTGTTAGC
CTCACTTTTTCAAGCTCGTG
ACATAAAGAAGTAAAAGCATAGTTGAAGGGGAAATATGAGAAAAAGACCCCTAAACTCAAGGCATTTTGCTCTTA
GACTTXXXXXXXXXXXCAAGGCCCAAGGTTTTGAGCCTTGGAGCTTCACAAAAAAAATGCGCCTTATTTTGTTAGC
CTCACTTTTTCAAGCTCGTG
>Marker779015
ACTCGGTAACTGACATAGTTGATTAAGAGTTTCATTTAACTGGGGAATCTACCTTCTCAGCTAAGTGCCCGGAGC
ATGAAXXXXXXXXXXXGCTTTTTGTTGAAAGGGCAGGCACATTTAAGTTGCAGAAGACCTATCTTATTCTTTTAGA
TGACTACATTGATAACTGTA
ACTCGGTAACTGACATAGTTGATTAAGAGTTTCATTTAACTGAGGAATCTACCTTCTCAGCTAAGTGCCCGGAGC
ATGAAXXXXXXXXXXXGCTTTTTGTTGAAAGGGCAGGCACATTTAAGTTGCAGAAGACCTATCTTATTCTTTTAGA
TGACTACATTGATAACTGTA
>Marker779481
TACACAGTTCATTTTTTCGCAGCACCTCCAAAGTCTTCCCCAAGTGTAACAAATGTTCCTCAAAATGCTTGTTGT
ATATTXXXXXXXXXXXGCTTTCTAAATGTTGTTTTCTCAATATCTTCCCCATACATGCAAATATGATGGTAGTTTT
CCTTTAAATCGATCTTAGTG
TACACAGTTCATTTTTTCGCAGCACCTCCAAAGTCTTCCCCAAGTGTAACAAATGTTCCTCAAAATGCTTGTTGT
ATATTXXXXXXXXXXXGCTTTCTAAATGTTGTTTTCTCAATATCGTCCCCATACATGCAAATATGATGGTAGTTTT
CCTTTAAATCGATCTTAGTG
>Marker779760

Create PDF with GO2PDF for free, if you wish to

TACACATCATCCAAGAGGTTTAGAAATAACTTTGCAATCCATCTTTGTTTTAAATAATATTTGAAAACAAAACCA
ATTTTXXXXXXXXXXXAACTTTTTTTTTTATATTATTATTATTTATTTACTTTTTAGAGAACTAAGCCAAAAATAA
CATAGCCAATCTTTTATGTT
TACACATCATCCAAGAGGTTTAGAAATAACTTTGCAATCCATCTTTGATTTAAATAATATTTGAAAACAAAACCA
ATTTTXXXXXXXXXXXAACTTTTTTTTTTATATTATTATTATTTATTTACTTTTTAGAGAACTAAGCCAAAAATAA
CATAGCCAATCTTTTAAGTT
>Marker780622
GACCTACCCTTTGGATGTAAAGCAAAAGACTGTAAATGGGTTTTGAGAAAGAAACTCAAACTTGATGGATCCGTA
GATAAXXXXXXXXXXXATATGAATGTTAAGACTGTTTTCCTAAATGGTGATTTAGAAGAAGAGATATATATGGAAC
AACCTGAAGGTTTCATAGTT
GACCTACCCTTTGGATGTAAAGCAAAAGACTGTAAATGGGTTTTGAGAAAGAAACTCAAACTTGATGGATCAGTA
GATAAXXXXXXXXXXXAGATGAATGTTAAGACTGTTTTCCTAAATGGTGATTTAGAAGAAGAGATATATATGGAAC
AACCTGAAGGTTTCATAGTT
>Marker780693
CACCATTCGAAACTCGTAATCACCTTCTCTGTTCGAAAACACGTAACACATTCACCTCTGTTCGAAAACGCGTGA
AGACAXXXXXXXXXXXTTCTTCTTTTCAATTTTACTTACTTGTAATCGAACTGTGTTACAGATAATCGAAGAAGGG
AGTCTTCAATTCAGAAAGGT
CACCATTCGAAACTCGTAATCACCTTCTCTGTTCGAAAACGCGTAACACATTCACCTCTGTTCGAAAACGCGTGA
AGACAXXXXXXXXXXXTTCTTCTTTTCAATTTTACTTACTTGTAATCGAACTGTGTTACAGATAATCGAAGAAGGG
AGTCTTCAATTCAGAAAGGT
>Marker781096
ACAAGAGCCTAAGTTTTTACATTCTATAAACTGACTTACGATGTGAGTCTTCAGTTTTTGTTCATACGGCTATAT
GGTCCXXXXXXXXXXXTGTTGCCACGTAGAGGGCACGAAGGGGTGGTCAGGGAGGCAGAGGAGTAGGCGTAATCAG
CTAGAAGGTCAGCCTGTTGT
ACAAGAGCCTAAGTTTTTACATTCTATAAACTGACTTACGATGTGAGTCTTCAGTTTTTGTCCGTACGGCTATAT
GGTCCXXXXXXXXXXXTGTTGCCACGTAGAGGGCACGAAGGGGTGGTCAGGGAGGCAGAGGAGTAGGCGTAATCAG
CTAGAAGGTCAGCCTGTTGT
>Marker782533
ACTTTTTCTTTGTGTTACAGTGGAAAAAGGAGATGGCATCCCACGACGAACCTCCTGGCTATGATCCTCCGACAA
CAGATXXXXXXXXXXXAGTTTATATATTGTGGGGATTTCTTTTGAAGAGCTTAATGGGAGTTAATTTTTTGTTTCT
TTTCCCTGGGTGATATGGTC
ACTTTTTCTTTGTGTTACAGTGGAAAAAGGAGATGGCATCCCACGACGAACCTCCTGGCTATGATCCTCCGACAA
CATATXXXXXXXXXXXAGTTTATATATTGTGGGGATTTCTTTTGAAGAGCTTAATGGGAGTTAATTTTTTGTTTCT
TTTCCCTGGGTGATATGGTC
>Marker783940
TACTCCATCTAGAGAAAACTTTTTGCAGATGAAAAGATAAAAGCATGTCGTATAGAGATAGCAAACCATATATTA
GATGTXXXXXXXXXXXTCAAGTATAAAGGGGTCAGGATTATGGTCTTGCCCAAAGTAATTTTGAGAATGAAATCCA
ACAAGTTGCTTAATTTGGGT
TACTCCATCTAGAGAAAACTTGTTGCAGATGAAAAGATAAAAGCATGTCGTATAGAGATAGCAAACCATATATTA
GATGTXXXXXXXXXXXTCAAGTATAAAGGGGTCAGGATTATGGTCTTGCCCAAAGTAATTTTGAGAATGAAATCCA
ACAAGTTGCTTAATTTGGGT
>Marker784324
AACGGTAAATATATATCTTGTCAAATATTACATTTTTTACAATTTTCATAATTTTTTTTAAATGCTTAATTCTAT
TACAAXXXXXXXXXXXTCCTAAGAAGAAAATGCTTAGCTGATTGCATATTTGCACCCTATTTCCCCCCTGAAGAGC
CAACCAAATTTGCCAATGTT

Create PDF with GO2PDF for free, if you wish to

AACGGTAAATACATATCTGGTCAAATATTACATTTTTTACAATTTTCATAATTTTTTTTAAATGCTTAATTCTAT
TACAAXXXXXXXXXXTCCTAAGAAGAAAATGCTTAGCTGATTGCATATTTGCACCCTATTTCCCCCCTGAAGAGC
CAACCAAATTTGCCAATGTT
>Marker784344
ACTGAACTGAAAGAATGATATGATGGTATCTTGAATTGTAAATTGATCAATTGACTATTTAAAATGTTAGAATGG
ACAAAXXXXXXXXXXCATCCTTCGGGAGCACAAGATTGATATGTGCATCCTTTGGGAGCGTCCTACGAGATCACG
AGACTGTTATGATGCAGGGT
ACTGAACTGAAAGAATGATATGATGGTATCTTGAATTGTAAATTGATCAATTGACTATTTAAAATGTTAGAATGG
ACAAAXXXXXXXXXXCATCCTTCGGGAGCACAAGATTGATATATGCATCCTTTGGGAGCGTCCTACGAGATCACG
AGACTGTTATGATGCAGGGT
>Marker784352
ACTTCTTGTTCTTCAATTTATTTCCTGGTCATTATTAGTTGTCCAGTTGTGATTTGTGATCTATTTTGTTATTTT
ACTTTXXXXXXXXXXCAAATAAGTTTGTTGCATTTATTATTATGATGAAAGTCAGGTCAATGTTGGGAAGATGTG
CATTTATTTTTCCCCAGAGT
ACTTCTTGTTCTTCAATTTATTTCTTGGTCATTATTAGTTGTCCAGTTGTGATTTGTGATCTATTTTGTTATTTT
ACTTTXXXXXXXXXXCAAATAAGTTTGTTGCATTTATTATTATGATGAAAGTCAGGTCAATGTTGGGAAGATGTG
CATTTATTTTTCCCCAGAGT
>Marker784859
AACCTAGGAAGAGTTCTTTTTAACTAAGTGATTGAATTTTAAAATAATTTATTTTAAAAATATTCATGTGTTTTA
CAACTXXXXXXXXXXTCAACCGACCTCCAACCACTATTTTTTTAAGCGGTCATCGTTGGCCAACCTCTGAGCATT
GTTTTACGATGAGCTCTGTT
AACCTAGGAAGAGTTCTTTTTAACTAAGTGATTGAATTTTAAAATAATTTATTTTAAAAATATTCATGTGTTTGA
CAACTXXXXXXXXXXTCAACCGACCTCCAACCACTATTTTTTTAAGCGGTCATCGTTGGCCAACCTCTGAGCATT
GTTTTACGATGAGCTCTGTT
>Marker785644
AACCCTAATTTGTAGATTTCATTCACAATTTTATAAATTTGTATTTTAAATTCTATAGCATTTGAAATTTCCAAT
ACTAAXXXXXXXXXXATTAACTTTATAAATTGGAATATAAGAAAATTTACATTTCCTTCCTTCTTTAATTGTTTT
TCAATCTAAAATGTCACGTC
AACCCTAATTTGTAGATTTCATTCACAATTTTATAAATTTGTATTTTAAATTCTATAGCATTTGAAATTTCCAAT
ACTAAXXXXXXXXXXATTAACTTTATAAATTGGAATATTAGAAAATTTACCTTTCCTTCCTTCTTTAATTGTTTT
TCAATCTAAAATGTCACGTC
>Marker785685
TACCTTGAATGCACTATATGAACTCTTGAAAGGAAGATGAGCATAGAACAAATATATCAACCACATGATAGTCAA
TATGTXXXXXXXXXXGTCGTGAAGGAATTCCAAAAAAGTCATACGATAAATGCGAAATTATTCAGCAAATTGAAA
ATAAATTCGAATATTGTTGT
TACCTTGAATGCACTATATGAACTCTTGAAAGGAAGATGAGCATAGAACAAATATATCAACCACATGATAGTCAA
TATGTXXXXXXXXXXGTCGTGAAGGAATTCCAAAACAGTCATACGATAAATGCAAAATTATTCAGCAAATTGAAA
ATAAATTCGAATATTGTTGT
>Marker785707
ACTCTGTATGGGAGCTTCAGAAATCACACTTCATTAGACACTCAACCCAATATGAGAGCTTTTCTGAATTCTTTT
GACTAXXXXXXXXXXTACAGCTTCCTAGTCACTCTAGCCCAAACACCACAATACAAAGCATCGATTACGTTTCAA
GCAACCGGGAGATGGATGTG
ACTCTGTATGGGAGCTTGAGAAATCACACTTCATTAGACACTCAACCCAATATGAGAGCTTTTCTGAATTCTTTT
GACTAXXXXXXXXXXAACAGCTTCCTAGTCACTCTAGCCCAAACACCACAATACAAAGCATCGATTACGTTTCAA
GCAACCGGGAGATGGATGTG
>Marker786357

Create PDF with GO2PDF for free, if you wish to

ACATTCGGTAATAGTTCTTCACTGCTAACACTGAACACTGAATCTCCATTATCAGACCCTTCATCTACTGCCCTT
GAAATXXXXXXXXXXCGAGAGTAAGAAGCTTCACACAACTCTCTTCCAATAAATCTGAGGTTTCCAAGGCTGTCC
ATGCATTGCGGAAGGATGTA
ACATTCGGTAATAGTTCTTCACTGCTAACACTGAACACTGAATCTCCATTATCAGACCCTTCATCTACTGCCCTT
GAAATXXXXXXXXXXCGAGAGTAAGAAGCTTCACACAACTCTCTTCCAATAAATCTGAGGTTTCCAAGGCTGTCC
ATGCATTGCGGAAGGACGTA
>Marker786725
AACAAAGTGTAAAGGTTGAAACATGGAATACTAAATTGAAACAAAATTTAAATTCAATGATAAAGTTGTAGCGTT
TTGGAXXXXXXXXXXXAGCATTATTGATGATGTTGAAGGTAGTAGGCCTATAAAACCTTACAACACAAACATAAAG
TCTAAATTTGAATGGTTGTT
AACAAAGTGTAAAGGTTGAAACATGGAATACTAAATTGAAACAAAATTTAAATTGAATGATAAAGTTGTAGCGTT
TTGGAXXXXXXXXXXXAGCATTATTGATGATGTTGAAGGTAGTAGGCCTATATAACCTTACAACACAAACATAAAG
TCTAAATTTGAATGGTTGTT
>Marker786998
TACTCCACCTGACATGTTCTCTACGTGTTTCACAAAGTTCTATGCTCCTCTAAGATATTCATTGGTTGCTCTATT
AAAAAXXXXXXXXXXXTATCTTCAAATTATTGATACAAGATTGTGAAATATAATTGAGAACCAACTCTATCCAAAG
TGAAATCGAGAATACTAGTA
TACTCCACCTGACATGTTCTCTACATGTTTCACAAAGTTCTATGCTCCTCTAAGATATTCATTGGTTGCTCTATT
AAAAAXXXXXXXXXXXTATCTTCAAATTATTGCTACAAGAATGTGAAATATAATTGAGAACCAACTCTATCCAAAG
TGAAATCGAGAATACTAGTA
>Marker787744
CACACTCTGTAAGACTCGTGTGAAAACTGCAACTACAACATTGTTAGGTTTTCAAGGAGATTTAACAATGATTGA
TTTTCXXXXXXXXXXAAGTTGAGAGCCGACAGAGATAAGAATGTTGTTCTAGTTTTAGGTTGTGAATTATGTGTC
CAATTATGAACCCGTTGGTT
CACACTCTGTAAGACTCGTGTGAAAACTGCAACTACAACATTGTTAGGTTTTCAAGGAGATTTAACAATGATTGA
TTTTCXXXXXXXXXXAAGTTGAGAGCCGACAAAGATAAGAATGTTGTTCTAGTTTTAGGTTGTGAATTATGTGTC
CAATTATGAACCCGTTGGTT
>Marker787810
CACCAGTTTGTATCTGTATTGATTCACTGCTTATTTATCTCTCCTGCTAGTTGTGGATTTACTTGCCATGATATG
TAGCTXXXXXXXXXXGGTGCAATTTGCGACAAACCACATAGGTAAGTTTATGTGATAGATCATTTATTGCATAAT
GAAAACTTCTAATCTCTGTG
CACCAGTTTGTATTTGTATTGATTCACTGCTTATTTATCTCTCCTGCTAGTTGTGGATTTACTTGCCATGATATG
TAGCTXXXXXXXXXXGGTGCAATTTGCGACAAACCACATAGGTAAGTTTATGTGATAGATCATTTATTGCATAAT
GAAAACTTCTAATCTCTGTG
>Marker788070
AACAATTTTAAAATTTGCAATAATATTTCTATCAATTTCCACCTAATTTGATTATTCCAAAATTACAAGACTAAT
CCAACXXXXXXXXXXTTGGATGATCTTGAATTCAAATACAAACTTAGATCCCACAACCTAAACCAAAATACAATA
ATGTCAAGTTAAAATGGGTT
AACAATTTTAAAATTTGCAATAATATTTCTATCAATTTCCACCTAATTTGATTATTCTAAAATTACAAGACTAAT
CCAACXXXXXXXXXXTTGGATGATCTTGAATTCAAATACAAACTTAGATCCCACAACCTAAACCAAAATACAATA
ATGTCAAGTTAAAATGGGTT
>Marker788114
AACAACACTAAACCAAATTCATTTTAGTAAAGAAAAAATCGTTATCAACTTCTAACATCCATTTATGTATAGCTA
ATTGTXXXXXXXXXXTAAAAAATAGTTGGATTAAATTAGTGAATAGAAATATAATTAAATTTACCTTAATCTATT
CAGTTTAAAAACACAGTGTT

Create PDF with GO2PDF for free, if you wish to

AACACCACTAAACCAAATTCATTTTAGTAAAGAAAAAATCGTTATCAACTTCTAACATCCATTTATGTATAGCTA
ATTGTXXXXXXXXXXXTAAAAAATAGTTGGATTAAATTAGTGAATAGAAATATAATTAAATTTACCTTAATCTATT
CAGTTTAAAAACACAGTGTT
>Marker788148
AACATTAAAAATATGAATTGGTAGCATGTTTTGGAGTGGGAAGTCTATGCATCATGAAATTTGCTATAGCTTGTC
AGTATXXXXXXXXXXXTCTAGAACTCAGTGGTGATAATTCATTTTTGGGGATAATGCAGAGACTAGGAATGCGACA
GGTGGCTTCAGACGGTGGTG
AACATTAAAAATATGAATTGGTAGCATGTTTTGGAGTGGGAAGTCTATGCATCATGAAATTTGCTATAGCTTGTC
AGTATXXXXXXXXXXXTCTAGAACTCAGTGGTGATAATTCATTTTTGGGGATAATGCAGAGACTAGGAATGCGTCA
GGTGGCTTCAGACGGTGGTG
>Marker788511
AACGTTTGAGGTTAATTTCAGTTGTATTCGTATCATTTTATAAAAGGTCGCTTCTAGCTAGCTACCTACCTACCT
AGCTAXXXXXXXXXXXGATATGACTGGTAAAGAAAAATAAATATCAAACATGAGAATTATACTAATAACGCCCTTC
ACTACCAACAGTTTTAAGTA
AACGTTTGAGGTTAATTTCAGTTGTATTCGTATCATTTTATAAAAGGTCGCTTCTAGCTACCTACCTACCTACCT
AGCTAXXXXXXXXXXXGATATGACTGGTAAAGAAAAATAAATATCAAACATGAGAATTATACTAATAACGCCCTTC
ACTACCAACAGTTTTAAGTA
>Marker788933
TACATGTTAGATGTTTCATGAATTGAATCCATATTTTTAACTTATAATAGTTAGGAAATAGGCGATGTTTTAATT
TGTTTXXXXXXXXXXXAATTTGCTTATAATAAATGTTGCAATTTTTTTCAATCCAATCATGACAACTAAGATAGAT
TAGACCCTCTCACTTTAAGT
TACATGTTAGATGTTTCATGAATTGAATCCATATTTTTAACTTATAATAGTTAGGAAATAGTCGATGTTCTAATT
TGTTTXXXXXXXXXXXAATTTGCTTATAATAAATGTTGCAATTTTTTTCAATCCAATCATGACAACTAAGATAGAT
TAGACCCTCCCACTTTAAGT
>Marker790327
AACAAGGATTGTTGATGAAGCTGGTCCCTCATCAAGAGTTGTTGAATCCAACACATCAGGTTAGTCTCATCTTTC
TTAATXXXXXXXXXXXATTGTCCTATAAACAGGCAATGAATGGTGTCGATAAAGATCAATGGGTCAAAGCCATGAA
CCTTGAAATGGAGTTTATGT
AACAAGGATTGTTGATGAAGCTGGTCCCTCAACAAGAGTTGTTGAATCCAACACATCAGGTTAGTCTCATCTTTC
TTAATXXXXXXXXXXXATTGTCCTATAAACAGACAATGAATGGTGTCGATAAAGATTAATGGGTCAAAGCCATGAA
CCTTGAAATGGAGTTTATGT
>Marker791000
ACATGCCTACCAAAGATTTCTACCACTTTCTGGAATTTAATTCTAAAAAATGATTCCTCTTAAGAATTTCTTTAA
ACTTTXXXXXXXXXXXTTTCTGATTGAAGCCAACTGCAGAAGACAGATTCCCTAAAATTGTTATTTTCATGTTCTG
GTGTTTGTTTTTGCAATGTT
ACATGCCTACCAAAGATTTCTACCACTTTCTGGAATTTAATTCTAAAAAATGATTCCTCTTAAGAATTTCTTAAA
ACTTTXXXXXXXXXXXTTTCTGATTGAAGCCAACTGCAGAAGACAGATTCCCTAAAATTGTTATTTTCATGTTCTG
GTGTTTGTTTTTGCAATGTT
>Marker792101
GACAAGAACTTTAGCTATCTCCAGGTAAAAGATTCTACTATTAGACCCTTGATATTATGTTTCCCCTTATTTTGC
ATTAAXXXXXXXXXXXATGAAATATACCATGGTTGAGATATATTTTGATTGAAATATGATATTTTTGCGATGTTAT
ATGTATATGCCTGTGTGGTC
GACAAGAACTTTAGCTATCTCCAGGTAAAAGATTCTACTATTAGACCCTTGATATTATGTTTCCCCTTATTTTGC
ATTAAXXXXXXXXXXXATGAAATATACCATGGTTGAGATATATTTTGATTGAGATATGATATTTTTGCGATGTTAT
ATGTATATGCCTGTGTGGTC
>Marker792146

Create PDF with GO2PDF for free, if you wish to

AACCACACATAAGTATGTAATGACCAACTGTATGTGTGTTCAAGGATATATATGCAATATGTGTTAGTTATTTGA
CAGAAXXXXXXXXXXXTATTTCAAATTGTATAATTTTTCTTTTGAAAAAAAAAAGCTCTTGACACGCAAAATGTGT
CAAGTATTACCTAAGTTGTC
AACCACACATAAGTATGTAATGACCAACTATATGTGTGTTCAAGGATATATATGCAATATGTGTTAGTTATTTGA
CAGAAXXXXXXXXXXXTATTTCAAATTGTATAATTTTTCTTTTGAAAAAAAAAAGCTCTTCACACGCAAAATGTGT
CAAGTATTACCTAAGTTGTC
>Marker792751
CACACTCCTGCGAGAGAAGCAATGATTCTCTCAGTTCTTGTCACTGCATCATGGATGTTCGCTGTGATACTTCTA
ACATGXXXXXXXXXXXACCGATATGCTGCAATTCGAGCAGCATTTGGAACAGCTTTTGTTACAAGTGAATTCCAAG
TAGGAACCTAGTATTTTGTG
CACACTCCTGCAAGAGAAGCAATGATTCTCTCAGTTCTTGTCACTGCATCATGGATGTTCGCTGTGATACTTCTA
ACATGXXXXXXXXXXXACCGATATGCTGCAATTCGAGCAGCATTTGGAACAGCTTTTGTTACAAGTGAATTCCAAG
TAGGAACCTAGTATTTTGTG
>Marker793882
AACCACAACCAAAAAAAGAAAACAACAACAACAAGTTAGCTTGCTTTTAAATATAAACAGTGAAAAATAGGAATT
AGACCXXXXXXXXXXXTTCGAGAGACGACCAGCAGAATAACTCATAGAACCAAAGCTAAACAATCTTAAACAAATA
TAGTCTTAGGAAAGAATGTA
AACCACAACCAAAAAAAGAAAACAACAACAACAAGTTAGCTTGCTTTTAAATATAAACAGTAAAAAATAGGAATT
AGACCXXXXXXXXXXXTTCGAGAGACGACCAGCAGAATAACTCATAGAACCAAAGCTAAACAATCTTAAACAAATA
TAGTCTTAGGAAAGAATGTA
>Marker794617
ACAATACTGCAAAATGCCCTCTAAGTTGAGAGTAGGTTCCATTTGTCAAAGACTCTCCATCATGTAAGAGGAGAT
GCCATXXXXXXXXXXXGAGGTTGAGGCATAATGCTACTTGATAAGGTGGTAGCCTAAGTAGCCTTAAGGAATAATC
CTCCACGAATCAATATCGTG
ACAATACTGCAAAATGCCCTCTAAGTTGAGAGTAGGTTCCATTTGTCAAAGACTCTCCATCATGTAAGAGGAGAT
GCCATXXXXXXXXXXXGAGGTTGAGGCATAATGCTACTTGATAAGGTGGTAGCCTAAGTAGCCTTAAGGAATAATC
CTCCACGAATCATTATCGTG
>Marker795409
ACAGTAGTAAAAGAACACAGACGAACGACTGCTTCGAACTCAAACTTTTGTCTAAAAGGTCAGAGGATATAACAA
GTCAAXXXXXXXXXXXTAAATATGACACCAAAATCAAATCGAAAAATAATATAAATAGGAAAAGAAATGTCTTGCA
AATTTTACAACTTTCACGTA
ACAGTAGTAAAAGAACACAGACGAACGAATGCTTCGAACTCAAACTTTTGTCTAAAAGGTCAGAGGATATAACAA
GTCAAXXXXXXXXXXXTAAATATGACACCAAAATCAAATCGAAAAATAATATAAATAGGAAAAGAAATGTCTTGCA
AATTTTACAACTTTCACGTA
>Marker795562
TACTTGATAACAACCTAGGCAGCCAGTGACCCAGAAAATTTAAACTTAAACAAGTCACGTAGCAAAACTATCAGT
CAAACXXXXXXXXXXXGCGAACACGAATGCAACCAGGATCCACAAATACAGTGTAATTGACCCCAAACAGATACAA
CATATAAAATGTTATAGGTA
TACTTGATAACAACCTAGGCAGCCAGTGACCCAGAAAATTTAAACTTAAACAAGTCACATAGCAAAACTATCAGT
CAAACXXXXXXXXXXXCCGAACACGAATGCAATCAGGATCCACAAATACAGTGTAATTGACCCCAAACAGATACAA
CATATAAAATGTTATAGGTA
>Marker796043
TACGGTTACTATGCAACAATTTAATTAGTCTCTACTATATACACTAGAATACTTTTTTTTTTAAAAAAAAATTTG
ATTAAXXXXXXXXXXXGATTAGAATATGTGACTAAATGATCAAATGGGTCGTGGGTTAGGTCAGGTCAAGGGTGTC
CAGTTCTACCAAAAGGATGT

Create PDF with GO2PDF for free, if you wish to

TACGGTTACTATGCAACAATTTAATTAGTCTCTACTATATACACTAGAATACTTTTTTTAAAAAAAAAAAAATTTG
ATTAAXXXXXXXXXXXGATTAGAATATGTGACTAAATGATCAAATGGGTCGTGGGTTAGGTCAGGTCAAGGGTGTC
CAGTTCTACCAAAAGGATGT
>Marker796513
CACTAACTAATCATGAGTATAATACTCTATACGAAGTTTGGCATTCATACACCGATATCAACTCATGAGTTTTGA
TTGCAXXXXXXXXXXXGGTTTTCCAAGTCGGTAAGAGAGAAAAGGACGACGACGCTCCAAAATTTCAACGTTTCAT
CTCCTTTGTCCAAGCTTGTG
CACTAACTAATCATGAGTATAATACTCTATACGAAGTTTGGCATTCATACACCGATATCAACTCATGAGTTTTGG
TTGCAXXXXXXXXXXXGGTTTTCCAAGTCGGTAAGAGAGAAAAGGACGACGACGCTCCAAAATTTCAACGTTTCAT
CTCCTTTGTCCAAGCTTGTG
>Marker797107
GACAAAAGCAGGAAGAGAATTTGCTTGGTTTGGTGACTAATATTTAGATGAATTTAGCTACTCTTAGGAAGTATC
CAATAXXXXXXXXXXXTGAAGATGATGGTGTGTATCAGTGTATGTGTTTCCAGCTTGTTCTAATGATGATAGGCGA
TAGTGTGGTATAGTTCATGT
GACAAAAGCAAGAAGAGAATTTGCTTGGTTTGGTGACTAATATTTAGATGAATTTAGCTACTCTTAGGAAGTATC
CAATAXXXXXXXXXXXTGAAGATGATGGTGTGTATCAGTGTATGCGTTTCCAGCTTGTTCTAATGATGATAGGCGA
TAGTGTGGTATAGTTCATGT
>Marker797510
ACAAAGGATTTAGGCTCCCAAAAACAACTGAAGTGCTAATATAAAAAATATAGTTATGTTAGATTCTGTGAAGTT
GGCAAXXXXXXXXXXXCAATAATTTTATCGGTCTTCTTCCTATTTCAGTGAGTGACCGTTTTGGAAAGTAGGGCAG
CTTCTTCATGATTCCATTGT
ACAAAGGATTTAGGCTCCCAAAAACAACTGAAGTGCTAATATAAAAAATATAGTTATGTTAGATTCTGTGAAGTT
GGCAAXXXXXXXXXXXCAATAATTTTATCGGTCTTATTCCTATTTCAGTGAGTGACCGTTTTGGAAAGTAGGGCAG
CTTCTTCATGATTCCATTGT
>Marker798351
ACCAAAAAGGAAAATACTAATGGAAAATACTAATAATCCATAGAACTCTAATTTTCCTTTAACACCATTCAATAT
TAAATXXXXXXXXXXXAATGAGAAAATGGTGAAAAAAGTTCGAAGAAAATGTTGTTAGGTAATCGATGTAGGGAGA
ATTATATTATTCTTTGGGTA
ACCAAAAAGGAAAATACTAATGGAAAATACTAATAATCCATAGAACTGTAATTTTCCTTTAACACCATTCAATAT
TAAATXXXXXXXXXXXAATGAGAAAATGGTGAAAAAAGTTCGAAGAAAATGTTGTTAGGTAATCGATGTAGGGAGG
ATTATATTATTCTTTGGGTA
>Marker799497
CACGTTTAATTCTTCTTTGATAATTTTTTCATAGACCTTTTTCCACCTTCTATCATTATCAGTTTGATGCAGAAA
AATTTXXXXXXXXXXXCTTTTTTTGCTTAGAAAATGGTGAAGAGAAGGAGGAGGTTAAACATTCTAACAATGACTT
CAAAGATGATTATGTAAGTA
CACGTTTAATTCTTCTTTGATAATTTTGTCACAGACCTTTTTCCACCTTCTATCATTATCAGTTTGATGCAGAAA
AATTTXXXXXXXXXXXCTTTTTTTGCTTAGAAAATGGTGAAGAGAAGGAGGAGGTTAAACATTCTAACAATGACTT
CAAAGATGATTATGTAAGTA
>Marker799604
TACCAAAGATAGAGGTCTATCACTGATATGGACTATGTTGAAAATATTGGTCTATCTGATAGACCATCAACGATA
GAAATXXXXXXXXXXXTCATATACCTAAGAATCCAAAAGCACTCTTAACTCCTATGTTTTTGGACTATTTTTCGCC
ATTTTCGATATTGATAAAGT
TACCAAAGATAGAGGTCTATCACTGATATGGACTATGTTGAAAATATTGGTCTATCTGATAGACCATCAACGATA
GAAATXXXXXXXXXXXTCATATACCTAAGAATCCTAAAGCACTCTTAACTCCTATGTTTTTGGACTATTTTTCGCC
ATTTTCGATATTGATAAAGT
>Marker799865

Create PDF with GO2PDF for free, if you wish to

TACCTTTTAGGAGGGTTCTCTCTACATTGACCCTTAGGTTGTTCCTCATTTTTTGTGGAATATGCTTCTCTGAAG
TTTCTXXXXXXXXXXCTCATGGCGCACTAAGGTGCAGTGGCCCTCTGGAGCCAGGCGCACAAAAGGCGCAAGGCT
TTTCTTCTCTTTTTGAGGTG
TACCTTTTAGGAGGGTTCTCTCTACATTGACCCTTAGGTTGTTCCTCATTTTTTGTGGAATATGCTTCTCTGAAG
TTTCTXXXXXXXXXXCTCATGGCACACTAAGGTGCAGTGGCCCTCTGGAGCCAGGCGCACAAAAGGCGCAAGGCT
TTTCTTCTCTTTTTGAGGTG
>Marker800054
ACCCGCCTCAAGAAGCAATATTACGCAGTAGCCAAATATTAATGCCAAAAGAAGTTTAAAGAATTGTAATTGTAA
TTTTCXXXXXXXXXXTTTGATATTTAAGTTATGCGAAAAGAAATAAGTTGTTTCGCTAATGGCTAGACGTTTAAT
GTGTTACCTCGCAAGAAGTG
ACCCGCCTCAAGAAGCAATATTACGCAGTAGCCAAATATTAGTGCCAAAAGAAGTTTAAAGAATTGTAATTGTAA
TTTTCXXXXXXXXXXTTTGATATTTAAGTTATGCGAAAAGAAATAAGTTGTTTCGCTAATGGCTAGACGTTTAAT
GTGTTACCTCGCAAGAAGTG
>Marker800342
TACTTGTATTTACTCTGCTTTTATTTATTTTCAGGGTTAAGTTGGATATATAACAAACTATATCATACATAAATG
ATTTCXXXXXXXXXXGGCACCCAAAAGTTACATTATAAAATGCTTTCGTAACAATTTGAAAATATCAATATCAAT
GTAAAAGGGAAATTTTGGTT
TACTTGTATTTACTCTGCTTTTATTTATTTTCAGGGTTAAGTTGGATATATAACAAACTATATCATACATAAATG
ATTTCXXXXXXXXXXGGCACCCAAAAGTGACATTAAAAAATGCTTTCGTAACAATTTGAAAATATCAATATCAAT
GTAAAAGGGAAATTTTGGTT
>Marker800481
CACGCGAAAAAAAACTGGAGACTCAAGAACAACTAGTGGTTTCTTCTTTATTGATTATCAAGAAAAATTAAAAGG
GTAGAXXXXXXXXXXGTAAAGGGAATATTCAAGAAGTTAGGGTGAAAATTCCTTCATCTATAATTTCTTATCAAG
TTGTTGTTCTTGTAGTTGTG
CACGCGAAAAAAAACTGGAGACTCAAGAACATCTAGTGGTTTCTTCTTTATTGATTATCAAGAAAAATTAAAAGG
GTAGAXXXXXXXXXXGTAAAGGGAATATTCAAGAAGTTAGGGTGAAAATTCCTTCATCTATAATTTCTTATCAAG
TTGTTGTTCTTGTAGTTGTG
>Marker801055
GACTCTAAATAGCAGATCATCTCTAAAACAAAAAACAAATAGAAGGTTTGGAAGGGGAACAATCATCTCATACCA
AATGGXXXXXXXXXXATTTTAAAATTTATGAACTACTGATTTTGAGAGAACTTTTTATTTGATAATCAATTTTGA
CTAAGATATGGTAGATTGGT
GACTCTTACTAGCAGATCATCTCTAAAACAAAAAACAAATAGAAGGTTTGGAAGGGGAACAATCATCTCATACCA
AATGAXXXXXXXXXXATTTTAAAATTTATGAACTACTGATTTTGAGAGAACTTTTTATTTGATAATCAATTTTGA
CTAAGATATGGTAGATTGGT
>Marker801403
CACTTCCACCAACTACACGATCCATTTGGATCATATTTGTATTGATATATGTTTATATTCTTCATATAGAAGAAG
AAGCTXXXXXXXXXXGCTCTACATGATATAATATAATAACAAATACATAGTCATACCGTTCATCTATACAATACT
AAAACATCACTCAAAACAGT
CACTTCCACCAACTACACGATCCATTTGGATCATATTTGTATTGATATATGTTTATATTCTTCATATAAAAGAAG
AAGCTXXXXXXXXXXGCTCTACATGATATAATATAATAACAAATACATAGTCATACCGTTCATCTATACAATACT
AAAACATCACTCAAAACAGT
>Marker801650
AACTCTTAGGGACGCTAGAGCTAAAAGTATGCTACTTTTCTCTAGCTAAATTGTTTTATAACGATTTTAAGGGTA
TTTTAXXXXXXXXXXCGAATTTTAATATACGATGTCTAGGTGATAAACCAGTTTTATTACCTCAGATCTCTCACC
CATGCTCATAAAATTAGGTT

Create PDF with GO2PDF for free, if you wish to

CACTCTTAGGGATGCTAGAGCTAAAAGTATGCTACTTTTCTCTAGCTAAATTGTTTTATAACGATTTTAAGGGTA
TTTTAXXXXXXXXXXCGAATTTTAATATACGATGTCTAGGTGATAAACCAGATTTATTACCTCAGATCTCTCACC
CATGCTCATAAAATTAGGTT
>Marker802044
AACCTATGCCTAATTTAACCTTTTCACTTACTTGCACATCTTCATTGTGTAACATTGTTGGTTGGACAATACTTG
GGGCGXXXXXXXXXXAATTGGAGATATAGATTGACAAATCTCGATGATGCAAATGTTTTCATCATGTTTGTTGGA
TTGAATAAAGTTAATTTGGT
AACCTCTGCCTAATTTAACCTTTTCACTTACTTGCACATCTTCATTGTGTAACATTGTTGGTTGGACAATACTTG
GGGCCXXXXXXXXXXAATTGGAGATATAGATTGACAAATCTCGATGATGCAAATGTTTTCATCATGTTTGTTGGA
TTGAATAAAGTTAATTTGGT
>Marker802377
TACAGTTGCCGAAGATTGGGTAGGGTGCCCAGTTCAGGAGGAATTTTCCCTGAAAGGCGATTTTCATTTAAATGG
AGATAXXXXXXXXXXTAGATTAAAATGCAAGAAATTGGTAAAGCAATTTATACCCGAAATTAAAATTACTTACAA
ATGAGTTAGGCCCTTTAGTG
TACAGTTGCCGAAGATTGGGTAGGGTGCCCAGTTCAGGAGGAATTTTCCCTGAAAGGCGATTTTCATTTAAATGG
AGATAXXXXXXXXXXTAGATTAAAATGCAAGAAATTGGTAAAACAATTTATACCCGAAATTAAAATTACTTACAA
ATGAGTTAGGCCCTTTAGTG
>Marker802693
GACTTTTCACTTTCGGGAGCATAATGATTCTAATATCTGAGATTCAAAAATGGAGGGTTACACTTACAAGAATTA
CTAAAXXXXXXXXXXAGGAGGAATAATCGACTACCCTTCGGCGGGGGTTATTCTAAACCTTTGAAATATCATTGC
AAAACTTTATAATGATGGGT
GACTTTTCACTTTCGGGAGCATAATGATTCTAATATCTAAGATTCAAAAATGGAGGGTTACACTTACAAGAATTA
CTAAAXXXXXXXXXXAGGAGGAATAATCGACTACCCTTCGGCGGGGGTTATTCTAAACCTTTGAAATATCATTGC
AAAACTTTATAATGATGGGT
>Marker803009
CACCCGAACCCATGTAAGGTGCAGAATAGGAGTAACCCTGAGGAGCTTGAACGGGTTTGGCAGCAGATTTCTGGA
GAGAGXXXXXXXXXXTAACTTTTATTTTATGAAAGAAATGTATAAGAAGTGTAGAGAGACTGGTCAAGGAATGTT
ATAAGTAACTGGCGAAAGTG
CACCCGAACCCATGTAAGGTGCAGAATAGGAGTAACCCTGAGGAGCTTGAACGGGTTTGGCAGCAGATTTCTGGA
GAGAGXXXXXXXXXXCAACATTTATTTTATGAAAGAAATGTATAAGAAGTGTAGAGAGACTGGTCAAGGAATGTT
ATAAGTAACTGGCGAAAGTG
>Marker803131
TACTCCTTTATAGTTTCTTTACTTATAAAAAGTTTGTATTATCAAAATCGATATTATTCTATCATATATTACAAA
TTGATXXXXXXXXXXGAAACACTAGTTGTTAGGAGTATTAAGATATAGTAAAGCGTTGAGTAAATGTTGGTAAAG
CATAATTCAAAATTTGGTGT
TACTCCTTTATAGTTTCTTTACTTATAAAAAGTTTGTATTATCAAAACCGATATCATTCTATCATATATTACAAA
TTGGTXXXXXXXXXXGAAACACTAGTTGTTAGGAGTATTAAGATATAGTAAAGCGTTGAGTAAATGTTGGTAAAG
CATAATTCAAAATTTGGTGT
>Marker803269
CACTTAAGCAGTTTTGAATATTTTAAAAATTAGAATTAATCCTTGCTTTGATAATTTTTTTTCACAAGTAGTCCT
CGTAGXXXXXXXXXXATCTTCCTCTCTAATGATGACAAGAAGAGGTTTAGGCCATTCTCACAAGTTTTTGATGAG
AATTTTAAATTTATTTTAGT
CACTTAAGCAGTTTTGAATATTTTAAAAATTAGAATTAATCCTTGCTTTGATAATTTTTTTTCACAAGTAGTCCT
CGTAGXXXXXXXXXXATCTTCCTCTCTAATGATGACAAGAAGAGGTTTAGGCCATTCTAACAAGTTTTTGATGAG
AATTTTAAATTTATTTTAGT
>Marker803274

Create PDF with GO2PDF for free, if you wish to

AACTTTAAAAAAATGTGCAAATTATTGTAAAAATTAAGAAAAATAGAATTTTATCTTAAAAATGAAAAAAAAAAG
AAAAAXXXXXXXXXXXATAGACGTGAAGGCACTGAAAACCCTAGCCGAGGGTGTGGATTCCAAAGAATAATCCCTT
GGAGGAACGTTATCGGATGT
AACTTTAAAAAAATGTGCAAATTATTGTAAAAATTAAGAAAAATAGAATTTTATCTTAAAAATGAAAAAAAAAAA
GAAAAXXXXXXXXXXXATAGACGTGAAGGCACTGAAAACCCTAGCCGAGGGTGTGGATTCCAAAGAATAATCCCTT
GGAGGAACGTTATCGGATGT
>Marker803500
TACTACCGGTTCCTGTTTTAATTTAGGCGATTATCTCATTTTCTAGTATAGTAAGAAACAAACTATTGTCTCTCG
TTTCAXXXXXXXXXXXTTGCTCACAATGATATTTTTCATGAGAGGACAAAACACATAAAAAATGACTGTCACCATC
TCCAAGGCTCTACCCTAGTC
TACTACCGGTTCCTGTTTTAATTTAGGCGATCCTCTCATTTTCTAGTATAGTAATAAACAAACTATTGTCTCTCG
TTTCAXXXXXXXXXXXTTGCTCACAATGATATTTTTCATGAGAGGACAAAACACATAAAAAATGACTGTCACCATC
TCCAAGGCTCTACCCTAGTC
>Marker804126
TACTTACTTTTCATCTCTGCATGCTTTGAGAACAAGGAAGTTTTAGCAAACTCTACTTCTTTGGAATTAGGCTCC
ACCTGXXXXXXXXXXXCAACTTTACCTTCCCGATTAATGTAATTTTTGCGCACGTCGGGTTTTCTGGATCAATCTT
GCCACAAGTTCCAATAGGGT
TACTTACTTTTCATCTCTGCATGCTTTGAGAACAAGGAAGTTTTAGCAAACTCTACTTCTTTGGAATTAGGCTCC
ATCTGXXXXXXXXXXXCAACTTTACCTTCCCGATTAACGTAATTTTTGCGCACGTCGGGTTTTCTGGATCAATCTT
GCCACAAGTTCCAATAGGGT
>Marker804473
AACTTATTCCACGTATGTCTCGTCGTGATGATAGATTTTCTTACTGCAAGCACTAACTCACCAACTTGAAATGAT
CGGAGXXXXXXXXXXXATAGTGTAGCATTGTCTTCAGTAGTTAGCCCTTCTTGGATTGCCATTCTCAAGGATGGAA
TTTCTCTCTCTAGCGGGAGT
AACTTATTCCACGTATGTCTCGTCGTGATGATAGATTTTCTTACTGCACGCACTAACTCACCAACTTGAAATTAT
CGGAGXXXXXXXXXXXATAGTGTAGCATTGTCTTCCGTAGTTAGCCCTTCTTGGATTGCCATTCTCAAGGATGGAA
TTTCTCTCTCTAGCGGGAGT
>Marker804513
ACCAAGCAAGAATCCATATGTTTAATTATTATGATAAGAGTTGAGATTAATTATGAGTGGAAAATGAAAATAATC
TATGGXXXXXXXXXXXATTTTGTTGGAAAACAATTCCATTTTTTTAAAAGAATAAATAATTTTGAAAAAATTTGTT
GTAGATCACAAATAAATGTT
ACCAAGCAAGAATCCATATGTTTAATTATTATGATAAGAGTTGAGATTAATTATGAGTGGAAAATGAAAATAATC
TATGAXXXXXXXXXXXATTTTGTTGGAAAACAATTCCATTTTTTTAAAAGAATAAATAATTTTGAAAAAATTTGTT
GTAGATCACAAATAAATGTT
>Marker804654
TACTCAGACATTTGGAGTCGATTACTCTTAAATTTTTCCACTAGTTGCAAAACTAAATACCGTTAGAGTTCTGCT
AACTTXXXXXXXXXXXAGTAGAGTTTGATCATCAGGCCCACAAACAACAATCCTTATATGCGCGAAAATGGTCACC
GAAGCACGGTTTGATAGGTT
TACTCAGACATTTGGAGTCGATTACTCTTAAATTTTTTCACTAGTTGCAAAACTAAATACCGTTAGAGTTCTGCT
AACTTXXXXXXXXXXXAGTAGAGTTTGATCATCAGGCCTACAAACAACAATCCTTATATGCACGAAAATGGTCACC
GAAGCACGGTTTGATAGGTT
>Marker805334
AACATCCCGATGTTAAGAATCCTCAAATTCACTCAACATTTGAGGTTGGTGAATATTCGGCATAGTCCAACCCTA
ATGTGXXXXXXXXXXXCTCGTTCCCTACTTTATCATTTTCCTATGTGAATGAGAATGTTAGTCCAACCACAATTGG
AGCTATTGTCCAGTCAGGTA

Create PDF with GO2PDF for free, if you wish to

AACATCCCGATGTTAAGAATCCTCAAATTCACTCAACATTTGAGGTTGGTGAATATTCGGCATAGTCCAACCCTA
ATGTGXXXXXXXXXXCTCGTTCCCTACTTTATCATTTTCCTATGTGAATGAGAATGCTAGTCCAACCACAATTGG
AGCTATTGTCCAGTCAGGTA
>Marker805469
CACCGCACAACCCTGGAGTTTTTGGAGGTTTTGTGATAACCTAAATTTTATCTTTGTTGCCTTATACCCTTCTGT
TTGACXXXXXXXXXXTCCTGAATAATTTTTGCAGTCTCTTTCCAGAAATAATAATTTTGTCATGCTATGGTTTTA
TTTTTATTATTTTCATTGTT
CACCGCACAAACCTGGAGTGTTTGGAGGTTTTGTGATAACCTAAATTTTATCTTTGTTGCCTTATACCCTTCTGT
TTGACXXXXXXXXXXTCCTGAATAATTTTTGCAGTCTCTTTCCAGAAATAATAATTTTGTCATGCTATGGTTTTA
TTTTTATTATTTTCATTGTT
>Marker805933
ACTTAGACTTGAGATTCTATGGATTACTTTGGCTACCAGATGTAGTAGGGTGATGGTGACTGTCTAGTTTCAATT
CAAGTXXXXXXXXXXCAAATCAGTCCAAACTCCCACATTGGACTAAATCAATTAGCAATCATCAAATGTAGTTTT
CATTTTGTTTCCTCTTATGT
ACTTAGACTTGAGATTCTATGGATTACTTTGGCTACCAGATGTAGTAGGGTGATGGTGACTGTCTAGTTTCAATT
CAAGTXXXXXXXXXXCAAATCAGTCCAAACACCCACATTGGACTAAATCAATTAGCAATCATCAAATGTAGTTTT
CATTTTGTTTCCTCTTATGT
>Marker806006
ACGTGATGACAGCCATACTAAAAATTGAAATGAAGGCACACAAATCTATAAGTTAAATATGTAAAAAAAACAAAA
TTAACXXXXXXXXXXAACCCTATCTAACTTAATTATAATACAACGATACTAGATGGACACATAGAATCCACTGAC
TACGCAACTACCATTGAGTG
ACGTGATGACAGCCATACTAAAAATTGAAATGAAGGCACACAAATCTATAAGTTAAATATGTAAAAAAAACAAAA
TTAACXXXXXXXXXXAACCCTATCTAACTTAATTATAATACAACGATACTAGATGGACACATAGAATCCACTGAC
TACGCAACTACCACTGAGTG
>Marker806228
TACCTATACTCATGGAAGCTCAGCCCTAATTGAAGACAATTAAACTGATGTCTTGTAGTTCATAAGTTAGCAACT
CTAGCXXXXXXXXXXGGTCCAAATACATCACAGATTAATTTTCAGTTTTTGATGTCTTATGGCTAGCATCCCGTC
ACTATTAATATAAGCAGAGT
TACCTATACTCATGGAAGCTCAGCCCTAATTGAAGACAATTAAACTGATGTCTTGTAGTTCATAAGTTAGCAACT
CTAGCXXXXXXXXXXGGTCCAAATACATCACAGATTAATTTTCAGTTTTTGATGTCTTATGGCTAGCATCCCGTC
ACTGTTAATATAAGCAGAGT
>Marker806257
GACAAAAGATAGACAAACCATACAAATTGAATGAAACTAAATCGGCAGAGTAGTCGCTACCTCTGATTTCTACAA
TATAGXXXXXXXXXXAAAAGCATGAATCGTTTGAGGTTGGGTTAAGTCTGTAAAGAAGATTTTGAAACATTTCAA
ATTTTTTGTTAAAGAGAGTA
GACAAAAGATAGACAAACCATACAAATTGAATGAAACTAAATCGGCAGAGTAGTCGCTACCTCTGATTTCTACAA
TATAGXXXXXXXXXXAAAAGCATGAATCGTTTGAGGTTGGGTTAAGTCTGTAAATAAGATTTTGAAACATTTCAA
ATTTTTTGTTAAAGAGAGTA
>Marker806793
ACCCTCCAACTATATTATTTGAATGCTTTACTCCAATCATCCGCTCTTCTCTATCAACCCTTTTCGTCAGTAAAT
GATCTXXXXXXXXXXGACCCCAGTCCAATACGTAAAGAAAAAGCAGAATAGAGACAAGCTAGTCATGAAATGAAA
TGAAAAGCTTGCCCTTTGTA
ACCCTCCAACTATATTATTTGAATGCTTTACTCCAATCATCCGCTCTTCTCTATCAACCCTTTTCGTCGGTAAAT
GATCTXXXXXXXXXXGACCCCAGTCCAATACGTAAAGAAAAAGCAGAATAGAGACAAGCTAGTCATGAAATGAAA
TGAAAAGCTTGCCCTTTGTA
>Marker806982

Create PDF with GO2PDF for free, if you wish to

CACTGGCTGATCTCGTTGTTCAATAAGCTCATGTTGTAGTTCTAGGAATGACGACTTGGACCTGCACCTATGGTT
ATTATXXXXXXXXXXXCCTGAAAATTGGCAGCTCAAGTAGTGAGCGCAAAGGAAGTGCCAATCATCCAAACGATTG
ACCAATTTGTTTGATGGGTT
CACTGGCTGATCTCGTTGTTCAATAAGCTCATGTTGTAGTTCTAGGAATGACGACTTGGACCTGCACCTATGGTT
ATTAGXXXXXXXXXXXCCTGAAAATTGGCAGCTCAAGTAGTGAGCGCAAAGGAAGTGCCAATCATCCAAACGATTG
ACCAATTTGTTCGATGGGTT
>Marker807213
CACAACCCTAAACACTTTATCGTTTCTCTTTCCATAGATCCCACAACAAAGCACATATCCTCACAAACCAAAGAA
AGCACXXXXXXXXXXXTTGCAAACTAGCCCTCTAAGAGAAAGTGATCTAGGTTTTCCTTTGCCTTCCGATAAAGAA
TGCAACAAAAAGGTTTTGTT
CACAACCCTAAACACTTTATCGTTTCTCTTTCCATAGATCCCACAACAAAGCACATATCCTCACAAACCAAAGAA
AGCGCXXXXXXXXXXXTTGCAAACTAGCCCTCTAAGAGAAAGTGATCTAGGTTTTCCTTTTCCTTCCGATAAAGAA
TGCAACAAAAAGGTTTTGTT
>Marker807594
AACGTTATATCCTGTGAAAGTAAAATTTGTAAATTGTTGGTTCGATTTGAAATATCTTGTTTTTCTCATAATATA
TATTTXXXXXXXXXXXCTGGCTCGATGTCTAGGTATCCAAAAAAGGGACCAAAAAATTCATGATTCTAATTTACAA
ATATCATTATGGGAATGAGT
AACGTTATATCCTGTGAAAGTAAAATTTGGAAATTGTTGGTTCGATTTGAAATATCTTGTTTTTCTCATAATATA
TATTTXXXXXXXXXXXCTGGCTCGATGTCTAGGTATCCAAAAAAGGGACCAAAAAATTCATGATTCTAATTTACAA
ATATCATTATGGGAATGAGT
>Marker807641
AACTGTGCGTTAATAACTAGTTGTGTCTTTTGTGTATTTTGTTATATTTATCAATTTAGTATAAGCTCTAAGAGA
CTAAAXXXXXXXXXXXXATAAAATGATATCTATAAATTTGTTTTAGATGGATTCTTTTCAAATTCAAGAAAACTTCA
AACGAATAATTGATCACAGT
AACTGTGCGTTAATAACTAGTTGTGTCTTTTGTGTATTTTGTTATATTTATCAATTTAGTATAAGCTCTAAGAGA
CTAAAXXXXXXXXXXXXATAAAATGATATCTATAACTTTGTTTTAGATGGATTCTTTTCAAATTCAAGAAAACTTCA
AACGAATAATTGATCACAGT
>Marker807643
TACTTATACCTTCTTCAATCCATCTATGTCTTTCTTGCTCATATGTCCTTATGTTATTTGTAGTTTATGTTTTTT
ATATGXXXXXXXXXXXAGATAACTTTTATCATTTGTATCCTGACAGGAGAACACATGTGGGTATCAAACGCTGAGA
AGTTGCTTCCGTTAAACGTG
TACTTATACCTTCTTCAATCCATCTATGTCTTTCTTGCTCATATGTCCTTATGTTATTTGTAGTTTATGTTTTTT
ATATGXXXXXXXXXXXAGATAACTTTTATCATTTGTATCCTGACAGGAGAACACATGTGGGTATCAAACGCTGAGA
GGTTGCTTCCGTTAAACGTG
>Marker808090
ACTTATTTATGAGTTATTTACATTTTTTACCCAGAAGTTCTTTCACTGAAGAAGAAAATTTGGAATAAAAGTGTG
ACTGAXXXXXXXXXXXGAGGGAAAAAAATTGATCCACAGTCTGCCCAAAATGGCCAAACATGTTTGATGTTAATAA
GAAAATTGAGCTTGATGGGT
ACTTATTTATGAGTTATTTACATTTTTTACCCAGAAGTTCTTTCACTGAAGAAGAAAATTTGGAATAAAAATGTG
ACTGAXXXXXXXXXXXGAGGGAAAAAAATTGATCCACAGTCTGCCCAAAATGGCCAAACATGTTTGATGTTAATAA
GAAAATCGAGCTTGATGGGT
>Marker808570
AACTCATGTTCATAATTCGTTTTGTTGTTTATTAATTTTGAAAGACCAGCACACTCGAGACCAGTGTATAATCTA
GGAGCXXXXXXXXXXXAGAACATAACTAAAAAATGATTCCCGCAGACCTATTCGAATCACAAAGTGTCCTCCATCA
TCAATAAACCCCCCTTTGTT

Create PDF with GO2PDF for free, if you wish to

AACTCGTGTTCATAATTCGTTTTGTTGTTTATTAATTTTGAAAGACCAGCACACTCGAGACCAGTGTATAATCTA
GGAGCXXXXXXXXXXAGAACATAACTAAAAAATGATTCCCGCAGACCTATTCGAATCACAAAGTGTCCTCCATCA
TCAATAAACCCCCCTTTGTT
>Marker808666
ACCCTACCTAATAATGACTGTTTTTTTCTCTATTCTCATTTATATCTTTTTCAAACATAATCAAAATCCTCTAA
CTTTAXXXXXXXXXXTTATATTATTATAATCTCCTCATGTAATCTTCACAATCTCCTCACGAAATCAAATTAAAA
TCAGGACTACCACTAGTGTT
ACCCTACCTAATAATGACTGTTTTTTTCTCTATTCTCATTTATATCTTTTTCAAACATAATCAAAATCCTCTAA
CTTTAXXXXXXXXXXTTATATTATTATAATCTCCTCACGTAATCTTCACAATCTCCTCACGAAATCAAATTAAAG
TCAGGACTACCACTAGTGTT
>Marker808823
AACGAATCCCAACGGATGATTCAAGAAACACATCAGTCTGTTGTGGCTGTTCTTTTGCTTGGAATAAAACAGTTC
GTCTTXXXXXXXXXXGAGGAGATGCTTGAAAAGTCCAAAGCAGCAGAAACTGTGGAAATTACTGAAGAAATATGT
TCATCTAGAGGCTCCTACGT
AACGAATCCCAACGGATGATTCAAGAAACACATCAGTCTGTTGTGGCTGCTCTTTTGCTTGGAATAAAACAGTTC
GTCTTXXXXXXXXXXGAGGAGATGCTTGAAAAGTCCAAAGCAGCAGAAACTGTGGAAATTACTGAAGAAATATGT
TCATCTAGAGGCTCCTACGT
>Marker809288
AACTGAACTTATATTATAGAAAGGTTCATTTTAGATAAATAAATTGCGTTTTGGGTAAATTTTAGGTTAAATTAA
ATAAAXXXXXXXXXXXTTACAATTCTCTAAGAAAATTTGAATCTTGAAAGGATGAGATAAATGGTATAGATCAAT
GGTGTAGTGATCATCATGTC
AACTGAACTTATATTATAGAAAGGTTCATTTTAGATAAATAAATTGCGTTTTGGGTAAATTTTAGGTTAAATTAA
ATAAAXXXXXXXXXXXTTACAATTCTCTAAGAAAATTTGAAACTTGAAAGGATGAGATAAATGGTATAGATCAAT
GGTGTAGTGATCATCATGTC
>Marker809442
ACCTCCTACTCTAATTCTTAATGTCTTGAAAGCTAATATCATACAAACCATGCACAACAGAATTGAGTTAGAATT
TTAATXXXXXXXXXXTTTCTTTCCATGTCCAAGATCAATAATTCTTACCATATTGAGCATTGATGCATGACCATC
CCAAACACAAAGGTTGGGTT
ACCTCCTACTCTAATTCTTAATGTCTTGAAAGCTAATATCATACAAACCATGCACAACAGCATTGAGTTAGAATT
TTAATXXXXXXXXXXTTTCTTTCCATGTCCAAGATCAATAATTCTTACCATATTGAGCATTGATGCATGACCATC
CCAAACACAAAGGTTGGGTT
>Marker809755
CACTATTTTTACATCCAACTGACATAGTATTAACAACGAGTTAAGAAACCTCAAGGGGACGAGGCAGAGGTTCTT
AAGAGXXXXXXXXXXGTAATCCGGGAGAGTTGATACGAATAACGCAAGTGAGAAATTTGAGTAATTTAGTGAGAA
AACATCTTTAATTATTGGTG
CACTATTTTTACATCCAACTGACATAGTATTAACAACGAGTTAAGAAACCTCAAGGGGACGAGGCAGAGGTTCTT
AAGAGXXXXXXXXXXGTAATCCGGGAGAGTTGATACGAATAACGCAAGTGAGAAATTTGAGTAATTTAGTGAGAA
AACATCCTTAATTATTGGTG
>Marker810384
TACTATTTCCTTCTCAAATTAAAATAGTTTGATATCTCAAAACATATACTATTATAACCCTAACTATAACAATCC
AAAACXXXXXXXXXXATCGGTTTCTATCACTCAGAGTATCATTGATAGACTTTTATTAATTTCTATCACTGATAG
ACGCTGGGGCTTTTGAGGTA
TACTATTTCCTTCTCAAATTAAAATAGTTTGATATCTCAAAACATATACTATTATAACCCTAACTATAACAATCC
AAAACXXXXXXXXXXATCGGTTTCTATCACTCAGAGTATCATTGATAGACTTTTATTAATTTCTATCACTGATAG
ACGCTGAGGCTTTTGAGGTA
>Marker810504

Create PDF with GO2PDF for free, if you wish to

TACTATTATTGTTACCATTTGACTCTAAGTAGGATCAAAATTGTGACAGTAACAGTTAAATGTAAAGATAAAATA
ACAAAXXXXXXXXXXXCCTTAATCAGATTATTTGATTTGATTACAAATTTGGAGGTAGGACCTACGATTGTTCATT
AAAATTACAAAACACAAGTA
TACTATTATTGTTACCATTTGACTCTAAGTAGGATCAAAATTGTGACTGTAACAATTAAATGTAAAGATAAAATA
ACAAAXXXXXXXXXXXCCTTAATCAGATTATTTGATTTGATTACAAATTTGGAGGTAGGACCTACGATTGTTCATT
AAAATTACAAAACACAAGTA
>Marker810508
ACTTTTGCTATGTAATGAAGAATGGTAAATGTGGATTCATAAAACCATAACATATTTGGAGTTGTAACACTTCAA
GCTGAXXXXXXXXXXXAAAATGGTCGTATAAATTAATTTTTTCTTAAAAGTAAAACAAAGAAACTAAATTAATGTT
TGATATATTACCCACTTGTA
ACTTTTGCTATGTAATGAAAAATGGTAAATGTAGATTCATAAAACCATAACATATTTGGAGTTGTAACACTTCAA
GCTGAXXXXXXXXXXXAAAATGGTCGTATAAATTAATTTCTTCTTAAAAGTAAAACAAAGAAACTAAATTAATGTT
TGATATATTACCCACTTGTA
>Marker812445
ACTTAAACTAAGAACCACTATAATTGTCTCTAGGGTTGTTTATCTTCTTCAATCAGCCTCTCCTTATTTTGATTA
CAAGTXXXXXXXXXXXTGTTTTTTGTTTTTTTTAAAAGTAGGGTGATACAACTTTGGATGAGAGATTATTTAACTC
GGTATCACTTATTTTCAAGT
ACTTAATCTAAGAACCACTATAATTGTCTCTAGGGTTGTTTATCTTCTTCAATCAGCCTCTCCTTATTTTGATTA
CAAGTXXXXXXXXXXXTGTTTTTTGTTTTTTTTAAAAGTAGGGTGATACAACTTTGGATGAGAGATTATTTAACTC
GGTATCACTTATTTTCAAGT
>Marker812478
AACACACACCTATAGAAATTTCTAAATGGCTAAATTATATATGTATATATATATAAAATAGCCTTGAACTTTTAT
AATCCXXXXXXXXXXXGTCTTTGTTTGAAATTCTAAAAATTGATATTACAAGTGAGACGGAGGATAGCTTTGAAAC
ACTGATGAAACTTGAGGGTT
AACACACACCTATAGAAATTTCTAAATGGCTAAATTATATATGTATATATATATAAAACAGCCTTGAACTTTTAT
AATCCXXXXXXXXXXXGTCTTTGTTTGAAATTCTAAAAATTGATATTACAAGTGAGACGGAGGATAGCTTTGAAAC
ACTGATGAAAGTTGAGGGTT
>Marker813231
ACTTGACCCTCCTTAAACCAATTGCCTGCCTTCCTCTCATCCCACTCCTATATTAATTCCTAGGTTAATAATTAA
CACGCXXXXXXXXXXXGAGAACTTCTAGAAGAGTGTTTGTTGATCAATGAAAGGTTGAAAGGAAAAGAGCATCCCA
GTTCAGTGAAGCACCTTGTA
ACTTGACCCTCCTTAAACCAATTGCCTGCCTTCCTCTTATCCCACTCATATATTAATTCCTAGGTTAATAATTAA
CACGCXXXXXXXXXXXGAGAACTTCTAGAAGAGTGTTTGTTGATCAATGAAAGGTTGAAAGGAAAAGAGCATCCCA
GTTCAGTGAAGCACCTTGTA
>Marker813438
CACACCCCAGCCAACCACAAAAAAAACCCTTTTTCTCTGAAGGGCGGATAGAGGAGGAACTCCTCAAGGATAGCT
CGAACXXXXXXXXXXXTCTTCCTTCACCTTCCGAAAAAGCAAACAAAAAGGTCCAACAAGAAGAGTCCTCCTCTTA
GCAAGCCTATCAACAGTGTT
CACACCCCAGCCAACCACAAAAAAAACCCTTTTTCTCTGAAGGGCGGATAGAGGAGGAACTCCTCAAGGATAGCT
CAAACXXXXXXXXXXXTCTTCCTTCACCTTCCGAAAAAGCAAACAAAAAGGTCCAACAAGAAGAGTCCTCCTCTTA
GCAAGCCTATCAACAGTGTT
>Marker813514
AACAACAACGTTGAGAGTTAGACATAAATTTTGCTATATCTAATTAATGTATTTGTAATTATTTTTTATTAAAAT
GATAAXXXXXXXXXXXTGGAGGTTATAATTTGGTGATATTTTCAATAGATTATCTGGTATGAAAGGAGTGAGATTT
TTGTTAAAGTAATAAAAGTC

Create PDF with GO2PDF for free, if you wish to

AACAACAACGTTGAGAGTTAGACATAAATTTTGCTATATCTAATTAATGTATTTGTAATTATTTTTTATTAAAAT
GATAAXXXXXXXXXXXTGGAGATTATAATTTGGTGATATTTTCAATAGATTATCTGGTATGAAAGGAGTGAGATTT
TTGTTAAAGTAATAAAAGTC
>Marker814305
AACAATTTAGCATCAATGTTTGACAAAAATGAGACCTCAATTATTCATCTACAATAATATTAAATTGCCGAGACC
CTAAGXXXXXXXXXXXGGGAATTCCAATATCGAACATCATATATCGTTATATATGGAAAGAAAACACTTAACGATA
ACTCAGAGATGCAACAAGTC
AACAATTTAGCATCAATGTTTGACAAAAATGAGACCTCAATTATTCATCTACAATAATATTAAATTGCCGAGACC
CTAAGXXXXXXXXXXXGGGAATTCCAATATCGAACATCATATATCGTTATATATGGAAAGAAAACACTTATCGATA
ACTCAGAGATGCAACAAGTC
>Marker814426
ACCTTCATTATTTTTGTGTTCTTTTAGTTTTCTCTAACAGTAATCACATGCAGCAAAATGAGAAATCGTTTTCTC
TCAAAXXXXXXXXXXXXTTTCTGTATAGAACTCGGTGAATGCTCGAGAGTCGAGGATTCCATATCCATGGATAGTTT
TCAATGAGAAGATTAAAGTG
ACCTTCATTATTTTTGTGTTCTTTTAGTTTTCTCTAACAGTAATCACATGCAGCAAAATGAGAAATCATTCTCTC
TCAAAXXXXXXXXXXXXTTTCTGTATAGAACTCGGTGAATGCTCGAGAGTCGAGGATTCCATATCCATGGATAGTTT
TCAATGAGAAGATTAAAGTG
>Marker814890
AACTACCACTATATAGTGAGAGTAACAACATGAACTACTATTTTTTTTCTTTTTTCTTTGTTTTTTTTGTGTGGT
CACTTXXXXXXXXXXXTCGAAAGATCTAGAATTTTTTGTAGTAGTTGTGTAAAGAATGGTATCTGTTGGCTTCACA
CCGTATCTTGGACTAAGAGT
AACTACCACTATATAGTGAGAGTAACAACATGAACTACTATTTTTTTTCTTTTTTCTTTGTTTTTTTTGTGTGGT
CACTTXXXXXXXXXXXTCGAAAGATCTAGAATTTTTTGTAGTAGTTGAGTAAAGAATGGTATCTGTTGGCTTCACA
CCGTATCTTGGACTAAGAGT
>Marker814903
CACTTTATTGATGCAAGTAGTTGGAAAAATAAAATATTTCAACAAAAAAACGAGTGATTTGAATCATACCTACAT
AATTCXXXXXXXXXXXATTGGATAACGACTGTGGATTTTTGTAGGTCTGTTTTATTAATTGTTACATTTAATTTTA
TCATATAGTATGGCATGAGT
CACTTTATTGATGCAAGTAGTTGGAAAAATAAAATATTTCAACAAAAAAACGAGTGATTTGAATCATACCTACAT
AATTCXXXXXXXXXXXATTGGATAACGACTGTGGATTTTTGTAGGTCTGTTTTATTAATTGTTAAATTTAATTTTA
TCATATAGTATGGCATGAGT
>Marker815084
CACACTCACGCTTTCAACCACAGCAGGATCTGTGTGCTTGTTTCTGTGGCCATAGCCAAATAGGAAAAGCTCCTA
AAACAXXXXXXXXXXXCCCCACCATCATCCATCAACTTCCGCACTTTCTGGACTCCATCCCACTCACCTTGTGATG
CATATATGTTAGAAAGGAGT
CACACTCACGCTTTCAACCACAGCTGGATCTGTGTGCTTGTTTCTGTGGCCATAGCCAAATAGGAAAAGATCCTA
AAACAXXXXXXXXXXXCCCCACCATCATCCATCAACTTCCGCACTTTCTGGACTCCATCCCACTCACCTTGTGATG
CATATATGTTAGAAAGGAGT
>Marker816259
CACTAATATTTAAAATGATATTGATTGTAGTGATGTTATTTTATTTATATATCATTGAGATGTTCTAACTATATA
TATTAXXXXXXXXXXXTGGGGTGAAATGACGAAAATATCCTTATTTAATTTTACTTCACATTTGCTCTTCTCTACC
AACCTCTCTCTCTTCAATGT
CACTAATATTTAAAATGATATTGATCGTAGTGATGTTATTTTATTTATATATCATTGAGATGTTCTAACTATATA
TATTAXXXXXXXXXXXTGGGGTGAAATGACGAAAATATCCTTATTTAATTTTACTTCACATTTGCTCTTCTCTACC
AACCTCTCTCTCTTCAATGT
>Marker816300

Create PDF with GO2PDF for free, if you wish to

ACTATTAATATTTTCACTTGTATCATAATTTTGATGCACAAAGAAGTTCGGACATAAATTAATATCAATTTATAT
GCTACXXXXXXXXXXXTTAGGAACATCATCTTTAACAAGATGGGGAAGCTCATTTCTATCCTCCTCCTTAACAAGA
AGGCATACTCCAGACACGTT
ACTATTAATATTTTCACTTGTATCATAATTTTGATGCACAAAGAAGTTTGGACATAAATTAATATCAATTTATAT
GCTACXXXXXXXXXXXTTAGGAACATCATCTTTAACAAGATGGGGAAGCTCATTTCTATCCTCCTCCTTAACAAGA
AGGCATACTCCAGACACGTT
>Marker816457
TACCAAAATGTATTGTAAAATGAAAATGGAACGCCCCAAAACATCAACTTGCAGAAAGGATGAGGTGAATAGTAT
TCCTCXXXXXXXXXXXGCAGAAATTCGAGACAAATTATTCAGCAAAAACAAACTACACGTCACAAATCAACAGGAA
GAAAGTCACAGAAACAGAGT
TACCAAAATGTATTGTAAAATGAAAATGGAACGCCCCAAAATATCAACTTGCAGATAGGATGAGGTGAATAGTAT
TCCTCXXXXXXXXXXXGCAGAAATTCGAGACAAATTATTCAGCAAAAACAAACTACACGTCACAAATCAACAGGAA
GAAAGTCACAGAAACAGAGT
>Marker816618
TACTTTCTTCAAGGGATAAGGCTTAAAAAAAAATCAAAATTTCAGTCTTATTAAATAAGATGTGATAAGAAAGGA
ATCTGXXXXXXXXXXXAGCATTGTCTGTTCAATCAACCATTTTTCTAAGCTTTTTACAAAATTTAATATTAGTGGG
AAACTTAACAGTAATTGGTA
TACTTTCTTCAAGGGATAAGGCTTAAAAAAAAATCAAAATTTCAGTCTTATTAAATAAGATGTGATAAGAAAGGA
ATCTGXXXXXXXXXXXAGCATTGTCTGTCCAATCAACCATTTTTCTAAGCTTTTTACAAAATTTAATATTAGTGGG
AAACTTAACAGTAATTGGTA
>Marker816761
AACATTCTGATGTGGATCATGCTTTTCGGGTCCCTAAATTGTGCAGAAATTCTTCAAAAGAAATCTCCAAGCAGG
TGCCTXXXXXXXXXXXTAGTTTCTTCTCCATCTTTGATATTGCTCGGGTATTCGACGCGTCCCTCAGCTCCTTGGT
GCTTTAGCTATTGGAGGGTC
AACATTCTGATGTGGATCATGCTTTTCGGGTCCCTAAATTGTGCAGAAATTCTTCAAAAGAAATCTCCAAGCAGG
TGCCTXXXXXXXXXXXTAGTTTCTTCTCCATCTTTGATATTGCTCGGGTATTCGACGCCTCCCTCAGCTCCTTGGT
GCTTTAGCTATTGGAGGGTC
>Marker818354
CACTCTCTCCTAACTTAGCTCACATTTTCGCTAATCCTATACTATATGACTGTCAAGAAAACTCGGAGAGCTTTT
AGAGTXXXXXXXXXXXTTGGTTTAAGTGATCCAAATAGCAGAAATTTTGAAAACAACGTTTTCATATCTGGATTTT
GAATTTGAAATCTTGATGTT
CACTCTCTCCTAACTTAGCTCACATTTTCGCTAATCCTATACTATTTGACTGTCAAGAAAACTCGGAGAGCTTTT
AGAGTXXXXXXXXXXXTTGGTTTAAGTGATCCAAATAGCAGAAATTTTGAAAACAACGTTTTCATATCTGGATTTT
GAATTTGAAATCTTGATGTT
>Marker818891
ACCATAAATTAGAAAAAAATAATCAAAATTCGATATTATATTAATAAAGTAAATCTCAGATGGCTACAAGGTATA
TCCCCXXXXXXXXXXXGTTAGTTCTATAATATGAATTTGTTGTATATAAATGTTTTTATTTGTAAAGAAAATTATT
ACAAAACATATTACTTTTGT
ACCATAAATTAGAAAAAAATAATCAAAATTCGATATTATATTAATAAAGTAAATCTCAGATGGCTACAAGGTATA
TCCCCXXXXXXXXXXXGTTAGTTCTATTATATGAATTTGTTGTATATAAATGTTTTTATTTGTAAAGAAAATTATT
ACAAAACATATTACTTTTGT
>Marker818975
AACTGCCTAGGCTTAGTGTTGTAGCTGAATATGGGATACGATCTCTGTTCAAGATAATTTGTTGGAGCAGATGAT
GTTGAXXXXXXXXXXXCTTTAATCAAGATTTACGTGGGTAGAGCAATATTTACTACCAAATAACTAAAACAAGCTA
ATTAACAACTAACAAATAGT

Create PDF with GO2PDF for free, if you wish to

AACTGCCTAGGCTTAGTGTTGTAGCTGAATATGGGATACGATCTCTGTTCAAGATAATTTGCAGGAGCAGATGAT
GTTGAXXXXXXXXXXCTTTAATCAAGATTTACGTGGGTAGAGCAATATTTACAACCAAATAACTAAAACAAGCTA
ATTAACAACTAACAAATAGT
>Marker819170
AACGTATGCCTAAAACGACCACCCAAATGAAGAGATGTGACCTAAAAACTGAAACTATAGATTTATATTTGCATG
ATGACXXXXXXXXXXGTCCATTTTAGCTCCATTTTCTTCTTAACATTCGATTTTGCCTATTTACTACTTGAGACG
TATAAAATAATAATGAAGTA
AACGTATGCCTAAAACGACCACCCAAATGAAGAGATGTGACCTAAAAAATGAAACTATAGATTTATATTTGCATG
ATGACXXXXXXXXXXGTCCATTTTAGCTCCATTTTCTTCTTAACATTCGATTTTGCCTATTTACTACTTGAGACG
TATAAAATAATAATGAAGTA
>Marker819280
GACTTGGTCTTATTTAAACTTAGGAATTAGAAAGATATGTGTGTTTGGTCTTTAAGTTCTAACAATCTATTTTTT
TTTCCXXXXXXXXXXTAATTTTGTTTTAAAAATCAAATAAAATAAATACTTGAGGATCCTTTTATACTTACTTTT
AAAAGCTCACCAACTTGAGT
GACTTGGTCTTATTTAAACTTAGGAATTAGAAAGATATGTGTGTTTGGTCTTTAAGTTCTAACAATCTATTTTTT
TTTCCXXXXXXXXXXTAATTTTGTTTTAAAAATCAAATAAAATAAATACTTGAGGATCCTTTTATACTTACTTTT
ACAAGCTCACCAACTTGAGT
>Marker819412
AACAAAAATGCAGCTTTTTGTTTTCAAATTCAGCGTCGGATCCAATTAGCTAAAGGTAGGGTTTGCCAATTGTAA
TTATTXXXXXXXXXXAATTTATAATTTTAAAAGTTTAACAATTTAATTAAATTAACACTAGGACTAAATTGGTAA
ATATAATCAAGGTTTATGTT
AACAAAAATGCAGCTTTTTGTTTTCAAATTCAGCGTCGGATCCAATTAGCTAATGGTAGGGTTTGCCAATTGTAA
TTATTXXXXXXXXXXAATTTATAATTTTAAAACTTTAACAATTTAATTAAATTAACACTAGGACTAAATTGGTAA
ATATAATCAAGGTTTATGTT
>Marker819788
TACTTACTTAAGAAAAATGTTAAAATGTAAGTATATGGTAAGAAAATGATAGAAAATAGACTTAATTTTTAAAAA
CTAAAXXXXXXXXXXAAAGGACAATACAACCATTTCTCATTTAAGAGATGGTTAAAAATAGTGTATAGATAGACA
ACAAAATAGCATTCAACGTA
TACTTACTTAAGAAAAATGTTAAAATGTAAGTATATGGTAAGAAAATGATAGAAAATAGACTTAATTTTTAAAAA
CTAAAXXXXXXXXXXAAAGGACAATACAACCATTTCTCATTTAAGAGATGGTTAAAAAAAGTGTATAGATAGACA
ACAAAATAGCATTCAACGTA
>Marker820585
AACTATATTCAACAAAATGGTGAAGGCCATTGGAGAGCTTTGCCTAAGAAAGCTGGTGAGTTTCCTTCTATTATT
CTAATXXXXXXXXXXCAACATAACAGCTGACGAAGATGACTTGATCATAAAGCTACATTCACTTCTCGGCAATCG
TTGGTCATTGATCGCTGGTA
AACTATATTCAACAAAATGGTGAAGGCCATTGGAGAGCTTTGCCTAAGAAAGCTGGTGAGTTTCCTTCTATTATT
CTAATXXXXXXXXXXCAGCATAACAGCTGACGAAGATGACTTGATCATAAAGCTACATTCACTTCTCGGCAATCG
TTGGTCATTGATCGCTGGTA
>Marker820596
AACAGAACAGCATGTATTGAGAAAGCAACATCATTTGCAGCAACAGGTATCATCTGAGAGATCCAAGAAAATCAA
CAACAXXXXXXXXXXGTAATCAGAAAACAAAAGGTGAAAGGATAGAAAACTCAATACATGAACATTTATCGACTA
ATGAAGTAATCAAGCTAGTG
AACAGAACAGCATGTATTGAGAAAGCAACATCACTTGCAGCAACAGGTATCATCTGAGAGATCCAAGAAAATCAA
CAACAXXXXXXXXXXGTAATCAGAAAACAAAAGGTGAAAGGATAGAAAACTCAATACATGAACATTTATCGACTA
ATGAAGTAATCAAGCTAGTG
>Marker820876

Create PDF with GO2PDF for free, if you wish to

CACCCCTGCCTGAGTTTGCAGCTCATCAGAAGAAACCACGCGTGAGCTTCATGCGCCACAAGAAGAGGTTGTCTC
ATCCCXXXXXXXXXXCTTGGCCCATAGGGAGGTTTGCTCTAAATAGTTTTCCTTGTTTATCTCACTGTATTATTC
TATTTATTCCCCCCTCTTGT
CACCCCTGCCTGAGTTTGCAGCTCATCAGAAGAAACCACGCGTGAGCTTCATGCGCCGTAAGAAGAGGTTGTCTC
GTCCCXXXXXXXXXXCTTGGCCCATAGGGAGGTTTGCTCTAAATAGTTTTCCTTGTTTATCTCACTGTATTATTC
TATTTATTCCCCCCTCTTGT
>Marker821351
AACGCGGCACTGACTCATCATTGGACGAGCTCAAATGTGTTCATACTTCTTTGGTTTGGAGCCCAAATAACATCC
TTTTAXXXXXXXXXXTAACAAATACACAATACACGAGATAGTATAAATATTCTTAATGAAAACAGAACATTCTCC
TTCTTTATCAGGCATGTGTA
AACGCGGCACTGACTCATCATTGGACGAACTCAAATGTGTTCATACTTCTTTGGTTTGGAGCCCAAATAACATCC
TTTTAXXXXXXXXXXTAACAAATACACAATACACGAGATAGTATAAATATTCTTAATGAAAACATAACATTCTCC
TTCTTTATCAGGCATGTGTA
>Marker822160
CACATTCCACCTTCAAGAAGGTGAATTTCAAGTCAAGCGGTAACGTTAGGCCTCTGATTATCCACGCATATAAAC
ACAGGXXXXXXXXXXTATATAAGGAATGTTTTAGGGATTGATGTAGGTATGTTTTATTTTGGTAGAAGCTCTTGT
GGCTTTTAGATGGAGAAGTT
CACATTCCACCTTCAAGAAGGTGAATTTCAAGTCAAGCGGTAATGTTAGGCCTCTGATTATCCACGCATATAAAC
ACAGGXXXXXXXXXXTATATAAGGAATGTTTTAGGGATTGATGTAGGTATGTTTTATTTTGGTAGAAGCTCTTGT
GGCTTTTAGATGGAGAAGTT
>Marker823459
ACTTAATGGACCCACATTCCCCCGCATTGCCTGCAACCTGGCTATGCTACCATAACAACTCTTGATAGTTGAAAA
AGTTGXXXXXXXXXXCTGATTTATAATCAGCCTTATTAATGCAAGTGCTTTATTCTCAAGTTCATCTCTTGTGCT
AGAGATCATATTGAAAAGTT
ACCTAATGGACCCACATTCCCCCGCATTGCCTGCAACCTGGCTATGCTACCATAACAACTCTTGATAGTTGAAAA
AGTTGXXXXXXXXXXCTGATTTATAATCAGCCTTATTAATGCAAGTGCTTTATTCTCAAGTTCATCTCTTGTGCT
AGAGATCATATTGAAAAGTT
>Marker823618
CACTGGTGGTCAAGAACTCATCCGGACTGGGATATCGAAACCTGTATCAAGCTTCCTGTCTTTGCAATCTATTCT
GAAGCXXXXXXXXXXCGATTTCTGGAGGGCAGTAGAAGAATGTGTAGCAATATCAGAGCCTTTCCTGAGAGTGTT
GAGAGAAGTGTGTGGGGGTA
CACTGGTGGTCAAGAACTCATCCGGACTGGGATATCAAAACCTGTATCGAGCTTCCTGTCTTTGCAATCTATTCT
GAAGCXXXXXXXXXXTGATTTCTGGAGGGCAGTAGAAGAATGTGTAGCAATATCAGAGCCTTTCCTGAGAGTGTT
GAGAGAAGTGTGTGGGGGTA
>Marker824091
ACTACATTCTCTTTTACTTTTATCCATTTACGTGCAAGACAAAGATAATAGAGCGGAATGAGTATAAAAAGTCTC
TTTATXXXXXXXXXXCGGCGCCTGACTTGCTTATCTCATCGTGTAGCAATGCACCTTATGCCTTTGCCATCCAAT
CTGTTTCTTTAAGACTGAGT
ACTACATTCTCTTTTACTTTTATCCATTTACGTGCAAGACAAAGATAATAGAGCGAAATGAGTATAAAAAGTCTC
TTTATXXXXXXXXXXCGGTGCCTGACTTGCTTATCTCATCGTGTAGCAATGCACCTTATGCCTTTGCCATCCAAT
CTGTTTCTTTAAGACTGAGT
>Marker824509
GACAGATGTTGCTTTACATCGAATGATCGCTCTTCAGCCCTCAAATGAGTGAATAGGAATAAGAAAATGAGAAAA
AAAATXXXXXXXXXXTTATTTGCCAATTTTGCCTTCCATCTTTTGATTGTGACTTTATTTGCCAATTTGCCCCCT
TATGAGTTCATAGGGTTGTA

Create PDF with GO2PDF for free, if you wish to

GACAGATGTTGCTTTACATCGAATGATCGCTCTTCAGCCCTCAAATGAGTGAATAGGAATAAGAAAATGAGAAGA
AAAATXXXXXXXXXXXTTATTTGCCAATTTTGCCTTCCATCTTTTGATTGTGACTTTATTTGCCAATTTGCCCCCT
TATGAGTTCATAGGGTTGTA
>Marker825640
GACACGGCTCATCAGATTAGCACTCGTTCGTCTTCTTTGTTTTTTTTATCTTACTTTTTTGCTTTTCTAATGTGG
GTCTCXXXXXXXXXXXCATCACTATTATGTATTTTGTTATCTATCTTTTATGATTATTTTCAAAATCAAAGTCAAA
TACAAGAAAGCTACAAGGTG
GACACGGCTCATCAGATTAGCACTCGTTCGTCTTCTTTGTTTTTTTTATCTTACTTTTTTGCTCTTCTAATGTGG
GTCTCXXXXXXXXXXXCATCACTTTTATGTATTTTGTTATCTATCTTTTATGATTATTTTCAAAATCAAAGTCAAA
TACAAGAAAGCTACAAGGTG
>Marker825812
CACCCTAAAGGGGGGAGCTGTGGTATGACGTAGAATCAAGTAAGGATGCATTACAGACTCTACTATGATGGCTGA
GTGCGXXXXXXXXXXXAAAAGTGGGGCATATTGTTGATCCATTTACAAAGACTCACATGGCTAAACTGTTCGAGGG
TCTTCTAGAAAGTCTAGGTC
CACCCTAAAGGGGGGAGCTGTGGTATGACGTAGAATCAAGTAAGGATGCATTACAGACTCTACTATGATGGTTGA
GTGCGXXXXXXXXXXXAAAAGTGGGGCATATTGTTGATCCATTTACACAGACTCACATGGCTAAACTGTTCGAGGG
TCTTCTAGAAAGTCTAGGTC
>Marker825841
TACTAGTGAAGCATAACAAGTTTATTAGAAGTGCATTAAGTGTATCAAGGCCTAAAGAATATCAAGTGTATAGAG
AAGAAXXXXXXXXXXXTGTATATCGAGTGTATCAAATGTAACAAGGAGTATTAGATGTATCAAGGGATATTAGATT
TATCGGATATATCAAATGTA
TACTAGTGAAGCATAACAAGTTTATTAGAAGTGCATTAAGTGTATCAAGGCCTAAAGAATATCAAGTGTATAGAG
AAGAAXXXXXXXXXXXTGTATATCGAGTGTATCGAATGTAACAAGGAGTATTAGATGTATCAAGGGATGTTAGATT
TATCGGATATATCAAATGTA
>Marker826474
CACCAGAGTTTAAAAGAATTACATGTATATAACTTATCTTTTCGACACAATATCCATAATTACAACAAAATCACG
TCACTXXXXXXXXXXXTAGATCTAACTCCTAGATAATAAAAGCATATCACTTATAGATTTTGAAATACTTTAATTT
TTTTTTAAAAAACGTTGGTC
CACTAGAGTTTAAAAGAATTACATGTATATAACTTATCTTTTCGACACAATATCCATAATTACAACAAAATCACG
TCACTXXXXXXXXXXXTAGATCTAACTCCTAGATAATAAAAGCATATCACTTATAGATTTTGAAATACTTTAATTT
TTTTTTAAAAAACGTTGGTC
>Marker827429
AACCTCTTGGTTCTCAGCATCAACTTATTCATTTTGCATTATATGATCTACCATGCCTTCAAACCCCGTGCAGAC
TCCTTXXXXXXXXXXXTCAAAAGAACATTAATAGTTTTTAAAAGTAAGACTTTGATTCGTAACATTTTTCTATGCT
CAATGTGTTTGAAAATTGTG
AACCTCTTGGTTCTCAGCATCAACTTATTCATTTTGCATTATATGATCTACCATGCCTTCAAACCCCGTGCAGAC
TCCTTXXXXXXXXXXXTCAAAAGAATATTAATAGTTTTTAAAAGTAAGACTTTGATTCGTAACATTTTTCTATGCT
CAATGTGTTTGAAAATTGTG
>Marker827560
ACCATGAATCATCTGATGAACAACTGGTTTAAGGTCCAACCTATAAACTGAATCCCCTCTCAAGCTAATGAGAGG
GTGGGXXXXXXXXXXXAGGGAAGACATATTGGGTCAGTGTTGATGAGCTGACCTCACCTATGCAGATCTAAGAATA
ATTTCGTCTGAATATGAGTT
ACCATGAATCATCTAATGAACAACTGGTTTAAGGTCCAACCTATAAACTGAATCCCCTCTCAAGCTAATGAGAGG
ATGGGXXXXXXXXXXXAGGGAAGACATATTGGGTCAGTGTTGATGAGCTGACCTCACCTATGCAGATCTAAGAATA
ATTTCGTCTGAATATGAGTT
>Marker827820

Create PDF with GO2PDF for free, if you wish to

CACTTGCTGCTCTCTACTATGACTATTTATAACATATGACTTTGTGTAGTAAATACTATTTTACAACACTTTTTC
TATAAXXXXXXXXXXXTACGAACATAAACTATATCATAATCCATACTAAAATATATAACATGTTCTTGAATATCAA
TTATTATAACTTGCTTATGT
CACTTGCTGCTCTCTACTATGACTATTTATAACATATGACTTTGTGTAGTAAATACTATTTTACAACACTTTTTC
TATAAXXXXXXXXXXXTACAAACATAAACTATATCATAATCCATACTAAAATATATAACATGTTCTTGAATATCAA
TTATTATAACTTGCTTATGT
>Marker828723
AACTTTGTAAGGTGTCTCTTTTAAGTTTATCACTTTCAAATTGGATCAAAATAAAAATTGGCCATTTTTTTTTTT
GCCAGXXXXXXXXXXXTCAACACATTTATTCAAACATCTCACATACTTGTCCTTTCATTTAAAGGACGACTTTACC
TAACTTAGCTTTATGTGGTG
AACTTTGTAAGGTGTCTCTTTTAAGTTTATCACTTTCAAATTGGATCAAAATAAAAATTGGCCATTTTTTTTTTT
TGCCAXXXXXXXXXXXTCAACACATTTATTCAAACATCTCACATACTTGTCCTTTCATTTAAAGGACGACTTTACC
TAACTTAGCTTTATGTGGTG
>Marker828775
CACTATGATGTCGAAAATTAATTTTTGATAGCTGATCAGTATTCATTACATTTTATGATTTCTTTTAGGTAACAA
CAAATXXXXXXXXXXXATGGGAGTCCTCACCCTTCTCTTCGTTCTAACGAAGAAACTAACCTTCAATATATACTAC
ATATGTATAGGTTCAAGGGT
CACTATGATGTCGAAAATTAATTTTTGATAGCTGATCAGTATTCATTACATTTTATGATTTCTTTTAGGTAACAA
CAAATXXXXXXXXXXXATGGGAGTCCTCACCCTTCTCTTTGTTCTAACGAAGAAACTAACCTTCAATATATACTAC
ATATGTATAGGTTCAAGGGT
>Marker832090
CACAATTTTGCTGTTTCTCATGGTCTTGAAACTTCATCGAGTAGAAATAACAAAGTTTTGTCTTTTGGTATAGAA
ATTGAXXXXXXXXXXXGCATTGTTTCAAATGGTGGCAAATTTGGTTTTAAGTGTAATACCAATGTTGTTGCTTCTC
ATGGTTTTGGAATTCAGGGT
CACAATTTTGCTGTTTCTCGTGGTCTTGAAACTTCATCGAGTAGAAATAACAAAGTTTTGTCTTTTGGTATAGAA
ATTGAXXXXXXXXXXXGCATTGTTTCAAATGGTGGCAAATTTGGTTTTAAGAGTAATACCAATGTTGTTGCTTCTC
ATGGTTTTGGAATTCAGGGT
>Marker832230
GACACATTTCTTAGTTTGTTTCCGAAGGAAAAGGTCATTTAATTAAATACTTTTTAGAAAACTTTTATACATGGT
TTTTGXXXXXXXXXXXCATGATATTACTGATTAGCTCATTTGCATATGCCATTCATTATCTTATGGTATCTAAAAC
AAAAGGATGTTTATAAAAGT
GACACATTTCTTAGTTTGATTCCGAAGGAAAAGGTCATTTAATTAAATACTTTTTAGAAAACTTTTATACATGGT
TTTTGXXXXXXXXXXXCATGATATTACTGATTAGCTAATTTGCATATGCCATTCATTATTTTATGGTATCTAAAAC
AAAAGGATGTTTATAAAAGT
>Marker832385
CACTAGATTAAACATTTGAATCAAGCAAGATAAGATTGGTTTCATTTTGTTGGACTGACATCTATTCCATAGTTG
GGGTTXXXXXXXXXXXATAAATAAACAATGTGTTTTCGAAAACCATAAAAAAATGTCATATAATCCAATACCAAAA
AAGATACTATTAACAATGTT
CACTAGATTAAACATTTGAATCAAGCAAGATAAGATTGGTTTCATTTTGTTGGACTGACATCTATTCCATAGTTG
GGGTTXXXXXXXXXXXATAAATAAACAATGTGTTTTCGAAAACCATAAAAAAATGTCATATAATCCAACACCAAAA
AAGATACTATTAACAATGTT
>Marker832625
TACTAATCAAAAGGAATTAGTTAGACCGGTGAAGAATCGCTTTACTACAACTTGCATGACATTATGATGGAAGCC
AACTAXXXXXXXXXXXAAGAAGCTCGACCATTTATGAGGTTATGGATAGAGCTAATGAGACCATCGCTAAATCCTT
TGATGGTAAGGAAGAAAAGT

Create PDF with GO2PDF for free, if you wish to

TACTAATCAAAAGGAATTAGTTAGACCAGTGAAGAATCGCTTTACTACAACTTGCATGACATTATGATGGAAGCC
AACTAXXXXXXXXXXXAAGAAGCTCGACCATTTATGAGGTTATGGATAGAGCTAATGAGACCATCGCTAAATCCTT
TGATGGTAAGGAAGAAAAGT
>Marker833154
AACTAAATTTTTGGACGGAGTTGGACCCAAGTGCTTTAAAGTCAATTTTTGGACGGAGTTGGATCAATTCAAACG
GTCTTXXXXXXXXXXXCTATTTGTAACATTAACAACATCAGTTTGGCTTATATGAACACCATCAGTGTTTGGGCTA
TTACCAGGAGCAACTATGTG
AACTAAATTTTTGGACGGAGTTGGACCCAAGTGCTTTAAAGTCAATTTTTGGACGGAGTTGGATCAATTCAAACG
GTCTGXXXXXXXXXXXCTATTTGTAACATTAACAACATCAGTTTGGCTTATATGAACACCATCAGTGTTTGGGCTA
TTACCAGGAGCAACTATGTG
>Marker833272
TACAAAAATTAATTTTAAAAAATAGAGATCCAGTAATAAAATTTGATTGAAAGAGAGTTAAAATAAGACATCTCT
TACTTXXXXXXXXXXXTCTACCAAAATTATTGCTTATAATGTTTTCACAATTTAAAACGAACATCTTTCATTATTG
TTCTTTTTAGATTTTAAGTG
TACAAAAATTAATTTTAAAAAATAGAGATCCAGTAATAAAATTTGATTGAAAGAGAGTAAAAATAAGACATCTCT
TACTTXXXXXXXXXXXTCTACCAAAATTATTGCTTATAATGTTTTCACAATTTAAAACGAACATCTTTCTTTATTG
TTCTTTTTAGATTTTAAGTG
>Marker833465
TACTCTATCAACTTTAATTCTTTAACAATTTATTTTATAATACTTATGCATACTTTTACTACACAACAATTTAGT
TTTTTXXXXXXXXXXXAAATGGCTACATTTGTTTTGTGAATAAAATTTGTCACTATTGTGGTAGAGAACATGAGTT
TAGTATGTATGTTAGTTTGT
TACTCTATCAACTTTAATTCTTTAACAATTTATTTTATAATACTTATGCATACTTTTACTACACAACAATTTAGT
TTTTTXXXXXXXXXXXAAATGGCTACATTTGTTTTGTGAATAAAATTTGTCACTATTGTGGTAGAGAACATGAGTT
TAGTATGTATGGTAGTTTGT
>Marker833705
ACATCGCCGTCGATTCCCATTTGTTCCTGATTCACTTCAATTTTCTCTACTTCAATCTCAAATCTCAATCCCAAC
TTCTCXXXXXXXXXXXTTTCTCACTGTATTCAAGGGTTTCCGCCCGGTTGCGAGCTGGCTATGGACTACGAGCCCT
ATGATAGTAGCGGTAAAGTT
ACATCGCCGTCGATTCCCATTTGTTCCTGATTCACTTCAATTTCCTCTACTTCAATCTCAAATCTCAATCCCAAC
TTCTCXXXXXXXXXXXTTTCTCACTGTATTCAAGGGTTTCCGCCCGGTTGCGAGCTGGCTATGGACTACGAGCCCT
ATGATAGTAGCGGTAAAGTT
>Marker833896
ACCTTTTATTTGTAATGTTGTATTTATTTGAAAAATTAATAGAACATTATATTTATTGTTTTCCCATTATTATTG
CGTCAXXXXXXXXXXXACATAAGATATAAGATTAATAATTGATGTGATTGTTCATTCTAAAGTGGAAGATTCGATC
TTCATCCTCACAATTGTTGT
ACCTTTTATTTGTAATGTTGTATTTATTTGAAAAATTAATAGAACATTATATTTATTGTTTTCCCATTATTATTG
CGTCAXXXXXXXXXXXACATAAGATATAAGATTAATAATTGATGTGATTGTTCATTCTAAAGTGGAAGATTGGATC
TTCATCCTCACAATTGTTGT
>Marker834143
TACACTAATTTTTCATTGTTATTAAAATTAATCAAACTTTTTCTGCATCATACTTATTTTAAGTTAGTCACTAGA
AAGTAXXXXXXXXXXXACCAATTCTAAACAAAATTTCAAAGTTAAGAGAAAACCAACACATTCTTTTGGCAAGCTA
TTGTTCATCATTTGATAGTT
TACACTAATTTTTCATTGTTATTAAAATTAATCAAACTTTTTCTGCATCATACTTATTTTAAGTTAGTCACTAGA
AAGTAXXXXXXXXXXXACCAATTCTAAACAAAATTTCAAAGTTAAGAGAAAACCAACACATTCTTTTTGCAAGCTA
TTGTTCATCATTTGATAGTT
>Marker834265

Create PDF with GO2PDF for free, if you wish to

CACCCCATTGTTTATATACCTCTAAATTCCTGGCAGAGATGCTATCATCTATACTCGTTAATTGTCCATTTACAC
TAGAAXXXXXXXXXXXCCAATGTTGAAAACAAGATTGTAAATGACATAAGCGGCAGCCATACAAACCAAACACAAG
AACCTATTTTAAAGAATGTA
CACCCCATTGTTTATATACCTCTAAATTCCTGGCAGAGATGCTATCATCTATACTCGTTAATTGTCCATTTACAC
TAGAAXXXXXXXXXXXCCAATGTTGAAAACAAGATTGTAAATGACATAAGCGGCAGCGATACAAACCAAACACAAG
AACCTATTTTAAAGAATGTA
>Marker834274
ACTATTCTGGTAAAACTATGGATACGACTCACTTTGTATTTGATACAAATGTCATGATTTAATACATTCATGTAG
GTGACXXXXXXXXXXXACATAAGTATTTAAAAATACATCTTACATAGATATCATGCCTGTTTTATGCGATTGCCAT
GTTTAAATGCAGGATAAGTG
ACTATTATGGTAAAACTATGGATACGACTCACTTTGTATTTGATACAAATGCCATGATTTAATACATTCATGTAG
GTGACXXXXXXXXXXXACATAAGTATTTAAAAATACATCTTACATAGATATCATGCCTGTTTTATGCGATTGCCAT
GTTTAAATGCAGGATAAGTG
>Marker834359
ACTTAGAATCTTGTCCTTTTGTATTTCTATGCTTCCATGTGAAATGTGCTGTATTCGACAGCTTTGGGTCTAGAG
ATCTTXXXXXXXXXXXGAGGGGACTCTTGTATGTGTTGGCATGTCGTGGTTTTGACTTCACACATCGACCAGGCAC
ACGGGACTTTCGGATCTGTT
ACTTAGAATCTTGTCCTTTTGTATTTCTATGCTTCCATGTGAAATGTGCTGTATTCGACAGCTTTGGGTCTAGAG
ATCTTXXXXXXXXXXXGAGGGGACTCTTGTATGTGTTGGCATGTCGTGGTTTTGACTTCACACATCGACCAGGCAC
ACGGGACTCTCGGATCTGTT
>Marker834593
CACTTGAGCCTCCAAGGCCGAAGAAGAACTATGCATGAAGGTCTAACTTCACTTCCTTGACTCATCATGATATTT
GTTTGXXXXXXXXXXXCCAAATTTGGAAGCATACTTATTCTAGTTTGTGTTCCTTCAATGACCTTGTTTGTTGATG
CTTGCTAAGGTTGAGTTGTT
CACTTGAGCCTCCAAGGCCGAAGAAGAACTATGCATGAAGGTCTAACTTCACTTCCTTGACTCATCATGATATTG
TTTGGXXXXXXXXXXXCCAAATTTGGAAGCATACTTATTCTAGTTTGTGTTCCTTCAATGACCTTGTTTGTTGATG
CTTGCTAAGGTTGAGTTGTT
>Marker835313
ACATCACTTGCTCTAGAGGAGAACCAATCATACTCATGGCATCATGGAAGCAGGGTTTCTTCTTTTATGATGCAT
GAACAXXXXXXXXXXXTTTCTATAATCATATTTTCACAGGAGTGTGTTAGGGGAATTCTAAGAGACAAGACTATCA
TACTTGTGACTCACCAAGTT
ACATCACTTGCTCTAGAGGAGAACCAATCATACTCATGGCATCATGGAAGCAGGGTTTCCTCTTTTATGATGCAT
GAACAXXXXXXXXXXXTTTCTATAATCATATTTTCACAGGAGTGTGTTAGGGGAATTCTAAGAGACAAGACTATCA
TACTTGTGACTCACCAAGTT
>Marker835808
CACCAAGATTCATTGATTGACTTATGGGTTATCTGCAGGTTGTATTGAAGGAAAGCAAAAATTCCCATGGAGAAT
TAGACXXXXXXXXXXXAATGAACCAAAAATCAGCGAATATGGGATCACAAAGTTTCTAGACGCTAAGAGAGTTCAT
CTTCTTTCCTCCAAGGGGTA
CACCAAGATTCATTGATTGACTTATGGGTTATCTGCAGGTTGTATTGAAGGAAAGCAAAAATTCCCATGGAGAAT
TAGACXXXXXXXXXXXAACGAACCAAAAATCAGCGAATATGGGATCACAAAGTTTCTAGACGCTAAGAGAGTTCAT
CTTCTTTCCTCCAAGGGGTA
>Marker835816
AACGTATCTTACATATAAAGTTTGTATAAGACTTGACAACAAAATAATTATTCTCTATTTGAAAATTTGATAATT
GAAGAXXXXXXXXXXXCAATGCCACAAGATTTCAATCAAACTCCATTCATTTATATATACTTCTGAAGTGAAGATG
CAAAAAGGTAACAATCAGTA

Create PDF with GO2PDF for free, if you wish to

AACGTATCTTACATATAAAGTTTGTATAAGACTTGACAACAAAATAATTATTCTCTATTTGAAAATTTGATAATG
GAAGAXXXXXXXXXXXCAATGCCACAAGATTTCAATCAAACTCCATTCATTTATATATACTTCTGAAGTGAAGATG
CAAAAAGGTAACAATCAGTA
>Marker836054
CACAATGCTATTAGTTGTAAATGTAATTCTTCCTGTCATTGTAGATTTCCAGTATGCCTTGGACACTTGAATGAT
CAATTXXXXXXXXXXXGTTAGAAGTGACTTTTTATTTTTTTAGTTTATATTGTGTCACAATTCATTTCTCTGCTAA
GTTATGTAAATAACTCAGTT
CACAATGCTATTAGTTGTAAATGTAATTCTTCCTGTCATTGTAGATTTCCAGTATGCCTTGGACACTTGAATGAT
CAATTXXXXXXXXXXXGTTAGAAGTGACTTTTTCTTTTTTTAGTTTATATTGTGTCACAATTCATCTCTCTGCTAA
GTTATGTAAATAACTCAGTT
>Marker836158
AACAACGAAACTTCATGTTTCAAATAACACTTTAAGTAATACTGCTTCCCATAAAACAAACTAGATGAGTGATGA
TTAGCXXXXXXXXXXXAGGCTTAAGAAACAGTTCTTTCAGAAATCCAAAACTTGAAGAATGAACACGGTAAAATGA
ATTTTGATATACAAAATGTT
AACAACGAAACTTCATGTTTCAAATAACACTTTAAGTAATACCGCTTCCCATAAAACAAACTAGATGAGTGATGA
TTAGCXXXXXXXXXXXAGGCTTAAGAAACAGTTCTTTCAGAAGTCCAAAACTTGAAGATTGAACACGGTAAAATGA
ATTTTGATATACAAAATGTT
>Marker836330
GACCAACCAAAAGGCTTCAAAAGGAGATCCCATAATTGTCGAGCAGAAGGACAATGAATAAAAAAATGAACATGA
GATTTXXXXXXXXXXXCCATCCAAATGATCGAATTAAAATCCCTTTCAGAAGGCTCGAGAGCACCTGTCAAGTCAT
CAATCATAGATTTCACAGTA
GACCAACCAAAAGGCTTCAAAAGGAGATCCCAAAATTGTCGAGCAGAAGGACAATGAATAAAAAAATGAACATGA
GATTTXXXXXXXXXXXCCATCCAAATGATCGAATTAAAATCCCTTTCAGAAGGCTCGAGAGCACCTGTCAAGTCAT
CAATCATAGATTTCACAGTA
>Marker836440
CACCTTTAACTAAAGGGAAGATGAAAAGCATTATCATAGAGACTAGGTAAGTTCTCTCCTTTCTCTCCTCTCTTT
TCCCTXXXXXXXXXXXCAAGATGAAGATTTTGAGGCAAATAAAATGCTGCCATTATCAAGCTCTGCTGATTTCAAA
AAAAGATGGATAGCTTTGTG
CACCTTTAACTAAAGGGAAGATGAAAAGCATTATCATAGAGACTAGGTAAGTTCTCTCCTTTCTCTCCTCTCTCT
TTTCCXXXXXXXXXXXCAAGATGAAGATTTTGAGGCAAATAAAATGCTGCCATTATCAAGCTCTGCTGATTTCAAA
AAAAGATGGATAGCTTTGTG
>Marker837129
CACTACATGTATTTACCTTTGTTTGTTTTGTATTAGAGTATGCCTTCTTGTCCTCCTTTAAGTGCAAGCACGTGT
AACTTXXXXXXXXXXXATGAACTGCCCAACTGCTTTGATCAAACAAGTGTGATTGTGAAACTTGATACAATGAGAT
GTAGAACATGAATTGGGGTG
CACTACATGTATTTACCTTTGTTTGTTTTGTATTAGAGTATGCCTTCTTGTCCTCCTTTAAGTGCAACCACGTGT
AACTTXXXXXXXXXXXATGAACTGCCCAACTGCTTTGATCAAACAAGTGTGATTGTGAAACTTGATACAATGAGAT
GTAGAACATGAATTGGGGTG
>Marker837272
CACATTCTTTAACTTTCTTTTCTGTTGTGGGGTGGGGGATGGTGCCAGCCAGCAAAGGGTGGATTTTAGTTCCCT
TGTCTXXXXXXXXXXXCCTAGTTCGTCCCATTATTTCAGCTTACTTAAAGATTACATTTGTATATGTTTCTGCATC
TTCATTAATTTCACCGAGTG
CACATTCTTTAACTTTCTTTTTTGTTGTGGGGTGGGGGTTGGTGCCAGCCAGCAAAGGGTGGATTTTAGTTCCCT
TGTCTXXXXXXXXXXXCCTAGTTCGTCCCATTATTTCAGCTTACTTAAAGATTACATTTGTATATGTTTCTGCATC
TTCATTAATTTCACCGAGTG
>Marker838006

Create PDF with GO2PDF for free, if you wish to

ACTGCATCACCAGCAATTTCATTGTCCAAGTAAAATCACAGTTTCATGGACTTTGCAAATGAAATAGAGTCGATC
TCAGAXXXXXXXXXXCAGGACAAAATTAACGATGACTTTTGTGTCAAAATCAGTTTCAAATGCAAAAATGGAAGT
TGAATGAAACAGTGTCTGTG
ACTGCATCACCAGCAATTTCATTGTCCAAGTAAAATCACAGTTTCATGGACTTTGCAAATGAAATAGAGTCGATC
TCAGAXXXXXXXXXXCAGGACAAAATTAACGATAACTTTTGTGTCAAAATCAGTTTCAAATGCAAAAATGGAAGT
TGAATGAAACAGTGTCTGTG
>Marker838431
AACGATTGTATGATGATGATGATCATTGTCCAAACCCTCACTATTTTCTTCTCTTTTCTTTAAATCAAGATTAGG
ATCAGXXXXXXXXXXGTTCTCTTCCGTATGTGGTTGGAGTATCTCGAACGACGTTGAACGATGGCACTATCCTTG
AAGTTGATTCAATTTGAGGT
AACGATTGTATGATGATGATGATCATTGTCCAAACCCTCACTATTTTCTTCTCTTTTCTTTAAATCAAGATTAGG
ATCAGXXXXXXXXXXGTTCTCTTCCGTATGTGGTTGGAGTATCTCGAACGACGTTGAACGATGGCTCTATCCTTG
AAGTTGATTCAATTTGAGGT
>Marker838436
AACAAATCGTGGGAGTGAAGAGCAGGGGATTGCGACGGCGCCAGATCAAAGATCGAAGCTTTGAGCTCTGCAATG
CTGCCXXXXXXXXXXAATTGGGGAAAAGAAGAGAGTTTAGAGAAACAGAGGTCTGGGAAGAGAGTGCCATTGCAA
AGGGATCGAGCAGTTTGGTG
AACAAATCGTGGGAGTGAAGAGCAGGGGATTGCGACGGCGCCAGATCGAAGATCGAAGCTTTGAGCTCTGCAATG
CTGCCXXXXXXXXXXAATTGGGGAAAAGAAGAGAGTTTAGAGAAACAGAGGTCTGGGAAGAGAGTGCCATTGCAA
AGGGATCGAGCAGTTTGGTG
>Marker838494
GACAAAATTGTAGAAGCCAATGGAAGCTATGAAGATGAACCAAATGCATATAATCGGAGCAAATGTGTAGCCAAC
TTTGTXXXXXXXXXXTCAAAAAACTTAATTAATTACTACTAATTGAAAAATCATTTGCGTATCAATCCTAAATGT
AATTGAATTATTTGTTAGTA
GACAAAATTGTAGAAGCCAATGGAAGCTATGAAGATGAACCAAATGCATATAATCGGAGCAAATGTGTAGCCAAC
TTTGTXXXXXXXXXXTCAAAAAACTTAATTAATTACTACTAATTGAAAAATCATCTGCCTATCAATCCTAAATGT
AATTGAATTATTTGTTAGTA
>Marker838801
AACATATCAAAATTTAAAAATTTATCTTCCATATGTCAATAAAAATTTAATACATATAGTATTCATAATGCTTTC
TAGTCXXXXXXXXXXCATTTTTGTCAATCATGAAATCACTAACTATGCAATGCAGAGAAGGGCCAAAGAAAGAAA
GAAGACCAAAAGATTTTGGT
AACATATCAAAATTTAAAAATTTATCTTCCATATGTCAATAAAAATTTAATACATATAGTATTCACAATGCTTTC
TAGTCXXXXXXXXXXCATTTTTGTCAATCATGAAATCACTAACTATGCAATGCAGAGAAGGGCCAAAGAAAGAAA
GAAGAACAAAAGATTTTGGT
>Marker838934
AACTACGTTCTCTTTGAAATGATTTCTTTTTCGTTTCGATGGGGCTCAGTCACCGCCACAATGTTAAACAAAAAA
AAAAAXXXXXXXXXXXXTAATGATTGAGTTTTGTTTTATTGGTTGAATTTAGGAATAATGATGATTTATGTGTATGA
ATATATACATATGATATGTT
AACTACATTCTCTTTGAAATGATTTCTTTTTCGTTTCGATGGGGCTCAGTCACCGCCACAATGTTAAACAAAAAA
GAAAAXXXXXXXXXXXXTAATGATTGAGTTTTGTTTTATTGGTTGAATTTAGGAATAATGATGATTTATGTGTATGA
ATATATACATATGATATGTT
>Marker839242
ACTTTTAAAAAAGAAAAAAAATATATGTAAAAATAGGGTATGGAATTTAGGTTTTTTCTAAGCGAATTAACAAAA
TTTTAXXXXXXXXXXCTAAAACTTACATTCACACATACATAATATTGCAAGTTTTATAAAGTTCAAAGTTTATTT
TATAATTTATATTTTTTAGT

Create PDF with GO2PDF for free, if you wish to

ACTTTTAAAAAAGAAAAAAAATATATGTAAAAATAGGGTATGGAATTTAGGTTTTTTCTAAGCGAATTAACAAAA
TTTTAXXXXXXXXXXXCTAAAACTTACATTCACACATACATAATATTGCAAGTTTTATAAAGTTCAAAGTTTATTT
TATAATTTTTATTTTTTAGT
>Marker839292
AACCTTAGCTCCAAGGGTTCAATTTATATAGCTCATAAACTCCTCATACCCAACCCAAGATCATATCTTACTTCC
AAACTXXXXXXXXXXXTGGTTATCATCATTTCTTAGAACTTGCTCGTTTAAACTTTACATAAGATCTCGGTAACTT
CTAATAAATCCAGAAAAGTT
AACCTTAGCTCCAAGGGTTCAATTTATATAGCTCATAAACTCCTCATACCCAACCCAATATCATATCTTACTTCC
AAACTXXXXXXXXXXXTGGTTATCATTATTTCTTAGAACTTGCTCGTTTAAACTTTACATAAGATCTCGGTAACTT
CTAATAAATCCAGAAAAGTT
>Marker839470
AACCAATGATAGATGGAGAAGCTGGAGATCTTAGAGTTAGTGTTTTTCCTATTATTCAACTTGTTCTTTTGTGAT
TGTTTXXXXXXXXXXXCAATTTAATGTTGTTAATAGTGTTCCCATGCAATACTTTATTTTTAACAACTAATCGATG
TGAGATTATTTGCCTTTGTG
AACCAATGATAGATGGAGAAGCTGGAGATCTTAGAGTTAGTGTTTTTCCTATTATTCAACTTGTTCTTTTGTGAT
TGTTTXXXXXXXXXXXCAATTTAATGTTGTTAATAGTGTCCCCATGCGATACTTTATTTTTAACAATTAATCGATG
TGAGATTATTTGCCTTTGTG
>Marker839540
TACTGGTGACATTTCTACAACAATGAATCTGATGGTTTTACATCTAAAAACTGATGAAGGCATGAACATGAACTA
CCAGAXXXXXXXXXXXAAGCAGTCTCAGATTATAACCACAAACACATATGCAACAAATCATAACATGTGGTATTAT
GCTTACTGCGGTAGGGGGTA
TACTGGTGACATTTCTACAACAATGAATCTGATGGTTTTACATCTAAAAACTGATGAAGGTATGAACATGAACTA
CCAGAXXXXXXXXXXXAAGCAGTCTCAGATTATAACCACAAACACATATGCAACAAATCATAACATGTGGTATTAT
TCTTACTGCGGTAGGGGGTA
>Marker839897
ACTTGGCCTCTGAAACATTCTTAAGCATAACCATCGATGTTCCTCGCTCTTTTACGATTCGCTCGTCATTTGGCA
AATTGXXXXXXXXXXXAAACTACTGGTCTCCACAAGCATTTTTAATAATCAACATTCAATTTCTTGGGGGATGATA
GTTCTCAATTCAGCGTAGTT
ACTTGGCCTCTGAAACATTCTTAAGCATAACCATTGATGTTCCTCGCTCTTTTACTATTCGCTCGTCATTTGGCA
AATTGXXXXXXXXXXXAAACTACTGGTCTCCACAAGCATTTTTAATAATCAACATTCAATTTCTTGGGGGATGATA
GTTCTCAATTCAGCGTAGTT
>Marker840166
CACTTGAATCCTTGTTGAGATATAAGATGCACTCTTGAATGTTAATACTTTATTGGCATTTTGGTTCTGGTTGCA
GAAGAXXXXXXXXXXXGCCAACTTCGTAACTAGTTTTGATTTTCCATCTTACTAAACTGACTTTGGATTGCTTTAA
ATTGTTTCCAAGAGGGTGTA
CACTTGAATCCTTGTTGAGATATAAGATGCACTCTTGAATGTTAATACTTTATTGGCATTTTGGTTCTGGTTGCA
GAAGAXXXXXXXXXXXGCCAACTTCGTAACTAGTTTTGATTTTCCGTCTTACTAAACTGACTTTGGATTGCTTTAA
GTTGTTTCCAAGAGGGTGTA
>Marker840548
ACAACATAGGTAGCGACCTTAGAGAACAATAGAGCAGACATGAAAATAGTATGCAAAGAGAAAATCAATGTAGTT
CTAGAXXXXXXXXXXXATGTTAAAATTATACTTCCTGCTAAACAAATATCCCAACACAAATATTCAAGAAATTCAC
AAGACAAACACCCTTGTGTA
ACAACATAGGTAGCGACCTTAGAGAACAATAGTGCAGACATGAAAATAGTATGCAAAGAGAAAATCAATGTAGTT
CTAGAXXXXXXXXXXXATGTTAAAATTATACTTCCTGCTAAACAAATATCCCAACACAAATATTCAAGAAATTCAC
AAGACAAACACCCTTGTGTA
>Marker840881

Create PDF with GO2PDF for free, if you wish to

CACATGGAGGCCACATTCACAAATTTTCTATCAGTGATAATTATAATAATAATTAGGGCAATTTAAAAAATGTCA
TACTTXXXXXXXXXXXACTTAAAATTCAATTAAAATACAAAAGAGCGATGGACTAGAAGAAAGGCTTCCAATTGAG
TGAAATACAAGATGCATTGT
CACATGGAGGCCACATTCACAAATTTTCTATCAGTGATAATTATAATAATAATTAGGGCAATTTAAAAAATGTCA
TACTTXXXXXXXXXXXACTTAAAATTCGATTAAAATACAAAAGAGAGATGGACTAGAAGAAAGGCTTCCAATTGAG
TGAAATACAAGATGCATTGT
>Marker841157
CACTTCTTCTATCACTATCAAACCCATAAAATATGAAAATATCGATAGAAATTTAAAACTATGAATAATTTAAAA
TCTTAXXXXXXXXXXXTGTTAATTATGTAATCATGTTTTACAAGATGATTGGAAGCCTTGTAATTACGCATATTAT
TGATTGTTAATAATTAGTGT
CACTTCTTCTATCACTATCAAACCCATAAAATATGAAAATATCGATAGAAATTTAAAACTATGAATAATTTAAAA
TCTTAXXXXXXXXXXXTGTTAATTATGTAATCATGTTTTACAAGATGATTGGAAGCCTTGTAATTACGCATATTAC
TGATTGTTAATAATTAGTGT
>Marker841294
AACCATGAAATGGTAATAAAAACTACCTTTAACATCAAAAGTAATAAAGGTGGAGCATACAGAAGAATGTTCATC
TTTATXXXXXXXXXXXAAAAGAAATAAGGCCAATGCAGCATGGAGAAGAGTCATGGCAAAGCAGTCATTGAATAGA
CGGAGCATAAAGATGGAGTG
AACCATGAAATGGTAACAAAAACTACCTTTAACATCAAAAGTAATAAAGGTGGAGCATACAGAAGAATGTTCATC
TTTATXXXXXXXXXXXAAAAGAAATAAGGCCAATGCAGCATGGAGAAGAGTCATGGCAAAGCAGTCATTGAATAGA
CGGAGCATAAAGATGGAGTG
>Marker841630
ACCATTCCAGCACAAATGTAAAGTCAAGGAACAGGGGGAGTTGCGAATATTCGTAGTGCAAAAATATAACGAAGT
GGAGAXXXXXXXXXXXTAAAGGGGAGGATCAAAGGAAAGGAAGTGATAGTATTAATCGATTGTGGTGCTACACAAA
ATTTTATATTTGAGAAGGTT
ACCATTCCAGCACAAATGTAAAGTCAAGGAACAGGGGGAGTTGCGAATATTCGTAGTGCAAAAATATAACGAAGT
GGAGAXXXXXXXXXXXTAAAGGGGAGGATCAAAGGAAAGGAAGTGATAGTATTAATCGATTGTGGTGCTACACAAA
ATTTTATATCTGAGAAGGTT
>Marker841829
ACAGACCATTGCAACAAATGGCATCACAGGCTGAAGAATGGCTACGATTGGCTTTGCATAAGAATTAAGAACGAG
TCCAAXXXXXXXXXXXATACACCATTTCTTGATCGTAGCGAGAGGAGATAAAATTTAGTTATAGCTCAGCAAGAGG
AAAGTATGCGATATAACAGT
ACAGACCATTGCAACAAATGGCATCACAGGCTGAAGAATGGCTACGATTGGCTTTGCATAAGAATTAAGAACGAG
TCCAAXXXXXXXXXXXATACACCATTTCTTGATCGTAGCAAGAGGAGATAAAATTTAGTTATAGCTCAGCAAGAGG
AAAGTATGCGATATAACAGT
>Marker841962
AACTACCTTCAGTTATTTATGGAAATGAACATGTTTGTTCATGATGTATAACTTTTCATTGTGGCATTTTTATCT
AAAGAXXXXXXXXXXXAATTGTCTCTTTGCCTATTCTGATTTCTAATGTTGTGATTTTTCTTGTTTCAGATTCCTC
AATTTGTTTTAGAAGCTGTT
AACTACCTTCAGTTATTTATGGAAATGAACATGTTTGTTCATGATGTATATCTTTTCATTGTGACATTTTTATCT
AAAGAXXXXXXXXXXXAATTGTCTCTTTGCCTATTCTGATTTCTAATGTTGTGATTTTTCTTGTTTCAGATTCCTC
AATTTGTTTTAGAAGCTGTT
>Marker842295
AACGTGTATAACGAGAGAGAGCTTTTGACATTTAGCATGAGTGACTCGAATGGCCTTGCTAGACAATGTAGAAGC
AGAATXXXXXXXXXXXATAGGTTCGTCCGGGGTATGATGAAGTTGATCTCATTGCCATGTTTGTCATTGGTAGGTT
GGAGAGGTAAGATTGTGGGT

Create PDF with GO2PDF for free, if you wish to

AACGTGTATAACGAGAGAGAGCTTTTGACATTTAGCATGAGTGACTCGAATGGCCTTGCTAGACAATGTAGAAGC
AGAATXXXXXXXXXXXATAGGTTCGTCCCGGGTATGATGAAGTTGATCTCATTGCCATGTTTGTCATTGGTAGGTT
GGAGAGGTAAGATTGTGGGT
>Marker843159
AACGACAAGAAGGATTCGTGACATGCCATATAGGAACACAATTTTACTTCTGCTTTTCATTGATCATTTTTAGTG
TTTTGXXXXXXXXXXXATCTTTAAAGTAATGAATTTAGCTTAGTATAAAAAGTTGAGTTGTTACATAAAAGGGCTA
AGTAGTTTGTTAAAAGTGTT
AACGACAAGAAGGATTCGTGACATGCCATATAGGAACACAATTTTACTTCTGCTTTTCATTGATCATTTTTAGTG
TTTTGXXXXXXXXXXXATCTTTAAAGTAATGAATTTAGCTTAGTATAAAGAGTTGAGTTGTTACATAAAAGGGCTA
AGTAGTTTGTTAAAAGTGTT
>Marker843667
AACTCAGTATTGGGTATTTCAGATATATATTTATAACTAAAATTCTGATTTTGATTGGTCTTTTTTAGAGAGAGA
GAAAAXXXXXXXXXXXTACCACGTTTGTGACGAGCTAAAAACTTAAAACAATGGGTTTGTATTGATATGTCATATT
GATGCTTTTGTATAATTGTT
AACTCAGTATTGGGTATTTCAGATATATATTTATAACTAAAATTCTGATTTTGATTGGTCTTTTTTAGAGAGAGA
GAAAAXXXXXXXXXXXTACCACGTTTGTGACGAGCTAACAACTTAAAACAATGGGTTTGTATTGATATGTCATATT
GATGCTTTTGTATAATTGTT
>Marker843736
TACCCTTTATAGTAAATCCATTAATAATGAATACATAAATTTTTAATCTTGACGTCATAATCTATAACAAATTAG
GAAATXXXXXXXXXXXAGATGTGATAAGTGATCCCATAACGAAATCGTGGCAATGAAATGTTACAGGAAGGTGACG
CGTGGCACTTGCCTTGCTGT
TACCCTTTATAGTAAATCCATTAATAATGAATACATAAATTTTTAATCTTGACGTCATAATATATAACAAATTAG
GAAATXXXXXXXXXXXAGATGTGATAAGTGATCCCATAACGAAATCGTGGCAATGAAATGTTACAGGAAGGTGACG
CGTGGCACTTGCCTTGCTGT
>Marker844453
TACTTTTTTCCCTTTTCCTTTCTGGCCACTCCGGCATGGATGTATTAAAATCGGTTCATCTTTTTCCAGCCTAAT
CATTTXXXXXXXXXXXTCCCTTGCCAAATATACAACAAGTCCAAGCTAGTTGAAGGAAATTCAGATAGATCATCAA
TCCTGACAAGATTGTTCGTA
TACTTTTTTCCCTTTTCCTTTCTGGCCACTCCGGCATGGATGTATTAAAATCGGTTCATCTTTTTCCAGCCTAAT
CATTTXXXXXXXXXXXTCCCTTGCCAAATATACAACAAGTCCAAGCTAGTTGAAGGAAATTCAGATAGATCATCGA
TCCTGACAAGATTGTTCGTA
>Marker844720
TACCTCAGTAAAGTTACGCCCCCACCTGGAACGTGTCCCTCCCTTGCGTTCCCAGGCGCGAACGCGACTTGGGCA
GCTCCXXXXXXXXXXXCTTGTCATATGATATAAGAACGCAACACTTAAGAACTCGAGGGAACTTAGCAATTTTATC
AACTCCTTAACAAACTTAGT
TACCTCAGTAAAGTTACGCCCCCACCTGGAACGTGTCCCTCCCTTGCGTTCCCAGGCGCGAACGCGACTTGGGCA
GCTCCXXXXXXXXXXXCTTGTCATATGATATAAGAACGCAACACTTAAGAACTCGAGGGAACTTAGCAATTTTATT
AACTCCTTAACAAACTTAGT
>Marker845139
CACTAAAACTCGTGCGCCTACACTTCTCAACAAAATAGGGTGGCTGAGAACCGTCATCTTCTGGAAGTAGCCCAC
TTCTTXXXXXXXXXXXAGACTCCATTAGATTGTCTTAAAGAGTCATATCCTACTACTCGTCTAATCTATGAGGTTC
CCTTTCGTGTGTTTGGGTGT
CACTAAAACTCGTGCGCCTACACTTCTCAACAAAATAGGGTGGCTGAGAACCGTCATCTTCTGGAAGTAGCCCAC
TTCTTXXXXXXXXXXXAGACTCCCTTAGATTGTCTTAAAGAGTCATATCCTACTACTCGTCTAATCTATGAGGTTC
CCTTTCGTGTGTTTGGGTGT
>Marker845219

Create PDF with GO2PDF for free, if you wish to

TACCTCCTAAAGGCACTCCCAAATATTGGATGGGAAGAAAGTTTATGGAGAAACCCCATTTTGATGTCACGCAAT
TTGTTXXXXXXXXXXCAGGAGGTGAGTGAGGTTGTGTTTCTTGTTGAAACTTACACCTTTAATCAAGTTATCTTT
TTCCAATTGATTGAGGAGTA
TACCTCCTAAAGGCACTCCCAAATATTGGATGGGAAGAAAGTTTATGGAGAAACCCTATTTTGATGTCACGCAAT
TTGTTXXXXXXXXXXCAGGAGGTGAGTGAGGTTGTGTTTCTTATTGAAACTTACACCTTTAATCAAGTTATCTTT
TTCCAATTGATTGAGGAGTA
>Marker845596
TACAAGAAAATAACAATTTGGAAATGATTGATCACTAAAGCTCTAAAACTTCTCAGAATGGAATTACTTACTTCA
TTGTAXXXXXXXXXXAATTTGATAGCAGCCAGAAGCAAGGACGAAAAATAAGCTAATAAGGAAAAAGATAACTGT
AAAGCAGAAGTCATGCCGTG
TACAAGAAAATAACAATTTGGAAATGATTGATCACTGAAGCTCTAAAACTTCTCAGAATGGAATTACTTACTTCA
TTGTAXXXXXXXXXXAATTTGATAGCAGCCAGAAGCAAGGACGAAAAATAAGCTAATAAGGAAAAAGATAACTGT
AAAGCAGAAGTCATGCCGTG
>Marker845883
GACTATTTTGAAACTTCTAGTCTAGTCGTGAAGAAATCAATAGTAAGAATTGTCCTTACCCTAGTTGCTACTTGT
AATTGXXXXXXXXXXTTGTTAAATAAATAAGTCCTTGTATGAATTTAAACAAGCACCTTGAGCATATAGTTTAAT
TATTTTACAACTCACTTGTT
GACTATTTTGAAACTTCTAGTCTAGTCGTGAAGAAATCAATAGAAAGAATTGTCCTTACCCTAGTTGCTACTTGT
AATTGXXXXXXXXXXTTGTTAAATAAATAAGTCCTTGTATGAATTTAAACAAGCACCTTGAGCATATAGTTTAAT
TATTTTACAACTCACTTGTT
>Marker846192
AACGATTTTCCTAACAAAATAGGAAGATGTGAATTCGGAAATGAAATATCTATTGCTACGTTGATATATCCGTAT
AATTAXXXXXXXXXXTTAGTTTGATTTTAAAAATAAATTAACTATTAATTTAAATAGTTATTATGAACTTATCAA
CACAAAAATATTTGTTGGGT
AACGATTTTCCTAACAAAATAGGAAGATGTGAATTCGGAAATGAAATATCTATTGCTACGTTGATATATCCGTAT
AATTAXXXXXXXXXXTTAGTTTGATTATAAAAATAAATTAACTATTAATTTAAATAGTTATTATGAACTTATCAA
CACAAAAATATTTGTTGGGT
>Marker846202
CACATTTAATTGTTTAAATATGTTGTTTGAAATGTTAATTAAGACATTAATTGGTTGAAGAAATAACATATATTT
TATATXXXXXXXXXXTTCATGTTAGCGGCTTCTTAAAGATGATAAAGGATCCACTCCCAATATATACATATATAT
GAATATAAAATCATAGTGTG
CACATTTAATTGTTCAAATATGTTGTTTGAAATGTTAATTAAGACATTAATTGGTTGAAGAAATAACATATATTA
TATATXXXXXXXXXXTTCATGTTAGCGGCTTCTTAAAGATGATAAAGGATCCACTCCCAATATATACATATATAT
GAATATAAAATCATAGTGTG
>Marker846328
CACTCTTCAAACTTGAACCTCAACTTCATCTAGAAAGAGAGAAAAAACCCCTAAGAACATGGGATCCAAATAAAT
AAGTGXXXXXXXXXXACATTTAGGCCTAGCTTGTGAGATGAGTTTGGTAGTCAAATCTCAAACACTTATGCCTGG
CTCGTGAGATGAGTCTGGTA
CACTCTTCAAACTTGAACCTCAACTTCATCTAGAAAGAGAGAAAAAACCCCTAAGAACATGGGATCCAAATAAAT
AAGTGXXXXXXXXXXACATTTAGGCCTAGCTTGTGAGATGAGTTTGGTAGTCGAATCTCAAACACTTATGCTTGG
CTCGTGAGATGAGTCTGGTA
>Marker848777
CACTATTTGAAAATTGACTAGAAAACGAAATTAGAATCTAGATATCATTACAACCTATCAATCAGTCTAAACTTT
CATCAXXXXXXXXXXACTTATTTTCCTTTTCATTCTTTTAAATCTTGTGTTTGATAGATGAATGATGTTGGATGT
ATTCTATATTTTTTAACGTT

Create PDF with GO2PDF for free, if you wish to

CACTATTTAAAAATTGACTAGAAAACGAAATTAGAATCTAGATATCATTACAACGTATCAATCAGTCTAAACTTT
CATCAXXXXXXXXXXACTTATTTTCCTTTTCATTCTTTTAAATCTTGTGTTTGATAGATGAATGATGTTGGATGT
ATTCTATATTTTTGAACGTT
>Marker849250
AACTCAAAAGATGATATTCTTTTCTTCTTTTCTGTTTTGATTATTGGATGGTGATTCAAAATATATATGTCGTTT
AACAAXXXXXXXXXXAATTTGATTTTTTGGGCACTAGTTCCTTTCTATCATGTCGAATATTCTTTGGTTGGTTTT
TGAGATTTTTGTATTTTGGT
AACTCAAAAGATGATATTCTTTTCTTCTTTTCTGTTTTGATTATTGGATGGTGATTCAAAATATATATGTCGTTT
AACAAXXXXXXXXXXAATTTGGTTTTTTGGGCACTAGTTCCTTTCTATCATGTCGAATATTCTTTGGTTGGTTTT
TGAGATTTTTGTATTTTGGT
>Marker849560
GACTTCAATGCCATAAATTAAAAGGTTTCAATATAACTTATTTTCTTATGTTTATAGTATTTTGTGAGAGTTGTA
TCAACXXXXXXXXXXATAGCATCCGCAGAGACTCAGTTCAACTTAAACTAGCCAACAGTATGAAGAATGACAATC
CAACTTCCTGTATGTTTGTT
GACTTCAATGCCATAAATTAAAAGGTTTCAATATAACTTATTTTCTTATGTTTATAGTATTTTGTGAGAGTTGTA
TCAACXXXXXXXXXXATAGCATCCGCAGAGACTCAGTTCAACTTAAACTAGCCAACAGTATGAAGCATGACAATC
CAACTTCCTGTATGTTTGTT
>Marker849628
GACTTAGGTATATATTTATGGAGTGACTTTTTTTATTTTGTTGAACAGCAAACATTGGAGGCATTGCTTAAAGCA
GGCTAXXXXXXXXXXCTTTTAAATTTATTTATGACATTTGTTCATATCAGATTTTTCATAGTTTTTAAAAAGCCA
ATTCTTGTATTTTTATTGTT
GACTTAGGTATATATATATGGAGTGACTTTTTTTATTTTGTTGAACAGCAAACATTGGAGGCATTGCTTAAAGCA
GGCTAXXXXXXXXXXCTTTTAAATTTATTTATGACATTTGTTCATATCAGATTTTTCATAGTTTTCAAAAAGCCA
ATTCTTGTATTTTTATTGTT
>Marker849953
AACATATATTCTATTAAATTTCACTTTTCAAGCTATTTTAATTTTAAAAATAAGTTATCTTGAAAATATTTGGGT
GTTCTXXXXXXXXXXTCAAAATCATTAAACATGAAACTCAATTATTCTCAAATATTAAGTAGTGACTAAAATATT
GAAATTATTTACAAACAGGT
AACATATATTCTATTAAATTTCACTTTTCAAACTATTTTAATTTTAAAAATAAGTTATCTTGAAAATATTTGGGT
GTTCTXXXXXXXXXXTCAAAATCATTAAAGATGAAACTCAATTATTCTCAAATATTAAGTAGTGACTAAAATATT
GAAATTATTTACAAACAGGT
>Marker850146
ACAGACGATGTCCAATGCTGCATTTCCATTTTTGATGCCTGTGTTCGTGATTCTTTCGAATATCATTTCTGACTC
TAGATXXXXXXXXXXTCTCGATTCAAGAACTGGCCGATAGTCATGTTTTAAAGCGTTAGCTAAATATCTGAAATC
ACATCCTGAAAGATGAAGGT
ACAGACGATGTCCAATGCTGCATTTCCATTTTTGATGCCTGTGTTCGTGATTCTTTCGAATATCATTTCTGATTC
TAGATXXXXXXXXXXTCTCGATTCAAGAACTGGCCGATAGTCATGTTTTAAAGCGTTAGCTAAATATCTGAAATC
ACATCCTGAAAGATGAAGGT
>Marker850466
CACTAACTATTCTTGAGATTTCCTGGTATTTTTTCTCACTAATGTTATTCCTTGCATTCGTTCAGTAAAATTCAT
CAATGXXXXXXXXXXTTTTCCCTACTCTTTTTAAGTAGCTTTTCGTTGGTCCGACCATGCAAAGATAATGGATTG
TAGTTTAATACAAGAAGGTT
CACTAACTATTCTTGAGATTTCCTGGTATTTTTTCTCACTAATGTTATTCCTTGCATTCGTTCAGTAAAATTCAT
CAATGXXXXXXXXXXTCTTCCCTACTCTTTTTAAGTAGCTTTTCGTTGGTCCGACCATGCAAAGATAATGGATTG
TAGTTTAATACAAGAAGGTT
>Marker851723

Create PDF with GO2PDF for free, if you wish to

ACTGGCATGAAATAGAGAATAAAGCAGTTGGTGCCCCTTCATATTTACCTTCAGAGGTAATTTTAACATCTCTCT
CTATTXXXXXXXXXXTTGGATAAATGATCGCCTAAAGGGCATTTGTATTTGGTAGTAACCAAGGGTCCCTTCTCG
TTTATTTCCGCCAAAACAGT
ACTGGCATGAAATAGAGAATAAAGCAGTTGGTGCCCCTTCATATTTACCTTCAGAGGTAATTTTAACATCTCTCT
CTATCXXXXXXXXXXTTGGATAAATGATCGGCTAAAGGGCATTTGTATTTGGTAGTAACCAAGGGTCCCTTCTCG
TTTATTTCCGCCAAAACAGT
>Marker852986
ACCAACCATATACTTGACCTGCTGATAGTTGTAAGATTCCTCAAATCATTAGTTCTGATAGTTCTGCCTTTATTC
ATGCTXXXXXXXXXXGGTTCTAATCCCTCCGTTTTTTATGTTCATGGAAAAACTCTGAAAGAAAAGGCATTGTTA
CTACGGAGTCTTCCAAGAGT
ACCAACCATATACTTGACCTGCTGATAGTTGTAAGATTCCTCAAATCATTAGTTCTGATAGTTCTGCCTCTATTC
ATGCTXXXXXXXXXXGGTTCTAATCCCTCCGTTTTTGATGTTCATGGAAAAACTCTGAAAGAAAAGGCATTGTTA
CTACGGAGTCTTCCAAGAGT
>Marker853155
AACCTTAACATTAATTTCACGTCTTCGTAACCTCTCTTATTGAAATATGATATAGTTTCATTAATGTCTCATTCC
TAAACXXXXXXXXXXATGTATGAGAGTCTAAAGTTTAAATTAATACAACTTCGAAATCAAGAATTTAAATAGATA
CAATTTCAAATTCAAAAGTT
AACCTTAACATTAATTTCACGTCTTCATAACCTCTCTTATTGAAATATGATATAGTTTCATTAATGTCTCATTTC
TAAACXXXXXXXXXXATGTATGAGAGTCTAAAGTTTAAATTAATACAACTTCGAAATCAAGAATTAAAATAGATA
CAATTTCAAATTCAAAAGTT
>Marker853236
AACCATTACTTCTTAACTTGGGTATGGGTCTTACAATTGGTCTCTGTAGAGGTTTCTGGTGTATTATTGAGGATT
TTAATXXXXXXXXXXGATCCTCTTTTGTTGCTTCTAAGTTTGATAGATTCCTTCTCTTCAAGCATTGGGTAGATT
TTTTTCAAAATGTTGTTGTG
AACCATTACTTCTTAACTTGGGTATGGGTCTTACAATTGGTCTCTGTAGAGGTTTCTGGTGTATTATTGGGGATT
TTAATXXXXXXXXXXGATCCTCTTTTGTTGCTTCTAAGTTTGATAGATTCCTTCTCTTCAAGCATTGGGTAGATT
TTTTTCAAAATGTTGTTGTG
>Marker853346
TACGTTGATGCAACGTCAAGAATTTTCAAACAATTAAAATGAACAGCAAGCACAACTATATGGCCATTAGGCATT
ATATCXXXXXXXXXXATAATGATAAATTGGCTATAAAGGCACCAACAAATTTGCACAGTTGGCTGTTGTATGCAA
GAGAAATACATTTACCTGTG
TACGTTGATGCAACGTCAAGAATTTTCAAACAATTAAAATGAACAGCAAGCACAACTATACGGCCATTAGGCATT
ATATCXXXXXXXXXXATAATGATAAATTGGCTATAAAGGCACCAACAAATTTGCACAGTTGGCTGTTGTATGCAA
GAGAAATACATTTACCTGTG
>Marker853570
ACTAGAAATCATATATACATCTTCCATCAGCATCATCTCATGTTTCTTGTTCCTGCCTCCGATCACCAAACTTGA
ATCTTXXXXXXXXXXGTGTCTGCACAGAAGCTGGAATGTATGCCATCCGAGCTCGAAGGAAGACTGTGACAGAGA
AGGACTTCCTTGACGCAGTG
ACTAGAAATCATATATACATCTTCCATTAGCATCATCTCATGTTTCTTGTTCCTGCCTCCGATCACCAAACTTGA
ATCTTXXXXXXXXXXGTGTCTGCACAGAAGCTGGAATGTATGCCATCCGAGCTCGAAGGAAGACTGTGACAGAGA
AGGACTTCCTTGACGCAGTG
>Marker854190
GACAAGAGGAGACGGTGTGGCCTGACATATGCCTCTAGACTGCATTGTGATAAGTGAAGTAATAGAGATGATATC
TAATTXXXXXXXXXXGGACAAGAAATATGGAATGTCCGGGGAAGTGGATAGAGATCAAACTTTTGGCTCCTGGTT
TGTTTTTATGTTTCTTTGTG

Create PDF with GO2PDF for free, if you wish to

GACAAGAGGAGACGGTGTGGCCTGACATATGCCTCTAGACTGCATTGTGATAAGTGAAGTAATAGTGATGATATC
TAATGXXXXXXXXXXXGGACAAGAAATATGGAATGTCCGGGGAAGTGGATAGAGATCAAACTTTTGGCTCCTGGTT
TGTTTTTATGTTTCTTTGTG
>Marker854440
ACTTCTAGATCTGAGGGAGGACAACCAGGCGGCGAGGGGAGCGAGGTCCCACACGAGCTAGCATCTCCAGAGAAG
TAAAGXXXXXXXXXXXGAGAAGAGGTTGGCCTATCGATACTATTCTTCCAACTACTTTGTAACCCAACCTTACAAC
AGTAGAGACATGTCGAGGTG
ACTTCTAGATCTGAGGGAGGACAACCAGGCGGCGAGGGGAGCGAGGTCCCACACGAGCTAGCATCTCCGGAGAAG
TAAAGXXXXXXXXXXXGAGAAGAGGTTGGCCTATCGATACTATTCTTCCAACTACTTTGTAACCCAACCTTACAAC
AGTAGAGACATGTCGAGGTG
>Marker854623
ACATTTGTTCGTCAATCCCAAGATTTTCTTTGATTATAATTTATCCTTTTGGTGGCTTCAACAGTTGCTCCCAAA
TGTTCXXXXXXXXXXXTGTCATGATTTCTTTAGATGTTCTTTAAAAGAGAAAGAGATGAAAGGTAGAGATTGTCCC
ACTAGGCGTGCCAAACTGTT
ACATTTGTTCGTGAATCCCAAGATTTTCTTTGATTATAATTTATCCTTTTGGTGGCTTCAACAGTTGCTCCCAAA
TGTTCXXXXXXXXXXXTGTCATGATTTCTTTAGATGTTCTTTAAAAGAGAAAGATATGAAAGGTAGAGATTGTCCC
ACTAGGCGTGCCAAACTGTT
>Marker854977
ACTTCTCAAGTCTTGCAATTCTAAAGATGAGTATAAAACAACTTGTCGTATTAAACACTATATATTCTCATTCAA
ACGTGXXXXXXXXXXXGCTTTTACCTACAACAATGACACAAATTAATTTTTTAATTTAAATAATGGTAAAAATATA
CTCACTTTTGTGAGATTGTA
ACTTCTCAAGTCTTGCAATTCTAAAGATGAGTATAAAACAACTTGTCGTATTAAACACTATATATTCTCATTCAA
ACGTGXXXXXXXXXXXGCTTTTACCTACAACAATGACACAAATTAATTTTTTAATTTAAATAATGGTAAAAATATA
CTCACTTTCGTGAGATTGTA
>Marker855505
TACATCTATTTTTAACCTCATTCGACACACGAGTTATTTTTCCCTTTACAATCCAAAACATTCATAATTTTCAAA
CTATTXXXXXXXXXXXAATCAACATTTATTCACAATATATTCTAATCATACCACAATGAAAACTTTATTTGCTAAT
TATAACGATCTCAATCAAGT
TACATCTATTTTTAACCTCATTCGACACACGAGTTATTTTTCCCTTTACAATCCAAAACATTCATAATTTTCAAA
CTATTXXXXXXXXXXXAATCAACATTTATTCACAATATATTCTAATCATACCACAATGAAAATTTTATTTGCTAAT
TATAACGATCTCAATCAAGT
>Marker855936
GACCCATGTGGTAGAAAGTGATCGAATTTTTGTTGCTGGTGTTGGTTTGCTTCAATGAAAAGTTTCTTTTATTTG
TTGTTXXXXXXXXXXXACCAATCTTAATGTGGTTTATGATCTTTTTTTTCTAAATTTACTTTCCAAATTGGATATA
TATAATTTTTTCATGATGTT
GACCCATGTGGTAGAAAGTGATCGAATTTTTGTTGCTGGTGTTGGTTTGCTTCAATGAAAAGTTTCTTTTATTTG
TTGTTXXXXXXXXXXXACCAATCTTAATGTGGTTTATGATTTTTTTTTTCTAAATTTACTTTCCAAATTGGATATA
TATAACTTTTTCATGATGTT
>Marker857256
CACAACATTACTTCGTATTTACTTTTTTGAATGAATGCATTCCTTTTATATTATATATTTAAGATGATGGTGCTA
ATTAGXXXXXXXXXXXTAACTAGCAAGTTTAAAATGTATTTAACGAAAATGACTTTGAAAGCAAAAGTCAAAGCTA
AGGTTTCTTAATTTCCTAGT
CACAACATTACTTCGTATTTACTTTTTTGAATGAATGCATTCCTTTTATATTATATACTTAAGATGATGGTGCTA
ATTAGXXXXXXXXXXXTAACTAGCAAGTTTAAAATGTATTTAACGAAAATGACTTTGAAAGCAAAAGTCAAAGCTA
AGGTTTCTTAATTTCCTAGT
>Marker857279

Create PDF with GO2PDF for free, if you wish to

TACATGGTTTGATAAAACCTTTATAAAAGTTCGAATATAAATGCTCCGGGAAGTAAATAAATTGAACAAGAATAA
GGGGCXXXXXXXXXXTATCAATAGGCTCTCCAAGCACGTTGAAATTCAGCCAAGAGTCGCTCCCCAATTGAAACA
CTTAGAGGAGTTCTCGTGTC
TACATGGTTTGATAAAACCTTTATAAAAGTTAGAATATAAATGCTCCAGGAAGTAAATAAATTGAACAAGAATAA
GGGGCXXXXXXXXXXTATCAATAGGCTCTCCAAGCACGTTGAAATTCAGCCAAGAGTCGCTCCCCAATTGAAACA
CTTAGAGGAGCTCTCGTGTC
>Marker857444
TACCTGTGGTTGTGAGCGACAACATCGAGCTTCCATTTGAAGACATGGTGGATTACTCAGAATTCTCTGTTTTTG
TAGCTXXXXXXXXXXTTGAATATGAAAATGGCCATCCGGGTGGTATTGGACCAGTTCCTCCAGATGGTGCTGTAA
ATCACATATGGAGAAAAGTG
TACCTGTGGTTGTGAGCGACAACATTGAGCTTCCATTTGAAGACATGGTGGATTACTCAGAATTCTCTGTTTTTG
TAGCTXXXXXXXXXXTTGAATATGAAAATGGCCATCCGGGTGGTATTGGACCAGTTCCTCCAGATGGTGCTGTAA
ATCACATATGGAGAAAAGTG
>Marker857599
GACAACATATTGATTCATTTAAATTATTGAAAGAAGGGAAAGGAGAAAATGAAAGACAACTATGGAAAATCTCAA
TTAATXXXXXXXXXXTGAAGAAAAGCAAAAATTACCTTTCAATTTTGTTTTGCATTGAAAAACCAAACAAAATGA
AATCGATCCAAGCGTATGTG
GACAACATATTGATTCATTTAAATTATTGAAAGAATGGAAAGGAGAAAATGAAAGACAACTATGGAAAATCTCAA
TTAATXXXXXXXXXXTGAAGAAAAGCAAAAATTACCTTTCAATTTTGTTTTGCATTGAAAAACCAAACAAAATGA
AATCGATCCAAGCGTATGTG
>Marker857893
ACAAGGAAAATGCGAAGGGAACCGACATGAGACTCGAGTTACATCAACAAGTTCAAGTTGAGCACACGATGAACT
TAGATXXXXXXXXXXATGTTCTCAATCAACGGAAATATGAACAGCTGGATAATTCAGAGGATTAACAATTTGACA
CAAGCTAGAGCAACAAAGTC
ACAAGGAAAATGCGAAGGGAACCCACATGAGACTCGAGTTACATCAACAAGTTCAAGTTGAGCACACGATGAACT
TAGATXXXXXXXXXXATGTTCTCAATCAACGGAAATATGAACAGCTGGATAATTCAGAGGATTAACAATTTGACA
CAAGCTAGAGCAACAAAGTC
>Marker858098
TACAGTAGCAGCGTATGAACAACATTCCATAACTATTGATATTTGCACAACCAAGACAACGTCAATCACAGGTCC
AATAGXXXXXXXXXXATTTTCAATATAATCTCAAAACTCTGCCTAACACGTGAGCTCTAATTTCCATCTCGACTA
ATCTCATGACACAAATAGTT
TACAGTAGCAGCGTATGAACAACATTCCATAACTATTGATATTTGCACAACCAAGACAACGTCAATCACAGGTCC
AATATXXXXXXXXXXATTTTCAATATAATCTCAAAACTCTGCCTAACACGTGAGCTCTAATTTCCATCTCGACTA
ATCTCATGACACAAATAGTT
>Marker858600
ACATCTACACTGGGTCTCACAATTCTTGTGTTTATATCTATGATTTGGTAAGTCACTTGACATTTGTTTTTGTGC
ATCTTXXXXXXXXXXATTTATAGTTCCGACCGTCCTTAAGAAGATAAAATAAGAATATTAGTCCAATTGCACACT
TGGAAAAATTAATTTTTGGT
ACATCTACACTGGGTCTCACAATTCTTGTGTTTATATCTATGATTTGGTAAGTCACTTGACATTTGTTTTTGTGC
ATCTTXXXXXXXXXXATTTATAGTTCTGACCGTCCTTAAGAAGATAAAATAAGAATATTAGTCCAATTGCACACT
TGGAAAAATTAATTTTTGGT
>Marker858629
CACCTATATTCATGAACCACCCTACTTCAAGAATATGACCATGGACCCTCCAGGAGCACATGGCGTTAAGGATGC
TTACTXXXXXXXXXXTTATTGACCGTGGAGTGGATCGCAAGGATTTTAATTCTTATGGAAGTAGGCGAGGCAATG
ATGAGGTGATGGCAAGGGGT

Create PDF with GO2PDF for free, if you wish to

CACCTATATTCATGAACCACCCTACTTCAAGAATATGACCATGGATCCTCCAGGAGCACATGGCGTTAAGGATGC
TTACTXXXXXXXXXXXTTCTTGACCGTGGAGTGGATCGCAAGGATTTTAATTCTTATGGAAGTAGGCGAGGCAATG
ATGAGGTGATGGCAAGGGGT
>Marker858828
ACGAGAGATCAAATAAGATCAAACCTCCAACTATAGAGTGGAAGAAAATGCCACTTGTCTTTGTAAGCTAAGTTT
TTTTAXXXXXXXXXXXCTTATTGTGTAGCAACTTGTTCTTTGTATTTTATAAACATATTTAGACCCTAATATATAC
ATATGAACAAAAAGAAAGTG
ACAAGAGATCAAATAAGATCAAACCTCCAACTATAGAGTGGAAGAAAATGCCACTTCTCTTTGTAAGCTAAGTTT
TTTTAXXXXXXXXXXXCTTATTGTGTAGCAACTTGTTCTTTGTATTTTATAAACATATTTAGACCCTAATATATAC
ATATGAACAAAAAGAAAGTG
>Marker859268
AACATTTACTGGGAGAACATATCTGGCGATGAAAATGGGATTTGTTAATGGACTATAAGGACATTATGGACTTAC
AATATXXXXXXXXXXXTTCAAGACTTTCGCTCAGCTTCATGATTCTTGCAAGTTACTTAAAGTTTAAAGATAGGCC
AGAACCATCTTACGATAGTG
AACATTTACTGGGAGAACATATCTGGCGATGAAAATGGGATTTGTTAATGGACTATAGGGACATTATGGACTTAC
AATATXXXXXXXXXXXTTCAAGACTTTCGCTCAGCTTCATGATTCTTGCAGGTTACTTAAAGTTTAAAGATAGGCC
AGAACCATCTTACGATAGTG
>Marker859618
ACTCCCATTGAATTGAACTGTAAACTGAGAAATTTTGATGATAAAGTTCTTGTTGATAAAGAACAATATCAACGC
CTTGTXXXXXXXXXXXCGACTCCTAGAAAAGGATTGATGTTCAGAAAGAAGGACAAAAACTATTGAGGCTTATACT
AGCTCAAACTGGACAGGGTT
ACTCCCACTGAATTGAACTGTAAACTGAGAAATTTTGATGATAAAGTTCTTGTTGATAAAGAACAATATCAACGC
CTTGTXXXXXXXXXXXCGACTCCTAGAAAAGGATTGATGTTCAGAAAGAAGGACAAAAACTATTGAGGCTTATACT
AGCTCAAACTGGACAGGGTT
>Marker859677
ACAGTGACCAAAGACCTATATTCTTTATATATAGACTGCATATTCATACCTCTTCAATCCACCATTCTTAACGTT
TCATCXXXXXXXXXXXAAGGTTAATGCACAAGAAGGAGTTTGGTGACAAATTTCACCTTAGCGAGGTAGCTTCCAA
CATTTGTATCCCGAAAGGTA
ACAGTGACCAAAGACCTATATTCTTTATATATAGACTACATATTCATACCTCTTCAATCCACCATTCTTAACGTT
TCATCXXXXXXXXXXXAAGGTTAATGCACAAGAAGGAGTTTGGTGACAAATTTCACCTTAGCGAGGTAGCTTCCAA
CATTTGTATCCCGAAAGGTG
>Marker859818
ACCAATACGCAAAGGCGAAACAAGACATTGTGATAGAGAGCCATGATTCTGCTTTCGAGATCAAGAAAGGGGAAA
TGATTXXXXXXXXXXXAAGCAGTGTCCGGCGAGGGATCTGGTGGTGCTGATGTGCAGAGTAGTGTTGGTGGAACTT
TTCCTCCGTTATGAGACGTT
ACCAATACGCAAAGGCGAAACAAGACATTGTGATAGAGAGCCATGATTCTGCTTTCGAGATCAAGAAAGGGGAAA
TGATTXXXXXXXXXXXAAGCAGTGTCCGGCGAGGGATCTGGTGGTGCTGATGTGCAGAGTAGTGTTGGTGGAACTT
TTCCTCCGTTATGATACGTT
>Marker861374
CACAATAATCCCTCGAAGCTCTTCCATTTTTAGAATCTCCAATCCAGTTCGTGTCTTCTCTACTAACCCTCCAAC
ACTATXXXXXXXXXXXAACCAACCCCATCCATTAATGATCCCTTCAACGTCTCCACTAGTCCTCCTATGACTGAAC
CAGGTTCGTTAAACATCGTA
CACAATAATCCCTCGAAGCTTTTCCATTTTTAGAATCTCCAATCCAGTTCGTGTCTTCTCTACTAACCCTCCAAC
ACTATXXXXXXXXXXXAACCAACCCCATCCATTAATGATCCCTTCAACGTCTCCACTAGTCCTCCTATGACTGAAC
CAGGTTCGTTAAACATCGTA
>Marker861474

Create PDF with GO2PDF for free, if you wish to

TACCAAGTTGCAGCTATGCGAAGTAGTCTCAACGCATTTACCAAAATTTCACCAATACCATCACGCATAATGCAT
CTTCCXXXXXXXXXXXATATCAGATCACATAGTGAGTATGAGATATCTCACACTAGAACACGACAAGTATAAATGA
GGCATCTCATCGCATAGGGT
TACCAAGTTGCAGCTATGCGAAGTAGTCTCAACGCATTTACCAAAATTTCACCAATACCATCACGCATAATGCAT
CTTCCXXXXXXXXXXXATATCAGATCACATAGCGAGTATGAGATATCTCACACTAGAACACGACAAGTATAAATGA
GGCATCTCATCGCATAGGGT
>Marker861565
ACTAGGATTGAACGGTTCGGCTGCAGCAGAAAGCTTGCTAGCATTCGTTTCTGCAGGCTTTTCCGAAGGAGATGA
ACAGGXXXXXXXXXXXTTATTACAGCTCTGGGGTTCAGCTACTTTCTCTTCTAACTCAATAACATTCTCCGTATCA
ACCAGTTGCCTTAGAACAGT
ACTAGGATTGAACGGTTCGGCTGCAGCAGAAAGCTTGCTAGCATTCGTTTCTGCAGGCTTTTCCGAAGGAGATGA
ACAGGXXXXXXXXXXXTGATTACAGCTCTGGGGTTCAGCTACTTTCTCTTCTAACTCAATAACATTCTCCGTATCA
ACCAGTTGCCTTAGAACAGT
>Marker862044
ACTCTATTTGTTGATGTAACTTTGAAGGCATTGTTTCTTCTCATCAATTGGTTAGCATAAGTATAGACACATTGG
CCCATXXXXXXXXXXXTTAATTGTTAATCGATATGCGAACCTATTGAGAGGAGTTATTGCTGTTGGAAGGGCTACA
CAAGTGCAACAAAGACATGT
ACTCTATTTGTTGATGTAACTTTGAAGGCATTGTTTCTTCTCATCAATTGGTTAGCATAAGTATGGACACATTGG
CCCATXXXXXXXXXXXTTAATTGTTAATCGATATGCGAACCTATTGAGAGGAGTTATTGCTGTTGGAAGGGCTACA
CAAGTGCAACAAAGACATGT
>Marker862349
GACAAAATGAAGGAGATAAATAGGGTTTAGACTTCCACATGCCCATGAAATTTACTCATTTGACTCTGAAGCAGC
CAAGAXXXXXXXXXXXATTGAAGTGAGAGCTACTAAACGGTAGATGGCAAGGATGCTTTCTCCTCTCTCTTTATTG
ACCATTGCTCTGTCGGTTGT
GACAAAATGAAGGAGATAAATAGGGTTTAGACTTCCACATGCCCATGAAATTTACTCATTTGTCTCTGAAGCAGC
CAAGAXXXXXXXXXXXATTGAAGTGAGAGCTACTAAACGGTAGATGGCAAGGATGCTTTCTCCTCTCTCTTTATTG
ACCATTGCTCTGTCGGTTGT
>Marker862569
GACTCAGCGCTCAGCTATGTTTTGACTTTAATTCTCAGAAGAACCTCATAATAAATGATGCTACTCTTCTCCTAT
TGGTCXXXXXXXXXXXAAATAGGTAATGCATAACTCTTTGAAATCGTGCAACTAGGAAAGTAGAGAATGTCATTTT
CCACGTTTCTCACAAACGTA
GACTCAGCGCTCAGCTATGTTTTGACTTTAATTCTCAGAAGAACCTTATAATAAATGATGCTACTCTTCTCCTAT
TGGTCXXXXXXXXXXXAAATAGGTAATGCATAACTCTTTGAAATCGTGCAACTAGGAAAGTAGAGAATGTCATTTT
CCACGTTTCTCACATACGTA
>Marker862658
TACTTCAGATGATCATTTTCTTGAAAACAAGCATAGCCACAAGGAAAGCATAATATTGTCTAGTAATTGTTCTAA
TCATGXXXXXXXXXXXAGTTAATAAATGCATCTTAGGTAATAGAAAAGTATTGTTGGGCATGCAAGAAAAAATATA
TAATAAATAAACATTGATGT
TACTTCAGATGATCATTTTCTTGAAAACAAGCATAGCCACAAGGAAAGCATAATATTGTCTAGTAATTGTTCTAA
TCATGXXXXXXXXXXXAGTTAATAAATGCATCTCAGGTAGTAGAAAAGTATTGTTGGGCATGCAAGAAAAAATATA
TAATAAATAAACATTGATGT
>Marker862930
AACATTAAAACAATAATGACAAATTTATATGCACAAAATGGGCGCATTAGCTGTCACCTTTTGGACTTAAATCAA
ATCCCXXXXXXXXXXXATATTCGCTTTAAAGTATTTTATTGGGGTTGTTTGATGCTCAAGCATGGATTATAGACCT
ATTAGATTTTATGATGTGTG

Create PDF with GO2PDF for free, if you wish to

AACATTAAAACAACAATGACAAATTTATATGCACAAAATGGGCGCATTAGCTGTCACCTTTTGGACTTAAATCAA
ATCCCXXXXXXXXXXXATCTTCGCTTTAAAGTATTTTATTGGGGTTGTTTGATGCTCAAGCATGGATTATAGACCT
ATTAGATTTTATGATGTGTG
>Marker863195
AACCTAACGAAGTAACCAAAATGATTCTGGTTGTTTTAAACTTGAGATGGAGAAGACACCCTACAACTGCAGATA
ATGGTXXXXXXXXXXXCTGCACATCTGTTCGTTGCCATCGAGGAATCTGTGAGGGAAAAGTTCGATCTCCATGCAT
AGGTGCAAATGCTGAAAGTT
AACCTAACGAAGTAACCAAAATGATTCTGGTTGTTTTAAACTTGAGATGGAGAAGACACCCTACAACTGCAGATA
AGGGTXXXXXXXXXXXCTGCACATCTGTTCGTTGCCATCGAGGAATCTGTGAGGGAAAAGTTCGATCTCCATGCAT
AGGTGCAAATGCTGAAAGTT
>Marker863215
ACTAGAACTCCACGGGATCTACTTTTAGTGAAGGAAGAATACCATGCTCGTTTCAAGCGGTCAATTGAAGAAGAT
GTCGCXXXXXXXXXXXGTTTGCTCGACACTATTTGATTCTTATTTCATTTTCAACAGTTATCTAAACCATTAGAAA
TTATTAACTATCCTGGTGTA
ACTAGAACTCCACGGGATCTACTTTTAGTGAAGGAAGAATACCATGCTCGTTTCAAGCGGTCAATTGAAGAAGAT
GTCGCXXXXXXXXXXXGTTTGCTCGACACTATTTGATTCTTATTTCATTTTCAACAGTTATCTAAACCATTAGAAA
TTATTAACTTTCCTGGTGTA
>Marker863532
GACCTCTAGAAATTGCAAATCTCAAAAGAAAGAGTTCTTGTGGTGTCAATGGCACACCTGATATCCAACTTGGAG
ATTTTXXXXXXXXXXXTTAGACATAAAATCATTTATAGATTTTCTTTTTTAATAATTCTTCCCTCGGATTGAATTC
AAATCTTCGAACAATGAGTA
GACCTCTAGAAATTGCAAATCTCAAAAGAAAGAGTTCTTGTGGTGTCAATGCCACACCTGATATCCAACTTGGAG
ATTTTXXXXXXXXXXXTTAGACATAAAATCATTTATAGATTTTCTTTTTTAATAATTCTTCCCTCGGATTGAATTC
AAATCTTCGAACAATGAGTA
>Marker863548
ACCTTATCCTAGTAACACTATTGATATGTCTCACTTTGTATTTGATACAAACGCAATGATCAATCGCGTTCGTGT
AATTGXXXXXXXXXXXCTAGGATGACCTACGTAACTTAGTCTTAATCCTAAGTGTGTTATGAACTTTTGTTCGCGA
GGGATTGTCCTTTGATTTGT
ACCTTATCCTAGTAACACTATTGATATGTCTCACTTTGTATTTGATACAAACGCAATGATCAATCGCGTTCGTGT
AATTGXXXXXXXXXXXCTAGGATGACCTACGTAACTTAGTCTTAATCCTGAGTGTGTTATGAACTTTTGTTCGCGA
GGGATTGTCCTTTGATTTGT
>Marker864137
TACCATCTGAACCTCGGCCTCCGGCTTGCTCAACGAGAAGGCTAAGAGGGTTTGCCTCATATACTAGACGAAGAT
GATCTXXXXXXXXXXXGGATTTTTGCCTTTCCCCTGTCTGATTGTGTCAATATATCGCCGCAATCCTTCTGGCCAG
TCGAAATATCTTGCATCGTT
TACCATCTGAACCTCGGCCTCCGGCTTGCTCAACGAGAAGGCTAAGAGGATTTGCCTCATATACTAGACGAAGAT
GATCTXXXXXXXXXXXGGATTTTTGCCTTTCCCCTGTCTGATTGTGTCAATATATCTCCGCAATCCTTCTGGCCAG
TCGAAATATCTTGCATCGTT
>Marker864245
AACCAGGCATATATCTTATGTAAAGTTTATTTCTAAATATTTATGCAATTAAAATATAAGCTACGCATTTTTACA
CTCTTXXXXXXXXXXXCTCTTCCTTAAAAACTTACCTTCCCTCAGCATTTTTAAATTTCTCAAAATGCGATCAAGT
TTTATTCATTATTTAGGGTT
AACCAGGCATATATCTTATGTAAAGTTTATTTCTAAATATTTATGCAATTAAAATATAAGCTACACATTTTTACA
CTCTTXXXXXXXXXXXCTCTTCCTTAAAAACTTACCTTCCCTCAGCATTTTTAAATTTCTCAAAATGCGATCAAGT
TTTATTCATTATTTAGGGTT
>Marker864248

Create PDF with GO2PDF for free, if you wish to

ACTATCTTTTCTAATTGCCTTATTTAAAACCCTTTTGTTTGAGGTTCTTCATATGATCATCACTCTCATGTGTAT
AAGATXXXXXXXXXXXATCATTTTACTGATGCAAATTTTGCTTCCTCTATACACTATTTTGTTTAGTGGGAAACAG
GAAGCAGAAAAACCCATGTA
ACTATCTTTTCTAATTGCCTTATTTAAAACCCTTTTGTTTGAGATTCTTCATATGATCATCACTCTCATGTGTAT
AAGATXXXXXXXXXXXATCATTTTACTGATGCAAATTTTGCTTCCTCTATACACTATTTTGTTTAGTGGGAAACAG
GAAGCAGAAAAACCCATGTA
>Marker864810
AACAACACTCCTACTCTTTTATAAATCATGATGACATCTTGTTTCTTATATATAAACTTAATTTCAATTTCCTAA
ATAGCXXXXXXXXXXXCTTCTAAATTAAAAACACACAAATGTAATACTATTTGATAGCGATATATAATTATTAATA
CGTGTTTTAATAAGATGGTA
AACAACACTCCTACTCTTTCATAAATCATGATGACATCTTGTTTCTTATATATAAACTTAATTTCAATTTCCTAA
ATAGCXXXXXXXXXXXCTTCTAAATTAAAAACACACAAATGTAATACTATTTGATAGCGATATATAATTATTAATA
CGTGTTTTAATAAGATGGTA
>Marker865008
ACATTATCTTTGATAAATACACTTATTGTTGGACCATTATTGATGATCTTTATTGTATATTATGTTAATAAAAGA
ACTATXXXXXXXXXXXTGAGCTCATTTATTTATTATTTCTGATTTTATCTTACGGTTTTGCTCTTATGATTGGTAT
ATACATAAATGAATTATGTA
ACATTATCTTTGATAAATACACTTATTGTTGGACCATTATTGATTATCTTTATTGTATATTATGTTAATAAAAGA
ACTATXXXXXXXXXXXTGAGCTCATTTATTTATTGTTTCTGATTTTATCTTACGGTTTTGCTCTTATGATTGGTAT
ATACATAAATGAATTATGTA
>Marker865263
TACTAATGGGATTCTGCATCGAATGCTTTGGTGGATTTGTAGGTAGATTTTCTCGTCGTCATCTACTGATGGGTT
TCGGTXXXXXXXXXXXGGCAGACTTCCATTTGCGGTCGGGGTTGAGCATGTTGAAGCCTTTGAAGACGTTGATGAT
GCCAAGAATGAGAATTGAGT
TACTAATGGGATTCTGCATCGAATGCTTTGGTGGATTTGTTGGTAGATTTTCTCGTCGTCATCTACTGATGGGTT
TCGGTXXXXXXXXXXXGGCAGACTTCCATTTGCGGTCGGGGTTGAGCATGTTGAAGCCTTTGAAGACGTTGATGAT
GCCAAGAATGAGAATTGAGT
>Marker865592
CACCATTCTTATCATGCTGTCAATGTTGTTAAAAGGCTATTTAGTGTAGCCTAGTGATAAAAGGAGATCAATAAA
ACCAAXXXXXXXXXXXATCCGTATCCTAATCAATCAAGGGAACTTATCCTAATTCTAATTAATCAAGGATTGTAAT
CTGATAGACCTCCTATCGTT
CACCATTCTTATCATGCTGTCAATGTTGTTAAAAGGCTATTTAGTGTAGCTTAGTGATAAAAGGAGATCAATAAA
ACCAAXXXXXXXXXXXATCCGTATCCTAATCAATCAAGGGAACTTATCCTAATTCTAATTAATCAAGGATTGTAAT
CTGATAGACCTCCTATCGTT
>Marker865924
TACCATTAAAACCTTTCTCTTTCTCATGAATAAATAGTCTTAAGAGAGTAATTTAATTAAGTTGCTCAAGCATTA
CTCCAXXXXXXXXXXXTATATATATTGCATAGCAATAATTAAAAAAGATCGTTACAAATATATCTAAATGCCGTCT
AACCTATTTGTAAATATAGT
TACCATTAAAACCTTTCTCTTTCTCATGAATAAATAGTCTTAAGAGAGTAATTTAATTAAGTTGCTCAAGCATTA
CTCCAXXXXXXXXXXXTATATATATTGCATAGCAAGAATTAAAAAAGATTGTTACAAATATATCTAAACGCCGTCT
AACCTATTTGTAAATATAGT
>Marker866023
ACAACCTATAAAACAAGTATGAGTAACAAAAAGGACTACTTGACCTAAATTTAAGATATAGAGAATTCTAACCTT
TCTATXXXXXXXXXXXTATTTTCTAGAAAAATTATTAGTCCAAGCTAAGTATGCTTAACTTTGATGTTCCTATGTC
ATTGAAAATTTTAAAAAGTG

Create PDF with GO2PDF for free, if you wish to

ACAACCTATAAAACAAGTATGAGTAACAAAAAGGACTATTTGACCTAAATTTAAGATATAGAGAATTCTAACCTT
TCTATXXXXXXXXXXTATTTTCTAGAAAAATTATTAGTCCAAGCTAAGTATGCTTAACTTTGATGTTCTTATGTC
ATTGAAAATTTTAAAAAGTG
>Marker866250
ACTTTATTAAAAGCAACATTTTCTTATTGTTGCAATAGCATGTCTTGTTTTCCATTTTATAAGTTGATTATGAAA
AGCAAXXXXXXXXXXXAATCCTGCTATAACATGTTATTCTCCCTATTTTCCCTAATTCAAATTAACATGTTTTGTG
CTTCTTATCAGCAAATAAGT
ACTTTATTAAAAGCAACATTTTCTTATTGTTGCAATAGCATGTCTTGTTTTCCATTTTATAAGTTGATTATGAAA
AGCAAXXXXXXXXXXXAATCCTGCTATTACATGTTATTCTCCCTATTTTCCCTAATTCAAATTAACATGTTTTGTG
CTTCTTATCAGCAAATAAGT
>Marker866252
ACTTACAATAGTGAAAAATTATCAGCTACATTGTGGCGGCTCAACAGAATAATTTCTCAATAAACACCAACTACC
TTTGTXXXXXXXXXXGAAAATAGAGGCAGAAATGAAAAAGAATGATTATCCAAAACAGCCATTCTTGCTATGGAG
TTTGAGTCTCGGTCCCTTGT
ACTTACAATAGTGAAAAATTATCAGCTACATTGTGGCGGCCCAACAGAATAATTTCTCAATAAACACCAACTACC
TTTGTXXXXXXXXXXGAAAATAGAGGCAGAAATGAAAAAGAATGATTATCCAAAACAGCCATTCTTGCTATGGAG
TTTGAGTCTCGGTCCCTTGT
>Marker866603
CACCTGCGAAATGATTTTTTGATATTTGGACATATTGAAGGCCTTTTAAACTACCAATCCAACTAGGAATTTTGC
CTGATXXXXXXXXXXTTCTAAAAAAGGGATGTAGTTGAACATAGAAATTTGTAAATCTCCAGAAAAGTTGTTATT
GGATAGATCCAACCAGTGTA
CACCTGCGAAATGATTTTTTGATATTTGGACATATTGAAGGCCTTTTAAACTACCAATCCAACTAGGAATTTTGC
CTGATXXXXXXXXXXTTCTAAAAAAGGAATGTAGTTGAACATAGAAATTTGTAAATCTCCAGAAAAGTTGTTATT
GGATAGATCCAACCAGTGTA
>Marker866618
AACCTTTAGGCCATTTAAGACTTCAGAGCCAAGATCTCTAGCAACAAAAGTGATGCTTTTGGAATACAGTTTCAA
TTTTCXXXXXXXXXXCAACTCACAAGCTCTTATATAAGCTTCCCTACTTATTTATCTTCACATAAATGCATTACT
CAGTTTCTTATCATAAAAGT
AACCTTTAGGCCATTTAAGACTTCAGAGCCAAGATCTCTAGCAACAAAAGTGATGCTTTTGGAATACAGTTTCAA
TTTTCXXXXXXXXXXCAACTCACAAGCTCTTATATAAGCTTCCTTACTTATTTATCTTCACATAAATGCATTACT
CAGTTTCTTATCATAAAAGT
>Marker866664
CACAAAAAAGATCAGAAAAGATTCATCATATTCATTGAGAAACTGTAAAACACAAACACAAAAGTGAAAAAGAAA
TACAAXXXXXXXXXXXTAAAAGGAGAAAAAAAGATAATGAATAATGAGGTTTAAAATTTGCTGAGCATTTACTGAA
AAACCAACAGAAACCATGTC
CACAAAAAAGATCAGAAAAGATTCATCATATTCATCGAGAAACTGTAAAACACAAACACAAAAGTGAAAAAGAAA
TACAAXXXXXXXXXXXTAAAAGGAGAAAAAAAGATAATGAATAATGAGGTTTAAAATTTGCTGAGCATTTACTGAA
AAACCAACAGAAACCATGTC
>Marker866753
AACTTTGAAGAAGGTAGAGAAGGGTGGAAGAGGTTGAAAGCCAATAGGCAAGATCGCTGATATTTTCTTGACTCT
GAAAAXXXXXXXXXXXGATCTGATTGTGTGAATAATTCTGTCAAATATATTTGTCCTCTCTGCTTCAAAAGATCTC
CATTGAAGAAGTGTCTTGTA
AACTTTGAAGAAGGTAGAGAAGGGTGGAAGAGGTTGAAAGCCAATAGGCAAGATCGCTGATATTTTCTTGACTCT
GAAAAXXXXXXXXXXXGATCTGATTGTGTGAATAATTCTGTCAAATATATTTGTCTTCTCTGCTTCAAAAGATCTC
CATTGAAGAAGTGTCTTGTA
>Marker867233

Create PDF with GO2PDF for free, if you wish to

TACACGTTCTTGATTAGGATGAAAGGAACCCGATGGAGTTATCAGATGAAAACTGGATGAATTGTTCTCCACAGA
ATCTGXXXXXXXXXXXTTGAGACAAAATAACTGTGTGCAGTTTCGTCCTGTTCAAGGCATTATCACAAACTCCAGC
TGTCACAACACGCATTAGGT
TACACGTTCTTTATTAGGATGAAAGGAACCCGATGGAGTTATCAGATGAAAACTGGATGAATTGTTCTCCACAGA
ATCTGXXXXXXXXXXXTTGAGACAAAATAACTGTGTGCAGTTTCGTCCTGTTCAAGGCATTATCACAAACTCCAGC
TGTCACAACACGCATTAGGT
>Marker867418
ACTTTCAGTAAATGCCAAATTGAGTTCTACACAAAAAATTTATTTCAAATTTAATTTCCAACAACATTTTGTCGG
TAACTXXXXXXXXXXXAACCTGAAACTATAATAAGCAACATTACAGAGGCTATACTGCTGGAAAACCTGCTTTAAT
ACGAATACTATGTCCATGTT
ACTTTCAGTAAATGCCAAATTGAGTTCTACACAAAAAATTTATTTCAAATTTAATTTCCAACAACATTTGGTCGG
TAACTXXXXXXXXXXXAACCTGAAACTATAATAAGCAACATTACAGAGGCTATACTGCTGGAAAACCTGCTTTAAT
ACGAATACTATGTCCATGTT
>Marker867459
AACAATATTCAGTTGTTCATGCAACCATTACAAAACTTAACACTAACTCTATGATAAGATCCTGAGGTTGAGAAT
GTGCAXXXXXXXXXXXAAAGTTTTCACTTGTTGGAGAAATTGAGAGTCCCACGTTGGAAAAACTAAGGAGACTCAT
ACTCCATATAAAATAGATGT
AACAATATTCAGTTGTTCATGCAACCATTACAAAACTTAACACTAACTCTATGATAAGATCCTGAGGTTGAGAAT
GTGCAXXXXXXXXXXXAAAGTTTTCACTTGTTGGAGAAATTGAGAGTCCCACGTTGGAAAAACTAAGGAGAATCAT
ACTCCATATAAAATAGATGT
>Marker867565
CACGTGCAAGCAAAGAGGAACATGCACACTGATGTGATGTAAGGTATAATCACTCCTATGCTTAAGTTAGAAGGT
TAAAGXXXXXXXXXXXTCCCTTTTTAACCTAGCCTATATTGGACTTTCGATCACTTAGGCCTCCCCTTGGGCTCGA
CCTTGATAGAGGTCAAGGTC
CACCTGCAAGCAAAGAGGAACATGCACACTGATGTGATGTAAGGTATAATCACTCCTATGCTTAAGTTAGAAGGT
TAAAGXXXXXXXXXXXTCCCTTTTTAACCCAGCCTATATTGGACTTTCGATCACTTAGGCCTCCTCTTGGGCTCGA
CCTTGATAGAGGTCAAGGTC
>Marker867783
TACTACCTTTATATGTGTTTGTCTAAACTGCATATTTTTCAATGAAAGGAAGCTTTCATAGATCTGAAACTTCAT
TTTTGXXXXXXXXXXXATTTTACTGTGCTTCATTTTAAGATAATTTTTCCGCTCTATCATCCAATATACAGAAGTA
GTATAGATTCAAAATCAGTC
TACTACCTTCATATGTGTTTGTCTAAACTGCATATTTTTCAATGAAAGGAAGCTTTCATAGATCTGAAACTTCAT
TTTTGXXXXXXXXXXXATTTTACTGTGCTTCATTTTAAGATAATTTTTCCACTCTATCATCCAATATACAGAAGTA
GTATAGATTCAAAATCAGTC
>Marker867870
CACATTACTGTGTTTAGAGTGCAGAGTTGTTTTGAGTTCGTGTTTGCGTTTGTGGTGCAGAGTTGTTTTGAGTTC
ATGTTXXXXXXXXXXXTATTCAATAATTTTACCATTCTTTTTTTTGAATGTTTGAATAAGATATGAATTGCATTTT
TTCTTTCTTATGGTGATGTA
CACATTACTGTGTTTAGAGTGCAGAGTTGTTTTGAGTTTGTGTTTGCGTTTGTGGTGCAGAGTTGTTTTGAGTTC
ATGTTXXXXXXXXXXXTATTCAATAATTTTACCATTCTTTTTTTTGAATGTTTGAATAAAATATGAATTGCATTTT
TTCTTTCTTATGGTGATGTA
>Marker868191
ACATAATTGCAAAACATTGTTTTCGTTCTACTGTTTCTTTTCAACTTTTCTCGAGTTGTCTATTTTTTACATCAA
TTTACXXXXXXXXXXXTTTTTAGGAGTTAATAATAAAAGAAACCAAAAACTAGAGGAATAGACAGGACTTGGATTG
AATGCAATATTATATATGTT

Create PDF with GO2PDF for free, if you wish to

ACATAATTGCAAAACATTGTTTTCATTCTACTGTTTCTTTTCAACTTTTCTCGAGTTGTCTATTTTTTACATCAA
TTTACXXXXXXXXXXTTTTTAGGAGTTAATAATAAAAGAAACCAAAATCTAGAGGAATAGACAGGACTTGGATTG
AATGCAATATTATATATGTT
>Marker868964
CACGTTTTTCTATACCTGGGAGTTTTAAACGTTGTCTTCCATTATATCTTATTTTCTAGATTTTTAATTTGTGTA
TCTTGXXXXXXXXXXCAGAATGGATGTAGAGAAATGAAATATATATATATATATTTGATGTTTTTCCATAACATT
TCTTATCCCTATTATTGGGT
CACGTTTTTCTATACTTGGGAGTTTTAAATGTTGTCTTCCATTATATCTTATTTTCTAGATTTTTAATTTGTGTA
TCTTGXXXXXXXXXXCAGAATGGATGTAGAGAAATGAAATATATATATATATAGTTGATGTTTTTCCATAACATT
TCTTATCCCTATTATTGGGT
>Marker869549
AACTTTTTAAGTGGTAATAATATTTTTGCTAATTTTGAGAATAATGATATAAACTTTACATCATTTAAGTTAAAC
ATAGAXXXXXXXXXXAATATATCGTGTCACACTTCCTATAAGACAAAAGCACGAGCTAAACAATGTTAGAGTTTG
AATTAGAATAAAAAGTAGTA
AACTTTTGAAGTGGTAATAATATTTTTGCTAATTTTGAGAATAATGATATAAACTTTACATCATTTAAGTTAAAC
ATAGAXXXXXXXXXXAATATATCGTGTCACACTTCCTATAAGACAAAAGCACGAGCTAAACAATGTTAGAGTTTG
AATTAGAATAAAAAGTAGTA
>Marker869818
AACATGTCCCGAACCGCCTTCTCCCAAAACATTGCTTTTGCTGAAGTTATCAGTTGCTGCTTCCAACAATTTATA
GTCGAXXXXXXXXXXAAGATTAAGTTGGTTTAAATTCTTGCTGATTATAAATAAAGCAAGAAAAGAGCATAATCA
AACTAATGGGAAAGGAAAGT
AACATGTCCCGAACCGCCTTCACCCAAAACATTGCTTTTGCTGAAGTTATCAGTTGCTGCTTCCAACAATTTATA
GTCGAXXXXXXXXXXAAGATTAAGTTGGTTTAAATTCTTGCTGATTATAAATAAAGCAAGAAAAGAGCATAATCA
AACTAATGGGAAAGGAAAGT
>Marker870650
CACTATCTGCTTTGCCATGAATGAAGATATAATTTAGTCACAACACTGTTGCTGAGCAAACAAAGGAACAAATTG
ATACGXXXXXXXXXXAACCACTTGGAATGTCTCTAACAATTTTCCGAGTTGGTGGGCATGTTGCTCCTCATCATT
CACTCCGTCAAGCATTATGT
TACTATCTGCTTTGCCATGAATGAAGATATAATTTAGTCACAACACTGTTGCTGAGCAAACAAAGGAACAAATTG
ATACGXXXXXXXXXXAACCACTTGGAATGTCTCTAACAATTTTCCGAGTTGGTGGGCATGTTGCTCCTCATCATT
CACTCCGTCAAGCATTATGT
>Marker870754
ACCTCAACATATCAACGGCTTACACATGATTTTATCGTTTAGTTATAGGACCGAAAGAAAAAAAAAACTAACTTA
AACAGXXXXXXXXXXGATGGGCAACTATCACAACAAATCAAGTCTCCACCATCTCCACAAATTCCACAAGTATCA
TCATTAGGATCTTCGACGTC
ACCTCAACATATCAACGGCATACACATGATTTTATCGTTTAGTTATAGGACCGAAAGAAAAAAAAAACTAACTTA
AACAGXXXXXXXXXXGATGGGCAACTATCACAACAAATCAAGTCTCCACCATCTCCACAAATTCCACAAGTATCA
TCATTAGGATCTTCGACGTC
>Marker870921
TACAAAATTGTTAATGGAAATCTTTGTGTCATCGATATCTCAAAAGACTGCTATAATTAATTGATGTGTGTGCGT
TACCCXXXXXXXXXXGGTTTAGTGTTTAACGATGAGAATGGAGGGAGAGCAAGGAGGCATATTGAGGAATGAAGA
TTAGGAACTGCAAATGGAGT
TACAAAATTGTTAATGGAAATCTTTGTGTCATCGATATCTCAAAAGACTGCTATAATTAATTGATGTGTGTGCGT
TACCCXXXXXXXXXXGGTTTAGTGTTTAACGATGAGAATGGAGGGAGAGCAAGGATGCATATTGAGGAATGAAGA
TTAGGAACTGCAAATGGAGT
>Marker871417

Create PDF with GO2PDF for free, if you wish to

ACCGAGAACAATAACGTCGTATTCTTCATCCATGGGAGCAGGAAGGAAGAGGAAAGGAGAGATCGAGAGAGAAAT
GAAAGXXXXXXXXXXCGCTACTATTATCAAATGACAAACCTCTTTTTCTTTTTCTTTTTTTAATATCAATATTTT
TGTAGAAATTTTGCTGTGTA
ACCGAGAACAATAACGTCGTATTCTTCATCCATGGGAGCAGGAAGGAAGAGGAAAGGAGAGATCGAGAGAGAAAT
GAAAGXXXXXXXXXXCGCTACTATTATCAAATGACAAACCTCTTTTTCTTTTTCTTTTTTTAATATCAATATTTT
TGTAGAAATTTTTCTGTGTA
>Marker871478
AACAAATAAGACTATATATATAAAAAAGAATAGGTTAAATGAATCAGACACATGTGCTTGAAAGTGTATGGATTC
CTAATXXXXXXXXXXCAAGTGGAAGCTGCTTGCAATGCAACAGTTTCATGTGCTGATATTCTTGCTCTAGCCACG
CGTGATGGAGTCGTTTTGGT
AACAAATAAGACTATATATATAAAAAAGAATAGGTTAAATGAATCAGACACATGTGCTTGAAAGTGTATGGATTC
CTAATXXXXXXXXXXCAAGTGGAAGCTGCTTGCAATGCAACAGTTTCATGTGCTGATATTCTTGCTCTAGCCACG
CGTGATGGGGTCGTTTTGGT
>Marker872336
ACCAACAGGACTAACACTTCCGTCAAACACCAACACGCCTCTTGATTTCAAATTATCCGGTTTAAACTCATGCAC
TGCTGXXXXXXXXXXGATTCGGATGCAAGGTCCCAGAAGGGAAAGGAGAGCGTGGGGGAAGGACGAACGGAGTTA
TAAGAAGTAATGTTGAAAGT
ACCAACAGGACTAACACTTCCGTCAAACACCAACACGCCTCTTGATTTCAAATTATCCGGTTTAAACTCATGCAC
TGCTGXXXXXXXXXXGATTGGGATGCAAGGTCCCAGAAGGGAAAGGAGAGCGTGGGGGAAGGACGAACGGAGTTA
TAAGAAGTAATGTTGAAAGT
>Marker872350
AACACAAGAGTATTAAGTTATGGCCTTCTCTTTTGTGTGTATATATATTGACTTCTTTTTTCTAAAATAATTTTA
AAAAGXXXXXXXXXXTAGATTTATTTGGTTCAAATACCATAATTCTAATAAGGATAATTTTTTTGTTTGAAAAAAA
AGAATCATGCTAGGGTTGTG
AACACAAGAGTATTAAGTTCTGGCCTTCTCTTTTGTGTATATATATATTGACTTCTTTTTTCTAAAATAATTTTA
AAAAGXXXXXXXXXXTAGATTTATTTGGTTCAAATACCATAATTCTAATAAGGATAATTTTTTTCTTTGAAAAAAA
AGAATCATGCTAGGGTTGTG
>Marker872560
AACTGATTCCAATTTCAGTTTCTCATTCTCAATCTCCTGCATGAAAAAACGGGCTTCAGACTATCAGACAAATAG
TCATTXXXXXXXXXXTATTATGGTCTCGTCCAATTATGATAGTGACGCGATAGATGAAGCATCAAGAACCAGTAA
TCTACAAATCTACTATAGTC
AACTGATTCCAATTTCAGTTTCTCATTCTCAATCTCCTGCATGAAAAAACGGGCTTCAGACAATCAGACAAATAG
TCATTXXXXXXXXXXTATTATGGTCTCGTCCAATTATGATAGTGACGCGATAGATGAAGCATCAAGAACCAGTAA
TCTACAAATCTACTATAGTC
>Marker872909
CACCAAGTTTGAGCAATCAGTTTGACGAGGTTTGGTTCCCTACCTCTTTTATCCTTATGTTCTTGAACTTCATTA
AGCAAXXXXXXXXXXXGCTGTGAAACTAGCATATCAAAATATAACAAATTTTTACAGGTAACGGATAAAGAATGTG
CTGATTCTACTATTTTAGTG
CACCAAGTTTGAGCAATCAGTTTGACGAGGTTTGGTTCCCTGCCTCTTTTATCCTTATGTTCTTGAACTTCATTA
AGCAAXXXXXXXXXXXGCTGTGAAACTAGCATATCAAAATATAACAAATTTTTACAGGTAACGGATAAAGAATGTG
CTGATTCTACTATTTTAGTG
>Marker872924
CACTTCAACGAATCAAATTTTTCGAGGATCTCGTCCAATAGGTATTTAAGACCAAAAGATTGGGAAATTGTTTAT
TTTATXXXXXXXXXXTTTAAAAACAATGTTCTTATTTTTCTTAGGCAAAGAAAAAGAGTAAAAATTAATTTTATT
TTTTACAAAATGAGAGAGTT

Create PDF with GO2PDF for free, if you wish to

CACTTCAACGAATCAAATTTTTCGAGGATCTCGTCCAATAGGTATTTAAGACCAAAAGATTGGGAAATTGTTTAT
TTTATXXXXXXXXXXTTTAAAAAGAATGTTCTTATTTTTCTTAGGCAAAGAAAAAGAGTAAAAATTAATTTTATT
TTTTACAAAATGAGAGAGTT
>Marker873124
CACCTTTTAAAGACGGACAAAAAACTCTTCTTCGCTCATTAATGACGGTTCATCCCTCCTTACCTTCCCATTTTC
TCTATXXXXXXXXXXCAAACTTGTTTTAAATCATTAGAAATTAATTTTGGTTCGTTTTAAAATGTATAAATAAAA
AATTGGGTTATTATAAATGT
CACCTTTTAAAGACGGACAAAAAACCCTTCTTCGCTCATTAATGACGGTTCATCCCTCCTTCCCTTCCCATTTTC
TCTATXXXXXXXXXXCAAACTTGTTTTAAATCATTAGAAATTAATTTTGGTTCGTTTTAAAATGTATAAATAAAA
AATTGGGTTATTATAAATGT
>Marker873382
ACTTGTGCAAAGCTTCGAAACACTAGATCATAATCCAGCGCACCGATCCCGGTTATTTTCAATATTTACGGTCAA
TCATCXXXXXXXXXXAAATCCCATATGAAGAGGTGGTTCTTGCAATTTGAATTTCGAGCTCAGACATCTGCACCA
AAATATGTTTTCATTATGGT
ACTTGTGTAAAGCTTCGAAACACTAGATCATAATCCAGCGCACCGATCCCGGTTATTTTCAATATTTACGGTCAA
TCATCXXXXXXXXXXAAATCCCATATGAAGAGGTGGTTCTTGCAATTTGAATTTCGAGCTCAGACATCTGCACCA
AAATATGTTTTCATTATGGT
>Marker874099
CACTTGCTTCACTCCCTTCATTCCTTCCAACGCTCTCTTCACTTTCCTCTCACATCCTTCGCAGTCTATTCGAAT
TTTTAXXXXXXXXXXCCCATTTTCGTTACTAACACATAGATAAGAATTCAAATTACTGACATTTATATATCATGA
AATAACTCCTTGTTTTTGGT
CACTTGCTTCACTCCCTTCATTCCTTCCAACGCTCTCTTCACTTTCCTCTCACATCCTTCGCAGTCTATTCGAAT
TTTTAXXXXXXXXXXCCCATTTTCGTTGCTAACACATGGATAAGAATTCAAATTACTGACATTTATATATCATGA
AATAACTCCTTGTTTTTGGT
>Marker874606
ACTCCATCTCCAAACGCATGTTCTCACTGATCTCGTGCTTGGATCCATTTAACTCGGACTTGTCTGCCTGAGAAT
CTTGTXXXXXXXXXXGCTATATCAAAGGGTAAGTCAATAAGTTGGCTGAGAAAATTATTCATAAAAACCACAATG
CAATACCTCGGCCAAAGGTG
ACTCCATCTCCAAACGCATGTTCTCACTGATCTCGTGCTTGGATCCATTTAACTCGGACTTGTCTGCCTGAGAAT
CTTGTXXXXXXXXXXGCTATATCAAAGGGTAAGTCAATAAGTTGGCTAAGAAAATTATTCATAAAAACCACAATG
CAATACCTCGGCCAAAGGTG
>Marker874996
TACCTGGTCTTTCTTTTAATCTAATTTAAACGAGAGTAACATATGGAGATTATTGCTTTTAATATGCTATTATTG
CCTTTXXXXXXXXXXGTGATATTGCAATTAGCTTTCGTTAGGTATGCGAGACAGCTTCCTCTCATTTAAAGATAT
ACCTTTCCTTTTCTATTGTG
TACCTGGTCTTTCTTTTAATCTAATTTAAACGAGAGTAACATATGGAGATTATTGCTTTTAATATGCTATTATTG
CCTTTXXXXXXXXXXGTGATATTGCAATTAGCTTTCGTTAGGTATGCGAGACAGCTTCCTCTCATTTAAAGATAT
ACCTTTCCTTTTTTATTGTG
>Marker875014
ACCGTTTCGAACAGACAAGGCCTGGAACGATTAGTGTCCGACCAAAATTTCGTAGGTAAATGGCTGAAGCATCCA
TCATTXXXXXXXXXXTATTCTTAACCTTAAATGTCAAAATGATGGAGTTTAACCCTCGAATAAGTATCGCCAAAA
AGTTCTATCCTATGTATGTA
ACCGTTTCGAACAGACAAGGCCTGGAACGATTAGTGTCCGACCAAAATTTCGTAGGTAAATGGCTGAAGCATCCA
TCATTXXXXXXXXXXTATTCTTAACCTTAAATGTCAAAATGATGGAGTTTAACCCTCTAATAAGTATCGCCAAAA
AGTTCTATCCTATGTATGTA
>Marker875148

Create PDF with GO2PDF for free, if you wish to

AACCCCATGTGGTGGATTAACAAAGACCTCTCCACTTAGCGAGGAGGGAATAATAGCTAAAATAGTGGAAAAGGG
GAGAAXXXXXXXXXXXCGCTTCATAAAAGGAAAATATATTGGACAACAAATAGAAGGAAGATCAACAGATACAAAA
AGCAATAGAGGCATCTTAGT
AACCCCATGTGGTGGATTAACAAAGACCTCTCCACTTAGCGAGGAGGGAGTAATAGCTAAAATAGTGGAAAAGGG
GAGAAXXXXXXXXXXXCGCTTCATAAAAGGAAAATATATTGGACAACAAATAGAAGGAAAATCAACAGATACAAAA
AGCAATAGAGGCATCTTAGT
>Marker875296
AACACTGACCTATCTGACTCTCCAGGAAGCAATGCGTAAAGCTGAAGCGGCTGGAAATGATGATTGTCCTGTTAA
AATTAXXXXXXXXXXXTGGATTTTCTCTAAAATTTAGTGACTTAAAAGCTTCTCAGGAATTACCGAAGGTGGGTCA
TGAGCACAGTGCGTGGAGTG
AACACTGACCTATCTGACTCTCCAGGAAGCAATGCGTAAAGCTGAAGCGGCTGGAAATGATGATTGTCCTGTTAA
AATTAXXXXXXXXXXXTGGATTTTCTCTAAAATTTAGTGACTTAAAAGCTTCTCAGGAATTACCGAAGGTGGGTCA
TGAGCACAGTGCCTGGAGTG
>Marker875298
TACTAACTCTCATTCATCGACATACTTAACTGCCAAGGACACATTTACTGCATAAGAAACCATGTTTGAGTTGTA
GTTTAXXXXXXXXXXXTGAGATATTATATTAGAAATATCATCATAATTATTTTAACATGGTTTAAGTTTAAAATAA
ATGTGATCTCATATTGAGGT
TACTAACTCTCATTCATCGACATACTTAACTGCCAAGGACACATTTACTGCATAAGAAACCATGTTTGAGTTGTA
GTTTAXXXXXXXXXXXTGAGATCTTATATTAGAAATATCATCATAATTATTTTAACATGGTTTAAGTTTAAAATAA
ATGTGATCTCATATTGAGGT
>Marker875641
AACAGTTATTTATGATAATCTTGGCTGTCAATGGTGAAGATTTTATACAAATTATAGCTTAAATTTCAAATTCCA
AATTTXXXXXXXXXXXTTTTGAAAACAGTGTATTTTCCTATATATATGTGAGACCTCCAGTTGTCTATGTGCCATT
GCGTGTGTGCTCCCAGAGTA
AACAGTTATTTATGATAATCTTGGCTGTCAATGGTGAAGATTTTATACAAATTATAGCTTAAATTTCAAATTCCA
AATTTXXXXXXXXXXXTTTTGAAAACAGTGTATTTTCCTATATATATGAGAGACCTCCAGTTGTCTATGTGCCATT
GCATGTGTGCTCCCAGAGTA
>Marker875676
ACATCATTTGATCTCTGGTAGCCATAACTATTTTTTCAACTAAACGAGCAATTGTCTCTAATATGTTTGTATCAA
ATTCTXXXXXXXXXXXTTTTAGTTACTATAACAAGAGCTCATTTTGATACCAATTAAACTATCGGTCTCAACATTT
CATATATAGGTTGTTCAAGT
ACATCATTCGATCTCTGGTAGCCATAACTATTTTTTCAACTAAACGAGCAATTGTCTCTAATATGTTTGTATCAA
ATTCTXXXXXXXXXXXTTTTAGTTACTATAATAAGAGCTCATTTTGATACCAATTAAACTATCGGTCTCAACATTT
CATATATAGGTTGTTCAAGT
>Marker875813
AACATAACCATGACAGCTTTTGGTGAAGAAATTCCTGCAGATTGCAAAACTGCTGGTCGTGATCCATCTCCATAG
AGAACXXXXXXXXXXXACGCTTCATGAAAATTACTGTCAAAATTAGAAAAAGGAACTACTTTACGAGAACATCATG
CCATTGCAAATAAAAGAAGT
AACATAACCATGACAGCTTTTGGTGAAGAAATTCCTGCAGATTGCAAAACTGCTGGTCGTGATCCATCTCCATAG
AGAACXXXXXXXXXXXACGCTTCATGAAAATTACTGTCAAACTTAGAAAAAGGAACTACTTTACGAGAACATCATG
CCATTGCAAATAAAAGAAGT
>Marker875819
AACACAGAGGGGGGAGGGGGGTAGAGTTTAGACAACGGGACCTTTTGCTTGGGTTATTTAGCATAATTGGTTAGA
ATGTTXXXXXXXXXXXGTGCATCATCGCTAAGAGGGACGTGTTCGACAATTGAGTGTGGGTAAGATTCTAAAATTA
ATTATATGATTAACAGGGTA

Create PDF with GO2PDF for free, if you wish to

AACACAGAGGGGGGAGGGGGGTAGAGTTTAGACAACGGGACCTTTTGCTTGGGTTATTTAGCATAATTGGTTAGA
ATGTTXXXXXXXXXXXGTGCATCATCGCTAAGAGGGACGTGTTCGACAATTGAGTGTGGGTAAGATTCTAAAATTT
ATTATATGATTAACAGGGTA
>Marker876105
ACATATCTCACAAGTGCAATCATCAGCAGGAAGCTGGTTTTGGCAAGCAATATTTCTACACCTTTTATACACAAA
AACCTXXXXXXXXXXXTCTTATCAATGTTTTTCCAGTTAAATCAGTTCTAGACTGAACAAGCTTTTGCAACATAAA
AATCTCGTCTCTCTGTTGTG
ACATATCTCACAAGTGCAATCATCAGCAGGAAGCTGGTTTTGGCAAGCAATATTTCTACACCTTTTATACACAAA
AACCTXXXXXXXXXXXTCTTATCAATGTTTTTCCAGTTAAATCAGTTCTAGGCTGAACAAGCTTTTGCAACATAAA
AATCTCGTCTCTCTGTTGTG
>Marker876119
ACTTTATTATTCTAATTCGTTGTTTTTATATAGTGTCCTCTACAAGTCAGAAGCATCGAATGTGGATACTATGCC
ATGAGXXXXXXXXXXXATCTAATTTGTTGAACCTTATTTTGCATGTTTATAGATTGATACACGAAAGTCATATTCA
CATGCCAAGTTAGATGAGGT
ACTTTATTATTCTAATTCGTTGTTTTTATATAGTGTCCTCTACAAGTCAGAAGCATTGAATGTGGATACTATGCC
ATGAGXXXXXXXXXXXATCTAATTTGTTGAACCTTATTTTGCATGTTTATAGATTGATACACGAAAGTCATATTCA
CATGCCAAGTTAGATGAGGT
>Marker876702
TACTCAACCAAAATGATCTCACAAGATGGGAGAAAATGGGAGAGAAGAATTTTCAAAGATTGAGATTTTTTTTTT
CCTTGXXXXXXXXXXXTCATGCAACCATGTGGAGGCATGATCACCTAGACACTCAACGCATATTTCCTTACGAGAT
AGCACGTTGAACACTTAGTA
TACTCAACCAAAATGATCTCACGAGATGGGAGAAAATGGGAGAGAAGAATTTTCAAAGATTGAGATTTTTTTTTT
CCTTGXXXXXXXXXXXTCATGCAACCATGTGGAGGCATGATCACCTAGACACTCAACGCATATTTCCTTACGAGAT
AGCACGTTGAACACTTAGTA
>Marker876790
AACAAAAGAACAAGTATCATAGAACCACTGCAGGAACTTAAACAACAGTGAAGGAATACTGCAAAGTATATTAGC
AATTGXXXXXXXXXXXCTTTTGTCACCTTCTTCATGTTAAATCCTACCTGCAGATGAAAGTATGATTTGAGTCGAA
CTGTAGGAACAAGCTATGGT
AACAAAAGAACAAGTATCATAGAACCACTGCAGGAACTTAAACAACAGTGAAGGAATACTGCAAAGTATATTAGC
AATTGXXXXXXXXXXXCTTTTGTCACCTTCTTCATGTTAAATCCTACCTGCAGATGAAAGTATGATTTGAGTTGAA
CTGTAGGAACAAGCTATGGT
>Marker877150
AACTTATGAACAAAAAATTATTGACACATTGTCCAAAAAATACCTCTAGACTTCTAAAAAAATTGCTTACGTTATA
CTAAAXXXXXXXXXXXXCAACATCGAAAAGTTGAATAAAAAACCAACACCATAAGTATTTTCATACATAGATAAGAG
ACAATTTGTATAATTTAGTA
AACGTATGAACAAAAAATTATTGACATATTGTCCAAAAAATACCTCTAGACTTCTAAAAAAATTGCTTACATTATA
CTAAAXXXXXXXXXXXXCAACATCGAAAAGTTGAATAAAAAACCAACACCATAAGTATTTTCATACATAGATAAGAG
ACAATTTGTATAATTTAGTA
>Marker877618
ACCTTCCATCTATATGCCATGATTATCGTGAGGTGGTCGCCTATGACGACTTTATTGAATATTTGTTTTCAACAT
GGAAGXXXXXXXXXXXGATTACATCAAGGAAACCTCGCAACCGATCCTCCACTACCAATAACTAACACATGGATCT
ATCATAAAATCAATGTTGTA
ACCTTCCATCTATATGCCATGATTATCGTGAGGTGGTCACCTATGACGACTTTATTGAATATTTGTTTTCAACAT
GGAAGXXXXXXXXXXXGATTACATCAAGGAAACCTCGCAACCGATCCTCCACTACCAATAACTAACAGATGGATCT
ATCATAAAATCAATGTTGTA
>Marker877773

Create PDF with GO2PDF for free, if you wish to

AACATGGTAAATTGATGTTTTATCATATAACTAAAAAAATTCGGTGTCTTTTAAATTTTGATCGTTGCCATCTTG
ACACAXXXXXXXXXXXTATTTTTTTGGGTTTTATGATTTGTTGTGGAAGTATTTGCTTACAAATTTGTAGAGTTTA
TTTTAAAAAATTGTTGTTGT
AACATGGTAAATTGATGTTTTATCATATAACTAAAAAAATTCGGTGTCTTTTAAATTTTGATCGTTGCCATCTTG
ACACAXXXXXXXXXXXTATTTTTTTGGGTTTTATGATTCGTTGTGGAAGTATTTGCTTACAAATTTGTAGAGTTTA
TTTTAAAAAATTGTTGTTGT
>Marker878205
AACCATTTGGTGATAATTAAATCACACGAGTTCGATCAATTTAAACCTTTCAACTAATAAGCCACCACTGGGAGA
TGATTXXXXXXXXXXXAAATTAATTGGTTTAATTGAAACCACAGTATAAATGGATATTTGGCTTCACATAAATTAA
TAAATTTAGTAGAATTTGTA
AACCATTTGGTGATAATTAAATCACACGAGTTCGATCAATTTAAACCTTTCAACTAATAATCCACCACTGGGAGA
TGATTXXXXXXXXXXXAAATTAATTGGTTTAATTGAAACCACAGTATAAATGGATATTTGGCTTCACATAAATTAA
TAAATTTAGTAGAATTTGTA
>Marker878733
ACTCAACCTAAGAGATTATCATAGTTGTTCAATCAAATCACATTCATTTGATGTCACAAAGAGATGGAGAGATTC
TTTCCXXXXXXXXXXXATGTGTAAAAATGTCACTCTTTTCATTTATGGCAATTTGAATATGTCAATACATGCACTT
GATGAAGGGATAATAGGGTA
ACTCAACCTAAGAGATTATCATAGTTGTTCAATCAAATCACATTCATTTGATGTCACAAAGAGATGGAGAGATTC
TTTCCXXXXXXXXXXXATGTGTAAAAATGTCACTCTTTTCATTTATGGAAATTTGAATATGTCAATACATGCACTT
GATGAAGGGATAATAGGGTA
>Marker879082
ACCTTTAGCTCATTAACGTGTATGATTGAGTGAATATTGGTTCTTTTGAGTTGCTTAGGACATTTCAAGAGGGTC
AATTGXXXXXXXXXXXGTTGTTTGGAATCATCGACAACTTTTTCTTTGAATTAGCTAATTGGAAGTTGAATGGATC
CTTTTTTGGTCCAAAATGTT
ACCTTTAGCTCATTAACGTGTATGATTGAGTGAATATTGGTTCTTTTGAGTTGCTTAGGACATTTCAAGAGGGTC
AATTGXXXXXXXXXXXGTTGTTTGGAATCATCGACAACTTTTTCTTTGAATTAGCTAATTGGAAGTTGAATGGATC
CTTTTTTGTTCCAAAATGTT
>Marker879268
TACTCTGCTCGGAGGCAGTGGGTTCCAGTGTATTTGCGGGACACGTTTTTTGCTGAAATGTCTATTACACAGCGA
AGTGAXXXXXXXXXXXTCTCCAATGGAAAAACAAGTTTCTGAGCTTTACACCAGGAAGCTATTCTCGAGGTTCCAA
GAGGAGTTAGTTGGGACGTT
TACTCTGCTCGGAGGCAGTGGGTTCCAGTGTATTTGCGGGATACGTTTTTTGCTGAAATGTCTATTACACAGCGA
AGTGAXXXXXXXXXXXTCTCCAATGGAAAAACAAGTTTCTGAGCTTTACACCAGGAAGCTATTCTCGAGGTTCCAA
GAGGAGTTAGTTGGGACGTT
>Marker879372
AACCTCTTTTATAAACGTGTCGATGAAGTGGGTAGAAATCGCTGTCATTATCGTAATGTTGTTATGTAGGAAACA
TTTTTXXXXXXXXXXXTTACGAAGATTTTAGTGTTAAATCATTTTCTTCGAATTGCTCTTCCAAGATTAACAAAAC
ATCATTTTTAGATCATAAGT
AACCTCTTTTATAAACGTGTCGATGAAGTGGGTAGAGATCGCAGTCATTATCGTAATGTTGTTATGTAGGAAACA
TTTTTXXXXXXXXXXXTTACGAAGACTTTAGTGTTAAATCATTTTCTTCGAATTGCTCTTCCAAGATTAACAAAAC
ATCATTTTTAGATCATAAGT
>Marker879670
AACCAAGAATAATCCAATCTCCATCTTATATGTTGTTAAATAAAAAAAAATTATACTCTTCTCAAATAGTATTAA
ATTTTXXXXXXXXXXXATATGAATTATAAGAAAATTTACAGAATATCGCTGATGTAAAGAGTAATATAAATAGTAT
AAGAGAAACTCTATAAAAGT

Create PDF with GO2PDF for free, if you wish to

AACCAAGAATAATCCAATCTCCATCTTATATGTTGTTAAATAAAAAAAAATTATACTCTTCTCATATAGTATTAA
ATTTTXXXXXXXXXXXATATGAATTATAAGAAAATTTACAGAATATCGCTGATGTAAAGAGTAATATAAATAGTAT
AAGAGAAACTCTATAAAAGT
>Marker879690
ACTTGTTCTGCTATTCCACTGAATAAAGATCACTTCCAGTTGGTGTTCTATCAGCCCATTAACCCAATCTTCTTC
TTCCAXXXXXXXXXXXTCTTTTCACCATTGTTATATAAGAACCCAACTTGCCCAACACTTTTCTTCTTATTTCGGA
CTATTTTTCCTCTTTTCGTT
ACTTGTTCTGCTATTCCATTGAATAAAGATCACTTCCAGTTGGTGTTCTATCAGCCCATTAACCCAATCTTCTTC
TTCCAXXXXXXXXXXXTCTTTTCACCATTGTTATATAAGAACCCAACTTGCCCAACACTTTTCTTCTTATTTCGGA
CTATTTTTCCTCTTTTCGTT
>Marker879992
AACAACTATATCTTTAATTATTTTTGTTATGTAGTCAATTCAAACAAGTCATTCTAATATCTTTCAATCAGTTAT
GGATTXXXXXXXXXXXCCATTTAGTCATTCATTATTTTATTTCAGTGTCTAATCATCTAAATATACAAATCACTTT
CATCATTTCATTAGAGAGTT
AACAACTATATCTTTAATTATTTTTGTTACGTAGTCAATTCAAACAAGTCATTCTAATATCTTTCAATCAGTTAT
GGATTXXXXXXXXXXXCCATTTAGTCATTCATTATCTTATTTCAGTGTCTAATCATCTAAATATACAAATCACTTT
CATCATTTCATTAGAGAGTT
>Marker880215
ACTTTATATGTAAATGCAATGCTTTTCCTGGCTGTCATGCAGTATTTGAAAGAACAAAGGCTTCCTCCTCAGACA
TTCATXXXXXXXXXXXAACACTTTTTACTTTCTTATTTTATCTTTCATGTTCTCTCTGGTTTATGTTTGAGAATCT
TTTTCACAAACGTGAAGGGT
ACTTTATATGTAAATGCAATGCTTTTCCTGGCTGTCATGCAGTATTTGAAAGAACAAAGGCTTCCTCCTCAGACA
TTCATXXXXXXXXXXXAACACCTTTTACTTTCTTATTTTATCTTCCATGTTCTCTCTGGTTTATGTTTGAGAATCT
TTTTCACAAACGTGAAGGGT
>Marker880282
ACCACCTGCTTTGTATTTGAGAGAGAACACCCATTTGCATCCCACATTTTTGTGTCCCTTGGGTAGAGCACAAAT
TCCCTXXXXXXXXXXXGAGTCAAGGCTTGCGGTAAAAGCTCTGAACTGTGGTGAGAGATTATCATAGGAAACATAG
TTACAAATGGGATGTTTAGT
ACCACCTGCTTTGTATTTGAGAGAGAACACCCATTTGCATCCCACATTTTTGTGTCCCTTGGGTAGAGCACAAAT
TCCCCXXXXXXXXXXXGAGTCAAGGCTTGCGGTAAAAGCTCTGAACTGTGGTGAGAGATTATCATAGGAAACATAG
TTACAAATGGGATGTTTAGT
>Marker880307
GACATACTTAGTCTTCATGTTGCATTGTTGTCATTTTAATTGCAGAGCCGTTTGGCCCGTTTCTTTATATATTGC
CTCTTXXXXXXXXXXXCCCCTGCTCCCAAACAAACTTGTGTATGTTTGTATGTAATTAATGTTTATTGTGTAATAT
TATCTAAACGAATGGTTGTA
GACATACTTAGTCTTCATGTTGCATTGTTGTCATTTTAATTGCATAGCCGTTTGGCCCGTTTCTTTATATATTGC
CTCTTXXXXXXXXXXXCCCCTGCTCCCAAACAAACTTGTGTATGTTTGTATGTAATTAATGTTTATTGTGTAATAT
TATCTAAACAAATGGTTGTA
>Marker880325
TACCATTGTGGATTTGAAAACATATTGGGCATAAATCAGACCCATTTATTTTAGCCTCCTAAGCAAAACAAGTAT
TCTATXXXXXXXXXXXTAGAGTTTAAAACCACATGATTGATGATACAACTTCATCATATATTAGACATGATTGTGA
GGGTAGATGAGTTGATAAGT
TACCATTGTGGATTTGAAAACATATTGGGCATAAATTAGACCCATTTATTTTAGCCTCCTAAGCAAAACAAGTAT
TCTATXXXXXXXXXXXTAGAGTTTAAAACCACATGATTGATGATACAACTTCATCATATATTAGACATGATTGTGA
GGGTAGATGGGTTGATAAGT
>Marker880503

Create PDF with GO2PDF for free, if you wish to

TACTGAATATATTTGCCCTGAAGAATAAGAAAGCAGTTGAAATAGGGTCCAAAAGATCCCCTTTATTGTCTCTCG
AAGTGXXXXXXXXXXXATAATCATTCTATTCACATTAGAAAAGATTCTTTTGGCTAATTCTCCAAATATTCATAAC
ATTTGAGTGCAGTGTAGGTC
TACTGAATATATTTGCCCTGAAGAATAAGAAAGCAGTTGAAATAGGGTCCAAAAGATCCCCCTTATTGTCTCTCG
AAGTGXXXXXXXXXXXATAATCATTCTATTCACATTAGAAAAGATTCTTTTGGCTAATTCTCCAAATATTCATAAC
ATTTGAGTGCAGTGTAGGTC
>Marker880935
ACTAAATCCTTGCTATTGGGTTTCTGGGTTATTCGATTTGGCTCATTTTAGGTTATTTTCCATTTTATGTTTGTG
TTTAAXXXXXXXXXXXTGTTCTTGTTTTTATCATTTTTTTGGAGTGTTTTTTTAAGTTTGTTTTGGCATTACCAAA
GTTGTTGTTTGGTTATTGGT
ACTAAATCTTTGCTATTGGGTTTCTGGGTTATTCGATTTGGCTCATTTTAGGTTATTTTCCATTTTATGTTTGTG
TTTAAXXXXXXXXXXXTGTTCTTGTTTTTATCATTTTTTTGGAGTGTTTTTTTAAGTTTGTTTTGGCATTACCAAA
GTTGTTGTTTGGTTATTGGT
>Marker881010
ACATGCCACCGTCTGACTAGAAATTGCCTCACACGCTGCCTCTGTTTGACGCATGGGCCCACGTGCCAACGTCGT
TTCATXXXXXXXXXXXCACTCTTTACAGCAACCGTTTTTCTATGGTAACGGGAATGGCCAACCCCATAACAAATCA
GACATTGAAGCAGGCAAGTC
ACATGTCACCGTCTGACTAGAAATTGCCTCACACGCTGCCTCTGTTTGACGCATGGGCCCACGTGCCAACGTCGT
TTCATXXXXXXXXXXXCACTCTTTACAGCAACCGTTTTTCTATGGTAACGGGACTGGCCAACCCCATAACAAATCA
GACATTGAAGCAGGCAAGTC
>Marker881047
AACCCAACCTCTTACTTCTAGCTACCCGACTCAACTTTTTTTCTCCAATTTTCTAACTCAATCCAATCCAACAAA
AAACTXXXXXXXXXXXATTCTCTTTTCACTTCTCATTAATTATTTGTATTGAAAATGATAAACTAACAAAACACCG
ACTCATGTAGAAACCCAAGT
AACCCAACCTCTTACTTCTGGCTACCCGACTCAACTTTTTTTCTCCAATTTTTTAACTCAATCCAATCCAACAAA
AAACTXXXXXXXXXXXATTCTCTTTTCACTTCTCATTAATTATTTGTATTGAAAATGATAAACTAACAAAACACCG
ACTCATGTAGAAACCCAAGT
>Marker881102
CACAAAACAATATATCACCTATGATATCAATAATAAATCATGGGTTTTTGAGAAATACGACATCACATCTACTAC
TCAGCXXXXXXXXXXXAAAAGATGAAAATGGCTATTTATCAAATTGAAACTTATCATACATGATACACTCAAAATT
TATGTTACTTTTCCTAAGTG
CACAAAACAATATATCACCTATGATATCAATAATAAATCATGGGTTTTTGAGAAATACGACATCACATCTACTAT
TCAGCXXXXXXXXXXXAAAAGATGAAAATGGCTATTTATCAAATTGAAACTTATCATACATGATACACTCAAAATT
TATGTTACTTTTCCTAAGTG
>Marker881385
GACTAATGCTCTTGAACTGTTACCCAATTTGTTATACACATGGAAGGTCGTCGTTGTAAAGTGAATATAATTACA
TGTAAXXXXXXXXXXXGTATCTAACCCTTTAGATAATTACTTTCCAAAATTTTAAAAGTCTTATTAAAAACCGTTT
AGACCCGACCATGAAGGAGT
GACTAATGTTCTTGAACTGTTACCCAATTTGTTATACACATGGAAGGTCGTCGTTGTAAAGTGAATATAATTACA
TGTAAXXXXXXXXXXXGTATCTAACCCTTTAGATAATTACTTTCCAAAATTTTAAAAGTCTTATTAAAAACCGTTT
AGACCCGACCATGAAGGAGT
>Marker882050
CACGAATATTGTAATATGGGACTGTATTTTATGGATGTGGTTTCCTAGAAAATGGATATTTGAGCATAACTGAGT
TTTTTXXXXXXXXXXXTTGGTCAAGACGTGTTTCACTGGCAAGCAACAATTATGGGTCCAGCAGATAGTCCTTATG
CGGGGGGTTTATTTTCAGTG

Create PDF with GO2PDF for free, if you wish to

CACGAATATTGTAATATGGGACTGTATTTTATGGATGTGGTTTCCTAGAAAATGGATATTTGAGCATAACTGAGT
TTTTTXXXXXXXXXXTTGGTCAAGACGTGTTTCACTGGCAAGCAACAATTATGGGTCCAGCAGATAGTCCTTATG
CTGGGGGTTTATTTTCAGTG
>Marker882098
ACTTATGAGATCGATGATGATATAATGGATGAATCGACTTCTTCATTAACAAAACTTAGTTGAAAAATAAGAAAA
GACAAXXXXXXXXXXXATAAGTTTTAATCTAACACGTATATTTTATCATTATATCCATTAAATGTTTCATAAAATT
GAGACCTCAAGATTTTGGTT
ACTTATGAGATCGATGATGATATAATGGATGAATCGACTTCTTCATTAACAAAACTTAGTTGAAAAATAAGAAAA
GACAAXXXXXXXXXXXATAAGTTTTAATCTAACACGTATATTTTATCAATATATCCATTAAATGTTTCATAAAATT
GAGACCTCAAGATTTTGGTT
>Marker882746
AACAGTTTGTGGAAAGAAAAGTATAGGTTGAAAGAAAAAGACACATATTTTTCTATTCTCAGGATTTAAATTCAA
AAATGXXXXXXXXXXXATGATTCATGATACTCTCTTTTAATATTATCATTAGTTATATCACACAAACTTACAAAAT
CATTATTTGTTTTGTTAGTT
AACAGTTTGTGGAAAGAAAAGTATAGGTTGAAAGAAAAAGACACATATTTTTCTATTCTCAGGATTTAAATTCAA
AAATGXXXXXXXXXXXATGATTCATGATACTCTCTTTCAATATTATCATTAGTTATATCACACAAACTTACAAAAT
CATTATTTGTTTTGTTAGTT
>Marker883223
TACAATGCAACTAATTGAAGCAAGTAGAGAAGAGTGCCTTGGTGTGATCAATGTTTGGTTGTTCGAAAGTAAATA
TAGATXXXXXXXXXXCTATCACAACGAAGAATAGTAGTAGAGTCTTGGGAAAACTCATGTTTTGCATACAACCTC
ATAAGTAGCATCAATAGGGT
TACAATGCAACTAATTGAAGCAAGTAGAGAAGAGTGCCTTGGTGTGATCAATGTTTGGTTGTTCGAAAGTAAATA
TAGATXXXXXXXXXXCTATCACAACGAAGAATAGTAGTAGAGTCTTGGGGAGCCTCATGTTTTGCATACAACCTC
ATAAGTAGCATCAATAGGGT
>Marker883543
TACCAGTAGGTAGAAGCTAGAGTAGTATATAATTGATCTCTTTTAGAATGAGTATTTTTAAATCCAAATCTATTT
GGAGTXXXXXXXXXXAGAAGGATGGTTTGCATAAGGATGGTAATTATTGAAAGGAGAGACAAAGAGCACACACTA
ATTTACGTGGAAACCTGAGT
TACCAGTAGGTAGAAGCTAGAGTTGTATATAATTGATCTCTTTTAGAATGAGTATTTTTAAATCCAAATCTATTT
GGAGTXXXXXXXXXXGGAAGGATGGTTTGCATAAGGATGGTAATTATTGAAAGGAGAGACAAAGAGCACACACTA
ATTTACGTGGAAACCTGAGT
>Marker883557
GACTTCAGATCTGTTGATGGATCAAACTCTGCAGCTGATGCAAAGGATTCCACAGATGGTGGAGCAGCTGAAGAA
GATTTXXXXXXXXXXAGAGAAGCAGAGCTATGAGAATCTATTGGTTGCAAGGGTTCCGGAGCGTCTTTTCTTGTT
CTGATGCTAAACTCATTTGT
GACTTCAGATCTGTTGTTGGATCAAACTCTGCAGCTGATGCAAAGGATTCCACAGATGGTGGAGCAGCTGAAGAA
GATTTXXXXXXXXXXAGAGAAGCAGAGCTATGAGAATCTATTGGTTGCAAGGGTTCCGGAGCGTCTTTTCTTGTT
CTGATGCTAAACTCATTTGT
>Marker883804
ACCTAAAAACAAGGTTTTAAATTGACATTATTGGCCTCTTTAATGTGTTTAAACCGATATCAAAGCCAACTAACA
TTAAAXXXXXXXXXXXAAAGATGTTGGTTGCCAACCAACATTAAAGGCTAAATTTCTTCTAGTGCTTCTTTTCAAG
TTGTTGTTCCTGTAGTTGTT
ACCTAAAAACAAGGTTTTAAATCGACATTATTGGCCTCTTTAATGTGTTTAAACCGATATCAAAGCCAACTGACA
TTAAAXXXXXXXXXXXAAAGATGTTGGTTGCCAACCAACATTAAAGGCTAAATTTCTTCTAGTGCTTCTTTTCAAG
TTGTTGTTCCTGTAGTTGTT
>Marker884498

Create PDF with GO2PDF for free, if you wish to

AACAAGGTTCATCTCTTATTATGTTGAGACATGGTTACCGAAGATGAAGAAGGAGAAGAATAAGAAGAAGATAGC
AGAGTXXXXXXXXXXXTTACCTGCATTAACAAAATAAATGACAGTAAAAGTAAGTTAATATTAACTAGATGAATCC
TTGCTTTAAGGAAACATGTA
AACAAGGTTCATCTCTTATTATGTTGAGACATGGTTACCGAAGATGAAGAAGGAGAAGAATAAGAAGAAGATAGC
AGAGTXXXXXXXXXXXTTACCTGCATTAACAAAATAAATGACAGTAAAAGTAAGTTAATATTAACTAGATGAATCC
TTGTTTTAAGGAAACATGTA
>Marker885005
ACTCTCTTGGATCCTTCTTCTATTCCTCACAACCAAATATATAATGCCTTAAGTTTTTATGATCATCTATATATA
CAATTXXXXXXXXXXXCTTTAAGTTGTGTATTTCTCATTTTCTCATCAAGAAATTTATGTGTAATATTCTTTTTAT
TTCTCACAATTGTATTAAGT
ACTCTCTTGGATCCTTCTTCTATTCCTCACAACCAAATATATAATGCCTTAAGTTTTTATGATCATCTATATATA
CAATTXXXXXXXXXXXATTTAAGTTGTGTATTTCTCATTTTCTCATCAAGAAATTTATGTGTAATATTCTTTTTAT
TTCTCACAATTGTATTAAGT
>Marker885447
AACCTAAACTCTGATTTGTTGGTTGAGTTTTCTTTTTAATTATTCTCTTTGCTTATTCTGATTGATTGAAGTCCT
TATTAXXXXXXXXXXXAAAACAGTGACATTTCTGGTTGGTTGGTTTTGTTTATAGAGAGATATAGCATCTGGAACT
TGATCGAATTTCCATCTGTA
AACCTAAACTCTGATTTGTTGGTTGAGTTTTCTTTTTAATTATTCTCTTTGCTTATTCTGATTGATTGGAGTCCT
TATTAXXXXXXXXXXXAAAACAATGACATTTCTGGTTGGTTGGTTTTGTTTATAGAGAGATATAGCATCTGGAACT
TGATCGAATTTCCATCTGTA
>Marker885600
GACGTTCAAGTTAATTAGATATTTGCTTTACCATCTATTGTTTTAACTTGATTGAATGAAAGGTGTTACAATTTT
TTTTCXXXXXXXXXXXTTTAGGAAAGGGATGTGACACAATTACCTACATTATATTATATGAATGAAACATATTTAA
ATGTAATGAAACATATAGTA
GACGTTCAAGTTAATTAGATATTTGCTTTACCATCTATTGTTTTAACTTGATTGAATGAAAGGTGTTACAATTTT
TTTCTXXXXXXXXXXXTTTAGGAAAGGGATGTGACACAATTACCTACATTATATTATATGAATGAAACATATTTAA
ATGTAATGAAACATATAGTA
>Marker885657
TACATTCATGATCACTATGTATAAACTCAATAATCGAATTGTGTTGTTTATTAATTACAAATTTGAGCGTCTTTA
TGTATXXXXXXXXXXXTGTAGTTAAACATTGACTTAGAAAAGGTTAAATTTTATTCCAACTACAAAATGATGGTGA
TGAGTTTGCAACCCTAAGTG
TACATTCATGATCACTATGTATAAACTCAATAATCGAATTGTGTTGTTTATTAATTACAAATTTGAGTGTCTTTA
TGTATXXXXXXXXXXXTGTAGTTAAACATTGACTTAGAAAAGGTTAAATTTTATTCCAACTACAAAATGATGGTGA
TGAGTTTGCAACCCTAAGTG
>Marker886137
AACCCTCTCATAGAGTTGGCCTTTTATCTTTTCTCAATTAATTCATTACAACTTTTCTAAATCAGAACCTAAATG
TGAAGXXXXXXXXXXXTTCTTCTTTAATTACTTGAGAGTTTGTATATATACTTAAAAACATTATGAAGGGGATGAC
TTATGTTTAATACATATGGT
AACCCTCTCATAGAGTTGGCCTTTTCTCTTTTCTCAATTAATTCATTACAACTTTTCTAAATCAGAACCTAAATG
TGAAGXXXXXXXXXXXTTCTTCTTTAATTACTTGAGAGTTTGTATATATACTTAAAAACATTATGAAGGGGATGAT
TTATGTTTAATACATATGGT
>Marker886149
CACAACCCACCATGTCATTGAGGAAGGCCACCCTCCATAAATGATACAAATGAAAAGATGGAATATATAGAGTGT
GGCTGXXXXXXXXXXXGAGAAAAAAAAAAATTGATTCAGAATTGATTACTTTGAAACTAGTTGCCAGCAAGTGTAT
TAAACATAAGAAAAGAGGGT

Create PDF with GO2PDF for free, if you wish to

CACAACCCACCATGTCATTGAGGAAGGCCACCCTCCATAAATGATACAAATGAAAAGATGGAATATATAGAGTGT
GGCTGXXXXXXXXXXCGAGAAAAAAAAAATTGATTCAGAATTGATTACTTTGAAACTAGTTGCCAGCAAGTGTAT
TAAACATAAGAAAAGAGGGT
>Marker887014
TACAATCTTGAACCAAATAACAGATTGAAAAGAAACTAAAAGAAAATTTACAGATGAGGAGAAAAGACGATGGAA
AGAAAXXXXXXXXXXTTACCGGTTTGTTATATTTATGAAAATTAACCAATAAATAAGTATATAGCCAACATGAGC
TTAACTCAACTGGCACCGGT
TACAATCTTGAACCAAATAACAGATTGAAAAGAAACTAAAAGAAAATTTGCAGATGAGGAGAAAAGACGATGGAA
AGAAAXXXXXXXXXXTTACCGGTTTGTTATATTTATGAAAATTAACCAATAAATAAGTATATAGCCAACATGAGC
TTAACTCAACTGGCACCGGT
>Marker887040
GACATCCGTTTGGAATAATATAACGGTAGAATTTAATAGCAAAAAATATATACAATTTCCAATAGTCATCAATTT
AAAGAXXXXXXXXXXTCAAAAACTGACCCACCATTCTCATTTAGATCAACATTTGCCTTAATGTCATTCACCCTG
CCGCCAATGTTGGGAAGAGT
GACATCCGTTTGGAATAATATAACGGTAGAATTTAATAGCAAAAAATATATACAATTTCCTATAGTCATCAATTT
AAAGAXXXXXXXXXXTCAAAAACTGACCCACCATTCTCATTTAGATCAACATTTGCCTTAATGTCATTCACCGTG
CCGCCAATGTTGGGAAGAGT
>Marker887225
GACTTTGACAGTATCTTTCGGTTCTATATATTTATTCTTCTTGTTTTAAGTCCTCCACAATAATGTTGTGTGCTC
CAATAXXXXXXXXXXTTTTATAGAAAGCATCAATAAAATATTGGATGCTGATGAATGTAATGTTCCTTGAGGTAA
AATATAAGTTTGTTGATGTC
GACTTTGACAGTATCTTTCGGTTCTATATATTTATTCTTCTTGTTTTAAGTCCTCCACAATAATGTTGTGTGCTC
CAATAXXXXXXXXXXTTTTATAGAAAGCATCAATAAAATATTGGATGCTGATGAATGTAATGTTCGTTGAGGTAA
AATATAAGTTTGTTGATGTC
>Marker887577
GACCTGGATAGTCCAGATAAACGGAAGAAGATGATGGTTGCATGCACTCAGCCTCGCAGGGTGGCAGCAATGTCA
GTTTCXXXXXXXXXXTCCTTGTGCCTGCTCATTGCCACTATCTTTTCTCTATATAATTTGAAATTTTATTCTTTA
CTTGATTGTAAATTTGTTGT
GACCTGGATAGTCCAGATAAACGGAAGAAGATGATGGTTGCATGCACTCAGCCTCGCAGGGTGGCAGCAATGTCA
GTTTCXXXXXXXXXXTCCTTGTGCCTGCTCATTGCCACTATCTTTTCTCTATATAATTTGAAATTTTATTCTCTA
CTTGATTGTAAATTTGTTGT
>Marker888279
TACCACATCACCAGTTGGTAAGTTTCTGAAATTGTTGTCTGTCAAACACATTCGATAACATCCCCATGGTGCCCA
TGCCGXXXXXXXXXXCACTACGTGAAGCATAGAAAATTCTTGGATCAATTGATTATGAGATCCATTCACAAACAA
TTGGGTTCCTCAAGGGCGTA
TACCACATCACCAGTTGGTAAGTTTCTGAAATTGTTGTCTGTCAAACACATTCGATAACATCCCCATGGTGCCCA
TGCCGXXXXXXXXXXCACTACGTGAAGCATAGAAAATTCTTGGATCAATTGATTATGGGATCCATTCACAAACAA
TTGGGTTCCTCAAGGGCGTA
>Marker888317
CACTAGTTTTGTTTTGGAAACTTTAGTCAAGTGATTGATTACTTTAGAACGCAAAAGAGCACCCAAATATCTCGA
CCTTTXXXXXXXXXXTGGGCATGTGGATAAATCCTCCTTTAGAGTTAAGTTAATAAGAAGCAAAAAAGGAATACT
GCTTTCACAAAGAAAATGTG
CACTAGTTTTGTTTTGGAAACTTTAGTCAAGTGATTGATTACTTTAGAACGCAAAAGAGCACCCAAATATCTCGA
CCTTTXXXXXXXXXXTGGGCATGTGGATAAATCCTCCTTTAGAGTTAAGTTAATAAGAAGCAAAAAAGGAATACT
GCTTTCACAAATAAAATGTG
>Marker888844

Create PDF with GO2PDF for free, if you wish to

ACTTGTTCCAACAACAATGGAACTTCTATTGAATCTCCACCAGTTTGTCGTGATTCAAACTCCCTTTGCTGCTCT
ACAGCXXXXXXXXXXCCAAGAAACGTTCAATCTTACCGTTATTCAATCATGGATCCAAAAACAGAAAAACAACTA
GATAATGCTTAGACAAAGTA
ACTTGTTCCAACAACAATGGAACTTCTATTGAATCTCCACCAGTTTGTCGTGATTCAAACTCCCTTTGCTGCTCT
ACAGCXXXXXXXXXXCCAAGAAACGTTCAATCTTACCGTTATTCAATCATGGATCCAAAAACAGAAAAACAACTA
AATAATGCTTAGACAAAGTA
>Marker888899
AACAGTTAAACAGAACTCAAAGGGAGAGAAAAAGGGTAAAAGACGATAGTAACCGACTTTCTGAACTCCCATTTC
TTTAGXXXXXXXXXXCTCCCTATTCAATAATTGTTATAATTATGGTAAATCTGAATGCCTTTCTATCTGACACCA
CTGCATCGATGGGAAGATGT
AACAGTTAAACAGAACTCAAAGGGAGAGAAAAAGGGTAAAAGACGATAGTAACCGACTTTCTGAACTCCCATTTC
TTTAGXXXXXXXXXXCTCCCTATTCAATAATTGTTATAATTATTGCAAATCTGAATGCCTTTCTATCTGACACCA
CTGCATCGATGGGAAGATGT
>Marker889235
ACCATACCCACTTGTAAGAGTTTGATAAGGACCTCAACTTTAAAATTTAATCAACAATCATTTATATAATCGACC
GCACGXXXXXXXXXXTTCTATTATATATTTTTACAATAGGATTAAGATCAAACACACGGATGAGATCAACTTATA
TGATGTAAAAGTTTAAAAGT
ACCATACCCACTTGTAAGAGTTTGATAAGGACCTCAACTTTAAAATTTAATTAACAATCATTTATATAATCGACC
GCACGXXXXXXXXXXTTCTATTATATATTTTTACAATAGGATTAAGATCAAACACACGGATGAGATCAACTTATA
TGATGTAAAAGTTTAAAAGT
>Marker889265
GACTCCTTTGGAAGTTCCTAATAGAGTTTGGGAAGATATATCCATGGATTTCGTGGAAGGGTTGTCGAAAGCCAA
TGGAAXXXXXXXXXXAATAGTGTCGGATATCTCTGTCCTTAAGTCACTTTTGGAGGGAGTTGTTCAGACAGGCTG
ATACATGGCTGAATTATAGT
GACTCCTTTGGAAGTTCCTAATAGAGTTTGGGAAGATATATCCATGGATTTCGTGGAAGGGTTGTCGAAAGCCAA
TGGAAXXXXXXXXXXAATAGTGTCGGATATCTCTGTCCTTAAGTCACTTTTGGAGGGAGTTGTTCAGACAGGCTG
ATACACGGCTGAATTATAGT
>Marker889605
ACGTTTATTTAAACCGTGTTTCAAAACTAACCACATAAGTAATTCTTGTATATATAAATTTAAAATAACCGATTT
TGAATXXXXXXXXXXATGAAGAAGGAGCGATCATGTTTCTCAATGACTTAAGTTAGACGTAAAATCAATGTCTCT
CAAAGTAAGTTGATATCCGT
ACGTTTATTTAAACCGTGTTTCAAAACTAACCACATAAGTAATTCTTGTATATATAAATTTAAAATAACCCATTT
TAAATXXXXXXXXXXATGAAGAAGGAGCGATCATGTTTCTCAATGACTTAAGTTGGACGTAAAATCAATGTCTCT
CAAAGTAAGTTGATATCCGT
>Marker889685
AACCTCCCTACTGATCTCAATGATCATGGAACGTGTGGCTTTCATAAAGTTCATGTTCGTGGAAACTGTTTTAAA
GGGTCXXXXXXXXXXATTGAGAATTAAATATGCTATCCTTCACAAGATTGTGTGTCACTGCTATATTTACTTATT
GTTTTCATTGTTGTTTTGTT
AACCTCCCTACTGATCTCAATGATCATGGAACGTGTGGCTTTCATAAAGTTCATGTTCGTGGAAACGGTTTTAAA
GGGTCXXXXXXXXXXATTGAGAATTAAATATGCTATCCTTCACAAGATTGTGTGTCACTACTATATTTATTTATT
GTTTTCATTGTTGTTTTGTT
>Marker889931
ACCTGATTTGTCTTCTTTAGTGGGATTGAGATTCCTGAGTTTGAATAACAGTGGGTTTTCTGGAGATTTTCCATG
GAAATXXXXXXXXXXGGATTGGGAACTTGTCTTTGCTTGAGAATCTTGAACTCTCACAAAATAAACTCACTGGTG
AAATTCCTTATGAGATTGTG

Create PDF with GO2PDF for free, if you wish to

ACCTGATTTGTCTTCTTTAGTGGGATTGAGATTCTTGAGTTTGAATAACAGTGGGTTTTCTGGAGATTTTCCATG
GAAATXXXXXXXXXXXGGATTGGGAACTTGTCTTTGCTTGAGAATCTTGAACTCTCACAAAATAAACTCACTGGTG
AAATTCCTTATGAGATTGTG
>Marker890314
TACAATAATTCCTTCTCTCCTAACGATGTTAATTAGGTGGGATTAACTCTTATCTAATAAAATACAAAAGCACTA
AACAAXXXXXXXXXXXXTTTCTCATGGCCAGAACAATAATAATACCTTATCAAAGCAGAAGCAGCATATGCTCATAA
TACTCTCTGCCACTATTGTA
TACAATAATTCCTTCTCTCCTAACGATGTTAATTAGGTGGGATTAACTCTTATCTAATAAAATACAAAAGCACTA
AACAAXXXXXXXXXXXXTTTCTCATGGCCAGAACAATAATAATACCTTATCAAAGCAGAAGCAGCATATGCTCATAA
TACTCTCTGCCGCTATTGTA
>Marker890937
CACTTAAACCCTCCTATCTTCAATAACTGCGTCAAAGGAGCCGCAATTGTCCCATAATTCTGGACAAACTTGCGA
TAGTAXXXXXXXXXXXTAATATGTCCCAAGTAATCTACTCGAGACTGAGCAAAGCTACATTTCTTCTTATTTGCAT
ATAGTTCATTATTCCTTAGT
CACTTAAACCCTCCTATCTTCAATAACTGCGTCAAAGGAGCCGCAATTGTCCCATAATTCTGGACAAACTTGCGA
TAGTAXXXXXXXXXXXTAATATGTCCCAAGTAATCTACTCGAGACTGAGCAAAGCTACATTTCTTCTTATTTGCAT
ATAGTTCATTCTTCCTCAGT
>Marker891982
ACCTGGATATTTAGTGCAAATGATCTCTTAAATAATAACAGCTTGAGTTAAAATTCTGGAAGAGCTTAAATTAGT
ATAAAXXXXXXXXXXXXTATTGTCCCCAAAATGTTTGCCATCGGAGATACTTCAGAAACCATTTAATATGAATAGGA
AAAGCTTGGATGCCTTTGTT
ACCTGGATATTTAGTGCAAATGATCTCTTAAATAATAACAGCTTGAGTTAAAATTCTGGAAGAGCTTAAATTAGT
ATAAAXXXXXXXXXXXXTATTGTCCCCAAAATGTTTGCCATCGGATATACTTCAGAAATCATTTAATATGAATAGGA
AAAGCTTGGATGCCTTTGTT
>Marker891997
GACCAAGACGCCGAAAAGTTGTTATCCTTAAATGAGAAGCACTCTCAGCAAAATATATAAGTATTACTAGATATA
AACCAXXXXXXXXXXXXATTGCAGTGAAGTGGTCATAGAACTTGCTCGCATGATAACATGGAACCTTCCTTCACCAC
CTTCTTAACGCAAGTTAGTT
GACCAAGACGCCGAAAAGTTGTTGTCCTTAAATGAGAAGCACTCTCAGCAAAATATATAAGTATTACTAGATATA
AACCAXXXXXXXXXXXXATTGCAGTGAAGTGGTCATAGAACTTGCTCGCATGATAACATGGAACCTTCCTTCACCAC
CTTCTTAACGCAAGTTAGTT
>Marker892052
CACCTTCCCGAGAAAGATATATATTGATATCCAAGAGATAATATGCCGAAGAAGTGATATGACAAGCAGGTGACT
CTTCTXXXXXXXXXXXTTTAGATTAAAGTGTGTTTTCTACACATTTACCACAAATTGGGCATACTATAGATCATGT
GTCATAACGTGATTGATGTA
CACCTTCCTGAGAAAGATATATATTGATATCCAAGAGATAATATGCGGAAGAAGTGATATGACAAGCAAGTGACT
CTTCTXXXXXXXXXXXTTTAGATTAAAGTGTGTTTTCTACACATTTACCACAAATTGGGCATACTATAGATCATGT
GTCATAACGTGATTGATGTA
>Marker892324
AACCAAAAATCTTAAAGAAGAAAATACTTCATTAACACGTAATAGAATTCTAAACGATCATATCTTATAGTCTAA
ATGAAXXXXXXXXXXXXATAATGTTGTTGATTATGGTAAGAGATCATATAGTTTAATGTAAATGATCATTAAAAACA
TAAAATCAAACGATTGTGTT
AACCAAAAATCTTAAAGAAGAAAATACTTCATTAACACGTAATAGAATTCTAAACGATCATATCTTATAGTCTAA
ATGAAXXXXXXXXXXXXATAATGTTGTTGATTATGGTAAGAGATCATATAGTCTAATGTAAATGATCATTAAAAACA
TAAAATCAAACGATTGTGTT
>Marker893216

Create PDF with GO2PDF for free, if you wish to

ACCTTCCAACAGGTAACTTTCATTATCACTACTTTCATCATCACCCCATTGTTTTCTAGCATTTTTAAGAGAGTA
AATTAXXXXXXXXXXXCACATGCTTACAATTATAACTGAATGATGCAGATGTGGATAACGAAGGCTGAATACGACG
AGTCGGGTCCAGCTATAGTT
ACCTTCCAACAGGTAACTTTCATTTTCACTACTTTCATCATCACCCCATTGTTTTCTAGCATTTTTAAGAGAGTA
GATTAXXXXXXXXXXXCACATGCTTACAATTATAACTGAATGATGCAGATGTGGATAACGAAGGCTGAATACGACG
AGTCGGGTCCAGCTATAGTT
>Marker893295
AACAAGTATGGATACTAGGCACCAAGAGGTTGTTGTCCGTAATTGTGAAGCTCTATACGTCTTATAAGTGAAATC
TTCGAXXXXXXXXXXXATTGCATCAAGGTTGTTGTGTTACTTAATTGAATTACAATGCATATACATTCATTCGCTT
TTTTCGCATACAAGTTAGTT
AACAAGTATGGATACTAGGCACCAAGAGGTTGTTGTCCGTAATTGTGAAGCTCTATACGTCTTATAAGTGAAATC
TTTCAXXXXXXXXXXXATTGCATCAAGGTTGTTGTGTTACTTAATTGAATTACAATGCATATACATTCATTCGCTT
TTTTCGCATACAAGTTAGTT
>Marker893313
CACTTCTCTTCCTTCCTATCTTTGGGGTGATGTCATTCTTACTGTCGCTCATCTCATAAATCACATGTCTTATCG
TGTCCXXXXXXXXXXXTAAAGCTCAAGCTTGTGTGTTTGTTGGATATCCTCTGCACCAATGAGACTATAAATGCTT
TCATCCATCTTCACGTTAGT
CACTTCTCTTCCTTCCTATCTTTGGGGTGATGTCATTCTTACTGTCGCTCATCTCATAAATCACATGCCTTATCG
TGTCCXXXXXXXXXXXTAAAGCTCAAGCTTGTGTGTTTGTTGGATATCCTCTGCACCAATGAGACTATAAATGCTT
TCATCCATCTTCACGTTAGT
>Marker893628
TACTTTTTACTAATGGTTTAAAAAAACCAAGCAAAAATTTGAAAACTAAAAGTAGCTTTTAAAAGAATGCTTTTG
ATTTTXXXXXXXXXXXCAGCAAATAGGCTAAGAGATTCCATAAACAGACCACACCTACTGAAGCAACTAGCTGTAA
ATTAAGGCCAGAGATATGTT
TACTTTTTACTAATGGTTTAAAAAAACCAAGCAAAAATTTGAAAACTAAAAGTAGCTTTTAAAAGAATGCTTTTG
ATTTTXXXXXXXXXXXCAGCAAATAGGCTAAGAGATTCCATAAACAGACCACACCCACTGAAGCAACTAGCTGTAA
ATTAAGGCCAGAGATATGTT
>Marker893695
CACATAAAAGTTAGCATCATCTCAATGAAACAACTAATTATTTGAAAACCATTTAATAGTATGGAAATCATAATC
ATCAAXXXXXXXXXXXAAAAGCACTCACCACACTTAAACAAAAATCCTCCTCTTCCTTCTCTTCTCGTGGAACCTT
CTTGCGGAACAAATCTTTGT
CACATAAAAGTTAGCATCATCTCAATGAAACAGCTAATTATTTGAAAACCATTTAATAGTATGGAAATCATAATC
ATCAAXXXXXXXXXXXAAAAGCACTCACCACACTTAAACAAAAATCCTCCTCTTCCTTCTCTTCTCGTGGAACCTT
CTTGCGGAACAAATCTTTGT
>Marker894288
AACCTAAACGTCAAGTTTAATAAATACCTACCAAAAAAGTTTATTAAAAAACAAAAAAATGAAACCCTTCAAAGA
CAAGTXXXXXXXXXXXAGATGTGAAATTTTGAAATTTTTAACATTGTTCTAAATTAGCTCATTTCTTTGTAAGTTA
TAGCATTGGTCATTTTTTGT
AACCTAAACGTCAAGTTTAATAAATACCTACCAAAAAAGTTTATTAAAAAACAAAAAAATGAAACCCTTCAAAGA
CATGTXXXXXXXXXXXAGATGTGAAATTTTGAAATTTTTAACATTGTTCTAAATTAGCTCATTTCTTTGTAGGCTA
TAGCATTGGTCATTTTTTGT
>Marker894760
GACTTGGAAGAGACCAAGGAGAGGGTAAAGATGTTAGAGGAATCTTGTCAATCTCTGTTGGGAGAGAAATCAACT
CTTTCXXXXXXXXXXXATTTTCTGATGCAATTGCTGAACTTGAAGCATTGAAGTTGAAATCAAAGGATCTAGAAGA
TTCCTGCCAATTGCTGGGTC

Create PDF with GO2PDF for free, if you wish to

GACTTGGAAGAGACCAAGGAGAGGGTAAAGATGTTAGAGGAATCTTGTCAATCTCTGTTGGGAGAGAAATCAACT
CTTTCXXXXXXXXXXXATTTTCTGATGCAATTGCTGAACTTGAAGCATTGAAGTTGAAATCAAAGGATCTGGAAGA
TTCCTGCCAATTGCTGGGTC
>Marker894969
AACAAATTATTGTTTCCAAATGCATCTGCAGCGAGGACACAACACTTCCAGAAATCTGTTCAACTCATGAAAACT
TGAGAXXXXXXXXXXXGTGGGTGGAGTGACTATTATAGCCCATCCCATGTTTGGGGCTCCAATTATAATAGTTAAT
ATTTTTAACCATTATAATGT
AACAAATTATTATTTCCAAATGCATCTGCAGCGAGGACACAACACTTCCAGAAATTTGTTCAACTCATGAAAACT
TGAGAXXXXXXXXXXXGTGGGTGGAGTGACTATTATAGCCCATCCCATGTTTGGGGCTCCAATTATAATAGTTAAT
ATTTTTAACCATTATAATGT
>Marker895118
CACGCTTCTCATCTGTCGGCTCAATCACTTGTATATGAACTTGAATTTGGAACATCAACCAACAAGTATGAGTTA
TTAAAXXXXXXXXXXXATCCCTATTTGATATGCAAATAATTGACATTGATTATCTAAAGCATAGAACAAGTAATTT
TCAAGCTAAGCTTTCCAGTA
CACGCTTCTCATCTGTCGGCTCAATCACTTGTATATGAACCTGAATTTGGAACATCAACCAACAAGTATGAGTTA
TTAAAXXXXXXXXXXXATCCCTATTTGATATGCAAATAATTGACATTGATTATCTAAAGCATAGAACAAGTAATTT
TCAAGCTAAGCTTTCCAGTA
>Marker895380
GACTGACCATTGACCTTTCGGAGATTAGAGATTCAAGGATTCTCTCACCACTTATGAAATTATTTCCTGGTTGTC
TTCAAXXXXXXXXXXXATCCATGTATAATTGAATTATTAGAATTACTACTATAAGTCTATAAGAAAGGAAAACTGA
CGTGGGTCTGTATCTCTGGT
GACTGACCATTGGCCTTTCGGAGATTAGAGATTCAAGGATTCTCTCACCACTTATGAAATTATTTCCTGGTTGTC
TTCAAXXXXXXXXXXXATCCATGTATAATTGAATTATTAGAATTACTACTATAAGTCTATAAGAAAGGAAAACTGA
CGTGGGTCTGTATCTCTGGT
>Marker895773
TACTCTTTCATCCTTTGGATCCATTAATTGGGGAAGATTTTGGTGTTCATCACTTTTTTTCAATTTATATTATTA
CCAAAXXXXXXXXXXXTGATTTTGCTATTTACTTACAATTTTGAAATCGAAGTAGCCCTTTCTTTTAGGTTATCCA
TAGAATAGGTGGCTGTTGTA
TACTCTTTCATCCTTTGGATCCATTAACTGGGGAAGATTTTAGTGTTCATCACTTTTTTTCAATTTATATTATTA
CCAAAXXXXXXXXXXXTGATTTTGCTATTTACTTACAATTTTGAAATCGAAGTAGCCCTTTCTTTTAGGTTATCCA
TAGAATAGGTGGCTGTTGTA
>Marker895918
CACCTGCACAAATTGGCTCTAAATTTTGATTGCAGAAGCATTTGTATAAGGAAACTGGACGACATGATAGTAATC
TTCATXXXXXXXXXXXCTGTAGATTATAAGGTAAAGGAAATCAAAGATCAACTGATAAGAGCAAAAGCCTACCTAA
GTTTTGCCCCACCAGGTAGT
CACCTGCACAAATTGGCTCTAAATTTTGATTGCAGAAGCATTCGTATAAGGAAACTGGACGACATGATAGTAATC
TTCATXXXXXXXXXXXCTGTAGATTATAAGGTAAAGGAAATCAAAGATCAACTGATAAGAGCAAAAGCCTACCTAA
GTTTTGCCCCACCAGGTAGT
>Marker895974
CACTAGGCTACTAGGCGGCGCAATTGATACACTGTTGTTGAAATTGTAAGAAGCTGATCCTCTTCGCTCTGTTCG
TTCGAXXXXXXXXXXXTTTGGCTGGAAATCTTCAAGGCAAACGGCGCATCTCTTGCCTTCTTCATCACCATTGCTG
TTGTGCCTCTCATTGGAGTT
CACTAGGCTACTAGGCGGCGCAATTGATACACTGTTGTTGAAATTGGAAGAAGCTGATCCTCTTCGCTCTGTTCG
TTCGAXXXXXXXXXXXTTTGGCTGGAAATCTTCAAGGCAAACGGCGCATCTCTTGCCTTCTTCATCACCATTGCTG
TTGTGCCTCTCATTGGAGTT
>Marker895980

Create PDF with GO2PDF for free, if you wish to

ACCCTCTCATCAATGATAAAATTGATAATATCTATGTAGGAAGGAGAGCAGAATTAGTCTCTCCCATAAATTTGT
GGGATXXXXXXXXXXXTAAATGAAAGTAACTATCTATACCAACAAAGACTCTTTCCTTTTTTGGTGGTTCAATTAT
AAGAATTTCAAAATTAAGTG
ACCCTCTCATCAATGATAAAATTGATAATATCTATGTAGGAAGGAGAGCAGAATTAGTCTCTCCCATAAATTTGT
GGGATXXXXXXXXXXXTAAATGAAAGTAACTATCTATACCAACAAAGACTCTTTCCGTTTTTGGTGGTTCAATTAT
AAGAATTTCAAAATTAAGTG
>Marker896082
CACCAATTTTGTTTCCATCTATTTCATTGCTTTTCCAAAGAAACCTAAGAATCAACTGATCCTTTGCAATTTGAA
AAGACXXXXXXXXXXXATTCGTGTAGATTGTGCGTATCAGTAAGGTAAAGATTTGGTTAAAGTGTTTATTTAACGC
ATTTGCAATCCATTTGAGTT
CACCAATTTTGTTTCCATCTATTTCATTGCTTTTCCAAAGAAACCTAAGAATCAACTGATTCTTTGAAATTTGAA
AAGACXXXXXXXXXXXATTCGTGTAGATTGTGTGTATCAGTAAGGTAAAGATTTGGTTAAAGTGTTTATTTAACGC
ATTTGCAATCCATTTGAGTT
>Marker896565
AACTTCATCTGTCCTGGACGTGCTTCACACTCTTCATGGTTAGAACAAATTTCAACACTTGCAGAATTGGCCTGC
ACTTCXXXXXXXXXXXAATGAGTTGTTCAATTTAAAATAATAGTCAGAATTGCAGCCAGATGGTGTTTTTAATTCT
CATGCGCCTCTTTTTTAGTC
AACTTCATCTGTCCTGGACGTGCTTCACACTCTTCATGGTTAGAACAAATTTCAACACTTGCAGAATTGGCCTGC
ACTTCXXXXXXXXXXXAATGAGTTGTGCAATTTAAAATAATAGTCAGAATTGCAGCCAGATGGTGTTTTTAATTCT
CATGCGCCTCTTTTTTAGTC
>Marker896955
ACAACAATTTAAACATTAGGGTCTCTTTCTCTTATATTTTTTCTCAATTCAATTCAATGTCAAATGTTTGTTTTC
TTCTTXXXXXXXXXXXTATGTTCCTCAAACAAAATTTTAGCCTCATACATTCAATGCTTGATGAAGCAAAGAGAAG
CACACTATATTCAGTTAGTT
ACAACAATTTAAACATTAGGGTCTCTTTCTCTTATATTTTTTCTCAATTCAATTCAATGTCAAATGTTTGTTTTC
TTCTTXXXXXXXXXXXTATGTTCCTCAAACAAAATTTTAGCCTCATACATCCAATGCTTGATGAAGCAAACAGAAG
CACACTATATTCAGTTAGTT
>Marker896999
ACCCGCCACAATAGTAACCTCCATCCAATCCAACCATGATAGCATACTAAATACACGGTCCGGAACAGCTCACAT
GTGATXXXXXXXXXXXATCCATAAGGAAACACGCGGCCAAATGAAGCTCACGCATACTTAGCACCTACCACAGTAA
CATCCATCCATTCCTATGTG
ACCCGCCACGATAGTAACCTCCATCCAATCCAACCATGATAGCATACTAAATACACGGTCCGGAACAGCTCACAT
GTGATXXXXXXXXXXXATCCATAAGGAAACACGCGGCCAAATGAAGCTCACGCATACTTAGCACCTACCACAGTAA
CATCCATCCATTCCTATGTG
>Marker897104
ACTCTTCTTTGTGCATAATCCTATTTGTAACGACAACATATATTTCAATAATATACACAAAATTAACAGATTCAT
TAGTTXXXXXXXXXXXTTGAACCCCCATTTTATTAAACCAATAACCATGTATGTAAAAAGTTTCTTTAGTTTTTGA
AGTTTGTAAGGAACTTTGGT
ACTCTTCTTTGTGCATAATCCTATTTGTAACGACAACATATATTTCAATAATATACACAAAATTAACAGATTCAT
TAGTTXXXXXXXXXXXTTGAACCCCCATTTTATTAAACCAATAACCATGTATGTAAAAAGTTTCTTTAGTTTTTGA
AGTTTGTAAGGAACTTTAGT
>Marker897156
TACTAAAGCATAGTTACATTTCTATGTGGAGATAGCTTTTTAGCATGATCAGAAATAAAAATTTCACTCGGATAT
ATTAGXXXXXXXXXXXGCACGGGAGCCTGTATAAAGAAGTTAATACATCAATTCATCTGATACATGATATTCATTT
GAAGAATGGAAACCACTGTT

Create PDF with GO2PDF for free, if you wish to

TACTAAAGCATAGTTACATTTCTATGTGGAGATAGCTTTTTAGCATGATCAGAAATAAAAATTTCACTAGGATAT
ATTAGXXXXXXXXXXXGCACGGAGCCTGTATAAAGAAGTTAATACATCAATTCATCTGATACATGATATTCATTT
GAAGAATGGAAACCACTGTT
>Marker897217
CACTTCATTTTGGGTGTGATGCAAAATTGACGAGAATTCCATTTGTTTTAAGAAGTTTATTATTTTCACTTGTTA
AGTTGXXXXXXXXXXATATATGCTTCATACTTTAATATAGTGAAGATTACTTCTTGTGGGTGGAAATGTTGCCCA
ACTTTTATATGAACACTGTT
TACTTCATTTTGGGTGTGATGCAAAATTGACGAGAATTCCATTTGTTTTAAGAAGTTTATTATTTTCACTTGTTA
AGTTGXXXXXXXXXXATATATGCTTCATACTTTAATATAGTGAAGATTACTTCTTGTGGGTGGAAATGTTGCCCA
ACTTTTATATGAACACTGTT
>Marker897719
ACCATTTTAACCTCTCTAGTGGTTCCTTCTTAAGCCTTTTTACGTTGAGAAATTGACAAAACTAGCACTTTTTTT
TTTATXXXXXXXXXXCCCAACTGGGTATTTCGAACGCTACATATTCTTCAAAAACATTAAACTCCAAACACTTTG
CTCATTTAGGACGAGAAGTG
ACCATTTTAACCTCTCTAGTGGTTCCTTCTTAAGCCTTTTTACGTTGAGAAATTGACAAAACTAGCACTTTTTTT
TTTATXXXXXXXXXXTCCAACTTGGTATTTCGAACGCTACATATTCTTCAAAAACATTAAACTCCAAACACTTTG
CTCATTTAGGACGAGAAGTG
>Marker898042
CACCTTCCACATCACGTCCATTTCTTTACTCTCATATACAAATCGAATCCTACACCTATCAATATCCCTCAACCC
AAGAAXXXXXXXXXXTCACTCTCCAACTCGAACCAAAACTTCTGGTTCCTCTCATATACTAACTGAATCAGGACT
TCATTAGAACATCTAAAAGT
CACCTTCCACATCACGTCCATTCCTTTACTCTCATATACAAATCGAATCCTACACCTATCAATATCCCTCAACCC
AAGAAXXXXXXXXXXTCACTCTCCAACTCGAACCAAAACTTCTGGTTCCTCTCATATACTAACTGAATCAGGACT
TCATTAGAACATCTAAAAGT
>Marker898220
CACGAACCTTGCTGCAATCGTCATCGAACACTCGCTGCTGTTGCTGCATGCTCGCTTCTTCAATTGCCTCCCTCT
CTAGTXXXXXXXXXXCTCATTGTGGTTAATGGGTCCAATCAGAGGGTTGATCGAGAAAGAAGAAGAAATTTGAGA
GAAAGCTCGATTCGAATGGT
CACGAACCTTGCTGCAATCGTCATCGAACACTCGCTGCTGTTGCTGCATGCTCGCTTCTTCAATTGCCTCCCTCT
CTAGTXXXXXXXXXXCTCATTGTGGTTAATGGGTCCAATCAGAGGGTTGATCGAGAAAGAAAAAGAAATTTGAGA
GAAAGCTCGATTCGAATGGT
>Marker898503
GACGTAAGTATATGTAACCACTAGAGCCTTGCAATTAAGAAAATAAATGCAACCACAAAGGTAAGAAACTTAACC
TTCACXXXXXXXXXXTCTTGCCAGCTTACCTATCTCAATTTATCTTTTACCAAGGATCAAATAGACATAAACATC
GTAAATTAAACTGGTAGGTT
GACGTAAGTATATGTAACCACTAGAGCCTTGCAATTAAGAAAATAAATGCAACCACAAAGGTAAGAAACTTAACC
TTCACXXXXXXXXXXTCTTGCCAGCTTACCTATCTCAATTTATCTTTTACCAAGGATCAAATAGACATAAACATC
GTAAATTAAACTAGTAGGTT
>Marker898509
CACCATCAACCCATCGTGGATTGCGAAAACCTGGCTCAGCTAGCCTACCTTGTCCTTTATATCTAGCCACCTGGG
ATAAAXXXXXXXXXXAGGAAGCTGGTATCTTTTTCCGACCTTTGCCCGAAATGCCACCTGTAAAACCATGACAAT
CATCAATACTACTCCACAGT
CACCATCAACCCATCGTGGATTGCGAAAACCTGGCTCAGCTAGCCTACCTTGTCCTTTATATCTAGCCACCTAGG
ATAAAXXXXXXXXXXAGGAAGCTGGTATCTTTTTCCGACCTTTGCCCGAAATGCCACCTGTAAAACCATGACAAT
CATCAATACTACTCCACAGT
>Marker898576

Create PDF with GO2PDF for free, if you wish to

CACAATTTGAACAAATTACGAAAAAATCTAAATTGTAAAATAAAACGAACTTCAAGATTGCATATCATAAAGAAG
GATTGXXXXXXXXXXAAGTAATGCACAATTTACGAAGATCATTGACACTCAATTGAAAGCCAAACCTTGTCCGGC
GATACAGAATCAAAAGCGTA
CACAATTTGAACAAATTACGAAAAAATCTAAATTGTAAAATAAAACGAACTTCAAGATTGCATATCATAAAGAAG
GATTGXXXXXXXXXXAAGTAATGCACAATTTACGAAGATCATTGACACCCAATTGAAAGCCAAACCTTGTCCGGC
GATACAGAATCAAAAGCGTA
>Marker898582
CACAACCTTTGCTTCCCCTCCTACTATACCTTTTACCATTTAATTTCTTCCATTCCTAATCAAAAGGGATCTCCT
TTCCTXXXXXXXXXXTCTGGAACAATAATGAATATAGCACATTTAGTCACCTGACACCTATTAATCCGTAAATAC
AATGGAGTGTAACAGAAGTC
CACAACCTTTGCTTCCCCTCCTACTATACCTTTTACCATTTAATTTCTTCCATTCCTAATCAAAAGGGATCTCCT
TTCCTXXXXXXXXXXTCTGGAACAATAATGAATATAGCACATTTAGTCACCTGACACTTATTAATCCGTAAATAC
AATGGAGTGTAACAGAAGTC
>Marker899077
AACGACGTATTGCGAGATTGTAGTTTTAGTTGAAGAAGTTAGTTTTTCGACTTGATTCCTCTAGTGTTAGATGAA
TTGGAXXXXXXXXXXTATCCAGTGGTTAAAGGAAGATTATCACTACATGCTTAATTTCAATGTTAAAGGATGAGA
AAATGTTGAAAAAGGATTGT
AACGACGTATTGCGAGATTGTAGTTTTAGTTGAAGAAGTTAGTTTTTCGACTTGATTCCTCTAGTGTTAGATGAA
TTGGAXXXXXXXXXXTATCTAGTGGTTAAAGGAAGATTATCACTAGATGCTTAATTTCAATGTTAAAGGATGAGA
AAATGTTGAAAAAGGATTGT
>Marker899128
ACTTTAAAATGCTACTGATAAAATTATACTTCAGGACAAAAACAAGTGGAAAAATGTGGCAAGACTAATTTTGAT
GCAAAXXXXXXXXXXATTCAAAAAACTTTTCATATTTATTTTGCATAAAATAGTATAGATTTAAAAAACAAAACT
TAGAAATAATAACTGATAGT
ACTTTAAAATGCTACTGATAAAATTATACTTCAGGACAAAAACAAGTGGAAAAATGTGGCAAGACTAATTTTGAT
GCAAAXXXXXXXXXXATTCAAAAAACTTTTCATATTTATTTTGCATAAAATAGTATAGATTTAAAAAACAACACT
TAGAAATAATAACTGATAGT
>Marker900174
AACAAAATGTGCGTGCATTAGTGCATTTTTAGTGATAAAGACTTGGTTGATTTTGTTGTTATTTAAATTAATATA
TTTGTXXXXXXXXXXAGTTCAAGTTTAGATGTTTGATTCTCCACATCATGCTTTAATACAATATTTTTGTTAAAA
AGATTAAGCCTTTGATTGTG
AACAAAATGTGCGTGCATTAGTGCATTTTTAGTGATAAAGACTTGGTTGATTTCGTTGTTATTTAAATTAATATA
TTTGTXXXXXXXXXXAGTTCAAGTTTAGATGTTTGATTCTCCACATCATGCTTTAATACAACATTTTTGTTAAAA
AGATTAAGCCTTTGATTGTG
>Marker900427
TACCTAGGGCCTTATGAGACAATGGAGCAGTCTAAGCATACGAGGAGTTTCTGAATGTAGGATATGAATTAAACA
ATGGAXXXXXXXXXXATGGAATATGATAGTTTCATTTTCAGATGCAAAATACCAACTTTTGTGTGAGTTAAAAGA
ATTGACTTGTTAAGAAAAGT
TACCTAGGGCCTTATGAGACAATGGAGCAGTCTAAGCATACGAGGAGTTTCTGAATGTAGGATATGAATTAAACA
ATGGAXXXXXXXXXXATGGCATATGATAGTTTCATTTTCAGATGCAAAATACCAACTTTTGTGTGAGTTAAAAGA
ATTGACTTGTTAAGAAAAGT
>Marker900613
AACAACTCTAAGCAATGAAAGTCAGTGACTATTCCTAAGACACATTGAGTATAATGCCATTTAATTAAACCTAAA
CCCTAXXXXXXXXXXTCCTTTATTTTCTACTCTTCAGGAATCAAGATTGTATGCATTTCTTTTCTATTATTTTAG
CTCTTAACGCTTTTGATAGT

Create PDF with GO2PDF for free, if you wish to

AACAACTCCAAGCAATGAAAGTCAGTGACTATTCCTAAGACACATTGAGTATAATGCCATTTAATTAAACCTAAA
CCCTAXXXXXXXXXXXTCCTTTATTTTCTACTCTTCAGGAATCAAGATTGTATGCATTTCTTTTCTATTATTTTAG
CTCTTAACGCTTTTGATAGT
>Marker900998
TACATTTAAAGTAATTATATTTCTTTAGAATCTTTTCTACATAGTGAGATTGATACAAAGAAATTTCCTTCTCAT
ACCCTXXXXXXXXXXXAAACACAATATTGTTTAAAGATTGTATTTTCGAACTTATGGTAGATGCATTTGTCACTTC
CATTAACTTTGAAACATGTA
TACATTTAAAGTAATTATATTTTTTTAGAATCTTTTCTACATAGTGAGATTGATACAAAGAAATTTCCTTCTCAT
ACCCTXXXXXXXXXXXAAACACAATATTGTGTAAAGATTGTATTTTCGAACTTATGGTAGATGCATTTGTCACTTC
CATTAACTTTGAAACATGTA
>Marker901233
ACTTAACCTCAACATTCTCCTCCTTCATCGCACTCTCAATCCTCTCCTCTGTTTCCATTTCGTCGTGTGAAACCT
CGTAGXXXXXXXXXXXATCACAAATGCGGCTCGGAACGGTCCAGTTTTATCAAACCCTGACGAGGATTTACCATAA
TCCCTCGGATCGAAACAGTA
ACTTAACCTCAACATTCTCCTCCTTCATCGCACTCTCAATCCTCTCCTCTGTCTCCATTTCGTCGTGTGAAACCT
CGTAGXXXXXXXXXXXATCACAAATGCGGCTCGGAACGGTCCAGTTTTATCAAACCCTGACGAGGATTTACCATAA
TCCCTCGGATCGAAACAGTA
>Marker901270
CACACCGTTCCATGGTTTTCTATTCATCAAGAAGTAGATTCCAACACTTTCATATCTTTTGCAGGTAGTGGTTCA
GCAACXXXXXXXXXXXCAAAATCTTATTATTTGGTGTCCTATCACATTATTCACAGGGAGAAAGCCCGGCAGTGGT
GCTGTTGATAACACAGGGTC
CACACCGTTCCATGGTTTTCTATTCATCAAGAAGTAGATTCCAACACTTTCATATCTTTGGCAGGTAGTGGTTCA
GCAACXXXXXXXXXXXCAAAATCTTATTATTTGGTGTCCTATCACATTATTCACAGGGAGAAAGCCCGGCAGTGGT
GCTGTTGATAACACAGGGTC
>Marker901522
GACTAATTACATAGATTAGGTTATTTAAGAGATGTTTTTCAAATGTTCAAAGACTTTTTTTGCTAATTGTTAGTT
TTTTCXXXXXXXXXXXGCTCATTCGTTTAATATGGCATTCTTAAATATAAGTTTAGGGTTTATAATTGAACACTTT
TCTTTTATAGTAGTGTAGTC
GACTAATTACATAGATTAGGTTATTTAAGAGATGTTTTTCAAATGTTCAAAGACTTTTTTTGCTAATTGTTAGTT
TTTTCXXXXXXXXXXXGCTCATTCGTTTAATATGGCATTCTTAAATATAAGTTTAGGGTTTATAATGGAACACTTT
TCTTTTATAGTAGTGTAGTC
>Marker901947
CACAATTTAATACTTTGATTCCAACTTCACTCCAACGCTGTCTCTGCCTCCATCTTCATCTGTTTAAACAAACTC
AATGTXXXXXXXXXXXCTTTTGCTCTTGCTTCGGCTCCTGCTCCTGCTCCTGCTAGGGCTTTTGCTAGCATCACGC
CTTTTGGGACTTTTACTAGT
CACAATTTAATACTTTGATTCCAACTTCACTCCAACGATGTCTCTGCCTCCATCTTCATCTGTTTAAACAAACTC
AATGTXXXXXXXXXXXCTCTTGCTCTTGCTTCGGCTCCTGCTCCTGCTCCTGCTAGGGCTTTTGCTAGCATCACGC
CTTTTGGGACTTTTACTAGT
>Marker902017
TACAATGAAACAGAACAACATGAACTATTCACAATATCAAACTTGCCCACATTAAGTTCGTAATAAAACAAAACA
AAAGGXXXXXXXXXXXTTAGCAAGTCTAGCAAATCTAATATGATAAGAAGCTAAACATAACCTCCATCCTAGGCAT
TTTATCCTCAAATATTATGT
TACAATGAAACAGAACAACATGAACTATTCACAATATCAAACTGGCCCACATTAAGGTCGTAATAAAACAAAACA
AAAGGXXXXXXXXXXXTTAGCAAGTCTAGCAAATCTAATATGATAAGAAGCTAAACATAACCTCCATCCTAGGCAT
TTTATCCTCAAATATTATGT
>Marker902247

Create PDF with GO2PDF for free, if you wish to

TACCGTGAGGTTTATCCCTTCCATATCGAAAATTTCTTTTTTTGTTTGACTCTGCTTTCCCCTTTCTTCTTATGT
ATTTGXXXXXXXXXXGGGGCACACTTCTAGGGTCAAAGAAATATATTTTATTCTTTGGAGAAGTGAAGGAAACCT
TTCTCTTTGAATTTTGAGTA
TACCGTGAGGTTTATCCCTTCCATATCGAAAATTTCTTTTTTTGTTTGACTCTGTTTTCCCCTTTCTTCTTATGT
ATTTGXXXXXXXXXXGGGGCACACTTCTAGGGTCAAAGAAATATATTTTATTCTTTGGAGAAGTGAAGGAAACCT
TTCTCTTTGAATTTTGAGTA
>Marker902875
CACAACATACCTCACTATCGTCTATCCTTATTGGTTCATCCTGGTTAGATTTCATATGTTTCCTCCTTGAACTCT
TACTTXXXXXXXXXXACATAAAAAGGTAATTTCATTTGTAAAGTATTCGTTTTTATAACCTAATGAAAATTCGTT
TTTATGTCACGGCCAGAAGT
CACAACATACCTCACTATCGTCTATCCTTATTGGTTAATCCTGGTTAGATTTCATATGTTTCCTCCTTGAACTCT
TACTTXXXXXXXXXXACATAAAAAGGTAATTTCATTTGTAGAGTATTCGTTTTTATAACCTAATGAAAATTCGTT
TTTATGTCACGGCCAGAAGT
>Marker903041
ACTTGAATAGTTCAAACATAAAAACAAACATGATAGTAAGTTGATAGGCTATGATTGCCATGTAGGTGAGTGCAG
GTAAAXXXXXXXXXXXAGCTATTGTTGGAAACGACCTGAATAATCTGCAACAGATTTTAAGAGAACGTCATCGTCA
AAGATCTGTATTACAGCGTC
ACTTGAATAGTTCAAACATAAAAACAAACATGATAGCAAGTTGTTAGGCTATGATTGCCATGTAGGTGAGTGCAG
GTAAAXXXXXXXXXXXAGCTATTGTTGGAAACGACCTGAATAATCTGCAACAGATTTTAAGAGAACGCCATCGTCA
AAGATCTGTATTACAGCGTC
>Marker903562
TACTAGTTCTCCATTTGAATTGACACATGAGACCTACATCCAAATTTGTAATGTAATAACGTAACCCGATTGCAC
CTGAGXXXXXXXXXXTATTGTTACTGTTACTGCTTGATCCTCAGAGGTAGAGGTAGCAGTAACAGTAAATTTGAC
AAAGTTCATAATTTTAAGTT
TACTAGTTCTGCATTTGAATTGACACATGAAACCTACATCCAAATTTGTAATGTAATAACGTAATCCGATTGCAC
CTGAGXXXXXXXXXXTATTGTTACTGTTACTGCTTGATCCTCAGAGGTAGAGGTAGCAGTAACAGTAAATTTGAC
AAAGTTCATAATTTTAAGTT
>Marker903830
AACTTCTTAACTTATTACTGACTATCCAAACGAAAAAAATAGACAAATTATAGCAGAAATGAAAACAGCAAAGGG
AAAGGXXXXXXXXXXCACATAAAAAACTCCACTATTCTTCCATTACATGAAAATTTTCATGTCGGAAGAAAGAAA
TAGATGGATATAGGGAGGGT
AACTTCTTAACTTATTACTGACTATCCAAACGAAAAAAATAGACAAATTATAGCAGAAATGAAAACAGCAAAGGG
AAAGGXXXXXXXXXXCACATACAAAACTCCACTATTCTTCCATTACATGAAAATTTTCATGTCGGAAGAAAGAAA
TAGATGGATATAGGGAGGGT
>Marker904159
CACACACTTCTGAGGGAGGGTGCATATGTTTATATACAGAAAGTGGTGCTGACATTCTGGCGTGCATCATTAACA
TTCTGXXXXXXXXXXGCTTGCTCTTTTTCACCTAATATCCTGCGATAAAACATGTTGAAACCTCGTAAAAACAGG
GATGAAGTTTTTTTGCGGTA
CACACACTTCTGAGGGAGGGTGCATATGTTTATATACAGAAAGTGGTGCTGACATTCTGGCGTGCATCATTAACA
TTCTGXXXXXXXXXXGCTTGCTCTTTTTCGCCTAATATCCTGCGATAAAACATGTTGAAACCTCGTAAAAACAGG
GATGAAGTTTTTTTGTGGTA
>Marker904187
TACGCGTAACTAAAGATGTGAAAGATTATCCCTTTGGAACGCCTGTTCAAATCTCTGTTGATGTCACAAGGAAAA
ATAAAXXXXXXXXXXXCTAAAATGCTAATCATTTTTGGTTCTCACAGGATTTTAATGAAGAAGGAAGTTTCTTCTA
TTCTATGTTTTGCATGTGTT

Create PDF with GO2PDF for free, if you wish to

TACGTGTAACTAAAGATGTGAAAGATTATCCCTTTGGAACGCCTGTTCAAATCTCTGTTGATGTCACAAGGAAAA
ATAAAXXXXXXXXXXXCTAAAATGCTAATCATTTTTGGTTCTCACAGGATTTTAATGAAGAAGGAAGTTTCTTCTA
TTCTATGTTTTGCATGTGTT
>Marker904654
ACCAACTTCCTTTATTTGAAGAGACAACTAACAAACAACCTATCGGCCATCCATACCAGAATCATCCATAAATAG
AGAAGXXXXXXXXXXXGCGCGCAACAAATTCATATATCCATAATCCATAAGCATTTAGATCATATTATAGGCCAAA
TCACATCAAAATATAAAGTC
ACCAACTTCCTTTATTTGAAGAGACAACTAACAAACAACCTATCGGCCATCCATACCAGAATCATCCCTAAATAG
AGAAGXXXXXXXXXXXGCGCGCAACAAATTCATATATCCATAATCCATAAGCATTTAGATCATATTATAGGCCAAA
TCACATCAAAATATAAAGTC
>Marker904669
ACCTGCAACCGTTGGTTGCCATAGAGTCATAGCATTGGACATCTGAGGGGTTGGTGATTGGCATTGATCATACTC
TGGCCXXXXXXXXXXXTGTCATTATATCATCAATACAAGAACTGAAACAAGGCTACAACACTGGAAACACGTCTGT
TTTCAATCACCAATTCAAGT
ACCTGCAACCGTTGGTTGCCATAGAGTCATAGCATTGGACATCTGAGGGGTTGGTGATTGGCATTGATCATACTC
TGGCCXXXXXXXXXXXTGTCATTATATCATCAATACAAGAACTGAAACAAGGCTACAACACTGGAAACACGTCTGT
TTTCAATCACCAACTCAAGT
>Marker905074
ACAACATACTAGTTTTTTATTTTGAAGATTTAGGGTTTTCCATCGATAAAAATCATTGGTAGGATGACAGACTCT
CATCAXXXXXXXXXXXAAGGGTAATCCAAATGTAATTCATTCCATCGGGTTGTTGTCAAATTGAATTGAGAGTTTT
AGAATTTGATATTAGGGGTT
ACAACATACTAGTTTTTTATTTTTAAGATTTAGGGTTTTCCATCAATAAAAATCATTGGTAGGATGACAGACTCT
CGTCAXXXXXXXXXXXAAGGGTAATCCAAATGTAATTCATTCCATCGGGTTGTTGTCAAATTGAATTGAGAGTTTT
AGAATTTGATATTAGGGGTT
>Marker905862
TACTCCTCTTGCTCCATCTTGAAAACATGCTGCAAGGAGAAGGCACATTTATTTTTGTGTAACTAAAAAGAAAAG
GTTTAXXXXXXXXXXXCAAGGATGTATTAGATATTGTAATCAAGATAATAATAGTAATCCGTGAGGGAATATCTTT
CATAATTTTATTGACCTGTT
TACTCCTCTTGCTCCATCTTGAAAACATGCTGCAAGGAGAAGGCACATTTATTTTTGTGTAACTAAAAAGAAAAG
GTTTAXXXXXXXXXXXAAGGAATGTATTAGATATTGTAATCAAGATAATAATAGTAATCCGTGAGGGAATATCTTT
CATAATTTTATTGACCTGTT
>Marker905983
CACCTACAAGAGTGAGGTCATCAACTATTTACTTCTTTTATGCGAGATAATCTTCCATTCTTATAGTGCCTTTCC
TTGTGXXXXXXXXXXXTTGTGGATCGTGCCATCGATCCAAACAACCAACCAAGTAAGGCTTTGATATGTGGTTATC
CAATCGTCATGTTCATTGTT
CACCTACAAGAGTGAGGTCATCAACTATTTACTTCTTTTATGCGAGATAATCTTCCATTCTTGTAGTGCCTTTCC
TTGTGXXXXXXXXXXXTTGTGGATCGTGCCATCAATCCAAACAACCAACCAAGTAAGGCTTTGATATGTGGTTATC
CAATCGTCATGTTCATTGTT
>Marker906409
ACAGGGAGTAACATCTGGGAGCAAACTTATTGTGCTAATGCTTTAAATGCCTCTAAATAATATTATGGCATGCAC
AAGAGXXXXXXXXXXXTTGACATGGAGAATATCGTCTAATTCAAACCAAGTAAGTTGTATTTAAAAAAATTTAGCA
CGTTGAAAATAAAATGAGTT
ACAGGGAGAAACATCTGGGAGCAAACTTATTGTGCTAATGCTTTAAATGCCTCTAAATAATATTATGGCATGCAC
AAGAGXXXXXXXXXXXTTGACATGGAGAATATAGTCTAATTCAAACCAAGTAAGTTGTATTTAAAAAAATTTAGCA
CGTTGAAAATAAAATGAGTT
>Marker906683

Create PDF with GO2PDF for free, if you wish to

ACCATACATTTTCTTGATTTTGTCATTTCTAATAAGGTTAATGTCAATTATACACTTTTGTCTAAAACTAACAGT
AGTTTXXXXXXXXXXTTCTACTATTGGTGATTCCAAATTAATAAAGTCTTGCTTTCAAACCTCTCCAATTAAGGC
TAATATTATTAATATAGAGT
ACCATACATTTTCTTGATTTTGTCATTTCTAATAATGTTAATGTCAATTATACACTTTTGTCTAAAACTAACAGT
AGTTTXXXXXXXXXXTTCTACTATTGGTGATTCCAAATTAATAAAGTCTTGCTTTCAAACCTCTCCAATTAAGGC
TAATATTATTAATATAGAGT
>Marker907288
ACAAAGACAAACCTCAGATAACAATTTCTCCTCTTTATCATAGAGATTTGAAAGCTCCCCTACCAGTTCAGAATG
CTTTCXXXXXXXXXXTTCTTCATCACAAAAAGACTAAAAATACAAATTCAATCCAAAATCTTCCCAATTCTCTAA
TTAGTCTTCAAAAATAAGTT
ACAAAGACAAACCTCAGATAACAATTTCTCCTCTTTATCATAGAGATTTGAAAGCTCCCCAACCAGTTCAGAATG
CTTTCXXXXXXXXXXTCCTTCACCACAAAAAGACTAAAAATACAAATTCAATCCAAAATCTTCCCAATTCTCTAA
TTAGTCTTCAAAAATAAGTT
>Marker908435
ACTTTTGGATTGGCATCCATTCCCATTCCCACTTCCCTTTTCCTCCTCTTCATCAAAAATCTCCATTTTTTCCCT
CTCTCXXXXXXXXXXTTTTCCATTGTTCATAACTACTGTTTGTGGATCATAACTCTATTCTTCAACAACTGCTTT
TCATCCTCATATCACATGTA
ACTTTTGGATTGGCATCCATTCCCATTCCCACTTCCCTTTTCCTCCTCTTCATCAAAAATCTCCATTTTTTCCCT
CTCTCXXXXXXXXXXTTTTCCATTGTTCATAACTACTGTTTGTGGATCACAACTCTATTCTTCAACAACTGCTTT
TCATCCTCATATCACATGTA
>Marker908483
AACTATAGATAGGAACCCATATTCAAATAATGCTAAAGAAGATATCATTGGTTTTCCTTCCGGTATAAAAAATAG
TAACTXXXXXXXXXXCAAATAGTGAAAATAAGGTTTTATAAAAGAAATGTCATGGCCAAAGTTATATGAGCATAT
CAGCCTTATAAAAGGTAGTA
AACTATAGATAGGAACCCATATTCAAATAATGCTAAAGAAGATATCATTGGTTTTCCTTCCGGTATAAAAAATAG
TAACTXXXXXXXXXXCAAATAGTGAAAATAAGGTTTTATAAAAGAAATGTCATGGCCAAAGTTATATGAGCATAC
CAGCCTTATAAAAGGTAGTA
>Marker909009
GACAGAGATCATACTAATCACAAGACTCGGAATGACAAACATCTCAGGCTCTCACCCAAGATATAGCATTTCTAA
ATTGTXXXXXXXXXXATAAAACTGTGCCCAAGTTCAGGCTCTCAGCCAAGGAACTAGATCATATTCCCAAAAAAC
TCACTCTCCCCAATTCAAGT
GACAGAGATCATACTAATCACAATACTCGGAATGACAAACATCTCAGGCTCTCACCCAAGATATAGCATTTCTAA
ATTGTXXXXXXXXXXATAAAACTGTGCCCAAGTTCAGGCTCTCAGCCAAGGAACTAGATCATATTCCCAAAAAAC
TCACTCTCCCCAATTCAAGT
>Marker909057
TACTATCAATGCTTAATTTTTTTAAAAAAGATCAATCCACTTAAAAGCCTTTTAAAGAATTGCTCAACTTTATGA
ACCTAXXXXXXXXXXGAAGTAGATGGATGATCATATGTGAATGCAGGGCTTTTTTGGGGAGTGTGAGTGACTATT
GCGCTCATCAAGCTTCATGT
TACTATCAATGCTTAATTTTTTAAAAAAAGATCAATCCACCTAAAAGTCTTTTAAAGAATTGCTCAACTTTATGA
ACCTAXXXXXXXXXXGAAGTAGATGGATGATCATATGTGAATGCAGGGCTTTTTTGGGGAGTGTGAGTGACTATT
GCGCTCATCAAGCTTCATGT
>Marker909152
AACTCAGTTGGAAGATTTATTAATTATTTGGGCTGTATATTTGTAATCTTGCTATAGAGAAGGTTTATACCATCA
GGTCAXXXXXXXXXXACAGTTTGCGTGGCGGCCGAGGCCACCATCCTTCTTGAGTCCAGAAAAAGAGGAAGAGAT
AGCGAAGAATTTGAAGAAGT

Create PDF with GO2PDF for free, if you wish to

AACTCAGTTGGAAGATTTATTAATTATTTGGGCTGTATATTTGTAATCTTGCTATAGAGAAGGTTTATACCATCA
GGTCAXXXXXXXXXXXACAGTTTGCGTGGCGGCCGAGGCCACCATCCTTTTTGAGTCCAGAAAAAGAGGAAGAGAT
AGCGAAGAATTTGAAGAAGT
>Marker909539
AACAAATTAAATACAAACCTTTTTTATATACCACAAGGGAAAAAAAGGAAAAAGGTGACCAAAATATATGAAAGG
GATGAXXXXXXXXXXXATTTAAAAAGATATATCAGATAGATGAACTGTTTAAATCGACAAAACTGCTAAAAATATT
TTTAAATATAGCAAAAGGTA
AACAAATTAAATACAAACCTTTTTTATATACCACAAGGGAAAAAAAGGAAAAAGGTGACCAAAATATATGAAAGG
GATGAXXXXXXXXXXXATTTAAAAGGATATATCAGATAGATGAACTGTTTAAATCAACAAAACTGCTAAAAATATT
TTTAAATATAGCAAAAGGTA
>Marker909553
CACTAGAAACCTTGCTTTGTAAATGGGCATGATCACTTTACAAGCAACGAACTTGATACACTTTTGAGAACTTCT
CAAAGXXXXXXXXXXXCGCCCGATCACAAAACTAAATCAAGCTTTCACCTCGAGAGACTAGACAAACCAGCCAACA
AGAAGAACGTCGAATTGGGT
CACTAGAAACCTTGCTTTGTAAATGGGCATGATAACTTTACAAGCAACGAACTTGATACACTTTTGAGAACTTCT
CAAAGXXXXXXXXXXXCGCCTGATCACAAAACTAAATCAAGCTTTCACCTCGAGAGACCAGACAAACCAGCCAACA
AGAAGAACGTCGAATTGGGT
>Marker909646
ACATTAGGCAAAGCTCTTAAGCATGATGCAATTTTAGGGCTACCAACACTGAATGTAAAGAGTCATTTATTCTGT
GAAGAXXXXXXXXXXXCTTCACTTGGATAAGATTCTTAAGGAGTAAATCTGAGACAGCTAAAGTATGTATAAATAT
ATGCCGAAGATTACAACGTG
ACATTAGGCAAAGCTCTTAAGCATGATGCAATTTTAGGGCTACCAACACTGAATGTAAAGAGTCATTTATTCTGT
GAAGAXXXXXXXXXXXCTTCACTTGGATAAGATTCTTAAGGAGTAAATCTGAGACAACTAAAGTATGTATAAATAT
ATGCCGAAGATTACAACGTG
>Marker910372
ACTCGTGGTTTAGTTTAATTTTAGGATACATGTGTATTTGGTCCCTATGACTTCAGTTTCTATCGTAGATACCAA
ACGTAXXXXXXXXXXXTTAAATTAAGTTATTAACAACCCAACTACCATTTTCATAGTTGAACCAATGATGACCAAA
TATTTCGAATCAATTATGGT
ACTCGTAGTTTAGTTTAATTTTAGGATACATGTGTATTTGGTCCCTATGACTTCAGTTTCTATCGTAGATACCAA
ACGTAXXXXXXXXXXXTTAAATTAAGTTATTAACAACCCAACTACCATTTTCATAGTTGAACCAATGATGACCAAA
TATTTCGAATCAATTATGGT
>Marker911000
AACAAGGATATTTCAACGGTGCTGGTTGGCATGAACTCTGTCGGACAGGTATCTCTCAATAATCTCATTGTTTGG
TATTCXXXXXXXXXXXCACTATTGTCCTTAAGATTTCAATAATGTTTAATTTTAGTCCTTAAAGTAACAGAATCGT
TTGTGACTAAACGGAAGGTT
AACAAGGATATTTCAGCGGTGCTGGTTGGCATGAACTCTGTCGGACAGGTATCTCTCAATAATCTCATTGTTTGG
TATTCXXXXXXXXXXXCACTATTGTCCTTAAGATTTCAATAATGTTTAATTTTAGTCCTTAAAGTAACAGAATCGT
TTGTGACTAAACGGAAGGTT
>Marker911069
AACTCCAAAGTTAATGGTAAATTGATATATGTGGAATTATGCAATTCAATAATTATTCTTTAAGGAAGGAGTTTA
GAGAGXXXXXXXXXXXTAACCTTACTCCTTCCTTCTCAACCAAAATCACCTATATACATATTTTCAAATAACATTC
ATAGTTACATATCCTTAAGT
AACTCCAAAGTTAATGGTAAATTGATATATGTGGAATTATGTAATTCAATAATTATTCTTTAAGGAAGGAGTTTA
GAGAGXXXXXXXXXXXTAACCTTACTCCTTCCTTCTTAACCAAAATCACCTATATACATATTTTCAAATAACATTC
ATAGTTACATATCCTTAAGT
>Marker911826

Create PDF with GO2PDF for free, if you wish to

AACGTGCTAGTTGTGTCCTACAAAAAGAGAGAGCAATCTTACTATTTGAGGGGAATACTAGAAAATATCAATACC
AATATXXXXXXXXXXTATGATTGAAGAATCGCCACCATCCAATTTGACTGAGCTTGAAATTTACATGAGCTCTAC
TACACTTTATCATAGTTGTT
AACGTGCTAGTTGTGTCCTACAAAAAGAGAGAGCAATCTTACTATTTGAGGGGAATACTAGAAAATATCAATACC
AATATXXXXXXXXXXTATGATAGAAGAATCGCCACCATCCAATTTGACTGAGCTTGAAATTTACATGAGCTCTAC
TACACTTTATCATAGTTGTT
>Marker912306
CACTGAGGAATGATTTGTTATTCTATAAACAGAGATTGATGTTACATCCGAATTTGGATTCTCTTGTAATTATAA
TCTGAXXXXXXXXXXACAGAAAAGTCGTCCGGTTCAAAATAGGGGAATTGTTGGCGACATTGAAGGTCGCTGCTG
TTCATAGATCTAGGAAGGTC
CACTGAGGAATGATTTGTTATTCTATAAACAGAGATTGATGTTACATCCGAATTTGGATTCTCTTGTAATTATAA
TCTGAXXXXXXXXXXACAGAAAAGTCGTCCGGTTCAAAATAGGGGAATTGTTGGCCACATTGAAGGTCGCTGCTG
TTCATAGATGTAGGAAGGTC
>Marker912670
AACCAGAAGAAATGTGTTATAGGACACTCAAGAATACAGTATTTGGGACATTGGATTTCAAGCCGATGGAGAGAA
ATCCAXXXXXXXXXXTTATTGCAAAATAAGGCTTTCAAATGGAGTGAAGAGGCCACCTCGACATTCGAAATACGG
AAGAGAGCTATGATAGTAGT
AACCAGAAGAAATGTGTTATAGGACACTCAACAATACAGTATTTGGGACATTGGATTTCAAGCCGATGGAGAGAA
ATCCAXXXXXXXXXXTTATTGCAAAATAACGCTTTCAAATGGAGTGAAGAGGCCACCTCGACATTCGAAATACGG
AAGAGAGCTATGATAGTAGT
>Marker912973
TACTGGATTTGGGTTATTAAGACAAAACTAATAATATAATCAATAACAATTATTACAATAATAACACTTTATTAA
TAACAXXXXXXXXXXTACTTCGATAATGATTTCGTTTTTACATCTATGTGTTTTCAAATTTATGTTTGTTTTCTT
CTAATTCTTCAATTATAGTT
TACTGGATTTGAGTTATTAAGACAAAACTAATAATATAATCAATAACAATTATTACAATAATAACACTTTATTAA
TAACAXXXXXXXXXXTACTTCGATAATGATTTCGTTTTTACATCTATATGTTTTCAAATTTATGTTTGTTTTCTT
CTAATTCTTCAATTATAGTT
>Marker913394
ACAATTATACTTGACATGTAAAAAACGTCAAGTATATGAAACTACTTGACGTTTTTTAAATGTCAAGTAATCTAG
TGTCAXXXXXXXXXXTTCCCTTTCAAATTGAAAATTGTATGATTTGAAATACCCATATAATTATATTGCATTTGT
TTTGAAGTCTAACAATGGTA
ACAATTATACTTGACATGTAAAAAGCGTCAAGTATATGAAACTACTTGACGTTTTTTAAATGTCAAGTAATCTAG
TGTCAXXXXXXXXXXTTCCCTTTCAAATTGAAAATTGTATGATTTGAAATACCCATATAATTATATTACATCTGT
TTTGAAGTCTAACAATGGTA
>Marker913627
TACATATGTGTGTAACCAATTGAAATCATCAATTGGTTAGGAAAGGAACCCAAACTGTGCAAAGATGTAGATGTA
ATAAAXXXXXXXXXXCCTCAACCGATTAAAAAACACCAGGAAATGAAATTCAGGAGCCCAATAAACAAATCCAAC
GACAGGTCCCCCTTTGATGT
TACATATGTGTGTAACCAATTGAAATCATCAATTGGTTAGGAAAGGAACCCAAACTGTGCAAAGATGTAGATGTT
ATAAAXXXXXXXXXXCCTCAACCGATTAAAAAACACCAGGAAATGAAATTCAGGAGCCCAATAAACAAATCCAAC
GACAGGTCCCCCTTTGATGT
>Marker913937
AACCAAACAAGAAAAGTTTATTCTGTGTGCAGATTTTCTGCTTGATTATTTAGTTGTATTTCTTTGCTATTTCTT
CATGCXXXXXXXXXXCTGATCTTGACATCCGAACTGCTATCCAGAATGCAACTGGTCCTAAATCTGCTTTATTTG
TTCCCGAAGTAGGTTATGGT

Create PDF with GO2PDF for free, if you wish to

AACCAAACAAGAAAAGTTTATTCTGTGTGCAGATTTTCTGCTTGATTATTTAGTTGTATTTCTTTGCTATTTCTT
CATGAXXXXXXXXXXXCTGATCTTGACATCCGAACTGCTATCCAGAATGCAACTGGTCCTAAATCTGCTTTATTTG
TTCCCGAAGTAGGTTATGGT
>Marker914897
TACAAAAGGTTTATGGCTTTAGGTTCTTGACTTATTCTTCTTCTTCCAATAAACCCCTTTTACGAGAAACACAAA
TTGATXXXXXXXXXXXTAAAAAATTTCAACTAGGGCTCAAAATATTCCCTTGTATAGAAAAAAAGAAACTTAACTG
GTTATTTTCTTGCTGCAGTG
TACAAAAGGTTTATGGCTTTAGGTTCTCGACTTATTCTTCTTCTTCCAATAAACCCCTTTTACGAGAAACACAAA
TTGATXXXXXXXXXXXTAAAAAATTTCAACTAGGGCTCAAAATATTCCCTTGTATAGAAAAAAAGAAACTTAACTG
GTTATTTTCTTGCTGCAGTG
>Marker914912
AACCTTAACTACCCATGGGCTTTCATCAAGGCCCAAGCCCACTATTTCTAAGAATTAATAATCAGAAAAAAGCCA
AAACAXXXXXXXXXXXCTTAAGGAACAAGAAGAAGTCTTATAATGCTATTTTAATGAAAATTTAAATAAATTCCCT
TAATATACAATTAGAGCGTG
AACCTTAACTACCCATGGGCTTTCATCAAGGCCCAAGCCCACTATTTCTAAGAATTAATAATCAGAACAAAGCCA
AAACAXXXXXXXXXXXCTTAAGGAACAAGAAGAAGTCTTATAATGCTATTTTAATGAAAATTTAAATAAATTCCCT
TAATATACAATTAGAGCGTG
>Marker914933
TACACATACATCACATTAAATGATTCTAATAGCGACCTAATCAATTCAACACAGATTAAATCTAGAAAACAAACC
AAGACXXXXXXXXXXXATTGAGATTTATGCACAAAGATGCTATTATCTCAAGTAAAACATAGAAGAATTAGTAAAG
TCCAACATGAATTCAAAGTA
TACACATACATCACATTAAATGATTCTAATAGCGACCTAATCAATTCAACACACATTAAGTCTAGAAAACAAACC
AAGAGXXXXXXXXXXXATTGAGATTTATGCACAAAGATGCTATTATCTCAAGTAAAACATAGAAGAATTAGTAAAG
TCCAACATGAATTCAAAGTA
>Marker915166
AACCTCTGCCTGTTTCCTCTTAACTCATTTAGGTGGGAGATAAATTGATTAAGTTCCTCGATCTTCCCTTTTCTT
TTCTTXXXXXXXXXXXAACAAACAAGAAGAAAACAAGAAACTAATGCATGAGCACTGGCAGATTGAAGAGATATAA
GTTCTATAATCTTTATCGTT
AACCTCTGCCTGTTTCCTCTTAACTCATTTAGGTGGGAGATAAATTGATTAAGTTCCTCGATCTTCCCTTTTCTT
TTTTTXXXXXXXXXXXAACAAACAAGAAGAAAACAAGAAACTAATGCATGAGCACTGGTAGATTGAAGAGATATAA
GTTCTATCATCTTTATCGTT
>Marker915267
GACTTTACCATTTTTCACAACATCACCACCATCTACGTTCCACATAATAACATTTTCTCCCCTTCCCTCACCAGA
TATCAXXXXXXXXXXXTAAAATTATGTTTCCTGAAATGGTGAATCAAGGGAGGTTTCATCAGAAAAACAATCTAAT
ATCTGAAAAGCAGGGAGAGT
GACTTTACCATTCTTCACAACATCACCACCATCTACGTTCCACATAATAACATTTTCTCCCCTTCCCTCACCAGA
TATCAXXXXXXXXXXXTAAAATTATGTTTCCTGAAATGGTGAATCAAGGGAGGTTTGATCAGAAAAACAATCTAAT
ATCTGAAAAGCAGGGAGAGT
>Marker915708
CACAACAACAATGATGGAATCAATTCAATAAACTTGATCTACATGATATTGTGACTGGAGCAGGGTATTTCAAAA
GGTAAXXXXXXXXXXXTCTCTGAAGAAATCTTCCCAGACATTTACCTTTTCTCCGAGAAACAAGACGCCACAGATG
CAGAATGTTCTAGACAGGGT
CACAACAACAATGATGGAATCAATTCAATAAACTTGATCTACTTGATATTGTGACTGGAGCAGGGTATTTCAAAA
GGTAAXXXXXXXXXXXTCTCTGAAGAAATCTTCCCAGACATTTACCTTTTCTCCGAGAAACAAGACGCCACAGATG
CAGAATGTTCTAGACAGGGT
>Marker915709

Create PDF with GO2PDF for free, if you wish to

ACCCTAGCCTTGTAATCCTTTATACCCAATGCTTCAGTCAAAATGTCTTCACTGTCATTTATTGTAGCTAATTCA
TCCTAXXXXXXXXXXCATCATCAAAATAAGTATTATTTTTGCTTTCCATGCTTCGTTCCATAGAGTTGAACGATA
GAAAAAATCACTCGGTAGTT
ACCCTAGCCTTGTGTTCCTTTATACCCAATGCTTCAGTCAAAATATCTTCACTGTCATTTATTGTAGCTAATTCA
TCCTAXXXXXXXXXXCATCATCAAAATAAGTATTATTTTTGCTTTCCATGCTTCGTTCCATAGAGTTGAACGATA
GAAAAAATCACTCGGTAGTT
>Marker915976
TACAGGTATGAACAAAAGTTAATTTTACCTCTTTACCCCACTACTCTATATTTATGATTGGCCTTTCTCCCTTGC
ACTAAXXXXXXXXXXAGTTAATTGTATGACTAGCAATATTTATTAAGAATTACAAATATAATAGAACTTATTAAC
TTTAGATTCAATGGTTGGTA
TACAGGTATGAACAAAAGTTAATTTTACCTCTTTACCCCACTACTCTATGTTTATGATTGGCCTTTCTCCCTTGC
ACTAAXXXXXXXXXXAGTTAATTGTATGACTAGCAATATTTATTAAGAATTACAAATATAATAGAACTTATTAAC
TTTAGATTCAATGGTTGGTA
>Marker916215
AACATAGCTTAAAGTTTTCAAAAACATAGGCCAGAATAGCTTCAGCTTAAGCACAATACTCTACAAGAAACCCTT
TCTAGXXXXXXXXXXCTGAAGCCCACAAATATATGAATGGGCTGCACATATGACTGAAGTGATGCAGTCTTCAAA
ATGTTAATTAATGCAATAGT
AACATAGCTTAAAGTTTTCAAAAACATAGGCCAGAATAGCTTCAGCTTAAGCACAATACTCTACAAGAAACCCTT
TCTAGXXXXXXXXXXCTGAAGCCCACAAATATATGAATGGGCTGCACACATGACTGAAGTGATGCAGTCTTCAAA
ATGTTAATTAATGCAATAGT
>Marker916567
TACCTCCAATGCAGATTCACATGGATGATATGGTCAAGAGCAAGATTTCAGGGTTTTGTCAAAACTATTCTCAAT
ATCGAXXXXXXXXXXATGGCTGGGGAGTTCAAAAATTGAAGGATTCTGAGAAAGTGCTGAAAGAAGAAAATGATT
CCCAGGAGGGGCATTTGAGT
TACCTCCAATGCAGATTCACATGGATGATATGGTCAAGAGCAAGATTTCAGGGTTTTGTCAAAACTATTCTCAAT
ATCGAXXXXXXXXXXATGGCTGGGGAGTTCAACAATTGAAGGATTCTGAGAAAGTGCTGAAAGAAGAAAATGATT
CCCAGGAGGGGCATTTGAGT
>Marker917078
ACGGGTGAGAGTGGCCAGATCGCTGACTCAATAAGCTTATCATTTTGCAGATAAGACCGAGTGGAGAGTTGGGAA
CATAAXXXXXXXXXXACGAGAGGGTTTCTGATTATTGGTAGAACTATAAATAGGTTGTTCATTGGAGGAGCACTG
ATATTTAAGTATTAAAGGTA
ACGGGTGAGAGTGGCCAGATCGCTGACTCAATAAGCTTATCATTTTGCAGACAAGACCGAGTGGAGAGTTGGGAA
CATAAXXXXXXXXXXACGAGAGGGTTTCTGATTATTGGTAGAACTATAAATAGGTTGTTCATTGGAGGAGCACTG
ATATTTAAGTATTAAAGGTA
>Marker917113
TACTTCAATGGAGGACATCAAATAAACTTAAATTCTTGAGAAAGCTCTAAACTTTTTAGTCATTTTCAAAAAAAA
AAAAAXXXXXXXXXXATAGGCATGGTTTTAAATGCTGAGAGAATGGCACCTTTGAAAGAAAATAGGCTAATTTCT
TATTGTTTATTATTTTCGTC
TACTTCAATGGAGGACATCAAATAAACTTAAATTCTTGAGAAAGCTCTAAACTTTTTAGTCATTTTCAAAAAAAA
AAAAAXXXXXXXXXXATAGGCATGGTTTTAAATGCTGAGAGAATGGCACCTTTGAAAGAAAATTGGCTAATTTCT
TATTGTTTATTATTTTCGTC
>Marker918253
GACATGCAATGGAATGAACATAATATTGCTTTTGAATTTACACTGACTCTACTACAAATCAGTCTTCCTTAGACT
AATACXXXXXXXXXXATTAATACTCTCTTTTGGTTTCTCCTCCATTGGAAATTTTGAATTACTCTCTTTTGGCTG
CTCCACTAAAATTGCAGGGT

Create PDF with GO2PDF for free, if you wish to

GACATGCAATGGAATGAACATAATATTGCTTTTGAATTTACACTGACTCTACTACAAATCAGTCTTCCTTAGACT
AATGCXXXXXXXXXXATTAATACTCTCTTTTGGTTTCTCCTCCATTGGAAATTTTGAATTACTCTCTTTTGGCTG
CTCCACTAAAATTGCAGGGT
>Marker919117
TACATATGGCAGGACAAAGAAGATGAAAAAAAGATCAGTGATATCAACAGGGACACATGATTAAACAAAACATCC
TTTCCXXXXXXXXXXATCTGGTTGGGACTCAGTTCTGTGTTATTCCTGTTAGTGAGGGCCTCAAGGTTTTGGTTG
GTTTCTGTTTTTTCTAATGT
TACATATGGCAGGACAAAGAAGATGATAAAAAGATCAGTGATATCAACAGGGACACATGATTAAACAAAACATCC
TTTCCXXXXXXXXXXATCTGGTTGGGACTCAGTTCTGTGTTATTCCTGTTAGTGAGGGCCTCAAGGTTTTGGTTG
GTTTCTGTTTTTTCTAATGT
>Marker919165
AACGGATTGGTATCAAAGGCACAGAAAGTTCCCGAAGAAGGATGGGTCATGCAAGATGGAACTCCATGGCCCGGA
AATAAXXXXXXXXXXGTAAAATGCGTGCAGTTTTCTTATTGAATACAAACTCATCGTCTCATTTACACGTCAGGT
TTTCCTAGGGCAAAACGGTG
AACGGATTGGTATCAAAGGCACAGAAAGTTCCCGAAGAAGGATGGGTCATGCAAGATGGAACTCCATGGCCCGGA
AATAAXXXXXXXXXXGTAAGATGCGTGCAGTTTTCTTATTGAATACAAACTCATCGTCTCATTTACACGTCAGGT
TTTCCTAGGGCAAAACGGTG
>Marker919808
ACTTTTAATTATGATATCCATTCTTTGAAACATATATAATCACATGAATAGTCTTTCCATCTTTTTTTCTTTTAG
AATTAXXXXXXXXXXTAGCATTGATAAATAAATAATTAGTTTCTCTTGATGTCAATATAATTATTCCAAATTAAA
ACTTTAGTTTAATTTTAGTC
ACTTTTAATTATGATATCCATTCTTTGAAACACATATAATCACATGAATAGTCTTTCCATCTTTTTTTCTTTTAG
AATTAXXXXXXXXXXTAGCATTGATAAATAAATAATTAGTTTCTCTTCATGTCAATATAATTATTCCAAATTAAA
ACTTTAGTTTAATTTTAGTC
>Marker919892
ACAACAATTATAATTATAAAAATAGAGATCCAACAATTCAATTTCTAAAAAAAAAAAAGAGAGAGAGAGAGAGAA
TTACAXXXXXXXXXXCAAATCAATAAAAGACAAAAGCTATAAAGATAAGAACTAATTCATCCATTGAGTTCTTCA
TTTCCAATCCAATCCACGTC
ACAACAATTATAATTATAAAAATAGAGATCCAACAATTCAATTTCTAAAAAAAAAAAAAAGAGAGAGAGAGAGAA
TTACAXXXXXXXXXXCAAATCAATAAAAGACAAAAGCTATAAAGATAAGAACTAATTCATCCATTGAGTTCTTCA
TTTCCAATCCAATCCACGTC
>Marker920343
ACAACCAACTAGAACCATCCAGCAAACAGCAATCCAATCCACTTCTTAGAAACATAACACATATTCGTAAAAATA
GAACAXXXXXXXXXXCTTCAACTACTGATGGTAGGGCAAGGAGATTTGTGCAACCAGCAATGAGTAGTTGAAAGA
TGCCCAATGGAGAGAGTGTG
ACAACCAACTAGAATCATCCAGCAAACAGCAATCCAATCCACTTCTTAGAAACATAACACATATTCGTAAAAATA
GAACAXXXXXXXXXXCTTCAACTACTGATGGTAGGGCAAGGAGATTTGTGCAACCAGCAATGAGTAGTTGAAAGA
TGCCCAATGGAGAGAGTGTG
>Marker920648
TACACCAATCTGATTAATGGACGACGATGATGTTAAATTTCTACTTTTTTGAGTCAGATCATACTAGACTTCAAA
TATTCXXXXXXXXXXGGGTGATAGAAATGAGCCAAATATTTTTTTCATCAGTGGTCTCAATGAAAAACAATAACA
TCAACTCATGAAATTTATGT
TACACCAATATGATTAATGGACGACGATGATGTTAAATTTCTACTTTTTTGAGTCAGATCATACTAGACTTCAAA
TATTCXXXXXXXXXXGGGTGATAGAAATGAGCCAAATATTTTTTTCATCAATGGTCTCAATGAAAAACAATAACA
TCAACTCATGAAATTTATGT
>Marker920862

Create PDF with GO2PDF for free, if you wish to

ACTGGAGTAACAGATGGATGACGCGACTGAGACAAAAGAAGGCAAGGACCGTGTGTGGTTGGGGATTATCGACGA
GTCAAXXXXXXXXXXXGCTCGGAAAGGAAAACGATTGAACGGTGGTGGTTCCAACCACTAAAACGAACATTGAAAG
CTTCAAAGGATCTGTAGGTC
ACTGGAGTAACGGATGGATGACGCGACTGAGACAAAAGAAGGCAAGGACCGTGTGTGGTTGGGGATTATCGACGA
GTCAAXXXXXXXXXXXGCTCGGAAAGGAAAACGATTGAACGGTGGTGGTTCCAACCACTAAAACGAACATTGAAAG
CTTCAAAGGATCTGTAGGTC
>Marker920983
AACTCAAAATTTTCATTTAACAATCCCCTACTTGATTTTTTTTTTACTATTAATCCCCCACCTGAAATTTTTTAA
ATGTGXXXXXXXXXXXATTTCACACACAACATTATTCGAGTGAAGTTCTAAATTTGCTAGTGCTTTAAGGCCTTGC
ACGTATATCCTAGTTTAGTG
AACTCAAAATTTTCATTTAACAATCCCCTACTTGATTTTTTTTTTACTATTAATCCCCCACGTGAAATTTTTTAA
ATGTGXXXXXXXXXXXATTTCACACACAACATTATTCGAGTGAAGTTCTAAATTTGCTAGTGCTTTAAGGCCTTGC
ACGTATATCCTAGTTTAGTG
>Marker921113
TACCATTCCTGAATTAAAAATTAACAACAAAATCACTACTTCGAGAACTTGCTACAACTTTTGTTCCTTAACTTT
CAACTXXXXXXXXXXXCTCTCTCTTTCTGAAGCAAGATATCAATTCTATTTTATAGCTCATACCAAACTATTTCAT
CCAATTGTCTGCTTCTTTGT
TACCATTCCTGAATTAAAAATTAACAACAAAATCACTACTTCGAGAACTTGCTACAACTTTTGTTTCTTAACTTT
CAACTXXXXXXXXXXXCTCTCTCTTTCTGAAGCAAGATATCAATTCTATTTTATAGCTCATACCAAACTATTTCAT
CCAATTGTCTGCTTCTTTGT
>Marker921439
ACTCCATTCCTTAGTTTTAGACAGAGTCTTATACAGAAAATTAAGCACAATCATGATACATGGTTCTAGAAAAGT
GAATTXXXXXXXXXXXATCCATTTTTTTTTGTAGTTTAGCTTCTACCGAACTTCCTGTTTCTATAAATTTCTTTCC
TGCCTCATCTGATTGTTGTG
ACTCCATTCCTTAGTTTTAGACAGAGTCTTATACAGAAAATTAAGCACAATCATGATACATGGTTCTAGAAAAGT
GAATTXXXXXXXXXXXATCCATATTTTTTTGTAGTTTAGCTTCTACCGAACTTCCTGTTTCTATAAATTTCTTTCC
TGCCTCATCCGATTGTTGTG
>Marker921663
AACAATAGTGTTATAAAATTCATCTTTCTAACCACTCACTTATTTCAACTATAAAGCATGAACTCTTGATGCTAC
TACTAXXXXXXXXXXXTGGTTGCTAGCATTTAACACTTGATAAGGAAAATGTATGACAGGATTCACTTTATATAAC
CCTTATGGATAAGTATCAGT
AACAATAGTGTTATAAAATTCATCTTTCTAACCACTCACTTATTTCAACTATAAAGCATGAACTCTTGATGCTAC
TACTAXXXXXXXXXXXTGGTTGCTAGCATTTAACACTTGATAAGGAAAATGTATGACAGGATTCACTTTATATAAT
CCTTATGGATAAGTATCAGT
>Marker923095
CACACACAAGAACTGCTAGATTTGCTGGTCAAATGCGAAAATAAAATTCAAACTCGTATTAAGATTGGGCTGAAT
TCAAAXXXXXXXXXXXTTTCTATACTCCCAAGGAAATTGGAGGTCTTGGCATGTTATCCATGGGTCACATATTGAT
CCCACAAAGTGATCTTCGGT
CACACACAAGAACTGCTAGATTTGCTGGTCAAATGCGAAAATAAAATTCAAACTCGTATTAAGATTGGGCTGAAT
TCAAAXXXXXXXXXXXTTTCTATACTCCCAAGGAAATTGGAGGTCTTGGCATGCTATCCATGGGTCACATATTGAT
CCCACAAAGTGATCTTCGGT
>Marker923283
ACCGTAATTTATCATTAATTTATCTTTCATTAGCATGCCAAGTTAAGTTTTAGCACATTCAACTCTTTTAAACAT
CCGTTXXXXXXXXXXXCAATTGCATTGGCATATTTCTTTCGATACAAGCAACAATCATTAAGGCATGCATGAATCT
TTTAGTAATCTATTCCTAGT

Create PDF with GO2PDF for free, if you wish to

ACCGTAATTTATCATTAATTTATCTTTCATTAGCATGCCAAGTTAAGTTTTAGCACATTCAACTCTTTTAAACAT
CCATTXXXXXXXXXXXGAATTGCATTGGCATATTTCTTTCGATACAAGCAACAATCATTAAGGCATGCATGAATCT
TTTAGTAATCCATTCCTAGT
>Marker923374
ACTATTTCAAGGTAACCAATAAATTTTCTTTTGATTTATGTAAGAAGATGAATATTGATGGGAAATGCTACATAC
CTACCXXXXXXXXXXXGCTGACTAGAAGACTTCAAAAAGATGTAAAACTAATTTTAACATACCTGTGAGGCAACAT
CAACGCCAATTTCATCGAGT
ACTATTTCAAGGTAACCAATAAATTTTCTTTTGATTTAAGTAAGAAGATGAATATTGATGGGAAATGCTACATAC
CTACCXXXXXXXXXXXGCTGACTAGAAGACTTCAAAAAGATGTAAAACTAATTTTAACATACCTGTGAGGCAACAT
CAACGCCAATTTCATCGAGT
>Marker923421
ACTTAATTAGTTAAATTTTACGCAAATTTTACTAATATCCATCAATAACATTTCAATACTTTACCGTTTCAGTGT
GGTGTXXXXXXXXXXXATTCGACCCTTTGAACTACCAACCCAAGCATAGCTCAAATGCTACCGATATAGCTAGACT
AAGAAGACTAAGAAGTAGTG
ACTTAATTAATTAAATTTTACGCAAATTTTACTAATATCCATCAATAACATTTCAATACTTTACAGTTTCAGTGT
GGTGTXXXXXXXXXXXATTCGACCCTTTGAACTACCAACCCAAGCATAGCTCAAATGCTACCGATATAGCTAGACT
AAGAAGACTAAGAAGTAGTG
>Marker924022
AACTAACAGAATTGTCGTCTTCTGATAGAAAAATTAATAAATAAATAATCAGATCCATAACAAATTAAATGAACT
TTTTGXXXXXXXXXXXTAACTAAACTTTAACAAAATATTAAAATTATGAAACTCAAACATAGTTAGATCTGTATAT
TAATGATTATAATGTAAGTT
AACTAACAGAATTGTCGTTTTCTGATAGAAAAACTAATAAATAAATAATCAGATCCATAACAAATTAAATGAACT
TTTTGXXXXXXXXXXXTAACTAAACTTTAACAAAATATTAAAATTATGAAACTCAAACATAGTTAGATCTGTATAT
TAATGATTATAATGTAAGTT
>Marker924187
ACTCAATTGATGTGAGTTTGGAAATGGTTGCGTTTGGAATTTTTCCACTGAATTGATTAGATGAGATATCAAGAA
GTTTAXXXXXXXXXXXAGTTTCTTAAACTCTAAAAGAAATCAAAAGTTAAAACTAAGCAATCTAAATTCATATTTC
TTAATAATATTTATGTAGTA
ACTCAATTGATGTGAGTTTGGAAATGGTTGCATTTGGAATTTTTCCACTGAATTGATTAGATGAGATATCAAGAA
GTTTAXXXXXXXXXXXAGCTTCTTAAACTCTAAAAGAAATCAAAAGTTAAAACTAAGCAATCTAAATTCATATTTC
TTAATAATATTTATATAGTA
>Marker924642
CACACTGAAAACATTTAAATGCCGCAAATCCTGTCGAATAGGGTTTGCAATATCAACAGGAGACACTGACCATGG
TAACGXXXXXXXXXXXGTCCAAAAATTTCAGTTAATGAGAAAAGGCACTGAAAGATTTTCCACCACCTCACCCCCA
CGTATTTCGGATTAACTAGT
CACACTGAAAACATTTAAATGCCGCAAATCCTGTCGAATAGGGTTTGCAATATCAACAGGAGACACTGACCATGG
TAACGXXXXXXXXXXXGTCCAAAAATTTCAGTTAATGAGAAAAGGCACTGAAAGATTTTCCACCTCCTCACCCCCA
CGTATTTCGGATTAACTAGT
>Marker925384
ACCTTTAAGTTCGTTGATTTCAACAGATTGTTCCGTTGTTAGTGTCTCTAAAATGTCATTTCTCATTGCTCATTT
CTGCAXXXXXXXXXXXCATTGAGCTCGTTGAAATCCATAAGCCGCATGGTTCGACTCTGGATCTCTGCCGTCCATC
AATTTTTTCCTATTAACGTC
ACCTTTAAGTTCGTTGATTTCAACAGATTGTTCCGTTGTTAGTGTCTCTAAAATGTCATTTCTCATTGCTCATTC
CTGCAXXXXXXXXXXXCATTGAGCTCGTTGAAATCCATAAGCCGCATGGTTCGACTCTGGATCTCTGCCGTCCATC
AATTTTTTCCTATTAACGTC
>Marker925607

Create PDF with GO2PDF for free, if you wish to

AACCATTTGGGAATTCGATTAAACTACCACCTCGAGCAGATGATGCTTCTTTGCTAGACTGAAGAGCTCTTTGAC
GTGTGXXXXXXXXXXTGATGCACCATAATTCTCCATACTAGAAATGCTGGAAAGTTTCCGCTGCTTCTTGCTTGG
TTCTTCACCATCATCACGGT
AACCATTTGGGAATTCGATTAAACTACCACCTCGAGCAGATGATGCTTCTTTGCTAGACTGAAGAGCTCTTTGAC
GTGTGXXXXXXXXXXTGATGCACCATAATTCTCCATATTAGAAATGCTGGAAAGTTTCCGCTGCTTCTTGCTTGG
TTCTTCACCATCATCACGGT
>Marker925750
ACTAACCCAGTGTTCTTGCTATTGACTTATGAATTGATTGAAAAATTTCTGTCCCAAGAACTTAGGCGAGAATTT
GTGGGXXXXXXXXXXAATACAGTAAAACACAACTTTGCATTCAAAGCTCCAAATTACATTCATGAATGGCACTTT
GAACAAAGGAAAGAAAAGTT
ACTAACCCAGTGTTCTTGCTATTGACTTATGAATTGATTGAAAAATTTCTGTCCCAAGAACTTAGGCGAGAATTT
GTGGGXXXXXXXXXXAATACAGTAAAACACGACTTTGCATTCAAAGCTCCAAATTACATTCATGAATGGCACTTT
GAACAAAGGAAAGAAAAGTT
>Marker925940
AACACTTAAAGAAGCTACCTTATCTACTTACTCAATAAGGAAAAGAAGGAATTACATGCATCTTGTGAAATTATA
TTCTCXXXXXXXXXXTCGTGTGAAATATTCTCTTTAATAAAATATATTTAGAAAAAATAACGACTCGAACAAAAA
TATGTATAGTTGAAGATGGT
AACACTCAAAGAAGCTACCTTATCTACTTACTCAATAAGGAAAAGAAGGAATTACATGCATCTTGTGAAATTATA
TTCTCXXXXXXXXXXTCGTGTGAAATATTCTCTTTAATAAAAGATATTTAGAAAAAATAACGACTCGAACAAAAA
TATGTATAGTTGAAGATGGT
>Marker926230
ACCAAGCACCAGACAAGACCTTGTAGCCTATTTTAGAGGGCTAGCCTCTCCTCTAAAAAATGCAGAATAGTCCAT
TTACTXXXXXXXXXXTTGAGACATTTGAGGATTGGAGATTTCTTCTGGACAGATGCATTGTTGAGAAAATATGAA
TTAGCTACTACCTTTCAAGT
ACCAAGCACTAGACAAGACCTTGTAGCCTATTTTAGAGGGCTAGCCTCTCCTCTAAAAAATGCAGAATAGTCCAT
TTACTXXXXXXXXXXTTGAGACATTTGAGGATTGGAGATTTCTTCTGGACAGATGCATTGTTGAGAAAATATGAA
TTAGCTACTACCTTTCAAGT
>Marker926750
TACCTGTCATGGATATTTATATATGCAAGATGGGTGTAATGTCATATAGATTATGAAAGAACAGCAACATGATCA
TAGTTXXXXXXXXXXTATTTTCAAAGATGAAAGGGTTAAATTCCCATTCTCCATATTTTCTACATTTAACATTAA
AGGAACTAATGCTAATTGTT
TACCTGTCATGGATATTTATATATGCAAGATGCGTGTAATGTCATATAGATTATGAAAGAACTGCAACATGATCA
TAGTTXXXXXXXXXXTATTTTCAAAGATGAAAGGGTTAAATTCCCATTCTCCATATTTTCTACATTTAACATTAA
AGGAACTAATGCTAATTGTT
>Marker927469
TACATAAAAATCCAAGGAATCAGAAGTATAACCTACCACAAATTACCAAGAAGTGACAAATCAGAGAGCAATAAC
TCTTCXXXXXXXXXXAATAGAGAGTGAAAAAGGGGACCTTCTTGTTTTGCATCTACTCCATGAACGATGGATGGC
TGGAACACGAGCAACGAGTA
TACATAAAAATCCAAGGAATCAGAAGTATAACCTACCACAAATTACCAAGAAGTGACAAATCAGAGAGCAATAAC
TCTTCXXXXXXXXXXAATAGAGAGTGAAAAAGGGGACCTTCTTGTTTTGCATCTACTCCATGAACGATGGATGGC
TGGAACACGAACAACGAGTA
>Marker927594
AACGTGGAAGCAAATATCTTAGAGACAGATGAGGCAACCGCAACATCACGTGCAGTGAGTTTGTCCGTTGTCGTA
TTGTCXXXXXXXXXXATTTTTTCATATGTTGGAAACTGAATCGCAACGTGACTTACACCGGCCAAAGCAGGCACA
AGACCACTGCAGAACACGGT

Create PDF with GO2PDF for free, if you wish to

AACGTGGAAGCAAATATCTTAGAGACAGATGAGGCAACCGCAACATCACGTGCAGTGAGTTTGTCCGTTGTCGTA
TTGTCXXXXXXXXXXXATTTTTTCATATGTTGGAAACTGAATCGCGACGTGACTTACACCGGCCAAAGCAGGCACA
AGACCACTGCAGAACACGGT
>Marker928144
ACCTTTCTTAAGCAGTTACTTCATAAGAGTTAATTAAGTTGGTAGTTATTAATTGTAGTTTATTGAGTATTTATT
TGGTTXXXXXXXXXXXTCGAAGGTGTCAAAATTATAGTGGATGGTCATGAAAGAGCAGTCACTGACAAGGAAGGAT
TCTACAAGCTCGATCAGGTA
ACCTTTCTTAAGCAGTTACTTCATAAGAGTTAATTAAGTTGGAAGTTATTAATTGTAGTTTATTGAGTATTTATT
TGGTTXXXXXXXXXXXTCGAAGGTGTCAAAATTATAGTGGATGGTCATGAAAGAGCAGTCACTGACAAGGAAGGAT
TCTACAAGCTCGATCAGGTA
>Marker928448
TACTTTTCAGAAATCTATTCTTGAATGGTAAGTGGATCATGATTCATGTGATAATTGTCATTTCATATGCTTTAA
TGGAAXXXXXXXXXXXGTTCATGAACTTCAGTTATTTATTACATGGTTCATCCAAAATAAAAAGGAGAAAAAGCAA
GAGGTTCTTAGTTGTTTGTT
TACTTTTCAGAAATCTATTCTTGAATGGTAAGTGGATCATGATTCATGTGATAATTGTCATTTCATATGCTTTAA
TGGAAXXXXXXXXXXXGTTCATGAACTTCAGTTATTTATTACATGGTTCATCCAAAATAAAAAAGAGAAAAAGCAA
GAGGTTCTTAGTTGCTTGTT
>Marker928838
TACAAATTTTCTAATTCTATTGTCTTTTTCTTTAATGATGACCAACATAGAGGAGGAGAAAAAGATGAATGCAAC
CTACAXXXXXXXXXXXTTTGCATTTATAAATTGTTAAAGAATGAATTGATAGGATGAAATTCAATAACAGCAAATC
AAAAGTTATAAGAATGTGTT
TACAAATTTTCTAATTCTATTGTCTTTTTCTTTAATGATGACCAACATAGAGGAGGAGAAAAAGATGAATGCAAC
CTACAXXXXXXXXXXXTTTGCATTTATAAATTGTTAAAGAATGAATTGATAGGATGAAATTCAATAACAGCAAATC
AAAAGTTATAAGAATATGTT
>Marker929060
ACAACACCAAAAAGATCTTGAAACTCAGATTAAAGAAAAGGCCTTCACTTTAATAATTGACCACTCACATAAGCT
CCACCXXXXXXXXXXXCTTTAAATTCACCCATTATATATCAATTGAGTTATGGTATGAGTTAGTTATTAAATGTAA
AAGAATGTTCTTGGTTGGTA
ACAACACCAAAAAGATCTTGAAACTCAGATTAAAGAAAAGACCTTCACTTTAATAATTGACCACTCACATAAGCT
CCACCXXXXXXXXXXXCTTTAAATTCACCCATTATATATCAATTGAGTTATGGTATGAGTTAGTTATTAAATGTAA
AAGAATGTTCTTGGTTGGTA
>Marker929082
ACTGAGCTTGTGAAGAAAATAAAGAGGCCTGTTTCTCAGTAATTAGATCCTTCTCTGTCTGAAACAAGGTATTGA
GATCAXXXXXXXXXXXGAATGGAGTTTGGGTAGGAGAACCTCTAGAAAGTTTTACATTTGAATTAAGCAAGGAGAA
AACTGATTATTTAAAATGTG
ACTGAGCTTGTGAAGAAAATAAAGAGGCCTGTTTCTCAGTAATTAGATCCTTCTCTGTCTGAAACAAGGTATTGA
GATCAXXXXXXXXXXXGAATGGAGTTTGGGTAGGAGAACCTCTAGAAAGTTTTACATTTGAATTAGGCAAGGAGAA
AACTGATTATTTAAAATGTG
>Marker929720
CACACTTCCATGCAGAATAGAGCAAAATCATTGAGCTCTTATATTGAGAATTTGCAAATGTTGAAGTTGAATTAT
GATCGXXXXXXXXXXXTATTGATTGAACCACTGTGATAGGAGTTTCTTGGTCTCACCATTGGGCGATACCTTCTTG
ACTTTGTGTTATGTTGTGTA
CACACTTCCATGCAGAATAGAGCAAAATCATTGAGCTCTTATATTGAGAATTTGCAAATGTTGAAGTTGAATTAT
GATCGXXXXXXXXXXXTATTGATTGAACCACTGTGATAGGAGTTTCTTGGTCTCACCATTGGGCGATACCTTCTTG
AATTTGTGTTATGTTGTGTA
>Marker929739

Create PDF with GO2PDF for free, if you wish to

TACTTAGGTCCAAGCTTGAATCACAAAGGGCATAGATGTTTGAGTTTTACTGTTTGGCTGTATATATCTCGAGAT
GTGCAXXXXXXXXXXXTAGCAAAATTTAGTATGGTTAAACTGGCGATTATAGAATTTTTTGGATTTTTTTTTACCC
GTTCCTTTAAGTAGGATAGT
TACTTAGGTCCAAGCTTGAATCACAAAGGGCATAGATGTTTGAGTTTTACTGTTTGGCTGTATATATCTTGAGAT
GTGCAXXXXXXXXXXXTAGCAAAATTTAGTATGGTTAAACTGGCGGTTATAGAATTTTTTGGATTTTTTTTTACCC
GTTCCTTTAAGTAGGATAGT
>Marker930068
CACCTATCTTGGACCGAATATCCGTTTGGACTTAACGGGCTTTGATACCATGTTTGATAGTATAGGGTTCCATCT
CAAAAXXXXXXXXXXXAACAATTTCACCACCAAGATAACAGACACTGTCCCAAAAAAACAAATAACAAAATGTTGC
AGAGATAGATAGAAACAAGT
CACCTATCTTGGACCGAATATCCGTTTGGACTTAACGGGCTTTGATACCATGTTTGATAATATAGGGTTCCATCT
CAAAAXXXXXXXXXXXAACAATTTCACCACCAAGATAACAGACACTGTCCCAAAAAAACAAATAACAAAATGTTGC
AGAGATAGATAGAAACAAGT
>Marker930379
TACAAATGCTGAAAGAAGAACATAGAACTGGGAAAAATGGACAAAACACAAGCCACAAGGATGTAGCAACAGAGG
ACTTTXXXXXXXXXXXCTTTTCATTATTTCTGTCAAACTTTTTCCAAATTATATCCAAAATGACAGAATCATTCTC
GAAAAACTTAATGCTCTAGT
TACAAATGCTGAAAGAAGAACATAGAACTGGGAAAAATGGACAAAACACAAGCCACAAGGATGTAGCAACAGAGG
ACTTTXXXXXXXXXXXCTTTTCATTATTTCTGTCAAACTTTTTCCAAATTATATCCAAAATGGCAGAATCATTCTC
GAAAAACTTAATGCTCTAGT
>Marker930388
CACCACGACCATTGAGATCTTCACCTAAGACTGATTTTTCTTCATAACTAGCTGTCTTTTCCCTCTGACTGGCTT
CATCAXXXXXXXXXXXAAACAGCCATCAGATTTAACACCTCTTCATTTCATGAAAGTAATAGTTTCATGATAAGTA
AATATGATTGAATCATCGTC
CACCATGACCATTGAGATCTTCACCTAAGACTGATTTTTCTTCATAACTAGCTGTCTTTTCCCTCTGACTGGCTT
CATCAXXXXXXXXXXXAAACAGCCATCAAATTTAACACCTCTTCATTTCATGAAAGTAATAGTTTCATGATAAGTA
AATATGATTGAATCATCGTC
>Marker931016
ACTAAAAATGAATTAAAAGAGAGAAAATGAAATAAAGGGAAGATTAGGTGATGTATGTTAGAAAAATAACCCAAA
TGAAAXXXXXXXXXXXTTGTGCCCTGGGCTGGACTCTAGGCCAACTGGGCTTTGGAGCCCAAAACTCCATTTTGAT
GGGCTTAGCCCAGTTTAGTT
ACTAAAAATGAATTAAAAGAGAGAAAATGAAATAAAGGGAAGATTAGGTGGTGTATGTTAGAAAAATAACCCAAA
TGAAAXXXXXXXXXXXTTGTGCCCTGGGCTGGACTCTAGGCCAACTGGGCTTTGGAGCCCAAAACTCCATTTTGAT
GGGCTTAGCCCAGTTTAGTT
>Marker931469
TACATTGGTTTAGAACTTGGGAGATTGGTGTCTTATGGTGGTGAAAATGTTATGAACTACAGAATGCATATGGAA
CAGGAXXXXXXXXXXXAGAGGTTCAAATTCCCAACAAGGCATGTTTCTCAACTAGAAAAAGGACATATATTTCGTT
TAGCTTCTCTTTTTTTGGGT
TACATTGGTTTAGAACTTGGGAGATTGGTGTCTTATGGTGGTGAAAATGTTATGAACTACACAATGCATATGGAA
CAGGAXXXXXXXXXXXAGAGGTTCAAATTCCCAACAAGGCATGTTTCTCAACTAGAAAAAGGACATATATTTCGTT
TAGCTTCTCTTTTTTTGGGT
>Marker932232
AACCACTACCACTCTAATGCTTAAGTCAGAAAATTGGAACGCTGAAGAGAGAATTAGTGTTGATTTTTAAAACCT
ACTTCXXXXXXXXXXXTTAGAATTTTATCGCTTTCTAACCAAGCCTATGTGCGACCTGTTTGCCCGTGGGTTTTGC
TAATGAGCTTGGTCAAGGTC

Create PDF with GO2PDF for free, if you wish to

AACCACTACCACTCTAATGCTTAAGTCAGAAAATTGGAACGCTGAAGAGAGAATTAGTGTTGATTTTTAAAACCT
ACTTCXXXXXXXXXXXTTAGAATTTTATCGCTTTCTAACCAAGCCTATGTTCGACCTGTTTGCCCGTGGGTTTTGC
TAATGAGCTTGGTCAAGGTC
>Marker932281
AACTATACAGATAACAGAGCAAAAATATTGTTTTCCACTACTCTGAACCCAATTATCGTCAAGTTCATTGGCCAA
TCAGTXXXXXXXXXXXGATAGTGATGATCCTTTTTTACATGGCAATGGGTAATGGGTAATGGGAAAAAAACTGAAC
TGGATAAGCTGAAGAGAAGT
AACTATACAGATAACAGAGCAAAAATATTCTTTTCCACTACTCTGAACCCAATTATCGTCAAGTTCATTGGCCAA
TCAGTXXXXXXXXXXXGATAGTGATGATCCTTTTTTACATGGCAATGGGTAATGGGTAATGGAAAAAAAACTGAAC
TGGATAAGCTGAAGAGAAGT
>Marker933498
ACCCTCCACTTGAACTTTCTATCTCGCTCTTCTTTACAGCCAAACAATTCTTTCGGTATGATCGAACAGGTGGCC
ATCCAXXXXXXXXXXXAGCTTGTTGATGATCTCTTATTAAAAAGATGAGTAGAACCATCATATAATTTTTGTAAGA
TAATAAATCATTATAAAGTT
ACCCTCCACTTGAACTTTCTATCTCGCTCTTCTTTACAGCCAAACAATTCTTTCGGTATGATCGAACAGGTGGCC
ATCCAXXXXXXXXXXXAGCTTGTTGATGATCTCTTATTAAAAAGATGAGTAGCACCATCATATAATTTTTGTAAGA
TAATAAATCATTATAAAGTT
>Marker933587
CACTTTTCTGCTTTTTTTTGTGAGGAAGTTATTGCATCAAAAGAAAGGAAAACAGACTGGGTATGGGCAAGGAAA
AGAAGXXXXXXXXXXXCGCATTGAGGCAATCTCTTCACGAAGTTGTCCCAGGATCTAAATGAACGAGTTCGATTAC
AATCAGACAGATAAAAATGT
CACTTTTCTGCTTTTTTTTGTGAGGAAGTTATTGCATCAAAAGAAAGGAAAACAGACTGGGTATGGGCAAGGAAA
AGAAGXXXXXXXXXXXCGCATTGAGGCAGTCTCTTCACGAAGTTGTCCCAGGATCTAAATGAACGAGTTCGATTAC
AATCAGACAGATAAAAATGT
>Marker933986
ACCTAACTTTCTTAATTCATGGTTATGTGTAGGCTCATATAACAATTTTAAGCTAAACAATGAAAAGTGAGCATA
CTTGAXXXXXXXXXXXTCATGTCACAGTCATTAAACATAATATGATTGAGTAATTGGTAGCTCTTTAGCTCTTTAG
TGGAGCATTTGAGATGGGTC
ACCTAACTTTCTTAATTCATGGTTATGTGTAGGCTCATATAACAATTTTAAACTAAACAATGAAAAGTGAGCATA
CTTGAXXXXXXXXXXXTCATGTCACAGTCATTAAACATAATGTGATTGAGTAATTGGTAGCTCTTTAGCTGTTTAG
TGGAGCATTTGAGATGGGTC
>Marker934157
CACACATACTCCAAATTGCGAAACCCTAAATGAAAAATCCACCAAAGCAAAATTAAATCGAAAACCCAAAGGTGG
CCGACXXXXXXXXXXXAAAATCTTCACTCTACCCTTAGCTACCAAACATTATTAATCTTATCATATATAATATAAT
AATGTAGCTCTATAATATGT
CACACATACTCCAAATTGCGAAACCCTAAATAAAAAATCCACCAAAGCAAAATGAAATGGAAAACCCAAAGGTGG
CCGACXXXXXXXXXXXAAAATCTTCACTCTACCCTTAGCTACCAAACATTATTAATCTTATCATATATAATATAAT
AATGTAGCTCTATAATATGT
>Marker934570
GACAATGTTCATCTGCACAAATGCCACGAGTGTCCGTGAAAAAAATGCATAAAGTCGTAAATTGGAAAAAAAAAA
ACAGAXXXXXXXXXXXACTACTACTATTCTTTAACAAGTGAAAACAAACCAAATTATACACAATCCAAACACCGAG
GTAAAGCTTGCTTCAAGGTT
GACAATGTTCATCTGCACAAATGCCACGAGTGTCCGTGAAAAAAATGCATAAAGTCGTAAATTGGAAAAAAAAAA
CAGAAXXXXXXXXXXXACTACTACTATTCTTTAACAAGTGAAAACAAACCAAATTATACACAATCCAAACACCGAG
GTAAAGCTTGTTTCAAGGTT
>Marker934821

Create PDF with GO2PDF for free, if you wish to

TACTGGAAGCTTCACTGTTGTAGAGAAACGAAGCCAACTTTAGGCCCACGAGGCGCGAGAAGAGAAAACCAAGTT
GAACAXXXXXXXXXXATTGGAGAACCGCAACTATTCTTTTGCCTTTAGGACACCGTGGTCTCCACATGATTATTA
ATATAGAATGCAATGTTTGT
TACTGGAAGCTTCACTGTTGTAGAGAAACGAAGCCAACTTTAGGCCCACGAGGCGCGAGAAGAGAAAACCAAGTT
GAATAXXXXXXXXXXATTGGAGAACCGCAACTATTCTTTTGCCTTTAGGACACCGTGGTCTCCACATGATTATTA
ATATAGAATGCAATGTTTGT
>Marker938653
CACAAATGTAGAATGAAAAAGGAAAATACAAGATAACAGAGACATACCTCTGAGACAGTAAGTCCAAGATCCCGA
GTTATXXXXXXXXXXCTTCTCTATCTGATCTTTTAGTTCATTCTAGTTTTTTCCATTGAAAAACTCAAACCCAGT
AACTGAAAAACGAACTAGTA
CACAAATGTAGAATGAAAAAGGAAAATACAAGATAACAGAGACATACCTCCGAGACAGTAAGTCCAAGATCCCGA
GTTATXXXXXXXXXXCTTCTCTATCTGATCTTTTAGTTCATTCTAGTTTTTTCCATTGAAAAACTCAAACCCAGT
AACTGAAAAACGAACTAGTA
>Marker938678
GACAAATTTCTTACATAAATTAAATGTTTATCATTTAGACGTCTTTAGAAACATTAATACCATGTTATGTGTGAG
GGGTTXXXXXXXXXXTTTTTTTTCTTTTAGTTTTAGACTTGGGAACATTCTACTTCAAACATGGTTTTTTTTTTC
AAGTTCAAACTTTGCGAGTA
GACAAATTTCTTACATAAATTAAATGTTTATCATTTAGACGTCTTTAGAAACATTAATCCCATGTTATGTGTGAG
GGGTTXXXXXXXXXXTTTTTTTTCTTTTAGTTTTAGACTTGGGAACATTCTACTTCAAACATGGTTTTTTTTTTC
AAGTTCAAACTTTGCGAGTA
>Marker938811
AACAGTCCCCATGCAGGTATCACCGCTTATTGTAGATTGATGATATGGAGTAGTCTAGAGAGCTCTACATAGAAA
ATTAAXXXXXXXXXXACTTATTTTCATTTTCACCTCACTCATTCCAAAAACTTAATCATATGAAATTATTCATTT
TCTTATTGTGACCTTTTGTG
AACAGTCCCCATGCAGGTATCACTGCTTATTGTAGATTGATGATATGGAGTAGTCTAGAGAGCTCTACGTAGAAA
ATTAAXXXXXXXXXXACTTATTTTCATTTTCACCTCACTCATTCCGAAAACTTAATCATATGAAATTATTCATTT
TCTTATTGTGACCTTTTGTG
>Marker939332
CACACCAAGATTTTTTATGTCTCATATTTCTAGCTTCTAATCTTGCTTCGTCACCATACCCATGAATACTTTGAA
GAATTXXXXXXXXXXTTCTCCACAGGATCCGGCTTCATTCTCACTTGCTCGGGCAGTCCATGCCCATATGATTGC
TTCCGGTTTCAAGCCTCGTG
CACACCAAGATTTTTTATGTCTCATATTTCTAGCTTCTAATCTTGCTTCGTCACCATACCCATGAATACTTTGAA
GAATTXXXXXXXXXXTTCTCCACAGGATCCGGCTTCATTCTCACTTGCTCGGGCGGTCCATGCCCATATGATTGC
CTCCGGTTTCAAGCCTCGTG
>Marker939580
ACTCTCTTGATAACATTCATTTTTACTCTCTTGATATTCTTTCCTTTGAGTTATTTGGAGAATATGGTGTATATT
TTTAAXXXXXXXXXXAACCGTCTAAACTACTATTTGTTTAGCTTTTTGCGAAAGCAAGAACAACTTTGAGCTCGG
TTTTGAGCATTGAAACTGGT
ACTCTCTTGATAACATTCATTTTTACTCTCTTGATATTCTTTCCTTTGAGTTGTTTGGAGAATATGGTGTATATT
TTTAAXXXXXXXXXXAACCGTCTAAACTACTATTTGTTTAGCTTTTTGCGAAAGCAAGAACAACTTTGAGCTCGG
TTTTGAGCATTGAAACTGGT
>Marker940705
ACCCTTTCCAAAAGCTCCATTCTGCTTCACTCGAAGACCACGACACTGCCTTGTCCATAAAAGCTGATCAAAATA
TTCCTXXXXXXXXXXAGCAACTTTGAAATTGAGAGAGATTACCTGAACTTGGTGAGGTGAATGAGGAGAAAGGAG
AGCAGAGATTTCATTGGGGT

ACCCTTTCCAAAAGCTCCATTCTGCTTCACTCGAAGACCACGACACTGCCTTGTCCATACAAGCTGATCAAAATA
TTCCTXXXXXXXXXXAGCAACTTTGAAATTGAGAGAGATTACCTGAACTTGGTGAGGTGAATGAGGAGAAAGGAG
AGCAGAGATTTCATTGGGGT
>Marker941505
CACAACTTCCATGCTCCTTGTCTGCAATATGGTCTAAACTTTTGTTTTCTGGTAACTTTTTATTATGTGTGGGGC
AGGTGXXXXXXXXXXTATCTTAACTGACTCTTCCGGCAAGAGAAGAAAGGTTATGCTGAACAGCTCAACTATTTC
AGAAGAGCGATTGTGTGGTT
CACAACTTCCATGCTCCTTGTCTGCAATATGGTCTAAACTTTTGTTTTCTGGTAACTTTTTATTATGTGTGGGGC
AGGTGXXXXXXXXXXTATCTTAACTGACTCTTCTGGCAAGAGAAGAAAGGTTATGCTGAACAGCTCAACTATTTC
AGAAGAGCGATTGTCTGGTT
>Marker942567
AACCCAATCGCGAACGCAAAAGATATGGTAAAAATACCCAAAAGAAGAAGATTGACGGGATGCCTCTGATAGTAA
CAAGAXXXXXXXXXXAAAGGTGTAAGAATGAGAAGGATGTATAAAGCCAAGCCCGCCCCAGTTGAGCTAAAGAAA
GTAGAAATTGGTCGGACGTA
AACCCAATCGCGAACGCAAAAGATATGGTAAAAATACCCAAAAGAAGAAGATTGACGGGATGCCTCTGATAGTAA
CAAGAXXXXXXXXXXAAAGGCGTAAGAATGAGAAGGATGTATAAAGCCAAGCCCGCCCCAGTTGAGCTAAAGAAA
GTAGAAATTGGTCGGACGTA
>Marker943459
ACTCTTCTGCCTTTCTTTTTTATGTGGAATCAAACCCAAAATCACTTCTAAGATTCTTCTTCCTTTGCTTTCCTT
TGATCXXXXXXXXXXCAATTTTCCACTATCATGTTTGTTTTCTTGTAGTAATGTCATTTCTATCAATTACCGAAC
ACAAACTTAGAGATAAGAGT
ACTCTTCTGCCTTTCTTTTTTATGTGGAATCAAACCCAAAATCACTTCTAAGATTCTTCTTCCTTTGCTTTCCTT
TGATCXXXXXXXXXXCAATTTTTCACTATCATGTTTGTTTTCTTGTGTTAATGTCATTTCTATCAATTACCGAAC
ACAAACTTAGAGATAAGAGT
>Marker943571
CACACTTTTCTGACCAAACAAACTTACCATTCTTATCCTCAATAAAATCTCTCCAATTCAATATCCAATTAGATA
TTGTTXXXXXXXXXXTGGCTCACAAAATTGACTTTCTATATTTATTTTAATGTTTCAATAATATGATATTTTAGT
GTGACAGTGTTAAACATAGT
CACACTTTTCTGACCAAACAAACTTACCATTCTTATCCTCAATAAAATCTCTCCAATTCAATATCCAATTAGATA
TTGTTXXXXXXXXXXTGGCTCACAAAATTGACTTTCTATATTTATTTTAATGTTCCAATAATATGATATTTTAGT
GTGACAGTGTTAAACATAGT
>Marker943594
CACCAAGTTCTTTAACCAAAAAAAAAAAGAAAAGAAAAGAAAACATCTGGTAGTCAAATTAGGGAAGCCATAGAA
ATAGTXXXXXXXXXXAAAATGGTGAGTATAAAAATACCCTACAAAGATGCACTACTAGTCTCGCTATGTGAATCG
TTGGCTTTATATTAGGAGGT
CACCAAGTTCTTTAACCAAAAAAAAAAAGAAAAGAAAAGAAAACATCTGGTAGTCAAATTAGGGAAGCCATAGAA
ATAGTXXXXXXXXXXAAAATAGTGAGTATAAAAATACCCTACAAAGATGCACTACTAGTCTCGCTATGTGAATCG
TTGGCTTTATATTAGGAGGT
>Marker943655
AACTCTCAAACGGAATGATGATGTGACTTTTACATGTGAATGACTAGTTAGACAGTCAAATAAGATGATAACTTT
TAAAAXXXXXXXXXXTTGAAGTATTGGCATTTTACTTAAATAAATTGTGCTTGTATTTACTTTTTTAATTATTGT
TTTACATTGTCGTGGATGTT
AACTCTCAAACGAAATGATGATGTGACTTTTACATGTGAATGACTAGTTAGACAGTCAAATAAGATGATAACTTT
TAAAAXXXXXXXXXXTTGAAGTATTGACATTTTACTTAAATAAATTGTGCTTGTATTTACTTTTTTAATTATTGT
TTTACATTGTCGTGGATGTT
>Marker944450

Create PDF with GO2PDF for free, if you wish to

TACAGAAGCATGCAGGAAGATGTAAGAGCCTCTTTGGAACACGTTACATCTAAGATCTCGGTCACTCTTGATTTC
TGGAAXXXXXXXXXXXCCTGTGGAGGTCTAGAGATCTTTCACTCTATTGTGAAAGTTCTTAAGATGTATAACATAG
AAAGTAGAATTCTTTCATGT
TACAGAAGCATGCAGGAAGATGTAAGAGCCTCTTTGGAACACGTTTCATCTAAGATCTCGGTCACTCTTGATTTC
TGGAAXXXXXXXXXXXCCTGTGGAGGTCTAGAGATCTTTCACTCTATTGTGAAAGTTCTTAAGATGTATAACATAG
AAAGTAGAATTCTTTCATGT
>Marker944695
ACCTTGTGCATGCTTCTGAAACTAAATGTTCATTGCATGACAGCATATTGGGACGATGTGAAAGGAGAAGTTGTT
TTCTGXXXXXXXXXXXTTTAAACTTCTATAGGGCTTAATGACTCCAATTGTGAGGAATGCCGATTTGAAGACCATA
TCGGCAATTTCTTCAGAGGT
ACCTTGTGCATGCTTCTGAAACTAAATGTTCATTGCATGACAGCATATTGGGACGATGTGAAAGGAGAAGTTGTT
TTCTGXXXXXXXXXXXTTTAAACTTCTGTAGGGCTTAATGACTCCAATTGTGAGGAATGCCGATTTGAAGACCATA
TCGGCAATTTCTTCAGAGGT
>Marker945474
GACCGGTCAATTCCAAAGGTTAATTCTACACTATACTATTCAACACACTCCATAATACTAAACCTGATAGTGAGA
AAGAAXXXXXXXXXXXAACAATAAGAAAAGGAGTAAGGGAAAAGGTCCAAGCAATACCGACCTCAGGTTTGGTAAT
ATTGTTAGTGATGTTTTGTC
GACCGGTCAATTCCCAAGGTTAATTCTACACTATACTATTCAACACACTCCATAATACTAAACTTGATAGTGAGA
AAGAAXXXXXXXXXXXAACAATAAGAAAAGGAGTAAGGGAAAAGGTCCAAGCAATACCGACCTCAGGTTTGGTAAT
ATTGTTAGTGATGTTTTGTC
>Marker945545
GACATTATTTTAGTATTCTAATTATAATGGTTAATCTTATTCTTCGTTTGTTGGCTTGAAATATATACGAAAAAG
AACCAXXXXXXXXXXXCTTAAACTAATAGGTTAGGATAAATTTAATTATATTAGGTTAAGAATTTCTTAAACAAAT
ACTTAATTCCCGATATTGTT
GACATTATTTTAGTATTCTAATTATAATGATTAATCTTATTCTTCGTTTGTTGGCTTGAAATATATACGAAAAAG
AACCAXXXXXXXXXXXCTTAAACTAATAGATTAGGATAAATTTAATTATATTAGGTTAAGAATTTCTTAAACAAAT
ACTTAATTCCCGATATTGTT
>Marker945800
GACTTTCTTAGTCGCCTCACCACCTAGCTGCATCTTTATCACAGCAGCTCCTACATACAACTTTTTCTTCCTACT
TCAAAXXXXXXXXXXXATCTCGTCAATAAATAGCAATTTCTCCTTACTAAAGAAATTATAATAATGAGATAGCTCA
TTAAAAAATAGCCTATGGTC
GACTTTCTTAGTCGCCTCACCACCTAGCTGCATCTTTATCACAGCAGCTCCTACATACAACTTTTTCTTCCTACT
TCAAAXXXXXXXXXXXATCTCGTCAATAAATAGCAATTTCTCCTTACTAAAGAAATCATAATAATGAGATAGCTCA
TTAAAAAATAGCCTATGGTC
>Marker945922
TACATAAAAACATAATGGTTGGAGACAGTGTAGTAGAGAAAGAGGACCAGAATTGAAAAGAATGTTAGGGATGGA
AGAGAXXXXXXXXXXXCACTTCCCATTATGGCATGAACCGAGTTCTTTCTCTCTACACGCATCAAAGGGCCCTTGG
ATATTCTCAAAATCTCAAGT
TACATAAAAACATAATGGTTGGAGACAGTGTAGTAGAGAAAGAGGACCAGAATTGAAAAGAATGTTAGGGATGGA
AGAGAXXXXXXXXXXXCACTTCCCATTATGGCATGAGCCGAGTTCTTTCTCTCTACACGCATCAAAGGGCCCTTGG
ATATTCTCAAAATCTCAAGT
>Marker946492
CACCGTGAATTAAATCTCATTTGGAAGGTGGAATTTCTTTGTTCGTTGGTTATATAGTCTTCAAAATCGAACCTC
TGATTXXXXXXXXXXXTTTTTGTTGGGCCTTATTAAAGACTTTTTGTTGTTTCCTGTAGCTAACTCAATAGATCGA
GGCCGGAGACTACATCTTGT

Create PDF with GO2PDF for free, if you wish to

CACCGTGAATTAAATCTCATTTGGAAGGTGGAATTTCTTTGTTCGTTGGTTATATTGTCTTCAAAATCGAACCTC
TGATTXXXXXXXXXXXTTTTTGTTGGGCCTTATTAAAGACTTTTTGTTGTTTCCTGTAGCTAACTCAATAGATCGA
GGCCGGAGACTACATCTTGT
>Marker946564
AACCTTGTCATTGCACTCTTCAGTTTGCGCACTCGGTCCAAGTTACGACTGCTAATCTGCATACAAATGCCAAAT
TTATTXXXXXXXXXXXGAGAACAATTATTTCCATTGATGTGAAGTCTTTTGGAGTGAAACCAAAAAGAAAATCCAG
GAGGGTATATGTCCAAAGTG
AACCTTGTCATTGCACTCTTCAGTTTGCGCACTCGGTCCAAGTTACGACTGCTAATCTGCATACAAATGCCAAAT
TTATTXXXXXXXXXXXGAGAACAATTATTTCCATTGATGTGAAGTCTTTTGGAGTGAAACCAAAAAGAAAATCTAG
GAGGGTATATGTCCAAAGTG
>Marker947710
ACATTCCGCAGTCTGTAAGGTTTGAAGATGAGAGTGAGACCCAACATTAACATAGACGGAAATATAACAATAATA
AAATGXXXXXXXXXXXAAACATCACCTGTATATTTGTCATCTATTGTGATATTTATTAATTGATTAATATTTTACT
TCATGCCGCTACATCAAGTA
ACATTCCGCAGTCTGTAAGTTTTGAAGATGAGAGTGAGACCCAACATTAACATAGACGGAAATACAACAATAACA
ATAATXXXXXXXXXXXAAACATCACCTGTATATTTGTCATCTATTGTGATATTTATTAATTGATTAATATTTTACT
TCATGCCGCTACATCAAGTA
>Marker948275
ACATTCATGAAACAATACTACTCATGGAATTGAGAGGATACAACCCTACTCATATTGTCACAACCATTTGTTCCA
ACAGTXXXXXXXXXXXCTTAATGATCTTCAAATGTATGTCAGGTTACCGTTAGTGGTTATGCCGTGGCAGGAGGTG
GGCGGGGAATAGAGAGAGTG
ACATTCATGAAACAATACTACTCATGGAATTGAGAGGATACAACCCTACTCATATTGTCACAACCATTTGTTCCA
ACAGTXXXXXXXXXXXCTTAATGATCTTCAAATGTATGTCAGGTTACCGTTAGTGGTTATGCCGTGGCAGGAGGTG
GGCGGGGAATAGAGAGAGTA
>Marker948282
ACAAGTCATTACTTGATAGGCAATTCAAGATGGTTCCAGCGACTTGCAATGTGTTGTTAGAGGTTTTGCTTAAGC
ATGAAXXXXXXXXXXXGAAGGTGGGAACTCAACCAAAGTCAAGGCCTTTTGCTATGGACGTTGCAGGGTATAATAA
TATCATTGCAAGGTTTTGTG
ACAAGTCGTTACTTGATAGGCAATTCAAGATGGTTCCAGCGACTTGCAATGTGTTGTTAGAGGTTTTGCTTAAGC
ATGAAXXXXXXXXXXXGAAGGTGGGAACTCAACCAAAGTCAAGGCCTTTTGCTATGGACGTTGCAGGGTATAATAA
TATCATTGCAAGGTTTTGTG
>Marker949256
GACGAATAAGTGCTCTATTGATAAAGGCAATGACAAGTATTATTATATGTTGATTCCTTTCAATCATGGAAAAAA
AGAATXXXXXXXXXXXTCTTGAGTCTATTTTAATTTTAGTCGGGACTTATTGTTACATGGAGCAATTAGGAGAAAC
TATCTTTTGCAGATCTAGTT
GACGAATAAGTGCTCTATTGATAAAGGCAATGACAAGTATTATTATATGTTGATTCCTTTCAATCATGGAAAAAA
AGAATXXXXXXXXXXXTGTTGAGTCTATTTTAATTTTAGTCGGGACTTATTGTTACATGGAGCAATTAGGAGAAAC
TATCTTTTGCAGATCTAGTT
>Marker949328
AACAAATTTAATGTGGAAAACTATAATAGAAAAAATGGGATGAATTTAAAGAAAACAAATAAAATAAAGATAGTT
ACAACXXXXXXXXXXXTAGATACGAAAATAAAGAAAAATCTTTATAGTTAAATTGTCTATGTTTGTATTTAATGAA
AACCTTGTTACATTATGGTG
AACAAATTTAATGTGGAAAACTATAATAGAAAAAATGGGATGAATTTAAAGAAAACAAATAAAATAAAGATAGTT
ACAACXXXXXXXXXXXTAGATACGAAAATAAAGAAAAATCTTTATAGTTAAATTGTCTATGTTTGTATTTAATGAA
AACCTTGTGACATTATGGTG
>Marker949757

Create PDF with GO2PDF for free, if you wish to

AACATGCTTCAGGGATGCAGAAGTGTTGATGAGTTTGAGAGACTAAACAAGATCGACGAAGGCACCTATGGCATT
GTGTTXXXXXXXXXXATTCCATCACCCATCCATTGTTGATGTGAAGGAAGTTGTGGTGGGGAATAGTCTTGATAG
CATTTTTATGGCCATGGAGT
AACATGCTTCAGGGATGCAGAAGTGTTGATGAGTTTGAGAGACTAAACAAGATCGACGAAGGCACCTATGGCATT
GTGTTXXXXXXXXXXATTCCATCACCCATCCATTGTTGATGTGAAGGAAGTTGTGGTGGGTAATAGTCTTGATAG
CATTTTTATGGCCATGGAGT
>Marker950067
TACTAGTAAAAGGAAATAGCCCAACCTAAATCAAACTACAAAATAAAAGAGAAATAAGTTCCTAATTTGGCAATT
TCAAAXXXXXXXXXXATCTCATAACTCATTCAGAGTAAAACTGGAGGCCCTATATTTGCTGGACCCTGCATATCT
ACTTCGATTCCTTCGCAGGT
TACTAGTAAAAGGAAATAGCCCAACCTAAATCAAACTACAAAATAAAAGAGAAATAAGTTCCTAATTTGGCAATT
TCAAAXXXXXXXXXXATCTCATAACTCATTCAGAGTAAAACTGGAGGCCCTATATTTGCTGGACCCTGCATATCT
ACTTCCATTCCTTCGCAGGT
>Marker950165
AACATTTTCAATTTCCATCATAATATATATGAAGATACCTTCATAATAAATTTACAGAGACGAAGCAACATGATT
GAGATXXXXXXXXXXCAGTTCCGGAATAGTATCGAATACTTCTAAGTTAGTGAAAACACTTTTCAAATTTATCAT
AATCTTTAGGCTAAAAAGTA
AACATTTTCAATTTCCATCATAATATATATGAAGATACCTTCATAATAAATTTACAGAGACGAAGCAACATGATT
GAGATXXXXXXXXXXCAGTTCCGGAATAGTATCGAATACTTCTAAGTTAGTGAAAACACTTTTCAAGTTTATCAT
AATCTTTAGGCTAAAAAGTA
>Marker950420
AACTCCATGTTTCAGCGGGTGAATAATGTATTTACTGGCCTCTCTTATTCACAAATTCTCTGCATGGAATCAATC
TAATCXXXXXXXXXXTACTTGAGACCTAATCCTGTTGTGTTTTCTATTTTATGCCATTTGCTGAAAACTGGCTGG
TTTCTCTTGGGACCCATGTC
AACTCCATGTTTCAGCGGGTGAATAATGTATTTACTGGCCTCTCTTATTCACAAATTCTCTGCATGGAATCAATC
TAATCXXXXXXXXXXTACTTGAGACCCAATCCTGTTGTGTTTTCTATTTTATGCCATTTGCTGAAAACTGGCTGG
TTTCTCTTGGGACCCATGTC
>Marker950565
CACGTCCTTGAGCGTTTAAATTTCTTCTAATTAATTAATGAAAACTTTGTATCTTGCTTAAAAAAAGACTATACA
TTCATXXXXXXXXXXTAATGCTAATATCTAGGAGACCTGCAAAACTGGTTCAATTAAATAAAGATAGCAAGACAA
TTTACAATTTACTGTTTGTT
CACATCCTTGAGCGTTTAAATTTCTTCTAATTAATTAATGAAAACTTTGTATCTTGCTTAAAAAAAGACTATACA
TTCATXXXXXXXXXXTAATGCTAATATCTAGGAGACCTGCAAAACTGGTTCAATTAAATAAAGATAGCAAGACAA
TTTACAATTTACTGTTTGTT
>Marker951177
AACGATTTCAATGACCTCCAAAGAATTTGAATCAATCCTTATTTTCCACTTGTTGAGTGTCGTCGTATAACGTAA
TTCATXXXXXXXXXXTAAGAAAGGTGGTGAAACAGATATATTAAATTAATGAACGAGAATATAAGCAGACAAATA
AATAAGCCAAATGCATAGTT
AACGATTTCAATGACCTCCAAAGAATATGAATCAATCCTTATTTTCCACTTGTTGAGTGTCGTCGTATAACGTAA
TTCATXXXXXXXXXXTAAGAAAGGTGGTGAAACAGATATATTAAATTAATGAACGAGAATATAAGCAGACAAATA
AAAAACCCAAATGCATAGTT
>Marker951516
ACTTGATGTTGAACGACCCGTTTAATAACGCAGGAGGATTGCCTTCGATGGGTGACACTTTAGGCTATTCTCATT
ACATAXXXXXXXXXXCTATATCAATCGCGTGCCCCAAGAGGGGACATTCCTATCTTACTATATGTCGTTCGATTC
AGCTGCATCATCAGTGTGTT

Create PDF with GO2PDF for free, if you wish to

ACTTGAAGTTGAACGACCCGTTTAATAACGCAGGAGGATTGCCTTCGATGGGTGACACTTTAGGCTATTCTCATT
ACATAXXXXXXXXXXXCTATATCAATCGCGTGCCCCAAGAGGGGACATTCCTATCTTACTATATGTCGTTCGGTTC
AGCTGCATCATCAGTGTGTT
>Marker953875
CACACTTCTAATGGATAGTCCTCCAGTCTTCATACTCCTAACTACGGGATAAAGACAGGTCTATTCTTGCCTTCT
AATGGXXXXXXXXXXXGACGAAGAGGTATCCCCCAGAAGGAAGTGTGGTATTCAATTCCCTTTTATTCAAGAAAAG
CAAGAAAGCCTATGGTTGGT
CACACTTCTAATGGATAGTCCTCCAGTCTTCATACTCCTAACTACGGGATAAAGACAGGTCTATTCTTGCCTTCT
AATGGXXXXXXXXXXXGACAAAGAGGTATCCCCCAGAAGGAAGTGTGGTATTCAATTCCCTTTTATTCAAGAAAAG
CAAGAAAGCCTATGGTTGGT
>Marker954192
ACTTAAAATTTTCAAAACAGTTCGCAATGACCCAAAATCATGTCATGACATTGCCTGACGCATATGGGTGGGCGG
CAGGGXXXXXXXXXXXTCTTGGTCGTGTGTCTGGCGTCTTACGCAACCTTCCTCTCTTGGTCGTGCATCTCGCGCA
TAGGTCATGTGTCCAATGTC
ACTTAAAATTTTCAAAACAGTTCGCAATGACCCAAAGTCATGTCATGACATTGCCTGACGCATATGGGTGGGCGG
CAGGGXXXXXXXXXXXTCTTGGTCGTGTGTCCGGCGTCTTACGCAACCTTCCTCTCTTGGTCGTGCATCTCGCGCA
TAGGTCATGTGTCCAATGTC
>Marker954519
AACTCCTGTATTTTCAAAATTCTCATCCCAAGTAGTATTGATGAAAAAGCAGTCGATTAGCGATCTTGCTAAGGA
AGAACXXXXXXXXXXXTCACTTCCTACACACCATGCCCCAATACAATAATTTGACAAGGAAAGGAGCTCGGGGCAA
ACAAATTTTCTTTTCTTGTA
AACTCCTGTATTTTCAAAATTCTCATCCCGAGTAGTATTGATGAAAAAGCAGTCGATTAGCGATCTTGCTAAGAA
AGAACXXXXXXXXXXXTCACTTCCTACACACCATGCCCCAATACAATAATTTGACAAGGAAAGGAGCTCGGGGCAA
ACAAATTTTCTTTTCTTGTA
>Marker954724
AACATGTTTAAATTATGAACATAATCACACATTTACATATTTAGATAATGGTAAAGTTCCAATATAATTTTGGCA
TGTTTXXXXXXXXXXXTTGGTCAAATAATCTTTGTAGGATGATATTAAAAAAATACCAATTATGGATAAATGCAAA
TGTGTTTTCCACCAAGGAGT
AACATGTTTAAATTATGAACATAATCACACATTTACATATTTAGATAATGGTAAAGTTCCAATATAATTTTGGCA
TTTTTXXXXXXXXXXXTTGGTCAAATAATCTTTGTAGGATGATATTAAAAAAATACCAATTATGGATAAATGCAAA
TGTGTTTTCCACCAAGGAGT
>Marker954810
AACAAATTATCCAATTTTATAACCTTATCTAAAACCTACTCCCTTTCAAACATTAGAGTTTAAATTTTGAATCTT
TCTTTXXXXXXXXXXXCAAATTTTCTCTCATTGGAGGTAAGCTTGACTATTTGCTATTTGTCTCTATCAATTATAT
GATTTTTAGGAGCATTTGTT
AACAAATTATCCAATTTCATAACCTTATCTAAAACCTACTCCCTTTCAAACATTAGAGTTTAAATTTTGAATTTT
TCTTTXXXXXXXXXXXCAAATTTTCTCTCATTGGAGGTAAGCTTGACTATTTGCTATTTGTCTCTATCGATTATAT
GATTTTTAGGAGCATTTGTT
>Marker955112
CACCTCCATCCATCCACAATGGTCCAAAATCCTTTGAAAAGGAAAGGCTTCCTAAGCTTATTTTGGGCAGATGTA
GATGGXXXXXXXXXXXTGTAATAGATATCTCCTTGAACTGAATTAAAATATGGTAAAATCAATCTATAAGCCACAT
AAGTTTTAACCAACAAAGTT
CACCTCCATCCATCCACAATTGTCCAAAATCCTTTGAAAAGGAAAGGCTTCCTAAGCTTATTTTGGGCAGATGTA
GATGGXXXXXXXXXXXTGTAATAGATATCTCCTTGAACTGAATTAAAATATGGTAAAATCAATCTATAAGCCACAT
AAGTTTTAACCAACAAAGTT
>Marker955654

Create PDF with GO2PDF for free, if you wish to

AACAATCTACTTCAAAAGCTACTAATAAAACATTGAAATTGAGAGTTTCATAAATGTCATTGAATTTAGAAACAA
GGGCAXXXXXXXXXXTTATGAACTTGAGTTTGCATTTTGGTCACATTTAGGGAGCTAGCTATTTTTATCTATGTT
TATTTAGACCATATGTTGTG
AACAATCTACTTCAAAAGATATTAATAAAACATTGAAATTGAGAGTTTCATAAATGTCATTGAATTTAGAAACAA
GGGCAXXXXXXXXXXTTATGAACTTGAGTTTGCATTTTGGTCACATTTAGGGAGCTAGCTATTTTTATCTATGTT
TATTTAGACCATATGTTGTG
>Marker956175
CACACACACTAATTGATTATTTAATTTCTCCGGAAAAGAGTGTCCTTTTAGCTTCCCATTTATATAAACATCACC
AAAGAXXXXXXXXXXTCTCATCAAACATCCTCTCCATAGAATCCTTCACAACCTCTCCAAAAGCTACGGACATGT
CTTCTCTCTTCGATTTGGTT
CACACACAGTAATTGATTATTTAATTTCTCCGGAAAAGAGTGTCCTTTTAGCTTCCCATTTATATAAACATCACC
AAAGAXXXXXXXXXXTCTCATCAAACATCCTCTCCATAGAATCCTTCACAACCTCTCCAAAAGCTACGGACATGT
CTTCTCTCTTCGATTTGGTT
>Marker956864
ACTTAGAAATATTTAGACCGATTCTACTGTATTTTTTGCAATTTGAACTGTGAGTTATGTTATAGAAGATGTGTT
GGGATXXXXXXXXXXTTTCAATTTTATTACAATTTAGTCCCTAAACCTTACTGCATAAAAATTTAAACAATTTAG
TTCCTACGGCAGAAATTAGT
ACTTAGAAATATTTAGACCGATTCTACTGTATTTTTTGCAATTTGAACTGTGAGTTATGTTATAGAAGATGTGTT
GGGATXXXXXXXXXXTTTCAATTTTATTACAATTTAGTCCCTAAACCTTACTGCATAACAATTTAAACAATTTAG
TTCCTACGGCAGAAATTAGT
>Marker957015
AACATATCATATCCATTATGATCAATATATATCTTCTCAAGTTCAAACAATTCATCATTTAAAGCATCTCTCAAC
CAATGXXXXXXXXXXAGTTGTAGAAAGTGCAACACACTTCTGCAACCTTGACTACCAAGGCCCTAACCACTTGTA
AATGTCATCAACCAAATGTA
AACATATCATATCCATTATGATCAATATATATCTTCTCAAGTTCAAACAATTCATCATTTAAAGCATCTCTCAAC
CAATGXXXXXXXXXXAGTTGTAGAAAGTGCAACACACTTCTGCAACCTTGACTACCAAGGCCCTAACCACCTGTA
AATGTCATCAACCAAATGTA
>Marker957075
GACTAGGGAGGTATTAATCAACGATTATTATGTATAAACTATTCAAACAAAGATAGCACATAAATCATCTAACAT
AAGATXXXXXXXXXXAATACGTCTGGAATATATAAGATAAAATCTGCCTGTAGTCGCCAGTCTTAATACCAGTAT
TGGGATCAATAAGGTAAGGT
GACTAGGGAGGTATTAATCAACGATTATTATGTATAAACTATTCAAACAAAGATAGCACATAAATCATCTAACAT
AAGATXXXXXXXXXXAATATGTCTGGAATATATAAGATAAAATCTGCCTGTAGTCGCCAGTCTTAATACCAGTAT
TGGGATCAATAAGGTAAGGT
>Marker957716
ACTTCTGTTTTCAATTTGATACATTCTATCTTGATTCATTTAATCTGCTGCTACAACTTTACTTGCACAATGTTG
TTTGTXXXXXXXXXXAATGTTTTTTTTCTTTAGAAAAAGACGAAACTTTTCATTAAATGAATGTAAATAGACTAA
TGCTCTGAGATACAAAAGTA
ACTTCTGTTTTCAATTTGATACATTCTATCTTGATTCATTTAATCTGCTGCTACAACTTTACTTGCACAATGTTG
TTTGTXXXXXXXXXXATGTTTTTTTTTCTTTAGAAAAAGACGAAACTTTTCATTAAATGAATGTAAATAGACTAA
TGCTCTGAGATACAAAAGTA
>Marker957867
AACTTCAATGTGCGTTGCCCTATCATGTAGAACTGGATTATGAGTTAGCAATAGCAGCCTTATTATCACAATAAA
CTCGTXXXXXXXXXXTTTTGTTTTTTACTACATCGTGTAACTAAATTTCCTCCAACAAATGAACAATAGCTAGAA
GTAAATCTTTTATCAGTAGT

Create PDF with GO2PDF for free, if you wish to

AACTTCAATGTGCGTTGCCCTATCATGTAGAACTGGATTATGAGTTAGCAATAGCAGCCTTATTATCACAATAAA
CTCGTXXXXXXXXXXTTTTGTTTTTTACTACGTCGTGTAACTAAATTTCCTCCAACAAATGAACAATAGCTAGAA
GTAAATCTTTTATCAGTAGT
>Marker957978
AACTCATAGATACGATAATTAGCAAGGAGCACATATAATTTTACTCTAATTCTAAATGTGCTTCCAGTTCATTGC
GAGGTXXXXXXXXXXCTATCAATAAATTATTTGACGTCAGTATTTATATAGAATATCCAGTTAAGAAAATGAGAA
GTTGAACAACAAACAAGGTG
AACTCATAGATACGATAATTAGCAAGGAGCACATATAATTTTACTCTAATTCTAAATGTGCTTCCAGTTCATTGC
GAGGTXXXXXXXXXXCTATCAATAAATTATTTGACGTCAGTATTTATACAGAATATCCAGTTAAGAAAATGAGAA
GTTGAACAACAAACAAGGTG
>Marker958864
ACTCTGGCAAGCCACCAAGAAATGCTTTTCATCCCATATTTTGAACTCGTATTTTCTTTGAATAGCATGTCTTTA
ATCCTXXXXXXXXXXATATTGAATTTATGTCAATTCAGAAGTTGTTTGGTCCTACCTGAATATTAGTGAATTTGC
CAAATAAATCGGACCCGGTA
ACTCTGGCAAGCCACCAAGAAATGCTTTTCATCCCATATTTTGAACTCGTATTTTCTTTGAATAGCATGTCTTTA
ATCCTXXXXXXXXXXATATTGAATTTATGTCAATTCATAAGTTGTTTGGTCCTACCTGAATATTAGTGAATTTGC
CAAATAAATCGGACCCGGTA
>Marker959409
ACCGTCAATACCCGCCGCCGCATCAGCTTAATGGCCAGCAGCACCAGCAGCCACACCAGGGATCCACCAGCGAGA
ACAAGXXXXXXXXXXTAGAATATTAATGCATACCAATTCATTTATTGACATTTTATTGATATCTACTGTTCTGGA
TTTGTTTGGCTAATTTTGTT
ACCGTCAATACCCGCCGCCGCATCAGCTTAATGGCCAGCAGCACCAGCAGCCACACCAGGGATCCACCAGCGAGA
ACAAGXXXXXXXXXXTAGAATATTAATGCATACCAATTCATTTATTGGCATTTTATTGATATCTACTGTTCTGGA
TTTGTTTGGCTAATTTTGTT
>Marker959943
TACCTTTTGCTTTGTAAGTCTTTCGCCGTCTCTTGTTTCTCCTTTATCTCATCTTTCGGTATTTTCTTACATTGC
ATCCGXXXXXXXXXXCGAGCACTGGAGTCGAGGTTCAATCTCACAGTGTTGCTTGTGTTTCTCTTTGTATTCGTT
CTAAATTCCTTTTGTTTGTA
TACCTTTTGCTTTGTAAGTCTTTCGCCGTCTCTTGTTTCTCCTTTATCTCATCTTTCGGTATTTTCTTACATTGC
ATCCGXXXXXXXXXXCGAGCACTGGAGTCGAGGTTCAATCTCACAGTGTTGCTTGTGTTTCTCTTTGTATTCGTT
CTAAATTCCTCGTGTTTGTA
>Marker960129
TACAATCTTAGTAACCAAACCTAAAAGCCCATGAGAAAATTATTCTTTGTGATTGTAATAATTTTTTTTTATCCA
TGGTTXXXXXXXXXXAATGTTTATAGCCTTTTGATGTATATACTGTTCTCAATTATGATGATAATTGGATTATTG
TATTTATGTTTTATGTAGTT
TACAATCTTAGTAACCAAACGTAAAAGCCCATGAGAAAATTATTCTTTGTGATTGTAATAATTTTTTTTTATCCA
TGGTTXXXXXXXXXXAATGTTTATAGCCTTTTGATGTATATACTGTTCTCAATTATGATGATAATTGGATTATTG
TATTTATGTTTTATGTAGTT
>Marker960518
CACCCATGTGTCTAATTTTATTTTAATTTAGATAAGAATCATATCTTTGATAAAAATGAATTAAGGGGAAACCCC
AACACXXXXXXXXXXCTCTGAAAAAACGTCCGTTGCGCTCGCCTCAAAAGTCCAAAAGAAAGAACACATAATTGC
CAACCAACCCATTTTTCAGT
CACCCATGTGTCTAATTTTATTTTAATTTAGATAAGAATCATATCTTTGATAAAAATGAATTAAGGGGAAACCCC
AACACXXXXXXXXXXCTCTGAAAAAACGTCCGTTGCGCTCGCCTCAAAAGTCCAAAAGAAAGCACACATAATTGC
CAACCAACCCATTTTTCAGT
>Marker961440

Create PDF with GO2PDF for free, if you wish to

AACACGTGAGATTCAAACACAAAAGTTCTCATACTTTAAAAACATTTCAACCAGAGTTTCTAAACTTTATTTTGT
AGATCXXXXXXXXXXACAGAACTTTTAAGAATCTATTTGATACTTTAAAAAATTCATAAATTATAAAATAAAAAA
CAAAAGTTAAAAGAAACGTT
AACACGTGAGATTCAAACACAAAAGTTCTCATACTTTAAAAACATTTCAACCAGAGTTTCTAAACTTTATTTCGT
AGATCXXXXXXXXXXACAGAACTTTTAAGAATCTATTTGATACTTTAAAAAATTCATAAATTATAAAATAAAAAA
CAAAAGTTAAAAGAAACGTT
>Marker962390
ACAGTGACACAATCTTTGCGGTTAGGATTGAACATCTACTTCCCTTCAAGAATATTCATAGTATGGAAGGTGAT
TTGGAXXXXXXXXXXAAATAAAGCAATTCGTATTAAAAAATAAAGTGATTCATGTTTAATAAAAAACTTCACATA
TTTAGTGATTGGAAGAAGTG
ACAGTGACACAATCTTTGCGGTTAGGATTGAACATCTACTTCCCTTCAAGAATATTCATAGCATGGAAGGTGAT
TTGGAXXXXXXXXXXAAATAAAGCAATTCGTATTAAAAAATAAAGTGATTCATGTTTAATAAAAAACTTCACATA
TTTAGTGATTGGAAGAAGTG
>Marker962710
ACGTATATTCAACGACCACTTTTTAATTATAAAAGAAAAGAATGATTAAATTGTTGGGTTTTGCAATATAATTAT
TATTAXXXXXXXXXXGAAGCTCAAAATGGACGGCTATGATTCTTCTCATCTTTGTCATACCTCTTGGGTCACTTC
CATGGGCTGCCCAGCAGGTG
ACGTATATTCAACGACCACTTTTAAATTATAAAAGAAAAGAATGATTAAATTGTTGGGTTTTGCAATATAATTAT
TATTAXXXXXXXXXXGAAGCTCAAAATGGACGGCTATGATTCTTCTCATCTTTGTCATACCTCTTGGGTCACTTC
CATGGGCTGCCCAGCAGGTG
>Marker963376
AACTATCTGGATCCTGTTTTTAACATTTTTGTTTTAGAATGGACAATTGATTAATTAATTATTATTTTCTTAAAA
AAAATXXXXXXXXXXGTAATTTACAACAATGAGTAATTAATTCGTTACTTTATGTTAGTGATGCTGCTGAATTAT
CAAACAAATTTCCGACAGTT
AACTATCTGGATCCTGTTTTTAACATTTTTGTTTTAGAATGGACAATCGATTAATTAATTATTATTTTCTTAAAA
AAAATXXXXXXXXXXGTAATTTACAACAATGAGTAATTAATTCGTTACTTTATGTTAGTGATGCTGCTGAATTAT
CAAACAAATTTCCGACAGTT
>Marker963381
AACATGTTATCCTATTGTGGAAGATAAATATTTTATTCTTCATAAACCCGAGAAATACTAGAAATGATTTGTCTT
GAAGCXXXXXXXXXXAAAAAAAAAGCGGCATAGGTCAAGAAAGCCAGTTCATAAATAAGTTCTGATGCTCGTGTT
GAGAGAGATGGAAGAAATGT
AACATGTTATCCTATTGTGGAAGATAAATATTTTATTCTTCATAAACCCGAGAAATACTAGCAATGATTTGTCTT
GAAGCXXXXXXXXXXAAAAAAAAAGCGGCATAGGTCAAGAAAGCCAGTTCATAAATAAGTTCTGATGCTCGTGTT
GAGAGAGATGGAAGAAATGT
>Marker964090
GACTCCTATTAGTGATGTGCTCTACCACTAATAGACTCCAAGAGTTAGATATAAATTTTGTTATATTTGCAAATT
CTTTTXXXXXXXXXXGCTATTTCTTTGAAATTTGACTAAAATGAAATCTTGTAATATAATATAGGCCTTTCAATT
TATAGGATTTAGACTTTGTT
GACTCCTATTAGTGATGTGCTCTACCACTAATAGACTCCAAGAGTTAGATATAAATTTTGTTATATTTGTAAATT
CTTTTXXXXXXXXXXGCTATTTCTTTGAAATTTGACTAAAATGAAATCTTGTAATATAATATAGGCCTTTTAATT
TATAGGATTTAGACTTTGTT
>Marker964869
AACTTTCAAGAGATCTGGGTTATTGACAAAAACTGAATGCTTGATGGTGTTATTTCGAAAAGAGTCAAAGGGGGA
ATTTTXXXXXXXXXXATATTCTTTCTTTCACCCGAGGAGGGCCATGTTACACATTCTACAAACCAATCTTTGGAT
GAATGGATCTTGGCAAGGTG

Create PDF with GO2PDF for free, if you wish to

AACTTTCAAGAGATCTGGGTTATTGACAAAAACTGAATGCTTGATGGTGTTATTTCGAAAAGAGTCAAAGGGGGA
ATTTTXXXXXXXXXXXATATTCTTTCTTTCACCCGAGGAGGGCCATATTACACATTCTACAAACCAATCTTTGGAT
GAATGGATCTTGGCAAGGTG
>Marker965203
AACTTGCTATTTGTGTCCAGGTATGTTATTCTAAATATATAAAGCCTTCTGTCCCAGTTCTCAAACAAGTTATAT
AATTAXXXXXXXXXXXAAAAATGATTAAGTTGATAAACATCAAATGTTGGGAAGGTAAAATCATGACATATGTGTA
AAGAAGCCAAATAAATGGTG
AACTTGCTATTTGTGTCCAGGTATGTTATTCTAAATATATAAAGCCTTCTGTCCCAGTTCTCAAACAAGTTATAT
AATTAXXXXXXXXXXXAAAAATCATTAAGTTGATAAACATCAAATGTTGGGAAGGTAAAATCATGACATATGTGTA
AAGAAGCCAAATAAATGGTG
>Marker965278
AACCAAGTCGTAGTTCTTCTCTGTTTTATTTACATTTCCATCCATTGTTCATATCACTTGTATTAGATATATTAG
ATTATXXXXXXXXXXXCTAAAGATGTAATTATTTTGTTTGACTTGCGGTGAAGCTACGCTTAACACATCAAAATAT
TAAGTTAGGTGATGGTAGTT
AACCAAGTCGTAGTTCTTCTCTGTTTTATTTACATTTCCATCCATTGTTCATATCACTTGTATTAGATATATTAG
ATTATXXXXXXXXXXXCTAAAGATGTAATTATTTTGTTTGACTTGCGGTGAAGCTACGCTTAACACATCAAAAGAT
TAAGTTAGGTGATGGTAGTT
>Marker965557
CACATGGATTTATGAGATGGAATAAATAGTAACAACTATCTTTACTTTCTGTATAGTTGTAAATAATAAAATAAA
ACATAXXXXXXXXXXXAAAATGTAATCTGAATTACTAGAAATTAGATATGTGTTGAAAAATTGAGTCTAAACATCT
CTCCCACATCTCTTGTTGTC
CACATGGATTTATGAGATGGAATAAATAGTAACAACTATCTTTACTTTCTGTATAGTTGTAAATAATAAAATAAA
ACATAXXXXXXXXXXXAAAATGTAATCTGAATTACTAGAAATTAGATATGTGTTGAAAAATTGAGTCTAAACATCT
CTCCCACATCTCTTATTGTC
>Marker965827
AACCTTGAATAGCAATAAACAAAGAACGATTCAACATAAAAAAGTTGGTTATCATCATTCAACAACACTACGATG
TTAATXXXXXXXXXXXATGAAAGGGTCAAATTAGAGACAAAACCCCCCATGGTCAAAAGCTTAATTGAGAATTTGT
CAAAAGCAAAACTTGTAGGT
AACCTTGAATAGCAATAAACAAAGAAGGATTCAACATAAAAAAGTTGGTTATCATCATTCAACAACACTACGATG
TTAATXXXXXXXXXXXATGAAAGGGTCAAATTAGAGACAAAACCCCCCATGGTCAAAAGCTTAATTGAGAATTTGT
CAAAAGCAAAACTTGTAGGT
>Marker966879
TACATTGATGCTGTTTAACTTATTTTCTCTCCAACATAAGTCGATCTTCCATTCTTCATGTTTAACTTCCTTAGT
TTTAGXXXXXXXXXXXATTGGTAAAGTAATATTAATTATTTCCATTTAAGATAAGATACAACGGGACTATCTTTCC
ATAATTTATAAAGGAAGGTT
TACATTGATGCTGTTTAACTTATTTTCTCTCCAACATAAGTGGATCTTCCATTCTTCATGTTTAACTTCCTTAGT
TTTAGXXXXXXXXXXXATTGGTAAAGTAATATTAATTATTTCCATTTAAGATAAGATACAACGGGACTATCTTTCC
ATAATTTATAAAGGAAGGTT
>Marker967273
GACCAACATGCGGAAGGAGAAGAGGACGAAAGGGGACGGCGAAGGCAACGCGGTGAGTGGGCCTCAAAATAGAAA
ACCTCXXXXXXXXXXXACCAACAATTCTAAAACTGCTTCCTTAACCTCCAATTCCATACTAACAATCTAAACCTTT
ATCCCCCATCCTCATTTGTA
GACCAACATGCGGAAGGAGAAGAGGACGAAAGGGGACGGCGAAGGCAACGCGGTGAGTGGGCCTCAAAATAGATA
ACCTCXXXXXXXXXXXACCAACAATTCTAAAACTGCTTCCTTAACCTCCAATTCCATACTAACTATCTAAACCTTT
ATCCCCCATCCTCATTTGTA
>Marker967856

Create PDF with GO2PDF for free, if you wish to

ACCATTTATTTCTGCATTATATTTTTACTAGGAAAGCATGGAACACTTTGTTTAGAATCTTTAATTTGGAGGTTT
GCCTTXXXXXXXXXXATAGTAGAATTTTTTAAGACAATTATAATTCTTTTGATTCTTTTTGGACTGTGTTGCAAC
GCACAACTTCTTGGTGGAGT
ACCATTTATTTCTGCATTATATTTTTACTAGGAAAGCATGGAACACTTTGTTTAGAATCTTTAATTTGGAGGTTT
GCCTTXXXXXXXXXXATAGTAGAATTTTTTAAGACAATTATAATTCTTTTGATTCTTTTTGGACTGTGTTGCAAT
GCACAACTTCTTGGTGGAGT
>Marker967911
AACGCTTCTCTCAGAAGAATGAATCAAAGGACCGAGCCCCGAATCTTGCTCGTCCTTTCAAGATCTTTTGCTTTC
CAACAXXXXXXXXXXTTTTAAAGAAATCATGATTCATTGTGTTTCGCAACGATATAATAGAAAGTATAAATTTTC
ATCACATATGTATAAGTGTG
AACGCTTCTCTCAGAAGAATGAATCAAAGGACCGAGCCCCGAATCTTGCTCGTCCTTTCAAGATCTTTTGCTTTC
CAACAXXXXXXXXXXTTTAAAGAAAATCATGATTCATTGTGTTTCGCAACGATATAATAGAAAGTATAAATTTTC
ATCACATAAGTATAAGTGTG
>Marker968847
TACTTAAACTAACAAAACCCAAAATTATTTATGACTATTGTTTTTCATAAATTTCATATTTATTCTTTGTAATTT
TTTAAXXXXXXXXXXCTTATATTACTAACTTTTCATATTATTTTTTCTTCTTATTCCCATATTTCATTGTTTGTA
ATTTCTTCATTTCCTTTTGT
TACTTAAACTAACAAAACCCAAAATTATTTATGACTATTGTTTTTCATAAATTTCATATTTATTCTTTGTAATTT
TTTAAXXXXXXXXXXCTTATATTATTAACTTTTCATATTATTTTTTCTTCTTATTCCCATATTTCATTGTTTGTA
ATTTCTTCATTTCTTTTTGT
>Marker968990
ACCATTTGACAGCAAAAATCAAGCCTCAACATCATCAATATCAGCTAGCCCGATACATAATTTGCTATTACACCC
AATTCXXXXXXXXXXAGAATAATAGGATCACTTTTTTCCTAAAAAAAATACATTTCAAGTTCATTGTTGATTGGT
ATAAGATTTAGATCTCTTGT
ACCATTTGACAGCAAAAATCAAGCCTCAACATCATCAATATCAGCTATCCCGATACATAATTTGCTATTACACCC
AATTCXXXXXXXXXXAGAATAATAGGATCACTTTTTTCCTAAAAAAAATACATTTCAAGTTCATTGTTGATTGGT
ATAAGATTTAGATCTCTTGT
>Marker969044
ACTATACGATGAGATACATGAGAGATATAATCACTAATGGGAGCATAGTAGTTACAGATTCGGTATGTTGTGTTT
AAATGXXXXXXXXXXAATGCAGCTAGCTAATTTCTTAGGTGGCCACATGTGATTATGGTGATTAGCTAAGTTTTT
TTTCTTTTTTGGGTTAGGTA
ACTATACGATGAGATACATGAGAGATATAATCACTAATGGGAGCATAGTAGTTACAAATTCGGTATGTTGTGTTT
AAATGXXXXXXXXXXAATGCATCTAGCTAATTTCTTAGGTGGCCACATGTGATTATGGTGATTAGCTAAGTTTTT
TTTCTTTTTTGGGTTAGGTA
>Marker969459
ACCAACATCGCCTGGGGGTGGATACAGAAGTGCAGTGGGTCGCATTTTTTTCCAGACAAAGGAAATGATTCAGGC
TGTGGXXXXXXXXXXTGAGCCCTCCATTGGTAAATATATCAGAAAATCAAATCAATGGCTGGAACATAGCTGACA
TAGCCTCTGGATTTTTGGTT
ACCAACATCGCCTGGGGGTGGATACAGAAGTGCAGTGGGTCGCATTTTTTTCAAGACAAAGGAAATGATTCAGGC
TGTGGXXXXXXXXXXTGAGCCCTCCATTGGTAAATATATCAGAAAATCAAATCAATGGCTGGAACATAGCTGACA
TAGCCTCTGGATTTTTGGTT
>Marker969891
AACCTAACTGTCTCACCGAGATTAGTGTCAAAATTAGCGGAAAATAAAATTAAAAAATTTAATTTAAATAAAGT
TTTTAXXXXXXXXXXTTATTATATAAATGTTACTTCACAAAAAAGTCCAATATGTAATTCCCAAAATTTGACTCC
ATACATTACCAATATTTGTC

Create PDF with GO2PDF for free, if you wish to

AACCTAACTGTCTCACCGAGATTAGTGTCAAAATTAGCGGGAAAATAAAATTAAAAATTTTAATTTAAATAAAGT
TTTTAXXXXXXXXXXTTATTATATAAATGTTACTTCACAAAAAAGTCCAATATGTAATTCCCAAAATTTGACTCC
ATACATTACCAATATTTGTC
>Marker970327
GACCAGAAACTAAATCAAGATCAATTCTTCATTCAAAGGACAAGAACAGAGATATGAAGAAAGGAATCTTATGAT
ATTGCXXXXXXXXXXAGTTTGAAAGCAAATAAAAACAGAGTAAGAACTAAAACTAAGAGGAAATTTCAAAAATTT
TCACCAGATTAAAATTAGTC
GACCAGAAACTAAATCAAGATCAATTCTTCATTCAAAGGACAAGAACAGAGATATGAAGAAAGGAATCTTATGAT
ATTGCXXXXXXXXXXAGTTTGAAAGCAAATAAAAACAGGGTAAGAACTAAAACTAAGAGGAAATTTCAAAAATTT
TCACCAGATTAAAATTAGTC
>Marker971129
ACAACTAATACTTTTTTACTTATTTTGAAAGAAAACTACTATGGAAATGAAAATAATTTGATATTTAATAGTAAT
TTATCXXXXXXXXXXCATGTGTAGAATTTGACGAGTGATAGAAGAGTCAATTGAAAACATTAATGCATAGGACAA
TATTGTTAATTTTTAAAGTT
ACAACTAATACTTTTTTACTTATTTTGAAAGAAAACTACTATGGAAATGAAAATAATTTGATATTTAATAGTAAT
TTATCXXXXXXXXXXCATGTTTAGAATTTGTCGAGTGATAGAAGAGTCAATTGAAAACATTAATGTATAGGACAA
TATTGTTAATTTTTAAAGTT
>Marker971696
AACTAGGAAATATCAAATATTTTATCTTTTTCTTGGTTCTGATGGATTTAAATTTCAACAGCTCCTTTGAGCGGC
CCCCTXXXXXXXXXXCCAAGTCACTTGAAAGTTTTTGCAGCTAATCAAGAGCAGGAAACTTCTACTGTTCCCTTA
TCTCTTGTTGCTGCGGAGTC
AACTAGGAAATGTCAAATATTTTATCTTTTTCTTGGTTCTGATGGATTTAAATTTCAACAGCTCCTTTGAGCGGC
CCCCTXXXXXXXXXXCCAAGTCACTTGAAAGTTTTTGCAGCTAATCAAGAGCAGGAAACTTCTACTGTTCCCTTA
TCTCTTGTTGCTGCGGAGTC
>Marker971868
ACGACGAAGGAACAAGACCAATGAGTGTAGCCAAAAGGAAAACATGAAAAGGTATATCGACAATGGGGGATGCCA
AGTTTXXXXXXXXXXTATGAAATGAGGTTACTTTTCAATTACCTCAGATTGGAAAGTTTTCAATTTTTCAGGCCA
CATCCAAGAAACAAAAGGTC
ACGACGAAGGAACAAGACCAATAAGTGTAGCCAAAAGGAAAACATGAAAAGGTATATCGACAATGGGGGATGCCA
AGTTTXXXXXXXXXXTATGAAATGAGGTTACTTTTCAATTACCTCAGATTGGAAAGTTTTCAATTTTTCAGGCCA
CATCCAAGAAACAAAAGGTC
>Marker972088
AACAAGAACAAATATATATACAAGTGACATTTAATACTTAACTATGTTCATTCCGTCTTTACTTACGGGATGCGT
AGAGTXXXXXXXXXXTATCATCTACAGTTGTATCGTCTTAAATACTGATATAATTAGTTTGACATTTTGACAAGT
TGTGTATATATATGCTTTGT
AACAAGAACAAATATATATACAAGTGACATTTAATACTTAACTATGTTCATTCCGTCTTTACTTACGGGATGCGT
AGAGTXXXXXXXXXXTATCATCTACAGTTGTATCGTCTTAAATACTGATATAATTAGTTTGACATTTTGACAACT
TGTGTATATATATGCTTTGT
>Marker972846
GACAGTTTTTAACCGTCATTACTCCTGTAAGAATTTATAACTACCCTAATGAAAACTGCAACATTAGATCATTAA
CACAGXXXXXXXXXXACAGTTTTTACCCCCATTAGTCTTTGTCAAATTAAGATGTCCCGTATTAATCTAAGGTTA
AGCCGTTGATGGTTTCTTGT
GACAGTTTTTAACCGTCATTACTCCTGTAAGAATTTATAACTACCCTAATGAAAACTGCAACATTAGATCATTAA
CACAGXXXXXXXXXXACAGTTTTTACCCCCATTAGTCTTTGTCAAATTAAGATGTCCCGTATTAATCTAAGGTTG
AGCCGTTGATGGTTTCTTGT
>Marker973662

Create PDF with GO2PDF for free, if you wish to

ACTGTGTTATACTTTTCTTCTGTGAGTTGTGACTCCAGGATAGATATGATCGGCATGCAAACAATTTATTCTGAA
AAATGXXXXXXXXXXCTAAAAGTTCGTAAATTCATCGGAATATGATGGCACCAATGCACTGTTCTATTGGTGGAG
ATAGAAATATCTTTGATGTT
ACTGTGTTATACTTTTCTTCTGTGAGTTGTGACTCCAGGATAGATATGATCGGCATGCAAACAAATTATTCTGAA
AAATGXXXXXXXXXXCTAAAAGTTTGTAAATTCATCGGAATATGATGGCACCAATGCACTGTTCTATTGGTGGAG
ATAGAAATATCTTTGATGTT
>Marker973722
ACCAAGACTGCTCCCGAATTATAGCAGATGGACGAAATTCAGAGGTCTTCCTCTAATTTTCGTTGAGCTTTGACT
CAAAGXXXXXXXXXXATAATTTTCTTTCGTAAGTCGGTTACTTGGAAGGAAACTAAATTGTTGTTTCCCATTTGC
AAAAAAACAATTTTTTTTGT
ACCAAGACTGCTCCCGAATTATAGCAGATGAACGAAATTCAGAGGTATTCCTCTAATTTTGGTTGAGCTTTGACT
CAAAGXXXXXXXXXXATAATTTTCTTTCGTAAGTCGGTTACTTGGAAGGAAACTAAATTGTTGTTTCCCATTTGC
AAAAAAACAATTTTTTTTGT
>Marker974690
GACTGGATTCATATACTTTTGGCTTTTGATCTTCTTGGCAAGTAAGGAGATTGATGCTTTGTTGTTCTGAGAAAA
CCAATXXXXXXXXXXGTTGATTTCAAACCAAACCTACCTCGTGAGTCTCTCTCTTCTCTCTATCTGGAAAATAAA
TGAAGTAGTTCAACTTTGTT
GACTGGATTCATATACTTTTGGCTTTTGATCTTCTTGGCAAGCAAGGACATTGATGCTTTGTTGTTCTGAGAAAA
CCAATXXXXXXXXXXGTTGATTTCAAACCAAACCTACCTCGTGAGTCTCTCTCTTCTCTCTATCTGGAAAATAAA
TGAAGTAGTTCAACTTTGTT
>Marker974881
CACTCAAGAATGCGGGTTGAGTTGAATTCTAGTGTGAAAATTGGGAAAGGAGGATCTTTGAGTTCTGGGACTGAG
GATACXXXXXXXXXXGCTTGCTAGGGAATGCCCTAGTAATGAGAGTCTAAAAAAGGAATACAAACTTGTCTGCGA
ATTGTCTTTGGATGAGAAGT
CACTCAAGAATGCGGGTTGAGTTGAACTCTAGTGTGAAAATTGGGAAAGGAGGATCTTTGAGTTCTGGGACTGAG
GATACXXXXXXXXXXGCTTGCTAGGGAATGCCCTAGTAATGAGAGTCTAAGAAAGGAATACAAACTTGTCTGCGA
ATTGTCTTTGGATGAGAAGT
>Marker974887
ACATGACTTTCAAGTTAAACATTGTATCAAGAAAAATATTTTTGTTGCATCAAGAAAGACCAAATTTGTAGTAGT
GATTGXXXXXXXXXXCTCTCACAAACCTTGCTTCAACAACAATGAAGAGGGCAACTTAAAGATCAACTATAATTT
TGAATCACTTTTCACACGTT
ACATGACTTTCAAGTTAAACATTGTATCAAAAAAAATATTTTTGCTGCATCAAGAAAGACCAAATTTGTAGTAGT
GATTGXXXXXXXXXXCTCTCACAAACCTTGCTTCAACAACAATGAAGAGGGCAACTTAAAGATCAACTATAATTT
TGAATCACTTTTCACACGTT
>Marker976464
ACTTTATTCTATTTTAGTTCAACTTTTCGAGTGAAACAAACCTAAATTAAAAAAGCTAATTGTTCTCTTTTCTCC
AAAATXXXXXXXXXXAAATATCCGAGACATTAAAATTAAATTTATATGTAGATGAATTGTATCTAGTAGATAATG
ACAATGAGCTAAAACACAGT
ACTTTATTCTATTTTAGTTCAACTTTTCGAGTGAAACAAACCTAAATTAAAAAAGCTAATTGTTCTCTTTTCTCC
AAAATXXXXXXXXXXAAATATCCGAGACATTAAAATTAAATTTATATGTAGATGAATTGTATCTAGTATATAATG
ACAATGAGCTAAAACACAGT
>Marker976681
ACTAAAAAGATAGCTATACCTATATAAGTTTCCTAAAACAAAAGTTAATAAACATACTTTTTTAGTTTTTTATTG
ACAATXXXXXXXXXXATAAATTAATAGTTGTCTATTTGTGTTTTAAAAATATTCATTTTGTTTCCATATTTTAAA
AAAGTGATAACTTAAAAGTG

Create PDF with GO2PDF for free, if you wish to

ACTAGAAAGATAGCTATACCTATATAAGTTTCCTAAAACAAAAGTTAATAAACATACTTTTTTAGTTTTTTATTG
ACAATXXXXXXXXXXXATAAATTAATAGTTGTCTATTTGTGTTTTAAAAATATTCATTTTGTTTCCATATTTTAAA
AAAGTGATAACTTAAAAGTG
>Marker976971
ACTTATTTTTTCAATCTAATATCAAAGTTGTCCAAGATTCCATTACCTCTTCCACTTTTCCTTCAACAATTCTAT
CTTTTXXXXXXXXXXXGCTGATATTGGAGTAAAAACCATTTTTTGATAAAGCAAGCTCAGGAAAATCCTCTACTTT
CGTAAATAAATCTCTAAGTT
ACTTATTTTTTCAATCTAATATCAAAGTTGTCCAAGATTCCATTACCTCTTCCTCTTTTCCTTTAACAATTCTAT
CTTTTXXXXXXXXXXXGCTGATATTGGAGTAAAAACCATTTTTTGATAAAGCAAGCTCAGGAAAATCCTCTACGTT
CGTAAATAAATCTCTAAGTT
>Marker978408
ACTTGTTGAGGAGATGGTTGGTGTTGGTTAATTCTTTTAATGGATTTTCTTTTCAAGAAAGATAAAAAAAGACAG
TCAATXXXXXXXXXXXCTTTAAATAAAATACTTGAAAACATACAATAAAATAAACACAACCAGTTATTTGATGGTA
TAAATTGTTGTATTAAGGTG
ACTTGCTGAGGAGATGGTTGGTGTTGGTTAATTCTTTTAATGGATTTTCTTTTCAAGAAAGATAAAAAAAGACGG
TCAATXXXXXXXXXXXCTTTAAATAAAATACTTGAAAACATACAATAAAATAAACACAACCAGTTATTTGATGGTA
TAAATTGTTGTATTAAGGTG
>Marker978433
ACATGAGGCATCGAGAAAGTTTTGTTTATTCATGTCGGATAGGTGTTCCATGCCAAATAAAATTATTTTTTTCCT
AATTAXXXXXXXXXXXTAGGAATTAAGGTAAAAGGAACAAGAAAAAGCTCGTATTTAGGTTGAATATCAGTTGGTT
GGAGTCACTTATTAAAGAGT
ACATGAGGCATCGAGAAAGTTTTGTTTATTCATGTCGGATAGGTGTTCCAGGCCAAATAAAATAATTTTTTTCCT
AATTAXXXXXXXXXXXTAGGAATTAAGGTAAAAGGAACAAGAAAAAGCTGGTATTTAGGTTGAATATCAGTTGGTT
GGAGTCACTTATTAAAGAGT
>Marker978636
AACTATGACAATTATGGTAACTATACTTTAACTATACTATACTACTTTCATCTCTCCTATTGTTTCTACTTTTCT
TGATTXXXXXXXXXXXTATACATTTAGGAATCGCAATGTATAATTAAGCATGAGAAGCTAAATTAGTTACACTATA
AATTTAGGTTTTATGTAGTA
AACTATGACAATTATGGTAACTATACTTTAACTATACTATACTACTTTCATCTCTCCTATTGTTTCTACTTTTCT
TGATTXXXXXXXXXXXTATACATTTAGGAATCGCTATGTATAATTAAGCACGAGAAGCTAAATTAGTTACACTATA
AATTTAGGTTTTATGCAGTA
>Marker978721
AACATTACTCTAAAGGAAAATTAAAGTTTATGTTGTTTTGAAAATTTCTACATTTGCATCTACTTTGGCAAGATT
CGAATXXXXXXXXXXXTTATAAAACTATCCCAAAATAAAAAATGTTTTGACTTACCATTTTAAAAGACTTAATAAG
GAATATAATAATTAATTGTA
AACATTACTCTAAAGGAAAATTAAAGTTTATGTTGTTTTGAAAATTTCTACATTTGCATCTACTTGGGCAAGATT
CGAATXXXXXXXXXXXTTATAAAACTATCCCAAAATAAAAAATGTTTTGACTTACCATTTTAATAGACTTAATAAG
GAATATAATAATTAATTGTA
>Marker979042
TACACATAACACGCGCAGACACACCCAACTTGTAATTCAGTATTACCTGTCGAACGGAAATGCTCACATCAGTTC
TAACCXXXXXXXXXXXTCATCATCACTACCACCCTCAAGCAATGCTTTTCCAGAAGTTCCAGCAACCTGCAAAACA
GAAACTAAGACACAGACGTA
TACACATAACACGCGCAGACACACCCAACTCGTAATTCAGTATTACCTGTCGAACGGAAATGCTCACATCAGTTC
TAACCXXXXXXXXXXXTCATCATCACTACCACCCTCAAGCAATGCTTTTCCAGAAGTTCCAGCAACCTGCAAAACA
GAAACTAAGACACAGACGTA
>Marker979154

Create PDF with GO2PDF for free, if you wish to

GACATAGTCGTAGAATTTTTCCTTCTTTCCATGGTATCTAACTCAAAAATTTTGGCAGTCATATAGTTTTCGCAG
AGGATXXXXXXXXXXGTTTTTCCTCTTGGATTTGCTATAAACCAAAGGTGACTATTATGCATCTGTTCTCTCTTC
AGAAATTCAGGAATGAGGTT
GACATAGTCGTAGAATTTTTCCTTCTTTCCATGGTATCTAACTCAAAAAATTTGGCAGTCATATAGTTTTCGCAG
AGGATXXXXXXXXXXGTTTTTCCTCTTGGATTTGCTATAAACCAAAGGTGACTATTATGCATCTGTTCTCTCTTC
AGAAATTCAGGAATGAGGTT
>Marker979256
AACCCACGCCGTTGGATTCCACCTACAAGTAATTTCAATGGAGAAATTAATTAGTAAAGTAATTAAATTTTTAGA
AAAGAXXXXXXXXXXXATGGGATAATATTTTGGAGAGATCAGTGATAGAAAGGGTTGGTTTTATAGCAAAAAAATC
TGAAGCTGTGTGGTTAAGTG
AACCCACGCCGTTGGATTCCACCTACAAGTAACTTCAATGGAGAAATTAATTAGTAAAGTAATTAAATTTTTAGA
AAAGAXXXXXXXXXXXATGGGATAATATTTTGGAGAGATCAGTGATAGAAAGGGTTGGTTTTATAGCAAAAAAATC
TGAAGCTGTGTGGTTAAGTG
>Marker979469
AACCAAAGTAAAAAAATTAGACCAATTATATGTTTTCTTTATTTTATTTTATTTAAACAAACATATACTATTTTA
AACTAXXXXXXXXXXXAAAATATTTGGTAGAGAGGAACAAAATACTTTATAATTATACTCTTTTAAATAAAACATT
GACTATCAACATAATACGTA
AACCAAAGTAAAAAAATTAGACCAATTATATGTTTTCTTTATTTTATTTTATTTAAACAAACATATACTATTTTA
AACTAXXXXXXXXXXXAAAATATTTGGTAGAGAGGAACAAAATACTTTATAATTATACTCTTTTAAATAAAACATT
GACTATCAACATAATATGTA
>Marker979715
ACCAAACAAGACAATGGAAAATAAAAGCAACAGAAATTACAGCATAAATTAAAATCAGTCTTACAATAATCTTCT
CTAAGXXXXXXXXXXGTCATCGTCCAACGATCAATTCAAGTATAGAGACTAAAATTATACTATATTATAACTATA
ATAGGGTATAAATTATAGTT
ACCAAACAAGACAATGGAAAATAAAAGCAACAGAAATTACAGCATAAATTAAAATCAGTGTTACAATAATCTTCT
CTAAGXXXXXXXXXXGTCATCGTCCAACGATCAATTCAAGTATAGAGACTAAAATTATACAATATTATAACTATA
ATAGGGTATAAATTATAGTT
>Marker979814
ACCAACAGTTGATTAAAAATTAAAAGAGGAAGTTAAGAAGAGGAAAGAAGGGGATACAGTTACTTGGAAGACAGT
TACTGXXXXXXXXXXXAAGGCTGGTGTTCTGTGTTTTGGTTTGGTTTTTAGTATTTTGTTTTGTGTGTTGTTTTTG
ATATTTTGGATTAGGAGGTT
ACCAACAGTTAATTAAAAATTAAAAGAGGAAGTTAAGAAGAGGAAAGAAGGGGATACAGTTACTTGGAAGACAGT
TACCGXXXXXXXXXXXAAGGCTGGTGTTCTGTGTTTTGGTTTGGTTTTTAGTATTTTGTTTTGTGTGTTGTTTTTG
ATATTTTGGATTAGGAGGTT
>Marker981747
ACTTTATTCAAGGTTCAACGTCATCTTTTTATAATTTTATGTGATTATCTTGGTGACTTTTATGTAAAATAATGT
TGCCTXXXXXXXXXXXAGGGGAGAAATTAAATACCCAATTAAGGTTTGCGACTTTCTGGTTTATTTTTCCAACCTG
GCTTTTGGAATTCCAACGTT
ACTTTATTCAAGGTTCAACGTCATCTTTTTATAATTTGATGTGATTATCTTGGTGACTTTTATGTAAAATAATGT
TGCCTXXXXXXXXXXXAGGGGAGAAATTAAATACCCAATTAAGGTTTGCGACTTTCTGGTTTATTTTTCCAACCTG
GCTTTTGGAATTCCAACGTT
>Marker982058
ACTTATCGCTTAGTAGTTGAACTTAAGTTAAGTGTTGAAAGCTAAAGGGTTAAGTATTTCCCTTACAATGAGTTG
TTAAAXXXXXXXXXXXTTGTAATATAGTTTATTAGTGATAGACTTTAACAAATTTTGTTATATTTGCAATTTTGTT
TTTGGAAATTTTGCTATGTA

Create PDF with GO2PDF for free, if you wish to

ACTTATCGTTTAGTAGTTGAACTTAAGTTAAGTGTTGAAAGCTAAAGGGTTAAGTATTTCCCTTACAATGAGTTG
TTAAAXXXXXXXXXXTTGTAATATAGTTTATTAGTGATAAACTTTAACAAATTTTGTTATATTTGCAATTTTGTT
TTTGGAAATTTTGCTATGTA
>Marker982219
CACTTGGGATTTAACTGACGATTGAGATGGCTGAATTGAACTCGATGTTGAATATATAGGAACTGTGATGGCGGG
ATGGTXXXXXXXXXXTCCCGGGCATGGAAAGTTCGTGGGTTTGATATAAACCCACCCTGATAGCCAAATTTTTCA
AGATTAATGAAAATACTGTG
CACTTGGGATTTAACTGACGATTGAGATGGCAGTATTGAACTCGATGTTGAATATATAGGAACTGTGATGGCGGG
ATGGTXXXXXXXXXXTCCCGGGCATGGAAAGTTCGTGGGTTTGATATAAACCCACCCGGATAGCCAAATTTTTCA
AGATTAATGAAAATACTGTG
>Marker982998
ACTTTAAACAACCTAAAAGCAAGACTTAAATAGCCACCCAATAAAACTCTAATGCATTTTAACAAGTTTAAATTA
ATAGTXXXXXXXXXXAAATTGATCCAACCGAAATTACGAACAACTTACGAAAGAGATTGACTTACTAATTAAGAG
AATAGCTACATATTATAGTG
ACTTTAAACAACCTAAAAGCAAGACTTAAATAGCCACCCAATAAAACTCTAATGCATTTTAACAAGTTTAAATTA
ATAGTXXXXXXXXXXAAATTGATCCAACCGAAATTACAAACAACTTACGAAAGAGATTGACTTACTAATTAAGAG
AATAGCTACATATTATAGTG
>Marker983042
ACAGCTTCATTTTTGTTGTTGAGTAATTATTCTTGGTTTAGCAGATTGCGTCCATTACCAATTCTGGGGGATTTA
TAGTTXXXXXXXXXXTTCTGAAGCAACGTTCTTTCTTACCCCTTTCTATACTTATTTTTTCAAATTTTCTTCTCA
TTTGGTTAGTTGGTTTCGTG
ACAGTTTCATTTTTGTTGTTGAGTAATTATTCTTGGTTTAGCAGATTGCGTCCATTACCAATTCTGGGGGATTTA
TAGTTXXXXXXXXXXTTCTGAAGCAACGTTCTTTCTTACCCCTTTCTATACTTATTTTTTCAATTTTTCTTCTCA
TTTGGTTAGTTGGTTTCGTG
>Marker983456
TACACCTTTTCAATCTATCATTTTGTGACTTCTCACCTTTTAATGTGTTATCAATAGTTAGAGTTGTGATAGGTT
CTTGAXXXXXXXXXXGGCTAATGCACGCAAGGAGGAGTTTGGTAAAAAATTTCACCTTAGCGAGGCAACTTTCAA
CATTTGTATCCCGAAAGGTG
TACACCTTTTCAATCTATCATTTTGTGACTTCTCACCTTTTAATGTGTTATCAATAGTTAGAGTTGTGATAGGTT
CTTGAXXXXXXXXXXGGCTAATGCACGCAAGGAGGAGTTTGGTAAAAAACTTCACCTTAGCGAGGCAACTTTCAA
CATTTGTATCCCGAAAGGTG
>Marker983474
GACTAAAAAACATATAGAATTAATCTGAATTAGTCATTATATATAATATATTGCAAAAAAATAAATAAATGAGAA
GTATAXXXXXXXXXXTCTATTCTTTTGTTCTCTAAAATAAGAAAACAAGAAAAGAGAAGAAAGGAAACAAGAATT
TCTCAACCCGTTCTGATTGT
GACTAAAAAACATATAGAATTAATCTGAATTAGTCATTATATATAATATATTGCAAGAAAATAAATAAATGAGAA
GTATAXXXXXXXXXXTCTATTCTTTTGTTCTCTAAAATAAGAAAACAAGAAAAGAGAAGAAAGGAAACAAGAATT
TCTCAACCCGTTCTGATTGT
>Marker983658
ACACAGAATTGGAAATTTTAATGTCTTTTTGTTATTGGGTTTTTCAAATAGATTGAAGAGTCAATTGGATGTTGA
GAAAAXXXXXXXXXXTTGGAGGTCGGTCTTAGACCCATGATAAAAGATTATATTGCTCGTGTAGTAATTGATCGT
TACAATGTTATGATTGAAGT
ACACAGAATTGGAAATTTTAATGTCTTTTTGTTATTGGGTTTTTCAAATAGATTGAAGAGTCAATTGGATGTTGA
GAAAAXXXXXXXXXXCTGGAAGTCGGTCTTAGACCCATGATAAAAGATTATATTGCTCGTGTAGTAATTGATCGT
TACAATGTTATGATTGAAGT
>Marker984050

Create PDF with GO2PDF for free, if you wish to

TACTTTACCAGACCTATACCTGAGCTTTGCTTGAAACTCAACGTTTCTGTTTTTACACTGAAACTAGATTCACTC
GAGCAXXXXXXXXXXXCCACTTTTCTTAAACTTTGTGCTGTTCTTTAAAATGTTCTCGTCTATATTCCTTCTTTCT
ATCATGTATATTAATCTGTG
TACTTTACCAGACCTATACCTGAGCTTTGCTTGAAACTCAACGTTTCTGTTTTTACACTGAAACTAGATTCACTC
GAGCAXXXXXXXXXXXCCACTTTTCTTAAACTTTGTGCTGTTCTTTAAAATGTTCCCGTCTATATTCCTTCTTTCT
ATCATGTATATTAATCTGTG
>Marker984240
TACTACTTTTAAAAATGGAAATCAAAATTATACTATTTTTTTAAAAATCAAATTTCCGGTAATGTTCCTATCTTT
CAATGXXXXXXXXXXXTTTTTTAAAAGAAAATGGTTAAAAATAGCACATTTTCGTTCTTTTTATTATTTCTAGAAA
AACCCTTAGTTGAATTCGTG
TACTACTTTTAAAAATGGAAATCAAAATTATACTATTTTTTTTATAATCAAATTTCCGGTAATGTTCCTATCTTT
CAATGXXXXXXXXXXXTTTTCTAAAAGAAAATGGTTAAAAATAGCACATTTTCGTTCTTTTTATTATTTCTAGAAA
AACCCTTAGTTGAATTCGTG
>Marker984728
AACCAAATAACTCGAGGAATTTATATAAATACAAAAAAATGGAAAGTATTTACATCAAATAACAAAAAAGAATTG
ATATTXXXXXXXXXXXAACAATGGTAATGGATTAACTTGTCATCAGCTTGGGGATGTAGCTCAGATGGTAGAGCGC
TCGCTTAGCATGCGAGAGGT
AACCAAATAACTCGAGGAATTTATATAAATACAAAAAAAAGAAAAGTATTTACATCAAATAACAAAAAAGAATTG
ATATTXXXXXXXXXXXAACAATGGTAATGGATTAACTTGTCATCAGCTTGGGGATGTAGCTCAGATGGTAGAGCGC
TCGCTTAGCATGCGAGAGGT
>Marker985193
AACCATATTCCTATTAAAATCATCAAGCACCTACAGCAGCTAATCATGACCAAGTAGCTCAGTGGGAAGAAAAGG
TAGGCXXXXXXXXXXXCAAAGCATGCCTAGACAAAATCGATGAAAGAAGAGTAATGACAAAGCGCACTGCATAAAG
CTCTACAACTAGATGTTGTG
AACCATATTCCTATTAAAATCATCAAGCACCTACAGCAGCTAATCATGACCAAGTAGCTCAGTGGGAAGAAAAGG
TAGGCXXXXXXXXXXXCAAAACATGCCTAGACAAAATCGATGAACGAAGAGTAATGACAAAGCGCACTGCATAAAG
CTCTACAACTAGATGTTGTG
>Marker985288
TACTGTTTTTTTAATAGGAAATCCCAACAAAGTTTGTGAGAGAATATGGTGTAAAAGCTTTTGCAACTAACCAAA
TATGTXXXXXXXXXXXACTCTGTTGGATTGGAGTATTTTCTCGTCTTTAAATACGAACGTCAATCTTCGAGCTTTT
ACGTGGTTATCTTTGATCGT
TACTGTTTTTTTAATAGGAAATCCCAACAAAGTTTGTGAGAGAATATGGTGTAAAAGCTTTTGCAACTAACCAAA
TATGTXXXXXXXXXXXACTCTGTTGGATTGGAGTATTTTCTCGTCTTTAAATACGAATGTCAATCTTCGAGCTTTT
ACGTGGTTATCTTTGATCGT
>Marker985590
ACCCAAAGCCCTATAATACCTCCTATTTGAAAGAGTTTTCAATGTCGATCACAAACCGATATTTAACATCAGCTT
TTGCAXXXXXXXXXXXTCTCTTTCAGCCCTCCAAAGTTGCTTCCAGCTACCACCCTCCTACCTATAAGCAAAGTTT
TTAAAAGGTCGTCATTTGTT
ACCCAAAGCCCTATAATACCTCCTATTTGAAAGAGTTTTCGATGTCGATCACAAACCGATATTTAACATCAGCTT
TTGCAXXXXXXXXXXXTCTCTTTCAGCCCTCCAAAGTTGCTTCCAGCTACCACCCTCCTACCTATAAGCAAAGTTT
TTAAAAGGTCGTCATTTGTT
>Marker985686
AACCATGATCATCCAACCCTTTCTTTCTCTCCCTGGCTTCTCTCTCTCACCTGAGAAAGTTCTCATCCATTTCCC
TTTGGXXXXXXXXXXXTATTCACAAAAATTTATCATTGATGTATATAATAATTATTTTGAAAATGTAATACCAAAC
TGTTCATATGTCTTAAAAGT

Create PDF with GO2PDF for free, if you wish to

AACCATGATCATCCAACCCTTTCTTTCTCTCCCTGGCTTCTCTCTCTCACCTGAGAAAGTTCTCATCCATTTCCC
TTTGGXXXXXXXXXXXTATTCAAAAAAATTTATCATTGATGTATATAATAATTATTTTGAAAATGTAATACCAAAC
TGTTCATATGTCTTAAAAGT
>Marker986034
TACCGTCAGGGGAACTTGAGTTTATATATTTAAGTTATGGTGATAACTTGTTAATATAACTTTAGGATTAAATTA
CAAACXXXXXXXXXXXGCTGAAGGATAATATTTATTGTCTCACCCATTTTTCATATTGCTTGTCATATTCATAACC
GTATCTCTCTTCGATCAAGT
TACCGTCAGGGGAGCTTGAGTTTATATATTTAAGTTATGGTGATAACTTGTTAATATAACTTTAGGATTAAATTA
CAAACXXXXXXXXXXXGCTGAAGGATAATATTTATTGTCTCACCCATTTTTCATATTGCTTGTCATATTCATAACC
GTATCTCTCTTCGATCAAGT
>Marker986273
ACTGTATAACGTTGGACAAAACTACTTGTTGTTTAGCATACCCGTTTATTTATCTCAACTTGCATTTTCATTTCT
AGATAXXXXXXXXXXXGTGGATGTGGAAAAAGGTATTGGGTTGAACTTTAGGTTGTAAGAATCATTATTTGATTGA
GTTGAATTGGGGAAATTAGT
ACTGTATAATGTTGGACAAAACTACTTGTTGTTTAGCATACCCGTTTATTTATCTCAACTTGCATTTTCATTTCT
AGATAXXXXXXXXXXXGTGGATGTGGAAAAAGGTATTGGGTTGAACTTTAGGTTGTAAGAATCATTATTTGATTGA
GTTGAATTGGGGAAATTAGT
>Marker986452
CACCTAAAGCCTGTTGAAAGTTGTTGGCAACCCCAGGATTATCTCCCAGACCCAGAATCTGACGGTTTCTACGAC
CAAGTXXXXXXXXXXXTGCAAGCCTCACCTCGTGGGCAATTTGGACAAGAGCATGGACTGCGGAAGAGAATAGACA
TGGTGACCTTTTGAATAAGT
CACCTAAAGCCTGTTGAAAGTTGTTGGCAACCTCAGGATTATCTCCCAGACCCAGAATCTGACGGTTTCTACGAC
CAAGTXXXXXXXXXXXTGCAAGCCTCACCTCGTGGGCAATTTGGACAAGAGCATGGACTGCGGAAGAGAATAGACA
TGGTGACCTTTTGAATAAGT
>Marker986467
ACTGTAGCCACAGATATCTGGGCCAAACATGATGCTGCAGGAAACAAAAAGCACATAATCATAAAGAAAGTCATG
AGATTXXXXXXXXXXXACCTCCACAATCAAGCTTCTGTTCGTTCTTGACGGAAAATTGGAAAACAAGAATGTTGTC
TTTGTTGCTGAATTCAGGGT
ACTGTAGCCACAGATATCTGGGCCAAACATGATGCTGCAGGCAACAAAAAACACATAATCATAAAGAAAGTCATG
AGATTXXXXXXXXXXXACCTCCACAATCAAGCTTCTGTTCGTTCTTGACGGAAAATTGGAAAACAAGAATGTTGTC
TTTGTTGCTGAATTCAGGGT
>Marker986903
ACAGTTGATATCAACTCATATTTTTTGTTACATTCTTATTTAATGGATGACCAAATATAATCTCTTTCTGTGTAG
GCGTAXXXXXXXXXXXGTGAAACTCTGCCAATACTGCTATGGGATGCACTTGTCAAGCTATTACCAGAAACCACTA
TTTTGAATTTGTATGGTAGT
ACAGTTGATATCAACTCATATTTTTTGTTACATTCTTATTTAATGGATGACCAAATATAATCTCTTTCTTTGTAG
GCGTAXXXXXXXXXXXGTGAAACTCTGCCAATACTGCTATGGGATGCACTTGTCAAGCTATTACCAGAAACCACTA
TTTTGAATTTGTATGGTAGT
>Marker987118
ACCTTTCAACCTCGCCATACACATAAAGATAATAATAAACTATGAAATAAAAAAAATAAAGATATGTTGTCATAT
GACCTXXXXXXXXXXXATTGTATAGATAAGTCTATCGAATAACTTAAGATTTTTTCAAGTCATAATGATTATAGAG
TCTCAAACACAAGTTTAGTT
ACCTTTCAACCTCGCCATACACATAAAGATAATAATAAACTATGAAATAAAAAAAATAAAGATATTTTATCATAT
GACCTXXXXXXXXXXXATTGTATAGATAAGTCTATCGAATAACTTAAGATTTTTTCAAGTCATAATGATTATAGAG
TCTCAAACACAAGTTTAGTT
>Marker987138

Create PDF with GO2PDF for free, if you wish to

ACTCTACTAATGACACCATATTTGTTATGTTGAGGGTGTGGCAAACTTAGTTCAATCACCAAAGGCTTCAATGTC
CCATCXXXXXXXXXXTTGCTTCATTGCCTATTATCACATAATTAAGAAAGAAAATTGATGATCCAAATCCAAATT
AGTATAGTTTAGCAGTAGTT
ACTCTACTAATGACACCATATTTGTTATGTTGAGGGTGTGGCAAACTTAGTTCAATCACCAAAGGCTTCAATGTC
CCATCXXXXXXXXXXTTGCTTCATTGCCTATTATCACATAATTAAGAAAGAAAATTGATGATCCAAATCCAAATT
AGTATAGTTTAGCAGTAGTC
>Marker987408
ACTATGGTTTGAGACCCTTTTTCTATTCATTTTCAAGTCGCATCAAATATCTAACAAGGCTGCTTTACTACAATA
ACTGCXXXXXXXXXXATGCATGCACGTATATAGAAAATCTGTCATCGTGGCCCTGAAAGACAAGAACTGGGTTAA
GCACCTAACTCAATAAAGTC
ACTATGGTTTGAGACCCTTTTTCTATTCATTTTCAAGTCGCATCAAATATCCAACAAGGCTGCTTTACTACAATA
ACTGCXXXXXXXXXXATGCATGCACGTATATAGAAAATCTGTCATCGTGGCCCTGAAAGACAAGAACTGGGTTAA
GCACCTAACTCAATAAAGTC
>Marker987443
AACAGCATGAAGGTAGGGGGAGAGACCGAGAGATACAGGAAAATTAAACTTACATTCGTCTTCCATGTCAATGAA
ATCTAXXXXXXXXXXGATAATTTTCAAGCAAGCAACCGCAACAGCTTCAAAAAAGTAGTTATGCTCAAATATCAA
CACAAAAAACGCAGAGGGTA
AACAGCATGAAGGTAGGGGGAGAGACCGAGAGATACAGGAAAATTAAACTTACATTCGTCTTCCATGTCAATGAA
ATCTAXXXXXXXXXXGATAATTTTCAAGCAAGCAACCGCAACAGCTTCAAAAAAGTAGTTATGCTCAAATATCAA
CACAAAAAAGGCAGAGGGTA
>Marker988287
ACATTCATGTTCATGCTTACTCACGTGAGATCACGTGCCCAAACGGAATTCTCATCTGCTTCAAAGTTTGGACTA
AAATTXXXXXXXXXXATTTGTTTGGTAACAATTTTAATTAGTTAGATTCTAATTTAAACAATTGTTTAAACTTTG
TTTGATGCTATGTATATGTA
ACATTCATGTTCATGCTTACTCACGTGAGATCACGTGCCCAAAGGTATTTCTCATCTGCTTCAAAGTTTGGACTA
AAATTXXXXXXXXXXATTTGTTTGGTAACAATTTTAATTAGTTAGATTCTAATTTAAACAATTGTTTAAACTTTG
TTTGATGCTATGTATATGTA
>Marker989047
ACATCAAATTGCAGGTGAGATACCTTTTGAGTTATTTTTTAGTTCTAGGTTCAGGTTCGATTCTGTTGGAGGAGT
TTATTXXXXXXXXXXGTATATTTTAGTCATTTATCAATGTCCTACAGATAATGGATCATTCCAGTCATTTAGTGG
CTTTTGTAATTTATGATGTG
ACATCAAATTGCAGGTGAGATACCTTTTGAGTTATTTTTTAGTTCTAGGTTCAGGTTCGATTCTGTTGGAGGAGT
TTATTXXXXXXXXXXGTATATTTTAGTCATTTATCAATGTCCTACAGATAATGGACCATTCCAGTCATTTAGTGG
CTTTTGTAATTTATGATGTG
>Marker989051
CACCGTGAAAAGGTCGTATGTTGCACAAGAGCCCAAAAAGAATCAAAAGAGCTAAAATTATCATCGACACAAAAC
TGAAAXXXXXXXXXXACATGCTATAAGAGCTCATGAAGTCTAAATAGGCATTTGGTTCGAAAAATTAAAAATTAG
GATCCAATGAAAGAATTGTG
CACCGTGAAAAGGTCGTATGTTGCACAAGAGCCCAAAAAGAATCAAAAGAGCTAAAATTATCATCGACACAAAAC
TGAAAXXXXXXXXXXACATGCCATAAGAGCTCATGAAGTCTAAATAGGCATTTGGTTCGAAAAATTAAAAATTAG
GATCCAATGAAAGAATTGTG
>Marker989345
AACATCATAGTCGTAAATTCTATCCCATTCTTGACGCTCTCCTTTTCCATCTCCTCTTAGATTCAATAGCTCTTC
TTCTCXXXXXXXXXXTTAAACAAACAAAAAATCAACTAATTAATTACCTTGTTGGCAAAGAAAATTCGATCTTTT
TTGTATCTTCTTTGAGGGTA

Create PDF with GO2PDF for free, if you wish to

AACATCATAGTCGTAAATTCTATCCCATTCTTGACGCTCTCCTTTTCCATCTCCTCTTAGATTCAATAGCTCTTC
TTCTCXXXXXXXXXXXTTAAACAAACAAAAAATCAACTAATTAATTACCTTGTTGGCAAAGAAAATTCGATCTTTT
CTGTATCTTCTTTGAGGGTA
>Marker989401
CACAACGATGTCATCCTCATGGCCATTGATCATACCCTCACACTCTTAACTACTGACACTAATTGGTGATTCTGT
TACAGXXXXXXXXXXXCTAAAGGATCAGAAACTGACCTGGTTTTTCCAGATGGTGCTTGAATGAAAGAGCTGCAGT
TTTCTAAAAGATCTGGGGGT
CACAACGATGTCATCCTCATGGCCATTGATCATACCCTCACACTCTTAACTACTGACACTAATTGGTGATTCTGT
TACATXXXXXXXXXXXCTAAAGGATCAGAAACTGACCTGGTTTTTCCAGATGGTGCTTGAATGAAAGAGTTGCAGT
TTTCTAAAAGATCTGGGGGT
>Marker991665
GACAACAACCATACAGGTTATTGAAAGTGAGATTGGATAATTAGTGAGAGTATTATAGTAATTTTACGCACATAC
AAATTXXXXXXXXXXXCAAACATTTTTAACTTTTTCTTTTTCAAAATTAAATGAAAACTAAATCAAATTAAACACA
CTAGATCTTTTTAAATTGGT
GACAACAACCATACAGGTTATTGAAAGTGAGATTGGAGAATTAGTGAGAGTATTATAGTAATTTTACGCACATAC
AAATTXXXXXXXXXXXCAAACATTTTTAACTTTTTCTTTTTCAAAATTAAACGAAAACTAAATCAAATTAAACACA
CTAGATCTTTTTAAATTGGT
>Marker992080
ACATTGGAAATGTGATACAAGTCATAATCCTAACTCACAAATTTACATGAGTGCCAATAGCAAGAAAAGTGCCAA
AAATGXXXXXXXXXXXTAACCAAACAACCACCCAATAATATGAAGTAACCATAAACTGAGAGAAACATTCTAAACA
AACTGAGAATGATGGAGAGT
ACATTGGAAATGTGATACAAATCATAATCCTAACTCACAAATTTACATGAGTGCCAATAGCAAGAAAAGTGCCAA
AAATGXXXXXXXXXXXTAACCAAACAACCACCCAATAATATGAAGTAACCATAAACTGAGAGAAACATTCTAAACA
AACTGAGAATGATGGAGAGT
>Marker992828
ACTGGGACGACAAATATGGCACCCATAGTGTTTCACCCGATGTAAGATTTTAAACATTGTATTTCATCTCTTGTA
GTTCGXXXXXXXXXXXGAACCATGCAATTAGCAATTCATTTCTATTAGACGACGATTCAAGGTTAGTATCTTTTTA
CCAGGATTTGAAGGACAGTA
ACTGGGACGACAAATATGGCACCCATAGTGTTTCACCCGATGTAAGATTTTAAACATTGTATATCATCTCTTGTA
GTTCGXXXXXXXXXXXGAACCATGCAATTAGCAATTCATTTCTATTGGACGACGATTCAAGGTTAGTATCTTTTTA
CCAGGATTTGAAGGACAGTA
>Marker993231
AACACTTTTGATAGACTTGCGAGGAGGAAAACTTTGCTTGTGGGGCCTTTTTGCTGCATTCTTTGTTGAAGGCAG
TGGAAXXXXXXXXXXXGTTTGTGCGACGATCGAGGAGCTCCTCTTCCATCCGCTTTTTCAGAGAGAAATGGTGTTT
TCTATAGCTTGTTGGATGTG
AACACTTTTGATAGACTTGCGAGGAGGAAAACTTTGCTTGTGGGGCCTTTTTGCTGCATTCTTTGTTGAAGGCAG
TGGAAXXXXXXXXXXXGTTTGTGTGACGATCAAGGAGCTCCTCTTCCATCCGCTTTTTCAGAGAGAAATGGTGTTT
TCTATAGCTTGTTGGATGTG
>Marker993527
AACATCCATCTTTCCTTCTAGATTTGTTTTAAGTTATTAGGTATATTTTGTCAATATTTTAAAAATTAAAGTTAA
AATTTXXXXXXXXXXXTAAGGAAATAACATTGTTTATTTATACTCCCAACCTAAGTTATTACATATGTTATTAGTA
TTTTTACACATATTGGAGTG
AACATCCATCTTTCCTTCTAGATTTGTTTTAAGTTATTAGGTATATTTTGTCAATATTTTAAAAATTAAAGTGAA
AATTTXXXXXXXXXXXTAAGGAAATAACATTGTTTATTTATACTCCCAACCTAAGTTATTACATATGTTATTAGTA
TTTTTACACATATTGGAGTA
>Marker994133

Create PDF with GO2PDF for free, if you wish to

CACAAAAGAATGAATCCCATCCTAAGACCACACCCGGCGACCTTCTCTGTTATTACCACACTCAATGCGTCCAGA
CAAGGXXXXXXXXXXXATATGAGATTACAGAATGGAGTCGGAACTACGAAAACAATTTCATTGGCTATAAGAGATG
CAAGACTAAACTTTGATAGT
CACAAAAGAATGAATCCCATCCTAAGACCACACCCGGCGACCTTCTCTGTTATTACCACACTCAATGCGTCCAGA
CAAGGXXXXXXXXXXXATATGAGATTACAGAAGGGATTCGGAACTACGAAAACAATTTCATTGGCTATAAGAGATG
CAAGACTAAACTTTGATAGT
>Marker995135
TACTTAATCAAACTTGTAATTATTGGTTAATATTTTGGTTTTAGATAGTATGAAGATTTCAAATTGTGGTAAGAG
TAGGTXXXXXXXXXXXAATATTAAACACAAGAAACCAAATCTAGAGATCAACCAAAATGTATCTTTAGTGCAATAT
CATCCATTTATGTTTGGGTG
TACTTAATCAAACTTGTAATTATTGGTTAATATTTTGGTTTTAAATAGTATGAAGATTTCAAATTGCGGTAAGAG
TAGGTXXXXXXXXXXXAATATTAAACACAAGAAACCAAATCTAGAGATCAACCAAAATGTATCTTTAGTGCCATAT
CATCCATTTATGTTTGGGTG
>Marker995289
GACATCCACCTGTATTTTGAGAAAGCTGGGGAGTAAGAACTCCGACTCTTTTCTTTGTTATTTATTCTTTTTGCT
TCCTCXXXXXXXXXXXGCTTGACTTAGCTTCATAATACTTTGTTCAATTTTCTGGTTAGAACTTAGTAACTATGGT
TCTGAACGAAATACAAAGTT
GACATCCATCTGTATTTTGAGAAAGCTGGGGAGTAAGAACTCCGACTCTTTTCTTTGTTATTTATTCTTTTTGCT
TCCTCXXXXXXXXXXXGCTTGACTTAGCTTCATAATACTTTGTTCAATTTTCTGGTTAGAACTTAGTAACTATGGT
TCTGAACGAAATACAAAGTT
>Marker995453
AACGCTTCTCTACTCCTTTTTCGATTTCAATTTCGTTTTCACCAAACCTCTCCATTTCTTCTCCACTACTCCTCT
CCGTTXXXXXXXXXXXTTAGATATACCATTAAATGTGTATGACCCATCTCTTTGATGCCATTGAGATTTTTCAGCA
AACAAATTGATGCATATGTC
AACGCTTCTCTACTCCTTTTTCGATTTCAATTTCGTTTTCACCAAACCTCTCCATTTCTTCTCCACTACTCCTCT
CCGTTXXXXXXXXXXXTTAGATATACCATTAAATGTGTCTGACCCATCTCTTTGATGCCATTGAGATTTTTCAGCA
AACAAATTGATGCATATGTC
>Marker995782
ACTGATCTTCGGATTAATTTTTGCTTTAGATTTTGAATTTAAATCATCATCAAACCCTTTGTCACATATTCAGTG
AAATAXXXXXXXXXXXTAATTAAAGTTTAATCAGAAGTTCCAAAAAAATTAGCCACGTGGTGCCGACGTGGGTTCGA
AATCGCGTGCAACGCACGTG
ACTGTTCTTCGGATTAATTTTTGCTTTAGATTTTGAATTTAAATCATCATCAAACCCTTTGTCACATATTCAATG
AAATAXXXXXXXXXXXTAATTAAAGTTTAATCAGAAGTTCCAAAAAAATTAGCCACGTGGTGCCGACGTGGGTTCGA
AATCGCGTGCAACGCACGTG
>Marker996141
TACAATCCCATGGTTTATTTCTTCCAACTAAACGGAGATTCAAACTTCACTGAAATCGAAATTAAAACCCACCTC
AAAACXXXXXXXXXXXCCTTTCTTAGAAACTCGCGTCAAATCCACACTCCACGACGTCTTTTCCATGTCCTTTCCG
GAGGATCTCAACTGCCTTGT
TACAATCCCATGGTCTATTTCTTCCAACTAAACGGAGATTCAAACTTCACTGAAATCGAAATTAAAACCCACCTC
AAAACXXXXXXXXXXXCCTTTCTTAGAAACTCGCATCAAATCCACACTCCACGACGTCTTTTCCATGTCCTTTCCG
GAGGATCTCAACTGCCTTGT
>Marker996745
TACCTTCAGAAAGAATGCCACAGCAACAATCAAAAGGCTAAGAGTCATTCCAACAGAGGAGATCTGAAATAAAAT
TATAAXXXXXXXXXXXTAAAAAACATACAAGTCGTTGCTGAAAAGCAGAGTTCAGATAGAGCAAATCAATCACTAA
GCACATCGATTTTAGATTGT

Create PDF with GO2PDF for free, if you wish to

TACCTTCAGAAAGAATGCCACAGCAACGATCAAAAGGCTAAGAGTCATTCCAACAGAGGAGATCTGAAATAAAAT
TATAAXXXXXXXXXXXTAAAAACATACAAGTCGTTGCTGAAAAGCAGAGTTCAGATAGAGCAAATCAATCACTAA
GCACATCGATTTTAGATTGT
>Marker997547
ACCAAAAAAAATGTTGTCCCATTCCTTCCTTCATCCAAATATTTATTACAAAAAAAAGTTCTCACTATTTGCATC
AAGATXXXXXXXXXXXTGAAATATATATATATATATCATCTCTCCCATTCTCCTTTCTTCTAGGGTTTTAAAGCTA
ATTAAAGTTACGATTTAGTC
ACCAAAAAAAATGTTGTCCCATTCCTTCCTTCATCCAAATATTTATTACAAAAAAAAGTTCTCACTATTTGCATC
AAGATXXXXXXXXXXXGATGAAATATATATATATATATCATCTCTCCCATTCTCCTTTCTTCTAGGGTTTTAAAGCTA
ATTAAAGTTACGATTTAGTC
>Marker998407
AACTAAAACCACCAAACATATTTGATTTACTAGAAGGAAGTTTGTTGGTGGTGCGCTAGACCCTTCCATTTTCAG
AGGAGXXXXXXXXXXXTTGAAAACGAAATGCAAGAATTTCAAGGATGTTTCTCATCTTTCGGTAGTTTTTTGCATT
TGATTTATTTCATACATTGT
AACTAAAACCACCAAACATATTTGATATACTAGAAGGAAGTTTGTTGGTGGTGCGCTAGACCCTTCCATTTTCAG
AGGGGXXXXXXXXXXXTTGAAAACGAAATGCAAGAATTTCAAGGATGTTTCTCATCTTTCGGTAGTTTTTTGCATT
TGATTTATTTCATACATTGT
>Marker998575
TACTTGGTCATGGTAGTGAAGTTAGTCATTGGATTCCACAAAAGGTAGGTGGTCAGATGGAGGGGATTCGTGTAT
CTTATXXXXXXXXXXXTTAGAACAACAAAAGTTGCCTGTGGTGTTTGGCACACTGCTGCTGCTGTTGAAGCAATTA
ATGAACTTTCTGATTCTGGT
TACTTGGTCATGGTAGTGAAGTTAGTCATTGGATTCCACAAAAGGTAAGTGGTCAGATGGAGGGGATTCGTGTAT
CTTATXXXXXXXXXXXTTAGAACAACAAAAGTTGCCTGTGGTGTTTGGCACACTGCTGCTGCTGTTGAAGCAATTA
ATGAACTTTCTGATTCTGGT
>Marker999994
ACATTTTGTCTGTCTTAAAAAATTAATTGCTCCTTGATTTGACCTAATTCTTGCAGCCTTTGGATGTCGTCCTCT
CTCAAXXXXXXXXXXXTCTTCTTTTTCTCAATTGTCGTAGTATGGCAAATATGAAAATATTAGAAGAATATTGAAT
TTTTATGTTTATAATATGTT
ACATTTTGTCTGTCTTAAAAAATTAATTGCTCCTTGATTTGACCTAATTCTTGCAGCCTTTGGATGTCGTCCTCT
CTCAAXXXXXXXXXXXTCTTCTTTTTCTCAATTGTCGTTGTATGGCAAATATGAAAATATTAGAAGAATATTGAAT
TTTTATGTTTATAATATGTT
>Marker1000252
AACAACAAGTAACAAAAAGAAAACCCAACTGGTATAAATATGAATTAGGCTACAATTACAGAAGAAAAGAAGAAA
GAGTAXXXXXXXXXXXAACATATTTAATGAAAGAGAAAGAGTGATATAAAACACAATAGATATGAAATGTGGAAGG
AAAAAGCAAGAGAGATTGTT
AACAACAAGTAACAAAAAGAAAACCCAACTGGTATAAATATGAATTAGGCTACAATTACAGAAGAAAAGAAGAAA
GAGTAXXXXXXXXXXXAACATATTTAATGAAAGAGAAAGAGTGATATAAAACACAATAGATATGAAATGAGGAAGG
AAAAAGCAAGAGAGATTGTT
>Marker1000474
TACTCTCTCCGTTTGCCTGGTGTTGGCCTTGTTGGATCGATTCCGGCTAATACAGTTGGGAAATTGACTCAGCTC
CGAGTXXXXXXXXXXXCGAGCTTGATTCGGCTGACTCGGCTGACTCGGCTGGATTTGTCGTCGAATGAATTTTCCG
GTCCGATTCCGGCTTCTGTT
TACTCTCTCCGTTTGCCTGGTGTTGGCCTTGTTGGATCGATTCCGGCTAATACAGTTGGGAAATTGACTCAGCTC
CGAGTXXXXXXXXXXXCGAGCTTGACTCGGCTGACTCGGCTGACTCGGCTGGATTTGTCGTCGAATGAATTTTCCG
GTCCGATTCCGGCTTCTGTT
>Marker1000814

Create PDF with GO2PDF for free, if you wish to

ACATTTCTTGTGCTTTTGTTATAACGCCCTAGGCCCAAGATTCAGAATTCGGATCCTCGACATTCCCTTTGTATC
CCCTAXXXXXXXXXXXAACACCCAACATATAATTACTCCAAACTAAGCACGATTAACTTTGGAGTTCTTATGATCG
AGCCATTGAAAAGGAAGGTG
ACATTTCTTGTGCTTTTGTTATAACGCCCTAGGCCCAAGATTCAGAATTCGGATCCTCGACATTCCCTTTGTATC
CCCTAXXXXXXXXXXXAACACCCAACATATAATTACTCCAAACTAAGCACAATTAACTTTGGAGTTCTTATGATCG
AGCCATTGAAAAGGAAGGTG
>Marker1001042
AACATAGGAAAAGTGTTGAGACTACTCTTGCGGAGCTTGGTGTTACTCTTAGCAATGCAGCTTACAAGTTGAATG
TTAGGXXXXXXXXXXXATCACATTTATACCGGGCCTAAAGACTCCATGATTTACAAAGCAATGAAAGAATGTGATC
CTTCTGGGCCTTTAATGGTT
AACATAGGAAAAGTGTTGAGACTACTCTTGCGGAGCTTGGTGTTACTCTTAGCAATGCAGCTTACAAGTTGAATG
TTAGGXXXXXXXXXXXATCATATTTATACCGGGCCTAAAGACTCCATGATTTACAAAGCAATGAAAGAATGTGATC
CTTCTGGGCCTTTAATGGTT
>Marker1001047
TACTCTGTCTTTCCCATTGTAAATATCCTATGCTCAAATGCATTATCTGGAAAAGAAATTGTTGTTCTTTTGGTT
TATTCXXXXXXXXXXXATTATCTGTAATTGAACCCTTTTTTCTCACATGGAAGCAAGCACTGCAATATATTATGAT
TTGATAGAGATTCTATGGTT
TACTCTGTCTTTCCCATTGTAAATATCCTATGCTCAAATGCATTATCTGGAAAAGAAATTGTTGTTCTTTTGGTT
TATTCXXXXXXXXXXXATTGTCTGTAATTGAACCCTTTTTTCTCACATGGAAGCAAGCACTGCAATATATTATGAT
TTGATAGAGATTCTATGGTT
>Marker1001741
ACATTAAAAAATCTATGTCATATGAATTTTAAAGCATTTCTTTTTGAAAATTTAAAATATAAGATTATTATGTTC
ATAAAXXXXXXXXXXXXATGGAAGGGTAATATTGTAGAAATATATACAATTTTAGAGAAAAAAAGGAAAACAAAAAA
TAAAAGACAGAAACTAAAGT
ACATTAAAAAATCTATGTCATATGAATTTTAAAGCATTTCATTTTGAAAATTTAAAATATAAGATTATTATGTTC
ATAAAXXXXXXXXXXXXATGGAAGGGTAATATTGTAGAAATATATACAATTTTAGAGAAAAAAAGGAAAACAAAAAA
TAAAAGACAGAAACTAAAGT
>Marker1002169
ACTTCAAATAGACTGAATATTGATGAAAACATTGTTTGAAATGTTTGAGTCATAGAGTCTTGGGTAGTAAATCAC
CTCTTXXXXXXXXXXXAACAGAACTTCACTAAGTTGGAAAAGTTTCCATTGAGGGAACCTTGATTTGAAGTTTCAA
TATTACCTTTTAGGTCTGTT
ACTTCAAATAGACTGAATATTGATGAAAACATTGTTTGAAATGTTTGAGTCATAGAGTCTTGGGTAGTAAATCAC
CTCTTXXXXXXXXXXXAACAGAACTTCACTAAGTTGGAAAAGTTTCCATTGAGGGAACCTTGATTTGAAGTTTCAA
TATTACCTTTTAAGTCTGTT
>Marker1002381
ACACGTATTTATTTTTAATTTTTGCAGATGTTTTCTTGTATGTGGTCATATATTTTCTTCCCATTGTGGTTTATT
AGTCTXXXXXXXXXXXTTTGACTGTAGATACCATGGGAGAACAAACTTTTATCAGTGGCTGGTCATACTAAGGATC
CTTCTGATGCTATTCAGGGT
ACACGTATTTATTTTTAATTTTTGCAGATGTTTTCTTTTATGTGGTCATATATTTTCTTCCCATTGTGGTTTATT
AGTCTXXXXXXXXXXXTTTGACTGTAGATACCATGGGAGAACAAACTTTTATCAGTAGCTGGTCATACTAAGGATC
CTTCTGATGCTATTCAGGGT
>Marker1002719
AACATATCACCAACAATAACATTATTATTAACATAATGAACATCAATCAAGGGTTGATCCAACAATATAATAATA
GATCCXXXXXXXXXXXGACTTTGTTTTTTCCAATAATTAATCCAACCAAGAATTTAATTGTTCAAATATTGTGTTG
TTGGATTTCAATTCGTAGTC

Create PDF with GO2PDF for free, if you wish to

AACATATCACCAACAACAACATTATTATTAACATAATGAACATCAATCAAGGGTTGATCCAACAATATAATAATG
GATCCXXXXXXXXXXGACTTTGTTTTTTCCAATAATTAATCCAACCAAGAATTTAATTGTTCAAATATTGTGTTG
TTGGATTTCAATTCGTAGTC
>Marker1002940
GACCAAGCTAAGTTTTGTGCTAACCACCGTATAAAAGCATTGGGTTGCATGCCATTCAAACGTAGTCTTTAAGAG
CAATAXXXXXXXXXXXCTAATTGTAGATTATCATAATATAAAATAATTTACAAATATAAAAAAGCTTAGGCAATGC
TCCAAGGGGTCGCTTGGAGT
GACCAAGCTAAGTTTTGTGCTAACCACCGTATAAAAGCATTGGGTTGCATGCCATTCAAACGTAGTCTTTAAGAG
CAATAXXXXXXXXXXXCTAATTGTAGATTATCATAATATAAAATAATTTACAAATATAAAAAAGGTTAGGCAATGC
TCCAAGGGGTCGCTTGGAGT
>Marker1003070
AACGAAAAAAGACTTACCACAACCAGAAACCGTCAGCTCAGTCCATGGCGCAGAGACAGGGAGAGAGTGGAAAAT
CAAAAXXXXXXXXXXXCCTCTTTTGAAGCCGACGACGACGATCATTGACATCTTTGAATATGTAATTACTATATTA
CCCTCACGGATGACTCCGTC
AACGAAAAAAGACTTACCACAACCAGAAACCGTCAGCTCAGTCCATGGCGCAGAGACAGGGAGAGAGTGGAAAAT
CAAAAXXXXXXXXXXXCCTCTTTTGAAGCCGACGACGACGATCATTGACATCTTTTAATATGTAATTACTATATTA
CCCTCACGGATGACTCCGTC
>Marker1003285
GACATGTTGTTCAACATTGATTAAGAAGAAGAGTTGTAATGTTTAGGGAAGTCTAATTATACTATAGTGCTGGAG
TTAAAXXXXXXXXXXXCAAGTATAATCTTGTCATTCATTGAGATTACCTACTAAAGATAAGTAGCATACTTGTAAT
CCATATTATATTCTCTAGTG
GACATGTTGTTCAACATTGATTAAGAAGAAGAGTTGTAATGTTTAGGGAAGCCTAATTATACTATAGTGCTGGAG
TTAAAXXXXXXXXXXXCAAGTATAATCTTGTCATTCATTGAGATTACCTACTAAAGATAAGTAGCATACTTGTAAT
CCATATTATATACTCTAGTG
>Marker1003363
GACCTAGAGTCATGTTCTCTAGGGTTTTCGAATCACCTTAGGATAATTATATAACCAATAAAACATAAGAATATA
TTTTTXXXXXXXXXXXTCTATGGTTTATTGGACATTTGACCAGGGTTGCCTCGACCTAGATTGAGGTTGCCTAAAC
CAACCACATTTTTTTAGGTC
GACCTAGAGTCATGTTCTCTAGGGTCTTCGAATCACCTTAGGATAATTATATAACCAATAAAACATAAGAATATA
TTTTTXXXXXXXXXXXTCTATGGTTTATTGGACATTTGACCAGGGTTGCCTCAACCTAGGTTGAGGTTGCCTAAAC
CAACCACATTTTTTTAGGTC
>Marker1003554
ACTTCATTCATTATATTACTTATTTTCTATTTTTCAATTTGTATTTTACTATATATGTTTCAAATTGATGGTATA
AATTTXXXXXXXXXXXCTACATAAAAACTTTAGCGAGTAAAGTAAAATTATACATTTTTTTGTCTTAAATTATTTG
ATTTCCAAGATATCTAAGTT
ACTTCATTCATTATATTACTTATTTTCTATTTTTCAATTTGTATTTTACTAAATATGTTTCAAATTGATGGTATA
AATTTXXXXXXXXXXXCTACATAAAAACTTTAGCGAGTAAAGTAAAATTATACATTTTTTTGTCTTAAATTATTTG
ATTTCCAAGATATCTAAGTT
>Marker1003574
TACAATGTCTGGCTTGATCTCCCGGAGTCTCGGAATATTGCATATTTGATTAGCCAAGTCACCGGTGACACGATC
CATGTXXXXXXXXXXXTTGGAGAACAATTAGAGGGGAAAATACTTGATGCAAAACATATTTCCTACAAATAATTTT
GGAGAAATATTAAATGGAGT
TACAATGTCTGGCTTGATCTCCCGGAGTCTCGGAATATTGCATATTTGATTAGCCAAGTCACCGGTGACACGGTC
CATGTXXXXXXXXXXXTTGGAGAACAATTAGAGGGGAAAATACTTGATGCAAAACATATTTCCTACAAATAATTTT
GGAGAAATATTAAATGGAGT
>Marker1003649

Create PDF with GO2PDF for free, if you wish to

ACTAGAGCTCTAAACAGGGAGCTAATACAACTTTTTCCTTATCTGCTTCGATCAATGTGCCTAGACCTACGATTT
CTTTGXXXXXXXXXXCTACATTGACAAAACCAACTATGGAATATTATCATTGTATTGGCCTTCACGAAAGGTATA
TTGGTGGAAGCAACCGTTGT
ACTAGAGCTCTAAACAGGGAGCTAATACAACTTTTTCCTTATCTGCTTCGATCAATGTGCCTAGACCTACGATTT
CTTTGXXXXXXXXXXCTGCATTGACAAAACCAACTATGGAATATTATCATTGTATTGGCCTTCACGAAAGGTATA
TTGGTGGAAGCAACCGTTGT
>Marker1004021
AACCAAGTTTTCACCAACGTTGTTGGGAGGGATGGACCTCATTTTTCAAGTCCTCAAATCAACACTTTAACAAAG
CTACAXXXXXXXXXXXAGATAATTTCTTAAATGTTAAATTTGACGTTTAATGACTATCGTTAATGCTGCTAATGGC
TATTAAATGATAACATTGTA
AACCAAGTTTTCACCTACGTTGTTGGGAGGGATGGACCTCATTTTTCAAGTCCTCAAAGCAACACTTTAACAAAG
CTACAXXXXXXXXXXXAGATAATTTCTTAAATGTTAAATTTGACGTTTAATGACTATCGTTAATGCTGCTAATGGC
TATTAAATGATAACATTGTA
>Marker1004028
TACTAACAAATAGCAAACAAAATACCTCTCATAGAGCACAGGGTGCAGATGAGGATGGCAAGAATTAACAGCTCT
AACAAXXXXXXXXXXXTGATTAGGGATAATTAAGAATTCAAAAGATTCTACATAACACCTTGAGAACCCCTGCCCT
TTTGTCCAAATTAAGTGGTA
TACTAACAAATAGCAAACAAAATACCTCTCATAGAGCACAGGGTGCAGATGAGGATGGCAAGAATTAACAGCTCT
AACAAXXXXXXXXXXXTGATTAGGGATAATTAAGAATTAAAAAGATTCTACATAACACCTTGAGAACCCCTGCCCT
TTTGTCCAAATTAAGTGGTA
>Marker1004061
AACATTACTAATTTTTGTGTGGTTTCTGCAGAAGGTCCGTGTAGAAACACTTTGGCAACTTTGCAGCAGGATTAC
AAACAXXXXXXXXXXXGCCACAAAAGAACCGCAAAGGGAGTGGCTGTTTGATCGGATTACATTGCCTAACACAGAA
TTCTGAGGTAAAGTTAATGT
AACATTACTAATTTTTGTGTTGTTTCTGCAGAAGGTCCGTGTAGAAACACTTTGGCAACTTTGCAGCAGGATTAC
AAACAXXXXXXXXXXXGCCACAAAAGAACCGCAAAGGGAGTGGCTGTTTGATCGGATTACATTGCCTAACACAGAA
TTCTGAGGTAAAGTTAATGT
>Marker1004685
GACTGTGTGCAAATATAGTATTAGAAATGGCCAACTCTCCAAGAGCCCTCAGCATCAATGATAAGTTTTTCTGTA
TTTTCXXXXXXXXXXGAAGCATTATCAAAAAATAAAGATAATAAAATAATTTTTGAAGTTTTCAAGTTTTTCTAA
AAAAACATTCATTTTAGGTT
GACTGTGTGCAAATATAGTATTAGAAATGGCCAACTCTCCAAGAGCCCTCAGCATCAATGATAAGTTCTTCTGTA
TTTTCXXXXXXXXXXGAAGCATTATCAAAAAATAAAGATAATAAAATAATTTTTGAAGTTTTCAAGTTTTTCTAA
AAAAACATTCATTTTAGGTT
>Marker1004780
ACATAAAATTAATCCAGACTTGGTTCTTATACAAGAAACCAAGAGAGCTTCCTTTAAGATATAGCCTTGATTAAG
AAGTTXXXXXXXXXXCAATACATTAGTAGAAAGCTAAATACAAAGCAAAACTTAAATGCTCTCTGAAAAACATCC
CACTCAAATCTAAATTCGTG
ACATAAAATTAATCCAGACTTGGTTCTTATACAAGAAACCAAGAGAGCTTCCTTTAAGATATAGCCTTGATTAAG
AAGTTXXXXXXXXXXCAATACATTGGTAGAAAGCTAAATACAAAGCAAAACTTAAATGCTCTCTGAAAAACATCC
CACTCAAATCTAAATTCGTG
>Marker1005178
ACTCAACAAGCACTCAAGAGGTGTATTGCCAAGCGAATTCTATCGAACCAGCAACCGTAACAATATATAACACAT
TACTCXXXXXXXXXXXATAGGTTCCAAATTTTTAGTATCCCTTTCCTATGCTTCACTTGAGCAGTTTGTGGATCTT
CTGTGATTGAGATTATAGTG

Create PDF with GO2PDF for free, if you wish to

ACTCAACAAGCACTCAAGAGGTGTATTGCCAAGCGAATTCTATCAAACCAGCAACCGTAACAATATATAATACAT
TACTCXXXXXXXXXXATAGGTTCCAAATTTTTAGTATCCCTTTCCTATGCTTCACTTGAGCAGTTTGTGGATCTT
CTGTGATTGAGATTATAGTG
>Marker1005320
ACAAAGTCTTTCACAACTAGTTCAATGTATTCACAAATATCACTTAATTAGCTCAATAGTATCTTTTACTATCCT
CGATCXXXXXXXXXXAAGATACCAACAATTGAACGAGATACCAACATTTTTTCTCTAACAAATTTAACCCTACAT
CAAAATACAGAAAATGGGTA
ACAAAGTCTTTCACAACTAGTTCAATGTATTCACAAATATCACTTAATTAGCTCAATAGTATCTTTTACTATCCT
CGATCXXXXXXXXXXAAGATACCAACAATTGAACGAGATACCAACATTTTTTCTCTAACAAATTTAACCCTACAT
CGAAATACAGAAAATGGGTA
>Marker1007299
TACCATATTTTCTTCGAAAGGAATAAAGAGCAGTATGACATTAGAAACTATACAAACTTGTGTCTCATACCTCTC
TCTTGXXXXXXXXXXTGAACATGTGGCTTCTTTCATATTTGGCAAGGTAGATGAAAACAAACTTTTAAATTTTTT
TTAAAAGAAAAGGTGTGGTA
TACCATATTTTCTTCAAAAGGAATAAAGAGCAGTATGACATTAGAAACTATACAAACTTGTGTCTCATACCTCTC
TCTTGXXXXXXXXXXTGAACATGTGGCTTCTTTCATATTTGGCAAGGTAGATGAAAACAAACTTTTAAATTTTTT
TTAAAAGAAAAGGTGTGGTA
>Marker1007361
ACTATCATACTACTATAAATTTTTCCAAAAAATTACTTGTGCTATGATCACACTATGCATACTCATATTTACTGT
TGGTCXXXXXXXXXXACACAACTCCATACCCTCCTTCTCCAATCAAATTCTCTTCTGAGAACAAATTAGTTGCCA
AATCCAGCTCTCTCAAAGTA
ACTATCATACTACTATAAATTTTTCCAAAAAATTACTTGTGCTATGATCACACTATGCATACTCATATTTACTGT
TGGTCXXXXXXXXXXATACAACTCCATACCCTCCTTCTCCAATCAAATTCTCTTCTGAGAACAAATTAGTTGCCA
AATCCAGCTCTCTCAAAGTA
>Marker1007754
AACTTTTTGATGAATTAGATTGCTTATAAGTCTTGGAATATAGATGATACCCTAATTTAAGCAATGGGCACTCCA
TCAATXXXXXXXXXXAAGATTGTCAATCTAAATTAATGAGATAACATTGTGTTTAACAAAAAGAATAGTGTAAAG
CACTACACGTAGAGGCTGTT
AACTTTTTGATGAATTAGATTGCTTATAAGTCTTGGAATATAGATGATACCCTAATTTAAGCAATGGGCACTCCA
TCAATXXXXXXXXXXAAGATTGCCAATCTAAATTAATGAGATAACATTGTGTATAACAAAAAGAATAGTGTAAAG
CACTACACGTAGAGGCTGTT
>Marker1008490
ACCATATTTAGTATTGGCTTACCTTTGTAATTTTTGTATTAAAGTGATTACTATTTTGTTCTCAAAGCCAAATAG
TTTTCXXXXXXXXXXGTTTTAAAATGTTTATTTTGATCCATAGTTAGAGCTATCAATTTAGCTGGTAGTGACAAC
ATATGAAGAAGCATAGTGTT
ACCGTATTTAGTATTGGCTTACCTTTGTAATTTTTGTATTAAAGTGATTACTATTTTATTCTCAAAGCCAAATAG
TTTTCXXXXXXXXXXGTTTTAAAATGTTTATTTTGATCCATAGTTAGAGCTATCAATTTAGCTGGTAGTGACAAC
ATATGAAGAAGCATAGTGTT
>Marker1008492
AACAATTCTTCCAAGTTTTCAACGAACTCACATTTTTTTCTTTTCTCTCATCAATGTTTGCACATTTTTCTTGTT
CATATXXXXXXXXXXTAATACAAAAGTAGAATAAAATATTACTTTGGGCTTTAATCGACTTCAAATGTATGCCCA
AAGTATGTCGGTATCAAGTT
AACAATTCTTCCAAGTTTTCAACGAACTCACATTTTTTTCTTTTCTCTCATCAATGTTTGCACATTTTTCTCGTT
CATATXXXXXXXXXXTAATACAAAAGTAGAATAAAATTTTACTTTGGGCTTTAATCGACTTCAAATGTATGCCCA
AAGTATGTCGGTATCAAGTT
>Marker1008595

Create PDF with GO2PDF for free, if you wish to

ACAAATTGTTAGCAAAAGCATTTAGTTTATGTTATTTTGTTTTATTGTAAGCACGCATACAGATTATGACACCAC
GTTGAXXXXXXXXXXXTTCATTAGTGAAGAAAGATATATTTTGTGACTTCGAAATGATAACAAAGAACTTCTTGGT
CCATCTTAGTGAAATTGGTG
ACAAATTGTTAGCAAAAGCATTTAGTTTATGTTATTTTGTTTTATTGTAAGCACGCATACAGATTGTGACACCAC
GTTGAXXXXXXXXXXXTTCATTAGTGAAGAAAGATATATTTTGTGACTTCGAAATGATAACAAAGAACTTCTTGGT
CCATCTTAGTGAAATTGGTG
>Marker1009348
AACACGTTTTAATCACTAAACTATGTTTGAAACTGACGTTTTCATTATTAAATAAATGTTTGTATTGAGAACTAA
TTAAAXXXXXXXXXXXXAATTTATTTAAAATATTAGAAACTCCAAGTGTAAGTATATTGGCATGAAAATTCGCTTAA
ATTTAGTAAATCGGAAAGTT
AACACGTTTTAATCACTAAACTATGTTTGAAACTGACGTTTTCATTATTAAATAAATGTTTGTATGGAGAACTAA
TTAAAXXXXXXXXXXXXAATTTATTTAAAATATTAGAAACTCCAAGTGTAAGTATATTGGCATGAAAATTCGCTTAA
ATTTAGTAAATCGGAAAGTT
>Marker1010011
TACTTTGTTTTTAATTTTCAAAATTTTTAAAATTATTGATAAAAAGTAGATAAACAGATAAAAAGAAAATTCTAT
TTAGAXXXXXXXXXXXAGTTAATTGTGGATTTCATAATTCATATTAACTCTACAGACAAAAATTGAAGATGTGTTT
GGATTATGTGGTGAATTGTT
TACTTTGTTTTTCATTTTCAAAATTTTTAAAATTATTGATAAAAAGTAGATAAACAGATAAAAAGAAAATTCTAT
TAAGAXXXXXXXXXXXAGTTAATTGTGGATTTCATAATTCATATTAACTCTACAGACAAAAATTGAAGATGTGTTT
GGATTATGTGGTGAATTGTT
>Marker1010387
TACTTTGTTTGAATTTTTTCTCTCTGAGAATCTGCACCCATATCATTTGAATTAATTATTGTTTTTCCATTCAAC
TTCATXXXXXXXXXXXAGTTAGCATGGTGTGATGAACGAATGTATTTTCGAAAATAACCATCTTAAAAAGAGGAGA
AGCAACTATTTGAATGTGTA
TACTTTGTTTGAATTTTTTCTCTATGAGAATCTGCACCCATATCATTTGAATTAATTATTGTTTTTCCATTCAAC
TTCATXXXXXXXXXXXAGTTAGCATGGTGTGATGAACGAATGTATTTTCGAAAATAACCATCTTAAAAAGAGGAGA
AGCAACTATTTGAATGTGTA
>Marker1011028
ACAGTCATCTCCCTTATCACTAGATTCTATGACCCTCTTCAGGGTATGCCACCACCAGCCCTTTTCCTTTTCATC
CCCTTXXXXXXXXXXXCCTCAGGGAAAACATTGGAATAGTTTCCCAGGAACCTGCACTCTTTGCTGGAACCATCAA
GGATAATATCAAAATGGGTA
ACAGTCATCTCCCTTATCACTAGATTCTATGACCCTCTTCAGGGTATGCCACCACCAGCCCTTTTCCTTTTCATC
CCCTTXXXXXXXXXXXCGTCAGGGAAAACATTGGAATAGTTTCCCAGGAACCTGCACTCTTTGCTGGAACCATCAA
GGATAATATCAAAATGGGTA
>Marker1011198
AACTTTCGTAACTAGATCAATGCATGTAAATAACTATTTTCACATCATTTTAGTTAAAATCAACTCAAAATCACT
TAAAAXXXXXXXXXXXXTAGGTGAAAAGCTAATGCTAATTTCATTCCACATTTTTTTCTCTAATTTACATTAATCCC
TATACTTAAAGAAGTCTTGT
AACTTTCGTAACTAAATCAATGCATGTAAATAACTATCTTCACATCATTTTAGTTAAAATCAACTCAAAATCACT
TAAAAXXXXXXXXXXXXTAGGTGAAAAGCTAATGCTAATTTCATTACACATTTTTTTCTCTAATTTACATTAATCCC
TATACTTAAAGAAGTCTTGT
>Marker1011283
TACTAAAAGCATAGTCTCAATTTTTATTTAAAAAACAAATCTCCTTGATAGTGCCTTGAATGCCTTGCTAGGAAA
GGCCAXXXXXXXXXXXATGGAATGATATAGTCAAGGATTAATTAGTTTTATTTATTTATTAACAAGATAGTAATAG
GAGAGAAGAATTACTAGGTG

Create PDF with GO2PDF for free, if you wish to

TACTAAAAGCATAGTCTCAATTTTTATTTAAAAAACAAATCTCCTTGATAGTGCCTTGAATGCCTTGCTAGGAAA
GGCCAXXXXXXXXXXXATGGAATGATATAGTCAAAGATTAATTAGTTTTATTTATTTATTAACAAGATAGTAATAG
GAGAGAAGAATTACTAGGTG
>Marker1011348
AACCAAACTTGCAAGTCACAAAAGCATTTCAATTTTATTGACTATTTTAAATATATAAAACACATATGATATACC
CAAATXXXXXXXXXXXCAACAACAAAAAGAAATGGTAATAAACTCATGAATTTTATAAAAATATACAATCCATCAC
TCGTTTTCTTGACTTATTGT
AACCAAACTTGCAAGTCACAAAAGCATTTCAATTTTATTGACTATTTTAAATATATAAAACACATATGATATACC
CAGATXXXXXXXXXXXCAACAACAAAAAGAAATGGTAATAAATTCATGAATTTTATAAAAATATACAATCCATCAC
TCGTTTTCTTGACTTATTGT
>Marker1011427
GACCTAAGTGACTTGGTGTTAATTTTAAGTTTATTATAGACTTATATGTTTATGATGGATTGTCCTCTTATTTGT
ATGAAXXXXXXXXXXXTCAACTTAAGACAAATAGATGAGTGTTCTCTTAAATGGTGATTTTCGGATTTGAACAAAA
GGTCTTACTCTCTTATTGGT
GACCTAAGTGACTTGGTGTTAATTTTAAGTTTATTATAGACTTATATGTTTATGATGGATTGTCCTCTTATTTGT
ATGAAXXXXXXXXXXXTCAACTTAAGACAAATAGATGAGTGTTCTCTTAAATGGTGATTTTCGGATTTGAACAAAA
GGTCTTACTCTCTCATTGGT
>Marker1011758
GACCATCCAGCTGTAGTTCATCATGATGACGAGTCTGATGATGGGGCTTTTTCTTTGCATGATGAACAAACTACA
AGAAAXXXXXXXXXXXGTTTTTCTGTTTCTTTTGGGGTGTAAGAACTAAGAAGGTAAATATTGATGCATTGGATAT
TTGATCTTCTTCTAAAATGT
GACCATCCAGCTGTAGTTCATCATGATGACGAGTCTGATGATGGGGCTTTTTCTTTGCATGATGAACAAACTACA
AGAAAXXXXXXXXXXXGTTTTTCTGTTTCTTTTGGGGTGTAAGAACTAAGAAGGTAAATATTGATGCATTGGAGAT
TTGATCTTCTTCTAAAATGT
>Marker1012064
AACAAAACCAACCAAAGGTTTCAACTAAAGTGAGAATTAACATACCAAGTGTCTTCTAAGGATGAGAGACAATAT
GAAATXXXXXXXXXXXCTATCATGTTTTTATGTGGTAAGAATTTTTTGTTTCAAGTGTAGAGTTAAAAAAGTAGAA
TTCCCAAGTAGAAACAGAGT
AACAAAACCAACCAAAGGTTTCAACTAAAGTGAGATTTAACATACCAAGTGTCTTCTAAGGATGAGAGACAATAT
GAAATXXXXXXXXXXXCTATCATGTTTTTATGTGGTAAGAATTTTTTGTTTCAAGTGTAGAGTTAAAAAAGTAGAA
TTCCCAAGTAGAAACAGAGT
>Marker1012536
ACATGTTCTATGCTTTCTTCTCTCCAACGTGGTTATAACTTTACACGCTCCCCAGTTTATTTAACTTTAGATACT
ATGAAXXXXXXXXXXXTTTCAAGGATGATTGTATATATATTTGAGAAATTCATGGAGTTTCTTCAAGGAACATAAG
AAAACATTGGAGTGGGCGTT
ACATGTTCTATGCTTTCTTCTCTCCAACGTGGTTATAACTTTACACGCTCCCCAGTATATTTAACTTTAGATACT
ATGAAXXXXXXXXXXXTTTCAAGGATGGTTGTATATATATTTGAGAAATTCATGGAGTTTCTTCAAGGAACATAAG
AAAACATTGGAGTGGGCGTT
>Marker1013153
GACCAATTGGTCATATCCTTAAACTCTATTAGTTCGCCAACTTCTACTTAGTGTTTTACACATGGTAGTTTAGTA
TCCTTXXXXXXXXXXXCTTCCCTTCTCAACCTTCTTTCCTCACCTGAATATTCCTTGCCTAGCTCAAAACACATAC
TACCCATATAGTCAAATGTA
GACCAATTGGTCATATCCTTAAACTCTATTAGCTCGCCAACTTCTACTTAGTGTTTTACACATGGTAGTTTAGTA
TCCTTXXXXXXXXXXXCTTCCCTTCTCAACCTTCTTTCCTCACCTGAATATTCCTTGCCTAGCTCAAAACACATAC
TACCCATATAGTCAAATGTA
>Marker1013728

Create PDF with GO2PDF for free, if you wish to

CACACCTGAGAGACTCTTGGAGCTTGTTGCCATGCAGGCTATTGATATATCTGGTGTTTCGTTGCTGGTAATTTG
ACAGCXXXXXXXXXXTAAAATATCGATTATAATGTTGTTGGAATTTGAATGTTGTGGATTATTTAGATATGTTGA
TATTGACGGATATTTTTGTA
CACACCTGAGAGACTCTTGGAGCTTGTTGCCATGCAGGCTATTGATATATCTGGTGTTTCGTTGCTGGTAATTTG
ACAGCXXXXXXXXXXTAAAATATCGATTATAATGTTGTTGGAATTTGAATGTCGTGGATTATTTAGATATGTTGA
TATTGACGGATATTTTTGTA
>Marker1013904
CACCTATCTTAACAAATAGATAGCATAAAATTTGGAATATCCATACTTGAAGAATTCTATTTGTTATAAATCAAC
GTTCCXXXXXXXXXXTCAGCGAAGGCAATGGTTCGAGATCAAAATTTTTCACTGTGGCCATAAGTATCATACCTC
TTAGACGAATCAATTTTAGT
CACCTATCTTAACAAATAGATAGCATAAAATTTGGAATATCCATACTTGAAGAATTCTATTTGTTATAAATCAAC
GTTCCXXXXXXXXXXTCAACGAAGGCAATGGTTCGAGATCAAAATTTTTCACTGTGGCCATAAGTATCATACCTC
TTAGACGAATCAATTTTAGT
>Marker1014237
CACATATTAGCATTTCAATAGTTTCTTTGTGAGAATTTAAGGCTTGCTTGGAAGCCAAAATAGATTAAAATTTTA
ACGTAXXXXXXXXXXCCAAGTAATCAAGTAATTAATGTTTGGTCATATTTTAAGTCTTATTTTTTTAGAAACTCT
ACCTACTCAATTCACTAGTA
CACATATTAGCATTTCAATAGTTTCTTTGTGAGAATTTAAGGCTTGCTTGGAAGCCAAAATAGATTAAAATTTTA
ACGTAXXXXXXXXXXCCAAGTAATCAAGTAATTAATGTTTGGTCATATTTTAAGTCTTATTTTTTTAGAAACTCT
ACCCACTCAATTCACTAGTA
>Marker1014874
ACTTTCAAAATCAATAATGATTTTGGACTTTTTGAAAACTTAACAATTTTGACCTCTTACTTATATCATATCTCA
TTGATXXXXXXXXXXAATTCACCTTTATTTTGCTTTTCTTCTTTGACAAAAACACACTAACCAACTTCGACTACT
TATTCGCACGGTTAGTGGTG
ACTTTCAAAATCAATAATGATTTTGGACTTTTTGAAAACTTAAGAATTTTGACCTCTTACTTATATCATATCTCA
TTGATXXXXXXXXXXAATTCACCTTTATTTTGCTTTTCTTCTTTGACAAAAACACACTAACCAACTTCGACTACT
TCTTCGCACGGTTAGTGGTG
>Marker1015029
ACCTAAAATAAAAATGCGATTAAAAATCTATAGAGGGTAATTGCTACAATTAAATAATTTCAGATAAAAAACACA
TTATTXXXXXXXXXXTAAATAAGAATAATTACTATGGAAAACATACCTCATTAAACGATAGTTCCATAAAAGGAT
TTTATTCCCCTAATTTAGGT
ACCTAAAATAAAAATGTGATTAAAAATCTATAGAGGGTAATTGCTACAATTAAATAATTTCAGATAAAAAACACA
TTATTXXXXXXXXXXTAAATAAGAATAATTACTATGGAAAACATACCTCATTAAATGATAGTTCCATAAAAGGAT
TTTATTCCCCTAATTTAGGT
>Marker1015430
GACCTCATTGGCAAATGTCTTCTTAACTTGACAGATAGCTGTTGGCGATGTCCTGTGTGAAATTGAAACCGACAA
AGCTAXXXXXXXXXXTACTAAGAAAGTTATCCTTGAATAACCCTCAGCTACAACAATTTTATAATTATTATAGAT
TTCAAAATTCATCATCAGTG
GACCTCATTGGCAAATGTCTTCTTAACTTGACAGATAGCTGTTGGCGATGTCCTGTGTGAAATTGAAACCGACAA
AGCTAXXXXXXXXXXTACTAAGAAAGTTATCCTTGAATAACCCTCAACTACAACAATTTTATAATTATTATAGAT
TTCAAAATTCATCATCAGTG
>Marker1015528
AACGGAAGAAATGGATAAGATGAAATTGCCCAAAGCAAAAAAGCACAAAAAGTTGATAGAAATAACACAAAAAAT
CATCAXXXXXXXXXXAAACACACCAAAGAAAACACTATATACATATAAGACACAACCTAATTAAACTACAAACAA
CAACAAGAAATTTCTAAGTT

Create PDF with GO2PDF for free, if you wish to

AACGGAAGAAATGGATAAGATGAAATTGCCCAAAGCAAAAAAGCACAAAAAGTTGATAGAAATAACACAAAAAAT
CATCAXXXXXXXXXXXAAACACACCAAAGAAAACACTATATACATATAAGACACAATCTAATTAAACTACAAACAA
CAACAAGAAATTTCTAAGTT
>Marker1015621
CACTGAATCTTAGAGTTGATCATGGAAAGATTCTCTTCTGCTCTTAATTGATTGAATCATAAGTAGCTTCTAGGT
TCAAAXXXXXXXXXXXAGAAAAAATGGTGGATAATGAAGAACTAGGAAGTAGGAGCTTGGAAATATGTGTTTGTCA
AATTTGCGTGGAAAACTGTG
CACTGAATCTTAGAGTTGATCATGGAAAGATTCTCTTCTGCTCTTAATTGATTGAATCATAACTAGCTTCTAGGT
TCAAAXXXXXXXXXXXAGAAAAAATGGTGGATAATGAAGAACTAGGAAGTAGGAGCTTGGAAATATGTGTTTGTCA
AATTTACGTGGAAAACTGTG
>Marker1015754
ACATAATGTGTATGGTTGAGATCAATTACTCACATCATATTCAAGAAACATGTGGTTTGTCTTTCAATAAAAATC
TACGAXXXXXXXXXXXTTCAACAAATTTCTTCAAAAGACAACTTGGCGAACTTATTCACAAACGCATTACCCACAC
CAACATTTGAAAAGCTAGTG
ACATAATGTGTATGGTTGAGATCAATTACTCACATCATATTCAAGAAACATGTGGTTTGTCTTTCAATAAAAATC
TACGAXXXXXXXXXXXTTCAACAAATTTCTTCAAAAGACAACTTGGCGAACTTATTCACAAAGGCATTACCCACAC
CAACATTTGAAAAGCTAGTG
>Marker1015945
AACAAACAAACCACTATTCAAACTATTTGACATCCGGAATACACATAATTTCACAAAGTAATTCTCTGCAACAAA
CCCATXXXXXXXXXXXTCCAAAGGAAGATTGAACTAAACCCATTGAACTAGAAAAAGTGAATTTCAACATCCCTAA
CATTCCAAATCCAAATCTGT
AACAAACAAACCACTATTCAAACTATTTGACATCCGGAATACACATAATTTCACAAAGTAATTCTCTGCAAGAAA
CCCATXXXXXXXXXXXTCCAAAGGAAGATTGAACTAAACCCATTGAACTAGAAAAAGTGAATTTCAACATCCCTAA
CATTCCAAATCCAAATCTGT
>Marker1016996
TACAATTTCCTAATGTTGCATGTTTTCATTATATGTGGTAGAGAGAAATTTAGGCAGATTTATTATAGTTGTATG
AGATGXXXXXXXXXXXATCCTAATATATGGAGAGTCCTATAAAATAAATCTTTGATATATTTTATACGCAAAAAAC
ATAAGTATTATTCGTAGGTC
TACAATTTCCTAATGTTGCGTGTTTTCATTATATGTGGTAGAGAGAAATTTAGGCAGATTTATTATAGTTGTATG
AGATGXXXXXXXXXXXATCCTAATATATGGAGAGTCCTATAAAATAAATCTTTGATATATTTTATACGCAAAAAAC
ATAAGTATTATTCGTAGGTC
>Marker1017050
TACTATTTTGGAGGACTTTTAAGAGAAAGATAATGGTATTGTGGTGAGTTTGAGGTAGTTATCTTAAATATAATC
ATGTGXXXXXXXXXXXTGTAATGTTTATTTGTTTTTTGGAAAAGAAATCATTATTTAATTTTAAATTTCTGAAAAT
ATGTCTTTTTAAACAAGAGT
TACTATTTTGGAGGACTTTTAAGAGAAAGATAATGGTATTGTGGTGAGTTTGAGGTAGTTATCTTAAATATAATC
ATGTGXXXXXXXXXXXTGTAATGTTTATTTGTTTTTTGGAAAAGAAATCATTATTTAATTTTAAATTTCTGAAAAT
ATGTCTTTCTAAACAAGAGT
>Marker1017240
AACGACCAAGGAATAGGTTGCGCATAACAATTGAGCCCATTATGGACTCAGAAGCTCAATCAGTAAATCCAGATG
CTCGAXXXXXXXXXXXAATCAAAATTGGCAAAGAAGTGGATAAGAACGCTAGAAAAGTATTTTAAAGTAATGCAGT
GCCCCGAGAAGAGGAAGGTG
AACGACCAAGGAATAGGTTGCGCATAACAATTGAGCCCATTATGGACTCAGAAGCTCAATCAGTAAATCCAGATG
CTCGAXXXXXXXXXXXAATCAAAATTGGCAAAGAAGTGGATAAGAACGCTAGAAAAGTATTTTAAAGTAATGCAGT
GCCCCGAGAAGAGAAAGGTG
>Marker1017378

Create PDF with GO2PDF for free, if you wish to

ACTAATATCTTATTTATGTAAAGTTTGTAATTTAATGATAATTACAAATGAGATGATTTTGAGTTCCAAACTTAT
TTGTGXXXXXXXXXXXACAATTATGGGGTTGGGATCGAACTTTCTGTTCTAAGGTGGAAGGTTGTGTAAATTACTC
TTTTAAGTTCTTTTGTAGTT
ACTAATATCTTATTTATGTAAAGTTTGTAATTTAATGATAATTACAAATGAGATGATTTTGAGTTCCGAACTTAT
TTGTGXXXXXXXXXXXACAATTATGGGGTTGGGATCGAACTTTCTGTTCTAAGGTGGAAGGTTGTGTAAATTACTC
TTTTAAGTTCTTTTGTAGTT
>Marker1017507
CACTTGAAATTGGGGTTGAGAGATGAAATTATTTTCGTGGGGTTGTAATAATTTTTCATATAATGAAAATTTTCT
AATCTXXXXXXXXXXXTGTTCCTAATTAATTAGGTCTTCAAAATTTATTTTGAAAAGATTAGTATGGATCAAATTG
ATATAATTTAACAAAATGTC
CACTTGAAATTGGGGTTGAGAGATGAAATTATTTTCGTGGGGTTGTAATAATTTTTCATATAATGAAAATTTTCT
AATCTXXXXXXXXXXXTGTTCCTAATTAATTAGGTCTTCAAAAGTTATTTTGAAAAGATTAGTATGGATCAAATTG
ATATAATTTAACAAAATGTC
>Marker1018030
ACATTATTAAAAATTGGAGATGTGTTATATTAAATACTAAAGTAAAAACTTAATTTTGGTTATACATAAAATATA
ATTTTXXXXXXXXXXXGGTTTAAATAAACTTTTTGACAAATAAAAAGTAAAATCAACTAATTAGATACTTTTTAAA
TTTAAATAAAAAACTTTGTA
ACATTATTAAAAATTGGAGATGTGTTATATTAAATACTAAAGTAAAAACTTAATTTTGGTTATACATAAAATATA
ATTTTXXXXXXXXXXXGGTTTAAATAAACTTTTTGACAAATAAAAAGTAAAATCAACTAATTAGATACTTTTTAAA
TTTAAATAAAGAACTTTGTA
>Marker1018557
ACTTTTTGTCGCTCTAAAAATATGCCATATTGAGTTGTTTCTTCTCTTAGTAATCATCAATTCTTTTAAACATTT
CTTACXXXXXXXXXXXCTTCACTCTTGTTGAGAGTAGCTTGACGTTGATACCATATGTTCTGCTCCTAACGACAGA
GTTCATGAGAATATGAAAGT
ACTTTTTGTCGCTCTAAAAATATGCCATATTGAGTTGTTTCTTCTCTTAGTAATCATCAATTCTTTTAAACATTT
CTTACXXXXXXXXXXXCTTCACTCTTGTTGAGAGTAGCTTGACTTTGATACCATATGTTCTGCTCCTAATGACAGA
GTTCATGAGAATATGAAAGT
>Marker1019327
AACATCGTGAAGTGATCATATATTGCAAAGTGCAGTTTGTAACCTGACACAGATGATTATTTTGATTTTTGCCAG
CAGAAXXXXXXXXXXXGTTGTATAGGTTTCCATATTTATATAAAGCATTGGGACGCTCAATATTTGTCACCCTGTT
GGTTTTTTGTGAATATGGTG
AACATCGTGAAGTGATCATATATTGCAAAGTGCAGTTTGTAACCTGACACAGATGATTATTTTGATTTTTGCCAG
CAGAAXXXXXXXXXXXGGTGTATAGGTTTCCATATTTATATAAAGCATTGGGACGCTCAATATTTGTCACCCTGTT
GGTTTTTTGTGAATATGGTG
>Marker1019718
CACAGCTTTCAGTTTTAAGCCATTAGTATCTTCAAAAGGTGATGGCTTTATTTCTTGCTTGAACTCATAATGGTT
CTTTAXXXXXXXXXXXAATCCCAATAGGAAGAGGTCCATTGAAAATATGTATGTTCAAATAGAAGAACTTGAGATT
ACTTTTCGAGCATTTCATGT
CACAGCTTTCAGTTTTAAGCCATTAGTATCTTCAAAAGGCGATGGCTTTATTTCTTGCTTGAACTCATAATGGTT
CTTTAXXXXXXXXXXXAATCCCAATGGGAAGAGGTCCATTGAAAATATGTATGTTCAAATAGAAGAACTTGAGATT
ACTTTTCGAGCATTTCATGT
>Marker1020178
CACTCTATGTTGTTCTTTTTTTTCCTTGAGAAAAGTTACTCCATGCTGTTGATTACGCGATTGCATTGTCGTTGCA
ATTCAXXXXXXXXXXXTCAGCCTTTTTTTTTCAGCTATATTAAAAATGTTAGGGCTACTCTATATTCATTGCTTTG
TTTTTAACTTCTATGTCTGT

Create PDF with GO2PDF for free, if you wish to

CACTCTATGTTGTTCTTTTTTTTCCTTGAGAAAAGTTACTCCATGCTGTTGATTACGCGATTGCATTGTCGTTGCA
ATTCAXXXXXXXXXXXCAGCCTTTTTTTTTTCAGCTATATTAAAAATGTTAGGGCTACTCTATATTCATTGCTTTG
TTTTTAACTTCTATGTCTGT
>Marker1020292
AACATGCTAGCAAAAAAGAATTGAACTAAAACACTCAAAATTTCATGTGAATTTTGAGGGCTAAACATGTTAAGA
AAATTXXXXXXXXXXXCAAGGTTGTGGAATTTGATGGAGTTTTGAGAAAGGAAATAATATGATCCGAAGTGAATTT
TCTCTGAAATTCAACAAAGT
AACATGCTAGCAAAAAAGAATCGAACTAAAACACTCAAAATTTCATGTGAATTTTGAGGGCTAAACATGTTAAGA
AAATTXXXXXXXXXXXCAAGGTTGTGGAATTTGATGGAGTTTTGAGAAAGGAAATAATATGATCCGAAGTGAATTT
TCTCTGAAATTCAACAAAGT
>Marker1020777
CACACTGCCACTCCACCATGTGCTAGACAAAAAACCGGTGCTTAAAAAGATAAACAAGATGCTAATCACGAGAAG
ACTCCXXXXXXXXXXXAATGCTTAGAAAATGTTCGACTGAAAACAAGCAAGAATTCCATTATCTTGTCAAAGAAAT
ATGAGAGATGATCGTCTGGT
CACAATGCCACTCCACCATGTGCTAGACAAAAAACCGGTGCTTAAAAAGATAAACAAGATGCTAATCACGAGAAG
ACTCCXXXXXXXXXXXAATGTTTAGAAAATGTTCGACTGAAAACAAGCAAGAATTCCATTATCTTGTCAAAGAAAT
ATGAGAGATGATCGTCTGGT
>Marker1021272
AACAACTCCATAATGTAGTAGATTGGAAACATCTTAAAATGGTAGATGAAGAGGAAACTTTGCTAAATTTTCCAT
GGGGTXXXXXXXXXXXAAAATTAGACATTGAAGCCCAAGAATCATAAATTGGAAATTAGAACAGCAACCTGTATGG
ACATATCTGCAATTGTAGTG
AACAACTCCATAATGTAGTAGATTGGAAACATCTTAAAATGGTAGATGAAGAGGAAACTTTGCTAAATTTTCCAT
GGGGTXXXXXXXXXXXAAAATTGGACATTGAAGCCCAAGAATCATAAATTGGAAATTAGAACAGCAACCTGTATGG
ACATATCTGCAATTGTAGTG
>Marker1021357
AACATCTTACTTTTGGGGGGCACACTGCCATGTGCCTTTTCTAGTATTCCTTAGACTTCATTAGTAGGACATTAT
TAGGCXXXXXXXXXXXCGAAAAAGAATCACAATCTGGCATTAAGAGGGGATGGGAAAGAATTTTGTGATGAATCTT
ATGGAAAATTAATTATGGGT
AACATCTTACTTTTGGGGGGCACACTGCCATGTGCCTTTTCTAGTATTCCTTAGACTTCATTAGTAGGACATTAT
TAGGCXXXXXXXXXXXCGAAAAAGAATCACAATCTGGCTTTAAGAGGGGATGGGAAAGAATTTTGTGATGAATCTT
ATGGAAAATTAATTATGGGT
>Marker1021435
CACATTTAAGCTAGAGCATGACCTTAATATTGTTGGTAGAATGATTGAAGTCATATCAGGTTGAAAGCTAAACGA
TTGTGXXXXXXXXXXXGCTCTTTTCATCTTTTTCTCATCTTCTTCATGTTACTCCCAACTCCGACCCCCTCAACCA
ATGTCGATCTTCTTCTCGTG
CACATTTAAGCTAGAGCATGACCTTAATATTGTTGGTAGAATGATTGAAGTCATATCGGGTTGAAAGCTAAGCGA
TTGTGXXXXXXXXXXXGCTCTTTTCATCTTTTTCTCATCTTCTTCATGTTACTCCCAACTCCGACCCCCTCAACCA
ATGTCGATCTTCTTCTCGTG
>Marker1021668
CACTTAATTATAATGATCATGTAAAAAGCTAGCCAAAAAGGTAATTTATTTTTAGAATATATAGGAGAAGTTTTG
ATTTGXXXXXXXXXXXAACCATTCATTAGATGGAGAAATTTTTAACATTTAATCATTAATTTGATGCCTAAGCTAA
ATATATATATCCCAAGTGTT
CACTTAATTATAATGATCATGTAAAAAGCTAGCCAAAAAGGTAATTTATTTTTAGAATATATAGGAGAAGTTTTG
ATTTGXXXXXXXXXXXAACCATTCATTAGATGGAGAGATTTTTAACATTTAATCATTAATTTGATGCCTAAGCTAA
ATATATATATCCCAAGTGTT
>Marker1021698

Create PDF with GO2PDF for free, if you wish to

AACAATTGTCAAGCGTGGGGTGGATTAAATGGTCAGTAAGAGTTGATATTATTCATAATCATGACACGTAGCAAA
AGGAAXXXXXXXXXXXTTGCGTATATGAAAATTGCTTCATTAAAACCTTACTAGTAAAAAACTCAATGGAAAAAAT
CATAGTGAAGGAAAAAGAGT
AACAATTGTCAAGCGTGGGGTGGATTAAATGGTCAGTAAGAGTTGATATTATTCATAATCATGACACGTAGCAAA
GGAATXXXXXXXXXXXTTACGTATATGAAAATTGCTTCGTTAAAACCTTACTAGTAAAAAACTCAATGGAAAAAAT
CATAGTGAAGGAAAAAGAGT
>Marker1021718
GACTAGTATGTGTATTAGAGTTTTTCCGTTTTGTCATAAATGCTTAGTGGCCTTGATTTTCAGTATGATGTTTCT
TGACAXXXXXXXXXXXTTGAACCATCTTAAGGTGTTAATATGAATGCTGCTTCTAGAACTCCTAGACACTTGTGTC
AGTCTGTTCTTTCTAAAGTT
GACTAGTATGTGTATTAGAGTTTTTCCGTTTTGTCATAAATGCTTAGTGGCCTTGATTTTCAGTATGATGTTTCT
TGACAXXXXXXXXXXXTTGAACCATCTTAAGGTGTTAATATGAATGCTGCTGCTAGAACTCCTAGACACTTGTGTA
AGTCTGTTCTTTCTAAAGTT
>Marker1022979
GACCATGACTAACACATCATTGAGATGTTTTATCATAGACTAAGATGAAACGATCATAGTTAGTGATAATTCGAG
GTAGAXXXXXXXXXXXGCATGTTTTGAATTTTTTTATTAGATATAAATACTTTCTTTTGGTCAAGTCAAATATCAT
AAACAAAACTATGTATTAGT
GACCATGACTAACACATCATTGAGATGTTTTATCACAGACTAAGATGAAACGATCATAGTTAGTGATAATTCGAG
GTAGAXXXXXXXXXXXGCATGTTTTGAATTTTTTTATTAAATATAAATACTTTCTTTTGGTCAAGTCAAATATCAT
AAACAAAACTATGTATTAGT
>Marker1023327
ACATCTAGCGAGCCTCATTGCACCAGCCATTTTAGACGTGGAAGTAGTGTGAAATGAGATCACTTCCGACGACTA
TCTCCXXXXXXXXXXXTCAGCCCATTACTCGTTGATTCCCACCATCCTCCATACATATCACAATTCGATCCTCAGA
GGGCATTTTGGGTATTTGTG
ACATCTAGCGAGCCTCATTGCACCAGCCATTTTAGACGTGAAAGTAGTGCGAAATGAGATCACTTCCGACGACTA
TCTCCXXXXXXXXXXXTCAGCCCATTACTCGTTGATTCCCACCATCCTCCATACATATCACAATTCGGTCCTCAGA
GGGCATTTTGGGTATTTGTG
>Marker1023421
AACCTCTAACCATCATTTTTTAGCATTGCCATATCAACCAAACTTTGTATCTAAACTTTTCACCAATTTTCTCAC
CAACTXXXXXXXXXXXCATGTATTTAATTATTAACGCACCATACATTTTTTAAAATATTCTCTCAACTTTAAAAAG
TTAAAAGAAATTAAAATAGT
AACCTCTAACCATCATTTTTTAGCATTGCCATATCAACCAAACTTTGTATCTAAACTTTTCACCAATTTTCTCAC
CAACTXXXXXXXXXXXCATGTATTCAATTATTAACGCACCATACATTTTTTAAAATATTCTCTCAACTTTAAAAAG
TTAAAAGAAATTAAAATAGT
>Marker1023594
ACCTTCCTTTTGGTAGAAGATCCTTCAATGAAGCTTCAGGAAAACAATAACATTTCTTTCTAATGTTATACAATA
CACGAXXXXXXXXXXXTAACCAGATCATCAACAACTTGATTAGATTCATAAATCCATCAAATACCAAAATTTGCAT
CAGAACATATCAATGGGGTT
ACCTTCCTTTTGGTAGAAGATCCTTCAATGAAGCTTCAGGAAAACAATAACATTTCTTTCTAATGTTATACAATA
CACGAXXXXXXXXXXXTAACCAGATCATCAACAACTTGATTAGATTCATAAATCCATCAAATACCAAAAATTGCAT
CAGAACATATCAATGGGGTT
>Marker1023740
GACCTCCATAACCACAACAAGCCATAATAGGTTGTTGAAACCCTGCCATTCACACACAAAATATAGCAACTCATC
AGATTXXXXXXXXXXXGCTATCAGTATATTGTCCCTGCAGTTTTTTACAGAGAGCATGAAGTTGTAGATTAAAGAG
CTTGGCTGCTTGGTTGTGTG

Create PDF with GO2PDF for free, if you wish to

GACCTCCATAACCACAACAAGCCATAATCGGTTGTTGAAACCCTGCCATTCACACACAAAATATAGCAACTCATC
AGATTXXXXXXXXXXGCTATCAGTATATTGTCCCTGCAGTTTTTTACAGAGAGCATGAAGTTGTAGATTAAAGAG
CTTGGCTGCTTGGTTGTGTG
>Marker1023924
ACTTTGGTTTGGGGTCAGAAATATTCTCAATTATGTGACATCAAAATACAGATGTCACACTGCTTATTTCTCTCT
ATAATXXXXXXXXXXTTGATCAATAATGCAAAATTAATTATTTAATAACTTTGTCAAATGTCAAAATATGTAACA
AAATTCTAAACATATTTGTT
ACTTTGGTTTGGGGTCAGAAATATTCTCAATTATGTGACATCAAAATACAGATGTCACACTGCTTATTTCTCTCT
ATAATXXXXXXXXXXTTGATCAATAATGCAAAATTAATTATTTAATAACTTTGTCAAATGTCAAAATATGTAACA
AAATTCTAACCATATTTGTT
>Marker1024444
ACCTCATTTGGAAAAGGATAAAATCGCTAAATGAGTCACCCTATCGACACACGTTGAATAGACCGTTCATAACAT
GCATGXXXXXXXXXXTATGTATCCTCTTGATCTTCTCTTAACCCATTAGTGTCAATATTTAAAAGAAAAGTAAAA
TAAACCATATTGTTTATGTC
ACCTCATTTGAAAAAGGATAAAATCGCTAAATGAGTCACCCTATCGACACACGTTGAATAGACCGTTCATAACAT
GCATGXXXXXXXXXXTATGTATCCTCTTGATCTTCTCTTAACCCATTAGTGTCAATATTTAAAAGAAAAGTAAAA
TAAACCATATTGTTTATGTC
>Marker1025046
AACTGTCTTCTTCCACGTATCTGATAATTATTTGTGTTGATATAAAAGATTATCAAAGAACAACTAAAATATATT
TAAAAXXXXXXXXXXACAGGTTAGGCGTTAAGTTTTAAATTACATAGGTTTTTTTTTTAATTATTAAATTACAAA
GAAAAAATAAGTTAATGGTT
AACTATCTTCTTCCACGTATCTGATAATTATTTGTGTTGATATAAAAGATTATCAAAGAACAACTAAAATACATT
TAAAAXXXXXXXXXXACAGGTTAGGCGTTAAGTTTTAAATTACATAGGATTTTTTTTTAATTATTAAATTACAAA
GAAAAAATAAGTTAATGGTT
>Marker1025196
GACCAACAGTGCAATTTGTTGGCTTTTCGATATAAGGAATTAAAGAAGTTTGAAGTTCCAAAGGTGAAGCAAAGT
ATATTXXXXXXXXXXACCAACATTCCCTTACGTAGTGTTAGAATTATAATAACCTACCAACACTAACGGCACAGT
AACCTACCAACATCATCGTT
GACCAACAGTGCAATTTGTTGGCTTTTCGATATAAGGAATTAAAGAAGTTTGAATTTCCAAAGGTGAAGCAAAGT
ATATTXXXXXXXXXXACCAACATTCCCTTACGTAGTGTTAGAGTTATAATAACCTACCAACATTAACGGCACAGT
AACCTACCAACATCATCGTT
>Marker1025679
TACCTTCCTATGAAATAAAAGCATACTTTAAATTATGAAGCTTGGCTGGATGCGCCCAATGACAGGGTGCTAAAA
GGAAAXXXXXXXXXXCCCAAGCAATTCACTATTAGAGAGATTGTGTTTTTAAGCCAGAGACCTTCTAACAAATAT
TAATAATCTCAAAAAGATGT
TACCTTCCTATGAAATAAAAGCATACTTTAAATTATGAAGCTTGGCTGGATGCGCCCAATGACAGGGTGCTAAAG
GAAACXXXXXXXXXXCCCAAGCAATTCACTATTAGAGAGATTGTGTTTTTAAGCCAGAGACCTTCTAACAAATAT
TAATAATCTCAAAAAGATGT
>Marker1025782
AACCATAAAGGTGAGGTCAAGATCACTGATTTTGGAGTAAGTGCAATGCTAGCAAGTTCTATGGGTCAGAGAGAT
ACATTXXXXXXXXXXACTTTCCTCTTTCAGCCTGAACGAATTAGCGGAGGAACATATGACTACAGCAGTGATATA
TGGAGTTTGGGTTTGGTAGT
AACCATAAAGGTGAGGTCAAGATCACTGATTTTGGAGTAAGTGCAATGCTAGCAAGTTCTATGGGTCAGAGAGAT
ACATTXXXXXXXXXXACTTCCCTCTTTCAGCCTGAACGAATTAGCGGAGGAACATATGACTACAGCAGTGATATA
TGGAGTTTGGGTTTGGTAGT
>Marker1026032

Create PDF with GO2PDF for free, if you wish to

TACTACTCCTCCATCTTATACAAGCTTTCAAATAAAAAACTCAAATTTACATAATGATAGAAATTGTAACAATGG
TATATXXXXXXXXXXTCTCTTTGTGTTGAATTAAAAATGGATTTATAAGAGGGTGAATAATAATGGGTCAATTAT
TGAAGGTTTGTAGGGTTGTA
CACTACTCCTCCATCTTATACAAGCTTTCAAATAAAAAACTCAAATTTACATAATGATAGAAATTGTAACAATGG
TATATXXXXXXXXXXTCTCTTTGTGTTGAATTAAAAATGGATTTATAAGAGGGTGAATAATAATGGGTCAATTAT
TGAAGGTTTGTAGGGTTGTA
>Marker1026563
ACTCTTAGCTTATATGTAATTTCACAGTCATCAACTAAATCTAAACCATCACTCATAAACCTCAACAACGTTGAA
CGAAGXXXXXXXXXXATTGTATCCTCTACCATATCGTCGATCGAAAACGGACATTAAAATTAGTTTTTGTTTTTT
TTCATCTTGTCTAACATTGT
ACTCTTAGCTTATATGTAATTTCACAGTCATCAACTAAATCTAAACCATCACTCATAAACCTCAACAACGTTGAA
CGAAGXXXXXXXXXXATTGTATCCTCTACCATATCGTCGATCGAAAACGGACATTAAAATCAGTTTTTGTTTTTT
TTCATCTTGTCTAACATTGT
>Marker1026691
ACTGTGTTTGAATGATTTACTGATGAAAGGTGTGGTGCTCAGATATATTCATGTAACTTTGAATGAGAACGTAAA
TGATAXXXXXXXXXXCTTTCCACTAACAATTTACATGGAGAAACTACTCTACATTGATGCTCTACTCTCAAGTCT
CAACTATGGCAGAAAAAATGT
ACTGTGTTTGAATGATTTACTGATGAAAGGTGTGGTGCTCAGATATATTCATGTAACTTTGAATGAGAACGTAAA
TGATAXXXXXXXXXXCTTTCCACTAACAATTTACATGGAGAAACTACTCTACATTGATGTTCTACTCTCAAGTCT
CAACTATGGCAGAAAAAATGT
>Marker1027585
CACATATCTCCCCATGATCTGATAATATAGCATTATATATATACATAGATGAAATTTAAGAATATGAAAGGAGGG
GTGACXXXXXXXXXXTTATTAAATGTCTTCTAACTCTCAATAAGTTATTGTTTGGAGAAAGATATTCTCTTGGAT
TGATATTAGCAGGTGAAGTA
CACATATCTCCCCATGATCTGATAATATAGCATTATATATATACATAGATGAAACTTAAGAATATGAAAGGAGGG
GTGACXXXXXXXXXXTTATTAAATGTCTTCTAACTCTCAATAAGTTATTGTTTGGAGAAAGATATTATCTTGGAT
TGATATTAGCAGGTGAAGTA
>Marker1028124
ACATTCATTGAATTGTGAGGGATTACTATAAGTTATAAATGTATTAGATTTCATGAGTTGTGTGGCATTATATAC
TGGCTXXXXXXXXXXTCAGATTATACTGATATCATTTAGTTGAGTTGATATAGACTTCATGGACCCTTAAAGCCT
ATCTTTTCTATTTTTCAGGT
ACATTCATTGAATAGTGAGGGATTACTATAAGTTATAAATGTATTAGATTTCATGAGTTGTGTGGCATTATATAC
TGGCTXXXXXXXXXXTCAGATTATACTGATATCATTTAGTTGAGTTGATATAGACTTCATGGACCCTTAAAGCCT
ATCTTTTCTATTTTTCAGGT
>Marker1028391
TACCAAGAGCCTGTTTTGTATTCTGGTTTATCAAATTAGTTCTCTGGTGCCATATGGAAAAACTGTCAAGGTTTT
AGTGCXXXXXXXXXXCCATCCCTATGCCTCTTGTATGAAATCATTACCTTTGGTTTGTCGTGGTATTTGTTAAAG
TCCAGATGTAATTTTACAGT
TACCAAGAGCCTGTTTTGTATTCTGGTTTATCAAATTAGTTCTCTGGTGCCATATGGAAAAACTGTCAAGGTTTT
AGTGCXXXXXXXXXXCCATCCCTATGCCTCTTGTATGAAATCATTACCTTTGGTTTGTCGTGGTATTTGTTAAAG
TCTAGATGTAATTTTACAGT
>Marker1029536
TACTTCTTCAAACGAGTAATATATATATAGGTAGATGCAAATTCAAAATCTCCATAACCCTAATATTCAATCAAA
GATAGXXXXXXXXXXGCAAAAAAATACAATTATCTGTTCTAATTTGGCCACAAGTTCTTTCTAATCTCAAAAACC
AATCTTTCTCAAATGTTGTA

Create PDF with GO2PDF for free, if you wish to

TACTTCTTCAAACGAGTTATATATATATATAGGTAGATGCAAATTCAAAATCTCCATAACCCTAATATTCAATCAAA
GATAGXXXXXXXXXXXGCAAAAAAATACAATTATCTGTTCTAATTTGGCCACAAGTTCTTTCTAATCTCAAAAACC
AATCTTTCTCAAATGTTGTA
>Marker1030162
AACTTGATCTAACGGTTATGATGAAAATACGTGGTATATCTGAATTCACCAATTTATCTATTTAATTTTCGTTTA
GATTTXXXXXXXXXXXACAGATTTACACAAATTAGGTCACTATATAATATAAACCTTTGCTACCTCAACACAAATA
TCAATATTAAAATTATCGTT
AACTTGATCTAACGGTTATGATGAAAACACGTGGTATATCTGAATTCACCAATTTATCTATTTAATTTTCGTTTA
GATTTXXXXXXXXXXXACAGATTTACACAAATTAGGTCACTATATAATATAAACCTTTGCTACCTCAACACAAATA
TCAATATTAAAATTATCGTT
>Marker1031035
AACCCATTTAGTTTAATATTCATATATCATAGTTTCTTCTTCCTTCATACATTCATATGTTTGGTTTAAATTTAT
ATGACXXXXXXXXXXXTGACAGCTCAGTCATCATTCACACTTGTCCTCTTCTTATTTACTCTCTATTTTTTATTTT
CTATTTTGTCTTTTATTGTA
AACCCATTTAGTTTAATAATCATATATCATAGTTTCTTCTTCCTTCATACATTCATATGTTTGGTTTAAATTTAT
ATGACXXXXXXXXXXXTGACAGCTCAGTCATCATTCACACTTGTCCTCTTCTTATTTACTCTCTATTTTTTATTTT
CTATTTTGTCTTTTATTGTA
>Marker1031143
AACAAAGATTCACAACGATTGGTAAAGACTTAATTAATTACGTAAGGATTTAATTATGCAAAAAACAGTAATGAA
ACCTAXXXXXXXXXXXATAATTGACTTGATTGTTGGAGTGTTGGTGGCCAAGCACCACACCGCAACAAATATTTTC
TATTTCTTAAGGTGTAAAGT
AACAAAGATTCACAACGATTGGTAAAGACTTAATTAATTACGTAAGGATTTAATTATGCAAAAAACAGTAATGAA
ACCTAXXXXXXXXXXXATAATTGACTTGATTGTTGGAGTGTCGGTGGCCAAGCACCACACCGCGACAAATATTTTC
TATTTCTTAAGGTGTAAAGT
>Marker1031170
ACTAAGTTAGATAACCACTGAGAAGTTCTCGTGTTATACCCCTTTTAAAAAAGAAGTGAAAGTTCTGACAATGTT
AGACTXXXXXXXXXXXTTCTGATTTTAATATTTTTTAAAAGTTCAACCATTGCGATCTAGAGAATCGGAGCATCGA
TACTTAGCATGATAATTAGT
ACTAAGTTAGATAACCACTGAGAAGTTCTCGTGTTATACCCCTTTTAAAAAAGAAGTGAAAGTTCTGACAATGTT
AGACTXXXXXXXXXXXTTCTGATTTTAATATTTTTTAAAAGTTCAACCATTGCGATCTAGAGAATCGGAGCATCGA
TACTTAGCATGATAATTGGT
>Marker1032207
GACATCCCCTTAGTATCCTTATCATTTCTAATAAAATAAAGGACCAAACAACAAATAAATAAAGTCCAATTGGGG
GCTAAXXXXXXXXXXXCTAAATCATCAATCAAGGAGGAAAGATGAAGGCCTGCAAATTTAGTAAGTTGTCCTGCAT
TGAAAAGTCTTTAAACTGTT
GACATCCCCTTAGTATCCTTATCATTTCTAATAAAATAAAGGACCAAACAACAAATAAATAAAGTCCAATTGGGG
GCTAAXXXXXXXXXXXCTAAATCATCAATCAAGGAGGAAAGATGAAGGCCTGCAAATTTAGTAAGTTGTCTTGCAT
TGAAAAGTCTTTAAACTGTT
>Marker1032341
GACTTCATTTATTGACTCTATAAAATAATCAAAACTTTGATCATAAGAAAATAAATTATCATGATTTTTTGACGA
AGTATXXXXXXXXXXXTGTTTCTTGTTCCGCAAATATAGAAAACACTAATTAAAGCTCCCACTCAAAATTGGCATT
ATTTGAGAAATGTAAAAGTG
GACTTCATTTATTGACTCTATAAAATAATCAAAACTTTGATCATAAGAAAATAAATTATCATGATTTTTTGACGA
AGTATXXXXXXXXXXXTGCTTCTTGTTCCGCAAATATAGAAAACACTAATTAAAGCTCCCACTCAAAATTGGCATT
ATTTGAGAAATGTAAAAGTG
>Marker1032720

Create PDF with GO2PDF for free, if you wish to

ACTTCTTTCTTCATTTTGTTTCTTTTCATAACAAATCACATGCTTCCTATAAACACTTATACGAGAGAATATATT
AATCCXXXXXXXXXXCTCTTTATCCTTTTTGAACTACATTTTATCATATATGGTAACACATTTATAGAATCTCTA
TTATTTATGTTGGAGTAAGT
ACTTCTTTCTTCATTTTGTTTCTTTTCATAACAAATCACATGCTTTCTATAAAGACTTATACGAGAGAATATATT
AATCCXXXXXXXXXXCTCTTTATCCTTTTTGAACTACATTTTATCATATATGGTAACACATTTATAGAATCTCTA
TTATTTATGTTGGAGTAAGT
>Marker1032722
ACGTAACCGGCGATGAAACACTAACACATTAATTTGAAGTTAATTAATGTAATAATAATAGGCTCAAGATTGGCT
TTATTXXXXXXXXXXCTCAAAATTATTTGATAAAACTATATAGCCAGGGCTTGAAATGACCTATGGAGATAGATT
TACAATAAAAAAGCAAGAGT
ACATAACCGGCGATGAAACACTAACACATTAATTTGAAGTTAATTAATGTAATAATAATAGGCTCAAGATTGGCT
TTATTXXXXXXXXXXCTCAAAATTATTTGATAAAACTATATAGCCAGGGCTTGAAATGACCTATGGAGATAGATT
TACAATAAAAAAGCAAGAGT
>Marker1032851
ACATGATATAGAGAAAATTTAAATAATTTAGTTAATTATTTAATTTTTCCCGTTATAGCCAATGTAGATGTCGGG
AAAAGXXXXXXXXXXAATCAAATTTAAAAAATCAAATTGGTGGATTAGTCCCGACGTTAAAATCAAGAAGTCGAG
ATTTCCCCTATACTGACGGT
ACATGATATAGAGAAAATTTAAATAATTTAGTTAATTATTTAATTTTTCCCGTTATAGCCAATGTAGATGTCGGG
AAAAGXXXXXXXXXXAATCAAATTTTAAAAATCAAATTGGTGGATTAGTCCCGACGTTAAAATCAAGAAGTCGAG
ATTTCCCCTATACTGACGGT
>Marker1033292
TACCCTTCATTTTCATTTCTTTCTTCTCTTCTTTTTCTGCAATTTTTCTTACCTTTCCATCGTCTTTATTCTCTT
CTTCTXXXXXXXXXXAAGCATCAATAATGAAATAATAATTAAAACGTTATAATCACATATTACTATAAATCACGA
GTATTTGCCTACAAACTTGT
TACCCTTCATTTTCATTTCTTTCTTCTCTTCTTTTTCTGCAATTTTTCTTACCTTTCCATCGTCTTTATTCTCTT
CTTCTXXXXXXXXXXAAGCATCAATAATGAAATAATAATTAAAACGTTATAATCACATATTACTATAAATCACGA
GTATTTTCCTACAAACTTGT
>Marker1033329
AACATTTTGCGGGTTTGATTATGGTTGCTTCTAAAACCCCAAGTATTGTTGGATTATAGTTCTCTTTTATTGAGG
TTTTCXXXXXXXXXXTTGGTTCGTATTAATAGAGTAATTGAAGACTATTAAAAGCGGTAAAGAAAATAGGCATCG
AGTTACGTGGAAATCCGAGT
AACATTTTGCGGGTTTGATTATGGTTGCTTCTAAAACCCCAAGTATTGTTGGATTATAGTTCTCTTTTATTGAGG
TTTTCXXXXXXXXXXTTGGTTCGTATTAATAGAGTAATTGAAGACTATTAAAAGCGGTAAAGAAAATAGGCATCG
AGTTACGAGGAAATCCGAGT
>Marker1033829
CACTGCATTAAAGGGTGATGGATAATTGTGAGGTTTCTAGCCCATTAATTGTAACTACAAAACCATCAACTTAAA
TTATTXXXXXXXXXXCAGAGATTGTCTTTCAGAATTAATAGAGTGATTATTTTCAAAGCTAATTCCAATGGATTG
TTTGAGTTGATTTACTTGTT
CACTGCATTAAAGGGTGATGGATAATTGTGAGGTTTCTAGCCCATTAATTGTAACTACAAAACCATCAACTTAAA
TTATTXXXXXXXXXXCAGAGATTGTCTTTCAGAATTAATAGAGTGATTATTTTGAAAGCTAATTCCAATGGATTG
TTTGAGTTGATTTACTTGTT
>Marker1033991
AACCAAAATTGAAATCAAACTTGGATGATGTTAGTTGAGTTAAGCTTATTTCAATTAAATTATATATAAAACTTA
TTTATXXXXXXXXXXTTTTTTCCCTTCTTTTTCTTTTTTGCTATTTTACCTCTACGGATATCAATAATCAAAATA
TAGACAGCTTTTGTAAAGTA

Create PDF with GO2PDF for free, if you wish to

AACCAAAATTGAAATCAAACTTGGATGATGTTAGTTGAGTTAAGCTTATTTTAATTAAATTATATATAAAACTTA
TTTATXXXXXXXXXXTTTTTTCCCTTCTTTTTCTTTTTTGCTATTTTACCTCTACGGATATCAATAATCAAAATA
TAGACAGCTTTTGTAAAGTA
>Marker1034245
TACTATGAATCCTGGTCCCTTCAATATAAAGGAGCATGTTCTGATTACCATTTTTGCCAATTCTGGTGCTGGCTC
TGTTTXXXXXXXXXXGGTGATATGAACGTTTTTTAATTGTGAAAAATATTGAAATGGTTGTTATGATCTGTTTGC
ATTATCATGTTGATAACGTT
TACTATGAATCCTGGTCCCTTCAATATAAAGGAGCATGTTCTGATTACCATTTTTGCCAATTCTGGTGCTGGCTC
TGTTTXXXXXXXXXXGGTGATATGAACGTTTTTTCATTGTGAAAAATATTAAAATGGTTGTTATGATCTGTTTGT
ATTATCATGTTGATAACGTT
>Marker1034815
CACAAGAACCAAAATAATAGAGCAAGCTCAAGGTGGATGATGCACGTTTCTAGCCTTTTCGACATTCCTTCGAAG
TCAGGXXXXXXXXXXCTTTTTCTTTTCTTTTCTTTTTTCCTTTTTTTTTTCTATCCCATCTTGGATGAACAGCAT
AGGATATGACATCACGAGGT
CACAAGAACCAAAATAATAGAGCAAGCTCAAGGTGGATGATGCAAGTTTCTAGCCTTTTCGACATTCCTTCGAAG
TCGGGXXXXXXXXXXCTTTTTCTTTTCTTTTCTTTTTTCCTTTTTTTTTTCTATCCCATCTTGGATGAACAGCAT
AGGATATGACATCACGAGGT
>Marker1035013
ACCAAAATTAGTTTCCACATTGAATATGATTATATTTGTTATAACTTTGACAAATTAATGCGGTAGATCTCAACT
TTTTTXXXXXXXXXXCCTACGTCAATCTTTGTATAATTTTCTAATTTGACTTACGTTCATGAGTAGGGAGGAATT
ATTATTATTATCAAAGAGTT
ACCAAAATTAGTTTCCACATTGAATATGATTATATTTGTTATAACTTTGACAAATTAATGCGGTAGATCTCAACT
TTTTTXXXXXXXXXXCCTACGTCAATCTTTATATAATTTTCTAATTTGACTTACGTTCATGAGTAGGGAGGAATT
ATTATTATTATCAAAGAGTT
>Marker1035095
ACTTAAGGCGGCGTTTCAGTCATCGGAGAAGATGGATAGTGGTGGGCAGGTGGCGCCTGTGTCGAAGCTGATGGA
GTTTCXXXXXXXXXXATGTGGAGTATTATGAGAATCAAAACGGCACTTCACAGGGCACTCTTCTCATTCGTTCTG
CTCTTCCATCTTCTCCCGTA
ACTTAAGGCGGCGTTTCAGTCATCAGAGAAGATGGATAGTGGTGGGCAGGTGGCGCCTGTGTCGAAGCTGATGGA
GTTTCXXXXXXXXXXATGTGGAGTATTATGAGAATCAAAACGGCACTTCACAGGGCACTCTTCTCATTCGTTCTG
CTCTTCCATCTTCTCCCGTA
>Marker1035180
ACTTTCTCACAACTTCATGAAACTTGTTAATGGTTTCTTCCTTCTCCATTTCCATTGCATTACTCTCTGCCCCAT
CATCAXXXXXXXXXXAAGCACGTCCAGCCCTCTAGCCTGTTCAGACAACCATTTATTTCATAATTGATTCAAACA
AAATAGGGGGGGTGAAAGTT
ACTTTCTCACAACTTCATGAAACTTGTTAATGGTTTCTTCCTTCTCCATTTCCATTGCATTACTCTGTGCCCCAT
CATCAXXXXXXXXXXAAGCACGTCCAGCCCTCTAGCCTGTTCAGACAACCATTTATTTCATAATTGATTCAAACA
AAATAGGGGGGGTGAAAGTT
>Marker1035842
TACTTTTAATTGGTTGGGCAGGAAAACCCCGGCAGTCTCCAAATTTCAAATAAACAAATGATCATTCATCATCTT
CAGTAXXXXXXXXXXATCTCAAATCAATACTCTTCATCTTTATATCAGCTTCAATTTTTCAATAAAGTCCTCTAA
AAACAATGTTCTAAAAAGTA
TACTTTTAATTGGTTGGGCAGGAAAACCCCGGCAGTCTCCAAATTTCAAATAAACAAATGATCATTCATCATCTT
CAGTAXXXXXXXXXXATCTCAAATCAATACTCTTCATCTTTATATCAGCTTCAAATTTTCAATAAAGTCCTCTAA
AAACAATGTTCTAAAAAGTA
>Marker1036026

Create PDF with GO2PDF for free, if you wish to

AACTTGTCATAAATAAACTTCCAAATGACTTTTGGATACCGAAATTAGAGCAACTGGATTCTATTGAAGTGCCCC
CAAAAXXXXXXXXXXTTAGTTTTCATATAATATTTTGTTATACTTATGATTATGAAAAAGGATGAAAAGGGAATG
ATTATTAATCTGTTAGAGTT
AACTTGTCATAAATAAACTTCCAAATGACTTTTGGATACTGAAATTAGAGCAACTGGATTCTATTGAAGTGCCCC
CAAAAXXXXXXXXXXTTAGTTTTCATATAATATTTTGTTATACTTATGATTATGAAAAAGGATGAAAAGGGAATG
ATTATTAATCTGTTAGAGTT
>Marker1036145
ACACCAATTAAATTGGTCTTGTCTGTGGATGCTACGGATTACCTTTTTCCAAAGAGAGTTCTCCTCATTAAAATG
TCTCCXXXXXXXXXXCCTCCCTTATGACCTTCCCAAAAAAACTTCGCATCAACCTTCCTATTGATTTAACCACCT
TTTCTGGAATCATAAAAGTG
ACACCAATTAAATTGGTCTTGTCTGTGGATGCTACGGATTACCTTTTTCCAAAGAGAGTTCTCCTCATTAAAATG
TCTCCXXXXXXXXXXCCTTCCTTATGACCTTCCCAAAAAAACTTCGCATCAACCTTCCTATTGATTTAACCACCT
TTTCTGGAATCATAAAAGTG
>Marker1036355
TACAAAGATATCCTATTGTATGCATTCACAAGTTACCCACAAGGCCACAGGCCCACAACACATACCAGCAATTAT
ATCAAXXXXXXXXXXTGACGGTTTTTCTTCTTTGTTTTTCATAACTGTGAGTTTCGAGGCTACCTTGCACGCACT
TCGACTTAATCTCAAGGGTC
TACAAAGATATCCTATTGTATGCATTCACAAGTTACCCACAAGGCCACAGGCCCACAACACATACCAGCAATTAT
ATCAAXXXXXXXXXXTGACGGTTTTTCTTCTTTGTTTTTCGTAACTGTGAGTTTCGAGGCTACCTTGCACGCACT
TCGACTTAATCTCAAGGGTC
>Marker1036520
CACACATTTTGTTGCTTAATTTTTTATGTTTCATCGATTTCTTTTGTGATACAATGGAAATGTTGATTCATCCAT
TATGTXXXXXXXXXXAAACCAAGGAAATTGAGAATTTGTAGGGTGAGCTAAACTATGACAAATGAAGATTTATCT
TTGTTTCTTTGCTATAGGTC
CACACATTTTGTTGCTTATTTTTTTATGTTTCATCGATTTCTTTTGTGATACAATGGAAATTTTGATTCATCCAT
TATGTXXXXXXXXXXAAACCAAGGAAATTGAGAATTTGTAGGGTGAGCTAAACTATGACAAATGAAGATTTATCT
TTGTTTCTTTGCTATAGGTC
>Marker1036551
ACTAAACAAGGACTCAGTAGTTTCACGATAAAGAAATACATAGTGACTACTACGTATGCAGTGATCATGACTAAT
GAATTXXXXXXXXXXTTCAGACTTTGATATTAGGCATAGCATGCGGTCTTGGTGAATCATGCGTTCAACTGAAGA
TTGCATGAAGAGGTTAAGGT
ACTAAACAAGGACTCAGTAGTTTCACGATAAAGAAATATATAGTGACTACTACGTATGCAGTGATCATGACTAAT
GAATTXXXXXXXXXXTTCAGACTTTGATATTAGGCATAGCATGCGGTCTTGGTGAATCATGCGTTCAACTGAAGA
TTGCATGAAGAGGTTAAGGT
>Marker1036790
AACATTCCCTGCCAAAGTCGTATTGATTGTTCTGATCCATGGGACTGTGATTGCAAGCTCAACCTATGTTTTTGT
CACCCXXXXXXXXXXAATTATATATATACCCTCCACCCCCCATTTCGATACACGTATATAGCTCGTAAATCGAAA
GAAATGTATTTTACTACAGT
AACATTCCCTGCCAAAGTCGTATTGATTGTTCTGATCCATGGGACTGTGATTGCAAGCTCAACCTATGTTTTTGT
CACCCXXXXXXXXXXAATTATATATATACCCTCCACCCCCCATTTCGATACACGTATATAGCTTGTAAATCGAAA
GAAATGTATTTTACTACAGT
>Marker1036917
AACCAATAATTGGAAGCATTTTTTTGTTGGGACCCTATAATTAATTAAGACATGGGTCTCTATCAACATGAACAAA
ATAAGXXXXXXXXXXGTGATTTCAAGTTTCAGCCAAGCACATATTAATCTCTCAATAGATTCTTTATATCATTCC
CTCCATGGGCCAACCTTGTG

Create PDF with GO2PDF for free, if you wish to

AACCAATAATTGGAAGCATTTTTTGTTGGGACCCTATAATTAATTAAGACATGGGTCTCTATCAACATAAACAAA
ATAAGXXXXXXXXXXGTGATTTCAAGTTTCAGCCAAGCACATATTAATCTCTCAATAGATTCTTTATATCATTCC
CTCCATGGGCCAACCTTGTG
>Marker1036940
CACCAGGGAAACCTTCTTGGCTGCAATGCAAAGCAAAAGCAAGTTTTGTGTTAAGGGGTGGTGGTGGTTGCATTC
AAACAXXXXXXXXXXAAACAGGTCAATAACATACTTAATCAAAATTCGAGGGACTTTTTACATATTTTAAAGTGA
GACCTATTCGACACAAAGTT
CACCAGGGAAACCTTCTTGGCTGCAATGCAAAGCAAAAGCAAGTTTTGTGTTAAGGGGTGGTGGTGGTTGCATTC
AAACAXXXXXXXXXXAAACAGGTCAATAACATACTTAATCAAAAGTCGAAGGACTTTTTACATATTTTAAAGTGA
GACCTATTCAACACAAAGTT
>Marker1037290
AACAAAAATACTCATGCTCACATATTCAACAAAGCAATTTGAGAATGATAGAAAGAGAAATCAAATTTTCTTTTG
CATAAXXXXXXXXXXTAGCGCTTGATAACGCTTAAAATGTGCCTAACTTATCCCGTTTAATTAACTAGAAACCAT
TTTCTAGTATTTTACGTGTT
AACAAAAATACTCATGCTCACATATTCAACAAAGCAATTTGAGAATGATAGAAAGAGAAATCAAATTTTCTTTTG
CATAAXXXXXXXXXXTAGCGCTTGATAACGCTTAAAATGTGCCTAACTTATCCCGTTTAATTAACTCGAAACCAT
TTTCTAGTATTTTACGTGTT
>Marker1037620
AACCTCTCACACTCATCCATTTGCACTCCCACATGTATATATAAATTTTTCCCCAAATGGACACTTTCAAACCGC
AATAGXXXXXXXXXXAATTTTCAAGGTTTTCTATTAGTTATATTTTCAACATTATTTGAAGGGCATAAGAAATGT
GTCTTTAAAGTTTTAAAGTT
AACCTCTCACACTCATCCATTTGCACTCCCACATGTATATATAAAATTTTCCCCAAATGGACACTTTCAAACCGC
AATAGXXXXXXXXXXAATTTTGAAGGTTTTCTATTAGTTATATTTTCAACATTATTTGAAGGGCATAAGAAATGT
GTCTTTAAAGTTTTAAAGTT
>Marker1038311
AACCATCTTTTTGTAATGCCTTATTTAGTTGAATTTGGACTATACTTAAGTTGGCACATTTGAACCATCTTGATT
GTTTGXXXXXXXXXXATCATAGCCTCTAAACAAATGTCCCTTTCTTTTTACCTCCTACACTAAAGAAATCACTCA
AAAGCTGAGATCAATAGGTG
AACCATCTTTTTGTATTGCCTTATTTAGTTGAATTTGGACTATACTTAAGTTGGCACATTTGAACCATCTTGATT
GTTTGXXXXXXXXXXATCATAGCCTCTAAACAAATGTCCCTTTCTTTTTACCTCCTACACTAAAGAAATCACTCA
AAAGCTGAGATCAATAGGTG
>Marker1038315
ACCATCTGAGTAGAAAGGAAGCAATTGCAATGTTTTAATTAACTAATAATATAGAGTTTAATTCGATGAAATTGA
AAGTTXXXXXXXXXXTGGTGGGTCATTTTTCACGTTTCGACTTGTTAAATAATGCAACATGTTTTACATAACACA
ATAAACATAAGCTTGTAGTT
ACCATCTGAGTAGAAAGGAAGCAATTGCAATGTTTTAATTAACTAATAATATAGAGTTTAATTCGATGAAATTGA
AAGTTXXXXXXXXXXTGGTGGGTCATTTTTCACGTTTTGACTTGTTAAATAATGCAACATGTTTTACATAACACA
ATAAACATAAGCTTGTAGTT
>Marker1038655
ACTAGATCGGGGAAGTTTTCCTCACCCATTCCCCTGATAAGGGATCCAATTGCCCGAGCAGCAACTGACCGAACT
TCTGGXXXXXXXXXXGTTACTAAGGAACACATATTTCCAGCAATCTGCGCTGCTTTTTTTTTGGTTTCGGCACTC
CTTTCCCTTAGACCTCTGTG
ACTAGATCGGGGAAGTTTTCCTCACCCATTCCCCTGATAAGGGATCCAATTGCCCGAGCAGCAACTGACCGAACT
TCTGGXXXXXXXXXXGTTACTAAGGAACACATATTTCCAGCAATCTGCGCTGCTTTTTTTTTGGTTTCAGCACTC
CTTTCCCTTAGACCTCTGTG
>Marker1039291

Create PDF with GO2PDF for free, if you wish to

GACATTTAAACAAAGTTGGTAAAAGTAGAGTATGCACATATCAATTAATCATTATTGTTATATTTATGATAATTC
TCAATXXXXXXXXXXXATTTGTCCTTCTTCACGAAGAAATTATCGATTTTTATTTCTATACCTAAATTTTAGGGTT
TGTATCAATTTAAACTATGT
GACATTTAAACAAAGTTGGTAAAAGTAGAGTATGCACATATCAATTAATCATTATTGTCATATTTATGATAATTC
TCAATXXXXXXXXXXXATTTGTCCTTCTTCACGAAGAAATTATCGATTTTTATTTCTATACCTAAATTTTAGGGTT
TGTATCAATTTAAACTATGT
>Marker1039967
TACAACAGGGTATTTTATGTTTGTTAAAGGCAACCTTGTAACATGGAAAAGTAAGAATTTCGAGGGATTGCAAAT
GGTATXXXXXXXXXXXATAATTGCACACGTAACCCGGTTCAACATGATCAAATGAAGCATGTAGAAATAGATCGGC
ATTTCATTAAAGAGAAAGTG
TACATCAGGGTATTTTATGTTTGTTAAAGGCAACCTTGTAACATGGAAAAGTAAGAATTTCGAGGGATTGCAAAT
GGTATXXXXXXXXXXXATAATTGCACACGTAATCCGGTTCAACATGATCAAATGAAGCATGTAGAAATAGATCGAC
ATTTCATTAAAGAGAAAGTG
>Marker1040196
ACTATCTAACCCCTTTAATACTTATAGTGGCCCTTACTGACCCTGGGACAACTCATGGTGGTTCTTTATTAACCT
TATTTXXXXXXXXXXXCTGGCTATTTTGTTTTATTGTTTCTTTCATCACTTCCAAAAAGAATTGGTATCCTTGAAA
TACTACTGCTCACAGCAGTG
ACTATCTAACCCCTTTAATACTTATAGTGGCCCTTACTGACCCTGGGACAACTCATGGTGGTTCTTTATTAACCT
TATCTXXXXXXXXXXXCTGGCTATTTCGTTTTATTGTTTCTTTCATCACTTCCAAAAAGAATTGGTATCCTTGAAA
TACTACTGCTCACAGCAGTG
>Marker1040282
CACTCTCATCTTGCAAGCACTCTTTCTCTATCGTCAGAGTTTCATTGCCTATTGTCCAATAAGAAACGAGACATT
ACAGTXXXXXXXXXXXTCCCTCTAATGTGTTTCAGAGGTGAAATTCTGCCCCCCAAAAAGTTAAAATTTTCATGTG
ATGGGCGTTCAAGAAAAGTT
CACTCTCATCTTGCGAGCACTCTTTCTCTATCGTCGGAGTTTCATTGCCTATTGTCCAATAAGAAACGAGACATT
ACAGTXXXXXXXXXXXTCCCTCTAATGTGTTTCAGAGGTGAAATTCTGCCCCCCAAAAAGTTAAAATTTTCATGTG
ATGGGCGTTCAAGAAAAGTT
>Marker1040715
ACTTTCCACCAATTTGATGAGTATTCTTCCGAGCATCAAGTATCCTTTTAGCATGAAAAACAAAGAAAGAATGTT
AGATTXXXXXXXXXXXCAATGCATTACATTGGAGGGGTGCTCTACTAAACAATCTGAAATACCAACCTCAGTCTCT
GTGTGCATGCAATTGATTGT
ACTTTCCACCAATTTGATGAGTATTCTTCCGAGCATCAAGTATCCTTTTAGCATGAAAAACAAAGAAAGAATGTT
AGATTXXXXXXXXXXXCAATGCATTACATTGGAGGGGTGCTCTACTAAACAATCTGAAATACCAACCTTAGTCTCT
GTGTGCATGCAATTGATTGT
>Marker1040957
TACCACTATGTCAATTGTTCAATATCTTTATGTTAAAATCATAAATAAATATCAGAACCTTGAAACTAGTAATTA
ATTGAXXXXXXXXXXXCAACAAGCCCTGCAAGAAAGTGAAACGTAGCTAAATATAAGGCTAAGATCGGTGCCTATG
CCTTCCCATGGAACCTTGTA
TACCACTATGTCAATTGTTCAATATCTTTATGTTAAAATCATAAATAAATATCAGAACCTTGAAACTAGTAATTA
ATTGAXXXXXXXXXXXCAACAAGCCCTGCAAGAAAGTGAAACGTAACTAAACATAAGGCTAAGATCGGTGCCTATG
CCTTCCCATGGAACCTTGTA
>Marker1040965
TACAAACACTACTTTGGTTTATTAGTTGTAGGCTTAGTTTTCAAAAACAAAAAATGAAATGGTTACCAAACTGTC
TATCAXXXXXXXXXXXTTTAGGATATAGAGTTACGTATCTGGAGTTTGAAAATAGGAAACATTAGAGAGGAGAAGA
TTAAGTTTATCGATAAATGT

Create PDF with GO2PDF for free, if you wish to

TACAAACACTACTTTGGTTTATTAGTTATAGGCTTAGTTTTCAAAAACAAAAAATGAAATGGTTACCAAACTGTC
TATCAXXXXXXXXXXTTTAGGATATAGAGTTACGTATCTGGAGTTTGAAAATAGGAAACATTAGAGAGGAGAATA
TTAAGTTTATCGATAAATGT
>Marker1042052
AACTTCGTCAATTACCAATGGAAGCCAAATTAAATTTAGTTTTAAAAAATTATTAGTATGAATGTATGAACTCTT
TGTGCXXXXXXXXXXTCGCTCGCTCGCTCGTTTCCACTCCCTGTTATCTTCCATTTTCAAACCTTTCAACCAACT
CTCTCAAATTCCCCATTGTT
AACTTCGTCAATTACCAATGGAAGCCAAATTAAATTTAGTTTTAAAACATTATTAGTATGAATGTATGAACTCTT
TGTGCXXXXXXXXXXTCGCTCGCTCGCTCGTTTCCACTCCCTGTTATCTTCCATTTTCAAACCTTTCAACCTACT
CTCTCAAATTCCCCATTGTT
>Marker1042795
CACTTTTCAGGTTAGATGGAGAGCACGTCGTCTTTGGCAAGGTTATCCAAGGAATGGACTACGTATATGCAATTG
AAGGCXXXXXXXXXXTTAACAACAGAAAATTTTCGTTTATTAATTCTTTTTTCCTTTTATGGTTTTCAAGTTTCT
TTTGATACGCCCCTAATGTC
CACTTTTCAGGTTAGATGGAGAGCACGTCGTCTTTGGCAAGGTTATCCAAGGAATGGACTACGTATATGCAATTG
AAGGCXXXXXXXXXXTTAACAACAGAAAATTTTCGTTTATTAATTCTTTTTTCCTTTTATGGTTTTCAAGTTTCT
TTTGATACGCCCCCAATGTC
>Marker1042938
TACTGGCCTAATAACTGTGGTTGATAACTATCTACAATCTGCCTGCAATCATCCTCGTTAATAAATCTTACATAC
GACTTXXXXXXXXXXTCATTGAGCTTACTTGACTGTTACATTGTTTCCAGGTGGATGTTAGAAAGCAGAGAACTG
CATTTCCGCCCAATTTTGTG
TACTGGCCTAATAACTGTGGTTGATAATTATCTACAATCTGCCTGCAATCATCCTCGTTAATAAATCTTACATAC
GACTTXXXXXXXXXXTCATTGAGCTTACTTGACTGTTACATTGTTTCCAGGTGGATGTTAGAAAGCAGAGAACTG
CATTTCCGCCCAATTTTGTG
>Marker1043928
ACAAGGGATGCACCTGTAAATCCTTTTACATCTCATTTTCCAATCTAAGATTGAATAAATTTGTTTTGTTGTAGA
TTTTAXXXXXXXXXXTTGCCTCTGTGATTGATAAAGCAACAACGGGTTCTAACTTAGTTGATTAGTGTCAAATAG
GAAACAATAGTCTGATAGGT
ACAAGGGATGCACCTTTAAATCCTTTTACATCTCATTTTCCAATCTAAGATTGAATAGATTTGTTTTGTTGTAGA
TTTTAXXXXXXXXXXTTGCCTCTGTGATTGATAAAGCAACAACGGGTTCTAACTTAGTTGATTAGTGTCAAATAG
GAAACAATAGTCTGATAGGT
>Marker1044100
CACGCGAGATTTTCAGATAAATTTAATTTGTGGAGTGTTGAATTTGTGTTAGTATTGTATTTATGTTGATTTGAT
GCGATXXXXXXXXXXAACTCTAAGAAAAAAATTTACTCGAAGAGGTCTAAAGATACTTCTTCCAAGAGCCATAAA
ACTTCCTAACTTCTGAAGTC
CACGCGAGATTTTAAGATAAATTTAATTTGTGGAGTGTTGAATTTGTGTTAGTATTGTATTTATGTTGATTTGAT
GCGATXXXXXXXXXXAACTCTAAGAAAAAAATTTACTCGAAGAGGTCTAAAGATACTTCTTCCAAGAGCCATAAA
ACTTCCTAACTTCTGAAGTC
>Marker1044273
TACCAAGCTGTGTTCCGCAATTAACTAAATTCTTTTTAGGGGAACAAGTTTTGGAAAAAGAGTGTTCAGTATATA
TGGTCXXXXXXXXXXTTTTATTTTTTTTGATAGATTCATTTTATCCAATTTATCTTTCTAGCACGTTGTCTTTCC
TCTATCTGTTCTTGAAGGTG
TACCAAGCTGTGTTCCGCAATTAACTAAATTCTTTTTAGGGGAACAAGTTTTGGAAAAAGAGTGTTCAGTATATA
TGGTCXXXXXXXXXXTTTTTTTTTTTTTGATAGATTCATTTTATCCAATTTATCTTTCTAGCACGTTGTCTTTCC
TCTATCTGTTCTTGAAGGTG
>Marker1045106

Create PDF with GO2PDF for free, if you wish to

TACACAAAATGAAATCTTAATATTGTGTTTGTATATACATTCTACAATAATACATGAAATAAGATCCTAATATAG
GATTTXXXXXXXXXXGATTAAATCCAAAATCTGCAAAATTATGTTGATAAAGGCCAACATTTGCACCATCCATAA
CAGCATCACAATAATTATGT
TACACAAAATGAAATCTTAATATTGTGTTTGTATATACATTCTACAATAATACATGAAATAAGATCCTAATATAG
GATTTXXXXXXXXXXGATTAAATCCAAAATCTGCAAAATTATGTTGATAAAGGCCAACATTTGCACCATCCATAA
CACCATCACAATAATTATGT
>Marker1045542
TACTTCTTTTTTTGGAAATTGTCATCATTGTTGTTAAGTTGAAATATTCATTATGATAAATGTTTGATTGCCAAT
AAAAAXXXXXXXXXXGAAGAGGACACCACGTGTTCCGATTTCATATTTAGAGGAAAGAGATAAAGGGGAAAATCA
TGCTTCTGGAAATAAATGTT
TACTTCTTTTTTTGGAAATTGTCATCATTGTTGTTAAGTTGAAATCTTCATTATGATAAATGTTTGATTGCCAAT
AAAAAXXXXXXXXXXGAAGAGGACACCACGTGTTGCGATTTCATATTTAGAGGAAAGAGATAAAGGGGAAAATCA
TGCTTCTGGAAATAAATGTT
>Marker1045696
CACCAACAACAGTTGGACCCCCTCATTGATAAACACTGCTCCAACTAAAATAAAAGGATACCCCTCTCCCAGGTC
GTGGCXXXXXXXXXXAGACAATCTAGAATCAGAACTTAACAAACCAGCAAAACGACTTAGAAAATCAAACTAATT
CTGTCCTAACGCAGAGTGTT
CACCAACAACAGTTGGACCCCCTCATTGATAAACACTGCTCCAACTAAAATAAAAGGATACCCCTCTCCCAGGTC
GTGGCXXXXXXXXXXAGACAATCTAGAATCAGAACTTAACAAACCAGAAAAACGACTAAGAAAATCAAACTAATT
CTGTCCTAACGCAGAGTGTT
>Marker1045858
GACTTTTCACTGGAACAGAACAAAGAAAACAACCGGTTTTATCTTTTGTGAGGGAGTTTTTTCAGAGCATTGACT
ATGAGXXXXXXXXXXCCATCGTCTTTGATCAACACATTGCTTGGTCCCTCGATGTTAGGAACTTGTCAGTTGGTG
CTGCTCTAATAGCAGTTGTG
GACTTTTCACTGGAACAGAACAAAGAAAACAACCGGTTTTATCTTTTGTGAGGGAGTTTTTTCAGAGCATTGACT
ATGAGXXXXXXXXXXCCATAGTCTTTGATCAACACATTGCCTGGTCCCTCGATGTTAGGAACTTGTCAGTTGGTG
CTGCTCTAATAGCAGTTGTG
>Marker1046248
AACAAATCGTAAAAACTACTCTAAATATTGAACTAAAAGTTACAATCAATAAGCTGATGATTTTAGAAAGCAGAA
CTGAAXXXXXXXXXXTTTGCACATACCTCAACTTAATCTTGCAAACATCTCATGTTTGATTCCACGTCTTTTACG
ATGTTAAATTTGAAGTAGTT
AACAAATCGTAAAAACTACTCTAAATATTGAACTAAAAGTTACAATCAATGAGCTGATGATTTTAGAAAGCAGAA
CTGAAXXXXXXXXXXTTTGCACATACCTCAACTTAATCTTGCAAACATCTCATGTTTGATTCCACGTCTTTTACG
ATGTTAAATTTGAAGTAGTT
>Marker1046649
GACTTATTGTTAGCTGTTGTTCTAATGGTTCCGAGGTTACCTGTGATGTTGATGTTTTTTCTTCGGACATTTATG
TTACTXXXXXXXXXXCAGGAATCCTAGACTCGTCTTTACTCCGAGAGCCCACGTCTTACCAAGATGGGTATGTGA
GATAGATTTTACATATTGTT
GACTTATTGTTAGCTGTTTTTCGAATGGTTCCGAGGTTACCTGTGATGTTGATGGTTTTTCTTCGGACATTTATG
TTACTXXXXXXXXXXCAGGAATCCTAGACTCGTCTTTACTCCGAGAGCCCACGTCTTACCAAGATGGGTATGTGA
GATAGATTTTACATATTGTT
>Marker1046677
ACCTTAAGTGTTGTAGTTTTCTCACAACACAACAATACAATTCTCTCAATTTCTTTTCAATCGTGATAAAAGTCA
AAATAXXXXXXXXXXTCTCCATTGATTATGAAATTAAACTTTCATCATTATGTATTTCAAACTTAAAAAAGGCAT
CACTCTAAAAAGAAAAGGTT

Create PDF with GO2PDF for free, if you wish to

ACCTTAAGTGTTGTAGTTTTCTCACAACACAACAATACAATTCTCTCAATTTATTTTCAATCGTGATAAAAGTCA
AAATAXXXXXXXXXXTCTCCATTGATTATGAAATTAAACTTTCATCATTATGTATTTCAAACTTAAAAAAGGTAT
CACTCTAAAAAGAAAAGGTT
>Marker1046808
ACCATACATCGTTTTCTTGAACTCTTCTTCCGCTTTAGCCTGTTCATCTGCAGCGGCTGAACTTTGTTTCATTAG
AGTTTXXXXXXXXXXGCGGGTGCAAAAGACTTGACCAGGCTGTCCTCACTTACTACTTCAACGATACTGCCTCTA
CCTTTAACTGAGCCAAGGTA
ACCATACATCGTTTTCTTGAACTCTTCTTCCGCTTTAGCCTGTTCATCTGCAGCGGCTGAACTTTGTTTCATAAG
AGTTTXXXXXXXXXXGCGGGTGCAAAAGACTTGACCAGGCTGTCCTCACTTACTACTTCAACGATACTGCCTCTA
CCTTTAACTGAGCCAAGGTA
>Marker1046943
ACTCTTATATATTACGGCATAACTAAAGAGGGGAGAAAATATATAAAATACATAGTTGGGGTTTGGGAGGAGTTG
ATGGGXXXXXXXXXXATTTGGATAGAGTATGGAAATGAAATTGAAATTAAATGATCCGTAGGGGTGGAAGGGATC
GAAACAAGAGAGGCTGCGTC
ACTCTTTTATATTACGGCATAACTAAAGAGGGGAGAAAATATATAAAATACATAGTTGGGGTTTGGGAGGAGTTG
ATGGGXXXXXXXXXXATTTGGATAGAGTATGGAAATGAAATTGAAATTAAATGATCCGTAGGGGTGGAAGGGATC
GAAACAAGAGAGGCTGCGTC
>Marker1047116
ACAAAGTCTAGGGACATTTCTTGTAATTTAACCTATAGGTTAAATCATAAAAGTATTCTTTTTTATATGGAAATT
CGATCXXXXXXXXXXTTTTTATACTGTTTGACACATTACCAGTTGTCTGCTTTCTACACTCTACTCATGCGTGCT
TGGCTGCAGATGGCAGCGTA
ACAAAGTCTAGGGACATTTCTTGTAATTTAACCTATAGGTTAAATCATAAAAGTATTCTTTTTTATATGGAAATT
CGAACXXXXXXXXXXTTTTTATACTGTTTGACACATTACCAGTTGTCTGCTTTCTACACTCTACTCATGCGTGCT
TGGCTGCAGATGGCAGCGTA
>Marker1047132
AACGAGCCTGTTAAATGTCCTCTCATATTTGGAGGTAAAACTTAAACAGTTTTGCTGTTTTTTGTTTGATATATT
TGAATXXXXXXXXXXGATGTTTGCTGAAAAGGAAATGGGGGAAAAACCGATACAGTCCTTCACTCCTGATTTATG
AGAAGGTTAAACTAGCTGTT
AACGAGCCTGTTAAATGTCCTCTCATATTTGGAGGTAAAACTTAAACAGTTTTGCTGTTTTTTGTTTGATATATT
TGAATXXXXXXXXXXGATGTTTGCTGAAAAGGAAATGGGGGAAAAACCGATACAGTCCTTCACTCCTGATTTATG
AAAAGGTTAAATTAGCTGTT
>Marker1047871
TACTAATTTGATAACTCTCGATAGCAAATTGAGACTTTTGAAATAGTGTTTAGTGTAGCTAATTGAAAACTATTT
AAATAXXXXXXXXXXGAGCTTAACACAATGTAATTGTTATGACATTTCCATCTTATAAGTCAAAGGTTCAAATAC
CCCAATTCATAAAAGCTTGT
TACTAATTTGATAACTCTTGATAGCAAATTGAGACTTTTGAAATAGTGTTTAGTGTAGCTAATTGAAAACTATTT
AAACAXXXXXXXXXXGAGCTTAACACAATGTAATTGTTATGACATTTCCATCTTATAAGTCAAAGGTTTAAATAC
CCCAATTCATAAAAGCTTGT
>Marker1048205
AACCAAAAGAACTCTTTAATAAGCCATCTACTAAATCTAAGCAACAAATCAGATTAAAAAACAACGAGAAATTAA
CGAAAXXXXXXXXXXAAAAAAAAAAAATCAAGATTTACGGTTTGTTATATTGCTATCTCGCCATGGAATGCGTTG
CATCGATTTTCAATCATGTA
AACCAAAAGAACTCTTTAATAAGCCATCTACTAAATCTAAGCAACAAATCAGATTAAAAAACAACGAGAAATTAA
CGAAAXXXXXXXXXXAAAAAAAAAAAATCAAGATTTACGTTTTGTTATATTGCTATCTCGCCATGGAATGCGTTG
CATCGATTTTCAATCATGTA
>Marker1048282

Create PDF with GO2PDF for free, if you wish to

AACAGATCCTCCATGGATCAGAAAACAGCTCGAGTTGCTCGACACCCAAATGAAAAAAAAAGATGGTGACGGTCA
TTTAGXXXXXXXXXXXGCCTGTATATATGTAATCTTCATAGAGACAAACCACTAATGCAGAAAGGTCCGATGAAAG
CAAATTGGTAGATTGAAGGT
AACAGATCCTCCATGGATCAGAAAACAGCTCGAGTTGCTCGACACCCAAATGAAAAAAAAAGATGGTGACGGTCA
TTTAGXXXXXXXXXXXGCCTGTATATACGTAATCTTCATAGAGACAAACCACTAATGCAGAAAGGTCCGATGAAAG
CAAATTGGTAGATTGAAGGT
>Marker1048738
GACAAGAATAAGATACAGCAACCTTCAAAATTGTTAAGTGTTAATTAGTTGATTTTTGTATATTTATATACGATG
TGAGTXXXXXXXXXXXAAAACATGTTACCACTTCATTTTGATGTTGCAAGGCCTCCACAAGCATTCTCTTATGAGA
AGGTTCAACCCTGAAAAGTG
GACAAGAATAAGATACAGCAACCTTCAAAATTGTTAAGTGTTAATTAGTTGATTTTTGTATATTTATATACGATG
TGAGTXXXXXXXXXXXAAAACGTGTTACCACTTCATTTTGATGTTGCAAGGCCTCCACAAGCATTCTCTTATGAGA
AGGTTCAACCCTGAAAAGTG
>Marker1048808
CACATTACGCAGCAAAGGAACAAGACAACATAACTTGTTGAGGAAAGAAACATGTTATTACTAAATAATTGTTTT
TTATTXXXXXXXXXXXAAACAAGAAGAGCCCTCCAACCGTTAATAATCATAAGACTAATTACAAGGGAAACCCTTG
TTTTTAGTTGAACTAAGAGT
CACATTACGCAGCAAAGGAACAAGACAACATAACTTGTTGAGGAAAGAAACATGTTATTACTAAATAATTGTTTT
TTATTXXXXXXXXXXXAAACAAGAAGAGCCCTCCAACCGTTAATAATCATAAGACTGATTACAAGGGAAACCCTTG
TTTTTAGTTGAACTAAGAGT
>Marker1048816
ACCTAAAGTTTAGGTCTTACAACTAGCTTATTTCAGACAAAACAACTAAATCATTTACAAAGTTAGTTACAACCC
AAAAAXXXXXXXXXXXTGAAGGGTAAGCTATAAAGCCTTGTGAGTGACAACTTTTAAAAACAAAACATGTTTATTG
AAAACTGTGAGCAATAAGTG
ACCTAAAGTTTAGGTCTTACAACTAGCTTATTTCAGACAAAACAACTAAATCTTTTACTAAGTTAGTTACAACCC
AAAAAXXXXXXXXXXXTGAAGGGTAAGCTATAAAGCCTTGTGAGTGACAACTTTTAAAAACAAAACATGTTTATTG
AAAACTGTGAGCAATAAGTG
>Marker1050122
CACAATGCTTTATCTTATAAAAATAATATTTTGATTGCATCCAACAGGTGGTGTTGCTAAAAGACCTTGCCCCTA
CAGGTXXXXXXXXXXXGGCCTCCTCATCCTTTTAGCTCTAGTGTTGAGTGTTGCACGTATGAAATATGTTGGTGGC
GATGAATTACCAGCCACGGT
CACAATGCTTTATCTTATAAAAATAATATTTTGATTGCATCCAACAGGTGGTGTTGCTAAAAGACCTTGCCCCTA
CAGGTXXXXXXXXXXXGGTCTCCTCATCCTTTTAGCTCTAGTGTTGAGTGTTGCACGTATGAAATATGTTGGTGGC
GATGAATTACCAGCCACGGT
>Marker1050291
AACCTAAAGGAAATGTAGGCAGCCATGTTTGATCATCTATGCGTTCAGATTGGAGTAAGGGCAAGATGGCTAACT
TCTAGXXXXXXXXXXXTTAAGAATCTTTTAACTTTAGTGGAAGACATGTGACAATTTAAGGTCAAGCATGGAGTTA
GTGTCCTAAAAAATAGGTGT
AACCTAAAGGAAATGTAGGCAGCCATGTTTGATCGTCTATGCGTTCAGATTGGAGTAAGGGCAAGATGGCTAACT
TCTAGXXXXXXXXXXXTTAAGAATCTTTTAACTTTAGTGGAAGACATGTGACAATTTAAGGTCAAGCATGGAGTTA
GTGTCCTAAAAAATAGGTGT
>Marker1050448
TACATCTTCAATTTATTATATCACCTTCTTATTAGGGATGAACAACTTGAATTTAGAACGAGATAATATCCTGCG
ATAAGXXXXXXXXXXXTCTTACATTTCTATGTGGTAAGGCTTCATTATTTTACATAAAAAGCCATAATAGCTTGTA
TTTCTACAAGTTATCAGAGT

Create PDF with GO2PDF for free, if you wish to

TACATCTTCAATTTATTATATCACCTTCTTATTAGGGATGAACAACTTGAATTTAGAACGAGATAATATCCTGCG
ATAAGXXXXXXXXXXXTCTTACATTTCTATGTGGTAAGGCTTCATTATTTTACATAAAAAGCCATAATAGCTTGTA
TTTCTACCAGTTATCAGAGT
>Marker1050540
CACTTTTCGTAGTTAAGTCAATGGTTATATCAATCTTGTCTATTTCAATCAACTCATTCAATATAGTTTCCTATT
GAGCAXXXXXXXXXXXTTCTTTTATTCACTAGTAGCTGAATCTCTCTAACCTGGTTGATGAAAGGAAACTGAGTGG
GAAGAAAAGAAAAAAAGGTT
CACTTTTCGTAGTTAAGTCAATGGTTATATCAATCTTGTCTATTTCAATCAACTCATTCAATATAGTTTCGTATT
GAGCAXXXXXXXXXXXTTCTTTTATTCACTAGTAGCTGAATCTCTCTAACCTGGTTGATGAAAGGAAACTGAGTGG
GAAGAAAAGAAAAAAAGGTT
>Marker1050568
ACGGTTGCGAGCATAACGATCTTAAAAATTTACACTACCTAGAGAGCCACTAGGGTTTTATTACTTTCTTAGTGT
AGATTXXXXXXXXXXXTCACATTGAGAGCAATGTAATATAAGTTGGGGGTGAGGTATATCTTGTGTTCTGGCTAAA
GTCCTCGAGCATCTAAGGTC
ACGGTTGCGAGCATAACGATCTTAAAAATTTACGCTACCTAGAGAGCCACTAGGGTTTTATTACTTTCTTAGTGT
AGATTXXXXXXXXXXXTCACATTGAGAGCAACGTAATATAAGTTGGGGGTGAGGTATATCTTGTGTTCTGGCTAAA
GTCCTCGAGCATCTAAGGTC
>Marker1050643
ACTGACCTTTTTTGTTTTTGCTATCAGATGTCGTGTTGGATCCTCTTCTTTCACTTAGAAAAGATCTTAAAGGTT
TGGGAXXXXXXXXXXXAGCAGTTGCTCTAAGAAATGTTTGTGGAGCCAATGGTAACTCTAATTCATCTTGCAAAAT
GTTATTGGGTGTCAAACGTA
ACTGACCTTTTTTGTTTTTGCTATCAGATGTCATGTTGGATCCTCTTCTTTCACTTAGAAAAGATCTTAAAGGTT
TGGAAXXXXXXXXXXXAGCAGTTGCTCTAAGAAATGTTTGTGGAGCCAATGGTAAGTCTAATTCATCTTGCAAAAT
GTTATTGGGTGTCAAACGTA
>Marker1050856
CACATGCTAAGCTTGGGAGGCAGGATGTTCTTCATTGTTTTCAAATCAAAAGTGAAACAAGAGTTTTTGAAATGG
AACTTXXXXXXXXXXXTAAAATTATTGATCTCTTCCTTTCTATGTGATGGGTATTGTTTCCTAAACAAGAGTATGG
CTTGTAATGAAGATGAGGTG
CACATGCTAGGCTTGGGAGGCAGGATGTTCTTCATTGTTTTCAAATCAAAAGTGAAACAAGAGTTTTTGAAATGG
AACTTXXXXXXXXXXXTAAAATTATTGATCTCTTCCTTTCTATGTGATGGGTATTGTTTCCTAAACAAGAGTATGG
CTTGTAATGAAGATGAGGTG
>Marker1051190
AACCAATCAGCATAAATTTATAATTACTCTTCTCTATTTTATCTGTTGATTTTTGCAGCGGGCTCCTGTATAACA
TGAGCXXXXXXXXXXXACACTTCTCCACAACACTTTAGAGCTTAATCCTGCCAAAGACCTAAAAGCAACAATTTAG
AGTGTATGTATTCTTGTAGT
AACCAATCAGCATAAATTTATAATTACTCTTCTCTATTTTATCTGTTGATTTTTGCAGCGGGCTCCTGTATAACA
TGAGCXXXXXXXXXXXACACTTCTCCACAACACTTTAGAGCTTAATCCTGCCAAAGACCTAAAAGCAACAATTTTG
AGTGTATGTATTCTTGTAGT
>Marker1051888
TACTTTAGTTGATTATTATCTGTTATTTCATATTTGTTTATTAATTTCCCAATAGTTTTATTACATAATTTAGTA
ATACTXXXXXXXXXXXATTATAATAAGGTGAGTTTTTAATTAAAGTTGTGCTAATTGAAGATGGATATATTAATTA
ACATGCCTTCAAAAATTGTC
TACTTTAGCTGATTATTATCTGTTATTTCATATTTGTTTATTAATTTCCCAATAGTTTTATTACATAATTTAGTA
ATACTXXXXXXXXXXXATTATAATAAGGTGAGTTTTTAATTAAAGTTGTGCTAATTGAAGATGGATATATTAATTA
ACATGCCTTCAAAAATTGTC
>Marker1052937

Create PDF with GO2PDF for free, if you wish to

AACTAAATTTAAAGCACCTCATTTCTCTCAGATTTAAATTGTGCTTTTTAGTTGAGTTTCCTCTACACACAACTA
ACTTTXXXXXXXXXXATTGTTCGTTTCTTAGTCTCGCATGCAACTAAAAGGAGTTGTTTTGTTATTTGTAAGGAA
TATAAAAACATGACAATTGT
AACTAAATTCAAAGCACCTCATTTCTCTCAGATTTAAATTGTGCTTTTTAGTTGAGTTTCCTCTACACACAACTA
ACTTTXXXXXXXXXXATTGTTCGTTTCTTAGTCTCGCATGCAACTAAAAGGAGTTGTTTTGTTATTTGTAAGGAA
TATAAAAACATGACAATTGT
>Marker1053604
AACCCATAATAATTGACTTTGCTCAAGCTTATGATCCGAAAAATGTGACACAAGATGTATGACTCGACACTAGAT
GATTGXXXXXXXXXXGTGTGGCAATCTTAACCATTTTGTATATGATCCTCAATCAGTTTGCAATCAAAAGGGTCC
CCAATAAAGTCTTAGCTGTA
AACCCATAATAATTGACTTTGCTCAAGCTTATGATCCGAAAAATGTGACACAAGATGTATGACTCGACACTAGAT
GATGGXXXXXXXXXXGTGTGGCAATCTTAACCATTTTGTATATGATCTTCAATCAGTTTGCAATCAAAAGGGTCC
CCAATAAAGTCTTAGCTGTA
>Marker1053673
ACCAATAAAAAATATATATAAGGCATATATACCTTAGAAATACTCAACCTTTAGTGCTATAAACTTTCTTCATAG
GGTCAXXXXXXXXXXTTGAAAACTCCAACTTGTTTAAGATTATCTGCTCTGGTTTCTAAATTTGGTAATAAGAAA
ATGTGTTATGGTTTTTAGTT
ACCAATAAAAAATATATATAAGGTATATATACCTTAGAAATACTCAACCTTTAGTGCTATAAACTTTCTTCATAG
GGTCAXXXXXXXXXXTTGAAAACTCCAACTTGTTTAAGATTATCTGCTCTGGTTTCTAAATTTGGTAATAAGAAA
ATGTGTTATGGTTTTTAGTT
>Marker1053846
ACAGCTCCGGTATCAGTCAAAAAACATGAAAAACAGAGTATAACTTCAACTTCTAAACTTCTTGGGTATTTTTTT
TAGCAXXXXXXXXXXATATTTACATGGATGCATGCAAAATAGTATCAACAATGAGTTACCCTCAGGATAGTAATG
ACAACGTTAAGTTTTAAAGT
ACAGCTCCGGTATCAGTCAAAAAACATGAAAAACAGAGTATAACTTCAACTTCTAAACTTCTTGGGTATTTTTTT
TAGCAXXXXXXXXXXATATTTACATGGATGCATGCAAAATAGTATCAACAATGAGTTACCCTCAGGATAGTAATG
ACAACGTTAAGTTTTGAAGT
>Marker1053917
TACCATCATTAAGCCATTATAATACATTGAAATGAACCTATCTGCTTGCCTTTTCACCTGTGACATTAAAAATTT
GTGATXXXXXXXXXXGCAATACCATATTCAATCCAAAATAAATTCAGGAGCCTTCTTGCTGTAAAACCACATATG
CATGTTCTAGAAAGAGCAGT
TACCATCATTAAGCCATTATAATACATTGAAATGAACCTATCTGCTTGCCTTTTCACCTGTGACATTAAAAATTT
GTGATXXXXXXXXXXGCAATACCAAATTCAATCCAAAATAAATTCAGGAGCCTTCTTGCTGTAAAACCACATATG
CATGTTCTAGAAAGAGCAGT
>Marker1054132
GACCAGTTCATTAGTCAAGTTTTTGGAATTTTATTTGGACCACTGCAGCCATGGCATCCATTACATGACTGCTGG
TATTTXXXXXXXXXXTAGTGAAATGTATTGTCCAAACTATAAGTAAGAGCAAAGTCTTAGACCTTTTTAAAAAAG
GAAATCTGGCTGAGTTAGTT
GACCAGTTCATTAGTCAAGTTTTTGGAATTTTATTTGGACCACTGCAGCCATGGCATCCATTACATGACTGCTGG
TATTTXXXXXXXXXXTAGTGAAATGTATTGTCCAAACTATAAGTAAGAGCAAAGTCTTAGACCTTTTTAAAAAAG
GAAATCTGGCTGATTTAGTT
>Marker1054392
TACTCATAAAGGTTGGTTTTACTTTTCCCCATTTCTCTACTCATTAAAAAGTAAATGTTTAAACTTTTTTTAAGG
GGTTTXXXXXXXXXXTTGTATAGTTGAATGGTGTATGGGGATGTGCTTCATATGGTTATAATCCCACCTTCCCCT
AATAATTCTGTGTGCTGGTA

Create PDF with GO2PDF for free, if you wish to

TACTCATAAAGTTTGGTTTTACTTTTCCCCATTTCTCTACTCATTAAAAAGTAAATGTTTAAACTTTTTTTAAGG
GGTTTXXXXXXXXXXXTTGTATAGTTGAATGGTGTATGGGGATGTGCTTCATATGGTTATAATCCCACCTTCCCCT
AATAATTCTGTGTGCTGGTA
>Marker1054527
ACTCTTTAACTTAGACATAGTAAATGTTAGCAACTTTAATTTTAAAACTTTGATTTGAAAGATATCCGTCAATAT
AAATAXXXXXXXXXXXATAAAAAAATGTTTAGGGTTTCATTTTAATATTGATCCCTAAACTTTCAATAATTGAAGA
GGGTCATCAATATCATGGTA
ACTCTTTAACTTAGACATAGTAAATGTTAGCAACTTTAATTTTAAAACTTTGATTTGAAAGATATCCGTCAATAT
AAATAXXXXXXXXXXXATAAAAAAATGTTTAGGGTTTCATTTTAATATTGGTCCCTAAACTTTCAATAATTCAAGA
GGGTCATCAATATCATGGTA
>Marker1054704
CACTAAGTTCAGTAATTCTTCATTTTTCAGATATTAATGATCAGGCTTAGTTTCCATTGCCATCTTCAGTGTTCC
ACTCTXXXXXXXXXXXCAAACGCAGTCCATGTTCCTTGCATCTGCGAAGACTAGCTGTTTTTCTATTCCTCAGGTG
TTCCTTAAGCTTCATACGTA
CACTAAGTTCAGTAATTCTTCATTTTTCAGATATTAATGATCAGGCTTAGTTTCCATTGCCATCTTCAGTGTTCC
ACTCTXXXXXXXXXXXCAAACGCAGTCCATGTTCCTTGCATCTGCGAAGACTAGCTGTTTTTCTATTCCTCAGGTG
TTCCTTAAGCTTCATATGTA
>Marker1055698
CACCATCAAGGCTAACCTTCACAAACCTAATCTTCTTCTTCATCACATTCTTCTTTTGGTCTTCATCATATTCTT
CCTCTXXXXXXXXXXXTTCTCGTATCAACGTAATCAACTTAGATTGGTCATACTTTTATCTCGTAGTTAATTATTA
ATTTTATTTCAATATTTGTG
CACCATCAAGGCTAACCTTCACAAACCTAATCTTCTTCTTCATCACATTCTTCTTTTGGTCTTCATCACATTCTT
CCTCTXXXXXXXXXXXTTCTCGTATCAACGTAATCAACTTAGATTGGTGATACTTTTATCTCGTAGTTAATTATTA
ATTTTATTTCAATATTTGTG
>Marker1055921
ACCAGATCCAGTTGCAGCAAGAACCAGACAATCTTGGTGATTTAACCATGCTTCTAGAGCTTCCTTTTGGAACTT
CTTCAXXXXXXXXXXXCTGAGTATCTTCCACGGGATAATGGGACACTTCTGGTTCAGAAAATGGAACAACTGGTTC
AGCTTCAGTCTTACTCCGTT
ACCAGATCCAGTTGCAGCAAGAACCAGACAATCTTGGTGATTTAACCATGCTTCTAGAGCTTCCTTTTGGAACTT
CTTCAXXXXXXXXXXXCTGAGTATCTTCCACGGGATAGTGGGACACTTCTGGTTCAGAAAATGGAACAACTGGTTC
AGCTTCAGTCTTACTCCGTT
>Marker1055984
TACCAAATGTGCCCAATATAGCTCTTTTTTATCGTTAAATAACTTAGCATAGATGTGGTTTGGTTTGAATTTCCT
ACATAXXXXXXXXXXXTGTTATACATTTGATACTTTTCTAATAGGCATTTCATACGTAGTTGATACACATGTTATA
CGTTTAATATACACTTTGTC
AACCAAATGTGCCCAATATAGCTCTTTTTTATCGTTAAATAACTTAGCATAGATGTGGTTTGGTTTGAATTTCCT
ACATAXXXXXXXXXXXTGTTATACATTTGATACTTTTCTAATAGGCATTTCATACGTAGTTGATACACATGTTATA
CGTTTAATATACACTTTGTC
>Marker1056430
AACTTGAAGATGCTCCATCTCTTGAAAGACATGTTGAGAATCCCACGTTGGAAAAATCAAAGGACTCACAATCTT
TATAAXXXXXXXXXXXCAATGAACAGTTCTGGTGTCTTTATTTATTTATTTTAATTATATATCAACAGAAAAGGGC
AGAGAAAATGAAAGCTGGTG
AACTTGAAGATGCTCCATCTCTTGAAAGACATGTTGAGAATCCCACGTTGGAAAAATCAAAGGACTCACAATCTT
TATAAXXXXXXXXXXXCAATGAACAGTTCTGGTGTCTTTATTTATTTATTTTAATTATATATGAACAGAAAAGGGC
AGAGAAAATGAAAGCTGGTG
>Marker1056657

Create PDF with GO2PDF for free, if you wish to

AACTTACCTCGTCAAATTCTATGCTTCATAAGAAACTATAAACATAAACTCACAAAGGAGCTCCAGAACCAGTTT
TTGCAXXXXXXXXXXXTTATTGAGGTGGAAGCCCTTCCGGCAATGGAAGCACATAGTTCAAGAATTATTTGGCTA
CACAGAAGGATGCACAAGGT
AACTTACCTCGTCAAATTCTATGCTTCATAAGAAACTATATACATAAACTCACAAAGGAGCTCCAGAACCAGTTT
TTGCAXXXXXXXXXXXTTATTGAGGTGGAAGCCCTTCCGGAAATGGAAGCACATAGTTCAAGAATTATTTGGCTA
CACAGAAGGATGCACAAGGT
>Marker1056942
ACATGAGGTGAAACCTAAACAAAATTTAAAAGAAAAAGGCATTAAATTACAGATGAAACAAAAAGTAAAAGAAGC
CAGAAXXXXXXXXXXXGAAGTGTATTTGGTTATTGATTGTTATGCCAATTTTTACCAAAAGGTCTGGATCTCTTA
AAGCCTGGAGATGGGACTGT
ACATGAGGTGAAACCTAAACAAAATTTAAAAGAAAAAGGCATTAAATTACAGATGAAACAAAAAGTAAAAGAAGC
CAGAAXXXXXXXXXXXGAAGTGTATTTGGTTATTGATTGTTATGCCAATTTTTACCAAAAGGTCTGGATCTCTTA
AAGCCTGGAGATGGGGCTGT
>Marker1057109
GACTTTGAGTTGTTGGGCTTTAGAATTGCATGGACATGCAATTTTGCATGGAAAATCCCTATAAATAGGGCATTT
GATGGXXXXXXXXXXXTTTTTCATCTTTTTCTCTCTCCCGATCCTTTCATCCAATAGGTCCAACAATCAAGTTTTA
AGTCCAAAGGATAGTAGGTC
GACTTTGAGTTGTTGGGTTTTAGAATTGCATGGACATGCAATTTTGCATGGAAAATCCCTATAAATAGGGCATTT
GATGGXXXXXXXXXXXTTTTTCATCTTTTTCTCTCTCCCGATCCTTTCATCCAACAGGTCCAACAATCAAGTTTTA
AGTCCAAAGGATAGTAGGTC
>Marker1057334
ACCTGTTCGACAGAGCTCTAGAATAGATTACTCCTTTCCTTTCCTGGAGTCATTGAATGAGGTGTGCCTTCAATT
TTTCCXXXXXXXXXXXGGTTTTGGAAACAAACCGAACAGAGGAATCCAAAAGTCACGTGCATAGAATGTATTTCAT
GGGCCCAAATAGTTTCAGTG
ACCTGTTCGACAGAGCTCTAGAATAGATTACTCCTTTCCTTTCCTGGAGTCACTGAATGAGGTGTGCCTTCAATT
TTTCCXXXXXXXXXXXGGTTTTGGAAACAAACCGAACAGAGGAATCCAAAAGTCACGTGCATAGAATGTATTTCAT
GGGCCCAAATAGTTTCAGTG
>Marker1057406
GACAATTCCTGAACAAAACAGATAAAATATTTTCCTTAAGAATGGAAAATCACTTCAAAGATATGATAAAAATGT
CAAGAXXXXXXXXXXXATTTAAAAAAGGTAACAAGTTTCTAACAGCTTTAACAAAAAACATTTATTTAATACTTTA
CCATATATTTTCCAACAAGT
GACAATTCCTGAACAAAACAGATAAAATATTTTCCTTAAGAATGGAAAATCACTTCAAAGATATGATAAAAATGT
CAAGAXXXXXXXXXXXATTTAAAGAAGGTAACAAGTTTCTAACAGCTTTAACAAAAAGCATTTATTTAATACTTTA
CCATATATTTTCCAACAAGT
>Marker1057468
TACCCAAATCAAGACAAGCAGCACACATCTCAATTCTTGTTCCTTCCTTCCACGTCTGTTTCTTTCCCTTTTCTG
AGTAAXXXXXXXXXXXCTTTTAACCCACCTGATGATGTTAGTTACCTGTCTGAACAAATGAATGACAGAGACATTT
GTTTAGAGTAAAAACAGTGT
TACCCAAATCAAGACAAGCAGCACACATCTCAATTCTTGTTCCTTCCTTCCACGTCTGTTTCTTTCCCTTTTCTG
AGTAAXXXXXXXXXXXTTTTTAACCCACCTGATGATGTTAGTTACCTGTCTGAACAAATGAATGACAGAGACATTT
GTTTAGAGTAAAAACAGTGT
>Marker1057882
ACTCTGCGCTGGGGAATTTGAAAATGGCTAGAGAAATATTCAATGAAACTCCATTGGATATGAGGGATACTGTTT
TCTACXXXXXXXXXXXATGACTTTACATTTGCAAGCGTGCTCAGTGCTTCAACGCTTATTTTTTATGATGAGCGCC
AGTGTGGCCAAATGCATGGT

Create PDF with GO2PDF for free, if you wish to

ACTCTGCGCTGGGGAATTTGAAAATGGCTAGAGAAATATTCAACGAAACTCCATTGGATATGAGGGACACTGTTT
TCTACXXXXXXXXXXATGACTTTACATTTGCAAGCGTGCTCAGTGCTTCAACGCTTATTTTTTATGATGAGCGCC
AGTGTGGCCAAATGCATGGT
>Marker1058717
TACATCTCTTCCTGTTTTGAGGAGTTTGCTCTCTTATATTTAGGTGTCCTGCTAAGGAGTTAGAGGACATTATGG
ACAACXXXXXXXXXXATACTCACCGATTAAGTTATATTATTTTGTGAATAGATATTTTTGTTTGATGGACACTTA
AATCGCTATCAATATAATGT
TACATGTCTTCCTGTTTTGAGGAGTTTGCTCTCTTATATTTAGGTGTCCTGCTAAGGAGTTAGAGGACATTATGG
ACAACXXXXXXXXXXATCCTCACCGATTAAGTTACATTATTTTGTGAATAGATATTTTTGTTTGATGGACACTTA
AATCGCTATCAATATAATGT
>Marker1058749
CACCCTCAAATGCATATCCTAGACGGTAAGACTCTTATGTCATTAGCTTGTAGCTACGAAAGTATTCTGTATTTT
ATTTTXXXXXXXXXXAGCAAGATGTATGATATTCACTTGTTTGTCATTAAAAAATAGATACATGTAATTTTAGGA
ACTATCTAGAAGATTAGGTC
CACCCTCAAATGCATATCCTAGACGGTAAGACTCTTATGTCATTAGCTTGTAGCTACGAAAGTATTCTGTATTTT
ATTTTXXXXXXXXXXAGCAAGATGTATGATATTCACTTGTTTGTCATTAAAAAATCGATACATGTAATTTTAGGA
ACTATCTAGAATATTAGGTC
>Marker1058833
GACCACAGTGCCTAAAATAAGCTACTTAACACAATTGGTGGATGCGATTATGGATCCATTCCGAACTAAATAGGG
TAGGTXXXXXXXXXXACCATCAAAATAATACTTCAAATGTCTATAACTATGCACACCCAAATTAAAGGAAGAAAT
TGAGGCCAGGAATCTTAGTC
GACCACAGTGCCTAAAATAAGCTACTTAACACAATTGGTGGATGCGATTATGGATCCATTCCGAACTAAACAGGG
TAGGTXXXXXXXXXXACCATCAAAATAATACTTCAAATGTCTATAACTATGCACACCCAAATTAAAGGAAGAAAT
TGAGGCCAGGAATCTTAGTC
>Marker1059258
GACTTTGGATGGGAAAAGGCCATATATGCCGGACCAGCTACCGGAAAAGTTGGACTTGTGCCTGGTTTGATAAGC
TTTTGXXXXXXXXXXGAAGATATAAGAACATTTTGAAATTGAAAAACACTACATAGATAATGAAATGAAAATAAT
TGGTTCGCTCGAAAAACAGT
GACTTTGGATGGGAAAAGGCCATATATGCCGGACCAGCTACCGGAAAAGTTGGACTTGTGCCTGGTTTGATAAGC
TTTTGXXXXXXXXXXGAAGATATAAGAACATTTTGAAATTGAAAAACACTACATAGATAATGAAATGAAAATAAT
TGGTTCCCTCGAAAAACAGT
>Marker1060160
ACATTAATGCGAAGCTTTATTAATTACAAATATGTTTAAAATGTGAGATTTTAGCATAATGTGTATATATATATA
TATGCXXXXXXXXXXCTAAATCATGCGTTGCTGAAACTAAAAGAAGGTTTTTTCTCAATATTTTCCCTTTTTTCT
TTTCTCAAGACTATGCAGTT
ACATTAATGTGAAGCTTTATTAATTACAAATATGTTTAAAATGTGAGATTTTAGCATAATGTGTATATATATATA
TATGCXXXXXXXXXXCTAAATCATGCGTTGCTGAAACTAAAAGAAGGTTTTTTCTCAACATTTTCCCTTTTTTCT
TTTCTCAAGACTATGCAGTT
>Marker1061565
GACTTATATACATTTTGCAAAGCTAAATCAAAGAAAAAGAGACAAAAACAAAGGTTTGTTGATATTTAAGAGGTC
AAAATXXXXXXXXXXAATCATTTATTCATTATGATCACACAAACAATAACCATAATCTAAAATTATTCCTAGAGA
TAAGATGCTAGTGAAGGGTA
GACTTATATACATTTTGCAAAGCTAAATCAAAGAAAAAGAGACAAAAACACAGGTTTGTTGATATTTAAGAGGTC
AAAATXXXXXXXXXXAATCATTTATTCATTATGATCACACAAACAATAACCATAATCTAAAATTATTCCTAGAGA
TAAGATGCTAGTGAAGGGTA
>Marker1061616

Create PDF with GO2PDF for free, if you wish to

TACTATGTTTTCTATTTATTATACCCAGAGTAAAATAGTCCAACCCCAAATCTAAACCATTATATTTAGATTTAA
GTAGGXXXXXXXXXXATTTCCTAAAATATCAACAAAAACATCAAAGAATTAATCGGAGATAGTTTTAAAGAAATT
AGTCGAGGCACGTCAAAGTC
TACTATGTTTTCTATTTATTATACCCAGACTAAAATAGTCCAACCCCAAATCTAAACCATTATATTTAGATTTAA
GTAGGXXXXXXXXXXATTTCCTAAAATATCAACAAAAACATCAAAGAATTAATCGGAGATAGTTTTAAAGAAATT
AGTCGAGGCACGTCAAAGTC
>Marker1061839
ACTTTAAATGTGTATTTAATATACTAATAGTTATTAGTATAGAGAGAATAATTTAGATTTTGTAACTACAAATTA
CTCTTXXXXXXXXXXAGCTATTTTGGCCCAACCGCTACAAACAAATTTTTTGTGCAAGGAAAAACTTACACCTAC
TTTCAATACATCAATTAGTG
ACTTTAAATGTGTATTTAATATACTAATAGTTATTAGTATAGAGAGAATAATTTAGATTTTGTAACTACAAATTA
CTCTTXXXXXXXXXXAGCTATTTTGGCCCAACCGCTACAAACAAATTTTTTGTGCAAGGAAAAACTTACACATAC
TTTCAATACATCAATTAGTG

Create PDF with GO2PDF for free, if you wish to
