# Supplementary material for: A High-Density Genetic Linkage Map for Cucumber (Cucumis sativus L.): Based on Specific Length Amplified Fragment (SLAF) Sequencing and QTL Analysis of Fruit Traits in Cucumber
Source: Front Plant Sci. 2016 Apr 19;7:437. doi: 10.3389/fpls.2016.00437 (PMC4835494; doi:10.3389/fpls.2016.00437)
Supplement: TABLE S2 — Sequence information of mapped SNPs. [file Table_2.PDF]

>Marker1000252 Chr2 SNP_site:14407158 Ref_Type:A
AACAACAAGTAACAAAAAGAAAACCCAACTGGTATAAATATGAATTAGGCTACAATTACAGAAGAAAAGAAGAAAGAGTAXXXXXXXXXXA
ACATATTTAATGAAAGAGAAAGAGTGATATAAAACACAATAGATATGAAATGAGGAAGGAAAAAGCAAGAGAGATTGTT
AACAACAAGTAACAAAAAGAAAACCCAACTGGTATAAATATGAATTAGGCTACAATTACAGAAGAAAAGAAGAAAGAGTAXXXXXXXXXXA
ACATATTTAATGAAAGAGAAAGAGTGATATAAAACACAATAGATATGAAATGTGGAAGGAAAAAGCAAGAGAGATTGTT
>Marker1000474 Chr2 SNP_site:953950 Ref_Type:T
TACTCTCTCCGTTTGCCTGGTGTTGGCCTTGTTGGATCGATTCCGGCTAATACAGTTGGGAAATTGACTCAGCTCCGAGTXXXXXXXXXXC
GAGCTTGACTCGGCTGACTCGGCTGACTCGGCTGGATTTGTCGTCGAATGAATTTTCCGGTCCGATTCCGGCTTCTGTT
TACTCTCTCCGTTTGCCTGGTGTTGGCCTTGTTGGATCGATTCCGGCTAATACAGTTGGGAAATTGACTCAGCTCCGAGTXXXXXXXXXXC
GAGCTTGATTCGGCTGACTCGGCTGACTCGGCTGGATTTGTCGTCGAATGAATTTTCCGGTCCGATTCCGGCTTCTGTT
>Marker1000814 Chr2 SNP_site:14270971 Ref_Type:G
ACATTTCTTGTGCTTTTGTTATAACGCCCTAGGCCCAAGATTCAGAATTCGGATCCTCGACATTCCCTTTGTATCCCCTAXXXXXXXXXXA
ACACCCAACATATAATTACTCCAAACTAAGCACAATTAACTTTGGAGTTCTTATGATCGAGCCATTGAAAAGGAAGGTG
ACATTTCTTGTGCTTTTGTTATAACGCCCTAGGCCCAAGATTCAGAATTCGGATCCTCGACATTCCCTTTGTATCCCCTAXXXXXXXXXXA
ACACCCAACATATAATTACTCCAAACTAAGCACGATTAACTTTGGAGTTCTTATGATCGAGCCATTGAAAAGGAAGGTG
>Marker1001042 Chr2 SNP_site:1609027 Ref_Type:T
AACATAGGAAAAGTGTTGAGACTACTCTTGCGGAGCTTGGTGTTACTCTTAGCAATGCAGCTTACAAGTTGAATGTTAGGXXXXXXXXXXA
TCACATTTATACCGGGCCTAAAGACTCCATGATTTACAAAGCAATGAAAGAATGTGATCCTTCTGGGCCTTTAATGGTT
AACATAGGAAAAGTGTTGAGACTACTCTTGCGGAGCTTGGTGTTACTCTTAGCAATGCAGCTTACAAGTTGAATGTTAGGXXXXXXXXXXA
TCATATTTATACCGGGCCTAAAGACTCCATGATTTACAAAGCAATGAAAGAATGTGATCCTTCTGGGCCTTTAATGGTT
>Marker1001047 Chr2 SNP_site:18416752 Ref_Type:G
TACTCTGTCTTTCCCATTGTAAATATCCTATGCTCAAATGCATTATCTGGAAAAGAAATTGTTGTTCTTTTGGTTTATTCXXXXXXXXXXA
TTATCTGTAATTGAACCCTTTTTTCTCACATGGAAGCAAGCACTGCAATATATTATGATTTGATAGAGATTCTATGGTT
TACTCTGTCTTTCCCATTGTAAATATCCTATGCTCAAATGCATTATCTGGAAAAGAAATTGTTGTTCTTTTGGTTTATTCXXXXXXXXXXA
TTGTCTGTAATTGAACCCTTTTTTCTCACATGGAAGCAAGCACTGCAATATATTATGATTTGATAGAGATTCTATGGTT
>Marker1001741 Chr2 SNP_site:11210674 Ref_Type:A
ACATTAAAAAATCTATGTCATATGAATTTTAAAGCATTTCATTTTGAAAATTTAAAATATAAGATTATTATGTTCATAAAXXXXXXXXXXA
TGGAAGGGTAATATTGTAGAAATATATACAATTTTAGAGAAAAAAAGGAAAACAAAAAATAAAAGACAGAAACTAAAGT
ACATTAAAAAATCTATGTCATATGAATTTTAAAGCATTTCTTTTTGAAAATTTAAAATATAAGATTATTATGTTCATAAAXXXXXXXXXXA
TGGAAGGGTAATATTGTAGAAATATATACAATTTTAGAGAAAAAAAGGAAAACAAAAAATAAAAGACAGAAACTAAAGT
>Marker1002169 Chr2 SNP_site:20997854 Ref_Type:G
ACTTCAAATAGACTGAATATTGATGAAAACATTGTTTGAAATGTTTGAGTCATAGAGTCTTGGGTAGTAAATCACCTCTTXXXXXXXXXXA
ACAGAACTTCACTAAGTTGGAAAAGTTTCCATTGAGGGAACCTTGATTTGAAGTTTCAATATTACCTTTTAAGTCTGTT
ACTTCAAATAGACTGAATATTGATGAAAACATTGTTTGAAATGTTTGAGTCATAGAGTCTTGGGTAGTAAATCACCTCTTXXXXXXXXXXA
ACAGAACTTCACTAAGTTGGAAAAGTTTCCATTGAGGGAACCTTGATTTGAAGTTTCAATATTACCTTTTAGGTCTGTT
>Marker1002381 Chr2 SNP_site:2980389 Ref_Type:T
ACACGTATTTATTTTTAATTTTTGCAGATGTTTTCTTGTATGTGGTCATATATTTTCTTCCCATTGTGGTTTATTAGTCTXXXXXXXXXXT
TTGACTGTAGATACCATGGGAGAACAAACTTTTATCAGTGGCTGGTCATACTAAGGATCCTTCTGATGCTATTCAGGGT
ACACGTATTTATTTTTAATTTTTGCAGATGTTTTCTTTTATGTGGTCATATATTTTCTTCCCATTGTGGTTTATTAGTCTXXXXXXXXXXT
TTGACTGTAGATACCATGGGAGAACAAACTTTTATCAGTAGCTGGTCATACTAAGGATCCTTCTGATGCTATTCAGGGT
>Marker1002381 Chr2 SNP_site:2980587 Ref_Type:A
ACACGTATTTATTTTTAATTTTTGCAGATGTTTTCTTGTATGTGGTCATATATTTTCTTCCCATTGTGGTTTATTAGTCTXXXXXXXXXXT
TTGACTGTAGATACCATGGGAGAACAAACTTTTATCAGTGGCTGGTCATACTAAGGATCCTTCTGATGCTATTCAGGGT
ACACGTATTTATTTTTAATTTTTGCAGATGTTTTCTTTTATGTGGTCATATATTTTCTTCCCATTGTGGTTTATTAGTCTXXXXXXXXXXT

Create PDF with GO2PDF for free, if you wish to

TTGACTGTAGATACCATGGGAGAACAAACTTTTATCAGTAGCTGGTCATACTAAGGATCCTTCTGATGCTATTCAGGGT
>Marker1002719 Chr2 SNP_site:12508747 Ref_Type:T
AACATATCACCAACAACAACATTATTATTAACATAATGAACATCAATCAAGGGTTGATCCAACAATATAATAATGGATCCXXXXXXXXXXG
ACTTTGTTTTTTCCAATAATTAATCCAACCAAGAATTTAATTGTTCAAATATTGTGTTGTTGGATTTCAATTCGTAGTC
AACATATCACCAACAATAACATTATTATTAACATAATGAACATCAATCAAGGGTTGATCCAACAATATAATAATAGATCCXXXXXXXXXXG
ACTTTGTTTTTTCCAATAATTAATCCAACCAAGAATTTAATTGTTCAAATATTGTGTTGTTGGATTTCAATTCGTAGTC
>Marker1002719 Chr2 SNP_site:12508805 Ref_Type:A
AACATATCACCAACAACAACATTATTATTAACATAATGAACATCAATCAAGGGTTGATCCAACAATATAATAATGGATCCXXXXXXXXXXG
ACTTTGTTTTTTCCAATAATTAATCCAACCAAGAATTTAATTGTTCAAATATTGTGTTGTTGGATTTCAATTCGTAGTC
AACATATCACCAACAATAACATTATTATTAACATAATGAACATCAATCAAGGGTTGATCCAACAATATAATAATAGATCCXXXXXXXXXXG
ACTTTGTTTTTTCCAATAATTAATCCAACCAAGAATTTAATTGTTCAAATATTGTGTTGTTGGATTTCAATTCGTAGTC
>Marker1002940 Chr2 SNP_site:20908379 Ref_Type:G
GACCAAGCTAAGTTTTGTGCTAACCACCGTATAAAAGCATTGGGTTGCATGCCATTCAAACGTAGTCTTTAAGAGCAATAXXXXXXXXXXC
TAATTGTAGATTATCATAATATAAAATAATTTACAAATATAAAAAAGCTTAGGCAATGCTCCAAGGGGTCGCTTGGAGT
GACCAAGCTAAGTTTTGTGCTAACCACCGTATAAAAGCATTGGGTTGCATGCCATTCAAACGTAGTCTTTAAGAGCAATAXXXXXXXXXXC
TAATTGTAGATTATCATAATATAAAATAATTTACAAATATAAAAAAGGTTAGGCAATGCTCCAAGGGGTCGCTTGGAGT
>Marker1003070 Chr2 SNP_site:3635554 Ref_Type:G
AACGAAAAAAGACTTACCACAACCAGAAACCGTCAGCTCAGTCCATGGCGCAGAGACAGGGAGAGAGTGGAAAATCAAAAXXXXXXXXXXC
CTCTTTTGAAGCCGACGACGACGATCATTGACATCTTTGAATATGTAATTACTATATTACCCTCACGGATGACTCCGTC
AACGAAAAAAGACTTACCACAACCAGAAACCGTCAGCTCAGTCCATGGCGCAGAGACAGGGAGAGAGTGGAAAATCAAAAXXXXXXXXXXC
CTCTTTTGAAGCCGACGACGACGATCATTGACATCTTTTAATATGTAATTACTATATTACCCTCACGGATGACTCCGTC
>Marker1003285 Chr2 SNP_site:11448697 Ref_Type:T
GACATGTTGTTCAACATTGATTAAGAAGAAGAGTTGTAATGTTTAGGGAAGCCTAATTATACTATAGTGCTGGAGTTAAAXXXXXXXXXXC
AAGTATAATCTTGTCATTCATTGAGATTACCTACTAAAGATAAGTAGCATACTTGTAATCCATATTATATACTCTAGTG
GACATGTTGTTCAACATTGATTAAGAAGAAGAGTTGTAATGTTTAGGGAAGTCTAATTATACTATAGTGCTGGAGTTAAAXXXXXXXXXXC
AAGTATAATCTTGTCATTCATTGAGATTACCTACTAAAGATAAGTAGCATACTTGTAATCCATATTATATTCTCTAGTG
>Marker1003285 Chr2 SNP_site:11448885 Ref_Type:T
GACATGTTGTTCAACATTGATTAAGAAGAAGAGTTGTAATGTTTAGGGAAGCCTAATTATACTATAGTGCTGGAGTTAAAXXXXXXXXXXC
AAGTATAATCTTGTCATTCATTGAGATTACCTACTAAAGATAAGTAGCATACTTGTAATCCATATTATATACTCTAGTG
GACATGTTGTTCAACATTGATTAAGAAGAAGAGTTGTAATGTTTAGGGAAGTCTAATTATACTATAGTGCTGGAGTTAAAXXXXXXXXXXC
AAGTATAATCTTGTCATTCATTGAGATTACCTACTAAAGATAAGTAGCATACTTGTAATCCATATTATATTCTCTAGTG
>Marker1003363 Chr2 SNP_site:9373618 Ref_Type:T
GACCTAGAGTCATGTTCTCTAGGGTCTTCGAATCACCTTAGGATAATTATATAACCAATAAAACATAAGAATATATTTTTXXXXXXXXXXT
CTATGGTTTATTGGACATTTGACCAGGGTTGCCTCAACCTAGGTTGAGGTTGCCTAAACCAACCACATTTTTTTAGGTC
GACCTAGAGTCATGTTCTCTAGGGTTTTCGAATCACCTTAGGATAATTATATAACCAATAAAACATAAGAATATATTTTTXXXXXXXXXXT
CTATGGTTTATTGGACATTTGACCAGGGTTGCCTCGACCTAGATTGAGGTTGCCTAAACCAACCACATTTTTTTAGGTC
>Marker1003363 Chr2 SNP_site:9373802 Ref_Type:G
GACCTAGAGTCATGTTCTCTAGGGTCTTCGAATCACCTTAGGATAATTATATAACCAATAAAACATAAGAATATATTTTTXXXXXXXXXXT
CTATGGTTTATTGGACATTTGACCAGGGTTGCCTCAACCTAGGTTGAGGTTGCCTAAACCAACCACATTTTTTTAGGTC
GACCTAGAGTCATGTTCTCTAGGGTTTTCGAATCACCTTAGGATAATTATATAACCAATAAAACATAAGAATATATTTTTXXXXXXXXXXT
CTATGGTTTATTGGACATTTGACCAGGGTTGCCTCGACCTAGATTGAGGTTGCCTAAACCAACCACATTTTTTTAGGTC
>Marker1003363 Chr2 SNP_site:9373809 Ref_Type:A
GACCTAGAGTCATGTTCTCTAGGGTCTTCGAATCACCTTAGGATAATTATATAACCAATAAAACATAAGAATATATTTTTXXXXXXXXXXT
CTATGGTTTATTGGACATTTGACCAGGGTTGCCTCAACCTAGGTTGAGGTTGCCTAAACCAACCACATTTTTTTAGGTC

Create PDF with GO2PDF for free, if you wish to

GACCTAGAGTCATGTTCTCTAGGGTTTTCGAATCACCTTAGGATAATTATATAACCAATAAAACATAAGAATATATTTTTXXXXXXXXXXT
CTATGGTTTATTGGACATTTGACCAGGGTTGCCTCGACCTAGATTGAGGTTGCCTAAACCAACCACATTTTTTTAGGTC
>Marker1003554 Chr2 SNP_site:17600245 Ref_Type:A
ACTTCATTCATTATATTACTTATTTTCTATTTTTCAATTTGTATTTTACTAAATATGTTTCAAATTGATGGTATAAATTTXXXXXXXXXXC
TACATAAAAACTTTAGCGAGTAAAGTAAAATTATACATTTTTTTGTCTTAAATTATTTGATTTCCAAGATATCTAAGTT
ACTTCATTCATTATATTACTTATTTTCTATTTTTCAATTTGTATTTTACTATATATGTTTCAAATTGATGGTATAAATTTXXXXXXXXXXC
TACATAAAAACTTTAGCGAGTAAAGTAAAATTATACATTTTTTTGTCTTAAATTATTTGATTTCCAAGATATCTAAGTT
>Marker1003574 Chr2 SNP_site:3680340 Ref_Type:A
TACAATGTCTGGCTTGATCTCCCGGAGTCTCGGAATATTGCATATTTGATTAGCCAAGTCACCGGTGACACGATCCATGTXXXXXXXXXXT
TGGAGAACAATTAGAGGGGAAAATACTTGATGCAAAACATATTTCCTACAAATAATTTTGGAGAAATATTAAATGGAGT
TACAATGTCTGGCTTGATCTCCCGGAGTCTCGGAATATTGCATATTTGATTAGCCAAGTCACCGGTGACACGGTCCATGTXXXXXXXXXXT
TGGAGAACAATTAGAGGGGAAAATACTTGATGCAAAACATATTTCCTACAAATAATTTTGGAGAAATATTAAATGGAGT
>Marker1003649 Chr2 SNP_site:10834230 Ref_Type:A
ACTAGAGCTCTAAACAGGGAGCTAATACAACTTTTTCCTTATCTGCTTCGATCAATGTGCCTAGACCTACGATTTCTTTGXXXXXXXXXXC
TACATTGACAAAACCAACTATGGAATATTATCATTGTATTGGCCTTCACGAAAGGTATATTGGTGGAAGCAACCGTTGT
ACTAGAGCTCTAAACAGGGAGCTAATACAACTTTTTCCTTATCTGCTTCGATCAATGTGCCTAGACCTACGATTTCTTTGXXXXXXXXXXC
TGCATTGACAAAACCAACTATGGAATATTATCATTGTATTGGCCTTCACGAAAGGTATATTGGTGGAAGCAACCGTTGT
>Marker1004021 Chr2 SNP_site:22665327 Ref_Type:T
AACCAAGTTTTCACCAACGTTGTTGGGAGGGATGGACCTCATTTTTCAAGTCCTCAAATCAACACTTTAACAAAGCTACAXXXXXXXXXXXA
GATAATTTCTTAAATGTTAAATTTGACGTTTAATGACTATCGTTAATGCTGCTAATGGCTATTAAATGATAACATTGTA
AACCAAGTTTTCACCTACGTTGTTGGGAGGGATGGACCTCATTTTTCAAGTCCTCAAAGCAACACTTTAACAAAGCTACAXXXXXXXXXXXA
GATAATTTCTTAAATGTTAAATTTGACGTTTAATGACTATCGTTAATGCTGCTAATGGCTATTAAATGATAACATTGTA
>Marker1004021 Chr2 SNP_site:22665370 Ref_Type:G
AACCAAGTTTTCACCAACGTTGTTGGGAGGGATGGACCTCATTTTTCAAGTCCTCAAATCAACACTTTAACAAAGCTACAXXXXXXXXXXXA
GATAATTTCTTAAATGTTAAATTTGACGTTTAATGACTATCGTTAATGCTGCTAATGGCTATTAAATGATAACATTGTA
AACCAAGTTTTCACCTACGTTGTTGGGAGGGATGGACCTCATTTTTCAAGTCCTCAAAGCAACACTTTAACAAAGCTACAXXXXXXXXXXXA
GATAATTTCTTAAATGTTAAATTTGACGTTTAATGACTATCGTTAATGCTGCTAATGGCTATTAAATGATAACATTGTA
>Marker1004028 Chr2 SNP_site:1274586 Ref_Type:A
TACTAACAAATAGCAAACAAAATACCTCTCATAGAGCACAGGGTGCAGATGAGGATGGCAAGAATTAACAGCTCTAACAAXXXXXXXXXXXT
GATTAGGGATAATTAAGAATTAAAAAGATTCTACATAACACCTTGAGAACCCCTGCCCTTTTGTCCAAATTAAGTGGTA
TACTAACAAATAGCAAACAAAATACCTCTCATAGAGCACAGGGTGCAGATGAGGATGGCAAGAATTAACAGCTCTAACAAXXXXXXXXXXXT
GATTAGGGATAATTAAGAATTCAAAAGATTCTACATAACACCTTGAGAACCCCTGCCCTTTTGTCCAAATTAAGTGGTA
>Marker1004061 Chr2 SNP_site:12643842 Ref_Type:T
AACATTACTAATTTTTGTGTGGTTTCTGCAGAAGGTCCGTGTAGAAACACTTTGGCAACTTTGCAGCAGGATTACAAACAXXXXXXXXXXXG
CCACAAAAGAACCGCAAAGGGAGTGGCTGTTTGATCGGATTACATTGCCTAACACAGAATTCTGAGGTAAAGTTAATGT
AACATTACTAATTTTTGTGTTGTTTCTGCAGAAGGTCCGTGTAGAAACACTTTGGCAACTTTGCAGCAGGATTACAAACAXXXXXXXXXXXG
CCACAAAAGAACCGCAAAGGGAGTGGCTGTTTGATCGGATTACATTGCCTAACACAGAATTCTGAGGTAAAGTTAATGT
>Marker100438 Chr4 SNP_site:4627215 Ref_Type:G
ACTTAATTAGCTGACCAAGAAAACAGAAAAGAAAAGTGAATTGTCGCATATAAGGAAAAAAAGAAAATCGTCCATATCCGXXXXXXXXXXA
AAGCTACGGTTTCTGGGAAAGAGGATAGGTAGAGAGGTAAAATTTTTGCCGTCCAATATACCGTTTCCAAAAGTAATGT
ACTTAATTAGCTGACCAAGAAAACAGAAAAGAAGAGTGAATTGTCGCATATAAGGAAAAAAAGAAAATCGTCCATATCCGXXXXXXXXXXA
AAGCTACGGTTTCTGGGAAAGAGGATAGGTAGAGAGGTAAAATTTTGGCCGTCGAATATACCGTTTCCAAAAGTAATGT
>Marker100438 Chr4 SNP_site:4627424 Ref_Type:G
ACTTAATTAGCTGACCAAGAAAACAGAAAAGAAAAGTGAATTGTCGCATATAAGGAAAAAAAGAAAATCGTCCATATCCGXXXXXXXXXXA

Create PDF with GO2PDF for free, if you wish to

AAGCTACGGTTTCTGGGAAAGAGGATAGGTAGAGAGGTAAAATTTTTGCCGTCCAATATACCGTTTCCAAAAGTAATGT
ACTTAATTAGCTGACCAAGAAAACAGAAAAGAAGAGTGAATTGTCGCATATAAGGAAAAAAAGAAAATCGTCCATATCCGXXXXXXXXXXA
AAGCTACGGTTTCTGGGAAAGAGGATAGGTAGAGAGGTAAAATTTTGGCCGTCGAATATACCGTTTCCAAAAGTAATGT
>Marker100438 Chr4 SNP_site:4627431 Ref_Type:G
ACTTAATTAGCTGACCAAGAAAACAGAAAAGAAAAGTGAATTGTCGCATATAAGGAAAAAAAGAAAATCGTCCATATCCGXXXXXXXXXXA
AAGCTACGGTTTCTGGGAAAGAGGATAGGTAGAGAGGTAAAATTTTTGCCGTCCAATATACCGTTTCCAAAAGTAATGT
ACTTAATTAGCTGACCAAGAAAACAGAAAAGAAGAGTGAATTGTCGCATATAAGGAAAAAAAGAAAATCGTCCATATCCGXXXXXXXXXXA
AAGCTACGGTTTCTGGGAAAGAGGATAGGTAGAGAGGTAAAATTTTGGCCGTCGAATATACCGTTTCCAAAAGTAATGT
>Marker1004685 Chr2 SNP_site:6551528 Ref_Type:T
GACTGTGTGCAAATATAGTATTAGAAATGGCCAACTCTCCAAGAGCCCTCAGCATCAATGATAAGTTCTTCTGTATTTTCXXXXXXXXXXG
AAGCATTATCAAAAAATAAAGATAATAAAATAATTTTTGAAGTTTTCAAGTTTTTCTAAAAAAACATTCATTTTAGGTT
GACTGTGTGCAAATATAGTATTAGAAATGGCCAACTCTCCAAGAGCCCTCAGCATCAATGATAAGTTTTTCTGTATTTTCXXXXXXXXXXG
AAGCATTATCAAAAAATAAAGATAATAAAATAATTTTTGAAGTTTTCAAGTTTTTCTAAAAAAACATTCATTTTAGGTT
>Marker1004780 Chr2 SNP_site:11127326 Ref_Type:A
ACATAAAATTAATCCAGACTTGGTTCTTATACAAGAAACCAAGAGAGCTTCCTTTAAGATATAGCCTTGATTAAGAAGTTXXXXXXXXXXC
AATACATTAGTAGAAAGCTAAATACAAAGCAAAACTTAAATGCTCTCTGAAAAACATCCCACTCAAATCTAAATTCGTG
ACATAAAATTAATCCAGACTTGGTTCTTATACAAGAAACCAAGAGAGCTTCCTTTAAGATATAGCCTTGATTAAGAAGTTXXXXXXXXXXC
AATACATTGGTAGAAAGCTAAATACAAAGCAAAACTTAAATGCTCTCTGAAAAACATCCCACTCAAATCTAAATTCGTG
>Marker1005178 Chr2 SNP_site:20611626 Ref_Type:A
ACTCAACAAGCACTCAAGAGGTGTATTGCCAAGCGAATTCTATCAAACCAGCAACCGTAACAATATATAATACATTACTCXXXXXXXXXXA
TAGGTTCCAAATTTTTAGTATCCCTTTCCTATGCTTCACTTGAGCAGTTTGTGGATCTTCTGTGATTGAGATTATAGTG
ACTCAACAAGCACTCAAGAGGTGTATTGCCAAGCGAATTCTATCGAACCAGCAACCGTAACAATATATAACACATTACTCXXXXXXXXXXA
TAGGTTCCAAATTTTTAGTATCCCTTTCCTATGCTTCACTTGAGCAGTTTGTGGATCTTCTGTGATTGAGATTATAGTG
>Marker1005178 Chr2 SNP_site:20611652 Ref_Type:T
ACTCAACAAGCACTCAAGAGGTGTATTGCCAAGCGAATTCTATCAAACCAGCAACCGTAACAATATATAATACATTACTCXXXXXXXXXXA
TAGGTTCCAAATTTTTAGTATCCCTTTCCTATGCTTCACTTGAGCAGTTTGTGGATCTTCTGTGATTGAGATTATAGTG
ACTCAACAAGCACTCAAGAGGTGTATTGCCAAGCGAATTCTATCGAACCAGCAACCGTAACAATATATAACACATTACTCXXXXXXXXXXA
TAGGTTCCAAATTTTTAGTATCCCTTTCCTATGCTTCACTTGAGCAGTTTGTGGATCTTCTGTGATTGAGATTATAGTG
>Marker1005320 Chr2 SNP_site:10983971 Ref_Type:G
ACAAAGTCTTTCACAACTAGTTCAATGTATTCACAAATATCACTTAATTAGCTCAATAGTATCTTTTACTATCCTCGATCXXXXXXXXXXA
AGATACCAACAATTGAACGAGATACCAACATTTTTTCTCTAACAAATTTAACCCTACATCAAAATACAGAAAATGGGTA
ACAAAGTCTTTCACAACTAGTTCAATGTATTCACAAATATCACTTAATTAGCTCAATAGTATCTTTTACTATCCTCGATCXXXXXXXXXXA
AGATACCAACAATTGAACGAGATACCAACATTTTTTCTCTAACAAATTTAACCCTACATCGAAATACAGAAAATGGGTA
>Marker100685 Chr4 SNP_site:11629029 Ref_Type:G
TACTGAAATCTTCTTTTTGATGCAACTTGTTGGTAGGGTGATGATGGGGTTAAAACCACAATTGAGCTTTTCATTAAAAGXXXXXXXXXXG
GTGGAGGGAAATATTGGAAAAAAAACCATGAAAAAGCCTTTTCCCTTCTTGTTCTTAATTGTGATGGACATTTTAAGTT
TACTGAAATCTTCTTTTTGATGCAACTTGTTGGTAGGGTGATGATGGGGTTAAAACCACAATTGAGCTTTTCATTAAAAGXXXXXXXXXXG
GTGGAGGGAAATATTGGGAAAAAAACCATGAAAAAGCCTTTTCCCTTCTTGTTCTTAATTGTGATGGACATTTTAAGTT
>Marker1007299 Chr2 SNP_site:20503191 Ref_Type:A
TACCATATTTTCTTCAAAAGGAATAAAGAGCAGTATGACATTAGAAACTATACAAACTTGTGTCTCATACCTCTCTCTTGXXXXXXXXXXT
GAACATGTGGCTTCTTTCATATTTGGCAAGGTAGATGAAAACAAACTTTTAAATTTTTTTTAAAAGAAAAGGTGTGGTA
TACCATATTTTCTTCGAAAGGAATAAAGAGCAGTATGACATTAGAAACTATACAAACTTGTGTCTCATACCTCTCTCTTGXXXXXXXXXXT
GAACATGTGGCTTCTTTCATATTTGGCAAGGTAGATGAAAACAAACTTTTAAATTTTTTTTAAAAGAAAAGGTGTGGTA
>Marker1007361 Chr2 SNP_site:3469590 Ref_Type:C

Create PDF with GO2PDF for free, if you wish to

ACTATCATACTACTATAAATTTTTCCAAAAAATTACTTGTGCTATGATCACACTATGCATACTCATATTTACTGTTGGTCXXXXXXXXXXXA
CACAACTCCATACCCTCCTTCTCCAATCAAATTCTCTTCTGAGAACAAATTAGTTGCCAAATCCAGCTCTCTCAAAGTA
ACTATCATACTACTATAAATTTTTCCAAAAAATTACTTGTGCTATGATCACACTATGCATACTCATATTTACTGTTGGTCXXXXXXXXXXXA
TACAACTCCATACCCTCCTTCTCCAATCAAATTCTCTTCTGAGAACAAATTAGTTGCCAAATCCAGCTCTCTCAAAGTA
>Marker1007754 Chr2 SNP_site:3213522 Ref_Type:T
AACTTTTTGATGAATTAGATTGCTTATAAGTCTTGGAATATAGATGATACCCTAATTTAAGCAATGGGCACTCCATCAATXXXXXXXXXXXA
AGATTGCCAATCTAAATTAATGAGATAACATTGTGTATAACAAAAAGAATAGTGTAAAGCACTACACGTAGAGGCTGTT
AACTTTTTGATGAATTAGATTGCTTATAAGTCTTGGAATATAGATGATACCCTAATTTAAGCAATGGGCACTCCATCAATXXXXXXXXXXXA
AGATTGTCAATCTAAATTAATGAGATAACATTGTGTTTAACAAAAAGAATAGTGTAAAGCACTACACGTAGAGGCTGTT
>Marker1007754 Chr2 SNP_site:3213552 Ref_Type:T
AACTTTTTGATGAATTAGATTGCTTATAAGTCTTGGAATATAGATGATACCCTAATTTAAGCAATGGGCACTCCATCAATXXXXXXXXXXXA
AGATTGCCAATCTAAATTAATGAGATAACATTGTGTATAACAAAAAGAATAGTGTAAAGCACTACACGTAGAGGCTGTT
AACTTTTTGATGAATTAGATTGCTTATAAGTCTTGGAATATAGATGATACCCTAATTTAAGCAATGGGCACTCCATCAATXXXXXXXXXXXA
AGATTGTCAATCTAAATTAATGAGATAACATTGTGTTTAACAAAAAGAATAGTGTAAAGCACTACACGTAGAGGCTGTT
>Marker100836 Chr4 SNP_site:8927103 Ref_Type:A
CACCCTAATAAGCATACAAAATTACCAAAAAAAAAGAAGAAAAAAACTTAAAGAACCCCTATTGTGACAATATGCAGAAGXXXXXXXXXXXA
GAATCCTCAAGAGATTCCTACAAACGCCTCCTTTATCATTCTTCATTAACTGTAGATGCGAAATTTGCTAACTCTCTGT
CACCCTAATAAGCATACAAAATTACCAAAAAAAAAGAAGAAAAAAACTTAAAGAACCCCTATTGTGACAATATGCGGAAGXXXXXXXXXXXA
GAATCCTCAAGAGATTCCTACAAACGCCTCCTTTATCATTCTTCATTAACTGTAGATGCGAAATTTGCTAACTCTCTGT
>Marker1008490 Chr2 SNP_site:14024547 Ref_Type:A
ACCATATTTAGTATTGGCTTACCTTTGTAATTTTTGTATTAAAGTGATTACTATTTTGTTCTCAAAGCCAAATAGTTTTCXXXXXXXXXXXG
TTTTAAAATGTTTATTTTGATCCATAGTTAGAGCTATCAATTTAGCTGGTAGTGACAACATATGAAGAAGCATAGTGTT
ACCGTATTTAGTATTGGCTTACCTTTGTAATTTTTGTATTAAAGTGATTACTATTTTATTCTCAAAGCCAAATAGTTTTCXXXXXXXXXXXG
TTTTAAAATGTTTATTTTGATCCATAGTTAGAGCTATCAATTTAGCTGGTAGTGACAACATATGAAGAAGCATAGTGTT
>Marker1008490 Chr2 SNP_site:14024601 Ref_Type:G
ACCATATTTAGTATTGGCTTACCTTTGTAATTTTTGTATTAAAGTGATTACTATTTTGTTCTCAAAGCCAAATAGTTTTCXXXXXXXXXXXG
TTTTAAAATGTTTATTTTGATCCATAGTTAGAGCTATCAATTTAGCTGGTAGTGACAACATATGAAGAAGCATAGTGTT
ACCGTATTTAGTATTGGCTTACCTTTGTAATTTTTGTATTAAAGTGATTACTATTTTATTCTCAAAGCCAAATAGTTTTCXXXXXXXXXXXG
TTTTAAAATGTTTATTTTGATCCATAGTTAGAGCTATCAATTTAGCTGGTAGTGACAACATATGAAGAAGCATAGTGTT
>Marker1008492 Chr2 SNP_site:10873143 Ref_Type:T
AACAATTCTTCCAAGTTTTCAACGAACTCACATTTTTTTCTTTTCTCTCATCAATGTTTGCACATTTTTCTCGTTCATATXXXXXXXXXXXT
AATACAAAAGTAGAATAAAATTTTACTTTGGGCTTTAATCGACTTCAAATGTATGCCCAAAGTATGTCGGTATCAAGTT
AACAATTCTTCCAAGTTTTCAACGAACTCACATTTTTTTCTTTTCTCTCATCAATGTTTGCACATTTTTCTTGTTCATATXXXXXXXXXXXT
AATACAAAAGTAGAATAAAATATTACTTTGGGCTTTAATCGACTTCAAATGTATGCCCAAAGTATGTCGGTATCAAGTT
>Marker1008492 Chr2 SNP_site:10873261 Ref_Type:A
AACAATTCTTCCAAGTTTTCAACGAACTCACATTTTTTTCTTTTCTCTCATCAATGTTTGCACATTTTTCTCGTTCATATXXXXXXXXXXXT
AATACAAAAGTAGAATAAAATTTTACTTTGGGCTTTAATCGACTTCAAATGTATGCCCAAAGTATGTCGGTATCAAGTT
AACAATTCTTCCAAGTTTTCAACGAACTCACATTTTTTTCTTTTCTCTCATCAATGTTTGCACATTTTTCTTGTTCATATXXXXXXXXXXXT
AATACAAAAGTAGAATAAAATATTACTTTGGGCTTTAATCGACTTCAAATGTATGCCCAAAGTATGTCGGTATCAAGTT
>Marker1008595 Chr2 SNP_site:21811014 Ref_Type:G
ACAAATTGTTAGCAAAAGCATTTAGTTTATGTTATTTTGTTTTATTGTAAGCACGCATACAGATTATGACACCACGTTGAXXXXXXXXXXXT
TCATTAGTGAAGAAAGATATATTTTGTGACTTCGAAATGATAACAAAGAACTTCTTGGTCCATCTTAGTGAAATTGGTG
ACAAATTGTTAGCAAAAGCATTTAGTTTATGTTATTTTGTTTTATTGTAAGCACGCATACAGATTGTGACACCACGTTGAXXXXXXXXXXXT
TCATTAGTGAAGAAAGATATATTTTGTGACTTCGAAATGATAACAAAGAACTTCTTGGTCCATCTTAGTGAAATTGGTG

Create PDF with GO2PDF for free, if you wish to

>Marker1009348 Chr2 SNP_site:3391184 Ref_Type:T
AACACGTTTTAATCACTAAACTATGTTTGAAACTGACGTTTTCATTATTAAATAAATGTTTGTATGGAGAACTAATTAAAXXXXXXXXXXA
ATTTATTTAAAATATTAGAAACTCCAAGTGTAAGTATATTGGCATGAAAATTCGCTTAAATTTAGTAAATCGGAAAGTT
AACACGTTTTAATCACTAAACTATGTTTGAAACTGACGTTTTCATTATTAAATAAATGTTTGTATTGAGAACTAATTAAAXXXXXXXXXXA
ATTTATTTAAAATATTAGAAACTCCAAGTGTAAGTATATTGGCATGAAAATTCGCTTAAATTTAGTAAATCGGAAAGTT
>Marker1010011 Chr2 SNP_site:22033873 Ref_Type:C
TACTTTGTTTTTAATTTTCAAAATTTTTAAAATTATTGATAAAAAGTAGATAAACAGATAAAAAGAAAATTCTATTTAGAXXXXXXXXXXA
GTTAATTGTGGATTTCATAATTCATATTAACTCTACAGACAAAAATTGAAGATGTGTTTGGATTATGTGGTGAATTGTT
TACTTTGTTTTTCATTTTCAAAATTTTTAAAATTATTGATAAAAAGTAGATAAACAGATAAAAAGAAAATTCTATTAAGAXXXXXXXXXXA
GTTAATTGTGGATTTCATAATTCATATTAACTCTACAGACAAAAATTGAAGATGTGTTTGGATTATGTGGTGAATTGTT
>Marker1010011 Chr2 SNP_site:22033937 Ref_Type:A
TACTTTGTTTTTAATTTTCAAAATTTTTAAAATTATTGATAAAAAGTAGATAAACAGATAAAAAGAAAATTCTATTTAGAXXXXXXXXXXA
GTTAATTGTGGATTTCATAATTCATATTAACTCTACAGACAAAAATTGAAGATGTGTTTGGATTATGTGGTGAATTGTT
TACTTTGTTTTTCATTTTCAAAATTTTTAAAATTATTGATAAAAAGTAGATAAACAGATAAAAAGAAAATTCTATTAAGAXXXXXXXXXXA
GTTAATTGTGGATTTCATAATTCATATTAACTCTACAGACAAAAATTGAAGATGTGTTTGGATTATGTGGTGAATTGTT
>Marker1010387 Chr2 SNP_site:2415674 Ref_Type:C
TACTTTGTTTGAATTTTTTCTCTATGAGAATCTGCACCCATATCATTTGAATTAATTATTGTTTTTCCATTCAACTTCATXXXXXXXXXXA
GTTAGCATGGTGTGATGAACGAATGTATTTTCGAAAATAACCATCTTAAAAAGAGGAGAAGCAACTATTTGAATGTGTA
TACTTTGTTTGAATTTTTTCTCTCTGAGAATCTGCACCCATATCATTTGAATTAATTATTGTTTTTCCATTCAACTTCATXXXXXXXXXXA
GTTAGCATGGTGTGATGAACGAATGTATTTTCGAAAATAACCATCTTAAAAAGAGGAGAAGCAACTATTTGAATGTGTA
>Marker1011028 Chr2 SNP_site:22327596 Ref_Type:G
ACAGTCATCTCCCTTATCACTAGATTCTATGACCCTCTTCAGGGTATGCCACCACCAGCCCTTTTCCTTTTCATCCCCTTXXXXXXXXXXC
CTCAGGGAAAACATTGGAATAGTTTCCCAGGAACCTGCACTCTTTGCTGGAACCATCAAGGATAATATCAAAATGGGTA
ACAGTCATCTCCCTTATCACTAGATTCTATGACCCTCTTCAGGGTATGCCACCACCAGCCCTTTTCCTTTTCATCCCCTTXXXXXXXXXXC
GTCAGGGAAAACATTGGAATAGTTTCCCAGGAACCTGCACTCTTTGCTGGAACCATCAAGGATAATATCAAAATGGGTA
>Marker1011198 Chr2 SNP_site:2735777 Ref_Type:A
AACTTTCGTAACTAAATCAATGCATGTAAATAACTATCTTCACATCATTTTAGTTAAAATCAACTCAAAATCACTTAAAAXXXXXXXXXXT
AGGTGAAAAGCTAATGCTAATTTCATTACACATTTTTTTCTCTAATTTACATTAATCCCTATACTTAAAGAAGTCTTGT
AACTTTCGTAACTAGATCAATGCATGTAAATAACTATTTTCACATCATTTTAGTTAAAATCAACTCAAAATCACTTAAAAXXXXXXXXXXT
AGGTGAAAAGCTAATGCTAATTTCATTCCACATTTTTTTCTCTAATTTACATTAATCCCTATACTTAAAGAAGTCTTGT
>Marker1011198 Chr2 SNP_site:2735800 Ref_Type:C
AACTTTCGTAACTAAATCAATGCATGTAAATAACTATCTTCACATCATTTTAGTTAAAATCAACTCAAAATCACTTAAAAXXXXXXXXXXT
AGGTGAAAAGCTAATGCTAATTTCATTACACATTTTTTTCTCTAATTTACATTAATCCCTATACTTAAAGAAGTCTTGT
AACTTTCGTAACTAGATCAATGCATGTAAATAACTATTTTCACATCATTTTAGTTAAAATCAACTCAAAATCACTTAAAAXXXXXXXXXXT
AGGTGAAAAGCTAATGCTAATTTCATTCCACATTTTTTTCTCTAATTTACATTAATCCCTATACTTAAAGAAGTCTTGT
>Marker1011198 Chr2 SNP_site:2735955 Ref_Type:A
AACTTTCGTAACTAAATCAATGCATGTAAATAACTATCTTCACATCATTTTAGTTAAAATCAACTCAAAATCACTTAAAAXXXXXXXXXXT
AGGTGAAAAGCTAATGCTAATTTCATTACACATTTTTTTCTCTAATTTACATTAATCCCTATACTTAAAGAAGTCTTGT
AACTTTCGTAACTAGATCAATGCATGTAAATAACTATTTTCACATCATTTTAGTTAAAATCAACTCAAAATCACTTAAAAXXXXXXXXXXT
AGGTGAAAAGCTAATGCTAATTTCATTCCACATTTTTTTCTCTAATTTACATTAATCCCTATACTTAAAGAAGTCTTGT
>Marker1011283 Chr2 SNP_site:16994395 Ref_Type:G
TACTAAAAGCATAGTCTCAATTTTTATTTAAAAAACAAATCTCCTTGATAGTGCCTTGAATGCCTTGCTAGGAAAGGCCAXXXXXXXXXXA
TGGAATGATATAGTCAAAGATTAATTAGTTTTATTTATTTATTAACAAGATAGTAATAGGAGAGAAGAATTACTAGGTG
TACTAAAAGCATAGTCTCAATTTTTATTTAAAAAACAAATCTCCTTGATAGTGCCTTGAATGCCTTGCTAGGAAAGGCCAXXXXXXXXXXA

Create PDF with GO2PDF for free, if you wish to

TGGAATGATATAGTCAAGGATTAATTAGTTTTATTTATTTATTAACAAGATAGTAATAGGAGAGAAGAATTACTAGGTG

>Marker1011348 Chr2 SNP_site:10998055 Ref_Type:A

AACCAAACTTGCAAGTCACAAAAGCATTTCAATTTTATTGACTATTTTAAATATATAAAACACATATGATATACCCAAATXXXXXXXXXXC

AACAACAAAAAGAAATGGTAATAAACTCATGAATTTTATAAAAATATACAATCCATCACTCGTTTTCTTGACTTATTGT

AACCAAACTTGCAAGTCACAAAAGCATTTCAATTTTATTGACTATTTTAAATATATAAAACACATATGATATACCCAGATXXXXXXXXXXC

AACAACAAAAAGAAATGGTAATAAATTCATGAATTTTATAAAAATATACAATCCATCACTCGTTTTCTTGACTTATTGT

>Marker1011348 Chr2 SNP_site:10998219 Ref_Type:C

AACCAAACTTGCAAGTCACAAAAGCATTTCAATTTTATTGACTATTTTAAATATATAAAACACATATGATATACCCAAATXXXXXXXXXXC

AACAACAAAAAGAAATGGTAATAAACTCATGAATTTTATAAAAATATACAATCCATCACTCGTTTTCTTGACTTATTGT

AACCAAACTTGCAAGTCACAAAAGCATTTCAATTTTATTGACTATTTTAAATATATAAAACACATATGATATACCCAGATXXXXXXXXXXC

AACAACAAAAAGAAATGGTAATAAATTCATGAATTTTATAAAAATATACAATCCATCACTCGTTTTCTTGACTTATTGT

>Marker1011427 Chr2 SNP_site:11040541 Ref_Type:C

GACCTAAGTGACTTGGTGTTAATTTTAAGTTTATTATAGACTTATATGTTTATGATGGATTGTCCTCTTATTTGTATGAAXXXXXXXXXXT

CAACTTAAGACAAATAGATGAGTGTTCTCTTAAATGGTGATTTTCGGATTTGAACAAAAGGTCTTACTCTCTCATTGGT

GACCTAAGTGACTTGGTGTTAATTTTAAGTTTATTATAGACTTATATGTTTATGATGGATTGTCCTCTTATTTGTATGAAXXXXXXXXXXT

CAACTTAAGACAAATAGATGAGTGTTCTCTTAAATGGTGATTTTCGGATTTGAACAAAAGGTCTTACTCTCTTATTGGT

>Marker1011758 Chr2 SNP_site:3128859 Ref_Type:T

GACCATCCAGCTGTAGTTCATCATGATGACGAGTCTGATGATGGGGCTTTTTCTTTGCATGATGAACAAACTACAAGAAAXXXXXXXXXXG

TTTTTCTGTTTCTTTTGGGGTGTAAGAACTAAGAAGGTAAATATTGATGCATTGGAGATTTGATCTTCTTCTAAAATGT

GACCATCCAGCTGTAGTTCATCATGATGACGAGTCTGATGATGGGGCTTTTTCTTTGCATGATGAACAAACTACAAGAAAXXXXXXXXXXG

TTTTTCTGTTTCTTTTGGGGTGTAAGAACTAAGAAGGTAAATATTGATGCATTGGATATTTGATCTTCTTCTAAAATGT

>Marker1012064 Chr2 SNP_site:14549424 Ref_Type:A

AACAAAACCAACCAAAGGTTTCAACTAAAGTGAGAATTAACATACCAAGTGTCTTCTAAGGATGAGAGACAATATGAAATXXXXXXXXXXC

TATCATGTTTTTATGTGGTAAGAATTTTTTGTTTCAAGTGTAGAGTTAAAAAAGTAGAATTCCCAAGTAGAAACAGAGT

AACAAAACCAACCAAAGGTTTCAACTAAAGTGAGATTTAACATACCAAGTGTCTTCTAAGGATGAGAGACAATATGAAATXXXXXXXXXXC

TATCATGTTTTTATGTGGTAAGAATTTTTTGTTTCAAGTGTAGAGTTAAAAAAGTAGAATTCCCAAGTAGAAACAGAGT

>Marker1012536 Chr2 SNP_site:21054120 Ref_Type:T

ACATGTTCTATGCTTTCTTCTCTCCAACGTGGTTATAACTTTACACGCTCCCCAGTATATTTAACTTTAGATACTATGAAXXXXXXXXXXT

TTCAAGGATGGTTGTATATATATTTGAGAAATTCATGGAGTTTCTTCAAGGAACATAAGAAAACATTGGAGTGGGCGTT

ACATGTTCTATGCTTTCTTCTCTCCAACGTGGTTATAACTTTACACGCTCCCCAGTTTATTTAACTTTAGATACTATGAAXXXXXXXXXXT

TTCAAGGATGATTGTATATATATTTGAGAAATTCATGGAGTTTCTTCAAGGAACATAAGAAAACATTGGAGTGGGCGTT

>Marker1012536 Chr2 SNP_site:21054261 Ref_Type:A

ACATGTTCTATGCTTTCTTCTCTCCAACGTGGTTATAACTTTACACGCTCCCCAGTATATTTAACTTTAGATACTATGAAXXXXXXXXXXT

TTCAAGGATGGTTGTATATATATTTGAGAAATTCATGGAGTTTCTTCAAGGAACATAAGAAAACATTGGAGTGGGCGTT

ACATGTTCTATGCTTTCTTCTCTCCAACGTGGTTATAACTTTACACGCTCCCCAGTTTATTTAACTTTAGATACTATGAAXXXXXXXXXXT

TTCAAGGATGATTGTATATATATTTGAGAAATTCATGGAGTTTCTTCAAGGAACATAAGAAAACATTGGAGTGGGCGTT

>Marker101271 Chr4 SNP_site:13072567 Ref_Type:A

GACTAAGAATTAAAAATGATGTCAAGTTCATTCTTTGTGTTGATGTCAAATTTATGAGAATATAATTATACTAATAGAAAXXXXXXXXXXC

AAGTATTAGATACTGTTTTTTTTAATTGTTAATTATTAAGAGTCAGTATGGAGACCTTGATCTCTCAAACTAAGTTGTG

GACTAAGAATTAAAAATGATGTCAAGTTCATTCTTTGTGTTGATGTCAAATTTATGAGAATATAATTATACTAATAGAAAXXXXXXXXXXC

AAGTATTAGATACTGTTTTTTTTAATTGTTAATTATTAAGAGTCAGTGTGGAGACCTTGATCTCTCAAACTAAGTTGTG

>Marker1013153 Chr2 SNP_site:7774535 Ref_Type:T

GACCAATTGGTCATATCCTTAAACTCTATTAGCTCGCCAACTTCTACTTAGTGTTTTACACATGGTAGTTTAGTATCCTTXXXXXXXXXXC

TTCCCTTCTCAACCTTCTTTCCTCACCTGAATATTCCTTGCCTAGCTCAAAACACATACTACCCATATAGTCAAATGTA

Create PDF with GO2PDF for free, if you wish to

GACCAATTGGTCATATCCTTAAACTCTATTAGTTCGCCAACTTCTACTTAGTGTTTTACACATGGTAGTTTAGTATCCTTXXXXXXXXXXC
TTCCCTTCTCAACCTTCTTTCCTCACCTGAATATTCCTTGCCTAGCTCAAAACACATACTACCCATATAGTCAAATGTA
>Marker101320 Chr4 SNP_site:1507394 Ref_Type:T
ACGAAGAAAGGTCAAGCAGTAGTCAGGAAAATCGCAACACCTTCATACGGCGATCAAGTATGAAGAAACTTCGATGCGAAXXXXXXXXXXC
TCTTGAAGCTAATGTATAAATCCATATATGCTAAAGAAACAAAAAATAATAATAATAATTAAAGGGGAACAACTTTGTG
ACGAAGAAAGGTCAAGCAGTTGTCAGGAAAATCGCAACACCTTCATACGGCGATCATGTATGAAGAAACTTCGATGCGAAXXXXXXXXXXC
TCTTGAAGCTAATGTATAAATCCATATATGCTAAAGAAACAAAAAATAATAATAATAATTAAAGGGGAACAACTTTGTG
>Marker101320 Chr4 SNP_site:1507430 Ref_Type:T
ACGAAGAAAGGTCAAGCAGTAGTCAGGAAAATCGCAACACCTTCATACGGCGATCAAGTATGAAGAAACTTCGATGCGAAXXXXXXXXXXC
TCTTGAAGCTAATGTATAAATCCATATATGCTAAAGAAACAAAAAATAATAATAATAATTAAAGGGGAACAACTTTGTG
ACGAAGAAAGGTCAAGCAGTTGTCAGGAAAATCGCAACACCTTCATACGGCGATCATGTATGAAGAAACTTCGATGCGAAXXXXXXXXXXC
TCTTGAAGCTAATGTATAAATCCATATATGCTAAAGAAACAAAAAATAATAATAATAATTAAAGGGGAACAACTTTGTG
>Marker1013728 Chr2 SNP_site:20201123 Ref_Type:C
CACACCTGAGAGACTCTTGGAGCTTGTTGCCATGCAGGCTATTGATATATCTGGTGTTTCGTTGCTGGTAATTTGACAGCXXXXXXXXXXT
AAAATATCGATTATAATGTTGTTGGAATTTGAATGTCGTGGATTATTTAGATATGTTGATATTGACGGATATTTTTGTA
CACACCTGAGAGACTCTTGGAGCTTGTTGCCATGCAGGCTATTGATATATCTGGTGTTTCGTTGCTGGTAATTTGACAGCXXXXXXXXXXT
AAAATATCGATTATAATGTTGTTGGAATTTGAATGTTGTGGATTATTTAGATATGTTGATATTGACGGATATTTTTGTA
>Marker1013904 Chr2 SNP_site:2931196 Ref_Type:A
CACCTATCTTAACAAATAGATAGCATAAAATTTGGAATATCCATACTTGAAGAATTCTATTTGTTATAAATCAACGTTCCXXXXXXXXXXT
CAACGAAGGCAATGGTTCGAGATCAAAATTTTTCACTGTGGCCATAAGTATCATACCTCTTAGACGAATCAATTTTAGT
CACCTATCTTAACAAATAGATAGCATAAAATTTGGAATATCCATACTTGAAGAATTCTATTTGTTATAAATCAACGTTCCXXXXXXXXXXT
CAGCGAAGGCAATGGTTCGAGATCAAAATTTTTCACTGTGGCCATAAGTATCATACCTCTTAGACGAATCAATTTTAGT
>Marker101419 Chr4 SNP_site:12393495 Ref_Type:C
AACCTTTTTTCTGGGTCAAAGATTGCTATATTATTGGTGAATGTCTTAAGAAATATATAATGATTTTGGGGGATTTTTTCXXXXXXXXXXT
ACTATTGCATCAGGAGAGATGGAGAATGCATGCATTGATGATGGTGATAGGATTGGAGGATTTTGATGGAGATATTTGT
AACCTTTTTTCTGGGTCAAAGATTGCTATATTATTGGTGAATGTCTTAAGAAATATATAATGATTTTGGGGGATTTTTTTXXXXXXXXXXT
ACTATTGCATCAGGAGAGATGGAGAATGCATGCATTGATGATGGTGATAGGATTGGAGAATTTTGATGGAGAGATTTGT
>Marker101419 Chr4 SNP_site:12393654 Ref_Type:G
AACCTTTTTTCTGGGTCAAAGATTGCTATATTATTGGTGAATGTCTTAAGAAATATATAATGATTTTGGGGGATTTTTTCXXXXXXXXXXT
ACTATTGCATCAGGAGAGATGGAGAATGCATGCATTGATGATGGTGATAGGATTGGAGGATTTTGATGGAGATATTTGT
AACCTTTTTTCTGGGTCAAAGATTGCTATATTATTGGTGAATGTCTTAAGAAATATATAATGATTTTGGGGGATTTTTTTXXXXXXXXXXT
ACTATTGCATCAGGAGAGATGGAGAATGCATGCATTGATGATGGTGATAGGATTGGAGAATTTTGATGGAGAGATTTGT
>Marker101419 Chr4 SNP_site:12393668 Ref_Type:T
AACCTTTTTTCTGGGTCAAAGATTGCTATATTATTGGTGAATGTCTTAAGAAATATATAATGATTTTGGGGGATTTTTTCXXXXXXXXXXT
ACTATTGCATCAGGAGAGATGGAGAATGCATGCATTGATGATGGTGATAGGATTGGAGGATTTTGATGGAGATATTTGT
AACCTTTTTTCTGGGTCAAAGATTGCTATATTATTGGTGAATGTCTTAAGAAATATATAATGATTTTGGGGGATTTTTTTXXXXXXXXXXT
ACTATTGCATCAGGAGAGATGGAGAATGCATGCATTGATGATGGTGATAGGATTGGAGAATTTTGATGGAGAGATTTGT
>Marker1014237 Chr2 SNP_site:3155039 Ref_Type:C
CACATATTAGCATTTCAATAGTTTCTTTGTGAGAATTTAAGGCTTGCTTGGAAGCCAAAATAGATTAAAATTTTAACGTAXXXXXXXXXXC
CAAGTAATCAAGTAATTAATGTTTGGTCATATTTTAAGTCTTATTTTTTTAGAAACTCTACCCACTCAATTCACTAGTA
CACATATTAGCATTTCAATAGTTTCTTTGTGAGAATTTAAGGCTTGCTTGGAAGCCAAAATAGATTAAAATTTTAACGTAXXXXXXXXXXC
CAAGTAATCAAGTAATTAATGTTTGGTCATATTTTAAGTCTTATTTTTTTAGAAACTCTACCTACTCAATTCACTAGTA
>Marker1014874 Chr2 SNP_site:14153583 Ref_Type:G
ACTTTCAAAATCAATAATGATTTTGGACTTTTTGAAAACTTAACAATTTTGACCTCTTACTTATATCATATCTCATTGATXXXXXXXXXXA

Create PDF with GO2PDF for free, if you wish to

ATTCACCTTTATTTTGCTTTTCTTCTTTGACAAAAACACACTAACCAACTTCGACTACTTATTCGCACGGTTAGTGGTG
ACTTTCAAAATCAATAATGATTTTGGACTTTTTGAAAACTTAAGAATTTTGACCTCTTACTTATATCATATCTCATTGATXXXXXXXXXXA
ATTCACCTTTATTTTGCTTTTCTTCTTTGACAAAAACACACTAACCAACTTCGACTACTTCTTCGCACGGTTAGTGGTG
>Marker1014874 Chr2 SNP_site:14153772 Ref_Type:C
ACTTTCAAAATCAATAATGATTTTGGACTTTTTGAAAACTTAACAATTTTGACCTCTTACTTATATCATATCTCATTGATXXXXXXXXXXA
ATTCACCTTTATTTTGCTTTTCTTCTTTGACAAAAACACACTAACCAACTTCGACTACTTATTCGCACGGTTAGTGGTG
ACTTTCAAAATCAATAATGATTTTGGACTTTTTGAAAACTTAAGAATTTTGACCTCTTACTTATATCATATCTCATTGATXXXXXXXXXXA
ATTCACCTTTATTTTGCTTTTCTTCTTTGACAAAAACACACTAACCAACTTCGACTACTTCTTCGCACGGTTAGTGGTG
>Marker101500 Chr4 SNP_site:7007083 Ref_Type:G
AACATCGTTAGAATGATGCTCTAGCCCTCTAAAGATCTTATTGTTCCTCTTTTCTTAAAGCTCCTCGACACAGTAGCACGXXXXXXXXXXC
TATAATCCTTCTTGATTCTAGGAGATGAGATACCTATCCTGATTCCTCTGGATTCTATTTCGGTGTGCAGTCTTCTGTG
AACATCGTTAGGATGATGCTCTAGCCCTCTAAAGATCTTATTGTTCCTCTTTTCTTAAAGCTCCTCGACACAGTAGCACGXXXXXXXXXXC
TATAATCCTTCTTGATTCTAGGAGATGAGATACCTATCCTGATTCCTCTGGATTCTATTTCGGTGTGCAGTCTTCTGTG
>Marker1015029 Chr2 SNP_site:9391544 Ref_Type:T
ACCTAAAATAAAAATGCGATTAAAAATCTATAGAGGGTAATTGCTACAATTAAATAATTTCAGATAAAAAACACATTATTXXXXXXXXXXT
AAATAAGAATAATTACTATGGAAAACATACCTCATTAAACGATAGTTCCATAAAAGGATTTTATTCCCCTAATTTAGGT
ACCTAAAATAAAAATGTGATTAAAAATCTATAGAGGGTAATTGCTACAATTAAATAATTTCAGATAAAAAACACATTATTXXXXXXXXXXT
AAATAAGAATAATTACTATGGAAAACATACCTCATTAAATGATAGTTCCATAAAAGGATTTTATTCCCCTAATTTAGGT
>Marker1015029 Chr2 SNP_site:9391734 Ref_Type:T
ACCTAAAATAAAAATGCGATTAAAAATCTATAGAGGGTAATTGCTACAATTAAATAATTTCAGATAAAAAACACATTATTXXXXXXXXXXT
AAATAAGAATAATTACTATGGAAAACATACCTCATTAAACGATAGTTCCATAAAAGGATTTTATTCCCCTAATTTAGGT
ACCTAAAATAAAAATGTGATTAAAAATCTATAGAGGGTAATTGCTACAATTAAATAATTTCAGATAAAAAACACATTATTXXXXXXXXXXT
AAATAAGAATAATTACTATGGAAAACATACCTCATTAAATGATAGTTCCATAAAAGGATTTTATTCCCCTAATTTAGGT
>Marker1015430 Chr2 SNP_site:20136595 Ref_Type:A
GACCTCATTGGCAAATGTCTTCTTAACTTGACAGATAGCTGTTGGCGATGTCCTGTGTGAAATTGAAACCGACAAAGCTAXXXXXXXXXXT
ACTAAGAAAGTTATCCTTGAATAACCCTCAACTACAACAATTTTATAATTATTATAGATTTCAAAATTCATCATCAGTG
GACCTCATTGGCAAATGTCTTCTTAACTTGACAGATAGCTGTTGGCGATGTCCTGTGTGAAATTGAAACCGACAAAGCTAXXXXXXXXXXT
ACTAAGAAAGTTATCCTTGAATAACCCTCAGCTACAACAATTTTATAATTATTATAGATTTCAAAATTCATCATCAGTG
>Marker1015528 Chr2 SNP_site:13511358 Ref_Type:T
AACGGAAGAAATGGATAAGATGAAATTGCCCAAAGCAAAAAAGCACAAAAAGTTGATAGAAATAACACAAAAAATCATCAXXXXXXXXXXA
AACACACCAAAGAAAACACTATATACATATAAGACACAACCTAATTAAACTACAAACAACAACAAGAAATTTCTAAGTT
AACGGAAGAAATGGATAAGATGAAATTGCCCAAAGCAAAAAAGCACAAAAAGTTGATAGAAATAACACAAAAAATCATCAXXXXXXXXXXA
AACACACCAAAGAAAACACTATATACATATAAGACACAATCTAATTAAACTACAAACAACAACAAGAAATTTCTAAGTT
>Marker1015621 Chr2 SNP_site:2730052 Ref_Type:G
CACTGAATCTTAGAGTTGATCATGGAAAGATTCTCTTCTGCTCTTAATTGATTGAATCATAACTAGCTTCTAGGTTCAAAXXXXXXXXXXA
GAAAAAATGGTGGATAATGAAGAACTAGGAAGTAGGAGCTTGGAAATATGTGTTTGTCAAATTTACGTGGAAAACTGTG
CACTGAATCTTAGAGTTGATCATGGAAAGATTCTCTTCTGCTCTTAATTGATTGAATCATAAGTAGCTTCTAGGTTCAAAXXXXXXXXXXA
GAAAAAATGGTGGATAATGAAGAACTAGGAAGTAGGAGCTTGGAAATATGTGTTTGTCAAATTTGCGTGGAAAACTGTG
>Marker1015621 Chr2 SNP_site:2730240 Ref_Type:G
CACTGAATCTTAGAGTTGATCATGGAAAGATTCTCTTCTGCTCTTAATTGATTGAATCATAACTAGCTTCTAGGTTCAAAXXXXXXXXXXA
GAAAAAATGGTGGATAATGAAGAACTAGGAAGTAGGAGCTTGGAAATATGTGTTTGTCAAATTTACGTGGAAAACTGTG
CACTGAATCTTAGAGTTGATCATGGAAAGATTCTCTTCTGCTCTTAATTGATTGAATCATAAGTAGCTTCTAGGTTCAAAXXXXXXXXXXA
GAAAAAATGGTGGATAATGAAGAACTAGGAAGTAGGAGCTTGGAAATATGTGTTTGTCAAATTTGCGTGGAAAACTGTG
>Marker1015754 Chr2 SNP_site:21338026 Ref_Type:C

Create PDF with GO2PDF for free, if you wish to

ACATAATGTGTATGGTTGAGATCAATTACTCACATCATATTCAAGAAACATGTGGTTTGTCTTTCAATAAAAATCTACGAXXXXXXXXXXXT
TCAACAAATTTCTTCAAAAGACAACTTGGCGAACTTATTCACAAACGCATTACCCACACCAACATTTGAAAAGCTAGTG
ACATAATGTGTATGGTTGAGATCAATTACTCACATCATATTCAAGAAACATGTGGTTTGTCTTTCAATAAAAATCTACGAXXXXXXXXXXXT
TCAACAAATTTCTTCAAAAGACAACTTGGCGAACTTATTCACAAAGGCATTACCCACACCAACATTTGAAAAGCTAGTG
>Marker1015945 Chr2 SNP_site:20615506 Ref_Type:C
AACAAACAAACCACTATTCAAACTATTTGACATCCGGAATACACATAATTTCACAAAGTAATTCTCTGCAACAAACCCATXXXXXXXXXXXT
CCAAAGGAAGATTGAACTAAACCCATTGAACTAGAAAAAGTGAATTTCAACATCCCTAACATTCCAAATCCAAATCTGT
AACAAACAAACCACTATTCAAACTATTTGACATCCGGAATACACATAATTTCACAAAGTAATTCTCTGCAAGAAACCCATXXXXXXXXXXXT
CCAAAGGAAGATTGAACTAAACCCATTGAACTAGAAAAAGTGAATTTCAACATCCCTAACATTCCAAATCCAAATCTGT
>Marker1016996 Chr2 SNP_site:4090966 Ref_Type:A
TACAATTTCCTAATGTTGCATGTTTTCATTATATGTGGTAGAGAGAAATTTAGGCAGATTTATTATAGTTGTATGAGATGXXXXXXXXXXXA
TCCTAATATATGGAGAGTCCTATAAAATAAATCTTTGATATATTTTATACGCAAAAAACATAAGTATTATTCGTAGGTC
TACAATTTCCTAATGTTGCGTGTTTTCATTATATGTGGTAGAGAGAAATTTAGGCAGATTTATTATAGTTGTATGAGATGXXXXXXXXXXXA
TCCTAATATATGGAGAGTCCTATAAAATAAATCTTTGATATATTTTATACGCAAAAAACATAAGTATTATTCGTAGGTC
>Marker1017050 Chr2 SNP_site:22029603 Ref_Type:T
TACTATTTTGGAGGACTTTTAAGAGAAAGATAATGGTATTGTGGTGAGTTTGAGGTAGTTATCTTAAATATAATCATGTGXXXXXXXXXXXT
GTAATGTTTATTTGTTTTTTGGAAAAGAAATCATTATTTAATTTTAAATTTCTGAAAATATGTCTTTCTAAACAAGAGT
TACTATTTTGGAGGACTTTTAAGAGAAAGATAATGGTATTGTGGTGAGTTTGAGGTAGTTATCTTAAATATAATCATGTGXXXXXXXXXXXT
GTAATGTTTATTTGTTTTTTGGAAAAGAAATCATTATTTAATTTTAAATTTCTGAAAATATGTCTTTTTAAACAAGAGT
>Marker1017240 Chr2 SNP_site:22215401 Ref_Type:A
AACGACCAAGGAATAGGTTGCGCATAACAATTGAGCCCATTATGGACTCAGAAGCTCAATCAGTAAATCCAGATGCTCGAXXXXXXXXXXXA
ATCAAAATTGGCAAAGAAGTGGATAAGAACGCTAGAAAAGTATTTTAAAGTAATGCAGTGCCCCGAGAAGAGAAAGGTG
AACGACCAAGGAATAGGTTGCGCATAACAATTGAGCCCATTATGGACTCAGAAGCTCAATCAGTAAATCCAGATGCTCGAXXXXXXXXXXXA
ATCAAAATTGGCAAAGAAGTGGATAAGAACGCTAGAAAAGTATTTTAAAGTAATGCAGTGCCCCGAGAAGAGGAAGGTG
>Marker1017378 Chr2 SNP_site:11149880 Ref_Type:G
ACTAATATCTTATTTATGTAAAGTTTGTAATTTAATGATAATTACAAATGAGATGATTTTGAGTTCCAAACTTATTTGTGXXXXXXXXXXXA
CAATTATGGGGTTGGGATCGAACTTTCTGTTCTAAGGTGGAAGGTTGTGTAAATTACTCTTTTAAGTTCTTTTGTAGTT
ACTAATATCTTATTTATGTAAAGTTTGTAATTTAATGATAATTACAAATGAGATGATTTTGAGTTCCGAACTTATTTGTGXXXXXXXXXXXA
CAATTATGGGGTTGGGATCGAACTTTCTGTTCTAAGGTGGAAGGTTGTGTAAATTACTCTTTTAAGTTCTTTTGTAGTT
>Marker1017507 Chr2 SNP_site:251585 Ref_Type:G
CACTTGAAATTGGGGTTGAGAGATGAAATTATTTTCGTGGGGTTGTAATAATTTTTCATATAATGAAAATTTTCTAATCTXXXXXXXXXXXT
GTTCCTAATTAATTAGGTCTTCAAAAGTTATTTTGAAAAGATTAGTATGGATCAAATTGATATAATTTAACAAAATGTC
CACTTGAAATTGGGGTTGAGAGATGAAATTATTTTCGTGGGGTTGTAATAATTTTTCATATAATGAAAATTTTCTAATCTXXXXXXXXXXXT
GTTCCTAATTAATTAGGTCTTCAAAATTTATTTTGAAAAGATTAGTATGGATCAAATTGATATAATTTAACAAAATGTC
>Marker1018030 Chr2 SNP_site:12489584 Ref_Type:G
ACATTATTAAAAATTGGAGATGTGTTATATTAAATACTAAAGTAAAAACTTAATTTTGGTTATACATAAAATATAATTTTXXXXXXXXXXXG
GTTTAAATAAACTTTTTGACAAATAAAAAGTAAAATCAACTAATTAGATACTTTTTAAATTTAAATAAAAAACTTTGTA
ACATTATTAAAAATTGGAGATGTGTTATATTAAATACTAAAGTAAAAACTTAATTTTGGTTATACATAAAATATAATTTTXXXXXXXXXXXG
GTTTAAATAAACTTTTTGACAAATAAAAAGTAAAATCAACTAATTAGATACTTTTTAAATTTAAATAAAGAACTTTGTA
>Marker1018557 Chr2 SNP_site:20018664 Ref_Type:T
ACTTTTTGTCGCTCTAAAAATATGCCATATTGAGTTGTTTCTTCTCTTAGTAATCATCAATTCTTTTAAACATTTCTTACXXXXXXXXXXXC
TTCACTCTTGTTGAGAGTAGCTTGACGTTGATACCATATGTTCTGCTCCTAACGACAGAGTTCATGAGAATATGAAAGT
ACTTTTTGTCGCTCTAAAAATATGCCATATTGAGTTGTTTCTTCTCTTAGTAATCATCAATTCTTTTAAACATTTCTTACXXXXXXXXXXXC
TTCACTCTTGTTGAGAGTAGCTTGACTTTGATACCATATGTTCTGCTCCTAATGACAGAGTTCATGAGAATATGAAAGT

Create PDF with GO2PDF for free, if you wish to

>Marker1018557 Chr2 SNP_site:20018690 Ref_Type:T
ACTTTTTGTCGCTCTAAAAATATGCCATATTGAGTTGTTTCTTCTCTTAGTAATCATCAATTCTTTTAAACATTTCTTACXXXXXXXXXXC
TTCACTCTTGTTGAGAGTAGCTTGACGTTGATACCATATGTTCTGCTCCTAACGACAGAGTTCATGAGAATATGAAAGT
ACTTTTTGTCGCTCTAAAAATATGCCATATTGAGTTGTTTCTTCTCTTAGTAATCATCAATTCTTTTAAACATTTCTTACXXXXXXXXXXC
TTCACTCTTGTTGAGAGTAGCTTGACTTTGATACCATATGTTCTGCTCCTAATGACAGAGTTCATGAGAATATGAAAGT
>Marker1019327 Chr2 SNP_site:19206770 Ref_Type:T
AACATCGTGAAGTGATCATATATTGCAAAGTGCAGTTTGTAACCTGACACAGATGATTATTTTGATTTTTGCCAGCAGAAXXXXXXXXXXG
GTGTATAGGTTTCCATATTTATATAAAGCATTGGGACGCTCAATATTTGTCACCCTGTTGGTTTTTTGTGAATATGGTG
AACATCGTGAAGTGATCATATATTGCAAAGTGCAGTTTGTAACCTGACACAGATGATTATTTTGATTTTTGCCAGCAGAAXXXXXXXXXXG
TTGTATAGGTTTCCATATTTATATAAAGCATTGGGACGCTCAATATTTGTCACCCTGTTGGTTTTTTGTGAATATGGTG
>Marker1019718 Chr2 SNP_site:4927688 Ref_Type:C
CACAGCTTTCAGTTTTAAGCCATTAGTATCTTCAAAAGGCGATGGCTTTATTTCTTGCTTGAACTCATAATGGTTCTTTAXXXXXXXXXXA
ATCCCAATGGGAAGAGGTCCATTGAAAATATGTATGTTCAAATAGAAGAACTTGAGATTACTTTTCGAGCATTTCATGT
CACAGCTTTCAGTTTTAAGCCATTAGTATCTTCAAAAGGTGATGGCTTTATTTCTTGCTTGAACTCATAATGGTTCTTTAXXXXXXXXXXA
ATCCCAATAGGAAGAGGTCCATTGAAAATATGTATGTTCAAATAGAAGAACTTGAGATTACTTTTCGAGCATTTCATGT
>Marker1019718 Chr2 SNP_site:4927871 Ref_Type:G
CACAGCTTTCAGTTTTAAGCCATTAGTATCTTCAAAAGGCGATGGCTTTATTTCTTGCTTGAACTCATAATGGTTCTTTAXXXXXXXXXXA
ATCCCAATGGGAAGAGGTCCATTGAAAATATGTATGTTCAAATAGAAGAACTTGAGATTACTTTTCGAGCATTTCATGT
CACAGCTTTCAGTTTTAAGCCATTAGTATCTTCAAAAGGTGATGGCTTTATTTCTTGCTTGAACTCATAATGGTTCTTTAXXXXXXXXXXA
ATCCCAATAGGAAGAGGTCCATTGAAAATATGTATGTTCAAATAGAAGAACTTGAGATTACTTTTCGAGCATTTCATGT
>Marker101999 Chr4 SNP_site:14151865 Ref_Type:A
ACCGATCATCGATCGTAGCAGAAGAATCATCTTAAAATTATTGCTGTTGCACTTTGGTGGAGCTAAGCGTCATAGTAGGAXXXXXXXXXXC
CCGTCATCCATAAAACAACCTCAGTTCTTCGTTCGGAGCTGCAGTGGAACCAAATGGAATACCAACAACTTTAAAGGGT
ACCGATCATCGATCGTAGCAGAAGAATCGTCTTAAAATTATTGTTGTTGCACTTTGGTGGAGCTAAGCGTCATAGTAGGAXXXXXXXXXXC
CCGTCATCCATAAAACAACCTCAGTTCTTCGTTCGGAGCTGCAGTGGAACCAAATGGAATACCAACAACTTTAAAGGGT
>Marker101999 Chr4 SNP_site:14151880 Ref_Type:C
ACCGATCATCGATCGTAGCAGAAGAATCATCTTAAAATTATTGCTGTTGCACTTTGGTGGAGCTAAGCGTCATAGTAGGAXXXXXXXXXXC
CCGTCATCCATAAAACAACCTCAGTTCTTCGTTCGGAGCTGCAGTGGAACCAAATGGAATACCAACAACTTTAAAGGGT
ACCGATCATCGATCGTAGCAGAAGAATCGTCTTAAAATTATTGTTGTTGCACTTTGGTGGAGCTAAGCGTCATAGTAGGAXXXXXXXXXXC
CCGTCATCCATAAAACAACCTCAGTTCTTCGTTCGGAGCTGCAGTGGAACCAAATGGAATACCAACAACTTTAAAGGGT
>Marker102009 Chr4 SNP_site:9828084 Ref_Type:G
ACATCATTATCAATCATAATCATCCTTTCTTATTTACCAATCTTTATTAGATTCTTTGTTCATTGTGTTTTTTCCCTTCCXXXXXXXXXXA
TTAAGCATCTCGTCCTATCTTCACATAAAAAAAAACTAGATCGAACAAACGTTCTAAGAGTAGCTCATGCAAGAGAGTA
ACATCATTATCAATGATAATCATCCTTTCTTATTTACCAATCTTTATTAGATTCTTTGTTCATTGTGTTTTTTCCCTTCCXXXXXXXXXXA
TTAAGCATCTCGTCCTATCTTCACATAAAAAAAAACTAGATCGAACAAACGTTCTAAGAGTAGCTCATGCAAGAGAGTA
>Marker1020292 Chr2 SNP_site:21852660 Ref_Type:C
AACATGCTAGCAAAAAAGAATCGAACTAAAACACTCAAAATTTCATGTGAATTTTGAGGGCTAAACATGTTAAGAAAATTXXXXXXXXXXC
AAGGTTGTGGAATTTGATGGAGTTTTGAGAAAGGAAATAATATGATCCGAAGTGAATTTTCTCTGAAATTCAACAAAGT
AACATGCTAGCAAAAAAGAATTGAACTAAAACACTCAAAATTTCATGTGAATTTTGAGGGCTAAACATGTTAAGAAAATTXXXXXXXXXXC
AAGGTTGTGGAATTTGATGGAGTTTTGAGAAAGGAAATAATATGATCCGAAGTGAATTTTCTCTGAAATTCAACAAAGT
>Marker1020777 Chr2 SNP_site:11354917 Ref_Type:A
CACAATGCCACTCCACCATGTGCTAGACAAAAAACCGGTGCTTAAAAAGATAAACAAGATGCTAATCACGAGAAGACTCCXXXXXXXXXXA
ATGTTTAGAAAATGTTCGACTGAAAACAAGCAAGAATTCCATTATCTTGTCAAAGAAATATGAGAGATGATCGTCTGGT
CACACTGCCACTCCACCATGTGCTAGACAAAAAACCGGTGCTTAAAAAGATAAACAAGATGCTAATCACGAGAAGACTCCXXXXXXXXXXA

Create PDF with GO2PDF for free, if you wish to

ATGCTTAGAAAATGTTCGACTGAAAACAAGCAAGAATTCCATTATCTTGTCAAAGAAATATGAGAGATGATCGTCTGGT
>Marker1020777 Chr2 SNP_site:11355098 Ref_Type:T
CACAATGCCACTCCACCATGTGCTAGACAAAAAACCGGTGCTTAAAAAGATAAACAAGATGCTAATCACGAGAAGACTCCXXXXXXXXXXA
ATGTTTAGAAAATGTTCGACTGAAAACAAGCAAGAATTCCATTATCTTGTCAAAGAAATATGAGAGATGATCGTCTGGT
CACACTGCCACTCCACCATGTGCTAGACAAAAAACCGGTGCTTAAAAAGATAAACAAGATGCTAATCACGAGAAGACTCCXXXXXXXXXXA
ATGCTTAGAAAATGTTCGACTGAAAACAAGCAAGAATTCCATTATCTTGTCAAAGAAATATGAGAGATGATCGTCTGGT
>Marker1021272 Chr2 SNP_site:8036488 Ref_Type:G
AACAACTCCATAATGTAGTAGATTGGAAACATCTTAAAATGGTAGATGAAGAGGAAACTTTGCTAAATTTTCCATGGGGTXXXXXXXXXXA
AAATTAGACATTGAAGCCCAAGAATCATAAATTGGAAATTAGAACAGCAACCTGTATGGACATATCTGCAATTGTAGTG
AACAACTCCATAATGTAGTAGATTGGAAACATCTTAAAATGGTAGATGAAGAGGAAACTTTGCTAAATTTTCCATGGGGTXXXXXXXXXXA
AAATTGGACATTGAAGCCCAAGAATCATAAATTGGAAATTAGAACAGCAACCTGTATGGACATATCTGCAATTGTAGTG
>Marker1021357 Chr2 SNP_site:3257689 Ref_Type:T
AACATCTTACTTTTGGGGGGCACACTGCCATGTGCCTTTTCTAGTATTCCTTAGACTTCATTAGTAGGACATTATTAGGCXXXXXXXXXXC
GAAAAAGAATCACAATCTGGCATTAAGAGGGGATGGAAAGAATTTTGTGATGAATCTTATGGAAAATTAATTATGGGT
AACATCTTACTTTTGGGGGGCACACTGCCATGTGCCTTTTCTAGTATTCCTTAGACTTCATTAGTAGGACATTATTAGGCXXXXXXXXXXC
GAAAAAGAATCACAATCTGGCTTTAAGAGGGGATGGAAAGAATTTTGTGATGAATCTTATGGAAAATTAATTATGGGT
>Marker1021435 Chr2 SNP_site:4332853 Ref_Type:A
CACATTTAAGCTAGAGCATGACCTTAATATTGTTGGTAGAATGATTGAAGTCATATCAGGTTGAAAGCTAAACGATTGTGXXXXXXXXXXG
CTCTTTTCATCTTTTTCTCATCTTCTTCATGTTACTCCCAACTCCGACCCCCTCAACCAATGTCGATCTTCTTCTCGTG
CACATTTAAGCTAGAGCATGACCTTAATATTGTTGGTAGAATGATTGAAGTCATATCGGGTTGAAAGCTAAGCGATTGTGXXXXXXXXXXG
CTCTTTTCATCTTTTTCTCATCTTCTTCATGTTACTCCCAACTCCGACCCCCTCAACCAATGTCGATCTTCTTCTCGTG
>Marker1021435 Chr2 SNP_site:4332867 Ref_Type:A
CACATTTAAGCTAGAGCATGACCTTAATATTGTTGGTAGAATGATTGAAGTCATATCAGGTTGAAAGCTAAACGATTGTGXXXXXXXXXXG
CTCTTTTCATCTTTTTCTCATCTTCTTCATGTTACTCCCAACTCCGACCCCCTCAACCAATGTCGATCTTCTTCTCGTG
CACATTTAAGCTAGAGCATGACCTTAATATTGTTGGTAGAATGATTGAAGTCATATCGGGTTGAAAGCTAAGCGATTGTGXXXXXXXXXXG
CTCTTTTCATCTTTTTCTCATCTTCTTCATGTTACTCCCAACTCCGACCCCCTCAACCAATGTCGATCTTCTTCTCGTG
>Marker1021668 Chr2 SNP_site:2591538 Ref_Type:A
CACTTAATTATAATGATCATGTAAAAAGCTAGCCAAAAAGGTAATTTATTTTTAGAATATATAGGAGAAGTTTTGATTTGXXXXXXXXXXA
ACCATTCATTAGATGGAGAAATTTTTAACATTTAATCATTAATTTGATGCCTAAGCTAAATATATATATATCCCAAGTGTT
CACTTAATTATAATGATCATGTAAAAAGCTAGCCAAAAAGGTAATTTATTTTTAGAATATATAGGAGAAGTTTTGATTTGXXXXXXXXXXA
ACCATTCATTAGATGGAGAGATTTTTAACATTTAATCATTAATTTGATGCCTAAGCTAAATATATATATATCCCAAGTGTT
>Marker1021698 Chr2 SNP_site:5551739 Ref_Type:C
AACAATTGTCAAGCGTGGGGTGGATTAAATGGTCAGTAAGAGTTGATATTATTCATAATCATGACACGTAGCAAAAGGAA-XXXXXXXXX
TTGCGTATATGAAAATTGCTTCATTAAAACCTTACTAGTAAAAAACTCAATGGAAAAAATCATAGTGAAGGAAAAAGAGT
AACAATTGTCAAGCGTGGGGTGGATTAAATGGTCAGTAAGAGTTGATATTATTCATAATCATGACACGTAGC-AAAGGAATXXXXXXXXXX
TTACGTATATGAAAATTGCTTCGTTAAAACCTTACTAGTAAAAAACTCAATGGAAAAAATCATAGTGAAGGAAAAAGAGT
>Marker1021698 Chr2 SNP_site:5551759 Ref_Type:T
AACAATTGTCAAGCGTGGGGTGGATTAAATGGTCAGTAAGAGTTGATATTATTCATAATCATGACACGTAGCAAAAGGAA-XXXXXXXXX
TTGCGTATATGAAAATTGCTTCATTAAAACCTTACTAGTAAAAAACTCAATGGAAAAAATCATAGTGAAGGAAAAAGAGT
AACAATTGTCAAGCGTGGGGTGGATTAAATGGTCAGTAAGAGTTGATATTATTCATAATCATGACACGTAGC-AAAGGAATXXXXXXXXXX
TTACGTATATGAAAATTGCTTCGTTAAAACCTTACTAGTAAAAAACTCAATGGAAAAAATCATAGTGAAGGAAAAAGAGT
>Marker1021718 Chr2 SNP_site:21891368 Ref_Type:T
GACTAGTATGTGTATTAGAGTTTTTCCGTTTTGTCATAAATGCTTAGTGGCCTTGATTTTCAGTATGATGTTTCTTGACAXXXXXXXXXXT
TGAACCATCTTAAGGTGTTAATATGAATGCTGCTGCTAGAACTCCTAGACACTTGTGTAAGTCTGTTCTTTCTAAAGTT

Create PDF with GO2PDF for free, if you wish to

GACTAGTATGTGTATTAGAGTTTTTCCGTTTTGTCATAAATGCTTAGTGGCCTTGATTTTCAGTATGATGTTTCTTGACAXXXXXXXXXXXT
TGAACCATCTTAAGGTGTTAATATGAATGCTGCTTCTAGAACTCCTAGACACTTGTGTCAGTCTGTTCTTTCTAAAGTT
>Marker1021718 Chr2 SNP_site:21891392 Ref_Type:C
GACTAGTATGTGTATTAGAGTTTTTCCGTTTTGTCATAAATGCTTAGTGGCCTTGATTTTCAGTATGATGTTTCTTGACAXXXXXXXXXXXT
TGAACCATCTTAAGGTGTTAATATGAATGCTGCTGCTAGAACTCCTAGACACTTGTGTAAGTCTGTTCTTTCTAAAGTT
GACTAGTATGTGTATTAGAGTTTTTCCGTTTTGTCATAAATGCTTAGTGGCCTTGATTTTCAGTATGATGTTTCTTGACAXXXXXXXXXXXT
TGAACCATCTTAAGGTGTTAATATGAATGCTGCTTCTAGAACTCCTAGACACTTGTGTCAGTCTGTTCTTTCTAAAGTT
>Marker1022979 Chr2 SNP_site:2320993 Ref_Type:T
GACCATGACTAACACATCATTGAGATGTTTTATCACAGACTAAGATGAAACGATCATAGTTAGTGATAATTCGAGGTAGAXXXXXXXXXXXG
CATGTTTTGAATTTTTTTTATTAAATATAAATACTTTCTTTTGGTCAAGTCAAATATCATAAACAAAACTATGTATTAGT
GACCATGACTAACACATCATTGAGATGTTTTATCATAGACTAAGATGAAACGATCATAGTTAGTGATAATTCGAGGTAGAXXXXXXXXXXXG
CATGTTTTGAATTTTTTTTATTAGATATAAATACTTTCTTTTGGTCAAGTCAAATATCATAAACAAAACTATGTATTAGT
>Marker1022979 Chr2 SNP_site:2321193 Ref_Type:G
GACCATGACTAACACATCATTGAGATGTTTTATCACAGACTAAGATGAAACGATCATAGTTAGTGATAATTCGAGGTAGAXXXXXXXXXXXG
CATGTTTTGAATTTTTTTTATTAAATATAAATACTTTCTTTTGGTCAAGTCAAATATCATAAACAAAACTATGTATTAGT
GACCATGACTAACACATCATTGAGATGTTTTATCATAGACTAAGATGAAACGATCATAGTTAGTGATAATTCGAGGTAGAXXXXXXXXXXXG
CATGTTTTGAATTTTTTTTATTAGATATAAATACTTTCTTTTGGTCAAGTCAAATATCATAAACAAAACTATGTATTAGT
>Marker1023327 Chr2 SNP_site:2949387 Ref_Type:A
ACATCTAGCGAGCCTCATTGCACCAGCCATTTTAGACGTGAAAGTAGTGCGAAATGAGATCACTTCCGACGACTATCTCCXXXXXXXXXXXT
CAGCCCATTACTCGTTGATTCCCACCATCCTCCATACATATCACAATTCGGTCCTCAGAGGGCATTTTGGGTATTTGTG
ACATCTAGCGAGCCTCATTGCACCAGCCATTTTAGACGTGGAAGTAGTGTGAAATGAGATCACTTCCGACGACTATCTCCXXXXXXXXXXXT
CAGCCCATTACTCGTTGATTCCCACCATCCTCCATACATATCACAATTCGATCCTCAGAGGGCATTTTGGGTATTTGTG
>Marker1023327 Chr2 SNP_site:2949396 Ref_Type:C
ACATCTAGCGAGCCTCATTGCACCAGCCATTTTAGACGTGAAAGTAGTGCGAAATGAGATCACTTCCGACGACTATCTCCXXXXXXXXXXXT
CAGCCCATTACTCGTTGATTCCCACCATCCTCCATACATATCACAATTCGGTCCTCAGAGGGCATTTTGGGTATTTGTG
ACATCTAGCGAGCCTCATTGCACCAGCCATTTTAGACGTGGAAGTAGTGTGAAATGAGATCACTTCCGACGACTATCTCCXXXXXXXXXXXT
CAGCCCATTACTCGTTGATTCCCACCATCCTCCATACATATCACAATTCGATCCTCAGAGGGCATTTTGGGTATTTGTG
>Marker1023327 Chr2 SNP_site:2949573 Ref_Type:G
ACATCTAGCGAGCCTCATTGCACCAGCCATTTTAGACGTGAAAGTAGTGCGAAATGAGATCACTTCCGACGACTATCTCCXXXXXXXXXXXT
CAGCCCATTACTCGTTGATTCCCACCATCCTCCATACATATCACAATTCGGTCCTCAGAGGGCATTTTGGGTATTTGTG
ACATCTAGCGAGCCTCATTGCACCAGCCATTTTAGACGTGGAAGTAGTGTGAAATGAGATCACTTCCGACGACTATCTCCXXXXXXXXXXXT
CAGCCCATTACTCGTTGATTCCCACCATCCTCCATACATATCACAATTCGATCCTCAGAGGGCATTTTGGGTATTTGTG
>Marker1023421 Chr2 SNP_site:4939884 Ref_Type:C
AACCTCTAACCATCATTTTTTAGCATTGCCATATCAACCAAACTTTGTATCTAAACTTTTCACCAATTTTCTCACCAACTXXXXXXXXXXXC
ATGTATTCAATTATTAACGCACCATACATTTTTTAAAATATTCTCTCAACTTTAAAAAGTTAAAAGAAATTAAAATAGT
AACCTCTAACCATCATTTTTTAGCATTGCCATATCAACCAAACTTTGTATCTAAACTTTTCACCAATTTTCTCACCAACTXXXXXXXXXXXC
ATGTATTTAATTATTAACGCACCATACATTTTTTAAAATATTCTCTCAACTTTAAAAAGTTAAAAGAAATTAAAATAGT
>Marker1023594 Chr2 SNP_site:3682163 Ref_Type:A
ACCTTCCTTTTGGTAGAAGATCCTTCAATGAAGCTTCAGGAAAACAATAACATTTCTTTCTAATGTTATACAATACACGAXXXXXXXXXXXT
AACCAGATCATCAACAACTTGATTAGATTCATAAATCCATCAAATACCAAAAATTGCATCAGAACATATCAATGGGGTT
ACCTTCCTTTTGGTAGAAGATCCTTCAATGAAGCTTCAGGAAAACAATAACATTTCTTTCTAATGTTATACAATACACGAXXXXXXXXXXXT
AACCAGATCATCAACAACTTGATTAGATTCATAAATCCATCAAATACCAAAATTTGCATCAGAACATATCAATGGGGTT
>Marker1023740 Chr2 SNP_site:2506890 Ref_Type:C
GACCTCCATAACCACAACAAGCCATAATAGGTTGTTGAAACCCTGCCATTCACACACAAAATATAGCAACTCATCAGATTXXXXXXXXXXXG

Create PDF with GO2PDF for free, if you wish to

CTATCAGTATATTGTCCCTGCAGTTTTTTACAGAGAGCATGAAGTTGTAGATTAAAGAGCTTGGCTGCTTGGTTGTGTG
GACCTCCATAACCACAACAAGCCATAATCGGTTGTTGAAACCCTGCCATTCACACACAAAATATAGCAACTCATCAGATTXXXXXXXXXXG
CTATCAGTATATTGTCCCTGCAGTTTTTTACAGAGAGCATGAAGTTGTAGATTAAAGAGCTTGGCTGCTTGGTTGTGTG
>Marker1023924 Chr2 SNP_site:5019267 Ref_Type:C
ACTTTGGTTTGGGGTCAGAAATATTCTCAATTATGTGACATCAAAATACAGATGTCACACTGCTTATTTCTCTCTATAATXXXXXXXXXXT
TGATCAATAATGCAAAATTAATTATTTAATAACTTTGTCAAATGTCAAAATATGTAACAAAATTCTAAACATATTTGTT
ACTTTGGTTTGGGGTCAGAAATATTCTCAATTATGTGACATCAAAATACAGATGTCACACTGCTTATTTCTCTCTATAATXXXXXXXXXXT
TGATCAATAATGCAAAATTAATTATTTAATAACTTTGTCAAATGTCAAAATATGTAACAAAATTCTAACCATATTTGTT
>Marker1024444 Chr2 SNP_site:13792019 Ref_Type:A
ACCTCATTTGAAAAAGGATAAAATCGCTAAATGAGTCACCCTATCGACACACGTTGAATAGACCGTTCATAACATGCATGXXXXXXXXXXT
ATGTATCCTCTTGATCTTCTCTTAACCCATTAGTGTCAATATTTAAAAGAAAAGTAAAATAAACCATATTGTTTATGTC
ACCTCATTTGGAAAAGGATAAAATCGCTAAATGAGTCACCCTATCGACACACGTTGAATAGACCGTTCATAACATGCATGXXXXXXXXXXT
ATGTATCCTCTTGATCTTCTCTTAACCCATTAGTGTCAATATTTAAAAGAAAAGTAAAATAAACCATATTGTTTATGTC
>Marker1025046 Chr2 SNP_site:20642995 Ref_Type:G
AACTATCTTCTTCCACGTATCTGATAATTATTTGTGTTGATATAAAAGATTATCAAAGAACAACTAAAATACATTTAAAAXXXXXXXXXXA
CAGGTTAGGCGTTAAGTTTTAAATTACATAGGATTTTTTTTTAATTATTAAATTACAAAGAAAAAATAAGTTAATGGTT
AACTGTCTTCTTCCACGTATCTGATAATTATTTGTGTTGATATAAAAGATTATCAAAGAACAACTAAAATATATTTAAAAXXXXXXXXXXA
CAGGTTAGGCGTTAAGTTTTAAATTACATAGGTTTTTTTTTTAATTATTAAATTACAAAGAAAAAATAAGTTAATGGTT
>Marker1025046 Chr2 SNP_site:20643062 Ref_Type:T
AACTATCTTCTTCCACGTATCTGATAATTATTTGTGTTGATATAAAAGATTATCAAAGAACAACTAAAATACATTTAAAAXXXXXXXXXXA
CAGGTTAGGCGTTAAGTTTTAAATTACATAGGATTTTTTTTTAATTATTAAATTACAAAGAAAAAATAAGTTAATGGTT
AACTGTCTTCTTCCACGTATCTGATAATTATTTGTGTTGATATAAAAGATTATCAAAGAACAACTAAAATATATTTAAAAXXXXXXXXXXA
CAGGTTAGGCGTTAAGTTTTAAATTACATAGGTTTTTTTTTTAATTATTAAATTACAAAGAAAAAATAAGTTAATGGTT
>Marker1025046 Chr2 SNP_site:20643195 Ref_Type:A
AACTATCTTCTTCCACGTATCTGATAATTATTTGTGTTGATATAAAAGATTATCAAAGAACAACTAAAATACATTTAAAAXXXXXXXXXXA
CAGGTTAGGCGTTAAGTTTTAAATTACATAGGATTTTTTTTTAATTATTAAATTACAAAGAAAAAATAAGTTAATGGTT
AACTGTCTTCTTCCACGTATCTGATAATTATTTGTGTTGATATAAAAGATTATCAAAGAACAACTAAAATATATTTAAAAXXXXXXXXXXA
CAGGTTAGGCGTTAAGTTTTAAATTACATAGGTTTTTTTTTTAATTATTAAATTACAAAGAAAAAATAAGTTAATGGTT
>Marker1025196 Chr2 SNP_site:10941460 Ref_Type:T
GACCAACAGTGCAATTTGTTGGCTTTTCGATATAAGGAATTAAAGAAGTTTGAAGTTCCAAAGGTGAAGCAAAGTATATTXXXXXXXXXXA
CCAACATTCCCTTACGTAGTGTTAGAATTATAATAACCTACCAACACTAACGGCACAGTAACCTACCAACATCATCGTT
GACCAACAGTGCAATTTGTTGGCTTTTCGATATAAGGAATTAAAGAAGTTTGAATTTCCAAAGGTGAAGCAAAGTATATTXXXXXXXXXXA
CCAACATTCCCTTACGTAGTGTTAGAGTTATAATAACCTACCAACATTAACGGCACAGTAACCTACCAACATCATCGTT
>Marker1025196 Chr2 SNP_site:10941627 Ref_Type:G
GACCAACAGTGCAATTTGTTGGCTTTTCGATATAAGGAATTAAAGAAGTTTGAAGTTCCAAAGGTGAAGCAAAGTATATTXXXXXXXXXXA
CCAACATTCCCTTACGTAGTGTTAGAATTATAATAACCTACCAACACTAACGGCACAGTAACCTACCAACATCATCGTT
GACCAACAGTGCAATTTGTTGGCTTTTCGATATAAGGAATTAAAGAAGTTTGAATTTCCAAAGGTGAAGCAAAGTATATTXXXXXXXXXXA
CCAACATTCCCTTACGTAGTGTTAGAGTTATAATAACCTACCAACATTAACGGCACAGTAACCTACCAACATCATCGTT
>Marker1025196 Chr2 SNP_site:10941647 Ref_Type:T
GACCAACAGTGCAATTTGTTGGCTTTTCGATATAAGGAATTAAAGAAGTTTGAAGTTCCAAAGGTGAAGCAAAGTATATTXXXXXXXXXXA
CCAACATTCCCTTACGTAGTGTTAGAATTATAATAACCTACCAACACTAACGGCACAGTAACCTACCAACATCATCGTT
GACCAACAGTGCAATTTGTTGGCTTTTCGATATAAGGAATTAAAGAAGTTTGAATTTCCAAAGGTGAAGCAAAGTATATTXXXXXXXXXXA
CCAACATTCCCTTACGTAGTGTTAGAGTTATAATAACCTACCAACATTAACGGCACAGTAACCTACCAACATCATCGTT
>Marker1025782 Chr2 SNP_site:445272 Ref_Type:C

Create PDF with GO2PDF for free, if you wish to

AACCATAAAGGTGAGGTCAAGATCACTGATTTTGGAGTAAGTGCAATGCTAGCAAGTTCTATGGGTCAGAGAGATACATTXXXXXXXXXXA
CTTCCCTCTTTCAGCCTGAACGAATTAGCGGAGGAACATATGACTACAGCAGTGATATATGGAGTTTGGGTTTGGTAGT
AACCATAAAGGTGAGGTCAAGATCACTGATTTTGGAGTAAGTGCAATGCTAGCAAGTTCTATGGGTCAGAGAGATACATTXXXXXXXXXXA
CTTTCCTCTTTCAGCCTGAACGAATTAGCGGAGGAACATATGACTACAGCAGTGATATATGGAGTTTGGGTTTGGTAGT
>Marker1026032 Chr2 SNP_site:3948152 Ref_Type:A
CACTACTCCTCCATCTTATACAAGCTTTCAAATAAAAAACTCAAATTTACATAATGATAGAAATTGTAACAATGGTATATXXXXXXXXXXT
CTCTTTGTGTTGAATTAAAAATGGATTTATAAGAGGGTGAATAATAATGGGTCAATTATTGAAGGTTTGTAGGGTTGTA
TACTACTCCTCCATCTTATACAAGCTTTCAAATAAAAAACTCAAATTTACATAATGATAGAAATTGTAACAATGGTATATXXXXXXXXXXT
CTCTTTGTGTTGAATTAAAAATGGATTTATAAGAGGGTGAATAATAATGGGTCAATTATTGAAGGTTTGTAGGGTTGTA
>Marker102614 Chr4 SNP_site:7807737 Ref_Type:A
ACTCCTGGTTTGATCGGGGAACTCTGTCTTTTTCTTTTGGGTTCTGTTGTCATTCATCCAAATGGGAAACAAAAGAGGTGXXXXXXXXXXT
GACTTTTGATGTGGTTTGGAGGATTAAGATATCTAAGAAAATTAGGTTCTTCACTTGGCAAGTTTTTCTCGTTCAGGTG
ACTCCTGGTTTGTTCGGGGAACTCTGTCTTTTTCTTTTGGGTTCTGTTGTCATTCATCCAAATGGGAAACAACAGTGGTGXXXXXXXXXXT
GACTTTTGATGTGGTTTGGAGGATTAAGATATCTAAGAAAATTAGGTTCTTCACTTGGCAAGTTTTTCTCGTTCAGGTG
>Marker102614 Chr4 SNP_site:7807797 Ref_Type:A
ACTCCTGGTTTGATCGGGGAACTCTGTCTTTTTCTTTTGGGTTCTGTTGTCATTCATCCAAATGGGAAACAAAAGAGGTGXXXXXXXXXXT
GACTTTTGATGTGGTTTGGAGGATTAAGATATCTAAGAAAATTAGGTTCTTCACTTGGCAAGTTTTTCTCGTTCAGGTG
ACTCCTGGTTTGTTCGGGGAACTCTGTCTTTTTCTTTTGGGTTCTGTTGTCATTCATCCAAATGGGAAACAACAGTGGTGXXXXXXXXXXT
GACTTTTGATGTGGTTTGGAGGATTAAGATATCTAAGAAAATTAGGTTCTTCACTTGGCAAGTTTTTCTCGTTCAGGTG
>Marker102614 Chr4 SNP_site:7807800 Ref_Type:A
ACTCCTGGTTTGATCGGGGAACTCTGTCTTTTTCTTTTGGGTTCTGTTGTCATTCATCCAAATGGGAAACAAAAGAGGTGXXXXXXXXXXT
GACTTTTGATGTGGTTTGGAGGATTAAGATATCTAAGAAAATTAGGTTCTTCACTTGGCAAGTTTTTCTCGTTCAGGTG
ACTCCTGGTTTGTTCGGGGAACTCTGTCTTTTTCTTTTGGGTTCTGTTGTCATTCATCCAAATGGGAAACAACAGTGGTGXXXXXXXXXXT
GACTTTTGATGTGGTTTGGAGGATTAAGATATCTAAGAAAATTAGGTTCTTCACTTGGCAAGTTTTTCTCGTTCAGGTG
>Marker1026563 Chr2 SNP_site:13911468 Ref_Type:T
ACTCTTAGCTTATATGTAATTTCACAGTCATCAACTAAATCTAAACCATCACTCATAAACCTCAACAACGTTGAACGAAGXXXXXXXXXXA
TTGTATCCTCTACCATATCGTCGATCGAAAACGGACATTAAAATCAGTTTTTGTTTTTTTTCATCTTGTCTAACATTGT
ACTCTTAGCTTATATGTAATTTCACAGTCATCAACTAAATCTAAACCATCACTCATAAACCTCAACAACGTTGAACGAAGXXXXXXXXXXA
TTGTATCCTCTACCATATCGTCGATCGAAAACGGACATTAAAATTAGTTTTTGTTTTTTTTCATCTTGTCTAACATTGT
>Marker1026691 Chr2 SNP_site:12578892 Ref_Type:C
ACTGTGTTTGAATGATTTACTGATGAAAGGTGTGGTGCTCAGATATATTCATGTAACTTTGAATGAGAACGTAAATGATAXXXXXXXXXXC
TTTCCACTAACAATTTACATGGAGAAACTACTCTACATTGATGCTCTACTCTCAAGTCTCAACTATGGCAGAAAAATGT
ACTGTGTTTGAATGATTTACTGATGAAAGGTGTGGTGCTCAGATATATTCATGTAACTTTGAATGAGAACGTAAATGATAXXXXXXXXXXC
TTTCCACTAACAATTTACATGGAGAAACTACTCTACATTGATGTTCTACTCTCAAGTCTCAACTATGGCAGAAAAATGT
>Marker1027585 Chr2 SNP_site:19335960 Ref_Type:C
CACATATCTCCCCATGATCTGATAATATAGCATTATATATATACATAGATGAAACTTAAGAATATGAAAGGAGGGGTGACXXXXXXXXXXT
TATTAAATGTCTTCTAACTCTCAATAAGTTATTGTTTGGAGAAAGATATTATCTTGGATTGATATTAGCAGGTGAAGTA
CACATATCTCCCCATGATCTGATAATATAGCATTATATATATACATAGATGAAATTTAAGAATATGAAAGGAGGGGTGACXXXXXXXXXXT
TATTAAATGTCTTCTAACTCTCAATAAGTTATTGTTTGGAGAAAGATATTCTCTTGGATTGATATTAGCAGGTGAAGTA
>Marker1027585 Chr2 SNP_site:19336138 Ref_Type:A
CACATATCTCCCCATGATCTGATAATATAGCATTATATATATACATAGATGAAACTTAAGAATATGAAAGGAGGGGTGACXXXXXXXXXXT
TATTAAATGTCTTCTAACTCTCAATAAGTTATTGTTTGGAGAAAGATATTATCTTGGATTGATATTAGCAGGTGAAGTA
CACATATCTCCCCATGATCTGATAATATAGCATTATATATATACATAGATGAAATTTAAGAATATGAAAGGAGGGGTGACXXXXXXXXXXT
TATTAAATGTCTTCTAACTCTCAATAAGTTATTGTTTGGAGAAAGATATTCTCTTGGATTGATATTAGCAGGTGAAGTA

Create PDF with GO2PDF for free, if you wish to

>Marker1028124 Chr2 SNP_site:17345323 Ref_Type:T
ACATTCATTGAATAGTGAGGGATTACTATAAGTTATAAATGTATTAGATTTCATGAGTTGTGTGGCATTATATACTGGCTXXXXXXXXXXXT
CAGATTATACTGATATCATTTAGTTGAGTTGATATAGACTTCATGGACCCTTAAAGCCTATCTTTTCTATTTTTCAGGT
ACATTCATTGAATTGTGAGGGATTACTATAAGTTATAAATGTATTAGATTTCATGAGTTGTGTGGCATTATATACTGGCTXXXXXXXXXXXT
CAGATTATACTGATATCATTTAGTTGAGTTGATATAGACTTCATGGACCCTTAAAGCCTATCTTTTCTATTTTTCAGGT
>Marker1028391 Chr2 SNP_site:15926498 Ref_Type:T
TACCAAGAGCCTGTTTTGTATTCTGGTTTATCAAATTAGTTCTCTGGTGCCATATGGAAAAACTGTCAAGGTTTTAGTGCXXXXXXXXXXXC
CATCCCTATGCCTCTTGTATGAAATCATTACCTTTGGTTTGTCGTGGTATTTGTTAAAGTCCAGATGTAATTTTACAGT
TACCAAGAGCCTGTTTTGTATTCTGGTTTATCAAATTAGTTCTCTGGTGCCATATGGAAAAACTGTCAAGGTTTTAGTGCXXXXXXXXXXXC
CATCCCTATGCCTCTTGTATGAAATCATTACCTTTGGTTTGTCGTGGTATTTGTTAAAGTCTAGATGTAATTTTACAGT
>Marker1029536 Chr2 SNP_site:15604595 Ref_Type:T
TACTTCTTCAAACGAGTAATATATATATATAGGTAGATGCAAATTCAAAATCTCCATAACCCTAATATTCAATCAAAGATAGXXXXXXXXXXXG
CAAAAAAATACAATTATCTGTTCTAATTTGGCCACAAGTTCTTTCTAATCTCAAAAACCAATCTTTCTCAAATGTTGTA
TACTTCTTCAAACGAGTTATATATATATATAGGTAGATGCAAATTCAAAATCTCCATAACCCTAATATTCAATCAAAGATAGXXXXXXXXXXXG
CAAAAAAATACAATTATCTGTTCTAATTTGGCCACAAGTTCTTTCTAATCTCAAAAACCAATCTTTCTCAAATGTTGTA
>Marker1030162 Chr2 SNP_site:8043862 Ref_Type:T
AACTTGATCTAACGGTTATGATGAAAACACGTGGTATATCTGAATTCACCAATTTATCTATTTAATTTTCGTTTAGATTTXXXXXXXXXXXA
CAGATTTACACAAATTAGGTCACTATATAATATAAACCTTTGCTACCTCAACACAAATATCAATATTAAAATTATCGTT
AACTTGATCTAACGGTTATGATGAAAATACGTGGTATATCTGAATTCACCAATTTATCTATTTAATTTTCGTTTAGATTTXXXXXXXXXXXA
CAGATTTACACAAATTAGGTCACTATATAATATAAACCTTTGCTACCTCAACACAAATATCAATATTAAAATTATCGTT
>Marker103074 Chr4 SNP_site:13127331 Ref_Type:T
ACTCTTTCTTATCTCTTTAGTTTTATGAGTAGTGTATGAAATAATAGAAGATGTTAAAATACTACTTTGGTTCGTATATTXXXXXXXXXXXA
CTACTTTACACCGCAAATACAACTTACAAATTGATAGGGTTTAATTTAGTGAGAGAAAGAAAGTGAAAATGGATTGGGT
ACTCTTTTTTATCTCTTTAGTTTTATGAGTAGTGTATGAAATAATAGAAGATGTTAAAATACTACTTTGGTTCGTATATTXXXXXXXXXXXA
CTACTTTACACCGCAAATACAACTTACAAATTGATAGGGTTTAATTTAGTGAGAGAAAGAAAGTGAAAATGGATTGGGT
>Marker1031035 Chr2 SNP_site:3395607 Ref_Type:A
AACCCATTTAGTTTAATAATCATATATCATAGTTTCTTCTTCCTTCATACATTCATATGTTTGGTTTAAATTTATATGACXXXXXXXXXXXT
GACAGCTCAGTCATCATTCACACTTGTCCTCTTCTTATTTACTCTCTATTTTTTATTTTCTATTTTGTCTTTTATTGTA
AACCCATTTAGTTTAATATTCATATATCATAGTTTCTTCTTCCTTCATACATTCATATGTTTGGTTTAAATTTATATGACXXXXXXXXXXXT
GACAGCTCAGTCATCATTCACACTTGTCCTCTTCTTATTTACTCTCTATTTTTTATTTTCTATTTTGTCTTTTATTGTA
>Marker1031143 Chr2 SNP_site:12517773 Ref_Type:C
AACAAAGATTCACAACGATTGGTAAAGACTTAATTAATTACGTAAGGATTTAATTATGCAAAAAACAGTAATGAAACCTAXXXXXXXXXXXA
TAATTGACTTGATTGTTGGAGTGTCGGTGGCCAAGCACCACACCGCGACAAATATTTTCTATTTCTTAAGGTGTAAAGT
AACAAAGATTCACAACGATTGGTAAAGACTTAATTAATTACGTAAGGATTTAATTATGCAAAAAACAGTAATGAAACCTAXXXXXXXXXXXA
TAATTGACTTGATTGTTGGAGTGTTGGTGGCCAAGCACCACACCGCAACAAATATTTTCTATTTCTTAAGGTGTAAAGT
>Marker1031143 Chr2 SNP_site:12517795 Ref_Type:G
AACAAAGATTCACAACGATTGGTAAAGACTTAATTAATTACGTAAGGATTTAATTATGCAAAAAACAGTAATGAAACCTAXXXXXXXXXXXA
TAATTGACTTGATTGTTGGAGTGTCGGTGGCCAAGCACCACACCGCGACAAATATTTTCTATTTCTTAAGGTGTAAAGT
AACAAAGATTCACAACGATTGGTAAAGACTTAATTAATTACGTAAGGATTTAATTATGCAAAAAACAGTAATGAAACCTAXXXXXXXXXXXA
TAATTGACTTGATTGTTGGAGTGTTGGTGGCCAAGCACCACACCGCAACAAATATTTTCTATTTCTTAAGGTGTAAAGT
>Marker1031170 Chr2 SNP_site:13930389 Ref_Type:A
ACTAAGTTAGATAACCACTGAGAAGTTCTCGTGTTATACCCCTTTTAAAAAAGAAGTGAAAGTTCTGACAATGTTAGACTXXXXXXXXXXXT
TCTGATTTTAATATTTTTTAAAAGTTCAACCATTGCGATCTAGAGAATCGGAGCATCGATACTTAGCATGATAATTAGT
ACTAAGTTAGATAACCACTGAGAAGTTCTCGTGTTATACCCCTTTTAAAAAAGAAGTGAAAGTTCTGACAATGTTAGACTXXXXXXXXXXXT

Create PDF with GO2PDF for free, if you wish to

TCTGATTTTAATATTTTTTAAAAGTTCAACCATTGCGATCTAGAGAATCGGAGCATCGATACTTAGCATGATAATTGGT
>Marker103169 Chr4 SNP_site:4057244 Ref_Type:A
ACCTAACAAAAAGCTTTTGATCATTTAGGACAAAAACATTTCAATGGCAGTTGATAATGGACTTAACATTAACAACTGAAXXXXXXXXXXT
GTTCTTGTGAAGACTTTAGATTGTTTCTGGAGGGATCATCTCATAAACATGAATAGACTCAGTTCTGCGGTATATAAGT
ACCTAACAAAAAGCTTTTGTTCATTTAGGACAAAAACATTTCAATGGCAGTTGATAATGGACTTAACATTAACAACTGAAXXXXXXXXXXT
GTTCTTGTGAAGACTTTAGATTGTTTCTGGAGGGATCATCTCATAAACATGAATAGACTCAGTTCTGCGGTATATAAGT
>Marker1032207 Chr2 SNP_site:20291267 Ref_Type:C
GACATCCCCTTAGTATCCTTATCATTTCTAATAAAATAAAGGACCAAACAACAAATAAATAAAGTCCAATTGGGGGCTAAXXXXXXXXXXC
TAAATCATCAATCAAGGAGGAAAGATGAAGGCCTGCAAATTTAGTAAGTTGTCCTGCATTGAAAAGTCTTTAAACTGTT
GACATCCCCTTAGTATCCTTATCATTTCTAATAAAATAAAGGACCAAACAACAAATAAATAAAGTCCAATTGGGGGCTAAXXXXXXXXXXC
TAAATCATCAATCAAGGAGGAAAGATGAAGGCCTGCAAATTTAGTAAGTTGTCTTGCATTGAAAAGTCTTTAAACTGTT
>Marker1032341 Chr2 SNP_site:20279224 Ref_Type:C
GACTTCATTTATTGACTCTATAAAATAATCAAAACTTTGATCATAAGAAAATAAATTATCATGATTTTTTGACGAAGTATXXXXXXXXXXT
GCTTCTTGTTCCGCAAATATAGAAAACACTAATTAAAGCTCCCACTCAAAATTGGCATTATTTGAGAAATGTAAAAGTG
GACTTCATTTATTGACTCTATAAAATAATCAAAACTTTGATCATAAGAAAATAAATTATCATGATTTTTTGACGAAGTATXXXXXXXXXXT
GTTTCTTGTTCCGCAAATATAGAAAACACTAATTAAAGCTCCCACTCAAAATTGGCATTATTTGAGAAATGTAAAAGTG
>Marker1032720 Chr2 SNP_site:13624885 Ref_Type:C
ACTTCTTTCTTCATTTTGTTTCTTTTCATAACAAATCACATGCTTCCTATAAACACTTATACGAGAGAATATATTAATCCXXXXXXXXXXC
TCTTTATCCTTTTTGAACTACATTTTATCATATATGGTAACACATTTATAGAATCTCTATTATTTATGTTGGAGTAAGT
ACTTCTTTCTTCATTTTGTTTCTTTTCATAACAAATCACATGCTTTCTATAAAGACTTATACGAGAGAATATATTAATCCXXXXXXXXXXC
TCTTTATCCTTTTTGAACTACATTTTATCATATATGGTAACACATTTATAGAATCTCTATTATTTATGTTGGAGTAAGT
>Marker1032720 Chr2 SNP_site:13624893 Ref_Type:C
ACTTCTTTCTTCATTTTGTTTCTTTTCATAACAAATCACATGCTTCCTATAAACACTTATACGAGAGAATATATTAATCCXXXXXXXXXXC
TCTTTATCCTTTTTGAACTACATTTTATCATATATGGTAACACATTTATAGAATCTCTATTATTTATGTTGGAGTAAGT
ACTTCTTTCTTCATTTTGTTTCTTTTCATAACAAATCACATGCTTTCTATAAAGACTTATACGAGAGAATATATTAATCCXXXXXXXXXXC
TCTTTATCCTTTTTGAACTACATTTTATCATATATGGTAACACATTTATAGAATCTCTATTATTTATGTTGGAGTAAGT
>Marker1032722 Chr2 SNP_site:15509204 Ref_Type:G
ACATAACCGGCGATGAAACACTAACACATTAATTTGAAGTTAATTAATGTAATAATAATAGGCTCAAGATTGGCTTTATTXXXXXXXXXXC
TCAAAATTATTTGATAAAACTATATAGCCAGGGCTTGAAATGACCTATGGAGATAGATTTACAATAAAAAAGCAAGAGT
ACGTAACCGGCGATGAAACACTAACACATTAATTTGAAGTTAATTAATGTAATAATAATAGGCTCAAGATTGGCTTTATTXXXXXXXXXXC
TCAAAATTATTTGATAAAACTATATAGCCAGGGCTTGAAATGACCTATGGAGATAGATTTACAATAAAAAAGCAAGAGT
>Marker1032851 Chr2 SNP_site:22663288 Ref_Type:A
ACATGATATAGAGAAAATTTAAATAATTTAGTTAATTATTTAATTTTTCCCGTTATAGCCAATGTAGATGTCGGGAAAAGXXXXXXXXXXA
ATCAAATTTAAAAAATCAAATTGGTGGATTAGTCCCGACGTTAAAATCAAGAAGTCGAGATTTCCCCTATACTGACGGT
ACATGATATAGAGAAAATTTAAATAATTTAGTTAATTATTTAATTTTTCCCGTTATAGCCAATGTAGATGTCGGGAAAAGXXXXXXXXXXA
ATCAAATTTTAAAAATCAAATTGGTGGATTAGTCCCGACGTTAAAATCAAGAAGTCGAGATTTCCCCTATACTGACGGT
>Marker1033292 Chr2 SNP_site:21190864 Ref_Type:G
TACCCTTCATTTTCATTTCTTTCTTCTCTTCTTTTTCTGCAATTTTTCTTACCTTTCCATCGTCTTTATTCTCTTCTTCTXXXXXXXXXXA
AGCATCAATAATGAAATAATAATTAAAACGTTATAATCACATATTACTATAAATCACGAGTATTTGCCTACAAACTTGT
TACCCTTCATTTTCATTTCTTTCTTCTCTTCTTTTTCTGCAATTTTTCTTACCTTTCCATCGTCTTTATTCTCTTCTTCTXXXXXXXXXXA
AGCATCAATAATGAAATAATAATTAAAACGTTATAATCACATATTACTATAAATCACGAGTATTTTCCTACAAACTTGT
>Marker1033329 Chr2 SNP_site:4592578 Ref_Type:A
AACATTTTGCGGGTTTGATTATGGTTGCTTCTAAAACCCCAAGTATTGTTGGATTATAGTTCTCTTTTATTGAGGTTTTCXXXXXXXXXXT
TGGTTCGTATTAATAGAGTAATTGAAGACTATTAAAAGCGGTAAAGAAAATAGGCATCGAGTTACGAGGAAATCCGAGT

Create PDF with GO2PDF for free, if you wish to

AACATTTTGCGGGTTTGATTATGGTTGCTTCTAAAACCCCAAGTATTGTTGGATTATAGTTCTCTTTTATTGAGGTTTTCXXXXXXXXXXT
TGGTTCGTATTAATAGAGTAATTGAAGACTATTAAAAGCGGTAAAGAAAATAGGCATCGAGTTACGTGGAAATCCGAGT
>Marker103351 Chr4 SNP_site:11418890 Ref_Type:A
ACAAGAATATATTTACAAATTTTTTTGAATGAAAAGCTAAAGGAACACTTTCATGTTTAATTTATCATTAAACATATGCTXXXXXXXXXXA
CCATTAAGTTAGTAGTATGAACACAAACACTACAGCACAGAAATTTCTCTACAATTTTTTCTCAAACTTTCTCTTAGTT
ACAAGAATATATTTACAATTTTTTTTGAATGAAAAGCTAAAGGAACACTTTCATGTTTAATTTATCATTAAACATATGCTXXXXXXXXXXA
CCATTAAGTTAGTAGTATGAACACAAACACTACAGCACAGAAATTTCTCTACAATTTTTTCTCAAACTTTCTCTTAGTT
>Marker1033829 Chr2 SNP_site:11571039 Ref_Type:G
CACTGCATTAAAGGGTGATGGATAATTGTGAGGTTTCTAGCCCATTAATTGTAACTACAAAACCATCAACTTAAATTATTXXXXXXXXXXC
AGAGATTGTCTTTCAGAATTAATAGAGTGATTATTTTCAAAGCTAATTCCAATGGATTGTTTGAGTTGATTTACTTGTT
CACTGCATTAAAGGGTGATGGATAATTGTGAGGTTTCTAGCCCATTAATTGTAACTACAAAACCATCAACTTAAATTATTXXXXXXXXXXC
AGAGATTGTCTTTCAGAATTAATAGAGTGATTATTTTGAAAGCTAATTCCAATGGATTGTTTGAGTTGATTTACTTGTT
>Marker1033991 Chr2 SNP_site:15996613 Ref_Type:C
AACCAAAATTGAAATCAAACTTGGATGATGTTAGTTGAGTTAAGCTTATTTCAATTAAATTATATATAAAACTTATTTATXXXXXXXXXXT
TTTTTCCCTTCTTTTTCTTTTTTGCTATTTTACCTCTACGGATATCAATAATCAAAATATAGACAGCTTTTGTAAAGTA
AACCAAAATTGAAATCAAACTTGGATGATGTTAGTTGAGTTAAGCTTATTTTAATTAAATTATATATAAAACTTATTTATXXXXXXXXXXT
TTTTTCCCTTCTTTTTCTTTTTTGCTATTTTACCTCTACGGATATCAATAATCAAAATATAGACAGCTTTTGTAAAGTA
>Marker1034245 Chr2 SNP_site:19081272 Ref_Type:A
TACTATGAATCCTGGTCCCTTCAATATAAAGGAGCATGTTCTGATTACCATTTTTGCCAATTCTGGTGCTGGCTCTGTTTXXXXXXXXXXG
GTGATATGAACGTTTTTTAATTGTGAAAAATATTGAAATGGTTGTTATGATCTGTTTGCATTATCATGTTGATAACGTT
TACTATGAATCCTGGTCCCTTCAATATAAAGGAGCATGTTCTGATTACCATTTTTGCCAATTCTGGTGCTGGCTCTGTTTXXXXXXXXXXG
GTGATATGAACGTTTTTTCATTGTGAAAAATATTAAAATGGTTGTTATGATCTGTTTGTATTATCATGTTGATAACGTT
>Marker1034245 Chr2 SNP_site:19081288 Ref_Type:G
TACTATGAATCCTGGTCCCTTCAATATAAAGGAGCATGTTCTGATTACCATTTTTGCCAATTCTGGTGCTGGCTCTGTTTXXXXXXXXXXG
GTGATATGAACGTTTTTTAATTGTGAAAAATATTGAAATGGTTGTTATGATCTGTTTGCATTATCATGTTGATAACGTT
TACTATGAATCCTGGTCCCTTCAATATAAAGGAGCATGTTCTGATTACCATTTTTGCCAATTCTGGTGCTGGCTCTGTTTXXXXXXXXXXG
GTGATATGAACGTTTTTTCATTGTGAAAAATATTAAAATGGTTGTTATGATCTGTTTGTATTATCATGTTGATAACGTT
>Marker1034245 Chr2 SNP_site:19081312 Ref_Type:C
TACTATGAATCCTGGTCCCTTCAATATAAAGGAGCATGTTCTGATTACCATTTTTGCCAATTCTGGTGCTGGCTCTGTTTXXXXXXXXXXG
GTGATATGAACGTTTTTTAATTGTGAAAAATATTGAAATGGTTGTTATGATCTGTTTGCATTATCATGTTGATAACGTT
TACTATGAATCCTGGTCCCTTCAATATAAAGGAGCATGTTCTGATTACCATTTTTGCCAATTCTGGTGCTGGCTCTGTTTXXXXXXXXXXG
GTGATATGAACGTTTTTTCATTGTGAAAAATATTAAAATGGTTGTTATGATCTGTTTGTATTATCATGTTGATAACGTT
>Marker1034815 Chr2 SNP_site:17079458 Ref_Type:C
CACAAGAACCAAAATAATAGAGCAAGCTCAAGGTGGATGATGCAAGTTTCTAGCCTTTTCGACATTCCTTCGAAGTCGGGXXXXXXXXXXC
TTTTTCTTTTCTTTTCTTTTTTCCTTTTTTTTTTCTATCCCATCTTGGATGAACAGCATAGGATATGACATCACGAGGT
CACAAGAACCAAAATAATAGAGCAAGCTCAAGGTGGATGATGCACGTTTCTAGCCTTTTCGACATTCCTTCGAAGTCAGGXXXXXXXXXXC
TTTTTCTTTTCTTTTCTTTTTTCCTTTTTTTTTTCTATCCCATCTTGGATGAACAGCATAGGATATGACATCACGAGGT
>Marker1034815 ā／r2 SNP_site:17079491 Ref_Type:G
CACAAGAACCAAAATAATAGAGCAAGCTCAAGGTGGATGATGCAAGTTTCTAGCCTTTTCGACATTCCTTCGAAGTCGGGXXXXXXXXXXC
TTTTTCTTTTCTTTTCTTTTTTCCTTTTTTTTTTCTATCCCATCTTGGATGAACAGCATAGGATATGACATCACGAGGT
CACAAGAACCAAAATAATAGAGCAAGCTCAAGGTGGATGATGCACGTTTCTAGCCTTTTCGACATTCCTTCGAAGTCAGGXXXXXXXXXXC
TTTTTCTTTTCTTTTCTTTTTTCCTTTTTTTTTTCTATCCCATCTTGGATGAACAGCATAGGATATGACATCACGAGGT
>Marker1035013 Chr2 SNP_site:12917018 Ref_Type:G
ACCAAAATTAGTTTCCACATTGAATATGATTATATTTGTTATAACTTTGACAAATTAATGCGGTAGATCTCAACTTTTTTXXXXXXXXXXC

Create PDF with GO2PDF for free, if you wish to

CTACGTCAATCTTTATATAATTTTCTAATTTGACTTACGTTCATGAGTAGGGAGGAATTATTATTATTATCAAAGAGTT
ACCAAAATTAGTTTCCACATTGAATATGATTATATTTGTTATAACTTTGACAAATTAATGCGGTAGATCTCAACTTTTTTXXXXXXXXXXC
CTACGTCAATCTTTGTATAATTTTCTAATTTGACTTACGTTCATGAGTAGGGAGGAATTATTATTATTATCAAAGAGTT
>Marker1035095 Chr2 SNP_site:13419566 Ref_Type:A
ACTTAAGGCGGCGTTTCAGTCATCAGAGAAGATGGATAGTGGTGGGCAGGTGGCGCCTGTGTCGAAGCTGATGGAGTTTCXXXXXXXXXXA
TGTGGAGTATTATGAGAATCAAAACGGCACTTCACAGGGCACTCTTCTCATTCGTTCTGCTCTTCCATCTTCTCCCGTA
ACTTAAGGCGGCGTTTCAGTCATCGGAGAAGATGGATAGTGGTGGGCAGGTGGCGCCTGTGTCGAAGCTGATGGAGTTTCXXXXXXXXXXA
TGTGGAGTATTATGAGAATCAAAACGGCACTTCACAGGGCACTCTTCTCATTCGTTCTGCTCTTCCATCTTCTCCCGTA
>Marker1035180 Chr2 SNP_site:20144499 Ref_Type:C
ACTTTCTCACAACTTCATGAAACTTGTTAATGGTTTCTTCCTTCTCCATTTCCATTGCATTACTCTCTGCCCCATCATCAXXXXXXXXXXA
AGCACGTCCAGCCCTCTAGCCTGTTCAGACAACCATTTATTTCATAATTGATTCAAACAAAATAGGGGGGGTGAAAGTT
ACTTTCTCACAACTTCATGAAACTTGTTAATGGTTTCTTCCTTCTCCATTTCCATTGCATTACTCTGTGCCCCATCATCAXXXXXXXXXXA
AGCACGTCCAGCCCTCTAGCCTGTTCAGACAACCATTTATTTCATAATTGATTCAAACAAAATAGGGGGGGTGAAAGTT
>Marker1035842 Chr2 SNP_site:21395784 Ref_Type:A
TACTTTTAATTGGTTGGGCAGGAAAACCCCGGCAGTCTCCAAATTTCAAATAAACAAATGATCATTCATCATCTTCAGTAXXXXXXXXXXA
TCTCAAATCAATACTCTTCATCTTTATATCAGCTTCAAATTTTCAATAAAGTCCTCTAAAAACAATGTTCTAAAAAGTA
TACTTTTAATTGGTTGGGCAGGAAAACCCCGGCAGTCTCCAAATTTCAAATAAACAAATGATCATTCATCATCTTCAGTAXXXXXXXXXXA
TCTCAAATCAATACTCTTCATCTTTATATCAGCTTCAATTTTTCAATAAAGTCCTCTAAAAACAATGTTCTAAAAAGTA
>Marker1036026 Chr2 SNP_site:4920696 Ref_Type:T
AACTTGTCATAAATAAACTTCCAAATGACTTTTGGATACCGAAATTAGAGCAACTGGATTCTATTGAAGTGCCCCCAAAAXXXXXXXXXXT
TAGTTTTCATATAATATTTTGTTATACTTATGATTATGAAAAAGGATGAAAAGGGAATGATTATTAATCTGTTAGAGTT
AACTTGTCATAAATAAACTTCCAAATGACTTTTGGATACTGAAATTAGAGCAACTGGATTCTATTGAAGTGCCCCCAAAAXXXXXXXXXXT
TAGTTTTCATATAATATTTTGTTATACTTATGATTATGAAAAAGGATGAAAAGGGAATGATTATTAATCTGTTAGAGTT
>Marker1036145 Chr2 SNP_site:6553772 Ref_Type:C
ACACCAATTAAATTGGTCTTGTCTGTGGATGCTACGGATTACCTTTTTCCAAAGAGAGTTCTCCTCATTAAAATGTCTCCXXXXXXXXXXC
CTCCCTTATGACCTTCCCAAAAAAACTTCGCATCAACCTTCCTATTGATTTAACCACCTTTTCTGGAATCATAAAAGTG
ACACCAATTAAATTGGTCTTGTCTGTGGATGCTACGGATTACCTTTTTCCAAAGAGAGTTCTCCTCATTAAAATGTCTCCXXXXXXXXXXC
CTTCCTTATGACCTTCCCAAAAAAACTTCGCATCAACCTTCCTATTGATTTAACCACCTTTTCTGGAATCATAAAAGTG
>Marker1036355 Chr2 SNP_site:1051542 Ref_Type:G
TACAAAGATATCCTATTGTATGCATTCACAAGTTACCCACAAGGCCACAGGCCCACAACACATACCAGCAATTATATCAAXXXXXXXXXXT
GACGGTTTTTCTTCTTTGTTTTTCATAACTGTGAGTTTCGAGGCTACCTTGCACGCACTTCGACTTAATCTCAAGGGTC
TACAAAGATATCCTATTGTATGCATTCACAAGTTACCCACAAGGCCACAGGCCCACAACACATACCAGCAATTATATCAAXXXXXXXXXXT
GACGGTTTTTCTTCTTTGTTTTTCGTAACTGTGAGTTTCGAGGCTACCTTGCACGCACTTCGACTTAATCTCAAGGGTC
>Marker1036520 Chr2 SNP_site:1109588 Ref_Type:T
CACACATTTTGTTGCTTAATTTTTTATGTTTCATCGATTTCTTTTGTGATACAATGGAAATGTTGATTCATCCATTATGTXXXXXXXXXXA
AACCAAGGAAATTGAGAATTTGTAGGGTGAGCTAAACTATGACAAATGAAGATTTATCTTTGTTTCTTTGCTATAGGTC
CACACATTTTGTTGCTTATTTTTTTATGTTTCATCGATTTCTTTTGTGATACAATGGAAATTTTGATTCATCCATTATGTXXXXXXXXXXA
AACCAAGGAAATTGAGAATTTGTAGGGTGAGCTAAACTATGACAAATGAAGATTTATCTTTGTTTCTTTGCTATAGGTC
>Marker1036520 Chr2 SNP_site:1109631 Ref_Type:T
CACACATTTTGTTGCTTAATTTTTTATGTTTCATCGATTTCTTTTGTGATACAATGGAAATGTTGATTCATCCATTATGTXXXXXXXXXXA
AACCAAGGAAATTGAGAATTTGTAGGGTGAGCTAAACTATGACAAATGAAGATTTATCTTTGTTTCTTTGCTATAGGTC
CACACATTTTGTTGCTTATTTTTTTATGTTTCATCGATTTCTTTTGTGATACAATGGAAATTTTGATTCATCCATTATGTXXXXXXXXXXA
AACCAAGGAAATTGAGAATTTGTAGGGTGAGCTAAACTATGACAAATGAAGATTTATCTTTGTTTCTTTGCTATAGGTC
>Marker1036551 Chr2 SNP_site:21300434 Ref_Type:T

Create PDF with GO2PDF for free, if you wish to

ACTAAACAAGGACTCAGTAGTTTCACGATAAAGAAATACATAGTGACTACTACGTATGCAGTGATCATGACTAATGAATTXXXXXXXXXXT
TCAGACTTTGATATTAGGCATAGCATGCGGTCTTGGTGAATCATGCGTTCAACTGAAGATTGCATGAAGAGGTTAAGGT
ACTAAACAAGGACTCAGTAGTTTCACGATAAAGAAATATATAGTGACTACTACGTATGCAGTGATCATGACTAATGAATTXXXXXXXXXXT
TCAGACTTTGATATTAGGCATAGCATGCGGTCTTGGTGAATCATGCGTTCAACTGAAGATTGCATGAAGAGGTTAAGGT
>Marker1036790 Chr2 SNP_site:13159887 Ref_Type:T
AACATTCCCTGCCAAAGTCGTATTGATTGTTCTGATCCATGGGACTGTGATTGCAAGCTCAACCTATGTTTTTGTCACCCXXXXXXXXXXA
ATTATATATATACCCTCCACCCCCCATTTCGATACACGTATATAGCTCGTAAATCGAAAGAAATGTATTTTACTACAGT
AACATTCCCTGCCAAAGTCGTATTGATTGTTCTGATCCATGGGACTGTGATTGCAAGCTCAACCTATGTTTTTGTCACCCXXXXXXXXXXA
ATTATATATATACCCTCCACCCCCCATTTCGATACACGTATATAGCTTGTAAATCGAAAGAAATGTATTTTACTACAGT
>Marker1036917 Chr2 SNP_site:3532186 Ref_Type:A
AACCAATAATTGGAAGCATTTTTTGTTGGGACCCTATAATTAATTAAGACATGGGTCTCTATCAACATAAACAAAATAAGXXXXXXXXXXG
TGATTTCAAGTTTCAGCCAAGCACATATTAATCTCTCAATAGATTCTTTATATCATTCCCTCCATGGGCCAACCTTGTG
AACCAATAATTGGAAGCATTTTTTGTTGGGACCCTATAATTAATTAAGACATGGGTCTCTATCAACATGAACAAAATAAGXXXXXXXXXXG
TGATTTCAAGTTTCAGCCAAGCACATATTAATCTCTCAATAGATTCTTTATATCATTCCCTCCATGGGCCAACCTTGTG
>Marker1036940 Chr2 SNP_site:16284343 Ref_Type:G
CACCAGGGAAACCTTCTTGGCTGCAATGCAAAGCAAAAGCAAGTTTTGTGTTAAGGGGTGGTGGTGGTTGCATTCAAACAXXXXXXXXXXA
AACAGGTCAATAACATACTTAATCAAAAGTCGAAGGACTTTTTACATATTTTAAAGTGAGACCTATTCAACACAAAGTT
CACCAGGGAAACCTTCTTGGCTGCAATGCAAAGCAAAAGCAAGTTTTGTGTTAAGGGGTGGTGGTGGTTGCATTCAAACAXXXXXXXXXXA
AACAGGTCAATAACATACTTAATCAAAATTCGAGGGACTTTTTACATATTTTAAAGTGAGACCTATTCGACACAAAGTT
>Marker1036940 Chr2 SNP_site:16284348 Ref_Type:A
CACCAGGGAAACCTTCTTGGCTGCAATGCAAAGCAAAAGCAAGTTTTGTGTTAAGGGGTGGTGGTGGTTGCATTCAAACAXXXXXXXXXXA
AACAGGTCAATAACATACTTAATCAAAAGTCGAAGGACTTTTTACATATTTTAAAGTGAGACCTATTCAACACAAAGTT
CACCAGGGAAACCTTCTTGGCTGCAATGCAAAGCAAAAGCAAGTTTTGTGTTAAGGGGTGGTGGTGGTTGCATTCAAACAXXXXXXXXXXA
AACAGGTCAATAACATACTTAATCAAAATTCGAGGGACTTTTTACATATTTTAAAGTGAGACCTATTCGACACAAAGTT
>Marker1036940 Chr2 SNP_site:16284383 Ref_Type:A
CACCAGGGAAACCTTCTTGGCTGCAATGCAAAGCAAAAGCAAGTTTTGTGTTAAGGGGTGGTGGTGGTTGCATTCAAACAXXXXXXXXXXA
AACAGGTCAATAACATACTTAATCAAAAGTCGAAGGACTTTTTACATATTTTAAAGTGAGACCTATTCAACACAAAGTT
CACCAGGGAAACCTTCTTGGCTGCAATGCAAAGCAAAAGCAAGTTTTGTGTTAAGGGGTGGTGGTGGTTGCATTCAAACAXXXXXXXXXXA
AACAGGTCAATAACATACTTAATCAAAATTCGAGGGACTTTTTACATATTTTAAAGTGAGACCTATTCGACACAAAGTT
>Marker1037290 Chr2 SNP_site:14344906 Ref_Type:C
AACAAAAATACTCATGCTCACATATTCAACAAAGCAATTTGAGAATGATAGAAAGAGAAATCAAATTTTCTTTTGCATAAXXXXXXXXXXT
AGCGCTTGATAACGCTTAAAATGTGCCTAACTTATCCCGTTTAATTAACTAGAAACCATTTTCTAGTATTTTACGTGTT
AACAAAAATACTCATGCTCACATATTCAACAAAGCAATTTGAGAATGATAGAAAGAGAAATCAAATTTTCTTTTGCATAAXXXXXXXXXXT
AGCGCTTGATAACGCTTAAAATGTGCCTAACTTATCCCGTTTAATTAACTCGAAACCATTTTCTAGTATTTTACGTGTT
>Marker103747 Chr4 SNP_site:2514087 Ref_Type:C
ACTTCCTTGAAGCTGCAATTTTTCTTCTCTTTTGGTATTATGGTGCTTGGGATGAGTCTCATGAATGAAACACATACACTXXXXXXXXXXA
ATGGGAAGTTGAGCAAAACTGTAGAATAAAATATTGTTGTTATTTCCAAAACACTCAAGAAAAAGGTATGGCCAACGTT
ACTTCCTTGAAGCTGCAATTTTTCTTCTCTTTTGGTATTATGGTGCTTGGGATGAGTCTCATGAATGAAACACATATACAXXXXXXXXXXA
ATGGGAAGTTGAGCAAAACTGTAGAATAAAAGATTGTTGTTATTTCCAAAACACTCAAGAAAAAGGTATGGCCAACGTT
>Marker103747 Chr4 SNP_site:2514090 Ref_Type:T
ACTTCCTTGAAGCTGCAATTTTTCTTCTCTTTTGGTATTATGGTGCTTGGGATGAGTCTCATGAATGAAACACATACACTXXXXXXXXXXA
ATGGGAAGTTGAGCAAAACTGTAGAATAAAATATTGTTGTTATTTCCAAAACACTCAAGAAAAAGGTATGGCCAACGTT
ACTTCCTTGAAGCTGCAATTTTTCTTCTCTTTTGGTATTATGGTGCTTGGGATGAGTCTCATGAATGAAACACATATACAXXXXXXXXXXA
ATGGGAAGTTGAGCAAAACTGTAGAATAAAAGATTGTTGTTATTTCCAAAACACTCAAGAAAAAGGTATGGCCAACGTT

Create PDF with GO2PDF for free, if you wish to

>Marker103747 Chr4 SNP_site:2514222 Ref_Type:T
ACTTCCTTGAAGCTGCAATTTTTCTTCTCTTTTGGTATTATGGTGCTTGGGATGAGTCTCATGAATGAAACACATACACTXXXXXXXXXXA
ATGGGAAGTTGAGCAAAACTGTAGAATAAAATATTGTTGTTATTTCCAAAACACTCAAGAAAAAGGTATGGCCAACGTT
ACTTCCTTGAAGCTGCAATTTTTCTTCTCTTTTGGTATTATGGTGCTTGGGATGAGTCTCATGAATGAAACACATATACAXXXXXXXXXXA
ATGGGAAGTTGAGCAAAACTGTAGAATAAAAGATTGTTGTTATTTCCAAAACACTCAAGAAAAAGGTATGGCCAACGTT
>Marker1037620 Chr2 SNP_site:20990050 Ref_Type:A
AACCTCTCACACTCATCCATTTGCACTCCCACATGTATATATAAAATTTTCCCCAAATGGACACTTTCAAACCGCAATAGXXXXXXXXXXA
ATTTTGAAGGTTTTCTATTAGTTATATTTTCAACATTATTTGAAGGGCATAAGAAATGTGTCTTTAAAGTTTTAAAGTT
AACCTCTCACACTCATCCATTTGCACTCCCACATGTATATATAAATTTTTCCCCAAATGGACACTTTCAAACCGCAATAGXXXXXXXXXXA
ATTTTCAAGGTTTTCTATTAGTTATATTTTCAACATTATTTGAAGGGCATAAGAAATGTGTCTTTAAAGTTTTAAAGTT
>Marker1037620 Chr2 SNP_site:20990235 Ref_Type:G
AACCTCTCACACTCATCCATTTGCACTCCCACATGTATATATAAAATTTTCCCCAAATGGACACTTTCAAACCGCAATAGXXXXXXXXXXA
ATTTTGAAGGTTTTCTATTAGTTATATTTTCAACATTATTTGAAGGGCATAAGAAATGTGTCTTTAAAGTTTTAAAGTT
AACCTCTCACACTCATCCATTTGCACTCCCACATGTATATATAAATTTTTCCCCAAATGGACACTTTCAAACCGCAATAGXXXXXXXXXXA
ATTTTCAAGGTTTTCTATTAGTTATATTTTCAACATTATTTGAAGGGCATAAGAAATGTGTCTTTAAAGTTTTAAAGTT
>Marker1038311 Chr2 SNP_site:1916168 Ref_Type:T
AACCATCTTTTTGTAATGCCTTATTTAGTTGAATTTGGACTATACTTAAGTTGGCACATTTGAACCATCTTGATTGTTTGXXXXXXXXXXA
TCATAGCCTCTAAACAAATGTCCCTTTCTTTTTACCTCCTACACTAAAGAAATCACTCAAAAGCTGAGATCAATAGGTG
AACCATCTTTTTGTATTGCCTTATTTAGTTGAATTTGGACTATACTTAAGTTGGCACATTTGAACCATCTTGATTGTTTGXXXXXXXXXXA
TCATAGCCTCTAAACAAATGTCCCTTTCTTTTTACCTCCTACACTAAAGAAATCACTCAAAAGCTGAGATCAATAGGTG
>Marker1038315 Chr2 SNP_site:261707 Ref_Type:T
ACCATCTGAGTAGAAAGGAAGCAATTGCAATGTTTTAATTAACTAATAATATAGAGTTTAATTCGATGAAATTGAAAGTTXXXXXXXXXXT
GGTGGGTCATTTTTCACGTTTCGACTTGTTAAATAATGCAACATGTTTTACATAACACAATAAACATAAGCTTGTAGTT
ACCATCTGAGTAGAAAGGAAGCAATTGCAATGTTTTAATTAACTAATAATATAGAGTTTAATTCGATGAAATTGAAAGTTXXXXXXXXXXT
GGTGGGTCATTTTTCACGTTTTGACTTGTTAAATAATGCAACATGTTTTACATAACACAATAAACATAAGCTTGTAGTT
>Marker1038655 Chr2 SNP_site:6533825 Ref_Type:A
ACTAGATCGGGGAAGTTTTCCTCACCCATTCCCCTGATAAGGGATCCAATTGCCCGAGCAGCAACTGACCGAACTTCTGGXXXXXXXXXXG
TTACTAAGGAACACATATTTCCAGCAATCTGCGCTGCTTTTTTTTTGGTTTCAGCACTCCTTTCCCTTAGACCTCTGTG
ACTAGATCGGGGAAGTTTTCCTCACCCATTCCCCTGATAAGGGATCCAATTGCCCGAGCAGCAACTGACCGAACTTCTGGXXXXXXXXXXG
TTACTAAGGAACACATATTTCCAGCAATCTGCGCTGCTTTTTTTTTGGTTTCGGCACTCCTTTCCCTTAGACCTCTGTG
>Marker1039291 Chr2 SNP_site:1065598 Ref_Type:C
GACATTTAAACAAAGTTGGTAAAAGTAGAGTATGCACATATCAATTAATCATTATTGTCATATTTATGATAATTCTCAATXXXXXXXXXXA
TTTGTCCTTCTTCACGAAGAAATTATCGATTTTTATTTCTATACCTAAATTTTAGGGTTTGTATCAATTTAAACTATGT
GACATTTAAACAAAGTTGGTAAAAGTAGAGTATGCACATATCAATTAATCATTATTGTTATATTTATGATAATTCTCAATXXXXXXXXXXA
TTTGTCCTTCTTCACGAAGAAATTATCGATTTTTATTTCTATACCTAAATTTTAGGGTTTGTATCAATTTAAACTATGT
>Marker1039967 Chr2 SNP_site:4338873 Ref_Type:A
TACAACAGGGTATTTTATGTTTGTTAAAGGCAACCTTGTAACATGGAAAAGTAAGAATTTCGAGGGATTGCAAATGGTATXXXXXXXXXXA
TAATTGCACACGTAACCCGGTTCAACATGATCAAATGAAGCATGTAGAAATAGATCGGCATTTCATTAAAGAGAAAGTG
TACATCAGGGTATTTTATGTTTGTTAAAGGCAACCTTGTAACATGGAAAAGTAAGAATTTCGAGGGATTGCAAATGGTATXXXXXXXXXXA
TAATTGCACACGTAATCCGGTTCAACATGATCAAATGAAGCATGTAGAAATAGATCGACATTTCATTAAAGAGAAAGTG
>Marker1039967 Chr2 SNP_site:4339059 Ref_Type:C
TACAACAGGGTATTTTATGTTTGTTAAAGGCAACCTTGTAACATGGAAAAGTAAGAATTTCGAGGGATTGCAAATGGTATXXXXXXXXXXA
TAATTGCACACGTAACCCGGTTCAACATGATCAAATGAAGCATGTAGAAATAGATCGGCATTTCATTAAAGAGAAAGTG
TACATCAGGGTATTTTATGTTTGTTAAAGGCAACCTTGTAACATGGAAAAGTAAGAATTTCGAGGGATTGCAAATGGTATXXXXXXXXXXA

Create PDF with GO2PDF for free, if you wish to

TAATTGCACACGTAATCCGGTTCAACATGATCAAATGAAGCATGTAGAAATAGATCGACATTTCATTAAAGAGAAAGTG
>Marker1039967 Chr2 SNP_site:4339101 Ref_Type:G
TACAACAGGGTATTTTATGTTTGTTAAAGGCAACCTTGTAACATGGAAAAGTAAGAATTTCGAGGGATTGCAAATGGTATXXXXXXXXXXA
TAATTGCACACGTAACCCGGTTCAACATGATCAAATGAAGCATGTAGAAATAGATCGGCATTTCATTAAAGAGAAAGTG
TACATCAGGGTATTTTATGTTTGTTAAAGGCAACCTTGTAACATGGAAAAGTAAGAATTTCGAGGGATTGCAAATGGTATXXXXXXXXXXA
TAATTGCACACGTAATCCGGTTCAACATGATCAAATGAAGCATGTAGAAATAGATCGACATTTCATTAAAGAGAAAGTG
>Marker1040196 Chr2 SNP_site:15685395 Ref_Type:C
ACTATCTAACCCCTTTAATACTTATAGTGGCCCTTACTGACCCTGGGACAACTCATGGTGGTTCTTTATTAACCTTATCTXXXXXXXXXXC
TGGCTATTTCGTTTTATTGTTTCTTTCATCACTTCCAAAAAGAATTGGTATCCTTGAAATACTACTGCTCACAGCAGTG
ACTATCTAACCCCTTTAATACTTATAGTGGCCCTTACTGACCCTGGGACAACTCATGGTGGTTCTTTATTAACCTTATTTXXXXXXXXXXC
TGGCTATTTTGTTTTATTGTTTCTTTCATCACTTCCAAAAAGAATTGGTATCCTTGAAATACTACTGCTCACAGCAGTG
>Marker1040196 Chr2 SNP_site:15685550 Ref_Type:C
ACTATCTAACCCCTTTAATACTTATAGTGGCCCTTACTGACCCTGGGACAACTCATGGTGGTTCTTTATTAACCTTATCTXXXXXXXXXXC
TGGCTATTTCGTTTTATTGTTTCTTTCATCACTTCCAAAAAGAATTGGTATCCTTGAAATACTACTGCTCACAGCAGTG
ACTATCTAACCCCTTTAATACTTATAGTGGCCCTTACTGACCCTGGGACAACTCATGGTGGTTCTTTATTAACCTTATTTXXXXXXXXXXC
TGGCTATTTTGTTTTATTGTTTCTTTCATCACTTCCAAAAAGAATTGGTATCCTTGAAATACTACTGCTCACAGCAGTG
>Marker1040282 Chr2 SNP_site:4520314 Ref_Type:A
CACTCTCATCTTGCAAGCACTCTTTCTCTATCGTCAGAGTTTCATTGCCTATTGTCCAATAAGAAACGAGACATTACAGTXXXXXXXXXXT
CCCTCTAATGTGTTTCAGAGGTGAAATTCTGCCCCCCAAAAAGTTAAAATTTTCATGTGATGGGCGTTCAAGAAAAGTT
CACTCTCATCTTGCGAGCACTCTTTCTCTATCGTCGGAGTTTCATTGCCTATTGTCCAATAAGAAACGAGACATTACAGTXXXXXXXXXXT
CCCTCTAATGTGTTTCAGAGGTGAAATTCTGCCCCCCAAAAAGTTAAAATTTTCATGTGATGGGCGTTCAAGAAAAGTT
>Marker1040282 Chr2 SNP_site:4520335 Ref_Type:A
CACTCTCATCTTGCAAGCACTCTTTCTCTATCGTCAGAGTTTCATTGCCTATTGTCCAATAAGAAACGAGACATTACAGTXXXXXXXXXXT
CCCTCTAATGTGTTTCAGAGGTGAAATTCTGCCCCCCAAAAAGTTAAAATTTTCATGTGATGGGCGTTCAAGAAAAGTT
CACTCTCATCTTGCGAGCACTCTTTCTCTATCGTCGGAGTTTCATTGCCTATTGTCCAATAAGAAACGAGACATTACAGTXXXXXXXXXXT
CCCTCTAATGTGTTTCAGAGGTGAAATTCTGCCCCCCAAAAAGTTAAAATTTTCATGTGATGGGCGTTCAAGAAAAGTT
>Marker1040715 Chr2 SNP_site:7811686 Ref_Type:T
ACTTTCCACCAATTTGATGAGTATTCTTCCGAGCATCAAGTATCCTTTTAGCATGAAAAACAAAGAAAGAATGTTAGATTXXXXXXXXXXC
AATGCATTACATTGGAGGGGTGCTCTACTAAACAATCTGAAATACCAACCTCAGTCTCTGTGTGCATGCAATTGATTGT
ACTTTCCACCAATTTGATGAGTATTCTTCCGAGCATCAAGTATCCTTTTAGCATGAAAAACAAAGAAAGAATGTTAGATTXXXXXXXXXXC
AATGCATTACATTGGAGGGGTGCTCTACTAAACAATCTGAAATACCAACCTTAGTCTCTGTGTGCATGCAATTGATTGT
>Marker1040957 Chr2 SNP_site:10959558 Ref_Type:A
TACCACTATGTCAATTGTTCAATATCTTTATGTTAAAATCATAAATAAATATCAGAACCTTGAAACTAGTAATTAATTGAXXXXXXXXXXC
AACAAGCCCTGCAAGAAAGTGAAACGTAACTAAACATAAGGCTAAGATCGGTGCCTATGCCTTCCCATGGAACCTTGTA
TACCACTATGTCAATTGTTCAATATCTTTATGTTAAAATCATAAATAAATATCAGAACCTTGAAACTAGTAATTAATTGAXXXXXXXXXXC
AACAAGCCCTGCAAGAAAGTGAAACGTAGCTAAATATAAGGCTAAGATCGGTGCCTATGCCTTCCCATGGAACCTTGTA
>Marker1040957 Chr2 SNP_site:10959564 Ref_Type:C
TACCACTATGTCAATTGTTCAATATCTTTATGTTAAAATCATAAATAAATATCAGAACCTTGAAACTAGTAATTAATTGAXXXXXXXXXXC
AACAAGCCCTGCAAGAAAGTGAAACGTAACTAAACATAAGGCTAAGATCGGTGCCTATGCCTTCCCATGGAACCTTGTA
TACCACTATGTCAATTGTTCAATATCTTTATGTTAAAATCATAAATAAATATCAGAACCTTGAAACTAGTAATTAATTGAXXXXXXXXXXC
AACAAGCCCTGCAAGAAAGTGAAACGTAGCTAAATATAAGGCTAAGATCGGTGCCTATGCCTTCCCATGGAACCTTGTA
>Marker1040965 Chr2 SNP_site:12551732 Ref_Type:G
TACAAACACTACTTTGGTTTATTAGTTATAGGCTTAGTTTTCAAAAACAAAAAATGAAATGGTTACCAAACTGTCTATCAXXXXXXXXXXT
TTAGGATATAGAGTTACGTATCTGGAGTTTGAAAATAGGAAACATTAGAGAGGAGAATATTAAGTTTATCGATAAATGT

Create PDF with GO2PDF for free, if you wish to

TACAAACACTACTTTGGTTTATTAGTTGTAGGCTTAGTTTTCAAAAACAAAAAATGAAATGGTTACCAAACTGTCTATCAXXXXXXXXXXXT
TTAGGATATAGAGTTACGTATCTGGAGTTTGAAAATAGGAAACATTAGAGAGGAGAAGATTAAGTTTATCGATAAATGT
>Marker1040965 Chr2 SNP_site:12551968 Ref_Type:G
TACAAACACTACTTTGGTTTATTAGTTATAGGCTTAGTTTTCAAAAACAAAAAATGAAATGGTTACCAAACTGTCTATCAXXXXXXXXXXXT
TTAGGATATAGAGTTACGTATCTGGAGTTTGAAAATAGGAAACATTAGAGAGGAGAATATTAAGTTTATCGATAAATGT
TACAAACACTACTTTGGTTTATTAGTTGTAGGCTTAGTTTTCAAAAACAAAAAATGAAATGGTTACCAAACTGTCTATCAXXXXXXXXXXXT
TTAGGATATAGAGTTACGTATCTGGAGTTTGAAAATAGGAAACATTAGAGAGGAGAAGATTAAGTTTATCGATAAATGT
>Marker10414 Chr4 SNP_site:18117841 Ref_Type:C
CACACGAGGTTTCGGAAAAATGTAATTCGTGGAATTGAAATTTGTATATTGATGAATATTGTGAATACAATTTCTAGTATXXXXXXXXXXXC
CTTCGATCTTATGGAATCCAAGGAAAGTTCAATCTTCCATGTCTTCAATTTTTCAGAAGTTGTTGATCTCGAGGTTGGT
CACACGAGGTTTCGGAAAAATGTAATTCGTGGAATTGAAATTTGTATATTGATGAATATTGTGAATACAATTTCTAGTATXXXXXXXXXXXC
CTTCGATCTTCTGGAATCCAAGGAAAGTTCAATCTTCCATGTCTTCAATTTTTCAGAAGTTGTTGATCTCGAGGTTGGT
>Marker1042052 Chr2 SNP_site:12647050 Ref_Type:C
AACTTCGTCAATTACCAATGGAAGCCAAATTAAATTTAGTTTTAAAAAATTATTAGTATGAATGTATGAACTCTTTGTGCXXXXXXXXXXXT
CGCTCGCTCGCTCGTTTCCACTCCCTGTTATCTTCCATTTTCAAACCTTTCAACCAACTCTCTCAAATTCCCCATTGTT
AACTTCGTCAATTACCAATGGAAGCCAAATTAAATTTAGTTTTAAAACATTATTAGTATGAATGTATGAACTCTTTGTGCXXXXXXXXXXXT
CGCTCGCTCGCTCGTTTCCACTCCCTGTTATCTTCCATTTTCAAACCTTTCAACCTACTCTCTCAAATTCCCCATTGTT
>Marker1042052 Chr2 SNP_site:12647286 Ref_Type:T
AACTTCGTCAATTACCAATGGAAGCCAAATTAAATTTAGTTTTAAAAAATTATTAGTATGAATGTATGAACTCTTTGTGCXXXXXXXXXXXT
CGCTCGCTCGCTCGTTTCCACTCCCTGTTATCTTCCATTTTCAAACCTTTCAACCAACTCTCTCAAATTCCCCATTGTT
AACTTCGTCAATTACCAATGGAAGCCAAATTAAATTTAGTTTTAAAACATTATTAGTATGAATGTATGAACTCTTTGTGCXXXXXXXXXXXT
CGCTCGCTCGCTCGTTTCCACTCCCTGTTATCTTCCATTTTCAAACCTTTCAACCTACTCTCTCAAATTCCCCATTGTT
>Marker1042795 Chr2 SNP_site:12863632 Ref_Type:T
CACTTTTCAGGTTAGATGGAGAGCACGTCGTCTTTGGCAAGGTTATCCAAGGAATGGACTACGTATATGCAATTGAAGGCXXXXXXXXXXXT
TAACAACAGAAAATTTTCGTTTATTAATTCTTTTTTCCTTTTATGGTTTTCAAGTTTCTTTTGATACGCCCCCAATGTC
CACTTTTCAGGTTAGATGGAGAGCACGTCGTCTTTGGCAAGGTTATCCAAGGAATGGACTACGTATATGCAATTGAAGGCXXXXXXXXXXXT
TAACAACAGAAAATTTTCGTTTATTAATTCTTTTTTCCTTTTATGGTTTTCAAGTTTCTTTTGATACGCCCCTAATGTC
>Marker1042938 Chr2 SNP_site:3739173 Ref_Type:T
TACTGGCCTAATAACTGTGGTTGATAACTATCTACAATCTGCCTGCAATCATCCTCGTTAATAAATCTTACATACGACTTXXXXXXXXXXXT
CATTGAGCTTACTTGACTGTTACATTGTTTCCAGGTGGATGTTAGAAAGCAGAGAACTGCATTTCCGCCCAATTTTGTG
TACTGGCCTAATAACTGTGGTTGATAATTATCTACAATCTGCCTGCAATCATCCTCGTTAATAAATCTTACATACGACTTXXXXXXXXXXXT
CATTGAGCTTACTTGACTGTTACATTGTTTCCAGGTGGATGTTAGAAAGCAGAGAACTGCATTTCCGCCCAATTTTGTG
>Marker1043928 Chr2 SNP_site:8058443 Ref_Type:T
ACAAGGGATGCACCTGTAAATCCTTTTACATCTCATTTTCCAATCTAAGATTGAATAAATTTGTTTTGTTGTAGATTTTAXXXXXXXXXXXT
TGCCTCTGTGATTGATAAAGCAACAACGGGTTCTAACTTAGTTGATTAGTGTCAAATAGGAAACAATAGTCTGATAGGT
ACAAGGGATGCACCTTTAAATCCTTTTACATCTCATTTTCCAATCTAAGATTGAATAGATTTGTTTTGTTGTAGATTTTAXXXXXXXXXXXT
TGCCTCTGTGATTGATAAAGCAACAACGGGTTCTAACTTAGTTGATTAGTGTCAAATAGGAAACAATAGTCTGATAGGT
>Marker1043928 Chr2 SNP_site:8058485 Ref_Type:G
ACAAGGGATGCACCTGTAAATCCTTTTACATCTCATTTTCCAATCTAAGATTGAATAAATTTGTTTTGTTGTAGATTTTAXXXXXXXXXXXT
TGCCTCTGTGATTGATAAAGCAACAACGGGTTCTAACTTAGTTGATTAGTGTCAAATAGGAAACAATAGTCTGATAGGT
ACAAGGGATGCACCTTTAAATCCTTTTACATCTCATTTTCCAATCTAAGATTGAATAGATTTGTTTTGTTGTAGATTTTAXXXXXXXXXXXT
TGCCTCTGTGATTGATAAAGCAACAACGGGTTCTAACTTAGTTGATTAGTGTCAAATAGGAAACAATAGTCTGATAGGT
>Marker1044100 Chr2 SNP_site:14422413 Ref_Type:A
CACGCGAGATTTTAAGATAAATTTAATTTGTGGAGTGTTGAATTTGTGTTAGTATTGTATTTATGTTGATTTGATGCGATXXXXXXXXXXXA

Create PDF with GO2PDF for free, if you wish to

ACTCTAAGAAAAAAATTTACTCGAAGAGGTCTAAAGATACTTCTTCCAAGAGCCATAAAACTTCCTAACTTCTGAAGTC
CACGCGAGATTTTCAGATAAATTTAATTTGTGGAGTGTTGAATTTGTGTTAGTATTGTATTTATGTTGATTTGATGCGATXXXXXXXXXXA
ACTCTAAGAAAAAAATTTACTCGAAGAGGTCTAAAGATACTTCTTCCAAGAGCCATAAAACTTCCTAACTTCTGAAGTC
>Marker1044273 Chr2 SNP_site:17062925 Ref_Type:A
TACCAAGCTGTGTTCCGCAATTAACTAAATTCTTTTTAGGGGAACAAGTTTTGGAAAAAGAGTGTTCAGTATATATGGTCXXXXXXXXXXT
TTTATTTTTTTTGATAGATTCATTTTATCCAATTTATCTTTCTAGCACGTTGTCTTTCCTCTATCTGTTCTTGAAGGTG
TACCAAGCTGTGTTCCGCAATTAACTAAATTCTTTTTAGGGGAACAAGTTTTGGAAAAAGAGTGTTCAGTATATATGGTCXXXXXXXXXXT
TTTTTTTTTTTTGATAGATTCATTTTATCCAATTTATCTTTCTAGCACGTTGTCTTTCCTCTATCTGTTCTTGAAGGTG
>Marker1045106 Chr2 SNP_site:19504142 Ref_Type:G
TACACAAAATGAAATCTTAATATTGTGTTTGTATATACATTCTACAATAATACATGAAATAAGATCCTAATATAGGATTTXXXXXXXXXXG
ATTAAATCCAAAATCTGCAAAATTATGTTGATAAAGGCCAACATTTGCACCATCCATAACACCATCACAATAATTATGT
TACACAAAATGAAATCTTAATATTGTGTTTGTATATACATTCTACAATAATACATGAAATAAGATCCTAATATAGGATTTXXXXXXXXXXG
ATTAAATCCAAAATCTGCAAAATTATGTTGATAAAGGCCAACATTTGCACCATCCATAACAGCATCACAATAATTATGT
>Marker1045542 Chr2 SNP_site:2947342 Ref_Type:A
TACTTCTTTTTTTGGAAATTGTCATCATTGTTGTTAAGTTGAAATATTCATTATGATAAATGTTTGATTGCCAATAAAAAXXXXXXXXXXG
AAGAGGACACCACGTGTTCCGATTTCATATTTAGAGGAAAGAGATAAAGGGGAAAATCATGCTTCTGGAAATAAATGTT
TACTTCTTTTTTTGGAAATTGTCATCATTGTTGTTAAGTTGAAATCTTCATTATGATAAATGTTTGATTGCCAATAAAAAXXXXXXXXXXG
AAGAGGACACCACGTGTTGCGATTTCATATTTAGAGGAAAGAGATAAAGGGGAAAATCATGCTTCTGGAAATAAATGTT
>Marker1045542 Chr2 SNP_site:2947519 Ref_Type:C
TACTTCTTTTTTTGGAAATTGTCATCATTGTTGTTAAGTTGAAATATTCATTATGATAAATGTTTGATTGCCAATAAAAAXXXXXXXXXXG
AAGAGGACACCACGTGTTCCGATTTCATATTTAGAGGAAAGAGATAAAGGGGAAAATCATGCTTCTGGAAATAAATGTT
TACTTCTTTTTTTGGAAATTGTCATCATTGTTGTTAAGTTGAAATCTTCATTATGATAAATGTTTGATTGCCAATAAAAAXXXXXXXXXXG
AAGAGGACACCACGTGTTGCGATTTCATATTTAGAGGAAAGAGATAAAGGGGAAAATCATGCTTCTGGAAATAAATGTT
>Marker1045696 Chr2 SNP_site:22640705 Ref_Type:C
CACCAACAACAGTTGGACCCCCTCATTGATAAACACTGCTCCAACTAAAATAAAAGGATACCCCTCTCCCAGGTCGTGGCXXXXXXXXXXA
GACAATCTAGAATCAGAACTTAACAAACCAGAAAAACGACTAAGAAAATCAAACTAATTCTGTCCTAACGCAGAGTGTT
CACCAACAACAGTTGGACCCCCTCATTGATAAACACTGCTCCAACTAAAATAAAAGGATACCCCTCTCCCAGGTCGTGGCXXXXXXXXXXA
GACAATCTAGAATCAGAACTTAACAAACCAGCAAAACGACTTAGAAAATCAAACTAATTCTGTCCTAACGCAGAGTGTT
>Marker1045696 Chr2 SNP_site:22640715 Ref_Type:T
CACCAACAACAGTTGGACCCCCTCATTGATAAACACTGCTCCAACTAAAATAAAAGGATACCCCTCTCCCAGGTCGTGGCXXXXXXXXXXA
GACAATCTAGAATCAGAACTTAACAAACCAGAAAAACGACTAAGAAAATCAAACTAATTCTGTCCTAACGCAGAGTGTT
CACCAACAACAGTTGGACCCCCTCATTGATAAACACTGCTCCAACTAAAATAAAAGGATACCCCTCTCCCAGGTCGTGGCXXXXXXXXXXA
GACAATCTAGAATCAGAACTTAACAAACCAGCAAAACGACTTAGAAAATCAAACTAATTCTGTCCTAACGCAGAGTGTT
>Marker1045858 Chr2 SNP_site:20594086 Ref_Type:C
GACTTTTCACTGGAACAGAACAAAGAAAACAACCGGTTTTATCTTTTGTGAGGGAGTTTTTTCAGAGCATTGACTATGAGXXXXXXXXXXC
CATAGTCTTTGATCAACACATTGCCTGGTCCCTCGATGTTAGGAACTTGTCAGTTGGTGCTGCTCTAATAGCAGTTGTG
GACTTTTCACTGGAACAGAACAAAGAAAACAACCGGTTTTATCTTTTGTGAGGGAGTTTTTTCAGAGCATTGACTATGAGXXXXXXXXXXC
CATCGTCTTTGATCAACACATTGCTTGGTCCCTCGATGTTAGGAACTTGTCAGTTGGTGCTGCTCTAATAGCAGTTGTG
>Marker1045858 Chr2 SNP_site:20594107 Ref_Type:T
GACTTTTCACTGGAACAGAACAAAGAAAACAACCGGTTTTATCTTTTGTGAGGGAGTTTTTTCAGAGCATTGACTATGAGXXXXXXXXXXC
CATAGTCTTTGATCAACACATTGCCTGGTCCCTCGATGTTAGGAACTTGTCAGTTGGTGCTGCTCTAATAGCAGTTGTG
GACTTTTCACTGGAACAGAACAAAGAAAACAACCGGTTTTATCTTTTGTGAGGGAGTTTTTTCAGAGCATTGACTATGAGXXXXXXXXXXC
CATCGTCTTTGATCAACACATTGCTTGGTCCCTCGATGTTAGGAACTTGTCAGTTGGTGCTGCTCTAATAGCAGTTGTG
>Marker1046248 Chr2 SNP_site:18553169 Ref_Type:A

Create PDF with GO2PDF for free, if you wish to

AACAAATCGTAAAAACTACTCTAAATATTGAACTAAAAGTTACAATCAATAAGCTGATGATTTTAGAAAGCAGAACTGAAXXXXXXXXXXT
TTGCACATACCTCAACTTAATCTTGCAAACATCTCATGTTTGATTCCACGTCTTTTACGATGTTAAATTTGAAGTAGTT
AACAAATCGTAAAAACTACTCTAAATATTGAACTAAAAGTTACAATCAATGAGCTGATGATTTTAGAAAGCAGAACTGAAXXXXXXXXXXT
TTGCACATACCTCAACTTAATCTTGCAAACATCTCATGTTTGATTCCACGTCTTTTACGATGTTAAATTTGAAGTAGTT
>Marker1046649 Chr2 SNP_site:4495421 Ref_Type:G
GACTTATTGTTAGCTGTTGTTCTAATGGTTCCGAGGTTACCTGTGATGTTGATGTTTTTTCTTCGGACATTTATGTTACTXXXXXXXXXXC
AGGAATCCTAGACTCGTCTTTACTCCGAGAGCCCACGTCTTACCAAGATGGGTATGTGAGATAGATTTTACATATTGTT
GACTTATTGTTAGCTGTTTTTCGAATGGTTCCGAGGTTACCTGTGATGTTGATGGTTTTTCTTCGGACATTTATGTTACTXXXXXXXXXXC
AGGAATCCTAGACTCGTCTTTACTCCGAGAGCCCACGTCTTACCAAGATGGGTATGTGAGATAGATTTTACATATTGTT
>Marker1046649 Chr2 SNP_site:4495425 Ref_Type:T
GACTTATTGTTAGCTGTTGTTCTAATGGTTCCGAGGTTACCTGTGATGTTGATGTTTTTTCTTCGGACATTTATGTTACTXXXXXXXXXXC
AGGAATCCTAGACTCGTCTTTACTCCGAGAGCCCACGTCTTACCAAGATGGGTATGTGAGATAGATTTTACATATTGTT
GACTTATTGTTAGCTGTTTTTCGAATGGTTCCGAGGTTACCTGTGATGTTGATGGTTTTTCTTCGGACATTTATGTTACTXXXXXXXXXXC
AGGAATCCTAGACTCGTCTTTACTCCGAGAGCCCACGTCTTACCAAGATGGGTATGTGAGATAGATTTTACATATTGTT
>Marker1046649 Chr2 SNP_site:4495457 Ref_Type:T
GACTTATTGTTAGCTGTTGTTCTAATGGTTCCGAGGTTACCTGTGATGTTGATGTTTTTTCTTCGGACATTTATGTTACTXXXXXXXXXXC
AGGAATCCTAGACTCGTCTTTACTCCGAGAGCCCACGTCTTACCAAGATGGGTATGTGAGATAGATTTTACATATTGTT
GACTTATTGTTAGCTGTTTTTCGAATGGTTCCGAGGTTACCTGTGATGTTGATGGTTTTTCTTCGGACATTTATGTTACTXXXXXXXXXXC
AGGAATCCTAGACTCGTCTTTACTCCGAGAGCCCACGTCTTACCAAGATGGGTATGTGAGATAGATTTTACATATTGTT
>Marker1046677 Chr2 SNP_site:10997727 Ref_Type:C
ACCTTAAGTGTTGTAGTTTTCTCACAACACAACAATACAATTCTCTCAATTTATTTTCAATCGTGATAAAAGTCAAAATAXXXXXXXXXXT
CTCCATTGATTATGAAATTAAACTTTCATCATTATGTATTTCAAACTTAAAAAAGGTATCACTCTAAAAAGAAAAGGTT
ACCTTAAGTGTTGTAGTTTTCTCACAACACAACAATACAATTCTCTCAATTTCTTTTCAATCGTGATAAAAGTCAAAATAXXXXXXXXXXT
CTCCATTGATTATGAAATTAAACTTTCATCATTATGTATTTCAAACTTAAAAAAGGCATCACTCTAAAAAGAAAAGGTT
>Marker1046677 Chr2 SNP_site:10997955 Ref_Type:C
ACCTTAAGTGTTGTAGTTTTCTCACAACACAACAATACAATTCTCTCAATTTATTTTCAATCGTGATAAAAGTCAAAATAXXXXXXXXXXT
CTCCATTGATTATGAAATTAAACTTTCATCATTATGTATTTCAAACTTAAAAAAGGTATCACTCTAAAAAGAAAAGGTT
ACCTTAAGTGTTGTAGTTTTCTCACAACACAACAATACAATTCTCTCAATTTCTTTTCAATCGTGATAAAAGTCAAAATAXXXXXXXXXXT
CTCCATTGATTATGAAATTAAACTTTCATCATTATGTATTTCAAACTTAAAAAAGGCATCACTCTAAAAAGAAAAGGTT
>Marker1046808 Chr2 SNP_site:13482917 Ref_Type:A
ACCATACATCGTTTTCTTGAACTCTTCTTCCGCTTTAGCCTGTTCATCTGCAGCGGCTGAACTTTGTTTCATAAGAGTTTXXXXXXXXXXG
CGGGTGCAAAAGACTTGACCAGGCTGTCCTCACTTACTACTTCAACGATACTGCCTCTACCTTTAACTGAGCCAAGGTA
ACCATACATCGTTTTCTTGAACTCTTCTTCCGCTTTAGCCTGTTCATCTGCAGCGGCTGAACTTTGTTTCATTAGAGTTTXXXXXXXXXXG
CGGGTGCAAAAGACTTGACCAGGCTGTCCTCACTTACTACTTCAACGATACTGCCTCTACCTTTAACTGAGCCAAGGTA
>Marker1046943 Chr2 SNP_site:21879751 Ref_Type:A
ACTCTTATATATTACGGCATAACTAAAGAGGGGAGAAAATATATAAAATACATAGTTGGGGTTTGGGAGGAGTTGATGGGXXXXXXXXXXA
TTTGGATAGAGTATGGAAATGAAATTGAAATTAAATGATCCGTAGGGGTGGAAGGGATCGAAACAAGAGAGGCTGCGTC
ACTCTTTTATATTACGGCATAACTAAAGAGGGGAGAAAATATATAAAATACATAGTTGGGGTTTGGGAGGAGTTGATGGGXXXXXXXXXXA
TTTGGATAGAGTATGGAAATGAAATTGAAATTAAATGATCCGTAGGGGTGGAAGGGATCGAAACAAGAGAGGCTGCGTC
>Marker1047116 Chr2 SNP_site:3030378 Ref_Type:A
ACAAAGTCTAGGGACATTTCTTGTAATTTAACCTATAGGTTAAATCATAAAAGTATTCTTTTTTATATGGAAATTCGAACXXXXXXXXXXT
TTTTATACTGTTTGACACATTACCAGTTGTCTGCTTTCTACACTCTACTCATGCGTGCTTGGCTGCAGATGGCAGCGTA
ACAAAGTCTAGGGACATTTCTTGTAATTTAACCTATAGGTTAAATCATAAAAGTATTCTTTTTTATATGGAAATTCGATCXXXXXXXXXXT
TTTTATACTGTTTGACACATTACCAGTTGTCTGCTTTCTACACTCTACTCATGCGTGCTTGGCTGCAGATGGCAGCGTA

Create PDF with GO2PDF for free, if you wish to

>Marker1047132 Chr2 SNP_site:19195020 Ref_Type:G
AACGAGCCTGTTAAATGTCCTCTCATATTTGGAGGTAAAACTTAAACAGTTTTGCTGTTTTTTGTTTGATATATTTGAATXXXXXXXXXXG
ATGTTTGCTGAAAAGGAAATGGGGGAAAAACCGATACAGTCCTTCACTCCTGATTTATGAAAAGGTTAAATTAGCTGTT
AACGAGCCTGTTAAATGTCCTCTCATATTTGGAGGTAAAACTTAAACAGTTTTGCTGTTTTTTGTTTGATATATTTGAATXXXXXXXXXXG
ATGTTTGCTGAAAAGGAAATGGGGGAAAAACCGATACAGTCCTTCACTCCTGATTTATGAGAAGGTTAAACTAGCTGTT
>Marker1047132 Chr2 SNP_site:19195030 Ref_Type:C
AACGAGCCTGTTAAATGTCCTCTCATATTTGGAGGTAAAACTTAAACAGTTTTGCTGTTTTTTGTTTGATATATTTGAATXXXXXXXXXXG
ATGTTTGCTGAAAAGGAAATGGGGGAAAAACCGATACAGTCCTTCACTCCTGATTTATGAAAAGGTTAAATTAGCTGTT
AACGAGCCTGTTAAATGTCCTCTCATATTTGGAGGTAAAACTTAAACAGTTTTGCTGTTTTTTGTTTGATATATTTGAATXXXXXXXXXXG
ATGTTTGCTGAAAAGGAAATGGGGGAAAAACCGATACAGTCCTTCACTCCTGATTTATGAGAAGGTTAAACTAGCTGTT
>Marker1047871 Chr2 SNP_site:12686888 Ref_Type:C
TACTAATTTGATAACTCTCGATAGCAAATTGAGACTTTTGAAATAGTGTTTAGTGTAGCTAATTGAAAACTATTTAAATAXXXXXXXXXXG
AGCTTAACACAATGTAATTGTTATGACATTTCCATCTTATAAGTCAAAGGTTCAAATACCCCAATTCATAAAAGCTTGT
TACTAATTTGATAACTCTTGATAGCAAATTGAGACTTTTGAAATAGTGTTTAGTGTAGCTAATTGAAAACTATTTAAACAXXXXXXXXXXG
AGCTTAACACAATGTAATTGTTATGACATTTCCATCTTATAAGTCAAAGGTTTAAATACCCCAATTCATAAAAGCTTGT
>Marker1047871 Chr2 SNP_site:12686948 Ref_Type:T
TACTAATTTGATAACTCTCGATAGCAAATTGAGACTTTTGAAATAGTGTTTAGTGTAGCTAATTGAAAACTATTTAAATAXXXXXXXXXXG
AGCTTAACACAATGTAATTGTTATGACATTTCCATCTTATAAGTCAAAGGTTCAAATACCCCAATTCATAAAAGCTTGT
TACTAATTTGATAACTCTTGATAGCAAATTGAGACTTTTGAAATAGTGTTTAGTGTAGCTAATTGAAAACTATTTAAACAXXXXXXXXXXG
AGCTTAACACAATGTAATTGTTATGACATTTCCATCTTATAAGTCAAAGGTTTAAATACCCCAATTCATAAAAGCTTGT
>Marker1047871 Chr2 SNP_site:12687104 Ref_Type:C
TACTAATTTGATAACTCTCGATAGCAAATTGAGACTTTTGAAATAGTGTTTAGTGTAGCTAATTGAAAACTATTTAAATAXXXXXXXXXXG
AGCTTAACACAATGTAATTGTTATGACATTTCCATCTTATAAGTCAAAGGTTCAAATACCCCAATTCATAAAAGCTTGT
TACTAATTTGATAACTCTTGATAGCAAATTGAGACTTTTGAAATAGTGTTTAGTGTAGCTAATTGAAAACTATTTAAACAXXXXXXXXXXG
AGCTTAACACAATGTAATTGTTATGACATTTCCATCTTATAAGTCAAAGGTTTAAATACCCCAATTCATAAAAGCTTGT
>Marker1048205 Chr2 SNP_site:3250622 Ref_Type:G
AACCAAAAGAACTCTTTAATAAGCCATCTACTAAATCTAAGCAACAAATCAGATTAAAAAACAACGAGAAATTAACGAAAXXXXXXXXXXA
AAAAAAAAAAATCAAGATTTACGGTTTGTTATATTGCTATCTCGCCATGGAATGCGTTGCATCGATTTTCAATCATGTA
AACCAAAAGAACTCTTTAATAAGCCATCTACTAAATCTAAGCAACAAATCAGATTAAAAAACAACGAGAAATTAACGAAAXXXXXXXXXXA
AAAAAAAAAAATCAAGATTTACGTTTTGTTATATTGCTATCTCGCCATGGAATGCGTTGCATCGATTTTCAATCATGTA
>Marker1048282 Chr2 SNP_site:17063713 Ref_Type:C
AACAGATCCTCCATGGATCAGAAAACAGCTCGAGTTGCTCGACACCCAAATGAAAAAAAAAGATGGTGACGGTCATTTAGXXXXXXXXXXG
CCTGTATATACGTAATCTTCATAGAGACAAACCACTAATGCAGAAAGGTCCGATGAAAGCAAATTGGTAGATTGAAGGT
AACAGATCCTCCATGGATCAGAAAACAGCTCGAGTTGCTCGACACCCAAATGAAAAAAAAAGATGGTGACGGTCATTTAGXXXXXXXXXXG
CCTGTATATATGTAATCTTCATAGAGACAAACCACTAATGCAGAAAGGTCCGATGAAAGCAAATTGGTAGATTGAAGGT
>Marker1048738 Chr2 SNP_site:16469470 Ref_Type:G
GACAAGAATAAGATACAGCAACCTTCAAAATTGTTAAGTGTTAATTAGTTGATTTTTGTATATTTATATACGATGTGAGTXXXXXXXXXXA
AAACATGTTACCACTTCATTTTGATGTTGCAAGGCCTCCACAAGCATTCTCTTATGAGAAGGTTCAACCCTGAAAAGTG
GACAAGAATAAGATACAGCAACCTTCAAAATTGTTAAGTGTTAATTAGTTGATTTTTGTATATTTATATACGATGTGAGTXXXXXXXXXXA
AAACGTGTTACCACTTCATTTTGATGTTGCAAGGCCTCCACAAGCATTCTCTTATGAGAAGGTTCAACCCTGAAAAGTG
>Marker1048808 Chr2 SNP_site:7763135 Ref_Type:A
CACATTACGCAGCAAAGGAACAAGACAACATAACTTGTTGAGGAAAGAAACATGTTATTACTAAATAATTGTTTTTTATTXXXXXXXXXXA
AACAAGAAGAGCCCTCCAACCGTTAATAATCATAAGACTAATTACAAGGGAAACCCTTGTTTTTAGTTGAACTAAGAGT
CACATTACGCAGCAAAGGAACAAGACAACATAACTTGTTGAGGAAAGAAACATGTTATTACTAAATAATTGTTTTTTATTXXXXXXXXXXA

Create PDF with GO2PDF for free, if you wish to

AACAAGAAGAGCCCTCCAACCGTTAATAATCATAAGACTGATTACAAGGGAAACCCTTGTTTTTAGTTGAACTAAGAGT
>Marker1048816 Chr2 SNP_site:21119224 Ref_Type:T
ACCTAAAGTTTAGGTCTTACAACTAGCTTATTTCAGACAAAACAACTAAATCATTTACAAAGTTAGTTACAACCCAAAAAXXXXXXXXXXXT
GAAGGGTAAGCTATAAAGCCTTGTGAGTGACAACTTTTAAAAACAAAACATGTTTATTGAAAACTGTGAGCAATAAGTG
ACCTAAAGTTTAGGTCTTACAACTAGCTTATTTCAGACAAAACAACTAAATCTTTTACTAAGTTAGTTACAACCCAAAAAXXXXXXXXXXXT
GAAGGGTAAGCTATAAAGCCTTGTGAGTGACAACTTTTAAAAACAAAACATGTTTATTGAAAACTGTGAGCAATAAGTG
>Marker1048816 Chr2 SNP_site:21119230 Ref_Type:T
ACCTAAAGTTTAGGTCTTACAACTAGCTTATTTCAGACAAAACAACTAAATCATTTACAAAGTTAGTTACAACCCAAAAAXXXXXXXXXXXT
GAAGGGTAAGCTATAAAGCCTTGTGAGTGACAACTTTTAAAAACAAAACATGTTTATTGAAAACTGTGAGCAATAAGTG
ACCTAAAGTTTAGGTCTTACAACTAGCTTATTTCAGACAAAACAACTAAATCTTTTACTAAGTTAGTTACAACCCAAAAAXXXXXXXXXXXT
GAAGGGTAAGCTATAAAGCCTTGTGAGTGACAACTTTTAAAAACAAAACATGTTTATTGAAAACTGTGAGCAATAAGTG
>Marker104898 Chr4 SNP_site:6321853 Ref_Type:C
ACCCCATCATGTCTATGATTTTGTCAGCAATTTAGAGGCTCTCTACTTTGACATAAACAGAAAATTCACAAGAATTTCCAXXXXXXXXXXXG
AATGAAAAGATAATGAAGAGTCTGTCAAGATTAGTATTGAGGTTCTTACTATCAAATCATTAAATAGTGAATAATTGGT
ACCCCATCATGTCTATGATTTTGTCAGCAATTTAGAGGCTCTCTACTTTGACATAAACAGAAAATTGACAAGAATTTCCAXXXXXXXXXXXG
AATGAAAAGATAATGAAGAGTCTGTCAAGATTAGTATTGAGGTTCTAACTATCAAATCATTAAATAGTGAATAATTGGT
>Marker104898 Chr4 SNP_site:6322017 Ref_Type:T
ACCCCATCATGTCTATGATTTTGTCAGCAATTTAGAGGCTCTCTACTTTGACATAAACAGAAAATTCACAAGAATTTCCAXXXXXXXXXXXG
AATGAAAAGATAATGAAGAGTCTGTCAAGATTAGTATTGAGGTTCTTACTATCAAATCATTAAATAGTGAATAATTGGT
ACCCCATCATGTCTATGATTTTGTCAGCAATTTAGAGGCTCTCTACTTTGACATAAACAGAAAATTGACAAGAATTTCCAXXXXXXXXXXXG
AATGAAAAGATAATGAAGAGTCTGTCAAGATTAGTATTGAGGTTCTAACTATCAAATCATTAAATAGTGAATAATTGGT
>Marker10491 Chr4 SNP_site:19302168 Ref_Type:G
ACAAGAAGTTTGAGTCAGTTATGGGGGAGTAAGGCTACACAAAAACTATTTCTGGTCACTGTGTTTTGTCCATAATTATTXXXXXXXXXXXT
AAAGAAAGTTCTTGGAATTTGAATAGTTCGAGATAAAACATCCAAGAAGTTATACATGTCACATGACCAAAAAGAAAGT
ACAAGAAGTTTGAGTCAGTTATGGGGGAGTAAGGCTACACAAAAACTATTTCTGGTCACTGTGTTTTGTCCATAATTATTXXXXXXXXXXXT
AAAGAAAGTTCTTGGAATTTGAATAGTTCGAGATAAAACATCCAAGAAGTTATACATGTCACATGACCAAAGAGAAAGT
>Marker104999 Chr4 SNP_site:14827869 Ref_Type:A
AACTAAATCGATGATCTGAATGGTTTCTTTCTTTTCTGGCGTGACATGATCTCCTAGAAGATCTAAGCGACCGAAACGAAXXXXXXXXXXXC
CCCTCTGGGCAAGATAAATTAAATACATTGCAAGAATCTTCCAAAACATCTGTAGTTTCTTGAACAGTATGTGAAGGTG
AACTAAATCGATGATCTGAATGGTTTCTTTCTTTTCTGGCGTGACATGATCTCCTAGAAGATCTAAGCGACCGAAACGAAXXXXXXXXXXXC
CCCTCTGGGCGAGATAAATTAAATACATTGCAAGAATCTTCCAAAACATCTGTAGTTTCTTGAACAGTATGTGAAGGTG
>Marker1050122 Chr2 SNP_site:375364 Ref_Type:C
CACAATGCTTTATCTTATAAAAATAATATTTTGATTGCATCCAACAGGTGGTGTTGCTAAAAGACCTTGCCCCTACAGGTXXXXXXXXXXXG
GCCTCCTCATCCTTTTAGCTCTAGTGTTGAGTGTTGCACGTATGAAATATGTTGGTGGCGATGAATTACCAGCCACGGT
CACAATGCTTTATCTTATAAAAATAATATTTTGATTGCATCCAACAGGTGGTGTTGCTAAAAGACCTTGCCCCTACAGGTXXXXXXXXXXXG
GTCTCCTCATCCTTTTAGCTCTAGTGTTGAGTGTTGCACGTATGAAATATGTTGGTGGCGATGAATTACCAGCCACGGT
>Marker1050291 Chr2 SNP_site:10919550 Ref_Type:G
AACCTAAAGGAAATGTAGGCAGCCATGTTTGATCATCTATGCGTTCAGATTGGAGTAAGGGCAAGATGGCTAACTTCTAGXXXXXXXXXXXT
TAAGAATCTTTTAACTTTAGTGGAAGACATGTGACAATTTAAGGTCAAGCATGGAGTTAGTGTCCTAAAAAATAGGTGT
AACCTAAAGGAAATGTAGGCAGCCATGTTTGATCGTCTATGCGTTCAGATTGGAGTAAGGGCAAGATGGCTAACTTCTAGXXXXXXXXXXXT
TAAGAATCTTTTAACTTTAGTGGAAGACATGTGACAATTTAAGGTCAAGCATGGAGTTAGTGTCCTAAAAAATAGGTGT
>Marker1050448 Chr2 SNP_site:21908007 Ref_Type:C
TACATCTTCAATTTATTATATCACCTTCTTATTAGGGATGAACAACTTGAATTTAGAACGAGATAATATCCTGCGATAAGXXXXXXXXXXXT
CTTACATTTCTATGTGGTAAGGCTTCATTATTTTACATAAAAAGCCATAATAGCTTGTATTTCTACAAGTTATCAGAGT

Create PDF with GO2PDF for free, if you wish to

TACATCTTCAATTTATTATATCACCTTCTTATTAGGGATGAACAACTTGAATTTAGAACGAGATAATATCCTGCGATAAGXXXXXXXXXXT
CTTACATTTCTATGTGGTAAGGCTTCATTATTTTACATAAAAAGCCATAATAGCTTGTATTTCTACCAGTTATCAGAGT
>Marker1050540 Chr2 SNP_site:3373857 Ref_Type:C
CACTTTTCGTAGTTAAGTCAATGGTTATATCAATCTTGTCTATTTCAATCAACTCATTCAATATAGTTTCCTATTGAGCAXXXXXXXXXXT
TCTTTTATTCACTAGTAGCTGAATCTCTCTAACCTGGTTGATGAAAGGAAACTGAGTGGGAAGAAAAGAAAAAAAGGTT
CACTTTTCGTAGTTAAGTCAATGGTTATATCAATCTTGTCTATTTCAATCAACTCATTCAATATAGTTTCGTATTGAGCAXXXXXXXXXXT
TCTTTTATTCACTAGTAGCTGAATCTCTCTAACCTGGTTGATGAAAGGAAACTGAGTGGGAAGAAAAGAAAAAAAGGTT
>Marker1050568 Chr2 SNP_site:22090014 Ref_Type:A
ACGGTTGCGAGCATAACGATCTTAAAAATTTACACTACCTAGAGAGCCACTAGGGTTTTATTACTTTCTTAGTGTAGATTXXXXXXXXXXT
CACATTGAGAGCAATGTAATATAAGTTGGGGGTGAGGTATATCTTGTGTTCTGGCTAAAGTCCTCGAGCATCTAAGGTC
ACGGTTGCGAGCATAACGATCTTAAAAATTTACGCTACCTAGAGAGCCACTAGGGTTTTATTACTTTCTTAGTGTAGATTXXXXXXXXXXT
CACATTGAGAGCAACGTAATATAAGTTGGGGGTGAGGTATATCTTGTGTTCTGGCTAAAGTCCTCGAGCATCTAAGGTC
>Marker1050568 Chr2 SNP_site:22090214 Ref_Type:T
ACGGTTGCGAGCATAACGATCTTAAAAATTTACACTACCTAGAGAGCCACTAGGGTTTTATTACTTTCTTAGTGTAGATTXXXXXXXXXXT
CACATTGAGAGCAATGTAATATAAGTTGGGGGTGAGGTATATCTTGTGTTCTGGCTAAAGTCCTCGAGCATCTAAGGTC
ACGGTTGCGAGCATAACGATCTTAAAAATTTACGCTACCTAGAGAGCCACTAGGGTTTTATTACTTTCTTAGTGTAGATTXXXXXXXXXXT
CACATTGAGAGCAACGTAATATAAGTTGGGGGTGAGGTATATCTTGTGTTCTGGCTAAAGTCCTCGAGCATCTAAGGTC
>Marker1050643 Chr2 SNP_site:20139886 Ref_Type:G
ACTGACCTTTTTTGTTTTTGCTATCAGATGTCATGTTGGATCCTCTTCTTTCACTTAGAAAAGATCTTAAAGGTTTGGAAXXXXXXXXXXA
GCAGTTGCTCTAAGAAATGTTTGTGGAGCCAATGGTAAGTCTAATTCATCTTGCAAAATGTTATTGGGTGTCAAACGTA
ACTGACCTTTTTTGTTTTTGCTATCAGATGTCGTGTTGGATCCTCTTCTTTCACTTAGAAAAGATCTTAAAGGTTTGGGAXXXXXXXXXXA
GCAGTTGCTCTAAGAAATGTTTGTGGAGCCAATGGTAACTCTAATTCATCTTGCAAAATGTTATTGGGTGTCAAACGTA
>Marker1050643 Chr2 SNP_site:20139932 Ref_Type:G
ACTGACCTTTTTTGTTTTTGCTATCAGATGTCATGTTGGATCCTCTTCTTTCACTTAGAAAAGATCTTAAAGGTTTGGAAXXXXXXXXXXA
GCAGTTGCTCTAAGAAATGTTTGTGGAGCCAATGGTAAGTCTAATTCATCTTGCAAAATGTTATTGGGTGTCAAACGTA
ACTGACCTTTTTTGTTTTTGCTATCAGATGTCGTGTTGGATCCTCTTCTTTCACTTAGAAAAGATCTTAAAGGTTTGGGAXXXXXXXXXXA
GCAGTTGCTCTAAGAAATGTTTGTGGAGCCAATGGTAACTCTAATTCATCTTGCAAAATGTTATTGGGTGTCAAACGTA
>Marker1050643 Chr2 SNP_site:20140107 Ref_Type:C
ACTGACCTTTTTTGTTTTTGCTATCAGATGTCATGTTGGATCCTCTTCTTTCACTTAGAAAAGATCTTAAAGGTTTGGAAXXXXXXXXXXA
GCAGTTGCTCTAAGAAATGTTTGTGGAGCCAATGGTAAGTCTAATTCATCTTGCAAAATGTTATTGGGTGTCAAACGTA
ACTGACCTTTTTTGTTTTTGCTATCAGATGTCGTGTTGGATCCTCTTCTTTCACTTAGAAAAGATCTTAAAGGTTTGGGAXXXXXXXXXXA
GCAGTTGCTCTAAGAAATGTTTGTGGAGCCAATGGTAACTCTAATTCATCTTGCAAAATGTTATTGGGTGTCAAACGTA
>Marker1050856 Chr2 SNP_site:2391375 Ref_Type:G
CACATGCTAAGCTTGGGAGGCAGGATGTTCTTCATTGTTTTCAAATCAAAAGTGAAACAAGAGTTTTTGAAATGGAACTTXXXXXXXXXXT
AAAATTATTGATCTCTTCCTTTCTATGTGATGGGTATTGTTTCCTAAACAAGAGTATGGCTTGTAATGAAGATGAGGTG
CACATGCTAGGCTTGGGAGGCAGGATGTTCTTCATTGTTTTCAAATCAAAAGTGAAACAAGAGTTTTTGAAATGGAACTTXXXXXXXXXXT
AAAATTATTGATCTCTTCCTTTCTATGTGATGGGTATTGTTTCCTAAACAAGAGTATGGCTTGTAATGAAGATGAGGTG
>Marker1051190 Chr2 SNP_site:441902 Ref_Type:A
AACCAATCAGCATAAATTTATAATTACTCTTCTCTATTTTATCTGTTGATTTTTGCAGCGGGCTCCTGTATAACATGAGCXXXXXXXXXXA
CACTTCTCCACAACACTTTAGAGCTTAATCCTGCCAAAGACCTAAAAGCAACAATTTAGAGTGTATGTATTCTTGTAGT
AACCAATCAGCATAAATTTATAATTACTCTTCTCTATTTTATCTGTTGATTTTTGCAGCGGGCTCCTGTATAACATGAGCXXXXXXXXXXA
CACTTCTCCACAACACTTTAGAGCTTAATCCTGCCAAAGACCTAAAAGCAACAATTTTGAGTGTATGTATTCTTGTAGT
>Marker1051888 Chr2 SNP_site:1796126 Ref_Type:T
TACTTTAGCTGATTATTATCTGTTATTTCATATTTGTTTATTAATTTCCCAATAGTTTTATTACATAATTTAGTAATACTXXXXXXXXXXA

Create PDF with GO2PDF for free, if you wish to

TTATAATAAGGTGAGTTTTTAATTAAAGTTGTGCTAATTGAAGATGGATATATTAATTAACATGCCTTCAAAAATTGTC
TACTTTAGTTGATTATTATCTGTTATTTCATATTTGTTTATTAATTTCCCAATAGTTTTATTACATAATTTAGTAATACTXXXXXXXXXXA
TTATAATAAGGTGAGTTTTTAATTAAAGTTGTGCTAATTGAAGATGGATATATTAATTAACATGCCTTCAAAAATTGTC
>Marker1052937 Chr2 SNP_site:4377296 Ref_Type:C
AACTAAATTCAAAGCACCTCATTTCTCTCAGATTTAAATTGTGCTTTTTAGTTGAGTTTCCTCTACACACAACTAACTTTXXXXXXXXXXA
TTGTTCGTTTCTTAGTCTCGCATGCAACTAAAAGGAGTTGTTTTGTTATTTGTAAGGAATATAAAAACATGACAATTGT
AACTAAATTTAAAGCACCTCATTTCTCTCAGATTTAAATTGTGCTTTTTAGTTGAGTTTCCTCTACACACAACTAACTTTXXXXXXXXXXA
TTGTTCGTTTCTTAGTCTCGCATGCAACTAAAAGGAGTTGTTTTGTTATTTGTAAGGAATATAAAAACATGACAATTGT
>Marker1053604 Chr2 SNP_site:296046 Ref_Type:T
AACCCATAATAATTGACTTTGCTCAAGCTTATGATCCGAAAAATGTGACACAAGATGTATGACTCGACACTAGATGATGGXXXXXXXXXXG
TGTGGCAATCTTAACCATTTTGTATATGATCTTCAATCAGTTTGCAATCAAAAGGGTCCCCAATAAAGTCTTAGCTGTA
AACCCATAATAATTGACTTTGCTCAAGCTTATGATCCGAAAAATGTGACACAAGATGTATGACTCGACACTAGATGATTGXXXXXXXXXXG
TGTGGCAATCTTAACCATTTTGTATATGATCCTCAATCAGTTTGCAATCAAAAGGGTCCCCAATAAAGTCTTAGCTGTA
>Marker1053604 Chr2 SNP_site:296216 Ref_Type:C
AACCCATAATAATTGACTTTGCTCAAGCTTATGATCCGAAAAATGTGACACAAGATGTATGACTCGACACTAGATGATGGXXXXXXXXXXG
TGTGGCAATCTTAACCATTTTGTATATGATCTTCAATCAGTTTGCAATCAAAAGGGTCCCCAATAAAGTCTTAGCTGTA
AACCCATAATAATTGACTTTGCTCAAGCTTATGATCCGAAAAATGTGACACAAGATGTATGACTCGACACTAGATGATTGXXXXXXXXXXG
TGTGGCAATCTTAACCATTTTGTATATGATCCTCAATCAGTTTGCAATCAAAAGGGTCCCCAATAAAGTCTTAGCTGTA
>Marker1053673 Chr2 SNP_site:19801816 Ref_Type:T
ACCAATAAAAAATATATATAAGGCATATATACCTTAGAAATACTCAACCTTTAGTGCTATAAACTTTCTTCATAGGGTCAXXXXXXXXXXT
TGAAAACTCCAACTTGTTTAAGATTATCTGCTCTGGTTTCTAAATTTGGTAATAAGAAAATGTGTTATGGTTTTTAGTT
ACCAATAAAAAATATATATAAGGTATATATACCTTAGAAATACTCAACCTTTAGTGCTATAAACTTTCTTCATAGGGTCAXXXXXXXXXXT
TGAAAACTCCAACTTGTTTAAGATTATCTGCTCTGGTTTCTAAATTTGGTAATAAGAAAATGTGTTATGGTTTTTAGTT
>Marker1053846 Chr2 SNP_site:18958022 Ref_Type:A
ACAGCTCCGGTATCAGTCAAAAAACATGAAAAACAGAGTATAACTTCAACTTCTAAACTTCTTGGGTATTTTTTTTAGCAXXXXXXXXXXA
TATTTACATGGATGCATGCAAAATAGTATCAACAATGAGTTACCCTCAGGATAGTAATGACAACGTTAAGTTTTAAAGT
ACAGCTCCGGTATCAGTCAAAAAACATGAAAAACAGAGTATAACTTCAACTTCTAAACTTCTTGGGTATTTTTTTTAGCAXXXXXXXXXXA
TATTTACATGGATGCATGCAAAATAGTATCAACAATGAGTTACCCTCAGGATAGTAATGACAACGTTAAGTTTTGAAGT
>Marker1053917 Chr2 SNP_site:13278841 Ref_Type:T
TACCATCATTAAGCCATTATAATACATTGAAATGAACCTATCTGCTTGCCTTTTCACCTGTGACATTAAAAATTTGTGATXXXXXXXXXXG
CAATACCAAATTCAATCCAAAATAAATTCAGGAGCCTTCTTGCTGTAAAACCACATATGCATGTTCTAGAAAGAGCAGT
TACCATCATTAAGCCATTATAATACATTGAAATGAACCTATCTGCTTGCCTTTTCACCTGTGACATTAAAAATTTGTGATXXXXXXXXXXG
CAATACCATATTCAATCCAAAATAAATTCAGGAGCCTTCTTGCTGTAAAACCACATATGCATGTTCTAGAAAGAGCAGT
>Marker105396 Chr4 SNP_site:10067623 Ref_Type:G
ACATTAATTAATTTGTATAAAAAATATTATTATGTAGCATGGGAATGTGCAACTCGGAACCACTAAATATTCTCATAGATXXXXXXXXXXA
CGACCCACCTTGAAGTGAGTATGGAAGAACCTTTTATCCTTCATTTGACAAAACACAATTAAGTTAATGATCCAATGTA
ACATTAATTAATTTGTATAAAAAATATTATTATGTAGCATGGGAATGTGCAACTCGGAACCACTAAATATTCTCATAGATXXXXXXXXXXA
CGACCCACCTTGAAGTGAGTATGGAAGAACCTTTTATCCTTCATTTTACAAAACACAATTAAGTTAATGATCCAATGTA
>Marker1054132 Chr2 SNP_site:1103325 Ref_Type:T
GACCAGTTCATTAGTCAAGTTTTTGGAATTTTATTTGGACCACTGCAGCCATGGCATCCATTACATGACTGCTGGTATTTXXXXXXXXXXT
AGTGAAATGTATTGTCCAAACTATAAGTAAGAGCAAAGTCTTAGACCTTTTTTAAAAAAGGAAATCTGGCTGAGTTAGTT
GACCAGTTCATTAGTCAAGTTTTTGGAATTTTATTTGGACCACTGCAGCCATGGCATCCATTACATGACTGCTGGTATTTXXXXXXXXXXT
AGTGAAATGTATTGTCCAAACTATAAGTAAGAGCAAAGTCTTAGACCTTTTTTAAAAAAGGAAATCTGGCTGATTTAGTT
>Marker1054392 Chr2 SNP_site:300579 Ref_Type:T

Create PDF with GO2PDF for free, if you wish to

TACTCATAAAGGTTGGTTTTACTTTTCCCCATTTCTCTACTCATTAAAAAGTAAATGTTTAAACTTTTTTTAAGGGGTTTXXXXXXXXXXT
TGTATAGTTGAATGGTGTATGGGGATGTGCTTCATATGGTTATAATCCCACCTTCCCCTAATAATTCTGTGTGCTGGTA
TACTCATAAAGTTTGGTTTTACTTTTCCCCATTTCTCTACTCATTAAAAAGTAAATGTTTAAACTTTTTTTAAGGGGTTTXXXXXXXXXXT
TGTATAGTTGAATGGTGTATGGGGATGTGCTTCATATGGTTATAATCCCACCTTCCCCTAATAATTCTGTGTGCTGGTA
>Marker1054527 Chr2 SNP_site:4627608 Ref_Type:G
ACTCTTTAACTTAGACATAGTAAATGTTAGCAACTTTAATTTTAAAACTTTGATTTGAAAGATATCCGTCAATATAAATAXXXXXXXXXXA
TAAAAAAATGTTTAGGGTTTCATTTTAATATTGATCCCTAAACTTTCAATAATTGAAGAGGGTCATCAATATCATGGTA
ACTCTTTAACTTAGACATAGTAAATGTTAGCAACTTTAATTTTAAAACTTTGATTTGAAAGATATCCGTCAATATAAATAXXXXXXXXXXA
TAAAAAAATGTTTAGGGTTTCATTTTAATATTGGTCCCTAAACTTTCAATAATTCAAGAGGGTCATCAATATCATGGTA
>Marker1054527 Chr2 SNP_site:4627629 Ref_Type:C
ACTCTTTAACTTAGACATAGTAAATGTTAGCAACTTTAATTTTAAAACTTTGATTTGAAAGATATCCGTCAATATAAATAXXXXXXXXXXA
TAAAAAAATGTTTAGGGTTTCATTTTAATATTGATCCCTAAACTTTCAATAATTGAAGAGGGTCATCAATATCATGGTA
ACTCTTTAACTTAGACATAGTAAATGTTAGCAACTTTAATTTTAAAACTTTGATTTGAAAGATATCCGTCAATATAAATAXXXXXXXXXXA
TAAAAAAATGTTTAGGGTTTCATTTTAATATTGGTCCCTAAACTTTCAATAATTCAAGAGGGTCATCAATATCATGGTA
>Marker1054704 Chr2 SNP_site:13579524 Ref_Type:T
CACTAAGTTCAGTAATTCTTCATTTTTCAGATATTAATGATCAGGCTTAGTTTCCATTGCCATCTTCAGTGTTCCACTCTXXXXXXXXXXC
AAACGCAGTCCATGTTCCTTGCATCTGCGAAGACTAGCTGTTTTTCTATTCCTCAGGTGTTCCTTAAGCTTCATACGTA
CACTAAGTTCAGTAATTCTTCATTTTTCAGATATTAATGATCAGGCTTAGTTTCCATTGCCATCTTCAGTGTTCCACTCTXXXXXXXXXXC
AAACGCAGTCCATGTTCCTTGCATCTGCGAAGACTAGCTGTTTTTCTATTCCTCAGGTGTTCCTTAAGCTTCATATGTA
>Marker105524 Chr4 SNP_site:4717001 Ref_Type:G
AACCGTAGGCTGAGCGAAGCGGTTCCCGACCTGCACCAAGCGAATTAGAGACCGCCGGTGTTATAACCGAAAAGAAACAAXXXXXXXXXXA
AGAAGATGACAACACGTGGCATGGTGACCAAGATAGGCTCATCGATCCAACGGGTGTTATAGGATGAAAATTAGGGGTC
AACCGTAGGCTGAGCGAAGCGGTTCCCGACCTGCACCAAGCGAATTAGAGACCGCCGGTGTTATAACCGAAAAGAAACAAXXXXXXXXXXA
AGAAGATGAGAACACGTGGCATGGTGACCAAGATAGGCTCATCGATCCAACGGGTGTTATAGGATGAAAATTAGGGGTC
>Marker1055698 Chr2 SNP_site:10950078 Ref_Type:T
CACCATCAAGGCTAACCTTCACAAACCTAATCTTCTTCTTCATCACATTCTTCTTTTGGTCTTCATCACATTCTTCCTCTXXXXXXXXXXT
TCTCGTATCAACGTAATCAACTTAGATTGGTGATACTTTTATCTCGTAGTTAATTATTAATTTTATTTCAATATTTGTG
CACCATCAAGGCTAACCTTCACAAACCTAATCTTCTTCTTCATCACATTCTTCTTTTGGTCTTCATCATATTCTTCCTCTXXXXXXXXXXT
TCTCGTATCAACGTAATCAACTTAGATTGGTCATACTTTTATCTCGTAGTTAATTATTAATTTTATTTCAATATTTGTG
>Marker1055698 Chr2 SNP_site:10950271 Ref_Type:G
CACCATCAAGGCTAACCTTCACAAACCTAATCTTCTTCTTCATCACATTCTTCTTTTGGTCTTCATCACATTCTTCCTCTXXXXXXXXXXT
TCTCGTATCAACGTAATCAACTTAGATTGGTGATACTTTTATCTCGTAGTTAATTATTAATTTTATTTCAATATTTGTG
CACCATCAAGGCTAACCTTCACAAACCTAATCTTCTTCTTCATCACATTCTTCTTTTGGTCTTCATCATATTCTTCCTCTXXXXXXXXXXT
TCTCGTATCAACGTAATCAACTTAGATTGGTCATACTTTTATCTCGTAGTTAATTATTAATTTTATTTCAATATTTGTG
>Marker1055921 Chr2 SNP_site:3015771 Ref_Type:G
ACCAGATCCAGTTGCAGCAAGAACCAGACAATCTTGGTGATTTAACCATGCTTCTAGAGCTTCCTTTTGGAACTTCTTCAXXXXXXXXXXC
TGAGTATCTTCCACGGGATAATGGGACACTTCTGGTTCAGAAAATGGAACAACTGGTTCAGCTTCAGTCTTACTCCGTT
ACCAGATCCAGTTGCAGCAAGAACCAGACAATCTTGGTGATTTAACCATGCTTCTAGAGCTTCCTTTTGGAACTTCTTCAXXXXXXXXXXC
TGAGTATCTTCCACGGGATAGTGGGACACTTCTGGTTCAGAAAATGGAACAACTGGTTCAGCTTCAGTCTTACTCCGTT
>Marker1055984 Chr2 SNP_site:20492352 Ref_Type:T
AACCAAATGTGCCCAATATAGCTCTTTTTTATCGTTAAATAACTTAGCATAGATGTGGTTTGGTTTGAATTTCCTACATAXXXXXXXXXXT
GTTATACATTTGATACTTTTCTAATAGGCATTTCATACGTAGTTGATACACATGTTATACGTTTAATATACACTTTGTC
TACCAAATGTGCCCAATATAGCTCTTTTTTATCGTTAAATAACTTAGCATAGATGTGGTTTGGTTTGAATTTCCTACATAXXXXXXXXXXT
GTTATACATTTGATACTTTTCTAATAGGCATTTCATACGTAGTTGATACACATGTTATACGTTTAATATACACTTTGTC

Create PDF with GO2PDF for free, if you wish to

>Marker1056430 Chr2 SNP_site:16028599 Ref_Type:G
AACTTGAAGATGCTCCATCTCTTGAAAGACATGTTGAGAATCCCACGTTGGAAAAATCAAAGGACTCACAATCTTTATAAXXXXXXXXXXXC
AATGAACAGTTCTGGTGTCTTTATTTATTTATTTTAATTATATATCAACAGAAAAGGGCAGAGAAAATGAAAGCTGGTG
AACTTGAAGATGCTCCATCTCTTGAAAGACATGTTGAGAATCCCACGTTGGAAAAATCAAAGGACTCACAATCTTTATAAXXXXXXXXXXXC
AATGAACAGTTCTGGTGTCTTTATTTATTTATTTTAATTATATATGAACAGAAAAGGGCAGAGAAAATGAAAGCTGGTG
>Marker1056657 Chr2 SNP_site:18962450 Ref_Type:T
AACTTACCTCGTCAAATTCTATGCTTCATAAGAAACTATAAACATAAACTCACAAAGGAGCTCCAGAACCAGTTTTTGCAXXXXXXXXXXXT
TATTGAGGTGGGAAGCCCTTCCGGCAATGGAAGCACATAGTTCAAGAATTATTTGGCTACACAGAAGGATGCACAAGGT
AACTTACCTCGTCAAATTCTATGCTTCATAAGAAACTATATACATAAACTCACAAAGGAGCTCCAGAACCAGTTTTTGCAXXXXXXXXXXXT
TATTGAGGTGGGAAGCCCTTCCGGAAATGGAAGCACATAGTTCAAGAATTATTTGGCTACACAGAAGGATGCACAAGGT
>Marker1056657 Chr2 SNP_site:18962605 Ref_Type:A
AACTTACCTCGTCAAATTCTATGCTTCATAAGAAACTATAAACATAAACTCACAAAGGAGCTCCAGAACCAGTTTTTGCAXXXXXXXXXXXT
TATTGAGGTGGGAAGCCCTTCCGGCAATGGAAGCACATAGTTCAAGAATTATTTGGCTACACAGAAGGATGCACAAGGT
AACTTACCTCGTCAAATTCTATGCTTCATAAGAAACTATATACATAAACTCACAAAGGAGCTCCAGAACCAGTTTTTGCAXXXXXXXXXXXT
TATTGAGGTGGGAAGCCCTTCCGGAAATGGAAGCACATAGTTCAAGAATTATTTGGCTACACAGAAGGATGCACAAGGT
>Marker1056942 Chr2 SNP_site:17593818 Ref_Type:A
ACATGAGGTGAAACCTAAACAAAATTTAAAAGAAAAAGGCATTAAATTACAGATGAAACAAAAAGTAAAAGAAGCCAGAAXXXXXXXXXXXG
AAGTGTATTTGGTTATTGATTGTTATGCCAATTTTTACCAAAAAGGTCTGGATCTCTTAAAGCCTGGAGATGGGACTGT
ACATGAGGTGAAACCTAAACAAAATTTAAAAGAAAAAGGCATTAAATTACAGATGAAACAAAAAGTAAAAGAAGCCAGAAXXXXXXXXXXXG
AAGTGTATTTGGTTATTGATTGTTATGCCAATTTTTACCAAAAAGGTCTGGATCTCTTAAAGCCTGGAGATGGGGCTGT
>Marker1057109 Chr2 SNP_site:11183148 Ref_Type:C
GACTTTGAGTTGTTGGGCTTTAGAATTGCATGGACATGCAATTTTGCATGGAAAATCCCTATAAATAGGGCATTTGATGGXXXXXXXXXXXT
TTTTCATCTTTTTCTCTCTCCCGATCCTTTCATCCAATAGGTCCAACAATCAAGTTTTAAGTCCAAAGGATAGTAGGTC
GACTTTGAGTTGTTGGGTTTTAGAATTGCATGGACATGCAATTTTGCATGGAAAATCCCTATAAATAGGGCATTTGATGGXXXXXXXXXXXT
TTTTCATCTTTTTCTCTCTCCCGATCCTTTCATCCAACAGGTCCAACAATCAAGTTTTAAGTCCAAAGGATAGTAGGTC
>Marker1057109 Chr2 SNP_site:11183346 Ref_Type:T
GACTTTGAGTTGTTGGGCTTTAGAATTGCATGGACATGCAATTTTGCATGGAAAATCCCTATAAATAGGGCATTTGATGGXXXXXXXXXXXT
TTTTCATCTTTTTCTCTCTCCCGATCCTTTCATCCAATAGGTCCAACAATCAAGTTTTAAGTCCAAAGGATAGTAGGTC
GACTTTGAGTTGTTGGGTTTTAGAATTGCATGGACATGCAATTTTGCATGGAAAATCCCTATAAATAGGGCATTTGATGGXXXXXXXXXXXT
TTTTCATCTTTTTCTCTCTCCCGATCCTTTCATCCAACAGGTCCAACAATCAAGTTTTAAGTCCAAAGGATAGTAGGTC
>Marker1057334 Chr2 SNP_site:375494 Ref_Type:C
ACCTGTTCGACAGAGCTCTAGAATAGATTACTCCTTTCCTTTCCTGGAGTCACTGAATGAGGTGTGCCTTCAATTTTTCCXXXXXXXXXXXG
GTTTTGGAAACAAACCGAACAGAGGAATCCAAAAGTCACGTGCATAGAATGTATTTCATGGGCCCAAATAGTTTCAGTG
ACCTGTTCGACAGAGCTCTAGAATAGATTACTCCTTTCCTTTCCTGGAGTCATTGAATGAGGTGTGCCTTCAATTTTTCCXXXXXXXXXXXG
GTTTTGGAAACAAACCGAACAGAGGAATCCAAAAGTCACGTGCATAGAATGTATTTCATGGGCCCAAATAGTTTCAGTG
>Marker1057406 Chr2 SNP_site:15495665 Ref_Type:A
GACAATTCCTGAACAAAACAGATAAAATATTTTCCTTAAGAATGGAAAATCACTTCAAAGATATGATAAAAATGTCAAGAXXXXXXXXXXXA
TTTAAAAAAGGTAACAAGTTTCTAACAGCTTTAACAAAAAACATTTATTTAATACTTTACCATATATTTTCCAACAAGT
GACAATTCCTGAACAAAACAGATAAAATATTTTCCTTAAGAATGGAAAATCACTTCAAAGATATGATAAAAATGTCAAGAXXXXXXXXXXXA
TTTAAAGAAGGTAACAAGTTTCTAACAGCTTTAACAAAAAGCATTTATTTAATACTTTACCATATATTTTCCAACAAGT
>Marker1057406 Chr2 SNP_site:15495699 Ref_Type:A
GACAATTCCTGAACAAAACAGATAAAATATTTTCCTTAAGAATGGAAAATCACTTCAAAGATATGATAAAAATGTCAAGAXXXXXXXXXXXA
TTTAAAAAAGGTAACAAGTTTCTAACAGCTTTAACAAAAAACATTTATTTAATACTTTACCATATATTTTCCAACAAGT
GACAATTCCTGAACAAAACAGATAAAATATTTTCCTTAAGAATGGAAAATCACTTCAAAGATATGATAAAAATGTCAAGAXXXXXXXXXXXA

Create PDF with GO2PDF for free, if you wish to

TTTAAAGAAGGTAACAAGTTTCTAACAGCTTTAACAAAAAGCATTTATTTAATACTTTACCATATATTTTCCAACAAGT

>Marker105743 Chr4 SNP_site:7542763 Ref_Type:G

AACCAACTTTCTATTGTTTCACTTCACCCTTTGTCTCGTTTTATGTATCGTTCCTGGCATAGTTAAATCACGTACTGATTXXXXXXXXXXG

ACTCTACTCCTTTGTGCAAACAGAATTATTGTCTCGTCGTCCCCATAATAAAATAATTATCAAGTATATGAATCAAGTT

AACCAACTTTCTATTGTTTCACTTCACCCTTTGTCTCGTTTTATGTATCGTTCCTGGCATAGTTAAATCACGTACTGATTXXXXXXXXXXG

ACTCTACTCCTTTGTGCAAACAGAATTATTGTCTCGTCGTCCCCATAATAAAATAATTATCAAGTATATGAATCGAGTT

>Marker1057468 Chr2 SNP_site:20598020 Ref_Type:T

TACCCAAATCAAGACAAGCAGCACACATCTCAATTCTTGTTCCTTCCTTCCACGTCTGTTTCTTTCCCTTTTCTGAGTAAXXXXXXXXXXC

TTTTAACCCACCTGATGATGTTAGTTACCTGTCTGAACAAATGAATGACAGAGACATTTGTTTAGAGTAAAAACAGTGT

TACCCAAATCAAGACAAGCAGCACACATCTCAATTCTTGTTCCTTCCTTCCACGTCTGTTTCTTTCCCTTTTCTGAGTAAXXXXXXXXXXT

TTTTAACCCACCTGATGATGTTAGTTACCTGTCTGAACAAATGAATGACAGAGACATTTGTTTAGAGTAAAAACAGTGT

>Marker1057882 Chr2 SNP_site:17761828 Ref_Type:T

ACTCTGCGCTGGGGAATTTGAAAATGGCTAGAGAAATATTCAACGAAACTCCATTGGATATGAGGGACACTGTTTTCTACXXXXXXXXXXA

TGACTTTACATTTGCAAGCGTGCTCAGTGCTTCAACGCTTATTTTTTATGATGAGCGCCAGTGTGGCCAAATGCATGGT

ACTCTGCGCTGGGGAATTTGAAAATGGCTAGAGAAATATTCAATGAAACTCCATTGGATATGAGGGATACTGTTTTCTACXXXXXXXXXXA

TGACTTTACATTTGCAAGCGTGCTCAGTGCTTCAACGCTTATTTTTTATGATGAGCGCCAGTGTGGCCAAATGCATGGT

>Marker1057882 Chr2 SNP_site:17761852 Ref_Type:T

ACTCTGCGCTGGGGAATTTGAAAATGGCTAGAGAAATATTCAACGAAACTCCATTGGATATGAGGGACACTGTTTTCTACXXXXXXXXXXA

TGACTTTACATTTGCAAGCGTGCTCAGTGCTTCAACGCTTATTTTTTATGATGAGCGCCAGTGTGGCCAAATGCATGGT

ACTCTGCGCTGGGGAATTTGAAAATGGCTAGAGAAATATTCAATGAAACTCCATTGGATATGAGGGATACTGTTTTCTACXXXXXXXXXXA

TGACTTTACATTTGCAAGCGTGCTCAGTGCTTCAACGCTTATTTTTTATGATGAGCGCCAGTGTGGCCAAATGCATGGT

>Marker105793 Chr4 SNP_site:13425848 Ref_Type:C

CACATTATATAGTCTATGTATTGGAAAAAAGTATGAGATTTTATGTATAATTTGAAATTGAAATTTTATGTGTATTGTATXXXXXXXXXXT

GAATTTGAATATGAATTTCTATTTAATTTCAAATTGAATTTGAATTTTGGAAAATGTTAGATATTTAATTAAAAAGGTT

CACATTATATAGTGTATGTATTGGAAAAAAGTATGAGATTTTATGTATAATTTGAAATTGAAATTTTATGTGTATTGTATXXXXXXXXXXT

GAATTTGAATATGAATTTCTATTTAATTTCAAATCGAATTTGAATTTTTGAAAATGTTAGATATTTAATTAAAAAGGTT

>Marker105793 Chr4 SNP_site:13426084 Ref_Type:T

CACATTATATAGTCTATGTATTGGAAAAAAGTATGAGATTTTATGTATAATTTGAAATTGAAATTTTATGTGTATTGTATXXXXXXXXXXT

GAATTTGAATATGAATTTCTATTTAATTTCAAATTGAATTTGAATTTTGGAAAATGTTAGATATTTAATTAAAAAGGTT

CACATTATATAGTGTATGTATTGGAAAAAAGTATGAGATTTTATGTATAATTTGAAATTGAAATTTTATGTGTATTGTATXXXXXXXXXXT

GAATTTGAATATGAATTTCTATTTAATTTCAAATCGAATTTGAATTTTTGAAAATGTTAGATATTTAATTAAAAAGGTT

>Marker105793 Chr4 SNP_site:13426098 Ref_Type:G

CACATTATATAGTCTATGTATTGGAAAAAAGTATGAGATTTTATGTATAATTTGAAATTGAAATTTTATGTGTATTGTATXXXXXXXXXXT

GAATTTGAATATGAATTTCTATTTAATTTCAAATTGAATTTGAATTTTGGAAAATGTTAGATATTTAATTAAAAAGGTT

CACATTATATAGTGTATGTATTGGAAAAAAGTATGAGATTTTATGTATAATTTGAAATTGAAATTTTATGTGTATTGTATXXXXXXXXXXT

GAATTTGAATATGAATTTCTATTTAATTTCAAATCGAATTTGAATTTTTGAAAATGTTAGATATTTAATTAAAAAGGTT

>Marker105850 Chr4 SNP_site:17045836 Ref_Type:A

AACCTCGGCACTAAGATCAACGTTAGGAGGTAATTTAAGAAGGTCAAATGTAAGATAAGTAGAATAAGTATAGATAACCTXXXXXXXXXXA

CTGAGTGAAGAGAGGTTGCCTAATGTTCTATTAATCACTTGTATTTGACCATCTGTTTGAGGATGGCTGGTCGAATAGT

AACCTCGGCACTAAGATCAACGTTAGGAGGTAATTTAAGAAGGTCAAATGTAAGATAAGTAGAATAAGTATAGATAACCTXXXXXXXXXXA

CTGAGTGAAGAGAGGTTGCCTAATGTTCTATTAGTCACTTGTATTTGACCATCTGTTTGAGGATGGCTGGCCGAATAGT

>Marker105850 Chr4 SNP_site:17045873 Ref_Type:T

AACCTCGGCACTAAGATCAACGTTAGGAGGTAATTTAAGAAGGTCAAATGTAAGATAAGTAGAATAAGTATAGATAACCTXXXXXXXXXXA

CTGAGTGAAGAGAGGTTGCCTAATGTTCTATTAATCACTTGTATTTGACCATCTGTTTGAGGATGGCTGGTCGAATAGT

Create PDF with GO2PDF for free, if you wish to

AACCTCGGCACTAAGATCAACGTTAGGAGGTAATTTAAGAAGGTCAAATGTAAGATAAGTAGAATAAGTATAGATAACCTXXXXXXXXXXXA
CTGAGTGAAGAGAGGTTGCCTAATGTTCTATTAGTCACTTGTATTTGACCATCTGTTTGAGGATGGCTGGCCGAATAGT
>Marker1058717 Chr2 SNP_site:17709218 Ref_Type:G
TACATCTCTTCCTGTTTTGAGGAGTTTGCTCTCTTATATTTAGGTGTCCTGCTAAGGAGTTAGAGGACATTATGGACAACXXXXXXXXXXXA
TACTCACCGATTAAGTTATATTATTTTGTGAATAGATATTTTTGTTTGATGGACACTTAAATCGCTATCAATATAATGT
TACATGTCTTCCTGTTTTGAGGAGTTTGCTCTCTTATATTTAGGTGTCCTGCTAAGGAGTTAGAGGACATTATGGACAACXXXXXXXXXXXA
TCCTCACCGATTAAGTTACATTATTTTGTGAATAGATATTTTTGTTTGATGGACACTTAAATCGCTATCAATATAATGT
>Marker1058717 Chr2 SNP_site:17709396 Ref_Type:C
TACATCTCTTCCTGTTTTGAGGAGTTTGCTCTCTTATATTTAGGTGTCCTGCTAAGGAGTTAGAGGACATTATGGACAACXXXXXXXXXXXA
TACTCACCGATTAAGTTATATTATTTTGTGAATAGATATTTTTGTTTGATGGACACTTAAATCGCTATCAATATAATGT
TACATGTCTTCCTGTTTTGAGGAGTTTGCTCTCTTATATTTAGGTGTCCTGCTAAGGAGTTAGAGGACATTATGGACAACXXXXXXXXXXXA
TCCTCACCGATTAAGTTACATTATTTTGTGAATAGATATTTTTGTTTGATGGACACTTAAATCGCTATCAATATAATGT
>Marker1058717 Chr2 SNP_site:17709413 Ref_Type:C
TACATCTCTTCCTGTTTTGAGGAGTTTGCTCTCTTATATTTAGGTGTCCTGCTAAGGAGTTAGAGGACATTATGGACAACXXXXXXXXXXXA
TACTCACCGATTAAGTTATATTATTTTGTGAATAGATATTTTTGTTTGATGGACACTTAAATCGCTATCAATATAATGT
TACATGTCTTCCTGTTTTGAGGAGTTTGCTCTCTTATATTTAGGTGTCCTGCTAAGGAGTTAGAGGACATTATGGACAACXXXXXXXXXXXA
TCCTCACCGATTAAGTTACATTATTTTGTGAATAGATATTTTTGTTTGATGGACACTTAAATCGCTATCAATATAATGT
>Marker1058749 Chr2 SNP_site:4919769 Ref_Type:C
CACCCTCAAATGCATATCCTAGACGGTAAGACTCTTATGTCATTAGCTTGTAGCTACGAAAGTATTCTGTATTTTATTTTXXXXXXXXXXXA
GCAAGATGTATGATATTCACTTGTTTGTCATTAAAAAATAGATACATGTAATTTTAGGAACTATCTAGAAGATTAGGTC
CACCCTCAAATGCATATCCTAGACGGTAAGACTCTTATGTCATTAGCTTGTAGCTACGAAAGTATTCTGTATTTTATTTTXXXXXXXXXXXA
GCAAGATGTATGATATTCACTTGTTTGTCATTAAAAAATCGATACATGTAATTTTAGGAACTATCTAGAATATTAGGTC
>Marker1058749 Chr2 SNP_site:4919800 Ref_Type:T
CACCCTCAAATGCATATCCTAGACGGTAAGACTCTTATGTCATTAGCTTGTAGCTACGAAAGTATTCTGTATTTTATTTTXXXXXXXXXXXA
GCAAGATGTATGATATTCACTTGTTTGTCATTAAAAAATAGATACATGTAATTTTAGGAACTATCTAGAAGATTAGGTC
CACCCTCAAATGCATATCCTAGACGGTAAGACTCTTATGTCATTAGCTTGTAGCTACGAAAGTATTCTGTATTTTATTTTXXXXXXXXXXXA
GCAAGATGTATGATATTCACTTGTTTGTCATTAAAAAATCGATACATGTAATTTTAGGAACTATCTAGAATATTAGGTC
>Marker1058833 Chr2 SNP_site:17008355 Ref_Type:C
GACCACAGTGCCTAAAATAAGCTACTTAACACAATTGGTGGATGCGATTATGGATCCATTCCGAACTAAACAGGGTAGGTXXXXXXXXXXXA
CCATCAAAATAATACTTCAAATGTCTATAACTATGCACACCCAAATTAAAGGAAGAAATTGAGGCCAGGAATCTTAGTC
GACCACAGTGCCTAAAATAAGCTACTTAACACAATTGGTGGATGCGATTATGGATCCATTCCGAACTAAATAGGGTAGGTXXXXXXXXXXXA
CCATCAAAATAATACTTCAAATGTCTATAACTATGCACACCCAAATTAAAGGAAGAAATTGAGGCCAGGAATCTTAGTC
>Marker1059258 Chr2 SNP_site:22356012 Ref_Type:G
GACTTTGGATGGGAAAAGGCCATATATGCCGGACCAGCTACCGGAAAAGTTGGACTTGTGCCTGGTTTGATAAGCTTTTGXXXXXXXXXXXG
AAGATATAAGAACATTTTGAAATTGAAAAACACTACATAGATAATGAAATGAAAATAATTGGTTCCCTCGAAAAACAGT
GACTTTGGATGGGAAAAGGCCATATATGCCGGACCAGCTACCGGAAAAGTTGGACTTGTGCCTGGTTTGATAAGCTTTTGXXXXXXXXXXXG
AAGATATAAGAACATTTTGAAATTGAAAAACACTACATAGATAATGAAATGAAAATAATTGGTTCGCTCGAAAAACAGT
>Marker1060160 Chr2 SNP_site:15107169 Ref_Type:C
ACATTAATGCGAAGCTTTATTAATTACAAATATGTTTAAAATGTGAGATTTTAGCATAATGTGTATATATATATATATGCXXXXXXXXXXXC
TAAATCATGCGTTGCTGAAACTAAAAGAAGGTTTTTTCTCAATATTTTCCCTTTTTTCTTTTCTCAAGACTATGCAGTT
ACATTAATGTGAAGCTTTATTAATTACAAATATGTTTAAAATGTGAGATTTTAGCATAATGTGTATATATATATATATGCXXXXXXXXXXXC
TAAATCATGCGTTGCTGAAACTAAAAGAAGGTTTTTTCTCAACATTTTCCCTTTTTTCTTTTCTCAAGACTATGCAGTT
>Marker1060160 Chr2 SNP_site:15107368 Ref_Type:T
ACATTAATGCGAAGCTTTATTAATTACAAATATGTTTAAAATGTGAGATTTTAGCATAATGTGTATATATATATATATGCXXXXXXXXXXXC

Create PDF with GO2PDF for free, if you wish to

TAAATCATGCGTTGCTGAAACTAAAAGAAGGTTTTTTCTCAATATTTTCCCTTTTTTCTTTTCTCAAGACTATGCAGTT

ACATTAATGTGAAGCTTTATTAATTACAAATATGTTTAAAATGTGAGATTTTAGCATAATGTGTATATATATATATATGCXXXXXXXXXXC

TAAATCATGCGTTGCTGAAACTAAAAGAAGGTTTTTTCTCAACATTTTCCCTTTTTTCTTTTCTCAAGACTATGCAGTT

>Marker1061565 Chr2 SNP_site:3780945 Ref_Type:C

GACTTATATACATTTTGCAAAGCTAAATCAAAGAAAAAGAGACAAAAACAAAGGTTTGTTGATATTTAAGAGGTCAAAATXXXXXXXXXXA

ATCATTTATTCATTATGATCACACAAACAATAACCATAATCTAAAATTATTCCTAGAGATAAGATGCTAGTGAAGGGTA

GACTTATATACATTTTGCAAAGCTAAATCAAAGAAAAAGAGACAAAAACACAGGTTTGTTGATATTTAAGAGGTCAAAATXXXXXXXXXXA

ATCATTTATTCATTATGATCACACAAACAATAACCATAATCTAAAATTATTCCTAGAGATAAGATGCTAGTGAAGGGTA

>Marker1061616 Chr2 SNP_site:6922945 Ref_Type:C

TACTATGTTTTCTATTTATTATACCCAGACTAAAATAGTCCAACCCCAAATCTAAACCATTATATTTAGATTTAAGTAGGXXXXXXXXXXA

TTTCCTAAAATATCAACAAAAACATCAAAGAATTAATCGGAGATAGTTTTAAAGAAATTAGTCGAGGCACGTCAAAGTC

TACTATGTTTTCTATTTATTATACCCAGAGTAAAATAGTCCAACCCCAAATCTAAACCATTATATTTAGATTTAAGTAGGXXXXXXXXXXA

TTTCCTAAAATATCAACAAAAACATCAAAGAATTAATCGGAGATAGTTTTAAAGAAATTAGTCGAGGCACGTCAAAGTC

>Marker1061839 Chr2 SNP_site:15455473 Ref_Type:A

ACTTTAAATGTGTATTTAATATACTAATAGTTATTAGTATAGAGAGAATAATTTAGATTTTGTAACTACAAATTACTCTTXXXXXXXXXXA

GCTATTTTGGCCCAACCGCTACAAACAAATTTTTTGTGCAAGGAAAAACTTACACATACTTTCAATACATCAATTAGTG

ACTTTAAATGTGTATTTAATATACTAATAGTTATTAGTATAGAGAGAATAATTTAGATTTTGTAACTACAAATTACTCTTXXXXXXXXXXA

GCTATTTTGGCCCAACCGCTACAAACAAATTTTTTGTGCAAGGAAAAACTTACACCTACTTTCAATACATCAATTAGTG

>Marker106267 Chr4 SNP_site:6165333 Ref_Type:C

TACATTCCTTATATATATATTACACGTTTATACCTTTTCAATCCATCATGTCTCTACTTTCCATCAGTTAATGTTTTATTXXXXXXXXXXA

CGCGTGCAAGGAAGAATTTTCTCTTCTTTTGCAAGATAACTTCCATCATTTGTATCCTGAAAGGTGAGCAAGTGAGGGT

TACATTCCTTATATATATATTACATGTTTATACCTTTTCAATCCATCATGTCTCTACTTTCCATCAGTTAATGTTTTATTXXXXXXXXXXA

CGCGTGCAAGGAAGAATTTTCTCTTCTTTTGCAAGATAACTTCCATCATTTGTATCCTGAAAGGTGAGCAAGTGAGGGT

>Marker106706 Chr4 SNP_site:14404261 Ref_Type:C

TACTCGCCCACAAACATCACAATCATCACATAGAAATCAATAGTTTTCTCTTTTCAAATAACATCAATATCAATGCAACAXXXXXXXXXXA

TAAAGGTCACTCATGAAAATACTTGATTCCCTTCTTCTCATAACGCCTAATTTCTTCAACAAGCAGCTCAACTCTTAGT

TACTCGCCCACAAACATCATAATCATCACATAGAAATCAATAGTTTTCTCTTTTCAAATAACATCAATATCAATGCAACAXXXXXXXXXXA

TAAAGGTCACTCATGAAAATACTTGATTCCCTTCTTCTCATAACGCCTAATTTCTTCAACAAGCAGCTCAACTCTTAGT

>Marker106789 Chr4 SNP_site:22223948 Ref_Type:A

AACAAATCAACTTAAAATTCTAAAGTCAATTACATGGGATTGTGGAAGGTCACACTATCCATTGCTTCGAAGCAGAAGTAXXXXXXXXXXA

CCTTCAAGGTGGCTGCTTATGTATGGGTCGGTGGTTGAGATGGTTGCTTGTCTTAGGCCAATACAGACATACAGGTGTT

AACAAATCAACTTAAAGTTCTAAAGTCAATTACATGGGATTGTGGAAGGTCACACTATCCATTGCTTCGAAGCAGAAGTAXXXXXXXXXXA

CCTTCAAGGTGGCTGCTTATGTATGGGTCGGTGGTTGAGATGGTTGCTTGTCTTAGGCCAATACAGACATACAGGTGTT

>Marker106971 Chr4 SNP_site:16429585 Ref_Type:A

CACACAAGAGTGAGATAATTGATTCAAGTGAAAAGTGTTTATCCCAAAAAATTGTTCATGTAATCTCTATCATTTAATTTXXXXXXXXXXA

AAATATATGAATGCTACAAATACATTTTCTTTTTAAATGAAAATGTATGACATATTTCAATATGAAATTACCATATGTT

CACACAAGAGTGAGATAATTGATTCAAGTGAAAAGTGTTTATCCCAACAAATTGTTCATGTAATCTCTATCATTTAATTTXXXXXXXXXXA

AAATATATGAATGCTACAAATACATTTTCTTTTTAAATGAAAATGTATGACATATTTCAATATGAAATTACTATATGTT

>Marker106971 Chr4 SNP_site:16429784 Ref_Type:C

CACACAAGAGTGAGATAATTGATTCAAGTGAAAAGTGTTTATCCCAAAAAATTGTTCATGTAATCTCTATCATTTAATTTXXXXXXXXXXA

AAATATATGAATGCTACAAATACATTTTCTTTTTAAATGAAAATGTATGACATATTTCAATATGAAATTACCATATGTT

CACACAAGAGTGAGATAATTGATTCAAGTGAAAAGTGTTTATCCCAACAAATTGTTCATGTAATCTCTATCATTTAATTTXXXXXXXXXXA

AAATATATGAATGCTACAAATACATTTTCTTTTTAAATGAAAATGTATGACATATTTCAATATGAAATTACTATATGTT

>Marker107254 Chr4 SNP_site:19254536 Ref_Type:A

Create PDF with GO2PDF for free, if you wish to

TACCTTTCTGAGACCTAGGCCTTTGATCCCTCATATCTGGTAGGCCAAGCATGGCTTCCAACTTGTTCATTAAATGGGGAXXXXXXXXXXC
CATTCTGATTTGATAACTGCTGAGGCAATAAATTGTTTAGGTGACTAGAATATTCTCCTGGAAGCATGCCAGTTTGGTT
TACCTTTCTGAGACCTAGGCCTTTGATCCCTCATATCTGGTAGGCCAAGCATGGCTTCCAACTTGTTCATTAAATGGGGAXXXXXXXXXXC
CATTCTGATTTGGTAACTGCTGAGGCAATAAATTGTTTAGGTGACTAGAATATTCTCCTGGAAGCATGCCAGTTTGGTT
>Marker107444 Chr4 SNP_site:3136669 Ref_Type:T
ACAATAAGATTTCTAACATTGATGAACAATAGAACAATTGAGACGCTCATACACACAAGAACTACATTTTATTTTAAGTTXXXXXXXXXXG
TAAAAAAGGAGTATTAATAATGATAACATATCTTTCCTTCCAGAAACTCCAAAAATTTCCATTTTCAAAACACACAGTG
ACAATAAGATTTCTAACATTGATGAACAATAGAACAATTGAGACGCTCATACACACAAGAACTACATTTTATTTTAAGTTXXXXXXXXXXG
TAAAAAAGGAGTATTAATAATGATAACATATCTTTCCTTCCAGAAACTCCAAAAATTTTCATTTTCAAAACACACAGTG
>Marker108079 Chr4 SNP_site:9706212 Ref_Type:A
CACTTTTAAGTAGAAGATCTTGGCACACTTAAGAAAAAAAGATTTTCATCTAAAACTTATGGAAAGCTAAATGATAATCAXXXXXXXXXXA
TATTACGCCCCAAATAATTTCAAGCTTGCTAACCCAATTTGAGGACGAGCCTAGTTTTAGGGAAGTGGATTATGGTGTA
CACTTTTAAGTAGAAGATCTTGGCACACTTAAGAAAAAAAGATTTTCATCTAAAACTTATGGAAAGCTGAATGATAATCAXXXXXXXXXXA
TATTACGCCCCAAATAATTTCAAGCTTGCTAACCCAATTTGAGGACGAGCCTAGTTTTAGGGAAGTGGATTATGGTGTA
>Marker10815 Chr4 SNP_site:19271922 Ref_Type:G
GACATTGCTGGGTTAGTGAAAGGAGCCAGTCAAGGAGAGGTAAAGTTTATTCTCACCTCAGTATTATCATCATCATCATCXXXXXXXXXXT
TATCATAATTTATTTTTTTCCATCATTTCAATTAGAATGCATACTTAAGTAGTAGCGGATTTAAAGAGTAATATTAGTT
GACATTGCTGGGTTAGTGAAAGGAGCCAGTCAAGGAGAGGTAAAGTTTATTCTCACCTCAGTATTATCATCATCATCATCXXXXXXXXXXT
TATCGTAATTTATTTTTTTCCATCATTTCAATTAGAATGCATACTTAAGTAGTAGCGGATTTAAAGAGTAATATTAGTT
>Marker108160 Chr4 SNP_site:16193400 Ref_Type:G
AACCCTCCGTGTGGGTGCCCTCTTTTTATAAAGATTAAACCCATCTAAACTCATAAACATTTATGATTATACTAAACCTTXXXXXXXXXXT
GGTAACATTTTAAGACTGTTTGTTGATTATTGCTTTAGAGGAAAAAAATTGTTAATTTCAAATCAAACATGAAATATGT
AACCCTCCTTGTGGGTGCCCTCTTTTTATAAAGATTAAACCCATCTAAACTCATAAACATTTATGATTATACTAAACCTTXXXXXXXXXXT
GGTAACATTTTAAGACTGTTTGTTGATTATTGCTTTAGAGGAAAAAAATTGTTAATTTCAAATCAAACATGAAATATGT
>Marker108661 Chr4 SNP_site:7604761 Ref_Type:G
CACCTTTCAAGACACAAATGATGGAAGTTATCTCGCAAATGTGGAGTTTCTCTCCAAACTCCTCCTTGCACGCATTAGTCXXXXXXXXXXA
TGAAAAGTCAAGAAATGATGGGTTGAAAAGGTATAAACATGTAATATATATATATAAAGAAAGTAGGCTTTAGGCCGTTGT
CACCTTTCAAGACACAAATGATGGAAGTTATCTCGCAAATGTGGAGTTTGTCTCCAAACTCCTCCTTGCACGCATTAGTCXXXXXXXXXXA
TGAAAAGTCAAGAAATGATGGGTTGAAAAGGTATAAACATGTAATATATATATATAAAGAAAGTAGGCTTTAGGCCGTTGT
>Marker108916 Chr4 SNP_site:1429984 Ref_Type:G
ACTGTTTAGTTGAAATTGATGATCTCCTGAAGAACAACATAATCTCTGGTAAAGAAAATTCTAATATAGGCTTAAACAATXXXXXXXXXXG
TCTAGATAGTGCTAAAAAGATTTGTCTCACAACATACTTGATGAAGTTTTGCAATGAACAAAACGTATGTCAATTAGTG
ACTGTTTAGTTGAAATTGATGATCTCCTGAAGAACAACATAATCTCTGGTAAAGAAAATTCTAATGTAGGCTTAAACAATXXXXXXXXXXG
TCTAGATAGTGCTAAAAAGATTTGTCTCACAACATACTTGATGAACTTTTGCAATGAACAAAACGTATGTCAATTAGTG
>Marker108916 Chr4 SNP_site:1430166 Ref_Type:C
ACTGTTTAGTTGAAATTGATGATCTCCTGAAGAACAACATAATCTCTGGTAAAGAAAATTCTAATATAGGCTTAAACAATXXXXXXXXXXG
TCTAGATAGTGCTAAAAAGATTTGTCTCACAACATACTTGATGAAGTTTTGCAATGAACAAAACGTATGTCAATTAGTG
ACTGTTTAGTTGAAATTGATGATCTCCTGAAGAACAACATAATCTCTGGTAAAGAAAATTCTAATGTAGGCTTAAACAATXXXXXXXXXXG
TCTAGATAGTGCTAAAAAGATTTGTCTCACAACATACTTGATGAACTTTTGCAATGAACAAAACGTATGTCAATTAGTG
>Marker109212 Chr4 SNP_site:21036011 Ref_Type:C
TACGTTCATACGCCAAAGTGAGGATTGGATGTTCTAAAAACACAAAATCAATTGGAACCATCTGATAAGACTTTACAAGTXXXXXXXXXXT
GAACACTCACATTACGCCTACTTCAGTCCTATGGCTTGAAGCCTCTTTAGTTCTTTTATGATGTAAGATTCTTCTTCGT
TACGTTCATACGCCAAAGTGAGGATTGGATGTTCTAAAAACACAAAATCAATTGGAACGATCTAATAAGACTTTACAAGTXXXXXXXXXXC
GAACACTCACATTACGCCTACTTCAGTCCTATGGCTTGAAGCCTCTTTAGTTCTTTTATGATGTAAGATTCTTCTTCGT

Create PDF with GO2PDF for free, if you wish to

>Marker109212 Chr4 SNP_site:21036016 Ref_Type:G

TACGTTCATACGCCAAAGTGAGGATTGGATGTTCTAAAAACACAAAATCAATTGGAACCATCTGATAAGACTTTACAAGTXXXXXXXXXXT

GAACACTCACATTACGCCTACTTCAGTCCTATGGCTTGAAGCCTCTTTAGTTCTTTTATGATGTAAGATTCTTCTTCGT

TACGTTCATACGCCAAAGTGAGGATTGGATGTTCTAAAAACACAAAATCAATTGGAACGATCTAATAAGACTTTACAAGTXXXXXXXXXXC

GAACACTCACATTACGCCTACTTCAGTCCTATGGCTTGAAGCCTCTTTAGTTCTTTTATGATGTAAGATTCTTCTTCGT

>Marker109212 Chr4 SNP_site:21036174 Ref_Type:C

TACGTTCATACGCCAAAGTGAGGATTGGATGTTCTAAAAACACAAAATCAATTGGAACCATCTGATAAGACTTTACAAGTXXXXXXXXXXT

GAACACTCACATTACGCCTACTTCAGTCCTATGGCTTGAAGCCTCTTTAGTTCTTTTATGATGTAAGATTCTTCTTCGT

TACGTTCATACGCCAAAGTGAGGATTGGATGTTCTAAAAACACAAAATCAATTGGAACGATCTAATAAGACTTTACAAGTXXXXXXXXXXC

GAACACTCACATTACGCCTACTTCAGTCCTATGGCTTGAAGCCTCTTTAGTTCTTTTATGATGTAAGATTCTTCTTCGT

>Marker109282 Chr4 SNP_site:6192605 Ref_Type:A

TACAGTTATCACTCAGTTTCTGGTCTTTTTGGGCATATTTATTAATGTTTGGATCCTATTGAAATGGATATCATAAGAATXXXXXXXXXXT

GTGAATGTGGAAAGCAGAGAATTATTAGTTTATTACATTGAAGATGTCGCCTGCTGATTCTTCACGGGAGGAAAATGTT

TACAGTTATCACTCAGTTTCTGGTCTTTTTGGGCATATTTATTAATGTTTGGATCCTATTGAAATGGATATCATAAGAATXXXXXXXXXXT

GTGATTGTGGAAAGCAGAGAATTATTAGTTTATTACATTGAAGATGTCGCCTGCTGATTCTTCACGGGAGGAAAATGTT

>Marker10958 Chr4 SNP_site:7949885 Ref_Type:T

GACGTTTTTTTCGTGGTTAGAAATATCTTTTATGACCACGATTTTAATTTGCAAGGTCATTTCTGCCGACACAACATAAAXXXXXXXXXXT

GCCCATGTTTTCCTGCATCGGTAAAGACAAAAATCTTTGTAGTGAAAACTCCCGACTAATTACTAATGGCTTCAAGAGT

GACGTTTTTTTTGTGGTTAGAAATATCTTTTATGACCACGATTTTAATTTGCAAGGTCATTTCTGCCGACACAACATAAAXXXXXXXXXXT

GCCCATGTTTTCCTGCATCGGTAAAGACAAAAATCTTTGTAGTGAAAACTCCCGACTAATTACTAATGGCTTCAAGAGT

>Marker109592 Chr4 SNP_site:16622181 Ref_Type:G

CACCAGAATTTAGTTAAGCAATTAGGTGGTGGGCGGAGATTCCTATCAAAACCTCTCTAGTTTTCATCGCCACCATTTCTXXXXXXXXXXT

CGCGCTCCCTCTCTCACTGTCAGTTTCTTTTCCACTCTGATCTGCATTTCCTTTTGCTTTATTCTCCGCATTTTTTGTT

CACCAGAATTTAGTTAAGCAATTAGGTGGTGGGCGGAGATTCCTATCAAAGCCTCTCTAGTTTTCATCGCCACCATTTCTXXXXXXXXXXT

CGCGCTCCCTCTCTCACTGTCAGTTTCTTTTCCACTCTGATCTGCATTTCCTTTTGCTTTATTCTCCGCATTTTTTGTT

>Marker11011 Chr4 SNP_site:14090514 Ref_Type:A

ACATGGACTTTCGATTCCAAGACTATTTGATAGAACATGAAATCCAGTTACAATTCTCTGCACCTAATACACCTTAGCAAXXXXXXXXXXT

GTCTATATTTTGAACAATGTTCCCACTAAAAGTGTTTTAGAAACAACTTATGAGCTTTAAAAAGGGCGTAAAGGAAGTT

ACATGGACTTTCGATTCCAAGACTATTTGATAGAGCATGAAATCCAATTACAATTCTCTGCACCTAATACACCTTAGCAAXXXXXXXXXXT

GTCTATATTTTGAACAATGTTCCCACTAAAAGTGTTTTAGAAACAACTTATGAGCTTTAAAAAGGGCGTAAAGGAAGTT

>Marker11011 Chr4 SNP_site:14090526 Ref_Type:G

ACATGGACTTTCGATTCCAAGACTATTTGATAGAACATGAAATCCAGTTACAATTCTCTGCACCTAATACACCTTAGCAAXXXXXXXXXXT

GTCTATATTTTGAACAATGTTCCCACTAAAAGTGTTTTAGAAACAACTTATGAGCTTTAAAAAGGGCGTAAAGGAAGTT

ACATGGACTTTCGATTCCAAGACTATTTGATAGAGCATGAAATCCAATTACAATTCTCTGCACCTAATACACCTTAGCAAXXXXXXXXXXT

GTCTATATTTTGAACAATGTTCCCACTAAAAGTGTTTTAGAAACAACTTATGAGCTTTAAAAAGGGCGTAAAGGAAGTT

>Marker110677 Chr4 SNP_site:12552096 Ref_Type:G

CACTCAAACATTGATAATTAGTATTAAGTCAATTAACATTTGCCTTTGCAATTCTTTTGAATAGATGAGTTGGTGGAAATXXXXXXXXXXC

ACCAAAAACCTTGTTTAACTTTATGGCTTAAACAATTAATTGCATTAAAATGACAAATTTGATTGAGTGATTTTGTGTG

CACTCAAACATTGATAATTAGTATTAAGTCAATTAACATTTGCCTTTGGAATTCTTTTGAATAGATGAGTTGGTGGAAATXXXXXXXXXXC

ACCAAAAACCTTGTTTAACTTTACGGCTTAAACAATTAATTACATTAAAATGACAAATTTGATTGAGTGATTTTGTGTG

>Marker110677 Chr4 SNP_site:12552293 Ref_Type:C

CACTCAAACATTGATAATTAGTATTAAGTCAATTAACATTTGCCTTTGCAATTCTTTTGAATAGATGAGTTGGTGGAAATXXXXXXXXXXC

ACCAAAAACCTTGTTTAACTTTATGGCTTAAACAATTAATTGCATTAAAATGACAAATTTGATTGAGTGATTTTGTGTG

CACTCAAACATTGATAATTAGTATTAAGTCAATTAACATTTGCCTTTGGAATTCTTTTGAATAGATGAGTTGGTGGAAATXXXXXXXXXXC

Create PDF with GO2PDF for free, if you wish to

ACCAAAAACCTTGTTTAACTTTACGGCTTAAACAATTAATTACATTAAAATGACAAATTTGATTGAGTGATTTTGTGTG
>Marker110677 Chr4 SNP_site:12552311 Ref_Type:A
CACTCAAACATTGATAATTAGTATTAAGTCAATTAACATTTGCCTTTGCAATTCTTTTGAATAGATGAGTTGGTGGAAATXXXXXXXXXXC
ACCAAAAACCTTGTTTAACTTTATGGCTTAAACAATTAATTGCATTAAAATGACAAATTTGATTGAGTGATTTTGTGTG
CACTCAAACATTGATAATTAGTATTAAGTCAATTAACATTTGCCTTTGGAATTCTTTTGAATAGATGAGTTGGTGGAAATXXXXXXXXXXC
ACCAAAAACCTTGTTTAACTTTACGGCTTAAACAATTAATTACATTAAAATGACAAATTTGATTGAGTGATTTTGTGTG
>Marker110834 Chr4 SNP_site:21180534 Ref_Type:G
CACTTAAAGCTAGGGATCAAGGCTAAGATTGTAAAACATGATACAATGAATAGATTATGAAATCCCACATTGTAAGATAAXXXXXXXXXXT
GACACACTAAAATCCTTGGTTCTGAATATTAAATACGTCATTAGCTCCACTAGCCAGTTGAATGAGTTACCAGTTCAGT
CACTTAAAGCTAGGGATCAAGGCTAAGATTGTAAAACATGATACAATGAATAGATTATGAAATCCCACATTGTAAGATAAXXXXXXXXXXT
GACACACTAAAATCCTTGGTTCTGAATATTAAATGCGTCATTAGCTCCACTAGCCAGTTGAATGAGTTACCAGTTCAGT
>Marker110882 Chr4 SNP_site:11203060 Ref_Type:G
AACTGTAGATTCATTATCAGAAGTCAGAAGTATGGAGTTTTATGTGCCACGAGATGAAGCATTCTCCATATCAAAACAGGXXXXXXXXXXT
TGTCCTAAACTTGTCCAAATCCTTACTGATAAGGCCATGGATATCTTACTCTTGCATTCTTCGCAGACATCCTATGGTA
AACTGTAGATTCATTATCAGAAGTCAGAAGTATGGAGTTTTATGTGCCACGAGATGAAGCGTTCTCCATATCAAAACAGGXXXXXXXXXXT
TGTCCTAAACTTGTTCAAATCCTTACTGATAAGGCCATGGATATCTTACTCTTGCATTCTTCGCAGACATCCTATGGTA
>Marker110882 Chr4 SNP_site:11203213 Ref_Type:T
AACTGTAGATTCATTATCAGAAGTCAGAAGTATGGAGTTTTATGTGCCACGAGATGAAGCATTCTCCATATCAAAACAGGXXXXXXXXXXT
TGTCCTAAACTTGTCCAAATCCTTACTGATAAGGCCATGGATATCTTACTCTTGCATTCTTCGCAGACATCCTATGGTA
AACTGTAGATTCATTATCAGAAGTCAGAAGTATGGAGTTTTATGTGCCACGAGATGAAGCGTTCTCCATATCAAAACAGGXXXXXXXXXXT
TGTCCTAAACTTGTTCAAATCCTTACTGATAAGGCCATGGATATCTTACTCTTGCATTCTTCGCAGACATCCTATGGTA
>Marker111659 Chr4 SNP_site:19459453 Ref_Type:A
ACAAGATACTTAAGTAGTTCCCCGGCCTGGCTCTTTAGTGACATGATCGCTTGTTTCAATCTTGGGTTACAGCATGCCACXXXXXXXXXXA
CCAACTAATGAGGAAAACTAAACATAATTGGCAGAGTTAAGACAAGAGCATCATTCTCATTCACCACTCTATAAAGGTC
ACAAGATACTTAAGTAGTTCCCCGGCCTGGCTCTTTAGTGACATGATCGCTTGTTTCAATCTTGGGTTACAGCATGCCACXXXXXXXXXXA
CCAACTAATGAGGAAAACTAGACATAATTGGCAGAGTTAAGACAAGAGCATCATTCTCATTCACCACTCTATAAAGGTC
>Marker111776 Chr4 SNP_site:14679026 Ref_Type:C
AACCAAACTCTAATATAATTCAACAAAATATAGTTTAAGTTTTGGAGTATGTGTGAAGGCTTACTACCATTTGTAGGTATXXXXXXXXXXT
TAGACGATAACTTCCAAATTTAATCCACGATCGAGCCTTTAATGTTTGTGTCAAATAATGTCACTCAATACAAATTGTC
AACCAAACTCTAATATAATTCAATAAAATATAGTTTAAGTTTTGGAGTATGTGTGAAGGCTTACTACGATTTGTAGGTATXXXXXXXXXXT
TAGACGATAACTTCCAAATTTAACCCACGATCGAGCCTTTAATGTTTGTGTCAAATAATGTCACTCAATACAAATTGTC
>Marker111776 Chr4 SNP_site:14679070 Ref_Type:C
AACCAAACTCTAATATAATTCAACAAAATATAGTTTAAGTTTTGGAGTATGTGTGAAGGCTTACTACCATTTGTAGGTATXXXXXXXXXXT
TAGACGATAACTTCCAAATTTAATCCACGATCGAGCCTTTAATGTTTGTGTCAAATAATGTCACTCAATACAAATTGTC
AACCAAACTCTAATATAATTCAATAAAATATAGTTTAAGTTTTGGAGTATGTGTGAAGGCTTACTACGATTTGTAGGTATXXXXXXXXXXT
TAGACGATAACTTCCAAATTTAACCCACGATCGAGCCTTTAATGTTTGTGTCAAATAATGTCACTCAATACAAATTGTC
>Marker111776 Chr4 SNP_site:14679231 Ref_Type:T
AACCAAACTCTAATATAATTCAACAAAATATAGTTTAAGTTTTGGAGTATGTGTGAAGGCTTACTACCATTTGTAGGTATXXXXXXXXXXT
TAGACGATAACTTCCAAATTTAATCCACGATCGAGCCTTTAATGTTTGTGTCAAATAATGTCACTCAATACAAATTGTC
AACCAAACTCTAATATAATTCAATAAAATATAGTTTAAGTTTTGGAGTATGTGTGAAGGCTTACTACGATTTGTAGGTATXXXXXXXXXXT
TAGACGATAACTTCCAAATTTAACCCACGATCGAGCCTTTAATGTTTGTGTCAAATAATGTCACTCAATACAAATTGTC
>Marker111975 Chr4 SNP_site:20362829 Ref_Type:G
CACAAAAATAATCTCAATTCTTATCCAATTATATTGACATAATTAAATTATCCATAAGTAGATAAATTGATCACATATAAXXXXXXXXXXT
TTTGTTACCATAATATAATTATAAGACCGATGTTTGATACTAAAAAAACAAAGTTTTAATTAATATAATTTCCAAAGTG

Create PDF with GO2PDF for free, if you wish to

CACAAAAATAATCTCAATTCTTATCCAATTATATTGACATAATTAAATTATCCATAAGTAGATAAATTGATCACATATAAXXXXXXXXXXT
TTTGTTACCATAATATAATTATAAGACCGATGTTTGATACTAAAAAAAGAAAGTTTTAATTAATATAATTTCCAAAGTG
>Marker112112 Chr4 SNP_site:13991130 Ref_Type:G
CACTTCAATCAAGAAAACATTGACCTTCAAATCACCAAACAGTCATTCGTCTCTTGTATTAGTATGTGGCACATGAAATTXXXXXXXXXXA
AAGGACTCAGGTCAGCAATATTGAAAGTAGAACTTACCGAATATTCTCATGGAAAGTCAATTTTGTATTCATTGTTGTT
CACTTCAATCAAGAAAACATTGACCTTCAAATCACCAAACGGTCATTCGTCTCTTGTATTAGTATGTGGCACATGAAATTXXXXXXXXXXA
AAGGACTCAGGTCAGCAATATTGAAAGTAGAACTTACCGAATATTCTCATGGAAAGTCAATTTTGTATTCATTGTTGTT
>Marker112853 Chr4 SNP_site:18298311 Ref_Type:T
TACCTAGCTAATAATGTTACAACTGTCAAGTAAGATTTTCATGATATAGATGTTGATGCCATTGAAATTGAAAAAGAAATXXXXXXXXXXG
GAAATGATTTGCCAATTGCAAAAACTAGAAATTAATTCTATATTGAAAATTACATAGTTTAAACAACAAAGATGACAGT
TACCTAGCTAATAATGTTATAACTGTCAAGTAAGATTTTCATGATATAGATGTTGATGCCATTGAAATTGAAAAAGAAATXXXXXXXXXXG
GAAATGATTTGCCAATTGCAAAAACTAGAAATTAATTCTATATTGAAAATTACATAGTTTAAACAACAAAGATGACAGT
>Marker112948 Chr4 SNP_site:18506912 Ref_Type:A
AACTACTTAAACAAATTGGCAGTGTGAAATGAAACATAGAATTGCAGTATTTTTCCATTTTCTTCAAATAAATTTACTAAXXXXXXXXXXXA
ATGGAATGGGGATTGAAAAGAAAAAGAAAGGAAAAATCGAATTTGAACTGAAGGGGCCAATAAAAGAAGGAAAAAGGTT
AACTACTTAAACAAATTGGCAGTGTGAAATGAAACATAGAATTGCAGTGTTTTTCCATTTTCTTCAAATAAATTTACTAAXXXXXXXXXXXA
ATGGAATGGAGATTGAAAAGAAAAAGAAAGGAAAAATCGAATTTGAACTGAAGGGGCCAATAAAAGAAGGAAAAAGGTT
>Marker112948 Chr4 SNP_site:18507048 Ref_Type:G
AACTACTTAAACAAATTGGCAGTGTGAAATGAAACATAGAATTGCAGTATTTTTCCATTTTCTTCAAATAAATTTACTAAXXXXXXXXXXXA
ATGGAATGGGGATTGAAAAGAAAAAGAAAGGAAAAATCGAATTTGAACTGAAGGGGCCAATAAAAGAAGGAAAAAGGTT
AACTACTTAAACAAATTGGCAGTGTGAAATGAAACATAGAATTGCAGTGTTTTTCCATTTTCTTCAAATAAATTTACTAAXXXXXXXXXXXA
ATGGAATGGAGATTGAAAAGAAAAAGAAAGGAAAAATCGAATTTGAACTGAAGGGGCCAATAAAAGAAGGAAAAAGGTT
>Marker11303 Chr4 SNP_site:841478 Ref_Type:G
TACTAGTCGTGTGCTCACGTATGAACTTAACTAGTGTTATATAGATTTGAGTATTATCCTCCTTAAGAAAATACTTTTTGXXXXXXXXXXA
GGAGATCTAAGTTCGAAAAGATTTTTCATGAGAAAAATTAATTTCGGCAAAAGTGTTGATAAACACTCATGAATGTGTA
TACTAGTCGTGTGCTCACGTATGAACTTAACTAGTGTTATATAGATTTGAGTATTATCCTCCTTAAGAAAATACTTTTTGXXXXXXXXXXA
GGATATCTAAGTTCGAAAAGATTTTTCATGAGAAAAATTAATTTCGGCAAAAGTGTTGATAAACACTCATGAATGTGTA
>Marker113318 Chr4 SNP_site:1692355 Ref_Type:C
GACTTAATTGTTTATAATAGCAATAATTGTATGAATACGAAGGTTTAATTATTATTTGTTAATAGTTAAAATCTATCTAAXXXXXXXXXXG
AGAGTCCAATTAGCGTGAGTTAATAGGTCAAATGTATAAAGTTAATAGGTCAAGTGTATAGTTATGTTGTTGGAAAGTT
GACTTAATTGTTTATAATAGCAATAATTGTATGAATATGAAGGTTCAATTATTATTTGTTAATAGTTAAAATCTATCTAAXXXXXXXXXXG
AGAGTCCAATTAGCGTGAGTTAATAGGTCAAATGTATAAAGTTAATAGGTCAAGTGTATAGTTATGTTGTTGGAAAGTT
>Marker113318 Chr4 SNP_site:1692363 Ref_Type:T
GACTTAATTGTTTATAATAGCAATAATTGTATGAATACGAAGGTTTAATTATTATTTGTTAATAGTTAAAATCTATCTAAXXXXXXXXXXG
AGAGTCCAATTAGCGTGAGTTAATAGGTCAAATGTATAAAGTTAATAGGTCAAGTGTATAGTTATGTTGTTGGAAAGTT
GACTTAATTGTTTATAATAGCAATAATTGTATGAATATGAAGGTTCAATTATTATTTGTTAATAGTTAAAATCTATCTAAXXXXXXXXXXG
AGAGTCCAATTAGCGTGAGTTAATAGGTCAAATGTATAAAGTTAATAGGTCAAGTGTATAGTTATGTTGTTGGAAAGTT
>Marker113401 Chr4 SNP_site:20987109 Ref_Type:A
ACCTTGGGCTTAGATTGGGCCCAAATCTTTGATTTAGGGAATTTATTCTGGGCTCTCACGATCTGCACTAACAAGTCGAAXXXXXXXXXXT
TAAATCATCATTTCCTTCTCATTCCATGAGACTTGATCAACTTTTTCTCCATGCTTTCAAATGTCCAAATTTGTATGTA
ACCTTGGGCTTAGATTGGGCCCAAATCTTTGATTTTGGGAATTTATTCTGGGCTCTCACGATCTGCACTAACAAGTCGAAXXXXXXXXXXT
TAAATCATCATTTCCTTCTCATTCCATGAGACTTGATCAACTTTTTCTCCATGCTTTCAAATGTCCAAATGTGTATGTA
>Marker113401 Chr4 SNP_site:20987326 Ref_Type:T
ACCTTGGGCTTAGATTGGGCCCAAATCTTTGATTTAGGGAATTTATTCTGGGCTCTCACGATCTGCACTAACAAGTCGAAXXXXXXXXXXT

Create PDF with GO2PDF for free, if you wish to

TAAATCATCATTTCCTTCTCATTCCATGAGACTTGATCAACTTTTTCTCCATGCTTTCAAATGTCCAAATTTGTATGTA
ACCTTGGGCTTAGATTGGGCCCAAATCTTTGATTTTGGGAATTTATTCTGGGCTCTCACGATCTGCACTAACAAGTCGAAXXXXXXXXXXXT
TAAATCATCATTTCCTTCTCATTCCATGAGACTTGATCAACTTTTTCTCCATGCTTTCAAATGTCCAAATGTGTATGTA
>Marker113531 Chr4 SNP_site:12697309 Ref_Type:G
TACATATTTTCTCTTCACATTCTCATAATCACTTTGTTGTTTGGGATTCAATTCCCACTTATCTTGATTATTCCATGGAAXXXXXXXXXXXC
CTTGAACCAACATTCCTTGTGATGGATATCTCCCCCCGATCGATGTTAGGTTTTTAAATACCCTTATTGGAACTTGGTC
TACATATTTTCTCTTCACATTCTCATAATCACTTTGTTGTTTGGGATTCAATTCCCACTTATCTTGATTATTCCATGGAAXXXXXXXXXXXC
CTTGAACCAACATTCCTTGTGATGGGTATCTCCCCCCGATCGATGTTAGGTTTTTAAATACCCTTATTGGAACTTGGTC
>Marker11362 Chr4 SNP_site:6254299 Ref_Type:T
CACTTCGCAAATCTTCCAATCAAGCTTCTTATGCCTACCTCTTTTACCAACATCACTGAAATTGCTCTTAAAACCATTCCXXXXXXXXXXXA
GGGATCTTGATCGCAAAGCCTGATTACAAGAAGGCCTATGTTACTCTCAGAAATCCCTTGTCAATATCTCCGGACATGT
CACTTCGCAAATCTTCCAATCAAGCTTCTTATGCCTACCTCTTTTACCAACATCACTGAAATTGCTCTTAAAACCATTCCXXXXXXXXXXXA
GGGATTTTGATCGCAAAGCCTGATTACAAGAAGGCCTATGTTACTCTCAGAAATCCCTTGTCAATATCTCCGGACATGT
>Marker113622 Chr4 SNP_site:11929187 Ref_Type:C
ACTTTGAATATATTTTCAAGTTTGAGATTAAGAATCCAACGTGATAAGTGGATACTATTTGATAGATTTGAAAGTTTGTTXXXXXXXXXXXA
CCATTTGTTCTTCTATGAAGACAGTCATATTTTAATTAGTTACATATGTTGTTGGCATAAATATAGTTCAACCGTGGTA
ACTTTGAATATATTTTCAAGTTTGAGATTAAGAATCCAATGTGATAAGTGGATACTATTTGATAGATTTGAAAGTTTGTTXXXXXXXXXXXA
CCATTTGTTCTTCTATGAAGACAGTCATATTTTAATTAGTTACATATGTTGTTGGCATAAATATAGTTCAACCGTGGTA
>Marker114255 Chr4 SNP_site:21598814 Ref_Type:T
ACTACCGTCTGAGCAAGACGTGAGGAACGAGAAACCGCAGCCACGGAAGTCGGCGAAGGGCACTTCACGGCGGAATCAACXXXXXXXXXXXT
TACAAATGAAGGGTTTATTAAATGCGACTGTTGCTCTAATCTGTTCCCCATCAGTAAATTTGAGGCTCATACAGGTAGT
ACTACCGTCTGAGCAAGACGTGTGGAACGAGAAACCGCAGCCACGGAAGGCGGCGAAGGGCACTTCACGGCGGAATCAACXXXXXXXXXXXT
TACAAATGAAGGGTTTATTAAATGCGACTGTTGCTCTAATCTGTTCCCCATCAGTAAATTTGAGGCTCATACAGGTAGT
>Marker114255 Chr4 SNP_site:21598841 Ref_Type:G
ACTACCGTCTGAGCAAGACGTGAGGAACGAGAAACCGCAGCCACGGAAGTCGGCGAAGGGCACTTCACGGCGGAATCAACXXXXXXXXXXXT
TACAAATGAAGGGTTTATTAAATGCGACTGTTGCTCTAATCTGTTCCCCATCAGTAAATTTGAGGCTCATACAGGTAGT
ACTACCGTCTGAGCAAGACGTGTGGAACGAGAAACCGCAGCCACGGAAGGCGGCGAAGGGCACTTCACGGCGGAATCAACXXXXXXXXXXXT
TACAAATGAAGGGTTTATTAAATGCGACTGTTGCTCTAATCTGTTCCCCATCAGTAAATTTGAGGCTCATACAGGTAGT
>Marker114460 Chr4 SNP_site:13814173 Ref_Type:C
CACTTATGTATCTTGCTAACAATACTAGACCAGATATTGCATTTTTCAATAAATCTATTTGCTAGATGTAGTTCATCTCCXXXXXXXXXXXT
GATCCACACAAAAGTAGATCTCAGACAGGTTATCTATTAACATGTGGAGAAACCGCTATATCTTGGCATCAATAAAGTA
CACTTATGTATCTTGCTAACAATACTAGACCCGATATTGCATTTTTCAATAAATCTATTTGCTAGATGTAGTTCATCTCCXXXXXXXXXXXT
GATCCACACAAAAGTAGATCTCAGACAGATTATCTATTAACATGTGGAGAAACCGCTATATCTTGGCATCAATAAAGTA
>Marker114460 Chr4 SNP_site:13814380 Ref_Type:A
CACTTATGTATCTTGCTAACAATACTAGACCAGATATTGCATTTTTCAATAAATCTATTTGCTAGATGTAGTTCATCTCCXXXXXXXXXXXT
GATCCACACAAAAGTAGATCTCAGACAGGTTATCTATTAACATGTGGAGAAACCGCTATATCTTGGCATCAATAAAGTA
CACTTATGTATCTTGCTAACAATACTAGACCCGATATTGCATTTTTCAATAAATCTATTTGCTAGATGTAGTTCATCTCCXXXXXXXXXXXT
GATCCACACAAAAGTAGATCTCAGACAGATTATCTATTAACATGTGGAGAAACCGCTATATCTTGGCATCAATAAAGTA
>Marker115048 Chr4 SNP_site:17092705 Ref_Type:C
CACAAAGATCAAATCGTGAGTGCTACATTGTAATATATTCCACATGAATCCTATGTCAATCTTGATCATGTTTGCCTCTTXXXXXXXXXXXA
GCAATACCATTATATCGGGTGTGTAAGGCGTCCAACTAATTTGTTAAAAAAACCATAAGTAGGCAATAAAACGTAAGTA
CACAAAGATCAAATCGTGAGTGCTACATTGTAATATATTCCATATGAATCCTATGTCAATCTTGATCATGTTTGCCTCTTXXXXXXXXXXXA
GCAATACCATTATATCGGGTGTGTAAGGCGTCCAACTAATTTGTTAAAAAAACCATAAGTAGGCAATAAAACGTAAGTA
>Marker11510 Chr4 SNP_site:17198949 Ref_Type:C

Create PDF with GO2PDF for free, if you wish to

AACCACAGTAACATAACAAACAAAGTAAATAAATATACAGGAGTTTAAAAAATAATATTAATAATAAAAAACCTTCTGAAXXXXXXXXXXXG
GAATTTTATTAGTTGGAAATGAATTTGACCTGGACTAAATTAAAATCCTGAATTTTAGTAGAAAACATGGTAGAGGGTC
AACCACAGTAACATAACAAACAAAGTAAATAAATATACAGGAGTTTAAAAAATAATATTAATAATAAAAAACCTTCTGAAXXXXXXXXXXXG
GAATTTTATTAGTTGGAAATGAATTTGACCTGGACTCAATTAAAATCCTGAATTTTAGTAGAAAACATGGTAGAGGGTC
>Marker115526 Chr4 SNP_site:15788389 Ref_Type:C
AACAAGTTGGATTTGGACACAATATTTGCATTTCCCTTAGACCAAAGTGTGGAAATTGTAGTGTTAGTGACCTATGCTCAXXXXXXXXXXXA
GTCCTACATTTTCCCCAAGTTTTTGGTTTTCTATCTTTTTTATTTGTTTTTGTTTTCTTTTTTGGCACTATAATGGTT
AACAAGTTGGATTTGGACAGAATATTTACATTTCCCTTAGACCAAAGTGTGGAAATTGTAGTGTTAGTGACCTATGCTCAXXXXXXXXXXXA
GTCCTACATTTTCCCCAAGTTTTTGGTTTTCTATCTTTTTTATTTGTTTTTGTTTTCTTTTTTGGCACTATAATGGTT
>Marker115526 Chr4 SNP_site:15788397 Ref_Type:G
AACAAGTTGGATTTGGACACAATATTTGCATTTCCCTTAGACCAAAGTGTGGAAATTGTAGTGTTAGTGACCTATGCTCAXXXXXXXXXXXA
GTCCTACATTTTCCCCAAGTTTTTGGTTTTCTATCTTTTTTATTTGTTTTTGTTTTCTTTTTTGGCACTATAATGGTT
AACAAGTTGGATTTGGACAGAATATTTACATTTCCCTTAGACCAAAGTGTGGAAATTGTAGTGTTAGTGACCTATGCTCAXXXXXXXXXXXA
GTCCTACATTTTCCCCAAGTTTTTGGTTTTCTATCTTTTTTATTTGTTTTTGTTTTCTTTTTTGGCACTATAATGGTT
>Marker11576 Chr4 SNP_site:1519062 Ref_Type:A
ACCAATATTTCAATGAAAAGCATCCATTCATCAAATTTCCGATACTTTAAGTTTCTTCTCAAGTTTTCGATTTAAGCAGCXXXXXXXXXXG
CACCTACGAATCAGAGCTCATCAAGTTATTTTGCTTTGAGTCTCTTTTGCATTTGTCCTCACATAGTCAAGGGTTTTGT
ACCAATATTTCAATGAAAAGCATCCATTCATCAAATTTCCGATACTTTAAGTTTCTTCTCAAGTTTTCGATTTAAGCAGCXXXXXXXXXXG
CACCTACGAATCAGAGCTCATCAGGTTATTTTGCTTTGAGTCTCTTTTGCATTTGTCCTCACATAGTCAAGGGTTTTGT
>Marker115981 Chr4 SNP_site:15080178 Ref_Type:T
AACAAGCAAGGGATTCTTGAGAATATGCTCGCCTTTGGATTTATGGAATGAAATCCCTCCTTGAAATGGTGTTGCATTCTXXXXXXXXXXXT
TATTTAATAAAATTTGACACTATTTTCATAAAACTGATCATCACGAACTTAATGTCATAAATAATCTATGGTTAGGGTA
AACAAGCAAGGGATTCTTGAGAATATGCTCGCCTTTGGATTTATGGAATGAAATCCCTCCTTGAAATGGTGTTGCATTCTXXXXXXXXXXXT
TATTTAATATAATTTGACACTATTTTCGTAAAACTGATCATCACGAACTTAATGTCATAAATAATCTATGGTTAGGGTA
>Marker115981 Chr4 SNP_site:15080196 Ref_Type:G
AACAAGCAAGGGATTCTTGAGAATATGCTCGCCTTTGGATTTATGGAATGAAATCCCTCCTTGAAATGGTGTTGCATTCTXXXXXXXXXXXT
TATTTAATAAAATTTGACACTATTTTCATAAAACTGATCATCACGAACTTAATGTCATAAATAATCTATGGTTAGGGTA
AACAAGCAAGGGATTCTTGAGAATATGCTCGCCTTTGGATTTATGGAATGAAATCCCTCCTTGAAATGGTGTTGCATTCTXXXXXXXXXXXT
TATTTAATATAATTTGACACTATTTTCGTAAAACTGATCATCACGAACTTAATGTCATAAATAATCTATGGTTAGGGTA
>Marker116003 Chr4 SNP_site:17988182 Ref_Type:C
AACTATCTTACCTCAAAAGTTTAGCTTGAAATTCGAAGAATTGAGTTTTGTGGCCTCCAGAAACTCAACACTGCTTAACAXXXXXXXXXXXT
GAATCACTCAATTTTCACAAGAAAAATGAGAGAAATTTAATTCTAAAGTGAGCCTATTTATAGAGTTTTCTGCAAAGTG
AACTATCTTACCTCAAAAGTTTAGCTTGAAATTCGAAGAATTGAGTTTTGTGGCCTCCAGAAACTCAACACTGCTTAACAXXXXXXXXXXXT
GAATCACTCAATTTTCACCAGAAAAATGAGAGAAATTTAATTCTAAAGTGAGCCTATTTATAGAGTTTTCTGCAAAGTG
>Marker116100 Chr4 SNP_site:17405341 Ref_Type:A
TACCTTTACTGTTTTGAATTATTTGATTTGTTATTCCTATATCATATCTTCAATGACTCCAAGGTGTTTTCCTTTCCTTTXXXXXXXXXXC
ACCTAGCTACAACTACGACTCCTACTTCTCGTCGCTCTAACTCTCTTAAACTTTCTAAAGGAAGGCATGAACAAACGTC
TACCTTTACTGTTTTGAATTATTTGATTTGTTATTCCTATATCATATCTTCAATGACTCCAAGGTGTTTTCCTTTCCTTTXXXXXXXXXXC
ACCTAGCTACAACTACGACTCCTACTTCTCGTCGCTCTGACTCTCTTAAACTTTCTAAAGGAAGGCATGAACAAACGTC
>Marker116274 Chr4 SNP_site:20049300 Ref_Type:T
TACGGTCTGAACTATCGTTTGAATTACCGTTATACTTATTCCCAGGCATACGCTTACTTTAGAGCCTACCCCCTTCAAGTXXXXXXXXXXG
GGAAGATTTTAAGACGGCTCCCAACAAAATTAAAAATTGAGAGAATATTCATATAATTTTTTTAAAGAAAAATGGGTC
TACGGTCTGAACTATCGTTTGAATTACCGTTATACTTATTCCCAGGCATACGCTTACTTTAGAGCCTACCCCCTTCAAGTXXXXXXXXXXG
GGAAGATTTTAAGACGGCTCCCAACAAAATTAAAAATTGAGAGAATATTCATATAATTTTTTTTAAGAAAAATGGGTC

Create PDF with GO2PDF for free, if you wish to

>Marker116475 Chr4 SNP_site:11539184 Ref_Type:T
AACATCGATTCAGAAGAAAGTCAATAATCCAATCTTACTACCAAAACTTATCTAGATCTGCCATCGTTCTTACCTAATATXXXXXXXXXXC
AGTAGCCTAACTAGATAACTTCCATTACATCCTGCACATTTCTTCATCAACACCTGATTGATCTCTATTATATAATGTA
AACATCGATTCAGAAGAAAGTCAATAATCCAATCTTACTACCAAAACTTATCTAGATCTGCTATCGTTCTTACCTAATATXXXXXXXXXXC
AGTAGCCTAACTAGATAACTTCCATTACATCCTGCACATTTCTTCATCAACACCTGATTGATCTCTATTATATAATGTA
>Marker116562 Chr4 SNP_site:14979097 Ref_Type:T
GACCCCAGTTCAATCTCAGCTGTTGATCCTAAACTCTATTCCAAGCGGTTCAGAGATTTTATTGGGAGAATATTTATTGAXXXXXXXXXXG
GAAGTAAGCTGCTGCACGGATGGGTGTAGCAAATGCTTCAATGTGGTCCAAAACTTCCATATCGCCATCCAGAGACGGT
GACCCCAGTTCAATCTCAGCTGTTGATCCTAAACTCTATTCCAAGCGGTTCAGAGATTTTATTGGGAGAATATTTATTGAXXXXXXXXXXG
GAAGTAAGCTGCTGCACGGATGGGTGTAGCAAATGCTTCAATGTGGTCCAAAACTTCCATATCGTCATCCAGAGACGGT
>Marker116923 Chr4 SNP_site:8379735 Ref_Type:C
ACCACCAATTCCTCTAATGAACAATAGTTATAGTCCCACTATAACTGAACTCTTCTCGGGCCAAGAGAAGGTGTGGCACCXXXXXXXXXXG
AATGGTAGGCCTATTGAGTCAGTGACAAAGGCCACTCTCACTAATGCAAATCAAAGGACAACCTTCATAGGCAGGAGTT
ACCACCAATTCCTCTAATGAACAATAGTTATAGTCCCACTATCACTGAACTCTTCTCGGGCCAAGAGAAGGTGTGGCACCXXXXXXXXXXG
AATGGTAGGCCTATTGAGTCAGTGACAAAGGCCACTCTCACTAATGCAAATCAAAGGACAACCTTCATAGGCAGGAGTT
>Marker117010 Chr4 SNP_site:1196872 Ref_Type:G
ACTAAAATAATGAATTAAATTAACTTACAAACTTCGGCAAGGTAGAAAGAAGTCTTGTCCTTGTAAAATTCTGGAGAGAAXXXXXXXXXXC
ACTTCTAGAACTACAATTTTTTCCATTGCTAAATTCAGATTAAAAAAAAGGATAAAGATAAAGATGGAGAGAGCAAGTA
ACTAAAATAATTAATTAAATTAACTTACAAACTTCGGCAAGGTAGAAAGAAGTCTTGTCCTTGTAAAATTCTGGAGAGAAXXXXXXXXXXC
ACTTCTAGAACTACAATTTTTTCCATTGCTAAATTCAGATTAAAAAAAAGGATAAAGATAAAGATGGAGAGAGCAAGTA
>Marker117183 Chr4 SNP_site:18061213 Ref_Type:A
CACATAAAGAGAAAGACAACTATATATAAAATGACTATATGGTGGAATAAACTCAGCTAGTGCATAAAAAATGTGTATTGXXXXXXXXXXA
TGTGAAATGCATGTCTATGTGATATGTGTGTTTATGTAAAAGACGTGTTTAAGTGTTTTTTATATGAACAAATGATGTT
CACATAAAGAGAAAGGCAACTATATATAAAATGACTATATGGTGGAATAAACTCAGCTAGTGCATAAAAAATGTGTATTGXXXXXXXXXXA
TGTGAAATGTATGTCTATGTGATATGTGTGTTTATGTAAAAGACGTGTTTAAGTGTTTTTGATATGAACAAATGATGTT
>Marker117183 Chr4 SNP_site:18061449 Ref_Type:C
CACATAAAGAGAAAGACAACTATATATAAAATGACTATATGGTGGAATAAACTCAGCTAGTGCATAAAAAATGTGTATTGXXXXXXXXXXA
TGTGAAATGCATGTCTATGTGATATGTGTGTTTATGTAAAAGACGTGTTTAAGTGTTTTTTATATGAACAAATGATGTT
CACATAAAGAGAAAGGCAACTATATATAAAATGACTATATGGTGGAATAAACTCAGCTAGTGCATAAAAAATGTGTATTGXXXXXXXXXXA
TGTGAAATGTATGTCTATGTGATATGTGTGTTTATGTAAAAGACGTGTTTAAGTGTTTTTGATATGAACAAATGATGTT
>Marker117183 Chr4 SNP_site:18061500 Ref_Type:T
CACATAAAGAGAAAGACAACTATATATAAAATGACTATATGGTGGAATAAACTCAGCTAGTGCATAAAAAATGTGTATTGXXXXXXXXXXA
TGTGAAATGCATGTCTATGTGATATGTGTGTTTATGTAAAAGACGTGTTTAAGTGTTTTTTATATGAACAAATGATGTT
CACATAAAGAGAAAGGCAACTATATATAAAATGACTATATGGTGGAATAAACTCAGCTAGTGCATAAAAAATGTGTATTGXXXXXXXXXXA
TGTGAAATGTATGTCTATGTGATATGTGTGTTTATGTAAAAGACGTGTTTAAGTGTTTTTGATATGAACAAATGATGTT
>Marker118052 Chr4 SNP_site:19291792 Ref_Type:G
ACTCAAGCAAAAACAAGACTGACAATGACTAGAGAATCGGGAGGGACTAGAGCACCACAGAGTTCAAAATCAACGACGAAXXXXXXXXXXA
ATACAGCTGAAAATTAATATAAATAGAACGGATTATAAGCATAAATTTCACTCAAGTAATGAAATTTGGATGAATGGTT
ACTCGAGCAAAAACAAGACTGACAATGACTAGAGAATCGGGAGGGACTAGAGCACCACATAGTTCAAAATCAACGACGAAXXXXXXXXXXA
CTACAGCTGAAAATTAATATAAATAGAACGGATTATAAGCATAAATTTCACTCAAGTAATGAAATTTGGATGAATGGTT
>Marker118052 Chr4 SNP_site:19291847 Ref_Type:T
ACTCAAGCAAAAACAAGACTGACAATGACTAGAGAATCGGGAGGGACTAGAGCACCACAGAGTTCAAAATCAACGACGAAXXXXXXXXXXA
ATACAGCTGAAAATTAATATAAATAGAACGGATTATAAGCATAAATTTCACTCAAGTAATGAAATTTGGATGAATGGTT
ACTCGAGCAAAAACAAGACTGACAATGACTAGAGAATCGGGAGGGACTAGAGCACCACATAGTTCAAAATCAACGACGAAXXXXXXXXXXA

Create PDF with GO2PDF for free, if you wish to

CTACAGCTGAAAATTAATATAAATAGAACGGATTATAAGCATAAATTTCACTCAAGTAATGAAATTTGGATGAATGGTT

>Marker118052 Chr4 SNP_site:19292006 Ref_Type:C

ACTCAAGCAAAAACAAGACTGACAATGACTAGAGAATCGGGAGGGACTAGAGCACCACAGAGTTCAAAATCAACGACGAAXXXXXXXXXXA

ATACAGCTGAAAATTAATATAAATAGAACGGATTATAAGCATAAATTTCACTCAAGTAATGAAATTTGGATGAATGGTT

ACTCGAGCAAAAACAAGACTGACAATGACTAGAGAATCGGGAGGGACTAGAGCACCACATAGTTCAAAATCAACGACGAAXXXXXXXXXXA

CTACAGCTGAAAATTAATATAAATAGAACGGATTATAAGCATAAATTTCACTCAAGTAATGAAATTTGGATGAATGGTT

>Marker118103 Chr4 SNP_site:11312740 Ref_Type:T

TACAAAGCTATTGTAATTAGAACAACAGGTTGGAGAATCAAAGATCTAACGTCGAGGTTGATAGTCTAAGCTATATAAAAXXXXXXXXXXG

CATTCCAATGCTAGATTTTGTTTTAATAAAGTCGAAAGTCATACACATACATCCACACCATATCATTCATTTTAACGTC

TACAAAGCTATTGTAATTAGAACAACAGGTTGGAGAATCAAAGATCTAACGTCGAGGTTGATAGTCTAAGCTATATAAAAXXXXXXXXXXG

CATTCCAATGCTTGATTTTGTTTTAATAAAGTCGAAAGTCATACACATACATCCACACCATATCATTCATTTTAACGTC

>Marker118476 Chr4 SNP_site:6236905 Ref_Type:T

ACCGTTTTTTCAACTCCGTTTTCTGTGCAAAATATGTCAAATAAAATAATAAGAAAAGTCAAGAGAGTGAATTACAATATXXXXXXXXXXG

AAATGAAGGTTTTGGTCGTGCAGATTATTTCCTTGTGGGAAAGGACTTCCCCAGTTATATTGAATGTCAAGAGAAGGTC

ACCGTTTTTTCAACTCCGTTTTCTTTGCAAAATATGTCAAATAAAATAATAAGAAAAGTCAAGAGAGTGAATTACAATATXXXXXXXXXXG

AAATGAAGGTTTTGGTCGTGCAGATTATTTCCTTGTGGGAAAGGACTTCCCCAGTTATATTGAATGTCAAGAGAAGGTC

>Marker118567 Chr4 SNP_site:11907660 Ref_Type:T

ACAAATATCGCTTTGCAGTTCAACTCACGAGTAAGCTTATACACATTAGATTATAGAAAATTTGGGGCACATACAACACAXXXXXXXXXXT

GATTAGTGGCACCAGAGTCTAGAATCCAGAATTTATAGATTTTTTCCATCAACACCAAAAAGACTGAAGGACTGAGGTA

ACAAATATCGCTTTGCAGTTCAACTCATGAGTAAGCTTATACACATTAGATTATAGAAAATTTGGGGCACATACAACACAXXXXXXXXXXT

GATTAGTGGCACCAGAGTCTAGAATCCAGAATTTATAGATTTTTTCCATCAACACCAAAAAGACTGAAGGACTGAGGTA

>Marker118751 Chr4 SNP_site:18096789 Ref_Type:A

ACTCTCAGTTCTTAATAATAAAGAAATTTTGTCTCCGTTTAAGAAAAAAAAACAAAACCCATGATTGGGTAGATCGATATGXXXXXXXXXXA

TCCTTAGTGATGTTTGTCCTTTGTGAAGTTTATCCTTAGTCAAGTTTTGTTCTTTATGCATTTTTGTTCTGGATCCTGT

ACTCTCAGTTCTTAATAATAAAGAAGTTTTGTCTCCGTTTAAGAAAAAAAAACAAAACCCATGATTGGGTAGATCGATATGXXXXXXXXXXA

TCCTTAGTGATGTTTGTCCTTTGTGAAGTTTATCCTTAGTCAAGTTTTGTTCTTTATGCATTTTTGTTCTGGATCCTGT

>Marker119299 Chr4 SNP_site:6069963 Ref_Type:G

TACATTAATTTGAACACATGAGTAAGTATCATATTTGAAGATAAAGAACCATACATCAACAGTTCAATGAAGGGCCACAAXXXXXXXXXXA

CACACAAAAGATCAATAGCAGAAAACACCTTTACCTTTTCCAGAGCTAGAGCAAGCTTTGAGAGATTGGGATAGATGGT

TACATTAATTTGAACACATGAGTAAGTATCATATTTGAAGATAAAGAACCATACATCAACAGTTCAATTAAGGGCCACAAXXXXXXXXXXA

CACACAAAAGATCAATAGCAGAAAACACCTTTACCTTTTCCAGAGCTAGAGCAAGCTTTGAGAGATTGGGATAGATGGT

>Marker119551 Chr4 SNP_site:18305239 Ref_Type:A

CACAAAACTGGTCAAGCTTGTTGAACATGATAATCGAACTGGTTGGAAGATAAATTTGTAGATTTACCTCTGTCACATGAXXXXXXXXXXA

AATGTGATCTCAATGCAAGCTTTCCTAATAAGAGAAATGAGAATGAGGTCCTAAAGTCCTAAGAGAACTGACAATAAGT

CACAAAACTGGTCAAGCTTGTTGAACATGATAATCGGACTGGTTGGAAGATAAATTTGTAGATTTACCTCTGTCACATGAXXXXXXXXXXA

AATGTGATCTCAATGCAAGCTTTCCTAATAAGAGAAATGAGAATGAGGTCCTAAAGTCCTAAGAGAACTGACAATAAGT

>Marker119618 Chr4 SNP_site:17249476 Ref_Type:T

ACCTTTGAGAATGCATCACTCATGATTCCATGGCCTTGATTTTCTTTTTCCTTTTTTCTTTTTGATGAAAAGCATGACTTXXXXXXXXXXC

ATAAAACCCCTTAATTTTAGAAGCTTAAAAAAGAAAGCGGATTCAATGTCAAAAATGGCCCTAAAATTCTTTCCGTGTG

ACCTTTGAGAATGTATCACTCATGATTCCATGGCCTTGATTTTCTTTTTCCTTTTTTCTTTTTGATGAAAAGCATGACTTXXXXXXXXXXC

ATAAAACCCCTTAATTTTAGAAGCTTAAAAAAGAAAGCGGATTCAATGTCGAAAATGGCCCTAAAGTTCTTTCCGTGTG

>Marker119618 Chr4 SNP_site:17249739 Ref_Type:G

ACCTTTGAGAATGCATCACTCATGATTCCATGGCCTTGATTTTCTTTTTCCTTTTTTCTTTTTGATGAAAAGCATGACTTXXXXXXXXXXC

ATAAAACCCCTTAATTTTAGAAGCTTAAAAAAGAAAGCGGATTCAATGTCAAAAATGGCCCTAAAATTCTTTCCGTGTG

Create PDF with GO2PDF for free, if you wish to

ACCTTTGAGAATGTATCACTCATGATTCCATGGCCTTGATTTTCTTTTTCCTTTTTTCTTTTTGATGAAAAGCATGACTTXXXXXXXXXXC
ATAAAACCCCTTAATTTTAGAAGCTTAAAAAAGAAAGCGGATTCAATGTCGAAAATGGCCCTAAAGTTCTTTCCGTGTG
>Marker119618 Chr4 SNP_site:17249754 Ref_Type:G
ACCTTTGAGAATGCATCACTCATGATTCCATGGCCTTGATTTTCTTTTTCCTTTTTTCTTTTTGATGAAAAGCATGACTTXXXXXXXXXXC
ATAAAACCCCTTAATTTTAGAAGCTTAAAAAAGAAAGCGGATTCAATGTCAAAAATGGCCCTAAAATTCTTTCCGTGTG
ACCTTTGAGAATGTATCACTCATGATTCCATGGCCTTGATTTTCTTTTTCCTTTTTTCTTTTTGATGAAAAGCATGACTTXXXXXXXXXXC
ATAAAACCCCTTAATTTTAGAAGCTTAAAAAAGAAAGCGGATTCAATGTCGAAAATGGCCCTAAAGTTCTTTCCGTGTG
>Marker119820 Chr4 SNP_site:16506122 Ref_Type:A
CACAACATAGCCAAAATCCAGCCAAGCCATCAATGCATTTCATCCAACTTTGTAGAAAATGATTCATTAAAAAGATATTAXXXXXXXXXXC
TGTTAGCCATGTGCAAAAAAATATTAGATCTCAAATTGTAATCAGTAAAAGTTTGTTAAATAATTTGAACAATAGTGTT
CACAACATAGCCAAAATCCAGCCAAGCCATCAATGCATTTCATCCAACTTTGTAGGAAATGATTCATTAAAAAGATATTAXXXXXXXXXXC
TGTTAGCCATGTGCAAAAAAATATTAGATCTCAAATTGTAATCAGTAAAAGTTTGTTAAATAATTTGAACAATAGTGTT
>Marker120304 Chr4 SNP_site:7609381 Ref_Type:T
CACCCACCTCTCTATATAGACATATCCCAAGAAGCTATATCTATAGTTTTTCCAAAATAAAGGGTTAATGAGCATCTAAGXXXXXXXXXXA
AGGGGTTACCGAGCATTGTTTTTGTGCCTTAACTAAAAAACTGTCATAAAACAAAACTGTAATGTGTTTTCAAAGGGTG
CACCCACCTCTCTATATAGACATATCCCAAGAAGCTATATCTATAGTTTTTCTAAAATAAAGGGTTAATGAGCATCTAAGXXXXXXXXXXA
AGGGGTTACCGAGCATTGTTTTTGTGCCTTAACTAAAAAACTGTCATAAAACAAAACTGTAATGTGTTTTCAAAGGGTG
>Marker120354 Chr4 SNP_site:18622182 Ref_Type:A
GACTTTGGGACAAACGTCATCCATGAGTTCTTTGATTAATGGAAAATGAGATTGGACATGACATCTTAAAGAACCCAAGAXXXXXXXXXXT
CTGGCTAGTGTTTGTGAAGAAAAAGATCATGCCTACTCGCCATGACAACAACATCTCCATGGAAAGGGTAATGTTGGTA
GACTTTGGGACAAACGTCATCCATGAGTTCTTTGGTTAAAGGAAAATGAGATTGGACATGACATCTTAAAGAACCCAAGAXXXXXXXXXXT
CTGGCTAGTGTTTGTGAAGAAAAAGATCATGCCTACTCGCCATGACAGCAACATCTCCATGGAAAGGGTAATGTTGGTA
>Marker120354 Chr4 SNP_site:18622187 Ref_Type:T
GACTTTGGGACAAACGTCATCCATGAGTTCTTTGATTAATGGAAAATGAGATTGGACATGACATCTTAAAGAACCCAAGAXXXXXXXXXXT
CTGGCTAGTGTTTGTGAAGAAAAAGATCATGCCTACTCGCCATGACAACAACATCTCCATGGAAAGGGTAATGTTGGTA
GACTTTGGGACAAACGTCATCCATGAGTTCTTTGGTTAAAGGAAAATGAGATTGGACATGACATCTTAAAGAACCCAAGAXXXXXXXXXXT
CTGGCTAGTGTTTGTGAAGAAAAAGATCATGCCTACTCGCCATGACAGCAACATCTCCATGGAAAGGGTAATGTTGGTA
>Marker120354 Chr4 SNP_site:18622391 Ref_Type:A
GACTTTGGGACAAACGTCATCCATGAGTTCTTTGATTAATGGAAAATGAGATTGGACATGACATCTTAAAGAACCCAAGAXXXXXXXXXXT
CTGGCTAGTGTTTGTGAAGAAAAAGATCATGCCTACTCGCCATGACAACAACATCTCCATGGAAAGGGTAATGTTGGTA
GACTTTGGGACAAACGTCATCCATGAGTTCTTTGGTTAAAGGAAAATGAGATTGGACATGACATCTTAAAGAACCCAAGAXXXXXXXXXXT
CTGGCTAGTGTTTGTGAAGAAAAAGATCATGCCTACTCGCCATGACAGCAACATCTCCATGGAAAGGGTAATGTTGGTA
>Marker120517 Chr4 SNP_site:1572211 Ref_Type:A
ACTATAGGCCTTTTATTAAGGACACTTTCATCGGTTATATCTCATTCTTACCCAATTTTTGGTGTAATTGTTCCTTTTCAXXXXXXXXXXC
CAGTAATCAGCATATTGTAAATATCATGCCAGAGCTAAGGAGCAACAAGTGGCAATAAACAATGGGAACTACTAGAGTA
ACTATAGGCCTTTTATTAAGGACACTTTCATCGGTTATATCTCATTCTTACCCAATTTTTGGTGTAATTGTTCCTTTTCAXXXXXXXXXXC
CAGTAATCAGCATATTGTAAATATCTTGCCAGAGCTAAGGAGCAACAAGTGGCAATAAACAATGGGAACTACTAGAGTA
>Marker120713 Chr4 SNP_site:18518230 Ref_Type:T
ACCTTTATGAATAATCTCCAACAACTTAGTGTTACTTTTTTTAAATAGAAACAAGACCTTTTCATTGGATTAACGAAATGXXXXXXXXXXA
ATCCTTACAACAAATGAAAATAACTAGTTAAACATTAAACACTAAGGGGTTGTTTGGCCCGACTTCATTCGTCGATGTT
ACCTTTATGAATAATCTCCAACAACTTAGTGTTACTTTTTTTAAATAGAAACAATACCTTTTCATTGGATTAACGAAATGXXXXXXXXXXA
ATCCTTACAACAAATGAAAATAACTAGTTAAACATTAAACACTAAGGGGTTGTTTGGCCCGACTTCATTCGTCGATGTT
>Marker120766 Chr4 SNP_site:23120943 Ref_Type:A
CACAGTAAATAGTTTCCTAGTCACCATCACCCTTATATATTTATATCTACATTTTCTGCAGGACCAGAAACTGAATAATTXXXXXXXXXXT

Create PDF with GO2PDF for free, if you wish to

TGGGGGTCAATCTTCGTCCGATGAAGCACAGAAATCCTGAGGTAAGGTATTTTATTTTTGGATATATGCTATCTTGGTG
CACAGTAAATGGTTTCCTAGTCACCATCACCCTTATATATTTATATCTACATTTTCTGCAGGACCAGAAACTGAATAATTXXXXXXXXXXT
TGGGGGTCAATCTTCGTCCGATGAAGCACAGAAATCCTGAGGTAAGGTATTTTATTTTTGGATATATGCTATCTTGGTG
>Marker120883 Chr4 SNP_site:12491056 Ref_Type:T
ACGAATAATGTGTGTGTGTGTGTATAACTATATATATAGTTTGCTGATGTGATGCTATTGGGCAGATTCATCAGCGCTCAXXXXXXXXXXXT
TTTAATTTCCTCATGCTAGGTGATCTAAGATTATTATTGCATTATGAAGATGAATTTTTCTCAAATTCCAATATTTGTA
ACGAATAATGTGTGTGTGTGTGTATAACTATATATATAGTTTGTTGATGTGATGCTATTGGGCAGATTCATCAGCGCTCAXXXXXXXXXXXT
TTTAATTTCCTCATGCTAGGTGATCTAAGATTATTATTGCATTATGAAGATGAATTTTTCTCAAATTCCAATATTTGTA
>Marker121698 Chr4 SNP_site:47798 Ref_Type:G
AACAAGAGCCTATCCAATCAAAGGGAGCGTTCCATCCAATTGAACAGTGAAGGCCTTGAGGTCAATTTCCTTATGATTTTXXXXXXXXXXA
ATGAAATTATTATTATATTCCACCATGAATATTGAAAGACTGGCTGGACATGCAAAATGAAAATCACAAATAATGGGTC
AACAAGAGCCTATCCAATCAAAGGGAGCGTTCCATGCAATTGAACAGTGAAGGCCTTGAGGTCAATTTCCTTATGATTTTXXXXXXXXXXA
ATGAAATTATTATTATATTCCACCATGAATATTGAAAGACTGGCTGGACATGCAAAATGAAAATCACAAATAATGGGTC
>Marker121748 Chr4 SNP_site:6820181 Ref_Type:C
AACCCCAAATCTGAATTATTGCTTCAAAAGTTATATTTCAGCATGTCATTCTTGCTCAATTATACCCAGCATGACTTTTAXXXXXXXXXXA
GCTTATCGGGAACCATTCCAATGTCATTAACTAATGTTAAATCCTTGCAAGTCCTGTGAGTGTTGTATTTATAAACGTT
AACCCCAAATCTGAATTATTGCTTCAAAAGTTATATTTCAGCATGTGATTCTTGCTCAATTATACCCAGCATGACTTTTAXXXXXXXXXXA
GCTTATCGGGAACCATTCCAATGTCATTAACTAATGTTAAATCCTTGCAAGTCCTGTGAGTGTTGTATTTATAAACGTT
>Marker121801 Chr4 SNP_site:1533335 Ref_Type:C
ACTTTCCCTAACACTCGCAAGTTCTGCTGAATCATTCGAGCTTTCCTTTTCACATGTGTATGATTTATCTATACAATTATXXXXXXXXXXG
TATCATCCGACACTCCTCGATCAGGAACTTGTAACGTTCCCTGCATGAAAAAATTTAACAGTCATTTCATTTGATAGTG
ACTTTCCCTAACACTTGCAAGTTCTGCTGAATCATTCGAGCTTTCCTTTTCACATGTGTATGATTTATCTATACAATTATXXXXXXXXXXG
TATCATCCGACACTCCTCGATCAGGAACTTGTAACGTTCCCTGCATGAAAAAACTTAACAGTCATTTCATTTGATAGTG
>Marker121801 Chr4 SNP_site:1533613 Ref_Type:C
ACTTTCCCTAACACTCGCAAGTTCTGCTGAATCATTCGAGCTTTCCTTTTCACATGTGTATGATTTATCTATACAATTATXXXXXXXXXXG
TATCATCCGACACTCCTCGATCAGGAACTTGTAACGTTCCCTGCATGAAAAAATTTAACAGTCATTTCATTTGATAGTG
ACTTTCCCTAACACTTGCAAGTTCTGCTGAATCATTCGAGCTTTCCTTTTCACATGTGTATGATTTATCTATACAATTATXXXXXXXXXXG
TATCATCCGACACTCCTCGATCAGGAACTTGTAACGTTCCCTGCATGAAAAAACTTAACAGTCATTTCATTTGATAGTG
>Marker122022 Chr4 SNP_site:7259665 Ref_Type:A
ACCAAGCAAGAACGTCCGCAGGGCGGGGTATATGATAATAAATGTTTTTCCTATTTTCTTTAATAGATAAAACAACTATAXXXXXXXXXXA
ATATTAAGCAAAATATCTGCAATTTGTAATTTATTGAGTATAATGAAAATGAAGAATGCTATCATAATTAATTTGAGTT
ACCAAGCAAGAACGTCCGCAGGGCGGGGTATATGATGATAAATGTTTTTCCCATTTTCTTTAATAGATAAAACAACTATAXXXXXXXXXXA
ATATTAAGCAAAATATCTGCAATTTGTAATTTATTGAGTATAATGAAAATGAAGAATGCTATCATAATTAATTTGAGTT
>Marker122022 Chr4 SNP_site:7259680 Ref_Type:T
ACCAAGCAAGAACGTCCGCAGGGCGGGGTATATGATAATAAATGTTTTTCCTATTTTCTTTAATAGATAAAACAACTATAXXXXXXXXXXA
ATATTAAGCAAAATATCTGCAATTTGTAATTTATTGAGTATAATGAAAATGAAGAATGCTATCATAATTAATTTGAGTT
ACCAAGCAAGAACGTCCGCAGGGCGGGGTATATGATGATAAATGTTTTTCCCATTTTCTTTAATAGATAAAACAACTATAXXXXXXXXXXA
ATATTAAGCAAAATATCTGCAATTTGTAATTTATTGAGTATAATGAAAATGAAGAATGCTATCATAATTAATTTGAGTT
>Marker122945 Chr4 SNP_site:6371700 Ref_Type:G
ACATTTCTTCTACTTCTACCTCTCCCATCTCTCTTCCAAAATTCTTTCAATATTCATTTTCAATCCATTTAATTAGTTTAXXXXXXXXXXA
TCGCCTAACAAATGAAAGCACGTATTTAACTAAACAAACGACGAAGAAATTGTAGCCTATATATATTTCTCCCTTTTGT
ACATTTCTTCTACTTCTACCTCTCCCATCTCTCTTCCAAAATTCTTTCAATATTCATTTTCAATCCATTTAATTAGTTTAXXXXXXXXXXA
TCGCCTAACAAGTGAAAGCACGTATTTAACTAAACAAACGACGAAGAAATTGTAGCCTATATATATTTCTCCCTTTTGT
>Marker123090 Chr4 SNP_site:6004664 Ref_Type:A

Create PDF with GO2PDF for free, if you wish to

CACATATCTCTCTACAGAAAACACAAAAGTGAAAACTTTAGCTTTGCTTATTATGGAGGGTAACGCGTGGAAGTGAGTCAXXXXXXXXXXXA
AAAAAAAAACCCTTTCCTCGAATATCCTAAACCACCCATTAGTTCCTATTTTCTTGTTCTATCATCATTCATATTAGTC
CACATATCTCTCTACAGAAAACACAAAAGTGAAAACTTTAGCTTTGCTTATTATGGAGGGTAACGCGTGGAAGTGAGTCAXXXXXXXXXXXA
AAATAAAAACCCTTTCCTCGAATATCCTAAACCACCCATTAGTTCCTATTTTCTTGTTCTATCATCATTCATATTAGTC
>Marker12313 Chr4 SNP_site:13445893 Ref_Type:G
ACCTGTCTTGATCTGTGATCAACGTTTGCTTGGCTTTCTGCATCTTGGGGCTTGAAACATTCAAAGCTGATGAGTTTTGAXXXXXXXXXXXT
TCCATTATGGTGAGCTTTCCTTACAGGAGAACTAGAGGAAGACAACATACCAATACCACCACCATGATTAACAGTTGGT
ACCTTTCTTGATCTGTGATCAACGTTTGCTTGGCTTTCTGCATCTTGGGGCTTGAAACATTCAAAGCTGATGAATTTTGAXXXXXXXXXXXT
TCCATTATGGTGAGCTTTCCTTACAGGAGAACTAGAGGAAGACAACATACCAATACCACCACCATGATTAACAGTTGGT
>Marker12313 Chr4 SNP_site:13445962 Ref_Type:G
ACCTGTCTTGATCTGTGATCAACGTTTGCTTGGCTTTCTGCATCTTGGGGCTTGAAACATTCAAAGCTGATGAGTTTTGAXXXXXXXXXXXT
TCCATTATGGTGAGCTTTCCTTACAGGAGAACTAGAGGAAGACAACATACCAATACCACCACCATGATTAACAGTTGGT
ACCTTTCTTGATCTGTGATCAACGTTTGCTTGGCTTTCTGCATCTTGGGGCTTGAAACATTCAAAGCTGATGAATTTTGAXXXXXXXXXXXT
TCCATTATGGTGAGCTTTCCTTACAGGAGAACTAGAGGAAGACAACATACCAATACCACCACCATGATTAACAGTTGGT
>Marker123378 Chr4 SNP_site:17582547 Ref_Type:G
ACTTGTAAATTGTTTCAAACTTGATTGGAAATGTTATAATTAAATCTATGGTTGTTCTTCTCTTCTGTTGTGTGTCATTTXXXXXXXXXXXT
GAGCAGTGAAGCCAACATGGAGCAATGGATTGATATAGGACTTTTTGTATGGTTGGTAGAAAAATCTTGATTCCAATGT
ACTTGTAAATTGTTTCAAACTTGATTGGAAATGTTATAATTAAATCTATGGTTGTTCTTCTCTTCTGTTGTGTGTCATTTXXXXXXXXXXXT
GAGCAGTGAAGCCAAGATGGAGCAATGGATTGATATAGGACTTTTTGTATGGTTGGTAGAAAAATCTTGATTCCAATGT
>Marker123715 Chr4 SNP_site:7563775 Ref_Type:C
TACTCCTCCTAATTCCAACGGTAAGAAATTAGCCGTTAATTTCCAGCCGCCCAGCTTCACTTCTACTGTTTCACAGACTCXXXXXXXXXXXG
AGATGAAATTGTGGGTAGCTCCACAATCGATCAGAATTATTACTTCCTTTTCTTTGATGCTTCCTTTGACGTTCATAGT
TACTCCTCCTAATTCTAACGGTAAGAAATTAGCCGTTAATTTCCAGCCGCCCAGCTTCACTTCTACTGTTTCACAGACTCXXXXXXXXXXXG
AGATGAAATTGTGGGTAGCTCCACAATCGATCAAAATTATTACTTCCTTTTCTTTGATGCTTCCTTTGACGTTCATAGT
>Marker123715 Chr4 SNP_site:7563966 Ref_Type:G
TACTCCTCCTAATTCCAACGGTAAGAAATTAGCCGTTAATTTCCAGCCGCCCAGCTTCACTTCTACTGTTTCACAGACTCXXXXXXXXXXXG
AGATGAAATTGTGGGTAGCTCCACAATCGATCAGAATTATTACTTCCTTTTCTTTGATGCTTCCTTTGACGTTCATAGT
TACTCCTCCTAATTCTAACGGTAAGAAATTAGCCGTTAATTTCCAGCCGCCCAGCTTCACTTCTACTGTTTCACAGACTCXXXXXXXXXXXG
AGATGAAATTGTGGGTAGCTCCACAATCGATCAAAATTATTACTTCCTTTTCTTTGATGCTTCCTTTGACGTTCATAGT
>Marker123749 Chr4 SNP_site:7958923 Ref_Type:A
TACTATCTATGGATGCTTAGTTTAAATACCTATTCTTATGTGGTTGAGTTTTATCGTAGAGAAACTATGTCAGCAACATAXXXXXXXXXXXC
ATCTATAGGTGAATTTAAAAGTTTATCAAGATGTTCTAATTGTAACCGTAAGGGTCACAATAGTAGGACTTGCAGAGTT
TACTATCTATGGATGCTTAGTTTAAATACCTATTCTTATGTGGTTGAGTTTTATCGTAGAGAAACTATGTCAGCAACATAXXXXXXXXXXXC
ATCTATAGGTGAATTTAAAAGTTTATCAAGATGTTCTAATTGTAACCGTAAGGGTCACAATAGTAGGTCTTGCAGAGTT
>Marker123923 Chr4 SNP_site:5982562 Ref_Type:T
CACAACCTAGGTTAGCATGCACAAATTTATATCATTAACTCTTTTAGCACAGAACATGAAAGGTCCAGTCAAGAGTGGCAXXXXXXXXXXXC
CAAAGGTCTTGGGTTCAAGACTCATACAATTATTGTGCCCCCTACATATGATTTCTGGATACACCACTAAAGAGTGGTA
CACAACCTAGTTTAGCATGCACAAATTTCTATCATTAACTCTTTTAGCACAGAACATGAAAGGTCCAGTCAAGAGTGGCAXXXXXXXXXXXC
CAAAGGTCTTGGGTTCAAGACTCATACAATTATTGTGCCCCCTACATATGATTTCTGGATACACCACTAAAGAGTGGTA
>Marker123923 Chr4 SNP_site:5982580 Ref_Type:C
CACAACCTAGGTTAGCATGCACAAATTTATATCATTAACTCTTTTAGCACAGAACATGAAAGGTCCAGTCAAGAGTGGCAXXXXXXXXXXXC
CAAAGGTCTTGGGTTCAAGACTCATACAATTATTGTGCCCCCTACATATGATTTCTGGATACACCACTAAAGAGTGGTA
CACAACCTAGTTTAGCATGCACAAATTTCTATCATTAACTCTTTTAGCACAGAACATGAAAGGTCCAGTCAAGAGTGGCAXXXXXXXXXXXC
CAAAGGTCTTGGGTTCAAGACTCATACAATTATTGTGCCCCCTACATATGATTTCTGGATACACCACTAAAGAGTGGTA

Create PDF with GO2PDF for free, if you wish to

>Marker123929 Chr4 SNP_site:3769223 Ref_Type:T
CACGATTCAGTTTGGTTCTATTACAATCTAAAACCGCACCGAACCGCAAAATGTAAAAAGATCTTATACTAAACCGAATCXXXXXXXXXXT
TCGAACAATCTCGGACTAGCAAAGCACTTAGTTCTATAAAATTTCCCTTTTTATTTTAGATATCAGTGGATCTCGGGTC
CACGATTCAGTTTGGTTCTATTTCAATCTAAAACCGCACCGAACCGCAAAATGTAAAAAGATCTTATACTAAACCGAATCXXXXXXXXXXT
TCGAACAATCTCGGACTAGCAAAGCACTTAGTTCTATAAAATTTCCCTTTTTATTTTAGATATCAGTGGATCTCGGGTC
>Marker124132 Chr4 SNP_site:5434983 Ref_Type:C
AACTGTTAAGAAAGAACTTTTCAACAGTTAAGCCGGGCTGTCAGCCTTGTTATATATGTGTTTAGGGTATTATTATTTGTXXXXXXXXXXT
TTGGAAGTTAGTGGATAAAGTGAGGGGGGCTTCCTCTTTTGCTATATACCTGACCGGCCCCACAATTTGATTGCACGTG
AACTGTTAAGAAAGAACTTTTCAACAGTTAAGCCGGGCTGTCAGCCTTGTTATATATGTGTTTAGGGTATTATTATTTGTXXXXXXXXXXT
TTGGAAGTTAGTGGATAAAGTGAGGGGGGCTTCCTTTTTTGCTATATACCTGACCGGCCCCACAATTTGATTGCACGTG
>Marker124221 Chr4 SNP_site:11875733 Ref_Type:A
ACATTCGGCAAGTTCCTTTATGACAACCTATTACGGCACCCTGCACATACATATAAGGATCCATTGATTTTTTTTTTTTAXXXXXXXXXXT
ATCTGGAATGTTCCATATCCTCACCTTTGCATCTAATGCTCCACTTATGAAATAATCATCATCCATTGGGTTGAACTGT
ACATTCGGCAAGTTCCTTTATGACAACCTATTACGGCACCCTGCACATACATATAAGGATCCATTGATTTTTTTTTTTTTXXXXXXXXXXT
ATCTGGAATGTTCCATATCCTCACCTTTGCATCTAATGCTCCACTTATGAAATAATCATCATCCATTGGGTTGAACTGT
>Marker124553 Chr4 SNP_site:22891305 Ref_Type:G
AACGCGCCATTGGATTTGAACTCAAATCAAATGCTCATTATGGCTACAAATCATTCCAAATCAAGGTTTGGATTCTCTAAXXXXXXXXXXT
GTAAGCAACTGTTAAGAGTAGAATACCCCAAATGCTATGAAATAGGATCAGTGCTATTGATGAGGTTTAGAAAATAAGT
AACGCGCCATTGGATTTGAACTCAAATCAAATGCTCATTATGGCTACAAATCATTCCAAATCAAGGTTTGGATTCTCTAAXXXXXXXXXXT
GTAAGCAACTGTTAAGAGTAGAATACCCCAAATGCTATGAAATAGGATCAGTGCTGTTGATGAGGTTTAGAAAATAAGT
>Marker124645 Chr4 SNP_site:4049790 Ref_Type:T
CACTCAAACTGTTGAAGGAAGCTCAGAGTAGAAGTATCTGGGCTTAGTGGAATGTCAAGGGGTTTGGGATCAGAAGAAAAXXXXXXXXXXA
GTTATAGGAACATCAGATGGCTTACTATTCGTTCCGTTGGTATTGCAAGGTGAATTGTTTCTTTCTGGAACGGGAAGTT
CACTCAAACTGTTGAAGGAAGCTCAGAGTAGAAGTATCTGGGCTTAGTGGAATGTCAAGGGGTTTGGGATCAGAAGAAAAXXXXXXXXXXA
GTTATAGGAACATCAGATGGCTTACTATTCGTTCTGTTGGTATTGCAAGGTGAATTGTTTCTTTCTGGAACGGGAAGTT
>Marker124955 Chr4 SNP_site:19210406 Ref_Type:A
ACGAGACTATTCATGCTTGTAACTATGACTGTGTATTGTTCTAGAAGAAGTTTGAGGAGTTGCAACAATGTCCTACTTGTXXXXXXXXXXA
TACATGTGATGACATAAGAATAAACGAGTGGAAACAGAGCATGTGTTGAGACATCCAATCGATGTAGAGGGATGAAAGT
ACGAGACTATTCATGCTTGTAACTATGACTGTGTATTGTTCTGGAAGAAGTTTGAGGAGTTGCAACAATGTCCTACTTGTXXXXXXXXXXA
TACATGTGATGACATAAGAATAAACGAGTGGAAACAGAGCATGTGTTGAGACATCCAATCGATGTAGAGGGATGAAAGT
>Marker125079 Chr4 SNP_site:4489840 Ref_Type:C
TACAGCGCCGCGATTTTAGCCCTCTTCTATCACCACATCCTTTCCATTCTTCGAACTTCCTCCATTTCATCTTCCTTAATXXXXXXXXXXC
AGAGGATTCCGATTTCCCGGCGGTTGATGTGTTTATTTGCACCACGGATCCTGAAAAGGAGCCACCCATGAGCGTGGTG
TACAGCGCCGCGATTTTCGCCCTCTTCTATCACCACATCCTTTCCATTCTTCGAAGTTCCTCCATTTCATCTTCCTTAATXXXXXXXXXXC
AGAGGATTCCGATTTCCCGGCGGTTGATGTGTTTATTTGCACCACGGATCCTGAAAAGGAGCCACCTATGAGCGTGGTG
>Marker125079 Chr4 SNP_site:4489878 Ref_Type:G
TACAGCGCCGCGATTTTAGCCCTCTTCTATCACCACATCCTTTCCATTCTTCGAACTTCCTCCATTTCATCTTCCTTAATXXXXXXXXXXC
AGAGGATTCCGATTTCCCGGCGGTTGATGTGTTTATTTGCACCACGGATCCTGAAAAGGAGCCACCCATGAGCGTGGTG
TACAGCGCCGCGATTTTCGCCCTCTTCTATCACCACATCCTTTCCATTCTTCGAAGTTCCTCCATTTCATCTTCCTTAATXXXXXXXXXXC
AGAGGATTCCGATTTCCCGGCGGTTGATGTGTTTATTTGCACCACGGATCCTGAAAAGGAGCCACCTATGAGCGTGGTG
>Marker125079 Chr4 SNP_site:4490086 Ref_Type:T
TACAGCGCCGCGATTTTAGCCCTCTTCTATCACCACATCCTTTCCATTCTTCGAACTTCCTCCATTTCATCTTCCTTAATXXXXXXXXXXC
AGAGGATTCCGATTTCCCGGCGGTTGATGTGTTTATTTGCACCACGGATCCTGAAAAGGAGCCACCCATGAGCGTGGTG
TACAGCGCCGCGATTTTCGCCCTCTTCTATCACCACATCCTTTCCATTCTTCGAAGTTCCTCCATTTCATCTTCCTTAATXXXXXXXXXXC

Create PDF with GO2PDF for free, if you wish to

AGAGGATTCCGATTTCCCGGCGGTTGATGTGTTTATTTGCACCACGGATCCTGAAAAGGAGCCACCTATGAGCGTGGTG
>Marker125099 Chr4 SNP_site:13625412 Ref_Type:G
GACACCAAAATAGAACAAAAATTTGATTTGAAGAGTAACAATTTAAAATTTTGATATGGAAAGACATTAATAAAATTTTAXXXXXXXXXXXT
TTGAGTGGGCCTTTAGAATGAAAAGGAGAAGCCCATGATTCATGGGCACTACCTGGACCAAAGCTACCCAATTGTGGTA
GACACCAAAATAGAACAAAAATTTGATTTGAAGAGTAACAATTTAAAATTTTGATATGGAAAGACATTAATAAAATTTTAXXXXXXXXXXXT
TTTAGTGGGCCTTTAGAATGAAAAGGAGAAGCCCATGATTCATGGGCACTACCTGGACCAAAACTACCCAATTGTGGTA
>Marker125099 Chr4 SNP_site:13625472 Ref_Type:G
GACACCAAAATAGAACAAAAATTTGATTTGAAGAGTAACAATTTAAAATTTTGATATGGAAAGACATTAATAAAATTTTAXXXXXXXXXXXT
TTGAGTGGGCCTTTAGAATGAAAAGGAGAAGCCCATGATTCATGGGCACTACCTGGACCAAAGCTACCCAATTGTGGTA
GACACCAAAATAGAACAAAAATTTGATTTGAAGAGTAACAATTTAAAATTTTGATATGGAAAGACATTAATAAAATTTTAXXXXXXXXXXXT
TTTAGTGGGCCTTTAGAATGAAAAGGAGAAGCCCATGATTCATGGGCACTACCTGGACCAAAACTACCCAATTGTGGTA
>Marker125459 Chr4 SNP_site:17203491 Ref_Type:T
CACTAAACAAAAATTCAGACTATTTCTTTTCCCAATTCATGTAAATTATAGTTAAACACCATTCTATCTTTAGAAATCAAXXXXXXXXXXXT
CAATAGAGAGAGAAAGAGCCATCATCTACTAACAAAAGGATATTTATCAATTCATGAATAATGTAACTCAGAACACGTT
CACTAAACAAAAATTCAGACTATTTCTTTTCCCAATTCATGTAAATTATAGTTAAACACCATTCTATCTTTAGAAATCAAXXXXXXXXXXXT
CAATAGAGAGAGAAAGAGCCATCATCTACTAACAAAAGGATATTTATCTATTCATGAATAATGTAACTCAGAACACGTT
>Marker126428 Chr4 SNP_site:6674936 Ref_Type:T
AACACTTACAAAACATTTAAAAACAACGAAAGTCTTTTATTAAAAATAAACATTTAAAATATACTTTGAAATCCGAAGTGXXXXXXXXXXXA
ACAAGACAATGTCCTTGCATAATGAAAACTAAGTTTGCCTAGTAGTAGTAAGTTCAACATACGAAGATCCACAATCTGT
AACACTTACAAAACATTTAAAAACAACGAAAGTCTTTTATTAAAAATAAACATTTAAAATATACTTTGAAATCCGAAGTGXXXXXXXXXXXA
ACAAGACAATGTCTTTGCATAATGAAAACTAAGTTTGCCTAGTAGTAGTAAGTTCAACATACGGAGATCCACAATCTGT
>Marker126428 Chr4 SNP_site:6674986 Ref_Type:G
AACACTTACAAAACATTTAAAAACAACGAAAGTCTTTTATTAAAAATAAACATTTAAAATATACTTTGAAATCCGAAGTGXXXXXXXXXXXA
ACAAGACAATGTCCTTGCATAATGAAAACTAAGTTTGCCTAGTAGTAGTAAGTTCAACATACGAAGATCCACAATCTGT
AACACTTACAAAACATTTAAAAACAACGAAAGTCTTTTATTAAAAATAAACATTTAAAATATACTTTGAAATCCGAAGTGXXXXXXXXXXXA
ACAAGACAATGTCTTTGCATAATGAAAACTAAGTTTGCCTAGTAGTAGTAAGTTCAACATACGGAGATCCACAATCTGT
>Marker126685 Chr4 SNP_site:23057049 Ref_Type:A
ACCAAATTACTATTTTTATTTGATGTGCTTGCATTGACATTTACACATGTATTTTGGGTATAACATATAAGACTACTTTAXXXXXXXXXXXA
GATGAGTTTTATCAAATTTAATATTACCATTGTGGTTTAGGTTATGTTATATGGTTATACAAATCATAACAGAAACGGT
ACCAAATTACTATTTTTATTTGATGTGCTTGCATTGACATTTACACATGTATTTTGGGTATAACATATAAGACTACTTTAXXXXXXXXXXXA
GATGAGTTTTATCATATTTAATATTACCATTGTGGTTTAGGTTATGTTATATGGTTATACAAATCATAACAGAAACGGT
>Marker126837 Chr4 SNP_site:23097070 Ref_Type:G
ACTTTTCCTCTTGGATTTGGTAAGAACATTTCGTCAAGACCATCGGTTGTGAATATTGGAGCTATTTTATCTCATAATTCXXXXXXXXXXXG
TGATCCTTTCCTTCTTTTCTTCTTTATTATTTTACCCCACTTGATTGCTTTTATTTTCTGATGTTCTTTTGCGTGTGTT
ACTTTTCCTCTTGGATTTGGTAAGAACGTTTCGTCAAGACCATCGGTTGTGAATATTGGAGCTATTTTATCTCATAATTCXXXXXXXXXXXG
TGATCCTTTCCTTCTTTTCTTCTTTATTATTTTACCCCACTTGATTGCTTTTATTTTCTGATGTTCTTTTGCGTGTGTT
>Marker126855 Chr4 SNP_site:4410742 Ref_Type:A
GACAACAAATATTTTGAACATGGAGCTTTTGTATTGTGTATAAACTCTTGGCTCTTAATACAACTGTATTAGTTTCTCTTXXXXXXXXXXXT
GTTAAAATCAGTAACTTTTAGATTTATTGCAACACATGCTTGAATTTTATAACGTTTTCTAATACATGAAATTGAGGTG
GACTACAAATATTTTGAACATGGAGCTTTTGTTTTGTGTATAAACTCTTGGCTCTTAATACAACTGTATTAGTTTCTCTTXXXXXXXXXXXT
GTTAAAATCAGTAACTTTTAGATTTATTGCAACACATGCTTGAATTTTATAACGTTTTCTAATACATGAAATTGAGGTG
>Marker126855 Chr4 SNP_site:4410771 Ref_Type:A
GACAACAAATATTTTGAACATGGAGCTTTTGTATTGTGTATAAACTCTTGGCTCTTAATACAACTGTATTAGTTTCTCTTXXXXXXXXXXXT
GTTAAAATCAGTAACTTTTAGATTTATTGCAACACATGCTTGAATTTTATAACGTTTTCTAATACATGAAATTGAGGTG

Create PDF with GO2PDF for free, if you wish to

GACTACAAATATTTTGAACATGGAGCTTTTGTTTTGTGTATAAACTCTTGGCTCTTAATACAACTGTATTAGTTTCTCTTXXXXXXXXXXT
GTTAAAATCAGTAACTTTTAGATTTATTGCAACACATGCTTGAATTTTATAACGTTTTCTAATACATGAAATTGAGGTG
>Marker126857 Chr4 SNP_site:1141526 Ref_Type:C
AACTTCATTATCTCAATTTAGTGTAATTTGGAAGTAATGTTCTTTCCGGCCAACCACTTAGAATTTTGTCACCACACTCGXXXXXXXXXXA
GAGCTCCATAAAAGGGAAACACATCCCAATGGATCAAATTTGAGAATAATATAAATATGGAAAGATATATTATGCAGTA
AACTTCATTATCTCAATTTAGTGTAATTTGGAAGTAATGTTCTTTCCGGCCAATCACTTAGAATTTTGTCACCACACTCGXXXXXXXXXXA
GAGCTCCATAAAAGGGAAACACATCCCAATGGATCAAATTTGAGAATAATATAAATATGGAAAGATATATTATGCAGTA
>Marker126948 Chr4 SNP_site:9669704 Ref_Type:A
TACTGCCACTAATGCTGATTATTCTTATTTTCTTACCATAATTGATGATGCCACTCGTTTCACATGTGTTTTTATGCTTAXXXXXXXXXXA
AATGCGTTCTGATAGTGCCCCAAAGTTAAAATTCATTGATTTCTTTAAGGAAAAAGGAGTTTTACTTCAGTTTTCATGT
TACTGCCACTAATGCTGATTATTCTTATTTTCTTACCATAATTGATGATGCCACTCGTTTCACATGTGTTTTTATGCTTAXXXXXXXXXXC
AATGCGTTCTGATAGTGCCCCAAAGTTAAAATTCATTGATTTCTTTAAGGAAAAAGGAGTTCTACTTCAGTTTTCATGT
>Marker126948 Chr4 SNP_site:9669766 Ref_Type:T
TACTGCCACTAATGCTGATTATTCTTATTTTCTTACCATAATTGATGATGCCACTCGTTTCACATGTGTTTTTATGCTTAXXXXXXXXXXA
AATGCGTTCTGATAGTGCCCCAAAGTTAAAATTCATTGATTTCTTTAAGGAAAAAGGAGTTTTACTTCAGTTTTCATGT
TACTGCCACTAATGCTGATTATTCTTATTTTCTTACCATAATTGATGATGCCACTCGTTTCACATGTGTTTTTATGCTTAXXXXXXXXXXC
AATGCGTTCTGATAGTGCCCCAAAGTTAAAATTCATTGATTTCTTTAAGGAAAAAGGAGTTCTACTTCAGTTTTCATGT
>Marker127087 Chr4 SNP_site:11007848 Ref_Type:C
ACTAATCAAATTCAATGCTGGACAAACCCCTTGATTTTTTAAAAATTTATGGATGAATATGCATACTTTATATAAACACAXXXXXXXXXXT
ATACGAAATTTGATGAAGCTATGAAGGTGAAAGAATAATAATAGATAGGAATAAGTAATTAGAGTGGCGGGCGGGTGTG
ACTAATCAAATTTAATTCTGGACAAACCCCTTGATTTTTTAAAAATTTATGGATGAATTTGCATACTTTATATAAACACAXXXXXXXXXXT
ATACGAAATTTGATGAAGCTATGAAGGTGAAAGAATAATAATAGATAGGAATAAGTAATTAGAGTGGCGGGCGGGTGTG
>Marker127087 Chr4 SNP_site:11007852 Ref_Type:G
ACTAATCAAATTCAATGCTGGACAAACCCCTTGATTTTTTAAAAATTTATGGATGAATATGCATACTTTATATAAACACAXXXXXXXXXXT
ATACGAAATTTGATGAAGCTATGAAGGTGAAAGAATAATAATAGATAGGAATAAGTAATTAGAGTGGCGGGCGGGTGTG
ACTAATCAAATTTAATTCTGGACAAACCCCTTGATTTTTTAAAAATTTATGGATGAATTTGCATACTTTATATAAACACAXXXXXXXXXXT
ATACGAAATTTGATGAAGCTATGAAGGTGAAAGAATAATAATAGATAGGAATAAGTAATTAGAGTGGCGGGCGGGTGTG
>Marker127087 Chr4 SNP_site:11007894 Ref_Type:A
ACTAATCAAATTCAATGCTGGACAAACCCCTTGATTTTTTAAAAATTTATGGATGAATATGCATACTTTATATAAACACAXXXXXXXXXXT
ATACGAAATTTGATGAAGCTATGAAGGTGAAAGAATAATAATAGATAGGAATAAGTAATTAGAGTGGCGGGCGGGTGTG
ACTAATCAAATTTAATTCTGGACAAACCCCTTGATTTTTTAAAAATTTATGGATGAATTTGCATACTTTATATAAACACAXXXXXXXXXXT
ATACGAAATTTGATGAAGCTATGAAGGTGAAAGAATAATAATAGATAGGAATAAGTAATTAGAGTGGCGGGCGGGTGTG
>Marker127300 Chr4 SNP_site:2667316 Ref_Type:C
AACACAGGCAAGAAAATTGGTAATTAGGGAAATATCTGTCATCGACATTACAAGCATTTTTGCATACTAGCACAAAGATGXXXXXXXXXXG
CAAAAGCCTAAGGAAAAAATGTCAAAAGAAATTTCACTCAGCTTCTCATACCTCCTAGGCTTTCAGCACCAATTCTGTC
AACACAGGCAAGAAAATTGGTAATTAGGGAAATATCTGTCATCGACATTACAAGCATTTTTGCATACTAGCACAAAGATGXXXXXXXXXXG
CAAAAGCCTACGGAAAAAATGTCAAAAGAAATTTCACTCAGCTTCTCATACCTCCGAGGCTTTCAGCACCAATTCTGTC
>Marker127300 Chr4 SNP_site:2667361 Ref_Type:G
AACACAGGCAAGAAAATTGGTAATTAGGGAAATATCTGTCATCGACATTACAAGCATTTTTGCATACTAGCACAAAGATGXXXXXXXXXXG
CAAAAGCCTAAGGAAAAAATGTCAAAAGAAATTTCACTCAGCTTCTCATACCTCCTAGGCTTTCAGCACCAATTCTGTC
AACACAGGCAAGAAAATTGGTAATTAGGGAAATATCTGTCATCGACATTACAAGCATTTTTGCATACTAGCACAAAGATGXXXXXXXXXXG
CAAAAGCCTACGGAAAAAATGTCAAAAGAAATTTCACTCAGCTTCTCATACCTCCGAGGCTTTCAGCACCAATTCTGTC
>Marker127316 Chr4 SNP_site:5429334 Ref_Type:A
ACTGAATTATTAAGATATAAGGAAGAGAATGGTTACAAAATTCCCATACCATTTGATACTATGAGACTCGGTGCCTATATXXXXXXXXXXT

Create PDF with GO2PDF for free, if you wish to

TTGCAAATCCTCAGTTTTCCCATAGAATTAAATAATTTAAGCCTCTTATCATAGTGATTGATTTGTATTTGTCAAAAGT
ACTGAATTATTAAGATATAAGGAAGAGAATGGTTACAAAATTCCCATACCATTTGATACTCTAAGACTCGGTGCCTATATXXXXXXXXXXT
TTGCAAATCCTCAGTTTTCCCATAGAATTAAATAATTTAAGCCTCTTATCATAGTGATTGATTTGTATTTGTCAAAAGT
>Marker127316 Chr4 SNP_site:5429336 Ref_Type:G
ACTGAATTATTAAGATATAAGGAAGAGAATGGTTACAAAATTCCCATACCATTTGATACTATGAGACTCGGTGCCTATATXXXXXXXXXXT
TTGCAAATCCTCAGTTTTCCCATAGAATTAAATAATTTAAGCCTCTTATCATAGTGATTGATTTGTATTTGTCAAAAGT
ACTGAATTATTAAGATATAAGGAAGAGAATGGTTACAAAATTCCCATACCATTTGATACTCTAAGACTCGGTGCCTATATXXXXXXXXXXT
TTGCAAATCCTCAGTTTTCCCATAGAATTAAATAATTTAAGCCTCTTATCATAGTGATTGATTTGTATTTGTCAAAAGT
>Marker127404 Chr4 SNP_site:5427421 Ref_Type:C
ACATCAGTGCTATAACATAATTGATTCGGAATGAAATAGCCCACTTTTAGATGAAAAAAAGGGAAAAATTTCGTCAGGAAXXXXXXXXXXT
AAAGTTAGATCTAACATTCACTTTTTTTCTTAGTTATGAAGTTTATGTTTTTAGTGTTGCTTTTAATTTGTAAGTTAGT
ACATCAGTGCTATAACATAATTGATTCGGAATGAAATAGCCCACTTTTAGATGAAAAAAAGGGAAAAATTTCGTCAGGAAXXXXXXXXXXT
AAAGTTAGATCTAACATTCACTTTTTTTTTTAGTTATGAAGTTTATGTTTTTAGTGTTGCTTTTAATTTGTAAGTTAGT
>Marker127422 Chr4 SNP_site:8809360 Ref_Type:A
ACTCCTTTCAAGGCTAATTGTTTTGATTTATTTAAGATACCTCTAGTGTTCCACGATATGACTTTCATGTTTCGCTTATTXXXXXXXXXXT
CCAGATTATTTGCCTCGAATTCAGCCAACACGTCGTCTCTCTCCTCTAAATTAACAGGTTCTAATTCTTCACTGCTGGT
ACTCCTTTCAAGGCTAATTGTTTTGATTTATTTAAGATACCTCTAGTGTTCCACGATATGACTTTCATGTTTCGCTTATTXXXXXXXXXXT
CCAGATTATTTGCCTCGTATTCAGCCAACACGTCGTCTCTCTCCTCTAAATTAACAGGTTCTAATTCTTCACTGCTGGT
>Marker127794 Chr4 SNP_site:4443519 Ref_Type:T
ACTGCACACCATCATTAAGGTAGTTCTAAACAATTTCTCTACAAAGATCTATTTCCTACCAAATGAGAGAAAGTTTGTTAXXXXXXXXXXA
TACAGCAATGGCTTGCCAATTTGTGGCTCTATATTGTTTATTAACATGACTTGAATTAGAGAATCATCCCCTTTCTAGT
ACTTCACACCATCATTAAGGTAGTTCTAAACAATTTCTCTACAAAGATCTATTTCCTACCAAATGAGAGAAAGTTTGTTAXXXXXXXXXXA
TACAGCAAGGGCTTGCCAATTTGTGGCTCTATATTGTTTATTAACATGACTTGAATTAGAGAATCATCCCCTTTCTAGT
>Marker127794 Chr4 SNP_site:4443742 Ref_Type:G
ACTGCACACCATCATTAAGGTAGTTCTAAACAATTTCTCTACAAAGATCTATTTCCTACCAAATGAGAGAAAGTTTGTTAXXXXXXXXXXA
TACAGCAATGGCTTGCCAATTTGTGGCTCTATATTGTTTATTAACATGACTTGAATTAGAGAATCATCCCCTTTCTAGT
ACTTCACACCATCATTAAGGTAGTTCTAAACAATTTCTCTACAAAGATCTATTTCCTACCAAATGAGAGAAAGTTTGTTAXXXXXXXXXXA
TACAGCAAGGGCTTGCCAATTTGTGGCTCTATATTGTTTATTAACATGACTTGAATTAGAGAATCATCCCCTTTCTAGT
>Marker128013 Chr4 SNP_site:6627075 Ref_Type:T
ACCATGCCCAACACCAACCACACATAAAATCCAATCAAGAAAATGGAAGCTCAGTAGAAATTTTTTCACACTGATTTGTCXXXXXXXXXXT
GCCAAAACCACTTCGCCAATAACTCCCTATTACAAGCCCTTTAAGTTCCGTAATTTCAACCTTCCTAGGTAAATGGGTT
ACCATGCCCAACACCAACCACACATAAAATCCAATCAAGAAAATGGAAGCTCAGTAGAAATTTTTTCACACTGATTTGTCXXXXXXXXXXT
GCCAAAACCACTTTGCCAATAACTCCCTATTACAAGCCCTTTAAGTTCTGTAATTTCAACCCTCCTAGGTAAATGGGTT
>Marker128013 Chr4 SNP_site:6627110 Ref_Type:T
ACCATGCCCAACACCAACCACACATAAAATCCAATCAAGAAAATGGAAGCTCAGTAGAAATTTTTTCACACTGATTTGTCXXXXXXXXXXT
GCCAAAACCACTTCGCCAATAACTCCCTATTACAAGCCCTTTAAGTTCCGTAATTTCAACCTTCCTAGGTAAATGGGTT
ACCATGCCCAACACCAACCACACATAAAATCCAATCAAGAAAATGGAAGCTCAGTAGAAATTTTTTCACACTGATTTGTCXXXXXXXXXXT
GCCAAAACCACTTTGCCAATAACTCCCTATTACAAGCCCTTTAAGTTCTGTAATTTCAACCCTCCTAGGTAAATGGGTT
>Marker128013 Chr4 SNP_site:6627123 Ref_Type:C
ACCATGCCCAACACCAACCACACATAAAATCCAATCAAGAAAATGGAAGCTCAGTAGAAATTTTTTCACACTGATTTGTCXXXXXXXXXXT
GCCAAAACCACTTCGCCAATAACTCCCTATTACAAGCCCTTTAAGTTCCGTAATTTCAACCTTCCTAGGTAAATGGGTT
ACCATGCCCAACACCAACCACACATAAAATCCAATCAAGAAAATGGAAGCTCAGTAGAAATTTTTTCACACTGATTTGTCXXXXXXXXXXT
GCCAAAACCACTTTGCCAATAACTCCCTATTACAAGCCCTTTAAGTTCTGTAATTTCAACCCTCCTAGGTAAATGGGTT
>Marker128890 Chr4 SNP_site:12160156 Ref_Type:G

Create PDF with GO2PDF for free, if you wish to

TACATTTCTACATAAAGTTCAAGTTTTACTCAAGATAGCCTCAAGACTTTAGTTTATTGGATTCAATATTATAGTATTCAXXXXXXXXXXT
TTCGATTCCACGCAATATTCTTTGCACTGCACCGTGGGAGTGGAGTCAAGGACCTTTCAATATTTCATTCATATTTGTA
TACATTTCTACATAAAGTTCAAGTTTTACTCAAGATAGCCTCGAGACTTTAGTTTATTGGATTCAATATTATAGTATTCAXXXXXXXXXXT
TTCGATTCCACGCAATATTCTTTGCACTGCACCGTGGGAGTGGAGTCAAGGACCTTTCAATATTTCATTCATATTTGTA
>Marker128991 Chr4 SNP_site:11741261 Ref_Type:C
CACGAAACTCTATGATAGAACCGAAATGTCATTCTTCGTTCTCATTCTTACTCCCCATTTCATTCTCCATGCTTGTAAAAXXXXXXXXXXT
ACTAGAAACTAATGAAGTTTTGAATATTCTAAGTATGTAATGTGTATAAATGAAGGGGCGCCGGTGGGGAGCTGGAGTT
CACGAAACTCTATGATAGAATCGAAATGTCATTCTTCGTTCTCATTCTTACTCCCCATTTCGTTCTCCATGCTTGTAAAAXXXXXXXXXXT
ACTAGAAACTAATGAAGTTTTGAATATTCTAAGTATGTAATGTGTATAAATGAAGGGGCGCCGGTGGGGAGCTGGAGTT
>Marker128991 Chr4 SNP_site:11741302 Ref_Type:A
CACGAAACTCTATGATAGAACCGAAATGTCATTCTTCGTTCTCATTCTTACTCCCCATTTCATTCTCCATGCTTGTAAAAXXXXXXXXXXT
ACTAGAAACTAATGAAGTTTTGAATATTCTAAGTATGTAATGTGTATAAATGAAGGGGCGCCGGTGGGGAGCTGGAGTT
CACGAAACTCTATGATAGAATCGAAATGTCATTCTTCGTTCTCATTCTTACTCCCCATTTCGTTCTCCATGCTTGTAAAAXXXXXXXXXXT
ACTAGAAACTAATGAAGTTTTGAATATTCTAAGTATGTAATGTGTATAAATGAAGGGGCGCCGGTGGGGAGCTGGAGTT
>Marker12900 Chr4 SNP_site:1530162 Ref_Type:T
ACTAAACAATGTCCGATAGAACTGTATTAGGGAATAGAGCTTAAACTAATATATTTCGTTGATGGCTAGGAAGAAAGAATXXXXXXXXXXG
GCGATTCGTTATAAGGATTGCTGCAACACAATAAACAGGAAGCTCATCATCACCTTGCTTAGTTGATGATATTGCAGTG
ACTAAACAATGTCCGATAGAACTGTATTAGGGAATAGATCTTAAACTAATATATTTCGTTGATGGCTAGGAAGAAAGAATXXXXXXXXXXG
GCGATTCGTTATAAGGATTGCTGCAACACAATAAACAGGAAGCTCATCATCACCTTGCTTAGTTGATGATATTGCAGTG
>Marker129475 Chr4 SNP_site:18318011 Ref_Type:C
ACTTCAAGAAGCAAGCCGAAACCTCTATCAACCCTAACAACTTTTGCAGAATCATAGATATCACCAATTCTCAAGTGCTAXXXXXXXXXXG
ACATATATAAAGAAATATGGAAACGTTTTGCATGGTTCAAGTTATCAACCACCCAATCTTATACACCCAGCGCAAAGTG
ACTTCAAGAAGCAAGCCGAAACCTCTATCAACCCTAACAACTTTTGCAGAATCATAGATATCACCAATTCTCACGTGCTAXXXXXXXXXXG
ACATATATAAAGAAATATGGAAACGTTTTGCATGGTTCAAGTTATCAACCACCCAATCTTATACACCCAGCGCAAAGTG
>Marker129505 Chr4 SNP_site:5790946 Ref_Type:A
ACTCTTATTTGCGATGATTTATTCATCTTCTCTTCCCAATTTTCTTAAAGATCAATTGTTTAAATTTATTATTTCATCTCXXXXXXXXXXA
CTTACCATGACCAACGCGATCGTTTAAAGTATATTATTTTACTACATGATCATTTTATTCGGACATAAACGATCGTGTA
ACTCTTATTTGCGATGATTTATTCATCTTCTCTTCCCAATTTTCTTTAAGATCAATTGTTTAAATTTATTATTTCATCTCXXXXXXXXXXA
CTTACCATGACCAACGCGATCGTTTAAAGTATATTATTTTACTACATGATCATTTTATTCGGACATAAACGATCGTGTA
>Marker13242 Chr4 SNP_site:22649051 Ref_Type:A
>Marker13242 Chr4 SNP_site:22649186 Ref_Type:A
TACCAACTTAAGTCGAGATGAGTGGGTTAAAACCTACTCATTTCGCAACTCTTAAATAACTTTATTCCACTAATTTTATAXXXXXXXXXXA
ATTACTCGATAAATGTGTAAACTTCAATAGTAAATACTATTGTATTGAAGTGAAGTTGTTACCAACTTAAAAGTGGGTG
>Marker13326 Chr4 SNP_site:21805497 Ref_Type:T
>Marker13330 Chr4 SNP_site:11392559 Ref_Type:A
CACTAAGTGATGGCATTTAAATTATATTGAATTACACGAATAACATTAGCCAACTTCCCATGCATAAGGTGAAGTTCGAGXXXXXXXXXXG
ATTTGATTTAATTTCTATTGATTGTTGTAAAATTTACAAAAATGATGCATTTACTGTTCAAGTTATGAAATAGATTTGT
>Marker13417 Chr4 SNP_site:12884596 Ref_Type:C
>Marker13650 Chr4 SNP_site:15413584 Ref_Type:A
AACACACAAAATTTCTCAAACCTAATTCCAAGCGTCTTTAAAGAAAATAAATCTTTGTTTAAAATACCAATTTGAGTATTXXXXXXXXXXA
CATTGAGCTAATGGATGATACAGCTTCAAATGCTTTTGTAGTCTATATGGGTATCGAAACAAACTAAATGCATAACGTT
>Marker13764 Chr4 SNP_site:15110045 Ref_Type:A
>Marker13842 Chr4 SNP_site:12835655 Ref_Type:T
GACTGTTCTAGGAATAACCTTAGATGCTCAGAATACAAGCCAGGTGCAATCAAGTCAGACTAACATGGTGGATTCAAGTAXXXXXXXXXXT

Create PDF with GO2PDF for free, if you wish to

CCTCACACAGACAAGATTTTTCTCCCAACAATCTTTTCTTCTAATGTCAGAAGGGAGCTTTCATTTTCAGCTGAGAGTA

>Marker13979 Chr4 SNP_site:18080633 Ref_Type:C

GACTAAGAGTAGGGAACAAAAAACAACGTATAACATTATACTAATACCCGATTCCTTAAAGCTGCAACCAAAAGTTCAGCXXXXXXXXXXT

AATGCTATTCCATTGTGAAATAAAAGAAAGTGAGATAGCAATAAAATATGGAACATGAATATTCACTATGAGTGAAGTA

GACTAAGAGTAGGGAACAAAAAACAATGTATAACATTATACTAATACCCGATTCCTTAAAGCTGCAACCAAAAGTTCAGCXXXXXXXXXXT

AATGCTATTCCATTGTGAAATAAAAGAAAGTGAGATAGCAATAAAATATGGAACATGAATATTCACTATGAGTGAAGTA

>Marker14084 Chr4 SNP_site:20870989 Ref_Type:T

AACAAATGACCTTCCTAATCCTGATCACCACATATGTGCAAACGACCTTTTCTGAAGGATATTAAGAATTTATCAGCCCAXXXXXXXXXXT

ATTTTCACAGTGGGCCTGCTTTTCAGAACACTACTTTTTTTCTCAGAACACTTACAATCTTTCTTCTTCCAAAACAGTG

AACAAATGACCTTCCTAATCCTGATCACCACATATGTGCAAACGACCTTTTCTGAAGGATATTAAGAATTTATCAGCCCAXXXXXXXXXXT

ATTTTCACAGTGGGCCTGCTTTTCAGAACACTACTTTTTTTCTCAGAACACTTATAATCTTTCTTCTTCCAAAACAGTG

>Marker142079 Chr6 SNP_site:16737258 Ref_Type:G

AACAAGTGAAAAGGCTAAGAGAATAAGAAAGGAAACGAAAACTTGGCAATTTACAGAACTTCAGAAACATTAAAGAGAGAXXXXXXXXXXG

AATGATTGGAAGATGTCATTAGCAGTTGCATCTTTTTGCACAATAACACTGATAGTTTCCTTCCTCTCCACTAGAAGGT

AACGAGTGAAAAGGCTAAGAGAATAAGAAAGGAAACGAAAACTTGGCAATTTACAGAACTTCAGAAACATTAAAGAGAGAXXXXXXXXXXG

AATGATTGGAAGATGTCATTAGCAGTTGCATCTTTTTGCACAATAACACTGATAGTTTCCTTCCTCTCCACTAGAAGGT

>Marker142319 Chr6 SNP_site:28533469 Ref_Type:T

AACAATAAAGATGGCTGGGAGATAAATATAAACACGAGCCTACACAGACCTACATCAATTAGTTTAAATTTTACATGGTTXXXXXXXXXXC

AGCTCTGCCCTTTCCCCTCCCACCCTCCATTATAAAAAATAAAAGGAAAAAGAATTTCCACTCTAGCAGTTGAGAAGTG

AACAATAAAGATGGCTGGGAGATAAATATAAACACGAGCCTACACAGACCTACATCAATTAGTTTAAATTTTACATGGTTXXXXXXXXXXC

AGTTCTGCCCTTTCCCCTCCCACCCTCCATTATAAAAAATAAAAGGAAAAAGAATTTCCACTCTAGCAGTTGAGAAGTG

>Marker142747 Chr6 SNP_site:10940806 Ref_Type:T

TACCTTTCTAACCAATTAAAAATGTTCTAGGACTAACTTTCTAATACTATGTTAATTTGAGCCTAGTTGAATGATTGCCAXXXXXXXXXXC

CACTTGAGTGCATGACCTGCAATGCATGCACACTATAAAAATGCTCATTCATATTTTCGACAGAGCCTTTATAAGTGTT

TACCTTTCTAACCAATTAAAAATGTTTTAGGACTAACTTTCTAATACTATGTTAATTTGAGCCTAGTTGAATGATTGCCAXXXXXXXXXXC

CACTTGAGTGCATGACCTGCAATGCATGCACACTATAAAAATGCTCATTCATATTTTCGACAGAGCCTTTATAAGTGTT

>Marker143500 Chr6 SNP_site:13653744 Ref_Type:G

ACAAAGAAACTAAGTGTAGGAAATCTATCGAAATTTTCCAAAGGGACAAATACAAGCAAAATATAAAATAAACGTTGGTAXXXXXXXXXXA

TCACTACAATAGATCAATCAGATAATAGCTTCAAACACACAAGGTCAGAGTCTGTAAAAATAAAAATATAAAATTAAGT

ACAAAGAAACTAAGTGTAGGAAATCTATCGAAATTTTCCAAAGGGACAAATACAAGCAAAATATAAAATAAACGTTGGTAXXXXXXXXXXA

TCACTACAATAGATCGATCAGATAATAGCTTCAAACACACAAGGTCAGAGTCTGTAAAAATAAAAATATAAAATTAAGT

>Marker14377 Chr4 SNP_site:1512386 Ref_Type:C

ACATTGACAAGTAAGAATATTTGGAAAACCTAAGGAGACCCTTACAAATAAGGGAGGAAAGTAAGCAAAAAACCAGTCTAXXXXXXXXXXG

ACTAATCTTATGAGACAACCCCGCCTAACACTACAATATTTGGGTATCATGAAAACTCCTAAGATATTAAATCCTAGGT

ACATTGACAAGTAAGAATATTTGGAAAACCTAAGGAGACCCTTACAAATAAGGGAGGAAAGTAAGCAAAAAACCAGTCTAXXXXXXXXXXG

ACTAATCTTATGAGACAACCCTGCCTAACACTACAATATTTGGGTATCATGAAAACTCCTAAGATATTAAATCCTAGGT

>Marker144100 Chr6 SNP_site:11507974 Ref_Type:C

TACTACCTGTCAGCATATTGTAACTCAAGTCAAGATGCACTAACGACACCAACTCAAAAACCTCGTTCGGAATTGCACCGXXXXXXXXXXT

TGAGTGAAGCAATTGAAGAAGAAGAGAGAATGAAGGTGAGGGAGATCAAAGATGAATGAAGGGAAAATAGCAGTAGGTT

TACTACCTGTCAGCATATTGTAACTCAAGTCAAGATGCACTAATGACACCAACTCAAAAACCTCGTTCGGAATTGCACCGXXXXXXXXXXT

TGAGTGAAGCAATTGAAGAAGAAGAGAGAATGAAGGTGAGGGAGATCAAAGATGAATGAAGGGAAAATAGCAGTAGGTT

>Marker144284 Chr6 SNP_site:9703461 Ref_Type:G

CACATTTTATGGTGGCTGCTATGTTATCTTGTAACCATGTGTTTTTCATTTCTGACGTTGTTGCTTATTCTAGACTACAAXXXXXXXXXXT

TTGGCTTCAACAACATTATAAACTTCCAACAAGCATCGTGATAGGCTATAATTTAAACGAAACAGTATTGTGGTTTGTA

Create PDF with GO2PDF for free, if you wish to

CACATTTTATGGTGGCTGCTATGTTATCTTGTAACCATGTGTTTTTCATTTCTGACGTTGTTGCTTATTCTAGACTACAAXXXXXXXXXXT
TTGGCTTCAACAACATTATAAACTTCCAACGAGCATCGTGATAGGCTATAATTTAAACGAAACAGTATTGTGGTTTGTA
>Marker144669 Chr6 SNP_site:17155408 Ref_Type:G
AACGTAATTGTGTTTAAGATTAAATTCTAAGGTATGCTTAAACTAATTTTAAGACTAAACAGTCACTTTTTTTTTCCTTTXXXXXXXXXXA
TTGTTTAAAATTGAGATATGTCAATAATCAGCAGAGATTCTAAAGAAACAACATATTTTTATCATTCATCCTTAATTGT
AACTTAATTGTGTTTAAGATTAAATTCTAAGGTATGCTTAAACTAATTTTAAGACTAAACAGTCACTTTTTTTTTCCTTTXXXXXXXXXXA
TTGTTTAAAATTGAGATATGTCAATAATCAGCAGAGATTCTAAAGAAACAACATATTTTTATCATTCATCCTTAATTGT
>Marker145011 Chr6 SNP_site:2348010 Ref_Type:A
CACAAATGGAGCTGAAGTTTCTTATAAATTTCCTATAGAAGGAAGCCAGCCCTAGGAAGACCTGTATTTCTTTGACAGTGXXXXXXXXXXT
TGATGTAAAGCTCCTTTTCTGTCAATACTTGGAACAACTTTCTAAGGTGGAGCAAGTGTTCATTGTTTCCACAGCTGTA
CACAAATGGAGCTGAAGTTTCTTATAAATTTCCTATAGAAGGAAGCCAGCCCTAGGAAGACCTGTATTTCTTTGACAGTGXXXXXXXXXXT
TGATGTAAAGCTCCTTTTCTGTCAATACTTGGAACAGCTTTCTAAGGTGGAGCAAGTGTTCATCGTTTCCACAGCTGTA
>Marker145011 Chr6 SNP_site:2348037 Ref_Type:T
CACAAATGGAGCTGAAGTTTCTTATAAATTTCCTATAGAAGGAAGCCAGCCCTAGGAAGACCTGTATTTCTTTGACAGTGXXXXXXXXXXT
TGATGTAAAGCTCCTTTTCTGTCAATACTTGGAACAACTTTCTAAGGTGGAGCAAGTGTTCATTGTTTCCACAGCTGTA
CACAAATGGAGCTGAAGTTTCTTATAAATTTCCTATAGAAGGAAGCCAGCCCTAGGAAGACCTGTATTTCTTTGACAGTGXXXXXXXXXXT
TGATGTAAAGCTCCTTTTCTGTCAATACTTGGAACAGCTTTCTAAGGTGGAGCAAGTGTTCATCGTTTCCACAGCTGTA
>Marker145082 Chr6 SNP_site:13244715 Ref_Type:C
ACCGACTCAGCTTTATCAGAATTAATTATAGTTCATGTTGTCCTGTCACATCCCAATTTGCCTATAAATAAAGGCTTCTTXXXXXXXXXXT
TAGATAAGAGTTTGAAAGAAAGAGAGTGGTCCTCAACCATTTTCTCAACGTAAAAGGCAAAAACCAGAGCCAAACGAGT
ACCGACTCAGCTTTATCAGAATTAATTATAGTTCATGTTGTTCTGTCACATCCCAATTTGCCTATAAATAAAGGCTTCTTXXXXXXXXXXT
TAGATAAGAGTTTGAAAGAAAGAGAGAGGTCCTCAACCATTTTCTCAACGTAAAAGGCAAAAACCAGAGCCAAACGAGT
>Marker145082 Chr6 SNP_site:13244852 Ref_Type:T
ACCGACTCAGCTTTATCAGAATTAATTATAGTTCATGTTGTCCTGTCACATCCCAATTTGCCTATAAATAAAGGCTTCTTXXXXXXXXXXT
TAGATAAGAGTTTGAAAGAAAGAGAGTGGTCCTCAACCATTTTCTCAACGTAAAAGGCAAAAACCAGAGCCAAACGAGT
ACCGACTCAGCTTTATCAGAATTAATTATAGTTCATGTTGTTCTGTCACATCCCAATTTGCCTATAAATAAAGGCTTCTTXXXXXXXXXXT
TAGATAAGAGTTTGAAAGAAAGAGAGAGGTCCTCAACCATTTTCTCAACGTAAAAGGCAAAAACCAGAGCCAAACGAGT
>Marker145136 Chr6 SNP_site:775189 Ref_Type:G
ACCATCCAAGTAAGTCTAATGTAGTAGCTGATGCTTTTAGCAGAAAAGTAACTCATCAACAGCTTTTATTATCAAATAGAXXXXXXXXXXA
GAAGCAGGACAAGTTGATGATTTCTCTATATTATTATTTAAGGATGGTCTCCCGTTTAAGAGACGTTTGTATGCTAGTA
ACCATCCAAGTAAGTCTAATGTAGTAGCTGATGCTTTTAGCAGAAAAGTAACTCATCAGTAGCTTTTATTATCAAATAGAXXXXXXXXXXA
GAAGCATGACAAGTTGATGATTTCTCTATATTATTATTTAAGGATGGTCTCCCGTTTAAGAGACGTTTGTATGCTAGTA
>Marker145136 Chr6 SNP_site:775190 Ref_Type:T
ACCATCCAAGTAAGTCTAATGTAGTAGCTGATGCTTTTAGCAGAAAAGTAACTCATCAACAGCTTTTATTATCAAATAGAXXXXXXXXXXA
GAAGCAGGACAAGTTGATGATTTCTCTATATTATTATTTAAGGATGGTCTCCCGTTTAAGAGACGTTTGTATGCTAGTA
ACCATCCAAGTAAGTCTAATGTAGTAGCTGATGCTTTTAGCAGAAAAGTAACTCATCAGTAGCTTTTATTATCAAATAGAXXXXXXXXXXA
GAAGCATGACAAGTTGATGATTTCTCTATATTATTATTTAAGGATGGTCTCCCGTTTAAGAGACGTTTGTATGCTAGTA
>Marker145136 Chr6 SNP_site:775374 Ref_Type:T
ACCATCCAAGTAAGTCTAATGTAGTAGCTGATGCTTTTAGCAGAAAAGTAACTCATCAACAGCTTTTATTATCAAATAGAXXXXXXXXXXA
GAAGCAGGACAAGTTGATGATTTCTCTATATTATTATTTAAGGATGGTCTCCCGTTTAAGAGACGTTTGTATGCTAGTA
ACCATCCAAGTAAGTCTAATGTAGTAGCTGATGCTTTTAGCAGAAAAGTAACTCATCAGTAGCTTTTATTATCAAATAGAXXXXXXXXXXA
GAAGCATGACAAGTTGATGATTTCTCTATATTATTATTTAAGGATGGTCTCCCGTTTAAGAGACGTTTGTATGCTAGTA
>Marker145156 Chr6 SNP_site:21948783 Ref_Type:A
TACATTTTCTTTGACATTTGAGGTTTTTGGTGGGTGAAATCCATGATCCAGCAACCTTCTTGGTAAAAGACACCGCATTTXXXXXXXXXXC

Create PDF with GO2PDF for free, if you wish to

TAGTCAGACCTACAGTCAGTTAAATATAATATTCATTGAACAGAATCTAAACTATGTAATGCAAAATGTAAAAATAGTT
TACATTTTCTTTGACATTTGAGGTTTTTGGTGGGTGAAATCCATGATCCAGCAACCTTCTTGGTAAAAGACACCGCATTTXXXXXXXXXXC
TAGTCAGACCTACGGTCAGTTAAATATAATATTCATTGAACAGAATCTAAACTATGAAATGCAAAATTTAAAAATAGTT
>Marker145156 Chr6 SNP_site:21948826 Ref_Type:T
TACATTTTCTTTGACATTTGAGGTTTTTGGTGGGTGAAATCCATGATCCAGCAACCTTCTTGGTAAAAGACACCGCATTTXXXXXXXXXXC
TAGTCAGACCTACAGTCAGTTAAATATAATATTCATTGAACAGAATCTAAACTATGTAATGCAAAATGTAAAAATAGTT
TACATTTTCTTTGACATTTGAGGTTTTTGGTGGGTGAAATCCATGATCCAGCAACCTTCTTGGTAAAAGACACCGCATTTXXXXXXXXXXC
TAGTCAGACCTACGGTCAGTTAAATATAATATTCATTGAACAGAATCTAAACTATGAAATGCAAAATTTAAAAATAGTT
>Marker145156 Chr6 SNP_site:21948837 Ref_Type:G
TACATTTTCTTTGACATTTGAGGTTTTTGGTGGGTGAAATCCATGATCCAGCAACCTTCTTGGTAAAAGACACCGCATTTXXXXXXXXXXC
TAGTCAGACCTACAGTCAGTTAAATATAATATTCATTGAACAGAATCTAAACTATGTAATGCAAAATGTAAAAATAGTT
TACATTTTCTTTGACATTTGAGGTTTTTGGTGGGTGAAATCCATGATCCAGCAACCTTCTTGGTAAAAGACACCGCATTTXXXXXXXXXXC
TAGTCAGACCTACGGTCAGTTAAATATAATATTCATTGAACAGAATCTAAACTATGAAATGCAAAATTTAAAAATAGTT
>Marker146044 Chr6 SNP_site:27882695 Ref_Type:A
ACCTCCTTATACCTTTTCAAATGGTGTTGGAACGGTTGCTGGAGAACATCCTTATGGAGAGCATCCTTCTAGAACGTTGTXXXXXXXXXXA
CACAACCTGATCAGCAAATTCATACTTGAATGTTTCTTAAAATGCCACAATCTAGAGATTTATTTGTGTATGCAAGGTC
ACCTCCTTATACCTTTTCAAATGGTGTTGGAACGGTTGCTGGAGAACATCCTTATGGAGAGCATCCTTCTAGAACGTTGTXXXXXXXXXXA
CGCAACCTGATCAGCAAATTCATACTTGAATGTTTCTTAAAATGCCACAATCTAGAGATTTATTTGCGTATGCAAGGTC
>Marker146044 Chr6 SNP_site:27882760 Ref_Type:T
ACCTCCTTATACCTTTTCAAATGGTGTTGGAACGGTTGCTGGAGAACATCCTTATGGAGAGCATCCTTCTAGAACGTTGTXXXXXXXXXXA
CACAACCTGATCAGCAAATTCATACTTGAATGTTTCTTAAAATGCCACAATCTAGAGATTTATTTGTGTATGCAAGGTC
ACCTCCTTATACCTTTTCAAATGGTGTTGGAACGGTTGCTGGAGAACATCCTTATGGAGAGCATCCTTCTAGAACGTTGTXXXXXXXXXXA
CGCAACCTGATCAGCAAATTCATACTTGAATGTTTCTTAAAATGCCACAATCTAGAGATTTATTTGCGTATGCAAGGTC
>Marker146191 Chr6 SNP_site:25460773 Ref_Type:G
ACATGAGAGCTGTGGCTGAGCCAATTCTTATAAGTCATTTTGGAGAAGCAATCATCGATGAATTGTTCATCAGATATGGGXXXXXXXXXXT
ATGTGGTAATTATTGTGTGTCTTAGTATAATATAGTATATTTACTATATGTTAAGCTTAGTTTAGTTCATAAAAATGTC
ACATGAGAGCTGTGGCTGAGCCAATTCTTATAAGTCATTTTGGAGAAGCAATCATCGATGAATTGTTCATCAGATATGGGXXXXXXXXXXT
ATGTGGTAATTATTGTGTGTCTTAGTATAATATAGTATATTTACTATATGTTAAGCTTAGTTTAGTTGATAAAAATGTC
>Marker146275 Chr6 SNP_site:5343506 Ref_Type:G
TACTTTCAATAATTATAAGTGTTTGTTTTGTTACTTGCTCATATAAGAGTTATGGACAATTTTTATGGTAAGTTTAATAAXXXXXXXXXXC
TTAGAAATTAGAAGTGTATGTTTCATTGGATAATGGATACAAAAGTCTATCTTTTAACTTGGAAGTGGATGGTTTAAGT
TACTTTCAATAATTATAAGTGTTTGTTTTGTTACTTGCTCGTATAAGAGTTATGGACAATTTTTATGGTAAGTTTAATAAXXXXXXXXXXC
TTAGAAATTAGAAGTGTATGTTTCATTGGATAATGGATACAAAAGTCTATCTTTTAACTTGGAAGTGGATGGTTTAAGT
>Marker146416 Chr6 SNP_site:24810092 Ref_Type:C
ACTCTTTATGCTGCTTGCTCTTTGTGGCGTAACATGAACATATAACAGCTCCTTAAAAGTTTTGCCTTCTTTCACCAATCXXXXXXXXXXC
CATATTATTTTGGCATCTCTTTCGATTGAATCTCAGGATAGTTTTGAACATATGATGTAGTTTTTCATCTTTCCCAGTT
ACTCTTTATGCTGCTTGCTCTTTGTGGCGTAACATGAACATATAACAGCTCCTTAAAAGTTTTGCCTTCTTTCACCAATCXXXXXXXXXXC
CATATTATTTTGGCATCTCTTTTGATTGAATCTCAGGATAGTTTTGAACATATGATGTAGTTTTTCATCTTTCCCAGTT
>Marker146512 Chr6 SNP_site:2125576 Ref_Type:C
TACGACAAGGCCATAGCACAACCAAGTAGGGTGGCATGCCCTCAGGCTACACGCGTGACTTTCCTAGAAGTGGTTAGCGTXXXXXXXXXXC
TTGAACAACCGAGACTTAGTGTTAGTAAATGGTGTATGAAAATTATATTCAGTCTAGGCGACATATCACTAATCATGTT
TACGACAAGGCCATAGCACAACCAAGTAGGGTGGCATGCCCTCAGGCTACATGCGTGACTTTCCTAGAAGTGGTTAGCGTXXXXXXXXXXC
TTGAACAACCGAGACTTAGTGTTAGTAAATGGTGTATGAAAATTATATTCAGTCTAGGCGACATATCACTAATCATGTT
>Marker146689 Chr6 SNP_site:15845794 Ref_Type:A

Create PDF with GO2PDF for free, if you wish to

ACATTACTTCTCTCTATCTAAAGTGTTCTATAATGGTTTTAAGAGTATTTAGCTACTTTAGGATTTTATTCATGCTAACTXXXXXXXXXXA
CACATGTTCATAAAGCTAGGTTAAATTAGGCTCAAGAACTAATTACGATCTTCAGGTAGAAGGTGTTCCGTAAATCGTC
ACATTACTTCTCTCTATCTAAAGTGTTCTATAATGGTTTTAAGGGTATTTAGCTACTTTAGGATTTTATTCATGCTAACTXXXXXXXXXXA
CACATGTTCATAAAGCTAGGTTAAATTAGGCTCAAGAACTAATTACGATCTTCAGGTAGAAGGTGTTCCGTAAATCGTC
>Marker146779 Chr6 SNP_site:21015139 Ref_Type:T
AACATCGATTCTCTGCAATTCTTCAGTGGTCGGCGCCGGAGAAAGCGCCCCCGATCTGCCGCCAGTGGCTCCGCCGTCGCXXXXXXXXXXA
GAAAATGAAGGGTTTCAAACGAAGATTGAGGCGAGGTTGGAAGATAAGGAGTGAAGAAGGCTTCGCAGCCATGGTGGTC
AACATCGATTCTCTGCAATTCTTCAGTGGTCGGCGCCGGAGAAAGCGCCCCCGATCTGCCGCCAGTGGTTCCGCCGTCGCXXXXXXXXXXA
GAAAATGAAGGGTTTCAAACGAAGATTGAGGCTAGGTTGGAAGATAAGGAGTGAAGAAGGCTTCGCAGCCATGGTGGTC
>Marker146779 Chr6 SNP_site:21015301 Ref_Type:T
AACATCGATTCTCTGCAATTCTTCAGTGGTCGGCGCCGGAGAAAGCGCCCCCGATCTGCCGCCAGTGGCTCCGCCGTCGCXXXXXXXXXXA
GAAAATGAAGGGTTTCAAACGAAGATTGAGGCGAGGTTGGAAGATAAGGAGTGAAGAAGGCTTCGCAGCCATGGTGGTC
AACATCGATTCTCTGCAATTCTTCAGTGGTCGGCGCCGGAGAAAGCGCCCCCGATCTGCCGCCAGTGGTTCCGCCGTCGCXXXXXXXXXXA
GAAAATGAAGGGTTTCAAACGAAGATTGAGGCTAGGTTGGAAGATAAGGAGTGAAGAAGGCTTCGCAGCCATGGTGGTC
>Marker146807 Chr6 SNP_site:18407041 Ref_Type:C
GACACCAATGGGGCAGGTATATGGCCTTCTTCTCTCTACTTTTAATTGGTAAATGCATGGAGATACTGTGTTACTTTTTTXXXXXXXXXXA
ACGTTTATTGGAATATTTTTGCATCCCCCATTTATCATATCATTTGTAGAGTTTAGACCATGAGGGCACACGTAGTTGT
GACACCAATGGGGCAGGTATATGGCCTTCTTCTCTCTACTTTTAATTGGTAAATGCATGGAGATACTGTGTTAGTTTTTTXXXXXXXXXXA
ACGTTTATTGGAATATTTTTGCATCCCCCATTTATCATATCATTTGTAGAGTTTAGACCATGAGGGCACACGTAGTTGT
>Marker146957 Chr6 SNP_site:2128393 Ref_Type:C
GACAAGTCTATAGCTCATAGGCCATTAGGAAGGGTATATATTTATGCCTTAAAGAAGGAGAATTACAATGAAAGTTGTTTXXXXXXXXXXA
CTGTTTATTGATAAATGATATTCAAGCTTCAGTTTTATGTTAAGCTATTTACAAAAACTTGTTTTATAAAATTGTTGTT
GACAAGTCTATAGCTCATAGGCCATTAGGAAGGGTATATATTTATGCCTTAAAGAAGGAGAATTACAATGAAAGTTGTTTXXXXXXXXXXA
CTGTTTCTTGATAAATGATATTCAAGCTTCAGTTTTATGTTAAGCTATTTACAAAAACTTGTTTTATAAAATTGTTGTT
>Marker147191 Chr6 SNP_site:9256287 Ref_Type:A
ACTTCAGGTTGTTGGACGCCATTATCATCATCAAGAATTGCTTTGGCCTTTCTGGCCAAAGCACCCCAGAATCCATGTTTXXXXXXXXXXG
ACAAGTGCAAAACAAAAACTGATAAGTAGCACTGTAGAAAAGTGAAAGCGCCACTGCAGAAGTAAAATGCAGAAACGGT
ACTTCAGGTTGTTGGACGCCATTATCATCATCAAGAATTGCTTTGGCCTTTCTGGCCAAAGCACCCCAGAATCCATGTTTXXXXXXXXXXG
ACGAGTGCAAAACAAAAACTGATAAGTAGCACTGTAGAAAAGTGAAAGCGCCACTGCAGAAGTAAAATGCAGAAACGGT
>Marker147821 Chr6 SNP_site:11662191 Ref_Type:C
ACTAACACCAAAGAGGCAGGGTGCAAGAAGTATGCAATAAGTTGATATTTGTGATAACAAATTACTAACCATAGATTTGTXXXXXXXXXXA
GGAATTCTCACTAGAGTGTCTGATCACCCAGTTGAGGATTTAGAAAGAAACTTGTAAGCGTTACCAAAAGTAGGAGGTG
ACTAACACCAAAGAGGCAGGGTGCAAGAAGTATGCAATAAGTTGATATTTGTGATAACAAATTACTAACCATAGATTTGTXXXXXXXXXXA
GGAATTCTCACTAGAGTGTCTGATCACCTAGTTGAGGATTTAGAAAGAAACTTGTAAGCGTTACCAAAAGTAGGAGGTG
>Marker147869 Chr6 SNP_site:27417249 Ref_Type:T
AACTCAGAAACAAATACTAGGGAAAAGAACTGCTGTAGTCAAGTAATCGAGCAAAAAGATTGGACTTTGAAAGTAAAAAAXXXXXXXXXXG
TCTCAAGATTGTTAAATAATCAAACGAAACACCCAATGAAGCATATTGAATAAACTATAATGTTAAAAATAAAGATGTA
AACTCAGAAACAAATACTAGGGAAAAGAACTGCTGTAGTCAAGTAATCGAGCAAAAAGATTGGACTTTGAAAGTAAAAAAXXXXXXXXXXG
TCTCAAGATTGTTAAATAATCAAACGAAATACCCAATGAAGCATATTGAATAAACTATAATGTTAAAAATAAAGATGTA
>Marker148176 Chr6 SNP_site:8756529 Ref_Type:A
CACGAGGAAGTGATGGGCAAACCGAGATGGGAAATTGAGCAGCCACAGCCACAGCAACCGAAAAACCAAGTGGGGTTCGAXXXXXXXXXXG
GTGATTTTGGGTCGTTGTCGTGATCATAATCATCATGTTACCTAATTGTTGTATAATTGGATTGGATGTGAAAAATGTT
CACGAGGAAGTGATGGGCAAACCGAGATGGGAAATTGAGCAGCCACAGCCACAGCAACCGAAAAACCAAGTGGGGTTCGAXXXXXXXXXXG
GTGATTTTGGGTCGTTGTCGTGATCATTATCATCATGTTACCTAATTGTTGTATAATTGGATTGGATGTGAAAAATGTT

Create PDF with GO2PDF for free, if you wish to

>Marker148257 Chr6 SNP_site:9781088 Ref_Type:A

TACTCATGGGACATCTTCTGCAAGGATACGCCGACTCGTCAATAAAGATCTTATCTCATTCTAATAAAGATCGTATTTTAXXXXXXXXXXT
TGCTTGCCTCCTTTAGTATTAGGATTATGCGTGATTTCTTTGACCTCTTTCAGTAGGAATACCTGTAACTTCAAGGGTG
TACTCATGGGACATCTTCTGCAAGGATACGCCGACTCGTCAATAAGGATCTTATCTCATTCTAATAAAGATCGTATTTTAXXXXXXXXXXT
TGCTTGCCTCCTTTAGTATTAGGATTATGCGTGATTTCTTTGACCTCTTTCAGTAGGAATACCTGCAACTTCAAGGGTG

>Marker148257 Chr6 SNP_site:9781313 Ref_Type:T

TACTCATGGGACATCTTCTGCAAGGATACGCCGACTCGTCAATAAAGATCTTATCTCATTCTAATAAAGATCGTATTTTAXXXXXXXXXXT
TGCTTGCCTCCTTTAGTATTAGGATTATGCGTGATTTCTTTGACCTCTTTCAGTAGGAATACCTGTAACTTCAAGGGTG
TACTCATGGGACATCTTCTGCAAGGATACGCCGACTCGTCAATAAGGATCTTATCTCATTCTAATAAAGATCGTATTTTAXXXXXXXXXXT
TGCTTGCCTCCTTTAGTATTAGGATTATGCGTGATTTCTTTGACCTCTTTCAGTAGGAATACCTGCAACTTCAAGGGTG

>Marker148272 Chr6 SNP_site:28345787 Ref_Type:A

AACACCCCACCAGGAACTCCAATTAGAATCATGAAGAATTTGAGAGTTTGTGCAGACTGTCATCTGGCAATCAAACTCATXXXXXXXXXXA
ATGAGTTTGCAACCTTTTAACGAGAGGTGACTTTCCATTGTATTATTGAACTATGAATAAAAGGTTGTGTTAGGAGGTT
AACACCCCACCAGGAACTCCAATTAGAATCATGAAGAATTTGAGAGTTTGTGCAGACTGTCATCTGGCAATCAAACTCATXXXXXXXXXXA
ATGAGTTTGCAACCTTTTAACGAGAGGTGACTTTCCATTGTATTATTGAGCTATGAATAAAAGGTTGTGTTAGGAGGTT

>Marker14837 Chr4 SNP_site:1187623 Ref_Type:C

AACCCTATAAAGGTTTTAGAGTAAAGTAGAATATAAATAGGGTGTGGCACAAAAGTTTAGGATCCTTTTGTGACCCAAATXXXXXXXXXXA
AAAATGCGAATGAATATTGTAGTTGAAGCTTGAGGCTTTAATTTCTTCGAGATAGTTCTCATTCTCTTTAATGATTAGT
AACCCTATAAAGGTTTTAGAGTAAAGTAGAATATAAATAGGGTGTGGCACAAAAGTTTAGGATCCTTTTGTGACCCAAATXXXXXXXXXXA
AAAATGTGAATGAATATTGTAGTTGAAGCTTGAGGCTTTAATTTCTTCGAGATAGTTCTCATTCTCTTTAATGATTAGT

>Marker148455 Chr6 SNP_site:19021360 Ref_Type:T

ACTTTGAATGTTGGAGCTAGTTCTAAGTGTTGTTGTTTCTTGTTTGTATCATCATATACTATGTATTTCTCACCTAATTTXXXXXXXXXXT
GTAGCTCAAATCTCATCTAAAGGCAGATTACTGTAATGTAATAAGGATTACTAACAATAGGAACACTATGTTTGAAGTG
ACTTTGAATGTTGGAGCTAGTTCTAAGTGTTGTTGTTTCTTGTTTGTATCATCATATACTATGTATTTCTCATCTAATTTXXXXXXXXXXT
GTAGCTCAAATCTCATCTAAAGGCAGATTACTGTAATGTAATAAGGATTACTAACAATAGGAATACTATGTTTGAAGTG

>Marker148455 Chr6 SNP_site:19021520 Ref_Type:T

ACTTTGAATGTTGGAGCTAGTTCTAAGTGTTGTTGTTTCTTGTTTGTATCATCATATACTATGTATTTCTCACCTAATTTXXXXXXXXXXT
GTAGCTCAAATCTCATCTAAAGGCAGATTACTGTAATGTAATAAGGATTACTAACAATAGGAACACTATGTTTGAAGTG
ACTTTGAATGTTGGAGCTAGTTCTAAGTGTTGTTGTTTCTTGTTTGTATCATCATATACTATGTATTTCTCATCTAATTTXXXXXXXXXXT
GTAGCTCAAATCTCATCTAAAGGCAGATTACTGTAATGTAATAAGGATTACTAACAATAGGAATACTATGTTTGAAGTG

>Marker148861 Chr6 SNP_site:14101315 Ref_Type:T

AACACCCACTGTCCCCTTTCTTCTTCCTCTTCTTCTTCTTCCTCTTCCACTCTCCCAACCCCCACTCTTCTCCAATATCAXXXXXXXXXXG
GCATAAATCTAACTGGGGCTTCTTTTTTGGTTTAGAGGGAACTTAATCTAGGTAGTTTTATCACTGTCCGTGGTTGGTT
AACACCCACTGTCCCCTTTCTTCTTCCTCTTCTTCTTCTTCCTCTTCCACTCTCCCAACCCCCACTCTTCTCCAATATCAXXXXXXXXXXG
GCATAAATCTAACTGGGGCTTCTTTTTTGGTTTAGAGGGAACTTAATCTTGGTAGTTTTATCACTGTCCATGGTTGGTT

>Marker148861 Chr6 SNP_site:14101335 Ref_Type:A

AACACCCACTGTCCCCTTTCTTCTTCCTCTTCTTCTTCTTCCTCTTCCACTCTCCCAACCCCCACTCTTCTCCAATATCAXXXXXXXXXXG
GCATAAATCTAACTGGGGCTTCTTTTTTGGTTTAGAGGGAACTTAATCTAGGTAGTTTTATCACTGTCCGTGGTTGGTT
AACACCCACTGTCCCCTTTCTTCTTCCTCTTCTTCTTCTTCCTCTTCCACTCTCCCAACCCCCACTCTTCTCCAATATCAXXXXXXXXXXG
GCATAAATCTAACTGGGGCTTCTTTTTTGGTTTAGAGGGAACTTAATCTTGGTAGTTTTATCACTGTCCATGGTTGGTT

>Marker149091 Chr6 SNP_site:26791072 Ref_Type:G

CACAAAGCAAGCAGCAATCTCCAATAATTACATTATGACCGATCTAAATAGCCAGGAAGAGTTTGAGAGTCACTATGATGXXXXXXXXXXC
AGAGCACTGTATTAGAAATTTAGAATGTCAGCTCATCCTATTCCCAAAAGCAATGACATTCAAGAAAATATAACTAGTC
CACAAAGCAAGCAGCAATCTCCGATAATTACATTATGACCGATCTAAATAGCCAGGAAGAGTTTGAGAGTCACTATGATGXXXXXXXXXXC

Create PDF with GO2PDF for free, if you wish to

AGAGCACTGTATTAGAAATTTAGAATGTCAGCTCATCCTATTCCCAAAAGCAATGACATTCAAGAAAATATAACTAGTC
>Marker149392 Chr6 SNP_site:13517029 Ref_Type:G
AACTTAAATATAAGTATATAGATTTAGGTATATATCCTCCATCCAGAAGCCAGTCATAATGCTCAAATATGAGTGTAGGAXXXXXXXXXXXA
TTAAGTTAAAGAGTAAATATTTTTTAACCAAAATCTTGTTATTTTCTTGACTTTGTTGCTTCTATTCTTATCTTATGTC
AACTTAAATATAAGTATATAGATTTAGGTATATATCCTCCATCCAGAAGCCATTCATAATGCTCAAATATGAGTGTAGGAXXXXXXXXXXXA
TTAAGTTAAAGAGTAAATTTTTTTTAACCAAAATCTTGTTATTTTCTTGACTTTGTTGCTTCTATTCTTATCTTATGTC
>Marker149392 Chr6 SNP_site:13517180 Ref_Type:A
AACTTAAATATAAGTATATAGATTTAGGTATATATCCTCCATCCAGAAGCCAGTCATAATGCTCAAATATGAGTGTAGGAXXXXXXXXXXXA
TTAAGTTAAAGAGTAAATATTTTTTAACCAAAATCTTGTTATTTTCTTGACTTTGTTGCTTCTATTCTTATCTTATGTC
AACTTAAATATAAGTATATAGATTTAGGTATATATCCTCCATCCAGAAGCCATTCATAATGCTCAAATATGAGTGTAGGAXXXXXXXXXXXA
TTAAGTTAAAGAGTAAATTTTTTTTAACCAAAATCTTGTTATTTTCTTGACTTTGTTGCTTCTATTCTTATCTTATGTC
>Marker150693 Chr6 SNP_site:977684 Ref_Type:G
ACTCCAATAATTCCAAACCACATTACCCATTGCTTGTAGCAAATGTGTGGATTCATACTTGTTTGAACACAACTTAACAAXXXXXXXXXXXA
ATGCATTGGGCATTGGAAAAGTTCTAAATAATGCTGTAAGTAATTCGATTAGACTTTTATCCAAGGATTCAACTTGGTA
ACTCCAATAATTCCAAACCACATTACCCATTGCTTGTAGCGAATGTGTGGATTCATACTTGTTTGAACACAACTTAACAAXXXXXXXXXXXA
ATGCGTTGGGCATTGGAAAAGTTCTAAATAATGCTGTAAGTAGTTCGATTAGACTTTTATCCAAGGATTCAACTTGGTA
>Marker150693 Chr6 SNP_site:977882 Ref_Type:G
ACTCCAATAATTCCAAACCACATTACCCATTGCTTGTAGCAAATGTGTGGATTCATACTTGTTTGAACACAACTTAACAAXXXXXXXXXXXA
ATGCATTGGGCATTGGAAAAGTTCTAAATAATGCTGTAAGTAATTCGATTAGACTTTTATCCAAGGATTCAACTTGGTA
ACTCCAATAATTCCAAACCACATTACCCATTGCTTGTAGCGAATGTGTGGATTCATACTTGTTTGAACACAACTTAACAAXXXXXXXXXXXA
ATGCGTTGGGCATTGGAAAAGTTCTAAATAATGCTGTAAGTAGTTCGATTAGACTTTTATCCAAGGATTCAACTTGGTA
>Marker150693 Chr6 SNP_site:977920 Ref_Type:G
ACTCCAATAATTCCAAACCACATTACCCATTGCTTGTAGCAAATGTGTGGATTCATACTTGTTTGAACACAACTTAACAAXXXXXXXXXXXA
ATGCATTGGGCATTGGAAAAGTTCTAAATAATGCTGTAAGTAATTCGATTAGACTTTTATCCAAGGATTCAACTTGGTA
ACTCCAATAATTCCAAACCACATTACCCATTGCTTGTAGCGAATGTGTGGATTCATACTTGTTTGAACACAACTTAACAAXXXXXXXXXXXA
ATGCGTTGGGCATTGGAAAAGTTCTAAATAATGCTGTAAGTAGTTCGATTAGACTTTTATCCAAGGATTCAACTTGGTA
>Marker151238 Chr6 SNP_site:20935814 Ref_Type:T
ACAAGAACATTTGAAATTGCAATGATTAAAATGGAAGAAAGAAATTACAAAAAATTAAAAAGAAAAAAGGGGAAGTCCAAXXXXXXXXXXXG
ATGTGGCTCCCATAACCCATCATAATGATAACCAATTGGTATATGACATGATTTGATGTGTATTTGTTACCATTATGGT
ACAAGAACATTTGAAATTGCAATGATTAAAATGGAAGAAAGAAATTTCAAAAAATTAAAAAGAAAAAAGGGGAAGTCCAAXXXXXXXXXXXG
ATGTGGCTCCCATAACCCATCATAATGATAACCAATTGGTATATGACATGATTTGATGTGTATTTGTTACCATTATGGT
>Marker151432 Chr6 SNP_site:7258764 Ref_Type:C
ACATGTAAGAACTTTCAAACACTTTTATTTTTTCTTGTAAGTGGTTAGTTAATTGATCCAAATAAATCCTTAATCGAAAAXXXXXXXXXXXA
TGATGTATTTACTATTTTGTTCAACTTGAAGAAAGTCTCTCAGTATTTCTTTTTTTACATCAATGCATGTATTTCCAGT
ACATGTAAGAACTTTCAAACACTTTTATTTTTTCTTGTAAGTGGTTAGTTAATTGATCCAAATAAATCCTTAATCGAAAAXXXXXXXXXXXA
TGATGTATTTACTATTTTGTTCAACTTGAAGAAAGTCTCTCAGTATTTCTTTTTTTACATCAATGCATGTATTTCGAGT
>Marker15147 Chr4 SNP_site:7981559 Ref_Type:G
CACCAAAGGATTGATCTGACAAATCAATACTATCATATGGAAAATAAAACTCAGAAGGGAAAGAGAAAAGAAAAAAAAAAXXXXXXXXXXT
CAGTGTGTAGAGAAAAGAATCTAAGGGGACCATTAATTGGTAAATCATCATCCTCTTACCTTTTACATATCAGTTAGTC
CACCAAAGGATTGATCTGACAAATCAATACTATCATATGGAAAATAAAACTCAGAAGGGAAAGAGAAAAGAAAAAAAAAGXXXXXXXXXXT
CAGTGTGTAGAGAAAAGAATCTAAGGGGACCATTAATTGGTAAATCATCATCCTCTTACCTTTTACATATCAGTTAGTC
>Marker15171 Chr4 SNP_site:20779525 Ref_Type:T
ACTGTCCTTTTGATTATTTGACACATTATAAATGATTTGTTTTTAATAGTTGATTAGTGGGAAGTGCTTAAATTTAAGATXXXXXXXXXXC
TCGGCCTCTTTCTGGCTACCCAATCACCAATCATCATGGAATTGGAAGCAAACGGACAATGAATGAATCAAAGTGTGTA

Create PDF with GO2PDF for free, if you wish to

ACTGTCTTTTTGATTATTTGACACATTATAAATGATTTGTTTTTAATAGTTGATTAGTGGGAAGTGCTTAAATTTAAGATXXXXXXXXXXC
TCGGCCTCTTTCTGGCTACCCAATCACCAATCATCATGGAATTGGAAGCAAACGGACAATGAATGAATCAAAGTGTGTA
>Marker151881 Chr6 SNP_site:13246338 Ref_Type:G
ACCACAAATCCTCTAATGAACAACTAGTTTAAGGTCTAACCTATAAACCAAATCCCTCTCGAGTTAATAAAAGGGTGTGGXXXXXXXXXXT
TCACCTTAAATGGGAGGCATATTGGGCCAACACTGATGAGTTACTCTCATCTAAGCAGATCTAAGGATAATCTTGTGTG
ACCACAAATCCTCTAATGAACAACTAGTTTAAGGTCTAACCTATAAACCAAATCCCTCTCGAGTTAATAAAAGGGTGTGGXXXXXXXXXXT
TCACCTTAAATGGGAGGCATATTGGGCCAACACTGATGAGTTGCTCTCACCTAAGCAGATCTAAGGATAATCTTGTGTG
>Marker151881 Chr6 SNP_site:13246345 Ref_Type:C
ACCACAAATCCTCTAATGAACAACTAGTTTAAGGTCTAACCTATAAACCAAATCCCTCTCGAGTTAATAAAAGGGTGTGGXXXXXXXXXXT
TCACCTTAAATGGGAGGCATATTGGGCCAACACTGATGAGTTACTCTCATCTAAGCAGATCTAAGGATAATCTTGTGTG
ACCACAAATCCTCTAATGAACAACTAGTTTAAGGTCTAACCTATAAACCAAATCCCTCTCGAGTTAATAAAAGGGTGTGGXXXXXXXXXXT
TCACCTTAAATGGGAGGCATATTGGGCCAACACTGATGAGTTGCTCTCACCTAAGCAGATCTAAGGATAATCTTGTGTG
>Marker151896 Chr6 SNP_site:13964774 Ref_Type:C
CACAAATTCAAAGCTCAAACTGCAAAATCTTCAAATAATAATAATGAAACTACAAAATTGGGCTAAAAGATTCTGTTTTGXXXXXXXXXXT
TCTCACCCTTTCAATTTTACCTTGTTTCCATTATTATTTTAAGTTATTTACTTTCAAATATCTAATTTTAGTTTGAGTA
CACAAATTCAAAGCTCAAACTGCAAAATCTTCAAATAATAATAATGAAACTACAAAATTGGGCTAAAAGATTCTGTTTTGXXXXXXXXXXT
TCTCACCCTTTTAATTTTACCTTGTTTCCATTATTATTTTAAGTTATTTACTTTCAAATATCTAATTTTAGTTTTAGTA
>Marker151896 Chr6 SNP_site:13964837 Ref_Type:G
CACAAATTCAAAGCTCAAACTGCAAAATCTTCAAATAATAATAATGAAACTACAAAATTGGGCTAAAAGATTCTGTTTTGXXXXXXXXXXT
TCTCACCCTTTCAATTTTACCTTGTTTCCATTATTATTTTAAGTTATTTACTTTCAAATATCTAATTTTAGTTTGAGTA
CACAAATTCAAAGCTCAAACTGCAAAATCTTCAAATAATAATAATGAAACTACAAAATTGGGCTAAAAGATTCTGTTTTGXXXXXXXXXXT
TCTCACCCTTTTAATTTTACCTTGTTTCCATTATTATTTTAAGTTATTTACTTTCAAATATCTAATTTTAGTTTTAGTA
>Marker152468 Chr6 SNP_site:14003967 Ref_Type:G
ACTAAGTTACCAACTTTACTTCTTTATCTATTCCAAAAAACAATAGGGTGAATCTCACTGCTAAATCTATCAAATTTACAXXXXXXXXXXA
TATAATAAGTGTTTGAATTAAAAAAGAAAGAGTATATTTAATAGATGGATACAAAATTAGTTTTATGTTAAAGTTTGTG
ACTAAGTTACCAACTTTACTTCTTTATCTATTCCAAAAAACAATAGGGTGAATCTCACTGCTAAATCTATCAAATTTACAXXXXXXXXXXA
TATAATAAGTGTTTGAATTAAAAAAGAGAGAGTATATTTAATAGATGGATACAAAATTAGTTTTATGTTAAAGTTTGTG
>Marker152487 Chr6 SNP_site:27550905 Ref_Type:T
ACCCACAAGTGACTAGGTGGGTTCGACACCAAGGATTTCGTGAAATCCCCAACCGCTCCGGCTAACATCATGTGCTCTTTXXXXXXXXXXC
AGAGGGAGCAGAGATTTGAACAGGAACACGCCTGACCGTAACCCATGGGAGAATGATTAGGAAGGTGATTGCTGCTGTG
ACCCACAAGTGACTAGGTGGGTTCGACACCAAGGATTTCGTGAAATCCCCTACCGCTCCGGCTAACATCATGTGCTCTTTXXXXXXXXXXC
AGAGGGAGCAGAGATTTGAACAGGAACACGCCTGACCGTAACCCATGGGAGAATGATTAGGAAGGTGATTGCTGCTGTG
>Marker152887 Chr6 SNP_site:3303448 Ref_Type:C
TACTTGAAAGAATTTTTTTTCCTGCAGCATGAAGTTCTTATACCCGGACTAGATTCTATTCTCACATCTGATGTTGCTGAXXXXXXXXXXG
TAATTCTTTTGTCACTTTCTAAATTCTTCTGTGTCTATACCCTAGAAGTTAATTTTAAAAAAACTTAATCCCATGTGTG
TACTTGAAAGAATTTTTTTTCCTGCAGCATGAAGTTCTTATACCCGGACTAGATTCTATTCTCACATCTGATGTTGCTGAXXXXXXXXXXG
TAATTCTTTTGTCACTTTCTAAATTTTTCTGTGTCTATACCCTAGAAGTTAATTTAAAAAAAACTTAATCCCATGTGTG
>Marker152887 Chr6 SNP_site:3303478 Ref_Type:T
TACTTGAAAGAATTTTTTTTCCTGCAGCATGAAGTTCTTATACCCGGACTAGATTCTATTCTCACATCTGATGTTGCTGAXXXXXXXXXXG
TAATTCTTTTGTCACTTTCTAAATTCTTCTGTGTCTATACCCTAGAAGTTAATTTTAAAAAAACTTAATCCCATGTGTG
TACTTGAAAGAATTTTTTTTCCTGCAGCATGAAGTTCTTATACCCGGACTAGATTCTATTCTCACATCTGATGTTGCTGAXXXXXXXXXXG
TAATTCTTTTGTCACTTTCTAAATTTTTCTGTGTCTATACCCTAGAAGTTAATTTAAAAAAAACTTAATCCCATGTGTG
>Marker152976 Chr6 SNP_site:26541713 Ref_Type:T
ACCTTTTGCCCGCAGACAGGAACTCACCCGAAAATCACTACTTTGAGGAATATTAATCGCTTTCGGTCTCAGGTGCAGTTXXXXXXXXXXT

Create PDF with GO2PDF for free, if you wish to

TATTATTTTTTATGATCTAAACTCCAAGCTGAAGCACATAAGAATGGCTCTATCTATAGCATATTTGGAATGAGTTGTG
ACCTTTTGCCCGCAGACAGGAACTCACCCGAAAATCACTACTTTGAGGAATATTAATCGCTTTCGGTCTCAGGTGCAGTTXXXXXXXXXXT
TTTTTTTTTTTATGATCTAAACTCCAAGCTGAAGCACATAAGAATGGCTCTATCTATAGCATATTTGGAATGAGTTGTG
>Marker152976 Chr6 SNP_site:26541716 Ref_Type:T
ACCTTTTGCCCGCAGACAGGAACTCACCCGAAAATCACTACTTTGAGGAATATTAATCGCTTTCGGTCTCAGGTGCAGTTXXXXXXXXXXT
TATTATTTTTTATGATCTAAACTCCAAGCTGAAGCACATAAGAATGGCTCTATCTATAGCATATTTGGAATGAGTTGTG
ACCTTTTGCCCGCAGACAGGAACTCACCCGAAAATCACTACTTTGAGGAATATTAATCGCTTTCGGTCTCAGGTGCAGTTXXXXXXXXXXT
TTTTTTTTTTTATGATCTAAACTCCAAGCTGAAGCACATAAGAATGGCTCTATCTATAGCATATTTGGAATGAGTTGTG
>Marker153181 Chr6 SNP_site:177107 Ref_Type:G
AACTTTCTTAGTGTTTGCCTATTTAGGTATTGTGAATGTTGGGGGTGGGTATTTTTTTTTGCTAAATGATTTTGGTATAGXXXXXXXXXXC
CTGGATTAAGTTTGGAGTCAAAGAAAATAAGAAAAACATAATTTTCCTCCCAGAAGCCACACAGCCCGAAGACAATAGT
AACTTTCTTAGTGTTTGCCTATTTAGGTATTGTGAATGTTGGGGGTGGGTATTTTTTTTTGCTAAATGATTTTGGTATAGXXXXXXXXXXC
CTGTATTAAGTTTGGAGTCAAAGAAAATAAGAAAAACATAATTTTCCTCCCAGAAGCCACACAGCCCGAAGACAATAGT
>Marker15346 Chr4 SNP_site:4645173 Ref_Type:A
CACATTAAATAATACTAAATAAATCTAAAATCTTTTTATTTAAAATATAAAACGATTCATCTTCATCTTTAATCACATTGXXXXXXXXXXA
TATCTTCTATTACAATATATAGCAAAATATCTTTATTTTATAAAAAAATTACTTGAAACTCCATTATTTACAAATTGTA
CACATTAAATAATACTAAATAAATCTAAAGTCTTTTTATTTAAAATATAAAACCATTCATCTTCATCTTTAATCACATTGXXXXXXXXXXA
TATCTTCTATTACAATATATAGCAAAATATCTTTATTTTATAAAAAAATTACTTGAAACTCCATTATTTACAAATTGTA
>Marker15346 Chr4 SNP_site:4645197 Ref_Type:G
CACATTAAATAATACTAAATAAATCTAAAATCTTTTTATTTAAAATATAAAACGATTCATCTTCATCTTTAATCACATTGXXXXXXXXXXA
TATCTTCTATTACAATATATAGCAAAATATCTTTATTTTATAAAAAAATTACTTGAAACTCCATTATTTACAAATTGTA
CACATTAAATAATACTAAATAAATCTAAAGTCTTTTTATTTAAAATATAAAACCATTCATCTTCATCTTTAATCACATTGXXXXXXXXXXA
TATCTTCTATTACAATATATAGCAAAATATCTTTATTTTATAAAAAAATTACTTGAAACTCCATTATTTACAAATTGTA
>Marker153569 Chr6 SNP_site:7072723 Ref_Type:C
CACTTGACTCTAAACTCATTTGTAATTACCACATTTCAACTATTCCTTTTACATTGGAATTATTTAGATATGGCGAGAAAXXXXXXXXXXG
CATAATTATTTTCAAAATTAAAGCAAATATATAGCTATCAAATAGAGCCTAAACGACCAATAATATGTCTTGGTTACGT
CACTTGACTCTAAACTCATTTGTAATTACCACATTTCAACTATTCCTTTTACATTGGAATTATTTAGATATGGCGAGAAAXXXXXXXXXXG
CATAATTATTTTCAAAATTAAAGCAAATATATAGCTATCAAATAGAGCCTAAACGACCAATAATATGTTTTGGTTACGT
>Marker153579 Chr6 SNP_site:16886358 Ref_Type:G
ACTACACTATAACAGTCTCAAACCAGATTTTGACAATTTAACTTATACCAAGTTTTTAGTGTTATAATTCGTTTGTTTTTXXXXXXXXXXA
AAATGTAAAATAGAAAAGGGGGAAATAAGTAGAGAGAACCGAAATAATATCTCCTTTTTAAAGCTCTTTCTGGGAGGTT
ACTGCACTATAACAGTCTCAAACCAGATTTTGACAATTTAACTTATACCAAGTTTTTAGTGTTATAATTCGTTTGTTTTTXXXXXXXXXXA
AAATGTAAAATAGAAAAGGGGGAAATAAGTAGAGAGAACCGAAATAATATCTCCTTTTTAAAGCTCTTTCTGGGAGGTT
>Marker153818 Chr6 SNP_site:15844507 Ref_Type:G
CACTTGTGCTTTTAGAGGACGATCCTTTTGAAAAGCTCCTTTTAGTGGTAGCAACATTTGCTTCCACCTTTTCCTTACCCXXXXXXXXXXT
TCAGAAGAGATTCTAAGATAAAACTAACTTGATTAGCCTCTTCAATAGAACCACCATTTAGTTCGACGATGTTGAAGTG
CACTTGTGCTTTTAGAGGACGATCCTTTTGAAAAGCTCCTTTTAGTGGTAGCAACATTTGCTTCCACCTTTTCCTTACCCXXXXXXXXXXT
TCAGAAGAGATTCTAAGATAAAGCTAACTTGATTAGCCTCTTCAATAGAACCACCATTTAGTTCGACGATGTTGAAGTG
>Marker153948 Chr6 SNP_site:10321183 Ref_Type:A
ACAAAACCTGAGATATAAATGATACAGCCATTTGCAGAGACCAACACAAAACATGTTAAAGCAAAAGGAACATCTCATAAXXXXXXXXXXT
CCAAACAAAGATAATGTTGAACGCTAAAACCATGTGAGGCTAGATAAAGTGGTGAAAATTCCATATGACAAGAGATGGT
ACAAAACCTGAGATATAAATGATACAGCCATTTGCAGAGACCAACACAAAACATGTTAAAGCAAAAGGAACATCTCATGAXXXXXXXXXXT
CCAAACAAAGATAATGTTGAACGCTAAAACCATGTGAGGCTAGATAAAGTGGTGAAAATTCCATATGACAAGAGATGGT
>Marker154034 Chr6 SNP_site:18988810 Ref_Type:A

Create PDF with GO2PDF for free, if you wish to

AACTGTGGCCTATAGTATAGGATAAGGAAAAAACACTAAAAGCTTATCTAACTTCTTCATACCATTACATTACCCTCTAGXXXXXXXXXXA
GTCATGAGTCCCATGACCAAACTTGTAAAGAGATGAACAATATGTCATAATCAGCACATGCTATGGGATAGAAACAGTA
AACTGTGGCCTATAGTATAGGATAAGGATAAAACACTAAAAGCTTATCTAACTTCTTCATACCATTACATTACCCTCTAGXXXXXXXXXXA
GTCATGAGTCCCATGACCAAACTTGTAAAGAGATGAACAATATGTCATAATCAGCACATGCTATGGGATAGAAACAGTA
>Marker154098 Chr6 SNP_site:3085570 Ref_Type:C
TACAGCTTCGGTGTAGTTCTACTTGAGCTTTTGACCGGTAGGAAACCTGTCGATCACACGATGCCTCGCGGACAGCAAAGXXXXXXXXXXC
CTTTGTGAGGCAATTCCTCGGTTCCAGCTCCATAAAGGCTGTTAAATATTCCTGCGTCTTGTGCTGATAGAACATTGTC
TACAGCTTCGGTGTAGTTCTACTTGAGCTTTTGACCGGTAGGAAACCTGTCGATCACACGATGCCTCGCGGACAGCAAAGXXXXXXXXXXC
CTTTGTGAGGCAATTCCTCGGTTTCAGCTCCATAAAGGCTGTTAAATATTCCTGCGTCTTGTGCTGATAGAACATTGTC
>Marker154708 Chr6 SNP_site:28163042 Ref_Type:C
AACAGTGAAATTTTCCAAAATACATGAACTGTAGAGCTGGGTCTGAAATTCCTTGGACATATAAGATTAGCTTCAGATTGXXXXXXXXXXT
CAACAGAGAAAGGTCTGTCATGAGTTCTATTTTCTACACTAAGTTAATGCAATTATCTGGTTCTTTTTATTACTCCGTA
AACAGTGAAATTTTCCAAAATACATGAACTGTAGAGCTGGGTCTGAAATTCCTTGGACATATAAGATTAGCTTCAGATTGXXXXXXXXXXT
CAACAGAGAAAGGTCTGTCATGAGTTCTATTTTCTACACTAAGTTAATGCAATTATGTGGTTCTTTTTATTACTCCGTA
>Marker155031 Chr6 SNP_site:13345963 Ref_Type:G
ACCAGTTGAGCTAAGCTTGTGTTGGCTTCACATAATTTATTTCATCGATGTGTTATTGAATCACAAAGAAAAATTAGAATXXXXXXXXXXT
TACATAAGATATAATTAAAATTTAGAGAAATGTTACAAAATTCTAAAAAGATCTTAATTCGGCTTGTGGGGATGGTGTA
ACCAGTTGAGCTAAGCTTGTGTTGGCTTCACATAATTTATTTCATCGATGTGTTATTGAATCGCAAAGAAAAATTAGAATXXXXXXXXXXT
TACATAAGATATAATTAAAATTTAGAGAAATGTTACAAAATTCTAAAAAGATCTTAATTCGGCTTGTGGGGATGGTGTA
>Marker156079 Chr6 SNP_site:22572016 Ref_Type:A
AACTTCCATTGATCCAATATTTCTTTCTTACAGAATACAAATACATCCAAAGAAGATATAAGCGCACTAACGACTACAGAXXXXXXXXXXA
TTCAATGCCTGCAATTAGACATTTCAATTAGCAATCAATTCTTCAGAAAATTAATATGTTAATAAATGTAATGAAACGT
AACTTCCATTGATCCAATATTTCTTTCTTACAGAATACAAATACATCCAAAGAAGATATAAGCGCACTAACGACTACAGAXXXXXXXXXXA
TTCAATGCCTGCAATTAGACATTTCAATTAGCAATCAATTCTTCAGAAAATTGATATGTTAATAAATGTAATGAAACGT
>Marker156199 Chr6 SNP_site:8872274 Ref_Type:A
ACAATTATTGTTGCAAATGAGATTATATATATATTGCTTACCAAAGTCATTCAATTTTTGCTGCCTTCCTAACTTTTACCXXXXXXXXXXT
AGATAAGTCAAATTATTAACCTTTATATGAACATAACATGCATTCAAAAAATATTTTTACTTAATTATATAATTAAGTA
ACAATTATTGTTGCAAATGAGATTATATATATATTGCTTACCAAAGTCATTCAATTTTTGCTGCCTTCCTAACTTTTACCXXXXXXXXXXT
AGATAAGTCAAATTATTAACCTTTATATGAACATAACATGCATTCAAAAAATATTTTTACTTAATTATATAATTATGTA
>Marker156835 Chr6 SNP_site:16892564 Ref_Type:C
ACTTTGTGACTCCGATGGCGAATAGATCCAATGTAGATCCAAATTTCTATTCACTCGTCAGATCGAATGACTCTACGTAAXXXXXXXXXXG
AGTCCAGTCCGCTCGAGCAGTTTAAGGTGCAAAAAACTTTAATATTACTCCAAATGAGACCACTCGGTAGATAGATGTG
ACTTTGTGACTCCGATGGCGAATAGATCCAATGTAGATCCAACTTTCTATTCACTCGTCAGATCGAATGACTCTACGTAAXXXXXXXXXXG
AGTCCAGTCCGCTCGAGCAGTTTAAGGTGCAAAAAACTTTAATATTACTCAAAATGAGACCACTCGGTAGATAGATGTG
>Marker156835 Chr6 SNP_site:16892759 Ref_Type:A
ACTTTGTGACTCCGATGGCGAATAGATCCAATGTAGATCCAAATTTCTATTCACTCGTCAGATCGAATGACTCTACGTAAXXXXXXXXXXG
AGTCCAGTCCGCTCGAGCAGTTTAAGGTGCAAAAAACTTTAATATTACTCCAAATGAGACCACTCGGTAGATAGATGTG
ACTTTGTGACTCCGATGGCGAATAGATCCAATGTAGATCCAACTTTCTATTCACTCGTCAGATCGAATGACTCTACGTAAXXXXXXXXXXG
AGTCCAGTCCGCTCGAGCAGTTTAAGGTGCAAAAAACTTTAATATTACTCAAAATGAGACCACTCGGTAGATAGATGTG
>Marker158579 Chr6 SNP_site:10284287 Ref_Type:T
AACAGAGAATAACAAAACTAACAAATTTTCCCTTGTAAGCAAAGATAATACATATAAATCCACACACTGTAAGAAAAATAXXXXXXXXXXA
TGTAACAGACTTCCATGGTCAGATAGACATAAATAAGGTTATTACAGAAATGACTGTGAATAGAAATTAGCATTTGAGT
AACAGAGAATAACAAAACTAACAAATTTTCCCTTGTAAGCAAAGATAATACATATAAATCCACACACTGTAAGAAAAATAXXXXXXXXXXA
TGTAACATACTTCCATGGTCAGATAGACATAAATAAGGTTATTACAGAAATGACTGTGAATAGAAATTAGCATTTGAGT

Create PDF with GO2PDF for free, if you wish to

>Marker15874 Chr4 SNP_site:6046342 Ref_Type:A
ACCTCCATTCAATCAGTCTTAGATAATATCAGGTCAGTATCTCCACGTCCCACTACTGTTCATTTTAATTACTCAAGCTTXXXXXXXXXXC
TTATAGTGTCAGCAGTGAAGATTCTCTAGGATTAACGCAAATTCAAGAAGACAGAGCAAATGTTGAAATTGAAGTAGTG
ACCTCCATTCAATCAGTCTTAGATAATATCAGGTCAGTATCTCCACGTCCCACTTCTGTTCATTTTAATTACTCAAGCTTXXXXXXXXXXC
TTATAGTGTCAGCAGTGAAGATTCTCTAGGATTAAGGCAAATTCAAGAAGACAGAGCAAATGTTGAAATTGAAGTAGTG
>Marker15874 Chr4 SNP_site:6046499 Ref_Type:C
ACCTCCATTCAATCAGTCTTAGATAATATCAGGTCAGTATCTCCACGTCCCACTACTGTTCATTTTAATTACTCAAGCTTXXXXXXXXXXC
TTATAGTGTCAGCAGTGAAGATTCTCTAGGATTAACGCAAATTCAAGAAGACAGAGCAAATGTTGAAATTGAAGTAGTG
ACCTCCATTCAATCAGTCTTAGATAATATCAGGTCAGTATCTCCACGTCCCACTTCTGTTCATTTTAATTACTCAAGCTTXXXXXXXXXXC
TTATAGTGTCAGCAGTGAAGATTCTCTAGGATTAAGGCAAATTCAAGAAGACAGAGCAAATGTTGAAATTGAAGTAGTG
>Marker159347 Chr6 SNP_site:1421923 Ref_Type:A
TACAGGATTACAAACTCGAGAATCGCCCTCTTACCAAGTGCAAGAATCTCCTAATCCTGCTGCATTGGGATGTCATGTCAXXXXXXXXXXC
CGGATGCATATAAGAGAGACACAGCAGAGGTCGATTTGATTTCTCTGGGTTCATTTTTCTCATTTACAATTCAGTTGTA
TACAGGATTACAAACTCGAGAATCGCCCTCTTACCAAGTGCAAGAATCTCCTAATCCTGCTGCATTGGGATGTCATGTCAXXXXXXXXXXC
CGGATGCATATAAGAGAGACACAGCAGAGGTCGATTTGATTTCTCTGGGTTCATTTTTCTCATTTACACTTCAGTTGTA
>Marker159460 Chr6 SNP_site:26754152 Ref_Type:G
CACTCACCTCACCACCTTTAAATTCATTCATCTGTTTTGTTTTATTTCTCAGACATTAGAAGAGCAAATAAATTGAAGAAXXXXXXXXXXA
TCAAAACGCTATTGAGTTTAGAGATAGAGTATATATATGTTCAATAAACTATAAAAGAAAAACGAAATCAAGAGATGTA
CACTCACCTCACCACCTTTAAATTCGTTCATCTGTTTTGTTTTATTTCTCAGACATTAGAAGAGCAAATAAATTGAAGAAXXXXXXXXXXA
TCAAAACGCTATTGAGTTTCGAGATAGAGTATATATATGTTCAATAAACTATAAAAGAAAAACGAAATCAAGAGATGTA
>Marker159460 Chr6 SNP_site:26754371 Ref_Type:C
CACTCACCTCACCACCTTTAAATTCATTCATCTGTTTTGTTTTATTTCTCAGACATTAGAAGAGCAAATAAATTGAAGAAXXXXXXXXXXA
TCAAAACGCTATTGAGTTTAGAGATAGAGTATATATATGTTCAATAAACTATAAAAGAAAAACGAAATCAAGAGATGTA
CACTCACCTCACCACCTTTAAATTCGTTCATCTGTTTTGTTTTATTTCTCAGACATTAGAAGAGCAAATAAATTGAAGAAXXXXXXXXXXA
TCAAAACGCTATTGAGTTTCGAGATAGAGTATATATATGTTCAATAAACTATAAAAGAAAAACGAAATCAAGAGATGTA
>Marker159685 Chr6 SNP_site:13352355 Ref_Type:G
AACGAAAGTGATAAGTGCATCTACTATAAGTTTGATAATGGCTTATGCACTATTGTATGCTTATATGTAGATGACATGTTXXXXXXXXXXT
CGGTCTGAAATAGGAATTTCTTTCGATCAATCCTCTTACATAGAGAAAATTGTGAAGAAATATAACTACCTTGAAAGTA
AACGAAAGTGATAAGTGCATCTACTATAAGTTTGATAATGGCTTATGCACTATTGTATGCTTATATGTAGATGACATGTTXXXXXXXXXXT
CGGTCTGAAATAGGAATTTCTTTGGATCAATCCTCTTACATAGAGAAAATTTTGAAGAAATATAACTACCTTGAAAGTA
>Marker159685 Chr6 SNP_site:13352383 Ref_Type:T
AACGAAAGTGATAAGTGCATCTACTATAAGTTTGATAATGGCTTATGCACTATTGTATGCTTATATGTAGATGACATGTTXXXXXXXXXXT
CGGTCTGAAATAGGAATTTCTTTCGATCAATCCTCTTACATAGAGAAAATTGTGAAGAAATATAACTACCTTGAAAGTA
AACGAAAGTGATAAGTGCATCTACTATAAGTTTGATAATGGCTTATGCACTATTGTATGCTTATATGTAGATGACATGTTXXXXXXXXXXT
CGGTCTGAAATAGGAATTTCTTTGGATCAATCCTCTTACATAGAGAAAATTTTGAAGAAATATAACTACCTTGAAAGTA
>Marker160266 Chr6 SNP_site:26946251 Ref_Type:G
ACTTGAAAAGTTAGAATAAAAGACCCAAAATTCCATGGACCTTTTGGGTGTTTTTAAAGAGAAAAAAAGAACGATAATTGXXXXXXXXXXT
ATATGAAGGGGGAAGGGGCAGTGAGGGCAATTTTGTGGAAGAAAGGGGAATTTTTGCATGGATTCACACCGGAGATGTG
ACTTGAAAAGTTAGAGTAAAAGACCCAAAATTCCATGGACCTTTTGGGTGTTTTTAAAGAGAAAAAAAGAACGATAATTGXXXXXXXXXXT
ATATGAAGGGGGAAGGGGCAGTGAGGGCAATTTTGTGGAAGAAAGAGGAATTTTTGCATGGATTCACACCGGAGATGTG
>Marker160266 Chr6 SNP_site:26946466 Ref_Type:A
ACTTGAAAAGTTAGAATAAAAGACCCAAAATTCCATGGACCTTTTGGGTGTTTTTAAAGAGAAAAAAAGAACGATAATTGXXXXXXXXXXT
ATATGAAGGGGGAAGGGGCAGTGAGGGCAATTTTGTGGAAGAAAGGGGAATTTTTGCATGGATTCACACCGGAGATGTG
ACTTGAAAAGTTAGAGTAAAAGACCCAAAATTCCATGGACCTTTTGGGTGTTTTTAAAGAGAAAAAAAGAACGATAATTGXXXXXXXXXXT

Create PDF with GO2PDF for free, if you wish to

ATATGAAGGGGGAAGGGGCAGTGAGGGCAATTTTGTGGAAGAAAGAGGAATTTTTGCATGGATTCACACCGGAGATGTG
>Marker160664 Chr6 SNP_site:17931361 Ref_Type:T
ACAATTTACAAACGATGATTTGAATCAAAGATGGAAGAAGATGGAGAAAGAAGAAGAAGATGATGATGAAGAAGAAAGATXXXXXXXXXXT
ACCATAACATTCCTCTATTATGAGGAATGATGAGACAAACCTACTGACTAACATCGTGTTTGAATGAAACGATACGGTT
ACAATTTACAAACGATGATTTGAATCAAAGATGGAAGAAGATGGAGAAAGAAGAAGAAGATGATGATGATGAAGAAAGATXXXXXXXXXXT
ACCATAACATTCCTCTATTATGAGGAATGATGAGACAAACCTACTGACTAACATCGTGTTTGAATGAAACGATACGGTT
>Marker160945 Chr6 SNP_site:1212445 Ref_Type:T
CACCAATTGATTTAGGTTTCCATTTCTCTGAGATTTTAATTGGAACCGTGAATGGATCATCAACAAACTCCTCAGTCAATXXXXXXXXXXA
ATCTAGAGAATGAGAAGGGGAGTTATAAAATATGTTGGAATGTCCTAAATCTAATATCTTGTGCTAACAACAACCAAGT
CACCAATTGATTTAGGTTTCCATTTCTCTGAGATTTTAATTGGAATCGTGAATGGATCATCAACAAACTCCTCAGTCAATXXXXXXXXXXA
ATCTAGAGAATGAGAAGGGGAGTTATAAAATATGTTGGAATGTCCTAAATCTAATATCTTGTGCTAACAACAACCAAGT
>Marker161457 Chr6 SNP_site:8260650 Ref_Type:G
GACTCATTTCTCACAATTTTCACTCCAAGGGCAAGGTAGAGAGAAGAGGGAGAGAGAATTTGAGTGGTTGGTGATTAAGTXXXXXXXXXXG
CTAATTTGACAAGCGTCACACCTATGTGTAATTCTTGTCTATTTTGCAGACAACATACTTTAACTAAAAATTTAAGGTC
GACTCATTTCTCACAATTTTCACTCCAAGGGCAAGGTAGAGAGAAGAGGGAGAGAGAATTTGAGTGGTTGGTGATTAAGTXXXXXXXXXXG
CTAATTTGACAAGCGTCACACCTATGTGTAATTCTTGTCTATTTTGCAGACAACATACTTTAACTAAAAATTTAGGGTC
>Marker16151 Chr4 SNP_site:8919430 Ref_Type:A
AACCTTCAAGAATTTGTTACATAAACATCTAAGTTATTTTCTTTGTGGTGGTTTATTTTAAATTGGAAATAGTAACAAGAXXXXXXXXXXG
AGAATTGGAAATAACAATATAAGTTTGAAGATCTAATTTTCAAATTTGAAATTCAAATAAAGAAGCTATCAATAAAGTC
AACCTTCAAGAATTTGTTACATAAACATCTAAGTTATTTTCTTTGTGGTGGTTTATTTTAAATTGGAAATAGTAACAAGAXXXXXXXXXXG
AGAATTGGAAATAACAATTTAAGTTTGAAGATCTAATTTTCAAATTTGAAATTCAAATAAAGAAGCTATCAATAAAGTC
>Marker16158 Chr4 SNP_site:20803832 Ref_Type:T
AACAATATCAGAAGACGCAAACGGTTGCTTTGATTGCAACATTTGCTTAGACTCAGCAGCTGACCCTGTTGTCACCCTCTXXXXXXXXXXT
CCTCGTTGGTTCCGCTCTATGGGCGTGGCACGTCGAACTCAGATTCCGAATCCAAGAAATCTCATTTGGGTATGGCTGT
AACAATATCAGAAGACGCAAACGGTTGCTTTGATTGCAACATTTGCTTAGACTCAGCAGCTGACCCTGTTGTCACCCTCTXXXXXXXXXXT
CCTCGTTGGTTCCGCTCTATGGGCGTGGCACGTCGAACTCAGATTCTGAATCCAAGAAATCTCATTTGGGTATGGCTGT
>Marker162062 Chr6 SNP_site:2686379 Ref_Type:T
ACTATCACGTAGGTCTCACCGTCCAAGTTCTCATCATCTGTGGTTGGTTAGGTCAAAACGCCGATTACTGAGCACTGTCCXXXXXXXXXXT
CTTCACAAGTTACCACATATAGTCTTTATCACGTAGCTTAAGAGGCGATTAGGGTCTCACAGAAACGGACCAAAAAGTT
ACTATCACGTAGGTCTTACCGTCCAAGTTCTCATCATCTGTGGTTGTTTAGGTCAAAACGCCGATTACTGAGCACTGCCCXXXXXXXXXXT
CTTCACAAGTTACCACATATAGTCTTTATCACGTAGCTTAAGAGGCGATTAGGGTCTCACAGAAACGGACCAAAAAGTT
>Marker162062 Chr6 SNP_site:2686409 Ref_Type:G
ACTATCACGTAGGTCTCACCGTCCAAGTTCTCATCATCTGTGGTTGGTTAGGTCAAAACGCCGATTACTGAGCACTGTCCXXXXXXXXXXT
CTTCACAAGTTACCACATATAGTCTTTATCACGTAGCTTAAGAGGCGATTAGGGTCTCACAGAAACGGACCAAAAAGTT
ACTATCACGTAGGTCTTACCGTCCAAGTTCTCATCATCTGTGGTTGTTTAGGTCAAAACGCCGATTACTGAGCACTGCCCXXXXXXXXXXT
CTTCACAAGTTACCACATATAGTCTTTATCACGTAGCTTAAGAGGCGATTAGGGTCTCACAGAAACGGACCAAAAAGTT
>Marker162062 Chr6 SNP_site:2686440 Ref_Type:C
ACTATCACGTAGGTCTCACCGTCCAAGTTCTCATCATCTGTGGTTGGTTAGGTCAAAACGCCGATTACTGAGCACTGTCCXXXXXXXXXXT
CTTCACAAGTTACCACATATAGTCTTTATCACGTAGCTTAAGAGGCGATTAGGGTCTCACAGAAACGGACCAAAAAGTT
ACTATCACGTAGGTCTTACCGTCCAAGTTCTCATCATCTGTGGTTGTTTAGGTCAAAACGCCGATTACTGAGCACTGCCCXXXXXXXXXXT
CTTCACAAGTTACCACATATAGTCTTTATCACGTAGCTTAAGAGGCGATTAGGGTCTCACAGAAACGGACCAAAAAGTT
>Marker162130 Chr6 SNP_site:13998825 Ref_Type:C
CACAAAAATGGGGGAAAACCCTTTCGATATTACAACAAAAAAAAAAAGGAAAGAAAACTCTGAAGAAGATGAAAACTTAGXXXXXXXXXXA
AGAGAGTGACAGATATAGAAAATATAAAGTTCCAGAAATAACCCTTTCCACAAATGAGAAAAAGCTTGAAGTTCTTAGT

Create PDF with GO2PDF for free, if you wish to

CACAAAAATGGGGGAAAACCCTTTTGATATTACAACAAAAAAAAAAAGGAAAGAAAACTCTGAAGAAGATGAAAACTTAGXXXXXXXXXXA
AGAGAGTGACAGATATAGAAAATATAAAGTTCCAGAAATAACCCTTTCCACAAATGAGAAAAAGCTTGAAGTTCTTAGT
>Marker162159 Chr6 SNP_site:26369804 Ref_Type:T
AACTCCGGCAATATACGAACATAACGTGTTCGCTCCATATCTAGTAAAGGACTGGATCTATAATCCCCCGCCACATAAAAXXXXXXXXXXG
AATATGAGACCACCCACAACGGAGAACATCCCTAAAAAGGGAAATCCATCAACATCGTTAGGAATTTTTTTACGACGTT
AACTCCGGCAATATACGAACATAACGTGTTCGCTCCATATCTAGTAAAGGATTGGATCTATAATCCCCCGCCACATAAAAXXXXXXXXXXG
AATATGAGACCACCCACAACGGAGAACATCCCTAAAAAGGGAAATCCATCAACATCGTTAGGAATTTTTTTACGACGTT
>Marker162526 Chr6 SNP_site:18441102 Ref_Type:A
GACAAATCTAGAGTTGCTATCATAAATAGTTAAATTTATCTAAATCCATCAACAAGGATATAACTTGGATGTTTGAAGACXXXXXXXXXXA
GAAGTTTTTTAGATTTTGAGTTTATGCATACAACATCAAATCATATATCTTGTCCATGCAAGAGGAGTAGTAATGCAGT
GACAAATCTAGAGTTGCTATCATAAATAGTTAAATTTATCTAAATCCATCAACAAGGATATAACTTGGATGTTTGAAGACXXXXXXXXXXA
GAAGTTTTTTAGATTTTGAGTTTATGCATACAACATCAAATCATATATCTTGTCCATGCAAGAGGTGTAGTAATGCAGT
>Marker163591 Chr6 SNP_site:22528181 Ref_Type:G
AACAATAACGGCTTGGCCATGTGTTTCTTTTTCAATCATTTTCTTTATTCTGTTGTTCTTGATTAGTTCTACTTTCCCTAXXXXXXXXXXG
GTAGAGTCTTTTAGTTCTAGAGAGTATTACTGAGGTGGCAGATATGTCTTTTAATTCTAGAGAGTTTTACCCCTTAAGT
AACAATAACGGCTTGGCCATGTGTTTCTTTTTCAATCATTTTCTTTATTCTGTTGTTCTTGATTAGTTCTACTTTCCCTAXXXXXXXXXXG
GTAGATTCTTTTAGTTCTAGAGAGTATTACTGAGGTGGCAGATATGTCTTTTAATTCTAGAGAGTTTTACCCCTTAAGT
>Marker163969 Chr6 SNP_site:26777381 Ref_Type:T
CACCAATATAAAAAGGAACGCAAATACATTATGGAAAAGCTTTGGTCTGGACACAACCAAATGAACCACAACAAAGTCACXXXXXXXXXXA
CAAGAAGATTCAGAAACTGTATGTATAATGAACGACTGAATGCAATAAAAAATTGAATATTATATTGGCCTAGCTAGTA
CACCAATATAAAAAGGATCGCAAATACATTATGGAAAAACTTTGGTCTGGACACAACCAAATGAACCACAACAAAGTCACXXXXXXXXXXA
CAAGAAGATTCAGAAACTGTATGTATAATGAACGACTGAATGCAATAAAAAATTGAATATTATGTTGGCCTAGCTAGTA
>Marker163969 Chr6 SNP_site:26777402 Ref_Type:A
CACCAATATAAAAAGGAACGCAAATACATTATGGAAAAGCTTTGGTCTGGACACAACCAAATGAACCACAACAAAGTCACXXXXXXXXXXA
CAAGAAGATTCAGAAACTGTATGTATAATGAACGACTGAATGCAATAAAAAATTGAATATTATATTGGCCTAGCTAGTA
CACCAATATAAAAAGGATCGCAAATACATTATGGAAAAACTTTGGTCTGGACACAACCAAATGAACCACAACAAAGTCACXXXXXXXXXXA
CAAGAAGATTCAGAAACTGTATGTATAATGAACGACTGAATGCAATAAAAAATTGAATATTATGTTGGCCTAGCTAGTA
>Marker163969 Chr6 SNP_site:26777601 Ref_Type:G
CACCAATATAAAAAGGAACGCAAATACATTATGGAAAAGCTTTGGTCTGGACACAACCAAATGAACCACAACAAAGTCACXXXXXXXXXXA
CAAGAAGATTCAGAAACTGTATGTATAATGAACGACTGAATGCAATAAAAAATTGAATATTATATTGGCCTAGCTAGTA
CACCAATATAAAAAGGATCGCAAATACATTATGGAAAAACTTTGGTCTGGACACAACCAAATGAACCACAACAAAGTCACXXXXXXXXXXA
CAAGAAGATTCAGAAACTGTATGTATAATGAACGACTGAATGCAATAAAAAATTGAATATTATGTTGGCCTAGCTAGTA
>Marker164070 Chr6 SNP_site:7479919 Ref_Type:G
AACAACTTGAAAAGAAGTTATAAATGAAGGAATTTCCACCTTAACCTCTTGACTTTTTACTTTACATGGTTATAAACTCCXXXXXXXXXXG
TTGTTCTTGAATCTAGTTTCCTTTCGAGTAAATTATATATAAATTCTTACTTATGCTTGACAACCTCGAACATGTAGGT
AACAACTTGAAAAGAAGTTATAAATGAAGGAATTTCCACCTTAACCTCTTGACTTTTTACTTTACGTGGTTATAAACTCCXXXXXXXXXXG
TTGTTCTTGAATCTAGTTTCCTTTCGAGTAAATTATATATAAATTCTTACTTATGCTTGACAACCTCGAACATGTAGGT
>Marker164287 Chr6 SNP_site:16161841 Ref_Type:T
TACATGGATATAAATATAAAATTGGTAGTAAGGTCCCATCTCCTTCTCCATATGGCTCTTTCCTTTGTTCGTTTTCTTCTXXXXXXXXXXA
AACATTCGTTACTACCGATTTCATAAATATAACAAACCATAACAAAATTAAAAAGTTCTAGAAATTGGCTAATATTTGT
TACATGGATATAAATATAAAATTGGTAGTAAGGTCCCATCTCCTTCTCCATATTGCTCTTTCCTTTGTTTGTTTTCTTCTXXXXXXXXXXA
AACATTCGTTACTACCGATTTCATAAATATAACAAACCATAACAAAATTAAAAAGTTCTAGAAATTGGCTAATATTTGT
>Marker164287 Chr6 SNP_site:16161857 Ref_Type:T
TACATGGATATAAATATAAAATTGGTAGTAAGGTCCCATCTCCTTCTCCATATGGCTCTTTCCTTTGTTCGTTTTCTTCTXXXXXXXXXXA

Create PDF with GO2PDF for free, if you wish to

AACATTCGTTACTACCGATTTCATAAATATAACAAACCATAACAAAATTAAAAAGTTCTAGAAATTGGCTAATATTTGT
TACATGGATATAAATATAAAATTGGTAGTAAGGTCCCATCTCCTTCTCCATATTGCTCTTTCCTTTGTTTGTTTTCTTCTXXXXXXXXXXA
AACATTCGTTACTACCGATTTCATAAATATAACAAACCATAACAAAATTAAAAAGTTCTAGAAATTGGCTAATATTTGT
>Marker164395 Chr6 SNP_site:15980153 Ref_Type:G
ACAGAAGTTCAACACAAATCATCAAAATCAAGTAGGCAAGATATAAAAGCAAACATGAGAAAATCAACAGACGGCATTCAXXXXXXXXXXT
TTTAGTTAGTCTGACGAATTTACATCACCACATATTTTAGCCTCATCTGTGACAATTTGAAAAGTGCAACTTCTCAAGT
ACAGAAGTTCAACACAAATCATCAAAATCAAGTAGGCAAGATATAAAAGCAAACATGAGAAAATCAACAGACGGCGTTCAXXXXXXXXXXT
TTTAGTTAGTCTGACGAATTTACATCACCACATATTTTAGCCTCATCTGTGACAATTTGAAAAGTGCAACTTCTCAAGT
>Marker164857 Chr6 SNP_site:2449838 Ref_Type:T
ACCAAATTTAGAAAAACATTAACTAAGCTTCTCAATACATAGAAAGAAAAAAGATAAATACAAGTTTAATCATTAACAACXXXXXXXXXXG
AAAGAACTATTCAAGTCATTCTTCTTCTAGCCCTAGTTTCAGTGTTTTCATCTTTTTCAGCTTATTCCTTACCCTTGTC
ACCAAATTTAGAAAAACATTAACTAAGCTTCTCAATACATAGAAAGAAAAAAGATAAATACAAGTTTTATCATTAACAACXXXXXXXXXXG
AAAGAACTATTCAAGTCATTCTTCTTCTAGCCCTAGTTTCAGTGTTTTCATCTTTTTCAGCTTATTCCTTACCCTTGTC
>Marker164858 Chr6 SNP_site:9819224 Ref_Type:A
AACTCATTAGACACCTATGCTTCCCTTTGACGTAAGACTTCTTCTCTTCAAAACTTAAGCTATCCGTAAGTGTGAATAGAXXXXXXXXXXG
GTAGAATTGGGTGTTAATATTGATACGAAACTTAAATAGGAAAAAACGAAATCAAGATGCTTCAACCGAAAGTCAAAGT
AACTCATTAGACACCTATGCTTCCCTTTGACGTAAGCCTTCTTCTCTTCAAAACTTAAGCTATCCGTAAGTGTGAATAGAXXXXXXXXXXG
GTAGAATTGGGTGTTAATATTGATACGAAACTTAAATAGGAAAAAACGAAATCAAGATGCTTCAACCGAAAGTCAAAGT
>Marker165285 Chr6 SNP_site:8403395 Ref_Type:G
ACACAACTTGTCCGGCAATATTCAAAACCAACAAAGGCATATATGAAATTTATGAAAAAAAAATAATCGGCTCCATAGGXXXXXXXXXXT
TAAAGTTAAATTCTATTAAATAAAGTAGTTATTAAAGACTTATTAGTGAAAATTGATTATTATAATCTTGAATCCAGTA
ACACAACTTGTCCGGCAATATTCAAAACCAACAAAGGCATATATGAAATTTATGAAAAAAAAATAATCGGCTCCATAGGXXXXXXXXXXT
TAAAGTTAAATTCTATTAAATAAAGTGGTTATTAAAGACTTATTAGTGAAAATTGATTATTATAATCTTGAATCCAGTA
>Marker165302 Chr6 SNP_site:26078274 Ref_Type:A
CACCATTAATGATGTTGGTGATGACACCATAGCCAGGGGATCTTCCTGCAGCATTGTCAGCACTCGAAGGTTGCCAATTGXXXXXXXXXXC
GGCGCTCCTATTGCATTCCCTGCTGGTCCGTAGTTGTAGTTGCTAAAAAAAGTCCAATCATTTTAATACGATGCTAGTT
CACCATTAATGATGTTGGTGATGACACCATAGCCAGGGGATCTTCCTGCAGCATTGTCAGCGCTCGAAGGTTGCCAATTGXXXXXXXXXXC
GGCGCTCCTATTGCATTCCCTGCTGGTCCGTAGTTGTAGTTGCTAAAAAAAGTCCAATCATTTTAATACGATGCTAGTT
>Marker16548 Chr4 SNP_site:7324440 Ref_Type:C
ACTACATATCAAAATATTACGTTAAAAGGAGAAAGCATGGTGAAAGTGGAAATATGAAAAAAAAGACCTCAAACACAAGAXXXXXXXXXXA
GCGCCTAATGAACGCCTTATTTTGTGAGCCTGGTCTTTCCAAGGTGAGGCACTAGACCTGTGCCTTGAGCCTAAACGTG
ACTACATATCAAAATATTACGTTAAAAGGAGAAAGCATGGTGAAAGTGGAAATATGAAAAAAAAGACCTCAAACACAAGAXXXXXXXXXXA
GCGCCTAATGCACGCCTTATTTTGTGAGCCTGGTCTTTCCAAGGTGAGGCACTAGACCTGTGCCTTGAGCCTAAACGTG
>Marker165671 Chr6 SNP_site:8735076 Ref_Type:T
GACGTAACCACATTATTTCACACACTGATTATGCCATTGCTTTGTATTCTGACTCAACACTTGATTGTGAAACTACATTTXXXXXXXXXXA
TTAAATATTCATGTGACCGTGATCTTTATATAGGATCCCACGTTCAGGTGCAACCTTAAGGTAACATAAAATTTGTTGT
GACGTAACCACATTATTTCACACACTGATTATGCCATTGCTTTGTATTCTTACTCAACACTTGATTGTGAAACTACATTTXXXXXXXXXXA
TTAAATATACATGTGACCGTGATCTTTATATAGGATCCCACGTTCAGGTGCAACCTTAAGGTAACATAAAATTTGTTGT
>Marker165671 Chr6 SNP_site:8735221 Ref_Type:A
GACGTAACCACATTATTTCACACACTGATTATGCCATTGCTTTGTATTCTGACTCAACACTTGATTGTGAAACTACATTTXXXXXXXXXXA
TTAAATATTCATGTGACCGTGATCTTTATATAGGATCCCACGTTCAGGTGCAACCTTAAGGTAACATAAAATTTGTTGT
GACGTAACCACATTATTTCACACACTGATTATGCCATTGCTTTGTATTCTTACTCAACACTTGATTGTGAAACTACATTTXXXXXXXXXXA
TTAAATATACATGTGACCGTGATCTTTATATAGGATCCCACGTTCAGGTGCAACCTTAAGGTAACATAAAATTTGTTGT
>Marker165778 Chr6 SNP_site:9033789 Ref_Type:A

Create PDF with GO2PDF for free, if you wish to

TACCTTTTTGCTAATCAAGAGTTATTCTCTGCTTTACTGGCATGTGGTTTGAAACAAAGTTTAGTGAAGGAGAAGTGTAGXXXXXXXXXXT
CTCTACAAGAAACTTGGGTTCCAAGTTGATAGCTTGATTAAAGGATACTATTCTGCGGATCGAGATGCCTACAGAATGT
TACCTTTTTGCTAATCAAGAGTTATTCTCTGCTTTACTGGCATGTGGTTTGAAACAAAGTTTAGTGAAGGAGAAGTGTAGXXXXXXXXXXT
CTCTACAAGAAACTTGGGTTCCAAGTTGATAGCTTGATTGAAGGATACTATTCTGCGGATCGAGATGCCTACAGAATGT
>Marker167669 Chr6 SNP_site:3183946 Ref_Type:A
ACATAAATCAGTTTAACAAATTGAGAGCATAACATCTACTTCATATAATATACAATTTATTGTTTCATATATACTATTCAXXXXXXXXXXT
ATATAATAGATAACAACTAATGTTTTAATGACATATCTTACATATACTCATCATATATTTTTTTATTCATGTTTATGTA
ACATAAATCAGTTTAACAAATTGAGAGCATAACATCTACTTCATATAATATACAATTTATTGTTTCATATATACTATTCAXXXXXXXXXXT
ATATAATAGATAACAACTAATGTTTTAATGACATATCTTACATATACTCATCATATTTTTTTTTATTAATGTTTATGTA
>Marker167669 Chr6 SNP_site:3183957 Ref_Type:C
ACATAAATCAGTTTAACAAATTGAGAGCATAACATCTACTTCATATAATATACAATTTATTGTTTCATATATACTATTCAXXXXXXXXXXT
ATATAATAGATAACAACTAATGTTTTAATGACATATCTTACATATACTCATCATATATTTTTTTATTCATGTTTATGTA
ACATAAATCAGTTTAACAAATTGAGAGCATAACATCTACTTCATATAATATACAATTTATTGTTTCATATATACTATTCAXXXXXXXXXXT
ATATAATAGATAACAACTAATGTTTTAATGACATATCTTACATATACTCATCATATTTTTTTTTATTAATGTTTATGTA
>Marker167750 Chr6 SNP_site:1147736 Ref_Type:G
CACAGCTTTCTAAGTTTTTATTTTTCTTTAAAAGGAAAAAAGTGTAGAGATTAGGAACTAAAAAGAAAAAGAAAGGAGTAXXXXXXXXXXC
CCAAATGAAAGCCTTATCCCTTTTAAGTAACTGATGGAAACACATTAATGCATTATATTTCATTTAAACTCATATCAGT
GACAGCTTTCTAAGTTTTTATTTTTCTTTAAAAGGAAAAAAGTGTAGAGATTAGGAACTAAAAAGAAAAAGAAAGGAGTAXXXXXXXXXXC
CCAAATGAAAGCCTTATCCCTTTTAAGTAACTGATGGAAACACATTAATGCATTATATTTCATTTAAACTCATATCAGT
>Marker167996 Chr6 SNP_site:9945363 Ref_Type:A
ACTGTCTTTCTCAGCAACCCAAATACAAGAGTCTCATAATTGCATTGTCTATCAACCAGCAAATTATAAATGTCGCTCTGXXXXXXXXXXA
ATTAAAATCTTCAACCCAAACCGAAAATGCAAACAAGGTTATTTATTTGTTTTCTTTTTTCTTTAACTATACTTTTGTT
ACTGTCTTTCTCAGCAACCCAAATACAAGAGTCTCGTAATTGCATTGTCTATCAACCAGCAAATTATAAATGCCGCTCTGXXXXXXXXXXA
ATTAAAATCTTCAACCCAAACCGAAAATGCAAACAAGGTTATTTATTTGTTTTCTTTTTTCTTTAACTATACTTTTGTT
>Marker167996 Chr6 SNP_site:9945400 Ref_Type:T
ACTGTCTTTCTCAGCAACCCAAATACAAGAGTCTCATAATTGCATTGTCTATCAACCAGCAAATTATAAATGTCGCTCTGXXXXXXXXXXA
ATTAAAATCTTCAACCCAAACCGAAAATGCAAACAAGGTTATTTATTTGTTTTCTTTTTTCTTTAACTATACTTTTGTT
ACTGTCTTTCTCAGCAACCCAAATACAAGAGTCTCGTAATTGCATTGTCTATCAACCAGCAAATTATAAATGCCGCTCTGXXXXXXXXXXA
ATTAAAATCTTCAACCCAAACCGAAAATGCAAACAAGGTTATTTATTTGTTTTCTTTTTTCTTTAACTATACTTTTGTT
>Marker168110 Chr6 SNP_site:26690308 Ref_Type:C
ACTTTATTCCACCAAATTTATAGGTTGGGTAATTTTCAGCCATCACCATTGTTTCATAAACATTAAAAAGAATAATAATAXXXXXXXXXXT
TTTAATTAATTTAGTGACACACTTTGATTGAAATCGCATCCTTAGGTCCAATTTTTTCATTTGGTAGGTCTTAAGTGGT
ACTTTATTCTACCAAATTTATAGGTTGGGTAATTTTCAGCCATCACCATTGTTTCATAAACATTAAAAAGAATAATAATAXXXXXXXXXXT
TTTAATTAATTTAGTGACACACTTTGATTGAAATCGCATCCTTAGGTCCAATTTTTTCATTTGGTAGGTCTTAAGTGGT
>Marker168182 Chr6 SNP_site:22640288 Ref_Type:A
TACTCGGCGACGATAGAAGATTAGGAGAGATATGTTCAAAGAGAGTGTATAGGATACTTATGGCTCAAAATGGAAACATGXXXXXXXXXXA
TTGCGTTTAACTGTCACAGACAGGTCCAAGGAGTATCATCGTTGGGCCATTACCTAATCACAAATGCCACTTGGTAGTC
TACTCGGCGACGATAGAAGATTAGGAGAGATATGTTCAAAGAGAGTGTATAGGATACTTATGGCTCAAAGTGGAAACATGXXXXXXXXXXA
TTGCGTTTAACTGTCACAGACAGGTCCAAGGAGTATCTTCGTTGGGCCATTACCTAATCACAAATGCCACTTGGTAGTC
>Marker168182 Chr6 SNP_site:22640443 Ref_Type:A
TACTCGGCGACGATAGAAGATTAGGAGAGATATGTTCAAAGAGAGTGTATAGGATACTTATGGCTCAAAATGGAAACATGXXXXXXXXXXA
TTGCGTTTAACTGTCACAGACAGGTCCAAGGAGTATCATCGTTGGGCCATTACCTAATCACAAATGCCACTTGGTAGTC
TACTCGGCGACGATAGAAGATTAGGAGAGATATGTTCAAAGAGAGTGTATAGGATACTTATGGCTCAAAGTGGAAACATGXXXXXXXXXXA
TTGCGTTTAACTGTCACAGACAGGTCCAAGGAGTATCTTCGTTGGGCCATTACCTAATCACAAATGCCACTTGGTAGTC

Create PDF with GO2PDF for free, if you wish to

>Marker168372 Chr6 SNP_site:17340904 Ref_Type:G
AACCTACCTCTCTATCTATCTATGAGATGGCTGGATTTTTTTGAACTTCACGGACAAGCAAAAGAGAAATAATATTTCCAXXXXXXXXXXT
AGAATTTAATTCTCGATGTAGTTGCTACATAGTTGGTTCTTATCCCTTAGAGACCACAAGTGTGTTAGGAGTATCTGTC
AACCTACCTCTCTATCTATCTATGAGATGTCTGGATTTTTTTGAACTTCATGGACAAGCAAAAGAGAAATAATATTTCCAXXXXXXXXXXT
AGAATTTAATTCTCGATGTAGTTGCTACATAGTTGGTTCTTATCCCTTAGAGACCACAAGTGTGTTAGGAGTATCTGTC
>Marker168372 Chr6 SNP_site:17340925 Ref_Type:C
AACCTACCTCTCTATCTATCTATGAGATGGCTGGATTTTTTTGAACTTCACGGACAAGCAAAAGAGAAATAATATTTCCAXXXXXXXXXXT
AGAATTTAATTCTCGATGTAGTTGCTACATAGTTGGTTCTTATCCCTTAGAGACCACAAGTGTGTTAGGAGTATCTGTC
AACCTACCTCTCTATCTATCTATGAGATGTCTGGATTTTTTTGAACTTCATGGACAAGCAAAAGAGAAATAATATTTCCAXXXXXXXXXXT
AGAATTTAATTCTCGATGTAGTTGCTACATAGTTGGTTCTTATCCCTTAGAGACCACAAGTGTGTTAGGAGTATCTGTC
>Marker168390 Chr6 SNP_site:26707675 Ref_Type:T
TACCTCTTTGTTCTTTCTAGGGATAATCTTTTATGATATTGTTTGAAACGGAATATTTTTAGAATGCCTTCACAGTTTTCXXXXXXXXXXT
TATTAGCAAGTCTTTAGATTTAAATATTGGTTGCCTATTGTGATCATCGTTGATTTTATTGACCCGTGGGGATTTCTGT
TACCTCTTTGTTCTTTCTAGGGATAATCTTTTATGTTATTGTTTGAAACGGAATATTTTTAGAATGCCTTCACAGTTTTCXXXXXXXXXXT
TATTAGCAAGTCTTTAGATTTAAATATTGGTTGCCTATTGTGATCATCGTTGATTTTATTGACCCGTGGGGATTTATGT
>Marker168390 Chr6 SNP_site:26707895 Ref_Type:A
TACCTCTTTGTTCTTTCTAGGGATAATCTTTTATGATATTGTTTGAAACGGAATATTTTTAGAATGCCTTCACAGTTTTCXXXXXXXXXXT
TATTAGCAAGTCTTTAGATTTAAATATTGGTTGCCTATTGTGATCATCGTTGATTTTATTGACCCGTGGGGATTTCTGT
TACCTCTTTGTTCTTTCTAGGGATAATCTTTTATGTTATTGTTTGAAACGGAATATTTTTAGAATGCCTTCACAGTTTTCXXXXXXXXXXT
TATTAGCAAGTCTTTAGATTTAAATATTGGTTGCCTATTGTGATCATCGTTGATTTTATTGACCCGTGGGGATTTATGT
>Marker168925 Chr6 SNP_site:10280702 Ref_Type:G
CACTTCTCAATTTTTATGGTATCTTTTTAACTACAGTTGTGCTTGCTGTTTCTATTTTTCCAATCTTTCTCTATCTGTGTXXXXXXXXXXG
AAGTTCCAAAATCTGCAAGAACTCTTCTTGATATATATTACAAATCCGCTGGAAGAAACTGTCTCTTGTTGCTAAATGT
CACTTCTCAATTTTTATGGTATCTTTTTAACTACAGTTGTGCTTGCTGTTTCTATTTTTCCGATCTTTCTCTCTCCGTGTXXXXXXXXXXG
AAGTTCCAAAATCTGCAAGAACTCTTCTTGATATATATTACAAATCCGCTGGAAGAAACTGTCTCTTGTTGCTAAATGT
>Marker168925 Chr6 SNP_site:10280713 Ref_Type:C
CACTTCTCAATTTTTATGGTATCTTTTTAACTACAGTTGTGCTTGCTGTTTCTATTTTTCCAATCTTTCTCTATCTGTGTXXXXXXXXXXG
AAGTTCCAAAATCTGCAAGAACTCTTCTTGATATATATTACAAATCCGCTGGAAGAAACTGTCTCTTGTTGCTAAATGT
CACTTCTCAATTTTTATGGTATCTTTTTAACTACAGTTGTGCTTGCTGTTTCTATTTTTCCGATCTTTCTCTCTCCGTGTXXXXXXXXXXG
AAGTTCCAAAATCTGCAAGAACTCTTCTTGATATATATTACAAATCCGCTGGAAGAAACTGTCTCTTGTTGCTAAATGT
>Marker168925 Chr6 SNP_site:10280716 Ref_Type:C
CACTTCTCAATTTTTATGGTATCTTTTTAACTACAGTTGTGCTTGCTGTTTCTATTTTTCCAATCTTTCTCTATCTGTGTXXXXXXXXXXG
AAGTTCCAAAATCTGCAAGAACTCTTCTTGATATATATTACAAATCCGCTGGAAGAAACTGTCTCTTGTTGCTAAATGT
CACTTCTCAATTTTTATGGTATCTTTTTAACTACAGTTGTGCTTGCTGTTTCTATTTTTCCGATCTTTCTCTCTCCGTGTXXXXXXXXXXG
AAGTTCCAAAATCTGCAAGAACTCTTCTTGATATATATTACAAATCCGCTGGAAGAAACTGTCTCTTGTTGCTAAATGT
>Marker169083 Chr6 SNP_site:28543664 Ref_Type:T
TACACCCAACAGAAAAACTAAAAACCATTCATTTTTATAACATAGTATTTATTTTCAATTTATTCTAGAAGGCTCTGATAXXXXXXXXXXT
GTAATAAAGGCATGAGATATGATATCTTAGTCATTAATTCTTTCCTTTTCAATATAAAAACTTCACACTCATTATAAGT
TACACCCAACAGAAAAACTAAAAACCATTCATTTTTATAATATAGTATTTATTTTCAATTTATTCTAGAAGGCTCTGATAXXXXXXXXXXT
GTAATAAAGGCATGAGATATGATATCTTAGTCATTAATTCTTTCCTTTTCAATATAAAAACTTCACACTCATTATAAGT
>Marker169181 Chr6 SNP_site:27997745 Ref_Type:A
ACTAAAATTACTGTTAGAAGTTGATTTAAAGACTCAAGTGTCCAAATGGAGAAAAAATCCACTTGGTATAAGATATTTTCXXXXXXXXXXG
ATAATGATTTTTCTTTTCAAATATGGAGGTAAAATTACGTATGTATGTATGCATACATTATTAATGTTAAGACGAGGTT
ACTAGAATTACTGTTAGAAGTTGATTTAAAGACTCAAGTGTCCAAATGGAGAAAAAATCCACTTGGTATAAGATATTTTCXXXXXXXXXXG

Create PDF with GO2PDF for free, if you wish to

ATAATGATTTTTCTTTTCAAATATGGAGGTAAAATTACGTATGTATGTATGCATACATTATTAATGTTAAGACGAGGTT
>Marker169379 Chr6 SNP_site:11579302 Ref_Type:C
TACTTTTCATATTAGTTGGCAAATATATGGGTGATTCCAATGCTCACTGAGGAAAATAATTGCTATTTCTGCAAGGCTATXXXXXXXXXXC
ATAGTCGTCCCCAGATAGAACAATGTCATCAACATAAACAATCAATACTGCAACCTTCCCAGCCTCAGAGATCTTTGTA
TACTTTTCATATTAGTTGGCAAATATATGGGTGATTTCAATGCTCACTGAGGAAAATAATTGCTATTTCTGCAAGGCTATXXXXXXXXXXC
ATAGTCGTCCCCAGATAGAACAATGTCATCAACATAAACAATCAATACTGCAACCTTCCCAGCCTCAGAGATCTTTGTA
>Marker169482 Chr6 SNP_site:28147129 Ref_Type:T
ACCCTGCTTGAAGAAGAGAACAAAAGAGCAGATGATATTTACAAGGTCTGCAGATATCTTACTTCAGGATGCATTCTATAXXXXXXXXXXT
CTTAGCTGTTGTAGAAATTACAGTGGCTGGAAGTTGCTCCGGATCACCAACCTATGACAAAGAAAACCAAATAAATGTG
ACCCTGCTTGAAGAAGAGAACAAAAGAGCAGATGATATTTACAAGGTCTGCAGATATCTTACTTCAGGATGCATTCTATAXXXXXXXXXXT
CTTAGCTGTTGTAGAAATTACAGTGGCTGGAAGTTGCTCTGGATCACCAACCTATGACAAAGAAAACCAAATAAATGTG
>Marker170100 Chr6 SNP_site:9773383 Ref_Type:A
AACCTTATCCATAGTGACGTTTGGGGTCCCTCAAAGGGTCATGACGTTCGGTGATTTGTGACCTTTATTGATGATCATACXXXXXXXXXXA
TTGCAACTCTTCGAAGTGATAATGGTCGTGAGTTTCAAAACATACCCTTAGTGAGTTTCTGTCCTCCAAAGGGATTGTT
AACCTTATCCATAGTGACGTTTGGGGTCCCTCAAAGGGTCGTGACGTTCGGTGATTTGTGACCTTTATTGATGATCATACXXXXXXXXXXA
TTGCAACTCTTCGAAGTGTTAATGGTCGTGAGTTTCAAAACATACCCTTAGTGAGTTTTTGTCCTCCAAAGGGATTGTT
>Marker170100 Chr6 SNP_site:9773538 Ref_Type:A
AACCTTATCCATAGTGACGTTTGGGGTCCCTCAAAGGGTCATGACGTTCGGTGATTTGTGACCTTTATTGATGATCATACXXXXXXXXXXA
TTGCAACTCTTCGAAGTGATAATGGTCGTGAGTTTCAAAACATACCCTTAGTGAGTTTCTGTCCTCCAAAGGGATTGTT
AACCTTATCCATAGTGACGTTTGGGGTCCCTCAAAGGGTCGTGACGTTCGGTGATTTGTGACCTTTATTGATGATCATACXXXXXXXXXXA
TTGCAACTCTTCGAAGTGTTAATGGTCGTGAGTTTCAAAACATACCCTTAGTGAGTTTTTGTCCTCCAAAGGGATTGTT
>Marker170100 Chr6 SNP_site:9773578 Ref_Type:C
AACCTTATCCATAGTGACGTTTGGGGTCCCTCAAAGGGTCATGACGTTCGGTGATTTGTGACCTTTATTGATGATCATACXXXXXXXXXXA
TTGCAACTCTTCGAAGTGATAATGGTCGTGAGTTTCAAAACATACCCTTAGTGAGTTTCTGTCCTCCAAAGGGATTGTT
AACCTTATCCATAGTGACGTTTGGGGTCCCTCAAAGGGTCGTGACGTTCGGTGATTTGTGACCTTTATTGATGATCATACXXXXXXXXXXA
TTGCAACTCTTCGAAGTGTTAATGGTCGTGAGTTTCAAAACATACCCTTAGTGAGTTTTTGTCCTCCAAAGGGATTGTT
>Marker170554 Chr6 SNP_site:28084580 Ref_Type:A
ACAGAAGCACATGATGATTTGTGTTTAAAATAGGGTCCCTCTATCTGACGCACCAATCCACTACGATGACTCATCAGCTAXXXXXXXXXXT
TTCTTATAATAGTGTGAAACTGAAACCCTTCACAAAGAGAGCATCTTGATAAAGAGAAAAGAAAAGGAGTTTCAGAGTA
ACAGAAGCACATGATGATTTGTGTTTAAAATAGGGTCCCTCTATCTGACGCACCAATCCACTACGATGACTCATCAGCTAXXXXXXXXXXT
TTCTTATAATAGTGTGAAACTGAAACCCTTCACAGAGAGAGCATCTTGATAAAGAGAAAAGAAAAGGAGTTTCAGAGTA
>Marker170613 Chr6 SNP_site:21615581 Ref_Type:T
TACCAGTGAAAGATAAGAAGGCCTTATTTTCTCAAATTTCCCCTCAACAAGCAATTTACAAATTGAGATAATCACAATTTXXXXXXXXXXC
AAAATCGTTATAATTTCAAGGCAACAATCAGAAAGTAACTTAAATACGCAATTACTAACTCAATCACTGTCTTACAGTG
TACCAGTGAAAGATAAGAAGGCCTTATTTTCTCAAATTTCCCCTCAACAAGCAATTTACAAATTGAGATAATCATAATTTXXXXXXXXXXC
AAAATCGTTATAATTTCAAGGCAACAATCAGAAAGTAACTTAAATACGCAATTACTAACTCAATCACTGTCTTACAGTA
>Marker170613 Chr6 SNP_site:21615757 Ref_Type:A
TACCAGTGAAAGATAAGAAGGCCTTATTTTCTCAAATTTCCCCTCAACAAGCAATTTACAAATTGAGATAATCACAATTTXXXXXXXXXXC
AAAATCGTTATAATTTCAAGGCAACAATCAGAAAGTAACTTAAATACGCAATTACTAACTCAATCACTGTCTTACAGTG
TACCAGTGAAAGATAAGAAGGCCTTATTTTCTCAAATTTCCCCTCAACAAGCAATTTACAAATTGAGATAATCATAATTTXXXXXXXXXXC
AAAATCGTTATAATTTCAAGGCAACAATCAGAAAGTAACTTAAATACGCAATTACTAACTCAATCACTGTCTTACAGTA
>Marker17092 Chr4 SNP_site:211146 Ref_Type:G
GACCCAATTAGCTCATGTTATCAAAACTAAATGACGATTCTTGATATGATAATTGTTGCTATGCAGGAAGGTTTTTTTTTXXXXXXXXXXC
TTCCTTAAACTTCAATGGATCGTATTTGCTGTTTATAACTTCTAAACTTTTTTCAAGACTGCTTCCTGATACCGCTTGT

Create PDF with GO2PDF for free, if you wish to

GACCCAATTAGCTCATGTTATCAAAACTAAATGACGATTCTTGATATGATAATTGTTGCTATGCAGGAAGTTTTTTTTTTXXXXXXXXXXC
TTCCTTAAACTTCAATGGATCGTGTTTGCTGTTTATAACTTCTAAACTTTTTTCAAGACTGCTTCCTGATACCGCTTGT
>Marker17092 Chr4 SNP_site:211317 Ref_Type:A
GACCCAATTAGCTCATGTTATCAAAACTAAATGACGATTCTTGATATGATAATTGTTGCTATGCAGGAAGGTTTTTTTTTXXXXXXXXXXC
TTCCTTAAACTTCAATGGATCGTATTTGCTGTTTATAACTTCTAAACTTTTTTCAAGACTGCTTCCTGATACCGCTTGT
GACCCAATTAGCTCATGTTATCAAAACTAAATGACGATTCTTGATATGATAATTGTTGCTATGCAGGAAGTTTTTTTTTTXXXXXXXXXXC
TTCCTTAAACTTCAATGGATCGTGTTTGCTGTTTATAACTTCTAAACTTTTTTCAAGACTGCTTCCTGATACCGCTTGT
>Marker171317 Chr6 SNP_site:5360181 Ref_Type:T
AACCACTTCAACGGTAGCGGTGGCTCTGGCGGTAGCGGCGATGTCATGGTCCGTCGCCCTAGGGGCCGCCCGGCCGGCTCXXXXXXXXXXC
GTGGGATCTGTGTGTTGAGTGGAAGTGGGATTGTTAATAATGTTAGCTTACGGCAACCCGCAGCTGCTGGATCGGTGTT
AACCATTTCAACGGTAGCGGTGGCTCTGGCGGTAGCGGCGATGTCATGGTCCGTCGCCCTAGGGGCCGCCCGGCCGGCTCXXXXXXXXXXC
GTGGGATCTGTGTGTTGAGTGGAAGTGGGATTGTTAATAATGTTAGCTTACGGCAACCCGCAGCTGCTGGATCGGTGTT
>Marker172483 Chr6 SNP_site:11254650 Ref_Type:C
ACCATCTGTTTTGTATTTAAGAGAGAACACTCATTTGCATCCCACAATTTTGTGTCCCTTGGGTAGAGCACAAATTTTCCXXXXXXXXXXG
AGTCAAGACTTGCTGTAAAAACTCTGAATTGTGGAGAGAGATTATCATAGGAAACATAGTTGCAAATTGGATGTTTAGT
ACCATCTGTTTTGTATTTCAGAGAGAACACTCATTTGCATCCCACAATTTTGTGTCCCTTGGGGAGAGCACAAATTTTCCXXXXXXXXXXG
AGTCAAGACTTGCTGTAAAAACTCTGAACTGTGGAGAGAGATTATCATAGGAAACATAGTTGCAAATTGGATGTTTAGT
>Marker172483 Chr6 SNP_site:11254695 Ref_Type:G
ACCATCTGTTTTGTATTTAAGAGAGAACACTCATTTGCATCCCACAATTTTGTGTCCCTTGGGTAGAGCACAAATTTTCCXXXXXXXXXXG
AGTCAAGACTTGCTGTAAAAACTCTGAATTGTGGAGAGAGATTATCATAGGAAACATAGTTGCAAATTGGATGTTTAGT
ACCATCTGTTTTGTATTTCAGAGAGAACACTCATTTGCATCCCACAATTTTGTGTCCCTTGGGGAGAGCACAAATTTTCCXXXXXXXXXXG
AGTCAAGACTTGCTGTAAAAACTCTGAACTGTGGAGAGAGATTATCATAGGAAACATAGTTGCAAATTGGATGTTTAGT
>Marker172483 Chr6 SNP_site:11254839 Ref_Type:C
ACCATCTGTTTTGTATTTAAGAGAGAACACTCATTTGCATCCCACAATTTTGTGTCCCTTGGGTAGAGCACAAATTTTCCXXXXXXXXXXG
AGTCAAGACTTGCTGTAAAAACTCTGAATTGTGGAGAGAGATTATCATAGGAAACATAGTTGCAAATTGGATGTTTAGT
ACCATCTGTTTTGTATTTCAGAGAGAACACTCATTTGCATCCCACAATTTTGTGTCCCTTGGGGAGAGCACAAATTTTCCXXXXXXXXXXG
AGTCAAGACTTGCTGTAAAAACTCTGAACTGTGGAGAGAGATTATCATAGGAAACATAGTTGCAAATTGGATGTTTAGT
>Marker172611 Chr6 SNP_site:7723322 Ref_Type:A
GACTAAGCATTCCAACAGTAAATTTACTTACCTTGAATTGATGTTTAAACAACTAGCACAAACCCTCTTAACTTGTAACAXXXXXXXXXXA
CCTCTAATTAATTACTTACCCCCTTAAGTCTTGATTCAAGGTCAATTTTAGGGTTTTTCTAATGGATTCGAACCTAAGT
GACTAAGCATTCCTACAGTAAATTTACTTACCTTGAATTGATGTTTAAACAACTAGCACAAACCCTCTTAACTTGTAACAXXXXXXXXXXA
CCTCTAATTAATTACTTACCCCCTTAAGTCTTGATTCAAGGTCAATTTTAGGGTTTTTCTAATGGATTTGAACCTAAGT
>Marker172611 Chr6 SNP_site:7723548 Ref_Type:C
GACTAAGCATTCCAACAGTAAATTTACTTACCTTGAATTGATGTTTAAACAACTAGCACAAACCCTCTTAACTTGTAACAXXXXXXXXXXA
CCTCTAATTAATTACTTACCCCCTTAAGTCTTGATTCAAGGTCAATTTTAGGGTTTTTCTAATGGATTCGAACCTAAGT
GACTAAGCATTCCTACAGTAAATTTACTTACCTTGAATTGATGTTTAAACAACTAGCACAAACCCTCTTAACTTGTAACAXXXXXXXXXXA
CCTCTAATTAATTACTTACCCCCTTAAGTCTTGATTCAAGGTCAATTTTAGGGTTTTTCTAATGGATTTGAACCTAAGT
>Marker173283 Chr6 SNP_site:13411005 Ref_Type:T
ACTAAATGAGAAGAGGAAGGAACTAGGCGTTATAAGGAAAATCCCAAGCATTTTCGTGATTTATCTTTTGTTTTATGAGTXXXXXXXXXXT
GATGTTATTGATGTTTTTGTTGGTGTGTTATTTGAAAAGAGAATATTATTGACTTATATGTGATGCTTGTGTATGTGTA
ACTAAATGAGAAGAGGAAGGAACTAGGCGTTATAAGGAAAATCCCAAGTATTTTCGTGATTTATCTTTTGTTTTATGAGTXXXXXXXXXXT
GATGTTATTGATGTTTTTGTTGGTGTGTTATTTGAAAAGAGAATATTATTGACTTATATGTGATGCTTGTGTATGTGTA
>Marker17423 Chr4 SNP_site:13506380 Ref_Type:A
CACACTAATCCTAGATTATAAATATTTCAATGTCTTTAAATTAATTGTGATAAAATATCGAACATAAATGTGTAGAATGAXXXXXXXXXXC

Create PDF with GO2PDF for free, if you wish to

TCAAAATTCATAAAATGAACAACTTAAAATATTGTTTGAAAATGTTACTCAAAACTCAACCATTTTTTTCTTATTTTGT
CACACTAATCCTAGATTATAAATATTTCAATGTCTTTAAATTAATTGTGATAAAATATCGAACATAAATGTGTAGAATGAXXXXXXXXXXC
TCAAAATTCATAAAATGAACAACTTAAAATATTGTTTGAAAATGTTACTCAAAACTCGACCATTTTTTTCTTATTTTGT
>Marker174765 Chr6 SNP_site:15845404 Ref_Type:G
ACCCATCATTATAGTTTTGCAACGATATTCCAAAGGTTTAGAATAATCTCCACCGAAGGGCAGTCAATTATTCCTTCTTTXXXXXXXXXXA
ACTTTATAATTTTGTAAGTGTAACCCTCCATTTTCGAATCTCAGAGATTAGAATCATTATACTCCTGAATGAGAAAGTC
ACCCATCATTATAGTTTTGCAACGATATTCCAAAGGTTTAGAATAATCTCCACCGAAGGGCAGTCAATTATTCCTTCTTTXXXXXXXXXXA
ACTTTATAATTTTGTAAGTGTAACCCTCCATTTTCGAATCTCAGAGATTAGAATCGTTATACTCCTGAATGAGAAAGTC
>Marker174885 Chr6 SNP_site:27488909 Ref_Type:G
CACAATGATGAACTTTGGGTTCCCACGGGAGAAGGTCTTACATCTGGATGAAACAATTGTTTTTTCTTATCTTTGCTCGTXXXXXXXXXXT
AATAATTCAATCCTGCCAGTGATGCTTATAGTTCAATCTAAACTCTTAACTCAGTTATATATGTAATCCATTTTGCAGT
CACAATGATGAACTTTGGGTTCCCACGGGAGAGGGTCTTACATCTGGATGAAACAATTGTTTTTTCTTATCTTTGCTCGTXXXXXXXXXXT
AATAATTCAATCCTGCCAGTGATGCTTTTAGTTCAATCTAAACTCTTAACTCAGTTATATATGTAATCCATTTTGCAGT
>Marker174885 Chr6 SNP_site:27489100 Ref_Type:T
CACAATGATGAACTTTGGGTTCCCACGGGAGAAGGTCTTACATCTGGATGAAACAATTGTTTTTTCTTATCTTTGCTCGTXXXXXXXXXXT
AATAATTCAATCCTGCCAGTGATGCTTATAGTTCAATCTAAACTCTTAACTCAGTTATATATGTAATCCATTTTGCAGT
CACAATGATGAACTTTGGGTTCCCACGGGAGAGGGTCTTACATCTGGATGAAACAATTGTTTTTTCTTATCTTTGCTCGTXXXXXXXXXXT
AATAATTCAATCCTGCCAGTGATGCTTTTAGTTCAATCTAAACTCTTAACTCAGTTATATATGTAATCCATTTTGCAGT
>Marker17503 Chr4 SNP_site:12841984 Ref_Type:C
TACTTATATTTTGGTATTTTTATTCTCAATCTCGGTTTCTTTTGGTGATTCTGTAGTTTAAACATATTGTATATGAAGAAXXXXXXXXXXT
TGAGTGTTATGTTTTCTTTTAAAAAAGAAAAAGAAAAAGAAAAACAGTTAAGAGAGAGTTTTGGTGGGTGTGGAGTGTG
TACTTATATTTTGGTATTTTTATTCTGAATCTCGGCTTCTTTTGGTGATTCTGTAGTTTAAACATATTGTATATGAAGAAXXXXXXXXXXT
TGAGTGTTATGTTTTCTTTTAAAAAAGAAAAAGAAAAAGAAAAACAGTTAAGAGAGAGTTTTGGTGGGTGTGGAGTGTG
>Marker17503 Chr4 SNP_site:12841993 Ref_Type:T
TACTTATATTTTGGTATTTTTATTCTCAATCTCGGTTTCTTTTGGTGATTCTGTAGTTTAAACATATTGTATATGAAGAAXXXXXXXXXXT
TGAGTGTTATGTTTTCTTTTAAAAAAGAAAAAGAAAAAGAAAAACAGTTAAGAGAGAGTTTTGGTGGGTGTGGAGTGTG
TACTTATATTTTGGTATTTTTATTCTGAATCTCGGCTTCTTTTGGTGATTCTGTAGTTTAAACATATTGTATATGAAGAAXXXXXXXXXXT
TGAGTGTTATGTTTTCTTTTAAAAAAGAAAAAGAAAAAGAAAAACAGTTAAGAGAGAGTTTTGGTGGGTGTGGAGTGTG
>Marker175174 Chr6 SNP_site:18047494 Ref_Type:A
ACTAAAAACTTCAGTTTAAATAATGTAAGAAAGTAGATGAATATAAATCATCTTACACTTGAGTTATCTGCCTGCTCTCAXXXXXXXXXXG
CAAACAATACAAGTGCATTGCACATGAGAGATAATTCAACATATCAACTACCTAGCACATCATTCAATAGAAGACAGTG
ACTAAAAACTTCAGTTTAAATAATGTAAGAAAGTAGATGAATATAAATCATCTTACACTTGAGTTATCTGCCTGCTCTCAXXXXXXXXXXG
CAAACAATACAAGTGCATTGCACATGAGAGATAATTCAACATATCAACTACCTAGCACATCATTCAATAGAAGACGGTG
>Marker17601 Chr4 SNP_site:3189846 Ref_Type:C
TACTCTTACTCCCTTCAATGGAGTCAAATAAATGATAATTCTGTAGGTGTATAAAGGTTCCTTAAAATAAACAGAGTAATXXXXXXXXXXC
AACATCCATCATAGTCAAAGATAATGACATAAATATAAAATCCAAACATTTGAAATTATCTAATATTCAAAAGAACGTC
TACTCTTACTCCCTTCAATGGAGTCAAATACATGATAATTCTGTAGGTGTATAAAGGTTCCTTAAAATAAACAGAGTAATXXXXXXXXXXC
AACATCCATCATAGTCAAAGATAATGACATAAATATAAAATCCAAACATTTGAAATTATCTAATATTCAAAAGAACGTC
>Marker176045 Chr6 SNP_site:2084538 Ref_Type:A
ACTCAAATCTTTCCTCTCCTCGTCCCTCTTCAAGATCCTATCAAAATTTGCTTCTACTATTGAGAAACTCTATAGAAGCTXXXXXXXXXXT
CTTTGGCAGTTTGTTCATCCAAAACTCAAGAGAAGCATTCCCAGGTTCATCAAGCTTACCAATCTTTCAAGTCTTTGTT
ACTCAAATCTTTCCTCTCCTCGTCCCTCTTCAAGATCCTATCAAAATTTGCTTCTACTATTGAGAAACTCTATAGAAGCTXXXXXXXXXXT
CTTTGGCAGTTTGTTCATCCATAACTCAAGAGAAGCATTCCCAGGTTCATCAAGCTTACCAATCTTTCAAGTCTTTGTT
>Marker176244 Chr6 SNP_site:13975643 Ref_Type:G

Create PDF with GO2PDF for free, if you wish to

ACTCTAAGAAATGTTCCTGTGGGTGGTGGATGTAGGAAAAACAAAAGGCCATCACCTTCTACTTCTTCTTCTTCATCTCCXXXXXXXXXXT
CCAACATCAATCAAATGGGCAAATGGGTCTTTTTGATACAACCCATAACAGCTTTCAAAATTTGTATTCTGGGTTTGGT
ACTCTAAGAAATGTTCCTGTGGGTGGTGGATGTAGGAAAAACAAAAGGCCATCACCTTCTACTTCTTCTTCTTCATCTCCXXXXXXXXXXT
CCAACATCAATCAAATGGGCAGATGGGTCTTTTTGATACAACCCATAACAGCTTTCAAAATTTGTATTCTGGGTTTGGT
>Marker176511 Chr6 SNP_site:16970690 Ref_Type:C
ACCAAAGAGACCAAACTTGATTATATATGAATATCCCTAAACCAGTGTTCAAATACAAAAAATACCCAAGCTTTCAAAAAXXXXXXXXXXG
AAGCACCATCCCAGTCAAAATGATAGTAAAAAATATATGCTTGATCATACTCTGTTGTCTTTCAACTATCTTTTTTGTT
ACCAAAGAGACCAAACTTGATTATATATGAATATCCCTAAACCAGTGTTCAAATACAAAAAATACCTAAGCTTTCAAAAAXXXXXXXXXXG
AAGCACCATCCCAGTCAAAATGATAGTAAAAAATATATGCTTGATCATACTCTGTTGTCTTTCAACTATCTTTTTTGTT
>Marker177307 Chr6 SNP_site:9379867 Ref_Type:T
TACTCTTTAATTATTTATTTTATCTCTCACGACGGAAAATATAATAAAAAAAAGTTTATACTTATTTTCCTTTTTTTAAAXXXXXXXXXXC
ATAATCTTTTTTCGTGTTTATTGTCTTTAAAAAATTAAAATTGATTCTTAAATTTTGGAGTTGGATTTCAAAAATGGGT
TACTCTTTAATTATTTATTTTATCTCTCACGACGGAAAATATAATAAAAAAAAGTTTATACTTATTTTCCTTTTTTTTAAXXXXXXXXXXC
ATAATCTTTTTTCGTGTTTATTGTCTTTAAAAAATTAAAATTGATTCTTAAATTTTGGAGTTGGATTTCAAAAATGGGT
>Marker178228 Chr6 SNP_site:16635108 Ref_Type:C
ACTGTCGAATAGAGTTTTAATCTCTTAAATATATCTAAATTAGTTGAATTACGTTAAGATTGATCGACTTGTGTTGGTTCXXXXXXXXXXT
GTGCTTCTATTCGAATATTGCTTAATTGTAGAAGAGTTAAATCTCACTTCTACAAATAATGGTTGGACTCAAAAAAGTA
ACTGTTGAATAGAGTTTTAATCTCTTAAATATATCTAAATTAGTTGAATTACGTTAAGATTGATCGACTTGTGTTGGTTCXXXXXXXXXXT
GTGCTTCTATTCGAATATTGCTTAATTATAGAAGAGTTAAATCTCACTTCTACAAATAATGGTTTGACTCAAAAAAGTA
>Marker178228 Chr6 SNP_site:16635326 Ref_Type:G
ACTGTCGAATAGAGTTTTAATCTCTTAAATATATCTAAATTAGTTGAATTACGTTAAGATTGATCGACTTGTGTTGGTTCXXXXXXXXXXT
GTGCTTCTATTCGAATATTGCTTAATTGTAGAAGAGTTAAATCTCACTTCTACAAATAATGGTTGGACTCAAAAAAGTA
ACTGTTGAATAGAGTTTTAATCTCTTAAATATATCTAAATTAGTTGAATTACGTTAAGATTGATCGACTTGTGTTGGTTCXXXXXXXXXXT
GTGCTTCTATTCGAATATTGCTTAATTATAGAAGAGTTAAATCTCACTTCTACAAATAATGGTTTGACTCAAAAAAGTA
>Marker178228 Chr6 SNP_site:16635363 Ref_Type:G
ACTGTCGAATAGAGTTTTAATCTCTTAAATATATCTAAATTAGTTGAATTACGTTAAGATTGATCGACTTGTGTTGGTTCXXXXXXXXXXT
GTGCTTCTATTCGAATATTGCTTAATTGTAGAAGAGTTAAATCTCACTTCTACAAATAATGGTTGGACTCAAAAAAGTA
ACTGTTGAATAGAGTTTTAATCTCTTAAATATATCTAAATTAGTTGAATTACGTTAAGATTGATCGACTTGTGTTGGTTCXXXXXXXXXXT
GTGCTTCTATTCGAATATTGCTTAATTATAGAAGAGTTAAATCTCACTTCTACAAATAATGGTTTGACTCAAAAAAGTA
>Marker178479 Chr6 SNP_site:15766304 Ref_Type:C
ACCCTGTGCTAGGAAAGTATTGATTCCTCACACATTGCAGTTCGCCATTCAAGATACTTGAAGGCTTCAGTGTATGATGTXXXXXXXXXXT
TGCAGCAACATCTATCTCATTTCCTATATCGACTGTAGATAAAACATGTAAAGACTGACAAGTGGTAACATCTAGTGTG
ACTCTGTGCTAGGAAAGTATTGATTCCTCACACATTGCAGTTCGCCATTCAAGATACTTGAAGGCTTCAGTGTATGATGTXXXXXXXXXXT
TGCAGCAACATCTATCTCATTTCCTATATCGACTGTAGATAAAACATGTAAAGACTGACAAGTGGCAACATCTAGTGTG
>Marker178479 Chr6 SNP_site:15766535 Ref_Type:T
ACCCTGTGCTAGGAAAGTATTGATTCCTCACACATTGCAGTTCGCCATTCAAGATACTTGAAGGCTTCAGTGTATGATGTXXXXXXXXXXT
TGCAGCAACATCTATCTCATTTCCTATATCGACTGTAGATAAAACATGTAAAGACTGACAAGTGGTAACATCTAGTGTG
ACTCTGTGCTAGGAAAGTATTGATTCCTCACACATTGCAGTTCGCCATTCAAGATACTTGAAGGCTTCAGTGTATGATGTXXXXXXXXXXT
TGCAGCAACATCTATCTCATTTCCTATATCGACTGTAGATAAAACATGTAAAGACTGACAAGTGGCAACATCTAGTGTG
>Marker178517 Chr6 SNP_site:13462612 Ref_Type:C
TACAATAGATACCCTTAGAACCTTAGCTTATTGGATTAAAGATTATAGTATTCTATTTTCAGTAATAAGTTCTCAATAACXXXXXXXXXXT
CAATTGACTTACGCTTTAAGTTTTCCCTCCCTAGGTTGTATGAGGTAAGTGATCGTCTAGAAAAGTAAGTGATTGGGTA
TACAATAGATACCCTTAGAACCTTAGCTTATTGGATTCAAGATTATAGTATTCTATTTTCAGTAATAAGTTCTCAATAACXXXXXXXXXXT
CATTTGACTTACGCTTTAAGTTTTCCCTCCCTAGGTTGTATGAGGTAAGTGATCGTCTAGAAAAGTAAGTGATTGGGTA

Create PDF with GO2PDF for free, if you wish to

>Marker178517 Chr6 SNP_site:13462810 Ref_Type:T
TACAATAGATACCCTTAGAACCTTAGCTTATTGGATTAAAGATTATAGTATTCTATTTTCAGTAATAAGTTCTCAATAACXXXXXXXXXXT
CAATTGACTTACGCTTTAAGTTTTCCCTCCCTAGGTTGTATGAGGTAAGTGATCGTCTAGAAAAGTAAGTGATTGGGTA
TACAATAGATACCCTTAGAACCTTAGCTTATTGGATTCAAGATTATAGTATTCTATTTTCAGTAATAAGTTCTCAATAACXXXXXXXXXXT
CATTTGACTTACGCTTTAAGTTTTCCCTCCCTAGGTTGTATGAGGTAAGTGATCGTCTAGAAAAGTAAGTGATTGGGTA
>Marker179016 Chr6 SNP_site:28196954 Ref_Type:T
ACCTAGAGCACGCCGAATATGATACTGGTAACTTGAATGAGGATCTGAAGACACAGTCCTTGATGAAACAAATGCTTTTTXXXXXXXXXXT
TAGGCCATTCCCTTTTTCCTTTGATCCTTTGATAAAATATTATGCAAGTTGTTACTCTCTTCTTTTATGTCATGCAGTG
ACCTAGAGCACGCCGAATATGATACTGGTAACTTGAATGAGGATCTGAAGACACAGTCCTTGATGAAACAAATGCTTTTTXXXXXXXXXXT
TAGGCCATTCCTTTTTTCCTTTGATCCTTTGATAAAATATTATGCAAGTTGTTACTCTCTTCTTTTATGTCATGCAGTG
>Marker179098 Chr6 SNP_site:16755432 Ref_Type:A
TACTCGAAATATACCTGAGCGTCAAGAAGTGAGCAAGTAAGTTCCAGTGCCAGCTCCAGTGCCCTAAATTCAAAGGGCAAXXXXXXXXXXT
GAAATACTATAATCAAACTATCCATTAAAGGATATGCTTGATAACCAGATTGCAATGCATCGCTGTGTTGAATAATTGT
TACTCGAAATATACCTGAGCGTCAAGAAGTGAGCAAGTAAGTTCCAGTGCCAGCTCCAGTGCCCTAAATTCAAAGGGCAAXXXXXXXXXXT
GAAATACTATGATCAAACTATCCATTAAATGATATGCTTGATAACCAGATTGCAATGCATCGCTGTGTTGAATAATTGT
>Marker179098 Chr6 SNP_site:16755451 Ref_Type:G
TACTCGAAATATACCTGAGCGTCAAGAAGTGAGCAAGTAAGTTCCAGTGCCAGCTCCAGTGCCCTAAATTCAAAGGGCAAXXXXXXXXXXT
GAAATACTATAATCAAACTATCCATTAAAGGATATGCTTGATAACCAGATTGCAATGCATCGCTGTGTTGAATAATTGT
TACTCGAAATATACCTGAGCGTCAAGAAGTGAGCAAGTAAGTTCCAGTGCCAGCTCCAGTGCCCTAAATTCAAAGGGCAAXXXXXXXXXXT
GAAATACTATGATCAAACTATCCATTAAATGATATGCTTGATAACCAGATTGCAATGCATCGCTGTGTTGAATAATTGT
>Marker179368 Chr6 SNP_site:1377084 Ref_Type:C
CACAATATACAGCATCCAGATCTTCATCCTTCACTTGCTTCTCTAAGCTCACTGGCATCACCATATGGCCAGCCTGAGTCXXXXXXXXXXC
ACCATGATCACATGTCTTCGATTCACCTTCTCCATCTTCAGTCTTAACGTTTAAGGAAACAAGTCGTGGCCTCATTGTG
CACAATATACAGCATCCAGATCTTCATCCTTCACTTGCTTCTCTAAGCTCACTGGCATCACCATATGGCCAGCCTGAGTCXXXXXXXXXXC
ACCATGATCACCGGTCTTCGATTCACCTTCTCCATCTTCAGTCTTCACGTTTAAGGAAACAAGTCGTGGCCTCATTGTG
>Marker179368 Chr6 SNP_site:1377085 Ref_Type:G
CACAATATACAGCATCCAGATCTTCATCCTTCACTTGCTTCTCTAAGCTCACTGGCATCACCATATGGCCAGCCTGAGTCXXXXXXXXXXC
ACCATGATCACATGTCTTCGATTCACCTTCTCCATCTTCAGTCTTAACGTTTAAGGAAACAAGTCGTGGCCTCATTGTG
CACAATATACAGCATCCAGATCTTCATCCTTCACTTGCTTCTCTAAGCTCACTGGCATCACCATATGGCCAGCCTGAGTCXXXXXXXXXXC
ACCATGATCACCGGTCTTCGATTCACCTTCTCCATCTTCAGTCTTCACGTTTAAGGAAACAAGTCGTGGCCTCATTGTG
>Marker179368 Chr6 SNP_site:1377118 Ref_Type:C
CACAATATACAGCATCCAGATCTTCATCCTTCACTTGCTTCTCTAAGCTCACTGGCATCACCATATGGCCAGCCTGAGTCXXXXXXXXXXC
ACCATGATCACATGTCTTCGATTCACCTTCTCCATCTTCAGTCTTAACGTTTAAGGAAACAAGTCGTGGCCTCATTGTG
CACAATATACAGCATCCAGATCTTCATCCTTCACTTGCTTCTCTAAGCTCACTGGCATCACCATATGGCCAGCCTGAGTCXXXXXXXXXXC
ACCATGATCACCGGTCTTCGATTCACCTTCTCCATCTTCAGTCTTCACGTTTAAGGAAACAAGTCGTGGCCTCATTGTG
>Marker179482 Chr6 SNP_site:9491404 Ref_Type:T
AACTTGGTTTTGTGATTTATTGAGGTTCCATTTTTTTTTCTATATGGAGTGTATTTATTTGTTTCTACCCTCTTTGATTTXXXXXXXXXXT
TAGGTAAGATTGTAACACATAGTTATTCATTGTATAAAGTATGATTGGAATGAGGGTTAAGAAGAAGCACATGGTTGTG
AACTTGGTTTTGTGATTTATTGAGGTTCCATTTTTTTTTCTATATGGAGTGTATTTATTTGTTTCTTCCCTCTTTGATTTXXXXXXXXXXT
TAGGTAAGATTGTAACACATAGTTATTCATTGTATAAAGTATGATTGAAATGAGGGTTAAGAAGAAGCACATGGTTGTG
>Marker179482 Chr6 SNP_site:9491558 Ref_Type:A
AACTTGGTTTTGTGATTTATTGAGGTTCCATTTTTTTTTCTATATGGAGTGTATTTATTTGTTTCTACCCTCTTTGATTTXXXXXXXXXXT
TAGGTAAGATTGTAACACATAGTTATTCATTGTATAAAGTATGATTGGAATGAGGGTTAAGAAGAAGCACATGGTTGTG
AACTTGGTTTTGTGATTTATTGAGGTTCCATTTTTTTTTCTATATGGAGTGTATTTATTTGTTTCTTCCCTCTTTGATTTXXXXXXXXXXT

Create PDF with GO2PDF for free, if you wish to

TAGGTAAGATTGTAACACATAGTTATTCATTGTATAAAGTATGATTGAAATGAGGGTTAAGAAGAAGCACATGGTTGTG

>Marker179503 Chr6 SNP_site:8897052 Ref_Type:A

TACGTATATGAATGTAAGGATTTCTCTTTATTCCTACACGTTAAGAAAGAAAAGAAAAATAAGGAAAGAAAGGAAAAATGXXXXXXXXXXXA

CTACAAGATTTACGTGGTTCGGTCAAAGAAAGCTTACATCCACGAAAATGATCTCTTTCCTTGATATTTGAAGATAAGT

TACGTATATGAATGTAAGGATTTCTCTTTATTCCTACACGTTAAGAAAGAAAAGAAAAATAAGGAAAGAAAGGAAAAATGXXXXXXXXXXXA

CTACAAGATTTGCGTGGTTCGGTCAAAGAAAGCTTACATCCACGAAAATGATCTCTTTCCTTGATATTTGAAGATAAGT

>Marker179536 Chr6 SNP_site:9225782 Ref_Type:G

ACTGTGAAAGCAAACCAAATAGGCATCTTGAGATTGAGGAAGGAATGATGAAGCCAAAATTTCTAAAAATATGCATTAAAXXXXXXXXXXXT

TTTGAACCACGTTCGTAGGACAAACCATACACACAGTGCAAGGAGTTTCATGAATACGATACCATAAAAACTTTTATGT

ACTGTGAAAGCAAACCAAATAGGCATCTTGAGATTGAGGAAGGAATGATGAAGCCAAAATTTCTAAAAATATGCATTAAAXXXXXXXXXXXT

TTTGAACCACGTTGGTAGGACAAACCATAAACACGGTGCAAGGAGTTTCATGAATACGATACCATAAAAACTTTTATGT

>Marker179536 Chr6 SNP_site:9225798 Ref_Type:A

ACTGTGAAAGCAAACCAAATAGGCATCTTGAGATTGAGGAAGGAATGATGAAGCCAAAATTTCTAAAAATATGCATTAAAXXXXXXXXXXXT

TTTGAACCACGTTCGTAGGACAAACCATACACACAGTGCAAGGAGTTTCATGAATACGATACCATAAAAACTTTTATGT

ACTGTGAAAGCAAACCAAATAGGCATCTTGAGATTGAGGAAGGAATGATGAAGCCAAAATTTCTAAAAATATGCATTAAAXXXXXXXXXXXT

TTTGAACCACGTTGGTAGGACAAACCATAAACACGGTGCAAGGAGTTTCATGAATACGATACCATAAAAACTTTTATGT

>Marker179536 Chr6 SNP_site:9225803 Ref_Type:G

ACTGTGAAAGCAAACCAAATAGGCATCTTGAGATTGAGGAAGGAATGATGAAGCCAAAATTTCTAAAAATATGCATTAAAXXXXXXXXXXXT

TTTGAACCACGTTCGTAGGACAAACCATACACACAGTGCAAGGAGTTTCATGAATACGATACCATAAAAACTTTTATGT

ACTGTGAAAGCAAACCAAATAGGCATCTTGAGATTGAGGAAGGAATGATGAAGCCAAAATTTCTAAAAATATGCATTAAAXXXXXXXXXXXT

TTTGAACCACGTTGGTAGGACAAACCATAAACACGGTGCAAGGAGTTTCATGAATACGATACCATAAAAACTTTTATGT

>Marker179581 Chr6 SNP_site:19810625 Ref_Type:G

CACTGTTGCAAATATTCACTTTGCCTACTTCACTCTGACCTCTCCAGAAAACATAGTCCATGAATTCGCGATTTTCCCTTXXXXXXXXXXXT

TAAGATTTCTGTCAATCAAGTATTTTCAACTCTTTTTCTATAAGCCTCTTTTCAGCTCTCGAGAAAGTTTGTGTAAGGT

CACTGTTGCAAATATTCACTTTGCCTACTTCACTCTGACCTCTCCAGAAAACATAGTCCATGAATTGGCGATTTTCCCTTXXXXXXXXXXXT

TAAGATTTCTGTCAATCAAGTATTTTCAACTCTTTTTCTATAAGCCTCTTTTCAGATCTCGAGAAAGTTTGTGTAAGGT

>Marker179581 Chr6 SNP_site:19810834 Ref_Type:A

CACTGTTGCAAATATTCACTTTGCCTACTTCACTCTGACCTCTCCAGAAAACATAGTCCATGAATTCGCGATTTTCCCTTXXXXXXXXXXXT

TAAGATTTCTGTCAATCAAGTATTTTCAACTCTTTTTCTATAAGCCTCTTTTCAGCTCTCGAGAAAGTTTGTGTAAGGT

CACTGTTGCAAATATTCACTTTGCCTACTTCACTCTGACCTCTCCAGAAAACATAGTCCATGAATTGGCGATTTTCCCTTXXXXXXXXXXXT

TAAGATTTCTGTCAATCAAGTATTTTCAACTCTTTTTCTATAAGCCTCTTTTCAGATCTCGAGAAAGTTTGTGTAAGGT

>Marker180347 Chr6 SNP_site:5004537 Ref_Type:C

AACTCCAAAGGATGGAATATGAAGAATGTGTGTTGATAGCAGGGTCATTAACTGAATAACAGTCAAATACCGCTTCCCTAXXXXXXXXXXXG

AAGGGTTGTTTGAATGACTTGTGATGCATTTTGGCCTTTCCAATGCCCCAAGCACATTTATGTGCCTAATGAACCAAGT

AACTCCAAAGGATGGAATATGAAGAATGTGTGTTGATAGCAGGGTCATTAATTGAATAACAGTCAAATACCGCTTCCCTAXXXXXXXXXXXG

AAGGGTTGTTTGAATGACTTGTGATGCATTTTGGCCTTTCGAATGCCCCAAGCACATTTATGTGCCTAATGAACCAAGT

>Marker180347 Chr6 SNP_site:5004731 Ref_Type:C

AACTCCAAAGGATGGAATATGAAGAATGTGTGTTGATAGCAGGGTCATTAACTGAATAACAGTCAAATACCGCTTCCCTAXXXXXXXXXXXG

AAGGGTTGTTTGAATGACTTGTGATGCATTTTGGCCTTTCCAATGCCCCAAGCACATTTATGTGCCTAATGAACCAAGT

AACTCCAAAGGATGGAATATGAAGAATGTGTGTTGATAGCAGGGTCATTAATTGAATAACAGTCAAATACCGCTTCCCTAXXXXXXXXXXXG

AAGGGTTGTTTGAATGACTTGTGATGCATTTTGGCCTTTCGAATGCCCCAAGCACATTTATGTGCCTAATGAACCAAGT

>Marker180350 Chr6 SNP_site:7917061 Ref_Type:G

AACTAAACAAGCGGTAGGAAAGTGATCACAATTCTATCTCATTGTCGTATTCATCCTTTGCATTCGTTACAGAGCTATTAXXXXXXXXXXXC

GTTGCTTGTCTTAATTTTGAAGTTAAGAAATGCTTTGTGTCGTCTTGAGTTATTAAACCGCTACTCATTTCAATAAGTT

Create PDF with GO2PDF for free, if you wish to

AACTAAACAAGCGGTAGGAAAGTGATCACAATTCTATCTCATTGTCGTATTCATCCTTTGCATTCGTTACAGAGCTGTTAXXXXXXXXXXC
GTTGCTTGTCTTAATTTTGAAGTTAAGAAATGCTTTGTGTCGTCTTGAGTTATTAAACCGCTACTCATTTCAATAAGTT
>Marker180451 Chr6 SNP_site:3357534 Ref_Type:A
AACATTCTGACAGGTATTAATTTTTTGACAATGATGAATACCTTTTCCTTTTATAGGAGGAAGATATTGGTTAGGCTCAAXXXXXXXXXXT
AAGATTTCTAGTAGTGGTCATCTTCCTGTAAAGGTTACGAATTTAGGATGTAATTAGTGATTAGCTTTGACACTTTGTA
AACATTCTGACAGGTATTAATTTTTTGACAATGATGAATACCTTTTCCTTTTATAGGAGGAAGATATTGGTTAGGCTCAAXXXXXXXXXXT
AAGATTTCTAGTAGTGGTCATCTTCCTGTTAAGGTTACGAATTTAGGATGTAATTAGTGATTAGCTTTGACACTTTGTA
>Marker181101 Chr6 SNP_site:17352345 Ref_Type:A
CACTATTAAATAATTGATTTTAGGTTTAACCTATAAACCAAATCCCACTCGAGCCAATAAGAGGGTAGGACGACTCATTGXXXXXXXXXXA
ACAAAAGTGTTTGGTAACGTTCCATGTTTCACGTTCTAAAAAAAGTAGAAAGTTTTCCATTTCATAAGAAATTATTGTG
CACTATTAAATAATTGATTTTAGGTTTAACCTATAAACCAAATCCCTCTCGAGCCAATAAGAGGGTAGGACGACCCATTGXXXXXXXXXXA
ACAAAAGTGTTTGGTAACGTTCCATGTTTCACGTTCTAAAAAAAGTAGAAAGTTTTCCATTTCATAAGAAATTATTGTG
>Marker181101 Chr6 SNP_site:17352373 Ref_Type:T
CACTATTAAATAATTGATTTTAGGTTTAACCTATAAACCAAATCCCACTCGAGCCAATAAGAGGGTAGGACGACTCATTGXXXXXXXXXXA
ACAAAAGTGTTTGGTAACGTTCCATGTTTCACGTTCTAAAAAAAGTAGAAAGTTTTCCATTTCATAAGAAATTATTGTG
CACTATTAAATAATTGATTTTAGGTTTAACCTATAAACCAAATCCCTCTCGAGCCAATAAGAGGGTAGGACGACCCATTGXXXXXXXXXXA
ACAAAAGTGTTTGGTAACGTTCCATGTTTCACGTTCTAAAAAAAGTAGAAAGTTTTCCATTTCATAAGAAATTATTGTG
>Marker181337 Chr6 SNP_site:8736822 Ref_Type:C
ACAACCAAATATTTAGGCTCAATAGAAAACAATGCTTTTGTTGGAAAGAGAACATGTTAAGGAATCTGACCTTGAGGAACXXXXXXXXXXA
TTCTTTCGTTCCACAATTCCATATTGGGATTGAGTATCAGCACAAGATGACCGGTGAATGATTGCATGTGTATAAAGGT
ACAACCAAATATTTAGGCTCAATAGAAAACAATGCTTTTGTTGGAAAGAGAACATGTTAAGGAATCTGACCTTGAGGAACXXXXXXXXXXA
TTCTTTCGTTCCACAATTCCATATTGGGATTGAGTATCAGCACAAGATGACTGGTGAATGATTGCATGTGTATAAAGGT
>Marker181480 Chr6 SNP_site:27988572 Ref_Type:C
AACCAAAAATCCTATTACGATACTTCCAAATTTCAACGTATGCTATACGTTAAAAGCTTGAATGATAACTAAAAAAAATCXXXXXXXXXXT
CTCGTGGATTTATTTGTTTAATTTAATTTAGATAATGATAGATATATTTGGTAACCGTTATGTTTGTGTTGTGGGTGTT
AACCAAAAATCCTATTACGATACTTCCAAATTTCAACGTATGCTATACGTTAAAAGCTTGAATGATAACTAAAAAAAATCXXXXXXXXXXT
CTTGTGGATTTATTTGTTTAATTTAATTTAGATAATGATAGATATATTTGGTAACCGTTATGTTTGTGTTGTGGGTGTT
>Marker181798 Chr6 SNP_site:28146171 Ref_Type:G
ACCTTTGTGTGGGGTAACCTAATTACTTCGAGAATTAAGAAGCAAAGTGTTGTTGCTAGAAACAATGCTGAAACTAAACAXXXXXXXXXXC
CTATACAACATGATAGAATGAAACATATGGAGATTGACAGACACATTATAAAACCAAAACTGGACAATGACATTATGTA
ACCTTTGTGTGGGGTAACCTAATTACTTCGAGAATTAAGAAGCAAAGTGTTGTTGCTAGAAACAATGCTGAAACTAAACAXXXXXXXXXXC
CTATACAACATGATAGAATGAAACATATGGAGATTGACAGACACATTGTAAAACCAAAACTGGACAATGACATTATGTA
>Marker181937 Chr6 SNP_site:378086 Ref_Type:T
CACAGGAAACTCATGCAGATATCTCACCATCATCACCTCAGTCACAGAACATCAACGGGGTAAGTTGGAGAACAGAGCAGXXXXXXXXXXT
CTAGTGGATATATGGATGTTGCATCTTCCAGGACTGAGGAAGATGCCTATGGACTAGGATTAAATTCTCCTCTTCTGTT
CACAGGAAACTCATGCAGATATCTCACCATCATCACCTCAGTCACAGAACATCAATGGGGTAAGTTGGAGAACAGAGCAGXXXXXXXXXXT
CTAGTGGATATATGGATGTTGCATCTTCCAGGACTGAGGAAGATGCCTATGGACTAGGATTAAATTCTCCTCTTCTGTT
>Marker181955 Chr6 SNP_site:12727985 Ref_Type:G
AACCTGAGAGACATATTGAAATAGATTGATATAACTCCTCACATGCATATATATATTATTATGATAATCAACACGACGCAXXXXXXXXXXA
ACTTATTAAAGCAAATACTCCTGACAAAAAAAATCAACATTATAAATATATTTAGCTAAGAATACATTATACCCACGTG
AACCTGAGAGACATATTGAAATAGATTGATATAACTCCTCACATGCATATATATATTATTATGATAATCAACACGACGCAXXXXXXXXXXA
ACTTATTAAAGCAAATACTCCTGACAAAAAAAATCAACATTGTAAATATATTTAGCTAAGAATACATTATACCCACGTG
>Marker182935 Chr6 SNP_site:22133890 Ref_Type:C
GACTGGTTTAATTCACAGATCAAATGAGATTAATAATGGAGAAAATCTCTCTTTTTGGAAAGGCATCTGGAGTAGTCTTAXXXXXXXXXXA

Create PDF with GO2PDF for free, if you wish to

ACAATGGAGGGAGATTAAAAGTGTGCTGACGAACCCTATTGAAAATAGAGGTCAAGATCTTCCATTATGGAATCTGAGT
GACTGGTTTAATTCACAGATCAAATGAGATTAATAATGGAGAAAATCTCTCTTTTTGGAAAGGCATCTGGAGTAGTCTTAXXXXXXXXXXA
ACAATGGAGGGAGATTAAAAGTGTGCTGACGACCCCTATTGAAAATAGAGGTCAAGATCTTCCATTATGGAATCTGAGT
>Marker18306 Chr4 SNP_site:6071694 Ref_Type:C
ACCCTCATCACATCCAAGAAACAAAAATAAACCCTTTAAACTAGGATCCTATTCCCAATACAAGATCAAAACAAAGTAAAXXXXXXXXXXC
GAAGACTTCAAGGCTATAAAGGCGAGCTAATTTAACAAAATTTCCTGCGAAGAGTAATCTTGAAAATATTTGGATGGTG
ACCCTCATCACATCCAAGAAACAAAAATAAACCCTTTAAACTAGGATCCTATTCCCAATACAAGATCAAAACAAAGTAAAXXXXXXXXXXT
GAAGACTTCGAGGCTATAAAGGCGAGCTAATTTAACAAAATTTCCTGCGAAGAGTAATCTTGAAAATATTTGGATGGTG
>Marker18306 Chr4 SNP_site:6071704 Ref_Type:A
ACCCTCATCACATCCAAGAAACAAAAATAAACCCTTTAAACTAGGATCCTATTCCCAATACAAGATCAAAACAAAGTAAAXXXXXXXXXXC
GAAGACTTCAAGGCTATAAAGGCGAGCTAATTTAACAAAATTTCCTGCGAAGAGTAATCTTGAAAATATTTGGATGGTG
ACCCTCATCACATCCAAGAAACAAAAATAAACCCTTTAAACTAGGATCCTATTCCCAATACAAGATCAAAACAAAGTAAAXXXXXXXXXXT
GAAGACTTCGAGGCTATAAAGGCGAGCTAATTTAACAAAATTTCCTGCGAAGAGTAATCTTGAAAATATTTGGATGGTG
>Marker183327 Chr6 SNP_site:7058332 Ref_Type:G
ACCATCATTACTCTATATTTTTCTCCCATAGTAAGCCCAACCTCATTCTCCAATCTAGCCCAACTACAAGCTTCAATTTTXXXXXXXXXXA
AAATCCCATTTATTTTTTATACAGAAGGCTTTCCCATCACATATTTTGGTGAGTTTTGGTATCCGCTATGTAACAAGTG
ACCATCGTTACTCTATATTTTTCTCCCATAGTAAGCCCAACCTCATTCTCCAATCTAGCCCAACTACAAGCTTCAATTTTXXXXXXXXXXA
AAATCCCATTTATTTTTTATACAGAAGGCTTTCCCATCACATATTTTGGTGAGTTTTGGTATCCGCTATGTAACAAGTG
>Marker183718 Chr6 SNP_site:7298779 Ref_Type:T
TACTATTGTCTCCACTTGCATCAACATGTCCTATGTCCTATGTCCTTTACCCTTGAATTTTGAATTTTTAAAAATGTGTTXXXXXXXXXXT
TCTGTTTTGTAATTTCTTCCTTGGATGATAATCATGAATATGAAAGAGATCTTAATTATAACTCCCCCCCTTCTTTGTT
TACTATTGTCTCCACTTGCATCAACATGTCCTATGTCCTATGTCCTTTACCCTTGAATTTTGAATTTTTTAAAATGTGTTXXXXXXXXXXT
TCTGTTTTGTAATTTCTTCCTTGGATGATAATCATGAATATGAAAGAGATCTTAATTATAACTCCCCCCCTTCTTTGTT
>Marker18424 Chr4 SNP_site:20794678 Ref_Type:G
CACTTCCCTATTGTTCAACGGCTTTGAAACATATGGTTTATCTCTTATTGCTAATGCTTTTCTGCTTGCAGGGTGCAGTCXXXXXXXXXXG
TGTATTAGTCCATCCGTTACCTTGAATTTTTTTGTGTCTAATTCAAGTTTCCTGTAAGGTGTGCAGGTATATTCTGGTG
CACTTCCCTATTGTTCAACGGCTTTGAAACGTATGGTTTATCTCTTATTGCTAATGCTTTTCTGCTTGCAGGGTGCAGTCXXXXXXXXXXG
TGTATTAGTCCATCCGTTACCTTGAATTTTTTTGTGTCTAATTCAAGTTTCCTGTAAGGTGTGCAGGTATATTCTGGTG
>Marker184419 Chr6 SNP_site:27436593 Ref_Type:A
ACAGATGTTACATTTTGTCGCAGTTCATCTTTTCTCCTCTTTCCTTTGTAACTGTGGCCCTTGATAGTTGTTGTATGTGAXXXXXXXXXXT
GAAAATGAATCTATCACTTGGCCAAAATCAGATTTTAACTTCCAATTAGATTGGATATGGGAATGATAGCAAAAAAGTG
ACAGATGTTACATTTTGTCGCAGTTCATCTTTTCTCCTCTTTCCTTTGTAACTGTGGCCCTTGATAGTTGTTGTATGTGAXXXXXXXXXXT
GAAAATGAATCTATCACTTGGCCAAAATCAGTTTTTAACTTCCAATTAGATTGGATATGGGAATGATAGCAAAAAAGTG
>Marker184837 Chr6 SNP_site:25371047 Ref_Type:C
CACATCCACGTAGGAATTACAAAGGCTTGGGAGATTCTAAGTCACTTAAAGTTGATTTAGAACAACCATTTCAAACATGGXXXXXXXXXXA
ATTTTGAAGAGATGAAACAAGACACGTCTCTTATGTAGTTAAGATATCAACTAAATATTATTAAATTGGTATATTTGTC
CACATCCAGGTAGGAATTACAAAGGCTTGGGAGATTCTAAGTCACTTAAAGTTGATTTAGAACAACCATTTCAAACATGGXXXXXXXXXXA
ATTTTGAAGAGATGAAACAAGACACGTCTCTTATGTAGTTAAGATATCAACTAAATATTATTAAATTGGTATATTTGTC
>Marker184907 Chr6 SNP_site:9245337 Ref_Type:T
TACGACCGAGCTGCTATAGAACTCCAGGGTCCAAACGCCGCAACGAACTTCTCCGGCGACGGAGCAGTTAAAACGGCGGTXXXXXXXXXXC
AGCTTTTTGACTCCGATTGAGGAGATGGGGTATTGTGGAGAGGTGGATGAGTTGGGATTGGAGATTGGAGCTGCAAGTT
TACGACCGAGCTGCTATAGAACTCCAGGGTCCAAACGCCGCAACGAACTTCTCCGGCGACGGAGCAGTTAAATCGGCGGTXXXXXXXXXXC
AGCTTTTTGACTCCGATTGAGGAGATGGGGTATTGTGGAGAGGTGGATGAGTTGGGATTGGAGATTGGAGCTGCAAGTT
>Marker185278 Chr6 SNP_site:18484199 Ref_Type:G

Create PDF with GO2PDF for free, if you wish to

CACCTCAAAAGAATAAAAAAATGAATATTATGTATATAGAGAGGTAAGTCATTATATCATTAAATTAATAAATGGTTATAXXXXXXXXXXT
ATGAATAAAGAAAAGAAAGACTGAATGAATCATTAAAATTAATTAATAAAGTTAGTGGTGACGTAAGAGTGAAATTGTG
CACCTCAAAAGGATAAAAAAATGAATATTATGTATATAGAGAGGTAAGTCATTATATCATTAAATTAATAAATGGTTATAXXXXXXXXXXT
ATGAATAAAGAAAAGAAAGATTGAATGAATCATTAAAATTAATTAATAAAGTTAGTGGTGACGTAAGAGTGAAATTGTG
>Marker185278 Chr6 SNP_site:18484382 Ref_Type:T
CACCTCAAAAGAATAAAAAAATGAATATTATGTATATAGAGAGGTAAGTCATTATATCATTAAATTAATAAATGGTTATAXXXXXXXXXXT
ATGAATAAAGAAAAGAAAGACTGAATGAATCATTAAAATTAATTAATAAAGTTAGTGGTGACGTAAGAGTGAAATTGTG
CACCTCAAAAGGATAAAAAAATGAATATTATGTATATAGAGAGGTAAGTCATTATATCATTAAATTAATAAATGGTTATAXXXXXXXXXXT
ATGAATAAAGAAAAGAAAGATTGAATGAATCATTAAAATTAATTAATAAAGTTAGTGGTGACGTAAGAGTGAAATTGTG
>Marker185477 Chr6 SNP_site:8041614 Ref_Type:C
CACAAATTTTCAATAAAAAACTTGACACAACAAAGATTTACATATCCATTTGTGTTTAGTGGAAGAAGCCCTAATTCTTTXXXXXXXXXXG
AGAAACAGCTTGAAAGTTCTCTTTAAAATTTTCGATTTTTCTGTGAGTAACACTTTAACCTTCTAACTTTACCATTTGT
CACAAATTTTCAATAAAAAACTTGACACAATAAAGATTTACATATCCATTTGTGTTTAGTGGAAGAAGCCCTAATTCTTTXXXXXXXXXXG
AGAAACAGCTTGAAAGTTCTCTTTAAAATTTTCGATTTTTCTATGAGTAACACTTTAACCTTCTAACTTTACCATTTGT
>Marker185477 Chr6 SNP_site:8041822 Ref_Type:G
CACAAATTTTCAATAAAAAACTTGACACAACAAAGATTTACATATCCATTTGTGTTTAGTGGAAGAAGCCCTAATTCTTTXXXXXXXXXXG
AGAAACAGCTTGAAAGTTCTCTTTAAAATTTTCGATTTTTCTGTGAGTAACACTTTAACCTTCTAACTTTACCATTTGT
CACAAATTTTCAATAAAAAACTTGACACAATAAAGATTTACATATCCATTTGTGTTTAGTGGAAGAAGCCCTAATTCTTTXXXXXXXXXXG
AGAAACAGCTTGAAAGTTCTCTTTAAAATTTTCGATTTTTCTATGAGTAACACTTTAACCTTCTAACTTTACCATTTGT
>Marker185507 Chr6 SNP_site:18816991 Ref_Type:C
ACTTGTATTTACAACCCTTTAGGAATCAAACCAATTCCTCCCGTGTCATCTGTAACCAAAATATAAGTAAGCTCCACTATXXXXXXXXXXG
AAAGCACTGAACAAGATATGAAAGTCATGGAAATGACAAGAATGATTTGGTTTGACTTTGGTTGCCTCTTCAATGAAGT
ACTTGTATTTACAACCCTTTAGGAATCAAACCAATTCCTCCCGTGTCATCTGTAACCAAAATATAAGTAAGCTCCATTATXXXXXXXXXXG
AAAGCACTGAACAAGATATGAAAGTCATGGAAATGACAAGAATGATTTGGTTTGACTTTGGTTGCCTCTTCAATGAAGT
>Marker185556 Chr6 SNP_site:4146226 Ref_Type:A
GACCCAATCTTTTAGTTTGCTCAATTTCCCCCATAAATGTGTTTAGATTGGACCCAATTAATTTAAATTACTTCGCAATCXXXXXXXXXXA
TTGTTCTTATCTCTTGTTGTGATAATATCACAATTTTGATTTTTATTAGACACAATTTCTTCTCCAAGTTGTTGGAGTG
GACCCAATCTTTTAGTTTGCTCAATTTCCCCCATAAATGTGTTTAGATTGGACCCAGTTAATTTAAATTACTTCGCAATCXXXXXXXXXXA
TAGTTCTTATCTTTTGTTGTGATAATATCACAATTTTGATTTTTATTAGACACAATTTCTTCTCCAAGTTGTTGGAGTG
>Marker185556 Chr6 SNP_site:4146339 Ref_Type:T
GACCCAATCTTTTAGTTTGCTCAATTTCCCCCATAAATGTGTTTAGATTGGACCCAATTAATTTAAATTACTTCGCAATCXXXXXXXXXXA
TTGTTCTTATCTCTTGTTGTGATAATATCACAATTTTGATTTTTATTAGACACAATTTCTTCTCCAAGTTGTTGGAGTG
GACCCAATCTTTTAGTTTGCTCAATTTCCCCCATAAATGTGTTTAGATTGGACCCAGTTAATTTAAATTACTTCGCAATCXXXXXXXXXXA
TAGTTCTTATCTTTTGTTGTGATAATATCACAATTTTGATTTTTATTAGACACAATTTCTTCTCCAAGTTGTTGGAGTG
>Marker185556 Chr6 SNP_site:4146350 Ref_Type:C
GACCCAATCTTTTAGTTTGCTCAATTTCCCCCATAAATGTGTTTAGATTGGACCCAATTAATTTAAATTACTTCGCAATCXXXXXXXXXXA
TTGTTCTTATCTCTTGTTGTGATAATATCACAATTTTGATTTTTATTAGACACAATTTCTTCTCCAAGTTGTTGGAGTG
GACCCAATCTTTTAGTTTGCTCAATTTCCCCCATAAATGTGTTTAGATTGGACCCAGTTAATTTAAATTACTTCGCAATCXXXXXXXXXXA
TAGTTCTTATCTTTTGTTGTGATAATATCACAATTTTGATTTTTATTAGACACAATTTCTTCTCCAAGTTGTTGGAGTG
>Marker185660 Chr6 SNP_site:18333856 Ref_Type:T
TACTTTTGTTTCTATAAACCTGAAACTTATCTTACCCTTTTTTACCTTCTCAAATCTTATCCATTCACACTTTTACATTAXXXXXXXXXXT
GAGTAACTAATAAAGGAGAATATCGTTACTAGAAAATTCTGAAGTTTTCAACAATGAAAAAACAAACCCAGTTCTTGTT
TACTTTTGTTTCTATAAACCTGAAACTTATCTTACCCTTTTTTACCTTCTCAAATCTTATCCATTCACACTTTTACATTAXXXXXXXXXXT
GAGTAACTAATAAAGGAGAATATCGTTACTAGAAAATTCTGAAGTTTTCAACAATTAAAAAACAAACCCAGTTCTTGTT

Create PDF with GO2PDF for free, if you wish to

>Marker186080 Chr6 SNP_site:1890506 Ref_Type:T
ACTTTATGACAAATTTTTCCTGTCAAATATATGTCAATCGTGACAACTTTTTTTATAAAATCAAATGTCATTGTTCTGCTXXXXXXXXXXT
GTCTTCTTGTTAAATATGCATAGCTAGCTGAGTTAGGAATGTCAAGGAATGAAATGCATCTTTACCCTCTTTTTAAGTG
ACTTTATGACAAATTTTTCCTGTCAAATATATGTCAATCGTGACAACTTTTTTTATAAAATCAAATGTCATTGTTTCGCTXXXXXXXXXXT
GTCTTCTTGTTAAATATGCATAGCTAGCTGAGTTATGAATGTCAAGGAATGAAATGCATCTTTACCCTCTTTTTAAGTG
>Marker186080 Chr6 SNP_site:1890507 Ref_Type:C
ACTTTATGACAAATTTTTCCTGTCAAATATATGTCAATCGTGACAACTTTTTTTATAAAATCAAATGTCATTGTTCTGCTXXXXXXXXXXT
GTCTTCTTGTTAAATATGCATAGCTAGCTGAGTTAGGAATGTCAAGGAATGAAATGCATCTTTACCCTCTTTTTAAGTG
ACTTTATGACAAATTTTTCCTGTCAAATATATGTCAATCGTGACAACTTTTTTTATAAAATCAAATGTCATTGTTTCGCTXXXXXXXXXXT
GTCTTCTTGTTAAATATGCATAGCTAGCTGAGTTATGAATGTCAAGGAATGAAATGCATCTTTACCCTCTTTTTAAGTG
>Marker186080 Chr6 SNP_site:1890667 Ref_Type:T
ACTTTATGACAAATTTTTCCTGTCAAATATATGTCAATCGTGACAACTTTTTTTATAAAATCAAATGTCATTGTTCTGCTXXXXXXXXXXT
GTCTTCTTGTTAAATATGCATAGCTAGCTGAGTTAGGAATGTCAAGGAATGAAATGCATCTTTACCCTCTTTTTAAGTG
ACTTTATGACAAATTTTTCCTGTCAAATATATGTCAATCGTGACAACTTTTTTTATAAAATCAAATGTCATTGTTTCGCTXXXXXXXXXXT
GTCTTCTTGTTAAATATGCATAGCTAGCTGAGTTATGAATGTCAAGGAATGAAATGCATCTTTACCCTCTTTTTAAGTG
>Marker186131 Chr6 SNP_site:13397637 Ref_Type:G
ACTAACGTAGCAAGAACCTTTGAAGTAAGAAACTAACACCTCCTTCTACTAATTTAGATCAATCAATAGAAATTAAAGTTXXXXXXXXXXC
ATTCTATCACAAGAATTACAAATTAAGGAAAAACTCTAAAAAATGAGATTCAAATTGCATTCATCAAAAGTCTTGTGTT
ACTAACGTAGCAAGAACCTTTGAAGTAAGAAACTAACACCTCCTTCTACTAATTTAGATCAATCAATAGAAATTAAAGTTXXXXXXXXXXC
ATTCTATCACAAGAATTGCAAATTAAGGAAAAACTCTAAAAAATGAGATTCAAATTGCATTCATCAAAAGTCTTGTGTT
>Marker18645 Chr4 SNP_site:17336145 Ref_Type:T
AACATTTTTACGGTGAGAAAAAGTATAAAGTGATTAGGAAATCAGGAGATTATATACAAATAAAGGAAGCATATTAAAAAXXXXXXXXXXC
ATGTAATTAGACCCTCTCAACAAAGATAGTATATAAAGACAAACATAAAGGCAAAAGGGGACTTAAGAAGTGAGTAGTA
AACTTTTTTAGGGTGAGAAAAAGTATAAAGTGATTAGGAAATCAGGAGATTATATACAAATAAAGGAAGCATATTAAAAAXXXXXXXXXXC
ATGTAATTAGACCCTCTCAACAAAGATAGTATATAAAGACAAACATAAAGGCAAAAGGGGACTTAAGAAGTGAGTAGTA
>Marker18645 Chr4 SNP_site:17336152 Ref_Type:G
AACATTTTTACGGTGAGAAAAAGTATAAAGTGATTAGGAAATCAGGAGATTATATACAAATAAAGGAAGCATATTAAAAAXXXXXXXXXXC
ATGTAATTAGACCCTCTCAACAAAGATAGTATATAAAGACAAACATAAAGGCAAAAGGGGACTTAAGAAGTGAGTAGTA
AACTTTTTTAGGGTGAGAAAAAGTATAAAGTGATTAGGAAATCAGGAGATTATATACAAATAAAGGAAGCATATTAAAAAXXXXXXXXXXC
ATGTAATTAGACCCTCTCAACAAAGATAGTATATAAAGACAAACATAAAGGCAAAAGGGGACTTAAGAAGTGAGTAGTA
>Marker186537 Chr6 SNP_site:17211512 Ref_Type:T
GACATGGATCGCCTCGAAAGTTCAGATTTGAGATTGGAATCGAGCATTTGGCCCTCCCTAGACATGCCTGAAACACCACAXXXXXXXXXXC
TCACATGTTCTACAATGGCTCGGGAGTTTGGCGAGTGACACAGTCCAATAGCATAGCTTTGACAATCACCAGATGGGGT
GACATGGATCGCCTCGAAAGTTCAGATTTGAGATTGGAATCGAGCATTTGGCCCTCCCTAGACATGCCTGAAACATCACAXXXXXXXXXXC
TCACATGTTCTACAATGGCTCTGGAGTTTGGCGAGTGACACAGTCCAATAGCATAGCTTTGACAATCACCAGATGGGGT
>Marker186537 Chr6 SNP_site:17211633 Ref_Type:T
GACATGGATCGCCTCGAAAGTTCAGATTTGAGATTGGAATCGAGCATTTGGCCCTCCCTAGACATGCCTGAAACACCACAXXXXXXXXXXC
TCACATGTTCTACAATGGCTCGGGAGTTTGGCGAGTGACACAGTCCAATAGCATAGCTTTGACAATCACCAGATGGGGT
GACATGGATCGCCTCGAAAGTTCAGATTTGAGATTGGAATCGAGCATTTGGCCCTCCCTAGACATGCCTGAAACATCACAXXXXXXXXXXC
TCACATGTTCTACAATGGCTCTGGAGTTTGGCGAGTGACACAGTCCAATAGCATAGCTTTGACAATCACCAGATGGGGT
>Marker18681 Chr4 SNP_site:6508643 Ref_Type:A
ACAGAGACAAAACATAATTGTCTAAACAACCCCTCTCAAACGAGGCAAGGGAGGGAATCGAAGTCGGAGGTTTGTCATGAXXXXXXXXXXC
AAGCATGATGAATCAGATTGCAAGTAATGGCTATAGAAAAAATGTTATAATACTATGTAGTTGAGGGAGAACAGAGGTT
ACAGAGACAAAACATAATTGTCTAAACAACCCCTCTCAAACGAGGCAAGGGAGGGAATCGAAGTCGGAGGTTTGTCATGAXXXXXXXXXXC

Create PDF with GO2PDF for free, if you wish to

AAGCATGATGAATCAGATTGCGAGTAATGGCTATAGAAAAAATGTTATAATACTATGTAGTTGAGGGAGAACAGAGGTT
>Marker18683 Chr4 SNP_site:7909895 Ref_Type:C
AACGAACCCACGACTATTAAAATTAACCTTGTGTGATTGATTTATATACCTTGTAAAAATAACGCATAGTGAAATTTCATXXXXXXXXXXC
TTAACTGAAATAGTTCTTGTAACTACTTATTGTAACCTTTCACTAATTGAATGCTTTATTTCTTTAAAAAAATGTAGGT
AACGAACCCACGACTATTAAAATTAACCTTGTGTGATTGATTTATATATCTTGTAAAAATAACGCATGGTGAAATTTCATXXXXXXXXXXC
TTAACTGAAATAGTTCTTGTAACTACTTATTGTAACCTTTCACTAATTGAATGTTTTATTTCTTTAAAAAAATGTAGGT
>Marker18683 Chr4 SNP_site:7909914 Ref_Type:A
AACGAACCCACGACTATTAAAATTAACCTTGTGTGATTGATTTATATACCTTGTAAAAATAACGCATAGTGAAATTTCATXXXXXXXXXXC
TTAACTGAAATAGTTCTTGTAACTACTTATTGTAACCTTTCACTAATTGAATGCTTTATTTCTTTAAAAAAATGTAGGT
AACGAACCCACGACTATTAAAATTAACCTTGTGTGATTGATTTATATATCTTGTAAAAATAACGCATGGTGAAATTTCATXXXXXXXXXXC
TTAACTGAAATAGTTCTTGTAACTACTTATTGTAACCTTTCACTAATTGAATGTTTTATTTCTTTAAAAAAATGTAGGT
>Marker18683 Chr4 SNP_site:7910116 Ref_Type:C
AACGAACCCACGACTATTAAAATTAACCTTGTGTGATTGATTTATATACCTTGTAAAAATAACGCATAGTGAAATTTCATXXXXXXXXXXC
TTAACTGAAATAGTTCTTGTAACTACTTATTGTAACCTTTCACTAATTGAATGCTTTATTTCTTTAAAAAAATGTAGGT
AACGAACCCACGACTATTAAAATTAACCTTGTGTGATTGATTTATATATCTTGTAAAAATAACGCATGGTGAAATTTCATXXXXXXXXXXC
TTAACTGAAATAGTTCTTGTAACTACTTATTGTAACCTTTCACTAATTGAATGTTTTATTTCTTTAAAAAAATGTAGGT
>Marker187297 Chr6 SNP_site:7302699 Ref_Type:G
GACTTAATTATATACTAGAGTCAAGAGTTTGTGCAGCAAGTATCTGCAAACTCTCTTCTTTTACCCAAACTAACTTCTTTXXXXXXXXXXA
AACATAGAAAATAGTGGATTAATTTGATTGAACAAGTGTGTGTGTGTGTGTGAAATAATGGGACCGTGAGGTAGAGT
GACTTAATTATATACTAGAGTCAAGAGTTTGTGCAGCAAGTATCTGCAAACTCTCTTCTTTTACCCAAACTAACTTCTTTXXXXXXXXXXA
AACATAGAAAGTAGTGGATTAATTTGATTGAACAAGTGTGTGTGTGTGTGTGAAATAATGGGACCGTGAGGTAGAGT
>Marker187399 Chr6 SNP_site:22090443 Ref_Type:T
GACTTTAAAAAAGAGAGATATAATGTAAATTGAAATATTCCTTAATGCATTGATAAAGAGAGGCCAGTTTTACATATTTAXXXXXXXXXXA
GTGGGCAAAAGAATTTAACCACAGTGATCTTAATAACATAAGCATCACCAACCCTGACGTATGGCTTCAACTTGAGGGT
GACTTTAAAAAAGAGATATATAATGTAAATTGAAATATTCCTTAATGCATTCATAAAGAGAGGCCAGTTTTACATATTTAXXXXXXXXXXA
GTGGGCAAAAGAATTTAACCACAGTGATCTTAATAACATAAGCATCACCAACCCTGACGTATGGCTTCAACTTAAGGGT
>Marker187399 Chr6 SNP_site:22090478 Ref_Type:C
GACTTTAAAAAAGAGAGATATAATGTAAATTGAAATATTCCTTAATGCATTGATAAAGAGAGGCCAGTTTTACATATTTAXXXXXXXXXXA
GTGGGCAAAAGAATTTAACCACAGTGATCTTAATAACATAAGCATCACCAACCCTGACGTATGGCTTCAACTTGAGGGT
GACTTTAAAAAAGAGATATATAATGTAAATTGAAATATTCCTTAATGCATTCATAAAGAGAGGCCAGTTTTACATATTTAXXXXXXXXXXA
GTGGGCAAAAGAATTTAACCACAGTGATCTTAATAACATAAGCATCACCAACCCTGACGTATGGCTTCAACTTAAGGGT
>Marker187399 Chr6 SNP_site:22090716 Ref_Type:A
GACTTTAAAAAAGAGAGATATAATGTAAATTGAAATATTCCTTAATGCATTGATAAAGAGAGGCCAGTTTTACATATTTAXXXXXXXXXXA
GTGGGCAAAAGAATTTAACCACAGTGATCTTAATAACATAAGCATCACCAACCCTGACGTATGGCTTCAACTTGAGGGT
GACTTTAAAAAAGAGATATATAATGTAAATTGAAATATTCCTTAATGCATTCATAAAGAGAGGCCAGTTTTACATATTTAXXXXXXXXXXA
GTGGGCAAAAGAATTTAACCACAGTGATCTTAATAACATAAGCATCACCAACCCTGACGTATGGCTTCAACTTAAGGGT
>Marker187904 Chr6 SNP_site:5012387 Ref_Type:A
AACTTCTACCTCTTATATGGTTCTTAATACGGTGCAGGGTTTGACGTGGCTTCTTCCTGAACGGTTTTCTGATTCAGAAAXXXXXXXXXXT
AAAAGTTATAAATTTACAGAAGCCTATATCAAAGATTCAAAACCCATTTTATTTTGATTTAAACAAAAACGAAGAGGTC
AACTTCTACCTCTTATATGGTTCTTAATACGGTGCAGGGTTTGACGTGGCTTCTTCCTGAACGGTTTTCTGATTCAGAAAXXXXXXXXXXT
AAAAGTTATAAATTTACAGAAGCCTATATCGAAGATTCAAAACCCATTTTATTTTGATTTAAACAAAAACGAAGAGGTC
>Marker188348 Chr6 SNP_site:7461500 Ref_Type:G
AACTCATGTTGTGACTAAGTAGATGAAAAGACTGACGTCTATAGAATTTTTCAATTCTTCTTCTTTTTTCTACTCGATCAXXXXXXXXXXC
TATAATTTAGTATTTTGGCATGTTTTAGATTAATATATTATAGTCTTCTATCACTGATAGTCAGTAATAGTCAATGGTA

Create PDF with GO2PDF for free, if you wish to

AACTCATGTTGTGACTAAGTAGATGAAAAGACTGACGTCTATAGAATTTTTCAATTCTTCTTCTTTTTTCTACTCGATCAXXXXXXXXXXC
TATAATTTGGTATTTTGGCATGTTTTAGATTAATATATTATAGTCTTCTATCACTGATAGTCAGTAATAGTCAATGGTA
>Marker188659 Chr6 SNP_site:13834310 Ref_Type:C
ACCCTGCACAGTCAAAGTCTTGTGATCTCGCAGGATACACATAATCAGTCTAACGCCCCGAAGGACGCATAAATCAGTCTXXXXXXXXXXT
TGGCATATAACTTATTCACTCGAAGGGTCTCCAACACTTGATGCAAATGCTCCTCATGCTCAGCCTCTGTCTTGGAGTA
ACCCTGCACAGTCAAAGTCTTGTGATCTCGTAGGATACACATAATCAGTCTAACGCCCCGAAGGACGCATAAATCAGTCTXXXXXXXXXXT
TGGCATATAACTTATTCACTCGAAGGGTCTCCAACACTTGATGCAAATGCTCCTCATGCTCAGCCTCTGTCTTGGAGTA
>Marker188663 Chr6 SNP_site:3542260 Ref_Type:G
TACTTTATTTGATTATAGTATTGCATTATATCAAATCATGAAATCGATTGGGGTAAGTGTTATGTTTGAACATATTTGTGXXXXXXXXXXA
TATGAAAATACAATGTAATTGTGAGGGGAAATATGGAACATACGGTTTCTCATCACTTGCATTTCTATGGTTAAAGAGT
TACTTTATTTGATTATAGTGTTACATTATATCAAATCATGAAATCGATTGGGGTAAGTGTCATGTTTGAACATATTTGTGXXXXXXXXXXA
TATGAAAATACAATGTAATTGTGAGGGGAAATATGGAACATACGGTTTCTCATCACTTGCATTTCTATGGTTAAAGAGT
>Marker188663 Chr6 SNP_site:3542263 Ref_Type:A
TACTTTATTTGATTATAGTATTGCATTATATCAAATCATGAAATCGATTGGGGTAAGTGTTATGTTTGAACATATTTGTGXXXXXXXXXXA
TATGAAAATACAATGTAATTGTGAGGGGAAATATGGAACATACGGTTTCTCATCACTTGCATTTCTATGGTTAAAGAGT
TACTTTATTTGATTATAGTGTTACATTATATCAAATCATGAAATCGATTGGGGTAAGTGTCATGTTTGAACATATTTGTGXXXXXXXXXXA
TATGAAAATACAATGTAATTGTGAGGGGAAATATGGAACATACGGTTTCTCATCACTTGCATTTCTATGGTTAAAGAGT
>Marker188663 Chr6 SNP_site:3542301 Ref_Type:C
TACTTTATTTGATTATAGTATTGCATTATATCAAATCATGAAATCGATTGGGGTAAGTGTTATGTTTGAACATATTTGTGXXXXXXXXXXA
TATGAAAATACAATGTAATTGTGAGGGGAAATATGGAACATACGGTTTCTCATCACTTGCATTTCTATGGTTAAAGAGT
TACTTTATTTGATTATAGTGTTACATTATATCAAATCATGAAATCGATTGGGGTAAGTGTCATGTTTGAACATATTTGTGXXXXXXXXXXA
TATGAAAATACAATGTAATTGTGAGGGGAAATATGGAACATACGGTTTCTCATCACTTGCATTTCTATGGTTAAAGAGT
>Marker189074 Chr6 SNP_site:16511935 Ref_Type:T
AACTAAATTTGTTGCAACAATTGTAGTCTTCATGTTCGTTTTACAATTCATGTTCGTTTTTGTTTTCAATCGCTATTTTTXXXXXXXXXXA
TCTGCCAGTTTTCTCTTATTTTGATAATTGAATTTAGATTAATGTTTAGAATATGATTGATTGATGACCATGTGTGGTT
AACTAAATTTGTTGCAACAATTGTAGTCTTCATGTTCGTTTTACAATTCATGTTCGTTTTTGTTTTCAATCGCTATTTTTXXXXXXXXXXA
TCTGCTAGTTTTCTCTTATTTTGATAATTGAATTTAGATTAATGTTTAGAATATGATTGATTGATGACCATGTGTGGTT
>Marker189162 Chr6 SNP_site:27429001 Ref_Type:G
ACTCCCGTTATTGAAGCTTGGAACCCTGGCTTTGAAAACTCTGAGCAAGCCACTCGGTAATAGACTCAAGAAACAAGCTGXXXXXXXXXXT
GATTGTTCTGTTCTGTTCTGATCTATACAAGAGCACATGTTGTTGAGCTGTTGTTGTTTTATAACAAATGGTCATGGTC
ACTCCCGTTATTGAAGCTTGGAACCCTGGCTTTGAAAACTCTGAGCAAGCCACTCGGTAATAGACTCAAGAAACAAGCTGXXXXXXXXXXT
GATTGTTCTGTTCTGTTCTGATCTATACAAGAGCACGTTTTGTTGAGCTGTTGTTGTTTTATAACAAATGGTCATGGTC
>Marker189162 Chr6 SNP_site:27429003 Ref_Type:T
ACTCCCGTTATTGAAGCTTGGAACCCTGGCTTTGAAAACTCTGAGCAAGCCACTCGGTAATAGACTCAAGAAACAAGCTGXXXXXXXXXXT
GATTGTTCTGTTCTGTTCTGATCTATACAAGAGCACATGTTGTTGAGCTGTTGTTGTTTTATAACAAATGGTCATGGTC
ACTCCCGTTATTGAAGCTTGGAACCCTGGCTTTGAAAACTCTGAGCAAGCCACTCGGTAATAGACTCAAGAAACAAGCTGXXXXXXXXXXT
GATTGTTCTGTTCTGTTCTGATCTATACAAGAGCACGTTTTGTTGAGCTGTTGTTGTTTTATAACAAATGGTCATGGTC
>Marker189293 Chr6 SNP_site:11839661 Ref_Type:G
GACTTCCATTCTCGGTTGATGATGGCTCTCTTGCAGATTGGGCATCGGATTTTCTCAAGGGAGAACAGTTGTTAAAAGATXXXXXXXXXXA
AAGAAATCACAGCATTGGAGCCTGCTGGAGCAACACCAAAACTATCTCCTCTGTGGTGGGCAGAACTAGAAATCTTGTC
GACTTCCATTCTCGGTTGATGATGGCTCTCTTGCAGATTGGGCATCGGATTTTCTCAAGGGAGAACAGTTGTTAAGAGATXXXXXXXXXXA
AAGAAATCACCGCATTGGAGCCTGCTGGAGCAACACCAAAACTATCTCCTCTGTGGTGGGCAGAACTAGAAATCTTGTC
>Marker189293 Chr6 SNP_site:11839803 Ref_Type:C
GACTTCCATTCTCGGTTGATGATGGCTCTCTTGCAGATTGGGCATCGGATTTTCTCAAGGGAGAACAGTTGTTAAAAGATXXXXXXXXXXA

Create PDF with GO2PDF for free, if you wish to

AAGAAATCACAGCATTGGAGCCTGCTGGAGCAACACCAAAACTATCTCCTCTGTGGTGGGCAGAACTAGAAATCTTGTC
GACTTCCATTCTCGGTTGATGATGGCTCTCTTGCAGATTGGGCATCGGATTTTCTCAAGGGAGAACAGTTGTTAAGAGATXXXXXXXXXXA
AAGAAATCACCGCATTGGAGCCTGCTGGAGCAACACCAAAACTATCTCCTCTGTGGTGGGCAGAACTAGAAATCTTGTC
>Marker189493 Chr6 SNP_site:28158125 Ref_Type:G
ACTCTTCTTGGTATCTAGGTGGGTAGTTAGTCATCAGTTTTCACTGTAAAATGGAAATAATCACCAGTGCTTCCAATGATXXXXXXXXXXT
CTTATTATCTGACTCTCGATCAACTTATAGATTCTTAACTTTCTGAGAAACAGGTCTCAAAGAACCAATGTGTTTTGTT
ACTCTTCTTGGTATCTAGGTGGGTAGTTAGTCATCAGTTTTCAGTGTAAAATGGAAATAATCACCAGTGCTTCCAATGATXXXXXXXXXXT
CTTATTATCTGACTCTCGATCAACTTATAGATTCTTAACTTTCTGAGAAACAGGTCTCAAAGAACCAATGTGTTTTGTT
>Marker189848 Chr6 SNP_site:2512601 Ref_Type:A
AACAAATTCAAAGTTTTTAAATGAAAAAGAAGTAATCAGACAACTAATTAAAACTATCTTTTAAACATCAAACAACAACAXXXXXXXXXXT
ATGCACCCCACTTGAGTAAAAATAAAAACTTTGGTATCCAACATTTCAAAATGAACCTTATTTACTTGCAAGTTGGTGT
AACAAATTCAAAGTTTTTATATGAAAAAGAAGTAATCAGACAACTAATTAAAACTATCTTTTAAACATCAAACAACAACAXXXXXXXXXXT
ATGCACCCCACTTGAGTAAAAATAAAAACTTTGGTATCCAACATTTCAAAATGAACCTTATTTACTTGCAAGTTGGTGT
>Marker189938 Chr6 SNP_site:9066388 Ref_Type:C
AACAATCATCAAATCAAGATCAAACATTTTCTCGCGTTGCATCAAAACACCTCTTCCTTGCATTATCAAACCAAAACGCAXXXXXXXXXXA
TTAACAATGATTGGATGTATGCATGGTTTCACTTTTTGATAATAGAGGAGGAAAAGAAAAAAGAAAAACTTTGAAGGTA
AACAATCATCAAATCAAGATCAAACATTTTCTCGTGTTGCATCAAAATACCTCTTCCTTGCATTATCAAACCAAAACGCAXXXXXXXXXXA
TTAACAATGATTGGATGTATGCATGGTTTCACTTTTTGATAATAGAGGAGGAAAAGAAAAAAGAAAAACTTTGAAGGTA
>Marker189938 Chr6 SNP_site:9066401 Ref_Type:C
AACAATCATCAAATCAAGATCAAACATTTTCTCGCGTTGCATCAAAACACCTCTTCCTTGCATTATCAAACCAAAACGCAXXXXXXXXXXA
TTAACAATGATTGGATGTATGCATGGTTTCACTTTTTGATAATAGAGGAGGAAAAGAAAAAAGAAAAACTTTGAAGGTA
AACAATCATCAAATCAAGATCAAACATTTTCTCGTGTTGCATCAAAATACCTCTTCCTTGCATTATCAAACCAAAACGCAXXXXXXXXXXA
TTAACAATGATTGGATGTATGCATGGTTTCACTTTTTGATAATAGAGGAGGAAAAGAAAAAAGAAAAACTTTGAAGGTA
>Marker190056 Chr6 SNP_site:13298309 Ref_Type:C
CACCCTTGATAGAGTGCTTGGTTGAGTTGGTGTTCGCTGGTGGGTGGAAATTGGATAACGAGACATTTGGACCCGGTTATXXXXXXXXXXT
TAGAAATGTGGGGTCCATCATGCAATGGGTTTGGTTGGAACGATGAGGTGAAGTGCATGATCGCTAAGAAGGAATTGTT
CACCCTTGATAGAGTGCTTGGTTGAGTTGGTGTTTGCTGGTGGGTGGAAATTGGATAACGAGACATTTGGACCCGGTTATXXXXXXXXXXT
TAGAAATGTAGGGTCCATCATGCAATGGGTTTGGTTGGAACGATGAGGTGAAGTGCATGATCGCTAAGAAGGAATTGTT
>Marker190056 Chr6 SNP_site:13298476 Ref_Type:G
CACCCTTGATAGAGTGCTTGGTTGAGTTGGTGTTCGCTGGTGGGTGGAAATTGGATAACGAGACATTTGGACCCGGTTATXXXXXXXXXXT
TAGAAATGTGGGGTCCATCATGCAATGGGTTTGGTTGGAACGATGAGGTGAAGTGCATGATCGCTAAGAAGGAATTGTT
CACCCTTGATAGAGTGCTTGGTTGAGTTGGTGTTTGCTGGTGGGTGGAAATTGGATAACGAGACATTTGGACCCGGTTATXXXXXXXXXXT
TAGAAATGTAGGGTCCATCATGCAATGGGTTTGGTTGGAACGATGAGGTGAAGTGCATGATCGCTAAGAAGGAATTGTT
>Marker190212 Chr6 SNP_site:28563924 Ref_Type:C
CACTTCCAGCACAATATCGGACACGGACCCGCAGTTGCTCTCGCCGCTCTTAGTAGCCAGAACCCTGGTGTTATTACCATXXXXXXXXXXA
TAACTATTACTTTTTTATGTTTTGAGTCTGTGGTGTTGTGTATGTGATGAAATCTTTGATTCTTTATTTGTTTGGATGT
CACTTCCAGCACAATATCGGACACGGACCCGCAGTTGCTCTCGCCGCTCTTAGTAGCCAGAACCCTGGTGTTATTACCATXXXXXXXXXXA
TAATTATTACTTTTTTATGTTTTGAGTCTGTGGTGTTGTGTATGTGATGAAATCTTTGATTCTTTATTTGTTTGGATGT
>Marker190287 Chr6 SNP_site:1430493 Ref_Type:G
ACTAGTTTAATTATGTGAGAACAACAACAACGTTGTGAGAGAAGTATTCTATGTAATTGTAACAAATTTTTACCTATGGAXXXXXXXXXXA
GTTTGTATATGAATGTGTTTTATACCATCAAATACAAATGTTATCTTACATTTTTTCTTTATATTTGTATTAAATTGTG
ACTAGTTTAATTATGTGAGAAGAACAACAACGTTGTGAGAGAAGTATTCTATGTAATTGTAACAAATTTTTACCTATGGAXXXXXXXXXXA
GTTCGTATATGAATGTGTTTTATACCATCAAATACAAATGTTATCTTACATTTTTTCTTTATATTTGTATTAAATTGTG
>Marker190287 Chr6 SNP_site:1430664 Ref_Type:C

Create PDF with GO2PDF for free, if you wish to

ACTAGTTTAATTATGTGAGAACAACAACAACGTTGTGAGAGAAGTATTCTATGTAATTGTAACAAATTTTTACCTATGGAXXXXXXXXXXA
GTTTGTATATGAATGTGTTTTATACCATCAAATACAAATGTTATCTTACATTTTTTCTTTATATTTGTATTAAATTGTG
ACTAGTTTAATTATGTGAGAAGAACAACAACGTTGTGAGAGAAGTATTCTATGTAATTGTAACAAATTTTTACCTATGGAXXXXXXXXXXA
GTTCGTATATGAATGTGTTTTATACCATCAAATACAAATGTTATCTTACATTTTTTCTTTATATTTGTATTAAATTGTG
>Marker190420 Chr6 SNP_site:26390597 Ref_Type:A
AACGATTGAACCAAATAAAATCACAACCAAACAAAATGACAGAATTCTCCCCTGAAGAAACTGACTAAGACTTAACCTTGXXXXXXXXXXC
AAGACAAAACTTATTAGAATCCAGAAACAAAACAAAGGGAATTGAGATTGACCCTGAGATAGTGAGTATAATAAAGGGT
AACGATTGAACCAAATAAAATCACAACCAAACAAAATGACAGAATTCTCCCCTGAAGAAACTGACTAAGACTTAACCTTGXXXXXXXXXXC
AAGACAAAACTTATTAGAATCCAGAAACAAAACAAAGGGAATTGAGATTGGCCCTGAGATAGTGAGTATAATAAAGGGT
>Marker190477 Chr6 SNP_site:29015880 Ref_Type:G
AACCTATACAGCATCTGATAAACCTTATAATTTGAAAAAATCGCAGAAACGTCATGGAGAGAAAGAAAAGCGCGGATATAXXXXXXXXXXG
CGATTTGTTTCAACCAGAAATTTATTTCCATATTTGGAAAGAGGATAAGAACACACCATTTCCCATCCCAATAATTAGT
AACCTATACAGCATCTGATAAACCTTATAATTTGAAAAAATCGCAGAAACGTCATGGAGAGAAAGAAAAGCGCGGATATAXXXXXXXXXXG
CGATTTGTTTCAACCAGAAATTTATTTCCATATTTTGAAAGAGGATAAGAACACACCATTTCCCATCCCAATAATTAGT
>Marker190733 Chr6 SNP_site:3363137 Ref_Type:G
AACCCGATTTAACCCAAACTCAATTCTTTTTTCATTTTTAGAAAAATAGATTTTTTAACTAAGGTAATTAGTTTGGTAATXXXXXXXXXXA
GAGAAAAAGAAAGAGTTATTAAATTTATGTATAAAATGGGAAAGCTTGGTCCTCAAGTAATTGACATCAGTTTCTTTGT
AACCCGATTTAACCCAAACTCAATTCTTTTTTCATTTTTAGAAAAATAGATTTTTTAACTAAGGTAATTAGTTTGGTAATXXXXXXXXXXA
GAGAAAAAGAAAGAGTTATTAAATTTATGTATAAAATGGGAAAGCTTGGTCCTCAAGTAATTGACATCATTTTCTTTGT
>Marker191036 Chr6 SNP_site:13813770 Ref_Type:C
ACCCGAAAACATGAAGAAAATGCTCGACTAATGGAAGAAATGAAGAAAATGATTGAGGAATTGAGTCGGATATAGAGAAGXXXXXXXXXXG
GAACTTGGCGTTTGCGACTATTAGGCGAATTAGTTTTTAGTTTTTATTTTTCATATAATTTTTGAACCTACAAATATGT
ACCCGAAAACATGAAGAAAATGCTCGACTAATGGAAGAAATGAAGAAAATGATTGAGGAATTGAGTTGGATATAGAGAAGXXXXXXXXXXG
GAACTTGGCGTTTGCGACTATTAGGCAAATTAGTTTTTAGTTTTTATTTTTCATATAATTTTTGAACCTACAAATATGT
>Marker191036 Chr6 SNP_site:13813924 Ref_Type:G
ACCCGAAAACATGAAGAAAATGCTCGACTAATGGAAGAAATGAAGAAAATGATTGAGGAATTGAGTCGGATATAGAGAAGXXXXXXXXXXG
GAACTTGGCGTTTGCGACTATTAGGCGAATTAGTTTTTAGTTTTTATTTTTCATATAATTTTTGAACCTACAAATATGT
ACCCGAAAACATGAAGAAAATGCTCGACTAATGGAAGAAATGAAGAAAATGATTGAGGAATTGAGTTGGATATAGAGAAGXXXXXXXXXXG
GAACTTGGCGTTTGCGACTATTAGGCAAATTAGTTTTTAGTTTTTATTTTTCATATAATTTTTGAACCTACAAATATGT
>Marker191269 Chr6 SNP_site:461353 Ref_Type:T
AACCGCAGAAACAGAGCTGACTTTGTATAGACTACATAACCTCCATTGTCTAATCAACTTAAAATTTGTAACTCTGTCATXXXXXXXXXXT
ATTTCATCAAACAGAACCTGATCATAAACTAAAACCCCACCAAAATTCTTCAACTGAAACGGAGACCTCTCCTCGGGGT
AACCGCAGAAACAGAGCTGACTTTGTATAGACTACATAACCTCCATTGTTTAATCAACTTAAAATTTGTAACTCTGTCATXXXXXXXXXXT
ATTTCATCAAACAGAACCTGATCATAAACTAAAACCCCACCAAAATTCTTCAACTGAAACGGAGACCTCTCCTCGGGGT
>Marker191350 Chr6 SNP_site:5407673 Ref_Type:T
TACCAAGCTTAATAAATTCTTTTAAATAGATTTTCTTTTTCTAGAAAAAAATCATCCAACCACCCAAATGATATCACAATXXXXXXXXXXA
AAGTAAAGAGGGAAGGATCCATAGGTAATTTTATGTTTCGTTTCTTCGATTCAAAACAATAACGTTATATAACGATGTC
TACCAAGCTTAATAAATTCTTTTAAATAGATTTTCTTTTTCTAGAAAAAAATCATCCAACCACCCAAATGATATCACAATXXXXXXXXXXA
AAGTAAAGAGGGAAGGATCCATAGGTAATTTTATGTTTCGTTTCTTCGTTTTAAAACAATAACGTTATATAACGATGTC
>Marker191350 Chr6 SNP_site:5407676 Ref_Type:T
TACCAAGCTTAATAAATTCTTTTAAATAGATTTTCTTTTTCTAGAAAAAAATCATCCAACCACCCAAATGATATCACAATXXXXXXXXXXA
AAGTAAAGAGGGAAGGATCCATAGGTAATTTTATGTTTCGTTTCTTCGATTCAAAACAATAACGTTATATAACGATGTC
TACCAAGCTTAATAAATTCTTTTAAATAGATTTTCTTTTTCTAGAAAAAAATCATCCAACCACCCAAATGATATCACAATXXXXXXXXXXA
AAGTAAAGAGGGAAGGATCCATAGGTAATTTTATGTTTCGTTTCTTCGTTTTAAAACAATAACGTTATATAACGATGTC

Create PDF with GO2PDF for free, if you wish to

>Marker191847 Chr6 SNP_site:16712483 Ref_Type:A
GACAGAACAGACTGACAAACCCCTTGAAACTAAGTAATTGAAACAAAATAGAGATTGAATGTGAAATACTTTGTTGTCTTXXXXXXXXXXA
TGTTGAGAAGCCAAATCTTGTTGCCAAATTGATTACACAATTTGTTCATGAAGATTCCAGAAAAGAGGTTCAGTTTGTT
GACAGAACAGACTGACAAACCCCTTGAAACTAAGTAATTGAAACAAAATAGAGATTGAATGTGAATGACTTTGTTGTCTTXXXXXXXXXXA
TGTTGAGAAGCCAAATCTTGTTGCCAAATTGATTACACAATTTGTTCATGAAGATTCCAGAAAAGAGGTTCAGTTTGTT
>Marker191847 Chr6 SNP_site:16712484 Ref_Type:T
GACAGAACAGACTGACAAACCCCTTGAAACTAAGTAATTGAAACAAAATAGAGATTGAATGTGAAATACTTTGTTGTCTTXXXXXXXXXXA
TGTTGAGAAGCCAAATCTTGTTGCCAAATTGATTACACAATTTGTTCATGAAGATTCCAGAAAAGAGGTTCAGTTTGTT
GACAGAACAGACTGACAAACCCCTTGAAACTAAGTAATTGAAACAAAATAGAGATTGAATGTGAATGACTTTGTTGTCTTXXXXXXXXXXA
TGTTGAGAAGCCAAATCTTGTTGCCAAATTGATTACACAATTTGTTCATGAAGATTCCAGAAAAGAGGTTCAGTTTGTT
>Marker192382 Chr6 SNP_site:8863561 Ref_Type:A
ACAGTAAATACATGGTAAATGTGTATTTAATTAAATATTAGCATCTAACGTAAATAGATAAGTCTAACATGGCTCATGCTXXXXXXXXXXC
CATTCCCAGGTTACAGAAAAACAACAATCAACCGAAATGTGGAATATATCAGATCACAGGGTTAGTCAGTATTGGAAGT
ACAGTAAATACATGGTAAATGTGTATTTAATTAAATATTGGCATCTAACGTAAATAGATAAGTCTAACATGGCTCATGCTXXXXXXXXXXC
CATTCCCAGGTTACAGAAAAACAACAATCAACCGAAATGTGGAATATATCAGATCACAGAGTTAGTCAGTATTGGAAGT
>Marker192382 Chr6 SNP_site:8863771 Ref_Type:G
ACAGTAAATACATGGTAAATGTGTATTTAATTAAATATTAGCATCTAACGTAAATAGATAAGTCTAACATGGCTCATGCTXXXXXXXXXXC
CATTCCCAGGTTACAGAAAAACAACAATCAACCGAAATGTGGAATATATCAGATCACAGGGTTAGTCAGTATTGGAAGT
ACAGTAAATACATGGTAAATGTGTATTTAATTAAATATTGGCATCTAACGTAAATAGATAAGTCTAACATGGCTCATGCTXXXXXXXXXXC
CATTCCCAGGTTACAGAAAAACAACAATCAACCGAAATGTGGAATATATCAGATCACAGAGTTAGTCAGTATTGGAAGT
>Marker19259 Chr4 SNP_site:5412572 Ref_Type:C
ACTTGCTTTTCTTTGTTTCTTTGGAACCATTGTAGTATTGAATTAAACAAACAACAACTTAGATGTTTTCAAGTTTCTGCXXXXXXXXXXT
GTTTAAAGAGGGCAGTAAAAAAAACAGAACAACGATATAATGGTGTTTAAAGAGGATAGTAAAAATGAAGAAAGATGGT
ACTTGCTTTTCTTTGTTTCTTTGGAACCATTGTAGTATTGAATTAAACAAACAACAACTTAGATGTTTTCAAGTTTCTGCXXXXXXXXXXT
GTTTAAAGAGGGCAGTAAAAAAAACAGAACAATGATATAATGGTGTTTAAAGAGGATAGTAAAAAGGAAGAAAGATGGT
>Marker19259 Chr4 SNP_site:5412605 Ref_Type:T
ACTTGCTTTTCTTTGTTTCTTTGGAACCATTGTAGTATTGAATTAAACAAACAACAACTTAGATGTTTTCAAGTTTCTGCXXXXXXXXXXT
GTTTAAAGAGGGCAGTAAAAAAAACAGAACAACGATATAATGGTGTTTAAAGAGGATAGTAAAAATGAAGAAAGATGGT
ACTTGCTTTTCTTTGTTTCTTTGGAACCATTGTAGTATTGAATTAAACAAACAACAACTTAGATGTTTTCAAGTTTCTGCXXXXXXXXXXT
GTTTAAAGAGGGCAGTAAAAAAAACAGAACAATGATATAATGGTGTTTAAAGAGGATAGTAAAAAGGAAGAAAGATGGT
>Marker192686 Chr6 SNP_site:24852803 Ref_Type:C
AACTAAGCAATCACTCTTCTAATTCTATAAATATATTAGTCCTCTCTCTATTTAATTTGTTCTATAACAAACAATAATAAXXXXXXXXXXT
CTACACATCAAAATTTGTTCAACGCCATGAGTTATATTATGTAAATGAATGATAATGTTATCGTTTTAAATTTAAGGTC
AACTAAGCAATCACTCTTTTAATTCTATAAATATATTAGTCCTCTCTCTATTTAATTTGTTCTATAACAAACAATAATAAXXXXXXXXXXT
CTACACATCAAAATTTGTTCAACGCCATGAGTTATATTATGTAAATGAATGATAATGTTATAGTTTTAAATTTAAGGTC
>Marker192686 Chr6 SNP_site:24853060 Ref_Type:C
AACTAAGCAATCACTCTTCTAATTCTATAAATATATTAGTCCTCTCTCTATTTAATTTGTTCTATAACAAACAATAATAAXXXXXXXXXXT
CTACACATCAAAATTTGTTCAACGCCATGAGTTATATTATGTAAATGAATGATAATGTTATCGTTTTAAATTTAAGGTC
AACTAAGCAATCACTCTTTTAATTCTATAAATATATTAGTCCTCTCTCTATTTAATTTGTTCTATAACAAACAATAATAAXXXXXXXXXXT
CTACACATCAAAATTTGTTCAACGCCATGAGTTATATTATGTAAATGAATGATAATGTTATAGTTTTAAATTTAAGGTC
>Marker192942 Chr6 SNP_site:3051076 Ref_Type:A
AACTGCCAACAGAATGAGTTGTGTGCATTGGAGCTGTTGAACTGATTTTCTGCAGTTGGTCCCTTCACAGCCCCATGGTGXXXXXXXXXXT
TGCTACTGGCTCTTTGTTCAGTGTTGTCCTCTTCTTCCTCTAGGTTTCGTTGGGGATATCTTCATTTTAATTTGGGGTG
AACTGCCAACAGGATGAGTTGTGTGCATTGGAGCTGTTGAACTGATTTTCTGCAGTTGGTCCCTTCACAGCCCCGTGGTGXXXXXXXXXXT

Create PDF with GO2PDF for free, if you wish to

TGCTACTGGCTCTTTGTTCAGTGTTGTCCTCTTCTTCCTCTAGGTTTCGTTGGGGATATCTTCATTTTAATTTGGGGTG

>Marker192942 Chr6 SNP_site:3051138 Ref_Type:A

AACTGCCAACAGAATGAGTTGTGTGCATTGGAGCTGTTGAACTGATTTTCTGCAGTTGGTCCCTTCACAGCCCCATGGTGXXXXXXXXXXT

TGCTACTGGCTCTTTGTTCAGTGTTGTCCTCTTCTTCCTCTAGGTTTCGTTGGGGATATCTTCATTTTAATTTGGGGTG

AACTGCCAACAGGATGAGTTGTGTGCATTGGAGCTGTTGAACTGATTTTCTGCAGTTGGTCCCTTCACAGCCCCGTGGTGXXXXXXXXXXT

TGCTACTGGCTCTTTGTTCAGTGTTGTCCTCTTCTTCCTCTAGGTTTCGTTGGGGATATCTTCATTTTAATTTGGGGTG

>Marker193059 Chr6 SNP_site:9286406 Ref_Type:C

ACTTCTTTTCCTATTGTCACTCTAAACACACCATATGATTGTCTCCTTCTCTTTACATAAAACTACAAGTATATATAAAAXXXXXXXXXXC

CAAGAACTAAATATACCAATGCAAATGATGCTAATGGGAATTGACAATAATTCAAGTTGTTAGATATTAGCGTTATGTC

ACTTCTTTTCCTATTGTCACTCTAAACACACCATATGATTGTCTCCTTCTCTTTACATAAAACTACAAGTATATCTAAAAXXXXXXXXXXC

CAAGAACTAAATATACCAATGCAAATGATGCTAATGGGAATTGACAATAATTCAAGTTGTTAGATATTAGCGTTATGTC

>Marker193227 Chr6 SNP_site:28249983 Ref_Type:T

ACTTATAACAGTCGATGTCGCAACAGCATGTAGTCATAGCATTAATGATTTTTTCCCCCTTTTTGATTTGAATTACCAAAXXXXXXXXXXA

AACAGCACAAGATTCAAACTGCAAACTTTGTGGCAAAGTTTATCATAAAACAAAAGAAACAAATAATTTTACCCTGAGT

ACTTATAACAGTCGATGTCGCAACAGCATGTAGTCATAGCATTAATGATTTTTTCCCCCTTTTTTATTTGAATTACCAAAXXXXXXXXXXA

AACAGCACAAGATTCAAACTGCAAACTTTGTGGCAAAGTTTATCATAAAACAAAAGAAACAAATAATTTTACCCTGAGT

>Marker193495 Chr6 SNP_site:13651413 Ref_Type:A

ACTATGTTTTATCTTGGAAAACTACTGCTCCTAAAATTAGTCACTGGACCGAAAAAAATTGTCATAAGAAGACAGATAGTXXXXXXXXXXC

TGTCGAGCTCCTTATAAACGTCGGACTCCAAAATATCGGAAGTGAGTTTTCGCCATGAAATGTTCTTCTTCTCCGCTGT

ACTATGTTTTATCTTGGAAAACTACTGCTCCTGAAATTAGTCACTGGACCGAAAAAAATTGTCATAAGAAGACAGATAGTXXXXXXXXXXC

TGTCGAGCTCCTTATAAACGTCGGACTCCAAAATATCGGAAGTGAGTTTTCGCCATGAAATGTTCTTCTTCTCCGCTGT

>Marker193566 Chr6 SNP_site:15649565 Ref_Type:T

ACTTTTCTATTATAATAATTAATGATATAAAGTGATGAAACCTTCCACCACCAGAGAAGAATATTATATTATATGTCACTXXXXXXXXXXA

GTTTATTTACTTTTCATTAATTTCTTACTTTCTTCATCTGTAATTTTTCTTTCATTAACCTAAAGATAATAATAATAGT

ACTTTTCTATTATAATAATTAATGATATAAAGTGATGAAACCTTCTACCACCAGAGAAGAATATTATATTATATGTCACTXXXXXXXXXXA

GTTTATTTACTTTTCATTAGTTTCTTACTTTCTTCATCTGTAATTTTTCTTTCATTAACCCAAAGATAATAATAATAGT

>Marker193566 Chr6 SNP_site:15649741 Ref_Type:G

ACTTTTCTATTATAATAATTAATGATATAAAGTGATGAAACCTTCCACCACCAGAGAAGAATATTATATTATATGTCACTXXXXXXXXXXA

GTTTATTTACTTTTCATTAATTTCTTACTTTCTTCATCTGTAATTTTTCTTTCATTAACCTAAAGATAATAATAATAGT

ACTTTTCTATTATAATAATTAATGATATAAAGTGATGAAACCTTCTACCACCAGAGAAGAATATTATATTATATGTCACTXXXXXXXXXXA

GTTTATTTACTTTTCATTAGTTTCTTACTTTCTTCATCTGTAATTTTTCTTTCATTAACCCAAAGATAATAATAATAGT

>Marker193566 Chr6 SNP_site:15649782 Ref_Type:C

ACTTTTCTATTATAATAATTAATGATATAAAGTGATGAAACCTTCCACCACCAGAGAAGAATATTATATTATATGTCACTXXXXXXXXXXA

GTTTATTTACTTTTCATTAATTTCTTACTTTCTTCATCTGTAATTTTTCTTTCATTAACCTAAAGATAATAATAATAGT

ACTTTTCTATTATAATAATTAATGATATAAAGTGATGAAACCTTCTACCACCAGAGAAGAATATTATATTATATGTCACTXXXXXXXXXXA

GTTTATTTACTTTTCATTAGTTTCTTACTTTCTTCATCTGTAATTTTTCTTTCATTAACCCAAAGATAATAATAATAGT

>Marker193617 Chr6 SNP_site:9081290 Ref_Type:G

GACATGCCTAGTGGAAATGTTTGTGTAGAATGATGCAAAATCACATTTTTTCTTATTTAGGTGGTTGCTTTTTTGTGTTTXXXXXXXXXXT

ACATATCTCTCTCTGATTGAACAAAAATATTGTAGTTTATTTTAAACCTAAGTGTCTTCTAGAAGAGTTGTTTAAAGTT

GACATGCCTAGTGGAAGTGTTTGTGTAGAATGATGCAAAATCACATTTTTTCTTATTTAGGTGGTTGCTTTTTTGTGTTTXXXXXXXXXXT

ACATATCTCTCTCTGATTGAACAAAAATATTGTAGTTTATTTTAAACCTAAGTGTCTTCTAGAAGAGTTGTTTAAAGTT

>Marker193623 Chr6 SNP_site:1173108 Ref_Type:T

AACCCTTTAAGTTTGATTCTATGAAAAATGCTTACATGGTTTCCTGAACAGTTATCATATTATCATTCGGACTAAAATATXXXXXXXXXXT

TAAGGACTATATATTTTATAGGCATGGTATAAAAGAGTTTTGCTATCTCATTTTGTGTTTATCCTTTTCACTTTTGGTT

Create PDF with GO2PDF for free, if you wish to

AACCCTTTAAGTTTGATTCTATGAAAAATGCTTACATGGTTTCCTGAACAGTTATCATATTATCATTTGGACTAAAATATXXXXXXXXXXT
TAAGGACTATATATTTTATAGGCATGGTATAAAAGAGTTTTGCTATCTCATTTTGTGTTTATCCTTTTCACTTTTGGTT
>Marker194300 Chr6 SNP_site:5282033 Ref_Type:C
ACAAACTTTCATAAACCGATCATTCTCACAATCCGAATGAAAAAAAGCTAAACTATAAATAAAGAAACTAGAAAGCCAAAXXXXXXXXXXT
CTAGGTTTTGATCTTGATGGGTCTCTGTCAAATGGGAATTCGAAGGCTCGGAGCCATTAATGGGTTTGTTGTTGATGTT
ACAAACTTTCATAAACCGATCATTCTCACAATCCGAATGAAAAAAAGCTAAACTATAAATAACGAAACTAGAAAGCCAAAXXXXXXXXXXT
CTAGGTTTTGATCTTGATGGGTCTCTGTCAAATGGGAATTCGAAGGCTCAGAGCCATTAATGGGTTTGTTGTTGATGTT
>Marker194300 Chr6 SNP_site:5282260 Ref_Type:A
ACAAACTTTCATAAACCGATCATTCTCACAATCCGAATGAAAAAAAGCTAAACTATAAATAAAGAAACTAGAAAGCCAAAXXXXXXXXXXT
CTAGGTTTTGATCTTGATGGGTCTCTGTCAAATGGGAATTCGAAGGCTCGGAGCCATTAATGGGTTTGTTGTTGATGTT
ACAAACTTTCATAAACCGATCATTCTCACAATCCGAATGAAAAAAAGCTAAACTATAAATAACGAAACTAGAAAGCCAAAXXXXXXXXXXT
CTAGGTTTTGATCTTGATGGGTCTCTGTCAAATGGGAATTCGAAGGCTCAGAGCCATTAATGGGTTTGTTGTTGATGTT
>Marker194505 Chr6 SNP_site:3897480 Ref_Type:T
AACCCACATAACACCTAAATATCACAGATTCAAATACGAGAATGAAAAACGATAAGACACGACACAGGCATGGCTACAAGXXXXXXXXXXA
ATGAGCTTGTGCATTTACATCATTAAAATTCATGATGCGTTTCGCTCTCATAATTGATTATTTTCGTCCCATATGTGTC
AACCCACATAACACCTAAATATCACAGATTCAAATACGAGAATGAAAAACGATATGACACGACACAGGCATGGCTACAAGXXXXXXXXXXA
ATGAGCTTGTGCATTTACATCATTAAAATTCATGATGTGTTTCGCTCTCATAATTGATTATTTTTGTCCCATATGTGTC
>Marker194505 Chr6 SNP_site:3897632 Ref_Type:T
AACCCACATAACACCTAAATATCACAGATTCAAATACGAGAATGAAAAACGATAAGACACGACACAGGCATGGCTACAAGXXXXXXXXXXA
ATGAGCTTGTGCATTTACATCATTAAAATTCATGATGCGTTTCGCTCTCATAATTGATTATTTTCGTCCCATATGTGTC
AACCCACATAACACCTAAATATCACAGATTCAAATACGAGAATGAAAAACGATATGACACGACACAGGCATGGCTACAAGXXXXXXXXXXA
ATGAGCTTGTGCATTTACATCATTAAAATTCATGATGTGTTTCGCTCTCATAATTGATTATTTTTGTCCCATATGTGTC
>Marker194505 Chr6 SNP_site:3897659 Ref_Type:T
AACCCACATAACACCTAAATATCACAGATTCAAATACGAGAATGAAAAACGATAAGACACGACACAGGCATGGCTACAAGXXXXXXXXXXA
ATGAGCTTGTGCATTTACATCATTAAAATTCATGATGCGTTTCGCTCTCATAATTGATTATTTTCGTCCCATATGTGTC
AACCCACATAACACCTAAATATCACAGATTCAAATACGAGAATGAAAAACGATATGACACGACACAGGCATGGCTACAAGXXXXXXXXXXA
ATGAGCTTGTGCATTTACATCATTAAAATTCATGATGTGTTTCGCTCTCATAATTGATTATTTTTGTCCCATATGTGTC
>Marker194651 Chr6 SNP_site:22589798 Ref_Type:T
CACTTGGACTTTGTTATTGTATGATGGGAGTTTGTTTAACTTGCCATACTACTTAGTTCATGATTTTGTTTTGTTTTGTTXXXXXXXXXXA
TGAGGGTTGCCGTTGGTATTCACAAAGATGATATTGATTCTGCTATTAAAACTTATCACACAATGTCACAACGTTGGTT
CACTTGGACTTTGTTATTGTATGATGGGAGTTTGTTTAACTTGCCATACTACTTAGTTCATGATTTTGTTTTGTTTTTCTXXXXXXXXXXA
TGAGGGTTGCCGTTGGTATTCACAAAGATGATATTGATTCTGCTATTAAAACTTATCACACAATGTCACAACGTTGGTT
>Marker194651 Chr6 SNP_site:22589799 Ref_Type:C
CACTTGGACTTTGTTATTGTATGATGGGAGTTTGTTTAACTTGCCATACTACTTAGTTCATGATTTTGTTTTGTTTTGTTXXXXXXXXXXA
TGAGGGTTGCCGTTGGTATTCACAAAGATGATATTGATTCTGCTATTAAAACTTATCACACAATGTCACAACGTTGGTT
CACTTGGACTTTGTTATTGTATGATGGGAGTTTGTTTAACTTGCCATACTACTTAGTTCATGATTTTGTTTTGTTTTTCTXXXXXXXXXXA
TGAGGGTTGCCGTTGGTATTCACAAAGATGATATTGATTCTGCTATTAAAACTTATCACACAATGTCACAACGTTGGTT
>Marker194920 Chr6 SNP_site:3934357 Ref_Type:G
TACTTTCAAATTGAAGAAGAAAAACCTGAAAATATAGCTGTCATTCTACACCAAACAACAAATTATTTCCATTTAAAAAAXXXXXXXXXXT
ATAACATCTGCCTCCATTACAGCGTAGCTCTATTATATGACATGATCTGAATGTTTCCAGGGTCCATTGATATATTAGT
TACTTTCAAATTGAAGAAGAAAAACCTGAAAATATAGCTGTCATTCTACACCAAACAACAAATTATTTCCATTTAAAAAAXXXXXXXXXXT
ATAACATCTGCCTCCATTACAGCGTAGCTCTGTTATATGACATGATCTGAATGTTTCCAGGGTCCATTGATATATTAGT
>Marker194932 Chr6 SNP_site:7927434 Ref_Type:A
GACAGCTTATGGCACAAAGAGAGGTTCAGTCTGAGGCTCTAGGCAATAATCACCAACAAGTTCAAAACTCTGATGTGCGTXXXXXXXXXXA

Create PDF with GO2PDF for free, if you wish to

ATGGGATAGTCATCCTACAGATGATGTGAAGGAATTAACCGATTCTAAAAGGGAACTAACGCATCATTAGCCACTTGTC

GACAGCTTATGGCACAAAGAGAGGTTCAGTCTGAGGCTCTAGGCAATAATCACCAACAAGTTCAAAACTCTGATGTGCGTXXXXXXXXXXA

ATGGGATAGTCATCCTACAGATGATGTGAAGGAATTAACCGATTCTAAAAGGGAACTAACGCGTCATTAGCCACTTGTC

>Marker195333 Chr6 SNP_site:19066059 Ref_Type:G

ACAAAGGATAATAAGTAGGGAGAAGAAGTTATCTCATTGTATATGACTTTTGGTTCTACTTTACTGGTGATAAGAAGGAAXXXXXXXXXXT

TTCCTTGTGAATGATGAGGCTCAAGTTCTCTTTTTTTAATGTCATGATCAAATCTAACATTTCAAACGAACGATCAAGT

ACAAAGGATAATAAGTAGGGAGAAGAAGTTATCTCATTGTATATGACTTTTGGTTCTACTTTACTGGTGATAAGAAGGAAXXXXXXXXXXT

TTCCTTGTGAATGATGAGGCTCAAGTTCTCTTTTTTTAATGTCATGATCAAATCTAACGTTTCAAACGAACCATCAAGT

>Marker195333 Chr6 SNP_site:19066072 Ref_Type:C

ACAAAGGATAATAAGTAGGGAGAAGAAGTTATCTCATTGTATATGACTTTTGGTTCTACTTTACTGGTGATAAGAAGGAAXXXXXXXXXXT

TTCCTTGTGAATGATGAGGCTCAAGTTCTCTTTTTTTAATGTCATGATCAAATCTAACATTTCAAACGAACGATCAAGT

ACAAAGGATAATAAGTAGGGAGAAGAAGTTATCTCATTGTATATGACTTTTGGTTCTACTTTACTGGTGATAAGAAGGAAXXXXXXXXXXT

TTCCTTGTGAATGATGAGGCTCAAGTTCTCTTTTTTTAATGTCATGATCAAATCTAACGTTTCAAACGAACCATCAAGT

>Marker195832 Chr6 SNP_site:2049155 Ref_Type:A

TACACAATGTTCAAAAAAGAAAAAAAAAAAAAAGAAAAAGAGGAAACTCACAAACCATAGCGAATGTCACTAAATTTAGTXXXXXXXXXXT

GCTTTCGCAGTCGGACCGTTGAAATTGTAAGAGTTAGATCGAGTTTGGAAACCGAACTCACCCGAGGTTGAGGAGGAGT

TACACAATGTTCAAAAAAGAAAAAAAAGAAAAAGAAAAAGAGGAAACTCACAAACCATAGCGAATGTCACTAAATTTAGTXXXXXXXXXXT

GCTTTCGCAGTCGGACCGTTGAAATTGTAAGAGTTAGATCGAGTTTGGAAACCGAACTCACCCGAGGTTGAGGAGGAGT

>Marker195883 Chr6 SNP_site:17127383 Ref_Type:T

AACTCCTGCCTATGAAGGTGATCATTTGATTTGCATGGGTGAGAGTGGCCTATGTCGCAGACTCAATATTTCTACCATTTXXXXXXXXXXG

CGCCACACCCTCTCTTGACCCTAGAGGAGTTCAGTTGCAACGGAACTATAACTTATTGTTCATTAGAGGAACCAACAGT

AACTCCTGCCTATGAAGGTGATCATTTGATTTGCATGGGTGAGAGTGGCCTATGTCGCAGACTCAATATTTCTACCATTTXXXXXXXXXXG

TGCCACACCCTCTCTTGACCCTAGAGGAGTTCAGTTACAACGGAACTATAACTTATTGTTCATTAGAGGAACCAACAGT

>Marker195883 Chr6 SNP_site:17127419 Ref_Type:A

AACTCCTGCCTATGAAGGTGATCATTTGATTTGCATGGGTGAGAGTGGCCTATGTCGCAGACTCAATATTTCTACCATTTXXXXXXXXXXG

CGCCACACCCTCTCTTGACCCTAGAGGAGTTCAGTTGCAACGGAACTATAACTTATTGTTCATTAGAGGAACCAACAGT

AACTCCTGCCTATGAAGGTGATCATTTGATTTGCATGGGTGAGAGTGGCCTATGTCGCAGACTCAATATTTCTACCATTTXXXXXXXXXXG

TGCCACACCCTCTCTTGACCCTAGAGGAGTTCAGTTACAACGGAACTATAACTTATTGTTCATTAGAGGAACCAACAGT

>Marker19633 Chr4 SNP_site:21623706 Ref_Type:T

ACTGCTCCTTGGTAAAACTATAGCTACTGAATCAGAATATTCCTTTCAGAACTGTTGGAGAGTTCTTATATGGTCTATATXXXXXXXXXXT

ATGTATTATATCTATCTTCTTTTCCATTAGCTACTTACCTCCTAGTCATCTTACTTCTAATAATAAGATATTTCAGAGT

ACTGCTCCTTGGTAAAACTATAGCTACTGAATCAGAATATTCCTTTCAGAACTGTTGGAGAGTTCTTATATGTTCTATATXXXXXXXXXXT

ATGTATTATATCTATCTTCTTTTCCATTAGCTACTTACCTCCTAGTCATCTTACTTCTAATAATAAGATATTTCAGAGT

>Marker196412 Chr6 SNP_site:18469997 Ref_Type:C

GACCTACACTCCTATTATTTATACGTATTTTGATTAAATTGGGTCGAATTTAAGTGAAGAACTTCCTCCAACTTACTACTXXXXXXXXXXA

TTTTACAATATATCTCTAGCGATAATATCGATAAATACACACAACAACAATGATGATGTTGGTATAAATAAGTTGTGTG

GACCTACACTCGTATTATTTATACGTATTTTGATTAAATTGGGTCGAATTTAAGTGAAGAACTTCCTCCAACTTACTACTXXXXXXXXXXA

TTTTACAATATATCTCTAGCGATAATATCGATAAATACACACAACAACAATGATGATGTTGGTATAAATAAGTTGTGTG

>Marker196480 Chr6 SNP_site:3452507 Ref_Type:G

ACCTTTTAATAAGAATGCAGTAAATGAGCTGCAAACCCAAAAACTAATGAACAGGGATATAACATCGTTAAAAAATGAGAXXXXXXXXXXA

TTTTTCAACTTTTTTTAGAGTCGATTGGAGTAATTTCTTAATTAACATGACCTTCTGTATATATGCTTGGCAAAACAGT

ACCTTTTAATAAGAATGCAGTAAATGAGCTGCAAAGCCAAAAACTAATGAACAGGGATATAACATCGTTAAAAAATGAAAXXXXXXXXXXA

TTTTTCAACTTTTTTTAGAGTCGATTGGAGTAATTTCTTAATTAACATGACCTTTTGTATATATGCTTGGCAAAACAGT

>Marker196480 Chr6 SNP_site:3452550 Ref_Type:A

Create PDF with GO2PDF for free, if you wish to

ACCTTTTAATAAGAATGCAGTAAATGAGCTGCAAACCCAAAAACTAATGAACAGGGATATAACATCGTTAAAAAATGAGAXXXXXXXXXXXA
TTTTTCAACTTTTTTTAGAGTCGATTGGAGTAATTTCTTAATTAACATGACCTTCTGTATATATGCTTGGCAAAACAGT
ACCTTTTAATAAGAATGCAGTAAATGAGCTGCAAAGCCAAAAACTAATGAACAGGGATATAACATCGTTAAAAAATGAAAXXXXXXXXXXXA
TTTTTCAACTTTTTTTAGAGTCGATTGGAGTAATTTCTTAATTAACATGACCTTTTGTATATATGCTTGGCAAAACAGT
>Marker196480 Chr6 SNP_site:3452753 Ref_Type:T
ACCTTTTAATAAGAATGCAGTAAATGAGCTGCAAACCCAAAAACTAATGAACAGGGATATAACATCGTTAAAAAATGAGAXXXXXXXXXXXA
TTTTTCAACTTTTTTTAGAGTCGATTGGAGTAATTTCTTAATTAACATGACCTTCTGTATATATGCTTGGCAAAACAGT
ACCTTTTAATAAGAATGCAGTAAATGAGCTGCAAAGCCAAAAACTAATGAACAGGGATATAACATCGTTAAAAAATGAAAXXXXXXXXXXXA
TTTTTCAACTTTTTTTAGAGTCGATTGGAGTAATTTCTTAATTAACATGACCTTTTGTATATATGCTTGGCAAAACAGT
>Marker196495 Chr6 SNP_site:12992596 Ref_Type:G
AACGCTTATTTTCTCCATTAAAATGTCATTTCCATTGCTCTATCTCGGGGTTATTTAAATTTCAAATGCACTTCTAATTAXXXXXXXXXXXA
TTAAAGCAGGGAATGAAAATAATATTTTAATCTTACTAGCTACTTATTCATATTTTATTTTGATCATATAATCAATGTC
AACGCTTATTTTCTCCATTAAAATGTCATTTCCATTGCTCTATCTCGGGGTTATTTAAATTTCAAATGCACTTCTAATTAXXXXXXXXXXXA
TTAAAGCAGGGAATGAAAATAATATTTTAATCTTACTAGCTACTTATTCATATTTTATTTTTATCATATAATCAATGTC
>Marker19658 Chr4 SNP_site:10851291 Ref_Type:T
ACATTTGACTTTGTTTTCAGTGTTCTTCAATGTTTAATTCTTGTTTCATATTTAAATCAGGATTTCATATTGAGGGGAGGXXXXXXXXXXXC
TAGATGGGATGACACTATTAATTAATCCATAGATTGATTGATCTTTCGTTTCGTCGTAGGAATTAAAAAACATAAAGTT
ACATTTGACTTTGTTTTCAGTGTTCTTCAATGTTTAATTCTTGTTTCATATTTAAATCAGGATTTCATATTGAGGGGAGGXXXXXXXXXXXC
TAGATGGGATGACACTATTAATTAATCCATAGATTGATTGATCTTTCGTTTCGTTGTAGGAATTAAAAAACATAAAGTT
>Marker196811 Chr6 SNP_site:12818649 Ref_Type:T
TACGACTAAATGAAGAAGAACCAGTATGTTAAGCTTGCAGACCTGCACCATTATCAACTTTGCTGTTATTGCCTCCATTGXXXXXXXXXXXA
TTTCAAGAAGTAAAGTTATTTCCATTCTGATACCCCATTATAAGGATAGTAACACCAAATAGCTGTGTGACCAAATGTC
TACGACTAAATGAAGAAGAACCAGTATTTTAAGCTTGCAGACCTGCACCATTATCAACTTTGCTGTTATTGCCTCCATTGXXXXXXXXXXXA
TTTCAAGAAGTAAAGTTATTTCCATTCTGATACCCCATTATAAGGATAGTAACACCAAATAGCTGTGTGACCAAATGTC
>Marker19684 Chr4 SNP_site:8112267 Ref_Type:T
TACATATAGAATCGTAAAATACTCTATTACCCTTAATTTTATATCCTTTCACATGTTTCATCAGTTACTTGAACCACAAAXXXXXXXXXXXG
GTAAATGTTGTTATAGATTTGGCTGCACTCGTAACAGATCAAATGCTTATACACAGATGCTCATATTTTGTGATTTGTG
TACATATAGAATCGTAAAATACTCTATTACCCTTAATTTTATATCCTTTCACATGTTTCATCATTTACTTGAACCACAAAXXXXXXXXXXXG
GTAAATGTTGTTATAGATTTGGCTGCACTCGTAACAGATCAGATGCTTATACACAGATGCTCATATTTTGTGATTTGTG
>Marker19684 Chr4 SNP_site:8112425 Ref_Type:G
TACATATAGAATCGTAAAATACTCTATTACCCTTAATTTTATATCCTTTCACATGTTTCATCAGTTACTTGAACCACAAAXXXXXXXXXXXG
GTAAATGTTGTTATAGATTTGGCTGCACTCGTAACAGATCAAATGCTTATACACAGATGCTCATATTTTGTGATTTGTG
TACATATAGAATCGTAAAATACTCTATTACCCTTAATTTTATATCCTTTCACATGTTTCATCATTTACTTGAACCACAAAXXXXXXXXXXXG
GTAAATGTTGTTATAGATTTGGCTGCACTCGTAACAGATCAGATGCTTATACACAGATGCTCATATTTTGTGATTTGTG
>Marker196896 Chr6 SNP_site:26467715 Ref_Type:T
ACCCTTACTGTTTTAGTGCAATTGAAGAGAGATATTTGAATTACAAATTTTATGATTATACATATAGTTATATACTCATTXXXXXXXXXXXT
CAAAGTATTCCCAAGTATGTGTTTTACTTCTAAAACTACTGTCAATCAAAATCAACGTGCGCATGCATCAACCATAGTC
ACCCTTACTGTTTTAGTGCAATTGAAGAGAGATATTTGAATTACAAATTTTATGATTATACATATAGTTATATACTCATTXXXXXXXXXXXT
CAAAGTATTCCCAAGTATGTGTTTTACTTCTAAAATTACTGTCAATCAAAATCAACGTGCGCATGCATCAACCATAGTC
>Marker197111 Chr6 SNP_site:15644303 Ref_Type:G
ACTTGAAAGAGTGTAGCAATAGAAGAAAGCACCAAAATTATATTTAGCCAATTTTGCCAGTGAGTCCTAGCAATAAAATTXXXXXXXXXXXA
AGTTAAAATAACTTTCAAAAGAATAAATTTATCTGTAACGATAGAGTTTATTTCATGTTGTATTAAAAATTAGAATGGT
ACTTGAAAGAGTGTAGCAATATAAGAAAGCACCAAAATTATATTTAGCCAATTTTGCCAGTGAGTCCTAGCACTAAAATTXXXXXXXXXXXA
AGTTAAAATAACTTTCAAAAGAATAAATTTATCTGTAACGATAGAGTTTATTTCATGTTGTATTAAAAATTAGAATGGT

Create PDF with GO2PDF for free, if you wish to

>Marker197111 Chr6 SNP_site:15644354 Ref_Type:A
ACTTGAAAGAGTGTAGCAATAGAAGAAAGCACCAAAATTATATTTAGCCAATTTTGCCAGTGAGTCCTAGCAATAAAATTXXXXXXXXXXA
AGTTAAAATAACTTTCAAAAGAATAAATTTATCTGTAACGATAGAGTTTATTTCATGTTGTATTAAAAATTAGAATGGT
ACTTGAAAGAGTGTAGCAATATAAGAAAGCACCAAAATTATATTTAGCCAATTTTGCCAGTGAGTCCTAGCACTAAAATTXXXXXXXXXXA
AGTTAAAATAACTTTCAAAAGAATAAATTTATCTGTAACGATAGAGTTTATTTCATGTTGTATTAAAAATTAGAATGGT
>Marker198038 Chr6 SNP_site:19037172 Ref_Type:C
CACTTTCTGTAATCTCATATAGCTTTCACAATGGTGGGCAGCCCTCACTTTTCTCTATTGTTTGTATCTTTTATGGACAGXXXXXXXXXXC
CTATGGATGTCAAATGAATATTAATGATATGGAGGTAGTTCTATCTATCATGAAAAAAGCTGGATACAGTGAAACTGTT
CACTTTCTGTAATCTCATATAGCTTTCACAATGGTGGGCAGCCCTCACTTTTCTCTATTGTTTGTATCTTTTATGGAGAGXXXXXXXXXXC
CTATGGATGTCAAATGAATATTAATGATATGGAGGTAGTTCTATCTATCATGAAAAAAGCTGGATACAGTGAAACTGTT
>Marker198455 Chr6 SNP_site:7335253 Ref_Type:C
CACTCATAGTTAATTCATTAATGATAGTTATTATCATTGATAAGACATGCATTAAAATTTTCAAACTTTAATAGATAATAXXXXXXXXXXT
TTTTGAAATGGTTTTAAGGGTATATGTTGTTTAATAAACAAAACTTTAATGTAACAATTAAAAAAGAACATAGTTAGTA
CACTCATAGTTAATTCATTAATGATAGTTATTATCATTGATAAGACATGTATTAAAATTTTCAAACTTTAATAGATAATAXXXXXXXXXXT
TTTTGAAATGGTTTTAAGGCTATATGTTGTTTAATAAACAAAACTTTAATGTAACAATTAAAAAAGAACATAGTTAGTA
>Marker198455 Chr6 SNP_site:7335408 Ref_Type:G
CACTCATAGTTAATTCATTAATGATAGTTATTATCATTGATAAGACATGCATTAAAATTTTCAAACTTTAATAGATAATAXXXXXXXXXXT
TTTTGAAATGGTTTTAAGGGTATATGTTGTTTAATAAACAAAACTTTAATGTAACAATTAAAAAAGAACATAGTTAGTA
CACTCATAGTTAATTCATTAATGATAGTTATTATCATTGATAAGACATGTATTAAAATTTTCAAACTTTAATAGATAATAXXXXXXXXXXT
TTTTGAAATGGTTTTAAGGCTATATGTTGTTTAATAAACAAAACTTTAATGTAACAATTAAAAAAGAACATAGTTAGTA
>Marker198515 Chr6 SNP_site:27552356 Ref_Type:G
AACAACAGTATCCAAATTCAGCTTCTTTTTTTTCTTAATTCAATAAGCATGGAGGAAAGAGAAAAAAAAAACCTTTTAATXXXXXXXXXXC
AGATACAAAGCACATATCATTTTAATTATACTCCCCCATTCTTCATATATTTGCTTAGAGAAGTGTTGAATTGAAAGTA
AACAACAGTATCCAAATTCAGCTTCTTTTTTTTCTTAATTCAATAAGCATGGAGGAAAGATAAAAAAAAAACCTTTTAATXXXXXXXXXXC
AGATACAAAGCACACATCATTTTAATTATACTCCCCCATTCTTCATATATTTGCTTAGAGAAGTGTTGAACTGAAAGTA
>Marker198515 Chr6 SNP_site:27552494 Ref_Type:T
AACAACAGTATCCAAATTCAGCTTCTTTTTTTTCTTAATTCAATAAGCATGGAGGAAAGAGAAAAAAAAAACCTTTTAATXXXXXXXXXXC
AGATACAAAGCACATATCATTTTAATTATACTCCCCCATTCTTCATATATTTGCTTAGAGAAGTGTTGAATTGAAAGTA
AACAACAGTATCCAAATTCAGCTTCTTTTTTTTCTTAATTCAATAAGCATGGAGGAAAGATAAAAAAAAAACCTTTTAATXXXXXXXXXXC
AGATACAAAGCACACATCATTTTAATTATACTCCCCCATTCTTCATATATTTGCTTAGAGAAGTGTTGAACTGAAAGTA
>Marker198515 Chr6 SNP_site:27552550 Ref_Type:T
AACAACAGTATCCAAATTCAGCTTCTTTTTTTTCTTAATTCAATAAGCATGGAGGAAAGAGAAAAAAAAAACCTTTTAATXXXXXXXXXXC
AGATACAAAGCACATATCATTTTAATTATACTCCCCCATTCTTCATATATTTGCTTAGAGAAGTGTTGAATTGAAAGTA
AACAACAGTATCCAAATTCAGCTTCTTTTTTTTCTTAATTCAATAAGCATGGAGGAAAGATAAAAAAAAAACCTTTTAATXXXXXXXXXXC
AGATACAAAGCACACATCATTTTAATTATACTCCCCCATTCTTCATATATTTGCTTAGAGAAGTGTTGAACTGAAAGTA
>Marker199175 Chr6 SNP_site:14043984 Ref_Type:A
ACACTGAGGGGCACTTTTATCACACTTTATTCACAAAATTATCTAAGTCATGAAAAATTACAGTTTTGTAGTCTATGTTGXXXXXXXXXXA
AATACTTCCTTGGGATGGAAATTGTCATATTTAGAGAGGGTGTCTCCGTATCCCAAAGAAAATACATAATTGGTTTGTT
ACACTGAGGGGCACTTTTATCACACTTTATTCACAAAATTATCTAAGTCATGAAAAATTACCGTTTTGTAGTCTATGATGXXXXXXXXXXA
AATACTTCCTTGGGATGGAAATTGTCATATTTAGAGAGGGTGTCTCCGTATCCCAAAGAAAATACATAATTGGTTTGTT
>Marker199175 Chr6 SNP_site:14044000 Ref_Type:T
ACACTGAGGGGCACTTTTATCACACTTTATTCACAAAATTATCTAAGTCATGAAAAATTACAGTTTTGTAGTCTATGTTGXXXXXXXXXXA
AATACTTCCTTGGGATGGAAATTGTCATATTTAGAGAGGGTGTCTCCGTATCCCAAAGAAAATACATAATTGGTTTGTT
ACACTGAGGGGCACTTTTATCACACTTTATTCACAAAATTATCTAAGTCATGAAAAATTACCGTTTTGTAGTCTATGATGXXXXXXXXXXA

Create PDF with GO2PDF for free, if you wish to

AATACTTCCTTGGGATGGAAATTGTCATATTTAGAGAGGGTGTCTCCGTATCCCAAAGAAAATACATAATTGGTTTGTT
>Marker199389 Chr6 SNP_site:13449503 Ref_Type:C
ACCTTGTTCAAGACTTGGATTCAGTCATTAAGGGAACAACCTATCTACTAACCCGAAAGCGGTTAGGAGTGAATTCCGTCXXXXXXXXXXXG
TGAATAGGAGTCCATAGTTGGCTCAAGGTTAAGATTAAGTTAAAAAGGATATCTATAAAATAGTAAGTTTTATTAAGTT
ACCTTGTTCAAGACTTGGATTCAGTCATTAAGGGAACAACCTATCTACTAACCCGAAAGCGGTTAGGAGTGAATTTCGTCXXXXXXXXXXXG
TGAATAGGAGTCCATAGTTGGCTCAAGGTTAAGATTAAGTTAAAAAGGATATCTATAAAATAGTAAGTTTTATTAAGTT
>Marker199401 Chr6 SNP_site:2677991 Ref_Type:C
AACCCAATTCCAACATAAATGGTAGCTGCCGCTTCCTGGACTCGTGCTTGGGCTGGGTTAAGTCTTCAATTTCAAAGGTAXXXXXXXXXXXA
TTTATTTTCTTTTAATTGTCCAAAATTTCATTCATGAAACGTTAATCCTAGATTTTGTTCTTCTTCTTACTTTATTGTT
AACCCAATTCCAACATAAATGGTAGCTGCCGCTTCCTGGACTCGTGCTTGGGCTGGGTTAAGTCTTCAATTTTAAAGGTAXXXXXXXXXXXA
TTTATTTTCTTTTAATTGTCCAAAATTTCATTCATGAAACGTTAATCCTAGATTTTGTTCTTCTTCTTACTTTATTGTT
>Marker200248 Chr6 SNP_site:8857724 Ref_Type:A
ACTGAAATTTTAATTTCTTGTTCAAAGCTTTAGATTATATTTGTAAGCTAGAAGTATTTTCTCTCTTAAAGAGAGAGAGGXXXXXXXXXXXA
CAAAGAAAAGGAGAACGACAAATCTTGGATGTAAAAAATAGATGAACAATGTGAAACTAATATTTAAGGATCTCTGGTC
ACTGAAATTTTAATTTCTTGTTCAAAGCTTTAGATTATATTTGTAAGCTAGAAGTATTTTCTCTCTTAAAGAGAGAGAGGXXXXXXXXXXXA
CAAAGAAAAGGAGAACGACCAATCTTGGATGTAAAAAATAGATGAACAATGTGAAACTAATATTTAAGGATCTCTGGTC
>Marker200270 Chr6 SNP_site:13461275 Ref_Type:T
ACAGATTGTGTATCTCCACGTGTTGAACTTACTGATGCCAAGCCAACTTAGTTTTGTTCATAAAAGGGAATTGATTTATTXXXXXXXXXXXA
TCTCTATGTTTAAAAGACTTCTGTTGTATAAAGTTTCATGCATTCATGTCATGTGTCAGTTGAGGTCATTACATAGGTG
ACAGATTGTGTATCTCCACGTGTTGAACTTACTGATGTCAGGCCAACTTAGTTTTGTTCATAAAAGGGAATTGATTTATTXXXXXXXXXXXA
TCTCTATGTTTAAAAGACTTCTGTTGTATAAAGTTTCATGCATTCATGTCATGTGTCAGTTGAGGTCATTACATAGGTG
>Marker200270 Chr6 SNP_site:13461278 Ref_Type:G
ACAGATTGTGTATCTCCACGTGTTGAACTTACTGATGCCAAGCCAACTTAGTTTTGTTCATAAAAGGGAATTGATTTATTXXXXXXXXXXXA
TCTCTATGTTTAAAAGACTTCTGTTGTATAAAGTTTCATGCATTCATGTCATGTGTCAGTTGAGGTCATTACATAGGTG
ACAGATTGTGTATCTCCACGTGTTGAACTTACTGATGTCAGGCCAACTTAGTTTTGTTCATAAAAGGGAATTGATTTATTXXXXXXXXXXXA
TCTCTATGTTTAAAAGACTTCTGTTGTATAAAGTTTCATGCATTCATGTCATGTGTCAGTTGAGGTCATTACATAGGTG
>Marker200273 Chr6 SNP_site:3716318 Ref_Type:A
TACATATATATACACACACATAATATTAGATTACAAGAATTTGTTGGATTTTGTTCTGGTTGAATCTTGGTATCTCTTTTXXXXXXXXXXXT
TAGACTAATTTTATTGTTTTAAATTTAGAGTATAAGATAGAAAGCATAGTTGAAAAGATTGATGCTGTGATGCTTAAGT
TACATATATATACACACACATGATATTAGATTACAAGAATTTGTTGGATTTTGTTCTGGTTGAATCTTGGTATCTCTTTTXXXXXXXXXXXT
TAGACTAATTTTATTGTTTTAAATTTAGAGTATAAGATAGAAAGCATAGTTGAAAAGATTGATGCTGTGATGCTTAAGT
>Marker200653 Chr6 SNP_site:7244913 Ref_Type:G
ACTCCTTTCAGAATGTTGCTACATTATGTCAATATACCTAGTGTATTTTGTCTAACTTTTGTTTAATCTTTGCAGGTTTCXXXXXXXXXXXA
TGTGATTGAGTTGCTTTCCTAGTAATGTATGTGTGTGTTTATATAGAAGCTCTCTTAACTCATTCCACTATACATTAGT
ACTCCTTTCAGAATGTTGCTACATTATGTCAATATACCTAGTGTATTTTGTCTAACTTTTGTTTAATCTTTGCAGGTTTCXXXXXXXXXXXA
TGTGATTGAGTTGCTTTCCTAGTGATGTATGTGTGTGTTTATATAGAAGCTCTCTTAACTCATTCCACTATACATTAGT
>Marker200695 Chr6 SNP_site:28357725 Ref_Type:G
CACCAAGCTATTTTCACTGATCCGGATGATCAAAGTGGTTGGTTTTATCATCTTTGGCTTCTGGACCAAACGGTGAAAACXXXXXXXXXXXC
CTCTCCATTCTGTAGTTTCTATTCGGATTCAGGAACAATACCCCTCATTCTTTACTTTGATCAACCTGTTCAAGGCGTT
CACCAAGCTATTTTCACTGATCCGGATGATCAAAGTGGTTGGTTTTATCATCTTTGGCTTCTGGACCAAACGGTGAAAGCXXXXXXXXXXXC
CTCTCCATTCTGTAGTTTCTATTCGGATTCAGGAACAATACCCCTCATTCTTTACTTTGATCAACCTGTTCAAGGCGTT
>Marker201297 Chr6 SNP_site:10466953 Ref_Type:C
ACAACATAATGTAGAAGTTCATAATGATTTATTTATTTTTATATGTTGAGGCTCGATTCAGTTTCGACTATGTTATGCAAXXXXXXXXXXXG
GTATTTCCATTAATTCGTGCTCGTAGGATGTATGCTTTGCTGACCCCTCTTCTTTTGTATTTGTTGGGTTAATAATAGT

Create PDF with GO2PDF for free, if you wish to

ACGACATAATGTAGAAGTTCATAATGATTTATTTATTTTTATATGTTGAGGCTCGATTCAGTTTCGACTATGTTATGCAAXXXXXXXXXXXG
GTATTTCCATTAATTCGTGCTCGTAGGATGTATGCTTTGCTGACCCCTCTTCTTTTGTATTTGTTGGGTTAATAATAGT
>Marker20138 Chr4 SNP_site:4186903 Ref_Type:T
ACCTAGAGACAAATCTAGCATGCAATCAGCCTAACATAACAAACGAGTCAGTTAAGATTCCAAAAAAACCTAACATACAAXXXXXXXXXXXA
GCCTCATCGAATCCTATATGCCTGGAAGGCGTGGATGCATTGTATGGTTTTCCAATTAAACTTGATTGTCGATAAAGTA
ACCTAGAGACAAATCTAGCATTCAATCAGCCTAACATAACAAACGAGTCAGTTAAGATTCCAAAAAAACCTAACATACAAXXXXXXXXXXXA
GCCTCATCGAATCCTATATGCCTGGAAGGCGTGGATGCATTGTATGGTTTTCCAATTAAACTTGATTGTCGATAAAGTA
>Marker201458 Chr6 SNP_site:21995677 Ref_Type:C
CACCCATGTAGTGTTCAATAGATGCAACCTTAAGTTCTGGCTTAAGACATACCCCATTCCTATTTATTCTAATATTTACTXXXXXXXXXXXT
AAGGGTGAGTGGCTTGCATTTGGTTGAACAGTCTATATTTAATCCTCTAGTCTTGGCGGCCTTGGGATAGAGTCCTGTG
CACCCATGTAGTGTTCAATAGATGCAACCTTAAGTTCTGGCTTAAGACATACCCCATTCCTATTTATTCTAATATTTACTXXXXXXXXXXXT
AAGGGTGAGTGGTTTGCATTTGGTTGAACAGTCTATATTTAATCCTCTAGTCTTGGCGGCCTTGGGATAGAGTCCTGTG
>Marker201469 Chr6 SNP_site:5336480 Ref_Type:T
ACTCCCGTTATTTTCGTCAGGGTCTTTGTCTTCTTTTTCTCTCTTCTCAATGATACTGGTATTCGGGTCCTTGTTATTTTXXXXXXXXXXXT
GAAAATGATTTTCCATGGAGGTAACTTAGTTCTGGCCTTGGATTTGTGAATAACTGCTTACATAAGATGAAATTTTAGT
ACTCCTGTTATTTTCGTCAGGGTCTTTGTCTTCTTTTTCTCTCTTCTCAATGATACTGGTATTCGGGTCCTTGTTATTTTXXXXXXXXXXXT
GAAAATGATTTTCCATGGAGGTAACTTAGTTCTGGCCTTGGATTTGTGAATAACTGCTTACATAAGATGAAATTTTAGT
>Marker201941 Chr6 SNP_site:9046042 Ref_Type:A
ACCAGGCACAGGACTACAATATTTGAACATTCCAGTATCAGACAGAAAGTAAATGACAATAAACAAATACACCAACATATXXXXXXXXXXXG
CATGAGATTAGCGGGTTGAAAGTTAGAAACAAAGAAGATTTGAACCTCCGTCTGCACTCACAATTTAGGCTATTGGGTA
ACCAGGCACAGGACTACAATATTTGAACATTCCAGTATCAGACAGAAAGTAAATGACAATAAACAAATACACCAACATATXXXXXXXXXXXG
CATGAGATTAGCGGGTTGAAAGTTAGAAACAAAGAAGATTTGAACCTCCGTCTGCGCTCACAATTTAGGCTATTGGGTA
>Marker201963 Chr6 SNP_site:26623772 Ref_Type:T
GACCATGCCCTCGAGACGGAACTGCACGACGTCCGCCATGGTCTAGCTGGGTAAGAGGGGAGTCGGAGGGAATGGAGCTGXXXXXXXXXXXA
GTTATTTACAAAAATGTTAGAGCCCACTCTCTCTTTTTTCAAAGATACAAATTACTTTCTGAAAATCTAGCTATTGGTT
GACCATGCCCTCGAGACGGAACTGCACGACGTCCGCCATGGTCTAGCTGGGTAAGAGGGGAGTCGGAGGGAATGGAGCTGXXXXXXXXXXXA
GTTATTTACAAAAATGTTAGAGCCCACTCTCTCTTTTTTTAAAGATACAAATTACTTTCTGAAAATCTAGCTATTGGTT
>Marker202612 Chr6 SNP_site:3534473 Ref_Type:T
TACCTGGCAATGATAAATAATGTTAAAGAGATCTGAGGGCATCTTGAAAATGGTTAATTTTTCATGAAACTGCAACTGTTXXXXXXXXXXXG
TTGTATTTTAAAATGGTTCTTTTTATTGTGTATGTAAGCTAATTTTGACACTTACAGATGTCAAAAAGAATAAAGTGGT
TACTTGGCAATGATAAATAATGTTAAAGAGATCTGAGGGCATCTTGAAAATGGTTAATTTTTCATGAAACTGCAACTGTTXXXXXXXXXXXG
TTGTATTTTAAAATGGTTCTTTTTATTGTATATGTAAGCTAGTTTTGACACTTACAGATGTCAAAAAGAATAAAGTGGT
>Marker202612 Chr6 SNP_site:3534669 Ref_Type:A
TACCTGGCAATGATAAATAATGTTAAAGAGATCTGAGGGCATCTTGAAAATGGTTAATTTTTCATGAAACTGCAACTGTTXXXXXXXXXXXG
TTGTATTTTAAAATGGTTCTTTTTATTGTGTATGTAAGCTAATTTTGACACTTACAGATGTCAAAAAGAATAAAGTGGT
TACTTGGCAATGATAAATAATGTTAAAGAGATCTGAGGGCATCTTGAAAATGGTTAATTTTTCATGAAACTGCAACTGTTXXXXXXXXXXXG
TTGTATTTTAAAATGGTTCTTTTTATTGTATATGTAAGCTAGTTTTGACACTTACAGATGTCAAAAAGAATAAAGTGGT
>Marker202612 Chr6 SNP_site:3534681 Ref_Type:G
TACCTGGCAATGATAAATAATGTTAAAGAGATCTGAGGGCATCTTGAAAATGGTTAATTTTTCATGAAACTGCAACTGTTXXXXXXXXXXXG
TTGTATTTTAAAATGGTTCTTTTTATTGTGTATGTAAGCTAATTTTGACACTTACAGATGTCAAAAAGAATAAAGTGGT
TACTTGGCAATGATAAATAATGTTAAAGAGATCTGAGGGCATCTTGAAAATGGTTAATTTTTCATGAAACTGCAACTGTTXXXXXXXXXXXG
TTGTATTTTAAAATGGTTCTTTTTATTGTATATGTAAGCTAGTTTTGACACTTACAGATGTCAAAAAGAATAAAGTGGT
>Marker205080 Chr6 SNP_site:27901706 Ref_Type:A
ACATTGTGATTCTTTGTTTGCTTTGCAACATATCTTTTACATTTTTGTATCAATTAAGGACTTATTTGATTGATATGAAGXXXXXXXXXXXC

Create PDF with GO2PDF for free, if you wish to

TTCACAGACAAACCATGCATGAATGATTCCTAAACAAAATCATAACTAGAAACCTTAAATTGCTTTACTTATTAAAGTG
ACATTGTGATTCTTTGTTTGCTTTGCAACCTATCTTTTACATTTTTGTATCAATTAAGGACTTATTTGATTGATATGAAGXXXXXXXXXXC
TTCACAGACAAACCATGCATGAATGATTCCTAAACAAAATCATAACTAGAAACCTTAAATTGCTTTACTTATTAAAGTG
>Marker205093 Chr6 SNP_site:12902580 Ref_Type:G
CACAATGCTCATCAGCGTCAGGAAAATACTCCAACTTGAATTACATCAGCAGCGACGAGTATGAGACCAGCATGAGTTCAXXXXXXXXXXC
AAGGAGTTGCCTCTGGTCGCAAAGACAAGAAAGGAACAGATGGTCGTAAATTAACTAGGAACAGATCACTAGTAGATGT
CACAATGCTCATCAGCGTCGGGAAAATACTCCAACTTGAATTACATCAGCAGCGACGAGTATGAGACCAGCATGAGTTCAXXXXXXXXXXC
AAGGAGTTGCCTCTGGTCGCAAAGACAAGAAAGGAACAGATGGTCGTAAATTAACTAGGAACAGATCACTAGTAGATGT
>Marker205268 Chr6 SNP_site:7064521 Ref_Type:T
AACCTTAGGTCTTGAAAACTAAAAAGTATTGGTTCTATTTAATAACTATTGACTTACCTTTCACAATTTTTTATTTATAAXXXXXXXXXXA
GATCGGAATTAGCCTTTGGGCTCTTTCATATGAACACACGATTAAGGGTAGATCGTATTCAATTTTTTATAAAAATGTA
AACCTTAGGTTTTGAAAACTTAAAAGTATTGGTTCTATTTAATAACTATTGACTTACCTTTCACAATTTTTTATTTATAAXXXXXXXXXXA
GATCGGAATTAGCCTTTGGGCTCTTTCATATGAACACACGATTAAGGGTAGATCGTATTCAATTTTTTATAAAAATGTA
>Marker205268 Chr6 SNP_site:7064531 Ref_Type:T
AACCTTAGGTCTTGAAAACTAAAAAGTATTGGTTCTATTTAATAACTATTGACTTACCTTTCACAATTTTTTATTTATAAXXXXXXXXXXA
GATCGGAATTAGCCTTTGGGCTCTTTCATATGAACACACGATTAAGGGTAGATCGTATTCAATTTTTTATAAAAATGTA
AACCTTAGGTTTTGAAAACTTAAAAGTATTGGTTCTATTTAATAACTATTGACTTACCTTTCACAATTTTTTATTTATAAXXXXXXXXXXA
GATCGGAATTAGCCTTTGGGCTCTTTCATATGAACACACGATTAAGGGTAGATCGTATTCAATTTTTTATAAAAATGTA
>Marker20552 Chr4 SNP_site:23091670 Ref_Type:G
CACTGATACTAACAGAATTACTGGAAGCCAACAATTAAATCTTTTTAAATAATCATATGGGTTGAACTTGTAGGGATTTGXXXXXXXXXXA
GCTCCAGGAGAAGTGAAATCTTCACAAACATGGATATGTTCAACAAGATCATCGATGCATATGTGCTCGTCTTCCATGT
CACTGATACTAACAGAATTACTGGAAGCCAACAATTAAGTCTTTTTAAATAATCATATGGGTTGAACTTCTAGGGATTTGXXXXXXXXXXA
GCTCCAGGAGAAGTGAAATCTTCACAAACATGGATATGTTCAACAAGATCATCGATGCATATGTGCTCGTCTTCCATGT
>Marker20552 Chr4 SNP_site:23091701 Ref_Type:C
CACTGATACTAACAGAATTACTGGAAGCCAACAATTAAATCTTTTTAAATAATCATATGGGTTGAACTTGTAGGGATTTGXXXXXXXXXXA
GCTCCAGGAGAAGTGAAATCTTCACAAACATGGATATGTTCAACAAGATCATCGATGCATATGTGCTCGTCTTCCATGT
CACTGATACTAACAGAATTACTGGAAGCCAACAATTAAGTCTTTTTAAATAATCATATGGGTTGAACTTCTAGGGATTTGXXXXXXXXXXA
GCTCCAGGAGAAGTGAAATCTTCACAAACATGGATATGTTCAACAAGATCATCGATGCATATGTGCTCGTCTTCCATGT
>Marker20562 Chr4 SNP_site:21249712 Ref_Type:T
CACTCTATCCTCCCATTCCATACATTGTCATTTTTGGTTACAGCCCAAACTTAGTCTAGATAATATGTTTTCACATGTTTXXXXXXXXXXA
ACTACATGGCCACATCATCACAAAATTACATTATCAACATAGAAGTGTTAAATTCAATAGAGTGCCAAACCTTACAAGT
CACTCTATCCTCCCATTCCATACATTGTCATTTTTGGTTACAGCCCAAACTTAGTCTAGATAATATGTTTTTACATGTTTXXXXXXXXXXA
ACTACATGGCCACATCATCACAAAATTACATTATCAACGTAGAAGTGTTAAATTCAATAGAGTGCCAAACCTTACAAGT
>Marker20562 Chr4 SNP_site:21249886 Ref_Type:G
CACTCTATCCTCCCATTCCATACATTGTCATTTTTGGTTACAGCCCAAACTTAGTCTAGATAATATGTTTTCACATGTTTXXXXXXXXXXA
ACTACATGGCCACATCATCACAAAATTACATTATCAACATAGAAGTGTTAAATTCAATAGAGTGCCAAACCTTACAAGT
CACTCTATCCTCCCATTCCATACATTGTCATTTTTGGTTACAGCCCAAACTTAGTCTAGATAATATGTTTTTACATGTTTXXXXXXXXXXA
ACTACATGGCCACATCATCACAAAATTACATTATCAACGTAGAAGTGTTAAATTCAATAGAGTGCCAAACCTTACAAGT
>Marker205962 Chr6 SNP_site:2218708 Ref_Type:C
AACAATTGCAAATTCTTTTAGTAATCACCTATAGGTGCTTATTTCATTTTATCAATGAGATGTTTCTTAACTAAACAAAAXXXXXXXXXXT
TATGAAAATGGAGGATTTGGAATCTCTCTAAACTCGGTGAGTTTTTTTCTTTTCTGAAAACTTTGAGCTCTTTTTTGTG
AACAATTGCAAATTCTTTTAGTAATCACCTATAGGTGCTTATTTCATTTTATCAATGAGATGTTTCTTAACTAAACAAAAXXXXXXXXXXT
TATGAAAATGGAGGATTTGGAATCTCTCTAAAGTCGGTGAGTTTTTTTCTTTTCTGAAAACTTTGAGCTCTTTTTTGTG
>Marker206194 Chr6 SNP_site:18083382 Ref_Type:C

Create PDF with GO2PDF for free, if you wish to

TACAGCAGTTGTCAAAATGCATACTAACTTATGTTCAACTTTGACAATGAAGCAATGCCTGTTTGATCACAAGAGGCCTCXXXXXXXXXXG
TTTTGCAGTGTGAGAACCAAATGTGTAGGTAGAAACAAAGTTGCTAGAATCTCCTAAAAGCTTGAACCGTTCCAAAGGT
TACAGCAGTTGTCAAAATGCATACTAACTTATGTTCAACTTTGACAATGAAGCAATGCCTGTTTGATCATAAGAGGCCTCXXXXXXXXXXG
TTTTGCAGTGTGAGAACCAAATGTGTAGGTAGAAACAAAGTTGCTAGAATCTCCTAAAAGCTTGAACCGTTCCAAAGGT
>Marker20640 Chr4 SNP_site:20868656 Ref_Type:T
CACCAAGTTCAACATGCTCAAGTAGCATGGCAATGGATAGATGTTTCCTGGTTCTAATCATCAACGAAATACAAGCCAAGXXXXXXXXXXT
ATGCCGCTGGCATTCATTCCATCACTTTAGAGAAAGAGCGAGTGATAAAGTAAATGGCAAGGGCCCCCATGTTGAGGTC
CACCAAGTTCAACATGCTTAAGTAGCATGGCAATGGATAGATGTTTCCTGGTTCTAATCATCAACGAAATACAAGCCAAGXXXXXXXXXXT
ATGCCGCTGGCATTCGTTCCATCACTTTAGAGAAAGAGCGAGTGATAAAGTAAATGGCAAGGGCCCCCATGTTGAGGTC
>Marker20640 Chr4 SNP_site:20868813 Ref_Type:G
CACCAAGTTCAACATGCTCAAGTAGCATGGCAATGGATAGATGTTTCCTGGTTCTAATCATCAACGAAATACAAGCCAAGXXXXXXXXXXT
ATGCCGCTGGCATTCATTCCATCACTTTAGAGAAAGAGCGAGTGATAAAGTAAATGGCAAGGGCCCCCATGTTGAGGTC
CACCAAGTTCAACATGCTTAAGTAGCATGGCAATGGATAGATGTTTCCTGGTTCTAATCATCAACGAAATACAAGCCAAGXXXXXXXXXXT
ATGCCGCTGGCATTCGTTCCATCACTTTAGAGAAAGAGCGAGTGATAAAGTAAATGGCAAGGGCCCCCATGTTGAGGTC
>Marker20644 Chr4 SNP_site:5252081 Ref_Type:G
CACCTCCAAAGGAACAATGAAGCTGAGAGGAAATGTGAGAGGGAAAGAGGTAGTGGTCCTTATTGATAGTAGAGCCACTAXXXXXXXXXXA
TTTTCTAGCAGTGGAATTGGGAAGGTATTGACCTAGTTCTGAGGATGCAGTGGCTGGATACCACCGGAACAATGAAGGT
CACCTCCAAAGGAACAATGAAGCTGAGAGGAAATGTGAGAGGGAAAGAGGTAGTGGTCCTTATTGATAGTAGAGCCACTAXXXXXXXXXXA
TTTTCTAGCGGTGGAATTGGGAAGGTATTGACCTAGTTTTGAGGATGCAGTGGCTGGATACCACCGGAACAATGAAGGT
>Marker20644 Chr4 SNP_site:5252110 Ref_Type:T
CACCTCCAAAGGAACAATGAAGCTGAGAGGAAATGTGAGAGGGAAAGAGGTAGTGGTCCTTATTGATAGTAGAGCCACTAXXXXXXXXXXA
TTTTCTAGCAGTGGAATTGGGAAGGTATTGACCTAGTTCTGAGGATGCAGTGGCTGGATACCACCGGAACAATGAAGGT
CACCTCCAAAGGAACAATGAAGCTGAGAGGAAATGTGAGAGGGAAAGAGGTAGTGGTCCTTATTGATAGTAGAGCCACTAXXXXXXXXXXA
TTTTCTAGCGGTGGAATTGGGAAGGTATTGACCTAGTTTTGAGGATGCAGTGGCTGGATACCACCGGAACAATGAAGGT
>Marker206975 Chr6 SNP_site:9111931 Ref_Type:C
TACCAATGAGGCATAACAAACAAGTTTATTAGCAAGACATCAATTGTCTTAAACTTATTAGTGAAGCATTTAAGTGTATCXXXXXXXXXXA
GGAGTATCAGGTATATCAGCTGTATCAAATGTGAATTAATAAAGAGGGCATTTGTGACATTTTACATATTGTATATGTG
TACCAATGAGGCATAACAAAGAAGTTTATTAGCAAGACATCAATTGTCTTAAACTTATTAGTGAAGCATTTAAGTGTATCXXXXXXXXXXA
GGAGTATCAGGTATATCAGCTGTATCAAATGTGAATTAATAAAGAGGGCATTTGTGACATTTTACATATTGTATATGTG
>Marker20723 Chr4 SNP_site:7936002 Ref_Type:G
CACATGAAATTGTCCTTAAATCTACATAGGTGAGGATAGCTCATTAGCACTAGCCAAATAAGCTTTCCATTTTAGGGGTAXXXXXXXXXXG
AATAATGGGCCCTACCCTCTCATTGTCTCGAGAGAAATTCGATTTATAGGTTAGATCTTAAACCAATTGTTCAACAGTG
CACATGAAATTGTCCTTAAATCTACGTAGGTGAGGATAGCTCATTAGCACTAGCCAAATAAGCTTTCCATTTTAGGGGTAXXXXXXXXXXG
AATAATGGGTCCTACCCTCTCATTGTCCCGAGAGAAATTCGATTTATAGGTTAGATCTTAAACCAATTGTTCAACAGTG
>Marker20723 Chr4 SNP_site:7936172 Ref_Type:T
CACATGAAATTGTCCTTAAATCTACATAGGTGAGGATAGCTCATTAGCACTAGCCAAATAAGCTTTCCATTTTAGGGGTAXXXXXXXXXXG
AATAATGGGCCCTACCCTCTCATTGTCTCGAGAGAAATTCGATTTATAGGTTAGATCTTAAACCAATTGTTCAACAGTG
CACATGAAATTGTCCTTAAATCTACGTAGGTGAGGATAGCTCATTAGCACTAGCCAAATAAGCTTTCCATTTTAGGGGTAXXXXXXXXXXG
AATAATGGGTCCTACCCTCTCATTGTCCCGAGAGAAATTCGATTTATAGGTTAGATCTTAAACCAATTGTTCAACAGTG
>Marker20723 Chr4 SNP_site:7936190 Ref_Type:C
CACATGAAATTGTCCTTAAATCTACATAGGTGAGGATAGCTCATTAGCACTAGCCAAATAAGCTTTCCATTTTAGGGGTAXXXXXXXXXXG
AATAATGGGCCCTACCCTCTCATTGTCTCGAGAGAAATTCGATTTATAGGTTAGATCTTAAACCAATTGTTCAACAGTG
CACATGAAATTGTCCTTAAATCTACGTAGGTGAGGATAGCTCATTAGCACTAGCCAAATAAGCTTTCCATTTTAGGGGTAXXXXXXXXXXG
AATAATGGGTCCTACCCTCTCATTGTCCCGAGAGAAATTCGATTTATAGGTTAGATCTTAAACCAATTGTTCAACAGTG

Create PDF with GO2PDF for free, if you wish to

>Marker207309 Chr6 SNP_site:9483604 Ref_Type:T
TACATGGTTTTTTTATTTTCTAGAATTTAAAAATATATATCTTCACTTAAAATAAGGATACGAACTGAGGGGAGGGAATAXXXXXXXXXXA
TCTCCGGATATTCTCTCACAGTCTCACTTTCCCTTAAATCCCTTCAATTTCTCTACCCTTTCCAGTTTCCACATCAAGT
TACATGGTTTTTTTATTTTCTAGAATTTAAAAATATATATCTTCACTTAAAATAAGGATACGAACTGAGGGGAGGGAATAXXXXXXXXXXA
TCTCCGGATATTCTCTCACAGTCTCACTTTCCCTTAAATCCCTTCAATTTCTCTACTCTTTCCAGTTTCCACATCAAGT
>Marker207411 Chr6 SNP_site:3414796 Ref_Type:T
AACCATGATTAATATTTGCATTTATTAGATATCTTATGTAAATATCGTAAGTGGTTAATTCTATGGTAATATTGTAATAAXXXXXXXXXXC
CTAATTATAGTATATATTGAATAATACGATGTATATTTTATTAATGAAAAATTATTATATCATTTGTTTATAGAAGGTG
AACCATGATTAATATTTGCATTTATTAGATATCTTATGTAAATATTGTAAGTGGTTAATTCTATGGTAATATTGTAATAAXXXXXXXXXXC
CTAATTATAGTATATATTGAATAATACGATGTATATTTTATTAATGAAAAATTATTATATCATTTGTTTATAGAAGGTG
>Marker207863 Chr6 SNP_site:17930764 Ref_Type:C
ACATTTATTACTCATCTTCTCAACTTTTTTGCTTTGTGGTTCATTTCATGGAGAATTTGCTTAATTTGAAACTTATTTTCXXXXXXXXXXT
ATTGTTATTTTAGCCACCGCATTTTAATTCTCTCATAGATCACTCGAGCTCCACTGTGAATCCTATGAGCATCTAGGTT
ACATTTCTTACTCATCTTCTCAACTTTTTTGCTTTGTGGTTCATTTCATGGAGAATTTGCTTAATTTGAAACTTATTTTCXXXXXXXXXXT
ATTGTTATTTTAGCCACCGCATTTTAATTCTCTCATAGATCACTCGAGCTCCACTGTGAATCCTATGAGCATCTAGGTT
>Marker208350 Chr6 SNP_site:1468416 Ref_Type:T
TACTGAACTTGTCATGTTCGAAGTAGTAAAATTTATCATTTCATTACATGAAAATAAGTTTTGGAGAGAAATTTCTCAAAXXXXXXXXXXT
TATCCTTCCTTTTTTTCCTTGGAAACGTGGCCTAGTTGTTTTATTTCCTGAATTCTATTTATCATGCCAAGAATCTGTT
TACTGAACTTGTCATGTTCGAAGTAGTAAAATTTATCATTTCATTACATGAAAATAAGTTTTGGAGAGAAATTTCTCAAAXXXXXXXXXXT
TATCCTTTCTTTTTTTCCTTGGAAACGTGGCCTAGTTGTTTTATTTCCTGAATTCTATTTATCATGCCAAGAATCTGTT
>Marker208544 Chr6 SNP_site:7997519 Ref_Type:C
ACTTGTGTTTGATATTTGAGATGTAACGTAACCCAACCAATGGCATTATTATTTTTTTGAGAGAACTAATTGCATTAATTXXXXXXXXXXT
AATTGCAGGTTGGAATGGCTGCTACTCTTGCTGGGGTTTGTCAGGTGCCTCTTACTGCTGTTTTGTTGCTTTTTGAGTT
ACTTGTGTTTGATATTTGAGATGTAACGTAACCCAACTAATGGCATTATTATTTTTTTGAGAGAACTAATTGCATTAATTXXXXXXXXXXT
AATTGCAGGTTGGAATGGCTGCTACTCTTGCTGGGGTTTGTCAGGTGCCTCTTACTGCTGTTTTGTTGCTTTTTGAGTT
>Marker209058 Chr6 SNP_site:15171563 Ref_Type:A
CACTATTTCAAAGCTTTCTTAAAAATTGGTTCTATCTGCAAAGCAATTTGATGGGATGGAAATGGATGTCTATCTATATGXXXXXXXXXXT
CACACCTATTTTAGACACCAATTAACCTCTAGATTCCATTCCCACTAACATCAATATAATAAAGTATAAAGAGGCATGT
CACTATTTCAAAGCTTTCTTAAAAATTGGTTCTATCTGCAAAGCAATTTGATGGGATGGAAATGGATGTCTATCTATATGXXXXXXXXXXT
CACACCTATTTTAGACACCAATTAACCTCTAGATTCCCTTCCCACTAACATCAATATAATAAAGTGTAAAGAGGCATGT
>Marker209058 Chr6 SNP_site:15171591 Ref_Type:A
CACTATTTCAAAGCTTTCTTAAAAATTGGTTCTATCTGCAAAGCAATTTGATGGGATGGAAATGGATGTCTATCTATATGXXXXXXXXXXT
CACACCTATTTTAGACACCAATTAACCTCTAGATTCCATTCCCACTAACATCAATATAATAAAGTATAAAGAGGCATGT
CACTATTTCAAAGCTTTCTTAAAAATTGGTTCTATCTGCAAAGCAATTTGATGGGATGGAAATGGATGTCTATCTATATGXXXXXXXXXXT
CACACCTATTTTAGACACCAATTAACCTCTAGATTCCCTTCCCACTAACATCAATATAATAAAGTGTAAAGAGGCATGT
>Marker209614 Chr6 SNP_site:7190632 Ref_Type:T
ACAAGCAATGGGAAACAATTTGAATATATGAATTTGATTATTCTATCTTCTATGAATGGGAGAGATGAAAAACAAAAAAGXXXXXXXXXXA
TTTTGAACCTCTACCATAATTTTATTTCTTTTTTAGAATTTAAAATTAGGGTTTTATGCATTTAATTGGGACAGTGGTT
ACAAGCAATGGGAAACAATTTGAATATATGAATTTGATTATTCTATCTTCTATGAATGGGAGAGATGAAAAACAAAAAAGXXXXXXXXXXA
TTTTGAATCTCTACCATAATTTTATTTCTTTTTTAGAATTTAAAATTAGGGTTTTATGCATTTAATTGGGACAGTGGTT
>Marker209644 Chr6 SNP_site:4922548 Ref_Type:C
GACGCAAACACTTGAAAATATTAAAAAGAACAAAATAAAATAAAACATATGTTCAAATACTGGTATCCTCCACTGCATGAXXXXXXXXXXA
AAATATTAATTATATGATATAATACATAATTGAAATACCATTCCTTGCTTTTAAAAATTATATAGCACACGAAATAAGT
GACGCAAACACTTGAAAATATTAAAAAGAACAAAATAAAATAAAACATATGTTCAAATACTGGTATCCTCCACTGCATGAXXXXXXXXXXA

Create PDF with GO2PDF for free, if you wish to

AAATATTAATTATATGATATAATACATAATTGAAATACCATTCTTTGCTTTTAAAAATTATATAGCACACGAAATAAGT
>Marker21074 Chr4 SNP_site:16529788 Ref_Type:C
GACACCTCAGTGCCCTCATCATATCCAAGAACGCACAAAACATGGAATAAGAGTGCCTAGACACTCCTAACAATACTGAGXXXXXXXXXXC
AGCAACTTCTTCATGGAGCTTGAGGATCTTGGCCTGCTAAATTAAGACGGAGATCAGAAATGCACCGAAAAAGCCAAGT
GACACCTCAGTGCCCTCATCATATCCAAGAACGCACCAAACATGGAATAAGAGTGCCTAGACACTCCTAACAATACTGAGXXXXXXXXXXC
AGCAACTTCTTCATGGAGCTTGAGGATCTTGGCCTGCTAAATTAAGATGGAGATCAGAAATGCACCGAAAAAGCCAAGT
>Marker21074 Chr4 SNP_site:16530002 Ref_Type:T
GACACCTCAGTGCCCTCATCATATCCAAGAACGCACAAAACATGGAATAAGAGTGCCTAGACACTCCTAACAATACTGAGXXXXXXXXXXC
AGCAACTTCTTCATGGAGCTTGAGGATCTTGGCCTGCTAAATTAAGACGGAGATCAGAAATGCACCGAAAAAGCCAAGT
GACACCTCAGTGCCCTCATCATATCCAAGAACGCACCAAACATGGAATAAGAGTGCCTAGACACTCCTAACAATACTGAGXXXXXXXXXXC
AGCAACTTCTTCATGGAGCTTGAGGATCTTGGCCTGCTAAATTAAGATGGAGATCAGAAATGCACCGAAAAAGCCAAGT
>Marker210840 Chr6 SNP_site:7316081 Ref_Type:C
AACTCTTGTTAGATAATGTAATTACATAATATATTATAATAGTTGAAAAAACCAATTGTGTTATAATGAAAGCATCAAAGXXXXXXXXXXT
ATGAATATGAAATTGATTATATAGCATCTAACGACAATTTTGTATCTACCATGTATGTAAATATGTTATATACATAGTA
AACTCTTGTTAGATAATGTAATTACATAATATATTATAATAGTTGAAAAAACCAATTGTGTTATAATGAAAGCATCAAAGXXXXXXXXXXT
ATGAATATGAAATTGATTATATAGCATCTAAGGACAATTTTGTATCTACCATGTATGTAAATATGTTATATACATAGTA
>Marker211004 Chr6 SNP_site:26256843 Ref_Type:C
TACATTCATGACCATGTGATACTGAATAAAACCATATTCAAAGTCAAGGCATACCCACCTTGCTCAATCTTTAACTCTAGXXXXXXXXXXA
AAATAACAAATAAGAGGCTGATGAAGTCAGTAGAAAGATAAGAGTAAAATAGGAAATAAAACAAACATTTAAAACCAGT
TACATTCATGACCATGTGATACTGAATAAAACCATATTCAAAGTCAAGGCATACCCACCTTGCTCAATCTTTAACTCTAGXXXXXXXXXXA
AAATAACAAATAAGAGGCTGATGAAGTCAGTAGAAAGATAAGAGTAAAATAGGAAATAAAACAAACATTTAAAACTAGT
>Marker211978 Chr6 SNP_site:26726263 Ref_Type:T
ACTTTGTATAACTATTAACTTCAACAAATAGCTCACTTTTATTTGCAAGTTAGGCATGTATTTTCCGTCGTTAGGTCAAAXXXXXXXXXXT
AATGGAATTGAACTCTTAATTTTTTAGATGGATAGTATGATTTTTATGTTGTTTGAATTATGACATGCTTTTCCTGGTT
ACTTTGTATAACTATTAACTTCAACAAATAGCTCACTTTTATTTGCAAGTTAGGCATGTATTTTCCGTCGTTAGGTCAAAXXXXXXXXXXT
AATGGAATTGAACTTTTAATTTTTTAGATGGATAGTATGATTTTTATGTTGTTTGAATTATGACATGCTTTTCCTGGTT
>Marker212876 Chr6 SNP_site:11589641 Ref_Type:T
ACCCCTATGTTACTTATAGGCCTTAAATAACAATGCTCCTCTTACGAACAACCTGTTTATGGTTCAACTAATAAACAAAAXXXXXXXXXXA
TGTTATCAGATCCCTTTTCGATCTTGTCTCTCCAAAATGATAAACTTATCGAGTCTGTAATCTGATCACTCTCACCTGT
ACCCCTATGTTACTTATAGGCCTTAAATAACAATGCTCCTCTTACGAACAACCTGTTTATGGTTCAACTAATAAACAAAAXXXXXXXXXXA
TGTTATCAGATCCCTTTTCGATCTTGTCTCTCCAATATGATAAACTTATCGAGTCTGTAATCTGATCACTCTCACATGT
>Marker212876 Chr6 SNP_site:11589681 Ref_Type:A
ACCCCTATGTTACTTATAGGCCTTAAATAACAATGCTCCTCTTACGAACAACCTGTTTATGGTTCAACTAATAAACAAAAXXXXXXXXXXA
TGTTATCAGATCCCTTTTCGATCTTGTCTCTCCAAAATGATAAACTTATCGAGTCTGTAATCTGATCACTCTCACCTGT
ACCCCTATGTTACTTATAGGCCTTAAATAACAATGCTCCTCTTACGAACAACCTGTTTATGGTTCAACTAATAAACAAAAXXXXXXXXXXA
TGTTATCAGATCCCTTTTCGATCTTGTCTCTCCAATATGATAAACTTATCGAGTCTGTAATCTGATCACTCTCACATGT
>Marker214163 Chr6 SNP_site:3454745 Ref_Type:T
ACTTTAACACATAAATTTGTATATTAATAACGTGGCTATGTTATGAAAATGTCCTTAAACTTTGTTATTTGTTTTAAAAAXXXXXXXXXXC
TGCTTAATAAAATTCAATTATAATGTTAGATGAAAAACAAAGATTTGAAATATATTGTAATTTTTATGTTGCATTTGGT
ACTTTAACACATAAATTTGTATATTAATAACGTGGCTATGTTATGAAAATGTCCTTAAACTTTGTTATTTGTTTTTAAAAXXXXXXXXXXC
TGCTTAATAAAATTCAATTATAATGTTAGATGAAAAAGAAAGAATTGAAATATATTGTAATTTTTATGTTGCATTTGGT
>Marker214163 Chr6 SNP_site:3454881 Ref_Type:G
ACTTTAACACATAAATTTGTATATTAATAACGTGGCTATGTTATGAAAATGTCCTTAAACTTTGTTATTTGTTTTAAAAAXXXXXXXXXXC
TGCTTAATAAAATTCAATTATAATGTTAGATGAAAAACAAAGATTTGAAATATATTGTAATTTTTATGTTGCATTTGGT

Create PDF with GO2PDF for free, if you wish to

ACTTTAACACATAAATTTGTATATTAATAACGTGGCTATGTTATGAAAATGTCCTTAAACTTTGTTATTTGTTTTTAAAAXXXXXXXXXXC
TGCTTAATAAAATTCAATTATAATGTTAGATGAAAAAGAAAGAATTGAAATATATTGTAATTTTTATGTTGCATTTGGT
>Marker214163 Chr6 SNP_site:3454887 Ref_Type:A
ACTTTAACACATAAATTTGTATATTAATAACGTGGCTATGTTATGAAAATGTCCTTAAACTTTGTTATTTGTTTTAAAAAXXXXXXXXXXC
TGCTTAATAAAATTCAATTATAATGTTAGATGAAAAACAAAGATTTGAAATATATTGTAATTTTTATGTTGCATTTGGT
ACTTTAACACATAAATTTGTATATTAATAACGTGGCTATGTTATGAAAATGTCCTTAAACTTTGTTATTTGTTTTTAAAAXXXXXXXXXXC
TGCTTAATAAAATTCAATTATAATGTTAGATGAAAAAGAAAGAATTGAAATATATTGTAATTTTTATGTTGCATTTGGT
>Marker214294 Chr6 SNP_site:11828326 Ref_Type:C
AACAAATATAATGTGTATTGATTAAATTAATGTTACATATGCAACAAAGATCTGTCACATCAAACCTAAGTCAGTGACTAXXXXXXXXXXT
TTCGTCTAGAGCCTGAAATGTGAAAACATGCTTGAGTTTTTAAACTTGCAGTGTGATCAGATTTTGGGTAAAAGTTGGT
AACAAATATAATGTGTATTGATTAAATTAATGTTACATATGCAACAAAGATCTGTCACATCAAACCTAAGTTAGTGACTAXXXXXXXXXXT
TTCGTCTAGAGCCTGAAATGTGAAAACATGCTTGAGTTTTTAAACTTGCAGTGTGATCAGATTTTGGGTAAAAGTTGGT
>Marker214382 Chr6 SNP_site:4921930 Ref_Type:G
CACAAGGATCTTCATGTTATGAGACAACATCTAGTAGTGACCATATGCTTGAAGATCACAATATAATATTATCAATTTATXXXXXXXXXXA
GATCCTAAGTGTGTGGTTTCAAATCGAACATTCTTTATGTATCAAACAAAGGTTTAAATAACCAACCAGGTGCAAATGT
CACAAGGATCTTCATGTTATGAGACAACATCTAGTAGTGACCATATGCTTGAAGATCACAATATAATATTATCAATTTATXXXXXXXXXXA
GATCCTAAGTGTGTGGTTTCAAATCGAACATTCTTTGTGTATCAAACAAAGGTTTAAATAACCAACCAGGTGCAAATGT
>Marker214524 Chr6 SNP_site:7188154 Ref_Type:G
ACCATTCAGGCATTCCACAACCCAAGCCAAACACCCACGCACGCTCCAAAACAAAATTCTACTTCTTTACTCTTATTCCTXXXXXXXXXXA
CTAACTTTTCTTCATATTTGGGACGACATTAGTAAATGGTTCCTACTTCGACGGCACTTCATAACGTATATACATCTGT
ACCATTCAGGCATTCCACAACCCAAGCCAAGCACCCACGCACGCTCCAAAACAAAATTCTACTTCTTTACTCTTATTCCTXXXXXXXXXXA
CTAACTTTTCTTCATATTTGGGACGACATTAGTAAATGGTTCCTACTTCGACGGCACTTCATAACGTATATACATCTGT
>Marker214693 Chr6 SNP_site:7898263 Ref_Type:C
ACTCCTCTTTGTGCATGTTAGCCTTATCTTTGCTACTTGCCCTCTTGAGTAATTGACGATATACATTGGCCAAGCAATAAXXXXXXXXXXG
AGAGAGAAGTCAAGAAATGATGGATTAAAAAGGTATAAACATGTAATATATATAAAGAATGTAGGCCTTAGGCCATTGT
ACTCCTCTTTGTGCATGTTAGCCTTATTTTTGCTACTTGCCCTCTTGAGTAATTGACGATATACATTGGCCAAGCAATAAXXXXXXXXXXG
AGAGAGAAGTCAAGAAATGATGGATTAAAAAGGTATAAACATGTAATATATATAAAGAATGTAGGCCTTAGGCCATTGT
>Marker214874 Chr6 SNP_site:2334628 Ref_Type:G
ACGGAGAATGATTGACATTATGATAGAATTGCTATTAATTCATCCATGTGCTATATGACACATGCTCCAAAGTATTTCTCXXXXXXXXXXA
TTCTCTCTTCTTAATATTTTTTTTTTTGGAATATGTTGGTTATCTTTGGTATTTGGTATTAGTTCTTACAGGTTTCTTGT
ACGGAGAATGATTGACATTATGATAGAATTGCTATTAATTCATCCATGTGCTATATGACACATGCTCCAAAGTATTTCTCXXXXXXXXXXA
TTCTCTCTTCTTAATATTTTTTTTTTTGGAATATGTTGGTTATCTTTGGTATTTGGTATTAGTTCTTACATGTTTCTTGT
>Marker214942 Chr6 SNP_site:13609808 Ref_Type:T
AACTTTCTCATATCCAAAATGTTCAACAACCTTCTTCAATAAAATAGACTCAAATTATGAACTTTCAAGACCAATTTTAGXXXXXXXXXXT
GAATTAATTAAACGTTAGCTAAAACGTTTGAATTAATTAAACGTTAGCTAAAACGCCCAACCTCTACGTTCCTTCTGTA
AACTTTCTCATATCCAAAATGTTCAACAACCTTCTTCAATAAAATAGACTCAAATTATGAACTTTCAAGACCAATTTTAGXXXXXXXXXXT
GAATTAATTAAACGTTAGCTAAAATGTTTGAATTAATTAAACGTTAGCTAAAACGCCCAACCTCTACGTTCCTTCTGTA
>Marker215173 Chr6 SNP_site:5296511 Ref_Type:C
GACCACCTTTTAGCCTAATCTAGAAGATGATCATCGGCTACAAACCTCAACAACAAGGCTAACACACCAGCTCTTACTGCXXXXXXXXXXG
TTAAGTTTCAATAACCGTGCATATTCAAACACAATTAACAATATCGATATCAGCGCCGTCCGATAAATGATTTAATGTA
GACCACCTTTTAGCCTAATCTAGAAGATGATCATCGGCTACAAACCTCAACAACAAGGCTAACACACCAGCTGTTACTGCXXXXXXXXXXG
TTAAGTTTCAATAACCGTGCATATTCAAACACAATTAACAACATCGATATCAGCGCCGTCCGAGAAATGATTTAATGTA
>Marker215173 Chr6 SNP_site:5296687 Ref_Type:T
GACCACCTTTTAGCCTAATCTAGAAGATGATCATCGGCTACAAACCTCAACAACAAGGCTAACACACCAGCTCTTACTGCXXXXXXXXXXG

Create PDF with GO2PDF for free, if you wish to

TTAAGTTTCAATAACCGTGCATATTCAAACACAATTAACAATATCGATATCAGCGCCGTCCGATAAATGATTTAATGTA
GACCACCTTTTAGCCTAATCTAGAAGATGATCATCGGCTACAAACCTCAACAACAAGGCTAACACACCAGCTGTTACTGCXXXXXXXXXXG
TTAAGTTTCAATAACCGTGCATATTCAAACACAATTAACAACATCGATATCAGCGCCGTCCGAGAAATGATTTAATGTA
>Marker215173 Chr6 SNP_site:5296709 Ref_Type:T
GACCACCTTTTAGCCTAATCTAGAAGATGATCATCGGCTACAAACCTCAACAACAAGGCTAACACACCAGCTCTTACTGCXXXXXXXXXXG
TTAAGTTTCAATAACCGTGCATATTCAAACACAATTAACAATATCGATATCAGCGCCGTCCGATAAATGATTTAATGTA
GACCACCTTTTAGCCTAATCTAGAAGATGATCATCGGCTACAAACCTCAACAACAAGGCTAACACACCAGCTGTTACTGCXXXXXXXXXXG
TTAAGTTTCAATAACCGTGCATATTCAAACACAATTAACAACATCGATATCAGCGCCGTCCGAGAAATGATTTAATGTA
>Marker215305 Chr6 SNP_site:2335047 Ref_Type:A
GACAACCTGAGAGAAAGACCTGATTAAAGAACCTCAAGGGGAGAGTGAGGGAATTGACCCCAACATTAAAAAATGGATAAXXXXXXXXXXG
TTTATAGGCAAACATTTCGGGAATAAATTCGGAGAAACTCACTTATTTTGAATGGAAGAAGCTCAATGAGTTTCAATGT
GACAACCTGAGAGAAAGACCTGATTAAAGAACCTCAAGGGGAGAGTGAGGGAATTGACCCCAACATTAAAAAATGGATAAXXXXXXXXXXG
TTTATAGGCAAACATTTCGGGAATAAATTCGGAGAAACTCACTTATTTTGTATGGAAGAAGCTCAATGAGTTTCAATGT
>Marker216324 Chr6 SNP_site:9508903 Ref_Type:A
ACCCTTTCTACAGCTCAGCCTCGCCGCCGCTTCTCTTCCGTTCAGTTCTACCGCCGCCTCTCTTTAGCCTAAACCTCCATXXXXXXXXXXT
CTCTCTGGTCTTTGCTGAAAACTGTCTTGTTTCCTCGACAATTAAACGTCCCAGCATTCCCTTTCTTCTTTATTTATGT
ACCCTTTCTACAGCTCAGCCTCGCCGCCGCTTCTCTTCCGTTCAGTTCTACCGCCGCCTCTCTTTCGCCTAAACCTCCATXXXXXXXXXXT
CTCTCTTGTCTTTGCTGAAAACTGTCTTGTTTCCTCGACAATTAAACGTCCCAGCATTCCCTTTCTTCTTTATTTATGT
>Marker216324 Chr6 SNP_site:9509057 Ref_Type:G
ACCCTTTCTACAGCTCAGCCTCGCCGCCGCTTCTCTTCCGTTCAGTTCTACCGCCGCCTCTCTTTAGCCTAAACCTCCATXXXXXXXXXXT
CTCTCTGGTCTTTGCTGAAAACTGTCTTGTTTCCTCGACAATTAAACGTCCCAGCATTCCCTTTCTTCTTTATTTATGT
ACCCTTTCTACAGCTCAGCCTCGCCGCCGCTTCTCTTCCGTTCAGTTCTACCGCCGCCTCTCTTTCGCCTAAACCTCCATXXXXXXXXXXT
CTCTCTTGTCTTTGCTGAAAACTGTCTTGTTTCCTCGACAATTAAACGTCCCAGCATTCCCTTTCTTCTTTATTTATGT
>Marker216436 Chr6 SNP_site:27789382 Ref_Type:C
AACTTTTTGTCAAATTTGAATAATTAACCTAATTTATTTTTCACATTTTTAAAATTGGTGTAGGCACTAAACTTAAAATTXXXXXXXXXXT
AAAAGAATTGAAAGTAATTGAATAATTGATTAGACAAAATTTTAAAAGTATTTTGAAAATAGAATAGACAGAAAATAGT
AACTTTTTGTCAAATTTGAATAATTAACCTAATTTCTTTTTCACATTTTTAAAATTGGCGTAGGCACTAAACTTAAAATTXXXXXXXXXXT
AAAAGAATTGAAAGTAATTGAATAATTGATTAGACAAAATTTTAAAAGTATTTTGAAAATAGAATAGACAGAAAATAGT
>Marker216436 Chr6 SNP_site:27789405 Ref_Type:C
AACTTTTTGTCAAATTTGAATAATTAACCTAATTTATTTTTCACATTTTTAAAATTGGTGTAGGCACTAAACTTAAAATTXXXXXXXXXXT
AAAAGAATTGAAAGTAATTGAATAATTGATTAGACAAAATTTTAAAAGTATTTTGAAAATAGAATAGACAGAAAATAGT
AACTTTTTGTCAAATTTGAATAATTAACCTAATTTCTTTTTCACATTTTTAAAATTGGCGTAGGCACTAAACTTAAAATTXXXXXXXXXXT
AAAAGAATTGAAAGTAATTGAATAATTGATTAGACAAAATTTTAAAAGTATTTTGAAAATAGAATAGACAGAAAATAGT
>Marker216706 Chr6 SNP_site:2931929 Ref_Type:A
ACAAAAAACTAGCAATAATTTACGTGAATTGGCAACACCAAAATATATTGAAATGAAAAATATTGTGTTTAATCATATTTXXXXXXXXXXT
ACTAATAAAGTAATGTTTGATGCTTCACCTCTTAATTCATTTGGAGTGCTTTTATGCATGTTTAGGAAGCCTTCTGAGT
ACAAAAAACTAGCAATAATTTACGTGAATTGGCAACACCAAAATATATTGAAATGAAAAATATTGTGTTTCATCATATTTXXXXXXXXXXT
ACTAATAAAGTAATGTTTGATGCTTCACCTCTTAATTCATTTGGAGTGCTTTTATGCATGTTTAGGAAGCCTTCTGAGT
>Marker217283 Chr6 SNP_site:26634049 Ref_Type:A
TACAAATTGAATAGAAAAATATAGTAGCGACTCTCGATATCTCGAACAGTTTCTGTGGGGGGAATAATAAAGTAATTATAXXXXXXXXXXT
CAATGGCTAAGAATAATTTCGTTTATGATATATATATAAAGAAAAAGGTGGAAATCGTTGAAGTATCGTCAGCACCGTT
TACATATTGAATAGAAAAATATAGTAGCGACTCTCGATATCTCGAACAGTTTCTGTGGGGGGAATAATAAAGTAATTATAXXXXXXXXXXT
CAATGGCTAAGAATAATTTCGTTTATGATATATATATAAAGAAAAAGGTGGAAATCGTTGAAGTATCGTCAGCACCGTT
>Marker218302 Chr6 SNP_site:8876874 Ref_Type:G

Create PDF with GO2PDF for free, if you wish to

TACATCAACACACACTTGACATATTTGTTTTTAAGTTGATGTTTTTTAAAAAAAATAAAAATAAGTAACTCTTTCGATCAXXXXXXXXXXT
ATCTTAAAAAATTTATGGAAAATAAAGTTCTTCATTTATTGTTTTCGATGTAGCTTTTGTTAATTGCAAGCAATAATGT
TACATCAACACACACTTGACATATTTGTTTTTAAGTTGATTTTTTTTTAAAAAAATAAAAATAAGTAACTCTTTCGATCAXXXXXXXXXXT
ATCTTAAAAAATTTATGAAAAATAAAGTTCTTCATTTATTGTTTTCGATGTAGCTTTTGTTAATTGCAAGCAATAATGT
>Marker218302 Chr6 SNP_site:8876881 Ref_Type:A
TACATCAACACACACTTGACATATTTGTTTTTAAGTTGATGTTTTTTAAAAAAAATAAAAATAAGTAACTCTTTCGATCAXXXXXXXXXXT
ATCTTAAAAAATTTATGGAAAATAAAGTTCTTCATTTATTGTTTTCGATGTAGCTTTTGTTAATTGCAAGCAATAATGT
TACATCAACACACACTTGACATATTTGTTTTTAAGTTGATTTTTTTTTAAAAAAATAAAAATAAGTAACTCTTTCGATCAXXXXXXXXXXT
ATCTTAAAAAATTTATGAAAAATAAAGTTCTTCATTTATTGTTTTCGATGTAGCTTTTGTTAATTGCAAGCAATAATGT
>Marker218302 Chr6 SNP_site:8877041 Ref_Type:G
TACATCAACACACACTTGACATATTTGTTTTTAAGTTGATGTTTTTTAAAAAAAATAAAAATAAGTAACTCTTTCGATCAXXXXXXXXXXT
ATCTTAAAAAATTTATGGAAAATAAAGTTCTTCATTTATTGTTTTCGATGTAGCTTTTGTTAATTGCAAGCAATAATGT
TACATCAACACACACTTGACATATTTGTTTTTAAGTTGATTTTTTTTTAAAAAAATAAAAATAAGTAACTCTTTCGATCAXXXXXXXXXXT
ATCTTAAAAAATTTATGAAAAATAAAGTTCTTCATTTATTGTTTTCGATGTAGCTTTTGTTAATTGCAAGCAATAATGT
>Marker218367 Chr6 SNP_site:18217960 Ref_Type:C
CACAGTTTCAAAGAATCGGGGTTTTCATTCGCCGTCCGTCTGCAAACCATCGCCGTCAGTTGAGAAATGGTCCTCCGATAXXXXXXXXXXG
TTCGAGAGATTTCTTTACGAAGGTGTATTTTCGCTTCAACCTAAGGTTACAATCCGGATCGGTCGGTTCTCAGACGGTT
CACAGTTTCAAAGAATCGGGGTTTTCATTCGCCGTCCGTCTGCAAACCATCGCCGTCAGTTGAGAAATGGTCCTCCGATAXXXXXXXXXXG
TTCGAGAGATTTCTTTACGAAGGTGTATTTTCGCTTCAACCTAAGGTTATAATCCGGATCGGTCGGTTCTCAGACGGTT
>Marker218451 Chr6 SNP_site:8031707 Ref_Type:A
CACTTCTTCTATCATTCTTCCATTTCTCTATTGTTATTCATATTCAATCTGTTTAATTAGTTCATGAGCAATGGAATGTGXXXXXXXXXXT
AACTAAGCAAGTGACGAGGAGATTGTAGTGATACAATATTATCATCGAATTCATCATTCGCATAAGTTGCAAAGATGTT
CACTTCTTCTATCATTCTTCCATTTCTCTATTGTTATTCATATTCAATCTGTTTAATTAGTTCATGAGCAATGGAATGTGXXXXXXXXXXT
AACTAAGCAAGTGACGAGGAGATTGTAGTGTTACAATATTATCATCGAATTCATCATTCGCATAAGTTGCAAAGATGTT
>Marker218807 Chr6 SNP_site:5291539 Ref_Type:T
CACAAATTTCTTAATTTCATATCTACTCGGACCACTGGGTGAAATTAGAATTTGACATAGTATTAATCTTTTTTCTGTTTXXXXXXXXXXG
GCTATATTTGGTGGAAGAGAAAAAGTGGGAATGCTTGAGATTCCAAAATTTCTATCCATTCATTTTGTGAAATCTGGTT
CACAAATTTCTTAATTTCATATCTACTTGGACCACTGGGTGAAATTAGAATTTGACATAGTATTAATCTTTTTTCTGTTTXXXXXXXXXXG
GCTATATTTGGTGGAAGAGAAAAAGTGGGAATGCTTGAGATTCCAAAATTTCTATCCATTCATTTTGTGAAATCTGGTT
>Marker218952 Chr6 SNP_site:4010119 Ref_Type:C
ACTATCTTCTTTGGCTGTAATTTACTGTATTTTCCAGCTGGAAGTCTTGTTTTTCAAAGATATATCATGACTAGATCTCCXXXXXXXXXXA
TGTTTTCGGATTCCATACTAAGATCAACCTTAGAAGGAATGTTAGCAAGATCAACAATTAGTCTAGGTAATTTAGTGTA
ACTATCTTCTTTGGCTGTAATTTACTGTATTTTCCAGCTGGAAGTCTTGTTTTTCAAAGATATATCATGACTAGATCTCCXXXXXXXXXXA
TGTTTTCGGCTTCCATACTACGATCAACCTTAGAAGGAATGTTAGCAAGATCAACAATTAGTCTAGGTAATTTAGTGTA
>Marker218952 Chr6 SNP_site:4010130 Ref_Type:C
ACTATCTTCTTTGGCTGTAATTTACTGTATTTTCCAGCTGGAAGTCTTGTTTTTCAAAGATATATCATGACTAGATCTCCXXXXXXXXXXA
TGTTTTCGGATTCCATACTAAGATCAACCTTAGAAGGAATGTTAGCAAGATCAACAATTAGTCTAGGTAATTTAGTGTA
ACTATCTTCTTTGGCTGTAATTTACTGTATTTTCCAGCTGGAAGTCTTGTTTTTCAAAGATATATCATGACTAGATCTCCXXXXXXXXXXA
TGTTTTCGGCTTCCATACTACGATCAACCTTAGAAGGAATGTTAGCAAGATCAACAATTAGTCTAGGTAATTTAGTGTA
>Marker219360 Chr6 SNP_site:12415995 Ref_Type:C
GACTTCCTAACTATCTTGGTGCTGCTTGTTGTATTTGATCCCAGATCTAGTGTTTTCAAGGCTTCCTCCACCAAACCAGCXXXXXXXXXXA
ATCGAATTGAATTGATCGGTGGCTTTAATTCCTCTTTTGGTTTTCATCTCTAGCCAGAGCTCTTCAGCGCGGCTTAGTA
GACTTCCTAACTATCTTGGTGCTGCTTGTTGTATTTGATCCCAGATCTAGTGTTTTCAAGGCTTCCTCCACCAAACCAGCXXXXXXXXXXA
ATCGAATTGAATTGATCGGTGGCTTTAATTCCTTTTTTGGTTTTCATCTCTAGCCAGAGCTCTTCAGCGCGGCTTAGTA

Create PDF with GO2PDF for free, if you wish to

>Marker219574 Chr6 SNP_site:21460334 Ref_Type:A

CACCAATCAACTGCAGCTCCATGACCCTCTCCTTTAATGATCTCAGGAGCCAAATATTCGTGAGTGCCAACAAAGGAATTXXXXXXXXXXG
CAGGTAGAAACCTGGGGCTGAAGCATGGGACTTGACATGATGGTTCAATGCAAAATGGTTCAGCACAACTAGAATCTGT
CACCAATCAACTGCAGCTCCATGACCCTCTCCTTTAATGATCTCAGGAGCCAAATATTCGTGAGTGCCAACAAAGGAATTXXXXXXXXXXG
CAGGTAGAAACCTGGGGCTGAAGCATGGGACTTGACATGATGGTTCAATGCAAAATGGTTCGGCACAACTAGAATCTGT

>Marker219688 Chr6 SNP_site:1892522 Ref_Type:C

TACCACACTTTTCTTGCTAGGTTGCAGTTGTGCGAGTGATCTCAATGCAACAACAATTCATCATCAATTCTATCACGCATXXXXXXXXXXA
GAAATAGGGTCTTCCCAAACGCCCCCACATCGATAAAAACCCCAAACATGTTGGGAAAGGTTACCCCGACGTCTTAGTT
TACCACACTTTTCTTGCTAGGTTGCAGTTGTGCGAGTGATCTCAATGCAACAACAATTCATCATCAATTCTATCACGCATXXXXXXXXXXA
GAAATAGGGTCTTCCCAAACGCCCCCACATCGATAAAAACCCCAAACATGTTGGGAAAGGTTACCTCGACGTCTTAGTT

>Marker22068 Chr4 SNP_site:18584217 Ref_Type:G

ACTTCGAGCATTAGTCTCATTAATTTTTAATAAAGAGACCTGTCATCGTTTCAAAAAAAAAAAATTTTTGTTAAGGTATTXXXXXXXXXXA
CAACACCTTGTTGGTGGTTGTGGAAATGCCATTGCTTGTAGAAAATGGCCTGATCACGTGAAGGAAGAAATTAAAGAGT
ACTTCGAGCATTAGTCTCATTAATTTTTAATAAAGAGACCTGTCATCGTTTCAAAAAAAAGAAAATTTTGTTAAGGTATTXXXXXXXXXXA
CAACACCTTGTTGGTGGTTGTGGAAATGCCATTGCTTGTAGAAAATAGCCTGATCACGTGAAGGAAGAAATTAAAGAGT

>Marker22068 Chr4 SNP_site:18584221 Ref_Type:A

ACTTCGAGCATTAGTCTCATTAATTTTTAATAAAGAGACCTGTCATCGTTTCAAAAAAAAAAAATTTTTGTTAAGGTATTXXXXXXXXXXA
CAACACCTTGTTGGTGGTTGTGGAAATGCCATTGCTTGTAGAAAATGGCCTGATCACGTGAAGGAAGAAATTAAAGAGT
ACTTCGAGCATTAGTCTCATTAATTTTTAATAAAGAGACCTGTCATCGTTTCAAAAAAAAGAAAATTTTGTTAAGGTATTXXXXXXXXXXA
CAACACCTTGTTGGTGGTTGTGGAAATGCCATTGCTTGTAGAAAATAGCCTGATCACGTGAAGGAAGAAATTAAAGAGT

>Marker22068 Chr4 SNP_site:18584443 Ref_Type:A

ACTTCGAGCATTAGTCTCATTAATTTTTAATAAAGAGACCTGTCATCGTTTCAAAAAAAAAAAATTTTTGTTAAGGTATTXXXXXXXXXXA
CAACACCTTGTTGGTGGTTGTGGAAATGCCATTGCTTGTAGAAAATGGCCTGATCACGTGAAGGAAGAAATTAAAGAGT
ACTTCGAGCATTAGTCTCATTAATTTTTAATAAAGAGACCTGTCATCGTTTCAAAAAAAAGAAAATTTTGTTAAGGTATTXXXXXXXXXXA
CAACACCTTGTTGGTGGTTGTGGAAATGCCATTGCTTGTAGAAAATAGCCTGATCACGTGAAGGAAGAAATTAAAGAGT

>Marker221483 Chr6 SNP_site:18775439 Ref_Type:T

GACATACAATGTTAGATAATGTTGGGTGTTTTATTCTAGTGACGGATAATTGTTACGAGAATTGTAAAATACTACGAACGXXXXXXXXXXT
ATAACACTGTTAATTGAAGAGATTAACATTTCAATAGAATGAGCAAATGTGATTCTAACCTAATCATGATTGAGTTGTA
GACATACAATGTTAGATAATGTTGGGTGTTTTATTCTAGTGACGGATAATTGTTACGAGAATTGTAAAATACTACGAACGXXXXXXXXXXT
ATAATACTGTTAATTGAAGAGATTAACATTTCAATAGAATGAGCAAATGTGATTCTAACATAATCATGATTGAGTTGTA

>Marker221483 Chr6 SNP_site:18775494 Ref_Type:A

GACATACAATGTTAGATAATGTTGGGTGTTTTATTCTAGTGACGGATAATTGTTACGAGAATTGTAAAATACTACGAACGXXXXXXXXXXT
ATAACACTGTTAATTGAAGAGATTAACATTTCAATAGAATGAGCAAATGTGATTCTAACCTAATCATGATTGAGTTGTA
GACATACAATGTTAGATAATGTTGGGTGTTTTATTCTAGTGACGGATAATTGTTACGAGAATTGTAAAATACTACGAACGXXXXXXXXXXT
ATAATACTGTTAATTGAAGAGATTAACATTTCAATAGAATGAGCAAATGTGATTCTAACATAATCATGATTGAGTTGTA

>Marker222357 Chr6 SNP_site:10323535 Ref_Type:C

AACTCACCTCTTCTAGAGGGGATTGAATATTCGGAGGTGGTGTTGAGTGCTTTTCAGAATATACAGCTGCTTGTCCTAATXXXXXXXXXX-
TAATGAAAAAAAAAGGCGGCGTTATTAATTCTAAGAAAGCTGAGACATAACCTGCAGCAATCTAATCAGATATAAAGGTT
AACTCACCTCTTCTAGAGGGGATTGAATATTCGGAGGTGGTGTTGAGTGCTTTTCAGAATATACAGCTGCTTGTCCTAATXXXXXXXXXXT
TAATG-AAAAAAAAGGCGACGTTATTAATTCTAAGAAAGCTGAGACATAACCTGCAGCAATCTAATCAGATATAAAGGTT

>Marker222398 Chr6 SNP_site:8676655 Ref_Type:A

AACGTTGAAGAAAAATAACTTCACATGTTATCACTTCCATTCTTTGATACTTTGCTTCTGCAGTTGAGTGGGATACAGTTXXXXXXXXXXA
TCTGCATCAGCAAAACCACAAATGGAGAAATTTTGCACTTTTGACGAGAACATTTCTTTTCCAGGGGAGCCTTTGATGT
AACGTTGAAGAAAAATAACTTCACATGTTATCACTTCCATTCTTTGATACTTTGCTTCTGCAGTTGAGTGGGATACAGTTXXXXXXXXXXA

Create PDF with GO2PDF for free, if you wish to

TCTGCATCAGCAAAACCACAAATGGAGAAATTTTGCACTTTTGACGAGAACATTTCTTTTCCTGGGGAGCCTTTGATGT
>Marker223472 Chr6 SNP_site:3427307 Ref_Type:C
ACCACTGATTCTTCTAATGAACAATAATTATAGTCCCACTATAACTGAACTCATCTCAGGCCAAGAGAGGGTATGGCGCCXXXXXXXXXXA
AACGGTAGGCATATTGAGTCGGCGACATAGACCACTCTCTCCCATGAAAATTAAAGGATCACCTTCTTAGGCAGAAGTT
ACCACTGATTCTTCTAATGAACAATAATTATAGTCCCACTATAACTGAATTCATCTCAGGCCAAGAGAGGGTATGGCGCCXXXXXXXXXXA
AACGGTAGGCATATTGAGTCGGCGACATAGACCACTCTCTCCCATGAAAATTAAAGGATCACCTTCTTAGGCAGAAGTT
>Marker224105 Chr6 SNP_site:27464066 Ref_Type:T
ACTAAAGAAATCTATCATCTTCCTAGTTCTCAACTAAAAGTAGTCTTTTTTAATTAAAAAGTTTCTCACTTTTTATGACCXXXXXXXXXXA
TTAAGTTTATTTTATCGTTGTGACATTTAATTTCTTTGAAACATCTCTTGTTCTAAAAAAAATTGAAAAATGAAGGAGT
ACTAAAGAAATCTATCATCTTCCTAGTTCTCAACTAAAAGTAGTCTTTTTTAATTAAAATGTTTCTCACTTTTTATGACCXXXXXXXXXXA
TTAAGTTTATTTTATCGTTGTGACATTTAATTTCTTTAAAACATCTCTTGTTCTAAAAAAAATTGAAAAATGAAGGAGT
>Marker224105 Chr6 SNP_site:27464232 Ref_Type:A
ACTAAAGAAATCTATCATCTTCCTAGTTCTCAACTAAAAGTAGTCTTTTTTAATTAAAAAGTTTCTCACTTTTTATGACCXXXXXXXXXXA
TTAAGTTTATTTTATCGTTGTGACATTTAATTTCTTTGAAACATCTCTTGTTCTAAAAAAAATTGAAAAATGAAGGAGT
ACTAAAGAAATCTATCATCTTCCTAGTTCTCAACTAAAAGTAGTCTTTTTTAATTAAAATGTTTCTCACTTTTTATGACCXXXXXXXXXXA
TTAAGTTTATTTTATCGTTGTGACATTTAATTTCTTTAAAACATCTCTTGTTCTAAAAAAAATTGAAAAATGAAGGAGT
>Marker22455 Chr4 SNP_site:6252151 Ref_Type:G
TACCTTGGCTAGGTGGGATTGTAAAATGGTGAGAAGGCCCTGACATTGCTTAGCATTCATGTTTGAAAGAGGGTCAGTGTXXXXXXXXXXT
GAGCGAGATTGTGGTGGATTTTGTGAAACATTTTTCACTAACATGGCTGAGTTTTCATAATTTATAGGAAGGTTAGGTG
TACCTTGGCTAGGTGGGATTGTAAAATGGTGAGAAGGCCCTGGCATTGCTTAGCATTCATGTTTGAAAGAGGGTCAGTGTXXXXXXXXXXT
GAGCGAGATTGTGGTGGATTTTATGAAACATTTTTCACTAACATGGCTGAGTTTTCATAATTTATAGGAAGGTTAGGTG
>Marker22455 Chr4 SNP_site:6252363 Ref_Type:A
TACCTTGGCTAGGTGGGATTGTAAAATGGTGAGAAGGCCCTGACATTGCTTAGCATTCATGTTTGAAAGAGGGTCAGTGTXXXXXXXXXXT
GAGCGAGATTGTGGTGGATTTTGTGAAACATTTTTCACTAACATGGCTGAGTTTTCATAATTTATAGGAAGGTTAGGTG
TACCTTGGCTAGGTGGGATTGTAAAATGGTGAGAAGGCCCTGGCATTGCTTAGCATTCATGTTTGAAAGAGGGTCAGTGTXXXXXXXXXXT
GAGCGAGATTGTGGTGGATTTTATGAAACATTTTTCACTAACATGGCTGAGTTTTCATAATTTATAGGAAGGTTAGGTG
>Marker224789 Chr6 SNP_site:3533741 Ref_Type:T
TACACATTACCATGGTTGCATCACTATTTTAAACTTGGAAGCCTCGATTGAGACTTAAGTGTGACTGGTAACTATGAATTXXXXXXXXXXG
TGAATTCTTGGGCTTTTCCTGTCTTGGTTGATATCAGTAACTTCGATTTTGCCCAATGATGGAAAATCACTATGAAGTG
TACACATTACCATGGTTGCATCACTATTTTAAACTTGGAAGCCTCGATTGAGACTTAAGTGTGACTGGTAACTATGAATTXXXXXXXXXXG
TGAATTCTTGGGCTTTTCCTGTCTTGGTTGATATCAGTAACTTTGATTTTGCCCAATGATGGAAAATCACTATGAAGTG
>Marker22480 Chr4 SNP_site:1901875 Ref_Type:G
CACTGCGCAGGGCTATCAATTTCATAAATGTTTTGCATTTTAACTTATCAAGCTCTGATAGTATCTGTATTGAACTAAAGXXXXXXXXXXT
TTTTCTCCAACTAAAAACCTGAGTTATATAGAAGGAAGAAGACATTCCGATTGGCAGAGGGAATAGGGCTTCGAAGGTT
CACTGCGCAGGGCTATCAATTTCATAAATGTTTTGCATTTTAACTTATCAAGCTCTGATAGTATCTGTATTGAACTAAAGXXXXXXXXXXT
TTTTCTCCAACTAAAAACCTGAGTTATATAGAGGGAAGAAGACATTCCGATTGGCAGAGGGAATAGGGCTTCGAAGGTT
>Marker224949 Chr6 SNP_site:25439046 Ref_Type:C
ACCCAAATATATATGTTTCAAAAAAAGGGAAAAAAGAAAAGACCTGAAGTAAGGAGTTTTTGGCATAGCCTTTGTCTCCAXXXXXXXXXXG
ACCCTTTTATCTCCAACTCTATCTCTTACGATCCTTCCCCCCATGCACATTTAATTTAATTTATATGCCTTTCCAGGTG
ACCCAAATATATATGTTTCAAAAAAAGGGAAAAAAGAAAAGACCTGAAGTAAGGAGTTTTTGGCATAGCCTTTGTCTCCAXXXXXXXXXXG
ACCCTTTTATCTCCAACTCTATCTCTTATGATCCTTCCCCCCATGCACATTTAATTTAATTTATATGCCTTTCCAGGTG
>Marker225276 Chr6 SNP_site:21945978 Ref_Type:A
ACTATCATCATCTATCGTTGATAAACAACTAAAAAATTGCTATAATGGTAAATATTTTCAACAATTTTACTATTTAAAACXXXXXXXXXXA
TATTAAGATGTTTGATAGTAACAGTGCCTAGCCAATGTCGAGATATAATTATTATAGGAGGAGAATAATTGGGAGAGTT

Create PDF with GO2PDF for free, if you wish to

ACTATCATCATCTATCGTTGATAAACAACTAAAAAATTGCTATAATGGTAAATATTTTCAACAATTTTACTATTTAAAACXXXXXXXXXXXA
TATTGAGATGTTTGATAGTAACAGTGCCTAGCCAATGTCGAGATATAATTATTATAGGAGGAGAATAATTGGGAGAGTT
>Marker225421 Chr6 SNP_site:5874891 Ref_Type:A
ACAACAGTTAGTTGATGACAATTTATTACCTCTACAAGCAGACTGCAAACGTATAGCCGACCTCCGAAGCAATACAAAACXXXXXXXXXXXT
CTAAGAAACACCTTTGTCTTACCAATCTGCCAGAATATGTTACATTTGTAACATAACACGCTATAGTTTATACTTTGTA
ACAACAGTTAGTTGATGACAATTTATTACCTCTACAAGCAGACTGCAGACGTATAGCCGACCTCCGAAGCAATACAAAACXXXXXXXXXXXT
CTAAGAAACACCTTTGTCTTACCAATCTGCCAGAATATGTTACATTTGTAACATAACACGCTATAGTTTATACTTTGTA
>Marker225895 Chr6 SNP_site:18988151 Ref_Type:C
GACACCTTGTGACAACATGATAAGAACATCAAAATCACGAAGTAATGGCAAAGTGATATATCACAAATTGAACCAAAAGAXXXXXXXXXXXT
TTTGAACGTAGGACCTTCTGATATGATGCCATTGTAAATCACCAATCAACTTAGAAACTTAGATCATTGGGTTGTGGTA
GACACCTTGTGATAACATGATAAGAACATCAAAATCACGAAGTAATGGCAAAGTGATATATCACAAATTGAACCAAAAGAXXXXXXXXXXXT
TTTGAACGTAGGACCTTCTGATATGATGCCATTGTAAATCACCAATCAACTTAGAAACTTAGATCATTGGGTTGTGGTA
>Marker226152 Chr6 SNP_site:3325185 Ref_Type:T
TACAGTTGAATTCAATAGGAGTATTAGCAAGATGATTTCCGAATATACTTGGCTTGGTTAGCAAATCAAGGGCGTATTTTXXXXXXXXXXXA
ACAAGCAACTTCTAAAAGGTGATGATTTTTTGTTCGGCAACCCCATTTTGTTGGGGGTGTAAGCATAGAACCTTTGGTG
TACAGTTGAATTCAATAGGAGTATTAGCAAGATGATTTCCGAATATACTTGGCTTGGTTAGCAAATCAAGGGTGTATTTTXXXXXXXXXXXA
ACAAGCAACTTCTAAAAGGTGATGATTTTTTGTTCGGCAACCCCATTTTGTTGGGGGTGTAAGCATAGAACCTTTGGTG
>Marker226221 Chr6 SNP_site:2525657 Ref_Type:A
TACAAATGTTAGGGACAATTCTAATAAACTCAAATTTAATCGGAAAAGATTTTAGAATAACATTATCGTCTAAATCTTGAXXXXXXXXXXXT
AACCATTTCAAAACTGTCATTGAAGCTAGCGTCAAGAAAGTTGAACTTCTTGACAGTTAATAAAACGTCAAGAATAGTG
TACAAATGTTAGGGACAATTCTAATAAACTCAAATTTAATCGGAAAAGATTTTAGAATAACGTTATCGTCTAAATCTTGAXXXXXXXXXXXT
AACCATTTCAAAACTGTCATTGAAGCTAGCATCAAGAAAGTTGAACTTCTTGACAGTTAATAAAACGTCAAGAATAGTG
>Marker226221 Chr6 SNP_site:2525811 Ref_Type:G
TACAAATGTTAGGGACAATTCTAATAAACTCAAATTTAATCGGAAAAGATTTTAGAATAACATTATCGTCTAAATCTTGAXXXXXXXXXXXT
AACCATTTCAAAACTGTCATTGAAGCTAGCGTCAAGAAAGTTGAACTTCTTGACAGTTAATAAAACGTCAAGAATAGTG
TACAAATGTTAGGGACAATTCTAATAAACTCAAATTTAATCGGAAAAGATTTTAGAATAACGTTATCGTCTAAATCTTGAXXXXXXXXXXXT
AACCATTTCAAAACTGTCATTGAAGCTAGCATCAAGAAAGTTGAACTTCTTGACAGTTAATAAAACGTCAAGAATAGTG
>Marker226758 Chr6 SNP_site:3977784 Ref_Type:A
TACTTAGGGAGACTTTTTTGTTTTAGTTATTACGAAATGGAGTTGTCTTCTAAGCATTAGTCCATGCCAAGGAATTTATTXXXXXXXXXXXA
TTTCGTTTATACGTGGATGCACTCTTAAAGTAGAACTCATTTGTCCTGTTCAAAGTGCGATTTTTTATTCAAAGTTGTT
TACTTAGGGAGTCTTTTTTGTTTTAGTTATTACGAAATGGAGTTGTCTTCTAAGCATTAGTCCATGCCAAGGAATTTATTXXXXXXXXXXXA
TTTCGTTTATACGTGGATGCACTCTTAAAGTAGAACTCATTTGTCCTGTTCAAAGTGCGATTTTTTATTCAAAGTTGTT
>Marker22683 Chr4 SNP_site:20988534 Ref_Type:G
CACTTTCGTAATCATTTTGATCTTAATTACTATTTTACTTTATTTTGTTTCAATTCTGTATTATAATTTTCAAAACCCTAXXXXXXXXXXXG
AAAAAAACCTAAATGAGACATTCAAACTTGAAAATAATTTATCAACTAGAATTATTTTAAACCCATATAATAATAAGTT
CACTTTGGTAATCATTTTGATCTTAATTACTATTTTACTTTCTTTTGTTTCAATTCTGTATTATAATTTTCAAAACCCTAXXXXXXXXXXXG
AAAAAAACCTAAATGAGACATTCAAACTTGAAAATAATTTATCAACTAGAATTATTTTAAACCCATATAATAATAAGTT
>Marker22683 Chr4 SNP_site:20988569 Ref_Type:C
CACTTTCGTAATCATTTTGATCTTAATTACTATTTTACTTTATTTTGTTTCAATTCTGTATTATAATTTTCAAAACCCTAXXXXXXXXXXXG
AAAAAAACCTAAATGAGACATTCAAACTTGAAAATAATTTATCAACTAGAATTATTTTAAACCCATATAATAATAAGTT
CACTTTGGTAATCATTTTGATCTTAATTACTATTTTACTTTCTTTTGTTTCAATTCTGTATTATAATTTTCAAAACCCTAXXXXXXXXXXXG
AAAAAAACCTAAATGAGACATTCAAACTTGAAAATAATTTATCAACTAGAATTATTTTAAACCCATATAATAATAAGTT
>Marker227459 Chr6 SNP_site:749048 Ref_Type:A
ACTAAAAAGCACATATACGGCTAGTTTCAGTAAAATCTTCCAATTGTTAAATCTACAAAACATCACCCGAGAATTTCATTXXXXXXXXXXXT

Create PDF with GO2PDF for free, if you wish to

GTGATTATAAAAAGCTCTATAACGGGACGGCTTAATTTTAAAAACAAATTATTAGAATGATTCATCTGTCAAAATTGTG
ACTAAAAAGCACATATACGGCTTGTTTCAGTAAAATCTTCCAATTGTTAAATCTACAAAACATCACCCGAGAATTTCATTXXXXXXXXXXT
GTGATTATAAAAAGCTCTATAACGGGACGGCTTAATTTTAAAAACAAATTATTAGAATGATTCATCTGTCAAAATTGTG
>Marker227774 Chr6 SNP_site:7191335 Ref_Type:G
TACTTATTTCTTTTTCTTTATTAATATTAACAAGATACATTAATGAGGAAGGTTAGTAGGTGGCATGGGAAAAATCTCAAXXXXXXXXXXG
CACTAAATTGAAATAACATTCAAAATATAAATTAGCTATTATAACGTATTTAACCTAGTTTGTTTGATGTATATGTGTG
TACTTATTTCTTTTTCTTTATTAATATTAACAAGATACATTAATGAGGAAGGTTAGTAGGTGGCATGGGAAAAATCTCAAXXXXXXXXXXG
GACTAAATTGAAATAACATTCAAAATATAAATTAGCTATTATAACGTATTTAACCTAGTTTGTTTGATGTATATGTGTG
>Marker22790 Chr4 SNP_site:9647583 Ref_Type:A
CACACGATGGAACTTAGTTTATTCATAACTTTGACATCCCTTAAAGAGTGGGATCTTAGTTATGATATTTTCAATGTAGAXXXXXXXXXXA
TTTTGGTAACTAGATAGTAGATCTAACTTGACGAAATAATCATACTACGTCTAAAAATGTCAAAAAGGGTTGTGGAGTG
CACACGATGGAACTTAGTTTGTTCATAACTTTGACATCCCTTAAAGAGTTGGATCTTAGTTATGATATTTTCAATGTAGAXXXXXXXXXXA
TTTTGGTAACTAGATAGTAGATCTAACTTGACGAAATAATCATACTACGTCTAAAAATGTCAAAAAGGGTTGTGGAGTG
>Marker22790 Chr4 SNP_site:9647612 Ref_Type:G
CACACGATGGAACTTAGTTTATTCATAACTTTGACATCCCTTAAAGAGTGGGATCTTAGTTATGATATTTTCAATGTAGAXXXXXXXXXXA
TTTTGGTAACTAGATAGTAGATCTAACTTGACGAAATAATCATACTACGTCTAAAAATGTCAAAAAGGGTTGTGGAGTG
CACACGATGGAACTTAGTTTGTTCATAACTTTGACATCCCTTAAAGAGTTGGATCTTAGTTATGATATTTTCAATGTAGAXXXXXXXXXXA
TTTTGGTAACTAGATAGTAGATCTAACTTGACGAAATAATCATACTACGTCTAAAAATGTCAAAAAGGGTTGTGGAGTG
>Marker228219 Chr6 SNP_site:26491251 Ref_Type:C
CACACTTGATTGACATAAAAATTTAAGGTTTTATTAACTCCATATGAAGCTAAAGAGTTGTTCCCATCTCAGACTTCAGAXXXXXXXXXXA
CTTAGGCCGAGAAAGCGGGCCTGGAGATGAGATGAAGACCCGCACCAGTAGAAACACAACCCACAGAATCAATCTCGGT
CACACTTGATTGACATAAAAATTTAAGGTTTTATTAACTCCATATGAAGCTAAAGAGTTGTTCCTATCTCAGACTTCAGAXXXXXXXXXXA
CTTAGGCCGAGAAAGCGGGCCTGGAGATGAGATGAAGACCCGCACCAGTAGAAACACAACCCACAGAATCAATCTCGGT
>Marker228352 Chr6 SNP_site:7137954 Ref_Type:T
ACTACAGATCGTAATGCGTAGAATCTATTGTTTAGTTTCTCCCTTCTCTGTCGTTCTGCTTCTACGTGATTCATTGCATTXXXXXXXXXXT
CCGCCACTTCGCTTATAAACCCAATATCCTCGAAGTTGAAGTCTTGATCCAAAAACGGAAGTGGTGCATCGGTGGTGGT
ACTACAGATCGTAATGCGTAGAATCTATTGTTTAGTTTCTCCCTTCTCTGTCGTTCTGCTTCTACGTGATTCATTGCATTXXXXXXXXXXT
CCGCCACTTCGCTTATAAACCCAATATCCTCGAAGTTGAAGTCTTGATCCAAAAACGGAAGTGGTGTATCGGTGGTGGT
>Marker228838 Chr6 SNP_site:28357315 Ref_Type:A
TACATGTCCATTTTGAAAGTAACCTCAGGTGTATATCTTTGTCTGTTCTCAGATTTGTGGCAGGGCTAATGAACATACCCXXXXXXXXXXT
CTTTTTTTTTTCTCTGTCGAAGATTTAGGAATTTGAGATTGTCTTGTAAGTGTAAATTCAATAATGTGACTTTTATAGT
TACATGTCCATTTTGAAAGTAACCTCGGGTGTATATCTTTGTCTGTTCTCAGATTTGTGGCAGGGCTAATGAACATACCCXXXXXXXXXXT
CTTTTTTTTTTCTCTGTCGAAGATTTAGGAATTTGAAATTGTCTTGTAAGTGTAAATTCAATAATGTGACTTTTATAGT
>Marker228838 Chr6 SNP_site:28357506 Ref_Type:G
TACATGTCCATTTTGAAAGTAACCTCAGGTGTATATCTTTGTCTGTTCTCAGATTTGTGGCAGGGCTAATGAACATACCCXXXXXXXXXXT
CTTTTTTTTTTCTCTGTCGAAGATTTAGGAATTTGAGATTGTCTTGTAAGTGTAAATTCAATAATGTGACTTTTATAGT
TACATGTCCATTTTGAAAGTAACCTCGGGTGTATATCTTTGTCTGTTCTCAGATTTGTGGCAGGGCTAATGAACATACCCXXXXXXXXXXT
CTTTTTTTTTTCTCTGTCGAAGATTTAGGAATTTGAAATTGTCTTGTAAGTGTAAATTCAATAATGTGACTTTTATAGT
>Marker229660 Chr6 SNP_site:10426100 Ref_Type:G
ACATCAAGAGAGTCTGTAACTTTCCTCTGCCAACATATTTTCATTTCAGCCATTATATATGACCTACTTTTTCTCACTAAXXXXXXXXXXT
CAATTATATTAGGTTCTATGTTTCATTGAATATTTTGATGATTTTTGTTTGAAATATTTAAACATATGCAACTAAGGTG
ACATCAAGAGAGTCTGTAACTTTCCTCTGCCAACATATTTTCATTTCAGCCATTATATATGACCTACTTTTTCTCACTAAXXXXXXXXXXT
CAATTATATTAGTTTCTATGTTTCATTTAATATCTTGATGATTTTTGTTTGAAATATTTAAACATATGCAACTAAGGTG
>Marker229660 Chr6 SNP_site:10426115 Ref_Type:G

Create PDF with GO2PDF for free, if you wish to

ACATCAAGAGAGTCTGTAACTTTCCTCTGCCAACATATTTTCATTTCAGCCATTATATATGACCTACTTTTTCTCACTAAXXXXXXXXXXT
CAATTATATTAGGTTCTATGTTTCATTGAATATTTTGATGATTTTTGTTTGAAATATTTAAACATATGCAACTAAGGTG
ACATCAAGAGAGTCTGTAACTTTCCTCTGCCAACATATTTTCATTTCAGCCATTATATATGACCTACTTTTTCTCACTAAXXXXXXXXXXT
CAATTATATTAGTTTCTATGTTTCATTTAATATCTTGATGATTTTTGTTTGAAATATTTAAACATATGCAACTAAGGTG
>Marker229660 Chr6 SNP_site:10426121 Ref_Type:T
ACATCAAGAGAGTCTGTAACTTTCCTCTGCCAACATATTTTCATTTCAGCCATTATATATGACCTACTTTTTCTCACTAAXXXXXXXXXXT
CAATTATATTAGGTTCTATGTTTCATTGAATATTTTGATGATTTTTGTTTGAAATATTTAAACATATGCAACTAAGGTG
ACATCAAGAGAGTCTGTAACTTTCCTCTGCCAACATATTTTCATTTCAGCCATTATATATGACCTACTTTTTCTCACTAAXXXXXXXXXXT
CAATTATATTAGTTTCTATGTTTCATTTAATATCTTGATGATTTTTGTTTGAAATATTTAAACATATGCAACTAAGGTG
>Marker229963 Chr6 SNP_site:5095834 Ref_Type:C
CACTTCATATTAGACATAATGTTAAGATGATTTAGTAGAGCAAAATGCTATATTTACGAACATAATGTTCGTATTTGAAAXXXXXXXXXXG
TAATTAATGTAATTTAATGTAAGTTATATGTAACTATGTATCTATCTAATATCTATAAACAAAAGGAGGTAGCTTCGTG
CACTTCATATTAGACATAATGTTAAGATGATTTAGTAGAGCAAAATGCTATATTTACGAACATAATGTTTGTATTTGAAAXXXXXXXXXXG
TAATTAATGTAATTTAATGTAAGTTATATGTAACTATGTATCTATCTAATATCTATAAACAAAAGGAGGTAGCTTCGTG
>Marker231632 Chr6 SNP_site:16744966 Ref_Type:A
TACGGCGACGTAAAATATCGCAATCGTGGCGTAAGAAAAGGAAATGAGAGGAATCAACTCGTGAGATTCCTCATACCGTTXXXXXXXXXXT
TCAATTCGAATCTCTTGCATTATAGTTCTTCTACAGGTAGTTCTTCTCATAGATTAATCTTCGTTAATCGTAATTGAGT
TACGGCGACGTAAATTATCGCAATCGTGGCGTAAGAAAAGGAAATGAGAGGAATCAACTCGTGAGATTCCTCATACCGTTXXXXXXXXXXT
TCAATTCGAATCTCTTGCATTATAGTTCTTCGACAGGTAGTTCTTCTCATAGATTAATCTTCGCTAATCGTAATTGAGT
>Marker231632 Chr6 SNP_site:16745198 Ref_Type:T
TACGGCGACGTAAAATATCGCAATCGTGGCGTAAGAAAAGGAAATGAGAGGAATCAACTCGTGAGATTCCTCATACCGTTXXXXXXXXXXT
TCAATTCGAATCTCTTGCATTATAGTTCTTCTACAGGTAGTTCTTCTCATAGATTAATCTTCGTTAATCGTAATTGAGT
TACGGCGACGTAAATTATCGCAATCGTGGCGTAAGAAAAGGAAATGAGAGGAATCAACTCGTGAGATTCCTCATACCGTTXXXXXXXXXXT
TCAATTCGAATCTCTTGCATTATAGTTCTTCGACAGGTAGTTCTTCTCATAGATTAATCTTCGCTAATCGTAATTGAGT
>Marker231632 Chr6 SNP_site:16745230 Ref_Type:T
TACGGCGACGTAAAATATCGCAATCGTGGCGTAAGAAAAGGAAATGAGAGGAATCAACTCGTGAGATTCCTCATACCGTTXXXXXXXXXXT
TCAATTCGAATCTCTTGCATTATAGTTCTTCTACAGGTAGTTCTTCTCATAGATTAATCTTCGTTAATCGTAATTGAGT
TACGGCGACGTAAATTATCGCAATCGTGGCGTAAGAAAAGGAAATGAGAGGAATCAACTCGTGAGATTCCTCATACCGTTXXXXXXXXXXT
TCAATTCGAATCTCTTGCATTATAGTTCTTCGACAGGTAGTTCTTCTCATAGATTAATCTTCGCTAATCGTAATTGAGT
>Marker231683 Chr6 SNP_site:21864900 Ref_Type:T
CACCGTGAAACAAGAGGGATACAAAAAGTAGAAGATTCCATTATATGAAAACTAGACACAATTGAAATGAATAACCACACXXXXXXXXXXT
GAGTTTAAAAAATAAGGGACCGCCCATTCCAAACATCGATCAGCGAAAGGACGTGCAACAAATTGGACGAATTGCAGTG
CACCGTTAAACAAGAGGGATACAAAAAGTAGAAGATTCCATTATATGAAAACTAGACACAATTGAAATGAATAACCACACXXXXXXXXXXT
GAGTTTCAAAAATAAGGGACCGCCCATTCCAAACATCGATCAGCGAAAGGACGTGCAACAAATTGGACGAATTGCAGTG
>Marker231683 Chr6 SNP_site:21865102 Ref_Type:C
CACCGTGAAACAAGAGGGATACAAAAAGTAGAAGATTCCATTATATGAAAACTAGACACAATTGAAATGAATAACCACACXXXXXXXXXXT
GAGTTTAAAAAATAAGGGACCGCCCATTCCAAACATCGATCAGCGAAAGGACGTGCAACAAATTGGACGAATTGCAGTG
CACCGTTAAACAAGAGGGATACAAAAAGTAGAAGATTCCATTATATGAAAACTAGACACAATTGAAATGAATAACCACACXXXXXXXXXXT
GAGTTTCAAAAATAAGGGACCGCCCATTCCAAACATCGATCAGCGAAAGGACGTGCAACAAATTGGACGAATTGCAGTG
>Marker231826 Chr6 SNP_site:53624 Ref_Type:C
TACCCCACACTGATCCCATCTCCAGCCCGATGTATCCAGCTCCAATTACAACAAGTTTCTTAGGGACCTCAGACAAAGCTXXXXXXXXXXA
CCACTGTATTACCACCATCAATGGTATCCACAGAAACTTCAGAAGGGGAAATCAATTTCCCATAGCCTTTAACATAGTT
TACCCCACACTGATCCCATCTCCAGCCCGATGTATCCAGCTCCAATTACAACAAGTTTCTTAGGGATCTCAGACAAAGCTXXXXXXXXXXA
CCACTGTATTACCACCATCAATGGTATCCACAGAAACTTCAGAAGGGGAAATCAATTTCCCATAGCCTTTAACATAGTT

Create PDF with GO2PDF for free, if you wish to

>Marker232023 Chr6 SNP_site:27670673 Ref_Type:G
ACAGCTGACAACTCTTTTGTTATTTGAAGCAAAGATTAATTCCATAAACAAACTGTATCCCATCATCAACTATTACTTTTXXXXXXXXXXG
CTTACTACCTTATTTAACTAAATTAAGCATATATTCTATTAATAATACAAAATAGATATGCTTGTAATGGAAGGAAAGT
ACAGCTGACAACTCTTTTGTTATTTGAAGCAAAGATTAATTCCATAAACAAACTGTATCCCATCATCAACTATTACTTTTXXXXXXXXXXT
CTTACTACCTTATTTAACTAAATTAAGCATATATTCTATTAATAATACAAAATAGATATGCTTGTAATGGAAGGAAAGT
>Marker232049 Chr6 SNP_site:16891909 Ref_Type:G
CACTGCATACATTGCCTTCTTCATAACTTGTATTTTCACTTTTAGTAAATCTCTTAAATCTCAAAAATAAATATTAATTTXXXXXXXXXXA
TCATTGGATTGAATAAGCTAAAATGGTTCGATTTGGAATTAATACTTGAAATCATGAGCCTCAAACACGCTCTAATGTT
CACTGCATACATTGCCTTCTTCATAACTTGTATTTTCACTTTTAGTGAATCTCTTAAGTCTCAAAAATAAATATTAATTTXXXXXXXXXXA
TCATTGGATTGAATAAGCTAAAATGGTTCGATTTGGAATTAATACTTGAAATCATGAGCCTCAAACACGCTTTAATGTT
>Marker232049 Chr6 SNP_site:16891920 Ref_Type:G
CACTGCATACATTGCCTTCTTCATAACTTGTATTTTCACTTTTAGTAAATCTCTTAAATCTCAAAAATAAATATTAATTTXXXXXXXXXXA
TCATTGGATTGAATAAGCTAAAATGGTTCGATTTGGAATTAATACTTGAAATCATGAGCCTCAAACACGCTCTAATGTT
CACTGCATACATTGCCTTCTTCATAACTTGTATTTTCACTTTTAGTGAATCTCTTAAGTCTCAAAAATAAATATTAATTTXXXXXXXXXXA
TCATTGGATTGAATAAGCTAAAATGGTTCGATTTGGAATTAATACTTGAAATCATGAGCCTCAAACACGCTTTAATGTT
>Marker232049 Chr6 SNP_site:16892108 Ref_Type:T
CACTGCATACATTGCCTTCTTCATAACTTGTATTTTCACTTTTAGTAAATCTCTTAAATCTCAAAAATAAATATTAATTTXXXXXXXXXXA
TCATTGGATTGAATAAGCTAAAATGGTTCGATTTGGAATTAATACTTGAAATCATGAGCCTCAAACACGCTCTAATGTT
CACTGCATACATTGCCTTCTTCATAACTTGTATTTTCACTTTTAGTGAATCTCTTAAGTCTCAAAAATAAATATTAATTTXXXXXXXXXXA
TCATTGGATTGAATAAGCTAAAATGGTTCGATTTGGAATTAATACTTGAAATCATGAGCCTCAAACACGCTTTAATGTT
>Marker232068 Chr6 SNP_site:18277442 Ref_Type:A
CACCAGTTTTTGACAAACTTGAAGCTGAGTTTGCTAAAGCTGTTTTGTCATTACCAGCATCGAAGGGCTTTGAAATTGGCXXXXXXXXXXT
TTGTGGTTGCTCGTTGCCTTAGTTTTGTTTTTCTTTTTTATTCAGCACTCACATGTTCAAAACTAAACCCCCTGCAGGT
CACCAGTTTTTGACAAACTTGAAGCTGAGTTTGCTAAAGCTGTTTTGTCATTACCAGCATCGAAGGGCTTTGAAATTGGCXXXXXXXXXXT
TTGTGGTTGCTCGTTGCCTTGGTTTTGTTTTTCTTTTTTATTCAGCACTCACATGTTCAAAACTAAACCCCCTGCAGGT
>Marker232072 Chr6 SNP_site:26861698 Ref_Type:A
AACCAAGACCCTGTCGTTTATCATGAAGGGAAACAACATTACCAAGCGGACAGCTCCTCTCAGGACTCTAATTCTCAGAGXXXXXXXXXXT
TAATGGAGAAATCCCTTGAGGAAAGCAATGGTCAGGTTCAACATGCTTCAAAAAGTTCATCAATTTCCTCAAACAATGT
AACCAAGACCCTGTCGTTTATCATGAAGGGAAACAACATTACCAAGCGGACAGCTCCTCTCAGGACTCTAATTCTCAGAGXXXXXXXXXXT
TAGTGGAGAAATCCCTTGAGGAAAGCAATGGTCAGGTTCAACATGCTTCAAAAAGTTCATCAATTTCCTCAAACAATGT
>Marker232308 Chr6 SNP_site:27355603 Ref_Type:C
AACCACTTTATTTGGAAATTAATAGAAACATTAAAGAAAATGAAAAGTTAGATATTAGTCTTTTTCTTTTCTTATAATTGXXXXXXXXXXT
CTCTAGACTTCCGGTGGCTAACCCAACCCCCCAATTAAAATATGTCTCATTTTGTAGGATTCTTTCATACATTTACGTT
AACCACTTTATTTGGAAATTAATAGAAACATTAAAGAAAATGAAAAGTTAGATATTAGTCTTTTTCTTTTCTTATAATTGXXXXXXXXXXT
CTCTAGACTTCCGGTGGCTAACCCAATCCCCCAATTAAAATATGTCTCATTTTGTAGGATTCTTTCATACATTTACGTT
>Marker232934 Chr6 SNP_site:10475208 Ref_Type:T
ACTGTGATTGATTATAGCTAAAATCACATATTATTCAATGATCACCCCATTGGCACATGAAATAATTGACTGCTTACTTTXXXXXXXXXXA
ATTTCTGGTGTTTTTGTCGTTCTATTGTATAGCAACATAAGCTCTTGCTCTCATGGAGTTTAGTTGTTTTTACACAAGT
ACTGTGATTGATTATAGCTAAAATCACATATTATTCAATGATCATCCCATTGGCACATGAAATAATTGACTGCTTACTTTXXXXXXXXXXA
ATTTCTGGTGTTTTTGTCGTTCTATTGTATAGCAACATAAGCTCTTGCTCTCACGGAGTTTAGTTGTTTTTACACAAGT
>Marker232934 Chr6 SNP_site:10475430 Ref_Type:C
ACTGTGATTGATTATAGCTAAAATCACATATTATTCAATGATCACCCCATTGGCACATGAAATAATTGACTGCTTACTTTXXXXXXXXXXA
ATTTCTGGTGTTTTTGTCGTTCTATTGTATAGCAACATAAGCTCTTGCTCTCATGGAGTTTAGTTGTTTTTACACAAGT
ACTGTGATTGATTATAGCTAAAATCACATATTATTCAATGATCATCCCATTGGCACATGAAATAATTGACTGCTTACTTTXXXXXXXXXXA

Create PDF with GO2PDF for free, if you wish to

ATTTCTGGTGTTTTTGTCGTTCTATTGTATAGCAACATAAGCTCTTGCTCTCACGGAGTTTAGTTGTTTTTACACAAGT

>Marker233002 Chr6 SNP_site:2218427 Ref_Type:G

CACCCTTAATCACCTAACCTCGCCTCTCACCAAGATTAAAAATAAATTTTCATAATTTGAATATACTGCATAGATGTAAAXXXXXXXXXXXT

TTATAAACTTGGTTTTGTATGCAGCTTTTTATTGGTCCGAAAATAGGCACATTTTTAAACACTTCAGTTTTTCTTAGTT

CACCCTTAATCACCTAACCTCGCCTCTCACCAAGATTAAAAATAAATTTTCATAATTTGAATATACTGCATAGATGTAAAXXXXXXXXXXXT

TTATAGACTTGGTTTTGTATGCAGCTTTTTATTGGTCCGAAAATAGGCACATTTTTAAACACTTCAGTTTTTCTTAGTT

>Marker233549 Chr6 SNP_site:16722620 Ref_Type:A

ACCCTATAGCAATGAGTCATGCCTTATATTAAGCGCTACCATTTTGATTTCTCTTAATACGATGCACCTATTTTTGTCATXXXXXXXXXXXA

GAAGCCTTTTTACAAGGGGAATGTTCCTCTTGAATAGAATCAACAGTAGCAAGAAAGATCTTCTTTGAAATACCAAGTT

ACCCTATAGCAATGAGTCATGCCTTATATTAAGCGCTACCATTTTGATTTCTCTTAATACGATGCACCTATTTTTGTCATXXXXXXXXXXXA

GAAGCCTTTTTACTAGGGGAATGTTCCTCTTGAATAGAATCAACAGTAGCAAGAAAGATCTTCTTTGAAATACCAAGTT

>Marker23361 Chr4 SNP_site:13094164 Ref_Type:T

ACAACAAAATGAAGAATAATTGTATGAGATTTTATCATTTTCAAACGAATTGAGTAAAATAATGTTGATGTAATATATATXXXXXXXXXXXT

TTCCATAGAGAATAAATACATGAATAATAATATATTTATTTGTGGTTATAGACATATATTTGATATATATTAATATGTT

ACAACAAAATGAAGAATAATTGTATGAGATTTTATCATTTTCAAACGAATTGAGTAAAATAATGTTGATGTAATATATATXXXXXXXXXXXT

TTCCATAGAGAATAAATACATGAATAATAATATATTTATTTGTTGTTATAGACATATATTTGATATATATTAATATGTT

>Marker233972 Chr6 SNP_site:16327852 Ref_Type:G

TACTGTTTTAATTTATCATACTTTTTGTTCAACCTTCTTAGAATAGCAAGTCCGGATTGGATAGAGGTTTTTATTGAATAXXXXXXXXXXXA

GGCTCTTAATTCATGGAGCAACGGACGTTGAGTTTTACCAAATTTTTGTAAAGCTGAGATTGTTGAAGCAGTTGAAGGT

TACTGTTTTAATTTATCATACTTTTTGTTCAACCTTCTTAGAATAGCAAGTCCGGATTGGATAGAGGTTTTTATTGAATAXXXXXXXXXXXA

GGCTCTTAATTCATGGAGCAACGGACGTTGAGTTTTGCCAAATTTTTGTAAAGCTGAGATTGTTGAAGCAGTTGAAGGT

>Marker234992 Chr6 SNP_site:3530454 Ref_Type:C

CACTCCGCCTTTAGTGAAACTCCATCTCTTTTTATGCTTCAAGTTTCTTTCATTTTCATTTCCATGGCTTCATTTTGAATXXXXXXXXXXXT

CTCTAACTATTTCACCTAAAACCTTGATGGGTGTGCCTTAAATTCTTTTTTGGTCCATTGAATGCCCTAGTTTCTTAGT

CACTCCGCCTTTAGTGAAACTCCATCTCTTTTTATGCTTCAAGTTTCTTTCATTTTCATTTCTATGGCTTCATTTTGAATXXXXXXXXXXXT

CTCTAACTATTTCACCTAAAACCTTGATGGGTGTGCCTTAAATTCTTTTTTGGTCCATTGAATGCCCTAGTTTCTTAGT

>Marker235121 Chr6 SNP_site:26555197 Ref_Type:T

AACCACTAAAATGGAGAATAAGGGATGGCATCCTATCACATTTACCTATGCTAATGCTGGACCTCGGCAGGTCAAGGAAAXXXXXXXXXXXT

ACTCTTTTTCACATTGTTTGTTTTGACTTGTTGTCTTATAATGACGCTATAAGGATCGGCGGCTATTTTATATGCCAGT

AACCACTAAAATGGAGAATAAGGGATGGCATCCTATCACATTTACCTATGCTAATGCTGGACCTCGGCAGGTCAAGGAAAXXXXXXXXXXXT

ACTCTTTTTCACATTGTTTGTTTTGACTTGTTGTCTTATAATGACGCTATAAGGATCGGCGTCTATTTTATATGCCAGT

>Marker235217 Chr6 SNP_site:3430078 Ref_Type:A

AACCTCAAGTTTGTGAGTGACTCATGCATTTAATGGGTTCTAAAGTATATTTCAAGCTTGATTTATTAATCACAATTTCAXXXXXXXXXXXT

GTTATAAAAGATGAAGTTTGAAAGCTTAAATGTTTTTCATTAGGCTATACTAGAACTATAACTACTACCTTTTAATGTG

AACCTCAAGTTTGTGAGTGACTCATGCATTTAATGGGTTCTAAAGTATATTTCAAGCTTGATTTATTAATCACGATTTCAXXXXXXXXXXXT

GTTATAAAAGATGAAGTTTGAAAGCTTAAATGTTTTTCATTAGGCTATACTAGAACTATAACTACTACCTCTTAATGTG

>Marker235217 Chr6 SNP_site:3430316 Ref_Type:T

AACCTCAAGTTTGTGAGTGACTCATGCATTTAATGGGTTCTAAAGTATATTTCAAGCTTGATTTATTAATCACAATTTCAXXXXXXXXXXXT

GTTATAAAAGATGAAGTTTGAAAGCTTAAATGTTTTTCATTAGGCTATACTAGAACTATAACTACTACCTTTTAATGTG

AACCTCAAGTTTGTGAGTGACTCATGCATTTAATGGGTTCTAAAGTATATTTCAAGCTTGATTTATTAATCACGATTTCAXXXXXXXXXXXT

GTTATAAAAGATGAAGTTTGAAAGCTTAAATGTTTTTCATTAGGCTATACTAGAACTATAACTACTACCTCTTAATGTG

>Marker235643 Chr6 SNP_site:27356089 Ref_Type:T

GACAACAATGATAGTGATGAAGCTGTTTTTATTCTTTGTCCAATAATGCATGGTTATTAGTGACAATAAATAGAGTCTTTXXXXXXXXXXXA

AGAGTTAAAAAGTAGTTGAAATTGGAACTAAAATTGAAACAAGAATAGGAAAGTGAGCAAAGACTAAACCACGAAAGTG

Create PDF with GO2PDF for free, if you wish to

GACAACAATGATAGTGATGAAGCTGTTTTTATTCTTTGTCCAATAATGCATGGTTATTAGTGACAATAAATAGAGTCTTTXXXXXXXXXXA
AGAGTTAAAAAGTAGTTGAAATTGGAACTAAAATTGAAACAAGAATAGGAAAGTGAGCAAAGACTAAACCATGAAAGTG
>Marker237194 Chr6 SNP_site:21625364 Ref_Type:A
GACTTCTGTTGCAATATGCCGAGGAGGAGGATGCGATGCAATATGATCCGATTCGATTGAATTCGGCCGATTTTACCATTXXXXXXXXXXT
ATGTATGAATGTGGTTGGTTTAGGTGATTTGTTAAATTTCTTCAAGTGTTTTTCATGGCAGCTGGCAATGGAGAATGTA
GACTTCTGTTGCAATATGCCGAGGAGGAGGATGCGATGCGATATGATCCGATTCGATTGAATTCGGCCGATTTTACCATTXXXXXXXXXXT
ATGTATGAATGTGGTTGGTTTAGGTGATTTGTTAAATTTCTTCAAGTGTTTTTCATGGCAGCTGGCAATGGAGAATGTA
>Marker237659 Chr6 SNP_site:21796226 Ref_Type:T
ACTGTAATATGATAGGGGGTCTATCATACGCCAAATCCTTTCTAGGGATATACAAGCAAGGATTTTGCAAGGCAGCGGTAXXXXXXXXXXA
TTGGAAACCATACCAAATATGTTTCCATAAACTGCCCACCATTGATCATTTAACCATATTATGCCAATTCATAGAGGTC
ACTGTAATATGATAGGGGGTCTATCATATGCCAAATCCTTTCTAGGGATATACAAGCAAGGATTTTGCGAGGCAGCGGTAXXXXXXXXXXA
TTGGAAACCATACCAAATATGTTTCCATAAACTGCCCACCATTGATCATTTAACCATATTATGCCAATTCATAGAGGTC
>Marker237659 Chr6 SNP_site:21796266 Ref_Type:G
ACTGTAATATGATAGGGGGTCTATCATACGCCAAATCCTTTCTAGGGATATACAAGCAAGGATTTTGCAAGGCAGCGGTAXXXXXXXXXXA
TTGGAAACCATACCAAATATGTTTCCATAAACTGCCCACCATTGATCATTTAACCATATTATGCCAATTCATAGAGGTC
ACTGTAATATGATAGGGGGTCTATCATATGCCAAATCCTTTCTAGGGATATACAAGCAAGGATTTTGCGAGGCAGCGGTAXXXXXXXXXXA
TTGGAAACCATACCAAATATGTTTCCATAAACTGCCCACCATTGATCATTTAACCATATTATGCCAATTCATAGAGGTC
>Marker237800 Chr6 SNP_site:26396874 Ref_Type:C
AACTGTGTGCTCGTGCAGGTTTTAATGGCAATCACAGCATCTTCTTTTTTTTTATTTTCAATAATTAATAGCAATTCAAGAXXXXXXXXXXG
AGACAAAACAATGATTTATGGCCGTATTTTATTAGTCTTGGTGGCTGTGGGATCTGAAATCAGATTCTCAGGTTCTGTT
AACTGTGTGCTCGTGCAGGTTTTAATGGCAATCACAGCATCTTCTTTTTTTTTCTTTTCAATAATTAATAGCAATTCAAGAXXXXXXXXXXG
AGGCAAAACAATGATTTATGGCCGTATTTTATTAGTCTTGGTGGCTGTGGGATCTGAAATCAGATTCTCAGGTTCTGTT
>Marker237800 Chr6 SNP_site:26397010 Ref_Type:G
AACTGTGTGCTCGTGCAGGTTTTAATGGCAATCACAGCATCTTCTTTTTTTTTATTTTCAATAATTAATAGCAATTCAAGAXXXXXXXXXXG
AGACAAAACAATGATTTATGGCCGTATTTTATTAGTCTTGGTGGCTGTGGGATCTGAAATCAGATTCTCAGGTTCTGTT
AACTGTGTGCTCGTGCAGGTTTTAATGGCAATCACAGCATCTTCTTTTTTTTTCTTTTCAATAATTAATAGCAATTCAAGAXXXXXXXXXXG
AGGCAAAACAATGATTTATGGCCGTATTTTATTAGTCTTGGTGGCTGTGGGATCTGAAATCAGATTCTCAGGTTCTGTT
>Marker238174 Chr6 SNP_site:15803553 Ref_Type:C
ACAAGTGGAAAGATCGGGCTGATCGATCTACCATAAATAAAAAGATTAGTTTCTCAAAATTAATTATTTTACTTAAAGTGXXXXXXXXXXT
GATCATCCAATTATCAATGTATAATTTTTAACTTCATACCTATATCTAGCCCAATTGATTACAAACACTAACCAATGGT
ACAAGTGGAAAGATCGGGCTGATCGATCTACCATAAATACAAAGATTAGTTTCTCAAAATTAATTATTTTACTTAAAGTGXXXXXXXXXXT
GATCATCCAATTATTAATGTATAATTTTTAACTTCATACCTATATCTAGCCCAATTGATTACAAAGACTAACCAATGGT
>Marker238174 Chr6 SNP_site:15803729 Ref_Type:T
ACAAGTGGAAAGATCGGGCTGATCGATCTACCATAAATAAAAAGATTAGTTTCTCAAAATTAATTATTTTACTTAAAGTGXXXXXXXXXXT
GATCATCCAATTATCAATGTATAATTTTTAACTTCATACCTATATCTAGCCCAATTGATTACAAACACTAACCAATGGT
ACAAGTGGAAAGATCGGGCTGATCGATCTACCATAAATACAAAGATTAGTTTCTCAAAATTAATTATTTTACTTAAAGTGXXXXXXXXXXT
GATCATCCAATTATTAATGTATAATTTTTAACTTCATACCTATATCTAGCCCAATTGATTACAAAGACTAACCAATGGT
>Marker238174 Chr6 SNP_site:15803780 Ref_Type:G
ACAAGTGGAAAGATCGGGCTGATCGATCTACCATAAATAAAAAGATTAGTTTCTCAAAATTAATTATTTTACTTAAAGTGXXXXXXXXXXT
GATCATCCAATTATCAATGTATAATTTTTAACTTCATACCTATATCTAGCCCAATTGATTACAAACACTAACCAATGGT
ACAAGTGGAAAGATCGGGCTGATCGATCTACCATAAATACAAAGATTAGTTTCTCAAAATTAATTATTTTACTTAAAGTGXXXXXXXXXXT
GATCATCCAATTATTAATGTATAATTTTTAACTTCATACCTATATCTAGCCCAATTGATTACAAAGACTAACCAATGGT
>Marker238349 Chr6 SNP_site:13980635 Ref_Type:G
AACAAGTGGTTTCATGAATGGTTGATAATCTATGTTAATAAAAGATGAATCTATAGCACATCACAGTTTTTAAGCACTACXXXXXXXXXXA

Create PDF with GO2PDF for free, if you wish to

TTCAAATTAACCATAACAGAATCTGTTTTGTTCGCAAAGAAAGTTATGCTTTATCATGGCTCAACAAAAAAGTTCCGTG
AACAAGTGGTTTCATGAATGGTTGATAATCTATGTTAATAAAAGATGAATCTATAGCACATCACAGTTTTTAAGCACTACXXXXXXXXXXXA
TTCAAATTAACCATAACAGAATCTGTTTTGTTCGCAAAGAAAGTTATGCTTTATCATGGCTCAACAAAAAGGTTCCGTG
>Marker239111 Chr6 SNP_site:26939936 Ref_Type:G
AACTTTAACTGCTCAACTGAGATTACAACTATATTCAACTTCTAATATAATTACAAAGTTTGTATTCCTCTAAACCTCCAXXXXXXXXXXXG
TTAAAATCAGTGTAAAGCATATCGATCTTTTAAACGATTACATTTTATATAGAATATCTAAATCTAGTAAGTCGAGGTG
AACTTTAACTGCTCAACTGAGATTACAACTATATTCAACTTCTAATATAATTACAAAGTTTGTATTCCTCTAAACCTCCAXXXXXXXXXXXG
TTAAAATCAGTGTAAAGCATATCGATCTTTTAAACGATTACATTTTATATAGAATATCTAAATCTAGTAAGTCGGGGTG
>Marker239176 Chr6 SNP_site:1169921 Ref_Type:A
ACCTCACTCAAGTCACAATTGCCTGACCCAACAACATTTAGGTGCTAAAGAAACTCATGAAATATTGAATTTTAGTAGGTXXXXXXXXXXXT
GCACAATTTTGTTAGCTGATAAATAATTTACCTACATAAGAGAATAGGAAAAGTCAATGAATTCTATCAATGTTTGGTA
ACCTCACTCAAGTCACAATTGCCTGACCCAACAACATTTAGGTGCTAAAGAAACTCATGGAATATTGAATTTTAGTAGGTXXXXXXXXXXXT
GCACAATTTTGTTAGCTGATAAATAATTTACCTACATAAGAGAATAGGAAAAGTCAATGAATTCTATCAATGTTTGGTA
>Marker239287 Chr6 SNP_site:1414627 Ref_Type:G
CACTAAACCACTCACAAGCGATCACATTCACCACCTCACAAGCAGACTCATGCTTCATCAACATTCATTTGAGGGGCATAXXXXXXXXXXXA
AATTAGTAGAAAACAACTCTCATTCTTGAGAATTTCTTCCATTATGTATTGTTTCAGTGATGACTTAAGAATTCATGTT
CACTAAACCACTCACAAGCGATCACATTCACCACCTCACAAGCATACTCATGCTTCATCAACATTCATTTGAGGGGCATAXXXXXXXXXXXA
AATTAGTAGAAAACAACTCTCATTCTTGAGAATTTCTTCCATTATGTATTGTTTCAGTGATGACTTAAGAATTCATGTT
>Marker239314 Chr6 SNP_site:28371380 Ref_Type:T
GACCAAAAATAAATAAATACCACAGAGATAAATTACACCAATAAATTTAAGTTTTGGTTATTGAAAATTGAAAAGGATTGXXXXXXXXXXXC
CATGTCTTCCTACCACGACTCACTGTCACTCAGAATTAAACATAAGATGCTCTTATTCGCAGGCACTCAACTCGTTGTG
GACCAAAAATAAATAAATACCACAGAGATAAATTACACCAATAAATTTAAGTTTTGGTTATTGAAAATTGAAAAGGATTGXXXXXXXXXXXC
CATGTCTTCCTACCACGACTCACTGTCACTCAGAATTAAACATAAGATGCTCTTATTCTCAGGCACTCAACTCGTTGTG
>Marker239498 Chr6 SNP_site:27425170 Ref_Type:A
ACATTGATGCAATGTTGTCACAAGTGCTCTAATGTGCTTTGAGCCAAATAATGAAAAGATCATATGTAAGAAGTTAGAAAXXXXXXXXXXXT
AATTGTATCAATGATTAAGTTGGCAACTAAATTTATTTGAAATGAAATCGACTATATCTTATATGTATATAGATGTGTG
ACATTGATGCAATGTTGTCACAAGTGCTCTAATGTGCTTTGAGCCAAATAATGAAAAGATCCTATGTAAGAAGTTAGAAAXXXXXXXXXXXT
AATTGTATCAATGATTAAGTTGGCAACTAAATTTATTTGAAATGAAATCGACTATATCTTATATGTATATAGATGTGTG
>Marker239624 Chr6 SNP_site:13560036 Ref_Type:C
GACTTCTTTCCAGTGCCAGATTGCTGTTTGATTCGTGTTCTGTCTTGGATTCTGTTTCTTGTAACATTATGATGTCTGGGXXXXXXXXXXXA
ATTGAGGTTTTTAAGGATATGAGGTCTGCAGGTGTAGCCCCAAATGAAGTGACAATGGCAAGTGTTATGTCAGCATGTT
GACTTCTTTCCAGTGCCAGATTGCTGTTTGATTCGTGTTCTGTTTTGGATTCTGTTTCTTGTAACATTATGATGTCTGGGXXXXXXXXXXXA
ATTGAGGTTTTTAAGGATATGAGGTCTGCAGGTGTAGCCCCAAATGAAGTGACAATGGCAAGTGTTATGTCAGCATGTT
>Marker239805 Chr6 SNP_site:416784 Ref_Type:T
ACTAGAGATTAAGATTCAAAATTTAATCAAAATTGTTGAATATCCCATGAGTTTCTTTGACATCCAAATGTTGCAAAGTCXXXXXXXXXXXA
ATCAAGATATCACAAAAAATCTCATCCATCACAGCAAAACAAAGCACATTGAGATAAATCGTTAATCCACAAGCATGTG
ACTAGAGATTAAGATTCAAAATTTAATCATAATTGTTGAATATCCCATGAGTTTCTTTGACATCCAAATGTTGCAAAGTCXXXXXXXXXXXA
ATCAAGATATCACAAAAAATCTCATCCATCACGGCAAAACAAAGCACATTGAGATAAATCGTTAATCCACAAGCATGTG
>Marker239805 Chr6 SNP_site:416977 Ref_Type:G
ACTAGAGATTAAGATTCAAAATTTAATCAAAATTGTTGAATATCCCATGAGTTTCTTTGACATCCAAATGTTGCAAAGTCXXXXXXXXXXXA
ATCAAGATATCACAAAAAATCTCATCCATCACAGCAAAACAAAGCACATTGAGATAAATCGTTAATCCACAAGCATGTG
ACTAGAGATTAAGATTCAAAATTTAATCATAATTGTTGAATATCCCATGAGTTTCTTTGACATCCAAATGTTGCAAAGTCXXXXXXXXXXXA
ATCAAGATATCACAAAAAATCTCATCCATCACGGCAAAACAAAGCACATTGAGATAAATCGTTAATCCACAAGCATGTG
>Marker239971 Chr6 SNP_site:16366267 Ref_Type:A

Create PDF with GO2PDF for free, if you wish to

AACGGGAACAAAAACTTCCTCTACATCACAATATCATCAAACAACAATTATTAACCTTCTAAAGGATGCAAAGTTGATAAXXXXXXXXXXXT
TATTCTGATTGCTAAACCAATAAAACTTGAACCACTTCTACAGATGAGATAAAAGAATGTAAAGATAAGAACTTCTGTA
AACGGGAACAAAAACTTCCTCTACATCACAATATCATCAAACAACGATTATTAACCTTCTAAAGGATGCAAAGTTGATAAXXXXXXXXXXXT
TATTCTGATTGCTAAACCAATAAAACTTGAACCACTTCTACAGATGAGATAAAAGAATGTAAAGATAAGAACTTCTGTA
>Marker240285 Chr6 SNP_site:10283904 Ref_Type:G
AACTATAGATAAACTTAGTTATATTGAATGACATTTCACATTCTATGCCCATAGGGGCTTTATAAAAATCTAATACAGCAXXXXXXXXXXXT
GACATGCAATGCTTTCACTTTCACAAATAGAAAGCATCTTCAAAATATGTTCGATTTGCCTAGGGTGTCATATGCTGTT
AACTATAGATAAACTTAGTTATATTGAATGACATTTCACATTCTATGCCCATAGGGGCTTTATAAAAATCTAATACAGGAXXXXXXXXXXXT
GACATGCAATGCTTTCACTTTCACAAATAGAAAGCATCTTCAAAATATGTTCAATTTGCCTAGGGTGTCATATGCTGTT
>Marker240285 Chr6 SNP_site:10284047 Ref_Type:A
AACTATAGATAAACTTAGTTATATTGAATGACATTTCACATTCTATGCCCATAGGGGCTTTATAAAAATCTAATACAGCAXXXXXXXXXXXT
GACATGCAATGCTTTCACTTTCACAAATAGAAAGCATCTTCAAAATATGTTCGATTTGCCTAGGGTGTCATATGCTGTT
AACTATAGATAAACTTAGTTATATTGAATGACATTTCACATTCTATGCCCATAGGGGCTTTATAAAAATCTAATACAGGAXXXXXXXXXXXT
GACATGCAATGCTTTCACTTTCACAAATAGAAAGCATCTTCAAAATATGTTCAATTTGCCTAGGGTGTCATATGCTGTT
>Marker240420 Chr6 SNP_site:1492190 Ref_Type:T
TACTTGAAATACTACGTTGGAATTTTTAGGATTTCAAGCTATTTTGGGAATCGTTAAACATTTTAAGTAGAGGAGATTTGXXXXXXXXXXXT
TTTTCCATGGCTTGAAGTATTGATATTATATTATGCTTTAATGTCAGGAACAGATGGTGTTTCGGAGCCCCATACAGTC
TACTTGAAATACTACGTTGGAATTTTTAGGATTTCAAGCTATTTTGGGATTCGTTAAACATTTTAAGTAGAGGAGATTTGXXXXXXXXXXXT
TTTTCCATGGCTTGAAGTATTGATATTATATTATGCTTTAATGTCAGGAACAGATGGTGTTTCGGAGCCCCATACAGTC
>Marker240739 Chr6 SNP_site:5869923 Ref_Type:T
ACTTTTGATGTATAACCTGTAATCAAGAGCATTAACAAGTAAGAAAACAATATGCTAAATATAACTATATGCATTTGTTGXXXXXXXXXXXA
TAGACGTCTGTGCTAAAATTGCACCAAATAAAAGACTAATCTCAAAATTTTACTTCTTTATCGAAAAGGGAAATTAAGT
ACTTTTGATGTATAACCTGTAATCAAGAGCATTAACAAGTAAGAAAACAATATGCTAAATATAACTATATGCATTTGTTGXXXXXXXXXXXT
TAGACGTCTGTGCTAAAATTGCACCAAATAAAAGACTAATCTCAAAATTTTACTTCTTTATCGAAAAGGGAAATTAAGT
>Marker240752 Chr6 SNP_site:720897 Ref_Type:G
ACAACAATTTGTTCCACGAGAAAGGAAAGAAGAGAAGAATTTTTTATTGAAGTGTGGTGAGTGGTTTTCTTTTTAAAAGTXXXXXXXXXXXT
TAAAACTTGATTTGATGATTACGATTTCAATGCTATGAAATGGTTTTCAAATCATTAGTTGATTCTTTAAGATGATGTT
ACAACAATTTGTTCCACGAGAAAGGAAAGAATAGAAGAATTTTTTATTGAAGTGTGGTGAGTGGTTTTCTTTTTAAAAGTXXXXXXXXXXXT
TAAAACTTGATTTGATGATTACGATTTCAATGCTATGAAATGGTTTTCAAATCATTAGTTGATTCTTTAAGATGATGTT
>Marker241415 Chr6 SNP_site:26854865 Ref_Type:T
ACTTTATCCGCAGACTGAAGTTGGAGAATAGCACGAGCCAAGCAGAGGAGGAAGGGATCATCTGCTGCAGTTTAGCAATCXXXXXXXXXXXT
CCCATTATGAAAAATGTCTCTGCAACTTGTTTGGACTGTGTTTGGTCGTTAAAGGATAGTGGTAGCAGTGTATGTAGTT
ACTTTATCCGCAGACTGAAGTTGGAGAATAGCACGAGCCAAGCAGAGGAGGAAGGGATCATCTGCTGCAGTTTAGCAATCXXXXXXXXXXXT
CCCATTATGAAAAATGTCTCTGCAACTTGTTTGGACTGTGTTTGGTCGTTTAAGGATAGTGGTAGCAGTGTATGTAGTT
>Marker24214 Chr4 SNP_site:7556187 Ref_Type:C
ACAAGCCTTCCTTGGCTTGGCATCCTTTTACAGAAGATTCATTCGAAACTTCAGCTCAATAGTGGCCCCCTTAACCGACTXXXXXXXXXXXA
TCACCATTTGAAGTAGCTGTTGATGCTTGCAGAACTGGGATTGGAGCTGTATTATCACAGCAAGGCCACCCTATTGAGT
ACAAGCCTTCCTTGGCTTGGCATCCTTTTACAGAAGATTCATTCGAAACTTCAGCTGAATAGTGGCCCCCTTAACCGACTXXXXXXXXXXXA
TCACCATTTGAAGTAGCTGTTGATGCTTGCAGAACTGGGATTGGAGCTGTATTATCACAGCAAGGCCACCCTATTGAGT
>Marker24279 Chr4 SNP_site:13135737 Ref_Type:A
ACTCTATAAATACATCCCATTTAATTTCTAATTTTCTATTAGGTGTCAAACCCTAATAAATGAATTTAGGAAAATAAAAAXXXXXXXXXXXT
AGTGATATCATATGAGTGTCAAACGATAATCAAAGGCTATTAGACAATAATCATAAGGTTATCGGCTTTTAAATTTGTT
ACTCTATAAATACATCCCATTTAATTTCTAATTTTCTATTAGGTGTCAAACCCTAATAAATGAATTTAGGAAAATAAAAAXXXXXXXXXXXT
AGTGATATCATATGGGTATCAAACGATAATCAAAGGCTATTAGACAATAATCATAAGATTATCGGCTTTTAAATTTGTT

Create PDF with GO2PDF for free, if you wish to

>Marker24279 Chr4 SNP_site:13135740 Ref_Type:G
ACTCTATAAATACATCCCATTTAATTTCTAATTTTCTATTAGGTGTCAAACCCTAATAAATGAATTTAGGAAAATAAAAAXXXXXXXXXXT
AGTGATATCATATGAGTGTCAAACGATAATCAAAGGCTATTAGACAATAATCATAAGGTTATCGGCTTTTAAATTTGTT
ACTCTATAAATACATCCCATTTAATTTCTAATTTTCTATTAGGTGTCAAACCCTAATAAATGAATTTAGGAAAATAAAAAXXXXXXXXXXT
AGTGATATCATATGGGTATCAAACGATAATCAAAGGCTATTAGACAATAATCATAAGATTATCGGCTTTTAAATTTGTT
>Marker24279 Chr4 SNP_site:13135780 Ref_Type:G
ACTCTATAAATACATCCCATTTAATTTCTAATTTTCTATTAGGTGTCAAACCCTAATAAATGAATTTAGGAAAATAAAAAXXXXXXXXXXT
AGTGATATCATATGAGTGTCAAACGATAATCAAAGGCTATTAGACAATAATCATAAGGTTATCGGCTTTTAAATTTGTT
ACTCTATAAATACATCCCATTTAATTTCTAATTTTCTATTAGGTGTCAAACCCTAATAAATGAATTTAGGAAAATAAAAAXXXXXXXXXXT
AGTGATATCATATGGGTATCAAACGATAATCAAAGGCTATTAGACAATAATCATAAGATTATCGGCTTTTAAATTTGTT
>Marker242800 Chr6 SNP_site:9266597 Ref_Type:A
GACGAATGCTCCATCCACCTTTCAATCTTTGATGAATACTATTTTTAAACCATACTTGAGGAGGTTCGTATTGGTGTTTCXXXXXXXXXXG
AGTATTTGGGCATATTTTATATCTGAAAAAGGAGTAGAGGTGGATCCCAAAAAAGTCAGAGCAATTAAGGAATGGCAGT
GACGAATGCTCCATCCACCTTTCAATCTTTGATGAATACTATTTTTAAACCATACTTGAGGTGGTTCGTATTGGTGTTTCXXXXXXXXXXG
AGTATTTGGGCATATTTTATATCTGAAAAAGGAGTAGAGGTGGATCCCAAAAAAGTCAGAGCAATTAAGGAATGTCAGT
>Marker242800 Chr6 SNP_site:9266820 Ref_Type:G
GACGAATGCTCCATCCACCTTTCAATCTTTGATGAATACTATTTTTAAACCATACTTGAGGAGGTTCGTATTGGTGTTTCXXXXXXXXXXG
AGTATTTGGGCATATTTTATATCTGAAAAAGGAGTAGAGGTGGATCCCAAAAAAGTCAGAGCAATTAAGGAATGGCAGT
GACGAATGCTCCATCCACCTTTCAATCTTTGATGAATACTATTTTTAAACCATACTTGAGGTGGTTCGTATTGGTGTTTCXXXXXXXXXXG
AGTATTTGGGCATATTTTATATCTGAAAAAGGAGTAGAGGTGGATCCCAAAAAAGTCAGAGCAATTAAGGAATGTCAGT
>Marker24310 Chr4 SNP_site:4054195 Ref_Type:A
CACCTCATTTGAAATTTTTAAATACGTAGATCAATTCTAATGGGATGAGTTCTAATGGGATGATAGACTGAAGCCAAGATXXXXXXXXXXT
CAATCCTACTAAAAGAGATCAAAATTTTGCATCCATTTTTGTTGTTCTATGATAATATAAGAATTTTGTTGTTTATTGT
CACCTCATTTGAAATTTTTATATACGTAGATCAATTCTAATGGGATGAGTTCTAATGGGATGATAGACTGAAGCCAAGATXXXXXXXXXXT
CAATCCTACTAAAAGAGATCAAAATTTTGCATCCATTTTTGTTGTTCTATGATAATATAAGAATTTTGTTGTTTATTGT
>Marker243412 Chr6 SNP_site:9792585 Ref_Type:A
ACATATGTCTAACACATGTTGAACAAACAAGTGCCCTTTACGTGCCTAACAAGTGTCGGAGTGCCTGACACTGACATGCTXXXXXXXXXXT
CAAAATGACCCAAGCAAATGAGAGAAACTAACTAGTTTCATACAAATGTCATAGAAGGATTTAATATACTTTAGGTGTG
ACATATGTCTAACACGTGTTGAACAAACAAGTGCCCTTTACGTGCCTAACAAGTGTCGGAGTGTCTGACACTGACATGCTXXXXXXXXXXT
CAAAATGACCCAAGCAAATGAGAGAAACTAACTAGTTTCATACAAATGTCATAGAAGGATATAATATACTTTAGGTGTG
>Marker243412 Chr6 SNP_site:9792633 Ref_Type:C
ACATATGTCTAACACATGTTGAACAAACAAGTGCCCTTTACGTGCCTAACAAGTGTCGGAGTGCCTGACACTGACATGCTXXXXXXXXXXT
CAAAATGACCCAAGCAAATGAGAGAAACTAACTAGTTTCATACAAATGTCATAGAAGGATTTAATATACTTTAGGTGTG
ACATATGTCTAACACGTGTTGAACAAACAAGTGCCCTTTACGTGCCTAACAAGTGTCGGAGTGTCTGACACTGACATGCTXXXXXXXXXXT
CAAAATGACCCAAGCAAATGAGAGAAACTAACTAGTTTCATACAAATGTCATAGAAGGATATAATATACTTTAGGTGTG
>Marker243412 Chr6 SNP_site:9792817 Ref_Type:T
ACATATGTCTAACACATGTTGAACAAACAAGTGCCCTTTACGTGCCTAACAAGTGTCGGAGTGCCTGACACTGACATGCTXXXXXXXXXXT
CAAAATGACCCAAGCAAATGAGAGAAACTAACTAGTTTCATACAAATGTCATAGAAGGATTTAATATACTTTAGGTGTG
ACATATGTCTAACACGTGTTGAACAAACAAGTGCCCTTTACGTGCCTAACAAGTGTCGGAGTGTCTGACACTGACATGCTXXXXXXXXXXT
CAAAATGACCCAAGCAAATGAGAGAAACTAACTAGTTTCATACAAATGTCATAGAAGGATATAATATACTTTAGGTGTG
>Marker243579 Chr6 SNP_site:26275979 Ref_Type:A
TACCTATTACATGGTCTAGCGACTGGAAATGATATTTCCAAGTATCTCGTTCGAATAATGATCAACAACTTTAGTTTCAAXXXXXXXXXXT
ATTAAAATTTTACTGAAATCCTTGTTTGGAGGTGATTTTTTATAGTTGTGAAGGTGACTTTTTGTAATTTCAAGAAGTT
TACCTATTACATGGTCTAGCGACTGGAAATGATATTTCCAAGTATCTCGTTCGAATAATGATCAACAACTTTAGTTTCAAXXXXXXXXXXT

Create PDF with GO2PDF for free, if you wish to

ATTAAAATTTTACTGAAATCCTTGTTTGGAGGTGATTTTTTATAGTTGTGAAGGTGACTTTTTGTGATTTCAAGAAGTT

>Marker24410 Chr4 SNP_site:168636 Ref_Type:C

ACAGTAATATTATATCATGAGGGGTGAAAAAAAATCACGAATCTTTAGTGACTAAATGCATACTTTTACTGAGGTTTGGTXXXXXXXXXXT

CTTGATCGTCTCTTCATCAGCTTCTTCTATAGGATCGAGAAATCCACTACCTTTTACGTTATTTTCGTTTGAGCGTGTA

ACAGTAATATTATATCATGAGGGGTGAAAAAAAATCACGAATCTTTAGTGACTAAATGCATATTTTTACTGAGGTTTGGTXXXXXXXXXXT

CTTGATCGTCTCTTCATCAGCTTCTTCTATAGGATCGAGAAATCCACTACCTTTTACGTTATTTTCGTTTGAGCGTGTA

>Marker244111 Chr6 SNP_site:13393908 Ref_Type:G

TACGACGACAAATATTTTTTGTCATAAATAAAATTCGTGATAATTTTTTATAGTCATAGGATCGCCTGTAAAGGAAAGACXXXXXXXXXXT

TAACTCATATTATTAGAGTAATTATGTCATCGTTTGCACTTACCACATTGGTGCAACTTCCACTATCAATTACTAAGGT

TACGGCGACAAATATTTTTTGTCATAAATAAAATTCGTGATAATTTTTTATAGTCATAGGATCGCCTGTAAAGGAAAGACXXXXXXXXXXT

TAACTCATATTATTAGAGTAATTATGTCATCGTTTGCACTTACCACATTGGTGCAACTTCCACTATCAATTACTAAGGT

>Marker24415 Chr4 SNP_site:20805087 Ref_Type:G

ACTCTCGTTTTCTATATAATTAATATATCTGTAATAAACGTGTCAATTTTTAATTTCTTCCATTTTAAAATATTATATTTXXXXXXXXXXT

ATTATCAATGGCTTCTAACAGATGTTTATTTAAGAAACAAAATCAAATTTTGTTTTAAATCTTTTAGAAATGGAAAGTA

ACTCTCGTTTTCTATATAGTTAATATATCTGTAATAAACGTGTCAATTTTTAATTTCTTCCATTTTAGAATATTATATTTXXXXXXXXXXT

ATTATCAATGGCTTCTAACAGATGTTTATTTAAGAAACAAAATCAAATTTTGTTTTAAATCTTTTAGAAATGGAAAGTA

>Marker24415 Chr4 SNP_site:20805136 Ref_Type:G

ACTCTCGTTTTCTATATAATTAATATATCTGTAATAAACGTGTCAATTTTTAATTTCTTCCATTTTAAAATATTATATTTXXXXXXXXXXT

ATTATCAATGGCTTCTAACAGATGTTTATTTAAGAAACAAAATCAAATTTTGTTTTAAATCTTTTAGAAATGGAAAGTA

ACTCTCGTTTTCTATATAGTTAATATATCTGTAATAAACGTGTCAATTTTTAATTTCTTCCATTTTAGAATATTATATTTXXXXXXXXXXT

ATTATCAATGGCTTCTAACAGATGTTTATTTAAGAAACAAAATCAAATTTTGTTTTAAATCTTTTAGAAATGGAAAGTA

>Marker245068 Chr6 SNP_site:26268639 Ref_Type:G

AACCTAGTAATAATCTAAAGCAACACTATCATAAACCGCTTGAAGATATACACAAGGAAAATGTAATGAAAAAATTAATGXXXXXXXXXXA

AGTATTATTTCAGATAAACAGTCATAAATAGTTGAAGTTGATCTACAAAAGGTTACGCCAAAGAGATAAAGCTGAAGTT

AACCTAGTAATAATCTAAAGCAACACTATCATAAACCGCTTGAAGATATACACAAGGAAAATGTAATGAGAAAATTAATGXXXXXXXXXXA

AGTATTATTTCAGATAAACAGTCGTAAATAGTTGAAGTTGATCTACAAAAGGTTACGCCAAAGAGATAAAGCTGAAGTT

>Marker245068 Chr6 SNP_site:26268762 Ref_Type:G

AACCTAGTAATAATCTAAAGCAACACTATCATAAACCGCTTGAAGATATACACAAGGAAAATGTAATGAAAAAATTAATGXXXXXXXXXXA

AGTATTATTTCAGATAAACAGTCATAAATAGTTGAAGTTGATCTACAAAAGGTTACGCCAAAGAGATAAAGCTGAAGTT

AACCTAGTAATAATCTAAAGCAACACTATCATAAACCGCTTGAAGATATACACAAGGAAAATGTAATGAGAAAATTAATGXXXXXXXXXXA

AGTATTATTTCAGATAAACAGTCGTAAATAGTTGAAGTTGATCTACAAAAGGTTACGCCAAAGAGATAAAGCTGAAGTT

>Marker24535 Chr4 SNP_site:853924 Ref_Type:A

AACAATATCTTTGATGTGCAAATTAACTAATAACATCAATTTGGAATTTCTATAGATTATGAATTGTAGTTAGACCATGAXXXXXXXXXXC

TCTACCTCAATCAATGTCATGAGATTGGCTTGAACCATTCCCCAATAACTTTATGAAATTATTCCACAAATTTTCTTGT

AACAATATCTTTGATGTGCAGATTAACTAATAACATCAATTTGGAATTTCTATAGATTATGAATTGTAGTTAGACCATCAXXXXXXXXXXC

TCTATCTCAATCAATGTCATGAGATTGGCTTGAACCATTCCCCAATAACTTTATGAAATTATTCCACAAATTTTCTTGT

>Marker24535 Chr4 SNP_site:853982 Ref_Type:G

AACAATATCTTTGATGTGCAAATTAACTAATAACATCAATTTGGAATTTCTATAGATTATGAATTGTAGTTAGACCATGAXXXXXXXXXXC

TCTACCTCAATCAATGTCATGAGATTGGCTTGAACCATTCCCCAATAACTTTATGAAATTATTCCACAAATTTTCTTGT

AACAATATCTTTGATGTGCAGATTAACTAATAACATCAATTTGGAATTTCTATAGATTATGAATTGTAGTTAGACCATCAXXXXXXXXXXC

TCTATCTCAATCAATGTCATGAGATTGGCTTGAACCATTCCCCAATAACTTTATGAAATTATTCCACAAATTTTCTTGT

>Marker24535 Chr4 SNP_site:854088 Ref_Type:C

AACAATATCTTTGATGTGCAAATTAACTAATAACATCAATTTGGAATTTCTATAGATTATGAATTGTAGTTAGACCATGAXXXXXXXXXXC

TCTACCTCAATCAATGTCATGAGATTGGCTTGAACCATTCCCCAATAACTTTATGAAATTATTCCACAAATTTTCTTGT

Create PDF with GO2PDF for free, if you wish to

AACAATATCTTTGATGTGCAGATTAACTAATAACATCAATTTGGAATTTCTATAGATTATGAATTGTAGTTAGACCATCAXXXXXXXXXXC
TCTATCTCAATCAATGTCATGAGATTGGCTTGAACCATTCCCCAATAACTTTATGAAATTATTCCACAAATTTTCTTGT
>Marker245400 Chr6 SNP_site:26948503 Ref_Type:A
AACTAATAAAACTATATAAATAAATATTTATTGTGGGCAAATGATAGAAATCTTAACTTGAAAACCCATGTGAGCGACGGXXXXXXXXXXT
ACGGAAAAGGGTTTTTTCTTCGTTTTCTTCTATAATTATTTTAATTGGATCCTTGGAATGCTAATAAATATTAATGGTC
AACTAATAAAACTGTATAAATAAATATTTATTGTGGGCAAATGATAGAAATCTTAACTTGAAAACCCATGTGAGCGACGGXXXXXXXXXXT
ACGGAAAAGGGTTTTTTCTTCGTTTTCTTCTATAATTATTTTAATTGGATCCTTGGAATGCTAATAAATATTAATGGTC
>Marker245488 Chr6 SNP_site:21986041 Ref_Type:T
GACAAACGTGAAAAGGCATGGATATCTTTATCTCTTTGCAATTTAAAAATTGCAGTCTGAAAGTGTTTTTTAATAGAAATXXXXXXXXXXT
GAGTGTATAGATGTGAATTACAAGAATAATTTTTACTGTTATGGGAATATATTTGTTGCAGGATCTAATTCGTGAAGTG
GACAAACGTGAAAATGCATGGATATCTTTATCTCTTTGCAGTTTAAAAATTGCAGTCTGAAAGTGTTTTTTAATAGAAATXXXXXXXXXXT
GAGTGTATAGATGTGAATTACAAGAATAATTTTTACTGTTATGGGAATATATTTGTTGCAGGATCTAATTCGTGAAGTA
>Marker245488 Chr6 SNP_site:21986067 Ref_Type:G
GACAAACGTGAAAAGGCATGGATATCTTTATCTCTTTGCAATTTAAAAATTGCAGTCTGAAAGTGTTTTTTAATAGAAATXXXXXXXXXXT
GAGTGTATAGATGTGAATTACAAGAATAATTTTTACTGTTATGGGAATATATTTGTTGCAGGATCTAATTCGTGAAGTG
GACAAACGTGAAAATGCATGGATATCTTTATCTCTTTGCAGTTTAAAAATTGCAGTCTGAAAGTGTTTTTTAATAGAAATXXXXXXXXXXT
GAGTGTATAGATGTGAATTACAAGAATAATTTTTACTGTTATGGGAATATATTTGTTGCAGGATCTAATTCGTGAAGTA
>Marker245488 Chr6 SNP_site:21986289 Ref_Type:A
GACAAACGTGAAAAGGCATGGATATCTTTATCTCTTTGCAATTTAAAAATTGCAGTCTGAAAGTGTTTTTTAATAGAAATXXXXXXXXXXT
GAGTGTATAGATGTGAATTACAAGAATAATTTTTACTGTTATGGGAATATATTTGTTGCAGGATCTAATTCGTGAAGTG
GACAAACGTGAAAATGCATGGATATCTTTATCTCTTTGCAGTTTAAAAATTGCAGTCTGAAAGTGTTTTTTAATAGAAATXXXXXXXXXXT
GAGTGTATAGATGTGAATTACAAGAATAATTTTTACTGTTATGGGAATATATTTGTTGCAGGATCTAATTCGTGAAGTA
>Marker245730 Chr6 SNP_site:9263837 Ref_Type:C
AACATTAATAACATCTCACCTTAAGCCCCCTTTCACCTACAACCGTAGAATACTTTTCAGGAAAGTTCATGATAGTATCGXXXXXXXXXXC
TCCTCCTCGGTAGCTGAAAGACGACCATAATGAATGTTATGGAAAATTGTATCATTGAAGAGAACCTGTTATTAACAGT
AACATTAATAACATCTCACCTTAAGCCCCCTTTCACCTACAACCGTAGAATACTTTTCAGGAAAGTTCATGATAGTATCGXXXXXXXXXXC
TCCTCCTCGGTAGCTGAAAGACGACCATAATGAATGTTATGGAAAATTGTATCATTGAAGAGAACCTGTTATTCACAGT
>Marker246176 Chr6 SNP_site:26764430 Ref_Type:A
AACTCATCGGTGGATCTTTTGTCAAGCATACACACGATACTTATAGTTCATGATTGAATGACATTTTGTTTCAAGGAGATXXXXXXXXXXA
CCATCTGATATTCATTAAGCTCATCATTTGTAAAGGTTTGCAAGTCTATTCATTATTCTGAATGGTATGCTTGTAGGTG
AACTCATCGGTGGATCTTTTGTCAAGCATACACTCGATACTTATAGTTCATGATTGAATGACATTTTGTTTCAAGGAGATXXXXXXXXXXA
CCATCTGATATTCATTAAGCTCATCATTTGTAAAGGTTTGCAAGTCTATTCATTATTCTGAATGGTATGCTTGTAGGTG
>Marker24691 Chr4 SNP_site:19292393 Ref_Type:C
ACATACACATACATACTGGTTGGTCCAGTATTCAATACACACCCTTTCAAATTTAAATACAAAGCTTGAATTCGTTATTTXXXXXXXXXXT
TAGGTCCAAACGCGCGTGAATTGAACTCATTTGTGGTAGGGCACGTAATGATGTAAAATAATTTTTTTCTATTTTGGTG
ACATACACATACATATTGGTTGGTCCAGTATTCAATACACACCCTTTCAAATTTAAATACAAAGCTTGAATTCGTTATTTXXXXXXXXXXT
TAGGTCCAAACGCGCGTGAATTGAACTCATTTGTGGTAGGGCACGTAATGATGTAAAATAATTTTTTTCTATTTTGGTG
>Marker24695 Chr4 SNP_site:9680202 Ref_Type:C
TACCATTTTTTCTCTCTCTAGACACTTCTCTCGTTTAAATGGCCATTGGCCAGTGACCTTAAGGTTGAAAAGGAAATGGGXXXXXXXXXXG
AGCCACATTGTTTTTCAATCTCTCTTTCTTCTCTTACTAAAGGTGGCGCCTCCTGAGATGAAAGGGACTTACTGGAAGT
TACCATTTTTTCTCTCTCTAGACACTTCTCTCGTTTAAATGGCCATTGGCCAGTGACCTTAAGGTTGAAAAGGAAATGGGXXXXXXXXXXG
AGCCATATTGTTTTTCAATCTCTCTTTCTTCTCTTACTAAAGGTGGCGCCTCCTGAGATGAAAGGGACTTACTGGAAGT
>Marker247042 Chr6 SNP_site:15092743 Ref_Type:T
TACTACTTTTTTTAGAAGAGAAGAAAAGCAGTGCTGGGATTGTTAGAACTTGTTTCTTACAAATTTAGTCTTTGAGTATTXXXXXXXXXXT

Create PDF with GO2PDF for free, if you wish to

CCGTTTGCAGGGAAGACCATGACAACACTGACATACCATAAGAAATCTGTTCGGGCAATGGCATTGCATCCTAAAGAGT
TACTATTTTTTTTAGAAGAGAAGAAAAGCAGTGCTGGGATTGTTAGAACTTGTTTCTTACAAATTTAGTCTTTGAGTATTXXXXXXXXXXT
CCGTTTGCAGGGAAGACCATGACAACACTGACATACCATAAGAAATCTGTTCGGGCAATGGCATTGCATCCTAAAGAGT
>Marker247319 Chr6 SNP_site:26495611 Ref_Type:G
TACCGACCAATTTCGATTCCACATACTGCTATGCATTGGGTTATGGTGCTGGAGCTCTCCTGCAGAGTGGAAAAACTGGAXXXXXXXXXXC
TTAGCTGCTCCTGTTGAAGAATGGACTGTTGGTGGAACTGCATTGACTTCATTAATGGACGTCGAGAGAAGACATGGTA
TACCGACCAATTTCGATTCCACATACTGCTATGCATTGGGTTATGGTGCTGGAGCTCTCCTGCAGAGTGGAAAAACTGGAXXXXXXXXXXC
TTAGCTGCTCCTGTTGAAGAATGGACTGTTGGTGGGACTGCATTGACTTCATTAATGGACGTCGAGAGAAGACATGGTA
>Marker247350 Chr6 SNP_site:3186705 Ref_Type:G
CACCAAGTAGTTGTGACAACACAATATTATCCACGTAAAAAAAAAGTATATTATCACGACATGCTAATGCTAATGCCACTXXXXXXXXXXT
GTAACTAAATAGGTAATTTTTACCCATTACCCTTTTTAATTTACTGGTATGATTAAAAGGCATGGTTTTTGTTTGGAGT
CACCAAGTAGTTGTGACAACACAATATTATCCACGTAAAAAAAAAGTATATTATCACGAGATGCTAATGCTAATGCCACTXXXXXXXXXXT
GTAACTAAATAGGTAATTTTTACCCATTACCCTTTTTAATTTACTGGTATGATTAAAAGGCATGGTTTTTGTTTGGAGT
>Marker24745 Chr4 SNP_site:20815094 Ref_Type:C
AACCTGCACCCGTGGTGACAATTGTGCCACCAGCCAAGCCAACAGATAATGACATAAAATCCAGTGGGGGATCAAATCAGXXXXXXXXXXT
CAGGAAACACTTCTATAATTGGAGTCTATACTTCCTTACATTGTTCGGACAATATATTACATTACAGGCAGTTTCATGT
AACCTGCACCCGTGGTGACAATTGTGCCACCAGCCAAGCCAACAGATAATGACATAAAATCCAGTGGGGGATCAAATCAGXXXXXXXXXXT
CAGGAAACACTTCTATAATTGGAGTCTATACTTCCTTACATTGTTCGGAGAATATATTACATTACAGGCAGTTTCATGT
>Marker247911 Chr6 SNP_site:3868743 Ref_Type:T
TACCAACCTTGAAAGTATATAGTTGATAGTAGAGTCTTTATTTAATTTTATTAATGTTTTTTTAAAATTTATTTTGCTACXXXXXXXXXXT
TTTAAATCCACGAGTATCCGAGCAATAGTCCTAGTGTTAATTTCATATATAAAAATAATCAAATAAGTTTTAACGAGTT
TACCAACCTTGAAAGTATATAGTTGATAGTAGAGTCTTTATTTAATTTTATTAATGTTTTTTTTAAATTTATTTTGCTACXXXXXXXXXXT
TTAAAATCCACGAGTATCCGAGCAGTAGTCCTAGTGTTAATTTCATATATAAAAATAATCAAATAAGTTTTAACGAGTT
>Marker247911 Chr6 SNP_site:3868847 Ref_Type:A
TACCAACCTTGAAAGTATATAGTTGATAGTAGAGTCTTTATTTAATTTTATTAATGTTTTTTTAAAATTTATTTTGCTACXXXXXXXXXXT
TTTAAATCCACGAGTATCCGAGCAATAGTCCTAGTGTTAATTTCATATATAAAAATAATCAAATAAGTTTTAACGAGTT
TACCAACCTTGAAAGTATATAGTTGATAGTAGAGTCTTTATTTAATTTTATTAATGTTTTTTTTAAATTTATTTTGCTACXXXXXXXXXXT
TTAAAATCCACGAGTATCCGAGCAGTAGTCCTAGTGTTAATTTCATATATAAAAATAATCAAATAAGTTTTAACGAGTT
>Marker247911 Chr6 SNP_site:3868869 Ref_Type:G
TACCAACCTTGAAAGTATATAGTTGATAGTAGAGTCTTTATTTAATTTTATTAATGTTTTTTTAAAATTTATTTTGCTACXXXXXXXXXXT
TTTAAATCCACGAGTATCCGAGCAATAGTCCTAGTGTTAATTTCATATATAAAAATAATCAAATAAGTTTTAACGAGTT
TACCAACCTTGAAAGTATATAGTTGATAGTAGAGTCTTTATTTAATTTTATTAATGTTTTTTTTAAATTTATTTTGCTACXXXXXXXXXXT
TTAAAATCCACGAGTATCCGAGCAGTAGTCCTAGTGTTAATTTCATATATAAAAATAATCAAATAAGTTTTAACGAGTT
>Marker248082 Chr6 SNP_site:8843209 Ref_Type:A
ACTTGATATTAAATTATCAACAGACTACAATAATGATAGAGATAAGTTGGAGGCATACCTTCAATTCTAGCCGCATTTAAXXXXXXXXXXT
GATGACAGCAGCCCTACTGGATTGCGTAAAGTGCCTCTCTTCCTGTCATCACCATTTGCCTACGAATTTTTTCTGAAGT
ACTTGATATTAAATTATCAACAGACTACAATAATGATAGAGATAAGTTGGAGGCATACCTTCAATTCTAGCCGCGTTTAAXXXXXXXXXXT
GATGACAGCAGCCCTACTGGATTGCGTAAAGTGCCTCTCTTCCTGTCATCACCATTTGCCTACGAATTTTTTCTGAAGT
>Marker248234 Chr6 SNP_site:27452625 Ref_Type:G
ACTTGAGACCAAAAGTTTAACTGTTACGTTTTTGTTAAGAAAATCTTCTCAAATCACCCTTATGATTCTTGTTTTCTAGGXXXXXXXXXXT
CAATCTCATAAAACCTAATAAATTTAAATAAAATTTTCAAAACATTTTTAATTTAGAGTTATCTTTTCGGTGAGATGTT
ACTTGAGACCAAAAGTTTAACTGTTAGGTTTTTGTTAAGAAAATCTTCTCAAATCACCCTTATGATTCTTGTTTTCTAGGXXXXXXXXXXT
CAATCTCATAAAACCTAATAAAGTTAAATAAAATTTTCAAAACATTTTTAATTTAGAGTTATCTTTTCGGTGAGATGTT
>Marker248234 Chr6 SNP_site:27452818 Ref_Type:G

Create PDF with GO2PDF for free, if you wish to

ACTTGAGACCAAAAGTTTAACTGTTACGTTTTTGTTAAGAAAATCTTCTCAAATCACCCTTATGATTCTTGTTTTCTAGGXXXXXXXXXXXT
CAATCTCATAAAACCTAATAAATTTAAATAAAATTTTCAAAACATTTTTAATTTAGAGTTATCTTTTCGGTGAGATGTT
ACTTGAGACCAAAAGTTTAACTGTTAGGTTTTTGTTAAGAAAATCTTCTCAAATCACCCTTATGATTCTTGTTTTCTAGGXXXXXXXXXXXT
CAATCTCATAAAACCTAATAAAGTTAAATAAAATTTTCAAAACATTTTTAATTTAGAGTTATCTTTTCGGTGAGATGTT
>Marker248786 Chr6 SNP_site:3510252 Ref_Type:C
CACATCTATGATGTTGAGAGACACAAATGGATAACAGGATAAGCCTTTACCAATGAATTTTCAAAAAGTTTGTTTGCTAAXXXXXXXXXXXG
AAAGTTACATATAACATGATTGATAGAGTTTGATATATTTACCATCAAATTGTAACCTATTATTATAATTCGTTTGGTT
CACATCTATGATGTTGAGAGACACAAATGGATAACAGGATAAGCCTTTACCAATGAATTTTCAAAAAGTTTGTTTGCTAAXXXXXXXXXXXG
AAAGTTATATATAACATGATTGATAGAGTTTGATATATTTACCATAAAATTGTAACCTATTATTACAATTCGTTTGGTT
>Marker248786 Chr6 SNP_site:3510290 Ref_Type:C
CACATCTATGATGTTGAGAGACACAAATGGATAACAGGATAAGCCTTTACCAATGAATTTTCAAAAAGTTTGTTTGCTAAXXXXXXXXXXXG
AAAGTTACATATAACATGATTGATAGAGTTTGATATATTTACCATCAAATTGTAACCTATTATTATAATTCGTTTGGTT
CACATCTATGATGTTGAGAGACACAAATGGATAACAGGATAAGCCTTTACCAATGAATTTTCAAAAAGTTTGTTTGCTAAXXXXXXXXXXXG
AAAGTTATATATAACATGATTGATAGAGTTTGATATATTTACCATAAAATTGTAACCTATTATTACAATTCGTTTGGTT
>Marker248786 Chr6 SNP_site:3510310 Ref_Type:T
CACATCTATGATGTTGAGAGACACAAATGGATAACAGGATAAGCCTTTACCAATGAATTTTCAAAAAGTTTGTTTGCTAAXXXXXXXXXXXG
AAAGTTACATATAACATGATTGATAGAGTTTGATATATTTACCATCAAATTGTAACCTATTATTATAATTCGTTTGGTT
CACATCTATGATGTTGAGAGACACAAATGGATAACAGGATAAGCCTTTACCAATGAATTTTCAAAAAGTTTGTTTGCTAAXXXXXXXXXXXG
AAAGTTATATATAACATGATTGATAGAGTTTGATATATTTACCATAAAATTGTAACCTATTATTACAATTCGTTTGGTT
>Marker249080 Chr6 SNP_site:2502494 Ref_Type:A
ACATTTAATAGACCTTCAGAAAGAGTGTCTTCCCCCCCTTCTCCAAAAACAAAATAAATGCTTAATTTCATAAATGATGGXXXXXXXXXXXC
CCATTTAGGTGAGATTAAGAATTCAAGTATGCTTACTTATTTCTAATAATGTCCATTTAAATGTGTGTGTGATTTAAGT
ACATTTAATAGACCTTCAGAAAGAGTGTCTTCCCCCCCTTCTCCAAAAACAAAATAAATGCTTAATTTCATAAATGATGGXXXXXXXXXXXC
CCATTTAGGTGAGATTAAGAATTCAAGTATGCTTACTTATTTCTAATAATGTCCCTTTAAATGTGTGTGTGATTTAAGT
>Marker249221 Chr6 SNP_site:7984120 Ref_Type:T
ACTAAAGAAGTTAGAGTAACTAAAGAAAATTAGCCCTATAGATTAGATTAACCCCAATGCAGTAGAGGGAGAGGCTGAAAXXXXXXXXXXXA
AAAAACCAGTTTAATCTTTTAAGACTCACATTTAAAGTTTAATCAGATTTTGAATTTTGCTTGCCATTTGAAGATCGTA
ACTAAAGAAGTTAGAGTAACTAAAGAAAATTAGCCCTATAGATTAGATTAACCCCAATGCAGTAGAGGGAGAGGCTGAAAXXXXXXXXXXXA
AAAAACCAGTTTAATCTTTTAAGACTCACATTTAAAGTTTAATCAGATTTTGAATTTTGTTTGCCATTTGAAGATCGTA
>Marker249274 Chr6 SNP_site:18040619 Ref_Type:G
AACCTATTTGATTTTCATTCAAATTTTAATATAATACATTCCTTTTAATATTCTATGTATCGAATATAATAAGATATAATXXXXXXXXXXXA
AATATTTCCTCATTTATTTTGTAGCCCTAATTCCTCTCTGTCCAAACTCAAATTTGAAGACGGTTTTTTAGTTCGAGTT
AACCTATTTGATTTTCATTCAAATTTTAATATAATGCATTCCTTTTAATATTCTATGTATCGAATATAATAAGATATAATXXXXXXXXXXXA
AATATTTCCTCATTTATTTTGTAGCCCTAATTCCTCTCTGTCCAAACTCAAATTTGAAGACGGTTTTTTAGTTCGAGTT
>Marker249671 Chr6 SNP_site:21529700 Ref_Type:T
ACCTAAAAATCATCTCTAAGTAAAGTTCCATCATTCTCATACACCAACAAGGACGTTACTTGGTTAGGAACATATCCGTAXXXXXXXXXXXT
ACAAGCATCAATTAATCTTTTGTAGAAAGATTTCCAATCCATCCATTCTTATTTGTTCCCCTTTGTTATAGAGCAAGTT
ACCTAAAAATCATCTCTAAGTAAAGTTCCATCATTCTCATACACCAACAAGGACGTTACTTGGTTAGGAACATATCTGTAXXXXXXXXXXXT
ACAAGCATCAATTAATCTTTTGTAGAAAGATTTCCAATCCATCCATTCTTATTTGTTCCCCTTTGTTATAGAGCAAGTT
>Marker249705 Chr6 SNP_site:13270097 Ref_Type:C
AACATGAATCTATTACGTTTCCACAAACCCACATATGGCCAATTCCACTGCTTTAAAGTAAGTAGATGAGTCTTCCCTTAXXXXXXXXXXXG
GGTAAAACGATAAATTGACCCAACTGGTGTTACGAACACTCGTGAAGGACTAACTTACTGTTATTGGTCTATATCCGTG
AACATGAATCTATTACGTTTCCACAAACCCACATATGGCCAATTCCATTGCTTTAAAGTAAGTAGATGAGTCTTCCCTTAXXXXXXXXXXXG
GGTAAAACGATAAATTGACCCAACTGGTGTTACGAACACTCGTGAAGGACTAACTTACTGTTATTGGTGTATATCCGTG

Create PDF with GO2PDF for free, if you wish to

>Marker249705 Chr6 SNP_site:13270333 Ref_Type:C
AACATGAATCTATTACGTTTCCACAAACCCACATATGGCCAATTCCACTGCTTTAAAGTAAGTAGATGAGTCTTCCCTTAXXXXXXXXXXXG
GGTAAAACGATAAATTGACCCAACTGGTGTTACGAACACTCGTGAAGGACTAACTTACTGTTATTGGTCTATATCCGTG
AACATGAATCTATTACGTTTCCACAAACCCACATATGGCCAATTCCATTGCTTTAAAGTAAGTAGATGAGTCTTCCCTTAXXXXXXXXXXXG
GGTAAAACGATAAATTGACCCAACTGGTGTTACGAACACTCGTGAAGGACTAACTTACTGTTATTGGTGTATATCCGTG
>Marker250030 Chr6 SNP_site:26385043 Ref_Type:G
ACTTATATTATGATTTAAGCTTTTAGTGATATATAATAATGGCTTTAAGAAAAAAGTTAATATGAACCCTCACCATATATXXXXXXXXXXXT
CATTTCTAAAAAATACACTTTCTTTATACATATTTAATATAAAGTTGAGTGTAAAATCTAGAATACTAAGTTTAGAGTC
ACTTATATTATGATTTAAGCTTTTAGTGATGTATAATAATGGCTTTAAGAAAAAAGTTAATATGAACCCTCACCATATATXXXXXXXXXXXT
CATTTCTAAAAAATACACTTTCTTTATACATATTTAATATAAAGTTGAGTGTAAAATCTAGAATACTAAGTTTAGAGTC
>Marker250064 Chr6 SNP_site:28010943 Ref_Type:C
CACTTGAACTGAAACTACTTTAACGAATTAGAAGTCATGGTCCTCGAATTACTCTGTTTATCTATTATCTTCTTTTTTGTXXXXXXXXXXXG
CTTTATCTGAAATGACAACCAGTGTAGTTATGGTATCCAATTTATTATCTGATAGCTGATAGAATATGAATGCATTGTA
CACTTGAACTGAAACTACTTTAACGAATTAGAAGTCATGGTCCTCGAATTACTCTGTTTATCTATTATCTTCTTTTTTGTXXXXXXXXXXXG
TTTTATCTGAAATGACAACCAGTGTAGTTATGGTATCCAATTTATTATCTGATAGCTGATAGAATATGAATGCATTGTA
>Marker250468 Chr6 SNP_site:3222292 Ref_Type:A
ACATTAGCGGCCACAGAACCTTGAAATATTGATTACATTTTAGATGGAGGTTGCATAGGAACAATAAGGGATGCAGCAGTXXXXXXXXXXXG
GCTCCACTGGAGAAACAAAAGATTGACCTGCAATAAAGTCAGACAATTAAACATAAAAGAATGTTGAAAGGACCAAGTG
ACATTAGCGGCCACAGAACCTTGAAATATTGATTACATTTTAGATGGAGGTTGCATAGGAACAATAAGGGATGCAGCAGTXXXXXXXXXXXG
GCTCCACTGGAGAAACAAAAGATTGACCTGCAATAAAGTCAGACAATTAAACATAAACGAATGTTGAAAGGACCAAGTG
>Marker250506 Chr6 SNP_site:2132157 Ref_Type:T
TACAAATCAATGTCGTCCAACTTCCATTTCCCTGCTTACAAGGCGGCCTGCCGGAAGGCTGTGAGAATTTGGACTTACTTXXXXXXXXXXXT
TGCTCATAAATTTCACATCCCTAGGATTGTTTTTTACAGCCTTTGTTGTTTCTCTCTCCTGTGTCAGCCCACTTTAGTA
TACAAATCAATGTCGTTCAACTTCCATTTCCCTGCTTACAAGGCGGCCTGCCGGAAGGCTGTGAGAATTTGGACTTACTTXXXXXXXXXXXT
TGCTCACAAATTTCACATCCCTAGGATTGTTTTTTACAGCCTTTGTTGTTTCTCTCTCCTGTGTCAGCCCACTTTAGTA
>Marker250506 Chr6 SNP_site:2132364 Ref_Type:C
TACAAATCAATGTCGTCCAACTTCCATTTCCCTGCTTACAAGGCGGCCTGCCGGAAGGCTGTGAGAATTTGGACTTACTTXXXXXXXXXXXT
TGCTCATAAATTTCACATCCCTAGGATTGTTTTTTACAGCCTTTGTTGTTTCTCTCTCCTGTGTCAGCCCACTTTAGTA
TACAAATCAATGTCGTTCAACTTCCATTTCCCTGCTTACAAGGCGGCCTGCCGGAAGGCTGTGAGAATTTGGACTTACTTXXXXXXXXXXXT
TGCTCACAAATTTCACATCCCTAGGATTGTTTTTTACAGCCTTTGTTGTTTCTCTCTCCTGTGTCAGCCCACTTTAGTA
>Marker250529 Chr6 SNP_site:4913502 Ref_Type:G
GACAGTGATCTTCCTCATTTTTGTTGATAGTTGGTGCATTAAGCGCATACACGCACTTAGACCATTGGAAGGGCCAAAAGXXXXXXXXXXXA
GAAGCAATATTTGACTGTTATTCGGTTAATGGCCCTACTATCAACATACATTCTCTAGCATCCATCCTTCTTTGGAGTT
GACAGTGATCTTCCTCATTTTTGTTGATAGTTGGTGCATTAAGCGCATACACGCACTTAGACCATTGGAAGGGCCAAAAGXXXXXXXXXXXA
GAAGCAATATTTGACTGTTATTCGGTTAATGGCCCTGCTATCAACATACATTCTCTAGCATCCATCCTTCTTTGGAGTT
>Marker250724 Chr6 SNP_site:442834 Ref_Type:A
TACAATGACAAGAACATTATTTAAGATGTAAATATATACTAATCAAAATGCATGGTATGAAAATCATTTCAAGATGGACTXXXXXXXXXXXT
TTATCACCACGTCATGCAAATCAACAATTTTAAATGAGTAAAGATCTAAATGCAAACAACATGAGCATGTATGTAAAGT
TACAATGACAAGAACATTATTTAAGATGTAAATATATACTAATCAAAATGCATGGTATGAAAATCATTTCAAGATGGACTXXXXXXXXXXXT
TTATCACCACGTCATGCGAATCAACAATTTTAAATGAGTAAAGATCTAAATGCAAACAACATGAGCATGTATGTAAAGT
>Marker250947 Chr6 SNP_site:10371189 Ref_Type:C
ACCAAAATAAAATTACTTCCCTACATCTACTTATATAAACTTGTTCCATTAGTGTTATAAGTTGTCTTTCTCTTTTTGCTXXXXXXXXXXXT
TTTCTCTAACCAATACTTGATAAAAGAGCAGTCTAAACTATTATTTATAATTGGAAAAGAAAAAGTCACTCATACTGTT
ACCAAAATAAAATTACTTCCCTACATCTACTTATATAAACTTGTTCCATTAGTGTTATAAGTTGTCTTTCTCTTTTTGCTXXXXXXXXXXXT

Create PDF with GO2PDF for free, if you wish to

TTTCTCTAACCAATACTTGATAAAAGAGCAGTCTAAACTATTATTTATAATTGGAAAAGAAAAAGTCAGTCATACTGTT
>Marker251021 Chr6 SNP_site:8849807 Ref_Type:A
GACCTATTAGACACAATTGGAAATTCAAAGACTATAAAGTTCAATAACCGATTAGATAAAAATTTGGTAGTTCAAGGTTGXXXXXXXXXXG
CTATAATAGATGCACTTGAGAATAAAAACAAAGGCCAATTACCTCCGAAAATGCTAATCTATCAGCAACATCATTTGTC
GACCTATTAGACACAATTGGAAATTCAAAGACTATAAAGTTCAATAACCGATTAGATAAAAATTTGGTAGTTCAAGGTTGXXXXXXXXXXG
CTATAATGGATGCACTTGAGAATAAAAACAAAGGCTAATTACCTCCGAAAATGCTAATCTATCAGCAACATCATTTGTC
>Marker251021 Chr6 SNP_site:8849835 Ref_Type:C
GACCTATTAGACACAATTGGAAATTCAAAGACTATAAAGTTCAATAACCGATTAGATAAAAATTTGGTAGTTCAAGGTTGXXXXXXXXXXG
CTATAATAGATGCACTTGAGAATAAAAACAAAGGCCAATTACCTCCGAAAATGCTAATCTATCAGCAACATCATTTGTC
GACCTATTAGACACAATTGGAAATTCAAAGACTATAAAGTTCAATAACCGATTAGATAAAAATTTGGTAGTTCAAGGTTGXXXXXXXXXXG
CTATAATGGATGCACTTGAGAATAAAAACAAAGGCTAATTACCTCCGAAAATGCTAATCTATCAGCAACATCATTTGTC
>Marker251666 Chr6 SNP_site:28295723 Ref_Type:C
AACTTGTAGAGATTACTCAACTTGTTACCTCCTTCCCAAAATATCTTCAAAATCCAAACCATCAGAAGCTATGAATAATAXXXXXXXXXXT
TTAACAGACAGTTCAAGAAATTCAGTCTAATAGTTTTCTCAAGTATAGATTATGTAGGGAGCTAGCTAGCTAGGAAGTA
AACTTGTAGAGATTACTCAACTTGTTACCTCCTTCCCAAAATATCTTCAAAATCCAAACCATCAGAAGCTATGAATAATAXXXXXXXXXXT
TTAACAGACAGTTCAAGAAATTCAGTCTAATAGTTTTCTCAAGTATAGCTTATGTAGGGAGCTAGCTAGCTAGGAAGTA
>Marker251722 Chr6 SNP_site:2684828 Ref_Type:A
GACTTTCATTATTTTTGTATGTATATACTCAAGTTTTGAACTGCATCATGAGACGGTTAGGAGTAGCGAGGGTTAAGATAXXXXXXXXXXG
TGAGACATACTCTAATATTATTTGGAGTATGAAGAATATTATCTAGAAGTAAAAAAATTCCCTCTTTTTAGGTTTAGTT
GACTTTCATTATTTTTGTATGTATATACTCAAGTTTTGAACTGCATCATGAGACGGTTAGGAGTAGCGAGGGTTAAGATAXXXXXXXXXXG
TGAGACATACTCTGATATTATTTGGAGTATGAAGAATATTATATAGAAGTAAAAAAATTCCCTCTTTTTAGGTTTGGTT
>Marker251722 Chr6 SNP_site:2684857 Ref_Type:A
GACTTTCATTATTTTTGTATGTATATACTCAAGTTTTGAACTGCATCATGAGACGGTTAGGAGTAGCGAGGGTTAAGATAXXXXXXXXXXG
TGAGACATACTCTAATATTATTTGGAGTATGAAGAATATTATCTAGAAGTAAAAAAATTCCCTCTTTTTAGGTTTAGTT
GACTTTCATTATTTTTGTATGTATATACTCAAGTTTTGAACTGCATCATGAGACGGTTAGGAGTAGCGAGGGTTAAGATAXXXXXXXXXXG
TGAGACATACTCTGATATTATTTGGAGTATGAAGAATATTATATAGAAGTAAAAAAATTCCCTCTTTTTAGGTTTGGTT
>Marker251722 Chr6 SNP_site:2684890 Ref_Type:G
GACTTTCATTATTTTTGTATGTATATACTCAAGTTTTGAACTGCATCATGAGACGGTTAGGAGTAGCGAGGGTTAAGATAXXXXXXXXXXG
TGAGACATACTCTAATATTATTTGGAGTATGAAGAATATTATCTAGAAGTAAAAAAATTCCCTCTTTTTAGGTTTAGTT
GACTTTCATTATTTTTGTATGTATATACTCAAGTTTTGAACTGCATCATGAGACGGTTAGGAGTAGCGAGGGTTAAGATAXXXXXXXXXXG
TGAGACATACTCTGATATTATTTGGAGTATGAAGAATATTATATAGAAGTAAAAAAATTCCCTCTTTTTAGGTTTGGTT
>Marker252770 Chr6 SNP_site:5345275 Ref_Type:C
TACCATGACTTGTTAATTATATAAAAAAAAAACTCGAAAACAAAGTATATTGTTTTGGTTTATATATTTATACAATTGTCXXXXXXXXXXT
TGATAGAGATGTTTAATTATTGGTATGATCTTCCACTTAATTCTTATATATAATTTAACTGATATAAAGAAGATTATGT
TACCATGACTTGTTAATTATATAAAAAAAAAACTCGAAAACAACGTATATTGTTTTGGTTTATATATTTATACAATTGTCXXXXXXXXXXT
TGATAGAGATGTTTAATTATTGGTATGATCTTCCACTTAATTCTTATATATAATTTAACTGATATAAAGAAGATTATGT
>Marker253564 Chr6 SNP_site:26240622 Ref_Type:G
ACTTCAGCATAAGCATATTTTATGTAGTCACCTCTTCAACATTCTGGGCAGTTTTGGCAGATGCCTCCATGAATATTAAAXXXXXXXXXXT
GTTTGCATTTGCATGCTGCCTTGCATCTTCTAACCAGCTAATCAAGTGATTGAAAGTCTCCCTTCTGAATAACGTAGTG
ACTTCAGCATAAGCATATTTTATGTAGTCACCTCTTCAACATTCTGGGCAGTTTTGGCAGATGCCTCCATGAATATTAAAXXXXXXXXXXT
GTTTGCGTTTGCATGCTGCCTTGCATCTTCTAACCAGCTAATCAAGTGATTGAAAGTCTCCCTTCTGAATAACGCAGTG
>Marker253564 Chr6 SNP_site:26240690 Ref_Type:C
ACTTCAGCATAAGCATATTTTATGTAGTCACCTCTTCAACATTCTGGGCAGTTTTGGCAGATGCCTCCATGAATATTAAAXXXXXXXXXXT
GTTTGCATTTGCATGCTGCCTTGCATCTTCTAACCAGCTAATCAAGTGATTGAAAGTCTCCCTTCTGAATAACGTAGTG

Create PDF with GO2PDF for free, if you wish to

ACTTCAGCATAAGCATATTTTATGTAGTCACCTCTTCAACATTCTGGGCAGTTTTGGCAGATGCCTCCATGAATATTAAAXXXXXXXXXXT
GTTTGCGTTTGCATGCTGCCTTGCATCTTCTAACCAGCTAATCAAGTGATTGAAAGTCTCCCTTCTGAATAACGCAGTG
>Marker253786 Chr6 SNP_site:28009514 Ref_Type:C
ACCTTAACTGTTCCCATTTTCTATTTTTCTCTATCGATCTTTGTTGTGGGTTGGACCACGAAGTGTCCAAAAACACACATXXXXXXXXXXT
CTCAGCTTTTAAGGGCAATCATGAATATTGCACCCTTAGCAATATCCACAACAGGCCGTGGTTCCTCTGGCGTTGGGTT
ACCTTAACTGTTCTCATTTTCTATTTTTCTTTATCGATCTTTGTTGTGGGTTGGACCACGAAGTGTCCAAAAACACACATXXXXXXXXXXT
CTCAGCTTTTAAGGGCAATCATGAATATTGCACCCTTAGCAATATCCACAACAGGCCGTGGTTCCTCTGGCGTTGGGTT
>Marker253786 Chr6 SNP_site:28009531 Ref_Type:C
ACCTTAACTGTTCCCATTTTCTATTTTTCTCTATCGATCTTTGTTGTGGGTTGGACCACGAAGTGTCCAAAAACACACATXXXXXXXXXXT
CTCAGCTTTTAAGGGCAATCATGAATATTGCACCCTTAGCAATATCCACAACAGGCCGTGGTTCCTCTGGCGTTGGGTT
ACCTTAACTGTTCTCATTTTCTATTTTTCTTTATCGATCTTTGTTGTGGGTTGGACCACGAAGTGTCCAAAAACACACATXXXXXXXXXXT
CTCAGCTTTTAAGGGCAATCATGAATATTGCACCCTTAGCAATATCCACAACAGGCCGTGGTTCCTCTGGCGTTGGGTT
>Marker254817 Chr6 SNP_site:3339280 Ref_Type:A
TACCTAGCTTCTAAACCTTGGTAAAAAGACTTGGAGTGTATCCTAACTTTAAACTAAATTGGAATATTTGTTCCCTGGGGXXXXXXXXXXT
CATTTTTTTTTCTAATTTATTTTAGGACACTCTTGCACACATGTTATGCATATGACTACTAAATGGAAAGATACGGGGT
TACCTAGCTTCTAAACCTTGGTAAAAAGACTTGGAGTGTATCCTAACTTTAAACTAATTTGGAATATTTGTTCCCTGGGGXXXXXXXXXXT
CATTTTTTTTTTAAATTTATTTTAGGACACTCTTGCACACATGTTATGCATATGACTACTAAATGGAAAGATACGGGGT
>Marker254817 Chr6 SNP_site:3339431 Ref_Type:C
TACCTAGCTTCTAAACCTTGGTAAAAAGACTTGGAGTGTATCCTAACTTTAAACTAAATTGGAATATTTGTTCCCTGGGGXXXXXXXXXXT
CATTTTTTTTTCTAATTTATTTTAGGACACTCTTGCACACATGTTATGCATATGACTACTAAATGGAAAGATACGGGGT
TACCTAGCTTCTAAACCTTGGTAAAAAGACTTGGAGTGTATCCTAACTTTAAACTAATTTGGAATATTTGTTCCCTGGGGXXXXXXXXXXT
CATTTTTTTTTTAAATTTATTTTAGGACACTCTTGCACACATGTTATGCATATGACTACTAAATGGAAAGATACGGGGT
>Marker254817 Chr6 SNP_site:3339432 Ref_Type:T
TACCTAGCTTCTAAACCTTGGTAAAAAGACTTGGAGTGTATCCTAACTTTAAACTAAATTGGAATATTTGTTCCCTGGGGXXXXXXXXXXT
CATTTTTTTTTCTAATTTATTTTAGGACACTCTTGCACACATGTTATGCATATGACTACTAAATGGAAAGATACGGGGT
TACCTAGCTTCTAAACCTTGGTAAAAAGACTTGGAGTGTATCCTAACTTTAAACTAATTTGGAATATTTGTTCCCTGGGGXXXXXXXXXXT
CATTTTTTTTTTAAATTTATTTTAGGACACTCTTGCACACATGTTATGCATATGACTACTAAATGGAAAGATACGGGGT
>Marker254911 Chr6 SNP_site:8450382 Ref_Type:G
ACAAAATCTTTAATTCCAAATACAAGCATAAATTTTGATGCTGATTCTAATGAAAGAGAGAATGAACCCAGAAAAAAGAAXXXXXXXXXXA
CAGTCTTCCCTGTCTTTGCCATAAACTTATCTTCTTGAGATTTTTTTGCCTTTCATTATGAAGACTCAAATATAGTGTT
ACAAAATCTTTAATTCCAAATACAAGCATAAATTTTGATGCTGATTCTAATGAAAGAGAGAATGAACCCGGAAAAAAGAAXXXXXXXXXXA
CAGTCTTCCCTGTCTTTGCCATAAACTTATCTTCTTGAGATTTTTTTGCCTTTCATTATGAAGACTCAAATATAGTGTT
>Marker255321 Chr6 SNP_site:5270798 Ref_Type:C
CACACTATAATGCGTCTTTTGGAATAAAAATTTATGATGATGTTAAGAGTCGTTGCTTATTATTTTGACACTCTTTATTGXXXXXXXXXXC
ATCCACAATTTACTCTTCATTGTTGTATATTGATGTGATTTTCACTCGCAGATTGTTTAATAAAAGTTTCATTGCTGTA
CACACTATAATGCGTCTTTTGGAATAAAAATTTATGATGATGTTAAGAGTCGTTGCTTATTATTTTGACACTCTTTATTGXXXXXXXXXXC
ATCCACAATTTACTCTTCATTGTTGTATATTGATGTGATTTTCAGTCGCAGATTGTTTAATAAAAGTTTCATTGCTGTA
>Marker255727 Chr6 SNP_site:16937515 Ref_Type:C
ACGGCTTCGAGGGCGGAAACAGTGCCTTTCAGTTTGTCAAGTTCATCATGGAAACCCCACAACAATCCAAGGTCTTGAAGXXXXXXXXXXT
GCTTGGAGAGAATTGGAACTTGAACCTTTATATTTCGACTCTTTTTGTAATTTCTAATCTAATCTTTAATTTGTTTGTT
ACGGCTTCGAGGGCGGAAACAGTGCCTTTCAGTTTGTCAAGTTCATCATGGAAACCCCACAACAATCCAAGGTCTTGAAGXXXXXXXXXXT
GCTTGGAGAGAATTGGAAGTTGAACCTTTATATTTCGACTCTTTTTGTAATTTCTAATCTAATCTTTAATTTGTTTGTT
>Marker255854 Chr6 SNP_site:22584226 Ref_Type:C
GACTCCTTTTCTTCCGCCATACCCTTCTTAAAATCAGTTAAAACCTACTTGAATCTCCGACGAATCAGACCCCAAACATCXXXXXXXXXXT

Create PDF with GO2PDF for free, if you wish to

GGCAAGCAGTTGCACTATGAAAATGACATTAGCACCAAAATGGGGTTAGTTGAAACCGCAAGCAAATGCCAGGCGAGTA
GACTCCTTTTCTTCCGCCATACCCTTCTTAAAATCAGTTAAAACCTACTTGAATCTGCGACGAATCAGACCCCAAACATCXXXXXXXXXXT
GGCAAGCAGTTGCACTATGAAAATGACATTAGCACCAAAATGGGGTTAGTTGAAACCGCAAGCAAATGCCAGGCGAGTA
>Marker256042 Chr6 SNP_site:4936971 Ref_Type:A
GACAACTGCATACATTTCATTATCAACTTTTTGAAGGTTTAAAGACTAGACAATTGTAATTCTATCGGTTGAAAAGTGATXXXXXXXXXXT
TACCGTTAATGAATTTGGCATGAAACAACATCTTTATAATATTTCATGAATTTGCTAGTTCTCATTGGATTTAAGTGTT
GACAACTGCATACATTTCATTATCAACTTTTTGAAGGTTTAAAGACTAGACAATTGTAATTCTATCGGTTGAAGAGTGATXXXXXXXXXXT
TACCGTTGATGAATTTGGCATGAAACAACATCTTTATAATATTTCATGAATTTGCTAGTTCTCATTGGATTTAAGTGTT
>Marker256042 Chr6 SNP_site:4937097 Ref_Type:A
GACAACTGCATACATTTCATTATCAACTTTTTGAAGGTTTAAAGACTAGACAATTGTAATTCTATCGGTTGAAAAGTGATXXXXXXXXXXT
TACCGTTAATGAATTTGGCATGAAACAACATCTTTATAATATTTCATGAATTTGCTAGTTCTCATTGGATTTAAGTGTT
GACAACTGCATACATTTCATTATCAACTTTTTGAAGGTTTAAAGACTAGACAATTGTAATTCTATCGGTTGAAGAGTGATXXXXXXXXXXT
TACCGTTGATGAATTTGGCATGAAACAACATCTTTATAATATTTCATGAATTTGCTAGTTCTCATTGGATTTAAGTGTT
>Marker256207 Chr6 SNP_site:9882886 Ref_Type:A
AACTAGTAGAAAAAGCTTTGAGATGACCAACTAATGAATCACTTATTTTATCAAAGTTTAACAGTAAATTGAATTTTATGXXXXXXXXXXA
GATATCAATCTACCAAAATGTTTAACTAATTGTGACCGAGACCCAGGTAACGAAAGGTGTTGTCTAAATTAAAATCAGT
AACTAGTAGAAAAGGCTTTGAGATGACCAACTAATGAATCACTTATTTTATCAAAGTTTAACAGTAAATTGAATTTTATGXXXXXXXXXXA
GATATCAATCTACCAAAATGTTTAACTAATTGTGACCGAGACCCAGGTAACGAAAGGTGTTGTCTAAATTAAAATCAGT
>Marker256255 Chr6 SNP_site:14365743 Ref_Type:A
ACCGAAGATTTGAAGATTCAAACTTCTACAAGCTCTCAAGACGTGCAAGTTCGTATCAACCTCGAGATCAACAACTTCTGXXXXXXXXXXT
ACTAGAGATTGTATTCACAATATTTATCAATATATCAAAGTTTATTTCCACGAATTACATTTCTTCGAAATCTCGTGTA
ACCGATGATTTGAAGATTCAAACTTCTACAAGCTCTCAAGACGTGCAAGTTCGTATCAACCTCGAGATCAACAACTTCTGXXXXXXXXXXT
ACTAGAGATTGTATTCACAATATTTATCAATATATCAAAGTTTATTTCCACGAATTACATTTCTTCGAAATCTCGTGTA
>Marker256666 Chr6 SNP_site:27804475 Ref_Type:C
TACCCTTGAACAAAATATACACCGACTATTACTTTAACTATTTTATTATTTTGGATGGGTGTGGATCAAATTATTACTTAXXXXXXXXXXA
AAGAAGATCGAATTGAGTATCACCTCTATCTCTAGTATGAATAATAAGATTGAATCTCAAACTTTGTGAGATCGACAGT
TACCCTTGAACAAAATATACACCGACTATTACTTTAACTATTTTATTATTTTGGATGGGTGTGGATCAAATTATTACTTAXXXXXXXXXXA
AAGAAGATCGAATTGAGTATCACCTCTATCTCTAGTATGAATAATAAGATTGAATCTCAAACTTTGTGAGATTGACAGT
>Marker256934 Chr6 SNP_site:8860058 Ref_Type:A
AACTGACACTACAATATCATACTTGAAAATGTATCTTAAAGTTTTGCCAGCAATTGATTTTGTTTGCTAGTTTAGATAAAXXXXXXXXXXG
AAACAATTACTAAGATTCTCTTTATAACAAAATCCTACATGCTACACCAGTTGTAGTTTAAAATGTTATCACTTTAGTG
AACTGACACTACGATATCATACTTGAAAATGTATCTTAAAGTTTTGCCAGCAATTGATTTTGTTTGCTAGTTTAGATAAAXXXXXXXXXXG
AAACAATTACTAAGATTCTCTTTATAACAAAATCCTACATGCTACACCAGTTGTAGTTTAAAATGTTATCACTTTAGTG
>Marker257104 Chr6 SNP_site:18780407 Ref_Type:T
AACATATTTAATGAGTCATGAGGTGTTGCTGCAATTGATGATGAAAACAACAAAATGATGAGGTAATGGGAAAATTCAATXXXXXXXXXXC
AATATCATGAGCTTAATTTCTCTAGGGATATATTTAACACTACGATACTCTTAAGGTTTTTATATAACTTGAGTTTGTG
AACATATTTAATGAGTCATGAGGTGTTGCTGCAATTGATGATGAAAACAACAAAATGATGAGGTAATGGGAAAATTCAATXXXXXXXXXXC
AATATCATGAGCTTAATTTCTCTAGGGATATATTTAACACTATGATACTCTTAAGGTTTTTATATAACTTGAGTTTGTG
>Marker257197 Chr6 SNP_site:8842085 Ref_Type:A
ACCAATTCCTTCCATTCATTTAATTACATATCACAAAATGATTTACTATTCATTTATTTTATAGACCTCAAATAATCTTTXXXXXXXXXXC
CCTAAAATGTGTTACACTTTATGACATTCTGAAGAAGACTAACAATGCATGCTCCATTTCTCTACACTACGCCTATGTC
ACCAATTCCTTCCATTCATTTAATTACATATCACTAAATGATTTACTATTCATTTATTTTATAGACCTCAAGTAATCTTTXXXXXXXXXXC
CCTAAAATGTGTTACACTTTATGACATACTGAAGAAGACTAACAATGCATGCTCCATTTCTCTACACTACGCCTATGTC
>Marker257197 Chr6 SNP_site:8842122 Ref_Type:A

Create PDF with GO2PDF for free, if you wish to

ACCAATTCCTTCCATTCATTTAATTACATATCACAAAATGATTTACTATTCATTTATTTTATAGACCTCAAATAATCTTTXXXXXXXXXXC
CCTAAAATGTGTTACACTTTATGACATTCTGAAGAAGACTAACAATGCATGCTCCATTTCTCTACACTACGCCTATGTC
ACCAATTCCTTCCATTCATTTAATTACATATCACTAAATGATTTACTATTCATTTATTTTATAGACCTCAAGTAATCTTTXXXXXXXXXXC
CCTAAAATGTGTTACACTTTATGACATACTGAAGAAGACTAACAATGCATGCTCCATTTCTCTACACTACGCCTATGTC
>Marker257197 Chr6 SNP_site:8842285 Ref_Type:T
ACCAATTCCTTCCATTCATTTAATTACATATCACAAAATGATTTACTATTCATTTATTTTATAGACCTCAAATAATCTTTXXXXXXXXXXC
CCTAAAATGTGTTACACTTTATGACATTCTGAAGAAGACTAACAATGCATGCTCCATTTCTCTACACTACGCCTATGTC
ACCAATTCCTTCCATTCATTTAATTACATATCACTAAATGATTTACTATTCATTTATTTTATAGACCTCAAGTAATCTTTXXXXXXXXXXC
CCTAAAATGTGTTACACTTTATGACATACTGAAGAAGACTAACAATGCATGCTCCATTTCTCTACACTACGCCTATGTC
>Marker257943 Chr6 SNP_site:22156026 Ref_Type:G
ACAATAATTTTACACAGGTAATTGTATGCTGAAAATGCCCCAACTCCAAGTGCAACGATAGATATTTATGTAGTTCCTTTXXXXXXXXXXC
AGCACTGTCATTGGATGACCTCATTGTTCCACAGGTTACTTCATATCTCCACCTCGTCATCCTCCTTAGTAATAGGAGT
ACAGTAATTTTACACAGGTAATTGTATGCTGAAAATGCCCCAACTCCAAGTGCAACGATAGTTATTTATGTAGTTCCTTTXXXXXXXXXXC
AGCACTGTCATTGGATGACCTCATTGTTCCACAGGTTACTTCATATCTCCACCTCGTCATCCTCCTTAGTAATAGGAGT
>Marker257943 Chr6 SNP_site:22156084 Ref_Type:T
ACAATAATTTTACACAGGTAATTGTATGCTGAAAATGCCCCAACTCCAAGTGCAACGATAGATATTTATGTAGTTCCTTTXXXXXXXXXXC
AGCACTGTCATTGGATGACCTCATTGTTCCACAGGTTACTTCATATCTCCACCTCGTCATCCTCCTTAGTAATAGGAGT
ACAGTAATTTTACACAGGTAATTGTATGCTGAAAATGCCCCAACTCCAAGTGCAACGATAGTTATTTATGTAGTTCCTTTXXXXXXXXXXC
AGCACTGTCATTGGATGACCTCATTGTTCCACAGGTTACTTCATATCTCCACCTCGTCATCCTCCTTAGTAATAGGAGT
>Marker25820 Chr4 SNP_site:11811556 Ref_Type:A
TACTACTTCAGTCCAACAACAACATTCAAATATAGGTAGAAAAGTTAGGAGAACATGTCCTTTTAAACTGATGAACACTAXXXXXXXXXXA
ATATTGCATCAAAATCTCCAAACCATATCTATTAATTGACAATGGGGGTTTCTAAACTCCATAATCAAAAATGCTTTGT
TACTACTTCAGTCCAACAACAACATTCAAATATAGGTAGAAAAGTTAGGAGAACATGTCCTTTTAAACTGATGAACACTAXXXXXXXXXXA
ATATTGCATCAAAATCTCCAAACCATATCTATTAATTGACAATGGGGGTTTCTGAACTCCATAATCAAAAATGCTTTGT
>Marker258632 Chr6 SNP_site:21804219 Ref_Type:G
CACTACAATCCGAGGAGTGTTTTGTGTAACAGAGTATATGAAATTAGTTAGAAATCGTTTTAACATGCTTAGAACTCAATXXXXXXXXXXA
AATCAACACAAACTCTCACCATTATTGTGTTGTAACTAAATTCTTAGACGATAATTTGATTCAAAAAGTCAATAATGTA
CACTACAATCCGAGGAGTGTTTTGTGTAACAGAGTATATGAAATTAGTTAGAAGTCGTTTTAACATGCTTAGAACTCAATXXXXXXXXXXA
AATCAACACAAACTCTCACCATTATTGTGTTGTAACTAAATTCTTAGACGATAATTTGATTAAAAAAGTCAATAATGTA
>Marker258632 Chr6 SNP_site:21804382 Ref_Type:A
CACTACAATCCGAGGAGTGTTTTGTGTAACAGAGTATATGAAATTAGTTAGAAATCGTTTTAACATGCTTAGAACTCAATXXXXXXXXXXA
AATCAACACAAACTCTCACCATTATTGTGTTGTAACTAAATTCTTAGACGATAATTTGATTCAAAAAGTCAATAATGTA
CACTACAATCCGAGGAGTGTTTTGTGTAACAGAGTATATGAAATTAGTTAGAAGTCGTTTTAACATGCTTAGAACTCAATXXXXXXXXXXA
AATCAACACAAACTCTCACCATTATTGTGTTGTAACTAAATTCTTAGACGATAATTTGATTAAAAAAGTCAATAATGTA
>Marker25864 Chr4 SNP_site:3605564 Ref_Type:C
TACCCTTTTCTTCTTCTTTTTTGTTAAATTATACAAAACCAAAATCAATTTTAGGGTATAATATTCCAACCAAAAGTTCTXXXXXXXXXXT
CATATAATTGGTTACAAATTTGGGTAATATATGGTTTTTCATAAGTATCAAACTTTAACTTTAACTTTATATCACAAGT
TACCCTTTTCTTCTTCTTTTTTGTTAAATTATACAAAACCAAAATGAATTTTAGGGTATAATATTCCAACCAAAAGTTCTXXXXXXXXXXT
CATATAATTGGTTACAAATTTGGGTAATATATGGTTTTTCATAAGTATCAAACTTTAACTTTAACTTTATATCACAAGT
>Marker258651 Chr6 SNP_site:3866409 Ref_Type:C
TACTTTGGTGCAATCCTTCATCTTCAATGAGGCATTGTATGCAACATATATCTCTACCAATATTAAATGCCTAATTTTAGXXXXXXXXXXA
GCACTCCGCTCATGTGAGAGAGATTTGTTTAGCTTATAGTTCTAATTTGATGATGAGAGAACTCGTGCATTATTGGGTA
TACTTTGGTGCAATCCTTCATCTTCAATGAGGCATTGTATGCAACATATATCTCTACCAATATTAAATGCCTAATTTTAGXXXXXXXXXXA
GCACTCCGCTCATGTGAGAGAGATTTGTTTAGCTTATAGTTCTAATTTGATGATGAGAGAACTCGTGCATTCTTGGGTA

Create PDF with GO2PDF for free, if you wish to

>Marker258945 Chr6 SNP_site:24504622 Ref_Type:G

AACTGAGAATAATTGTTGAGATAATAGATTTGATGATGATTTAATTTGGTTAGATATTTGAAAGATTTGAAATTTATGATXXXXXXXXXXT

TCATTTCTTTGTTCTTCTGCGGGCACACGAATTTTCATTTCTTTGAGTCCTTGGTTTATTGGACGTTTTTGTGTTGAGT

AACTGAGAATAATTGTTGAGATAATAGATTTGATGATGATTTAATTTGGTTAGATATTTGAAAGATTTGAAATTTATGATXXXXXXXXXXT

TCATTTCTTTGTTCTTCTGCGGGCACACGAATTTTCATTTCTTTGAGTCGTTGGTTTATTGGACGTTTTTGTGTTGAGT

>Marker259162 Chr6 SNP_site:5875868 Ref_Type:C

AACAAATTCATCAAAAGTTTTCCTTGTTGGAAAACCAGCACAACTTATCCTTATTGCCTCCATGACTCCCTAATGAAAAGXXXXXXXXXXA

ATGCCAATTTCTCTCCCATTACAGTGTGCCCAGCAGTGGTAATCACAACCAAAGATAACTTAAAGTTTGCTTCAGGGTA

AACAAATTCATCAAAAGTTTTCCTTGTTGGAAAACCAGCACAACTTATCCTTATTGCCTCCATGACTCCCTAATGAAAAGXXXXXXXXXXA

ATGCCAATTTCTCTCCCATTACAGTGTGTCTAGCAGTGGTAATCACAACCAAAGATAACTTAAAGTTTGCTTCAGGGTA

>Marker259162 Chr6 SNP_site:5875870 Ref_Type:C

AACAAATTCATCAAAAGTTTTCCTTGTTGGAAAACCAGCACAACTTATCCTTATTGCCTCCATGACTCCCTAATGAAAAGXXXXXXXXXXA

ATGCCAATTTCTCTCCCATTACAGTGTGCCCAGCAGTGGTAATCACAACCAAAGATAACTTAAAGTTTGCTTCAGGGTA

AACAAATTCATCAAAAGTTTTCCTTGTTGGAAAACCAGCACAACTTATCCTTATTGCCTCCATGACTCCCTAATGAAAAGXXXXXXXXXXA

ATGCCAATTTCTCTCCCATTACAGTGTGTCTAGCAGTGGTAATCACAACCAAAGATAACTTAAAGTTTGCTTCAGGGTA

>Marker259639 Chr6 SNP_site:19057937 Ref_Type:G

ACTGTTATGAAAAATTGAATATTCAAACCTCTAAAGTTCGTATCAACCTCGAGATCAACATCTTCTAAAAAATTGAAGACXXXXXXXXXXA

TATTAGAGATTGTATCCGCTATATCAATCAATACACAATGCTCAATTCCACGAACTACATTTCTTCGAAATCACGTGTG

ACTGTTATGAAAAATTGAATATTCAAACCTCTAAAGTTCGTATCAACCTCGAGATCAACATCTTCTAAAAAATTGAAGACXXXXXXXXXXA

TATTAGAGATTGTATCCGCTATATCAATCAATACACAATGCTCAATTCCACGAACTACATTTCTTCGAAATCGCGTGTG

>Marker260236 Chr6 SNP_site:24206984 Ref_Type:A

ACTACCCGTTCGTAAATTCATACATCTAATTGCTAGTGGAACTATAATGAACAAGGAAGCTCTTAATACATACTCATATGXXXXXXXXXXC

CTCAAATAATTACCTATTTAAGGTTAAATTGCGTTGTTTTTATTTTGAAAAATTGTCCCCCTGTTGCTTGTAGAAAGTA

ACTACCCGTTCGTGAATTCATACATCTAATTGCTAGTGGAACTATAATGAACAAGGAAGCTCTTAATACATACTCATATGXXXXXXXXXXC

CTCAAATAATTACCTATTTAAGGTTAAATTGCGTTGTTTTTATTTTGAAAAATTGTCCCCCTGTTGCTCGTAGAAAGTA

>Marker260236 Chr6 SNP_site:24207231 Ref_Type:T

ACTACCCGTTCGTAAATTCATACATCTAATTGCTAGTGGAACTATAATGAACAAGGAAGCTCTTAATACATACTCATATGXXXXXXXXXXC

CTCAAATAATTACCTATTTAAGGTTAAATTGCGTTGTTTTTATTTTGAAAAATTGTCCCCCTGTTGCTTGTAGAAAGTA

ACTACCCGTTCGTGAATTCATACATCTAATTGCTAGTGGAACTATAATGAACAAGGAAGCTCTTAATACATACTCATATGXXXXXXXXXXC

CTCAAATAATTACCTATTTAAGGTTAAATTGCGTTGTTTTTATTTTGAAAAATTGTCCCCCTGTTGCTCGTAGAAAGTA

>Marker261597 Chr6 SNP_site:3584558 Ref_Type:C

CACTTTAGCAAGCATTAGTTTTGCATGATGATATGGCCTCTTTTGATTTTTATCATCGTGCCATAACTATGCTCTTGAAAXXXXXXXXXXA

TGGTCCCCATTGCTGCTCAACTGCTTCTTTAGTGATAATGACCATTCTTTGACATGCATATTGATTTTTAGTGGTAGTT

CACTTTAGCAAGCCTTAGTTTTGCATGATGATATGGCCTCTTTTGATTTTTATCATCGTGCCATAACTATGCTCTTGAAAXXXXXXXXXXA

TGGTCCCCATTGCTGCTCAACTGCTTCTTTAGTGATAATGACCATTCTTTGACATGCATATTGATTTTTAGTGGTAGTT

>Marker262609 Chr6 SNP_site:18585460 Ref_Type:G

CACCTCTTCAGCATCTCTAATGCAACTTTGGGCTGATCCATGGGGACCATGTGCCCCGCATCATGAACCTGTTGTGTTGAXXXXXXXXXXT

TGAATTATAGAGGAAGAACTACAAATCAAACTAAGTATAAACCTCCCTCTTGAGTATGCTAACACTAGGCCCAATTGTC

CACCTCTTCAGCATCTCTAATGCAGCTTTGGGCTGATCCATGGGGACCATGTGCCCCGCATCATGAACCTGTTGTGTTGAXXXXXXXXXXT

TGAATTATAGAGGAAGAACTACAAATCAAACTAAGTATAAACCTCCCTCTTGAGTATGCTAACACTAGGCCCAATTGTC

>Marker26265 Chr4 SNP_site:19886139 Ref_Type:A

ACTTAAATTGTTGCAAGGTATTTATCGTTTCTTGTTTTTGCTTCTGGTTTTGATTCAAAGCCACTATGTTCTTCATTATTXXXXXXXXXXC

TTCTTTCTTTTACTTGCTATGTAATAATGATAAGAGGGAGAATTTCTAAATTTTAATTAGACGAGAATCTGTTTTAGTG

ACTTAAATTGTTGCAAGGTATTTATCGTTTCTTGTTTTTGCTTCTGGTTTTGATTCAAAGCCACTATGTTCTTCATTATTXXXXXXXXXXC

Create PDF with GO2PDF for free, if you wish to

TTCTTTCTTTTACTTGCTATGTAATAATGATAAGAGGGAGAATTTCTAAATTTTAATTAGACGAGGATCTGTTTTAGTG
>Marker262939 Chr6 SNP_site:12912802 Ref_Type:C
CACTTCAAGGCTCAACCAAAATTTAATAATTTTGATTTCAATTTATATCCTCCTGGAGAGATTGGGAAGGACAAAAGGAGXXXXXXXXXXC
TCATAGTTCAAGGATTGTTTTTGCATCTTTTTCTGTTGTTTTTGTTCTTATTGAGGCCATGTTTAAAAGTGCAGGGGTA
CACTTCAAGGCTCAACCAAAATTTAATAATTTTGATTTCAATTTATATCCTCTTGGAGAGATTGGGAAGGACAAAAGGAGXXXXXXXXXXC
TCATAGTTCAAGGATTGTTTTTGCATCTTTTTCTGTTGTTTTTGTTCTTATTGAGGCCATGTTTAAAAGTGCAGGGGTA
>Marker263600 Chr6 SNP_site:2446325 Ref_Type:C
TACCTGATCACTTATCTTTCTAAACGATCACTTACCTCTTACAATCTGAGGAGGGAAAACTTTCAAATATAAGTCAAAAGXXXXXXXXXXT
TTCACCAAGACATGAAACATTCTCAACATGGACTACTGTGATGGTCTTTCATCTATAGTATCATTTAGAATTTTTAGTT
TACCTGATCACTTATCTTTCTAAACGATCACTTACCTCTTACAATCTGAGGAGGGAAAACTTTCAAATATAAGTCAAAAGXXXXXXXXXXT
TTCACCAAGACATGAACCATTCTCAACATGGACTACTGTGATGGTCTTTCATCTATAGTATCATTTAGAATTTTTAGTT
>Marker26363 Chr4 SNP_site:12683547 Ref_Type:G
TACTGTAAAAACTCGATCTGCAGAACGAGAAAAATGATGTTGGTATCTAACTGCTTCACCCATGATATTTTGTTTCCAAAXXXXXXXXXXC
CATAATGCATGGCAGTGTCAATGTCACAGTTGGAGAACAAGAAGAACGGATGATGATCACAGGATTGCACACCGTTGTT
TACTGTAAAAGCTCGATCTGCAGAACGAGAAAAATGATGTTGGTATCTAACTGCTTCACCCATGATATTTTGTTTCCGAAXXXXXXXXXXC
CATAATGCATGGCAGTGTCAATGTCACAGTTGGAGAACAAGAAGAACGGATGATGATCACAGGATTGCACACCGTTGTT
>Marker26363 Chr4 SNP_site:12683614 Ref_Type:G
TACTGTAAAAACTCGATCTGCAGAACGAGAAAAATGATGTTGGTATCTAACTGCTTCACCCATGATATTTTGTTTCCAAAXXXXXXXXXXC
CATAATGCATGGCAGTGTCAATGTCACAGTTGGAGAACAAGAAGAACGGATGATGATCACAGGATTGCACACCGTTGTT
TACTGTAAAAGCTCGATCTGCAGAACGAGAAAAATGATGTTGGTATCTAACTGCTTCACCCATGATATTTTGTTTCCGAAXXXXXXXXXXC
CATAATGCATGGCAGTGTCAATGTCACAGTTGGAGAACAAGAAGAACGGATGATGATCACAGGATTGCACACCGTTGTT
>Marker263814 Chr6 SNP_site:1158185 Ref_Type:A
CACCTCAGGGCCCAGCAATTCCTCATCATTGATTTCTCCATCTACCATATGAATATACAATGGATACACAGAGAGCATCAXXXXXXXXXXT
GCAAATATGTGACATTGGATAGTCTATTCTTAGATGCATATAAACCATCTTTCTCCCATTTTCCACTCTTTTGTTATGT
CACCTCAGGGCCCAGCAATTCCTCATCATTGATTTCTCCATCTACCATATGAATATACAATGGATACACAGAGAGCATCAXXXXXXXXXXT
GCAAATATGTGACATTGGGTAGTCTATTCTTAGATGCATATAAACCATCTTTCTCCCATTTTCCACTCTTTTGTTATGT
>Marker26389 Chr4 SNP_site:22657589 Ref_Type:G
ACAATCGAAAAAAATGGACTCCGATTTTGGGTTCGATCGGTGAGAAAACTGAGAATTGGGATTTAAATCATTTTCTTTCTXXXXXXXXXXC
GTGTCTTCTCAATGAGGTGGTAAGTTCTTATTGGATGCAATGAAGTCTTCTTACCGGTCTTAAGGTCTGATGTGACGTG
ACAATCGAAAAAAATGGACTCCGATTTTTGGTTCGATCGGTGAGAAAACTGAGAATTGGGATTTAAATCATTTTCTTTCTXXXXXXXXXXC
GTGTCTTCTCAATGAGGTGGTAAGTTCTTATTGGATGCAATGAAGTCTTCTTACCGGTCTTAAGGTCTGATGTGACGTG
>Marker26415 Chr4 SNP_site:11537079 Ref_Type:G
TACTTCAGATTATAAAACTAACTGCTCCCTTAATAAAAATTATATATACTCTACTTTTTCCTTCATATCAGTTTTCATTTXXXXXXXXXXT
TGGATATCTGGATCATCCCCAGCTTCTTTATCCTAAAGTTTAGAAAACAATAATATTAATAGTAATAATAACAAAAGTT
TACTTCAGATTATAAAACTAACTGCTCCCTTGATAAAAATTATATATACTCTACTTTTTCCTTCATATCAGTTTTCATTTXXXXXXXXXXT
TGGATATCTGGATCATCCCCAGCTTCTTTATCCTAAAGTTTAGAAAATAATAATATTAATAGTAATAATAACAAAAGTT
>Marker26415 Chr4 SNP_site:11537313 Ref_Type:T
TACTTCAGATTATAAAACTAACTGCTCCCTTAATAAAAATTATATATACTCTACTTTTTCCTTCATATCAGTTTTCATTTXXXXXXXXXXT
TGGATATCTGGATCATCCCCAGCTTCTTTATCCTAAAGTTTAGAAAACAATAATATTAATAGTAATAATAACAAAAGTT
TACTTCAGATTATAAAACTAACTGCTCCCTTGATAAAAATTATATATACTCTACTTTTTCCTTCATATCAGTTTTCATTTXXXXXXXXXXT
TGGATATCTGGATCATCCCCAGCTTCTTTATCCTAAAGTTTAGAAAATAATAATATTAATAGTAATAATAACAAAAGTT
>Marker264223 Chr6 SNP_site:2142241 Ref_Type:G
GACCATAGAAGGCGTTTTGACCAGACGAGATACTTACACTTTACGCGAGATACAAGGCAAGGAAACACACATTTTGTCTAXXXXXXXXXXT
CCTCTCACCTCGAGCGCCCTTTTCGCTTTGAATGAGCACATTTCAAAAGATGGAAGCAAAAGATGTTTTTCCTCTTGTT

Create PDF with GO2PDF for free, if you wish to

GACCATAGAAGGCGTTTTGACCAGACGAGATACTTACACTTTACGCGAGATACAAGGCAAGGAAACACACATTTTGTCTAXXXXXXXXXXT
CCTCTGACCTCGAGCGCCCTTTTCGCTTTGAATGAGCACATTTCAAAAGATGGAAGCAAAAGATGTTTTTCCTCTTGTT
>Marker264617 Chr6 SNP_site:8841645 Ref_Type:A
AACTTTCATTGGAAACAAATTAAAGAATACAATCGTCATACAAAATAACAAAACCCACAACCACCCTAAACAAAAGGAAAXXXXXXXXXXT
CATTGCATTATTAATGCAAAAGCTACATCAGAAACGAGGAATATTATACTTTGTTGAGACACGATCTTTGGACTTTGGT
AACTTTCATTGGAAACAAATTAAAGAATACAATCGTCATACAAAATAACAAAACCCACAACCACCCTAAACAAAAGGAAAXXXXXXXXXXT
CATTGCATTTTTAATGCAAAAGCTACATCAGAAACGAGGAATATTATACTTTGTTGAGACACGATCTTTGGACTTTGGT
>Marker265447 Chr6 SNP_site:11676041 Ref_Type:A
ACTATGAAACCCTAAACATTATGTTTCATCAATTTCTCTCTTTATACCTCAATAAATTAGTTTAAATGCTGTTTTCTAAGXXXXXXXXXXT
TCAAAGTGAATTAAAATATCACTTAAATAAGTGCATTTTCTTTTACTAAAAAACTAAGTAACAAAAAACAAAATTAGTA
ACTATGAAACCCTAAACGTTATGTTTCATCAATTTCTCTCTTTATACCTCAATAAATTAGTTTAAATGCTGTTTTCTAAGXXXXXXXXXXT
TCAAAGTGAATTAAAATATCACTTAAATAAGTGCATTTTCTTTTACTAAAAAACTAAGTAACAAAAAACAAAATTAGTA
>Marker265707 Chr6 SNP_site:21545488 Ref_Type:G
TACACCAATCGTGAAAATGGAACTACTTCCTCATACAAATATGCATTATATGAATCTGGTAATATTCATGATGTAACCATXXXXXXXXXXT
CAAATTTGTTGTCATTGGTATGTATAGAAAGAAACTTCTGCTACACAAGAAAAACTTGTTAATGGCTTCAAGTTTTTGT
TACACCAATCGTGAAAATGGAACTACTTCCTCATACAAATATGCATTATATGAATCTGGTAATATTCATGATGTAACCATXXXXXXXXXXT
CAAATTTGTTGTCATTGGTATGTATAGAAGGAAACTTCTGCTACACAAGAAAAACTTGTTAATGGCTTCAAGTTTTTGT
>Marker266233 Chr6 SNP_site:7322929 Ref_Type:A
ACTGAATTGACTAGCATAAGGTAGCTTTTACATGGCTAATGTGGGAGTTTGAGGCTTAATAGAAGTGGGAGTCCAAGATTXXXXXXXXXXT
TTGGAATTGGATCTCTTTCTAAGCAGTAGATGACTACATTTGAGGGATAAAAAGGTTTATGAATTTTGGCCACTTTGTG
ACTGAATTGACTAGCATAAGGTAGCTTTTACATGGCTAATGTGGGAGTTTGAGGCTTAATGGAAGTGGGAGTCCAAGATTXXXXXXXXXXT
TTGGAATTGGATCTCTTTCTAAGCAGTAGATGACTACATTTGAGGGATAAAAAGGTTTATGAATTTTGGCCACTTTGTG
>Marker266525 Chr6 SNP_site:12707079 Ref_Type:T
ACAAACGAAGGGGGCTCTTTGGTTGGTGAAGCCTAAAGAGAGAGCCTTAGACCTAGCAAGGATGAATTGATTTTTATATGXXXXXXXXXXT
AGTGATGCTATCCACACTCCATGGATGTCTCATTTAACTTGTATCAGTATCCATGTCTGTTTCTTATTTATTTGTTAGT
ACAAACGAAGGGGGCTCTTTGGTTGGTGAAGCCTAAAGAGAGAGCCTTAGACCTAGCAAGGATGAATTGATTTTTATATGXXXXXXXXXXT
AGTGATGCTATCCACACTCCATGGATGTCTCATTTAATTTGTATCAGTATCCATGTCTGTTTCTTATTTATTTGTTAGT
>Marker267025 Chr6 SNP_site:2799358 Ref_Type:G
AACCGGACAGGTCAGAGATTCAGAAATTATCACAAACCTCTCCCTCATGAGCACCTTCTAAATAGGTCTTCTGCTGTAGGXXXXXXXXXXA
CATTTAACATACTGATCTGATTAGAAACATAGAAACTCTTTCATATCCAACGAACATAACAAAAATACTACAGTAGGGT
AACCGGACAGGTCAGAGATTCAGAAATTATCACAAACCTCTCCCTCATGAGCACCTTCTAAATAGGTCTTCTGCTGTAGGXXXXXXXXXXA
CATTTAACATACTGATCTGATTAGAAACATAGAAACTCTTTCATATCCAACGAACATAACAAAAATACTAGAGTAGGGT
>Marker267262 Chr6 SNP_site:15145529 Ref_Type:T
ACTCAAACCATAGATATGCGGCCTGAAAAGTAACAAAACGGCTTGAACTAGAGGAAGGTAAAACCAGGACAAGACTGCGCXXXXXXXXXXT
AAAGGAAAATGGACGATAAGATGGCTTATGTTGCCACGAAAGAACTAGAATTTTCAGGTATTAAAAACACGAAGATAGT
ACTCAAACCATAGATATGCGGCCTGAAAAGTAACAAAACGGCTTGAACTAGAGGAAGGTAAAACCAGGACAAGACTGCGCXXXXXXXXXXT
AAAGGAAAATGGACGATAAGATGGCTTATGTTGCCACGAAAGAACTAGAATTTTCAGGTATTAAAAACATGAAGATAGT
>Marker267530 Chr6 SNP_site:17257600 Ref_Type:C
TACATTCTTTATATATATTACATGTTTATACCTTTTCAATCCATCATTTCTTGTCTTCTTACTTGTCAATGTGTTATTAAXXXXXXXXXXG
GCTAATGCGTGCAAGGAAGAATTTGACAATAAACTTCAACTTAGCGAGATAACTTCCAACATTTGTATCTCGAAAGGTG
TACATTCTTTATATATATTACATGTTTATACCTTTTCAATCCATTATTTCTTGACTTCTTACTTGTCAATGTGTTATTAAXXXXXXXXXXG
GCTAATGCGTGCAAGGAAGAATTTGACAATAAACTCCAACTTAGCGAGATAACTTCCAACATTTGTATCTCGAAAGGTG
>Marker267530 Chr6 SNP_site:17257609 Ref_Type:T
TACATTCTTTATATATATTACATGTTTATACCTTTTCAATCCATCATTTCTTGTCTTCTTACTTGTCAATGTGTTATTAAXXXXXXXXXXG

Create PDF with GO2PDF for free, if you wish to

GCTAATGCGTGCAAGGAAGAATTTGACAATAAACTTCAACTTAGCGAGATAACTTCCAACATTTGTATCTCGAAAGGTG

TACATTCTTTATATATATTACATGTTTATACCTTTTCAATCCATTATTTCTTGACTTCTTACTTGTCAATGTGTTATTAAXXXXXXXXXXG

GCTAATGCGTGCAAGGAAGAATTTGACAATAAACTCCAACTTAGCGAGATAACTTCCAACATTTGTATCTCGAAAGGTG

>Marker267530 Chr6 SNP_site:17257804 Ref_Type:T

TACATTCTTTATATATATTACATGTTTATACCTTTTCAATCCATCATTTCTTGTCTTCTTACTTGTCAATGTGTTATTAAXXXXXXXXXXG

GCTAATGCGTGCAAGGAAGAATTTGACAATAAACTTCAACTTAGCGAGATAACTTCCAACATTTGTATCTCGAAAGGTG

TACATTCTTTATATATATTACATGTTTATACCTTTTCAATCCATTATTTCTTGACTTCTTACTTGTCAATGTGTTATTAAXXXXXXXXXXG

GCTAATGCGTGCAAGGAAGAATTTGACAATAAACTCCAACTTAGCGAGATAACTTCCAACATTTGTATCTCGAAAGGTG

>Marker26880 Chr4 SNP_site:215536 Ref_Type:T

AACTCCATCTCATAGTATGTTATTCATGTAGTTAAGTTTTATAAATCAAAGTAATATATGTTCAAAAGATGTTAATTCAAXXXXXXXXXXG

TGTTATGAAGTATAACCGACAAAGAGCTGAGTTGAAGCCTTGATAGTATTTAACATTATTTAACAAGGAAAGATGAGGT

AACTCCATCTCATAGTATGTTATTCATGTAGTTAAGTTTTATAAATCAAAGTAATATATGTTCAAAAGATGTTAATTCAAXXXXXXXXXXG

TGTTATGAAGTATAACTGACAAAGAGCCGAGTTGAAGCCTTGATAGTATTTAACATTATTTAACAAGGAAAGATGAGGT

>Marker26880 Chr4 SNP_site:215547 Ref_Type:C

AACTCCATCTCATAGTATGTTATTCATGTAGTTAAGTTTTATAAATCAAAGTAATATATGTTCAAAAGATGTTAATTCAAXXXXXXXXXXG

TGTTATGAAGTATAACCGACAAAGAGCTGAGTTGAAGCCTTGATAGTATTTAACATTATTTAACAAGGAAAGATGAGGT

AACTCCATCTCATAGTATGTTATTCATGTAGTTAAGTTTTATAAATCAAAGTAATATATGTTCAAAAGATGTTAATTCAAXXXXXXXXXXG

TGTTATGAAGTATAACTGACAAAGAGCCGAGTTGAAGCCTTGATAGTATTTAACATTATTTAACAAGGAAAGATGAGGT

>Marker269979 Chr6 SNP_site:28159913 Ref_Type:T

AACTAGAGCTGATATCCGTATTTCTAAGGGCGACAAACTTAAGTGTGCTTCTTCCAGTGATGAACTTGTTGAGGTTTGCTXXXXXXXXXXG

ATTGTGTTGAGGCTTCGTGATGATGCTTTGAAAGAGAGGGATGTTGGTCATAACTCTGCTATCGTTACTGATTCAATGT

AACTAGAGCTGATATCCGTATTTCTAAGGGCGACAAACTTAAGTGTGCTTCTTCCAGTGATGAACTTGTTGAGGTTTGCTXXXXXXXXXXG

ATTGTGTTGAGGCTTCGTGATGATGCTTTGAAAGAGAGGGATGTTGGTCATAACTCTGCTATTGTTACTGATTCAATGT

>Marker270085 Chr6 SNP_site:26873501 Ref_Type:A

CACGTGTCCGATGGTGGCATAATGACACGTGATCCACGGCACTTTTCCACCATTCTTAGGGGGCCGACCATGTCTGACTCXXXXXXXXXXA

AAACGACGTGTCTAAAATTATCGTAGAAAATGTAATTATAAGATGGTAACAAAGTTAATAACTCATCCAGAACTTGGTT

CACGTGTCCGATGGTGGCATAATGACACGTGATCCACGGCACTTTTCCACCATTCTTAGGGGGCCGACCATGTCTGACTCXXXXXXXXXXA

AAACGACGTGTCTAAAATTATCGTAGAAGATGTAATTATAAGATGGTAACAAAGTTAATAACTCATCCAAAACTTGGTT

>Marker270085 Chr6 SNP_site:26873542 Ref_Type:G

CACGTGTCCGATGGTGGCATAATGACACGTGATCCACGGCACTTTTCCACCATTCTTAGGGGGCCGACCATGTCTGACTCXXXXXXXXXXA

AAACGACGTGTCTAAAATTATCGTAGAAAATGTAATTATAAGATGGTAACAAAGTTAATAACTCATCCAGAACTTGGTT

CACGTGTCCGATGGTGGCATAATGACACGTGATCCACGGCACTTTTCCACCATTCTTAGGGGGCCGACCATGTCTGACTCXXXXXXXXXXA

AAACGACGTGTCTAAAATTATCGTAGAAGATGTAATTATAAGATGGTAACAAAGTTAATAACTCATCCAAAACTTGGTT

>Marker270206 Chr6 SNP_site:5353493 Ref_Type:T

ACTTCAGAATGATCCTGAACCGTGAGAATGTTGCTAATTCTGTTGTAATGATCCAACCGTCATTGATTTCTTACACATTTXXXXXXXXXXC

TGGGTATCACAATCAACCGGAACATCAGGTATTTCTTTTCATTTTCCTTTTTAGTAACTAAATGTTACTGCAGAAGAGT

ACTTCAGAATGATCCTGAACCGTGAGAATGTTGCTAATTCTGTTGTAATGATCCAACCGTCATTGATTTCTTACACATTTXXXXXXXXXXC

TGGGTATCACAATCAACCGGAACATCAGGTATTTCTTTTCATTTTCCTTTTTAGTAACTAATTGTTACTGCAGAAGAGT

>Marker270228 Chr6 SNP_site:10193543 Ref_Type:G

CACTATAACTCAATATATCCCGAAGGAGGTGAGTTCCCATTATTCATTAGTTTAATAATTGACTGTTTCATTTACTTCTTXXXXXXXXXXT

TTGTCTTCCCTAATTCTATGTTATTTGTTCAGCTAGATTTTGTTTTTTGCACTGATAGCATCTTAAAATAGGTTTCAGT

CACTATAACTCAATATATCCCGAAGGAGGTGAGTTCCCATTATTCATTAGTTTAATAATTGACTGTTTCATTTACTTCTTXXXXXXXXXXT

TTGTCTTCCCTAATTGTATGTTATTTGTTCAGCTAGATTTTGTTTTTTGCTCTGATAGCATCTTAAAATAGGTTTCAGT

>Marker270228 Chr6 SNP_site:10193578 Ref_Type:T

Create PDF with GO2PDF for free, if you wish to

CACTATAACTCAATATATCCCGAAGGAGGTGAGTTCCCATTATTCATTAGTTTAATAATTGACTGTTTCATTTACTTCTTXXXXXXXXXXT
TTGTCTTCCCTAATTCTATGTTATTTGTTCAGCTAGATTTTGTTTTTTGCACTGATAGCATCTTAAAATAGGTTTCAGT
CACTATAACTCAATATATCCCGAAGGAGGTGAGTTCCCATTATTCATTAGTTTAATAATTGACTGTTTCATTTACTTCTTXXXXXXXXXXT
TTGTCTTCCCTAATTGTATGTTATTTGTTCAGCTAGATTTTGTTTTTTGCTCTGATAGCATCTTAAAATAGGTTTCAGT
>Marker270264 Chr6 SNP_site:15841957 Ref_Type:A
ACCATATTTAACATTCCTTAGACACGTATTAAGGCTTTTTTGAGTATATATATTGAATTATGATAGCAAAAACAACTAGTXXXXXXXXXXA
TAGCAGAAAGAAATTCAAATGGAGATCTATGGGTGTTATATGAAAGTTTTTAACTGAGTGGATCCTTGAAAATTAAGTC
ACCATATTTAACATTCCTTAGACACGTATTAAGGCTTTTTTGAGTATATATATTGAATTATGATAGCAAAAACAACTAGTXXXXXXXXXXA
TAGCAGAAATAAATTCAAATGGAGATCTATGGGTGTTATATGAAAGTTTTTAACTGAGTGGATCCTTGAAAATTAAGTA
>Marker270264 Chr6 SNP_site:15842026 Ref_Type:T
ACCATATTTAACATTCCTTAGACACGTATTAAGGCTTTTTTGAGTATATATATTGAATTATGATAGCAAAAACAACTAGTXXXXXXXXXXA
TAGCAGAAAGAAATTCAAATGGAGATCTATGGGTGTTATATGAAAGTTTTTAACTGAGTGGATCCTTGAAAATTAAGTC
ACCATATTTAACATTCCTTAGACACGTATTAAGGCTTTTTTGAGTATATATATTGAATTATGATAGCAAAAACAACTAGTXXXXXXXXXXA
TAGCAGAAATAAATTCAAATGGAGATCTATGGGTGTTATATGAAAGTTTTTAACTGAGTGGATCCTTGAAAATTAAGTA
>Marker270861 Chr6 SNP_site:27323620 Ref_Type:G
AACACATAGGTATTTTCATGTTTTGTAAAGGAATTTTGTAGGACGATTTGTTTTTCTCTTGGTTCAATTTATGGCAATTCXXXXXXXXXXG
GTAAAGAGAAAAGTTCTAAAGTTGTGGGATCATAGAGTAAGAATATTATAGAACATAAAAGAGTAGAAAGAGTAGAGTT
AACACATAGGTATTTTCATGTTTTGTAAAGGAATTTTGTAGGACGATTTGTTTTTCTCTTGGTTCAATTTATGGCAATTCXXXXXXXXXXG
GTAAAGAGAAAAGTTCTAAAGTTGTGGGATCATAGAGTAAGAATATTATAGAACATAAAATAGTAGAAAGAGTAGAGTT
>Marker27176 Chr4 SNP_site:4590953 Ref_Type:C
AACAGTGAAATGGTGTTGTTTAACTATGCTTGCTTCATGAAGATGGTGGGATTAATCCCTTGCCCGCAACAACACCCAAGXXXXXXXXXXA
AATTTTGGAATCTCTACATTATTTCCTTTTCAATATTCATTTTCCAAAAGAAATTGAAAGTAGTGGAAGATTGTGTGTG
AACAGTGAAATGGTGTTGTTTAACTATGCTTGCTTCATGAAGATGGTGGGATTAATCCCTTGCCCGCAACAACACTCAAGXXXXXXXXXXA
AATTTTGGAATCTCTACATTATTTCCTTTTCAATATTCATTTTCCAAAAGAAATTGAAAGTAGTGGATGATTGTGTGTG
>Marker27176 Chr4 SNP_site:4591156 Ref_Type:A
AACAGTGAAATGGTGTTGTTTAACTATGCTTGCTTCATGAAGATGGTGGGATTAATCCCTTGCCCGCAACAACACCCAAGXXXXXXXXXXA
AATTTTGGAATCTCTACATTATTTCCTTTTCAATATTCATTTTCCAAAAGAAATTGAAAGTAGTGGAAGATTGTGTGTG
AACAGTGAAATGGTGTTGTTTAACTATGCTTGCTTCATGAAGATGGTGGGATTAATCCCTTGCCCGCAACAACACTCAAGXXXXXXXXXXA
AATTTTGGAATCTCTACATTATTTCCTTTTCAATATTCATTTTCCAAAAGAAATTGAAAGTAGTGGATGATTGTGTGTG
>Marker271836 Chr6 SNP_site:28523853 Ref_Type:C
ACCTCAAGGTTTTGGATCTATAAAGTTGGTAAGTCTTTCTAAAGCTATTTCTACATGGTTCTCCGTGCTTAACTGATCATXXXXXXXXXXA
ATTCTTGTATGATTATGTTTGAATGACTCAAGTGTTCTTAAATTCTTGTATGATTAGTTCGTTAGCACGTTAGTTAGTT
ACCTCAAGGTTTTGGATCTATAAAGTTGGTAAGTCTTTCTAAAGCTATTTCTACATGGTTCTCCGTGCTTAACTGATCATXXXXXXXXXXA
ATTCTTGTATGATTATGTTTGAATGACTTAAGTGTTCTTAAATTCTTGTATGATTAGTTCGTTAGCACGTTAGTTAGTT
>Marker272239 Chr6 SNP_site:15669495 Ref_Type:G
ACTATTTTGATTCTTTGGTAGGTGAGAACAAGCATTGTTGATGCCACAAAAGCGAAAACAAGTGCTAAAAAATGGCTTTTXXXXXXXXXXA
AATAACTGCAAAATACAACCAAGCACAATTAAAATGCTTATTCAACTGCAATCCAGAAGGCGAAGAGGAAGGTTATGTT
ACTATTTTGATTCTTTGGTAGGTGAGAACAAGCATTGTTGATGCCACAAAAGCGAAAACAAGTGCTAAAAAATGGCTTTTXXXXXXXXXXA
AATAACTGCAAAATACAACCAAGCACAATTAAAATGCTTATTCAGCTGCAATCCAGAAGGCGAAGAGGAAGGTTATGTT
>Marker27245 Chr4 SNP_site:210065 Ref_Type:A
TACAACCTAATAGATCCTAATAGATCTTCTCTCTCTCTCTTTTGGCCTAGTAGCTAGAGGGCCTGGAAGGTGCAACGTAAXXXXXXXXXXG
TTGTAACAGGATAATGGGGAGGTTAAATGTAAATGTCCTCCAGGATTTAAAGGTGATGGTGTCAAAACATGTGAAGGTA
TACAACCTAATAGATCCTAATAGATCTTCTCTCTCTCTCTTTTGGCCTAGTAGCTAGAGGGCCTGGAAGGTGCAACGTAAXXXXXXXXXXG
TTGTAACAGGATAATGGGGAGGTTAAATGTAAATGTCCTCCAGGATTTAAAGGTGATGGTGTCAAAACTTGTGAAGGTA

Create PDF with GO2PDF for free, if you wish to

>Marker272585 Chr6 SNP_site:10208236 Ref_Type:C
ACTTGAAAGTATAATTTGTTTTAGAATGTTTTAAATCAAGCACACCTGCCCTTTTTCTTCTGACAGGGTAGCAGGTTAGGXXXXXXXXXXG
TAGCTGTTGCTGAAGCTAAGGTGAAAAGAAAAGCAAAAAAGTTGGCAGGCAATCCTCATTCACATCTGAAGGATGAAGT
ACTTGAAAGTATAATTTGTTTTAGAATGTTTTAACTCAAGCACACCTGCCCTTTTTCTTCTGACAGGGTAGCAGGTTAGGXXXXXXXXXXG
TAGCTGTTGCTGAAGCTAAGGTGAAAAGAAAAGCAAAAAAGTTGGCAGGCAATCCTCATTCACATCTGAAGGATGAAGT
>Marker272619 Chr6 SNP_site:5007434 Ref_Type:G
ACTGCCACATTATTTATTTTCCTTCTTTTTAGTTTTGGTGGGGAGGAGGAAACAGAGCCACTACAAATGGAGTAAAAACAXXXXXXXXXXT
AGAAGTTGAAAATGATGAAGAACTCATTATAGCTATCCAACTTATATACAATCCTATGAGATAACAACTGAGCGTCGTT
ACTGCCACATTATTTATTTTCCTTCTTTTTAGTTTTGGTGGGGAGGAGGAAACAGAGGCACTACAAATGGAGTAAAAACAXXXXXXXXXXT
AGAAGTTGAAAATGATGAAGAACTCATTATAGCTATCCAACTTATATACAATCCTATGAGATAACAACTGAGCGTCGTT
>Marker273003 Chr6 SNP_site:12180234 Ref_Type:G
AACCATTGAAGAAGACTTGAATTTAAGAGAGTGTTAGTATGTGCTATTACTACTGTTATTTATTTTTTTTATGGTTGAAAXXXXXXXXXXT
CCAGCGTCAGTCTTTATTCACGGGATAGGCTCTCAATTTAGTTCAGCAGTTCGAACAATTTCCTTTTGCTAGAGAGGTG
AACCATTGAAGAAGACTTGAATTTAAGAGAGTGTTAGTATGTGCTGTTACTACTGTTATTTATTTTTTTTATGGTTGAAAXXXXXXXXXXT
CCAGCGTCAGTCTTTATTCACGGGATAGGCTCTCAATTTAGTTCAGCAGTTCGAACAATTTCCTTTTGCTAGAGAGGTG
>Marker273170 Chr6 SNP_site:2466398 Ref_Type:G
GACCTTATCCGACGCAGTTACACATTGAAAAAACTTCAATTTGTGTCATGCAAGGTATTTCCGACGTTTGAATCAACGTCXXXXXXXXXXC
GGGGAAGGCTTTTTCCGACGTGATGCGAATAAAGCCTTCCCCGACGTCGTGCAATATACGTCAGGAAGGCTTTCGTAGT
GACCTTATCCGACGCAGTTACACGTTGAAAAAACTTCAATTTGTGTCATGCAAGGTATTTCCGATGTTTGAATCAACGTTXXXXXXXXXXC
GGGGAAGGCTTTTTCCGACGTGATGCGAATAAAGCCTTCCCCGACGTCGTGCAATATACGTCAGGAAGGCTTTCGTAGT
>Marker273170 Chr6 SNP_site:2466439 Ref_Type:C
GACCTTATCCGACGCAGTTACACATTGAAAAAACTTCAATTTGTGTCATGCAAGGTATTTCCGACGTTTGAATCAACGTCXXXXXXXXXXC
GGGGAAGGCTTTTTCCGACGTGATGCGAATAAAGCCTTCCCCGACGTCGTGCAATATACGTCAGGAAGGCTTTCGTAGT
GACCTTATCCGACGCAGTTACACGTTGAAAAAACTTCAATTTGTGTCATGCAAGGTATTTCCGATGTTTGAATCAACGTTXXXXXXXXXXC
GGGGAAGGCTTTTTCCGACGTGATGCGAATAAAGCCTTCCCCGACGTCGTGCAATATACGTCAGGAAGGCTTTCGTAGT
>Marker273170 Chr6 SNP_site:2466454 Ref_Type:T
GACCTTATCCGACGCAGTTACACATTGAAAAAACTTCAATTTGTGTCATGCAAGGTATTTCCGACGTTTGAATCAACGTCXXXXXXXXXXC
GGGGAAGGCTTTTTCCGACGTGATGCGAATAAAGCCTTCCCCGACGTCGTGCAATATACGTCAGGAAGGCTTTCGTAGT
GACCTTATCCGACGCAGTTACACGTTGAAAAAACTTCAATTTGTGTCATGCAAGGTATTTCCGATGTTTGAATCAACGTTXXXXXXXXXXC
GGGGAAGGCTTTTTCCGACGTGATGCGAATAAAGCCTTCCCCGACGTCGTGCAATATACGTCAGGAAGGCTTTCGTAGT
>Marker273390 Chr6 SNP_site:2801507 Ref_Type:C
AACCCTAATCCTCACTTAACTGAACTTTGCTGTCTTAATAGTAAGAATGCACTTTCTCTGGGAAAAAATTCTGCTTGGCAXXXXXXXXXXC
ACAACCGGATGTGACCTTAGTATTGAATGAGTATATGACTAAAAGAATGATGAGAAATTATCAATGGCCATCCTTCGTT
AACCCTAATCCTCATTTAACTGAACTTTGCTGTCTTAATAGTAAGAATGCACTTTCTCTGGGAAAAATTTCTGCTTGGCAXXXXXXXXXXC
ACAACCGGATGTGACCTTAGTATTGAATGAGTATATGACTAAAAGAATGATGAGAAATTATCAATGGCCATCCTTCGTT
>Marker273390 Chr6 SNP_site:2801560 Ref_Type:T
AACCCTAATCCTCACTTAACTGAACTTTGCTGTCTTAATAGTAAGAATGCACTTTCTCTGGGAAAAAATTCTGCTTGGCAXXXXXXXXXXC
ACAACCGGATGTGACCTTAGTATTGAATGAGTATATGACTAAAAGAATGATGAGAAATTATCAATGGCCATCCTTCGTT
AACCCTAATCCTCATTTAACTGAACTTTGCTGTCTTAATAGTAAGAATGCACTTTCTCTGGGAAAAATTTCTGCTTGGCAXXXXXXXXXXC
ACAACCGGATGTGACCTTAGTATTGAATGAGTATATGACTAAAAGAATGATGAGAAATTATCAATGGCCATCCTTCGTT
>Marker273529 Chr6 SNP_site:11262827 Ref_Type:T
ACCCTTAGTGTAGCATAATTATTCTCTAGCATCTTATTCATGTTGCTTCCGTTTCCTTGCAAGAACATTGGGATTAGAGGXXXXXXXXXXT
GTGAAAATAAATGTTTATAGTTATCAATGTTTAAATCAATTAACAATCTTTGGCATAAGAACTATTGCAAACCAAGGGT
ACCCTTAGTGTAGCATAATTATTCTCTAGCATCTTATTCATGTTGCTTCCGTTTCCTTGCAAGAACATTGGGATTAGAGGXXXXXXXXXXT

Create PDF with GO2PDF for free, if you wish to

GTGAAAATAAATGTTTATAGTTATCAATGTTTAAATCAATTAACAATCTTTTGCATAAGAACTATTGCAAACCAAGGGT
>Marker273620 Chr6 SNP_site:1991772 Ref_Type:T
ACAGACAATGGGTTGTGAAGATGTCCTTAAAGTAACTAGTTAAGTTACGTGTAAGGTAAGACTAGTTAGTAAATGCTACAXXXXXXXXXXXA
AACTATGATTTCTGCATCATGCAAATAAAATCCCAAGTCATATATTGCTATATGAATAAGAAAAGTGGATATAGCTGTT
ACAGACAATGGGTTGTGAAGATGTCCTTAAAGTAACTAGTTAAGTTACGTGTAAGGTAAGACTAGTTAGTAAATGCTACAXXXXXXXXXXXA
AACTATGATTTCTGCATTATGCAAATAAAATCCCAAGTCATATATTGCTATATGAATAAGAAAAGTGGATATAGCTGTT
>Marker273652 Chr6 SNP_site:8765934 Ref_Type:T
ACCCATGAACTTACGAACAATTCAAATATCGCTTTTTTTAACACAGAAGCACATAAATACCATGGATAGGACGAATCATTXXXXXXXXXXXT
TAGTTCTGAACATTACTCCCATCTTAAAAGGTAATCGAGCATGTAGTGAATCAAACGCATCAACGTCAATAATCTCTGT
ACCCATGAACTTATGAACAATTCAAATATCGCTTTTTTTAACACAGAAGCACATAAATACCATGGATAGGACGAATCATTXXXXXXXXXXXT
TAGTTCTGAACATTACTCCCATCTTAAAAGGTAATCGAGCATGTAGTGAATCAAACGCATCAACGTCAATAATCTCTGT
>Marker27377 Chr4 SNP_site:5316852 Ref_Type:A
AACTAAAACAATAAATATCACACATCTTCTCATTATAACAAAGTTTGTTTTCATGATTGTGTTCGTTTTGGCCTTGAACAXXXXXXXXXXXT
TAAAAGCATAATGCTTACCCTAAAACCACACTAGCACTACTTCGTCATTAGTAGAATGGGTGAAAGATTTCAATGTAGT
AACTAAAACAATAGATATCACACATCTTCTCATTATAACAAAGTTTGTTTTCATGATTGTGTTCGTTTTGGCCTTGAACAXXXXXXXXXXXT
TAAAAGCATAATGCTTACCCTAAAACCACACTAGCACTACTTCGTCATTAGTAGAATGGGTGAAAGATTTCAATGTAGT
>Marker27416 Chr4 SNP_site:6188913 Ref_Type:G
ACTCCATTAACTAGTTAGGTTTCTATTTTACTAGGATAACCTAAGTAACTTAGTTTTGCTTATAATTCTTATTGGATTAAXXXXXXXXXXXA
GGAGAACAAGTGTGGGCGTTAGGTGCCAAGAGGTTACTATCCTTAAACACGAAGCTCTATAAGCCTTATAAGTGAAGTA
ACTCCATTAACTAGTTAGGTTTCTATTTTACTAGGATGACCTAAGTAACTTAGTTTTGCTTATAATTCTTATTGGATTAAXXXXXXXXXXXA
GGAGAACAAGTGTAGGCGTTAGGTGCCAAGAGGTTACTATCCTTAAACACGAAGCTCTATAAGCCTTATAAGTGAAGTA
>Marker27416 Chr4 SNP_site:6189113 Ref_Type:A
ACTCCATTAACTAGTTAGGTTTCTATTTTACTAGGATAACCTAAGTAACTTAGTTTTGCTTATAATTCTTATTGGATTAAXXXXXXXXXXXA
GGAGAACAAGTGTGGGCGTTAGGTGCCAAGAGGTTACTATCCTTAAACACGAAGCTCTATAAGCCTTATAAGTGAAGTA
ACTCCATTAACTAGTTAGGTTTCTATTTTACTAGGATGACCTAAGTAACTTAGTTTTGCTTATAATTCTTATTGGATTAAXXXXXXXXXXXA
GGAGAACAAGTGTAGGCGTTAGGTGCCAAGAGGTTACTATCCTTAAACACGAAGCTCTATAAGCCTTATAAGTGAAGTA
>Marker274882 Chr6 SNP_site:17969005 Ref_Type:A
AACATTAATTCCTTTCAACAAAATGGCACATGCCAAGAAAAATCCCCGATCATACAAAAATACACATGACAGGAACTAGCXXXXXXXXXXXA
TCAATAATTGGAGTAGGGTCTGCACAAGCTCTTCTAGCAGACTCTGGATCCCGGAAAGTCACCTAAAACCCTCCATGTA
AACATTAATTCCTTTCAACAAAATGGCACATGCCAAGAAAAGTCCCCGATCATACAAAAATACACATGACAGGAACTAGCXXXXXXXXXXXA
TCAATAATTGGAGTAGGGTCTGCACAAGCTCTTCTAGCAGACTCTGGATCCCGGAAAGTCACCTAAAACCCTCCATGTA
>Marker274933 Chr6 SNP_site:22566227 Ref_Type:A
CACCATAATCATAATATCCCCCTGATTGGCGACCATAACCACGTCCCCTTCCACGAAATGCACGACCTCTTCCACGTCCTXXXXXXXXXXXT
TAATAATCAAGTGAAACAACATTTTAAAAAATTTACAAAAATATCAAAATCTCAATATAGACCAATTTTGCTATATGTG
CACCATAATCATAATATCCCCCTGATTGGCGACCATAACCACGTCCCCTTCCACGAAATGCACGACCTCTTCCACGTCCTXXXXXXXXXXXT
TAATAATCAAGTGAAACAACATTTTAAAAAATTTACAAATATATCAAAATCTCAATATAGACCAATTTTGCTATATGTG
>Marker27562 Chr4 SNP_site:15429060 Ref_Type:G
ACATTTGGTAGTGTCTTGCCATTGCGTCTAGCACACATAAGTTATAAGTTATTTGTGAGAGAGTTCAATTCAAGGTTGGGXXXXXXXXXXXT
TTGCTATATAAATTTTCTAGGATTATGAAATTCTATAATATTTTCTTTAATAAAATTGTCGGTTTTCCTCTATCTCGTA
ACATTTGGTAGTTTCTTGCCATTGCGTCTAGCACACATAAGTTATAAGTTATTTGTGAGAGAGTTCAATTCAAGGTTGGGXXXXXXXXXXXT
TTGCTATATAAATTTTCTAGGATTATGAAATTCTATAATATTTTCTTTAATAAAATTGTCGGTTTTCCTCTATCTCGTA
>Marker275699 Chr6 SNP_site:2863431 Ref_Type:T
TACCAAAGTTTGAATTGTCTAAAGATGCATCTAATCTATTAATGCCTAGCCCAACATGATCAATCCTAGATAAGAAGTCTXXXXXXXXXXXA
TAACTTTCATGTATCTTTTGAAGGGATACATCCATTTTAGATATATGGGCCAAAAAAGTTTGACTTTTCTAACAATGTG

Create PDF with GO2PDF for free, if you wish to

TACCAAAGTTTGAATTGTCTAAAGATGCATCTAATCTATTAATGTCTAGCCCAACATGATCAATCCTTGATAAGAAGTCTXXXXXXXXXXA
GAACTTTCATGTATCTTTTGAAGGGATACATCCATTTTAGATATATGGGCCAAAAAAGTTTGACTTTTCTAACAATGTG
>Marker275699 Chr6 SNP_site:2863454 Ref_Type:T
TACCAAAGTTTGAATTGTCTAAAGATGCATCTAATCTATTAATGCCTAGCCCAACATGATCAATCCTAGATAAGAAGTCTXXXXXXXXXXA
TAACTTTCATGTATCTTTTGAAGGGATACATCCATTTTAGATATATGGGCCAAAAAAGTTTGACTTTTCTAACAATGTG
TACCAAAGTTTGAATTGTCTAAAGATGCATCTAATCTATTAATGTCTAGCCCAACATGATCAATCCTTGATAAGAAGTCTXXXXXXXXXXA
GAACTTTCATGTATCTTTTGAAGGGATACATCCATTTTAGATATATGGGCCAAAAAAGTTTGACTTTTCTAACAATGTG
>Marker275699 Chr6 SNP_site:2863552 Ref_Type:G
TACCAAAGTTTGAATTGTCTAAAGATGCATCTAATCTATTAATGCCTAGCCCAACATGATCAATCCTAGATAAGAAGTCTXXXXXXXXXXA
TAACTTTCATGTATCTTTTGAAGGGATACATCCATTTTAGATATATGGGCCAAAAAAGTTTGACTTTTCTAACAATGTG
TACCAAAGTTTGAATTGTCTAAAGATGCATCTAATCTATTAATGTCTAGCCCAACATGATCAATCCTTGATAAGAAGTCTXXXXXXXXXXA
GAACTTTCATGTATCTTTTGAAGGGATACATCCATTTTAGATATATGGGCCAAAAAAGTTTGACTTTTCTAACAATGTG
>Marker275952 Chr6 SNP_site:26226771 Ref_Type:C
TACATATATATATCACCATTCAAAAATTGAAGAAGAATTAAAATTTCATCTGTAAATGTGAGAAAATAGAATGATGTTTGXXXXXXXXXXT
ATTTTACTAAAATTATTTGAGTTTAGGGAAATTTACAACTTTGGTGAGATTTTAAGTTAAAATTAGTTTAGACCACGTT
TACATATATATATCACCATTCAAAAATTGAAGAAGAATTAAAATTTCATCTGTAAATGTGAGAAAATAGAATGATGTTTGXXXXXXXXXXT
ATTTTAGTAAAATTATTAGAGTTTAGGGAAATTTACAACTTTTGTGAGATTTTAAGTTAAAATTAGTTTAGACCACGTT
>Marker275952 Chr6 SNP_site:26226782 Ref_Type:T
TACATATATATATCACCATTCAAAAATTGAAGAAGAATTAAAATTTCATCTGTAAATGTGAGAAAATAGAATGATGTTTGXXXXXXXXXXT
ATTTTACTAAAATTATTTGAGTTTAGGGAAATTTACAACTTTGGTGAGATTTTAAGTTAAAATTAGTTTAGACCACGTT
TACATATATATATCACCATTCAAAAATTGAAGAAGAATTAAAATTTCATCTGTAAATGTGAGAAAATAGAATGATGTTTGXXXXXXXXXXT
ATTTTAGTAAAATTATTAGAGTTTAGGGAAATTTACAACTTTTGTGAGATTTTAAGTTAAAATTAGTTTAGACCACGTT
>Marker275952 Chr6 SNP_site:26226807 Ref_Type:G
TACATATATATATCACCATTCAAAAATTGAAGAAGAATTAAAATTTCATCTGTAAATGTGAGAAAATAGAATGATGTTTGXXXXXXXXXXT
ATTTTACTAAAATTATTTGAGTTTAGGGAAATTTACAACTTTGGTGAGATTTTAAGTTAAAATTAGTTTAGACCACGTT
TACATATATATATCACCATTCAAAAATTGAAGAAGAATTAAAATTTCATCTGTAAATGTGAGAAAATAGAATGATGTTTGXXXXXXXXXXT
ATTTTAGTAAAATTATTAGAGTTTAGGGAAATTTACAACTTTTGTGAGATTTTAAGTTAAAATTAGTTTAGACCACGTT
>Marker276124 Chr6 SNP_site:3585054 Ref_Type:T
ACCAGCATGACAGAATTACTTGATAGATAATTTTTACCAGAGCACTCAAGGTTTTAATTTCTCTTTTACACAACGGGATGXXXXXXXXXXT
TTGTGCTGTTACACCCATTATTACTTGTTAGAGATAGAGATATATGTGGCATCTGATATAATATTCAATTGGAAGAGTT
ACCAGCATGACAGAATTACTTGATAGATAATTTTTACCAGAGCACTCAAGGTTTTAATTTCTCTTTTACACAACGGGATGXXXXXXXXXXT
TTGTGCTGTTACACCCATTATTACTTGTTAGAGATAGATATATATGTGGCATCTGATATAATATTCAATTGGAAGAGTT
>Marker276235 Chr6 SNP_site:28159551 Ref_Type:T
ACCCTTTATTTTATTTGAAACATTGGCTTCGAGCCATCCAACAATTTGTATCATGTTATTTAACTTGCTTAATGTATGTGXXXXXXXXXXA
GTCCTTGGATGATCTGAAATCAATGGCTGTTGAAACTGTGTTATTGCTGCAAGAGAAGATTAATGATGACGAAGGTGGT
ACCCTTTATTTTATTTGAAACATTGGCTTCGAGCCATCCAACAATTTGTATCATGTTATTTAACTTGCTTAATGTATGTGXXXXXXXXXXA
GTCCTTGGATGATCTGAAATCAATGGCTGTTGAAACTGTGTTATTGCTGCAAGAGAAGATTAATGATGATGAAGGTGGT
>Marker276715 Chr6 SNP_site:2127965 Ref_Type:A
ACTGTGAGTAACACATTGATTTGCAAGCTACTAATTAAACATGGTTTCAACCTACACAAGCAATAACTTGTGAAATGATTXXXXXXXXXXG
CAAAGAAAATATTTCAATGTTTTCAATACATTTTCTCCTAGCTCGAATATTCTATGAGACCTATTGTATATATGTGGTT
ACTGTGAGTAACACATTGATTTGCAAGCTACTAATTAAACATGGTTTCCACCTACACAAGCAATAACTTGTGAAATGATTXXXXXXXXXXG
CAAAGAAAATATTTCAATGTTTTCAATACATTTTCTCCTAGCCCGAATATTCTATGAGACCTATTGTATATATGTGGTT
>Marker276715 Chr6 SNP_site:2128132 Ref_Type:T
ACTGTGAGTAACACATTGATTTGCAAGCTACTAATTAAACATGGTTTCAACCTACACAAGCAATAACTTGTGAAATGATTXXXXXXXXXXG

Create PDF with GO2PDF for free, if you wish to

CAAAGAAAATATTTCAATGTTTTCAATACATTTTCTCCTAGCTCGAATATTCTATGAGACCTATTGTATATATGTGGTT
ACTGTGAGTAACACATTGATTTGCAAGCTACTAATTAAACATGGTTTCCACCTACACAAGCAATAACTTGTGAAATGATTXXXXXXXXXXG
CAAAGAAAATATTTCAATGTTTTCAATACATTTTCTCCTAGCCCGAATATTCTATGAGACCTATTGTATATATGTGGTT
>Marker276855 Chr6 SNP_site:18449654 Ref_Type:T
CACCATTTGGTTCATCAACAATTAAAGATGTTTTATCATTGGTTATTCATATCAACACATATTTTTCACTGAAACACTCAXXXXXXXXXXA
ATCACATTTTAAAATATCATAATGTATTTGTGATAAATTAAGATAGTTTCTAATATTATTAACATATTATGAATAAAGT
CACCATTTGGTTCATCAATAATTAAAGATGTTTTATCATTCGTTATTCATATCAACACATATTTTTCACTGAAACACTCAXXXXXXXXXXA
ATCACATTTTAAAATATCATAATGTATTTGTGATAAATTAAGATAGTTTCTAATATTATTAACATATTATGAATAAAGT
>Marker276855 Chr6 SNP_site:18449676 Ref_Type:C
CACCATTTGGTTCATCAACAATTAAAGATGTTTTATCATTGGTTATTCATATCAACACATATTTTTCACTGAAACACTCAXXXXXXXXXXA
ATCACATTTTAAAATATCATAATGTATTTGTGATAAATTAAGATAGTTTCTAATATTATTAACATATTATGAATAAAGT
CACCATTTGGTTCATCAATAATTAAAGATGTTTTATCATTCGTTATTCATATCAACACATATTTTTCACTGAAACACTCAXXXXXXXXXXA
ATCACATTTTAAAATATCATAATGTATTTGTGATAAATTAAGATAGTTTCTAATATTATTAACATATTATGAATAAAGT
>Marker276974 Chr6 SNP_site:21978838 Ref_Type:T
GACTAATTAGAGATCCTTTTTGTAACTCCACATTCTCTATTGTAATGTTATCTTGTTTCTTAAGAAACCATGAAAAGAAAXXXXXXXXXXA
ATTACGCACCCACTAGCTAAACTCTCCGTATTTTTTGGTTGCTGCCTATATAATAGTAACAATTAGCTCAATGTTGGTG
GACTAATTAGAGATCCTTTTTGTAACTCCACATTCTCTATTGTAATGTTATCTTGTTTCTTAATAAACCATGAAAAGAAAXXXXXXXXXXA
ATTACGCACCCACTAGCTAAACTCTCCGTATTTTTTGGTTGCTGCCTATATAATAGTAACAATTAGCTCAATGTTGGTG
>Marker277158 Chr6 SNP_site:27319205 Ref_Type:G
GACTTTACACCCTTGAAACTAAGTATCTTTGTCTGGCATGAGAGGATCAATTAAAACTTAGTCCCATGTTATACTTCATTXXXXXXXXXXG
AACAGAGGAAACTAAGAAAGAGAGCAGATTCTTTCATGCTGAATAAGTCCAAAGCCTGTGTGTCGATCCAGGATGGGTA
GACTTTACACCCTTGAAACTAAGTATCTTTGTCTGGCATGAGAGGATCAATTAAAACTTAGTCCCATGTTATACTTGATTXXXXXXXXXXG
AACAGAGGAAACTAAGAAAGAGAGCAGATTCTTTCATGCTGAATAAGTCCAAAGCCTGTGTGTCGATCCAGGATGGGTA
>Marker277271 Chr6 SNP_site:11497141 Ref_Type:T
ACTAAAGATTTCAAGTGAATTTATACATGTAATATTTTTCTTTGTTTCCAAAACTATACAGATTATGTTAAATCTTCTATXXXXXXXXXXT
ATATAAACCTAATTAACACTCTGGTCGAGATAATTACAACATTAAAAGTATGATTAAGGAATTACAGAGAACAAGTGTA
ACTAAAGATTTCAAGTGAATTTATACATGTAATATTTTTCTTTGTTTCCAAAACTATACAGATTATGTTAAATCTTTTATXXXXXXXXXXT
ATATAAACCTAATTAACACTCTAGTCGAGATAATTACAACATTAAAAGTATGATTAAGGAATTACAGAGAACAAGTGTA
>Marker277271 Chr6 SNP_site:11497279 Ref_Type:A
ACTAAAGATTTCAAGTGAATTTATACATGTAATATTTTTCTTTGTTTCCAAAACTATACAGATTATGTTAAATCTTCTATXXXXXXXXXXT
ATATAAACCTAATTAACACTCTGGTCGAGATAATTACAACATTAAAAGTATGATTAAGGAATTACAGAGAACAAGTGTA
ACTAAAGATTTCAAGTGAATTTATACATGTAATATTTTTCTTTGTTTCCAAAACTATACAGATTATGTTAAATCTTTTATXXXXXXXXXXT
ATATAAACCTAATTAACACTCTAGTCGAGATAATTACAACATTAAAAGTATGATTAAGGAATTACAGAGAACAAGTGTA
>Marker277404 Chr6 SNP_site:10321451 Ref_Type:C
ACTTAAGTCATCAAAATCAATCCTTAATATCCTTAGTAAAAAAAATCATGAACAAATATAAAACACAGCAAAGCTGAAATXXXXXXXXXXA
GGGAAGAAATCTGAAACTGACAAAAATAGCAAAAATTTTACCTCTCGACGAACATTAAGTTCTCTTTGGATAAGTGGTT
ACTTAAGTCATCAAAATCAATCCTTAATATCCTTAGTAAAAAAAATCATGAATAAATATAAAACACAGCAAAGCTGAAATXXXXXXXXXXA
GGGAAGAAATCTGAAACTGACAAAAATAGCAAAAATTTTACCTCTCGACGAACATTAAGTTCTCTCTGGATAAGTGGTT
>Marker277404 Chr6 SNP_site:10321666 Ref_Type:T
ACTTAAGTCATCAAAATCAATCCTTAATATCCTTAGTAAAAAAAATCATGAACAAATATAAAACACAGCAAAGCTGAAATXXXXXXXXXXA
GGGAAGAAATCTGAAACTGACAAAAATAGCAAAAATTTTACCTCTCGACGAACATTAAGTTCTCTTTGGATAAGTGGTT
ACTTAAGTCATCAAAATCAATCCTTAATATCCTTAGTAAAAAAAATCATGAATAAATATAAAACACAGCAAAGCTGAAATXXXXXXXXXXA
GGGAAGAAATCTGAAACTGACAAAAATAGCAAAAATTTTACCTCTCGACGAACATTAAGTTCTCTCTGGATAAGTGGTT
>Marker27820 Chr4 SNP_site:6820449 Ref_Type:T

Create PDF with GO2PDF for free, if you wish to

CACTCTGAGGACCAGTATTTTTGCTCTTTTCCTATCATTTTCATTTTTCTGTTTTAGGTAACATAATCATTCAACTGTTCXXXXXXXXXXXT
TACGATAACCCATTAAGTTGATGACCATCCTTATCCCTGCAGTTTTGTTCATAATGACCTTAATGAATCTACTGTGCGT
CACTCTGAGGACCAGTATTTTTGCTCTTTTCCTATCATTTTCATTTTTCTGTTTTAGGTAACATTATCATTCAACTGTTCXXXXXXXXXXXT
TACGATAACCCATTAAGTTGATGACCATCCTTATCCCTGCAGTTTTGTTCATAATGACCTTAATGAATCTACTGTGCGT
>Marker278549 Chr6 SNP_site:16630093 Ref_Type:T
TACTTTTTAGCACCCCAAACGATTTCCAAGTGAAATTCAAATATTTCCTTCTCGAAGATTAAGATTACAGAGAGGAACAAXXXXXXXXXXXC
AATGATTTTCCACCCAATAAATTTGTCACACATCAACACTCTCAACCATATCCTATCAATTTTTTAGATTATCAAAGTA
TACTTTTTAGCACCCCAAACGATTTCCTAGTGAATTTCAAATATTTCCTTCTCGAAGATTAAGATTACAGAGAGGAACAAXXXXXXXXXXXC
AATGATTTTCCACCCAATAAATTTGTCACACATCAACACTCTCAACCATATCCTATCAATTTTTTAGATTATCAAAGTA
>Marker278549 Chr6 SNP_site:16630100 Ref_Type:T
TACTTTTTAGCACCCCAAACGATTTCCAAGTGAAATTCAAATATTTCCTTCTCGAAGATTAAGATTACAGAGAGGAACAAXXXXXXXXXXXC
AATGATTTTCCACCCAATAAATTTGTCACACATCAACACTCTCAACCATATCCTATCAATTTTTTAGATTATCAAAGTA
TACTTTTTAGCACCCCAAACGATTTCCTAGTGAATTTCAAATATTTCCTTCTCGAAGATTAAGATTACAGAGAGGAACAAXXXXXXXXXXXC
AATGATTTTCCACCCAATAAATTTGTCACACATCAACACTCTCAACCATATCCTATCAATTTTTTAGATTATCAAAGTA
>Marker279757 Chr6 SNP_site:2457655 Ref_Type:C
AACCAGCAAATAGAACAACCTAAATGAAGACAAAGAATCAAAAAGAGAGGGAGGAAGAAACACAAGCCACAAGATAATAAXXXXXXXXXXXA
AGCAACATCCAACACTATTGGGAATGTATGAAAATTTTCTTGATTCTAAAATATATTGTTGAAGTAGTGAATGAATGTA
AACCAGCAAATAGAACAACCTAAATGAAGACAAAGAATCAAAAAGAGAGGGAGGAAGAAACACAAGCCACAAGATAATAAXXXXXXXXXXXA
AGCAACATCCAACACTATTGGGAATGTATGAAAATTTTGTTGATTCTAAAATATATTGTTGAAGTAGTGAATGAATGTA
>Marker27982 Chr4 SNP_site:20637519 Ref_Type:G
AACGTCAAACCGTTAAGCCTTTGAAACAAGCACATCCACACATGATTATCAAAGATGAAACATGAAGGTCACCTGTATAAXXXXXXXXXXXT
CAACTTTAACTCCATCGACACCAGCAGATGCAAGATACGAGTGTTGTTCATTGTAAAAGTTGAAAACTTTTTCAGGGTT
AACGTCAAGCCGTTAAGCCTTTGAAACAAGCACATTCACACATGATTATCAAAGATGAAACATGAAGGTCACCTGTATAAXXXXXXXXXXXT
CAACTTTAACTCCATCGACACCAGCAGATGCAAGATACGAGTGTTGTTCATTGTAAAAGTTGAAAACTTTTTCAGGGTT
>Marker27982 Chr4 SNP_site:20637546 Ref_Type:T
AACGTCAAACCGTTAAGCCTTTGAAACAAGCACATCCACACATGATTATCAAAGATGAAACATGAAGGTCACCTGTATAAXXXXXXXXXXXT
CAACTTTAACTCCATCGACACCAGCAGATGCAAGATACGAGTGTTGTTCATTGTAAAAGTTGAAAACTTTTTCAGGGTT
AACGTCAAGCCGTTAAGCCTTTGAAACAAGCACATTCACACATGATTATCAAAGATGAAACATGAAGGTCACCTGTATAAXXXXXXXXXXXT
CAACTTTAACTCCATCGACACCAGCAGATGCAAGATACGAGTGTTGTTCATTGTAAAAGTTGAAAACTTTTTCAGGGTT
>Marker279846 Chr6 SNP_site:26359059 Ref_Type:G
ACAAAAGTGAGAAATTATAATAACTTTATGAAATCAGTGGGCATTTGAATTCTAATCTCTGACCAAACAGCTTCTCTTTTXXXXXXXXXXXT
TTTACAATTGGAAATAAACAAAACAAACATTTAATTGACTGATCAATTTGTTGTATTTTCATCATTTAAATCCCAAGTC
ACAAAAGTGAGAAATTATAATAACTTTATGAAATCAGTGGGCGTTTGAATTCTAATCTCTGACCAAACAGCTTCTCTTTTXXXXXXXXXXXT
TTTACAATTGGAAATAAACAAAACAAACATTTAATTGACTGATCAATTTGTTGTATTTTCATCATTTAAATCCCAAGTC
>Marker280361 Chr6 SNP_site:9040006 Ref_Type:A
GACTTAATGAATAGAGTGTTTCTCGTTACTTGGATCGATTTATCATTGTCTTTATTGATGACATGCATTTTGGTTTATTCXXXXXXXXXXXA
CAAAAAATTGAGGCAATTGAAAGTTGGGAACATCCTAACACAGTTTCTGAAGTTCGGAGCTTCCTTGGTTTGGCATGGT
GACTTAATGAATAGAGTGTTTCTCGTTACTTGGATCGATTTATCATTGTCTTTATTGATGACATGCATTTTGGTTTATTCXXXXXXXXXXXA
CGAAAAATTGAGGCAATTGAAAGTTGGGAACATCCTAACACAGTTTCTGAAGTTCGGAGCTTCCTTGGTTTGGCATGGT
>Marker280799 Chr6 SNP_site:25354818 Ref_Type:C
ACCCCAACTTCACTTCTCTACTCATCAGAAATTGACTCTCCATCATATACACAAACAACACACAAGAGACCATCCTGAAGXXXXXXXXXXXC
ATTTGCAGGTGCAACAGACAACAATATACCTTCATCGGTTATTCGATACCGAGAATGTTTGAAAAACCACGCAGCAAGT
ACCCCAACTTCACTTCTCTACTCATCAGAAATTGACTCTCCATCATATACATAAACAACACACAAGAGACCATCCTGAAGXXXXXXXXXXXC
ATTTGCAGGTGCAACAGACAACAATATACCTTCATCGGTTATTCGATACCGAGAATGTTTGAAAAACCACGCAGCAAGT

Create PDF with GO2PDF for free, if you wish to

>Marker280893 Chr6 SNP_site:8770198 Ref_Type:T
AACACCGGCACACTAGCAAGTGAGCTACAAACGTGGTTGTCAATACTTTTGCCACTAACCCTTTTTTGTGATAATATTTAXXXXXXXXXXXT
TTTCTTTGCACATCACTTTCCTCGCAAACTAACACCCTTATTTCCCATCATTTTCTTTATAGACTTTTGGTTCAATTGT
AACACGGACACACTAGCAAGTGAGCTACAAACGTGGTTGTCAATACTTTTGCCACTAACCCTTTTTTGTGATAATATTTAXXXXXXXXXXXT
TTTCTTTGCACATCACTTTCCTCGCAAACTAACACCCTTATTTCCCATCATTTTCTTTATAGACTTTTGGTTCAATTGT
>Marker280893 Chr6 SNP_site:8770200 Ref_Type:G
AACACCGGCACACTAGCAAGTGAGCTACAAACGTGGTTGTCAATACTTTTGCCACTAACCCTTTTTTGTGATAATATTTAXXXXXXXXXXXT
TTTCTTTGCACATCACTTTCCTCGCAAACTAACACCCTTATTTCCCATCATTTTCTTTATAGACTTTTGGTTCAATTGT
AACACGGACACACTAGCAAGTGAGCTACAAACGTGGTTGTCAATACTTTTGCCACTAACCCTTTTTTGTGATAATATTTAXXXXXXXXXXXT
TTTCTTTGCACATCACTTTCCTCGCAAACTAACACCCTTATTTCCCATCATTTTCTTTATAGACTTTTGGTTCAATTGT
>Marker281759 Chr6 SNP_site:8846996 Ref_Type:A
ACTTTCTCTGTTTTGATTTGTTGGGATTTTTCTGCTTTTCAGGTTTGGGGTCCTAACAAATTGGTATCAAAGCCTCAACAXXXXXXXXXXXA
GTGTGGAACAGCTTGCGAAAGACATGAAAGAGAGTGCCTCCAGTCAAGGAGGAATATCAAGGACGTCTGATGCCTTGGT
ACTTTCTCTGTTTTGATTTGTTGGGATTTTTCTGCTTTTCAGGTTTGGGGTCCTAACAAATTGGTATCAAAGCCTCAACAXXXXXXXXXXXA
GTGTGGAACAGCTTGCGGAAGACATGAAAGAGAGTGCCTCCAGTCAAGGAGGAATATCAAGGACGTCTGATGCCTTGGT
>Marker281931 Chr6 SNP_site:16753372 Ref_Type:T
GACTCTTCAACAATATGTTGCTGGAGATGTGCCTCTATGCATCAAAAAGTTGTAGAAAAAGCATACTCATTCAAAATGAAXXXXXXXXXXXA
CAAAGTATGCTTCAAGCAGCATCTCCAATTGTTCGATGTTATCTCCGCTGGTCGAGGAACTCATCAAGCTGCTTGAGTT
GACTTTTCAACAATATGTTGCTGGAGATGTGCCTCTATGCATCAAAAAGTTGTAGAAAAAGCATACTCATTCAAAATGAAXXXXXXXXXXXA
CAAAGTATGCTTCAAGCAGCATCTCCAATTGTTCGATGTTATCTCCGCTGGTCGAGGAACTCATCAAGCTGCTTGAGTT
>Marker282253 Chr6 SNP_site:17383910 Ref_Type:T
ACCAACATTTATTGTTAGATATGAATAATAGAGGTGATATGTTGATATTTTTTTAAAAAATAATTAGATTTATTTGGTATXXXXXXXXXXXA
GCATGAGAATGTGGTGTAATAGGTTTTAGGCGCAACATCATTATCAATTCCAACTTACAAGAAGTCATAATAATGAGTG
ACCAACATTTATTGTTAGATATGAATAATAGAGGTGATATGTTGATATTTTTTTTAAAAATAATTAGATTTATTTGGTATXXXXXXXXXXXA
GCATGAGAATGTGGTGTAATAGGTTTTAGGCGCAACATCATTATCAATTCCAACTTACAAGAAGTCATAATAATGAGTG
>Marker282266 Chr6 SNP_site:4901729 Ref_Type:T
CACATCATATTTTATGCGCCTCATCTTGAGGTGCCAGACCTGCACTTAATGCCTAGGCATGTTCCTGAGATCGTTTTTTAXXXXXXXXXXXT
TATGGAAGCCGTGCATACACGTTGGCCTAAAGCTATTGTTCAGGTTTATTGGTTAAGATATCAAATTTTTAACTGTGTT
CACATCATATTTTATGTGCCTCATCTTGAGGTGCCAGACCTGCACTTAATGCCTAGGCATGTTCCCGAGATCGTTTTTTAXXXXXXXXXXXT
TATGGAAGCCGTGCATACACGTTGGCCTAAAGCTATTGTTCAGGTTTATTGGTTAAGATATCAAATTTTTAACTGTGTT
>Marker282266 Chr6 SNP_site:4901778 Ref_Type:C
CACATCATATTTTATGCGCCTCATCTTGAGGTGCCAGACCTGCACTTAATGCCTAGGCATGTTCCTGAGATCGTTTTTTAXXXXXXXXXXXT
TATGGAAGCCGTGCATACACGTTGGCCTAAAGCTATTGTTCAGGTTTATTGGTTAAGATATCAAATTTTTAACTGTGTT
CACATCATATTTTATGTGCCTCATCTTGAGGTGCCAGACCTGCACTTAATGCCTAGGCATGTTCCCGAGATCGTTTTTTAXXXXXXXXXXXT
TATGGAAGCCGTGCATACACGTTGGCCTAAAGCTATTGTTCAGGTTTATTGGTTAAGATATCAAATTTTTAACTGTGTT
>Marker282299 Chr6 SNP_site:10599233 Ref_Type:C
TACAAGTGAATAAAAAAGAAAAAGATTTAGGAAATAAGGAAAATATTCTCATAATCTTTCCATAAATATTATAGGATTCTXXXXXXXXXXXA
ATGAACAAAGAGAACTCTCCCCCACTTACTGATCCTCCCAAAAGCACGTGTTTATACCATCCCCCACAAACAACGAGTA
TACAAGTGAATAAAAAAGAAAAAGATTTAGGAAATAAGGAAAATATTCTCATAATCTTTCCATAAATATTATAGGATTCTXXXXXXXXXXXA
ATGAACAAAGAGAACTCTCCCCCACTTACTGATGCTCCCAAAAGCACGTGTTTATACCATCCCCCACAAACAACGAGTA
>Marker283567 Chr6 SNP_site:3643599 Ref_Type:C
ACGTAAAAACATAGAATATTTATGCTTAATTACTTTTGTTATTTCTTTCACTGTCTTATATTCATGCAAATGTGTTCTCTXXXXXXXXXXXG
GTGAAAACTGGGTTCCCGGAGTAGGGAAGTAAAAGATTTCATTTTCTTGCGATGCTTCCGATCTGGTGCTTACATTGTT
ACGTAAAAACATAGAATATTTATGCTTAATTATTTTTGTTATTTCTTTCACTGTCTTATATTCATGCAAATGTGTTCTTCXXXXXXXXXXXG

Create PDF with GO2PDF for free, if you wish to

GTGAAAACTGGGTTCCCGGAGTAGGGAAGTAAAAGATTTCATTTTCTTGCGATGCTTCCGATCTGGTGCTTACATTGTT
>Marker283567 Chr6 SNP_site:3643645 Ref_Type:C
ACGTAAAAACATAGAATATTTATGCTTAATTACTTTTGTTATTTCTTTCACTGTCTTATATTCATGCAAATGTGTTCTCTXXXXXXXXXXG
GTGAAAACTGGGTTCCCGGAGTAGGGAAGTAAAAGATTTCATTTTCTTGCGATGCTTCCGATCTGGTGCTTACATTGTT
ACGTAAAAACATAGAATATTTATGCTTAATTATTTTTGTTATTTCTTTCACTGTCTTATATTCATGCAAATGTGTTCTTCXXXXXXXXXXG
GTGAAAACTGGGTTCCCGGAGTAGGGAAGTAAAAGATTTCATTTTCTTGCGATGCTTCCGATCTGGTGCTTACATTGTT
>Marker283567 Chr6 SNP_site:3643646 Ref_Type:T
ACGTAAAAACATAGAATATTTATGCTTAATTACTTTTGTTATTTCTTTCACTGTCTTATATTCATGCAAATGTGTTCTCTXXXXXXXXXXG
GTGAAAACTGGGTTCCCGGAGTAGGGAAGTAAAAGATTTCATTTTCTTGCGATGCTTCCGATCTGGTGCTTACATTGTT
ACGTAAAAACATAGAATATTTATGCTTAATTATTTTTGTTATTTCTTTCACTGTCTTATATTCATGCAAATGTGTTCTTCXXXXXXXXXXG
GTGAAAACTGGGTTCCCGGAGTAGGGAAGTAAAAGATTTCATTTTCTTGCGATGCTTCCGATCTGGTGCTTACATTGTT
>Marker28424 Chr4 SNP_site:9629923 Ref_Type:T
AACCTTCAAAGCCTTACCTCTCTATAACTATATGTTAGACCTCCAATATTATATCACTACAGGAGGAAGGGGAAAAAGTTXXXXXXXXXXA
GGGAGGGAGGGATATGATTATTTTTTCAGTATTTGAATCTTTCTGTTATTTCTTGGAAGAGAGGGAAACAGAGGATTGT
AACCTTCAAAGCCTTACCTCTCTATAACTATATGTTAGACCTCCAATATTATATCACTACAGGAGGAAGGGGAATAAGTTXXXXXXXXXXA
GGGAGGGAGGGATATGATTATTTTTTCAGTATTTGAATCTTTCTGTTATTTCTTGGGAGAGAGGGAAACAGAGGATTGT
>Marker28424 Chr4 SNP_site:9630062 Ref_Type:G
AACCTTCAAAGCCTTACCTCTCTATAACTATATGTTAGACCTCCAATATTATATCACTACAGGAGGAAGGGGAAAAAGTTXXXXXXXXXXA
GGGAGGGAGGGATATGATTATTTTTTCAGTATTTGAATCTTTCTGTTATTTCTTGGAAGAGAGGGAAACAGAGGATTGT
AACCTTCAAAGCCTTACCTCTCTATAACTATATGTTAGACCTCCAATATTATATCACTACAGGAGGAAGGGGAATAAGTTXXXXXXXXXXA
GGGAGGGAGGGATATGATTATTTTTTCAGTATTTGAATCTTTCTGTTATTTCTTGGGAGAGAGGGAAACAGAGGATTGT
>Marker284407 Chr6 SNP_site:12417539 Ref_Type:G
ACTGACCGCTGAAGGAATGCTGGAAACTGTTCGGCGAAATGCTTGAATGAGCCTATACCCAGAGAAAAAGCTCCCCGTTGXXXXXXXXXXG
AACCAAATTAGCAAAAATTCTCTCTTCAAATTTTACATTTTCTAACCTTTATTATTTACCAAATACTCATTTCCATGTT
ACTGACCGCTGAAGGAGTGCTGGAAACTGTTCGGCGAAATGCTTGAATGAGCCTATACCCAGAGAAAAAGCTCCCCGTTGXXXXXXXXXXG
AACCAAATTAGCAAAAATTCTCTCTTCAAATTTTACATTTTCTAACCTTTATTATTTACCAAATACTCATTTCCATGTT
>Marker284491 Chr6 SNP_site:8897200 Ref_Type:C
TACTTGTAAAAGTGAGTGTATGTAAAATTTGATTAATTTTCTAATACACGTTTATATTTATAGGTGTTTTACAGAGACAAXXXXXXXXXXC
AGTTATACTATATGTTTATACGGAGTCACTAGCAAGACTAGGGGTGTTTCGAAGGAATCACTCGCGCAGTTAGAGTTGT
TACTTGTAAAAGTGAGTGTATGTAAAATTTGATTAATTTTTTAATACACCTTTATATTTATAGGTGTTTTACAAAGACAAXXXXXXXXXXC
AGTTATACTATATGTTTATACGGAGTCACTAGCAAGACTAGGGGTGTTTCGAAGGAATCACTCGCGCAGTTAGAGTTGT
>Marker284491 Chr6 SNP_site:8897209 Ref_Type:G
TACTTGTAAAAGTGAGTGTATGTAAAATTTGATTAATTTTCTAATACACGTTTATATTTATAGGTGTTTTACAGAGACAAXXXXXXXXXXC
AGTTATACTATATGTTTATACGGAGTCACTAGCAAGACTAGGGGTGTTTCGAAGGAATCACTCGCGCAGTTAGAGTTGT
TACTTGTAAAAGTGAGTGTATGTAAAATTTGATTAATTTTTTAATACACCTTTATATTTATAGGTGTTTTACAAAGACAAXXXXXXXXXXC
AGTTATACTATATGTTTATACGGAGTCACTAGCAAGACTAGGGGTGTTTCGAAGGAATCACTCGCGCAGTTAGAGTTGT
>Marker284491 Chr6 SNP_site:8897233 Ref_Type:G
TACTTGTAAAAGTGAGTGTATGTAAAATTTGATTAATTTTCTAATACACGTTTATATTTATAGGTGTTTTACAGAGACAAXXXXXXXXXXC
AGTTATACTATATGTTTATACGGAGTCACTAGCAAGACTAGGGGTGTTTCGAAGGAATCACTCGCGCAGTTAGAGTTGT
TACTTGTAAAAGTGAGTGTATGTAAAATTTGATTAATTTTTTAATACACCTTTATATTTATAGGTGTTTTACAAAGACAAXXXXXXXXXXC
AGTTATACTATATGTTTATACGGAGTCACTAGCAAGACTAGGGGTGTTTCGAAGGAATCACTCGCGCAGTTAGAGTTGT
>Marker285192 Chr6 SNP_site:26728760 Ref_Type:C
AACGACGACTTCACCATAATTCTCCACCTTCGACCTTCCACGTAATGGATGGTTTGATTTTATATGTGCTGAGTAGCAAAXXXXXXXXXXG
CCGGACGGGATGACCCATTTTGCTCTTCTGGACGACAATCAATCAAACTAACCATTTTAATGGGCCGGGCTTTCATGTA

Create PDF with GO2PDF for free, if you wish to

AACGACGACTTCACCATAATTCTCCACCTTCGACCTTCCACGTAATGGATGGTTTGATTTTATATGTGCTGAGTAGCAAAXXXXXXXXXXXG
CCGGACGGGATGACCCATTTTGCTCTTCTGGACGACAATCAATCAAACTAACCATTTTAATGGGCCGGGCTTTGATGTA
>Marker285317 Chr6 SNP_site:5875396 Ref_Type:T
AACAGTGACAAATAGTCTGCCAATTGCAACACAAGGTCACTTCAAGACCTAACTTTGATCTCTCATCAAACAATTACCTGXXXXXXXXXXXA
TACTCTGCAGTCTGAAGAAAGCTAAGAACCAATTATACCTCCCATCCAAAACTTCTGGAGCAAGGAGTCCAAAACGGTC
AACAGTGACAATTAGTCTGCCAATTGCAACACAAGGTCACTTCAAGACCTAACTTTGATCTCTCATCAAACAATTACCTGXXXXXXXXXXXA
TACTCTGCAGTCTGAAGAAAGCTAAGAACCAATTATACCTCCCATCCAAAACTTCTGGAGCAAGGAGTCCAAAACGGTC
>Marker285351 Chr6 SNP_site:7336065 Ref_Type:G
TACAAATGTCACAAAGATTATTGATTTTTAATTTTATGCCGGTGAGCCGTTTGTGTAGAATTAGCAAATATATTAAACAAXXXXXXXXXXXA
CAAAAAGAAAAAAAAAACAAAGAGAGAAATTAACCGAACACAAAATTAAAAATTTAGAAATGTATTAATTAACACATGT
TACAAATGTCACAAATATTATTGATTTTTAATTTTATGCCGGTGAGCCGTTTGTATAGAATTAGCAAATATATTAAACAAXXXXXXXXXXXA
CAAAAAGAAAAAAAAAACAAAGAGAGAAATTAACCGAACACAAAATTAAAAATTTAGAAATGTATTAATTAACACATGT
>Marker285351 Chr6 SNP_site:7336104 Ref_Type:G
TACAAATGTCACAAAGATTATTGATTTTTAATTTTATGCCGGTGAGCCGTTTGTGTAGAATTAGCAAATATATTAAACAAXXXXXXXXXXXA
CAAAAAGAAAAAAAAAACAAAGAGAGAAATTAACCGAACACAAAATTAAAAATTTAGAAATGTATTAATTAACACATGT
TACAAATGTCACAAATATTATTGATTTTTAATTTTATGCCGGTGAGCCGTTTGTATAGAATTAGCAAATATATTAAACAAXXXXXXXXXXXA
CAAAAAGAAAAAAAAAACAAAGAGAGAAATTAACCGAACACAAAATTAAAAATTTAGAAATGTATTAATTAACACATGT
>Marker285890 Chr6 SNP_site:9701090 Ref_Type:G
ACCCATTGATTTTCGTGAATCAATCTTTCTTGATCACGTTTTATAACCAAAATAGTAATTTTGAGTCTTAAACATTCTTAXXXXXXXXXXXA
CCCTTTTTCGATGTTGCTATTTTGTCCCGAACTACCTTAGAAACATCATCGAAATCACTAGAATCAAATTTCCAATGTG
ACCCATTGATTTTCGTGAATCAATCTTTCTTGATCACGTTTTATAACCAAAATAGTAATTTTGAGTCTTAAACGTTCTTAXXXXXXXXXXXA
CCCTTTTTCGATGTTGCTATTTTGTCCCGAACTACCTTAGAAACATCATCGAAATCACTAGAATCAAATTTCCAATGTG
>Marker285969 Chr6 SNP_site:1496071 Ref_Type:G
GACTCGGTCATTAGATTGTTGGATATTGTTCTCATAGTTATGTTTCCTTTGTGGGCTCTCCAGATTGTAGCCATGATTTGXXXXXXXXXXXT
GTGGTGTAAAACAGAAGTGTGTCAATTCTATGCGAAGTTTGTATTATTTTTTGATTGTGATAGTTTTGTGGATTTGGTG
GACTCGGTCATTAGATTGTTGGATATTGTTCTCATAGTTATGTTTCCTTTTTGGGCTCTCCAGATTGTAGCCATGATTTGXXXXXXXXXXXT
GTGGTGTAAAACAGAAGTGTGTCAATTCTATGCGAAGTTTGTATTATTTTTTGATTGTGATAGTTTTGTGGATTTGGTG
>Marker287115 Chr6 SNP_site:4897232 Ref_Type:G
CACGGGCTTGGTTTGGAAAATTTATCGAGGTGATAGAAAGTTTTGGAATGCAAAAGAGCACGTCAGATCATTCAAACTTTXXXXXXXXXXXG
TTGAAATACCTCCTAGGAATTTGAGGTAAAAACAGGCAAGAAAGCAATACTATTATCACAGAAAAAATATGGACTTGTT
CACGGGCTTGGTTTTGAAAATTTATCGAGGTGATAGAAAGTTTTGGAATGCAAAAGAGCACGTCAGATCATTCAAACTTTXXXXXXXXXXXG
TTGAAATACCTCCTAGGAATTTGAGGTAAAAACAGGCAAGAAAGCAATACTATTATCACAGAAAAAATATGGACTTGTT
>Marker287666 Chr6 SNP_site:20979442 Ref_Type:G
ACTATTTTCAGAAGATAGTATGTGTAGTCTCTTTTGGTTAGGTCTTTTATTTTGGCAATTTATACTTTCTGTTGAAATTGXXXXXXXXXXXT
TCCTTGTGGGCACTTGTATGGCATGTCTTGTATCAATAGATGGTTGCAACAACGTAAAAATGCAGGGAAGGTATTTGTC
ACTATTTTCAGAAGATAGTATGTGTAGTCTCTTTTGGTTAGGTCTTTTATTTTGGCAATTTATACTTTCTGTTGAAATTGXXXXXXXXXXXT
TCCTTGTGGGCACTTGTATGGCATTTCTTGTATCAATAGATGGTTGCAACAACGTAAAAATGCAGGGAAGGTATTTGTC
>Marker287892 Chr6 SNP_site:28590833 Ref_Type:T
AACTATGTTATGCTATGTTTCAAGGCTGTGGGGATGGATAAACAATGGATTTGGGTTTTAAGGTAGCAAATTTTCTTGGTXXXXXXXXXXXA
TAAATTCAGAGGAAATTGCTTAGTTATGTTTGTCCTTCCATAGGTTGGCCATCTTGGAGAAGCATATCAAGAGTGGGTT
AACTATGTTATGCTATGTTTCAAGGCTGTGGGGATGGATAAACAATGGATTTGGGTTTTAAGGTAGCAAATTTTCTTGGTXXXXXXXXXXXA
TAAATTCAGAGGAAATTGCTTAGTTATGTTTGTTCTTCCATAGGTTGGCCATCTTGGAGAAGCATATCAAGAGTGGGTT
>Marker288011 Chr6 SNP_site:8159371 Ref_Type:T
ACCCTTCGAGTCATCCAAGAGGGAGTTCGAGGATAGCAAAAAGCCTTTAGGTTTTGGATTTTCTTTAAAGACCTTATAACXXXXXXXXXXXA

Create PDF with GO2PDF for free, if you wish to

CAACATATTTGAGATTGCCTATGATGCTTGCATACTCGAATTGGTTTTTGAACAACTTTACACTAGGGTCATAAGGAGT
ACCCTTCGAGTCATCCAAGAGGGAGTTCGAGGATAGCAAAAAGCCTTTAGGTTTTGGATTTTCTTTAAATACCTTATAACXXXXXXXXXXA
CAACATATTTGAGATTGCCTATGATGCTTGCATACTCGAATTGGTTTTTGAACAACTTTACACTAGGGTCATAAGGAGT
>Marker28855 Chr4 SNP_site:4794819 Ref_Type:G
AACTATTTCATTAAGAAGATGATAAAGTTCGAGCAATGAAAGAAAAACTAAACCTTAGGATCTAATTAAACATGGAAAGAXXXXXXXXXXA
AGAATGGTTTAATTTGGATAAATAAACTCAAACCAGTCATCCAACGACCCTTGACCAAGGATAAGGTAGAGGTTGAGTA
AACTATTTCATTAAGAAGATGATAAAGTTCGAGCAATGAAAGAAAAACTAAACCTTGGGATCTAATTAAACATGGAAAGAXXXXXXXXXXA
AGAATGGTTTAATTTGGATAAATAAACTCAAACCAGTCATCCAACGACCCTTGACCAAGGATAAGGTAGAGGTTGAGTA
>Marker289051 Chr6 SNP_site:7042665 Ref_Type:A
GACTCAACAATCTTTTGCAACTGCCTTCACTAAAACATCCAATTTTATACACATTTTCACCCCACAAAGAGGCAAAAGAGXXXXXXXXXXA
AGAAAAAAAAAAAAGGAAAAAAGACTAATGGTGTCTTGTATTTGAGCTACGTAGGGTGACACCATGTAAAAACTTTGTA
GACTCAACAATCTTTTGCAACTGCCTTCACTAAAACATCCAATTTTATACACATTTTCACCCCACAAAGAGGCAAAAGAGXXXXXXXXXXA
AGAAGAAAAAAAAAAGGAAAAAAGACTAATGGTGTCTTGTATTTGAGCTACGTAGGGTGACACCATGTAAAAACTTTGTA
>Marker28924 Chr4 SNP_site:8705618 Ref_Type:A
ACAAAGGAGGACTTTGAGTCAAAGGCCAGATGCAGTGTTTGTGATTGTGAGCGTGATTATTTTTAGTAAAAAGGATTTCTXXXXXXXXXXA
TGAAATGATCATAACTTGCATTAGAAATGAAGGCAAAATAATTTAAAGGATGCATGTGAATAAGAAAATGGTGCTTAGT
ACAAAGGAGGACTTTGAGTCAAAGGCCAGATGCAGTGTTTGTGATTGTGAGCGTGATTATTTTTAGTAAAAAGGATTTCTXXXXXXXXXXT
TGAAATGATCGTAACTTGCATTAGAAATGAAGGCAAAATAATTTAAAGGATGCATGCGAATAAGAAAATGGTGCTTAGT
>Marker28924 Chr4 SNP_site:8705629 Ref_Type:A
ACAAAGGAGGACTTTGAGTCAAAGGCCAGATGCAGTGTTTGTGATTGTGAGCGTGATTATTTTTAGTAAAAAGGATTTCTXXXXXXXXXXA
TGAAATGATCATAACTTGCATTAGAAATGAAGGCAAAATAATTTAAAGGATGCATGTGAATAAGAAAATGGTGCTTAGT
ACAAAGGAGGACTTTGAGTCAAAGGCCAGATGCAGTGTTTGTGATTGTGAGCGTGATTATTTTTAGTAAAAAGGATTTCTXXXXXXXXXXT
TGAAATGATCGTAACTTGCATTAGAAATGAAGGCAAAATAATTTAAAGGATGCATGCGAATAAGAAAATGGTGCTTAGT
>Marker28924 Chr4 SNP_site:8705675 Ref_Type:T
ACAAAGGAGGACTTTGAGTCAAAGGCCAGATGCAGTGTTTGTGATTGTGAGCGTGATTATTTTTAGTAAAAAGGATTTCTXXXXXXXXXXA
TGAAATGATCATAACTTGCATTAGAAATGAAGGCAAAATAATTTAAAGGATGCATGTGAATAAGAAAATGGTGCTTAGT
ACAAAGGAGGACTTTGAGTCAAAGGCCAGATGCAGTGTTTGTGATTGTGAGCGTGATTATTTTTAGTAAAAAGGATTTCTXXXXXXXXXXT
TGAAATGATCGTAACTTGCATTAGAAATGAAGGCAAAATAATTTAAAGGATGCATGCGAATAAGAAAATGGTGCTTAGT
>Marker289818 Chr6 SNP_site:16623403 Ref_Type:T
ACATTAGCGTAAATGGGTTATGTTTCTCTATTTTATCAATTAGGAGAATTACAAATTCACCTTTAGGAAGAGTGAAGCCAXXXXXXXXXXA
ACTGCTTGAAAAACCTGCATATCAAGAGCCAATATATTACTACATAATACCAACGGAGAGAGGGAGGAAAAAGAAGGGT
ACATTAGCGTAAATGGGTTATGTTTCTCTATTTTATCAATTAGGAGAATTACAAATTCACCTTTAGGAAGAGTGAAGCCAXXXXXXXXXXA
ACTGCTTGAAAAACCTGCATATCAAGAGCCAATATATTACTACATAATTCCAACGGAGAGAGGGAGGAAAAAGAAGGGT
>Marker289826 Chr6 SNP_site:13266140 Ref_Type:G
ACAAGTAAAAACGAAGACATATTGAGTTGGTTCCTGTTAAAGAGTTGGTTCCTATTAAAGAGGCAGTTTCAGTATGTGAGXXXXXXXXXXG
GGGGATGATCTCTATACGATAAAAAGGGAAAAGAGCGGAAGTGATTATGACAATCTCCTTTGGCAGCCTCATGGAGGTG
ACAAGTAAAAACGAAGACATATTGAGTTGGTTCCTGTTAAAGAGTTGGTTCCTATTAAAGAGGCAGTTTCGGTATGTGAGXXXXXXXXXXG
GGGGATGATCTCTATACGATAAAAAGGGAAAAGAGCGGAAGTGATTATGACAATCTCCTTTGGCAGCCTCATGGAGGTG
>Marker290224 Chr6 SNP_site:26302765 Ref_Type:C
ACAAATGAGTGTCTATCAGCCACACTGCTTTTCTTTGATTTCTTGGCTGGAGGCTCCTCATCATTTCTTCCTGTGGCCACXXXXXXXXXXG
AAGAAAAAAGAAACAGATAAAGTAGATTGGATACTTTGAGAAGTTTTGTTGGGCTTTTTCTTCAACAAGAAATCTCGTT
ACAAATGAGTGTGTATCAGCCACACTGCTTTTCTTTGATTTCTTGGCTGGAGGCTCCTCATCATTTCTTCCTGTGGCCACXXXXXXXXXXG
AAGAAAAAAGAAACAGATAAAGTAGATTGGATACTTTGAGAAGTTTTGTTGGGCTTTTTCTTCAACAAGAAATCTCGTT
>Marker29108 Chr4 SNP_site:77998 Ref_Type:A

Create PDF with GO2PDF for free, if you wish to

ACATGAGGGACATACAAAGAGCAACCGGGGAGGGGGAGGATCAGCAATTGAATTGAAACATTTAATTGATTAATGAAATAXXXXXXXXXXT
TTGACTTTTAGGTATATAATTTCCCCGTATATGTTAGGTTCTATTTCTTTCTCTGACAAACTGTCTTGTCCTTTTCGTT
ACATGAGGGGCATACAAAGAGCAACCGGGGAGGGGGAGGATCAGCAATTGAATTGAAACATTTAATTGATTAATGAAATAXXXXXXXXXXT
TTGACTTTTAGGTATATAATTTCCCCGTATATGTTAGGTTCTATTTCTTTCTCTGACAAACTGTCTTGTCCTTTTCGTT
>Marker29116 Chr4 SNP_site:9760177 Ref_Type:T
AACCCTATTGCACTCCTAGTCCTATTTGTAAAAGAACATTACATACCACAATGAGTCGTAACAATCAAATCATGGTGGGCXXXXXXXXXXT
ATTCTACCTCAAGCCAATTTAAAACCAACTTTTAGTTAATCTGATCATGCCGTCTGCCTGATCACTAGTCAGGGTAAGT
AACCCTATTGCACTCCTAGTCCTATTTGTAAAAGAACATTACATACCACAATGAGTCGTAACAATCAAATCATGGTGGGTXXXXXXXXXXT
ATTCTACCTCAAGCCAATTTAAAACCAACTTTTAGTTAATCTGATCATGCCGTCTGCCTGATCACTAGTCAGGGTAAGT
>Marker291339 Chr6 SNP_site:6153537 Ref_Type:C
AACAATAGGCTTCTCCATCAGAATTTCCATGCCATTTGACACCTTTTTTGTAGTCCCATCCCATTGCGCTGAACTTATTGXXXXXXXXXXA
TTATATATATTATACTGCAAAAGTAATATGAACAGTTACTACTAGTAGTAGCGTTGTGTATAAACTATTTAACTATAGT
AACAATAGGCTTCTCCATCAGAATTTCCCTGCCATTTGACACCTTTTTTGTAGTCCCATCCCATTGCGCTGAACTTATTGXXXXXXXXXXA
TTATATATATTATACTGCAAAAGTAATATGAACAGATACTACTAGTAGTAGCGTTGTGTATAAACTATTTAACTATAGT
>Marker291339 Chr6 SNP_site:6153726 Ref_Type:A
AACAATAGGCTTCTCCATCAGAATTTCCATGCCATTTGACACCTTTTTTGTAGTCCCATCCCATTGCGCTGAACTTATTGXXXXXXXXXXA
TTATATATATTATACTGCAAAAGTAATATGAACAGTTACTACTAGTAGTAGCGTTGTGTATAAACTATTTAACTATAGT
AACAATAGGCTTCTCCATCAGAATTTCCCTGCCATTTGACACCTTTTTTGTAGTCCCATCCCATTGCGCTGAACTTATTGXXXXXXXXXXA
TTATATATATTATACTGCAAAAGTAATATGAACAGATACTACTAGTAGTAGCGTTGTGTATAAACTATTTAACTATAGT
>Marker291532 Chr6 SNP_site:22530341 Ref_Type:T
CACCTGATGATAGCCATTTTGAGGAGCCTTTTGCTGGGGTGAGTCGCCATCAGCTGTCATTGACAGTAGAAACCATTCTTXXXXXXXXXXT
GCTCCCCAGGCTGATTCAACCTTACCCATTCATCAACAATCTCCTTCCATTTTCTGACGAAAAACAAATTGATATCGTT
CACCTGATGATAGCCATTTTGAGGAGCTTTTTGCTGGGGTGAGTCGCCATCAGCTGTCATTGACAGTAGAAACCATTCTTXXXXXXXXXXT
GCTCCCCAGGCTGATTCAACCTGACCCATTCATCAACAATCTCCTTCCATTTTCTGTCGAAAAACAAATTGATATCGTT
>Marker291532 Chr6 SNP_site:22530516 Ref_Type:G
CACCTGATGATAGCCATTTTGAGGAGCCTTTTGCTGGGGTGAGTCGCCATCAGCTGTCATTGACAGTAGAAACCATTCTTXXXXXXXXXXT
GCTCCCCAGGCTGATTCAACCTTACCCATTCATCAACAATCTCCTTCCATTTTCTGACGAAAAACAAATTGATATCGTT
CACCTGATGATAGCCATTTTGAGGAGCTTTTTGCTGGGGTGAGTCGCCATCAGCTGTCATTGACAGTAGAAACCATTCTTXXXXXXXXXXT
GCTCCCCAGGCTGATTCAACCTGACCCATTCATCAACAATCTCCTTCCATTTTCTGTCGAAAAACAAATTGATATCGTT
>Marker291532 Chr6 SNP_site:22530550 Ref_Type:T
CACCTGATGATAGCCATTTTGAGGAGCCTTTTGCTGGGGTGAGTCGCCATCAGCTGTCATTGACAGTAGAAACCATTCTTXXXXXXXXXXT
GCTCCCCAGGCTGATTCAACCTTACCCATTCATCAACAATCTCCTTCCATTTTCTGACGAAAAACAAATTGATATCGTT
CACCTGATGATAGCCATTTTGAGGAGCTTTTTGCTGGGGTGAGTCGCCATCAGCTGTCATTGACAGTAGAAACCATTCTTXXXXXXXXXXT
GCTCCCCAGGCTGATTCAACCTGACCCATTCATCAACAATCTCCTTCCATTTTCTGTCGAAAAACAAATTGATATCGTT
>Marker291778 Chr6 SNP_site:13270024 Ref_Type:A
AACCTCGCGAATAGCCATGTCAAACTCCTCCATATCCCCAGGATTTGGAGCTCCACCAAAAGCTTTAGAATGAAGTGTAAXXXXXXXXXXA
AAGAACCTCTAACCTATCCCCCGAGTGCAAATCTTTTACCAAACCAGAGATAAATTGATATGACACCTCACTAGGCGTA
AACCTCGCGAATAGCCATGTCAAACTCCTCCATATCCCCAGGATTTGGAGCTCCACCAAAAGCTTTAGAATGAAGTGTAAXXXXXXXXXXA
AAGAACCTCTAACCTATCCCCCGAGTGCAAATCTTTTACCAAACCAGAGATAATTTGATATGACACCTCACTAGGCGTA
>Marker292153 Chr6 SNP_site:13156011 Ref_Type:G
TACATGTCTTAAGAAAAAGTCAAATCGCCATTGATGGTTAGATTATGTATCGAGTTCATATTGGTGGCATAGCGCGACGTXXXXXXXXXXA
ATGCTGAACAAACGGTTCCTTCAACTGCTAGATAAAAAGAGAAAGTCGAAAGTCGAGAAGGAAGGAATTGAGGCTAAGT
TACATGTCTTAAGGAAAAGTCAAATCGCCATTGATGGTTAGATTATGTATCGAGTTCATATTGGTGGCATAGCGCGACGTXXXXXXXXXXA
ATGCTGAACAAACGGTTCCTTCAACTGCTAGATAAAAAGAGAAAGTCGAAAGTCGAGAAGGAAGGAATTGAGGCTAAGT

Create PDF with GO2PDF for free, if you wish to

>Marker292404 Chr6 SNP_site:2712887 Ref_Type:C

GACCAAAAAAGCTGATGGCCTAACCATATCAAGATAGGCATATATGAATTTGATTGGCATTTTCCACTACCATACCCAACXXXXXXXXXXXT

CGAAATTAAATTTGAACGAGAGAAATCATGAGTTTATGAAAGTATTTTTAAGACAAACTTTAAATAATTGAAAGTTAGT

GACCAAAAAAGCTGATGGCCTAACCATATCAAGATAGGCATATATGAATTTGATTGGCATTTTCCACTACCATACCCAACXXXXXXXXXXXT

CGAAATTACATTTGAACGAAAGAAATCATGAGTTTATGAAAGTATTTTTAAGACAAACTTTAAATAATTGAAAGTTAGT

>Marker292404 Chr6 SNP_site:2712898 Ref_Type:A

GACCAAAAAAGCTGATGGCCTAACCATATCAAGATAGGCATATATGAATTTGATTGGCATTTTCCACTACCATACCCAACXXXXXXXXXXXT

CGAAATTAAATTTGAACGAGAGAAATCATGAGTTTATGAAAGTATTTTTAAGACAAACTTTAAATAATTGAAAGTTAGT

GACCAAAAAAGCTGATGGCCTAACCATATCAAGATAGGCATATATGAATTTGATTGGCATTTTCCACTACCATACCCAACXXXXXXXXXXXT

CGAAATTACATTTGAACGAAAGAAATCATGAGTTTATGAAAGTATTTTTAAGACAAACTTTAAATAATTGAAAGTTAGT

>Marker293319 Chr6 SNP_site:26840071 Ref_Type:C

ACTCATAGACTAGAAGTTTTTCTCCTCCTTCAACACAACATCCCAAGAGCCTGACCAGATTCTTATGTTGAAGCTTCCAAXXXXXXXXXXXT

CATATGGTTGTTCTAACACACCTTACAAATAATAGTAATAAAAACTTATATCTCAAACTTCTGATTCACTAACCTTGTA

ACTCATAGACTAGAAGTTTTTCTTCTCCTTCAACACAACATCCCAAGAGCCTGACCAGATTCTTATGTTGAAGCTTCCAAXXXXXXXXXXXT

CATATGGTTGTTCTAACACACCTTACAAATAATAGTAATAAAAACTTATATCTCAAACTTCTGATTCACTAACCTTGTA

>Marker293539 Chr6 SNP_site:15127865 Ref_Type:A

TACTTGATTGAAACCATTTTTTTATTGGGCTTCCTTTTGTTAATATTTTATTTCTATATGTCCCATGTAATCTTTCATTTXXXXXXXXXXXA

TTTGATACAAAAAGGTGTTGGTATGAAATTGACTATTGAGATAGCCATGTATTGATTATGCTGCCAAATACCCCATGTT

TACTTGATTGAAACCATTTTTTTATTGGGCTTCCTTTTGTTAATATTTTATTTCTATATGTCCCATGTAATCTTTCATTTXXXXXXXXXXXA

TTTGATACAAAAAGGTGTTGGTATGAAATTGACTATTGAGATAGCCATGTATTGATTATGCTGCCAAATTCCCCATGTT

>Marker293969 Chr6 SNP_site:7737537 Ref_Type:T

AACTAGGTTGGGTTGCCTCTTTGAGTAACTTCAAATTATCGACTTTAATCTCATTCTATTTTATGAACTTTCAGCCAACTXXXXXXXXXXXT

CATCGTATATGTCGAGACTAATTACCATTATACATAATATAACGGGCCCTTCTAATAGTTATCTGGCTATCACTATGTA

AACTAGGTTGGGTTGCCTCTTTGAGTAACTTCAAATTATCGACTTTAATCTCATTCTATTTTATGAACTTTCAGCCAACTXXXXXXXXXXXT

CATCGTATATGTCGAGACTAATTACCATTATACATAATATAACGGGTCCTTCTAATAGTTATCTGGCTATCACTATGTA

>Marker294808 Chr6 SNP_site:27716638 Ref_Type:G

ACTAATTTGTGTATTTGGTCTCTAGTCCTCAGGAAGATATCACACTTTGATGTCAGACATTAAGCATGGGCAGATCTTGTXXXXXXXXXXXG

AATTTGATCGTTGGAAGTTGATGTCTATAAATACTAACTTGGAAATGCATCAAGCAAACCCAACCACACCGAAAATGGT

ACTAATTTTTGTATTTGGTCTCTAGTCCTCAGGAAGATATCACACTTTGATGTCAGACATTAAGCATGGGCAGATCTTGTXXXXXXXXXXXG

AATTTGATCGTTGGAAGTTGATGTCTATAAATACTAACTTGGAAATGCATCAAGCAAACCCAACCACACCGAAAATGGT

>Marker294940 Chr6 SNP_site:4465788 Ref_Type:C

CACCTTTGAAGAAATGTAAAAAGCTCAAACATTGAGCTGATAGATCAAACAATGGTGGCCTATACAGCAAAGTTAAAAAGXXXXXXXXXXXT

GCCTAAAAATATAATCATAATTATTAATTTCAGAGGTCCATAGTTTATTTTGATGATCTTTTGTGAAATAGGTCACGTT

CACCTTTGAAGAAATGTAAAAAGCTCAAACATTGAGCTGATAGATCAAACAATGGTGGCCTATACAGCAAAGTTAAAAAGXXXXXXXXXXXT

GCCTAAAAATATAATCATCATTATTAATTTCAGAGGTCCATAGTTTATTTTGATGATCTTTTGTGAAATAGGTCACGTT

>Marker295115 Chr6 SNP_site:2171561 Ref_Type:A

ACTTGGATCATCATGCTTCATACGAACGTTACCTCGAATAATGACCCCTTGTTTGATCTCATCAGAAGGAACCTTAAACCXXXXXXXXXXXT

TAAATCTTACTCCTCATCTCCTAGATGAGTTGATAAAGTAGTCTCTTGAATATCATACTCCTCAACATGCTAAGAGGTT

ACTTGGATCATCATGCTTCATACGAACGTTACCTCGAATAATGACCCCTTGTTTGATCTCATCAGAAGGAACCTTAAACCXXXXXXXXXXXT

TAAATCTTACTCCTCATCTCCTAGGTGAGTTGATAAAGTAGTCTCTTGAATATCATACTCCTCAACATGCTAAGAGGTT

>Marker29547 Chr4 SNP_site:209512 Ref_Type:T

TACCATCTGTCCCAAATTACAAGTAGCCATTACTTTACAAACCTACAACATTTGCAGAGACCACTGCTTAAACCACTTGCXXXXXXXXXXXC

AAGCTTAGATTTTTCAACAAATATGTTTTAGTCATGGACTTCAAGTTCTATTTAATCTTGTTTCTAAATTTTAAATGTG

TACCATCTGTCCCAAATTACAAGTAGCCATTACTTTATAAACCTACAACATTTGCAGAGACCACTGCTTAAACCACTTGCXXXXXXXXXXXC

Create PDF with GO2PDF for free, if you wish to

AAGCTTAGATTTTTCAACAAATATGTTTTAGTCATGGACTTCAAGTTCTATTTAATCTTGTTTCTAAATTTTAAATGTG

>Marker295485 Chr6 SNP_site:10299684 Ref_Type:T

ACTGAATATGAAATTGTTCGAATACTGAACAAATTAGTGTCACAAAGGAAACGTTGAGAGCATTCATAGGTCTATTATAAXXXXXXXXXXT

TAGCGAATGTATTCCTAAATAATATGTTTGAATATTCTACTATTTTCTTTAATATCTAGTTACTCAGAAACACAATGTT

ACTGAATATGAAATTGTTCGAATACTGAACAAATTTGTGTCACAAAGGAAACGTTGAGAGCATTCATAGGTCTATTATAAXXXXXXXXXXT

TAGCGAATGTATTCCTAAATAATATGTTTGAATATTCTACTATTTTCTTTAATATCTAGTTACTCAGAAACACAATGTT

>Marker295589 Chr6 SNP_site:27901990 Ref_Type:A

CACATTTAATCTTCACAATGACAAGTCTCACATAACACTCATTCAAAACAAACCCAATAGATGAACAACTGATATATTCAXXXXXXXXXXT

TATAGATATAATCGAACATTTCTCACATTATTTCGAAGGATATGAAGTAGAAGCAAAAGAAATTAACATTGGGCCAGTG

CACATTTAATCTTCACAATGACAAGTCTCACATGACACTCATTCAAAACAAACCCAATAGATGAACAACTGATATATTCAXXXXXXXXXXT

TATAGATATAATCGAACATTTCTCACATTATTTCGAAGGATATGAAGTAGAAGCAAAAGAAATTAACATTGGGCCAGTG

>Marker295987 Chr6 SNP_site:15835160 Ref_Type:C

GACAAATGACAGCTCCTCCGACTCTGGCTCTCATAGATAACTCCCCTCCGATTTCAAAGGCGTAAAGAAGGAAGAAGCACXXXXXXXXXXA

ACAGCTGGACCTTAGCATCTCTTATAAACCCTTCCATGCAGAAAAGGTTGTGGTATCGATCGAAGATAAAAATTTGGTG

GACAAATGACAGCTCCTCCGACTCTGGCTCTCCTAGATCACTCCCCTCCGATTTCAAAGGCGTAAAGAAGGAAGAAGCACXXXXXXXXXXA

ACAGCTGGACCTTAGCATCTCTTATAAACCCTTCCATGCAGAAAAGGTTGTGGTATCGATCGAAGATAAAAATTTGGTG

>Marker295987 Chr6 SNP_site:15835166 Ref_Type:C

GACAAATGACAGCTCCTCCGACTCTGGCTCTCATAGATAACTCCCCTCCGATTTCAAAGGCGTAAAGAAGGAAGAAGCACXXXXXXXXXXA

ACAGCTGGACCTTAGCATCTCTTATAAACCCTTCCATGCAGAAAAGGTTGTGGTATCGATCGAAGATAAAAATTTGGTG

GACAAATGACAGCTCCTCCGACTCTGGCTCTCCTAGATCACTCCCCTCCGATTTCAAAGGCGTAAAGAAGGAAGAAGCACXXXXXXXXXXA

ACAGCTGGACCTTAGCATCTCTTATAAACCCTTCCATGCAGAAAAGGTTGTGGTATCGATCGAAGATAAAAATTTGGTG

>Marker296234 Chr6 SNP_site:13383430 Ref_Type:G

GACATGTTCTCAGAATTCAGGTTCGGTGTGTATGGCCGCAGAAGAAGCTGAGATTCATTTTCTGCAAACTGAAGCAGACGXXXXXXXXXXG

AAGTTAGTGCTCAAAAGCCCATCCACATTAAAAGCCAACCTAGGATAATTATTAGAGATATTTCTCAAGCCCTTGTGTA

GACATGTTCTCAGAATTCAGGTTCGGTGTGTATGGCCGCAGAAGAAGCTGAGGTTCATTTTCTGCAAACTGAAGCAGACGXXXXXXXXXXG

AAGTTAGTGCTCAAAAGCCCATCCACATTAAAAGCCAACCTAGGATAATTATTAGAGATATTTCTCAAGCCCTTGTGTA

>Marker296240 Chr6 SNP_site:28553980 Ref_Type:C

ACCTTCAATTTTTTAGTAGATAGATTAAATTTTTTTGGTATCGAAATATAAAATTAGAGAAGCATTTACTAGACATGATTXXXXXXXXXXA

TAGATAAGAGTATTTCGGTTCTTTATTTTTCCTCATTTCTGTTTTATAGAAAAAAATTGCTTTTTTGAATGCATGAGTC

ACCTTCAATTTTTTAGTAGATAGATTAAATTTTTTTGGTATCGAAATATAAAATTAGAGAAGGATTTACTAGACATGATTXXXXXXXXXXA

TAGATAAGAGTATTTCGGTTCTTTATTTTTCCTCATTTCTGTTTTATAGAAAAAAATTGATTTTTTGAATGCATGAGTC

>Marker296240 Chr6 SNP_site:28554186 Ref_Type:C

ACCTTCAATTTTTTAGTAGATAGATTAAATTTTTTTGGTATCGAAATATAAAATTAGAGAAGCATTTACTAGACATGATTXXXXXXXXXXA

TAGATAAGAGTATTTCGGTTCTTTATTTTTCCTCATTTCTGTTTTATAGAAAAAAATTGCTTTTTTGAATGCATGAGTC

ACCTTCAATTTTTTAGTAGATAGATTAAATTTTTTTGGTATCGAAATATAAAATTAGAGAAGGATTTACTAGACATGATTXXXXXXXXXXA

TAGATAAGAGTATTTCGGTTCTTTATTTTTCCTCATTTCTGTTTTATAGAAAAAAATTGATTTTTTGAATGCATGAGTC

>Marker296284 Chr6 SNP_site:24231374 Ref_Type:C

CACATTTGAGGGATACATTTGTGATATGGAATTGAAAAAAATGATAAAATAGGTAGGGATAAGTAGTATATATATGGAACXXXXXXXXXXC

TGTAATTAAAGGTTATAAAAACAATACCACAAACATTATTCATGATCAATTAATATATATGAATATATTATATTGAGTC

CACATTTGAGGGATACATTTGTGATATGGAATTGAAAAAAATGATAAAATAGGTAGGGATAAGTAGTATATATATGGAACXXXXXXXXXXC

TGTAATTAAAGGTTATAAAAATAATACCACAAACATTATTCATGATCAATTAATATATATGAATATATTATATTGAGTC

>Marker297311 Chr6 SNP_site:12839181 Ref_Type:T

ACTACAAATTTCTTTCAAATACTTTAAGCATAAACTAATTAATTTCCAATGCCACAACCTAGTAAATTAATGTGAGAAATXXXXXXXXXXT

AATCACAAAGGAAAAAAGAGAAATTATTACCTTTTACAAAGGATTTGGAGACAAATGGAGGTTTGACGGTTAATGTGTT

Create PDF with GO2PDF for free, if you wish to

ACTACAAATTTCTTTCAAATACTTTAAGCATAAACTAATTAATTTCCAATGCCACAACCTAGTAAATTAATGTGAGAAATXXXXXXXXXXT
AATCACAAAGGAAAAAAGAGAAATTATTACCTTTTACAAAGGATTTGGAGACAAATGGAGGTTTGTCGGTTAATGTGTT
>Marker297473 Chr6 SNP_site:16878433 Ref_Type:T
CACAATTTCACTACTTTTGCGTTGAACAACTTCTTATATAGAATCACATTTCTTCTACGAAACAATGCCTTTTATCATTCXXXXXXXXXXT
ATGAGAACACTCGAATGATTGGCATGAAAATACAACATTGTTACACGATGGGGTATTGTTGGTGTGATAAAACATTGTT
CACAATTTCACTACTTTTGCGTTGAACAACTTCTTATATAGAATCACATTTCTTCTATGAAACAATGCCTTTTATCATTCXXXXXXXXXXT
ATGAGAACACTCGAATGATTGGCATGAAAATACGACATTGTTACACGATGGGGTGTTGTTGGTGTGATAAAACATTGTT
>Marker297473 Chr6 SNP_site:16878570 Ref_Type:G
CACAATTTCACTACTTTTGCGTTGAACAACTTCTTATATAGAATCACATTTCTTCTACGAAACAATGCCTTTTATCATTCXXXXXXXXXXT
ATGAGAACACTCGAATGATTGGCATGAAAATACAACATTGTTACACGATGGGGTATTGTTGGTGTGATAAAACATTGTT
CACAATTTCACTACTTTTGCGTTGAACAACTTCTTATATAGAATCACATTTCTTCTATGAAACAATGCCTTTTATCATTCXXXXXXXXXXT
ATGAGAACACTCGAATGATTGGCATGAAAATACGACATTGTTACACGATGGGGTGTTGTTGGTGTGATAAAACATTGTT
>Marker297473 Chr6 SNP_site:16878591 Ref_Type:G
CACAATTTCACTACTTTTGCGTTGAACAACTTCTTATATAGAATCACATTTCTTCTACGAAACAATGCCTTTTATCATTCXXXXXXXXXXT
ATGAGAACACTCGAATGATTGGCATGAAAATACAACATTGTTACACGATGGGGTATTGTTGGTGTGATAAAACATTGTT
CACAATTTCACTACTTTTGCGTTGAACAACTTCTTATATAGAATCACATTTCTTCTATGAAACAATGCCTTTTATCATTCXXXXXXXXXXT
ATGAGAACACTCGAATGATTGGCATGAAAATACGACATTGTTACACGATGGGGTGTTGTTGGTGTGATAAAACATTGTT
>Marker297511 Chr6 SNP_site:28687496 Ref_Type:T
ACCCTCTCACGCTACACAAGTTCAACTGAAATCACATCCAAAATAGTCTAAGTTGGTTTCCAAAGTCGAAATAAAGAATAXXXXXXXXXXG
TAACATAGTGACAGATCGCACATTTGACAGATGGAGCTCCATAAGGAAAGACAAGTGTTGTTCGGCAGTTTCCACAGTT
ACCCTCTTACGCTACACAAGTTCAACTGAAATCACATCCAAAATAGGCTAAGTTGGTTTCCAAAGTCGAAATAAAGAATAXXXXXXXXXXG
TAACATAGTGACAGATCGCACATTTGACAGATGGAGCTCCATAAGGAAAGACAAGTGTTGTTCGGCAGTTTCCACAGTT
>Marker297511 Chr6 SNP_site:28687535 Ref_Type:G
ACCCTCTCACGCTACACAAGTTCAACTGAAATCACATCCAAAATAGTCTAAGTTGGTTTCCAAAGTCGAAATAAAGAATAXXXXXXXXXXG
TAACATAGTGACAGATCGCACATTTGACAGATGGAGCTCCATAAGGAAAGACAAGTGTTGTTCGGCAGTTTCCACAGTT
ACCCTCTTACGCTACACAAGTTCAACTGAAATCACATCCAAAATAGGCTAAGTTGGTTTCCAAAGTCGAAATAAAGAATAXXXXXXXXXXG
TAACATAGTGACAGATCGCACATTTGACAGATGGAGCTCCATAAGGAAAGACAAGTGTTGTTCGGCAGTTTCCACAGTT
>Marker297846 Chr6 SNP_site:8002672 Ref_Type:G
ACATTCATAAATCAACATATAATGACTTGTTACTAGTTACATGTAGCCCTATCACATTATGCCATCTGTGTCATCGGTTTXXXXXXXXXXA
GATGAGATGAAAGGTGTATGTGATGAGAGAGCTTATTTATTTGAGTGAATGGAAAAAGGAGTAAGAGTGAGAGGCAGTG
ACATTCATAAATCAACATATAATGACTTGTTACTAGTTACATGTAGCCCTATCACATTATGCCATCTGTGTCATCTGTTTXXXXXXXXXXA
GATGAGATGAAAGGTGTATGTGATGAGAGAGGTTATTTATTTGAGTGAATGGAAAAAGGAGTAAGAGTGAGAGGCAGTG
>Marker297846 Chr6 SNP_site:8002829 Ref_Type:C
ACATTCATAAATCAACATATAATGACTTGTTACTAGTTACATGTAGCCCTATCACATTATGCCATCTGTGTCATCGGTTTXXXXXXXXXXA
GATGAGATGAAAGGTGTATGTGATGAGAGAGCTTATTTATTTGAGTGAATGGAAAAAGGAGTAAGAGTGAGAGGCAGTG
ACATTCATAAATCAACATATAATGACTTGTTACTAGTTACATGTAGCCCTATCACATTATGCCATCTGTGTCATCTGTTTXXXXXXXXXXA
GATGAGATGAAAGGTGTATGTGATGAGAGAGGTTATTTATTTGAGTGAATGGAAAAAGGAGTAAGAGTGAGAGGCAGTG
>Marker297909 Chr6 SNP_site:28597224 Ref_Type:G
ACATCCATCCCATCTATGAGCCATTACAGACAATATGAAGCCAACTAATTAGCCCACCAAATCACAGATTCAATCCAACCXXXXXXXXXXT
AAACAGAAGCTCTGAATGTGTTCTTTGGTTTTGAATAAATGGGTTGCAACAAAAGTCTCATAAAAAGATCGCTGTCGGT
ACATCCATCCCATCTATGAGCCATTACAGACAATATGAAGCCAACTAATTAGCCCACCAAATCACAGATTCAATCCAACCXXXXXXXXXXT
AAACAGAAGCTCTGAGTGTGTTCTTTGGTTTTGAATAAATGGGTTGCAACAAAAGTCTCATAAAAAGATCGCTGTCGGT
>Marker298441 Chr6 SNP_site:22354297 Ref_Type:A
CACTCTCTTATGAAACTATGAAAGAGGAAAAGGGAATTGACACTCCATTAATTCCAACTTTTCTTCTTTCATGTCAAAATXXXXXXXXXXT

Create PDF with GO2PDF for free, if you wish to

ATTGAACATTTAATTATGAAAAACATGCCTATTAAAGAAACATTTTTTTTAAAAAAAAATCAACTAAAACAAATGAGTT
CACTCTCTTATGAAACTATGAAAGAGGAAAAGGGAATTGACACTCCATTAATTCCAACTTTTCTTCTTTCATGTCAAAATXXXXXXXXXXT
ATTGAACATTTAATTATGAAAAACATGCCTATTAAAGAAACATTTTTTTTAAAAAAAAATCAACTAAAACAAATGAGTT
>Marker298632 Chr6 SNP_site:10519628 Ref_Type:T
ACTGTTTTTCTTCTACTAATGGAGAAACAAACTTTAGGATCCCAGATTTTTTTTTAAAAAAATGCAATAAATTTCATTTTXXXXXXXXXXT
TGTATCTGCTATCGTTATCTGTTTTTGGATTCTTATTAAAAGAATGTTTGATGCTTACTGCTTTAGGTAATGCATTTGT
ACTGTTTTTCTTCTACTAATGGAGAAACAAACTTTAGGATCCCAGATTTTTTTTTATAAAAATGCAATAAATTTCATTTTXXXXXXXXXXT
TGTATCTGCTATCGTTATCTGTTTTTGGATTCTTATTAAAAGAATGTTTGATGCTTACTGCTTTAGGTAAGGCATTTGT
>Marker298632 Chr6 SNP_site:10519883 Ref_Type:G
ACTGTTTTTCTTCTACTAATGGAGAAACAAACTTTAGGATCCCAGATTTTTTTTTAAAAAAATGCAATAAATTTCATTTTXXXXXXXXXXT
TGTATCTGCTATCGTTATCTGTTTTTGGATTCTTATTAAAAGAATGTTTGATGCTTACTGCTTTAGGTAATGCATTTGT
ACTGTTTTTCTTCTACTAATGGAGAAACAAACTTTAGGATCCCAGATTTTTTTTTATAAAAATGCAATAAATTTCATTTTXXXXXXXXXXT
TGTATCTGCTATCGTTATCTGTTTTTGGATTCTTATTAAAAGAATGTTTGATGCTTACTGCTTTAGGTAAGGCATTTGT
>Marker298968 Chr6 SNP_site:18815024 Ref_Type:A
ACTCATCATCAAGAAGTTTGGAAAAACCACCTCCCAAACCACCTCTCATCCCGACAAAACCATCGACTCCTTCGTCAATAXXXXXXXXXXC
ACCTGCTGTTCCTGAAAAATCCAATTCCTCTGCTAAATTCCGCCGGAGTTTGGATTCAAATTCACGGACAAAATCCGTA
ACTCATCATCAAGAAGTTTGGAAAAACCACCTCCCAAACCACCTCTCATCCCGACAAAACCATCGACTCCTTCGTCAATAXXXXXXXXXXC
ACCTGCTGTTCCTGAAAAATCCAATTCCTCTGCTAAATTCCGCCGGAGTTTGGATTCCAATTCACGGACAAAATCCGTA
>Marker299092 Chr6 SNP_site:26358915 Ref_Type:C
ACAAGCTGGGCCACTGATTAGAGTCAAATTCAATAATTAATTGAAACCACATAAGCTTTAAACTTTGAAGTCTTCCTTTTXXXXXXXXXXC
CCCCTCACTTTCAATTTGGGGTATTTTTAATTAAATAAAAACAGCGTTGTTTTTTTGGAGGTTAGTTGAAATTGTAGGT
ACAAGCTGGGCCACTGATTAGAGTCAAATTCAATAATTAATTGAAACCACATAAGCTTTAAACTTTGAAGTCTTCCTTTTXXXXXXXXXXC
TCCCTCACTTTCAATTTGGGGTATTTTTAATTAAATAAAAACAGCGTTGTTTTTTTGGAGGTTAGTTGAAATTGTAGGT
>Marker299383 Chr6 SNP_site:18437247 Ref_Type:G
AACTTTTTGGGCGTGGTCGAACATCCAAATCTTGTCAAGTTACTAGGCTACTGTGCCGAGGACGATGAAAGGGGGATCCAXXXXXXXXXXA
TGAAGGAATGGATTTCCAGGTAACTTGTCATTTTGCAAGATTTTTATAGCATGAGATATGTTTTGAGCCAAAACTAGTG
AACTTTTTGGGCGTGGTCGAACATCCAAATCTTGTCAAGTTACTAGGCTACTGTGCCGAGGACGATGAAAGGGGGATCCAXXXXXXXXXXA
TGAAGGAATGGATTTCCAGGTAACTTGTCATTTTGCAAGATTTTTATAGCATGAGATATTTTTTGAGCCAAAACTAGTG
>Marker304726 Chr1 SNP_site:25853811 Ref_Type:G
AACCAAAAAATCGTTAAGATTTGGTTACTCAAATTTAAACGATTAAATATAAACAACAAACAACGTGTAAGAAAGAATAGXXXXXXXXXXT
ATTTTTTAAAAGACCCCTATTATTGTTATTATTGTATGACATTTAATTTACCCTACATAACCTAGTGGAAAAAAAAGTG
AACCAAAAAATCGTTGATATTTGGTTACTCAAATTTAAACGATTAAATATAAACAACAAACAACGTGTAAGAAAGAATAGXXXXXXXXXXT
ATTTTTTAAAAGACCCCTATTATTGTTATTATTGTATGACATTTAATTTACCCTACATAACCTAGTGGAAAAAAAAGTG
>Marker304726 Chr1 SNP_site:25853813 Ref_Type:T
AACCAAAAAATCGTTAAGATTTGGTTACTCAAATTTAAACGATTAAATATAAACAACAAACAACGTGTAAGAAAGAATAGXXXXXXXXXXT
ATTTTTTAAAAGACCCCTATTATTGTTATTATTGTATGACATTTAATTTACCCTACATAACCTAGTGGAAAAAAAAGTG
AACCAAAAAATCGTTGATATTTGGTTACTCAAATTTAAACGATTAAATATAAACAACAAACAACGTGTAAGAAAGAATAGXXXXXXXXXXT
ATTTTTTAAAAGACCCCTATTATTGTTATTATTGTATGACATTTAATTTACCCTACATAACCTAGTGGAAAAAAAAGTG
>Marker304912 Chr1 SNP_site:27688299 Ref_Type:C
ACTATTCTTTAAGTGATTATATCTTCTTTACAACTTTTTTGGAACAGTAACTATATGATCCATATGGATATATGTCAGTTXXXXXXXXXXG
TGATTAACTTTGGTATTTCTCATTTCACAGACCTTGTAAATGTTTTTCGAGAGTTTCTTCCAAAAGTCGTGCAAGAGTG
ACTATTCTTTAAGTGATTATATCTTCTTTACAATTTTTTTGGAACAGTAACTATATGATCCATATGGATATTTGTCAGTTXXXXXXXXXXG
TGATTAACTTTGGTATTTCTCATTTCACAGACCTTGTAAATGTTTTTCGAGAGTTTCTTCCAAAAGTCGTGCAAGAGTG
>Marker304912 Chr1 SNP_site:27688337 Ref_Type:A

Create PDF with GO2PDF for free, if you wish to

ACTATTCTTTAAGTGATTATATCTTCTTTACAACTTTTTTGGAACAGTAACTATATGATCCATATGGATATATGTCAGTTXXXXXXXXXXG
TGATTAACTTTGGTATTTCTCATTTCACAGACCTTGTAAATGTTTTTCGAGAGTTTCTTCCAAAAGTCGTGCAAGAGTG
ACTATTCTTTAAGTGATTATATCTTCTTTACAATTTTTTTGGAACAGTAACTATATGATCCATATGGATATTTGTCAGTTXXXXXXXXXXG
TGATTAACTTTGGTATTTCTCATTTCACAGACCTTGTAAATGTTTTTCGAGAGTTTCTTCCAAAAGTCGTGCAAGAGTG
>Marker305536 Chr1 SNP_site:2574331 Ref_Type:A
TACTTCTTCTCCCTCTTTTGCATTATTGATTGATGCAGGTAAAAATTTCTTGCTTTGGAATCTCTCGGCTCTAATTTATTXXXXXXXXXXT
CACAATTCCTGAATTTCCATTCATTTCAATCTTAACACCTATCGCTGCAATATTTCAAAATAGGTTTTTGCTCGATGGT
TACTTCTTCTCCCTCTTTTGCATTATTGATTGTTGCAGGTAAAAATTTCTTGCTTTGGAATCTCTCGACTCTAATTTATTXXXXXXXXXXT
CACAATTCCTGAATTTCCATTCATTTCAATCTTAACACCTGTCGCTGCAATATTTCAAAATAGGTTTTTGCTCGATGGT
>Marker305536 Chr1 SNP_site:2574366 Ref_Type:G
TACTTCTTCTCCCTCTTTTGCATTATTGATTGATGCAGGTAAAAATTTCTTGCTTTGGAATCTCTCGGCTCTAATTTATTXXXXXXXXXXT
CACAATTCCTGAATTTCCATTCATTTCAATCTTAACACCTATCGCTGCAATATTTCAAAATAGGTTTTTGCTCGATGGT
TACTTCTTCTCCCTCTTTTGCATTATTGATTGTTGCAGGTAAAAATTTCTTGCTTTGGAATCTCTCGACTCTAATTTATTXXXXXXXXXXT
CACAATTCCTGAATTTCCATTCATTTCAATCTTAACACCTGTCGCTGCAATATTTCAAAATAGGTTTTTGCTCGATGGT
>Marker305536 Chr1 SNP_site:2574553 Ref_Type:A
TACTTCTTCTCCCTCTTTTGCATTATTGATTGATGCAGGTAAAAATTTCTTGCTTTGGAATCTCTCGGCTCTAATTTATTXXXXXXXXXXT
CACAATTCCTGAATTTCCATTCATTTCAATCTTAACACCTATCGCTGCAATATTTCAAAATAGGTTTTTGCTCGATGGT
TACTTCTTCTCCCTCTTTTGCATTATTGATTGTTGCAGGTAAAAATTTCTTGCTTTGGAATCTCTCGACTCTAATTTATTXXXXXXXXXXT
CACAATTCCTGAATTTCCATTCATTTCAATCTTAACACCTGTCGCTGCAATATTTCAAAATAGGTTTTTGCTCGATGGT
>Marker306063 Chr1 SNP_site:27392454 Ref_Type:C
AACTTCTTATGCACTACTTTTTCCAAAAATGCCCAATCCACTCTGTCGAAAGCCTTTTCAAGGTCTAATTTAATGATCGAXXXXXXXXXXT
ATTTGTATTAAAAGAGAGTTACTTGTTTTTTAAATGAGGAAAGTTATAAAGTTCATTAGAAGGGTAGTTTGGACCTTGT
AACTTCTTATGCACTACTTTTTCTAAAAATGCCCAATCCACTCTGTCGAAAGCCTTTTCAAGGTCTAATTTAATGATCGAXXXXXXXXXXT
ATTTGTATTAAAAGAGAGTTACTTGTTTTTTAAATGAGGAAAGTTAGAAAGTTCATTAGAAGGGTAGTTTGGACCTTGT
>Marker306063 Chr1 SNP_site:27392657 Ref_Type:T
AACTTCTTATGCACTACTTTTTCCAAAAATGCCCAATCCACTCTGTCGAAAGCCTTTTCAAGGTCTAATTTAATGATCGAXXXXXXXXXXT
ATTTGTATTAAAAGAGAGTTACTTGTTTTTTAAATGAGGAAAGTTATAAAGTTCATTAGAAGGGTAGTTTGGACCTTGT
AACTTCTTATGCACTACTTTTTCTAAAAATGCCCAATCCACTCTGTCGAAAGCCTTTTCAAGGTCTAATTTAATGATCGAXXXXXXXXXXT
ATTTGTATTAAAAGAGAGTTACTTGTTTTTTAAATGAGGAAAGTTAGAAAGTTCATTAGAAGGGTAGTTTGGACCTTGT
>Marker306066 Chr1 SNP_site:23958358 Ref_Type:T
TACCCACCATTTCTTGGAATTGCCCACCATGTTGCATTCTGAAATGACTCTACAAAGGTCAATATCTGCAACACCATCTGXXXXXXXXXXT
TTGAACCTTCACCAGAAAACTTTTTTTTCTTGTTACTCTATAGTCTATTTTCTAGAATGTTGGCCTTGGGATCTATTGT
TACCCACCATTTCTTGGAATTGCCCACCATGTTGCATTCTGAAATGACTCTACAAAGGTCAATATCTGCAACACCATCTGXXXXXXXXXXT
TTGAACCTTCACCAGAAAACTTTTTTTTCTTGTTACTCTATAGTTTATTTTCTAGAATGTTGGCCTTGGGATCTATTGT
>Marker306703 Chr1 SNP_site:2783849 Ref_Type:A
ACAAGCTCTCAAGAAGTGCAAGTTTGTATCAATCTCAAGATTAACATCTTATGAAATATCGGAGACTTTGAAGATTAAAAXXXXXXXXXXA
TACTAGAGACTATATTCACAATATTTATCAATATACAAAGTTCAATTCCACGAAATACATTTTCTTGAAATCTCGTGTG
ACAAGCTCTCAAGAAGTGCAAGTTTGTATCAATCTCAAGATTAACATCTTATGAAATATCGGAGACTTTGAAGATTAAAAXXXXXXXXXXA
TACTAGAGACTGTATTCACAATATTTATCAATATATAAAGTTCAATTCCACGAAATACATTTTCTTGAAATCTCGTGTG
>Marker306703 Chr1 SNP_site:2783873 Ref_Type:C
ACAAGCTCTCAAGAAGTGCAAGTTTGTATCAATCTCAAGATTAACATCTTATGAAATATCGGAGACTTTGAAGATTAAAAXXXXXXXXXXA
TACTAGAGACTATATTCACAATATTTATCAATATACAAAGTTCAATTCCACGAAATACATTTTCTTGAAATCTCGTGTG
ACAAGCTCTCAAGAAGTGCAAGTTTGTATCAATCTCAAGATTAACATCTTATGAAATATCGGAGACTTTGAAGATTAAAAXXXXXXXXXXA
TACTAGAGACTGTATTCACAATATTTATCAATATATAAAGTTCAATTCCACGAAATACATTTTCTTGAAATCTCGTGTG

Create PDF with GO2PDF for free, if you wish to

>Marker307032 Chr1 SNP_site:3695401 Ref_Type:G
AACAGATCATTTGCAAGTTGGAGCTTCAAAATTTGGGAACACTATAAAAGCAATGGACAATGGAGCTATAACTATAGGAGXXXXXXXXXXG
ATATGGACAAATCTGATGCCTTGTTTCCAACCCAACTTACTGTTTTCTTAATCCCTTGCATGAAAGAGCTCCCTTTGTG
AACAGATCATTTGCAAGTTGGAGCTTCAAAATTTGGGAACACTATAAAAGCAATGGACAATGGAGCTATAACTATAGGAGXXXXXXXXXXG
ATATGGACAAATCTGATTCCTTGTTTCCAACCCAACTTACTGTTTTCTTAATCCCTTGCATGAAAGAGCTCCCTTTGTG
>Marker30762 Chr4 SNP_site:17149858 Ref_Type:A
CACAAAGAAGAATATCACGTTATCGATATTGAGTATGTTCCATGACAACAATGCAACCGTAAGTGTCGAGCATACCCCACXXXXXXXXXXG
CCCTTCCACAACGTAATAAAGTTAAGCCAGTGATTCTAAGTCACGAGTTACTTTGCTAGGGATACTTGCCTAGAGTAGT
CACAAAGAAGAATATCACGTTGTCGATATTGAGTATGTTCCATGACAACAATGCAACCGTAAGTGTCGAGCATACCCCACXXXXXXXXXXG
CCCTTCCACAACGTAATAAAGTTAAGCCAGTGATTCTAAGTCATGAGTTACTTTGCTAGGGATACTTGCCTAGAGTAGT
>Marker30762 Chr4 SNP_site:17150048 Ref_Type:C
CACAAAGAAGAATATCACGTTATCGATATTGAGTATGTTCCATGACAACAATGCAACCGTAAGTGTCGAGCATACCCCACXXXXXXXXXXG
CCCTTCCACAACGTAATAAAGTTAAGCCAGTGATTCTAAGTCACGAGTTACTTTGCTAGGGATACTTGCCTAGAGTAGT
CACAAAGAAGAATATCACGTTGTCGATATTGAGTATGTTCCATGACAACAATGCAACCGTAAGTGTCGAGCATACCCCACXXXXXXXXXXG
CCCTTCCACAACGTAATAAAGTTAAGCCAGTGATTCTAAGTCATGAGTTACTTTGCTAGGGATACTTGCCTAGAGTAGT
>Marker307653 Chr1 SNP_site:25067174 Ref_Type:C
AACATGATCATGACCTTGGTCAAACATAATCTAATGCAACCATGCTATAATTAGATTTCACTATTTATCAAAGTATTCCTXXXXXXXXXXA
ATATTTATTTAGTAACACACGATAAATTATATTCATTGGATTAAAACAATTAAACAAAATAAAAGTAACTAGCTAGAGT
AACATGATCATGACTTTGGTCAAACATAATCTAATGCAACCATGCTATAATTAGATTTCACTATTTATCAAAGTATTCCTXXXXXXXXXXA
ATATTTATTTAGTAACACACGATAAATTATATTCATCGGATTAAAACAATTAAACAAAATAAAAGTAACTAGCTAGAGT
>Marker307653 Chr1 SNP_site:25067369 Ref_Type:T
AACATGATCATGACCTTGGTCAAACATAATCTAATGCAACCATGCTATAATTAGATTTCACTATTTATCAAAGTATTCCTXXXXXXXXXXA
ATATTTATTTAGTAACACACGATAAATTATATTCATTGGATTAAAACAATTAAACAAAATAAAAGTAACTAGCTAGAGT
AACATGATCATGACTTTGGTCAAACATAATCTAATGCAACCATGCTATAATTAGATTTCACTATTTATCAAAGTATTCCTXXXXXXXXXXA
ATATTTATTTAGTAACACACGATAAATTATATTCATCGGATTAAAACAATTAAACAAAATAAAAGTAACTAGCTAGAGT
>Marker30833 Chr4 SNP_site:18256593 Ref_Type:C
ACCACATTGGTCATCCCTCAATGTGGGACAAAAGCTTCCCGTAACTACTTTGGTTCCTAACAATACCCCATCCCTAGAAAXXXXXXXXXXT
TTGACATTTTGGAGTGGAACTGAGAGGGTTTTGATTGGGAGCGGCTGATAAGTATTGTCAAAGTAGCTGCTGGGACGTT
ACCACATTGGTGATCCCTCAATGTGGGACAAAAGCTTCCCGTAACTACTTTGGTTCCTAACAATACCCCATCCCTAGAAAXXXXXXXXXXT
TTGACATTTTGGAGTCGAACTGAGAGGGTTTTGATTGGGAGCGGCTGATAAGTATTGTCAAAGTAGCTGCTGGGACGTT
>Marker30833 Chr4 SNP_site:18256764 Ref_Type:C
ACCACATTGGTCATCCCTCAATGTGGGACAAAAGCTTCCCGTAACTACTTTGGTTCCTAACAATACCCCATCCCTAGAAAXXXXXXXXXXT
TTGACATTTTGGAGTGGAACTGAGAGGGTTTTGATTGGGAGCGGCTGATAAGTATTGTCAAAGTAGCTGCTGGGACGTT
ACCACATTGGTGATCCCTCAATGTGGGACAAAAGCTTCCCGTAACTACTTTGGTTCCTAACAATACCCCATCCCTAGAAAXXXXXXXXXXT
TTGACATTTTGGAGTCGAACTGAGAGGGTTTTGATTGGGAGCGGCTGATAAGTATTGTCAAAGTAGCTGCTGGGACGTT
>Marker308380 Chr1 SNP_site:14744127 Ref_Type:C
AACAGATTATATACTTATAACATAAACATTTAATTTTCATCTTTAAACTGTAAATGAGTAACAGTATTGATTTCAACCCTXXXXXXXXXXA
AAAAATGTTATAATTGGAGATTGATTTGTAATCCTTGTTTGTAAAACGGTTAGCTTGGTTTTAGGTTTTGAATTGAGTC
AACAGATTATATACTTATAATATAAACATTTAATTTTCATCTTTAAACTGTAAATGAGTAACAGTATTGATTTCAACCCTXXXXXXXXXXA
AAAAATGTTATAATTGGAGATTGATTTGTAATCCTTGTTTGTAAAACCGTTAGCTTGGTTTTAGGTTTTGAATTGAGTC
>Marker308380 Chr1 SNP_site:14744343 Ref_Type:G
AACAGATTATATACTTATAACATAAACATTTAATTTTCATCTTTAAACTGTAAATGAGTAACAGTATTGATTTCAACCCTXXXXXXXXXXA
AAAAATGTTATAATTGGAGATTGATTTGTAATCCTTGTTTGTAAAACGGTTAGCTTGGTTTTAGGTTTTGAATTGAGTC
AACAGATTATATACTTATAATATAAACATTTAATTTTCATCTTTAAACTGTAAATGAGTAACAGTATTGATTTCAACCCTXXXXXXXXXXA

Create PDF with GO2PDF for free, if you wish to

AAAAATGTTATAATTGGAGATTGATTTGTAATCCTTGTTTGTAAAACCGTTAGCTTGGTTTTAGGTTTTGAATTGAGTC
>Marker30896 Chr4 SNP_site:16551298 Ref_Type:C
TACAAGGGAATAAAAAAGGAAAATATTTAGGAAATAAGGAAAATATTCCCATAATCTATCCATAAATATTCTAGGATTCTXXXXXXXXXXC
CATGCAAATCAACAATTTTGAGCATAATTGCACTCTACAAGGATGTGCTAAATGTCTTCCTCTTCTCCTTTGCACGGTG
TACAAGGGAATAAAAAAGGAAAATATTTAGGAAATAAGGAAAATATTCCCATAATCTATCCATAAATATTCTAGGATTCTXXXXXXXXXXC
CATGCAAATCAACAATTTTGAGCATAATTGCACTCTACAAGGATGTGCTAAATGTCTTGCTCTTCTCCTTTGCACGGTG
>Marker309055 Chr1 SNP_site:6956650 Ref_Type:G
ACATTTGTTTAATAAGCCATTTAATTTATCAAATTAAAACATTTTAATTATCAAACAAAATGATATTTTATTTGCAGCCTXXXXXXXXXXT
AAAGGTCCTTGTAAAAGTCCTATTGAACTACAAATTGAAGGAACACTACAAGCTTCGCCTGACCCAAAAGGAGATGGTT
ACATTTGTTTAATAAGCCATTTAATTTATCAAATTAAAACATTTTAATTATCAAACAAAATTATATTTTATTTGCAGCCTXXXXXXXXXXT
AAAGGTCCTTGTAAAAGTCCTATTGAACTACAAATTGAAGGAACACTACAAGCTTCGCCTGACCCAAAAGGAGATGGTT
>Marker309411 Chr1 SNP_site:9364373 Ref_Type:G
AACCATAGGAAGCAACTGACTTTTAACGATTTAACACCAAGGTCACATTGATTTTCCTTTTAAACACAATAGTTTCCACAXXXXXXXXXXT
CCTTATACGATATATGGCAACCTCGATATTCACATGATCTTACTTGTTCTAAATATCTTGGCATTTTCACAATAGTGTA
AACCATAGGAAGCAACTGACTTTTAACGATTTAACACCAAGGTCACATTGATTTTCCTTTTAAACACAGTAGTTTCCACAXXXXXXXXXXT
CCTTATACGATATATGGCAAGCTCGATATTCACATGATCTTACTTGTTCTAAATATCTTGGCATTTTCACAATAGTGTA
>Marker309411 Chr1 SNP_site:9364531 Ref_Type:G
AACCATAGGAAGCAACTGACTTTTAACGATTTAACACCAAGGTCACATTGATTTTCCTTTTAAACACAATAGTTTCCACAXXXXXXXXXXT
CCTTATACGATATATGGCAACCTCGATATTCACATGATCTTACTTGTTCTAAATATCTTGGCATTTTCACAATAGTGTA
AACCATAGGAAGCAACTGACTTTTAACGATTTAACACCAAGGTCACATTGATTTTCCTTTTAAACACAGTAGTTTCCACAXXXXXXXXXXT
CCTTATACGATATATGGCAAGCTCGATATTCACATGATCTTACTTGTTCTAAATATCTTGGCATTTTCACAATAGTGTA
>Marker309520 Chr1 SNP_site:5016673 Ref_Type:T
AACTACGTGACTTATATTGACTGAACCAAAATCTCAAATAGAATATAAAGACAGACTCAAAAAATGCCAAACGATTCAGGXXXXXXXXXXT
GAGGAGATTGATGAATGTCTATAGACTATATGAAACTAATAGTTACAACACTCTTCACTTACAGAGCTCTGAGCTGAGT
AACTACGTGACTTATATTGACTGAACCAAAATCTCAAATAGAATATAAAGACAGATTCAAAAAATGCCAAACGATTCAGGXXXXXXXXXXT
GAGGAGATTGATGAATGTCTATAGACTATATGAAACTAATAGTTACAACACTCTTCACTTACAGAGCTCTGAGCTGAGT
>Marker309762 Chr1 SNP_site:25519967 Ref_Type:G
CACCTCCTTGCATGTAAGTTTCCCCATTCTCTCTGTCTCTCTCTTTTTTTTTGTTTTGACAAGAAATAGTTCTTTGATGTAXXXXXXXXXXG
ATCGATAAGATAAAATGTTCAAATTTCCACCGACATGTTGAAGTTAAAATAAACTTCTCTGTCCCTCATTTATACTTGT
CACCTCCTTGCATGTAAGTTTCCCCATTCTCTCTGTCTCTCTCTTTTTTTTTGTTTTGACAAGAAATAGTTCTTTGATGTAXXXXXXXXXXG
ATCGATAAGATAAGATGTTCAAATTTCCACCGACATGTTGAAGTTAAAATAAACTTCTCTGTCCCTCATTTATACTTGT
>Marker309769 Chr1 SNP_site:25357415 Ref_Type:G
AACTTAAGGTGGTTGAAAAATTCAAGTTCTTACTATCCACTTTTAAACAACAAACAGGTCACCAGCTCAGAGTGGAAGATXXXXXXXXXXC
ATATCTTCTCAACAAACAAAAAGGTGTATTAAATTTATAGGAAAGGATGCTTGTAACTACCTAGGATTATGGTGTAGTA
AACTTAAGGTGGTTGAAAAATTCAAGTTCTTACTATCCACTTTTAAACAACAAACAGGTCACCAGCTGAGAGTCGAAGATXXXXXXXXXXC
ATATCTTCTCAACAAACAAAAAGGTGTATTAAATTTATAGGAAAGGATGCTTGTAACTACCTAGGATTATGGTGTAGTA
>Marker309769 Chr1 SNP_site:25357421 Ref_Type:C
AACTTAAGGTGGTTGAAAAATTCAAGTTCTTACTATCCACTTTTAAACAACAAACAGGTCACCAGCTCAGAGTGGAAGATXXXXXXXXXXC
ATATCTTCTCAACAAACAAAAAGGTGTATTAAATTTATAGGAAAGGATGCTTGTAACTACCTAGGATTATGGTGTAGTA
AACTTAAGGTGGTTGAAAAATTCAAGTTCTTACTATCCACTTTTAAACAACAAACAGGTCACCAGCTGAGAGTCGAAGATXXXXXXXXXXC
ATATCTTCTCAACAAACAAAAAGGTGTATTAAATTTATAGGAAAGGATGCTTGTAACTACCTAGGATTATGGTGTAGTA
>Marker309837 Chr1 SNP_site:25907873 Ref_Type:A
CACCGGTTGCATCTTTTTTGCTAGTCTCTAGATCCGAATGATTTCCTTTTGAGAAAGTTATTTTGTTATGCTTTGTATTTAXXXXXXXXXXC
TTTTATTGACCGAGGTCGTCTTTGGGCAGTTTGTTTGGTTGTTTGTCTTTATTTTTTGCTCTTTGTATAATTCTCTTGT

Create PDF with GO2PDF for free, if you wish to

CACCGGTTGCATCTTTTTGCTAGTCTCTAGATCCGAATGGTTTCCTTCTGAGAAAGTTATTTTGTTATGCTTTGTATTTAXXXXXXXXXXC
TTGTATTGACCGAGGTCGTCTTTGGGCAGTTTGTTTGGTTGTTTGTCTTTATTTTTTGCTCTTTGTATAATTCTCTTGT
>Marker309837 Chr1 SNP_site:25907881 Ref_Type:T
CACCGGTTGCATCTTTTTGCTAGTCTCTAGATCCGAATGATTTCCTTTTGAGAAAGTTATTTTGTTATGCTTTGTATTTAXXXXXXXXXXC
TTTTATTGACCGAGGTCGTCTTTGGGCAGTTTGTTTGGTTGTTTGTCTTTATTTTTTGCTCTTTGTATAATTCTCTTGT
CACCGGTTGCATCTTTTTGCTAGTCTCTAGATCCGAATGGTTTCCTTCTGAGAAAGTTATTTTGTTATGCTTTGTATTTAXXXXXXXXXXC
TTGTATTGACCGAGGTCGTCTTTGGGCAGTTTGTTTGGTTGTTTGTCTTTATTTTTTGCTCTTTGTATAATTCTCTTGT
>Marker309837 Chr1 SNP_site:25908015 Ref_Type:T
CACCGGTTGCATCTTTTTGCTAGTCTCTAGATCCGAATGATTTCCTTTTGAGAAAGTTATTTTGTTATGCTTTGTATTTAXXXXXXXXXXC
TTTTATTGACCGAGGTCGTCTTTGGGCAGTTTGTTTGGTTGTTTGTCTTTATTTTTTGCTCTTTGTATAATTCTCTTGT
CACCGGTTGCATCTTTTTGCTAGTCTCTAGATCCGAATGGTTTCCTTCTGAGAAAGTTATTTTGTTATGCTTTGTATTTAXXXXXXXXXXC
TTGTATTGACCGAGGTCGTCTTTGGGCAGTTTGTTTGGTTGTTTGTCTTTATTTTTTGCTCTTTGTATAATTCTCTTGT
>Marker310501 Chr1 SNP_site:28579849 Ref_Type:T
AACTAACTTGCATGCAAGAGAAACGTGAAGGAAGGTCTATGCATTATTAGGCAACTAAGCAGTGTGAGAACTCTGATGCGXXXXXXXXXXA
GGTATATCTAGAAGACTTGACTTATAGAACTTCTCACTTAAGGACAACAACCGCTTAGTGCCTAATGTATGCACTTGTT
AACTAACTTGCATGCAAGAGAAACGTGAAGGAAGGTCTATGCATTATTAGGCAACTAAGCAGTGTGAGAACTCTGATGCGXXXXXXXXXXA
GGTATATCTAGAAGACTTGACTTATAGAACTTCTCACTTAAGGACAACAACCTCTTAGTGCCTAATGTATGCACTTGTT
>Marker311295 Chr1 SNP_site:10350256 Ref_Type:A
TACGGATATATTTCCTTTCTAGTATATATATTGTGACAAATATCAAATTATAATCCTAAACAAAGAATTGTATCTATTAAXXXXXXXXXXA
TACATCGTAACTACAAGAAAATTATTTATGCATTAACTCTTAATATTTTACAAAATGAACCATTAGAATTAATGGTGTG
TACGGATATATTTCCTTTCTAGTATATATATTGTGACAAATATCAAATTATAATCCTAAACAAAGAATTGTATCTATTAAXXXXXXXXXXT
TAGATCGTAACTACAAGAAAATTATTTATGCATTAACTCTTAATATTTTACAAAATGAACCATTAGAATTAATGGCGTG
>Marker311295 Chr1 SNP_site:10350259 Ref_Type:C
TACGGATATATTTCCTTTCTAGTATATATATTGTGACAAATATCAAATTATAATCCTAAACAAAGAATTGTATCTATTAAXXXXXXXXXXA
TACATCGTAACTACAAGAAAATTATTTATGCATTAACTCTTAATATTTTACAAAATGAACCATTAGAATTAATGGTGTG
TACGGATATATTTCCTTTCTAGTATATATATTGTGACAAATATCAAATTATAATCCTAAACAAAGAATTGTATCTATTAAXXXXXXXXXXT
TAGATCGTAACTACAAGAAAATTATTTATGCATTAACTCTTAATATTTTACAAAATGAACCATTAGAATTAATGGCGTG
>Marker311295 Chr1 SNP_site:10350332 Ref_Type:T
TACGGATATATTTCCTTTCTAGTATATATATTGTGACAAATATCAAATTATAATCCTAAACAAAGAATTGTATCTATTAAXXXXXXXXXXA
TACATCGTAACTACAAGAAAATTATTTATGCATTAACTCTTAATATTTTACAAAATGAACCATTAGAATTAATGGTGTG
TACGGATATATTTCCTTTCTAGTATATATATTGTGACAAATATCAAATTATAATCCTAAACAAAGAATTGTATCTATTAAXXXXXXXXXXT
TAGATCGTAACTACAAGAAAATTATTTATGCATTAACTCTTAATATTTTACAAAATGAACCATTAGAATTAATGGCGTG
>Marker311335 Chr1 SNP_site:7242158 Ref_Type:C
AACTTCATCCCTAGCTGTCCCCACCCTTTTTTTTCTCTCTTCTTATTCCTAATTTAAAATACCAAAAGTATATCATTACCXXXXXXXXXXT
TATGAATGAAACTTAATGTTAGTTGTTGGGTTTAGAAAGATTTGAAACTTAAAAGCAAGGAATAGCGTTGTAGAAGGTT
AACTTCATCCCTAGCTGTCCCCACCCTTTTTTTTCTCTCTTCTTATTCCTAATTTAAAATACCAAAAGTATATCATTACTXXXXXXXXXXT
TGGGAATGAAACTTAATGTTAGTTGTTGGGTTTAGAAAGATTTGAAACTTAAAAGCAAGGAATAGCGTTGTAGAAGGTT
>Marker311335 Chr1 SNP_site:7242289 Ref_Type:A
AACTTCATCCCTAGCTGTCCCCACCCTTTTTTTTCTCTCTTCTTATTCCTAATTTAAAATACCAAAAGTATATCATTACCXXXXXXXXXXT
TATGAATGAAACTTAATGTTAGTTGTTGGGTTTAGAAAGATTTGAAACTTAAAAGCAAGGAATAGCGTTGTAGAAGGTT
AACTTCATCCCTAGCTGTCCCCACCCTTTTTTTTCTCTCTTCTTATTCCTAATTTAAAATACCAAAAGTATATCATTACTXXXXXXXXXXT
TGGGAATGAAACTTAATGTTAGTTGTTGGGTTTAGAAAGATTTGAAACTTAAAAGCAAGGAATAGCGTTGTAGAAGGTT
>Marker311335 Chr1 SNP_site:7242290 Ref_Type:T
AACTTCATCCCTAGCTGTCCCCACCCTTTTTTTTCTCTCTTCTTATTCCTAATTTAAAATACCAAAAGTATATCATTACCXXXXXXXXXXT

Create PDF with GO2PDF for free, if you wish to

TATGAATGAAACTTAATGTTAGTTGTTGGGTTTAGAAAGATTTGAAACTTAAAAGCAAGGAATAGCGTTGTAGAAGGTT
AACTTCATCCCTAGCTGTCCCCACCCTTTTTTTTCTCTCTTCTTATTCCTAATTTAAAATACCAAAAGTATATCATTACTXXXXXXXXXXT
TGGGAATGAAACTTAATGTTAGTTGTTGGGTTTAGAAAGATTTGAAACTTAAAAGCAAGGAATAGCGTTGTAGAAGGTT
>Marker311736 Chr1 SNP_site:1153621 Ref_Type:T
ACTTCTACGACTAGTGTTTCCCGTCTTGTTGCTTTCAAATGGTCATCGCCGTTCTCCCTTCGTCTATTGTGTTAAAAAATXXXXXXXXXXG
GACTCAAGCGTGGATCGCCTCCAGGATGCAACGTTTGATCGACCTCATCTTTGGTATCCTACTTCTGAAGTAATTAGTG
ACTTCTACGACTAGTGTTTCCTGTCTTGTTGCTTTCAAATGGTCATCGCCGTTCTCCCTTCGTCTATTGTGTTAAAAAATXXXXXXXXXXG
GACTCAAGCGTGGATCGCCTCCAGGATGCAACGTTTGATCGACCTCATCTTTGGTATCCTACTTCTGAAGTAATTAGTG
>Marker312290 Chr1 SNP_site:23911948 Ref_Type:G
AACCGATATCGCTCCCTTTTTTAACAACACCAGCTGATCCTTAACCTTTCACCTTTAACTAAAACGCAGACGGAAGCTAAXXXXXXXXXXT
CTAACTTCAACCCTCTTAAATCCAAGTCCGTTACAATTAAAAGACCAACTAAATGTAACTTCTAAACAGCAAGACATGT
AACCGATATCGCTCCCTTTTTTAACAACACCAGCTGATCCTTAACCTTTCACCTTTAACTAAAACGCAGGCGGAAGCTAAXXXXXXXXXXT
CTAACTTCAACCCTCTTAAATCCAAGTCCGTTACAATTAAAAGACCAACTAAATGTAACTTCTAAACAGCAAGACATGT
>Marker312526 Chr1 SNP_site:4667414 Ref_Type:A
CACAGATAAATGCATTCAAGAGTTCGATCAAATTTTTTCCTCTGTGAAATACAAAATACAATGATATTTTGGCAAGGGTTXXXXXXXXXXA
TTTAAAATTACAGATTGGTTAAAATAATAAGCAAAGCTACCCATTTAGTAATCATTACATCTCTACTACACGTTTTGGT
CACAGATAAATGCATTCAAGCGTTCGATCAAATTTTTTCCTCTGTGAAATACAAAATACAATGATATTTTGGCAAGGGTTXXXXXXXXXXA
TTTAAAATTACAGATTGGTTAAAATAATAAGCAAAGCTACCCATTTAGTAATCATTACATCTCTACTACACGTTTTGGT
>Marker312708 Chr1 SNP_site:27401953 Ref_Type:A
ACTTGAAAATTCCTGGTCTTTGATTTGGAAATCTTCTGCTAACTTTTTCTTCACGAAAGTCATTTCTGTCTCATCATTGCXXXXXXXXXXG
CTTTTCGAAGTGTTTTTTTTTAACTCGGGACCTCATGTCCGCAGATACATACAGGTGTCATTTGAGCTAAGCTCATGTT
ACTTGAAAATTCCTGGTCTTTGATTTGGGAATCTTCTGCTAACTTTTTCTTCGCGAAAGTCATTTCTGTCTCATCATTGCXXXXXXXXXXG
CTTTTCGAAGTGTTTTTTTTTAACTCAGGACCTCATGTCCGCAGATACATACAGGTGTCATTTGAGCTAAGCTCATGTT
>Marker312708 Chr1 SNP_site:27401977 Ref_Type:A
ACTTGAAAATTCCTGGTCTTTGATTTGGAAATCTTCTGCTAACTTTTTCTTCACGAAAGTCATTTCTGTCTCATCATTGCXXXXXXXXXXG
CTTTTCGAAGTGTTTTTTTTTAACTCGGGACCTCATGTCCGCAGATACATACAGGTGTCATTTGAGCTAAGCTCATGTT
ACTTGAAAATTCCTGGTCTTTGATTTGGGAATCTTCTGCTAACTTTTTCTTCGCGAAAGTCATTTCTGTCTCATCATTGCXXXXXXXXXXG
CTTTTCGAAGTGTTTTTTTTTAACTCAGGACCTCATGTCCGCAGATACATACAGGTGTCATTTGAGCTAAGCTCATGTT
>Marker312708 Chr1 SNP_site:27402149 Ref_Type:G
ACTTGAAAATTCCTGGTCTTTGATTTGGAAATCTTCTGCTAACTTTTTCTTCACGAAAGTCATTTCTGTCTCATCATTGCXXXXXXXXXXG
CTTTTCGAAGTGTTTTTTTTTAACTCGGGACCTCATGTCCGCAGATACATACAGGTGTCATTTGAGCTAAGCTCATGTT
ACTTGAAAATTCCTGGTCTTTGATTTGGGAATCTTCTGCTAACTTTTTCTTCGCGAAAGTCATTTCTGTCTCATCATTGCXXXXXXXXXXG
CTTTTCGAAGTGTTTTTTTTTAACTCAGGACCTCATGTCCGCAGATACATACAGGTGTCATTTGAGCTAAGCTCATGTT
>Marker313097 Chr1 SNP_site:9590998 Ref_Type:C
TACAACAAGAGACCTGAAATGATTGTGTATTCCAACTCTCTTATCTTCAAAAATGCAATGATTAAGGAGTCCGAAGAAACXXXXXXXXXXC
CAAAATGATTGTGCAAAGTCACAGAAAACAAAATTATCCTCCAAAACTATTGTGCAAAGTCACAGAAAACCAAGATGTT
TACAACAAGAGACCTGAAATGATTGTGTATTCCAACTCTCTTATCTTCAAAAATGCAATGATTAAGGAGTCCGAAGAAACXXXXXXXXXXC
CAAAATGATTGTGCAAAGTCACAGAAAACAAAATTATCCTCCAAAATTATTGTGCAAAGTCACAGAAAACCAAGATGTT
>Marker313525 Chr1 SNP_site:21860967 Ref_Type:G
ACTGCTTTCATAGGAAAACTAATCTATTGTATCCTGTGAGACAATTATTGGCAACACAATTATTCCCAACTCAGCACTAAXXXXXXXXXXT
CAGTTTCAAAACCATCCCCACAATCACAATCAAACAAAAGAAACAGAAATGAAGACTGAGATTAAAAAGTTTTGGGGTA
ACTGCTTTCATAGGAAAGCTAATCTATTGTATCCTGTGAGACAATTATTGGCAACACAATTATTCCCAACTCAGCACTAAXXXXXXXXXXT
CAGTTTCAAAACCATCCCCACAATCACAATCAAACAAAAGAAACAGAAATGAAGACTGAGATTAAAAAGTTTTGGGGTA
>Marker313528 Chr1 SNP_site:4643276 Ref_Type:A

Create PDF with GO2PDF for free, if you wish to

ACTGTTAAAAACACCGACAAACAAACAGCTTCTTCAGGTATAAAATTCGAATTCCTTAGAACTCACCCAATGATAATAATXXXXXXXXXXC
ACTTTAAATTTAATGTTATACCTTGTGTCTTGCATGGAAATTAATTCTCATTCTCACATGCATCCATTATAAATATAGT
ACTGTTAAGAACACCGACAAACAAACAGCTTCTTCAGGTATAAAATTCGAATTCCTTAGAACTCACCCAATGATAATAATXXXXXXXXXXC
ACTTTAAATTTAATGTTATACCTTGTGTCTTGCATGGAAATTAATTCTCATTCTCACATGCATCCATTATAAATATAGT
>Marker313585 Chr1 SNP_site:262904 Ref_Type:T
AACCCAAGACTTTGAATTTCTTTTTTCAATTGAACATGGGTTATAGGACTATATGTTTCTTTTGATCATTAAATTATGTCXXXXXXXXXXT
AATCTCTGATCAGGCATTATTAACATCCATAATTGCAGTCCCATCAAGATTTCTCAGTGTTCAAAACTTCTGATTAGTT
AACCCAAGACTTTGAATTTCTTTTTTCAATTGAACATGGGTTATAGGTCTATATGTTTCTTTTGATCATTAAATTATGTCXXXXXXXXXXT
AATCTCTGATCAGGCATTATTAACATCCATAATTGCAGTCCCATCAAGATTTCTCAGTGTTCAAAACTTCTGATTAGTT
>Marker313855 Chr1 SNP_site:26072734 Ref_Type:G
CACATCAATGAGAAGGTGTTGGAGGAAGATTTCAGTAGCTCGTGGGTAGTCTCAAGACTATTTGTGTATAATGAGAGGGAXXXXXXXXXXT
GGGTGGATTTCCATCAAAAATCTACCACTTGATTACTGGAAAAATGACGTCTTTATTACATTTGGAAATTACTTCGGTG
CACATCAATGAGAAGGTGTTGGAGGAAGATTTCAGTAGCTCGTGGGTAGTCTCAAGACTATTTGTGTATAATGAGAGGGAXXXXXXXXXXT
GGGTGGATTTCCATCAAAAATCTACCACTTGATTACTGGAAAAATGGCGTCTTTATTACATTTGGAAATTACTTCGGTG
>Marker314131 Chr1 SNP_site:12294912 Ref_Type:T
CACATGAAATTTCTGGAGAAATTTGATTCGTGGAGTTGAACTTGAGTTGATGTTTTATTTTTGTTGATTTGATGCGATGTXXXXXXXXXXA
AGTCTTGTAAAGCTTGTAGAAATTGGAGTCTTGGAAGAAAGCTTGTATCCTAAAGGAGCTTCAATCTTCAAGGATGGTT
CACATGAAATTTCTGGAGAAATTTGATTCGTGGAGTTGAACTTGAGTTGATGTTTTATTTTTGTTGATTTGATGCGATGTXXXXXXXXXXA
AGTCTTGTAAAGCTTGTAGAAATTGGAGTCTTGGAAGAAAGCTTGTATCTTAAAGGAGCTTCAATCTTCAAGGATGGTT
>Marker314609 Chr1 SNP_site:20952619 Ref_Type:G
GACCTTGAGGCAAGCTGTCACAAATCTTTTAGTGCTTTGGTCAAGGAATTCCTCTTCTATCCGCCGTTTTGGGGTAAGAGXXXXXXXXXXT
TGAATTTTTTTATTTTCTCAATTAAAGTTGGTATATTGTCTAAAAAGCTACAAATGTAGTCATATTCTAGGTTGGTGTA
GACCTTGAGGCAAGCTGTCACAAATCTTTTAGTGCTTTGGTCGAGGAATTCCTCTTCTATCCGCCGTTTTGGGGTAAGAGXXXXXXXXXXT
TGAATTTTTTTATTTTCTCAATTAAAGTTGGTATATTGTCTAAAAAGCTACAAATGTAGTCATATTCTAGGTTGGTGTA
>Marker315017 Chr1 SNP_site:21844752 Ref_Type:T
CACAAAGATGGATGGATGCAATTTGAAATGCTCCCAACACCAATCCATTATTATGTATGTTCTCAAAGCTCTAGGATCCAXXXXXXXXXXA
TCCTAAGCTTTTCAGAAAGAATCAATTTAACTTAATTTGTTAGTAATTTGTAACTTTGTTTGATTCTGTCCAATCAGGT
CACAAAGATGGATGGATGCAATTTGAAATGCTCCCAACACCAATCCATTATTATGTATGTTCTCAAAGCTCTAGGATCCAXXXXXXXXXXA
TCCTAAGCTTTTCAGAAAGAATCAATTTAACTTATTTTGTTAGTAATTTGTAACTTTGTTTGATTCTGTCCAATCAGGT
>Marker315368 Chr1 SNP_site:9680833 Ref_Type:A
TACCCAAATCTTTCATGGTCAAAGTGATGGATTGCTGGGTAGCTCCCTCAGCTCGAAGGTCAGATAGTTTGTGATCCACAXXXXXXXXXXA
CTAACATAAGCTAACCAAACAAAATCAACAATTAAATTTCAAATATTCATTCAGTATAGTCAATATTGAACAAATTAGT
TACCCAAATCTTTCATGGTCAAAGTGATGGATTGCTGGGTAGCTCCCTCAGCTCGAAGGTCAGATAGTTTGTGATCCACAXXXXXXXXXXA
CTGACATAAGCTAACCAAACAAAATCAACAATTAAATTTCAAATATTCATTCAGTATAGTCAATATTGAACAAATTAGT
>Marker31602 Chr4 SNP_site:5956007 Ref_Type:C
ACATCACAATAAAAAATATTTTAATATTTCAAAATATTCTCCATCAACATACTTTACAAAGATCTATTTGATGAAAATTTXXXXXXXXXXA
TTTCTTTCCATAAAAGATTTAAGAATAGTGTGTATAGTCTAACTTGGGTGTGGGAGTTGGGGTTTCCATATGAGTGGTG
ACATCACAATAAAAAATATTTTAATATTTCAAAATATTCTCCATCAACATACTTTAGAAAGATCTATTTGATGAAAATTTXXXXXXXXXXA
TTTCTTTCCATAAAAGATTTAAGAATAGTGTGTATAGTCTAACTTGGGTGTGGGAGTTGGGGTTTCCATATTAGTGGTG
>Marker31602 Chr4 SNP_site:5956189 Ref_Type:G
ACATCACAATAAAAAATATTTTAATATTTCAAAATATTCTCCATCAACATACTTTACAAAGATCTATTTGATGAAAATTTXXXXXXXXXXA
TTTCTTTCCATAAAAGATTTAAGAATAGTGTGTATAGTCTAACTTGGGTGTGGGAGTTGGGGTTTCCATATGAGTGGTG
ACATCACAATAAAAAATATTTTAATATTTCAAAATATTCTCCATCAACATACTTTAGAAAGATCTATTTGATGAAAATTTXXXXXXXXXXA
TTTCTTTCCATAAAAGATTTAAGAATAGTGTGTATAGTCTAACTTGGGTGTGGGAGTTGGGGTTTCCATATTAGTGGTG

Create PDF with GO2PDF for free, if you wish to

>Marker316167 Chr1 SNP_site:96660 Ref_Type:C
AACATGATAACCTCACCAAGTTTTTCAAATTTATCTATTTCGTTGAACAAGCAGAATGAGAAATTTGAATTTTTGACCTCXXXXXXXXXXT
TAATTTCATAATTTTAGACCATAGGAATTGAATTCCCCATCAAATTGGTTTCTTTTTTGAAATTTTAAAGCTCCTAGTA
AACATGATAACCTCACCAAGTTTTTCAAATTTATTTATTTCGTTGAACAAGCGGAATGAGAAATTTGAATTTTTGACCTCXXXXXXXXXXT
TAATTTCATAATTTTAGACCATAGGAATTGAATTCCCCATCAAATTGGTTTCTTTTTTGAAATTTTAAAGCTCCTAGTA
>Marker316167 Chr1 SNP_site:96678 Ref_Type:A
AACATGATAACCTCACCAAGTTTTTCAAATTTATCTATTTCGTTGAACAAGCAGAATGAGAAATTTGAATTTTTGACCTCXXXXXXXXXXT
TAATTTCATAATTTTAGACCATAGGAATTGAATTCCCCATCAAATTGGTTTCTTTTTTGAAATTTTAAAGCTCCTAGTA
AACATGATAACCTCACCAAGTTTTTCAAATTTATTTATTTCGTTGAACAAGCGGAATGAGAAATTTGAATTTTTGACCTCXXXXXXXXXXT
TAATTTCATAATTTTAGACCATAGGAATTGAATTCCCCATCAAATTGGTTTCTTTTTTGAAATTTTAAAGCTCCTAGTA
>Marker316802 Chr1 SNP_site:21362745 Ref_Type:C
TACTTTTGTTGGAAGGAGATCAATTATTAAGGAACGGTTCTCAAAGAAATATAGACATCCATTACTGGATTCCAAGCTTAXXXXXXXXXXC
TGGAACTTCATGTTAGGAAGCAAGGTGCATGACCAAAGCTAGGCGACTCGGAGTAGCCACACCAGTGTTGTATGCTGTT
TACTTTTGTTGGAAGGAGATCAATTATTAAGGAACGGTTCTCAAAGAAATATAGACATCCATTACTGGATTCCAAGCTTAXXXXXXXXXXC
TGGAACTTCATGTTAGGAAGCAAGGTGCATGACCAAAGCTAGGCGACTCGGAGTAGCTACACCAGTGTTGTATGCTGTT
>Marker317240 Chr1 SNP_site:1208854 Ref_Type:G
TACACCGCCGGATGCCTCACCTGCGACGGCAGCGAGAAGGAGAAGCTCTTTGAGCGGTGGTGATAAGAAAGCAAGTTTGTXXXXXXXXXXG
AAAACAAAGATTTCGAAACCAGAAACAAAACGCGCACTCTTCAACAAGATTTCTAACGATAAGACTATTAAATTCGGTG
TACACCGCCGGATGCCTCACCTGCGACGGCAGCGAGAAGGAGAAGCTCTTTGAGCGGTGGTGATAAGAAAGCAAGTTTGTXXXXXXXXXXG
AAAACAAAGATTTCGAAACCAGAAACAAAACGCGCACTCTTCAACAAGATTTCTGACGATAAGACTATTAAATTCGGTG
>Marker31740 Chr4 SNP_site:16207681 Ref_Type:C
TACAACTGTCATAATAGGACATTTTTGTTGCAGTATTCACTAGCCATTTACCCGCATAGACCTAAGATTCACTTCCACCAXXXXXXXXXXA
AATTTTTTACATACAACACATCGCCTCACATTAGTAACATTCTCAAATGTTAATAATCAAACATAAGATTACAAAGAGT
TACAACTGTCATAATAGGATATTTTTGTTGCAGTATTCACTAGCCATTTACCCGCCTAGACCTAAGATTCACTTCCACCAXXXXXXXXXXA
AATTTTTTACATACAACACATCGCCTCACATTAGTAACATTCTCAAATGTTAATAATCAAACATAAGATTACAAAGAGT
>Marker31740 Chr4 SNP_site:16207717 Ref_Type:A
TACAACTGTCATAATAGGACATTTTTGTTGCAGTATTCACTAGCCATTTACCCGCATAGACCTAAGATTCACTTCCACCAXXXXXXXXXXA
AATTTTTTACATACAACACATCGCCTCACATTAGTAACATTCTCAAATGTTAATAATCAAACATAAGATTACAAAGAGT
TACAACTGTCATAATAGGATATTTTTGTTGCAGTATTCACTAGCCATTTACCCGCCTAGACCTAAGATTCACTTCCACCAXXXXXXXXXXA
AATTTTTTACATACAACACATCGCCTCACATTAGTAACATTCTCAAATGTTAATAATCAAACATAAGATTACAAAGAGT
>Marker31765 Chr4 SNP_site:5261204 Ref_Type:A
ACAAACAAGAAAATTAACAGAATGAAAGAATAAATAAGATTAAAACTTTGCTACGCAATAACGATTTGAACTGAGATCTTXXXXXXXXXXT
CAATTCATTACGTTAAGATATGAAAAAACCAAAAGTTATATGCTTACCAATGCCATATCCAAAGAGATTAATAACGGTC
ACAAACAAGAAAATTAACAGAATGAAAGAATAAATAAGATTAAAACTTTGCTACGCGATAACGATTTGAACTGAGATCTTXXXXXXXXXXT
CAATTCATTACGTTAAGATATGAAAAAACCAAAAGTTATATGCTTACCAATGCCATATCCAAAGAGATTAATAACGGTC
>Marker31803 Chr4 SNP_site:22892831 Ref_Type:C
ACTTTTAATCAATGTTTTTTTAGCATATTTTTACAGAGCTCGAAGTCTTGAAGTATAATCACCCATTCACTCTACCATTTXXXXXXXXXXT
CAGAATAACCAAAAAACAATGAACAAAACAAAAACTCATCCAAGATTGAATTAACCTCTTTATTGCATTAAAACAAGTA
ACTTTTAATCAATGTTTTTTTAGCATATTTTTACAGAGCTCGAAGTCTTGAAGTATAATCACCCATTCACTCTACCATTTXXXXXXXXXXT
CAGAATAACCAAAAAACAATGAACAAAACAAAAATTCATCCAAGATTGAATTAACCTCTTTATTGCATTAAAACAAGTA
>Marker318357 Chr1 SNP_site:19520576 Ref_Type:C
CACCTTGTAGATTTTCAATGCCTACAAACAATTGATTACGTATTAAGATATTAGTGTGTTTTGATTTTCAATGCCTACAAXXXXXXXXXXT
GAACCAATTGCATTTCTAATAAAAAATAATC-AAAAGTAGAAACTTGAAAGAAAATTGTTTTGAGTTTTTTGAAAAATGT
CACCTTGTAGATTTTCAATGCCTACAAACAATTGATTACGTATTAAGATATTAGTGTGTTTTGATTTTGAATGCCTACAAXXXXXXXXXXT

Create PDF with GO2PDF for free, if you wish to

GAACCAATTGCATTTCTAAT-AAAAATAATCAAAAAGTAGAAACTTGAAAGAAAATTGTTTTGAGTTTTTTGAAAAATGT

>Marker318361 Chr1 SNP_site:7520677 Ref_Type:C

CACTCCAATTGATCAAATATATCAACTAAACAAATTATATAGAGATATTTGGTCCATCAACTTCATTAGTATTAAATAACXXXXXXXXXXXA

AAATCTAACCTATTAACTCAATTGGATTTAGATATTTTGATTGTAACCTATCAACTCGCGACTTTAACTCAATTTAGTA

CACTCCAATTGATCAAATATATCAACTAAACAAATTATATAGAGATATTTGGTCCATCAACTTCATTAGTATTAAATAACXXXXXXXXXXXA

AAATCTAACCTATTAACTCAATTGGATTTAGATATTTTGATTGTAACCTATCAACTCGTGACTTTAACTCAATTTAGTA

>Marker319000 Chr1 SNP_site:13799483 Ref_Type:T

CACAAATAAATGTGATTAACACTTTAGGATTCGTTACGAAATGTAATTGTATTGCAATGTTATAAAATAGAAAACTCAAAXXXXXXXXXXXG

TGATATATTTGTTGTTTCCACCGTAAAATATTGTTGTTCTTATCATGATTGCATTATATTATTTTTCTTCAAAACGGTC

CACAAATAAATGTGATTAACACTTTAGGATTCGTTATGAAATGTAATTGTATTGCAATGTTATAAAATAGAAAACTCAAAXXXXXXXXXXXG

TGATATATTTGTTGTTTCCACGGTAAAATATTGTTGTTCTTATCATGATTGCATTATATTATTTTTCTTCAAAACGGTC

>Marker319000 Chr1 SNP_site:13799651 Ref_Type:G

CACAAATAAATGTGATTAACACTTTAGGATTCGTTACGAAATGTAATTGTATTGCAATGTTATAAAATAGAAAACTCAAAXXXXXXXXXXXG

TGATATATTTGTTGTTTCCACCGTAAAATATTGTTGTTCTTATCATGATTGCATTATATTATTTTTCTTCAAAACGGTC

CACAAATAAATGTGATTAACACTTTAGGATTCGTTATGAAATGTAATTGTATTGCAATGTTATAAAATAGAAAACTCAAAXXXXXXXXXXXG

TGATATATTTGTTGTTTCCACGGTAAAATATTGTTGTTCTTATCATGATTGCATTATATTATTTTTCTTCAAAACGGTC

>Marker319607 Chr1 SNP_site:795674 Ref_Type:A

ACTGAAGTTATTGCCACAGTTAAGGTATGAAATGTTGAGCTTCTTTCTCTTTGAACTATGCTATTCACTACATTTTTACAXXXXXXXXXXXT

CCAATGCAACCTGACAAAGGGAAGGTCTCGATCTCTGTTGATTGTAGTCCGACTGCAGAGCCGGCATTTGAGGTGGGGT

ACTGAAGTTATTGCCACAGTTAAGGTATGAAATGTTGAGCTTCTTTCTCTTTGAACTATGCTTTTCACTACATTTTTACAXXXXXXXXXXXT

CCAATGCAACCTGACAAAGGGAAGGTCTCGATCTCTGTTGATTGTAGTCCGACTGCAGAGCCGGCATTTGAGGTGGGGT

>Marker319654 Chr1 SNP_site:12598044 Ref_Type:C

GACAGTGATAGAAAGAAGTATCAACAATACAGATAACTGTGACATTGAAATATTTAAAACTAGGTAGCAGCTGTCAGTGCXXXXXXXXXXXA

TATCCCAAGAACAACCAAAAGTTAAAAACTTTCATTTGATTATGAATTCAGCTTACGAGAAATTGAGAAAGAGGAAGTA

GACAGTGATAGAAAGAAGTATCAACAATACAGATAACTGTGACATTGAAATATTTAAAACTAGGTAGCAGCTGTCAGTGCXXXXXXXXXXXA

TATGCCAAGAACAACCAAAAGTTAAAAACTTTCATTTGATTATGAATTCAGCTTACGAGAAATTGAGAAAGAGGAAGTA

>Marker320100 Chr1 SNP_site:12861440 Ref_Type:C

ACTAAATAATGCTTCTTAAAACATAAAAATCACCTGCCTTCTCCTTAAACATGAAGTTAGTAAAAGGTTAGAATTAATACXXXXXXXXXXXA

ATCTTTATTGAGGACTGTAGAAGTTTGGCTTATGAACAGGAGATTGAAGGAGTTTATTCCCCCCTCCCCCCAAGATGTA

ACTAAATAATGCTTTTTAAAACATAAAAATCACCTGCCTTCTCCTTAAACATGAAGTTAGTAAAAGGTTAGAATTAATACXXXXXXXXXXXA

ATCTTTATTGAGGACTGTAGAAGTTTGGCTTATGAACAGGAGATTGAAGGAGTTTATTCCCCCCTCCCCCCAAGATGTA

>Marker320187 Chr1 SNP_site:24674268 Ref_Type:C

CACTATGATATTTGGGGGCCTTGGGGCCTTTTCAAACACCCACACATGCTGGTTGTTCCTATTTTGTTACTTTGGTTGATXXXXXXXXXXXA

GTTTTCAAAAGTTATTAAAACCTTTAGGACTTATAATGCCCATGAGCTTGGTTTCAAAGAATTTCTTGCTTCTATTGGT

CACTATGATATTTGGGGGCCTTGGGGCCTTTTCAAACACCCACACATGTTGGTTGTTCCTATTTTGTTACTTTGGTTGATXXXXXXXXXXXA

GTTTTCAAAAGTTATTAAAACATTTAGGACTTATAATGCCCATGAGCTTGGTTTCAAAGAATTTCTTGCTTCTATTGGT

>Marker320187 Chr1 SNP_site:24674413 Ref_Type:C

CACTATGATATTTGGGGGCCTTGGGGCCTTTTCAAACACCCACACATGCTGGTTGTTCCTATTTTGTTACTTTGGTTGATXXXXXXXXXXXA

GTTTTCAAAAGTTATTAAAACCTTTAGGACTTATAATGCCCATGAGCTTGGTTTCAAAGAATTTCTTGCTTCTATTGGT

CACTATGATATTTGGGGGCCTTGGGGCCTTTTCAAACACCCACACATGTTGGTTGTTCCTATTTTGTTACTTTGGTTGATXXXXXXXXXXXA

GTTTTCAAAAGTTATTAAAACATTTAGGACTTATAATGCCCATGAGCTTGGTTTCAAAGAATTTCTTGCTTCTATTGGT

>Marker320278 Chr1 SNP_site:14323229 Ref_Type:A

CACTTAAAATCAACCCGGCCAACATGAAATTCACAACTTACCCAAGTTGAATGAAGCATAAACTTCCAATGAAGCTTGATXXXXXXXXXXXA

ATAAACTTACAACAAGACAGCGCAGCGCGGATAGATGGAATCGGTGTGTGCAAAATAGATATGAACGAAAATGGATGTG

Create PDF with GO2PDF for free, if you wish to

CACTTAAAATCAACCCGGCCAACGTGAAATTCACAACTTACCCAAGTTGAATGAAGCATAAACTTCCAATGAAGCTTGATXXXXXXXXXXA
ATAAACTTACAACAAGACAGCGCAGCGCGGATAGATGGAATCGGTGTGTGCAAAATAGATATGAACGAAAATGGATGTG
>Marker320417 Chr1 SNP_site:8133023 Ref_Type:G
GACTTAATTATTAACCCTACTAATACTACAATTTTAGTTAGGTTTCAAGTTTATAATTTATTTGGTGATTAGAAAGTATTXXXXXXXXXXT
CTCCAGCTATATTTAAAGCAATTAACTATAGAAATAACTCAAGCACACAAAAGTAATAAATAACACATAAGACTTTGTT
GACTTAATTATTAACCCTACTAATACTACAATTTTAGTTAGGTTTCAAGTTTATAATTTATTTGGTGATTAGAAAGTATTXXXXXXXXXXT
CTCCGGCTATATTTAAAGCAATTAACTATAGAAATAACTCAAGCACACAAAAGTAATAAATAACACATAAGACTTTGTT
>Marker320680 Chr1 SNP_site:9090844 Ref_Type:T
ACTTTTAATTTTTTAGTAAAATTACTTCTCAATTTATTTCGCAAATTTGGAACCTTTCTCACTTTTAATCACACTTTAATXXXXXXXXXXT
CCATTAAATAAACCTTTCATCTTTAATTTTAACTTTCGTATTAGAACTTGGATTGTATTTCATTTGATTAAACAAAGTG
ACTTTTAATTTTTTAGTAAAATTACTTCTCAATTTATTTCTGAAATTTGGAACCTTTCTCACTTTTAATCACACTTTAATXXXXXXXXXXT
CCATTAAATAAACCTTTCATCTTTAATTTTAACTTTCGTATTAGAACTTGGATTGTATTTCATTTGATTAAACAAAGTG
>Marker320680 Chr1 SNP_site:9090845 Ref_Type:G
ACTTTTAATTTTTTAGTAAAATTACTTCTCAATTTATTTCGCAAATTTGGAACCTTTCTCACTTTTAATCACACTTTAATXXXXXXXXXXT
CCATTAAATAAACCTTTCATCTTTAATTTTAACTTTCGTATTAGAACTTGGATTGTATTTCATTTGATTAAACAAAGTG
ACTTTTAATTTTTTAGTAAAATTACTTCTCAATTTATTTCTGAAATTTGGAACCTTTCTCACTTTTAATCACACTTTAATXXXXXXXXXXT
CCATTAAATAAACCTTTCATCTTTAATTTTAACTTTCGTATTAGAACTTGGATTGTATTTCATTTGATTAAACAAAGTG
>Marker321070 Chr1 SNP_site:16816186 Ref_Type:A
ACCAATATTCGTTATATTCTCCACAACCACGCTTGTGACCATGCACGTAAGAAGATGATGGATCGCTCCCTAAAACATAGXXXXXXXXXXA
GCATCATCATCATTATACTAACGTAACCAGGCCTACTTTGTGTTTGTGTGATGGGAGGCAATTATTTCACGTGTTGGTC
ACCAATATTCGTTATGTTCTCCACAACCACGCTTGTGACCATGCACGTAAGAAGATGATGGATCGCTCCCTAACACATAGXXXXXXXXXXA
GCATCATCATCATTAGACTAACGTAACCAGGCCTACTTTGTGTTTGTGTGATGGGAGGCAATTATTTCACGTGTTGGTC
>Marker321070 Chr1 SNP_site:16816244 Ref_Type:A
ACCAATATTCGTTATATTCTCCACAACCACGCTTGTGACCATGCACGTAAGAAGATGATGGATCGCTCCCTAAAACATAGXXXXXXXXXXA
GCATCATCATCATTATACTAACGTAACCAGGCCTACTTTGTGTTTGTGTGATGGGAGGCAATTATTTCACGTGTTGGTC
ACCAATATTCGTTATGTTCTCCACAACCACGCTTGTGACCATGCACGTAAGAAGATGATGGATCGCTCCCTAACACATAGXXXXXXXXXXA
GCATCATCATCATTAGACTAACGTAACCAGGCCTACTTTGTGTTTGTGTGATGGGAGGCAATTATTTCACGTGTTGGTC
>Marker321070 Chr1 SNP_site:16816378 Ref_Type:T
ACCAATATTCGTTATATTCTCCACAACCACGCTTGTGACCATGCACGTAAGAAGATGATGGATCGCTCCCTAAAACATAGXXXXXXXXXXA
GCATCATCATCATTATACTAACGTAACCAGGCCTACTTTGTGTTTGTGTGATGGGAGGCAATTATTTCACGTGTTGGTC
ACCAATATTCGTTATGTTCTCCACAACCACGCTTGTGACCATGCACGTAAGAAGATGATGGATCGCTCCCTAACACATAGXXXXXXXXXXA
GCATCATCATCATTAGACTAACGTAACCAGGCCTACTTTGTGTTTGTGTGATGGGAGGCAATTATTTCACGTGTTGGTC
>Marker321528 Chr1 SNP_site:28383882 Ref_Type:C
ACTCCTCGCCGGATAGCCATAGGTCCCAAGAGTGTAATGATTTGATACCTATTTGGTATAAACTACCAAAGTGATTTTTGXXXXXXXXXXA
CAGTAATTAACAATCAACCTTTACCTTTAAATATAGTATATAACAATACAGCTACTATAGCTTTATTTACAGCTTTGTT
ACTCCTCGCCGGATAGCCATAGGTCCCAAGAGTGTAATGCTTTGATACCTATTTGGTATAAACTACCAAAGTGATTTTTGXXXXXXXXXXA
CAGTAATTAACAATCAACCTTTACCTTTAACTATAGTATATAACAATACAGCTACTATAGCTTTATTTACAGCTTTGTT
>Marker321528 Chr1 SNP_site:28384056 Ref_Type:C
ACTCCTCGCCGGATAGCCATAGGTCCCAAGAGTGTAATGATTTGATACCTATTTGGTATAAACTACCAAAGTGATTTTTGXXXXXXXXXXA
CAGTAATTAACAATCAACCTTTACCTTTAAATATAGTATATAACAATACAGCTACTATAGCTTTATTTACAGCTTTGTT
ACTCCTCGCCGGATAGCCATAGGTCCCAAGAGTGTAATGCTTTGATACCTATTTGGTATAAACTACCAAAGTGATTTTTGXXXXXXXXXXA
CAGTAATTAACAATCAACCTTTACCTTTAACTATAGTATATAACAATACAGCTACTATAGCTTTATTTACAGCTTTGTT
>Marker321924 Chr1 SNP_site:25090593 Ref_Type:T
CACCTCATCCTTTTCCACAGCTGAAACCATCTTTGTCAATTTCCACATTCCCTCCAACTCCAACTCCTTGCCCAGTGTCCXXXXXXXXXXT

Create PDF with GO2PDF for free, if you wish to

TTGGACTGATCCCATTTCCTAATTGTAACAGCTTTAATACCAAGATAACAGAATTTGATGCTACTAAGAACAAAAGGGT
CACCTCATCCTTTTCCACAGCTGAAACCATCTTTGTCAATTTCCACATTCCCTCCAACTCCAACTCCTTGCCCAGTGTCCXXXXXXXXXXT
TTGGACTGATCCCATTTCCTAATTGTAACAGCTTTAATACCAAGATAACAGAATTTGATGTTACTAAGAACAAAAGGGT
>Marker321979 Chr1 SNP_site:2461656 Ref_Type:A
AACAGTAATTTCTGTGGTAAGAAATTTGAATCACACACTTTTTCATCAATAATACCTACTCTACATATGTGGAATTTGAAXXXXXXXXXXT
ATTTACAAAGGTGGGATATGGATTATGAGGGTAAATGCATATTCATATCATAAACAAAACAAAACTATGCATCATTGGT
AACAGTAATTTCTGTGGTAAGAAATTTGAATCACACACTTTTTCATCAATAATACCTACTCTACATATGTGGAATTTGAAXXXXXXXXXXT
ATTTACAAAGGTGGGATATGGATTATGGGGGTAAATGCATATTCATATCATAAACAAAACAAAACTATGCATCATTGGT
>Marker322036 Chr1 SNP_site:10069855 Ref_Type:G
TACTTTTGTCGAACAATATGATTGTTGATGTCAAATTTTGGTTAAAAAGATAAATTTACATGACTCTTACGTGACACCAAXXXXXXXXXXG
ATTTAATATATGAGTTTTCAAATCTTAGATAAGATAGGTTATCTAACAAGCTGAGTTGGGCCTTCTAATGGAGAAGGTG
TACTTTTGTCGAACAATATGATTGTTGATGTCAAATTTTGGTTAAAAGGATAAATTTACATGACTCTTACGTGACATCAAXXXXXXXXXXG
ATTTAATATATGAGTTTTCAAATCTTAGATAAGATAGGTTATCTAACAAGCTGAGTTGGGCCTTCTAATGGAGAAGGTG
>Marker322036 Chr1 SNP_site:10069884 Ref_Type:T
TACTTTTGTCGAACAATATGATTGTTGATGTCAAATTTTGGTTAAAAAGATAAATTTACATGACTCTTACGTGACACCAAXXXXXXXXXXG
ATTTAATATATGAGTTTTCAAATCTTAGATAAGATAGGTTATCTAACAAGCTGAGTTGGGCCTTCTAATGGAGAAGGTG
TACTTTTGTCGAACAATATGATTGTTGATGTCAAATTTTGGTTAAAAGGATAAATTTACATGACTCTTACGTGACATCAAXXXXXXXXXXG
ATTTAATATATGAGTTTTCAAATCTTAGATAAGATAGGTTATCTAACAAGCTGAGTTGGGCCTTCTAATGGAGAAGGTG
>Marker322040 Chr1 SNP_site:7408424 Ref_Type:C
AACAATTACTTCTAATTTGAGAAACTTGCAAATAAAAACTTGTAATATTGATAAACAGAAAGTTATCAACTGATAGCAACXXXXXXXXXXA
GCTTTTAATTTGAGAAGTTTAAAAAGAAACGTTTAAAATGACTACACATGAGTTTTTTAGTCTTTTATTAATTTTTGTA
AACAATTACTTCTAATTTGAGAAACTTGTAAATAAAAACTTGTAATATTGATAAACAGAAAGTTATCAACTGATAGCAACXXXXXXXXXXA
GCTTTTAATTTGAGAAGTTTAAAAAGAAACGTTTAAAATGACTACACATGAGTTTTTTAGTCTTTTATTAATTTTTGTA
>Marker322318 Chr1 SNP_site:18664144 Ref_Type:T
ACCAATTTGTTTCAATCATTCAATTTCAGCACGAGCTTACTTGCCCTGTGAAGACGGATTCTTCTTTCTGGACCTTAGGTXXXXXXXXXXA
GAGGATGATATAATGCACATTACACTGCAGAAGAGGGACAAGGGTCAGACATGGGCTTCCCCAATACAGGGTCAGGGTC
ACCTATTTGTTTCAATCATTCAATTTCAGCACGAGCTTACTTGCCCCGTGAAGACGGATTCTTCTTTCTGGACCTTAGGTXXXXXXXXXXA
GAGGATGATATAATGCACATTACACTGCAGAAGAGGGACAAGGGTCAGACATGGGCTTCCCCAATACAGGGTCAGGGTC
>Marker322318 Chr1 SNP_site:18664187 Ref_Type:T
ACCAATTTGTTTCAATCATTCAATTTCAGCACGAGCTTACTTGCCCTGTGAAGACGGATTCTTCTTTCTGGACCTTAGGTXXXXXXXXXXA
GAGGATGATATAATGCACATTACACTGCAGAAGAGGGACAAGGGTCAGACATGGGCTTCCCCAATACAGGGTCAGGGTC
ACCTATTTGTTTCAATCATTCAATTTCAGCACGAGCTTACTTGCCCCGTGAAGACGGATTCTTCTTTCTGGACCTTAGGTXXXXXXXXXXA
GAGGATGATATAATGCACATTACACTGCAGAAGAGGGACAAGGGTCAGACATGGGCTTCCCCAATACAGGGTCAGGGTC
>Marker322333 Chr1 SNP_site:25757039 Ref_Type:C
TACCTTGTGATAAGATCTTCAAACTTAGTTCCACTATCTTTTAGCCATTTGGAAAAGTTGCGTGATGCTGGACTTTGATGXXXXXXXXXXA
TGTCTTTCGAGTGCGCGTCGATCTTTGATAGATAGTTTGTTCGCAGTGCATAGTTTCTTACTGCTTGAAGCGAGATGTT
TACCTTGTGATAAGATCTTCAAACTTAGTTCCACTATCTTTTAGCCATTTGGAAAAGTTGCGTGATGCTGGACTTTGATGXXXXXXXXXXA
TGTCTTTTGAGTGCGCGTCGATCTTTGATAGATAGTTTGTTCGCAGTGCATAGTTTCTTACTGCTTGAAGCGAGATGTT
>Marker322422 Chr1 SNP_site:7063929 Ref_Type:C
AACCTAGTTCGATGAATTTGACCTATGTCTAGGGAGTTTCTCCGCTCAAATAAGAAATATTATTAAGATTCAAACGAGTAXXXXXXXXXXC
AACTCTTATAAATTCATCAACTGCAATTTAACTATTTCAGTATTCTTTCATATTTTTTATCAACAAGTGCAGCGTAAGT
AACCTAGTTCGATGAATTTGACCTATGTCTAGGGAGTTTCTCCGCTCAAATAAGAAATATTATTAAGATTCAAACGAGTAXXXXXXXXXXC
AACTCTTATAAATTCATCAATTGCAATTTAACTATTTCAGTCTTCTTTCATATTTTTTATCAACAAGTGCAGCGTAAGT
>Marker322422 Chr1 SNP_site:7063950 Ref_Type:A

Create PDF with GO2PDF for free, if you wish to

AACCTAGTTCGATGAATTTGACCTATGTCTAGGGAGTTTCTCCGCTCAAATAAGAAATATTATTAAGATTCAAACGAGTAXXXXXXXXXXXC
AACTCTTATAAATTCATCAACTGCAATTTAACTATTTCAGTATTCTTTCATATTTTTTATCAACAAGTGCAGCGTAAGT
AACCTAGTTCGATGAATTTGACCTATGTCTAGGGAGTTTCTCCGCTCAAATAAGAAATATTATTAAGATTCAAACGAGTAXXXXXXXXXXXC
AACTCTTATAAATTCATCAATTGCAATTTAACTATTTCAGTCTTCTTTCATATTTTTTATCAACAAGTGCAGCGTAAGT
>Marker322442 Chr1 SNP_site:17155082 Ref_Type:T
AACCACTCTTTCCTTCCTCTTTCTAGTTATATGGATCGGCTCTGGCGACTTGTATCCAAGTCCTTTTCTCAACACAGGTAXXXXXXXXXXXT
CATTCACCTTTGGGGCAAGTTTGCTTCCATCATATCTATCTTTACCTTCTATTTCGTTATCTTAGTTAGTAATGTAGTG
AACCACTTTTTCCTTCCTCTTTCTAGTTATATGGATCGGCTCTGGCGACTTGTATCCAAGTCCTTTTCTCAACACGGGTAXXXXXXXXXXXT
CATTCACCTTTGGGGCAAGTTTGCTTCCATCATATCTATCTTTACCTTCCATTTCGTTATCTTAGTTAGTAATGTAGTG
>Marker322442 Chr1 SNP_site:17155150 Ref_Type:G
AACCACTCTTTCCTTCCTCTTTCTAGTTATATGGATCGGCTCTGGCGACTTGTATCCAAGTCCTTTTCTCAACACAGGTAXXXXXXXXXXXT
CATTCACCTTTGGGGCAAGTTTGCTTCCATCATATCTATCTTTACCTTCTATTTCGTTATCTTAGTTAGTAATGTAGTG
AACCACTTTTTCCTTCCTCTTTCTAGTTATATGGATCGGCTCTGGCGACTTGTATCCAAGTCCTTTTCTCAACACGGGTAXXXXXXXXXXXT
CATTCACCTTTGGGGCAAGTTTGCTTCCATCATATCTATCTTTACCTTCCATTTCGTTATCTTAGTTAGTAATGTAGTG
>Marker322442 Chr1 SNP_site:17155352 Ref_Type:C
AACCACTCTTTCCTTCCTCTTTCTAGTTATATGGATCGGCTCTGGCGACTTGTATCCAAGTCCTTTTCTCAACACAGGTAXXXXXXXXXXXT
CATTCACCTTTGGGGCAAGTTTGCTTCCATCATATCTATCTTTACCTTCTATTTCGTTATCTTAGTTAGTAATGTAGTG
AACCACTTTTTCCTTCCTCTTTCTAGTTATATGGATCGGCTCTGGCGACTTGTATCCAAGTCCTTTTCTCAACACGGGTAXXXXXXXXXXXT
CATTCACCTTTGGGGCAAGTTTGCTTCCATCATATCTATCTTTACCTTCCATTTCGTTATCTTAGTTAGTAATGTAGTG
>Marker322788 Chr1 SNP_site:27080529 Ref_Type:C
TACATACAATGCCATCATTCGAGGTATTTGCAAGGAAGGAATGGAGGATCGAGCCTTGGATTTTGTTCGACATTTATCAGXXXXXXXXXXXA
TTTTAATTAGTTCGTTTTGTCGCGAAGGGAGAGTAAGGGAAGCCGTGAATGTGTTGGAGGTGATGAAGGAGAAAGGGTT
TACATACAATGCTATCATTCGAGGTATTTGCAAGGAAGGAATGGAGGATCGAGCCTTGGATTTTGTTCGACATTTATCAGXXXXXXXXXXXA
TTTTAATTAGTTCGTTTTGTCGCGAAGGGAGAGTAAGGGAAGCCGTGAATGTGTTGGAGATGATGAAGGAGAAAGGGTT
>Marker322788 Chr1 SNP_site:27080797 Ref_Type:G
TACATACAATGCCATCATTCGAGGTATTTGCAAGGAAGGAATGGAGGATCGAGCCTTGGATTTTGTTCGACATTTATCAGXXXXXXXXXXXA
TTTTAATTAGTTCGTTTTGTCGCGAAGGGAGAGTAAGGGAAGCCGTGAATGTGTTGGAGGTGATGAAGGAGAAAGGGTT
TACATACAATGCTATCATTCGAGGTATTTGCAAGGAAGGAATGGAGGATCGAGCCTTGGATTTTGTTCGACATTTATCAGXXXXXXXXXXXA
TTTTAATTAGTTCGTTTTGTCGCGAAGGGAGAGTAAGGGAAGCCGTGAATGTGTTGGAGATGATGAAGGAGAAAGGGTT
>Marker322919 Chr1 SNP_site:28618769 Ref_Type:A
ACATGTGAAAAAGAGAAGGAATCTTTAAATATTTTTGAATTAGAACATTGTAAGAATATTAAAGTTGTGTTCTCTCCACAXXXXXXXXXXXA
CAAGAGTATAGGGTTAGATGGTTAATTTATATCTACCAAGCTAATTGTTAAACATATTTAAATAATACATGAAAGTGTG
ACATGTGAAAAAGAGAAGGAATCTTTAAATATTTTTGAATTAGAACATTGTAAGAATATTAAAGTTGTGTTCTCTCCACAXXXXXXXXXXXT
CAAGAGTATAGGGTTAGATGGTTAATTTATATCTACCAAGCTAATTGTTAAACATATTTAAATAATACATGAAAGTGTG
>Marker322944 Chr1 SNP_site:6578826 Ref_Type:A
AACCTGCAGTGCTAGAAGAGATCTCTCATCATGGATATGCCATGAGACATCTCCCCCTTTGGCCTTGAAGGCTTTCATATXXXXXXXXXXXT
CCAGGAGCAAGCCCAGCAACATCAGCAACAACAAGTTTCTCGAGGAAAGCGATAGAATCCTTCCCCTTAAGGCTCAGTC
AACCTGCAGTGCTAGAAGAGATCTCTCATCGTGGATATGCCACGAGACATCTCCCCCTTTGGCCTTGAAGGCTTTCATATXXXXXXXXXXXT
CCAGGAGCAAGCCCAGCAACATCAGCAACAACAAGTTTCTCGAGGAAAGCGATAGAATCCTTCCCCTTAAGGCTCAGTC
>Marker322944 Chr1 SNP_site:6578838 Ref_Type:T
AACCTGCAGTGCTAGAAGAGATCTCTCATCATGGATATGCCATGAGACATCTCCCCCTTTGGCCTTGAAGGCTTTCATATXXXXXXXXXXXT
CCAGGAGCAAGCCCAGCAACATCAGCAACAACAAGTTTCTCGAGGAAAGCGATAGAATCCTTCCCCTTAAGGCTCAGTC
AACCTGCAGTGCTAGAAGAGATCTCTCATCGTGGATATGCCACGAGACATCTCCCCCTTTGGCCTTGAAGGCTTTCATATXXXXXXXXXXXT
CCAGGAGCAAGCCCAGCAACATCAGCAACAACAAGTTTCTCGAGGAAAGCGATAGAATCCTTCCCCTTAAGGCTCAGTC

Create PDF with GO2PDF for free, if you wish to

>Marker323131 Chr1 SNP_site:27394197 Ref_Type:A
TACAAAATGGTCATTATGTAAGAAAATTGTTCTAATTGTGTATGTAGATAATATTCTCTTGTCAGGAGATGATACGACTTXXXXXXXXXXA
AAATCAAGGGAAAGGATCTTTGTTACACAGTAGAAATACACCCTTGGCCTGTTAAAAGGAACAAGTATGATTGGATGTA
TACAAAATGGTCATTATGTAAGAAAATTGTTCTAATTGTGTATGTAGATAATATTCTCTTGTCAGGTGATGATACGACTTXXXXXXXXXXA
AAATCAAGGGAAAGGATCTTTGTTACACAGCAGAAATACACCCTTGGCCTGTTAAAAGGAACAAGTATGATTGGATGTA
>Marker323131 Chr1 SNP_site:27394328 Ref_Type:T
TACAAAATGGTCATTATGTAAGAAAATTGTTCTAATTGTGTATGTAGATAATATTCTCTTGTCAGGAGATGATACGACTTXXXXXXXXXXA
AAATCAAGGGAAAGGATCTTTGTTACACAGTAGAAATACACCCTTGGCCTGTTAAAAGGAACAAGTATGATTGGATGTA
TACAAAATGGTCATTATGTAAGAAAATTGTTCTAATTGTGTATGTAGATAATATTCTCTTGTCAGGTGATGATACGACTTXXXXXXXXXXA
AAATCAAGGGAAAGGATCTTTGTTACACAGCAGAAATACACCCTTGGCCTGTTAAAAGGAACAAGTATGATTGGATGTA
>Marker323148 Chr1 SNP_site:7750412 Ref_Type:G
TACATACCTTGTATTTGCACTAATGTGATAAGAATAACTATAACTATAGATAAGACCCACAAATATGTTATAATGTGAAAXXXXXXXXXXT
AGTGAAAAATACCTTAACTATCTATTTTGTTGGTGCATCATCAATTGTCAAATTAAGAGGTAGGCACATCACTCTTGTA
TACATACCTTGTATTTGCACTAATGTGATAAGAATAACTATAGCTATAGATAAGACCCACAAATATGTTATGATGTGAAAXXXXXXXXXXT
AGTGAAAAATACCTTAACTATCTATTTTGTTGGTGCATCATCAATTGTCAAATTAAGAGGTAGGCACATCGCTCTTGTA
>Marker323148 Chr1 SNP_site:7750441 Ref_Type:A
TACATACCTTGTATTTGCACTAATGTGATAAGAATAACTATAACTATAGATAAGACCCACAAATATGTTATAATGTGAAAXXXXXXXXXXT
AGTGAAAAATACCTTAACTATCTATTTTGTTGGTGCATCATCAATTGTCAAATTAAGAGGTAGGCACATCACTCTTGTA
TACATACCTTGTATTTGCACTAATGTGATAAGAATAACTATAGCTATAGATAAGACCCACAAATATGTTATGATGTGAAAXXXXXXXXXXT
AGTGAAAAATACCTTAACTATCTATTTTGTTGGTGCATCATCAATTGTCAAATTAAGAGGTAGGCACATCGCTCTTGTA
>Marker323148 Chr1 SNP_site:7750624 Ref_Type:A
TACATACCTTGTATTTGCACTAATGTGATAAGAATAACTATAACTATAGATAAGACCCACAAATATGTTATAATGTGAAAXXXXXXXXXXT
AGTGAAAAATACCTTAACTATCTATTTTGTTGGTGCATCATCAATTGTCAAATTAAGAGGTAGGCACATCACTCTTGTA
TACATACCTTGTATTTGCACTAATGTGATAAGAATAACTATAGCTATAGATAAGACCCACAAATATGTTATGATGTGAAAXXXXXXXXXXT
AGTGAAAAATACCTTAACTATCTATTTTGTTGGTGCATCATCAATTGTCAAATTAAGAGGTAGGCACATCGCTCTTGTA
>Marker323950 Chr1 SNP_site:6831301 Ref_Type:C
CACAATCAAACAAGATGATGGTGCATTGCCTCAAAACTTGAACAAAGCTGATATTGTTCTTGCTGGGGTATCTCGAACAGXXXXXXXXXXT
GACTATAAATCCAATTGTGTTGCAAACAATAAGAAGAGCAAGAGCAAAGAGTTTGGGATTTAGTGAAGAAATGAGAAGT
CACAATCAAACAAGATGATGGTGCATTGCCTCAAAACTTGAACAAAGCTGATATTGTTCTTGCTGGGGTATCTCGAACAGXXXXXXXXXXT
GACTATAAATCCAATTGTGTTGCAAACAATCAGAAGAGCAAGAGCAAAGAGTTTGGGATTTAGTGAAGAAATGAGAAGT
>Marker323973 Chr1 SNP_site:25612382 Ref_Type:T
AACCTAGAGAATCTGTAAAGATAGTTTAATGAGATGGTTGAGGCAGTAAAAGCATGAATGAATTAAGTTGCATTAAATAAXXXXXXXXXXA
ATCATTAAACGCCACACTGGATCTCCTTCTGGGATCCAAATGACTAAACCTAACTAAACAAGGTATATTTAGAAGTGTT
AACCTAGAGAATCTGTAAAGATAGTTTAATGAGATGGTTGAGGCAGTAAAAGCATGAATGAATTAAGTTGCATTAAATAAXXXXXXXXXXA
ATCATTAAACGCCACACTGGATCTCCTTCTGGGATCCAAATGACTAAATCTAACTAAACAAGGTATATTTAGAAGTGTT
>Marker324914 Chr1 SNP_site:1520948 Ref_Type:G
AACTCCCGTGATTAATTATTTAAAATGAAATCAAGCTTAATTCAACAAATTAAGAGAGGAATAGAAAGCCACGAAATTACXXXXXXXXXXT
TTTTTCCTTAGTCAAATTGATGAATGATGAGATTTTTTTTGGAGAATAATCAGTAAATGATCGAATTAACGGATCATGT
AACTCCGGTGATTAATTATTTAAAATGAAATCAAGCTTAATTCAACAAATTAAGAGAGGAATAGAAAGCCACGAAATTACXXXXXXXXXXT
TTTTTCCTTAGTCAAATTGATGAATGATGAGATTTTTTTTGGAGAATAATCGGTAAATGATGGAATTAACGGATCATGT
>Marker324914 Chr1 SNP_site:1521179 Ref_Type:G
AACTCCCGTGATTAATTATTTAAAATGAAATCAAGCTTAATTCAACAAATTAAGAGAGGAATAGAAAGCCACGAAATTACXXXXXXXXXXT
TTTTTCCTTAGTCAAATTGATGAATGATGAGATTTTTTTTGGAGAATAATCAGTAAATGATCGAATTAACGGATCATGT
AACTCCGGTGATTAATTATTTAAAATGAAATCAAGCTTAATTCAACAAATTAAGAGAGGAATAGAAAGCCACGAAATTACXXXXXXXXXXT

Create PDF with GO2PDF for free, if you wish to

TTTTTCCTTAGTCAAATTGATGAATGATGAGATTTTTTTTGGAGAATAATCGGTAAATGATGGAATTAACGGATCATGT
>Marker324914 Chr1 SNP_site:1521189 Ref_Type:G
AACTCCCGTGATTAATTATTTAAAATGAAATCAAGCTTAATTCAACAAATTAAGAGAGGAATAGAAAGCCACGAAATTACXXXXXXXXXXT
TTTTTCCTTAGTCAAATTGATGAATGATGAGATTTTTTTTGGAGAATAATCAGTAAATGATCGAATTAACGGATCATGT
AACTCCGGTGATTAATTATTTAAAATGAAATCAAGCTTAATTCAACAAATTAAGAGAGGAATAGAAAGCCACGAAATTACXXXXXXXXXXT
TTTTTCCTTAGTCAAATTGATGAATGATGAGATTTTTTTTGGAGAATAATCGGTAAATGATGGAATTAACGGATCATGT
>Marker324932 Chr1 SNP_site:28822883 Ref_Type:C
CACAAAAGGGCCCTATAAAAGGGAATACAAAAGATGTTTCCAAATTGGAAATGAGAAAAAAGTAACTGTGGCTCTTGATAXXXXXXXXXXG
AATAATCTTGCATTTTTATCGTTCCAACCCTTCCACAAAATCACTGAAACAATATTTTTTCCCAAATACGTTCTTGGTT
CACAAAAGGGCCCTATAAAAGGGAATACAAAAGATGTTTCCAAATTGGAAATGAGAAAAAAGTAACTGTGGCTCTTGATAXXXXXXXXXXG
AATAATCTTGCATTTTTATCGTTCCAACCCTTCCACAAAATCATTGAAACAATATTTTTTCCCAAATACGTTCTTGGTT
>Marker324977 Chr1 SNP_site:18938666 Ref_Type:T
ACTGCAGTTAAAATATATATTAAAGGAAAAACACTGGAAGATGTGATGGGAAAGCACCTTTATACGCTCGTATTCTCGCAXXXXXXXXXXC
TTGTATGCAATGAGGACAAGTCAGACAATAGAGTTTGCTCAGTCAGTAAAGCACTTGAGCGTTCTGAGAATGCACCGTG
ACTGCAGTTAAAATATATATTAAAGGAAAAACACTGGTAGATGTGATGGGAAAGCACCTTTATACGCTCGTATTCTCGCAXXXXXXXXXXC
TTGTATGCAATGAGGACAAGTCAGACAATAGAGTTTGCTCGGTCAGTAAAGCACTTGAGCGTTCTGAGAATGCACCGTG
>Marker324977 Chr1 SNP_site:18938869 Ref_Type:G
ACTGCAGTTAAAATATATATTAAAGGAAAAACACTGGAAGATGTGATGGGAAAGCACCTTTATACGCTCGTATTCTCGCAXXXXXXXXXXC
TTGTATGCAATGAGGACAAGTCAGACAATAGAGTTTGCTCAGTCAGTAAAGCACTTGAGCGTTCTGAGAATGCACCGTG
ACTGCAGTTAAAATATATATTAAAGGAAAAACACTGGTAGATGTGATGGGAAAGCACCTTTATACGCTCGTATTCTCGCAXXXXXXXXXXC
TTGTATGCAATGAGGACAAGTCAGACAATAGAGTTTGCTCGGTCAGTAAAGCACTTGAGCGTTCTGAGAATGCACCGTG
>Marker32525 Chr4 SNP_site:13097234 Ref_Type:G
AACGTAATTGTTTAATTTATAGCTTGATGTATATTCTAGGGCTTAAATTCATAATATCATACAATGTATTGATATGTAAAXXXXXXXXXXT
TAAACTTACAAGTGTATGCATATTTTGGAATGAAAACTTAGATTGTTTAAATTTGAGATTTTGACTGATTAGGCAAGTA
AACGTAATTGTTTAATTTATAGCTTGATGTATATTCTAGGGCTTAAATTCATAATATCATACAGTGTATTGATATGTAAAXXXXXXXXXXT
TAAACTTACAAGTGTATGCATATTTTAGAATGAAAACTTAGATTGTTTAAATTTGAGATTTTGACTGATTAGGCAAGTA
>Marker32525 Chr4 SNP_site:13097407 Ref_Type:A
AACGTAATTGTTTAATTTATAGCTTGATGTATATTCTAGGGCTTAAATTCATAATATCATACAATGTATTGATATGTAAAXXXXXXXXXXT
TAAACTTACAAGTGTATGCATATTTTGGAATGAAAACTTAGATTGTTTAAATTTGAGATTTTGACTGATTAGGCAAGTA
AACGTAATTGTTTAATTTATAGCTTGATGTATATTCTAGGGCTTAAATTCATAATATCATACAGTGTATTGATATGTAAAXXXXXXXXXXT
TAAACTTACAAGTGTATGCATATTTTAGAATGAAAACTTAGATTGTTTAAATTTGAGATTTTGACTGATTAGGCAAGTA
>Marker326141 Chr1 SNP_site:23267360 Ref_Type:C
AACATTTTCCCTTTTCAATTCTTTTCTTTTTATATTCTACTTTGTGTCATCAATGAATCCTACCTTCACATTTTAAATTAXXXXXXXXXXA
ACGATCTAATTTATAATCATCCATCTATAAGAAATATAACTAATATTGTGAGTCGGCAGTGAAATTACAGGTTTGGGTT
AACATTTTCCCTTTTCAATTCTTTTCTTTTTATATTCTACTTTGTGTCATGAATGAATCCTACCTTCACATTTTAAATTAXXXXXXXXXXA
ACGATCTAATTTATAATCATCCATCTATAAGAAATATAACTAATATTGTGAGTCGGCAGTGAAATTACAGGTTTGGGTT
>Marker32628 Chr4 SNP_site:10878308 Ref_Type:T
AACAATTTGTTTAGATTTGACTATCCAATATTTTTACGATAGACATGAAAAGAAGTAATATTGTTGAAGATGGTAAAGAAXXXXXXXXXXT
TTTCTTAAATTGTTGTATGGACGGTAAATATTTTACCATTTTGTTATATTTATGAAAATTAATCTAATTTTTATTAGTA
AACAATTTGTTTAGATTTGACTATCTAATATTTTTACGATAGACATGAAAAGAAGTAATATTGTTGAAGATGGTAAAGAAXXXXXXXXXXT
TTTCTTAAATTGTTGTATGGACGGTAAATATTTTACCATTTTGTTATATTTATGAAAATTAATCTAATTTTTATTAGTA
>Marker326633 Chr1 SNP_site:10630397 Ref_Type:A
TACTCTTGCAGTGTCTCCTCTCAAGCAAACCAATATCTTCCCTTTCACTTTTGAATGATCCAATGTTTTTGGTTTGCAAAXXXXXXXXXXA
GAGAGTGAAAGCTGAGACGAACCATATAACGGTGACCATTTTGGAGCTCAACAGGAGCCTGAAACTCACGATCCATAGT

Create PDF with GO2PDF for free, if you wish to

TACTCTTGCAGTGTCTCCTCTCAAGCAAACCAATATCTTCCCTTTCACTTTTGAATGATCCAATGTTTTTGGTTTGCAAAXXXXXXXXXXA

GAGAGTGAAAGCTGAGACGAACCATATAACGGTGACCATTTTGGAGCTCAACAGGAGCCTGGAACTCACGATCCATAGT

>Marker326838 Chr1 SNP_site:25223312 Ref_Type:T

AACAATAACCAACCATTTTTTAAGCAAAATGCATAGTGATATAAAAATGATGAAACTTTGAAATAGTAGTGTCACCACATXXXXXXXXXXC

AAAAAATAAAGTTGAATTCGGAAGTAATTTGTGTATAGGGAAGGTATTAGCACCCACAACGCTCATTTAAAAAAACGGT

AACAATAACCAACCATTTTTTAAGCAAAATGCATAGTGATATAAAAATGATGAAATTTTGAAATAGTAGTGTCACCACGTXXXXXXXXXXC

AAAAAATAAAGTTGAATTCGGAAGTAATTTGTGTATAGGGAAGGTATTAGCACCCACAACACTCATTTAAAAAAACGGT

>Marker326838 Chr1 SNP_site:25223335 Ref_Type:G

AACAATAACCAACCATTTTTTAAGCAAAATGCATAGTGATATAAAAATGATGAAACTTTGAAATAGTAGTGTCACCACATXXXXXXXXXXC

AAAAAATAAAGTTGAATTCGGAAGTAATTTGTGTATAGGGAAGGTATTAGCACCCACAACGCTCATTTAAAAAAACGGT

AACAATAACCAACCATTTTTTAAGCAAAATGCATAGTGATATAAAAATGATGAAATTTTGAAATAGTAGTGTCACCACGTXXXXXXXXXXC

AAAAAATAAAGTTGAATTCGGAAGTAATTTGTGTATAGGGAAGGTATTAGCACCCACAACACTCATTTAAAAAAACGGT

>Marker326838 Chr1 SNP_site:25223545 Ref_Type:A

AACAATAACCAACCATTTTTTAAGCAAAATGCATAGTGATATAAAAATGATGAAACTTTGAAATAGTAGTGTCACCACATXXXXXXXXXXC

AAAAAATAAAGTTGAATTCGGAAGTAATTTGTGTATAGGGAAGGTATTAGCACCCACAACGCTCATTTAAAAAAACGGT

AACAATAACCAACCATTTTTTAAGCAAAATGCATAGTGATATAAAAATGATGAAATTTTGAAATAGTAGTGTCACCACGTXXXXXXXXXXC

AAAAAATAAAGTTGAATTCGGAAGTAATTTGTGTATAGGGAAGGTATTAGCACCCACAACACTCATTTAAAAAAACGGT

>Marker328363 Chr1 SNP_site:4697174 Ref_Type:G

TACAAGGGGTCCTCATTGCTTTTTGAGGGAATTTTGTCATCAATTTGCTAGATATAGGAAATAAGACTTGTTCAGTTGTGXXXXXXXXXXC

ATTGTATAATTCTCTTGTCCATTGAGCTCTTATTATTATTAATAAAGAAGTTTATCTCCATTTCAAAAAAATTGCATGT

TACAAGGGGTCCTCATTGCTTTTTGAGGGAATTTTGTCATCAATTTGCTAGATATAGGAAATAAGACTTGTTCAGTTGTGXXXXXXXXXXC

ATTGTATAATTCTCTTGTCCATTGAGCTCTTATTATTATTAATAAAGAAGTTTGTCTCCATTTCAAAAAAATTGCATGT

>Marker328495 Chr1 SNP_site:27406919 Ref_Type:C

CACCTTACCCAAGTTATTACTAGTATGGATGAAGATCTCCCAAATGGCTGGATGACTAATGAATTCAGAAAGGACTGTGGXXXXXXXXXXA

CATACTCTATTTATAGTTGGTCGTAAGGCTGTCTTCCATAAAAAGGCTTACAAATATTTTGAAGCTTTGACCAACAAGT

CACCTTACCCAAGTTATTACTAGTATGGATGAAGATCTCCCAAATGGCTGGATGACTAATGAATTCAGAAAGGACTGTGGXXXXXXXXXXA

CATACTCTATTTATAGTTGGTCGTAAGGCTGTCTTTCATAAAAAGGCTTACAAATATTTTGAAGCTTTGACCAACAAGT

>Marker3285 Chr4 SNP_site:16235195 Ref_Type:G

ACTCTCTTGTCTTGTGATGTATGCTTATATTCATGTTGTCTTTGTAGGTTCAACTAAAGCCAACAACTTCATGAGTTTTTXXXXXXXXXXA

TTCTGATGGTATTTACACTAGTTTTGATTAAGGGCTAAAATTCCATATTTGTTAATTGTTCAAACTTGATTGGAAATGT

ACTCTCTTGTCTTGTGATGTATGCTTATATTCATGTTGTCTTTGTAGGTTCAACTAAAGCCAACAACTTCATGAGTTTTTXXXXXXXXXXA

TTCTGATGGTATTTACACTAGTTTTGATTAAGGGCTGAAATTCCATATTTGTTAATTGTTCAAACTTGATTGGAAATGT

>Marker328659 Chr1 SNP_site:2745009 Ref_Type:T

CACTGCGTTGGGAAATCCTTGTGTCTCCAAACAAAGACCTGCATGCTTTCCATAAGCAGCCCCTCCCTTGCCAACAACACXXXXXXXXXXT

GCTTCAGACCTGATTTTTCGTCTCCACAGTCGAGAACATAATTGTGGTCGTATCCCATGCCAACCTCATGAATAGAGGT

CACTGCGTTGGGAAATCCTTGTGTCTCCAAACAAAGACCTGCATGCTTTCCATAAGCAGCCCCTCCTTTGCCAACAACACXXXXXXXXXXT

GCTTCAGACCTGATTTTTCGTCACCACAGTCGAGAACATAATTGTGGTCGTATCCCATGCCAACCTCATGAATAGAGGT

>Marker328659 Chr1 SNP_site:2745141 Ref_Type:A

CACTGCGTTGGGAAATCCTTGTGTCTCCAAACAAAGACCTGCATGCTTTCCATAAGCAGCCCCTCCCTTGCCAACAACACXXXXXXXXXXT

GCTTCAGACCTGATTTTTCGTCTCCACAGTCGAGAACATAATTGTGGTCGTATCCCATGCCAACCTCATGAATAGAGGT

CACTGCGTTGGGAAATCCTTGTGTCTCCAAACAAAGACCTGCATGCTTTCCATAAGCAGCCCCTCCTTTGCCAACAACACXXXXXXXXXXT

GCTTCAGACCTGATTTTTCGTCACCACAGTCGAGAACATAATTGTGGTCGTATCCCATGCCAACCTCATGAATAGAGGT

>Marker329746 Chr1 SNP_site:28657885 Ref_Type:T

AACAAGTGTATTAGCAATGCATCAAGTGTGTCAAACTTATCAGCGAAGCATTTATGTGTATCAAGGCATAAGGGGTATCAXXXXXXXXXXA

Create PDF with GO2PDF for free, if you wish to

TGTATCTATCAAATGTATAAAGTGTATCAAAAAGCATCAAATATATCAATGAGTATTAAGTGTATCAGTATTAAGTGTA
AACAAGTGTATTAGCAATGCATCAAGTGTGTCAAACTTATCAGTGAAGCATTTATGTGTATCAAGGCATAAGGGGTATCAXXXXXXXXXXXA
TGTATCTATCAAATGTATAAAGTGTATCAAAAAGCATCAAATATATCAATGAGTATTAAGTGTATAAATATTAAGTGTA
>Marker329746 Chr1 SNP_site:28658097 Ref_Type:A
AACAAGTGTATTAGCAATGCATCAAGTGTGTCAAACTTATCAGCGAAGCATTTATGTGTATCAAGGCATAAGGGGTATCAXXXXXXXXXXXA
TGTATCTATCAAATGTATAAAGTGTATCAAAAAGCATCAAATATATCAATGAGTATTAAGTGTATCAGTATTAAGTGTA
AACAAGTGTATTAGCAATGCATCAAGTGTGTCAAACTTATCAGTGAAGCATTTATGTGTATCAAGGCATAAGGGGTATCAXXXXXXXXXXXA
TGTATCTATCAAATGTATAAAGTGTATCAAAAAGCATCAAATATATCAATGAGTATTAAGTGTATAAATATTAAGTGTA
>Marker329746 Chr1 SNP_site:28658099 Ref_Type:A
AACAAGTGTATTAGCAATGCATCAAGTGTGTCAAACTTATCAGCGAAGCATTTATGTGTATCAAGGCATAAGGGGTATCAXXXXXXXXXXXA
TGTATCTATCAAATGTATAAAGTGTATCAAAAAGCATCAAATATATCAATGAGTATTAAGTGTATCAGTATTAAGTGTA
AACAAGTGTATTAGCAATGCATCAAGTGTGTCAAACTTATCAGTGAAGCATTTATGTGTATCAAGGCATAAGGGGTATCAXXXXXXXXXXXA
TGTATCTATCAAATGTATAAAGTGTATCAAAAAGCATCAAATATATCAATGAGTATTAAGTGTATAAATATTAAGTGTA
>Marker329937 Chr1 SNP_site:15299792 Ref_Type:C
AACCTCTTTAAGCTAGAGGTATAATCAATGCACATACGTCATCCGTTTTTAATTCGAGCCGGTGTCATTGCTCCTTTCTCXXXXXXXXXXXA
GAGTGATAAAGTGCATGCAAAGAGAAGCCTATATTTCTGTTTAATGTAGTCTAAGATTCATCTAATTGATGGTTTTGTT
AACCTCTTTAAGCTAGAGGTATAATCAATGCACATACGTCATCCGTTTTTAATTCGAGTCGGTGTCATTGCTCCTTTCTCXXXXXXXXXXXA
GAGTGATAAAGTGCATGCAAAGAGAAGCCTATATTTCTGTTTAATGTAGTCTAAGATTCATCTAATTGATGGTTTTGTT
>Marker330324 Chr1 SNP_site:14851014 Ref_Type:C
GACTAAGTAGAACAATTTTCGCAAAACAGCTCGTCCGTGTTTGAAAGATTTCGATTGTGACATCGTGCGCCTTGTAGCCCXXXXXXXXXXXC
TTAGAAATGTCTTTATTATGTTACCTCAAATGTTCAACCCTTAATTCTAAGTTATGTGTCCTGGAGCCTCTAGAAAGTG
GACTAAGTAGAACAATTTTCGCACAACAGCTCGTCCGTGTTTGAAAGATTTCGATTGTGACATCGTGCGCCTTGTAGCCCXXXXXXXXXXXC
TTAGAAATGTCTTTATTATGTTACCTCAAATGTTCAATCCTTAATTCTAAGTTATGTGTCCTGGAGCCTCTAGAAAGTG
>Marker330324 Chr1 SNP_site:14851235 Ref_Type:T
GACTAAGTAGAACAATTTTCGCAAAACAGCTCGTCCGTGTTTGAAAGATTTCGATTGTGACATCGTGCGCCTTGTAGCCCXXXXXXXXXXXC
TTAGAAATGTCTTTATTATGTTACCTCAAATGTTCAACCCTTAATTCTAAGTTATGTGTCCTGGAGCCTCTAGAAAGTG
GACTAAGTAGAACAATTTTCGCACAACAGCTCGTCCGTGTTTGAAAGATTTCGATTGTGACATCGTGCGCCTTGTAGCCCXXXXXXXXXXXC
TTAGAAATGTCTTTATTATGTTACCTCAAATGTTCAATCCTTAATTCTAAGTTATGTGTCCTGGAGCCTCTAGAAAGTG
>Marker330843 Chr1 SNP_site:6145238 Ref_Type:A
ACAAAGAGCAATTTCCTGTTTGCAACTAAAACTGCTAATAAACACTAAACCAAACGCTTTTTGACAATCTCAAATGCCTAXXXXXXXXXXXT
ATGATGCGAAAATGGTTTTACCAATTAGTATAACCAAGAAATGGAAAAAGATCACAAGCTATTGCGTTTGCTTGTTGGT
ACAAAGAGCAATTTCCTGTTTGCAACTAAAACTGCTAATATACACTAAACCAAACGCTTTTTGACAATCTCAAATGCCTAXXXXXXXXXXXT
ATGATGCGAAAATGGTTTTACCAATTAGTATAACCAAGAAATGGAAAAAGATCACAAGCTATTGCGTTTGCTTGTTGGT
>Marker331025 Chr1 SNP_site:26069326 Ref_Type:C
ACACGATATTACAAGGGGACAATAACAAAACAAAAACAAAGGAACAGGAGATGCACCCGAGCATATGAACTAAGGTGACAXXXXXXXXXXXT
TTAGACCGGAAATATAACAGCATGCCAATTTACATTTAAATCTTGTATAGAAAAGTCTTCAAAACTCTTAGATACGGTG
ACAGGATATTACAAGGGGACAATAACAAAACAAAAACAAAGGAACAGGAGATGCACCCGAGCATATGAACTAAGGTGACAXXXXXXXXXXXT
TTAGACCGGAAATATAACAGCATGCCAATTTACATTTAAATCTTGTATAGAAAAGTCTTCAAAACTCTTAGATACGGTG
>Marker332030 Chr1 SNP_site:23845186 Ref_Type:A
GACCTTTACTCGAACATTACAACCGCAATACTAGAAAATGAAAATGAAAATGGGAAATATTGAAGAAAATGAACATGGAAXXXXXXXXXXXT
GATAACAATAATTAAAATAATTATTGACCTTAATAAATACCAAAATTTAGACTACCAACTTTTACAATTATGGTATAGT
GACCTTTACTCGAACATTACAACCGCAATACTAGAAAATGAAAATGAAAATGGGAAATATTGAAGAAAATGAACGTGGAAXXXXXXXXXXXT
GATAACAATAATTAAAATAATTATTGACCTTAATAAATACCAAAATTTAGACTACCAACTTTTACAATTATGGTATAGT
>Marker33207 Chr4 SNP_site:12222616 Ref_Type:G

Create PDF with GO2PDF for free, if you wish to

AACCATAAGATTCATAACTCTAACCCAAGCTCCAAACAACTTTAATGTATAACTATGAGTTGGATATGTTTTGAGCCAGGXXXXXXXXXXC
ACAAGTATTCCCCTTTCTTCTAGTTCTCTCTCTTTATTTCCATGCTAAGGATGGAACAGGTCCTCCCTTCAGATTCGTT
AACCATAGGATTCATAACTCTAACCCAAGCTCCAAACAACTTTAATGTATAACTATGAGTTGGATATGTTTTGAGCCAGGXXXXXXXXXXC
ACAAGTATTCCCCTTTCTTCTAGTTCTCTCTCTTTATTTCCATGCTAAGGATGGAACAGGTCCTCCCTTCAGATTCGTT
>Marker332198 Chr1 SNP_site:8795475 Ref_Type:G
CACTTCAGACTTGAAAGCAACAACATTTTTTAATGAACTACGGTTGTGGCAGGGGAAAAAAGATGTTAAAAAATATTCAGXXXXXXXXXXG
TAATTAACCGCCCGATCGGTTTAGAGGTCTTTGTAAGTAACTTGGTGTTCCCATTGTTATTATTGTTGAAATCAAGGTT
CACTTCAGACTTGAAAGCAACAACATTTTTTAATGAACTACGGTTGTGGCAGGGGAAAAAAGATGTTAAAAAATATTCAGXXXXXXXXXXG
TAATTAACCTCCCGATCGGTTTAGAGGTCTTTGTAAGTAACTTGGTGTTCCCATTGTTATTATTGTTGAAATCAAGGTT
>Marker332201 Chr1 SNP_site:1131790 Ref_Type:A
TACTACCCTTTCAAAAGGGACTAAGTGGAGGATAGGGAATGTATGGATGCTTTTTTAAAATTTTGTTCCTAAAGAACATTXXXXXXXXXXT
AAGCAGCGTGAAACATTCTCCAGAACTCTTCTTTCCCAAAAAGAACATGCGCCTTCAATAGATACTCATAGAATGAGTC
TACTACCCTTTCAAAAGGGACTAAGTGGTGGATAGGGAATGTATGGATGCTTTTTTAAAATTTTGTTCCTAAAGAACATTXXXXXXXXXXT
AAGCAGCGTGAAACATTCTCCAGAACTCTTCTTTCCCAAAAAGAACATGCGCCTTCAATAGATACTCATAGAATGAGTC
>Marker332785 Chr1 SNP_site:12737418 Ref_Type:A
AACAGAATATGCATATCTCCCCTTCCAACTACGACAATCGCAATGAACTTTAATGGGCCTTTCACAACGTCCCCCACTCCXXXXXXXXXXA
AAATTTGTTGTTTCCTTCTTATTAGGAATTGGTGGATTTTCAAAATGTCACTAATTTAAAATTATCAATTGGAAATGTA
AACAGAATATGCATATCTCCCCTTCCAACTACGACAATCGCAATGAACTTTAATGGGCCTTTCACAACGTCCCCCACTCCXXXXXXXXXXA
TAATTTGTTGTTTCCTTCTTATTAGGAATTGGTGGATTTTCAAAATGTCACTAATTTAAAATTATCAATTGGAAATGTA
>Marker332882 Chr1 SNP_site:6530620 Ref_Type:A
ACTAACATTTTAAAGAAAATGAGAATGAATAAATAAGAGGTCTTTAAATAAAATGTCTAAAATTTAAAATACTAAAAAAAXXXXXXXXXXA
ATATTAAACATAGGAAATAGTGAGTATATAAAATATATCTAGTAAGGGATAGACTACTAGTCCCGCTAGGTGATCTGTT
ACTAACATTTTAAAGAAAATGAGAATGAATAAATAAGAGGTCTTTAAATAAAATGTCTAATATTTAAAATACTAAAAAAAXXXXXXXXXXA
ATATTAAACATAGGAAATAGTGAGTATATAAAATATATCTAGTAAGGGATAGACTACTAGTCCCGCTAGGTGATCTGTT
>Marker333003 Chr1 SNP_site:23450106 Ref_Type:A
GACCAGTTGTAAATTAGTTTTATCGATCTCCTCTAAACCTAGGTTTAAGCCCTACTCTATACAGTGTAATGCCACACACTXXXXXXXXXXA
AAATATATATGTGGGAAAAAAAAAATTTCCAATGCATGGTATGTTTCGTGGGAAGAATTGCATTTTTTACCCACGTGTT
GACCAGTTGTAAATTAGTTTTATCGATCTCCTCTAAACCTAGGTTTAAGCCCTACTCTATACAGTGTAATGCCACACACTXXXXXXXXXXA
AAATATATATGTGGGAAAAAAAAAGATTCCAATGCATGGTATGTTTCGTGGGAAGAATTGCATTTTTTACCCACGTGTT
>Marker333003 Chr1 SNP_site:23450107 Ref_Type:T
GACCAGTTGTAAATTAGTTTTATCGATCTCCTCTAAACCTAGGTTTAAGCCCTACTCTATACAGTGTAATGCCACACACTXXXXXXXXXXA
AAATATATATGTGGGAAAAAAAAAATTTCCAATGCATGGTATGTTTCGTGGGAAGAATTGCATTTTTTACCCACGTGTT
GACCAGTTGTAAATTAGTTTTATCGATCTCCTCTAAACCTAGGTTTAAGCCCTACTCTATACAGTGTAATGCCACACACTXXXXXXXXXXA
AAATATATATGTGGGAAAAAAAAAGATTCCAATGCATGGTATGTTTCGTGGGAAGAATTGCATTTTTTACCCACGTGTT
>Marker333213 Chr1 SNP_site:25998302 Ref_Type:A
TACTCCTCCAGCAATTTTTTGAGTAAGGGCAAGTAAAAAGGAGATGCAGCAGGTTCTCACTGTCCTTCATACATAGTGGGXXXXXXXXXXT
AGGTCTTTCCTCTTTTTTGCATTAAGGAATCTATGAAAAAAGCCAAAATTTTCATCTCCTACAGCAAGCCAATTTAGTT
TACTCCTCCAGCAATTTTTTGAGTAAGGGCAGGTAAAAAGGAGATGCAGCAGGTTCTCACTGTCCTTCATACATAGTGGGXXXXXXXXXXT
AGGTCTTTCCTCTTTTTTGCATTAAGGAATCTACGAAAAAAGCCAGAATTTTCATCTCCTACAGCAAGCCAATTTAGTT
>Marker333213 Chr1 SNP_site:25998544 Ref_Type:T
TACTCCTCCAGCAATTTTTTGAGTAAGGGCAAGTAAAAAGGAGATGCAGCAGGTTCTCACTGTCCTTCATACATAGTGGGXXXXXXXXXXT
AGGTCTTTCCTCTTTTTTGCATTAAGGAATCTATGAAAAAAGCCAAAATTTTCATCTCCTACAGCAAGCCAATTTAGTT
TACTCCTCCAGCAATTTTTTGAGTAAGGGCAGGTAAAAAGGAGATGCAGCAGGTTCTCACTGTCCTTCATACATAGTGGGXXXXXXXXXXT
AGGTCTTTCCTCTTTTTTGCATTAAGGAATCTACGAAAAAAGCCAGAATTTTCATCTCCTACAGCAAGCCAATTTAGTT

Create PDF with GO2PDF for free, if you wish to

>Marker333213 Chr1 SNP_site:25998556 Ref_Type:A

TACTCCTCCAGCAATTTTTTGAGTAAGGGCAAGTAAAAAGGAGATGCAGCAGGTTCTCACTGTCCTTCATACATAGTGGGXXXXXXXXXXT

AGGTCTTTCCTCTTTTTTGCATTAAGGAATCTATGAAAAAAGCCAAAATTTTCATCTCCTACAGCAAGCCAATTTAGTT

TACTCCTCCAGCAATTTTTTGAGTAAGGGCAGGTAAAAAGGAGATGCAGCAGGTTCTCACTGTCCTTCATACATAGTGGGXXXXXXXXXXT

AGGTCTTTCCTCTTTTTTGCATTAAGGAATCTACGAAAAAAGCCAGAATTTTCATCTCCTACAGCAAGCCAATTTAGTT

>Marker33376 Chr4 SNP_site:8017021 Ref_Type:C

AACCCATGTCAAATCAAATTTGACATCTTCTCGCCAAACCTTTCTTCTCGCTTAACTCCATTTCGTGAATAGCTTTAACAXXXXXXXXXXT

AACTTCTTAATAGCAAAATTTATACTTAATCATATAAATTTTTATCACTCAATCCTTTTCACTAATTGATTCAAACGTC

AACCCATGTCAAATCAAATTTGACATCTTCTCGCCAAACCTTTCTTCTCGCTTAACTCCATTTCGTGAATAGCTTTAACAXXXXXXXXXXT

AACTTCTTAATCGCAAAACTTATACTTAATCATATAAATTTTTATCACTCAATCATTTTCACTAATTGATTCAAACGTC

>Marker33376 Chr4 SNP_site:8017028 Ref_Type:C

AACCCATGTCAAATCAAATTTGACATCTTCTCGCCAAACCTTTCTTCTCGCTTAACTCCATTTCGTGAATAGCTTTAACAXXXXXXXXXXT

AACTTCTTAATAGCAAAATTTATACTTAATCATATAAATTTTTATCACTCAATCCTTTTCACTAATTGATTCAAACGTC

AACCCATGTCAAATCAAATTTGACATCTTCTCGCCAAACCTTTCTTCTCGCTTAACTCCATTTCGTGAATAGCTTTAACAXXXXXXXXXXT

AACTTCTTAATCGCAAAACTTATACTTAATCATATAAATTTTTATCACTCAATCATTTTCACTAATTGATTCAAACGTC

>Marker33376 Chr4 SNP_site:8017064 Ref_Type:A

AACCCATGTCAAATCAAATTTGACATCTTCTCGCCAAACCTTTCTTCTCGCTTAACTCCATTTCGTGAATAGCTTTAACAXXXXXXXXXXT

AACTTCTTAATAGCAAAATTTATACTTAATCATATAAATTTTTATCACTCAATCCTTTTCACTAATTGATTCAAACGTC

AACCCATGTCAAATCAAATTTGACATCTTCTCGCCAAACCTTTCTTCTCGCTTAACTCCATTTCGTGAATAGCTTTAACAXXXXXXXXXXT

AACTTCTTAATCGCAAAACTTATACTTAATCATATAAATTTTTATCACTCAATCATTTTCACTAATTGATTCAAACGTC

>Marker334549 Chr1 SNP_site:17083989 Ref_Type:C

TACACATATATATGCCAAATGAGTAAAAAAACAGTTAAACACATTGAAAATTATAATAATAAAAATAATCCAAATGAAACXXXXXXXXXXT

ATTCATGTATAATACATAGAATATTTATCAATAAAATATCCCCCACGTGGTATCAGAGCTTTAAACCTACATCTATTGT

TACACATATATATGCCAAATGAGTAAAAAAACAGTTAAACACATTGAAAATTATAATAATAAAAATAATCCAAATGAAACXXXXXXXXXXT

ATTCATGTATAATACATAGAATATTTATCAATAAAATATCCCCCACGTGGTATCAGAGCTTTAAACCTAGATCTATTGT

>Marker335524 Chr1 SNP_site:6147617 Ref_Type:C

AACCTTCAGCTTCAGCGATAGAAATAGAGGAGAGCCGGAACCTGTCCCTCAAGAGTTCGGAAGCGGAGTCGTCTTCTTCCXXXXXXXXXXT

TCTTTTTCTCTTTTTTGTCCTAATAAAAATTAGAATAAAATAAATAAACAAAAAATATTAGTATAAAGCATGTAGAGTC

AACCTTCAGCTTCAGCGATAGAAATAGAGGAGAGCCGGAACCTGTCCCTCAAGAGTTCGGAAGCGGAGTCGTCTTCTTCCXXXXXXXXXXT

TTTTTTTCTCTTTTTTGTCCTAATAAAAATTAGAATAAAATAAATAAACAAAAAATATTAGTATAAAGCATGTAGAGTC

>Marker336156 Chr1 SNP_site:3578088 Ref_Type:T

AACATTCATGCAGATATTGCAATTACTTGAAGCAATTTTGATATTGCAATAACATGATCTAAACCTTGTCTTCGGGATTCXXXXXXXXXXG

AAGGTATGATGCTATTGCTATTGCTGGACCTCTAGCTGCCCAATCTCTTTCACAAGTTTTAGATTATATGGGCCATAGT

AACATTCATGCAGATATTGCAATTACTTGAATCAATTTTGATATTGCAATAACATGATCTAAACCTTGTCTTCGGGATTCXXXXXXXXXXG

AAGGTATGATGCTATTGCTATTGCTGGACCTCTAGCTGCCCAATCTCTTTCACAAGTTTTAGATTATATGGGCCATAGT

>Marker336282 Chr1 SNP_site:28673 Ref_Type:C

GACTCCATTGCACACATGGGGTCTGTTGTTGTCATCCTAGGTTTGGGTAGAGTAATTTGGGGTAGAGTGTGACACTTCTTXXXXXXXXXXT

GGCATTCATGAAAGTAGTGTCTATTCAAGCTCAACTTGATTTTTCTCCGATCAATGTATATCCATCGACTTGTTTGGTC

GACTCCATTGCACACATGGGGTCTGTTGTTGTCATCCTAGGTTTGGGTAGAGTAATTTGGGGTAGAGTGTGACACTTCTTXXXXXXXXXXT

GGCATTCATGAAAGTAGTGTCTATTCAAGCTCAACTTGATTTTTCTTCGATCAATGTATATCCATCGACTTGTTTGGTC

>Marker336478 Chr1 SNP_site:6251235 Ref_Type:A

ACTCCACTCTCCTTAATTACATCTTTTATAGTAATGCCACTGATACAAGCATTGCACATTAATATGCAGACAATAATGAGXXXXXXXXXXC

AATTACCTGCATTATTTTGTAGTTGATTATAACGAAATTTGATAACATAGAAATGATGTCATTATCAGAACTAATAGTG

ACTCCACTCTCCTTAATTACATCTTTTATAGTAATGCCACTGATACAAGCATTGCACGTTAATATGCAGACAATAATGAGXXXXXXXXXXC

Create PDF with GO2PDF for free, if you wish to

AATTACCTGCATTATTTTGTAGTTGATTATAACGAAATTTGACAACATAGAAATGATGTCATTATCAGAACTAATAGTG
>Marker336478 Chr1 SNP_site:6251443 Ref_Type:T
ACTCCACTCTCCTTAATTACATCTTTTATAGTAATGCCACTGATACAAGCATTGCACATTAATATGCAGACAATAATGAGXXXXXXXXXXC
AATTACCTGCATTATTTTGTAGTTGATTATAACGAAATTTGATAACATAGAAATGATGTCATTATCAGAACTAATAGTG
ACTCCACTCTCCTTAATTACATCTTTTATAGTAATGCCACTGATACAAGCATTGCACGTTAATATGCAGACAATAATGAGXXXXXXXXXXC
AATTACCTGCATTATTTTGTAGTTGATTATAACGAAATTTGACAACATAGAAATGATGTCATTATCAGAACTAATAGTG
>Marker337374 Chr1 SNP_site:24592231 Ref_Type:T
TACAAGATCTGATATATTTCTGTTTAAAACGAATGTCATGGTTTTATGAAATGATTTATGAAAATGTATTACGAAAAGGAXXXXXXXXXXT
TTTAAGTTTTAGTTTAGGTTGTATTGTATAATATGTATGAAACGCATGTTGATAATAAAGTTTAAGTATTTGTTATGTT
TACAAGATCTGATATATTTCTGTTTAAAACGAATGTCATGGTTTTATGAAATGATTTATGAAAATGTATTACGAAAATGAXXXXXXXXXXT
TTTAAGTTTTAGTTTAGGTTGTATTGTATAATATGTATGAAACGCATGTTGATAATAAAGTTTAAGTATTTGTTATGTT
>Marker337742 Chr1 SNP_site:6450953 Ref_Type:T
GACGACGACAATAAAACCATATTTCATGTAGCAGCTGAAAATCGACAAGAAGATGTGTTTAGTCTTATACATGAGATCGGXXXXXXXXXXG
TGGTTTAAGATAAGTTCTTTGATGCATACATTGCATGTTTTTTCAAACAATAATTGAGAGCGTTATTTTGAAATCTTGT
GACGACGACAATAAAACCATATTTCATGTAGCAGCTGAAAATCGACAAGAAGATGTGTTTAGTCTTATACATGAGATCGGXXXXXXXXXXG
TGGTTTAAGATAAGTTCTTTGATGCATACATTGCATGTTTTTTTAAACAATAATTGAGAGCGTTATTTTGAAATGTTGT
>Marker337742 Chr1 SNP_site:6450984 Ref_Type:G
GACGACGACAATAAAACCATATTTCATGTAGCAGCTGAAAATCGACAAGAAGATGTGTTTAGTCTTATACATGAGATCGGXXXXXXXXXXG
TGGTTTAAGATAAGTTCTTTGATGCATACATTGCATGTTTTTTCAAACAATAATTGAGAGCGTTATTTTGAAATCTTGT
GACGACGACAATAAAACCATATTTCATGTAGCAGCTGAAAATCGACAAGAAGATGTGTTTAGTCTTATACATGAGATCGGXXXXXXXXXXG
TGGTTTAAGATAAGTTCTTTGATGCATACATTGCATGTTTTTTTAAACAATAATTGAGAGCGTTATTTTGAAATGTTGT
>Marker337853 Chr1 SNP_site:21027465 Ref_Type:A
ACTTTTATTTATTTCCTCTTAGTGGTATAATCTACTAATCACAATCTTAATACATTTCAAAAAAATAAATAAATAAAACAXXXXXXXXXXT
CATATAAACCTTATCATATTTTTAATAAATGAACTCAAATCTCAAATTGTTAAATTCAATTATGATGAGTGGATGAGTT
ACTTTTATTTCTTTCCTCTTAGTGGTATAATCTACTAATCACAATCTTAATACATTTCAAAAAAATAAATAAATAAAACAXXXXXXXXXXT
CATATAAACCTTATCATATTTTTAATAAATGAACTCAAATCTCAAATTGTTAAATTCAATTATGATGAGTGGATGAGTT
>Marker33791 Chr4 SNP_site:13258372 Ref_Type:C
GACTAATTAAAGAAAGATAAGTGTAGCATCACAGCCAACATTTAAATATGTATTTTAGAATAGTTAAATGGATTGGATGGXXXXXXXXXXC
CTCTCAAATCATGCTATCCTTTTGACACGTCTGATTCATACTTAACATAAAACATCTTAAATATTATCTTCATTATGTT
GACTAATTAAAGAAAGATAAGTGTAGCATCACAGCCAACATTTAAATATGTATTTTAGAATAGTTAAATGGATTGGATGGXXXXXXXXXXC
CTCTTAAATCATGCTATCCTTTTGACACGTCTGATCCATACTTAACATAAAACATCTTAAATATTATCTTCATTATGTT
>Marker33791 Chr4 SNP_site:13258403 Ref_Type:T
GACTAATTAAAGAAAGATAAGTGTAGCATCACAGCCAACATTTAAATATGTATTTTAGAATAGTTAAATGGATTGGATGGXXXXXXXXXXC
CTCTCAAATCATGCTATCCTTTTGACACGTCTGATTCATACTTAACATAAAACATCTTAAATATTATCTTCATTATGTT
GACTAATTAAAGAAAGATAAGTGTAGCATCACAGCCAACATTTAAATATGTATTTTAGAATAGTTAAATGGATTGGATGGXXXXXXXXXXC
CTCTTAAATCATGCTATCCTTTTGACACGTCTGATCCATACTTAACATAAAACATCTTAAATATTATCTTCATTATGTT
>Marker337939 Chr1 SNP_site:20460543 Ref_Type:G
TACACTTATTGGAAGGAAAAGGTATGAATAAACCCCTAGTATTATTGGGGTTTTGTTTAGTATTTTGACATATTGCTTTTXXXXXXXXXXA
GTTTTGCTTGTAATTTTTATGGCGGAACACAGAAAGGAAGATGAGACATTCTCTAACACAAAGAATGCTTGCTTAAGTG
TACACTTATTGGAAGGAAAAGGTATGAATAAACCCCTAGTATTATTGGGGTTTTGTTTAGTATTTTGACATATTGCTTTTXXXXXXXXXXA
GTTTTGCTTGTAATTTTTATGGCGGAACACAGAAAGGAAGATGAGACATTCTCTAACACGAAGAATGCTTGCTTAAGTG
>Marker337990 Chr1 SNP_site:25832812 Ref_Type:A
GACCTCGCTGCACTGTTTGTTACTGGGATACTATACCACACTTGTTTTATTAGCGGTTGCTGGTCTGTTGGAGGTTGGAGXXXXXXXXXXG
AAAATTGTGTATTTACACCCGTTAAAATGTCCTGCCTTCAACAAAACTAGAAAGAAATGATATTTTTATGTGCTTTAGT

Create PDF with GO2PDF for free, if you wish to

GACCTCGCTGCACTGTTTGTTACTGGGATACTATACCACACTTGTTTTATTAGCGGTTGCTGGTCTGTTGGAGGTTGGAGXXXXXXXXXXG
AAAATTGTGTATTTACACCCGTTAATATGTCCTGCCTTCAACAAAACTAGAAAGAAATGATATTTTTATGTGCTTTAGT
>Marker338400 Chr1 SNP_site:12354401 Ref_Type:C
AACAAGGTTGAATTTAATTTATTAAAAACTCTAGCTTACGCTCTTGAAATACTTTCCCTCGTTTTGGATACCTCCACTCAXXXXXXXXXXT
AGATTATTGCTTGAACGAAATCTACTACGTCTATACATAGTTACCAGGATAACAATTATTTTTTCAATCAATCATTGTT
AACAAGGTTGAATTTAATTTATTAAAAACTCTAGCTTACGCTCTTGAAATACTTTCCCTTGTTTTGGATACCTCCACTCAXXXXXXXXXXT
AGATTATTGCTTGAACGAAATCTACTACGTCTATACATAGTTACCAGGATAACAATTATTTTTTCAATCAATCATTGTT
>Marker338605 Chr1 SNP_site:4604141 Ref_Type:T
AACGTCTTCGTCACACACCAATTTTAGTTTTTCCCGTTTTGAGTTTTTCCTTTTAAATGTGGTTTTACGATTTTGCTTTTXXXXXXXXXXT
ATGAACAAATGAGTTTTTTCGATAAAAGTGTAAAGTTTAATAATGTTTTCTATTGAAATCGAGTTGTGTGAAGTTGGTG
AACGTCTTCGTCACACACCAATTTTAGTTTTTCCCGTTTTGAGTTTTTCCTTTTAAATGTGGTTTTACGATTTTGCTTTTXXXXXXXXXXT
ATGAACAAATGAGTTTTTTCGATAAAAGTGTAAAGTTTAATAATGTTTTCTATTGAAATTGAGTTGTGTGAAGTTGGTG
>Marker338748 Chr1 SNP_site:28147142 Ref_Type:G
CACTCACAATATCACTAAGTATGAAGTCTGCATTATGGGACTTCAAGTAAAATGCGACGTGAGTATTAAAAAATTGAAGGXXXXXXXXXXA
CCGGTTTATAGGGAGGACAATCGAATAGCAGATGCATTAGCCACCCTAACAATGATGTTTGATCTTAATCTTGAATGTG
CACTCACAATATCACTAAGTATGAAGTCTGCATTATGGGACTTCAAGTAGAATGCGACGTGAGTATTAAAAAATTGAAGGXXXXXXXXXXA
CCGGTTTATAGGGAGGACAATCGAATAGCAGATGCATTAGCCACCCTAACAATGATGTTTGATCTTAATCTTGAATGTG
>Marker338812 Chr1 SNP_site:2746326 Ref_Type:A
AACTGCCAAAATTCCCACCATGGTAGATTTGGGGTAATAATAAGTGAGAAACGACAAACTCGATGCATGCTATGAGATTTXXXXXXXXXXT
GAAAATTCCCAATTCCTTACCATGGAGACTATTTGGAGGTTTATTAATAGGCAAAGAGTATTGTTCCCCATCGAGTGTG
AACTGCCAAAATTCCCACCATGGTAGATTTGGGGTAATAATAAGTGAGAAACGACAAACTCGATGCATGCTATGAGATTTXXXXXXXXXXT
GAAAATTCCCAATTCCTTACCATGGAGACTGTTTGGAGGTTTATTAATAGGCAAAGAGTATTGTTCCCCATCGAGTGTG
>Marker33896 Chr4 SNP_site:5350623 Ref_Type:A
CACTTGGAGAGATAAGTATGAAAAGCATGGGGATGCCCATCACGTGACCCAATGGCAAATATAAGATATAACCTGAAGGCXXXXXXXXXXA
CAACTTTACAATATTGACTTCTGAAGTTTGACCATGCTTTCCTTTTACTCTTTTCAGAGAACTTCTTTCTTAACATTGT
CACTTGGAGAGATAAGTATGAAAAGCATGGGGATGCCCATCACGTGACCCAATGGCAAATATAAGATATAACCTGAAGGCXXXXXXXXXXA
CAACTTTACAATATTGACTTCTGAAGTTTGACCATGCTTTCCTTTTCCTCTTTTCAGAGAACTTCTTTCTTAACATTGT
>Marker339038 Chr1 SNP_site:11103802 Ref_Type:G
AACTAATGTTGTGAGCTAAGATTATTTTGATCAAATAGTCACTACTTACATCAAACAACATTACAAAAAAGTTTTCAACAXXXXXXXXXXT
TCTACTTTGCATTAGGTTGGATTGAGTTAGGATAAATTTTATTTTGAATTTTAGGGTTCATAATTCTTTTCGTCATGTT
AACTAATGTTGTGAGCTAATATTATTTTGATCAAATAGTCACTACTTACATCAAACAACATTACAAAAAAATTTTCAACAXXXXXXXXXXT
TCTACTTTGCATTAGGTTTGATTGAGTTAGGATAAATTTTATTTTGAATTTTAGGGTTCATAATTCTTTTCGTCATGTT
>Marker339038 Chr1 SNP_site:11103853 Ref_Type:G
AACTAATGTTGTGAGCTAAGATTATTTTGATCAAATAGTCACTACTTACATCAAACAACATTACAAAAAAGTTTTCAACAXXXXXXXXXXT
TCTACTTTGCATTAGGTTGGATTGAGTTAGGATAAATTTTATTTTGAATTTTAGGGTTCATAATTCTTTTCGTCATGTT
AACTAATGTTGTGAGCTAATATTATTTTGATCAAATAGTCACTACTTACATCAAACAACATTACAAAAAAATTTTCAACAXXXXXXXXXXT
TCTACTTTGCATTAGGTTTGATTGAGTTAGGATAAATTTTATTTTGAATTTTAGGGTTCATAATTCTTTTCGTCATGTT
>Marker339038 Chr1 SNP_site:11103996 Ref_Type:G
AACTAATGTTGTGAGCTAAGATTATTTTGATCAAATAGTCACTACTTACATCAAACAACATTACAAAAAAGTTTTCAACAXXXXXXXXXXT
TCTACTTTGCATTAGGTTGGATTGAGTTAGGATAAATTTTATTTTGAATTTTAGGGTTCATAATTCTTTTCGTCATGTT
AACTAATGTTGTGAGCTAATATTATTTTGATCAAATAGTCACTACTTACATCAAACAACATTACAAAAAAATTTTCAACAXXXXXXXXXXT
TCTACTTTGCATTAGGTTTGATTGAGTTAGGATAAATTTTATTTTGAATTTTAGGGTTCATAATTCTTTTCGTCATGTT
>Marker339217 Chr1 SNP_site:25503418 Ref_Type:A
AACCATTTCAAAGATCTTTACAATGGCAGTGGTAAGGAGTCCTTTCTCATAGAAAATCTGGAATAGAACAAAATTGTAGAXXXXXXXXXXA

Create PDF with GO2PDF for free, if you wish to

TAAGATTTTTAGAGTCATCTCAAGAAGAACATCATGAAAGTCTTTAAAGATTAGTAAAGACATCATCAAGAAAAATGTA
AACCATTTCAAAGATCTTTACAATGGCAGTGGTAAGGAGTCCTTTCTCATAGAAAATCTGGAATAGAACAAAATTGTAGAXXXXXXXXXXA
TAAGATTTTTAGAGTCATCTCAAGAAGAACATCATGGAAGTCTTTAAAGATTAGTAAAGGCATCATCAAGAAAAATGTA
>Marker339217 Chr1 SNP_site:25503441 Ref_Type:A
AACCATTTCAAAGATCTTTACAATGGCAGTGGTAAGGAGTCCTTTCTCATAGAAAATCTGGAATAGAACAAAATTGTAGAXXXXXXXXXXA
TAAGATTTTTAGAGTCATCTCAAGAAGAACATCATGAAAGTCTTTAAAGATTAGTAAAGACATCATCAAGAAAAATGTA
AACCATTTCAAAGATCTTTACAATGGCAGTGGTAAGGAGTCCTTTCTCATAGAAAATCTGGAATAGAACAAAATTGTAGAXXXXXXXXXXA
TAAGATTTTTAGAGTCATCTCAAGAAGAACATCATGGAAGTCTTTAAAGATTAGTAAAGGCATCATCAAGAAAAATGTA
>Marker339234 Chr1 SNP_site:924288 Ref_Type:T
ACTTGATTGTTATTCTGTTTTACAGACTGTCTTAATGAATTTTGATTTGGAAGTTCTTTGTGTTATCTGGAAAAAATCATXXXXXXXXXXA
CAGATACCGACAACTTGCAAAATCAGGAAATGATGATTTGCGACAAGATGCTGTAAGATTTCAGTGATGAAATGATGGT
ACTTGATTGTTATTCTGTTTTACAGACTTTCTTAATGAATTTTGATTTGGAAGTTCTTTGTGTTATCTGGAAAAAATCATXXXXXXXXXXA
CAGATACCGACAACTTGCAAAATCAGGAAATGATGATTTGCGACAAGATGCTGTAAGATTTCAGTGATGAAATGATGGT
>Marker339297 Chr1 SNP_site:28788511 Ref_Type:G
AACCAATTCCATACATTTTAGGAATTTGAGAAATATAATAATAATAATAATCATTGAACTTAAGAAATTCAAAGAGAATAXXXXXXXXXXT
GTAATTTCTTGAAAATGGTAGCTCACCAAAAATCTTTGCAACTCCTTCAGCTTTTCTTAAGTAAATACAAGGTAAGAGT
AACCAATTCCATGCATTTTAGGAATTTGAGAAATATAATAATAATAATAATCATTGAACTTAAGAAATTCAAAGAGAATAXXXXXXXXXXT
GTAATTTCTTGAAAATGGTAGCTCACCAAAAATCTTTGCAACTCCTTCAGCTTTTCTTAAGTAAATACAAGGTAAGAGT
>Marker340443 Chr1 SNP_site:28788217 Ref_Type:C
TACCACACTTTTTTGCTGAGTTACGGCTGTGCGGAGTAATCTCCACACATACAGTAAATTTTTCATCAATTCCATTATGCXXXXXXXXXXA
ACAAGTATGAGACATCATATTTTAGAACACGCAACAAGAATAAGGCATCCTACAACATATCACAAAGCAAGTGTGAGGT
TACCACACTTTTTTGCTGAGTTACGGCTGTGCGGAGTAATCTCCACAGATACAGTAAATTTTTCATCAATTCCATTATGCXXXXXXXXXXA
ACAAGTATGAGACATCATATTTTAGAACACGCAACAAGAATAAGGCATCCTACAACATATCACAAAGCAAGTGTGAGGT
>Marker340733 Chr1 SNP_site:8689260 Ref_Type:T
ACTTTGATTAATATATCTCAATTTAGTCTGTTTGACTGTTTAAATTGAAAGCTTGTAAAATAACCTTCTTCATCATTGACXXXXXXXXXXT
TATTGTTTTACTTGGATTTACTTTCTTAAACATCGTTCTAACTTATCTCAAACCTTAAATTGATTTTGCAAATATGGTT
ACTTTGATTAATATATCTCAATTTAGTCTGTTTGACTGTTTAAATTGAAAGCTTGTAAAATAACCTTCTTTATCATTGACXXXXXXXXXXT
TATTGTTTTACTTGGATTTACTTTCTTAAACATCGTTCTAACTTATCTCAAACCTTAAATTGATTTTGCAAATATGGTT
>Marker340770 Chr1 SNP_site:4603259 Ref_Type:T
TACTAGCACATAAATTTTTTGATAGATTCATTCTTAATTTTAATTTTATAAATCTATAATTGGATCATTTGTTGAAAAGAXXXXXXXXXXA
GTTTTTCTTTTCAAACACCTTTTCTCTCTGTTTTCTTTTGCTTTTTCCCACAATTGATTGTTCAAATAGAAATTTAGTT
TACTAGCACATAAATTTTTTGATAGATTCATTCTTAATTTTAATTTTATAAATCTATAATTGGATCATTTGTTGAAAAGAXXXXXXXXXXA
GTTTTTCTTTTCAAATACCTTTTCTCTCTGTTTTCTTTTTCTTTTTCCCACAATTGATTGTTCAAATAGAAATTTAGTT
>Marker340770 Chr1 SNP_site:4603283 Ref_Type:T
TACTAGCACATAAATTTTTTGATAGATTCATTCTTAATTTTAATTTTATAAATCTATAATTGGATCATTTGTTGAAAAGAXXXXXXXXXXA
GTTTTTCTTTTCAAACACCTTTTCTCTCTGTTTTCTTTTGCTTTTTCCCACAATTGATTGTTCAAATAGAAATTTAGTT
TACTAGCACATAAATTTTTTGATAGATTCATTCTTAATTTTAATTTTATAAATCTATAATTGGATCATTTGTTGAAAAGAXXXXXXXXXXA
GTTTTTCTTTTCAAATACCTTTTCTCTCTGTTTTCTTTTTCTTTTTCCCACAATTGATTGTTCAAATAGAAATTTAGTT
>Marker340833 Chr1 SNP_site:1258545 Ref_Type:C
ACCCCAAACTAATCAACAATCCAACCTAGTCTTCACAAATTTTTTGTATTTTCTGACTTTGTCAATAATCTTAGTTCTTAXXXXXXXXXXG
ATTCTTAGTCGGCACCCAAACTTTCCTACAACTATATATTGAACTCAAATTCTCTACTTCCCTTAACCAACTCTTCAGT
ACCCCAAACTAATTAACAATCCAACCTAGTCTTCACAAATTTTTTGTATTTTCTGACTTTGTCAATAATCTTAGTTCTTAXXXXXXXXXXG
ATTCTTAGTCGGCACCCAAACTTTCCTACAACTATATATTGAACTCAAATTCTCTACTTCCCTTAACCAACTCTTCAGT
>Marker341596 Chr1 SNP_site:28362490 Ref_Type:T

Create PDF with GO2PDF for free, if you wish to

AACACATAGACAAATATATATGTTTAGGAAAACCCTATTTGGTCATTTATTGGATGTTAAGTTGGTGTTTATTAGACCCAXXXXXXXXXXA
CAGGAGAAAACTCTTAGATTGAAACTGTTATAATTGGACAATAAGACAAATATGAATGAGTTTGAGTTGGATAAAGAGT
AACACATAGACAAATATATATGTTTAGGAAAACCCTATTTGGTCATTTATTGGATGTTAAGTTGGTGTTTATTAGACCTAXXXXXXXXXXA
CAGGAGAAAACTCTTAGATTGAGACTGTTATAATTGGACAATAAGACAAATATGAATGGGTTTGAGTTGGATAAAGAGT
>Marker341596 Chr1 SNP_site:28362668 Ref_Type:G
AACACATAGACAAATATATATGTTTAGGAAAACCCTATTTGGTCATTTATTGGATGTTAAGTTGGTGTTTATTAGACCCAXXXXXXXXXXA
CAGGAGAAAACTCTTAGATTGAAACTGTTATAATTGGACAATAAGACAAATATGAATGAGTTTGAGTTGGATAAAGAGT
AACACATAGACAAATATATATGTTTAGGAAAACCCTATTTGGTCATTTATTGGATGTTAAGTTGGTGTTTATTAGACCTAXXXXXXXXXXA
CAGGAGAAAACTCTTAGATTGAGACTGTTATAATTGGACAATAAGACAAATATGAATGGGTTTGAGTTGGATAAAGAGT
>Marker341596 Chr1 SNP_site:28362704 Ref_Type:G
AACACATAGACAAATATATATGTTTAGGAAAACCCTATTTGGTCATTTATTGGATGTTAAGTTGGTGTTTATTAGACCCAXXXXXXXXXXA
CAGGAGAAAACTCTTAGATTGAAACTGTTATAATTGGACAATAAGACAAATATGAATGAGTTTGAGTTGGATAAAGAGT
AACACATAGACAAATATATATGTTTAGGAAAACCCTATTTGGTCATTTATTGGATGTTAAGTTGGTGTTTATTAGACCTAXXXXXXXXXXA
CAGGAGAAAACTCTTAGATTGAGACTGTTATAATTGGACAATAAGACAAATATGAATGGGTTTGAGTTGGATAAAGAGT
>Marker34185 Chr4 SNP_site:12822618 Ref_Type:A
GACCTTGTTGATGAAGAGAAGGATGAATTACTAAAACTGGCATTTGTGCGAATTGTTGAGGCATATAAGCAAATAGCAGTXXXXXXXXXXT
GGTCTGGTTAAATGATCCATATTTTGTTCAACGCTCTCTGTTCATATCATACACTTAACTGTTACTAATGCAGCATGTT
GACCTTGTTGATGAGGAGAAGGATGAATTACTAAAACTGGCATTTGTGCGAATTGTTGAGGCATATAAGCAAATAGCAGTXXXXXXXXXXT
GGTCTGGTTAAATGATCCATATTTTGTTCAACGCTCTCTGTTCATATCATACACTTAACTGTTACTAATGCAGCATGTT
>Marker342411 Chr1 SNP_site:15244752 Ref_Type:A
CACCATTTCTTTTTTTATTACTCCTAATAGGATGAGTTTGTAACCTAAATGTATGTTTGATTTTAGTGTTATTTTTTACTXXXXXXXXXXG
CAAATGTGTATAATATAATATTACTATGGCATAGACTCAAATCAATTGAATTTTTCAGATCTCAATCATCGCCTATGTA
CACCCTTTCTTTTTTTATTACTCCTAATAGGATGAGTTTGTAACCTAAATGTATGTTTGATTTTAGTGTTATTTTTTACTXXXXXXXXXXG
CAAATGTGTATAATATAATATTACTATGGCATAGACTCAAATCAATTGAATTTTTCAGATCTCAATCATCGCCTATGTA
>Marker343313 Chr1 SNP_site:2037850 Ref_Type:A
CACAATAATGTAATTCATAATTCATGATTAGATAGAACGTTTTTGAGCTCGAATTGTAATTCTAAACTCATTATGTTTCTXXXXXXXXXXC
TACTATAACAATTGATTCTTCAACCATTCAAATGTTTTAAGAAAATAAAATTAAAAGAGAAAGAAAAGTTGGGTGTGTG
CACAATAATGTGATTCATAATTCATGATTAGATAGAGCGTTTTTGAGCTCGAATTGTAATTCTAAACTCATTATGTTTCTXXXXXXXXXXC
TACTATAACAATTGATTCTTCAACCATTCAAATGTTTTAAGAAAATAAAATTAAAAGAGAAAGAAAAGTTGGGTGTGTG
>Marker343313 Chr1 SNP_site:2037875 Ref_Type:A
CACAATAATGTAATTCATAATTCATGATTAGATAGAACGTTTTTGAGCTCGAATTGTAATTCTAAACTCATTATGTTTCTXXXXXXXXXXC
TACTATAACAATTGATTCTTCAACCATTCAAATGTTTTAAGAAAATAAAATTAAAAGAGAAAGAAAAGTTGGGTGTGTG
CACAATAATGTGATTCATAATTCATGATTAGATAGAGCGTTTTTGAGCTCGAATTGTAATTCTAAACTCATTATGTTTCTXXXXXXXXXXC
TACTATAACAATTGATTCTTCAACCATTCAAATGTTTTAAGAAAATAAAATTAAAAGAGAAAGAAAAGTTGGGTGTGTG
>Marker343315 Chr1 SNP_site:10727422 Ref_Type:G
CACAAACTTTTGGTTTCCTTAGCATTACAAGACTAGCTCCACCTTTCAGCTACTGGCACTTGTAAAAGACATGTCATCATXXXXXXXXXXC
AATTTTAACAAATTGGAATCTAAATGTAGAGCTAGGTTAAACTACCACACTTTCCTGAAAAATTAAAAAATAGATCGTA
CACAAACTTTTGGTTTCCTTAGCATTACAAGACTGGCTCCACCTTTCAGCTACTGGCACTTGTAAAAGACATGTCATCATXXXXXXXXXXC
AATTTTAACAAATTGGAATCTAAATGTAGAGCTAGGTTAAACTACCACACTTTCCTGAAAAATTAAAAAATAGATCGTA
>Marker343318 Chr1 SNP_site:26086432 Ref_Type:G
ACAAAGGAAGAATACCTTGAAAAAGCAAATAATATATAAGAAAATAAAAGACCAACAAGGACAATGCCAAGAAACCAAGAXXXXXXXXXXA
GGTCATCTAAGTAATTAGCTAAAGAGTTTGTTAGGGTTTTTAGTTATAAAAAGAGGGAGTGGGTTGGGAGCAAGGGGTG
ACAAGGGAAGAATACCTTGAAAAAGCAAATAATATATAAGAAAATAAAAGACCAACAAGGACAATGCCAAGAAACCAAGAXXXXXXXXXXA
GGTCATCTAAGTAATTAGCTAAAGAGTTTGTTAGGGTTTTTAGTTATAAAAAGAGGGAGTGGGTTGGGAGCAAGGGGTG

Create PDF with GO2PDF for free, if you wish to

>Marker343885 Chr1 SNP_site:25909175 Ref_Type:C
TACTTCTATATTGTTCTGTATTTTGAGTTCAATTAATAAAGAAGTTTTTGTCTCCATTTCAAAAAACAAAAAAACAAAACXXXXXXXXXXA
AGAAATGGAAACTTAAACCATAAGTCAGATACTTAGTGAGAGATGTTTTGGAAAATCTTCCTAGGAAAACAATAGAGTC
TACTTCTATATTGTTCTGTATTTTGAGTTCAATTAATAAAGAAGTTTTTGTCTCCATTTTAAAAAACAAAAAAACAAAACXXXXXXXXXXA
AGAAATGGAAACTTAAACCATAAGTCAGATACTTAGTGAGAGATGTTTTGGAAAATCTTCCTAGGAAAACAATAGAGTC
>Marker344372 Chr1 SNP_site:23875447 Ref_Type:A
CACGAGTTTTCAGACTTGACTCGAACTCCTTTCTTCTCAGAAACAATCTGTTCTTTTTTTCACAACCACACACCCACAAAXXXXXXXXXXA
GTAGAACGACCCATGCCACAAGCTCCATTCACACGATTCTTGTGTATGTTGTTTGAGTCGTTCGCTTGCTTCAAATGTT
CACGAGTTTTCAGACTTGACTCGAACTCCTTTCTTCTCAGAAACAATCTGTTCTTTTTTTCACAACCACACACCCACAAAXXXXXXXXXXA
GTAGAACGACCCATGCCACAAGCTCCATTCACGCGATTCTTGTGTATGTTGTTTGAGTCGTTCGCTTGCTTCAAATGTT
>Marker345442 Chr1 SNP_site:19737531 Ref_Type:C
CACAACTTATTCTGTCTGGCCACAGTAAGATTTCACTTTAATCTACCAATGATGGTTTGATGAAGTTTGTGCATTGATGAXXXXXXXXXXT
TGCTAACTGGGGGGATACTAAACTCTGGATGATACTACATGAATACTTATTTTTTATATATTGACTTCACAAACGGAGT
CACAATTTATTCTGTCTGGCCACAGTAAGATTTCACTTTAATCTACCAATGATGGTTTGATGAAGTTTGTGCATTGATGAXXXXXXXXXXT
TGCTAACTGGGGGGATACTAAACTCTGGATGATACTACATGAATACTTATTTTTTATACATTGACTTCACAAACGGAGT
>Marker345442 Chr1 SNP_site:19737826 Ref_Type:T
CACAACTTATTCTGTCTGGCCACAGTAAGATTTCACTTTAATCTACCAATGATGGTTTGATGAAGTTTGTGCATTGATGAXXXXXXXXXXT
TGCTAACTGGGGGGATACTAAACTCTGGATGATACTACATGAATACTTATTTTTTATATATTGACTTCACAAACGGAGT
CACAATTTATTCTGTCTGGCCACAGTAAGATTTCACTTTAATCTACCAATGATGGTTTGATGAAGTTTGTGCATTGATGAXXXXXXXXXXT
TGCTAACTGGGGGGATACTAAACTCTGGATGATACTACATGAATACTTATTTTTTATACATTGACTTCACAAACGGAGT
>Marker34548 Chr4 SNP_site:8590653 Ref_Type:G
ACATAAATTGAATAAAGTAATTATGTAAAGAGAGAAAAAGAAAAGAAAGAAAAATGAGAAATGAAGAGAGAAATGATTGGXXXXXXXXXXA
CTTACTAAAACCTTCATACACTAACTCTTTCTTTCAAAATACTTACCTTTTTATTACTATTATATTATTAAATGGAGTG
ACATAAATTGAATAAAGTAATTATGTAAAGAGAGAAAAAGAAAAGAAAGAAAAATGAGAAATGAAGAGAGAAATGATTGGXXXXXXXXXXA
CTTACTAAAACCTTCATACACTAACTCTTTCTTTCAAAATACTTACCTTTTTATTGCTATTATATTATTAAATGGAGTG
>Marker345789 Chr1 SNP_site:26186427 Ref_Type:A
TACTAAAAAAACAAACACATCGACTTATGTGATATACTAATTAATATCCACACATTAGTCATTTGAATTCTCATTATCCGXXXXXXXXXXG
AAGAAGAATTTGGTGTCAAAATTTCAATTGACATTAGTAGGCAACTTCGTTGTGTTTTGGACCTGATGGTGTCAAGGTC
TACTAAAAAAGCAAACACATCGACTTATGTGATATACTAATTAATATCCACACATTAGTTATTTGAATTCTCATTATCCGXXXXXXXXXXG
AAGAAGAATTTGGTGTCAAAATTTCAATTGACATTAGTAGGCAACTTCGTTGTGTTTTGGACCTGATGGTGTCAAGGTC
>Marker345789 Chr1 SNP_site:26186476 Ref_Type:C
TACTAAAAAAACAAACACATCGACTTATGTGATATACTAATTAATATCCACACATTAGTCATTTGAATTCTCATTATCCGXXXXXXXXXXG
AAGAAGAATTTGGTGTCAAAATTTCAATTGACATTAGTAGGCAACTTCGTTGTGTTTTGGACCTGATGGTGTCAAGGTC
TACTAAAAAAGCAAACACATCGACTTATGTGATATACTAATTAATATCCACACATTAGTTATTTGAATTCTCATTATCCGXXXXXXXXXXG
AAGAAGAATTTGGTGTCAAAATTTCAATTGACATTAGTAGGCAACTTCGTTGTGTTTTGGACCTGATGGTGTCAAGGTC
>Marker345832 Chr1 SNP_site:17478097 Ref_Type:A
GACGTATAGTTGGATTTGAAAGATGTGAAAAAAAGAAGAGATTTGATGATATATGAAGATGAATAATATAAATGGGGAAAXXXXXXXXXXC
TATAAACATATCTAATGGAAAAAACACACAACACTCGACTCATAAAACCTCACTATTATCTTTGTTTTCATCATCTGTT
GACGTATAGTTGGATTTGAAAGATGTGAAAAAAAGAAGAGATTTGATGATATATGAAGATGAATAATATAAATGGGGAAAXXXXXXXXXXC
TATAAACATATCTAATGGAACAAACACACAACACTCGACTCATAAAACCTCACTATTATCTTTGTTTTCATCATCTGTT
>Marker345840 Chr1 SNP_site:3371305 Ref_Type:T
ACTTGAACACTGTAACCACACCAGCCAAAATTTAACTGACTACAAACTATTATGAATAAACGTGGTTTTGAAATGGTTTAXXXXXXXXXXC
GGATAACATTCACTATTAAAGAAGGATTGTATTTGTATTTACAAATGTAGCATCAAAGAGACTTAGAAATGGGACAGTT
ACTTGAACACTGTAACCACACCAGCCAAAATTTAACTGACTACAAACTATTATGAATAAACGTGGTTTTGAAATGGTTTAXXXXXXXXXXC

Create PDF with GO2PDF for free, if you wish to

GGATAACATTCACTATTAAAGAAGGATTGTATTTGTTTTTACATATGTAGCATCAAAGAGACTTAGAAATGGGACAGTT
>Marker345840 Chr1 SNP_site:3371312 Ref_Type:T
ACTTGAACACTGTAACCACACCAGCCAAAATTTAACTGACTACAAACTATTATGAATAAACGTGGTTTTGAAATGGTTTAXXXXXXXXXXXC
GGATAACATTCACTATTAAAGAAGGATTGTATTTGTATTTACAAATGTAGCATCAAAGAGACTTAGAAATGGGACAGTT
ACTTGAACACTGTAACCACACCAGCCAAAATTTAACTGACTACAAACTATTATGAATAAACGTGGTTTTGAAATGGTTTAXXXXXXXXXXXC
GGATAACATTCACTATTAAAGAAGGATTGTATTTGTTTTTACATATGTAGCATCAAAGAGACTTAGAAATGGGACAGTT
>Marker346373 Chr1 SNP_site:20940033 Ref_Type:G
ACCATAAATTTACTTTTACTCAATTTCTTTGTTTTGTTATATCAGTTTCTAGAGTCCTAATTACAAAAGCAAAAATGAAAXXXXXXXXXXXA
TATACTTCTAACATAATAACACAAATTTGTATATCAAATACAAATTTTTCAACTTACAAATATATTAACTTAATACGTA
ACCATAAATTTACTTTTGCTCAATTTCTTTGTTTTGTTATATCAGTTTCTAGAGTCCTAATTACAAAAGCAAAAATGAAAXXXXXXXXXXXA
TATACTTCTAACATAATAACACAAATTTGTATATCAAATACAAATTTTTCAACTTACAAATATATTAACTTAATACGTA
>Marker346744 Chr1 SNP_site:10846095 Ref_Type:G
ACAAGTGCTGAGTGTTTCGACAACTCCTACCAATGACAGTGACAATAACAAGCACAACATAAAACCAATTCCTGTCTGTGXXXXXXXXXXXA
CCTTCTCAACCACCTGATCCACATGAAATACATACCAATCCCAGTCCCGTTACTTTAGGTGTCATTGTCCAACCAGGTA
ACAAGTGCTGAGTGTTTCGACAACTCCTACCAATGACAGTGACAATAACAAGCACAACATAAAACCAATTCCTGTCTGTGXXXXXXXXXXXA
CCTTCTCAACCACCTGATCCACATGAAATACATACCAATCCCGGTCCCGCTACTTTAGGTGTCATTGTCCAACCAGGTA
>Marker346744 Chr1 SNP_site:10846102 Ref_Type:C
ACAAGTGCTGAGTGTTTCGACAACTCCTACCAATGACAGTGACAATAACAAGCACAACATAAAACCAATTCCTGTCTGTGXXXXXXXXXXXA
CCTTCTCAACCACCTGATCCACATGAAATACATACCAATCCCAGTCCCGTTACTTTAGGTGTCATTGTCCAACCAGGTA
ACAAGTGCTGAGTGTTTCGACAACTCCTACCAATGACAGTGACAATAACAAGCACAACATAAAACCAATTCCTGTCTGTGXXXXXXXXXXXA
CCTTCTCAACCACCTGATCCACATGAAATACATACCAATCCCGGTCCCGCTACTTTAGGTGTCATTGTCCAACCAGGTA
>Marker346856 Chr1 SNP_site:25488475 Ref_Type:T
ACAATGGTCGAACGCTTATATTCTTTAATATATTACATGGCTATACTTTTTCAATCCATCATTTCTCTACCTGCCATCAAXXXXXXXXXXXC
TGAGAGAATAAGTAACAAGAACGTGGCTAATGCCCACAATTTTGCAAGATAACTTCCATCATTTAAATCCCGAAAGGTG
ACAATGGTCGAACGCTTATATTCTTTAATATATTACATGGCTATACTTTTTCAATCCATCATTTCTCTACCTTCCATCAAXXXXXXXXXXXC
CGAGAGAATAAGTAACAAGAACGTGGCTAATGCCCACAATTTTGCAAGATAACTTCCATCATTTATATCCCGAAAGGTG
>Marker346856 Chr1 SNP_site:25488603 Ref_Type:C
ACAATGGTCGAACGCTTATATTCTTTAATATATTACATGGCTATACTTTTTCAATCCATCATTTCTCTACCTGCCATCAAXXXXXXXXXXXC
TGAGAGAATAAGTAACAAGAACGTGGCTAATGCCCACAATTTTGCAAGATAACTTCCATCATTTAAATCCCGAAAGGTG
ACAATGGTCGAACGCTTATATTCTTTAATATATTACATGGCTATACTTTTTCAATCCATCATTTCTCTACCTTCCATCAAXXXXXXXXXXXC
CGAGAGAATAAGTAACAAGAACGTGGCTAATGCCCACAATTTTGCAAGATAACTTCCATCATTTATATCCCGAAAGGTG
>Marker346856 Chr1 SNP_site:25488668 Ref_Type:T
ACAATGGTCGAACGCTTATATTCTTTAATATATTACATGGCTATACTTTTTCAATCCATCATTTCTCTACCTGCCATCAAXXXXXXXXXXXC
TGAGAGAATAAGTAACAAGAACGTGGCTAATGCCCACAATTTTGCAAGATAACTTCCATCATTTAAATCCCGAAAGGTG
ACAATGGTCGAACGCTTATATTCTTTAATATATTACATGGCTATACTTTTTCAATCCATCATTTCTCTACCTTCCATCAAXXXXXXXXXXXC
CGAGAGAATAAGTAACAAGAACGTGGCTAATGCCCACAATTTTGCAAGATAACTTCCATCATTTATATCCCGAAAGGTG
>Marker347385 Chr1 SNP_site:6436498 Ref_Type:C
TACGGCGACAACTTGCATATTTCACGTAGCAGTTGAAAATCGACAAAAAAATGTATGCAGTATATATCAGATAGCATTTGXXXXXXXXXXXA
TTCTTTCTCCTTGCTTCCTATACTTATCTATGTTTTATAGTTAATGAAGCGGAAATGACGAGGATGCTAAGAGGATGTC
TACGGCGACAACTTGCATATTTCACGTAGCAGTTGAAAATCGACAAAAAAATGTATGCAGTATATATCAGATAGCATTTGXXXXXXXXXXXC
TTCTTTCTCCTTGCTTCCTATACTTATCTATGTTTTATAGTTAATGAAGCGGAAATGACGAGGATGCTAAGAGGATGTC
>Marker347603 Chr1 SNP_site:24115196 Ref_Type:A
AACCTAATAATTATCATTGTGAATGGAATTGCTGCTTTTGAGAACCATCAAACACAAATATCTGAGAACAGCCAAAAGTCXXXXXXXXXXXG
ACACAAAACAGAAACTCAAGGACTGTGAGGTGCTAATCGGAGAGAGAATTTGCACTAATAAATATGGATTAAAAGATGT

Create PDF with GO2PDF for free, if you wish to

AACCTAATAATTATCATTGTGAATGGACTTGCTGCTTTTGAGAACCATCAAACACAAATATCTGAGAACAGCCAAAAGTCXXXXXXXXXXG
ACACAAAACAGAAACTCAAGGACTGTGAGGTGCTAATCGGAGAGAGAATTTGCAGTAATAAATATGGATTAAAAGATGT
>Marker347603 Chr1 SNP_site:24115424 Ref_Type:C
AACCTAATAATTATCATTGTGAATGGAATTGCTGCTTTTGAGAACCATCAAACACAAATATCTGAGAACAGCCAAAAGTCXXXXXXXXXXG
ACACAAAACAGAAACTCAAGGACTGTGAGGTGCTAATCGGAGAGAGAATTTGCACTAATAAATATGGATTAAAAGATGT
AACCTAATAATTATCATTGTGAATGGACTTGCTGCTTTTGAGAACCATCAAACACAAATATCTGAGAACAGCCAAAAGTCXXXXXXXXXXG
ACACAAAACAGAAACTCAAGGACTGTGAGGTGCTAATCGGAGAGAGAATTTGCAGTAATAAATATGGATTAAAAGATGT
>Marker347961 Chr1 SNP_site:9858058 Ref_Type:G
CACATATATTGAATTTAATTGTGTGTGATGGTCTGAAATATGTGAATGATACATTGATTCAAATTCGAAATGTCATTAGGXXXXXXXXXXC
ACATACTTTAAACTATCATAGTCATGAATCATCTTCAAGCTCGATTGCATCATCACAACTTCAAGTGTTTAAGGATTGT
CACATATATTGAATTTAATTGTGTGTGATGGTCTGAAATATGTGAATGATACATTGATTCAAATTCGAAATGTCGTTAGGXXXXXXXXXXC
ACATACTTTAAACTATCATAGTCATGAATCATCTTCAAGCTCGATTGCATCATCACAACTTCAAGTGTTTAAGGATTGT
>Marker348286 Chr1 SNP_site:14315471 Ref_Type:C
ACATGTTTTGAATCTCACACCATGTGTTCCTTTGGGATCAAAAGTTCCAAACAGAATATGGTTAGGTAAGGATATATCTTXXXXXXXXXXA
ACTAAGAAAAAGCTTATAAGAAGTCGAGATGTTGTATTTGTTGAAGACCAAACAATAGCAAACATTGAGAAAATAGGTG
ACATGTTTTGAATCTCACACGATGTGTTCCTTTGGGATCAAAAGTTCCAAACAGAATATGGTCAGGTAAGGATATATCTTXXXXXXXXXXA
ACTAAGAAAAAGCTTATAAGAAGTCGAGATGTTATATTTGTTGAAGACCAAACAATAGCAAACATTGAGAAAATAGGTG
>Marker348286 Chr1 SNP_site:14315513 Ref_Type:T
ACATGTTTTGAATCTCACACCATGTGTTCCTTTGGGATCAAAAGTTCCAAACAGAATATGGTTAGGTAAGGATATATCTTXXXXXXXXXXA
ACTAAGAAAAAGCTTATAAGAAGTCGAGATGTTGTATTTGTTGAAGACCAAACAATAGCAAACATTGAGAAAATAGGTG
ACATGTTTTGAATCTCACACGATGTGTTCCTTTGGGATCAAAAGTTCCAAACAGAATATGGTCAGGTAAGGATATATCTTXXXXXXXXXXA
ACTAAGAAAAAGCTTATAAGAAGTCGAGATGTTATATTTGTTGAAGACCAAACAATAGCAAACATTGAGAAAATAGGTG
>Marker348286 Chr1 SNP_site:14315703 Ref_Type:G
ACATGTTTTGAATCTCACACCATGTGTTCCTTTGGGATCAAAAGTTCCAAACAGAATATGGTTAGGTAAGGATATATCTTXXXXXXXXXXA
ACTAAGAAAAAGCTTATAAGAAGTCGAGATGTTGTATTTGTTGAAGACCAAACAATAGCAAACATTGAGAAAATAGGTG
ACATGTTTTGAATCTCACACGATGTGTTCCTTTGGGATCAAAAGTTCCAAACAGAATATGGTCAGGTAAGGATATATCTTXXXXXXXXXXA
ACTAAGAAAAAGCTTATAAGAAGTCGAGATGTTATATTTGTTGAAGACCAAACAATAGCAAACATTGAGAAAATAGGTG
>Marker34851 Chr4 SNP_site:1572947 Ref_Type:T
AACAATAAAGAACATGTTGATATTAATTATTTAAGATAATCAGACCAAAATTATTCGGTATAAACATTATGCTTCGTTTCXXXXXXXXXXT
TTCACATAGAAATATGCAAGAACTCTAATGAAGATATGAAGTTCAAATAGAAAACACTGATAAACATCAAACCTTTGTT
AACAATAAAGAACATGTTGATATTAATTATTTAAGATAATCAGACCAAAATTATTCGGTATAAACATTATGCTTTGTTTCXXXXXXXXXXT
TTCACATAGAAATATGCAAGAACTCTAATGAAGATATGAAGTTCAAATAGAAAACACTGATAAACATCAAACCTTTGTT
>Marker349000 Chr1 SNP_site:9292508 Ref_Type:G
GACCCTTCAAGTCTAGATTCCTACGAGGAAGATTCATAATCTTTCTAGGTTGAAGGGTTGAGTTTTCCCATAAGCTTCTTXXXXXXXXXXC
TTTAAGGTTTCAGACAAGTGATTTGACGTTTCAAACGAGGTTGAGTTTTCCAGTGAAAGTGAAGAACACACACAAAGTG
GACCCTTCAAGTCTAGATTCCTACGAGGAAGATTCATAATCTTTCTAGGTTGAAGGGTTGAGTTTTCCCATAAGCTTCTTXXXXXXXXXXC
TTTGAGGTTTCAGACAAGTGATTTGACGTTTCAAACGAGGTTGAGTTTTCCAGTAAAAGTGAAGAACACACACAAAGTG
>Marker349000 Chr1 SNP_site:9292559 Ref_Type:A
GACCCTTCAAGTCTAGATTCCTACGAGGAAGATTCATAATCTTTCTAGGTTGAAGGGTTGAGTTTTCCCATAAGCTTCTTXXXXXXXXXXC
TTTAAGGTTTCAGACAAGTGATTTGACGTTTCAAACGAGGTTGAGTTTTCCAGTGAAAGTGAAGAACACACACAAAGTG
GACCCTTCAAGTCTAGATTCCTACGAGGAAGATTCATAATCTTTCTAGGTTGAAGGGTTGAGTTTTCCCATAAGCTTCTTXXXXXXXXXXC
TTTGAGGTTTCAGACAAGTGATTTGACGTTTCAAACGAGGTTGAGTTTTCCAGTAAAAGTGAAGAACACACACAAAGTG
>Marker349127 Chr1 SNP_site:8436048 Ref_Type:T
CACCTCTTACCTCGACCTCCCATCAATGGGGTTACGTTGGACATATATATTATACTTCTTAGCTTTAGTGGATCGTGCTTXXXXXXXXXXT

Create PDF with GO2PDF for free, if you wish to

TGAACCTCAAACTTAGTAAATGTTACGATGAATACACACGTATGGATTTACAATTAAAAAAGCAAATCATGAAGAGAGT
CACCTCTTACCTCGACCTCCCATCAATGGGGTTACGTTGGACATATATATTATACTTCTTAGCTTTAGTGGATCGTGCTTXXXXXXXXXXT
TGAACCTCAAACTTAGTAAATGTTATGATCAATACAGACGTATGGATTTACAATTAAAAAAGCAAATCATGAAGAGAGT
>Marker349127 Chr1 SNP_site:8436052 Ref_Type:C
CACCTCTTACCTCGACCTCCCATCAATGGGGTTACGTTGGACATATATATTATACTTCTTAGCTTTAGTGGATCGTGCTTXXXXXXXXXXT
TGAACCTCAAACTTAGTAAATGTTACGATGAATACACACGTATGGATTTACAATTAAAAAAGCAAATCATGAAGAGAGT
CACCTCTTACCTCGACCTCCCATCAATGGGGTTACGTTGGACATATATATTATACTTCTTAGCTTTAGTGGATCGTGCTTXXXXXXXXXXT
TGAACCTCAAACTTAGTAAATGTTATGATCAATACAGACGTATGGATTTACAATTAAAAAAGCAAATCATGAAGAGAGT
>Marker349127 Chr1 SNP_site:8436059 Ref_Type:G
CACCTCTTACCTCGACCTCCCATCAATGGGGTTACGTTGGACATATATATTATACTTCTTAGCTTTAGTGGATCGTGCTTXXXXXXXXXXT
TGAACCTCAAACTTAGTAAATGTTACGATGAATACACACGTATGGATTTACAATTAAAAAAGCAAATCATGAAGAGAGT
CACCTCTTACCTCGACCTCCCATCAATGGGGTTACGTTGGACATATATATTATACTTCTTAGCTTTAGTGGATCGTGCTTXXXXXXXXXXT
TGAACCTCAAACTTAGTAAATGTTATGATCAATACAGACGTATGGATTTACAATTAAAAAAGCAAATCATGAAGAGAGT
>Marker349789 Chr1 SNP_site:4905867 Ref_Type:T
AACAATTTTTAGACACCCACATTAGAGAGAGAAATAATCACCCATCTTACATAAAAACTTCCATTATCCAATAAAACAATXXXXXXXXXXA
CTCAGGAGTCGAGACACACTCGGGGACCGTTTTACTATTTCTATTATAGTTGTTGTTTCAACTGTTTAGTTAGACAGTC
AACAATTTTTAGACACCCACATTAGAGATAGAAATAATCACCCATCTTACATAAAAACTTCGATTATCCAATAAAACAATXXXXXXXXXXA
CTCAGGAGTCGAGACACACTCGGGGACCGTTTTACTATTTCTATTATAGTTGTTGTTTCAACTGTTTAGTTAGACAGTC
>Marker349789 Chr1 SNP_site:4905900 Ref_Type:G
AACAATTTTTAGACACCCACATTAGAGAGAGAAATAATCACCCATCTTACATAAAAACTTCCATTATCCAATAAAACAATXXXXXXXXXXA
CTCAGGAGTCGAGACACACTCGGGGACCGTTTTACTATTTCTATTATAGTTGTTGTTTCAACTGTTTAGTTAGACAGTC
AACAATTTTTAGACACCCACATTAGAGATAGAAATAATCACCCATCTTACATAAAAACTTCGATTATCCAATAAAACAATXXXXXXXXXXA
CTCAGGAGTCGAGACACACTCGGGGACCGTTTTACTATTTCTATTATAGTTGTTGTTTCAACTGTTTAGTTAGACAGTC
>Marker350326 Chr1 SNP_site:24173751 Ref_Type:G
ACTTCTCTTTTACTCCTTTAGGAACTGTAGCTTGGGTTTATTAAGTCCGGGGTTTTAAGCGGGTGCTTAGATTGTGGAAAXXXXXXXXXXA
TAATTGTTTCATGTAATTCATGAATTGAACTTTCAGGCGTTTATCTTTGTTCTTGTTCGACCCATTTCAAAGTGTTTGT
ACTTCTCTTTTACTCCTTTAGGAACTGTAGCTTGGGTTTATTAAGTCCTGGGTTTTAAGCGGGTGCTTAGATTGTGGAAAXXXXXXXXXXA
TAATTGTCTCATGTAATTCATGAATTGAACTTTCAGGCGTTTATCTTTGTTCTTGTTCGACCCATTTCAAAGTGTTTGT
>Marker350326 Chr1 SNP_site:24173897 Ref_Type:T
ACTTCTCTTTTACTCCTTTAGGAACTGTAGCTTGGGTTTATTAAGTCCGGGGTTTTAAGCGGGTGCTTAGATTGTGGAAAXXXXXXXXXXA
TAATTGTTTCATGTAATTCATGAATTGAACTTTCAGGCGTTTATCTTTGTTCTTGTTCGACCCATTTCAAAGTGTTTGT
ACTTCTCTTTTACTCCTTTAGGAACTGTAGCTTGGGTTTATTAAGTCCTGGGTTTTAAGCGGGTGCTTAGATTGTGGAAAXXXXXXXXXXA
TAATTGTCTCATGTAATTCATGAATTGAACTTTCAGGCGTTTATCTTTGTTCTTGTTCGACCCATTTCAAAGTGTTTGT
>Marker350824 Chr1 SNP_site:7716278 Ref_Type:G
CACATCCAACCTCACTCTTTCCTCATAGCTAAGCAAGAAGAAGAAGAAGAAGAATTGGAGGAGGAGGAGGAAGAAAGCCAXXXXXXXXXXC
TCTCTTTCCTCACCTCGTCCTCGTGAGCCTCCTCCTCCTCCTCGCTCTCTCTCTCTCTATTTCTCTCTCATTGCCGGTT
CACATCCAACCTCACTCTTTCCTCATAGCTAAGCAAGAAGAAGAAGAAGAAGAATTGGAGGAGGAGGAGGAAGAAAGCCAXXXXXXXXXXC
TCTCTTTCCTCAGCTCGTCCTCGTGAGCCTCCTCCTCCTCCTCGCTCTCTCTCTCTCTATTTCTCTCTCATTGCCGGTT
>Marker351292 Chr1 SNP_site:21325722 Ref_Type:C
ACTCTTTAATCAAGCTTTTTCACAAGTGAAGTTAATCCTTTTAATTTTAGGATTTAAATTTCGTTTTATTGTAAATTGAAXXXXXXXXXXC
GAATATTTATAGTTCTTATAAGGGGTATATTATAAAATATAATTCTATATGTTATTTGGATATTTTTTGTCATGTAGTC
ACTCTTTAATCAAGCTTTTTCATAAGTGAAGTTAATCCTTTTAATTTTAGGATTTAAATTTCGTTTTATTGTAAATTGAAXXXXXXXXXXC
GAATATTTATAGTCCTTATAAGGGGCATATTATAAAATATAATTCTATATGTTATTTGGATATTTTTTGTCATGTAGTC
>Marker351292 Chr1 SNP_site:21325894 Ref_Type:T

Create PDF with GO2PDF for free, if you wish to

ACTCTTTAATCAAGCTTTTTCACAAGTGAAGTTAATCCTTTTAATTTTAGGATTTAAATTTCGTTTTATTGTAAATTGAAXXXXXXXXXXC
GAATATTTATAGTTCTTATAAGGGGTATATTATAAAATATAATTCTATATGTTATTTGGATATTTTTTGTCATGTAGTC
ACTCTTTAATCAAGCTTTTTCATAAGTGAAGTTAATCCTTTTAATTTTAGGATTTAAATTTCGTTTTATTGTAAATTGAAXXXXXXXXXXC
GAATATTTATAGTCCTTATAAGGGGCATATTATAAAATATAATTCTATATGTTATTTGGATATTTTTTGTCATGTAGTC
>Marker351292 Chr1 SNP_site:21325906 Ref_Type:T
ACTCTTTAATCAAGCTTTTTCACAAGTGAAGTTAATCCTTTTAATTTTAGGATTTAAATTTCGTTTTATTGTAAATTGAAXXXXXXXXXXC
GAATATTTATAGTTCTTATAAGGGGTATATTATAAAATATAATTCTATATGTTATTTGGATATTTTTTGTCATGTAGTC
ACTCTTTAATCAAGCTTTTTCATAAGTGAAGTTAATCCTTTTAATTTTAGGATTTAAATTTCGTTTTATTGTAAATTGAAXXXXXXXXXXC
GAATATTTATAGTCCTTATAAGGGGCATATTATAAAATATAATTCTATATGTTATTTGGATATTTTTTGTCATGTAGTC
>Marker351762 Chr1 SNP_site:5958041 Ref_Type:C
ACTTGAGGTTAGTCTTTTATACTACCGCTCTATTACTTGCACCTTCATTGATGAGGAATAACCAACGGATAACCAGTAATXXXXXXXXXXC
TACATTTAAATTATGCTTCATTCCCACTTTGATTAACAACATCTAAATATTGTAGAGTATTTTGTCCATTTTGTTTGTT
ACTTGAGGTTAGTCTTTTATACTACCGCTCTATTACTTGCACCTTCATTGATGAGGAATAACCAATGGATAACCAGTAATXXXXXXXXXXC
TACATTTAAATTATGCTTCATTCCCACTTTGATTAACAACATCTAAATATTGTAGAGTATTTTGTCCATTTTGTTTGTT
>Marker351845 Chr1 SNP_site:1745912 Ref_Type:C
ACCCAACACATGGTTTGCAGTAAAATCACTTACAATCTTAGCCCAAAGAAATGAATCTACACATAAAACAAGAGTATAAAXXXXXXXXXXC
TTTAACACTATTCAAATCTTCAATCTAACGTCCAAAACATCATGGGTAACATCAAATCAATGTCCAATAATAATGGGTA
ACCCAACACATGGTTTGCAGTAAAATCACTTACAATCTTAGCCCAAAGAAATGAATCTACACATAAAACAAGAGTATAAAXXXXXXXXXXC
TTTAACACTCTCCAAATCTTCAATCTAACGTCCAAAACATCATGGGTAACATCAAATCAATGTCCAATAATAATGGGTA
>Marker351845 Chr1 SNP_site:1745914 Ref_Type:C
ACCCAACACATGGTTTGCAGTAAAATCACTTACAATCTTAGCCCAAAGAAATGAATCTACACATAAAACAAGAGTATAAAXXXXXXXXXXC
TTTAACACTATTCAAATCTTCAATCTAACGTCCAAAACATCATGGGTAACATCAAATCAATGTCCAATAATAATGGGTA
ACCCAACACATGGTTTGCAGTAAAATCACTTACAATCTTAGCCCAAAGAAATGAATCTACACATAAAACAAGAGTATAAAXXXXXXXXXXC
TTTAACACTCTCCAAATCTTCAATCTAACGTCCAAAACATCATGGGTAACATCAAATCAATGTCCAATAATAATGGGTA
>Marker352813 Chr1 SNP_site:7279707 Ref_Type:T
AACTGATTTGAACCAATCGAAAGCTTACCCCCAAATTTAAAAAACGCTGCTGCAATGGACCCAGCCTCAAGCGGAGAAGAXXXXXXXXXXG
TGAGGTCGCCGGCGAAGAGATCTGAGCTGACGGTAAGTGATCGGCGGGTGGAAGTGGAGGGGAGGAGATGATGAAGGGT
AACTGATTTGAACCAATCGAAAGCTTACCCCCAAATTTAAAAAACGCTGCTGCAATGGACCCAGTCTCAAGCGGAGAAGAXXXXXXXXXXG
TGAGGTCGCCGGCGAAGAGATCTGAGCTGACGGTAAGTGATCGGCGGGTGGAAGTGGAGGGGAGGAGATGATGAAGGGT
>Marker352948 Chr1 SNP_site:25837696 Ref_Type:G
GACCTATGATAGACATAGATAGATTGCTATTTGTGTCTATTTTATATATATCGAGATTGAAATCTATTTGTGTCTGTCTCXXXXXXXXXXT
GGAAATCAACAATTTATTGTGAAGTTAAATTTAGGATCTTGTAACTGTCGAGTGTGGAATTTTGAGAAAATTCTATGTG
GACCTATGATAGACATGGATAGATTGCTATTTGTGTCTATTTTATATATATCGAGATTGAAATCTATTTGTGTCTGTCTCXXXXXXXXXXT
GGAAATCAACAATTCATTGTGAAGTTAAATTTAGGATCTTGTAACTGTCGAGTGTGGAATTTTGAGAAAATTCTATGTG
>Marker352948 Chr1 SNP_site:25837898 Ref_Type:C
GACCTATGATAGACATAGATAGATTGCTATTTGTGTCTATTTTATATATATCGAGATTGAAATCTATTTGTGTCTGTCTCXXXXXXXXXXT
GGAAATCAACAATTTATTGTGAAGTTAAATTTAGGATCTTGTAACTGTCGAGTGTGGAATTTTGAGAAAATTCTATGTG
GACCTATGATAGACATGGATAGATTGCTATTTGTGTCTATTTTATATATATCGAGATTGAAATCTATTTGTGTCTGTCTCXXXXXXXXXXT
GGAAATCAACAATTCATTGTGAAGTTAAATTTAGGATCTTGTAACTGTCGAGTGTGGAATTTTGAGAAAATTCTATGTG
>Marker353204 Chr1 SNP_site:1628363 Ref_Type:C
AACTTGAATGAGCTTAACAAGTTCAACATCTCATACAATCCTCTCATAACTGGGGAAGTAATTCCAAGTGGGCAATTCTCXXXXXXXXXXC
AGGATCATCAAAAAGGAATTCAAGGCTAGTTGGAATGTTGGCTTCTTTATCATTGATCCTTGCTTTTTTGGTATTTGGT
AACTTGAATGAGCTTAACAAGTTCAACATCTCATACAATCCTCTCATAACTGGGGAAGTAATTCCAAGTGGGCAATTCTCXXXXXXXXXXC
AGGATCATCAAAAAGGAATTCAAGGCTAGTTGGAATGTTGGCTTCTTTATCCTTGATCCTTGCTTTTTTGGTATTTGGT

Create PDF with GO2PDF for free, if you wish to

>Marker354216 Chr1 SNP_site:23013935 Ref_Type:T
CACCAACAAAGAGCAAATGAACTATTTTCTCCCATTTATCATCGGCCGATCTCTTTCTTGGCGTAATTATAAGATTCCCTXXXXXXXXXXT
TTGCCCAAGAACAAAAAAGAAAGAAAAGGTGAAAAGGAAAATATAATAACGATAATATTTACCCCTTCTTTTTCATTGT
CACCAACAAAGAGCAAATGAACTATTTTCTCCCATTTATCATCGGCCTATCTCTTTCTTGGCGTAATTATAAGATTCCCTXXXXXXXXXXT
TTGCCCAAGAACAAAAAAGAAAGAGAAGGTGAAAAGGAAAATATACTAACGATAATATTTACCCCTTCTTTTTCATTGT
>Marker354216 Chr1 SNP_site:23014105 Ref_Type:G
CACCAACAAAGAGCAAATGAACTATTTTCTCCCATTTATCATCGGCCGATCTCTTTCTTGGCGTAATTATAAGATTCCCTXXXXXXXXXXT
TTGCCCAAGAACAAAAAAGAAAGAAAAGGTGAAAAGGAAAATATAATAACGATAATATTTACCCCTTCTTTTTCATTGT
CACCAACAAAGAGCAAATGAACTATTTTCTCCCATTTATCATCGGCCTATCTCTTTCTTGGCGTAATTATAAGATTCCCTXXXXXXXXXXT
TTGCCCAAGAACAAAAAAGAAAGAGAAGGTGAAAAGGAAAATATACTAACGATAATATTTACCCCTTCTTTTTCATTGT
>Marker354216 Chr1 SNP_site:23014126 Ref_Type:C
CACCAACAAAGAGCAAATGAACTATTTTCTCCCATTTATCATCGGCCGATCTCTTTCTTGGCGTAATTATAAGATTCCCTXXXXXXXXXXT
TTGCCCAAGAACAAAAAAGAAAGAAAAGGTGAAAAGGAAAATATAATAACGATAATATTTACCCCTTCTTTTTCATTGT
CACCAACAAAGAGCAAATGAACTATTTTCTCCCATTTATCATCGGCCTATCTCTTTCTTGGCGTAATTATAAGATTCCCTXXXXXXXXXXT
TTGCCCAAGAACAAAAAAGAAAGAGAAGGTGAAAAGGAAAATATACTAACGATAATATTTACCCCTTCTTTTTCATTGT
>Marker354508 Chr1 SNP_site:11709245 Ref_Type:C
ACTCCTAGACTACTAGCTAAGGAGAGTTCAAATGACCCATGAAGGAAGACCTCTGAGCCATTGAATGTGAAATAATTTGGXXXXXXXXXXT
TCAAGAGGCACAATTATAATATAAGCATCGGATGCACATCACCATCTTATATAAATAACCAAGGCCACTGGTCGAAAGT
ACTCCTAGACTACTAGCTAAGGAGAGTTCAAATGACCCATGAAGGAAGACCTTTGAACCATTGAATGTGAAATAATTTGGXXXXXXXXXXT
TCAAGAGGCACAATTATAATATAAGCATCGGATGCACACCACCATCTTATATAAATAACCAAGGCCACTGGTCGAAAGT
>Marker354508 Chr1 SNP_site:11709249 Ref_Type:G
ACTCCTAGACTACTAGCTAAGGAGAGTTCAAATGACCCATGAAGGAAGACCTCTGAGCCATTGAATGTGAAATAATTTGGXXXXXXXXXXT
TCAAGAGGCACAATTATAATATAAGCATCGGATGCACATCACCATCTTATATAAATAACCAAGGCCACTGGTCGAAAGT
ACTCCTAGACTACTAGCTAAGGAGAGTTCAAATGACCCATGAAGGAAGACCTTTGAACCATTGAATGTGAAATAATTTGGXXXXXXXXXXT
TCAAGAGGCACAATTATAATATAAGCATCGGATGCACACCACCATCTTATATAAATAACCAAGGCCACTGGTCGAAAGT
>Marker354508 Chr1 SNP_site:11709419 Ref_Type:T
ACTCCTAGACTACTAGCTAAGGAGAGTTCAAATGACCCATGAAGGAAGACCTCTGAGCCATTGAATGTGAAATAATTTGGXXXXXXXXXXT
TCAAGAGGCACAATTATAATATAAGCATCGGATGCACATCACCATCTTATATAAATAACCAAGGCCACTGGTCGAAAGT
ACTCCTAGACTACTAGCTAAGGAGAGTTCAAATGACCCATGAAGGAAGACCTTTGAACCATTGAATGTGAAATAATTTGGXXXXXXXXXXT
TCAAGAGGCACAATTATAATATAAGCATCGGATGCACACCACCATCTTATATAAATAACCAAGGCCACTGGTCGAAAGT
>Marker355094 Chr1 SNP_site:3715197 Ref_Type:T
AACGGAACAAAGTAATCCAATAGGTTAATGGTTTTTTCCTGAAAAAGCAAGGCACCACAATCATGTCAGCAGAAGAAACAXXXXXXXXXXG
GATACAAATTGATCTTATAAGAAGAAATAGATGGATGTAGATCAGAACAGTTAATGGCACGATCGAAAACTAAAAGGGT
AACGGAACAAAGTAATCCAATAGGTTAATGGTTTTTTCCTGAAAAAGCAAGGCACCACAATCATGTCAGCAGAAGAAATAXXXXXXXXXXG
GATACAAATTGATTTAATAAGAAGAAATAGATGGATGTAGATCAGAACAGTTAATGGCACGATCGAAAACTAAAAGGGT
>Marker355094 Chr1 SNP_site:3715323 Ref_Type:T
AACGGAACAAAGTAATCCAATAGGTTAATGGTTTTTTCCTGAAAAAGCAAGGCACCACAATCATGTCAGCAGAAGAAACAXXXXXXXXXXG
GATACAAATTGATCTTATAAGAAGAAATAGATGGATGTAGATCAGAACAGTTAATGGCACGATCGAAAACTAAAAGGGT
AACGGAACAAAGTAATCCAATAGGTTAATGGTTTTTTCCTGAAAAAGCAAGGCACCACAATCATGTCAGCAGAAGAAATAXXXXXXXXXXG
GATACAAATTGATTTAATAAGAAGAAATAGATGGATGTAGATCAGAACAGTTAATGGCACGATCGAAAACTAAAAGGGT
>Marker355094 Chr1 SNP_site:3715325 Ref_Type:A
AACGGAACAAAGTAATCCAATAGGTTAATGGTTTTTTCCTGAAAAAGCAAGGCACCACAATCATGTCAGCAGAAGAAACAXXXXXXXXXXG
GATACAAATTGATCTTATAAGAAGAAATAGATGGATGTAGATCAGAACAGTTAATGGCACGATCGAAAACTAAAAGGGT
AACGGAACAAAGTAATCCAATAGGTTAATGGTTTTTTCCTGAAAAAGCAAGGCACCACAATCATGTCAGCAGAAGAAATAXXXXXXXXXXG

Create PDF with GO2PDF for free, if you wish to

GATACAAATTGATTTAATAAGAAGAAATAGATGGATGTAGATCAGAACAGTTAATGGCACGATCGAAAACTAAAAGGGT
>Marker355366 Chr1 SNP_site:1131526 Ref_Type:C
TACCTTTCCGGTAGAACTCCATACTTCTCCCATACATAAAAGAACTCACGATGTGATGAATTAGCTGCCGGAATATCCCCXXXXXXXXXXG
TCCTCATATCGGCCTCATGATACCTACATTACCAAATAATTTCAAACAGCCCCCCAACTTAAGATAATATATTTTTGTA
TACCTTTCCGGTAGAACTCCATACTTCTCCCATACATAAAAGAACTCACGATGTGATGAATTAGCTGCTGGAATATCCCCXXXXXXXXXXG
TCCTCATATCGGCCTCATGATACCTACATTACCAAATAATTTCAAACAGCCCCCCAACTTAAGATAATATATTTTTGTA
>Marker355607 Chr1 SNP_site:2459536 Ref_Type:A
ACCTCCATTCTCAACTTCTTCCACAGAAGACGGATTTGGGGGATTCGACGGATTCGACGGATTCAATGAAATGCTTCGTTXXXXXXXXXXA
GCTCCAACACCACTGCATACCCCTGATACGAATCAAAACAAGAAAACTTTTAATCAATCTTCAAAGAGGCAAACGTGTG
ACCTCCATTCTCAACTTCTTCCACAGGAGACGGATTTGGGGGATTCGACGGATTCGACGGATTCAATGAAATGCTTCGTTXXXXXXXXXXA
GCTCCAACACCACTGCATACCCCTGATACGAATCAAAACAAGAAAACTTTTAATCAATCTTCAAAGAGGCAAACATGTG
>Marker355607 Chr1 SNP_site:2459750 Ref_Type:G
ACCTCCATTCTCAACTTCTTCCACAGAAGACGGATTTGGGGGATTCGACGGATTCGACGGATTCAATGAAATGCTTCGTTXXXXXXXXXXA
GCTCCAACACCACTGCATACCCCTGATACGAATCAAAACAAGAAAACTTTTAATCAATCTTCAAAGAGGCAAACGTGTG
ACCTCCATTCTCAACTTCTTCCACAGGAGACGGATTTGGGGGATTCGACGGATTCGACGGATTCAATGAAATGCTTCGTTXXXXXXXXXXA
GCTCCAACACCACTGCATACCCCTGATACGAATCAAAACAAGAAAACTTTTAATCAATCTTCAAAGAGGCAAACATGTG
>Marker356416 Chr1 SNP_site:5931666 Ref_Type:T
AACATCACATAATTTTACAACTAAAACAAACAAACACTTCTCCTCCTCCACTTCTTAATTCCAAAAGTATCCATTAAAAAXXXXXXXXXXT
TATGCTAGATGGGCTAGGATGTGATCGAGAATGTGTAGTTTCTTACAAATATCATTGATTATTTGAACATGCTTAGGTG
AACATCACATAATTTTACAACTAAAACAAACAAACACTTCTCCTCCTCCACTTTTTAATTCCAAAAGTATCCATTAAAAAXXXXXXXXXXT
TATGCTAGATGGGCTAGGATGTGATCGAGAATGTGTAGTTTCTTACAAATATCATTGATTATTTGAACATGCTTAGGTG
>Marker357085 Chr1 SNP_site:20693616 Ref_Type:A
CACAGGCAATAATTTGTGGAAGGGTTGGGCAAGCCCCTGTGCAGAAGATCTTGAGAAATGGGCGGACGATAACAATCTTTXXXXXXXXXXG
TTCAAAAACTTTGTAGAAAGTATGATTTGAACCTCTCATAATAACTGATTTGACTGTATAATTTCACTGAATCTCTGTG
CACAGGCAATAATTTGTGGAAGGGTTGGGCAAGCCCCTGTGCAGAAGATCTTGAGAAATGGGCGGACGATAACAATCTTTXXXXXXXXXXG
TTCAAAAACTTTGTAGAAAGTATGATTTGAACCTCTCATAATAACTGATTTGACTGTATAATTTCACTGACTCTCTGTG
>Marker357159 Chr1 SNP_site:22987425 Ref_Type:G
AACCAAACGGTGAGTTATCTTTTTTACCCACTCAATGGAACTCTTCTAAATTAACAAGTTGTCGATACTCCTAAATCATAXXXXXXXXXXA
AAAAAAAAAAAAGATCCCAAGTAAAGTAAAGTAAGGGAAATTTGGGATGTGATGTGCATGCTAGTGATAGTGAATGGGT
AACCAAAGGGTGAGTTATCTTTTTTACCCACTCAATGGAACTCTTCTAAATTAACAAGTTGTCGATACTCCTAAATCATAXXXXXXXXXXA
AAAAAAAAAAAAGATCCCAAGTAAAGTAAAGTAAGGGAAATTTGGGATGTGATGTGCATGCTAGTGATAGTGAATGGGT
>Marker35761 Chr4 SNP_site:6059124 Ref_Type:C
ACCCTCACTGATAGATATGAGTGTGGTTGAGAAGGATATTTAGGCCGATGAAAAGTTGAAAGGGATTTATGATCAAGATGXXXXXXXXXXT
GGGACATTTGCCCACTTACAAGCGTATTGCAGCGGAGTTATTTTGGGAAGGGATGAGCAATGACATTAAAAAGTATGTG
ACCCTCACTGCTAGATATGAGTGTGGTTGAGAAGGAGATTTAGGCCGATGAAAAGTTGAAAGGGATTTATGATCAAGATGXXXXXXXXXXT
GGGACATTTGCCCACTTACAAGCGTATTGCGGCGGAGTTATTTTGGGAAGGGATGAGCAATGACATTAAAAAGTATGTG
>Marker35761 Chr4 SNP_site:6059150 Ref_Type:G
ACCCTCACTGATAGATATGAGTGTGGTTGAGAAGGATATTTAGGCCGATGAAAAGTTGAAAGGGATTTATGATCAAGATGXXXXXXXXXXT
GGGACATTTGCCCACTTACAAGCGTATTGCAGCGGAGTTATTTTGGGAAGGGATGAGCAATGACATTAAAAAGTATGTG
ACCCTCACTGCTAGATATGAGTGTGGTTGAGAAGGAGATTTAGGCCGATGAAAAGTTGAAAGGGATTTATGATCAAGATGXXXXXXXXXXT
GGGACATTTGCCCACTTACAAGCGTATTGCGGCGGAGTTATTTTGGGAAGGGATGAGCAATGACATTAAAAAGTATGTG
>Marker35761 Chr4 SNP_site:6059359 Ref_Type:G
ACCCTCACTGATAGATATGAGTGTGGTTGAGAAGGATATTTAGGCCGATGAAAAGTTGAAAGGGATTTATGATCAAGATGXXXXXXXXXXT
GGGACATTTGCCCACTTACAAGCGTATTGCAGCGGAGTTATTTTGGGAAGGGATGAGCAATGACATTAAAAAGTATGTG

Create PDF with GO2PDF for free, if you wish to

ACCCTCACTGCTAGATATGAGTGTGGTTGAGAAGGAGATTTAGGCCGATGAAAAGTTGAAAGGGATTTATGATCAAGATGXXXXXXXXXXT
GGGACATTTGCCCACTTACAAGCGTATTGCGGCGGAGTTATTTTGGGAAGGGATGAGCAATGACATTAAAAAGTATGTG
>Marker357839 Chr1 SNP_site:847000 Ref_Type:T
TACAAATTCGTAGAGTTTTAAACCAAGTATTGTGCTCGTTTTAATTTTTTGGTTTATTTTACCGTCATAAAGGTTAAACAXXXXXXXXXXT
AAGTTTGAAAGAAAAATAATTAAATTTGTTTTGAATTTAGATTTATTTAACCCAAGTGTCTTAATTATATTTATAGGTT
TACAAATTTGTAGAGTTTTAAACCAAGTATTGTGCTCGTTTTAATTTTTTGGTTTATTTTACCGTCGTAAAGGTTAAACAXXXXXXXXXXT
AAGTTTGAAAGAAAAATAATTAAATTTGTTTTGAATTTAGATTTATTTAACCCAAGTGTCTTAATTATATTTATAGGTT
>Marker357839 Chr1 SNP_site:847058 Ref_Type:G
TACAAATTCGTAGAGTTTTAAACCAAGTATTGTGCTCGTTTTAATTTTTTGGTTTATTTTACCGTCATAAAGGTTAAACAXXXXXXXXXXT
AAGTTTGAAAGAAAAATAATTAAATTTGTTTTGAATTTAGATTTATTTAACCCAAGTGTCTTAATTATATTTATAGGTT
TACAAATTTGTAGAGTTTTAAACCAAGTATTGTGCTCGTTTTAATTTTTTGGTTTATTTTACCGTCGTAAAGGTTAAACAXXXXXXXXXXT
AAGTTTGAAAGAAAAATAATTAAATTTGTTTTGAATTTAGATTTATTTAACCCAAGTGTCTTAATTATATTTATAGGTT
>Marker358029 Chr1 SNP_site:10677314 Ref_Type:G
GACCTAGAGCCTAACTCACCGCTGCCTATTTGCTTGAGAGTTTTAGTTTCCATTCCAGTATGTCTTATATATCTAAAACAXXXXXXXXXXC
GTGGTAGAACTTAGTTGTTCATGGTATGGGCCACACTACATTTATATTAAGTTTGGTTTCATCTTCAAGCATGAGCGTG
GACCTAGAGCCTGACTCACCGCTGCCTATTTGCTTGAGAGTTTTAGTTTCCATTCCAGTATGTCTTATATATCTAAAACAXXXXXXXXXXC
GTGGTAGAACTTAGTTGTTCATGGTATGGGCCACACTACATTTATATTAAGTTTGGTTTCATCTTCAAGCATGAGCGTG
>Marker35867 Chr4 SNP_site:115432 Ref_Type:A
AACTTTTAATAAGTTAAACTGATTCATTACGTTTGATAGACCAATATATTTGACATTCATAAAAAAAAATACAATAAAATXXXXXXXXXXC
AAAGCAGTTGTTGGTCACCTATAAAGCACCAACCCTCTTGGAATGACAAAGTGCAGGCCTCGTCTAACACTGACACGTT
AACTTTTAATAAGTTAAACTGATTCATTACGTTTGATAGACCAATATATTTGACATTCATAGAAAAAAATACAATAAAATXXXXXXXXXXC
AAAGCAGTTGTTGGTCACCTATAAAGCACCAACCCTCTTGGAATGGCAAAGTGCAGGCCTCGTCTAACACTGACACGTT
>Marker35867 Chr4 SNP_site:115578 Ref_Type:A
AACTTTTAATAAGTTAAACTGATTCATTACGTTTGATAGACCAATATATTTGACATTCATAAAAAAAAATACAATAAAATXXXXXXXXXXC
AAAGCAGTTGTTGGTCACCTATAAAGCACCAACCCTCTTGGAATGACAAAGTGCAGGCCTCGTCTAACACTGACACGTT
AACTTTTAATAAGTTAAACTGATTCATTACGTTTGATAGACCAATATATTTGACATTCATAGAAAAAAATACAATAAAATXXXXXXXXXXC
AAAGCAGTTGTTGGTCACCTATAAAGCACCAACCCTCTTGGAATGGCAAAGTGCAGGCCTCGTCTAACACTGACACGTT
>Marker358824 Chr1 SNP_site:6590050 Ref_Type:G
CACCTCCAAATCCACCACCTGCTCCTCCGCCAGCTCCACCACCTAAAATGCCACCACCACCGCCGCCGCCAAACCCTCCGXXXXXXXXXXG
AAAGGCAATGTCTTTTCACTCTCAATCTCTCCCCCATTTGTAATTACAAGTTTTCTGGGCTCTGAGGCCCAAATTAGTG
CACCTCCAAATCCACCACCTGCTCCTCCGCCAGCTCCGCCACCTAAAATGCCACCACCACCGCCGCCGCCAAACCCTCCGXXXXXXXXXXG
AAAGGCAATGTCTTTTCACTCTCAATCTCTCCCCCATTTGTAATTACAAGTTTTCTGGGCTCTGAGGCCCAAATTAGTG
>Marker359007 Chr1 SNP_site:26044232 Ref_Type:T
ACCTCTTGTTTCAAAAAAGCAGAGAACCCAGAAGATGAAATTATGGAAGTTGAATCAGTTATCAGTGTGAGTAGTGCAGAXXXXXXXXXXA
AGACAAAAGACTCCTTCCCCCTCAAGAGTTAATCCCATTCTTGGCAGCCTTTGAAATTAAATTGATCTAAGGCAGGGGT
ACCTCTTGTTTCAAAAAAGCAGAGAACCCAGAAGATGAAATTATGGAAGTTGAATCAGTTATCAGTGTGAGTAGTGCAGAXXXXXXXXXXA
AGACAAAAGACTCCTTCCCCCTCAAGAGTTAATCCCATTCTTGGCAGCCTTTTAAATTAAATTGATCTAAGGCAGGGGT
>Marker359019 Chr1 SNP_site:257575 Ref_Type:T
AACCATTATCTCCAATTTTATAACTTTAAAAAGTGACTCTTAAGTTGTGAGAGTGGTGCAGTAATGATGTGAAATAAAAAXXXXXXXXXXT
GAATTTACACAATTTGAACAAATTCAAACTTCGTTTATACCAAAGGATAATATATATTAAATTGTTCATTCTTGAAAGT
AACCATTATCTCCAATTTTATAACTTTAAAAAGTGACTCTTAAGTTGTGAGAGTGGTGCAGTAATGATGTGAAATAAAAAXXXXXXXXXXT
GAATTTACACAATTTGAACAAATTCAAACTTTGTTTATACCTAAGGATAATATATATTAAATTGTTCATTCTTGAAAGT
>Marker359019 Chr1 SNP_site:257585 Ref_Type:T
AACCATTATCTCCAATTTTATAACTTTAAAAAGTGACTCTTAAGTTGTGAGAGTGGTGCAGTAATGATGTGAAATAAAAAXXXXXXXXXXT

Create PDF with GO2PDF for free, if you wish to

GAATTTACACAATTTGAACAAATTCAAACTTCGTTTATACCAAAGGATAATATATATTAAATTGTTCATTCTTGAAAGT
AACCATTATCTCCAATTTTATAACTTTAAAAAGTGACTCTTAAGTTGTGAGAGTGGTGCAGTAATGATGTGAAATAAAAAXXXXXXXXXXT
GAATTTACACAATTTGAACAAATTCAAACTTTGTTTATACCTAAGGATAATATATATTAAATTGTTCATTCTTGAAAGT
>Marker35963 Chr4 SNP_site:4973033 Ref_Type:A
TACAATCACTCTGAATCGTCTTCTCATTGTTTTTATTATTATTTTCCGTCCATTTCTGTGTTGCCTGACTTCGGCTAATTXXXXXXXXXXT
CTGTTATGGTGTTTGATTGTTGTTATTGAAGTTCGTTCTGTTATATGAAATTTTGTAGGGATTTTTGAAGATTTAAGTG
TACAATCACTCTGAATCGTCTTCTCATTGTTTTTATTCTTATTTTCCGTCCATTTCTGTGTTGCCTGACTTCGGCTAATTXXXXXXXXXXT
CTGTTATGGTGTTTGATTGTTGTTATTGAAGTTCGTTCTGTTATATGAAATTTTGTAGGGATTTTTGAAGATTTAAGTG
>Marker359692 Chr1 SNP_site:16842432 Ref_Type:T
ACTGTTACAGATGCCCCACCTTTTGACCCTACGAATATTGTGTCACTGCAGGCAAGATTGCTTTTCGTTTAGTTTCTTCTXXXXXXXXXXC
CAACTTTTGTTGAAGGTCCATTTCAACTTCCATTATCAAGTATAAGGGCTTATATAAAATATCCTCTAACTCCCAGGTT
ACTGTTACAGATGCCCCACCTTTTGATCCTACGAATATTGTGTCACTGCAGGCAAGATTGCTTTTCTTTTAGTTTCTTCTXXXXXXXXXXC
CAACTTTTGTTGAAGGTCCATTTCAACTTCCACTATCAAGTATAAGGGCTTATATAAAATATCCTCTAACTCCCAGGTT
>Marker359692 Chr1 SNP_site:16842472 Ref_Type:T
ACTGTTACAGATGCCCCACCTTTTGACCCTACGAATATTGTGTCACTGCAGGCAAGATTGCTTTTCGTTTAGTTTCTTCTXXXXXXXXXXC
CAACTTTTGTTGAAGGTCCATTTCAACTTCCATTATCAAGTATAAGGGCTTATATAAAATATCCTCTAACTCCCAGGTT
ACTGTTACAGATGCCCCACCTTTTGATCCTACGAATATTGTGTCACTGCAGGCAAGATTGCTTTTCTTTTAGTTTCTTCTXXXXXXXXXXC
CAACTTTTGTTGAAGGTCCATTTCAACTTCCACTATCAAGTATAAGGGCTTATATAAAATATCCTCTAACTCCCAGGTT
>Marker359692 Chr1 SNP_site:16842610 Ref_Type:C
ACTGTTACAGATGCCCCACCTTTTGACCCTACGAATATTGTGTCACTGCAGGCAAGATTGCTTTTCGTTTAGTTTCTTCTXXXXXXXXXXC
CAACTTTTGTTGAAGGTCCATTTCAACTTCCATTATCAAGTATAAGGGCTTATATAAAATATCCTCTAACTCCCAGGTT
ACTGTTACAGATGCCCCACCTTTTGATCCTACGAATATTGTGTCACTGCAGGCAAGATTGCTTTTCTTTTAGTTTCTTCTXXXXXXXXXXC
CAACTTTTGTTGAAGGTCCATTTCAACTTCCACTATCAAGTATAAGGGCTTATATAAAATATCCTCTAACTCCCAGGTT
>Marker359713 Chr1 SNP_site:7529003 Ref_Type:G
CACTGCGCTGTTCTTTTTTGCTCTGGTTCAGTTATTTTTTTGCTACATTCCCATATCGTTTATTTATTCGTGTGTTTCTTXXXXXXXXXXC
TTATTTGGAATTTAAAAGAATGCCCTGGAGGAATTTAACCCTCAACCTTTTTCACTAGGAAATGGTAAAGTTTAGTAGT
CACTTCGCTGTTCTTTTTTGCTCTGGTTCAGTTATTTTTTTGCTACATTCCCATATCGTTTATTTATTTGTGTGTTTCTTXXXXXXXXXXC
TTATTTGGAATTTAAAAGAATGCCCTGGAGGAAATTAACCCTCAACCTTTTTCACTAGGAAATGGTAAAGTTTAGTAGT
>Marker359713 Chr1 SNP_site:7529067 Ref_Type:C
CACTGCGCTGTTCTTTTTTGCTCTGGTTCAGTTATTTTTTTGCTACATTCCCATATCGTTTATTTATTCGTGTGTTTCTTXXXXXXXXXXC
TTATTTGGAATTTAAAAGAATGCCCTGGAGGAATTTAACCCTCAACCTTTTTCACTAGGAAATGGTAAAGTTTAGTAGT
CACTTCGCTGTTCTTTTTTGCTCTGGTTCAGTTATTTTTTTGCTACATTCCCATATCGTTTATTTATTTGTGTGTTTCTTXXXXXXXXXXC
TTATTTGGAATTTAAAAGAATGCCCTGGAGGAAATTAACCCTCAACCTTTTTCACTAGGAAATGGTAAAGTTTAGTAGT
>Marker359713 Chr1 SNP_site:7529233 Ref_Type:T
CACTGCGCTGTTCTTTTTTGCTCTGGTTCAGTTATTTTTTTGCTACATTCCCATATCGTTTATTTATTCGTGTGTTTCTTXXXXXXXXXXC
TTATTTGGAATTTAAAAGAATGCCCTGGAGGAATTTAACCCTCAACCTTTTTCACTAGGAAATGGTAAAGTTTAGTAGT
CACTTCGCTGTTCTTTTTTGCTCTGGTTCAGTTATTTTTTTGCTACATTCCCATATCGTTTATTTATTTGTGTGTTTCTTXXXXXXXXXXC
TTATTTGGAATTTAAAAGAATGCCCTGGAGGAAATTAACCCTCAACCTTTTTCACTAGGAAATGGTAAAGTTTAGTAGT
>Marker359924 Chr1 SNP_site:24170666 Ref_Type:T
AACTTTAATTTCTCTCGATATTGGAAAGATTTTGAACAACAAATGCTTTAATCCAAGAACAAGACTGTTGCTCCATCTTCXXXXXXXXXXT
CAAACCGAAATATGGGAATAATATTTGGACGATGGTGTTGGTAATCAACTCTTTCCCATTAAAGAGCTCCATCGAAGTA
AACTTTAATTTCTCTCGATATTGGAAAGATTTTGAATAACAAATGCTTTAATCCAAGAACAAGACTGTTGCTCCATCTTCXXXXXXXXXXT
CAAACCGAAATATGGGAATAATATTTGGACGATGGTGTTGGTAATCAACTCTTTCTCATTAAAGAGCTCCATCGAAGTA
>Marker359924 Chr1 SNP_site:24170910 Ref_Type:T

Create PDF with GO2PDF for free, if you wish to

AACTTTAATTTCTCTCGATATTGGAAAGATTTTGAACAACAAATGCTTTAATCCAAGAACAAGACTGTTGCTCCATCTTCXXXXXXXXXXT
CAAACCGAAATATGGGAATAATATTTGGACGATGGTGTTGGTAATCAACTCTTTCCCATTAAAGAGCTCCATCGAAGTA
AACTTTAATTTCTCTCGATATTGGAAAGATTTTGAATAACAAATGCTTTAATCCAAGAACAAGACTGTTGCTCCATCTTCXXXXXXXXXXT
CAAACCGAAATATGGGAATAATATTTGGACGATGGTGTTGGTAATCAACTCTTTCTCATTAAAGAGCTCCATCGAAGTA
>Marker359941 Chr1 SNP_site:27863348 Ref_Type:C
AACCGACAATGTCACTAAGGATGGTGTTTGGAGTTGGTCGATGAATTTTCTAGATTGAATTAGAAAAAAAAATTAATCCAXXXXXXXXXXG
GGCATCACTATGATCGCTTGTTAAGAGGCATGAGATTGATAATTAACGATAACGATTGGTTGTTGTCGATCAAGATGTT
AACCGACAATGTCACTAAGGATGGTGTTTGGAGTTGGTTGATGAATTTTCTAGATTGAATTAGAAAAAAAAAATAATCCAXXXXXXXXXXG
GGCATCACTATGATCGCTTGTTAAGAGGCATGGGATTGATAATTAACGATAACGATTGGTTGTTGTCGATCAAGATGTT
>Marker359941 Chr1 SNP_site:27863382 Ref_Type:T
AACCGACAATGTCACTAAGGATGGTGTTTGGAGTTGGTCGATGAATTTTCTAGATTGAATTAGAAAAAAAAATTAATCCAXXXXXXXXXXG
GGCATCACTATGATCGCTTGTTAAGAGGCATGAGATTGATAATTAACGATAACGATTGGTTGTTGTCGATCAAGATGTT
AACCGACAATGTCACTAAGGATGGTGTTTGGAGTTGGTTGATGAATTTTCTAGATTGAATTAGAAAAAAAAAATAATCCAXXXXXXXXXXG
GGCATCACTATGATCGCTTGTTAAGAGGCATGGGATTGATAATTAACGATAACGATTGGTTGTTGTCGATCAAGATGTT
>Marker359941 Chr1 SNP_site:27863584 Ref_Type:A
AACCGACAATGTCACTAAGGATGGTGTTTGGAGTTGGTCGATGAATTTTCTAGATTGAATTAGAAAAAAAAATTAATCCAXXXXXXXXXXG
GGCATCACTATGATCGCTTGTTAAGAGGCATGAGATTGATAATTAACGATAACGATTGGTTGTTGTCGATCAAGATGTT
AACCGACAATGTCACTAAGGATGGTGTTTGGAGTTGGTTGATGAATTTTCTAGATTGAATTAGAAAAAAAAAATAATCCAXXXXXXXXXXG
GGCATCACTATGATCGCTTGTTAAGAGGCATGGGATTGATAATTAACGATAACGATTGGTTGTTGTCGATCAAGATGTT
>Marker360007 Chr1 SNP_site:5902074 Ref_Type:C
AACAACTCTGGTGACCCATTAACATTTAAATGTCAAAAAAACCTGTAAAATACTAAATCCTATACAGATTGCCTCTATAGXXXXXXXXXXA
GGAATCAAATGAGTTTGTTTAGAAAATCTACTACCAATCTACCTCCTGTTTCTAAGATCTTTCCTTCATGTTGAATGTG
AACAACTCTGGTGACCCATTAACATTTAAATGTCAAAAAAACCTGTAAAATACTAAATCCTATATAGATTGCCCCTATAGXXXXXXXXXXA
GGAATCAAATGAGTTTGTTTAGAAAATCTACTACCAATCTACCTCCTGTTTCTAAGATCTTTCCTTCATGTTGAATGTG
>Marker360007 Chr1 SNP_site:5902083 Ref_Type:T
AACAACTCTGGTGACCCATTAACATTTAAATGTCAAAAAAACCTGTAAAATACTAAATCCTATACAGATTGCCTCTATAGXXXXXXXXXXA
GGAATCAAATGAGTTTGTTTAGAAAATCTACTACCAATCTACCTCCTGTTTCTAAGATCTTTCCTTCATGTTGAATGTG
AACAACTCTGGTGACCCATTAACATTTAAATGTCAAAAAAACCTGTAAAATACTAAATCCTATATAGATTGCCCCTATAGXXXXXXXXXXA
GGAATCAAATGAGTTTGTTTAGAAAATCTACTACCAATCTACCTCCTGTTTCTAAGATCTTTCCTTCATGTTGAATGTG
>Marker360132 Chr1 SNP_site:28178773 Ref_Type:G
TACTTGAAATGTATATACATTTTCAAGGTTGTTCTATTTTTGATAGAATATTATCAATTTTCTTATTTAAAACAATTAAAXXXXXXXXXXC
AATTCACAAGTATTCTAACAAAGTCTTTAGTTACATCATGTTTTTTAAGATACAAGAACATATTTTTTTAAGAAAAGTT
TACTTGAAATGTATATACATTTTCAAGGTTGTTCTATTTTTGATAGAATATTATCAATTTTCTTATTTAAAACAGTTAAAXXXXXXXXXXC
AATTCATAAGTATTCTAACAAAGTCTTTAGTTACATCATGTTTTTTAAGATACAAGAACATATTTTTTTAAGAAAAGTT
>Marker360132 Chr1 SNP_site:28178889 Ref_Type:T
TACTTGAAATGTATATACATTTTCAAGGTTGTTCTATTTTTGATAGAATATTATCAATTTTCTTATTTAAAACAATTAAAXXXXXXXXXXC
AATTCACAAGTATTCTAACAAAGTCTTTAGTTACATCATGTTTTTTAAGATACAAGAACATATTTTTTTAAGAAAAGTT
TACTTGAAATGTATATACATTTTCAAGGTTGTTCTATTTTTGATAGAATATTATCAATTTTCTTATTTAAAACAGTTAAAXXXXXXXXXXC
AATTCATAAGTATTCTAACAAAGTCTTTAGTTACATCATGTTTTTTAAGATACAAGAACATATTTTTTTAAGAAAAGTT
>Marker360566 Chr1 SNP_site:6463454 Ref_Type:C
CACCTAGTTGAGATGTTTGATGCGCTCACTGATTCCTCCACCACCAATTCCCATCTCAAAAAATACAGAAATATGACTAAXXXXXXXXXXA
TCTTAACTAGATTGACACCTCTATAGCTCCATCATCCGTGCCAAATTCAAAAGCCATGTCATTAATTGATGGAAAGGTA
CACCTAGTTGAGATGTTTGATGCGCTCACTGATTCCTCCACCACCAATTCCCATCTCAAAAAATACAGAAATATGACTAAXXXXXXXXXXA
TCTTAACTAGATTGACACCTCTATAGCTCCCTCATCCGTGCCAAATTCAAAAGCCATGTCATTAATTGATGGAAAGGTA

Create PDF with GO2PDF for free, if you wish to

>Marker360845 Chr1 SNP_site:10053784 Ref_Type:G
TACTAAAAAGGTTTCTTTATACATTGGTCTATGAACAAGCATATGAATGCTAAGTTTATGAAATTATTTAAGTTAAATGTXXXXXXXXXXG
ATTATTTGAGCATGTTTTAGTATATACGTGAGAGATGTCCAGAGTTTTGTGGCAACTCTATAGGGGAGTTTTTCTTGTG
TACTAAAAATGTTTCTTTATACATTGGTCTATGAACAAGCATATGAATGCTAAGTTTATGAAATTATTTAAGTTAAATGTXXXXXXXXXXG
ATTATTTGAGCATGTTTTAGTATATACGTGAGAGATGTCCAGAGTTTTGTGGCAACTCTATAGGGGAGTTTTTCTTGTG
>Marker360930 Chr1 SNP_site:16247231 Ref_Type:G
ACTAATATGAAGAAGATGTATAAGCATTCAATGTGAAATTCGAAACCAACCAAAAAATGACAAGCAAATATGTGTCATTTXXXXXXXXXXA
ATGCTAGCTAACAGTAAATATAGAGACTTTAAAACAGAAAATGGTGATGAATTCAAAGAGATGAACTTCGATGGTTGTG
ACTAATATGAAGAAGATGTATAAGCATTCAATGTGAAATTCGAAACCAACCAAAAAATGACAATCAAATATGTGTCATTTXXXXXXXXXXA
ATGCTAGCTAACAGTAAATATAGAGACTTTAAAACAGAAAATGGTGATGAATTCAAAGAGATGAACTTCGATGGTTGTG
>Marker361157 Chr1 SNP_site:4706293 Ref_Type:G
TACCATCAATATCTTGATATGAAATTCATTTACTTGTTTCTTGTTATCTATTTCAGTTCAGGCTTCTATTGCGGCAAACAXXXXXXXXXXA
ATTTTTTCTTGCAAATTTTGGTCATACGTTTTAAAAAGAACAAATAGTCCCTAAATGAAAATGACACTACAGAGATGTC
TACCATCAATATCTTGATATGAAATTCATTTAGTTGTTTCTTGTTATCTATTTCAGTTCAGGCTTCTATTGCGGCAAACAXXXXXXXXXXA
ATTTTTTCTTGCAAATTTTGGTCATACGTTTTAAAAAGAACAAATAGTCCCTAAATGAAAATGACACTACAGAGATGTC
>Marker361920 Chr1 SNP_site:25825007 Ref_Type:A
ACTCGTAGTTTCTTTAAATATAATTGCAGCATCTGAATGCAGATATTTTGTTTTCGTGCATTACTAACTAGGTGTATATAXXXXXXXXXXC
TCGGAGTTATATCATCACAGCCCTTGAAGAATCAATACAACGAGTCAATTCAGCTATTCATCTTTTACTGATGGAGCGT
ACTCGTAGTTTCTTTAAATATAATTGCAGCATCTGAATGCAGATATTTTGTTTTCGTGCATTACTAACTAGGTGTATATAXXXXXXXXXXC
TCGGAGTTATATCATCACAGCCCTTGAAGAATCAATACAGCGAGTCAATTCAGCTATTCATCTTTTACTGATGGAGCGT
>Marker362183 Chr1 SNP_site:1968953 Ref_Type:A
ACTCTAGAGTTAGGCAATGGACCGTTGGTTTATCAGGAGGTGGAGACCAAATAGCTGTGATCAGAGCTGGAGGAAGCATTXXXXXXXXXXT
TTAAATATCGTTGAATGGTGTATTGGCACGGTGCCATCTTTTATTTATCTCATTACGATCATCGAGCTCATACCTTGTG
ACTCTAGAGTTAGGCAATGGACCGTTGGTTTATCAGGAGGTGGAGACCAAATAGCTGTGATCAGAGCTGGAGGAAGCATTXXXXXXXXXXT
TTAAATATCGTTGAATGGTGTATTGGCACGGTGCCGTCTTTTATTTATCTCATTACGATCATCGAGCTCATACCTTGTG
>Marker3624 Chr4 SNP_site:4034926 Ref_Type:G
TACAAATCTGAGCTTTCAGGTTAGTTTATGTTTCTTGAAAGTGGGTATCAGTATCAAAATTTGAACAGAAAGCCTTCAAAXXXXXXXXXXT
GTCCATTTCCTGAAGTTTTCTGTTAGTGAAGAGTTGCAAGGCACTCGACCCTCCAACAGAATTGAAGAAAAAGTCAGTT
TACAAATCTGAGCTTTCAGGTTAGTTTATGTTTCTTGAAAGTGGGTATCAGTATCAAAATTTGAACAGAAAGCCTTCAGAXXXXXXXXXXT
GTCCATTTCCTGAAGTTTTCTGTTAGTGAAGAGTTGCAAGGCACTCGACCCTCCAACAGAATTGAAGAAAAAGTCAGTT
>Marker36295 Chr4 SNP_site:4636127 Ref_Type:C
AACGAGGCTCAAGATCGCCATTGGAGCAGCAAAGGGGCTCGACTTCCTTCATGGGGCAGAGAAACCTGTCATTTATAGGGXXXXXXXXXXC
AGCAAAGTTGTCTGACTTTGGATTGGCAAAGATGGGACCTGAGGGATCAGACACTCATGTAACCACCAGAGTAATGGGT
AACGAGGCTCAAGATCGCCATTGGAGCAGCAAAGGGGCTGGACTTCCTTCATGGGGCAGAGAAACCTGTCATTTATAGGGXXXXXXXXXXC
AGCAAAGTTGTCTGACTTTGGATTGGCAAAGATGGGACCTGAGGGATCAGACACTCATGTAACCACCAGAGTAATGGGT
>Marker363661 Chr1 SNP_site:21215240 Ref_Type:T
AACGCACCCCATGGATTGAAATCCACTATCTTAACACTTTCATTTCTCGTCACATACACATCCAACGTGTAATTCTCCAAXXXXXXXXXXA
ACCTCACGCTGAGAAATGCCAATCAGGTTCCGATTCTTAACAAAGCAACGAAATTCCATCTCCGGCCGAAGGGATGGGT
AACGCACCCCATGGATTGAAATCCACTATCTTAACACTTTCATTTCTTGTCACATACACATCCAACGTGTAATTCTCCAAXXXXXXXXXXA
ACCTCACGCTGAGAAATGCCAATCAGGTTCCGATTCTTAACAAAGCAACGAAATTCCATCTCCGGCCGAAGGGATGGGT
>Marker363991 Chr1 SNP_site:17094800 Ref_Type:G
TACATCTAATTTAAAATTTTCACCAGAAATATAAATATAAATCCATATCTTAGTAAAGAAAAAGATTTGAAATTGGAAGAXXXXXXXXXXA
CTCTCTTCTTCCAAAATGCCATATTCTACTTTTATTTTGTTTTCTACTTCAATTCCTACACTCAACTAAACCATTTGTT
TACATCTAATTTAAAATTTTCACCAGAAATATAAATATAAATCGATATCTTAGTAAAGAAAAAGATTTGAAATTGGAAGAXXXXXXXXXXA

Create PDF with GO2PDF for free, if you wish to

CTCTCTTCTTCCAAAATGCCATATTCTACTTTTATTTTGTTTTCTACTTCAATTCCTACACTCAACTAAACCATTTGTT
>Marker364209 Chr1 SNP_site:1305728 Ref_Type:T
CACATTTTTATTTCATTCTGTCTCAGCCGTTGGATTGCAAAGCACCATTCCCAAGTAGAGATAAACATTACATCAGAAAAXXXXXXXXXXG
AGGCTACCGATCACTCATTGGATAGGTAATAACACAACCTGTATAATTTTGGTTTGCTTCTTATATCTTCAAGTAAGTA
CACATTTTTATTTCATTTTGTCTCAGCCGTTGGATTGCAAAGCACCATTCCCAAGTAGAGATAAACATTACATCAGAAAAXXXXXXXXXXG
AGGCTACCGATCACTCATTGGATAGGTAATAACACAACCTGTATAATTTTGGTTTGCTTCTTATATCTTCAAGTAAGTA
>Marker365332 Chr1 SNP_site:9452821 Ref_Type:C
TACATGATTCTAATTCTTTTGTTAATTGCTTGGTCATGTCCTCATCGTTCAGATTGTTTGCTGGGCGGTCTCTGAAGATTXXXXXXXXXXT
CAAATTTTTCCTCTTAGTCCGAGTTCTAGGGTGAATAATTTTTTATTTATGTTTTGAAGATGCCTGTGAAAATGATTGT
TACATGATTCTAATTCTTTTGTTAATTGCTTGGTCATGTCCTCATCGTTCAGATTGTTTGCTGGGCGGTCTCTGAAGATTXXXXXXXXXXT
CAAATTTTTCCTCTTAGTTCGAGTTCTAGGGTGAATAATTTTTTATTTATGTTTTGAAGATGCCTGTGAAAATGATTGT
>Marker365520 Chr1 SNP_site:25585555 Ref_Type:C
CACATAGCACTCACAAAAGGTAGGAAAAATATGCTCCAGGCTTCCAGCTTCCTAACATAAGTGAAGGACCCCTGCAGGGCXXXXXXXXXXT
CCTTAGAGAAAAGCAATATGCACGCATCTATGATTGACTAAGAAATCCAACTTCAGACAGCAACTTAAATGACAATGTA
CACATAGCACTCACAAAAGGTAGGAAAAATATGCTCCAGGCTTCCAGCTTCCTAACATAAGTGAAGGACCCCTGCAGGGCXXXXXXXXXXT
CCTTAGAGAAAAGCAATATGCACGCATCTATGATTGACTAAGAAATTCAACTTCAGACAGCAACTTAAATGACAATGTA
>Marker365870 Chr1 SNP_site:15826837 Ref_Type:T
CACATCCATCTAAAGAAAAGAGGAGTTTCAATGCTATTTGGAAAAAGATTTTTTTCTCTTGATGGTATGTCAAAGCCACTXXXXXXXXXXT
ATAAGAGCATGTCACATCTTTGAAGAATAACCAACACATGGTCTAGCTCCTCCCACGAACAAACCATAGTAAAGGTGTG
CACATCCATCTAAAGAAAAGAGGAGTTTCAATGCTATTTGGAAAAAGATTTTTTTCTTTTGATGGTATGTCAAAGCCACTXXXXXXXXXXT
ATAAGAGCATGTCACATCTTTGAAGAATAATCAACACATGGTCTAGCTCCTCCCACGAACAAACCATAGTAAAGGTGTG
>Marker365870 Chr1 SNP_site:15827019 Ref_Type:T
CACATCCATCTAAAGAAAAGAGGAGTTTCAATGCTATTTGGAAAAAGATTTTTTTCTCTTGATGGTATGTCAAAGCCACTXXXXXXXXXXT
ATAAGAGCATGTCACATCTTTGAAGAATAACCAACACATGGTCTAGCTCCTCCCACGAACAAACCATAGTAAAGGTGTG
CACATCCATCTAAAGAAAAGAGGAGTTTCAATGCTATTTGGAAAAAGATTTTTTTCTTTTGATGGTATGTCAAAGCCACTXXXXXXXXXXT
ATAAGAGCATGTCACATCTTTGAAGAATAATCAACACATGGTCTAGCTCCTCCCACGAACAAACCATAGTAAAGGTGTG
>Marker366028 Chr1 SNP_site:1172758 Ref_Type:T
CACGACGACTTCTGTAAATTGAGATCTATTTAAAATTGGCTACTGCTTTGATTCAATTATGCAAAATACAGACAATAACTXXXXXXXXXXA
GCCAACGTATAAGGACCCTATGGCTTCAAAAATTAGTTCATCAAAAGAATGACTTGAATGCTATACTTAGATGTTGGTT
CACGACGACTTCTGTAAATTGAGATCTATTTAAAATTGGCTACTGCTTTGATTCAATTTTGCAAAATACAGACAATAACTXXXXXXXXXXA
GCCAACGTATAAGGACCCTATGGCTTCAAAAATTAGTTCATCAAAAGAATGACTTGAATGCTATACTTAGATGTTGGTT
>Marker366572 Chr1 SNP_site:12927578 Ref_Type:C
ACCATGGTTGGCTTGGGAAATTAACAGCGCAGGCTGTGCTTTCATGCAATGATGAACTAATTCCATCTGAACTAACCTCTXXXXXXXXXXA
ATCATATTTTACCCTCACATGGTGCATTATGTGTGAATCTAGTGCTAAATATTTAGGTTCTTTATTTGACCGTCAGGTT
ACCATGGTTGGCTTGGGAAATTAACAGCGCAGGCTGTGCTTTCATGCAATGATGAACTAATTCCATCTGAACTAACCTCTXXXXXXXXXXA
ATCATATTTTACCTTCACATGGTGCATTATGTGTGAATCTAGTGCTAAATATTTAGGTTCTTTATTTGACCGTCAGGTT
>Marker367053 Chr1 SNP_site:19549860 Ref_Type:G
TACACCATCGACAGGGATATTGACCCTCAAAACTCCCTTTGTCAATGTCTCAAAAGAGGCATCGATACATGACAGGATCCXXXXXXXXXXT
TGCTGGCAGCAAGGAAAAGGGGAAAAAGAAGAAAGAAATATGGAAAATATCATTAATATGAAATCACAAAAGAATTGTA
TACACCATCGACAGGGATATTGACCCTCAAAACTCCCTTTGTCAATGTCTCAAAAGAGGCATCGATACATGACAGGATCCXXXXXXXXXXT
TGCTGGCAGCAAGGAAAAGGGGAAAAAGAAGAAAGAAATATGGAAAATATCATTAATATGAAATCACAGAAGAATTGTA
>Marker367544 Chr1 SNP_site:23281899 Ref_Type:G
CACCACCTCGTAAGAATCAAATTAAACAACAATGACATGGGAGAAGGAAGAAACAATAGAATTGAGATGTTAAGACCCTCXXXXXXXXXXC
CCACTTCACCCACCATGACACTTTTCAAGGTTTTGCAAAGCATCAATATTCACTTTCCATTCATTCATATAAAGGGAGT

Create PDF with GO2PDF for free, if you wish to

CACCACCTCGTAAGAATCAAATTAAACAACAGTGACATGGGAGAAGGAAGAAACAATAGAATTGAGATGTTAAGACCCTCXXXXXXXXXXC
CCACTCCACCCACCATGACACTTTTCAAGGTTTTGCAAAGCATCAATATTCACTTTCCATTCATTCATATAAAGGGAGT
>Marker367544 Chr1 SNP_site:23282048 Ref_Type:C
CACCACCTCGTAAGAATCAAATTAAACAACAATGACATGGGAGAAGGAAGAAACAATAGAATTGAGATGTTAAGACCCTCXXXXXXXXXXC
CCACTTCACCCACCATGACACTTTTCAAGGTTTTGCAAAGCATCAATATTCACTTTCCATTCATTCATATAAAGGGAGT
CACCACCTCGTAAGAATCAAATTAAACAACAGTGACATGGGAGAAGGAAGAAACAATAGAATTGAGATGTTAAGACCCTCXXXXXXXXXXC
CCACTCCACCCACCATGACACTTTTCAAGGTTTTGCAAAGCATCAATATTCACTTTCCATTCATTCATATAAAGGGAGT
>Marker367708 Chr1 SNP_site:8397576 Ref_Type:G
AACAAGAAAAAAAATAATCTCCACTCTCTCCATTTCCCGTTTGGAATGGGTTCCATATGGGTTAAATAAATATCTCTATTXXXXXXXXXXA
AAGGATAATCCAAAAATCTAACAATTTTTCTTGGATTGTGTCGAATTGATTGAAGTTTTAGAATTTGATATTAGAAGTT
AACAAGAAAAAAAATAATCTCCACTCTCTCCATTTCCCGTTTGGAATGGGTTCCATATGGGTTAAATAAATATCTCTATTXXXXXXXXXXA
AAGGATAATCCAAAAATCTAACAATTTTTCTTGGATTGTGTCGAATTGATTGAAGTTTTAGAATTTGATATTAGAGGTT
>Marker367764 Chr1 SNP_site:21183707 Ref_Type:C
AACTGGAAGAGAAATTAGGATCCTACTGTTCTATTTGTAGTGTTTTTCTTTCTTTGCCAATTGTTTTTGAGTTTGAACTCXXXXXXXXXXT
CAGCAGCAGTGATCGTTGGAGGACAAATTGGACTCCGGCAATGGAGCAATATTTTATTGATCTTATGTTGAATCAAGTG
AACTGGAAGAGAAATTAGGATCCTACTGTTCTATTTGTAGTGTTTTTCTTTCTTTGTCAATTGTTTTTGAGTTTGAACTCXXXXXXXXXXT
CAGCAGCAGTGATCGTTGGAGGACAAATTGGACTCCGGCAATGGAGCAATATTTTATTGATCTTATGTTGAATCAAGTG
>Marker368497 Chr1 SNP_site:21242603 Ref_Type:C
TACTAAACATGCTTTATGTGGGTTCAAATGAAAATAATGTTTTACTAGCTATTAAACACGATTTCTGAGTCATAATAATCXXXXXXXXXXT
TTAGCAACTCTAACTTTGAAGACCGTTACTATGCTTGCTTCTAAAACAGGCGTGCCTAAATATCTGAAACCATCATGTG
TACTAAACATGCTTTATGTGGGTTCAAATGAAAATAATGTTTTACTAGCTATTAAACATGATTTCTGAGTCATAATAATCXXXXXXXXXXT
TTAGCAACTCTAACTTTGAAGACCGTTACTATGCTTGCTTCTAAAACAGGCGTGCCTAAATATCTGAAACCATCAAGTG
>Marker368497 Chr1 SNP_site:21242837 Ref_Type:T
TACTAAACATGCTTTATGTGGGTTCAAATGAAAATAATGTTTTACTAGCTATTAAACACGATTTCTGAGTCATAATAATCXXXXXXXXXXT
TTAGCAACTCTAACTTTGAAGACCGTTACTATGCTTGCTTCTAAAACAGGCGTGCCTAAATATCTGAAACCATCATGTG
TACTAAACATGCTTTATGTGGGTTCAAATGAAAATAATGTTTTACTAGCTATTAAACATGATTTCTGAGTCATAATAATCXXXXXXXXXXT
TTAGCAACTCTAACTTTGAAGACCGTTACTATGCTTGCTTCTAAAACAGGCGTGCCTAAATATCTGAAACCATCAAGTG
>Marker369382 Chr1 SNP_site:10047380 Ref_Type:A
ACTTCAGACATAACTCCATCAATTCGGTTCTTCAAAGGGTCAATCTAAAATGCAACACTCTTTGTAGGATTTATAACAGCXXXXXXXXXXA
TGCACTTACCCGGAGTTGTAAGGGAACAATACTAGTTGGTCATACTCGGCTTCGAGCAATCAAGCATTCAACAACTGTG
ACTTCAGACATAACTCCATCAATTCGGTTCTTCAAAGGGTCAATCTAAAATGCAACACTCTTTGTAGGATTTATAACGGCXXXXXXXXXXA
TGCACTTACTCGGAGTTGTAAGGGAACAATACTAGTTGGTCATACTCGGCTCCGAGCAATCAAGCATTCAACAACTGTG
>Marker369382 Chr1 SNP_site:10047487 Ref_Type:C
ACTTCAGACATAACTCCATCAATTCGGTTCTTCAAAGGGTCAATCTAAAATGCAACACTCTTTGTAGGATTTATAACAGCXXXXXXXXXXA
TGCACTTACCCGGAGTTGTAAGGGAACAATACTAGTTGGTCATACTCGGCTTCGAGCAATCAAGCATTCAACAACTGTG
ACTTCAGACATAACTCCATCAATTCGGTTCTTCAAAGGGTCAATCTAAAATGCAACACTCTTTGTAGGATTTATAACGGCXXXXXXXXXXA
TGCACTTACTCGGAGTTGTAAGGGAACAATACTAGTTGGTCATACTCGGCTCCGAGCAATCAAGCATTCAACAACTGTG
>Marker369382 Chr1 SNP_site:10047529 Ref_Type:T
ACTTCAGACATAACTCCATCAATTCGGTTCTTCAAAGGGTCAATCTAAAATGCAACACTCTTTGTAGGATTTATAACAGCXXXXXXXXXXA
TGCACTTACCCGGAGTTGTAAGGGAACAATACTAGTTGGTCATACTCGGCTTCGAGCAATCAAGCATTCAACAACTGTG
ACTTCAGACATAACTCCATCAATTCGGTTCTTCAAAGGGTCAATCTAAAATGCAACACTCTTTGTAGGATTTATAACGGCXXXXXXXXXXA
TGCACTTACTCGGAGTTGTAAGGGAACAATACTAGTTGGTCATACTCGGCTCCGAGCAATCAAGCATTCAACAACTGTG
>Marker369636 Chr1 SNP_site:1234547 Ref_Type:T
CACACGTTATTGTAATTCTTATTGGCTCAACATCGAGCTGTAATGCGTTAAATAAAAAACAAATTATAAATAAAATCTTAXXXXXXXXXX-

Create PDF with GO2PDF for free, if you wish to

ATATTTAAAAAGGTAGGTTGTAGATTCTGTTGTCTTAAATATCTTATTTAGTTCAAATCAATGGCTGATTTTGAAATGGT

CACACGTTATTGTAATTCTTATTGGCTCAACATTGAGCTGTAATGCGTTAAATAAAAAACAAATTATAAATAAAATCTTAXXXXXXXXXXXT

ATATTT-AAAAGGTAGGTTGTAGATTCTGTTGTCTTAAATATCTTATTTAGTTCAAATTAATGGCTGATTTTGAAATGGT

>Marker369636 Chr1 SNP_site:1234765 Ref_Type:A

CACACGTTATTGTAATTCTTATTGGCTCAACATCGAGCTGTAATGCGTTAAATAAAAAACAAATTATAAATAAAATCTTAXXXXXXXXXXX-

ATATTTAAAAAGGTAGGTTGTAGATTCTGTTGTCTTAAATATCTTATTTAGTTCAAATCAATGGCTGATTTTGAAATGGT

CACACGTTATTGTAATTCTTATTGGCTCAACATTGAGCTGTAATGCGTTAAATAAAAAACAAATTATAAATAAAATCTTAXXXXXXXXXXXT

ATATTT-AAAAGGTAGGTTGTAGATTCTGTTGTCTTAAATATCTTATTTAGTTCAAATTAATGGCTGATTTTGAAATGGT

>Marker369850 Chr1 SNP_site:2045464 Ref_Type:A

AACAGGATGATGTTAATGAACAACAGTTATATTAAATTTGTGTTGTTAATGTTTAATGAAAACAGTTTGCTGAATTTTGAXXXXXXXXXXXG

AGAAATTAAAATAGAAAGAAGATATTACAGAAGTCTAACCAATTGAGAGAGGAAACTTCATGCAGGTGGTGTTAGTGGT

AACAGGATGATGTTAATGAACAACAGTTATATTCAATTTGTGTTGTTAATGTTTAATGAAAACAGTTTGCTGAATTTTGAXXXXXXXXXXXG

ATAAATTAAAATAGAAAGAAGATATTACAGAAGTCTAACCAATTGAGAGAGGAAACTTCATGCAGGTGGTGTTAGTGGT

>Marker369850 Chr1 SNP_site:2045619 Ref_Type:G

AACAGGATGATGTTAATGAACAACAGTTATATTAAATTTGTGTTGTTAATGTTTAATGAAAACAGTTTGCTGAATTTTGAXXXXXXXXXXXG

AGAAATTAAAATAGAAAGAAGATATTACAGAAGTCTAACCAATTGAGAGAGGAAACTTCATGCAGGTGGTGTTAGTGGT

AACAGGATGATGTTAATGAACAACAGTTATATTCAATTTGTGTTGTTAATGTTTAATGAAAACAGTTTGCTGAATTTTGAXXXXXXXXXXXG

ATAAATTAAAATAGAAAGAAGATATTACAGAAGTCTAACCAATTGAGAGAGGAAACTTCATGCAGGTGGTGTTAGTGGT

>Marker369853 Chr1 SNP_site:19398345 Ref_Type:G

TACTCTATGGCCTTACTCCTCACCGGTGACCTTTTCCTCTTTTCCCACCATGTAGTGGGATTTTGATCTCAATGAGTCTCXXXXXXXXXXXA

CAACTCGCAACCCAAATTAATATGCTTAAAACTAAAGTCATGTTAGATGGTTTGATAGATTAATAAGGGTTGGAGAGGT

TACTCTATGGCCTTACTCCTCACGGGTGACCTTTTCCTCTTTTCCCACCATGTAGTGGGATTTTGATCTCAATGAGTCTCXXXXXXXXXXXA

CAACTCGCAACCCAAATTAATATGCTTAAAACTAAAGTCATGTTAGATGGTTTGATAGATCAATAAGGGTTGGAGAGGT

>Marker369853 Chr1 SNP_site:19398593 Ref_Type:C

TACTCTATGGCCTTACTCCTCACCGGTGACCTTTTCCTCTTTTCCCACCATGTAGTGGGATTTTGATCTCAATGAGTCTCXXXXXXXXXXXA

CAACTCGCAACCCAAATTAATATGCTTAAAACTAAAGTCATGTTAGATGGTTTGATAGATTAATAAGGGTTGGAGAGGT

TACTCTATGGCCTTACTCCTCACGGGTGACCTTTTCCTCTTTTCCCACCATGTAGTGGGATTTTGATCTCAATGAGTCTCXXXXXXXXXXXA

CAACTCGCAACCCAAATTAATATGCTTAAAACTAAAGTCATGTTAGATGGTTTGATAGATCAATAAGGGTTGGAGAGGT

>Marker37014 Chr4 SNP_site:16629507 Ref_Type:A

CACTTATCACTATTGGATGTCTATATTATATGAAAAAGATTAATATATTTATAAGTTTCTTCATATTCCTTGCATTATGAXXXXXXXXXXXT

CTTGAAAAGTATGTCCAAAATCCAAGGCATATTGAGTTTCAGGTAATTATTCCAAAATCTCAGGAAATTATTTTTTAGT

CACTTATCACTATTGGATGTCTATATTATATGAAAAAGATTAATATATTTGTAAGTTTCTTCATATTCCTTGCATTATGAXXXXXXXXXXXT

CTTGAAAAGTATGTCCAAAATCCAAGGCATATCGAGTTTCAGGTAATTATTCCAAAATCTCAGGAAATTATTTTTTAGT

>Marker37014 Chr4 SNP_site:16629723 Ref_Type:T

CACTTATCACTATTGGATGTCTATATTATATGAAAAAGATTAATATATTTATAAGTTTCTTCATATTCCTTGCATTATGAXXXXXXXXXXXT

CTTGAAAAGTATGTCCAAAATCCAAGGCATATTGAGTTTCAGGTAATTATTCCAAAATCTCAGGAAATTATTTTTTAGT

CACTTATCACTATTGGATGTCTATATTATATGAAAAAGATTAATATATTTGTAAGTTTCTTCATATTCCTTGCATTATGAXXXXXXXXXXXT

CTTGAAAAGTATGTCCAAAATCCAAGGCATATCGAGTTTCAGGTAATTATTCCAAAATCTCAGGAAATTATTTTTTAGT

>Marker370245 Chr1 SNP_site:8252210 Ref_Type:A

TACGTTGTATCTCTTTATATCAACCATGAATTGGTGCAACAAATATTTCTTTCTAATCTGTCCATGATATAACAACTCAGXXXXXXXXXXXC

ATAAACGAGCTATTTTGGAAAAGTAAAAGAAAATGATATGTTAGAACTTTTTTGGTGTGTATTGAGAGAATGGTAAGTG

TACGTTGTATCTCTTTATATCAACCATGAATTGGTGCAACAAATATTTCTTTCTAATCTGTCCATGATATAACAACTCAGXXXXXXXXXXXC

ATAAACGTGCTATTTTGGAAAAGTAAAAGAAAATGATATGTTAGAACTTTTTTGGTGTGTATTGAGAGAATGGTAAGTG

>Marker370370 Chr1 SNP_site:6424704 Ref_Type:G

Create PDF with GO2PDF for free, if you wish to

ACCATTGCTAGAATAACCTTGAGCAAAATCACTGCTATAGGTTCCAATATCATTCCATAATTTCTTGAGCTTTTTGAAGTXXXXXXXXXXG
AGCCCATTAAATCATCTATTGAGTTTCCTTTGAGGATGTCGTCAAGGTCTTTACCTACACACATGTTATTTTTTTCGTC
ACCATTGCTAGAATAACCTTGAGCAAAATCACTGCTATAGGTTCCAATGTCATTCCATAATTTCTTGAGCTTTTTGAAGTXXXXXXXXXXG
AGCCCATTAAATCATCTATTGAGTTTCCTTTGAGGATGTCGTCAAGGTCTTTACCTACACACATGTTATTTTTTTCGTC
>Marker372328 Chr1 SNP_site:25895066 Ref_Type:C
GACTTGAAATAAGATAAAGGTCCAACAAGTAGAAATCAATATTTTGTTTTTGAAACGAGGACAAAAGTAGAAATCAATATXXXXXXXXXXT
TGACCCAAAAAGCTTAAGTTAGTGGGTAACTATTGAAAGTAGAGACAAAAAACACACAAATTCACGTGAAAACCCGAGT
GACTTGAAATAAGATAAAGGTCCAACAAGTAGAAATCAATATTTTGTTTTTGAAACGAGGACAAAAGTAGAAATCAATATXXXXXXXXXXT
TGACCCAAAAAGCTTAAGTTAGTGGGTAACTATTGAAAGTAGAGACAAAAAACACACAAATTCACGTGAAAACCTGAGT
>Marker373811 Chr1 SNP_site:4196873 Ref_Type:C
ACTTCAATTCATACCTTTTGTTGGTATGGTTATGGAAGGAAATACCAGAGTTTCCAGCACTCCAATTGCAAATATTATGGXXXXXXXXXXT
TTTCATAAAACCCCCAGTTGTATTTTTTCCTATAGTCGAGTATGTGCTTGCCCAGATCGTCATCTTTCGACTTACAGTG
ACTTTAATTCATACCTTTTGTTAGTATGGTTATGGAAGGAAATACCAGAGTTTCCAGCACTCCAATTGCAAATATTATGGXXXXXXXXXXT
TTTCATAAAACCCCCAGTTGTATTTTTTCCTATAGCCGAGTATGTGCTTGCCCAGATCGTCATCTTTCGACTTACAGTG
>Marker373811 Chr1 SNP_site:4196891 Ref_Type:G
ACTTCAATTCATACCTTTTGTTGGTATGGTTATGGAAGGAAATACCAGAGTTTCCAGCACTCCAATTGCAAATATTATGGXXXXXXXXXXT
TTTCATAAAACCCCCAGTTGTATTTTTTCCTATAGTCGAGTATGTGCTTGCCCAGATCGTCATCTTTCGACTTACAGTG
ACTTTAATTCATACCTTTTGTTAGTATGGTTATGGAAGGAAATACCAGAGTTTCCAGCACTCCAATTGCAAATATTATGGXXXXXXXXXXT
TTTCATAAAACCCCCAGTTGTATTTTTTCCTATAGCCGAGTATGTGCTTGCCCAGATCGTCATCTTTCGACTTACAGTG
>Marker373811 Chr1 SNP_site:4197096 Ref_Type:T
ACTTCAATTCATACCTTTTGTTGGTATGGTTATGGAAGGAAATACCAGAGTTTCCAGCACTCCAATTGCAAATATTATGGXXXXXXXXXXT
TTTCATAAAACCCCCAGTTGTATTTTTTCCTATAGTCGAGTATGTGCTTGCCCAGATCGTCATCTTTCGACTTACAGTG
ACTTTAATTCATACCTTTTGTTAGTATGGTTATGGAAGGAAATACCAGAGTTTCCAGCACTCCAATTGCAAATATTATGGXXXXXXXXXXT
TTTCATAAAACCCCCAGTTGTATTTTTTCCTATAGCCGAGTATGTGCTTGCCCAGATCGTCATCTTTCGACTTACAGTG
>Marker374325 Chr1 SNP_site:6614348 Ref_Type:G
TACAAAATGGTTGAAAATGTAAGGGCCAAGGGTGGTGAGAATATGATCATTAGAGAACCTTTTGAGCTGAAAGGCAGCCCXXXXXXXXXXA
ACGCTCTATTAACTTCTACCAAAAGGTAAAGTCACAAATTTTCAAAAAGTTTCGAACAATTTAGCCCTCAAACTTTAGT
TACAAAATGGTTGAAAATGTAAGGGCCAAGGGTGGTGAGAATATGATCATTAGAGAACCTTTTGAGCTGAAAGGCAGCCCXXXXXXXXXXA
ACGCTCTATTGACTTCTACCAAAAGGTAAAATCACAAATTTTCAAAAAGTTTCGAACAATTTAGCCCTCAAACTTTGGT
>Marker374325 Chr1 SNP_site:6614368 Ref_Type:A
TACAAAATGGTTGAAAATGTAAGGGCCAAGGGTGGTGAGAATATGATCATTAGAGAACCTTTTGAGCTGAAAGGCAGCCCXXXXXXXXXXA
ACGCTCTATTAACTTCTACCAAAAGGTAAAGTCACAAATTTTCAAAAAGTTTCGAACAATTTAGCCCTCAAACTTTAGT
TACAAAATGGTTGAAAATGTAAGGGCCAAGGGTGGTGAGAATATGATCATTAGAGAACCTTTTGAGCTGAAAGGCAGCCCXXXXXXXXXXA
ACGCTCTATTGACTTCTACCAAAAGGTAAAATCACAAATTTTCAAAAAGTTTCGAACAATTTAGCCCTCAAACTTTGGT
>Marker374325 Chr1 SNP_site:6614414 Ref_Type:G
TACAAAATGGTTGAAAATGTAAGGGCCAAGGGTGGTGAGAATATGATCATTAGAGAACCTTTTGAGCTGAAAGGCAGCCCXXXXXXXXXXA
ACGCTCTATTAACTTCTACCAAAAGGTAAAGTCACAAATTTTCAAAAAGTTTCGAACAATTTAGCCCTCAAACTTTAGT
TACAAAATGGTTGAAAATGTAAGGGCCAAGGGTGGTGAGAATATGATCATTAGAGAACCTTTTGAGCTGAAAGGCAGCCCXXXXXXXXXXA
ACGCTCTATTGACTTCTACCAAAAGGTAAAATCACAAATTTTCAAAAAGTTTCGAACAATTTAGCCCTCAAACTTTGGT
>Marker375483 Chr1 SNP_site:16916602 Ref_Type:A
CACAAAGCGCTACTTCCTTTTCCCTTTCATCTGCATTTAGCAGAGCATACGCTGTTTTTGGAGAGTAACCTTCTTCTTTCXXXXXXXXXXA
ACCGCGTTGTCCGATTTTCTTCCTTAATTTTTGAACTTCTTCCCATTTCTCTGCCTCCGCATAAATGTTTGCCAAGAGT
CACAAAGCGCTACTTCCTTTTCCCTTTCATCTGCATTTAGCAGAGCATACGCTGTTTTTGGAGAGTAACCTTCTTCTTTCXXXXXXXXXXA
ACCGCGTTGTCCGATTTTCTTCCTTAGTTTTTGAACTTCTTCCCATTTCTCTGCCTCCGCATAAATGTTTGCCAAGAGT

Create PDF with GO2PDF for free, if you wish to

>Marker375691 Chr1 SNP_site:22658561 Ref_Type:C
TACAACGTTGGACAACGAAAAGAAATCAACTTTGAAAATTTTAGATTGGTTAAAGAACATTGTTTGAAGAATCAATTCAAXXXXXXXXXXG
AAAATAAAAAGTAATTACAAAGTAGATCGAACTCATCTTTTGAAGTCTAGCATAGCATACAAATCTCTCGAGAAGTGTG
TACAATGTTGGACAACGAAAAGAAATCAACTTTGAAAGTTTTAGATTGGTTAAAGAACATTGTTTGAAGAATCAATTCAAXXXXXXXXXXG
AAAATAAAAAGTAATTACAAAGTAGATCGAACTCATCTTTTGAAGTCTAGCATAGCATACAAATCTCTCGAGAAGTGTG
>Marker375691 Chr1 SNP_site:22658593 Ref_Type:A
TACAACGTTGGACAACGAAAAGAAATCAACTTTGAAAATTTTAGATTGGTTAAAGAACATTGTTTGAAGAATCAATTCAAXXXXXXXXXXG
AAAATAAAAAGTAATTACAAAGTAGATCGAACTCATCTTTTGAAGTCTAGCATAGCATACAAATCTCTCGAGAAGTGTG
TACAATGTTGGACAACGAAAAGAAATCAACTTTGAAAGTTTTAGATTGGTTAAAGAACATTGTTTGAAGAATCAATTCAAXXXXXXXXXXG
AAAATAAAAAGTAATTACAAAGTAGATCGAACTCATCTTTTGAAGTCTAGCATAGCATACAAATCTCTCGAGAAGTGTG
>Marker375837 Chr1 SNP_site:25491670 Ref_Type:G
AACAAGCATAGTTGTCAGCTCCTATACAAAGCAGACGGTATTTATGAGTGGCATATAGATTTTCATTTTCCTATCGAGATXXXXXXXXXXT
TTTCATCATGATTCATCTGTTGCTGCAATTGCTTTAACCACACAGCTATGCTATTTGAAATTTTTTCTAGAAGAATGTC
AACAAGCATAGTTGTCAGCTCCTATACAAAGCAGACGGTATTTATGAGTGGCATATAGATTTTCATTTTCCTATCGAGATXXXXXXXXXXT
TTTCATCATGATTCATCTGTTGCTGCAGTTGCTTTAACCACACAGCTATGCTATTTGAAATTTTTTCTAGAAGAATGTC
>Marker376067 Chr1 SNP_site:4924790 Ref_Type:G
TACTCTTTTCCACACTAACTAGGGTAGCTAGGTTAATGTTTATATGCTTTTATATATTTAATTTGTTAATATATTACGGTXXXXXXXXXXA
CTTTTTAAATTAATGATGAATCCTTAATAATATTTTTCGTTAATTATCAATCTTAATTGCGTAGTTATGTCTTGATGTA
TACTCTTTTCCACACTAACTAGGGTAGCTAGGTTAATGTTTATATGCTTTTATATATTTAATTTGTTAATATATTACGGTXXXXXXXXXXA
CTTTTTAAATTAATGATGAATCCTTGATAATATTTTTCGTTAATTATCAATCTTAATTGCGTAGTTATGTCTTGATGTA
>Marker376344 Chr1 SNP_site:6852332 Ref_Type:G
AACCCTTGGCTTTGCAAATTTCTTTGGGTTTTATAGATCCAGCATCTTCTGTTTTCTTTTCTGTTGTTTGAGATTTGTAGXXXXXXXXXXA
TAGTTTAAAATATAAACTTATAAATCAAGTGTAGCTGAAAAAACTAAAACTTAATCGAACAAAACTTTGATTTGTTGTC
AACCCTTGGCTTTGCAAATTTCTTTGGGTTTTATAGATCCAGCATCTTCTGTTTTCTTTTCTGTTGTTTGAGATTTGTAGXXXXXXXXXXA
TAGTTTAAGATATAAACTTATAAATCAAGTGTAGCTGAAAAAACTAAAACTTAATCGAACAAAACTTTGATTTGTTGTC
>Marker37640 Chr4 SNP_site:1224158 Ref_Type:G
ACATAACAGCGAATGTGGTAGATAGAAACAATGGACGAGTCGTTGCAACTGCATCCACAGTGGAACACTCCATAAAAAACXXXXXXXXXXG
AAGAGGGATTCACGCAGACATAAAAAAAGAAATTGAGAAGAAAGGCTTCAAAAACCACACAAAAATCTGGGCTATAGTG
ACATAACAGCGAATGTGGTAGATAGAAACAATGGACGAGTCGTTGCAACTGCATCCACAGTGGAGCACTCCATAAAAAACXXXXXXXXXXG
AAGAGGGATTCACGCAGACATAAAAAAAGAAATTGAGAAGAAAGGCTTCAAAAACCACACAAAAATCTGGGCTATAGTG
>Marker377265 Chr1 SNP_site:8258414 Ref_Type:G
CACACACGGCTTATAAATTCTACAATTATTAGTCAATCAGTGAGGTTAGCTTCTTGAGACTTTTAAATTAATATCTTATAXXXXXXXXXXT
TGTGGGAGTTGAATGACAAGATATCTACTCCAAAAGAAGCTTTTGATGCCTTATGCCTTTGTCTCATCATGTTCAAGGT
CACACACGGCTTATAAATTCTACAATTATTAGTCAATCGGTGAGGTTAGCTTCTTGAGACTTTTAAATTAATATCTTATAXXXXXXXXXXT
TGTGGGAGTTGAATGACAAGATATCTACTCCAAAAGAAGCTTTTGATGCCTTATGCCTTTGTCTCATCATGTTCAAGGT
>Marker378110 Chr1 SNP_site:26087437 Ref_Type:A
ACAATAGATATATACTAAAAATAAAAATTAAGAGACCTAAAGATTAGGAGACACACCCAAACATCTCAACTAGGTTGCCAXXXXXXXXXXT
CCAGATATCTTAGCATGTAAAAGCCAACCAATTTGAACACAAATCTTGAATAGATGTGCTAAACTCCTTGAATAAAGTG
ACAATAGATATATACTAAAAATAAAAATTAAGAGACCTAAAGATTAGGAGACACACCCAAACATCTCAACTAGGTTGCCAXXXXXXXXXXT
CCAGATATCTTAGCATGTAAAAGCCGACCAATTTGAACACAAATCTTGAATAGATGTGCTAAACTCCTTGAATAAAGTG
>Marker378872 Chr1 SNP_site:27311503 Ref_Type:T
ACTTCTCCATTTTTGGTTAAACAAAATGAAACATTCATGGCTTTGAATTGTAAAATTTTAAACGACATTGTTGTGGTCACXXXXXXXXXXT
TACGTGCAAGAAGGACAACACGATAAAGGATCTGATTTTGTTGCTCGATTCTAAGAAGATCAACCGTGTGTATGTGGTG
ACTTCTCCATTTTTGGTTAAACAAAATGAAACATTCATGGCTTTGAATTGTAAAATTTTAATCGACATTGTTGTGGTCACXXXXXXXXXXT

Create PDF with GO2PDF for free, if you wish to

TACGTGCAAGAAGGACAACACGATAAAGGATCTGATTTTGTTGCTCGATTCTAAGAAGATCAACCGTGTGTATGTGGTG
>Marker379207 Chr1 SNP_site:14581646 Ref_Type:G
CACCTTGCGCCTAGGCTTCAGAGGGCCATTGTGCCTTAGGCATTTAAAAACACTGAGTATTCAGCTTTTTCGTGGTTCCAXXXXXXXXXXT
TAAAAATTCACAATCTGCAAATCCCCCGAATAGAGCCAATTCTCTTACTCTTTCAACAAGTTCCCTGCAGAAAATGGTA
CACCTTGCGCCTAGGCTTCAGAGGGCGATTGTGCCTTAGGCATTTAAAAACATTGAGTATTCAGCTTTTTCGTGGTTCCAXXXXXXXXXXT
TAAAAATTCACAATCTGCAAATCCCCCGAATAGAGCCAATTCTCTTACTCTTTCAACAAGTTCCCTGCAGAAAATGGTA
>Marker379207 Chr1 SNP_site:14581672 Ref_Type:T
CACCTTGCGCCTAGGCTTCAGAGGGCCATTGTGCCTTAGGCATTTAAAAACACTGAGTATTCAGCTTTTTCGTGGTTCCAXXXXXXXXXXT
TAAAAATTCACAATCTGCAAATCCCCCGAATAGAGCCAATTCTCTTACTCTTTCAACAAGTTCCCTGCAGAAAATGGTA
CACCTTGCGCCTAGGCTTCAGAGGGCGATTGTGCCTTAGGCATTTAAAAACATTGAGTATTCAGCTTTTTCGTGGTTCCAXXXXXXXXXXT
TAAAAATTCACAATCTGCAAATCCCCCGAATAGAGCCAATTCTCTTACTCTTTCAACAAGTTCCCTGCAGAAAATGGTA
>Marker379621 Chr1 SNP_site:15228487 Ref_Type:G
CACATCGCCGGCATCCCCCAACAGTCCAACATCGTGTCGCTGGAAGGCCCACACTTTCAGCCACCACCGTTCTGACACTCXXXXXXXXXXT
CGCAACAGTTGGATTTCGACTCAAGTGGGTTTCAGCTTGGAACCGAAGGGAATTGTAACATGAGGTGAATACAGAAGTG
CACATCGCCGGCATCCCCCAACAGTCCAACATCGTGTCGCTGGAAGGCCCACACTTTCAGCCACCACCGTTCTGACACTCXXXXXXXXXXT
GGCAACAGTTGGATTTCGACTCAAGTGGGTTTCAGCTTGGAACCGAAGGGAATTGTAACATGAGGTGAATACAGAAGTG
>Marker380169 Chr1 SNP_site:6878179 Ref_Type:G
ACAAAAAAAGAACGTCAAATTACTATAAAAGAAGACGATTAAGACAAAGACTAGTCGTCACAAAAATTGATGGTTGGAGGXXXXXXXXXXT
GAGAGTTACTTTTCATTCATATATATACATTTCATCGTCTAGTAAAATTATTCAAAACAAACTATAAGACCTTGAGGTT
ACAAAAAAAGAACGTCGAATTACTATAAAAGAAGACGATTAAGACGATGACTAGTCGTCACAAAAATTGATGGTTGGAGGXXXXXXXXXXT
GAGAGTTACTTTTCATTCATATATATACATTTCATCGTCTAGTAAAATTATTCAAAACAAACTATAAGACCTTGAGGTT
>Marker380169 Chr1 SNP_site:6878208 Ref_Type:G
ACAAAAAAAGAACGTCAAATTACTATAAAAGAAGACGATTAAGACAAAGACTAGTCGTCACAAAAATTGATGGTTGGAGGXXXXXXXXXXT
GAGAGTTACTTTTCATTCATATATATACATTTCATCGTCTAGTAAAATTATTCAAAACAAACTATAAGACCTTGAGGTT
ACAAAAAAAGAACGTCGAATTACTATAAAAGAAGACGATTAAGACGATGACTAGTCGTCACAAAAATTGATGGTTGGAGGXXXXXXXXXXT
GAGAGTTACTTTTCATTCATATATATACATTTCATCGTCTAGTAAAATTATTCAAAACAAACTATAAGACCTTGAGGTT
>Marker380169 Chr1 SNP_site:6878210 Ref_Type:T
ACAAAAAAAGAACGTCAAATTACTATAAAAGAAGACGATTAAGACAAAGACTAGTCGTCACAAAAATTGATGGTTGGAGGXXXXXXXXXXT
GAGAGTTACTTTTCATTCATATATATACATTTCATCGTCTAGTAAAATTATTCAAAACAAACTATAAGACCTTGAGGTT
ACAAAAAAAGAACGTCGAATTACTATAAAAGAAGACGATTAAGACGATGACTAGTCGTCACAAAAATTGATGGTTGGAGGXXXXXXXXXXT
GAGAGTTACTTTTCATTCATATATATACATTTCATCGTCTAGTAAAATTATTCAAAACAAACTATAAGACCTTGAGGTT
>Marker380306 Chr1 SNP_site:1089678 Ref_Type:A
CACCTCCATGGCGTGAGTGTTGCTTTTTGCTTATTTTTTCTTCCTCCTTCCAAATTGAGTTCCATTTTCAGAACATTAGTXXXXXXXXXXA
TTTTATTTCCATTCTCCCGGTTTTCCTCTTTATTTTGTTAGAATCTGCTGTTTGGAAAGTAAATTTGTATTACTTTGTA
CACCTCCATGGCGTGAGTGTTGCTTTTTGCTTATTTTTTCTTCCTCCTTCCAAATTGAGTTCCATTTTCAGAACATTAGTXXXXXXXXXXA
TTTTATTTCCATTCTCCCGGTTTTCCTCTTTATTTTGTTAGAATCTGCTGTTTGGAAAGTTAATTTGTATTACTTTGTG
>Marker380306 Chr1 SNP_site:1089696 Ref_Type:A
CACCTCCATGGCGTGAGTGTTGCTTTTTGCTTATTTTTTCTTCCTCCTTCCAAATTGAGTTCCATTTTCAGAACATTAGTXXXXXXXXXXA
TTTTATTTCCATTCTCCCGGTTTTCCTCTTTATTTTGTTAGAATCTGCTGTTTGGAAAGTAAATTTGTATTACTTTGTA
CACCTCCATGGCGTGAGTGTTGCTTTTTGCTTATTTTTTCTTCCTCCTTCCAAATTGAGTTCCATTTTCAGAACATTAGTXXXXXXXXXXA
TTTTATTTCCATTCTCCCGGTTTTCCTCTTTATTTTGTTAGAATCTGCTGTTTGGAAAGTTAATTTGTATTACTTTGTG
>Marker38062 Chr4 SNP_site:13333902 Ref_Type:G
TACTAGAATACTATCAACCAACTTATAATATGGTTGTGTGATGATTAACTAATTAATAACTTATTTTAGTTAATGAGTCCXXXXXXXXXXG
CTTAGCCTTCATACTTAGCTGAACAAAAATCTTCTCTTATTCCTAATTAGTCCTTGACACAATAGTTTCTCTCTTAGTC

Create PDF with GO2PDF for free, if you wish to

TACTAGAATACTATCAACCAACTTATAATATGGTTGTGTGATGATTAACTAATTAATAACTTATTTTAGTTAATTAGTCCXXXXXXXXXXG
CTTAGCCTTCATACTTAGCTGAACAAAAATCTTCTCTTATTCCTAATTAGTCCTTGACACAATAGTTTCTCTCTTAGTC
>Marker380691 Chr1 SNP_site:20691792 Ref_Type:G
AACCAATACGAAACAATTTTTTTTTACTTAAAAAGATGTTTAAAAATAATAATAAAGAAGCAGTTGCCTGTAAAAAAAATGXXXXXXXXXXA
AAATAGGAATAAAAAAAAGCCTAAAACCCTCCTTCTACCCTGATCGAATCGTCGCTTCCATTTCTGCTCTTTCACAGTC
AACCAATACGAAACAATTTTTTTTTACTTAAAAAGATGTTTAAAAATAATAATAAAGAAGCAGTTGCCTGTAAAAAAAATGXXXXXXXXXXA
AAATAGGAATGAAAAAAAGCCTAAAACCCTCCTTCTACCCTGATCGAATCGTCGCTTCCATTTCTGCTCTTTCACAGTC
>Marker381527 Chr1 SNP_site:22417150 Ref_Type:C
ACTTTAGTGGGCAGACTAGTGCTGCCATGTAGGCATTTCACACTGCCATTCTTTATAAACCATGCACCTCCTTAAGTTGAXXXXXXXXXXG
TTTGTTAAGTTAAACATTTGATTGTTTTGTCAGAGTCGGGATGACAGGAGAATTTTTATTTAGAGTAAAAAATGCTGTT
ACTTTAGTGGGCAGACTAGTGCTGCCATGTAGGCATTTCACACTGTCATTCTTTATAAACCATGCACCTCCTTAAGTTGAXXXXXXXXXXG
TTTGTTAAGTTAAACATTTGATTGTTTTGTCAGAGTCGGGATGACAGGAGAATTTTTATTTAGAGTAAAAAATGCTGTT
>Marker382136 Chr1 SNP_site:26024787 Ref_Type:C
TACCACATCTCTATATCTTCTAAGGCAAGCATGATGGCAGAAGCATTTTGATCATCGTTCCCTAGATCAATTTCAAATCTXXXXXXXXXXC
AAACCCATGATTTTCTGTTAAATTATTCAAATACAAAGCAAGCTCCAAAGCTGCACCATATATTGATGGAGAGAAATGT
TACCACATTTCTATATCTTCTAAGGCAAGCATGATGGCAGAAGCATTTTGATCATCGTTCCCTAGATCAATTTCAAATCTXXXXXXXXXXC
AAACCCATGATTTTCTGTTAAATTATTCAAATACAAAGCAAGCTCCAAAGCTGCACCATATATTGATGGAGAGAAATGT
>Marker382154 Chr1 SNP_site:16477060 Ref_Type:C
ACTCCAGGGGTAATGTAGAAAAGAAGGAGGCACTTGAGAACTTGGACCTCATTTTACTGTGTCTCGATGAAATTATCGACXXXXXXXXXXT
CTGTATCTTGATTGTGCCACTCTTTTTACTCACCCTCTCTAAGTTGATGCCTGTGTCCATTCTTGCTAGACAAATTGTG
ACTCCAGGGGTAATGTAGAAAAGAAGGAGGCACTTGAGAACTTGGACCTCATTTTACTGTGTCTCGATGAAATTATCGATXXXXXXXXXXT
CTGTATCTTGATTGTGCCACTCTTTTTACTCACCCTCTCTAAGTTGATGCCTGTGTCCATTCTTGCTAGACAAATTGTG
>Marker382229 Chr1 SNP_site:8978766 Ref_Type:A
TACAATAGGTCTCATGGAAAATTCGAGCATGCATAAAAACAGTTAAAAACATGAAAACATTTTCTTGAACTTGATCATGAXXXXXXXXXXT
TACAAACTATTACTTGTAAAGCTTGAAATCATGTTTCACAATATCATTTAGAAATCAATGTGTTACTCACAATATTTGT
TACAATAGGTCTCATGGAATATTCGAGCATGCATAAAAACAGTTAAAAACATGAAAACATTTTCTTGAACTTGATCATGAXXXXXXXXXXT
TACAAACTATTACTTGTAAAGCTTGAAATCATGTTTCACAATATCATTTAGAAATCAATGTGTTACTCACAATATTTGT
>Marker382262 Chr1 SNP_site:16833782 Ref_Type:G
ACATGCTTCCAAGATTGGATAAGATGATGAAAGAATGAAAAATAGAAATGCAATTAAGAGATCAAACATGGACATGTGTTXXXXXXXXXXT
CAAATAATATACCATAAAATATACACTATTATTGGCCACATCCAATATATACCATATGCTTTATTTTGATATTTGTGTA
ACATGGTTCCAAGATTGGATAAGATGATGAAAGAATGAAAAATAGAAATGCAATTAAGAGATCAAACATGGACATGTGTTXXXXXXXXXXT
CAAATAATATACCATAAAATATACACTATTATTGGCCACATCCAATATATACCATATGCTTTATTTTGATATTTGTGTA
>Marker382626 Chr1 SNP_site:20470167 Ref_Type:A
ACCTTTATGATGACTTATAAAACCTACCTTGTCCTTTTTAGTGCTATAGCACCATGCTATAACTGTTCTAGCCTGGTTACXXXXXXXXXXA
TTGTGCAGGATCCTTTGGAACCAGTTTTGTTGTTTGCAAAACCTCTGATTACAGAGAAGTTGGCAGCTGCAACTATAGT
ACCTTTATGATGACTTATAAAACCTACCTTGTCCTTTTTAGTGCTATAGCACCATGCTATAACTGTTCTAGCCTGGTTACXXXXXXXXXXA
TTGTGCAGGATCCTTTGGAACCAGTTTTGTTGTTTGCAAAACCTCTGATTACAGAGAAGTTGGCAGCTGCAGCTATAGT
>Marker382913 Chr1 SNP_site:23513525 Ref_Type:A
ACAATAATTTTAATAATATAAGTTTGTAATTATATGTTTTCTATTACATGGGCACACAAAAAAAATAAATTAATGAAAACXXXXXXXXXXA
AAGATTAGTGCAAAAGAAAGATTGAAGCTTAATTTATGATATAGACGTAGTAATTAAAAAACTTTTTTCATTCTGATGT
ACAATAATTTTAATAATATAAGTTTGTAATTATATGTTTTCTATTACATGGGCACACAAAAAAAATAAATTAATGAAAACXXXXXXXXXXA
AAGATTAGTGCAAAAGGAAGATTGAAGCTTAATTTATGATATAGACGTAGTAATTAAAAAACTTTTTTCATTCTGATGT
>Marker383257 Chr1 SNP_site:15142888 Ref_Type:G
CACAAAATCACTAGAAAGAAACATTTAACTTTCCTTTCAAATAAGGTATTATTTACTTATTATTATTATAGGAAATGGAGXXXXXXXXXXA

Create PDF with GO2PDF for free, if you wish to

GAAAAGGGTGAGGAGTAAGAAGGTGGTGCAGTCTAGAGGAAGCAAATGCTTGGGGGAATGGAAAAAGACCACAATGAGT
CACAAAATCACTAGAAAGAAACATTTAACTTTCCTTTCAAATAAGGTGTTATTTACTTATTATTATTATAGGAAATGGAGXXXXXXXXXXA
GAAAAGGGTGAGGAGTAAGAAGGTGGTGCAGTCTAGAGGAAGCAAATGCTTGGGGGAATGGAAAAAGACCACAATGAGT
>Marker383305 Chr1 SNP_site:19018251 Ref_Type:C
ACATAAAATTGTATTTTAAAATAGTGTCCATATAGTAATGATATATTTGAACGAATGCATTTTTTAAAAAAGTGTAGATGXXXXXXXXXXA
TTAATTTTGATTGATGCAATAATAATGCCATATTTTACATAAAATTTGAATATATACTAGTGCTAACAAAATAATTGGT
ACATAAAATTGTATTTTAAAATAGTGTCGATATAGTAATGATATATTTGAACGAATGCATTTTTTAAAAAAGTGTAGATGXXXXXXXXXXA
TTAATTTTGATTGATGCAATAATAATGTCATATTTTACATAAAATTTGAATATATACTAGTGCTAACAAAATAATTGGT
>Marker383305 Chr1 SNP_site:19018470 Ref_Type:C
ACATAAAATTGTATTTTAAAATAGTGTCCATATAGTAATGATATATTTGAACGAATGCATTTTTTAAAAAAGTGTAGATGXXXXXXXXXXA
TTAATTTTGATTGATGCAATAATAATGCCATATTTTACATAAAATTTGAATATATACTAGTGCTAACAAAATAATTGGT
ACATAAAATTGTATTTTAAAATAGTGTCGATATAGTAATGATATATTTGAACGAATGCATTTTTTAAAAAAGTGTAGATGXXXXXXXXXXA
TTAATTTTGATTGATGCAATAATAATGTCATATTTTACATAAAATTTGAATATATACTAGTGCTAACAAAATAATTGGT
>Marker384086 Chr1 SNP_site:26585664 Ref_Type:C
CACATGATACCTTTTGTCCCCTAGATATATCCAAAACATCTACGACATGACAAAACATCTAATCACCGACCCTTCTTCTCXXXXXXXXXXA
ACAACTTCCTTTCAACTTTCATTAAACTTAGCTCTCTCTTAGAACATAGAAATTCTCAAGTAAGGTTAGATTTAGGGTT
CACATGATACCTTTTGTCCCCTAGATATATCCAAAACATCTACGACATGACAAAACATCTAATCACCGACCCTTCTTCTCXXXXXXXXXXA
ACAACTTCCTTTCAACTTTCATTAAACTTAGCTCTCTTTTAGAACATAGAAATTCTCAAGTAAGGTTAGATTTAGGGTT
>Marker384336 Chr1 SNP_site:1763397 Ref_Type:G
ACTCTTCTGTCAATGAAGGATGACGGTGAAGATGAGAATGAGAAGCTCAATGATACTGAGATTAAGGCTTTGTTGTTGGTXXXXXXXXXXA
AACAATTTCATTACATAATTAGTAATTACAATGTTAATCAATTTCTCTATGATCGTTATTATTTCATACAGAACATGTT
ACTCTTCTGTCAATGAAGGATGACGGTGAAGATGAGAATGAGAAGCTCAATGATACTGAGATTAAGGCTTTGTTGTTGGTXXXXXXXXXXA
AACAATTTCATTACATAATTAGTAATTACAATGTTAATCAATTTCTGTATGATCGTTATTATTTCATACAGAACATGTT
>Marker384917 Chr1 SNP_site:11100449 Ref_Type:G
AACTCCCATTGTGATTTGAACTTTGAACTGCGCACACCTACATGCTTGCATGCATTTTCAACAGCAATTGATTATAAATCXXXXXXXXXXC
TCAGACTTTCTTTGAAGATGTCCTTGATGTTCATCATTCTTGTTGATGCTACTACAAGGATTATAAAGAACTAAAAGGT
AACTCCCATTGTGATTTGAACTTTGAAGTGCGCACACCTACATGCTTGCATGCATTTTCAACAGCAATTGATTATAAATCXXXXXXXXXXC
TCAGCCTTTCTTTGAAGATGTCCTTGATGTTCATCATTCTTGTTGATGCTACTACAAGGATTATAAAGAACTAAAAGGT
>Marker384917 Chr1 SNP_site:11100614 Ref_Type:C
AACTCCCATTGTGATTTGAACTTTGAACTGCGCACACCTACATGCTTGCATGCATTTTCAACAGCAATTGATTATAAATCXXXXXXXXXXC
TCAGACTTTCTTTGAAGATGTCCTTGATGTTCATCATTCTTGTTGATGCTACTACAAGGATTATAAAGAACTAAAAGGT
AACTCCCATTGTGATTTGAACTTTGAAGTGCGCACACCTACATGCTTGCATGCATTTTCAACAGCAATTGATTATAAATCXXXXXXXXXXC
TCAGCCTTTCTTTGAAGATGTCCTTGATGTTCATCATTCTTGTTGATGCTACTACAAGGATTATAAAGAACTAAAAGGT
>Marker385878 Chr1 SNP_site:16904098 Ref_Type:A
CACATTGGTTGGAGAGAGAGAAAGGAAAGATAAGTGGAAAAGAACAGGTGATAGAAAAGTTTTTACTATTTGGTTACTTTATXXXXXXXXXXT
TGGAAGATTCACAGACTCTGGGTTGCTTGTGTTTGTGGGGTTGCTTTTCCAGTTTCTTCTGTAGCGGTTTGGGTTGGTG
CACATTGGTTGGAGAGAGAGAAAGGAAAGATAAGTGGAATAGAACAGGTGATAGAAAAGTTTTTACTATTTGGTTACTTTATXXXXXXXXXXT
TGGAAGATTCACAGACTCTGGGTTGCTTGTGTTTGTGGGGTTGCTTTTCCAGTTTCTTCTGTAGCGGTTTGGGTTGGTG
>Marker386072 Chr1 SNP_site:24823020 Ref_Type:A
ACATAATCGTATGATATAAAGAAATTTAATTTCTATTTGAAAAATGTTAGAAACATACGTAACAAATAATATCAAATGAAXXXXXXXXXXT
TGAAAGAAGTATATTATTAAGTTTCACTTTTTTTTCAACCACGATGAATTGACCTAGTGGTGAATACTTGGGATGTGTT
ACATAATCGTATGATATAAAGAAATTTAATTTCTATTTGAAAAATGTTAGAAACGTACGTAACAAATAATATCAAATGAAXXXXXXXXXXT
TGAAAGAAGTATATTATTAAGTTTCACTTTTTTTTCAACCACGATGAATTGACCTAGTGGTGAATACTTGGGATGTGTT
>Marker386167 Chr1 SNP_site:25118291 Ref_Type:C

Create PDF with GO2PDF for free, if you wish to

TACTATACGTTGGGATATTGTGGGCTTCTTTTGTTTTGGACTTAATTTTTAATTTCTTACTATTTGGGGAGTTTGGTTTTXXXXXXXXXXT
AGGGTTTTGGTTCTGTTTTGAAGCTGACTGAAGATTCTTGGAATTGCAGTGGGTTACTGTCAGATTGTTCAGATCAGGT
TACTATACGTTGGGATATTGTGGGCTTGTTTTGTTTTGGACTTGATTTTTAATTTCTTACTATTTGGGGAGTTTGGTTTTXXXXXXXXXXT
AGGGTTTTGGTTCTGTTTTGAAGCTGACTGAAGATTCTTGGAATTGCAGTGGGTTACTGTCAGATTGTTCAGATCAGGT
>Marker386167 Chr1 SNP_site:25118307 Ref_Type:A
TACTATACGTTGGGATATTGTGGGCTTCTTTTGTTTTGGACTTAATTTTTAATTTCTTACTATTTGGGGAGTTTGGTTTTXXXXXXXXXXT
AGGGTTTTGGTTCTGTTTTGAAGCTGACTGAAGATTCTTGGAATTGCAGTGGGTTACTGTCAGATTGTTCAGATCAGGT
TACTATACGTTGGGATATTGTGGGCTTGTTTTGTTTTGGACTTGATTTTTAATTTCTTACTATTTGGGGAGTTTGGTTTTXXXXXXXXXXT
AGGGTTTTGGTTCTGTTTTGAAGCTGACTGAAGATTCTTGGAATTGCAGTGGGTTACTGTCAGATTGTTCAGATCAGGT
>Marker38619 Chr4 SNP_site:23400851 Ref_Type:G
AACTCACACGTGTGATATAGAACTCACACACGTGCAACCGACGCCTTTTCGCTTCCCCTTCCTTGAATCCAACACGGAATXXXXXXXXXXG
TAAATGAGAATCAGCTCATGCAGCTGGAAACAGATCCAAAATGAGACTCGGCTAAATGGTAAAGTAGCTTGGAAAAGTG
AACTCACACGTGTGATATAGAACTCACACACGTGCAACCGACGCCTTTTCGCTTCCCCTTCCTTGAATCCAACACGGAATXXXXXXXXXXG
TAAATGGGAATCAGCTCATGCAGCTGGAAACAGATCCAAAATGAGACTCGGCTAAAAGGTAAAGTAGCTTGGAAAAGTG
>Marker38619 Chr4 SNP_site:23400901 Ref_Type:A
AACTCACACGTGTGATATAGAACTCACACACGTGCAACCGACGCCTTTTCGCTTCCCCTTCCTTGAATCCAACACGGAATXXXXXXXXXXG
TAAATGAGAATCAGCTCATGCAGCTGGAAACAGATCCAAAATGAGACTCGGCTAAATGGTAAAGTAGCTTGGAAAAGTG
AACTCACACGTGTGATATAGAACTCACACACGTGCAACCGACGCCTTTTCGCTTCCCCTTCCTTGAATCCAACACGGAATXXXXXXXXXXG
TAAATGGGAATCAGCTCATGCAGCTGGAAACAGATCCAAAATGAGACTCGGCTAAAAGGTAAAGTAGCTTGGAAAAGTG
>Marker386227 Chr1 SNP_site:8258983 Ref_Type:T
CACTGACATATGGTTGTAGTATTTTCCATGACAACATCATAGGTCCAATCCTTTCATGTTTAGGTTAAATTGCAAATTTGXXXXXXXXXXA
AGTTTTATCAAACCATAATTATTAAATTCTAACTTTTAAAATCATAGGGACTCAATTCCAATATTCTTCAAACCATGTG
CACTGACATATGGTTGTAGTATTTTCCATGACAACATCATAGGTCCAATCCTTTCATGTTTAGGTTAAATTGCAAATTTGXXXXXXXXXXA
AGTTTTATCAAACCATAATTATTAAATTCTAACTTTTAAAATCATAGGGATTCAATTCCAATATTCTTCAAACCATGTG
>Marker38717 Chr4 SNP_site:20454878 Ref_Type:G
ACTTTTAACTATTGTTTTTGGTATTTTGCAATATCGCGAACATCTCGTAATTGTCAAACTTTAAATGCCCAAGGCTGAATXXXXXXXXXXA
ATCAAATTTTGCAGGAATTTAAAAAGTTAAAGGTGGTTTGAGGATGTCACATGTTCGAATTTTAGTGTGTTACGGTGGT
ACTTTTAACTATTGTTTTTGGTATTTTGCAATATCGCGAACATCTCGTAGCTGTCAAACTTTAAATGCCCAAGGCTGAATXXXXXXXXXXA
ATCAAATTTTGCAGGAATTTAAAAAGTTAAAGGTGGTTTGAGGATGTCACATGTTCGAATTTTAGTGTGTTACGGTGGT
>Marker38717 Chr4 SNP_site:20454879 Ref_Type:C
ACTTTTAACTATTGTTTTTGGTATTTTGCAATATCGCGAACATCTCGTAATTGTCAAACTTTAAATGCCCAAGGCTGAATXXXXXXXXXXA
ATCAAATTTTGCAGGAATTTAAAAAGTTAAAGGTGGTTTGAGGATGTCACATGTTCGAATTTTAGTGTGTTACGGTGGT
ACTTTTAACTATTGTTTTTGGTATTTTGCAATATCGCGAACATCTCGTAGCTGTCAAACTTTAAATGCCCAAGGCTGAATXXXXXXXXXXA
ATCAAATTTTGCAGGAATTTAAAAAGTTAAAGGTGGTTTGAGGATGTCACATGTTCGAATTTTAGTGTGTTACGGTGGT
>Marker387434 Chr1 SNP_site:12321037 Ref_Type:C
CACCTTTCGAGATACAAATTTTAGAAGATGCCTCGCTAAGGAAAAGTTTCTTACCAAACTCCTCTTTACATGCATTAGTCXXXXXXXXXXT
GAAAAGTTAAGAAATGGTGGATTGCAATGGGACAAACATTCAGTTATGTATAAAAAGAATATAGGTCTTCAGCCACTGT
CACCTTTCGAGATACAAATTTTAGAAGATGCCTCGCTAAGGAAAAGTTTCTTACCAAACTCCTTTTTACATGCATTAGTCXXXXXXXXXXT
GAAAAGTTAAGAAATGGTGGATTGCAAAGGGACAAACATTCAGTTATGTATAAAAAGAATATAGGTCTTCAGCCACTGT
>Marker387434 Chr1 SNP_site:12321230 Ref_Type:T
CACCTTTCGAGATACAAATTTTAGAAGATGCCTCGCTAAGGAAAAGTTTCTTACCAAACTCCTCTTTACATGCATTAGTCXXXXXXXXXXT
GAAAAGTTAAGAAATGGTGGATTGCAATGGGACAAACATTCAGTTATGTATAAAAAGAATATAGGTCTTCAGCCACTGT
CACCTTTCGAGATACAAATTTTAGAAGATGCCTCGCTAAGGAAAAGTTTCTTACCAAACTCCTTTTTACATGCATTAGTCXXXXXXXXXXT
GAAAAGTTAAGAAATGGTGGATTGCAAAGGGACAAACATTCAGTTATGTATAAAAAGAATATAGGTCTTCAGCCACTGT

Create PDF with GO2PDF for free, if you wish to

>Marker387747 Chr1 SNP_site:28666894 Ref_Type:C
CACTCTTTAGCCAAACTTTTTTGTTTCTTTGCAAGGATTGACTGATGCTTACTCTGTTTAACTTTCTCAGGTTGTGGGGCXXXXXXXXXXT
TAGATCACTTAAAATTAGTTATTCGAAAATGACTCTCAAATATATCTTAAATTGGTGTTGAATAGAAATCTAAAGGGTT
CACTCTTTAGCCAAACTTTTTTGTTTCTTTGCAAGGATTGATTGATGCTTACCCTGTTTAACTTTCTCAGGTTGTGGGGCXXXXXXXXXXT
TAGATCACTTAAAATTAGTTATTCGAAAATGACTCTCAAATATATCTTAAATTGGTGTTGAATAGAAATCTAAAGGGTT
>Marker387747 Chr1 SNP_site:28666905 Ref_Type:T
CACTCTTTAGCCAAACTTTTTTGTTTCTTTGCAAGGATTGACTGATGCTTACTCTGTTTAACTTTCTCAGGTTGTGGGGCXXXXXXXXXXT
TAGATCACTTAAAATTAGTTATTCGAAAATGACTCTCAAATATATCTTAAATTGGTGTTGAATAGAAATCTAAAGGGTT
CACTCTTTAGCCAAACTTTTTTGTTTCTTTGCAAGGATTGATTGATGCTTACCCTGTTTAACTTTCTCAGGTTGTGGGGCXXXXXXXXXXT
TAGATCACTTAAAATTAGTTATTCGAAAATGACTCTCAAATATATCTTAAATTGGTGTTGAATAGAAATCTAAAGGGTT
>Marker38800 Chr4 SNP_site:12671958 Ref_Type:C
ACTCAAAACCTTTGTTTTCTCTCTAGAGCTAGCCTTGGTGTGGGGAAGGCCTCTTGGCAACTAGCAGGGTTGGTTCTATAXXXXXXXXXXG
TCTGTAAATGCATTTTTAAGGTTACATGCTCACTTGTGGGTCATTTGTGTTTTTTGAATATTGAAATATATTTGAACGT
ACTCAAAACCTTTGTTTTCTCTCTAGAGCTAGCCTTGGTGTGGGGAAGGCCTCTTGGCAATTAGCAGGGTTGGTTCTATAXXXXXXXXXXG
TCTGTAAATGCATTTTTAAGGTTACATGCTCACTTGTGGGTCATTTGTGTTTTTTGAATATTGAAATATTTTTGAACGT
>Marker38800 Chr4 SNP_site:12672157 Ref_Type:A
ACTCAAAACCTTTGTTTTCTCTCTAGAGCTAGCCTTGGTGTGGGGAAGGCCTCTTGGCAACTAGCAGGGTTGGTTCTATAXXXXXXXXXXG
TCTGTAAATGCATTTTTAAGGTTACATGCTCACTTGTGGGTCATTTGTGTTTTTTGAATATTGAAATATATTTGAACGT
ACTCAAAACCTTTGTTTTCTCTCTAGAGCTAGCCTTGGTGTGGGGAAGGCCTCTTGGCAATTAGCAGGGTTGGTTCTATAXXXXXXXXXXG
TCTGTAAATGCATTTTTAAGGTTACATGCTCACTTGTGGGTCATTTGTGTTTTTTGAATATTGAAATATTTTTGAACGT
>Marker388130 Chr1 SNP_site:16930248 Ref_Type:A
CACTGTGATCTAAAACCTAACAATATACTGTTAGATGGAGATATGGTTGCACACTTGACAGATTTTGGGATTTCAAAACTXXXXXXXXXXC
ATACCGTATAACATGCTTCTTTGTTGTGCTGAATGCAGAATTGGGATTGGATGGAATTGTTTCTAGAAAGTGTGATGTT
CACTGTGATCTAAAACCTAACAATATACTGTTAGATGGAGATATGGTTGCACACTTGACAGATTTTGGGATTTCAAAACTXXXXXXXXXXC
ATACCGTATAACATGCTTCTTTGTTGTGCTGAGTGCAGAATTGGGATTGGATGGAATTGTTTCTAGAAAGTGTGATGTT
>Marker388243 Chr1 SNP_site:3868186 Ref_Type:G
CACTATAGAATCCCTTGAGGGCGTTCTTGATAAAAGTATCTGATCGAATGAGGACGCTTGCGACGGAGCTGTTTTGTTGTXXXXXXXXXXA
AGATTCATAATTTAAGATCCAGGAAGAACTCAAAATTTCCATACACAACTTAAATGCTTAACATAATTACAATGTTGTA
CACTATAGAATCCCTTGAGGGCGTTCTTGATAAAAGTATCTGATCGAATGAGGACGCTTGCGACGGAGCTGTTTTGTTGTXXXXXXXXXXA
AGATTCATAGTTTAAGATCCAGGAAGAACTCAAAATTTCCATACACAACTTAAATGCTTAACATAATTACAATGTTGTA
>Marker388496 Chr1 SNP_site:24193586 Ref_Type:C
ACTTAACAATGGATGATCTTGCTGATCAATTTGGAATTGGAACACTTCGTCTAACCACCAGGCAAACATTTCAGCTACATXXXXXXXXXXG
TTCTTGCCCCAGCTGCTCCACTTGTGAGAAAAGATTACCTTTTTGCACAGCAAACTGCAGAAAACATTGCTGCTTTGTT
ACTTAACAATGGATGATCTTGCTGATCAATTTGGAATTGGAACACTTCGTCTAACCACCAGGCAAACATTTCAGCTACATXXXXXXXXXXG
TTCTTGCTCCAGCTGCTCCACTTGTGAGAAAAGATTACCTTTTTGCACAGCAAACTGCAGAAAACATTGCTGCTTTGTT
>Marker388601 Chr1 SNP_site:17318870 Ref_Type:A
TACATTCAAGACAATGTCAGCTGGGGTTGGGGCTTTGGCATTTGCCTCGCCACTAACATCATTTCTCTTGCTATTTTCTTXXXXXXXXXXT
CAAGCACGGAGAGGGATAATGGTTGCTACTATCAAGGTAAGGACCATCATTTGAGGAATCAAGCTGGAGATGGAGTGTT
TACATTCAAGACAGTGTCAGCTGGGGTTGGGGCTTTGGCATTTGCCTCGCCACTAACATCATTTCTCTTGCTATTTTCTTXXXXXXXXXXT
CAAGCACAGAGAGGGATAATGGTTGCTACTATCAAGGTAAGGACCATCATTTGAGGAATCAAGCTGGAGATAGAGTGTT
>Marker388601 Chr1 SNP_site:17319051 Ref_Type:G
TACATTCAAGACAATGTCAGCTGGGGTTGGGGCTTTGGCATTTGCCTCGCCACTAACATCATTTCTCTTGCTATTTTCTTXXXXXXXXXXT
CAAGCACGGAGAGGGATAATGGTTGCTACTATCAAGGTAAGGACCATCATTTGAGGAATCAAGCTGGAGATGGAGTGTT
TACATTCAAGACAGTGTCAGCTGGGGTTGGGGCTTTGGCATTTGCCTCGCCACTAACATCATTTCTCTTGCTATTTTCTTXXXXXXXXXXT

Create PDF with GO2PDF for free, if you wish to

CAAGCACAGAGAGGGATAATGGTTGCTACTATCAAGGTAAGGACCATCATTTGAGGAATCAAGCTGGAGATAGAGTGTT

>Marker388601 Chr1 SNP_site:17319115 Ref_Type:G

TACATTCAAGACAATGTCAGCTGGGGTTGGGGCTTTGGCATTTGCCTCGCCACTAACATCATTTCTCTTGCTATTTTCTTXXXXXXXXXXT

CAAGCACGGAGAGGGATAATGGTTGCTACTATCAAGGTAAGGACCATCATTTGAGGAATCAAGCTGGAGATGGAGTGTT

TACATTCAAGACAGTGTCAGCTGGGGTTGGGGCTTTGGCATTTGCCTCGCCACTAACATCATTTCTCTTGCTATTTTCTTXXXXXXXXXXT

CAAGCACAGAGAGGGATAATGGTTGCTACTATCAAGGTAAGGACCATCATTTGAGGAATCAAGCTGGAGATAGAGTGTT

>Marker388606 Chr1 SNP_site:961414 Ref_Type:G

TACAGTATGAGCATTGGCATCAAGAAGAGTCTCAAGTGATCTCGCCTTTCGGCTCCGAACTCTACCAGCTTCTTTCAACAXXXXXXXXXXA

GAGTCATGAGAGGAGAACTAATCAATTGCATCATTCGTTGCTCAAAGAAAACTTTCAGCGACTTCCTACTCTAAACGTA

TACAGTATGAGCATTGGCATCAAGAAGAGTCTCGAGTGATCTCGCCTTTCGGCTCCGAACTCTACCAGCTTCTTTCAACAXXXXXXXXXXA

GAGTCATGAGAGGAGAACTAATCAATTGCATCATTCGCTGCTCAAAGAAAACTTTCAGCGACTTCCTACTCTAAACGTA

>Marker388606 Chr1 SNP_site:961612 Ref_Type:C

TACAGTATGAGCATTGGCATCAAGAAGAGTCTCAAGTGATCTCGCCTTTCGGCTCCGAACTCTACCAGCTTCTTTCAACAXXXXXXXXXXA

GAGTCATGAGAGGAGAACTAATCAATTGCATCATTCGTTGCTCAAAGAAAACTTTCAGCGACTTCCTACTCTAAACGTA

TACAGTATGAGCATTGGCATCAAGAAGAGTCTCGAGTGATCTCGCCTTTCGGCTCCGAACTCTACCAGCTTCTTTCAACAXXXXXXXXXXA

GAGTCATGAGAGGAGAACTAATCAATTGCATCATTCGCTGCTCAAAGAAAACTTTCAGCGACTTCCTACTCTAAACGTA

>Marker389209 Chr1 SNP_site:26248943 Ref_Type:A

AACAAGATTCACTAGCCAAACATATTAATTCTAAATCAGGTTTAAAAAAGAGACTTCAAACCGCATTCCTTGAACAGAAAXXXXXXXXXXG

ATTCCAAATTGAGAAACGCCGTAGCACCACGGCACCGCTCACCGGCAGCTTGAACTGCTTGAAGCTCAGCAGATTCAGT

AACAAGATTCACTAGCCAAACATATTCATTCTAAATCAGGTTTAAAAAAGAGACTTCAAACCGCATTCCTTGAACAGAAAXXXXXXXXXXG

ATTCCAAATTGAGAAACGCCGTAGCACCACGGCACCGCTCACCGGCAGCTTGAACTGCTTGAAGCTCAGCAGATTCAGT

>Marker389841 Chr1 SNP_site:26018509 Ref_Type:T

ACAGTCAGGGGCATTTTGATCATACTTTATTTACAAAGGTTTCCAAGATATGGAAGGTTGTTGTTCAGATAGAGCATGTTXXXXXXXXXXA

AAAAGGCATCTCCATATCTCAAAGAAAATACACCCTTGATTTCCTGGCCGAGACTAGTATGCTGAGATGTCGTCTTGTT

ACAGTCAGGGGCATTTTGATCATACTTTATTTACAAAGGTTTCCAAGATATGGAAGGTTGTTTTTCAGATAGAGCATGTTXXXXXXXXXXA

AAAAGGCATCTCCATATCTCAAAGAAAATACACCCTTGATTTCCTGGCCGAGACTAGTATGCTGAGATGTCGTCTTGTT

>Marker390130 Chr1 SNP_site:27846294 Ref_Type:C

AACAAAAGACGAACTATTTATGGTTTTCATTGTTCAACTGATTATGCAATTGAAGAATGTCACACTCACACGTGCCTTTTXXXXXXXXXXT

TGGTTGTTACAACAATCTTTTTCGTTTAATGAAAAAGGTGAAGACTGTTTGCCTCTTTGATAAACAAATATAACATGTG

AACAAAAGACGAACTATTTATGGTTTTCATTGTTCAACTGATTATGCAATTGAAGAATGTCACACTCACACGTGCCTTTTXXXXXXXXXXT

TGGTTGTTACAACAATCTTTTTCGTTTAATGAAAAAGGTGAAGACTGTTTGCCTTTTTGATAAACAAATATAACATGTG

>Marker39046 Chr4 SNP_site:22700315 Ref_Type:C

CACCTATTCAATCGAAAAAATAGAATTAGTTAAAAGTATATTTTAAATTAAGATACAAAATTTTAGCTATATATGGCTATXXXXXXXXXXT

AAAGATAACATCTTTTATAAGGAGAGACGGATCGAGACCATATAAAAACTCAGGAAAATAAATTGAGACAAAAAAAGTC

CACCTATTCAATTGAAAAAATAGAATTACTTAAAAGTATATTTTAAATTAAGATACAAAATTTTAGCTATATAGGGCTATXXXXXXXXXXT

AAAGATAACATCTTTTATAAGGAGAGACGGATCGAGACCATATAAAAACTCAGGAAAATAAATTGAGACAAAAAAAGTC

>Marker39046 Chr4 SNP_site:22700331 Ref_Type:G

CACCTATTCAATCGAAAAAATAGAATTAGTTAAAAGTATATTTTAAATTAAGATACAAAATTTTAGCTATATATGGCTATXXXXXXXXXXT

AAAGATAACATCTTTTATAAGGAGAGACGGATCGAGACCATATAAAAACTCAGGAAAATAAATTGAGACAAAAAAAGTC

CACCTATTCAATTGAAAAAATAGAATTACTTAAAAGTATATTTTAAATTAAGATACAAAATTTTAGCTATATAGGGCTATXXXXXXXXXXT

AAAGATAACATCTTTTATAAGGAGAGACGGATCGAGACCATATAAAAACTCAGGAAAATAAATTGAGACAAAAAAAGTC

>Marker39046 Chr4 SNP_site:22700376 Ref_Type:T

CACCTATTCAATCGAAAAAATAGAATTAGTTAAAAGTATATTTTAAATTAAGATACAAAATTTTAGCTATATATGGCTATXXXXXXXXXXT

AAAGATAACATCTTTTATAAGGAGAGACGGATCGAGACCATATAAAAACTCAGGAAAATAAATTGAGACAAAAAAAGTC

Create PDF with GO2PDF for free, if you wish to

CACCTATTCAATTGAAAAAATAGAATTACTTAAAAGTATATTTTAAATTAAGATACAAAATTTTAGCTATATAGGGCTATXXXXXXXXXXXT
AAAGATAACATCTTTTATAAGGAGAGACGGATCGAGACCATATAAAAACTCAGGAAAATAAATTGAGACAAAAAAAGTC
>Marker390710 Chr1 SNP_site:6945393 Ref_Type:T
CACCCATTTAAAATCATAAATACTGTGAGTTTAAGTAATTTATTAGTTTGTGATCCTCATCTTATCTTTACAAATTTCTGXXXXXXXXXXXT
CCAAAGAAGATGAAATCAAGGATTAGAATTAGAAATATGAGAGATATTTATCTTTAGGTTCATAAAATCATACTGTTGT
TACCCATTTAAAATCATAAATATTGTGAGTTTAAGTAATTTATTAGTTTGTGATCCTCATCTTATCTTTACAAATTTCTGXXXXXXXXXXXT
CCAAAGAAGATGAAATCAAGGATTAGAATTAGAAATATGAGAGATATTTATCTTTAGGTTCATAAAATCATACTGTTGT
>Marker390710 Chr1 SNP_site:6945415 Ref_Type:T
CACCCATTTAAAATCATAAATACTGTGAGTTTAAGTAATTTATTAGTTTGTGATCCTCATCTTATCTTTACAAATTTCTGXXXXXXXXXXXT
CCAAAGAAGATGAAATCAAGGATTAGAATTAGAAATATGAGAGATATTTATCTTTAGGTTCATAAAATCATACTGTTGT
TACCCATTTAAAATCATAAATATTGTGAGTTTAAGTAATTTATTAGTTTGTGATCCTCATCTTATCTTTACAAATTTCTGXXXXXXXXXXXT
CCAAAGAAGATGAAATCAAGGATTAGAATTAGAAATATGAGAGATATTTATCTTTAGGTTCATAAAATCATACTGTTGT
>Marker390747 Chr1 SNP_site:28825700 Ref_Type:G
AACATGAAAGAACGTTTGAAGTTTTGATACTATGTAATGTTATTTGAAATAACTAGGTTGATATTTACGATAGTAAATGGXXXXXXXXXXXT
AAACCATAACATTGTTTGAACATAGTAAATGAAGAGGATGGGTATTTTTTATTTTATGGGTATGGTGTTTAGATATGTT
AACGTGAAAGAACGTTTGAAGTTTTGATACTATGTAATGTTATTTGAAATAACTAGTTTGATATTTACGATAGTAAATGGXXXXXXXXXXXT
AAACCATAACATTGTTTGAACATAGTAAATGAAGAGGATGGGTATTTTTTATTTTATGGGTATGGTGTTTAGATATGTT
>Marker390747 Chr1 SNP_site:28825753 Ref_Type:T
AACATGAAAGAACGTTTGAAGTTTTGATACTATGTAATGTTATTTGAAATAACTAGGTTGATATTTACGATAGTAAATGGXXXXXXXXXXXT
AAACCATAACATTGTTTGAACATAGTAAATGAAGAGGATGGGTATTTTTTATTTTATGGGTATGGTGTTTAGATATGTT
AACGTGAAAGAACGTTTGAAGTTTTGATACTATGTAATGTTATTTGAAATAACTAGTTTGATATTTACGATAGTAAATGGXXXXXXXXXXXT
AAACCATAACATTGTTTGAACATAGTAAATGAAGAGGATGGGTATTTTTTATTTTATGGGTATGGTGTTTAGATATGTT
>Marker391279 Chr1 SNP_site:21891386 Ref_Type:G
ACTTTTACTCTATCTGATTTTAGTAATTTTACATTTAAACTCAGTTAGACTCTTTTAAATTCACTTACAAATCAATTTCAXXXXXXXXXXXA
ATCAATTCAAAGTATGATTTCAAACACATTACTTTTGAAGGTAGATGATATTTCTTCGGACCGAATGACGAATTTTTGT
ACTTTTACTCTATCTGATTTTAGTGATTTTACATTTAAACTCAGTTAGACTCTTTTAAATTCACTTACAAATCAATTTCAXXXXXXXXXXXA
ATCAATTCAAAGTATGATTTCAAACACATTACTTTTGAAGGTAGATGATATTTCTTCGGACCGAATGACGAATTTTTGT
>Marker392028 Chr1 SNP_site:20451187 Ref_Type:T
CACCAAAAGAAAGTAATAATACCATTCATTTTTGTATGTTGTGGAATTATTCCTTTCGGTGAAATATGGGGAAGTTTTGGXXXXXXXXXXXT
CTATTGGAGATGACCTTTGCCGAAGTGCCAAAAATTCTCCCAAAAGAACTCTTTTCAACGTTCCTCCCACTCCATCGTC
CACCAAAAGAAAGTAATAATACCATTCATTTTTGTATGTTGTGGAATTATTCCTTTTGGTGAAATATGGGGAAGTTTTGGXXXXXXXXXXXT
CTATTGGAGATGACCTTTGCCGAAGTGCCAAAAATTCTCCCAAAAGAACTCTTTTCAACGTTCCTCCCACTCCATCGTC
>Marker392382 Chr1 SNP_site:27393163 Ref_Type:G
ACTTCAAGAAAGGCTTTCAAGAATTCAAAGACAAGTCCAAGAAAATCCAAGAAAGCAACAAAATATTCAAGAAATGCTCCXXXXXXXXXXXC
TCCGGATCATAAGAAGGTAAAGTTGATAGCTCTCAAGTTGAAAGGGGGAGCCTCGACATGGTGGGTGCAATTAGAGGTA
ACTTCAAGAAAGGCTTTCAAGAATTCAAAGACAAGTCCAAGAAAATCCAATAAAGCAACAAAATATTCAAGAAATGCTCCXXXXXXXXXXXC
TCCGGATCATAAGAAGGTAAAGTTAATAGCTCTCAAGTTGAAAGGGGGAGCCTCGACATGGTGGGTGCAATTAGAGGTA
>Marker392382 Chr1 SNP_site:27393309 Ref_Type:G
ACTTCAAGAAAGGCTTTCAAGAATTCAAAGACAAGTCCAAGAAAATCCAAGAAAGCAACAAAATATTCAAGAAATGCTCCXXXXXXXXXXXC
TCCGGATCATAAGAAGGTAAAGTTGATAGCTCTCAAGTTGAAAGGGGGAGCCTCGACATGGTGGGTGCAATTAGAGGTA
ACTTCAAGAAAGGCTTTCAAGAATTCAAAGACAAGTCCAAGAAAATCCAATAAAGCAACAAAATATTCAAGAAATGCTCCXXXXXXXXXXXC
TCCGGATCATAAGAAGGTAAAGTTAATAGCTCTCAAGTTGAAAGGGGGAGCCTCGACATGGTGGGTGCAATTAGAGGTA
>Marker393565 Chr1 SNP_site:28177547 Ref_Type:T
ACATAGAACTGGTGAAGAATGTGTGTAGTTATCGGCCATTTCTTTTAGACATTCTTCCAATGGGTTTGGTCACCCCAGTTXXXXXXXXXXXA

Create PDF with GO2PDF for free, if you wish to

AGTTTGTCACATATAAAACGATGTTCCAAAAGTTTGGATCTTTTTTCCCCTCTTGCAAATGTCACTGTGGATTTATGTT
ACATAGAACTGGTGAAGAATGTGTGTAGTTATCGGCCATTTCTTTTAGACATTCTTCCAATGGGTTTGGTCACCCCATTTXXXXXXXXXXA
AGTTTGTCACATATAAAACGATGTTCCAAAAGTTTGGATCTTTTTTCCCCTCTTGCAAATGTCACTGTGGATTTATGTT
>Marker394231 Chr1 SNP_site:4170925 Ref_Type:A
AACAGAGCCCTAAGACACATACCAATTCTTAATCGAGGGAAATCACGACTGCCTTTGAAATGATCAATAATACTCCTCATXXXXXXXXXXG
ATCCTTGAACTGAGTTTTCTTGGATCAGGCGCACACATGACACCGTTAAATCTTTTGGCAACTTTCACATTTACTTGTC
AACAGAGCCCTAAGACACATACCAATTCTTAATCGAGGGAAATCACGACTGCCTTTGAAATGATCAATAATACTCCTCATXXXXXXXXXXG
ATCCTTGAACTGAGTTTTCTTGGATCAGGCGCACACATGACACCGTTTAATCTTTTGGCAACTTTCACATTTACTTGTC
>Marker395182 Chr1 SNP_site:3692528 Ref_Type:A
AACAATTTAAACTAATTCGTATAAATATTTGATATTTTTTAAAAAAAATTGACCATTTCTCATAATATAATTCATTAAGAXXXXXXXXXXG
ATTTTGATAGATTAATAATAATTTAGTTGTATGATTTCCCATAATCACTTTCTAAACGAGGCTGACGCCGACGCCGTA
AACAATTTAAACTAATTCGTATAAATATTTGATATTTTTTTAAAAAAATTTGACCATTTCTCATAATATAATTCATTAAGAXXXXXXXXXXG
ATTTTGATAGATTAATAATAATTTAGTTGTATGATTTCCCATAATCACTTTCTAAACGAGGCTGACGCCGACGCCGTA
>Marker395182 Chr1 SNP_site:3692535 Ref_Type:A
AACAATTTAAACTAATTCGTATAAATATTTGATATTTTTTAAAAAAAATTGACCATTTCTCATAATATAATTCATTAAGAXXXXXXXXXXG
ATTTTGATAGATTAATAATAATTTAGTTGTATGATTTCCCATAATCACTTTCTAAACGAGGCTGACGCCGACGCCGTA
AACAATTTAAACTAATTCGTATAAATATTTGATATTTTTTTAAAAAAATTTGACCATTTCTCATAATATAATTCATTAAGAXXXXXXXXXXG
ATTTTGATAGATTAATAATAATTTAGTTGTATGATTTCCCATAATCACTTTCTAAACGAGGCTGACGCCGACGCCGTA
>Marker39553 Chr4 SNP_site:11946878 Ref_Type:C
ACTTTAGATTCTTTGGATTTTGTTGATGGGGGCTTGTTGCTTTTGTCTGATTTTTCTGTTTAGCTTTAGTTTGGGGTTACXXXXXXXXXXC
CATTTGAAGACTATAATTTCTTCTGTTCATTTGTTTCTTTGCTTTGATGAATCATTTTCACAATTTATTTGAAGGGGTA
ACTTTAGATTCTTTGGATTTTGTTGATGGGGGCTTGTTGCTTTTGTTTGATTTTTCTGTTTAGCTTTAGTTTGGGGTTACXXXXXXXXXXC
CATTTGCAGACTATAATTTCTTCTGTTCATTTGTTTCTTTGCTTTGATGAATCATTTTCACAATTTATTTGAAGGGGTA
>Marker39553 Chr4 SNP_site:11947035 Ref_Type:A
ACTTTAGATTCTTTGGATTTTGTTGATGGGGGCTTGTTGCTTTTGTCTGATTTTTCTGTTTAGCTTTAGTTTGGGGTTACXXXXXXXXXXC
CATTTGAAGACTATAATTTCTTCTGTTCATTTGTTTCTTTGCTTTGATGAATCATTTTCACAATTTATTTGAAGGGGTA
ACTTTAGATTCTTTGGATTTTGTTGATGGGGGCTTGTTGCTTTTGTTTGATTTTTCTGTTTAGCTTTAGTTTGGGGTTACXXXXXXXXXXC
CATTTGCAGACTATAATTTCTTCTGTTCATTTGTTTCTTTGCTTTGATGAATCATTTTCACAATTTATTTGAAGGGGTA
>Marker395557 Chr1 SNP_site:21015977 Ref_Type:C
AACGAAGGTGACAAAATTTGAGAATTAAATAGCAAAAGGAAGAAACCTTTGCAAGAAAAAAAATGAAGAAGCTGTTCCACXXXXXXXXXXG
AGGCACGCCTACGAAGAAACGTTCGATGCAAAAGCGTTATCCAACCGAGTCGAAGAAGTTCTAGATCAGCTAGAAAGTT
AACGAAGGTGACAAAATTTGAGAATTAAATAGCAAAAGGAAGAAACCTTTGCAAGAAAAAAAATGAAGAAGCTGTTCCACXXXXXXXXXXG
AGGCACGCCTACGAAGAAACGTTTGATGCAAAAGCGTTATCCAACCGAGTCGAAGAAGTTCTAGATCAGCTAGAAAGTT
>Marker395618 Chr1 SNP_site:6656715 Ref_Type:A
ACATTTCTTTCTTCTTTTTTCCATTCTCTGTTTTGCTTTTGCCCAAAACTAGTAAGGATTCCTCCCCTTGTCTGATTTAGXXXXXXXXXXC
AATGAAAGCATGTCTGTGGTTCTTGAAAGATTTGGTGCCATGGGTATTGACACCTCTGGAGTGGTTGCTTTACTAGGTA
ACATTTCTTTCTTCTTTTTTCCATTCTCTGTTTTGCTTTTGCCCAAAACTAGTAAGGATTCCTCCCCTTGTCTGATTTTGXXXXXXXXXXC
AATGAAAGCATGTCTGTGGTTCTTGAAAGATTTGGTGCCATGGGTATTGACACCTCTGGAGTGGTTGCTTTACTAGGTA
>Marker395727 Chr1 SNP_site:23991054 Ref_Type:G
CACAGCATCAAACTAAATTGCTTCACACAAATCCTTTTGTAATTACATCAAGCAAGGAAACAACTAGGATAATGATCTTGXXXXXXXXXXA
AGTCGAGCTGTTCATTTAGGGAAAGGTGCAGTAATTGTTTGTTCTCGTCTTCACTCAAGCCTATTAGTAAAAGGAAGGT
CACAGCATCAGACTAAATTGCTTCACACAAATCCTTTTGTAATTACATCAAGCAAGGAAACAACTAGGATAATGATCTTGXXXXXXXXXXA
AGTCGAGCTGTTCATTTAGGGAAAGGTGCAGTAATTGTTTGTTCTCGTTTTCACTCAAGCCTATTAGTAAAAGGAAGGT
>Marker395727 Chr1 SNP_site:23991312 Ref_Type:T

Create PDF with GO2PDF for free, if you wish to

CACAGCATCAAACTAAATTGCTTCACACAAATCCTTTTGTAATTACATCAAGCAAGGAAACAACTAGGATAATGATCTTGXXXXXXXXXXA
AGTCGAGCTGTTCATTTAGGGAAAGGTGCAGTAATTGTTTGTTCTCGTCTTCACTCAAGCCTATTAGTAAAAGGAAGGT
CACAGCATCAGACTAAATTGCTTCACACAAATCCTTTTGTAATTACATCAAGCAAGGAAACAACTAGGATAATGATCTTGXXXXXXXXXXA
AGTCGAGCTGTTCATTTAGGGAAAGGTGCAGTAATTGTTTGTTCTCGTTTTCACTCAAGCCTATTAGTAAAAGGAAGGT
>Marker395787 Chr1 SNP_site:16841351 Ref_Type:C
GACGCTCTGTATGACCAAACAGAGGGTCTAGTGGCACCTCTAGATTGCGTTATTTGATATTTATCTCTAATAATAAATTGXXXXXXXXXXG
GGTGCTTTAGACGATGTGGTAATGGGTGGAGTAAGTGAAAGTTCATTTCAAATCGACATGAACGGTGGTGAAAATGGTG
GACGCTCTGTATGACCAAACAGAGGGTCTAGTGGCACCTCTAGATTGCGTTATTTGATATTTATCTCTAATAATAAATTGXXXXXXXXXXG
GGTGTTTTAGACGATGTGGTAATGGGTGGAGTAAGTGAAAGTTCATTTCAAATCGACATGAACGGTGGTGAAAATGGTG
>Marker397147 Chr1 SNP_site:24151638 Ref_Type:A
GACTCACGAGGCTTTTCAAAGCCACCAGTATGATACACATACAATGCAAAAACCAACAGGTTGATGCCAAACGCAACTGAXXXXXXXXXXG
ATTTTAATGATTTTCCAGTTTCTTGAGCGTTATCTTCTCCCATCCTTTGCTAATTCATTTGCCTATGAATTAATTAGTG
GACTCACGAGGCTTTTCAAAGCCACCAGTGAGATACACATACAATGCAAAAACCAACAGGTTGATGCCAAACGCAACTGAXXXXXXXXXXG
ATTTTAATGATTTTCCAGTTTCTTGAGCGTTATCTTCTCCCATCCTTTGCTAATTCATTTGCCTATGAATTAATTAGTG
>Marker397147 Chr1 SNP_site:24151639 Ref_Type:T
GACTCACGAGGCTTTTCAAAGCCACCAGTATGATACACATACAATGCAAAAACCAACAGGTTGATGCCAAACGCAACTGAXXXXXXXXXXG
ATTTTAATGATTTTCCAGTTTCTTGAGCGTTATCTTCTCCCATCCTTTGCTAATTCATTTGCCTATGAATTAATTAGTG
GACTCACGAGGCTTTTCAAAGCCACCAGTGAGATACACATACAATGCAAAAACCAACAGGTTGATGCCAAACGCAACTGAXXXXXXXXXXG
ATTTTAATGATTTTCCAGTTTCTTGAGCGTTATCTTCTCCCATCCTTTGCTAATTCATTTGCCTATGAATTAATTAGTG
>Marker39728 Chr4 SNP_site:12631826 Ref_Type:C
GACCCATTAGAGGAAAAGCACGAAGAAGGCAAAATCAATGGGAAAGAAGAAGACCCAGATGGAAAAAAGAAGACCCAAGAXXXXXXXXXXT
TTTGAGTAGTGTGAGAGGTTGTGTTATGTAAGGAGGAATGAAATGCAGAGAGAATTAAAGAGAGTGAAAATGAAAGGTC
GACCCATTAGAGGAAAAGCACGAAGAAGGCAAAATCAATGGGAAAGAAGAAGAGCCAGATGGAAAAAAGAAGACCCAAGAXXXXXXXXXXT
TTTGAGTAGTGTGAGAGGTTGTGTTATGTAAGGAGGAATGAAATGCAGAGAGAATTAAAGAGAGTGAAAATGAAAGGTC
>Marker397435 Chr1 SNP_site:69409 Ref_Type:G
TACATTGTATCTTGGAGTATTAGTCTCTTTTCATTTTCTTCATGAAAAGTGTGTAATATCCTTTGGAAAAAAATGGAAAGXXXXXXXXXXA
AACCAGAATCACGATGCTTGTTAAGTTCTAAATTTTCATCACGGTAGCATACTTTAAACTACGGTTTTCTATATTATGT
TACATTGTATCTTGGAGTATTAGTCTCTTTTCATTTTCTTCATGAAAAGTGTGTAATATCCTTTGGAAAAAAATGGAAAGXXXXXXXXXXA
AACCAGAATCGTGATGCTTGTTAAGTTCTAAATTTTCATCACGGTAGCATACTTTAAACTACGGTTTTCTATATTATGT
>Marker397435 Chr1 SNP_site:69410 Ref_Type:T
TACATTGTATCTTGGAGTATTAGTCTCTTTTCATTTTCTTCATGAAAAGTGTGTAATATCCTTTGGAAAAAAATGGAAAGXXXXXXXXXXA
AACCAGAATCACGATGCTTGTTAAGTTCTAAATTTTCATCACGGTAGCATACTTTAAACTACGGTTTTCTATATTATGT
TACATTGTATCTTGGAGTATTAGTCTCTTTTCATTTTCTTCATGAAAAGTGTGTAATATCCTTTGGAAAAAAATGGAAAGXXXXXXXXXXA
AACCAGAATCGTGATGCTTGTTAAGTTCTAAATTTTCATCACGGTAGCATACTTTAAACTACGGTTTTCTATATTATGT
>Marker397523 Chr1 SNP_site:20612376 Ref_Type:G
GACCTAGCAAGTAGAAATAAATATTAATTGGAGAGAATAACACTGCAAAGGTTTGATCACAACACCTCCTAGACCACCTGXXXXXXXXXXC
AATTTCACTTTCAAATCAATTGGAAGACTTCTATTCCTCTTTTTTACATTACATTCTTGATTCATCGAAAAAAAAGGTT
GACCTAGCAAGTAGAAATAAATATTAATTGGAGAGAATAACACTGCAAAGGTTTGGTCACAACACCTCCTAGACCACCTGXXXXXXXXXXC
AATTTCACTTTCAAATCAATTGGAAGACTTCTCTTCCTCTTTTTTACATTACATTCTTGATTCATCGAAAAAAAAGGTT
>Marker397523 Chr1 SNP_site:20612563 Ref_Type:C
GACCTAGCAAGTAGAAATAAATATTAATTGGAGAGAATAACACTGCAAAGGTTTGATCACAACACCTCCTAGACCACCTGXXXXXXXXXXC
AATTTCACTTTCAAATCAATTGGAAGACTTCTATTCCTCTTTTTTACATTACATTCTTGATTCATCGAAAAAAAAGGTT
GACCTAGCAAGTAGAAATAAATATTAATTGGAGAGAATAACACTGCAAAGGTTTGGTCACAACACCTCCTAGACCACCTGXXXXXXXXXXC
AATTTCACTTTCAAATCAATTGGAAGACTTCTCTTCCTCTTTTTTACATTACATTCTTGATTCATCGAAAAAAAAGGTT

Create PDF with GO2PDF for free, if you wish to

>Marker397643 Chr1 SNP_site:16175909 Ref_Type:G
CACACAAGATTTCTAGAGAAATGTCTAATTGGCTGAAATTTCTTACTCAATAGATATGATGCATAAGTATGTCGAGTGGTXXXXXXXXXXG
AACCTTCTCATGTGCGCGTTTGGTGTGACCTTAGAAAATTTTCTCGGTTTCATTGTAAGGTATTGGGGGATTGAAGGTA
CACACAAGATTTCTAGAGAAATGTGTAATTGGCTGAAATTTCTTACTCAATAGATATGATGCATAAGTATGTCGAGTGGTXXXXXXXXXXG
AACCTTCTCATGTGCGCGTTTGGTGTGACCTTAGAAAATTTTCTCGGTTTCATTGTAAGGTATTGGGGGATTGAAGGTA
>Marker397653 Chr1 SNP_site:5892445 Ref_Type:G
TACTTTGGCTAGAAGAGAGGCTGGTGAAAGAACTTCGCCATTGACAGAAACAGGGAGACCTCCCACAATTCCTATTTCCAXXXXXXXXXXA
TGAATTGAGAAAGTATTATCTTAATATTACAACTCTAATATTGAATTATTAAAAGTGCCTCTCTTTTTCAAACTATGTA
TACTTTGGCTAGAAGAGAGGCTGGTGAAAGAACTTCGCCATTGACAGAAACAGGGAGACCTCCCACGATTCCTATTTCCAXXXXXXXXXXA
TGAATTGAGAAAGTATTATCTTAATATTACAACTCTAATATTGAATTATTAAAAGTGCCTCTATTTTTCAAACTATGTA
>Marker397653 Chr1 SNP_site:5892622 Ref_Type:A
TACTTTGGCTAGAAGAGAGGCTGGTGAAAGAACTTCGCCATTGACAGAAACAGGGAGACCTCCCACAATTCCTATTTCCAXXXXXXXXXXA
TGAATTGAGAAAGTATTATCTTAATATTACAACTCTAATATTGAATTATTAAAAGTGCCTCTCTTTTTCAAACTATGTA
TACTTTGGCTAGAAGAGAGGCTGGTGAAAGAACTTCGCCATTGACAGAAACAGGGAGACCTCCCACGATTCCTATTTCCAXXXXXXXXXXA
TGAATTGAGAAAGTATTATCTTAATATTACAACTCTAATATTGAATTATTAAAAGTGCCTCTATTTTTCAAACTATGTA
>Marker398034 Chr1 SNP_site:26585792 Ref_Type:C
AACGAAAGAGAGGTTGTCGGTCATCCAAAGGAAAAGGAAAGCAAATTGTTTATTTTGATCAGTGATCCCATTTTTACCAAXXXXXXXXXXG
ATTTTTGGGTTAATTTGTAGCAGTTTAGTATTTAGAAGATCATGAAGTGTAGCAAACAAATTAGAAGAGTTAAAAAGTG
AACGAAAGAGAGGTTGTCGGTCATCCAAAGGAAAAGGAAAGCAAATTGTTTATTTTGATCAGTGATCTCATTTTTACCAAXXXXXXXXXXG
ATTTTTGGGTTAATTTGTAGCAGTCTAGTATTTAGAAGATCTTGAAGTGTAGCAAACAAATTAGAAGAGTTAAAAAGTG
>Marker398034 Chr1 SNP_site:26585953 Ref_Type:T
AACGAAAGAGAGGTTGTCGGTCATCCAAAGGAAAAGGAAAGCAAATTGTTTATTTTGATCAGTGATCCCATTTTTACCAAXXXXXXXXXXG
ATTTTTGGGTTAATTTGTAGCAGTTTAGTATTTAGAAGATCATGAAGTGTAGCAAACAAATTAGAAGAGTTAAAAAGTG
AACGAAAGAGAGGTTGTCGGTCATCCAAAGGAAAAGGAAAGCAAATTGTTTATTTTGATCAGTGATCTCATTTTTACCAAXXXXXXXXXXG
ATTTTTGGGTTAATTTGTAGCAGTCTAGTATTTAGAAGATCTTGAAGTGTAGCAAACAAATTAGAAGAGTTAAAAAGTG
>Marker398034 Chr1 SNP_site:26585970 Ref_Type:A
AACGAAAGAGAGGTTGTCGGTCATCCAAAGGAAAAGGAAAGCAAATTGTTTATTTTGATCAGTGATCCCATTTTTACCAAXXXXXXXXXXG
ATTTTTGGGTTAATTTGTAGCAGTTTAGTATTTAGAAGATCATGAAGTGTAGCAAACAAATTAGAAGAGTTAAAAAGTG
AACGAAAGAGAGGTTGTCGGTCATCCAAAGGAAAAGGAAAGCAAATTGTTTATTTTGATCAGTGATCTCATTTTTACCAAXXXXXXXXXXG
ATTTTTGGGTTAATTTGTAGCAGTCTAGTATTTAGAAGATCTTGAAGTGTAGCAAACAAATTAGAAGAGTTAAAAAGTG
>Marker398547 Chr1 SNP_site:21025045 Ref_Type:T
ACAAATATAGATCGAAAAGAACTTTGATTGTTTCCCTATATATATAAACACATAATTCAACGCAAATGAATAAACCAAGCXXXXXXXXXXG
ATTCTGTCAGGTTCAATGCATTATCCACGCAGCACTGAAGCAATGTGGCCTGATCTTATTCAAAAAGCTAAAGATGGTG
ACAAATATAGATCGAAAAGAACTTTGATTGTTTCCCTATATATATAAACACATAATTCAACGCAAATGAATAAACCAAGCXXXXXXXXXXG
ATTTTGTCAGGTTCAATGCATTATCCACGCAGCACTGAAGCAATGTGGCCTGATCTTATTCAAAAAGCTAAAGATGGTG
>Marker398718 Chr1 SNP_site:25884204 Ref_Type:C
ACATGTGTTCAGTTGTGTTGTATATGTTGGTTGTTCATATATACTAATTGAATACTATGTAACTAGTCTCTGTAAGGCTTXXXXXXXXXXT
GATATTCACTATTCATCCATCACGCTCTCCTTCAAAACAATGTGGATCATTCACAAGAGGGTGTGACACTCAAGTGGTC
ACATGTGTTCAGTTGTGTTGTATATGTTGGTTGTTGATATATACTAATCGAATACTATGTAACTAGTCTCTGTAAGGCTTXXXXXXXXXXT
GATATTCACTATTCATCCATCACGCTCTCCTTCAAAACAATGTGGATCATTCACAAGAGGGTGTGACACTCAGGTGGTC
>Marker398718 Chr1 SNP_site:25884217 Ref_Type:T
ACATGTGTTCAGTTGTGTTGTATATGTTGGTTGTTCATATATACTAATTGAATACTATGTAACTAGTCTCTGTAAGGCTTXXXXXXXXXXT
GATATTCACTATTCATCCATCACGCTCTCCTTCAAAACAATGTGGATCATTCACAAGAGGGTGTGACACTCAAGTGGTC
ACATGTGTTCAGTTGTGTTGTATATGTTGGTTGTTGATATATACTAATCGAATACTATGTAACTAGTCTCTGTAAGGCTTXXXXXXXXXXT

Create PDF with GO2PDF for free, if you wish to

GATATTCACTATTCATCCATCACGCTCTCCTTCAAAACAATGTGGATCATTCACAAGAGGGTGTGACACTCAGGTGGTC
>Marker398718 Chr1 SNP_site:25884441 Ref_Type:A
ACATGTGTTCAGTTGTGTTGTATATGTTGGTTGTTCATATATACTAATTGAATACTATGTAACTAGTCTCTGTAAGGCTTXXXXXXXXXXT
GATATTCACTATTCATCCATCACGCTCTCCTTCAAAACAATGTGGATCATTCACAAGAGGGTGTGACACTCAAGTGGTC
ACATGTGTTCAGTTGTGTTGTATATGTTGGTTGTTGATATATACTAATCGAATACTATGTAACTAGTCTCTGTAAGGCTTXXXXXXXXXXT
GATATTCACTATTCATCCATCACGCTCTCCTTCAAAACAATGTGGATCATTCACAAGAGGGTGTGACACTCAGGTGGTC
>Marker398763 Chr1 SNP_site:16094544 Ref_Type:G
CACCGTAAAATAAATTTTAGAAGATAGTCTGAGAAATAATTCAAATACAGCACCAACAACACGTCTAAGTTTTTAAATTCXXXXXXXXXXC
TCCCCAAATCAGCCAATTTCTGGTTTAACTCAAACCAGTCACAGTTTTGAAGTAAAGCTAAGGTCATTGGATTAATGTT
CACCGTAAAATAAATTTTAGAAGATGGTCTGAGAAATAATTCAAATACAGCACCAACAACACGTCTAAGTTTTTAAATTCXXXXXXXXXXC
TCCCCAAATCAGCCAATTTCTGGTTTAACTCAAACCGGTCACAGTTTTGAAGTAAAGCTAAGGTCATTGGATTAATGTT
>Marker398763 Chr1 SNP_site:16094778 Ref_Type:G
CACCGTAAAATAAATTTTAGAAGATAGTCTGAGAAATAATTCAAATACAGCACCAACAACACGTCTAAGTTTTTAAATTCXXXXXXXXXXC
TCCCCAAATCAGCCAATTTCTGGTTTAACTCAAACCAGTCACAGTTTTGAAGTAAAGCTAAGGTCATTGGATTAATGTT
CACCGTAAAATAAATTTTAGAAGATGGTCTGAGAAATAATTCAAATACAGCACCAACAACACGTCTAAGTTTTTAAATTCXXXXXXXXXXC
TCCCCAAATCAGCCAATTTCTGGTTTAACTCAAACCGGTCACAGTTTTGAAGTAAAGCTAAGGTCATTGGATTAATGTT
>Marker398880 Chr1 SNP_site:6200097 Ref_Type:C
ACCTTATTAGGTTCATCAATCTTAGATCGAAATACAAAAATCGAAATGATTATCATACGAAACCTTAATAACTAGGGTGGXXXXXXXXXXC
ATGTAACTATGACTTTGTTTTATACTAATAAGTTAATTTAGAAGTGCCATAAATTAGGAAGTTCATTAATAACAAGGTC
ACCTTCTTAGGTTCATCAATCTTAGATCGAAATACAAAAATCGAAATGATTATCATACGAAACCTTAATAACTAGGGTGGXXXXXXXXXXC
ATGTAACTATGACTTTGTTTTATACTAATAAGTTAATTTAGAAGTGCCATAAATTAGGAAGTTCATTAATAACAAGGTC
>Marker399152 Chr1 SNP_site:25747190 Ref_Type:T
CACGACCTGGAAAAATTAGCTTCCAATGAGATGATTTAAAATTAAATCAAATACATATAATTTTAAGACTAAAAATTGATXXXXXXXXXXC
TCATAATTGAAAAATAATATAATTCAATAAGTTGAAATAAACAGCATTTAGGAATTAATTAAATTGTATCAATACAAGT
CACGACCTGGAAAAATTAGCTTCCAATGAGATGATTTAAAATTAAATCAAATACATATAATTTTAAGACTAAAAATTTATXXXXXXXXXXC
TCATAATTGAAAAATAATATAATTCAATAAGTTGAAATAAACAGCATTTAGGAATTAATTAAATTGTATCAATACAAGT
>Marker39931 Chr4 SNP_site:14137242 Ref_Type:G
AACAACAGGGTAAGAAGAAGCTCACCCTTTAACACAACATGTTAAGCAAGAACTTCAACATAACACAGACTAAAATACAGXXXXXXXXXXG
TGAAAATCCACCCTTAAACACAACAAGTTAAGCAAAAACTCCAACATAACACTTAGGACTAAAAAATAAGTAAAAAAGT
AACAACAGGGTAAGAAGAAGCTCACCCTTTAACACAACATGTTAAGCAAGAACTTCAACATAACACATACTAAAATACAGXXXXXXXXXXG
TGAAAATCCACCCTTAAACACAACAAGTTAAGCAAAAACTCCAACATAACACTTAGGGCTAAAAAATAAGTAAAGAAGT
>Marker39931 Chr4 SNP_site:14137400 Ref_Type:A
AACAACAGGGTAAGAAGAAGCTCACCCTTTAACACAACATGTTAAGCAAGAACTTCAACATAACACAGACTAAAATACAGXXXXXXXXXXG
TGAAAATCCACCCTTAAACACAACAAGTTAAGCAAAAACTCCAACATAACACTTAGGACTAAAAAATAAGTAAAAAAGT
AACAACAGGGTAAGAAGAAGCTCACCCTTTAACACAACATGTTAAGCAAGAACTTCAACATAACACATACTAAAATACAGXXXXXXXXXXG
TGAAAATCCACCCTTAAACACAACAAGTTAAGCAAAAACTCCAACATAACACTTAGGGCTAAAAAATAAGTAAAGAAGT
>Marker39931 Chr4 SNP_site:14137417 Ref_Type:A
AACAACAGGGTAAGAAGAAGCTCACCCTTTAACACAACATGTTAAGCAAGAACTTCAACATAACACAGACTAAAATACAGXXXXXXXXXXG
TGAAAATCCACCCTTAAACACAACAAGTTAAGCAAAAACTCCAACATAACACTTAGGACTAAAAAATAAGTAAAAAAGT
AACAACAGGGTAAGAAGAAGCTCACCCTTTAACACAACATGTTAAGCAAGAACTTCAACATAACACATACTAAAATACAGXXXXXXXXXXG
TGAAAATCCACCCTTAAACACAACAAGTTAAGCAAAAACTCCAACATAACACTTAGGGCTAAAAAATAAGTAAAGAAGT
>Marker399422 Chr1 SNP_site:4556834 Ref_Type:A
TACACCCCACGCTGTATCTATCACAAAAGGGATTGAGGATATCGGAGCAGTTTTCTAGTCCAAAAAGTCCTTAAGAAATGXXXXXXXXXXC
ATGAGTTCTCGTTATCTTCGACTTGTTTTTTGTCTCAGTCTTTTGAAAGCCATAAAGGTTACAGCAGCTGAGAGAGAGT

Create PDF with GO2PDF for free, if you wish to

TACACCCCACGCTGTATCTATCACAAAAGGGATTGAGGATATCGGAGCAGTTTTCTAGTCCAAAAAGTCCTTGAGAAATGXXXXXXXXXXXC
ATGAGTTCTCGTTATCTTCGACTTGTTTTTTGTCTCAGTCTTTTGAAAGCCATAAAGGTTACAGCAGCTGAGAGAGAGT
>Marker399658 Chr1 SNP_site:27924351 Ref_Type:C
GACATGCCTGAAGACATTATATTATTTGCCTGATACACATAAAAGGGAAGTTAGTGTCTAGATCACGAGCCCCTGACTCAXXXXXXXXXXXA
TGTTAGCATACCAAGGAAAGAGTTTCATAAGATATTAAAATTTTATATTTGGAAGATACTACCTGATGAAGTTGCGGTA
GACATGCCTGAAGACATTATATTATTTGCCTGATACACATAAAAGGGAAGTTAGTGTCTAGATCACGAGCCCCTGACTCAXXXXXXXXXXXA
TGTTAGCATACCAAGGAAAGAGTTTCATCAGATATTAAAATTTTATATTTGGAAGATACTACCTGATGAAGTTGCGGTA
>Marker399909 Chr1 SNP_site:27151663 Ref_Type:A
ACCTCTTTATCGCAAGTCATTGTTGAATCCAACTTTTCATACTGCTTGTCCAAGTGCAAGCGAAACAATAGATTTACCTGXXXXXXXXXXXT
AGAAAAACTAAAGTTATTTACACTGGCCTAGAGACTCATGCATAGAAAGATAAATTATGATAAAATGGATGATTAAGTG
ACCTCTTTATCGCAAGTCATTGTTGAATCCAACTTTTCATACTGCTTGTCCAAGTGCAAGCGAAACAATAGATTTACCTGXXXXXXXXXXXT
AGAAAAACTAAAGTTATTTACACTGGCCTAGAGACTCATGCATAGAGAGATAAATTATGATAAAATGGATGATTAAGTG
>Marker400277 Chr1 SNP_site:25524044 Ref_Type:T
ACATTGCTACGTTGGACAGCAAGTCTTGCTTGTATTGGATGACCTTGAGTTATTTCTCCTCTTTGCTTCAGCCAGTATATXXXXXXXXXXXT
TGACTTCTGTAGTCGCCACCTTCTTTAAAGTTACAGTCTAACTTTGTTTGGTATTGTGTCTGGCTGTCTGTTTCGAAGT
ACATTGCTACGTTGGACAGCAAGTCTTGCTTGTATTGGATGACCTTGAGTTATTTCTCCTCTTTGCTTCAGCCAGTTTATXXXXXXXXXXXT
TGACTTCTGTAGTCGCCACCTTCTTTAAAGTTACAGTCTAACTTTGTTTGGTATTGTGTCTGGCTGTCTGTTTCGAAGT
>Marker400734 Chr1 SNP_site:25080581 Ref_Type:C
TACCACTCTTTTGCTCAGTTGCGACTAAACTCAACGCATTTAGCAAGTTTCATCAATTCCACTATGAATTATGCAGTCTTXXXXXXXXXXXT
ATCCAAAATCACACAACAAGGATAAGGCATCCTATACCATATCACATGACAAGTGTGAGACATTCTCATTGCATAGGGT
TACCACTCTTTTGCTCAGTTGCGACTAAACTCAACGCATTTAGCAAGTTTCATCAATTCCACTATGAATTATGCAGTCTTXXXXXXXXXXXT
ATCCCAAATCACACAACAAGGATAAGGCATCCTATACCATATCACATGACAAGTGTGAGACATTCTCATTGCATAGGGT
>Marker401062 Chr1 SNP_site:19398862 Ref_Type:C
AACATTATTTCAAAACATCTAATTTACTACAACTTTTACTACGTTACGTTCTTCTATAAGAAAGCAAAATAACTGATGTTXXXXXXXXXXXA
ATGTATGAAAGAAACATAAGAAATAGTGAGGAGACAGATTGGACAGATAATAGATCCCCACATGGCCAAAAACAACAGT
AACATTATTTCAAAACATCTAATTTACTACAACTTTTACTACGTTACGTTCTTCTATAAGAAAGCAAAATAAGTCATGTCXXXXXXXXXXXA
ATGTATGAAAGAAACATAAGAAATAGTGAGGAGACAGATTGGACAGATAATAGATCCCCACATGGCCAAAAACAACAGT
>Marker401062 Chr1 SNP_site:19398864 Ref_Type:G
AACATTATTTCAAAACATCTAATTTACTACAACTTTTACTACGTTACGTTCTTCTATAAGAAAGCAAAATAACTGATGTTXXXXXXXXXXXA
ATGTATGAAAGAAACATAAGAAATAGTGAGGAGACAGATTGGACAGATAATAGATCCCCACATGGCCAAAAACAACAGT
AACATTATTTCAAAACATCTAATTTACTACAACTTTTACTACGTTACGTTCTTCTATAAGAAAGCAAAATAAGTCATGTCXXXXXXXXXXXA
ATGTATGAAAGAAACATAAGAAATAGTGAGGAGACAGATTGGACAGATAATAGATCCCCACATGGCCAAAAACAACAGT
>Marker401062 Chr1 SNP_site:19398869 Ref_Type:T
AACATTATTTCAAAACATCTAATTTACTACAACTTTTACTACGTTACGTTCTTCTATAAGAAAGCAAAATAACTGATGTTXXXXXXXXXXXA
ATGTATGAAAGAAACATAAGAAATAGTGAGGAGACAGATTGGACAGATAATAGATCCCCACATGGCCAAAAACAACAGT
AACATTATTTCAAAACATCTAATTTACTACAACTTTTACTACGTTACGTTCTTCTATAAGAAAGCAAAATAAGTCATGTCXXXXXXXXXXXA
ATGTATGAAAGAAACATAAGAAATAGTGAGGAGACAGATTGGACAGATAATAGATCCCCACATGGCCAAAAACAACAGT
>Marker401174 Chr1 SNP_site:11923697 Ref_Type:T
AACAAACACAAAAAAATGCCCGTCTATGATCTATAATATCAATGATTTAATCTTTTTAAAAAAATTATCAATCAAATTAGXXXXXXXXXXXA
CTGAAACTAGCATATATTAACTAGTTTATGTCATGAACGATTCATAATTAATGATCAAAGTTAGTAACCCATAAAAGTT
AACAAACACAAAAAAATGCCCGTCTATGATCTATAATATCAATGATTTAATCTTTTTAAAAAAATTATCAATCAAATTAGXXXXXXXXXXXA
CTGAAACTAGCATATATTAACTAGTTTATGTCATGAACGATTCATAATTAATTATCAAAGTTAGTAACCCATAAAAGTT
>Marker401300 Chr1 SNP_site:4200333 Ref_Type:C
ACTTTCGATTCTTCATTTGATGGATTTAAACATGATGACCTAATTATCATGTTTAATTCCAAAGCTACGAATAAGAGAAAXXXXXXXXXXXT

Create PDF with GO2PDF for free, if you wish to

TCTTGTCTCTATTATTAAAAGATTTGTTGTTGTTGTTATAATTACTAAATCTTTGTTAGATCATTTCAATTGTTATTGT
ACTTTCGATTCTTCATTTGATGGATTTAAACATGATGACCTAATTATCATGTTTAATTCCAAAGCTACGAATAAGAGAAAXXXXXXXXXXT
TCTTGTCTCTATTATTAAAAGATTTGTTGTTGTTGTTATAATTACTAAATCTTTGTTAGATTATTTCAATTGTTATTGT
>Marker401428 Chr1 SNP_site:16851932 Ref_Type:T
AACTTTAATTCTGGTTTTCAAATTTTAAATATAGATTTCTTGTGATGGCATTCTTTCCTTTTTTAAATACAGGGTAAGGTXXXXXXXXXXC
TTTATTTTCTTGTGTGATAAAATGATTTAGTTACCTTTTCCCCTCCATCCATGATCTATCTAGTATCCCATTCATTGTT
AACTTTAATTCTGGTTTTCAAATTTTAAATATAGATTTCTTGTGATGGCATTCTTTCCTTTTTTAAATACAGGGTAAGGTXXXXXXXXXXC
TTTATTTTCTTGTGTGATAAAATGATTTAGTTACCTTTTCCCCTCCATCCATGATCTATCTAGTATCTCATTCATTGTT
>Marker40151 Chr4 SNP_site:384010 Ref_Type:A
ACTTATAAATTTTTTCAAGAAGTCACACCAAACTTTAGAGTTTCCACGACGAACGAACCACAAAGAAAAGAAGGTGTATCXXXXXXXXXXC
ATGTATCTCTTAAACTTACATTTCCAAGTTGAGAATATTTTCAAAATATTCTTTGGATGAGTGGAAGGTATAAGAAGTA
ACTTATAAATTTTTTCAAGAAGTCACACCAAACTTTAGAGTTTCCACGACGAACGAACCACAAAGAAAAGAAGGTGTATCXXXXXXXXXXC
ATGTCTCTCCTAAACTTACATTTCCAAGTTGAGAATATTTTCAAAATATTCTTTGGATGAGTGGAAGGTATAAGAAGTA
>Marker40151 Chr4 SNP_site:384015 Ref_Type:T
ACTTATAAATTTTTTCAAGAAGTCACACCAAACTTTAGAGTTTCCACGACGAACGAACCACAAAGAAAAGAAGGTGTATCXXXXXXXXXXC
ATGTATCTCTTAAACTTACATTTCCAAGTTGAGAATATTTTCAAAATATTCTTTGGATGAGTGGAAGGTATAAGAAGTA
ACTTATAAATTTTTTCAAGAAGTCACACCAAACTTTAGAGTTTCCACGACGAACGAACCACAAAGAAAAGAAGGTGTATCXXXXXXXXXXC
ATGTCTCTCCTAAACTTACATTTCCAAGTTGAGAATATTTTCAAAATATTCTTTGGATGAGTGGAAGGTATAAGAAGTA
>Marker4017 Chr4 SNP_site:12468519 Ref_Type:C
AACTCCATCCACTGTGTCCCATCGGTGCCATAAAATTCAGGTCAAATCTTGTAGTCTGTAGGGATTGTATAAACAATCCTXXXXXXXXXXA
GCCAAGCAAGGTTACTTATGTCAGTTGCAATTGCAGCTAGTTCAAAACTGGGGAAGGATGGTGGAGGCTCTGAGAAGTC
AACTCCATCCACTGTGTCCCATTGGTGCCATAAAATTCAGGTCAAATCTTGTAGTCTGTAGGGATTGTATAAACAATCCTXXXXXXXXXXA
GCCAAGCAAGGTTACTTATGTCAGTTGCAATTGCAGCTAGTTCAAAACTGGGGAAGGATGGTGGAGGCTCTGAGAAGTC
>Marker402275 Chr1 SNP_site:25954189 Ref_Type:A
TACACCAGGAAATTGTGATATTAATAACCTGATCAACAAAATTATTAAAGTCATTCAATTTGTTGTTGAAAATATGGTCAXXXXXXXXXXA
GATGAAACCTCATACTATCAAGTTTAACATGGTATCAGAGCCCAGAAAACGCAAACAGATATTTGGTCCAAGATAGGTG
TACACCAGGAAATTGTGATATTAATAACCTGATCAACAAAATTATTAAAGTCATTCAATTTGTTGTTGAAAATATGGTCAXXXXXXXXXXA
GATGAAACCTCATACTATCAAGTTTAACATGGTATCAGAGCCCAGAAAACGCAAACGGATATTTGGTCCAAGATAGGTG
>Marker40288 Chr4 SNP_site:11649971 Ref_Type:C
ACAGCATTAAGGTTCATTTTATCATTTTTTTTAAAAAAGTGTAAGTTGAGAACAAAAGAGAGTGACTGTTTTAGATAACAXXXXXXXXXXA
ATGGCTTATGTTCCCTTACCTTTTATCTTCTTTTTGTTATATTAGCTTTAAGCATAGCTCAATTGATATAAAGTTTGGT
ACAGCATTAAGGTTCATTTTATCATTTTTTTTAAAAAAGTGTAAGTTGAGAACAAAAGAGAGTGACTGTTTTAGATAACAXXXXXXXXXXA
ATGGCTTATGTTCCCTTACCTTTTATCTTCTTTTTGTTATATTAGCTTTAAGTATAGCTCAATTGATATAAAGTTTGGT
>Marker403330 Chr1 SNP_site:25988543 Ref_Type:T
CACATAAAGAGCCAAGGCAACCATGGGGTGAAAGGAGCTACATGGTGAAGAGAAGCTAGCTAATAGGTTGAAAGAAGCATXXXXXXXXXXT
CCTTGACAAAAGGAATGCTTGGTAACTAATGTGTGTTTATGCAAGATGAAACATTTATGCAAAATGATTTATTTGTGTG
CACATAATGAGTCAAGGCAACCATGGGGTGAAAGGAGCTACATGGTGAAGAGAAGCTAGCTAATAGGTTGAAAGAAGCATXXXXXXXXXXT
CCTTGACAAAAGGAATGCTTGGTAACTAATGTGTGTTTATGCAAGATGAAACATTTATGCAAAATGATGTATTTGTGTG
>Marker403330 Chr1 SNP_site:25988547 Ref_Type:T
CACATAAAGAGCCAAGGCAACCATGGGGTGAAAGGAGCTACATGGTGAAGAGAAGCTAGCTAATAGGTTGAAAGAAGCATXXXXXXXXXXT
CCTTGACAAAAGGAATGCTTGGTAACTAATGTGTGTTTATGCAAGATGAAACATTTATGCAAAATGATTTATTTGTGTG
CACATAATGAGTCAAGGCAACCATGGGGTGAAAGGAGCTACATGGTGAAGAGAAGCTAGCTAATAGGTTGAAAGAAGCATXXXXXXXXXXT
CCTTGACAAAAGGAATGCTTGGTAACTAATGTGTGTTTATGCAAGATGAAACATTTATGCAAAATGATGTATTTGTGTG
>Marker403330 Chr1 SNP_site:25988796 Ref_Type:G

Create PDF with GO2PDF for free, if you wish to

CACATAAAGAGCCAAGGCAACCATGGGGTGAAAGGAGCTACATGGTGAAGAGAAGCTAGCTAATAGGTTGAAAGAAGCATXXXXXXXXXXT
CCTTGACAAAAGGAATGCTTGGTAACTAATGTGTGTTTATGCAAGATGAAACATTTATGCAAAATGATTTATTTGTGTG
CACATAATGAGTCAAGGCAACCATGGGGTGAAAGGAGCTACATGGTGAAGAGAAGCTAGCTAATAGGTTGAAAGAAGCATXXXXXXXXXXT
CCTTGACAAAAGGAATGCTTGGTAACTAATGTGTGTTTATGCAAGATGAAACATTTATGCAAAATGATGTATTTGTGTG
>Marker40343 Chr4 SNP_site:10507430 Ref_Type:T
ACCTTTTATTTATAGGCATAAAATCGTAGGAGCCCAAACCAAAGATTTCTCTAAAATTCTAACTATTCTAGATATATATTXXXXXXXXXXC
TTCAAAATTATGATGGCATTTCTTTTTTGATTCAAGTGGCTTTCATGATAATGCTTCATCATGTGTAATTTTAAAGGTA
ACCTTTTATTTATAGGCATAAAATCGTAGGAGCCCAAACCAAAGATTTCTCTAAAATTCTAACTATTCTAGATATATATTXXXXXXXXXXC
TTCAAAATTATGATGGCATTTCTTTTTTGATTCAAGTGGCTTTCATGATAATGCTTCATCATGTGTAATTTTAAATGTA
>Marker403491 Chr1 SNP_site:6921896 Ref_Type:C
ACTAAATAATTGATATTAATTTATAAACATGGACACGAATGTTTGTGAGTGAATAAATAATGAAAATTAAGGTTAAAGATXXXXXXXXXXT
ATATATCATTTTAAAATATAAGAACTAAATAGAAAAGGCAATGACCGAAACTTATTTTTCTCAATGTTTAAAACTGGTT
ACTAAATAATTGATATTAATTTATAAACATGGACACGAATGTTTGTGAGTGAATAAATAATGAAAATTAAGGTTAAAGATXXXXXXXXXXT
ATATATCATTTTAAAATATAAGAACTAAATAGAAAAGGCAATGACGGAAACTTATTTTTCTCAATGTTTAAAACTGGTT
>Marker404055 Chr1 SNP_site:1981060 Ref_Type:C
ACTTGCTATCTAGTTTATTAATACTTGACTGAACTGAAAATCATCGAATTAAGTCAAGATATAAACAAAAACCACCACCAXXXXXXXXXXC
ATATGGAGACTGCCTATTGTATTACCATCAGTTAGACTTCTTGTTTAGTGAAATATGGGAAGGAGACAATTTAGTGGTG
ACTTGCTATCTAGTTTATTAATACTTGACTGAACTGAAAATCATCGAATTAAGTCAAGATATAAACAAAAACCACCACCAXXXXXXXXXXT
ATATGGAGACTGCCTATTGTATTACCATCAGTTAGACTTCTTGTTTAGTGAAATATGGGAAGGAGACAATTTAGTGGTG
>Marker404292 Chr1 SNP_site:4447143 Ref_Type:C
ACTCATACTAGAATAAAAAGAACCCTAACAAGACAATTAGCGAGAATTGAAGTGTTAAAATGATTGGATATGCAAATATTXXXXXXXXXXG
GGTTGAAAGAGAAAAGGTTGGGTGAAAGGGGAGAGTGGGCCAAAAGAGGCACGCGGGTTTTTGAACAATAATAATAGTA
ACTCATACTAGAATAAAAAGAACCCTAACAAGACAATTAGGGAGAATTGAAGTGTTAAAATGATTGGATATGCAAATATTXXXXXXXXXXG
GGTTGAAAGAGAAAAGGTTGGGTGAAAGGGGAGAGTGGGCCAAAAGAGGCACGCGGGTTTTTGAACAATAATAATAGTA
>Marker404396 Chr1 SNP_site:19180334 Ref_Type:A
ACTGTTCCTTTTGAAGTTGATGATGATAAATTTGGGGAAAGAATGCAAGTTGTGTAGAATTGTCTCCTGAGTCTATATCAXXXXXXXXXXT
CTAGAAGTGTCTATTTTATGCCAATTAGTTGATCCATGATGGTATTATTTTCAGATTTATGAGGTTGCTCCATCACTGT
ACTGTTCCTTTTGAAGTTGATGGTGATAAATTTGGGGAAAGAATGCAAGTTGTGTAGAATTGTCTCCTGAGTCTATATCAXXXXXXXXXXT
CTAGAAGTGTCTATTTTATGCCAATTAGTTGATCCATGATGGTATTATTTTCAGATTTATGAGGTTGCTCCATCACTGT
>Marker404849 Chr1 SNP_site:858564 Ref_Type:A
TACATACTTGTGTATCTTGTTTAATATAAGTTAATGTATTTGTCTTCCATCAACATTTAGCCACTTGAAAATTTGTTAAGXXXXXXXXXXT
TAAAAATGCATATAAATCACATAGTTGTGTATGTTAGCTATAGTTCGAATCTCCTACTAATCTCGAACTAAGATTATGT
TACATACTTGTGTATCTTGTTTAATATAAGTTAATGTATTTGTCTTCCATCAACGTTTAGCCACTTGAAAATTTGTTAAGXXXXXXXXXXT
TAAAAATGCATATAAATCACATAGTTGTGTATGTTAGCTATAGTTCGAATCTCCTACTAATCTCGAACTAAGATTATGT
>Marker405296 Chr1 SNP_site:4189480 Ref_Type:T
TACGCATCTGAATTCTGGTCATTTTGTGTCGATAGCATACAGTGTTTGTCATGATCGTGGAGATGCCTCTATTTACACTAXXXXXXXXXXA
CATGGGTTCAATGTGACGCTCCTTGCAGCAGTTGTGGCAAGGTAATCTTCCAATGTTATTGAGGAATTTATGCTCTTGT
TACGCATCTGAATTCTGGTCATTTTGTGTCGATAGCATACAGTGTTTGTCATGATCTTGGAGATGCCTTTATTTACACTAXXXXXXXXXXA
CATGGGTTCAATGTGACGCTCCTTGCAGCAGTTGTGGCAAGGTAATCTTCCAATGTTATTGAGGAATTTATGCTCTTGT
>Marker405296 Chr1 SNP_site:4189492 Ref_Type:T
TACGCATCTGAATTCTGGTCATTTTGTGTCGATAGCATACAGTGTTTGTCATGATCGTGGAGATGCCTCTATTTACACTAXXXXXXXXXXA
CATGGGTTCAATGTGACGCTCCTTGCAGCAGTTGTGGCAAGGTAATCTTCCAATGTTATTGAGGAATTTATGCTCTTGT
TACGCATCTGAATTCTGGTCATTTTGTGTCGATAGCATACAGTGTTTGTCATGATCTTGGAGATGCCTTTATTTACACTAXXXXXXXXXXA
CATGGGTTCAATGTGACGCTCCTTGCAGCAGTTGTGGCAAGGTAATCTTCCAATGTTATTGAGGAATTTATGCTCTTGT

Create PDF with GO2PDF for free, if you wish to

>Marker405828 Chr1 SNP_site:25227287 Ref_Type:A
AACCACAATGCAAATATGTTTCTAAGTTCTCATTTTCCTCTTAAAATAAACAACAATCATTTACACATGCATGAATGTTTXXXXXXXXXXC
TAAAACTTATATCAGTCCAACCACACAAAACTTTAAGTTTATATAAGGTCACCATAGCTGACATCTTTATGAATTTTGT
AACCACAATGCAAATATGTTTCTAAGTTCTCATTTTCCTCTTAAAATAAACAACAATCATTTACACATGCATGAATGTTTXXXXXXXXXXC
TAAAACTTTTATCAGTCCAACCACACAAAACTTTAAGTTTATATAAGGTCACCGTAGCTGACATCTTTATGAATTTTGT
>Marker405828 Chr1 SNP_site:25227332 Ref_Type:A
AACCACAATGCAAATATGTTTCTAAGTTCTCATTTTCCTCTTAAAATAAACAACAATCATTTACACATGCATGAATGTTTXXXXXXXXXXC
TAAAACTTATATCAGTCCAACCACACAAAACTTTAAGTTTATATAAGGTCACCATAGCTGACATCTTTATGAATTTTGT
AACCACAATGCAAATATGTTTCTAAGTTCTCATTTTCCTCTTAAAATAAACAACAATCATTTACACATGCATGAATGTTTXXXXXXXXXXC
TAAAACTTTTATCAGTCCAACCACACAAAACTTTAAGTTTATATAAGGTCACCGTAGCTGACATCTTTATGAATTTTGT
>Marker405968 Chr1 SNP_site:23114004 Ref_Type:T
AACAACGTTAGTGTTAATGGTAGAGATTTCACGAGTTTTGACACTTCAAAGTTTGGAAAGTTTCTACAGTATGATATAACXXXXXXXXXXA
AATGACAATTCAGAAGAATATAATTTTTCTCAATGGATCTATTTTATATTGATATTACTGTAATCAAACTTTTTTTGTT
AACAACGTTAGTGTTAATGGTAGAGATTTCATGAGTTTTGACACTTCAAAGTTTGGAAAGTTTCTACAGTATGATATAACXXXXXXXXXXA
AATGACAATTCAGAAGAATATAATTTTTCTCAATGGATCTATTTTATATTGATATTACTGTAATCAAACTTTTTTTGTT
>Marker406537 Chr1 SNP_site:13441987 Ref_Type:T
TACAACTACAGATTCCTTTGTGGTAATTGGTATTGTTATTTGGATTTTGCAACATTTTCACTTAACAATCCTTGTGTGTTXXXXXXXXXXT
AAACTCTTCGTCTTTGTCTCTTTTTGTCTCCACTCTACTACAAAAGCTGGCTTACTTGACGCCAATACAGTATCAAGTA
TACAACTACAGATTCCTTTGTGGTAATTGGTATTGTTATTTGGATTTTGCAACATTTTCACTTAACAATCCTTGTGTGTTXXXXXXXXXXT
AAACTCTTCGTCTTTGTCTCTTTTTGTCTCCACTCTACTACAAAAGCTGGCTTACTTGACGTCAATACAGTATCAAGTA
>Marker406971 Chr1 SNP_site:6593110 Ref_Type:T
CACTTGCAGTTAAGTTCCATCTGATTGCAGAAGAGATTGAAACCATATTTTAACAAGCTATGACACAAAATCTTGCAAACXXXXXXXXXXC
AGATGAAGGTAACTTAAATCGTCAATAATATTTCAAACAATACTAGACTAAAAACCAATTGAAATGCTAATAAGGAGTT
CACTTGCAGTTAAGTTCCATCTGATTGCAGAAGAGATTGAAACCATATTTTAACAAGCTATGACACAAAATCTTGCAAACXXXXXXXXXXC
AGATTAAGGTAACTTAAATCGTCAATAATATTTCAAACAATACTAGACTAAAAACCAATTGAAATGCTAATAAGGAGTT
>Marker407091 Chr1 SNP_site:9570905 Ref_Type:G
CACCTTTCAGGACACAAATGATGGAAGTTATCTCACAAGGTGGTGTTTGTCTCAAAACTCCTCTTTGCGCATGTTAGTCTXXXXXXXXXXG
AATGGAAAGTTAAGAAATGATGGATTGAAAAGGTATAACCATGTAATATATATAAAGAATGCAGGCCTTAGGCCATTGT
CACCTTTCAGGACACAAATGATTGAAGTTATCTCACAAGGTGGTGTTTGTCTCAAAACTCCTCTTTGCGCATGTTAGTCTXXXXXXXXXXG
AATGGAAAGTTAAGAAATGATGGATTGAAAAGGTATAACCATGTAATATATATAAAGAATGCAGGCCTTAGGCCATTGT
>Marker407772 Chr1 SNP_site:14692490 Ref_Type:T
AACCAATTTTTGTAGTAGTGTTTTTTGAAAAATACATTTATATCTAGACCACATTTTGTTTATATAAGATGCTTGAGACAXXXXXXXXXXC
CTATGTCATTTCCAATGAGTATAATATCATCTACATACAAAACTAAGAATGCTATAATGGAATTGACGACCTTTTTGTA
AACCAATTTTTGTAGTAGTGTTTTTTGAAAAATACATTTATATCTAGACCACATTTTGTTTATATAAGATGCTTGAGACAXXXXXXXXXXC
CTATGTCATTTCCAATTAGTATAATATCATCTACATACAAAACTAAGAATGCTATAATGGAATTGACGACCTTTTTGTA
>Marker408295 Chr1 SNP_site:15243423 Ref_Type:C
AACTGCAACATTCTTTCCCTGAAGACGATTTCAAGCTGGGAGAGAACATATTTTGGGGGAGTGGATCGGCATGGAGGCCAXXXXXXXXXXG
GGTTGCGCTCGTGTCGTTTGTGACAATGGGGATATCTTCATGACTTGTAACTATGATCCGCCTGGTAACTATCTGGGTG
AACTGCAACATTCTTTCCCTGAAGACGATTTCAAGCTGGGAGAGAACATATTTTGGGGGAGTGGATCGGCATGGAGGCCAXXXXXXXXXXG
GGTTGCGCTCGTGTCGTTTGTGACAATGGGGATATCTTCATGACTTGTAACTATGATCCGCCTGGTAACTATGTGGGTG
>Marker408458 Chr1 SNP_site:21029064 Ref_Type:G
ACATTCTTCAAATCATTGTTTGATTGTATCACGATATTAGTTTGAGTTTCGTTGCTATTAGTTCTCTTTTTTCTCCCTACXXXXXXXXXXC
CAGGCACATACAAAATAACAAAAACAAACTTTTTAAAAAGAAAATGTTATACAGCAATTTTATGATTGAACACTTTGTG
ACATTCTTCAAATCATTGTTTGATTGTATCACGATATTAGTTTGAGTTTCGTTGCTATTAGTTCTCTTTTTTCTCCCTACXXXXXXXXXXC

Create PDF with GO2PDF for free, if you wish to

CATGCACATACAAAATAACAAAAACAAACTTTTTAAAAAGAAAATGTTATACAGCAATTTTATGATTGAACACTTTGTG

>Marker409297 Chr1 SNP_site:17048618 Ref_Type:G

TACATCATAGCCAGAATATGGTGTTGGGGAGGTATTTGAAAGGAAGAGTTATAGCCTGATAAAACTTCTCCGGATGGAGAXXXXXXXXXXXA

ACATTGGATATAGAACTGATGCTAGGTATCAAGAAACTGGTCAAGTAAAGCCAAATTCTGCTTTAATTTTATCTGGGTA

TACATCATAGCCAGAATATGGTGTTGGGGAGGTATTTGAAAGGAAGAGTTATAGCCTGATAAAACTTCTCCGGGTGGAGAXXXXXXXXXXXA

ACATTGGATATAGAACTGATGCTAGGTATCAAGAAACTGGTCGAGTAAAGCCAAATTCTGCTTTAATTTTATCTGGGTA

>Marker409297 Chr1 SNP_site:17048796 Ref_Type:A

TACATCATAGCCAGAATATGGTGTTGGGGAGGTATTTGAAAGGAAGAGTTATAGCCTGATAAAACTTCTCCGGATGGAGAXXXXXXXXXXXA

ACATTGGATATAGAACTGATGCTAGGTATCAAGAAACTGGTCAAGTAAAGCCAAATTCTGCTTTAATTTTATCTGGGTA

TACATCATAGCCAGAATATGGTGTTGGGGAGGTATTTGAAAGGAAGAGTTATAGCCTGATAAAACTTCTCCGGGTGGAGAXXXXXXXXXXXA

ACATTGGATATAGAACTGATGCTAGGTATCAAGAAACTGGTCGAGTAAAGCCAAATTCTGCTTTAATTTTATCTGGGTA

>Marker410571 Chr1 SNP_site:25526194 Ref_Type:C

GACTACACTCACCAAGAAGAAGACCCCGGGCTTTTCTCTCTCTAAACTTACAGAAAATTCGCTCTCTCAGTTTGTGTTATXXXXXXXXXXXA

ATGGTATTAGAGCTATGTTGCTGATGTTCTAGAGTGGAAACGACAGAGGCTTCTGACCAGGTGAGGCCTTGAGTAAAGT

GACTACACTCACCAAGAAGAAGACCCCGGGCTTTTCTCTCTCTAAACTTATAGAAAATTCGCTCTCTCAGTTTGTGTTATXXXXXXXXXXXA

ATGGTATTAGAGCTATGTTGCTGATGTTCTAGAGTGGAAACGACAGAGACTTCTGACCAGGTGAGGCCTTGAGTAAAGT

>Marker410571 Chr1 SNP_site:25526394 Ref_Type:G

GACTACACTCACCAAGAAGAAGACCCCGGGCTTTTCTCTCTCTAAACTTACAGAAAATTCGCTCTCTCAGTTTGTGTTATXXXXXXXXXXXA

ATGGTATTAGAGCTATGTTGCTGATGTTCTAGAGTGGAAACGACAGAGGCTTCTGACCAGGTGAGGCCTTGAGTAAAGT

GACTACACTCACCAAGAAGAAGACCCCGGGCTTTTCTCTCTCTAAACTTATAGAAAATTCGCTCTCTCAGTTTGTGTTATXXXXXXXXXXXA

ATGGTATTAGAGCTATGTTGCTGATGTTCTAGAGTGGAAACGACAGAGACTTCTGACCAGGTGAGGCCTTGAGTAAAGT

>Marker410782 Chr1 SNP_site:9172776 Ref_Type:T

CACGTTATATTAGTCCTTATGAGATTCTAGCAAGCGTAGGACCAATGACTTATAAGCTAAGATTGCCTGCAAAACTATCTXXXXXXXXXXXC

CCCATGGTCAAAATTTTGCGAGACAACCATGGCGTTGAAGAAGCTACTTGAGAAAGTGGGGAGCTAATAAAAACCAAGT

CACGTTATATTAGTCCTTATGAGATTCTAGCAAGCGTAGGACCAATGACTTATAAGCTAAGATTGCCTGCAAAACTATCTXXXXXXXXXXXC

CCCATGGTCAAAATTTTGCGAGACAACCATGGCGTTGAAGAATCTACTTGAGAAAGTGGGGAGCTAATAAAAACCAAGT

>Marker411001 Chr1 SNP_site:7069243 Ref_Type:A

GACTTAGTAAAGTGATGATTTCCATTTACTAAAAGAGTTGAGTTGTATTTTTTATATTTATTGAAGAAATTAAAAACCTAXXXXXXXXXXXG

TCTCTAATATAATCATTTTTGTTATTCATATCCACATTTTAGACTGATACTGCCTCAGTTCTACATGAAGCAATTGAGT

GACTTAGTAAAGTGATGATTTCCATTTACTAAAAGAGTTGAGTTGTATTTTTTATATTTATTGAAGAAATTAAAAACCTAXXXXXXXXXXXG

TCTCTATTATAATCATTTTTGTTATTCATATCCACATTTTAGACTGATACTGCCTCAGTTCTACATGAAGCAATTGAGT

>Marker411361 Chr1 SNP_site:28771101 Ref_Type:A

AACTAAAAATAGGAAAAGAAATCACTGGAGATAGAAGGTTTTCCTTAAAAGAATAAAGGAAAGGGGAAAAGGAAAAGGAGXXXXXXXXXXXA

CTTCTTGTCGAGGGTAGAGTAGATATATGTTAATTAACACCAAAAATTCACTAAATTAAAACCTTAGAAGTTTGGAGTT

AACTAAAAATAGGAAAAGAAATCACTGGAGATAGAAGGTTTTCCTTAAAAGAATAAAGGAAAGGGGAAAAGGAAAAGGAGXXXXXXXXXXXA

CTTCTTGTCGAGGGTAGAGTAGATATATGTTAATTAACACCAAATATTCACTAAATTAAAACCTTAGAAGTTTGGAGTT

>Marker411449 Chr1 SNP_site:1089189 Ref_Type:C

TACTTTCTGTTGTTTTTGTGCTATTTATTTTATTGTGTGGAGTCCCATCTATAGTTTTTCCACCTAGTATTCTTGTGTGGXXXXXXXXXXXA

TTACCGCATTTTCATCATCTGGGGTTTTTGTTTTGGATGATTTAGGAGATGGGTTGTGCTTAGATTTGAGTTTTGTTGT

TACTTTCTGTTGTTTTTGTGCTATTTATTTTATTGTGTGGAGTCCCATCTATAGTTTTTCCACCTAGTATTCTTGTGTGGXXXXXXXXXXXA

TTACTGCATTTTCATCATTTGGGGTTTTTGTTTTGGATGATTTAGGAGATGGGTTGTGCTTAGATTTGAGTTTTGTTGT

>Marker411449 Chr1 SNP_site:1089203 Ref_Type:C

TACTTTCTGTTGTTTTTGTGCTATTTATTTTATTGTGTGGAGTCCCATCTATAGTTTTTCCACCTAGTATTCTTGTGTGGXXXXXXXXXXXA

TTACCGCATTTTCATCATCTGGGGTTTTTGTTTTGGATGATTTAGGAGATGGGTTGTGCTTAGATTTGAGTTTTGTTGT

Create PDF with GO2PDF for free, if you wish to

TACTTTCTGTTGTTTTGTGCTATTTATTTTATTGTGTGGAGTCCCATCTATAGTTTTCCACCTAGTATTCTTGTGTGGXXXXXXXXXXA
TTACTGCATTTTCATCATTTGGGGTTTTTGTTTTGGATGATTTAGGAGATGGGTTGTGCTTAGATTTGAGTTTTGTTGT
>Marker411882 Chr1 SNP_site:4104657 Ref_Type:T
ACGAAGATCCCCTCAAATAGAAATCTTGGAATTCAAAGTTAGGTAAGGGGTAGCATATTACATCTTGCTGCAAGTTGCCTXXXXXXXXXXT
TTGACTTTCCATGTATCTCCATTCCTTTCAGGTCTTGAGCAAAGACAATCCTCCCCGCAAAACTTTTGTGTATAGGGTA
ACGAAGATCCCCTCAAATAGAAATCTTGGAATTCTAAGTTTGGTAAGGGGTAGCATATTACATCTTGCTGCAAGTTGCCTXXXXXXXXXXT
TTGACTTTCCATGTATCTCCATTCCTTTCAGGTCTTGAGCAAAGACAATCCTCCCCGCAAAACTTTTGTGTATAGGGTA
>Marker411882 Chr1 SNP_site:4104663 Ref_Type:T
ACGAAGATCCCCTCAAATAGAAATCTTGGAATTCAAAGTTAGGTAAGGGGTAGCATATTACATCTTGCTGCAAGTTGCCTXXXXXXXXXXT
TTGACTTTCCATGTATCTCCATTCCTTTCAGGTCTTGAGCAAAGACAATCCTCCCCGCAAAACTTTTGTGTATAGGGTA
ACGAAGATCCCCTCAAATAGAAATCTTGGAATTCTAAGTTTGGTAAGGGGTAGCATATTACATCTTGCTGCAAGTTGCCTXXXXXXXXXXT
TTGACTTTCCATGTATCTCCATTCCTTTCAGGTCTTGAGCAAAGACAATCCTCCCCGCAAAACTTTTGTGTATAGGGTA
>Marker412858 Chr1 SNP_site:25964333 Ref_Type:G
CACTTTAACCATGCTTTCTCATGAATGTAGTATGTTTATATATAATGCGCCTCATTAAAGAACTATTGCGTTGAGCCCTAXXXXXXXXXXT
TCGTATGAAAGGAAGTTTATATATTAATGCTGTTATGAACCTGTTTGTGATGTTACTTGTTACAGTCTCCAGACGAGTA
CACTTTAACCATGCTTTCTCATGAATGTAGTATGTTTATATATAATGCGCCTCATTGAAGAACTATTGCGTTGAGCCCTAXXXXXXXXXXT
TCGTATGAAAGGAAGTTTATATATAAATGCTGTTATGAACCTGTTTGTGATGTTACTTGTTACAGTCTCCAGACGAGTA
>Marker412858 Chr1 SNP_site:25964535 Ref_Type:A
CACTTTAACCATGCTTTCTCATGAATGTAGTATGTTTATATATAATGCGCCTCATTAAAGAACTATTGCGTTGAGCCCTAXXXXXXXXXXT
TCGTATGAAAGGAAGTTTATATATTAATGCTGTTATGAACCTGTTTGTGATGTTACTTGTTACAGTCTCCAGACGAGTA
CACTTTAACCATGCTTTCTCATGAATGTAGTATGTTTATATATAATGCGCCTCATTGAAGAACTATTGCGTTGAGCCCTAXXXXXXXXXXT
TCGTATGAAAGGAAGTTTATATATAAATGCTGTTATGAACCTGTTTGTGATGTTACTTGTTACAGTCTCCAGACGAGTA
>Marker412990 Chr1 SNP_site:15312216 Ref_Type:T
CACTTATTTTGGGACACTATTTTTATCCTTCACAATTTTTATTAAAATTTGCCCTGTTTGTAGATCCTTGGTAGTGGTATXXXXXXXXXXG
GACTGATAACAGGGGAGCAATACTTTCCTGCTGATTCTCTGTCTGATAGCCAGAAGGTAAATAAACCCATGTTTGTTGT
CACTTATTTTGGGACACTATTTTTATCCTTCACAATTTTTATTAAAATTTTCCCTGTTTGTAGATCCTTGGTAGTGGTATXXXXXXXXXXG
GACTGATAACAGGGGAGCAATACTTTCCTGCTGATTCTCTGTCTGATAGCCAGAAGGTAAATAAACCCATGTTTGTTGT
>Marker413009 Chr1 SNP_site:16002781 Ref_Type:A
CACTTTAATCGTCCAAGTCTTCAATCTTTCGGAAGTTGTTGATCTCGAGGTTGATACGAACAGAGCTTCAATTCTTTCTTXXXXXXXXXXT
CTCCTCACAAACCAACACAAGTCTTTTCAAGATGCTTTGTCCTCATTCACATGTATCATAGAAAAATTTCTAGAAGGTT
CACTTTAATCGTCCAAGTCTTCAATCTTTCGGAAGTTGTTGATCTCGAGGTTGATACGAACAGAGCTTCAATTCTTTCTTXXXXXXXXXXT
CTCCTCACAAACCAACACAAGTCTTTTCAGGATGCTTTGTCCTCATTCACATGTATCGTAGAAAAATTTCTAGAAGGTT
>Marker413009 Chr1 SNP_site:16002809 Ref_Type:A
CACTTTAATCGTCCAAGTCTTCAATCTTTCGGAAGTTGTTGATCTCGAGGTTGATACGAACAGAGCTTCAATTCTTTCTTXXXXXXXXXXT
CTCCTCACAAACCAACACAAGTCTTTTCAAGATGCTTTGTCCTCATTCACATGTATCATAGAAAAATTTCTAGAAGGTT
CACTTTAATCGTCCAAGTCTTCAATCTTTCGGAAGTTGTTGATCTCGAGGTTGATACGAACAGAGCTTCAATTCTTTCTTXXXXXXXXXXT
CTCCTCACAAACCAACACAAGTCTTTTCAGGATGCTTTGTCCTCATTCACATGTATCGTAGAAAAATTTCTAGAAGGTT
>Marker413445 Chr1 SNP_site:20524385 Ref_Type:C
ACTCCCAAATTATGGAATCCCAATGACCTTTATTTTAGACTTGAAATTTCCACTGCTAAATTGAAAATTGGTAGCAAGTTXXXXXXXXXXT
AAGAATTAAAAAGTTGAACTATGAATTCATATACGTCTTTCCCAGTTTGAAAGATCTATTCATCCTCCAGCTTTTTTGT
ACTCCCAAATTATGGAATCCCAATGACCTTTATTTTAGACTTGAAATTTCCACTGCTAAATTGAAAATTGGTAGCAAGTTXXXXXXXXXXT
AAGAATTAAAAAGTTGAACTATGAATTCATATACGTTTTTCCCAGTTTGAAAGATCTATTCATCCTCCAGCTTTTTTGT
>Marker414230 Chr1 SNP_site:16883162 Ref_Type:T
AACAATAGCTCAGACTTGATCATTGCTACACCTTGTGTGAGGAGATATCCCTTTCTCCCGAAGTCAGCAGAATGCTATTTXXXXXXXXXXT

Create PDF with GO2PDF for free, if you wish to

AGTTTATATACTTTAAAATGCTTGAAATTTGGTCAATTTTAGTTCTTATATTTTCAAATATACAATTTTAGTCGTTGTA
AACAATAGCTCAGACTTGATCATTGCTACACCTTGTGTGAGGAGATATCCCTTTCTCCCGAAGTCAGTAGAATGCTATTTXXXXXXXXXXT
AGTTTATATACTTTAAAATGCTTGAAATTTGGTCAATTTTAGTTCTTATATTTTCAAATATACAATTTTGGTGGTTGTA
>Marker414230 Chr1 SNP_site:16883332 Ref_Type:A
AACAATAGCTCAGACTTGATCATTGCTACACCTTGTGTGAGGAGATATCCCTTTCTCCCGAAGTCAGCAGAATGCTATTTXXXXXXXXXXT
AGTTTATATACTTTAAAATGCTTGAAATTTGGTCAATTTTAGTTCTTATATTTTCAAATATACAATTTTAGTCGTTGTA
AACAATAGCTCAGACTTGATCATTGCTACACCTTGTGTGAGGAGATATCCCTTTCTCCCGAAGTCAGTAGAATGCTATTTXXXXXXXXXXT
AGTTTATATACTTTAAAATGCTTGAAATTTGGTCAATTTTAGTTCTTATATTTTCAAATATACAATTTTGGTGGTTGTA
>Marker414230 Chr1 SNP_site:16883335 Ref_Type:C
AACAATAGCTCAGACTTGATCATTGCTACACCTTGTGTGAGGAGATATCCCTTTCTCCCGAAGTCAGCAGAATGCTATTTXXXXXXXXXXT
AGTTTATATACTTTAAAATGCTTGAAATTTGGTCAATTTTAGTTCTTATATTTTCAAATATACAATTTTAGTCGTTGTA
AACAATAGCTCAGACTTGATCATTGCTACACCTTGTGTGAGGAGATATCCCTTTCTCCCGAAGTCAGTAGAATGCTATTTXXXXXXXXXXT
AGTTTATATACTTTAAAATGCTTGAAATTTGGTCAATTTTAGTTCTTATATTTTCAAATATACAATTTTGGTGGTTGTA
>Marker414421 Chr1 SNP_site:11767072 Ref_Type:T
AACCACAAGACAATTTGTCGCATATTGAACTATTACAGCATATGACCTGGTAAATGTCACTCTATGTTATGAAATCATATXXXXXXXXXXT
ATAGTCATTTGTATATAATATTTTGTTACCTAAGTGTCATTAGTCGGTTAAATATCTGTTGATATGTTACCAACATGTT
AACCACAAGACAATTTGTCGCATATTGAACTATTACAGCATATGACCTGGTAAATGTCACTCTATGTTATGAAATCATATXXXXXXXXXXT
ATATTCATTTGTATATAATATTTTGTTACCTAAGTGTCATTAGTCGGTTAAATATCTGTTGATATGTTACCAACATGTT
>Marker414452 Chr1 SNP_site:9000625 Ref_Type:T
ACTCTCAAAATTGGCTCACTCCCTCAATTTCTCCTTCATTTCATAACATTGCACGTTATTGTCAGATTCTTCTTTTAACCXXXXXXXXXXA
ATTCTTCTTGGTAAATTTGCGTTTTCTTCTTTGGGTGATCGATCATGCTTTGGTTTGCATGTTTGTTCCATTGAGGGTT
ACTCTCAAAATTGGCTCACTCCCTCAATTTCTCCTTCATTTCATAACATTGCACGTTATTGTCAGATTCTTCTTTTAACCXXXXXXXXXXA
ATTCTTCTTGGTAAATTTGCGTTTTCTTCTTTGGGTGATCGATTATGCTTTGGTTTGCATGTTTGTTCCATTGAGGGTT
>Marker414867 Chr1 SNP_site:7542041 Ref_Type:A
ACAAGATATTTATGTCAAACATCAATAATTATAACTTTTTCAACTAACATGTCTATTTCTTTCAACTTTCTTTGAAGTTCXXXXXXXXXXT
TCAGTAAAGACAGCAAATCCACCCCTTTCACTTGAATTATTTAAATCTCACCCATTTTCCATCAACAGTTTAAAATGTT
ACAAGATATTTATGTCAAACATCAATAATTATAACTTTTTCAACTAACATGTCTATTTCTTTCAACTTTCTTTGAAGTTCXXXXXXXXXXT
TCAGTAAAGACAGCAAATCCACCCCTTTCACTTGAATTATTTAAATCTCACCCATTTTCCATCAACAGTTTTAAATGTT
>Marker415046 Chr1 SNP_site:22224730 Ref_Type:C
ACCCTGTTTGGAGCTGGGAATAAGGGAATCCTAGCTCTAACAATGATACCAAATTGGCCTAAACCACCAAGGACTGAGTTXXXXXXXXXXA
TATAATTATTATTTTGTCTTCTGAAAAATACGATCTCTTTTTAATTTTAAATATAAATAACTTTGCTACGTGTGATTGT
ACCCTGTTTGGAGCTGGGAATAAGGGAATCCTAGCTCTAACAATGATACCAAATTGGCCTAAACCACCCAGGACTGAGTTXXXXXXXXXXA
TATAATTATTATTTTGTCTTCTGAAAAATACGATCTCTTTTTAATTTTAAATATAAATAACTTTGCTACGTGTGATTGT
>Marker415086 Chr1 SNP_site:19818061 Ref_Type:A
GACGTTTATGGGATACCCACAGGCCACAGCAGATTGTAAGTCACTCCATGTGAGATGCTCCACAAAACAAATACGAGGTTXXXXXXXXXXA
TCATACACAATACAAAAAGCAGTCATAGCCCTTAGTAATCAATCCAGTAGGAAAGCACTAAAATCTAAGACTAGCAGTT
GACGTTTATGGGATACCCACAGGCCACAGCAGATTGTAAGTCACTCCATGTGAGATGCTCCACAAAACAAATACGAGGTTXXXXXXXXXXA
TCATACACAATACAAAAAGCAGTCATGGTCCTTAGTAATCAATCCAGTAGGAAAGCACTAAAATCTAAGACTAGCAGTT
>Marker415086 Chr1 SNP_site:19818063 Ref_Type:T
GACGTTTATGGGATACCCACAGGCCACAGCAGATTGTAAGTCACTCCATGTGAGATGCTCCACAAAACAAATACGAGGTTXXXXXXXXXXA
TCATACACAATACAAAAAGCAGTCATAGCCCTTAGTAATCAATCCAGTAGGAAAGCACTAAAATCTAAGACTAGCAGTT
GACGTTTATGGGATACCCACAGGCCACAGCAGATTGTAAGTCACTCCATGTGAGATGCTCCACAAAACAAATACGAGGTTXXXXXXXXXXA
TCATACACAATACAAAAAGCAGTCATGGTCCTTAGTAATCAATCCAGTAGGAAAGCACTAAAATCTAAGACTAGCAGTT
>Marker415440 Chr1 SNP_site:27129916 Ref_Type:A

Create PDF with GO2PDF for free, if you wish to

TACAAGTTAGCACGCATCTCCATTTCGCATCCATCTAGTCCCACTTTACAGATTATCTAGGCATACATAAGTAGTGATTCXXXXXXXXXXC
TATTGTTTCACTTATACATGGGCAAGCAATAGGAAAACTTGTATTCGACAAGGATTTAGTGATTACACGGTAAAGAGGT
TACAAGTTAGCACGCATCTCCATTTCGCATCCATCTAGTCCCACTTTACAGATTATCTAGGCATACATAAGTAGTGATTCXXXXXXXXXXC
TATTGTTTCGCTTATACATGGGCAAGCAATAGGAAAACTTGTATTCGACAAGGATTTAGTGATTACACGGTAAAGAGGT
>Marker415659 Chr1 SNP_site:27851157 Ref_Type:C
ACTTCGAGGACTAGTTTTTTATCAACGCACTTTACTACTTTTGAAAAAGATTGTATGTTGTGGTATTTCCAGCTGGTCAXXXXXXXXXXXA
GTCTCCTTTCCCTCACAATCATATAAACGAACCAACCTTTCACCTCTATCCATAGTGATGTTTAGGGGCCATCCAAGTT
ACTTCGAGGACTAGTTTTTTATCAACGCACTTTACTACTTTTGAAAAAGATTGTATGTTGTGGTATTTCCAGCTGGTCAXXXXXXXXXXXA
GTCTCCTTTTCCTCACAATCATATAAACGAACCAACCTTTCACCTCTATCCATAGTGATGTTTAGGGGCCATCCAAGTT
>Marker416016 Chr1 SNP_site:953022 Ref_Type:G
TACAAATCTACCTACCAGACAGCATGCTTTGACGCGGTAATATACTTTTCAACAAAATAAAGTCATTGTCCAGAAGTGACXXXXXXXXXXT
GATGCTAGTATTTCAAAACAAAAAACACCATACAAGAGCACTTAAACTGCTAATAACAACCTGGCTGCAAGCTAGGGTC
TACAAATCTACCTACCAGACAGCATGCTTTGACGCGGTAATATACTTTTCAACAAAATAAAGTCATTGTCCAGAAGTGACXXXXXXXXXXT
GATGCTAGTGTTTCAAAACAAAAAACACCATACAAGAGCACTTAAACTGCTAATAACAACCTGGCTGCAAGCTAGGGTC
>Marker416188 Chr1 SNP_site:23922582 Ref_Type:A
CACGTCCTTTTGGAGATAAAATGAGTGAAAAAAAGTCAATAGGCATCAACCGGGGTGAAGCATGAAGAAAAGAAGCCAAAXXXXXXXXXXC
TTTTCCAATTGAATTTGGGTTAGATGAAGCCAACCCTTGAACCTAGTAATAAGAAACGCACATGAATGAATGCATTGTA
CACGTCCTTTTGGAGATAAAATGAGTGAAAAAAAGTCAATAGGCATCAACCGGGGTGAAGCATGAAGAAAAGAAGCCAAAXXXXXXXXXXC
TTTTCCAATTGAATTTGGGTTAGGTGAAGCCAACCCTTGAACCTAGTAATAAGAAATGCACATGAATGAATGCATTGTA
>Marker416188 Chr1 SNP_site:23922615 Ref_Type:C
CACGTCCTTTTGGAGATAAAATGAGTGAAAAAAAGTCAATAGGCATCAACCGGGGTGAAGCATGAAGAAAAGAAGCCAAAXXXXXXXXXXC
TTTTCCAATTGAATTTGGGTTAGATGAAGCCAACCCTTGAACCTAGTAATAAGAAACGCACATGAATGAATGCATTGTA
CACGTCCTTTTGGAGATAAAATGAGTGAAAAAAAGTCAATAGGCATCAACCGGGGTGAAGCATGAAGAAAAGAAGCCAAAXXXXXXXXXXC
TTTTCCAATTGAATTTGGGTTAGGTGAAGCCAACCCTTGAACCTAGTAATAAGAAATGCACATGAATGAATGCATTGTA
>Marker41649 Chr4 SNP_site:446689 Ref_Type:G
ACATTTTAAAGCTTATGTTTATTTGATCTAACATTTAAAAGAGTCTTAAATATAAATTTATCCATTATAACGAAAAACTTXXXXXXXXXXA
ATAAAAAACGTTAATTGTTAGAGATTTCTTAAACGACTTTAGAGTTCAAAATTACTACCAAAGTTGATGGATTGATGTT
ACATTTTAAAGCTTATGTTTATTTGATCTAACATTTAAAAGAGTCTTAAATATAAATTTATCCATTATAACGGAAAACTTXXXXXXXXXXA
ATAAAAAACGTTAATTGTTAGAGATTTTTTAAACGACTTTAGAGTTCAAAATTACTACCAAAGTTGATGGATTGAAGTT
>Marker41649 Chr4 SNP_site:446859 Ref_Type:T
ACATTTTAAAGCTTATGTTTATTTGATCTAACATTTAAAAGAGTCTTAAATATAAATTTATCCATTATAACGAAAAACTTXXXXXXXXXXA
ATAAAAAACGTTAATTGTTAGAGATTTCTTAAACGACTTTAGAGTTCAAAATTACTACCAAAGTTGATGGATTGATGTT
ACATTTTAAAGCTTATGTTTATTTGATCTAACATTTAAAAGAGTCTTAAATATAAATTTATCCATTATAACGGAAAACTTXXXXXXXXXXA
ATAAAAAACGTTAATTGTTAGAGATTTTTTAAACGACTTTAGAGTTCAAAATTACTACCAAAGTTGATGGATTGAAGTT
>Marker41649 Chr4 SNP_site:446907 Ref_Type:A
ACATTTTAAAGCTTATGTTTATTTGATCTAACATTTAAAAGAGTCTTAAATATAAATTTATCCATTATAACGAAAAACTTXXXXXXXXXXA
ATAAAAAACGTTAATTGTTAGAGATTTCTTAAACGACTTTAGAGTTCAAAATTACTACCAAAGTTGATGGATTGATGTT
ACATTTTAAAGCTTATGTTTATTTGATCTAACATTTAAAAGAGTCTTAAATATAAATTTATCCATTATAACGGAAAACTTXXXXXXXXXXA
ATAAAAAACGTTAATTGTTAGAGATTTTTTAAACGACTTTAGAGTTCAAAATTACTACCAAAGTTGATGGATTGAAGTT
>Marker417014 Chr1 SNP_site:17968994 Ref_Type:G
CACGCTTTCAAAACTTCCGTATTATACCGATATAAGTTGGGTTAATAATCATGTCAGGCTTATGTTTATTTTAGTAACGAXXXXXXXXXXA
AGCTGTTCATGGGTTTGTTCATTTTAAATTTAACGATTTTGTTTAATCATGATAAAGCAAATAAAATTAAAAATTTGTC
CACGCTTTCAAAACTTCCGTATTATACCGGTATAAGTTGGGTTAATAATCATGTCAGGCTTATGTTTATTTTAGTAACGAXXXXXXXXXXA
AGCTGTTCATGGGTTTGTTCATTTTAAATTTAACGATTTTGTTTAATCATGATAAAGCAAATAAAATTAAAAATTTGTC

Create PDF with GO2PDF for free, if you wish to

>Marker417104 Chr1 SNP_site:18748071 Ref_Type:C
TACTAGAATTGATAAGGTATAGAGAACAAAAGGTTCCAATTGCAACTCAAACAATGAGATGATAGATTTGATTCAAAAAAXXXXXXXXXXA
AAGAAAAGGATGCATGCATGCTCAAAAACTGCTAACACGTAAGAAGAATTAGGTCATACCTGAAATTGGCAAATACAGT
TACTAGAATTGATAAGGTATAGAGAACAAAAGGTTCCCATTGCAACTCAAACAATGAGATGATAGATTTGATTCAAAAAAXXXXXXXXXXA
AAGAAAAGGATGCATGCATGCTCAAAAACTGCTAACACGTAAGAAGAATTAGGTCATACCTGAAATTGGCAAATACAGT
>Marker417138 Chr1 SNP_site:19559124 Ref_Type:C
TACGTTTGTTCCATTTTTCAAATTCAAGATGAAATGTTTAAGTTTGTTCCATTTTTCAAATTTGAGATGAAATGAACCCAXXXXXXXXXXA
GCGATTTCCTGGTCATTCGCCAATCATCAAATGCAAATAGTTAGGTAACTATCTAAAGACTTTCAAAGTTGGTTGTGTC
TACGTTTGTTCCATTTTTCAAATTCAAGATGAAATGTTTAAGTTTGTTCCATTTTTCAAATTTGAGATGAAATGAACCCAXXXXXXXXXXA
GCGATTTCCTGGTCATTCGCCAATCATCAAATGCAAATAGTTAGGTAACTATCTAAAGACTTTCCAAGTTGGTTGTGTC
>Marker417839 Chr1 SNP_site:25827920 Ref_Type:T
AACACTTAGCCCAATAAGTTATCTAATGGTTAGTGGATTTTTAGGTGTTGAGATTTTATAAACTAATATTTTGCATGTAAXXXXXXXXXXC
TTCAATTTCCATCTCTCTCTCAATTTTCTTCTCTTATCGGGTCTCATAATTTGGTTCTAAGTTTGAAGAATAAAGGGTC
AACACTTAGCCCAATAAGTTATCTAATGGTTAGTGGATTTTTAGGTGTTGAGTTTTTATAAACTAATATTTTGCATGTAAXXXXXXXXXXC
TTCAATTTCCATCTCTCTCTCAATTTTCTTCTCTTATCGGGTCTCATAATTTGGTTCTAAGTTTGAAGAATAAAGGGTC
>Marker417930 Chr1 SNP_site:14609733 Ref_Type:T
AACCAACTTCCAATCGTCCTATATCTTATTCTGATCGTCCGACTTTGGTTTGGTCAGTTATGGTCGGTCGTTTCAATTTTXXXXXXXXXXT
ATATTTCATCAACCACAAATTAAATGTGTGCTAACTAAGCAAACAAAATTTTGTATTGTCCAATAGATCAAGTAGTAGT
AACCAACTTCCAATCGTCCTATATCTTATTCTGATCGTCCGACTTTGGTTTGGTCAGTTATGGTTGGTCGTTTCAATTTTXXXXXXXXXXT
ATATTTCATCAACCACAAATTAAATGTGTGCTAACTAAGCAAACAAAATTTTGTATTGTCCAATAGATCAAGTAGTAGT
>Marker418013 Chr1 SNP_site:21592219 Ref_Type:A
TACTTGTTTAAACTCCAAGCCAGTTTTGAAATCTAAAAAGTAGGTTTTCAATGAACTTGTTGTGTTTCAAACTTGGCTGAXXXXXXXXXXA
GGTCATTTGATGTTTCTTTAGGAAAGATGAAAATCATTGTAATGAATTGCAAGAAAACAAACGCAATTTTCAAAATGTA
TACTTGTTTAAACTCCAAGCCAGTTTTGAAATCTAAAAAGTAGGTTTTCAATGAACTTGTTGTGTTTCAAACTTGGCTGAXXXXXXXXXXA
GGTCATTTGATGTTTCTTTAGGAAAGATGAAAATCATTGTAATGAATTGCGAGAAAACAAACGCAATTTTCAAAATGTA
>Marker41805 Chr4 SNP_site:7968193 Ref_Type:G
ACTAAATAATGAGAATGGCCAAGTTAGTTAGTATATTTTATGTTATTGGGAAATCAATGATTAAGTTATAAATAAGTAGGXXXXXXXXXXA
ATCCCCTTATTATTCTACTGTAAGCCTTTTCAACTGATAGGTGGAGTGCCTCTTTGTGGTTGGACCTATACTATGTTGT
ACTAAATAATGAGAATGGCCAAGTTAGTTGGTATATTTTATGTTATTGGGAAATCAATGATTAAGTTATAAATAAGTAGGXXXXXXXXXXA
ATCCCCTTATTACTCTACTGTAAGCCTTTTCAACTGATAGGTGGAGTGCCTCTTTGTGGTTGGACCTATACTATGTTGT
>Marker41805 Chr4 SNP_site:7968373 Ref_Type:C
ACTAAATAATGAGAATGGCCAAGTTAGTTAGTATATTTTATGTTATTGGGAAATCAATGATTAAGTTATAAATAAGTAGGXXXXXXXXXXA
ATCCCCTTATTATTCTACTGTAAGCCTTTTCAACTGATAGGTGGAGTGCCTCTTTGTGGTTGGACCTATACTATGTTGT
ACTAAATAATGAGAATGGCCAAGTTAGTTGGTATATTTTATGTTATTGGGAAATCAATGATTAAGTTATAAATAAGTAGGXXXXXXXXXXA
ATCCCCTTATTACTCTACTGTAAGCCTTTTCAACTGATAGGTGGAGTGCCTCTTTGTGGTTGGACCTATACTATGTTGT
>Marker418227 Chr1 SNP_site:25181219 Ref_Type:A
TACCTACTAGATAGAAAATATTTTGTAATCATTAGATAAAAAATGTGTTATGTTAGCAAAATCCTATACGTAGAATTATGXXXXXXXXXXA
TGTGACAGACAAGATACATGTGATAAGCCAATCGATCATTATATATGTGTATATAATGCTCAATTATCAAAACGTCAGT
TACCTACTAGATAGAAAATATTTTGTAATCATTAGATAAAAAATGTGTTATGTTAGCAAAATCCTATACGTAGAATTATGXXXXXXXXXXA
TGTGACAGCCAAGATACATGTGATAAGCCAATCGATCATTATATATGTGTATATAATGCTCAATTATCAAAACGTCAGT
>Marker418577 Chr1 SNP_site:959716 Ref_Type:T
AACAACACAACTGCAAATGAAAACTAATGGGCGTTGAAAAGCAGAAACATATAATAGAAGCATAAACATTGAAAGAAAGAXXXXXXXXXXC
TCCAATTCATTAAGAAACTGCAGAACATCGAACAGACGAGATTGATTTCAAGCTTACAACTGGCTGATCAACGAGAGTA
AACAACACAACTGCAAATGAAAACTAATGGGTGTTGAAAAGCAGAAACATATAATAGAAGCATAAACATTGAAAGAAAGAXXXXXXXXXXC

Create PDF with GO2PDF for free, if you wish to

TCCAATTCATTAAGAAACTGCAGAACATCGAACAGACGAGATTGATTTCAAGCTTACAACTGGCTGATCAACGAGAGTA

>Marker418615 Chr1 SNP_site:24667292 Ref_Type:T

ACCTGAGATCTTAAGACGCATAACAAACTCCATTTCTGGCAAAAAAAATTGAAAAGCACATGGAGCTTTTCGCCGAGAAXXXXXXXXXXA

AACCATGATTATGTGAATTTCCTTATGCTTCCCAATTCCTAAAATGCTCGTGATTTATGAATGAATTGTATTTATTGTG

ACCTGAGATCTTAAGACGCATAACAAACTCCATTTCTGGCAAAAAAAATTGAAAAGCACATGGATCTTTTCGCCGAGAAXXXXXXXXXXA

AACCATGATTATGTGAATTTCCTTATGCTTCCCAATTCCTAAAATGCTCGTGATTTATGAATGAATTGTATTTATTGTG

>Marker418696 Chr1 SNP_site:16463832 Ref_Type:A

GACATTATTTACCTGAAGAAGTAACGTAGTGCAGGTGAAATCGAATGCTTCAGGAGCTAACTTATTGAAATAATGACGAAXXXXXXXXXXG

TGGATGTGCAAGAGAGGAGGAGATATATTGAGAATGATACCCTCTTTTTATGGTGATTGCGGAAATATATTTTCTGGTA

GACATTATTTACCTGAAGAAGTAACGTAGTGCAGGTGAAATCGAATGCTTCAGGAGCTAACTTATTGAAATAATGACGAAXXXXXXXXXXG

TGGATGTGCAAGAGAGGAGGAGATATATTGAGTATGATACCCTCTTTTTATGGTGATTGCGGAAATATATTTTCTGGTA

>Marker419492 Chr1 SNP_site:4800652 Ref_Type:T

GACATCCTCTTTCTCATTTTAAGCAGCCTCAACGAGGAAGTAAAGCAAGTAAAGAACTCTACAATTGCCCGGCATCTGCAXXXXXXXXXXC

TTATCATTAAAAAACCATTTCAACTTTTTAAGTAAATTATCTAACAAAAAACTAACATAAACGTTATGAAATGCTTGTG

GACATCCTTTTTCTCATTTTAAGCAGCCTCAACGAGGAAGTAAAGCAAGTAAAGAACTCTACAATTGCCCGGCATCTGCAXXXXXXXXXXC

TTATCATTAAAAAACCATTTCAACTTTTTAAGTAAATTATCTAACAAAAAACTAACATAAACGTTATGAAATGCTTGTG

>Marker419531 Chr1 SNP_site:757509 Ref_Type:T

ACTAAAATTTCTGCTTGAATATGATCTTTTTGGTTCTGATTGTCCCAGCTTAATTTTCATATGTATTCGTTTTTGTCTAAXXXXXXXXXXA

GAGTCTTGTTCTTGCATCTCTTATTACTTGTGTGGGGAGTAGTTCTTCAATGAATTGTTCCATTTTATTGTTATTGGTA

ACTAAAATTTCTGCTTGAATATGATCTTTTTGGTTCTGATTGTCCCAGCTTAATTTTCATATGTATTCTTTTTTGTCTAAXXXXXXXXXXA

GAGTCTTGTTCTTGCATCTCTTATTACTTGTGTGGGGAGTAGTTCTTCAATGAATTGTTCCATTTTATTGTTATTGGTA

>Marker420414 Chr1 SNP_site:21583110 Ref_Type:C

ACTTGCAAATTGCTATTGTTTCTGCTAATCTATAAATTATGGGGAAATTTTAGGTCTTGACATCTGGATTATCAATTGCTXXXXXXXXXXA

AACTGAAAGCATGGAAGAGATCAAGGGTGGAATCAAGAAAGATGTCACTGAAGTAATTTTCTCTTTCTATGAGTTGGTT

ACTTGCAAATTGCTATTGTTTCTGCTAATTTATAAATTATGGGGAAATTTTAGGTCTTGACATCTGGATTATCAATTGCTXXXXXXXXXXA

AACTGAAAGCATGGAAGAGATCAAGGGTGGAATCAAGAAAGATGTCACTGAAGTAATTTTCTCTTTCTATGAGTTGGTT

>Marker420443 Chr1 SNP_site:21192067 Ref_Type:T

TACTCTTTTGTTAATAAATTAATAATTGGAAAGAAGCACACAAAGGAATAGATTGGGCCAATACATTTACGGCCCAAAAAXXXXXXXXXXT

CCCACTTTTCATGATCGGAGAAATTTGTCACACTGATTTTCAGATTTGGGATTTAAAATGAATTTCATTACCTTACGTT

TACTCTTTTGTTAATAAATTAATAATTGGAAAGAAGCACACAAAGGAATAGATTGGGCCAATACATTTACGGCCCAAAAAXXXXXXXXXXT

CCCACTTTTCATGATCGGAGAAATTTGTCACACTGATTTTCAGATTTGGGATTTAAAATGAATTTTATTACCTTACGTT

>Marker420651 Chr1 SNP_site:22642975 Ref_Type:A

ACATCAAACCAATTGTGGAACTATCCAATGGATTTCAGTGAGTTCTGTCGAAGTTCATTCATTTATCAATTTCGTTTTCTXXXXXXXXXXG

AGCAGCTCATCTATTCTAATTATATGTTTTACTCCTTATGACTCTGCAACATTGTCACCCAACAATTCTGCTTTATGTT

ACATCAACCCAATTGTGGAACTATCCAATGGATTTCAGTGAGTTCTGTCGAAGTTCATTCATTTATCAATTTCGTTTTCTXXXXXXXXXXG

AGCAGCTCATCTATTCTAATTATATGTTTTACTCCTTATGACTCTGCAACATTGTCACCCAACAATTCTGCTTTATGTT

>Marker421111 Chr1 SNP_site:25962734 Ref_Type:C

TACCTCTCTCTCACAGCCATCTGCAGAAATCTCCAACGGACAGCCTCTCCGTCGTCGTTGGCCGCCGTTCGACCACTCCCXXXXXXXXXXG

TGGTCTTCTCCGGAATTTCGTTTGGGTGAGTTTTTGTGTTAGTTGGAAAGATGTTTTTGACGCATCTAGAATGCCTGTT

TACCTCTCTCTCACAGCGATCTGCAGAAATCTCCAACGGACAGCCTCTCCGTCGTCGTTGGCCGCCGTTCGACCACTCCCXXXXXXXXXXG

TGGTCTTCTCCGGAATTTCGTTTGGGTGAGTTTTTGTGTTAGTTGGAAAGATGTTTTTGACGCATCTAGAATGCCTGTT

>Marker422019 Chr1 SNP_site:6053297 Ref_Type:C

ACTTGTAGCAAGACGACAGAATCTACTTGAAGAAGTTGCAGATATAGCTCGCTATTATGGATCACCAGGTGTAATCACCAXXXXXXXXXXA

AATTTCAATTTAGACAACCAAAAACAGAGTAGTTTTTTGCCAATGATTTTGTAATGCAAATGCAGTGGATCATTTGGTG

Create PDF with GO2PDF for free, if you wish to

ACTTGTAGCAAGACGACAGAATCTACTTGAAGAAGTTGCAGATATAGCTCGGTATTATGGATCACCAGGTGTAATCACCAXXXXXXXXXXA
AATTTCAATTTAGACAACCAAAAACAGAGTAGTTTTTTGCCAATGATTTTGTAATGCAAATGCAGTGGATCATTTGGTG
>Marker42259 Chr4 SNP_site:18851838 Ref_Type:G
GACTAATCAGGCTCCTCCTTGCCTACCTTGTCACCTGATAATTGGTTAAGTGTTTCCTGTCACCTTCCCTTCTTGTGTTAXXXXXXXXXXT
ATCCGAACTTCAAAAATTAATTTACAAGAAACCTCTTTATTAGGATCCAAGTTTCACACATGGGTAGCCAATTGTTGTT
GACTAATCAGGCTCCTCCTTGCCTACCTTGTCACCTGATAATTGGTTAAGTGTTTCCTGTCACCTTCCCTTCTTGTGTTAXXXXXXXXXXT
ATCCGAACTTCAAAAATTAATTTACAAGAAACCTCTTTATTAGGATCCAGGTTTCACACATGGGTAGCCAATTGTTGTT
>Marker422656 Chr1 SNP_site:9305632 Ref_Type:A
ACATAACTTTCTCCATCAAATTTATTACGAAACATCGCACTTATTTAAAGTTCAGTGTTTGGTTCTTAAAATTAATGAAAXXXXXXXXXXT
CACCTTATCCTTGCAGGTTGCACCTAGTGAAATGGTGGGAAAGTGGATCTTCACAATTGATCTGAAGAAAGCTAGGAGT
ACATAACTTTCTCCATCAAATTTATTACGAAACTTCGCACTTATTTAAAGTTCAGTGTTTGGTTCTTAAAATTAATGAAAXXXXXXXXXXT
CACCTTATCCTTGCAGGTTGCACCTAGTGAAATGGTGGGAAAGTGGATCTTCACAATTGATCTGAAGAAAGCTAGGAGT
>Marker422733 Chr1 SNP_site:28352268 Ref_Type:G
GACCTTGTGTTCTTCCATTGTCAAGCTATAGATTATTATGTCATATAGGTAGATCACAATAAACTTGTCAAGATATTCATXXXXXXXXXXT
TGGGCTTATCTCCCTAAACAATCATTGCTTGATAATGTCCTGATCGCAAGTCTAGTTTCGGGAAATATTTCACTCAAGT
GACCTTGTGTTCTTCCATTGTCAAGCTATAGATTATTATGTCATATAGGTAGATCACGATAAACTTGTCAAGGTATTCATXXXXXXXXXXT
TGGGCTTATCTCCCTAAACAATCATTGCTTGATAATGTCCTGATCGCAAGTCTAGTTTCGGGAAATATTTCGCTCAAGT
>Marker422733 Chr1 SNP_site:28352283 Ref_Type:G
GACCTTGTGTTCTTCCATTGTCAAGCTATAGATTATTATGTCATATAGGTAGATCACAATAAACTTGTCAAGATATTCATXXXXXXXXXXT
TGGGCTTATCTCCCTAAACAATCATTGCTTGATAATGTCCTGATCGCAAGTCTAGTTTCGGGAAATATTTCACTCAAGT
GACCTTGTGTTCTTCCATTGTCAAGCTATAGATTATTATGTCATATAGGTAGATCACGATAAACTTGTCAAGGTATTCATXXXXXXXXXXT
TGGGCTTATCTCCCTAAACAATCATTGCTTGATAATGTCCTGATCGCAAGTCTAGTTTCGGGAAATATTTCGCTCAAGT
>Marker422733 Chr1 SNP_site:28352467 Ref_Type:G
GACCTTGTGTTCTTCCATTGTCAAGCTATAGATTATTATGTCATATAGGTAGATCACAATAAACTTGTCAAGATATTCATXXXXXXXXXXT
TGGGCTTATCTCCCTAAACAATCATTGCTTGATAATGTCCTGATCGCAAGTCTAGTTTCGGGAAATATTTCACTCAAGT
GACCTTGTGTTCTTCCATTGTCAAGCTATAGATTATTATGTCATATAGGTAGATCACGATAAACTTGTCAAGGTATTCATXXXXXXXXXXT
TGGGCTTATCTCCCTAAACAATCATTGCTTGATAATGTCCTGATCGCAAGTCTAGTTTCGGGAAATATTTCGCTCAAGT
>Marker422747 Chr1 SNP_site:16010775 Ref_Type:G
AACCTTGTTAAATGGTTTTCGCAATTACCCTTTTGTTTCCTAAGTTTGAAGGAGCAATGGTTTTATGGAGAATGTCCTCGXXXXXXXXXXT
CCCTAGTGATATGGTAAGAAGCATGGCCACATACACATGCTACAAATACGACAGATGGCACAAAGATATTGGGCATGTA
AACCTTGTTAAATGGTTTTCGCAATTACCCTTTTGTTTCCTAAGTTTGAAGGAGCAATGGTTTTATGGAGAATGTCCTCGXXXXXXXXXXT
CCCTAGTGATATGGTAAGAAGCATGGCCACATACACATGCTACAAATACGACAGATGGCACAAAGATATTGGGGATGTA
>Marker423145 Chr1 SNP_site:28933059 Ref_Type:G
CACCTTGCACTACCAAGCACAATAGTGTAAATATCTAGGATCAATATTATGTTGTTAATATCTTTACTATATAGTGTATTXXXXXXXXXXT
TCTCATGATAGCAACGCATGAATATAAATGCACCTTTAATTTTATTGCATAACCCTTTTTTAGTTGCATAATAGTCGTA
CACCTTGCACTGCCAAGCACAATAGTGTAAATATCTAGGATCAATATTATGTTGTTAATATCTTTACTATATAGTGTATTXXXXXXXXXXT
TCTCATGATAGCAACGCATGAATATAAATGCACCTTTAATTTTATTGCATAACCCTTTTTTAGTTGCATAATAGTCGTA
>Marker423446 Chr1 SNP_site:6971401 Ref_Type:A
ACCTCAACATTACATAAGCGTGTGAGGCGTTCAAGAATTTGTCTTCTTTTTTGCTTAAGGCTAAGATCATTTTTATTGCCXXXXXXXXXXA
TACCAAGAAGGTAGCATTATGTAAGATCCAAATGGAGTTAAGATTTCACAATAACAAAACAGAAAATGACCAGAAGGTC
ACCTCAACATTACATGAGCGTGTGAGGCGTTCAAGAATTTGTCTTCTTTTTTGCTTAAGGCTAAGATCATTTTTATTGCCXXXXXXXXXXA
TACCACGAAGGTAGCATTATGTAAGATCCAAATGGAGTTAAGATTTCACAATAACAAAACAGAAAATGACCAGAAGGTC
>Marker423446 Chr1 SNP_site:6971628 Ref_Type:A
ACCTCAACATTACATAAGCGTGTGAGGCGTTCAAGAATTTGTCTTCTTTTTTGCTTAAGGCTAAGATCATTTTTATTGCCXXXXXXXXXXA

Create PDF with GO2PDF for free, if you wish to

TACCAAGAAGGTAGCATTATGTAAGATCCAAATGGAGTTAAGATTTCACAATAACAAAACAGAAAATGACCAGAAGGTC
ACCTCAACATTACATGAGCGTGTGAGGCGTTCAAGAATTTGTCTTCTTTTTTGCTTAAGGCTAAGATCATTTTTATTGCCXXXXXXXXXXA
TACCACGAAGGTAGCATTATGTAAGATCCAAATGGAGTTAAGATTTCACAATAACAAAACAGAAAATGACCAGAAGGTC
>Marker424356 Chr1 SNP_site:25187328 Ref_Type:A
CACAATGAAGTGAATTTCAATTCCACAGAAAAAAGGGAAAAAAAAGATTCGTAAAAAGCATTATCACTAATCTATAGTAAXXXXXXXXXXC
CAATTCACCAACTGACTCTGCAATCTTCTCAATACAAGAAATGGAATCAATGAGCAAAGAAGCCACAGTCGCCATCGGT
CACAATGAAGTGAATTTCAATTCCACAGAGAAAAGGGAAAAAAAAGATTCGTCAAAAGCATTATCACTAATCTATAGTAAXXXXXXXXXXC
CAATTCACCAACTGACTCTGCAATCTTCTCAATACAAGAAATGGAATCAATGAGCAAAGAAGCCACAGTCGCCATCGGT
>Marker424356 Chr1 SNP_site:25187351 Ref_Type:A
CACAATGAAGTGAATTTCAATTCCACAGAAAAAAGGGAAAAAAAAGATTCGTAAAAAGCATTATCACTAATCTATAGTAAXXXXXXXXXXC
CAATTCACCAACTGACTCTGCAATCTTCTCAATACAAGAAATGGAATCAATGAGCAAAGAAGCCACAGTCGCCATCGGT
CACAATGAAGTGAATTTCAATTCCACAGAGAAAAGGGAAAAAAAAGATTCGTCAAAAGCATTATCACTAATCTATAGTAAXXXXXXXXXXC
CAATTCACCAACTGACTCTGCAATCTTCTCAATACAAGAAATGGAATCAATGAGCAAAGAAGCCACAGTCGCCATCGGT
>Marker425076 Chr1 SNP_site:10113458 Ref_Type:T
AACAATAGACTGAATTACACAAACAATGAAAAGGAACATGATCAAATTGATCGAGATATCAATTTGAGAAACTATGAAAAXXXXXXXXXXC
CCCCTTTTACACGTGCTATTCCTTTCTTTCCCTTTCTAATGTATTGCATAATAATAGGTGGTCTAACGTAAGCCAAAGT
AACAATAGACTGAATTACACAAACAATGAAAAGGAACATGATCAAATTGATCGAGATATCAATTTGAGAAACTATGAAAAXXXXXXXXXXC
CCCCTTTTACATGTGCTATTCCTTTCTTTCCCTTTCTAATGTAATGCATAATAATAGGTGGTCTAACGTAAGCCAAAGT
>Marker425076 Chr1 SNP_site:10113490 Ref_Type:A
AACAATAGACTGAATTACACAAACAATGAAAAGGAACATGATCAAATTGATCGAGATATCAATTTGAGAAACTATGAAAAXXXXXXXXXXC
CCCCTTTTACACGTGCTATTCCTTTCTTTCCCTTTCTAATGTATTGCATAATAATAGGTGGTCTAACGTAAGCCAAAGT
AACAATAGACTGAATTACACAAACAATGAAAAGGAACATGATCAAATTGATCGAGATATCAATTTGAGAAACTATGAAAAXXXXXXXXXXC
CCCCTTTTACATGTGCTATTCCTTTCTTTCCCTTTCTAATGTAATGCATAATAATAGGTGGTCTAACGTAAGCCAAAGT
>Marker425363 Chr1 SNP_site:8707909 Ref_Type:T
CACAAGGACGGATTTATCAAGAGGCGTGTCCCCCTTCACATTATCGATTTTTTTTTAATATTAATTTTGAAGTGTCAAACXXXXXXXXXXC
GCCTATTACAACTAAAAAATAGTTTTTATTTCACCAAATTTATTGTTTTGCTCAATCTATTTATAGTTTAGAATTAGTT
CACAAGGACGGATTTATCAAGAGGCGTGTCCCCCTTCACATTATTGATTTTTTTTTAATATTAATTTTGAAGTGTCAAACXXXXXXXXXXC
GCCTATTACAACTAAAAAATAGTTTTTATTTCACCAAATTTATTGTTTTGCTCAATCTATTTATAGTTTAGAATTAGTT
>Marker425488 Chr1 SNP_site:26122382 Ref_Type:G
ACTCATGGTGAAGAAGGAAGATGGTTATCATCAAATTAGGATGTGTAGCCAAGACATAGAAAAAACAGCCTTTAGAACAGXXXXXXXXXXT
TGATTTACAGTAGGGGATTGGAGGAGCACTGCCAACATATAGAATTGGTATTGGAGGTGTTGAGGAGTCATAAACTGTT
ACTCATGGTGAAGAAGGAAGATGGTTATCATCAAATTAGGATGTGTAGCCAAGACATAGAAAAAACAGCCTTTAGAACAGXXXXXXXXXXT
TGATTTACAGTAGGGGATTGGAGTAGCACTGCCAACATATAGAATTGGTATTGGAGGTGTTGAGGAGTCATAAACTGTT
>Marker425579 Chr1 SNP_site:10900733 Ref_Type:A
ACCCATATGTGGCTACGTCCATGTTCCATTGTAGCTAGGTCATAAATAGTTGATTTGATTTGAAATTACTAATTGATATTXXXXXXXXXXG
GTTGAAGTTATTTGCGGAAGGATATTAGGCGAGAAGAAAGGTTTGGCAAGAAGATGTCAAATCTGACTTGACAAGGGTT
ACCCATATGTGGCTACGTCCATGTTCCATTGTAGCTAGGTCATAAATAGTTGATTTGATTTGAAATTACTAATTGATATTXXXXXXXXXXG
GTTGAAGTTATTTGCGGAAGGATATTAGGCGAGAAGAAAGGTTTGGCGAGAAGATGTCAAATCTGACTTGACAAGGGTT
>Marker426713 Chr1 SNP_site:28253469 Ref_Type:T
AACTTGTGTGTTATTTTTGTTGTTACTTTCAATATTTACCAAACTTGTAAAAACACAAGAATAAAACTAAAAGTAATATCXXXXXXXXXXG
GTTCAAGCTCGGTTGCTGAAAGCAACTTCTCTTTTGTAACCAGATTAGGATACAGACTAGTTAGAGCAGTCAGCCTTGT
AACTTGTGTGTTATTTTTGTTGTTACTTTCAATATTTACCAAACTTGTAAAAACACAAGAATAAAACTAAAAGTAATATCXXXXXXXXXXG
GTTCAAGCTCGGTTGTTGAAAGCAACTTCTCTTTTGTAACCAGATTAGGATACAGACTAGTTAGAGCAGTCAGCCTTGT
>Marker426791 Chr1 SNP_site:17483445 Ref_Type:G

Create PDF with GO2PDF for free, if you wish to

TACAAGAGTCACTAGAGAGGGAGTCTATCAAATAAGTCATTTCGTTGTAATTGTTAAACTCTTTATATAGTGATATTTCAXXXXXXXXXX-
CTGTTTTTTTTTATTTCTATCAAGTATATTGACTACCTTGTTCATTGAATCTTCAATAAAGTCGATTAGGGAAGGTGGGTT
TACAAGAGTCACTAGAGAGGGGGTCTATCAAATAAGTCATTTCGTTGTAATTGTTAAACTCTTTATATAGTGATATTTCAXXXXXXXXXXG
CTG-TTTTTTTATTTCTATCAAGTATATTGACTACCTTGTTCATTGAATCTTCAATATAGTTGATTAGGGAAGGTGGGTT
>Marker426791 Chr1 SNP_site:17483723 Ref_Type:A
TACAAGAGTCACTAGAGAGGGAGTCTATCAAATAAGTCATTTCGTTGTAATTGTTAAACTCTTTATATAGTGATATTTCAXXXXXXXXXX-
CTGTTTTTTTTTATTTCTATCAAGTATATTGACTACCTTGTTCATTGAATCTTCAATAAAGTCGATTAGGGAAGGTGGGTT
TACAAGAGTCACTAGAGAGGGGGTCTATCAAATAAGTCATTTCGTTGTAATTGTTAAACTCTTTATATAGTGATATTTCAXXXXXXXXXXG
CTG-TTTTTTTATTTCTATCAAGTATATTGACTACCTTGTTCATTGAATCTTCAATATAGTTGATTAGGGAAGGTGGGTT
>Marker426791 Chr1 SNP_site:17483727 Ref_Type:G
TACAAGAGTCACTAGAGAGGGAGTCTATCAAATAAGTCATTTCGTTGTAATTGTTAAACTCTTTATATAGTGATATTTCAXXXXXXXXXX-
CTGTTTTTTTTTATTTCTATCAAGTATATTGACTACCTTGTTCATTGAATCTTCAATAAAGTCGATTAGGGAAGGTGGGTT
TACAAGAGTCACTAGAGAGGGGGTCTATCAAATAAGTCATTTCGTTGTAATTGTTAAACTCTTTATATAGTGATATTTCAXXXXXXXXXXG
CTG-TTTTTTTATTTCTATCAAGTATATTGACTACCTTGTTCATTGAATCTTCAATATAGTTGATTAGGGAAGGTGGGTT
>Marker426825 Chr1 SNP_site:24631146 Ref_Type:G
TACTGCTAAGGACATATTTGGAAATGATTTTGAAATGGTTAAAATCAACTTGACGTGTTTAAAATCACTCGGAAACACACXXXXXXXXXXC
AAACATTAATTAAATGAAATAAAAGATAATACAAGTCCAAGTAGTTGTAGAGTTATGCACATAAACATTACACTCTTGT
TACTGCTAAGGACATATTTGGAAGTGATTTTGAAATGGTTAAAATCAACTTGACGTGTTTAAAATCACTCGGAAACACACXXXXXXXXXXC
AAACATTAATTAAATGAAATAAAAGATAATACAAGTCCAAGTAGTTGTAGAGTTATGCACATAAACATTACACTCTTGT
>Marker427323 Chr1 SNP_site:28603819 Ref_Type:G
ACCCAACCTTTTCCCAAAACTAGTCTTCTTGAAAAAGTAGGTTCCCTTTTTTTCCAACTTTTGCCATTATGAAAACCTCAAXXXXXXXXXXC
TTCTAGCCAACTCACTTGCAATAGTTGCACAACCGACTTCGGGTCCTTCCTATGTAATGTTATAAGGATGAACTTGGTA
ACCCAACCTTTTCCCAAAACTAGTCTTCTTGAAAAAGTAGGTTCCCTTTTTTTCCAACTTTTGCCATTATGAAAACCTCAAXXXXXXXXXXC
TTCTAGCCAACTCACTTGCAATAGTTGCACAGCCGACTTCGGGTCCTTCCTATGTAATGTTATAAGGATGAACTTGGTA
>Marker427665 Chr1 SNP_site:775441 Ref_Type:G
TACTTTCTGTCAATCGAGCAATATGTGGCCATTATTGTGTTACGGGCAGTTATTTGAAGATTGGGCAGCAACTTTACATCXXXXXXXXXXT
GTGTTTAATTTCTGTCAATCGAGCAATATGTGGCCATTATTGTGTAACAGGAAGGTATTTGAAGATTGAGCAGCAGGTT
TACTTTCTGTCAATCGAGCAATATGTGGCCATTATTGTGTTACGGGCAGTTATTTGAAGATTGGGCAGCAACTTTACATCXXXXXXXXXXT
GTGTTTAATTTCTGTCAATCGAGCAATATGTGGCCATTATTGTGTAACAGGAAGGTATTTGAAGGTTGAGCAGCAGGTT
>Marker428001 Chr1 SNP_site:6501188 Ref_Type:C
ACTGAAAGCCTTGCATCAGTGCGAAGCTTGGTATTTTTCCATCATATGCAAAGGCTTGGCATGAGTCCTTTAGTTTGTTTXXXXXXXXXXA
TTTTTTATTTTTAAAAAAGGATACTCAGCATCGTCAATGATTATATTCAAAATAGAGCTTGGAGGAGATTGATTAAGTT
ACTGAAAGCCTTGCATCAGTGCGAAGCTTGGTATTTTTCCATGATATGCAAAGGCTTGGCATGAGTCCTTTAGTTTGTTTXXXXXXXXXXA
TTTTTTATTTTTAAAAAAGGATACTCAGCATCGTCAATGATTATATTCAAAATAGAGCTTGGAGGAGATTGATTAAGTT
>Marker428067 Chr1 SNP_site:7533279 Ref_Type:T
TACTCTGTTGAGAAAACATCTTGTTTTCCCTCTATGTTATATTCAAGATTATCATTTTTCTCTGATAAATCAGTTCATTAXXXXXXXXXXA
GGAAGAAGAAAAGCATGGGAAGATTATGGGTAATCACGTGGTTGATTACGCAGATGCTGTTGTAATGCCTGGCTTGGTT
TACTCTGTTGAGAAAACATCTTGTTTTCCCTCTATGTTATATTTAAGATTATCATTTTTCTCTGATAAATCAGTTCATTAXXXXXXXXXXA
GGAAGAAGAAAAGCATGGGAAGATTATGGGTAATCACGTGGTTGATTACGCAGATGCTGTTGTAATGCCTGGCTTGGTT
>Marker4282 Chr4 SNP_site:13606661 Ref_Type:A
GACCTAACCATATTTTTCTTTTACTTATTATAAACTTCATTTTGATATTAAATATGGATCTTACCTTCTTATTCTTCTTAXXXXXXXXXXT
TTATATATATAACAATTAAGTGAAAGAATTGGAGATGAGTGTGGTGCAGATATTTTTTGAAATTTTTTAATAGAAATGT
GACCTAACCATATTTTTCTTTTACTTATTATAAACTTCATTTTGATATTAAATATGGATCTTACCTTCTTATTCTTCTTAXXXXXXXXXXT
TTATATATATAACAATTTAGTGAAAGAATTGGAGATGAGTGTGGTGCAGATATTTTTTGAAATTTTTTAATAGAAATGT

Create PDF with GO2PDF for free, if you wish to

>Marker428201 Chr1 SNP_site:17419853 Ref_Type:T
ACATTTTTTAAAGTTATACTATTCAACACTTTGTGATTACTAACACATTCAAACATTAAATTTATCGTCAAACATGCGATXXXXXXXXXXC
CAACAAAGAAAGAGGCAAGCCAATCAAACACAACCATATAAGGCCCTTCCTCCAAAGGCCAAACAATTAATACTTTTGT
ACATTTTTTAAAGTTATACTATTCAACACTTTTTGATTACTAACACATTCAAACATTAAATTTATCGTCAAACATGCGATXXXXXXXXXXC
CAACAAAGAAAGAGGCAAGCCAATCAAACACAACCATATAAGGCCCTTCCTCCAAAGGCCAAACAATTAATACTTTTGT
>Marker428573 Chr1 SNP_site:6507024 Ref_Type:G
GACATCATTAAAAAAAGGACAATAACGCAACAATTTATTAAAAATAAAATTGTAAAAACGGACCCAAAAACAAGGCTTTAXXXXXXXXXXT
TGTGCATCATTGAATGAAATTTTGATGGAAGATAATAAGGGTGCTAAAAGGGTGGCCATCTAATAGGGATGCTTGGGTG
GACATCATTAAAAAAAGGACAATAACGCAACAATTTATTAAAAATAAAATTGTAAAAACGGACCCAAAAACAAGGCTTTAXXXXXXXXXXT
TGTGCATCATTGAATGAAATTTTGATGGAAGATAATAAGGGTGCTAAAAGGGTGGCCATCTAGTAGGGATGCTTGGGTG
>Marker429225 Chr1 SNP_site:5506390 Ref_Type:G
CACTTCATTGTTCCATAGCTGTAATAATGAGCAATTGTTTAAAGCAAACTTTTACAATCGTTGCAGAGGAGGAGAAAACCXXXXXXXXXXT
TATCTGCAGCATTCCTTTCTCAGACCATGAATCTGGCGAGAGAGACGACCGCTCAGAAAACGATGCTTCCGTCGCAGTG
CACTTCATTGTTCCATGGCTGTAATAATGAGCAATTGTTTAAAGCAAACTTTTACAATCGTTGCAGAGGAGGAAAAAACCXXXXXXXXXXT
TATCTGCAGCATTCCTTTCTCAGACCATGAATCTGGCGAGAGAGACGACCGCTCAGAAAACGATGCGTCCGTCGCAGTG
>Marker429225 Chr1 SNP_site:5506447 Ref_Type:A
CACTTCATTGTTCCATAGCTGTAATAATGAGCAATTGTTTAAAGCAAACTTTTACAATCGTTGCAGAGGAGGAGAAAACCXXXXXXXXXXT
TATCTGCAGCATTCCTTTCTCAGACCATGAATCTGGCGAGAGAGACGACCGCTCAGAAAACGATGCTTCCGTCGCAGTG
CACTTCATTGTTCCATGGCTGTAATAATGAGCAATTGTTTAAAGCAAACTTTTACAATCGTTGCAGAGGAGGAAAAAACCXXXXXXXXXXT
TATCTGCAGCATTCCTTTCTCAGACCATGAATCTGGCGAGAGAGACGACCGCTCAGAAAACGATGCGTCCGTCGCAGTG
>Marker429225 Chr1 SNP_site:5506616 Ref_Type:G
CACTTCATTGTTCCATAGCTGTAATAATGAGCAATTGTTTAAAGCAAACTTTTACAATCGTTGCAGAGGAGGAGAAAACCXXXXXXXXXXT
TATCTGCAGCATTCCTTTCTCAGACCATGAATCTGGCGAGAGAGACGACCGCTCAGAAAACGATGCTTCCGTCGCAGTG
CACTTCATTGTTCCATGGCTGTAATAATGAGCAATTGTTTAAAGCAAACTTTTACAATCGTTGCAGAGGAGGAAAAAACCXXXXXXXXXXT
TATCTGCAGCATTCCTTTCTCAGACCATGAATCTGGCGAGAGAGACGACCGCTCAGAAAACGATGCGTCCGTCGCAGTG
>Marker430872 Chr1 SNP_site:5354191 Ref_Type:C
ACATTTCTTGATCTTCTTATGAATGTTGTTGATGGATCAATACCATTTCGATTTAATTAATTATAGAAGAGACCTCCTCCXXXXXXXXXXA
GCTAGAATCTAAATTTGAGAGGACAATTTTTTTGAAAATAGACATGATTTTAATTAGTGATTCTGTAGGGTATCAGGGT
ACATTTCTTGATCTTCTTATGAATGTTGTTGATGGATCAATACCATTTGGATTTAATTAATTATAGAAGAGACCTCCTCCXXXXXXXXXXA
GCTAGAATCTAAATTTGAGAGGACAAATTTTTTGAAAATAGACATGATTTTAATTAGTGATTCTGTAGGGTATCAGGGT
>Marker430872 Chr1 SNP_site:5354346 Ref_Type:T
ACATTTCTTGATCTTCTTATGAATGTTGTTGATGGATCAATACCATTTCGATTTAATTAATTATAGAAGAGACCTCCTCCXXXXXXXXXXA
GCTAGAATCTAAATTTGAGAGGACAATTTTTTTGAAAATAGACATGATTTTAATTAGTGATTCTGTAGGGTATCAGGGT
ACATTTCTTGATCTTCTTATGAATGTTGTTGATGGATCAATACCATTTGGATTTAATTAATTATAGAAGAGACCTCCTCCXXXXXXXXXXA
GCTAGAATCTAAATTTGAGAGGACAAATTTTTTGAAAATAGACATGATTTTAATTAGTGATTCTGTAGGGTATCAGGGT
>Marker431451 Chr1 SNP_site:24231483 Ref_Type:T
ACCAATGTTTCCTGTGATCATACTTTCCTAATGTCTTCACTAAATTATCTACAAATATTATTGTATCGTCATCTGTCATTXXXXXXXXXXA
AAACATAGAAAGCTGATCAATTCATGAGTTATAACTTTTAGATGTTTATACATATTTAGTCTCTCACAACTTTGAAGGT
ACCAATGTTTCTTGTGATCATACTTTCCTAATGTCTTCACTAAATTATCTACAAATATTATTGTATCGTCATCTGTCATTXXXXXXXXXXA
AAACATAGAAAGCTGATCAATTCATGAGTTATAACTTTTAGATGTTTATACATATTTAGTCTCTCACAACTTTGAAGGT
>Marker431768 Chr1 SNP_site:14228357 Ref_Type:C
ACCTCAGCGAACAAAAAATGGGATGTGTTTATTGATTTGTAGCCTAGTTCCTTGTGCATCCTAGGTTTTTTGTTTTTGTTXXXXXXXXXXC
TATTTTGTGTTGTGCGTCGGAAATCACGCTCTAACGAGTGAAATCGTACGCCACCAATAATGCATCCACTGTAAGAGTG
ACCTCCGCGAACAAAAAATGGGATGTGTTTATTGATTTGTAGCCTAGTTCCTTGTGCATCCTAGGTTTTTTGTTTTTGTTXXXXXXXXXXC

Create PDF with GO2PDF for free, if you wish to

TATTTTGTGTTGTGCGTCGGAAATCACGCTCTAACAAGTGAAATCGTACGCCACCAATAATGCATCCACTGTAAGAGTG
>Marker431768 Chr1 SNP_site:14228604 Ref_Type:A
ACCTCAGCGAACAAAAAATGGGATGTGTTTATTGATTTGTAGCCTAGTTCCTTGTGCATCCTAGGTTTTTTGTTTTTGTTXXXXXXXXXXC
TATTTTGTGTTGTGCGTCGGAAATCACGCTCTAACGAGTGAAATCGTACGCCACCAATAATGCATCCACTGTAAGAGTG
ACCTCCGCGAACAAAAAATGGGATGTGTTTATTGATTTGTAGCCTAGTTCCTTGTGCATCCTAGGTTTTTTGTTTTTGTTXXXXXXXXXXC
TATTTTGTGTTGTGCGTCGGAAATCACGCTCTAACAAGTGAAATCGTACGCCACCAATAATGCATCCACTGTAAGAGTG
>Marker431956 Chr1 SNP_site:27420530 Ref_Type:A
TACTAACCAACAACAAATTGCAAGCTAATTTTTGAACATGAAGGGATGACCTTCAAATTTTGGAGCAGTTAGTATCTTTAXXXXXXXXXXG
CCAACTTCAGAAAATAGGTTATTAATAGATGGAAGAGAGGTTTTACCAAGTATTGGACCTCTCTAACCTCATAAAAGTC
TACTAACCGACAACAAATTGCAAGCTAATTTTTGAACATGAAGGGATGACCTTCAAATTTTGGAGCAGTTAGTATCTTTAXXXXXXXXXXG
CCAACTTCAGAAAATAGGTTATTAATAGATGGAAGAGAGGTTTTACCAAGTATTGGACCTCTCTAACCTCATAAAAGTC
>Marker432214 Chr1 SNP_site:25987527 Ref_Type:C
CACTAAGTGTTAGTAGAATGTGAGGTGTTAGATTTTGACGAACTAATTAATGTATTTATGCATGTAAATAGATTATTTAAXXXXXXXXXXT
CAAATTTAAAAACTTTTTGCACTCTAATTATTTTTCTTTCAAAAAATAAGAAAGAAAAATCTTACCAAAGATTTTGGTG
CACTAAGTGTTAGTAGAATGTGAGGTGTTAGATTTTGACGAACTAATTAATGTATTTATGCATGTAAATAGATTATTTAAXXXXXXXXXXT
CAAATTTAAAAACTTTTTGCACTTTAATTATTTTTCTTTTAAAAAATAAGAAAGAAAAATCTTACCAAAGATTTTGGTG
>Marker432214 Chr1 SNP_site:25987543 Ref_Type:C
CACTAAGTGTTAGTAGAATGTGAGGTGTTAGATTTTGACGAACTAATTAATGTATTTATGCATGTAAATAGATTATTTAAXXXXXXXXXXT
CAAATTTAAAAACTTTTTGCACTCTAATTATTTTTCTTTCAAAAAATAAGAAAGAAAAATCTTACCAAAGATTTTGGTG
CACTAAGTGTTAGTAGAATGTGAGGTGTTAGATTTTGACGAACTAATTAATGTATTTATGCATGTAAATAGATTATTTAAXXXXXXXXXXT
CAAATTTAAAAACTTTTTGCACTTTAATTATTTTTCTTTTAAAAAATAAGAAAGAAAAATCTTACCAAAGATTTTGGTG
>Marker432892 Chr1 SNP_site:16100882 Ref_Type:T
ACCATCCAAGAGCGCTCCACATCTAAAGGATTACTGATAAGTAATGAATAATGTGTTAGAAATATAAAATTTTCAACAATXXXXXXXXXXA
TTATAGTTTGATTAGAATAGTTTGTTTTTGGGTGTAGATTATTTTAGTTTGGGTTATAATAGTATGTATCTGGTGTGTA
ACCATCCAAGAGCGCTCCACATCTAAAGGATTACTGATAAGTAATGAATAATGTGTTAGAAATATAAAATTTTCAACAATXXXXXXXXXXA
TTATAGTTTGATTAGAATAGTTTGTTTTTTGGTGTAGATTATTTTAGTTTGGGTTATAATAGTATGTGTCTGGTGTGTA
>Marker432892 Chr1 SNP_site:16100920 Ref_Type:G
ACCATCCAAGAGCGCTCCACATCTAAAGGATTACTGATAAGTAATGAATAATGTGTTAGAAATATAAAATTTTCAACAATXXXXXXXXXXA
TTATAGTTTGATTAGAATAGTTTGTTTTTGGGTGTAGATTATTTTAGTTTGGGTTATAATAGTATGTATCTGGTGTGTA
ACCATCCAAGAGCGCTCCACATCTAAAGGATTACTGATAAGTAATGAATAATGTGTTAGAAATATAAAATTTTCAACAATXXXXXXXXXXA
TTATAGTTTGATTAGAATAGTTTGTTTTTTGGTGTAGATTATTTTAGTTTGGGTTATAATAGTATGTGTCTGGTGTGTA
>Marker433210 Chr1 SNP_site:25568932 Ref_Type:C
AACCCAAAAATAATGCTATAGAAACTAATGTTGAACATTGATGGGAAGGGTATAGAATATAGATACCGCATTTTCATCATXXXXXXXXXXT
TGAGAACAGAACAACAGCTAGAAACTGTTCATCAAAGAGAACGTGTCAGGAAAAAAACCAGAAAGAAAAATAATTTGTT
AACCCAAAAATAATGCTATAGAAACTAATGTTGAACATTGATGGGAAGGGTATAGAATATAGATACCGCATTTTCATCATXXXXXXXXXXT
TGAGACCAGAACAACAGCTAGAAACTGTTCATCAAAGAGAACGTGTCAGGAAGAAAACCAGAAAGAAAAATAATTTGTT
>Marker433210 Chr1 SNP_site:25568979 Ref_Type:G
AACCCAAAAATAATGCTATAGAAACTAATGTTGAACATTGATGGGAAGGGTATAGAATATAGATACCGCATTTTCATCATXXXXXXXXXXT
TGAGAACAGAACAACAGCTAGAAACTGTTCATCAAAGAGAACGTGTCAGGAAAAAAACCAGAAAGAAAAATAATTTGTT
AACCCAAAAATAATGCTATAGAAACTAATGTTGAACATTGATGGGAAGGGTATAGAATATAGATACCGCATTTTCATCATXXXXXXXXXXT
TGAGACCAGAACAACAGCTAGAAACTGTTCATCAAAGAGAACGTGTCAGGAAGAAAACCAGAAAGAAAAATAATTTGTT
>Marker433546 Chr1 SNP_site:28859199 Ref_Type:T
ACTTTTATTTTATCTTTATTGTGCAGTTTAAAAAAATGAAAACCTTGCGTCTTATCTTTCTTTCCCCCCACTTTTTGCTGXXXXXXXXXXA
GAAAAGAAAATAATCAAGCATAGACACACACACACGCACACAACCACAAAATAGTAGTTTCTACACCCATAAATATGTT

Create PDF with GO2PDF for free, if you wish to

ACTTTTATTTTATCTTTATTGTGCAGTTTAAAAAAATGAAAACCTTTCGTCCTATCTTTCTTTCCCCCCACTTTTTGCTGXXXXXXXXXXA
GAAAAGAAAATAATCAAGCATAGACACACACACACGCACACAACCACAAAATAGTAGTTTCTACACCCATAAATATGTT
>Marker433546 Chr1 SNP_site:28859204 Ref_Type:C
ACTTTTATTTTATCTTTATTGTGCAGTTTAAAAAAATGAAAACCTTGCGTCTTATCTTTCTTTCCCCCCACTTTTTGCTGXXXXXXXXXXA
GAAAAGAAAATAATCAAGCATAGACACACACACACGCACACAACCACAAAATAGTAGTTTCTACACCCATAAATATGTT
ACTTTTATTTTATCTTTATTGTGCAGTTTAAAAAAATGAAAACCTTTCGTCCTATCTTTCTTTCCCCCCACTTTTTGCTGXXXXXXXXXXA
GAAAAGAAAATAATCAAGCATAGACACACACACACGCACACAACCACAAAATAGTAGTTTCTACACCCATAAATATGTT
>Marker43411 Chr4 SNP_site:7281438 Ref_Type:G
AACGTGTTAGATTAAGAAAATGTCTATTCCCTCTCTACGAGAACAAAAGGAAGAGGAGAATTTTTTGTTAAAGTGTCGTGXXXXXXXXXXA
GTTTGTGTATTCCAACATTATAAAAATCATGTTAAAGCTTGATTTAATGATTCCTTAAGATGATGCTAACTTTAATGTG
AACGTGTTAGATTAAGAAAATGTCTATTCCCTCTCTACGAGAACAAAAGGAAGAGGAGAATTTTTTGTTAAAGTGTCGTGXXXXXXXXXXA
GTTTGTGTATTCCAGCATTATAAAAATCATGTTAAAGCTTGATTTAATGATTCCTTAAGATGATGCTAACTTTAATGTG
>Marker434603 Chr1 SNP_site:28387966 Ref_Type:C
AACCTTTGCATTGCAGCTTCAAGCCTGTCACGGAAATTTTTTTAAGACTGACTGCATTTATGATAAAACTAACAACTTATXXXXXXXXXXA
TAAAAATTTCCAGACAACATCCTAGCAACAGATTACATGGACAAAATAATCCGTTGCAAAACCACTTCAGGTTAATGTT
AACCTTTGCATTGCAGCTTCAAGCCTGTCAGAGAAATTTTTTTAAGACTGACCGCATTTATGATAAAACTAACAACTTATXXXXXXXXXXA
TAAAAATTTCCAGACAACATCCTAGCAACAGATTACATGGACAAAATAATCCGTTGCAAAACCACTTCAGGTTAATGTT
>Marker434603 Chr1 SNP_site:28387967 Ref_Type:A
AACCTTTGCATTGCAGCTTCAAGCCTGTCACGGAAATTTTTTTAAGACTGACTGCATTTATGATAAAACTAACAACTTATXXXXXXXXXXA
TAAAAATTTCCAGACAACATCCTAGCAACAGATTACATGGACAAAATAATCCGTTGCAAAACCACTTCAGGTTAATGTT
AACCTTTGCATTGCAGCTTCAAGCCTGTCAGAGAAATTTTTTTAAGACTGACCGCATTTATGATAAAACTAACAACTTATXXXXXXXXXXA
TAAAAATTTCCAGACAACATCCTAGCAACAGATTACATGGACAAAATAATCCGTTGCAAAACCACTTCAGGTTAATGTT
>Marker434603 Chr1 SNP_site:28387988 Ref_Type:T
AACCTTTGCATTGCAGCTTCAAGCCTGTCACGGAAATTTTTTTAAGACTGACTGCATTTATGATAAAACTAACAACTTATXXXXXXXXXXA
TAAAAATTTCCAGACAACATCCTAGCAACAGATTACATGGACAAAATAATCCGTTGCAAAACCACTTCAGGTTAATGTT
AACCTTTGCATTGCAGCTTCAAGCCTGTCAGAGAAATTTTTTTAAGACTGACCGCATTTATGATAAAACTAACAACTTATXXXXXXXXXXA
TAAAAATTTCCAGACAACATCCTAGCAACAGATTACATGGACAAAATAATCCGTTGCAAAACCACTTCAGGTTAATGTT
>Marker434739 Chr1 SNP_site:16103183 Ref_Type:C
AACAGCCTGCATATAATACATCAATTGGTTGAAAGACCTCAGATACGGATAAACAGAAGGAAAAAGAATCAGACACTCTAXXXXXXXXXXC
TTTGCCCATGTATCCTCCTCAAAATTAGCAGGGAGTGTCGGTTTTGCTTCATAGAGAAAACGGAAAATGAGATATTGTA
AACAGCCTGCATATAATACATCAATTGGTTGAAAGACCTCAGATAGGGATAAACAGAAGGAAAAAGAATCAGAAACTCTAXXXXXXXXXXC
TTTGCCCATGTATCCTCCTCAAAATTAGCAGGGAGTGTCGGTTTTGCTTCATAGAGAAAACGGAAAATGAGATATTGTA
>Marker434739 Chr1 SNP_site:16103211 Ref_Type:C
AACAGCCTGCATATAATACATCAATTGGTTGAAAGACCTCAGATACGGATAAACAGAAGGAAAAAGAATCAGACACTCTAXXXXXXXXXXC
TTTGCCCATGTATCCTCCTCAAAATTAGCAGGGAGTGTCGGTTTTGCTTCATAGAGAAAACGGAAAATGAGATATTGTA
AACAGCCTGCATATAATACATCAATTGGTTGAAAGACCTCAGATAGGGATAAACAGAAGGAAAAAGAATCAGAAACTCTAXXXXXXXXXXC
TTTGCCCATGTATCCTCCTCAAAATTAGCAGGGAGTGTCGGTTTTGCTTCATAGAGAAAACGGAAAATGAGATATTGTA
>Marker434832 Chr1 SNP_site:22777377 Ref_Type:G
TACTGATACAATAGATTTTTATAAGGAGAAAAGAAGATACGAAGTATATATGTGTTGTTAAGAATAATGATCGTTAAAATXXXXXXXXXXT
AAAAATTGTTTCAGTAAATCGATGTATTGATATTTTAAGTTGAAACTTTACAATAAAAATTAATATGACGCGATAAGTT
TACTGATACAATAGATTTTTATAAGGAGAAAAGAAGATACGAAGTATATATGTGTTGTTAAGAATAATGATCGTTAAAGTXXXXXXXXXXT
AAAAATTGTTTAAGTAAATCGATGTATTGATATTTTAAGTTGAAACTTTACAATAAAAATTAATATGACGCGATAAGTT
>Marker434832 Chr1 SNP_site:22777480 Ref_Type:A
TACTGATACAATAGATTTTTATAAGGAGAAAAGAAGATACGAAGTATATATGTGTTGTTAAGAATAATGATCGTTAAAATXXXXXXXXXXT

Create PDF with GO2PDF for free, if you wish to

AAAAATTGTTTCAGTAAATCGATGTATTGATATTTTAAGTTGAAACTTTACAATAAAAATTAATATGACGCGATAAGTT
TACTGATACAATAGATTTTTATAAGGAGAAAAGAAGATACGAAGTATATATGTGTTGTTAAGAATAATGATCGTTAAAGTXXXXXXXXXXT
AAAAATTGTTTAAGTAAATCGATGTATTGATATTTTAAGTTGAAACTTTACAATAAAAATTAATATGACGCGATAAGTT
>Marker435658 Chr1 SNP_site:2567611 Ref_Type:T
AACAGTATCCACAGTTCTGTATCTGTTCTTGTAGATGATACAATACCAAATGCTTTTTAATCAATAGATGGTTTAATATGXXXXXXXXXXT
GGAGTAATTCAAGGGACCTTATAATATTTATTCCATTGATGGAGTAACCACCTATTGTCAATTGTCAGTTGTTTTCGTG
AACAGTATCCACAGTTCTGTATCTGTTCTTGTAGATGATACAATACCAAATGCTTTTTAATCAATAGATGGTTTATTATGXXXXXXXXXXT
GGAGTAATTCAAGGGACCTTATAATATTTATTCCATTGATGGAGTAACCACCTATTGTCAATTGTCAGTTGTTTTCGTG
>Marker43570 Chr4 SNP_site:11704772 Ref_Type:T
ACCGAAGCATTGCTTCTCTGATGCACATATATCTATATAGAATATCATGAAATATTGGTTCTCATCTCTACATGCTTGTTXXXXXXXXXXA
TGATTTATGTATATATAATCTGATTATATTGGAACAAACAGAAAATTCCTTACTTCAACGTGCAAAGTCATTGCGCGTG
ACCGAAGCATTGCTTCTCTGATGCACATATATCTATATAGAATATCATGAAATATTGGTTCTCATCTCTATATGCTTGTTXXXXXXXXXXA
TGATTTATGTATATATAATCTGATTATATTGGAACAAACAGAAAATTCCTTACTTCAACGTGCAAAGTCATTGCGCGTG
>Marker436008 Chr1 SNP_site:904329 Ref_Type:G
ACTTTTAGGGAATAATCCCAACTCCAGTTATAGTCTTTTCAGTTCATCTTGTTTTAATTTGAATCTCTATTAGTTACATAXXXXXXXXXXG
TTTTGTTACCTATGGTTAATTTGCAGGTGCAAGAACACATATTTGTAGAATCAAACAAGTTGATCAAGTCAATTCAAGT
ACTTTTAGGGAATAATCCCAACTCCAGTTATAGTGTTTTCAGTTCATCTTGTTTTAATTTGAATCTCTATTAGTTACATAXXXXXXXXXXG
TTTTGTTACCTATGGTTAATTTGCAGGTGCAAGAACACATATTTGTAGAATCAAACAAGTTGATCAAGTCAATTCAAGT
>Marker436670 Chr1 SNP_site:3536666 Ref_Type:T
ACTTGTTCCAAATTGTTAGGAAATTCACAGGCTTTATTGTTGGCTAACTAGCTGAGAGACATTAAAAAAGATATTAAAAGXXXXXXXXXXT
ACTCTTAACCCTTGCCCTGTTCATCTCAAGAATGTAGAACAAGATTTCCATCATCCTATATGCATGCAGTGGAACCGTG
ACTTGTTCCAAATTGTTAGGAAATTCACAGGCTTTATTGTTGGCTAACTAGCTGAGAGATATTAAAAAAGATATTAAAAGXXXXXXXXXXT
ACTCTTAACCCTTGCCCTGTTCATCTCAAGAATGTAGAACAAGATTTCCATCATCCTATACGCATGCAGTGCAACCGTG
>Marker436670 Chr1 SNP_site:3536868 Ref_Type:C
ACTTGTTCCAAATTGTTAGGAAATTCACAGGCTTTATTGTTGGCTAACTAGCTGAGAGACATTAAAAAAGATATTAAAAGXXXXXXXXXXT
ACTCTTAACCCTTGCCCTGTTCATCTCAAGAATGTAGAACAAGATTTCCATCATCCTATATGCATGCAGTGGAACCGTG
ACTTGTTCCAAATTGTTAGGAAATTCACAGGCTTTATTGTTGGCTAACTAGCTGAGAGATATTAAAAAAGATATTAAAAGXXXXXXXXXXT
ACTCTTAACCCTTGCCCTGTTCATCTCAAGAATGTAGAACAAGATTTCCATCATCCTATACGCATGCAGTGCAACCGTG
>Marker436670 Chr1 SNP_site:3536879 Ref_Type:C
ACTTGTTCCAAATTGTTAGGAAATTCACAGGCTTTATTGTTGGCTAACTAGCTGAGAGACATTAAAAAAGATATTAAAAGXXXXXXXXXXT
ACTCTTAACCCTTGCCCTGTTCATCTCAAGAATGTAGAACAAGATTTCCATCATCCTATATGCATGCAGTGGAACCGTG
ACTTGTTCCAAATTGTTAGGAAATTCACAGGCTTTATTGTTGGCTAACTAGCTGAGAGATATTAAAAAAGATATTAAAAGXXXXXXXXXXT
ACTCTTAACCCTTGCCCTGTTCATCTCAAGAATGTAGAACAAGATTTCCATCATCCTATACGCATGCAGTGCAACCGTG
>Marker436867 Chr1 SNP_site:25912416 Ref_Type:G
AACCCAACACAAGAAATCCTCAAGTCATCTAAGGAACTCAGGAGGAGTTGAGGTTAGTTGGAGAAAGATGATTCAAGATTXXXXXXXXXXA
AGAAGGATTGTTTTGGTGTGACCAAGAAGTGTCAAGATAACCTCTATAGCACAAGGCTAGGTGGCCAAAGACATTGGTT
AACCCAACACAAGAAATCCTCAAGTCATCTAAGGAACTCAGGAGGAGTTGAGGTTAGTTGGAGAAAGATGATTCAAGATTXXXXXXXXXXA
AGAAGGATTGTTTTGGTGTGACCAAGAGGTGTCAAGATAACCTCTATAGCACAAGGCTAGGTGGCCAAAGACATTGGTT
>Marker436936 Chr1 SNP_site:9008569 Ref_Type:A
ACTCACACAACAGTAGTCATACCTTAAAAACATTGATTAACATTGATTGCCATAATCTAAACTAAAGAAAATAGTGAAGCXXXXXXXXXXA
ATAGAGAGGTGTTTGGAAGCAAAAGATCTGACGGTCCAGATTGAATAAAAAAACCCGGTTTTGTGATTCCCAAAACAGT
ACTCACACAACAGTAGTCATACCTTAACAACATTGATTAACATTGATTGTGATAATCTAAACTAAAGAAAATAGTGAAGCXXXXXXXXXXA
ATAGAGAGGTGTTTGGAAGCAAAAGATCTGACGGTCCAGATTGAATAAAAAAACCCGGTTTTGTGATTCCCAAAACAGT
>Marker436936 Chr1 SNP_site:9008591 Ref_Type:C

Create PDF with GO2PDF for free, if you wish to

ACTCACACAACAGTAGTCATACCTTAAAAACATTGATTAACATTGATTGCCATAATCTAAACTAAAGAAAATAGTGAAGCXXXXXXXXXXA
ATAGAGAGGTGTTTGGAAGCAAAAGATCTGACGGTCCAGATTGAATAAAAAAACCCGGTTTTGTGATTCCCAAAACAGT
ACTCACACAACAGTAGTCATACCTTAACAACATTGATTAACATTGATTGTGATAATCTAAACTAAAGAAAATAGTGAAGCXXXXXXXXXXA
ATAGAGAGGTGTTTGGAAGCAAAAGATCTGACGGTCCAGATTGAATAAAAAAACCCGGTTTTGTGATTCCCAAAACAGT
>Marker436936 Chr1 SNP_site:9008592 Ref_Type:C
ACTCACACAACAGTAGTCATACCTTAAAAACATTGATTAACATTGATTGCCATAATCTAAACTAAAGAAAATAGTGAAGCXXXXXXXXXXA
ATAGAGAGGTGTTTGGAAGCAAAAGATCTGACGGTCCAGATTGAATAAAAAAACCCGGTTTTGTGATTCCCAAAACAGT
ACTCACACAACAGTAGTCATACCTTAACAACATTGATTAACATTGATTGTGATAATCTAAACTAAAGAAAATAGTGAAGCXXXXXXXXXXA
ATAGAGAGGTGTTTGGAAGCAAAAGATCTGACGGTCCAGATTGAATAAAAAAACCCGGTTTTGTGATTCCCAAAACAGT
>Marker437180 Chr1 SNP_site:19556839 Ref_Type:G
TACATGAGCCCCCAACTAGATTTGAAACCCAATTTGGTAAGCAGGTATGGAATCTCCAGAAATCCCTAATGGTCTGAAACXXXXXXXXXXC
AAAACAGGGAAGATTGCAGTTCTTATAGTTTATATGGATGACATCGTTTTGTCTTGAGATGAACAGACAGAAATCAGTC
TACATGAGCCCCCAACTGGATTTGAAACCCAATTTGGTAAGCAGGTATGGAATCTCCAGAAATCCCTAATGGTCTGAAACXXXXXXXXXXC
AAAACAGGGAAGATTGCAGTTCTTATAGTTTATGTGGATGACATCGTTTTGTCTTGAGATGAACAGACAGAAATCAGTC
>Marker437180 Chr1 SNP_site:19557033 Ref_Type:G
TACATGAGCCCCCAACTAGATTTGAAACCCAATTTGGTAAGCAGGTATGGAATCTCCAGAAATCCCTAATGGTCTGAAACXXXXXXXXXXC
AAAACAGGGAAGATTGCAGTTCTTATAGTTTATATGGATGACATCGTTTTGTCTTGAGATGAACAGACAGAAATCAGTC
TACATGAGCCCCCAACTGGATTTGAAACCCAATTTGGTAAGCAGGTATGGAATCTCCAGAAATCCCTAATGGTCTGAAACXXXXXXXXXXC
AAAACAGGGAAGATTGCAGTTCTTATAGTTTATGTGGATGACATCGTTTTGTCTTGAGATGAACAGACAGAAATCAGTC
>Marker437639 Chr1 SNP_site:19721147 Ref_Type:T
ACTAAAATCCTTTTGTGCTTCCATTAAGCTGTTTCTTCCTTCATCTTTCCACCTTGATTAATGATTCAGTTTGGGCCACGXXXXXXXXXXA
TTGTTGTTTGGTTCTCTATTTTGTTTGGATGTGATGAGGGTGTTAAAGGAGATGTCAATCTAGTTGAGATATTTGGGTG
ACTAAAATCCTTTTGTGCTTCCATTAATCTGTTTCTTCCTTCATCTTTCCACCTTGATTAATGATTCAGTTTGGGCCACGXXXXXXXXXXA
TTGTTGTTTGGTTCTCTATTTTGTTTGGATGTGATGAGGGTGTTAAAGGAGATGTCAATCTAGTTGAGATATTTGGGTG
>Marker437657 Chr1 SNP_site:2611259 Ref_Type:T
GACATCATTTCTTTCTTTGGAACTGTATTTTGTATTATATCAAAACAAGGATAAGTTTCTATCCAAAATTTCACAAACTTXXXXXXXXXXC
AATGGGCCATCAAGCATGTGGAGCTCTCTCTCTCTGGAACTATCCATCATGGATGAGAAAACTTATTGCCCACGATGTT
GACATCATTTCTTTCTTTGGAACTGTATTTTGTATTATATCAAAACAAGGATAAGTTTCTATCCAAATTTTCACAAACTTXXXXXXXXXXC
AATGGGCCATCAAGCATGTGGAGCTCTCTCTCTCTGGAACTATCCATCATGGATGAGAAAACTTATTGCCCACGATGTT
>Marker438324 Chr1 SNP_site:22073869 Ref_Type:C
GACGGCACCCTTCTGGGGCAGTGTTTCAAAAGCTGAGACAGTGTTCTCAAATTTAATACGATGAAGAGGAGCTCCACTCTXXXXXXXXXXA
TATAGAAGAACGGGTTCATGAAGCAGAAAACTGAAACAAAGTCATGAGGAAAAACATGGCAAATACAATCGTCATAGTG
GACGGCACCCTTCTGGGGCAGTGTTTCAAAAGCTGAGACAGTGTTCTCAAATTTAATACGATGAAGAGGAGCTCCACTCTXXXXXXXXXXA
TATAGAAGAACGGGTTCATGAAGCAGAAAACTGAAACAAAGTCATGAGGAAAAACATGGCAAATACAATCGTCCTAGTG
>Marker438395 Chr1 SNP_site:7469843 Ref_Type:A
TACAACGTTATTATCCATGATAATATTATCACCGCCACTCCCGGTCCCCTGTGTGACAAACACACAATAGATTAATTTAGXXXXXXXXXXA
CCCTTAATAACTCGAGCGCCCGATTGTAATATTTAGGGCTCAAATTTCATCATTATGGGTATAAGTGCTTTTTACCGTT
TACAACGTTATTATCCATGATAATATTATCACCGCCACTCCCGGTCCCCTGTGTGACAAACACACAATAGATTAATTTAGXXXXXXXXXXA
CCCTTAATAACTCGAGCGCCCGATTGTAATGTTTATGGCTCAAATTTCATCATTATGGGTATAAGTGCTTTTTACCGTT
>Marker438395 Chr1 SNP_site:7469848 Ref_Type:G
TACAACGTTATTATCCATGATAATATTATCACCGCCACTCCCGGTCCCCTGTGTGACAAACACACAATAGATTAATTTAGXXXXXXXXXXA
CCCTTAATAACTCGAGCGCCCGATTGTAATATTTAGGGCTCAAATTTCATCATTATGGGTATAAGTGCTTTTTACCGTT
TACAACGTTATTATCCATGATAATATTATCACCGCCACTCCCGGTCCCCTGTGTGACAAACACACAATAGATTAATTTAGXXXXXXXXXXA
CCCTTAATAACTCGAGCGCCCGATTGTAATGTTTATGGCTCAAATTTCATCATTATGGGTATAAGTGCTTTTTACCGTT

Create PDF with GO2PDF for free, if you wish to

>Marker43843 Chr4 SNP_site:19904766 Ref_Type:G

AACAGAGAACCAATTTTCTGGAAATACAAGGTCATGGACAAGGAGAAAGATCCATCAACATTGAAACATCTGCAGTCACTXXXXXXXXXXA
TTAGGTTATGATTGAACATTCTTCAAACTATTTCATTTGACCACAGCTCCAAGTTGTGTATTGTGTTTAGAATGAAGTG
AACAGAGAACCAATTTTCTGGAAATACAAGGTCATGGACAAGGAGAAAGATCCATCAACATTGAAACATCTGCAGTCACTXXXXXXXXXXA
TTAGGTTATGATTGAACATTCTTCAAACTATTTCATTTGGCCACAGCTCCAAGTTGTGTATTGTGTTTAGAATGAAGTG

>Marker438701 Chr1 SNP_site:28593686 Ref_Type:C

ACTTTTCTTTGAGAATGCTAGTTCTACCACTAATTGCTTTCTAATTTCAATCTTTAGTTGAAATTTCAATTTTGATTTTAXXXXXXXXXXA
AAATGGAAGTAAATTCTAGCATGAAGATTAATTCAATCAACAACTAATTAGTTCAAGAAAATTTATCAACGTGTTTGTT
ACTTTTCTTTGAGAATGCTAGTTCTACCACTAATTGCTTTCTAATTTCAATCTTTAGTTGAAATTTCAATTTTGCTTTTAXXXXXXXXXXA
AAATGGAAGTAAATTCTAGCATGAAGATTAATTCAATCAACAACTAATTAGTTCAAGAAAATTTATCAACGTATTTGTT

>Marker438701 Chr1 SNP_site:28593848 Ref_Type:A

ACTTTTCTTTGAGAATGCTAGTTCTACCACTAATTGCTTTCTAATTTCAATCTTTAGTTGAAATTTCAATTTTGATTTTAXXXXXXXXXXA
AAATGGAAGTAAATTCTAGCATGAAGATTAATTCAATCAACAACTAATTAGTTCAAGAAAATTTATCAACGTGTTTGTT
ACTTTTCTTTGAGAATGCTAGTTCTACCACTAATTGCTTTCTAATTTCAATCTTTAGTTGAAATTTCAATTTTGCTTTTAXXXXXXXXXXA
AAATGGAAGTAAATTCTAGCATGAAGATTAATTCAATCAACAACTAATTAGTTCAAGAAAATTTATCAACGTATTTGTT

>Marker438950 Chr1 SNP_site:15466961 Ref_Type:G

CACCTTTGATTTTCAAGTAAAGTAATGAAAATCAATTAATTATAAATTTTAGTAGAAATTAGCAATGAAATTAAGAAGTGXXXXXXXXXXA
TTCAAAATCTACTAAAAGAATAAATTTTCCGAAGAGAAAAGTGAATAGCTAAGAAAATAAATAAATTGGTGATGTAGTC
CACCTTTGATTTTGAAGTAAAGTAATGAAAATCAATTAATTATAAATTTTAGTAGAAATTAGCAATGAAATTAAGAAGTGXXXXXXXXXXA
TTCAAAATCTACTAAAAGAATAAATTTTCCGAAGAGAAAAGTGAATAGCTAAGAAAATAAATAAATTGGTGATGTAGTC

>Marker439021 Chr1 SNP_site:8226356 Ref_Type:T

GACAACACTTTTCACCGATAGATATAGTCAAAAGTCATAAAATATTAGTGAAGGAAACATAAAACACTCTGAAGGAAAAAXXXXXXXXXXA
ACCAGTATCAGATAATGAATTGATCTCGCCTCCTGTTGATGGAATATGTTGTCTTGAATTTAATCCCTTGGAGGTTGTG
GACAACACTTTTCACCGATAGATATAGTCAAAAGTCATAAAATATTAGTGAAGGAAACATAAAACACTCTGAAGTAAAAAXXXXXXXXXXA
ACCAGTATCAGATAATGAATTGATCTCGCCTCCTGTTGATGGAATATGTTGTCTTGAATTTAATCCCTTGGAGGTTGTG

>Marker439130 Chr1 SNP_site:13146686 Ref_Type:C

ACCCTTTTAGTGATTAGTTTGAATTATATTTTGGCATAACCACTTTATTGGCGAAAATGAGATTTAATCACTTATTGTTGXXXXXXXXXXG
GTAGTTTATAAATGGTTAATATTCATGAATTAGTGTCTGATGAGCATTTACTACAAAGCTAAAGCTAAAGACTCAGGTG
ACCCTTTTAGTGATTAGTTTGAATTATATTTTGGCATAACCACTTTATTGGTGAAAATGAGATTTAATCACTTATTGTTGXXXXXXXXXXG
GTAGTTTATAAATGGTTAATATTCATGAATTAGTGTCTGATGAGCATTTACTACAAAGCTAAAGCTAAAGACTCAGGTG

>Marker439186 Chr1 SNP_site:17173482 Ref_Type:G

TACCAATTCTTCAATTTTTTCAAAGGTCATCTAAAGATCTATCAAAACTTTAAAATCTTGAAAGTCGTGTAAATGAGTGTXXXXXXXXXXG
TGATGATTTTTGTATAATTTAAATATATACACATATACATACATTATTTTAGTCTCCATATGCTAAGACTACTTATGTT
TACCAATTCTTCAATTTTTTCAAAGGTCATCTAAAGATCTATCAAAACTTTAAAATCTTGAAAGTCGTGTAAATGAGTGTXXXXXXXXXXG
TGATTATTTTTGTATCATTTAAATATATACACATATACATACATTATTTTAGTCTCCATATGCTAAGACTACTTATGTT

>Marker439186 Chr1 SNP_site:17173493 Ref_Type:A

TACCAATTCTTCAATTTTTTCAAAGGTCATCTAAAGATCTATCAAAACTTTAAAATCTTGAAAGTCGTGTAAATGAGTGTXXXXXXXXXXG
TGATGATTTTTGTATAATTTAAATATATACACATATACATACATTATTTTAGTCTCCATATGCTAAGACTACTTATGTT
TACCAATTCTTCAATTTTTTCAAAGGTCATCTAAAGATCTATCAAAACTTTAAAATCTTGAAAGTCGTGTAAATGAGTGTXXXXXXXXXXG
TGATTATTTTTGTATCATTTAAATATATACACATATACATACATTATTTTAGTCTCCATATGCTAAGACTACTTATGTT

>Marker439505 Chr1 SNP_site:18936072 Ref_Type:T

TACATCTGGGCCCCGCCTTCCACTAATTGATTATGGCTGCTTGGAAACTAAAAATGCCTCCACGAATCTTTAAAACCAATXXXXXXXXXXA
ATCATGTTTCTTCACGCTTGAATTTCGGAGGCATTTTGTAACTATTTTATGGACAGTATTTCGCATAGTTGGAATCGTG
TACATCTGGGCCCCGCCTTCCACTAATTGATTATGGCTGCTTGGAAACTAAAAATGCCTCCACGAATCTTTTAAACCAATXXXXXXXXXXA

Create PDF with GO2PDF for free, if you wish to

ATCATGTTTCTTCACGCTTGAATTTCGGAGGCATTTTGTAACTATTTTATGGGCAGTATTTCGCATAGTTGGAATCGTG
>Marker439505 Chr1 SNP_site:18936232 Ref_Type:G
TACATCTGGGCCCCGCCTTCCACTAATTGATTATGGCTGCTTGGAAACTAAAAATGCCTCCACGAATCTTTAAAACCAATXXXXXXXXXXA
ATCATGTTTCTTCACGCTTGAATTTCGGAGGCATTTTGTAACTATTTTATGGACAGTATTTCGCATAGTTGGAATCGTG
TACATCTGGGCCCCGCCTTCCACTAATTGATTATGGCTGCTTGGAAACTAAAAATGCCTCCACGAATCTTTTAAACCAATXXXXXXXXXXA
ATCATGTTTCTTCACGCTTGAATTTCGGAGGCATTTTGTAACTATTTTATGGGCAGTATTTCGCATAGTTGGAATCGTG
>Marker439635 Chr1 SNP_site:4120723 Ref_Type:T
ACCCTTTTAACTTCTCTTCTTCTGATCTTCTGAACTTCTTTTTCCCTTTAATCATTTGTTTCTTATACAGTTTTAGTCTAXXXXXXXXXXT
ACTGTTACATGCAAATGGGTTGCTTCATATTGGAATTCCATATACACTTCTCAAATATATATATTTTTATACAAAAGTA
ACCCTTTTAACTTCTCTTCTTCTGATCTTCTGAACTTCTTTTTCCCTTTAATCATTTGTTTCTTATACAGTTTTAGTCTAXXXXXXXXXXT
ACTGTTACATGCAAATGGGTTGCTTCATATTGGAATTCCATATACACTTCTCAAATATATATTTTTTTATACAAAAGTA
>Marker439885 Chr1 SNP_site:17914920 Ref_Type:A
ACATCAAAACGACGTCGGATTATATCCACAATACCAAAACAATTCAATGGTGATTCAAAGCGGTGGGATTGAGACGAAAAXXXXXXXXXXC
TGGGGATTTACAAGCCAGCCCGCAGTCGCCACCGCCACCGTCGATGGTGCCCACTTCGGCCTCTGATGAAATCTCTGTT
ACATCAAAACGACGTCGGATTATATCCACAATACCAAAACAATTCAATGGTGATTCAAAGCGGTGGGATTGTGAAGAAAAXXXXXXXXXXC
TGGGGATTTACAAGCCAGCCCGCAGTCGCCACCGCCACCGTCGATGGTGCCCACTTCGGCCTCTGATGAAATCTCTGTT
>Marker439885 Chr1 SNP_site:17914923 Ref_Type:C
ACATCAAAACGACGTCGGATTATATCCACAATACCAAAACAATTCAATGGTGATTCAAAGCGGTGGGATTGAGACGAAAAXXXXXXXXXXC
TGGGGATTTACAAGCCAGCCCGCAGTCGCCACCGCCACCGTCGATGGTGCCCACTTCGGCCTCTGATGAAATCTCTGTT
ACATCAAAACGACGTCGGATTATATCCACAATACCAAAACAATTCAATGGTGATTCAAAGCGGTGGGATTGTGAAGAAAAXXXXXXXXXXC
TGGGGATTTACAAGCCAGCCCGCAGTCGCCACCGCCACCGTCGATGGTGCCCACTTCGGCCTCTGATGAAATCTCTGTT
>Marker440466 Chr1 SNP_site:17021159 Ref_Type:A
TACTAAAGTGGGAATTTTCAGATTTTTTCTCATTCGCTTTAGATATAGAACTAGCTGCCTTGGAAAATCGCCTCACAGACXXXXXXXXXXA
GAAAGAAGAGAGTAAATAAAAGTCACGAACACCGCCAATTTTCGACCGGAAAATAAAGCCAAACTGAATAAACCCTTGT
TACTAAAGTGGGAATTTTCAGATTTTTTCTCATTCGCTTTAGATATAGAACTAGCTGCCTTGGAAAATCGCCTCACAGACXXXXXXXXXXA
GAAAGAAGAGAGTAAATAAAAGTCACGAACACCGCCAATTTTCGACCGGAAAATAAAGCCAATCTGAATAAACCCTTGT
>Marker441336 Chr1 SNP_site:22766613 Ref_Type:G
ACTCCGACACAATAGGTAAAACAAAAAAAAATGCAATCGAGTCTAAAATCATAGTCTTCATGCAATTGAGTCTAAAACCAXXXXXXXXXXA
AAATTAATTTCCTACTGGTTTCCTTGACACCCAAATGTTGTAGGATTAGATGGATTGATCCATGAAATTAGTCGATGTG
ACTCCGACACAATAGGTAAAACAAAAAAAAATGCAATCGAGTCTAAAATCATAGTCTTCATGCAATTGAGTCTAAAACCAXXXXXXXXXXA
AAATTAATTTCCTACTGGTTTCCTTGACACCCAAATGTTGTAGGATTAGATGGGTTGATCCATGAAATTAGTCGATGTG
>Marker44177 Chr4 SNP_site:4112499 Ref_Type:A
AACATTTAGCTATCATGTAACTAAAATATTAATTTTTTTACTTATTCTCCATAATCTTTTGATTAATTTTTTTAAAATTTTXXXXXXXXXXT
AGACTATAACTTGTCAATCTTTGGTAGAGCTTAAATCTGAAATTAATTTCCATATATTGAATTAATCGTGGTTATAAGT
AACATTTAGCTATCATGTAACTAAAATATTAATTTTTTTACTTATTCTCCATAATCTTTTGATTAATTTTTTTTAAATTTTXXXXXXXXXXT
AGACTATAACTTGTCAATATTTGGTAGAGCTTAAATCTGAAATTAATTTCCATATATTGAATTAATCGTGGTTATAAGT
>Marker44177 Chr4 SNP_site:4112666 Ref_Type:A
AACATTTAGCTATCATGTAACTAAAATATTAATTTTTTTACTTATTCTCCATAATCTTTTGATTAATTTTTTTAAAATTTTXXXXXXXXXXT
AGACTATAACTTGTCAATCTTTGGTAGAGCTTAAATCTGAAATTAATTTCCATATATTGAATTAATCGTGGTTATAAGT
AACATTTAGCTATCATGTAACTAAAATATTAATTTTTTTACTTATTCTCCATAATCTTTTGATTAATTTTTTTTAAATTTTXXXXXXXXXXT
AGACTATAACTTGTCAATATTTGGTAGAGCTTAAATCTGAAATTAATTTCCATATATTGAATTAATCGTGGTTATAAGT
>Marker44211 Chr4 SNP_site:4899119 Ref_Type:G
AACCAGATCCCCCTTTAGATTGATCATGCCTGTAATTGCAAAGTAGAATATGTTCCAAACCATACTGTCATCAGAGCATTXXXXXXXXXXA
GCCGACCCTGGTATTATGTCTAAATTTGATAATCGGGTAACAGCCCCCAACAATAATCAGGGTTTATCATCAAAGGGTT

Create PDF with GO2PDF for free, if you wish to

AACCAGATCCCCCTTTAGATTGATCATGCCTGTAATTGCAAAGTGGAATATGTTCCAAACCATACTGTCATCAGAGCATTXXXXXXXXXXA
GCCGACCCTGGTATTATGTCTAAATTTGATAATCGGGTAACAGCCCCCAACAATAATCAGGGTTTATCATCAAAGGGTT
>Marker443082 Chr1 SNP_site:13455468 Ref_Type:A
TACTCATTATTGGAAACTCCAAAGAATCTAGTTCCAATATTTCGTGGTCATTCATTACTACTGAAGATGATTTAAGCTCTXXXXXXXXXXC
TATCCCTCATGCACATGTTTGATATCATTTTGCACTCCGACCGGATAAATCACCATATGCTAGAAAGTCATTAATTGTC
TACTCATTATTGGAAACTCCAAAGAATCTAGTTCCAATATTTCGTGGTCATTCATTACTACTGAAGATGATTTAAGCTCTXXXXXXXXXXC
TATCCCTCATGCACATGTTTGATATCATTTTGCACTCCGACCGGATAAATCACCATATGCTGGAAAGTCATTAATTGTC
>Marker443322 Chr1 SNP_site:27127418 Ref_Type:T
CACAACTCACTCAGAATTAACGTCAAGTCTCCTATGGTCATCTTAGTGAAATATTTGTTCCTTCGAATAACAATGTTACAXXXXXXXXXXA
TTTACATCTCATGTAACAATTAAAGAGTGAGTTGCATCCACAATGTTATCAGAATAAGGCACTCGGCCTCTATTCAAGT
CACAACTCACTCAGAATTAATGTCAAGTCTCCTATGGTCATCTTAGTGAAATATTTGTTCCTTCGAATAACAATGTTACAXXXXXXXXXXA
TTTACATCTCATGTAACAATTAAAGAGTGAGTTGCATCCACAATGTTATCAGAATAAGGCACTCGACCTCTATTCAAGT
>Marker443322 Chr1 SNP_site:27127652 Ref_Type:A
CACAACTCACTCAGAATTAACGTCAAGTCTCCTATGGTCATCTTAGTGAAATATTTGTTCCTTCGAATAACAATGTTACAXXXXXXXXXXA
TTTACATCTCATGTAACAATTAAAGAGTGAGTTGCATCCACAATGTTATCAGAATAAGGCACTCGGCCTCTATTCAAGT
CACAACTCACTCAGAATTAATGTCAAGTCTCCTATGGTCATCTTAGTGAAATATTTGTTCCTTCGAATAACAATGTTACAXXXXXXXXXXA
TTTACATCTCATGTAACAATTAAAGAGTGAGTTGCATCCACAATGTTATCAGAATAAGGCACTCGACCTCTATTCAAGT
>Marker443736 Chr1 SNP_site:20469059 Ref_Type:C
TACCATAATAAGTGCATTCTAATTACAGCTACAAATATTAAAGAGGACAAATCAACATAAAGAAATCATGTTACCGTAACXXXXXXXXXXT
TTTATTACTACTTTTGGAGGGATATTATTCTTACAGGAATCGTTGAGGTTCGTATGATTTAGTAGATGAATAGGAGGTG
TACCATAATAAGTGCATTCTAATTACAGCTACAAATATTAAAGAGGACAAATCAACATAAAGAAATCATGTTACCGTAACXXXXXXXXXXT
TTTATTACTACTTTTGGAGGGATATTATTCTTACAGGAATCGTTGAGGTTCGTATGATTTCGTAGATGAATAGGAGGTG
>Marker44411 Chr4 SNP_site:18999950 Ref_Type:A
CACCGGATGGCCCTCCCAATCCTTGACTCTCACCTCTAGCAACCCCTTCACTTCCCTTACTGTGGTCATTGTCGTCCCTTXXXXXXXXXXT
GAAGTTCATATACACACAATTAGATAAGTAACTTATAGTTTAAAGATCATGCTTACCTCATATTCGGTTGTCTTATGTG
CACCGGATGGCCCTCCCAATCCTTGGCTCTCAGCTCTAGCAACCCCTTCACTTCCCTTACTGTGGTCATTGTCGTCCCTTXXXXXXXXXXT
GAAGTTCATATACACACAATTAGATAAGTAACTTATAGTTTAAAGATCATGCTTACCTCATATTCGGTTGTCTTATGTG
>Marker44411 Chr4 SNP_site:18999957 Ref_Type:C
CACCGGATGGCCCTCCCAATCCTTGACTCTCACCTCTAGCAACCCCTTCACTTCCCTTACTGTGGTCATTGTCGTCCCTTXXXXXXXXXXT
GAAGTTCATATACACACAATTAGATAAGTAACTTATAGTTTAAAGATCATGCTTACCTCATATTCGGTTGTCTTATGTG
CACCGGATGGCCCTCCCAATCCTTGGCTCTCAGCTCTAGCAACCCCTTCACTTCCCTTACTGTGGTCATTGTCGTCCCTTXXXXXXXXXXT
GAAGTTCATATACACACAATTAGATAAGTAACTTATAGTTTAAAGATCATGCTTACCTCATATTCGGTTGTCTTATGTG
>Marker445371 Chr1 SNP_site:12354687 Ref_Type:T
TACAAAATTACGAATCACGAGTTTAGGGCATAAACCCCGACAAGAGTGACACCAAATTCCTGATTTAATCATGTTAGACAXXXXXXXXXXT
CCTCCAGCTATATCGAAAGTAATTAACTGAGAGAATAGCTAAAGAACTAGAAAAAACAAATAACACATAAGGCTTTGTT
TACAAAATTACGAATCACGAGTTTAGGGCATAAACCCCGACAAGAGTGACACCAAATTCTCGATTTAATCATGTTAGACAXXXXXXXXXXT
CCTCCAGCTATCTCGAAAGTAATTAACTGAGAGAATAGCTAAAGAACTAGAAAAAACAAATAACACATAAGGCTTTGTT
>Marker445371 Chr1 SNP_site:12354688 Ref_Type:C
TACAAAATTACGAATCACGAGTTTAGGGCATAAACCCCGACAAGAGTGACACCAAATTCCTGATTTAATCATGTTAGACAXXXXXXXXXXT
CCTCCAGCTATATCGAAAGTAATTAACTGAGAGAATAGCTAAAGAACTAGAAAAAACAAATAACACATAAGGCTTTGTT
TACAAAATTACGAATCACGAGTTTAGGGCATAAACCCCGACAAGAGTGACACCAAATTCTCGATTTAATCATGTTAGACAXXXXXXXXXXT
CCTCCAGCTATCTCGAAAGTAATTAACTGAGAGAATAGCTAAAGAACTAGAAAAAACAAATAACACATAAGGCTTTGTT
>Marker445371 Chr1 SNP_site:12354836 Ref_Type:C
TACAAAATTACGAATCACGAGTTTAGGGCATAAACCCCGACAAGAGTGACACCAAATTCCTGATTTAATCATGTTAGACAXXXXXXXXXXT

Create PDF with GO2PDF for free, if you wish to

CCTCCAGCTATATCGAAAGTAATTAACTGAGAGAATAGCTAAAGAACTAGAAAAAACAAATAACACATAAGGCTTTGTT
TACAAAATTACGAATCACGAGTTTAGGGCATAAACCCCGACAAGAGTGACACCAAATTCTCGATTTAATCATGTTAGACAXXXXXXXXXXT
CCTCCAGCTATCTCGAAAGTAATTAACTGAGAGAATAGCTAAAGAACTAGAAAAAACAAATAACACATAAGGCTTTGTT
>Marker445616 Chr1 SNP_site:22472649 Ref_Type:G
AACTTTTTCCATTCAACTCTAAATTTCTCCATCTCTCTTCATTTATGCCCAAATGAATCTCTATTCAATCACAACATTTTXXXXXXXXXXC
CCTCTTAATAGGTGAGACTCAAAGGATAAATTTAAAATTACTACACACAAGGAAGATTTTGAGATAAATTTGTTAAAGT
AACTTTTTCCATTCAACTCTAAATTTCTCCATCTCTCTTCATTTATGCCCAAATGAATCTCTATTCAATCACAACATTTTXXXXXXXXXXC
CCTCTTAATAGGTGAGACTCAAATGATAAATTTAAAATTACAACACACAAGGAAGATTTTGAGATAAATTTGTTAAAGT
>Marker445616 Chr1 SNP_site:22472667 Ref_Type:T
AACTTTTTCCATTCAACTCTAAATTTCTCCATCTCTCTTCATTTATGCCCAAATGAATCTCTATTCAATCACAACATTTTXXXXXXXXXXC
CCTCTTAATAGGTGAGACTCAAAGGATAAATTTAAAATTACTACACACAAGGAAGATTTTGAGATAAATTTGTTAAAGT
AACTTTTTCCATTCAACTCTAAATTTCTCCATCTCTCTTCATTTATGCCCAAATGAATCTCTATTCAATCACAACATTTTXXXXXXXXXXC
CCTCTTAATAGGTGAGACTCAAATGATAAATTTAAAATTACAACACACAAGGAAGATTTTGAGATAAATTTGTTAAAGT
>Marker446762 Chr1 SNP_site:19368604 Ref_Type:C
AACAAAATCACCACAGCATAATTAGAATAAAAGCTTTATAGTCCAATTCAAAAACATATAGAAACAATTATACCTTAAGAXXXXXXXXXXT
TAAAATCTGCATCATACATTTATACATGACATTATATGATATCATCTCATATCATCCTGGAGATATCTAGAAATTAGTT
AACAAAATCACCACAGCATAATTAGAATAAAAGCTTTATAGTCCAATTCAAAAACATATAGAAACAATTATACCTTAAGAXXXXXXXXXXT
TAAAATCTGCATCATACATTTATACATGACATTATATGATATCATCTCATATCATCCTGGAGATATGTAGAAATTAGTT
>Marker447064 Chr1 SNP_site:7433428 Ref_Type:T
GACTAGGCTTCAGACCCTGATGATAGAAAATCCACTTTTGGCTTTTGTGTCTATCTTGGTGGTAACTTAATCTCGTAGGGXXXXXXXXXXT
CTTAGTATTATCTTACGTCACCCCCTATTTTATGGTGTGACAAATTAAGTGTAGTTCATCTAAGTGTTAATAATCCTGT
GACTAGGTTTCAGACCCTGATGATAGAAAATCCACTTTTGGCTTTTGTGTCTATCTTGGTGGTAACTTAATCTCGTAGGGXXXXXXXXXXT
CTTAGTATTATCTTACGTCACCCCCTATTTTATGGTGTGACAACTTAAGTGTAGTTCATCTAAGTGTTAATAATCATGT
>Marker447064 Chr1 SNP_site:7433652 Ref_Type:C
GACTAGGCTTCAGACCCTGATGATAGAAAATCCACTTTTGGCTTTTGTGTCTATCTTGGTGGTAACTTAATCTCGTAGGGXXXXXXXXXXT
CTTAGTATTATCTTACGTCACCCCCTATTTTATGGTGTGACAAATTAAGTGTAGTTCATCTAAGTGTTAATAATCCTGT
GACTAGGTTTCAGACCCTGATGATAGAAAATCCACTTTTGGCTTTTGTGTCTATCTTGGTGGTAACTTAATCTCGTAGGGXXXXXXXXXXT
CTTAGTATTATCTTACGTCACCCCCTATTTTATGGTGTGACAACTTAAGTGTAGTTCATCTAAGTGTTAATAATCATGT
>Marker447064 Chr1 SNP_site:7433684 Ref_Type:C
GACTAGGCTTCAGACCCTGATGATAGAAAATCCACTTTTGGCTTTTGTGTCTATCTTGGTGGTAACTTAATCTCGTAGGGXXXXXXXXXXT
CTTAGTATTATCTTACGTCACCCCCTATTTTATGGTGTGACAAATTAAGTGTAGTTCATCTAAGTGTTAATAATCCTGT
GACTAGGTTTCAGACCCTGATGATAGAAAATCCACTTTTGGCTTTTGTGTCTATCTTGGTGGTAACTTAATCTCGTAGGGXXXXXXXXXXT
CTTAGTATTATCTTACGTCACCCCCTATTTTATGGTGTGACAACTTAAGTGTAGTTCATCTAAGTGTTAATAATCATGT
>Marker447104 Chr1 SNP_site:11132041 Ref_Type:T
ACTCCTTTGGAAAAAAAGCCATCGGCCAAGCCCCAATAGAACTCTATAAAACCAGACTAGCTTAGAAATTACCTTCTGAAXXXXXXXXXXT
CCTGAGAATGGCACCGAAACCCGTAAATCATAATAAGGTTGGAGTAGAACAGCACAATCAATAAAGGGGACAGAAAGTT
ACTCCTTTGGATAAAAAGCCATCGGCCAAGCCCCAATAGAACTCTATAAAACCAGACTAGCTTAGAAATTACCTTCTGAAXXXXXXXXXXT
CCTGAGAATGGCACCGAAACCCGTAAATCATAATAAGGTTGGAGTAGAACAGCACAATCAATAAAGGGGACAGAAAGTT
>Marker447791 Chr1 SNP_site:2667823 Ref_Type:T
AACTTTCGGTGTCTAATAAATCTTTTTAGTTTTAGTATCACCAAAGAAAATGCTAGGCAAAATGATTAAAAGTATGCTTAXXXXXXXXXXT
TTTCCTTTCAGCAAGAACGTTTGTGTATATTTAAACAAACCCAAGATATGGTCTAGATCCTCAGGGTGGAAATGATAGT
AACTTTCTGTGTCTAATAAATCTTTTTAGTTTTAGTATCACCAAAGAAAATGCTAGGCAAAATGATTAAAAGTATGCTTAXXXXXXXXXXT
TTTCCTTTCAGCAAGAACGTTTGTGTATATTTAAACAAACCCAAGATATGGTCTAGATCCTCAGGGTGGAAATGATAGT
>Marker447888 Chr1 SNP_site:26040901 Ref_Type:A

Create PDF with GO2PDF for free, if you wish to

ACTCCAAGCTTTAAACTCGTTCATTACATCAATAAATAGTCTTGTTTCCGTTTAAAATAAATAAATAAAGGCTTGTAACTXXXXXXXXXXG
TTTCAACAAAAGGTGGAACATTTTGTGTTAGTTGTCACACTAAACAACTAGTCCAACTTTTTTGCTCAATTTCAAAGTC
ACTCCAAGCTTTAAACTCGTTCATTACATCAATAAATAGTCTTGTTTCCGTTTAAAATAAATAAATAAAGGCTTGTAACTXXXXXXXXXXG
TTTCAACAAAAGGTGGAACATTTTGTGTTAGTTGTCACACTAAACCACTAGTCCAACTTTTTTGCTCAATTTCAAAGTC
>Marker448346 Chr1 SNP_site:8681162 Ref_Type:T
CACACAACAAGGTCATCCAAATAGATTAGTGAATTCAAAGAACTCGCACTAGATCCTGGCTTCTCAACCTCCCTGACAATXXXXXXXXXXA
GTTAAAATCTTTTTTTAAAGAAATATTTTCCACAGTGACGAACGGCTCAAATTTCCTGTGAAACATTTTTTTCTTCGTT
CACACAACAAGGTCATCCAAATAGATTAGTGAATTTAAAGAACTCGCACTAGATCCTGGCTTCTCATCCTCCCTGACAATXXXXXXXXXXA
GTTAAAATCTTTTTTTAAAAAAATATTTTCCACAGTGACGAACGGCTCAAATTTCCTGTGAAACATTTTTTTCTTCGTT
>Marker448346 Chr1 SNP_site:8681193 Ref_Type:T
CACACAACAAGGTCATCCAAATAGATTAGTGAATTCAAAGAACTCGCACTAGATCCTGGCTTCTCAACCTCCCTGACAATXXXXXXXXXXA
GTTAAAATCTTTTTTTAAAGAAATATTTTCCACAGTGACGAACGGCTCAAATTTCCTGTGAAACATTTTTTTCTTCGTT
CACACAACAAGGTCATCCAAATAGATTAGTGAATTTAAAGAACTCGCACTAGATCCTGGCTTCTCATCCTCCCTGACAATXXXXXXXXXXA
GTTAAAATCTTTTTTTAAAAAAATATTTTCCACAGTGACGAACGGCTCAAATTTCCTGTGAAACATTTTTTTCTTCGTT
>Marker448346 Chr1 SNP_site:8681380 Ref_Type:A
CACACAACAAGGTCATCCAAATAGATTAGTGAATTCAAAGAACTCGCACTAGATCCTGGCTTCTCAACCTCCCTGACAATXXXXXXXXXXA
GTTAAAATCTTTTTTTAAAGAAATATTTTCCACAGTGACGAACGGCTCAAATTTCCTGTGAAACATTTTTTTCTTCGTT
CACACAACAAGGTCATCCAAATAGATTAGTGAATTTAAAGAACTCGCACTAGATCCTGGCTTCTCATCCTCCCTGACAATXXXXXXXXXXA
GTTAAAATCTTTTTTTAAAAAAATATTTTCCACAGTGACGAACGGCTCAAATTTCCTGTGAAACATTTTTTTCTTCGTT
>Marker448560 Chr1 SNP_site:3714885 Ref_Type:A
ACTTGTTCCTTTCTGACCAAGTTGTTGAACTTGAAGAAGAGGGGGCATGTCCATGATCATCATCAAAAGGATTGGAACTAXXXXXXXXXXT
GTTTTCCTAACCTAAAATGAACCTTCCTTGAACCAAACATTTTCCCGATAGCTAGAATGTCAAAATTGGCTTTAATGTA
ACTTGTTCCTTTCTGACCAAGTTGTTGAACTTGATGAAGAGGGGGCATGTCCATGATCATCATCAAAAGGATTGGAACTAXXXXXXXXXXT
GTTTTCCTAACCTAAAATGAACCTTCCTTGAACCAAACATTTTCCCGATAGCTAGAATGTCAAAATTGGCTTTAATGTA
>Marker449218 Chr1 SNP_site:3416249 Ref_Type:C
TACCTTTCTAATGGCATCCAGCTCATGCAACAAAACTTGGTAGAACTGTCCTTCGCTAACCCCATCCCTACAAGAAAAAGXXXXXXXXXXT
CCAGTTGCTCGACGGAAGGAGATGAGGAGTTCCCTGCATCACCGGGAACAAGGAAAGACAAAAGATAAGGACACAAGTT
TACCTTTCTAATGGCATCGAGCTCATGCAACAAAACTTGGTAGAACTGTCCTTCGCTAACCCCATCCCTACAAGAAAAAGXXXXXXXXXXT
CCAGTTGCTCGACGGAAGGAGATGAGGAGTTCCCTGCATCACCGGGAACAAGGAAAGACAAAAGATAAGGACACAAGTT
>Marker449445 Chr1 SNP_site:1110737 Ref_Type:A
ACTAAGCAGTGGATTAATGTTAGGTTTCAATGCAATTTTTATTAGTATAAACGTTATGTTATTCTTCACTTGTAATGGAAXXXXXXXXXXG
AGATGACTTGTTGAATGAATTAAAAAGGTATGATGGAAGATGAGAATTTTTGAATAAAAAACTATATGGCTTTTGAGTA
ACTAAGCAGTGGATTAATGTTAGGTTTCAATGCAATTTTTATTAGTATAAACGTTATGTTATTCTTCACTTGTAATGGAAXXXXXXXXXXG
AGATGACTTGTTGAATGAATTTAAAAGGTATGATGGAAGATGAGAATTTTTGAATAAAAAACTATATGGCTTTTGAGTA
>Marker450697 Chr1 SNP_site:869028 Ref_Type:A
AACCTCAAAGTAAATGTAGATTGACATAACACCTAAAAGTTGCAGAAAACGTTAGCGAATGAGGCTGAAAAAAGTGTTGAXXXXXXXXXXT
AAGTGGAAGAGGATGAGGTTGAAAAAAAAACATTGGGGGAAAATAGGTAAGAAAACCCAGAATCTGTAACAAAATTGTA
AACCTCAAAGTAAATGTAGATTGACATAACACCTAAAAGTTGCAGAAAACGTTAGCGAATGAGGCTGAAAAAAGTGTTGAXXXXXXXXXXT
AAGTGGAAGAGGATGGGGTTGAAAAAAAAACATTGGGGGAAAATAGGTAAGAAAACCCAGAATCTGTAACAAAATTGTA
>Marker45070 Chr4 SNP_site:10696212 Ref_Type:A
ACAAGAGAATAAAATTAAAATTGAAAAAAAAAAGAAAAAAAGTTAGTTGTGAAGTTATGAAAGAATATCCTTATCATTTAXXXXXXXXXXT
TTACGCCCTTCCTCTTCTCCCTTCTCATTCCTCCATTTCTGTTCTATACTGTGTTTGATCTTCCATGGCCGACCGTGTT
ACAAGAGAATAAAATTAAAATTGAAAAAAAAAAGAAAAAACGTTAGTTGTGAAGTTATGAAAGAATATCCTTATCATTTAXXXXXXXXXXT
TTACGCCCTTCCTCTTCTCCCTTCTCATTCCTCCATTTCTGTTCTATACTGTGTTTGATCTTCCATGGCCGACCGTGTT

Create PDF with GO2PDF for free, if you wish to

>Marker45074 Chr4 SNP_site:20759168 Ref_Type:A
ACTAATCGAAGCAGTATCTTTACTTTCAAAGTTAATAGAATAATATTAACTATATGATTTTTATATTAACTGCGTCAGCAXXXXXXXXXXC
CATTTATAGCTTCCCTTTTTACACGAATCCGATAAAAAAGTTAATTCATGATCTACCTCTTTAACATAAATTAGTAGTG
ACTAATCGAAGCAGTATCTTTACTTTCAAAGTTAATAGAATAATATTAACTATATGATTTTTATATTAACTGCGTCAGCAXXXXXXXXXXC
CATTTATAGCTTCCCTTTTTACACGAATCCGATAAAAAAGTTATTTCATGATCTATCTCTTTAACATAAATTAGTAGTG
>Marker45074 Chr4 SNP_site:20759180 Ref_Type:C
ACTAATCGAAGCAGTATCTTTACTTTCAAAGTTAATAGAATAATATTAACTATATGATTTTTATATTAACTGCGTCAGCAXXXXXXXXXXC
CATTTATAGCTTCCCTTTTTACACGAATCCGATAAAAAAGTTAATTCATGATCTACCTCTTTAACATAAATTAGTAGTG
ACTAATCGAAGCAGTATCTTTACTTTCAAAGTTAATAGAATAATATTAACTATATGATTTTTATATTAACTGCGTCAGCAXXXXXXXXXXC
CATTTATAGCTTCCCTTTTTACACGAATCCGATAAAAAAGTTATTTCATGATCTATCTCTTTAACATAAATTAGTAGTG
>Marker45093 Chr4 SNP_site:10845954 Ref_Type:T
ACAAAACGTGGCTGTGTATTTATGCTCTGTTTCGGCATCCAAATGAATGTGGGTTATAGTATTGGTTGATTATGAATGCAXXXXXXXXXXA
TCATCAAAAGAACTGTTGGAGGCTACTTCGTTGTTCAAGGTAGGGCTGATGACACCATGAACCTTGGCGGCATCAAGGT
ACAAAACTTGGCTGTGTATTTATGCTCTGTTTCGGCATCCAAATGAATGTGGGTTATAGTATTGGTTGATTATGAATGCAXXXXXXXXXXA
TCATCAAAAGAACTGTTGGAGGCTACTTCGTTGTTCAAGGTAGGGCTGATGACACCATGAACCTTGGCGGCATCAAGGT
>Marker45133 Chr4 SNP_site:1797651 Ref_Type:G
TACTTGCTTTCTAGGTGGCACCTATAAACCTTTTACACAACCATTTGGCTTATATGTTTTAGTGTTTTGAGATTTCACTTXXXXXXXXXXA
ACTAGGAAAAATCTTAAGCAATAAAATAAAAGTTAAATAATGCATTTGATTTCAAATTTTTGTTTAGTTCTTAAGAAGT
TACTTGCTTTCTAGGTGGCACCTATAAACCTTTTACACAACCATTTGGCTTATATGTTTTAGTGTTTTGAGATTTCACTTXXXXXXXXXXA
AGTAGGAAAAATCTTAAGCAATAAAATAAAAGTTAAATAATGCATTTGATTTCAAATTTTTGTTTAGTTTTTAAAAAGT
>Marker45133 Chr4 SNP_site:1797719 Ref_Type:T
TACTTGCTTTCTAGGTGGCACCTATAAACCTTTTACACAACCATTTGGCTTATATGTTTTAGTGTTTTGAGATTTCACTTXXXXXXXXXXA
ACTAGGAAAAATCTTAAGCAATAAAATAAAAGTTAAATAATGCATTTGATTTCAAATTTTTGTTTAGTTCTTAAGAAGT
TACTTGCTTTCTAGGTGGCACCTATAAACCTTTTACACAACCATTTGGCTTATATGTTTTAGTGTTTTGAGATTTCACTTXXXXXXXXXXA
AGTAGGAAAAATCTTAAGCAATAAAATAAAAGTTAAATAATGCATTTGATTTCAAATTTTTGTTTAGTTTTTAAAAAGT
>Marker45133 Chr4 SNP_site:1797724 Ref_Type:A
TACTTGCTTTCTAGGTGGCACCTATAAACCTTTTACACAACCATTTGGCTTATATGTTTTAGTGTTTTGAGATTTCACTTXXXXXXXXXXA
ACTAGGAAAAATCTTAAGCAATAAAATAAAAGTTAAATAATGCATTTGATTTCAAATTTTTGTTTAGTTCTTAAGAAGT
TACTTGCTTTCTAGGTGGCACCTATAAACCTTTTACACAACCATTTGGCTTATATGTTTTAGTGTTTTGAGATTTCACTTXXXXXXXXXXA
AGTAGGAAAAATCTTAAGCAATAAAATAAAAGTTAAATAATGCATTTGATTTCAAATTTTTGTTTAGTTTTTAAAAAGT
>Marker451353 Chr1 SNP_site:6953397 Ref_Type:C
ACCTTCGTTATGATCTCAAGACAATCATTTCTGCTAAGCCTAAGGAAGAGAAGCAGGCCCTTAAGGACCTCACCGGAAAAXXXXXXXXXXG
GGAAATTTTTGCAGTTGGATTATGCAGCAAAGATTAAGAGCAGCTCAGAAGCAGAGAAGTATTATGCTCAGACTGTGTC
ACCTTCGTTATGATCTCAAGACAATCATTTCTGCTAAGCCTAAGGAAGAGAAGCAGGCGCTTAAGGACCTCACCGGAAAAXXXXXXXXXXG
GGAAATTTTTGCAGTTGGATTATGCAGCAAAGATTAAGAGCAGCTCAGAAGCAGAGAAGTATTATGCTCAGACTGTGTC
>Marker451366 Chr1 SNP_site:23247068 Ref_Type:A
AACATAGTCTATATAGGAGACCACCAATTTATAAGCAGACTCAAGGGATTTTTCTTTTGGCCTAGTGATAATTATATGAAXXXXXXXXXXA
GCCATAGATTGGGAGCATGCTAATATTGAGAATGCATGTAGGCAAAAACATCACAAACTATGTATTCCATGGTTTTGTG
AACATAGTCTATATAGGAGACCACCAATTTATAAGCTGACTCAAGGGATTTTTCTTTTGGCCTAGTGATAATTATATGAAXXXXXXXXXXA
GCCATAGATTGGGAGCATGCTAATATTGAGAATGCATGTAGGCAAAAACATCACAAACTATGTATTCCATGGTTTTGTG
>Marker45220 Chr4 SNP_site:7176402 Ref_Type:C
GACTTATCCCATAGTCGATGTTGAAAGTGTTGAGTATTCCAAGCCTGAAATGGATTGGACTGATGCTAAAGATGAAACTGXXXXXXXXXXA
GTTAAGATGTCTCGCCTTTATATCATAACCTCTCAGTTTGAGGCTCTTAAAATGTTGGAAGGTGAATCCATAGTTGAGT
GACTTATCCCATAGTCGATGTTGAAAGTGTTGAGTATTCCAAGCCTGAAATGGATTGGACTGATGCTAAAGATGAAACTGXXXXXXXXXXA

Create PDF with GO2PDF for free, if you wish to

GTTAAGATGTCTCGTCTTTATATCATAACCTCTCAGTTTGAGGCTCTTAAGATGTTGGAAGGTGAATCCATAGTTGAGT

>Marker45220 Chr4 SNP_site:7176438 Ref_Type:A

GACTTATCCCATAGTCGATGTTGAAAGTGTTGAGTATTCCAAGCCTGAAATGGATTGGACTGATGCTAAAGATGAAACTGXXXXXXXXXXXA

GTTAAGATGTCTCGCCTTTATATCATAACCTCTCAGTTTGAGGCTCTTAAAATGTTGGAAGGTGAATCCATAGTTGAGT

GACTTATCCCATAGTCGATGTTGAAAGTGTTGAGTATTCCAAGCCTGAAATGGATTGGACTGATGCTAAAGATGAAACTGXXXXXXXXXXXA

GTTAAGATGTCTCGTCTTTATATCATAACCTCTCAGTTTGAGGCTCTTAAGATGTTGGAAGGTGAATCCATAGTTGAGT

>Marker452204 Chr1 SNP_site:14708389 Ref_Type:A

TACAACAAGGAAAGTAATACCATTATCATATGTCCTTTTCTCTTACCAAACCATGCACGAGGACTTTTGTTTTAGTCCATXXXXXXXXXXXG

AAAAATATTTTTAATGTTGAGTTAATGCAATGACTAATTGTGAATAGCAACCATAGAAATAAATAGTTGAATGGAAGTT

TACAACAAGGAAAGTAATACCATTATCATATGTCCTTTTCTCTTACCAAACCATGCACGAGGACTTTTGTTTTAGTCCATXXXXXXXXXXXG

AAAAATATTTTTAATGTTGAGTTAATGCAATGACTAATTGTGAGTAGCAACCATAGAAATAAATAGTTGAATGGAAGTT

>Marker452248 Chr1 SNP_site:4550077 Ref_Type:T

TACACTTGCACGTCTAATCATATTCCACATTCACTAATTTAAATCAACAAACTTAACATCCTCTAAACTAAAGGTCTCAAXXXXXXXXXXXT

TAATTGTATGATATGATGATTGAAAACATCAACTATGGTAACTATAGCACTAAATATGGAGTGTGATATAATAATAGTC

TACACTTGCACGTCTAATCATATTCCACATTCACTAATTTAAATCAACAAACTTAACATCCTCTAAACTAAAGGTCTCAAXXXXXXXXXXXT

TAATTGTATGTTATGATGATTGAAAACATCATCTATGGTAACTATAGCACTAAATATGGAGTGTGATATAATAATAGTC

>Marker452248 Chr1 SNP_site:4550098 Ref_Type:T

TACACTTGCACGTCTAATCATATTCCACATTCACTAATTTAAATCAACAAACTTAACATCCTCTAAACTAAAGGTCTCAAXXXXXXXXXXXT

TAATTGTATGATATGATGATTGAAAACATCAACTATGGTAACTATAGCACTAAATATGGAGTGTGATATAATAATAGTC

TACACTTGCACGTCTAATCATATTCCACATTCACTAATTTAAATCAACAAACTTAACATCCTCTAAACTAAAGGTCTCAAXXXXXXXXXXXT

TAATTGTATGTTATGATGATTGAAAACATCATCTATGGTAACTATAGCACTAAATATGGAGTGTGATATAATAATAGTC

>Marker452468 Chr1 SNP_site:3584139 Ref_Type:T

AACATGCATGCATCCAATTAACATCATGAGTCAAAAGTCATAATGTCGTTGTGTGCTTATATTGACTCCGAAACTTTTATXXXXXXXXXXXA

AAAATAATAGTGAATAAGAATACTGGCATGTGCATTAAATGAACACAAAAACATATATGTTATAGGGTATTGTGATTGT

AACATGCATGCATCCAATTAACATCATGAGTCAAAAGTCATAATGTTGTTGTGTGCTTATATTGACTCCAAAACTTTTATXXXXXXXXXXXA

AAAATAATAGTGAATAAGAATAGTGGCATGTGCATTAAATGAACACAAAAACATATATGTTATAGGGTATTGTGATTGT

>Marker452468 Chr1 SNP_site:3584162 Ref_Type:A

AACATGCATGCATCCAATTAACATCATGAGTCAAAAGTCATAATGTCGTTGTGTGCTTATATTGACTCCGAAACTTTTATXXXXXXXXXXXA

AAAATAATAGTGAATAAGAATACTGGCATGTGCATTAAATGAACACAAAAACATATATGTTATAGGGTATTGTGATTGT

AACATGCATGCATCCAATTAACATCATGAGTCAAAAGTCATAATGTTGTTGTGTGCTTATATTGACTCCAAAACTTTTATXXXXXXXXXXXA

AAAATAATAGTGAATAAGAATAGTGGCATGTGCATTAAATGAACACAAAAACATATATGTTATAGGGTATTGTGATTGT

>Marker452468 Chr1 SNP_site:3584294 Ref_Type:G

AACATGCATGCATCCAATTAACATCATGAGTCAAAAGTCATAATGTCGTTGTGTGCTTATATTGACTCCGAAACTTTTATXXXXXXXXXXXA

AAAATAATAGTGAATAAGAATACTGGCATGTGCATTAAATGAACACAAAAACATATATGTTATAGGGTATTGTGATTGT

AACATGCATGCATCCAATTAACATCATGAGTCAAAAGTCATAATGTTGTTGTGTGCTTATATTGACTCCAAAACTTTTATXXXXXXXXXXXA

AAAATAATAGTGAATAAGAATAGTGGCATGTGCATTAAATGAACACAAAAACATATATGTTATAGGGTATTGTGATTGT

>Marker452637 Chr1 SNP_site:7295419 Ref_Type:A

AACCAAACTGCGGAAAGAGCGCAGTAGAAAACATACCGACCAAAAGGGGTTTCTAATTGTCAAACAAAAACGGAGAGTGAXXXXXXXXXXXC

AGGTGGCAAATTGTTTCCCAGTGCCGCCGTAACTGACCGACCGTCCATGCTTCCATTGCCTGACACGTGTCGCCTAGTG

AACCAAACTGCGGAAAGAGCGCAGTAGAAAACATACCGACCAAAAGGGGTTTCTAATTGTCAAACAAAAACGGAGAGTGAXXXXXXXXXXXC

AGGTGGCAAGTTGTTTCCCAGTGCCGCCGTAACTGACCGACCGTCCATGCTTCCATTGACTGACACGTGTCGCCTAGTG

>Marker452637 Chr1 SNP_site:7295468 Ref_Type:C

AACCAAACTGCGGAAAGAGCGCAGTAGAAAACATACCGACCAAAAGGGGTTTCTAATTGTCAAACAAAAACGGAGAGTGAXXXXXXXXXXXC

AGGTGGCAAATTGTTTCCCAGTGCCGCCGTAACTGACCGACCGTCCATGCTTCCATTGCCTGACACGTGTCGCCTAGTG

Create PDF with GO2PDF for free, if you wish to

AACCAAACTGCGGAAAGAGCGCAGTAGAAAACATACCGACCAAAAGGGGTTTCTAATTGTCAAACAAAAACGGAGAGTGAXXXXXXXXXXC
AGGTGGCAAGTTGTTTCCCAGTGCCGCCGTAACTGACCGACCGTCCATGCTTCCATTGACTGACACGTGTCGCCTAGTG
>Marker452900 Chr1 SNP_site:11948096 Ref_Type:C
ACTCTGTATCATAACAGTCTCGTGATCTCGTAGAATGCACATAATCAGTCGAGTGCTCCGAAGGATGCACATATCAATCTXXXXXXXXXXA
CTCAAAATACAATCCAAGCTACCATCATTATATCATCATTTCAGTCCCACACATAAACAGTCAAATTACCCATACTAGT
ACTCTGTATCATAACAGTCTTGTGATCTCGTAGAATGCACATAATCAGTCGAGTGCTCCGAAGGATGCACATATCAATCTXXXXXXXXXXA
CTCAAAATACAATCCAAGCTACCATCATTATATCATCATTTCAGTCCCACACATAAACAGTCAAATTACCCATACTAGT
>Marker452938 Chr1 SNP_site:7311807 Ref_Type:T
ACTCTAATGACTAATTAATCCATACTCTCCATAGAAAGAGTATCCATACTCTTTAATTTATTTTAGTATTAATAGATTTTXXXXXXXXXXG
CTTATCAACTTCTTCATTAACCATCTAAACTATATATGGTTAAGTTGTCAAAAATATATGTATGATTAAATTTTAAGTA
ACTCTAATGACTAATTAATCCATACTCTCCATAGAAAGAGTATCCATACTCTTTAATTTATTTTAGTATTAATAGATTTTXXXXXXXXXXG
CTTATCAACTTCTTCATTAACTATCTAAACTATATATGGTTAAGTTGTCAAAAATATATGTATGATTAAATTTTAAGTA
>Marker45298 Chr4 SNP_site:18028406 Ref_Type:C
AACTAGTTTGTTACAAATCAGATTATTGTTGAATAAAATTTGCATGTATTTATTGGTTATGATGGAATTAAATATTCTTGXXXXXXXXXXA
TACTTGGATTACTCTCATTGCCTATTAGTTTTTAGATGAACCCTTGTTTATCTAATGATTCTACCATGATATCAAAGTA
AACTAGTTTGTTACAAATCAGATTATTGTTGAATAAAATTTGCATGTATTTATTGGTTATGATGGAATTAAATATTCTTGXXXXXXXXXXA
TACTTGGATTACTTTCATTGCCTATTAGTTTTTAGATGAACCCTTGTTTATCTAATGATTCTACCATGATATCAAAGTA
>Marker453414 Chr1 SNP_site:24657944 Ref_Type:C
TACTGCTACGGACATGTGTTGGGAGCGATTTTGAAATGGTTAAAACCAATTTTTCGTATTTAAAATCACTCTGAAACACAXXXXXXXXXXG
ATTTTAATCATTTCAGAATCACTCTCAAACATGTCATGAAATAAAAGATAATACAAATACAAGTAGTCGTAGAGTTGTG
TACTGCTACGGACATGTGTTGGGAGTGATTTTGAAATGGTTAAAACCAATTTTTCGTATTTAAAATCACTCTGAAACACAXXXXXXXXXXG
ATTTTAATCATTTCAGAATCACTCTCAAACATGTCATGAAATAAAAGATAATACAAATACAAGTAGTTGTAGAGTTGTG
>Marker453414 Chr1 SNP_site:24658192 Ref_Type:C
TACTGCTACGGACATGTGTTGGGAGCGATTTTGAAATGGTTAAAACCAATTTTTCGTATTTAAAATCACTCTGAAACACAXXXXXXXXXXG
ATTTTAATCATTTCAGAATCACTCTCAAACATGTCATGAAATAAAAGATAATACAAATACAAGTAGTCGTAGAGTTGTG
TACTGCTACGGACATGTGTTGGGAGTGATTTTGAAATGGTTAAAACCAATTTTTCGTATTTAAAATCACTCTGAAACACAXXXXXXXXXXG
ATTTTAATCATTTCAGAATCACTCTCAAACATGTCATGAAATAAAAGATAATACAAATACAAGTAGTTGTAGAGTTGTG
>Marker453692 Chr1 SNP_site:8693681 Ref_Type:A
AACCAAGTGAGGTTGAGAAGATTTTGGCTGAGCAATGGAAATTAGAGTCTAAAAGAGATTCTAGAGATGCAATTACTTGGXXXXXXXXXXT
TTTCTGTTCGTAGTATTTAAATATTAGCCTTGTGTTTATTGTAAAAGTAGCAATAAAGACATATGTTGTCAGTCTTTGT
AACCAAGTGAGGTTGAGAAGATTTTGGCTGAGCAATGGAAATTAGAGTCTAAAAGAGATTCTAGAGATGCAATTACTTGGXXXXXXXXXXT
TTTCTGTTCGTAGTATTTAAATATTAGCCTTGTGTTTATTGTAAGAGTAGCAATAAAGACATATGTTGTCAGTCTTTGT
>Marker453787 Chr1 SNP_site:22621929 Ref_Type:C
TACAACCACAGCAGCGAGGCATCTCTAGGTAGATAGAGCACTCGGTGCAAGAGCAGCAGCATAGCCATGACAAATTAAAGXXXXXXXXXXT
TCACCCTGCAACTGCTTCAAGAAAAGACGCATGAGCTTGGATCATAATCAATGCAGTTTTAAATTTATGCAGATTAGTT
TACAACCACAGCAGCGAGGCATCTCTAGGTAGATAGCGCACTCGGTGCAAGAGCAGCAGCATAGCCATGACAAATTAAAGXXXXXXXXXXT
TCACCCTGCAACTGCTTCAAGAAAAGACGCATGAGCTTGGATCATAATCAATGCAGTTTTAAATTTATGCAGATTAGTT
>Marker454089 Chr1 SNP_site:11976807 Ref_Type:G
CACCGGCTAAGATGAAGTTGAATCAAAATTCAGGTATGAACAAGACATCTTTCAAAATCAGACTAGATAGTAAGTGAATAXXXXXXXXXXA
AGCATATGTGGGTTGTGGCACCAAAATCTATAATCTAGCAGCTCATTTCAAAAATGGACAATGGCAAAAAATGACAAGT
CACCGGCTAAGATTAAGTTGAATCAAAATTCAGGTATGAACAAGACATCTTTCAAAATCAGACTAGATAGTAAGTGAATAXXXXXXXXXXA
AGCATATGTGGGTTGTGGCACCAAAATCTATAATCTAGCAGCTCATTTCAAAAATGGACAATGGCAAAAAATGACAAGT
>Marker454349 Chr1 SNP_site:25712986 Ref_Type:G
CACCCCTAACCTCAAATGGACAGTCAAACCTAACAAAGCTTTGCTCAAGGTTGCCCTTTTCTTAAATTAAACATGTAGCGXXXXXXXXXXA

Create PDF with GO2PDF for free, if you wish to

AAACAAGTTGGGTAAGTCAATCCAAAATTGGGTTGATAAAAACTCAAAAGAAGACAGATGTCTCAAGGCAAAGCTAAGT
CACCCCTAACCTCAAATGGACAGTCGAACCTAACAAAGCTTTGCTCAAGGTTGCCCTTTTCTTAAATTAAACATGTAGCGXXXXXXXXXXA
AAACAAGTTGGGTAAGTCAATCCAAAATTGGGTTGATAAAAACTCAAAAGAAGACAGATGTCTCAAGGCAAAGCTAAGT
>Marker454484 Chr1 SNP_site:9766504 Ref_Type:C
ACTTAACACTTCTTGCGAATAACCTTATCGCATCGTTATATTTTTACTACAAGAAACCTTTCTCATAAACTAACCAACTTXXXXXXXXXXT
TGTCTAACACTTAGTCAAAACTTCACTTCTTAATTTAGACGAAGACTTTTTCTCTTCTTATCGGATAACTTATTTGGTG
ACTTAACACTTGTTGCGAATAACCTTATCGCATCGTTATATTTTTACTACAAGAAACCTTTCTCATAAACTAACCAACTTXXXXXXXXXXT
TGTCTAACACTTAGTCAAAACTTCACTTCTTAATTTAGACGAAGACTTTTTCTCTTCTTATCGGATAACTTATTTGGTG
>Marker454627 Chr1 SNP_site:8512534 Ref_Type:T
GACAACGACTCAATCGGATGTCGAATTATTTTCATCATCCGACAATAAGAAGATGAAAAACTGTGATGAAGAACAAGATCXXXXXXXXXXT
TTATTTTTAAGCAACAAGGTTCTTGGAAAGGTCAAATACCATAAAAAATTTATTGTGATGGTAATATATGCAATGTGTA
GACAACGACTCAATCGGATGTCGAATTATTTTCATCATCCGACAATAAGAAGATGAAAAACTGTGATGAAGAACAAGATCXXXXXXXXXXT
TTATTTTTAAGCAACAAGGTTCTTGGAAATGTCAAATACCATACAAATTTTATTGTGATGGTAATATATGCAATGTGTA
>Marker454627 Chr1 SNP_site:8512548 Ref_Type:C
GACAACGACTCAATCGGATGTCGAATTATTTTCATCATCCGACAATAAGAAGATGAAAAACTGTGATGAAGAACAAGATCXXXXXXXXXXT
TTATTTTTAAGCAACAAGGTTCTTGGAAAGGTCAAATACCATAAAAAATTTATTGTGATGGTAATATATGCAATGTGTA
GACAACGACTCAATCGGATGTCGAATTATTTTCATCATCCGACAATAAGAAGATGAAAAACTGTGATGAAGAACAAGATCXXXXXXXXXXT
TTATTTTTAAGCAACAAGGTTCTTGGAAATGTCAAATACCATACAAATTTTATTGTGATGGTAATATATGCAATGTGTA
>Marker454627 Chr1 SNP_site:8512552 Ref_Type:T
GACAACGACTCAATCGGATGTCGAATTATTTTCATCATCCGACAATAAGAAGATGAAAAACTGTGATGAAGAACAAGATCXXXXXXXXXXT
TTATTTTTAAGCAACAAGGTTCTTGGAAAGGTCAAATACCATAAAAAATTTATTGTGATGGTAATATATGCAATGTGTA
GACAACGACTCAATCGGATGTCGAATTATTTTCATCATCCGACAATAAGAAGATGAAAAACTGTGATGAAGAACAAGATCXXXXXXXXXXT
TTATTTTTAAGCAACAAGGTTCTTGGAAATGTCAAATACCATACAAATTTTATTGTGATGGTAATATATGCAATGTGTA
>Marker45484 Chr4 SNP_site:20311306 Ref_Type:G
AACACAAGAACAGAGGGATGCAGGACAAAAACTACAATAAACGGGATTTCAAGCTCAAGAACATCCCATAAATGAACCACXXXXXXXXXXG
TAGAGAAGGAAATGGAGAGGCTCCCAGTAATTGAAAACTTCGTCGCAATCGTGAATGATGTTGGATGTGGCACTCATGT
AACACAAGAACAGGGGGATGCAGGACAAAAACTACAATAAACGGGATTTCAAGCTCAAGAACATCCCATAAATGAACCACXXXXXXXXXXG
TAGAGAAGGAAATGGAGAGGCTCCCAGTAATTGAAAACTTCGTCGCAATCGTGAATGATGTTGGATGTGGCACTCATGT
>Marker454949 Chr1 SNP_site:26141920 Ref_Type:A
CACCTTTCTAGATACAAATGTTGGGAGTTACCTCGCTAAGTTGAAACTTGTCTCCAAATTTCTTCTTGTGCGCGCTAGCCXXXXXXXXXXA
TGAGATGTCAAGAAATGGTAGATTGAAAAGGCATAAATATGCAGTCTATGTATAAAGAATATAGGTCTTTGGCCACTGT
CACCTTTCTAGATACAAATGTTGGGAGTTACCTCGCTAAGTTGAAACTTGTCTCCAAATTTCTTCTTGTGCGCGCTAGCCXXXXXXXXXXA
TGAGATGTCAAGAAATGGTAGATTGAAAAGGCATAAATATGCGGTCTATGTATAAAGAATATAGGTCTTTGGCCACTGT
>Marker455594 Chr1 SNP_site:17483766 Ref_Type:T
AACAAAGCAATTAAGTAAGGTAATGAAATGTTTGAATAAATATCCTAGTAATGAAAACTCCCCACATATCAGAGACATCAXXXXXXXXXXC
TTGAGATATAGAGCATGAGAAGGGTTTTATCATTACAAAATTAAATGTCCAAACTTCATGAGAAGACAAAGTAAAGGGT
AACAAAGCAATTAAGTAAGGTTATGAAATGTTTGAATAAATATCCTAGTAATGAAAACTCCCCACATATCAGAGACATCAXXXXXXXXXXC
TTGAGATATAGAGCATTAGAAGGGTTTTATCATTACAAAATTAAATGTCCAAACTTCATGAGAAGACAAAGTAAAGGGT
>Marker455594 Chr1 SNP_site:17483936 Ref_Type:T
AACAAAGCAATTAAGTAAGGTAATGAAATGTTTGAATAAATATCCTAGTAATGAAAACTCCCCACATATCAGAGACATCAXXXXXXXXXXC
TTGAGATATAGAGCATGAGAAGGGTTTTATCATTACAAAATTAAATGTCCAAACTTCATGAGAAGACAAAGTAAAGGGT
AACAAAGCAATTAAGTAAGGTTATGAAATGTTTGAATAAATATCCTAGTAATGAAAACTCCCCACATATCAGAGACATCAXXXXXXXXXXC
TTGAGATATAGAGCATTAGAAGGGTTTTATCATTACAAAATTAAATGTCCAAACTTCATGAGAAGACAAAGTAAAGGGT
>Marker456289 Chr1 SNP_site:7822494 Ref_Type:C

Create PDF with GO2PDF for free, if you wish to

ACATGAATTGCTAACCGGTTGTGACGGCCTATTAAAGAAAATAAATAATCCATATTCAAAAAATCAATTCCCAAAGTCTCXXXXXXXXXXA
AATATTTTCAGATTTTTCATGTTCCATCAGGTAGAAATCCTCATATAGTTTATATATGTTTTAATTCTTCTCTATTGTT
ACATGAATTGCTAACCGGTTGTGACGGCCTATTAAAGAAAATAAATAATCCATATTCAAAAAATCAATTCCCAAAGTCTCXXXXXXXXXXA
AATATTTTCAGATTTTTCATGTTCCCTCAGGTAGAAATCCTCATATAGTTTATATATGTTTTAATTCTTCTCTATTGTT
>Marker456658 Chr1 SNP_site:21314112 Ref_Type:C
AACAAGGTAGAAATTGTGAGGGTAAAAATGCTAGAACTATCAAGCATCAAAGTTTTAATTCAGTTATATGTAAGACACCCXXXXXXXXXXC
CTCTCGTAACTTTTCTTATGAAATTATAGTATCTTTTCATACATTTCCATACTTGAGTGTTCTTGAGTTTTAGGAGGTA
AACAAGGTAGAAATTGTGAGGGTAAAAATGCTAGAACTATCAAGCATCAAAGTTTTAATTCAGTTATATGTAAGACACTCXXXXXXXXXXC
CTCTCGTAACTTTTCTTATGAAATTATAGTATCTTTTCATACATTTCCATACTTGAGTGTTCTTGAGTTTTAGGAGGTA
>Marker457128 Chr1 SNP_site:20469566 Ref_Type:T
TACTGGTGTGTTTTCATTTTTGTTGAGTTCTTTTATATCCGGGATAATTGCTTTGATCTTACTTCAATGATCTATATTTTXXXXXXXXXXC
CCACCACGAATACGAACTTCTGGATCTTTGGTGGAGGCAAACGCAATTATGCTCCCTACTGAAAGCGTGAAGCAAGAGT
TACTTGTGTGTTTTCATTTTTGTTGAGTTCTTTTATATCCGGGATAATTGCTTTGATCTTACTTCAATGATCTATATTTTXXXXXXXXXXC
CCACCACGAATACGAACTTCTGGATCTTTGGTGGAGGCAAACGCAATTATGCTCCCTACTGAAAGCGTGAAGCAAGAGT
>Marker457517 Chr1 SNP_site:24209312 Ref_Type:T
CACAAGGTAAAACTGTGTTTTTCCAGACAAATTTTGAGAATTCCAAGGTTGCTGACCAAAAGGTCTCACAATGGGATGAAXXXXXXXXXXC
TACTATTACACAGTATATATACTATTTTGATCAAATTTAGGAAGAGGATGTGTAATATGATGCTCATTTGGTTTGAAGT
CACAAGGTAAAACTGTGTTTTTCCAGACAAATTTTGAGAATTCCAAGGTTGCTGACCAAAAGGTCTCACAATGGGATGAAXXXXXXXXXXC
TACTATTACACAGTATATATACTATTTTGATCAAATTTAGGAAGAGGATGTGTAATATGATGTTCATTTGGTTTGAAGT
>Marker457608 Chr1 SNP_site:21261580 Ref_Type:T
CACAGCAATCCAAGGTCTTGGAGTGCTAAAGAACCCAATTTGGTTGCAATATTTGCTGCAAGGCTGAACAGAATTGATTCXXXXXXXXXXA
AGTGATAAGATTTCGTGTGGTATCATCGTTGCAAGCTGTCACTATAATGCCATAAATTTCCAACCAACCCATTTTCGTT
CACAGCAATCCAAGGTCTTGGAGTGCTAAAGAACCCAATTTGGTTGCAATATTTGCTGCAAGGCTGAACAGAATTGATTCXXXXXXXXXXA
AGTGATAAGATTTCGTGTGGTATCATCGTTGCAAGCTGTCACTATAATGCCATAAATTTCCAACCAACCCATTTTTGTT
>Marker457847 Chr1 SNP_site:6146235 Ref_Type:A
ACCATTGCCATTTAATAAGTTAGCCCTGTGATTCCAAACATAAGCATGACCCTACACCAAGATATGAACTTAAGGTCTAAXXXXXXXXXXG
AAAGAATAGAATATTCATTGGTAACAGAAAACCGACTTTTTTAAAAAAAGAAGCTTCTTTATTAAATAGAACTCGAAGT
ACCATTGCCATTTAATAAGTTAGCCCTGTGATTCCAAACATAAGCATGACCCTACACCAAGATATGAACTTAAGGTCTAAXXXXXXXXXXG
AAAGAATAGAATATTCATTGGTAACAGAAAACCGACTTTTTTTAAAAAAGAAGCTTCTTTATTAAATAGAACTCGAAGT
>Marker458117 Chr1 SNP_site:25923620 Ref_Type:A
GACTAGGAAAGCGTGTCAAAACTAGGGCATGCCATCAAAAAGTAGGTGATAGATGTCAATCTTGAGTCATGCAACCTTTCXXXXXXXXXXT
ATTGAGGAGCAAAAAAAGGTTTGAGTTTGAGTTGAGAAAAGGGAGAAAGGTTAGTTTTCAGGGCAATATGACCAAGGTG
GACTAGGAAAGCGTGTCAAGACTAGGGCATGCCATCAAAAAGTAGGTGATAGATGTCAATCTTGAGTCATGCAACCTTTCXXXXXXXXXXT
ATTGAGGAGCAAAAAAAGGTTTGAGTTTGAGTTGAGAAAAGGGAGAAAGGTTAGTTTTCAGGGCAATATGACCAAGGTG
>Marker458277 Chr1 SNP_site:227568 Ref_Type:G
ACCTTTGCGGGTAATAATGGTATTCATTCTTCTTCACTTCCACCATTTTCGGGCCTTCTTCTGCTCCTGCTTGGCCGGCGXXXXXXXXXXA
CGACTTCTGATTCATTATTCTCCGGCGAAACTTCCGGCTCTTGCTTCACAATCGCGGCGTGTTTTCCTGTCCGGCGGTG
ACCTTTGCGGGTAATAATGGTATTCATTCTTCTTCACTTCCACCATTTTCGGGCCTTCTTCTGCTGCTGCTTGGCCGGCGXXXXXXXXXXA
CGACTTCTGATTCATTATTCTCCGGCGTAACTTCCGGCTCTTGCTTCACAATCGCGGCGTGTTTTCCCGTCCGGCGGTG
>Marker458277 Chr1 SNP_site:227761 Ref_Type:T
ACCTTTGCGGGTAATAATGGTATTCATTCTTCTTCACTTCCACCATTTTCGGGCCTTCTTCTGCTCCTGCTTGGCCGGCGXXXXXXXXXXA
CGACTTCTGATTCATTATTCTCCGGCGAAACTTCCGGCTCTTGCTTCACAATCGCGGCGTGTTTTCCTGTCCGGCGGTG
ACCTTTGCGGGTAATAATGGTATTCATTCTTCTTCACTTCCACCATTTTCGGGCCTTCTTCTGCTGCTGCTTGGCCGGCGXXXXXXXXXXA
CGACTTCTGATTCATTATTCTCCGGCGTAACTTCCGGCTCTTGCTTCACAATCGCGGCGTGTTTTCCCGTCCGGCGGTG

Create PDF with GO2PDF for free, if you wish to

>Marker458277 Chr1 SNP_site:227801 Ref_Type:C
ACCTTTGCGGGTAATAATGGTATTCATTCTTCTTCACTTCCACCATTTTCGGGCCTTCTTCTGCTCCTGCTTGGCCGGCGXXXXXXXXXXA
CGACTTCTGATTCATTATTCTCCGGCGAAACTTCCGGCTCTTGCTTCACAATCGCGGCGTGTTTTCCTGTCCGGCGGTG
ACCTTTGCGGGTAATAATGGTATTCATTCTTCTTCACTTCCACCATTTTCGGGCCTTCTTCTGCTGCTGCTTGGCCGGCGXXXXXXXXXXA
CGACTTCTGATTCATTATTCTCCGGCGTAACTTCCGGCTCTTGCTTCACAATCGCGGCGTGTTTTCCCGTCCGGCGGTG
>Marker458436 Chr1 SNP_site:6469719 Ref_Type:T
AACTTGCAATATTAATTGTGGATCTTGGCGAAGGAGTATTAGCAGGAACTCGATATTTCCAACTTCTGCTGCATCGCGCAXXXXXXXXXXT
GATGGGCTAATGTCTGCATTAGAGCTTTATTGTAAATTCCATTGAACCCTATAGCAAATACACAAAAGATTTAGTTGTA
AACTTGCAATATTAATTGTGGATCTTGGCGAAGGAGTATTAGCAGGAACTCGATATTTCCAATTTCTGCTGCATCGCGCAXXXXXXXXXXT
GATGGGCGAATGTCTGCATTAGAGCTTTATTGTAAATTCCATTGAACCCTATAGCAAATACACAAAAGATTTAGTTGTA
>Marker458436 Chr1 SNP_site:6469831 Ref_Type:T
AACTTGCAATATTAATTGTGGATCTTGGCGAAGGAGTATTAGCAGGAACTCGATATTTCCAACTTCTGCTGCATCGCGCAXXXXXXXXXXT
GATGGGCTAATGTCTGCATTAGAGCTTTATTGTAAATTCCATTGAACCCTATAGCAAATACACAAAAGATTTAGTTGTA
AACTTGCAATATTAATTGTGGATCTTGGCGAAGGAGTATTAGCAGGAACTCGATATTTCCAATTTCTGCTGCATCGCGCAXXXXXXXXXXT
GATGGGCGAATGTCTGCATTAGAGCTTTATTGTAAATTCCATTGAACCCTATAGCAAATACACAAAAGATTTAGTTGTA
>Marker459111 Chr1 SNP_site:18955779 Ref_Type:C
AACTTTATCTGAATGTTTTTGTTTGATTCTTTTGAAATTCTTAGTTTAAACAAATACTCCAAAAGTCCAAAAGCAAAGCAXXXXXXXXXXT
AAAAAGGATAAGGGTTTTTCATCAAGATTTAGTATGGAAGAACCTGTAAAGCTCAGGAGGGATAGCGCCAATTAATAGT
AACTTTATGTGAATGTTTTTGTTTGATTCTTTTGAAATTCTTAGTTTAAACAAATACTCCAAAAGTCCAAAAGCAAAGCAXXXXXXXXXXT
AAAAAGGATAAGGGTTTTTCATCAAGATTTAGTATGGAAGAACCTGTAAAGCTCAGGAGGGATAGCGCCAATTAATAGT
>Marker459336 Chr1 SNP_site:6577410 Ref_Type:T
ACATGTGTTTTACTTTTATCTTTTTGAAAGGTTAGAAACTAAATGCGGGTAGTGTAATTTATAAACAGAAGTGCAGAACGXXXXXXXXXXC
ATTTTGGAAATACAGAATTTGGTCAGAAGAGAAGGGTTTCAGATCAGAAAAATCTTGACAGGACATGACAACACAAGTA
ACATGTGTTTTACTTTTATCTTTTTGAAAGGTTAGAAACTAAATGCGGGTAGTGTAATTTATAAACAGAAGTGCAGAATGXXXXXXXXXXC
ATTTTGGAAATACAGAATTTGGTCAGAAGAGAAGGGTTTCAGATCAGAAAAATCTTGACAGGACATGACAACATAAGTA
>Marker459336 Chr1 SNP_site:6577592 Ref_Type:T
ACATGTGTTTTACTTTTATCTTTTTGAAAGGTTAGAAACTAAATGCGGGTAGTGTAATTTATAAACAGAAGTGCAGAACGXXXXXXXXXXC
ATTTTGGAAATACAGAATTTGGTCAGAAGAGAAGGGTTTCAGATCAGAAAAATCTTGACAGGACATGACAACACAAGTA
ACATGTGTTTTACTTTTATCTTTTTGAAAGGTTAGAAACTAAATGCGGGTAGTGTAATTTATAAACAGAAGTGCAGAATGXXXXXXXXXXC
ATTTTGGAAATACAGAATTTGGTCAGAAGAGAAGGGTTTCAGATCAGAAAAATCTTGACAGGACATGACAACATAAGTA
>Marker459435 Chr1 SNP_site:25574171 Ref_Type:A
GACCAATATCATCACCTTTTGATTCTCCCCCACCCTTACAACTTTGAATGCTAACAGTTTCAAATAGCTACATCACAAGTXXXXXXXXXXC
ATCTAAATAGTTGTCACAAAAAACCTGCAAAAAACCAATGAAACGTATTAGCAAAACCCACAAGAATTGTGGTAACAGT
GACCATAATCATCACCTTTTGATTCTCCCCCACCCTTACAACTTTGAATGCTAACAGTTTCAAATAGCTACATCACAAGTXXXXXXXXXXC
ATCTAAATAGTTGTCACAAAAAACCTGCAAAAAACCAATGAAACGTATTAGCAAAACCCACAAGAATTGTGGTAACAGT
>Marker459435 Chr1 SNP_site:25574172 Ref_Type:T
GACCAATATCATCACCTTTTGATTCTCCCCCACCCTTACAACTTTGAATGCTAACAGTTTCAAATAGCTACATCACAAGTXXXXXXXXXXC
ATCTAAATAGTTGTCACAAAAAACCTGCAAAAAACCAATGAAACGTATTAGCAAAACCCACAAGAATTGTGGTAACAGT
GACCATAATCATCACCTTTTGATTCTCCCCCACCCTTACAACTTTGAATGCTAACAGTTTCAAATAGCTACATCACAAGTXXXXXXXXXXC
ATCTAAATAGTTGTCACAAAAAACCTGCAAAAAACCAATGAAACGTATTAGCAAAACCCACAAGAATTGTGGTAACAGT
>Marker460711 Chr1 SNP_site:12178722 Ref_Type:C
TACAAAAAGGTCGTCAATTCCATTGTAGCATTCTTAGTTTTGTATGTAGATGATATTCTATTGGAAACGACGTAGGTTATXXXXXXXXXXT
GTCACAAGCATCTTATATAGACAAAATGTTGTCTATGTGTTAAATGTAGATTCTAACAAGGGTTTGTTGTCATACAGGT
TACAAAAAGGTCGTCAATTCCATTGTAGCATTCTTAGTTTTGTATGTAGATGATATTCTATTGGAAACGATGTAGGTTATXXXXXXXXXXT

Create PDF with GO2PDF for free, if you wish to

GTCACAAGCATCTTATATAGACAAAATGTTGTCTATGTGTTAAATGTAGATTCTAACAAGGGTTTGTTGTCATACAGGT
>Marker460809 Chr1 SNP_site:10460019 Ref_Type:A
AACCTTGCTCATAAATTAACTGAGAAATAACATTCTTGCCTACTCTTGATCAAAATACAAATTTCCTTGAATCTAAAATGXXXXXXXXXXG
AGAAATCAACTGCTAAAATTGACCTATTTATAGAGTCCCTTGCATGCAATTAAGATGACACTTAATCATTGATTAGGTT
AACCTTGCTCATAAATTAACTGAGAAATAACATTCTTGCCTACTCTTGATCAAAATACAAATTTCCTTGAATCTAAAATGXXXXXXXXXXG
GGAAATCAACTGCTAAAATTGACCTATTTATAGAGTCCCTTGCATGCAATTAAGATGACACGTAATCATTGATTAGGTT
>Marker460809 Chr1 SNP_site:10460080 Ref_Type:T
AACCTTGCTCATAAATTAACTGAGAAATAACATTCTTGCCTACTCTTGATCAAAATACAAATTTCCTTGAATCTAAAATGXXXXXXXXXXG
AGAAATCAACTGCTAAAATTGACCTATTTATAGAGTCCCTTGCATGCAATTAAGATGACACTTAATCATTGATTAGGTT
AACCTTGCTCATAAATTAACTGAGAAATAACATTCTTGCCTACTCTTGATCAAAATACAAATTTCCTTGAATCTAAAATGXXXXXXXXXXG
GGAAATCAACTGCTAAAATTGACCTATTTATAGAGTCCCTTGCATGCAATTAAGATGACACGTAATCATTGATTAGGTT
>Marker461092 Chr1 SNP_site:8459397 Ref_Type:A
ACCAAGCACCTAACCCATGTATCTCATCCTCAAACTTGATATTCATCCTAGATAATTGATTTAAAATACCTTGGAAAGTAXXXXXXXXXXA
GAGCTTATTCTACATAATTCGTGCATGAATTTCTTCACAAATATAGTTTAGAAAATTATCTTCTGCCCATAGCATCGTA
ACCAAGCACCTAACCCATGTATCTCATCCTCAAACTTGATATTCATCCTAGATAATTGATTTAAAATACCTTGGAAAGTAXXXXXXXXXXA
GAGCTTATTCTACATAATTCGTGCATGAGTTTCTTCACAAATATAGTTTAGAAAATTATCTTCTGCCCATAGCATCGTA
>Marker46113 Chr4 SNP_site:13261660 Ref_Type:T
AACTTCTTTCTATGAAGGCAGTCATTTGGTTTGCATGGGTGAGAGTGACCTATGTTGCTGACTCAATATGCCAACTATTTXXXXXXXXXXA
CCACACACTCTCTCTTGGCTCGAGAGGAGTTCAATTATAGTGAAACTATAACTTATTGTTCATTAGATGAATTAGTGGT
AACTTCTTTCTATGAAGGCAGTCATTTGGTTTGCATGGGTGAGAGTGACCTATGTTGCTGACTTAATATGCCAACTATTTXXXXXXXXXXA
CCACACACTCTATCTTGGCTCGAGAGGAGTTCAATTATAGTGAAACTATAACTTATTGTTCATTAGATGAATTAGTGGT
>Marker46113 Chr4 SNP_site:13261817 Ref_Type:A
AACTTCTTTCTATGAAGGCAGTCATTTGGTTTGCATGGGTGAGAGTGACCTATGTTGCTGACTCAATATGCCAACTATTTXXXXXXXXXXA
CCACACACTCTCTCTTGGCTCGAGAGGAGTTCAATTATAGTGAAACTATAACTTATTGTTCATTAGATGAATTAGTGGT
AACTTCTTTCTATGAAGGCAGTCATTTGGTTTGCATGGGTGAGAGTGACCTATGTTGCTGACTTAATATGCCAACTATTTXXXXXXXXXXA
CCACACACTCTATCTTGGCTCGAGAGGAGTTCAATTATAGTGAAACTATAACTTATTGTTCATTAGATGAATTAGTGGT
>Marker461184 Chr1 SNP_site:14581036 Ref_Type:A
AACCGTGACAGTCAGGATTAATGTAAATATTTAATCACAAAATGCAAATTATCGATGAAAAACTTCGTGTAAGAACAAAAXXXXXXXXXXT
TACCATGATTCAATGTTGAAAATTGGTTTCATCATATTCTAAGAAATCCAAGTTGGATCTAATCAAATCAAAGTCTAGT
AACCGTGACGGTCAGGATTAATGTAAATATTTAATCACAAAATGCAACTTATCGATGAAAAACTTCGTGTAAGAACAAAAXXXXXXXXXXT
TACCATGATTCAATGTTGAAAATTGGTTTCATCATATTCTAACAAATCCAAGTTGGATCTAATCAAATCAAAGTCTAGT
>Marker461184 Chr1 SNP_site:14581074 Ref_Type:A
AACCGTGACAGTCAGGATTAATGTAAATATTTAATCACAAAATGCAAATTATCGATGAAAAACTTCGTGTAAGAACAAAAXXXXXXXXXXT
TACCATGATTCAATGTTGAAAATTGGTTTCATCATATTCTAAGAAATCCAAGTTGGATCTAATCAAATCAAAGTCTAGT
AACCGTGACGGTCAGGATTAATGTAAATATTTAATCACAAAATGCAACTTATCGATGAAAAACTTCGTGTAAGAACAAAAXXXXXXXXXXT
TACCATGATTCAATGTTGAAAATTGGTTTCATCATATTCTAACAAATCCAAGTTGGATCTAATCAAATCAAAGTCTAGT
>Marker461184 Chr1 SNP_site:14581246 Ref_Type:G
AACCGTGACAGTCAGGATTAATGTAAATATTTAATCACAAAATGCAAATTATCGATGAAAAACTTCGTGTAAGAACAAAAXXXXXXXXXXT
TACCATGATTCAATGTTGAAAATTGGTTTCATCATATTCTAAGAAATCCAAGTTGGATCTAATCAAATCAAAGTCTAGT
AACCGTGACGGTCAGGATTAATGTAAATATTTAATCACAAAATGCAACTTATCGATGAAAAACTTCGTGTAAGAACAAAAXXXXXXXXXXT
TACCATGATTCAATGTTGAAAATTGGTTTCATCATATTCTAACAAATCCAAGTTGGATCTAATCAAATCAAAGTCTAGT
>Marker461861 Chr3 SNP_site:18495241 Ref_Type:T
AACTCTTTACATAGGATGTCCCACTTTCATGTCTCTACATAAACGATTCAAGATTACATCGTTTGTATTAACTACAAAGCXXXXXXXXXXG
GACTCACAGTTCCCTTCAACGTTTTCTTCTTCCTCTCATTTTTTCTACTTTAAAATCATATAAGTAGATTGTCGTTGTT

Create PDF with GO2PDF for free, if you wish to

AACTCTTTACATAGGATGTCCCACTTTCATGTCTCTACATAAACGATTCAAGATTACATCGTTTGTATTAACTACAAAGCXXXXXXXXXXG
GACTTACAGTTCCCTTTAACATTTTCTTCTTCCTCTCATTTTTTCTACTTTAAAATCATATAAGTAGATTGTCGTTGTT
>Marker461861 Chr3 SNP_site:18495253 Ref_Type:T
AACTCTTTACATAGGATGTCCCACTTTCATGTCTCTACATAAACGATTCAAGATTACATCGTTTGTATTAACTACAAAGCXXXXXXXXXXG
GACTCACAGTTCCCTTCAACGTTTTCTTCTTCCTCTCATTTTTTCTACTTTAAAATCATATAAGTAGATTGTCGTTGTT
AACTCTTTACATAGGATGTCCCACTTTCATGTCTCTACATAAACGATTCAAGATTACATCGTTTGTATTAACTACAAAGCXXXXXXXXXXG
GACTTACAGTTCCCTTTAACATTTTCTTCTTCCTCTCATTTTTTCTACTTTAAAATCATATAAGTAGATTGTCGTTGTT
>Marker461861 Chr3 SNP_site:18495257 Ref_Type:A
AACTCTTTACATAGGATGTCCCACTTTCATGTCTCTACATAAACGATTCAAGATTACATCGTTTGTATTAACTACAAAGCXXXXXXXXXXG
GACTCACAGTTCCCTTCAACGTTTTCTTCTTCCTCTCATTTTTTCTACTTTAAAATCATATAAGTAGATTGTCGTTGTT
AACTCTTTACATAGGATGTCCCACTTTCATGTCTCTACATAAACGATTCAAGATTACATCGTTTGTATTAACTACAAAGCXXXXXXXXXXG
GACTTACAGTTCCCTTTAACATTTTCTTCTTCCTCTCATTTTTTCTACTTTAAAATCATATAAGTAGATTGTCGTTGTT
>Marker46197 Chr4 SNP_site:8676871 Ref_Type:G
AACTCTAAAAAGCAAAGCAAAGATCGGATACGTTAGTTTATGTGTAAGGGTTGAATAGCTCACCTCCATACTTTCTAGTTXXXXXXXXXXT
GGGATGGGTTATCTATATCGACAATTCATATGGACTAACAATATCGACTTAGTTAATGAAATCAGAACAAAAGAGAGTG
AACTCTAAAAAGCAAAGCAAAGATCGGATACGTTAGTTTATGTGTAAGGGTTGAATAGCTCACCTCCATACTTTCTAGTTXXXXXXXXXXT
GGGATGGGTTATCTATATCGACAATTCATATTGACTAACAATATCGACTTAGTTAATGAAATCAGAACAAAAGAGAGTG
>Marker462042 Chr3 SNP_site:31904127 Ref_Type:G
ACTTTTGATATCAAAAGAAATGAGTTGATACATTTATTATCATCATCATATACGTGTCTTTTTAACATACTGAACATTTCXXXXXXXXXXT
TTTCA-TTTTTTTTGGGTGTGATTCACAAGACATATCCAAATTTGATTAAACCAATCATCAAGGGCTTGAAGAACCAAGT
ACTTTTTATATCAAAAGAAATGAGTTGATACATTTATTATCATCATCATATACGTGTCTTTTTAACATACTGAACATTTCXXXXXXXXXX-
TTTCATTTTTTTTTGGGTGTGATTCACAAGACATATCCAAATTTGATTAAACCAATCATCAAGGGCTTGAAGAACCAAGT
>Marker462243 Chr3 SNP_site:38732267 Ref_Type:G
ACTATGTTTTTCTTAGAATTAGAAGGAATGCTATATGGCTTTCATGCTTTATCTACCAAGACCTCTTTTTTGGTTTATCCXXXXXXXXXXA
AATTATGATATAAAGAGAATGCTATTACTATCAATTGGGATTAAATAAATCCTTTATTTTTGGCTAATTTATTTAAAGT
ACTATGTTTTTCTTAGAATTAGAAGGAATGCTATATGGCTTTCATGCTTTATCTACCAAGACCTCTTTTTTGGTTTATGCXXXXXXXXXXA
AATTATGATATAAAGAGAATGCTATTACTATCAATTGGGATTAAATAAATCCTTTATTTTTGGCTAATTTATTTAAAGT
>Marker462725 Chr3 SNP_site:11857911 Ref_Type:G
AACTCAGATGGATACTTATGAGTATCTATCACTTAAACACAATAAAATTTTACTATATTTATAGATATTTACAACCGTATXXXXXXXXXXC
AGAATGCAAAGAGGAGAGAAGAAGTAGTTTCCTTGCTTCTTCCTTCCTTAACAATCAAACATTAGTATAAAACTATAGT
AACTCAGATGGATACTTATGAGTATCTATCACTTAAACACAATAAGATTTTACTATATTTATAGATATTTACAACAGTATXXXXXXXXXXC
AAAATGCAAAGAGGAGAGAAGAAGTAGTTTCCTTGCTTCTTCCTTCCTTAACAATCAAACATTAGTATAAAACTATAGT
>Marker462725 Chr3 SNP_site:11857941 Ref_Type:A
AACTCAGATGGATACTTATGAGTATCTATCACTTAAACACAATAAAATTTTACTATATTTATAGATATTTACAACCGTATXXXXXXXXXXC
AGAATGCAAAGAGGAGAGAAGAAGTAGTTTCCTTGCTTCTTCCTTCCTTAACAATCAAACATTAGTATAAAACTATAGT
AACTCAGATGGATACTTATGAGTATCTATCACTTAAACACAATAAGATTTTACTATATTTATAGATATTTACAACAGTATXXXXXXXXXXC
AAAATGCAAAGAGGAGAGAAGAAGTAGTTTCCTTGCTTCTTCCTTCCTTAACAATCAAACATTAGTATAAAACTATAGT
>Marker462725 Chr3 SNP_site:11858065 Ref_Type:A
AACTCAGATGGATACTTATGAGTATCTATCACTTAAACACAATAAAATTTTACTATATTTATAGATATTTACAACCGTATXXXXXXXXXXC
AGAATGCAAAGAGGAGAGAAGAAGTAGTTTCCTTGCTTCTTCCTTCCTTAACAATCAAACATTAGTATAAAACTATAGT
AACTCAGATGGATACTTATGAGTATCTATCACTTAAACACAATAAGATTTTACTATATTTATAGATATTTACAACAGTATXXXXXXXXXXC
AAAATGCAAAGAGGAGAGAAGAAGTAGTTTCCTTGCTTCTTCCTTCCTTAACAATCAAACATTAGTATAAAACTATAGT
>Marker462748 Chr3 SNP_site:29652867 Ref_Type:T
GACGTTATAAACAAAAGATGTGTCGAATATATGTCGGGTTCTTATTAGATAATTGATAAAAACTTAGACAATGACTAAAAXXXXXXXXXXT

Create PDF with GO2PDF for free, if you wish to

TGTTTTTATTAAATACCTCTCAAAGGCAAAATAAAGTTTTCATTACTTTTAACATTTAGCTTTATTAAAAAAGATATGT
GACGTTATAAACAAAAGATGTGTCGAATATATGTCGGGTTCTTATTAGATAATTGATAAAAACTTAGACAATGACTAAAAXXXXXXXXXXT
TGTTTTTATTAAATACCTCTCAAAGGCAAAATAAAGTTTTCATTACTTTTAACATTTAGTTTTATTAAAAAAGATATGT
>Marker462897 Chr3 SNP_site:20565849 Ref_Type:G
AACTCAGGCTTTCTACAAAAGGAAAAAGATGACAGATTGATTTCACAAGAACTTACAGAAATTACAAAACCAAAAGCATAXXXXXXXXXXT
TATAGAAGGTAACATTCAAATCTCTATTCTCAAACTTCAGATTTAAAAATCTCTTCCTTCACTTAGTCGAAGGGTCGTG
AACTCAGGCTTTCTACAAAAGGAAAAAGATGACAGATTGATTTCACAAGAACTTACAGAAATTACAAAACCAAAAGCATAXXXXXXXXXXT
TATAGAAGGTAACATTCAAATGTCTATTCTCAAACTTCAGATTTAAAAATCTCTTCCTTCACTTAGTCGAAGGGTCGTG
>Marker46302 Chr4 SNP_site:7926946 Ref_Type:C
ACAACATATCCATTTTAGACTTCGTATTTCTTCACCTTTTCCATTTTGATTTCTGTTCTGTCCATTTTTGCTTTGCATACXXXXXXXXXXG
TCAAAATCGCCATATTTTAGAAAGTGTAGTTCTAAAACAAACATTTTAAAAGTAAATGTAATAAAATTAATAGAAGGTT
ACAACATATCCATTTTAGACTTCGTATTTCTTCACCTTTTCCATTTTGATTTCTGTTCTGTCCATTTTTGCTTTGCATACXXXXXXXXXXG
TTAAAATCGCCATATTTTAGAAAGTGTAGTTCTAAAACAAACATTTTAAAAGTAAATGTAATAAAATTAATAGAAGGTT
>Marker463038 Chr3 SNP_site:38772813 Ref_Type:T
TACCCTTACAATGAACAATCTTCTCTTATAGGTCTTTGAAATGTATAAAATCTCACACAAAAGAAATGTTTTGGCTGCAGXXXXXXXXXXT
TTTCTTGTAATACTTTTAAAGATTTTAAATTTTCTGTAAGTTGGGGGGCGTATGCTCTCTCTCAGTTGTAATTTTATGT
TACCCTTATAATGAACAATCTTCTCTTATAGGTCTTTGAAATGTATAAAATCTCACACAAAAGAAATGTTTTGCCTGCAGXXXXXXXXXXT
TTTCTTGTAATACTTTTAAAGATTTTAAATTTTCTGTAAGTTGGGGGTCGTATGCTCTCTCTCAGTTGTAATTTTATGT
>Marker463038 Chr3 SNP_site:38772878 Ref_Type:C
TACCCTTACAATGAACAATCTTCTCTTATAGGTCTTTGAAATGTATAAAATCTCACACAAAAGAAATGTTTTGGCTGCAGXXXXXXXXXXT
TTTCTTGTAATACTTTTAAAGATTTTAAATTTTCTGTAAGTTGGGGGGCGTATGCTCTCTCTCAGTTGTAATTTTATGT
TACCCTTATAATGAACAATCTTCTCTTATAGGTCTTTGAAATGTATAAAATCTCACACAAAAGAAATGTTTTGCCTGCAGXXXXXXXXXXT
TTTCTTGTAATACTTTTAAAGATTTTAAATTTTCTGTAAGTTGGGGGTCGTATGCTCTCTCTCAGTTGTAATTTTATGT
>Marker463038 Chr3 SNP_site:38773054 Ref_Type:T
TACCCTTACAATGAACAATCTTCTCTTATAGGTCTTTGAAATGTATAAAATCTCACACAAAAGAAATGTTTTGGCTGCAGXXXXXXXXXXT
TTTCTTGTAATACTTTTAAAGATTTTAAATTTTCTGTAAGTTGGGGGGCGTATGCTCTCTCTCAGTTGTAATTTTATGT
TACCCTTATAATGAACAATCTTCTCTTATAGGTCTTTGAAATGTATAAAATCTCACACAAAAGAAATGTTTTGCCTGCAGXXXXXXXXXXT
TTTCTTGTAATACTTTTAAAGATTTTAAATTTTCTGTAAGTTGGGGGTCGTATGCTCTCTCTCAGTTGTAATTTTATGT
>Marker463106 Chr3 SNP_site:1800416 Ref_Type:G
GACTGGTTTTGCTTCGATTCCAAACTCCTTCTTCATGGACTTGAAGTATTGAACCCCAAGATCAACGAATCCAGCATGGTXXXXXXXXXXG
GCATTCCAGGAAATCACATCCTTTCGTGGCATCTCGAGAAACAACTTCCATGCACTGTCGAGATCTCCACACTTGCAGT
GACTGGTTTTGCTTCGATTCCAAACTCCTTCTTCATGGACTTGAAGTATTGAACCCCAAGATCGACGAATCCAGCATGGTXXXXXXXXXXG
GCATTCCAGGTAATCACATCCTTTCGTGGCATCTCGAGAAACAACTTCCATGCACTGTCGAGATCTCCACACTTGCAGT
>Marker463106 Chr3 SNP_site:1800565 Ref_Type:T
GACTGGTTTTGCTTCGATTCCAAACTCCTTCTTCATGGACTTGAAGTATTGAACCCCAAGATCAACGAATCCAGCATGGTXXXXXXXXXXG
GCATTCCAGGAAATCACATCCTTTCGTGGCATCTCGAGAAACAACTTCCATGCACTGTCGAGATCTCCACACTTGCAGT
GACTGGTTTTGCTTCGATTCCAAACTCCTTCTTCATGGACTTGAAGTATTGAACCCCAAGATCGACGAATCCAGCATGGTXXXXXXXXXXG
GCATTCCAGGTAATCACATCCTTTCGTGGCATCTCGAGAAACAACTTCCATGCACTGTCGAGATCTCCACACTTGCAGT
>Marker463148 Chr3 SNP_site:21052051 Ref_Type:G
ACCAAAATAATATATGAAATAACATAACAGGTTAGGAATATACGTCCCTTGAAAGTTGAAAGCAGTCAGACTCTTGAGCTXXXXXXXXXXT
AGTATGCGGCCCACCAACAAGAAATATTGAGCATCATCATTTGGATTCATATATTCAATACAGCTACAGATTTTTAGTG
ACCAAAATAATATATGAAATAACATAACAGGTTAGGAATATACGTCCCTTGAAAGTTGAAAGCAGTCAGACTCTTGAGCTXXXXXXXXXXT
AGTATGCGGCCCACCAACAAGAAATATTGAGCATCATCATTTGGATTCATATATTCAATACAGCTACAGATTTTTGGTG
>Marker463807 Chr3 SNP_site:1162138 Ref_Type:A

Create PDF with GO2PDF for free, if you wish to

AACGTGGTCATCAAAGTTGGCTTTTTTTGGAGTTTCCTATTGGAGATAGTTACCATAGCCCAAAATTAGTTGTTGGAGGTXXXXXXXXXXA
TCTGAGTGTGGTAATGGTAATTGATCGTTGAATCTATTAGAAAAACTGGAGAGAGCTTCATCAACCTATAAAGTTAGTG
AACGTGGTCATCAAAGTTGGCTTTTTTTGGAGTTTCCTATTGGAGATAGTTACCATAGCCCAAAATTAGTTGTTGGAGGTXXXXXXXXXXC
TCTGAGTGTGGTAATGGTAATTGATCGTTGAATCTATTAGAAAAACTGGAGAGAGCTTCATCAACCTATAAAGTTAGTG
>Marker463935 Chr3 SNP_site:28501257 Ref_Type:T
CACGTTGGTTGATTTTTTTTTTAATCATGGAGTGCTTTTTATTTTTTAAGGCTTTATTGAAAAACCAATATATTTTATTTTXXXXXXXXXXT
AAATATCATTCACATCAATAAAAAAAAAAATTTAATTTTAATTGAATTATGCTGGAAATAGTTAATTAAAATAGTGGTA
CACGTTGGTTGATTTTTTTTTTAATCATGGAGTGCTTTTTATTTTTTAAGGCTTTATTGAAAAACCAATATATTTTATTTTXXXXXXXXXXT
AAATATCATTCACATCAATAAAAAAATATATTTAATTTTAATTGAATTATGCTGGAAATAGTTAATTAAAATAGTGGTA
>Marker463935 Chr3 SNP_site:28501259 Ref_Type:T
CACGTTGGTTGATTTTTTTTTTAATCATGGAGTGCTTTTTATTTTTTAAGGCTTTATTGAAAAACCAATATATTTTATTTTXXXXXXXXXXT
AAATATCATTCACATCAATAAAAAAAAAAATTTAATTTTAATTGAATTATGCTGGAAATAGTTAATTAAAATAGTGGTA
CACGTTGGTTGATTTTTTTTTTAATCATGGAGTGCTTTTTATTTTTTAAGGCTTTATTGAAAAACCAATATATTTTATTTTXXXXXXXXXXT
AAATATCATTCACATCAATAAAAAAATATATTTAATTTTAATTGAATTATGCTGGAAATAGTTAATTAAAATAGTGGTA
>Marker465079 Chr3 SNP_site:4128209 Ref_Type:G
ACTCAACATTAAGTGGATGATGTTCATGAGAAATGGAGAGAAAGCGCTCATGAATTAGGGTGATACATAGGTAGCTGCCTXXXXXXXXXXA
GACTTATGAATTAGGAGAAGGGCTTTGTTATTTGAATCATCCGATTATGTCATTTTTAAATCACGGTTTAAGATATAGT
ACTCAACATTAAGTGGATGATGTTCATGAGAAATGGAGAGAGAGCGCTCATGAATTAGGGTGATACATAGGTAGCTGCCTXXXXXXXXXXA
GACTTATGAATTAGGAGAAGGGCTTTGTTATTTGAATCATCCGATTATGTCATTTTTAAATCACGGTTTAAGATATAGT
>Marker465315 Chr3 SNP_site:11275236 Ref_Type:G
GACCTCAAATGCCAGACGAAGATTTGATTGAGCTTAAGTTCCGACTTTATGATGGATTAGATATTGGTCCATTTTGTTATXXXXXXXXXXG
CGATCTTCCTAGAGGAGTTTTTACTATGCATGTCGTGGTGCAGCCAACTATAGCAAAAGCAAAAGCAGAAAATAAGGTT
GACCTCAAATGCCGGACGAAGATTTGATTGAGCTTAAGTTCCGACTTTATGATGGATTAGATATTGGTCCATTTTGTTCTXXXXXXXXXXG
CGATCTTCCTAGAGGAGTTTTTACTATGCATGTCGTGGTGCAGCCAACTATAGCAAAAGCAAAAGCAGAAAATAAGGTC
>Marker465315 Chr3 SNP_site:11275301 Ref_Type:C
GACCTCAAATGCCAGACGAAGATTTGATTGAGCTTAAGTTCCGACTTTATGATGGATTAGATATTGGTCCATTTTGTTATXXXXXXXXXXG
CGATCTTCCTAGAGGAGTTTTTACTATGCATGTCGTGGTGCAGCCAACTATAGCAAAAGCAAAAGCAGAAAATAAGGTT
GACCTCAAATGCCGGACGAAGATTTGATTGAGCTTAAGTTCCGACTTTATGATGGATTAGATATTGGTCCATTTTGTTCTXXXXXXXXXXG
CGATCTTCCTAGAGGAGTTTTTACTATGCATGTCGTGGTGCAGCCAACTATAGCAAAAGCAAAAGCAGAAAATAAGGTC
>Marker465315 Chr3 SNP_site:11275541 Ref_Type:C
GACCTCAAATGCCAGACGAAGATTTGATTGAGCTTAAGTTCCGACTTTATGATGGATTAGATATTGGTCCATTTTGTTATXXXXXXXXXXG
CGATCTTCCTAGAGGAGTTTTTACTATGCATGTCGTGGTGCAGCCAACTATAGCAAAAGCAAAAGCAGAAAATAAGGTT
GACCTCAAATGCCGGACGAAGATTTGATTGAGCTTAAGTTCCGACTTTATGATGGATTAGATATTGGTCCATTTTGTTCTXXXXXXXXXXG
CGATCTTCCTAGAGGAGTTTTTACTATGCATGTCGTGGTGCAGCCAACTATAGCAAAAGCAAAAGCAGAAAATAAGGTC
>Marker465562 Chr3 SNP_site:27118810 Ref_Type:T
TACACCTCAAACACATATTATTATAGTTTAAACTAAAATAATATACAACTCAAATACAAACAATTATGATTTAACTACAAXXXXXXXXXXT
TTCATTGTCAGAGACAAGCTTGGACAAGCCAAAATCTGCAACTTTTGCATTTAAATGTTCATCCAATAGAATATTGGTG
TACACCTCAAACACATATTATTATAGTTTAAACTAAAATAATATACAACTCAAATACATACAATTATGATTTAACTACAAXXXXXXXXXXT
TTCATTGTCAGAGACAAGCTTGGACAAGCCAAAATCTGCAACTTTTGCATTTAAATGTTCATCTAATAGAATATTGGTG
>Marker465562 Chr3 SNP_site:27119054 Ref_Type:C
TACACCTCAAACACATATTATTATAGTTTAAACTAAAATAATATACAACTCAAATACAAACAATTATGATTTAACTACAAXXXXXXXXXXT
TTCATTGTCAGAGACAAGCTTGGACAAGCCAAAATCTGCAACTTTTGCATTTAAATGTTCATCCAATAGAATATTGGTG
TACACCTCAAACACATATTATTATAGTTTAAACTAAAATAATATACAACTCAAATACATACAATTATGATTTAACTACAAXXXXXXXXXXT
TTCATTGTCAGAGACAAGCTTGGACAAGCCAAAATCTGCAACTTTTGCATTTAAATGTTCATCTAATAGAATATTGGTG

Create PDF with GO2PDF for free, if you wish to

>Marker466288 Chr3 SNP_site:11332758 Ref_Type:C
CACGAAAAATTATTATGACGTATAACTGTAAAGCAATTATTGTGATTTTTATGTGATTCTATGATTTTTTAATGCATCATXXXXXXXXXXA
GTTTATTGTCATCACATCTCATTCTAAGTTCAATACAGTAGTTTGGGGTGGGGAATGAGTTGAATTTTTTTCTCCAGTA
CACGAAAAATTATTATGACGTATAACTGTAAAGCAATTATTGTGATTTTTATGTGATTCTATGATTTTTTAATGCATCATXXXXXXXXXXA
GTTTATTGTCATCACATCTCATTGTAAGTTCAATACAGTAGTTTGGGGTGGGGAATGAGTTGAATTTTTTTCTCCAGTA
>Marker466763 Chr3 SNP_site:2169792 Ref_Type:T
AACCAAGCTCCTTGAACATGCTTCAATGTTGAAACTGAATCAACAAGTAAATTACACCCTACATTTTGTTTTAGAAGTCTXXXXXXXXXXA
CAAACCAGAACCTGAAGGCAAAATGAAGTTGAAATCACTCAAACTGATGCCATTTTAAAAATGAAGCAAACTACAAGTG
AACCAAGCTCCTTGAACATGCTTCAATGTTGAAACTGAATCAACAAGTAAATTACACCCTACATTTTGTTTTAGAAGTCTXXXXXXXXXXA
CAAACCAGAACCTGAAGGCAAAATGAAGTTGAAATCACTCAAACTGATGTCATTTTAAAAATGAAGCAAACTACAAGTG
>Marker467020 Chr3 SNP_site:38366662 Ref_Type:G
GACACTACCTCAAATGACTAATAATCTATAATGTTAATTCTTCTCTCTATTTTATTGTTTAGGTTTTTGCTGCTTATTGTXXXXXXXXXXT
TATCTTGTTCGGACTAACTTAGATTTATTTTAAAAAGTAGTCATAATTTAATTAATCTCGTTCCTATAAAAAAAATTGTT
GACACTACCTCGAATGACTAATAATCTATAATGTTAATTCTTCTCTCTATTTTTTTGTTTAGGTTTTTGCTGCTTATTGTXXXXXXXXXXT
TATCTTGTTCGAACTAACTTAGATTTATTTTAAAAAGTAGTCATAATTTAATTAATCTCGTTCCTATAAAAAAAATTGTT
>Marker467020 Chr3 SNP_site:38366704 Ref_Type:T
GACACTACCTCAAATGACTAATAATCTATAATGTTAATTCTTCTCTCTATTTTATTGTTTAGGTTTTTGCTGCTTATTGTXXXXXXXXXXT
TATCTTGTTCGGACTAACTTAGATTTATTTTAAAAAGTAGTCATAATTTAATTAATCTCGTTCCTATAAAAAAAATTGTT
GACACTACCTCGAATGACTAATAATCTATAATGTTAATTCTTCTCTCTATTTTTTTGTTTAGGTTTTTGCTGCTTATTGTXXXXXXXXXXT
TATCTTGTTCGAACTAACTTAGATTTATTTTAAAAAGTAGTCATAATTTAATTAATCTCGTTCCTATAAAAAAAATTGTT
>Marker467020 Chr3 SNP_site:38366863 Ref_Type:A
GACACTACCTCAAATGACTAATAATCTATAATGTTAATTCTTCTCTCTATTTTATTGTTTAGGTTTTTGCTGCTTATTGTXXXXXXXXXXT
TATCTTGTTCGGACTAACTTAGATTTATTTTAAAAAGTAGTCATAATTTAATTAATCTCGTTCCTATAAAAAAAATTGTT
GACACTACCTCGAATGACTAATAATCTATAATGTTAATTCTTCTCTCTATTTTTTTGTTTAGGTTTTTGCTGCTTATTGTXXXXXXXXXXT
TATCTTGTTCGAACTAACTTAGATTTATTTTAAAAAGTAGTCATAATTTAATTAATCTCGTTCCTATAAAAAAAATTGTT
>Marker467252 Chr3 SNP_site:21674529 Ref_Type:C
ACCTTTGTATTTATGTAACCAAATATGTCGTATATCCTTCACTGAAAAAATAAAGAAAAGAATAATGCTATTGCTAGCAAXXXXXXXXXXT
TTCTCACTCCTAATTAATAAATATCATTTTTAATAGTCATTATAAACCTGTATTATGAACACCTGTTGCTTAATTTGTT
ACCTTTGTATTTATGTAACCAAATATGTCGTATATCCTTCATTGAAAAAATAAAGAAAAGAATAATGCTATTGCTAGCAAXXXXXXXXXXT
TTCTCACTCCAAATTAATAAATATCATTTTTAATAGTCATTATAAACCTGTATTATGAACACGTGTTGCTTAATTTGTT
>Marker467252 Chr3 SNP_site:21674709 Ref_Type:T
ACCTTTGTATTTATGTAACCAAATATGTCGTATATCCTTCACTGAAAAAATAAAGAAAAGAATAATGCTATTGCTAGCAAXXXXXXXXXXT
TTCTCACTCCTAATTAATAAATATCATTTTTAATAGTCATTATAAACCTGTATTATGAACACCTGTTGCTTAATTTGTT
ACCTTTGTATTTATGTAACCAAATATGTCGTATATCCTTCATTGAAAAAATAAAGAAAAGAATAATGCTATTGCTAGCAAXXXXXXXXXXT
TTCTCACTCCAAATTAATAAATATCATTTTTAATAGTCATTATAAACCTGTATTATGAACACGTGTTGCTTAATTTGTT
>Marker467252 Chr3 SNP_site:21674761 Ref_Type:C
ACCTTTGTATTTATGTAACCAAATATGTCGTATATCCTTCACTGAAAAAATAAAGAAAAGAATAATGCTATTGCTAGCAAXXXXXXXXXXT
TTCTCACTCCTAATTAATAAATATCATTTTTAATAGTCATTATAAACCTGTATTATGAACACCTGTTGCTTAATTTGTT
ACCTTTGTATTTATGTAACCAAATATGTCGTATATCCTTCATTGAAAAAATAAAGAAAAGAATAATGCTATTGCTAGCAAXXXXXXXXXXT
TTCTCACTCCAAATTAATAAATATCATTTTTAATAGTCATTATAAACCTGTATTATGAACACGTGTTGCTTAATTTGTT
>Marker467328 Chr3 SNP_site:33692874 Ref_Type:G
GACCATTTTGGTTTAGAAATGTAATGGATGACCTTTGTTTTTCCATGAATAGGCAATCCTTTATATTCAATATCTCATGCXXXXXXXXXXC
ACACTCATCTCTGATCTTCAAATCAGTTCCACAGCTTTGTTGGACTTCACATGTTTAAGATATTTGCGTATGTTTTTGT
GACCATTTTGGTTTAGAAATTTAATGGATGACCTTTGTTTTTCCATGAATAGGCAATCCTTTATATTCAATATCTCATGCXXXXXXXXXXC

Create PDF with GO2PDF for free, if you wish to

ACACTCATCTCTGATCTTCAAATCAGTTCCACAGCTTTGTTGGACTTCACATGTTTAAGATATTTGCGTATGTTTTTGT
>Marker467394 Chr3 SNP_site:32530697 Ref_Type:C
CACTAAATAATAATTAATAGATGGGTGTATATCTCATTAACAGATGAGAGATGAGATAGAATCTAATGTATTAAATCGTTXXXXXXXXXXXT
TAGTATCATTAATCATATTCATTCTCACTCCTTAATTCACCTAAATGGATACAATGATCTTTTTTTTTCTTCACTAGTG
CACTAAATAATAATTAATAGATGGGTGTATATCTCATTAACAGATGAGAGATGAGATAGAATCTAATGTATTAAATCGTTXXXXXXXXXXXT
TAGTATCATTAATCCTATTCATTCTCACTCCTTAATTCACCTAAATGGATACAATGATCTTTTTTTTTCTTCACTAGTG
>Marker467397 Chr3 SNP_site:29795377 Ref_Type:A
ACTCATATCGTTATTGATTCGAGCAATAGCTCAATTGGCATAAATGTGTGGTATCATCCGCAAAGAAAAAGGCATCGATAXXXXXXXXXXXA
CAACTTATACAATTGGTTCGGCTTTTAAGTATAAAACAGACTAACAGAGAATCTGTGACAAAATAAGTCCTTGAACTGT
ACTCTTATCGTTATTGATTCGAGCAATAGCTCAATTGGCATAAATGTGTGGTATCATCCGCAAAGAAAAAGGCATCGATAXXXXXXXXXXXA
CAACTTATACAATTGGTTCGGTTTTTAAGTATAAAACAGACTAACAGAGAATCTGTGACAAAATAAGTCCTTGAACTGT
>Marker467397 Chr3 SNP_site:29795602 Ref_Type:T
ACTCATATCGTTATTGATTCGAGCAATAGCTCAATTGGCATAAATGTGTGGTATCATCCGCAAAGAAAAAGGCATCGATAXXXXXXXXXXXA
CAACTTATACAATTGGTTCGGCTTTTAAGTATAAAACAGACTAACAGAGAATCTGTGACAAAATAAGTCCTTGAACTGT
ACTCTTATCGTTATTGATTCGAGCAATAGCTCAATTGGCATAAATGTGTGGTATCATCCGCAAAGAAAAAGGCATCGATAXXXXXXXXXXXA
CAACTTATACAATTGGTTCGGTTTTTAAGTATAAAACAGACTAACAGAGAATCTGTGACAAAATAAGTCCTTGAACTGT
>Marker467835 Chr3 SNP_site:29277467 Ref_Type:C
CACAATAACCACTTACTCGAGCAAAAGTTGATTGTTTCTTTGGATAACCAGAGATGTGTATGCTTCCTTCAATATAACCTXXXXXXXXXXXA
TTTGGAAGATATAGATGTGTAGTGATCAAACAGTTTCCATTGTTGATAACATCTTTGCAAAAATTTACAAACTTCAGTT
CACAATAACCACTTACTCGAGCAAAAGTTGATTGTTTCTTTGGATAACCAGAGATGTGTATGCTTCCTTCAATATAACCTXXXXXXXXXXXA
TTTGGAAGATATAGATGTGTAGTGATCAAACAGTTTCCATTGTTGATAACATCTTTGCAAAAATTTACCAACTTCAGTT
>Marker467875 Chr3 SNP_site:8262584 Ref_Type:A
AACGCTAACCTCTCGATATCGGCGTAACACTCCTGCTTCTTTTGCCGGGTTTCAGCATCCTGAATAATTGAAATACAATTXXXXXXXXXXXG
TGAGCAATGAGAAGATTAACCCTAGAAGGATCAAGGGTTTGAGTGTGTTGAGAAGCTTTGAATTTCTCGAAGAATTGTT
AACGCTAACCTCTCGATATCGGCGTAACACTCCTGCTTCTTTTGCCGGGTTTCAGCATCCTGAATAATTGAAATGCAATTXXXXXXXXXXXG
TGAGCAATGAGAAGATTAACCCTAGAAGGATCAAGGGTTTGAGTGTGTTGAGAAGCTTTGAATTTCTCGAAGAATTGTT
>Marker468180 Chr3 SNP_site:39244618 Ref_Type:A
AACCAATGACGACATCCAAATTAGAATCAAAAGGTCAAGGGTTTATCAGTATAAGTAGGTAGGCAAAAAGATGCCACTTAXXXXXXXXXXXA
AAGATATTTGAGTGGCTAGAAATATTATTAGGGAATTAAAGACAATAATACTTAGCCAAGGAGTTTGTTAGTGGGAGTT
AACCAATGACGACATCCAAATTAGAATCAAAAGGTCAAGGGTTTATCAGTATAAGTAGGTAGGCAAAAAGATGCCACTTAXXXXXXXXXXXA
AAGATATTTGGGTGGCTAGAAATATTATTAGGGAATTAAAGACAATAATACTTAGCCAAGGAGTTTGTTAGTGGGAGTT
>Marker468272 Chr3 SNP_site:13016312 Ref_Type:C
CACAATGTTGTATCATGAAAAACAGAGAGCTATTTGCATCTGCATATTTCTTATTCGAGATGCATATTGTAGCATTCAGAXXXXXXXXXXXT
TGCTGAAAGAGATTACAGATTCATAGCTCCCATGATAGCTAGAAAAGCCAGTCCAACAAGGAAGAAAATCATCATAAGT
CACAATGTTGTATTATAAAAAACAGAGAGCTATTTGCATCTGCATATTTCTTATTCGAGATGCATATTGTAGCATTCAGAXXXXXXXXXXXT
TGCTGAAAGAGATTACAGATTCATAGCTCCCATGATAGCTAGAAAAGCCAGTCCAACAAGGAAGAAAATCATCATAAGT
>Marker468272 Chr3 SNP_site:13016315 Ref_Type:G
CACAATGTTGTATCATGAAAAACAGAGAGCTATTTGCATCTGCATATTTCTTATTCGAGATGCATATTGTAGCATTCAGAXXXXXXXXXXXT
TGCTGAAAGAGATTACAGATTCATAGCTCCCATGATAGCTAGAAAAGCCAGTCCAACAAGGAAGAAAATCATCATAAGT
CACAATGTTGTATTATAAAAAACAGAGAGCTATTTGCATCTGCATATTTCTTATTCGAGATGCATATTGTAGCATTCAGAXXXXXXXXXXXT
TGCTGAAAGAGATTACAGATTCATAGCTCCCATGATAGCTAGAAAAGCCAGTCCAACAAGGAAGAAAATCATCATAAGT
>Marker468306 Chr3 SNP_site:26167117 Ref_Type:G
TACCTTTTCCAGCGTCAATCCATGTAAATGATATCTAAGTTGCCAGCTTCATATTTTGAATTGTGCATGGTGCTGAAGAAXXXXXXXXXXXC
TAATAGGATTTCCTACCTCACCAGCCAAGAGGGTATTAGATCATCAGCCCAAAGAAATTGAAGTCGCTGATCTGTCAGT

Create PDF with GO2PDF for free, if you wish to

TACCTTTTCCAGCGTCAATCCATGTAAATGATATCTAAGTTGCCGGCTTCATATTTTGAATTGTGCATGGTGCTGAAGAAXXXXXXXXXXC
TAATAGGATTTCCTACCTCACCAGCCAAGAGGGTATTAGATCATCAGCCCAAAGAAATTGAAGTCGCTGATCTGTCAGT
>Marker468478 Chr3 SNP_site:9075999 Ref_Type:A
AACTTCGAGAACTACCTTCCTGAGGACCAGAGTCACCTGAAGGGCACGATGAGCGTTGTCGTCTAGATCCCTTCTCCGAGXXXXXXXXXXC
TATTACCAGTTCATCAAAATGCAATAAATCAAACAACATTTTACATCACCAATAAGGTAAAATAGAGAGTAGTTTTGGT
AACTTCGAGAACTACCTTCCTGAGGACCAGAGTCACCTGAAGGGCACGATGAGCGTTGTCGTCTAGATCCCTTCTCCGAGXXXXXXXXXXC
TATTACCAGTTCATCAAAATGCAATAAATCAAACAACATTTTACATCACCAATAAGGTAGAATAGAGAGTAGTTTTGGT
>Marker469102 Chr3 SNP_site:31732709 Ref_Type:T
TACATCTATCTGGGTTGGATCTTGAATTGGCAATTATGTTAGTCTCTGTATGGGTTTTTCGTTTTTTGTATTTCTTGTAAXXXXXXXXXXT
TCTTTAAAAAAATGTCAATTATGCTAGAGGTGGATTGAGAAAAAGTTTCAGGAGTCTGTTGATTATCTTTGTCTTTGTG
TACATCTATCTGGGTTGGATCTTGAATTGGCAATTATGTTAGTCTCTTTATGGGTTTTTCGTTTTTTGTATTTCTTGTAAXXXXXXXXXXT
TCTTTAAAAAAATGTCAATTATGCTAGAGGTGGATTGAGAAAAAGTTTCAGGAGTCTGTTGATTATCTTTGTCTTTGTG
>Marker469128 Chr3 SNP_site:34021592 Ref_Type:A
TACCCTCCAGAGAAAGCCCTAGACATAGTCAAGGAAAGAAACCAACTTCACACCCAAAGGCATCTGCCCGTCTCCTTATCXXXXXXXXXXA
CTGGAGGTGTGAAATGGCTAACTCTTTTGAACAAAATAAAATTTTATGATACTTCCTTCCACCGCTTTCATAATATGTC
TACCCTCCAGAGAAAGCCCTAGACATAGTCAAGGAAAGAAACCAGCTTCACACCCAAAGGCATCTGCCCGTCTCCTTATCXXXXXXXXXXA
CTGGAGGTGTGAAATGGCTAACTCTTTTGAAGAAAATAAAATTTTATGATACTTCCTTCCACCGCTTTCATAATAAGTC
>Marker469128 Chr3 SNP_site:34021808 Ref_Type:C
TACCCTCCAGAGAAAGCCCTAGACATAGTCAAGGAAAGAAACCAACTTCACACCCAAAGGCATCTGCCCGTCTCCTTATCXXXXXXXXXXA
CTGGAGGTGTGAAATGGCTAACTCTTTTGAACAAAATAAAATTTTATGATACTTCCTTCCACCGCTTTCATAATATGTC
TACCCTCCAGAGAAAGCCCTAGACATAGTCAAGGAAAGAAACCAGCTTCACACCCAAAGGCATCTGCCCGTCTCCTTATCXXXXXXXXXXA
CTGGAGGTGTGAAATGGCTAACTCTTTTGAAGAAAATAAAATTTTATGATACTTCCTTCCACCGCTTTCATAATAAGTC
>Marker469128 Chr3 SNP_site:34021852 Ref_Type:T
TACCCTCCAGAGAAAGCCCTAGACATAGTCAAGGAAAGAAACCAACTTCACACCCAAAGGCATCTGCCCGTCTCCTTATCXXXXXXXXXXA
CTGGAGGTGTGAAATGGCTAACTCTTTTGAACAAAATAAAATTTTATGATACTTCCTTCCACCGCTTTCATAATATGTC
TACCCTCCAGAGAAAGCCCTAGACATAGTCAAGGAAAGAAACCAGCTTCACACCCAAAGGCATCTGCCCGTCTCCTTATCXXXXXXXXXXA
CTGGAGGTGTGAAATGGCTAACTCTTTTGAAGAAAATAAAATTTTATGATACTTCCTTCCACCGCTTTCATAATAAGTC
>Marker469434 Chr3 SNP_site:38755605 Ref_Type:A
CACAATAATTTTGATTTAACGAAGAAAAAAACCGTTAAAATAAGCATAAGTCAATTACATAATAGTGCAAGTTTTATACAXXXXXXXXXXA
AAATCAAAACTCTTCAATATTTATAGGAGAAAAGAAAAACTTCAAACTCAGCTTGGTCAAAATTTTTGAAGTCAAAGTG
CACAATAATTTTGATTTAACGAAGAAAAAAACCGTTAAAATAAGCATAAGTCAATTACATAATAGTGCAAGTTTTATACAXXXXXXXXXXA
AAATCAAAACTCTTCAATATTTATAGGAGAAAAGAAAATCTTCAAATTCAGCTTGGTCAAAATTTTTGAAGTCAAAGTG
>Marker469434 Chr3 SNP_site:38755613 Ref_Type:C
CACAATAATTTTGATTTAACGAAGAAAAAAACCGTTAAAATAAGCATAAGTCAATTACATAATAGTGCAAGTTTTATACAXXXXXXXXXXA
AAATCAAAACTCTTCAATATTTATAGGAGAAAAGAAAAACTTCAAACTCAGCTTGGTCAAAATTTTTGAAGTCAAAGTG
CACAATAATTTTGATTTAACGAAGAAAAAAACCGTTAAAATAAGCATAAGTCAATTACATAATAGTGCAAGTTTTATACAXXXXXXXXXXA
AAATCAAAACTCTTCAATATTTATAGGAGAAAAGAAAATCTTCAAATTCAGCTTGGTCAAAATTTTTGAAGTCAAAGTG
>Marker469720 Chr3 SNP_site:29091996 Ref_Type:T
ACATTTCTTCTCAAACTTCAATGGATATCCCTTTTCACAAGTTGGATTGTTTTGGAACGCCATTCCAAAATCCTAAATTTXXXXXXXXXXT
GAGAAGTTAGGAGATATTCTGAAGGGGAAAACAGCTTCAAAAGCTACCAAGAAACCAATATTCAGAATTGGTGATAGGT
ACATTTCTTCTCAAACTTCAATGGATATCCCTTTTCACAAGTTGGATTGTTTTGTAACGCCATTCCAAAATCCTAAATTTXXXXXXXXXXT
GAGAAGTTAGGAGATATTCTGAAGGGGAAAACAGCTTCAAAAGCTACCAAGAAACCAATATTCAGAATTGGTGATAGGT
>Marker469731 Chr3 SNP_site:35048963 Ref_Type:G
ACAAAACTTGGCAATTTTAAAAATTCAAATTTAGTTTCAGGAATTCATTGTGTGTTTCATTTATAGCCTCATTGCATGGGXXXXXXXXXXA

Create PDF with GO2PDF for free, if you wish to

ATATCCTTTAGATGATAAAGAACTTAATGCGGAAAGATAAAACAACTTTAAACTTTTATTCCTTAACAAGGCGTTTAGT
ACAAAACTTGGCAATTTTAAAAATTCAAATTTAGTTTCAGGAATTCATTGTGTGTTTCATTTGTAGCCTCATTGCATGGGXXXXXXXXXXA
ATATCCTTTAGACGATAAAGAACTTAATGCGGAAAGATAAAACAACTTTAAACTTTTATTCCTTAACAAGGCGTTTAGT
>Marker469731 Chr3 SNP_site:35049141 Ref_Type:C
ACAAAACTTGGCAATTTTAAAAATTCAAATTTAGTTTCAGGAATTCATTGTGTGTTTCATTTATAGCCTCATTGCATGGGXXXXXXXXXXA
ATATCCTTTAGATGATAAAGAACTTAATGCGGAAAGATAAAACAACTTTAAACTTTTATTCCTTAACAAGGCGTTTAGT
ACAAAACTTGGCAATTTTAAAAATTCAAATTTAGTTTCAGGAATTCATTGTGTGTTTCATTTGTAGCCTCATTGCATGGGXXXXXXXXXXA
ATATCCTTTAGACGATAAAGAACTTAATGCGGAAAGATAAAACAACTTTAAACTTTTATTCCTTAACAAGGCGTTTAGT
>Marker469831 Chr3 SNP_site:32455386 Ref_Type:A
CACTTCTGAAACTGATTCAAACGGTTCATTACTTACTTCTTGCTTGCATATTGTGTTGGAATATAAATTACGTTGTTTAAXXXXXXXXXXT
GATATATCTGATACATGTGCAAGGACTCGCCGAACATGTTCATTCAACTTTAAATTCCAAATTCACACTTCTCCTTGTG
CACTTCTGAAACTGATTCAAACGGTTCATTACTTACTTCTTGCTTGCATATTGTGTTGGATTATAAATTACGTTGTTTAAXXXXXXXXXXT
GATATATCTGATACATGTGCAAGGACTCGCCGAACATGTTCATTCAACTTTAAATTCCAAATTCACACTTCTCCTTGTG
>Marker469899 Chr3 SNP_site:34759779 Ref_Type:T
GACCTAACTAATGATTAATGAGGGTCCATGCATAAATAATAATAGATTTTATTGAGAATGAAAGTGAAGTTACCAACATCXXXXXXXXXXA
AGTGTGTATTTAATTACCTATTAAATTTGAAACGTTTTTTTAAAAATTTTTTAATTTAGATTGAAAAAACGTTTTCGTT
GACCTAACTAATGATTAATGAGGGTCCATGCATAAATAATAATAGATTTTATTGAGAATGAAAGTTAAGTTACCAACATCXXXXXXXXXXA
AGTGTGTATTTAATTACCTATTAAATTTGAAACGTTTTTTTAAAAATTTTTTAATTTAGATTGAAAAAACGTTTTCGTT
>Marker469939 Chr3 SNP_site:25605564 Ref_Type:A
ACCAACAATCCGCCTTTACGTGACCAAACTTCTTGCAATGGTAACACTGAATACTAGCTTTGCTGCTTGATTGCTCTCCTXXXXXXXXXXC
TTCGTTCCTTTTCGTCGAACTGTTAATTCTCGACTCATGTGCTTGAAGAGATCCCATCAATTCATTAAATGAAAAAGTG
ACCAACGATCCGCCTTTACGTGACCAAACTTCTTGCAATGGTAACACTGAATACTAGCTTTGCTGCTTGATTGCTCTCCTXXXXXXXXXXC
TTCGTTCCTTTTCGTCGAACTGTTAATTCTCGACTCATGTGCTTGAAGAGATCCCATCAATTCATTAAATGAAAAAGTG
>Marker470406 Chr3 SNP_site:9998300 Ref_Type:T
AACATTAACGATTATTTTTTGAAAAGTTAATGAACTTAAACAAAAGATCACTTTGTGTGGCAAAATTCATGAAATAAATGXXXXXXXXXXA
ACACAACTAATGATTATAATCATATTTTAAATAAATTATAAACAACTAATAGTTTTAAACAAAATACTTTAAATTGAGT
AACATTAACGATTATTTTTTGAAAAGTTAATGAACTTAAACAAAATATCACTTTGTGTGGCAAAATTCATGAAATAAATGXXXXXXXXXXA
ACACAACTAATGATTATAATCATATTTTAAATAAATTATAAACAACTAATAGTTTTAAACAAAATACTTTAAATTGAGT
>Marker471326 Chr3 SNP_site:29343970 Ref_Type:C
ACTAAAGTTCTAATTTAGGTTAGATAAAAATTTAAATGAAATTAGAGTATGGGAAGAAAAATAAGTAGGAGACTGAAAGTXXXXXXXXXXA
GTCCTACATATTTGTATGAATGTTTATATATTGTTTCATCCACTAAACTCAAATCTTAAATTCTAAACCATGATGGGTA
ACTAAAGTTCTAATTTAGGTTAGATAAAAATTTAAATGAAATTAGAGTATGGGAAGAAAAATAAGTAGGAGACTGAAAGTXXXXXXXXXXA
GTCCTACATATTTGTATGAATGTTTATATATTGTTTCCTCCACTAAACTCAAATCTTAAATTCTAAACCATGATGGGTA
>Marker471809 Chr3 SNP_site:11501862 Ref_Type:A
ACCCTGACTTAGTAATTATCCTTGATGTTCTTCGGCTTTGATTTCGAAAATATTTTGAAGTTATCTTCTAGACTTGATTTXXXXXXXXXXG
CCCGAATATCCTAATAACACCCTAATATCATTCCAATTACCTTCTTTATTTAGGCAAAAATTAAAAAAAAATCCATGTA
ACCCTGACTTAGTAATTATCCTTGATGTTCTTCGGCTTTGATTTCGAAAATATTTTGAAGTTATCTTCTAGACTTGATTTXXXXXXXXXXG
CCCGAATATCCTAATAACACCCTAATATCATTCCAATTACCTTCTTTATTTAGGCAAAAATTTAAAAAAAATCCATGTA
>Marker471870 Chr3 SNP_site:36467206 Ref_Type:C
TACATATTACCCCTCCGTATTTGGTATAATAGACAAAACATTTGTATTTGGCTTGCAGACCAATTGTGAAAATTATACTGXXXXXXXXXXT
TATACCGTGTTGTTTCTTTTAGTTTTATAAATTCATTTTGTTTTTTCTTTTCTTCTATCTAAACTAGGGTCCGCATGTA
TACATATTACCTCTCCGTATTTGGTATAATAGACAAAACATTTGTATTTGGCTTGCAGACCAATTGTGAAAATTATACTGXXXXXXXXXXT
TATACCGTGTTGTTTCTTTTAGTTTTATAAATTCATTTTGTTTTTTCTTTTCTTCTATCTAAACTAGGGTCCGCATGTA
>Marker471936 Chr3 SNP_site:29254236 Ref_Type:T

Create PDF with GO2PDF for free, if you wish to

AACCCTACCTGAAGCACCCACAAAGAAAGAAAAAGAGCATTCTTGTCGTAAGCCAAAGCATGAGTGAAATATTACAAATCXXXXXXXXXXT
ACAGCTTTCCTAAGGAAAGAAAGCCTTGCACCTTGCCCCATCTCGTGAGATAAACTCCCCAACCCTCATCAAATAAAGT
AACCCTACCTGAAGCACCCACAAAGAAAGAAAAAGAGCATTCTTGTCGTAAGCCAAAGCATGAGTGAAATATTACAAATCXXXXXXXXXXT
ACAGCTTTCCTAAGGAAAGAAAGCCTTGCACCTTGCCCCATCTCGTGAGATAAATTCCCCAACCCTCATCAAATAAAGT
>Marker472427 Chr3 SNP_site:35803834 Ref_Type:T
TACTGGACAAGATGAAGTATCCGCCAGGCCTTAAAATTCTATTCATTTCTAGGAGAAGCTTCCCATCTAGAGAAAGAGAGXXXXXXXXXXC
TAGGAAAAGCAAGTCTTCTATTCCCAAAAGGGCTAACCAGAGTTGGAAACCCCCGCTCAAGTGCAACTTGAGCCAAGTC
TACTGGACAAGATGAAGTATCCGCCAGGCCTTAAAATTCTATTCATTTCTAGGAGAAGCTTCCCATCTAGAGAAAGAGAGXXXXXXXXXXC
TAGGAAAAGCAAGTCTTCTATTCCCAAAAGGGCTAACCAGAGTTGGAAACCCTCGCTCAAGTGCAACTTGAGCCAAGTC
>Marker472756 Chr3 SNP_site:26378396 Ref_Type:C
CACTGAAAATTTATAATGAAACCAAAAGACACATAAAATCACATTAAGTTAAAGTTAAACAAAATATCTTGAAACAGTAAXXXXXXXXXXA
TGTAAGGTAGATGGGCTGGACGTGTTAAATTCATCGTCTTGTCTCCTTGCTTAAACGACACTTCAGGTTCCTTTGTTGT
CACTGAAAATTTATAATGAAACCAAAAGACATATAAAATCACATTAAGTTAAAGTTAAACAAAATATCTTGAAACAGTAAXXXXXXXXXXA
TGTAAGGTAGATGGGCTGGACGTGTTAAATTCATCGTCTTGTCTCCTTGCTCAAACGACACTTCAGGTTCCTTTGTTGT
>Marker472756 Chr3 SNP_site:26378615 Ref_Type:T
CACTGAAAATTTATAATGAAACCAAAAGACACATAAAATCACATTAAGTTAAAGTTAAACAAAATATCTTGAAACAGTAAXXXXXXXXXXA
TGTAAGGTAGATGGGCTGGACGTGTTAAATTCATCGTCTTGTCTCCTTGCTTAAACGACACTTCAGGTTCCTTTGTTGT
CACTGAAAATTTATAATGAAACCAAAAGACATATAAAATCACATTAAGTTAAAGTTAAACAAAATATCTTGAAACAGTAAXXXXXXXXXXA
TGTAAGGTAGATGGGCTGGACGTGTTAAATTCATCGTCTTGTCTCCTTGCTCAAACGACACTTCAGGTTCCTTTGTTGT
>Marker473697 Chr3 SNP_site:28780935 Ref_Type:A
CACTTCCTTGTATCACTCTTCTTAAATAACCACTTCTTGGTTCAAATAGCATGTGTGCATTAATGTGGAAAGTAACAAAAXXXXXXXXXXA
AGTTTTGATTTTGAATCATCTTTCACATAATTTCAAGTCCTAATTTTATAGAAGCTCTACGAAAATTTAATAAAAAGTG
CACTTCCTTGTATCACTCTTCTTAAATAACCACTTCTTGGTTCAAATGGCATGTGTGCATTAATGTGGAAAGTAACAAAAXXXXXXXXXXA
AGTTTTGATTTTGAATCATCTTTCACATAATTTCAAGTCCTAATTTTATAGAAGCTCTACGAAAATTTAATAAAAAGTG
>Marker473709 Chr3 SNP_site:12905321 Ref_Type:T
CACCTCCCTTAGCGTGCCATCCGTGCGTGCGACCACCCTGTGTGGCATGCGCCCTTTTTGCGCATCATCCTTGGTGCATGXXXXXXXXXXC
CCCAAGATCGAGGCACTTGTTGCCTGTCTCGCCTAGCTATCCGCCATCAGGTGGGGTTGTGGCATGTCTTGACTTGGTC
CACCTCCCTTAGCGTGCCATCCGTGCGTGCGACCACCCTGTGTGGCATGCGCCCTTTTTGCGCATCATCCTTGGTTCATGXXXXXXXXXXC
CCCAAGATCGAGGCACTTGTTGCCTATCTCGCCTAGCTATCCGCCATTAGGTGGGGTTGTGGCATGTCTTGACTTGGTC
>Marker473709 Chr3 SNP_site:12905483 Ref_Type:A
CACCTCCCTTAGCGTGCCATCCGTGCGTGCGACCACCCTGTGTGGCATGCGCCCTTTTTGCGCATCATCCTTGGTGCATGXXXXXXXXXXC
CCCAAGATCGAGGCACTTGTTGCCTGTCTCGCCTAGCTATCCGCCATCAGGTGGGGTTGTGGCATGTCTTGACTTGGTC
CACCTCCCTTAGCGTGCCATCCGTGCGTGCGACCACCCTGTGTGGCATGCGCCCTTTTTGCGCATCATCCTTGGTTCATGXXXXXXXXXXC
CCCAAGATCGAGGCACTTGTTGCCTATCTCGCCTAGCTATCCGCCATTAGGTGGGGTTGTGGCATGTCTTGACTTGGTC
>Marker473709 Chr3 SNP_site:12905505 Ref_Type:T
CACCTCCCTTAGCGTGCCATCCGTGCGTGCGACCACCCTGTGTGGCATGCGCCCTTTTTGCGCATCATCCTTGGTGCATGXXXXXXXXXXC
CCCAAGATCGAGGCACTTGTTGCCTGTCTCGCCTAGCTATCCGCCATCAGGTGGGGTTGTGGCATGTCTTGACTTGGTC
CACCTCCCTTAGCGTGCCATCCGTGCGTGCGACCACCCTGTGTGGCATGCGCCCTTTTTGCGCATCATCCTTGGTTCATGXXXXXXXXXXC
CCCAAGATCGAGGCACTTGTTGCCTATCTCGCCTAGCTATCCGCCATTAGGTGGGGTTGTGGCATGTCTTGACTTGGTC
>Marker474374 Chr3 SNP_site:23542729 Ref_Type:G
TACTCGGGACTGGGATTGGTCTAGTTTCCATATGGATCAAATGGTTGAGAAGATCTAGCAGACTCACTCACATAAGCCCGXXXXXXXXXXT
GCTTGTCACTGCCACTGGTAGAGGACCTCACATTAAAAGTAGCAGAGTCAATAGCATGAGTCGCCGAAATATTCATAGT
TACTCGGGACTGGGATTGGTCTGGTTTCCATATGGATCAAATGGTTGAGAAGATCTAGCAGACTCACTCACATAAGCCCGXXXXXXXXXXT
GCTTGTCACTGCCACTGGTAGAGGACCTCGCATTAAAAGTAGCAGAGTCAATAGCATGAGTCGCCGAAATATTCATAGT

Create PDF with GO2PDF for free, if you wish to

>Marker474374 Chr3 SNP_site:23542946 Ref_Type:G
TACTCGGGACTGGGATTGGTCTAGTTTCCATATGGATCAAATGGTTGAGAAGATCTAGCAGACTCACTCACATAAGCCCGXXXXXXXXXXXT
GCTTGTCACTGCCACTGGTAGAGGACCTCACATTAAAAGTAGCAGAGTCAATAGCATGAGTCGCCGAAATATTCATAGT
TACTCGGGACTGGGATTGGTCTGGTTTCCATATGGATCAAATGGTTGAGAAGATCTAGCAGACTCACTCACATAAGCCCGXXXXXXXXXXXT
GCTTGTCACTGCCACTGGTAGAGGACCTCGCATTAAAAGTAGCAGAGTCAATAGCATGAGTCGCCGAAATATTCATAGT
>Marker474803 Chr3 SNP_site:32795285 Ref_Type:A
TACGTATACGTGCATACATATAAATATAAGCTATACAATACATATATGTATAGACATATACACATATAAATATGTCACACXXXXXXXXXXXG
CAGCAGCCACGATGCAGATAGAACTCGTGGATGCATCGACAAAAATAAAGGCAATTTAATGGTCAAGATTGAGAGGGTT
TACGTATACGTGCATACATATAAATATAAGCTATACAATACATATATGTATAGACATATACACATATATATATGTCACACXXXXXXXXXXXG
CAGCAGCCACGATGCAGATAGAACTCGTGGATGCATCGACAAAAATAAAGGCAATTTAATGGTCAAGATTGAGAGGGTT
>Marker474904 Chr3 SNP_site:33941716 Ref_Type:T
CACACCCTAGCAAGGCATAAGAAGCACCCTTTCCAAAACAAAGACAAATTGAGGAGGAACTCCCTGATCGAGTTTATAGCXXXXXXXXXXXT
TCTTCCGCTTTCAACAAAGAATACAAAAGAAAGGTCCCAGCAACTAAAGTGACATACTCGTGAGCCGATCCAAGGTGTT
CACACCCTAGCAAGGCATAAGAAGCACCCTTTCCAAAACAAAGACAAATTGAGGAGGAACTCCCTGATTGAGTTTATAGCXXXXXXXXXXXT
TCTTCCGCTTTCAACAAAGAATACAAAAGAAAGGTCCCAGCAACTAAAGTGACATACTCGTGAGCCGATCCAAGGTGTT
>Marker475386 Chr3 SNP_site:38350819 Ref_Type:C
AACTTTTGTATCCATATGCTATCTCTATGACTTTAGGCCTCAGTAGTTTTTGTCTAAGTGACTCTTAATCAACCAGGAGCXXXXXXXXXXXG
TCACATGTGATATGAACATCGAAGCTGCTGTATAATTTTGTATATTTCTCTGAACTCTATGTATTAGTTCATGACGGTT
AACTTTTGTATCCATCTGCTATCTCTATGACTTTAGGCCTCAGTAGTTTTTGTCTAAGTGACTCTTAATCAACCAGGAGCXXXXXXXXXXXG
TCACATGTGATATGAACATCGAAGCTGCTGTATAATTTTGTATATTTCTCTGAACTCTATGTATTAGTTCATGACGGTT
>Marker475426 Chr3 SNP_site:36742896 Ref_Type:A
GACATTCGGTGCATTTGCAGTTTGTTCAACTACAAAGGTTACCTTTGCAGGCATGCATTAAATGTTTTTAATTATAACGGXXXXXXXXXXXA
AGTAATACAAATGTGTATAGTTCGACATACCAATATAGTCATATTCTTAATTTTGCACTTCCAGTTGTAGAAGAAGGTG
GACATTCGGTGCATTTGCAGTTTGTTCAACTACAAAGGTTACCTTTGCAGGCATGCGTTAAATGTTTTTAATTATAACGGXXXXXXXXXXXA
AGTAATACAAATGTGTATAGTTCGACATACCAATATAGTCATATTCTTAATTTTGCACTTCCAGTTGTAGAAGAAGGTG
>Marker475476 Chr3 SNP_site:31525334 Ref_Type:A
TACAATACACTATTACACAAAGATACTATTAGTATTATTTATTCAGATACTATGAATTATAGGCATCTCATTTTATACAAXXXXXXXXXXXC
TAATACCAAATAGGAACTAAACTCAAAATTTAAGGCTATAAGGATTTAATGGTTACTAAATTCAAAACTTGGGTAATGT
TACAATACACTATTACACAAAGATACTATTAGTGTTATTTATTCAGATACTATGAATTATAGGCATCTCATTTTATACAAXXXXXXXXXXXC
TAATACCAAATAGGAACGAAACTCAAAATTTAAGGCTATAAGGATTTAATGGTTACTAAATTCAAAACTTGGGTAATGT
>Marker475476 Chr3 SNP_site:31525499 Ref_Type:T
TACAATACACTATTACACAAAGATACTATTAGTATTATTTATTCAGATACTATGAATTATAGGCATCTCATTTTATACAAXXXXXXXXXXXC
TAATACCAAATAGGAACTAAACTCAAAATTTAAGGCTATAAGGATTTAATGGTTACTAAATTCAAAACTTGGGTAATGT
TACAATACACTATTACACAAAGATACTATTAGTGTTATTTATTCAGATACTATGAATTATAGGCATCTCATTTTATACAAXXXXXXXXXXXC
TAATACCAAATAGGAACGAAACTCAAAATTTAAGGCTATAAGGATTTAATGGTTACTAAATTCAAAACTTGGGTAATGT
>Marker475627 Chr3 SNP_site:34397290 Ref_Type:G
TACCCATCTTCCGACGTTACACTTTTCACGTCAGAAGAAGGGGGTTATTTCGACACATTTTTTGGGACGTTGGAGGAACCXXXXXXXXXXXT
ACAGTGACGTCGCAGACAATGTCGATAGGCACATTGAGGAAAGCACGTCGGGGATACTTTTCTCGATGCGGCTCATGTT
TACCCATCTTCCGACGTTACACTTTTCACGTCAGAAGAAGGGGGTTATTTCGACACATTTTTTGGGACGTTGGAGGAACCXXXXXXXXXXXT
ACAGTGACGTCGCGGACAATGTCGATAGGCACATTGAGGAAAGCACGTCGGGGATACTTTTCTTGATGCGGCTCATGTT
>Marker475627 Chr3 SNP_site:34397340 Ref_Type:T
TACCCATCTTCCGACGTTACACTTTTCACGTCAGAAGAAGGGGGTTATTTCGACACATTTTTTGGGACGTTGGAGGAACCXXXXXXXXXXXT
ACAGTGACGTCGCAGACAATGTCGATAGGCACATTGAGGAAAGCACGTCGGGGATACTTTTCTCGATGCGGCTCATGTT
TACCCATCTTCCGACGTTACACTTTTCACGTCAGAAGAAGGGGGTTATTTCGACACATTTTTTGGGACGTTGGAGGAACCXXXXXXXXXXXT

Create PDF with GO2PDF for free, if you wish to

ACAGTGACGTCGCGGACAATGTCGATAGGCACATTGAGGAAAGCACGTCGGGGATACTTTTCTTGATGCGGCTCATGTT

>Marker475640 Chr3 SNP_site:36743344 Ref_Type:A

CACAATCTCAAGAACGTTATAAGCTTGCATTGCAAGAATTGGAGGAGTTATTACATAAATTAAATCTTGTAGAGGATGATXXXXXXXXXXC

TTCCCTCCCTCAATCATCATTTCCTAGATCCATATGTATCTGTTGAGTCCCTCTATAAAACTGATACATAATGCAGGTA

CACAATCTCAAGAACGTTATAAGCTTGCATTGCAAGAATTGGAGGAGTTATTACATAAATTAAATCTTGTAGAGGATGATXXXXXXXXXXC

TTCCCTCCCTCAATCATCATTTCCTAGATCCATATGTATCTGTTGAGTCCCTCTATAAAACTGATTCATAATGCAGGTA

>Marker475834 Chr3 SNP_site:34121626 Ref_Type:A

AACCTAAAGGTAAAATTTGGGCTTCAAGAAGTTTCAGACAGGGACATCTTCTTTCCCCGTTTCTTTTCATTATCGTGGTGXXXXXXXXXXT

TTGCTTGAACCACATTATCATCTTCTTTGAAGAGATGTTGGGTTTGAAAATTAATAGAGGTAAGAGTTGTGTTATGGGT

CACCTAAAGGTAAAATTTGGGCTTCAAGAAGTTTCAGACAGGGACATCTTCTTTCCCCGTTTCTTTTCATTATCGTGGTGXXXXXXXXXXT

TTGCTTGAACCACATTATCATCTTCTTTGAAGAGATGTTGGGTTTGAAAATTAATAGAGGTAAAAGTTGTGTTATGGGT

>Marker475834 Chr3 SNP_site:34121888 Ref_Type:G

AACCTAAAGGTAAAATTTGGGCTTCAAGAAGTTTCAGACAGGGACATCTTCTTTCCCCGTTTCTTTTCATTATCGTGGTGXXXXXXXXXXT

TTGCTTGAACCACATTATCATCTTCTTTGAAGAGATGTTGGGTTTGAAAATTAATAGAGGTAAGAGTTGTGTTATGGGT

CACCTAAAGGTAAAATTTGGGCTTCAAGAAGTTTCAGACAGGGACATCTTCTTTCCCCGTTTCTTTTCATTATCGTGGTGXXXXXXXXXXT

TTGCTTGAACCACATTATCATCTTCTTTGAAGAGATGTTGGGTTTGAAAATTAATAGAGGTAAAAGTTGTGTTATGGGT

>Marker476305 Chr3 SNP_site:15413904 Ref_Type:G

ACCCTCCCATTCATCAGGTTTGTCTTTGTTGCCCTTAGTTACTTTCAGAATGTAGAGGGGCTGATTTGATTTTCAATTTAXXXXXXXXXXC

CATCATTCGATGTCGGCTGTAAGTTTGGGGATATGAACTGTTTTGGATGGATGTGTCAAATGGGAACTTGTTATTGAGT

ACCCTCCCATTCATCAGGTTTGTCTTTGTTGCCCTTAGTTACTTTCAGAATGTAGAGGGGCTGATTTGATTTTCAATTTAXXXXXXXXXXC

GATCATTCGATGTCGGCTGTAAGTTTGGGGATATGAACTGTTTTGGATGGATGTGTCAAATGGGAACTTGTTATTGAGT

>Marker477183 Chr3 SNP_site:38883834 Ref_Type:C

AACTCACCTAGAAGTTCAAATTTTGTCTCCATTTCTTTTCATTTCAAACCTCATTTCCTTCCAATAATTTGCATAAACTAXXXXXXXXXXG

AATTCCAAAACTATTACCAATCGGGACCTTAGTTTTTTTTCCAGGTAAAGACAACAAAAATGAATCAAGGTGGGAAAGT

AACTCACCTAGAAGTTCAAATTTTGTCTCCATTTCTTTTCATTTCAAATCTCATTTCCTTCCAATAATTTGCATAAACTAXXXXXXXXXXG

AATTCCAAAACGATTACCAATCGGGACCTTAGTTTTTTTTCCAGGTAAAGACAACAAAAATGAATCAAGGTGGGAAAGT

>Marker477183 Chr3 SNP_site:38883974 Ref_Type:T

AACTCACCTAGAAGTTCAAATTTTGTCTCCATTTCTTTTCATTTCAAACCTCATTTCCTTCCAATAATTTGCATAAACTAXXXXXXXXXXG

AATTCCAAAACTATTACCAATCGGGACCTTAGTTTTTTTTCCAGGTAAAGACAACAAAAATGAATCAAGGTGGGAAAGT

AACTCACCTAGAAGTTCAAATTTTGTCTCCATTTCTTTTCATTTCAAATCTCATTTCCTTCCAATAATTTGCATAAACTAXXXXXXXXXXG

AATTCCAAAACGATTACCAATCGGGACCTTAGTTTTTTTTCCAGGTAAAGACAACAAAAATGAATCAAGGTGGGAAAGT

>Marker477622 Chr3 SNP_site:35675284 Ref_Type:A

GACTCCTAAAAAGGAGCTGAGCACCAAACTGAGGCTGTAAGGTTATGAACAGCATGGGGGAATGAGTATAGGTTGGAGAAXXXXXXXXXXG

CTAAATGTTTCCGAGGCTCGCAAAATCTCAAAGGATTGAAGCAGTTTGCATCCTCTCCCCAAAACTCTTTGTCATGGTG

GACTCCTAAAAAGGAGCTGAGCACCAAACTGAGGCTGTAAGGTTATGAACAGCATGGGGGAATGAGTATAGGTTGGAGAAXXXXXXXXXXG

CTAAATGTTTCCGAGGCTCGCCAAATCTCAAAGGATTGAAGCAGTTTGCATCCTCTCCCCAAAACTTTTTGTCGTGGTG

>Marker477622 Chr3 SNP_site:35675329 Ref_Type:C

GACTCCTAAAAAGGAGCTGAGCACCAAACTGAGGCTGTAAGGTTATGAACAGCATGGGGGAATGAGTATAGGTTGGAGAAXXXXXXXXXXG

CTAAATGTTTCCGAGGCTCGCAAAATCTCAAAGGATTGAAGCAGTTTGCATCCTCTCCCCAAAACTCTTTGTCATGGTG

GACTCCTAAAAAGGAGCTGAGCACCAAACTGAGGCTGTAAGGTTATGAACAGCATGGGGGAATGAGTATAGGTTGGAGAAXXXXXXXXXXG

CTAAATGTTTCCGAGGCTCGCCAAATCTCAAAGGATTGAAGCAGTTTGCATCCTCTCCCCAAAACTTTTTGTCGTGGTG

>Marker477622 Chr3 SNP_site:35675336 Ref_Type:A

GACTCCTAAAAAGGAGCTGAGCACCAAACTGAGGCTGTAAGGTTATGAACAGCATGGGGGAATGAGTATAGGTTGGAGAAXXXXXXXXXXG

CTAAATGTTTCCGAGGCTCGCAAAATCTCAAAGGATTGAAGCAGTTTGCATCCTCTCCCCAAAACTCTTTGTCATGGTG

Create PDF with GO2PDF for free, if you wish to

GACTCCTAAAAAGGAGCTGAGCACCAAACTGAGGCTGTAAGGTTATGAACAGCATGGGGGAATGAGTATAGGTTGGAGAAXXXXXXXXXXXG
CTAAATGTTTCCGAGGCTCGCCAAATCTCAAAGGATTGAAGCAGTTTGCATCCTCTCCCCAAAACTTTTTGTCGTGGTG
>Marker478460 Chr3 SNP_site:6568982 Ref_Type:T
ACCAGACAGCTTGCACTGGTGGGAAAAAACTAACAGAATAATGAATTATAGAGGAATGAGTGAAGTTCAGGCAACAATCAXXXXXXXXXXXA
AATTAAGAACTGCATCAAAGTATAGACACATAAAAATATTGATAAATCAACGGGTTAAGTTCAATTTTATAGAAATGTA
ACCAGATAGCTTGCACTGGTGGGAAAAAACTAACAGAATAATGAATTATAGAGGAATGAGTGAAGTTCAGGCAACAATCAXXXXXXXXXXXA
AATTAAGAACTGCATCAAAGTATAGACACATAAAAATATTGATAAATCAACGGGTTAAGTTCAATTTTATAGAAATGTA
>Marker478488 Chr3 SNP_site:11467262 Ref_Type:A
ACTAATGCACCAATCATCTCCTCACACACACATACACAAAGCAATCATTTAAACTGCTGCAATGGAAAGGCCAGAAGCAAXXXXXXXXXXXG
ATTGCTCAGGAAAAGTATAAAAAAGATGAACTGATATTAATTAAAGACTAACCTCCTCGAGGCTAGGACTTATTCGGTG
ACTAATGCACCAATCATCTCCTCACACACACATACACACAGCAATCATTTAAACTGCTGCCATGGAAAGGCCAGAAGCAAXXXXXXXXXXXG
ATTGCTCAGGAAAAGTATAAAAAAGATGAACTGATATTGATTAAAGACTAACCTCCTCGAGGCTAGGACTTATTCGGTG
>Marker478488 Chr3 SNP_site:11467284 Ref_Type:A
ACTAATGCACCAATCATCTCCTCACACACACATACACAAAGCAATCATTTAAACTGCTGCAATGGAAAGGCCAGAAGCAAXXXXXXXXXXXG
ATTGCTCAGGAAAAGTATAAAAAAGATGAACTGATATTAATTAAAGACTAACCTCCTCGAGGCTAGGACTTATTCGGTG
ACTAATGCACCAATCATCTCCTCACACACACATACACACAGCAATCATTTAAACTGCTGCCATGGAAAGGCCAGAAGCAAXXXXXXXXXXXG
ATTGCTCAGGAAAAGTATAAAAAAGATGAACTGATATTGATTAAAGACTAACCTCCTCGAGGCTAGGACTTATTCGGTG
>Marker478488 Chr3 SNP_site:11467447 Ref_Type:A
ACTAATGCACCAATCATCTCCTCACACACACATACACAAAGCAATCATTTAAACTGCTGCAATGGAAAGGCCAGAAGCAAXXXXXXXXXXXG
ATTGCTCAGGAAAAGTATAAAAAAGATGAACTGATATTAATTAAAGACTAACCTCCTCGAGGCTAGGACTTATTCGGTG
ACTAATGCACCAATCATCTCCTCACACACACATACACACAGCAATCATTTAAACTGCTGCCATGGAAAGGCCAGAAGCAAXXXXXXXXXXXG
ATTGCTCAGGAAAAGTATAAAAAAGATGAACTGATATTGATTAAAGACTAACCTCCTCGAGGCTAGGACTTATTCGGTG
>Marker478618 Chr3 SNP_site:2462167 Ref_Type:C
AACATCGTAGATGCGATAAAAATTTAAACTCTTGACATTGTGATCAAAAGTATAATAATGTCCTAATCAATTAAGCTTCAXXXXXXXXXXXC
ATGAAAAAATCATACTATATCAAAATAATTTTACATTGTCTCTCTCACCTAATTAAGCATTCAACAATGTAAAATTGTC
AACATTGTAGATGCGATAAAAATTTAAACTTTTGACATTGTGATCAAAAGTATAATAATGTCCTAATCAATTAAGCTTCAXXXXXXXXXXXC
ATGAAAAAATCATACTATATCAAAATAATTTTACATTTTCTCTCTCACCTAATTAAGCATTCAACAATGTAAAATTGTC
>Marker478618 Chr3 SNP_site:2462192 Ref_Type:C
AACATCGTAGATGCGATAAAAATTTAAACTCTTGACATTGTGATCAAAAGTATAATAATGTCCTAATCAATTAAGCTTCAXXXXXXXXXXXC
ATGAAAAAATCATACTATATCAAAATAATTTTACATTGTCTCTCTCACCTAATTAAGCATTCAACAATGTAAAATTGTC
AACATTGTAGATGCGATAAAAATTTAAACTTTTGACATTGTGATCAAAAGTATAATAATGTCCTAATCAATTAAGCTTCAXXXXXXXXXXXC
ATGAAAAAATCATACTATATCAAAATAATTTTACATTTTCTCTCTCACCTAATTAAGCATTCAACAATGTAAAATTGTC
>Marker478618 Chr3 SNP_site:2462400 Ref_Type:G
AACATCGTAGATGCGATAAAAATTTAAACTCTTGACATTGTGATCAAAAGTATAATAATGTCCTAATCAATTAAGCTTCAXXXXXXXXXXXC
ATGAAAAAATCATACTATATCAAAATAATTTTACATTGTCTCTCTCACCTAATTAAGCATTCAACAATGTAAAATTGTC
AACATTGTAGATGCGATAAAAATTTAAACTTTTGACATTGTGATCAAAAGTATAATAATGTCCTAATCAATTAAGCTTCAXXXXXXXXXXXC
ATGAAAAAATCATACTATATCAAAATAATTTTACATTTTCTCTCTCACCTAATTAAGCATTCAACAATGTAAAATTGTC
>Marker478667 Chr3 SNP_site:811463 Ref_Type:A
GACCCTATTGCTTTTGTATATATATGGTTTGAAAGGGGATAAAATCAAATGAAATCATATATATAAAGTGCTTGCTTTGCXXXXXXXXXXXT
TTAAATTATAGCATCATGGTTTAAGTAACATGACAACAACAACTCCCACACTAATTATGTTATTTTATTTAAGAATAGT
GACCCTATTGCTTTTGTGTATATATGGTTTGAAAGGGGATAAAATCAAATGAAATCATGTATATAAAGTGCTTGCTTTGCXXXXXXXXXXXT
TTAAATTATAGCATCATGGTTTAAGTAACATGACAACAACAACTCCCACACTAATTATGTTATTTTATTTAAGAATAGT
>Marker478667 Chr3 SNP_site:811504 Ref_Type:A
GACCCTATTGCTTTTGTATATATATGGTTTGAAAGGGGATAAAATCAAATGAAATCATATATATAAAGTGCTTGCTTTGCXXXXXXXXXXXT

Create PDF with GO2PDF for free, if you wish to

TTAAATTATAGCATCATGGTTTAAGTAACATGACAACAACAACTCCCACACTAATTATGTTATTTTATTTAAGAATAGT
GACCCTATTGCTTTTGTGTATATATGGTTTGAAAGGGGATAAAATCAAATGAAATCATGTATATAAAGTGCTTGCTTTGCXXXXXXXXXXT
TTAAATTATAGCATCATGGTTTAAGTAACATGACAACAACAACTCCCACACTAATTATGTTATTTTATTTAAGAATAGT
>Marker47870 Chr4 SNP_site:5433305 Ref_Type:T
ACTACATCTGTATGTTTAAAAATGGGGCACTTGCTTTACTGTAAAATGCCTTCTCTGATGTGAGTTACTTCATGTTTTCTXXXXXXXXXXG
ATAAACATTTTTTTTTTAAATACTGCATGTTACTGTTGAAACCGGTGTAAATATTTTATGTTGGGTGGCTTCCTTTGTT
ACTACATCTGTATGTTTAAAAATGGGGCACTTGCTTTACTGTAAAATGCCTTCTCTGATGTGAGTTACTTCATGTTTTCTXXXXXXXXXXG
ATAAACATTTTTTTTTTAAATACTGCATGTTACTGTTGAAACCGGTGTAAATATTTTATGTTGGGTGGCTTCTTTTGTT
>Marker479285 Chr3 SNP_site:10265459 Ref_Type:A
AACCTTATTCTCAAGTAATGGGGTAGTTGCTCCTTGTTTTTATGCTATCATGTTATGTTTCTCACTCTTCACACTGTAACXXXXXXXXXXG
AAGTTTTGAGATCTTTACTGTCATAAATAGAATTGAGAAGTCTTTCATTTTGCTAAAATGTTGAGGATCCTGAGGAGTG
AACCTTATTCTCAAGTAATGGGGTAGTTGCTCCTTGTTTTTATGCTATCATGTTATGTTTCTCTCTCTTCACACTGTAACXXXXXXXXXXG
AAGTTTTGAGATCTTTACTGTCATAAATAGAATTGAGAAGTCTTTCATTTTGCTAAAATGTTGAGGATCCTGAGGAGTG
>Marker480039 Chr3 SNP_site:22125775 Ref_Type:G
ACTTTAAGCGTTGTATTTATTATGTATCAAAGTTGGATAAATTTAAGAGATCGAGCTTCTAGTGAATATTCTAATGATGTXXXXXXXXXXA
ACACATTGATATTGTTATGTAATTATGGTGTAGGATGAAATGATGAAATTAAAAACCACTTCATCACAAGAAGGGAGTG
ACTTTAAGCGTTGTATTTATTATGTATCAAAGTTGGGTAAATTTAAGAGGTCGAGCTTCTAGTGAATATTCTAATGATGTXXXXXXXXXXA
ACACATTGATATTGTTATGTAATTATGGTGTAGGATGAAATGATGAAATTAAAAACCACTTCATCACAAGAAGGGAGTG
>Marker480039 Chr3 SNP_site:22125788 Ref_Type:G
ACTTTAAGCGTTGTATTTATTATGTATCAAAGTTGGATAAATTTAAGAGATCGAGCTTCTAGTGAATATTCTAATGATGTXXXXXXXXXXA
ACACATTGATATTGTTATGTAATTATGGTGTAGGATGAAATGATGAAATTAAAAACCACTTCATCACAAGAAGGGAGTG
ACTTTAAGCGTTGTATTTATTATGTATCAAAGTTGGGTAAATTTAAGAGGTCGAGCTTCTAGTGAATATTCTAATGATGTXXXXXXXXXXA
ACACATTGATATTGTTATGTAATTATGGTGTAGGATGAAATGATGAAATTAAAAACCACTTCATCACAAGAAGGGAGTG
>Marker48024 Chr4 SNP_site:4874254 Ref_Type:C
ACCAACCTCTAGATCCAAGGGCACCTTCTTCTCCTCCGTCTCTAAAACGCTTGGGAAATTAAATCCCTTCCGTCACGGATXXXXXXXXXXT
CTGCTCGTTTTGTATAAAGTTGCTTTCTTTTACTCCTTTTAAAGTTGGTGTTTCAATGTTTTGTTGAAGTTGTGTTGTG
ACCAACCTCTAGATCCAAGGGCACCTTCTTCTCCTCCGTCTCTAAAACGCTTGGGAAATTAAATCCCTTCCGTCACGGATXXXXXXXXXXT
CTGTTCGTTTTGTATAAAGTTGCTTTGTTTTACTCCTTTTAAAGTTGGTGTTTCAATGTTTTGTTGAAGTTGTGTTGTG
>Marker48024 Chr4 SNP_site:4874277 Ref_Type:C
ACCAACCTCTAGATCCAAGGGCACCTTCTTCTCCTCCGTCTCTAAAACGCTTGGGAAATTAAATCCCTTCCGTCACGGATXXXXXXXXXXT
CTGCTCGTTTTGTATAAAGTTGCTTTCTTTTACTCCTTTTAAAGTTGGTGTTTCAATGTTTTGTTGAAGTTGTGTTGTG
ACCAACCTCTAGATCCAAGGGCACCTTCTTCTCCTCCGTCTCTAAAACGCTTGGGAAATTAAATCCCTTCCGTCACGGATXXXXXXXXXXT
CTGTTCGTTTTGTATAAAGTTGCTTTGTTTTACTCCTTTTAAAGTTGGTGTTTCAATGTTTTGTTGAAGTTGTGTTGTG
>Marker48054 Chr4 SNP_site:3930245 Ref_Type:C
ACCCTTGAAGACAAAAGAGTGTAGGGTCTGAGTGTCCCACACTAACACATTTTTTCTCAAAACAGTCCCCACTCCGAATAXXXXXXXXXXG
TTTCTCTTTGTTTAGTCTCTTTTGTTTTTCAAACTAACATCTCTTAAACATCTTTACATTGGTGTGATTTCGATAAGTT
ACCCTTGAAGACAAAAGAGTGTAGGGTCTGAGTGTCCCACACTAACACATTTTTTCTCAAAACAGTCCCCACTCCGAATAXXXXXXXXXXG
TTTGTCTTTGTTTAGTCTCTTTTGTTTTTCAAACTAACATCTCTTAAACATCTTTACATTGGTGTGATTTCGATAAGTT
>Marker480875 Chr3 SNP_site:34654161 Ref_Type:G
TACTCGAGTAAGCACATAAAGAATATTGCTTAATCTCATCAAGTTGTTGGTGTGGTCATTTACAAAGTCAATCATCTAACXXXXXXXXXXT
GGTCGATTTGAAACTAAAGGACTTGAACCCTTTTATCAAGCTTTTCCCAAGTCTTCTAGGTTTGAACTAGATTGATAGT
TACTCGAGTAAGCACATAAAGAATATTGCTTAATCTCATCAAGTTGTTGGTGTGGTCATTTACAAAGTCAATCATCTAACXXXXXXXXXXT
GGTCGATTTGAAACTAAAGGACTTGAACCCTTTTATCAAGCTTTTCCCAAGTCTTCTAGGTTTGAGCTAGATTGATAGT
>Marker481167 Chr3 SNP_site:37087231 Ref_Type:T

Create PDF with GO2PDF for free, if you wish to

ACTGGTTTTGTGCATCAAACACTGGTATTGTGTATCTTAAGTGCCATGTTATATAAGCAATTTTCGGTGTAAGATTTGTAXXXXXXXXXXT

ATAAAGGATGCTCACTCTGTTGATTAGGTGAAGAAACGTAGAGAGGCAATCAACAACTTTTTTCTAGGAATTAGAGGTA

ACTGGTTTTGTGCATCAAACACTGGTATTGTGTATCTTAAGTGCCATGTTATATAAGCAATTTTCGGTGTAAGATTTGTAXXXXXXXXXXT

ATTAAGGATGCTCACTCTGTTGATTAGGTGAAGAAACGTAGAGAGGCAATCAACAACTTTTTTCTAGGAATTAGAGGTA

>Marker481176 Chr3 SNP_site:3114139 Ref_Type:T

GACCTTTTAACCATTTCCTGCTTCAATTGACTTATTCAGATGCCAAAACTTCACATAACAACAGGAAACCTATTCTGGCCXXXXXXXXXXT

AATAATTAATAAATCAATAATTAACAAAACAAAAACTAGCATCAACGACTTCCCAACTCAAACTTCCTCAGGGGAAGTT

GACCTTTTAACCATTTCCTGCTTCTAATGACTTATTCAGATGCCAAAACTTCACATAACAACAGGAAACCTATTCTGGCCXXXXXXXXXXT

AATAATTAATAAATCAATAATTAACAAAACAAAAACTAGCATCAACGACTTCCCAACTCAAACTTCCTCAGGGGAAGTT

>Marker481176 Chr3 SNP_site:3114141 Ref_Type:A

GACCTTTTAACCATTTCCTGCTTCAATTGACTTATTCAGATGCCAAAACTTCACATAACAACAGGAAACCTATTCTGGCCXXXXXXXXXXT

AATAATTAATAAATCAATAATTAACAAAACAAAAACTAGCATCAACGACTTCCCAACTCAAACTTCCTCAGGGGAAGTT

GACCTTTTAACCATTTCCTGCTTCTAATGACTTATTCAGATGCCAAAACTTCACATAACAACAGGAAACCTATTCTGGCCXXXXXXXXXXT

AATAATTAATAAATCAATAATTAACAAAACAAAAACTAGCATCAACGACTTCCCAACTCAAACTTCCTCAGGGGAAGTT

>Marker481511 Chr3 SNP_site:1047136 Ref_Type:G

AACATCAATCTTAAGACCAAAATGGATGGGATCAAATGACCATCTTATGTAATTCTTTTTTACACATTTTTTTTATATCAXXXXXXXXXXG

TGAATTCGAAAAAAAGAAAAAAGATAAATTGACTTGTCAAATTTAACCGTATGTTAAAATCAAGCTAATATAATTTGTC

AACATCAATCTTAAGACCAAAATGGATGGGATCAAATGACCATCTTATGTAATTCTTTTTTACACGTTTTTTTTATATCAXXXXXXXXXXG

TGAATTCGAAAAAAAGAAAAAAGATAAATTAACTTGTCAAATTTAACCGTATGTTAAAATCAAGCTAATATAATTTGTC

>Marker481511 Chr3 SNP_site:1047286 Ref_Type:A

AACATCAATCTTAAGACCAAAATGGATGGGATCAAATGACCATCTTATGTAATTCTTTTTTACACATTTTTTTTATATCAXXXXXXXXXXG

TGAATTCGAAAAAAAGAAAAAAGATAAATTGACTTGTCAAATTTAACCGTATGTTAAAATCAAGCTAATATAATTTGTC

AACATCAATCTTAAGACCAAAATGGATGGGATCAAATGACCATCTTATGTAATTCTTTTTTACACGTTTTTTTTATATCAXXXXXXXXXXG

TGAATTCGAAAAAAAGAAAAAAGATAAATTAACTTGTCAAATTTAACCGTATGTTAAAATCAAGCTAATATAATTTGTC

>Marker481579 Chr3 SNP_site:34580809 Ref_Type:A

AACTTTTTTTAGGAGTGTGCAATCCAAACAAGCAGCTAAAATAATTTTTGTTCATTTAAAGAAAAACTAAAACAAATTTTXXXXXXXXXXT

ACAAATGTATTATACTCTCGATTGTATTTTCGACTAAATAGCACGTTAGACTTATACATTTTTATCCCATTAATTCGTT

AACTTTTTTTGGGAGTGTGCAATCCAAACAAGCAGCTAAAATAATTTTTGTTAATTTAAAGAAAAACTAAAACAAATTTTXXXXXXXXXXT

ACAAATGTATTATACTCTCGATTGTATTTTCGACTAAATAGCACGTTAGACTTATACATTTTTATCCCATTAATTCGTT

>Marker481579 Chr3 SNP_site:34580851 Ref_Type:C

AACTTTTTTTAGGAGTGTGCAATCCAAACAAGCAGCTAAAATAATTTTTGTTCATTTAAAGAAAAACTAAAACAAATTTTXXXXXXXXXXT

ACAAATGTATTATACTCTCGATTGTATTTTCGACTAAATAGCACGTTAGACTTATACATTTTTATCCCATTAATTCGTT

AACTTTTTTTGGGAGTGTGCAATCCAAACAAGCAGCTAAAATAATTTTTGTTAATTTAAAGAAAAACTAAAACAAATTTTXXXXXXXXXXT

ACAAATGTATTATACTCTCGATTGTATTTTCGACTAAATAGCACGTTAGACTTATACATTTTTATCCCATTAATTCGTT

>Marker481853 Chr3 SNP_site:25578156 Ref_Type:C

AACACATTTTTTAAACATAAATGAACGTTATCATAAAAATGTTAGGTCATGTTAGCTTTCCAAGTAATATCATAAAATAGXXXXXXXXXXC

CTAAAACAACGATTAAGGTCCCTTAGTTATCTTCTCATGTCAAGATATTACAATTATTTCCAAAATAAGTTATAAGGTT

AACACATTTTTTAAACATAAATGAACGTTATCATAAAAATGTTAGGTCATGTTAGCTTTCCAAGTAATATCATAAAATAGXXXXXXXXXXC

TTGAAACAACGATTAAGGTCCCTTAGTTATCTTCTCACGTCAAGATATTACAATTATTTCCAAAATAAGTTATAAGGTT

>Marker481853 Chr3 SNP_site:25578158 Ref_Type:A

AACACATTTTTTAAACATAAATGAACGTTATCATAAAAATGTTAGGTCATGTTAGCTTTCCAAGTAATATCATAAAATAGXXXXXXXXXXC

CTAAAACAACGATTAAGGTCCCTTAGTTATCTTCTCATGTCAAGATATTACAATTATTTCCAAAATAAGTTATAAGGTT

AACACATTTTTTAAACATAAATGAACGTTATCATAAAAATGTTAGGTCATGTTAGCTTTCCAAGTAATATCATAAAATAGXXXXXXXXXXC

TTGAAACAACGATTAAGGTCCCTTAGTTATCTTCTCACGTCAAGATATTACAATTATTTCCAAAATAAGTTATAAGGTT

Create PDF with GO2PDF for free, if you wish to

>Marker481853 Chr3 SNP_site:25578193 Ref_Type:T
AACACATTTTTTAAACATAAATGAACGTTATCATAAAAATGTTAGGTCATGTTAGCTTTCCAAGTAATATCATAAAATAGXXXXXXXXXXC
CTAAAACAACGATTAAGGTCCCTTAGTTATCTTCTCATGTCAAGATATTACAATTATTTCCAAAATAAGTTATAAGGTT
AACACATTTTTTAAACATAAATGAACGTTATCATAAAAATGTTAGGTCATGTTAGCTTTCCAAGTAATATCATAAAATAGXXXXXXXXXXC
TTGAAACAACGATTAAGGTCCCTTAGTTATCTTCTCACGTCAAGATATTACAATTATTTCCAAAATAAGTTATAAGGTT
>Marker481912 Chr3 SNP_site:1663014 Ref_Type:T
CACAAAATATCAAATTAAAATTATAGGAAACTCTCAAAAATAAAAAGATTGACAAGAGATTTAAATTTAAAAAAAGTGTAXXXXXXXXXXT
TGTTATAGGAGATTCTAGGAAATTAGGTTCTTGTGAGAATTATTTCCCATTTTTAGGAAGTAAATTTTTTATTAAGGTT
CACAAAATATCAAATTAAAATTATAGGAAACTTTCAAAAATAAAAAAATTGACAAGATATTTAAATTTAAAAAAAGTGTAXXXXXXXXXXT
TGTTATAGGAGATTCTAGGAAATTAGGTTCTTGTGAGAATTATTTCCCATTTTTAGGAAGTAAATTTTTTATTAAGGTT
>Marker481912 Chr3 SNP_site:1663028 Ref_Type:A
CACAAAATATCAAATTAAAATTATAGGAAACTCTCAAAAATAAAAAGATTGACAAGAGATTTAAATTTAAAAAAAGTGTAXXXXXXXXXXT
TGTTATAGGAGATTCTAGGAAATTAGGTTCTTGTGAGAATTATTTCCCATTTTTAGGAAGTAAATTTTTTATTAAGGTT
CACAAAATATCAAATTAAAATTATAGGAAACTTTCAAAAATAAAAAAATTGACAAGATATTTAAATTTAAAAAAAGTGTAXXXXXXXXXXT
TGTTATAGGAGATTCTAGGAAATTAGGTTCTTGTGAGAATTATTTCCCATTTTTAGGAAGTAAATTTTTTATTAAGGTT
>Marker481912 Chr3 SNP_site:1663039 Ref_Type:T
CACAAAATATCAAATTAAAATTATAGGAAACTCTCAAAAATAAAAAGATTGACAAGAGATTTAAATTTAAAAAAAGTGTAXXXXXXXXXXT
TGTTATAGGAGATTCTAGGAAATTAGGTTCTTGTGAGAATTATTTCCCATTTTTAGGAAGTAAATTTTTTATTAAGGTT
CACAAAATATCAAATTAAAATTATAGGAAACTTTCAAAAATAAAAAAATTGACAAGATATTTAAATTTAAAAAAAGTGTAXXXXXXXXXXT
TGTTATAGGAGATTCTAGGAAATTAGGTTCTTGTGAGAATTATTTCCCATTTTTAGGAAGTAAATTTTTTATTAAGGTT
>Marker48194 Chr4 SNP_site:20851703 Ref_Type:G
ACCATCAATTTTCAAACACAAAAACTATTAAGTATCCTATCATTATCTATCTCAAATAGACTCTAATAGTATTTTATCTTXXXXXXXXXXG
CATATCTCTATCTAGCTATCTAGGATAGAAAATGATACATCACTATGATTGTCTAAGTCTCGTTGAGTCGCACAGGGTA
ACCATCAATTTTCAAGCACAAAAACTATTAAGTATCCTATCATTATCTATCTCAAATAGACTCTAATAGTATTTTATCTTXXXXXXXXXXG
CATATCTCTATCTAGCTATCTAGGATAGAAAATGATACATCACTATGATTGTCTAAGTCTCGTTGAGTCGCACAGGGTA
>Marker482420 Chr3 SNP_site:36065832 Ref_Type:T
GACTAATGTATAAACTGAGAATTCATTGATTAAAAATGAAAGTAAAGAGAACTAATAAGCTCAAAATAACTTGCCACTCCXXXXXXXXXXC
TGAAAATGTTATAGATATAAGAAGTTTAGAAGAGTGATAAAAACAACAGACTATTCAACTGAAATCCAAGAAATGAGTT
GACTAATGTATAAACTGAGAATTCATTGATTAAAAATGAAAGTAAAGAGAACTAATAAGCTCAAAATAACTTGCCACTCCXXXXXXXXXXC
TGAAAATGTTATAGATATAATAAGTTTAGAAGAGTGATAAAAACAACAGACTATTCAACTGAAATCCAAGAAATGAGTT
>Marker482547 Chr3 SNP_site:11470422 Ref_Type:C
AACCTTTTCACTCATGAAAACTTTAACTAATCCACCTATGAAATTCATTTCAATCTAATTTAGAATACAAGAAATTCATTXXXXXXXXXXT
TACGAAATAACACAGGATACCAAGAGACAGGATATAGAAAATAACAGCTTCTCTAAACACTAAGTTTTATATATTAGTG
AACCTTTTCACTCATGAAAACTTTAACTAATCCACCTATGAAATTCATTTCAATCTAATTTAGAATACAAGAAATTCATTXXXXXXXXXXT
TACGAAATAACATAGGATACCAGGAGACAGGATATAGAAAATAACAGCTTCTCTAAACACTAAGTCTTATATATTAGTG
>Marker482547 Chr3 SNP_site:11470432 Ref_Type:A
AACCTTTTCACTCATGAAAACTTTAACTAATCCACCTATGAAATTCATTTCAATCTAATTTAGAATACAAGAAATTCATTXXXXXXXXXXT
TACGAAATAACACAGGATACCAAGAGACAGGATATAGAAAATAACAGCTTCTCTAAACACTAAGTTTTATATATTAGTG
AACCTTTTCACTCATGAAAACTTTAACTAATCCACCTATGAAATTCATTTCAATCTAATTTAGAATACAAGAAATTCATTXXXXXXXXXXT
TACGAAATAACATAGGATACCAGGAGACAGGATATAGAAAATAACAGCTTCTCTAAACACTAAGTCTTATATATTAGTG
>Marker482547 Chr3 SNP_site:11470475 Ref_Type:T
AACCTTTTCACTCATGAAAACTTTAACTAATCCACCTATGAAATTCATTTCAATCTAATTTAGAATACAAGAAATTCATTXXXXXXXXXXT
TACGAAATAACACAGGATACCAAGAGACAGGATATAGAAAATAACAGCTTCTCTAAACACTAAGTTTTATATATTAGTG
AACCTTTTCACTCATGAAAACTTTAACTAATCCACCTATGAAATTCATTTCAATCTAATTTAGAATACAAGAAATTCATTXXXXXXXXXXT

Create PDF with GO2PDF for free, if you wish to

TACGAAATAACATAGGATACCAGGAGACAGGATATAGAAAATAACAGCTTCTCTAAACACTAAGTCTTATATATTAGTG
>Marker482665 Chr3 SNP_site:12993275 Ref_Type:A
CACAACATTGAAATGCGACGACGGAGAGATCTCAAGTGATGTTACCATGAGGGAAGTAAATTTTACAACATTCTTCTTATXXXXXXXXXXG
ATTTTGACTTTTTTTAAGATACTCTTGATAAAAAGTTTTAAAAGAACATTAGGTGAAAGTCATATTTGCAGGTAATGTT
CACAACATTGAAATGCGGCGACTGAGAGATCTCAAGTGATGTTACCATGAGGGGAGTAAATTTTACAACATTCTTCTTATXXXXXXXXXXG
ATTTTGACTTTTTTTAAGATACTCTTGATAAAAAGTTTTAAAAGAACATTAGGTGAAAGTCATATTTGCAGGTAATGTT
>Marker482665 Chr3 SNP_site:12993280 Ref_Type:G
CACAACATTGAAATGCGACGACGGAGAGATCTCAAGTGATGTTACCATGAGGGAAGTAAATTTTACAACATTCTTCTTATXXXXXXXXXXG
ATTTTGACTTTTTTTAAGATACTCTTGATAAAAAGTTTTAAAAGAACATTAGGTGAAAGTCATATTTGCAGGTAATGTT
CACAACATTGAAATGCGGCGACTGAGAGATCTCAAGTGATGTTACCATGAGGGGAGTAAATTTTACAACATTCTTCTTATXXXXXXXXXXG
ATTTTGACTTTTTTTAAGATACTCTTGATAAAAAGTTTTAAAAGAACATTAGGTGAAAGTCATATTTGCAGGTAATGTT
>Marker482665 Chr3 SNP_site:12993311 Ref_Type:A
CACAACATTGAAATGCGACGACGGAGAGATCTCAAGTGATGTTACCATGAGGGAAGTAAATTTTACAACATTCTTCTTATXXXXXXXXXXG
ATTTTGACTTTTTTTAAGATACTCTTGATAAAAAGTTTTAAAAGAACATTAGGTGAAAGTCATATTTGCAGGTAATGTT
CACAACATTGAAATGCGGCGACTGAGAGATCTCAAGTGATGTTACCATGAGGGGAGTAAATTTTACAACATTCTTCTTATXXXXXXXXXXG
ATTTTGACTTTTTTTAAGATACTCTTGATAAAAAGTTTTAAAAGAACATTAGGTGAAAGTCATATTTGCAGGTAATGTT
>Marker482814 Chr3 SNP_site:21694519 Ref_Type:T
AACCTCATATCTAATAAAAATAATACATTCTATTCAAAGTTCCTCGATCTATAAAAACTATTAATCATATCTATAGAAAGXXXXXXXXXXC
CTTTTGAGAATGAAAGCAACACATTGTTAGCTTACCATACATCAAAACCTTTCATGAATAAAAATTTCTTACTAATGTC
AACCTCATATCTAATAAAAATAATACATTCTATTCAAAGTTTCTCGATCTATAAAAACTATTAATCATATCCATAGAAAGXXXXXXXXXXC
CTTTTGAGAATGAAAGCAACACATTGTTAGCTTACCATACATCAAAACCTTTCATGAATAAAAATTTCTTACTAATGTC
>Marker482814 Chr3 SNP_site:21694549 Ref_Type:C
AACCTCATATCTAATAAAAATAATACATTCTATTCAAAGTTCCTCGATCTATAAAAACTATTAATCATATCTATAGAAAGXXXXXXXXXXC
CTTTTGAGAATGAAAGCAACACATTGTTAGCTTACCATACATCAAAACCTTTCATGAATAAAAATTTCTTACTAATGTC
AACCTCATATCTAATAAAAATAATACATTCTATTCAAAGTTTCTCGATCTATAAAAACTATTAATCATATCCATAGAAAGXXXXXXXXXXC
CTTTTGAGAATGAAAGCAACACATTGTTAGCTTACCATACATCAAAACCTTTCATGAATAAAAATTTCTTACTAATGTC
>Marker482838 Chr3 SNP_site:14131555 Ref_Type:T
AACATCCTGTAATATGCATTTACCCGTGTCCTTCCTGATTGTAACCTTTGTTCATATTAAGTATTTAGAGAAGGAGGGGCXXXXXXXXXXA
TAAAATTTTCTGCATTTATTCTTCTGGGAGCTCTGTAGTGGTCTGTCATTCAGAGGGGGGAAATTTCTGTGAAACTGTG
AACATCCTGTAATATGCATTTACCCGTGTCCTTCCTGATTGTAACCTTTGTTCATATTAAGTATTTAGAGAAGGAGGGGCXXXXXXXXXXA
TAAAATTTTCTGCATTTATTCTTCTGGGAGCTTTGTAGTGGTCTGTCATTCAGAGGGGGGAAATTTCTGTGAAACTGTG
>Marker483092 Chr3 SNP_site:33727131 Ref_Type:C
CACATTCATCATTTATATCCATTAACTATACCCATTCAAAATGATGAGTTTCTTTCTTCTCCACTTTTTCTCTTTTAATAXXXXXXXXXXG
CCACCGTTAAAAAAAATGAATTAATTATATTATATTAGTTTTCTAGACTAAATTTTATAAACTTTTAGAAAATAGAGTT
CACATTCATCATTTATATCCATTAACTATACCCATTCAAAATGATGAGTTTCTTTCTTTTCCACTTTTTCTCTTTTAATAXXXXXXXXXXG
CCACCGTTAAAAAAAATGAATTAATTATATTATATTAGTTTTCTAGACTAAATTTTATAAACTTTTAGAAAATAGAGTT
>Marker483785 Chr3 SNP_site:1165965 Ref_Type:A
ACAACAATACCCACAACTCATTTTTGCACTCCATAGCAAAAAATAATATATTATGTTAAGGACAACCCCTTAGGTAAATTXXXXXXXXXXT
AGGATTCATTTCTTGTAGCAAATATTCACTTGTAGCATACGATATCTAAAGGGACTGGCGTAAGTTTTCTACTGCGGGT
ACAACAATACCCACAACTCATTTTTGCACTCCATAGCAAAAAATAATATATTATGTTAAGGACGACCCCTTACGTAAATTXXXXXXXXXXT
AGGATTCATTTCTTGTAGCAAATATTCACTTGTAGCATACGATATCTAAAGGGACTGGCGTAAGTTTTCTACTGCGGGT
>Marker483785 Chr3 SNP_site:1165974 Ref_Type:G
ACAACAATACCCACAACTCATTTTTGCACTCCATAGCAAAAAATAATATATTATGTTAAGGACAACCCCTTAGGTAAATTXXXXXXXXXXT
AGGATTCATTTCTTGTAGCAAATATTCACTTGTAGCATACGATATCTAAAGGGACTGGCGTAAGTTTTCTACTGCGGGT

Create PDF with GO2PDF for free, if you wish to

ACAACAATACCCACAACTCATTTTTGCACTCCATAGCAAAAAATAATATATTATGTTAAGGACGACCCCTTACGTAAATTXXXXXXXXXXT
AGGATTCATTTCTTGTAGCAAATATTCACTTGTAGCATACGATATCTAAAGGGACTGGCGTAAGTTTTCTACTGCGGGT
>Marker484086 Chr3 SNP_site:34756146 Ref_Type:G
CACCGAATTGGTAGAACAGGGAGAGCTGGAACAAAAGGAACTGCATACACTTTCTTTACTGCTGCTAATGCCAGATTTGCXXXXXXXXXXC
AACACCAGGGATAAAAGGAAAAGTAATCAAGAAGCTTGGTCCATTACTTACATTTCTCTGGTGCATGCTCGGTGGAGTT
CACCGAATTGGTAGAACAGGGAGAGCTGGAGCAAAAGGAACTGCATACACTTTCTTTACTGCTGCTAATGCCAGATTTGCXXXXXXXXXXC
AACACCAGGGATAAAAGGAAAAGTAATCAAGAAGCTTGGTCCATTACTTACATTTCTCTGGTGCATGCTCGGTGGAGTT
>Marker484381 Chr3 SNP_site:39114078 Ref_Type:T
CACAGCTAAACTAAGACAACACTTTCAATAACACTTTGAAGTAATTAGAATCATAAATAATCTCTAACTCTCAACTTTAAXXXXXXXXXXA
TAAAACTGATATTAGTGTTACAAAATCACCCAACAAAAGAATATAAAAATTTATTTAGTTCATTACATTAGCTATGGTT
CACAGCTAAACTAAGACAACACTTTCAATAACACTTTGAAGTAATTAGAATCATAAATAATCTCTAACTCTCAACTTTAAXXXXXXXXXXA
TAAAACTGATATTAGTGTTACAAAATCACCCAACAAAAGAATATAAAAATTTATTTAGTTCATTATATTAGCTATGGTT
>Marker484392 Chr3 SNP_site:9590323 Ref_Type:T
CACCTTGAATTGAATTGAATTTGATGTGACTTTTGAAAGAGTTGAATGTGTCTTTCCTCCATCAATGACGGTTTTAACAAXXXXXXXXXXT
CATACTAATTGTTTATCAATTTTGAAAAATACTTATTTTAAGTAACATTTATACTACTTTAAAACCACTTTAAGCTGTT
CACCTTGAATTGAATTGAATTTGATGTGACTTTTGAAAGAGTTGAATGTGTCTTTCCTCCATCAATGACGGTTTTAACAAXXXXXXXXXXT
CATACTAATTGTTTATCAATTTTGAAAAATACTTATTTTAAGTAACATTTATACTACTTTAAAATCACTTTAAGCTGTT
>Marker484437 Chr3 SNP_site:33185100 Ref_Type:G
ACCCTAGGGAAATGGCTGATCTCAAATGACACAAACTTATCCTTGAGCTCCTTAGCCACTTTACAAAATTTAGCCATATTXXXXXXXXXXT
GTGATTGTGCGTAGTTTAAGTAAAATTTGACAGAAGTCATGTTTAACAGCTTCCTTGATCTTATCTATCTATATGTGTA
ACCCTAGGGAAATGGCTGATCTCAAATGACACAAACTTATCCTTGAGCTCCTTAGCCACTTTACAAAATTTAGCCATATTXXXXXXXXXXT
GTGATTGTGCGTAGTTTAAGTAGAATTTGACAGAAGTCATGTTTAACAGCTTCCTTGATCTTATCTATCTATATGTGTA
>Marker484510 Chr3 SNP_site:5816097 Ref_Type:A
CACCTAAGTATTATTTTTCCTTTTTGCCGGGTTAGAATTTAGTGTATGAATTTATGTATTATCTAAATATATGAGAAATAXXXXXXXXXXA
CATATATATATTTCAAGCTATATCACTTATGTAAGAAATACGTCACTCCAATTTGTTGTTTTTCTAAAATAATATTGTA
CACCTAAGTATTATTTTTCCTTTTTGCCGGGTTAGAATTTAGTGTATGAATTTATGTATTATCTAAATATATGAGAAATAXXXXXXXXXXA
CATATGTATATTTCAAGCTATATCACTTATGTAAGAAATACGTCACTCCAATTTGTTGTTTTTCTAAAATAATATTGTA
>Marker484859 Chr3 SNP_site:7017533 Ref_Type:G
AACCAGAAGTTACAGGGTTGAAAAGGGTATGTCTTATTTTCATGGATTGTGTTATTTAATTATTGGGTAAGTCAAGATTTXXXXXXXXXXT
CTATCATTAAATTGCCCTCTCTATGTGTAAATGCATGTATTATGAAAAGTAATATGCTGCTGTCTCTATATGCAATGGT
AACCAGAAGTTACAGGGTTGAAAAGGGTATGTCTTATTTTCATGGATTGTGTTGTTTAATTATTGGGTAAGTCAAGATTTXXXXXXXXXXT
CTATCATTAAATTGCCCTCTCTATGTGTAAATGCATGTATTATGAAAAGTAATATGCTGCTGTCTCTATATGCAATGGT
>Marker485276 Chr3 SNP_site:4300033 Ref_Type:C
ACATATTCATGAATATTAAATATATATGATGGTAAGCGAAATTATTAGCAAAATTTGACTTGTTTGTTTCAACTAGATCAXXXXXXXXXXA
CTTAAATCCATCGTTCTCAAGACGGATGGTCTTCGGCATCGGAACACCCGGCGGCTTAATTCCGAATTGCGTTCCTGTG
ACATATTCATGAATATTAAATATATATGATGGTAAGCGAAATTATTAGCAAAATTTGACTTGTTTGTTTCAACTAGATCAXXXXXXXXXXA
CTTAAATCCATCGTTCTCAAGACGGATGGTCTTCGGCATCGGAACAGCCGGCGGCTTAATTCCGAATTGCGTTCCTGTG
>Marker486515 Chr3 SNP_site:36949858 Ref_Type:C
AACAACATAAAGAAAAAGGGAGTTGAGATATCAACTTCCTTAAGCTTTGTTGAGCCTCGAAGCCAACAATAATACCTCACXXXXXXXXXXT
TAATTTTCATTCATCAAAACTAACTTTTAAGTGGCATTCAACCTTTGACTTTGGAAGGTAACTGAAATGAAGGCAGAGT
AACAACATAAAGAAAAAGGGAGTTGAGATATCAACTTCCTTAAGCTTTGTTGAGCCTTGAAGCCAACAATAATACCTCACXXXXXXXXXXT
TAATTTTCATTCATCAAAACTAACTTTTAAGTGGCATTCAACCTTTGACTTTGGAAGGTAACTGAAATGAAGGCAGAGT
>Marker48653 Chr4 SNP_site:16264173 Ref_Type:A
AACAGAAGCATTAGAGATTGACGGAAGTTTGAAAGCAGGGGTTAAGTTATTCCATTTTAAAATTACAGAAACCAAATCGTXXXXXXXXXXA

Create PDF with GO2PDF for free, if you wish to

ATTTACTAAACTTCGTGTGTTTAGTTAAGCACATTTTGTTTTTTAAGTTAAATTTACATGATTTTATCTATAAAGAAGT
AACAGAAGCATTAGAGATTGACGGAAGTTTGAAAGCAGGGGTTAAGTTATTCCATTTTAAAATTACAGGAACCAAATCGTXXXXXXXXXXA
ATTTACTAAACTTCGTGTGTTTAGTTAAGCACATTTTGTTTTTTAAGTTAAATTTACATGATTTTATCTATAAAGAAGT
>Marker486679 Chr3 SNP_site:12533680 Ref_Type:A
AACACAAAAGCAAAGTTATTTGTTATCCAAACCTATCCTAATAGTAATTATAATTAACTTTAAAACAAGTTTTTAAATATXXXXXXXXXXT
TTTAGTTTAGGCATCAATTTAGTTAAATTGAATCTTGCATTCATCGGAAACATAATTGTTGAATAAAGCAAACTTTGTT
TACACAAAAGCAAAGTTATTTGTTATCCAAACCCATCCTAATAGTAATTATAATTAACTTTAAAACAAGTTTTTAAATATXXXXXXXXXXT
TTTAGTTTAGGCATCAATTTAGTTAAATTGAATCTTGCATTCATCGGAAACATAATTGTTGAATAAAGCAAACTTTGTT
>Marker486679 Chr3 SNP_site:12533713 Ref_Type:A
AACACAAAAGCAAAGTTATTTGTTATCCAAACCTATCCTAATAGTAATTATAATTAACTTTAAAACAAGTTTTTAAATATXXXXXXXXXXT
TTTAGTTTAGGCATCAATTTAGTTAAATTGAATCTTGCATTCATCGGAAACATAATTGTTGAATAAAGCAAACTTTGTT
TACACAAAAGCAAAGTTATTTGTTATCCAAACCCATCCTAATAGTAATTATAATTAACTTTAAAACAAGTTTTTAAATATXXXXXXXXXXT
TTTAGTTTAGGCATCAATTTAGTTAAATTGAATCTTGCATTCATCGGAAACATAATTGTTGAATAAAGCAAACTTTGTT
>Marker486720 Chr3 SNP_site:26105947 Ref_Type:C
TACAAAAGGGACCTGATCACTTTATTAATTTATATAATTTAGAAGACCCAAAATTTCAAAGAAATTAAGCAAAACAGCTTXXXXXXXXXXT
GATCCAACTCATAGGAATGGGCAGACATCTCTCTCCATCTAAAAAACACTTCTTCTAATGACATTAAATCAGAATTGTT
TACAAAAGGGACCTGATCACTTTATTAATTTATATAATTTAGAAGACCCAAAATTTCAAAGAAATTAAGCAAAACAGCTTXXXXXXXXXXT
GATCCAACTGATAGGAATGGGCAGACATCTCTCTCCATCTAAAAAACACTTCTTCTAATGACATTAAATCAGAATTGTT
>Marker487192 Chr3 SNP_site:13906987 Ref_Type:T
ACCATTTCTTTTAAAAGTTGTAATACATGATCTTTTATTGGTATTTAAAAAAAAAACCCTTCAAAATTTTGAAGAGAGCTXXXXXXXXXXT
TTAGACTAAATTATTAAAAATGTGGTTCAACTTTGTGTTTTATGTAAAAAATATCTTAAGCTTTCAATCATTTCTGGTG
ACCATTTCTTTTAAAAGTTGTAATACATGATCTTTTATTGGTATTTTAAAAAAAAACCCTTCAAAATTTTGAAGAGAGCTXXXXXXXXXXT
TTAGACTAAATTATTAAAAATGTGGTTCAACTTTGTGTTTTATGTAAAAAATATCTTAAGCTTTCAATCATTTCTGGTG
>Marker487258 Chr3 SNP_site:30643296 Ref_Type:G
AACTTATCACTGAAGCTCTATTAGTTGGGTCATACTTTTAGAAGCAAACATGTGGTTTTTATTTATTATGTGTGTTATAAXXXXXXXXXXG
TATTGGTGTAATTTATAAGATTGTTAATGGATGGTTACTATAACTAAGCTTAATTATGAGCTATTTGGTATATTTGGTG
AACTTATCACTGAAGCTCTATTAGTTGGGTCATACTTTTAGAAGCAAACATGTGGTTTTTATTTATTATGTGTGTTATAAXXXXXXXXXXG
TATTGGTGTAATTTGTGAGATTGTTAATGGATGGTTACTATAACTAAGCTTAATTATGAGCTATTTGGTATATTTGGTG
>Marker487258 Chr3 SNP_site:30643298 Ref_Type:G
AACTTATCACTGAAGCTCTATTAGTTGGGTCATACTTTTAGAAGCAAACATGTGGTTTTTATTTATTATGTGTGTTATAAXXXXXXXXXXG
TATTGGTGTAATTTATAAGATTGTTAATGGATGGTTACTATAACTAAGCTTAATTATGAGCTATTTGGTATATTTGGTG
AACTTATCACTGAAGCTCTATTAGTTGGGTCATACTTTTAGAAGCAAACATGTGGTTTTTATTTATTATGTGTGTTATAAXXXXXXXXXXG
TATTGGTGTAATTTGTGAGATTGTTAATGGATGGTTACTATAACTAAGCTTAATTATGAGCTATTTGGTATATTTGGTG
>Marker487508 Chr3 SNP_site:8162634 Ref_Type:A
ACAAGGCTGAAAATTCTGCGACTCAAATGCTATTAGATCTAGAGTTGAAGAAAGGATTCGCTTTAAGAAAACTGACAAACXXXXXXXXXXA
ACTCCATTCTTAGACTAGGGTTGGGAGCATTTCTATGTGGATCACACTAATCAGTGAAAATTTTCTTTTAATGCAGAGT
ACAAGGCTGAAAATTCTGCGACTCAAATGCTATTAGATCTAGTGTTGAAGAAAGGATTCGCTTTAAGAAAACTGACAAACXXXXXXXXXXA
ACTCCATTCTTAGACTAGGGTTGGGAGCATTTCTATGTGGATCACACTAATCAGTGAAAATTTTCTTTTAATGCAGAGT
>Marker487609 Chr3 SNP_site:37362959 Ref_Type:C
CACTAGCAAGAGACTCTTAAAAACATTTTATTTTTGGTGTAATACATTTACATTTATATTTCAAAGTTAAAAAAGGAATXXXXXXXXXXC
AAAAATGTTCCCTTAAATAGTAAGAAATATCTAACTATGTTTCATTTTAGGTTAAAGTATCGGTTTAAGCACTAAGGTG
CACTAGCAAGAGACTCTTAAAAACATTTTATTTTTGGTGTAATACATTTACATTTATATTTCAAAGTTCAAAAAGGAATXXXXXXXXXXC
AAAAATGTTCCCTTAAATAGTAAGAAATATCTAACTATGTTTCATTTTAGGTTAAAGTATCGGTTTAAGCACTAAGGTG
>Marker487940 Chr3 SNP_site:34216211 Ref_Type:C

Create PDF with GO2PDF for free, if you wish to

ACGTAAGCCTATTTGTTTATATGAATAGTTTCTCCGGGAGTGTTATCATAATGGATACTTCTTGCAATGAGAGATTAGATXXXXXXXXXXA
AAGTTTTAAAGTTGATGAGTTTTGGGAGTGGCTTGTTTTTGTTTTATAGACTACTGTGGTCTTCGAAGATGCTTTGGTT
ACGTAAGCCTATTTGTTTATATGAATAGTTTCTCCGGGAGTGTTATCATAATGGATACTTCTTGCAATGAGAGATTAGATXXXXXXXXXXA
AAGTTTTAAAGTTGATGAGTTTTGGGAGTGGCTTGTTTTTGTTTTATAGACTATTGTGGTCTTCGAAGATGCTTTGGTT
>Marker487999 Chr3 SNP_site:16427586 Ref_Type:G
CACATCAATAGACTTCGTTATTTTACATAATGCAATTCTAGTCTATCCTGCATTCTAGCATTTCTTCTCTTCTTCACTACXXXXXXXXXXC
CTTTCTTTTTCTTGCAACACTTTTATTAGCTAAGTTTATTTATTTACATACATTTGGCGTTGAGTGGAAAATCATAGGT
CACATCGATAGACTTCGTTATTTTACATAATGCAATTCTAGTCTATCCTGCATTCTAGCATTTCTTCTCTTCTTCACTACXXXXXXXXXXC
CTTTCTTTTTCTTGCAACGCTTTTATTAGCTAAGTTTATTTATTTACATACATTTGGCGTTGAGTGAAAAATCATAGGT
>Marker487999 Chr3 SNP_site:16427806 Ref_Type:G
CACATCAATAGACTTCGTTATTTTACATAATGCAATTCTAGTCTATCCTGCATTCTAGCATTTCTTCTCTTCTTCACTACXXXXXXXXXXC
CTTTCTTTTTCTTGCAACACTTTTATTAGCTAAGTTTATTTATTTACATACATTTGGCGTTGAGTGGAAAATCATAGGT
CACATCGATAGACTTCGTTATTTTACATAATGCAATTCTAGTCTATCCTGCATTCTAGCATTTCTTCTCTTCTTCACTACXXXXXXXXXXC
CTTTCTTTTTCTTGCAACGCTTTTATTAGCTAAGTTTATTTATTTACATACATTTGGCGTTGAGTGAAAAATCATAGGT
>Marker487999 Chr3 SNP_site:16427854 Ref_Type:A
CACATCAATAGACTTCGTTATTTTACATAATGCAATTCTAGTCTATCCTGCATTCTAGCATTTCTTCTCTTCTTCACTACXXXXXXXXXXC
CTTTCTTTTTCTTGCAACACTTTTATTAGCTAAGTTTATTTATTTACATACATTTGGCGTTGAGTGGAAAATCATAGGT
CACATCGATAGACTTCGTTATTTTACATAATGCAATTCTAGTCTATCCTGCATTCTAGCATTTCTTCTCTTCTTCACTACXXXXXXXXXXC
CTTTCTTTTTCTTGCAACGCTTTTATTAGCTAAGTTTATTTATTTACATACATTTGGCGTTGAGTGAAAAATCATAGGT
>Marker48805 Chr4 SNP_site:9755443 Ref_Type:C
ACCAACATAACACTTCCCCAAACACTTATCATAATACTATCATAACACTACCCACATAACTCTTTCCCTAAACACATCTTXXXXXXXXXXC
CAAATATAAAGTCGTTTTATGATTTAGACAAAAGAATTATTGGTTTTGGTTTTTGGTTGTAAGTGTAAATATTTTATGT
ACCAACATAACACTTCCCCAAACACTTATCATAATACTATCATAACACTACTCACATAACCCTTTCCCTAAACACATCTTXXXXXXXXXXC
CAAATATAAAGTCGTTTTATGATTTGGACAAAAGAATTATTGGTTTTGGTTTTTGGTTGTAAGTGTAAATATTTTATGT
>Marker48805 Chr4 SNP_site:9755452 Ref_Type:T
ACCAACATAACACTTCCCCAAACACTTATCATAATACTATCATAACACTACCCACATAACTCTTTCCCTAAACACATCTTXXXXXXXXXXC
CAAATATAAAGTCGTTTTATGATTTAGACAAAAGAATTATTGGTTTTGGTTTTTGGTTGTAAGTGTAAATATTTTATGT
ACCAACATAACACTTCCCCAAACACTTATCATAATACTATCATAACACTACTCACATAACCCTTTCCCTAAACACATCTTXXXXXXXXXXC
CAAATATAAAGTCGTTTTATGATTTGGACAAAAGAATTATTGGTTTTGGTTTTTGGTTGTAAGTGTAAATATTTTATGT
>Marker48805 Chr4 SNP_site:9755641 Ref_Type:A
ACCAACATAACACTTCCCCAAACACTTATCATAATACTATCATAACACTACCCACATAACTCTTTCCCTAAACACATCTTXXXXXXXXXXC
CAAATATAAAGTCGTTTTATGATTTAGACAAAAGAATTATTGGTTTTGGTTTTTGGTTGTAAGTGTAAATATTTTATGT
ACCAACATAACACTTCCCCAAACACTTATCATAATACTATCATAACACTACTCACATAACCCTTTCCCTAAACACATCTTXXXXXXXXXXC
CAAATATAAAGTCGTTTTATGATTTGGACAAAAGAATTATTGGTTTTGGTTTTTGGTTGTAAGTGTAAATATTTTATGT
>Marker488542 Chr3 SNP_site:26742920 Ref_Type:G
AACGCGTTAAATTTAATTCTGGACTTTCTGTGACGTCTTATTCCTCTACGTCGCAGAAAGCCCCTTTCTGCAACGTAATAXXXXXXXXXXA
TCTTTATAAAATGATATTTTCAATCAAGCTAAAGGCCCTCATATATACATAATATGTCATCAAATTGGGTTACCAAAGT
AACGCGTTAAATTTAATTCTGGACTTTCTGTGACGTCTTATTCCTCTACGTCGCAGAAAGCCCCTTTCTGCAACGTAATAXXXXXXXXXXA
TCTTTATAAAGTGATATTTTCAATCGAGCTAAAGGCCCTCATATATACATAATGTGTCATCAAATTGGGTTACCAAAGT
>Marker488542 Chr3 SNP_site:26742935 Ref_Type:G
AACGCGTTAAATTTAATTCTGGACTTTCTGTGACGTCTTATTCCTCTACGTCGCAGAAAGCCCCTTTCTGCAACGTAATAXXXXXXXXXXA
TCTTTATAAAATGATATTTTCAATCAAGCTAAAGGCCCTCATATATACATAATATGTCATCAAATTGGGTTACCAAAGT
AACGCGTTAAATTTAATTCTGGACTTTCTGTGACGTCTTATTCCTCTACGTCGCAGAAAGCCCCTTTCTGCAACGTAATAXXXXXXXXXXA
TCTTTATAAAGTGATATTTTCAATCGAGCTAAAGGCCCTCATATATACATAATGTGTCATCAAATTGGGTTACCAAAGT

Create PDF with GO2PDF for free, if you wish to

>Marker488542 Chr3 SNP_site:26742963 Ref_Type:G
AACGCGTTAAATTTAATTCTGGACTTTCTGTGACGTCTTATTCCTCTACGTCGCAGAAAGCCCCTTTCTGCAACGTAATAXXXXXXXXXXA
TCTTTATAAAATGATATTTTCAATCAAGCTAAAGGCCCTCATATATACATAATATGTCATCAAATTGGGTTACCAAAGT
AACGCGTTAAATTTAATTCTGGACTTTCTGTGACGTCTTATTCCTCTACGTCGCAGAAAGCCCCTTTCTGCAACGTAATAXXXXXXXXXXA
TCTTTATAAAGTGATATTTTCAATCGAGCTAAAGGCCCTCATATATACATAATGTGTCATCAAATTGGGTTACCAAAGT
>Marker488627 Chr3 SNP_site:39607238 Ref_Type:T
GACCAAGAGGAAGGCTTTGTTCTCACATAATGTGGATGATATTATGTTGTTAAATATATAACAGATCCAAGCTTTTCCATXXXXXXXXXXT
CAGGCTTTTGCCTAATTCATCTCATTAATCAGATCATTGATGTCATCTTCTCTATTACCACTATCTCCACCTTCCTTGT
GACCAAGTGGAAGGCTTTGTTCTCACATAATGTGGATGATATTATGTTGTTAAATATATAACAGATCCAAGCTTTTCCATXXXXXXXXXXT
CAGGCTTTTGCCTAATTCATCTCATTAATCAGATCATTGATGTCATCTTCTCTATTACCACTATCTCCACCTTCCTTGT
>Marker488638 Chr3 SNP_site:27742075 Ref_Type:T
AACTATCTTCAAATACGTCTCAATATGGACAATGTAACTGCTTCAAATAGTGTCACTAGTTGGTCAGGAACTTGCTACCTXXXXXXXXXXA
CAATTTTTCTTTAATTAGCGGCTGCATTGCGTAATGATATTATCTTTTGATGTATGGTCTTGCAGGGCATGACGTTGTT
AACTATCTTCAAATACGTCTCAATATGGACAATGTAACTGCTTCAAATAGTGTCACTAGTTGGTCAGGAACTTGCTACCTXXXXXXXXXXA
CAATTTTTTTTTAATTAGCGGCTGCATTGCGTAATGATATTATCTTTTGATGTATGGTCTTGCAGGGCATGACGTTGTT
>Marker488718 Chr3 SNP_site:34355167 Ref_Type:C
AACTATAAGTTCGCATGAAAGGCCTCAAAGCTACAGAGGTAATGATAATATTAGTATTTACAAACCTGTGCATTTGGGACXXXXXXXXXXC
CAAACCCTGCCGGGCTGCCGGCAGGAAGAGGCTTGAACTGCTGGATTCCATACTTCAAGTTATTTGCATTCATGTGAGT
AACTATAAGTTCGCATGAAAGGCCTCAAAGCTACAGAGGTAATGATAATATTAGTATTTACAAACCTGTGCATTTGGGACXXXXXXXXXXC
CAAACCCTGCTGGGCTGCCGGCAGGAAGAGGCTTGAACTGCTGGATTCCATACTTCAAGTTATTTGCATTCATGTGAGT
>Marker48929 Chr4 SNP_site:16648648 Ref_Type:G
ACCATTTACCAAGAGTAGATGAGAATTTTAAGAGATTAGGATCAAATGGAGAATAACCTAAAAGATAAGTAACCAACCTCXXXXXXXXXXG
AAGAACATTCAGCACCTACAGAACATGGATAATGTTGCATTTTTCAAAACTAAGTTATAAAAGTCTGTTTTCAGTAGTT
ACCATTTACCAATAGTAGATGAGAATTTTAAGAGATTAGGATCAAATGGAGAATAACCTAAAAGATAAGTAACCAACCTCXXXXXXXXXXG
AAGAACATTCAGCACCTACAGAACATGGATAATGTTGCATTTTTCAAAACTAAGTTATAAAAGTCTGTTTTCAGTAGTT
>Marker48930 Chr4 SNP_site:20456765 Ref_Type:G
AACATATGCATCAATTTGTTTGCTGAAAAATCTCAAATGACATCAAACAAAGGGGTCATAAAAATTTAATGGTATATCTAXXXXXXXXXXT
TTGATTACTCACTTGACTTCAAAGAGGATGAATATGATACTGAAAACAAAGAGGAATGGAGAAGAAATGGAGAGGCGTT
AACATATGCATCAATTTGTTTGCTGAAAAATCTCAAATGGCATCAAACAAAGGGGTCATAAAAATTTAATGGTATATCTAXXXXXXXXXXT
TTGATTACTCACTTGACTTCAAAGAGGATGAATATGATACTGAAAACAAAGAGGAATGGAGAAGAAATGGAGAGGCGTT
>Marker489458 Chr3 SNP_site:8419255 Ref_Type:A
ACAAGGTAGATTATCCAGAGATTATCAAATAATATTGTTTATTGAAAAAAGAATCATATTTGACTGCTGTCATTTGTGTTXXXXXXXXXXG
TTGGAAAACTCTTTATTTTCTGTTTTTACTGTATCCAGTGTCCCTATTTGGTTAGATTGATGCTGTAGGGATTTTGGTA
ACAAGGTAGATTATCCAGAGATTATCTAATAATATTGTTTATTGAAAAAAGAATCATATTTGACTGCTGTCATTTGTGTTXXXXXXXXXXG
TTGGAAAACTCTTTATTTTCTGTTTTTACTGTATCCAGTGTCCCTATTTGGTTAGATTGATGCTGTAGGGATTTTGGTA
>Marker489772 Chr3 SNP_site:3328298 Ref_Type:G
ACATTTTTTTTTGAAAAAAAAATCAATATTGCACTTACAACATCAATTTTAAATAAATATATATAATTTCTTTAGCACGTXXXXXXXXXXA
AATTTTGGCAAATTAAAAATATGAATATTTGTTTATTTGACTTACAATACGATTTTTTTTAAAAGAAAAATACAAGAGT
ACGTTTTTTTTTGAAAAAAAAATCAATATTGCACTTACAACATCAATTTTAAATAAATATATATAATTTCTTTAGCACGTXXXXXXXXXXA
AATTTTGGCAAATTAAAAATATGAATATTTGTTTATTTGACTTACAATACGATTTTTTTTAAAAGAAAAATACAAGAGT
>Marker489878 Chr3 SNP_site:35845389 Ref_Type:G
AACCGATATCACCCCCTTCAAGAACAACACCAACTAACCTTTTAACCTAAACCGTTAAACTGAAAAACGTTAACGGAGACXXXXXXXXXXT
ATTTACATAAAACAAAACCTAACAGCAACCTTCCCAAAAACCTAAGTCTAACATTACAAAAGACTAACTGATCGCAAGT
AACCGATATCGCCCCCTTCAAGAACAACACCAACTAACCTTTTAACCTAAACCTTTAAACTGAAAAACGTTAACGGAGACXXXXXXXXXXT

Create PDF with GO2PDF for free, if you wish to

ATTTACATAAAACAAAACCTAACAGCAACCTTCCCAAAAACCTAAGTCTAACATTACAAAAGACTAACTGATCGCAAGT
>Marker489878 Chr3 SNP_site:35845432 Ref_Type:T
AACCGATATCACCCCCTTCAAGAACAACACCAACTAACCTTTTAACCTAAACCGTTAAACTGAAAAACGTTAACGGAGACXXXXXXXXXXT
ATTTACATAAAACAAAACCTAACAGCAACCTTCCCAAAAACCTAAGTCTAACATTACAAAAGACTAACTGATCGCAAGT
AACCGATATCGCCCCCTTCAAGAACAACACCAACTAACCTTTTAACCTAAACCTTTAAACTGAAAAACGTTAACGGAGACXXXXXXXXXXT
ATTTACATAAAACAAAACCTAACAGCAACCTTCCCAAAAACCTAAGTCTAACATTACAAAAGACTAACTGATCGCAAGT
>Marker489943 Chr3 SNP_site:27270747 Ref_Type:G
AACGAGAAGCAACAAATTTCTTCACACACCATAAACGATAAGACTAAAAGAAATGAAAGTAAATAATTGAAACGACAGTAXXXXXXXXXXC
ACAAGACAAAAGATAGCGTTGATCAGCAATCGCAGGTTATAAGATTATGATATATAAACACTTATAATCGAGTTAATGT
AACGAGAAGCAACAAATTTCTTCACACACCATAAACGATAAGACTAAAAGAAATGAAAGTAAATAATTGAAAGGACAGTAXXXXXXXXXXC
ACAAGACAAAAGATAGCGTTGATCAGCAATCGCAGGTTATAAGATTATGATATATAAACACTTATAATCGAGTTAATGT
>Marker489996 Chr3 SNP_site:3228814 Ref_Type:C
ACTGTTAATGACTTAGTAAGCTGAACCCCACGAAATACTTGATGAATGGAAGAATGGCAATAATAAAGCATGCAGACAAAXXXXXXXXXXC
TCAATAGGTAACTGAACATTACAGAGCTTTCTATTGGAAGTAATGATCAATCGTAGCCAGCAATAGCATCTTAAAGGTA
ACTGTTAATGACTTAGTAAGCTGAACCCCACGAAATACTTGATGAATGGAAGACTGGCAATAATAAAGCATGCAGACAAAXXXXXXXXXXC
TCAATAGGTAACTGAACATTACAGAGCTTTCTATTGGAAGTAATGATCAATCGTAGCCAGCAATAGCATCTTAAAGGTA
>Marker490029 Chr3 SNP_site:7000719 Ref_Type:A
GACATGCTTCTACTAGTTGATAAGATTTTAATCCCGCACTAAGTCTGTTATTAAGTTTCTTGGCTGAATTTTCCTTTCCTXXXXXXXXXXA
ATCTCTAATAAGCAATTTCTTGTATTGATATTTGGTATGGGTGAATTAAGGTCAAACCTGGATCTGTTTCAATGTTGTT
GACATGCTTCTACTAGTTGATAAGTTTTTAATCCCGCACTAAGTCTGTTATTAAGTTTCTTGGCTGAATTTTCCTTTCCTXXXXXXXXXXG
ATCTCTAATAAGCAATTTCTTGTATTGATATTTGGTATGGATGAATTAAGGTCAAACCTGGATCTGTTTCAATGTTGTT
>Marker490029 Chr3 SNP_site:7000862 Ref_Type:A
GACATGCTTCTACTAGTTGATAAGATTTTAATCCCGCACTAAGTCTGTTATTAAGTTTCTTGGCTGAATTTTCCTTTCCTXXXXXXXXXXA
ATCTCTAATAAGCAATTTCTTGTATTGATATTTGGTATGGGTGAATTAAGGTCAAACCTGGATCTGTTTCAATGTTGTT
GACATGCTTCTACTAGTTGATAAGTTTTTAATCCCGCACTAAGTCTGTTATTAAGTTTCTTGGCTGAATTTTCCTTTCCTXXXXXXXXXXG
ATCTCTAATAAGCAATTTCTTGTATTGATATTTGGTATGGATGAATTAAGGTCAAACCTGGATCTGTTTCAATGTTGTT
>Marker490029 Chr3 SNP_site:7000903 Ref_Type:G
GACATGCTTCTACTAGTTGATAAGATTTTAATCCCGCACTAAGTCTGTTATTAAGTTTCTTGGCTGAATTTTCCTTTCCTXXXXXXXXXXA
ATCTCTAATAAGCAATTTCTTGTATTGATATTTGGTATGGGTGAATTAAGGTCAAACCTGGATCTGTTTCAATGTTGTT
GACATGCTTCTACTAGTTGATAAGTTTTTAATCCCGCACTAAGTCTGTTATTAAGTTTCTTGGCTGAATTTTCCTTTCCTXXXXXXXXXXG
ATCTCTAATAAGCAATTTCTTGTATTGATATTTGGTATGGATGAATTAAGGTCAAACCTGGATCTGTTTCAATGTTGTT
>Marker49009 Chr4 SNP_site:5383215 Ref_Type:C
TACCTTGCAGAGTCATATATATCTCACTTGCTCCACACGACGATAATTCACAAATAAAATCAAAAGAATATAAATGTTTCXXXXXXXXXXC
AATATATAAGAATATAAATTTCATTCTATTTTTAAATCTTTCATCTGGCAAACAAAATTGTTGAATTAATTATTTTAGT
TACCTTGCAGAGTCATATATATCTCACTTGCTCCACACGACGATAATTCACAAATAAAATCAAAAGAATATAAATGTTTCXXXXXXXXXXC
AATATATAAGAATATAAATTTTATTCTATTTTTAAATCTTTCATCTTGCAAACAAAATTGTTGAATTAATTATTTTAGT
>Marker49009 Chr4 SNP_site:5383240 Ref_Type:G
TACCTTGCAGAGTCATATATATCTCACTTGCTCCACACGACGATAATTCACAAATAAAATCAAAAGAATATAAATGTTTCXXXXXXXXXXC
AATATATAAGAATATAAATTTCATTCTATTTTTAAATCTTTCATCTGGCAAACAAAATTGTTGAATTAATTATTTTAGT
TACCTTGCAGAGTCATATATATCTCACTTGCTCCACACGACGATAATTCACAAATAAAATCAAAAGAATATAAATGTTTCXXXXXXXXXXC
AATATATAAGAATATAAATTTTATTCTATTTTTAAATCTTTCATCTTGCAAACAAAATTGTTGAATTAATTATTTTAGT
>Marker490165 Chr3 SNP_site:19280908 Ref_Type:T
AACCAAAAGTGACCTATTTATACTAAACCTTGCATGAATATTGATTGATACCTCATACTTGCCAAAATCTAAATTAAGCAXXXXXXXXXXA
TTATAGTAAATGCAGTTGCAATACATCACCGATTCAGATCATTACGCATAATGCGCTCATTAAAATTCTTTGTATTGTA

Create PDF with GO2PDF for free, if you wish to

AACCAAAAGTGACCTATTTATACTAAACCTTGCATGAATATTGATTGATACCTCATACTTGCCAAAATCTAAATTAAGCAXXXXXXXXXXA
TTATAGTAAATGCAGTTGCAATACATCACCGATTCAGATCATTATGCATAATGCGCTCATTAAAATTCTTTGTATTGTA
>Marker49019 Chr4 SNP_site:20870021 Ref_Type:C
CACTAAGAAACTCTCATCATCTACATCAGAAACATTGCAAATAAAATCCCTCAGACTGTCTTCAAGAACTTCTTACAAAAXXXXXXXXXXT
TACCCTTGACCTTTAAAATTTGTTTTAAAAAATAACCTTGTAATTTGAAAAATACACTTCCTATTAAAGCAAATAGGTT
CACTAAGAAACTCTCATTATCTACATCAGAAACATTGCAAATAAAATCCCTCAGACTGTCTTCAAGAACTTCTTACAAAAXXXXXXXXXXT
TACCCTTGACCTTTAAAATTTGTTTTAAAAAATAACCTTGTAATTTGAAAAATACACTTCCTATTAAAGCAAATAGGTT
>Marker490272 Chr3 SNP_site:9386571 Ref_Type:T
ACTTTCTGATCAAAATTTATAATCCTTGATTGCTGATATACTCCAGCAATTTCTGTGTAATGATCTATTTTTCTCGTTTGXXXXXXXXXXT
ATGAATGTATTTTAGGCTACCTTCTTATTACCAGCACACTTGATATTTTCATCTGTTCAGTTACTTAAATGAATTTGTT
ACTTTCTGATCAAAATTTATAATCCTTGATTGCTGATATACTCTAGCAATTTCTGTGTAATGATCTATTTTTCTCGTTTGXXXXXXXXXXT
ATGAATGTATTTTAGGCTACCTTCTTATTACCAGCACACTTGATATTTTCATCTGTTCAGTTACTTAAATGAATTTGTT
>Marker49131 Chr4 SNP_site:14703892 Ref_Type:T
ACTCTATTTTGGCTCCTCTGATTTCATTCATACTTGATGAAGCATCTAGAATTATAATATTGTTTATACTTGCAGGTTGGXXXXXXXXXXT
CGATGAGCTCTTTGTTGATACTCAGGTAATTGAGAATTTTGTAAATCTAGTATCCTGTGACATATTATAATATGCTGTG
ACTCTATTTTGGCTCCTCTGATTTCATTCATACTTGATGAAGCATCTAGAATTATAATATTGTTTTTACTTGCAGGTTGGXXXXXXXXXXT
CGATGAGCTCTTTGTTGATACTCAGGTAATTGAGAATTTTGTAAATCTAGTATCCTGTGACATATTATAATATGCTGTG
>Marker491693 Chr3 SNP_site:11191903 Ref_Type:C
AACACCAAAATAGGATTTAAAGTGTTCTTTTTTTCTTTCTAAAAGACTTTTGAATCATTTTGAAAAGAGAGATTCCATTTXXXXXXXXXXT
CTTTGATTGAACAAGAACACAACGAAATCATTCACAACAATAACACTGAAGAATTCCCCTATAATCTTTCACAGCTGTT
AACACCAAAATAGGATTTAAAGTGTTCTTTTTTTCTTTCTAAAAGACTTTTGAATCATTTTGAAAAGAGAGATTCCATTTXXXXXXXXXXT
CTTTGATTGAATAAGAACACAACGAAATCATTCACAACAATAACACTGAAGAATTCCCCTATAATCTTTCACAGCTGTT
>Marker49202 Chr4 SNP_site:18603575 Ref_Type:A
AACAAATCAATGGTGTATTTTCTCTGAGATATAGAGATGCCTTCTTTAGATTTGGCCACCACCATGCCAAGGAAATATTCXXXXXXXXXXC
CAACATACACTATCAGAACTGCAATCTTTCCATCTTGGAAACCTTTGTAAATAAATATGATCAAAATGCCCCTGACTGT
AACAAATCAATGGTGTATTTTCTCTGAGATATAGAGATGCCTTCTTTAGATTTGGCCACCACCATGCCAAGGAAATATTCXXXXXXXXXXC
CAACATACACTATCAGAACTGCAATCTTTCCATCTTGGAAACCTTTGTAAATAAATATGATCAGAATGCCCCTGACTGT
>Marker492316 Chr3 SNP_site:38572202 Ref_Type:T
AACAATAGCCTTCTTCAACTAAGACATCACCATATAGAAAATGTGTAATTTTAGTTAATTTTCCTTATTATTCTACGTAAXXXXXXXXXXT
AATAATGCCAAAAGTATAGGGACTTTCTTTTTGTAGGCAGTAACAACAAATTAAATAGGTCCAACTTTGTCTCTATTGT
AACAATAGCCTTCTTCAACTAAGACATCACCATATAGAAAATGTGTAATTTTAGTTAATTTTTCTTATTATTCTACGTAAXXXXXXXXXXT
AATAATGCCAAAAGTATAGGGACTTTCTTTTTGTAGGCAGTAACAACAAATTAAATAGGTCCAACTTTGTCTCTATTGT
>Marker492820 Chr3 SNP_site:31348891 Ref_Type:T
TACTCTCTTCATTTTTTATCCATCTGGTTCAAATCGTAGATAATACTTTGTTGAAAGTTCATACTTTGTTTGTCATATCCXXXXXXXXXXT
TCTTGTCGAATTTTGATATCTAGATCTTTTGTAACTATTCCATAGGCATTATTTTACTTGGTTCAAATTCCCTTTGGTG
TACTCTCTTCATTTTTTATCCATCTGGTTCAAATCGTAGATAATACTTTGTTGAAAGTTCATACTTTGTTTGTCATATCCXXXXXXXXXXT
TCTTGTCGAATTTTGATTTCTAGATCTTTTGTAACTATTCCATAGGCATTATTTTACTTGGTTCAAATTCCCTTTGGTG
>Marker493115 Chr3 SNP_site:33810390 Ref_Type:C
ACTAACCTCAACCTGGCGGCTCATGAAGGATGGCATGTTTCCTCTAACATCTCTATTTGGGGCTGCTGGAGGATTCCCCGXXXXXXXXXXC
CAAATGGAAAGAGACTAATACTAAAAAATACAAAACATTCAATTAAAAAGAAAAATAAAGACACAAAATTAATGAAGTG
ACTAACCTCAACCTGGCGGCTCATGAAGGATGGCATGTTTCCTCTAACATCTCTATTTGGGGCTGCTGGAGGATTCCCCGXXXXXXXXXXC
CAAATGGAAAGAGACTAATACTAAAAAATACAAAACATTCAATTAAAAAGACAAATAAAGACACAAAATTAATGAAGTG
>Marker494048 Chr3 SNP_site:5341556 Ref_Type:A
ACAATCAATAAATTTTTATCCTCGCATCTACCATCACCATTTCTTTTTATTTATGGTGACTTTCCGAGTGCTTTACGATTXXXXXXXXXXA

Create PDF with GO2PDF for free, if you wish to

TTCAGAAACTAATGTCATATTTGATCTTTGATATTGTTTTCCAAAATTAGAATGACTCAACTAATATACAATTTATGTT
ACAGTCAATAAATTTTTATCCTCGCATCTACCATCACCATTTCTTTTTATTTATGGTGACTTTCCGAGTGCTTTACGATTXXXXXXXXXXA
TTCAGAAACTAATGTCATATTTGATCTTTGATATTGTTTTCCAAAATTAGAATAACTCAACTAATATACAATTTATGTT
>Marker494048 Chr3 SNP_site:5341798 Ref_Type:G
ACAATCAATAAATTTTTATCCTCGCATCTACCATCACCATTTCTTTTTATTTATGGTGACTTTCCGAGTGCTTTACGATTXXXXXXXXXXA
TTCAGAAACTAATGTCATATTTGATCTTTGATATTGTTTTCCAAAATTAGAATGACTCAACTAATATACAATTTATGTT
ACAGTCAATAAATTTTTATCCTCGCATCTACCATCACCATTTCTTTTTATTTATGGTGACTTTCCGAGTGCTTTACGATTXXXXXXXXXXA
TTCAGAAACTAATGTCATATTTGATCTTTGATATTGTTTTCCAAAATTAGAATAACTCAACTAATATACAATTTATGTT
>Marker494292 Chr3 SNP_site:27718826 Ref_Type:C
AACTTATCATTGGACAATATGGCCCGTGTTACTATATACTCTTTCGTAAAGTATCATAAGAATATTTATTTTTTGTTTCAXXXXXXXXXXA
GCCTATTATTGATGATTTTGATAATAATCTAGTCAAATTTTGAGTTAAGTGTCCTTGGGATAAAAGAATGAATAGAGTA
AACTTATCATTGGACAATATGGTCCGTGTTACTATATACTCTTTCGTCAAGTATCATAAGAATATTTATTTTTTGTTTCAXXXXXXXXXXA
GCCTATTATTGATGATTTTGATAATAATCTAGTCAAATTTTGAGTTAAGTGTCCTTGGGATAAAAGAATGAATAGAGTA
>Marker494292 Chr3 SNP_site:27718851 Ref_Type:A
AACTTATCATTGGACAATATGGCCCGTGTTACTATATACTCTTTCGTAAAGTATCATAAGAATATTTATTTTTTGTTTCAXXXXXXXXXXA
GCCTATTATTGATGATTTTGATAATAATCTAGTCAAATTTTGAGTTAAGTGTCCTTGGGATAAAAGAATGAATAGAGTA
AACTTATCATTGGACAATATGGTCCGTGTTACTATATACTCTTTCGTCAAGTATCATAAGAATATTTATTTTTTGTTTCAXXXXXXXXXXA
GCCTATTATTGATGATTTTGATAATAATCTAGTCAAATTTTGAGTTAAGTGTCCTTGGGATAAAAGAATGAATAGAGTA
>Marker494320 Chr3 SNP_site:3113300 Ref_Type:A
ACATTCAATTTTATCATCAATTGGGGCATTTGGATGGAATACAGATAATGCACGAGCAAACTCTTCGAAGCCCAATATTCXXXXXXXXXXA
TGTAGTCCATCTAAGAAAGAGCAATTTTAATATCCTGCAGATATACATTATTAAATAAATAATACTTAGAAAAAATAGT
ACATTCAATTTTATCATCAATTGGGGCATTTGGATGGAATACAGATAATGCACGAGCAAACTCTTCGAAGCCCAATATTCXXXXXXXXXXA
TGTAGTCCATCTAAGAAAGAGCAATTTTAATATCCTGCAGATATACATTATTAAATACATAATACTTAGAAAAAATAGT
>Marker495686 Chr3 SNP_site:7277903 Ref_Type:G
TACCTTAATACTTGTATCATCATGATATTCAGGTGCTACTTTGCATGTGAAGTCTTAAATGAAAAGCTCTATACCATTGGXXXXXXXXXXA
AATTGAAGATTCTATTGTAATGGATGGAAAGATATATATCCGTCTTCGATCTGCAGATTCTCAAGTGTATGCTCTTGTT
TACCTTAATACTTGTATCATCATGATATTCAGGTGCTACTTTGCATGTGAAGTCTTAAATGAAAAGCTCTATACCATTGGXXXXXXXXXXA
AATTGAAGATTCTATTGTAATGGATGGAAAGATATATATCCGTCTTCGATCTGCGGATTCTCAAGTGTATGCTCTTGTT
>Marker495734 Chr3 SNP_site:37879038 Ref_Type:C
TACTTGGCATTCAATGCTCACGAGTATTTTGTCAATGTTTCGTTGCCCTCTCCTCCAACAAAGCGAAAATGGTTTCGTGTXXXXXXXXXXA
ACAGTTCAGTTTATGAACTTTATTTGACAATGATACAGTTTAGAAAAACTATTTTAAATAACAAAATTCTGTAAATGTT
TACTTGGCATTCAATGCTCACGAGTATTTTGTCAATGTTTCGTTGCCCTCTCCTCCAACAAAGCGAAAATGGTTTCGTGTXXXXXXXXXXA
ACAGTTTAGTTTATGAACTTTATTTGACAATGATACAGTTTAGAAAAATTATTTTAAATAACAAAATTCTGTAAATGTT
>Marker495734 Chr3 SNP_site:37879080 Ref_Type:C
TACTTGGCATTCAATGCTCACGAGTATTTTGTCAATGTTTCGTTGCCCTCTCCTCCAACAAAGCGAAAATGGTTTCGTGTXXXXXXXXXXA
ACAGTTCAGTTTATGAACTTTATTTGACAATGATACAGTTTAGAAAAACTATTTTAAATAACAAAATTCTGTAAATGTT
TACTTGGCATTCAATGCTCACGAGTATTTTGTCAATGTTTCGTTGCCCTCTCCTCCAACAAAGCGAAAATGGTTTCGTGTXXXXXXXXXXA
ACAGTTTAGTTTATGAACTTTATTTGACAATGATACAGTTTAGAAAAATTATTTTAAATAACAAAATTCTGTAAATGTT
>Marker495856 Chr3 SNP_site:27997594 Ref_Type:A
CACTGGTTTCAAATAGTTGTAAGCATTTATCCGCGATGGCGGATGAAGAATCATTCTACATATGAAGAATCATCGTCAGAXXXXXXXXXXG
TCAAAACACTAGCCACCAATACTCAATTTGAACCGAGCTTAACTTGGCTAAAAGAACGAACTAAAACCCTAGGAAAGTT
CACTGGTTTCAAATTGTTGTAAGCATTTATCCGCGATGGCGGATGAAGAATCATTCTACATATGAAGAATCATCGTCAGAXXXXXXXXXXG
TCAAAACACTAGCCACCAATAATCAATTTGAACCGAGCTTAACTTGGCTAAAAGAACGAACTAAAACCCTAGGAAAGTT
>Marker495856 Chr3 SNP_site:27997821 Ref_Type:C

Create PDF with GO2PDF for free, if you wish to

CACTGGTTTCAAATAGTTGTAAGCATTTATCCGCGATGGCGGATGAAGAATCATTCTACATATGAAGAATCATCGTCAGAXXXXXXXXXXG
TCAAAACACTAGCCACCAATACTCAATTTGAACCGAGCTTAACTTGGCTAAAAGAACGAACTAAAACCCTAGGAAAGTT
CACTGGTTTCAAATTGTTGTAAGCATTTATCCGCGATGGCGGATGAAGAATCATTCTACATATGAAGAATCATCGTCAGAXXXXXXXXXXG
TCAAAACACTAGCCACCAATAATCAATTTGAACCGAGCTTAACTTGGCTAAAAGAACGAACTAAAACCCTAGGAAAGTT
>Marker495863 Chr3 SNP_site:8776414 Ref_Type:G
ACTACATCTAGACAGCTTCCCAACATCAGAGTCCCCACCATTTACAAAACTAGAAGTGCAGATCCAGCATATACAAATAAXXXXXXXXXXC
ATTATCCAGAAGTTATTGAATTTCCATAAATAATCATTACACTGAAGATGCAAGAAATCGTCGGGAAGGCCTACCTGTA
ACTACATCTAGACAGCTTCCCAACATCAGAGTCCCCACCATTTGCAAAACTAGAAGTCCAGATCCAGCATATACAAATAAXXXXXXXXXXC
ATTATCCAGAAGTTATTGAATTTCCATAAATAATCATTACACTGAAGATGCAAGAAATCGTCGGGAAGGCCTACCTGTA
>Marker495863 Chr3 SNP_site:8776428 Ref_Type:C
ACTACATCTAGACAGCTTCCCAACATCAGAGTCCCCACCATTTACAAAACTAGAAGTGCAGATCCAGCATATACAAATAAXXXXXXXXXXC
ATTATCCAGAAGTTATTGAATTTCCATAAATAATCATTACACTGAAGATGCAAGAAATCGTCGGGAAGGCCTACCTGTA
ACTACATCTAGACAGCTTCCCAACATCAGAGTCCCCACCATTTGCAAAACTAGAAGTCCAGATCCAGCATATACAAATAAXXXXXXXXXXC
ATTATCCAGAAGTTATTGAATTTCCATAAATAATCATTACACTGAAGATGCAAGAAATCGTCGGGAAGGCCTACCTGTA
>Marker495966 Chr3 SNP_site:354993 Ref_Type:A
ACTATTTTTCTTCCTACCCACTTTATTTTACTAATCTTTCTTTCTACCCCTTTATTCATTATCTGATTATACACTTACTTXXXXXXXXXXA
ACTTCTGACTTCGAAGTTTGGAGGTAAGTTTTTCACCCACCAATGGAATCACCTTGTTGGAGTTGCCACCAATGTTGTT
ACTATTTTTCTTCCTACCCACTTTATTTTACTAATCTTTCTTTCTTCCCCTTTATTCATTATCTGATTACACACTTACTTXXXXXXXXXXA
ACTTCTGACTTCGAAGTTTGGAGGTAAGTTTTTCACCCACCAATAGAATCACCTTGTTGGAGTTGCCACCAATGTTGTT
>Marker495966 Chr3 SNP_site:355017 Ref_Type:C
ACTATTTTTCTTCCTACCCACTTTATTTTACTAATCTTTCTTTCTACCCCTTTATTCATTATCTGATTATACACTTACTTXXXXXXXXXXA
ACTTCTGACTTCGAAGTTTGGAGGTAAGTTTTTCACCCACCAATGGAATCACCTTGTTGGAGTTGCCACCAATGTTGTT
ACTATTTTTCTTCCTACCCACTTTATTTTACTAATCTTTCTTTCTTCCCCTTTATTCATTATCTGATTACACACTTACTTXXXXXXXXXXA
ACTTCTGACTTCGAAGTTTGGAGGTAAGTTTTTCACCCACCAATAGAATCACCTTGTTGGAGTTGCCACCAATGTTGTT
>Marker495966 Chr3 SNP_site:355158 Ref_Type:G
ACTATTTTTCTTCCTACCCACTTTATTTTACTAATCTTTCTTTCTACCCCTTTATTCATTATCTGATTATACACTTACTTXXXXXXXXXXA
ACTTCTGACTTCGAAGTTTGGAGGTAAGTTTTTCACCCACCAATGGAATCACCTTGTTGGAGTTGCCACCAATGTTGTT
ACTATTTTTCTTCCTACCCACTTTATTTTACTAATCTTTCTTTCTTCCCCTTTATTCATTATCTGATTACACACTTACTTXXXXXXXXXXA
ACTTCTGACTTCGAAGTTTGGAGGTAAGTTTTTCACCCACCAATAGAATCACCTTGTTGGAGTTGCCACCAATGTTGTT
>Marker49608 Chr4 SNP_site:12798568 Ref_Type:T
ACCTTCCCAATTCAATACATCTATGTCCTTGGATCCAAGACGAGTTTTGAACCAACCATTCTGCAAATGCAAAAGTCCCCXXXXXXXXXXA
AAAAACAAAACCAACTCCTAAACATAAACAAAACATCAAGAACTGGCAAATAGCATTACAATTTGAACCATTCTAAGTA
ACCTTCCCAATTCAATACATCTATGTCCTTGGATCCAAGACGAGTTTTGAACCAACCATTCTGCAAATGCAAAAGTCCCCXXXXXXXXXXA
AAAAACAAAACCAACTCCTAAACATAAACAAAACATCAAGAACTGGCATATAGCATTACAATTTGAACCATTCTAAGTA
>Marker496362 Chr3 SNP_site:265642 Ref_Type:T
CACTCTTTAGGTGTGATCATGTTGAGATGCTGACGATACTAACTAGGCTACTGCAATCACATGTTCGATTTTTCTTGCAGXXXXXXXXXXT
CCTATGCTTGTTAAAATGTCTGCTATTTTTCCGTATCTTTTGAAAATTATACTTGTTTCGGTGCAAAGATAAGTAAGTG
CACTCTTTAGGTGTGATCATGTTGAGATGCTGACGATACTAACTAGGCTACTGCAATCACATGTTCGATTTTTCTTGCAGXXXXXXXXXXT
CCTATGCTTGTTAAAATGTCTGCTATTTTTCCGTATCTTTTGAAAATTATACTTGTTTTGGTGCAAAGATAAGTAAGTG
>Marker496412 Chr3 SNP_site:29584286 Ref_Type:C
ACTTTTATTGGATCCATCATGGGGCAAACTCATGTGGATTTGAGAAATACAGTGAGTATTTTATATAAACCATGTTTCGAXXXXXXXXXXT
ATTTCACCAATCTTAATTAAATTTTGCTATTTATCCAAAATAAACCGTTTTGCTATTTTTCTGGTCAGCCCTTACCGGT
ACTTTTATTGGATCCATCATGGGGCAAACTCATGTGGATTTGAGAAATACAGTGAGTATTTTATATAAACCATGTTTCGAXXXXXXXXXXT
ATTTCACCAATCTTAATTAAATTTTGCTATTTATCCAAAATAAACCGTTTTGCTATTTTTCTGGTCAGCCCTTACTGGT

Create PDF with GO2PDF for free, if you wish to

>Marker496483 Chr3 SNP_site:34812786 Ref_Type:A
TACAAGTGGTAGTTGCTTGTGCTCTCTTGGTTTCTAACTGAACAATTTCACATATTGAGTTGTGAATTTTAACCTCTTGTXXXXXXXXXXT
AGACATCTCACCAAGAATACCAGTGGAAAGCTGGATATGATCCCACACACCTGCCTTGATCATGGTGGGAAATCTTTGT
TACAAGTGGTAGTTGCTTGTGCTCTCTTGGTTTCTAACTGAACAATTTCACATATTGAGTTGTGAATTTTAACCTCTTGTXXXXXXXXXXT
AGACATCTCACCAAGAATACCAGTGGAAAGCTGGATATGGCCCCACACACCTGCCTTGATCATGGTGGGAAATCTTTGT
>Marker496483 Chr3 SNP_site:34812787 Ref_Type:T
TACAAGTGGTAGTTGCTTGTGCTCTCTTGGTTTCTAACTGAACAATTTCACATATTGAGTTGTGAATTTTAACCTCTTGTXXXXXXXXXXT
AGACATCTCACCAAGAATACCAGTGGAAAGCTGGATATGATCCCACACACCTGCCTTGATCATGGTGGGAAATCTTTGT
TACAAGTGGTAGTTGCTTGTGCTCTCTTGGTTTCTAACTGAACAATTTCACATATTGAGTTGTGAATTTTAACCTCTTGTXXXXXXXXXXT
AGACATCTCACCAAGAATACCAGTGGAAAGCTGGATATGGCCCCACACACCTGCCTTGATCATGGTGGGAAATCTTTGT
>Marker496610 Chr3 SNP_site:1780832 Ref_Type:T
ACCTTCTTACCTTGCCACGCAGAAGCTAGGAGATGGAATAAATAACATCACTAACACATAGCATAGTGACTTGTCTAAATXXXXXXXXXXT
ATAGTGCCACACTTGAGTTCTAAGACCTTTTAGGCATTGTTAAGTGACTAAATCTTGAACAGCTGTGCATAGGATGAGT
ACCTTCTTACCTTGCCACGCAGAAGCTAGGAGATGGAATAAATAACATCACTAACACATAGCATAGTGACTTGTCTAAATXXXXXXXXXXT
ATAGTGCCACACTTGAGTTCTAAGACCTTTTAGGCATTGTTAAGTGACTAAATCTTGAACAGCTGTGCATAGGTTGAGT
>Marker496735 Chr3 SNP_site:31413231 Ref_Type:A
ACCAAAATATTATATAAATCAGAGCTGAACAATTTTTTACTCCCCATAACCAAAAGTAATGAACTCCTAGAGAATGTCACXXXXXXXXXXG
ACTTACCTTATGAACCCATTCATATTCCCCGCCTTTTGAATCGAAAGTTCCATGCTGCAAACTGATCAATTTCATTGTG
ACCAAAATGTTATATAAATCAGAGCTGAACAATTTTTTACTCCCCATAACCAAAAGTAATGAACTCCTAGAGAATGTCACXXXXXXXXXXG
ACTTACCTTATGAACCCATTCATATTCCCCGCCTTTTGAATCGAAAGTTCCATGCTGCAAACTGATCAATTTCATTGTG
>Marker496895 Chr3 SNP_site:21711979 Ref_Type:T
AACATATCGACAAATATTTGACTAACTAACACACACTCAGGTATCAATTGAGTAATATATGAATGAATATTACTACTTATXXXXXXXXXXG
TTGCGACAATTTGGTATGTTGCAGATCATACTATCATCATACTTTGCATACTCAACGCTACATATTGATTTAAGAGGTA
AACATATCGACAAATATTTGACTAACTAACACACACTCAGGTATCAATTGAGTAATATATGAATGAATATTACTACTTATXXXXXXXXXXG
TTGCGACAATTTGGTATGTTGCATATCATACTGTCATCATACTTTGCATACTCAACGCTACATATTGATTTAAGAGGTA
>Marker496895 Chr3 SNP_site:21711988 Ref_Type:G
AACATATCGACAAATATTTGACTAACTAACACACACTCAGGTATCAATTGAGTAATATATGAATGAATATTACTACTTATXXXXXXXXXXG
TTGCGACAATTTGGTATGTTGCAGATCATACTATCATCATACTTTGCATACTCAACGCTACATATTGATTTAAGAGGTA
AACATATCGACAAATATTTGACTAACTAACACACACTCAGGTATCAATTGAGTAATATATGAATGAATATTACTACTTATXXXXXXXXXXG
TTGCGACAATTTGGTATGTTGCATATCATACTGTCATCATACTTTGCATACTCAACGCTACATATTGATTTAAGAGGTA
>Marker496898 Chr3 SNP_site:5583100 Ref_Type:G
GACAACATGAGATCTCTTCAGACTATTAATAAGACTGCTCTTACCAACATTAGGCAAGCCAATGACGCCCACGGTAATTGXXXXXXXXXXT
AAAGAATACAATAATATGCTAGGAAACTAATCCTATGGAGGCAAAAAGAAAATACAACTTCTTCATGCATATATATGTC
GACAACATGAGATCTCTTCAGACTATTAATAAGACTGCTCTTACCAACATTAGGCAAGCCAATGACGCCCACGGTAATTGXXXXXXXXXXT
AAATAATACAATAATATGCTAGGAAACTAATCCTACGGAGGCAAAAAGAAAATACAACCTCTTCATGCATATATATGTC
>Marker496898 Chr3 SNP_site:5583132 Ref_Type:T
GACAACATGAGATCTCTTCAGACTATTAATAAGACTGCTCTTACCAACATTAGGCAAGCCAATGACGCCCACGGTAATTGXXXXXXXXXXT
AAAGAATACAATAATATGCTAGGAAACTAATCCTATGGAGGCAAAAAGAAAATACAACTTCTTCATGCATATATATGTC
GACAACATGAGATCTCTTCAGACTATTAATAAGACTGCTCTTACCAACATTAGGCAAGCCAATGACGCCCACGGTAATTGXXXXXXXXXXT
AAATAATACAATAATATGCTAGGAAACTAATCCTACGGAGGCAAAAAGAAAATACAACCTCTTCATGCATATATATGTC
>Marker496898 Chr3 SNP_site:5583155 Ref_Type:T
GACAACATGAGATCTCTTCAGACTATTAATAAGACTGCTCTTACCAACATTAGGCAAGCCAATGACGCCCACGGTAATTGXXXXXXXXXXT
AAAGAATACAATAATATGCTAGGAAACTAATCCTATGGAGGCAAAAAGAAAATACAACTTCTTCATGCATATATATGTC
GACAACATGAGATCTCTTCAGACTATTAATAAGACTGCTCTTACCAACATTAGGCAAGCCAATGACGCCCACGGTAATTGXXXXXXXXXXT

Create PDF with GO2PDF for free, if you wish to

AAATAATACAATAATATGCTAGGAAACTAATCCTACGGAGGCAAAAAGAAAATACAACCTCTTCATGCATATATATGTC

>Marker49726 Chr4 SNP_site:4979472 Ref_Type:A

GACGGTGAATTTTAAAAAGTAAAAATAAGTAACAATTTTGTAAGTTTAGAAATTTAAGAATCATATAGGCATGAATACAAXXXXXXXXXXA

TGATGAACACTAGTAGATTTTTATTAGTGTAATGTTATTAATAGAAATCGATTGAAATCTATATTTTGTAGGTTTGGTA

GACGGTGAATTTTAAAAAGTAAAAATAAGTAACAATTTTGTAAGTTTAGAAATTTAAGAATCATATAGGCATGAATACAAXXXXXXXXXXA

TGATGAACACTAGTAGATTTTTATTAGTGTAATGTTATTAATAGAAATCGATTGAAGTCTATATTTTGTAGGTTTGGTA

>Marker497563 Chr3 SNP_site:9433184 Ref_Type:T

AACTGTGACCGCAAACGCCCCTTGGTAGTCGGTTTCTCTCTCACTCTGCTAATCCTTCCCCTCTCAATCCATTTCTCCCTXXXXXXXXXXT

TTTATGCTGAGCGGTTGAATAAAGCAGTTCTTAATGGATTTGAGGGATATAAGGTATGTGGGTTTCTTTGATTCTTGTT

AACTGTGACCGCAAACGCCCCTTGGTAGTCGGTTTCTCTCTCACTCTGCTAATCCTTCTCCTCTCAATCCATTTCTCCCTXXXXXXXXXXT

TTTATGCTGAGCAGTTGAATAAAGCAGTTCTTAATGGATTTGAGGGATATAAGGTATGTGGGTTTCTTTGATTCTTGTT

>Marker497563 Chr3 SNP_site:9433325 Ref_Type:A

AACTGTGACCGCAAACGCCCCTTGGTAGTCGGTTTCTCTCTCACTCTGCTAATCCTTCCCCTCTCAATCCATTTCTCCCTXXXXXXXXXXT

TTTATGCTGAGCGGTTGAATAAAGCAGTTCTTAATGGATTTGAGGGATATAAGGTATGTGGGTTTCTTTGATTCTTGTT

AACTGTGACCGCAAACGCCCCTTGGTAGTCGGTTTCTCTCTCACTCTGCTAATCCTTCTCCTCTCAATCCATTTCTCCCTXXXXXXXXXXT

TTTATGCTGAGCAGTTGAATAAAGCAGTTCTTAATGGATTTGAGGGATATAAGGTATGTGGGTTTCTTTGATTCTTGTT

>Marker49820 Chr4 SNP_site:18580746 Ref_Type:G

TACTGCATGAGGGAAATTGAAAAAGATGTGCTGAAGGTCTTCCTGATTATTACAGCATAGATGGCACATATGGGGTGAGAXXXXXXXXXXG

TGAGATGATTAACCAAAGATTTAACAGTGAAGGAACCTTCTGGTTCTAGGGACCAAACTCTCTTATTTGTGGAGAAGTT

TACTGCATGAGGGAAATTGAAAAAGATGTGCTGAAGGTCTTCCTGATTGTTACAGCATAGATGGCACATATGGGGTGAGAXXXXXXXXXXG

TGAGATGATTAACCAAAGATTTAACAGTGAAGGAACCTTCTGGTTCTAGGGACCAAACTCTCTTATTTGTGGAGAAGTT

>Marker498763 Chr3 SNP_site:30652727 Ref_Type:A

CACATCATTATAGATTGCTAATTGCTATTAGTGATGGTATTTCTTATTTTTCATCTTGAACCTTGTATAAGAGGAAGCCAXXXXXXXXXXT

TTTTATACCAAGCTTCTGATTGACATGTTCTATTAGTATTTCTCCTTCCTGATTGTGACAGCTTTGGAAATGTAGATGT

CACATCGTTATAGATTGCTAATTGCTATTAGTGATGGTATTTCTTATTTTTCATCTTGAACCTTGTATAAGAGGAAGCCAXXXXXXXXXXT

TTTTATACCAAGCTTCTGATTGACATGTTCTATTAGTATTTCTCCTTCCTGATTGTGACAGCTTTGGAAATGTAGATGT

>Marker499631 Chr3 SNP_site:27372788 Ref_Type:C

CACTACTATAAACTTATCCCCTTCCTTCTACATCTCTGGAATTTGGTTCATATTCGAGAATCCAATGTGTTCATGTTATTXXXXXXXXXXG

ACGATAAGACATGGCCATGCGCTAAAAAATGCTAACTATGCCTATGTGTTGCTACTTCTAATGATATTCTACGTTGAGT

CACTACTATAAACTTATCCCCTTCCTTCTACATCTCTGGAATTTGGTTCATATTCGAGAATCCAATGTGTTCATGTTATTXXXXXXXXXXG

ACGATAAGACATGGCTATGCGCTAAAAAATGCTAACTATGCCTATGTGTTGCTACTTCTAATGATATTCTACGTTGAGT

>Marker499767 Chr3 SNP_site:2353365 Ref_Type:A

ACTATTTTCGTCCAACTATGTTTAACTGTGAAATTTTGGTGGAATCTGATGCATGTATTAGTGTTTTGATGTCGTCACACXXXXXXXXXXT

CTAAACCAATTCAAGTTAATGCTTTCAACCAAATGAAAACTTTATTGATTGTTTAGAAAGATTTAATCAAAATTCAGGT

ACTATTTTCGTCCAGCTATGTTTAACTGTGAAATTTTGGTGGAATATGATGCATGTATTAGTGTTTTGATGTCGTCACACXXXXXXXXXXT

CTAAACCAATTCAAGTTAATGCTTTCAACCAAATGAAAACTTTATTGATTGTTTAGAAAGATTTAATCAAAATTCAGGT

>Marker499767 Chr3 SNP_site:2353396 Ref_Type:C

ACTATTTTCGTCCAACTATGTTTAACTGTGAAATTTTGGTGGAATCTGATGCATGTATTAGTGTTTTGATGTCGTCACACXXXXXXXXXXT

CTAAACCAATTCAAGTTAATGCTTTCAACCAAATGAAAACTTTATTGATTGTTTAGAAAGATTTAATCAAAATTCAGGT

ACTATTTTCGTCCAGCTATGTTTAACTGTGAAATTTTGGTGGAATATGATGCATGTATTAGTGTTTTGATGTCGTCACACXXXXXXXXXXT

CTAAACCAATTCAAGTTAATGCTTTCAACCAAATGAAAACTTTATTGATTGTTTAGAAAGATTTAATCAAAATTCAGGT

>Marker500044 Chr3 SNP_site:30428005 Ref_Type:T

AACTCTGTTAAAGGTTTGGATTATGATAAGAACTTGAACTCAGAGGTAGAAATCTTCAAATCTCTGGCTATCCAGAAAGGXXXXXXXXXXT

GTTCAGGTGTAGAAGAAGTTATTGTCACTTTGGTTAATACCTTAGTTAGACGTGGTAGAGTCAGAGAAGCACTTCGGGT

Create PDF with GO2PDF for free, if you wish to

AACTCTGTTAAAGGTTTGGATTATGATAAGAACTTGAACTCAGAGGTAGAAATCTTCAAATCTCTGGCTATCCAGAAAGGXXXXXXXXXXT
GTTCAGGTGTAGAAGAAGTTATTGTCACTTTGGTTAATACCTTAGTTAGACGTGGTAGAGTTAGAGAAGCACTTCGGGT
>Marker500175 Chr3 SNP_site:29783282 Ref_Type:T
CACCTAATATTTGTTGTTAGGTAATGCCCCAAATTTACTCCTCCAATTTCATTCTCAACTTTCTTTAAGCTTAGTTTAAGXXXXXXXXXXA
CATGTTCAAATCACTTCCATATAATCGACGAGTTATAAATATCTTAGTTGCATATTATTTTAGTTTGAATTAAACAGTA
CACCTAATATTTGTTGTTAGGTAATGCCCCAAATTTACTCCTCCAATTTCATTCTCAACTTTCTTTAAGCTTAGTTTAAGXXXXXXXXXXA
CATGTTCAAATCACTTTCATATAATCGAGGAGTTATAAATATCTTAGTTGCATATTATTTTAGTTTGAATTAAACAGTA
>Marker500175 Chr3 SNP_site:29783294 Ref_Type:G
CACCTAATATTTGTTGTTAGGTAATGCCCCAAATTTACTCCTCCAATTTCATTCTCAACTTTCTTTAAGCTTAGTTTAAGXXXXXXXXXXA
CATGTTCAAATCACTTCCATATAATCGACGAGTTATAAATATCTTAGTTGCATATTATTTTAGTTTGAATTAAACAGTA
CACCTAATATTTGTTGTTAGGTAATGCCCCAAATTTACTCCTCCAATTTCATTCTCAACTTTCTTTAAGCTTAGTTTAAGXXXXXXXXXXA
CATGTTCAAATCACTTTCATATAATCGAGGAGTTATAAATATCTTAGTTGCATATTATTTTAGTTTGAATTAAACAGTA
>Marker500976 Chr3 SNP_site:31510573 Ref_Type:C
ACAATTTGTCTCTACTTTTGATGGTTCCATCGGTTAGCTTTCACATGGATAGTTTCTTGCGTTCTTTCTTTTTGAGATGGXXXXXXXXXXA
ATGAATGAAGCCCATATGGAACGTTATATCTTGAAATTAATTTGTCAAGTTGATGCTAAAATACACAAAGGTTTTGGTG
ACAATTTGTCTCTACTTTTGATGGTTCCATCGGTTAGCTTTCACATGGATCGTTTCTTGCGTTCTTTCTTTTTGAGATGGXXXXXXXXXXA
ATGAATGAAGCCCATATGGAACGTTATATCTTGAAATTAATTTGTCAAGTTGATGCTAAAATACACAAAGGTTTTGGTG
>Marker500988 Chr3 SNP_site:33683400 Ref_Type:A
ACAACACTATAGTTTAAAAAGGTCACCATTTGGGTCACATCAAAGTAATGTATGGAAGCTGTTGGAGGGGGAGAGAGAGGXXXXXXXXXXC
TTTCTCTCTTTTTCAATCAATCCCTACGGCGGAGATTTTTTTATAAAGATTTTAATATTTCCTTTTATGAATTGTTGTG
ACAACACTATAGTTTAATAAGGTCACCATTTGGGTCACATCAAAGTAATGTATGGAAGCTGTTGGAGGGGGAGAGAGAGGXXXXXXXXXXC
TTTCTCTCTTTTTCAATCAATCCCTACGGCGGAGATTTTTTTATAAAGATTTTAATATTTCCTTTTATGAATTGTTGTG
>Marker501246 Chr3 SNP_site:27114937 Ref_Type:C
CACTTGTCATATTAAAAAAAAAACCTATAATAAACATGGCTAAATAGGGGTAACAAACAGCCTATACAACATAAGATAACXXXXXXXXXXC
TCC-CCCTCTCTCTCTCTTAATGCACCAACCAACGAACCTATCAACAAGCATAAACCCAATTTTGTTTCCTAGACTAGTC
CACTTGTCATATTAAAAAAAAAACCTATAATAAACATGGCTAAATAGGGGTAACAAACAGCCTATACAACATAAGATAACXXXXXXXXXX-
TCCTCCCCCTCTCTCTCTTAATGCACCAACCAACGAACCTATCAACAAGCATAAACCCAATTTTGTTTCCTAGACTAGTC
>Marker501347 Chr3 SNP_site:9939173 Ref_Type:G
TACTGTCGTTTTACACCATCCTGGGTTCAGTTGGGTAATTTGGGGCGGGATGTGACAACAATAATCCGATAACATGTATGXXXXXXXXXXG
GCCTCAGACACCCCTTTCAATCTGAATGCTGATGATTTGGACCAGAGAATCAAACAAAGAATCACAGGGGAAAACAAGT
TACTGTCGTTTTACACCATCCTGGGTTCAGTTGGGTAATTTGGGGCGGGGTGTGACAACAATAATCCGATAACATGTATGXXXXXXXXXXG
GCCTCAGACACCCCTTTCAATCTGAATGCTGATGATTTGGACCAGAGAATCAAACAAAGAATCACAGGGGAAAACAAGT
>Marker501399 Chr3 SNP_site:17992324 Ref_Type:A
ACTCCAAATAGTAGGCACCAAAACAGGATTGTGTTGCACCGCTATGACTTTTATTATAATTTTCTTTATTAGTAAATAGGXXXXXXXXXXT
TATGTGATATATCTACGGCCTCCGTCGATCGATAGCCGATTTTTTGACAATGATGAAGTCTGGAGCTTTCAAAGGAGTG
ACTCCAAATAGTAGGCACCAAAACAGGATTGTGTTGCACCGCTGTGACTTTTATTATAATTTTCTTTATTAGTAAATAGGXXXXXXXXXXT
TATGTGATATATCTACGGCCTCCGTCGATCGATAGCCGATTTTTTGACAATGATGAAGTCTGGAGCTTTCAAAGGAGTG
>Marker501742 Chr3 SNP_site:12035579 Ref_Type:C
AACCACAATTTTCAACCTTGTTCAGAGAAAAGGGGATAGGAATATGATAGATAGCCTCAATTATATTTGACAACAGCATTXXXXXXXXXXC
ACCACCTTGGGCCCCACCTAAACACGTAAAAATTTACCCTTTAACTCTGTTTGCACACCTTCCTCCCTTCCAACGTGTA
AACCACAATTTTCAACCTTGTTCAGAGAAAAGGGGATAGGAATATGATAGATAGCCTTAATTATATTTGACAACAGCATTXXXXXXXXXXC
ACCACCTTGGGCCCCACCTAAACACGTAAAAATTTACCCTTTAACTCTGTTTGCACACCTTCCTCCCTTCCAACGTGTA
>Marker501782 Chr3 SNP_site:27628800 Ref_Type:T
AACATGTATTTCTTCCTGTTCTAGTGAGATTGTTTTGAGAAATATCAAAGACTTGAAGGCAGAGGTTTTTTGGGCAAAAGXXXXXXXXXXA

Create PDF with GO2PDF for free, if you wish to

GTTGTATCAAGTATTGAAAGCGACGTTATACTTGTCGAATCATTCCAAGAGCTCACAATTTCAAGTGGGTCATTGAGTA
AACATGTATTTCTTCCTGTTCTAGTGAGATTGTTTTGAGAAATATCAAAGACTTGAAGGCAGAGGTTTTTTGGGCAAAAGXXXXXXXXXXA
GTTGTATTAAGTATTGAAAGCGACGTTATACTTGTCGAATCATTCCAAGAGCTCACAATTTCAAGTGGGTCATTGAGTA
>Marker501793 Chr3 SNP_site:12891265 Ref_Type:C
AACCACTTAAAAGGGTCGAGAGCTCTCTTCAACATTCTAGTGAGTCATATTCAAAGTGAAAATACATGGTGTATTTTTCTXXXXXXXXXXT
CGAGGATTTCTTTCAATCTATATCGATGTAGTAGAACTTGTTGTTGCAAAAGACTAAGAGTAGTCCCGAGTTTGGGGTG
AACCACTTAAAAGGGTCGAGAGCTCTCTTCAACATTCTAGTGAGTCATATTCAAAGTGAAAATACATGGTGTATTTTTCTXXXXXXXXXXT
CGCGGATTTCTTTCAATCTATATCGATGTAGTAGAACTTGTTGTTGCAAAAGACTAAGAGTAGTCCCGAGTTTGGGGTG
>Marker501939 Chr3 SNP_site:11187300 Ref_Type:C
AACATTGCCTATTATCCTTGCAGGCGATCTCTGTCATGGCCAAGTCTGGGTTTGCAGAGCTGATTGACTTCAACCCTATTXXXXXXXXXXA
TTTTCATCTACTGTTAATATTGGACACTAGCTGTCTTTTTATTTTGAGTTGTATAATTTGTTTGATTCTTTACAATGTG
AACATTGCCTATTATCCTTGCAGGCGATCTCTGTCATGGCCAAGTCTGGGTTTGCAGAGCTGATTGACTTCAACCCTATTXXXXXXXXXXA
TTTTCATCTATTGTTAATATAGGACACTAGCTGTCTTTTTATTTTGAGTTGTATAATTTGTTTGATTCTTTACAATGTG
>Marker501939 Chr3 SNP_site:11187310 Ref_Type:T
AACATTGCCTATTATCCTTGCAGGCGATCTCTGTCATGGCCAAGTCTGGGTTTGCAGAGCTGATTGACTTCAACCCTATTXXXXXXXXXXA
TTTTCATCTACTGTTAATATTGGACACTAGCTGTCTTTTTATTTTGAGTTGTATAATTTGTTTGATTCTTTACAATGTG
AACATTGCCTATTATCCTTGCAGGCGATCTCTGTCATGGCCAAGTCTGGGTTTGCAGAGCTGATTGACTTCAACCCTATTXXXXXXXXXXA
TTTTCATCTATTGTTAATATAGGACACTAGCTGTCTTTTTATTTTGAGTTGTATAATTTGTTTGATTCTTTACAATGTG
>Marker502910 Chr3 SNP_site:26014269 Ref_Type:C
AACACTTGAATGCGGATACCCATTCGAGTCTCTGACTAATTTCTCTTCCACATTAATGCAGTTGGAGGCTTTGATTCATCXXXXXXXXXXA
CTACTGGTCCATCAGCTGTGATATATATGCCTAACTCTAGTTTATTTCAATTGTGTTGCCAACCAGAATTGAAGGGAGT
AACACTTGAATGCGGATACCCATTCGAGTCTCTGACTAATTTCTCTTCCACATTAATGCAGTTGGAGGCTTTGATTCATCXXXXXXXXXXA
CTACTGGTCCATCAGCTGTGATATATATGCCTAACTTTAGTTTATTTCAATTGTGTTGCCAACCAGAATTGAAGGGAGT
>Marker503065 Chr3 SNP_site:1017203 Ref_Type:A
ACTCTTGGGGAAATACCACTTGGCTTTGAAAGGTCTTTAGAGGTTTTCTCACACTCGGTTGCTGTAGGGACTTAAAAAAGXXXXXXXXXXC
TCTTTTAACATGATATACACAAGTATGGTTCTATATGGTTTGTAGCATTGTTTTCTCTAATATGATATTGTGATATGTT
ACTCTTGGGGAAATACCACTTGGCTTTGAAAGGTCTTTAGAGGTTTTCTCACACTCGGTTGCTGTAGGGACTTGAAAAAAXXXXXXXXXXC
TCTTTTAACATGATATACACAAGTATGGTTCTATATGATTTGTAGCATTGTTTTCTCTAATATGATATTGTGATATGTT
>Marker503065 Chr3 SNP_site:1017209 Ref_Type:G
ACTCTTGGGGAAATACCACTTGGCTTTGAAAGGTCTTTAGAGGTTTTCTCACACTCGGTTGCTGTAGGGACTTAAAAAAGXXXXXXXXXXC
TCTTTTAACATGATATACACAAGTATGGTTCTATATGGTTTGTAGCATTGTTTTCTCTAATATGATATTGTGATATGTT
ACTCTTGGGGAAATACCACTTGGCTTTGAAAGGTCTTTAGAGGTTTTCTCACACTCGGTTGCTGTAGGGACTTGAAAAAAXXXXXXXXXXC
TCTTTTAACATGATATACACAAGTATGGTTCTATATGATTTGTAGCATTGTTTTCTCTAATATGATATTGTGATATGTT
>Marker503065 Chr3 SNP_site:1017383 Ref_Type:G
ACTCTTGGGGAAATACCACTTGGCTTTGAAAGGTCTTTAGAGGTTTTCTCACACTCGGTTGCTGTAGGGACTTAAAAAAGXXXXXXXXXXC
TCTTTTAACATGATATACACAAGTATGGTTCTATATGGTTTGTAGCATTGTTTTCTCTAATATGATATTGTGATATGTT
ACTCTTGGGGAAATACCACTTGGCTTTGAAAGGTCTTTAGAGGTTTTCTCACACTCGGTTGCTGTAGGGACTTGAAAAAAXXXXXXXXXXC
TCTTTTAACATGATATACACAAGTATGGTTCTATATGATTTGTAGCATTGTTTTCTCTAATATGATATTGTGATATGTT
>Marker503126 Chr3 SNP_site:25303591 Ref_Type:C
ACCCTCTTCTGACCAATTAGCTTACTACCTCACAAAAACCCTCACGCATTCTCAATTTCAATATCTTCGTTCCAAACGCGXXXXXXXXXXT
GTATCGATTTTTTATCAATAAGAAAAGTGAGAAAGATTTTCTCCCAAGATTTCAGTATTCTTCAGACCCTACTTCTGTG
ACCCTCTTCTGACCAATTAGCTTACTACCTCACAAAAACCCTCACGCATTCTCAATTTCAATATCTTCGTTCCAAAGGCGXXXXXXXXXXT
GTATCGATTTTTTATCAATAAGAAAAGTGAGAAAGATTTTCTCCCAAGATTTCAGTATTCTTCAAACCCTACTTCTGTG
>Marker503126 Chr3 SNP_site:25303780 Ref_Type:G

Create PDF with GO2PDF for free, if you wish to

ACCCTCTTCTGACCAATTAGCTTACTACCTCACAAAAACCCTCACGCATTCTCAATTTCAATATCTTCGTTCCAAACGCGXXXXXXXXXXT
GTATCGATTTTTTATCAATAAGAAAAGTGAGAAAGATTTTCTCCCAAGATTTCAGTATTCTTCAGACCCTACTTCTGTG
ACCCTCTTCTGACCAATTAGCTTACTACCTCACAAAAACCCTCACGCATTCTCAATTTCAATATCTTCGTTCCAAAGGCGXXXXXXXXXXT
GTATCGATTTTTTATCAATAAGAAAAGTGAGAAAGATTTTCTCCCAAGATTTCAGTATTCTTCAAACCCTACTTCTGTG
>Marker503189 Chr3 SNP_site:21077904 Ref_Type:G
ACTCTTTTGAGTTTTTTAGATGAGGTTGATACAAGATTACGTGAAAGCAGAAGTTATAATATAAAGACAAATATAAGTAAXXXXXXXXXXA
ACGGGTCCGGAATTTCTTGAAACCCTAAACCCTCGCAATATAAACTCTGCCGCCTACTTCCTCCTTCTCTTTGGACGGT
ACTCTTTTGAGTTTTTTAGATGAGGTTGATACAAGATTACGTGAAAGCAGAAGTTATAATATAAAGACAAATATAAGTAAXXXXXXXXXXA
ACGGGTCCGGAATTTCTTGGAACCCTAAACCCTCGCAATATAAACTCTGCCGCCTACTTCCTCCTTCTCTTTGGACGGT
>Marker50323 Chr4 SNP_site:4448214 Ref_Type:G
CACTATAAAAAGTGGAACACTTTGGATTCCATTGTCAATAAGACAAAAAAAGAGACGATGTTAAATGTGTTAAAAATAAAXXXXXXXXXXG
ATAAAAACTTATATAAGTTTATTGATAAAACTAATAGAATTTTATCAGTGATGGAAATCAATAGAAACTAGCATAAGTC
CACTATAAAAAGTGGAACACTTTGGATTCCATTGTCAATAAGACAAAAAAAGAGACGATGTTAAATGTGTTAAAAATAAAXXXXXXXXXXG
ATAAAAACTTATATAAGTTTATTGATAAAACTAATAGAATTTTATCAGTGATGGGAATCAATAGAAACTAGCATAAGTC
>Marker503234 Chr3 SNP_site:9178230 Ref_Type:A
GACCCAAAAATTTCAACGAGAAAAATTGATTGTGACTTTATATAAATATTGAATTCTTTCCAC-TTTTTTTTCTTAAAAATXXXXXXXXXX
AAAATTAGAAATTAAGCTTTTAAACTTATTCACTGAACTATAAATTGATTAACTTTAAAAAAGAATTATCAAATGAAGTG
GACCCAAAAATTTCAACGAGAAAAATTGATTGTGACTTTATATAAATATTGAATTCTTTCCACTTTTTTTTTCTTAAAAA-XXXXXXXXXX
AAAATCAGAAATTAAGCTTTTAAACTTATCCACTGAACTATAAATTGATTAACTTTAAAAAAGAATTATCAAATGAAGTG
>Marker503234 Chr3 SNP_site:9178254 Ref_Type:C
GACCCAAAAATTTCAACGAGAAAAATTGATTGTGACTTTATATAAATATTGAATTCTTTCCAC-TTTTTTTTCTTAAAAATXXXXXXXXXX
AAAATTAGAAATTAAGCTTTTAAACTTATTCACTGAACTATAAATTGATTAACTTTAAAAAAGAATTATCAAATGAAGTG
GACCCAAAAATTTCAACGAGAAAAATTGATTGTGACTTTATATAAATATTGAATTCTTTCCACTTTTTTTTTCTTAAAAA-XXXXXXXXXX
AAAATCAGAAATTAAGCTTTTAAACTTATCCACTGAACTATAAATTGATTAACTTTAAAAAAGAATTATCAAATGAAGTG
>Marker504217 Chr3 SNP_site:16450434 Ref_Type:C
ACTAAAGTTTAGTCATACCTCTTTGCTATCTTAACATTCAGCTACACAAATACAACTTGTGTGCTAATCAAGTCCTAAAGXXXXXXXXXXT
TAACCATATTAGGAGTCTTAGAGGTAATCTCCCTCCCTTTGATAAGATGATATCAACATCAAATACTGAAACTGACTGT
ACTAAAGTTTAGTCATACCTCTTTGCTATCTTAACATTCAGCTACACAAATCCAACTTGTGTGCTAATCAAGTCCTAAAGXXXXXXXXXXT
TAACCATATTAGGAGTCTTAGAGGTAATCTCCCTCCCTTTGATAAGATGATATCAACATCAAATACTGAAACGGACTGT
>Marker504217 Chr3 SNP_site:16450645 Ref_Type:G
ACTAAAGTTTAGTCATACCTCTTTGCTATCTTAACATTCAGCTACACAAATACAACTTGTGTGCTAATCAAGTCCTAAAGXXXXXXXXXXT
TAACCATATTAGGAGTCTTAGAGGTAATCTCCCTCCCTTTGATAAGATGATATCAACATCAAATACTGAAACTGACTGT
ACTAAAGTTTAGTCATACCTCTTTGCTATCTTAACATTCAGCTACACAAATCCAACTTGTGTGCTAATCAAGTCCTAAAGXXXXXXXXXXT
TAACCATATTAGGAGTCTTAGAGGTAATCTCCCTCCCTTTGATAAGATGATATCAACATCAAATACTGAAACGGACTGT
>Marker504251 Chr3 SNP_site:15967354 Ref_Type:C
ACCAATTTTATTGGCAAAGCCTCAAACTTACATGAATTTCTGTGGAGAATCGGTTTGTCCTCTTGTCTCTCTTTTCTTCTXXXXXXXXXXA
TATTCATAGGGATAGATTGTTAGTTTCTTTCCATTGAAGCAATGACATAGCATTAATATCGCATTTTGATAAGTATGTG
ACCAATTTTATTGGCAAAGCCTCAAACTTACATGAATTTCTGTGGAGAATCGGTTTGTCCTCTTGTCTCTCTTTTCTTCTXXXXXXXXXXA
TATTCATAGGGATAGATTGTTAGTTTTTTTCCATTGAAACAATGACATAGCATTAATGTCGCATTTTGATAAGTATGTG
>Marker504251 Chr3 SNP_site:15967366 Ref_Type:G
ACCAATTTTATTGGCAAAGCCTCAAACTTACATGAATTTCTGTGGAGAATCGGTTTGTCCTCTTGTCTCTCTTTTCTTCTXXXXXXXXXXA
TATTCATAGGGATAGATTGTTAGTTTCTTTCCATTGAAGCAATGACATAGCATTAATATCGCATTTTGATAAGTATGTG
ACCAATTTTATTGGCAAAGCCTCAAACTTACATGAATTTCTGTGGAGAATCGGTTTGTCCTCTTGTCTCTCTTTTCTTCTXXXXXXXXXXA
TATTCATAGGGATAGATTGTTAGTTTTTTTCCATTGAAACAATGACATAGCATTAATGTCGCATTTTGATAAGTATGTG

Create PDF with GO2PDF for free, if you wish to

>Marker504251 Chr3 SNP_site:15967385 Ref_Type:A
ACCAATTTTATTGGCAAAGCCTCAAACTTACATGAATTTCTGTGGAGAATCGGTTTGTCCTCTTGTCTCTCTTTTCTTCTXXXXXXXXXXA
TATTCATAGGGATAGATTGTTAGTTTCTTTCCATTGAAGCAATGACATAGCATTAATATCGCATTTTGATAAGTATGTG
ACCAATTTTATTGGCAAAGCCTCAAACTTACATGAATTTCTGTGGAGAATCGGTTTGTCCTCTTGTCTCTCTTTTCTTCTXXXXXXXXXXA
TATTCATAGGGATAGATTGTTAGTTTTTTTCCATTGAAACAATGACATAGCATTAATGTCGCATTTTGATAAGTATGTG
>Marker504376 Chr3 SNP_site:5259596 Ref_Type:C
CACTGATTGCCTAAGGCATCATCTCGGGTAATAATATATGGTCAATGTATTAAGTCGTTAGAAATTTTAAAGCTCAAGCTXXXXXXXXXXG
ATTCATCCCAACTTAGTATCACTGAATTTTATATTCAGATAAAAGAAAATGCAAGGATATGGAATGAATATTGACAAGT
CACTGATTGCCTAAGGCATCATCTCGGGTAATAATATATGGTCAATGTATTAAGTCGTTAGAAATTTTAAAGCTCAAGCTXXXXXXXXXXG
ATTCATCCCAACTTAGTATCACTGAATTTTATATTCAGATCAAAGAAAATGCAAGGATATGGAATGAATATTGACAAGT
>Marker505000 Chr3 SNP_site:12790195 Ref_Type:G
ACAGGTGATTCTCGTTTAATAGTTATTTTCCAACAGAATGTGTTTTAGGTTTCAAATGACATTTATCAATGGGGACTGTXXXXXXXXXXC
TATAGGTAGAACATCAACGCCTGTAGCTAATTATTGATATGTTATGCCATTCAGAATGAAGATGGAACAAAAATTGAGT
ACAGGTGATTCTCGTTTAATAGTTATTTTCCAACAGAATGTGTTTTAGGTTTCAAATGACATTTATCAATGGGGACTGTXXXXXXXXXXC
TATAGGTAGAACATCAACGCCTGTAGCTAATTGTTGATATGTTATGCCATTCAGAATGAAGATGGAACAAAAATTGAGT
>Marker505432 Chr3 SNP_site:22320381 Ref_Type:T
CACTTTATGTCAATACCTAACCATAGAAATCAAATTAATTCCATAAACCAACATCGTAAATTAATTTTTAACAATCCTTAXXXXXXXXXXA
AAACACTTTCAAATTCAAATACTTATGCCCCTTTAAGTAACCATCTGGCTTTGGCTTTTTGGTTTTTGAAAATGTAGTC
CACTTTATGTCAATACCTAACCATAGAAATCAAATTAATTCCATAAACCAACATCGTAAATTAATTTTTAACAATCCTTAXXXXXXXXXXA
AAACACTTTCAAATTCAAATACTTATGCCTCTTTAAGTAACCATCTGGCTTTGGCTTTTTGGTTTTTGAAAATGTAGTC
>Marker505510 Chr3 SNP_site:2710850 Ref_Type:T
ACTCTAATCAAATGCATCCCTGACAGAATTCTAATTTCTGTTCAGCATCTTCAAAACTACATAGTTCATGTCACACAAAGXXXXXXXXXXC
TTTGTAGCATTTGTTTAATTCTGAACTCTCATAAGACACAATAACGCCTCCTTTATGCTCTTATAACTTGGAATTTGTT
ACTCTAATCAAATGCATCCCTGACAGAATTCTAATTTCTGTTCAGCATCTTCAAAACTACATAGTTCATGTCACACAAAGXXXXXXXXXXC
TTTGTAGCATTTGTTTAATTCTGAACTCTCATAAGACATAATAACGCCTCCTTTATGCTCTTATAACTTGGAATTTGTT
>Marker506791 Chr3 SNP_site:13145835 Ref_Type:C
CACACGACTCACTTGACACAACCTAGCTAGCCATAATAAAGACTTATGACAACTAGTTAAGAATGAGCCAAACAAGCGTCXXXXXXXXXXG
CTTGTAGAAATACAAGTTATTGTAGCCTCTTTTATAGAATAATGAGGCCAAAATATTAAAGTAGTGTATCAAAACATGT
CACACGATTCACTTGACACAACCTAGCTAGCCATAATAAAGACTTATGACAACTAGTTAAGAATGAGCCAAACAAGCGTCXXXXXXXXXXG
CTTGTAGAAATACAAGTTATTGTAGCCTCTTTTATAGAATAATGAGGCCAAAATATAAAAGTAGTGTATCAAAACATGT
>Marker506791 Chr3 SNP_site:13146117 Ref_Type:T
CACACGACTCACTTGACACAACCTAGCTAGCCATAATAAAGACTTATGACAACTAGTTAAGAATGAGCCAAACAAGCGTCXXXXXXXXXXG
CTTGTAGAAATACAAGTTATTGTAGCCTCTTTTATAGAATAATGAGGCCAAAATATTAAAGTAGTGTATCAAAACATGT
CACACGATTCACTTGACACAACCTAGCTAGCCATAATAAAGACTTATGACAACTAGTTAAGAATGAGCCAAACAAGCGTCXXXXXXXXXXG
CTTGTAGAAATACAAGTTATTGTAGCCTCTTTTATAGAATAATGAGGCCAAAATATAAAAGTAGTGTATCAAAACATGT
>Marker507292 Chr3 SNP_site:34923002 Ref_Type:T
ACTAAAAATTATGAAATCGTTAAGGTAATAGGGGAAAAATAAGAACGAACCTTTCCTGTTTGACTTCGCAATGTCAATGAXXXXXXXXXXG
AGAGGGCAGGAGCATTCAAAGAGAAAGAAATCACATCCCCGGAAAACTCAAGCCCTGATAAACAGGGAGCGTCAATGTC
ACTAAAAATTATGAAATCGTTAAGGTAATAGGGGAAAAATAAGAACGAACCTTTCCTGTTTGACTTTGCAATGTCAATGAXXXXXXXXXXG
AGAGGGCAGGAGCATTCAAAGAGAAAGAAATCACATCCCCGGAAAACTCAAGCCCTGATAAACAGGGAGCGTCAATGTC
>Marker507325 Chr3 SNP_site:4872087 Ref_Type:C
AACACCCCTATCCCTTCTTCCATCTCTGTCTTTGCCGAGAACCCGTTACGTATGATCCTACAAACGCAGCATTTCTCCGAXXXXXXXXXXT
TCTTTGGGAGCTTGCTGGCTTTGATCTTAACTGTCTCGCGATACTCTTCGAAACGAGCTAGGGTTCGTTGCATATTGTG
AACACCCCTATCCCTTCTTCCATCTCTGTCTTTGCTGAGAACCCGTTACGTATGATCCTACAAACGCAGCATTTCTCCGAXXXXXXXXXXT

Create PDF with GO2PDF for free, if you wish to

TCTTTGGGAGCTTGCTGGCTTTGATCTTAACTGTCTCGCGATACTCTTCGAAACGAGCTAGGGTTCGTTGCATATTGTG

>Marker507486 Chr3 SNP_site:35092966 Ref_Type:A

CACATTGAAATAAAATTTTAAGTGGGTGCTCAACCTTGTTATCTTCCCAAAGTAGTTCTTGCACCATTTCTAACCTCCATXXXXXXXXXXC
ATTCTAGTGATTTTTTCTTATTTTATTTATTTATCTTATTGGAAATATTTCATTAATGATATGAAATATTTCCACCTGT
CACGTTGAAATAAAATTTTAAGTGGGTGCTCAACCTTGTTATCTTCCCAAAGTAGTTCTTGCACCATTTCTAACCTCCATXXXXXXXXXXC
ATTCTAGTGATTTTTTCTTATTTTATTTATTTATCTTATTGGAAATATTTCATTAATGATATGAAATATTTCCACCTGT

>Marker508178 Chr3 SNP_site:36542929 Ref_Type:T

ACAGTTTTGTTTAAGATCAATTTATCACTCAGCCCCATCCGCTCTTCCGCCATACTTTATTCGTTTCTCCTGAATCTTAAXXXXXXXXXXA
TTCAGAAAGTATTATAGTCCAAACTGAAACAGATGACTAGAGAATTACAAACTTTTGACCTGGTTGATTGCAATGTGTT
ACAGTTTTGTTTAAGATCAATTTATCACTCAGCCCCATCTGCTCTTCCGCCATACTTTATTCGTTTCTCCTGAATCTTCAXXXXXXXXXXA
TTCAGAAAGTATTATAGTCCAAACTGAAACAGATGACTAGAGAATTACAAACTTTTGACCTGGTTGATTGCAATGTGTT

>Marker508178 Chr3 SNP_site:36542968 Ref_Type:C

ACAGTTTTGTTTAAGATCAATTTATCACTCAGCCCCATCCGCTCTTCCGCCATACTTTATTCGTTTCTCCTGAATCTTAAXXXXXXXXXXA
TTCAGAAAGTATTATAGTCCAAACTGAAACAGATGACTAGAGAATTACAAACTTTTGACCTGGTTGATTGCAATGTGTT
ACAGTTTTGTTTAAGATCAATTTATCACTCAGCCCCATCTGCTCTTCCGCCATACTTTATTCGTTTCTCCTGAATCTTCAXXXXXXXXXXA
TTCAGAAAGTATTATAGTCCAAACTGAAACAGATGACTAGAGAATTACAAACTTTTGACCTGGTTGATTGCAATGTGTT

>Marker508338 Chr3 SNP_site:32752086 Ref_Type:C

AACCATATAGACATCTCTTAATACTTCATCATCTTGTTTCATTAATATATTTTGATCAATATTTTATTTCTTCGTCGTGAXXXXXXXXXXG
TGACTTTTTCACCTGCAGGATCCATCCTAAGATCTAAGGATAAGTAAGTAGATCTATAAGAAACTCTTTTTGATTAAGT
CACCATATAGACATCTCTTAATACTTCATCATCTTGTTTCATTAATATATTTTGATCAATATTTTATTTCTTCGTCGTGAXXXXXXXXXXG
TGACTTTTTCACCTGCAGGATCCATCCTAAGATCTAAGGATAAGTAAGTAGAGCTATAAGAAACTCTTTTTGATTAAGT

>Marker508338 Chr3 SNP_site:32752335 Ref_Type:G

AACCATATAGACATCTCTTAATACTTCATCATCTTGTTTCATTAATATATTTTGATCAATATTTTATTTCTTCGTCGTGAXXXXXXXXXXG
TGACTTTTTCACCTGCAGGATCCATCCTAAGATCTAAGGATAAGTAAGTAGATCTATAAGAAACTCTTTTTGATTAAGT
CACCATATAGACATCTCTTAATACTTCATCATCTTGTTTCATTAATATATTTTGATCAATATTTTATTTCTTCGTCGTGAXXXXXXXXXXG
TGACTTTTTCACCTGCAGGATCCATCCTAAGATCTAAGGATAAGTAAGTAGAGCTATAAGAAACTCTTTTTGATTAAGT

>Marker508390 Chr3 SNP_site:21738962 Ref_Type:T

AACTTTAATATTATTTATGATTGAAATTGTCCATATTAGAAAACTGTTGATATATGAACATGAAATTTGAAAAAAAAGCTXXXXXXXXXXA
AAGGGTTGAGAAGTAGGAGTTTGGGAATAAAGTGTATGAATCGCACATTTCACTCCCTATTTGGTCTAAAGATTTTGTG
AACTTTAATATTATTTATGATTGAAATTGTCCATATTAGAAAACTGTTGATATATGAACATGAAATTTGAAAAAAAAGTTXXXXXXXXXXA
AAGGGTTGAGAAGTAGGAGTTTGGGAATAAAGTGTATGAATCGCATATTTCACTCTCTATTTGGTCTAAAGATTTTGTG

>Marker508390 Chr3 SNP_site:21739101 Ref_Type:T

AACTTTAATATTATTTATGATTGAAATTGTCCATATTAGAAAACTGTTGATATATGAACATGAAATTTGAAAAAAAAGCTXXXXXXXXXXA
AAGGGTTGAGAAGTAGGAGTTTGGGAATAAAGTGTATGAATCGCACATTTCACTCCCTATTTGGTCTAAAGATTTTGTG
AACTTTAATATTATTTATGATTGAAATTGTCCATATTAGAAAACTGTTGATATATGAACATGAAATTTGAAAAAAAAGTTXXXXXXXXXXA
AAGGGTTGAGAAGTAGGAGTTTGGGAATAAAGTGTATGAATCGCATATTTCACTCTCTATTTGGTCTAAAGATTTTGTG

>Marker508390 Chr3 SNP_site:21739111 Ref_Type:T

AACTTTAATATTATTTATGATTGAAATTGTCCATATTAGAAAACTGTTGATATATGAACATGAAATTTGAAAAAAAAGCTXXXXXXXXXXA
AAGGGTTGAGAAGTAGGAGTTTGGGAATAAAGTGTATGAATCGCACATTTCACTCCCTATTTGGTCTAAAGATTTTGTG
AACTTTAATATTATTTATGATTGAAATTGTCCATATTAGAAAACTGTTGATATATGAACATGAAATTTGAAAAAAAAGTTXXXXXXXXXXA
AAGGGTTGAGAAGTAGGAGTTTGGGAATAAAGTGTATGAATCGCATATTTCACTCTCTATTTGGTCTAAAGATTTTGTG

>Marker50847 Chr4 SNP_site:19883059 Ref_Type:G

CACATCCAGGCTTTATAGTTTTCTTCCCTATCTTGCATTGTGATAAAATCTTTAACTTAACTTTTGATTAATGGAGAAGCXXXXXXXXXXA
ACCAGAGGGAGCATTTGCTTCTACTCCTTGCTAACATGCAAGCTCGACTAACAAATAAGCAGAAGTCTGGCTCAAAGGT

Create PDF with GO2PDF for free, if you wish to

CACATCCAGGCTTTGTAGTTTTCTTCCCTATCTTGCATTGTGATAAAATCTTTAACTTAACTTTTGATTAATGGAGAAGCXXXXXXXXXXA
ACCAGAGGGAGCATTTGCTTCTACTCCTTGCTAACATGCAAGCTCGACTAACAAATAAGCAGAAGTCTGGCTCAAAGGT
>Marker509256 Chr3 SNP_site:24969550 Ref_Type:C
TACTTGTTTGTATGGAGGAGTTTCCAAAGGCAGCCAAATATCTTGTCTTAAATCTGGTGTTGTATGATGTTTCTGCTCCCXXXXXXXXXXA
CTACTCATACCTCTCTCATGTTTTTTACTTCCTTTTGGTTAATTCAAGTCTATGTTTCCCCTTTTAGTGATAATAGGTT
TACTTGTTTGTATGGAGGAGTTTCCAAAGGCAGCCAAATATCTTGTCTTAAATCTGGTGTTGTATGATGTTTCTGCTCCCXXXXXXXXXXC
ATACTCATACCTCTCTCATGTTTTTTACTTCCTTTTGGTTAATTCAAGTCTATGTTTCCCCTTTTAGTGATAATAGGTT
>Marker509256 Chr3 SNP_site:24969551 Ref_Type:A
TACTTGTTTGTATGGAGGAGTTTCCAAAGGCAGCCAAATATCTTGTCTTAAATCTGGTGTTGTATGATGTTTCTGCTCCCXXXXXXXXXXA
CTACTCATACCTCTCTCATGTTTTTTACTTCCTTTTGGTTAATTCAAGTCTATGTTTCCCCTTTTAGTGATAATAGGTT
TACTTGTTTGTATGGAGGAGTTTCCAAAGGCAGCCAAATATCTTGTCTTAAATCTGGTGTTGTATGATGTTTCTGCTCCCXXXXXXXXXXC
ATACTCATACCTCTCTCATGTTTTTTACTTCCTTTTGGTTAATTCAAGTCTATGTTTCCCCTTTTAGTGATAATAGGTT
>Marker509450 Chr3 SNP_site:3906991 Ref_Type:T
ACCCTTTCTATTGACATAAACAACAAAATAACAGTTAGGGGTTTGCAGTTATCATAGCTTTGATGCAAATTCTAACATTAXXXXXXXXXXT
TAAAACTTGTGGAATGCAAAGATACCGTTACCCGGTCCTCTTCTGTTTAAGGACTAGATAACTCTAATTAAATATTAGT
ACCCTTTCTATTGACATAAACAACAAAATAACAGTTAGGGGTTTGCAGTTATCATAGCTTTGATGCAAATTCTAACATTAXXXXXXXXXXT
TAAAACTTGTGGAATGCAAAGATACCGTTACCCGGTCTTCTTCTGTTTAAGGACTAGATAACTCTAATAAAATATTAGT
>Marker509450 Chr3 SNP_site:3907022 Ref_Type:A
ACCCTTTCTATTGACATAAACAACAAAATAACAGTTAGGGGTTTGCAGTTATCATAGCTTTGATGCAAATTCTAACATTAXXXXXXXXXXT
TAAAACTTGTGGAATGCAAAGATACCGTTACCCGGTCCTCTTCTGTTTAAGGACTAGATAACTCTAATTAAATATTAGT
ACCCTTTCTATTGACATAAACAACAAAATAACAGTTAGGGGTTTGCAGTTATCATAGCTTTGATGCAAATTCTAACATTAXXXXXXXXXXT
TAAAACTTGTGGAATGCAAAGATACCGTTACCCGGTCTTCTTCTGTTTAAGGACTAGATAACTCTAATAAAATATTAGT
>Marker509818 Chr3 SNP_site:17065447 Ref_Type:C
CACTCATCTCACTATAGATATATTTGTGTCCATCTAATATGACCAGTATCAGTAAGTTAATCCTTCACAGGTTGTTCGTAXXXXXXXXXXT
GGAGGGGCCCCTTGTTCAATGCTTGGATTCAGTCCTTATGGAAATCACCTATCTACTAACCCTAAAGTGGGTAGGAGTG
CACTCATCTCACTATAGATATATTTGTGTGCATCTAATATGACCAGTATCAGTAAGTTAATCCTTCACAGGTTGTTCGTAXXXXXXXXXXT
GGAGGGGCCCCTTGTTCAATGCTTGGATTCAGTCCTTATGGAAACCACCTATCTACTAACCCTAAAGTGGGTAGGAGTG
>Marker509818 Chr3 SNP_site:17065672 Ref_Type:T
CACTCATCTCACTATAGATATATTTGTGTCCATCTAATATGACCAGTATCAGTAAGTTAATCCTTCACAGGTTGTTCGTAXXXXXXXXXXT
GGAGGGGCCCCTTGTTCAATGCTTGGATTCAGTCCTTATGGAAATCACCTATCTACTAACCCTAAAGTGGGTAGGAGTG
CACTCATCTCACTATAGATATATTTGTGTGCATCTAATATGACCAGTATCAGTAAGTTAATCCTTCACAGGTTGTTCGTAXXXXXXXXXXT
GGAGGGGCCCCTTGTTCAATGCTTGGATTCAGTCCTTATGGAAACCACCTATCTACTAACCCTAAAGTGGGTAGGAGTG
>Marker509971 Chr3 SNP_site:37246902 Ref_Type:G
ACTCGACGACGACGGCGATCTAGAATCGAGCTTCGAGCTAAAATTCATGACAAAGATTCGACGGCTAGCTGATTTCACGGXXXXXXXXXXT
AGTTCATCGTCTTTAATTTCTTTTTTCTAATTTTATTTTCCGTTTTGTAGAGATTTAAGCGCTTAGATAATGACACGTG
ACTCGACGACGACGGCGATCTAGAATCGAGCTTCGAGCTAGAATTCATGACAAAGATTCGACGGCTAGCTGATTTCACGGXXXXXXXXXXT
AGTTCATCGTCTTTAATTTCTTTTTTCTAATTTTATTTTCCGTTTTGTAGAGATTTAAGCGCTTAGATAATGACACGTG
>Marker510562 Chr3 SNP_site:36092364 Ref_Type:C
ACTCCATCAAGCATATATAAATATATGTTGTAGTTTGAATGACTTCCCAATTTATGTAACATTTTCCGTCATTTTCTCCAXXXXXXXXXXC
CAAAATTTACCCAATCCCCAGATAGCTCTGTATATATAATTGTTCTAATTATGGGTCAGATTCTGGTTCAGCATAGGGT
ACTCCATCAAGCATATATAAATATATGTTGTAGTTTGAATGACTTTCCAATTTATGTAACATTTTCCGTCACTTTCTCCAXXXXXXXXXXC
CAAAATTTACCCAATCCCCAGATAGCTCTGTATATATAATTGTTCTAATTATGGGTCAGATTCTGGTTCAGCATAGGGT
>Marker510562 Chr3 SNP_site:36092390 Ref_Type:T
ACTCCATCAAGCATATATAAATATATGTTGTAGTTTGAATGACTTCCCAATTTATGTAACATTTTCCGTCATTTTCTCCAXXXXXXXXXXC

Create PDF with GO2PDF for free, if you wish to

CAAAATTTACCCAATCCCCAGATAGCTCTGTATATATAATTGTTCTAATTATGGGTCAGATTCTGGTTCAGCATAGGGT
ACTCCATCAAGCATATATAAATATATGTTGTAGTTTGAATGACTTTCCAATTTATGTAACATTTTCCGTCACTTTCTCCAXXXXXXXXXXXC
CAAAATTTACCCAATCCCCAGATAGCTCTGTATATATAATTGTTCTAATTATGGGTCAGATTCTGGTTCAGCATAGGGT
>Marker510748 Chr3 SNP_site:39496489 Ref_Type:C
ACAAATCAATCAAATTATCAAAAAAAGTTATGATAAAAACAACAAACCATTTCTATATTTGTTTTCATCTAATTGAAAAAXXXXXXXXXXXA
TCAGAAGTTTAAATTTAAATTTTCGTCCCAATCCATGGAAATTATAAAAGTCTTATACATTTCAATTGCTTTTTGCGTT
ACAAATCAATCCAATTATCAAAAAAAGTTATGATAAAAACAACAAACCATTTCTATATTTGTTTTCATCTAATTGAAAAAXXXXXXXXXXXA
TCAGAAGTTTAAATTTAAATTTTCGTCCCAATCCATGGAAATTATAAAAGTCTTATACATTTCAATTGCTTTTTGCGTT
>Marker510802 Chr3 SNP_site:28197064 Ref_Type:A
CACTATATGGAGAGACAACAACAACCTTTCTAGGATTCTTGCATTTCTCAGAGATTCTACAGATTTCTCGAGACCCAGCCXXXXXXXXXXXC
TCCTCTGTTAAACTATCACCTTATGCTTTAGATAAACTCCTTTTAGACTTTTACTACGAAGGTTTCAATATTATAAGTA
CACTATGTGGAGAGACAACAACAACCTTTCTAGGATTCTTGCATTTCTCAGAGATTCTACAGATTTCTCGAGACCCAGCCXXXXXXXXXXXC
TCCTCTGTTAAACTATCACCTTATGGTTTAGATAAACTTCTTTTAGACTTTTACTACGAAGGTTTCAATATTATAAGTA
>Marker510802 Chr3 SNP_site:28197299 Ref_Type:C
CACTATATGGAGAGACAACAACAACCTTTCTAGGATTCTTGCATTTCTCAGAGATTCTACAGATTTCTCGAGACCCAGCCXXXXXXXXXXXC
TCCTCTGTTAAACTATCACCTTATGCTTTAGATAAACTCCTTTTAGACTTTTACTACGAAGGTTTCAATATTATAAGTA
CACTATGTGGAGAGACAACAACAACCTTTCTAGGATTCTTGCATTTCTCAGAGATTCTACAGATTTCTCGAGACCCAGCCXXXXXXXXXXXC
TCCTCTGTTAAACTATCACCTTATGGTTTAGATAAACTTCTTTTAGACTTTTACTACGAAGGTTTCAATATTATAAGTA
>Marker510802 Chr3 SNP_site:28197312 Ref_Type:C
CACTATATGGAGAGACAACAACAACCTTTCTAGGATTCTTGCATTTCTCAGAGATTCTACAGATTTCTCGAGACCCAGCCXXXXXXXXXXXC
TCCTCTGTTAAACTATCACCTTATGCTTTAGATAAACTCCTTTTAGACTTTTACTACGAAGGTTTCAATATTATAAGTA
CACTATGTGGAGAGACAACAACAACCTTTCTAGGATTCTTGCATTTCTCAGAGATTCTACAGATTTCTCGAGACCCAGCCXXXXXXXXXXXC
TCCTCTGTTAAACTATCACCTTATGGTTTAGATAAACTTCTTTTAGACTTTTACTACGAAGGTTTCAATATTATAAGTA
>Marker510939 Chr3 SNP_site:9873839 Ref_Type:A
ACCTAAATAAGATCAACTGTAAATTAAGGATCTCTAAAAATAGTAGAATGATTACTATGAAAGCAATAAAAAAGGTTACCXXXXXXXXXXXT
AGAACTTCAGTGAGTCCCCTTTGAGGTCCAGCCACTTGGTTTCATCTAAACTTCCAAAATTTCAGAGTTCTAACCTGTA
ACCTAAATAAGATCAACTGTAAATTAAGGATCTCTAAAAATAGTAGAATGATTACTATGACAGCAATAAAAAAGGTTACCXXXXXXXXXXXT
AGAACTTCAGTGAGTCCCCTTTGAGGTCCAGTCACTTGGTTTCATCTAAACTTCCAAAATTTCAGAGTTCTAACCTGTA
>Marker510939 Chr3 SNP_site:9874000 Ref_Type:C
ACCTAAATAAGATCAACTGTAAATTAAGGATCTCTAAAAATAGTAGAATGATTACTATGAAAGCAATAAAAAAGGTTACCXXXXXXXXXXXT
AGAACTTCAGTGAGTCCCCTTTGAGGTCCAGCCACTTGGTTTCATCTAAACTTCCAAAATTTCAGAGTTCTAACCTGTA
ACCTAAATAAGATCAACTGTAAATTAAGGATCTCTAAAAATAGTAGAATGATTACTATGACAGCAATAAAAAAGGTTACCXXXXXXXXXXXT
AGAACTTCAGTGAGTCCCCTTTGAGGTCCAGTCACTTGGTTTCATCTAAACTTCCAAAATTTCAGAGTTCTAACCTGTA
>Marker510951 Chr3 SNP_site:34112986 Ref_Type:T
AACACCTCAACTACGAAGGTTAGGACCCCAGAACAAAGGCCCCTTCTCTCCATAGTATAGAAAGATACCCAAATACAAACXXXXXXXXXXXA
TTCAATAATTTCAGCTCCTGTGAGATGCCATCTCAAAATAAGATCCCAAGAAGCATTCTATTTCCAACAATTGGCCGTG
AACACCTCAACTACGAAGGTTAGGACCCCAGAACAAAGGCTCCTTCTCTCCATAGTATAGAAAGATACCCAAATACAAACXXXXXXXXXXXA
TTCAATAATTTCAGCTCCTGTGAGATGCCATCTCAAAATAAGATCCCAAGAAGCATTCTATTTCCAACAATTGGCCGTG
>Marker511002 Chr3 SNP_site:39120488 Ref_Type:G
CACCACCGCCGACTAGTTTGTGTGCCTATGTTGAAGCAAATAACAAATCACATCACTAAACCATCAAGAACTGAATTCGAXXXXXXXXXXXC
AAAGATATAACTATTACCCAAACAATCCTCAAAACTCTTTTTTGCTTTCTGAGTTCCCAAAAAGAGACTTGAATAAAGT
CACCACCGCCGACTAGTTTGTGTGCCTATGTTGAAGCAAATAACAAATCACATCACTAAACCATCAAGAACTGAATTCGAXXXXXXXXXXXC
AAAGATATAACTATTACCGAAACAATCCTCAAAACTCTTTTTTGCTTTCTGAGTTCCCAAAAAGAGACTTGAATAAAGT
>Marker511188 Chr3 SNP_site:39323118 Ref_Type:A

Create PDF with GO2PDF for free, if you wish to

AACTTCACTCCTGTTTTGGAGAAAATAGTTTCAGAAAATGGGGTTAGAGAAAAAGGTTTCATAAACGTTTGAAAGCATTTXXXXXXXXXXA
ATACATATACCTATCACCGATCAACAAGAAAGTCTGCAGTCATATTATTTCTCTCATTTGCATTTTACAGGCATGCTGT
AACTTCTCTCCTGTTTTGGAGAAAATAGTTTCAGAAAATGGGGTTAGAGAAAAAGGTTTCATAAACGTTTGAAAGCATTTXXXXXXXXXXA
ATACATATACCTATCACCGATCAACAAGAAAGTCTGCAGTCATATTATTTCTCTCATTTGCATTTTACTGGCATGCTGT
>Marker511188 Chr3 SNP_site:39323399 Ref_Type:A
AACTTCACTCCTGTTTTGGAGAAAATAGTTTCAGAAAATGGGGTTAGAGAAAAAGGTTTCATAAACGTTTGAAAGCATTTXXXXXXXXXXA
ATACATATACCTATCACCGATCAACAAGAAAGTCTGCAGTCATATTATTTCTCTCATTTGCATTTTACAGGCATGCTGT
AACTTCTCTCCTGTTTTGGAGAAAATAGTTTCAGAAAATGGGGTTAGAGAAAAAGGTTTCATAAACGTTTGAAAGCATTTXXXXXXXXXXA
ATACATATACCTATCACCGATCAACAAGAAAGTCTGCAGTCATATTATTTCTCTCATTTGCATTTTACTGGCATGCTGT
>Marker511940 Chr3 SNP_site:35786151 Ref_Type:G
ACATTGAGCTTGGGCCTCTCCATGATCTTTAGGAAAAGGAAATTGAAAATTTAAAGTTGCAGCCTTTTATGAGAGATCTTXXXXXXXXXXG
TCTTTCAAACATAAGCTGAAGATAACTTTAGCTTCATTATTGTTATTGAGATTTACAACAAGAAATAAGGAACTCTTGT
ACATTGAGCTTGGGCCTCTCCATGATCTTTAGGGAAAGGAAATTGAAAATTTAAAGTTGCAGCCTTTTATGAGAGATCTTXXXXXXXXXXG
TCTTTCAAACATAAGCTGAAGATAACTTTAGCTTCATTATTGTTATTGAGATTTACAACAAGAAATAAGGAACTCTTGT
>Marker512118 Chr3 SNP_site:39295291 Ref_Type:A
AACTTGGACTTCAATCCAAAAGCCTGACAGCTAAAAAAAAATTGGCGTCTTTATACAAATGATAAGAGAAACATGCTCAAXXXXXXXXXXA
AAAGGCATAGGTTATATACCCCCCATGGAGATGCTCCTTGAACACCAGGTCAAATGCACGGCAAAGTTCTAGGATGGTA
AACTTGGACTTCAATCCAAAAGCCTGACAGCTAAAATAAAATTGGCGTCTTTATACAAATGATAAGAGAAACATGCTCAAXXXXXXXXXXA
AAAGGCATAGGTTATATACCCCCCATGGAGATGCTCCTTGAACACCAGGTCAAATGCACGGCAAAGTTCTAGGATGGTA
>Marker512622 Chr3 SNP_site:14049976 Ref_Type:C
CACAACATTTTGTCTGCCTCCAACAGAACAAATGGTTCTCCTTTAACTTTGTTTTGGCAGGAGGGTCATGTCACAGGTCCXXXXXXXXXXT
GCAGACTCGACAAGAAATGATTTTGAGCTCCACTTCAGTGTTGCATGTATCTTTCCCTCTTGTGAGAATCAATGTTGGT
CACAACATTTTGTCTGCCTCCAACAGAACAAATGGTTCTCCTTTAACTTTGTTTTGGCAGGAGGGTCATGTCACAGGTCCXXXXXXXXXXT
GCAGACTCGACAAGAAATGATTTTGAGCTCCACTTCAGTGTTGCATGTATCTTTCCCTCTTGTGCGAATCAATGTTGGT
>Marker513707 Chr3 SNP_site:11574691 Ref_Type:T
AACCTACTGTTTGTTGTCATCGATAGTTTATTTAATATATATAAAAGAAGAGAGATGACCCACTGTCTAAAACTTTTTGTXXXXXXXXXXT
CAAGGCCCTTCCCCTCTCCACAAGCATATTGTTTGCTTTCCTGCGTCTGTTAAATTATGTATAATGTTCAATTGTTGTA
AACCTACTGTTTGTTGTCATCGATAGTTTATTTAATATATATAAAAGAAGAGAGATGACCCACTGTCTAAAACTTTTTGTXXXXXXXXXXT
CAAGGTCCTTCCCCTCTCCACAAGCATATTGTTTGCTTTCCTGCGTCTGTTAAATTATGTATAATGTTCAATTGTTGTA
>Marker51406 Chr4 SNP_site:22913990 Ref_Type:T
ACCAATGGTGATGAAGTATTAGTGAATTGCTACCCTTGAATCTAGTGTCACAACAATGGGATTTTATTTGCCATGAGACAXXXXXXXXXXT
GCTGCAATCAGAGAATTGTTATATATTTGAAAGAGCATTCCAGCTTCCAAGCTTAGCTCATATTTTTGCCGTGTATGTA
ACCAATGGTGATGAAGTATTAGTGAATTGCTACCCTTGAATCTAGTGTCACAACAATGGGATTTTATTTGCCATGAGACAXXXXXXXXXXT
GCTGCAATCAGAGAATTGTTATATATTTGAAAGAGCATTCCAGCTTCCTAGCTTAGCTCATATTTTTGCCGTGTATGTA
>Marker514226 Chr3 SNP_site:27996221 Ref_Type:G
AACATGCCTATTTTGCTATAACATGTGATTTTAGGAGAGAGTTTAACCATAATCAGGGTGCGATTTGATCAGTTCATCTGXXXXXXXXXXT
CAAGCCTCGAGCTAAACGCTTAGAATCACTAAAATGCTTGATCAATACCTCCAGCTATTGCTGCTATGTCACATCTGTG
AACATGCCTATTTTGCTATAACATGTGATTTTAGGAGAGAGTTTAACCATAATCAGGGTGCGATTTGATCAGTTCATCTGXXXXXXXXXXT
CAAGCCTCGAGCTAAACGCTTAGAATGACTAAAATGCTTGATCAATACCTCCAGCTATTGCTGCTATGTCACATCTGTG
>Marker514917 Chr3 SNP_site:443971 Ref_Type:G
ACAGAGGGCTACTACAAAAACAAAAAATCAGCCTAACCAACAGAGGGAGGACCTACAAAAAACATGTTAGCCATATGCCCXXXXXXXXXXT
GCTAATTGTCAAAGTCTCATATAACCGATAGATGATTCAAAACCCACCACCAAACCATACAATAAAGAAGTTTCATGTA
ACAGAGGGCTACTACAAAAACAAAAAATCAGCGTAACCAACAGAGGGAGGACCTACAAAAAACATGTTAGCCATATGCCCXXXXXXXXXXT
GCTAATTGTCAAAGTCTCATATAACCGATAGATGATTCAAAACCCACCACCAAACCATACAATAAAGTAGTTTCATGTA

Create PDF with GO2PDF for free, if you wish to

>Marker514917 Chr3 SNP_site:444178 Ref_Type:T
ACAGAGGGCTACTACAAAAACAAAAAATCAGCCTAACCAACAGAGGGAGGACCTACAAAAAACATGTTAGCCATATGCCCXXXXXXXXXXXT
GCTAATTGTCAAAGTCTCATATAACCGATAGATGATTCAAAACCCACCACCAAACCATACAATAAAGAAGTTTCATGTA
ACAGAGGGCTACTACAAAAACAAAAAATCAGCGTAACCAACAGAGGGAGGACCTACAAAAAACATGTTAGCCATATGCCCXXXXXXXXXXXT
GCTAATTGTCAAAGTCTCATATAACCGATAGATGATTCAAAACCCACCACCAAACCATACAATAAAGTAGTTTCATGTA
>Marker51550 Chr4 SNP_site:6067671 Ref_Type:A
TACTAGTTCATAACATTGGTTGATGTCTTCTCATGCAGCAAGACCAATTTGGTCGGCTACTTCCAACCTTCATTCAGGTCXXXXXXXXXXXT
AGCATCAATACGAAGGTTTTGTTCTAATTACTAAACAAAATTACCTGTTTGAAAAACCTTTTTAATAAATCTACAAAGT
TACTAGTTCATAACATTGGTTGATGTCTTCTCATGCAGCAAGACCAATTTGGTCGGCTACTTCCAACCTTCATTCAGGTCXXXXXXXXXXXT
AGCATCAATACGAAGGTTTTGTTCTAATTACTAGACAAAATTACCTGTTTGAAAAACCTTTTTAATAAATCTACAAAGT
>Marker515722 Chr3 SNP_site:30993926 Ref_Type:G
GACAACAAAACGAAGAAATCAATTGGTAGATGTAGAGTTCTTTATAATTTATAAGCTTAATTTTCAATACTCAAAAAACCXXXXXXXXXXXC
AATAAACGGTCAAGAAAAATGATCCAAGAAATAAAAGTCACGAAAGGCATTCAATTGATCAGAGCATTGACAATGAAGT
GACAACAAAACGAAGAAATCAATTGGTAGATGTAGAGTTGTTTATAATTTATAAGCTTAATTTTCAAAACTCAAAAAACCXXXXXXXXXXXC
AATAAACGGTCAAGAAAAATGATCCAAGAAATAAAAGTCACGAAAGGCATTCAATTGATCAGAGCATTGACAATGAAGT
>Marker515722 Chr3 SNP_site:30993954 Ref_Type:A
GACAACAAAACGAAGAAATCAATTGGTAGATGTAGAGTTCTTTATAATTTATAAGCTTAATTTTCAATACTCAAAAAACCXXXXXXXXXXXC
AATAAACGGTCAAGAAAAATGATCCAAGAAATAAAAGTCACGAAAGGCATTCAATTGATCAGAGCATTGACAATGAAGT
GACAACAAAACGAAGAAATCAATTGGTAGATGTAGAGTTGTTTATAATTTATAAGCTTAATTTTCAAAACTCAAAAAACCXXXXXXXXXXXC
AATAAACGGTCAAGAAAAATGATCCAAGAAATAAAAGTCACGAAAGGCATTCAATTGATCAGAGCATTGACAATGAAGT
>Marker515764 Chr3 SNP_site:18485370 Ref_Type:C
TACACACACAATTTATCTAATAAATTGACAAAAACTACCATGTGCAATTGAGTAAGATTAAGCATGCTCTGATACCATATXXXXXXXXXXXT
AATAGAAAAACTCACATTTGATGCTTTTCTATTGTCATAATCTCGTCATGTTCTTGAAGTCGAAACTACCACTAGCGTT
TACACACACAATTTATCTAATAAATTGACAAAAACTACCATGTGCAATTGAGTAAGATTAAGCATGCTCTGATACCATATXXXXXXXXXXXT
AATAGAAAAACTCACATTTGATGCTTTTCTATTGTCATAATCTCGTCATGTTCTTGAAGTTGAAACTACCACTAGCGTT
>Marker515791 Chr3 SNP_site:8135347 Ref_Type:A
AACAAAAGTTTTAAACAAGAAAAGTGGTAGGTGCTCTACTATTTAACAGTTTTCTTCCTAGTCTTGAAATATTGAAAGATXXXXXXXXXXXT
TGCATATTTTGTTCTGTTTGCAATACCAATTATGACGGCAGTGTTTAGTGGAACAATGTCGGTGGGAGCTTATGTTGTT
AACAAAAGTTTTAAACAAGAAAAGTGGTAGGTGCTCTACTCTTTAACAGTTTTCTTCCTAGTCTTGAAATATTGAAAGATXXXXXXXXXXXT
TGCATATTTTGTTCTGTTTGCAATACCAATTATGACGGCAGTGTTTAGTGGAACAATGTCGGTGGGAGCTTATGTTGTT
>Marker515978 Chr3 SNP_site:30657449 Ref_Type:A
AACAGTAATTGATCATTCATGAACTAGGATGTGATCATTTTTTAATTAACAAAAGAATAAGAAATCAGCTTCTTTAGGGAXXXXXXXXXXXT
TCATGCAAAAAAGGCCTTCTGCAGATAGTAGCTAGAAATTAGAAAAAAGGTCATTGGAAAAGGAATTATAGAATTAAGT
AACAGTAATTGATCATTCATGAACTAGGATGTGATCGTTTTTTAATTAACAAAAGAATAAGAAATCAGCTTCTTTAGGGAXXXXXXXXXXXT
TCATGCAAAAAAGGCCTTCTGCAGATAGTAGCTAGAAATTAGAAAAAAGGTCATTGGAAAAGGAATTATAGAATTAAGT
>Marker516527 Chr3 SNP_site:7244271 Ref_Type:C
TACTCATGGAAAGGGTGTTGGAGATTTAGACGGCGGTTCTGATGTTCCCGAGCGGCCAAGCCCTGTTTCAGTTCTTGAGCXXXXXXXXXXXA
CATTCCCCTGGCTAATATCAGTTTATTCTATCAGCAGGCTTACCAATACAACCAGTTCATATTGAATTTGATGATCGTG
TACTCCTGGAAAGGGTGTTGGAGATTTAGACGGCGGTTCTGATGTTCCCGAGCGGCCAAGCCCTGTTTCAGTTCTTGAGCXXXXXXXXXXXA
CATTCCCCTGGCTAATATCAGTTTATTCTATCAGCAGGCTTACCAATACAACCAGTTCATATTGAATTTGATGATCGTG
>Marker516546 Chr3 SNP_site:6693731 Ref_Type:C
GACGTCGAAGAGAACCTCTTATCTTTGAACATCTGGTTAAATCGTGAATCAATCACAACCTTGGCTTTGTGCTTGGGGGCXXXXXXXXXXXA
GTTGCTCAAGTTTTTGGAGCCCATGACGACGGCCTACAATCAGAGTGCAACTGGCGAAACGCAGTGATAATCGGTCGTA
GACGTCGAAGAGAACCTCTTATCTTTGAACATCTGGTTAAATCGTGAATCAATCACAACCTTGGCTTTGTGCTTGGGGGCXXXXXXXXXXXA

Create PDF with GO2PDF for free, if you wish to

GTTGCTCAAGTTTTTGGAGCCCATGATGACGGCCTACAATCAGACTGCAACTGGCGAAACGCAGTGATAATCGGTCGTA
>Marker516546 Chr3 SNP_site:6693749 Ref_Type:G
GACGTCGAAGAGAACCTCTTATCTTTGAACATCTGGTTAAATCGTGAATCAATCACAACCTTGGCTTTGTGCTTGGGGGCXXXXXXXXXXA
GTTGCTCAAGTTTTTGGAGCCCATGACGACGGCCTACAATCAGAGTGCAACTGGCGAAACGCAGTGATAATCGGTCGTA
GACGTCGAAGAGAACCTCTTATCTTTGAACATCTGGTTAAATCGTGAATCAATCACAACCTTGGCTTTGTGCTTGGGGGCXXXXXXXXXXA
GTTGCTCAAGTTTTTGGAGCCCATGATGACGGCCTACAATCAGACTGCAACTGGCGAAACGCAGTGATAATCGGTCGTA
>Marker516646 Chr3 SNP_site:33857054 Ref_Type:G
GACATCTCAAAGCAAATGCCTAACACAGACGTGTGCTCAAACATACATAACAATTATTATATGATTGGAAAAACCATTAAXXXXXXXXXXG
ACTTTTTCTTGTGGCTCAACACTTAGTTAATCTTCCGGAATCAGGAATGACCTTGACGAGGCCAGGTCCAGGAATAAGT
GACGTCTCAAAGCAAATGCCTAACACAGACGTGTGCTCAAACATACATAACAATTATTATATGATTGGAAAAACCATTAAXXXXXXXXXXG
ACTTTTTCTTGTGGCTCAACACTTAGTTAATCTTCCGGAATCAGGAATGACCTTGACGAGGCCAGGTCCAGGAATAAGT
>Marker517095 Chr3 SNP_site:31350381 Ref_Type:C
CACTACATTCAGGTTATATAAACAACTACTTTTTTTTCTTCTCTTTTGTGTATATGTATTGAAACAAAAATTCAGGTTTTXXXXXXXXXXT
GGTTAAGAGCAACCGTAGAAATTGGCTTCCGTAGAAGTTTCTTGTATTCACTCACGAGCTTTTTTCAAGATACATGGTT
CACTACATTCAGGTTATATAAACAACTACTTTTTTTTCTTCTCTTTTGTGTATATGTATTGAAACAAAAATTCAGGTTTTXXXXXXXXXXT
GGTTAAGAGCAACTGTAGAAATTGCCTTCCGTAGAAGTTTCTTGTATTCACTCACGAGCTTTTTTCAAGATACATGGTT
>Marker517095 Chr3 SNP_site:31350392 Ref_Type:G
CACTACATTCAGGTTATATAAACAACTACTTTTTTTTCTTCTCTTTTGTGTATATGTATTGAAACAAAAATTCAGGTTTTXXXXXXXXXXT
GGTTAAGAGCAACCGTAGAAATTGGCTTCCGTAGAAGTTTCTTGTATTCACTCACGAGCTTTTTTCAAGATACATGGTT
CACTACATTCAGGTTATATAAACAACTACTTTTTTTTCTTCTCTTTTGTGTATATGTATTGAAACAAAAATTCAGGTTTTXXXXXXXXXXT
GGTTAAGAGCAACTGTAGAAATTGCCTTCCGTAGAAGTTTCTTGTATTCACTCACGAGCTTTTTTCAAGATACATGGTT
>Marker517322 Chr3 SNP_site:11865285 Ref_Type:C
ACAAACTCTCTCATAACCCTGCAAACAAAACATCTTCAAATTCAGACTTGACTTGTTTAGCCATTTTTGAGCCTGTGCTTXXXXXXXXXXT
TTATGAATGAATATGAAGAGAGAATTGGCAGCATAACATTAATTTGATTGATATTATGAAGGAATAATAATGGTTGGTA
ACAAACTCTCTCATAACCCTGCAAACAAAACATTTTCAAATTCAGACTTGACTTGTTTAGCCATTTTTGAGCCTGTGCTTXXXXXXXXXXT
TTATGAATGAATATGAAGAGAGAATTGGCAGCATAACATTAATTTGATTGATATTATGAAGGAATAATAATGGTTGGTA
>Marker518023 Chr3 SNP_site:37866988 Ref_Type:C
TACTGCTTTGTCCAATGACATTTCGGAAATCTTCAACTCTCTGTGGGCCACCTATTATTGGTTGCAAGAAGGTTATTTGAXXXXXXXXXXA
AAATTTGGCACCTTTTTAAGAACTTTAGTTTATTGGTCTTAGATGCCAATTGGCGCTCTTTTTGTAATCACCTTTAGGT
TACTGCTTTGTCCAATGACATTTCGGACATCTTCAACTCTCTGTGGGCCACCTATTATTGGTTGCAAGAAGGTTATTTGAXXXXXXXXXXA
AAATTTGGCACCTTTTTAAGAACTTTAGTTTATCGGTCTTAGTTGCCAATTGGCGCTCTTTTTGTAATCACCTTTAGGT
>Marker518023 Chr3 SNP_site:37867202 Ref_Type:C
TACTGCTTTGTCCAATGACATTTCGGAAATCTTCAACTCTCTGTGGGCCACCTATTATTGGTTGCAAGAAGGTTATTTGAXXXXXXXXXXA
AAATTTGGCACCTTTTTAAGAACTTTAGTTTATTGGTCTTAGATGCCAATTGGCGCTCTTTTTGTAATCACCTTTAGGT
TACTGCTTTGTCCAATGACATTTCGGACATCTTCAACTCTCTGTGGGCCACCTATTATTGGTTGCAAGAAGGTTATTTGAXXXXXXXXXXA
AAATTTGGCACCTTTTTAAGAACTTTAGTTTATCGGTCTTAGTTGCCAATTGGCGCTCTTTTTGTAATCACCTTTAGGT
>Marker518023 Chr3 SNP_site:37867211 Ref_Type:T
TACTGCTTTGTCCAATGACATTTCGGAAATCTTCAACTCTCTGTGGGCCACCTATTATTGGTTGCAAGAAGGTTATTTGAXXXXXXXXXXA
AAATTTGGCACCTTTTTAAGAACTTTAGTTTATTGGTCTTAGATGCCAATTGGCGCTCTTTTTGTAATCACCTTTAGGT
TACTGCTTTGTCCAATGACATTTCGGACATCTTCAACTCTCTGTGGGCCACCTATTATTGGTTGCAAGAAGGTTATTTGAXXXXXXXXXXA
AAATTTGGCACCTTTTTAAGAACTTTAGTTTATCGGTCTTAGTTGCCAATTGGCGCTCTTTTTGTAATCACCTTTAGGT
>Marker518033 Chr3 SNP_site:39586650 Ref_Type:T
AACTTAAGGATGAAGAAATTGGATTGTTAAGACGAAAAGATACAACAACCATCACTCAACCAAGCACATTTCTTTAGGATXXXXXXXXXXG
AACTCGACACGAGGCCGAGTAGGAGAAGTCTTCTATCATGTTAAGTTTTTGTTGATGTGATAGTATTAAGACATATGTT

Create PDF with GO2PDF for free, if you wish to

AACTTAAGGATGAAGAAATTGGATTGTTAAGACGAAAAGTTACAACAACCATCACTCAACCAAGCACATTTCTTTAGGATXXXXXXXXXXG
AACTCGACACGAGGCCGAGTAGGAGAAGTCTTCTATCATGTTAAGTTTTTGTTGATGTGATAGTATTAAGACATATGTT
>Marker518134 Chr3 SNP_site:1045766 Ref_Type:T
GACAAGATTGCACAGTTCATGCAGATCTACAGAAGTAGAGAAGTAGGACAATCGTATTTCACTTCCGTTTGGACAACTTTXXXXXXXXXXT
CCTTTTAGAGAACCTATTGTGGTGTGTGTTTTCTTTGACCAGTTTTGTTTTGTTGCAGATTCTATGCAATGGCCCTGGT
GACAAGATTGCACAGTTCATGCAGATCTACAGAAGTAGAGAAGTAGGACAATCGTATTTCACTTCTGTTTGGACAACTTTXXXXXXXXXXT
CCTTTTAGAGAACCTATTGTGGTGTGTGTTTTCTTTGACCAGTTTTGTTTTGTTGCAGATTCTATGCAATGGCCCTGGT
>Marker518171 Chr3 SNP_site:2522708 Ref_Type:G
CACATTTTCTTTTTTGTTCCACATTGGATGTTTTTAGTTTCGAGTAACTTTTGAGATCTACGTTTTGAAGGCAAACGAAGXXXXXXXXXXA
CACAACAATTCAATATTTACATAAAAAGTGTTTGAATCAAACCTGGAGCGCCATCACTCCTATATATGCAGAAAAAAGT
CACATTTTCTTTTTTGTTCCACATTGGGTGTTTTTAGTTTCGAGTAACTTTTGAGATCTACGTTTTGAAGGCAAACGAAGXXXXXXXXXXA
CACAACAATTCAATATTTACATAAAAAGTGTCTGAATCAAACCTGGAGCGCCATCACTCCTATATATGTAGAAAAAAGT
>Marker518171 Chr3 SNP_site:2522876 Ref_Type:C
CACATTTTCTTTTTTGTTCCACATTGGATGTTTTTAGTTTCGAGTAACTTTTGAGATCTACGTTTTGAAGGCAAACGAAGXXXXXXXXXXA
CACAACAATTCAATATTTACATAAAAAGTGTTTGAATCAAACCTGGAGCGCCATCACTCCTATATATGCAGAAAAAAGT
CACATTTTCTTTTTTGTTCCACATTGGGTGTTTTTAGTTTCGAGTAACTTTTGAGATCTACGTTTTGAAGGCAAACGAAGXXXXXXXXXXA
CACAACAATTCAATATTTACATAAAAAGTGTCTGAATCAAACCTGGAGCGCCATCACTCCTATATATGTAGAAAAAAGT
>Marker518171 Chr3 SNP_site:2522913 Ref_Type:T
CACATTTTCTTTTTTGTTCCACATTGGATGTTTTTAGTTTCGAGTAACTTTTGAGATCTACGTTTTGAAGGCAAACGAAGXXXXXXXXXXA
CACAACAATTCAATATTTACATAAAAAGTGTTTGAATCAAACCTGGAGCGCCATCACTCCTATATATGCAGAAAAAAGT
CACATTTTCTTTTTTGTTCCACATTGGGTGTTTTTAGTTTCGAGTAACTTTTGAGATCTACGTTTTGAAGGCAAACGAAGXXXXXXXXXXA
CACAACAATTCAATATTTACATAAAAAGTGTCTGAATCAAACCTGGAGCGCCATCACTCCTATATATGTAGAAAAAAGT
>Marker518212 Chr3 SNP_site:861558 Ref_Type:G
ACTAAATGCCCACCTTTCGATGATAATTAATGGAATGAAATAATGAAATTAAACTGCTGATCATTTCACTTTTTAAATATXXXXXXXXXXC
TGTATAGCTTTTGATAGAATAAGGATATTCTTGAAACAAGGGACAGTTCATAGTTTATCTTTTTTAGCCAATTTGTGTA
ACTAAATGCCCACCTTTCGATGATAATTAATGGAATGAAATAATGAGATTAAACTGCTGATCATTTCACTTTTTAAATATXXXXXXXXXXC
TGTATAGCTTTTGATAGAATAAGGACATTCTTGAAACAAGGGACAGTTCATAGTTTATCTTTTTTAGCCAATTTGTGTA
>Marker518212 Chr3 SNP_site:861713 Ref_Type:C
ACTAAATGCCCACCTTTCGATGATAATTAATGGAATGAAATAATGAAATTAAACTGCTGATCATTTCACTTTTTAAATATXXXXXXXXXXC
TGTATAGCTTTTGATAGAATAAGGATATTCTTGAAACAAGGGACAGTTCATAGTTTATCTTTTTTAGCCAATTTGTGTA
ACTAAATGCCCACCTTTCGATGATAATTAATGGAATGAAATAATGAGATTAAACTGCTGATCATTTCACTTTTTAAATATXXXXXXXXXXC
TGTATAGCTTTTGATAGAATAAGGACATTCTTGAAACAAGGGACAGTTCATAGTTTATCTTTTTTAGCCAATTTGTGTA
>Marker518645 Chr3 SNP_site:35528777 Ref_Type:C
AACTTACTCTATCTAAAGTTCTTTACAAGGCTAGTGTAGGAGATTGGATGTCAGCCACTGTAGTTCTCTTGTTGGTGGTCXXXXXXXXXXA
ATTCTGTAGTTAGGCTGAGGTAATTCTGTGTGATGTTTTGAACAACTAATCAATTTTCGACGTTTGATGAATTATTGGT
AACTTACTCTATCTAAAGTTCTTTACAAGGCTAGTGTAGGAGATTGGATGTCAGCCACTGTAGTTCTCTTGTTGGTGGTCXXXXXXXXXXA
ATTCTGTAGTTAGGCTGAGGTAATTCTGTGTGATGTTTTGAACAAGTAATCAATTTTCGACGTTTGATGAATTATTGGT
>Marker5195 Chr4 SNP_site:10887086 Ref_Type:C
TACATCATCAGCCTCTAAAGTTGCATTTAATTTTCATTTTTTCGAGAGAGTGAAAAGTGAGACAATGAGAGAGCGAGAGGXXXXXXXXXXA
TGATAGCGAGAAAAGCTAATTCACATAAGTGAGTTAATTTTGCACAAATATGATGTAAGAAAAGGGTTAAAAAGACGTC
TACATCATCAGCCTCTAAAGTTGCATTTAATTTTCATTTTTTGGAGAGAGTGAAAAGTGAGACAATGAGAGAGCGAGAGGXXXXXXXXXXA
TGATAGCGAGAAAAGCTAATTCACGTAAGTGAGTTAATTTTGCACAAATATGATGTAAGAAAAGGGTTAAAAAGACGTC
>Marker5195 Chr4 SNP_site:10887313 Ref_Type:A
TACATCATCAGCCTCTAAAGTTGCATTTAATTTTCATTTTTTCGAGAGAGTGAAAAGTGAGACAATGAGAGAGCGAGAGGXXXXXXXXXXA

Create PDF with GO2PDF for free, if you wish to

TGATAGCGAGAAAAGCTAATTCACATAAGTGAGTTAATTTTGCACAAATATGATGTAAGAAAAGGGTTAAAAAGACGTC
TACATCATCAGCCTCTAAAGTTGCATTTAATTTTCATTTTTTGGAGAGAGTGAAAAGTGAGACAATGAGAGAGCGAGAGGXXXXXXXXXXA
TGATAGCGAGAAAAGCTAATTCACGTAAGTGAGTTAATTTTGCACAAATATGATGTAAGAAAAGGGTTAAAAAGACGTC
>Marker519569 Chr3 SNP_site:39753082 Ref_Type:T
AACCTAGTGTTATTTTTTTCTACTGTTTAACATATGCACACACATGTATATATGCACATATACTGATATACACATGTATGXXXXXXXXXXG
AGAAGGTTACATATAATATTTTTTTGTAAAAGGAAAGTTACATATAATCTGCTGTAACTTGCTAATGGAAAGAGCCGTT
AACCTAGTGTTATTTTTTTCTACTGTTTAACATATGCACACACATGTATATATGCACATATACTGATATACACATGTATTXXXXXXXXXXG
AGAAGGTTACATATAATATTTTTTTGTAAAAGGAAAGTTACATATAATCTGCTGTAACTTGCTAATGGAAAGAGCCGTT
>Marker519662 Chr3 SNP_site:767977 Ref_Type:G
GACCCCCAACTAAAAACTTCAATTTTCTGCCTTATTAAGGCCTTACACTATGGACCCAAGAGTTCATAACTCTCAAGATTXXXXXXXXXXG
AAGAAATGGAATTCAATTTGAGATGCTTTTTCAATTTTAGCATGATATGCAATTAGGACCCATGCAAACAAGATCTGTT
GACCCCCAACTAAAAACTTCAATTTTCTGCCTTATTAAGGCCTTACACTATGGACCCAAGAGTTCATAACTCTCAAGATTXXXXXXXXXXG
AAGAAATGGAATTCAATTTGAGATGCTTTTTCAATTTTAGCATGATATGCAATTAGGGCCCATGCAAACAAGATCTGTT
>Marker519948 Chr3 SNP_site:26769564 Ref_Type:G
CACCAATCTTTGTGAATGGAATGTCGATTATTCCGACCTTTGCTGTCGCTGGTCCATTGAAGGGGAAGAAAATCCATGTTXXXXXXXXXXA
AACCATCCGGGTTGATCGGAACTGGATTTGACTCGTTGGATTTATCATACAACAGAGCCCCGTTGGAGAAAAACCAGTC
CACCAATCTTTGTGAATGGAATGTCGATTATTCCGACCTTTGCTGTCGCTGGTCCATTGAAGGGGAAGAAAATCCATGTTXXXXXXXXXXA
AACCGTCCGGGTTGATCGGAACTGGATTTGACTCGTTGGATTTATCATACAACAGAGCCCCGTTGGAGAAAAACCAGTC
>Marker520039 Chr3 SNP_site:36446146 Ref_Type:T
ACAGGCCTTTTGCTGTGCATAGATCGTATCGAGGGAAATTTAAGAAATATGGTGATAAGTGATACGGTTGTGTTAAAGCAXXXXXXXXXXA
TGTTCAAAATCCATATTATTTGATTATTTTGCCAGAGAGAATCCTAAGGTAGTTTTGAATGTGATTGTATCAAAAGGTT
ACAGGCCTTTTGCTGTGCATAGATTGTATCGAGGGAAATTTAAGAAATATGGTGATAAGTGATACGGTTGTGTTAAAGCAXXXXXXXXXXA
TGTTCAAAATCCATATTATTTGGTTATTTTGCCAGAGAGAATCCTAAGGTAGTTTTGAATGTGATTGTATCAAAAGGTT
>Marker520039 Chr3 SNP_site:36446353 Ref_Type:G
ACAGGCCTTTTGCTGTGCATAGATCGTATCGAGGGAAATTTAAGAAATATGGTGATAAGTGATACGGTTGTGTTAAAGCAXXXXXXXXXXA
TGTTCAAAATCCATATTATTTGATTATTTTGCCAGAGAGAATCCTAAGGTAGTTTTGAATGTGATTGTATCAAAAGGTT
ACAGGCCTTTTGCTGTGCATAGATTGTATCGAGGGAAATTTAAGAAATATGGTGATAAGTGATACGGTTGTGTTAAAGCAXXXXXXXXXXA
TGTTCAAAATCCATATTATTTGGTTATTTTGCCAGAGAGAATCCTAAGGTAGTTTTGAATGTGATTGTATCAAAAGGTT
>Marker520519 Chr3 SNP_site:38931575 Ref_Type:C
ACAGTTTAAGTATGCTTAGTTTAGAACAATTCTTTATCGGATGTTCTTCTGAAAGATTTTATAGAAAGCATACTATGATTXXXXXXXXXXG
CATTACACGTCTTTTTTCTTTTTAAACTATAGAAAAAAGTCTATTAGGTTCCTTGACACAAATTTTCTCCGGAATAGGT
ACAGTTTAAGTATGCTTAGTTTAGAACAATTCTTTATTGGATGTTCTTCTGAAAGTTTTTATAGAAAGCATACTATGATTXXXXXXXXXXG
CATTACACGTCTTTTTTCTTTTTAAACTATAGAAAAAAGTCTATTAGGTTCCTTGACACAAATTTTCTCCGGAATAGGT
>Marker520519 Chr3 SNP_site:38931593 Ref_Type:A
ACAGTTTAAGTATGCTTAGTTTAGAACAATTCTTTATCGGATGTTCTTCTGAAAGATTTTATAGAAAGCATACTATGATTXXXXXXXXXXG
CATTACACGTCTTTTTTCTTTTTAAACTATAGAAAAAAGTCTATTAGGTTCCTTGACACAAATTTTCTCCGGAATAGGT
ACAGTTTAAGTATGCTTAGTTTAGAACAATTCTTTATTGGATGTTCTTCTGAAAGTTTTTATAGAAAGCATACTATGATTXXXXXXXXXXG
CATTACACGTCTTTTTTCTTTTTAAACTATAGAAAAAAGTCTATTAGGTTCCTTGACACAAATTTTCTCCGGAATAGGT
>Marker521131 Chr3 SNP_site:28346994 Ref_Type:A
TACATGGCAAGAAGAATTAGAATGCCTTGTTCGTGGGGGGTTGCCAAAAGATCTGAGGGGAGAGGTAAAGTTGAAAATTTXXXXXXXXXXA
TTGAGAAATATTACCAAGATTTGTTGGATCAAGAAACTAATTGTAGTGCAGATAATGAGAACAATATTCCATCTGGTGT
TACATGGCAAGAAGAATTAGAATGCCTTGTTCGTGGGGGGTTGCCAAAAGATCTGAGGGGAGAGGTAAAGTTGAAATTTTXXXXXXXXXXA
TTGAGAAATATTACCAAGATTTGTTGGATCAAGAAACTAATTGTAGTGCAGATAATGAGAACAATATTCCATCTGGTGT
>Marker522251 Chr3 SNP_site:2328681 Ref_Type:G

Create PDF with GO2PDF for free, if you wish to

CACAGCAATAGAAGAGATTCAATGAACTTTGATTATTTGAATTTGAGCTCTAAAGTTAGTATTGGCAACATTTGTGATTCXXXXXXXXXXG
AATCAAAATTTCAATCAAGCATGTGTGATCATACTTTCTTATTTTAAGATGTCACAACCCTCCATATTGCATTAATGTC
CACAGCAATAGAAGAGATTCAATGAACTTTGGTTATTTGAATTTGAGCTCTAAAGTTAGTATTGGCAACATTTGTGATTCXXXXXXXXXXG
AATCAAAATTTCAATCAAGCATGTGTGATCATACTTTCTTATTTTAAGATGTCACAACCCTCCATATTGCATTAATGTC
>Marker523145 Chr3 SNP_site:32321183 Ref_Type:G
AACATATAAGATCAATCAATAACAATATTTATAACAAAACCATAATTATAATCACAGAAAATTGAGCATAAATGGAGTCTXXXXXXXXXXA
AAATGAAAATGCCCTAAAAGAAGAAGAGTAAGCATTTAGTAATTGTTTAGAATAATGGAGAAATAATGGAATTAACGTC
AACATATAAGATCAATCAATAACAATATTTATAACAAAACGATAATTATAATCACGGAAAATTGAGCATAAATGGAGTCTXXXXXXXXXXA
AAATGAAAATGCTCTAAAAGAAGAAGAGTAAGCATTTAGTAATTGTTTAGAATAATGGAGAAATAATGGAATTAACGTC
>Marker523145 Chr3 SNP_site:32321198 Ref_Type:G
AACATATAAGATCAATCAATAACAATATTTATAACAAAACCATAATTATAATCACAGAAAATTGAGCATAAATGGAGTCTXXXXXXXXXXA
AAATGAAAATGCCCTAAAAGAAGAAGAGTAAGCATTTAGTAATTGTTTAGAATAATGGAGAAATAATGGAATTAACGTC
AACATATAAGATCAATCAATAACAATATTTATAACAAAACGATAATTATAATCACGGAAAATTGAGCATAAATGGAGTCTXXXXXXXXXXA
AAATGAAAATGCTCTAAAAGAAGAAGAGTAAGCATTTAGTAATTGTTTAGAATAATGGAGAAATAATGGAATTAACGTC
>Marker523145 Chr3 SNP_site:32321336 Ref_Type:T
AACATATAAGATCAATCAATAACAATATTTATAACAAAACCATAATTATAATCACAGAAAATTGAGCATAAATGGAGTCTXXXXXXXXXXA
AAATGAAAATGCCCTAAAAGAAGAAGAGTAAGCATTTAGTAATTGTTTAGAATAATGGAGAAATAATGGAATTAACGTC
AACATATAAGATCAATCAATAACAATATTTATAACAAAACGATAATTATAATCACGGAAAATTGAGCATAAATGGAGTCTXXXXXXXXXXA
AAATGAAAATGCTCTAAAAGAAGAAGAGTAAGCATTTAGTAATTGTTTAGAATAATGGAGAAATAATGGAATTAACGTC
>Marker52338 Chr4 SNP_site:16493126 Ref_Type:C
ACCACACACTGAAAACACACTTTTTATTAAGTCTAACATCATGTTAAAGGACTTGTTACTCCAACCATTTAAAACCTTTAXXXXXXXXXXA
ATATTGTAGGGTATCTCATTCTCCAAACCTTCCTCTATTTCCCGTTCATGTTCAATCAGAGCTTGTAAATCATTAAGTA
ACCACACATTGAAAACACACTTTTTATTAAGTCTAACATCATGTTAAAGGACTTGTTACTCCAACCATTTAAAACCTTTAXXXXXXXXXXA
ATATTGTAGGGTATCTCATTCTCCAAACCTTCCTCTATTTCCCGTTCGTGTTCAATCAGAGCTTGTAAATCGTTAAGTA
>Marker52338 Chr4 SNP_site:16493369 Ref_Type:A
ACCACACACTGAAAACACACTTTTTATTAAGTCTAACATCATGTTAAAGGACTTGTTACTCCAACCATTTAAAACCTTTAXXXXXXXXXXA
ATATTGTAGGGTATCTCATTCTCCAAACCTTCCTCTATTTCCCGTTCATGTTCAATCAGAGCTTGTAAATCATTAAGTA
ACCACACATTGAAAACACACTTTTTATTAAGTCTAACATCATGTTAAAGGACTTGTTACTCCAACCATTTAAAACCTTTAXXXXXXXXXXA
ATATTGTAGGGTATCTCATTCTCCAAACCTTCCTCTATTTCCCGTTCGTGTTCAATCAGAGCTTGTAAATCGTTAAGTA
>Marker52338 Chr4 SNP_site:16493393 Ref_Type:A
ACCACACACTGAAAACACACTTTTTATTAAGTCTAACATCATGTTAAAGGACTTGTTACTCCAACCATTTAAAACCTTTAXXXXXXXXXXA
ATATTGTAGGGTATCTCATTCTCCAAACCTTCCTCTATTTCCCGTTCATGTTCAATCAGAGCTTGTAAATCATTAAGTA
ACCACACATTGAAAACACACTTTTTATTAAGTCTAACATCATGTTAAAGGACTTGTTACTCCAACCATTTAAAACCTTTAXXXXXXXXXXA
ATATTGTAGGGTATCTCATTCTCCAAACCTTCCTCTATTTCCCGTTCGTGTTCAATCAGAGCTTGTAAATCGTTAAGTA
>Marker523560 Chr3 SNP_site:33805782 Ref_Type:G
ACTGCATATGGTCAGCTAGGCAACCCAAGTTCAGATGGAGTTATTCCTTGCTTAGTGCAAGATAGATTAGTCGGTGAATTXXXXXXXXXXC
CCAACACTCATAGAAGCTTTGAAGGATAGGCATGTTAAAAGTATATCTTGTGGGTCAAATTTTACTGCAAGTATATGTA
ACTGCATATGGTCAGCTAGGCAACCCAAGTTCAGATGGAGTTATTCCTTGCTTAGTGCAGGATAGATTAGTCGGTGAATTXXXXXXXXXXC
CCAACACTCATAGAAGCTTTGAAGGATAGGCATGTTAAAAGTATATCTTGTGGGTCAAATTTTACTGCAAGTATATGTA
>Marker523763 Chr3 SNP_site:34101281 Ref_Type:G
GACCATTGGGACCCAAGACATTATGCAATGCATGTGGAGTAAGATACAAAAAGTCAGGAAGATTATTACCAGAGTATAGAXXXXXXXXXXC
TACCTTTCCCTCCTCTTCGTAAAACCGACTACCGACGCACCAAACCCACAGTTGCAAGACAATTTGGTTCTCCAATGTA
GACCATTGGGACCCAAGACATTATGCAATGCATGTGGAGTAAGATACAAAAAGTCAGGAAGATTATTACCAGAGTATAGAXXXXXXXXXXC
TACCTTTCCCTCCTCTTCGTAAAACCGACTACCGACGCACCAAACCCGCAGTTGCAAGACAATTTGGTTCTCCAATGTA

Create PDF with GO2PDF for free, if you wish to

>Marker524093 Chr3 SNP_site:30417483 Ref_Type:G
ACTCCTATAATATCAGAAAATGTTAAAATACATTTTCTCATATAATGAACCTGTCTTAGAGTTTGTGAAGTCAATTCTATXXXXXXXXXXG
CACTCCATATCATACATTACTTGTTGAAGGTTTTGTAGATGGGCCTGTTGATATTTGTAAGTGTTACTGATAAATAGTT
ACTCCTATAATATCAGAAAATGTTAAAATACATTTTCTCGTATAATGAACCTGTCTTAGAGTTTGTGAAGTCAATTCTATXXXXXXXXXXG
CACTCCATATCATACATTACTTGTTGAAGGTTTTGTAGATGGGCCTGTTGATATTTGTAAGTGTTACTGATAAATAGTT
>Marker524214 Chr3 SNP_site:12057266 Ref_Type:G
ACCTATACTCTATTATCCTACTACAATATTTGATGAAACAACTTGAATTATAATTGCAATTACAAAGGGGTTGTTCGAAAXXXXXXXXXXXA
GCCTAAAGCAGTGATTGCATGGAACTTGGGACGAATGCTCTTCTTAATCCTTAAAATAAACACCTCATAAAATCAAGTT
ACCTATACTCTATTATCCTACTACAATATTTGATGAAACAACTTGAATTATAATTGCAATTACAAAGGGGTTGTTCGAAAXXXXXXXXXXXA
GCCTAAAGCAGTGATTGCATGGAACTTGGGACGAATGCTCTTCTTAATCCTTAAAATAAACACCTGATAAAATCAAGTT
>Marker524307 Chr3 SNP_site:33229566 Ref_Type:T
GACCAACTTTTTTTGTAATGTAATCATATTATTTGGGATTTTGAAATATTAAAACGTGGTGGGCATGCTACCTTGAATTTXXXXXXXXXXT
TTCTTAAAATAAACAAAAAATTTTTTTGATGGGTTTGCATATATAATATATAGAAACTCATTTGATCAGTTAGTGAAGT
GACCAACTTTTTTTGTAATGTAATCATATTATTTGGGATTTTGAAATATTAAAACGTGGTGGGCATGCTACCTTGAATTTXXXXXXXXXXT
TTCTTAAAATAAACAAAAATTTTTTTTGATGGGTTTGCATATATAATATATAGAAACTCATTTGATCAGTTAGTGAAGT
>Marker524425 Chr3 SNP_site:11227702 Ref_Type:C
CACCTAAATGTAGCCTCGTTGGCACTCCTATAGTAATCTAATATTAACAACTAGACTTTAACAAGATTTCTGCCATATTTXXXXXXXXXXG
AAAATGTTTATCTAAAACATAACTCAACGAAGTAACGTTTGTAAAGTAATTTGAAAGTAATTTTGAAATAGCTAAAAGT
TACCTAAATGTAGCCTCGTTGGCACTCCTATAGTAATCTAATATTAACAACTAGACTTTAACAAGATTTCTGCCATATTTXXXXXXXXXXG
AAAATGTTTATCTAAAACATAACTCAACGAAGTAACGTTTGTAAAGTAATTTGAAAGTAATTTTGAAATAGCTAAAAGT
>Marker524719 Chr3 SNP_site:36982928 Ref_Type:T
AACCCTCCAAAGCAGACCTTGATATAAGTATAACTACATAGATATTAATGTGTCTTTCTACTTCCAACAATTAGCTTCAAXXXXXXXXXXC
GAATGAAGATTATTAAAGCTGATAATTATCTATACGACCTAACTTAGAATAATAATCTAAACCCTTTTAAAAAGTAGGT
AACCTTCCAAAGCAGACCTTGATATAAGTATAACTACATAGATATTAATGTGTCTTTCTACTTCCAACAATTAGCTTCAAXXXXXXXXXXC
GAATGAAGATTATTAAAGCTGATAATTATCTATACGACCTAACTTAGAATAATAATCTAAACCCTTTTAAAAAGTAGGT
>Marker524868 Chr3 SNP_site:4221841 Ref_Type:A
TACAGTTTTGGTGTTGTGTTCTTGGAGATTATAACTGGAAGAAGGGTTATAGACAATGCAAGACCAACAGCAGAACAAAAXXXXXXXXXXT
AGATGTTGTTATCTTGTGTTCCAAACTATCTAAGCTTGAACACTGAATAATTTGTTGAGACCAAAACTTATTAATTGTC
TACAGTTTTGGTGTTGTGTTCTTGGAGATTATAACTGGAAGAAGGGTTATAGACAATGCAAGACCAACAGCAGAACAGAAXXXXXXXXXXT
AGATGTTGTTATCTTGTGTTCCAAACTATCTAAGCTTGAACACTGAATAATTTGTTGAGACCAAAACTTATTAATTGTC
>Marker524981 Chr3 SNP_site:39018355 Ref_Type:G
ACATTAATAGACTTACTGTTAAAAAAGAATATAAATGATCCAATCTTAGAGAAAGTGTCATTGAAGAAGAGGAAGAGAAAXXXXXXXXXXT
TTTTTTTACCAAGTCAATCTGTTTTTTCATGAGAGTGAAGGGCACCTCAAAAGTGTATAATATATAGGTAATAAATGGT
ACATTAATAGACTTACTGTTAAAAAAGAATATAAATGATCCAATCTTAGAGAAAGTGTCATTGAAGAAGAGGAAGAGAAGXXXXXXXXXXT
TTTTTTTACCAAGTCAATCTGTTTTTTCATGAGAGTGAAGGGCACCTCAAAAGTGTATAATATATAGGTAATAAATGGT
>Marker525127 Chr3 SNP_site:18502083 Ref_Type:A
AACAAAAATCCTGAAAGCACAACCAAAATAAAACTCTTAATAAGATGCGATAAGCACTGTTCCAGAACCTAGAAACAAAAXXXXXXXXXXT
TTCCTTTACTTCCCATTTCCCTGTGAGTTGATTGGGAAAAGGAATATATTATAGATAATGAAATTCAGGAAACGAAGGT
AACGAAAATCCTGAAAGCACAACCAAAATAGAACTCTTAATAAGATGCGATAAGCACTGTTCCAGAACCTAGAAACAAAAXXXXXXXXXXT
TTCCTTTACTTCCCATTTCCCTGTGAGTTGATTGGGAAAAGGAATATATTTTAGATAATGAAATTCAGGAAACGAAGGT
>Marker525127 Chr3 SNP_site:18502110 Ref_Type:A
AACAAAAATCCTGAAAGCACAACCAAAATAAAACTCTTAATAAGATGCGATAAGCACTGTTCCAGAACCTAGAAACAAAAXXXXXXXXXXT
TTCCTTTACTTCCCATTTCCCTGTGAGTTGATTGGGAAAAGGAATATATTATAGATAATGAAATTCAGGAAACGAAGGT
AACGAAAATCCTGAAAGCACAACCAAAATAGAACTCTTAATAAGATGCGATAAGCACTGTTCCAGAACCTAGAAACAAAAXXXXXXXXXXT

Create PDF with GO2PDF for free, if you wish to

TTCCTTTACTTCCCATTTCCCTGTGAGTTGATTGGGAAAAGGAATATATTTTAGATAATGAAATTCAGGAAACGAAGGT

>Marker525127 Chr3 SNP_site:18502309 Ref_Type:A

AACAAAAATCCTGAAAGCACAACCAAAATAAAACTCTTAATAAGATGCGATAAGCACTGTTCCAGAACCTAGAAACAAAAXXXXXXXXXXT

TTCCTTTACTTCCCATTTCCCTGTGAGTTGATTGGGAAAAGGAATATATTATAGATAATGAAATTCAGGAAACGAAGGT

AACGAAAATCCTGAAAGCACAACCAAAATAGAACTCTTAATAAGATGCGATAAGCACTGTTCCAGAACCTAGAAACAAAAXXXXXXXXXXT

TTCCTTTACTTCCCATTTCCCTGTGAGTTGATTGGGAAAAGGAATATATTTTAGATAATGAAATTCAGGAAACGAAGGT

>Marker52515 Chr4 SNP_site:12191782 Ref_Type:A

AACTTTTCCTTGCTTCACTAATAAAGCTGTAGATGAATAATGCCGTGACCTCATTAACTATGACAAGAGTCTCTATTTAAXXXXXXXXXXT

AGTATTCTACTACTTAATGGAACTCCTTTTTTCTTTTTCATTCTTCCTATAATTTAGGTTGCTTAGTTTAGTTGATGTC

AACTTTTCCTTGCTTCACTAATAAAGCTGTAGATGAATAATGCCGTGACCTCTTTAACTATGACAAGAGTCTCTATTTAAXXXXXXXXXXT

AGTATTCTACTACTTAATGGAACTCCTTTTTTCTTTTTCATTCTTCCTATAATTTAGGTTGCTTAGTTTAGTTGATGTC

>Marker525237 Chr3 SNP_site:21724971 Ref_Type:A

CACGAACTTGTTCAGTTCTCCTTATTATCTTCCCTCAGTATGGATTTGGTTTATGCGTTGGCTATTGTGTGTTGTGTGACXXXXXXXXXXG

GCTAGAAAAGTAGTTTTTATTTTATTTTATTGTGTTATTTATTAATCTTTGCCATTTTTTCAATTAAGAATTTTTTGTG

CACGAACTTGTTCAGTTCTCCTTATTATCTTCCCTCAGTATGGATTTGGTTTGTGCGTTGGCTATTGTGTGTTGTGTGACXXXXXXXXXXG

GCTAGAAAAGTAGTTTTTATTTTATTTTATTGTGTTATTTATTAATCTTTGCCATTTTTTCAATTAAGAATTTTTTGTG

>Marker525428 Chr3 SNP_site:23537704 Ref_Type:A

ACTTTAGCAGTCCTTGTTGCCACGTATAATTCTTCACTTCTTCACCTTTCTACAGTCTGTTGATGATTTCCATCAAAAATXXXXXXXXXXC

ATCGGCTGCCTTTTTTTCTAGACGGGTTTATACACTACCTCAAATGAATAGCCGAGAAACTTGGCAATCCATCTTTGGT

ACTTTAGCAGTCCTTGTTGCCACGTATAATTCTTCACTTCTTCACCTTTCTGCAGTCAGTTGATGATTTCCATCAAAAATXXXXXXXXXXC

ATCGGCTGCCTTTTTTTCTAGACGGGTTTATACACTACCTCAAATGAATAGCCGAGAAACTTGGCAATCCATCTTTGGT

>Marker525428 Chr3 SNP_site:23537710 Ref_Type:T

ACTTTAGCAGTCCTTGTTGCCACGTATAATTCTTCACTTCTTCACCTTTCTACAGTCTGTTGATGATTTCCATCAAAAATXXXXXXXXXXC

ATCGGCTGCCTTTTTTTCTAGACGGGTTTATACACTACCTCAAATGAATAGCCGAGAAACTTGGCAATCCATCTTTGGT

ACTTTAGCAGTCCTTGTTGCCACGTATAATTCTTCACTTCTTCACCTTTCTGCAGTCAGTTGATGATTTCCATCAAAAATXXXXXXXXXXC

ATCGGCTGCCTTTTTTTCTAGACGGGTTTATACACTACCTCAAATGAATAGCCGAGAAACTTGGCAATCCATCTTTGGT

>Marker526334 Chr3 SNP_site:34248541 Ref_Type:G

AACACAAACCGTCACGCTCAACCCAGCAGATCGTGTCTCCAATGGAAATGTTTCACTAGGAACTAACCATGCAAGGGGACXXXXXXXXXXA

GTATAATGGCGATCATAAGTTGGGAGAGGAACATTTGAACTCCGGCTTCTAACAAAAGCATTCGTCGCCCGATCCTGTC

AACACAAACCGTCACGCTCAACCCAGCAGATCGTGTCTCCAATGGAAATGTTTCACTAGGAACTAACCATGCAAGGGGGCXXXXXXXXXXA

GTATAATGGCGATCATAAGTTGGGAGAGGAACATTTGAACTCCGGCTTCTAACAAAAGCATTCGTCGCCCGATCCTGTC

>Marker526476 Chr3 SNP_site:11558486 Ref_Type:G

ACCCCGAAACACATACACCTTAGACATATCAGAGAATCCCAATTCATGAACCTGAACCTGAGTCCCAAACGAAACAAATCXXXXXXXXXXG

GGAGGAATTCGCCTGTTGGGCTGAATCCAGAGCAGGATTAGGATGAGTGGATTGAGGAGGAGCATATTGAACAGTGGTA

ACCCCGAAACACATACACCTTAGACATATCAGAGAATCCCAATTCATGAACCTGAACCTGAGTCCCAAACGAAACAAATCXXXXXXXXXXG

GGAGGGATTCGCCTGTTGGGCTGAATCCAGAGCAGGATTAGGATGAGTGGATTGAGGAGGAGCATATTGAACAGTGGTA

>Marker52675 Chr4 SNP_site:8391127 Ref_Type:A

TACCTCAATCCCTACCTTGCTCGGAACCACCAAAGCCACAACTGAGGTAGTATTCGTCACTCATCAATAAACACTACTACXXXXXXXXXXA

CAAAATTGAAAAAATGCATAATTTTTTTAAAAAAAATTCAATACTTAACACATTAAAATCGTCAAGTCTAGTTTAATGT

TACCTCAATCCCTACCTTGCTCGGAACCACCAAAGCCACAACTGAGGTAGTATTCGTCACTCATCAATAAACACTACTACXXXXXXXXXXA

CAAAATTGAAAAAATGCATAATTTTTTTTAAAAAAATTCAATACTTGACACATTAAAATCGTCAAGTCTAGTTTAATGT

>Marker52675 Chr4 SNP_site:8391145 Ref_Type:A

TACCTCAATCCCTACCTTGCTCGGAACCACCAAAGCCACAACTGAGGTAGTATTCGTCACTCATCAATAAACACTACTACXXXXXXXXXXA

CAAAATTGAAAAAATGCATAATTTTTTTAAAAAAAATTCAATACTTAACACATTAAAATCGTCAAGTCTAGTTTAATGT

Create PDF with GO2PDF for free, if you wish to

TACCTCAATCCCTACCTTGCTCGGAACCACCAAAGCCACAACTGAGGTAGTATTCGTCACTCATCAATAAACACTACTACXXXXXXXXXXA
CAAAATTGAAAAAATGCATAATTTTTTTTAAAAAAATTCAATACTTGACACATTAAAATCGTCAAGTCTAGTTTAATGT
>Marker527100 Chr3 SNP_site:35051138 Ref_Type:A
ACTTTTACGAAACTATTTAAAAAAATGCTACCATTAAAGATTTATTATTGTAATAACAAAGTTTTTACACATCATTAATTXXXXXXXXXXT
TCAATTGATACACGTCTTTATATGCTCCAACCTTAAAAGAATGCATGGCAAGAAAACGTGGAGGCAGAATATAAATGTA
ACTTTTACGAAACTATTTTAAAAAATGCTACCATTCAAGATTTATTATTGTAATAACAAAGTTTTTACACATCATTAATTXXXXXXXXXXT
TCAATTGATACACGTCTTTATATGCTCCAACCTTAAAAGAATGCATGGCAAGAAAACGTGGAGGCAGAATATAAATGTA
>Marker527100 Chr3 SNP_site:35051155 Ref_Type:A
ACTTTTACGAAACTATTTAAAAAAATGCTACCATTAAAGATTTATTATTGTAATAACAAAGTTTTTACACATCATTAATTXXXXXXXXXXT
TCAATTGATACACGTCTTTATATGCTCCAACCTTAAAAGAATGCATGGCAAGAAAACGTGGAGGCAGAATATAAATGTA
ACTTTTACGAAACTATTTTAAAAAATGCTACCATTCAAGATTTATTATTGTAATAACAAAGTTTTTACACATCATTAATTXXXXXXXXXXT
TCAATTGATACACGTCTTTATATGCTCCAACCTTAAAAGAATGCATGGCAAGAAAACGTGGAGGCAGAATATAAATGTA
>Marker527481 Chr3 SNP_site:9547830 Ref_Type:G
AACCAGTTGTTTCCTTACCAAAAATGTGCAAGTATAGAACAAAGCAAAATAAAGGTGGTGATTTTATTTATATATATCTTXXXXXXXXXXA
TATTTGGTATTGAAATATGGAAATATACGAAGGTTGGTCTCAAAATTAAAATTAAAGTTTCTGCTCAAACATGCATGTC
AACCAGTTGTTTCCTTACCAAAAATGTGCAAGTATAGAAGAAAGCAAAATAAAGGTGGTGATTTTATTTATATATATCTTXXXXXXXXXXA
TATTTGGTATTGAAATATAGAAATATACGAAGGTTGGTCTCAAAATTAAAATTAAAGTTTCTGCTCAAACATGCATGTC
>Marker527481 Chr3 SNP_site:9547990 Ref_Type:A
AACCAGTTGTTTCCTTACCAAAAATGTGCAAGTATAGAACAAAGCAAAATAAAGGTGGTGATTTTATTTATATATATCTTXXXXXXXXXXA
TATTTGGTATTGAAATATGGAAATATACGAAGGTTGGTCTCAAAATTAAAATTAAAGTTTCTGCTCAAACATGCATGTC
AACCAGTTGTTTCCTTACCAAAAATGTGCAAGTATAGAAGAAAGCAAAATAAAGGTGGTGATTTTATTTATATATATCTTXXXXXXXXXXA
TATTTGGTATTGAAATATAGAAATATACGAAGGTTGGTCTCAAAATTAAAATTAAAGTTTCTGCTCAAACATGCATGTC
>Marker527832 Chr3 SNP_site:37243693 Ref_Type:G
GACAATATAATATATCAGAAATAACATTAAATAAACATTCAATTTAAGTTATGGACAATATAAATTCAGTCGTCGAAGACXXXXXXXXXXG
CGCTTCAAATGATGTATAATAAATGATTGAAATACATTTTTCAGATGAAATACGCCATGGGAGAAGGAGAAAAAAAGTA
GACAATATAATATATCAGAAATAACATTAAATAAACATTCAATTTAAGTTATGGACAATATAAATTCAGTCGTCGAAGACXXXXXXXXXXG
CGCTTCAAATGATGTATAATAAATTATTGAAATACATTTTTCAGATGAAATACGCCATGGGAGAAGGAGAAAAAAAGTA
>Marker527959 Chr3 SNP_site:19111797 Ref_Type:A
ACAAATTGTGGTTCTTCGCATGCTGAACATACTGTTGCCAAGCCAACTTAGTTATCAATTATGAAATAAAGAGGTTTGTTXXXXXXXXXXA
GTTTAAAGACTTCCACTGGCAAATGTTTTAATGTTTCACATTTTCAAGTCAAGTGTCAGTTGAGGTTGTTTCAAAGGTG
ACAGATTGTGGTTCTTCGCATGCTGAACATACTGTTGCCAAGCCAACTTAGTTATCAATTATGAAATAAAGAGGTTTGTTXXXXXXXXXXA
GTTTAAAGACTAACACTGGCAAATGTTTTAATGTTTCACATTTTCAAGTCAAGTGTCAGTTGAGGTTGTTTCAAAGGTG
>Marker527959 Chr3 SNP_site:19111998 Ref_Type:T
ACAAATTGTGGTTCTTCGCATGCTGAACATACTGTTGCCAAGCCAACTTAGTTATCAATTATGAAATAAAGAGGTTTGTTXXXXXXXXXXA
GTTTAAAGACTTCCACTGGCAAATGTTTTAATGTTTCACATTTTCAAGTCAAGTGTCAGTTGAGGTTGTTTCAAAGGTG
ACAGATTGTGGTTCTTCGCATGCTGAACATACTGTTGCCAAGCCAACTTAGTTATCAATTATGAAATAAAGAGGTTTGTTXXXXXXXXXXA
GTTTAAAGACTAACACTGGCAAATGTTTTAATGTTTCACATTTTCAAGTCAAGTGTCAGTTGAGGTTGTTTCAAAGGTG
>Marker527959 Chr3 SNP_site:19111999 Ref_Type:C
ACAAATTGTGGTTCTTCGCATGCTGAACATACTGTTGCCAAGCCAACTTAGTTATCAATTATGAAATAAAGAGGTTTGTTXXXXXXXXXXA
GTTTAAAGACTTCCACTGGCAAATGTTTTAATGTTTCACATTTTCAAGTCAAGTGTCAGTTGAGGTTGTTTCAAAGGTG
ACAGATTGTGGTTCTTCGCATGCTGAACATACTGTTGCCAAGCCAACTTAGTTATCAATTATGAAATAAAGAGGTTTGTTXXXXXXXXXXA
GTTTAAAGACTAACACTGGCAAATGTTTTAATGTTTCACATTTTCAAGTCAAGTGTCAGTTGAGGTTGTTTCAAAGGTG
>Marker528224 Chr3 SNP_site:31462061 Ref_Type:C
CACAAAATATCAAAATTTTCCCATAGGAACTATGAACACACAACCATAGATGTGATGCTAAAAAAGCATTCAATTATGAAXXXXXXXXXXC

Create PDF with GO2PDF for free, if you wish to

ACCAAAAAAAGAAAAAAAAATGGTATGCGTAAATTCATGAGATTTAAGTTCGGGAGTCCGTTGTGTGTAGGGAAGGTGTT
CACAAAATATCAAAATTTTCCCATAGGAACTATGAACACACAATCATAGATGTGATGCTAAAAAAGCATTCAATTATGAAXXXXXXXXXXC
ACCAAAAAAAGAAAAAAAAATGGTATGCTTAAATTCATGAGATTTAAGTTCGGGAGTCCGTTGTGTGTAGGGAAGGTGTT
>Marker528224 Chr3 SNP_site:31462228 Ref_Type:G
CACAAAATATCAAAATTTTCCCATAGGAACTATGAACACACAACCATAGATGTGATGCTAAAAAAGCATTCAATTATGAAXXXXXXXXXXC
ACCAAAAAAAGAAAAAAAAATGGTATGCGTAAATTCATGAGATTTAAGTTCGGGAGTCCGTTGTGTGTAGGGAAGGTGTT
CACAAAATATCAAAATTTTCCCATAGGAACTATGAACACACAATCATAGATGTGATGCTAAAAAAGCATTCAATTATGAAXXXXXXXXXXC
ACCAAAAAAAGAAAAAAAAATGGTATGCTTAAATTCATGAGATTTAAGTTCGGGAGTCCGTTGTGTGTAGGGAAGGTGTT
>Marker529413 Chr3 SNP_site:31461198 Ref_Type:G
GACCTTTTCTAACATAGTTTATAACTTAACTCACGCATTTTTAAATTTGTTATAATTTAGGTCTCTTTTGATAATTTTTTXXXXXXXXXXG
TTAATTATAAGTTTTGAGAACTTCTTTCATTAGTTTTAAAATTTGGAAGGTTCGAATTGTAAAATACTTCTAAAATGTA
GACCTTTTCTAACATAGTTTATAACTTAACTCACGCATTTTTAAATTTGTTATAATTTAGGTCTCTTTTGATAATTTTTTXXXXXXXXXXG
TTAATTATAAGTTTTGAGAACTTCTTTCATTAGTTTTAAAATTTTGAAGGTTCGAATTGTAAAATACTTCTAAAATGTA
>Marker529842 Chr3 SNP_site:33555259 Ref_Type:G
GACTTTTTTACAGGGTTGTTATCAATTATGATTTCCCATCCGGAGTGGAGGATTATGTCCATAGGATTGGTCGAACTGGAXXXXXXXXXXT
AGACGTTGGGGTTCTGGCTCTGATGGTCGTGATGGTGGTCGAGGTGGGCGCAATGATTCAAACTCTGGTGGGAGGGGTG
TACTTTTTTACAGGGTTGTTATCAATTATGATTTCCCATCCGGAGTGGAGGATTATGTCCATAGGATTGGTCGAACTGGAXXXXXXXXXXT
AGACGTTGGGGTTCTGGCTCTGATGGTCGTGATGGTGGTCGAGGTGGGCGCAATGATTCAAACTCTGGTGGGAGGGGTG
>Marker529918 Chr3 SNP_site:35533409 Ref_Type:G
ACTAAAACTAAAATATCTTCTTATCCAAAACGCCAAGTTACATACTAAGTCAAAATTTTATCTTATAACTTTTAATTTAGXXXXXXXXXXC
TAATCTATAATTTTTTTAAGCATGCTTAACCCTAGTTTAAATTATAAGAAAATTTGTTTATGTAATTGTTTGCCAATGT
ACTAAAACTAAAATATCTTCTTGTCCAAAACGCCAAGTTACATACTAAGTCAAAATTTTATCTTATAACTTTTAATTTAGXXXXXXXXXXC
TAATCTATAATTTTTTTAAGCATGCTTAACCTTAGTTTAAATTATAAGAAAATTTGTTTATGTAATTGTTTGCCAATGT
>Marker529918 Chr3 SNP_site:35533605 Ref_Type:T
ACTAAAACTAAAATATCTTCTTATCCAAAACGCCAAGTTACATACTAAGTCAAAATTTTATCTTATAACTTTTAATTTAGXXXXXXXXXXC
TAATCTATAATTTTTTTAAGCATGCTTAACCCTAGTTTAAATTATAAGAAAATTTGTTTATGTAATTGTTTGCCAATGT
ACTAAAACTAAAATATCTTCTTGTCCAAAACGCCAAGTTACATACTAAGTCAAAATTTTATCTTATAACTTTTAATTTAGXXXXXXXXXXC
TAATCTATAATTTTTTTAAGCATGCTTAACCTTAGTTTAAATTATAAGAAAATTTGTTTATGTAATTGTTTGCCAATGT
>Marker530311 Chr3 SNP_site:32652364 Ref_Type:T
CACACACATATAATTTATTATTGAAAATTGTGAAATATTAGAGATGAAAAGTATATACAGATGAAATAATAATAAGAAGAXXXXXXXXXXC
AATAGACTTTTAATCCTATATCTAATTAAGTTCCATATTAGATCATTTTCAAATTTTTTGAAGTTTGTGAATCTTATGT
CACACACATATAATTTATTATTGAAAATTGTGAAATATTAGAGATGAAAAGTATATACAGATGAATTAATAATAATAAGAXXXXXXXXXXC
AATAGACTTTTAATCCTATATCTAATTAAGTTCCATATTAGATCATTTTCAAATTTTTTGAAGTTTGTGAATCTTATGT
>Marker530311 Chr3 SNP_site:32652374 Ref_Type:T
CACACACATATAATTTATTATTGAAAATTGTGAAATATTAGAGATGAAAAGTATATACAGATGAAATAATAATAAGAAGAXXXXXXXXXXC
AATAGACTTTTAATCCTATATCTAATTAAGTTCCATATTAGATCATTTTCAAATTTTTTGAAGTTTGTGAATCTTATGT
CACACACATATAATTTATTATTGAAAATTGTGAAATATTAGAGATGAATTAATAATAATAAGAXXXXXXXXXXC
AATAGACTTTTAATCCTATATCTAATTAAGTTCCATATTAGATCATTTTCAAATTTTTTGAAGTTTGTGAATCTTATGT
>Marker530667 Chr3 SNP_site:30446006 Ref_Type:T
AACAATTATGGGACCCTTTCACCATTAGCTTCATCCCAAACCTCTCTTCTATAATTATGTTCCCTAAAATTTACTTTTTGXXXXXXXXXXT
TATGTATTTTTTAGTCATCTTATATCGTGTTTATTTTCATTTATCAATGTAACTTGAAAAGTGAATTACATTTGTTGTT
AACAATTATGGGACCCTTTCACCATTAGCTTCATCCCAAACCTCTCTTCTATAATTATGTTCCCTAAAATTTACTTTTTGXXXXXXXXXXT
TATGTATTTTTTAGTCATCTTATATCGTGTTTATTTTCATTTATCAATGTAATTTGAAAAGTGAATTACATTTGTTGTT
>Marker530745 Chr3 SNP_site:14040187 Ref_Type:A

Create PDF with GO2PDF for free, if you wish to

AACATTGTCTTCAATAATTGATGGTGTTATGGTAATATGTGTTTGGAGGAAGAAATATGAATGGTAGTGTTATGATAATAXXXXXXXXXXA
GTAGAAAAATTTGTAGGGTTACGAGAAAATGAAGTGGAGAAATTTGTATGGTTATTTAACCATAATCCCCAAAAATGTA
AACATTGTCTTCAATAATTGGTGGTGTTATGGTAATATGTGTTTGGAGGAAGAAATATGAATGGTAGTGTTATGATAATAXXXXXXXXXXA
GTAGAAAAATTTGTAGGGTTACGAGAAAATGAAGTGGAGAAATTTGTATGGTTATTTAACCATAATCCCCAAAAATGTA
>Marker531029 Chr3 SNP_site:35111370 Ref_Type:A
CACATTTGTGTTCAACCAATGAATGAATGATACATTGTGAAATTTTGAATCTGTTGCCAAACTTGTTGATGGCATGTATTXXXXXXXXXXA
GGGAGGTCTCATTTAACTACATGTTGATATTTTAGGCTCTTGAAAAGCTGAAACGTCAATTGGCTGAAGCCGAGGCTGT
CACATTTGTGTTCAACCACTGAATGAATGATACATTGTGAAATTTTGAATCTGTTGCCAAACTTGTCGATGGCATGTATTXXXXXXXXXXA
GGGAGGTCTCATTTAACTACATGTTGATATTTTAGGCTCTTGAAAAGCTGAAACGTCAATTGGCTGAAGCCGAGGCTGT
>Marker531029 Chr3 SNP_site:35111418 Ref_Type:T
CACATTTGTGTTCAACCAATGAATGAATGATACATTGTGAAATTTTGAATCTGTTGCCAAACTTGTTGATGGCATGTATTXXXXXXXXXXA
GGGAGGTCTCATTTAACTACATGTTGATATTTTAGGCTCTTGAAAAGCTGAAACGTCAATTGGCTGAAGCCGAGGCTGT
CACATTTGTGTTCAACCACTGAATGAATGATACATTGTGAAATTTTGAATCTGTTGCCAAACTTGTCGATGGCATGTATTXXXXXXXXXXA
GGGAGGTCTCATTTAACTACATGTTGATATTTTAGGCTCTTGAAAAGCTGAAACGTCAATTGGCTGAAGCCGAGGCTGT
>Marker531800 Chr3 SNP_site:39389271 Ref_Type:A
TACCGCAGCCCACTTGAGTGATAATATTATTATAATCACTTTCATGTGCTTATTATTGGCTTTTAGGGAGCATTTAGTGTXXXXXXXXXXA
ATAAATTGAACCCATTTTCAAAACAATTATTGTCTTTTTTTTCAATCATATGTTTTTGAAGAAATGAATTAAATAGGTT
TACCGCAGCCCACTTGAGTGATAATATTATTATAATCACTTTCATGTGCTTATTATTGGCTTTTAGGGAGCATTTAGTGTXXXXXXXXXXA
ATAAATTGAACCCATTTTCAAAACAATTATTGTCTTTTTTTTCAATCCTATGTTTTTGAAGAAATGAATTAAATAGGTT
>Marker532061 Chr3 SNP_site:34127996 Ref_Type:C
CACCTTAACTAATCTTACAGAACAACCTGTATGACACTATAACATTTAGGTGTCAAGGAAACTTGTAGGAAATTAATTTCXXXXXXXXXXC
CCAATGACTTGAAACAAATAAGTTGGAATGTCAAAGACATGAAAGCACAGAAAGTAGTCCTCAAAAGACCGGCCAGGTA
CACCTTAACTAATCTTACAGAACAACCTGTCTGACACTATAACATTTAGGTGTCAAGGAAACCTGTAGGAAATTAATTTCXXXXXXXXXXC
CCAGTGACTTGAAACAAATAAGTTGGAATGTCAAAGACATGAAAGCACAGAAAGTAGTCCTCAAAAGACCGGCCAGGTA
>Marker532061 Chr3 SNP_site:34128028 Ref_Type:C
CACCTTAACTAATCTTACAGAACAACCTGTATGACACTATAACATTTAGGTGTCAAGGAAACTTGTAGGAAATTAATTTCXXXXXXXXXXC
CCAATGACTTGAAACAAATAAGTTGGAATGTCAAAGACATGAAAGCACAGAAAGTAGTCCTCAAAAGACCGGCCAGGTA
CACCTTAACTAATCTTACAGAACAACCTGTCTGACACTATAACATTTAGGTGTCAAGGAAACCTGTAGGAAATTAATTTCXXXXXXXXXXC
CCAGTGACTTGAAACAAATAAGTTGGAATGTCAAAGACATGAAAGCACAGAAAGTAGTCCTCAAAAGACCGGCCAGGTA
>Marker532061 Chr3 SNP_site:34128149 Ref_Type:G
CACCTTAACTAATCTTACAGAACAACCTGTATGACACTATAACATTTAGGTGTCAAGGAAACTTGTAGGAAATTAATTTCXXXXXXXXXXC
CCAATGACTTGAAACAAATAAGTTGGAATGTCAAAGACATGAAAGCACAGAAAGTAGTCCTCAAAAGACCGGCCAGGTA
CACCTTAACTAATCTTACAGAACAACCTGTCTGACACTATAACATTTAGGTGTCAAGGAAACCTGTAGGAAATTAATTTCXXXXXXXXXXC
CCAGTGACTTGAAACAAATAAGTTGGAATGTCAAAGACATGAAAGCACAGAAAGTAGTCCTCAAAAGACCGGCCAGGTA
>Marker532147 Chr3 SNP_site:24992176 Ref_Type:C
CACCTCTAAGCACATTCGAATGTTGGAGTGACAACCTGACAAAGTCAAAAAATCACAACATAGCATCCATCCAACTTTGGXXXXXXXXXXA
CTGAAAACCTTCTTTACGCTTCCACATCTTCTACTTGGAATTTTCCATCTTCAAACCATATACAGTAGTGTGCTTGGTG
CACCTCTAAGCACATTCGAATGTTGGAGTGACAACCTGACAAAGTCAAAAAATCACAACATAGCATCCATCCAACTTTGGXXXXXXXXXXA
CTGACAACCTTCTTTGCGCTTCCACATCTTCTACTTGGAATTTTCCATCTTCAAACCATATACAGTAGTGTGCTTGGTG
>Marker532147 Chr3 SNP_site:24992187 Ref_Type:G
CACCTCTAAGCACATTCGAATGTTGGAGTGACAACCTGACAAAGTCAAAAAATCACAACATAGCATCCATCCAACTTTGGXXXXXXXXXXA
CTGAAAACCTTCTTTACGCTTCCACATCTTCTACTTGGAATTTTCCATCTTCAAACCATATACAGTAGTGTGCTTGGTG
CACCTCTAAGCACATTCGAATGTTGGAGTGACAACCTGACAAAGTCAAAAAATCACAACATAGCATCCATCCAACTTTGGXXXXXXXXXXA
CTGACAACCTTCTTTGCGCTTCCACATCTTCTACTTGGAATTTTCCATCTTCAAACCATATACAGTAGTGTGCTTGGTG

Create PDF with GO2PDF for free, if you wish to

>Marker532201 Chr3 SNP_site:9148544 Ref_Type:C
ACCTTAGACAGAGATGGAGAAAATCGACGAGGATCCGCCGGAGACAATGCAGGAACAGAGAGAGAGAAGAACCTGGAACAXXXXXXXXXXA
GAGAAATACGATTGTAACTACCTCTTTCCTTTTTCTTGTTTATTATTTATTAGAGAAGTTGGGCTGGGCTGAACACGTA
ACCTTAGACAGAGATGGAGAAAATCGACGAGGATCCGCCGGAGACAATGCAGGAACAGAGAGAGAGAAGAACCTGGAACAXXXXXXXXXXA
GAGAAATACGATTGTAACTACCTTTTTCCTTTTTCTTGTTTATTATTTATTAGAGAAGTTGGGCTGGGCTGAACACGTA
>Marker533186 Chr3 SNP_site:8813733 Ref_Type:T
AACACTTAGGCTTCATGTCAATATCTGAAGTTGTAATTGTTAGAATTATGATATTTTCACATTTTAATTTTCTATGCTCGXXXXXXXXXXC
GTTCCTTAGCCTTGTAAGGCCGATACCAAATAATGCAATATTCCCCTTTATAACTTGTGTGTTCATTTACATATTGGTT
AACACTTAGGCTTCATGTCAATATCTGAAGTTGTAATTGTTAGAATTATGATATTTTCACATTTTAATTTTCTATGCTCGXXXXXXXXXXT
GTTCCTTAGCCTTGTAAGGCCGATACCAAATAATGCAATATTCCCCTTTATAACTTGTGTGTTCATTTACATATTGGTT
>Marker533223 Chr3 SNP_site:26235436 Ref_Type:A
CACAAAAAACCAACTTACCTGTGCTAAAATCATTATCTCCTACACCATACCGGCTGCAACTCTGTAAAAAGAAGGGAAAGXXXXXXXXXXA
TTCTTCACTTCTATATATCGAAAAAATATTTAGCCTAGAAAGTTGAAAATTTTATCATTTCTAAACCCAATTGCAAGTT
CACCAAAAACCAACTTACCTGTGCTAAAATCATTATCTCCTACACCATACCGGCTGCAACTCTGTAAAAAGAAGGGAAAGXXXXXXXXXXA
TTCTTCACTTCTATATATCGAAAAAATATTTAGCCTAGAAAGTTGAAAATTTTATCATTTCTAAACCCAATTGCAAGTT
>Marker533275 Chr3 SNP_site:5862903 Ref_Type:T
AACATATCCGTTATTATAACCTCTTATATAGAACAATGAGGCCAAAATGCATGAAGAATGTCAAAATGCATGGCTTGTATXXXXXXXXXXC
GAGTGAATCACAGAGGTGCTCGCAAGAAGACCAGGTGAAAAGACCAAGTATTTACGTGAGAAGAACTCATCTGACAAGT
AACATATCCGTTATTATAACCTCTTATATAGAACAATGAGGCCAAAATGCATGAAGAATGTCAAAATGCATGGCTTGTATXXXXXXXXXXC
TAGTGAATCACAGAGGTGCTCACAAGAAGACCAGGTGAAAAGACCAAGTATTTACGTGAGAAGAACTCATCTGACAAGT
>Marker533275 Chr3 SNP_site:5862924 Ref_Type:A
AACATATCCGTTATTATAACCTCTTATATAGAACAATGAGGCCAAAATGCATGAAGAATGTCAAAATGCATGGCTTGTATXXXXXXXXXXC
GAGTGAATCACAGAGGTGCTCGCAAGAAGACCAGGTGAAAAGACCAAGTATTTACGTGAGAAGAACTCATCTGACAAGT
AACATATCCGTTATTATAACCTCTTATATAGAACAATGAGGCCAAAATGCATGAAGAATGTCAAAATGCATGGCTTGTATXXXXXXXXXXC
TAGTGAATCACAGAGGTGCTCACAAGAAGACCAGGTGAAAAGACCAAGTATTTACGTGAGAAGAACTCATCTGACAAGT
>Marker533698 Chr3 SNP_site:37333696 Ref_Type:G
ACATATTATTGTGGGTAGATTTTGGACCAACGATAAGATAGTTAAAACATAACAAATGAAAGGGAGAAGATCAAGATCGAXXXXXXXXXXA
GTTTTATATTGTTTAAGATGAATGTGAAATTGTATTCAAATCATTAGTATTTTCCTATCAAAGTCCTAACTGTTATGTG
ACATATTATTGTGGGTAGATTTTGGACCAACGATAAGATAGTTAAAACATAACAAATGAAAGGGAGAAGATCAAGATCGAXXXXXXXXXXA
GTTTTATATTGTTTAAGATGAATGTGGAATTGTATTCAAATCATTAGTATTTTCCTATCAAAGTCCTAACTGTTATGTG
>Marker534270 Chr3 SNP_site:8156934 Ref_Type:G
CACTATGCCAACCCATGGGTGTTGTAGTCGATATGATCTTCCTTCCAAAACCTTTTACAAAAATGAACATATATGTTTCAXXXXXXXXXXA
GCAAAACCTCAAAACTAATACAGGATAACAACTTACATCCAAAGCATTAGTTCCTTTGTCCATCAAGTCAAGCTTCGTC
CACTATGCCAACCCATGGGTGTTGTAGTCGATATGATCTTCCTTCCAAAACCTTTTACAAAAATGAACATATATGTTTCAXXXXXXXXXXA
GCAAGACCTCAAAACTAATACAGGATAACAACTTACATCCAAAGCATTAGTTCCTTTGTCCATCAAGTCAAGCTTCGTC
>Marker534880 Chr3 SNP_site:37882677 Ref_Type:A
AACACCACTATGGCTTCTAATCCCACCACCTTCCTTAAACAAATATTAAACAATCAAACAAAGTTTGACTATAATATTGGXXXXXXXXXXC
CAAGAAGAAGAAAAAAGATTGTTTTATGAAAACAATCAAGATCTACATATTGAGGAAATTCAAGGAATTAATTAACGTA
AACACCACTATGGCTTCTTATCCCACCACCTTCCTTAAACAAATATTAAACAATCAAACAAAGTTTGACTATAATATTGGXXXXXXXXXXC
CAAGAAGAAGAAAAAAGATTGTTTTATGAAAACAATCAAGATCTACATATTGAGGAAATTCAAGGAATTAATTAACGTA
>Marker53505 Chr4 SNP_site:7167903 Ref_Type:A
AACTAAAACTCAGTGAATGACAAGTTTTTAAAAGTAAAAGCTCATAAAACAATACTTTCAAACTATTTAAATGTGTCATCXXXXXXXXXXG
ACGCCTAAATATACGACCAAGAATAGCTCGCACATGATTAAAGAATTATGCGACCACAACTAGCTCACACATATTTAGT
AACTAAAACTCAGTGAATGGCAAGTTTTTAAAAGTAAAAGCTCATAAAACAATACTTTCAAACTATTTAAATATGTCATCXXXXXXXXXXG

Create PDF with GO2PDF for free, if you wish to

ACGCCTAAATATACGACCAAGAATAGCTCGCACATGATTAAAGAATCATGCGACCACAACTAGCTCACACATATTTAGT
>Marker53505 Chr4 SNP_site:7167956 Ref_Type:G
AACTAAAACTCAGTGAATGACAAGTTTTTAAAAGTAAAAGCTCATAAAACAATACTTTCAAACTATTTAAATGTGTCATCXXXXXXXXXXG
ACGCCTAAATATACGACCAAGAATAGCTCGCACATGATTAAAGAATTATGCGACCACAACTAGCTCACACATATTTAGT
AACTAAAACTCAGTGAATGGCAAGTTTTTAAAAGTAAAAGCTCATAAAACAATACTTTCAAACTATTTAAATATGTCATCXXXXXXXXXXG
ACGCCTAAATATACGACCAAGAATAGCTCGCACATGATTAAAGAATCATGCGACCACAACTAGCTCACACATATTTAGT
>Marker53505 Chr4 SNP_site:7168103 Ref_Type:T
AACTAAAACTCAGTGAATGACAAGTTTTTAAAAGTAAAAGCTCATAAAACAATACTTTCAAACTATTTAAATGTGTCATCXXXXXXXXXXG
ACGCCTAAATATACGACCAAGAATAGCTCGCACATGATTAAAGAATTATGCGACCACAACTAGCTCACACATATTTAGT
AACTAAAACTCAGTGAATGGCAAGTTTTTAAAAGTAAAAGCTCATAAAACAATACTTTCAAACTATTTAAATATGTCATCXXXXXXXXXXG
ACGCCTAAATATACGACCAAGAATAGCTCGCACATGATTAAAGAATCATGCGACCACAACTAGCTCACACATATTTAGT
>Marker535635 Chr3 SNP_site:35018877 Ref_Type:A
CACTACAATTACTAAGGATATTTAGGACATAGCCCATAAACTATATTTTAAACATTAGGACGCCTCTCACCTATAAACACXXXXXXXXXXC
TCTAGGATTTAAGAGATTGACATGATTTATGACTTTATTGTTGGTTTTATTCCTAGGTTTGATGTAATTTGAGGGCGTA
CACTACAATTACTAAGGATATTTAGGACATAGCCCATAAACTATATTTTAAACGTTAGGACGCCTCTCACCTATAAACACXXXXXXXXXXC
TCTAGGATTTAAGAGATTGACATGATTTATGACTTTATTGTTGGTTTTATTCCTAGGTTTGATGTAATTTGAGGGCGTA
>Marker536247 Chr3 SNP_site:39014415 Ref_Type:T
ACCAAAAAGTGAAATCACTACTTCAACTATATATCATTGAATATATCAAGAAAATGCACAATATCAATAACACAATCTCC-XXXXXXXXX
ATTACCTCATAATGGCTCTTTTCTTTTAATTAGCCACAAGGGGTATAACCAATATGCATTTAGAAGCATATATATAAGTG
ACTAAAAAGTGAAATCACTACTTCAACTATATATCATTGAATATCTCAAGAAAATGCACGATATCAATAACACAAT-TCCGXXXXXXXXXX
ATTACCTCATAATGGCTCTTTTCTTTTAATTAGCCACAAGGGGTATAACCAATATGCATTTAGAAGCATATATATAAGTG
>Marker536247 Chr3 SNP_site:39014457 Ref_Type:C
ACCAAAAAGTGAAATCACTACTTCAACTATATATCATTGAATATATCAAGAAAATGCACAATATCAATAACACAATCTCC-XXXXXXXXX
ATTACCTCATAATGGCTCTTTTCTTTTAATTAGCCACAAGGGGTATAACCAATATGCATTTAGAAGCATATATATAAGTG
ACTAAAAAGTGAAATCACTACTTCAACTATATATCATTGAATATCTCAAGAAAATGCACGATATCAATAACACAAT-TCCGXXXXXXXXXX
ATTACCTCATAATGGCTCTTTTCTTTTAATTAGCCACAAGGGGTATAACCAATATGCATTTAGAAGCATATATATAAGTG
>Marker536247 Chr3 SNP_site:39014472 Ref_Type:G
ACCAAAAAGTGAAATCACTACTTCAACTATATATCATTGAATATATCAAGAAAATGCACAATATCAATAACACAATCTCC-XXXXXXXXX
ATTACCTCATAATGGCTCTTTTCTTTTAATTAGCCACAAGGGGTATAACCAATATGCATTTAGAAGCATATATATAAGTG
ACTAAAAAGTGAAATCACTACTTCAACTATATATCATTGAATATCTCAAGAAAATGCACGATATCAATAACACAAT-TCCGXXXXXXXXXX
ATTACCTCATAATGGCTCTTTTCTTTTAATTAGCCACAAGGGGTATAACCAATATGCATTTAGAAGCATATATATAAGTG
>Marker536779 Chr3 SNP_site:11920067 Ref_Type:T
CACAATTAATATAATCCAAGACAGTGTTATGAAATAGATGGTGTATATCAAACACCCTATAATGTGAATAAAAGCTAATGXXXXXXXXXXT
TTTATTTCCTAACACCTAACTCTTCTAATCAATACAGAGTTTATGTAATAATAAGGTTGGTCATCATAAATAAAACGTT
CACAATTAATATAATCCAAGACAGTGTTATGAAATAGATGGTGTATATCAAACACCCTATAATGTGAATAAAAGCTATTGXXXXXXXXXXT
TTTATTTCCTAACACCTAACTCTTCTAATCAATACACAGTTTATGTAATAATAAGGTTGATCATCATAAATAAAACGTT
>Marker536779 Chr3 SNP_site:11920215 Ref_Type:C
CACAATTAATATAATCCAAGACAGTGTTATGAAATAGATGGTGTATATCAAACACCCTATAATGTGAATAAAAGCTAATGXXXXXXXXXXT
TTTATTTCCTAACACCTAACTCTTCTAATCAATACAGAGTTTATGTAATAATAAGGTTGGTCATCATAAATAAAACGTT
CACAATTAATATAATCCAAGACAGTGTTATGAAATAGATGGTGTATATCAAACACCCTATAATGTGAATAAAAGCTATTGXXXXXXXXXXT
TTTATTTCCTAACACCTAACTCTTCTAATCAATACACAGTTTATGTAATAATAAGGTTGATCATCATAAATAAAACGTT
>Marker536779 Chr3 SNP_site:11920238 Ref_Type:A
CACAATTAATATAATCCAAGACAGTGTTATGAAATAGATGGTGTATATCAAACACCCTATAATGTGAATAAAAGCTAATGXXXXXXXXXXT
TTTATTTCCTAACACCTAACTCTTCTAATCAATACAGAGTTTATGTAATAATAAGGTTGGTCATCATAAATAAAACGTT

Create PDF with GO2PDF for free, if you wish to

CACAATTAATATAATCCAAGACAGTGTTATGAAATAGATGGTGTATATCAAACACCCTATAATGTGAATAAAAGCTATTGXXXXXXXXXXT
TTTATTTCCTAACACCTAACTCTTCTAATCAATACACAGTTTATGTAATAATAAGGTTGATCATCATAAATAAAACGTT
>Marker536810 Chr3 SNP_site:31242262 Ref_Type:A
ACTCACTTTAGTGAGTTTCTCAAGAGCTCTCTAAAAATTACTAAATCAAATAACTATTTTAGACGTATGTGATATATTATXXXXXXXXXXT
TTTCATTCACTTACTTTGGTGTGTTAATTTTAGATAATGACAACATGGAAAAGAATAGGGAAAAATTACAAGATATTGT
ACTCACTTTAGTGAGTTTCTCAAGAGCTCTCTAAGAATTACTAAATCAAATAACTATTTTAGACGTATGTGATATATTATXXXXXXXXXXT
TTTCATTCACTTACTTTGGTGTGTTAATTTTAGATAATGACAACATGGAAAAGAATAGGGAAAAATTACAAGATATTGT
>Marker536941 Chr3 SNP_site:8135164 Ref_Type:A
GACTAATACTAATATTAGTTGGTGAAAAGGTTTTATGACATGATAGTTTGCAGGCAAAATTTTCTAGCAACGATGTGAGCXXXXXXXXXXT
AAAAAAAATTAAATGTGTGAAAAGAAAGGAATGCATGAGAATAGATGGCATTAAGAAGTAAAACATAAACACAAAAGTC
GACTAATACTAATATTAGTTGGTGAAAAGGTTTTATGACATGATAGTTTGCAGGCAAAATTTTCTAGCAACGATGTGAGCXXXXXXXXXXT
AAAAAAAATTCAATGTGTGAAAAGAAATGAATGCATGAGAATAGATGGCATTAAGAAGTAAAACATAAACACAAAAGTC
>Marker536941 Chr3 SNP_site:8135181 Ref_Type:G
GACTAATACTAATATTAGTTGGTGAAAAGGTTTTATGACATGATAGTTTGCAGGCAAAATTTTCTAGCAACGATGTGAGCXXXXXXXXXXT
AAAAAAAATTAAATGTGTGAAAAGAAAGGAATGCATGAGAATAGATGGCATTAAGAAGTAAAACATAAACACAAAAGTC
GACTAATACTAATATTAGTTGGTGAAAAGGTTTTATGACATGATAGTTTGCAGGCAAAATTTTCTAGCAACGATGTGAGCXXXXXXXXXXT
AAAAAAAATTCAATGTGTGAAAAGAAATGAATGCATGAGAATAGATGGCATTAAGAAGTAAAACATAAACACAAAAGTC
>Marker537256 Chr3 SNP_site:4734830 Ref_Type:G
AACCTTTCCGATATTTCCTTCTCCGGCGCCCCCAGAAGAGGCCACCGGATTTCCCCCGACGTCTCCCTCATTGCCCTCCCXXXXXXXXXXA
TATACACACAATTTTCTTTTGGGTTACGGGAACACTTGGCGCGTATGGCCCACCATTGTCCATAAGGATGTTGGGTGTT
AACCTTTCCGATATTTCCTTCTCCGGCGCCCCCAGAAGAGGCCACCGGATTTCCCCCGACGTCTCCCTCATTGCCCTCCCXXXXXXXXXXA
TATACACACAATTTTCTTTTGGGTTACGGGAACACTTGGCGCGTATGGCCCACCATTGTCCATGAGGATGTTGGGTGTT
>Marker537365 Chr3 SNP_site:35374039 Ref_Type:G
TACATATATGCAAAAGCTTGAAAGAAATGATTCGATTGTAGAGGGATGGTTGGATTATTGGTGAAAGATATGGATGGAAAXXXXXXXXXXT
GCTCAATTTTGGTGTTTCTTTTCATGCCACGTGGAAATTGAGTATAGTGGATATAATTCATGGACGGCTGTTAAATGTT
TACATATATGCAAAAGCTTGAAAGAAATGATTCGGTTGTAGAGGGATGGTTGGATTATTGGTGAAAGATATGGATGGAAAXXXXXXXXXXT
GCTCAATTTTGGTGTTTCTTTTCATGCCACGTGGAAATTGAGTATAGTGGATATAATTTATGGACGGCTGTGAAATGTT
>Marker537365 Chr3 SNP_site:35374275 Ref_Type:T
TACATATATGCAAAAGCTTGAAAGAAATGATTCGATTGTAGAGGGATGGTTGGATTATTGGTGAAAGATATGGATGGAAAXXXXXXXXXXT
GCTCAATTTTGGTGTTTCTTTTCATGCCACGTGGAAATTGAGTATAGTGGATATAATTCATGGACGGCTGTTAAATGTT
TACATATATGCAAAAGCTTGAAAGAAATGATTCGGTTGTAGAGGGATGGTTGGATTATTGGTGAAAGATATGGATGGAAAXXXXXXXXXXT
GCTCAATTTTGGTGTTTCTTTTCATGCCACGTGGAAATTGAGTATAGTGGATATAATTTATGGACGGCTGTGAAATGTT
>Marker537365 Chr3 SNP_site:35374288 Ref_Type:G
TACATATATGCAAAAGCTTGAAAGAAATGATTCGATTGTAGAGGGATGGTTGGATTATTGGTGAAAGATATGGATGGAAAXXXXXXXXXXT
GCTCAATTTTGGTGTTTCTTTTCATGCCACGTGGAAATTGAGTATAGTGGATATAATTCATGGACGGCTGTTAAATGTT
TACATATATGCAAAAGCTTGAAAGAAATGATTCGGTTGTAGAGGGATGGTTGGATTATTGGTGAAAGATATGGATGGAAAXXXXXXXXXXT
GCTCAATTTTGGTGTTTCTTTTCATGCCACGTGGAAATTGAGTATAGTGGATATAATTTATGGACGGCTGTGAAATGTT
>Marker538060 Chr3 SNP_site:30688846 Ref_Type:A
ACCAAGTTGAATTATAGGGACAAAACCTGGAAAAAATATTAAGAAAAAATGAATTGATTTTTTTTCAAAAAGAAAAAGAAXXXXXXXXXXT
TAAATTCAAATAGTCGGAAATGCAACCAGTAAAAGAATTACACGTGGAACAAACAATAATGTCGTTGAAGAAAAAGGTA
ACCCAGTTGAATTATAGGGACAAAACCTGGAAAAAATATTAAGAAAAAATGAATTGATTTTTTTTCAAAAAGAAAAAGAAXXXXXXXXXXT
TAAATTCAAATAGTCGGAAATGCAACCAGTAAAAGAATTACACGTGGAACAAACAATAATGTCGTTGAAGAAAAAGGTA
>Marker538133 Chr3 SNP_site:34537706 Ref_Type:G
ACCTACAAAAGTTCATTCATAAAAAGTATCGAATCATATTTTAAATTTTTAGAAATTATATCAATTTAAACCTTACAAAGXXXXXXXXXXT

Create PDF with GO2PDF for free, if you wish to

GAATCAATTTAAACTCTTAAAAATATTTTTTTAGAATTGTTAAATAAACTATTTTATTAGTCTGATAACAAAATAAGTT

ACCTACAAAAGTTCATTCATAAAAAGTATCGAATCGTATTTTAAATTTTTAGAAATTATATCAATTTAAACCTTACAAAGXXXXXXXXXXT

GAATCAATTTAAACTCTTAAAAATATTTTTTTAGAATTGTTAAATAAACTATTTTATTAGTCTGATAACAAAATAAGTT

>Marker538988 Chr3 SNP_site:34680371 Ref_Type:G

ACTTTTTTATCTATTTCTTTTTCTATGGTGCTTAATGGAAGTTCTTGTCTCATATTATGTTACAAACGAATTCTATGCTTXXXXXXXXXXA

TAAGTTCTTGAAACATACAAATTTTGATTTAAATGGGTTTCTCTTCCTAGTTCATGGTATTATGAAAATACAATGATGT

ACTTTTTTGTCTATTTCTTTTTCTATGGTGCTTAATGGAAGTTCTTGTCTCATATTATGTTACAAACGAATTCTATGCTTXXXXXXXXXXA

TAAGTTCTTGAAACATACAAATTTTGATTTAAATGGGTTTCTCTTCCTAGTTCATGGTATTATGAAAATACAATGATGT

>Marker539333 Chr3 SNP_site:3397458 Ref_Type:G

ACCAACAAAACAACTTTGGGCCTATCCAAAGAAATGTTGTGTTCTGCAAATATTAAATCAAAGATCAGATATCAATTTACXXXXXXXXXXC

CAAACTGAGGCATGTATTTCTATGAAGGGTTATTATTTTGGACGAGCTTACTTCAACAACGACTGTTCTATACAGTTGT

ACCAACAAAACAACTTTGGGCCTATCCAAAGAAATGTTGTGTTCTGCAAATATTAAATCAAAGATCAGATATCAATTTACXXXXXXXXXXC

CAAACTGAGGCATGTATTTCTATGAAGGGTTATTATTTTGGACGAGCTTACTTGAACAACGACTGTTCTATACAGTTGT

>Marker539358 Chr3 SNP_site:30514183 Ref_Type:A

TACTTTATATCTGAACAGATTTTTTTTAGAGTAAATAAAATGTTCCAATATTGGCAGCACAAATAAAAATCATTAAGAATXXXXXXXXXXT

CCATTCCGTGACTGCATGCACCGAGCATATAAACTTACAAAGGACTTCGTGGGCTGTTAGTCTTTTCTTGGGATCTCGT

TACTTTATATCTGAACAGATTTTTTTTAGAGTAAATAAAATGTTCCAATATTGGCAGCACAAATAAAAATCATTAAGAATXXXXXXXXXXT

CCGTTCCGTGACTGCATGCACCGAGCATATAAACTTACAAAGGACTTCGTGGGCTGTTAGTCTTTTCTTGGGATCTCGT

>Marker53941 Chr4 SNP_site:10783518 Ref_Type:A

CACACATAATGTTCGGCTCAAACTTAATTTCCTGCAATAAAGCAAGTCTTAAGAATATCTACAAGTATCATTAACAAGGCXXXXXXXXXXT

CAACCAACTAACAGTGCTACAAAGGCATTTAATCATACTGACCATAAGAACATCAAAAGCTGATTCTTAAAGTTAAAGT

CACACATAATGTTCGGCTCAAACTTAATTTCCTGCAATAAAGCAAGTCTTAGGAATATCTACAAGTATCATTAACAAGGCXXXXXXXXXXT

CAACCAACTAACAGTGCTACAAAGGCATTTAATCATACTGACCATAAGAACATCAAAACCTGATTCTTTAAGTTAAAGT

>Marker53941 Chr4 SNP_site:10783754 Ref_Type:G

CACACATAATGTTCGGCTCAAACTTAATTTCCTGCAATAAAGCAAGTCTTAAGAATATCTACAAGTATCATTAACAAGGCXXXXXXXXXXT

CAACCAACTAACAGTGCTACAAAGGCATTTAATCATACTGACCATAAGAACATCAAAAGCTGATTCTTAAAGTTAAAGT

CACACATAATGTTCGGCTCAAACTTAATTTCCTGCAATAAAGCAAGTCTTAGGAATATCTACAAGTATCATTAACAAGGCXXXXXXXXXXT

CAACCAACTAACAGTGCTACAAAGGCATTTAATCATACTGACCATAAGAACATCAAAACCTGATTCTTTAAGTTAAAGT

>Marker53941 Chr4 SNP_site:10783764 Ref_Type:A

CACACATAATGTTCGGCTCAAACTTAATTTCCTGCAATAAAGCAAGTCTTAAGAATATCTACAAGTATCATTAACAAGGCXXXXXXXXXXT

CAACCAACTAACAGTGCTACAAAGGCATTTAATCATACTGACCATAAGAACATCAAAAGCTGATTCTTAAAGTTAAAGT

CACACATAATGTTCGGCTCAAACTTAATTTCCTGCAATAAAGCAAGTCTTAGGAATATCTACAAGTATCATTAACAAGGCXXXXXXXXXXT

CAACCAACTAACAGTGCTACAAAGGCATTTAATCATACTGACCATAAGAACATCAAAACCTGATTCTTTAAGTTAAAGT

>Marker539519 Chr3 SNP_site:12865545 Ref_Type:G

TACATCACAATCCAAATAATGAAAACAACACATTAAAAATGTATCACTTTATTTTATGGAATGTGAGAAATCTGAAAATGXXXXXXXXXXG

AAGGATAACAAAACTAGAGTTATTCAATATTCGTCAAATTAATAGAACAACTAAAAATGTAATAATTTCCCGAACTGTA

TACATCACAATCCAAATAATGAAAACAACACATTAAAAATGTATCACTTTATTTTATTGAATGTGAGAAATCTGAAAATGXXXXXXXXXXG

AAGGATAACAAAACTAGAGTTATTCAATATTCGTCAAATTAATAGAACAACTAAAAATGTAATAATTTCCCGAACTGTA

>Marker539528 Chr3 SNP_site:25243876 Ref_Type:A

ACTTTTATAATGGTAGAGGGATGGGTTGGTTGTCAATGTCAATGTAAATACAAAGTGAAGGCACAGTAAGCAATGATTGGXXXXXXXXXXT

CTCTTTTCTTTTAGCAGGGAAGAAGCAAACATAAATAAAATGATGATGACATATGAGCATGTTAGTAATATTGTTGGTT

ACTTTTATAATGGTAGAGGGATGGGTTGGTTGTCAATGTCAATGTAAATACAAAGTGAAGGCACAGTAAGCAATGATTGGXXXXXXXXXXT

CTCTTTTCTTTTAGCAGGGAAGAAGCAAACATAAATAAAATGATGGTGACATATGAGCATGTTAGTAATATTGTTGGTT

>Marker539563 Chr3 SNP_site:13410014 Ref_Type:T

Create PDF with GO2PDF for free, if you wish to

GACCATTGTCAATCACTTTTTGTTACCGTTTTTCAAGGAGCATTGCGGCCAACTACAAACAACTGTTTTTTGTGACTACCXXXXXXXXXXT
TCTTTTATTATAATTTGAAATTATAAAACATTTTTAGCATATAACAATTCAAACAATTTTTTTTACAAAAAAGTTGGTA
GACCATTGTCAATCACTTTTTGTTACCGTTTTTCAATGAGCATTGCGGCCAACTACAAACAACTGTTTTTTGTGACTACCXXXXXXXXXXT
TCTTTTATTATAATTTGAAATTATAAAACATTTTTAGCATATAACAATTCAAACAATTTTTTTTACAAAAAAGTTGGTA
>Marker53990 Chr4 SNP_site:13545104 Ref_Type:A
TACCCATTTAACAAAATGCCAATGATTTAACCTAATGATAATAAACACAAAATACTAATATATTGAGAACATTTTAACTCXXXXXXXXXXG
TATCATATTATTTAAAATATGTAATGTATAAAATTCAAATCCACTAATTGGCTTAAAAGGGAATATGCTCTGTTTGGTA
TACCCATTTAACAAAATGCCAATGATTTAGCCTAATGATAATAAACACAAAATACTAATATATCGAGAACATTTTAACTCXXXXXXXXXXG
TATCGTATTATTTAAAATATGTAATGTATAAAATTCAAATCCACTAATTGGCTTAAAAGGGAATATGCTCTGTTTGGTA
>Marker53990 Chr4 SNP_site:13545138 Ref_Type:T
TACCCATTTAACAAAATGCCAATGATTTAACCTAATGATAATAAACACAAAATACTAATATATTGAGAACATTTTAACTCXXXXXXXXXXG
TATCATATTATTTAAAATATGTAATGTATAAAATTCAAATCCACTAATTGGCTTAAAAGGGAATATGCTCTGTTTGGTA
TACCCATTTAACAAAATGCCAATGATTTAGCCTAATGATAATAAACACAAAATACTAATATATCGAGAACATTTTAACTCXXXXXXXXXXG
TATCGTATTATTTAAAATATGTAATGTATAAAATTCAAATCCACTAATTGGCTTAAAAGGGAATATGCTCTGTTTGGTA
>Marker53990 Chr4 SNP_site:13545279 Ref_Type:A
TACCCATTTAACAAAATGCCAATGATTTAACCTAATGATAATAAACACAAAATACTAATATATTGAGAACATTTTAACTCXXXXXXXXXXG
TATCATATTATTTAAAATATGTAATGTATAAAATTCAAATCCACTAATTGGCTTAAAAGGGAATATGCTCTGTTTGGTA
TACCCATTTAACAAAATGCCAATGATTTAGCCTAATGATAATAAACACAAAATACTAATATATCGAGAACATTTTAACTCXXXXXXXXXXG
TATCGTATTATTTAAAATATGTAATGTATAAAATTCAAATCCACTAATTGGCTTAAAAGGGAATATGCTCTGTTTGGTA
>Marker539995 Chr3 SNP_site:32182993 Ref_Type:C
CACAACCTTATTTAAATATTGAATGCTAAGAAAATTGAAATTAGAGACAAGATATTTAATTTAAGCTTGGGAAGCTATCTXXXXXXXXXXG
TTTCTATTTAATTAATCATTAGTCTTAGGAAGTGTCAATTACAAATTTAAATTTATAATTATCCATCCATTAACGAGTT
CACAACGTTATTTAAATATTGAATGCTAAGAAAATTGAAATTAGAGACAAGATATTTAATTTAAGCTTGGGAAGCTATCTXXXXXXXXXXG
TTTCTATTTAATTAATCATTAGTCTTAGGAAGTGTCAATTACAAATTTAAATTTATAATTATCCATCCATTAACGAGTT
>Marker540057 Chr3 SNP_site:31873992 Ref_Type:A
GACACAAAGAATGAGTAGATAAAACTAAGTCACTTTTAAAACTTTGAATAACAAATTTAACTCGAAGATGTATCTATATTXXXXXXXXXXA
TTATTTTGTTCAAATGCCAAATGGTTACAAATAGTGTTAAATATTTTAAACATTTCTAAAAACATTATTCATGATTGTT
GACACAAAGAATGAGTAGATAAAACTAAGTCACTTTTAAAACTTTGGATAACAAATTTAACTCGAAGATGTATCTATATTXXXXXXXXXXA
TTATTTTGTTCAAATGCCAAATGGTTACAAATAGTGTTAAATATTTTAAACATTTCTAAAAACATTATTCATGATTGTT
>Marker540256 Chr3 SNP_site:17350876 Ref_Type:A
AACTTGCTTTATGACGTATTGCAATTGTATTACACGCATACAAAAGTCTAAGTTCTCCAATATAAACACCCATCATTCCAXXXXXXXXXXXC
TTACTAAACATTCCACATCATACACCCTGAATTTTTTTCTCCATCTCCAATATCCAATCCCCTCCTTCTTCTCGTTGTT
AACTTGCTTTGTGACGTATTGCAATTGTATTACACGCATACAAAAGTCTAAGTTCTCCAATATAAACACCCATCATTCCAXXXXXXXXXXXC
TTACTAAACATTCCACATCATACACCCTGAATTTTTTTCTCCATCTCCAATATCCAATCCCCTCCTCCTTCTCGTTGTT
>Marker540256 Chr3 SNP_site:17351125 Ref_Type:T
AACTTGCTTTATGACGTATTGCAATTGTATTACACGCATACAAAAGTCTAAGTTCTCCAATATAAACACCCATCATTCCAXXXXXXXXXXXC
TTACTAAACATTCCACATCATACACCCTGAATTTTTTTCTCCATCTCCAATATCCAATCCCCTCCTTCTTCTCGTTGTT
AACTTGCTTTGTGACGTATTGCAATTGTATTACACGCATACAAAAGTCTAAGTTCTCCAATATAAACACCCATCATTCCAXXXXXXXXXXXC
TTACTAAACATTCCACATCATACACCCTGAATTTTTTTCTCCATCTCCAATATCCAATCCCCTCCTCCTTCTCGTTGTT
>Marker540786 Chr3 SNP_site:5446167 Ref_Type:G
ACTAAACTCAAAGAAGACGTTTTCATTTTAGGGTTTCTATGAATTCTTTAGAGTTTGCATTAACCTTCTAGGTTTAAGTGXXXXXXXXXXA
AAAAATGTTTAATTAACATCAAACTAAGAATTTTCCAAGCAATCTTGCTTCAAGAATCATGCTTCCCATCATTAAAGTA
ACTAAACTCAAAGAAGACGTTTTCATTTTAGGGTTTCTATGAATTCTTTAGAGTTTGCATTAACCTTCTAGGTTTAAGTGXXXXXXXXXXA
AAAAATGTTTAATTAACATCAAACTAAGAATTTTCCAATCAATCTTGCTTCAAGAATCATGCTTAGCATCATTAAAGTA

Create PDF with GO2PDF for free, if you wish to

>Marker540786 Chr3 SNP_site:5446193 Ref_Type:C
ACTAAACTCAAAGAAGACGTTTTCATTTTAGGGTTTCTATGAATTCTTTAGAGTTTGCATTAACCTTCTAGGTTTAAGTGXXXXXXXXXXA
AAAAATGTTTAATTAACATCAAACTAAGAATTTTCCAAGCAATCTTGCTTCAAGAATCATGCTTCCCATCATTAAAGTA
ACTAAACTCAAAGAAGACGTTTTCATTTTAGGGTTTCTATGAATTCTTTAGAGTTTGCATTAACCTTCTAGGTTTAAGTGXXXXXXXXXXA
AAAAATGTTTAATTAACATCAAACTAAGAATTTTCCAATCAATCTTGCTTCAAGAATCATGCTTAGCATCATTAAAGTA
>Marker540786 Chr3 SNP_site:5446194 Ref_Type:C
ACTAAACTCAAAGAAGACGTTTTCATTTTAGGGTTTCTATGAATTCTTTAGAGTTTGCATTAACCTTCTAGGTTTAAGTGXXXXXXXXXXA
AAAAATGTTTAATTAACATCAAACTAAGAATTTTCCAAGCAATCTTGCTTCAAGAATCATGCTTCCCATCATTAAAGTA
ACTAAACTCAAAGAAGACGTTTTCATTTTAGGGTTTCTATGAATTCTTTAGAGTTTGCATTAACCTTCTAGGTTTAAGTGXXXXXXXXXXA
AAAAATGTTTAATTAACATCAAACTAAGAATTTTCCAATCAATCTTGCTTCAAGAATCATGCTTAGCATCATTAAAGTA
>Marker540931 Chr3 SNP_site:39009145 Ref_Type:G
ACATTGAGTGGTGTGGATAATTGGTAGTTCATGCCATAAATCATGAGTGTTCATCCTTCAGTCTATTCAAAATGTAGGCGXXXXXXXXXXA
AATCGTAATAAGCAAAAGACTTGGTGATAACAACCATAATAAAATATTACTTAAACTTGACATTAATTCTATTTCACGT
ACATTGAGTGGTGTGGATGATTGGTAGTTCATGCCATAAATCATGAGTGTTCATCCTTCAGTCTATTCAAAATGTAGGCGXXXXXXXXXXA
AATCGTAATAAGCAAAAGACTTGGTGATAACAACCATAATAAAATATTACTTAAACTTGACATTAATTCTATTTCACGT
>Marker54098 Chr4 SNP_site:16333611 Ref_Type:A
ACATGGATTTGAGATTTCAGAACTATATGATAGAACATGAAATCTAATTCCAACTCTCGGCTCCTGATGCACCTCAACAAXXXXXXXXXXA
GAGACTGCAATTCATATCTTGAACAATGTTCCCTCGAAAAGTGTTTCTGAAACACCTTTTGAGTTATGGAGATGACGTA
ACATGGATTTGAGATTTCAGGACTATATGATAGAACATGAAATCTAATTCCAACTCTCGGCTCCTGATGCACCTCAACAAXXXXXXXXXXA
GAGACTGCAATTCATATCTTGAACAATGTTCCCTCGAAAAGTGTTTCTGAAACACCTTTTGAGTTATGGAGATGACGTA
>Marker541042 Chr3 SNP_site:25200108 Ref_Type:T
AACTAAGTGGCATCTAAGTTCATACCATAATATTTTTTTATAAGAAAGTTCGTGCCGTAACATAAGTTCGGAAAATGAAAXXXXXXXXXXA
AAGGTGTTGATCTAAAGATTTCTTTTGGAAGGAGGTATGGTCTCTTTTTGTCTTAAGAGATATTTGATACCGAAGTGTT
AACTAAGTGGCATCTAAGTTCATACCATAATATTTTTTTATAAGAAAGTTCGTGCCGTAACATAAGTTTGGAAAATGAAAXXXXXXXXXXA
AAGGTGTTGATCTAAAGATTTCTTTTGGAAGGAGGTATGGTCTCTTTTTGTCTTAAGAGATATTTGATACCAAAGTGTT
>Marker541042 Chr3 SNP_site:25200290 Ref_Type:A
AACTAAGTGGCATCTAAGTTCATACCATAATATTTTTTTATAAGAAAGTTCGTGCCGTAACATAAGTTCGGAAAATGAAAXXXXXXXXXXA
AAGGTGTTGATCTAAAGATTTCTTTTGGAAGGAGGTATGGTCTCTTTTTGTCTTAAGAGATATTTGATACCGAAGTGTT
AACTAAGTGGCATCTAAGTTCATACCATAATATTTTTTTATAAGAAAGTTCGTGCCGTAACATAAGTTTGGAAAATGAAAXXXXXXXXXXA
AAGGTGTTGATCTAAAGATTTCTTTTGGAAGGAGGTATGGTCTCTTTTTGTCTTAAGAGATATTTGATACCAAAGTGTT
>Marker541267 Chr3 SNP_site:29743823 Ref_Type:T
TACTTGGTTGGAAATATATAAAAAGTTAGTTACGGGATAGATGGATAGACCGCAGGATGATATGTTGAGATTGATTTACTXXXXXXXXXXT
CTATTTATCTTGGAAACCTTCAACGTTAGCCTCATTGTTTTCCCACATGGACTTGGGTTCCACATTTCAGCTTTCAGTG
TACTTGGTTGGAAATATATAAAAAGTTAGTTACGGGATAGATGGATAGACCGCAGGATGATATGTTGAGATTGATTTACTXXXXXXXXXXT
CTATTTATCTTGGAAACCTTCAACTTTAGCCTCATTGTTTTCCCACATGGACTTGGGTTCCACATTTCAGCTTTCAGTG
>Marker541489 Chr3 SNP_site:38741913 Ref_Type:T
ACTTCTAAAATTAGGTTACTAGACTTAGGTGATATCATTCTATGACATTGTATCAAGTAAATAGACATAGACGTGGTAGAXXXXXXXXXXG
AGAAGATTTTTTTCTAAAACCATTTCTAAAGTCAAACTAGACATAAAAACCAGTTTTGGACTATTAAGGGAATTTTGGT
ACTTCTAAAATTAGGTTACTAGACTTAGGTGATATCATTCTATGACATTGTATCAAGTAAATAGACATAGACGTGGTAGAXXXXXXXXXXG
AGAAGATTTTTTTCTAAAACCATTTCTAAAGTCAAACTTGACATAAAAACCAGTTTTGGACTATTAAGGGAATTTTGGT
>Marker541694 Chr3 SNP_site:29807712 Ref_Type:G
CACATATAATCCTGCTTTATTGGTTGATGACCCCCTGTTTATGTGGTTTGCAGTATAATCCCCCCTTTACTCTTCCATTTXXXXXXXXXXG
GTGTATAGCCACATATAATGAACTGTATATATGTCACTTTCACATTTAAATACAGCTCCATCACGTATTATTGATGGTG
CACATATAATCCTGCTTTATTGGTTGATGACCCCCTGTTTATGTGGTTTGCAGTATAATCCCCCCTTTACTCTTCCATTTXXXXXXXXXXG

Create PDF with GO2PDF for free, if you wish to

GTGTATAGCCACATATAATGAACTGTATATATGTCACTTTCACATTTAAATACAGCTCCATCAGGTATTATTGATGGTG
>Marker541750 Chr3 SNP_site:12491346 Ref_Type:C
AACCGTTGCTATCATTCTATCAAAGTTATATTGGTTTGAAATGAATCCATTTTAAAAAGATCAGGTTTGGTCCTAAAACTXXXXXXXXXXA
TCTTAGAAAATGAAGTAAAGATTTTGAATCACTTTATGAAGTGATATATCACAATCAAAGATGACATTGTTTGGTTGGT
AACCGTTGCTATCATTCTATCAAAGTTATATTGGTTTGAAATGAATCCATTTTAAAAAGATCAGGTTTGGTCCTAAAACTXXXXXXXXXXA
TCTTAGAAAATGAAGTAAAGATTTTGAATCACTTTATGAAGTGATATCTCACAATCAAAGATGACATTGTTTGGTTGGT
>Marker542398 Chr3 SNP_site:39350552 Ref_Type:A
ACTTTCAACTTTCCAGCCACCTAATGAGACAAATATGTGGAGCAACAGAAGGTGATTCTCAACTCATTTGTAATCTTTTTXXXXXXXXXXT
TCAATGATATGCAGTTACTCAAGTGTTGTATTTGTAGAAAAATTCCCTAAACACAAACTTCTTTTTTCCTTCAAAAGTT
ACTTTCAACTTTCCAGCCACCTAATGAGACAAATATGTGGAGCAACAGAAGGTGATTCTCAACTCATTTGTAATCTTTTTXXXXXXXXXXT
TCAATGATTTGCAGTTACTCAAGTGTTGTATTTGTAGAAAAATTCCCTAAACAAAAACTTCTTTTTTCCTTCAAAAGTT
>Marker542398 Chr3 SNP_site:39350597 Ref_Type:C
ACTTTCAACTTTCCAGCCACCTAATGAGACAAATATGTGGAGCAACAGAAGGTGATTCTCAACTCATTTGTAATCTTTTTXXXXXXXXXXT
TCAATGATATGCAGTTACTCAAGTGTTGTATTTGTAGAAAAATTCCCTAAACACAAACTTCTTTTTTCCTTCAAAAGTT
ACTTTCAACTTTCCAGCCACCTAATGAGACAAATATGTGGAGCAACAGAAGGTGATTCTCAACTCATTTGTAATCTTTTTXXXXXXXXXXT
TCAATGATTTGCAGTTACTCAAGTGTTGTATTTGTAGAAAAATTCCCTAAACAAAAACTTCTTTTTTCCTTCAAAAGTT
>Marker54247 Chr4 SNP_site:7320056 Ref_Type:G
AACAATCTTTAGTGATAATACAAAAACTTCTCTACGTCATACTTTATGCTGTTCTGAATATTGCACATCTTAACAAACTTXXXXXXXXXXA
AAATGAAGAAGGCTGGAAAACTCATAAAGACTACCTCAAATATCTTTTTTGCAATTTCAGGATAACATCTTGTGCTGTC
AACAATCTTTAGTGATAATACAAAAACTTCTCTACGTCATACTTTATGCTGTTCTGAATATTGCACATCTTAACAAACTTXXXXXXXXXXA
AAATGAAGAAGGCTGGAAAACTCATAAAGACTACCTCAAATATCTTTTTTGCAATTTCAGGATAACATCTTGTTCTGTC
>Marker542722 Chr3 SNP_site:18419359 Ref_Type:G
CACAACCTACCACTATTACAAAACTTTTCTTCGATTTTTTTAAATTGAAAGCTACAGGTAGTAAGGATATCTACTATCACXXXXXXXXXXT
AATTTTTCAATGATGATAAAATTTTATTAATGGACTGTTTTTAATTGAGTGTTTAAGATATCAATTAGAATGTTTGAGT
CACAACCTACCACTATTACAAAACTTTTCTTCGATTTTTTTAAATTGAAAGCTACAGGTAGTAAGGATATCTACTATCACXXXXXXXXXXT
AATTTTTCAATGATGATAAAATTTTATTAATGGACTGTTTTTAATTGAGTGTTTAAGATATCAATTAGAATGTTTGGGT
>Marker542894 Chr3 SNP_site:5549212 Ref_Type:T
TACTTGAATACAACATTTAAAGAGGTAAAAACTGGTCATCAAAGTTCAACATATGATTTCTTTATCCCACTTTCTTGCAAXXXXXXXXXXC
ACATCGGGTGCTTGTGCAAAGTTAGAGGAGGATGCATATTCAGCTATGAAATTCCCAGCAACCTGCAACAAGAATAGTT
TACTTGAATACAACATTTAAAGAGGTAAAAACTGGTCTTCAAAGTTCAACATATGATTTCTTTATCCCACTTTCTTGCAAXXXXXXXXXXC
ACATCGGGTGCTTGTGCAAAGTTAGAGGAGGATGCATATTCAGCTATGAAATTCCCAGCAACCTGCAACAAGAATAGTT
>Marker543451 Chr3 SNP_site:11821616 Ref_Type:C
GACATCCATGCATGTCATTACCAGCAAAGAAAGCTAACGACAGGGAAACAGTCTATTACGGCCACTTTTTTAACATAAACXXXXXXXXXXA
AAAATAAATAAATAAAAATTGGTATTGGTATTATCACATTAGTTCCACCCATGTCTTCTAATTGTTCATGGTATGGAGT
GACATCCATGCATGTCATTACCAGCAAAGAAAGCTAATGACAGGGAAACAGTCTATTACGGCCACTTTTTTAACATAAACXXXXXXXXXXA
AAAATAAATAAATAAAAATTGGTATTGGTATTATCACATTAGTTCCACCCATGTCTTCTAATTGTTCATGGTATGGAGT
>Marker543731 Chr3 SNP_site:9532587 Ref_Type:A
AACATTTCCCCCCACTCATCTCTATCACTTTAATTTCAGTGCATATTAAGTCTAAGTCTTCCTCAAAAGAGGCTCAAGTCXXXXXXXXXXC
TGTTATCAAAACATAATATAATCGTATAGGACAATTCATTTAATTTTTATTTATTCTGATATTGAAAAACATTATATGT
AACATTTCCCCCCACTCATCTCTATCACTTTAATTTCAGTGCATATTAAGTCTAAGTCTTCCTCAAAAGAGGCTCAAGTCXXXXXXXXXXC
TGTTATCAAAACATAATATAATCGTATAGGACAATTCATTTAATTTTTATTTATTCTGATATTGAAAATCATTATATGT
>Marker544133 Chr3 SNP_site:32780572 Ref_Type:G
TACAGTGATTGATTGATTGATTTGGAGTTTAATTAAATAAAGTTGGAGAGATAATGCACTTTTTCATGTTCATCTCCATAXXXXXXXXXXT
GAGTATAGTTCATATTTACTCAAAATTTCTAGGCAGCAAAATTTGAGATTAGAAATTTGATTCTTTTGTATGGATATGT

Create PDF with GO2PDF for free, if you wish to

TACAGTGATTGATTGATTGATTTGGAGTTTAATTAAATAAAGTTGGAGAGATAATGCACTTTTTCATGTTCATCTCCATAXXXXXXXXXXT
GAGTATAGTTCATATTTACTCAAAATTTCTATGCAGCAAAATTTGAGATTAGAAATTTGATTCTTTTGTATGCATATGT
>Marker544133 Chr3 SNP_site:32780613 Ref_Type:G
TACAGTGATTGATTGATTGATTTGGAGTTTAATTAAATAAAGTTGGAGAGATAATGCACTTTTTCATGTTCATCTCCATAXXXXXXXXXXT
GAGTATAGTTCATATTTACTCAAAATTTCTAGGCAGCAAAATTTGAGATTAGAAATTTGATTCTTTTGTATGGATATGT
TACAGTGATTGATTGATTGATTTGGAGTTTAATTAAATAAAGTTGGAGAGATAATGCACTTTTTCATGTTCATCTCCATAXXXXXXXXXXT
GAGTATAGTTCATATTTACTCAAAATTTCTATGCAGCAAAATTTGAGATTAGAAATTTGATTCTTTTGTATGCATATGT
>Marker545182 Chr3 SNP_site:33909129 Ref_Type:A
ACCTCAAAATGTTATTGCATTATATTATTGCGCCATCTTAGATTACATCATTTGCTTATTATAGGAAGTGATCAATAATTXXXXXXXXXXT
AGAAACAATTGAGGTTTAATATTAGTAATTTGGTGACTGTTATATAAATACTTACAAGCTAGTATATATTTCATTTAGT
ACCTCAAAATGTTATTGCATTATATTATTGCGCCATCTTAGATTACATCATTTGCTTATTATAGGAAGTGATCAATAATTXXXXXXXXXXT
AGAAACAATTGAGGTTTAATATTAGTAATTTGGTGACTGTTATATATATACTTACAAGCTAGTATATATTTCATTTAGT
>Marker545801 Chr3 SNP_site:32652008 Ref_Type:T
ACATATTTGATGCTCCCACTTCGTTTTAAACACATCCAATTGCTATCAAACGTAATCACTTCCTCCATTCTATTATATATXXXXXXXXXXA
AAACAAATTTTTGCTTGATTTTTTAAACATGTTTCTACATTGATGTAGGCAACAAATAAAAAATCATTAGAACGACGTA
ACATATTTGATGCTCCCACTTTGTTTTAAACACATCCAATTGCTATCAAACGTAATCACTTCCTCCATTCTATTATATATXXXXXXXXXXA
AAACAAATTTTTGCTTGATTTTTTAAACATGTTTCTACATTGATGTAGGCAACAAATAAAAAATCATTAGAACGACGTA
>Marker545827 Chr3 SNP_site:6715342 Ref_Type:T
AACAAGCCATTGGACATGGAGTTTCAAAAGAACATCCAATCATTTTTGCTTTCTTCTCTCTAAATATGTAGTTGGTTTCCXXXXXXXXXXA
CAATTTCATTCATATTCCCTCCAAATTTACTCTAAAGTTAAGTCTCGAGTAAGAATTTCGAATATTCGATCAATTGGTA
AACAAGCCATTGGACATGGAGTTTCAAAAGAACATCCAATCATTTTTGCTTTCTTCTCTCTAAATATGTAGTTGGTTTCCXXXXXXXXXXA
TAATTTCATTCATATTCCCTCCAAATTTACTCTAAAGTTAAGTCTCGAATAAGAATTTCGAATATTCGATCAATTGGTA
>Marker545827 Chr3 SNP_site:6715390 Ref_Type:A
AACAAGCCATTGGACATGGAGTTTCAAAAGAACATCCAATCATTTTTGCTTTCTTCTCTCTAAATATGTAGTTGGTTTCCXXXXXXXXXXA
CAATTTCATTCATATTCCCTCCAAATTTACTCTAAAGTTAAGTCTCGAGTAAGAATTTCGAATATTCGATCAATTGGTA
AACAAGCCATTGGACATGGAGTTTCAAAAGAACATCCAATCATTTTTGCTTTCTTCTCTCTAAATATGTAGTTGGTTTCCXXXXXXXXXXA
TAATTTCATTCATATTCCCTCCAAATTTACTCTAAAGTTAAGTCTCGAATAAGAATTTCGAATATTCGATCAATTGGTA
>Marker54630 Chr4 SNP_site:11394765 Ref_Type:A
AACCTTAAAATTTTCCTATTGATTTTGTTAATTTTAATCCATTAGTTTTCTACACCTAATCCTACCTGCTTGTAAAAAATXXXXXXXXXXT
ACCATTTAAGCTCAGACTTGACTAAGTTGTGTTACAATGGAAATGGGATTTGAAAAAATTAAAGGGAAAGTGATGAAGT
AACCTTAAAATTTTCCTATTGGTTTTGTTAATTTTAATCCATTAGTTTTCTACACCTAATCCTACCTGCTTGTAAAAAATXXXXXXXXXXT
ACCATTTAAGCTCAGACTTGACTAAGTTGTGTTACAATGGAAATGGGATTTGAAAAAATTAAAGGGAAAGTGATGAAGT
>Marker546457 Chr3 SNP_site:22018858 Ref_Type:A
AACTTCCTCTTCACGTCCTCAACAACACCACACCAATTTTTCTTCCCATTAACCTTTTGCAAAACCCTCTTCATTTCATTXXXXXXXXXXT
AATTTGTGCCTCCAAATAATTAGAAAGGATCAAAAGAAAATATTAGTAACATTACCCGTGTAAATCAATGCATAAAAGT
AACTTCCTCTTCACGTCCTCAACAACACCACACCAATTTTTCTTCCCATTAACCTTTTGCAAAACCCTCTTCATTTCATTXXXXXXXXXXT
AATTTGTGCCTCCAAATAATTAGAAAGGATCAAATGAAAATATTAGTAACGTTACCCGTGTAAATCAATGCATAAAAGT
>Marker546457 Chr3 SNP_site:22018874 Ref_Type:A
AACTTCCTCTTCACGTCCTCAACAACACCACACCAATTTTTCTTCCCATTAACCTTTTGCAAAACCCTCTTCATTTCATTXXXXXXXXXXT
AATTTGTGCCTCCAAATAATTAGAAAGGATCAAAAGAAAATATTAGTAACATTACCCGTGTAAATCAATGCATAAAAGT
AACTTCCTCTTCACGTCCTCAACAACACCACACCAATTTTTCTTCCCATTAACCTTTTGCAAAACCCTCTTCATTTCATTXXXXXXXXXXT
AATTTGTGCCTCCAAATAATTAGAAAGGATCAAATGAAAATATTAGTAACGTTACCCGTGTAAATCAATGCATAAAAGT
>Marker54662 Chr4 SNP_site:6285742 Ref_Type:T
GACCTATCATTTCTATTGATGGCAAGTTTTTGAAGTTTAAGTTTGGTAACCTCCTATTAACAGCCTAATCACAGGATGGTXXXXXXXXXXT

Create PDF with GO2PDF for free, if you wish to

TAGTCATTGTTTCTTATAGACATCTTAGCATCTCTAAAGGAGTTTTATGAGTGTTTCCTAATGTTGAGTATTGTGTGTG
GACCTATCATTTCTATTGATGGCAAGTTTTTGAAGTTTAAGTTTGGTAACCTCCTATTAATAGCCTAATCACAGGATGGTXXXXXXXXXXT
TAGTCATTGTTTCTTATAGACATTTTAGCATCTCTAAAGGAGTTTTATGAGTGTTTCCTAATGTTGAGTATTGTGTGTG
>Marker54662 Chr4 SNP_site:6285883 Ref_Type:T
GACCTATCATTTCTATTGATGGCAAGTTTTTGAAGTTTAAGTTTGGTAACCTCCTATTAACAGCCTAATCACAGGATGGTXXXXXXXXXXT
TAGTCATTGTTTCTTATAGACATCTTAGCATCTCTAAAGGAGTTTTATGAGTGTTTCCTAATGTTGAGTATTGTGTGTG
GACCTATCATTTCTATTGATGGCAAGTTTTTGAAGTTTAAGTTTGGTAACCTCCTATTAATAGCCTAATCACAGGATGGTXXXXXXXXXXT
TAGTCATTGTTTCTTATAGACATTTTAGCATCTCTAAAGGAGTTTTATGAGTGTTTCCTAATGTTGAGTATTGTGTGTG
>Marker546960 Chr3 SNP_site:16372792 Ref_Type:G
AACTCAAAGACATTTAAATTAATTCTGCTACATGTAAAATTATCAACTAGAAATAGACTTCATTAAATCGTTTCCTTGACXXXXXXXXXXA
CTCTAAGGATGAGATTATCGTAACCATATCCATTAAGCTTGTTAATTGATCTTTGTGCATCCTCACGATTCACAAAGTT
AACTCAAAGACATTTAAATTAATTCTGCTACATGTAAAATTATCAAGTAGAAATAGACTTCATTAAATCGTTTCCTTGACXXXXXXXXXXA
CTCTAAGGATGAGATTATCGTAACCATATCCATTAAGCTTGTTAATTGATCTTTGTGCATCCTCACGATTCACAAAGTT
>Marker547027 Chr3 SNP_site:11356340 Ref_Type:C
AACTATAAAATTATTGTGTTTGTAAAGCAAGGGAATAAAAGTTTTTTGATAAGGAGAAGTAGATAAACATAGTAAGATAAXXXXXXXXXXT
CAAACTTCAAGTTACAAGATTGAAAGTTTAATAATTTAAAACCTTTATTGCACTTACTATCAAGTTCTAAAATTTAAGT
AACTATAAAATTATTGTGTTTGTAACGCAAGGGAATAAAAGTTTTTTGATAAGGAGAAGTAGATAAACATAATAAGATAAXXXXXXXXXXT
CAAACTTCAAGTTACAAGATTGAAAGTTTAATAATTTAAAACCTTTATTGCACTTACTATCAAGTTCTAAAATTTAAGT
>Marker547027 Chr3 SNP_site:11356386 Ref_Type:A
AACTATAAAATTATTGTGTTTGTAAAGCAAGGGAATAAAAGTTTTTTGATAAGGAGAAGTAGATAAACATAGTAAGATAAXXXXXXXXXXT
CAAACTTCAAGTTACAAGATTGAAAGTTTAATAATTTAAAACCTTTATTGCACTTACTATCAAGTTCTAAAATTTAAGT
AACTATAAAATTATTGTGTTTGTAACGCAAGGGAATAAAAGTTTTTTGATAAGGAGAAGTAGATAAACATAATAAGATAAXXXXXXXXXXT
CAAACTTCAAGTTACAAGATTGAAAGTTTAATAATTTAAAACCTTTATTGCACTTACTATCAAGTTCTAAAATTTAAGT
>Marker547319 Chr3 SNP_site:31637823 Ref_Type:G
CACCGGACTCGGTATGGCCACGCCGAAGAAACCCAAAAGACTTTACCAAGTTTGGAGGGGAAGTAATGTGAGTTCTTAGCXXXXXXXXXXA
CTCTGTTTATGAGCTGAAAATGTTGATGGGATGTCTAAGTTGTATGTGAGAGTGTTTCTGCCTTCTATGATGTCATGTA
CACCGGACTCGGTATGGCCACGCCGAAGAAACCCAAAAGACTTTACCAAGTTTGGAGGGGAAGTAATGTGAGTTCTTAGCXXXXXXXXXXA
CTCTGTTTATGAGCTGAGAATGTTGATGGAATGTCTAAGTTGTATGTGAGAGTGTTTCTGCCTTCTATGATGTCATGTA
>Marker547319 Chr3 SNP_site:31637835 Ref_Type:A
CACCGGACTCGGTATGGCCACGCCGAAGAAACCCAAAAGACTTTACCAAGTTTGGAGGGGAAGTAATGTGAGTTCTTAGCXXXXXXXXXXA
CTCTGTTTATGAGCTGAAAATGTTGATGGGATGTCTAAGTTGTATGTGAGAGTGTTTCTGCCTTCTATGATGTCATGTA
CACCGGACTCGGTATGGCCACGCCGAAGAAACCCAAAAGACTTTACCAAGTTTGGAGGGGAAGTAATGTGAGTTCTTAGCXXXXXXXXXXA
CTCTGTTTATGAGCTGAGAATGTTGATGGAATGTCTAAGTTGTATGTGAGAGTGTTTCTGCCTTCTATGATGTCATGTA
>Marker547393 Chr3 SNP_site:32631458 Ref_Type:C
TACTTTTGTAACCACAAGTGTTGTAATTTATTCAAGAAAACAAATAGCTACAAACAACTTTTAGATTGATGCTATATTATXXXXXXXXXXT
ATATTTGGATATGCATCAGCTTGAACTTTACTCATATGTCTTTTTCATCTCTTTTCAATCATACAACACTAGTGTATGT
TACTTTTGTAATGACAAGTGTTGTAATTTATTCAAGAAAACAAATAGCTACAAACAACTTTTAGATTGATGCTATATTATXXXXXXXXXXT
ATATTTGGATATGCATCAACTTGAACTTTACTCATATGTCTTTTTCATCTCTTTTCAATCATACAACACTAGTGTATGT
>Marker547393 Chr3 SNP_site:32631459 Ref_Type:C
TACTTTTGTAACCACAAGTGTTGTAATTTATTCAAGAAAACAAATAGCTACAAACAACTTTTAGATTGATGCTATATTATXXXXXXXXXXT
ATATTTGGATATGCATCAGCTTGAACTTTACTCATATGTCTTTTTCATCTCTTTTCAATCATACAACACTAGTGTATGT
TACTTTTGTAATGACAAGTGTTGTAATTTATTCAAGAAAACAAATAGCTACAAACAACTTTTAGATTGATGCTATATTATXXXXXXXXXXT
ATATTTGGATATGCATCAACTTGAACTTTACTCATATGTCTTTTTCATCTCTTTTCAATCATACAACACTAGTGTATGT
>Marker547393 Chr3 SNP_site:32631670 Ref_Type:G

Create PDF with GO2PDF for free, if you wish to

TACTTTTGTAACCACAAGTGTTGTAATTTATTCAAGAAAACAAATAGCTACAAACAACTTTTAGATTGATGCTATATTATXXXXXXXXXXT
ATATTTGGATATGCATCAGCTTGAACTTTACTCATATGTCTTTTTCATCTCTTTTCAATCATACAACACTAGTGTATGT
TACTTTTGTAATGACAAGTGTTGTAATTTATTCAAGAAAACAAATAGCTACAAACAACTTTTAGATTGATGCTATATTATXXXXXXXXXXT
ATATTTGGATATGCATCAACTTGAACTTTACTCATATGTCTTTTTCATCTCTTTTCAATCATACAACACTAGTGTATGT
>Marker54753 Chr4 SNP_site:1156832 Ref_Type:T
ACCTCATAAAAACCCTAAAATTGAAAATCTTGAACTAACCTAACACAACTAATTAATTAAAAAAACAACTTAAAATTTATXXXXXXXXXXA
TATCTAAATGCTTTGGTGATCATTGAAAGGATTTGATTCGATGGCAACTTATTGTGTTAAGGACAATTTATTAAAAGTA
ACCTCATAAAAACCCTAAAATTGAAAATCTTGAACTAACCTAACACAACTAATTAATTAAAAAAACAACTTAAAATTTATXXXXXXXXXXA
TATCTAAATGCTTTGGTGATCATTGAAAGGATTTGATTCGATGGCAACTTATTGTGTTAATGACAATTTATTAAAAGTA
>Marker547611 Chr3 SNP_site:27264719 Ref_Type:T
TACCACATTCGGACTTGGAATATGCAAAGTATATCCATATGATATGGATAAGGATTGTAATATAAGCCTCCGATCAGTAGXXXXXXXXXXA
AGATATATTGGTTAGGAAGTTAATAAGTTAAGTAATATGCTATTAACTCTCAACAGGTTCTAAATAATGCACGGAAAGT
TACCACATTCGGACTTGGAATATGCAAAGTATATCCATATGATATGGATAAGGATTGTAATATAAGCCTCCGATCAGTAGXXXXXXXXXXA
AGATATATTGGTTAGGAATTTAATAAGTTAAGTAATATGCTATTAACTCTCAACAGGTTCTAAATAATGCACGGAAAGT
>Marker547729 Chr3 SNP_site:20472905 Ref_Type:A
TACTTTTTATGGTCCATCTTTTACGACTAAATTGCAATCAACGACTCTAAATTAGTCATTTCTTTTTAAAAACGACTACTXXXXXXXXXXA
ATGTATTGGACGGTGAAATTGAGACTTGGACACAACCACATCGTTTTAAGATTTTGCCACAATATTCTTATCGAGTTGT
TACTTTTTATGGTCCATCTTTTACGACTAAATTGCAATCAACGACTCTAAATTAGTCATTTCTTTTTAAAAACGGCTACTXXXXXXXXXXA
ATGTATTGGACGGTGAAATTGAGACTTGGACACAACCACATCGTTTTAAGATTTTGCCACAATATTCTTATCGAGTTGT
>Marker548207 Chr3 SNP_site:29781878 Ref_Type:C
AACATAGGATGACAATTACGATAATCGTATAGCAATGATAGATATATGAAGGTGAGTATGTGTCAGCCAAATGTTTGAGTXXXXXXXXXXT
ATTATCAATACGGTGATTCATATCTCATTAAATAAATGAATTAGACTAAATTAAATTGATCAAACATTGACTTTGAGTC
AACATAGGATGACAATTACGATAATCGTATAGCAATGATAGATATATGAAGGTGAGTATGTGTCAGCCAAATGTTTGAGTXXXXXXXXXXT
ATTATCAATACGGTGATTCATATTTCATTAAATAAATGAATTAGACTAAATTAAATTGATCAAACATTGACTTTGAGTC
>Marker54856 Chr4 SNP_site:21341573 Ref_Type:G
TACCAAATTAACCACTCAGCAGAAACATAAAAACCTTCACTGGCAAGTAAAAGTAGGATTTCGTGTCTATATGCAGCATGXXXXXXXXXXT
TCATTTCCCTAGTTTCCAGTTCCTTTTCCCATAATTGGCACCTGAAAAAAATAAGCAAAGTGATGCTTGAAAATAAGTA
TACCAAATTAACCACTCGGCAGAAACATAAAAACCTTCACTGGCAAGTAAAAGTAGGATTTCGTGTCTATATGCAGCATGXXXXXXXXXXT
TCATTTCCCTAGTTTCCAGTTCCTTTTCCCATAATTGGCACCTGAAAAAAATAAGCAAAGTGATGCTTGAAAATAAGTA
>Marker548945 Chr3 SNP_site:34499846 Ref_Type:T
TACTCTGTCTGGAGTAGTCTTTTCAAGTTTTTGTTTTGTTTGTCTCTAAATTAGTTAAAAACTTAAAATGCTATGCATTCXXXXXXXXXXT
TCTGCTTCAGATTAACACATTTAATGGATTTTACTGGGAGAACTTTGAACCAAACAAGGCTTATAAACCCAAAATTGTG
TACTCTTTCTGGAGTAGTCTTTTCAAGTTTTTGTTTTGTTTGTCTCTAAATTAGTTAAAAACTTAAAATGCTATCCATTCXXXXXXXXXXT
TCTGCTTCAGATTAACACATTTAATGGATTTTACTGGGAGAACTTTGAACCAAACAAGGCTTATAAACCCAAAATTGTG
>Marker548945 Chr3 SNP_site:34499914 Ref_Type:C
TACTCTGTCTGGAGTAGTCTTTTCAAGTTTTTGTTTTGTTTGTCTCTAAATTAGTTAAAAACTTAAAATGCTATGCATTCXXXXXXXXXXT
TCTGCTTCAGATTAACACATTTAATGGATTTTACTGGGAGAACTTTGAACCAAACAAGGCTTATAAACCCAAAATTGTG
TACTCTTTCTGGAGTAGTCTTTTCAAGTTTTTGTTTTGTTTGTCTCTAAATTAGTTAAAAACTTAAAATGCTATCCATTCXXXXXXXXXXT
TCTGCTTCAGATTAACACATTTAATGGATTTTACTGGGAGAACTTTGAACCAAACAAGGCTTATAAACCCAAAATTGTG
>Marker549515 Chr3 SNP_site:6917481 Ref_Type:G
AACAACTTTTTCGGAATATTATGACAATGACAAGCCTTGCTATGTTTCTTGTAGTTCTACCTTTTCTTTCAAGTCTGTGAXXXXXXXXXXC
AAATGATAGGTCGGGAAAATCCGATAAATTTTAGGCGTAAAGGATTAAAATCAAGAAACTAATGTGCCCAGTGAAAGTT
AACAACTTTTTCGGAATATTATGACAATGACAAGCCTTGCTATGTTTCTTGTAGTTCTACCTTTTCTTTCAAGTCTGTGAXXXXXXXXXXC
AAATGATAGGTCGGGAAAATCCGATAAATTTTAGGCGTAAAGGATTAAAATCAAGAAACTAATGTGCCCAGTGGAAGTT

Create PDF with GO2PDF for free, if you wish to

>Marker549828 Chr3 SNP_site:499805 Ref_Type:A
CACCACCTGGAAGATACCCCATTCCTCGCAAGCTTCAACAATGCTGTGGCGAATCATGGCCCTGCGTTCGCCGTTGTCGTXXXXXXXXXXA
GAAAAAGTCATTGTTGGTTAAGATTTTGTTAGTTTGAGGAGTGGGAAAATGAATATGATATTTATTAGTGTTTTTGGTA
CACCACCTGGAAGATACCCCATTCCTCGCAAGCTTCAACGATGCTGTGGCGAATCATGGCCCTGCGTTCGCCGTTGTCGTXXXXXXXXXXA
GAAAAAGTCATTGTTGTTTAAGATTTTGTTAGTTTGAGGAGTGGGAAAATGAATATGATATTTATTAGTGTTTTTGGTA
>Marker549828 Chr3 SNP_site:499981 Ref_Type:G
CACCACCTGGAAGATACCCCATTCCTCGCAAGCTTCAACAATGCTGTGGCGAATCATGGCCCTGCGTTCGCCGTTGTCGTXXXXXXXXXXA
GAAAAAGTCATTGTTGGTTAAGATTTTGTTAGTTTGAGGAGTGGGAAAATGAATATGATATTTATTAGTGTTTTTGGTA
CACCACCTGGAAGATACCCCATTCCTCGCAAGCTTCAACGATGCTGTGGCGAATCATGGCCCTGCGTTCGCCGTTGTCGTXXXXXXXXXXA
GAAAAAGTCATTGTTGTTTAAGATTTTGTTAGTTTGAGGAGTGGGAAAATGAATATGATATTTATTAGTGTTTTTGGTA
>Marker549995 Chr3 SNP_site:27951466 Ref_Type:A
GACCTTAAAACCAAAAAACCCAAATTCAGAATTTCGAAAAAAACTCATTTGGGTTTGTTTAAAAAATAAAAAAAATTAAAXXXXXXXXXXA
TCACAAACAGAGGCAGTAACTTTGAAGCCTTTTTGTTGCCATTCCTGAAGCCTTTGGTCAATCTCTGTTTGGTTACGTG
GACCTTAAAACCAAAAAACCCAAATTCAGCATTTCGAAAAAAACTCATTTGGGTTTGTTTAAAAAATAAAAAAAATTAAAXXXXXXXXXXA
TCACAAACAGAGGCAGTAACTTTGAAGCCTTTTTGTTGCCATTCCTGAAGCCTTTGGTCAATCTCTGTTTGGTTACGTG
>Marker550134 Chr3 SNP_site:37550828 Ref_Type:C
ACCATTTATCGTTTGATACAGTTCAAATATAGTCTAATAAAATACGATTACGTGATATTTTTAAGTTTTTGAAATTCTTTXXXXXXXXXXA
TTAGTCACCGATAAATTTGGAGTAGAAAACTTATGTAAATGAGTTTTACTACATCACCTCATTTGATGTTGGGGATGGT
ACCATTTATCGTTTGATACAGTTCAAATATAGTCTAATAAAATACGATTATGTGATATTTTTAAGTTTTTGAAATTCTTTXXXXXXXXXXA
TTAGTCACCGATAAATTTGGAGTAGAAAACTTATGTAAATGAGTTTTACTACATCACCTCATTTGATGTTGGGGATGGT
>Marker55043 Chr4 SNP_site:15752552 Ref_Type:T
AACAATTATTTCTACAAAAGGCCGGTAGTGAAAGTTGTAAGATCGAGTGTTGATCCACGCGGAATCTATGCAAAGTTTCTXXXXXXXXXXT
ATAGATAAAATAAGTTCTACAGTTTGAACCTTGTCACAACATATCAGAAAATGCGAGTGCGAGTGTGTAGCATTTGGGT
AACAATTATTTCTACAAAAGGTCGGTAGTGAAAGTTGTAAGATCGAGTGTTGATCCACGCGGAATCTATGCAAAGTTTCTXXXXXXXXXXT
ATAGATAAAATAAGTTCTACAGTTTGAACCTTGTCACAACATATCAGAAAATGCAAGTGCGAGTGTGTAGCATTTGGGT
>Marker55043 Chr4 SNP_site:15752784 Ref_Type:A
AACAATTATTTCTACAAAAGGCCGGTAGTGAAAGTTGTAAGATCGAGTGTTGATCCACGCGGAATCTATGCAAAGTTTCTXXXXXXXXXXT
ATAGATAAAATAAGTTCTACAGTTTGAACCTTGTCACAACATATCAGAAAATGCGAGTGCGAGTGTGTAGCATTTGGGT
AACAATTATTTCTACAAAAGGTCGGTAGTGAAAGTTGTAAGATCGAGTGTTGATCCACGCGGAATCTATGCAAAGTTTCTXXXXXXXXXXT
ATAGATAAAATAAGTTCTACAGTTTGAACCTTGTCACAACATATCAGAAAATGCAAGTGCGAGTGTGTAGCATTTGGGT
>Marker55063 Chr4 SNP_site:11199773 Ref_Type:C
AACCTAGTTGAGATATCCGAGTGTGCTCGTTGATCTTTCAACCTCTAGTTCGATCTCCTTTCAAAAAAGAAAATAAATCAXXXXXXXXXXT
TGGCTCAACGATAATTGATATGATCTTTCATTTTAAAGGTGGAAGGTTTGAATCCCTCAACCTCAAATGTTGTGTCGTA
AACCTAGTTGAGATATCCGAGTGTGCTCGTTGATCTTTCAACCTCTAGTTCGATCTCTTTTCAAAAAAGAAAATAAATCAXXXXXXXXXXT
TGGCTCAACGATAATTGACATGATCTTTCATTTTAAAGGTGGAAGGTTTGAATCCCTCAACCTCAAATGTTGTGTCGTA
>Marker55063 Chr4 SNP_site:11199947 Ref_Type:T
AACCTAGTTGAGATATCCGAGTGTGCTCGTTGATCTTTCAACCTCTAGTTCGATCTCCTTTCAAAAAAGAAAATAAATCAXXXXXXXXXXT
TGGCTCAACGATAATTGATATGATCTTTCATTTTAAAGGTGGAAGGTTTGAATCCCTCAACCTCAAATGTTGTGTCGTA
AACCTAGTTGAGATATCCGAGTGTGCTCGTTGATCTTTCAACCTCTAGTTCGATCTCTTTTCAAAAAAGAAAATAAATCAXXXXXXXXXXT
TGGCTCAACGATAATTGACATGATCTTTCATTTTAAAGGTGGAAGGTTTGAATCCCTCAACCTCAAATGTTGTGTCGTA
>Marker55242 Chr4 SNP_site:22330339 Ref_Type:C
TACCTGCAAAGAACAATCCAAGTGATTTTCTTTCAAGAGAAAATAAACTTTGAAAGCTCAATGGTAGCCAAACACTAAAAXXXXXXXXXXG
CAAAATCTCAATGTTGCAATTGATAGTAAATTTCAGGATTGCTGCCAATCGCAGTAAATCTTTCACCCGAAACTCAGTA
TACCTGCAAAGAACAATCCAAGTGATTTTCTTTCAAGAGAAAATAAACTTTGAAAGCTCAATGGTAGCCAAACACTAAAAXXXXXXXXXXG

Create PDF with GO2PDF for free, if you wish to

CAAAATCTCAATGTTGCAATTGATAGTAAATTTCAGGATTGCTGCCAATCGCAGTAAATCTTTCATCCGAAACTCAGTA

>Marker553007 Chr3 SNP_site:2109259 Ref_Type:C

TACTATTTTTTATTATAGTTCGTTTTACCTACTCATTAACTCAAAATAAACTATTATAACCAATTTAATATATGTTCGAGXXXXXXXXXXC

TAGTGCTCACATTTACGGATTAATTTTATCCTATTTCTTTATCTTCCTCCAATATTTCTAATGACTCTAACTGCTCGTT

TACTATTTTTTATTATAGTTCGTTTTACCTACTCATTAACTCAAAATAAACTATTATAACCAATTTAATATATGTTCGAGXXXXXXXXXXC

TAGTGCTCACATTTACGGATTAATTTTATCCTATTTCTTTATCTTCCTCCAATATTTCTAATGACTCTAATTGCTCGTT

>Marker553301 Chr3 SNP_site:13202306 Ref_Type:G

GACAACATGATTCCTCCTTTGCCAAGTAAATTCAGTGATGTATTCGATTGGTCAAAAGAACTACCCCTAAAAAGGGATATXXXXXXXXXXT

CATTAGACCAAGCACTAGTCCCTATTCCAGGTCGGTCCTATTAGTTAAAAAGAAGGATGGAAGGTGGAGATTTTGCGTG

GACAACATGATTCCTCCTTTGCCAAGTAAATTCAGTGATGTATTCGATTGGTCAGAAGAACTACCCCTAAAAAGGGATATXXXXXXXXXXT

CATTAGACCAAGCACTAGTCCCTATTCCAGGTCGGTCCTATTAGTTAAAAAGAAGGATGGAAGGTGGAGATTTTGCGTG

>Marker553857 Chr3 SNP_site:8127181 Ref_Type:C

AACAACCCATTTCTTCACAGAACTACTCTGATCGTTAATCCAGTGCAATGCTATAAAAAATTTAGAAAAAAAAATTGCAGXXXXXXXXXXC

CCAAGTTCTCTTGAGAGGCAAACTTACAAAAGTGGCTTATGCCCTTTCTTATTCTCTGTGCCAAAGAGGCATTCAGGTC

AACAACTCATTTCTTCACAGAACTACTCTGATCGTTAATCCAGTGCAATGCTATAAAAAATTTAGAAAAAAAAACTGCAGXXXXXXXXXXC

CCAAGTTCTCTTGAGAGGCAAACTTACAAAAGTGGCTTATGCCCTTTCTTATTCTCTGTGCCAAAGAGGCATTCAGGTC

>Marker553857 Chr3 SNP_site:8127249 Ref_Type:T

AACAACCCATTTCTTCACAGAACTACTCTGATCGTTAATCCAGTGCAATGCTATAAAAAATTTAGAAAAAAAAATTGCAGXXXXXXXXXXC

CCAAGTTCTCTTGAGAGGCAAACTTACAAAAGTGGCTTATGCCCTTTCTTATTCTCTGTGCCAAAGAGGCATTCAGGTC

AACAACTCATTTCTTCACAGAACTACTCTGATCGTTAATCCAGTGCAATGCTATAAAAAATTTAGAAAAAAAAACTGCAGXXXXXXXXXXC

CCAAGTTCTCTTGAGAGGCAAACTTACAAAAGTGGCTTATGCCCTTTCTTATTCTCTGTGCCAAAGAGGCATTCAGGTC

>Marker553874 Chr3 SNP_site:38769846 Ref_Type:A

AACTTTGACAGTGGAGTAGATGACCTTCATAATCCAAGTCAATATTTAGTTTCAAATGTTAATATGAAAAGCCACATTTTXXXXXXXXXXA

TGCCACATGTATCTTAAGGAAAAATTGATACTGAACAGGATGCATGAATGCAATGTTTCTTTTTATTATTACTATTTGT

AACTTTGACAGTGGAGTAGATGACCTTCATAATCCAAGTCAATATTTAGTTTCAAATGTTAATGTGAAAAGCCACATTTTXXXXXXXXXXA

TGCCACATGTATCTTAAGGAAAAATTGATACTGAACAGGATGCATGAATGCAATGTTTCTTTTTATTATTACTATTTGT

>Marker553910 Chr3 SNP_site:34126238 Ref_Type:A

ACAAGAGAATTATAAAAATAGCTAAATCTCTAAGGAAAAAGAATCCAGAATCCAAAAATGCGGGCAATAAATGAAGATCTXXXXXXXXXXT

ATTAGGTTAAATCTCCGTCACTACTATTTAATTGTTTTTTTTTAAAAGAGACAAACTTCTTTATTTAGAACTCAAAAGT

ACAAGAGAATTATAAAAATAGCTAAATCTCTAAGGAAAAAGAATCCAGAATCCAAAAATGCGGGCAATAAATGAAGATCTXXXXXXXXXXT

ATTAGGTTAAATCTCCGTCACTACTATTTAATTGTTTTTTTTTAAAGGAGACAAACTTCTTTATTTAGAACTCAAAAGT

>Marker553914 Chr3 SNP_site:5878934 Ref_Type:A

AACCAATGAAAAACCCTACCACGAAAACCTTGACAACTGGCCCATGCACAAGGTATTACTGATTCTAAAAACAATAATTTXXXXXXXXXXT

CAAATGACTCGGATAAACCAAGGAGTTACGATCCCTCTCTTGACATACCCATTGCCTTGAGAAAGGGCACGAGGTTGTG

AACCAATGAAAAACCCTACCACGAAAACCTTGACAACTGGCCCATGCACAAGGTATTACTGATTCTAAAAACAATAATTTXXXXXXXXXXT

CAAATGACTCGGATAAACCAAGGAGTTACGATCCCTCTCTTGACATACCCATTGCCTTGAGAAAGGGCGCGAGGTTGTG

>Marker554070 Chr3 SNP_site:31349216 Ref_Type:C

AACTTCCCTTAATTTCTCTCTTGTTCCTACATTATTGTTCTTTAGGTGACTCTGAATGGTTACAACCACAAATTAAGGATXXXXXXXXXXT

GAAACCTAAATTTTATCTATTAATTAAATTTGGAACCTGAATAATATCTATCAACTGTTGATATATTTAAAATTTCAGT

AACTTCCCTTAATTTCTCTCTTGTTCTTACATTATTGTTCTTTAGGTGACTCTGAATGGTTACAACCACAAATTAAGGATXXXXXXXXXXT

GAAACCTAAATTTTATCTATTAATTAAATTTGGAACCTGAATAATATCTATCAACTGTTGATATATTTAAAATTTCAGT

>Marker554170 Chr3 SNP_site:663766 Ref_Type:C

ACCTAAAAGAAATGAAATGCAATCAAGATTATTCAAAGTTAGGAAAATTGTTGGCATGCATATCTAATTAAGGGCCGAACXXXXXXXXXXA

TTACTTTACAATGTATTTTATTCTCAATACAAGTTCTTAAAACCCTATAGTTTGCTCCCTTTTATCTTCCTTATTTTGT

Create PDF with GO2PDF for free, if you wish to

ACCTAAAAGAAATGAAATGCAATCAAGATTATTCAAAGTTAGGAAAATTGTTGGCATGCATATCTAATTAAGGGCCGAACXXXXXXXXXXA
TTACTTTACAATGTATTTTATTTTCAATACAAGTTCTTAAAACCCTATAGTTTGCTCCCTTTTATCTTCCTTATTTTGT
>Marker554229 Chr3 SNP_site:518738 Ref_Type:A
AACTTCGTTTGAGAAAGATATAAACAAACTTAACATAACATAGCATCAATGGCAGAAAGAAACTAACCACGACAAAGAGTXXXXXXXXXXT
TTAGAAGCTAAACTAAAATTTGAAAACTAAAAAAGGTAGCTTGTTGAAATGTTGTTTTTGTTTTTGCTAGAGCCTTTGT
AACTTCGTTTGAGAAAGATATAAACAAACTTAACATACCATAGCATCAATGGCAGAAAGAAACTAACCACGACAAAGAGTXXXXXXXXXXT
TTAGAAGCTAAACTAAAATTTGAAAACTAAAAAAGGTAGCTTGTTGAAATGTTGTTTTTGTTTTTGCTAGAGCCTTTGT
>Marker554667 Chr3 SNP_site:5369194 Ref_Type:A
AACCTCTTATTTTGTTATTCTTATTGTAAGACCAGGAATAATTAACATGCTTTTTAAGAGTCATTTTGAAAAATGACAAAXXXXXXXXXXG
CATTATGGACCTTTAATTATTTTCGTGACGTGTAAAGATATAACAACCCATATCATAACTTCTTTCTAATATTTTCAGT
AACCTCTTATTTTGTTGTTCTTGTTGTAAGACCAGGAATAATTAACATGCTTTTTAAGAGTCATTTTGAAAAATGACAAAXXXXXXXXXXG
CATTATGGACCTTTAATTATTTTCGTGACGTGTAAAGATATAACAACCCATATCATAACTTCTTTCTAATATTTTCAGT
>Marker554667 Chr3 SNP_site:5369200 Ref_Type:A
AACCTCTTATTTTGTTATTCTTATTGTAAGACCAGGAATAATTAACATGCTTTTTAAGAGTCATTTTGAAAAATGACAAAXXXXXXXXXXG
CATTATGGACCTTTAATTATTTTCGTGACGTGTAAAGATATAACAACCCATATCATAACTTCTTTCTAATATTTTCAGT
AACCTCTTATTTTGTTGTTCTTGTTGTAAGACCAGGAATAATTAACATGCTTTTTAAGAGTCATTTTGAAAAATGACAAAXXXXXXXXXXG
CATTATGGACCTTTAATTATTTTCGTGACGTGTAAAGATATAACAACCCATATCATAACTTCTTTCTAATATTTTCAGT
>Marker554972 Chr3 SNP_site:34406405 Ref_Type:A
ACTACATATCACGTTTTCAGAGATCCAAGTGCCACTTTTTCCCTTACGAAGTGATAGTCAAGTTCTATGTATTTGGATCTXXXXXXXXXXA
TCCATGTTATCTCAATAGCTGCATTAGCCATAACTTCAACTTTTGAACTTGAACAAGGCACAGTTGGTTGCTTATTAGT
ACTACATATCACGTTTTCAGAGATCCAAGTGCCACTTTTTCCCTTACGAAGTGATAGTCAAGTTCTATGTATTTGGATCTXXXXXXXXXXA
TCCATGTTATCTCAATAGCTGCATTAGCCATAACTTCAACTTTTGAACTTGAACGAGGCACAGTTGGTTGCTTATTAGT
>Marker555227 Chr3 SNP_site:3861338 Ref_Type:A
AACAGCCACATCTATTCAAATTTACTGCGTGATATCTACAGAATACTCAAATACCTGCTCAATCAAATCAAGAGCAAGAGXXXXXXXXXXA
GCTGAGTTGCCAATTCTTGAAGCTGTATAAGACAAAATACAAAATAATTCAATGTTAAAAAGCTCCAAATAAACAAGTT
AACAGCCACATCTATTCAAATTTACTGCGTGATATCTACAGAATACTCAAATACCTGCTCAATCAAATCAAGAGCAAGTGXXXXXXXXXXA
GCTGAGTTGCCAATTCTTGAAGCTGTATAAGACAAAATACAAAATAATTCAATGTTAAAAAGCTCCAAATAAACAAGTT
>Marker55523 Chr4 SNP_site:13706922 Ref_Type:G
AACCTTTGTAATTTAGTTTAAAAGATAAAATTTGGTAGGTTTTTTTTATATATTTTTGGGTGACCAATCAGTTAGTGGAGXXXXXXXXXXC
TTTTATTACTTAAATTCTTAAAAGTTTTCAGATAAATTCCATTGATATCTAAAGCCTAATCTTGTCTCTTCTAAACGTG
AACCTTTGTAATTTAGTTTAAAAGATAAAATTTGGTAGGTTTTTTTTATATATTTTTGGGTGACCAATCGGTTAGTGGAGXXXXXXXXXXC
TTTTATTACTTAAATTCTTAAAAGTTTTCAGATAAATTCCATTGATATCTAAAGCCTAATCTTGTCTCTTCTAAACGTG
>Marker555296 Chr3 SNP_site:31430480 Ref_Type:G
ACAATAATATTAAAGAAAAAGAGAAATAAAAGTATTATAAAAATTCAAAGGTAAAGAAGTGTTCATTTAAGTTTTATAATXXXXXXXXXXT
TTCTAGTAATTCTAAAAAGTGATAAAATGGATAATATTATTTTCATTTTATATTCCAACTCTAAGAGATAAAAATGGTC
ACAATAATATTAAAGAAAAAGAGAAATAAAAGTATTATAAAAATTCAAAGGTAAAGAAGTGTTCATTTAAGTTTTATAATXXXXXXXXXXT
TTCTAGTAATTCTAAAAAGTGATAAAATGGATAATATTGTTTTCATTTTATATTCCAACTCTAAGAGATCAAAATGGTC
>Marker555296 Chr3 SNP_site:31430511 Ref_Type:C
ACAATAATATTAAAGAAAAAGAGAAATAAAAGTATTATAAAAATTCAAAGGTAAAGAAGTGTTCATTTAAGTTTTATAATXXXXXXXXXXT
TTCTAGTAATTCTAAAAAGTGATAAAATGGATAATATTATTTTCATTTTATATTCCAACTCTAAGAGATAAAAATGGTC
ACAATAATATTAAAGAAAAAGAGAAATAAAAGTATTATAAAAATTCAAAGGTAAAGAAGTGTTCATTTAAGTTTTATAATXXXXXXXXXXT
TTCTAGTAATTCTAAAAAGTGATAAAATGGATAATATTGTTTTCATTTTATATTCCAACTCTAAGAGATCAAAATGGTC
>Marker556320 Chr3 SNP_site:30999041 Ref_Type:C
TACACTCTGGCAGAGCTTCAATCCGCTACCAACAACTTTAGTCAAGAGAATCTTCTAGGAGAGGGATCTCTTGGTGCTGTXXXXXXXXXXT

Create PDF with GO2PDF for free, if you wish to

TTCTATGAACTTTTTTCTGACTTCTAATTTCTAACCAGGTTTTGGCTGTGAAAAATATCCACATGGGAACGCTCTTGTT
TACACTCTGGCAGAGCTTCAATCCGCTACTAACAACTTTAGTCAAGAGAATCTTCTAGGAGAGGGATCTCTTGGTGCTGTXXXXXXXXXXT
TTCTATGAACTTTTTTCTGACTTCTAATTTCTAACCAGGTTTTGGCTGTGAAAAATATCCACATGGGAACGCTCTTGTT
>Marker557049 Chr3 SNP_site:9732386 Ref_Type:G
AACGTCAAAGTATAAAAGAATTAAAAAAGTTGAACTTAGTGCAAAATCTAAAATTTGTATTTTTTTGGAAAAGACAATGAXXXXXXXXXXT
AACGATCGTTATCTAAAATTTCTTCATAATTCAAATATATGTGTGATTGTATGTATAAGAAAAAAAAACACTTTAGGGT
AACGTCAAAGTATAAAAGAATTAAAAAAGTTGAAGTTAGTGCAAAATCTAAAATTTGTATTTTTTTGGAAAAGACAATGAXXXXXXXXXXT
AACGATCGTTATCTAAAATTTCTTCATAATTCAAATATATGTGTGATTGTATGTATAAGAAAAAAAAACACTTTAGGGT
>Marker557614 Chr3 SNP_site:11420742 Ref_Type:T
TACCTTGAAACCATCAATTTCAAAATTCAAAATTTTGGTAATTTGTTTAAACAATCAATTTGGATTTCAAAATTCCAAAAXXXXXXXXXXA
AACACATGATTCTTATTGTTAAACAATAATTAAATACTAAACAACCAGTAAAGATTTTTATAGTATAAAAATTTATGGT
TACCTTGAAACCATCAATTTCAAAATTCTAAATTTTGGTAATTTGTTTAAACAATCAATTTGGATTTCAAAATTCCAAAAXXXXXXXXXXA
AACACATGATTCTTATTGTTAAACAATAATTAAATACTAAACAACCATTAAAGATTTTTATAGTATAAAAATTTATGGT
>Marker557614 Chr3 SNP_site:11420936 Ref_Type:T
TACCTTGAAACCATCAATTTCAAAATTCAAAATTTTGGTAATTTGTTTAAACAATCAATTTGGATTTCAAAATTCCAAAAXXXXXXXXXXA
AACACATGATTCTTATTGTTAAACAATAATTAAATACTAAACAACCAGTAAAGATTTTTATAGTATAAAAATTTATGGT
TACCTTGAAACCATCAATTTCAAAATTCTAAATTTTGGTAATTTGTTTAAACAATCAATTTGGATTTCAAAATTCCAAAAXXXXXXXXXXA
AACACATGATTCTTATTGTTAAACAATAATTAAATACTAAACAACCATTAAAGATTTTTATAGTATAAAAATTTATGGT
>Marker557775 Chr3 SNP_site:969017 Ref_Type:T
AACATCATTAGCAGCAGTAAGAGAAATCTTGGTAAATACTCGTGCAATAAACCAAAAATGTCAAAACAAAAAGAATCCACXXXXXXXXXXT
TCTTTTAATTATACACGAGCTTCACAAATTTGTTATGCATTGCACTTCACAATTAAGCTCAATTGCTCAAGTGTCAGTG
AACATCATTAGCAGCAGTAAGAGAAATCTTGGTAAATACTCGTGCAATAAACCAAAAATGTCAAAACAAAAAGAATCCACXXXXXXXXXXT
TCTTTTAATTATACATGAGCTTCACAAATTTGTTATGCATTGCACTCCACAATTAAGCTCAATTGCTCAAGTGTCAGTG
>Marker557775 Chr3 SNP_site:969048 Ref_Type:C
AACATCATTAGCAGCAGTAAGAGAAATCTTGGTAAATACTCGTGCAATAAACCAAAAATGTCAAAACAAAAAGAATCCACXXXXXXXXXXT
TCTTTTAATTATACACGAGCTTCACAAATTTGTTATGCATTGCACTTCACAATTAAGCTCAATTGCTCAAGTGTCAGTG
AACATCATTAGCAGCAGTAAGAGAAATCTTGGTAAATACTCGTGCAATAAACCAAAAATGTCAAAACAAAAAGAATCCACXXXXXXXXXXT
TCTTTTAATTATACATGAGCTTCACAAATTTGTTATGCATTGCACTCCACAATTAAGCTCAATTGCTCAAGTGTCAGTG
>Marker557893 Chr3 SNP_site:17391220 Ref_Type:T
CACCAAAACTCGTGTGCCTACACTCCTCAACAAAATGGGGTGGCTGAGCGAAAAAATCATCTTCTGGAAGTAGCACATTCXXXXXXXXXXA
GACTCCCTTAGATTGTCTTAAGGAGTCCTACCTCTCTACTCGTCTAATTGCTGAGGTTCCTCTTTGCGTGTTTGGGTGT
CACCAAAACTCGTGTGCCTACACTCCTCAACAAAATGGGGTGGCTGAGCGAAAAAATCATCTTCTGGAAGTAGCACATTCXXXXXXXXXXA
GACTCCCTTAGATTGTCTTAAGGAGTCCTACCTCTCTACTCGTCTAATTTCTGAGGTTCCTCTTTACGTGTTTGGGTGT
>Marker557893 Chr3 SNP_site:17391236 Ref_Type:A
CACCAAAACTCGTGTGCCTACACTCCTCAACAAAATGGGGTGGCTGAGCGAAAAAATCATCTTCTGGAAGTAGCACATTCXXXXXXXXXXA
GACTCCCTTAGATTGTCTTAAGGAGTCCTACCTCTCTACTCGTCTAATTGCTGAGGTTCCTCTTTGCGTGTTTGGGTGT
CACCAAAACTCGTGTGCCTACACTCCTCAACAAAATGGGGTGGCTGAGCGAAAAAATCATCTTCTGGAAGTAGCACATTCXXXXXXXXXXA
GACTCCCTTAGATTGTCTTAAGGAGTCCTACCTCTCTACTCGTCTAATTTCTGAGGTTCCTCTTTACGTGTTTGGGTGT
>Marker558233 Chr3 SNP_site:16327468 Ref_Type:A
CACTTAGGGTTTCAAGCTTAGGGATTATTGTGTGAGCCCCAACATTCATTTTTGTAATAAAACCTTTTTGTTTTTTTCTTXXXXXXXXXXC
ACTACAAACAACAAATTAGCCTATAGGAAGAATAAACACTAATCTTATATACATAGAATGACCCAGACAAAATGGAGTG
CACTTAGGGTTTCAAGCTTAGGGATTATTGTGTGAGCCCCAACATTCATTTTTGTAATAAAACCTTTTTGTTTTTTTCTTXXXXXXXXXXC
ACTACAAACAACAAGTTAGCCTATAGGAAGAATAAACACTAATCTTATATAGATAGAATGACCCAGACAAAATGGAGTG
>Marker558233 Chr3 SNP_site:16327505 Ref_Type:C

Create PDF with GO2PDF for free, if you wish to

CACTTAGGGTTTCAAGCTTAGGGATTATTGTGTGAGCCCCAACATTCATTTTTGTAATAAAACCTTTTTGTTTTTTTCTTXXXXXXXXXXC
ACTACAAACAACAAATTAGCCTATAGGAAGAATAAACACTAATCTTATATACATAGAATGACCCAGACAAAATGGAGTG
CACTTAGGGTTTCAAGCTTAGGGATTATTGTGTGAGCCCCAACATTCATTTTTGTAATAAAACCTTTTTGTTTTTTTCTTXXXXXXXXXXC
ACTACAAACAACAAGTTAGCCTATAGGAAGAATAAACACTAATCTTATATAGATAGAATGACCCAGACAAAATGGAGTG
>Marker558401 Chr3 SNP_site:10739550 Ref_Type:T
ACCATCCCTCCCACTCTCGCAAAGAACCAAAACACCACAAAGCTCATAACTACCTTGATCATATGCTTATGACATATATAXXXXXXXXXXA
ACACCGAAATTCCCCTTTTACCCTTTCGATAATATTGGAATGGAGTTGGGGGCCTAACAGATTCTTAGAGAGGAAAGTC
ACCATCCCTCCCACTCTCGCAAAGAACCAAAACACCACAAAGCTCATAACTACCTTGATCATATGCTTATGACATATATAXXXXXXXXXXA
ACACTGAAATTCCCCTTTTACCCTTTCAATAATATTGGAATGGAGTTGGGGGCCTAACAGATTCTTAGAGAGGAAAGTC
>Marker558401 Chr3 SNP_site:10739573 Ref_Type:A
ACCATCCCTCCCACTCTCGCAAAGAACCAAAACACCACAAAGCTCATAACTACCTTGATCATATGCTTATGACATATATAXXXXXXXXXXA
ACACCGAAATTCCCCTTTTACCCTTTCGATAATATTGGAATGGAGTTGGGGGCCTAACAGATTCTTAGAGAGGAAAGTC
ACCATCCCTCCCACTCTCGCAAAGAACCAAAACACCACAAAGCTCATAACTACCTTGATCATATGCTTATGACATATATAXXXXXXXXXXA
ACACTGAAATTCCCCTTTTACCCTTTCAATAATATTGGAATGGAGTTGGGGGCCTAACAGATTCTTAGAGAGGAAAGTC
>Marker558477 Chr3 SNP_site:36551109 Ref_Type:T
CACATCAAGATAGAGATTTGAACAAAAGGATAAAACAGTGTTGATTATGTTTATACTCAAAATTCTTTCTACCGAAGATCXXXXXXXXXXT
TAAATAGGAAGAACACGTATATGATAGTCTAAGATTTAAAATGGGGACGTGTTATTAATCATGACCATGAAAATGGGTG
CACATCAAGATAGAGATTTGAACAAAAGGATAAAACAGTGTTTATTATGCTTATACTCAAAATTCTTTCTACAGAAGATCXXXXXXXXXXT
TAAATAGGAAGAACACGTATATGATAGTCTAAGATTTAAAATGGGGACGTGTTATTAATCATGACCATGAAAATGGGTG
>Marker558477 Chr3 SNP_site:36551116 Ref_Type:C
CACATCAAGATAGAGATTTGAACAAAAGGATAAAACAGTGTTGATTATGTTTATACTCAAAATTCTTTCTACCGAAGATCXXXXXXXXXXT
TAAATAGGAAGAACACGTATATGATAGTCTAAGATTTAAAATGGGGACGTGTTATTAATCATGACCATGAAAATGGGTG
CACATCAAGATAGAGATTTGAACAAAAGGATAAAACAGTGTTTATTATGCTTATACTCAAAATTCTTTCTACAGAAGATCXXXXXXXXXXT
TAAATAGGAAGAACACGTATATGATAGTCTAAGATTTAAAATGGGGACGTGTTATTAATCATGACCATGAAAATGGGTG
>Marker558477 Chr3 SNP_site:36551139 Ref_Type:A
CACATCAAGATAGAGATTTGAACAAAAGGATAAAACAGTGTTGATTATGTTTATACTCAAAATTCTTTCTACCGAAGATCXXXXXXXXXXT
TAAATAGGAAGAACACGTATATGATAGTCTAAGATTTAAAATGGGGACGTGTTATTAATCATGACCATGAAAATGGGTG
CACATCAAGATAGAGATTTGAACAAAAGGATAAAACAGTGTTTATTATGCTTATACTCAAAATTCTTTCTACAGAAGATCXXXXXXXXXXT
TAAATAGGAAGAACACGTATATGATAGTCTAAGATTTAAAATGGGGACGTGTTATTAATCATGACCATGAAAATGGGTG
>Marker558518 Chr3 SNP_site:3795194 Ref_Type:C
TACAACATAGAGGTTCTTTTGACACATCCTTAGCCTTATAGCTCACACTTGCATACAAAGCAAACACTTAGTCCAATCCTXXXXXXXXXXA
CTTAGTCACTTGAATAAAATAACCTCACACTAAGGAAATTTCATCATTCTATAATCAACTACATAGAAAGTCTAGGGTT
TACAACATAGAGGTTCTTTTGACACATCCTTAGCCTTATAGCTCACACTTGCATACAAAGCAAACACTTAGTCCAATCCTXXXXXXXXXXA
CTTAGTCACTTGAATAAAATAACCTCACACTAAGGAAATTTCATCCTTCTATAATCAACTACATAGAAAGTCTAGGGTT
>Marker559418 Chr3 SNP_site:16392998 Ref_Type:C
AACAAGTATCACGTTTGAATTAAAGGAATTTAATGGACATGAAAGTTAAAATGATTACTAGCTATAATCTAATACCAACGXXXXXXXXXXG
ACATTTTGGTGGGTGGAAAAGTGTTTAGTCGTATAGAGAGAAGACTGTAAAAAGTAAAAAGAAATGAAAGGACATTGTG
AACAAGTATCACGTTTGAATTAAAGGAATTTAATGGACATGAAAGTTAAAATGATTACTAGCTATAATCTACTACCAACGXXXXXXXXXXG
ACATTTTGGTGGGTGGAAAAGTGTTCAGTCGTATAGAGAGAAGACTGTAAAAAGTAAAAAGAAATGAGAGGACATTGTG
>Marker559418 Chr3 SNP_site:16393143 Ref_Type:C
AACAAGTATCACGTTTGAATTAAAGGAATTTAATGGACATGAAAGTTAAAATGATTACTAGCTATAATCTAATACCAACGXXXXXXXXXXG
ACATTTTGGTGGGTGGAAAAGTGTTTAGTCGTATAGAGAGAAGACTGTAAAAAGTAAAAAGAAATGAAAGGACATTGTG
AACAAGTATCACGTTTGAATTAAAGGAATTTAATGGACATGAAAGTTAAAATGATTACTAGCTATAATCTACTACCAACGXXXXXXXXXXG
ACATTTTGGTGGGTGGAAAAGTGTTCAGTCGTATAGAGAGAAGACTGTAAAAAGTAAAAAGAAATGAGAGGACATTGTG

Create PDF with GO2PDF for free, if you wish to

>Marker559418 Chr3 SNP_site:16393185 Ref_Type:G
AACAAGTATCACGTTTGAATTAAAGGAATTTAATGGACATGAAAGTTAAAATGATTACTAGCTATAATCTAATACCAACGXXXXXXXXXXG
ACATTTTGGTGGGTGGAAAAGTGTTTAGTCGTATAGAGAGAAGACTGTAAAAAGTAAAAAGAAATGAAAGGACATTGTG
AACAAGTATCACGTTTGAATTAAAGGAATTTAATGGACATGAAAGTTAAAATGATTACTAGCTATAATCTACTACCAACGXXXXXXXXXXG
ACATTTTGGTGGGTGGAAAAGTGTTCAGTCGTATAGAGAGAAGACTGTAAAAAGTAAAAAGAAATGAGAGGACATTGTG
>Marker559494 Chr3 SNP_site:29733803 Ref_Type:T
ACTCATGAAATTTTAATTAACACAGTTGTAACTAAATTTTTATTACCGAACTTATACGCTAAGAACTAAAAATATATAAAXXXXXXXXXXA
AATGAAAGAAAAGAAAGGAGGCTTTTCATTCGGATTCTATCTGAGGAAGAAAGTGATGGGAGGATCTAATCAGTAGGTA
ACTCATGAAATTTTAATTTACACAGTTGTAACTAAATTTTTATTACCGAACTTATACGCTAAGAACTAAAAATATATAAAXXXXXXXXXXA
AATGAAAGAAAAGAAAGGAGGCTTTTCATTCGGATTCTATCTGAGGAAGAAAGTGATGGGAGGATCTAATCAGTAGGTA
>Marker559541 Chr3 SNP_site:28404424 Ref_Type:T
ACCTTCATACAGGCTGTATTCCAGCAATTATTCACAGGGACTTGAAAAGCAGTAATATTCTTCTTGACAGGCACATGAAAXXXXXXXXXXG
ATCCGGAGTAAGCTTTTCCCCTTTCTTGAGAATTTAAATATATCCTGAAGTAAAGTTTGATGGCTATAATGTATCTGTA
ACCTTCATACAGGCTGTATTCCAGCAATTATTCACAGGGACTTGAAAAGCAGTAATATTCTTCTTGACAGGCACATGAAAXXXXXXXXXXG
ATCCGGAGTAAGCTTTTCCTCTTTCTTGAGAATTTAAATATATCCTGAAGTAAAGTTTGATGGCTATAATGTATCTGTA
>Marker559641 Chr3 SNP_site:6785509 Ref_Type:T
TACCTACCCCGACAATTTGCGCTCGTGTTTTCTGGATGGTGCTAGAACGTATTTGTTTGTTGGGGTTGTTTGAATTTGATXXXXXXXXXXG
ACAGCATTTGGCATTTGCAATGGCAATGGAGCCATCTATGCGTATGGACTAATTGAACCGTTTATGTTTTCCTGGTTGT
TACCTACCCCGACAATTTGCGCTCGTGTTTTCTGGATGGTGCTAGAACGTATTTGTTTGTTGGGGTTGTTTGAATTTGATXXXXXXXXXXG
ACAGTATTTGGCATTTGCAATGGCAATGGAGCCATCTATGCGTATGGACTAATTGAACCGTTTATGTTTTCCTGGTTGT
>Marker559707 Chr3 SNP_site:3179627 Ref_Type:T
GACTGTCTGGTAAAGCTGGTAAGACAAAGTTTGCCATCGAAAATGCCACCAAGACGAGGATAGTCATTGCAGATACTAAGXXXXXXXXXXA
GAAAGGTTTTGATCTGTAATTTAATGTTTGTTTGTCTTGATAAATGAATTTTGCTTACACTAGTTGTTTGAGGAAGAGT
GACTGTCTGGTAAAGCTGGTAAGACAAAGTTTGCCATCGAAAATGCCACCAAGACGAGGATAGTTATTGCAGATACTAAGXXXXXXXXXXA
GAAAGGTTTTGATCTGTAATTTAATGTTTGTTTGTCTTGATAAATGAATTTTGCATACACTAGTTGTTTGAGGAAGAGT
>Marker559707 Chr3 SNP_site:3179820 Ref_Type:A
GACTGTCTGGTAAAGCTGGTAAGACAAAGTTTGCCATCGAAAATGCCACCAAGACGAGGATAGTCATTGCAGATACTAAGXXXXXXXXXXA
GAAAGGTTTTGATCTGTAATTTAATGTTTGTTTGTCTTGATAAATGAATTTTGCTTACACTAGTTGTTTGAGGAAGAGT
GACTGTCTGGTAAAGCTGGTAAGACAAAGTTTGCCATCGAAAATGCCACCAAGACGAGGATAGTTATTGCAGATACTAAGXXXXXXXXXXA
GAAAGGTTTTGATCTGTAATTTAATGTTTGTTTGTCTTGATAAATGAATTTTGCATACACTAGTTGTTTGAGGAAGAGT
>Marker559740 Chr3 SNP_site:32167122 Ref_Type:T
ACTCTTACTGATATATATTCCTAGATATGTGGATGCGCATCATCCATTACATATTCATGATCGAAATAGAAGCAATTTGGXXXXXXXXXXT
CTAGAATCTGTTATTTCAGAGCATAGTTCTCAAACTTTTGCATCTTGTGATAAATGAAACATGCATATGATGTGAAAGT
ACTCTTACTGATATATATTCCTAGATATGTGGATGTGCATCATCCATTACATATTCATGATCGAAATAGAAGCAATTTGGXXXXXXXXXXT
CTAGAATCTGTTATTTCAGAGCAGAGTTCTCAAACTTTTGCATCTTGTGATAAATGAAACATGCATATGATGTGAAAGT
>Marker559740 Chr3 SNP_site:32167296 Ref_Type:G
ACTCTTACTGATATATATTCCTAGATATGTGGATGCGCATCATCCATTACATATTCATGATCGAAATAGAAGCAATTTGGXXXXXXXXXXT
CTAGAATCTGTTATTTCAGAGCATAGTTCTCAAACTTTTGCATCTTGTGATAAATGAAACATGCATATGATGTGAAAGT
ACTCTTACTGATATATATTCCTAGATATGTGGATGTGCATCATCCATTACATATTCATGATCGAAATAGAAGCAATTTGGXXXXXXXXXXT
CTAGAATCTGTTATTTCAGAGCAGAGTTCTCAAACTTTTGCATCTTGTGATAAATGAAACATGCATATGATGTGAAAGT
>Marker55977 Chr4 SNP_site:8771519 Ref_Type:T
CACTAAACAATGAAGTTACATTTGATATTAAAAGACTTTGAACCACAAATATTGACAACTAAGAAGGAAAATTAAAAGAAXXXXXXXXXXC
AGAGAGAATCCTAAAAAGTTTTCATTGGCAGGGGCACAATGGCAGAAAGATTAACCATTTGGTAAAATGGTTAATTGTT
CACTAAACAATGAAGTTATATTTGATATCAAAAGACTTTGAACCACAAATATTGACAACTAAGAAGGAAAATTAAAAGAAXXXXXXXXXXC

Create PDF with GO2PDF for free, if you wish to

AGAGAGAATCCTAAAAAGTTTTCATTGGCAGGGGCACAATGGCAGAAAGATTAACCATTTGGTAAAATGGTTAATTGTT

>Marker55977 Chr4 SNP_site:8771529 Ref_Type:C

CACTAAACAATGAAGTTACATTTGATATTAAAAGACTTTGAACCACAAATATTGACAACTAAGAAGGAAAATTAAAAGAAXXXXXXXXXXC

AGAGAGAATCCTAAAAAGTTTTCATTGGCAGGGGCACAATGGCAGAAAGATTAACCATTTGGTAAAATGGTTAATTGTT

CACTAAACAATGAAGTTATATTTGATATCAAAAGACTTTGAACCACAAATATTGACAACTAAGAAGGAAAATTAAAAGAAXXXXXXXXXXC

AGAGAGAATCCTAAAAAGTTTTCATTGGCAGGGGCACAATGGCAGAAAGATTAACCATTTGGTAAAATGGTTAATTGTT

>Marker56001 Chr4 SNP_site:17035928 Ref_Type:A

TACTTGTAAAATAACATATTTATCTTTAGTCTTTAAAATATGTGGATAATTTTTTGCCATGTTTTCGAGTGGATTACTTTXXXXXXXXXXT

CAATTTAAGCTTTGGTTTGATAGCTATTTTGTTTTTAAGTTTTTAAAAATTAACCTAACGATTGACATCTTTCTAAAGT

TACTTGTAAAATAACATATTTATCTTTAGTCTTTAAAATGTGTGGATAATTTTTTGCCATGTTTTCGAGTGGATTACTTTXXXXXXXXXXT

CAATTTAAGCTTTGGTTTGATAACTATTTTGTTTTTAAGTTTTTAAAAATTAATCTAACGATTGACATCTTTCTAAAGT

>Marker56001 Chr4 SNP_site:17036087 Ref_Type:G

TACTTGTAAAATAACATATTTATCTTTAGTCTTTAAAATATGTGGATAATTTTTTGCCATGTTTTCGAGTGGATTACTTTXXXXXXXXXXT

CAATTTAAGCTTTGGTTTGATAGCTATTTTGTTTTTAAGTTTTTAAAAATTAACCTAACGATTGACATCTTTCTAAAGT

TACTTGTAAAATAACATATTTATCTTTAGTCTTTAAAATGTGTGGATAATTTTTTGCCATGTTTTCGAGTGGATTACTTTXXXXXXXXXXT

CAATTTAAGCTTTGGTTTGATAACTATTTTGTTTTTAAGTTTTTAAAAATTAATCTAACGATTGACATCTTTCTAAAGT

>Marker56001 Chr4 SNP_site:17036118 Ref_Type:C

TACTTGTAAAATAACATATTTATCTTTAGTCTTTAAAATATGTGGATAATTTTTTGCCATGTTTTCGAGTGGATTACTTTXXXXXXXXXXT

CAATTTAAGCTTTGGTTTGATAGCTATTTTGTTTTTAAGTTTTTAAAAATTAACCTAACGATTGACATCTTTCTAAAGT

TACTTGTAAAATAACATATTTATCTTTAGTCTTTAAAATGTGTGGATAATTTTTTGCCATGTTTTCGAGTGGATTACTTTXXXXXXXXXXT

CAATTTAAGCTTTGGTTTGATAACTATTTTGTTTTTAAGTTTTTAAAAATTAATCTAACGATTGACATCTTTCTAAAGT

>Marker560135 Chr3 SNP_site:36096278 Ref_Type:T

CACCACATGAACCAGTAATGACGATTCTGATATCTCCTCTAATGTTGCTCTGAATGCAGCAACCTGTAGGACTTAACCAGXXXXXXXXXXG

GGTAGTTGAGATTTACTTTGCCCTAATTTTTTATTATTCTAATTAGGCCCATGTTGTCTATAAATATTTTTCCCTTTGT

CACCACATGAACCAGTAATGACGATTCTGATATCTCCTCTAATGTTGCTCTGAATGCAGCAACCTGTAGGACTTAACCAGXXXXXXXXXXG

GGTAGTTGAGATTTACTTTGCCCTAATTTTTTATTATTTTAATTAGGCCCATGTTGTCAATAAATATTTTTCCCTTTGT

>Marker560135 Chr3 SNP_site:36096298 Ref_Type:A

CACCACATGAACCAGTAATGACGATTCTGATATCTCCTCTAATGTTGCTCTGAATGCAGCAACCTGTAGGACTTAACCAGXXXXXXXXXXG

GGTAGTTGAGATTTACTTTGCCCTAATTTTTTATTATTCTAATTAGGCCCATGTTGTCTATAAATATTTTTCCCTTTGT

CACCACATGAACCAGTAATGACGATTCTGATATCTCCTCTAATGTTGCTCTGAATGCAGCAACCTGTAGGACTTAACCAGXXXXXXXXXXG

GGTAGTTGAGATTTACTTTGCCCTAATTTTTTATTATTTTAATTAGGCCCATGTTGTCAATAAATATTTTTCCCTTTGT

>Marker560488 Chr3 SNP_site:27601985 Ref_Type:G

CACACTTGATATGCGTCCATTTGATATTCTTTGGTGATGGCAAACCGAAATTTAGTGTGGAACGTTGTGAAGAAATCTTTXXXXXXXXXXT

TAATCCTATTGCGACTTCTCTGACAGGACCAATGACTGGTAATTTTCTAAACCTAGCATATTTATTATGGCGAGATGGT

CACACTTGATATGCTTCCATTTGATATTCTTTGGTGATGGCAAACCGAAATTTAGTGTGGAACGTTGTGAAGAAATCTTTXXXXXXXXXXT

TAATCCTATTGCGACTTCTCTGACAGGACCAATGACTGGTAATTTTCTAAACCTAGCATATTTATTATGGCGAGATGGT

>Marker560554 Chr3 SNP_site:37837958 Ref_Type:G

CACCAAGCGTCGGAGTGTCCAGGTGTCCAACATGTCAGGCATGGACATGCTAGCCAAACGAGAGTGTTTGTGCTTCTTAAXXXXXXXXXXC

TTCATTTTGCCTAAATCCTGCTTATGCGGTAATGGTTGTGCCTATGCACAATACTTTGGTTATGAGTTTAAATTTTGTC

CACCAAGCGTCGGAGTGTCCAGGTGTCCAACATGTCAGGCATGGACATGCTAGCGAAACGAGAGTGTTTGTGCTTCTTAAXXXXXXXXXXC

TTCATTTTGCCTAAATCCTGCTTATGCGGTAATGGTTGTGCCTCTGCACAATCCTTTGGTTATGAGTTTAAATTTTGTC

>Marker560554 Chr3 SNP_site:37838161 Ref_Type:C

CACCAAGCGTCGGAGTGTCCAGGTGTCCAACATGTCAGGCATGGACATGCTAGCCAAACGAGAGTGTTTGTGCTTCTTAAXXXXXXXXXXC

TTCATTTTGCCTAAATCCTGCTTATGCGGTAATGGTTGTGCCTATGCACAATACTTTGGTTATGAGTTTAAATTTTGTC

Create PDF with GO2PDF for free, if you wish to

CACCAAGCGTCGGAGTGTCCAGGTGTCCAACATGTCAGGCATGGACATGCTAGCGAAACGAGAGTGTTTGTGCTTCTTAAXXXXXXXXXXXC
TTCATTTTGCCTAAATCCTGCTTATGCGGTAATGGTTGTGCCTCTGCACAATCCTTTGGTTATGAGTTTAAATTTTGTC
>Marker560554 Chr3 SNP_site:37838170 Ref_Type:C
CACCAAGCGTCGGAGTGTCCAGGTGTCCAACATGTCAGGCATGGACATGCTAGCCAAACGAGAGTGTTTGTGCTTCTTAAXXXXXXXXXXXC
TTCATTTTGCCTAAATCCTGCTTATGCGGTAATGGTTGTGCCTATGCACAATACTTTGGTTATGAGTTTAAATTTTGTC
CACCAAGCGTCGGAGTGTCCAGGTGTCCAACATGTCAGGCATGGACATGCTAGCGAAACGAGAGTGTTTGTGCTTCTTAAXXXXXXXXXXXC
TTCATTTTGCCTAAATCCTGCTTATGCGGTAATGGTTGTGCCTCTGCACAATCCTTTGGTTATGAGTTTAAATTTTGTC
>Marker560910 Chr3 SNP_site:18767148 Ref_Type:C
AACCCCATGCCTGATAGGTAAAATCTTTCTTAGAGTTCTGCATCGAATATCAAACCAACATTTTGTCTATATAGGTTGCCXXXXXXXXXXXA
TTGGGTTGCTAGCCAATTTTTAACGTCAGTTAGATATCCTATATCATTCCTAATGAGAAGGATATCGTCCATATAAAGT
AACCCCATGCCTGATAGGTAAAATCTTTCTTAGAGTTCTGGATCGAATATCGAACTAACATTTTGTCTATATAGGTTGCCXXXXXXXXXXXA
TTGGGTTGCTAGCCAATTTTTAACGTCAGTTAGATATCCTATATCATTCCTAATGAGAAGGATATCGTCCATATAAAGT
>Marker560910 Chr3 SNP_site:18767159 Ref_Type:A
AACCCCATGCCTGATAGGTAAAATCTTTCTTAGAGTTCTGCATCGAATATCAAACCAACATTTTGTCTATATAGGTTGCCXXXXXXXXXXXA
TTGGGTTGCTAGCCAATTTTTAACGTCAGTTAGATATCCTATATCATTCCTAATGAGAAGGATATCGTCCATATAAAGT
AACCCCATGCCTGATAGGTAAAATCTTTCTTAGAGTTCTGGATCGAATATCGAACTAACATTTTGTCTATATAGGTTGCCXXXXXXXXXXXA
TTGGGTTGCTAGCCAATTTTTAACGTCAGTTAGATATCCTATATCATTCCTAATGAGAAGGATATCGTCCATATAAAGT
>Marker560910 Chr3 SNP_site:18767163 Ref_Type:C
AACCCCATGCCTGATAGGTAAAATCTTTCTTAGAGTTCTGCATCGAATATCAAACCAACATTTTGTCTATATAGGTTGCCXXXXXXXXXXXA
TTGGGTTGCTAGCCAATTTTTAACGTCAGTTAGATATCCTATATCATTCCTAATGAGAAGGATATCGTCCATATAAAGT
AACCCCATGCCTGATAGGTAAAATCTTTCTTAGAGTTCTGGATCGAATATCGAACTAACATTTTGTCTATATAGGTTGCCXXXXXXXXXXXA
TTGGGTTGCTAGCCAATTTTTAACGTCAGTTAGATATCCTATATCATTCCTAATGAGAAGGATATCGTCCATATAAAGT
>Marker561011 Chr3 SNP_site:778372 Ref_Type:A
CACTAACCATTGACATTGAAATATGGATATGCAACCAAAAAAAGCTCATCTTCAGGTTTCACACGGTCAGCCATTTTCACXXXXXXXXXXXA
AAAAGAGCTTTTATATGGTCAGAAAAGAGAAAAAAGAACAGCAGACAGTAAGCATATTACAAACAAAAGCATGATTGTT
CACTAACCATTGACATTGAAATATGGATATGCAACCAAAAAAAGCTCATCTTCAGGTTTCACACGGTCAGCCATTTTCACXXXXXXXXXXXT
AAAAGAGTTTTTATATGGTCAGAAAAGAGAAAAAAGAACAGCAGACAGTAAGCATATTACAAACAAAAGCATGATTGTT
>Marker561011 Chr3 SNP_site:778380 Ref_Type:C
CACTAACCATTGACATTGAAATATGGATATGCAACCAAAAAAAGCTCATCTTCAGGTTTCACACGGTCAGCCATTTTCACXXXXXXXXXXXA
AAAAGAGCTTTTATATGGTCAGAAAAGAGAAAAAAGAACAGCAGACAGTAAGCATATTACAAACAAAAGCATGATTGTT
CACTAACCATTGACATTGAAATATGGATATGCAACCAAAAAAAGCTCATCTTCAGGTTTCACACGGTCAGCCATTTTCACXXXXXXXXXXXT
AAAAGAGTTTTTATATGGTCAGAAAAGAGAAAAAAGAACAGCAGACAGTAAGCATATTACAAACAAAAGCATGATTGTT
>Marker561048 Chr3 SNP_site:30983761 Ref_Type:T
ACTATATATCATCTATTATTGCCCAATATTGTTTCAACTTTTTAAAATTAAAAAGAAAAAGTGGAAAAATAATTTACAAAXXXXXXXXXXXA
TTTAGCTTCACCACAAAATGCTTGTTGGTGTTGAGAATTAGACATTATGATTATAAATAAAAATTAATAGTCAAAAGTC
ACTATATTTCATCTATTATTGCCCAATATTGTTTCAACTTTTTAAAATTAAAAAGAAAAAGTGGAAAAATAATTTACAAAXXXXXXXXXXXA
TTTAGCTTCACCACAAAATGCTTGTTGGTGTTGAGAATTAGACATTATGATTATAAATAAAAATTAATAGTCAAAAGTC
>Marker561793 Chr3 SNP_site:30634029 Ref_Type:A
TACCAGATTGGTGACCTTTTATAGAAAACCAAAATTACATACACCATAGGTTGGTCTGGGCTCATTTGTTTAGGACCCAAXXXXXXXXXXXT
ATTTTTTTAAGCTCTAGTTGCCTTTTTTTTCACTTGCACTTAATCAGGCTCAAAAAATCATCGTAGATTTTTACATGTG
TACCAGATTGGTGACCTTTTATAGAAAACCAAAATTACATACACCATAGGTTGGTCTGGGCTCATTTGTTTAGGACCCAAXXXXXXXXXXXT
TTTTTTTTAAGCTCTAGTTGCCTTTTTTTTCACTTGCACTTAATCAGGCTCAAAAAATCATCGTAGATTTTTACATGTG
>Marker561935 Chr3 SNP_site:31716778 Ref_Type:C
CACTTTGCTCTTCATTTCTTTATGGCACCTTCAAAGTTCTATAGATCTTTTGTCCGAGTTTTATGTATGGCTTGCTGCATXXXXXXXXXXXA

Create PDF with GO2PDF for free, if you wish to

CTAGGAGAGGCCCCCATCTGCCATGCCCCATCTTCTGAACTTTCTCATCCTATAGATATATCCGGTCTTTGATAAAGTT
CACTTTGCTCTTCATTTCTTTATGGCACCTTCAAAGTTCTATAGATGTTTTGTCCGAGTTTTATGTATGGCTTGCTGCATXXXXXXXXXXA
CTAGGAGAGGCCCCCATCTGCCATGCCCCATCTTCTGAACTTTCTCATCGTATAGATATATCCGGTCTTTGATAAAGTT
>Marker561935 Chr3 SNP_site:31716970 Ref_Type:C
CACTTTGCTCTTCATTTCTTTATGGCACCTTCAAAGTTCTATAGATCTTTTGTCCGAGTTTTATGTATGGCTTGCTGCATXXXXXXXXXXA
CTAGGAGAGGCCCCCATCTGCCATGCCCCATCTTCTGAACTTTCTCATCCTATAGATATATCCGGTCTTTGATAAAGTT
CACTTTGCTCTTCATTTCTTTATGGCACCTTCAAAGTTCTATAGATGTTTTGTCCGAGTTTTATGTATGGCTTGCTGCATXXXXXXXXXXA
CTAGGAGAGGCCCCCATCTGCCATGCCCCATCTTCTGAACTTTCTCATCGTATAGATATATCCGGTCTTTGATAAAGTT
>Marker562045 Chr3 SNP_site:792262 Ref_Type:C
ACTGTGAATAGGACTACTTACAGGGAGTGGCAAAATCTTTTCTCCCAAACCTCTCTTCACAAAATTCTGAATTGCTTCTTXXXXXXXXXXC
CATGTATCTTCACTTGGACCATAGCCTTTCCAACGCACCTGATGAAATAGAATATTTTATTATGAATGTAAAGAAAAGT
ACTGTGAATAGGACTACTTACAGGGAGTGGCAAAATCTTTTCTCCCAAACTTCTCTTCACAAAATTCTGAATTGCTTCTTXXXXXXXXXXC
CATGTATCTTCACTTGGACCATAGCCTTTCCAACGCACCTGATGAAATAGAATATTTTATTATGAATGTAAAGAAAAGT
>Marker562210 Chr3 SNP_site:34120480 Ref_Type:T
ACTAATTCAAGAATCTAGAAGAGAATACTTCGATAAACATTCATAAAAAGCATCTAGAGCTCAAAAGATGTTGGCTGGAGXXXXXXXXXXG
AGTATAGTCTTTGTTTAAGTTTTAGCTTGTCGCCTTGTATTTCCCTAAGTTCTTTTTGCTCTTAGTATAATACTCTTGT
ACTAATTCAAGAATCTAGAAGAGAATACTTCGATAAACATTCATAAAAAGCATCTAGAGCTCAAAAGATGTTGGCTGGAGXXXXXXXXXXG
AGTATAGTCTTTGTTTAAGTTTTAGCTTGTCGCCTTGTATTTCCCTAAGTTTTTTTTGCTCTTAGTATAATACTCTTGT
>Marker562355 Chr3 SNP_site:4702049 Ref_Type:G
AACCGACTCAACATCTTTGTTAGACACACTATGTTAAGACGTAGGCTATGTTGGGCCTTTGACCCTCAAACTAAGATAAGXXXXXXXXXXC
TAGAATCAACGGGTAAGACGGACAATAAACTAGTTTTGAAGTGTCAATTTAAGTTTAATAAAAATTTAAATTCAAAGGT
AACCGACTCAACATCTTTGTTAGACAGACTATGTTAAGACGTAGGCTATGTTGGGCCTTTGACCCTCAAACTAAGATAAGXXXXXXXXXXC
TAGAATCAACGGGTAAGACGGACAATAAACTAGTTTTGAAGTGTCAATTTAAGTTTAATAAAAATTTAAATTCAAAGGT
>Marker562720 Chr3 SNP_site:27996332 Ref_Type:G
CACCTTTGATTGTCCTCCCCTGTCTTAAAGAGAATAGGCTATTCAGTTGAAATTAATTGTTCTTTTTTTCAAAAAAGGATXXXXXXXXXXG
AAATGTAACTAAAAAGAAATGCTTCTATACACTATGAAGATTTGAATATGATCTGTAATTGAATTGCATAGGGCAAGTA
CACCTTTGATTGTCCTCCCCTGTCTTAAAGAGAATAGGCTATTCAGTTGAAATTAATTTTTCTTTTTTTCAAAAAAGGATXXXXXXXXXXG
AAATGTAACTAAAAAGAAATGCTTCTATACACTATGAAGATTTGAATATGATCTGTAATTGAATTGCATAGGGCAAGTA
>Marker562727 Chr3 SNP_site:11177390 Ref_Type:G
TACATATGCAACCCAAAATTACTTTTAAATTGATACCTCTTGTTGGAACACTAGGTGTATTCATTGATTTGTTAGGTTGTXXXXXXXXXXT
TGAAATATCTAATTTGATTCTCGACATTACATTTAGATGCTCGTATATGACTTTTATGGAGATACCTAAATTTATAGTG
TACATATGCAACCCAAAATTACTTTTAAATTGATACCTCTTGTTGGAACACTAGGTGTATTCATTGATTTGTTAGGTTGTXXXXXXXXXXT
TGAAATATCTAATTTGATTCTCGACATTACATTTAGATGCTCGTATATGACTTTTATGGAGATACCTAAATTTATGGTG
>Marker56294 Chr4 SNP_site:17613040 Ref_Type:T
TACACATTTTATTACATTTGTTTATACTTAACAAATTTCAACAATATTTACATTGTATTAACAAAATCAAAGTATTAGTCXXXXXXXXXXT
AGGACAAATTTAGGTCGAAATGACAAAAATACCCTTATTTAATTTTACTTCACATTTACTCTTCTCTTCAAGGTAGAGT
TACACATTTTATTACATTTGTTTATACTTAACAAATTTCAACAATATTTACATTGTATTAACAAAATCAAAGTATTAGTCXXXXXXXXXXT
AGGACAAATTTAGGTCGAAATGACAAAAATACCCTTATTTAATTTTACTTCACATTTACTCTTCTCTTCAATGTAGAGT
>Marker562951 Chr3 SNP_site:346534 Ref_Type:A
AACTAAACAAGAAACAATTAGACATCAGAAAACAAAATCAGAATGAGCATACCTCACTTCTAGATGAGGTGTCCATCATAXXXXXXXXXXA
TCCATCAAACTTCGACCTCATGAAGACATACATGCAAAAAAATAATTACAAAAGTAATGTTGATCGGGATTGAATGTT
AACTAAACAAGAAACAATTAGACATCAGAAAACAAAATCAGAATGAGCATACCTCACTTCTAGATGAGGTGTCCATCATAXXXXXXXXXXA
TCCGTCAAACTTCGACCTCATGAAGACATACATGCAAAAAAATAATTACAAAAGTAATGTTGATCGGGATTGAATGTT
>Marker562962 Chr3 SNP_site:39119074 Ref_Type:A

Create PDF with GO2PDF for free, if you wish to

GACTTTAGCACAAATAATAATATTTAATTGAATCAAAATAGTTGATTTAATTCAATGATCACGATAAAAACACTTTGTATXXXXXXXXXXT
GCTAGAATGCCTCTAAGTGAGGTCAAGGAGTTTTCGACGTTAATAAGGATTATGGTTGGATATTCTCCCCATCTTGGTT
GACTTTAGCACAAATAATAATATTTAATTGAATCAAAATAGTTGATTTAATTCAATGATCACGATAAAAACACTTTGTATXXXXXXXXXXT
GCTAGAATGCCTCTAAGTGAGGTCAAGGGGTTTTCAACGTTAATAAGGATTATGGTTGGATATTCTCCCCATCTTGGTT
>Marker562962 Chr3 SNP_site:39119081 Ref_Type:G
GACTTTAGCACAAATAATAATATTTAATTGAATCAAAATAGTTGATTTAATTCAATGATCACGATAAAAACACTTTGTATXXXXXXXXXXT
GCTAGAATGCCTCTAAGTGAGGTCAAGGAGTTTTCGACGTTAATAAGGATTATGGTTGGATATTCTCCCCATCTTGGTT
GACTTTAGCACAAATAATAATATTTAATTGAATCAAAATAGTTGATTTAATTCAATGATCACGATAAAAACACTTTGTATXXXXXXXXXXT
GCTAGAATGCCTCTAAGTGAGGTCAAGGGGTTTTCAACGTTAATAAGGATTATGGTTGGATATTCTCCCCATCTTGGTT
>Marker563008 Chr3 SNP_site:25821221 Ref_Type:C
ACTACAAATCGTGATCGGATTTTATCTTCGACCTCAGAAATTGGAGAGTGGCTGAAGCTCTGTTGGTGTTGTTTTCATGTXXXXXXXXXXC
TTATTTCACATTTTTTTTACTAGAAACATATTTTTTGGGGGTTAAATTTGCTTTATTTGTCTTCTGTCGTCTCGATGTA
ACTACAAATCGTGATCGGCTTTTATCTTCGACCTCAGAAATTGGAGAGTGGGTGAAGCTCTGTTGGTGTTGTTTTCATGTXXXXXXXXXXC
TTATTTCACATTTTTTTTACTAGAAACATATTTTTTGGGGGTTAAATTTGCTTTATTTGTCTTCTGTCGTCTCGATGTA
>Marker563008 Chr3 SNP_site:25821254 Ref_Type:G
ACTACAAATCGTGATCGGATTTTATCTTCGACCTCAGAAATTGGAGAGTGGCTGAAGCTCTGTTGGTGTTGTTTTCATGTXXXXXXXXXXC
TTATTTCACATTTTTTTTACTAGAAACATATTTTTTGGGGGTTAAATTTGCTTTATTTGTCTTCTGTCGTCTCGATGTA
ACTACAAATCGTGATCGGCTTTTATCTTCGACCTCAGAAATTGGAGAGTGGGTGAAGCTCTGTTGGTGTTGTTTTCATGTXXXXXXXXXXC
TTATTTCACATTTTTTTTACTAGAAACATATTTTTTGGGGGTTAAATTTGCTTTATTTGTCTTCTGTCGTCTCGATGTA
>Marker563087 Chr3 SNP_site:31349907 Ref_Type:T
ACTATTTTCTCTTTCGTCTTGATATATCTCAGGGATTACAAGCTTTTGGCATTAATGGTTCATTACTCAGGAAACATTGAXXXXXXXXXXT
TGTGAAACTTGAGAGGGGCATTGGTTATTTCTATCCAGCAGAGGGTCTCAATTCAAATGACGAGAATTCCGCTCTTGTT
ACTATTTTCTCTTTTGTCTTGATATATCTCAGGGATTACAAGCTTTTGGCATTAATGGTTCATTACTCAGGAAACATTGAXXXXXXXXXXT
TGTGAAGCTTGAGAGGGGCATTGGTTATTTCTATTCAGCAGAGGGTCTCAATTCAAATGACGAGAATTCCGCTCTTGTT
>Marker563087 Chr3 SNP_site:31350087 Ref_Type:G
ACTATTTTCTCTTTCGTCTTGATATATCTCAGGGATTACAAGCTTTTGGCATTAATGGTTCATTACTCAGGAAACATTGAXXXXXXXXXXT
TGTGAAACTTGAGAGGGGCATTGGTTATTTCTATCCAGCAGAGGGTCTCAATTCAAATGACGAGAATTCCGCTCTTGTT
ACTATTTTCTCTTTTGTCTTGATATATCTCAGGGATTACAAGCTTTTGGCATTAATGGTTCATTACTCAGGAAACATTGAXXXXXXXXXXT
TGTGAAGCTTGAGAGGGGCATTGGTTATTTCTATTCAGCAGAGGGTCTCAATTCAAATGACGAGAATTCCGCTCTTGTT
>Marker563087 Chr3 SNP_site:31350115 Ref_Type:T
ACTATTTTCTCTTTCGTCTTGATATATCTCAGGGATTACAAGCTTTTGGCATTAATGGTTCATTACTCAGGAAACATTGAXXXXXXXXXXT
TGTGAAACTTGAGAGGGGCATTGGTTATTTCTATCCAGCAGAGGGTCTCAATTCAAATGACGAGAATTCCGCTCTTGTT
ACTATTTTCTCTTTTGTCTTGATATATCTCAGGGATTACAAGCTTTTGGCATTAATGGTTCATTACTCAGGAAACATTGAXXXXXXXXXXT
TGTGAAGCTTGAGAGGGGCATTGGTTATTTCTATTCAGCAGAGGGTCTCAATTCAAATGACGAGAATTCCGCTCTTGTT
>Marker563110 Chr3 SNP_site:35676896 Ref_Type:A
CACACAATTAATCTCATGGAACAACCTGTTTGACTTTGCTAGACTTGTGGCATATTAAATCCTAAGAAGGTAACTATAATXXXXXXXXXXA
TGCGCTTTCCTTCTTCAAAGTTACTTCCAAATGCAGTTCTCGAAATAACGTCTGCTGAGAGACGACGAACTTCTTTGTG
CACACGATTAATCTCATGGAACAACCTGTTTGACTCTGCTAGACTTGTGGCATATTAAATCCTAAGAAGGTAACTATAATXXXXXXXXXXA
TGCGCTTTCCTTCTTCAAAGTTACTTCCAAATGCAGTTCTTGAAATAACGTCTGCTGAGAGACGACGAACTTCTTTGTG
>Marker563110 Chr3 SNP_site:35676926 Ref_Type:T
CACACAATTAATCTCATGGAACAACCTGTTTGACTTTGCTAGACTTGTGGCATATTAAATCCTAAGAAGGTAACTATAATXXXXXXXXXXA
TGCGCTTTCCTTCTTCAAAGTTACTTCCAAATGCAGTTCTCGAAATAACGTCTGCTGAGAGACGACGAACTTCTTTGTG
CACACGATTAATCTCATGGAACAACCTGTTTGACTCTGCTAGACTTGTGGCATATTAAATCCTAAGAAGGTAACTATAATXXXXXXXXXXA
TGCGCTTTCCTTCTTCAAAGTTACTTCCAAATGCAGTTCTTGAAATAACGTCTGCTGAGAGACGACGAACTTCTTTGTG

Create PDF with GO2PDF for free, if you wish to

>Marker563110 Chr3 SNP_site:35677128 Ref_Type:C
CACACAATTAATCTCATGGAACAACCTGTTTGACTTTGCTAGACTTGTGGCATATTAAATCCTAAGAAGGTAACTATAATXXXXXXXXXXA
TGCGCTTTCCTTCTTCAAAGTTACTTCCAAATGCAGTTCTCGAAATAACGTCTGCTGAGAGACGACGAACTTCTTTGTG
CACACGATTAATCTCATGGAACAACCTGTTTGACTCTGCTAGACTTGTGGCATATTAAATCCTAAGAAGGTAACTATAATXXXXXXXXXXA
TGCGCTTTCCTTCTTCAAAGTTACTTCCAAATGCAGTTCTTGAAATAACGTCTGCTGAGAGACGACGAACTTCTTTGTG
>Marker563120 Chr3 SNP_site:33700094 Ref_Type:G
ACTGTTATTTATGTGGTGCGGATTATATGAGTGAAATGCATCAAATGTAGATAACTGTTTTATTGGTGTTGCGATCTTCCXXXXXXXXXXG
TGAGGGATGATTATTCTGCCCACTGGCTTGATAACTCAGATAACCTTTTGACAGATGCAACACGCTTACAGGTATGGTC
ACTGTTATTTATGTGGTGCGGATTATATGGGTGAAATGCATCAAATGTAGATAACTGTTTTATTGGTGTTGCGATCTTCCXXXXXXXXXXG
TGAGGGATGATTATTCTGCCCACTGGCTTGATAACTCAGATAACCTTTTGACAGATGCAACACGCTTACAGGTATGGTC
>Marker563164 Chr3 SNP_site:36702814 Ref_Type:A
ACTGGCAACACTTGTCCGGGCAAGTGCAGCATTTGAAAGATACTGCAAAAGGAGAATGTATTAGGAACTGCGATTGAGAGXXXXXXXXXXT
TCCAGATTAACCTGATCCCATGTTAATTTGAGATTAACTACATCATCCACCAACTTTCCTCAAACTACAACTAGTGAGT
ACTGGCCACACTTGTCCGGGCAAGTGCAGCATTTGAAAGATACTGCAAAAGGAGAATGTATTAGGAACTGCGATTGAGAGXXXXXXXXXXT
TCCAGATTAACCTGATCCCATGTTAATTTGAGATTAACTACATCATCCACCAACTTTCCTCAAACTACAACTAGTGAGT
>Marker563668 Chr3 SNP_site:1546294 Ref_Type:G
TACATGTAAATATTAGATATTGAAAAAAATTTAAGAATAACTTAGAAGATACATTCTTTATTTTCTTAGCATAGTTATTAXXXXXXXXXXA
ATTTAGGAAATTTTTTGAAAAGTAAGGTAGTTATAATGTTGCATTCATTTGAATGATAGAAAGTTATATATATATGAGT
TACATGTAAATATTAGATATTGAAAAAAATTTAAGAATAACTTAGAAGATACATTCTTTATTTTCTTAGCATAGTTATTAXXXXXXXXXXA
ATTTAGGAAATTTTTTGAAAAGTAAGGTAGTTATAATGTTGCATTCATTTGGATGATAGAAAGTTATATATATATGAGT
>Marker564514 Chr3 SNP_site:34868834 Ref_Type:C
AACTCTACTTCACCAGAATTAGGCTTGAGTTTGGAGTGTGTGGTAGAGATATCATGCTCACTGCCAAAAGTATCCATTTTXXXXXXXXXXA
GAAGCCTTGGCTTTCTCAATAAATTCATCATACTCCCCACATCGAAATGCCTTCACTCTTTCAGACCTTTCAAGATTGT
AACTCTACTTCACCAGAATTAGGCTTGAGTTTGGAGTGTGTGGTAGAGATATCATGCTCACTGCCAAAAGTATCCATTTTXXXXXXXXXXA
GAAGCCTTGGCTTTCTCAATAAATTCATCATACTCCCCACATCGAAATGCCTTCACTCTTTTAGACCTTTCAAGATTGT
>Marker565646 Chr3 SNP_site:6622502 Ref_Type:T
AACGACCAAATAAAATTTTGGTCTTCTTTCACATCATGGCGTGCTTGAAGAATGGAAAGAGAGACCCTTTTAAATGTCTCXXXXXXXXXXC
TCGGTTCATAGAAATGGGCATATACGATATGAAAAGAAAAGGGCTAAAATAATTTCACACGTTGAATTTTAATCTATGT
AACGACCAAATAAAATTTTGGTCTTCTTTCACATCATGGCGTGCTTGAAGAATGGAAAGAGAGACTCTTTTAAATGTCTCXXXXXXXXXXC
TCGGTTCATAGAAATGGGCATATACGATATGAAAAGAAAAGGGCTAAAATAATTTCACACGTTGAATTTTAATCTATGT
>Marker566171 Chr3 SNP_site:13060531 Ref_Type:A
AACCTTCTTTTTACTAGTATTTACATCTGGTTTAGTGGCCAGATTTTTTTGGTGGAATGTCATCTTTCTTGACAAGCTGTXXXXXXXXXXT
CTTGCTCTTTTGAGTGACTTCAATCTTAGATGCAAAGGAAGTAGCATTTGAAAGAAAAGTGAGAAGATACATGTAAGTT
AACCTTCTTTTTACTAGTATTTACATCTGGTTTAGTGGCCGGATTTTTTTGGTGGAATGTCATCTTTCTTGACAAGCTGTXXXXXXXXXXT
CTTGCTCTTTTGACTGACTTCAATCTTAGATGCAAAGGAAGTAGCATTTGAAAGAAAAGTGAGAAGATACATGTAAGTT
>Marker566171 Chr3 SNP_site:13060686 Ref_Type:G
AACCTTCTTTTTACTAGTATTTACATCTGGTTTAGTGGCCAGATTTTTTTGGTGGAATGTCATCTTTCTTGACAAGCTGTXXXXXXXXXXT
CTTGCTCTTTTGAGTGACTTCAATCTTAGATGCAAAGGAAGTAGCATTTGAAAGAAAAGTGAGAAGATACATGTAAGTT
AACCTTCTTTTTACTAGTATTTACATCTGGTTTAGTGGCCGGATTTTTTTGGTGGAATGTCATCTTTCTTGACAAGCTGTXXXXXXXXXXT
CTTGCTCTTTTGACTGACTTCAATCTTAGATGCAAAGGAAGTAGCATTTGAAAGAAAAGTGAGAAGATACATGTAAGTT
>Marker566653 Chr3 SNP_site:9619211 Ref_Type:T
AACTAAAATGAAGAGATTTGTTAAGAATGAAGTTATTGTGTTAAGTTAGAAATTGATAGGAAGAGTAGTAGAAAGCAATGXXXXXXXXXXG
GTGGAATTTAGGACCCACCACACGCACACAACTTTAATTTAATGTTGTTTTATTTGCCTTTGAGCAAATGGTTGAAGTG
AACTAAAATGAAGAGATTTGTTAAGAATGAAGTTATTGTGTTAAGTTATAAATTGATAGGAAGAGTAGTAGAAAGCAATGXXXXXXXXXXG

Create PDF with GO2PDF for free, if you wish to

GTGGAATTTAGGACCCACCACACGCACACAACTTTAATTTAATGTTGTTTTATTTGCCTTTGAGCAAATGGTTGAAGTG

>Marker566778 Chr3 SNP_site:18481733 Ref_Type:C

CACTCTTAATTAATCTCCCAACTGCATTAAGACTTATGTGATCAAGTCTTAAGTGCCATAAATAGGCGTTAGAAGAAACCXXXXXXXXXXA

TAATTTCAAAATATATTCAATACATTTGTTCTAAAATACAAGAAATAGATATTAAATTTCTCAAGATTTTTGAGAAGTA

CACTCTTAATTAATCTTCCAACCGCATTAAGACTTATGTGATCAAGTCTTAAGTGCCATAAATAGGCGTTAGAAGAAACCXXXXXXXXXXA

TAATTTCAAAATATATTCAATACATTTGTTCTAAAATACAAGAAATAGATATTAAATTTCTCAAGATTTTTGAGAAGTA

>Marker566778 Chr3 SNP_site:18481739 Ref_Type:T

CACTCTTAATTAATCTCCCAACTGCATTAAGACTTATGTGATCAAGTCTTAAGTGCCATAAATAGGCGTTAGAAGAAACCXXXXXXXXXXA

TAATTTCAAAATATATTCAATACATTTGTTCTAAAATACAAGAAATAGATATTAAATTTCTCAAGATTTTTGAGAAGTA

CACTCTTAATTAATCTTCCAACCGCATTAAGACTTATGTGATCAAGTCTTAAGTGCCATAAATAGGCGTTAGAAGAAACCXXXXXXXXXXA

TAATTTCAAAATATATTCAATACATTTGTTCTAAAATACAAGAAATAGATATTAAATTTCTCAAGATTTTTGAGAAGTA

>Marker566930 Chr3 SNP_site:35808055 Ref_Type:A

AACCCTCAGCTGCCACCGTCTCTTCATTCCGACGAAGTCACCGCCATTCTCTTCTTCTTTCACTCTCTCGCCTCTCTGATXXXXXXXXXXG

AAGAAAATGAAGAACTGTTTGTTCTCTTCATTGGATTTCTTCTTCAAATTAGTGTTAATGAAAATATAAAAATAATGTA

AACCCTCAGCTGCCACCGTCTCTTCATTCCGACGAAGTCACCGCCATTCTCTTCTTCTTTCACTCTCTCGCCTCTCTGATXXXXXXXXXXG

AAGAAAATGAAGAACTGTTTGTTCTCTTCATTGGATTTCTTCTTCAAATTAGTGTTAATGAAAATATAAAAGCAATGTA

>Marker566930 Chr3 SNP_site:35808056 Ref_Type:T

AACCCTCAGCTGCCACCGTCTCTTCATTCCGACGAAGTCACCGCCATTCTCTTCTTCTTTCACTCTCTCGCCTCTCTGATXXXXXXXXXXG

AAGAAAATGAAGAACTGTTTGTTCTCTTCATTGGATTTCTTCTTCAAATTAGTGTTAATGAAAATATAAAAATAATGTA

AACCCTCAGCTGCCACCGTCTCTTCATTCCGACGAAGTCACCGCCATTCTCTTCTTCTTTCACTCTCTCGCCTCTCTGATXXXXXXXXXXG

AAGAAAATGAAGAACTGTTTGTTCTCTTCATTGGATTTCTTCTTCAAATTAGTGTTAATGAAAATATAAAAGCAATGTA

>Marker566944 Chr3 SNP_site:39012697 Ref_Type:T

ACTCATAGAAAACATAATGCATATTTTTTATATATATCCTCTTTACCTGAAATTGAGAAACTTGTGAAGTTGTTCCATTGXXXXXXXXXXG

TTGTTAATGATGAAGGATTTGAGGAGCTTCCTGATTCCATCTCCAAACATAGCTGCATCCCCAAATCAAATATAGTGTT

ACTCATAGAAAACATAATGCATATTTTTTATATATATCCTCTTTACCTGAAATTGAGAAACTTGTGAAGTTGTTCCATTGXXXXXXXXXXG

TTGTTAATGATGAAGGATTTGAGGAGCTTCCTGATTCCATCTCCAAACATAGCTGCATCTCCAAATCAAATATAGTGTT

>Marker56699 Chr4 SNP_site:16506465 Ref_Type:C

TACCATCAATACTAAATGCAGATTCAAAAGTTGGGAACAACTAGAACATCTTTTGCAATTTCATACAAAACAAGATACTAXXXXXXXXXXC

CTGCTTTCCTCTTTTGCCCAAATGATGTTCCCTTATCAAACCTCTTTGACTGAACCTCATCCTTCTGAATACTCATGTC

TACCATCAATACTAAATGTAGATTCAAAAGTTGGGAACAAGTATAACATCTTTTGCAATTTCATACAAAACAAGATACTAXXXXXXXXXXC

CTGCTTTCCTCTTTTGCCCAAATGATGTTCCCTTATCAAACCTCTTTGACTGAACCTCATCCTTCTGAATACTCATGTC

>Marker56699 Chr4 SNP_site:16506487 Ref_Type:C

TACCATCAATACTAAATGCAGATTCAAAAGTTGGGAACAACTAGAACATCTTTTGCAATTTCATACAAAACAAGATACTAXXXXXXXXXXC

CTGCTTTCCTCTTTTGCCCAAATGATGTTCCCTTATCAAACCTCTTTGACTGAACCTCATCCTTCTGAATACTCATGTC

TACCATCAATACTAAATGTAGATTCAAAAGTTGGGAACAAGTATAACATCTTTTGCAATTTCATACAAAACAAGATACTAXXXXXXXXXXC

CTGCTTTCCTCTTTTGCCCAAATGATGTTCCCTTATCAAACCTCTTTGACTGAACCTCATCCTTCTGAATACTCATGTC

>Marker56699 Chr4 SNP_site:16506490 Ref_Type:G

TACCATCAATACTAAATGCAGATTCAAAAGTTGGGAACAACTAGAACATCTTTTGCAATTTCATACAAAACAAGATACTAXXXXXXXXXXC

CTGCTTTCCTCTTTTGCCCAAATGATGTTCCCTTATCAAACCTCTTTGACTGAACCTCATCCTTCTGAATACTCATGTC

TACCATCAATACTAAATGTAGATTCAAAAGTTGGGAACAAGTATAACATCTTTTGCAATTTCATACAAAACAAGATACTAXXXXXXXXXXC

CTGCTTTCCTCTTTTGCCCAAATGATGTTCCCTTATCAAACCTCTTTGACTGAACCTCATCCTTCTGAATACTCATGTC

>Marker567290 Chr3 SNP_site:33920269 Ref_Type:C

GACATGGTTGGTATTCGTATTTCAATTACTTTCCCTTATCATTGTCCTATCGCATTGATAAACATTTTCCAGTCTCAGTTXXXXXXXXXXG

AGAATATTGAGAGTCAAATTTGTTATGGGTTTGTTTGAGAACCCATTAGCTGATTTAAGCTTGATTAATGAGCTTGGTA

Create PDF with GO2PDF for free, if you wish to

GACATGGTTGGTATTCGTATTTCAATTACTTTCCTTTATCATTGTCCTATCGCATTGATAAACATTTTCCAGTGTCAGTTXXXXXXXXXXG
AGAATATTGAGAGTCAAATTTGTTATGGGTTTGTTTGAGAACCCATTAGCTGATTTAAGCTTGATTAATGAGCTTGGTA
>Marker567290 Chr3 SNP_site:33920308 Ref_Type:C
GACATGGTTGGTATTCGTATTTCAATTACTTTCCCTTATCATTGTCCTATCGCATTGATAAACATTTTCCAGTCTCAGTTXXXXXXXXXXG
AGAATATTGAGAGTCAAATTTGTTATGGGTTTGTTTGAGAACCCATTAGCTGATTTAAGCTTGATTAATGAGCTTGGTA
GACATGGTTGGTATTCGTATTTCAATTACTTTCCTTTATCATTGTCCTATCGCATTGATAAACATTTTCCAGTGTCAGTTXXXXXXXXXXG
AGAATATTGAGAGTCAAATTTGTTATGGGTTTGTTTGAGAACCCATTAGCTGATTTAAGCTTGATTAATGAGCTTGGTA
>Marker567482 Chr3 SNP_site:29384099 Ref_Type:T
AACCACTCAAAATTTTGCAACATTTTGAGTCTTACTTATTCGGCAATATGAGAGTCTTATATATCATTATCGCTTTTCAAXXXXXXXXXXA
AAACCAATATTCTAAGCAAAAAAAACAATTCCACACTACACCTCATCACAACGCCAAGATGACGTTCTTACAAACAGTA
AACCACTCAAATTTTTGCAACATTTTGAGTCTTACTTATTGGGCAATATGAGAGTCTTATATATCATTATCGCTTTTCAAXXXXXXXXXXA
AAACCAATATTCTAAGCAAAAAAAACAATTCCACACTACACCTCATCACAACGCCAAGATGACGTTCTTACAAACAGTA
>Marker567482 Chr3 SNP_site:29384128 Ref_Type:G
AACCACTCAAAATTTTGCAACATTTTGAGTCTTACTTATTCGGCAATATGAGAGTCTTATATATCATTATCGCTTTTCAAXXXXXXXXXXA
AAACCAATATTCTAAGCAAAAAAAACAATTCCACACTACACCTCATCACAACGCCAAGATGACGTTCTTACAAACAGTA
AACCACTCAAATTTTTGCAACATTTTGAGTCTTACTTATTGGGCAATATGAGAGTCTTATATATCATTATCGCTTTTCAAXXXXXXXXXXA
AAACCAATATTCTAAGCAAAAAAAACAATTCCACACTACACCTCATCACAACGCCAAGATGACGTTCTTACAAACAGTA
>Marker567527 Chr3 SNP_site:5748316 Ref_Type:G
AACCATTATTTATGTAATGACTACATATCCTAGCGGGAATAAGCGGAGTATGCATTGACAATTGTCTTGAACATACAATAXXXXXXXXXXT
AGAGGAAATCCTGGAATTGAATCTACACATTACTGTTTATCCAGAAAAAGAAAAGGCACATATATTTATGGAAACAGTC
AACCATTATTTATGTAATGACTACATATCCTAGCGGGAATAAGCGGAGTATGCATTGACAATTGTCTTGAACATACAGTAXXXXXXXXXXT
AGAGGAAATCCTGGAATTGAATCTACACATTACTGTTTATCCAGAAAAAGAAAAGGCACATATATTTATGGAAACAGTC
>Marker567739 Chr3 SNP_site:10740601 Ref_Type:T
TACTTGAATCTTTGGTTTAACACATCCTCACAATTTGTCCCCATTGCTCAGTTGATATGTCCATCAAAGAGGGAGGTGATXXXXXXXXXXT
CAACATGGGGCTCTGAGGGTGGCTGCTGTTGTGCAGCTTGTTTGAGCAATGGTATGTATGTATTTATTTTTGTGATGGT
TACTTGAATCTTTGGTTTAACACATCCTCACAATTTTTCCCCATTGCTCAGTTGATATGTCCATCAAAGAGGGAGGTGATXXXXXXXXXXT
CAACATGGGGCTCTGAGGGTGGCTGCTGTTGTGCAGCTTGTTTGAGCAATGGTATGTATGTATTTATTTTTGTGATGGT
>Marker567944 Chr3 SNP_site:30646285 Ref_Type:T
CACGCTTACACCCACTCATACACATCCTTTTATCTACATTCTTAATCACGAAAGCTTTGCTTTACAATTGAACCTAGAACXXXXXXXXXXC
AATTGGGTTGGGATTTTCAGATTTCTATTTCTGTGTGAGTATTTGAGTGGTTCTATTCGTGATTGTGAAGACATGTGGT
CACGCTTACACCCACTCATACACATCCTTTTATCTACATTTTTAATCACGAAAGCTTTGCTTTACAATTGAACCTAGAACXXXXXXXXXXC
AATCGGGTTGGGATTTTCAGATTTCTATTTCTGTGTGAGTATTTGAGTGGTTCTATTCGTGATTGTGAAGACATGTGGT
>Marker567944 Chr3 SNP_site:30646437 Ref_Type:C
CACGCTTACACCCACTCATACACATCCTTTTATCTACATTCTTAATCACGAAAGCTTTGCTTTACAATTGAACCTAGAACXXXXXXXXXXC
AATTGGGTTGGGATTTTCAGATTTCTATTTCTGTGTGAGTATTTGAGTGGTTCTATTCGTGATTGTGAAGACATGTGGT
CACGCTTACACCCACTCATACACATCCTTTTATCTACATTTTTAATCACGAAAGCTTTGCTTTACAATTGAACCTAGAACXXXXXXXXXXC
AATCGGGTTGGGATTTTCAGATTTCTATTTCTGTGTGAGTATTTGAGTGGTTCTATTCGTGATTGTGAAGACATGTGGT
>Marker568090 Chr3 SNP_site:34256397 Ref_Type:G
AACAAAAGTTTTTCTTCTTGAACTAACACATCAATTTTAGTTGCTAAATGTTGTAAATTAATCTTTTTCGAAATTTATAAXXXXXXXXXXG
TCTTACACTCTCATAGTTTGTAATATTTTGATACATTCTTTAGTTTCTTATGTTTTGTATTAAAGCACTCTTGTTTGTG
AACAAGAGTTTTTCTTTTTGAACTAACACATCAATTTTAGTTGCTAAATGTTGTAAATTAATCTTTTTCAAAATTTATAAXXXXXXXXXXG
TCTTACACTCTCATAGTTTGTAATATTTTGATACATTCTTTAGTTTCTTATGTTTTGTATTAAAGCACTCTTGTTTGTG
>Marker568090 Chr3 SNP_site:34256408 Ref_Type:T
AACAAAAGTTTTTCTTCTTGAACTAACACATCAATTTTAGTTGCTAAATGTTGTAAATTAATCTTTTTCGAAATTTATAAXXXXXXXXXXG

Create PDF with GO2PDF for free, if you wish to

TCTTACACTCTCATAGTTTGTAATATTTTGATACATTCTTTAGTTTCTTATGTTTTGTATTAAAGCACTCTTGTTTGTG
AACAAGAGTTTTTCTTTTTGAACTAACACATCAATTTTAGTTGCTAAATGTTGTAAATTAATCTTTTTCAAAATTTATAAXXXXXXXXXXXG
TCTTACACTCTCATAGTTTGTAATATTTTGATACATTCTTTAGTTTCTTATGTTTTGTATTAAAGCACTCTTGTTTGTG
>Marker568090 Chr3 SNP_site:34256461 Ref_Type:A
AACAAAAGTTTTTCTTCTTGAACTAACACATCAATTTTAGTTGCTAAATGTTGTAAATTAATCTTTTTCGAAATTTATAAXXXXXXXXXXXG
TCTTACACTCTCATAGTTTGTAATATTTTGATACATTCTTTAGTTTCTTATGTTTTGTATTAAAGCACTCTTGTTTGTG
AACAAGAGTTTTTCTTTTTGAACTAACACATCAATTTTAGTTGCTAAATGTTGTAAATTAATCTTTTTCAAAATTTATAAXXXXXXXXXXXG
TCTTACACTCTCATAGTTTGTAATATTTTGATACATTCTTTAGTTTCTTATGTTTTGTATTAAAGCACTCTTGTTTGTG
>Marker568273 Chr3 SNP_site:26384404 Ref_Type:T
CACTTGGCTGTGATTCGTTGTTCCTTTTTTCTGACTTTTTCATCTTTTGAATAAGAATTTCTAGTTTATTTTTTTAAAGAXXXXXXXXXXXT
TGATGGAAGAGAACTTCTTTGGAGGAACTCATTTGGGCTACAATCATAAATCATCTTAGACCAGGAATTCTGCTTGGTC
CACTTGGCTGTGATTCTTTGTTCCTTTTTTCTGACTTTTTCATCTTTTGAATAAGAATTTCTAGTTTATTTTTTTAAAGAXXXXXXXXXXXT
TGATGGAAGAGAACTTCTTTGGAGGAACTCATTTGGGCTACAATCATAAATCATCTTAGACCAGGAATTCTGCTTGGTC
>Marker568490 Chr3 SNP_site:25139435 Ref_Type:A
CACTGCGATTCTTGCCCTTTAAGGTGGAAAAAATGTTTAGGAGGGAACATTAAGGAAGAAGTATCTTATTTTAATGAACAXXXXXXXXXXXC
CCAACACCAAAAAGTGCATTACAAGAAAATCTTCAATTTAAAAGAAGAGAGGAAAGATGGTAATTGGTAAAAGGGTGTA
CACTGCGATTCTTGCCCTTTAAGGTGGAAAAAATGTTTAGGAGGGAACATTAAGGAAGAAGTATCTTATTTTAATGAACAXXXXXXXXXXXC
CCAACACCAAAAAGTGCATTACAAGAAAATCTTCAATTTAAAAGAAGAGAGGAAAGATGGTAATTGGTAAAGGGGTGTA
>Marker56865 Chr4 SNP_site:8412100 Ref_Type:C
ACCCATGCAATAAGACGCCTTACACTTGTTGTGTGCTTTAGTGTGGGATGTCTTATCTTTGTTGTTTGGTTTGGTATAAGXXXXXXXXXXXC
GCAACTCAACAAAAGAGTGTGGTCAATGCTTGAGATGAGGAAAGACCACAAAGATATAATTAAACATGCATAATGAGTC
ACCCATGCAATAAGACGCTTTACACTTGTTGTGTGCTTTAGTGTGGGATGTCTTATCTTTGTTGTTTGGTTTGGTATAAGXXXXXXXXXXXC
GCAACTCAACAAAAGAGTGTGGTCAATGCTTGAGATGAGGAAAGACCACAAAGATATAATTAAACATGCATAATGAGTC
>Marker568991 Chr3 SNP_site:28104793 Ref_Type:T
AACATCAGCTTCCAAAGGACATTGAGATGTGACGAATGTTTCCTTCAATATAGCCTTCTCTCTCTCCATGTTTGCATCCAXXXXXXXXXXXT
TAAAGCAACCGTAAATGGTATTCTCCTGCAAATGGCCAAGCTCATCCCTTGAGAAAACTTTATTAGGAGGTTTAATGTT
AACATCAGCTTCCAAAGGACATTGAGATGTGATGAATGTTTCCTTCAATATAGCCTTCTCTCTCTCCATGTTTGCATCCAXXXXXXXXXXXT
TAAAGCAACCGTAAATGGTATTCTCCTGCAAATGGCCAAGCTCATCCCTTGAGAAAACTTTATTAGGAGGTTTAATGTT
>Marker56917 Chr4 SNP_site:19447197 Ref_Type:C
ACCTTAAAGTGAGCATTTTGTTAAATAAAAATATCCCTTGTAACGTTTAAAATGATTATGTATATGAAATGTTTGATATGXXXXXXXXXXXT
AAAACATAATAAAAAATATTCTTTGAGTTAATTTCATCATTTGAACTTCTAATCATTTTAAAATGATACTTCACATGTT
ACCTTAAAGTGAGCATTTTGTTAAATAAAAATATCCCTTGTAACGTTTAAAATGATTATGTATATGAAATGTTTGATATGXXXXXXXXXXXT
AAAACATAATAAAAAATATTCTTTGAGTTAATTTGATCATTTGAACTTCTAATCATTTTAAAATGATACTTCACATGTT
>Marker56919 Chr4 SNP_site:14971488 Ref_Type:T
AACCCAACAACCCAAACCATTTTTCCCTCAATATTTCTTTAACCCAACCAAATCCAAAACATAATTTAACCCAATCCAATXXXXXXXXXXXT
ACACAGTCCTAAAAAGCTTCATCTTCGATGCACAATGAGATAAAACAACCATGGAAGAAACTTCCATGTTTCAGATGTC
AACCCAACAACCCAAACCATTTTTCCCTCAATATTTCTTTAACCCAACCAAATCCAAAACATAATTTAACCCAATCTAATXXXXXXXXXXXT
ACACAGTCCTAAAAAGCTTCATCTTCGATGCACAATGAGATAAGACAACCATGGAAGAAACTTCCATGTTTCAGATGTC
>Marker56919 Chr4 SNP_site:14971636 Ref_Type:G
AACCCAACAACCCAAACCATTTTTCCCTCAATATTTCTTTAACCCAACCAAATCCAAAACATAATTTAACCCAATCCAATXXXXXXXXXXXT
ACACAGTCCTAAAAAGCTTCATCTTCGATGCACAATGAGATAAAACAACCATGGAAGAAACTTCCATGTTTCAGATGTC
AACCCAACAACCCAAACCATTTTTCCCTCAATATTTCTTTAACCCAACCAAATCCAAAACATAATTTAACCCAATCTAATXXXXXXXXXXXT
ACACAGTCCTAAAAAGCTTCATCTTCGATGCACAATGAGATAAGACAACCATGGAAGAAACTTCCATGTTTCAGATGTC
>Marker56931 Chr4 SNP_site:21826473 Ref_Type:A

Create PDF with GO2PDF for free, if you wish to

AACCAGGTATTTAGGAACAAGGATGGGACTCTCTGTGGCTCCCATTCCCATCCTCTCCTTACTTTACACACAAAAGATATXXXXXXXXXXT
CGATCTCTCTTTTTCTTTTACCCGAATATTACACATTTATCCACTCTTTGCGTCCAATCCATCAACATTTATTTATGTG
AACCAGGTATTTAGGGACAAGGATGGGACTCTCTGTGGCTCCCATTCCCATCCTCTCCTTACTTTACACACAAAAGATATXXXXXXXXXXT
CGATCTCTCTTTTTCTTTTACCCGAATATTACACATTTATCCACTCTTTGCGTCCAATCCATCAACATTTATTTATGTG
>Marker569378 Chr3 SNP_site:30751275 Ref_Type:A
TACCACAATTTTCATTGTTCTTGTGGATTTACTCCTCTACTTGAGGACTGTTTTCTTTTTCTCTCCATATAAATCTACCCXXXXXXXXXXT
TTAGAGTGAAAATGTGAATATAATTACACTTGCACATTTCTATTAAACATATATTAAGAACGTAACCATTTTTATTTGT
TACCACAATTTTCATTGTTCTTGTGGATTTACTCCTCTACTTGAGGACTGTTTTCTTTTTCTCTCCATATAAATCTACCCXXXXXXXXXXT
TTAGAGTGAAAATGTGAATATAATTACACTTGCACATTTCTATTTAACATATATTAAGAACGTAACCATTTTTATTTGT
>Marker56960 Chr4 SNP_site:7922503 Ref_Type:G
AACAGAAAATATGAAAATGTAAAAGAAATAACAAACTTTCTCGTTTTTTGTTTATCCTATCTCTCAAGGATGAAAACAGAXXXXXXXXXXC
TTCTTCCCTTTTCTACTATATTGTAAAATAATAGGTGGTCTAACATATTGATAGGCTTCCAACTTGTTATACAAAAGGT
AACAGAAAATATGAAAATGTAAAAGAAATAACAAACTTTCTCGTTTTTTGTTTATCCTATCTCTCAAGGATGAAAACAGAXXXXXXXXXXC
TTCTTCCCTTTTCTACTATATTGTAAAATAATAGGTGGTCTAACGTATTGATAGGCTTCCAACTTGTTATACGAAAGGT
>Marker56960 Chr4 SNP_site:7922531 Ref_Type:G
AACAGAAAATATGAAAATGTAAAAGAAATAACAAACTTTCTCGTTTTTTGTTTATCCTATCTCTCAAGGATGAAAACAGAXXXXXXXXXXC
TTCTTCCCTTTTCTACTATATTGTAAAATAATAGGTGGTCTAACATATTGATAGGCTTCCAACTTGTTATACAAAAGGT
AACAGAAAATATGAAAATGTAAAAGAAATAACAAACTTTCTCGTTTTTTGTTTATCCTATCTCTCAAGGATGAAAACAGAXXXXXXXXXXC
TTCTTCCCTTTTCTACTATATTGTAAAATAATAGGTGGTCTAACGTATTGATAGGCTTCCAACTTGTTATACGAAAGGT
>Marker570073 Chr3 SNP_site:33972104 Ref_Type:C
TACGCATATCTCGACTAATCTCACAGGACAACCTGCTTAACCCTACAACATTAGGGTGTCAAGGTAACTCATAGGAAATTXXXXXXXXXXT
GAAAATAGGCCAATGCTCCAAGATAGGAGAGGAAAATCAATTACACAAAACTATTAATCCATTTGGTGATGAAAATGTT
TACGCATATCTCGACTAATCTCACAGGACAACCTGCTTAACTCTACAACATTAGGGTGTCAAGGTAACTCATAGAAAATTXXXXXXXXXXT
GAAAATAGGCCAATGCTCCAAGATAGGAGAGGAAAATCAATTACACAAAACTATTAATCCATTTGGTGATGAAAATGTT
>Marker570073 Chr3 SNP_site:33972137 Ref_Type:G
TACGCATATCTCGACTAATCTCACAGGACAACCTGCTTAACCCTACAACATTAGGGTGTCAAGGTAACTCATAGGAAATTXXXXXXXXXXT
GAAAATAGGCCAATGCTCCAAGATAGGAGAGGAAAATCAATTACACAAAACTATTAATCCATTTGGTGATGAAAATGTT
TACGCATATCTCGACTAATCTCACAGGACAACCTGCTTAACTCTACAACATTAGGGTGTCAAGGTAACTCATAGAAAATTXXXXXXXXXXT
GAAAATAGGCCAATGCTCCAAGATAGGAGAGGAAAATCAATTACACAAAACTATTAATCCATTTGGTGATGAAAATGTT
>Marker570172 Chr3 SNP_site:31232424 Ref_Type:A
ACTTTTGTTTGAAAGCCTTGAGAATCTCACCCACAAAATTGTTTCTACAACCATAGCTTTGATATTAATTGATTTGGATAXXXXXXXXXXT
GATCTTCTGCTACGAACTTAAATCTTTAACATCTTAACCTCTATCTAGGAGAAAGCATCGATTGGTCAGAGTCGAAGTC
ACTTTTGTTTGAAAGCCTTGAGAATCTCACCCACAAAATTGTTTCTACAACCATAGCTTTGATATTAATTGATTTGGATAXXXXXXXXXXT
GATCTTCTGCTACGAACTTAAATCTTTAACATCTTAACCTCTATCTAGGAGCAAGCATCGATTGGTCAGAGTCGAAGTC
>Marker570325 Chr3 SNP_site:36538248 Ref_Type:T
AACTTGATTTGATTCCTTTATTGTTTTGTTCTGATTTGATTCCTTTATTGTTTTGTTCTCTTTTCGAAATCACAGTTTAAXXXXXXXXXXT
GACAAAGTTTTCATCAATTATCAGCTTTTTTATTTTATGATTGTCTGAGGGTATATCCAATTACAATGCCTTTTCTGTT
AACTTGATTTGATTCCTTTATTGTTTTGTTCTGATTTTATTCCTTTATTGTTTTGTTCTCTTTTCGAAATCACAGTTTAAXXXXXXXXXXT
GACAAAGTTTTCGTCAATTATCAGCTTTTTTATTTTATGATTGTCTGAGGGTATATCCATTTACAATGCCTTTTCTGTT
>Marker570325 Chr3 SNP_site:36538437 Ref_Type:G
AACTTGATTTGATTCCTTTATTGTTTTGTTCTGATTTGATTCCTTTATTGTTTTGTTCTCTTTTCGAAATCACAGTTTAAXXXXXXXXXXT
GACAAAGTTTTCATCAATTATCAGCTTTTTTATTTTATGATTGTCTGAGGGTATATCCAATTACAATGCCTTTTCTGTT
AACTTGATTTGATTCCTTTATTGTTTTGTTCTGATTTTATTCCTTTATTGTTTTGTTCTCTTTTCGAAATCACAGTTTAAXXXXXXXXXXT
GACAAAGTTTTCGTCAATTATCAGCTTTTTTATTTTATGATTGTCTGAGGGTATATCCATTTACAATGCCTTTTCTGTT

Create PDF with GO2PDF for free, if you wish to

>Marker570325 Chr3 SNP_site:36538484 Ref_Type:T
AACTTGATTTGATTCCTTTATTGTTTTGTTCTGATTTGATTCCTTTATTGTTTTGTTCTCTTTTCGAAATCACAGTTTAAXXXXXXXXXXT
GACAAAGTTTTCATCAATTATCAGCTTTTTTATTTTATGATTGTCTGAGGGTATATCCAATTACAATGCCTTTTCTGTT
AACTTGATTTGATTCCTTTATTGTTTTGTTCTGATTTTATTCCTTTATTGTTTTGTTCTCTTTTCGAAATCACAGTTTAAXXXXXXXXXXT
GACAAAGTTTTCGTCAATTATCAGCTTTTTTATTTTATGATTGTCTGAGGGTATATCCATTTACAATGCCTTTTCTGTT
>Marker5704 Chr4 SNP_site:17528225 Ref_Type:C
ACTTAGATTATATAACCCTATTCTTATATAATAAATTGACGAATCAATCTGTATGATCTATCTCAAGAATCTTTCAATTGXXXXXXXXXXC
CTTAGAGTATTATTAAACACGAAGCTTCTTGTGTGGTTAAATCGGACTTCGCTACTCTTACTATCAAGAAGTTGTGGTT
ACTTAGATTATATAACCCTATTCTTATATAATAAATTGACGAATCAATCTGTATGATCTATCTCAAGAATCTTTGAATTGXXXXXXXXXXC
CTTAGAGTATTATTAAACACGAAGCTTCTTGTGTGGTTAAATCGGACTTCGCTACTCTTACTATCAAGAAGTTGTGGTT
>Marker570797 Chr3 SNP_site:21725676 Ref_Type:C
CACTTTAATTTTGGGCCAGACTCTCAATTTATGTTTCTGATTTTGACGCTCATGCTCTTATCAAGGATCTGCCTTTGTAAXXXXXXXXXXA
TGTTTCTTTCTACATCACTAGGGTTGTGATCAGCCATTGATTGTTCGTCTGGCAGACCCTAAGAAACCCAGGATTGGTG
CACTTTAATTTTGGGCCAGACTCTCAATTTATGTTTCTGATTTTGACGCTCATGTTCTTATCAAGGATCTGCCTTTGTAAXXXXXXXXXXA
TGTTTCTTTCTACATCACTAGGGTTGTGATCAGCCATTGATTGTTCGTCTGGCAGACCCTAAGAAACCCAGGATTGGTG
>Marker570971 Chr3 SNP_site:2687120 Ref_Type:T
AACTCATGTTTCTTTAAAATCCAAATAATCTTAAATAGGTCTCATTACTTTGCATCATTACTCCAAATCTAGCTAAACATXXXXXXXXXXA
GATAAACATCGTTTGATTTTTATCTAAAATAAGAAATTATTTAGTCGTATAATAAATCAATTCTATCTAATTAAGTGTA
AACTCATGTTTCTTTATAATCCAAATAATCTTAAATAGGTCTCATTACTTTGCATCATTACTCCAAATCTAGCTAAACATXXXXXXXXXXA
GATAAACATCGTTTGATTTTTATCTAAAATAAGAAATTATTTAGTCGTATAATAAATCAATTCTATCTAATTAAGTGTA
>Marker57114 Chr4 SNP_site:5425941 Ref_Type:C
AACCTCTGCAAATCTTGGTTCTTGCCTTCATAAACATGCTCATATTTCCAGTGAATGCTTGTTGGTAGTATTATAAATTTXXXXXXXXXXT
CATCTTTAGCTTCCCCCGACGATCTGTTTAACTTCTTCAGTGAGATGCGAGGTTAGTTCTGCAAATTTGTATTGTTGTT
AACCTCTGCAAATCTTGGTTCTTGCCTTCATAAACATGCTCATATTTCCCGTGAATGCTTGTTGGTAGTATTATAAATTTXXXXXXXXXXT
CATCTTTAGCTTCCCCCGACGATCTGTTTAACTTCTTCAGTGAGATGCGAGGTTAGTTCTGCAAATTTGTATTGTTGTT
>Marker571673 Chr3 SNP_site:1038011 Ref_Type:G
ACTATAGATTCTCAATGGTGCTTTGATTTTACTATGTTGTTTATATGCCCTTATAATATTGCACAAAATGGGAAGAGTGTXXXXXXXXXXC
AAAATGCAGATTGGGAGACATGTGTAGCTGTGACCGATTTTGACTGGAAACAGATTGCAGAACCCGTTATGCAATTGTA
ACTATAGATTCTCAATGGTGCTTTGATTTTACTATGTTGTTTATATGCCCTTATAATATTGCACAAAGTGGGAAGAGTGTXXXXXXXXXXC
AAAATGCAGATTGGGAGACATGTGTAGCTGTGACCGATTTTGACTGGAAACAGATTGCAGAACCCGTTATGCAATTGTA
>Marker572333 Chr3 SNP_site:23801945 Ref_Type:C
GACCAATACTTCGAAGACCTAATGTTCAACAATCCCATCTACAAGAAAATTGAAAATGGATAATGTTTTGAGGGGCCATGXXXXXXXXXXG
GTAGATGGCTCGTCTCCCTTCCTTCAATTCGTGCATTTTGGCTCCTTTTGATTACCAATGAATCCTTGTTTGTTCAGTA
GACCAATACTTCGAAGACCTAATGTTCAACAATCCCATCTACAAGAAAATTGAAAATGGATAATGTTTTGAGGGGCCATGXXXXXXXXXXG
GTAGATGGCTCGTCTCCCTTCCTTCAATTCGTGCATTTTGGCTCCTTTTGATTACTAATGAATCCTTGTTTGTTCAGTA
>Marker572516 Chr3 SNP_site:27133001 Ref_Type:T
AACTCTTCTAGATTTCCATATGCCCCTAATGAACTGAGAATCAAATTTGCCCCTTTAAGTCTCTGTTGGGATCACGTGTGXXXXXXXXXXA
ATACAAGATTATCTAATTTTGGTGACTCCCTTCCTAATCTCACTCTTTGTTGAGTTTTATTTTCCTTTTCTGAAACGTA
AACTCTTCTAGATTTCCATATGCCCCTAATGAACTGAGAATCAAATTTGCCCCTTTAAGTCTCTGTTGGGATCACGTGTGXXXXXXXXXXA
ATACAAGATTATCTAATTTTGGTGACTCCCTTCCTAATTTCACTCTTTGTTGAGTTTTATTTTCCTTTTCTGAAACGTA
>Marker572606 Chr3 SNP_site:12687958 Ref_Type:A
TACCCAGCAATGTATATAGAGAATGTTACCGAGCTTGCTTTTGATGATATTACACCCCAAGACCCTGATTTTGCATCTATXXXXXXXXXXT
CTTCTTCATTGGACGAAGACCAGGGTCCCTTATATTTCTCTCCTGAAAGGTTATTTCCATTTTATCCTTTATTCTGGTT
TACCCAGCAATGTATATAGAGAATGTTACCGAGCTTGCTTTTGATGATATTACACCCCAAGACCCTGATTTTGCATCTATXXXXXXXXXXT

Create PDF with GO2PDF for free, if you wish to

CTTCTTCATTGGACGAAGACCAGGGTCCCTTATATTTCTCTCCTGAAAGGTTATTTCCTCTTTATCCTTTATTCTGGTT
>Marker572606 Chr3 SNP_site:12687959 Ref_Type:T
TACCCAGCAATGTATATAGAGAATGTTACCGAGCTTGCTTTTGATGATATTACACCCCAAGACCCTGATTTTGCATCTATXXXXXXXXXXT
CTTCTTCATTGGACGAAGACCAGGGTCCCTTATATTTCTCTCCTGAAAGGTTATTTCCATTTTATCCTTTATTCTGGTT
TACCCAGCAATGTATATAGAGAATGTTACCGAGCTTGCTTTTGATGATATTACACCCCAAGACCCTGATTTTGCATCTATXXXXXXXXXXT
CTTCTTCATTGGACGAAGACCAGGGTCCCTTATATTTCTCTCCTGAAAGGTTATTTCCTCTTTATCCTTTATTCTGGTT
>Marker572638 Chr3 SNP_site:17288990 Ref_Type:T
ACCGAAGCTCTAAAGAATTGAAGATTCAAACTTCTACAAGCTTTCTAGACGTGCAACTTCGTATCAACCTCGAGATCAACXXXXXXXXXXT
ACTAGAGACTGTATTCACAATATTCATCAATATAACAAAGTTCAATTTCACGAAATACGTTTCTTTGAAATCTCGTGTG
ACCGAAGCTCTAAAGAATTGAATATTCAAACTTCTACAAGCTTTCTAGACGTGCAAGTTCGTTTCAACCTCGAGATCAACXXXXXXXXXXT
ACTAGAGACTGTATTCACAATATTCATCAATATAACAAAGTTCAATTTCACGAAATACGTTTCTTTGAAATCTCGTGTG
>Marker572638 Chr3 SNP_site:17289024 Ref_Type:G
ACCGAAGCTCTAAAGAATTGAAGATTCAAACTTCTACAAGCTTTCTAGACGTGCAACTTCGTATCAACCTCGAGATCAACXXXXXXXXXXT
ACTAGAGACTGTATTCACAATATTCATCAATATAACAAAGTTCAATTTCACGAAATACGTTTCTTTGAAATCTCGTGTG
ACCGAAGCTCTAAAGAATTGAATATTCAAACTTCTACAAGCTTTCTAGACGTGCAAGTTCGTTTCAACCTCGAGATCAACXXXXXXXXXXT
ACTAGAGACTGTATTCACAATATTCATCAATATAACAAAGTTCAATTTCACGAAATACGTTTCTTTGAAATCTCGTGTG
>Marker572638 Chr3 SNP_site:17289030 Ref_Type:T
ACCGAAGCTCTAAAGAATTGAAGATTCAAACTTCTACAAGCTTTCTAGACGTGCAACTTCGTATCAACCTCGAGATCAACXXXXXXXXXXT
ACTAGAGACTGTATTCACAATATTCATCAATATAACAAAGTTCAATTTCACGAAATACGTTTCTTTGAAATCTCGTGTG
ACCGAAGCTCTAAAGAATTGAATATTCAAACTTCTACAAGCTTTCTAGACGTGCAAGTTCGTTTCAACCTCGAGATCAACXXXXXXXXXXT
ACTAGAGACTGTATTCACAATATTCATCAATATAACAAAGTTCAATTTCACGAAATACGTTTCTTTGAAATCTCGTGTG
>Marker57282 Chr4 SNP_site:7169891 Ref_Type:A
ACTCCAAAATCGCATTATTTGAAGCCTCCTACTTTTCTCCTAAATTTGAACATATTTCTAATCAGAATTGGCTAATGAGTXXXXXXXXXXA
TTTTTCTACTTCAAAAATCATTCACTTAACCTCAGAATTTACTCAAGCTTTATTCACGAAACTTGTTAGAAGTTGAGTT
ACTCCAAAATCGCATTATTTGAAGCCTCCTACTTTTCTCCTAAATTTGAACATATTTCTAATCAGAATTGGCTAATGAGTXXXXXXXXXXA
TTTTTCTACTTCAAAAATCATTCACTTAACCTCAGAATTTGCTCAAGCTTTATTCACGAAACTTGTTAGAAGTTGAGTT
>Marker57308 Chr4 SNP_site:21067425 Ref_Type:G
CACCTCGAAGGCCATGAATAAAAACAACATCTACCAAAGGAGAATCTGGCCTAGAATCATTCTGAGACACATGAGATAAAXXXXXXXXXXG
AACCTTCCAGTGAGGCAGTTCAGGATTTATTAAAAAAACCATATCATCATAACGGGGACATTTCTTTTTCCTGTTTGTA
CACCTCGGAGGCCATGAATAAAAACAACATCTACCAAAGGAGAATCTGGCCTAGAATCATTCTGAGACACATGAGATAAAXXXXXXXXXXG
AACCTTCCAGTGAGGCAGTTCAGGATTTATTAAAAAAACCATATCATCATAACGGGGACAATTCTTTTTCCTGTTTGTA
>Marker57308 Chr4 SNP_site:21067671 Ref_Type:A
CACCTCGAAGGCCATGAATAAAAACAACATCTACCAAAGGAGAATCTGGCCTAGAATCATTCTGAGACACATGAGATAAAXXXXXXXXXXG
AACCTTCCAGTGAGGCAGTTCAGGATTTATTAAAAAAACCATATCATCATAACGGGGACATTTCTTTTTCCTGTTTGTA
CACCTCGGAGGCCATGAATAAAAACAACATCTACCAAAGGAGAATCTGGCCTAGAATCATTCTGAGACACATGAGATAAAXXXXXXXXXXG
AACCTTCCAGTGAGGCAGTTCAGGATTTATTAAAAAAACCATATCATCATAACGGGGACAATTCTTTTTCCTGTTTGTA
>Marker573381 Chr3 SNP_site:451153 Ref_Type:C
ACTTCTTCATCACATATTCCGAGAGCTTAACCTGAGCTGTGGTTATTTCAGATGTGGAATTCAAAACACTCCACATCCGTXXXXXXXXXXG
AGTATTGTTAGCAGCCCAATCAAAATAGCTAGCATCACCTGTATTTTAATGTAATAAATCATCAAAGCCAGTATTAGTG
ACTTCTTCATCACATATTCTGAGAGCTTAACCTGAGCTGTGGTTATTTCAGATGTGGAATTCAAAACACTCCACATCCGTXXXXXXXXXXG
AGTATTGTTAGCAGCCCAATCAAAATAGCTAGCATCACCTGTATTTTAATGTAATAAATCATCAAAGCCAGTATTAGTG
>Marker573624 Chr3 SNP_site:7252142 Ref_Type:C
GACTATTTGGAATGCCTTGTGGAGGAATTTTTTTCAAAAAGCAATTCCCATTTGACTCCACCAAACAAAGACCGGTCTCCXXXXXXXXXXC
TGTTGCTGTGGCATCAGGCTGAAGGGCTTCTGCTTCCATCCCTCATTGTTGATTGGGTGCTGGGACAACTTGAGGTGTG

Create PDF with GO2PDF for free, if you wish to

GACTATTTGGAATGCCTTGTGGAGGAATTTTTTTCAAAAAGCAATTCCCATTTGACTGCACCAAACAAAGACCGGTCTCCXXXXXXXXXXC
TGTTGCTGTGGCATCAGGCTGAAGGGCTTCTGCTTCCATCCCTCATTGTTGATTGGGTGCTGGGACAACTTGAGGTGTG
>Marker573723 Chr3 SNP_site:33389958 Ref_Type:G
ACAAAAGTTCAAGGTATATTTCTATCGTTTATTCCAAATACCAAAACTTAAAAAAAATTGTATTTATGTGATTACTTAATXXXXXXXXXXT
AAAACGAACATTTAAGTTCAAAGATTAACCTTGTAGTTTAATCTGTAATTTAAAGTATCTTTGCTACTTTACATAGGTA
ACAAAAGTTCAAGGTATATTTCTATCGTTTATTCCAAATACCAAAACTTAAAAAAAATTGTATTTATGTGATTACTTAATXXXXXXXXXXT
AAAAGGGACATTTAAGTTCAAAGATTAACCTTGTAGTTTAATCTGTATTTTAAAGTATCTTTGCTACTTTACATAGGTA
>Marker573723 Chr3 SNP_site:33389960 Ref_Type:G
ACAAAAGTTCAAGGTATATTTCTATCGTTTATTCCAAATACCAAAACTTAAAAAAAATTGTATTTATGTGATTACTTAATXXXXXXXXXXT
AAAACGAACATTTAAGTTCAAAGATTAACCTTGTAGTTTAATCTGTAATTTAAAGTATCTTTGCTACTTTACATAGGTA
ACAAAAGTTCAAGGTATATTTCTATCGTTTATTCCAAATACCAAAACTTAAAAAAAATTGTATTTATGTGATTACTTAATXXXXXXXXXXT
AAAAGGGACATTTAAGTTCAAAGATTAACCTTGTAGTTTAATCTGTATTTTAAAGTATCTTTGCTACTTTACATAGGTA
>Marker573723 Chr3 SNP_site:33390001 Ref_Type:T
ACAAAAGTTCAAGGTATATTTCTATCGTTTATTCCAAATACCAAAACTTAAAAAAAATTGTATTTATGTGATTACTTAATXXXXXXXXXXT
AAAACGAACATTTAAGTTCAAAGATTAACCTTGTAGTTTAATCTGTAATTTAAAGTATCTTTGCTACTTTACATAGGTA
ACAAAAGTTCAAGGTATATTTCTATCGTTTATTCCAAATACCAAAACTTAAAAAAAATTGTATTTATGTGATTACTTAATXXXXXXXXXXT
AAAAGGGACATTTAAGTTCAAAGATTAACCTTGTAGTTTAATCTGTATTTTAAAGTATCTTTGCTACTTTACATAGGTA
>Marker57386 Chr4 SNP_site:12704420 Ref_Type:G
ACTAATGTTTTAAATCAGTCAAGTTAAGTTTTATGTATTTATTTGAGAGGTAGAAAACACAATTGAGAACTTGATAAGTAXXXXXXXXXXT
AATCTAATTGAAAGATTAACACGAACGCGATAATCAAGACGTCTCACTAGAGATTTTGGACTTCCTTTACCAAAGTTGT
ACTAATGTTTTAAATCAGTCAAGTTAAGTTTTATGTGTTTATTTGAGAGGTAGAAAACACAATTGAGAACTTGATAAGTAXXXXXXXXXXT
AATCTAATTGAAAGATTAACACGAACGCGATAATCAAGACGTCTCACTAGAGATTTTGGACTTCCTTTACCAAAGTTGT
>Marker574117 Chr3 SNP_site:30095089 Ref_Type:T
AACCGATGAATTTTATCGTAATTATGATACTGATCTTTTGAGTATCGGTTTCTAAATAAAATATATAATATTGATGCTTTXXXXXXXXXXC
TTAAATTTTAGTAGGGATCAGGCCAAATGGAGTAATTAACACGAGAATTTCTGTATGGATGATCAAGTTTAGAGTTGTT
AACCGATGAATTTTATCGTAATTATGATACTGATCTTTTGAGTATCGGTTTCTAAATAAAATATATAATATTGATGCTTTXXXXXXXXXXC
TTAAATTTTAGTAGGGATCAGGCCAAATGGAGTAATTAACATGAGAATTTCTGTATGGATGATCAAGTTTAGAGTTGTT
>Marker574220 Chr3 SNP_site:2578929 Ref_Type:T
ACTAAAAAAGATTCTACCCCACGTATTGAAGTTAGTTTGTAGCAATCTAAAAGTAATGTGACAAAAAAAATGTATCAAACXXXXXXXXXXT
CTTGATGGTTAAATTTGTCACCCTTAACAAAAAAACTTTGTTATGTTAGTGTTAATTAACTTTTGTATATTAGGTTGTA
ACTAAAAAAGATTCTACCCCACGTATTGAAGTTAGTTTGTAGCAATCTAAAAGTAATGTGATAAAAAAAATGTATCAAACXXXXXXXXXXT
CTTGATGGTTAAATTTGTCACCCTTAACAAAAAAACTTTGTTATGTTAGTGTTAATTAACTTTTGTATATTAGGTTGTA
>Marker575257 Chr3 SNP_site:33429166 Ref_Type:C
AACTTTATAAATAAACAAACATCTTGTAAAAGGAAAAAGAAAAAAGAAAAGCTACTTGATGATATAAGCTAAGTGAGAACXXXXXXXXXXT
CAACGAAGTTTCTGTTCTTACTTACATACAAAAGGATCTTCCCTACATTTAACTAATCTGGAAACCATGTGCAGTTGTA
AACTTTATAAATAAACAAACATCTTGTAAAAGGAAAAAGAAAAAAGAAAAGCTACTTGATGATATAAGCTAAGTGAGAACXXXXXXXXXXT
CAACGAAGTTTCTGTTCTTACTTACATACAAAAGGATCTTCCCTATATTTAACTAATCTGGAAACCATGTGCAGTTGTA
>Marker575607 Chr3 SNP_site:30796554 Ref_Type:G
AACTTTTAATTTTGTTCTATTCTTTACTTATCTCCTTCCCATTGCTCTGATATGTAGCATACCTTAGGCAACATTTACCGXXXXXXXXXXT
TTGTCAGGGATTGAACCAAATCTTTGAAATTGTTGTAGTGAAATTTTATTACTAATGAAACCATATCAGTAACGATGTC
AACTTTTAATTTTTTTCTATTCTTTACTTATCTCCTTCCCATTGCTCTGATATGTAGCATACCTTAGGCAACATTTACCGXXXXXXXXXXT
TTGTCAGGGATTGAACCAAATCTTTGAAATTGTTGTAGTGAAATTTTATTACTAATGAAACTATATCAGTAACGATGTT
>Marker575607 Chr3 SNP_site:30796825 Ref_Type:C
AACTTTTAATTTTGTTCTATTCTTTACTTATCTCCTTCCCATTGCTCTGATATGTAGCATACCTTAGGCAACATTTACCGXXXXXXXXXXT

Create PDF with GO2PDF for free, if you wish to

TTGTCAGGGATTGAACCAAATCTTTGAAATTGTTGTAGTGAAATTTTATTACTAATGAAACCATATCAGTAACGATGTC

AACTTTTAATTTTTTTCTATTCTTTACTTATCTCCTTCCCATTGCTCTGATATGTAGCATACCTTAGGCAACATTTACCGXXXXXXXXXXT

TTGTCAGGGATTGAACCAAATCTTTGAAATTGTTGTAGTGAAATTTTATTACTAATGAAACTATATCAGTAACGATGTT

>Marker575607 Chr3 SNP_site:30796842 Ref_Type:C

AACTTTTAATTTTGTTCTATTCTTTACTTATCTCCTTCCCATTGCTCTGATATGTAGCATACCTTAGGCAACATTTACCGXXXXXXXXXXT

TTGTCAGGGATTGAACCAAATCTTTGAAATTGTTGTAGTGAAATTTTATTACTAATGAAACCATATCAGTAACGATGTC

AACTTTTAATTTTTTTCTATTCTTTACTTATCTCCTTCCCATTGCTCTGATATGTAGCATACCTTAGGCAACATTTACCGXXXXXXXXXXT

TTGTCAGGGATTGAACCAAATCTTTGAAATTGTTGTAGTGAAATTTTATTACTAATGAAACTATATCAGTAACGATGTT

>Marker57561 Chr4 SNP_site:3158860 Ref_Type:T

TACACTAATTGAGTAATTATGTTGATGTGGTTATTCAAATGCTTGAATCATTCCAAATATGTCCAAAATCCTAATAAACTXXXXXXXXXXT

GCTTCCAATTTTTGAAGGAAAAATTAAGATACGTAAGCATTTACTATTTACTATTTTATGATTGACATTTGATTGAAGT

TACACTAATTGAGTAATTATGTTGATGTGGTTATTCAAATGTTTGAATCATTCCAAATATGTCCAAAATCCTAATAAACTXXXXXXXXXXT

GCTTCCAATTTTTGAAGGAAAAATTAAGATACGTAAGCATTTACTATTTACTATTTTATGATTGACATTTGATTGAAGT

>Marker575770 Chr3 SNP_site:36602371 Ref_Type:T

AACAAGTATGAAAAAACATACATAAAATCATACCATAACACACACCCAAAGAGTTATATTTTGATTTCCTTAGTTGTTGGXXXXXXXXXXA

TACTGGGATAGACGTTTATGATTGTTTGGGCATTATTGAGTGTTCATTTGGCACGAGCAGCACGTGGCAAATTGGTTGT

AACAAGTATGAAAAAACATACATAAAATCATACCATAACACACACCTAAAGAGTTATATTTTGATTTCCTTAGTTGTTGGXXXXXXXXXXA

TACTGGGATAGACGTTTATGATTGTTTGGGCATTATTGAGTGTTCATTTGGCACGAGCAGCACGTGGCAAATTGGTTGT

>Marker575863 Chr3 SNP_site:5616082 Ref_Type:G

ACTACCCTTCTGAAAAGAGTCATTTTCATATTAACAAAATTACTTTATCATTTTCAAAACCATTCCAAACATATTCACACXXXXXXXXXXA

TATTGAATAGAAAGGTAATAATTTGAAAAGAAACATAAGAGTTTGGGTATTAGTGATGAAGAATTGTTCAAAAGTGGTG

ACTACCCTTCTGAAAAGAGTCATTTTCATATTAACAAAATTACTTTATCATTTTCAAAACCATTCCAAACATATTCACACXXXXXXXXXXA

TATTGAGTAGAAAGGTAATAATTTGAAAAGAAACATAAGAGTTTGGGTATTAGTGATGAAGAATTGTTCAAAAGTGGTG

>Marker576004 Chr3 SNP_site:35323473 Ref_Type:A

ACATCAAAATGTGAATATTTACAAACTTATCGTCTTATAATTAAAAGTATAATTAGAAACGGTTGAAATTTTGTATATCAXXXXXXXXXXT

TGTAAATTTATCCTGAAGTTAGTTTAGTGGTATTAGACATGCTTCTAGTTGTTGGCTCAATTTTTCATTCCATGGTGGT

ACATCAAAATGTGAATATTTACAAACTTATCGTCTTATAATTAAAAGTATAATTAGAAACGGTTGATATTTTGTATATCAXXXXXXXXXXT

TGTAAATTTATCCTGAAGTTAGTTTAGTGGTATTAGACATGCTTCTAGTTGTTGGCTCAATTTTTCATTCCATGGTGGT

>Marker576482 Chr3 SNP_site:1742855 Ref_Type:T

CACATTTAATTTTTTTTCTTATACAAATTTGATTTTTTAATACATGTGTTGGTGAAACAAATTTATTACCTAAAAGATATXXXXXXXXXXA

CTTAGGTTTTTGAGACATGAAATTCAACCGATTTACTAGCAAAATATATTTTTACGTGTTTGAATGCCAAAAACATGTT

CACATTTAATTTTTTTTCTTATACAAATTTGATTTTTTAATACATGTGTTGGTGAAACAAATTTATTACCTAAAAGATATXXXXXXXXXXA

CTTAGGTTTTTGAGACATGAAATTCAACCGATTTACTAGTAAAATATATTTTTACGTGTTTGAATGCCAAAAACATGTT

>Marker576684 Chr3 SNP_site:27429515 Ref_Type:G

AACATTGGATTTTCGTGATTTATAGCCTGTTGCTGTAGTCTGAGAAGTATTTAGTATTCCTCTTCAATGGCGCCATATATXXXXXXXXXXT

TGTCTTACCAAAACTTAGCACCTCCAAGATACCATGTGGTTTTCTGTTGCTATTTTTGGATTGGAATACAAACAATGTG

AACATTGGATTTTCGTGATTTATAGCCTGTTGCTGTAGTCTGAGAAGTATTTAGTATTCCTCTTCAATGGCGCCATATATXXXXXXXXXXT

TGTCTTACCGAAACTTAGCACCTCCAAGATACCATGTGGTTTTCTGTTGCTATTTTTGGATTGGAATACAAACAATGTG

>Marker576880 Chr3 SNP_site:7391718 Ref_Type:G

AACGCGTCAAGAGAAGTTAAATATATTAAAAAAGTTATAATCTTCTTTCAACACCATACATAACAACATCCCGAGAAGTTXXXXXXXXXXT

CGTATGTCAAAAAATACCTATTTTCTTATAGTGGTGTTGGGTGAGAAAATTGAAACCTTGGTTGTTTAAGTTTGATAGT

AACGCGTCAAGAGAAGTTAAATATATTAAAAAAGTTATAATCTTCTTTCAACGCCATACATAACAACATCCCGAGAAGTTXXXXXXXXXXT

CGTATGTCGAAAAATACCTATTTTCTTATAGTGGTGTTGGGAGAGAAAATTGAAACCTTGGTTGTTTAAGTTTGATAGT

>Marker576880 Chr3 SNP_site:7391874 Ref_Type:G

Create PDF with GO2PDF for free, if you wish to

AACGCGTCAAGAGAAGTTAAATATATTAAAAAAGTTATAATCTTCTTTCAACACCATACATAACAACATCCCGAGAAGTTXXXXXXXXXXT
CGTATGTCAAAAAATACCTATTTTCTTATAGTGGTGTTGGGTGAGAAAATTGAAACCTTGGTTGTTTAAGTTTGATAGT
AACGCGTCAAGAGAAGTTAAATATATTAAAAAAGTTATAATCTTCTTTCAACGCCATACATAACAACATCCCGAGAAGTTXXXXXXXXXXT
CGTATGTCGAAAAATACCTATTTTCTTATAGTGGTGTTGGGAGAGAAAATTGAAACCTTGGTTGTTTAAGTTTGATAGT
>Marker576880 Chr3 SNP_site:7391907 Ref_Type:A
AACGCGTCAAGAGAAGTTAAATATATTAAAAAAGTTATAATCTTCTTTCAACACCATACATAACAACATCCCGAGAAGTTXXXXXXXXXXT
CGTATGTCAAAAAATACCTATTTTCTTATAGTGGTGTTGGGTGAGAAAATTGAAACCTTGGTTGTTTAAGTTTGATAGT
AACGCGTCAAGAGAAGTTAAATATATTAAAAAAGTTATAATCTTCTTTCAACGCCATACATAACAACATCCCGAGAAGTTXXXXXXXXXXT
CGTATGTCGAAAAATACCTATTTTCTTATAGTGGTGTTGGGAGAGAAAATTGAAACCTTGGTTGTTTAAGTTTGATAGT
>Marker577667 Chr3 SNP_site:14119014 Ref_Type:A
GACCTAAAATGTGAAGCGCTGGAGCAAAATCGACATCACCAGATATGAGCATTATGGATGATGGGGGAGGGTTATCTAGGXXXXXXXXXXC
CTTCTCTCAGTCGTCTAGGAAAAGCATTGAAATCCCCATATGCAGAAAACATCATAACTGCTCCTTGTATTACAGGGTG
GACCTAAAATGTGAAGCGCTGGAGCAAAATCGACATCTCCAGATATGAGCATTATGGATGATGGGGGAGGGTTATCTAGGXXXXXXXXXXC
CTTCTCTCAGTCGTCTAGGAAAAGCATTGAAATCCCCATATGCAGAAAACATCATAACTGCTCCTTTTATTACAGGGTG
>Marker577667 Chr3 SNP_site:14119217 Ref_Type:G
GACCTAAAATGTGAAGCGCTGGAGCAAAATCGACATCACCAGATATGAGCATTATGGATGATGGGGGAGGGTTATCTAGGXXXXXXXXXXC
CTTCTCTCAGTCGTCTAGGAAAAGCATTGAAATCCCCATATGCAGAAAACATCATAACTGCTCCTTGTATTACAGGGTG
GACCTAAAATGTGAAGCGCTGGAGCAAAATCGACATCTCCAGATATGAGCATTATGGATGATGGGGGAGGGTTATCTAGGXXXXXXXXXXC
CTTCTCTCAGTCGTCTAGGAAAAGCATTGAAATCCCCATATGCAGAAAACATCATAACTGCTCCTTTTATTACAGGGTG
>Marker577769 Chr3 SNP_site:470429 Ref_Type:T
ACCTCAGGACAATACTCTCGTATCCCATCGTTATTGTGCTCCCTTGGAACAGAACTGTTCAAGCTTTGAGTTGCAATGAAXXXXXXXXXXA
ACAATCCAGTGACCCATCAACAACGAAAGAAGTCCAAAGAGCATCAGCTCTGCTCAAGAAAACAAAACCCAGAAACGTA
ACCTCAGGACAATACTCTCGTATCCCATCGTTATTGTGCTCCCTTGGAACAGAACTGTTCAAGCTTTGAGTTGCAATGAAXXXXXXXXXXA
ACAATCCAGTGACCCATCAACAACGAAAGAAGTCCAAAGAGCATCAGCTCTGCTCAAGAAAACAAAACCCATAAACGTA
>Marker577846 Chr3 SNP_site:34454835 Ref_Type:T
TACACGTGCAATTGTTGCACGTAATTTGTCTATTTAAAGGGTGAATTTTCAATCGTAGGTAACTTCTTTACTCACTATTTXXXXXXXXXXT
CATGCTAGGGTGGAGGTTTGAGTGATTATGGTCCTACGAATGTGAGTTATCCCTAACTCTAATTGTAATCTAATTAAGT
TACATGTGCAATTGTTGCACGTAATTTGTCTATTTAAAGGGTGAATTTTCAATCGTAGGTAACTTCTTTACTCATTATTTXXXXXXXXXXT
CATGCTAGGGTGGAGGTTTGAGTGATTATAGTCCTACGAATGTGAGTTATCCCTAACTCTAATTGTAATCTAATTAAGT
>Marker577846 Chr3 SNP_site:34454905 Ref_Type:T
TACACGTGCAATTGTTGCACGTAATTTGTCTATTTAAAGGGTGAATTTTCAATCGTAGGTAACTTCTTTACTCACTATTTXXXXXXXXXXT
CATGCTAGGGTGGAGGTTTGAGTGATTATGGTCCTACGAATGTGAGTTATCCCTAACTCTAATTGTAATCTAATTAAGT
TACATGTGCAATTGTTGCACGTAATTTGTCTATTTAAAGGGTGAATTTTCAATCGTAGGTAACTTCTTTACTCATTATTTXXXXXXXXXXT
CATGCTAGGGTGGAGGTTTGAGTGATTATAGTCCTACGAATGTGAGTTATCCCTAACTCTAATTGTAATCTAATTAAGT
>Marker577846 Chr3 SNP_site:34455092 Ref_Type:A
TACACGTGCAATTGTTGCACGTAATTTGTCTATTTAAAGGGTGAATTTTCAATCGTAGGTAACTTCTTTACTCACTATTTXXXXXXXXXXT
CATGCTAGGGTGGAGGTTTGAGTGATTATGGTCCTACGAATGTGAGTTATCCCTAACTCTAATTGTAATCTAATTAAGT
TACATGTGCAATTGTTGCACGTAATTTGTCTATTTAAAGGGTGAATTTTCAATCGTAGGTAACTTCTTTACTCATTATTTXXXXXXXXXXT
CATGCTAGGGTGGAGGTTTGAGTGATTATAGTCCTACGAATGTGAGTTATCCCTAACTCTAATTGTAATCTAATTAAGT
>Marker577879 Chr3 SNP_site:11468669 Ref_Type:A
CACAATTTATGTATAGGAGTTCTCCAGAGTTGAAAAAATGAACAATATTCACCTTCCCCATTAATTTAGGCTGGAAGCTTXXXXXXXXXXC
CTTTCTTAACCATAATAATAATAATAACCAATAAATAAAAATTATGGTTCCAAATAAATTGATAAATCATGGAATTGTT
CACAATTTATGTATAGGAGTTCTCCAGAGTTGAAAAAATGAACAATATTCACCTTCCCCATTAATTTAGGCTGGAAGCTTXXXXXXXXXXC
CTTTCTTAACCATAATAATAATAATAACCAATAAATAAAAATTATGGTTCCAAATACATTGATAAATCATGGAATTGTT

Create PDF with GO2PDF for free, if you wish to

>Marker578350 Chr3 SNP_site:31368065 Ref_Type:T
ACTTAATTTCAGTTTTTCTCCTTACATTTTAAACATTTTTATTACAATAGAAATTGCTTAAAGAGACCCTTTCTTGTGTAXXXXXXXXXXXA
ATATATCTTCTCATGACCATTCATCAAAATGTTACAAAGTCTATTGAAAGAAAATGAAAAATTACATTTTATTTACTGT
ACTTAATTTCAGTTTTTCTCCTTACATTTTAAACATTTTTATTATAATAGAAATTGCTTAAAGAGACCCTTTCTTGTGTAXXXXXXXXXXXA
ATATATCTTCTCATGACCATTCATCAAAATGTTACAAAGTCTATTGAAAGAAAATGAAAAATTACATTTTATTTACTGT
>Marker579205 Chr3 SNP_site:5551550 Ref_Type:G
TACTTTTACTTCTCAATTATATTACACATATTCGTGTAATTGATATATATGCAACAATGAACCTGAAGTTCATTGATCCAXXXXXXXXXXXT
TAAACTCTCACTAGCGAAAATTGAAATTAAAACTTTCATGATTTTGGAAGATCAGATTCATGGTTTATTAATTCTTGTT
TACTTTTACTTCTCAATTATATTACACATATTCGTGTAATTGATATATATGCAACAATGAACCTGGAGTTCATTGATCCAXXXXXXXXXXXT
TAAACTCTCACTAGCGAAAATTGAAATTAAAACTTTCATGATTTTGGAAGATCAGATTCATGGTTTATTAATTCTTGTT
>Marker579342 Chr3 SNP_site:36525033 Ref_Type:T
TACTATGAAATCTATGATTGATGACTTGACAGGTGCTCTCGAGCCGTCTGAAAGGGATTTTTATTTGATCATTTGGATGGXXXXXXXXXXXT
GAATCCTCTGCTCATCTATTTATTCAATGGCCTTTTGCTCGACAATTTTGGGATCTCATTTTGGGGCTTTTTGTTGGTC
TACTATGAAATCTATGATTGATGACTTGATAGGTGCTCTCGAGCCTTCTGAAAGGGATTTTTATTTGATCATTTGGATGGXXXXXXXXXXXT
GAATCCTCTGCTCATCTATTTATTCAATGGCCTTTTGCTCGACAATTTTGGGATCTCATTTTGGGGCTTTTTGTTGGTC
>Marker579342 Chr3 SNP_site:36525049 Ref_Type:T
TACTATGAAATCTATGATTGATGACTTGACAGGTGCTCTCGAGCCGTCTGAAAGGGATTTTTATTTGATCATTTGGATGGXXXXXXXXXXXT
GAATCCTCTGCTCATCTATTTATTCAATGGCCTTTTGCTCGACAATTTTGGGATCTCATTTTGGGGCTTTTTGTTGGTC
TACTATGAAATCTATGATTGATGACTTGATAGGTGCTCTCGAGCCTTCTGAAAGGGATTTTTATTTGATCATTTGGATGGXXXXXXXXXXXT
GAATCCTCTGCTCATCTATTTATTCAATGGCCTTTTGCTCGACAATTTTGGGATCTCATTTTGGGGCTTTTTGTTGGTC
>Marker579512 Chr3 SNP_site:6620609 Ref_Type:C
CACCCTTGTCCCTATTTGATTTACCAGTAGAAAATTAGAAAAAAATAGATTTTGCCAAGTGGATCACCGTAACATTTTTCXXXXXXXXXXXA
CTTTTTCCACTGTTCCTATCACACAACGATATCCACAGTTAATTTTGATAATTCTAGAAAATTCCTTAACTCAAAGGTT
CACCCTTGTCCCTATTTGATTTACCAGTAGAAAATTAGAAAAAAATAGATTTTGCCAAGTGGATCACCGTAACATTTTTCXXXXXXXXXXXA
CTTTTTCCACTGTTTCTATCACACAACGATATCCACAGTTAATTTTGATAATTCTAGAAAATTCCTTAACTCAAAGGTT
>Marker579731 Chr3 SNP_site:39310585 Ref_Type:A
AACTTCTTGAGTTAGGGTTTGTGTTAATTTCTATGGTTTATCAATTGATACGGGTTTATGAATTGATGGTTCGTGAAATTXXXXXXXXXXXA
GTGGATTTATTTTTTCCGAGCTCAATCCTCTGAAATCTAGTTTTCATGGATTTCCATTATGTAGAGTGTTTTCCTTTGT
AACTTCTTGAGTTAGGGTTTGTGTTAATTTCTATGGTTTATCGATTGATACGGGTTTATGAATTGATGGTTCGTGAAATTXXXXXXXXXXXA
GTGGATTTCTTTTTTCCGAGCTCAATCCTCTGAAATCTAGTTTTCATGGATTTCCATTATGTAGAGTGTTTTCCTTTGT
>Marker579731 Chr3 SNP_site:39310744 Ref_Type:A
AACTTCTTGAGTTAGGGTTTGTGTTAATTTCTATGGTTTATCAATTGATACGGGTTTATGAATTGATGGTTCGTGAAATTXXXXXXXXXXXA
GTGGATTTATTTTTTCCGAGCTCAATCCTCTGAAATCTAGTTTTCATGGATTTCCATTATGTAGAGTGTTTTCCTTTGT
AACTTCTTGAGTTAGGGTTTGTGTTAATTTCTATGGTTTATCGATTGATACGGGTTTATGAATTGATGGTTCGTGAAATTXXXXXXXXXXXA
GTGGATTTCTTTTTTCCGAGCTCAATCCTCTGAAATCTAGTTTTCATGGATTTCCATTATGTAGAGTGTTTTCCTTTGT
>Marker579929 Chr3 SNP_site:37202127 Ref_Type:C
AACCAATTGAAAGAGAGATCTACCAATTTTGAGACAAAGGTTGAGCAAAGCCGGTCAAATTAGGAGTCATCCTCTTCAAAXXXXXXXXXXXA
ACTCAAACTTCAAAGCATTCATTCATTTAAAATACTAAATGAGTTGATTATTACAGTCTTAGATAATTTTTTAGTCGTA
AACCAATTGAAAGAGAGATTTACCAATTTTGAGACAAAGGTTGAGCAAAGCCGGTCAAATTAGGAGTCATCCTCTTCAAAXXXXXXXXXXXA
ACTCAAACTTCAAAGCATTCATTCATTTAAAATACTAAATGAGTTGATTATTACAGTCTTAGATAATTTTTTAGTCGTA
>Marker580275 Chr3 SNP_site:8609490 Ref_Type:C
CACAAAATCAAACTTTCAGTTACACATGGTGGATATGAGCAATGTAATTATGGATTACTTGGGTAATTGAATAAATCTGAXXXXXXXXXXXG
GGTTTTGTGTTGGAGAAAGGGAGTGGGAAGAAATGTTATATGATAGGAGCAAGAGATCTGGAGATTGTTTGGGGGAGTT
CACAAAATCAAACTTTCAGTTAGACATGGTGGATATGAGCAATGTAATTATGGATTACTTGGGTAATTGAATAAATCTGAXXXXXXXXXXXG

Create PDF with GO2PDF for free, if you wish to

GGTTTTGTGTTGGAGAAAGGGAGTGGGAAGAAATGTTATATGATAGGAGCAAGAGATCTGGAGATTGTTTGGGGGAGTT
>Marker580667 Chr3 SNP_site:31481941 Ref_Type:G
GACAATTTTATATCTTGAATGCATGCATATCATACATGCAGCTCCATCATGCTATAAAGATTATAAAAAGGAATATGAGGXXXXXXXXXXA
TCCTTTGTTCGAATTGTCAGAGACGCTGTAAAATCTGCTTTCTGCAGAATGATGGTGATTGAGTGTGCTAAAGAATGTC
GACAATTTTATATCTTGAATGCATGCATATCATACATGCAGCTCCATCATGCTATGAAGATTATAAAAAGGAATATGAGGXXXXXXXXXXA
TCCTTTGTTCGAATTGTCAGAGACGCTGTAAAATCTGCTTTCTGCAGAATGATGGTGATTGAGTGTGCTAAAGAATGTC
>Marker580760 Chr3 SNP_site:37874482 Ref_Type:A
AACTCTACCCTACAGACTGTTCTTACCTTTGAAAAGATATATATTTGATACGATATTAAGCATTACTTCCTTTTAACTCTXXXXXXXXXXT
TCTTTCAAGTTAGTCTTGGTGAGAGTAGCTCTAGAGAGAGAGAGAAAGAGAGAAAAATCTTGGTGAGAATAGATTAAGT
AACTCTACCCTACAGACTGTTCTTCCCTTTGAAAAGATATATATTTGATACGATATTAAGCATTACTTCCTTTTAACTCTXXXXXXXXXXT
TCTTTCAAGTTAGTCTTGGTGAGAGTAGCTCTAGAGAGAGAGAGAAAGAGAGAAAAATCTTGGTGAGAATAGATTAAGT
>Marker58122 Chr4 SNP_site:12815516 Ref_Type:G
CACGATATGGACAAAGCTACTATATATCATTTTGTTTGGCAAAATAGGGAGATGAGGAACAATTTGTTGTTTGAGGGACTXXXXXXXXXXT
GTGGAAGTTGAATTTTGATGCCAATTGGAATTCAAAATAAACAATAGAATATTGGTTGGTAGGGTGAGTTTGTTAGGTT
CACGATATGGACAAAGCTACTATATATCATTTTGTTTGGCAAAATAGGGAGATGAGGAACAGTTTGTTGTTTGAGGGACTXXXXXXXXXXT
GTGGAAGTTGAATTTTGATGCCAATTGGAATTCAAAATAAACAATAGAATATTGGTTGGTAGGGTGAGTTTGTTAGGTT
>Marker581311 Chr3 SNP_site:36740991 Ref_Type:G
ACACGTTGGCTGGCACTGTATGATTAGATTTATGATGGATTTCATGATACCATATATAACCACAAAATTTAATTATTGAAXXXXXXXXXXT
GGAGGAAGTTTGTCTTAATAGTGAGCCTATCTTTGATGAATGTGACGAATATGAGGTGGATGGTGATGATTCCTTTGTG
ACACGTTGGCTGGCACTGTGTGATTAGATTTATGATGGATTTCATGATACCATATATAACCACAAAATTTAATTATTGAAXXXXXXXXXXT
GGAGGAAGTTTGTCTTAATAGTGAGCCTATCTTTGATGAATGTGACGAATATGAGGTGGATGGTGATGATTCCTTTGTG
>Marker581709 Chr3 SNP_site:39679242 Ref_Type:G
AACAGATATGGGGTAGAACTTCGTTGGTATTTTGTCATGATAATGGTTTAAGATTTTTTTTTAATTAAAATTTGAAACTAXXXXXXXXXXG
GCTGCAGTGTGATAATTTATTTAGAGGTCAATAAACCTTAGAGTAATAACAAGCAAATTCAATCTTCAATAATTATGTG
AACAGATATGGGGTAGAACTTCGTTGGTATTTTGTCATGATAATGGTTTAAGATTTTTTTTTAATTAAAATTTGAAACTAXXXXXXXXXXG
GCTGCAGTGTGATAATTTATTTAGAGGTCAATAAACCTTAGAGTAATAGCAAGCAAATTCAATCTTCAATAATTATGTG
>Marker582056 Chr3 SNP_site:24798698 Ref_Type:A
ACCATCTAAGTTTTAAAAAGATTAGGGTAAATTTTAGAACTATTCCTAAAGTATGTTAATAGTTACAATTACACTCTCAAXXXXXXXXXXA
ATTGAGGGTTCAATTTTTACATTTATACACCTTTTAAAATTTGATTCCTAGAAACTGTCAAATGATTTTGCATTTTGTT
ACCATCTAAGTTTTAAAAAGATTAGGGTAAATTTTAGAACTATTCCTAAAGTATGTTAATAGTTACAATTACACTCTCAAXXXXXXXXXXA
ATTGAGGGTTCAATTTTTACCTTTATACACCTTTTAAAATTTGATTCCTAGAAACTGTCAAATGATTTTGCATTTTGTT
>Marker582197 Chr3 SNP_site:25369648 Ref_Type:C
ACTTACTGGACTCAATTTTATCAAGAAAGATGCACAATTCAATTGGTTATCACTGCTCACTTAGCAAGACTCGATTTGCTXXXXXXXXXXA
AGTATCATTGTTCATGCATGTAGTATAATTATTATGTTTTAGAATGAGTTGAGGAAGTAAGAATTTCGTGGGGGGAGTG
ACTTACTGGACTCAATTTTATCAAGAAAGATGCACAATTCAATTGGTTATCACTGCTCACTTAGCAAGACTCGATTTGCTXXXXXXXXXXA
AGTATCATTGTTGATGCATGTAGTATAATTATTATGTTTTAGAATGAGTTGAGGAAGTAAGAATTTCGTGGGGGGAGTG
>Marker582904 Chr3 SNP_site:5449947 Ref_Type:T
ACTTCTTATCCTTTACTGATGATTTTTCTAGTAAAAGTTGGATTTATTTTCTAAAAACAAAAGACCAAGTTTTTGAAAATXXXXXXXXXXC
AAAACTGTGAGATACACACCTCAACAAAATGTGGTGGCAGAAAGACTTAATAAAACTATAATGGAAAGGGTAAGATGTC
ACTTTTTATCCTTTACTGATGATTTTTCTAGTAAAAGTTGGATTTATTTTCTAATAACAAAAGACCAAGTTTTTGAAAATXXXXXXXXXXC
AAAACTGTGAGATACACACCTCAACAAAATGTGGTGGCAGAAAGACTTAATAGAACTATAATGGAAAGGGTAAGATGTC
>Marker582904 Chr3 SNP_site:5449997 Ref_Type:T
ACTTCTTATCCTTTACTGATGATTTTTCTAGTAAAAGTTGGATTTATTTTCTAAAAACAAAAGACCAAGTTTTTGAAAATXXXXXXXXXXC
AAAACTGTGAGATACACACCTCAACAAAATGTGGTGGCAGAAAGACTTAATAAAACTATAATGGAAAGGGTAAGATGTC

Create PDF with GO2PDF for free, if you wish to

ACTTTTTATCCTTTACTGATGATTTTTCTAGTAAAAGTTGGATTTATTTTCTAATAACAAAAGACCAAGTTTTTGAAAATXXXXXXXXXXC
AAAACTGTGAGATACACACCTCAACAAAATGTGGTGGCAGAAAGACTTAATAGAACTATAATGGAAAGGGTAAGATGTC
>Marker582904 Chr3 SNP_site:5450207 Ref_Type:G
ACTTCTTATCCTTTACTGATGATTTTTCTAGTAAAAGTTGGATTTATTTTCTAAAAACAAAAGACCAAGTTTTTGAAAATXXXXXXXXXXC
AAAACTGTGAGATACACACCTCAACAAAATGTGGTGGCAGAAAGACTTAATAAAACTATAATGGAAAGGGTAAGATGTC
ACTTTTTATCCTTTACTGATGATTTTTCTAGTAAAAGTTGGATTTATTTTCTAATAACAAAAGACCAAGTTTTTGAAAATXXXXXXXXXXC
AAAACTGTGAGATACACACCTCAACAAAATGTGGTGGCAGAAAGACTTAATAGAACTATAATGGAAAGGGTAAGATGTC
>Marker583204 Chr3 SNP_site:36058124 Ref_Type:T
GACGGAAGACTGGGCACATCCGAGAACGATGGCATTGCCGTAATAGCCGTGAGGGATTTTGGGGGAAAGTTTGTTACGGAXXXXXXXXXXT
GGAGGAGGGGATCAACAAGGGATGAGATTGGAGGGATTTAGTGATGTCGAGGTGCCAGTTGGGGCGTGGGGTTTTGGTA
GACGGAAGATTGGGCACATCCGAGAACGATGGCATTGCCGTAATAGCCGTGAGGGATTTTGGGGGAAAGTTTGTTACGGAXXXXXXXXXXT
GGAGGAGGGGATCAACAAGGGATGAGATTGGAGGGATTTAGTGATGTCGAGGTGCCAGTTGGGGCGTGGGGTTTTGGTA
>Marker583236 Chr3 SNP_site:12603880 Ref_Type:T
CACCTTCATTACAAATAAAACAACTGGCGAATTTAGTTCCCCCTGCAGATCCAAGATGGAAAACAATACACATAATTCAGXXXXXXXXXXA
ATATTCTACATATTATGCAAAGCACTGATAAATATGAGTTTGAATTTGTAATAAAATTTACCATCTTGAAGGGGTTGTG
CACTTTCATTACAAATAAAACAACTGGCGAATTTAGTTCCCCCTGCAGATCCAAGATGGAAAACAATACACATAATTCAGXXXXXXXXXXA
ATATTCTACATATTATGCAAAGCACTGATAAATATGAGTTTGAATTTGTAATAAAATTTACCATCTTGAAGGGGTTGTG
>Marker583828 Chr3 SNP_site:18099655 Ref_Type:T
ACTGAGGAAGAATGAACTGTATGCAAATAAGAAGAAATGCACCTTTGCTCAGTCTCGAGTAGATTACTTGAGACATATTAXXXXXXXXXXT
ACTATCGCAAGTTTGTCCAGAATTATGGGACAATTGCGGCTCCTTTGACGCAATTATTGAAGATAGGAGGGTTTAAGTG
ACTGAGGAAGAATGAACTGTATGCAAATAAGAAGAAATGCACCTTTGCTCAGTCTTGAGTAGATTACTTGAGACATATTAXXXXXXXXXXT
ACTATCGCAAGTTTGTCCAGAATTATGGGACAATTGCGGCTCCTTTGACGCAATTATTGAAGATAGGAGGGTTTAAGTG
>Marker583852 Chr3 SNP_site:27462320 Ref_Type:T
TACAACCAATAAACTATTTTTCCCTTGAACATTTTTACATTAAAACAGTTCAACTTTTCAACTAGCGCCAATAATTAGCAXXXXXXXXXXT
GTTATTTTCAAGTAGAAAGTTTTAAAATTAATTCTCCTCTTTGAATATATATATTGAAAAATATATGGACACTTTTGT
TACAACCAATAAACTATTTTTCCCTTGAACATTTTTACATTAAAACAGTTCAACTTTTCAACTAGCGTCAATAATTAGCAXXXXXXXXXXT
GTTATTTTCAAGTAGAAAGTTTTAAAATTAATTCTCCTCTTTGAATATATATATTGAAAAATATATGGAACACTTTTGT
>Marker583852 Chr3 SNP_site:27462519 Ref_Type:A
TACAACCAATAAACTATTTTTCCCTTGAACATTTTTACATTAAAACAGTTCAACTTTTCAACTAGCGCCAATAATTAGCAXXXXXXXXXXT
GTTATTTTCAAGTAGAAAGTTTTAAAATTAATTCTCCTCTTTGAATATATATATTGAAAAATATATGGACACTTTTGT
TACAACCAATAAACTATTTTTCCCTTGAACATTTTTACATTAAAACAGTTCAACTTTTCAACTAGCGTCAATAATTAGCAXXXXXXXXXXT
GTTATTTTCAAGTAGAAAGTTTTAAAATTAATTCTCCTCTTTGAATATATATATTGAAAAATATATGGAACACTTTTGT
>Marker583938 Chr3 SNP_site:8407627 Ref_Type:C
ACCATCTCGATGCATTATATGTTCGATGTGGAATTAATTAAAGGTATGATTATTTCATCAGGAGCCATTGTATGCTTGAGXXXXXXXXXXA
AAAAAAACTATGGTAATTAGTAAAATGGAAATTTTGTTAAGAAAAGCTACAAATACAATAAAATGACACAGTCTACGTA
ACCATCTCGATGCATTATATGTTCGATGTGGAATTAATTAAAGGTATGATTATTTCATCAGGAGCCATTGTATGCTTGAGXXXXXXXXXXA
AAAAAAACTATGGTAATTAGTAAAATGGAAATTTTGTTAAGAAAAGCTACAAATACAATAAAATGCCACAGTCTACGTA
>Marker584206 Chr3 SNP_site:37502485 Ref_Type:A
TACGGAGTTCAATTAATCAAGTTTCAATTGTTTCTTTTTCTCCTTTCACGTTCCTGTTTCCCTGCCTCTCATTCTCCTGGXXXXXXXXXXA
TTTCACTCAGAAAACAAATGGAACGTCCAACTTGCTTCTGCAAAAATATTGTAGGAGGCGATATTTACAAAATATCGTA
TACGGAGTTCAATTAATCAAGTTTCAATTGTTTCTTTTTCTCCTTTCACGTTCCTGTTTCCCTGCCTCTCATTCTCCTGGXXXXXXXXXXA
TTTCACTCAGAAAACAAATGGAACGTCCGACTTGCTTCTGCAAAAATATTGTAGGAGGCGATATTTACAAAATATCGTA
>Marker584428 Chr3 SNP_site:15245762 Ref_Type:C
ACACCTTTTTCCCAGTGCCAGCGAGGAAAAGTTTTAAAATGGTGATCCAAGCCTAGACTGAGGATTTGACGAATGCCATTXXXXXXXXXXT

Create PDF with GO2PDF for free, if you wish to

AACATAACAATTCTTTTTAATACCTACTGGTGCGGGAGCAGGAACAACTACAGTTTGAGGCACATTTCTCACAGGGGTT
ACATCTTTTTCCCAGTGCCAGCGAGGAAAAGTTTTGAAATGGTGATCCAAGCCTAGACTGAGGATTTGACGAATGCCATTXXXXXXXXXXT
AACATAACAATTCTTTTTAATACCTACTGGTGCGGGAGCAGGAACAACTACAGTTTGAGGCACATTTCTCACAGGGGTT
>Marker584428 Chr3 SNP_site:15245794 Ref_Type:A
ACACCTTTTTCCCAGTGCCAGCGAGGAAAAGTTTTAAAATGGTGATCCAAGCCTAGACTGAGGATTTGACGAATGCCATTXXXXXXXXXXT
AACATAACAATTCTTTTTAATACCTACTGGTGCGGGAGCAGGAACAACTACAGTTTGAGGCACATTTCTCACAGGGGTT
ACATCTTTTTCCCAGTGCCAGCGAGGAAAAGTTTTGAAATGGTGATCCAAGCCTAGACTGAGGATTTGACGAATGCCATTXXXXXXXXXXT
AACATAACAATTCTTTTTAATACCTACTGGTGCGGGAGCAGGAACAACTACAGTTTGAGGCACATTTCTCACAGGGGTT
>Marker584592 Chr3 SNP_site:33901752 Ref_Type:T
GACCATCAATACCAATTCACCAACACCCACAACTTCTTCACAATGAATTGATTGAGACCTCCCAAAATAATTTCAAATTAXXXXXXXXXXT
AAACCTTCAAATTAAGAATGTTTCAGTCAAGAAATTTTCAAACAAAAAATATGAAAAAACATAAAGAAAAAAAGTAGTC
GACCATCAATACCAATTCACCAACACCCACAACTTCTTCACAATGAATTGATTGAGACCTCCCAAAATAATTTCAAATTAXXXXXXXXXXT
AAACCTTCAAATTAAGAATGTTTCAGTCAAGAAATTTTCAAACAAAAAATATGAAAAAACATAAAGAAAAAAATTAGTC
>Marker584642 Chr3 SNP_site:37500354 Ref_Type:T
GACATTTTTGACAAAAAAGAAGCAATAAATGGATATATATATATATGTAGATAGTTATGACTTGTGAGTAGGCTAACATGXXXXXXXXXXT
TGAGTGGGAAATGGATTCAGCATCATTGGCTGCTCTCGCATCTCTTCACCCCCAGCAGAATCTTCACACAGTCTTCGTC
GACATTTTTGACAAAAAAGAAGCAATAAATGGATATATATATATATGTAGATAGTTATGACTTGTGAGTAGGCTAACATGXXXXXXXXXXT
TGAGTGGGAAATGGATTCAGCATCATTGGCTGCTCTCGCATCTCTTCACTCCCAGCAGAATCTTCACACAGTCTTCGTC
>Marker584877 Chr3 SNP_site:784633 Ref_Type:T
AACTTAACTTGGGCCATTGCATTAATTCGCTGCAAAAATAGATGCAAGTGCAACGAATACAATTTACAAATCATGAGATTXXXXXXXXXXA
ATGAAGAAATTTCGCTTTCATTTTTCCAGTGCTGTTCTCAATGAATTCTAACCAGCCCCATTTGATCAAATTAAATGTC
AACTTAACTTGGGCCATTGCATTAATTCGCTGCAAAAATAGATGCAAGTGCAACGAATACAATTTACAAATCATGAGATTXXXXXXXXXXA
ATGAAGAAATTTCGCTTTCATTTTTCCAGTGCTGTTCTCATTGAATTCTAACCAGCCCCATTTGATCAAATTAAATGTC
>Marker58493 Chr4 SNP_site:11601789 Ref_Type:C
AACGTAGCTCTGTTGTTTTTAGAAACCTTCAAATTCTGCACCTCTTGTTTTCTTTTAATTTTGCCTCTTTTACTTAGATTXXXXXXXXXXC
GAATTTGTGACTGTGGCTCTACCGACCTGTCCATGTGTTCTTTTCCAAAATTGGTTACTTGATTATTGCAGTTACCAGT
AACGTAGCTCTGTTGTTTTTAGAAACCTTCAAATTCTGCACCTCTTGTTTTCTTTTAATTTTGCCTCTTTTACTTAGATTXXXXXXXXXXC
GAATTTGTGACTGTGGCTCTATCGACTTGTCCATGTGTTCTTTTCCAAAATTGGTTACTTGATTATTGCAGTTACCAGT
>Marker58493 Chr4 SNP_site:11601794 Ref_Type:C
AACGTAGCTCTGTTGTTTTTAGAAACCTTCAAATTCTGCACCTCTTGTTTTCTTTTAATTTTGCCTCTTTTACTTAGATTXXXXXXXXXXC
GAATTTGTGACTGTGGCTCTACCGACCTGTCCATGTGTTCTTTTCCAAAATTGGTTACTTGATTATTGCAGTTACCAGT
AACGTAGCTCTGTTGTTTTTAGAAACCTTCAAATTCTGCACCTCTTGTTTTCTTTTAATTTTGCCTCTTTTACTTAGATTXXXXXXXXXXC
GAATTTGTGACTGTGGCTCTATCGACTTGTCCATGTGTTCTTTTCCAAAATTGGTTACTTGATTATTGCAGTTACCAGT
>Marker585484 Chr3 SNP_site:35630579 Ref_Type:T
ACTTGGGCCTTGGCCTTTTTGTCAATAATTGCTTTCTGTTTCTCCATTTCTTGTAGTTGTTTCAGTCTTGGGTCGTTTGGXXXXXXXXXXT
TCCAAATTAAAAATTTATTCGGTTCTTCTTGACGTGACGTGAAAGATTTGAACCTTAAATTTAGTTTTAGTGAGGAGTT
ACTTGGGCCTTGGCCTTTTTGTCAATAATTGCTTTCTGTTTCTCCATTTCTTGTAGTTGTTTCAGTCTTGGGTCGTTTGGXXXXXXXXXXT
TCTAAATTAAAAATTTATTCGGTTCTTCTTAACGTGACGTGAAAGATTTGAACCTTAAATTTAGTTTTAGTGAAGAGTT
>Marker585484 Chr3 SNP_site:35630607 Ref_Type:A
ACTTGGGCCTTGGCCTTTTTGTCAATAATTGCTTTCTGTTTCTCCATTTCTTGTAGTTGTTTCAGTCTTGGGTCGTTTGGXXXXXXXXXXT
TCCAAATTAAAAATTTATTCGGTTCTTCTTGACGTGACGTGAAAGATTTGAACCTTAAATTTAGTTTTAGTGAGGAGTT
ACTTGGGCCTTGGCCTTTTTGTCAATAATTGCTTTCTGTTTCTCCATTTCTTGTAGTTGTTTCAGTCTTGGGTCGTTTGGXXXXXXXXXXT
TCTAAATTAAAAATTTATTCGGTTCTTCTTAACGTGACGTGAAAGATTTGAACCTTAAATTTAGTTTTAGTGAAGAGTT
>Marker585484 Chr3 SNP_site:35630650 Ref_Type:A

Create PDF with GO2PDF for free, if you wish to

ACTTGGGCCTTGGCCTTTTTGTCAATAATTGCTTTCTGTTTCTCCATTTCTTGTAGTTGTTTCAGTCTTGGGTCGTTTGGXXXXXXXXXXT
TCCAAATTAAAAATTTATTCGGTTCTTCTTGACGTGACGTGAAAGATTTGAACCTTAAATTTAGTTTTAGTGAGGAGTT
ACTTGGGCCTTGGCCTTTTTGTCAATAATTGCTTTCTGTTTCTCCATTTCTTGTAGTTGTTTCAGTCTTGGGTCGTTTGGXXXXXXXXXXT
TCTAAATTAAAAATTTATTCGGTTCTTCTTAACGTGACGTGAAAGATTTGAACCTTAAATTTAGTTTTAGTGAAGAGTT
>Marker585795 Chr3 SNP_site:29345834 Ref_Type:C
TACATATTGCAGGCACCGATTTAGACTAATTGGAAACATCTTCTAAGAAATTTCAAAGTCATTTTGCTTCTTCTTTAGCCXXXXXXXXXXA
TATATTACACACTAATGATTCCCATTTAATTTTTTGTCAAATCCACCACTATAATCATTATATAATGTGTTTTCTAGTC
TACATATTGCAGGCACCGATTTAGACTAATTGGAAATATCTTCTAAGAAATTTCAAAGTCATTTTGCTTCTTCTTCAGCCXXXXXXXXXXA
TATATTACACACTAATGATTCCCATTTAATTTTTTGTCAAATCCACCACTATAATCATTATATAATGTGTTTTCTAGTC
>Marker585795 Chr3 SNP_site:29345873 Ref_Type:T
TACATATTGCAGGCACCGATTTAGACTAATTGGAAACATCTTCTAAGAAATTTCAAAGTCATTTTGCTTCTTCTTTAGCCXXXXXXXXXXA
TATATTACACACTAATGATTCCCATTTAATTTTTTGTCAAATCCACCACTATAATCATTATATAATGTGTTTTCTAGTC
TACATATTGCAGGCACCGATTTAGACTAATTGGAAATATCTTCTAAGAAATTTCAAAGTCATTTTGCTTCTTCTTCAGCCXXXXXXXXXXA
TATATTACACACTAATGATTCCCATTTAATTTTTTGTCAAATCCACCACTATAATCATTATATAATGTGTTTTCTAGTC
>Marker58638 Chr4 SNP_site:13828681 Ref_Type:G
ACTCTACTGGCTCTCTGGCCTTACGTGTTCGGATGAAAATTTTATAATCAAAACGGGGGCACAACGCACTGCTTCCAGTGXXXXXXXXXXT
AATAATTCTTTTAAGTTCTAATATATTATAAATTAACAAATTTTGCATTCTAAGATATGTGTTTTGATGATATCTAGTT
ACTCTACTGGCTCTCTGGCCTTACGTGTTCGGATGAAAATTTTATAATCAAAGCGGGGGCACAACGCACTGCTTCCAGTGXXXXXXXXXXT
AATAATTCTTTTAAGTTCTAATATATTATAAATTAACAAATTTTGCATTCTAAGATATGTGTTTTGATGATATCTAGTT
>Marker586437 Chr3 SNP_site:9625027 Ref_Type:A
TACCCATCATCCCCATCGAGATTTTCGTTTCTAATAGCCAGACAACACTAGTAAATAGGTTTGTCTTATGGGTATTTCCGXXXXXXXXXXT
TTTTGGACTCTTCTCCTTGCATATGGGAATTTTCTAGCCGTCGGAGATGGGTTTTCTTTTATGCTATGTAGTATTGGTT
TACCCATCATCCCCATCGAGATTTTCGTTTCTGATAGCCAGACAACACTAGTAAATAGGTTTGTCTTATGGGTATTTCCGXXXXXXXXXXT
TTTTGGACTCTTCTCCTTGCATATGGGAATTTTCTAGCCGTCGGAGATGGGTTTTCTTTTATGCTATGTAGTATTGGTT
>Marker586703 Chr3 SNP_site:34081014 Ref_Type:G
TACACCCTAGTGAAATAATCAGCGGCTACACAAAAGGAATCAATAAGGTTTCTCACTTTCTACAATGATAATGGAATATAXXXXXXXXXXG
TCAAGAAGATTTTATTTGTTCTCTAGTTGCTGATGTATATTTTTGCCTACTCCTGTTATAATTGAGTAATTGTCCTGTA
TACACCCTAGTGAAATAATCAGCGGCTACACAAAAGGAATCAATAAGGTTTCTCACTTTCTACAATGATGATGGAATATAXXXXXXXXXXG
TCAAGAAGATTTTATTTGTTCTCTAGTTGCTGATGTATATTTTTGCCTACTCCTGTTATAATTGAGTAATTGTCCTGTA
>Marker586874 Chr3 SNP_site:18500994 Ref_Type:C
AACTCTTGCCAAGTAAAACACCTCTCTTTGGCCATTTGTGACTAAAGATTCTTTTGTAACATTGTTTAACCCTTATTATTXXXXXXXXXXA
GGGTGTTTATATTTCATTTTGCATGTGACTGAATGCACGTTATGTATTGTTTTGAGAACACCTTTTTGCATTGAATGTA
AACTCTTGCCAAGTAAAACACCTCTCTTTGGCCATTTGTGACTAAAGATTCTTTTGTAACATTGTTTAACCTTTATTATTXXXXXXXXXXA
GGGTGTTTATATTTCATTTTGCATGTGACTGAATGCACGTTATGTATTGTTTTGAGAACACCTTTTTGCATTGAATGTA
>Marker586878 Chr3 SNP_site:29338753 Ref_Type:G
ACAAGAGAATGACATCTGGTGTTCTAAACTCCACTCCTTGACCTTGGAACTTCTTGTGCTCTTCCAATATTCAAACTGTTXXXXXXXXXXG
CCAAACTAAGGCTATGTAACATATTATACATACCCTTGACCTAGGATAGCAATCCCTGGTCCCAAATAAAATATCGGTA
ACAAGAGAATGACATCTGGTGTTCTAAACTCCACTCCTTGACCTTGGAACTTCTTGTGCTCTTCCAATATTCAAACTGTTXXXXXXXXXXG
CCAAACTGAGGCTATGTAACATATTATACATACCCTTGACCTAGGATAGCAATCCCTGGTCCCAAATAAAATATCGGTA
>Marker586926 Chr3 SNP_site:11265625 Ref_Type:A
TACTATTTTAACAAGGTGCTTGGCTTGGTCTTCTTTACTGCATGCAGGAAGCAGAATCTGCAACTCTTGAAGCCATCTCAXXXXXXXXXXT
TGGCTTGAAGAGAACTTTCATTTTATGCACATTTTTTGGATATGCTGCTTGGTCAGTTTCCTGTAGCAGGTGCTTTGTC
TACTATTTTAACAAGGTGCTTGGCTTGGTCTTCTTTACTGCATGCAGGAAGCGGAATCTGCAACTCTTGAAGCCATCTCAXXXXXXXXXXT
TGGCTTGAAGAGAACTTTCATTTTATGCACATTTTTTGGATATGCTGCTTGGTCAGTTTCCTGTAGCAGGTGCTTTGTC

Create PDF with GO2PDF for free, if you wish to

>Marker587075 Chr3 SNP_site:17509144 Ref_Type:C
CACGTTTTCACAGGCGAAGATTAGCAGGGCGGCAAGCATTTCTAAGAATGCTAAAAAGGTCTCTCATGAAACAAAACTACXXXXXXXXXXC
TTTCGACGATTGGAACGAAATAGCTGAACTTTTAGAAAACTTTTTTCAAGTTAAAATTATCATCAATCCTTTGTTTGTA
CACGTTTTCACAGGCGAAGATTAGCAGGGCGGCAAGCATTTCTAAGAATGCTAAAAAGGTCTCTCATGAAACAAAACTACXXXXXXXXXXC
TTTTGACGATTGGAACGAAATAGCTGAACTTTTGGAAAACTTTTTTCAAGTTAAAATTATCATCAATCCTTTGTTTGTA
>Marker587075 Chr3 SNP_site:17509174 Ref_Type:A
CACGTTTTCACAGGCGAAGATTAGCAGGGCGGCAAGCATTTCTAAGAATGCTAAAAAGGTCTCTCATGAAACAAAACTACXXXXXXXXXXC
TTTCGACGATTGGAACGAAATAGCTGAACTTTTAGAAAACTTTTTTCAAGTTAAAATTATCATCAATCCTTTGTTTGTA
CACGTTTTCACAGGCGAAGATTAGCAGGGCGGCAAGCATTTCTAAGAATGCTAAAAAGGTCTCTCATGAAACAAAACTACXXXXXXXXXXC
TTTTGACGATTGGAACGAAATAGCTGAACTTTTGGAAAACTTTTTTCAAGTTAAAATTATCATCAATCCTTTGTTTGTA
>Marker587087 Chr3 SNP_site:9495820 Ref_Type:A
AACAAATCCCACTATTTAATGTCAAAACTTGAGCCATTCCTAAAGTTGAAAGTGTATGTTATTAATATATATATATACACXXXXXXXXXXT
TTTTACTTTTTGTTTCATGGAATTATTCGTATTTTAATTGATCCTTTTTCTTTTTCTTTTCTATTGAAATTGCAGAGTG
AACAAATCCCACTATTTAATGTCAAAACTTGAGCCATTCCTAAAGTTGAAAGTGTATGTTATTAATGTATATATATATACXXXXXXXXXXT
TTTTACTTTTTGTTTCATGGAATTATTCGTATTTTAATTGATCCTTTTTCTTTTTCTTTTCTATTGAAATTGCAGAGTG
>Marker587087 Chr3 SNP_site:9495831 Ref_Type:C
AACAAATCCCACTATTTAATGTCAAAACTTGAGCCATTCCTAAAGTTGAAAGTGTATGTTATTAATATATATATATACACXXXXXXXXXXT
TTTTACTTTTTGTTTCATGGAATTATTCGTATTTTAATTGATCCTTTTTCTTTTTCTTTTCTATTGAAATTGCAGAGTG
AACAAATCCCACTATTTAATGTCAAAACTTGAGCCATTCCTAAAGTTGAAAGTGTATGTTATTAATGTATATATATATACXXXXXXXXXXT
TTTTACTTTTTGTTTCATGGAATTATTCGTATTTTAATTGATCCTTTTTCTTTTTCTTTTCTATTGAAATTGCAGAGTG
>Marker587385 Chr3 SNP_site:7140601 Ref_Type:A
AACATAGATTGAACAGCCACACCAGATGCCTCGGCCAAAGCCATCAATCCAATAGCAAATAGGGCAACAGATGAAACTTTXXXXXXXXXXG
CTACTGCAGTTGGTGCAACTATCACGCCACTGCAAGTCAAAGTTTGAACAAATAATGTTAGGGAAGATAAAGTTAAAGT
AACATAGATTGAACAGCCACACCAGATGCCTCGGCCAAAGCCATCAATCCAATAGCAAATAGGGCAACAGATGAAACTTTXXXXXXXXXXG
CTACTGCAGTTGGTGCAACTATCACGCCACTGCAAGTCAAAGTTTGAACAAATAATGTTAGGGGAGATAAAGTTAAAGT
>Marker587816 Chr3 SNP_site:27472274 Ref_Type:C
AACTTGTTTATCATTCTTTCTTTTTTATGTAAAACTATTGTTCTTCATTGCAGCTTGATGAGTTCGTCATTCCACTACTGXXXXXXXXXXT
ATTTCTCTAATATTGTCTTTTACTTTATCTCACTTTAGAGTCATACTGATTGTCCCATGAGATTAGTTGAGTGCATGTA
AACTTGTTTATTATTCTTTCTTTTTTATGTAAAACTATTGTTCTTCATTGCAGCTTGATGAGTTCGTCATTCCACTACTGXXXXXXXXXXT
ATTTCTCTAATATTGTCTTTTACTTTATCTCACTTTAGAGTCATACTAATTGTCCCATGAGATTAGTTGAGTGCATGTA
>Marker587816 Chr3 SNP_site:27472541 Ref_Type:G
AACTTGTTTATCATTCTTTCTTTTTTATGTAAAACTATTGTTCTTCATTGCAGCTTGATGAGTTCGTCATTCCACTACTGXXXXXXXXXXT
ATTTCTCTAATATTGTCTTTTACTTTATCTCACTTTAGAGTCATACTGATTGTCCCATGAGATTAGTTGAGTGCATGTA
AACTTGTTTATTATTCTTTCTTTTTTATGTAAAACTATTGTTCTTCATTGCAGCTTGATGAGTTCGTCATTCCACTACTGXXXXXXXXXXT
ATTTCTCTAATATTGTCTTTTACTTTATCTCACTTTAGAGTCATACTAATTGTCCCATGAGATTAGTTGAGTGCATGTA
>Marker587880 Chr3 SNP_site:37866901 Ref_Type:A
TACTGTGAAATCTATGATTGATGACTTGACAAGTGCTCTCAATCCTTCTGAAAGGAGTTTTTATTCGATCATTTGGATGGXXXXXXXXXXG
AATCCCATGCTCATCTTTTAATTCATTGTCCTTTTGCTTGACAATTTTGGAATCTCATTTTGGAGGTTTTTTGTTGGTC
TACTGTGAAATCTATGATTGATGACTTGACAAGTGCTCTCAATCCTTCTGAAAGGAGTTTTTATTCGATCATTTGGATGGXXXXXXXXXXG
AATCCCATGCTCATCTTTTTATTCATTGTCCTTTTGCTTGACAATTTTGGAATCTCATTTTGGAGGTTTTTTGTTGGTC
>Marker588078 Chr3 SNP_site:3794577 Ref_Type:A
CACATCAAAGCCTCAAAATTTCCCTATTTATTAATATGGTTGCATGACTAAGATTAACCAAAAACCAACACCTCCTACTTXXXXXXXXXXT
TCTTTTTTTTTTAACCAAACTCTTTTAACAAACTTTAAAACATACCAAGGCTTAAATAAATAAGAAAAGATCAAGGGTT
CACATCAAAGCCTCAAAATTTCCCTATTTATTAATATGGTTGCATGACTAAGATTAACCAAAAACCAACTCCTCCTACTTXXXXXXXXXXT

Create PDF with GO2PDF for free, if you wish to

TCTTTTTTTTTTAACCAAACTCTTTTAACAAACTTTAAAACATACCAAGGCTTAAATAAATAAGAAAAGATCAAGGGTT
>Marker588496 Chr3 SNP_site:26323458 Ref_Type:A
CACCAATTCCACATCCAACATCCAACACCTGGAATCTAGACAAAGTTATTCTCCATAGTTTGAAAAAAATGCAGAAAATTXXXXXXXXXXA
AAATATGCACGTTTAGATTTGAGATTGAAAAAAACAGAGACACATTCTGGTCTGTCGAATGCAGGAGGAGGAAGATAGT
CACCAATTCCACATCCAACATCCAACACCTGGAATCTAGACAAAGTTATTCTCCATAGTTTGAAATAAATGCAGAAAATTXXXXXXXXXXA
AAATATGCACGTTTAGATTTGAGATTGAAAAAAACAGAGACACATTCTGGTCTGTCGAATGCAGGAGGAGGAAGATAGT
>Marker590648 Chr3 SNP_site:3111648 Ref_Type:A
CACACAATTCAACCCTTTCGCTATATATTTCGACCATTTACTTTTTTGGCTCAAGAGATGCTTCATTGACAAACACTTCCXXXXXXXXXXA
GACCCAAAATAACCCAAAGAACAACCCATGCGAAGTGAAAATTCGATGTTTGCCCCTCTAAGAAGTGGCCTATAGAGTT
CACATATTTCAACCCTTTCGCTATATATTTCGACCATTTACTTTTTTGGCTCAAGAGATGCTTCATTGACAAACACTTCCXXXXXXXXXXA
GACCCAAAATAACCCAAAGAACAACCCATGCGAAGTGAAAATTCGATGTTTGCCCCTCTAAGAAGTGGCCTATAGAGTT
>Marker590648 Chr3 SNP_site:3111650 Ref_Type:C
CACACAATTCAACCCTTTCGCTATATATTTCGACCATTTACTTTTTTGGCTCAAGAGATGCTTCATTGACAAACACTTCCXXXXXXXXXXA
GACCCAAAATAACCCAAAGAACAACCCATGCGAAGTGAAAATTCGATGTTTGCCCCTCTAAGAAGTGGCCTATAGAGTT
CACATATTTCAACCCTTTCGCTATATATTTCGACCATTTACTTTTTTGGCTCAAGAGATGCTTCATTGACAAACACTTCCXXXXXXXXXXA
GACCCAAAATAACCCAAAGAACAACCCATGCGAAGTGAAAATTCGATGTTTGCCCCTCTAAGAAGTGGCCTATAGAGTT
>Marker591057 Chr3 SNP_site:29682215 Ref_Type:C
AACGGTAATACCAAAGTAACCGATTCACTGTCGTCATGTTTCAGAAAAAGAAAGGAGCTTTCCACCACTCAAGTTTCTTGXXXXXXXXXXG
GGGTTGGTAGTATTGGCTTAACTAGACCTTTTCGTTCTCTGATGGCTTTGATATCTTCAGGCCATCTCAGCATATAGTG
AACGGTAATACCAAAGTAACCGATTCACTGTCGTCATGTTTCAGAAAAAGAAAGGAGCTTTCCACCACTCAAGTTTCTTGXXXXXXXXXXG
GGGTTGGTAGTATTGGCTTAACTAGACTTTTTCGTTCTCTGATGGCTTTGATATCTTCAGGCCATCTCAGCATATAGTG
>Marker591355 Chr3 SNP_site:35172992 Ref_Type:G
CACTTTCTTTTACTTTTTCCATCTTTTAATCTCAGATAAGAAGAATCAACACAATTATCTAGGAAGATAGAAACCACAGAXXXXXXXXXXT
TGAATTTTGGTTGTTTCCAGAATCATCATGTGAAGTGGAATAAGCAAAAACTGCACTTATATGACAGCATTTTTCATGT
CACTTTCTTTTACTTTTTCCATCTTTTAATCTCAGATAAGAAGAATCAACACAATTGTCTAGGAAGATAGAAACCACAGAXXXXXXXXXXT
TGAATTTTGGTTGTTTCCAGAATCATCATGTGAAGTGGAACAAGCAAAAACTGCACTTATATGACAGCATTTTTCATGT
>Marker591355 Chr3 SNP_site:35173153 Ref_Type:C
CACTTTCTTTTACTTTTTCCATCTTTTAATCTCAGATAAGAAGAATCAACACAATTATCTAGGAAGATAGAAACCACAGAXXXXXXXXXXT
TGAATTTTGGTTGTTTCCAGAATCATCATGTGAAGTGGAATAAGCAAAAACTGCACTTATATGACAGCATTTTTCATGT
CACTTTCTTTTACTTTTTCCATCTTTTAATCTCAGATAAGAAGAATCAACACAATTGTCTAGGAAGATAGAAACCACAGAXXXXXXXXXXT
TGAATTTTGGTTGTTTCCAGAATCATCATGTGAAGTGGAACAAGCAAAAACTGCACTTATATGACAGCATTTTTCATGT
>Marker591491 Chr3 SNP_site:31213558 Ref_Type:T
AACTACCTCCACCGAAACAACTCTCACTGCTGTTGTGCGACGAGATGATCGAGCTGCATTTGTTCCAGCCATGCTCCTAAXXXXXXXXXXT
TTCACAAGTGATTGAACCCATGATAAATCTGGCTCTTCTCCATTGTTCCCGAGCTCAAAAGACGACGATCGCCGAAGTT
AACTACCTCCACCGAAACAACTTTCACTGCTGTTGTGCGACGAGATGATCGAGCTGCATTTGTTCCAGCCATGCTCCTAAXXXXXXXXXXT
TTCACAAGTGATTGAACCCATGATAAATCTGGCTCTTCTCCATTGTTCCCGAGCTCAAAAGACGACGATCGCCGAAGTT
>Marker591557 Chr3 SNP_site:3846700 Ref_Type:G
CACTTCCTTCAATTTATCGTAGTTTTGGGACTGGTTCTCCTCATCAAAGACATAGTGTTTTCCTAGTGAAATGGCTTTAGXXXXXXXXXXT
CTTCGGTATGTCTAGAAAGAAACAAATAGTGGTTTATGAAGATATGTGTTCCTTTTTTTGCTCCTCTTAACTTTCAGTG
CACTTCCTTCAATTTATCGTAGTTTTGGGACTGGTTCTCCTCATCAAAGACATAGTGTTTTCCTAGTGAAATGGCTTTAGXXXXXXXXXXT
CTTCGGTATGTCTAGAAAGAAACAAATAGTGGTTTATGAAGATATGTGTTCCTTTTTTTGCTCCTCTTAACTTTCGGTG
>Marker591571 Chr3 SNP_site:38930425 Ref_Type:C
AACAAATTTGAAGACTTGTTTCAGTATAGAATTACAGATGGAGATGAAGATGAAGAGTTTCAACTCACCTCTTCTATCTAXXXXXXXXXXT
CGGCTTGGTTTTAGATGAAGTCACTCCTCCTCTATGGGCTCCCATTAAGTAGGGTGGTTACCCTTAGCCCAATGAGGTT

Create PDF with GO2PDF for free, if you wish to

AACAAATTTGAAGACTTGTTTCAGTATAGAATTACAGATGGAGATGAAGATGAAGAGTTTCAACTCATCTCTTCTATCTAXXXXXXXXXXT
CGGCTTGGTTTTAGATGAAGTCACTCCTCCTCTATGGGCTCCCATTAAGTAGGGTGGTTACCCTTAGCCCAATGAGGTT
>Marker591591 Chr3 SNP_site:8250163 Ref_Type:A
AACAACCAGCTTTAACTGCGAATGGTCATTCTGAAGCTACGTCCAAAGAACATCTACAAGATAAATCCATAGCAGCGATTXXXXXXXXXXT
GCAAATTAGAAACACATGAATTGGAATATATCGTTTATGATTTTGTGCTATAGTGGATTTTGTATGTATATATATCGTT
AACAGCCAGCTTTAACTGCGAATGGTCATTCTGAAGCTACGTCCAAAGAACATCTACAAGATAAATCCATAGCAGCGATTXXXXXXXXXXT
GCAAATTAGAAACACATGAATTGGAATATATCGTTTATGATTTTGTGCTATAGTGGATTTTGTATGTCTATATATCGTT
>Marker591591 Chr3 SNP_site:8250414 Ref_Type:A
AACAACCAGCTTTAACTGCGAATGGTCATTCTGAAGCTACGTCCAAAGAACATCTACAAGATAAATCCATAGCAGCGATTXXXXXXXXXXT
GCAAATTAGAAACACATGAATTGGAATATATCGTTTATGATTTTGTGCTATAGTGGATTTTGTATGTATATATATCGTT
AACAGCCAGCTTTAACTGCGAATGGTCATTCTGAAGCTACGTCCAAAGAACATCTACAAGATAAATCCATAGCAGCGATTXXXXXXXXXXT
GCAAATTAGAAACACATGAATTGGAATATATCGTTTATGATTTTGTGCTATAGTGGATTTTGTATGTCTATATATCGTT
>Marker592359 Chr3 SNP_site:7122366 Ref_Type:T
GACTAATTCTCTAATGTTATTATCGTCGATGTTGTAAATTTACTTACTTTGCTTCTGTAACTCGAGTGTCCTGTTTGGTTXXXXXXXXXXC
TACAGTTTTAGGCGTTTCTGATTTGGGATGCTCTTGGTTTGAAGATTATATAGATGCTGAAGTTTTAGTCTATTTTTGT
GACTAATTCTCTAATGTTATTATCGTCGATGTTGTAAATTTACTTACTTTTCTTCTGTAACTCGAGTGTCCTGTTTGGTTXXXXXXXXXXC
TACAGTTTTAGGCGTTTCTGATTTGGGATGCTCTTGGTTTGAAGATTATATAGATGCTGAAGTTTTAGTCTATTTTTGT
>Marker592420 Chr3 SNP_site:36515904 Ref_Type:A
AACGATTTTTTCTTATTTTTTCAAAGTTGATTGGATGTTGAAACATGGAATAGAAGTTTTTGGAAAAGAGGTTTCCAAGCAAXXXXXXXXXXG
AGAAATACTCTGAATCGCTTCAAAATGCTTTGAAACTGGGGAAGAGGAGTGGTTTCGCAAAAGGCTTTGGCGTGGGGTT
AACGATTTTTTCTTATTTTTTCAAAGTTGATTGGATGTTGAAACATGGAATAGAAGTTTTTGGAAAAGAGGTTTCCAAGCAAXXXXXXXXXXG
AGAAATACTCTGAGTCGCTTCAAAATGCTTTGAAACTGGGGAAGAGGAGTGGTTTCGCAAAAGGCTTTGGCGTGGGGTT
>Marker592492 Chr3 SNP_site:2817898 Ref_Type:T
ACCTATCCATTCTTTTTCTAATGAAAGGAGGAATATCAGAGGAGGTATTGGGAGCAAAGAACTGCAGGGTTTCACCAAGTXXXXXXXXXXG
CTCCAATCAAAAGAAGCCACATTTTGGTTATAAATAGGAACAGAGAAGTTCCAAAGAAGGAATATATATATGATTGGTG
ACCTATCCATTCTTTTTCTAATGAATGGAGGAATATCAGAGGAGGTATTGGGAGCAAAGAACTGCAGGGTTTCACCAAGTXXXXXXXXXXG
CTCCAATCAAAAGAAGCCACATTTTGGTTATAAATAGGAACAGAGAAGTTCCAAAGAAGGAATATATATATGATTGGTG
>Marker592553 Chr3 SNP_site:13273850 Ref_Type:T
AACTACTCGAGAGAGTAAGTAGCAAGGACCTCACTAAACACCTGCATAATGAATTCTGGCATTGTGCCTCCAATCATTTAXXXXXXXXXXT
TGTGTCCCGAAAAGAGAACAAGTGGGGTGTTTAGGCACCGAGAGGTTGCTCGCCTTAAATAGAAGATGGTTAAATGGTC
AACTACTCGAGAGAGTAAGTAGCAAGGACCTCACTAAACATCTGCATAATGAATTCTGGCATTGTGCCTCCAATCATTTAXXXXXXXXXXT
TGTGTCCAGAAAAGAGAACAAGTGGGGTGTTTAGGCACCGAGAGGTTGCTCGCCTTAAATAGAAGATGGTTAAATGGTC
>Marker592553 Chr3 SNP_site:13274023 Ref_Type:A
AACTACTCGAGAGAGTAAGTAGCAAGGACCTCACTAAACACCTGCATAATGAATTCTGGCATTGTGCCTCCAATCATTTAXXXXXXXXXXT
TGTGTCCCGAAAAGAGAACAAGTGGGGTGTTTAGGCACCGAGAGGTTGCTCGCCTTAAATAGAAGATGGTTAAATGGTC
AACTACTCGAGAGAGTAAGTAGCAAGGACCTCACTAAACATCTGCATAATGAATTCTGGCATTGTGCCTCCAATCATTTAXXXXXXXXXXT
TGTGTCCAGAAAAGAGAACAAGTGGGGTGTTTAGGCACCGAGAGGTTGCTCGCCTTAAATAGAAGATGGTTAAATGGTC
>Marker593205 Chr3 SNP_site:36668033 Ref_Type:C
GACTGTTTTGAAACATTGATGAACATACCATGTAAATATGAACCAAAAAAGAAAGATCCAAATTCTGACGTGATTTATACXXXXXXXXXXT
AATAGTAATATGTTGTAATTTGCGACCACATCCTCAAATTAACATCTCCACGTAATAGTTAAACTAATCAAATTCTTGT
GACTGTTTTGAAACATTGATGAACATCCCATGTAAATATGAACCAAAAAAGAAAGATCCAAATTCTGACGTGATTTATACXXXXXXXXXXT
AATAGTAATATGTTGTAATTTGCGACCACATCCTCAAATTAACATCTCCACGTAATAGTTAAACTAATCAAATTCTTGT
>Marker593866 Chr3 SNP_site:2743931 Ref_Type:C
ACAAAAACCAAGTAGAGAACCATGGAGATGCAATTCTTCATTCATACTACAGTAAAATATCAAATGAACCTTCTTAACAAXXXXXXXXXXC

Create PDF with GO2PDF for free, if you wish to

TAAAATATGAGCTCAAAAGCTGCCACCAAACAATTGGGTCCGGTCGGCTAGCCCAGAAATGAGCTCAAAAGTTGAAGTG
ACAAAAACCCAGTAGAGAACCATGGAGATGCAATTCTTCATTCATACTACAGTAAAATATCAAATGAACCTTCTTAACAAXXXXXXXXXXC
TAAAATATGAGCTCAAAAGCTGCCACCAAACAATTGGGTCCGGTCGGCTAGCCCAGAAATGAGCTCAAAAGTTGAAGTG
>Marker5942 Chr4 SNP_site:12015744 Ref_Type:T
ACTGCTCAAACTCTTGATCAATGGTTGGTTTGATAGGCTTGGATTCTGTCTCTGCATATTTGAATGATTCAGTATTAGTTXXXXXXXXXXG
CTGTATTGGTCTGGTTTAAATCTTCGTTCCATCACTAAATTTTGCCACTTATTTCATTCAAATTTTCATATAATATGTG
ACTGCTCAAACTCTTGATCAATGGTTGGTTTGATAGGCTTGGATTCTGTCTCTGCATATTTGAATGATTCAGTATTAGTTXXXXXXXXXXG
CTGTATTGGTCTGGTTTAAATCTTCGTTCCATCATTAAATTTTGCCACTTATTTCATTCAAATTTTCATATAATATGTG
>Marker594418 Chr3 SNP_site:37344804 Ref_Type:T
ACCCATAAAAGAAAAAATATTGTAAATAAAAAAACTGCTGAAAATATTTAGAATCTATAGCAAAATTTATTTTGGTTGTGXXXXXXXXXXG
GAGTGATGGTCCAATTAAGCATGATTTATATTACCTAATATTTGGAGAGGGAAAGGATTAGGTTAAGAGAGAAAATGTT
ACCCATAAAAGAAAAAATATTGTAAATAAAAAAACTGCTGAAAATATTTAGAATCTATAGCAAAATTTATTTTGGTTGTGXXXXXXXXXXG
GTGTGATGGTCCAATTAAGCATGATTTATATTACCTAATATTTGGAGAGGGAAAGGATTAGGTTAAGAGAGGAAATGTT
>Marker594418 Chr3 SNP_site:37344874 Ref_Type:G
ACCCATAAAAGAAAAAATATTGTAAATAAAAAAACTGCTGAAAATATTTAGAATCTATAGCAAAATTTATTTTGGTTGTGXXXXXXXXXXG
GAGTGATGGTCCAATTAAGCATGATTTATATTACCTAATATTTGGAGAGGGAAAGGATTAGGTTAAGAGAGAAAATGTT
ACCCATAAAAGAAAAAATATTGTAAATAAAAAAACTGCTGAAAATATTTAGAATCTATAGCAAAATTTATTTTGGTTGTGXXXXXXXXXXG
GTGTGATGGTCCAATTAAGCATGATTTATATTACCTAATATTTGGAGAGGGAAAGGATTAGGTTAAGAGAGGAAATGTT
>Marker594469 Chr3 SNP_site:21130454 Ref_Type:A
CACTAAGCAATATGACAATCTCATACGTAAAGACCTACCAATTTAATTAATGTAACATAATAAACAGAACAACCCTTATTXXXXXXXXXXA
TCCATTGATTGACTCTATATATAAACTACTTAATGTCTTCAAAAAAATCATATTGCCCAAATCGTAGCTCCATAAAGTT
CACTAAGCAATATGACAATCTCATACGTCAAGACCTACCAATTTAATTAATGTAACATAATAAACAGAACAACCCTTATTXXXXXXXXXXA
TCCATTGATTGACTCTATATATAAACTACTTAATGTCTTCAAAAAAATCATATTGCCCAAATCGTAGCTCCACAAAGTT
>Marker594469 Chr3 SNP_site:21130702 Ref_Type:T
CACTAAGCAATATGACAATCTCATACGTAAAGACCTACCAATTTAATTAATGTAACATAATAAACAGAACAACCCTTATTXXXXXXXXXXA
TCCATTGATTGACTCTATATATAAACTACTTAATGTCTTCAAAAAAATCATATTGCCCAAATCGTAGCTCCATAAAGTT
CACTAAGCAATATGACAATCTCATACGTCAAGACCTACCAATTTAATTAATGTAACATAATAAACAGAACAACCCTTATTXXXXXXXXXXA
TCCATTGATTGACTCTATATATAAACTACTTAATGTCTTCAAAAAAATCATATTGCCCAAATCGTAGCTCCACAAAGTT
>Marker594509 Chr3 SNP_site:37106054 Ref_Type:C
CACTCCGATCTGCATAAACATATTAAAGAGATGGAAGAATCTCTGTAGTTTTGTATTTTTTGTTCATCACATAACTGCCAXXXXXXXXXXA
ACTGATGGATCTTGAGCTGTGACAACTGAAAGATGTGGAAACTTACAAGCACTTGCCAACTGATTCTTTATCTGGTAGT
CACTCCGATCTGCATAAACATATTAAAGAGATGGAAGAATCTCTGTAGTTTTGTATTTTTTGTTCATCACATAACTGCCAXXXXXXXXXXA
ACTGATGGATCTTGAGCTGTGATAACTGAAAGATGTGGAAACTTACAAGCACTTGCCAACTGATTCTTTATCTGGTAGT
>Marker594644 Chr3 SNP_site:28547870 Ref_Type:C
ACTTCAAAAGGTAGTTGGCTCAAGAGTCTTTCCAAACATCGTTAAGACTATTTTAGTTTCGTCTCATTTTAATTTAGTGAXXXXXXXXXXA
TCATTAACCATAACAACCGAAGATAAAAGCACATGAAAATGATTGAATTTTTCCTCACCTTAGGTTTCAAAACCAAGTC
ACTTCAAAAGGTAGTTGGCTCAAGAGTCTTTCCAAACATCGTTAAGACTATTTTAGTTTCGTCTCATTTTAATTTAGTGAXXXXXXXXXXA
TCATTAACTATAACAACCGAAGATAAAAGCACATGAAAATGATTGAATTTTTCCTCACCTTAGGTTTCAAAACCAAGTC
>Marker595412 Chr3 SNP_site:35620622 Ref_Type:G
ACATATGTTAGTTACTAAGATGCATTCTAAACTTTATGAATAGATTGTCTATATTCGGTTATTTTCTTTTCCTTATACTGXXXXXXXXXXA
TTTGTAAATACTACATGACAATATTATTCTTTTGAAATTAGTTCAACAGTGTTTGACAGGTTGTATAGCATATGTTGTT
ACATATGTTAGTTACTAAGATGCATTCTAAACTTTATGAATAGATTGTCTATATTCGGTTATTTTCTTTTCCTTATACTGXXXXXXXXXXA
TTTGTAAATACTACATGACAATATTATTCTTTTGAAATTAGTTCAACGGTGTTTGACAGGTTGTATAGCATATGTTGTT
>Marker595939 Chr3 SNP_site:496469 Ref_Type:G

Create PDF with GO2PDF for free, if you wish to

ACAGAACATGCAAATGTTCTGTGAATGATTACTTATTTTTTTCCGAGTATCGAAGATCTTTAAGGATTTCTTTTATTAGTXXXXXXXXXXC
GTTGAATTTAAAAAGTATTTATTTAAATTCTTTACCTTCTAGGTGTTGAGTATTCCAGTTGTTTTGGTCGAGAACAGTG
ACAGAACATGGAAATGTTCTGTGATTGATTACTTATTTTTTTCCGAGTATCGAAGATCTTTAAGGATTTCTTTTATTAGTXXXXXXXXXXC
GTTGAATTTAAAAAGTATTTATTTAAATTCTTTACCTTCTAGGTGTTGAGTATTCCAGTTGTTTTGGTCGAGAACAGTG
>Marker595939 Chr3 SNP_site:496483 Ref_Type:T
ACAGAACATGCAAATGTTCTGTGAATGATTACTTATTTTTTTCCGAGTATCGAAGATCTTTAAGGATTTCTTTTATTAGTXXXXXXXXXXC
GTTGAATTTAAAAAGTATTTATTTAAATTCTTTACCTTCTAGGTGTTGAGTATTCCAGTTGTTTTGGTCGAGAACAGTG
ACAGAACATGGAAATGTTCTGTGATTGATTACTTATTTTTTTCCGAGTATCGAAGATCTTTAAGGATTTCTTTTATTAGTXXXXXXXXXXC
GTTGAATTTAAAAAGTATTTATTTAAATTCTTTACCTTCTAGGTGTTGAGTATTCCAGTTGTTTTGGTCGAGAACAGTG
>Marker59598 Chr4 SNP_site:12817413 Ref_Type:A
ACTTAGGTGTTTCACGCACATGAACTATTGAACTCTACAAATTTTGGTTGTAGTTGCTCGTTATAATTTAGAAATACTCGXXXXXXXXXXT
GATTGGTTATCTCTCAATGGTATTAAAATTTATATCTTGATGCCATATTAATGGTCTTATGCATCAATTTTGGTAGGTG
ACTTAGGTGTTTCACGCACATGAACTATTGAACTCTACCAATTTTGGTTGTAGTTGCTCGTTATAATTTAGAAATACTCGXXXXXXXXXXT
GATTGGTTATCTCTCAATGGTATTAAAATTTATATCTTGATGCCATATTAATGGTCTTATGCATCAATTTTGGTAGGTG
>Marker596193 Chr3 SNP_site:35862835 Ref_Type:A
ACATAAGTAAGCTTTCACATCCAACTTTCTTTCTGGGATCGTTGTGCTTCTTTACGTATTGGTCTTTTTTATTATATAAAXXXXXXXXXXT
GGAGGCTTCTTATGATGGGGGATAGCATAGGTCACCAGTGTTGAATCAGAGTCACCAGCTAAATTTGATATAGGAATGT
ACATAAGTAAGCTTTCACATCCAACTTTCTTTCTGGGATCGTTGTGCTTCTTTACGTATTGGTCTTTTTTATTATATAAAXXXXXXXXXXT
GGAGGCTTCTTCTGATGGGGGATAGCATAGGTCACCAGTGTTGAATCAGAGTCACCAGCTAAATTTGATATAGGAATGT
>Marker596591 Chr3 SNP_site:23542436 Ref_Type:G
AACTTTGCAATTTAACTCATGAGTAAAATCTTACTTATAGCTAGCAAATTATAAGAAATTTTGGGCACATGCAAAACATTXXXXXXXXXXA
TCAAATGATCTATGGCACCAGAGTCTAGAATCCAAGGGTTCTTTCCCATCAACACTAATAAGACCGAAGGACTGAGGTA
AACTTTGCAATTTAACTCATGAGTAAAATCTTACTTATAGCTAGCAGATTATAAAAAATTTTGGGCACATGCAAAACATTXXXXXXXXXXA
TCAAATGATCTATGGCACCAGAGTCTAGAATCCAAGGGTTCTTTCCCATCAACACTAATAAGACCGAAGGACTGAGGTA
>Marker596591 Chr3 SNP_site:23542444 Ref_Type:A
AACTTTGCAATTTAACTCATGAGTAAAATCTTACTTATAGCTAGCAAATTATAAGAAATTTTGGGCACATGCAAAACATTXXXXXXXXXXA
TCAAATGATCTATGGCACCAGAGTCTAGAATCCAAGGGTTCTTTCCCATCAACACTAATAAGACCGAAGGACTGAGGTA
AACTTTGCAATTTAACTCATGAGTAAAATCTTACTTATAGCTAGCAGATTATAAAAAATTTTGGGCACATGCAAAACATTXXXXXXXXXXA
TCAAATGATCTATGGCACCAGAGTCTAGAATCCAAGGGTTCTTTCCCATCAACACTAATAAGACCGAAGGACTGAGGTA
>Marker596602 Chr3 SNP_site:3294214 Ref_Type:C
AACGATTTACCGTCTTTGATATTTTACGAAGAGTTCTTGATATATCTTCATTATTGGTTCAAGTGTATTTGAGAGAAGAGXXXXXXXXXXT
ATTTTTAGTTTCTATTCATAACTAAAAAGAGTTCTTATACTAGAAGGAATCTCTTCTGGGTTATTAAAAATTGTTTAGT
AACGATTTACCGTCTTTGATATTTTACGAAGAGTTCTTGATATATTTTCATTATTGGTTCAAGTGTATTTGAGAGAAGAGXXXXXXXXXXT
ATTTTTAGTTTCTATTCATAACTAAAAAGAGTTCTTATACTAGAAGGAATCTCTTCTGGGTTATTAAAAATTGTTTAGT
>Marker597071 Chr3 SNP_site:28024737 Ref_Type:T
GACGACGGTGAGAGAATGGTTGGCGATGGAGAAGAAGAGTTCATCGTTGAGTGCAGCGTTGATTAGTCGGAGTAAGTATGXXXXXXXXXXT
AGGTATCAGCTTAAACTAAAATTAGTTTAGTGAGCTTTTAAAAAATGGAGTCAAACAAAGTAAGATTAATAGTGAGAGT
GACGACGGTGAGAGAATGGTTGGCGATGGAGAAGAAGAGTTCATCGTTGAGTGCAGCGTTGATTAGTCGGAGTAAGTATGXXXXXXXXXXT
AGGTATTAACTTAAACTAAAATTAGTTTAGTGAGCTTTTAAAAAATGGAGTCAAACAAAGTAAGATTAATAGTGAGAGT
>Marker597071 Chr3 SNP_site:28024739 Ref_Type:A
GACGACGGTGAGAGAATGGTTGGCGATGGAGAAGAAGAGTTCATCGTTGAGTGCAGCGTTGATTAGTCGGAGTAAGTATGXXXXXXXXXXT
AGGTATCAGCTTAAACTAAAATTAGTTTAGTGAGCTTTTAAAAAATGGAGTCAAACAAAGTAAGATTAATAGTGAGAGT
GACGACGGTGAGAGAATGGTTGGCGATGGAGAAGAAGAGTTCATCGTTGAGTGCAGCGTTGATTAGTCGGAGTAAGTATGXXXXXXXXXXT
AGGTATTAACTTAAACTAAAATTAGTTTAGTGAGCTTTTAAAAAATGGAGTCAAACAAAGTAAGATTAATAGTGAGAGT

Create PDF with GO2PDF for free, if you wish to

>Marker597166 Chr3 SNP_site:34389705 Ref_Type:T
ACCATGATTTCTTTATGATTTTCTTCCATATTGGATTTTCTTTAATTACATAACTGATCCCTCTCAGATCTCTAGCCTAGXXXXXXXXXXT
CAATAATTATTCATTTTAAAGTTGAATAATAGGATATTATTATAAGGAGCTGAGTGTCTCCTTCTTATGAACGGGTGTT
ACCATGATTTCTTTATGATTTTCTTCCATATTGGATTTTCTTTAATTACATAACTGATCCCTCTCAGATCTCTAGCCTAGXXXXXXXXXXT
CATTAATTATTCATTTTAAAGTTGAATAATAGGATATTATTATAAGGAGCTGAGTGTCTCCTTCTTATGAACGGGTGTT
>Marker597456 Chr3 SNP_site:12353796 Ref_Type:C
TACAGGACCTTACGATTTCACACTTTTACCTCTCTTTCTTTAATTTCTTCCTGCCCCCGTTCATAATTTGTTGTTGTTTGXXXXXXXXXXA
GGGTAAGTAATATTAATCTATGTTGTTAGTTGTGAAAAGTAGGTAGATCTAACATAGAACACAATATCAACTACGATGT
TACAGGACCTTACGATTTCACACTTTTACCTCTCTTTCTTTAATTTCTTCCTGCCCCCGTTCATAATTTGTTGTTGTTTGXXXXXXXXXXA
GGGTAAGTAATATTAATCTATGTTGTTAGTTGTGAAAAGTAGGTAGATCTAACATAGAACACAATATCATCTAGGATGT
>Marker597456 Chr3 SNP_site:12353800 Ref_Type:A
TACAGGACCTTACGATTTCACACTTTTACCTCTCTTTCTTTAATTTCTTCCTGCCCCCGTTCATAATTTGTTGTTGTTTGXXXXXXXXXXA
GGGTAAGTAATATTAATCTATGTTGTTAGTTGTGAAAAGTAGGTAGATCTAACATAGAACACAATATCAACTACGATGT
TACAGGACCTTACGATTTCACACTTTTACCTCTCTTTCTTTAATTTCTTCCTGCCCCCGTTCATAATTTGTTGTTGTTTGXXXXXXXXXXA
GGGTAAGTAATATTAATCTATGTTGTTAGTTGTGAAAAGTAGGTAGATCTAACATAGAACACAATATCATCTAGGATGT
>Marker598057 Chr3 SNP_site:23243434 Ref_Type:A
AACTCGGTTGTATATATTTGCATAGAGACACATAATTTAATTATTCTACAATGAAGAAGAGGTTGAGTTTATGGAAGGATXXXXXXXXXXT
TTTCTATATACTTTTTTTTCATCCAGTTTGCTAGTAATTATTGTCATTTCTTTTTTCTTTTTAAGAAAAACAATAAGTG
AACTCGGTTGTATATATTTGCATAGAGACACATAATTTAATTATTCTACAATGAAGAAGAGGTTGAGTTTATGGAAGGATXXXXXXXXXXT
TTTCTATATACTTTTTTTTCATCCAGTTTGCTAGTAATTATTGTCTTTTCTTTTTTCTTTTTAAGAAAAACAATAAGTG
>Marker598064 Chr3 SNP_site:33421466 Ref_Type:T
TACTGGCAGTTTGAAATGAAGAAAGTTGTAGGGAATGAAATGAAAAACAAAAGGTTCAAGGAATTATTACATATACACATXXXXXXXXXXA
GAATATTAAGTTAATGTAATATGTGATTTATGGTAACCTATTGACTTATGAGCCACAAGGGAAATGGGAGAGATGGGTG
TACTGGCAGTTTGAAATGAAGAAAGTTGTAGGGAATGAAATGAAAAACAAAAGGTTTAAGGAATTATTACATATACACATXXXXXXXXXXA
GAATATTAAGTTAATGTAATATGTGATTTATGGTAACCTATTGACTTATGAGCCACAAGGGAAATGGGAGAGATGGGTG
>Marker598416 Chr3 SNP_site:38845049 Ref_Type:G
AACAAATACCAACTTTCAAGAATTTTGGATGTTACTGAATTGATTAGTAGCATGCAATCCACTAGAAGTGTATCCGTGATXXXXXXXXXXA
ATGCAATAAATGTAACCAAGAGCAACAAGAGAAAAACAGTAGTTTATAAAGCCGATAAGTTGGAATAGATAAACAAGGT
AACAAATACCAACTTTCAAGAATTTTGGATGTTACTGAATTGATTAGTAGCATGCAATCCACTAGAAGTGTATCCGTGATXXXXXXXXXXA
ATGCAATAAATGTAACCAAGAGCAACAAGAGAAGAACAGTAGTTTATAAAGCCGATAAGTTGGAATAGATAAACAAGGT
>Marker599312 Chr3 SNP_site:29366966 Ref_Type:T
TACATTTTCTATGCAAAAACAACACTTTTTTAGACTAGCATAAAGCTTTTCCCTTCTAAATGCATCTAGAACACACCTAAXXXXXXXXXXC
AGTGGAATGACCTTGTTTAGAAACTAGGGTGTGTTTGAATTTTGGTTTGCATTTTAAAGTCTAGAAACTTAGATTAGTT
TACATTTTCTATGCAAAAACAACACTTTTTTAGATTAGCATAAAGCTTTTCCCTTCTAAATGCATCTAGAACACACCTAAXXXXXXXXXXC
AGTGGAATGACCTTGTTTAGAAACTAGGGTGTGTTTGAATTTTGGTTTGCATTTTAAAGTCTAGAAACTTAGATTAGTT
>Marker599688 Chr3 SNP_site:31485119 Ref_Type:G
ACTCACATAATTATATAATAGCAAAAAACTTTTTGAGGAATTCTACCAATTTCTAAATTATAACGCTGTAAGTGGGTTAGXXXXXXXXXXA
ACTAGTGTAAGGACTCAAATGTTAGTTATTTTTGTAAAAGCTGTAAAGTTTATTGCGAACAGAGAATCTTGAAAACGTC
ACTCACATAATTATATAATAGCAAAAAACTTTTTGAGGAATTCTACCAATTTCTAAATTATAACGCTGTAAGTGGGTTAGXXXXXXXXXXA
ACTAGTGTAAGGACTCAGATGTTAGTTATTTTTGTAAAAGCTGTAAAGTTTATTGCGAACAGAGAATCTTGAAAACGTC
>Marker599751 Chr3 SNP_site:4705376 Ref_Type:A
ACAAGTATTTATTACTACTTGGAAGTTTGGTCCTTACAATGTGTTGCTTAGGCTGTTGGTCATGCAACTCAATGTTAGCAXXXXXXXXXXC
ATGCACTAGTATCAGTGTTTTGACTTACAACCCTTGAGTTCTCCACCGGCTTCTCCTTTTCACATAGAAGAAGAATGGT
ACAAGTATTTATTACTACTTGGAAGTTTGGTCCTTACTATGTGTTGCTTAGGCTGTTGGTCATGCAACTTAATGTTAGCAXXXXXXXXXXC

Create PDF with GO2PDF for free, if you wish to

ATGCACTAGTATCAGTGTTTTGACTTACAACCCTTGAGTTCTCCACCGGCTTCTCCTTTTCACATAGAAGAAGAATGGT
>Marker599751 Chr3 SNP_site:4705408 Ref_Type:C
ACAAGTATTTATTACTACTTGGAAGTTTGGTCCTTACAATGTGTTGCTTAGGCTGTTGGTCATGCAACTCAATGTTAGCAXXXXXXXXXXXC
ATGCACTAGTATCAGTGTTTTGACTTACAACCCTTGAGTTCTCCACCGGCTTCTCCTTTTCACATAGAAGAAGAATGGT
ACAAGTATTTATTACTACTTGGAAGTTTGGTCCTTACTATGTGTTGCTTAGGCTGTTGGTCATGCAACTTAATGTTAGCAXXXXXXXXXXXC
ATGCACTAGTATCAGTGTTTTGACTTACAACCCTTGAGTTCTCCACCGGCTTCTCCTTTTCACATAGAAGAAGAATGGT
>Marker600016 Chr3 SNP_site:34198383 Ref_Type:T
TACTGAAGTTTCAACTTTAACTCTTCCAATATGTTATTTTGCATGCTGATGCATGGCATAAAGTAGTATTTATTTTGTTAXXXXXXXXXXXT
TTCTTTTGACGTTTGGCGTTAATTTGTGGGTTCTTTTATGAAATCTAATAAAATCTAATACTCTGCTTATATTAAAGTC
TACTGAAGTTTCAACTTTAACTCTTCCAATATGTTATTTTGCATGCTGATGCATGGCATAAAGTAGTATTTATTTTGTTAXXXXXXXXXXXT
TTCTTTTGACGTTTGGCGTTAATTTGTGGGTTCTTTTATGAAATTTAATAAAATCTAATACTCTGCTTATATTAAAGTC
>Marker600097 Chr3 SNP_site:8169940 Ref_Type:C
AACAATATTTAAAATTTTATGGCAGATTTAATTCTTCCTTAATGAAAAAAAAAAACCTTAGAGGAGATCTCTTACTGAATXXXXXXXXXXXG
TTTAGATCATGAATACAATGAGAACAAGTCAATAAAATTGTCGTCACTGATAGATAGCAACCTTAACTCACTTTATAGT
AACAATATTTAAAATTTTATGGCAGATTTAATTCTTCCTTAATGAAAAAAAAACACCTTAGAGGAGATCTCTTACTGAATXXXXXXXXXXXG
TTTAGATCATGAATACAATGAGAACAAGTCAATAAAATTGTCGTCACTGATAGATAGCAACCTTAACTCACTTTATAGT
>Marker600518 Chr3 SNP_site:27163051 Ref_Type:A
GACTTTGAATACCGTAACAGCTTTAAAAAGTCATGTCAGGTTTTGCATTTCATCTGTTGTTACTTGTTAGTTGCTTTAAAXXXXXXXXXXXA
ACTACAATCTAGATCAGTTAAAGAGTGCTAATGGAGAAATAGTGAGAGAATTATTAAGAATGTAGTTATGTGAGTTGTT
GACTTTGAATACCGTAACAGCTTTAAAAAGTCATGTCAGGTTTTGCATTTCATCTGTTGTTACTTGTTAGTTGCTTTAAAXXXXXXXXXXXC
AATAAAATCTAGATCAGTTAAAGAGTGCTAATGGAGAAATAGTGAGAGAATTATTAAGAATGTAGTTATGTGAGTTGTT
>Marker600518 Chr3 SNP_site:27163053 Ref_Type:C
GACTTTGAATACCGTAACAGCTTTAAAAAGTCATGTCAGGTTTTGCATTTCATCTGTTGTTACTTGTTAGTTGCTTTAAAXXXXXXXXXXXA
ACTACAATCTAGATCAGTTAAAGAGTGCTAATGGAGAAATAGTGAGAGAATTATTAAGAATGTAGTTATGTGAGTTGTT
GACTTTGAATACCGTAACAGCTTTAAAAAGTCATGTCAGGTTTTGCATTTCATCTGTTGTTACTTGTTAGTTGCTTTAAAXXXXXXXXXXXC
AATAAAATCTAGATCAGTTAAAGAGTGCTAATGGAGAAATAGTGAGAGAATTATTAAGAATGTAGTTATGTGAGTTGTT
>Marker600518 Chr3 SNP_site:27163056 Ref_Type:C
GACTTTGAATACCGTAACAGCTTTAAAAAGTCATGTCAGGTTTTGCATTTCATCTGTTGTTACTTGTTAGTTGCTTTAAAXXXXXXXXXXXA
ACTACAATCTAGATCAGTTAAAGAGTGCTAATGGAGAAATAGTGAGAGAATTATTAAGAATGTAGTTATGTGAGTTGTT
GACTTTGAATACCGTAACAGCTTTAAAAAGTCATGTCAGGTTTTGCATTTCATCTGTTGTTACTTGTTAGTTGCTTTAAAXXXXXXXXXXXC
AATAAAATCTAGATCAGTTAAAGAGTGCTAATGGAGAAATAGTGAGAGAATTATTAAGAATGTAGTTATGTGAGTTGTT
>Marker601090 Chr3 SNP_site:6654960 Ref_Type:T
ACTTTGTAGTTTCCAGTTATTAACATCTTGTTTTCTTCAGCACCCTCTCTAACATTCTTTGCTTCATAGAACAAGAACGCXXXXXXXXXXXT
CTTTGCCACTCACCTGAAACCCTTTTGAAATTATGTCATCGTGTCTTGATATTTGATTCCTGCAATTATGGCAACTGTA
ACTTTGTAGTTTCCAGTTATTAACATCTTGTTTTCTTCAGCTCCCTCTCTAACATTCTTTGCTTCATAGAACAAGAACGCXXXXXXXXXXXT
CTTTGCCACTCACCTGAAACCCTTTTGAAATTATGTCATCGTGTCTTGATATTTGATTCCTGCAATTATGGCAACTGTA
>Marker601502 Chr3 SNP_site:22858104 Ref_Type:T
CACTTATGAAGTTTAGCATGTAACAATTTATTTCGATAGTTTAAGATTTGTAATAACCTAGTTTTTGAATTTTAGCTTTCXXXXXXXXXXXA
AAACTAAATCATTACAAAATTTAAAAACTGCATATTCCGTCTAGATTTTGACTTCGGGTTTTTTCCAGGTTCTTGGGTG
CACTTATGAAGTTTAGCATGTAACAATTTATTTCGATAGTTTAAGATTTGTAATAACCTAGTTTTTGAATTTTAGCTTTCXXXXXXXXXXXA
AAACTAAATCATTACAAAATTTAAAAACTGCATATTCCGTCTAGATTTTGACTTTGGGTTTTTTCCAGGTTCTTGGGTG
>Marker601688 Chr3 SNP_site:10075921 Ref_Type:T
AACTCAAATGAATGTGGATTGAAAGTGTTCGTCCAAATTTCAATTTCTATAAATTTCCCTAAATTTTTTGTTTTGACTTTXXXXXXXXXXXT
TCAAATTCGTTACAATTTAACCTCTACAATGAAGAATTTCATTAAAATTGTTTGCCAATTTTTCTAATTCAGTGATGTA

Create PDF with GO2PDF for free, if you wish to

AACTCAAATGAATGTGGATTGAAAGTGTTCGTCCAAATTTCAATTTCTATAAATTTCCCTAAATTTTTTGTTTTGACTTTXXXXXXXXXXT
TCAAATTTGTTACAATTTAACCTCTACAATGAAGAATTTCATTAAAATTGTTTGCCAATTTTTCTAATTCAGTGATGTA
>Marker6029 Chr4 SNP_site:1178676 Ref_Type:A
CACAATTACATAACAATGAACTCATGGTTTTGGTTAGTTGGCGTTTTTGTAATGGTGGTATTGGAACAAAACTTTAGGGTXXXXXXXXXXT
TTCTAGGTCACAGGGTGAGTATCCAAATCCTGTTTTCAATAGATTCAATTAATTTGAAAACTAGAAATTTAACTTCGGT
CACAATTACATAACAATGAACTCATGGTTTTGGTTAGTTGGCGTTTTTGTAATGGTGGTATTGGAACAAAACTTTAGGGTXXXXXXXXXXT
TTCTAGGTCACAGGGTGAGTATCCAAATCCTGTTTTCTATAGATTCAATTAATTTGAAAACTAGAAATTTAACTTTGGT
>Marker6029 Chr4 SNP_site:1178714 Ref_Type:C
CACAATTACATAACAATGAACTCATGGTTTTGGTTAGTTGGCGTTTTTGTAATGGTGGTATTGGAACAAAACTTTAGGGTXXXXXXXXXXT
TTCTAGGTCACAGGGTGAGTATCCAAATCCTGTTTTCAATAGATTCAATTAATTTGAAAACTAGAAATTTAACTTCGGT
CACAATTACATAACAATGAACTCATGGTTTTGGTTAGTTGGCGTTTTTGTAATGGTGGTATTGGAACAAAACTTTAGGGTXXXXXXXXXXT
TTCTAGGTCACAGGGTGAGTATCCAAATCCTGTTTTCTATAGATTCAATTAATTTGAAAACTAGAAATTTAACTTTGGT
>Marker602924 Chr3 SNP_site:34371844 Ref_Type:T
GACAATAATTTTAAATATGGATGGTTGAATTTTCTACCTCTAAGGAGAATTGATTTTGAAAATTGAAAAAATATGGAGACXXXXXXXXXXG
CTTAATAATAGTGTTTATTTACATCCGCCATCCGGAAACAAATTTAGTCCGGTGAAATATCCTTAAATAGAGCGCAGTT
TACAATAATTTTAAATATGGATGGTTGAATTTTCTACCTGTAAGGAGAATTGATTTTGAAAATTGAAAAAATATGGAGACXXXXXXXXXXG
CTTAATAATAGTGTTTATTTACATCCGCCATCCGGAAACAAATTTAGTCCGGTGAAATATCCTTAAATAGAGCGCAGTT
>Marker602924 Chr3 SNP_site:34371883 Ref_Type:G
GACAATAATTTTAAATATGGATGGTTGAATTTTCTACCTCTAAGGAGAATTGATTTTGAAAATTGAAAAAATATGGAGACXXXXXXXXXXG
CTTAATAATAGTGTTTATTTACATCCGCCATCCGGAAACAAATTTAGTCCGGTGAAATATCCTTAAATAGAGCGCAGTT
TACAATAATTTTAAATATGGATGGTTGAATTTTCTACCTGTAAGGAGAATTGATTTTGAAAATTGAAAAAATATGGAGACXXXXXXXXXXG
CTTAATAATAGTGTTTATTTACATCCGCCATCCGGAAACAAATTTAGTCCGGTGAAATATCCTTAAATAGAGCGCAGTT
>Marker603112 Chr3 SNP_site:9119214 Ref_Type:C
ACAAAATTGTAAATTCCATGAAATTTTAAACCAATATCTGAATTCAATAAATATTAGGGTAAGAGTAATATCTAGTTTTCXXXXXXXXXXC
CAAGATAGATCTTGAAAATCATTTCCTCTCCGCTGTGAATTATAGTTGCTTTTTAGCTCTCAGAAACAGGCTTATTGTG
ACAAAATTGTAAATTCCATGAAATTTTAAACCAATATCTGAATTCAATAAATATTAGGGTAAGAGTAATATCTCGTTTTCXXXXXXXXXXC
CAAGATAGATCTTGAAAATCATTTCCTCTCCGCTGTGAATTATAGTTGCTTTTTAGCTCTCAGAAACAGGCTTATTGTG
>Marker60345 Chr4 SNP_site:7007010 Ref_Type:C
CACATGAAATTTTTGTTCCATGTATAATGGTGGGGATTTTCTCTCTCTTCTTCATGTTCTTTCTTCTTTCTTTTCTTCCTXXXXXXXXXXA
AGCAAAACAATACCCAACGAGTAATTAGAAAAAGAAAACCTTTGCAACGGATATCCAAATGGAACCTAGCAAGGGAGTA
CACATGAAATTTTTGTTCCATGTATAATGGTGGGGATTTTCTCTCTCTTCTTCATGTTCTTTCTTCTTTCTTTTCTTCCTXXXXXXXXXXA
AGCAAAACAATACCCAATGAGTAATTAGAAAAAAAAAACCTTTGCAACGGATATCCAAATGGAACCTAGCAAGGGAGTA
>Marker60345 Chr4 SNP_site:7007026 Ref_Type:G
CACATGAAATTTTTGTTCCATGTATAATGGTGGGGATTTTCTCTCTCTTCTTCATGTTCTTTCTTCTTTCTTTTCTTCCTXXXXXXXXXXA
AGCAAAACAATACCCAACGAGTAATTAGAAAAAGAAAACCTTTGCAACGGATATCCAAATGGAACCTAGCAAGGGAGTA
CACATGAAATTTTTGTTCCATGTATAATGGTGGGGATTTTCTCTCTCTTCTTCATGTTCTTTCTTCTTTCTTTTCTTCCTXXXXXXXXXXA
AGCAAAACAATACCCAATGAGTAATTAGAAAAAAAAAACCTTTGCAACGGATATCCAAATGGAACCTAGCAAGGGAGTA
>Marker603540 Chr3 SNP_site:221292 Ref_Type:T
CACACTTCACTTTCGTAAGATTTCAGACAATACATCTTGTAATCCATTTTAATTTTTCATTCATCCAAGTGTTCTCCATTXXXXXXXXXXA
CTTTACCCTCTATGTAACATAACAATCTTTTCCAAATAATGGAAAGTGTATTTTTAAACTAATTGCTTCAGAGGACGTC
CACACTTCACTTTCGTAAGATTTCAGACAATACATCTTGTATTCCCTTTTAATTTTTCATTCATCCAAGTGTTCTCCATTXXXXXXXXXXA
CTTTACCCTCTATGTAACATAACAATCTTTTCCAAATAATGGATAGTGTATTTTTAAACTAATTGCTTCAGAGGACGTC
>Marker603540 Chr3 SNP_site:221296 Ref_Type:C
CACACTTCACTTTCGTAAGATTTCAGACAATACATCTTGTAATCCATTTTAATTTTTCATTCATCCAAGTGTTCTCCATTXXXXXXXXXXA

Create PDF with GO2PDF for free, if you wish to

CTTTACCCTCTATGTAACATAACAATCTTTTCCAAATAATGGAAAGTGTATTTTTAAACTAATTGCTTCAGAGGACGTC
CACACTTCACTTTCGTAAGATTTCAGACAATACATCTTGTATTCCCTTTTAATTTTTCATTCATCCAAGTGTTCTCCATTXXXXXXXXXXA
CTTTACCCTCTATGTAACATAACAATCTTTTCCAAATAATGGATAGTGTATTTTTAAACTAATTGCTTCAGAGGACGTC
>Marker603540 Chr3 SNP_site:221488 Ref_Type:T
CACACTTCACTTTCGTAAGATTTCAGACAATACATCTTGTAATCCATTTTAATTTTTCATTCATCCAAGTGTTCTCCATTXXXXXXXXXXA
CTTTACCCTCTATGTAACATAACAATCTTTTCCAAATAATGGAAAGTGTATTTTTAAACTAATTGCTTCAGAGGACGTC
CACACTTCACTTTCGTAAGATTTCAGACAATACATCTTGTATTCCCTTTTAATTTTTCATTCATCCAAGTGTTCTCCATTXXXXXXXXXXA
CTTTACCCTCTATGTAACATAACAATCTTTTCCAAATAATGGATAGTGTATTTTTAAACTAATTGCTTCAGAGGACGTC
>Marker603611 Chr3 SNP_site:29265746 Ref_Type:C
AACCGTCTAATAGAATGGAACCGTCAGATTCTATGTTTGGATGACTGGATGTCAAATAAGATAGTTTGTGGTTTTAATTTXXXXXXXXXXT
CACAAGTTTGGAATTTTTTTGTGATTTTGTTTTGAATTTTGGTTTATTTAATTGAATTATATTGATTATGTTTCTAGGTT
AACCGTCTAATAGAATGGAACCGTCAGATTCTATGTTTGGATGATTGGATGTCAAATAAGATAGTTTGTGGTTTTAATTTXXXXXXXXXXT
CACAAGTTTGGAATATTTTTGTGATTTTGTTTTGAATTTTGGTTTATTTAATTGAATTATATTGATTATGTTTGTAGGTT
>Marker603611 Chr3 SNP_site:29265920 Ref_Type:T
AACCGTCTAATAGAATGGAACCGTCAGATTCTATGTTTGGATGACTGGATGTCAAATAAGATAGTTTGTGGTTTTAATTTXXXXXXXXXXT
CACAAGTTTGGAATTTTTTTGTGATTTTGTTTTGAATTTTGGTTTATTTAATTGAATTATATTGATTATGTTTCTAGGTT
AACCGTCTAATAGAATGGAACCGTCAGATTCTATGTTTGGATGATTGGATGTCAAATAAGATAGTTTGTGGTTTTAATTTXXXXXXXXXXT
CACAAGTTTGGAATATTTTTGTGATTTTGTTTTGAATTTTGGTTTATTTAATTGAATTATATTGATTATGTTTGTAGGTT
>Marker603611 Chr3 SNP_site:29265978 Ref_Type:C
AACCGTCTAATAGAATGGAACCGTCAGATTCTATGTTTGGATGACTGGATGTCAAATAAGATAGTTTGTGGTTTTAATTTXXXXXXXXXXT
CACAAGTTTGGAATTTTTTTGTGATTTTGTTTTGAATTTTGGTTTATTTAATTGAATTATATTGATTATGTTTCTAGGTT
AACCGTCTAATAGAATGGAACCGTCAGATTCTATGTTTGGATGATTGGATGTCAAATAAGATAGTTTGTGGTTTTAATTTXXXXXXXXXXT
CACAAGTTTGGAATATTTTTGTGATTTTGTTTTGAATTTTGGTTTATTTAATTGAATTATATTGATTATGTTTGTAGGTT
>Marker603662 Chr3 SNP_site:11186077 Ref_Type:G
CACAGATCAAGGGAGGTAACTTGTTTGGTTTTTATAAGTCATTGGACTTTGAACCTACAGAAACTTGCATTATGCAACTGXXXXXXXXXXA
ACTTAAAGTAGGAGGCAATATATGGTGGTAGATTCCGGATCCCTTATGTTTTAGTTCAAAATCTGGAATCAATTCAGTG
CACAGATCAAGGGAGGTAACTTGTTTGGTTTTTATAAGTCATTGGACTTTGAACCTACAGAAACTTGCATTATGCAACTGXXXXXXXXXXA
ACTTAAAGTAGGAGGCAATATGTGGTGGTAGATTCCGGATCCCTTATGTTTTAGTTCAAAATCTGGAATCAATTCAGTG
>Marker60423 Chr4 SNP_site:9978107 Ref_Type:C
AACCCAGTTCCCCTAGACCGCGGGTTCAACAAGGTCCGCCTCTCGAATAGCCATGTCAAACTCCTCCATGTCCCCCATGCXXXXXXXXXXA
CAAAACCTCTACCTTCTCCCCAGAGATCAAATCTGTTACCACACCATAAATAAACTGGTCGGCAACCTCATAAGTCGTG
AACCCAGTTCCCCTAGACCGCGGGTTCAACAAGGTCCGCCTCTCGAATAGCCATGTCAAACTCCTCCATGTCCCCCATGTXXXXXXXXXXA
CAAAACCTCTACCTTCTCCCCAGAGATCAAATCTGTTACCACACCATAAATAAACTGGTCGGCAACCTCATAAGTCGTG
>Marker604284 Chr3 SNP_site:39740391 Ref_Type:A
AACAACATGAGAAAAGATTGTCATTCAAAGACAATAGATTTAAGATTTAAATATCACGATAACACATGAACAATAAAATCXXXXXXXXXXA
AAGGAGGAGGAGAAGAAGAAGAAACCCAGAGGTGAAGTGCCGAAGAAGATGGAATCGTTAATCAATTCGCATGTGAAGT
AACAACATGAGAAAAGATTGTCATTCGAAGACAATAGATTTAAGATTTAAATATCACGATAACACATGAACAATAAAATCXXXXXXXXXXA
AAGGAGGAGGAGAAGAAGAAGAAACCCAGAGGTGAAGTGCCGAAGAAGATGGAATCGTTAATCAACTCGCATGTGAAGT
>Marker604284 Chr3 SNP_site:39740641 Ref_Type:T
AACAACATGAGAAAAGATTGTCATTCAAAGACAATAGATTTAAGATTTAAATATCACGATAACACATGAACAATAAAATCXXXXXXXXXXA
AAGGAGGAGGAGAAGAAGAAGAAACCCAGAGGTGAAGTGCCGAAGAAGATGGAATCGTTAATCAATTCGCATGTGAAGT
AACAACATGAGAAAAGATTGTCATTCGAAGACAATAGATTTAAGATTTAAATATCACGATAACACATGAACAATAAAATCXXXXXXXXXXA
AAGGAGGAGGAGAAGAAGAAGAAACCCAGAGGTGAAGTGCCGAAGAAGATGGAATCGTTAATCAACTCGCATGTGAAGT
>Marker604311 Chr3 SNP_site:27974426 Ref_Type:A

Create PDF with GO2PDF for free, if you wish to

TACAAAGGTGTAACGAGAGACAGACCTTCAATTGCCATTAAAGTAATAGACTTGGAACAAATGGATTTCTTATCGAATGTXXXXXXXXXXG
CAGATCAGAACAGAACCATGGCTGACTCTGAAATGAAGAATAGACATAGCTTTAGATGTAGCAGCAGCAATAATGGAGT
TACAAGGGTGTAACGAGAGACAGACCTTCAATTGCCATTAAAGTAATAGACTTGGAACAAATGGATTTCTTATCGAATGTXXXXXXXXXXG
CAGATCAGAACAGAACCATGGCTGACTCTGAAATGAAGAATAGACATAGCTTTAGATGTAGCAGCAGCAATAATGTAGT
>Marker604311 Chr3 SNP_site:27974693 Ref_Type:G
TACAAAGGTGTAACGAGAGACAGACCTTCAATTGCCATTAAAGTAATAGACTTGGAACAAATGGATTTCTTATCGAATGTXXXXXXXXXXG
CAGATCAGAACAGAACCATGGCTGACTCTGAAATGAAGAATAGACATAGCTTTAGATGTAGCAGCAGCAATAATGGAGT
TACAAGGGTGTAACGAGAGACAGACCTTCAATTGCCATTAAAGTAATAGACTTGGAACAAATGGATTTCTTATCGAATGTXXXXXXXXXXG
CAGATCAGAACAGAACCATGGCTGACTCTGAAATGAAGAATAGACATAGCTTTAGATGTAGCAGCAGCAATAATGTAGT
>Marker605112 Chr3 SNP_site:14086285 Ref_Type:A
AACAGTATCGGAACTTCGTGACCGAATTCCAAGATTGCTTGTTTGAGAATGTTTATGGGCTTCAAGCGACTGATGAGAACXXXXXXXXXXT
GATAGAGGAGTTCGAGGAGGCTGCTAATGGAGGAGTGCAGAGCTTGACATTAGGGGCGCTGGATAACAGCTTCTTGGTG
AACAGTATCGGAACTTCGTGACCGAATTCCAGGATTGCTTGTTTGAGAATGTTTATGGGCTTCAAGCGACTGATGAGAACXXXXXXXXXXT
GATAGAGGAGTTCGAGGAGGCTGCTAATGGAGGAGTGCAGAGCTTGACATTAGGGGCGCTGGATAACAGCTTCTTGGTG
>Marker60513 Chr4 SNP_site:1814860 Ref_Type:G
GACAACGGTATATCCCTTCTCAACACAGTTCTCTACAGTCACAAGGGGCCTGCTCTGGTAGCTTTCGCTGCAACTCGTGTXXXXXXXXXXA
CATAGAGGAGAAGCTGTTTAATCTTAGCCAACCAATCGGGGTCATCGATCGGCATTCCACTCGTATCATCGGTGATGTA
GACAACGGTATATCCCTTCTCAACACAGTTCTCTACAGTCACAAGGGGCCTGCTCTGGTAGCTTTCGCTGCAACTCGTGTXXXXXXXXXXA
CATAGAGGAGAAGCTGTTTAATCTTAGCCAACCAATCGGGGTCATCGATCGGCATTCCACTCGTGTCATCGGTGATGTA
>Marker605191 Chr3 SNP_site:11123973 Ref_Type:C
AACCGCATTCAACATTGATAAACACTAGACATCCATGTCTATCATGAAATATAGATTCTAGTGTTCTTTATTTCCCACCTXXXXXXXXXXC
AATTTAGCTAGGTTGAGATAACCTGACAACTTCTGATTAGTGAAATTTTCTATTTAATATTTTATCAAAATGGGTCGTT
AACCGCATTCAACATTGATAAACACTAGACATCCATGTCTATCATGAAATATAGATTCTAGTGTTCTTTATTTCCCACCTXXXXXXXXXXC
AATTTAGCTAGGTTGAGATAACTTGACAACTTCTGATTAGTGAAATTTTCTATTTAATATTTTATCAAAATGGGTCGTT
>Marker605371 Chr3 SNP_site:6918057 Ref_Type:A
AACATATTTACAGATATTGTCCGAAATCGGTTTGAAAAACTTTTCATATCTAACTTCTGGATGCTATATCACAGCACAACXXXXXXXXXXC
CAATCACATGAACAACAATTAACAATTTTCAAACTAACACAATGATGGTATATATAGACTTATAGTCTATGCTCCTGTT
AACATATTTACAGATATTGTCCGAAATCGGTTTGAAAAACTTTTCATATCTAACTTCTGGATGCTATATCACAGCACAACXXXXXXXXXXC
CAATCACATGAACAACAATTAACAATTTTCGAACTAACACAATGATGGTATATATAGACTTATAGTCTATGCTCCTGTT
>Marker605521 Chr3 SNP_site:35110659 Ref_Type:T
AACTGCCTACTTGTTTGAATCTCATTTCAAACCAATGTGAAATCTTCATCTCTATGATTAATTAATTAGAATGTGAGTAAXXXXXXXXXXT
CGGTTGTCATGACCTTTCTCTTTTTCCTGTTGAACAGTTTGTGCGTGTGGGTAATGATAGTATTTTCAATTCATAAGTG
AACTGCCTACTTGTTTGAATCTCATTTCAAACCAATGTGAAATCTTCATCTCTATGATTAATTAATTAGAATGTGAGTAAXXXXXXXXXXT
CGGTTGTCATGTCCTTTCTCTTTTTCCTGTTGAACAGTTTTTGCGTGTGGGTAATGATAGTATTTTCAATTCATAAGTG
>Marker605521 Chr3 SNP_site:35110688 Ref_Type:T
AACTGCCTACTTGTTTGAATCTCATTTCAAACCAATGTGAAATCTTCATCTCTATGATTAATTAATTAGAATGTGAGTAAXXXXXXXXXXT
CGGTTGTCATGACCTTTCTCTTTTTCCTGTTGAACAGTTTGTGCGTGTGGGTAATGATAGTATTTTCAATTCATAAGTG
AACTGCCTACTTGTTTGAATCTCATTTCAAACCAATGTGAAATCTTCATCTCTATGATTAATTAATTAGAATGTGAGTAAXXXXXXXXXXT
CGGTTGTCATGTCCTTTCTCTTTTTCCTGTTGAACAGTTTTTGCGTGTGGGTAATGATAGTATTTTCAATTCATAAGTG
>Marker606557 Chr3 SNP_site:30583624 Ref_Type:A
CACACATGACACAAGGCTCAAACTTTATTTCCTGAAGATCAACAAATGAACTTATGATTAAGAATGATTTATAGATGAATXXXXXXXXXXG
AACTCTTCACAAAAAGCCTACCTACGTATCTGGCATGAGAAAAGGCATGTGAAAATGTCATTATCCAGAATCCGAAGTT
CACACATGACACAAGGCTCAAACTTTATTTCCTGAAGATCAACAAATGAACTTATGATTAAGAATGATTTATAGATGAGTXXXXXXXXXXG
AACTCTTCACAAAAAGCCTACCTACGTATCTGGCATGAAAAAAGGCATGTGAAAATGTCATTATCCAGAATCCGAAGTT

Create PDF with GO2PDF for free, if you wish to

>Marker606557 Chr3 SNP_site:30583802 Ref_Type:G
CACACATGACACAAGGCTCAAACTTTATTTCCTGAAGATCAACAAATGAACTTATGATTAAGAATGATTTATAGATGAATXXXXXXXXXXG
AACTCTTCACAAAAAGCCTACCTACGTATCTGGCATGAGAAAAGGCATGTGAAAATGTCATTATCCAGAATCCGAAGTT
CACACATGACACAAGGCTCAAACTTTATTTCCTGAAGATCAACAAATGAACTTATGATTAAGAATGATTTATAGATGAGTXXXXXXXXXXG
AACTCTTCACAAAAAGCCTACCTACGTATCTGGCATGAAAAAAGGCATGTGAAAATGTCATTATCCAGAATCCGAAGTT
>Marker606636 Chr3 SNP_site:39119543 Ref_Type:G
ACTTAGTATCAATTTTTTTGTGTCCATGGGCAAAATTTTTGAAGCAGACAACCACCCCTATCATTTCTTTTCCCCTTCAAXXXXXXXXXXT
GATCACCATCACCACACCAGCCTTCATTGTTTGTATTGCTTAATGTCACACTTCGATACCTTCTTTTCTTCGTCTGGTG
ACTTAGTATCAATTTTTTTGTGTCCATGGGCAAAATTTTTGAAGCAGACAACCACCCCTATCATTTCTTTTCCCCTTCAAXXXXXXXXXXT
GATCACCATCACCACACCAGCCTTCATTGTTTGTATTGCTTAATGTCACACTTCTATACCTTCTTTTCTTCGTCTGGTG
>Marker60722 Chr4 SNP_site:15671352 Ref_Type:C
ACCAATATAGGCAAGAAAGGAGAATATATTATCTAAGTAGGGCTGGTGAAAGTTGAGGAATAAACAATACTGTGGTCATGXXXXXXXXXXC
ACAGTGAAGTAATAAAAAAGTAAGCTAATTTTCTCTTCCAATTTATTTGTGGAAAAAAAGGTAAAGTATATACGTGTC
ACCAATATAGGCAAGAAAGGAGAATATATTATCTAAGTAGGGCTGGTGAAAGTTGAGGAATAAACAATACTGTGGTCATGXXXXXXXXXXC
ACAGTGAAGTAATAAAAAAGTAAGCTAATTTTCTCTTCCAATTTATTTGTGGAAAAAAAGGTAAAGTATATATGTGTC
>Marker607262 Chr3 SNP_site:22127024 Ref_Type:A
ACTTATTTATTATGTTATGTTACACATTTTAGATTTGACACACAAATGGTCTTCTATACTAGATTTTGATGGTTTTTGGTXXXXXXXXXXT
GTAACTTCATGGTTATAACTTTGGAACTAACTTTAGACATTCATTTATTTGATGTAACTTTATACTTATAACTTAGGTT
ACTTATTTATTATGTTATGTTACACATTTTAGATTTGACACACAAATGGTCTTCTATACTAGATTTTGATGGTTTTTGGTXXXXXXXXXXT
GTAACTTCATGGTTATAACTTTGGAACTAACTTTGGACATTCATTTATTTGATGTAACTTTATACTTATAACTTAGGTT
>Marker607357 Chr3 SNP_site:19445879 Ref_Type:C
CACAATGCAAGTGGCAATGGTGTATCATACTTTCATTCTTGCAATTTGGAGAACCACAGTTATTATGATGGATTGAACTCXXXXXXXXXXA
TTTTGGATTATTATTAAAGAAAAATGGCTCTTGCTTATTGTGAATAGTCACCTATTTTATTCTCTGTTTCTTTATTGTT
CACAATGCAAGTGGCAATGGTGTATCATACTTTCATTCTTGCAATTTGGAGAACCACAGTTATTATGATGGATTGAAGTCXXXXXXXXXXA
TTTTGGATTATTATTAAAGAAAAATGGCTCTTGCTTATTGTGAATAGTCACCTATTTTATTCTCTGTTTCTTTATTGTT
>Marker607363 Chr3 SNP_site:27157064 Ref_Type:G
CACACTCCTTATAAGTAGTAAAAGTTGGAAAAATTTCACACATTGTCTCGAGCTCAGTGCAATTTTCTTTACCGCTTGTAXXXXXXXXXXG
CTCAACTTCAATTTATGGATGAAATAGATTCTAACACTACTCGAATAACCCAATGAACGCTCGACCCAATTTGGGTTGT
CACACTCCTTATAAGTAGTAAAAGTTGGAAAAATTTCACACATTGTCTCGAGCTCAGTGCAATTTTCTTTACCGCTTGTAXXXXXXXXXXG
CTCAACTTCAGTTTATGGATGAAATAGATTCTAACACTACTCGAATAACCCAATGAACGCTCGACCCAATTTGGGTTGT
>Marker607722 Chr3 SNP_site:32188863 Ref_Type:A
ACTTTTATTATATTTTTTCTTTTTATGTTTAAGTGATTTATTTGACATTTTTTTAAATTTTGAATTAATGGGTGTTTTTCXXXXXXXXXXT
TTAAAATAATAATAAAATAAACGTGTGAAATTAAATGAAAAAATATTGTTTAAAATGAATTTGATTTAGTGGTATTTGT
ACTTTTATTATATTTTTTCTTTTTATGTTTAAGTGATTTGTTTGACATTTTTTTAAATTTTGAATTAATGGGTGTTTTTCXXXXXXXXXXT
TTAAAATAATAAAAAAATAAACGTGTGAAATTAAATGAAAAAATATTGTTTAAAATGAATTTGATTTAGTGGTATTTGT
>Marker607722 Chr3 SNP_site:32189018 Ref_Type:T
ACTTTTATTATATTTTTTCTTTTTATGTTTAAGTGATTTATTTGACATTTTTTTAAATTTTGAATTAATGGGTGTTTTTCXXXXXXXXXXT
TTAAAATAATAATAAAATAAACGTGTGAAATTAAATGAAAAAATATTGTTTAAAATGAATTTGATTTAGTGGTATTTGT
ACTTTTATTATATTTTTTCTTTTTATGTTTAAGTGATTTGTTTGACATTTTTTTAAATTTTGAATTAATGGGTGTTTTTCXXXXXXXXXXT
TTAAAATAATAAAAAAATAAACGTGTGAAATTAAATGAAAAAATATTGTTTAAAATGAATTTGATTTAGTGGTATTTGT
>Marker608040 Chr3 SNP_site:26764221 Ref_Type:G
ACTAATACAACCCCTTGTTAATACATTTTACAATATTTTAATGATTCGATTGCATATATATCTTAGATGAAGGTGAGAGTXXXXXXXXXXA
TGAATGAAAGGAGAGTTCTAAAATTGGAAGAATACTTATTGAAATTTTAGAATGTTGAGAGATGCTTTTGAAACAAAGT
ACTAATACAACCCCTTGTTAATACATTTTACAATATTTTAATGATTCGGTTGCATATATATCTTAGATGAAGGTGAGAGTXXXXXXXXXXA

Create PDF with GO2PDF for free, if you wish to

TGAATGAAAGGAGAGTTCTAAAATTGGAAGAATACTTATTGAAATTTTAGAATGTTGAGAGATGCTTTTGAAACAAAGT
>Marker60817 Chr4 SNP_site:3112226 Ref_Type:C
TACAAATTTAATTCTAAAAAACGAAATGTTTTTCAAAATAGGCCTAACACATCCTAAGACTTAATAGCTATCCTCAAGCTXXXXXXXXXXT
TTTCCAATAGAATTTGCCTCGTCTTCCAATGATTTGAAAAGTGTTATCAGGTTGAAATTGTGTTTTTCTTTTGACTTGT
TACAAATTTAATTCTCAAAAACGAAATGTTTTTCAAAATAGGCCTAACACATCCTAAGACTTAATAGCTATCCTCAAGCTXXXXXXXXXXT
TTTCCAATAGAATTTGCCTCGTCTTCCAATGATTTGAAAAGTGTTATCAGGTTGAAATTGTGTTTTTCTTTTGACTTGT
>Marker608258 Chr3 SNP_site:31243443 Ref_Type:C
ACAAATATATGATCCATTTTTACGGTAGGTAAGAAAACTCTTGACACTCAAGCTTATTTCTCCTCCAACAAGATATACATXXXXXXXXXXT
TTGTTTGAAAATATTTGATTGTAACGAGCATTTTTATCTCTTTAAGAGTTGTTTGTCTCTTGCTTCTTTCAATATTGTT
ACAAATATATGATCCATTTTTATGGTAGGTAAGAAAACTCTTGACACTCGAGCTTATTTCTCCTCCAACAAGATATACATXXXXXXXXXXT
TTGTTTGAAAATATTTGATTGTAACGATCATTTTTATCTCTTTAAGAGTTGTTTGTCTCTTGCTTCTTTCAATATTGTT
>Marker608258 Chr3 SNP_site:31243470 Ref_Type:A
ACAAATATATGATCCATTTTTACGGTAGGTAAGAAAACTCTTGACACTCAAGCTTATTTCTCCTCCAACAAGATATACATXXXXXXXXXXT
TTGTTTGAAAATATTTGATTGTAACGAGCATTTTTATCTCTTTAAGAGTTGTTTGTCTCTTGCTTCTTTCAATATTGTT
ACAAATATATGATCCATTTTTATGGTAGGTAAGAAAACTCTTGACACTCGAGCTTATTTCTCCTCCAACAAGATATACATXXXXXXXXXXT
TTGTTTGAAAATATTTGATTGTAACGATCATTTTTATCTCTTTAAGAGTTGTTTGTCTCTTGCTTCTTTCAATATTGTT
>Marker608258 Chr3 SNP_site:31243628 Ref_Type:G
ACAAATATATGATCCATTTTTACGGTAGGTAAGAAAACTCTTGACACTCAAGCTTATTTCTCCTCCAACAAGATATACATXXXXXXXXXXT
TTGTTTGAAAATATTTGATTGTAACGAGCATTTTTATCTCTTTAAGAGTTGTTTGTCTCTTGCTTCTTTCAATATTGTT
ACAAATATATGATCCATTTTTATGGTAGGTAAGAAAACTCTTGACACTCGAGCTTATTTCTCCTCCAACAAGATATACATXXXXXXXXXXT
TTGTTTGAAAATATTTGATTGTAACGATCATTTTTATCTCTTTAAGAGTTGTTTGTCTCTTGCTTCTTTCAATATTGTT
>Marker608260 Chr3 SNP_site:30303578 Ref_Type:G
AACATACAACCACCTTTCAAGGAATAAAACTACATTGAGACCAAAATTTAAGGACTTGACACAACATAAAACTCAAGGTTXXXXXXXXXXC
TATCTGTGACTGTGTTGCCTACATATACTTGCTACTAACCAAGAGCTAACTCTCTAAGTTCATTACTAGTTGCTAGGTA
AACATACAACCACCTTTCAAGGAATAAAACTACATTGAGACCAAAATTTAAGGACTTGACACAACATAAAACTCAAGGTTXXXXXXXXXXC
TATCTGTGACTGTGTTGCCTACATATACTTGCTACTAACCAAGAGCTAGCTCTCTAAGTTCATTACTAGTTGCTAGGTA
>Marker608481 Chr3 SNP_site:31370117 Ref_Type:G
TACTCTCTTCATCTTTGTTCCATCTGGTTCAAATAGTAGATAATACTTTGTAGAAAGTTCATTATTTGTTTGTCATATCCXXXXXXXXXXT
CTTGTCAAATTTTGATTTCAAAGATATTTTGAAACTATTCCATGGGCATTATTTTACTTGGTTCAAATTCCCTTTTGTG
TACTCTCTTCATCTTTGTTCCATCTGGTTCAAATAGTAGATAATACTTTGTAGAAAGTTCATTATTTGTTTGTCATATCCXXXXXXXXXXT
CTTGTCGAATTTTGATTTCAAAGATATTTTGAAACTATTCCATGGGCATTATTTTACTTGGTTCAAATTCCCTTTTGTG
>Marker6085 Chr4 SNP_site:596366 Ref_Type:A
AACCAAAACCAACCCCTTGAATGTTTCTATCCCTAAACCCACACAGAATCCCAACCTACTAACTAATCAAATATAAGCAAXXXXXXXXXXT
CTCTAGAAGCTAACTCTTACTCACCCAACTTTATGGATGTGTATTAGCATTGAGTATGATCTCCAAAGCATACAAAGTA
AACCAAAACCAACCCCTTGAATGTTTCTATCCCTAAACCCACACAGAATCCCAACCTACTAACTAATCAAATATAAGCAAXXXXXXXXXXT
CTCTGGAAGCTAACTCTTACTCACCCAACTTTATGGATGTGTATTAGCATTGAGTATGATCTCCAAAGCATACAAAGTA
>Marker608501 Chr3 SNP_site:11810878 Ref_Type:A
ACCCCACAGCCATTTTACCTGAAATCGTAGGGGAGATTTTTGTTAAATGATCGACGTCTTTTTTTATATATACTTATTATXXXXXXXXXXT
ATTAATTAACTCATAACATTTTGTTAAGCTATTTTATTGAATTTTAGATCGAAACTGAAAACGAAATAGCACCGAGGTA
ACCCCACAGCCATTTTACCTGAAATCGTAGGGGAGATTTTTGTTAAATGATCGACGTCTTTTTTTATATATACTTATTATXXXXXXXXXXT
ATTAATTAACTCATAACATTTTGTTCAGCTATTTTATTGAATTTTAGATCGAAACTGAAAACGAAATAGCACCGAGGTA
>Marker608768 Chr3 SNP_site:15219804 Ref_Type:C
ACATTGCATTAACAAATGGATAATGATACGTGTCTGACAATGAAATTAACCACGCAAAAAAATTAAACTTTTGTGAATTGXXXXXXXXXXT
CAACTTACTCTAACTCCGTTTGCAGTTAGATAAAAGACTAGTGATCAATTTCGTATCTCTTACATGCAGAACTATCGTG

Create PDF with GO2PDF for free, if you wish to

ACATTGCATTAACAAATGGATAATGATACGTGTCTGACAATGAAATTAACCACGCAAAAAAATTAAACTTTTGTGAATTGXXXXXXXXXXT
GAACTTACTCTAACTCCGTTTGCAGTTAGATAAAAGACTAGTGATCAATTTCGTATCTCTTACATGCAGAACTATCGTG
>Marker60883 Chr4 SNP_site:1428186 Ref_Type:A
AACTTTCTTTTAAAAATAGCATGAAGAGGTTTGTTATCCATTAAAGAGTTGTTTAAGATGAGACAAGAATATATAGTGAGXXXXXXXXXXC
TTCTCAACACAGTCCCACCATATATATCTTTTCTCCCTTTTCTATCATTATTTCCATCCATTAAATTTGCAACATATGT
AACTTTCTTTTAAAAATAGCATGAAGAGGTTTGTTATCCATTAAAGAGTTGTTTATGATGAGACAAGAATATATAGTGAGXXXXXXXXXXC
TTCTCAACACAATCCCACCATATATATCTTTTCTCCCTTTTCTATCATTATTTCCATCCATTAAATTTGCAACATATGT
>Marker60883 Chr4 SNP_site:1428327 Ref_Type:G
AACTTTCTTTTAAAAATAGCATGAAGAGGTTTGTTATCCATTAAAGAGTTGTTTAAGATGAGACAAGAATATATAGTGAGXXXXXXXXXXC
TTCTCAACACAGTCCCACCATATATATCTTTTCTCCCTTTTCTATCATTATTTCCATCCATTAAATTTGCAACATATGT
AACTTTCTTTTAAAAATAGCATGAAGAGGTTTGTTATCCATTAAAGAGTTGTTTATGATGAGACAAGAATATATAGTGAGXXXXXXXXXXC
TTCTCAACACAATCCCACCATATATATCTTTTCTCCCTTTTCTATCATTATTTCCATCCATTAAATTTGCAACATATGT
>Marker60959 Chr4 SNP_site:23333082 Ref_Type:T
GACCCTAAATTTTGAAAAAGGGGTGGATTCCATTCTTTAAAATGGTCCATTTAATCCGTAAGTTAATAAGTTTAAAATGTXXXXXXXXXXA
TGTGGGACCTACGAATTTGTGGATATTTTGTTAATCCTGACTATGTTCGTGATTATAGGTTAATTCGTAGATTCAAAGT
GACCCTAAATTTTGAAAAAGGGGTGGATTCCATTCTTTAAAATGGTCCATTTAATCCGTAAGTTAATAAGTTTAAAATGTXXXXXXXXXXA
TGTGGGACCTACGAATTTGTGGATATTTTGTTAATCCTGATTATGTTCGTGATTATAGGTTAATTCGTAGATTCAAAGT
>Marker609817 Chr3 SNP_site:34972587 Ref_Type:C
GACTTAGTTAATATCTACTAGATCCCTAGTTGCATCTTCAATTAACCACAACCATATCATCCATTCTGTAGTTTTAAACCXXXXXXXXXXA
TGATGTCTTATACATGTGTTTGACATTCGAATATTTTTTAAGAAATTTGAAAAAAAATTAAAATTTGTATTTGTTTTGT
GACTTAGTTAATATCTAGTAGATCCCTAGTTGCATCTTCAATTAACCACAACCATATCATCCATTCTGTAGTTTTAAACCXXXXXXXXXXA
TGATGTCTTATACATGTGTTTGACATTCGAATATTTTTTAAGAAATTTGAAAAAAAATTAAAATTTGTATTTGTTTTGT
>Marker60989 Chr4 SNP_site:16624202 Ref_Type:T
AACTTCCTCCCTGATTTAAAACACAATATACTAGCAGTTTGGGCTGATTACCAAGTAAAGACACCTATTCTATGAAGTTTXXXXXXXXXXG
TTTGGTTACCGGCTTTTGACATTCAATATGGGCTGAAGAGATGAAAGATCCAATTTCAGAATAGAAGCATGGTTTGAGT
AACTTCCTCCCTGATTTAAAACACAATATACTAGCAGTTTGGGCTGATTACCAAGTAAAGACACCTATTCTATGAAGTTTXXXXXXXXXXG
TTTGGTTACCGGCTTTTGACATTCAATATGGGCTGAAGAGATGAAAGATCCAATTTTAGAATAGAAGCATGGTTTGAGT
>Marker61010 Chr4 SNP_site:18325636 Ref_Type:G
TACCAGAGAGGCTAATCGGCAATGAAGTTGAAGGGAAGGAGGAGAAGGAGAGAAAGGGAGAGAGAGAGAACTTCCCTAGCXXXXXXXXXXA
GGTAAATGAATAAGAACTCACAGTTCCATTAAAATAAGTGAGAAAGGATAGCAAGATCCCATTTTCAAGGGTTGATTGT
TACCAGAGAGGCTAATCGGCAATGAAGTTGAAGGGAAGGAGGAGAAGGAGAGAAAGGGAGAGAGAGAGAACTTCCCTAGCXXXXXXXXXXA
GGTAGATGAATAAGAACTCACAGTTCCATTAAAATAAGTGAGAAAGGATAGCAAGATCCCATTTTCAAGGGTTGATTGT
>Marker610420 Chr3 SNP_site:33192802 Ref_Type:C
CACACCATGGAATTGAAAATTAGGGGTTTTGGTCATGGTTTCTCTTTTCTTGCACTTATAAGTTGTCTTCGTATCCAATCXXXXXXXXXXA
TTGAAACATACATTATTGTATTAAAAGGTTTCAGGTTTTAAGTTTTAATTTTCGTATGTTGTTTAGTTTAAAAGAAGTG
CACACCATGGAATTGAAAATTAGGGGTTTTGGTCATGGTTTCTCTTTTCTTGCACTTATAAGTTGTCTTCGTATCCAATCXXXXXXXXXXA
TTGAAACATATATTATTGTATTAAAAGGTTTCAGGTTTTAAGTTTTAATTTTCGTATGTTGTTTAGTTTAAAAGAAGTG
>Marker610758 Chr3 SNP_site:39075253 Ref_Type:C
CACTATAAAAACATTCCAATGTGTAATCCCTCTTCATCTGTGTAAGCATATCTTTCTTGTTTGAAAAATTACCCTACACTXXXXXXXXXXA
CATGTCCTCGATGTTCTTAATCCGCATAGAAACATCTTGAATATCACTCGCGTTAGCTATGACGAAAAGGATCAGCGTG
CACTATAAAAACATTCCAATGTGTAATCCCTCTTCATCTGTGTAAGCATATCTTTCTTGTTTGAAAAATTACCCTACACTXXXXXXXXXXA
CATGTCCTCGATGTTCTTAATCCGCATAGAAACATCTTGAATATCACTCGGGTTAGCTATGACGAAAAGGATCAGCGTG
>Marker611143 Chr3 SNP_site:19988070 Ref_Type:C
CACTAGAGTGTCTATGATTCCATCAGTGTGATTCGTGATTAGTAATGAAAAATACAAAAATATTTTTTAATGATATGTTTXXXXXXXXXXT

Create PDF with GO2PDF for free, if you wish to

GTAAGCCTGACCGTTGTCTCCAAGTCTATTGTGTAAAGTTATAGATAGTTTTACTACTCTATTGGTTAATACTTAGGTT

CACTAGAGTGTCTATGATTCCATCAGTGTGATTCGTGATTAGTAATGAAAAATACAAAAATATTTTTTAATGATATGTTTXXXXXXXXXXT

GTAAGCCTGACCGTTGTCTCCAAGTCTATTGTGTAAAGTTATAGATAGTTTTACTACTTTATTGGTTAATACTTAGGTT

>Marker611176 Chr3 SNP_site:9328317 Ref_Type:A

AACCATTTGGTCAATAATACACTCTATATATTTGTCAAATTATATTTATTTTGGCATACTTTCTCTACTAATTTTTATTTXXXXXXXXXXT

ATTTTAACAATATTTTGAATCCTCAAATATAACAATGATTTATGTTTCTTTTCATTTCGAAGACATTTTATTTTTATGT

AACCATTTGGTCAATAATACACTCTATATATTTGTCAAATTATATTTATTTTGGCATACTTTCTCTACTATTTTTTATTTXXXXXXXXXXT

ATTTTAACAATATTTTGAATCCTCAAATATAACAATGATTTATGTTTCTTTTCATTTCGAAGACATTTTATTTTTATGT

>Marker611413 Chr3 SNP_site:6922605 Ref_Type:A

AACAACTAGGTTTTGATAAATATACGTAACTCCCCGGAAGAAGCCGATTGATAAAACAAGTGTAATTATTAGACTATATGXXXXXXXXXXG

TGGTCAGTATCAAAGAATCTTAAAATTAAATAATTATTTTACATGGAGGACAAATTCTAGGCCAGAATTCGAACTTGTT

AACAACTAGGTTTTGATAAATATACGTAACTCCCCGGAAGAAGCCGATTGATAAAACAAGTGTAATTATTAGACTATATGXXXXXXXXXXG

TGGTCTGTATCAAAGAATCTTAAAATTAAATAATTATTTTACATGGAGGACAAATTCTAGGCGAGAATTCGAACTTGTT

>Marker611413 Chr3 SNP_site:6922662 Ref_Type:C

AACAACTAGGTTTTGATAAATATACGTAACTCCCCGGAAGAAGCCGATTGATAAAACAAGTGTAATTATTAGACTATATGXXXXXXXXXXG

TGGTCAGTATCAAAGAATCTTAAAATTAAATAATTATTTTACATGGAGGACAAATTCTAGGCCAGAATTCGAACTTGTT

AACAACTAGGTTTTGATAAATATACGTAACTCCCCGGAAGAAGCCGATTGATAAAACAAGTGTAATTATTAGACTATATGXXXXXXXXXXG

TGGTCTGTATCAAAGAATCTTAAAATTAAATAATTATTTTACATGGAGGACAAATTCTAGGCGAGAATTCGAACTTGTT

>Marker611432 Chr3 SNP_site:30939579 Ref_Type:A

ACATAAGATGAAACTCTACTTAAATTTTTTCTTTTGGGTTAGAAAAATAAATTGATTCATTGACACGATGAAATCATACAXXXXXXXXXXG

CAAATTCAGCATCCCAGCCTGTTGAACACAGATAGAGCATTAATCATTTTTAAAAGAAACCAAAAGTAATCACAAATGT

ACATAAGATGAAACTCTACTTAAATTTTTTCTTTTGGGTTAGAAAAATAAATTGATTCATTGACACGATGAAATCATACAXXXXXXXXXXG

CAAATTCAGCATCCCAGCCTGTTGAACACAGATAGAGCATTAATCATTTTTAAAAGAAACCAAAAGTAATCACAGATGT

>Marker61156 Chr4 SNP_site:4656548 Ref_Type:C

ACTTAGTTTGAGCAATTTTGGGGTGGCCTTTTGAGAATTTTTCTAGTAGTATATAGGTGGAAACAAATCATGTGAAAAAAXXXXXXXXXXA

AATTTCATGGTTGAAAAGTTACAATACTAATCATTTGCCTATTCAATTGGAGAAAATTTTAAAATCTCATTTGGTTAGT

ACTTAGTTTGAGCAATTTTGGGGTGGTCTTTTGAGAATTTTTCTAGTAGTATATAGGTGGAAACAAATCATGTGAAAAAAXXXXXXXXXXA

AATTTCATGGTTGAAAAGTTACAATACTAATCATTTGCCTATTCAATTGGAGAAAATTTTAAAATCTCATTTGGTTAGT

>Marker611822 Chr3 SNP_site:2500824 Ref_Type:T

ACTCAATTTGAATTGATTTTCATAGGTCCTGTGGTTTCCCTGAAACTGCATTAATTGGCCATTATAAGCCTCTAATTGGTXXXXXXXXXXC

TCCGGGTTCAATCTCTCTCTCCCTCTCTCATTTTTTTTTATTCCGAGGCTTTGGTTGAATTCTAAGAATTTGCAATGGTC

ACTCAATTTGAATTGATTTTTATAGGTCCTGTGGTATCCCTGGAACTGCATTAATTGGCCATTATAAGCCTCTAATTGGTXXXXXXXXXXC

TCCGGGTTCAATCTCTCTCTCCCTCTCTCATTTTTTTTTATTCCGAGGCTTTGGTTGAATTCTAAGAATTTGCAATGGTC

>Marker611822 Chr3 SNP_site:2500839 Ref_Type:A

ACTCAATTTGAATTGATTTTCATAGGTCCTGTGGTTTCCCTGAAACTGCATTAATTGGCCATTATAAGCCTCTAATTGGTXXXXXXXXXXC

TCCGGGTTCAATCTCTCTCTCCCTCTCTCATTTTTTTTTATTCCGAGGCTTTGGTTGAATTCTAAGAATTTGCAATGGTC

ACTCAATTTGAATTGATTTTTATAGGTCCTGTGGTATCCCTGGAACTGCATTAATTGGCCATTATAAGCCTCTAATTGGTXXXXXXXXXXC

TCCGGGTTCAATCTCTCTCTCCCTCTCTCATTTTTTTTTATTCCGAGGCTTTGGTTGAATTCTAAGAATTTGCAATGGTC

>Marker611822 Chr3 SNP_site:2500846 Ref_Type:G

ACTCAATTTGAATTGATTTTCATAGGTCCTGTGGTTTCCCTGAAACTGCATTAATTGGCCATTATAAGCCTCTAATTGGTXXXXXXXXXXC

TCCGGGTTCAATCTCTCTCTCCCTCTCTCATTTTTTTTTATTCCGAGGCTTTGGTTGAATTCTAAGAATTTGCAATGGTC

ACTCAATTTGAATTGATTTTTATAGGTCCTGTGGTATCCCTGGAACTGCATTAATTGGCCATTATAAGCCTCTAATTGGTXXXXXXXXXXC

TCCGGGTTCAATCTCTCTCTCCCTCTCTCATTTTTTTTTATTCCGAGGCTTTGGTTGAATTCTAAGAATTTGCAATGGTC

>Marker612162 Chr3 SNP_site:2719871 Ref_Type:T

Create PDF with GO2PDF for free, if you wish to

GACGTTGGCGTTGTAGGTCGAACCAAACCTCCAACTAGATGGAGCAACGTTCCAGGCTATGATGGTCTCTTTGGTTGTGTXXXXXXXXXXC

CCACATCCCCGACACGTCGCCGCCGCCACCGACATTCATCACGTACGCCAAAAGCCAGTAGCCATTTCCTTGTAATGTG

GACGTTGGCGTTGTAGGTTGAACCAAACCTCCAACTAGATGGAGCAACGTTCCAAGCTATGATGGTCTCTTTGGTTGTGTXXXXXXXXXXC

CCACATTCCCGACACGTCGCCGCCGCCACCGACATTCATCACGTACGCCAAAAGCCAGTAGCCATTTCCTTGTAATGTG

>Marker612162 Chr3 SNP_site:2719907 Ref_Type:A

GACGTTGGCGTTGTAGGTCGAACCAAACCTCCAACTAGATGGAGCAACGTTCCAGGCTATGATGGTCTCTTTGGTTGTGTXXXXXXXXXXC

CCACATCCCCGACACGTCGCCGCCGCCACCGACATTCATCACGTACGCCAAAAGCCAGTAGCCATTTCCTTGTAATGTG

GACGTTGGCGTTGTAGGTTGAACCAAACCTCCAACTAGATGGAGCAACGTTCCAAGCTATGATGGTCTCTTTGGTTGTGTXXXXXXXXXXC

CCACATTCCCGACACGTCGCCGCCGCCACCGACATTCATCACGTACGCCAAAAGCCAGTAGCCATTTCCTTGTAATGTG

>Marker612162 Chr3 SNP_site:2720048 Ref_Type:T

GACGTTGGCGTTGTAGGTCGAACCAAACCTCCAACTAGATGGAGCAACGTTCCAGGCTATGATGGTCTCTTTGGTTGTGTXXXXXXXXXXC

CCACATCCCCGACACGTCGCCGCCGCCACCGACATTCATCACGTACGCCAAAAGCCAGTAGCCATTTCCTTGTAATGTG

GACGTTGGCGTTGTAGGTTGAACCAAACCTCCAACTAGATGGAGCAACGTTCCAAGCTATGATGGTCTCTTTGGTTGTGTXXXXXXXXXXC

CCACATTCCCGACACGTCGCCGCCGCCACCGACATTCATCACGTACGCCAAAAGCCAGTAGCCATTTCCTTGTAATGTG

>Marker612325 Chr3 SNP_site:27751568 Ref_Type:T

TACGTTTCTTATTAATCAATAATTGAATGCTTCATTAATCATAACCAAACACCACACCCCATCAACCAATTCCTATTTTGXXXXXXXXXXT

AAACTCTTTCATTTCTACTATTAAATAGGAAGAAGAAGTAAGACAAATTAAAAGGTTGATAATGTAATATAATATATGT

TACGTTTCTTATTAATCAATAATTGAATGCTTCATTAATCATAACCAAACACCACACCCTATCAACCAATTCCTATTTTGXXXXXXXXXXT

AAACTCTTTCATTTCTACTATTAAATAGGAAGAAGAAGTAAGACAAATTAAAAGGTTGATAATGTAATATAATATATGT

>Marker612644 Chr3 SNP_site:12992982 Ref_Type:C

TACAATGTTGCATAGAGGATTCCTCGACCTTCTCCGACGCAACCCTATTTTATGTCAGCTTATCTCCATGAATCCCCTACXXXXXXXXXXA

ACCTTTGGATTGAGGATTATGATTGCCTTATCATTTATATACAAAATCACCACCTACCGCAACCATTAAAAAATTGGTG

TACAATGTTGCATAGAGGATTGCTCGACCTTCTTCGACGCAACCCTATTTTATGTCAGCTTATCTCCATGAATCCCCTACXXXXXXXXXXA

ACTTTTGGATTGAGGATTATGATTGCCTTATCATTTATATACAAAATCACCACCTACCGCAACCATTAAAAAATTGGTG

>Marker612644 Chr3 SNP_site:12992994 Ref_Type:C

TACAATGTTGCATAGAGGATTCCTCGACCTTCTCCGACGCAACCCTATTTTATGTCAGCTTATCTCCATGAATCCCCTACXXXXXXXXXXA

ACCTTTGGATTGAGGATTATGATTGCCTTATCATTTATATACAAAATCACCACCTACCGCAACCATTAAAAAATTGGTG

TACAATGTTGCATAGAGGATTGCTCGACCTTCTTCGACGCAACCCTATTTTATGTCAGCTTATCTCCATGAATCCCCTACXXXXXXXXXXA

ACTTTTGGATTGAGGATTATGATTGCCTTATCATTTATATACAAAATCACCACCTACCGCAACCATTAAAAAATTGGTG

>Marker612644 Chr3 SNP_site:12993181 Ref_Type:C

TACAATGTTGCATAGAGGATTCCTCGACCTTCTCCGACGCAACCCTATTTTATGTCAGCTTATCTCCATGAATCCCCTACXXXXXXXXXXA

ACCTTTGGATTGAGGATTATGATTGCCTTATCATTTATATACAAAATCACCACCTACCGCAACCATTAAAAAATTGGTG

TACAATGTTGCATAGAGGATTGCTCGACCTTCTTCGACGCAACCCTATTTTATGTCAGCTTATCTCCATGAATCCCCTACXXXXXXXXXXA

ACTTTTGGATTGAGGATTATGATTGCCTTATCATTTATATACAAAATCACCACCTACCGCAACCATTAAAAAATTGGTG

>Marker612827 Chr3 SNP_site:8807053 Ref_Type:C

GACTTGGCATTCGGAGAATTTGTAGAGTGGTTGGGAGTGTTTGAAGAAGTAATCGGAGAGAAATTGTTGGAATCGGGCTCXXXXXXXXXXG

CAACATCGTTCTGCGTTGAATTCATCCAATGATATGAAGAACGTTCTTGGGTTTTTGGAGCTCTTTCGATGTTTTTGTG

GACTTGGGATTCGGAGAATTTGTAGAGTGGTTGGGAGTGTTTGAAGAAGTAATCGGAGAGAAATTGTTGGAATCGGGCTCXXXXXXXXXXG

CAACATCGTTCTGCGTTGAATTCATCCAATGATATGAAGAACGTTCTTGGGTTTTTGGAGCTCTTTCGATGTTTTTGTG

>Marker61303 Chr4 SNP_site:5671655 Ref_Type:G

ACATGATTTTGGGTAAAACAGAGCAAGTTTGTTTTGAGGTTGATTCTGATTGGAAGAAGCAATTTTAGAGTTCCAAAACCXXXXXXXXXXA

AAATGATAGGTTTTACGCATCGAAAGTTTGGTTGATTCAAGGAGTTCAAGAGTGTTTCAAATGTCCAAATTTTGAGGTA

ACATGATTTTGGGTAAAACAGAGCAAGTTTGTTTTGAGGTTGATTCTGATTGGAAGAAGCAATTTTAGAGTTCCAAAACCXXXXXXXXXXA

AAATGATAGGTTTTACGCATCGAAAGTTTGGTTGATTCAAGGAGTTCGAGAGTGTTTCAAATGTCCAAATTTTGAGGTA

Create PDF with GO2PDF for free, if you wish to

>Marker613118 Chr3 SNP_site:33945725 Ref_Type:A
ACAAAGGAGAATAAATAGAGAATACAAGGGAATAAAAAAGGAAAATATTTAGGAAATAAGGAAAATATTCCCATAATCTTXXXXXXXXXXT
CAATGAGACCTAACTTGCTAACACAAAAATGGAAGTTTTGGTCTGAGAAGCCCCTTGGTAAGAACATCAGCAACTTGTT
ACAAAGGAGAATAAATAGAGAATACAAGGGAATAAAAAAGGAAAATATTTAGGAAATAAGGAAAATATTCCCATAATCTTXXXXXXXXXXT
CAATGAGGCCCAACTTGCTAACACAAAAATGGAAGTTTTGGTCTGAGAAGCCCCTTGGTAAGAACATCAGCAACTTGTT
>Marker613118 Chr3 SNP_site:33945728 Ref_Type:T
ACAAAGGAGAATAAATAGAGAATACAAGGGAATAAAAAAGGAAAATATTTAGGAAATAAGGAAAATATTCCCATAATCTTXXXXXXXXXXT
CAATGAGACCTAACTTGCTAACACAAAAATGGAAGTTTTGGTCTGAGAAGCCCCTTGGTAAGAACATCAGCAACTTGTT
ACAAAGGAGAATAAATAGAGAATACAAGGGAATAAAAAAGGAAAATATTTAGGAAATAAGGAAAATATTCCCATAATCTTXXXXXXXXXXT
CAATGAGGCCCAACTTGCTAACACAAAAATGGAAGTTTTGGTCTGAGAAGCCCCTTGGTAAGAACATCAGCAACTTGTT
>Marker613716 Chr3 SNP_site:9890591 Ref_Type:T
TACACATTTGGTCTTGTTTTAACTATACCATTTTCATCTTCTCTCTCAATTATTTAAGGTTTTATTTGGAGTATGTGTTGXXXXXXXXXXC
TCTCAACTTCATTCATCTCAAGATAGAGTAAGCTTTGCCAATTTGGCAAATAGCTAGGGAGATAATAGAGGTGTGTGTA
TACACATTTGGTCTTGTTTTAACTATACCATTTTCATTTTCTCTCTCAATTATTTAAGGTTTTATTTGGAGTATGTGTTGXXXXXXXXXXC
TCTCAACTTCATTCATCTCAAGATAGAGTAAGCTTTGCCAATTTGGCAAATAGCTAGGGAGATAATAGAGGTGTGTGTA
>Marker614301 Chr3 SNP_site:33464287 Ref_Type:T
ACTAAAAAAAATTGTTGCTAATTTTTTTTTCTAACATAACCGTCCGTTCAATCTTGAAGTATTTTCACAATAATATATCAATXXXXXXXXXXT
ATACTTTAATTGATGGGACAATAATGTTATATGAATAATATAAAAAGAAAATTAATTAGGAAATATACGTCGTTGTGTA
ACTAAAAAAAATTGTTGCTAATTTTTTTTTCTAACATAACCGTCTGTTCAATCTTGAAGTATTTTCACAATAACATATCAATXXXXXXXXXXT
ATACTTTAATTGATGGGACAATAATGTTATATGAATAATATAAAAAGAAAATTAATTAGAAAATATACGTCGTTGTGTA
>Marker614301 Chr3 SNP_site:33464316 Ref_Type:C
ACTAAAAAAAATTGTTGCTAATTTTTTTTTCTAACATAACCGTCCGTTCAATCTTGAAGTATTTTCACAATAATATATCAATXXXXXXXXXXT
ATACTTTAATTGATGGGACAATAATGTTATATGAATAATATAAAAAGAAAATTAATTAGGAAATATACGTCGTTGTGTA
ACTAAAAAAAATTGTTGCTAATTTTTTTTTCTAACATAACCGTCTGTTCAATCTTGAAGTATTTTCACAATAACATATCAATXXXXXXXXXXT
ATACTTTAATTGATGGGACAATAATGTTATATGAATAATATAAAAAGAAAATTAATTAGAAAATATACGTCGTTGTGTA
>Marker614301 Chr3 SNP_site:33464498 Ref_Type:A
ACTAAAAAAAATTGTTGCTAATTTTTTTTTCTAACATAACCGTCCGTTCAATCTTGAAGTATTTTCACAATAATATATCAATXXXXXXXXXXT
ATACTTTAATTGATGGGACAATAATGTTATATGAATAATATAAAAAGAAAATTAATTAGGAAATATACGTCGTTGTGTA
ACTAAAAAAAATTGTTGCTAATTTTTTTTTCTAACATAACCGTCTGTTCAATCTTGAAGTATTTTCACAATAACATATCAATXXXXXXXXXXT
ATACTTTAATTGATGGGACAATAATGTTATATGAATAATATAAAAAGAAAATTAATTAGAAAATATACGTCGTTGTGTA
>Marker61467 Chr4 SNP_site:1077145 Ref_Type:T
CACATCTTGTCATATAGTGCATAAGTTGCAATTATATACCTCTGGTGGAGTCACAGATATATAATTAATTCCTAGTTTGAXXXXXXXXXXT
AAATGTATTAAGGGTAACTAAATAGATTATAATGGATGATGTATTTACTATAAATAGCTACAATTCATCACATGAGGTA
CACATCTTGTCATATAGTGCATAAGTTGCAATTATATACCTTTGGTGGAGTCACAGATATATAATTAATTCCTAGTTTGAXXXXXXXXXXT
AAATGTATTAAGGGTAACTAAATAGATTATAATGGATGATGTATTTACTATAAATAGCTATAATTCATCACATGAGGTA
>Marker61467 Chr4 SNP_site:1077345 Ref_Type:T
CACATCTTGTCATATAGTGCATAAGTTGCAATTATATACCTCTGGTGGAGTCACAGATATATAATTAATTCCTAGTTTGAXXXXXXXXXXT
AAATGTATTAAGGGTAACTAAATAGATTATAATGGATGATGTATTTACTATAAATAGCTACAATTCATCACATGAGGTA
CACATCTTGTCATATAGTGCATAAGTTGCAATTATATACCTTTGGTGGAGTCACAGATATATAATTAATTCCTAGTTTGAXXXXXXXXXXT
AAATGTATTAAGGGTAACTAAATAGATTATAATGGATGATGTATTTACTATAAATAGCTATAATTCATCACATGAGGTA
>Marker615555 Chr3 SNP_site:18538646 Ref_Type:A
CACTTAAAGAAGAGACATGAAACATAAAAGCATATTTATAATTGTATTGTATAACTGCTTCATCTAATTTTTTTCTTATAXXXXXXXXXXT
TGGTAAAGTTATGTTTTTTTTTCATAAATATTTGATGATTGACCTTACTTAAATGTCATCAATGATACCAGATTGTTGT
CACTTAAAGAAGAGACATGAAACATAAAAGCATATTTGTAATTGTATTGTATAACTGCTTCATATAATTTTTTTCTTATAXXXXXXXXXXT

Create PDF with GO2PDF for free, if you wish to

TGGTAAAGTTATGTTTTTTTTTCATAAATATTTGATGATTGGCCTTACTTAAATGTCATCAATGATACCAGATTGTTGT
>Marker615555 Chr3 SNP_site:18538672 Ref_Type:C
CACTTAAAGAAGAGACATGAAACATAAAAGCATATTTATAATTGTATTGTATAACTGCTTCATCTAATTTTTTTCTTATAXXXXXXXXXXT
TGGTAAAGTTATGTTTTTTTTTCATAAATATTTGATGATTGACCTTACTTAAATGTCATCAATGATACCAGATTGTTGT
CACTTAAAGAAGAGACATGAAACATAAAAGCATATTTGTAATTGTATTGTATAACTGCTTCATATAATTTTTTTCTTATAXXXXXXXXXXT
TGGTAAAGTTATGTTTTTTTTTCATAAATATTTGATGATTGGCCTTACTTAAATGTCATCAATGATACCAGATTGTTGT
>Marker615555 Chr3 SNP_site:18538836 Ref_Type:A
CACTTAAAGAAGAGACATGAAACATAAAAGCATATTTATAATTGTATTGTATAACTGCTTCATCTAATTTTTTTCTTATAXXXXXXXXXXT
TGGTAAAGTTATGTTTTTTTTTCATAAATATTTGATGATTGACCTTACTTAAATGTCATCAATGATACCAGATTGTTGT
CACTTAAAGAAGAGACATGAAACATAAAAGCATATTTGTAATTGTATTGTATAACTGCTTCATATAATTTTTTTCTTATAXXXXXXXXXXT
TGGTAAAGTTATGTTTTTTTTTCATAAATATTTGATGATTGGCCTTACTTAAATGTCATCAATGATACCAGATTGTTGT
>Marker615712 Chr3 SNP_site:24230391 Ref_Type:T
CACCTTTTATAAACAAATTAATATAAGAACATTTCATTCAAAAAATATACACTGCATTATTCAATATATACGATAATTAGXXXXXXXXXXT
TATAATATCACCATAATAGAATTAATCACTTACCATATTTACAAAGAATATTTAATAAATGCAAATATCAATCATGGTC
CACCTTTTATAAACAAATTAATATAAGAACATTTCATTCAAAAAATATACACTGCATTATTCAATATATACGATAATTAGXXXXXXXXXXT
TATAATATTACCATAATAGAATTAATCACTTACCATATTTACAAAGAATATTTAATAAATGCAAATATCAATCATGGTC
>Marker61597 Chr4 SNP_site:22689417 Ref_Type:A
GACCCAACTCAACTTTAATGTTTATATTATTTTTATTTTATTTTTTCATCCATTAAAATTATTCCTTTCTCTTTTTCTTAXXXXXXXXXXC
TTTTAACTTTTAAAACTGATAAGAAAATTGAAAAAAAATATAACTTACACAAATATTTTTAATATTGTATTTGTTTTGT
GACCCAACTCAACTTTAATGTTTATATTATTTTTATTTTATTTTTTCATCCATTAAAATTATTCCTTTCTCTTTTTCTTAXXXXXXXXXXC
TTTTAACTTTTAAAACTGATAAGAAAATTGAAAAAAAATATAACTTACACAAATATTTTTAATCTTGTATTTGTTTTGT
>Marker616129 Chr3 SNP_site:31718784 Ref_Type:G
ACATTTTCAAGTATAGAGAACTTCTAATTGGAATTTGGGAGGCAAAATTATATAGCTTTATCAGTGGTTGTTCTGGGAGTXXXXXXXXXXC
TGTGAGATTAATGATAATGTAAGATAAATTGCACAAAAAGAAGGTGCATTCCCATAAACAGTATAAAGTCATCTCTGTA
ACATTTTCAAGTATAGAGAACTTCTAATTGGAATTTGGGAGGCAAAATTATATAGCTTTATCGGTGGTTGTTCTGGGAGTXXXXXXXXXXC
TGTGAGATTAATGATAATGTAAGATAAATTGCACAAAAAGAAGGTGCATTCCCATAAACAGTATAAAGTCATCTCTGTA
>Marker616200 Chr3 SNP_site:6688690 Ref_Type:A
AACTAGAAGCATTACCACGATGAGCACCCACCATACAACATGACAAATTATTGCTTGATGTGAAGAAAGAAAGCCAACCAXXXXXXXXXXC
CCTCTCTTCTACATTTATTTTACTGGAGAAAAAAGGCACAAAACTCAAAGAAAAATAAATCAATAATACAGTAAATGTC
AACTAGAAGCATTACCACGATGAGCACCCACCATACAACATGACAAATTATTGCTTGATGTGAAGAAAGAAAGCCAACCAXXXXXXXXXXC
CCTCTCTTCTACATTTATTTTACTGGAGAAAGAAGGCACAAAACTCAAAGAAAAATAAATCAATAATACAGTAAATGTC
>Marker616226 Chr3 SNP_site:27263478 Ref_Type:C
ACTTTATAAATACCAATCTTCTTTTAAACCTTCACAAATAACAATATTAGCAACGCTTATAAAATCTATTATTAAAATACXXXXXXXXXXT
GAAGTTTTCAAACGGCGAACATAAGGAGTTTGTTGTGCTGTATGGTTGGTGTATGTGAATTATCAGAGGCAAAGTAAGT
ACTTTATAAATACCAATCTTCTTTTAAACCTTTACAAATAACAATATTAGCAACGCTTATAAAATCTATTATTAAAATACXXXXXXXXXXT
GAAGTTTTCAAACGGCGAACATAAGGAGTTTGTTGTGCTGTATGGTTGGTGTATGTGAATTATCAGAGGCAAAGTAAGT
>Marker616317 Chr3 SNP_site:24283388 Ref_Type:C
CACTAGAAGTAAGCTTTATATATAACTTTCTTCAGCTCATTTCTGCTGTCTAGTTTTCGGTTAGATGATTCATTTCATTCXXXXXXXXXXA
CTTTCATTTCTGCGCTACCAATCAAAATATTGAGCTGTTCTATTGAGTCTAAATTTCCTATTTTGGCACATCTTTTGTG
CACTAGAAGTAAGCTTTATATATAACTTTCTTCAGCTCATTTCTGCTGTCTAGTTTTCGGTTAGATGATTCATTTCATTCXXXXXXXXXXA
CTTTCATTTCTGCGCTACCAATCAAAATATTGAGCTGTTCTATTGAGTCTAAATTTCGTATTTTGGCACATCTTTTGTG
>Marker616617 Chr3 SNP_site:926239 Ref_Type:G
TACATTGGTGGGTTGTTTTTTTGATGGGATTAAGAAAATGGTTTGATTGGCAATAACACATGCTTTGCTTTGGAGTCTTTXXXXXXXXXXT
CACCCATAGGCGTTGGGGTTTTCCCTACTTCATTTGTTAATGAAATATTTCTTATTCCAGAAAAGTTCCGAGAGGATGT

Create PDF with GO2PDF for free, if you wish to

TACATTGGTGGGTTGTTTTTTTTATGGGATTAAGAAAATGGTTTGATTGGCAATAACACATGCTTTGCTTTGGAGTCTTTXXXXXXXXXXT
CACCCATAGGCGTTGGGGTTTTCCCTACTTCATTTGTCAATGAAATATTTCTTATTCCAGAAAAGTTCCAAGAGGATGT
>Marker616617 Chr3 SNP_site:926484 Ref_Type:C
TACATTGGTGGGTTGTTTTTTTGATGGGATTAAGAAAATGGTTTGATTGGCAATAACACATGCTTTGCTTTGGAGTCTTTXXXXXXXXXXT
CACCCATAGGCGTTGGGGTTTTCCCTACTTCATTTGTTAATGAAATATTTCTTATTCCAGAAAAGTTCCGAGAGGATGT
TACATTGGTGGGTTGTTTTTTTTATGGGATTAAGAAAATGGTTTGATTGGCAATAACACATGCTTTGCTTTGGAGTCTTTXXXXXXXXXXT
CACCCATAGGCGTTGGGGTTTTCCCTACTTCATTTGTCAATGAAATATTTCTTATTCCAGAAAAGTTCCAAGAGGATGT
>Marker616617 Chr3 SNP_site:926516 Ref_Type:A
TACATTGGTGGGTTGTTTTTTTGATGGGATTAAGAAAATGGTTTGATTGGCAATAACACATGCTTTGCTTTGGAGTCTTTXXXXXXXXXXT
CACCCATAGGCGTTGGGGTTTTCCCTACTTCATTTGTTAATGAAATATTTCTTATTCCAGAAAAGTTCCGAGAGGATGT
TACATTGGTGGGTTGTTTTTTTTATGGGATTAAGAAAATGGTTTGATTGGCAATAACACATGCTTTGCTTTGGAGTCTTTXXXXXXXXXXT
CACCCATAGGCGTTGGGGTTTTCCCTACTTCATTTGTCAATGAAATATTTCTTATTCCAGAAAAGTTCCAAGAGGATGT
>Marker616638 Chr3 SNP_site:18759587 Ref_Type:G
TACTCGCATGATGAAGAAAATAATGGAGAGCATTTTGCATGCAGTGATCCATATTTAATAACACTTCATGAGGAGTTAAAXXXXXXXXXXA
TCAATAATTAGGTTGTAGGCGTAGTTGAGTGGGTTTCAGTGGTGCAAACCCTCAAACCAACCTTCAATCAAGCTAAGTA
TACTCGCATGATGAAGAGAATAATGGAGAGCATTTTGCATGCAGTGATCCATATTTAATAACACTTCATGAGGAGTTAAAXXXXXXXXXXA
TCAATAATTAGGTTGTAGGCGTAGTTGAGTGGGTTTCAGTGGTGCAAACCCTCAAACCAACCTTCAATCAAGCTAAGTA
>Marker61746 Chr4 SNP_site:12557831 Ref_Type:T
CACACTACCTTGAGGTGACGCTCGCTTAGGGAAATTTGAGATAGGAAGGCGTAATGTTTAGATCGAGCTTGCATTTTTCAXXXXXXXXXXG
AAGGAATTCATTTCGTTTATTAATCCCTTGTGTTTTTCTTTTGTTTTTATTTTATTCGAGTTTGAGCTCTGTTTAAGTC
CACACTACCTTGAGGTGACGCTCGCTTAGGGAAATTTGAGATAGGAAGGCGTAATGTTTAGATCGAGCTTGCATTTTTCAXXXXXXXXXXT
AGGGAATTCATTTCGTTTATTAATCCCTTGTGTTTTTCTTTTGTTTTTATTTTATTCGAGTTTGAGCTCTGTTTAAGTC
>Marker61746 Chr4 SNP_site:12557833 Ref_Type:G
CACACTACCTTGAGGTGACGCTCGCTTAGGGAAATTTGAGATAGGAAGGCGTAATGTTTAGATCGAGCTTGCATTTTTCAXXXXXXXXXXG
AAGGAATTCATTTCGTTTATTAATCCCTTGTGTTTTTCTTTTGTTTTTATTTTATTCGAGTTTGAGCTCTGTTTAAGTC
CACACTACCTTGAGGTGACGCTCGCTTAGGGAAATTTGAGATAGGAAGGCGTAATGTTTAGATCGAGCTTGCATTTTTCAXXXXXXXXXXT
AGGGAATTCATTTCGTTTATTAATCCCTTGTGTTTTTCTTTTGTTTTTATTTTATTCGAGTTTGAGCTCTGTTTAAGTC
>Marker617589 Chr3 SNP_site:1551191 Ref_Type:C
ACTACTTTTAACAATCAAAATTGATTTTATATAACCCTAATTTAAGGAAAATCAATTTCTACTCCTACATGTGTGGTTTGXXXXXXXXXXG
TTACATATGTGTGTTTAATTTATTATTGAATATTGGAAGCGAATGAAAAGAGATACGAAAAGAATGCTATGGATTTGTT
ACTACTTTTAACAATCAAAATTGATTTTATATAACCCTAATTTAAGGAAAATCAATTTCTACTCCTACATGTGTGGTTTGXXXXXXXXXXG
TTATATATGTGTGTTTAATTTATTATTGAATATTGGAAGCGAATGAAAAGAGATACGAAAAGAATGCTATGGATTTGTT
>Marker617610 Chr3 SNP_site:29494817 Ref_Type:A
AACTTTCTTTGGGTTCATAATTATAAGATGAAAGAAAAGATGTAATACACATTCTATAATCAACTTGGGTGTTAGAGAATXXXXXXXXXXC
CTTCTTTGCATAGGATTCATGTTTGGCCAATGAAGAAATTAAGAATTGTTCAACCCATCAACTTAAGATCTACATGGTC
AACTTTCTTTGGGTTCATAATTATAAGATGAAAGAAAAGATGTAATACACATTCTGTAATCAACTTGGGTGTTAGAGAATXXXXXXXXXXC
CTTCTTTGCATAGGATTCATGTTTGGCCAATGAAGAAATTAAGAATTGTTCAACCCATCAACTTAAGATCTACATGGTC
>Marker618214 Chr3 SNP_site:34399279 Ref_Type:G
CACTAAGTTCCACTAATTATTAGTGGATTATTAGATGTTGAGATTTGACCAACTAATTACATGCAGGAATTAGGTTATAAXXXXXXXXXXC
TTCACCAAATTGGATCCCACAACTCGGTTCTAAGTCTGAAGATTAGCGGGTCAATTCTAGTGGTTGTCTCGATCTGAGT
CACTAAGTTCCACTAATTATTAGTGGATTATTAGATGTTGAGATTTGACCAACTAATTACATGCATGAATTAGGTTATAAXXXXXXXXXXC
TTCACCAAATTGGATCCCACAACTCGATTCTAAGTCCGAAGATTAGCGGGTCAATTCTAGTGGTTGTCTCGATCTGAGT
>Marker618214 Chr3 SNP_site:34399450 Ref_Type:G
CACTAAGTTCCACTAATTATTAGTGGATTATTAGATGTTGAGATTTGACCAACTAATTACATGCAGGAATTAGGTTATAAXXXXXXXXXXC

Create PDF with GO2PDF for free, if you wish to

TTCACCAAATTGGATCCCACAACTCGGTTCTAAGTCTGAAGATTAGCGGGTCAATTCTAGTGGTTGTCTCGATCTGAGT
CACTAAGTTCCACTAATTATTAGTGGATTATTAGATGTTGAGATTTGACCAACTAATTACATGCATGAATTAGGTTATAAXXXXXXXXXXC
TTCACCAAATTGGATCCCACAACTCGATTCTAAGTCCGAAGATTAGCGGGTCAATTCTAGTGGTTGTCTCGATCTGAGT
>Marker618214 Chr3 SNP_site:34399460 Ref_Type:T
CACTAAGTTCCACTAATTATTAGTGGATTATTAGATGTTGAGATTTGACCAACTAATTACATGCAGGAATTAGGTTATAAXXXXXXXXXXC
TTCACCAAATTGGATCCCACAACTCGGTTCTAAGTCTGAAGATTAGCGGGTCAATTCTAGTGGTTGTCTCGATCTGAGT
CACTAAGTTCCACTAATTATTAGTGGATTATTAGATGTTGAGATTTGACCAACTAATTACATGCATGAATTAGGTTATAAXXXXXXXXXXC
TTCACCAAATTGGATCCCACAACTCGATTCTAAGTCCGAAGATTAGCGGGTCAATTCTAGTGGTTGTCTCGATCTGAGT
>Marker618323 Chr3 SNP_site:6102904 Ref_Type:A
TACTGTCAAATTCAACTCCAAAAACTTGATTCTTTGAGGAATCAAAAGCCAAATCCGAGCTAAATAATCCAAGAAATCCCXXXXXXXXXXA
CCTGTGATGCAGAGGATTTTGTGATTAGGCCAAATTCATGATTGAATTTAAAGAGAAAACTATTTATGTTATTGATTGT
TACTGTCAAATTCAACTCCAAAAACTTGATTCTTTGAGGGATCAAAAGCCAAATCCGAGCTAAATAATCCAAGAAATCCCXXXXXXXXXXA
CCTGTGATGCAGAGGATTTTGTGATTAGGCCAAATTCATGATTGAATTTAAAGAGAAAACTATTTATGTTATTGATTGT
>Marker618462 Chr3 SNP_site:5614556 Ref_Type:T
GACATACGTTTATCAACCACCAACACTTTCCCTTCCTTTCCCTCACTCATATCCCTATAGCTATTTTAATATTCTCTATGXXXXXXXXXXA
TTATAGACCACGTTTGAAAATAACTTTATTTGTTTTTTCATTATTTCGTATAAATCTTATAATTTCGTTTGAATTGGTA
GACATACGTTTATCAACCACCAACACTTTCCCTTCCTTTCCCTCACTCATATCCCTATAGCTATTTTAATATTCTCTATGXXXXXXXXXXA
TTATAGACCACGTTTGAAAATAACTTTATTTGTTTTTTCATTATTTCGTATAAATCTTATAATTTCGTTTGAATTGGTG
>Marker618738 Chr3 SNP_site:36032533 Ref_Type:G
CACTTGAATTGGCACCTAGATCAATTGCAAAAAGTGATACGATTCAGATACCACAGCACATAATCACGCCCAAATTTTGAXXXXXXXXXXA
TATTTATGCACTTGCAGGCAATTCAATGCCACTAACTGAAATATTTCAACAGTTATTTTGGCCACTTAAGGAAGTAGTT
CACTTGAATTGGCACCTAGATCAATTGCAAGAAGTGATACGATTCAGATACCACAGCACATAATCACGCCCAAATTTTGAXXXXXXXXXXA
TATTTATGCACTTGCAGGCAATTCAATGCCACTAACTGAAATATTTCAACAGTTCTTTTGGCCACTTAAGGAAGTAGTT
>Marker618738 Chr3 SNP_site:36032721 Ref_Type:C
CACTTGAATTGGCACCTAGATCAATTGCAAAAAGTGATACGATTCAGATACCACAGCACATAATCACGCCCAAATTTTGAXXXXXXXXXXA
TATTTATGCACTTGCAGGCAATTCAATGCCACTAACTGAAATATTTCAACAGTTATTTTGGCCACTTAAGGAAGTAGTT
CACTTGAATTGGCACCTAGATCAATTGCAAGAAGTGATACGATTCAGATACCACAGCACATAATCACGCCCAAATTTTGAXXXXXXXXXXA
TATTTATGCACTTGCAGGCAATTCAATGCCACTAACTGAAATATTTCAACAGTTCTTTTGGCCACTTAAGGAAGTAGTT
>Marker618984 Chr3 SNP_site:7266080 Ref_Type:G
CACAACTTTTGGTCCATTATAATATGTGATTTGATGATGGAATCAATTAATTCTTCTGCTCTTACACTTGACCCTGTGAGXXXXXXXXXXG
CTTATAGCGATTACATTGGACAAGAATTGGTGCCGAGGCTATTGTGACTTAAGATTTAGTAGCTAAAGTAATAGTTGTT
CACGACTTTTGGTCCATTATAATATGTGATTTGATGATGGAATCAATTAATTCTTCTGCTCTTACACTTGACCCTGTGAGXXXXXXXXXXG
CTTATAGCGATTACATTGGACAAGAATTGGTGCCGAGGCTATTGTGACTTAAGATTTAGTAGCTAAAGTAATAGTTGTT
>Marker619176 Chr3 SNP_site:27645803 Ref_Type:T
CACGAAGGTAGATGTAATTCCTCCAAACCATCACATAGATTGTTGTTTCCAAGGATGGAAAACATGGTTGAATTGGAAAAXXXXXXXXXXT
GAGAGATTTAGTTCTTCTAGACCTTTCAAAGCTTTCAAAGACTCGGGAATTGTTCCTTCAAATTGGTTGCCTCCTAAGT
CACGAAGGTAGATGTAATTCCTCCAAACCATCACATAGATTGTTGTTTCCAAGGATGGAAAACATGGTTGAATTGGAAAAXXXXXXXXXXT
GAGAGATTTAGTTCTTCTAGACCTTTCAAATCTTTCAAAGACTCGGGAATTGTTCCTTCAAATTGGTTGCCTCCTAAGT
>Marker619259 Chr3 SNP_site:9502703 Ref_Type:C
CACTTAAAAGGAACCAATTGCTGTGCAAAGAAAAGATAATAGATTGATAAAACAAACGCAACAGAAACTTCTCGTGAAAGXXXXXXXXXXA
TTCTTTGGAATGCCAGAAAGCTGCTTAAATAAGGGTTACTAACCTTCAATAAATACTCGTAGAAGGAATCAATGCTCGT
CACTTAAAAGGAACCAATTGCTGTGCAAAGAAAAGATAATAGATTGATAAAACAAATGCAACAGAAACTTCTCGTGAAAGXXXXXXXXXXA
TTCTTTGGAATGCCAGAAAGCTGCTTAAATAAGGGTTACTAACCTTCAATAAATACTCGTAGAAGGAATCAATGCTCGT
>Marker61945 Chr4 SNP_site:12795801 Ref_Type:G

Create PDF with GO2PDF for free, if you wish to

ACAGTTGGATTAGGGAGATACGAACCAAGTCACTAATACATACAAATGTCAGTTGAACTATGTTCTCTATGCTCTCTTTGXXXXXXXXXXC
AAAAGTTCCATGAAAATGAAAAAGACTACAAACTCAATGTATTTGAGTAGATTTAAAAGCTTTAAAAGTTATCAGTAGT
ACAGTTGGATTAGGGAGATACGAACCAGGTCACTAATACATGCAAATATCAGTTGAACTATGTTCTCTATGCTCTCTTTGXXXXXXXXXXC
AAAAGTTCCATGAAAATGAAAAAGACTACAAACTCAATGTATTTGAGTAGATTTAAAAGCTTTAAAAGTTATCAGTAGT
>Marker61945 Chr4 SNP_site:12795815 Ref_Type:G
ACAGTTGGATTAGGGAGATACGAACCAAGTCACTAATACATACAAATGTCAGTTGAACTATGTTCTCTATGCTCTCTTTGXXXXXXXXXXC
AAAAGTTCCATGAAAATGAAAAAGACTACAAACTCAATGTATTTGAGTAGATTTAAAAGCTTTAAAAGTTATCAGTAGT
ACAGTTGGATTAGGGAGATACGAACCAGGTCACTAATACATGCAAATATCAGTTGAACTATGTTCTCTATGCTCTCTTTGXXXXXXXXXXC
AAAAGTTCCATGAAAATGAAAAAGACTACAAACTCAATGTATTTGAGTAGATTTAAAAGCTTTAAAAGTTATCAGTAGT
>Marker61945 Chr4 SNP_site:12795821 Ref_Type:A
ACAGTTGGATTAGGGAGATACGAACCAAGTCACTAATACATACAAATGTCAGTTGAACTATGTTCTCTATGCTCTCTTTGXXXXXXXXXXC
AAAAGTTCCATGAAAATGAAAAAGACTACAAACTCAATGTATTTGAGTAGATTTAAAAGCTTTAAAAGTTATCAGTAGT
ACAGTTGGATTAGGGAGATACGAACCAGGTCACTAATACATGCAAATATCAGTTGAACTATGTTCTCTATGCTCTCTTTGXXXXXXXXXXC
AAAAGTTCCATGAAAATGAAAAAGACTACAAACTCAATGTATTTGAGTAGATTTAAAAGCTTTAAAAGTTATCAGTAGT
>Marker620644 Chr3 SNP_site:34372316 Ref_Type:C
TACCCAAATTTTCCCTTTCTCACACCCGAAGATCTTTGGTGCCATCACTTCGAGACCGCATCGACCCCTCTCTTTCTACCXXXXXXXXXXC
CCCATCTCCATCTCCACCTCCCTGTTTTTCTTAGCTAATTTTATCGATTCCTCGCGTCTATCCAGGTAAGGATCATGTT
TACCCAAATTTTCCCTTTCTCACACCCGAAGATCTTTGGTGCCATCACTTCGAGACCGCATCGACCCCTCTCTTTCTACCXXXXXXXXXXC
TCCATCTCCACCTCCACCTCCCTGTTTTTCTTAGCTAATTTTATCGATTCCTCGCGTCTATCCAGGTAAGGATCATGTT
>Marker620644 Chr3 SNP_site:34372326 Ref_Type:T
TACCCAAATTTTCCCTTTCTCACACCCGAAGATCTTTGGTGCCATCACTTCGAGACCGCATCGACCCCTCTCTTTCTACCXXXXXXXXXXC
CCCATCTCCATCTCCACCTCCCTGTTTTTCTTAGCTAATTTTATCGATTCCTCGCGTCTATCCAGGTAAGGATCATGTT
TACCCAAATTTTCCCTTTCTCACACCCGAAGATCTTTGGTGCCATCACTTCGAGACCGCATCGACCCCTCTCTTTCTACCXXXXXXXXXXC
TCCATCTCCACCTCCACCTCCCTGTTTTTCTTAGCTAATTTTATCGATTCCTCGCGTCTATCCAGGTAAGGATCATGTT
>Marker620650 Chr3 SNP_site:217618 Ref_Type:G
GACAAGGATATGTATTCAAGGAAAAATCTGTGTTAGTAATTGCACAATGATTCAGTTTTAGTTCCATTATTCTACTGATCXXXXXXXXXXA
TTGTTGTTGGAAAGTTTGTTTGATTCTTCTACCTTGTTTTAAGCTTTGTCCATTGGTGATTGAAATGATAGTGTTGGTT
GACAAGGATATGTATTCAAGGAAAAATCTGTGTTAGTAATTGCACAATGATTCAGTTTTAGTTCCATTATTCTACTGATCXXXXXXXXXXA
TTGTTGTTGGAGAGTTTGTTTGATTCTTCTACCTTGTTTTAAGCTTTGTCCATTGGTGATTGAAATGATAGTGTTGGTT
>Marker621508 Chr3 SNP_site:38625848 Ref_Type:G
AACAACGACATCCTGCCGAATATCCTGCACAAAATGAAGAGACACATCAAATTGGTGATAAAAAATTATCATAAAAATAAXXXXXXXXXXG
CATTAGAAAGTTGATTATTCACAAATTACATAATGATATATATATTTTTTATAACCGAGAGTTGGAGCTTAGCTCAGATGT
AACAACGACATCCTGCCGAATATCCTGCACAAAATGAAGAGACACATCAAATTGGTGATAAAAAATTATCATAAAAGTAAXXXXXXXXXXG
CATTAGAAAGTTGATTATTCACAAATTACATTATGATATATATATTTTTTATAACCGAGAGTTGGAGCTTAGCTCAGATGT
>Marker621508 Chr3 SNP_site:38626002 Ref_Type:T
AACAACGACATCCTGCCGAATATCCTGCACAAAATGAAGAGACACATCAAATTGGTGATAAAAAATTATCATAAAAATAAXXXXXXXXXXG
CATTAGAAAGTTGATTATTCACAAATTACATAATGATATATATATTTTTTATAACCGAGAGTTGGAGCTTAGCTCAGATGT
AACAACGACATCCTGCCGAATATCCTGCACAAAATGAAGAGACACATCAAATTGGTGATAAAAAATTATCATAAAAGTAAXXXXXXXXXXG
CATTAGAAAGTTGATTATTCACAAATTACATTATGATATATATATTTTTTATAACCGAGAGTTGGAGCTTAGCTCAGATGT
>Marker621666 Chr3 SNP_site:35664761 Ref_Type:A
ACATTGACAAAAACCCCGGAAAAAGGAAAGAACAGTTAATGTTTTGATGTGTTTTTCTGAAGAGACTATCAAAATGGATCXXXXXXXXXXT
GTCTGGATGGTAATTTATATGAACAATGACAGGTTTCAACTGTTTCAATTTTGGATCCTTTCGAACCGTCTTGAAGAGT
ACATTGACAAAAACCCCGGAAAAAGGAAAGAACAGTTAATGTTTTGATGTGTTTTTCTGAAGAGACTATCAAAATGGATCXXXXXXXXXXT
GTCTGGATGGTAATTTATATGAACAATGACGGGTTTCAACTGTTTCAATTTTGGATCCTTTCGAACCGTCTTGAAGAGT

Create PDF with GO2PDF for free, if you wish to

>Marker621798 Chr3 SNP_site:32791270 Ref_Type:A
CACTGCTTTTTTATATGTAATTTTTGGCAGATAGGAGAGCTTGGAGGCGTAGCTGTGGGGTCAGCTGTATGTATCTCGTCXXXXXXXXXXT
TGAATTCCAAATTTGGACCCTATAGTTTGGAGAAAATTTGTTTATTTATGAGATTTTATCAAACTATAAAGCTGGGGTT
CACTGCTTTTTTATATGTAATTTTTGGCAGATAGGAGAGCTTGGAGGCGTAGCTGTGGGGTCAGCTGTATGTATCTCGTCXXXXXXXXXXT
TGAATTCCAAATTTGGACCCTTTAGTTTGGAGAAAATTTGTTTATTTATGAGATTTTATCAAACTATAAAGCTGAGGTT
>Marker621798 Chr3 SNP_site:32791323 Ref_Type:G
CACTGCTTTTTTATATGTAATTTTTGGCAGATAGGAGAGCTTGGAGGCGTAGCTGTGGGGTCAGCTGTATGTATCTCGTCXXXXXXXXXXT
TGAATTCCAAATTTGGACCCTATAGTTTGGAGAAAATTTGTTTATTTATGAGATTTTATCAAACTATAAAGCTGGGGTT
CACTGCTTTTTTATATGTAATTTTTGGCAGATAGGAGAGCTTGGAGGCGTAGCTGTGGGGTCAGCTGTATGTATCTCGTCXXXXXXXXXXT
TGAATTCCAAATTTGGACCCTTTAGTTTGGAGAAAATTTGTTTATTTATGAGATTTTATCAAACTATAAAGCTGAGGTT
>Marker621930 Chr3 SNP_site:30569714 Ref_Type:G
ACATTACACTTGCCTTATATGCTAGGAGACTAGGAGTTATATCGTTATTTGAAACAGAAGTGAGATTCACAAATTACAATXXXXXXXXXXC
TAGGTCTTTGTAGTTTGGTATGGTGTGCATATTTTATTGGAGGACAACCCTAAGGAGAGTGTTTTACGACAGGTTAGTG
ACATTACACTTGCCTTATATGCTAGGAGACTAGGAGTTATATCGTTATTTGAAACAGAAGTGAGATTCACAAATTACAGTXXXXXXXXXXC
TAGGTCTTTGTAGTTTGGTATGGTGTGCATATTTTATTGGAGGACAACCCTAAGGAGAGTGTTTTACGACAGGTTAGTG
>Marker622285 Chr3 SNP_site:3244749 Ref_Type:A
CACGTCGTTCCGACTAGTTGTATAACATGAGTCGGGGTGGAAATAGATTTAATTTTTAAAAACTAAAAAAAAAACAAAAXXXXXXXXXXXA
GCAAAAAAAAAGTTGAATAAAGAAATAATATTATGTATATTTTGTTTGTTTTAGAGAAGAAAATAAATAAAAAGTGAGT
CACGTCGTTCCGACTAGTTGTATAACATGAGTCGGGGTGGAAATAGATTTAATTTTTAAAAACTAAAAAAAAAACAAAAAXXXXXXXXXXXA
GCAAAAAAAAAGTTGAATAAAGAAATAATATTATGTATATTTTGTTTGTTTTAGAGAAGAAAATAAATAAAAAGTGAGT
>Marker622285 Chr3 SNP_site:3244750 Ref_Type:C
CACGTCGTTCCGACTAGTTGTATAACATGAGTCGGGGTGGAAATAGATTTAATTTTTAAAAACTAAAAAAAAAACAAAAXXXXXXXXXXXA
GCAAAAAAAAAGTTGAATAAAGAAATAATATTATGTATATTTTGTTTGTTTTAGAGAAGAAAATAAATAAAAAGTGAGT
CACGTCGTTCCGACTAGTTGTATAACATGAGTCGGGGTGGAAATAGATTTAATTTTTAAAAACTAAAAAAAAAACAAAAAXXXXXXXXXXXA
GCAAAAAAAAAGTTGAATAAAGAAATAATATTATGTATATTTTGTTTGTTTTAGAGAAGAAAATAAATAAAAAGTGAGT
>Marker622452 Chr3 SNP_site:23772588 Ref_Type:T
AACAAAGACTTGTCTCCCAGCTCGTTTGAGAAGGTTGGATTCTTCTCCCAAAAAGCCCTCGGCAGCTTTTCGCAAAATATXXXXXXXXXXC
CCAACATGAGCTCCCCTGATTTTTTCCTTTTATATGTATAAATAATTGGGGGCCTAACAATACCGCTTGCCTTGAAGTG
AACAAAGACTTGTCTCCCAGCTCGTTTGAGAAGGTTGGATTCTTCTCCCAAAAAGCCCTCGGCAGCTTTTCGCTAAATATXXXXXXXXXXC
CCAACATGAGCTCCCCTGATTTTTTCCTTTTATATGTATAAATAATTGGGGGCCTAACAATACCGCTTGCCTTGAAGTG
>Marker622564 Chr3 SNP_site:34039592 Ref_Type:C
ACTCCAATAAATTGCCTGAGAAGTAAGTCGTTTTGTTTATCTTGATCTTGATGTTTTGCTTATTAATTGATGGTTGGCTGXXXXXXXXXXA
ATGCGGTTGTTACGATGTAAAGGTGTAGCCTCCAACTTCTCCAACGTTTGCTCTAAAATTGAAGTCCTCACTGATAGGT
ACTCCAATAAATTGCCTGAGAAGTAAGTCGTTTTGTTTATCTTGATCTTGATGTTTTGCTTATTAATTGATGGTTGGCTGXXXXXXXXXXA
ATGCGGTTGTTACGATGTAAAGGTGTAGCCTCCAACTTCTCCCACGTTTGCTCTAAGATTGAAGTCCTCACTGATAGGT
>Marker622564 Chr3 SNP_site:34039606 Ref_Type:G
ACTCCAATAAATTGCCTGAGAAGTAAGTCGTTTTGTTTATCTTGATCTTGATGTTTTGCTTATTAATTGATGGTTGGCTGXXXXXXXXXXA
ATGCGGTTGTTACGATGTAAAGGTGTAGCCTCCAACTTCTCCAACGTTTGCTCTAAAATTGAAGTCCTCACTGATAGGT
ACTCCAATAAATTGCCTGAGAAGTAAGTCGTTTTGTTTATCTTGATCTTGATGTTTTGCTTATTAATTGATGGTTGGCTGXXXXXXXXXXA
ATGCGGTTGTTACGATGTAAAGGTGTAGCCTCCAACTTCTCCCACGTTTGCTCTAAGATTGAAGTCCTCACTGATAGGT
>Marker622723 Chr3 SNP_site:33714044 Ref_Type:A
ACTTAGAGTCATGCTTTCATAAGTGATATTAGACCTGTAAAGGTAGTGTGGCTCTTGATAGAGACTCTTAGATTTTTGTAXXXXXXXXXXT
TCAATCTTATCGTTTTTTTCTTAATATAAACAGTTTTTTTTTTTAATATAAACAGTTTTCATTTAATAGACTTTAGGTA
ACTTAGAGTCATGCTTTCATAAGTGATATTAGACCTGTAAAGGTAGTGTGGCTCTTGATAGAGACTCTTAGATTTTTGTAXXXXXXXXXXT

Create PDF with GO2PDF for free, if you wish to

TCAATCTTATCGTTTTTTTCTTAATATAAACAGTTTTTTTTTTTAATATGAACAGTTTTCATTTAATAGACTTTAGGTA
>Marker623146 Chr3 SNP_site:14289020 Ref_Type:A
CACAAGAAGAACATTGTAAAAATGGAACAACAAGCTCATGTCATGTTATGAAATAAAAGACAAAAATTAAGTGATGCTTTXXXXXXXXXXA
GCTAGAGCCTTTGCCTTAGAAGAACTGATTTAGAAGGCTCCCTCTTCCAAAAATATTTAACATGACCTTTAAGATAGTG
CACAAGAAGAACATTGTAAAAATGGAACAACAAGCTCATGTCATGTTATGAAATAAAAGACAAAAATTAAGTGATGCTTTXXXXXXXXXXT
GCTAGAGCCTTTGCCTTAGAAGAACTGATTTAGAAGGCTCCCTCTTCCAAAAATATTTAACATGACCTTTAAGATAGTG
>Marker6235 Chr4 SNP_site:1161519 Ref_Type:T
TACAACACGAGATGATAGTAAGCATCGTAACCACGGTGGAGGATCCTCTCCAGATGCTGAGAGATGAGGAAAGCGCCTCCXXXXXXXXXXA
ATGGTTCCAGGTTTTGTTAATAATTATATATGGTGAGGCTGTATCTTTGATCATTAGCAATGTAAATGGTTAATAGGTA
TACAACACGAGATGATAGTAAGCATCGTAACCACGGTGGAGGATCCTCTCCAGATGCTGAGAGATGAGGAAAGCGCCTCTXXXXXXXXXXA
ATGGTTCCAGGTTTTGTTAATAATTATATATGGTGAGGCTGTATCTTTGATCATTAGCAATGTAAATGGTTAATAGGTA
>Marker624332 Chr3 SNP_site:26757130 Ref_Type:C
AACACAAAAAGATTTAGACTTGAAAATTAGAACAATTGTGTGCCTTTTAGACAAAAATAATAGTTAGATGTGAATGGAGGXXXXXXXXXXT
ATAATGACCTGTTGAATTTTTATTGAACACTTTATAAAGTTGAGAGTGGAGACTTTGAACACAGATTCGAAAATTTGGT
AACACAAAAAGATTTAGAGTTGAAAATTAGAACAATTGTGTGCCTTTTAGACAAAAATAATAGTTAGATTTGAATGGAGGXXXXXXXXXXT
ATAATGACCTGTTGAATTTTTATTGAACACTTTATAAAGTTGAGAGTGGAGACTTTGAACACAGATTCGAAAATTTGGT
>Marker624332 Chr3 SNP_site:26757181 Ref_Type:G
AACACAAAAAGATTTAGACTTGAAAATTAGAACAATTGTGTGCCTTTTAGACAAAAATAATAGTTAGATGTGAATGGAGGXXXXXXXXXXT
ATAATGACCTGTTGAATTTTTATTGAACACTTTATAAAGTTGAGAGTGGAGACTTTGAACACAGATTCGAAAATTTGGT
AACACAAAAAGATTTAGAGTTGAAAATTAGAACAATTGTGTGCCTTTTAGACAAAAATAATAGTTAGATTTGAATGGAGGXXXXXXXXXXT
ATAATGACCTGTTGAATTTTTATTGAACACTTTATAAAGTTGAGAGTGGAGACTTTGAACACAGATTCGAAAATTTGGT
>Marker624471 Chr3 SNP_site:14281605 Ref_Type:C
CACGAGCACACATATGAATTTCTTCTTCTCCTTGCTTTATTGCTTCCTGATGCGCCAATTTGGCATTCTTCACAGAACCAXXXXXXXXXXC
TAGTTTAGGTGCAAGACAAGATACAATGATATGGTGCAATGCCTACAGAAATCTTCAAGAAAACTGACTTGGTTGTGTT
CACGAGCACACATATGAATTTCTTCTTCTCCTTGCTTTATTGCTTCCTGATGCGCCAATTTGGCATTCTTCACAGAACCAXXXXXXXXXXC
TAGTTTAGGTGCAAGACAAGATACAATGATATGGTGCAATGCTTACAGAAATCTTCAAGAAAACTGACTTGGTTGTGTT
>Marker62496 Chr4 SNP_site:14066800 Ref_Type:G
ACTGTTACACCTAATTATTTATGTTATTGTATTGTTACTACATGTAGAAGAAAGAAGATAAAAAAATTAGAACCATAGTGXXXXXXXXXXC
CAAGGACGTAGAACTCAAAAAGTTACATGGAATCAACTAACTAGTAGTCTTAGTAGCAAGTTGAACCACAAATATTGGT
ACTGTTACACCTAATTATTTATGTTATTGTATTGTTACTGCATGTAGAAGAAAGAAGATAAAAAAATTAGAACCATAGTGXXXXXXXXXXC
CAAGGACGTAGAACTCAAAAAGTTACATGGAATCAACTAACTAGTAGTCTTAGTAGCAAGTTGAACCACAAATATTGGT
>Marker625238 Chr3 SNP_site:8010746 Ref_Type:G
ACTGTTGTATCCATAAAATGACACTCCTTTATATTTTCTGCACGTAGCTCACTATGTTATTCAAGACAAAAGTGCAGAATXXXXXXXXXXA
GAGTCTTTTGCACCACATATAGACAGGCACACAAGGCCAATAAAATGGGAGGAAAGGAATGCATAATTTGATTGCAGTT
ACTGTTGTATCCATAAAATGACACTCCTTTATATTTTCTGCACGTAGCTCACTATGTTATTCAAGACAAGAGTGCAGAATXXXXXXXXXXA
GAGTCTTTTCCACCACATATAGACAGGCACACAAGGCCAATAAAATGGGAGGAAAGGAATGCATAATTTGATTGCAGTT
>Marker625238 Chr3 SNP_site:8010887 Ref_Type:C
ACTGTTGTATCCATAAAATGACACTCCTTTATATTTTCTGCACGTAGCTCACTATGTTATTCAAGACAAAAGTGCAGAATXXXXXXXXXXA
GAGTCTTTTGCACCACATATAGACAGGCACACAAGGCCAATAAAATGGGAGGAAAGGAATGCATAATTTGATTGCAGTT
ACTGTTGTATCCATAAAATGACACTCCTTTATATTTTCTGCACGTAGCTCACTATGTTATTCAAGACAAGAGTGCAGAATXXXXXXXXXXA
GAGTCTTTTCCACCACATATAGACAGGCACACAAGGCCAATAAAATGGGAGGAAAGGAATGCATAATTTGATTGCAGTT
>Marker625516 Chr3 SNP_site:4193011 Ref_Type:G
CACAGGCCAGTAGGGACGAGAGTCAGTTTTGATGATGATGGCAACCCACTTGCCCCTCTTGCTAAGCTTGCTGACATCAAXXXXXXXXXXC
CTTTGCTTTTTGATAAGCGCTGCTTTCTTTGATATGGTTTCTTCTTTCTTGTCTATTCAATCTTCCCACGTAAAGCTGT

Create PDF with GO2PDF for free, if you wish to

CACAGGCCAGTAGGGACGAGAGTCAGTTTTGATGATGATGGCAACCCACTTGCCCCTCTTGCTAAGCTTGCTGACATCAAXXXXXXXXXXXC
CTTTGCTTTTTGATAGGCGCTGCTTTCTTTGATATGGTTTCTTCTTTCTTGTCTATTCAATCTTCCCACGTAAAGCTGT
>Marker625603 Chr3 SNP_site:9576514 Ref_Type:G
ACCCTACACATAAAGTAAAATTGGTATAACATTTTATTTCGCATCTACTCAAACGTTGTCCTTTCTTTTGTCCATTGAAGXXXXXXXXXXXG
AAAATGAGAAAAATAAGTTTTATTTTCCTTCTAATGAATGTTTTAACTACGAAATACCTTGCCATATTTGTTGTTTGTA
ACCCTACACATAAAGTAAAATTGGTATAACATTTTATTTCGCATCTACTCAAACGTTGTCCTTTCTTTTGTCCATTGAAGXXXXXXXXXXXG
AAAATGAGAAAAATAAGTTTTATTTTCCTTCTAATTAATGTTTTAACTACGAATTACCTTGCCATATTTGTTGTTTGTA
>Marker625603 Chr3 SNP_site:9576532 Ref_Type:A
ACCCTACACATAAAGTAAAATTGGTATAACATTTTATTTCGCATCTACTCAAACGTTGTCCTTTCTTTTGTCCATTGAAGXXXXXXXXXXXG
AAAATGAGAAAAATAAGTTTTATTTTCCTTCTAATGAATGTTTTAACTACGAAATACCTTGCCATATTTGTTGTTTGTA
ACCCTACACATAAAGTAAAATTGGTATAACATTTTATTTCGCATCTACTCAAACGTTGTCCTTTCTTTTGTCCATTGAAGXXXXXXXXXXXG
AAAATGAGAAAAATAAGTTTTATTTTCCTTCTAATTAATGTTTTAACTACGAATTACCTTGCCATATTTGTTGTTTGTA
>Marker625673 Chr3 SNP_site:6868080 Ref_Type:T
AACCAAAATGGTCCTCAAAATGAGATGTATTATTTGGATTATGTATCGACGGATAATAGCCTTGAGTGGGCATTTCAACTXXXXXXXXXXXA
TAGGCCAAGCACCAGAGTGATAAAATGGTTTAGTAGGTTTCGCCCCACTATTGACCGGAGCCATTTGACTGGGATTGTG
AACCAAAATGGTCCTCAAAATGAGATGTATTATTTGGATTATGTATCGACGGATAATAGCCTTGAGTGGGCATTTCAACTXXXXXXXXXXXA
TAGGCCAAGCACCAGAGTGATAAAATGGTTTAGTAGGTTTCGCCCCACTATTGACTGGAGCCATTTGACTGGGATTGTG
>Marker625755 Chr3 SNP_site:12020241 Ref_Type:T
CACTTCCACATTTGATCACACACAAAAACCATTCACATTTATGCAAAAAGAATTACAGCACCAATAATATTACTGCCTACXXXXXXXXXXXT
CTCACTATATTTAATTTTCACCATAATTTATGTCACCTCCTCATTGAAAACAGATAAAGAACTTATTGTTCTGGTGGTT
CACTTCCACATTTGATCACACACAAAAACCATTCACATTTATGCAAAAATAATTACAGCACCAATATTATTACTGCCTACXXXXXXXXXXXT
CTCACTATATTTAATTTTCACCATAATTTATGTCACCTCCTCATTGAAAACAGATAAAGAACTTATTGTTCTGGTGGTT
>Marker625755 Chr3 SNP_site:12020258 Ref_Type:T
CACTTCCACATTTGATCACACACAAAAACCATTCACATTTATGCAAAAAGAATTACAGCACCAATAATATTACTGCCTACXXXXXXXXXXXT
CTCACTATATTTAATTTTCACCATAATTTATGTCACCTCCTCATTGAAAACAGATAAAGAACTTATTGTTCTGGTGGTT
CACTTCCACATTTGATCACACACAAAAACCATTCACATTTATGCAAAAATAATTACAGCACCAATATTATTACTGCCTACXXXXXXXXXXXT
CTCACTATATTTAATTTTCACCATAATTTATGTCACCTCCTCATTGAAAACAGATAAAGAACTTATTGTTCTGGTGGTT
>Marker626989 Chr3 SNP_site:6859472 Ref_Type:A
GACACAGTTGTAGATAAGTCATGGATATATAAATCTGTGGTGTCATAATTTTACTTACCAAACAGATATATAAATCATAAXXXXXXXXXXXA
CTAATGTTTTATACAAAACTCTATACATATGGATTTACTAACCACAAATTAACATAAAATTACATATTGAAAGAGAGTT
GACACAGTTGTAGATAAGTCATGGATATATAAATCTGTGGTGTCATAGTTTTACTTACCAAACAGATATATAAATCATAAXXXXXXXXXXXA
CTAATGTTTTATACAAAACTCTATACATATGGATTTACTAACCACAAATTAACATAAAATTACATATTGAAAGAGAGTT
>Marker627082 Chr3 SNP_site:650178 Ref_Type:G
ACCAGCACATTCACTTGCACTGCCGCCAATTACCTCATCACCTTTTGTCCCCTTTCAGATAGGTGAGTCACTTCTATTCTXXXXXXXXXXXA
GAGATGGGTAGGGACTTGTAATTTTATTTTGATCGAAATTTGTTCTTTATGCAGAAACGCACAATCCCCAAAGGGAGTG
ACCAGCACATTCACTTGCACTGCCGCCAATTACCTCATCACCTTTTGTCCCCTTTCAGATAGGTGAGTCACTTCTGTTCTXXXXXXXXXXXA
GAGATGGGTAGGGACTTGTAATTGTATTTTGATCGAAATTTGTTCTTTATGCAGAAACGCACAATCCCCAAAGGGAGTG
>Marker627082 Chr3 SNP_site:650330 Ref_Type:G
ACCAGCACATTCACTTGCACTGCCGCCAATTACCTCATCACCTTTTGTCCCCTTTCAGATAGGTGAGTCACTTCTATTCTXXXXXXXXXXXA
GAGATGGGTAGGGACTTGTAATTTTATTTTGATCGAAATTTGTTCTTTATGCAGAAACGCACAATCCCCAAAGGGAGTG
ACCAGCACATTCACTTGCACTGCCGCCAATTACCTCATCACCTTTTGTCCCCTTTCAGATAGGTGAGTCACTTCTGTTCTXXXXXXXXXXXA
GAGATGGGTAGGGACTTGTAATTGTATTTTGATCGAAATTTGTTCTTTATGCAGAAACGCACAATCCCCAAAGGGAGTG
>Marker627366 Chr3 SNP_site:32665683 Ref_Type:T
TACTTAATTTAGTGATGCTGCTACTTTTTTGCTGTAATTTCCATAATTGAGTATGAAGGCTTTTTAGAAATGTTGTTTTCXXXXXXXXXXXT

Create PDF with GO2PDF for free, if you wish to

AGGATGCTGTGAGCGAGAAATCAGGTGGGGCTAAGCTGGAAAATGGAGATGGAGGGTTGTCTGTCTCGACTGAAGAGTT
TACTTAATTTAGTGATGCTGCTACTTTTTTGCTGTAATTTCCATAATTGAGTATGAAGGCTTTTTAGAAATGTTGTTTTCXXXXXXXXXXT
AGGATGCTGTGAGCGAGAAATCAGTTGGGGCTAAGCTGGAAAATGGAGATGGAGGGTTGTCTGTCTCGACTGAAGAGTT
>Marker627448 Chr3 SNP_site:37871679 Ref_Type:C
ACCATATTGTTCTTGTAATCTGAGTTTCATTCCATATTGTTCTTGTGTTTTCAAATCTTCAAAATCAATCTTTGCACTTTXXXXXXXXXXA
ATTTTGATAAGAATTAACTTCAAGTTATTAAAGTTAAGATTATAAAAAAGATGAAGAGTAAATTTGGGCATTTGAAAGT
ACCATATTGTTCTTGTAATTTGAGTTTCATTCCATATTGTTCTTGTGTTTTCAAATCTTCAAAATCAATCTTTGCACTTTXXXXXXXXXXA
ATTTTGATAAGAATTAACTTCAAGTTATTAAAGTTAAGATTATAAAAAAGATGAAGAGTAAATTTGGGCATTTGAAAGT
>Marker627676 Chr3 SNP_site:5489879 Ref_Type:T
CACGCCACAATGAGTCTCAACCTTGCACTCAAAAGGATGGCAGAGAAGGAAAAGATGGGAGATAAAAAGAAACAGAAAAAXXXXXXXXXXT
GCACATCCGAGTATTATGCATACCTCACCTCTTAGAACAAAACCTGCCAACAATTTTTTTCATATGAAATACCAGCAGT
CACGCCACAATGAGTCTCAACCTTGCACTTAAAAGGATGGCAGAGAAGGAAAAGATGGGAGATAAAAAGAAACAGAAAAAXXXXXXXXXXT
GCACATCCGAGTATTATGCATACCTCACCTCTTAGAACAAAACCTGCCAACAATTTTTTTCATATGAAATACCAGCAGT
>Marker628294 Chr3 SNP_site:11575759 Ref_Type:A
GACAGAGTATCAACTATAGTGCCATTAGATTTTTTTCTCTTTCCTTGATTGAGAATTACTTATTTGTCTATTCAGTTTCCXXXXXXXXXXT
GAAAGCAAGAGGAAACATTGAAAGAACAAATAAGAAATGAGATCACAATAGTGAAAAGTAAAAGTGACAAATGTTTGTT
GACAGGGTATCAACTATAGTGCCATTAGATTTTTTTCTCTTTCCTTGATTGAGAATTACTTATTTGTCTATTCAGTTTCCXXXXXXXXXXT
GAAAGCAAGAGGAAACATTGAAAGAACAAATAAGAAATGAGATCACAATAGTGAAAAGTAAAAGTGACAAATGTTTGTT
>Marker628577 Chr3 SNP_site:2626067 Ref_Type:G
GACAAATTTTAAATAATTTTGTATTTTTATAGATTAAATTTATAGTTTTGAAGGTTGAAGAATTAAATAGGATACATCCAXXXXXXXXXXA
ATGACAAAATTTCTAAAAATATTTATTAAGGTAAAGATAGTAAATATCTCAATTTTCTTTTATAAACAGTTATTTATGT
GACAAATTTTAAATAATTTTGTATTTTTATAGATTAAATTTATAGTTTTGAAGGTTGAAGAATTAAATAGGATACATCCAXXXXXXXXXXA
ATGACAAAATTTCTAAAAATATTTATTAAGGTAAAGATAGTAAATATCTCAATTTTCTTTTATAGACAGTTATTTATGT
>Marker628988 Chr3 SNP_site:34325348 Ref_Type:C
ACGTAGTTTCCGGTATTCTCAGGTTCCATTTCAAATAGATGTTTAGCAGCAATCTCCCCCACCTCCAAATTACCATGGATXXXXXXXXXXC
AATGCCAAAACCCTTCCTCATAAGTTCAAAATACTTGCATCCTTGTTCAATCAACCCTGCATGGGCACATGCATTTAGT
ACGTAGTTTCCGGTATTCTCAGGTTCCATTTCAAATAGATGTTTAGCAGCAATCTCCCCCACCTCCAAATTACCATGGATXXXXXXXXXXT
AATGCCAAAACCCTTCCTCATAAGTTCAAAATACTTGCATCCTTGTTCAATCAACCCTGCATGGGCACATGCATTTAGT
>Marker628994 Chr3 SNP_site:31337345 Ref_Type:G
TACTTGGGACATTATCTTGTTATATTCGTCCTTTTTAGATTGTTCAATTAAAAATTGTCCCTTTTAATTTTCATAAAAATXXXXXXXXXXC
GGTTTCCAATTGTTTAGGCTTTGATGATGATGTATAGAAGGCCACTAGTTTCCTTATTGTGCCGCATCTTTTTACTTGT
TACTTGGGACATTATCTTGTTATATTCGTCCTTTTTAGATTGTTCAATTAAGAATTGTCCCTTTTAATTTTCATAAAAATXXXXXXXXXXC
GGTTTCCAATTGTTTAGGCTTTGATGATGATGTATAGAAGGCCACTAGTTTCCTTATTGTGCCACATCTTTTTACTTGT
>Marker628994 Chr3 SNP_site:31337542 Ref_Type:A
TACTTGGGACATTATCTTGTTATATTCGTCCTTTTTAGATTGTTCAATTAAAAATTGTCCCTTTTAATTTTCATAAAAATXXXXXXXXXXC
GGTTTCCAATTGTTTAGGCTTTGATGATGATGTATAGAAGGCCACTAGTTTCCTTATTGTGCCGCATCTTTTTACTTGT
TACTTGGGACATTATCTTGTTATATTCGTCCTTTTTAGATTGTTCAATTAAGAATTGTCCCTTTTAATTTTCATAAAAATXXXXXXXXXXC
GGTTTCCAATTGTTTAGGCTTTGATGATGATGTATAGAAGGCCACTAGTTTCCTTATTGTGCCACATCTTTTTACTTGT
>Marker629012 Chr3 SNP_site:9840637 Ref_Type:C
ACCCAATGATATTCGTTTGATATGGTTTATTGGGACTTTCTCAATGACTTATTGATTGCAAAGGGTTTGGCCCAAAATGGXXXXXXXXXXG
TGGGTCTATCTATTTTCCTCTATACAATTGTGGTTAAGCTTAGTAAGATGTTATTGCAAGAACTAAAAGAAGATCCGTA
ACCTAATGATATTCGTTTGATATGGTTTATTGGGACTTTCTCAATGACTTATTGATTGCAAAGGGTTTGGCCCAAAATGGXXXXXXXXXXG
TGGGTCTATCTATTTTCCTCTATACAATTGTGGTTAAGCTTAGTAAGATGTTATTGCAAGAACTAAAAGAAGATCCGTA
>Marker629770 Chr3 SNP_site:11143777 Ref_Type:C

Create PDF with GO2PDF for free, if you wish to

GACTTAGTGATTTTTCAATCCTTTTATTAGAATACATTTTTATTTACTCCTCACTAGTGAAGTGTTCGTTTGAATTCACTXXXXXXXXXXG
GAAAGTAAATTTTAAAATGTAATATTAGTTTTTTTTCAACCAAAAATAACAGCACAATACACCGAGGATCAAAAGGGTT
GACTTAGTGATTTTTCAATCCTTTTATTAGAATACATTTTTATTTACTCCTCACTAGTGAAGTGTTCGTTTGAATTCACTXXXXXXXXXXG
GAAAGTAAATTTTAAAATGTAATATTAGTTTTTTTTCAACCAAAAATAACAGCACAATACACTGAGGATCAAAAGGGTT
>Marker629903 Chr3 SNP_site:31501990 Ref_Type:C
AACACATCAACCCATTTAGTGGAACTTTCTTTTAATTAAGAATGACCTTATTGTCCAAAATGCATAATTTAATCAATGTGXXXXXXXXXXG
ACACTTTACTTATATAATTTAGGGTTAGAGAAGTCCATGGGTTTGGAACAAACACACTACTATTTCAATCTCCATTGTC
AACACATCAACCCATTTAGTGGAACTTTCTTTTAATTAAGAATGACGTTATTGTCCAAAATGCATAATTTAATCAATGTGXXXXXXXXXXG
ACACTTTACTTATATAATTTAGGGTTAGAGAAGTCCATGGGTTTGGGACAAACACACTACTATTTCAATCTCCATTGTC
>Marker629903 Chr3 SNP_site:31502206 Ref_Type:A
AACACATCAACCCATTTAGTGGAACTTTCTTTTAATTAAGAATGACCTTATTGTCCAAAATGCATAATTTAATCAATGTGXXXXXXXXXXG
ACACTTTACTTATATAATTTAGGGTTAGAGAAGTCCATGGGTTTGGAACAAACACACTACTATTTCAATCTCCATTGTC
AACACATCAACCCATTTAGTGGAACTTTCTTTTAATTAAGAATGACGTTATTGTCCAAAATGCATAATTTAATCAATGTGXXXXXXXXXXG
ACACTTTACTTATATAATTTAGGGTTAGAGAAGTCCATGGGTTTGGGACAAACACACTACTATTTCAATCTCCATTGTC
>Marker629937 Chr3 SNP_site:34818524 Ref_Type:C
AACTTTTCTTTCAAGGATATAATCCTAACATTTTTCAACTCATGTCATTGTTATCATTATTTGGGATAAAAAAAGAAATTXXXXXXXXXXA
AGGTTGAAATTATTTCAACCTCTAACTTTTATTATATAGTTAAAAAGGAAATTTTGGTATGTTAAAATAGTATATGCGT
AACTTTTCTTTGAAGGATATAATCCTAACATTTTTCAACTCATGTCATTGTTATCATTATTTGGGATAAAAAAAGAAATTXXXXXXXXXXA
AGGTTGAAATTATTTCAACCTCTAACTTTTATTATATAGTTAAAAAGGAAATTTTGGTATGTTAAAATAGTATATGCGT
>Marker630326 Chr3 SNP_site:34404930 Ref_Type:A
TACTATTGTCACTTTCATTGGCCTCAATGGATGTATGAATTTGGATACTCCAAAACAAATGTCAATGTTAATAGAGAAAGXXXXXXXXXXA
TATGAACCATTAATATATAATCCATATGGAGTTTTTAGATGGAAAGATTTAACCACACATGCATTTTTTAACAGGTGTT
TACTATTGTCACTTTCATTGGCCTCAATGGATGTATGAATTTGGATGCTCCAAAACAAATGTCAATGCTAATAGAGAAAGXXXXXXXXXXA
TATGAACCATTAATATATAATCCATATGGAGTTTTTAGATGGAAAGATTTAACCACACATGCATTTTTTAACAGGTGTT
>Marker630326 Chr3 SNP_site:34404951 Ref_Type:T
TACTATTGTCACTTTCATTGGCCTCAATGGATGTATGAATTTGGATACTCCAAAACAAATGTCAATGTTAATAGAGAAAGXXXXXXXXXXA
TATGAACCATTAATATATAATCCATATGGAGTTTTTAGATGGAAAGATTTAACCACACATGCATTTTTTAACAGGTGTT
TACTATTGTCACTTTCATTGGCCTCAATGGATGTATGAATTTGGATGCTCCAAAACAAATGTCAATGCTAATAGAGAAAGXXXXXXXXXXA
TATGAACCATTAATATATAATCCATATGGAGTTTTTAGATGGAAAGATTTAACCACACATGCATTTTTTAACAGGTGTT
>Marker631189 Chr3 SNP_site:8221224 Ref_Type:A
GACATGGTGATGCTAAGATCAGGGAATAAGAATACTGTAAATGCCATGTTCTTCCCTCATGGTCAAGGACCCTCTTAAGTXXXXXXXXXXT
TGCCGATAAATTAAGGTGGCGTTTTCATGTTATCATCAATTCTTAAGTTTATATTTCTTTCTCGGTTCTCGCATCCGTA
GACATGGTGATGCTAAGATCAGGGAATAAGAATACTGTAAATGCCATGTTCTTCCCTCATGGTCAAGGACCCTCTTAAGTXXXXXXXXXXT
TGCCGATAAATTAAGGTGGCGTTTTCATGTTATCATCATTTCTTAAGTTTATATTTCTTTCTCGGTTCTCGCATCCGTA
>Marker631603 Chr3 SNP_site:2463960 Ref_Type:A
TACCTTTTAATTCCTTACAGAGACGAGAGCTTCTAGTATAGCTAAAACAAAACCAATCAAATCTTAGTAAAGTTATTTCTXXXXXXXXXXT
TACCATAACTCATAAGGTAATTAAGTAAGGTTTTATTGATGACATAAACAATTCAATCTTCAATTCTTTTGATCGAGTA
TACCTTTTAATTCCTTACAGAGACGAGAGCTTCTGGTATAGCTAAAACAAAACCAATCAAATCTTAGTAAAGTTATTTCTXXXXXXXXXXT
TACCATAACTCATAAGGTAATTAAGTAAGGTTTTATTGATGACATAAACAATTCAATCTTCAATTCTTTTGATCGAGTA
>Marker632582 Chr3 SNP_site:36104536 Ref_Type:A
GACCATCCTTATGCAGGAAAAAATTCACAAGCACACAACTTTATGAAAGAATGAAAACAAACTACAGATATCACTCAAGCXXXXXXXXXXT
TTTGTTGTCACTGAACTAGAGTGTGGTGTTTCGGCATTTTCATATGCAAGTATATCAAAGCTAAAAATGTTTAAAATGT
GACCATCCTTATGCAGGAAAAAATTCACAAGCACACAACTTTATGAAAGAATGAAAGCAAACTACAGATATCACTCAAGCXXXXXXXXXXT
TTTGTTGTCACTGAACTGGAGTGTGGTGTTTCGGCATTTTGATATGCAAGTATATCAAAGCTAAAAATGTTTAAAATGT

Create PDF with GO2PDF for free, if you wish to

>Marker632582 Chr3 SNP_site:36104710 Ref_Type:A
GACCATCCTTATGCAGGAAAAAATTCACAAGCACACAACTTTATGAAAGAATGAAAACAAACTACAGATATCACTCAAGCXXXXXXXXXXT
TTTGTTGTCACTGAACTAGAGTGTGGTGTTTCGGCATTTTCATATGCAAGTATATCAAAGCTAAAAATGTTTAAAATGT
GACCATCCTTATGCAGGAAAAAATTCACAAGCACACAACTTTATGAAAGAATGAAAGCAAACTACAGATATCACTCAAGCXXXXXXXXXXT
TTTGTTGTCACTGAACTGGAGTGTGGTGTTTCGGCATTTTGATATGCAAGTATATCAAAGCTAAAAATGTTTAAAATGT
>Marker632582 Chr3 SNP_site:36104733 Ref_Type:C
GACCATCCTTATGCAGGAAAAAATTCACAAGCACACAACTTTATGAAAGAATGAAAACAAACTACAGATATCACTCAAGCXXXXXXXXXXT
TTTGTTGTCACTGAACTAGAGTGTGGTGTTTCGGCATTTTCATATGCAAGTATATCAAAGCTAAAAATGTTTAAAATGT
GACCATCCTTATGCAGGAAAAAATTCACAAGCACACAACTTTATGAAAGAATGAAAGCAAACTACAGATATCACTCAAGCXXXXXXXXXXT
TTTGTTGTCACTGAACTGGAGTGTGGTGTTTCGGCATTTTGATATGCAAGTATATCAAAGCTAAAAATGTTTAAAATGT
>Marker632698 Chr3 SNP_site:13996586 Ref_Type:A
CACAACAAACTTCAAAAGTTCGATAAAAGGGAGACTTCTTATACTTCTTTTCCAGGTAAGTCACATTATTAAGAAAAGAGXXXXXXXXXXT
AGTGATTTGAGTTTGAGCTTTGAACCAACTCTTCTTAAGGAAAACAAATTATATGATGGGTTGTCAATCCAATTAGGTT
CACAACAAACTTCAAAAGTTCGATAAAAGGGAGACTTCTTATACTTCTTTTCCAGGTAAGTCACATTATTAAGAAAAGAGXXXXXXXXXXT
AGTGATTTGAGTTTGAGCTTTGAACCATCTCTTCTTAAGGAAAACAAATTATATGATGGGTTGTCAATCCAATTAGGTT
>Marker633407 Chr3 SNP_site:38772327 Ref_Type:C
TACCAGAAGGAAGGATAGTGATTCCTGCTTTCGTAACTGATGTTATACATCTATTAAAAGGAAGGATACTGTTTCCTGCCXXXXXXXXXXA
GCTAACCTTTTAATCTCACACCCAACTATAGGAAGTCCCTGAACCACTTCAAGAAAGCGTTACTTCATGTCATGGTGGT
TACTAGAAGGAAGGATAGTGATTCCTGCTTTCGTAACTGATGTTATACATCTATTAAAAGGAAGGATACTGTTTCCTGCCXXXXXXXXXXA
GCTAACCTTTTAATCTCACACCCAACTATAGGAAGTCCCTGAACCACTTCAAGAAAGCGTTACTTCATGTCGTGGTGGT
>Marker633407 Chr3 SNP_site:38772577 Ref_Type:A
TACCAGAAGGAAGGATAGTGATTCCTGCTTTCGTAACTGATGTTATACATCTATTAAAAGGAAGGATACTGTTTCCTGCCXXXXXXXXXXA
GCTAACCTTTTAATCTCACACCCAACTATAGGAAGTCCCTGAACCACTTCAAGAAAGCGTTACTTCATGTCATGGTGGT
TACTAGAAGGAAGGATAGTGATTCCTGCTTTCGTAACTGATGTTATACATCTATTAAAAGGAAGGATACTGTTTCCTGCCXXXXXXXXXXA
GCTAACCTTTTAATCTCACACCCAACTATAGGAAGTCCCTGAACCACTTCAAGAAAGCGTTACTTCATGTCGTGGTGGT
>Marker633918 Chr3 SNP_site:36812878 Ref_Type:G
ACATATGATGCATTAGATTTCCATTAGTAGTGAGAGAGCAAAGCTTCTAAATCAATTTAGATGATAGAAAGCATTCACTAXXXXXXXXXXT
CAGTATGATCTATTGTTGAAAAGGCATTGAGATAGTAACAAAGGTCATTTGAATCATGTGAATATCTTCATGGAAGGTT
ACATATGATGCATTAGATTTCCATTAGTAGTGAGAGAGCAAAGCTTCTAAATCAATTTAGATGATAGAAAGCATTCACTGXXXXXXXXXXT
CAGTATGATCTATTGTTGAAAAGGCATTGAGATAGTAACAAAGGTCATTTGAATCATGTGAATATCTTCATGGAAGGTT
>Marker63448 Chr4 SNP_site:14229845 Ref_Type:C
AACACAACACAACAAACTCTAATATAAACTTGGTGCAATTATGGATGCAAACCAATGAACTCAATATATATATATAACTTXXXXXXXXXXT
AAAATGTTTAATTAAATCTAAGTATCAATATCTCTTTCGGAATGAACTGAGAAAAAATGTAACAAATCGTTTAGAAGTG
AACACAACACAACAAACTGTAATATAAACTTGGTGCAATTATGGATGCAAACCAATGAACTCAATATATATATATAACTTXXXXXXXXXXT
AAAATGTTTAATTAAATCTAAGTATCAATATCTCTTTCGGAATGAACTGAGAAAAAATGTAACAAATCGTTTAGAAGTG
>Marker635075 Chr3 SNP_site:34399787 Ref_Type:C
AACAAAATCAAATAATTTTCATCACGTGACAAATAAATAACGTCCAATAAATTAAATCTAATTAAATAAAACTAACATAAXXXXXXXXXXG
TTCTACTCAATAGGAGTTTCAACTATCCTCTAACCAAGTGAATATGTTTCTTTCAATAAATCAAGAATCTTCTTTAGTT
AACAAAATCAAATAATTTTCATCATGTGACAAATAAATAACGTCCAATAAATTAAATCTAATTAAATAAAACTAAGATAAXXXXXXXXXXG
TTCTACTCAATAGGAGTTTCAACTATCCTCTAACCAAGTAAATATGTTTCTTTCAATAAATCAAGAATCTTCTTTAGTT
>Marker635075 Chr3 SNP_site:34399838 Ref_Type:C
AACAAAATCAAATAATTTTCATCACGTGACAAATAAATAACGTCCAATAAATTAAATCTAATTAAATAAAACTAACATAAXXXXXXXXXXG
TTCTACTCAATAGGAGTTTCAACTATCCTCTAACCAAGTGAATATGTTTCTTTCAATAAATCAAGAATCTTCTTTAGTT
AACAAAATCAAATAATTTTCATCATGTGACAAATAAATAACGTCCAATAAATTAAATCTAATTAAATAAAACTAAGATAAXXXXXXXXXXG

Create PDF with GO2PDF for free, if you wish to

TTCTACTCAATAGGAGTTTCAACTATCCTCTAACCAAGTAAATATGTTTCTTTCAATAAATCAAGAATCTTCTTTAGTT

>Marker635075 Chr3 SNP_site:34399979 Ref_Type:G

AACAAAATCAAATAATTTTCATCACGTGACAAATAAATAACGTCCAATAAATTAAATCTAATTAAATAAAACTAACATAAXXXXXXXXXXXG

TTCTACTCAATAGGAGTTTCAACTATCCTCTAACCAAGTGAATATGTTTCTTTCAATAAATCAAGAATCTTCTTTAGTT

AACAAAATCAAATAATTTTCATCATGTGACAAATAAATAACGTCCAATAAATTAAATCTAATTAAATAAAACTAAGATAAXXXXXXXXXXXG

TTCTACTCAATAGGAGTTTCAACTATCCTCTAACCAAGTAAATATGTTTCTTTCAATAAATCAAGAATCTTCTTTAGTT

>Marker635205 Chr3 SNP_site:33389493 Ref_Type:C

TACACTAGTAATAATGAAAAGGAACCAATTTATTTATTAATCTAATTACAAAGTTGATTTTTCTTTAGTGCAAATGTTGAXXXXXXXXXXXG

TGGTGAGACAGAGCCCCACTGGCACCAAGCCTCCAAAAACGAGAAATGTTTTAACATATTTTGAAAATATTTTTTTAGT

TACACTAGTAATAATGAAAAGGAACCAATTTATTTATTAATCTAATTACAAAGTTGATTTTTCTTTAGTGCAAATGTTGAXXXXXXXXXXXG

TGGTGAGACAGAGCCCCACTGGCACCAAGCCTCCAAAAAGGAGAAATGTTTTAACATATTTTGAAAATATTTTTTTAGT

>Marker635432 Chr3 SNP_site:38768637 Ref_Type:A

GACTTCCCCAGAGGTTTTTATATTACGAAGATGGGGATTGGGTAGATTACCCACAAATAATTGTCAAGTTAATTAGGGAAXXXXXXXXXXXG

CAGCACATTGCTTGGATTGATGACAATGATCAATGTTTCTTTCCAGAATTTTATTCTGGTGACAGAATGATGCATCAGT

GACTTCCCCAGAGGTTTTTATATTACGAAGATGGGGATTGGGTAGATTACCCACAAATAATTGTCAAGTTAATTAGGGAAXXXXXXXXXXXG

CAGCACATTGCTTGGATTGATGACAATGATCAATGTTTCTTTCCAGAATTTTATTCTGGTGACAGTATGATGCATCAGT

>Marker635888 Chr3 SNP_site:16372484 Ref_Type:T

TACCTTTACGATTTGATTTCGAAGACTTATTGTCATTACTATTTAGGCAACATTATATTTTCTCAATCGCATTTCTTTTAXXXXXXXXXXXT

TTGAAGCAAATAACTACTCAAAACTATCCCTACAAAACTTGTCCCAACCAATAAAAGAATCTCTCAGCCTAAATGCAGT

TACCTTTACGATTTGATTTCGAAGACTTATTGTCATTACTATTTAGGCAACATTATATTTTCTCAATCGCATTTCTTTTAXXXXXXXXXXXT

TTGAAGCAAATAACTTCTCAAAACTATCCCTACAAAACTTGTCCCAACCAATAAAAGAATCTCTCAGCCTAAATGCAGT

>Marker637283 Chr3 SNP_site:27615156 Ref_Type:C

ACATCAGTAGATGAAGTTGAATCAAGCATAGCTACTGCAGGGTGTGCTTTTTGTGAATATATTGGCCAAGAAGTGTTTAAXXXXXXXXXXXT

GAAGAGGTAAAATGTAGTGCATAATTTATTATTAAGCACTTAGTAAATTATTTCAAACAAACTCTTGAAAAGCTATGTC

ACATCAGTAGATGAAGTTGAATCAAGCATAGCTACTGCAGGGTGTGCTTTTTGTGAATATATTGGCCAAGAAGTGTTTAAXXXXXXXXXXXT

GAAGAGGTAAAATGTAGTGTATAATTTATTGTTAAGCACTTAGTAAATTATTTCAAACAAACTCTTGAAAAGCTATGTC

>Marker637283 Chr3 SNP_site:27615167 Ref_Type:A

ACATCAGTAGATGAAGTTGAATCAAGCATAGCTACTGCAGGGTGTGCTTTTTGTGAATATATTGGCCAAGAAGTGTTTAAXXXXXXXXXXXT

GAAGAGGTAAAATGTAGTGCATAATTTATTATTAAGCACTTAGTAAATTATTTCAAACAAACTCTTGAAAAGCTATGTC

ACATCAGTAGATGAAGTTGAATCAAGCATAGCTACTGCAGGGTGTGCTTTTTGTGAATATATTGGCCAAGAAGTGTTTAAXXXXXXXXXXXT

GAAGAGGTAAAATGTAGTGTATAATTTATTGTTAAGCACTTAGTAAATTATTTCAAACAAACTCTTGAAAAGCTATGTC

>Marker63740 Chr4 SNP_site:16041007 Ref_Type:G

TACTCTTACCTTTCGAACCAAAAAAGTTGTTCAATTCAAAGCAACATAAATTGGCTTTTACAACAATAGACTCAAATCGTXXXXXXXXXXXA

AAAAAAAAGAAAGGAAAGTTTGTAAGGTCCTCTTCTTCATCTCTAAATAAATGCACTTACCTCTTAAACCAGCATTGTT

TACTCTTACCTTTCGAACCAAAAAAGTTGTTCAATTGAAAGCAACATAAATTGGCTTTTACAACAATAGACTCAAATCGTXXXXXXXXXXXA

AAAAAAAAAAAAGGAAAGTTTGTAAGGTCCTCTTCTTCATCTCTAAATAAATGCACTTACCTCTTAAACCAGCATTGTT

>Marker63740 Chr4 SNP_site:16041159 Ref_Type:A

TACTCTTACCTTTCGAACCAAAAAAGTTGTTCAATTCAAAGCAACATAAATTGGCTTTTACAACAATAGACTCAAATCGTXXXXXXXXXXXA

AAAAAAAAGAAAGGAAAGTTTGTAAGGTCCTCTTCTTCATCTCTAAATAAATGCACTTACCTCTTAAACCAGCATTGTT

TACTCTTACCTTTCGAACCAAAAAAGTTGTTCAATTGAAAGCAACATAAATTGGCTTTTACAACAATAGACTCAAATCGTXXXXXXXXXXXA

AAAAAAAAAAAAGGAAAGTTTGTAAGGTCCTCTTCTTCATCTCTAAATAAATGCACTTACCTCTTAAACCAGCATTGTT

>Marker637432 Chr3 SNP_site:27145451 Ref_Type:G

TACTGAAAATCAGGAATTTTAATTTGATGTTAAAAGTCTTAGGTTAAATTGTAAGGTTTATTACGTGAAAACAATTATTAXXXXXXXXXXXT

ATAAGATAGCAATTTGATAACAAACAATGTCAATATAAGATGATAGTTAGATAAGAATCACACATTTTTAACACTTGTG

Create PDF with GO2PDF for free, if you wish to

TACTGAAAATCAGGAATTTTAATTTGATGTTAAAAGTCTTAGGTTAAATTGTAAGTTTTATTACGTGAAAACAATTATTAXXXXXXXXXXT
ATAAGATAGCAATTTGATAACAAACAATGTCAATATAAGATGATAGTTAGATAAGAATCACACATTTTTAACACTTGTG
>Marker637509 Chr3 SNP_site:32349941 Ref_Type:G
ACTTCTTACACCTTCTCAAAATCTCAACACTCGGCCCCATCTTTCTTTCTAGTCTTTTGGAAATGGGGATTGTGCTTTGTXXXXXXXXXXT
GTAACATTCTAAAGAACTTCCATCATCTTCCTCTCTTCTTATTTTAAACGGGAACAAAACTTTTCATTGATAAAACGTC
ACTTCTTACGCCTTCTCAAAATCTCAACACTCGGCCCCATCTTTCTTTCTAGTCTTTTGGAAATGGGGATTGTGCTTTGTXXXXXXXXXXT
GTAACATTCTAAAGAACTTCCATCATCTTCCTCTCTTCTTATTTTAAACGGGAACAAAACTTTTCATTGATAAAACGTC
>Marker637791 Chr3 SNP_site:34001727 Ref_Type:C
ACTCACCGTCTTGTTTCAATTGCAGTCTATGTAATTTTATATGTTAGAATGTTGAATTTTAATCCCTTTTAATAAATCTTXXXXXXXXXXG
ATTTATTGAATATGAACTTACGTTACACATTTAAATAGACTCGAGTGAAATGAAATCAAAATATAATATAAAATTAGTA
ACTCACCGTTTTGTTTCAATTGCAGTCTATGTAATTTTATATGTTAGAATGTTGAATTTTAATCCCTTTTAATAAATCTTXXXXXXXXXXG
ATTTATTGAATATGAACTTACGTTACACATTTAAATAGACTCGAGTGAAATGAAATCAAAATATAATATAAAATTAGTA
>Marker638811 Chr3 SNP_site:5767680 Ref_Type:A
ACCAAAAAAAACACAAAATTACACAGGACAAAAACCACTATATCTTGAGACTTGAAAGAGATTGCAATCATTGAGTTTGTXXXXXXXXXXC
CAACAAAATCACATTCATCCATGACTTCCAACTCAATATCAAATCAAATGTTTATAAGGAAATTTGGATTACGAAAAGT
ACCAAAAAAAACACAAAATTACACAGGACAAAAACCACTATATCTTGAGACTTGAAAGAGATTGCAATCATTGAGTTTGTXXXXXXXXXXC
CAACAAAGTCACATTCATCCATGACTTCCAACTCAATATCAAATCAAATGTTTATAAGGAAATTTGGATTACGAAAAGT
>Marker639000 Chr3 SNP_site:39386865 Ref_Type:T
AACGACAAGCTTAGCAAAATGGCCAATGTGAATGAGTGCCTCAGCTTGAGAAAATAAAGGGTTACTATAACAACTATTTTXXXXXXXXXXT
ACTATATTTGAAAGTATTTTCAAAAGTTTTGTCATCTAAAAGAACTTCCCTTATCATAATATCACCCAACCCAAAAGTT
AACGACAAGCTTAGCAAAATGGCCAATGTGAATGAGTGCCTCAGCTTGAGAAAATAAAGGGTTTCTATAACAACTATTTTXXXXXXXXXXT
ACTATATTTGAAAGTATTTTCAAAAGTTTTGTCATCTAAAAGAACTTCCCTTATCATAATATCACCCAACCCAAAAGTT
>Marker63965 Chr4 SNP_site:4712064 Ref_Type:A
GACTTCCTTACTTATCATTTTCTTTAAATTGTGGGAAGGAGTTAGATAATTGACATAGTTTATGGTAGTAAGAGTTTGAAXXXXXXXXXXA
TGCTTTGTAAGATGAAGAATTTAGGGAATAAAGCCTTCTAAGATGGACCTATATGGCTTTACCCAGATGAAGCTGGGTG
GACTTCCTTACTTATCATTTTCTTTAAGTTGTGGGAAGGAGTTAGATAATTGAAATAGTTTATGGTAGTAAGAGTTTGAAXXXXXXXXXXA
TGCTTTGTAAGATGAAGAATTTAGGGAATAAAGCCTTCTAAGATGGACCTATATGGCTTTACCCAGATGAAGCTGGGTG
>Marker63965 Chr4 SNP_site:4712090 Ref_Type:C
GACTTCCTTACTTATCATTTTCTTTAAATTGTGGGAAGGAGTTAGATAATTGACATAGTTTATGGTAGTAAGAGTTTGAAXXXXXXXXXXA
TGCTTTGTAAGATGAAGAATTTAGGGAATAAAGCCTTCTAAGATGGACCTATATGGCTTTACCCAGATGAAGCTGGGTG
GACTTCCTTACTTATCATTTTCTTTAAGTTGTGGGAAGGAGTTAGATAATTGAAATAGTTTATGGTAGTAAGAGTTTGAAXXXXXXXXXXA
TGCTTTGTAAGATGAAGAATTTAGGGAATAAAGCCTTCTAAGATGGACCTATATGGCTTTACCCAGATGAAGCTGGGTG
>Marker639661 Chr3 SNP_site:11258080 Ref_Type:G
AACATATAACAACCGAGCTTCTCAAACCTTTTTGTTATTTGAAACTATTATCATGTTACTCTTACCGTCCTCCATAAGTTXXXXXXXXXXG
GCCTACTCTATGGTCTTATTTCCTTTGATTCATCACCCCATAGAGATAAAGACAGCAAGTGGCATATTAGTGAGGAGGT
AACATATAACAACCGAGCTTCTCAAACCTTTTTGTTATTTGAAACTATTATCATGTTACTCTTACCGTCCTGCATAAGTTXXXXXXXXXXG
GCCTACTCTATGGTCTTATTTCCTTTGATTCATCACCCCATAGAGATAAAGACAGCAAGTGGCATATTAGTGAGGAGGT
>Marker639704 Chr3 SNP_site:33392799 Ref_Type:T
TACCTTGAAGAAATGGATCCTGGAAAACATGCACAAGAAGAGAGAAAAGATTAGCGACAACAAATTTACCACAACTACGGXXXXXXXXXXG
TCCTCCTCTTTTAGCTGCACTCCACCATTTTTCTTAACAATGATATCACAAACAAGCTAAATTCAGACTCTACTTTGTT
TACCTTGAAGAAATGGATCCTGGAAAACATGCACAAGAAGAGAGAAAAGATTAGCGACAACAAATTTACCACAACTACTGXXXXXXXXXXG
TCCTCCTCTTTTAGCTGCACTCCACCATTTTTCTTAACAATGATATCACAAACAAGCTAAATTCAGACTCTACTTTGTT
>Marker639761 Chr3 SNP_site:31430697 Ref_Type:A
ACTATAGGTAAAAAGAACACTAAAAAAATCTGGAAAGAAAGCTTACACGTCTAAGATTGAGATATTTCAAAGGGATATTGXXXXXXXXXXA

Create PDF with GO2PDF for free, if you wish to

CCTAAATTGATCACAATGGATCCTATGCCATAGCATTAACTAAAGTTTGCTACCCCTATTCAATTTCCCCTCAACAGTC
ACTGTAGGTAAAAAGAACACTAAAAAAATCTGGAAAGAAAGCTTACACGTCTAAGATTGAGATATTTCAAAGGGATATTGXXXXXXXXXXA
CCTAAATTGATCACAATGGATCCTATGCCATAGCATTAACTAAAGTTTGCTACCCCTATTCAATTTCCCCTCAACAGTC
>Marker639956 Chr3 SNP_site:38733889 Ref_Type:G
CACAAAAGACTTTGATGAACCGATACCAGCAAATCAACTCTGGGATCCTGATTCCTTGATAAGGATTGCACCTGATGATGXXXXXXXXXXG
ACACTTCAAAGATATGCAACATTGAGATCCAAAGAAGCTATTTCTTTGATCGAAAAGCAGACTCAATCATTGTTTGGTA
CACAAAAGACTTTGATGAACCGATACCGGCAAATCAACTCTGGGATCCTGATTCCTTGATAAGGATTGCACCTGATGATGXXXXXXXXXXG
ACACTTCAAAGATATGCAACATTGAGATCCAAAGAAGCTATTTCTTTGATCGAAAAGCAGACTCAATCATTGTTTGGTA
>Marker640351 Chr3 SNP_site:25156998 Ref_Type:T
CACCAAAATTCTTGTGCTTACACCCTCCAACAAAATGGGGTTGCCGAACGAAAAAACCGTTACATTTTGGAAGTTGCATCXXXXXXXXXXA
GACATCGTTAGATTGTGTTAAAGAATCCTATCCCTCCACTCGTCACATCCCTGATGTTCCCCTTCGGGTGTTCGGATGT
CACCAAAATTCTTGTGCTTACACCCTCCAACAAAATGGGGTTGCCGAACGAAAAAACTGTTACATTTTGGAAGTTGCATCXXXXXXXXXXA
GACATCGTTAGATTGTGTTAAAGAATCCTATCCCTCCACTCGTCACATCCCTGATGTTCCCCTTCGGGTGTTTGGATGT
>Marker640351 Chr3 SNP_site:25157197 Ref_Type:T
CACCAAAATTCTTGTGCTTACACCCTCCAACAAAATGGGGTTGCCGAACGAAAAAACCGTTACATTTTGGAAGTTGCATCXXXXXXXXXXA
GACATCGTTAGATTGTGTTAAAGAATCCTATCCCTCCACTCGTCACATCCCTGATGTTCCCCTTCGGGTGTTCGGATGT
CACCAAAATTCTTGTGCTTACACCCTCCAACAAAATGGGGTTGCCGAACGAAAAAACTGTTACATTTTGGAAGTTGCATCXXXXXXXXXXA
GACATCGTTAGATTGTGTTAAAGAATCCTATCCCTCCACTCGTCACATCCCTGATGTTCCCCTTCGGGTGTTTGGATGT
>Marker640656 Chr3 SNP_site:12035487 Ref_Type:C
AACATCTGAATTACTTTTAAAATCTGACTACTTTTTCCCACGTCCCATTCCAGCATAATTTATGATCTCTTCCATTGTATXXXXXXXXXXT
TATGGCACTAGGCTTGGGGCATTGCCCATATGCTTTTGGAATGTCCGTATGCTTAATTGCTAACAATCATGGCGAAGTG
AACATCTGAATTACTTTTAAAATCTGACTACTTTTTCCCACGTCCCATTCCAGCATAATTTATGATCTCTTCCATTGTATXXXXXXXXXXT
TATGGCACTAGGCTTGGGGCATTGCCCATATGCTTTTGGAATGTTCGTATGCTTAATTGCTAACAATCATGGCGAAGTG
>Marker640996 Chr3 SNP_site:6740240 Ref_Type:C
TACTTTGATTAATTTCTTTGTAGTCAGATATTTTGGTTTTGGTCCTTTTGCTATCCCTCTCCCTTCATGTCAAATTTGCAXXXXXXXXXXT
AAAGCAATGAGATTAATCTGGGTTTGAATATTGGTTTGGCTTTCTGATATTTTCATCATCATCATTATTTATTCCTGTT
TACTTTGATTAATTTCTTTGTAGTCAGATATTTTGGTTTTGGTCCTTTTGCTATCCCTCTCCCTTCATGTCAAATTTGCAXXXXXXXXXXT
AAAGCAATGAGATTAATCTGGGTTTGAATATTGGTTTGGGTTTCTGATATTTTCATCATCATCATTATTTATTCCTGTT
>Marker64118 Chr4 SNP_site:18447211 Ref_Type:T
TACCTCAGCCAGTGAGTATGATCTTCGTGTATTAATCTATAAACATACAAAATAAATTAAATGAATTAACTCACAATCCAXXXXXXXXXXC
TCGGTGCTCTCAACTTGTAGAGAACGCTATATAAAAACAACAAATTAGAATAACGAAGTAATCCTAATTGCTAGTAGTC
TACCTCAGCCTGTGAGTATGATCTTCGTGTATCAATCTATAAACATGCAAAATAAATTAAATGAATTAACTCACAATCCAXXXXXXXXXXC
TCGGTGCTCTCAACTTGTAGAGAACGCTATATAAAAACAACAAATTAGAATAACGAAGTAATCCTAATTGCTAGTAGTC
>Marker64118 Chr4 SNP_site:18447233 Ref_Type:C
TACCTCAGCCAGTGAGTATGATCTTCGTGTATTAATCTATAAACATACAAAATAAATTAAATGAATTAACTCACAATCCAXXXXXXXXXXC
TCGGTGCTCTCAACTTGTAGAGAACGCTATATAAAAACAACAAATTAGAATAACGAAGTAATCCTAATTGCTAGTAGTC
TACCTCAGCCTGTGAGTATGATCTTCGTGTATCAATCTATAAACATGCAAAATAAATTAAATGAATTAACTCACAATCCAXXXXXXXXXXC
TCGGTGCTCTCAACTTGTAGAGAACGCTATATAAAAACAACAAATTAGAATAACGAAGTAATCCTAATTGCTAGTAGTC
>Marker64118 Chr4 SNP_site:18447247 Ref_Type:G
TACCTCAGCCAGTGAGTATGATCTTCGTGTATTAATCTATAAACATACAAAATAAATTAAATGAATTAACTCACAATCCAXXXXXXXXXXC
TCGGTGCTCTCAACTTGTAGAGAACGCTATATAAAAACAACAAATTAGAATAACGAAGTAATCCTAATTGCTAGTAGTC
TACCTCAGCCTGTGAGTATGATCTTCGTGTATCAATCTATAAACATGCAAAATAAATTAAATGAATTAACTCACAATCCAXXXXXXXXXXC
TCGGTGCTCTCAACTTGTAGAGAACGCTATATAAAAACAACAAATTAGAATAACGAAGTAATCCTAATTGCTAGTAGTC
>Marker641490 Chr3 SNP_site:27248352 Ref_Type:C

Create PDF with GO2PDF for free, if you wish to

TACAGTGCCATTCCATGAGTATCAATGAAGCACGACGTAGCACATTTTACTTGTAAGACAAATTAAATCGGCATGCATATXXXXXXXXXXT
CTTCTCTAGCAAGTCCAAAGTCTGCAAGCTTCACAGACCTTTGATTTGCAGTAAGCAACAAATTATCTACATTGCAAGT
TACAGTGCCATTCCATGAGTATCAATGAAGCACGACGTAGCACATTTTACTTGTAAGACAAATTAAATTGGCATGCATATXXXXXXXXXXT
CTTCTCTAGCAAGTCCAAAGTCTGCAAGCTTCACAGACCTTTGATTTGCAGTAAGCAACAAATTATCTACATTGCAAGT
>Marker641519 Chr3 SNP_site:31164870 Ref_Type:G
TACCTTTAATTTTTTCATTTAGACATTACATCGATCAACATTATGCCAACTTCTCGTCTTTTAAGTAAAGGGCAAAGGTTXXXXXXXXXXG
AATATATTTTATCTTACATTTATTCTCATGGCAAGTGCCGGCAATATTTATTGATGCCTATCAATTGACATAACTTGGT
TACCTTTAATTTTTTCATTTATACATTACATCGATCAACATTATGCCAACTTCTTGTCTTTTAAGTAAAGGGCAAAGGTTXXXXXXXXXXG
AATATATTTTATCTTACATTTATTCTCATGGCAAGTGCCGGCTATATTTATTGATGCCTATCAATTGACATAACTTGGT
>Marker641519 Chr3 SNP_site:31164903 Ref_Type:C
TACCTTTAATTTTTTCATTTAGACATTACATCGATCAACATTATGCCAACTTCTCGTCTTTTAAGTAAAGGGCAAAGGTTXXXXXXXXXXG
AATATATTTTATCTTACATTTATTCTCATGGCAAGTGCCGGCAATATTTATTGATGCCTATCAATTGACATAACTTGGT
TACCTTTAATTTTTTCATTTATACATTACATCGATCAACATTATGCCAACTTCTTGTCTTTTAAGTAAAGGGCAAAGGTTXXXXXXXXXXG
AATATATTTTATCTTACATTTATTCTCATGGCAAGTGCCGGCTATATTTATTGATGCCTATCAATTGACATAACTTGGT
>Marker641519 Chr3 SNP_site:31165098 Ref_Type:A
TACCTTTAATTTTTTCATTTAGACATTACATCGATCAACATTATGCCAACTTCTCGTCTTTTAAGTAAAGGGCAAAGGTTXXXXXXXXXXG
AATATATTTTATCTTACATTTATTCTCATGGCAAGTGCCGGCAATATTTATTGATGCCTATCAATTGACATAACTTGGT
TACCTTTAATTTTTTCATTTATACATTACATCGATCAACATTATGCCAACTTCTTGTCTTTTAAGTAAAGGGCAAAGGTTXXXXXXXXXXG
AATATATTTTATCTTACATTTATTCTCATGGCAAGTGCCGGCTATATTTATTGATGCCTATCAATTGACATAACTTGGT
>Marker641536 Chr3 SNP_site:24376709 Ref_Type:T
TACTAGGCCAAAGAACGATTTCCTCCCTAATGGAAGTCTATTCTGAAATACACCTCAAGGAGGATCACACAAGTGCTCTGXXXXXXXXXXA
AGATCAGTGTTGAAAGTTACATGGTCGTCCCCCAAGAGGTAAAAAACGCTTTCCCAACGACAAATACTGAGAGGCTGTA
TACTTGGCCAAAGAACGATTTCCTCCCTAATGGAAGTCTATTCTGAAATACACCTCAAGGAGGATCACACAAGTGCTCTGXXXXXXXXXXA
AGATCAGTGTTGAAAGTTACATGGTCGTCCCCCAAGAGGTAAAAAACGCTCTCCCAACGACAAATACTGAGAGGCTGTA
>Marker641536 Chr3 SNP_site:24376963 Ref_Type:C
TACTAGGCCAAAGAACGATTTCCTCCCTAATGGAAGTCTATTCTGAAATACACCTCAAGGAGGATCACACAAGTGCTCTGXXXXXXXXXXA
AGATCAGTGTTGAAAGTTACATGGTCGTCCCCCAAGAGGTAAAAAACGCTTTCCCAACGACAAATACTGAGAGGCTGTA
TACTTGGCCAAAGAACGATTTCCTCCCTAATGGAAGTCTATTCTGAAATACACCTCAAGGAGGATCACACAAGTGCTCTGXXXXXXXXXXA
AGATCAGTGTTGAAAGTTACATGGTCGTCCCCCAAGAGGTAAAAAACGCTCTCCCAACGACAAATACTGAGAGGCTGTA
>Marker64188 Chr4 SNP_site:11653426 Ref_Type:A
ACAAGTTCATGGATTAGCATTTTCCAAATAAGACTAGGTCACAAAAACTCCATTCAACTATACACACACAGCAAAACCCAXXXXXXXXXXT
CTTCATAGTCATGCCTTCCTTGGTGGAATACTGCAACAACTCCTTCACAATAGCCAAATGCCCCTTCTCGGCTGCGGTA
ACAAGTTCATGGATTAGCATTTTCCCAATAAGACTAGGTCACAAAAACTCAATTCAACTATACACACACAGCAAAACCCAXXXXXXXXXXT
CTTCATAGTCATGCCTTCCTTGGTGGAATACTGCAACAACTCCTTCACAATAGCCAAATGCCCCTTCTCGGCTGCGGTA
>Marker64188 Chr4 SNP_site:11653451 Ref_Type:C
ACAAGTTCATGGATTAGCATTTTCCAAATAAGACTAGGTCACAAAAACTCCATTCAACTATACACACACAGCAAAACCCAXXXXXXXXXXT
CTTCATAGTCATGCCTTCCTTGGTGGAATACTGCAACAACTCCTTCACAATAGCCAAATGCCCCTTCTCGGCTGCGGTA
ACAAGTTCATGGATTAGCATTTTCCCAATAAGACTAGGTCACAAAAACTCAATTCAACTATACACACACAGCAAAACCCAXXXXXXXXXXT
CTTCATAGTCATGCCTTCCTTGGTGGAATACTGCAACAACTCCTTCACAATAGCCAAATGCCCCTTCTCGGCTGCGGTA
>Marker642193 Chr3 SNP_site:818607 Ref_Type:G
ACTTCAAGTCTAGTTGTTGAAAAGAATCTTCACCAACATGTAATCTGATATTTTGATGCATTTCTTCATGACTTGCTGATXXXXXXXXXXG
GAAACGTTAGAGAGAGAGAAAGATTGGATAGAAAAATGTGCAAAGTTCTTCTGTGACCACTTTTGAAGTCGATAGAGTT
ACTTCAAGTCTAGTTGTTGAAAAGAATCTTCACCAACATGTAATCTGATATTTTGATGCATTTCTTCATGACTTGCTGATXXXXXXXXXXG
GAAACGTTAGAGAGAGAGAAAGATTGGATAGAAAAATGTGCAAAGTTCTTCTGTGACCACTTTTGAAGTCGGTAGAGTT

Create PDF with GO2PDF for free, if you wish to

>Marker642234 Chr3 SNP_site:7100166 Ref_Type:G
AACTCAGAAGAACCAAAAGTTTCGTGGTTGTATGAATCATTAGCATTCCCTCGTTGATGGGCTGCAGCCTGATTAATAAAXXXXXXXXXXA
TAACCTAGGGATAGAAATTTAATTTAAATTATGTTTGCAAGTGAGGAGATTGGAAAACACAATGTAATTGTGAAATGTG
AACTCAGAAGAACCAAAAGTTTCGTGGTTGTATGAATCATTAGCATTCCCTCGTTGATGGGCTGCAGCCTGATTAATAAAXXXXXXXXXXA
TAACCTAGGGATAGAAATTTAATTTAAATTATGTTTGCAAGTGAGGAGATTGGGAAACATAATGTAATTGTGAAATGTG
>Marker642234 Chr3 SNP_site:7100172 Ref_Type:T
AACTCAGAAGAACCAAAAGTTTCGTGGTTGTATGAATCATTAGCATTCCCTCGTTGATGGGCTGCAGCCTGATTAATAAAXXXXXXXXXXA
TAACCTAGGGATAGAAATTTAATTTAAATTATGTTTGCAAGTGAGGAGATTGGAAAACACAATGTAATTGTGAAATGTG
AACTCAGAAGAACCAAAAGTTTCGTGGTTGTATGAATCATTAGCATTCCCTCGTTGATGGGCTGCAGCCTGATTAATAAAXXXXXXXXXXA
TAACCTAGGGATAGAAATTTAATTTAAATTATGTTTGCAAGTGAGGAGATTGGGAAACATAATGTAATTGTGAAATGTG
>Marker642491 Chr3 SNP_site:36432208 Ref_Type:C
TACCTTTAAAAAAAATCCAAACAAGCAGCAAGGTTATCAAATGTCATTATTATTTGGTTACTCAATAGCAGAATAGATCTXXXXXXXXXXA
AATCCCATATTTTGAGTTTGTGTTTAAAAAAATGCAAAACAAATGGTTGATGAATTAAAACTAAAAAAAGTTTGCGGTG
TACCTTTAAAACAAATCCAAACAAGCAGCAAGGTTATCAAATGTCATTATTATTTGGTTACTCAATAGCAGAATAGATCTXXXXXXXXXXA
AATCCCATATTTTGAGTTTGTGTTTAAAAAAATGCAAAACAAATGGTTGATGAATTAAAACTAAAAAAAGTTTGCGGTG
>Marker643000 Chr3 SNP_site:38198705 Ref_Type:G
CACCCACTTTGATATCACTGCCTACATACTCAAGGAGATAAAAACTCTAATTATACACTCAATGAAAGCATGAACATAATXXXXXXXXXXC
ATTATTCAAAGAGAAACTTCCATTCTGATCTGCTCTTGTCCAAAATTGGTAGCCCTGAGTTGCCAAACGTATTGAAGTC
CACCCACTTTGATATCACTGCCTACATACTCAAGGAGATAAAAACTCTAATTATACACTCAATGAAAGCATGAACATAATXXXXXXXXXXC
ATTATTCAAAGAGAAACTTCCATTCTGATCTGCTCTTGTCCAAAATTGGTAGCCCTGAGTTGCCAAGCGTATTGAAGTC
>Marker643752 Chr3 SNP_site:4126715 Ref_Type:T
TACACTACCACAACCGAGAAAGAGAGTCGAAAGAAGAACTAAAGAGAACAAATCGAAACTAACTCTAAGCTTATATGCTTXXXXXXXXXXT
ATGAGAAAATGAGAAGTGAATTGTGAAAGAGGTAACTGAATTTGTGGTAATCAAATAGTGGAACTTCGATTCTGGTGTC
TACACTACCACAACCGAGAAAGAGAGTCGAAAGAAGAACTAAAGAGAACAAATCGAAACTAACTCTAAGCTTATATGCTTXXXXXXXXXXT
ATGAGAAAATGAGAAGTGAATTGTGAAAGAGGTAACTGAATTTGTGGTAATCAAATAGTGGAACTTCGATTTTGGTGTC
>Marker643781 Chr3 SNP_site:33183548 Ref_Type:A
AACAAAATCAAGATTTGTTAATTATACATCAACCTTTTATAATGGAAAAAAAAAATCATAAATATAAGGGTTTAGGGTATXXXXXXXXXXA
ACAAAAATTTTCAATTTCACCATTTTAAGAGGAAATTTTAGGGAGCTTGAACCCAGAGCAAGGATTACCAGTCACAAGT
AACAAAATCAAGATTTGTTAATTATACATCAACCTTTTATAATGGAAAAAATAAATCATAAATATAAGGGTTTAGGGTATXXXXXXXXXXA
ACAAAAATTTTCAATTTCACCATTTTAAGAGGAAATTTTAGGGAGCTTGAACCCAGAGCAAGGATTACCAGTCACAAGT
>Marker643861 Chr3 SNP_site:9546066 Ref_Type:C
ACAATAATGTAGCCAAACTTGACCCATACAACACTCTATCTCAATCTATCTTTAAATGAATTCTCTTTTTTACCAATCCCXXXXXXXXXXG
ATATAGAATTAGAAAGAAATAGTAAAAGAGAAGCATTAATGTGAGGTTAATTAAGGTAGGACCAATGGAGACAATGGGT
ACAATAATGTAGCCAAACTTGACCCATAGAACACTCTATCTCAATCTATCTTTAAATGAATTCTCTTTTTTACCAATCCCXXXXXXXXXXG
ATATAGAATTAGAAAGAAATAGTAAAAGAGAAGCATTAATGTGAGGTTAATTAAGGTAGGACCAATGGAGACAATGGGT
>Marker644003 Chr3 SNP_site:36438791 Ref_Type:T
GACAACTTCCGCAAAACTTCTTATATCTAACAAAGAAAACTGCAACTACATTTTACATCATACTTACGAATCTTTTCAAAXXXXXXXXXXA
CCGCGGCGCCCCAAGGTGAGGTCCTCTCGGCCTCCTGAGCCGACCTGCTCTTAGTTCTCATGGAAGCAGAACCAACTGT
GACAACTTCCGCAAAACTTCTTATATCTAACAAAGAAAACTGCAACTACATTTTACATCATACTTACGAATCTTTTCAAAXXXXXXXXXXA
CCGCGGCGCCCCAAGGTGAGGTCCTCTCTGCCTCCTGAGCCGACCTGCTCTTAGTTCTCATGGAAGCAGAACCAACTGT
>Marker644563 Chr3 SNP_site:24635989 Ref_Type:C
CACCTGTTAGGGAACTTTGATAAAAGGGTTATAACCATATTGAAGTGATTTAATTAATGTGTATATAATATCAAAAAGATXXXXXXXXXXT
ATTATAGCATAGTAAAGAAAGATGAAATTAAAAAAGTAAAAAAGGAGTCAAGTGGATTAATAATTCTTGTTTAATTGTT
CACCTGTTAGGGAACTTTGATAAAAGGGTTATAACCATATTGAAGTGATTTAATTAATGTGTATATAATATTAAAAAGATXXXXXXXXXXT

Create PDF with GO2PDF for free, if you wish to

ATTATAGCATAGTAAAGAAAGATGAAATTAAAAAAGTAAAAAAGGAGTCAAGTGGATTAATAATTCTTGTTTAATTGTT
>Marker644836 Chr3 SNP_site:29778180 Ref_Type:G
AACCTGAAATAACACAATACCATTTGATTGCCGAAGTTAGTTTATTAACGGAAAGTAGTTTGCGACAAGAAAAATTGTTTXXXXXXXXXXA
CGCACATTACTCCTTTGTGTGAAAAGCTATCTCAAACAATGTCATAATGTTAATCCATCTTCAAAACATTGAGCAAAGT
AACCTGAAATAACACAATACCATTTGATTGCCGAAGTTAGTTTATTAACGGAAAGTAGTTTGCGACAAGAAAAATTGTTTXXXXXXXXXXA
CGCACATTACTCCTTTGTGTGAAAAGCTATCTCAAACAATGTCATAATGTTAATCCATCTTCAAAGCATTGAGCAAAGT
>Marker644852 Chr3 SNP_site:4059458 Ref_Type:C
ACTCTTATTTAGAGTTGTTTTTATTAGATTTAACGCGTTTGCTAGTTTATTTTTGAAGAATTATTAAAATAAATGAGTATXXXXXXXXXXC
ATTGTGGGCACTGCAACATTATACATGTTGTGCTATATATCGAAGCTGAGGCATCCGGTCATGCTGTGCGTGAGAGGTT
ACTCTTATTTAGAGTTGTTTTTATTAGATTTAACGTGTTTGCTAGTTTATTTTTGAAGAATTATTAAAATAAATGAGTATXXXXXXXXXXC
ATTGTGGGCACTGCAACATTATACATGTTGTGCTATATATCGAAGCTGAGGCATCCGGTCATGCTGTGCGTGAGAGGTT
>Marker644887 Chr3 SNP_site:2585784 Ref_Type:A
ACTCAAGCACAATTACTCTGGATGTTGTTTGAATGTCAAATTTTAATGTTCTTGACGTTTTTTCAAAACATTTTTTAAACXXXXXXXXXXA
GTTTATATTGCTACAAAATAGGATTTCCTAACCAAATATTTAGATATATTTAGTGTATATCATTATTAATATAGTTGTA
ACTCAAGCACAATTACTCTGGATGTTGTTTGAATGTCAAATTTTAATGTTCTTGACGTTTTTTCAATACATTTTTTAAACXXXXXXXXXXA
GTTTATATTGCTACAAAATAGGATTTCCTAACCAAATATTTAGATATATTTAGTTTATATCATTATTAATATAGTTGTA
>Marker644887 Chr3 SNP_site:2585934 Ref_Type:T
ACTCAAGCACAATTACTCTGGATGTTGTTTGAATGTCAAATTTTAATGTTCTTGACGTTTTTTCAAAACATTTTTTAAACXXXXXXXXXXA
GTTTATATTGCTACAAAATAGGATTTCCTAACCAAATATTTAGATATATTTAGTGTATATCATTATTAATATAGTTGTA
ACTCAAGCACAATTACTCTGGATGTTGTTTGAATGTCAAATTTTAATGTTCTTGACGTTTTTTCAATACATTTTTTAAACXXXXXXXXXXA
GTTTATATTGCTACAAAATAGGATTTCCTAACCAAATATTTAGATATATTTAGTTTATATCATTATTAATATAGTTGTA
>Marker645135 Chr3 SNP_site:5881581 Ref_Type:T
AACAAGAAACTCCTTTGCTGAAACACCAACCGTAGCAGTAGAGTTGATAGAAAACCAGGGAATTCCACTAAAAAGAAAAAXXXXXXXXXXT
GTAATACTTTCTTCTAATCTTTTTTCCATTCTCTGTTTTCCAAATTTCAGCGGAAGACATATAGGCGAAGGTGCTTGTT
AACAAGAAACTCCTTTGCTGAAACACCAACCGTAGCAGTAGAGTTGATAGAAAACCAGGGAATTCCACTAAAAAGAAAAAXXXXXXXXXXT
GTAATACTTTCTTCTAATCTTTTTTCCATTCTCTGTTTTCCAAATTTCAGTGGAAGACATATAGGCGAAGGTGCTTGTT
>Marker645205 Chr3 SNP_site:35808928 Ref_Type:A
ACAAACTTCTTATTGTCTCCGTCTTTTTAATTGTTGTTTCATTTGTATTATCCAGGACTCTACTTTTGAATTTGAGAGGTXXXXXXXXXXG
TTAAAAATTCCTTCCATTCCCATTTGTTATATGCCTATATTTTTTGCAAATGCTATTTCAGTTGTAACTCAGGATCGTT
ACAAACTTCTTATTGTCTCCGTCTTTTTAATTGTTGTTTCATTTGTATTATCCAGGACTCTACTTTTGAATTTGAGAGGTXXXXXXXXXXG
TTGAAAATTCCTTCCATTCCCATTTGTTATATGCCTATATTTTTTGCAAATGCTATTTCAGTTGTAACTCAGGATCGTT
>Marker645938 Chr3 SNP_site:35196975 Ref_Type:A
TACTTTTAAAATTCATACACCAAAAGCATATATTCATCTAAACTTCAAGACTAAAAATGTAGTTTTTTTTTTAATTTTTGXXXXXXXXXXT
AAAAACATTTTAGATTTTACCTCTATTTCTTTTCTTTTTTGTTTCTTCTAAAAAAATCAATGACATAGCGGAGTATGTA
TACTTTTAAAATTCATACACCAAAAGCATATATTCATCTAAACTTCAAGACTAAAAATGTAGTTTTTTTTTTAATTTTTGXXXXXXXXXXT
AAAAACATTTTAGATTTTACCTCTATTTCTTTTCTTTTTTGTTTCTTCTAAAAAAATCATTGACATAGCGGAGTATGTA
>Marker645957 Chr3 SNP_site:38989725 Ref_Type:G
ACTCCTTACTTGGGTTATATGTATCAATCTCTGAATCTTACCGGGGTTTGTAGATTGGGAGATATTTCAATGAAACCATCXXXXXXXXXXT
AACAAAGATACGAATATATTGATTCAATACCTTCACTTCTTAGGTTGCTTTTAATTAAGAAAACATACATTCTTAGGTT
ACTCCTTACTTGGGTTATATGTATCAATCTCTGAATCTTACCGGGGTTTGTAGATTGGGAGATATTTCAATGAAACCGTCXXXXXXXXXXT
AACAAAGATACGAATATATTGATTCAATACCTTCACTTCTTAGGTTGCTTTTAATTAAGAAAACATACATTCTTAGGTT
>Marker646096 Chr3 SNP_site:27717018 Ref_Type:A
CACCCTCAAACCTTAACCATATAAGGATTTAGATTGTGAAACGAATGGTCTTTAACTTCATCTCACTTCTCCAAAGAAATXXXXXXXXXXT
CATTTGGATTTTGGTTAAAAATTCTAAAGCTTCCTTTAGAAAGATGAAAACTATGGTAACAAATTGTAATAAAACCAGT

Create PDF with GO2PDF for free, if you wish to

CACCCTCAAACCTTAACCATATAAGGATTTAGATTGTGAAACGAATGGTCTTTAACTTCATCTCTCTTCTCCAAAGAAATXXXXXXXXXXT
CATTTGGATTTTGGTTAAAAATTCTAAAGCTTCCTTTAGAAAGATGAAAACTATGGTAACAAATTGTAATAAAACCAGT
>Marker646395 Chr3 SNP_site:26099928 Ref_Type:A
AACAAAAGAAAAAAATAATAGATTTTGAGCAAATTTTCTTTTTGCTAATTACCATCTCTTTCTCCACCAATGCTGAAAGCXXXXXXXXXXC
AAATTTTCCTCTTCAATTCAACTCCTGCCATTTAAAATTACCTGAAAGTAGCATACTACGTATAACATACCAAGTGGTT
AACAAAGGAAAAAAATAATAGATTTTGAGCAAATTTTCTTTTTGCTAATTACCATCTCTTTCTCCACCAATGCTGAAAGCXXXXXXXXXXC
AAATTTTCCTCTTCAATTCAACTCCTGCCATTTAAAATTACCTGAAAGTAGCATACTACGTATAACATACCAAGTGGTT
>Marker646407 Chr3 SNP_site:36711565 Ref_Type:C
ACATGACACCGTGAATCCTGTCTGGAATCAAACGTTCGACTTTCTGGTGGAGGATGCATTACATGATATGCTGATTGTAGXXXXXXXXXXT
GGTTTGAATATTACTTCGATTAATAAAACCCAACATATAAATTATCGAAGACACCGACTGAGCAACTAAAAAACATGTA
ACATGACACCGTGAATCCTGTCTGGAATCAAACGTTCGACTTTCTGGTGGAGGATGCATTACATGATATGCTGATTGTAGXXXXXXXXXXT
GGTTTGAATATTACTTTGATTAATAAAACCCAACATATAAATTATCGAAGACACTGACTCAGCAACTAAAAAACATGTA
>Marker646407 Chr3 SNP_site:36711603 Ref_Type:C
ACATGACACCGTGAATCCTGTCTGGAATCAAACGTTCGACTTTCTGGTGGAGGATGCATTACATGATATGCTGATTGTAGXXXXXXXXXXT
GGTTTGAATATTACTTCGATTAATAAAACCCAACATATAAATTATCGAAGACACCGACTGAGCAACTAAAAAACATGTA
ACATGACACCGTGAATCCTGTCTGGAATCAAACGTTCGACTTTCTGGTGGAGGATGCATTACATGATATGCTGATTGTAGXXXXXXXXXXT
GGTTTGAATATTACTTTGATTAATAAAACCCAACATATAAATTATCGAAGACACTGACTCAGCAACTAAAAAACATGTA
>Marker646407 Chr3 SNP_site:36711608 Ref_Type:G
ACATGACACCGTGAATCCTGTCTGGAATCAAACGTTCGACTTTCTGGTGGAGGATGCATTACATGATATGCTGATTGTAGXXXXXXXXXXT
GGTTTGAATATTACTTCGATTAATAAAACCCAACATATAAATTATCGAAGACACCGACTGAGCAACTAAAAAACATGTA
ACATGACACCGTGAATCCTGTCTGGAATCAAACGTTCGACTTTCTGGTGGAGGATGCATTACATGATATGCTGATTGTAGXXXXXXXXXXT
GGTTTGAATATTACTTTGATTAATAAAACCCAACATATAAATTATCGAAGACACTGACTCAGCAACTAAAAAACATGTA
>Marker646410 Chr3 SNP_site:35812603 Ref_Type:G
CACATCTTCACCTTTTTTCGGCAATGTTTCAGATTGGTGACCATGTTTCTTGAATACCACAGCAATGTGACTATGGCCCTXXXXXXXXXXG
AATAGAAGCAATGAACATGCTCTTACCGTGGAATTTTTCTAATAATCATTTTTTTAAGGGAAACTCTATCTTCTGGGTC
CACATCTTCACCTTTTTTGGGCAATGTTTCAGATTGGTGACCATGTTTCTTGAATACCACAGCAATGTGACTATGGCCCTXXXXXXXXXXG
AATAGAAGCAATGAACATGCTCTTACCGTGGAATTTTTCTAATAATCATTTTTTTAAGGGAAACTCTATCTTCTGGGTC
>Marker646428 Chr3 SNP_site:20531034 Ref_Type:T
CACATAAAAGTTAGCATCATCTCAAGGAATCAACTAATGGTTTGAAAACCATTTCATAGCATTGAAATCATAATCATCAAXXXXXXXXXXA
ACTTTTAAGAAGAAAATCATTCACCACACTTTAACAAAAATCCTCCTATTCCTTCTCTTCCCGCACAACAAATCGTCGT
CACATAAAAGTTAGCATCATCTCAAGGAATCAACTAATGGTTTGAAAACCATTTCATAGCATTGAAATCATAATCATCAAXXXXXXXXXXA
ACTTTTAAGAAGAAAATCATTCACCACACTTTAACAAAAATCCTCCTATTCCTTCTCTTCTCGCACAACAAATCGTCGT
>Marker64666 Chr4 SNP_site:11873247 Ref_Type:C
ACCCTTGGTTTTCAATTGATGAAGTCTAGATCAAGTTTATAGGGTGATATGTGCTGTCTAATTCCTCATAACTAACAAACXXXXXXXXXXA
TGTCTAAAATAAATTTTGTCTAGGAAAAGGGTTACTGCTTCATCTACAGTTTTTTGTTCTTCCTATATATTCTAATGTT
ACCCTTGGTTTTCAATTGATGAAGTCTAGATCAAGTTTATAGGGTGATATGTGCTGTCTAATTCCTCATAACTAACAAACXXXXXXXXXXA
TGTCTAAAATAAATTTTGTCTAGGAAAAGGGTTACTGCTTCATCTACAGTTTTTTGTTCTTTCTATATATTCTAATGTT
>Marker646840 Chr3 SNP_site:35706759 Ref_Type:T
CACCATTCCTGCCAGAAATGTAAAGTTATCCTCAGCAGAGACCTATGCAGAAGGACTTTAATCTTCAGACCACAAGAGGAXXXXXXXXXXA
TTAAAACAAACATTTTTAATTTGGAGACTAAATGAGACAAGATTTGAGAAAAATGGTGCTTTGCAACAATACAAGCAGT
CACCATTCCTGCCAGAAATGTAAAGTTATCCTCAGCAGAGACCTATGCAGAAGGACTTTAATCTTCAGACCACAAGAGGAXXXXXXXXXXA
TTAAAACAAACATTTTTAATTTGGAGACTAAATGAGACAATATTTGAGAAAAATGGTGCTTTGCAACAATACAAGCAGT
>Marker647047 Chr3 SNP_site:35627670 Ref_Type:A
ACTTTAAGAAGATGATGTGTGACATTTAAGTGATCATTAGTTGGTTTTTATAGAAATTGGCTAAGGCGATGTATAGCAAAXXXXXXXXXXC

Create PDF with GO2PDF for free, if you wish to

AGTTTAATGTTCGAATCCATGGGCAACGAAATAGGCTTGATATCTAGATAACTAGTGTCTTCCAGGATCTATAGTGAGT
ACTTTAAGTAGATGATGTGTGACATTTAAGTGATCATTAGTTGGTTTTTATAGAAATTGGCTAAGGCGATGTATAGCAAAXXXXXXXXXXXC
AGTTTAATGTTCGAATCTATGGGCAACGAAATAGGCTTGATATCTAGATAACTAGTGTCTTCGAGGATCTATAGTGAGT
>Marker647047 Chr3 SNP_site:35627863 Ref_Type:C
ACTTTAAGAAGATGATGTGTGACATTTAAGTGATCATTAGTTGGTTTTTATAGAAATTGGCTAAGGCGATGTATAGCAAAXXXXXXXXXXXC
AGTTTAATGTTCGAATCCATGGGCAACGAAATAGGCTTGATATCTAGATAACTAGTGTCTTCCAGGATCTATAGTGAGT
ACTTTAAGTAGATGATGTGTGACATTTAAGTGATCATTAGTTGGTTTTTATAGAAATTGGCTAAGGCGATGTATAGCAAAXXXXXXXXXXXC
AGTTTAATGTTCGAATCTATGGGCAACGAAATAGGCTTGATATCTAGATAACTAGTGTCTTCGAGGATCTATAGTGAGT
>Marker647047 Chr3 SNP_site:35627908 Ref_Type:C
ACTTTAAGAAGATGATGTGTGACATTTAAGTGATCATTAGTTGGTTTTTATAGAAATTGGCTAAGGCGATGTATAGCAAAXXXXXXXXXXXC
AGTTTAATGTTCGAATCCATGGGCAACGAAATAGGCTTGATATCTAGATAACTAGTGTCTTCCAGGATCTATAGTGAGT
ACTTTAAGTAGATGATGTGTGACATTTAAGTGATCATTAGTTGGTTTTTATAGAAATTGGCTAAGGCGATGTATAGCAAAXXXXXXXXXXXC
AGTTTAATGTTCGAATCTATGGGCAACGAAATAGGCTTGATATCTAGATAACTAGTGTCTTCGAGGATCTATAGTGAGT
>Marker64707 Chr4 SNP_site:4270790 Ref_Type:A
AACAAAATTCACAAATTATAACAAACATATATTAGCTCTAATGTCATTTTTTTCTAAAATTTTATTACCAATTAATTCCAXXXXXXXXXXXA
TTTACTTAGTTAAAGTCTATATTGTGACACTTCTCTATTTAATCTTGCAATGCGAATTTAGTGAAATCAAATTTACGTT
AACAAAATTCACAAATTATAACAAACATATATTAGCTCTAATGTCATTTTTTTCTAAAGTTTTATTACCAATTAATTCCAXXXXXXXXXXXA
TTTACTTAGTTAAAGTCTATATTGTGACACTTCTCTATTTAATCTTGCAATGCGAATTTAGTGAAATCATATTTACGTT
>Marker64707 Chr4 SNP_site:4270978 Ref_Type:A
AACAAAATTCACAAATTATAACAAACATATATTAGCTCTAATGTCATTTTTTTCTAAAATTTTATTACCAATTAATTCCAXXXXXXXXXXXA
TTTACTTAGTTAAAGTCTATATTGTGACACTTCTCTATTTAATCTTGCAATGCGAATTTAGTGAAATCAAATTTACGTT
AACAAAATTCACAAATTATAACAAACATATATTAGCTCTAATGTCATTTTTTTCTAAAGTTTTATTACCAATTAATTCCAXXXXXXXXXXXA
TTTACTTAGTTAAAGTCTATATTGTGACACTTCTCTATTTAATCTTGCAATGCGAATTTAGTGAAATCATATTTACGTT
>Marker647268 Chr3 SNP_site:28943420 Ref_Type:C
ACCATTATTCAAATGATACATAGCATAGGAAACTTTTGTGTTGTTTGACAAACTGGGTGACTTGGAAAAGATGGATTGGAXXXXXXXXXXXA
TCAAGACTCGTAGGACAAGACAATAATTCACAAAAGCAAGAGCAACCCGATAAAAGATCTGATAATTCACTAATAAAGT
ACCATTATTCAAATGATACATAGCATAGGAAACTTTTGTGTTGTTTGACAAACTGGGTGACTTGGAAAAGATGGATTGGAXXXXXXXXXXXA
TTTAGACTCGTAGGATAAGACAATAATTCACAAAAGCAAGAGCAACCCGATAAAAGATCTGATAATTCACTAATAAAGT
>Marker647268 Chr3 SNP_site:28943421 Ref_Type:A
ACCATTATTCAAATGATACATAGCATAGGAAACTTTTGTGTTGTTTGACAAACTGGGTGACTTGGAAAAGATGGATTGGAXXXXXXXXXXXA
TCAAGACTCGTAGGACAAGACAATAATTCACAAAAGCAAGAGCAACCCGATAAAAGATCTGATAATTCACTAATAAAGT
ACCATTATTCAAATGATACATAGCATAGGAAACTTTTGTGTTGTTTGACAAACTGGGTGACTTGGAAAAGATGGATTGGAXXXXXXXXXXXA
TTTAGACTCGTAGGATAAGACAATAATTCACAAAAGCAAGAGCAACCCGATAAAAGATCTGATAATTCACTAATAAAGT
>Marker647268 Chr3 SNP_site:28943434 Ref_Type:C
ACCATTATTCAAATGATACATAGCATAGGAAACTTTTGTGTTGTTTGACAAACTGGGTGACTTGGAAAAGATGGATTGGAXXXXXXXXXXXA
TCAAGACTCGTAGGACAAGACAATAATTCACAAAAGCAAGAGCAACCCGATAAAAGATCTGATAATTCACTAATAAAGT
ACCATTATTCAAATGATACATAGCATAGGAAACTTTTGTGTTGTTTGACAAACTGGGTGACTTGGAAAAGATGGATTGGAXXXXXXXXXXXA
TTTAGACTCGTAGGATAAGACAATAATTCACAAAAGCAAGAGCAACCCGATAAAAGATCTGATAATTCACTAATAAAGT
>Marker647283 Chr3 SNP_site:15567696 Ref_Type:A
ACATAAAAATGAAATAGATAGTATAAAATGAATCCACAAAATAGTTAATTTAAATTTTGTTGTATTGCGTGTTCAAGAAAXXXXXXXXXXXA
AGAAAATTCTTTTGGAAAGGACATTCTAGAGGTAAATTTATTAATGGGAAGGTGGTGTCTTGTTTGCAAGAGGATGGTG
ACATAAAAATGAAATAGATAGTATAAAATGAATCCACAAAATAGTTAATTTAAATTTTGTTGTATTGCGTGTTCAAGAAAXXXXXXXXXXXA
AGAAAATTCTTTTGGAATGGACATTCTAGAGGTAAATTTATTAATGGGAAGGTGGTGTCTTGTTTGCAAGAGGATGGTG
>Marker647434 Chr3 SNP_site:22772984 Ref_Type:A

Create PDF with GO2PDF for free, if you wish to

ACCATCAAGCCTCCTTCAAACAGTCATTTACCCTCCTAAATCTATGCTAACTTTCTTTCTCGGAGAAAACCGGCGATCCAXXXXXXXXXXXA
TTAAACTAAGAACGTAGCTTTCATATCACTTGTTAGAGCATGAACCCTTTAAATATTTCTGCAATAGTATGATATTGTT
ACCATCAAGCCTCCTTCAAACAGTCATTTACCCTCCTAAATCTATGCTAACTTTCTTTCTCGGAGAAAACCGGCGATCCAXXXXXXXXXXXA
TTAAACTAAGAACGTAGCTTTCATATCACTTGTTAGGGCATGAACCCTTTAAATATTTCTGCAATAGTATGATATTGTT
>Marker648037 Chr3 SNP_site:277545 Ref_Type:G
GACATTTTTGCATCTGTAAAATCAAATCAACAAAAATTATTCACCATAGTTGAGTTTCCAAAACAGGGTTTTCAGTTTCTXXXXXXXXXXXG
CTCTAAACTTTGTAATTATGTATTATAAGGCAAGGATGTTTTTCAATTGGGTTTCAACATTTTAGTGTTAGGTGTGGGT
GACATTTTTGCATCTGTAAAATCAAATCAACAAAAATTATTCACCATAGTTGAGTTTCCAAAACAGGGTTTTCAGTTTCTXXXXXXXXXXXG
CTCTAAACTTTGTGATTATGTATTATAAGGCAAGGATGTTTTTCAATTGGGTTTCAACATTTTAGTGTTAGGTGTGGGT
>Marker6481 Chr4 SNP_site:4841019 Ref_Type:T
GACTGAATTGAATAGATTCCTCCATTGGCTTTGTAATTTCATAGTTCTGCAGATTTATCATTTATGGCCTTGACTTTCTTXXXXXXXXXXXA
ACTACCTAAACAATTCATAATTCGAAAAGGTAACTTTTCATTTATTCTTCATTTTCTTGTTCCACAGATGCCCCCGGTG
GACTGAATTGAATAGATTCCTCCATTGGCTTTGTAATTTCATAGTTCTGCAGATTTATCATTTATGTCCTTGACTTTCTTXXXXXXXXXXXA
ACTACCTAAACAATTCATAATTCGAAAAGGTAACTTTTCATTTATTCTTCATTTTCTTGTTCCACAGATGCCCCGGGTG
>Marker6481 Chr4 SNP_site:4841208 Ref_Type:G
GACTGAATTGAATAGATTCCTCCATTGGCTTTGTAATTTCATAGTTCTGCAGATTTATCATTTATGGCCTTGACTTTCTTXXXXXXXXXXXA
ACTACCTAAACAATTCATAATTCGAAAAGGTAACTTTTCATTTATTCTTCATTTTCTTGTTCCACAGATGCCCCCGGTG
GACTGAATTGAATAGATTCCTCCATTGGCTTTGTAATTTCATAGTTCTGCAGATTTATCATTTATGTCCTTGACTTTCTTXXXXXXXXXXXA
ACTACCTAAACAATTCATAATTCGAAAAGGTAACTTTTCATTTATTCTTCATTTTCTTGTTCCACAGATGCCCCGGGTG
>Marker648233 Chr3 SNP_site:7277392 Ref_Type:C
TACTGCTACGATACTTCCATAAATAGTTGGACTCCAGTTGCTCAATTGTCATCGGCTAGGTAATGGTTATTCTTATACACXXXXXXXXXXXT
TGGTTTATATTTTTTTTTATAAGATATTGACCATTGGTTTATTAGTAACATATTTGCCTATGCACAATTTTCTTTGGTT
TACTGCTACGATACTTCCATAAATAGTTGGAGTCCAGTTGCTCAATTGTCATCGGCTAGGTAATGGTTATTCTTATACACXXXXXXXXXXXT
TGGTTTATATTTCTTTTTATAAGATATTGACCATTGGTTTATTAGTAACATATTTGCCTATGCACAATTTTCTTTGGTT
>Marker648233 Chr3 SNP_site:7277600 Ref_Type:T
TACTGCTACGATACTTCCATAAATAGTTGGACTCCAGTTGCTCAATTGTCATCGGCTAGGTAATGGTTATTCTTATACACXXXXXXXXXXXT
TGGTTTATATTTTTTTTTATAAGATATTGACCATTGGTTTATTAGTAACATATTTGCCTATGCACAATTTTCTTTGGTT
TACTGCTACGATACTTCCATAAATAGTTGGAGTCCAGTTGCTCAATTGTCATCGGCTAGGTAATGGTTATTCTTATACACXXXXXXXXXXXT
TGGTTTATATTTCTTTTTATAAGATATTGACCATTGGTTTATTAGTAACATATTTGCCTATGCACAATTTTCTTTGGTT
>Marker648578 Chr3 SNP_site:9995954 Ref_Type:G
ACCTCAACTCTTCTATCACCATCAATACTTTAAACATTATTTAGTGTGTTTTTTAAATTTAGGTTGAATGAAAAATATGAXXXXXXXXXXXA
TTTAATAGTTCCAAGTTTTGCAAAACATATATTTATATTGCCTGCCTTATCTAACCTAGTTCTTTTAGTATATTGAAGT
ACCTCAACTCTTCTATCACCATCAATACTTTAAACATTATTTAGTGTGTTTTTTAAATTTAGGTTGAGTGAAAAATATGAXXXXXXXXXXXA
TTTAATAGTTCCAAGTTTTGCAAAACATATATTTATATTGCCTGCCTTATCTAACCTAGTTCTTTTAGTATATTGAAGT
>Marker648596 Chr3 SNP_site:5645636 Ref_Type:T
TACAGTAGCATATCATCTGTCATATAGTCTGGCTTCAATATACAGAATCCATGTCTAACTTAGGATGCTATCTTGTTAAAXXXXXXXXXXXA
CAATTGTTTGATCATAAAGAAGCGGCAATAACTTGGGAAGAAGCCCCTTTACAAGATTAGGCTTATTGTGGGCAAATGT
TACAGTAGCATATCATCTGTCATATAGTCTGGCTTCAATATACAGAATCTATGTCTAACTTAGGATGCTATCTTGTTAAAXXXXXXXXXXXA
CAATTGTTTGATCATAAAGAAGCGGCAATAACTTGGGAAGAAGCCCCTTTACAAGATTAGGCTTATTGTGGGCAAATGT
>Marker648667 Chr3 SNP_site:9440432 Ref_Type:T
CACACAAATCATTTAGCCCGAGATGGGCTTTCCAATGAAACAAAGAAATACCGAACTGTAACAATACCGTCACGTTTTCAXXXXXXXXXXXC
CTTGACGTTTTGGTTGAGAATAAAAGTTAAATGTTTGCTTAGCTATATGAATAGATACCTGGACAATTATTCTCTATGT
CACACAAATCATTTAGCCCGAGATGGGCTTTCCAATGAAACAAAGAAATACCGAACTGTAACAATACCGTCACGTTTTTAXXXXXXXXXXXC
CTTGACGTTTCGGTTGAGAATAAAAATTAAATGTTTGCTTAGCTATATGAATAGATACCTGGACAATTATTCTCTATGT

Create PDF with GO2PDF for free, if you wish to

>Marker648667 Chr3 SNP_site:9440552 Ref_Type:C
CACACAAATCATTTAGCCCGAGATGGGCTTTCCAATGAAACAAAGAAATACCGAACTGTAACAATACCGTCACGTTTTCAXXXXXXXXXXC
CTTGACGTTTTGGTTGAGAATAAAAGTTAAATGTTTGCTTAGCTATATGAATAGATACCTGGACAATTATTCTCTATGT
CACACAAATCATTTAGCCCGAGATGGGCTTTCCAATGAAACAAAGAAATACCGAACTGTAACAATACCGTCACGTTTTTAXXXXXXXXXXC
CTTGACGTTTCGGTTGAGAATAAAAATTAAATGTTTGCTTAGCTATATGAATAGATACCTGGACAATTATTCTCTATGT
>Marker648667 Chr3 SNP_site:9440567 Ref_Type:A
CACACAAATCATTTAGCCCGAGATGGGCTTTCCAATGAAACAAAGAAATACCGAACTGTAACAATACCGTCACGTTTTCAXXXXXXXXXXC
CTTGACGTTTTGGTTGAGAATAAAAGTTAAATGTTTGCTTAGCTATATGAATAGATACCTGGACAATTATTCTCTATGT
CACACAAATCATTTAGCCCGAGATGGGCTTTCCAATGAAACAAAGAAATACCGAACTGTAACAATACCGTCACGTTTTTAXXXXXXXXXXC
CTTGACGTTTCGGTTGAGAATAAAAATTAAATGTTTGCTTAGCTATATGAATAGATACCTGGACAATTATTCTCTATGT
>Marker648932 Chr3 SNP_site:25487608 Ref_Type:T
ACTTTCCTTGAATTAACTTAATCTGTAATTCTCTAATCGACTAGGGCCCACAAACAAATCTATAATCAACAAAACACAATXXXXXXXXXXG
CTTCCGTTAGTTACCATTATAGATATCAAGAAGATAATGATACTAGACAAAGTAAAGTAAGCATTATGTATAAGGAAGT
ACTTTCCTTGAATTAACTTAATCTGTAATTCTCTAATCGACTAGGGCCCACAAACAAATCTATAATCAACAAAACACAATXXXXXXXXXXG
TTTCCGTTAGTTACCATTATAGATATCAAGAAGATAATGATACTAGACAAAGTAAAGTAAGCATTATGTATAAGGAAGT
>Marker648999 Chr3 SNP_site:30484182 Ref_Type:C
GACTAGTGTTTTCCTTATTCCTTTCAAATAAAAAAGGGGATAGCAGAAAAGCATTTCCCGAGGGAAGGAGATAACAGAAAXXXXXXXXXXA
GTTTCTAAAAAAGTGAAGCTGAAAAATCTTCTCATATTTTTGAAGCAATGCTCCATCATTTTAATGAAAAAACTGGGTT
GACTAGTGTTTTCCTTATTCCTTTCAAATAAAAAAGGGGATAGCAGAAAAGCATTTCCCGAGGGAAGGAGATAACAGAAAXXXXXXXXXXA
GTTTCTAAAAAAGTGAAGCTGAAAAATCTTCTCATATTTTTGAAGCAATGTTCCATCATTTTAATGAAAAAACTGGGTT
>Marker649099 Chr3 SNP_site:27531413 Ref_Type:T
CACTCTTTTGATTCCAAAAGCCATTAAACAAAAACAAACCCTACTGTTCATTCCCTCTTTCTTTTTCTAATTCTCTTAATXXXXXXXXXXT
GCCTCCTGATATTTTTCACTATCTTAACCCTTCCTTTTTCTACCTGTGGTTTTTGTATTGTTTGTGTTAGATTTTTGTT
CACTCTTTTGATTCCAAAAGCCATTAAACAAAAACAAACCCTACTGTTCATTCCCTCTTTCTTTTTCTAATTCTCTTAATXXXXXXXXXXT
GCCTCCTGATATTTTTCACTATCTTAACCCTTCCTTTTTTTACCTGTGGTTTTTGTATTGTTTGTGTTAGATTTTTGTT
>Marker649324 Chr3 SNP_site:2708835 Ref_Type:G
ACTTACCTGATCAAACAATTAAATCTCTCCGTTATTACTTAATTCAATTGAAGATTGATCAGATACTTACAAAAATTTTAXXXXXXXXXXT
AATTTTAAACTCTATTGGGAAGCTATCTAATCTTATTCCATGTTTTAGAGTAGAAAACGACGTGAAGATAATAGTGGTT
ACTTACCTGATCAAACAATTAAATCTCTCCGTTATTACTTAATTCAATTGAAGATTGATCAGATACTTACAAAAATTTTAXXXXXXXXXXT
AGTTTTAAACTCTATTGGGAAGCTATCTAATCTTATTCCATGTTTTAGAGTAGAAAACGACGTGAAGATAATAGTGGTT
>Marker649430 Chr3 SNP_site:31461291 Ref_Type:A
GACTTAATTTTTTAAAACAAAATTGATAATGAAATAGTTCCCCATAAGTCTTCGTTTTTCAATTTTAGTAAGCAATGGTTXXXXXXXXXXA
AATTTTATCTCTGATTAAGTCATCAATCTTTATGCAGACTGTAAAATAAATGGTGATAGCATAGTAAATTGTTCATAGT
GACTTAATTTTTTTAAAAAAAATTGATAATGAAATAGTTCCCCATAAGTCTTCGTTTTTCAATTTTAGTAAGCAATGGTTXXXXXXXXXXA
AATTTTATCTCTGATTAAGTCATCAATCTTTATGCAGACTGTAAAATAAATGGTGATAGCATAGTAAATTGTTCATAGT
>Marker649430 Chr3 SNP_site:31461295 Ref_Type:C
GACTTAATTTTTTAAAACAAAATTGATAATGAAATAGTTCCCCATAAGTCTTCGTTTTTCAATTTTAGTAAGCAATGGTTXXXXXXXXXXA
AATTTTATCTCTGATTAAGTCATCAATCTTTATGCAGACTGTAAAATAAATGGTGATAGCATAGTAAATTGTTCATAGT
GACTTAATTTTTTTAAAAAAAATTGATAATGAAATAGTTCCCCATAAGTCTTCGTTTTTCAATTTTAGTAAGCAATGGTTXXXXXXXXXXA
AATTTTATCTCTGATTAAGTCATCAATCTTTATGCAGACTGTAAAATAAATGGTGATAGCATAGTAAATTGTTCATAGT
>Marker649971 Chr3 SNP_site:39614389 Ref_Type:A
CACAGATTTATTATTATCTTTATATTTTACAGAATTATCGTTTTGTGTAATAGTAAATCCCATATGACTTTTCCATTTGGXXXXXXXXXXC
TTGTTCCTTAACTAATTCCTTCTGTTTTCCAAATCAGGATGATTCGTATCTGGATAGCTACATTAGCACTATTGGAGTT
CACAGATTTATTATTATCTTTATATTTTACAGAATTCTCGTTTTGTGTAATAGTAAATCCCATATGACTTTTCCATTTGGXXXXXXXXXXC

Create PDF with GO2PDF for free, if you wish to

TTGTTCCTTAACTAATTCCTTCTGTTTTCCAAATCAGGATGATTCGTATCTGGATAGCTACATTAGCACTATTGGAGTT
>Marker650408 Chr3 SNP_site:10733085 Ref_Type:G
ACTGATGACAAAAATGAACAAAATAAATGTTTCATATCAAGAAAAATGAATTCATAATTAAGAAAGATAAATGTCTATTCXXXXXXXXXXG
GGTAGTCATTAAAAGCCCAAGAAAGTTCTTCACTTCACAAATTTCAAATTTCCAGACTGCAAATGTGATGCTGAAAGTG
ACTGATGACAAAAATGAACAAAATAAATGTTTCATATCAAGAAAAATGAATTCATAATTAAGAAAGATAAATGTCTGTTCXXXXXXXXXXG
GGTAGTCATTAAAAGCCCAAGAAAGTTCTTCACTTCACAAATTTCAAATTTCCAGACTGCAAATGTGATGCTGAAAGTG
>Marker65056 Chr4 SNP_site:7359259 Ref_Type:G
ACTAAATGAAGACTTAGAAGCAACTATGGATATCAAAATTGCTAAAATATCTCTTGCAATTCGACTAAAAACCTTATATCXXXXXXXXXXT
CCATATTTTCAAAATGGATACGAAAAACGTCTGAAGCATAAGAAGAAGTTCTAGATGTAGTGGAAGTAGTTATAGATGT
ACTAAATGAAGACTTAGAAGCAACTATGGATATCAGAATTGCTAAAATATCTCTTGCAATTCGACTAAAAACCTTATATCXXXXXXXXXXT
CCATATTTTCAAAATGGATACGAAAAACGTCTGAAGCATAAGAAGAAGTTCTAGATGTAGCGGAAGTAGTTATAGATGT
>Marker65056 Chr4 SNP_site:7359478 Ref_Type:C
ACTAAATGAAGACTTAGAAGCAACTATGGATATCAAAATTGCTAAAATATCTCTTGCAATTCGACTAAAAACCTTATATCXXXXXXXXXXT
CCATATTTTCAAAATGGATACGAAAAACGTCTGAAGCATAAGAAGAAGTTCTAGATGTAGTGGAAGTAGTTATAGATGT
ACTAAATGAAGACTTAGAAGCAACTATGGATATCAGAATTGCTAAAATATCTCTTGCAATTCGACTAAAAACCTTATATCXXXXXXXXXXT
CCATATTTTCAAAATGGATACGAAAAACGTCTGAAGCATAAGAAGAAGTTCTAGATGTAGCGGAAGTAGTTATAGATGT
>Marker650951 Chr3 SNP_site:2969337 Ref_Type:T
ACTTATTGTTCAATACCCGAAATCAACTCTTAGTGGAGAAATTCATTTACTTACCCTAAATGGAAAGAAGTAGATTTCATXXXXXXXXXXT
TAGAATGAAGCTCATATAACTCATTGAGGTTTGAAGACCAGTTATATATGGTCATCCTATGAAATGTTAATATTTAGTC
ACTTATTGTTCAATACCCGAAATCAACTCTTAGTGGATAAATTCATTTACTTATCCTAAATGGAAAGAAGTAGATTTCATXXXXXXXXXXT
TAGAATGAAGTTCATATAACTCATTGAGGTTTGAAGACCAGTTATATATGGTCATCCTATGAAATGTTAATATTTAGTC
>Marker650951 Chr3 SNP_site:2969353 Ref_Type:T
ACTTATTGTTCAATACCCGAAATCAACTCTTAGTGGAGAAATTCATTTACTTACCCTAAATGGAAAGAAGTAGATTTCATXXXXXXXXXXT
TAGAATGAAGCTCATATAACTCATTGAGGTTTGAAGACCAGTTATATATGGTCATCCTATGAAATGTTAATATTTAGTC
ACTTATTGTTCAATACCCGAAATCAACTCTTAGTGGATAAATTCATTTACTTATCCTAAATGGAAAGAAGTAGATTTCATXXXXXXXXXXT
TAGAATGAAGTTCATATAACTCATTGAGGTTTGAAGACCAGTTATATATGGTCATCCTATGAAATGTTAATATTTAGTC
>Marker650951 Chr3 SNP_site:2969509 Ref_Type:T
ACTTATTGTTCAATACCCGAAATCAACTCTTAGTGGAGAAATTCATTTACTTACCCTAAATGGAAAGAAGTAGATTTCATXXXXXXXXXXT
TAGAATGAAGCTCATATAACTCATTGAGGTTTGAAGACCAGTTATATATGGTCATCCTATGAAATGTTAATATTTAGTC
ACTTATTGTTCAATACCCGAAATCAACTCTTAGTGGATAAATTCATTTACTTATCCTAAATGGAAAGAAGTAGATTTCATXXXXXXXXXXT
TAGAATGAAGTTCATATAACTCATTGAGGTTTGAAGACCAGTTATATATGGTCATCCTATGAAATGTTAATATTTAGTC
>Marker651103 Chr3 SNP_site:24212510 Ref_Type:A
TACAAACACCATTTTTACTTTTATGGATTATCGGCAATTGAACAAAGTGACAATCAGGAACAAATATCTTTTGCCGCAAAXXXXXXXXXXC
AACCTTTAGAATGCATTATGGGCATTATGAATTCTTGGTAATGTCTTTTGGATTGACGAATGCCCCAACTGCTTTTGTG
TACAAACACCATTTTTACTTTTATGGATTATCGGCAATTGAACAAAGTGACAATCAGGAACAAATATCTTTTGCCGCAAAXXXXXXXXXXC
AACCTTTAGAATGCATTATGGGCATTGTGAATTCTTGGTAATGTCTTTTGGATTGACGAATGCCCCAACTGCTTTCGTG
>Marker651103 Chr3 SNP_site:24212559 Ref_Type:T
TACAAACACCATTTTTACTTTTATGGATTATCGGCAATTGAACAAAGTGACAATCAGGAACAAATATCTTTTGCCGCAAAXXXXXXXXXXC
AACCTTTAGAATGCATTATGGGCATTATGAATTCTTGGTAATGTCTTTTGGATTGACGAATGCCCCAACTGCTTTTGTG
TACAAACACCATTTTTACTTTTATGGATTATCGGCAATTGAACAAAGTGACAATCAGGAACAAATATCTTTTGCCGCAAAXXXXXXXXXXC
AACCTTTAGAATGCATTATGGGCATTGTGAATTCTTGGTAATGTCTTTTGGATTGACGAATGCCCCAACTGCTTTCGTG
>Marker651738 Chr3 SNP_site:12522760 Ref_Type:A
ACCCCTTTAGTCCAGTGCCGGAGCTAGAACTGTCAGGTCGTCTAATTGCGGCTCGGACGCCGCTGTTGGTATTATCGGGGXXXXXXXXXXG
CAAATTCCTGTATATTGAAAGAATTTGTAGCAACATAGCGTGAAGAGGTGGTTGCTTGGTTGTTTGTGGGGGATTTGTA

Create PDF with GO2PDF for free, if you wish to

ACCCCTTTAGTCCAGTGCCGGAGCTAGAACTGTCAGGTCGTCTAATTGCGGCTCGGACGCCGCTGTTGGTATTATCGGGGXXXXXXXXXXG
CAAGTTCCTGTATATTGAAAGAATTTGTAGCAACATAGCGTGAAGAGGTGGTTGCTTGGTTGTTTGTGGGGGATTTGTA
>Marker6518 Chr4 SNP_site:7403320 Ref_Type:C
AACAAATGATGGATCATTAAATTTCTTTGTTTGCACTTGTTATGAATTACCAAGTATATAGTTTGAAAATTTGACGAAGTXXXXXXXXXXT
TTAAAAGCATATATATGCATCAACTAATTCAATGCTAACGCATTTGTTTTTCATTTTCATTTTCTTCGGTTTTAAGTT
AACAAATGATGGATCATTAAATTTCTTTGTTTGCACTTGTTATGAATTACCAAGTATATAGTTTGAAAATTTGAGGAAGTXXXXXXXXXXT
TTAAAAGCATATATATGCATCAACTAATTCAATGCTAACGCATTTGTTTTTCATTTTCATTTTCTTCGGTTTTAAGTT
>Marker652039 Chr3 SNP_site:11900068 Ref_Type:T
TACAAGTATTTGACTTTTGACAGCTATATAATGTATAGTATGAAATTCATATGATGAAATGCGGTATATACACATTGCCAXXXXXXXXXXG
AAACTTTATTCTAGATGACAAAGCTGCCATGTCATGTCAGTAAGTTTGGTGGAGTTGGAATGGGAGAAATATGCAGGTT
TACAAGTATTTGACTTTTGACAGCTATATAATGTATAGTATGAAATTCATATGATGAAATGCGGTATATACACATTGCCAXXXXXXXXXXG
AAATTTTATTCTAGATGACAAAGCTGCCATGTCATGTCAGTAAGTTTGGTGGAGTTGGAATGGGAGAAATATGCAGGTT
>Marker652066 Chr3 SNP_site:36438506 Ref_Type:A
CACCTCTAGTTTCAAGGTCCCTAAACTGAAGAACGAAATCAATTTGTAGATTTGCTAGTGTGTTACTTATAGCTGTGTGTXXXXXXXXXXT
GTCTGTTTTAGGAAACTTTTAACATAAAATAGTAGTATTGTGGGCATATTGATAATATGCCGTCAGCCTCTATAGAGTA
CACCTCTAGTTTCAAGGTCCCTAAACTGAAGAACGAAATCAATTTGTAGATTTGCTAGTGTGTTACTTATAGCTGTGTGTXXXXXXXXXXT
GTCTGTTTTAGGAAACTTTTAACATAAAATAGTAGTGTTGTGGGCATATTGATAATATGCCGTCAGCCTCTATAGAGTA
>Marker652562 Chr3 SNP_site:5686775 Ref_Type:G
ACTTGGGTTACGGTGGTCTTCAGCTAGTCCAAAAAATGTATCAAAAGTAGTTTTGTAATTATTTTCAAAATTAACCGATATXXXXXXXXXXT
TACCAAAATCTTGTTAAAAAAAATAAATTGGTTAAGAATTTAAGTTTTGTTTTAATTTGAGTTTTTTAAAAATAAATGT
ACTTGGGTTACGGTTGTCTTCAGCTAGTCCAAAAAATGTATCAAAAGTAGTTTTGTAATTATTTTCAAAATTAACCGTTATXXXXXXXXXXT
TACCAAAATCTTGTTAAAAAAAATAAATTGGTTACGAATTTAAGTTTTGTTTTAATTTGAGTTTTTTAAAAATAAATGT
>Marker652562 Chr3 SNP_site:5686837 Ref_Type:A
ACTTGGGTTACGGTGGTCTTCAGCTAGTCCAAAAAATGTATCAAAAGTAGTTTTGTAATTATTTTCAAAATTAACCGATATXXXXXXXXXXT
TACCAAAATCTTGTTAAAAAAAATAAATTGGTTAAGAATTTAAGTTTTGTTTTAATTTGAGTTTTTTAAAAATAAATGT
ACTTGGGTTACGGTTGTCTTCAGCTAGTCCAAAAAATGTATCAAAAGTAGTTTTGTAATTATTTTCAAAATTAACCGTTATXXXXXXXXXXT
TACCAAAATCTTGTTAAAAAAAATAAATTGGTTACGAATTTAAGTTTTGTTTTAATTTGAGTTTTTTAAAAATAAATGT
>Marker652562 Chr3 SNP_site:5687006 Ref_Type:A
ACTTGGGTTACGGTGGTCTTCAGCTAGTCCAAAAAATGTATCAAAAGTAGTTTTGTAATTATTTTCAAAATTAACCGATATXXXXXXXXXXT
TACCAAAATCTTGTTAAAAAAAATAAATTGGTTAAGAATTTAAGTTTTGTTTTAATTTGAGTTTTTTAAAAATAAATGT
ACTTGGGTTACGGTTGTCTTCAGCTAGTCCAAAAAATGTATCAAAAGTAGTTTTGTAATTATTTTCAAAATTAACCGTTATXXXXXXXXXXT
TACCAAAATCTTGTTAAAAAAAATAAATTGGTTACGAATTTAAGTTTTGTTTTAATTTGAGTTTTTTAAAAATAAATGT
>Marker653248 Chr3 SNP_site:33421173 Ref_Type:C
TACAAGATTCAATTTCCCCAATAAAAAACTTAGCAACAAAAAAGAAGTATATATACACACATACATATACATATACATATXXXXXXXXXXA
TAGATTAAAAACAAAATTGTTACCATACAAGTCTTTAAGTTTCAAAACTGTGAAATAAAATGTAAATTATGGAAATGTT
TACAAGATTCAATTTCCCCAATAAAAAACTTAGCAACAAACAAGAAGTATATATATACACATACATATACATATACATATXXXXXXXXXXA
TAGATTAAAAACAAAATTGTTACCATACAAGCCTTTAAGTTTCAAAACTGTGAAATAAAATGTAAATTATGGAAATGTT
>Marker653248 Chr3 SNP_site:33421188 Ref_Type:T
TACAAGATTCAATTTCCCCAATAAAAAACTTAGCAACAAAAAAGAAGTATATATACACACATACATATACATATACATATXXXXXXXXXXA
TAGATTAAAAACAAAATTGTTACCATACAAGTCTTTAAGTTTCAAAACTGTGAAATAAAATGTAAATTATGGAAATGTT
TACAAGATTCAATTTCCCCAATAAAAAACTTAGCAACAAACAAGAAGTATATATATACACATACATATACATATACATATXXXXXXXXXXA
TAGATTAAAAACAAAATTGTTACCATACAAGCCTTTAAGTTTCAAAACTGTGAAATAAAATGTAAATTATGGAAATGTT
>Marker653248 Chr3 SNP_site:33421362 Ref_Type:C
TACAAGATTCAATTTCCCCAATAAAAAACTTAGCAACAAAAAAGAAGTATATATACACACATACATATACATATACATATXXXXXXXXXXA

Create PDF with GO2PDF for free, if you wish to

TAGATTAAAAACAAAATTGTTACCATACAAGTCTTTAAGTTTCAAAACTGTGAAATAAAATGTAAATTATGGAAATGTT
TACAAGATTCAATTTCCCCAATAAAAAACTTAGCAACAAACAAGAAGTATATATATACACATACATATACATATACATATXXXXXXXXXXA
TAGATTAAAAACAAAATTGTTACCATACAAGCCTTTAAGTTTCAAAACTGTGAAATAAAATGTAAATTATGGAAATGTT
>Marker653522 Chr3 SNP_site:32351416 Ref_Type:T
AACTGACTTCCCAGCAGATGCATGGAGATCTAAACTTTCATAAGCACCTTTGAAAGCTACAGCAGGCTAATGGTGGCAAAXXXXXXXXXXC
GTCAAGAGGGATGTAATGCAATAAATTGACTACAAAACAAGGGTAAAGAAAAAACCAGCTCATACTTTAAAGGGTAGTC
AACTGACTTCCCAGCAGATGCATGGAGATCTAAACTTTCATAAGCACCTTTGAAAGCTACAGCAGGCTAATGGTGGCAAAXXXXXXXXXXT
GTCAAGAGGGATGTAATGCAATAAATTGACTACGAAACAAGGGTAAAGAAAAAACCAGCTCATACTTTAAAGGGTAGTC
>Marker653522 Chr3 SNP_site:32351450 Ref_Type:G
AACTGACTTCCCAGCAGATGCATGGAGATCTAAACTTTCATAAGCACCTTTGAAAGCTACAGCAGGCTAATGGTGGCAAAXXXXXXXXXXC
GTCAAGAGGGATGTAATGCAATAAATTGACTACAAAACAAGGGTAAAGAAAAAACCAGCTCATACTTTAAAGGGTAGTC
AACTGACTTCCCAGCAGATGCATGGAGATCTAAACTTTCATAAGCACCTTTGAAAGCTACAGCAGGCTAATGGTGGCAAAXXXXXXXXXXT
GTCAAGAGGGATGTAATGCAATAAATTGACTACGAAACAAGGGTAAAGAAAAAACCAGCTCATACTTTAAAGGGTAGTC
>Marker653752 Chr3 SNP_site:15934231 Ref_Type:C
CACTTTCCTGTCATCTAATTGGACAAGCCTATTGTAATTCCATATAGTTTATCCCTTTTTATAAATTCTATAAATCAACAXXXXXXXXXXA
GGTTATTGTGAATCAAATTGCTTTATATTGCTGAAGCAGTTACATTGAGCTAGATTAATTATTAATGCATCTAAATGGT
CACTTTCCTGTCATCTAATTGGACAAGCCTATTGTAATTCCATATAGTTTATCCCTTTTTATAAATTCTATACATCAACAXXXXXXXXXXA
GGTTATTGTGAATCAAATTGCTTTATATTGCTGAAGCAGTTACATTGAGCTAGATTAATTATTAATGCATCTAAATGGT
>Marker653766 Chr3 SNP_site:12388360 Ref_Type:C
TACCGGGGACAAACATAAACTAACAGAGAACTACAAGTGATGGAGGAACATGTATCATCCCAAAAGGGAGAGACATGAATXXXXXXXXXXA
TCCACTTTGTTCCGAGCAAGGAATCATTTATTGTTTGACAAGGGGCAGGATTGAACTGGGACAGAGCGAGGAACGGGTA
TACCGGGGACAAACATAAACTAACAGAGAACTACAAGTGATGGAGGAACATGTATCATCTCAAAAGGGAGAGACATGAATXXXXXXXXXXA
TCCACTTTGTTCCGAGCAAGGAATCATTTATTGTTTGACAAGGGGCAGGATTGAACTGGGACAGAGCGAGGAACGGGTA
>Marker653778 Chr3 SNP_site:21694276 Ref_Type:A
ACAGACCTCAAGTTAAAGGCAAAAGAAAAAGAAAAAAAAAACCACAAACAACGACAAAAACTTGGATCTTTTTGTGTCCAXXXXXXXXXXT
TACAAATCCATATCAAAGAAAAAATATAAAAATAACATGACTACTAAAAAAAGAAAAGAAGCTATATGCAACAAATGTA
ACAGACCTCAAGTTAAAGGCAAAAGAAAAAGAAAAAAAAAACCACAAACAACGACAAAAACTTGGATCTTTTTGTGTCCAXXXXXXXXXXT
TACAAATCCATATCAAAGAAAAAATATAAAAATAACATGACTACTAAAAAATGAAAAGAAGCTATATGCAACAAATGTA
>Marker65404 Chr4 SNP_site:12716744 Ref_Type:A
ACTCTCGGAGTCCCTCTCCCCTTGGTCTTGTCCCGAGGCCTTGCCTGAACATTTTCCCCCCGAAAAAAAATTCAAAATATXXXXXXXXXXC
TCTCCGTTTTCTTATCGCCTCTGCCACAATATACTGAAAAAAAAGTAAAGACAGAACAGAAATTAAGCAATGAAAAGTG
ACTCTCGGAGTCCCTCTCCCCTTGGTCTTGTCCCGAGGCCTTGCCTGAACATTTTCCCCCCGAAAAAAAATTCAAAATATXXXXXXXXXXC
TCTCCGTTTTCTTATCGCCTCTGCCACAATATACTGAAAAAAAAGTAAAGACAGAACAGAAATTAAGCATTGAAAAGTG
>Marker654184 Chr3 SNP_site:21720042 Ref_Type:A
ACTTAAACACACAAAGAGGAACCTCAGAAATAAAACGAGTAGAGGGGTAGGATTCCTTAAGAAAATCTAAGGGAGTTTGAXXXXXXXXXXC
GGGCTACTTCCAGAAGGTGACAGTTTTTTCGCTTGGCCACTTCATTTTGTTAAGGAGTGTAGGTGCATGAGTTTTGGTG
ACTTAAACACACGAAGAAGAACCTCAGAAATAAAACGAGTAGAGGGGTAGGATTCCTTAAGACAATCTAAGGGAGTTTGAXXXXXXXXXXC
GGGCTACTTCCAGAAGGTGACAGTTTTTTCGCTTGGCCACTTCATTTTGTTAAGGAGTGTAGGTGCATGAGTTTTGGTG
>Marker654184 Chr3 SNP_site:21720047 Ref_Type:G
ACTTAAACACACAAAGAGGAACCTCAGAAATAAAACGAGTAGAGGGGTAGGATTCCTTAAGAAAATCTAAGGGAGTTTGAXXXXXXXXXXC
GGGCTACTTCCAGAAGGTGACAGTTTTTTCGCTTGGCCACTTCATTTTGTTAAGGAGTGTAGGTGCATGAGTTTTGGTG
ACTTAAACACACGAAGAAGAACCTCAGAAATAAAACGAGTAGAGGGGTAGGATTCCTTAAGACAATCTAAGGGAGTTTGAXXXXXXXXXXC
GGGCTACTTCCAGAAGGTGACAGTTTTTTCGCTTGGCCACTTCATTTTGTTAAGGAGTGTAGGTGCATGAGTTTTGGTG
>Marker654184 Chr3 SNP_site:21720092 Ref_Type:A

Create PDF with GO2PDF for free, if you wish to

ACTTAAACACACAAAGAGGAACCTCAGAAATAAAACGAGTAGAGGGGTAGGATTCCTTAAGAAAATCTAAGGGAGTTTGAXXXXXXXXXXXC
GGGCTACTTCCAGAAGGTGACAGTTTTTTCGCTTGGCCACTTCATTTTGTTAAGGAGTGTAGGTGCATGAGTTTTGGTG
ACTTAAACACACGAAGAAGAACCTCAGAAATAAAACGAGTAGAGGGGTAGGATTCCTTAAGACAATCTAAGGGAGTTTGAXXXXXXXXXXXC
GGGCTACTTCCAGAAGGTGACAGTTTTTTCGCTTGGCCACTTCATTTTGTTAAGGAGTGTAGGTGCATGAGTTTTGGTG
>Marker655093 Chr3 SNP_site:28954097 Ref_Type:A
CACTTTATTTTCTTTCATATTTTTAGGAACAAACATTTGCCTTTCACGAAAAGCATGTATATAAACTCATTTTATTTTTAXXXXXXXXXXXA
TGGGAGGGGTTCTGCCATATCCTTTCCCGTCGGCGCAATACATGCAATGCAAGGACACTATTTTCCTTCACTGCTAAGT
CACTTTATTTTCTTTCATATTTTTAGGAACAAACATTTGCCTTTCACGAAAAGCATGTATATAAACTCATTTTATTTTTAXXXXXXXXXXXA
TGGGAGGGGTTCTGCCATATCCTTTCCCGTCGGCGCAATACATGCAGTGCAAGGACACTATTTTCCTTCATTGCTAAGT
>Marker655093 Chr3 SNP_site:28954121 Ref_Type:C
CACTTTATTTTCTTTCATATTTTTAGGAACAAACATTTGCCTTTCACGAAAAGCATGTATATAAACTCATTTTATTTTTAXXXXXXXXXXXA
TGGGAGGGGTTCTGCCATATCCTTTCCCGTCGGCGCAATACATGCAATGCAAGGACACTATTTTCCTTCACTGCTAAGT
CACTTTATTTTCTTTCATATTTTTAGGAACAAACATTTGCCTTTCACGAAAAGCATGTATATAAACTCATTTTATTTTTAXXXXXXXXXXXA
TGGGAGGGGTTCTGCCATATCCTTTCCCGTCGGCGCAATACATGCAGTGCAAGGACACTATTTTCCTTCATTGCTAAGT
>Marker656252 Chr3 SNP_site:21703454 Ref_Type:T
TACTTACTACTTTTCCAGAAGCATTTCTTCAAATTAAGCAGTTCTGATAAAACAAAAATTTAACGTAAAACAGTCATTTCXXXXXXXXXXXT
CATTTCCCAGCCAAAAACATATGAACCAAGCCATACCTTTAGTTTATGGGTTATCATAGTGGGATTTTGGAAAAGAGTT
TACTTACTACTTTTCCAGAAGCATTTCTTCAAATTAAGCAGTTCTGATAATACAAAAATTTAACGTAAAACAGTCATTTCXXXXXXXXXXXT
CATTTCCCAGCCAAAAACATATGAACCAAGCCATACCTTTAGTTTATGGGTTATCATAGTGGGATTTTGGAAAAGAGTT
>Marker657231 Chr3 SNP_site:34407446 Ref_Type:G
ACTATGAAACCTTCAGGTTGTTCCGTATAAATCTCTTCTTCTAATTCACCATTTAGAAAAGCAGTTTTGACTTCCATTTGXXXXXXXXXXXT
TATCTACTGATCCATCAGGTTTGAGTTTGTTCCTTAAAACCCATTTACATTCTATTGCTTTGCATTCAGGGGGTAAGTT
ACTATGAAACCTTCAGGTTGTTCCGTATAAATCTCTTCTTCTAATTCACCATTTAGAAAAGCAGTTTTGACTTCCATTTGXXXXXXXXXXXT
TATCTACTGGTCCATCAGGTTTGAGTTTGTTCCTTAAAACCCATTTACATTCTATTGCTTTGCATTCAGGGGGTAAGTT
>Marker657322 Chr3 SNP_site:11829897 Ref_Type:T
GACTCCGATTCCTCTCAGAATTATCAAACACAACGGAGACAGTTGCTTTGGTAATACCCGCTTGCCCCTGCTTATACACTXXXXXXXXXXXC
AGTCCTCGTGGCGTAGGATTTGAACCCCTCCAGACAGATTTCCTTGATGTGCATCGTTCCCAAAAATGCTTGGAAAGGT
GACTCCTATTCCTCTCAGAATTATCAAACACAACGGAGACAGTTGCTTTGGTAATACCCGCTTGCCCCTGCTTATACACTXXXXXXXXXXXC
AGTCCTCGTGGCGTAGGATTTGAACCCCTCCAGACAGATTTCCTTGATGTGCATCGTTCCCAAAAATGCTTGGAAAGGT
>Marker657555 Chr3 SNP_site:38586304 Ref_Type:T
ACTCACCTTCCTTCCAGATCTTGAATTATTTCATGTAAGTGAAGCAGTGGCATCTAGATAATTCGTCACGAACCATTTGTXXXXXXXXXXXC
ATCTTCCACGATTTGTTCGATGAAGTCGCTTGCCTAAAAGAACAGTTCCATTAGGTGGTAGTTTTTAAGGTGCTAAGGT
ACTCACCTTCCTTCTAGATCTTGAATTATTTCATGTAAGTGAAGCAGTGGCATCTAGATAATTCGTCACGAACCATTTGTXXXXXXXXXXXC
ATCTTCCACGATTTGTTCGATGAAGTCGCTTGCCTAAAAGAACAGTTCCATTAGGTGGTAGTTTTTAAGGTGCTAAGGT
>Marker657659 Chr3 SNP_site:27079952 Ref_Type:A
TACCATGTAAATTTTACAACATATGAAAAAAAAATTAGGTGTTCAACTTATGTTTCAATATATGCATTATTTTATGTTATXXXXXXXXXXXA
ATTGCCCAAATCAATAATCTTCCTCATGATTCTTCTTTGGTTGATTTATAACATTATTCACTATTAAATGCAAATAGTT
TACCATGTAAATTTTACAACATATGAAAAAAAAATTAGGTGTTCAGCTTATGTTTCAATATATGCCTTATTTTATGTTATXXXXXXXXXXXA
ATTGCCCAAATCAATAATCTTCCTCATGATTCTTCTTAGGTTGATTTATAACATTATTCACTATTAAATGCAAATAGTT
>Marker657659 Chr3 SNP_site:27079972 Ref_Type:A
TACCATGTAAATTTTACAACATATGAAAAAAAAATTAGGTGTTCAACTTATGTTTCAATATATGCATTATTTTATGTTATXXXXXXXXXXXA
ATTGCCCAAATCAATAATCTTCCTCATGATTCTTCTTTGGTTGATTTATAACATTATTCACTATTAAATGCAAATAGTT
TACCATGTAAATTTTACAACATATGAAAAAAAAATTAGGTGTTCAGCTTATGTTTCAATATATGCCTTATTTTATGTTATXXXXXXXXXXXA
ATTGCCCAAATCAATAATCTTCCTCATGATTCTTCTTAGGTTGATTTATAACATTATTCACTATTAAATGCAAATAGTT

Create PDF with GO2PDF for free, if you wish to

>Marker657659 Chr3 SNP_site:27080172 Ref_Type:A
TACCATGTAAATTTTACAACATATGAAAAAAAAATTAGGTGTTCAACTTATGTTTCAATATATGCATTATTTTATGTTATXXXXXXXXXXXA
ATTGCCCAAATCAATAATCTTCCTCATGATTCTTCTTTGGTTGATTTATAACATTATTCACTATTAAATGCAAATAGTT
TACCATGTAAATTTTACAACATATGAAAAAAAAATTAGGTGTTCAGCTTATGTTTCAATATATGCCTTATTTTATGTTATXXXXXXXXXXXA
ATTGCCCAAATCAATAATCTTCCTCATGATTCTTCTTAGGTTGATTTATAACATTATTCACTATTAAATGCAAATAGTT
>Marker659576 Chr3 SNP_site:39287012 Ref_Type:G
AACTATTTTAGCTAAGCTCACTCTAACAACATAGTTAATTAATAATTAAGTATTTGTTTATATAATTTCAAAAAAACATTXXXXXXXXXXXA
ACAAAATTATAAATACTAAAAAAGTATTAGACTTATATAGTTGATAGATTTATACCGATAGATTGCCAACGACATAGTA
AACTATTTTGGCTAAGCTCACTCTAACAACATAGTTAATTAATAATTAAGTATTTGTTTATATAATTTCAAAAAAACATTXXXXXXXXXXXA
ACAAAATTATAAATACTAAAAAAGTATTAGACTTATATAGTTGATAGATTTATACTGATAGATTGCCAACGACATAGTA
>Marker659576 Chr3 SNP_site:39287261 Ref_Type:T
AACTATTTTAGCTAAGCTCACTCTAACAACATAGTTAATTAATAATTAAGTATTTGTTTATATAATTTCAAAAAAACATTXXXXXXXXXXXA
ACAAAATTATAAATACTAAAAAAGTATTAGACTTATATAGTTGATAGATTTATACCGATAGATTGCCAACGACATAGTA
AACTATTTTGGCTAAGCTCACTCTAACAACATAGTTAATTAATAATTAAGTATTTGTTTATATAATTTCAAAAAAACATTXXXXXXXXXXXA
ACAAAATTATAAATACTAAAAAAGTATTAGACTTATATAGTTGATAGATTTATACTGATAGATTGCCAACGACATAGTA
>Marker659904 Chr3 SNP_site:34561489 Ref_Type:T
TACAACAATCTTCATTTTTTTTTTACCGTTTATTGTGTTTTTCCTATTTTCTCACAGTTTGTTATGTTTTCATGAATATTTXXXXXXXXXXXA
AAATGTGGTAGTGCATTATGATGTAAGCAGAAGATTATATTTTGTATGCTTGTGGGGACGAGTCATTAATCATAAATGT
TACAACAATCTTCATTTTTTTTTTACCGTTTATTGTGTTTTTCCTATTTTCTTACAGTTTGTTATGTTTTCATGAATATTTXXXXXXXXXXXA
AAATGTGGTAGTGCATTCTGATATAAGCAGAAGATTATATTTTGTATGCTTGTGGGGACGAGTCATTAATCATAAATGT
>Marker659904 Chr3 SNP_site:34561690 Ref_Type:C
TACAACAATCTTCATTTTTTTTTTACCGTTTATTGTGTTTTTCCTATTTTCTCACAGTTTGTTATGTTTTCATGAATATTTXXXXXXXXXXXA
AAATGTGGTAGTGCATTATGATGTAAGCAGAAGATTATATTTTGTATGCTTGTGGGGACGAGTCATTAATCATAAATGT
TACAACAATCTTCATTTTTTTTTTACCGTTTATTGTGTTTTTCCTATTTTCTTACAGTTTGTTATGTTTTCATGAATATTTXXXXXXXXXXXA
AAATGTGGTAGTGCATTCTGATATAAGCAGAAGATTATATTTTGTATGCTTGTGGGGACGAGTCATTAATCATAAATGT
>Marker659904 Chr3 SNP_site:34561695 Ref_Type:A
TACAACAATCTTCATTTTTTTTTTACCGTTTATTGTGTTTTTCCTATTTTCTCACAGTTTGTTATGTTTTCATGAATATTTXXXXXXXXXXXA
AAATGTGGTAGTGCATTATGATGTAAGCAGAAGATTATATTTTGTATGCTTGTGGGGACGAGTCATTAATCATAAATGT
TACAACAATCTTCATTTTTTTTTTACCGTTTATTGTGTTTTTCCTATTTTCTTACAGTTTGTTATGTTTTCATGAATATTTXXXXXXXXXXXA
AAATGTGGTAGTGCATTCTGATATAAGCAGAAGATTATATTTTGTATGCTTGTGGGGACGAGTCATTAATCATAAATGT
>Marker660916 Chr3 SNP_site:25057615 Ref_Type:A
CACCTAGAATAAGGGGAATTCTTAAACGTGATTGAGGTAGTTTCGTTTAGCGCTTCTACCACTGTTGAACCAAACTAGCCXXXXXXXXXXXA
ATTGATTGGGACTTTCTGGATGCAGTTCTTCAAGCTAAGGATTTTGGTATTCTCCGGCATTAGAAGTTGTATGTCTGGT
CACCTAGAATAAGGGGAATTCTTAAACGTGATTGAGGTAGTTTCGTTTAGCGCTTCTACCGCTGTTGAACCAAACTAGCCXXXXXXXXXXXA
ATTGATTGGGACTTTCTGGATGCAGTTCTTCAAGCTAAGGATTTTGGTATTCTCCGGCATTAGAAGTTGTATGTCTGGT
>Marker6611 Chr4 SNP_site:18579322 Ref_Type:C
ACTTGTCGGGCATTCAGGTTAAGTATTCTAAATCTAGTTTTGATGATTTACATCATGGTTCATTTAAGATTTTAATTTTAXXXXXXXXXXXA
TATGCTCTTTTTGCTCTCCAATTATTTTGTTTGTTTGCCTCAAGTATTTTTTTTCCTTGTATAATTGGATTTGTATGTA
ACTTGTCGGGCATTCAGGTTAAGTATTCTAAATCTAGTTTTGATGATTTACATCATGGTTCATTTAAGATTTTAATTTTAXXXXXXXXXXXA
TATGCTCTTTTTGTTCTCCAATTATTTTGTTTGTTTGCCTCAAGTATTTTTTTTCCTTGTATAATTGGATTTGTATGTA
>Marker661197 Chr3 SNP_site:24994742 Ref_Type:C
TACTCGTAGATGCGAAGTGGGCTCAATATGCACTGTTTGAATAAAAAATTATGAAGAATTTTGTTCATAGGCTTGCTATCXXXXXXXXXXXA
TTTTTTTTGGAAATTTTCCTCACTACAATTTTGATTGCTCAATAAAAGTCTGGAGGTTTTTTGGCTTAACATTTTCAGT
TACTTGTAGATGCGAAGTGGGCTCAATATGCACTGTTTGAATAAAAAATTATGAAGAATTTTGTTCATAGGCTTGCTATCXXXXXXXXXXXA

Create PDF with GO2PDF for free, if you wish to

TTTTTTTTGGAAATTTTCCTCACTACAATTTTGATTGCTCAATAAAAGTCTGGAGGTTTTTTGGCTTAACATTTTCAGT
>Marker661381 Chr3 SNP_site:38620379 Ref_Type:G
AACAAGTTGAGAACTAGATCTTACGATATATTCTTTCTTCTTTCAGGCTTATGCATCGAGGGGATATGTAGCTATTGCCAXXXXXXXXXXXA
CCTGATGTCATAATCTGGTGCAGGCTCTTATTTCTGCATGGAAAAAAGGTGATACCATGCCATTCATATTTGACACGGT
AACAAGTTGAGAACTAGATCTTACGATATATTCTTTCTTCTTTCAGGCTTATGCATCTAGGGGATATGTAGCTATTGCCAXXXXXXXXXXXA
CCTGATGTCATAATCTGGTGCAGGCTCTTATTTCTGCATGGAAAAAAGGTGATACCATGCCATTCATATTTGACACGGT
>Marker661591 Chr3 SNP_site:14023423 Ref_Type:G
AACCTCCTTGGTTTGAGTGAAATTTCCTGTGAAAGGGCATGAAAGTTGAAACCATAAATTTTGAAGAAAAATGGTCACTAXXXXXXXXXXXT
TTCCACAAAAGTTCAAAATCTAGCAGTTATTTGTTGTGTCCCAAATTCCAGCTATGGAAGCATTCTTTCTTTCAGCAGT
AACCTCCTTGGTTTGAGTGAAATTTCCTGTGAAAGGGCATGAAAGTTGAAACCATAAATTTTGAAGAAAAATGGTCACTGXXXXXXXXXXXT
TTCCACAAAAGTTCAAAATCTAGCAGTTATTTGTTGTGTCCCAAATTCCAGCTATGGAAGCATTCTTTCTTTCAGCAGT
>Marker662447 Chr3 SNP_site:35075211 Ref_Type:C
GACCCGGACTACTCGCCCGACGACGTCTGAAGCTAGTGGAGTAGTTTTTCCATGGCGACGGCGGTGGTGGCAGCTCTCTCXXXXXXXXXXXA
CTAAGTTACGTGAAAATCCGAGAACCGAGAGAAAAACCATGATCTGACTTTCGTATTATTTTCTCACATTAACAAAGGT
GACCCGGACTACTCGCCCGACGACGTCTGAAGCTAGTGGAGTAGTTTTTCCATGGCGACGGCGGTGGTGGCAGCTCTCTCXXXXXXXXXXXA
TTGAGTTACGTGAAAATCCGAGGACCGAGAGAAAAACCATGATCTGACTTTCGTATTATTTTCTCACATTAACAAAGGT
>Marker662447 Chr3 SNP_site:35075213 Ref_Type:A
GACCCGGACTACTCGCCCGACGACGTCTGAAGCTAGTGGAGTAGTTTTTCCATGGCGACGGCGGTGGTGGCAGCTCTCTCXXXXXXXXXXXA
CTAAGTTACGTGAAAATCCGAGAACCGAGAGAAAAACCATGATCTGACTTTCGTATTATTTTCTCACATTAACAAAGGT
GACCCGGACTACTCGCCCGACGACGTCTGAAGCTAGTGGAGTAGTTTTTCCATGGCGACGGCGGTGGTGGCAGCTCTCTCXXXXXXXXXXXA
TTGAGTTACGTGAAAATCCGAGGACCGAGAGAAAAACCATGATCTGACTTTCGTATTATTTTCTCACATTAACAAAGGT
>Marker662447 Chr3 SNP_site:35075233 Ref_Type:A
GACCCGGACTACTCGCCCGACGACGTCTGAAGCTAGTGGAGTAGTTTTTCCATGGCGACGGCGGTGGTGGCAGCTCTCTCXXXXXXXXXXXA
CTAAGTTACGTGAAAATCCGAGAACCGAGAGAAAAACCATGATCTGACTTTCGTATTATTTTCTCACATTAACAAAGGT
GACCCGGACTACTCGCCCGACGACGTCTGAAGCTAGTGGAGTAGTTTTTCCATGGCGACGGCGGTGGTGGCAGCTCTCTCXXXXXXXXXXXA
TTGAGTTACGTGAAAATCCGAGGACCGAGAGAAAAACCATGATCTGACTTTCGTATTATTTTCTCACATTAACAAAGGT
>Marker66302 Chr4 SNP_site:16632776 Ref_Type:C
GACTATAAAAATTTGAACCCAATCCAACCCATATCATTTTTGTTTGGATTAAATTTAATTTTGGGTAGAAGAATTGGGCTXXXXXXXXXXXT
TGCTTTTTAATTATAAATACAGCACTTGTCTTCTTAGATTTGCAACTTTTAAACATTAATAAAATTTTCTGATTTGGTA
GACTATCAAAATTTGAACCCAATCCAACCCATATCATTTTTGTTTGGATTAAATTTAATTTTGGGTAGGAGAATTGGGCTXXXXXXXXXXXT
TGCTTTTTAATTATAAATACAGCACTTGTCTTCTTAGATTTGCAACTTTTAAACATTAATAAAATTTTCTGATTTGGTA
>Marker66302 Chr4 SNP_site:16632838 Ref_Type:G
GACTATAAAAATTTGAACCCAATCCAACCCATATCATTTTTGTTTGGATTAAATTTAATTTTGGGTAGAAGAATTGGGCTXXXXXXXXXXXT
TGCTTTTTAATTATAAATACAGCACTTGTCTTCTTAGATTTGCAACTTTTAAACATTAATAAAATTTTCTGATTTGGTA
GACTATCAAAATTTGAACCCAATCCAACCCATATCATTTTTGTTTGGATTAAATTTAATTTTGGGTAGGAGAATTGGGCTXXXXXXXXXXXT
TGCTTTTTAATTATAAATACAGCACTTGTCTTCTTAGATTTGCAACTTTTAAACATTAATAAAATTTTCTGATTTGGTA
>Marker66303 Chr4 SNP_site:18061179 Ref_Type:C
AACTATTAAATGTTGTTACTCTAGAAAGAGAAGTATTTGGATCGGTTGTTTGAATGTTTTCTTCAAAAGCATTTCATATGXXXXXXXXXXXA
TGTTTTATATGATTCAAATGATTGTGAATGATGTTTAAACTATTAGTCTTCTTCTTATCACAGGACTAACTGTTGTGTG
AACTATTAAATGTTGTTACTCTAGAAAGAGAAGTATTTGGATCGGTTGTTTGAATGTTTTCTTCAAAAGCATTTCATATGXXXXXXXXXXXA
TGTTTTATATGATTCAAATGATTGTGAATGATGTTTAAACTATTAGTCTTCTTCTTATCATAGGACTAACTGTTGTGTG
>Marker663910 Chr3 SNP_site:18776109 Ref_Type:G
TACACAAAATGAAATCTTAATATTGTGTTTGTATATACATTCTACAATAATACATGAAACAAGATCCTAATACAGGATTTXXXXXXXXXXXT
TAAATCCAGAGAATCTACAAAATTATGTTGATAAAGGCCAACATTTTCTTTATCCACAGTAGCATCACAATGATTATGT

Create PDF with GO2PDF for free, if you wish to

TACACAAAATGAAATCTTAATATTGTGTTTGTATATACATTCTACGATAATACATGAAACAAGATCCTAATACAGGATTTXXXXXXXXXXT
TAAATCCAGAGAATCTACAAAATTATGTTGATAAAGGCCAACATTTTCTCTATCCACAATAGCATCACAATGATTATGT
>Marker663910 Chr3 SNP_site:18776317 Ref_Type:C
TACACAAAATGAAATCTTAATATTGTGTTTGTATATACATTCTACAATAATACATGAAACAAGATCCTAATACAGGATTTXXXXXXXXXXT
TAAATCCAGAGAATCTACAAAATTATGTTGATAAAGGCCAACATTTTCTTTATCCACAGTAGCATCACAATGATTATGT
TACACAAAATGAAATCTTAATATTGTGTTTGTATATACATTCTACGATAATACATGAAACAAGATCCTAATACAGGATTTXXXXXXXXXXT
TAAATCCAGAGAATCTACAAAATTATGTTGATAAAGGCCAACATTTTCTCTATCCACAATAGCATCACAATGATTATGT
>Marker663910 Chr3 SNP_site:18776326 Ref_Type:A
TACACAAAATGAAATCTTAATATTGTGTTTGTATATACATTCTACAATAATACATGAAACAAGATCCTAATACAGGATTTXXXXXXXXXXT
TAAATCCAGAGAATCTACAAAATTATGTTGATAAAGGCCAACATTTTCTTTATCCACAGTAGCATCACAATGATTATGT
TACACAAAATGAAATCTTAATATTGTGTTTGTATATACATTCTACGATAATACATGAAACAAGATCCTAATACAGGATTTXXXXXXXXXXT
TAAATCCAGAGAATCTACAAAATTATGTTGATAAAGGCCAACATTTTCTCTATCCACAATAGCATCACAATGATTATGT
>Marker663980 Chr3 SNP_site:9648584 Ref_Type:T
ACTGGAGGCTCCATACGATCACACAAATACTGAAGAATTAATCAAAGATTTGTTAAGTCCGAGAAAATATAAGCCACACAXXXXXXXXXXT
AGCTTTAATAGAACGAAGAGACTTCTCACAGATATCGGTAAAAAGAATATCCTCATAATTATCTACTGTCAATTTGGTG
ACTGGAGGCTCCATACGATCACACAAATACTGAAGAATTAATCAAAGATTTGTTAAGTCCGAGAAAATATAAGCCACTCAXXXXXXXXXXT
AGCTTTAATAGAACGAAGAGACTTCTCACAGATATCGGTAAAAAGAATATCCTCATAATTATCCACCGTCAATTTGGTG
>Marker663980 Chr3 SNP_site:9648784 Ref_Type:C
ACTGGAGGCTCCATACGATCACACAAATACTGAAGAATTAATCAAAGATTTGTTAAGTCCGAGAAAATATAAGCCACACAXXXXXXXXXXT
AGCTTTAATAGAACGAAGAGACTTCTCACAGATATCGGTAAAAAGAATATCCTCATAATTATCTACTGTCAATTTGGTG
ACTGGAGGCTCCATACGATCACACAAATACTGAAGAATTAATCAAAGATTTGTTAAGTCCGAGAAAATATAAGCCACTCAXXXXXXXXXXT
AGCTTTAATAGAACGAAGAGACTTCTCACAGATATCGGTAAAAAGAATATCCTCATAATTATCCACCGTCAATTTGGTG
>Marker663980 Chr3 SNP_site:9648787 Ref_Type:C
ACTGGAGGCTCCATACGATCACACAAATACTGAAGAATTAATCAAAGATTTGTTAAGTCCGAGAAAATATAAGCCACACAXXXXXXXXXXT
AGCTTTAATAGAACGAAGAGACTTCTCACAGATATCGGTAAAAAGAATATCCTCATAATTATCTACTGTCAATTTGGTG
ACTGGAGGCTCCATACGATCACACAAATACTGAAGAATTAATCAAAGATTTGTTAAGTCCGAGAAAATATAAGCCACTCAXXXXXXXXXXT
AGCTTTAATAGAACGAAGAGACTTCTCACAGATATCGGTAAAAAGAATATCCTCATAATTATCCACCGTCAATTTGGTG
>Marker664198 Chr3 SNP_site:12024392 Ref_Type:C
TACAGCTCAAAACCTCATAACTCATCAATGTTCGAAGAAATTTCACTTCCATGTCATGCTCATAGAAAATAAATTTATAAXXXXXXXXXXT
TATAACGGTTTTCATCCTGTATAAGTTGAAAAGTAACGAAAACTAAGAACAGAAATGTATTTAGCAATCGGATGGTGTG
TACAGCTCAAAACCTCATAACTCATCAATGTTCGAAGAAATTTCACTTCCATGTCATGCTCATAGAAAATAAATTTATAAXXXXXXXXXXT
TATAACGGTTTTCATCCTGTATAAGTTGAAAAGTAATGAAAACTAAGAACAGAAATGTATTTAGCAATCGGATGGTGTG
>Marker665023 Chr3 SNP_site:27948933 Ref_Type:C
GACTTCAACGACGACAACGTTCTCCCAGCATTATTCACCTAACTCAAATAATCGATCAAAATCCAAAATCCAAAATCCAAXXXXXXXXXXG
AGCCACTCACTTTGAAGCCCATTGCTTCCCATTCTTTCAAACATTTGTCTAAATCTTCTTGGCTTCGACAGCATGTGTG
GACTTTAACGACGACAACGTTCTCCCAGCATTATTCACCTAACTCAAATAATCGATCAAAATCCAAAATCCAAAATCCAAXXXXXXXXXXG
AGCCACTCACTTTGAAGCCCATTGCTTCCCATTCTTTCAAACATTTGTCTAAATCTTCTTGGCTTCGACAGCATGTGTG
>Marker665331 Chr3 SNP_site:35127660 Ref_Type:T
CACATGACCGAGAGAGAAAGTGTGATGACCTAGACTCTCAATGTTTATCTCATTGAAAAATAACACGTTGGACATCTAATXXXXXXXXXXT
TATACCACTTAAACAATTTCTGCTTAAGATATCATAACATATTATGCATTTGGAAGTTGGACAACATTAGGAGGAAAGT
CACATGATCGAGAGAGAAAGTGTGATGACCTAGACTCTCAATGTTTATCTCATTGAAAAATAACACGTTGGACATCTAATXXXXXXXXXXT
TATACCACTTAAACAATTTCTGCTTAAGATATCATAACATATTATGCATTTGGAAGTTGGACAACATTAGGAGGAAAGT
>Marker666034 Chr3 SNP_site:22296018 Ref_Type:T
GACCATTGTCAAGTGGAGTTACTAACATACCTGAATGAGAGCTTTGACATCAAGCTCATCGATATGTTTGGGAGAATACTXXXXXXXXXXA

Create PDF with GO2PDF for free, if you wish to

CCGAAGTAGATCAAAAAACCAAAAATGGATTCAAGATGAACACAACAAAACCTTCATAGCTTGGTTACGAGAAGAGGTA
GACCATTGTCAAGTGGAGTTACTAACATACCTGAATGAGAGCTTTTACATCAAGCTCATCGATATGTTTGGGAGAATACTXXXXXXXXXXA
CCGAAGTAGATCAAAAAACCAAAAATGGATTCAAGATGAACACAACAAAACCTTCATAGCTTGGTTACGAGAAGAGGTA
>Marker666166 Chr3 SNP_site:34518279 Ref_Type:T
CACTTTTAAGGCTGTGTGTGATAAGCTGGAAAAACATGCGTTTCACTTTCTTTACCTCACTAAGGAGGGATTCAGGAATAXXXXXXXXXXT
TTACTCATAACTTTAGATCATCATCCTTAAAATAATAACAGTATGTTCCTACACTGTCAATCTCTTCTAAGTTAATAGT
CACTTTTAAGGCTGTGTGTGATAAGCTGGAAAAACATGCGTTTCACTTTCTTTACCTCACTAAGGAGGGATTCAGGAATAXXXXXXXXXXT
TTACTCATAACTTTAGATCATTATCCTTAAAATAATAACAGTATGTTCCTACACTGTCAATCTCTTCTAAGTTAATAGT
>Marker666780 Chr3 SNP_site:34834848 Ref_Type:G
AACAAATGAACCCAATACCGACGCAGCATATCCTGCATCAATGTAGGTCGTATTCCCCAACGTGGGAATCCACTGCGTCAXXXXXXXXXXT
CTAACGTTTGAGAGAAAGACGTCGGGGAAGGTCTGACACATTGATGCGACCGTTGGATATACCCTGATGCCATAGCAGT
AACAAATGAACCCAATACCGACGCAGCATATCCTGCATCAGTGTAGGTCGTATTCCCCAACGTGGGAATCCACTGCGTCAXXXXXXXXXXT
CTAACGTTTGAGAGAAAGACGTCGGGGAAGGTCTGACACATTGATGCGACCGTTGGATATACCCCGATGCCATAGCAGT
>Marker666780 Chr3 SNP_site:34835061 Ref_Type:C
AACAAATGAACCCAATACCGACGCAGCATATCCTGCATCAATGTAGGTCGTATTCCCCAACGTGGGAATCCACTGCGTCAXXXXXXXXXXT
CTAACGTTTGAGAGAAAGACGTCGGGGAAGGTCTGACACATTGATGCGACCGTTGGATATACCCTGATGCCATAGCAGT
AACAAATGAACCCAATACCGACGCAGCATATCCTGCATCAGTGTAGGTCGTATTCCCCAACGTGGGAATCCACTGCGTCAXXXXXXXXXXT
CTAACGTTTGAGAGAAAGACGTCGGGGAAGGTCTGACACATTGATGCGACCGTTGGATATACCCCGATGCCATAGCAGT
>Marker667073 Chr3 SNP_site:4028860 Ref_Type:G
TACAATCTCCTGCTCTAAAGATGATGTATTAGGAAATCTAAACATAGTGAAGTATTTAATTGGTTTAATCAATTTTCATCXXXXXXXXXXA
AGCTGAAGTTTTGAAAAACAACAGATCGGTCACTACCAATGAACTATACTCCTTTTACAGTGCTAGGAGTGACTGCTGT
TACAATCTCCTGCTCTAAAGATGATGTATTAGGAAATCTAAACATAGTGAAGTGTTTAATTGGTTTAATCAATTTTCATCXXXXXXXXXXA
AGCTGCAGTTTTGAAAAACAACAGATCGGTCACTACCAATGAACTATACTCCTTTTACAGTGCTAGGAGTGACTGCTGT
>Marker667073 Chr3 SNP_site:4029000 Ref_Type:C
TACAATCTCCTGCTCTAAAGATGATGTATTAGGAAATCTAAACATAGTGAAGTATTTAATTGGTTTAATCAATTTTCATCXXXXXXXXXXA
AGCTGAAGTTTTGAAAAACAACAGATCGGTCACTACCAATGAACTATACTCCTTTTACAGTGCTAGGAGTGACTGCTGT
TACAATCTCCTGCTCTAAAGATGATGTATTAGGAAATCTAAACATAGTGAAGTGTTTAATTGGTTTAATCAATTTTCATCXXXXXXXXXXA
AGCTGCAGTTTTGAAAAACAACAGATCGGTCACTACCAATGAACTATACTCCTTTTACAGTGCTAGGAGTGACTGCTGT
>Marker667459 Chr3 SNP_site:34675632 Ref_Type:G
AACCATTCCAGATTATTGGTGGTGACCTGCATTATTTCAGAACTCTTCCTGAGGTATTTATTATGCACTTATTTCCTTTCXXXXXXXXXXT
TTATAGTGTCATTCTTCATCTTTCGGTTCAGTTCAGACTAGTCAGAAATGGATTTTGATACAATACTTTGAAGCAGGTC
AACCATTCCAGATTATTGGTGGTGACCTGCATTATTTCAGAACTCTTCCTGAGGTATTTATTATGCACTTATTTCCTTTCXXXXXXXXXXT
TTATAGTGTCATTCTTCATCTTTCGGTTCAGTTCAGACTAGTCGGAAATGGATTTTGATACAATACTTTGAAGCAGGTC
>Marker667496 Chr3 SNP_site:8356424 Ref_Type:G
ACTTAAAAAAGAGTTTTTAAATTTTGAAAAATAAGTTAAATCTAAGCCCTTGTTTTGTAAATCATTTTCTTTTTGTTCTTXXXXXXXXXXT
ATTTCTTCAAAGATAGTGAAACCAGGTAATAATATTGATTGATAAAACAAGTTTAAATTTAAAAAATCAAGTCAGGGTT
ACTTAAAAAAGAGTTTTTAAATTTTGAAAAATAAGTTAAATCTAAGCCCTTGTTTTGTAAATCATTTTCTTTTTGTTCTTXXXXXXXXXXT
GTTTCTTCAAAGATAGTGAAACCAGGTAATAATATTGATTGATAAAACAAGTTTAAATTTAAAAAATCAAGTCAGGGTT
>Marker667506 Chr3 SNP_site:3685293 Ref_Type:C
ACCCTATTGATTCGATATAACAGCAACAAACATAATTATGAATGATCATGAAAACCATTGACAATAGCACTTATAATTGAXXXXXXXXXXT
TAAATTGTCGTAATTGCTCTTTAATTGTTCAATTTAATGTTTGTCATTTTAAGTTTTCTTTTGAGTTATATAAGTTTGT
ACCCTATTGATTTGATATAACAGCAACAAACATAATTATGAATGATCATGAAAACCATTGATAATAGCACTTATAATTGAXXXXXXXXXXT
TAAATTGTCGTAATTGCTCTTTAATTGTTCAATTTAATGTTTGTCATTTTAAGTTTTCTTTTGAGTTATATAAGTTTGT
>Marker667506 Chr3 SNP_site:3685342 Ref_Type:C

Create PDF with GO2PDF for free, if you wish to

ACCCTATTGATTCGATATAACAGCAACAAACATAATTATGAATGATCATGAAAACCATTGACAATAGCACTTATAATTGAXXXXXXXXXXXT
TAAATTGTCGTAATTGCTCTTTAATTGTTCAATTTAATGTTTGTCATTTTAAGTTTTCTTTTGAGTTATATAAGTTTGT
ACCCTATTGATTTGATATAACAGCAACAAACATAATTATGAATGATCATGAAAACCATTGATAATAGCACTTATAATTGAXXXXXXXXXXXT
TAAATTGTCGTAATTGCTCTTTAATTGTTCAATTTAATGTTTGTCATTTTAAGTTTTCTTTTGAGTTATATAAGTTTGT
>Marker668750 Chr3 SNP_site:31680437 Ref_Type:G
TACAAACAATTGGTTAAAATCAGAAGCTCATGGATATCTTTTAATAGGAGATGATTGAAAATCTAAATAGGAATCAAATAXXXXXXXXXXXC
ATCGATCAACTCTAGCTACTACCATTGGCTGCCACCTACATCCAATTCAGCCAACTTTGATGACCTCTACCTAGAAGTC
TACAAACAATTGGTTGAAATCAGAAGCTCATGGATATCTTTTAATAGGAGATGATTGAAAATCTAAATAGGAATCAAATAXXXXXXXXXXXC
ATCGATCAACTCTAGCTACTACCGTTGGCTGCCACCTACATCCAATTCAGCCAACTTTGATGACCTCTACCTAGAAGTC
>Marker668750 Chr3 SNP_site:31680652 Ref_Type:G
TACAAACAATTGGTTAAAATCAGAAGCTCATGGATATCTTTTAATAGGAGATGATTGAAAATCTAAATAGGAATCAAATAXXXXXXXXXXXC
ATCGATCAACTCTAGCTACTACCATTGGCTGCCACCTACATCCAATTCAGCCAACTTTGATGACCTCTACCTAGAAGTC
TACAAACAATTGGTTGAAATCAGAAGCTCATGGATATCTTTTAATAGGAGATGATTGAAAATCTAAATAGGAATCAAATAXXXXXXXXXXXC
ATCGATCAACTCTAGCTACTACCGTTGGCTGCCACCTACATCCAATTCAGCCAACTTTGATGACCTCTACCTAGAAGTC
>Marker668985 Chr3 SNP_site:4132365 Ref_Type:T
ACAAATGTAAATCAACATTGTTGTTTTTATTTAATTCTAGTTCATATTAGATATAAAGTAAAGATTGTATAGTTTTTATGXXXXXXXXXXXA
AGGCTACAAAATTATCATTGATTTTCTTTTTCTTGCTTTCCTCTCTTATTCTCTATTTTGTATTAATCCATTTGTGGTT
ACAAATGTAAATCAACATTGTTGTTTTTATTTAATTCTAGTTCATATTAGATATAAAGTAATGATTGTATAGTTTTTATGXXXXXXXXXXXA
AGGCTACAAAATTATCATTGATTTTCTTTTTCTTGCTTTCCTCTCTTATTCTCTATTTTGTATTAATCCATTTGTGGTT
>Marker669000 Chr3 SNP_site:12576296 Ref_Type:T
GACTGGGTGATGATTATTAAATGAAGTTTTAGAAAAAAATATGTGCAAAATACCGAATCAAACCAAATTCGTTTATGTTTXXXXXXXXXXXA
ATGGGCGGTTATATTTGTCAGGCAATGGCCATTGGGAAAGCAGCAAGCAAGTTGATTGAAGAGGAGCTGAAAATGGAGT
GACTTGGTGATGATTATTAAATGAAGTTTTAGAAAAAAATATGTGCAAAATACCGAATCAAACCAAATTCGTTTATGTTTXXXXXXXXXXXA
ATGGGCGGTTATATTTGTCAGGCAATGGCCATTGGGAAAGCAGCAAGCAAGTTGATTGAAGAGGAGCTGAAAATGGAGT
>Marker669551 Chr3 SNP_site:12840396 Ref_Type:A
CACGTTTTTCATGAATTCCGTTTTCCCACTAGCGAAAAAGAGAGGTTTTATCACCAATTATGTGTAGGAAGAAATAGGCCXXXXXXXXXXXA
AGCACTGAACAGAGTATATGGAATATGGATGGAACCAACATTTCTTTTCACTCTTCAATGTCTATTTCAATGCAAAGTG
CACGTTTTTCATGAATTCCGTTTTCCCACTAGCGGAAAAGAGAGGTTTTATCACCAATTATGTGTAGGAAGAAATAGGCCXXXXXXXXXXXA
AGCACTGAACAGAGTATATGGAATATGGATGGAACCAACATTTCTTTTCACTCTTCAATGTCTATTTCAATGCAAAGTG
>Marker669588 Chr3 SNP_site:36710101 Ref_Type:A
ACTATAACGTGGCCAGTCCGGAAGATTGTGCCCATCCTAGCAGGAGATTACAGGTATGTTAAAAAAGCAGAACATGAAGAXXXXXXXXXXXG
AAGTGAAGCTTGTGCAGGCAAAGGAATTAACCAATAAAGACATTATAGGAAAGTCGGATCCTTATGCTGTGCTGTTTGT
ACTATAACGTGGCCCGTTCGGAAGATTGTGCCCATCCTAGCAGGAGATTACAGGTATGTTAAAAAAGCAGAACATGAAGAXXXXXXXXXXXG
AAGTGAAGCTTGTGCAGGCAAAGGAATTAACCAATAAAGACATTATAGGAAAGTCGGATCCTTATGCTGTGCTGTTTGT
>Marker669588 Chr3 SNP_site:36710104 Ref_Type:C
ACTATAACGTGGCCAGTCCGGAAGATTGTGCCCATCCTAGCAGGAGATTACAGGTATGTTAAAAAAGCAGAACATGAAGAXXXXXXXXXXXG
AAGTGAAGCTTGTGCAGGCAAAGGAATTAACCAATAAAGACATTATAGGAAAGTCGGATCCTTATGCTGTGCTGTTTGT
ACTATAACGTGGCCCGTTCGGAAGATTGTGCCCATCCTAGCAGGAGATTACAGGTATGTTAAAAAAGCAGAACATGAAGAXXXXXXXXXXXG
AAGTGAAGCTTGTGCAGGCAAAGGAATTAACCAATAAAGACATTATAGGAAAGTCGGATCCTTATGCTGTGCTGTTTGT
>Marker669849 Chr3 SNP_site:34677936 Ref_Type:A
TACCCAACAGTTTCAAGATCTACTTTTTTCTTAATACATGGTATAACTGATTATAGGTTGAAAGATGGTGGGGATTATTXXXXXXXXXXXT
TGTCAAGAACTTTTTAGGCTTCCTACAATACTGGAAACATTCTGTATTTTTTTAGGGTCCTTTAGGTAGTCTCTTGGTC
TACCCAACAGTTTCAAGATCTACTTTTTTCTTAATACTTGGTATAACTGATTATAGGTTGAAAGATGGTGGGGATTATTXXXXXXXXXXXT
TGTCAAGAACTTTTTAGGCTTCCTACAATACTGGAAACATTCTGTATATTTTTAGGGTCCTTTGGGTAGTCTCTTGGTC

Create PDF with GO2PDF for free, if you wish to

>Marker669849 Chr3 SNP_site:34678160 Ref_Type:T
TACCCAACAGTTTCAAGATCTACTTTTTTCTTAATACATGGTATAACTGATTATAGGTTGAAAGATGGTGGGGGATTATTXXXXXXXXXXT
TGTCAAGAACTTTTTAGGCTTCCTACAATACTGGAAACATTCTGTATTTTTTTAGGGTCCTTTAGGTAGTCTCTTGGTC
TACCCAACAGTTTCAAGATCTACTTTTTTCTTAATACTTGGTATAACTGATTATAGGTTGAAAGATGGTGGGGGATTATTXXXXXXXXXXT
TGTCAAGAACTTTTTAGGCTTCCTACAATACTGGAAACATTCTGTATATTTTTAGGGTCCTTTGGGTAGTCTCTTGGTC
>Marker669849 Chr3 SNP_site:34678176 Ref_Type:A
TACCCAACAGTTTCAAGATCTACTTTTTTCTTAATACATGGTATAACTGATTATAGGTTGAAAGATGGTGGGGGATTATTXXXXXXXXXXT
TGTCAAGAACTTTTTAGGCTTCCTACAATACTGGAAACATTCTGTATTTTTTTAGGGTCCTTTAGGTAGTCTCTTGGTC
TACCCAACAGTTTCAAGATCTACTTTTTTCTTAATACTTGGTATAACTGATTATAGGTTGAAAGATGGTGGGGGATTATTXXXXXXXXXXT
TGTCAAGAACTTTTTAGGCTTCCTACAATACTGGAAACATTCTGTATATTTTTAGGGTCCTTTGGGTAGTCTCTTGGTC
>Marker670015 Chr3 SNP_site:8814305 Ref_Type:C
TACCTGCAAATGACTTGGTCGATGTTATTGCTCCATCTTGCTACAGGTATTTTTTCTCATTGTTTCCCATTTCATTTGGTXXXXXXXXXXA
AATCAAATGTAGTCATTAAGGAATCATTCTTAAACACTTAAACCAAGATGCAATCATGACAGGAAAAAGATAAAAAGTG
TACCTGCAAATGACTTGGTCGATGTTATTGCTCCATCTTGCTACAGGTATTTTTTCTCATTGTTTCCCATTTCATTTGGTXXXXXXXXXXA
AATTAAATGTAGTCATTAAGGAATCATTCTTAAACACTTAAACCAAGATGCAATCATGACAGGAAAAAGATAAAAAGTG
>Marker67015 Chr4 SNP_site:1808147 Ref_Type:C
TACACCATATTCTCAATCAACATGTAATTTGTGTATATCTACTGGTAAAAAAGCATCTTCAAATCTTAAGCTACAATATTXXXXXXXXXXG
ATTTAAGTGGGTGTAATTTTGTTTATATAACAGTTGCAATGAAATTGGAAAAGGTAATTGGATTGTTTTCTAAAATGTG
TACACCATATTCTCAATCAACATGTAATTTGTGTATATCTATTGGTAAAAAAGCATCTTCAAATCTTAAGCTACAATATTXXXXXXXXXXG
ATTTAAGTGGGTGTAATTTTGTTTATATAACAGTTGCAATGAAATTGGAAAAGGTAATTGGATTGTTTTCTAAAATGTG
>Marker671749 Chr3 SNP_site:2683593 Ref_Type:T
AACTAAACATATTCCGTGTATTATCTAATTCTTTTACCTCTTTTTGTGAAGAATCTAAAACTTTGAGTAATTGTCATGAAXXXXXXXXXXT
AAGATAAAAGAAGTGAATTGTATTGGTATGTTTTTATTCTACAAGAGACATACTATTTTGATACACAGAAAAATACGTG
AACTAAACATATTCCGTGTATTATCTAATTCTTTTACCTCTTTTTGTGATGAATCTAAAACTTTGAGTAATTGTCATGAAXXXXXXXXXXT
AAGATAAAAGAAGTGAATTGTATTGGTATGTTTTTATTCTACAAGAGACATACTATTTTGATACACAGAAAAATACGTG
>Marker67607 Chr4 SNP_site:19683288 Ref_Type:G
ACTTCATTGGAACATAGCCTTCATATAGTTTTCTGAATTTCTTTAACTGGCGTGGGAGGAAACGAAGAAATTATGCAATAXXXXXXXXXXA
TGAAGCACAATACTGGTCTGAATGGGCAAGAACAATGTTCAATAAAATTTACTGCACGAATTTGGTATAAAGTAACAGT
ACTTCATTGGAACATAGCCTTCATATAGTTTTCTGAATTTCTTTAACTGGCGTGGGAGGAAACGAAGAAATTATGCAATAXXXXXXXXXXA
TGAAGCGCAATACTGGTCTGAATGGGCAAGAACAATGTTCAATAAAATTTACTGCACGAATTTGGTATAAAGTAACAGT
>Marker677182 Chr5 SNP_site:14591486 Ref_Type:C
CACATAAGAGTATAGGCAGCCATGTAGAAAATGCATGCATGGTGGAGTAAAGGCAATCAATGCATAAAAGATGTGCATTGXXXXXXXXXXG
GAATGCAACTAATGTGTGTTATGTATATTCATTGTTTCTTGAAAGTTTAAGAACTTTAAGGCTTAACCAGACGATTCGT
CACATAAGAGTATAGGCAGCCATGTAGAAAATGCATGCATGGTGGAGTAAAGGCAATCAATGCATAAAAGATGTGCATTGXXXXXXXXXXG
GAATGCAACTAATGTGTGTTATGTATATTCATTGTTTCTTGAAAGTTTAAGAACTTTAAGGCTTAACTAGACGATTCGT
>Marker677609 Chr5 SNP_site:702491 Ref_Type:G
CACCAAACATTAGAATCTTTGTCCGACCATAAACTGAAAGAGAATGTCCCAGTCTAGATGGGGGTGTCCCAGATGTGGGAXXXXXXXXXXA
GCTATGCCAGGATCTAGGCAATGGAGGAGCTCCACCAGAGATTTCCTTCCATGTTGGTTGTTGGGCATCCAAATCGAGT
CACCAAACATTAGAATCTTTGTCCGACCATAAACTGAAAGAGAATGTCCCAGTCTAGATGGGGGTGTCCCAGATGTGGGAXXXXXXXXXXA
GCTGTGCCAGGATCTAGGCAATGGAGGAGCTCCACCAGAGATTTCCTTCCATGTTGGTTGTTGGGCATCCAAATCGAGT
>Marker677698 Chr5 SNP_site:7562213 Ref_Type:T
ACCTTATTAACCATAATACTGAAAAAATCATGAAGTCAATTGCTAATGGATGAAAATATGAAAATCAAATAACTAAATCTXXXXXXXXXXG
CTTTCAATCTTGACTAAATCATACCAGTAAAAGATGAGCATTTTTTCAAGTAGATATCATGTAAAAAAACAACAAAAGT
ACCTTATTAACCATAATACTGAAAAAATCTTGAAGTCAATTGGTAATGGATGAAAATATGAAAATCAAATAACTAAATCTXXXXXXXXXXG

Create PDF with GO2PDF for free, if you wish to

CTTTCAATCTTGACTAAATCATACCAGTAAAAGATGAGCATTTTTTCAAGTAGATATCATGTAAAAAAACAACAAAAGT
>Marker677698 Chr5 SNP_site:7562226 Ref_Type:G
ACCTTATTAACCATAATACTGAAAAAATCATGAAGTCAATTGCTAATGGATGAAAATATGAAAATCAAATAACTAAATCTXXXXXXXXXXG
CTTTCAATCTTGACTAAATCATACCAGTAAAAGATGAGCATTTTTTCAAGTAGATATCATGTAAAAAAACAACAAAAGT
ACCTTATTAACCATAATACTGAAAAAATCTTGAAGTCAATTGGTAATGGATGAAAATATGAAAATCAAATAACTAAATCTXXXXXXXXXXG
CTTTCAATCTTGACTAAATCATACCAGTAAAAGATGAGCATTTTTTCAAGTAGATATCATGTAAAAAAACAACAAAAGT
>Marker677844 Chr5 SNP_site:15495708 Ref_Type:A
AACGTGAAATTACTAATGCCAACCAGAGCTTGACTCAAATGTATGTTATATTGAGGATTACAAGATCCATAGTTTGAATCXXXXXXXXXXA
TTAAAATTTAAGTCATGAAAATTAACATCAAGTAATGTTAGTTCATCCAATCGAATTGTGTCATGTGTCAATCAAAGTC
AACGTGAAGTTACTAATGCCAACCAGAGCTTGACTCAAATGTATGTTATGTTGAGGATTACAAGATCCATAGTTTGAATCXXXXXXXXXXA
TTAAAATTTAAGTCATGAAAATTAACATCAAGTAATGTTAGTTCATCCAATCGAATTGTTTCATGTGTCAATCAAAGTC
>Marker677844 Chr5 SNP_site:15495749 Ref_Type:A
AACGTGAAATTACTAATGCCAACCAGAGCTTGACTCAAATGTATGTTATATTGAGGATTACAAGATCCATAGTTTGAATCXXXXXXXXXXA
TTAAAATTTAAGTCATGAAAATTAACATCAAGTAATGTTAGTTCATCCAATCGAATTGTGTCATGTGTCAATCAAAGTC
AACGTGAAGTTACTAATGCCAACCAGAGCTTGACTCAAATGTATGTTATGTTGAGGATTACAAGATCCATAGTTTGAATCXXXXXXXXXXA
TTAAAATTTAAGTCATGAAAATTAACATCAAGTAATGTTAGTTCATCCAATCGAATTGTTTCATGTGTCAATCAAAGTC
>Marker677844 Chr5 SNP_site:15495929 Ref_Type:G
AACGTGAAATTACTAATGCCAACCAGAGCTTGACTCAAATGTATGTTATATTGAGGATTACAAGATCCATAGTTTGAATCXXXXXXXXXXA
TTAAAATTTAAGTCATGAAAATTAACATCAAGTAATGTTAGTTCATCCAATCGAATTGTGTCATGTGTCAATCAAAGTC
AACGTGAAGTTACTAATGCCAACCAGAGCTTGACTCAAATGTATGTTATGTTGAGGATTACAAGATCCATAGTTTGAATCXXXXXXXXXXA
TTAAAATTTAAGTCATGAAAATTAACATCAAGTAATGTTAGTTCATCCAATCGAATTGTTTCATGTGTCAATCAAAGTC
>Marker677903 Chr5 SNP_site:14037590 Ref_Type:G
GACCAAAATATTTACAAATTATAACAAAGTTTCAGTTTCTATTCGATGATAGAATCTGAAATTTTGCTATATTTTATAAAXXXXXXXXXX-
GTGTCCCATAATGTTGATAAAATTTCTTAGAATTTTTTCTTTTGAAATCATACACTTGTTATCCGCAGAGCTTAGCAGTA
GACCAAAATATTTACAAATTATAACAAAGTTTCAGTTTCTATTCGATGATAGAATCTGAAATTTTGCTATATTTTGTAAAXXXXXXXXXXA
GTG-CCCATAATGTTGATAAAATTTCTTAGAATTTTTTCTTTTGAAATCATACACTTGTTATCCGTAGAGCTTAGCAGTA
>Marker677903 Chr5 SNP_site:14037777 Ref_Type:A
GACCAAAATATTTACAAATTATAACAAAGTTTCAGTTTCTATTCGATGATAGAATCTGAAATTTTGCTATATTTTATAAAXXXXXXXXXX-
GTGTCCCATAATGTTGATAAAATTTCTTAGAATTTTTTCTTTTGAAATCATACACTTGTTATCCGCAGAGCTTAGCAGTA
GACCAAAATATTTACAAATTATAACAAAGTTTCAGTTTCTATTCGATGATAGAATCTGAAATTTTGCTATATTTTGTAAAXXXXXXXXXXA
GTG-CCCATAATGTTGATAAAATTTCTTAGAATTTTTTCTTTTGAAATCATACACTTGTTATCCGTAGAGCTTAGCAGTA
>Marker678056 Chr5 SNP_site:9340163 Ref_Type:G
ACTCGTTGAAGTGCTTTATGAATGAATTCTCTTCCAACTTATAAGAAAACATGAATAGTCACAACAATAGTGTGCTCACAXXXXXXXXXXT
TTTGATTGCACATATTATAGTTTTGAATTTAATAATAATTGTATAGGGTATTAGAAATGATCGTAAAAATCAAAATGTT
ACTCGTTGAAGTGCTTTATGAATGAGTTCTCTTCCAACTTATAAGAAAACATGAATAGTCACAACAATAGTGTGCTAACAXXXXXXXXXXT
TTTGATTGCACATATTATAGTTTTGAATTTAATAATAATTGTATAGGGTATTAGAAATGATTGTAAAAATCAAAATGTT
>Marker678056 Chr5 SNP_site:9340214 Ref_Type:C
ACTCGTTGAAGTGCTTTATGAATGAATTCTCTTCCAACTTATAAGAAAACATGAATAGTCACAACAATAGTGTGCTCACAXXXXXXXXXXT
TTTGATTGCACATATTATAGTTTTGAATTTAATAATAATTGTATAGGGTATTAGAAATGATCGTAAAAATCAAAATGTT
ACTCGTTGAAGTGCTTTATGAATGAGTTCTCTTCCAACTTATAAGAAAACATGAATAGTCACAACAATAGTGTGCTAACAXXXXXXXXXXT
TTTGATTGCACATATTATAGTTTTGAATTTAATAATAATTGTATAGGGTATTAGAAATGATTGTAAAAATCAAAATGTT
>Marker678056 Chr5 SNP_site:9340383 Ref_Type:T
ACTCGTTGAAGTGCTTTATGAATGAATTCTCTTCCAACTTATAAGAAAACATGAATAGTCACAACAATAGTGTGCTCACAXXXXXXXXXXT
TTTGATTGCACATATTATAGTTTTGAATTTAATAATAATTGTATAGGGTATTAGAAATGATCGTAAAAATCAAAATGTT

Create PDF with GO2PDF for free, if you wish to

ACTCGTTGAAGTGCTTTATGAATGAGTTCTCTTCCAACTTATAAGAAAACATGAATAGTCACAACAATAGTGTGCTAACAXXXXXXXXXXT
TTTGATTGCACATATTATAGTTTTGAATTTAATAATAATTGTATAGGGTATTAGAAATGATTGTAAAAATCAAAATGTT
>Marker678115 Chr5 SNP_site:23709802 Ref_Type:T
ACCAATTATTCTACCAACAATTTTATCCTGTTCATCCAGAGAAAAATATGTATGGAACAACGGCAACAATTCTGATTCCTXXXXXXXXXXA
TGAAACTTCATGCAACTTGAATTATTACACTTTTTATTACCTTTCAAGTCTCTGCGAAGGCCTGCCAGCTTAGATAGTG
ACCAATTATTCTACCAACAATTTTATCCTGTTCATCCAGAGAAAAATATGTATGGAACAACGGCAACAATTCTGATTCCTXXXXXXXXXXA
TGAAACTTCATGCAACTTGAATTATTACACTTTTTATTACCTTTCAAGTCTCTGCGAAGGTCTGCCAGCTTAGATAGTG
>Marker678436 Chr5 SNP_site:690386 Ref_Type:T
TACCAAAGGAAGGGTAAGAAGGGAAATTCAACCTAGACAACATGGTTGTTAGAATGGCCAGTGGTCCCAGCAACAAGGTAXXXXXXXXXXA
AAAACTATCGTAGAACTCTTCTGGAATTCTTGTTGAATATACCTATATCTCATCTCTTCTCATATTGGTGTTATGTGTG
TACCAAAGGAAGGGTAAGAAGGGAAATTCAACCTATGCAACATGGTTGTTAGAATGGCCAGTGGTCCCAGCAACAAGGTAXXXXXXXXXXA
AAAACTATCGTAGAACTCTTTTGGAATTCTTGTTGAATATACCTATATCTCATCTCTTCTCATATTGGTGTTATGTGTG
>Marker678436 Chr5 SNP_site:690387 Ref_Type:G
TACCAAAGGAAGGGTAAGAAGGGAAATTCAACCTAGACAACATGGTTGTTAGAATGGCCAGTGGTCCCAGCAACAAGGTAXXXXXXXXXXA
AAAACTATCGTAGAACTCTTCTGGAATTCTTGTTGAATATACCTATATCTCATCTCTTCTCATATTGGTGTTATGTGTG
TACCAAAGGAAGGGTAAGAAGGGAAATTCAACCTATGCAACATGGTTGTTAGAATGGCCAGTGGTCCCAGCAACAAGGTAXXXXXXXXXXA
AAAACTATCGTAGAACTCTTTTGGAATTCTTGTTGAATATACCTATATCTCATCTCTTCTCATATTGGTGTTATGTGTG
>Marker678436 Chr5 SNP_site:690574 Ref_Type:T
TACCAAAGGAAGGGTAAGAAGGGAAATTCAACCTAGACAACATGGTTGTTAGAATGGCCAGTGGTCCCAGCAACAAGGTAXXXXXXXXXXA
AAAACTATCGTAGAACTCTTCTGGAATTCTTGTTGAATATACCTATATCTCATCTCTTCTCATATTGGTGTTATGTGTG
TACCAAAGGAAGGGTAAGAAGGGAAATTCAACCTATGCAACATGGTTGTTAGAATGGCCAGTGGTCCCAGCAACAAGGTAXXXXXXXXXXA
AAAACTATCGTAGAACTCTTTTGGAATTCTTGTTGAATATACCTATATCTCATCTCTTCTCATATTGGTGTTATGTGTG
>Marker678453 Chr5 SNP_site:25568698 Ref_Type:A
CACAGTTCTTTACAAAGGAAAAATAGGAATACTGAAAACTGATTCAGAAGATAAAGAAAATTATTATTATAAACACTTCAXXXXXXXXXXT
GTAGAAAGATTCATGGTTCATATCTAAATCATTATTTTAAAACTATTTAAACCACGTGGAACACATGCAGAAACCAAGT
CACAGTTCTTTACGAAGGAAAAATAGGAATACTGAAAACTGATTCAGAAGATAAAGAAAATTATTATTATAAACACTTCAXXXXXXXXXXT
GTAGAAAGATTCATGGTTCATATCTAAATCATTATTTTAAAACTATTTAAACCACGTGGAACACATGCAGAAACCAAGT
>Marker678914 Chr5 SNP_site:14645204 Ref_Type:G
ACAATATTTGGGTTATTGCATCTCTAGTAAAGGAGTGGCAGCAGATGGGGAATAGGTCAAAGGTATGGTCCAACGGCCTCXXXXXXXXXXC
ACTAAACTCTTATCAAAAATGCATTTAGATGGAGTGAGGAAGCTACAGAGTCCTTTGAACAATTGAAGCAAGCCATGGT
ACAATATTTGGGTTATTGGATCTCTAGTAAAGGAGTGGCAGCAGATGGGGAATAGGTCAAAGGTATGGTCCAACGGCCTCXXXXXXXXXXC
ACTAAACTCTTATAAAAAATGCATTTAGATGGAGTGAGGAAGCTACAGAGTCCTTTGAACAATTGAAGCAAGCCATGGT
>Marker678914 Chr5 SNP_site:14645373 Ref_Type:A
ACAATATTTGGGTTATTGCATCTCTAGTAAAGGAGTGGCAGCAGATGGGGAATAGGTCAAAGGTATGGTCCAACGGCCTCXXXXXXXXXXC
ACTAAACTCTTATCAAAAATGCATTTAGATGGAGTGAGGAAGCTACAGAGTCCTTTGAACAATTGAAGCAAGCCATGGT
ACAATATTTGGGTTATTGGATCTCTAGTAAAGGAGTGGCAGCAGATGGGGAATAGGTCAAAGGTATGGTCCAACGGCCTCXXXXXXXXXXC
ACTAAACTCTTATAAAAAATGCATTTAGATGGAGTGAGGAAGCTACAGAGTCCTTTGAACAATTGAAGCAAGCCATGGT
>Marker679435 Chr5 SNP_site:1386428 Ref_Type:T
ACCGTGTTTCGCCATCTTGGGCCATCTTATATGCGTATAATGTTCTACTTTAGGAAACCTTGTAGGCAATCAACATATCCXXXXXXXXXXC
ACGAAAGAATTCAAATGCAACGTATCATAAATCAAGCAATATACCCTTTCTAAAGACATCAAAAGTTATAACTAATGTG
ACCGTGTTTCGCCATCTTGGGCCATCTTATATGCGTATAATGTTTTACTTTAGGAAACCTTGTAGGCAATCAACATATCCXXXXXXXXXXC
ACGAAAGAATTCAAATGCAACGTATCATAAATCAAGCAATATACCCTTTCTAAAGACATCAAAAGTTATAACTAATGTG
>Marker67953 Chr4 SNP_site:11294985 Ref_Type:G
ACTGATCCCTGCATAATTCAGGTCAAACGGAAATTAGTTAGTTCCCCATGGAAACGAAACCACTACTCAACAAGCAGAGAXXXXXXXXXXA

Create PDF with GO2PDF for free, if you wish to

AAGATTTTAAAACAATTGTGTTTTCACTGCTCAGCTTGGGATCAAGCAAAGATGGAAATCTAAATCTTTTATCCCTGTC
ACTGATCCCTGCATAATTCAGGTCAAACGGAAATTAGTTAGTTCCCCATGGAAACGAAACCACTACTCAACAGGCAGAGAXXXXXXXXXXXA
AAGATTTTAAAACAATTGTGTTTTCACTGCTCAGCTTGGGATCAAGCAAAGATGGAAATCTAAATCTTTTATCCCTGTC
>Marker680014 Chr5 SNP_site:9713406 Ref_Type:T
AACCGCTCATTCTTTTGCAAATATTTTCAAAAACCACCCTATTATCACACTAAATCCCTAGACTTGATGTATAAACGTAGXXXXXXXXXXXT
TCTAAGGGGTCTTTCCTTTTAGAACCTTCATAATGACTCAATAATGTGTGATGTTCATGACATGAATAGCACCTGATGT
AACCGCTCATTCTTTTGCAAATATTTTCAAAAACCACCCTATTATCACACTAAATCCCTAGACTTGATGTATAAACGTAGXXXXXXXXXXXT
TCTAAGGGGTCTTTCCTTTTAGAACCTTCATAATGACTCAATAATGTGTGATGTTCATGACATGAATAGCACTTGATGT
>Marker68063 Chr4 SNP_site:16269911 Ref_Type:A
ACCCGATTGTTGGACAAAAAGGTGACTGAAATCAGAATTCAAACAAAAACTAATTTGCACTTTTCTACAATCGACATTTCXXXXXXXXXXXG
TAGAGCTTCAAGCCCATCGCTTTCTTAACCCTTTCAGGTGTGAGATTACTCTCTTGTAATATTTTGCCAAAAAAATAGT
ACCCGATTGTTGGACAAAAAGGTGACTGAACTCAGAATTCAAACAAAAACTAATTTGCACTTTTCTACAATCGACATTTCXXXXXXXXXXXG
TAGAGCTTCAAGCCCATCGCTTTCTTAACCCTTTCAGGTGTGAGATTACTCTCTTGTAATATTTTGCCAAAAAAATAGT
>Marker68142 Chr4 SNP_site:1647934 Ref_Type:A
CACCTTTTCTGATTTTGATGAACAAACAGACCTCTCTAGTCCTACGTCAATGCAATATTTGAAGAACAACAAAGGGTTTGXXXXXXXXXXXA
CTTATTGAAGTTAATGATAATCAAAGCCATGCCCGAGTCCATGGTGGAATTTGCTATTATAGCTCAACGTTTCCTTGGT
CACCTTTTCTGATTTTGATGAACATACAGACCTCTCTAGTCCTACGTCAATGCAATATTTGAAGAACAACAAAGGGTTTGXXXXXXXXXXXA
CTTATTGAAGTTAATGATAATCAAAGCCATGCCGGAGTCCATGGTGGAATTCGCTATTATAGCTCAACGTTTCCTTGGT
>Marker68142 Chr4 SNP_site:1648147 Ref_Type:C
CACCTTTTCTGATTTTGATGAACAAACAGACCTCTCTAGTCCTACGTCAATGCAATATTTGAAGAACAACAAAGGGTTTGXXXXXXXXXXXA
CTTATTGAAGTTAATGATAATCAAAGCCATGCCCGAGTCCATGGTGGAATTTGCTATTATAGCTCAACGTTTCCTTGGT
CACCTTTTCTGATTTTGATGAACATACAGACCTCTCTAGTCCTACGTCAATGCAATATTTGAAGAACAACAAAGGGTTTGXXXXXXXXXXXA
CTTATTGAAGTTAATGATAATCAAAGCCATGCCGGAGTCCATGGTGGAATTCGCTATTATAGCTCAACGTTTCCTTGGT
>Marker68142 Chr4 SNP_site:1648165 Ref_Type:T
CACCTTTTCTGATTTTGATGAACAAACAGACCTCTCTAGTCCTACGTCAATGCAATATTTGAAGAACAACAAAGGGTTTGXXXXXXXXXXXA
CTTATTGAAGTTAATGATAATCAAAGCCATGCCCGAGTCCATGGTGGAATTTGCTATTATAGCTCAACGTTTCCTTGGT
CACCTTTTCTGATTTTGATGAACATACAGACCTCTCTAGTCCTACGTCAATGCAATATTTGAAGAACAACAAAGGGTTTGXXXXXXXXXXXA
CTTATTGAAGTTAATGATAATCAAAGCCATGCCGGAGTCCATGGTGGAATTCGCTATTATAGCTCAACGTTTCCTTGGT
>Marker681840 Chr5 SNP_site:695517 Ref_Type:G
TACTAAAGAAAGGGTAAGAAGGAAAATTCAACCTAGGCAACATGGTTGTTAGGATGGCCAGTGGTCCCAACAACGTGGCAXXXXXXXXXXXA
AAAACTATCATAAAACTCTTGTGGAATTCTTATTAAATATACCTATATCTCATATCTTCTCATATTGGTGTTGTGTGTG
TACTAAAGAAAGGGTAAGAAGGAAAATTCAACCTAGGCAACATGGTTGTTAGGATGGCCAGTGGTCCCAACAACGTGGCAXXXXXXXXXXXA
AAAACTATCATAGAACTCTTTTGGAATTCTTATTAAATATACCTATATCTCATCTCTTCTCATATTGGTGTTGTGTGTG
>Marker681840 Chr5 SNP_site:695525 Ref_Type:T
TACTAAAGAAAGGGTAAGAAGGAAAATTCAACCTAGGCAACATGGTTGTTAGGATGGCCAGTGGTCCCAACAACGTGGCAXXXXXXXXXXXA
AAAACTATCATAAAACTCTTGTGGAATTCTTATTAAATATACCTATATCTCATATCTTCTCATATTGGTGTTGTGTGTG
TACTAAAGAAAGGGTAAGAAGGAAAATTCAACCTAGGCAACATGGTTGTTAGGATGGCCAGTGGTCCCAACAACGTGGCAXXXXXXXXXXXA
AAAACTATCATAGAACTCTTTTGGAATTCTTATTAAATATACCTATATCTCATCTCTTCTCATATTGGTGTTGTGTGTG
>Marker681840 Chr5 SNP_site:695558 Ref_Type:C
TACTAAAGAAAGGGTAAGAAGGAAAATTCAACCTAGGCAACATGGTTGTTAGGATGGCCAGTGGTCCCAACAACGTGGCAXXXXXXXXXXXA
AAAACTATCATAAAACTCTTGTGGAATTCTTATTAAATATACCTATATCTCATATCTTCTCATATTGGTGTTGTGTGTG
TACTAAAGAAAGGGTAAGAAGGAAAATTCAACCTAGGCAACATGGTTGTTAGGATGGCCAGTGGTCCCAACAACGTGGCAXXXXXXXXXXXA
AAAACTATCATAGAACTCTTTTGGAATTCTTATTAAATATACCTATATCTCATCTCTTCTCATATTGGTGTTGTGTGTG
>Marker681975 Chr5 SNP_site:6605243 Ref_Type:T

Create PDF with GO2PDF for free, if you wish to

CACCAGCCAATCAAGAGGAAAAATCCAGGTGAGGAGAAGAAAATGAGTCAGGAAGAAATGCTTCTTGAAGCAGCTCAAACXXXXXXXXXXT
CATGAACTTGAGGAACTTGGAGCGTGTTTTGGCAAGGGAAGAAGAAGTCAAAAAGAGAGCAATTGTGCATAAAGCTGTG
CACCAGCCAATCAAGAGGAAAAATCCAGGTGAGGAGAAGAAAATGAGTCAGGAAGAAATGCTTCTTGAAGCTGCTCAAACXXXXXXXXXXT
CATGAACTTGAGGAACTTGGAGCGTGTTTTGGCAAGGGAAGAAGAAGTCAAAAAGAGAGCAATTGTGCATAAAGCTGTG
>Marker682242 Chr5 SNP_site:2543425 Ref_Type:G
TACCTCCACAGTTTTAACCAAATCAATTTCATTCTCTAAAAGCATCAAGGAAATGAAGGAAGAAAGGCAACAAGGAAAAGXXXXXXXXXXA
ATCACAACCCCTCCATTCCCTTTAGGAAACCCATCTCTTAATTTCTCCCCTACTTTCTCACAAGAATTCAAATCCCAGT
TACCTCCACAGTTTTAACCAAATCAATTTCATTCTCTAAAAGCATCAAGGAAATGAAGGAAGAAATGCAACATGGAAAAGXXXXXXXXXXA
ATCACAACCCCTCCATTCCCTTTCGGAAACCCATCTCTTAATTTCTCCCCTACTTTCTCACAAGAATTCAAATCCCAGT
>Marker682242 Chr5 SNP_site:2543432 Ref_Type:A
TACCTCCACAGTTTTAACCAAATCAATTTCATTCTCTAAAAGCATCAAGGAAATGAAGGAAGAAAGGCAACAAGGAAAAGXXXXXXXXXXA
ATCACAACCCCTCCATTCCCTTTAGGAAACCCATCTCTTAATTTCTCCCCTACTTTCTCACAAGAATTCAAATCCCAGT
TACCTCCACAGTTTTAACCAAATCAATTTCATTCTCTAAAAGCATCAAGGAAATGAAGGAAGAAATGCAACATGGAAAAGXXXXXXXXXXA
ATCACAACCCCTCCATTCCCTTTCGGAAACCCATCTCTTAATTTCTCCCCTACTTTCTCACAAGAATTCAAATCCCAGT
>Marker682242 Chr5 SNP_site:2543586 Ref_Type:A
TACCTCCACAGTTTTAACCAAATCAATTTCATTCTCTAAAAGCATCAAGGAAATGAAGGAAGAAAGGCAACAAGGAAAAGXXXXXXXXXXA
ATCACAACCCCTCCATTCCCTTTAGGAAACCCATCTCTTAATTTCTCCCCTACTTTCTCACAAGAATTCAAATCCCAGT
TACCTCCACAGTTTTAACCAAATCAATTTCATTCTCTAAAAGCATCAAGGAAATGAAGGAAGAAATGCAACATGGAAAAGXXXXXXXXXXA
ATCACAACCCCTCCATTCCCTTTCGGAAACCCATCTCTTAATTTCTCCCCTACTTTCTCACAAGAATTCAAATCCCAGT
>Marker682555 Chr5 SNP_site:2447451 Ref_Type:A
CACTAGAGTCGGTGGCATTATCCAACAAAATGTTGGATTTTTCCACTAACAAAGTCAAATGGCAAAATGGTCATTTGGTTXXXXXXXXXXT
TTGACTTTTCAAAGTAAAAAAAATCAATATTTTAACTTTTTACAACTTTGACTATTTCCATCATTTTTTAGCTTCGAGT
CACTAGAGTCGGTGGCATTATCCAACAAAATGTTGGATTTTTCCACTAACAAAGTCAAATGGCAAAATGGTCATTTGGTTXXXXXXXXXXT
TTGACTTTTCAAAGTAAAAAAAGTCAATATTTTAACTTTTTACAACTTTGACTATTTCCATCATTTTTTAGCTTCGAGT
>Marker684046 Chr5 SNP_site:25276951 Ref_Type:C
TACCTAGAACACAAAGGGGTCATAATTCTGTTTTTGTAGTAGTAGATCGCTTTAGTAAAATGGCCCATTTCATCCCTTGTXXXXXXXXXXT
GTCAAATCACGATGTAAAGTATTTGAGTCATTTTTGGAGATCCCTATGGAAGAAATTCGATACCAACCTTCTATTTGGT
TACCTAGAACACAAAGGGGTCATAATTCTGTTTTTGTAGTAGTAGATCGCTTTAGTAAAATGGTCCATTTCATCCCTTGTXXXXXXXXXXT
GTCAAATCACGATGTAAAGTATTTGAGTCATTTTTGGAGATCCCTATGGAAGAAATTCGATACCAACCTTCTATTTGGT
>Marker684273 Chr5 SNP_site:1354393 Ref_Type:G
ACTTAGTGTAATGAGTTAAGTGAATAAAGCGAAAAAGTTAAGCATATTCAGCTACATCATTGTTATTATTTGCCATATCCXXXXXXXXXXA
AAATAGAAGCTTTGCGAAGAGATAAGTTCAAGAAGTTGCTCTGCTTACTAGGCATTTAGCGTGTTATCTCGCGATTGGT
ACTTAGTGTAATGAGTTAAGTGAATAAAGCGAAAAAGTTAAGCATATTCAGCTACATCATTGTTATTATTTGCCATATCCXXXXXXXXXXA
AAGTAGAAGCTTTGCGAAGAGATAAGTTCAAGAAGTTGCTCTGCTTACTAGGCATTTAGCGTGTTATCTCGCGATGGGT
>Marker684273 Chr5 SNP_site:1354466 Ref_Type:G
ACTTAGTGTAATGAGTTAAGTGAATAAAGCGAAAAAGTTAAGCATATTCAGCTACATCATTGTTATTATTTGCCATATCCXXXXXXXXXXA
AAATAGAAGCTTTGCGAAGAGATAAGTTCAAGAAGTTGCTCTGCTTACTAGGCATTTAGCGTGTTATCTCGCGATTGGT
ACTTAGTGTAATGAGTTAAGTGAATAAAGCGAAAAAGTTAAGCATATTCAGCTACATCATTGTTATTATTTGCCATATCCXXXXXXXXXXA
AAGTAGAAGCTTTGCGAAGAGATAAGTTCAAGAAGTTGCTCTGCTTACTAGGCATTTAGCGTGTTATCTCGCGATGGGT
>Marker684358 Chr5 SNP_site:15915398 Ref_Type:T
ACATCAATATTATGTTAATACATGAAATAACAAAAAATTAAAATAATATCAAACTCAAACTTCAAAAGTAGATTATCAAGXXXXXXXXXXC
GTGTGTTTGAGGTGTAAATTATGTTGATTTGAGAATTAAGGAAGGAAGAATAAAAAAAAAGATGAACCAAGTGAAAGTA
ACATCAATATTATGTTAATACATGAAATAACAAAAAATTAAAATAATATCAAACTCAAACTTCAAAAGTAGATTATCAAGXXXXXXXXXXT
GTGTGTTTGAGGTGTAAATTATGTTGATTTGAGAATTAAGGAAGGAAGAATAAAAAAAAAGATGAACCAAGTGAAAGTA

Create PDF with GO2PDF for free, if you wish to

>Marker684533 Chr5 SNP_site:18202986 Ref_Type:A
ACTCGCCGTTTTCATTTACCCCTTTCAGCTTGCGAACTGATGAAAGAGCAGCAAGAAGAAAGGAGGCAAGCTTTTGTTGCXXXXXXXXXXC
ACAAACGAGTCACAAAAAAAGGTGCAACTTCAAACAAAACTAAAGGTAACAAATAAAAAATATTACTTGGTAATCTGTA
ACTCGCCGTTTTCATTTACCCCTTTCAGCTTGCGAACTGATGAAAGAGCAGCAAGAAGAAAGGAGGCGAGCTTTTGTTGCXXXXXXXXXXC
ACAAACGAGTCACAAAAAAAGGTGCAACTTCAAACAAAACTAAAGGTAACAAATAAAAAATATTACTTGGTAATCTGTA
>Marker684690 Chr5 SNP_site:6181673 Ref_Type:A
AACATGTTAGTTTTTATTGTTTTCATAATCTAGATGATTTGAAATTAGCAAAATCAAATTTAGATCATCTGTCAAAATAAXXXXXXXXXXC
AGGTTTCAATTAAGCAATGTTTCTGATAATAGTGAGTTTGAAAAGGTTTTCTCAGGGCACCAAGAGCTTTTGGATAGTG
AACATGTTAGTTTTTATTGTTTTCATAATCTAGATGATTTGAAATTAGCAAAATCAAATTTAGATCATCTGTCAAAATAAXXXXXXXXXXC
AGGTTTCAGTTAAGCAATGTTTCTGATAATAGTGAGTTTGAAAAGGTTTTCTCAGGGCACCAAGAGCTTTTGGATAGTG
>Marker685403 Chr5 SNP_site:16576550 Ref_Type:A
TACACTAGATAGCCCCATGACTTTAGTTTATTGAATTCAAAATTATAATATTCAATTTTCAGAAATAAGTCGTAATTAATXXXXXXXXXXA
AATTTACAATAATATATCTTCTATCCATCCCTACCAAATATGATAATTCCAAAATTACAAGACCAATCCACACATGGTA
TACACTAGATAGCCCCATGACTTTAGTTTATTGGATTCAAAATTATAATATTCAATTTTCAGAAATAAGTCGTAATTAATXXXXXXXXXXA
AATTTACAATAATATATCTTCTATCCATCCCTACCAAATATGATAATTCCAAAATTACAAGACCAATCCACACATGGTA
>Marker68666 Chr4 SNP_site:16456803 Ref_Type:C
ACTATTACAGCAGGTGTTTAATATCACCATTGAAGTATGTTTTTGAGTAGTCATCCAAAATACGTTGAAGTATGTGTGCTXXXXXXXXXXC
TCATGCTCATGGGAATTACCCAAGTGTGAGTCTGTTTGAAAGGACCTCAAACATGGGTGCGATCATACCAGCCCTAGTG
ACTATTACAGCAGGTGTTTAATATCACCATTGAAGTATGTTTTTGAGTAGTCATCCAAAATACGTTGAAGTATGTGTGCTXXXXXXXXXXC
TCATGCTCATGGGAATTACCCAAGTGTGAGTCTGTTTGAAAGGACCTCAAACATGGGTGCGATCATACCAGTCCTAGTG
>Marker687102 Chr5 SNP_site:23191736 Ref_Type:C
ACTCATAATTTATAAAATTGGGAGAAGACTAATTGTAAAGAAACATGATGTGGTTATGACTTGTTGGGGAGTATATTCTCXXXXXXXXXXG
TCTAAACTATCGATAATCATTTATTTTTTCATTTTCTAAAATTCCATCCATATGTCTAAACTATTAGTGATAAATCAGT
ACTCATAATTTATAAAATTGGGAGAAGACTCATTGTAAAGAAACATGATGTGGTTATGACTTGTTGGGGAGTATATTCTCXXXXXXXXXXG
TCTAAACTATCGATAATCATTTGTTTTTTCATTTTCTAAAATTCCATCCATATATCTAAACTATTAGTGATAAATCAGT
>Marker687102 Chr5 SNP_site:23191958 Ref_Type:G
ACTCATAATTTATAAAATTGGGAGAAGACTAATTGTAAAGAAACATGATGTGGTTATGACTTGTTGGGGAGTATATTCTCXXXXXXXXXXG
TCTAAACTATCGATAATCATTTATTTTTTCATTTTCTAAAATTCCATCCATATGTCTAAACTATTAGTGATAAATCAGT
ACTCATAATTTATAAAATTGGGAGAAGACTCATTGTAAAGAAACATGATGTGGTTATGACTTGTTGGGGAGTATATTCTCXXXXXXXXXXG
TCTAAACTATCGATAATCATTTGTTTTTTCATTTTCTAAAATTCCATCCATATATCTAAACTATTAGTGATAAATCAGT
>Marker687102 Chr5 SNP_site:23191989 Ref_Type:A
ACTCATAATTTATAAAATTGGGAGAAGACTAATTGTAAAGAAACATGATGTGGTTATGACTTGTTGGGGAGTATATTCTCXXXXXXXXXXG
TCTAAACTATCGATAATCATTTATTTTTTCATTTTCTAAAATTCCATCCATATGTCTAAACTATTAGTGATAAATCAGT
ACTCATAATTTATAAAATTGGGAGAAGACTCATTGTAAAGAAACATGATGTGGTTATGACTTGTTGGGGAGTATATTCTCXXXXXXXXXXG
TCTAAACTATCGATAATCATTTGTTTTTTCATTTTCTAAAATTCCATCCATATATCTAAACTATTAGTGATAAATCAGT
>Marker68731 Chr4 SNP_site:11623258 Ref_Type:T
CACACAAATACGTTTTCAATCTTTTATATTTGATAGAATAAAATCACTATGTTTTTCTATATAACCTGGAGCTTGTCATTXXXXXXXXXXA
TTAATGAAGCTTGTAAGTTTTTCTTTTGATCTTTCATCAATGATTGAAAAATTATAAAAGACTTCAAGAATTTAAGGTC
CACACAAATACGTTTTCAATCTTTTATATTTGATAGAATAAAATCACTATGTTTTTCTATATAACCTGGAGCTTGTCATTXXXXXXXXXXA
TTAATGAAGCTTGTAAGTTTTTCTTTTGATCTTTCATTAATGATTGAAAAATTATAAAAGACTTCAAGAATTTAAGGTC
>Marker687344 Chr5 SNP_site:13784259 Ref_Type:A
CACTTGTCTAAGGAACAGTGTCCTAAGACACCTCAAGTTGTGGGCAACTTAATATATGTTATGCTCTACATTAGGCCAGAXXXXXXXXXXG
ATTTGATCCTTATGGGATACACTGACTCTAATTTTCAAACCAATCAGGATTCTAGAAAATTCATGTCGGGATTAGTGTT
CACTTGTCTAAGGAACAGTGTCCTAAGACACCTCAAGTTGTGGGCAGCTTAATATATGTTATGCTCTACATTAGGCCAGAXXXXXXXXXXG

Create PDF with GO2PDF for free, if you wish to

ATTTGATCCTTATGGGATACACTGACTCTAATTTTCAAACCAATCAGGATTCTAGAAAATTCATGTCGGGATTAGTGTT

>Marker687426 Chr5 SNP_site:14703379 Ref_Type:A

ACCACTTTGAATCATAATCTTTCTTGAACCATTTAGGTTATCTGTTGTTGGGCCATTGTGACTAAGAGATGCACTGAGTTXXXXXXXXXXC

TTCGGCTAAAGTTGTCCTGAAATGTATGGAAGAAAAAATCAAGCAATTATGTAGCATTGTGTAGTATGATCTAGGTTGT

ACCACTTTGAATCATAATCTTTCTTGAACCATTTAGGTTATCTGTTGTTGGGCCATTGTGACTATGAGATGCACTGAGTTXXXXXXXXXXC

TTGGGCTAAAGTTGTCCTGAAATGTATGGAAGAAAAAATCAAGCAATTATGTAGCATTGTGTAGTATGATCTAGGTTGT

>Marker687426 Chr5 SNP_site:14703513 Ref_Type:C

ACCACTTTGAATCATAATCTTTCTTGAACCATTTAGGTTATCTGTTGTTGGGCCATTGTGACTAAGAGATGCACTGAGTTXXXXXXXXXXC

TTCGGCTAAAGTTGTCCTGAAATGTATGGAAGAAAAAATCAAGCAATTATGTAGCATTGTGTAGTATGATCTAGGTTGT

ACCACTTTGAATCATAATCTTTCTTGAACCATTTAGGTTATCTGTTGTTGGGCCATTGTGACTATGAGATGCACTGAGTTXXXXXXXXXXC

TTGGGCTAAAGTTGTCCTGAAATGTATGGAAGAAAAAATCAAGCAATTATGTAGCATTGTGTAGTATGATCTAGGTTGT

>Marker687707 Chr5 SNP_site:202434 Ref_Type:T

ACATAGAACTAAAAGCGAAAATTCATAGGACCTTATGGAAGTTCAAGATAATGTCTAAACTATAAAATATGTGATAGTTTXXXXXXXXXXT

GCAGTAGAACCTTAAAAATAGTCATGGTATTATCCATGGTTGTGCTTTATTGGTTAAGCAGAATGCATTAGAGAGGAGT

ACATAGAACTAAAAGCGAAAATTCATAGGACCTTATGGAAGTTCAAGATAATGTCTAAACTATAAAATATGTGATAGTTTXXXXXXXXXXT

GTAGTAGAACCTTAAAAATAGTCATGGTATTATCCATGGTTGTGCTTTATTGGTTAAGCAGAATGCATTAGAGAGGAGT

>Marker687846 Chr5 SNP_site:24178770 Ref_Type:C

AACAGGAACCAGTAAATTTTTCTCAAGAACCATTCCTCAAATGCCGCTCCAAGGCCTTGTGCAATGGATGATCATCGATGXXXXXXXXXXT

CATGAATTCGAGAATCACCTGTTTTTCAATATTCATTCGATTCGATCTCAGAGCTGATTATGAATGTATGAGTGCTTGT

AACAGGAACCAGTAAATTTTTCTCAAGAACCATTTCTCAAATGCCGCTCCAAGGCCTTGTGCAATGGATGATCATCGATGXXXXXXXXXXT

CATGAATTCGAGAATCACCTGTTTTTCAATATTCATTCGATTCGATCTCAGAGCTGATTATGAATGTATGAGTGCTTGT

>Marker68878 Chr4 SNP_site:5424985 Ref_Type:C

CACATGGTTGTTTTTGGACGGAATTAAACTTTCACTTTAAAACTAATTTACTTGATTTAAGTTCATGCTTTCCAATCTCAXXXXXXXXXXG

TATGACAATTTTTTTTAAATGGTCCATAAATGTTTAATTATAATGTAATTAGCTAATGCAGAAGTAGAGAAATTATGTC

CACATGGTTGTTTTTGGAGGGAATTAAACTTTCACTTTAAAACTAATTTACTTGATTTAAGTTCATGCTTTCCAATCTCAXXXXXXXXXXG

TATGACAATTTTTTTTAAATGGTCCATAAATGTTTAATTATAATGTAATTAGCTAATGCAGAAGTAGAGAAATTATGTC

>Marker688861 Chr5 SNP_site:14700172 Ref_Type:A

AACCGCCCTTCCCCAAAACGGTCCTACAATGTTAATGTTTTAGTTGGACGAAGAGCAAGAGTGGGAGGGATGAAAAAAAAXXXXXXXXXXT

ATCCCCGCCACCCCTTGTCGTGAAAAAAGCTAGAAGGTATGGATTGAAACCCTAATTTGAAACCCATAAAATTTGAAGT

AACCGCCCTTCCCCAAAACGGTCCTACAATGTTAATGTTTTAGTTGGACGAAGAGCAAGAGTGGGAGGGATGGAAAAAAAXXXXXXXXXXT

ATCCCCGCCACCCCTTGTCGTGAAAAAAGCTAGAAGGTATGGATTGAAACCCTAATTTGAAACCCATAAAATTTGAAGT

>Marker689014 Chr5 SNP_site:6830190 Ref_Type:T

ACCTTGTTTCATGTCGTCTGGACTCTTTCTGCTTCCCATTGTAAATAAGCATCTCTGTGCTGGCACAACCTCCTCCTATTXXXXXXXXXXA

ATATTGGAAACGATTACTGATATACAAAAGAAAACGAATAAAAGAAAAATTCATGGCAAGTGGAACCAAGAAGGACGTA

ACCTTGTTTCATGTCGTCTGGACTCTTTCTGCTTCCCATTGTAAATAAGCATTTCTGTGCTGGCACAACCTCCTCCTATTXXXXXXXXXXA

ATATTGGAAACGATTACTGATATACAAAAGAAAACGAATAAAAGAAAAATTCATGGCAAGTGGAACCAAGAAGGACGTA

>Marker689843 Chr5 SNP_site:8224281 Ref_Type:A

TACCAACACAACTGTTAGAAAACAGTAGCATAGCCTGAGAAGAATATTTCAAAATTCCAGTTTCTACCACGTGTTGCATGXXXXXXXXXXA

TCAAGGGTGGAAATAATCAAGAGTTACTGTTTGTCTCATTAAGAACAGTTTGCTTCTTGCTAAACACGAGATAAAAGGT

TACCAACACAACTGTTAGAAAACAGTAGCATAGCCTGAGAAGAATATTTCAAAATTCCAGTTTCTACCACGTGTTGCATGXXXXXXXXXXA

TCAAGGGTGGAAATAATCAAGAGTTACTGTTTGTCTCTTTAAGAACAGTTTGCTTCTTGCTAAACACGAGATAAAAGGT

>Marker689933 Chr5 SNP_site:23793417 Ref_Type:T

AACAGAAACATTGATTAGTTGGATGATAACCTTATTGTTTGGACTAAGTCTTGCTCTTTCAACATGGGGAAGTATTATAGXXXXXXXXXXA

TCTCACTAATTATCACATAATTTATTTTCCCAAGTCCTAATTTTAAATCAAGCATAAATGATTGATTTTCAATGTTGTT

Create PDF with GO2PDF for free, if you wish to

AACAGAAACATTGATTAGTTGGATGATAACCTTATTGTTTGGACTAAGTCTTGCTCTTTCAACATGGGGAAGTATTATAGXXXXXXXXXXA
TCTCACTAATTATCACATAATTTATTTTCCCAAGTCCTAATTTTAAATCAAGCATAAATGATTGATTTTTAATGTTGTT
>Marker690064 Chr5 SNP_site:10019251 Ref_Type:C
GACAAAATGGGATTGCTGAAAGAAAAAAATAGAAATTTACTTGAAATGGCACACGCCCTAATGTTTTCCATGCATGTTTCXXXXXXXXXXT
CAAAAATGTGAAAAATTAATTTGATCAAAGTAACATTTAGCTATATCAAAAGATATTAGCGGTAAAACTAGCTAAAGTA
GACAAAATGGGATTGCTGAAAGAAAAAAATAGAAATTTACTTGAAATGGCACACGCCGTAATGTTTTCCATGCATGTTTCXXXXXXXXXXT
CAAAAATGTGAAAAATTAATATGATCAAAGTAACATTTAGCTATATCAAAAGATATTAGCTGTAAAACTAGCTAAAGTA
>Marker690064 Chr5 SNP_site:10019398 Ref_Type:T
GACAAAATGGGATTGCTGAAAGAAAAAAATAGAAATTTACTTGAAATGGCACACGCCCTAATGTTTTCCATGCATGTTTCXXXXXXXXXXT
CAAAAATGTGAAAAATTAATTTGATCAAAGTAACATTTAGCTATATCAAAAGATATTAGCGGTAAAACTAGCTAAAGTA
GACAAAATGGGATTGCTGAAAGAAAAAAATAGAAATTTACTTGAAATGGCACACGCCGTAATGTTTTCCATGCATGTTTCXXXXXXXXXXT
CAAAAATGTGAAAAATTAATATGATCAAAGTAACATTTAGCTATATCAAAAGATATTAGCTGTAAAACTAGCTAAAGTA
>Marker690064 Chr5 SNP_site:10019438 Ref_Type:G
GACAAAATGGGATTGCTGAAAGAAAAAAATAGAAATTTACTTGAAATGGCACACGCCCTAATGTTTTCCATGCATGTTTCXXXXXXXXXXT
CAAAAATGTGAAAAATTAATTTGATCAAAGTAACATTTAGCTATATCAAAAGATATTAGCGGTAAAACTAGCTAAAGTA
GACAAAATGGGATTGCTGAAAGAAAAAAATAGAAATTTACTTGAAATGGCACACGCCGTAATGTTTTCCATGCATGTTTCXXXXXXXXXXT
CAAAAATGTGAAAAATTAATATGATCAAAGTAACATTTAGCTATATCAAAAGATATTAGCTGTAAAACTAGCTAAAGTA
>Marker690094 Chr5 SNP_site:23411956 Ref_Type:G
GACCTAATTAACGAAAGAAAATGGTTCATTATTGTATGTGCCTAGCTTTATTAGTTTAGTATGGATTGTAAGTGTTGGAGXXXXXXXXXXA
TTGATAAGAGCAATTATATATTTAACTGTTCCAATTTTGAAATTGGGCACTAGGGTTTTGACGTTAGACTTTTTTCGTT
GACCTAATTAACGAAAGAAAGTGGTTCATTATTGTATGTGCCTAGCTTTATTAGTTTAGTATGGATTGTAAGTGTTGGAGXXXXXXXXXXA
TTGATAAGAGCAATTATATATTTAACTGTTCCAATTTTGAAATTGGGCACTAGGGTTTTGACGTTAGACTTTTTTCGTT
>Marker69017 Chr4 SNP_site:1632847 Ref_Type:G
ACCGACGCTGAATTCGGTATTTGACCATTGCCAACAAAGAAAAAGGGCCTACAAAAAAATACAGATGTCATTATAATAGAXXXXXXXXXXT
ACCCATTGATTCACACACGAACAAGATGAAGTTTCAACACTATGGGGCGTTTTGGTTTATCCAGCAAGAAGAAGAAAGT
ACCGACGCTGAATTCGGTATTTGACCATTGCCAACAAAGAAAAAGGGCCTACAAAAAGATACAGACGTCATTATAATAGAXXXXXXXXXXT
ACCCATTGATTCACACACGAACAAGATGAAGTTTCAACACTATGGGGCGTTTTGGTTTATCCAGCAAGAAGAAGAAAGT
>Marker69017 Chr4 SNP_site:1632855 Ref_Type:C
ACCGACGCTGAATTCGGTATTTGACCATTGCCAACAAAGAAAAAGGGCCTACAAAAAAATACAGATGTCATTATAATAGAXXXXXXXXXXT
ACCCATTGATTCACACACGAACAAGATGAAGTTTCAACACTATGGGGCGTTTTGGTTTATCCAGCAAGAAGAAGAAAGT
ACCGACGCTGAATTCGGTATTTGACCATTGCCAACAAAGAAAAAGGGCCTACAAAAAGATACAGACGTCATTATAATAGAXXXXXXXXXXT
ACCCATTGATTCACACACGAACAAGATGAAGTTTCAACACTATGGGGCGTTTTGGTTTATCCAGCAAGAAGAAGAAAGT
>Marker690249 Chr5 SNP_site:6440806 Ref_Type:G
TACATCAACTTGCATTTAATCGTTCAATTTTAAGTTCCAGCATGAAAATTCTTTAATATTTAGGAGTTAGATTAAAAATTXXXXXXXXXXA
CCCAACAAAGGATAGAGAAAAATAGGGAGGACGCTCCCTGTTTCGATGTTATATTCAAAAATGAATTCCACCACTAGTT
TACATCAACTTGCATTTAATCGTTCAATTTTAAGTTCCAGCATGAAAATTCTTTAATATTTAGGAGTTAGATTAAAAATTXXXXXXXXXXA
CCGAACAAAGGATAGAGAAAAATAGGGAGGACGCTCCCTGTTTCGATGTTATATTCAAAAATGAATTCCACCACTAGTT
>Marker690717 Chr5 SNP_site:11639896 Ref_Type:T
ACTTAAAATTGACCTTCTCACCTTCTCTTGGTCCAGACGCCAAACTTCTCGATTCCAATTTACACAATAGCCTAGGTCTAXXXXXXXXXXG
CTTGCATGATTTAGTCTTAACACCACCTACTTGACACCTTATGCTAACATTTTCACACTTTAAACCAAACATCCAATGT
ACTTAAAATTGACCTTCTCACCTTCTCTTGGTCCAGACGCCAAACTTCTCGATTCCAATTTACACAATAGCCTAGGTCTAXXXXXXXXXXG
CTTGCATGATTTAGTCTTAACACCACCTACTTGACACCTTATGCTAACATTTTCACACTTTAAACCAAACTTCCAATGT
>Marker691632 Chr5 SNP_site:1106315 Ref_Type:C
CACATATGTTGCCTCCTCTCCCTCCCCTTCATTGCTAACTAGATGTAATAGAATAACTAGCTAGGGATATCTTAATTGCAXXXXXXXXXXT

Create PDF with GO2PDF for free, if you wish to

ATCTTGTTCAAACTCAATACGACATCTCGACCTATACCTCAACCATATAATAATGTTAAAATATTCCCGTCCCAAAGTA
CACATATGTTGCCTCCTCTCCCTCCCCTTCATTGCTAACTAGATGTAATAGAATAACTAGCTAGGGATATCTTAATTGCAXXXXXXXXXXT
ATCTTGTTCAAACTCAATACGACATCTCGACCTATACCTCAACCATATAATAATGTTAAAATATTCTCGTCCCAAAGTA
>Marker691798 Chr5 SNP_site:25460404 Ref_Type:G
AACAAGATGCGAGTCCTGCAGCAGAAACAAAAGACCAATCCCCACAGTTCATTTGAAGGAGAAGTTAAAGCAATTCCGAAXXXXXXXXXXC
ACCACAACTTGTTTAACAATGCCAACCAACATTCATTTTTGACAATTTTCTAGCTTCTCAAAGAATCATCAATTCAAGT
AACAAGATGCGAGTCCTGCAGCAGAAAGAAAAGACCAATCCCCACAGTTCATTTGAAGGAGAAGTTAAAGCAATTCCGAAXXXXXXXXXXC
ACCACAACTTGTTTAACAATGCCAACCAACATTCATTTTTGACAATTTTCTAGCTTCTCAAAGAATCATCAATTCAAGT
>Marker692010 Chr5 SNP_site:24802587 Ref_Type:G
ACTGAACGTTATATTTTCAAGTGTTGAGAGTCATGTTTGGAAGACTTATTATGCAAATTGTTGATATAAATATTATATTTXXXXXXXXXXA
AACAACCCATCTCTCAGTCAATTACCATATACATATACACTGAATTCATCTCTTTCTCAAGTAAATTATATTATAGGTT
ACTGAACGTTATATTTTCAAGTGTTGGGAGTCATGTTTGGAAGACTTATTATGAAAATTGTTGATATAAATATTATATTTXXXXXXXXXXA
AACAACCCATCTCTCAGTCAATTACCATATACATATACACTGAATTCATCTCTTTCTCAAGTAAATTATATTATAGGTT
>Marker692010 Chr5 SNP_site:24802614 Ref_Type:A
ACTGAACGTTATATTTTCAAGTGTTGAGAGTCATGTTTGGAAGACTTATTATGCAAATTGTTGATATAAATATTATATTTXXXXXXXXXXA
AACAACCCATCTCTCAGTCAATTACCATATACATATACACTGAATTCATCTCTTTCTCAAGTAAATTATATTATAGGTT
ACTGAACGTTATATTTTCAAGTGTTGGGAGTCATGTTTGGAAGACTTATTATGAAAATTGTTGATATAAATATTATATTTXXXXXXXXXXA
AACAACCCATCTCTCAGTCAATTACCATATACATATACACTGAATTCATCTCTTTCTCAAGTAAATTATATTATAGGTT
>Marker692722 Chr5 SNP_site:2604787 Ref_Type:G
AACTCATGGATTGCCTCACGTGAGGATGATGCCCTTAATGAAATGAAAAGGGAGGCTAACCCCTGAACTTGGGGGAAGGCXXXXXXXXXXG
ATCAACCCCACGTAGCTGTTTTGGCTGGAACACGTTCGTCGCCGGACAGCCTCTATTCGCTCTCTCCCTGGTGTGGGTC
AACTCATGGATTGCCTCACGTGAGGATGATGCCCTTAATGAAATGAAAATGGAGGCTAACCCCTGAACTTGGGGGAAGGCXXXXXXXXXXG
ATCAACCCCACGTAGCTGTTTTGGCTGGAACACGTTCGTCGCCGGACAGCCTCTATTCGCTCTCTCCCTGGTGTGGGTC
>Marker693409 Chr5 SNP_site:11391050 Ref_Type:C
AACCTTCTCCAAAATTCAGCAAGATTAATCCTTTTATTACCCAACTAATGTATGCATTATATAGTTAATATACATACAAAXXXXXXXXXXA
ATGGACTAATTCCACTAACTATGGAATTATCTACTAACCATGGTCAAAGTCATCATTTGACTTTCAAGAGTCAGAAGTC
AACCTTCTCCAAAATTCAGCAAGATTAATCCTTTTATTACCCAACTAATGTATGCATTATATAGTTAATATACATACAAAXXXXXXXXXXA
ATGGATTAATTCCACTAACTATGGAATTATCTACTAACCATGGTCAAAGTCATCATTTGACTTTCAAGAGTCAGAAGTC
>Marker693655 Chr5 SNP_site:15878831 Ref_Type:A
AACTAGGCTAACATACCAAAATTTTCTAAAATAAAAGAAAGAATGTGCAATTGGACCCAAACCTTAAAATCAAACCAACAXXXXXXXXXXT
TTGCTATCTATTTTTACCATCATAGTTTATAAAACCAAACAATACTTTGAAAAAAAAAACTAGCTTTTAAAATTTTGTT
AACTAGGCTAACATACCAAAATTTTCTAAAATAAAAGAAAGAATGTGCAATTGGACCCGAACCTTAAAATCAAACCAACAXXXXXXXXXXT
TTGCTATCTATTTTTACCATCATACTTTATAAAACCAAACAATACTTTGAAAAAAAAAACTAGCTTTTAAAATTTTGTT
>Marker693655 Chr5 SNP_site:15878990 Ref_Type:G
AACTAGGCTAACATACCAAAATTTTCTAAAATAAAAGAAAGAATGTGCAATTGGACCCAAACCTTAAAATCAAACCAACAXXXXXXXXXXT
TTGCTATCTATTTTTACCATCATAGTTTATAAAACCAAACAATACTTTGAAAAAAAAAACTAGCTTTTAAAATTTTGTT
AACTAGGCTAACATACCAAAATTTTCTAAAATAAAAGAAAGAATGTGCAATTGGACCCGAACCTTAAAATCAAACCAACAXXXXXXXXXXT
TTGCTATCTATTTTTACCATCATACTTTATAAAACCAAACAATACTTTGAAAAAAAAAACTAGCTTTTAAAATTTTGTT
>Marker69461 Chr4 SNP_site:6887668 Ref_Type:G
ACTCTATAAATCTGAATTGAATAAAAATAAGATTACTTGACTATAATATGAACATTGAACTTTATTTAGTAACATAAAAGXXXXXXXXXXT
TAATATAAACGAATAAATACAAGAAGCAAGAAAGAAAACAAACTAAGGACCGGAGGAAAGCCGAAGAGAGACCCAAAGT
ACTCTATAAATCTGAATTGAATAAAAATAAGATTACTTGACTATAATATGAACATTGAACTTTATTTAGTAACATAAAAGXXXXXXXXXXT
TAATATAAACGAATAAATACAAGAAGCAAGAAAGAAAACAAACTAAGGACCGGAGGAAAGCCGAGGAGAGACCCAAAGT
>Marker694909 Chr5 SNP_site:6964916 Ref_Type:C

Create PDF with GO2PDF for free, if you wish to

AACCTTTGCAGATAAGCGAAGATGAAATTACTTTCTTGACTGGTGGAGATGATCCCTATGATGACAATGTGGTGTTGAAAXXXXXXXXXXC
TCTCTTTTCCACTCTTTGTTAAATCACCAATAGACCCAAATACTTAACCTGTAGCATCCAACACTTCTATTTTCTTGTG
AACCTTTGCAGATAAGCGAAGATGAAATTACTTTCTTGACTGGTGGAGATGATCCCTATGATGATAATGTGGTGTTGAAAXXXXXXXXXXC
TCTCTTTTCCACTCTTTGTTAAATCACCAATAGACCCAAATACTTAACCTGTAGCATCCAACACTTCTATTTTCTTGTG
>Marker695531 Chr5 SNP_site:24329979 Ref_Type:G
AACGGATGGTTTCTTTTACCATTTTTTTCTCTTGTTTCTGTGTAGGAAATGTTGAACATTAGAAAATGAAAAGTTTGCTGXXXXXXXXXXC
TCCTGTCAGTTTGTGTGCCTACCTTGTTTAATACTGTTGCATCCATTCTATTTAAACTTTTTACACAAGTATTTGAGTG
AACGGATGGTTTCTTTTACCATTTTTTTCTCTTGTTTCTGTGTAGGAAATGTTGAACATTAGAAAATGAAAAGTTTGCTGXXXXXXXXXXC
TCCTGTCAGTTTGTGTGCCTACCTTGTTTAATACTGTTGCATCCATTCTGTTTAAACTTTTTACACAAGTATTTGAGTG
>Marker696234 Chr5 SNP_site:23286219 Ref_Type:C
AACCAATTTGTGATACTTTTGACACCCTGCACACTGAGCACACAATAATAATTGGAAGCATTAAGAACACATTAAACAGCXXXXXXXXXXA
ATAACACACACTATATGTTTTTAATAAAAAAAACTTTAATCTGCTAGACGTTGCAAACATGAAATTTAGTCTATTTAGT
AACCAATTTGTGATACTTTTGACACCCTGCACACTGAGCACAGAATAATAATTGGAAGCATTAAGAACACATTAAACAGCXXXXXXXXXXA
ATAACACACACTATATGTTTTTAATAAAAAAAACTTTAATCTGCTAGACGTTGCAAACATGAAATTTAGTCTATTTAGT
>Marker698195 Chr5 SNP_site:24719743 Ref_Type:T
TACGTATATATATCATCTACCAAGAGGTTAAAGGTTCAAATTTCATCCCCAATATGTTGCTGAACCTTTTTCAGAATAGAXXXXXXXXXXG
AATAATGTTGGCCGGAATTCACCGTGGGTTGGTCTGGCAGTCATTACAACTAAAGAATAGAGATTGGTTCAGTATAGTA
TACGTATATATATCATCTACCAAGAGGTTAAAGGTTCAAATTTCATCCCCAATATGTTGCTGAACCTTTTTCAGAATAGAXXXXXXXXXXG
AATAATGTTGGCTGGAATTCACCGTGGGTTGGTCTGGCAGTCATTACAACTAAAGAATAGAGATTGGTTCAGTATAGTA
>Marker698374 Chr5 SNP_site:11652210 Ref_Type:T
CACAACGAGGAGTTTGGTAACAAACTTCACCTTAGTGAGGTAACTTCATTGGTCTATTTTCTTTCATTAAATACACTAGAXXXXXXXXXXT
TCTTGCAAATACTTGTCGAATTCAAGCTCGTGATGGATCTTTGAAAATCTTTGAAATTCTTTCAATTCTTTCTTAAGTG
CACAACGAGGAGTTTGGTAACAAACTTCACCTTAGTGAGGTAACTTCATTGGTCTATTTTCTTTCATTAAATACACTATAXXXXXXXXXXT
TCTTGCAAATACTTGTCGAATTCAAGCTCGTGATGGATCTTTGAAAATCTTTGAAATTCTTTCAATTCTTTCTTAAGTG
>Marker698681 Chr5 SNP_site:8918362 Ref_Type:T
CACGCTAAAGCTTTTGAAAAATGTTTGAAAATCCTGTGGCATCTTTTGAAAAACAAGGCATTTTAACAAAAGTTAAAACTXXXXXXXXXXC
TTCAAAAGTAAAAACCAGAGAGTTAAGTAATAAGAGGCGATGAGAGATACAATTGAGGAGAGAAACATATCCATAGAGT
CACGCTAAAGCTTTTGAAATATGTTTGAAAATCCTGTGGCATCGTTTGAAAAACAAGGCATTTTAACAAAAGTTAAAACTXXXXXXXXXXC
TTCAAAAGTAAAAACCAGAGAGTTAAGTAATAAGAGGCGATGAGAGATACAATTGAGGAGAGAAACATATCCATAGAGT
>Marker698681 Chr5 SNP_site:8918386 Ref_Type:G
CACGCTAAAGCTTTTGAAAAATGTTTGAAAATCCTGTGGCATCTTTTGAAAAACAAGGCATTTTAACAAAAGTTAAAACTXXXXXXXXXXC
TTCAAAAGTAAAAACCAGAGAGTTAAGTAATAAGAGGCGATGAGAGATACAATTGAGGAGAGAAACATATCCATAGAGT
CACGCTAAAGCTTTTGAAATATGTTTGAAAATCCTGTGGCATCGTTTGAAAAACAAGGCATTTTAACAAAAGTTAAAACTXXXXXXXXXXC
TTCAAAAGTAAAAACCAGAGAGTTAAGTAATAAGAGGCGATGAGAGATACAATTGAGGAGAGAAACATATCCATAGAGT
>Marker698721 Chr5 SNP_site:25438307 Ref_Type:C
ACCCACATAACACCATCCAATCTTCTGCACTCCCCCCACATCCCACTTCCCACTGACTAATTGTCAATTTGTTAAATAACXXXXXXXXXXT
TTTGTTTGAAGGGTTGTGAATTTTTAATCGCATTTATATAATTATGTTTCTTTCTTCTATCTGCTGTGTTTCATGCGGT
ACCCACATAACACCATCCAATCTTCTGCACTCCCCCCACATCCCACTTCCCACTGACTAATTGTCAATTTGTTAAATAACXXXXXXXXXXT
TTTGTTTGAAGGGTTGTGAATTTTTAATCGCATTTATATAATTATGTTTCTTTCTTCTATGTGCTGTGTTTCATGCGGT
>Marker698853 Chr5 SNP_site:25318239 Ref_Type:A
ACAATGTTTTAAGACACCTCAAGAAGTTGAGAATATAAGACATGTTTCCTATGCATCAACTGATTGGAGTCTGATATATGXXXXXXXXXXT
ATGGAGGTGGAAATTTGATACACTGATTTCGATTTCTAGTTAGTAAATATTTTAGAAAATCAACACCAGGATCAGTGTT
ACAATGTTTTGAGACACCTCAAGAAGTTGAGAATATAAGACATGTTTCCTATGCATCAACTGATTGGAGTTTGATATATGXXXXXXXXXXT
ATGGAGGTGGAAATTTGATACACTGATTTCGATTTCTAGTTAGTAAATATTTTAGAAAATCAACACCAGGATCAGTGTT

Create PDF with GO2PDF for free, if you wish to

>Marker698853 Chr5 SNP_site:25318299 Ref_Type:C
ACAATGTTTTAAGACACCTCAAGAAGTTGAGAATATAAGACATGTTTCCTATGCATCAACTGATTGGAGTCTGATATATGXXXXXXXXXXT
ATGGAGGTGGAAATTTGATACACTGATTTCGATTTCTAGTTAGTAAATATTTTAGAAAATCAACACCAGGATCAGTGTT
ACAATGTTTTGAGACACCTCAAGAAGTTGAGAATATAAGACATGTTTCCTATGCATCAACTGATTGGAGTTTGATATATGXXXXXXXXXXT
ATGGAGGTGGAAATTTGATACACTGATTTCGATTTCTAGTTAGTAAATATTTTAGAAAATCAACACCAGGATCAGTGTT
>Marker699409 Chr5 SNP_site:14087901 Ref_Type:T
CACTTCGGTTCCAGTATAGGTCTCGATTGATAAAAATGCTGGAACTAGCATATGTATCCCGGTATGTGACTAAGATAGTTXXXXXXXXXXA
CGACCACTATCTTGTTTCATCAGTTTCTTCTTAAGATTAGCAATCGCCTCCTAATGAGAACTTAAATCCTCTGGTTGGT
CACTTCGGTTCCAGTATAGGTCTCGATTGATAAAAATGCTGGAACTAGCATATGTATCCCGGTATGTGACTAAGATAGTTXXXXXXXXXXA
CGACCACTATCTTGTTTCATCAGTTTTTTCTTAAGATTAGCAATCGCCTCCTAATGAGAACTTAAATCCTCTGGTTGGT
>Marker700067 Chr5 SNP_site:3236403 Ref_Type:A
AACTAGATTTGTATGCCTAATGGCCAACTAGTTTTGGTTTAAAATTTGCATGCATAGCAGTAATGACCCTAGTTAGGGACXXXXXXXXXXC
TTCGAATTTTGTAGTTTGTCTTTCTTTTTTTCTATCTACACTTGATTATATCTTCTAGTGGGCTCATTGTTGAAGAGTA
AACTAGATTTGTATGCCTAATGGCCAACTAGTTTTGGTTTAAAATTTGCATGCATAGCAGTAATGACCCTGGTTAGGGACXXXXXXXXXXC
TTCGAATTTTGTAGTTTGTCTTTCTTTTTTTCTATCTACACTTGATTATATCTTCTAGTGGGCTCATTGTTGAAGAGTA
>Marker700069 Chr5 SNP_site:8862251 Ref_Type:T
CACAATTCATAAATGCCTAAATCCAATATCTACATAGATGTTAAAGCAATACTGATAACTAATAATGGCCCCAAGTCCAAXXXXXXXXXXT
CATATAGTTGTATATTTATTATGCTTAGCAAACGTTAAGTAAGCGACTAGCTAATTGAACTCATAGACTTTGCACAGTA
CACAATTCATAAATGCCTAAATCCAATATCTACATAGATGTTAAAGCAATACTGATAACTAATAATGGCCTCAAGTCCAAXXXXXXXXXXT
CATATAGTTGTATATTTATTATGCTTAACAAACGTTAAGTAAGCGACTAGCTAATTGAACTCATAGACTTTGCACAGTA
>Marker700069 Chr5 SNP_site:8862388 Ref_Type:A
CACAATTCATAAATGCCTAAATCCAATATCTACATAGATGTTAAAGCAATACTGATAACTAATAATGGCCCCAAGTCCAAXXXXXXXXXXT
CATATAGTTGTATATTTATTATGCTTAGCAAACGTTAAGTAAGCGACTAGCTAATTGAACTCATAGACTTTGCACAGTA
CACAATTCATAAATGCCTAAATCCAATATCTACATAGATGTTAAAGCAATACTGATAACTAATAATGGCCTCAAGTCCAAXXXXXXXXXXT
CATATAGTTGTATATTTATTATGCTTAACAAACGTTAAGTAAGCGACTAGCTAATTGAACTCATAGACTTTGCACAGTA
>Marker7005 Chr4 SNP_site:4271242 Ref_Type:A
CACAAGTAAAGAAAAAAACAACTTTATAAAAAAAAAATTAATGATACAAATCAAATTATGCACGTCTAGGGTTATAGATTXXXXXXXXXXT
ATATGTATATTTCCAAGTTAATAAGTCTAACTCCTTAAGATGTTTGGGACAACGGTTGAGGTTATGAAAATTGAAAAGT
CACAAGTAAAGAAAAAAACAACTTTATAAAAGAAAAATTAATGATACAAATCAAATTATGCACGTCTAGGGTTAAAGATTXXXXXXXXXXT
ATATGTATATTTCCAAGTTAATAAGTCTAACTCCTTAAGATGTTTGGGACAACGGTTGAGGTTATGAAAATTGAAAAGT
>Marker7005 Chr4 SNP_site:4271285 Ref_Type:T
CACAAGTAAAGAAAAAAACAACTTTATAAAAAAAAAATTAATGATACAAATCAAATTATGCACGTCTAGGGTTATAGATTXXXXXXXXXXT
ATATGTATATTTCCAAGTTAATAAGTCTAACTCCTTAAGATGTTTGGGACAACGGTTGAGGTTATGAAAATTGAAAAGT
CACAAGTAAAGAAAAAAACAACTTTATAAAAGAAAAATTAATGATACAAATCAAATTATGCACGTCTAGGGTTAAAGATTXXXXXXXXXXT
ATATGTATATTTCCAAGTTAATAAGTCTAACTCCTTAAGATGTTTGGGACAACGGTTGAGGTTATGAAAATTGAAAAGT
>Marker700742 Chr5 SNP_site:26763321 Ref_Type:T
ACAATTTTTCGGAAGAGACTGTCATGTAGTTTGAGGATATTAAAGCTTCTTGCTGACCTCTCGAAGAAATTTCCATATATXXXXXXXXXXT
TGAGAACACCATTCTGTTACTCTTGGAAGAATTTTTGGAGCTAGTTCAGATTGTATTTCGCAACAGCTACGTTTGTGTG
ACAATTTTTCGGAAGAGACTGTCATGTAGTTTGAGGATATTAAAGCTTCTTGCTGACCTCTCGAAGAAATTTCCATATATXXXXXXXXXXT
TGAGAACACCATTCTGTTACTCTTGGAAGAATTTTTGGAGCTAGTTCAGATTGTATTTCGCAACATCTACGTTTGTGTG
>Marker700797 Chr5 SNP_site:8673506 Ref_Type:G
ACTGTGTTTTTCAAAGTCGTCGATCCATCCGGCAAATTCGAATCATAATAACCAGAAAGCACTCCCCAACTGTTTTCAGCXXXXXXXXXXA
AACTATGTTGAGTTCTTTTCCAATGAAGATGTCAATCCCTAAAGAATTCAAAGTAGTGGTCTCCGATACTATCCACAGT
ACTGTGTTTTTCAAAGTCGTCGATCCATCCGGCAAATTCGAATCATAATAACCAGAAAGCACTCCCCAACTGTTTTCAGCXXXXXXXXXXA

Create PDF with GO2PDF for free, if you wish to

AACTATGTTGAGTTCTTTTCCAATGAAGATGTCAGTCCCTAAAGAATTCAAAGTAGTGGTCTCCGAAACTATCCACAGT
>Marker700797 Chr5 SNP_site:8673538 Ref_Type:A
ACTGTGTTTTTCAAAGTCGTCGATCCATCCGGCAAATTCGAATCATAATAACCAGAAAGCACTCCCCAACTGTTTTCAGCXXXXXXXXXXA
AACTATGTTGAGTTCTTTTCCAATGAAGATGTCAATCCCTAAAGAATTCAAAGTAGTGGTCTCCGATACTATCCACAGT
ACTGTGTTTTTCAAAGTCGTCGATCCATCCGGCAAATTCGAATCATAATAACCAGAAAGCACTCCCCAACTGTTTTCAGCXXXXXXXXXXA
AACTATGTTGAGTTCTTTTCCAATGAAGATGTCAGTCCCTAAAGAATTCAAAGTAGTGGTCTCCGAAACTATCCACAGT
>Marker700806 Chr5 SNP_site:13873453 Ref_Type:G
ACTCTTAAAGAGTTCTATGAATTAAGCATCAATGTAAAAGTTCTTTTCCTGCATTAACCTTTAATAAACTTCGGCAATCTXXXXXXXXXXC
CAAGAAAAAGAGTAAAAAGAAGAATTTAACCACAAAGAAAGGTCACCTTATAGGAGGGTCAAAACAGTTCGTGCATAGT
ACTCTTAAAGAGTTCTATGAATTAAGCATCAATGTAAAAGTTCTTTTCCTGCATTAACCTTTAATAAGCTTCGGCAATCTXXXXXXXXXXC
CAAGAAAAAGAGTAAAAAGAAGAATTTAACCACAAAGAAAGGTCACCTTATAGGAGGGTCAAAACAGTTCGTGCATAGT
>Marker70097 Chr4 SNP_site:20124372 Ref_Type:A
ACCTTCGTCGGCCGGCAGCCGAGCAGCGGCGTAGGCGAGAGAGAAGAAAAGAAAAACGGCGAGGAATCGAGCCATGAAAAXXXXXXXXXXG
TTTTATTAACTTCCTTTTTTTAGTTACAAAACAAAACCAAATTATTTTAAAATTAAGTTAAGAATTGTATATTTTGGTA
ACCTTCGTCGGCCGGCAGCCGAGCAGCGGCGTAGGCGAGAGAGAAGAAAAGAAAAACGGCGAGGAATCGAGCCATGAAAAXXXXXXXXXXG
TTTTATTAACTTCCTTTTTTTAGTTACAAAACAAAACCAAATTATTTTTAAATTAAGTTAAGAATTGTATATTTTGGTA
>Marker701302 Chr5 SNP_site:24561409 Ref_Type:G
AACAAACAAAAACAGAGCTATCATTGAATAATGATATCCAAAGAAGCCATTGAGTTCAAGTAATTCTAGTGCAACTTGATXXXXXXXXXXA
CTAATTAAAATATGATTGGATGATGGAGTTGGAGTGAGTCAAAGACGTGGGGAAAGATTTATTATATCCTACAAATGTG
AACAAACAAAAACAGAGCTATCATTGAATAATGATATCCAAAGAAGCCATTGAGTTCAAGTAATTCTAGTGCAACTTGATXXXXXXXXXXA
CTAATTAAAATATGATTGGATGGTGGAGTTGGAGTGAGTCAAAGACGTGGGGAAAGATTTATTATATCCTACAAATGTG
>Marker702073 Chr5 SNP_site:3921453 Ref_Type:T
ACTATTTTTGGTATCATCATCTAATCATTTTCATAACGCTCTGGATTTCAACTTGCATATTAAGCATAGCAATACTGTGCXXXXXXXXXXT
ACATTACATGTCTTTATAAAAATGCTTTTCTTTTGTTTTTGTCCCAAATTTCGTGGAGGTTTTCTTGGTGTTCTGGGTA
ACTATTTTTGGTATCATCATCTAATCATTTTCATAACGCTCTGGATTTCAACTTGCATATTAAGCATAGCAATACTGTGCXXXXXXXXXXT
ACATTACATGTCTTTATAAAATTGCTTTTCTTTTGTTTTTGTCCCAAATTTCGTGGAGGTTTTCTTGGTGTTCTGGGTA
>Marker70216 Chr4 SNP_site:18447711 Ref_Type:T
TACAAAATAAAATGACTTTAAATGTTTGTTTCTTGCTTTTTTTAATGTTCTTTTGTGATTGAAACATCGGATCAATAAACXXXXXXXXXXC
ATTTGTGACACCCTCCAAACTTCTAATCCAACCCAAAAATGAAATCAAACCAATTATTAGGCAAAAACTTTAATGGGTA
TACAAAATAAAATGACTTTAAATGTTTGTTTCTTGCTTTTTTTAATGTTCTTTTGTGATTGAAACATCGGATCAATAAACXXXXXXXXXXC
ATTTGTGACACCCTCCAAACTTCTAATCCAACCCAAAAATGAATTCAAACCAATTATTAGGCAAAAACTTTAATGGGTA
>Marker702308 Chr5 SNP_site:13878683 Ref_Type:G
AACTTAAAACTTAAACTTGGAATTTATATGCATTGAAATCCATTGTTAATTTATATTCATTATTAAACCTAAACAATTAGXXXXXXXXXXA
CAATAAGCATTGAGAGAAAGTAAAACAATTAGCATAACAAATTTCTTGCATTCGTGTAATACATAATACTATAATGGTG
AACTTAAAACTTAAACTTGGAATTTATATGCATTGAAATCCATTGTTAATTTATATTCATTATTAAACCTAAACAATTAGXXXXXXXXXXA
CAATAAGCATTGAGAGAAAGTAAAACAATTAGCATAACAAATTTCTTGCATTCGTGTAATACATAATACTGTAATGGTG
>Marker702976 Chr5 SNP_site:201316 Ref_Type:A
CACAAAGGACTTCCATTATCAACTTATGAGAATTGGCATTTCTCTTGGTTCAAGAGATAATTTTGGTTGCGTTATCTACTXXXXXXXXXXG
GATGTGAATAATAGAATGGAGACTATGAGATTTTGTTATTTACTTGATGATGAATTGGCAGCTTTCATTGAAATAAGGT
CACAAAGGACTTCCATTATCAACTTATGGGAATTGGCATTTCTCTTGGTTCAAGAGATAATTTTGGTTGCGTTATCTACTXXXXXXXXXXG
GATGTGAATAATAGAATGGAGACTATGAGATTTTGTTATTTACTTGATGATGAATTGGCAGCTTTCATTGAAATAAGGT
>Marker703429 Chr5 SNP_site:191261 Ref_Type:A
ACTTGACTTCGCAAATAAATCCATCTTCAATGAGCTTATTTACCTCTTCCTCGATTTGAGAAATGAGTTTTGATTGAAATXXXXXXXXXXT
TTACTAAGCTCACATACTTGTTTTCGTTACCATCAGAAAGTTGAGCGCTTATGAAGGTTGGACGAGGATCTTTTATCGT

Create PDF with GO2PDF for free, if you wish to

ACTTGACTTCGCAAATAAATCCATCTTCAATGAGCTTATTTACCTCTTCCTCGATTTGAGAAATGAGTTTTGGTTGAAATXXXXXXXXXXT
TTACTAAGCTCACATACTTGTTTTCGTTACCATCAGAAAGTTGAGCGCTTATGAAGGTTGGACGAGGATCTTCTATCGT
>Marker703429 Chr5 SNP_site:191462 Ref_Type:T
ACTTGACTTCGCAAATAAATCCATCTTCAATGAGCTTATTTACCTCTTCCTCGATTTGAGAAATGAGTTTTGATTGAAATXXXXXXXXXXT
TTACTAAGCTCACATACTTGTTTTCGTTACCATCAGAAAGTTGAGCGCTTATGAAGGTTGGACGAGGATCTTTTATCGT
ACTTGACTTCGCAAATAAATCCATCTTCAATGAGCTTATTTACCTCTTCCTCGATTTGAGAAATGAGTTTTGGTTGAAATXXXXXXXXXXT
TTACTAAGCTCACATACTTGTTTTCGTTACCATCAGAAAGTTGAGCGCTTATGAAGGTTGGACGAGGATCTTCTATCGT
>Marker704358 Chr5 SNP_site:13968304 Ref_Type:C
AACTCAACAATATATTCTCATGAACAAACTTTTATGAGTTCTGTCCAATATAATGATAGATGAGATGAAAACAATTGTTAXXXXXXXXXXT
TGTCGAATACAACAAAGATGTGTTGTAGTTGTTGTCCTTTCTTTCTAATTCCTCATTTGATGATGTTTCTGTTGTTGTC
AACTCAACAATATATTCTCATGAACAAACTTTTATGAGTTCTGTCCAATATAATGATAGATGAGATGAAAACAATTGTTAXXXXXXXXXXT
TGTCGAATACAACAAAGATGTGTTGTAGTTGTTGTCCTTTCTTTCTAATTCCTCCTTTGATGATGTTTCTGTTGTTGTC
>Marker704781 Chr5 SNP_site:2431488 Ref_Type:C
ACTCCTTTTATTCCTTTCAGTATGCTTCCAACAGGAAATGGAAATCTTGATATTGGCAATGTTTGATTGAGAAACTCGTTXXXXXXXXXXA
TGCCATACTTGCCTACACTAAATCATATGTAAGAAGCTTGTTGTGGATGTTTAATCCTTAAAGAGGGGGATGGTAAGGT
ACTCCTTTTATTCCTTTCAGTATGCTTCCAACAGGAAATGGAAATCTTGATATTGGCACTGTTTGATTGAGAAACTCGTTXXXXXXXXXXA
TGCCATACTTGCCTACACTAAATCATATGTAAGAAGCTTGTTGTGGATGTTTAATCCTTAAAGAGGGGGATGGTAAGGT
>Marker704856 Chr5 SNP_site:25499112 Ref_Type:A
ACCTAAGCAAAGAGTTACAAGGGAATTATGTTCCGGCCATACAAGCTTCCTTTCTTGATGTTGAGTAGGGCCATACACGTXXXXXXXXXXT
CAAACTCCATAGAACCTTAATTAAGGAGTTGTCAATAGTCTCTTGTTTTGTCTCCAAAATTAGAACATCTGGATTGGTA
ACCTAAGCAAAGAGTTGCAAGGGGATTATGTTCCGGCCATACAAGCTTCCTTTCTTGATGTTGAGTAGGGCCATACACGTXXXXXXXXXXT
CAAACTCCATAGAACCTTAATTAAGGAGTTGTCAATAGTCTCTTGTTTTGTCTCCAAAATTAGAACATCTGGATTGGTA
>Marker704856 Chr5 SNP_site:25499119 Ref_Type:A
ACCTAAGCAAAGAGTTACAAGGGAATTATGTTCCGGCCATACAAGCTTCCTTTCTTGATGTTGAGTAGGGCCATACACGTXXXXXXXXXXT
CAAACTCCATAGAACCTTAATTAAGGAGTTGTCAATAGTCTCTTGTTTTGTCTCCAAAATTAGAACATCTGGATTGGTA
ACCTAAGCAAAGAGTTGCAAGGGGATTATGTTCCGGCCATACAAGCTTCCTTTCTTGATGTTGAGTAGGGCCATACACGTXXXXXXXXXXT
CAAACTCCATAGAACCTTAATTAAGGAGTTGTCAATAGTCTCTTGTTTTGTCTCCAAAATTAGAACATCTGGATTGGTA
>Marker704897 Chr5 SNP_site:12104745 Ref_Type:A
ACTCAAAATGGAAGATTAACACCCTCAGTAATACGATATCTGCAACAAAAGAACAAGAGATTATATAAGATATAGGAGAAXXXXXXXXXXT
CATAAAAAAAAAAACGGATAACTTATTAGATGAAACCAAAACGGGGGAATCATTAACTCTGTATTATAGTAATTATTGT
ACTCAAAATGGAAGATTAACACCCTCAGTAATACGATATCTGCAACAAAAGAACAAGAGATTATATAAGATATAGGAGAAXXXXXXXXXXT
CATAAAACAAAAAACGGATAACTTATTAGATGAAACCAAAACGGGGGAATCATTAACTCTGTATTATAGTAATTATTGT
>Marker705185 Chr5 SNP_site:2714595 Ref_Type:T
GACCGAATTTGTGCTTCTCTATAAGAATAATATGAAAAAGGTGCATTGTATTAACAATATATGTTTATCGATATGAAAAGXXXXXXXXXXG
AGGGATTTAATATATGAATGGTATTGAGTAGTGGGGAGTGTATGTTAAAAGGAGAAGGGAATAGATGCCAAGTTTGGTT
GACCGAATTTGTGCTTCTCTATAAGAATAATATGAAAAAGGTGCATTGTATTAACAATATATGTTTATTGATATGAAAAGXXXXXXXXXXG
AGGGATTTAATATATGAATGGTATTGAGTAGTGGGGAGTGTATGTTAAAAGGAGAAGGGAATAGATGCCAAGTTTGGTT
>Marker705235 Chr5 SNP_site:17109508 Ref_Type:A
ACCATCTAAAGACACTGTGAAATTGAAAATAAAGCAATCAGAGAACTCAACAATAACAACAACTAACAAATACACTATAAXXXXXXXXXXG
GGACTAGGAAGTCCTTGTATTTATAGAGAATGGATGCTTCATTAAAAATTCATTCGGTTTAATACTAATTAATGTAGTA
ACCATCTAAAGACACTGTGAAATTGAAACTAAAGCAATCAGAGAACTCAACAATAACAACAACTAACAAATACACTGTAAXXXXXXXXXXG
GGACTAGGAAGTCCTTGTATTTATAGAGAATGGATGCTTCATTAAAAATTCATTCGGTTTAATACTAATTAATGTAGTA
>Marker705235 Chr5 SNP_site:17109556 Ref_Type:A
ACCATCTAAAGACACTGTGAAATTGAAAATAAAGCAATCAGAGAACTCAACAATAACAACAACTAACAAATACACTATAAXXXXXXXXXXG

Create PDF with GO2PDF for free, if you wish to

GGACTAGGAAGTCCTTGTATTTATAGAGAATGGATGCTTCATTAAAAATTCATTCGGTTTAATACTAATTAATGTAGTA
ACCATCTAAAGACACTGTGAAATTGAAACTAAAGCAATCAGAGAACTCAACAATAACAACAACTAACAAATACACTGTAAXXXXXXXXXXG
GGACTAGGAAGTCCTTGTATTTATAGAGAATGGATGCTTCATTAAAAATTCATTCGGTTTAATACTAATTAATGTAGTA
>Marker705259 Chr5 SNP_site:2346201 Ref_Type:A
CACGCTCCCGCACTTCGCGAGATATTTTCTAATATTATGTTAAAATTATTGATTGATTTCAGAATTTAGTTGATGGATTTXXXXXXXXXXA
AGATTATAAAACTGCACTACATATTTATGTATTTGAGGTGAATGCAATATATATGATGAACAAACAAATTTGGCCTGTG
CACGCTCCCGCACTTCGCGAGATATTTTCTAATATTATGTTAAAATTATTGATTGATTTCAGAATTTAGTTGATGGATTTXXXXXXXXXXA
AGATTATAAAACTGCACTACATATTTATGTATTTGAGGTGAATGCGATATATATGATGAACAAACAAATTTGGCCTGTG
>Marker70541 Chr4 SNP_site:6116977 Ref_Type:T
ACAGCCTGAACGATGCTCGATGCCAGGTATATATGCTTTTTAACTGGTATGTAATCTATATGCACAAACTAGACAAAACAXXXXXXXXXXA
ACTTATCAAGAATTCATACAAAGTTATCAGGTGCGGTCCAACCCCAAACCCCACATCCCTCTTTTCCTTTTCCATGGTT
ACAGCCTGAACGATGCTCGATGCCAGGTATATATGCTTTTTAACTGGTATTTAATCTATATGCACAAACTAGACAAAACAXXXXXXXXXXA
ACTTATCAAGAATTCATACAAAGTTATCAGGTGCGGTCCAACCCCAAACCCCACATCCCTCTTTTCCTTTTCCATGGTT
>Marker705418 Chr5 SNP_site:11440765 Ref_Type:G
AACCAAGATTTCGTCAATGACAACTAACACAAAAGTGTCTAAGAAATCCTTAAATATCATGTTCATTAAGCCCATGAATAXXXXXXXXXXC
CACTATTATATCCCTTTTGATTAGAATAATGGGATTAAAACGTATAAGGAGATGCTCAGAACGCAAAACTTATGTTGTA
AACCAAGATTTCGTCAATGACAACTAACACAAAAGTGTCTAAGAAATCCTTAAATATCATGTTCATTAAGCCCATGAATAXXXXXXXXXXC
CACTATTATATCCCTTTTTATTAGAATAATGGGATTAAAACATATAAGGAGATGCTCAGAACACAAAACTTATGTTGTA
>Marker705418 Chr5 SNP_site:11440788 Ref_Type:G
AACCAAGATTTCGTCAATGACAACTAACACAAAAGTGTCTAAGAAATCCTTAAATATCATGTTCATTAAGCCCATGAATAXXXXXXXXXXC
CACTATTATATCCCTTTTGATTAGAATAATGGGATTAAAACGTATAAGGAGATGCTCAGAACGCAAAACTTATGTTGTA
AACCAAGATTTCGTCAATGACAACTAACACAAAAGTGTCTAAGAAATCCTTAAATATCATGTTCATTAAGCCCATGAATAXXXXXXXXXXC
CACTATTATATCCCTTTTTATTAGAATAATGGGATTAAAACATATAAGGAGATGCTCAGAACACAAAACTTATGTTGTA
>Marker705418 Chr5 SNP_site:11440809 Ref_Type:G
AACCAAGATTTCGTCAATGACAACTAACACAAAAGTGTCTAAGAAATCCTTAAATATCATGTTCATTAAGCCCATGAATAXXXXXXXXXXC
CACTATTATATCCCTTTTGATTAGAATAATGGGATTAAAACGTATAAGGAGATGCTCAGAACGCAAAACTTATGTTGTA
AACCAAGATTTCGTCAATGACAACTAACACAAAAGTGTCTAAGAAATCCTTAAATATCATGTTCATTAAGCCCATGAATAXXXXXXXXXXC
CACTATTATATCCCTTTTTATTAGAATAATGGGATTAAAACATATAAGGAGATGCTCAGAACACAAAACTTATGTTGTA
>Marker705808 Chr5 SNP_site:6226139 Ref_Type:G
GACCAATTGTAGAAGGCACATTAAGGTTGATATCCCAAGCACGTTAAACATGAACTTGACATAAGGGAAGCATTTTAAGAXXXXXXXXXXG
CATAATCTGTCAGCATAATATGAGCAAGAAAATTAACAGAGGTGATACTTGAAGAGATACAATAAATATGAAAGAAGTT
GACCAATTGTAGAAGGCACGTTAAGGTTGATATCCCAAGCACGTTAAACATGAACTTGACATAAGGGAAGCATTTTAAGAXXXXXXXXXXG
CATAATCTGTCAGCATAATATGAGCAAGAAAATTAACAGAGGTGATACTTGAAGAGATACAATAAATATGAAAGAAGTT
>Marker705844 Chr5 SNP_site:5015615 Ref_Type:T
AACAGGGTAGATTGCCACAGAAAATCAAAACCCAAGTCACTTAGTTTATAGCTACCGTTTCGAATTGAAACGCAACACAAXXXXXXXXXXT
GGTTCCGGCTTCTTATCAAGCAAGTTCCGCAGCTCATCTTCTTGAATACACTCTTCCCCAACACTTCTAACAATTTGGT
AACAGGGTAGATTGCCACAGAAAATCAAAACCCAATTCACTTAGTCTATAGCTACCGTTTCGAATTAAAACGCAACACAAXXXXXXXXXXT
GGTTCCGGCTTCTTATCAAGCAAGTTCCGCAGCTCATCTTCTTGAATACACTCTTCCCCAACACTTCTAACAATTTGGT
>Marker705844 Chr5 SNP_site:5015625 Ref_Type:C
AACAGGGTAGATTGCCACAGAAAATCAAAACCCAAGTCACTTAGTTTATAGCTACCGTTTCGAATTGAAACGCAACACAAXXXXXXXXXXT
GGTTCCGGCTTCTTATCAAGCAAGTTCCGCAGCTCATCTTCTTGAATACACTCTTCCCCAACACTTCTAACAATTTGGT
AACAGGGTAGATTGCCACAGAAAATCAAAACCCAATTCACTTAGTCTATAGCTACCGTTTCGAATTAAAACGCAACACAAXXXXXXXXXXT
GGTTCCGGCTTCTTATCAAGCAAGTTCCGCAGCTCATCTTCTTGAATACACTCTTCCCCAACACTTCTAACAATTTGGT
>Marker705844 Chr5 SNP_site:5015646 Ref_Type:A

Create PDF with GO2PDF for free, if you wish to

AACAGGGTAGATTGCCACAGAAAATCAAAACCCAAGTCACTTAGTTTATAGCTACCGTTTCGAATTGAAACGCAACACAAXXXXXXXXXXXT
GGTTCCGGCTTCTTATCAAGCAAGTTCCGCAGCTCATCTTCTTGAATACACTCTTCCCCAACACTTCTAACAATTTGGT
AACAGGGTAGATTGCCACAGAAAATCAAAACCCAATTCACTTAGTCTATAGCTACCGTTTCGAATTAAAACGCAACACAAXXXXXXXXXXXT
GGTTCCGGCTTCTTATCAAGCAAGTTCCGCAGCTCATCTTCTTGAATACACTCTTCCCCAACACTTCTAACAATTTGGT
>Marker706001 Chr5 SNP_site:24823238 Ref_Type:T
CACAATTAACAACAACAGGACAAATTGATTACATTTCTTTCTTGAATGACCGGCGGATGAGATTGCTTTTCTCCAAGGTGXXXXXXXXXXXA
AGCAATATGCAGTAACTGTAGGCTGAGTCACAGCCACAGGTGTATTGTAGTCCATCCAAGGCCATAATGGCTGAGCTGT
CACAATTAACAACAACAGGACAAATTGATTACATTTCTTTCTTGAATGACCGGCGGATGAGATTGCTTTTCTCCAAGGTGXXXXXXXXXXXA
AGCAATATGCAGTAACTGTAGGCTGAGTCACAGCCACAGGTGTATTGTATTCCATCCAAGGCCATAATGGCTGAGCTGT
>Marker706072 Chr5 SNP_site:1411161 Ref_Type:A
AACTTACGAACCATCCATGCCCCACCTTATGCACGTAAATACCAACCACAAGACAAATCCTTACAATGAATATCAAATAAXXXXXXXXXXXA
TTGTGAGGATTCAAAAGCTAAAAAATCAATTGTTTTGGAATTTAGATTTGACTGTTAGACACAATGCACAATTCTTGTT
AACTTACGAACCATCCATGCCCCACCTTATGCACGTAAATACCAACCACAAGACAAATCCTTACAATGAATATCAAATAAXXXXXXXXXXXA
TTGTGAGGATTCAAAAGCTAAAAATTCAATTGTTTTGGAATTTAGATTTGACTGTTAGACACAATGCACAATTCTTGTT
>Marker70626 Chr4 SNP_site:18020074 Ref_Type:G
TACTAAGAGCACAAAGGACTAACAGAACAAATACAACATAGTCCAAGACAAGAGAGAGCATAAAGAAATTATAATGAGCAXXXXXXXXXXXC
ACCATGAATAGGCAACCAGCCATGGAATCCTGTGCGCTTGAAGACTTTCTGAAATCTTTCATATCATTAATAAAAAGTG
TACTAAGAGCACAAAGGACTAAGAGAACAAATACAACATAGTCCAAGACAAGAGAGAGCATAAAGAAATTATAATGAGCAXXXXXXXXXXXC
ACCATGAATAGGCAACCAGCCATGGAATCCTGTGCGCTTGAAGACTTTCTGAAATCTTTCATATCATTAATAAAAAGTG
>Marker70639 Chr4 SNP_site:14682691 Ref_Type:C
TACAAAAATACGTGACAAAAAACAAGTCTAATCAAAAATTGAAATTTGCATACACATGTGGTTCCGACTTCAACACATCTXXXXXXXXXXXA
TGTAACTCTAGGCTTCGCTAGTAAGTTCGGCTTAATTATAATCTTTTAACAAAATCATATAGTCTAGCTCAACTAGGTT
TACAAAAATACGTGACAAAAAACAAGTCTAATCAAAAATTGAAATTTGCATCCACATGTGGTTCCGACTTGAACACATCTXXXXXXXXXXXA
TGTAACTCTAGGCTTCGCTAGTAAGTTCGGCTTAATTATAATCTTTTAACAAAATCATATAGTCTAGCTCAACTAGGTT
>Marker70639 Chr4 SNP_site:14682710 Ref_Type:G
TACAAAAATACGTGACAAAAAACAAGTCTAATCAAAAATTGAAATTTGCATACACATGTGGTTCCGACTTCAACACATCTXXXXXXXXXXXA
TGTAACTCTAGGCTTCGCTAGTAAGTTCGGCTTAATTATAATCTTTTAACAAAATCATATAGTCTAGCTCAACTAGGTT
TACAAAAATACGTGACAAAAAACAAGTCTAATCAAAAATTGAAATTTGCATCCACATGTGGTTCCGACTTGAACACATCTXXXXXXXXXXXA
TGTAACTCTAGGCTTCGCTAGTAAGTTCGGCTTAATTATAATCTTTTAACAAAATCATATAGTCTAGCTCAACTAGGTT
>Marker706405 Chr5 SNP_site:752132 Ref_Type:T
TACAAGCACAGTAAAACCATAACCAAAGAACACCAACATAGAAAAGAAGAAATAGGAAAATAGAAAGTGATGACTAGAATXXXXXXXXXXXC
TTCTGTCTATTTCCTAACATACCTTCCCCCGTCTTCCTCCTTCTGTAGGTGTTAATAATTGGGGGCCTAACATGTTGTT
TACAAGCACAGTAAAACCATAACCAAAGAACACCAACATAGAAAAGAAGAAATAGGAAAATAGAAAGTGATGACTAGAATXXXXXXXXXXXC
TTCTGTCTATTTCCTAACATACCTTCCCCCGTCTTCCTCCTTCTGTAGGTGTTAATTATTGGGGGCCTAACATGTTGTT
>Marker706661 Chr5 SNP_site:10488332 Ref_Type:T
ACAATGGCCGAAAGGCCTACAACTTTCAATACATTGCATCTTAATTCTCTGTTAATCTATCATTTCTTTACTATTCATCTXXXXXXXXXXXC
ATGGCCAACGTTTATCGCCAACTACTCGAGAGGGTAAATAGCAAGGACATGGCTAAACGCCCACAAGAAGAAGTTTGTA
ACAATGGCCGAAAGGCCTACAACTTTCAATACATTGCATCTTAATTCTCTGTTAATCTATCATTTTTTTACTATTCATCTXXXXXXXXXXXC
ATGGCCAACGTTTATCGCCAACTACTCGAGAGGGTAAATAGCAAGGACATGGCTAAACGCCCACAAGAAGAAGTTTGTA
>Marker708861 Chr5 SNP_site:2706625 Ref_Type:C
ACTTAAAATTGATACCTGATCCATGTTATTACAATTAAAAGGAAAAGCAATAGTGTGGTAGAGCATCAGAACAAGTATGCXXXXXXXXXXXT
CCTAAATTAGTAATGAAACTCCTATTTATGAGAATTGATCATCCCTTGGTTTGCATGGGTGAGAATGACTCAAGTTGTC
ACTTAAAATTGATACCTGATCCATGTTATTACAATTAAAAGGAAAAGCAATAGTGTGGTAGAGCATCAGAACAAGTATGCXXXXXXXXXXXT
CCTAAATTAGTAATGAAACTTCTATTTATGAGAATTGATCATCCCTTGGTTTGCATGGGTGAGAATGACTCAAGTTGTC

Create PDF with GO2PDF for free, if you wish to

>Marker709393 Chr5 SNP_site:1356064 Ref_Type:C
TACATGGACTATTTTCATAAATTACATGCTTATTAGTTCTGTATATATAGTTATTTGCTGAAAATATTATATAAATATTTXXXXXXXXXXC
AATTGAAAATACCAAAAATGCCCCGATCAATACGCCATTAGCCGGTCACACGTGTGTGTAGTTCTTCTAAATGATCGTG
TACATGGACTATTTTCATAAATTACATGCTTATTAGTTCTGTATATATAGTTATTTGCTGAAAATATTATATAAATATTTXXXXXXXXXXC
AATTGAAAATACCAAAAATGCTCCGATCAATACGCCATTAGCCGGTCACACGTGTGTGTAGTTCTTCTAAATGATCGTG
>Marker709994 Chr5 SNP_site:25729882 Ref_Type:T
CACTGTTTCTGCCTAATAATATTCTCAGATATTATATTATCATCAACAGTGATTAATAAGTTTATTGAACAAAAGGTTATXXXXXXXXXXXA
CTGTATTGCCTAGGTAGTTGTGTCGGTTATTATTTTATTTTATTATTTGTTTTTTGAGTTTAGGGTTAGGGATTCCAGT
CACTGTTTCTGCCTAATAATATTCTCAGATATTATATTATCATCAATAGTGATTAATAAGTTTATCGAACAAAAGGTTATXXXXXXXXXXXA
CTGTATTGCCTAGGTAGTTGTGTCAGTTATTATTTTATTTTATTATTTGTTTTTTGAGTTTAGGGTTAGGGATTCCAGT
>Marker709994 Chr5 SNP_site:25729901 Ref_Type:C
CACTGTTTCTGCCTAATAATATTCTCAGATATTATATTATCATCAACAGTGATTAATAAGTTTATTGAACAAAAGGTTATXXXXXXXXXXXA
CTGTATTGCCTAGGTAGTTGTGTCGGTTATTATTTTATTTTATTATTTGTTTTTTGAGTTTAGGGTTAGGGATTCCAGT
CACTGTTTCTGCCTAATAATATTCTCAGATATTATATTATCATCAATAGTGATTAATAAGTTTATCGAACAAAAGGTTATXXXXXXXXXXXA
CTGTATTGCCTAGGTAGTTGTGTCAGTTATTATTTTATTTTATTATTTGTTTTTTGAGTTTAGGGTTAGGGATTCCAGT
>Marker709994 Chr5 SNP_site:25730037 Ref_Type:A
CACTGTTTCTGCCTAATAATATTCTCAGATATTATATTATCATCAACAGTGATTAATAAGTTTATTGAACAAAAGGTTATXXXXXXXXXXXA
CTGTATTGCCTAGGTAGTTGTGTCGGTTATTATTTTATTTTATTATTTGTTTTTTGAGTTTAGGGTTAGGGATTCCAGT
CACTGTTTCTGCCTAATAATATTCTCAGATATTATATTATCATCAATAGTGATTAATAAGTTTATCGAACAAAAGGTTATXXXXXXXXXXXA
CTGTATTGCCTAGGTAGTTGTGTCAGTTATTATTTTATTTTATTATTTGTTTTTTGAGTTTAGGGTTAGGGATTCCAGT
>Marker710266 Chr5 SNP_site:13813532 Ref_Type:G
TACACAAGGTTCATCAACATTTTGGTTAAAACCGTAAGATTTGATCGCAATATCAAATCTAATCTTCTAAGATCTAGATGXXXXXXXXXXT
AATCATAAAAAGTGGCTATGGACAAGAGATTCCTTATAAACTTTAACATAGCAACAGGGGAAAAAGTTTCCTCATAGTA
TACACAAGGTTCATCAACATTTTGGTTAAAACCGTAAGATTTGATCGCAGTATCAAATCTAATCTTCTAAGATCTAGATGXXXXXXXXXXT
AATCATAAAAAGTGGCTATGGACAAGAGATTCCTTATAAACTTTAACATAGCAACAGGGGAAAAAGTTTCCTCATAGTA
>Marker710347 Chr5 SNP_site:13966339 Ref_Type:T
GACTTCTCTTTTTTTTACCTAATTCAAACTTTTTGAGCTAATCCAACACATTGTTCTAAGTTAATTCAATATGAGATAGTXXXXXXXXXXC
ACTAAAATCTCGTATTTGTGCTCCCTTCAGTATAGATATATTTGTGTCCATCTGATATAACTATGATTAGTAAGTTAGT
GACTTCTCTTTTTTTTACCTAATTCAAACTTTTTGAGCTAATCCAACATATTGTTCTAAGTTAATTCAATATGAGATAGTXXXXXXXXXXC
ACTAAAATCTCGTATTTGTGCTCCCTTCAGTATAGATATATTTGTGTCCATCTGATATAACTATGATTAGTAAGTTAGT
>Marker710770 Chr5 SNP_site:23734836 Ref_Type:G
ACTCTTCTTCACTGATCTCCCCAACTCTTAATTCATCTGGTCTCTTTCTTTCACCCACCATCTCAAGAACCAACATTCCAXXXXXXXXXXXA
CAGAAGTCATCGTCCAAAAGAATGTTGTGTGGTTTGATGTCAAAATGAAAAATCCTCGTGTTGCAGCCTCGATGCAGGT
ACTCTTCTTCGCTGATCTCCCCAACTCTTAATTCATCTGGTCTCTTTCTTTCACCCACCATCTCAAGAACCAACATTCCAXXXXXXXXXXXA
CAGAAGTCATCGTCCAAAAGAATGTTGTGTGGTTTGATGTCAAAATGAAAAATCCTCGTGTTGCAGCCTCGATGCAGGT
>Marker710926 Chr5 SNP_site:13477858 Ref_Type:C
AACGACAAAGTGTATCATTACATCGAAGAACGCAGGTGGGAATATTTTTTCCAATTTACAAAGCATGATTAGGATATCTTXXXXXXXXXXT
AAACACCAATAGAAACAAATCCATTGGGAAATTTAAATGATTTCAGATACTTGCAAACATAACTTTCTCGCTGCTAGTC
AACGACAAAGTGTATCATTACATCGAAGAACGCAGGTGGGAATATTTTTTCCAATTTACAAAGCATGATTAGGATATCTTXXXXXXXXXXT
AAACACCAATAGAAACAAATCCATTGGGAAATTTAACTGATTTCAGATACTTGCAAACATAACTTTCTCGCTGCTAGTC
>Marker71093 Chr4 SNP_site:6109542 Ref_Type:A
CACAAACTCAGGTTTCTTTAATTAATCTGCGTTTCATTAGAGCTTTTATTTGTTATTTAATTTTTAATTTTAAATTTACCXXXXXXXXXXXA
TTTTGTTTCTTTATATATGTTTCACAGGATCTTGTTTGTAAGACATTCTATGAGTTCTGGTTTGAGGAGCCTTCCAGTT
CACAAACTCAGGTTTCTTTAATTAATCTGCGTTTCATTAGAGCTTTTATTTGTTATTTACTTTTTAATTTTAAATTTACCXXXXXXXXXXXA

Create PDF with GO2PDF for free, if you wish to

TTTTGTTTCTTTATATATGTTTCACAGGATCTTGTTTGTAAGACATTCTATGAGTTCTGGTTTGAGGAGCCTTCCAGTT
>Marker71094 Chr4 SNP_site:8295006 Ref_Type:C
ACCACAACAGCAGCCTTTCAACCTTTTTAGATTCCAACTCAAAAACATACAACCAACCACATAACCACACTCCACCTTGCXXXXXXXXXXT
GGTAAGATCTGCTATAACCTCGTTCTAATCTTGAATGGAAACACTCAGAGACCGAGACTCATAAGATAGGAGTTGTGTA
ACCACAACAGCAGCCTTTCAACCTTTTTAGATTCCAACTCAAAAACATACAACCAACCACATAATCACACTCCACCTTGCXXXXXXXXXXT
GGTAAGATCTGCTATAACCTCGTTCTAATCTTGAATGGAAATACTCAGAGACCGAGACTCATAAGATAGGAGTTGTGTA
>Marker71094 Chr4 SNP_site:8295169 Ref_Type:C
ACCACAACAGCAGCCTTTCAACCTTTTTAGATTCCAACTCAAAAACATACAACCAACCACATAACCACACTCCACCTTGCXXXXXXXXXXT
GGTAAGATCTGCTATAACCTCGTTCTAATCTTGAATGGAAACACTCAGAGACCGAGACTCATAAGATAGGAGTTGTGTA
ACCACAACAGCAGCCTTTCAACCTTTTTAGATTCCAACTCAAAAACATACAACCAACCACATAATCACACTCCACCTTGCXXXXXXXXXXT
GGTAAGATCTGCTATAACCTCGTTCTAATCTTGAATGGAAATACTCAGAGACCGAGACTCATAAGATAGGAGTTGTGTA
>Marker711008 Chr5 SNP_site:15532697 Ref_Type:T
ACCTTAACTGTTGAATGAAAAATTCTTTATGTGAGAAGACCAAGCTATGCGATATAGCTATGTTTTAAACGCTAATTATTXXXXXXXXXXA
ATATTGATCGTGACTATGCGATAACATGAGCTATGCGAGATAGCTATGTGAAAGGTTAGAATATCTCATCGCATATGTT
ACCTTAACTGTTGAATGAAAAATTCTTTATGTGAGAAGACCAAGCTATGCGATATAGCTATGTTTTAAATGCTAATTATTXXXXXXXXXXA
ATATTGATCGTGACTATGCGATAACATGAGTTATGCGAGATAGCTATGTGAAAGGTTAGAATATCTCATCGCATATGTT
>Marker711008 Chr5 SNP_site:15532837 Ref_Type:T
ACCTTAACTGTTGAATGAAAAATTCTTTATGTGAGAAGACCAAGCTATGCGATATAGCTATGTTTTAAACGCTAATTATTXXXXXXXXXXA
ATATTGATCGTGACTATGCGATAACATGAGCTATGCGAGATAGCTATGTGAAAGGTTAGAATATCTCATCGCATATGTT
ACCTTAACTGTTGAATGAAAAATTCTTTATGTGAGAAGACCAAGCTATGCGATATAGCTATGTTTTAAATGCTAATTATTXXXXXXXXXXA
ATATTGATCGTGACTATGCGATAACATGAGTTATGCGAGATAGCTATGTGAAAGGTTAGAATATCTCATCGCATATGTT
>Marker711270 Chr5 SNP_site:18500379 Ref_Type:A
ACTAAGGGGAAATAAATAATCGCATCCTTATTAATGTATTATTGAGGAAGGTAAGGAAATGGAGTGAATAGGAGTCGATAXXXXXXXXXXT
TGAAAGTTCCACTTTACTTGGGGAGATGCAAAATGAATGAAGCCACAACATTAACACGTTTTATGAACTAATTTTAAGT
ACTAAGGGGAAATAAATAATCGCATCCTTATTAATGTATTATTGAGGAAGGTAAGGAAATGGAGTGAATAGGAGTCGATAXXXXXXXXXXT
TGCAAGTTCCACTTTACTTGGGGAGATGCAAAATGAATGAAGCCACAACATTAACACGTTTTATGAACTAATTTTAAGT
>Marker711829 Chr5 SNP_site:8278723 Ref_Type:T
GACAAGTCTGGCCATATATTAAACAGTAATTGAACATTAGGGTAATGTTTAACTATCAATGTAATGAGAGATACACCAATXXXXXXXXXXA
ATTTTGTCACTTCCTAGACATTATTTACCTAAAAATCTTATTAACTTTACCAATAAAACCAAAAGATAACATGAAGGTG
GACAAGTCTGGCCATATATTAAACAGTAATTGAACATTAGGGTAATGTTTAACTATCAATGTAATGAGATATACACCAATXXXXXXXXXXA
ATTTTGTCACTTCCTAGACATTATTTACCTAAAAATCTTATTAACTTTACCAATAAAACCAAAAGATAACATGAAGGTG
>Marker712279 Chr5 SNP_site:15574584 Ref_Type:G
AACTTAGTGTATTTGAATTCAGAGAGTAGAGCAAATATGGTAGAATGTTGAGAAGCATCTCGCCTTAATTTGAAGTTTAAXXXXXXXXXXT
TCCATTATTCATCTGTTGTCATCTCGCCTTGTCATCTTGCGCCGCCACACACACCACATGTATATAATAGATCAGAGTA
AACTTAGTGTATTTGAATTCAGAGAGTAGAGCAAATATGGTAGAATGTTGAGAAGCATCTCGCCTTAATTTGGAGTTTAAXXXXXXXXXXT
TCCATTATTCATCTGTTGTCATCTCGCCTTGTCATCTTGCGCCGCCACACACACCACATGTATATAATAGATCAGAGTA
>Marker712437 Chr5 SNP_site:24012956 Ref_Type:T
ACTCATCGTCCGAGAATTGCTCCGCCGCGTAGTGATACGAACATCGCCAGACTCGTGAAACAGAATAGGAACGAGACTGCXXXXXXXXXXG
TTTGATATTATGCATTCTAACAAAGAAGACCTAATCATGGCGGCTGATATGGTGTCCCTTCATCACACTGATCATTTGT
ACTCATCGTCCGAGAATTGCTCCGCCGCGTAGTGATACGAACATCGCCAGACTCGTGAAACAGAATAGGAACGAGACTGCXXXXXXXXXXG
TTTGATATTATGCATTCTAACAAAGAAGACTTAATCATGGCGGCTGATATGGTGTCCCTTCATCACACTGATCATTTGT
>Marker71244 Chr4 SNP_site:8158565 Ref_Type:C
CACTATAGCCACCCTACCTAATGAGCTCCAGCGATTTCAGAATCTTACCATGGGTAAAGTGAAGGAAGTAGAAGCAAATGXXXXXXXXXXT
AGTAAGAGAAAGAATATTGCGAAAGGTAAATGTTACCACAGCAACAACAATAGGCATTGGTTGAGAAATTACCCGTAGT

Create PDF with GO2PDF for free, if you wish to

CACTATAGCCACCCTACCTAATGAGCTCCAGCGATTTCAGAATCTTACCATGGGTAAAGTGAAGGAAGTAGAAGCAAATGXXXXXXXXXXT
AGTAAGAGAAAGAATATTGCGAAAGGTAAATGTTACCATTGCAACAACAATAGGCATTGGTTGAGAAATTACCCGTAGT
>Marker71244 Chr4 SNP_site:8158566 Ref_Type:A
CACTATAGCCACCCTACCTAATGAGCTCCAGCGATTTCAGAATCTTACCATGGGTAAAGTGAAGGAAGTAGAAGCAAATGXXXXXXXXXXT
AGTAAGAGAAAGAATATTGCGAAAGGTAAATGTTACCACAGCAACAACAATAGGCATTGGTTGAGAAATTACCCGTAGT
CACTATAGCCACCCTACCTAATGAGCTCCAGCGATTTCAGAATCTTACCATGGGTAAAGTGAAGGAAGTAGAAGCAAATGXXXXXXXXXXT
AGTAAGAGAAAGAATATTGCGAAAGGTAAATGTTACCATTGCAACAACAATAGGCATTGGTTGAGAAATTACCCGTAGT
>Marker712609 Chr5 SNP_site:14036823 Ref_Type:C
TACCAAAACTTCTCAGCTTTAATTCCTTTCTCGAGATACCACCTACTTGCAGCTAAAAGTTAAAATTTTCTTCCCAAGACXXXXXXXXXXC
CATGGCAGAAGCCTGAGGTTTATATTAAGACGAATCTAATAATTTATGTCATTGGATTCAAAGCAGTGTCGCAAGGGTT
TACCAAAACTTCTCAGCTTTAATTCCTTTCTCGAGATACCACCTACTTGCAGCTAAAAGTTAAAATTTTCTTCCCAAGACXXXXXXXXXXC
CATGGCAGAAGGCTGAGGTTTATATTAAGACGAATCTAATAATTTATGTCATTGGATTCAAAGCAGTGTCGCAAGGGTT
>Marker712691 Chr5 SNP_site:6551117 Ref_Type:A
TACATTTTATTGACTCAATACTTTATTTACCTATTTTTTTTACTATTGGTTCAATTTCCATTCCTGTATAAACCATTTCCXXXXXXXXXXA
CTAAAGCAGAGAGTTCCAATATATGATAGTTTCCGATGCGAAAGTCAGAGGTTTGCTCACATAATTGTTGAATCGTGTG
TACATTTTATTGACTCAATACTTTATTTACCTATTTTTTTTACTGTTGGTTCAATTTCCATTCCTGTATAAACCATTTCCXXXXXXXXXXA
CTAAAGCAGAGAGTTCCAATATATGATAGTTTCCGATGCGAAAGTCAGAGGTTTGCTCACATAATTGTTGAATCGTGTG
>Marker712860 Chr5 SNP_site:3182650 Ref_Type:C
AACTTGTCACCAAAGAGCTAACAATTCTTACAAAATGTGTTTGATGCTCTATTAAGTGGATAAGTGCTTGCAAGAACAACXXXXXXXXXXA
ACAGATTCATATTCTGGGCGTAATCCCATAACACTACAGGAAAAAAGGTCTATGTTAGCTCATTAATAACACTATCGTT
AACTTGTGACCAAAGAGCTAACAATTCTTACAAAATGTGTTTGATGCTCTATTAAGTGGATAAGTGCTTGCAAGAACAAAXXXXXXXXXXA
ACAGATTCATATTCTGGGCGTAATCCCATAACACTACAGGAAAAAAGGTCTATGTTAGCTCATTAATAACACTATCGTT
>Marker712860 Chr5 SNP_site:3182722 Ref_Type:C
AACTTGTCACCAAAGAGCTAACAATTCTTACAAAATGTGTTTGATGCTCTATTAAGTGGATAAGTGCTTGCAAGAACAACXXXXXXXXXXA
ACAGATTCATATTCTGGGCGTAATCCCATAACACTACAGGAAAAAAGGTCTATGTTAGCTCATTAATAACACTATCGTT
AACTTGTGACCAAAGAGCTAACAATTCTTACAAAATGTGTTTGATGCTCTATTAAGTGGATAAGTGCTTGCAAGAACAAAXXXXXXXXXXA
ACAGATTCATATTCTGGGCGTAATCCCATAACACTACAGGAAAAAAGGTCTATGTTAGCTCATTAATAACACTATCGTT
>Marker71378 Chr4 SNP_site:14046566 Ref_Type:T
ACTTACAACAGCACGAAAGTTCTTAAACATGAAACATTCGAGTTGTTGAAGGGTCGATGGGCAATCCTACGAGGAAAGCCXXXXXXXXXXT
CTCCTTATTATGATGACCTGTCATATGTTTTTGAAAAAGATTAGGCTACGGGAGCACGATTAGAGACCTTCGCTGATGT
ACTTACAACAGCACGAAAGTTCTTAAACATGAAACATTCGAGTTGTTGAAGGGTCGATGGGCAATCCTACGAGGAAAGCCXXXXXXXXXXT
CTCCTTATTATGATGACCTGTCATATGTTTTTGAAAAAGATTAGGCTACGGGAGCACGATTAGAGACCTTTGCTGATGT
>Marker713920 Chr5 SNP_site:13981730 Ref_Type:A
TACATAGTGATAGCCAGGCAACTATTGGAAAGACATAGAATATTATGTATAATAATGTTAAGTCTCGACATATATGACGAXXXXXXXXXXA
GGTGGTTGAAAGTTCATAAAAAAGAATGAGATTAAAGCCTACAATATGAAGTTACTCAAAGAGACAATCCAACCTAGTT
TACGTAGTGATAGCCAGGCAACTATTGGAAAGACATAGAATATTATGTATAATAATGTTAAGTCTCGACATATATGACGAXXXXXXXXXXA
GGTGGTTGAAAGTTCATAAAAAAGAATGAGATTAAAGCCTACAATATGAAGTTACTCAAAGAGACAATCCAACCTAGTT
>Marker71461 Chr4 SNP_site:19138432 Ref_Type:G
ACCAAAATTGCCAGCAGGGAAGCAGAACTGTTGCTGATTACATAGAAGAATTCCACCGATTGGGAGCAAGAACTAATTTGXXXXXXXXXXC
ATCACTCATGCAGAATCTGTAGAAGAGATTAATGAATTGAATTCAAGAAAAAAATACAAGAAGAGGTCCGTGGAATGTC
ACCAAAATTGCCAGCAGGGAAGCAGAACTGTTGCTGATTACATAGAAGAATTCCACCGATTGGGAGCAAGAACTAATTTGXXXXXXXXXXC
ATCACTCATGCAGAATCTGTAGAAGAGATTAATGAATTGAATTCAAGAAAAAAATACAAGAAGATGTCCGTGGAATGTC
>Marker714792 Chr5 SNP_site:12884537 Ref_Type:A
ACTCATATTACAGGAACTAATTAATTAATTTTATAATTGTCATAAAATTTGAGGATTAGAAACCGTCACCCTATATTAAAXXXXXXXXXXT

Create PDF with GO2PDF for free, if you wish to

TTAATGAAACTAAGTAGGAAAAATAGGATTTAAGGTTATAAGGAAACTCCATCAGTAAAGTTCTGTCTAAGGCTGGAGT
ACTCATATTACAGGAACTAATTAATTAATTTTATTATTGTCATAAAATTTGAGGATTAGAAACCGTCACTCTATATTAAAXXXXXXXXXXT
TTAATGAAACTAAGTAGGAAAAATAGGATTTAAGGTTATAAGGAAACTCCATCAGTAAAGTTCTGTCTAAGGCTGGAGT
>Marker714792 Chr5 SNP_site:12884572 Ref_Type:C
ACTCATATTACAGGAACTAATTAATTAATTTTATAATTGTCATAAAATTTGAGGATTAGAAACCGTCACCCTATATTAAAXXXXXXXXXXT
TTAATGAAACTAAGTAGGAAAAATAGGATTTAAGGTTATAAGGAAACTCCATCAGTAAAGTTCTGTCTAAGGCTGGAGT
ACTCATATTACAGGAACTAATTAATTAATTTTATTATTGTCATAAAATTTGAGGATTAGAAACCGTCACTCTATATTAAAXXXXXXXXXXT
TTAATGAAACTAAGTAGGAAAAATAGGATTTAAGGTTATAAGGAAACTCCATCAGTAAAGTTCTGTCTAAGGCTGGAGT
>Marker714810 Chr5 SNP_site:15986700 Ref_Type:T
TACCAAGGCTTGTTATTAATTACAAGCCCCTCAACAAAGTTCTCAAATGGATTCGGTATCCAAGTCCTAATCGTCAAGACXXXXXXXXXXT
CGGATTGAAGAATGCTCCGTCCGAATTTCATAAGACAATGAACGATATCTTCAACCCGTATCAAGATTTCATAATAGTA
TACCAAGGCTTGTTATTAATTACAAGCCCCTCAACAAAGTTCTCAAATGGATTTGGTATCCAAGTCCTAATCGTCAAGACXXXXXXXXXXT
CGGATTGAAGAATGCTCCGTCCGAATTTCATAAGACAATGAACGATATCTTCAACCCGTATCAAGATTTCATAATAGTA
>Marker715122 Chr5 SNP_site:16916795 Ref_Type:C
CACAGAAGGTCAAGTGATGATAAACAAATTGAAAATTATCATGAATGTGGATAAAAAATGAATAAACCCAAAAATCAAAAXXXXXXXXXXA
TAGGTTACAGAAAGTTTAAATAATGGGATTATATAAGGTAAGGTCCGGTAGCTTTACAAATGAACCAGATTATACTGTA
CACAGAAGGTCAAGTGATGATAAACAAATTGAAAATTATCATGAATGTGGATAAAAAATGAATAAACCCAAAAATCAAAAXXXXXXXXXXA
TAGGTTACCGAAAGTTTAAATAATGGGATTATATAAGGTAAGGTCCGGTAGCTTTACAAATGAACCAGATTATACTGTA
>Marker715172 Chr5 SNP_site:16076783 Ref_Type:T
AACTTCTTTTAATGAATTATGGGTAGCTTTGTTGAGAGCTGGAGACTTGAGTTGTTTTCTTAACAACTGCAAGTTTGTTTXXXXXXXXXXT
GAAGCCAGAGCAGGCGAACAAGTTATCGAAACTAGAAACTTGGCGCTCCGAACTAATGCTTCTAGAAGAACAAAGGGTA
AACTTCTTTTAATGAATTATGGGTAGCTTTGTTGAGAGCTGGAGACTTGAGTTGTTTTCTTAACATCTGCAAGTTTGTTTXXXXXXXXXXT
GAAGCCAGAGCAGGCGAACAAGTTATCGAAACTAGAAACTTGGCGCTCCGAACTAATGCTTCTAGAAGAACAAAGGGTA
>Marker715406 Chr5 SNP_site:30508 Ref_Type:C
TACAAATTTCAACTTATATGGCTATACAGACTCTTATGCATAACTCCACACACATACAAAAAATTTGTCGGGAAACTTCTXXXXXXXXXXT
TATATATGTTAGCAAGTGATAAAGTTTTCAAACATCTAGATAAATCTGTAAAGCAAAGCAACCTCGTGATTTTAATGTA
TACAAATTTCAACTTATATGGCTATACAGACTCTTATGCATAACTCTACACACATACAAAAAATTTGTCGGGAAACTTCTXXXXXXXXXXT
TATATATGTTAGCAAGTGATAAACTTTTCAAACATCTAGATAAATCTGTAAAGCAAAGCAACCTCGTGATTTTAATGTA
>Marker715406 Chr5 SNP_site:30676 Ref_Type:G
TACAAATTTCAACTTATATGGCTATACAGACTCTTATGCATAACTCCACACACATACAAAAAATTTGTCGGGAAACTTCTXXXXXXXXXXT
TATATATGTTAGCAAGTGATAAAGTTTTCAAACATCTAGATAAATCTGTAAAGCAAAGCAACCTCGTGATTTTAATGTA
TACAAATTTCAACTTATATGGCTATACAGACTCTTATGCATAACTCTACACACATACAAAAAATTTGTCGGGAAACTTCTXXXXXXXXXXT
TATATATGTTAGCAAGTGATAAACTTTTCAAACATCTAGATAAATCTGTAAAGCAAAGCAACCTCGTGATTTTAATGTA
>Marker715740 Chr5 SNP_site:8726498 Ref_Type:G
GACGAGGAGCTTATCTCCCAATTTCATAGTTTGCAGTTCTTATGTTACTGAATGAAAGGGTTTAAATTCTTTTCCTATTTXXXXXXXXXXG
AAATATGGCCTTATGTGGTTGAAATGTCGGTTCTTCTTGCAACTAGCATTCACAATATGAATCCTCGTAGCTCATGGTA
GACGAGGAGCTTATCTCCCAATTTCATAGTTTGCAGTTCTTATGTTACTGAATGAAAGGGTTTAAATTCTTTTCCTATTTXXXXXXXXXXG
AAATATGGCCTTGTGTGGTTGAAATGTCGGTTCTTCTTGCAACTAGCATTCACAATATGAATCCTCGTAGCTCATGGTA
>Marker716247 Chr5 SNP_site:32913 Ref_Type:G
AACAAGCATGCAAGAAAGCATGTGCTAAGAGGATAATTGTTGTGAGTAGCCATTCAAATTTATAAGTTG-AAAAAGGTTCTXXXXXXXXXX
GCTTATAGTGCAAATTTATGTGGATGATATTATATTTGGTTCTACTAATTCATTTTTGTGTGAAGAATTTTCCAAGTGTA
AACAAGCATGCAAGAAAGCATTGGCTAAGAGGATAATTGTTGTGAGTAGCCATTCAAATTTATAAGTTGAAAAAAGGTTC-XXXXXXXXXX
GCTTATAGTGCAAATTTATGTGGATGATATTATATTTGGTTCTACTAATTCATTTTTGTGTGAAGAATTTTCCAAGTGTA
>Marker716247 Chr5 SNP_site:32914 Ref_Type:T

Create PDF with GO2PDF for free, if you wish to

AACAAGCATGCAAGAAAGCATGTGCTAAGAGGATAATTGTTGTGAGTAGCCATTCAAATTTATAAGTTG-AAAAAGGTTCTXXXXXXXXXX
GCTTATAGTGCAAATTTATGTGGATGATATTATATTTGGTTCTACTAATTCATTTTTGTGTGAAGAATTTTCCAAGTGTA
AACAAGCATGCAAGAAAGCATTGGCTAAGAGGATAATTGTTGTGAGTAGCCATTCAAATTTATAAGTTGAAAAAAGGTTC-XXXXXXXXXX
GCTTATAGTGCAAATTTATGTGGATGATATTATATTTGGTTCTACTAATTCATTTTTGTGTGAAGAATTTTCCAAGTGTA
>Marker716345 Chr5 SNP_site:9887269 Ref_Type:T
GACTGTATCGTAGTCTTCTAACCTCTCAAATGCCTTTTCAAACTTTCAGCTGCATCAAGCATCAAGTATGTGGAATTTCAXXXXXXXXXXC
ATAACTGCATTTCGAATTCGAATTAATATATCATTTAGATTTTTCAGACCATCGCACACAATTAAATTAAATATATGTG
GACTGTATCGTAGTCTTCTAACCTCTCAAATGCCTTTTCAAACTTTCAGTTGCATCAAGCATCAAGTATGTGGAATTTCAXXXXXXXXXXC
ATAACTGCATTTCGAATTCGAATTAATGTATCATTTAGATTTTTCAGACCATCGCACACAATTAAATTAAATATATGTG
>Marker716345 Chr5 SNP_site:9887464 Ref_Type:G
GACTGTATCGTAGTCTTCTAACCTCTCAAATGCCTTTTCAAACTTTCAGCTGCATCAAGCATCAAGTATGTGGAATTTCAXXXXXXXXXXC
ATAACTGCATTTCGAATTCGAATTAATATATCATTTAGATTTTTCAGACCATCGCACACAATTAAATTAAATATATGTG
GACTGTATCGTAGTCTTCTAACCTCTCAAATGCCTTTTCAAACTTTCAGTTGCATCAAGCATCAAGTATGTGGAATTTCAXXXXXXXXXXC
ATAACTGCATTTCGAATTCGAATTAATGTATCATTTAGATTTTTCAGACCATCGCACACAATTAAATTAAATATATGTG
>Marker716783 Chr5 SNP_site:2099498 Ref_Type:C
AACGAAAATGTCTCGAATTAAATATGCTGAGAATAAAACATTTAAATAAGAGAAATTGCATCAAATAACAAAAATATTTTXXXXXXXXXXA
CTATCAGTAACCATAGGGCTATCGACTTTTAAATTTGCTACTTTTACAATTTAGAAAATGTAGAGATATGGATCATGTT
AACGAAAATGTCTCGAATTAAATATGCTGAGAATAAAACATTTAAATAAGAGAAATTGCATCAAATAACAAAAATATTTTXXXXXXXXXXA
CTATCAGTAACCATAGGGCTATCGACTTTTAAATTTGCTACTTTTACAATTTAGAAAATGTAGAGATATGGATCCTGTT
>Marker716860 Chr5 SNP_site:26456095 Ref_Type:T
ACCTGAAGAAATTCCAGATTCCTCGTCGAATGATTTCCAACACAGCGACAATGGCAATCAAGGCTTGTCTATGAATAAATXXXXXXXXXXC
TAAGTGACACCATATAGTTTCCTCTTCCAAGCTAAACGAAATGAAAAAGGTTGATTATCCTTACAATTGCTACAAAGTA
ACCTGAAGAAATTCCAGATTCCTCGTCTAATGATTTCCAACACAGCGACAATGGCAATCAAGGCTTGTCTATGAATAAATXXXXXXXXXXC
TAAGTGACATCATATAGTTTCCTCTTCCAAGCTAAACGAAATGAAAAAGGTTGATTATCCTTACAATTGCTACAAAGTA
>Marker716860 Chr5 SNP_site:26456253 Ref_Type:T
ACCTGAAGAAATTCCAGATTCCTCGTCGAATGATTTCCAACACAGCGACAATGGCAATCAAGGCTTGTCTATGAATAAATXXXXXXXXXXC
TAAGTGACACCATATAGTTTCCTCTTCCAAGCTAAACGAAATGAAAAAGGTTGATTATCCTTACAATTGCTACAAAGTA
ACCTGAAGAAATTCCAGATTCCTCGTCTAATGATTTCCAACACAGCGACAATGGCAATCAAGGCTTGTCTATGAATAAATXXXXXXXXXXC
TAAGTGACATCATATAGTTTCCTCTTCCAAGCTAAACGAAATGAAAAAGGTTGATTATCCTTACAATTGCTACAAAGTA
>Marker717560 Chr5 SNP_site:25573408 Ref_Type:A
ACCATTGCTCATCCACAAATGGATGGACAAACTGAGGTAACTAACCGGTCCTTGGAAAATTTAATTTGTTGCCTTAGTGGXXXXXXXXXXC
TTCACACAGAAGTCATTGACCATATCACTAAGACTGTCGAGTCTTACAAAGAAGAGAATAATAAGAAGAGAAGGGAAGT
ACCATTGCTCATCCACAAATGGATGGACAAACTGAGGTAACTAACCGGTCCTTGGAAAATTTAATTTGTTGCCTTAGTGGXXXXXXXXXXC
TTCACACAGAAGTCATTGACCATGTCACTAAGACTGTCGAGTCTTACAAAGAAGAGAATAATAAGAAGAGAAGGGAAGT
>Marker717647 Chr5 SNP_site:11532654 Ref_Type:A
CACCTATAAGACTTATAGAGCTTCTCTTTTAAGGGTGACAACCTTTCGGCACCAAATATCCACACTTGTTCTCCTTTCATXXXXXXXXXXA
ACACTCGGTCTTAGAACCCTTAAGGTTTTGCTTAATCGCCTAGTAACTTTCCTTTGTCTTAACTTACAACTTAACAAGT
CACCTATAAGACTTATAGAGCTTCTCTTTTAAGGGTGACAACCTTTCGGCACCAAATATCCACACTTGTTCTCCTTTCATXXXXXXXXXXA
ACACTCGGTCTTAGATCCCTTAAGGTTTTGCTTAATCGCCTAGTAACTTTCCTTTGTCTTAACTTACAACTTAACAAGT
>Marker717837 Chr5 SNP_site:20144523 Ref_Type:T
AACTATAAGCATAAGAAATAAAATTATGATTTAATTTTTTGCAGTTGTGACCCCAATGATGAAAAATGTCAATTTCTTTTXXXXXXXXXXC
CACTATGGTGTTTATTTGGATTCGGATCAAGTAAAGCCCAATGAGAAACATTGATTGCCTTTTGGAATAATAGCCGAGT
AACTATAAGCATAAGAAATAAAATTATGATTTAATTTTTTGCAGTTGTGACCCCAATGATGAAAAATGTCAATTTCTTTTXXXXXXXXXXC
CACTATGGTGTTTATTTGGATTTGGATCAAGTAAAGCCCAATGAGAAACATTGATTGCCTTTTGGAATAATAGCCGAGT

Create PDF with GO2PDF for free, if you wish to

>Marker718339 Chr5 SNP_site:13238675 Ref_Type:G
GACAATCCCAAAGTAACGTAGTTCTACGTCATTAAAGGGTATTTACTTGCTTTTCAGGTAGGGGAAGATTATGAAAAGCTXXXXXXXXXXA
GTGGTGGTGCTTATTATTGCCTATCACAATTGTTTTCAAAGGAATATAATTTGAACCTACCTTATTTACTTGCCTTGTG
GACAATCCCAAAGTAACGTAGTTCTACGTCATTAAAGGGTATTTACTTGCTTTTCAGGTAGGGGAAGATTATGAAGAGCTXXXXXXXXXXA
GTGGTGGTGCTTATTATTGCCTATCACAATTGTTTTCAATGGAATATAATTTGAACCTACCTTATTTACTTGCCTTGTG
>Marker718339 Chr5 SNP_site:13238836 Ref_Type:T
GACAATCCCAAAGTAACGTAGTTCTACGTCATTAAAGGGTATTTACTTGCTTTTCAGGTAGGGGAAGATTATGAAAAGCTXXXXXXXXXXA
GTGGTGGTGCTTATTATTGCCTATCACAATTGTTTTCAAAGGAATATAATTTGAACCTACCTTATTTACTTGCCTTGTG
GACAATCCCAAAGTAACGTAGTTCTACGTCATTAAAGGGTATTTACTTGCTTTTCAGGTAGGGGAAGATTATGAAGAGCTXXXXXXXXXXA
GTGGTGGTGCTTATTATTGCCTATCACAATTGTTTTCAATGGAATATAATTTGAACCTACCTTATTTACTTGCCTTGTG
>Marker718491 Chr5 SNP_site:13327438 Ref_Type:T
ACTTTGGACAATTTCTTAACCAATGTCCGTCTTGGTTGCAGTGGTAACATTTACCTTTAGCAACTCTTTTTCCCTTACTAXXXXXXXXXXA
CATTTTCTTTCGCTTCCTTTCCCTTACCTTGATAAGGTTTTGGAATCGCTGGAGCTTATTTAGAAGAGTGGTCAAAGTG
ACTTTGGACAATTTCTTAACCAATGTCCGTCTTGGTTGCAGTGGTAACATTTACCTTTAGCAACTTTTTTTCCCTTACTAXXXXXXXXXXA
CATTTTCTTTCACTTCCTTTCCCTTACCTTGATAAGGTTTTGGAATCGCTGGAGCTTATTTAGAAGAGTGGTCAGAGTG
>Marker718491 Chr5 SNP_site:13327557 Ref_Type:A
ACTTTGGACAATTTCTTAACCAATGTCCGTCTTGGTTGCAGTGGTAACATTTACCTTTAGCAACTCTTTTTCCCTTACTAXXXXXXXXXXA
CATTTTCTTTCGCTTCCTTTCCCTTACCTTGATAAGGTTTTGGAATCGCTGGAGCTTATTTAGAAGAGTGGTCAAAGTG
ACTTTGGACAATTTCTTAACCAATGTCCGTCTTGGTTGCAGTGGTAACATTTACCTTTAGCAACTTTTTTTCCCTTACTAXXXXXXXXXXA
CATTTTCTTTCACTTCCTTTCCCTTACCTTGATAAGGTTTTGGAATCGCTGGAGCTTATTTAGAAGAGTGGTCAGAGTG
>Marker718491 Chr5 SNP_site:13327620 Ref_Type:G
ACTTTGGACAATTTCTTAACCAATGTCCGTCTTGGTTGCAGTGGTAACATTTACCTTTAGCAACTCTTTTTCCCTTACTAXXXXXXXXXXA
CATTTTCTTTCGCTTCCTTTCCCTTACCTTGATAAGGTTTTGGAATCGCTGGAGCTTATTTAGAAGAGTGGTCAAAGTG
ACTTTGGACAATTTCTTAACCAATGTCCGTCTTGGTTGCAGTGGTAACATTTACCTTTAGCAACTTTTTTTCCCTTACTAXXXXXXXXXXA
CATTTTCTTTCACTTCCTTTCCCTTACCTTGATAAGGTTTTGGAATCGCTGGAGCTTATTTAGAAGAGTGGTCAGAGTG
>Marker718697 Chr5 SNP_site:23768505 Ref_Type:G
CACAGTATGAACTTAAGCTTTTTTTTAACCTTTGATGTTCCATTTTTTCACTGAGGTCATTTGGATTGTAAATCCATCGCXXXXXXXXXXA
GATTATCATAAAAAGTAACTATAGGAAACTGATTATAGGATATTCATAGTCAAGTTCCTTTAAGCATCAGCTCGTTGGT
CACAGTATGAACTTAAGCTTTTTTTTAACCTTTGATGTTCCATTTTTTCACTGAGGTCATTTGGATTGTAAATCCGTCGCXXXXXXXXXXA
GATTATCATAAAAAGTAACTATAGGAAACTGATTATAGGATATTCATAGTCAAGTTCCTTTAAACATCAGCTCGTTGGT
>Marker718697 Chr5 SNP_site:23768656 Ref_Type:A
CACAGTATGAACTTAAGCTTTTTTTTAACCTTTGATGTTCCATTTTTTCACTGAGGTCATTTGGATTGTAAATCCATCGCXXXXXXXXXXA
GATTATCATAAAAAGTAACTATAGGAAACTGATTATAGGATATTCATAGTCAAGTTCCTTTAAGCATCAGCTCGTTGGT
CACAGTATGAACTTAAGCTTTTTTTTAACCTTTGATGTTCCATTTTTTCACTGAGGTCATTTGGATTGTAAATCCGTCGCXXXXXXXXXXA
GATTATCATAAAAAGTAACTATAGGAAACTGATTATAGGATATTCATAGTCAAGTTCCTTTAAACATCAGCTCGTTGGT
>Marker718888 Chr5 SNP_site:27644781 Ref_Type:T
CACCTACCTAAGTCACAATTTGACACTTTAAATTGGTTGGAATTTTAGATTGTGATATCAATGATTCAATATTTCGTGTAXXXXXXXXXXA
CTCTAATTCACTAACCTCACGTATTATCGTTTAGAATCCTAAGGTTATACTTTAGCTTCTCGAACACTCAAGACTTGTT
CACCTACCTAAGTCACAATTTGACACTTTAAATTGGTTGGAATTTTAGATTGTGATATCAATGATTCAATATTTTGTGTAXXXXXXXXXXA
CTCTAATTCACTAACCTCACGTATTATCGTTTAGAATCCTAAGGTTATACTTTAGCTTCTCGAACACTCAAGACTTGTT
>Marker718978 Chr5 SNP_site:9802042 Ref_Type:G
AACGACCGAGTTACTCAAAAGGTCTTGGTTGGGGCCCAAAACCCAAGTCTAAGACTACCGCTTCATCTTCTTCCTCTAATXXXXXXXXXXC
ATGCACGACAAATTGAGGAGATGAAGAAGATGCTCGAAGAGATCAGTCAGGCACAAAGAGGCCCGTGATCACTTGGAGT
AACGACCGGGTTACTCAAAAGGTCTTGGTTGGGGCCCAAAACCCAAGTCTAAGACTACCGCTTCATCTTCTTCCTCTAATXXXXXXXXXXC

Create PDF with GO2PDF for free, if you wish to

ATGCACGACAAATTGAGGAGATGAAGAAGATGCTCGAGGAGATCAGTCAGGCACAAAGAGGCCCGTGATCACTTGGAGT
>Marker718978 Chr5 SNP_site:9802296 Ref_Type:G
AACGACCGAGTTACTCAAAAGGTCTTGGTTGGGGCCCAAAACCCAAGTCTAAGACTACCGCTTCATCTTCTTCCTCTAATXXXXXXXXXXC
ATGCACGACAAATTGAGGAGATGAAGAAGATGCTCGAAGAGATCAGTCAGGCACAAAGAGGCCCGTGATCACTTGGAGT
AACGACCGGGTTACTCAAAAGGTCTTGGTTGGGGCCCAAAACCCAAGTCTAAGACTACCGCTTCATCTTCTTCCTCTAATXXXXXXXXXXC
ATGCACGACAAATTGAGGAGATGAAGAAGATGCTCGAGGAGATCAGTCAGGCACAAAGAGGCCCGTGATCACTTGGAGT
>Marker719020 Chr5 SNP_site:27067496 Ref_Type:G
AACCCCAAAAATTATCAATTCTGATTAAGAGTCAGTTCAGTTCTTGAGTAGAGGCAAGATAAAACTCTAAGGGGACGTTTXXXXXXXXXXC
TATTTTTGTTTGGGTTGTAAATAGCAAACAGTTTAGCTAAGAAGAAAAAAGAGGGTGGATGATAAATGGTAAATAATGT
AACCCCAAAAGTTATCAATTCTGATTAAGAGTCAGCTCAGTTCTTGAGTAGAGGCAAGATAAAACTCTAAGGGGACGTTTXXXXXXXXXXC
TATTTTTGTTTGGGTTGTAAATAGCAAACAGTTTAGCTAAGAAGAAAAAAGAGGGTGGATGATAAATGGTAAATAATGT
>Marker719020 Chr5 SNP_site:27067521 Ref_Type:C
AACCCCAAAAATTATCAATTCTGATTAAGAGTCAGTTCAGTTCTTGAGTAGAGGCAAGATAAAACTCTAAGGGGACGTTTXXXXXXXXXXC
TATTTTTGTTTGGGTTGTAAATAGCAAACAGTTTAGCTAAGAAGAAAAAAGAGGGTGGATGATAAATGGTAAATAATGT
AACCCCAAAAGTTATCAATTCTGATTAAGAGTCAGCTCAGTTCTTGAGTAGAGGCAAGATAAAACTCTAAGGGGACGTTTXXXXXXXXXXC
TATTTTTGTTTGGGTTGTAAATAGCAAACAGTTTAGCTAAGAAGAAAAAAGAGGGTGGATGATAAATGGTAAATAATGT
>Marker719378 Chr5 SNP_site:1499073 Ref_Type:A
CACAAAGTTGCTTTCACAATGCTACAAATAAATTTTGGATGTTCATGAAATGTTTTCTCTTCTTTTCTTTCTTGAAGAAGXXXXXXXXXXG
ATTTCATGAAATCAGCCCTATGTTTGAATTCTATTTCGTGACTTTGAACTTCAAGCCGTATTAATTCAAATAACGATGT
CACAAAGTTGCTTTCACAATGCTACAAATAAATTTTGGATGTTCATGAAATGTTTTCTCTTCTTTTCTTTCTTGGAGAAGXXXXXXXXXXG
ATTTCATGAAATCAGCCCTATGTTTGAATTCTATTTCGTGACTTTGAACTTCAAGCCGTATTAATTCAAATAACGATGT
>Marker720050 Chr5 SNP_site:2553613 Ref_Type:T
TACCACCAAGCGGGAAACCCAAGTAGTGGAAGAGCTTGGAAAGTTGAAATATTTCCTTGGAATCGAAGTTGCCTATTCTAXXXXXXXXXXXT
ATGTGCAGCCGATGAGAGTCCTTCAGTAAATAAGGAAACATAACAAAGGTTAGTTGGCAAACTAATATATCTCTTTCGT
TACCACCAAGCGGGAAACTCAAGTAGTGGAAGAGCTTGGAAAGTTGAAATATTTCCTTGGAATCGAAGTTGCCTATTCTAXXXXXXXXXXXT
ATGTGCAGCCGATGAGAGTCCTTCAGTAAATAAGGAAACATAACAAAGGTTAGTTGGCAAACTAATATATCTCTTTCGT
>Marker720409 Chr5 SNP_site:16759612 Ref_Type:G
AACTACGCGTCAAGGCCTTTTGTTGAGGTCTTGTGAAGGAATATGAGGTGCTAGGCCTTTTGAATGACCCTTAGAAATTCXXXXXXXXXXC
ATAAAACTCTATAAATAGACCATTTTGAAGGCTGCTTTCAATTTCCTTGTGCATTTATGTAATTCTTGTCTCAAAAGTT
AACTAGGCGTCAAGGCCTTTTGTTGAGGTCTTGTGAAGGAAGATGAGGTGCTAGGCCTTTTGAATGACCCTTAGAAATTCXXXXXXXXXXC
ATAAAACTCTATAAATAGACCATTTTGAAGGCTGCTTTCAATTTCCTTGTGCATTTATGTAATTCTTGTCTCAAAAGTT
>Marker720409 Chr5 SNP_site:16759648 Ref_Type:G
AACTACGCGTCAAGGCCTTTTGTTGAGGTCTTGTGAAGGAATATGAGGTGCTAGGCCTTTTGAATGACCCTTAGAAATTCXXXXXXXXXXC
ATAAAACTCTATAAATAGACCATTTTGAAGGCTGCTTTCAATTTCCTTGTGCATTTATGTAATTCTTGTCTCAAAAGTT
AACTAGGCGTCAAGGCCTTTTGTTGAGGTCTTGTGAAGGAAGATGAGGTGCTAGGCCTTTTGAATGACCCTTAGAAATTCXXXXXXXXXXC
ATAAAACTCTATAAATAGACCATTTTGAAGGCTGCTTTCAATTTCCTTGTGCATTTATGTAATTCTTGTCTCAAAAGTT
>Marker72078 Chr4 SNP_site:14862679 Ref_Type:A
ACTCTGATATAGTGATATTTCCCACTTATAGTAAATCTATTGGCCATATTATTACACACAGGGTGGAGACTTGAACCTATXXXXXXXXXXA
GTTATGCCATATTATTTCCATATATTTTTGTGATTACATCCTTTTCCTTTGATTCTTGCCAGCTGAGATACATATTTGT
ACTCTGATATAGTGATATTTCCCACTTGTAGTAAATCTATTGGCCATATTATTACACACAGGGTGGAGACTTGAACCTATXXXXXXXXXXA
GTTATGCCATATTATTTCCATATATTTTTGTGATTACATCCTTTTCCTTTGATTCTTGCCAGCTGAGATACATATTTGT
>Marker72110 Chr4 SNP_site:20047683 Ref_Type:T
ACAAGCACTTACGAAATGACGCGCATAATCATATGTGTGAAAACTGCAAAATTATAGTCATTTCGTAAAGGTCGCACAAAXXXXXXXXXXT
TATTCTTGTAAAAGGTTGGAGGAAACAAAACTTTCATACTATGCTCCTATATATAAAGGTTCAGGAAGGCAAATTCAGT

Create PDF with GO2PDF for free, if you wish to

ACAAGCACTTACGAAATGACGCGCATAATCATATGTGTGAAAACTGCAAAATTATAGTCATTTTGTAAAGGTCGCACAAAXXXXXXXXXXXT
TATTCTTGTAAAAGGTTGGAGGAAACAAAACTTTCATACTATGCTCCTATATATAAAGGTTCAGGAAGGCAAATTCAGT
>Marker721308 Chr5 SNP_site:5013006 Ref_Type:A
ACCACTAAACCTTGCCTTTACTTCAATAAAACCTCAAATCTTCTAAAAGTTGCAGTATTACCATTTACATACATATTTCAXXXXXXXXXXXC
ACGAGACATTTATGGATCAAGAGTAATACTACAACTTCTGAAAAATATGAGTATTTTTAAGCAAATGGCAAAGTGAGTT
ACCACTAAACCTTGCCTTTACTTCAATAAAACCTCAAATCTTCTAAATGTTGCAGTATTACCATTTACATACATACTTCAXXXXXXXXXXXC
ACGAGACATTTATGGATCAAGAGTAATACTACAACTTCTGAAAAATATGAGTATTTTTAAGCAAATGGCAAAGTGAGTT
>Marker721308 Chr5 SNP_site:5013034 Ref_Type:T
ACCACTAAACCTTGCCTTTACTTCAATAAAACCTCAAATCTTCTAAAAGTTGCAGTATTACCATTTACATACATATTTCAXXXXXXXXXXXC
ACGAGACATTTATGGATCAAGAGTAATACTACAACTTCTGAAAAATATGAGTATTTTTAAGCAAATGGCAAAGTGAGTT
ACCACTAAACCTTGCCTTTACTTCAATAAAACCTCAAATCTTCTAAATGTTGCAGTATTACCATTTACATACATACTTCAXXXXXXXXXXXC
ACGAGACATTTATGGATCAAGAGTAATACTACAACTTCTGAAAAATATGAGTATTTTTAAGCAAATGGCAAAGTGAGTT
>Marker721331 Chr5 SNP_site:15446842 Ref_Type:A
TACAGTAAATGAAGAAATTCAACCATTTTTTTCATAAGAATATAACTTCATTCTTTCAACACAATCAAACTAAAAATCATXXXXXXXXXXXA
CTTTACATTCTCAACAACAAAATCCAATAACGATAATTTTGCAACCATAAAAATAACAATTTAAAATAGTATGGAAAGT
TACAGTAAATGAAGAAATTCAACCATTTTTTTCTTAAGAATATAACTTCATTCTTTCAACACAATCAAACTAAAAATCATXXXXXXXXXXXA
CTTTACATTCTCAACAACAAAATCCAATAACGATAATTTTGCAACCATAAAAATAACAATTTAAAATAGTATGGAAAGT
>Marker721588 Chr5 SNP_site:12838777 Ref_Type:A
ACTTACGCCATGTATGCCACCGATGTGAAAGTATTGACTTCTCGTATGTTACAGAAATCAATAATATCTTCGCGGAGAACXXXXXXXXXXXA
CTCACAAGTAACTTCTTTTTAATTAGGAACCTAAGAAAGTCATAAACCCGCATTCAGCTCATGGTCTCACAAGAATGTA
ACTTACGCCATGTATGCCACCGATGTGAAAGTATTGACTTCTCGTATGTTACAGAAATCAATAATATCTTCGCGGAGAACXXXXXXXXXXXA
CTCACAAGTATCTTCTTTTTAATTAGGAACCTAAGAAAGTCATAAACCCGCATTCAGCTCATGGTCTCACAAGAATGTA
>Marker721680 Chr5 SNP_site:13858929 Ref_Type:C
GACCCTTTCTTAGATCTGCATCATCAGTATTTCGAAATGGCAAAATCTGGAGCAAACAAATGCCAACCTCTAACAGATTTXXXXXXXXXXXG
ACATTGAGGAAAGGAGTGTTCATTGCTGGGGCAGTGTTTGTGGTTGCTACCATGATTCTTAATGTGTATTATTACATGT
GACTCTTTCTTAGATCTGCATCATCAGTATTTCGAAATGGCAAAATCTGGAGCAAACAAATGCCAACCTCTAACAGATTTXXXXXXXXXXXA
ACATTGAGGAAAGGAGTGTTCATTGCTGGGGCAGTGTTTGTGGTTGCTACCATGATTCTTAATGTGTATTATTACATGT
>Marker721680 Chr5 SNP_site:13859142 Ref_Type:G
GACCCTTTCTTAGATCTGCATCATCAGTATTTCGAAATGGCAAAATCTGGAGCAAACAAATGCCAACCTCTAACAGATTTXXXXXXXXXXXG
ACATTGAGGAAAGGAGTGTTCATTGCTGGGGCAGTGTTTGTGGTTGCTACCATGATTCTTAATGTGTATTATTACATGT
GACTCTTTCTTAGATCTGCATCATCAGTATTTCGAAATGGCAAAATCTGGAGCAAACAAATGCCAACCTCTAACAGATTTXXXXXXXXXXXA
ACATTGAGGAAAGGAGTGTTCATTGCTGGGGCAGTGTTTGTGGTTGCTACCATGATTCTTAATGTGTATTATTACATGT
>Marker721705 Chr5 SNP_site:2377298 Ref_Type:G
AACTCTTTGACTTTTTACAACTTTGGTCATTTCCATTCGAGCTTACGAATATGAATATGCATTCATATTTTTAATATTTAXXXXXXXXXXXA
TGAACCTATAGATCATGGGCTTGAACGATTCAAGATTAACTAACTAAACTCTTTTACACTGAGCTAATCAACATTTGTT
AACTCTTTGACTTTTTACAACTTTGGTCATTTCCATTCGAGCTTACGAATATGAATATGCATTCATATTTTTAATATTTAXXXXXXXXXXXA
TGGACCTATAGATCATGGGCTCGAACGATTCAAGATTAACTAACTAAACTCTTTTAGACTGAGCTAATCAACATTTGTT
>Marker721705 Chr5 SNP_site:2377317 Ref_Type:C
AACTCTTTGACTTTTTACAACTTTGGTCATTTCCATTCGAGCTTACGAATATGAATATGCATTCATATTTTTAATATTTAXXXXXXXXXXXA
TGAACCTATAGATCATGGGCTTGAACGATTCAAGATTAACTAACTAAACTCTTTTACACTGAGCTAATCAACATTTGTT
AACTCTTTGACTTTTTACAACTTTGGTCATTTCCATTCGAGCTTACGAATATGAATATGCATTCATATTTTTAATATTTAXXXXXXXXXXXA
TGGACCTATAGATCATGGGCTCGAACGATTCAAGATTAACTAACTAAACTCTTTTAGACTGAGCTAATCAACATTTGTT
>Marker721705 Chr5 SNP_site:2377352 Ref_Type:G
AACTCTTTGACTTTTTACAACTTTGGTCATTTCCATTCGAGCTTACGAATATGAATATGCATTCATATTTTTAATATTTAXXXXXXXXXXXA

Create PDF with GO2PDF for free, if you wish to

TGAACCTATAGATCATGGGCTTGAACGATTCAAGATTAACTAACTAAACTCTTTTACACTGAGCTAATCAACATTTGTT
AACTCTTTGACTTTTTTACAACTTTGGTCATTTCCATTCGAGCTTACGAATATGAATATGCATTCATATTTTTAATATTTAXXXXXXXXXXA
TGGACCTATAGATCATGGGCTCGAACGATTCAAGATTAACTAACTAAACTCTTTTAGACTGAGCTAATCAACATTTGTT
>Marker721921 Chr5 SNP_site:14490183 Ref_Type:C
ACAGTTATTATGTGATCAATATGGAAATGTTGCTGCTTTGCACAGTCGTGATTGCAGTGTTCAGAGACGCCACCAAAAGGXXXXXXXXXXA
GGAGGGCCCAATTACTGTTGCGCAACTGGAGACAGTAAAAAAGCTAGAACAGGCAGCTAGAAGACTGGCTAAATGTGTG
ACAGTTATTATGTGATCAATATGGAAATGTTGCTGCTTTGCACAGTCGTGATTGCAGTGTTCAGAGACGCCACCAAAAGGXXXXXXXXXXA
GGAGGGCCCAATTACTGTTGCGCCACTGGAGACAGTAAAAAAGCTAGAACAGGCAGCTAGAAGACTGGCTAAATGTGTG
>Marker722017 Chr5 SNP_site:2192195 Ref_Type:C
TACTTAAAATAGCTTTGAGACGGATTTAAGATTAGAGTATTCAAGTTCTCAATAACGACTTTATTGAATAGAATATGATTXXXXXXXXXXA
ATTTTATTTTTGAATTTTGAATTTTTAAAAAATAGTCAATATGTTTTCATTCTGTCAACTGAGCTAATTTACTGTCGTG
TACTTAAAATAGCTTTGAGATGGATTTAAGATTAGAGTATTCAAGTTCTCAATAACGACTTTATTGAATAGAATATGATTXXXXXXXXXXA
ATTTTATTTTTGAATTTTGAATTTTTAAAAAATAGTCAATATGTTTTCATTCTGTCAACTGGGCTAATTTCCTGTCGTG
>Marker722017 Chr5 SNP_site:2192457 Ref_Type:A
TACTTAAAATAGCTTTGAGACGGATTTAAGATTAGAGTATTCAAGTTCTCAATAACGACTTTATTGAATAGAATATGATTXXXXXXXXXXA
ATTTTATTTTTGAATTTTGAATTTTTAAAAAATAGTCAATATGTTTTCATTCTGTCAACTGAGCTAATTTACTGTCGTG
TACTTAAAATAGCTTTGAGATGGATTTAAGATTAGAGTATTCAAGTTCTCAATAACGACTTTATTGAATAGAATATGATTXXXXXXXXXXA
ATTTTATTTTTGAATTTTGAATTTTTAAAAAATAGTCAATATGTTTTCATTCTGTCAACTGGGCTAATTTCCTGTCGTG
>Marker722017 Chr5 SNP_site:2192466 Ref_Type:A
TACTTAAAATAGCTTTGAGACGGATTTAAGATTAGAGTATTCAAGTTCTCAATAACGACTTTATTGAATAGAATATGATTXXXXXXXXXXA
ATTTTATTTTTGAATTTTGAATTTTTAAAAAATAGTCAATATGTTTTCATTCTGTCAACTGAGCTAATTTACTGTCGTG
TACTTAAAATAGCTTTGAGATGGATTTAAGATTAGAGTATTCAAGTTCTCAATAACGACTTTATTGAATAGAATATGATTXXXXXXXXXXA
ATTTTATTTTTGAATTTTGAATTTTTAAAAAATAGTCAATATGTTTTCATTCTGTCAACTGGGCTAATTTCCTGTCGTG
>Marker722265 Chr5 SNP_site:13203643 Ref_Type:A
GACCAAATGTTATGAATTTAAGATAGTGACATGCAATGAATCATAAACTTTTATGTAGTCATTGGTATCACCTTCGCCTAXXXXXXXXXXT
CAATAACAAAGGTCCTACCCATCAACTACTCAAACAACATCCATAAGAAATTTAAATCAACAAGATCATGTGCCTTAGT
GACCAAATGTTATGAATTTAAGATAGTGACATGCAATGAATCATAAACTTTTATGTAGTCATTGGTATCACCTTCGCCTAXXXXXXXXXXT
CAATAACAAAGGTCCTACCCATCAACTACTCAAACAACATCCATAAGGAATTTAAATCAACAAGATCATGTGCCTTAGT
>Marker722284 Chr5 SNP_site:2936544 Ref_Type:C
ACAGAGATAATGCTTTCAAAATCGATTTACCACCACACATCCACATACACCCAGTCTTCAATGTTGCTGATCTAAAGCCAXXXXXXXXXXA
GTTAAAACCCTTAATTAGTTAAAGAACAGCTATAATCAATAACTAACTAAAACAGTTAGACACTCAACTGTTTCAAGTA
ACAGAGATAATGCTTTCAAAATCGATTTACCACCACACATCCACATACACCCAGTCTTCAATGTTGTTGATCTAAAGCCAXXXXXXXXXXA
GTTAAAACCCTTAATTAGTTAAAGAACAGCTATAATCAATAACTAACTAAAACAGTTAGACACTCAACTGTTTCAAGTA
>Marker722787 Chr5 SNP_site:2601862 Ref_Type:G
ACAAGGGCTGTAGATTCCATCTGGCAGCCTTAAAAGCTCAATTTCTCATTCAACTTATTAGCCGACAAAGGAAAAGAAGAXXXXXXXXXXT
TTTATGAAAGTCGGTAAAGGTATGGTTAGTATCAAGGGATGGATAACTTTCTGAGAGTGAGACTTTCCATTTTCTTGTT
ACAAGGGCTGTAGATTCCATCTGGCAGCCTTGAAAGCTCAATTTCTCATTCAACTTATTAGCCTACAAAGGAAAAGAAGAXXXXXXXXXXT
TTTATGAAAGTCGGTAAAGGTATGGTTAGTATCAAGGGATGGATAACTTTCTGAGAGTGAGACTTTCCATTTTCTTGTT
>Marker722787 Chr5 SNP_site:2601894 Ref_Type:G
ACAAGGGCTGTAGATTCCATCTGGCAGCCTTAAAAGCTCAATTTCTCATTCAACTTATTAGCCGACAAAGGAAAAGAAGAXXXXXXXXXXT
TTTATGAAAGTCGGTAAAGGTATGGTTAGTATCAAGGGATGGATAACTTTCTGAGAGTGAGACTTTCCATTTTCTTGTT
ACAAGGGCTGTAGATTCCATCTGGCAGCCTTGAAAGCTCAATTTCTCATTCAACTTATTAGCCTACAAAGGAAAAGAAGAXXXXXXXXXXT
TTTATGAAAGTCGGTAAAGGTATGGTTAGTATCAAGGGATGGATAACTTTCTGAGAGTGAGACTTTCCATTTTCTTGTT
>Marker72286 Chr4 SNP_site:2503789 Ref_Type:C

Create PDF with GO2PDF for free, if you wish to

CACTTCAATATGGATGGATACATATAGGTGCATTCGTGTTTATATAGGTCAATTTAAGTGTTACGGTGATCTCTGCTTTAXXXXXXXXXXXA
GCACTACCTAGATTTTAGATAGAAAAGCTTCTGTGCAAGGCTGCAAATTTCATATATTTCACAAAAATGATGTGAGAGT
CACTTCAATATGGATGGATACATATAGGTGCATTTGTGTTTATATAGGTCAATTTAAGTGTTACGGTGATCTCTGCTTTAXXXXXXXXXXXA
GCACTACCTAGATTTTAGATAGAAAAGCTTCTGTGCAAGGCTGCAAATTTCATATATTTCACAAAAATGATGTGAGAGT
>Marker72295 Chr4 SNP_site:22687705 Ref_Type:T
ACAGATTATAGTAAGAAAACTTCATACATTTTTCCTAAGTAGCCAAGCTTTAAAATAGAGATCTAATAAAATTAGTAAATXXXXXXXXXXXC
AAGACTTACTAATGACAGGCAGGTCCATCTCCAGCCCAACAAGCTCTAGCAGCTTAAATCCATCCATGTCAGGCATGTG
ACAGATTATAGTAAGAAAACTTCATACATTTTTCCTAAGTAGCTAAGCTTTAAAATAGAGATCTAATAAAATTAGTAAATXXXXXXXXXXXC
AAGACTTACTAATGACAGGCAGGTCCATCTCCAGCCCAACAAGCTCTAGCAGCTTAAATCCATCCATGTCAGGCATGTG
>Marker724768 Chr5 SNP_site:7436906 Ref_Type:A
CACTACTCTCTAACATGAACTACCTCAATAGATTTTGCAACTGATACGGTATTGCAGAAGAAACTGAAAAAGTGTTGCAAXXXXXXXXXXXC
TATATTTACTATTATAGTTGCCTTTAGGGGTCAAACGGTGATAGCAACATTAGATGTGTTAAATTCTTAAATTGAAGTA
CACTACTCTCTAACATGAACTACCTCAGTAGCTTTTGCAACTGATACGGTATTGCAGAAGAAACTGAAAAAGTGTTGCAAXXXXXXXXXXXC
TATATTTACTATTATAGTTGCCTTTAGGGGTCAAACGGTGATAGCAACATTAGATGTGTTAAATTCTTAAATCGAAGTA
>Marker724768 Chr5 SNP_site:7436910 Ref_Type:A
CACTACTCTCTAACATGAACTACCTCAATAGATTTTGCAACTGATACGGTATTGCAGAAGAAACTGAAAAAGTGTTGCAAXXXXXXXXXXXC
TATATTTACTATTATAGTTGCCTTTAGGGGTCAAACGGTGATAGCAACATTAGATGTGTTAAATTCTTAAATTGAAGTA
CACTACTCTCTAACATGAACTACCTCAGTAGCTTTTGCAACTGATACGGTATTGCAGAAGAAACTGAAAAAGTGTTGCAAXXXXXXXXXXXC
TATATTTACTATTATAGTTGCCTTTAGGGGTCAAACGGTGATAGCAACATTAGATGTGTTAAATTCTTAAATCGAAGTA
>Marker724768 Chr5 SNP_site:7437163 Ref_Type:T
CACTACTCTCTAACATGAACTACCTCAATAGATTTTGCAACTGATACGGTATTGCAGAAGAAACTGAAAAAGTGTTGCAAXXXXXXXXXXXC
TATATTTACTATTATAGTTGCCTTTAGGGGTCAAACGGTGATAGCAACATTAGATGTGTTAAATTCTTAAATTGAAGTA
CACTACTCTCTAACATGAACTACCTCAGTAGCTTTTGCAACTGATACGGTATTGCAGAAGAAACTGAAAAAGTGTTGCAAXXXXXXXXXXXC
TATATTTACTATTATAGTTGCCTTTAGGGGTCAAACGGTGATAGCAACATTAGATGTGTTAAATTCTTAAATCGAAGTA
>Marker725009 Chr5 SNP_site:25020576 Ref_Type:T
ACCTTAAGTTTGAGTCGTCAGCTAAAAAGAAAAGAAAGAGATGTTAAACAAAAAGGAATTCTGAGTCGGTTAGAGAGAGAXXXXXXXXXXXT
TTTTTAACAAGGAAGAAAAATAATATACTTTGAAAATTAAGGCAAAACTTACAAAAGTGTAATAACCAAAGTTGATGTT
ACCTTAAGTTTGAGTCGTCAGCTAAAAAGAAAAGAAAGAGATGTTAAACAAAAAGGAATTCTGAGTCGGTTAGAGAGAGAXXXXXXXXXXXT
TTTTTAACAATGAAGAAAAATAATATACTTTGAAAATTAAGGCAAAACTTACAAAAGTGTAATAACCAAAGTTGATGTT
>Marker725043 Chr5 SNP_site:27822741 Ref_Type:A
CACATATTTAATCATTTATTCATATTCTTTAACCAGATTCAAGCAAGATCCCAGAAAAATTTGTTTAGAGTTACCAACGAXXXXXXXXXXXT
GATGTCAAGATATTAAAGCAAGAAAGAATTATTGAGAGTCATTCATGCAGTGAAGAAGAGGTAGTTAAAGTGTTTGGTG
CACATATTTACTCATTTATTCATATTCTTTAACCAGATTCAAGCAAGATCCCAGAAAAATTTGTTTAGAGTTACCAACGAXXXXXXXXXXXC
GATGTCAAGATATTAAAGCAAGAAAGAATTATTGAGAGTCATTCATGCAGTGAAGAAGAGGTAGTTAAAGTGTTTGGTG
>Marker725043 Chr5 SNP_site:27822938 Ref_Type:T
CACATATTTAATCATTTATTCATATTCTTTAACCAGATTCAAGCAAGATCCCAGAAAAATTTGTTTAGAGTTACCAACGAXXXXXXXXXXXT
GATGTCAAGATATTAAAGCAAGAAAGAATTATTGAGAGTCATTCATGCAGTGAAGAAGAGGTAGTTAAAGTGTTTGGTG
CACATATTTACTCATTTATTCATATTCTTTAACCAGATTCAAGCAAGATCCCAGAAAAATTTGTTTAGAGTTACCAACGAXXXXXXXXXXXC
GATGTCAAGATATTAAAGCAAGAAAGAATTATTGAGAGTCATTCATGCAGTGAAGAAGAGGTAGTTAAAGTGTTTGGTG
>Marker725050 Chr5 SNP_site:23838960 Ref_Type:C
AACTGCTCTTGACGCTAGTGGAATTGATGTGGGAAATTTATTCCTCTAGATGGCTGTTTGAATAGCTACGAGCTTTCTTTXXXXXXXXXXXT
AAGGCTCCTTGTAGGGTTGATTCCTTATGTTTGGCAAAGGGGATAGATCTCACATGCTTCAATGAAACGTTTTCTTGTT
AACTGCTCTTGACGCTAGTGGAATTGATGTGGGAAATTTATTCCTCTAGATGGCTGTTTGAATAGCTACGAGCTTTCTTTXXXXXXXXXXXT
AAGGCTCCTTGTAGGGTTGATTCCTTATGTTTGGCAAAGGGGATAGATCTCACATGCTTCAATGAAATGTTTTCTTGTT

Create PDF with GO2PDF for free, if you wish to

>Marker72525 Chr4 SNP_site:23398669 Ref_Type:A
ACAGTAAAATTTAAAAATTTTAAATAGAAAGGATGATAAACCACCAAAGCTGAACTCACAAATATATCACTCTCTTGGTTXXXXXXXXXXT
GCTCTGAAGCTCAAAAGTGCCTCACCTACAATAAACAAGAGACACCAAGCACTGAGAAAGAGCGTCATAATATGAAGGT
ACAGTAAAATTTAAAAATTTTAAATAGAAAGGATGATAAACCACCAAAGCTGAACTCACAGATATATCACTCTCTTGGTTXXXXXXXXXXT
GCTCTGAAGCTCAAAAGTGCCTCACCTACAATAAACAAGAGACACCAAGCACTGAGAAAGAGCGTCATAATATGAAGGT
>Marker725429 Chr5 SNP_site:2788342 Ref_Type:C
AACTAAACAAATACAATATCTAATAATGATGATCAAAGATCAAGAAGAAAAAACAACACAACTTTGTTCAATTTCTTTAGXXXXXXXXXXC
TCAATTTGAACCATGTGAATTAAATAAAATGACACAACTTTTGATCATCTTTCGACTGCAACACATAAAATAAATAGGT
AACTAAACAAATACAATATCTAATAATGATGATCAAAGATCAAGAAGAAAAAACAACAGAACTTTGTTCAATTTCTTTAGXXXXXXXXXXC
TCAATTTGAACTATGTGAATTAAATAAAATGACACAACTTTTGATCATCTTTCGACTGCAACACATAAAATAAATAGGT
>Marker725429 Chr5 SNP_site:2788475 Ref_Type:C
AACTAAACAAATACAATATCTAATAATGATGATCAAAGATCAAGAAGAAAAAACAACACAACTTTGTTCAATTTCTTTAGXXXXXXXXXXC
TCAATTTGAACCATGTGAATTAAATAAAATGACACAACTTTTGATCATCTTTCGACTGCAACACATAAAATAAATAGGT
AACTAAACAAATACAATATCTAATAATGATGATCAAAGATCAAGAAGAAAAAACAACAGAACTTTGTTCAATTTCTTTAGXXXXXXXXXXC
TCAATTTGAACTATGTGAATTAAATAAAATGACACAACTTTTGATCATCTTTCGACTGCAACACATAAAATAAATAGGT
>Marker725567 Chr5 SNP_site:3827777 Ref_Type:A
ACATGAAATCACACAAATATGAAAGGGGAAAGGAGGGTGGAATTTCATCTTAGGTTTAGAACTTTGCCAAGGAATGGATGXXXXXXXXXXT
TCTTCAATGCTAATGCATCTAGGAGGAGGGTAGAAAACTGGTGGATTGCAATAGAGATAATTATAAGCTTGATATTGTT
ACATGAAATCACACAAATATGAAAGGGGAAAGGAGGGTGGAATTTCATCTTAGGTTTAGAACTTTGCCAAGGAATGGATGXXXXXXXXXXT
TCTTCAATGCTAATGCATCTAGGAGGAGGGTAGAAAACTGGTGGATTGCAATAGAGATTATTATAAGCTTGATATTGTT
>Marker725579 Chr5 SNP_site:26085429 Ref_Type:C
ACTCTGATTGAACCACTTGTAAAAATTTAGTCTTGATGTAAGTAAAAAACAGAAAATTTCAATGTAACTACTTTCTTTTTXXXXXXXXXXT
TGCAATTGGAACCTCAGAGAGGAATAGCAAAAAAGCGCTCCATCTTAATCAAAACAAGAAACCTTTAGTTAGGAGAGTG
ACTCTGATTGAACCACTTGTAAAAATTTAGTCTTGATGTAAGTAAAAAACAGAAAATTTCAATGTAACTACTTTCTTTTTXXXXXXXXXXT
TGCAATTGGAACCTCAGAGAGGAATAGCAAAAAAGCGCTCCATCTTAATCAAAACAAGAAACGTTTAGTTAGGAGAGTG
>Marker725592 Chr5 SNP_site:2438154 Ref_Type:G
CACCTAGTCGTGTGATATCGTGTTGTATAACGAGCATGCTTGTCCATGTCATTGTTTGTCTATGGTTATGTGCCCAAATAXXXXXXXXXXC
AAGCCTACCAAAGCTAAAAAAGTCCAAAATATATTATAAGGGAGGCATAATAGTTGAATCCCCGCCCAAGCATTGTTGT
CACCTAGTCGTGTGATATCGTGTTGTATAACGAGCATGCTTGTCCATGTCATTGTTTGTCTATGGTTATGTGCCCAAATAXXXXXXXXXXC
AAGCCTACCAGAGCTAAAAAAGTCCAAAATATATTATAAGGGAGGCATAATAGTTGAATCCCCGCCCAAGCATTGTTGT
>Marker725820 Chr5 SNP_site:25641091 Ref_Type:T
ACCATAAGACCCTTCCACCCAAAAAAATAATAATAACAACAACAAAATGTAGATTTGCTAGGATTCACTATATGCCTAGGXXXXXXXXXXG
AAGCTCTTCTAACACAATAGTTGTGGCAGTTTTTCATGGAGCACAATGCTTTGTAGCACAGAGTTATTGTGAGAAGGTT
ACCATAAGACCCTTCCACCCAAAAAAATAATAATAATAACAACAAAATGTAGATTTGCTAGGATTCACTATATGCCTAGGXXXXXXXXXXG
AAGCTCTTCTAACACAATAGTTGTGGCAGTTTTTCATGGAGCACAATGCTTTGTAGCACAGAGTTATTGTGAGAAGGTT
>Marker725906 Chr5 SNP_site:27782321 Ref_Type:T
TACTCTACCAAAGGTAAAACCCAATTTTTTAATTATACTTCATTCCTTTAGTTTCACTACATGCAACACTCTTAATTACTXXXXXXXXXXT
ATGGCTTTCAATTCCTGGCATGGATATGTGATCTTTTAGTTAAAGTTTGTGTTTGTATTTCTGTTTGTTCAATAATAGT
TACTCTACCAAAGGTAAAACCCAATTTTTTAATTATACTTCATTCCTTTAGTTTCTCTACATGCAACACTCTTAATTACTXXXXXXXXXXT
ATGGCTTTCAATTCCTGGCATGGATATATGATCTTTTAGTTAAAGTTTGTGTTTGTATTTCTGTTTGTTCAATAATAGT
>Marker725906 Chr5 SNP_site:27782510 Ref_Type:A
TACTCTACCAAAGGTAAAACCCAATTTTTTAATTATACTTCATTCCTTTAGTTTCACTACATGCAACACTCTTAATTACTXXXXXXXXXXT
ATGGCTTTCAATTCCTGGCATGGATATGTGATCTTTTAGTTAAAGTTTGTGTTTGTATTTCTGTTTGTTCAATAATAGT
TACTCTACCAAAGGTAAAACCCAATTTTTTAATTATACTTCATTCCTTTAGTTTCTCTACATGCAACACTCTTAATTACTXXXXXXXXXXT

Create PDF with GO2PDF for free, if you wish to

ATGGCTTTCAATTCCTGGCATGGATATATGATCTTTTAGTTAAAGTTTGTGTTTGTATTTCTGTTTGTTCAATAATAGT
>Marker72609 Chr4 SNP_site:5357179 Ref_Type:T
CACTAATTTGGTTCTCCTTGGGCTGCCATCTTGCAGCCAAATGCCAAGATTATTCCAATTACACACAGCAAGAACTTCAGXXXXXXXXXXC
AGCAACTGAAAGAATCTTTTGAAAGATATCCCATTCATGATAAGTTGCTCCATTCGGTTTTAACTTTGGCAGAACCGGT
CACTAATTTGGTTCTCCTTGGGCTGCCATCTTGCAGCCAAATGCCAAGATTATTCCAATTACACACAGCAAGAACTTCAGXXXXXXXXXXC
AGCAACTGAAAGAATCTTTTGAAAGATATCCCATTCATGATAAGTTGCTCCATTCGGTTTTAACTTTGGCAGAACTGGT
>Marker727206 Chr5 SNP_site:26782153 Ref_Type:A
ACCCTCTTGGATATATTGTGAGAATTTCACATCGAAAATGGACTTCACTCTTCATGAGATAGATAGACTACTCTTCATTCXXXXXXXXXXG
TCTAATGCACATAAATTTCTTCCATTTTTGCTGATGTTGTGTATGTGGGTGTTCCTCCTGGAGGGAGAGGCGTTTAGTC
ACCCTCTTGGATGTATTGTGAGAATTTCACATCGAAAATGGACTTCACTCTTCATGAGATAGATAGACTACTCTTCATTCXXXXXXXXXXG
TCTAATGCACATAAATTTCTTCCATTTTTGCTGATGTTGTGTATGTGGGTGTTCCTCCTGGAGGGAGAGGCGTTTAGTC
>Marker727462 Chr5 SNP_site:11810310 Ref_Type:G
GACTAGAAATAGCAAAAGATATGCCATAATGTTTTGTCGATGATTGTTCTAATTTTACTTTTATTTACTTGCTTAGAAATXXXXXXXXXXG
ATAAGGGAACTAAATATGATTCAGCGTTGCCTTTAATGAGTTCTACAACTCAAAAGGAATAATTCGTGAGAAAATTGTG
GACTAGAAATAGCAAAAGATATGCCATAATGTTTTGTCGATGATTGTTCTAATTTTACTTTTATTTACTTGCTTAGAAATXXXXXXXXXXG
ATAAGGGAACTAAATATGATTCAGCGTTTCCTTTAATGAGTTCTACAACTCAAAAGGAATAATTCGTGAGAAAATTGTG
>Marker72798 Chr4 SNP_site:18156862 Ref_Type:A
TACTACTTCAAAGCGACATTTTTCTGTTGAACGATTCTTTGTATTGTTGAAAGCAAACTCAGCTTGAGAAACTACTAGATXXXXXXXXXXT
TGTGTCAAAATTATTCCATAGGGTTTGCCAGAAATGGCTTAGAAACTTAACATCTTTGTCGGACACAATAGTTTTAGGT
TACTACTTCAAAGCGACATTTTTCTGTTGAACGATTCTTTGTATTGTTGAAAGCAAACTCAGCTTGAGAATCTACTAGATXXXXXXXXXXT
TGTGTCAAAATTATTCCATAGGGTTTGCCAGAAATGGCTTAGAAACTTAACATCTTTGTCGGACACAATAGTTTTAGGT
>Marker728101 Chr5 SNP_site:17228010 Ref_Type:C
TACATCTTGTAGTCCCATCGCGGTTGAATTTTCGTCATTCGATTCTCGCCATATGCATAATCAGCTCGAGCTCGTTTCGTXXXXXXXXXXG
AATCGCAGTCGTAGCACAGTATGTTGGGTGCCCCGTTCCATCGGTAGCAATCAGGATCTTGCACGTTCTCGCTGCATGT
TACATCTTGTAGTCCCATCGTGGTTGAATTTTCGTCATTCGATTCTCGCCATATGCATAATCAGCTCGAGCTCGTTTCGTXXXXXXXXXXG
AATCGCAGTCGTAGCACAGTATGTTGGGTGCCCCGTTCCATCGGTAGCAATCAGGATCTTGCACGTTCTCGCTGCATGT
>Marker728297 Chr5 SNP_site:1460841 Ref_Type:C
ACCTTATGTGATGTCTTACACTTGTTGTGTGTTCTGGTGTGGGATGCCTTATCATTGCTATGTGATCTGGGATAATAAGAXXXXXXXXXXA
TGCTTGATGAAATTGATGGAAAATTGTTGTGGCGTTGAGATTACTCTGCATAATCGCAACTCAACAAAAGCGTATGGTA
ACCTTATGTGATGTCTTACACTTGTTGTGTGTTCTGGTGTGGGATGCCTTATCCTTGCTATGTGATCTGGGATAATAAGAXXXXXXXXXXA
TGCTCGATGAAATTGATGGAAAATTGTTGTGCCGTTGAGATTACTCTGCATAATCGCAACTCAACAAAAGCGTATGGTA
>Marker728297 Chr5 SNP_site:1460983 Ref_Type:C
ACCTTATGTGATGTCTTACACTTGTTGTGTGTTCTGGTGTGGGATGCCTTATCATTGCTATGTGATCTGGGATAATAAGAXXXXXXXXXXA
TGCTTGATGAAATTGATGGAAAATTGTTGTGGCGTTGAGATTACTCTGCATAATCGCAACTCAACAAAAGCGTATGGTA
ACCTTATGTGATGTCTTACACTTGTTGTGTGTTCTGGTGTGGGATGCCTTATCCTTGCTATGTGATCTGGGATAATAAGAXXXXXXXXXXA
TGCTCGATGAAATTGATGGAAAATTGTTGTGCCGTTGAGATTACTCTGCATAATCGCAACTCAACAAAAGCGTATGGTA
>Marker728297 Chr5 SNP_site:1461010 Ref_Type:C
ACCTTATGTGATGTCTTACACTTGTTGTGTGTTCTGGTGTGGGATGCCTTATCATTGCTATGTGATCTGGGATAATAAGAXXXXXXXXXXA
TGCTTGATGAAATTGATGGAAAATTGTTGTGGCGTTGAGATTACTCTGCATAATCGCAACTCAACAAAAGCGTATGGTA
ACCTTATGTGATGTCTTACACTTGTTGTGTGTTCTGGTGTGGGATGCCTTATCCTTGCTATGTGATCTGGGATAATAAGAXXXXXXXXXXA
TGCTCGATGAAATTGATGGAAAATTGTTGTGCCGTTGAGATTACTCTGCATAATCGCAACTCAACAAAAGCGTATGGTA
>Marker728330 Chr5 SNP_site:13592194 Ref_Type:T
AACAAGATAAAATATATTTTCACGATTACAGTTATCTTGTGATCATCAAGCTATGCTAACTCTGACACCAAAATATGGAAXXXXXXXXXXG
TTTATCAATGATGAGAGTAGAAGATTTGAATTTACTGGGAAAATTAACACCTTTTTTGTTTTATATTTCAAAAGTAGTA

Create PDF with GO2PDF for free, if you wish to

AACAAGATAAAATATATTTTCACGATTACAGTTATCTTGTGATCATCAAGCTATGCTAACTCTGACATCAAAATATGGAAXXXXXXXXXXXG
TTTATCAATGATGAGAGTAGAAGATTTGAATTTACTGGGAAAATTAACACTTTTTTTGTTTTATATTTCAAAAGTAGTA
>Marker728330 Chr5 SNP_site:13592375 Ref_Type:T
AACAAGATAAAATATATTTTCACGATTACAGTTATCTTGTGATCATCAAGCTATGCTAACTCTGACACCAAAATATGGAAXXXXXXXXXXXG
TTTATCAATGATGAGAGTAGAAGATTTGAATTTACTGGGAAAATTAACACCTTTTTTGTTTTATATTTCAAAAGTAGTA
AACAAGATAAAATATATTTTCACGATTACAGTTATCTTGTGATCATCAAGCTATGCTAACTCTGACATCAAAATATGGAAXXXXXXXXXXXG
TTTATCAATGATGAGAGTAGAAGATTTGAATTTACTGGGAAAATTAACACTTTTTTTGTTTTATATTTCAAAAGTAGTA
>Marker728332 Chr5 SNP_site:15896557 Ref_Type:T
ACTCTTTTATCTTTGATGGACGATTGATATGAAATTGTTTTATTCAAATAAATCAAACTACGATCTAACTGGTTATGCAAXXXXXXXXXXXA
GCTAAAAGAGCATGTGTGTGGTTGGGGTCAATGACTCATCATATTCGAGAAAGATATGGTTTGTCTTTTAGTGAAAGTT
ACTCTTTTATCTTTGATGGACGATTGATATGAAATTGTTTTATTCAAATAAATCAAACTACGATCTAATTGGTTATGCAAXXXXXXXXXXXA
GCTAAAAGAGCATGTGTGTGGTTGGGGTCAATGACTCATCATATTCGAGAAAGATATGGTTTGTCTTTTAGTGAAAGTT
>Marker728444 Chr5 SNP_site:24804312 Ref_Type:G
ACTTTCAATTTTGGTAATTTGAGTGTAAATTTTACATATTTGTTATAATTAGATATCACCCTTACTTCAATTTCAACTATXXXXXXXXXXXC
TAGGAAGAAACTATCAAATTTCTATATAGATGTTGCACTAATTATGTATGTTATGTCTAATAACAATATGGTATAAGTT
ACTTTCAATTTTGGTAATTTGAGTGTAAATTTTACATATTTGTTATAATTATATATCACCCTTACTTCAATTTCAACTATXXXXXXXXXXXC
TAGGAAGAAACTATCAAATTTCTATATAGATGTTGCACTAATTATGTATGTTATGTCTAATAACAATATGGTATAAGTT
>Marker728952 Chr5 SNP_site:9584340 Ref_Type:G
TACAATGAGAATAGCTCTGCAAAAACAAACTCTGCCTACGCCCACCCTGTCAAAAGCATAGAATTGAAGAGCTCATCAGCXXXXXXXXXXXA
CATACTTGCAGGAAGTCGGCAGCAGTTATATCTCCAGCAGTAGCCATTTAGCTAACCTCCAAAACGAACTCCGCCTGTA
TACAATGAGAATAGCTCTGCAGAAACAAACTCTGCCTACGCCCACCCTGTCAAAAGCATAGAATTGAAGAGCTCATCAGCXXXXXXXXXXXA
CATACTTGCAGGAAGTCGGCAGCAGTTATATCTCCAGCAGTAGCCATTTAGCTAACCTCCAAAACGAACTCCGCCTGTA
>Marker729262 Chr5 SNP_site:1594792 Ref_Type:C
TACATTTGTATATCTTATTACCCCACTATAATTATAGGTGTCCCTTCGAACCCTAACCCATTACTCAACGTATGAATTTGXXXXXXXXXXXT
GAAAAAAAATGTGAAATTCCTCCCATGGAAAATCACTAACTCTTTGTCTTAATACCGTGGTAGGTTACAGAAGCGATGT
TACATTTGTATATCTTATTACCCCACTATAATTATAGGTGTCCCTTCGAACCCTAACCCATTACTGAACGTATGAATTTGXXXXXXXXXXXT
GAAAAAAAATGTGAAATTCCTCCCATGGAAAATCACTAACTCTTTGTCTTAATACCGTGGTAGGTTACAGAAGCGATGT
>Marker7294 Chr4 SNP_site:21621731 Ref_Type:C
AACAAGTGTTGGCATATGTGTATTGTGGTGTGTATGAGATGAGATGGACCGTTTGGGAATAATAAATCAAACC-TTAGGCTXXXXXXXXXX
CAGAGTGAAGAACAGAACAGAATGACGAGCAACCACCATAGCCTATCAATCATTTTTTCATTCCTACATCGTGGGTCGTT
AACAAGTGTTGGCATATGTGTATTGTGGTGTGTATGAGATGAGATGGACCGTTTGGGAATAATAACTCAAACCTTTAGGC-XXXXXXXXXX
CAGAGTGAAGAACAGAACAGAATGACGAGCAACCACCATAGCCTATCAATCATTTTTTCATTCCTACATCGTGGGTCGTT
>Marker729432 Chr5 SNP_site:8862651 Ref_Type:A
AACATACCTATTGTGCTACATTGTGAAAAAATGTGAGCGTCTATGCGAACAATATACATTATGGTTATCAATCATTCAACXXXXXXXXXXXA
ATTCATATTTAAAGTGGCCTCGTGATATTATGCAATTAAAGTATCATCCATATGGAAAATTTAGAGAGTGACCAATGTA
AACATACCTATTGTGCTACATTGTGAAAAAATGTGAGCGTCTATGCGAACAATATACATTATGGTTATCAATCATTCAACXXXXXXXXXXXA
ATTCATATTTAAAGTGGCCTCGTGATATTATGCAATTGAAGTATCATCCATATGGAAAATTTAGAGAGTGACCAATGTA
>Marker729637 Chr5 SNP_site:5992887 Ref_Type:A
TACATTGTCATAGATTTTAATGGATAAGATACTAATACTTAAATAAAAAATGATATAATAAATAGATGAAATATTTAATTXXXXXXXXXXXT
ATACTTTGTGGGTAGGGGTTCATTTGCACTCTTCACTAGGAACTTTATTTTGCCTCTTCTTGTGTTCTCAATTTGAGTT
TACATTGTCATAGATTTTAGTGGATAAGATACTAATACTTAAATAAAAAATGATATAATAAATAGATGAAATATTTGATTXXXXXXXXXXXT
ATACTTTGTGGGTAGGGGTTCATTTGCACTCTTCACTAGGAACTTTATTTTGCCTCTTCTTGTGTTCTCAATTTGAGTT
>Marker729637 Chr5 SNP_site:5992944 Ref_Type:A
TACATTGTCATAGATTTTAATGGATAAGATACTAATACTTAAATAAAAAATGATATAATAAATAGATGAAATATTTAATTXXXXXXXXXXXT

Create PDF with GO2PDF for free, if you wish to

ATACTTTGTGGGTAGGGGTTCATTTGCACTCTTCACTAGGAACTTTATTTTGCCTCTTCTTGTGTTCTCAATTTGAGTT
TACATTGTCATAGATTTTAGTGGATAAGATACTAATACTTAAATAAAAAATGATATAATAAATAGATGAAATATTTGATTXXXXXXXXXXT
ATACTTTGTGGGTAGGGGTTCATTTGCACTCTTCACTAGGAACTTTATTTTGCCTCTTCTTGTGTTCTCAATTTGAGTT
>Marker729669 Chr5 SNP_site:9804056 Ref_Type:C
AACCCTATATATCATGCCCTAATAAATCATTTTAGCCCAAAAATATGGCAAATATAAGATACTTGGCATTGCCTTTGACCXXXXXXXXXXC
ATACAACCCACCAAACAAGGGATGTTGTGCTGTAGGTCTAACCCAAGAAAGGTGGATAAGATAAAAAGATAGGTGTGTA
AACCCTATATATCATGCCCTAATAAATCATTTTAGCCCAAAAATATGGCAAATATAAGATACTTGGCATTGCCTTTGACCXXXXXXXXXXC
ATAGAACCCACCAAACAAGGGATGTTGTGCTGTAGGTCTAACCCAAGAAAGGTGGATAAGATAAAAAGATAGGTGTGTA
>Marker73031 Chr4 SNP_site:12829085 Ref_Type:C
ACAATGAACTTCCAATAAATCTGGATTAGTTTCTAAAGATTTCTTGAAACTGCTTGACTGTAATGAGAGATTTGAATTTGXXXXXXXXXXG
ACCAATGGCTATAGAAGAAAACTAGAGATTATAAAAAGCGTTCCAGATAATCAATAGGTTGTTTAGACTGCAATTTGTG
ACAATGAACTTCCAATAAATCTGGATTAGTTTCTAAAGATTTCTTGAACCTGCTTGACTGTAATGAGAGATTTGAATTTGXXXXXXXXXXG
ACCAATGGCTATAGAAGAAAACTAGAGATTATAAAAAGCGTTCCAGATAATCAATAGGTTGTTTAGACTGCAATTTGTG
>Marker730364 Chr5 SNP_site:2817001 Ref_Type:G
ACCAACCTACCAACGAAGGAAACATCCCAATGATTAAGAAAATGATAAGAGAAAAGAGAGATAAAGAAAGAAGAATGAGAXXXXXXXXXXA
TAAAGATTCTTCTTTCTTTTTGCATGGCAATCATATGGCAAGCAAATTTGTGAGGCCATAAATACTCTCCCCCTTTGTA
ACCAACCTACCAACGAAGGAAACATCCCAATGATTAAGAAAATGATGAGAGAAAAGAGAGATAAAGAAAGAAGAATGAGAXXXXXXXXXXA
TAAAGATTCTTCTTTCTTTTTGCATGGCAATCATATGGCAAGCAAATTTGTGAGGCCATAAATACTCTCCCCCTTTGTA
>Marker730753 Chr5 SNP_site:6170394 Ref_Type:T
TACATCAACAAGAAATCTCAGGTAAATAATTCAAAACTCAAGCAAAGATGATTCGTGAAGAACAAAACTCAAGCAACGATXXXXXXXXXXT
CATCCTGTTTATCACCTAGAAAACGACTTGTCCTAACCGTTCTGTGAAACCGTGGTTCTGTTGTGTTCTAGGTGTGGTT
TACATCAACAAGAAATCTCAGGTAAATAATTCAAAACTCAAGCAAAGATGATTCGTGAAGAACAAAACTCAAGCAACGATXXXXXXXXXXT
CATCCTGTTTATCTCCTAGAAAACGACTTGTCCTAACCGTTCTGTGAAACCGTGGTTCTGTTGTGTTCTAGGTGTGGTT
>Marker730915 Chr5 SNP_site:11483785 Ref_Type:G
CACAAGATTATCAAGCATTTTCATCATCAAACTTGCAGAAAATTAAGGTATCATCAGCGAATTGGAGAATAGAGACATACXXXXXXXXXXC
CTTGACTATATTGAACAGTAATATTTGACATTGAAGATAATATGGGGCCAACCAAGATTTGTTGTAAATCTTCACAGTT
CACAAGATTATCAAGCATTTTCATCATCAAACTTGCAGAAAATTAAGGTGTCATCAGCGAATTGGAGAATAGAGACATACXXXXXXXXXXC
CTTGACTATATTGAACAGTAATATTTGACATTGAAGATAATATGGGGCCAACCAAGATTTGTTGTAAATCTTCACAGTT
>Marker730936 Chr5 SNP_site:24847905 Ref_Type:T
AACGTATGAATTAAATTAGGGTTTAAAACGTTTTTTTTAATTGATTATCGAGATTTGAGGTAAGAATTTTAAGTTTAAAAXXXXXXXXXXG
TAATATAGTGAAATCAACACGTTTATTAAAGTGGAAAGTTGGTGTATAAAAAGGTCTATCTAGTTTAGTAGATATGGTA
AACGTATTAATTAAATTAGGGTTTAAAACGTTTTTTTTAATTGATTATCGAGATTTGAGGTAAGAATTTTAAGTTTAAAAXXXXXXXXXXG
TAATATAGTGAAATCAACACGTTTATTAAAGTGGAAAGTTGGTGTATAAAAAGGTCTATCTAGTTTAGTAGATATGGTA
>Marker73145 Chr4 SNP_site:16762088 Ref_Type:C
AACGCCTAGCTCACCAACACTTAGCCAAAATTTCACTTAACTTCCTCTCTTTCCTTCTCTTGGCTGCCAATCCTAAGTCCXXXXXXXXXXA
ATAAGTTTAGGATTTATAATATTTAATTGATTCAATTTCCTTTGCCAGCGAGTGCATGTTTGAGATTAGAGGTTGTGTA
AACGCCTAGCTCACCAACAGTTAGCCAAAATTTCACTTAACTTCCTCTCTTTCCTTCTCTTGGCTGCCAATCCTAAGTCCXXXXXXXXXXA
ATAAGTTTAGGGTTTATAATATTTAATTGATTCAATTTCCTTTGCCAGCGAGTGCATGTTTGAGATTAGAGGTTGTGTA
>Marker73145 Chr4 SNP_site:16762257 Ref_Type:A
AACGCCTAGCTCACCAACACTTAGCCAAAATTTCACTTAACTTCCTCTCTTTCCTTCTCTTGGCTGCCAATCCTAAGTCCXXXXXXXXXXA
ATAAGTTTAGGATTTATAATATTTAATTGATTCAATTTCCTTTGCCAGCGAGTGCATGTTTGAGATTAGAGGTTGTGTA
AACGCCTAGCTCACCAACAGTTAGCCAAAATTTCACTTAACTTCCTCTCTTTCCTTCTCTTGGCTGCCAATCCTAAGTCCXXXXXXXXXXA
ATAAGTTTAGGGTTTATAATATTTAATTGATTCAATTTCCTTTGCCAGCGAGTGCATGTTTGAGATTAGAGGTTGTGTA
>Marker731989 Chr5 SNP_site:12146908 Ref_Type:G

Create PDF with GO2PDF for free, if you wish to

AACTTTGGTGCAACTCATAACTTCATGACAAAGACGGAAGCCAGACAGTTGAACCTCCATCGAGAAAGTGTATCATGGTTXXXXXXXXXXT
TCTTTGGAGACTAGAGAGCACTATTTTCTTAACCGGCAATCAAAATAGACATCATCAAGACTAGCTCTATATCAAATGT
AACTTTGGTGCAACTCATAACTTCATGACAAAGACGGAAGCCAGACGGTTGAACCTCCATCAAGAAAGTGTATCATGGTTXXXXXXXXXXT
TCTTTGGAGACTAGAGAGCACTATTTTCTTAACCGGCAATCAAAATAGACATCATCAAGACTAGCTCTATATCAAATGT
>Marker731989 Chr5 SNP_site:12146923 Ref_Type:A
AACTTTGGTGCAACTCATAACTTCATGACAAAGACGGAAGCCAGACAGTTGAACCTCCATCGAGAAAGTGTATCATGGTTXXXXXXXXXXT
TCTTTGGAGACTAGAGAGCACTATTTTCTTAACCGGCAATCAAAATAGACATCATCAAGACTAGCTCTATATCAAATGT
AACTTTGGTGCAACTCATAACTTCATGACAAAGACGGAAGCCAGACGGTTGAACCTCCATCAAGAAAGTGTATCATGGTTXXXXXXXXXXT
TCTTTGGAGACTAGAGAGCACTATTTTCTTAACCGGCAATCAAAATAGACATCATCAAGACTAGCTCTATATCAAATGT
>Marker732418 Chr5 SNP_site:2288093 Ref_Type:C
AACTAATATTTTATAAACATTTTCTCAAATTGATCCCTTCTGAGTCTAAGTTGTGCATAACTCGACCAGTATCTCTATTGXXXXXXXXXXT
GTGCATAATGTGAATGACAACGATCAATGCAACAAAAAAATGAATGAATGGAAACTTCCCAAATGCTATGGATTGAGTG
AACTAATATTTTATAAATATTTTCTCAAATTGATCCCTTCTGAGTCTGAGTTGTGCATAACTCGACCAGTATCTCTATTGXXXXXXXXXXT
GTGCATAATGTGAATGGCAACGATCAATGCAACAAAAAAATGAATGAATGGAAACTTCCCAAATGCTATGGATTGAGTG
>Marker732418 Chr5 SNP_site:2288123 Ref_Type:A
AACTAATATTTTATAAACATTTTCTCAAATTGATCCCTTCTGAGTCTAAGTTGTGCATAACTCGACCAGTATCTCTATTGXXXXXXXXXXT
GTGCATAATGTGAATGACAACGATCAATGCAACAAAAAAATGAATGAATGGAAACTTCCCAAATGCTATGGATTGAGTG
AACTAATATTTTATAAATATTTTCTCAAATTGATCCCTTCTGAGTCTGAGTTGTGCATAACTCGACCAGTATCTCTATTGXXXXXXXXXXT
GTGCATAATGTGAATGGCAACGATCAATGCAACAAAAAAATGAATGAATGGAAACTTCCCAAATGCTATGGATTGAGTG
>Marker732418 Chr5 SNP_site:2288299 Ref_Type:A
AACTAATATTTTATAAACATTTTCTCAAATTGATCCCTTCTGAGTCTAAGTTGTGCATAACTCGACCAGTATCTCTATTGXXXXXXXXXXT
GTGCATAATGTGAATGACAACGATCAATGCAACAAAAAAATGAATGAATGGAAACTTCCCAAATGCTATGGATTGAGTG
AACTAATATTTTATAAATATTTTCTCAAATTGATCCCTTCTGAGTCTGAGTTGTGCATAACTCGACCAGTATCTCTATTGXXXXXXXXXXT
GTGCATAATGTGAATGGCAACGATCAATGCAACAAAAAAATGAATGAATGGAAACTTCCCAAATGCTATGGATTGAGTG
>Marker732574 Chr5 SNP_site:1097081 Ref_Type:C
TACATGGCTGATGCAACAAAATAAAACCTTACCCAATCAAGTAGATTCTCCGCATCCATAAATGAAGTAAATGACGAATTXXXXXXXXXXA
GTGAATCGCTTCAGTTAGGATTCAGAAATTTGACAAGTTTTCTCAATGAGCAAAGCAAAGATTCAGAAACTTACTTTGT
TACATGGCTGATGCAACAAAATAAAACCTTACCCAATCAAGTAGATTCTCCGCATCCATAAATGAAGTAAATGACGAATTXXXXXXXXXXA
GTGAATTGCTTCAGTTAGGATTCAGAAATTTGACAAGTTTTCTCAATGAGCAAAGCAAAGATTCAGAAACTTACTTTGT
>Marker73273 Chr4 SNP_site:13852138 Ref_Type:C
AACACGTTCGAAGCCTTAGTAAGGAGCTTCGTATTGCTAAAGAATCCACGGATAGAGCTAAGAGAGAGGTTGAGCGAAATXXXXXXXXXXG
CATTTTTCCATCTATCTTTGGGTTCTCATTTGAGTTGTAATTCTTTGTGTTCTATGGTTAATTCAAAATGTTTTAGGTT
AACACGTTCGAAGCCTTAGTAAGGAGGTTCGTATTGCTAAAGAATCCACGGATAGAGCTAAGAGAGAGGTTGAGTGAAATXXXXXXXXXXG
CATTTTTCCATCTATCTTTGGGTTCTCATTTGAGTTGTAATTCTTTGTGTTCTATGATTAATTCAAAATGTTTTAGGTT
>Marker73273 Chr4 SNP_site:13852186 Ref_Type:C
AACACGTTCGAAGCCTTAGTAAGGAGCTTCGTATTGCTAAAGAATCCACGGATAGAGCTAAGAGAGAGGTTGAGCGAAATXXXXXXXXXXG
CATTTTTCCATCTATCTTTGGGTTCTCATTTGAGTTGTAATTCTTTGTGTTCTATGGTTAATTCAAAATGTTTTAGGTT
AACACGTTCGAAGCCTTAGTAAGGAGGTTCGTATTGCTAAAGAATCCACGGATAGAGCTAAGAGAGAGGTTGAGTGAAATXXXXXXXXXXG
CATTTTTCCATCTATCTTTGGGTTCTCATTTGAGTTGTAATTCTTTGTGTTCTATGATTAATTCAAAATGTTTTAGGTT
>Marker73273 Chr4 SNP_site:13852368 Ref_Type:G
AACACGTTCGAAGCCTTAGTAAGGAGCTTCGTATTGCTAAAGAATCCACGGATAGAGCTAAGAGAGAGGTTGAGCGAAATXXXXXXXXXXG
CATTTTTCCATCTATCTTTGGGTTCTCATTTGAGTTGTAATTCTTTGTGTTCTATGGTTAATTCAAAATGTTTTAGGTT
AACACGTTCGAAGCCTTAGTAAGGAGGTTCGTATTGCTAAAGAATCCACGGATAGAGCTAAGAGAGAGGTTGAGTGAAATXXXXXXXXXXG
CATTTTTCCATCTATCTTTGGGTTCTCATTTGAGTTGTAATTCTTTGTGTTCTATGATTAATTCAAAATGTTTTAGGTT

Create PDF with GO2PDF for free, if you wish to

>Marker733093 Chr5 SNP_site:9979531 Ref_Type:A
AACATTTAAGAATATTATTGATAAATATAGTATTTTTTGAGCCCATTAACAAGGATTCGAATTTCGTGTTTCTTGAAAAAXXXXXXXXXXT
TTTTCTTGATGGAATTTGCCCATGATTTATTTTTGGTCTTTATCCATCTAGAAACTCTAGACCTATTAAGAAAATTGTA
AACATTTAAGAATATTATTGATAAATATAGTATTTTTTGAGCCCATTAACAAGGGTTCGAATTTCGTGTTTCTTGAAAAAXXXXXXXXXXT
TTTTCTTGATGGAATTTGCCCATGATTTATTTTTGGTCTTTATCCATCTAGAAACTCTAGACCTATTAAGAAAATTGTA
>Marker733414 Chr5 SNP_site:2389522 Ref_Type:T
ACCCATCATTATAGTTTTGCAATGATATTCCAAAGGTTTAGAATAACCCCCACCGAAAGGCAGTCGATTATTCCTCCTTTXXXXXXXXXXA
GCTTAGTAATTTTGTAAGTATAACCCTCCATTTTCGAATCTCATAGGTTAGAATCATTATGCTCCCAAAGGAAAAAGTC
ACCCATCATTATAGTTTTGCAATGATATTCCAAAGGTTTAGAATAACCCCCATCGAAAGGCAGTCGATTATTCCTCCTTTXXXXXXXXXXA
GCTTAGTAATTTTGTAAGTATAACCCTCCATTTTCGAATCTCATAGGTTAGAATCATTATGCTCCCAAAGGAAAAAGTC
>Marker733437 Chr5 SNP_site:3830855 Ref_Type:C
CACTTACTTAATTGACTAATTATCTATCTTATAATAATTAGAATTTTGTGAGATGTTCTAAACTATGTGATAATAGAGAGXXXXXXXXXXA
ATTGATGTCATTCTTTATACATGAACGTCAGTTATGACCCAATCACTTACTAATAATCATAATTTGAATGGTAAGAGGT
CACTTACTTAATTGACTAATTATCTATCTTATAATAATTAGAATTTTGTGAGATGTTCTAAACTATGTGATAATAGAGAGXXXXXXXXXXA
ATTGATGTCATTCTTTATACATGAAGGTCAGTTATGACCCAATCACTTACTAATAATCATAATTTGAATGGTAAGAGGT
>Marker733522 Chr5 SNP_site:7793169 Ref_Type:A
AACCACACGTGATTGGGAGAATAGAAGGGTTTTATGTGGAAAACAGTGAAGAGAATAGTGGTGGGCATATGATGGGTATGXXXXXXXXXXC
TAATGGTTATGCAACTTTGAAGAAATCATTTAGAACAAAGTTTGGTGGCAAATATAATAAGTTCATAGAATTCAATGTT
AACCTCACGTGATTGGGAGAATAGAAGGGTTTTATGTGGAAAATAGTGAAGAGAATAGTGGTGGGCATATGATGGGTATGXXXXXXXXXXC
TAATGGTTATGCAACTTTGAAGAAATCATTTAGAACAAAGTTTGGTGGCAAATATAATAAGTTCATAGAATTCAACGTT
>Marker733522 Chr5 SNP_site:7793208 Ref_Type:C
AACCACACGTGATTGGGAGAATAGAAGGGTTTTATGTGGAAAACAGTGAAGAGAATAGTGGTGGGCATATGATGGGTATGXXXXXXXXXXC
TAATGGTTATGCAACTTTGAAGAAATCATTTAGAACAAAGTTTGGTGGCAAATATAATAAGTTCATAGAATTCAATGTT
AACCTCACGTGATTGGGAGAATAGAAGGGTTTTATGTGGAAAATAGTGAAGAGAATAGTGGTGGGCATATGATGGGTATGXXXXXXXXXXC
TAATGGTTATGCAACTTTGAAGAAATCATTTAGAACAAAGTTTGGTGGCAAATATAATAAGTTCATAGAATTCAACGTT
>Marker733522 Chr5 SNP_site:7793442 Ref_Type:T
AACCACACGTGATTGGGAGAATAGAAGGGTTTTATGTGGAAAACAGTGAAGAGAATAGTGGTGGGCATATGATGGGTATGXXXXXXXXXXC
TAATGGTTATGCAACTTTGAAGAAATCATTTAGAACAAAGTTTGGTGGCAAATATAATAAGTTCATAGAATTCAATGTT
AACCTCACGTGATTGGGAGAATAGAAGGGTTTTATGTGGAAAATAGTGAAGAGAATAGTGGTGGGCATATGATGGGTATGXXXXXXXXXXC
TAATGGTTATGCAACTTTGAAGAAATCATTTAGAACAAAGTTTGGTGGCAAATATAATAAGTTCATAGAATTCAACGTT
>Marker733718 Chr5 SNP_site:10017735 Ref_Type:G
GACCCTCATGACTGCAAAATGTTCGTATTCACACCTGTAGCCACATTCCGTGCTGAAATGTAAATTATCCATCCTCAACTXXXXXXXXXXG
CATTGTAGCTATTTTGTGCAAGTTGTATGGTTTCTGATGGTTCAACTTTGTAAAAGCACTAAACAACGTTTGTGAAAGT
GACCCTCATGACTGCAAAATGTTCGTATTCACACCTGTAGCCACGTTCCGTGCTGAAATGTAAATTATCCATCCTCAACTXXXXXXXXXXG
CATTGTAGCTATTTTGTGCAAGTTGTATGGTTTCTGATGGTTCAACTTTGTAAAAGCACTAAACAATGTTTGTGAAAGT
>Marker733718 Chr5 SNP_site:10017961 Ref_Type:T
GACCCTCATGACTGCAAAATGTTCGTATTCACACCTGTAGCCACATTCCGTGCTGAAATGTAAATTATCCATCCTCAACTXXXXXXXXXXG
CATTGTAGCTATTTTGTGCAAGTTGTATGGTTTCTGATGGTTCAACTTTGTAAAAGCACTAAACAACGTTTGTGAAAGT
GACCCTCATGACTGCAAAATGTTCGTATTCACACCTGTAGCCACGTTCCGTGCTGAAATGTAAATTATCCATCCTCAACTXXXXXXXXXXG
CATTGTAGCTATTTTGTGCAAGTTGTATGGTTTCTGATGGTTCAACTTTGTAAAAGCACTAAACAATGTTTGTGAAAGT
>Marker733958 Chr5 SNP_site:14771113 Ref_Type:A
GACTCTTGATTTCTCTGACTCAAGCTCCAACACCAATTGCTTGAGACGTCCAACTTCTGCCTTCATTTTCTCAAACTCTAXXXXXXXXXXG
ATCCAGATCCAACTTAAGCTTCATTGATTCCTCAACTTTTTCATGTAATGACTCAAGTAAAGTTTTTTTCTCCTTTGTT
GACTCTTGATTTCTCTGACTCAAGCTCCAACACCAATTGCTTGAGACGTCCAACTTCTGCCTTCATTTTCTCAAACTCTAXXXXXXXXXXG

Create PDF with GO2PDF for free, if you wish to

ATCCAGATCCAACTTAAGCTTCATTGATTCCTCAACTTTTTCTTGTAATGACTCAAGTAAAGTTTTTTTCTCCTTTGTT
>Marker734081 Chr5 SNP_site:13485731 Ref_Type:T
ACCAAACATCAGATTGTCACACTGGTTTAGGTCATTGCTACATCTTTGAAGTTCCAAGTTCCAATTTTTGTTAAATAATTXXXXXXXXXXC
AAAGGTTAAGAATATATAATATTGCACCTTTTAAGATAAGCATTGCACTTTCTCAAAAACAAGTTGGATTATAAAAGTG
ACCAAACATCAGATTGTCACACTGGTTTAGGTCATTGCTACATCTTTGAAGTTCCAAGTTCCAATTTTTGTTAAATAATTXXXXXXXXXXC
AAAGGTTAAGAATATATAATATTGCACCTTTTAAGATAAGCATTGCACTTTCTCAAAAATAAGTTGGATTATAAAAGTG
>Marker734308 Chr5 SNP_site:24592054 Ref_Type:G
ACTTTTTTTATTCTTTCTCCGAAATCTTTCATATATAAAAGTATTGTTAAGATTAATTTTCATAAATATAACAAAGAGAAXXXXXXXXXXT
TTGGTTGTTCCAAATATAAATAATAATTAGATATTGAATAGTCAAATCTGAACGACCCTTTTTCCAAATATAAACGAGT
ACTTTTTTTATTCTTTCTCCGAAATCTTTCATATATAAAAGTATTGTTAAGATTAATTTTCATAAATATAACAAAGAGAAXXXXXXXXXXT
TTGGTTGTTCCAAATATAAATAATAATTAGATATTGGATAGTCAAATCTGAACGACCCTTTTTCCAAATATAAACGAGT
>Marker734324 Chr5 SNP_site:15121073 Ref_Type:A
CACGGAATGAAGAATCGAGTCGTCAAAAGGGATTTGGTGCAGGTCCGATGAAACAATTTCCCGATATTTCAACCTCGTATXXXXXXXXXXA
GTCCTACTACTGCAATCTACAATTTTTTTAACTCTGCAGAATTACCGTAGGTTATTGGACAGGACTTACATAAGATGTC
CACGGAATGAAGAATCGAGTCGTCAAAAGGGATTTGGTGCAGGTCCGATGAAACAATTTCCCGATATTTCAACCTCGTATXXXXXXXXXXA
GTCCTACTACTGCGATCTACAATTTTTTTAACTCTGCAGAATTACCGTAGGTTATTGGACAGGACTTACATAGGATGTC
>Marker734324 Chr5 SNP_site:15121132 Ref_Type:A
CACGGAATGAAGAATCGAGTCGTCAAAAGGGATTTGGTGCAGGTCCGATGAAACAATTTCCCGATATTTCAACCTCGTATXXXXXXXXXXA
GTCCTACTACTGCAATCTACAATTTTTTTAACTCTGCAGAATTACCGTAGGTTATTGGACAGGACTTACATAAGATGTC
CACGGAATGAAGAATCGAGTCGTCAAAAGGGATTTGGTGCAGGTCCGATGAAACAATTTCCCGATATTTCAACCTCGTATXXXXXXXXXXA
GTCCTACTACTGCGATCTACAATTTTTTTAACTCTGCAGAATTACCGTAGGTTATTGGACAGGACTTACATAGGATGTC
>Marker735039 Chr5 SNP_site:15886326 Ref_Type:A
TACTAATAACCTAATTTAATATCTTATAAGATGTTGAAAAACAACAAAATAGAGTATGAGTTAGAAAATGAGGTTTCACAXXXXXXXXXXA
ATTTAGAGACAATCATTATGCTTCAAGGCTAAATGACAAGCTAGAGCTAAAAGACTTCATGATTGATTAAATACTTGTT
TACTAATAACCTAATTTAATATCTTATAAGATGTTGAAAAACAACAAAATAGAGTATGAGTTAGAAAATGAGGTTTCACAXXXXXXXXXXA
ATTTAGAGACAATCATTATGCTTCAAGGCTAAATGACAAGCTAGAGCTAAAAGACTTCGTGATTGATTAAATACTTGTT
>Marker735101 Chr5 SNP_site:25383559 Ref_Type:T
AACTAATGTGTGTTAATGCAAGATGTTGCATTTACGCGAAAGGAAATGTTTATGTGAAATTGTTATGTTATAAACGTGTTXXXXXXXXXXT
CAACAAACTTTACAAGCTACATAACACTTCCAACAAACAAAACTACTAACAAATTAAAAACTAAGTTTAAAATAAAGTC
AACTAATGTGTGTTAATGCAAGATGTTGCATTTACGCGAAAGGATATGTTTATGTGAAATTGTTATGTTATAAACGTGTTXXXXXXXXXXT
CAACAAACATTACAAGCTACATAACACTTCCAACAAACAAAACTACTAACAAATGAAAAACTAAGTTTAAAATAAAGTC
>Marker735101 Chr5 SNP_site:25383730 Ref_Type:A
AACTAATGTGTGTTAATGCAAGATGTTGCATTTACGCGAAAGGAAATGTTTATGTGAAATTGTTATGTTATAAACGTGTTXXXXXXXXXXT
CAACAAACTTTACAAGCTACATAACACTTCCAACAAACAAAACTACTAACAAATTAAAAACTAAGTTTAAAATAAAGTC
AACTAATGTGTGTTAATGCAAGATGTTGCATTTACGCGAAAGGATATGTTTATGTGAAATTGTTATGTTATAAACGTGTTXXXXXXXXXXT
CAACAAACATTACAAGCTACATAACACTTCCAACAAACAAAACTACTAACAAATGAAAAACTAAGTTTAAAATAAAGTC
>Marker735101 Chr5 SNP_site:25383776 Ref_Type:G
AACTAATGTGTGTTAATGCAAGATGTTGCATTTACGCGAAAGGAAATGTTTATGTGAAATTGTTATGTTATAAACGTGTTXXXXXXXXXXT
CAACAAACTTTACAAGCTACATAACACTTCCAACAAACAAAACTACTAACAAATTAAAAACTAAGTTTAAAATAAAGTC
AACTAATGTGTGTTAATGCAAGATGTTGCATTTACGCGAAAGGATATGTTTATGTGAAATTGTTATGTTATAAACGTGTTXXXXXXXXXXT
CAACAAACATTACAAGCTACATAACACTTCCAACAAACAAAACTACTAACAAATGAAAAACTAAGTTTAAAATAAAGTC
>Marker735127 Chr5 SNP_site:6227775 Ref_Type:G
AACAATTTACAAAGCGTGGAAGAAGCCTCCATCGAAATTCAAAATCAATAATACCCGAAGGATAGTAAAACCCTTAATCAXXXXXXXXXXT
AAACCCTAGCAGGATCCAGAATCAAACTTAAAATTGGAAACATCAAAATACGAATCAAAGAAGAAGAGAGCACCTGGTA

Create PDF with GO2PDF for free, if you wish to

AACAATTTACAAAGCGTGGAAGAAGCCTCCATCGAAATTCAAAATCAATGATACCCGAAGGATAGTAAAACCCTTAATCAXXXXXXXXXXT
AAACCCTAGCAGGATCCAGAATCAAACTTAAAATTGGAAACATCAAAATACGAATCAAAGAAGAAGAGAGCACCTGGTA
>Marker735397 Chr5 SNP_site:24541206 Ref_Type:A
AACCCCTCCTATTTGATTGCCACCTTTCCTTTACCTCGGGTTTCGGGTGACAGATCTAAAGGATGTTTCATTACTAGAAAXXXXXXXXXXC
ATGTCCTTAAAGAGTCAGCTAATGTTCAGGATGAAAATAGAAATTTTGGTGACAAAAAGAAATAATCTATGAATATTGT
AACCCCTCCTATTTGATTGCCACCTTTCCTTTACCTCGGGTTTCGGGTGACAGATCTAAAGGATGTTTCATTACTAGAAAXXXXXXXXXXC
ATGTCCTTAATGAGTCAGCTAATGTTCAGGATGAAAATAGAAATTTTGGTGACAAAAAGAAATAATCTATGAATATTGT
>Marker735652 Chr5 SNP_site:16060929 Ref_Type:A
TACTAGCCCAATGTGCAGCCACCTAGTGCCAAAAACCATTAATTTTGAAAAATGAATTCCAAAAGAACACTTACCACATTXXXXXXXXXXA
AATTCATGAGCACGACAGGTCTCTATAATCTCTGAGATCACCTAAGAAACAACAAACCATTTTAATCAAACAAAAGGTC
TACTAGCCCAATGTGCAGCCACCTAGTGCCAAAAACCATTAATTTTGAAAAATGGATTCCAAAAGAACACTTACCACATTXXXXXXXXXXA
AATTCATGAGCACGGCAGGTCTCTATAATCTCTGAGATCACCTAAGAAACAACAAACCATTTTAATCAAACAAAAGGTC
>Marker735652 Chr5 SNP_site:16061066 Ref_Type:G
TACTAGCCCAATGTGCAGCCACCTAGTGCCAAAAACCATTAATTTTGAAAAATGAATTCCAAAAGAACACTTACCACATTXXXXXXXXXXA
AATTCATGAGCACGACAGGTCTCTATAATCTCTGAGATCACCTAAGAAACAACAAACCATTTTAATCAAACAAAAGGTC
TACTAGCCCAATGTGCAGCCACCTAGTGCCAAAAACCATTAATTTTGAAAAATGGATTCCAAAAGAACACTTACCACATTXXXXXXXXXXA
AATTCATGAGCACGGCAGGTCTCTATAATCTCTGAGATCACCTAAGAAACAACAAACCATTTTAATCAAACAAAAGGTC
>Marker735777 Chr5 SNP_site:2118447 Ref_Type:C
ACTCTTTTTTAATGGATCTTAGAATCATCGTGACTTAGCGAAGAATTCATTTGTCACACCCCGCCCGCAGACCTTCTTGTXXXXXXXXXXT
GCTTTAGGCGATAAAGCTTAATGCAATCAACATAACAAAAACTTAAACTTTACTAACAACAGACATTTTATACATAGTA
ACTCTTTTTTAATGGATCTTAGAATCATCGTGACTTAGCGAAGAATTCATTTGTCACACCCCGCTCGCAGACCTTCTTGTXXXXXXXXXXT
GCTTTAGGCGATAAAGCTTAATGCAATCAACATAACAAAAACTTAAACTTTATTAACAACAGACATTTTATACATAGTA
>Marker735777 Chr5 SNP_site:2118625 Ref_Type:C
ACTCTTTTTTAATGGATCTTAGAATCATCGTGACTTAGCGAAGAATTCATTTGTCACACCCCGCCCGCAGACCTTCTTGTXXXXXXXXXXT
GCTTTAGGCGATAAAGCTTAATGCAATCAACATAACAAAAACTTAAACTTTACTAACAACAGACATTTTATACATAGTA
ACTCTTTTTTAATGGATCTTAGAATCATCGTGACTTAGCGAAGAATTCATTTGTCACACCCCGCTCGCAGACCTTCTTGTXXXXXXXXXXT
GCTTTAGGCGATAAAGCTTAATGCAATCAACATAACAAAAACTTAAACTTTATTAACAACAGACATTTTATACATAGTA
>Marker735986 Chr5 SNP_site:24726772 Ref_Type:T
ACTAGATCTACGACTCATTTAAAGTATAAAAATAAAATTTGATCATTCAAATATAACAATAACAAAAATAATATGAAGTTXXXXXXXXXXG
TTAGATTCACTTTGGTCGTTGTTATCTTAAATATTAATTGCTTTCTATTTAAGAGTTGTATTTTAAGGAAGAAAATGTG
ACTAGATCTACGATTCATTTAAAGTATAAAAATAAAATTTGATCATTCAAATATAACAATAACAAAAATAATATGAAGTTXXXXXXXXXXG
TTAGATTCACTTTGGTCGTTGTTATCTTAAATATTAATTGCTTTCTATTTAAGAGTTGTATTTTAAGGAAGAAAATGTG
>Marker736094 Chr5 SNP_site:10585671 Ref_Type:T
CACCTTGAGAACAAAATGAGGGCTATCAGAGGCTCCACCTTGTGTTTAATTTGGTATAGCAAGTAAATTGATTTAATTGTXXXXXXXXXXC
ATTGTCTCCAAGTCCGCTGTGTAAAAATTGTGAATAGTTTAGTTACTAAGGGTCAGTTTGTGTTTAAACCTTATCATGT
CACCTTGAGAACAAAATGAGGGCTATCAGAGGCTCCACCTTGTGTTTAATTTGGTATAGCAAGTAAATTGATTTAATTGTXXXXXXXXXXC
ATTGTCTCCAAGTCCGTTGTGTAAAAATTGTGAATAGTTTAGTTACTAAGGGTCAGTTTGTGTTTAAACCTTATCATGT
>Marker736587 Chr5 SNP_site:25371104 Ref_Type:A
ACCATAGTTTAGGTTACTCAAAACTTGTTTTGTTTTTCTGCTGAAAAAAAGATAATGGAAAAGAAAAGAAACCCTCTCCTXXXXXXXXXXT
AGCATTCCGCCGCCATCATAGTTTAACTTGGTCTTTGCATAGATGTGATTCCAACCAGCAAATATCTCAACCACTTGTG
ACCATAGTTTAGGTTACTCAAAACTTGTTTTGTTTTTCTGCTGAAAAAAAGATAATGGAAAAGAAAAGAAACCCTCTCCTXXXXXXXXXXT
AGCATTCCGCCGCCATCATGGTTTAACTTGGTCTTTGCATAGATGTGATTCGAACCAGCAAATATCTCAACCACTTGTG
>Marker736587 Chr5 SNP_site:25371136 Ref_Type:C
ACCATAGTTTAGGTTACTCAAAACTTGTTTTGTTTTTCTGCTGAAAAAAAGATAATGGAAAAGAAAAGAAACCCTCTCCTXXXXXXXXXXT

Create PDF with GO2PDF for free, if you wish to

AGCATTCCGCCGCCATCATAGTTTAACTTGGTCTTTGCATAGATGTGATTCCAACCAGCAAATATCTCAACCACTTGTG
ACCATAGTTTAGGTTACTCAAAACTTGTTTTGTTTTTCTGCTGAAAAAAAGATAATGGAAAAGAAAAGAAACCCTCTCCTXXXXXXXXXXT
AGCATTCCGCCGCCATCATGGTTTAACTTGGTCTTTGCATAGATGTGATTCGAACCAGCAAATATCTCAACCACTTGTG
>Marker73671 Chr4 SNP_site:17223111 Ref_Type:T
AACAATCTCCTTGGAGGCTAGAAATTCACTAAGGTTATGGTTTTGGAATTCCAGACCATTATCAATTCTAAAAACAGCAAXXXXXXXXXXC
AATGAAAGTTACAAATCACCGTTTCTCAGATGAGGTGGTGACCTTGGAGGGACCCCAAATGTCACTATGGATAAGGGTA
AACAATCTCCTTGGAGGCTAGAAATTCACTAAGGTTATGGTTTTGGAATTCCAGACCATTATCAATTCTAAAAATAGCAAXXXXXXXXXXC
AATGTAAGTTACAAATCACCGTTTCTCAGATGAGGTGGTGACCTTGGAGGGACCCCAAATGTCACTATGGATAAGGGTA
>Marker73671 Chr4 SNP_site:17223229 Ref_Type:T
AACAATCTCCTTGGAGGCTAGAAATTCACTAAGGTTATGGTTTTGGAATTCCAGACCATTATCAATTCTAAAAACAGCAAXXXXXXXXXXC
AATGAAAGTTACAAATCACCGTTTCTCAGATGAGGTGGTGACCTTGGAGGGACCCCAAATGTCACTATGGATAAGGGTA
AACAATCTCCTTGGAGGCTAGAAATTCACTAAGGTTATGGTTTTGGAATTCCAGACCATTATCAATTCTAAAAATAGCAAXXXXXXXXXXC
AATGTAAGTTACAAATCACCGTTTCTCAGATGAGGTGGTGACCTTGGAGGGACCCCAAATGTCACTATGGATAAGGGTA
>Marker736933 Chr5 SNP_site:6363064 Ref_Type:G
GACTTCATGATCAATCAACTTCACAGTTGATGACCGATCGCCGATCGTTTGGACCATGACGATCAACCACCAATCGTCTTXXXXXXXXXXA
AGTGATGACCAAACACCATGACCTTGAGGACCAAAAACTATGGAATGAACTTCTTATGAAATTTAATTTCAAAGCTGTT
GACTTCATGATCAATCAACTTCACAGTTGATGACCGATCGCCGATCGTTTGGACCATGACGATCAACCACCAATCGTCTTXXXXXXXXXXA
AGTGATGACCAAACACCATGACCTTGAGGACCAAAAACTATGGAATGAACTTGTTATGAAATTTAATTTCAAAGCTGTT
>Marker73728 Chr4 SNP_site:13080263 Ref_Type:A
AACACCAATAAAACAAACACGCACGTATATCAACAGGACTTGAATCTATAGATTATTTATTTTAAAAAAAAAAAAAAAAXXXXXXXXXXT
ATTCTGCAAACTTCTTAAGGTATTAAGGTATGTCTTTCAAAATGATTAAAAATGTTAAGAGTGTTAAACCAATAAAAGT
AACACCAATGAAACAAACACGCACGTATATCAACAGGACTTGAATCTATAGATTATTTATTTTAAAAAAAAAAAAAAAAXXXXXXXXXXT
ATTCTGCAAACTTCTTAAGGTATTAAGGTATGTCTTTCAAAATGATTAAAAATGTTAAGAGTGTTAAACCAATAAAAGT
>Marker737383 Chr5 SNP_site:6416487 Ref_Type:G
CACGCTGCTTTATTTCTTGATCTCATGTGCATATTTCCCTTCAGGTAGGCTCAAGGGTGTCGGTGCTACACAAGCTCACGXXXXXXXXXXA
AAATGACAGAGAACGACGAACTCGAACTTATTCGATTACCAATCAGACTACATTTGATGCTGTATGGCTTCTTTCGGTA
CACGCTGCTTTATTTCTTTATCTCATGTGCATATTTCCCTTCAGGTAGGCTCAAGGGTGTCTGTGCTACACAAGCTCACGXXXXXXXXXXA
AAATGACAGAGAACGACGAACTCCAACTTATTCGATTACCAATCAGACTACATTTGATGCTGTATGGCTTCTTTCGGTA
>Marker737383 Chr5 SNP_site:6416530 Ref_Type:G
CACGCTGCTTTATTTCTTGATCTCATGTGCATATTTCCCTTCAGGTAGGCTCAAGGGTGTCGGTGCTACACAAGCTCACGXXXXXXXXXXA
AAATGACAGAGAACGACGAACTCGAACTTATTCGATTACCAATCAGACTACATTTGATGCTGTATGGCTTCTTTCGGTA
CACGCTGCTTTATTTCTTTATCTCATGTGCATATTTCCCTTCAGGTAGGCTCAAGGGTGTCTGTGCTACACAAGCTCACGXXXXXXXXXXA
AAATGACAGAGAACGACGAACTCCAACTTATTCGATTACCAATCAGACTACATTTGATGCTGTATGGCTTCTTTCGGTA
>Marker737383 Chr5 SNP_site:6416690 Ref_Type:G
CACGCTGCTTTATTTCTTGATCTCATGTGCATATTTCCCTTCAGGTAGGCTCAAGGGTGTCGGTGCTACACAAGCTCACGXXXXXXXXXXA
AAATGACAGAGAACGACGAACTCGAACTTATTCGATTACCAATCAGACTACATTTGATGCTGTATGGCTTCTTTCGGTA
CACGCTGCTTTATTTCTTTATCTCATGTGCATATTTCCCTTCAGGTAGGCTCAAGGGTGTCTGTGCTACACAAGCTCACGXXXXXXXXXXA
AAATGACAGAGAACGACGAACTCCAACTTATTCGATTACCAATCAGACTACATTTGATGCTGTATGGCTTCTTTCGGTA
>Marker737981 Chr5 SNP_site:15563742 Ref_Type:G
TACTGGTTCAGACTTTTTATTCTTGAGTAGCTTTCAAAGTAATTTGCTGAGGGAAGGTTTGAAGAACCAGATTGGCTGGGXXXXXXXXXXT
CCCATTCCTCGAGACATTAAGAGAATGGAGAATTATGGAGTTCATTTAACGAGCTTTGGGACTGTTGATGAAGATAGTC
TACTGGTTCAGACTTTTTATTCTTGAGTAGCTTTCAAAGTAATTTGCTGAGGGAAGGTTTGAAGAACCAGATTGGCTGGGXXXXXXXXXXT
CCCATTCCTCGAGACGTTAAGAGAATGGAGAATTATGGAGTTCATTTAACGAGCTTTGGGACTGTTGATGAAGATAGTC
>Marker738061 Chr5 SNP_site:15805568 Ref_Type:A

Create PDF with GO2PDF for free, if you wish to

AACGATGTATTTCTGTTACGTGAATGTAAATTGTGTGAGGTGTGATACATTTTATGGGCCCATAGGAATTTTGTAAGTTGXXXXXXXXXXC
CTTGGCAAGTAAATTCACAATTCGAGAACTATCATTCTACAAGCTCGAACAGTTCTTTTCTTTTCAATTTTCTATTGTT
AACGATGTATTTCTGTTACGTGAATGTAAATTGTGTGAGGTGTGATACATTTTATGGGCCCATAGGAATTTTGTAAGTTGXXXXXXXXXXC
CTTGGCAAGTAAATTCACAATTCGAGAACTATCATTCTACAAGCTCGAACAGTTCTTTTCTTTTCAATTTTCTCTTGTT
>Marker73855 Chr4 SNP_site:13249397 Ref_Type:C
TACCCAATAAGTGCAATCCTACCGATACTCATCTCATTCTTCTACCTAATATCCAAGTGATCATTTTTCCTTTCCCCCTTXXXXXXXXXXA
CTAAGTAAAAGATAAGCAACATGTTTATCTTTCCTTTAAATAATGTTGTTTAATATTGCTGGTCATACAAAGAGCCTGT
TACCCAATAAGTGCAATCCTACTGATACTCATCTCATTCTTCTACCTAATATCCAAGTGATCATTTTTCCTTTCCCCCTTXXXXXXXXXXA
CTAAGTAAAAGATAAGCAACATGTTTATCTTTCCTTTAAATAATGTTGTTTAATATTGCTGGTCATACAAAGAGCCTGT
>Marker738568 Chr5 SNP_site:3839483 Ref_Type:A
AACGTCGCACATAAATTCCTAACGATGTTGATGGACTTCCTTTTTAAGGACGTTCTCCGTTGTGGGTGGTCTCATATTCCXXXXXXXXXXG
TTTATGTGATGGGGGATTATAGATCCAATCCTTTACTAGATATGGAGCGAACGCGTTATGTTCGTATATTACCGGAGTT
AACGTCGCACATAAATTCCTAACGATGTTGATGGACTTCCTTTTTAAGGCCGTTCTCCGTTGTGGGTGGTCTCATATTCCXXXXXXXXXXG
TTTATGTGATGGGGGATTATAGATCCAATCCTTTACTAGATATGGAGCGAACGCGTTATGTTCGTATATTACCGGAGTT
>Marker738900 Chr5 SNP_site:14519087 Ref_Type:G
CACTCAATGACAATTACGTAATGAATAGATTCTATTTGTCTATTTAGTATCGATGTATTCTAACTAAGTTATATATGGTTXXXXXXXXXXT
AATTTTTCTTTTTTACATATTTAACTTACTGATTTATGTGCCTTCATTTCAATTTTTAGTATGGCAACTTCGTCATGTG
CACTCAATGACAATTACGTAATGAGTAGATTCTATTTGTCTATTTAGTATCGATGTATTCTAACTAAGTTATATATGGTTXXXXXXXXXXT
AATTTTTCTTTTTTACATATTTAACTTACTGATTTATGTGCCTTCATTTCAATTTTTAGTATGGCAACTTCGTCATGTG
>Marker739775 Chr5 SNP_site:2083023 Ref_Type:G
AACATTTTTTGATGTGCTCATCAAAGAAGTTCAAATTTGTTGTAGTGATTAGGTTAAAGTGTAAAGGATAATTAATTGCAXXXXXXXXXXT
TCCCATCAAATTTGAATTGGTTTGGAACTGAATTTTTGGTAAGTTAAAATTAGGAAAAACTAGATACGAGCTATTGGTA
GACATTTTTTGATGTGCTCATCAAAGAAGTTCAAATTTGTTGTAGTGATTAGGTTAAAGTGTAAAGGATAATTAATTGCAXXXXXXXXXXT
TCCCATCAAATTTGAATTGGTTTGGAACTGAATTTTTGGTAAGTTAAAATTAGGAAAAACTAGATACGAGCTATTGGTA
>Marker739850 Chr5 SNP_site:5235294 Ref_Type:T
GACTGTTTGCCCCGGCCAAAGGCAGTCCCTATTGTTCTCTGCAACCATTCCAAGGGAGGTAAGTCCATTTTCTCTTCTTTAXXXXXXXXXXT
ATCTCAGCTAGTTTTGAAAAGGGAACATGTTTTTGTTAATAATGTGGGAATTGGTTGTGTAGAAACGCCTGCTCAGGTA
GACTGTTTGCCCCGGCCAAAGGCAGTCCCTATTGTTCTCTGCAACCATTCCAAGGGAGGTAAGTCCATTTTCTCTTCTTTAXXXXXXXXXXT
ATCTCAGCTAGTTTTGAAAAGGGAACATGTTTTTGTTAATAATGTGGGAATTGGTTGTGTAGAAACGCCTGTTCAGGTA
>Marker739889 Chr5 SNP_site:14909667 Ref_Type:C
TACCCTATCCTTGCGCTCCTTGTAAGTCGCTTCACTTTGCACCTCTACACTTTCTCATTTGTTTTAGATGGTTCATGGTGXXXXXXXXXXG
TTTGTCTATGATTTGGTTCTTCTCTTCCTCTTCATATGTTTTATCCCAAAGAAAGGAGTTGAGGTCTAAGCACTCTTGT
TACCCTATCCTTGCGCTCCTTGTAAGTCGCTTCACTTTGCACCTCTACACTTTCTGATTTGTTTTAGATGGTTCATGGTGXXXXXXXXXXG
TTTGTCTATGATTTGGTTCTTCTCTTCCTCTTCATATGTTTTATCCCAAAGAAAGGAGTTGAGGTCTAAGCACTCTTGT
>Marker739933 Chr5 SNP_site:18135539 Ref_Type:T
CACCTACTATTTCGTAATTTCAAGTTTTGATTTTAAATTCAAATCAAATAAAATCGTAACTTACTCTAGTCTTAAAATTTXXXXXXXXXXT
TCTGATTTTTGGAAGAATTCTTTCTAAACTTCTCTAACGAGCCTTGTGAGCTTGGACTTGTTTGGCCTATGGACTTAGT
CACCTACTATTTCTTAATTTCAAGTTTTGATTTTAAATTCAAATCAAATAAAATCGTAACTTACTCTAGTCTTAAAATTTXXXXXXXXXXT
TCTGATTTTTGGAAGAATTCTTTCTAAACTTCTCTAACGAGCCTTGTGAGCTTGGACTTGTTTGGCCTATGGACTTAGT
>Marker74018 Chr4 SNP_site:16709097 Ref_Type:G
CACTGGAGTTCTGTGTAAATCAGTTATTATTGACTGAGTCCATCTGAGTTGGCTAACGGAAATAGAAATGCTTCTGCAGAXXXXXXXXXXT
ACAAGAGAAAGAGAGAGGAGAGAGAAGGGAAAGAACTTACCATTCAACCAAGTAGTAGGAGGAATTGGTTTTAATCAGT
CACTGGAGTTCTGTGTAAATCAGTTATTATTGACTGAGTCCATCTGAGTTGGCTAACGGAAATAGAAATGCTTCTGCAGAXXXXXXXXXXT
ACGAGAGAAAGAGAGAGGAGAGAGAAGGGAAAGAACTTACCATTCAACCAAGTAGTAGGAGGAATTGGTTTTAATCAGT

Create PDF with GO2PDF for free, if you wish to

>Marker740314 Chr5 SNP_site:21008876 Ref_Type:A
ACCGTTCTGTGTCCTCTTTTCAGATTTGTTAGAATTGAACTAAAACATTGCTGTAAAATTCCATGGCCACAGCATGGAATXXXXXXXXXXC
AAAACTTTGTATGATAGAGAATAGTCAATGGGATCATATTTGGTTAAAACTCTCTTAATATCAAGTTGAAGTGGGTGTG
ACCGTTCTGTGTCCTCTTTTCAGATTTGTTAGAATTGAACTAGAACATTGCTGTAAAATTCCATGGCCACAGCATGGAATXXXXXXXXXXC
AAAACTTTGTATGATAGATAATAGTCAATGGGATCATATTTGGTTAAAACTCTCTTAATATCAAGTTGAAGTGGGTGTG
>Marker740314 Chr5 SNP_site:21009063 Ref_Type:G
ACCGTTCTGTGTCCTCTTTTCAGATTTGTTAGAATTGAACTAAAACATTGCTGTAAAATTCCATGGCCACAGCATGGAATXXXXXXXXXXC
AAAACTTTGTATGATAGAGAATAGTCAATGGGATCATATTTGGTTAAAACTCTCTTAATATCAAGTTGAAGTGGGTGTG
ACCGTTCTGTGTCCTCTTTTCAGATTTGTTAGAATTGAACTAGAACATTGCTGTAAAATTCCATGGCCACAGCATGGAATXXXXXXXXXXC
AAAACTTTGTATGATAGATAATAGTCAATGGGATCATATTTGGTTAAAACTCTCTTAATATCAAGTTGAAGTGGGTGTG
>Marker740874 Chr5 SNP_site:2807142 Ref_Type:T
ACTCAAATGAATGTCAAATAATTATAGCAAGATGCATGCTGATTAAGGAATGCAAATAATTCGAATGAGTAATTGACCCAXXXXXXXXXXT
AATTAATTAATCATGGATAATTATTAACGAACTATGTGATATGAATACAAGTAATTAACACATAATGTAAATACATGGT
ACTCAAATGAATGTCAAATAATTATAGCAAGATGCATGCTGATTAATGAATGCAAATAATTCGAATGAGTAATTGACTCAXXXXXXXXXXT
AATTAATTAATCATGGATAATTATTAACGAACTATGTGATATGAATACAAGTAATTAACACATAATGTAAATACATGGT
>Marker740874 Chr5 SNP_site:2807173 Ref_Type:T
ACTCAAATGAATGTCAAATAATTATAGCAAGATGCATGCTGATTAAGGAATGCAAATAATTCGAATGAGTAATTGACCCAXXXXXXXXXXT
AATTAATTAATCATGGATAATTATTAACGAACTATGTGATATGAATACAAGTAATTAACACATAATGTAAATACATGGT
ACTCAAATGAATGTCAAATAATTATAGCAAGATGCATGCTGATTAATGAATGCAAATAATTCGAATGAGTAATTGACTCAXXXXXXXXXXT
AATTAATTAATCATGGATAATTATTAACGAACTATGTGATATGAATACAAGTAATTAACACATAATGTAAATACATGGT
>Marker740883 Chr5 SNP_site:3526135 Ref_Type:T
TACTCATCTAGTAGTATTTTAAAATTCAAAATTATATCTTTTCGAGATTTAATATTAATTATATAAGATTTTATTCCAATXXXXXXXXXXA
TTATTATATAAAATTAAATAACAAGAAGAGTTTAAAAGTAATCTGGACTGTAAAAATAGTTAATTAGATTATTAATGTG
TACTCATCTAGTAGTATTTTAAAATTCAAAATTATATCTTTTCGAGATTTAATATTAATTATATAAGATTTTTTTCCAATXXXXXXXXXXA
TTATTATATAAAATTAAATAACAAGAAGAGTTTAAAAGTAATCTGGACTGTAAAAATAGTTAATTAGATTATTAATGTG
>Marker741148 Chr5 SNP_site:18145418 Ref_Type:C
ACTCGTCTAATCTCATTTTCTAAATACTCATTTAATTCGAATTTTATCATTTTAGCTGAGAAATAGTTGTTTGGTATGTTXXXXXXXXXXC
TCATTTACTTTCCGGAATATATTACTTTCGAAAAAAATAACAATAACTTTAAAAAACTTCCTTTATCTTCAATAATGTT
ACTCGTCTAATCTCATTTTCTAAATATTCATTTAATTCGAATTTTATCATTTTAGCTGAGAAATAGTTGTTTGGTATGTTXXXXXXXXXXC
TCATTTACTTTCCGGAATATATTACTTTCGAAAAAAATAACAATAACTTTAAAAAACTTCCATTATCTTCAATAATGTT
>Marker741148 Chr5 SNP_site:18145673 Ref_Type:T
ACTCGTCTAATCTCATTTTCTAAATACTCATTTAATTCGAATTTTATCATTTTAGCTGAGAAATAGTTGTTTGGTATGTTXXXXXXXXXXC
TCATTTACTTTCCGGAATATATTACTTTCGAAAAAAATAACAATAACTTTAAAAAACTTCCTTTATCTTCAATAATGTT
ACTCGTCTAATCTCATTTTCTAAATATTCATTTAATTCGAATTTTATCATTTTAGCTGAGAAATAGTTGTTTGGTATGTTXXXXXXXXXXC
TCATTTACTTTCCGGAATATATTACTTTCGAAAAAAATAACAATAACTTTAAAAAACTTCCATTATCTTCAATAATGTT
>Marker741454 Chr5 SNP_site:11821322 Ref_Type:G
TACTTCACAAGCAAAATTTTACAAATATCTTAAACTTTAATCATATTTCACAACTTAATTCACATGGTTTTTTTTTTGGAXXXXXXXXXXXG
ATAAAGATATGATGTGTTGATGTAATTTTATTGTGTGAGCAAATATATGATTTAGTATGTTCTTATTCATTTTAAATGT
TACTTCACAAGCAAAATTTTACAAATATCTTAAACTTTAATCATATTTCACAACTTAATTCACATGGTTTTTTTTTTTTTXXXXXXXXXXG
ATAAAGATATGATGTGTTGATGTAATTTTATTGTGTGAGCAAATATATGATTTAGTATGTTCTTATTCATTTTAAATGT
>Marker741454 Chr5 SNP_site:11821323 Ref_Type:G
TACTTCACAAGCAAAATTTTACAAATATCTTAAACTTTAATCATATTTCACAACTTAATTCACATGGTTTTTTTTTTGGAXXXXXXXXXXXG
ATAAAGATATGATGTGTTGATGTAATTTTATTGTGTGAGCAAATATATGATTTAGTATGTTCTTATTCATTTTAAATGT
TACTTCACAAGCAAAATTTTACAAATATCTTAAACTTTAATCATATTTCACAACTTAATTCACATGGTTTTTTTTTTTTTXXXXXXXXXXG

Create PDF with GO2PDF for free, if you wish to

ATAAAGATATGATGTGTTGATGTAATTTTATTGTGTGAGCAAATATATGATTTAGTATGTTCTTATTCATTTTAAATGT
>Marker741454 Chr5 SNP_site:11821324 Ref_Type:A
TACTTCACAAGCAAAATTTTACAAATATCTTAAACTTTAATCATATTTCACAACTTAATTCACATGGTTTTTTTTTTGGAXXXXXXXXXXG
ATAAAGATATGATGTGTTGATGTAATTTTATTGTGTGAGCAAATATATGATTTAGTATGTTCTTATTCATTTTAAATGT
TACTTCACAAGCAAAATTTTACAAATATCTTAAACTTTAATCATATTTCACAACTTAATTCACATGGTTTTTTTTTTTTTXXXXXXXXXXG
ATAAAGATATGATGTGTTGATGTAATTTTATTGTGTGAGCAAATATATGATTTAGTATGTTCTTATTCATTTTAAATGT
>Marker741497 Chr5 SNP_site:14690329 Ref_Type:G
TACCATTATGTATCAACATTTATTAATGATATTTTGTATAATTAAAAAACAACCTTAAAATATTGGATGGAAGAAGCATAXXXXXXXXXXA
CACTTTCATCAACCTTACCAAACATATAAATTATTGTCATCTACCCCTAGTTTAATTTACCTGATTCCATGTCCGGGTC
TACCATTATGTATCAACATTTATTAATGATATTTTGTATAATTAAAAAGCAACCTTAAAATATTGGATGGAAGAAGCATAXXXXXXXXXXA
CACTTTCATCAACCTTACTAAACATATAAATTATTGTCATCTACCCCTAGTTTAATTTACCTGATTCCATGTCCGGGTC
>Marker741497 Chr5 SNP_site:14690535 Ref_Type:T
TACCATTATGTATCAACATTTATTAATGATATTTTGTATAATTAAAAAACAACCTTAAAATATTGGATGGAAGAAGCATAXXXXXXXXXXA
CACTTTCATCAACCTTACCAAACATATAAATTATTGTCATCTACCCCTAGTTTAATTTACCTGATTCCATGTCCGGGTC
TACCATTATGTATCAACATTTATTAATGATATTTTGTATAATTAAAAAGCAACCTTAAAATATTGGATGGAAGAAGCATAXXXXXXXXXXA
CACTTTCATCAACCTTACTAAACATATAAATTATTGTCATCTACCCCTAGTTTAATTTACCTGATTCCATGTCCGGGTC
>Marker741797 Chr5 SNP_site:13156889 Ref_Type:G
ACTGTATATGGTCGATTTACAAATTTTCAAACGAAATTGTAGTATATTGACGCTATTTTAAGATCCATGACACTTATCCTXXXXXXXXXXG
ATAAAAGGAGGCTTATTGGAAAATTTGACGCGATGAACTGATAGACATTGGGCTCATTTCACCCAAAAATGGCAACGTA
ACTGTATATGGTCGATTTACAAATTTTCAAACGAAATTGTAGTATATTGACGCTATTTTAAGATCCATGACACTTATCCTXXXXXXXXXXG
ATAAAAGGAGGCTTATTGGAAAATTTGACGCGATGAACTGATAGACATTGGGCTCATTTCACCCAAAAATGGCAGCGTA
>Marker742081 Chr5 SNP_site:23748977 Ref_Type:A
TACTTTGATTGTTGTTTATGAGTCTCATGTAATCAACAATAAATTAGCAATATGTGTAGTTAATTATCTAAATTTTTTTTXXXXXXXXXXA
ATACAGAAATTAATTAAAAGAGAGCATGCCACACACAACTAATTTCAATCCACTCTTCAAATGAGTGTTTAAATAAGTT
TACTTTGATTGTTGTTTATGAGTCTCATGTAATCAACAATAAATTAGCAATATGTGTAGTTAATTATCTTAAAATTTTTTXXXXXXXXXXA
ATACAGAAATTAATTAAAAGAGAGCATGCCACACACAACTAATTTCAATCCACTCTTCAAATGAGTGTTTAAATAAGTT
>Marker742081 Chr5 SNP_site:23748980 Ref_Type:T
TACTTTGATTGTTGTTTATGAGTCTCATGTAATCAACAATAAATTAGCAATATGTGTAGTTAATTATCTAAATTTTTTTTXXXXXXXXXXA
ATACAGAAATTAATTAAAAGAGAGCATGCCACACACAACTAATTTCAATCCACTCTTCAAATGAGTGTTTAAATAAGTT
TACTTTGATTGTTGTTTATGAGTCTCATGTAATCAACAATAAATTAGCAATATGTGTAGTTAATTATCTTAAAATTTTTTXXXXXXXXXXA
ATACAGAAATTAATTAAAAGAGAGCATGCCACACACAACTAATTTCAATCCACTCTTCAAATGAGTGTTTAAATAAGTT
>Marker742081 Chr5 SNP_site:23748981 Ref_Type:T
TACTTTGATTGTTGTTTATGAGTCTCATGTAATCAACAATAAATTAGCAATATGTGTAGTTAATTATCTAAATTTTTTTTXXXXXXXXXXA
ATACAGAAATTAATTAAAAGAGAGCATGCCACACACAACTAATTTCAATCCACTCTTCAAATGAGTGTTTAAATAAGTT
TACTTTGATTGTTGTTTATGAGTCTCATGTAATCAACAATAAATTAGCAATATGTGTAGTTAATTATCTTAAAATTTTTTXXXXXXXXXXA
ATACAGAAATTAATTAAAAGAGAGCATGCCACACACAACTAATTTCAATCCACTCTTCAAATGAGTGTTTAAATAAGTT
>Marker742447 Chr5 SNP_site:25089428 Ref_Type:C
CACCCCGCTGCTCCGCAACCCCCGACGGTCCTCGTTGTATTACGTGAATTTAATTTCCATCAGAGTCGGCCGCAAAATCGXXXXXXXXXXG
AAAAACAATAGCCAAAATTTGAATGTATAAAAATGGTTGTTGAGTTGTTGAGTTGTTGTTGTTGTTCAGGGACGACGTT
CACCCCGCTGCTCCGCAACCCCCGACGGTCCTCGTTGTATTATGTGAATTTAATTTCCATCAGAGTCGGCCGCAAAATCGXXXXXXXXXXG
AAAAACAATAGCCAAAATTTGAATGTATAAAAATGGTTGTTGAGTTGTTGAGTTGTTGTTGTTGTTCAGGGACGACGTT
>Marker742682 Chr5 SNP_site:25729802 Ref_Type:C
ACTGAAATTAATCAAGTGAAGTATGGGAACAGTTTGGAGTTCATTGTGATAGGACTAAGCTTGGCATGGGGGACAAGAACXXXXXXXXXXC
TTATGCTTCAGAACCGAACAATAATGAAAATTGGAAATATAGAAACTATTCTTAATATATCACATTTCCAGGGTTGGTT

Create PDF with GO2PDF for free, if you wish to

ACTGAAATTAATCAAGTGAAGTATGGGAACAGTTTGGAGTTCATTGTGATAGGACTAAGCTTGGCATGGGGGACAAGAACXXXXXXXXXXC
TTATGCTTCAGAACCGAACAATAATGAAAATTGGAAATATAGAAAGTATTCTTAATATATCACATTTCCAGGGTTGGTT
>Marker742828 Chr5 SNP_site:16034625 Ref_Type:G
AACAAAATCTATTAAAGTTACACTTAGGTCATCGTAGATCACAACAAAATAGAATGGAACACTAACCAAAAACTAAAACTXXXXXXXXXXT
ACATATTAAAATACTATCAATCCCACTTTAGATTCAAAACTTTGTTTCATTACTATAGATCTATAAATCTTATCATGGT
AACAAAATCTATTAAAGTTACACTTAGGTCATCGTAGGTCACAACAAAATAGAATGGAACACTAACCAAAAACTAAAACTXXXXXXXXXXT
ACATATTAAAATACTATCAATCCCACTTTAGATTCAAAACTTTGTTTCATTACTATAGATCTATAAATCTTATCATGGT
>Marker742930 Chr5 SNP_site:25809115 Ref_Type:A
AACAGAAAGCTCCATTCATTCATACCATTATACCTCTGATAATTTCGAACCCCCTCCTACTCACACTATTACATCCCAATXXXXXXXXXXT
GTAAAATGAAAAGAATTTTCAGCCTATGCTTTTTCTAAATTTGAAAACCATTTATGGGACATATCATTTCAATAATGTG
AACAGAAAGCTCCATTCATTCATACCATTATACCTCTGATAATTTCGAACCCCCTCCTACTCACACTATTTCATCCCAATXXXXXXXXXXT
GTAAAATGAAAAGAATTTTCAGCCTATGCTTTTTCTAAATTTGAAAACCATTTATGGGACATATCATTTCAATAATGTG
>Marker743423 Chr5 SNP_site:3920063 Ref_Type:A
ACATCATCAATAGAGAAGGACTGATTGCTGCTCTCTTTTGTAGTGGATGATAGGAATTTGATGGTCGAGTCATTCTTGTTXXXXXXXXXXG
TCAATGTCTCGTCACGACGGCACCAATTTTCGTATCGTGAAGGCATCCGATTTGACAAAATCAATGATCAATCAGGGTC
ACATCATCAATAGGGAAGGACTGATTGCTGCTCTCTTTTGTAGTGGATGATAGGAATTTGATGGTCGAGTCATTCTTGTTXXXXXXXXXXG
TCAATGTCTCGTCACGACGGCACCGATTTTCATATCGTGAAGGCATCCGATTTGACAAAATCAATGATCAATCAGGGTC
>Marker743423 Chr5 SNP_site:3920268 Ref_Type:A
ACATCATCAATAGAGAAGGACTGATTGCTGCTCTCTTTTGTAGTGGATGATAGGAATTTGATGGTCGAGTCATTCTTGTTXXXXXXXXXXG
TCAATGTCTCGTCACGACGGCACCAATTTTCGTATCGTGAAGGCATCCGATTTGACAAAATCAATGATCAATCAGGGTC
ACATCATCAATAGGGAAGGACTGATTGCTGCTCTCTTTTGTAGTGGATGATAGGAATTTGATGGTCGAGTCATTCTTGTTXXXXXXXXXXG
TCAATGTCTCGTCACGACGGCACCGATTTTCATATCGTGAAGGCATCCGATTTGACAAAATCAATGATCAATCAGGGTC
>Marker743423 Chr5 SNP_site:3920275 Ref_Type:G
ACATCATCAATAGAGAAGGACTGATTGCTGCTCTCTTTTGTAGTGGATGATAGGAATTTGATGGTCGAGTCATTCTTGTTXXXXXXXXXXG
TCAATGTCTCGTCACGACGGCACCAATTTTCGTATCGTGAAGGCATCCGATTTGACAAAATCAATGATCAATCAGGGTC
ACATCATCAATAGGGAAGGACTGATTGCTGCTCTCTTTTGTAGTGGATGATAGGAATTTGATGGTCGAGTCATTCTTGTTXXXXXXXXXXG
TCAATGTCTCGTCACGACGGCACCGATTTTCATATCGTGAAGGCATCCGATTTGACAAAATCAATGATCAATCAGGGTC
>Marker743513 Chr5 SNP_site:18125162 Ref_Type:C
TACAACAAATTCTGCCTATCTACTACTTTTGGAGTTTTTCCCTGGATTTTCCTCCTAGGTTATGATTTGCCTTACTCTAGXXXXXXXXXXG
CTATAGACAAAAACCGACCATCTGGAGAACACAACCTTGTGGAATGGGCAAAACCTTACCTCACTAGCAAACGAAGAGT
TACAACAAATTCTGCTTATCTACTACTTTTGGAGTTTTTCCCTGGATTTTCCTCCTAGGTTATGATTTGCCTTACTCTAGXXXXXXXXXXG
CTATAGACAAAAACCGACCATCTGGAGAACACAACCTTGTGGAATGGGCAAAACCTTACCTCACTAGCAAACGAAGAGT
>Marker74354 Chr4 SNP_site:19147485 Ref_Type:C
CACCATGATAGAACTAACCATGTTGAGATTGATCGACAATTCATCAAAGAAAGACTTAACCATCAGAGCATATGCATTCTXXXXXXXXXXA
AGGGATTTGCCCTAAATATTTTTTTTCTTTTTTTTCCTCACTATATTTTTTCTATTTATTCTCCTCTTGTAACAATTGT
CACCATGATAGAACTAACCATGTTGAGATTGATCGACAATTCATCAAAGAAAGACTTAACCATCAGAGCATATGCATTTTXXXXXXXXXXA
AGGGATTTGCCCTAAATATTTTTTTTCTTTTTTTTCCTCACTATATTTTTTCTATTTATTCTCCTCTTGTAACAATTGT
>Marker743804 Chr5 SNP_site:12584186 Ref_Type:A
TACGTAACCAGCATGTAAATCATTTTGCCTCGACTTTCTTTTTCTTGGCTTCCGATTTCTTCATCACGTCTAACAGCTAGXXXXXXXXXXT
GATGGTCATGAATTTTGTGCTTTACACATGTTGTCTTAGCATGCACCGCGTTAAGCTACCAATCCAAACTTGACATGTT
TACGTAACCAGCATGTAAATCATTTTGCCTCGACTTTCTTTTTCTTGGCTTCCGATTTCTTCATCACGTCTGACAGCTAGXXXXXXXXXXT
GATGGTCATGAATTTTGTGCTTTACACATGTTGTCTTAGCATGCACCGCGTTAAGCTACCAATCCAAACTTGACATGTT
>Marker743899 Chr5 SNP_site:2257250 Ref_Type:C
ACCCAAAGCTATCGTGTGTCCGATATGGAACACACTCAATCTAGTGATCCCCAAGGATCACTATCATTTATCTGTGAATCXXXXXXXXXXA

Create PDF with GO2PDF for free, if you wish to

TAATATGACAGACTAGTGATCGATGCACACAGTCATAAATCTGTGAAAGGTGGTGATCAAGAGGAACACCCATGTAGGT
ACCCAAAGCTATCGTGTGTCCGATATGGAACACACTCAATCTAGTGATCCCCAAGGATCACTATCATTTATCTGTGAATCXXXXXXXXXXA
TAATATGACAGACTAGTGATCGATGCACACAGTCATAAATCTGTGAAAGGTGGTGATCCAGAGGAACACCCATGTAGGT
>Marker7439 Chr4 SNP_site:11753063 Ref_Type:T
CACGAAAGGAACTCATGATTACTAAAATTGAAGATTCCTTTATCAGAAAAAAGGACAAAATGATTCCAGTGCCTCTCTCTXXXXXXXXXXA
TTAAATTAGGCCCAATTTAGGCGTTTGAATTTAACTAATTTTATAATTTTAACCTTCAAAAAATCCTATTCATTCAAGT
CACGAAAGGAACTCATGATTACTAAAATTGAAGATTCCTTTATCAGAAAAAAGGACAAAATGATTCCAGTGCTTCTCTCTXXXXXXXXXXA
TTAAATTAGGCTCAATTTAGGCGTTTGAATTTAACTAATTTTATAATTTTAACCTTAAAAAAATCCTATTCATTCAAGT
>Marker7439 Chr4 SNP_site:11753173 Ref_Type:T
CACGAAAGGAACTCATGATTACTAAAATTGAAGATTCCTTTATCAGAAAAAAGGACAAAATGATTCCAGTGCCTCTCTCTXXXXXXXXXXA
TTAAATTAGGCCCAATTTAGGCGTTTGAATTTAACTAATTTTATAATTTTAACCTTCAAAAAATCCTATTCATTCAAGT
CACGAAAGGAACTCATGATTACTAAAATTGAAGATTCCTTTATCAGAAAAAAGGACAAAATGATTCCAGTGCTTCTCTCTXXXXXXXXXXA
TTAAATTAGGCTCAATTTAGGCGTTTGAATTTAACTAATTTTATAATTTTAACCTTAAAAAAATCCTATTCATTCAAGT
>Marker7439 Chr4 SNP_site:11753218 Ref_Type:A
CACGAAAGGAACTCATGATTACTAAAATTGAAGATTCCTTTATCAGAAAAAAGGACAAAATGATTCCAGTGCCTCTCTCTXXXXXXXXXXA
TTAAATTAGGCCCAATTTAGGCGTTTGAATTTAACTAATTTTATAATTTTAACCTTCAAAAAATCCTATTCATTCAAGT
CACGAAAGGAACTCATGATTACTAAAATTGAAGATTCCTTTATCAGAAAAAAGGACAAAATGATTCCAGTGCTTCTCTCTXXXXXXXXXXA
TTAAATTAGGCTCAATTTAGGCGTTTGAATTTAACTAATTTTATAATTTTAACCTTAAAAAAATCCTATTCATTCAAGT
>Marker74393 Chr4 SNP_site:11776587 Ref_Type:G
TACAGCAAGCTAAGTAAGAAATAAATTTTGAACTAAATAAAATAAATACAGGTAGGCTGATAAATCCAATGGAAAAACATXXXXXXXXXXA
TAGGAATGTAATTAAATCAAGATAAGGTTTGGGTTTTTTATTCTTTCCTCAATTATATTTTTCCTATAAAAAGATAGGT
TACAGCAAGCTAAGTAAGAAATAAATTTTGAACTAAATAAAATAAATACAGGTGGGCTGATAAATCCAATGGAAAAACATXXXXXXXXXXA
TAGGAATGTAATTAAATCAAGATAAGGTTTGGGTTTTTTATTCTTTCCTCAATTATATTTTTCCTATAAAAAGATAGGT
>Marker744016 Chr5 SNP_site:13497301 Ref_Type:G
GACCACGTCGCCAAAGGTTCAACCTTTAAATAATTATTGCAACATTTTTGCAATGACAAAAAGTGACACGTTAGGATTTTXXXXXXXXXXG
TAGTCACTTATAGCATTGCGATTGGGGAGTTCTCACGATGTTTTCGAGTTGCGTCGGAAGAACCTTAGTCATTAAAGTT
GACCACGTCGCCAAAGGTTCAACCTTTAAATAATTATTGCAACATTTTTGCAATGACAAAAAGTGACACGTTAGGATTTTXXXXXXXXXXG
TAGTCACTTATAGCATTGCGATTGGGGAGTTCTCACGATGTTTTCGAGTTGCGTCGGGAGAACCTTTGTCATTAAAGTT
>Marker744016 Chr5 SNP_site:13497310 Ref_Type:T
GACCACGTCGCCAAAGGTTCAACCTTTAAATAATTATTGCAACATTTTTGCAATGACAAAAAGTGACACGTTAGGATTTTXXXXXXXXXXG
TAGTCACTTATAGCATTGCGATTGGGGAGTTCTCACGATGTTTTCGAGTTGCGTCGGAAGAACCTTAGTCATTAAAGTT
GACCACGTCGCCAAAGGTTCAACCTTTAAATAATTATTGCAACATTTTTGCAATGACAAAAAGTGACACGTTAGGATTTTXXXXXXXXXXG
TAGTCACTTATAGCATTGCGATTGGGGAGTTCTCACGATGTTTTCGAGTTGCGTCGGGAGAACCTTTGTCATTAAAGTT
>Marker744336 Chr5 SNP_site:3535730 Ref_Type:A
GACACTTACCTTTCCTCGAATTGCAGCATGGACTTGCAGAATATATAGCTGAGGTGAGAATACCAGCATCTGTTTTTCACXXXXXXXXXXA
TTTTAAACTGTAATTGGACATTTTCACAATTTTATGAATATTTTCTTCTTTCAGGAGGAAATGGATCGGAAAAATGGGT
GACACTTACCTTTCCTCGAATTGCAGCATGGACTTGCAGAATATATAGCTGAGGTGAGAATACCAGCATCTGTTTTTCACXXXXXXXXXXA
TTTTAAACTGTAGTTGGACATTTTCACAATTTTATGAATATTTTCTTCTTTCAGGAGGAAATGGATCGGAAAAATGGGT
>Marker7446 Chr4 SNP_site:2502756 Ref_Type:G
ACTGATGGTAAGTTCCTGAATTTTGTCCATTTAGTTTATATAATGTCAATGAGTATTCTACAGGCTTGAGCTCTCTGCCAXXXXXXXXXXT
AACCGTGCAAAGACAACAGTCAAGCAGGGTTATTCCACATATTTTGGTTAGGTGTGCCGATTATCTCGTCTTGTCAGGT
ACTGATGGTAAGTTCCTGAATTTTGTCCATTTAGTTTATATAGTGTCAATGAGTATTCTACAGGCTTGAGCTCTCTGCCAXXXXXXXXXXT
AACCGTGCAAAAACAACAGTCAAGCAGGGTTATTCCACATATTTTGGTTAGGTGTGCCGATTATCTCGTCTTGTCAGGT
>Marker7446 Chr4 SNP_site:2502902 Ref_Type:A

Create PDF with GO2PDF for free, if you wish to

ACTGATGGTAAGTTCCTGAATTTTGTCCATTTAGTTTATATAATGTCAATGAGTATTCTACAGGCTTGAGCTCTCTGCCAXXXXXXXXXXXT
AACCGTGCAAAGACAACAGTCAAGCAGGGTTATTCCACATATTTTGGTTAGGTGTGCCGATTATCTCGTCTTGTCAGGT
ACTGATGGTAAGTTCCTGAATTTTGTCCATTTAGTTTATATAGTGTCAATGAGTATTCTACAGGCTTGAGCTCTCTGCCAXXXXXXXXXXXT
AACCGTGCAAAAACAACAGTCAAGCAGGGTTATTCCACATATTTTGGTTAGGTGTGCCGATTATCTCGTCTTGTCAGGT
>Marker744951 Chr5 SNP_site:10101996 Ref_Type:A
ACCATTTTTAGAAAATTGTAGAGATGCAGATTTCACTAAAATATTAAAATCTAAACTAATTCTTCCAAACTTAAACCAATXXXXXXXXXXXT
ACTGTATAATATTTGAAAAGGATTATGTTGATCTCGGTTCATCTTGTATAAATTTGTATCCAGAAAAACCAAAATTAGT
ACCATTTTTAGAAAATTGTAGAGATGCAGATTTCACTCAAACATTAAAATCTAAACTAATTCTTCCAAACTTAAACCAATXXXXXXXXXXXT
ACTGTATAATATTTGAAAAGGATTATGTTGATCTCGGTTCATCTTGTATAAATTTGTATCCAGAAAAACCAAAATTAGT
>Marker744951 Chr5 SNP_site:10102000 Ref_Type:T
ACCATTTTTAGAAAATTGTAGAGATGCAGATTTCACTAAAATATTAAAATCTAAACTAATTCTTCCAAACTTAAACCAATXXXXXXXXXXXT
ACTGTATAATATTTGAAAAGGATTATGTTGATCTCGGTTCATCTTGTATAAATTTGTATCCAGAAAAACCAAAATTAGT
ACCATTTTTAGAAAATTGTAGAGATGCAGATTTCACTCAAACATTAAAATCTAAACTAATTCTTCCAAACTTAAACCAATXXXXXXXXXXXT
ACTGTATAATATTTGAAAAGGATTATGTTGATCTCGGTTCATCTTGTATAAATTTGTATCCAGAAAAACCAAAATTAGT
>Marker745914 Chr5 SNP_site:25195237 Ref_Type:G
TACCCTGAGGCCCCAGCACAAGCCAAAAGAACCTCATATGCTGTTTCTCGAAGATCGTCATCTGTGATTCCTACAACAAAXXXXXXXXXXXA
ATTGGAATATAGTCTTTTTGCTTAACAACGGAAACAAGACCTTTCATTGCATTAATGAAATGTGTCTAATGCTCGAAGT
TACCCTGAGGCCCCAGCACAAGCCAAAAGAACCTCATATGCTGTTTCTCGAAGATCGTCATCTGTGATTCCTACAACAAAXXXXXXXXXXXA
ATTGGAATATAGTCTTTTTTCTTAACAACGGAAACAAGACCTTTCATTGCATTAATGAAATGTGTCTAATGCTCGAAGT
>Marker745959 Chr5 SNP_site:20906207 Ref_Type:A
AACCCAGTTGGAACGTGAATCACTCTAACAGCACTATTTGTGGTATTTGCATGCTGACCTCCTGAACCGCCTGATCTGAAXXXXXXXXXXXT
TCATTTTCTAGGAAGAAAATGGCTTGGGTTGGACCTCGTCTGCTTGAGGGAGAATGGCTACAGAAATAGCACTCGTGTG
AACCCAGTTGGCACGTGAATCACTCTAACAGCACTATTTGTGGTATTTGCATGCTGACCTCCTGAACCGCCTGATCTGAAXXXXXXXXXXXT
TCATTTTCTAGGAAGAAAATGGCTTGGGTTGGACCTCGTCTGCTTGAGGGAGAATGGCTACAGAAATAGCACTCGTGTG
>Marker746815 Chr5 SNP_site:6073660 Ref_Type:T
TACCACACAAGCCAGCTACCACCCAAATGTGTTCTCTCCACTGTAAGTAACATACAGAAATCCATCTTCATCCTTCTTCTXXXXXXXXXXXA
GTTTTTGAAATTTTGAACATGACCTTTCATTAAATTTTCTGCTATCTTTGATATAATTGTTATTTGAGGTGCCATGTGT
TACCACATAAGCCAGCTACCACCCAAATGTGTTCTCTCCACTGTAAGTAACATACAGAAATCCATCTTCATCCTTCTTCTXXXXXXXXXXXA
GTTTTTGAAATTTTGAACATGACCTTTCATTAAATTTTCTGTTATCTTTGATATAATTGTTATTTGAGGTGCCATGTGT
>Marker746815 Chr5 SNP_site:6073889 Ref_Type:T
TACCACACAAGCCAGCTACCACCCAAATGTGTTCTCTCCACTGTAAGTAACATACAGAAATCCATCTTCATCCTTCTTCTXXXXXXXXXXXA
GTTTTTGAAATTTTGAACATGACCTTTCATTAAATTTTCTGCTATCTTTGATATAATTGTTATTTGAGGTGCCATGTGT
TACCACATAAGCCAGCTACCACCCAAATGTGTTCTCTCCACTGTAAGTAACATACAGAAATCCATCTTCATCCTTCTTCTXXXXXXXXXXXA
GTTTTTGAAATTTTGAACATGACCTTTCATTAAATTTTCTGTTATCTTTGATATAATTGTTATTTGAGGTGCCATGTGT
>Marker747621 Chr5 SNP_site:10012059 Ref_Type:C
ACTCTTGTTCTTCTAAACATCTTTGCTGATTTTTAAACTTTCTTTGTAATTTTTTTTCCTTATCGTCTTTGAATTTATTGXXXXXXXXXXXG
TTTTAGGCCTTCCCATCAATCATTCTTCTCTACTGGAGATTTTTAATCTTCTGTAATTTGCCTCAAGTCATTCTATGTT
ACTCTTGTTCTTCTAAACATCTTTGCTGATTTTTAAACTTTCTTTGTAATTTTTTTTCCTTATCGTCTTTGAATTTATTGXXXXXXXXXXXG
TTTTAGGCCTTCCCATCAATCATTCTTCTCTACTGGAGATTTTTAATCTTCTGTAATTTGCCTCAAGTCATTCTCTGTT
>Marker74771 Chr4 SNP_site:21894075 Ref_Type:T
AACTAAACACTTAATTATTAATTAAACAGAAAACTAAAAACGGAATTGTTATAAAAACAGAACACTTCCGTTTGTTAAAAXXXXXXXXXXXC
TATGATGAGAGAAGAATTAAGTGTGTGTGTGTGTATATATATATACCTGACATTTTGCAGCTTTTGATGAAGTGCTTGT
AACTAAACACTTAATTATTAATTAAACAGAAAACTAAAAACGGAATTGTTATAAAAACAGAACACTTCCGTTTGTTATAAXXXXXXXXXXXC
TATGATGAGAGAAGAATTAAGTGTGTGTGTGTGTATATATATATACCTGACATTTTGCAGCTTTTGATGAAGTGCTTGT

Create PDF with GO2PDF for free, if you wish to

>Marker748292 Chr5 SNP_site:14003259 Ref_Type:A
AACGTGTCTGTAACGGATAGACCAAGGTATCGAACAAGAAAGGGAAAAGTGGCATTGAATGTCCTCGACGTATGCGATACXXXXXXXXXXG
AAGGTTCCAAAGGGTGAGATATATGTTCCAACTAACCCTCGTTAAAACAATTTGGAATTTGTTCATTCTTTCTTGGGTT
AACGTGTCTGTAACGGATCGACCAAGGTATCGAACAAGAAAGGGAAAAGTGGCATCGAATGTCCTCGACGTATGCGATACXXXXXXXXXXG
AAGGTTCCAAAGGGTGAGATATATGTTCCAACTAACCCTCGTTAAAACAATTTGGAATTCGTTCATTCTTTCTTGGGTT
>Marker748292 Chr5 SNP_site:14003296 Ref_Type:T
AACGTGTCTGTAACGGATAGACCAAGGTATCGAACAAGAAAGGGAAAAGTGGCATTGAATGTCCTCGACGTATGCGATACXXXXXXXXXXG
AAGGTTCCAAAGGGTGAGATATATGTTCCAACTAACCCTCGTTAAAACAATTTGGAATTTGTTCATTCTTTCTTGGGTT
AACGTGTCTGTAACGGATCGACCAAGGTATCGAACAAGAAAGGGAAAAGTGGCATCGAATGTCCTCGACGTATGCGATACXXXXXXXXXXG
AAGGTTCCAAAGGGTGAGATATATGTTCCAACTAACCCTCGTTAAAACAATTTGGAATTCGTTCATTCTTTCTTGGGTT
>Marker748292 Chr5 SNP_site:14003474 Ref_Type:T
AACGTGTCTGTAACGGATAGACCAAGGTATCGAACAAGAAAGGGAAAAGTGGCATTGAATGTCCTCGACGTATGCGATACXXXXXXXXXXG
AAGGTTCCAAAGGGTGAGATATATGTTCCAACTAACCCTCGTTAAAACAATTTGGAATTTGTTCATTCTTTCTTGGGTT
AACGTGTCTGTAACGGATCGACCAAGGTATCGAACAAGAAAGGGAAAAGTGGCATCGAATGTCCTCGACGTATGCGATACXXXXXXXXXXG
AAGGTTCCAAAGGGTGAGATATATGTTCCAACTAACCCTCGTTAAAACAATTTGGAATTCGTTCATTCTTTCTTGGGTT
>Marker748554 Chr5 SNP_site:12056533 Ref_Type:C
AACAACTTTGAGTCACTTACTTGAATTTAGATAGATACAACATGCAAATTTTTATTTACAAATTATTTGAAGCAAAGGCTXXXXXXXXXXT
CCTACCTAGGGGAATAAGAAGAAGGAAAAAGGTTTGGCTTTAAATGGTCCTTTAATTTACCAACATGACATGTTGAGTT
AACAACTTTGAGTCACTTCCTTGAATTTAGATAGATACAACATGCAAATTTTTATTTACAAATTATTTGAAGCAAAGGCTXXXXXXXXXXT
CCTACCTAGGGGAATAAGAAGAAGGAAAAAGGTTTGGCTTTAAATGGTCCTTTAATTTACCAACATGACATGTTGAGTT
>Marker748718 Chr5 SNP_site:12744907 Ref_Type:A
TACAACGTTGCCACCATTGGATGTGCTCCTGAGCAGCTAATTGTTCAATTTGTTCCCTCAATAAACGACTTGTGGATAATXXXXXXXXXXA
TGAACGAAAGGAACATTTTAATGCTTTGAGCTTCATCATCATTCCATGTCTAGGCCAACCAGCAACCCTATTGTTTGTC
TACAACGTTGCCACCATTGGATGTGCTCCTGAGCAGCTAATTGTTCAATTTGTTCCCTCAATGAACGACTTGTGGATAATXXXXXXXXXXA
TGAACGAAAGGAACATTTTAATGCTTTGAGCTTCATCATCATTCCATGTCTAGGCCAACCAGCAACCCTATTGTTTGTC
>Marker749101 Chr5 SNP_site:25479814 Ref_Type:A
AACATAATAATGAAGAAAGGGTTGCAAACAATTTGAAAATTGAAGATTGTTCATATCATAAAAAAAACTGTTTCTTATGAXXXXXXXXXXT
TTAGATAAGCTTGTATTAGTAAATACCTTTTGTTTGCCCTTGACTCACTTCTGTGCCCCCATTTTATCCAAAGACAAGT
AACATAATAATGAAGAAAGGGTTGCAAACAATTTGAAAATTGAAGATTGTTCATATCATCAAAAAAACTGTTTCTTATGAXXXXXXXXXXT
TTAGATAAGCTTGTATTAGTAAATACCTTTTGTTTGCCCTTGACTCACTTCTGTGCCCCCATTTTATCCAAAGACAAGT
>Marker750084 Chr5 SNP_site:8794186 Ref_Type:T
ACCTTTCTATGAGACGACCTTGTAACTTGGAGGAGTAAGAAGCAAAGTGTTGTGACTAGAAATAATGCTAAGGTCGAATAXXXXXXXXXXT
CAATGTGATAGAATTAAACATGTTGAGATTGATTGACATTTCATCAAAGAAAGACTTGACAGTGGGAGCATATGATAGT
ACCTTTCTATGAGACGATCTTGTAACTTGGAGGAGTAAGAAGCAAAGTGTTGTGACGAGAAATAATGCTAAGGTCGAATAXXXXXXXXXXT
CAATGTGATAGAATTAAACATGTTGAGATTGATTGGCATTTCATCAAAGAAAGACTTGACAGTGGGAGCATATGATAGT
>Marker750084 Chr5 SNP_site:8794225 Ref_Type:G
ACCTTTCTATGAGACGACCTTGTAACTTGGAGGAGTAAGAAGCAAAGTGTTGTGACTAGAAATAATGCTAAGGTCGAATAXXXXXXXXXXT
CAATGTGATAGAATTAAACATGTTGAGATTGATTGACATTTCATCAAAGAAAGACTTGACAGTGGGAGCATATGATAGT
ACCTTTCTATGAGACGATCTTGTAACTTGGAGGAGTAAGAAGCAAAGTGTTGTGACGAGAAATAATGCTAAGGTCGAATAXXXXXXXXXXT
CAATGTGATAGAATTAAACATGTTGAGATTGATTGGCATTTCATCAAAGAAAGACTTGACAGTGGGAGCATATGATAGT
>Marker750084 Chr5 SNP_site:8794424 Ref_Type:G
ACCTTTCTATGAGACGACCTTGTAACTTGGAGGAGTAAGAAGCAAAGTGTTGTGACTAGAAATAATGCTAAGGTCGAATAXXXXXXXXXXT
CAATGTGATAGAATTAAACATGTTGAGATTGATTGACATTTCATCAAAGAAAGACTTGACAGTGGGAGCATATGATAGT
ACCTTTCTATGAGACGATCTTGTAACTTGGAGGAGTAAGAAGCAAAGTGTTGTGACGAGAAATAATGCTAAGGTCGAATAXXXXXXXXXXT

Create PDF with GO2PDF for free, if you wish to

CAATGTGATAGAATTAAACATGTTGAGATTGATTGGCATTTCATCAAAGAAAGACTTGACAGTGGGAGCATATGATAGT
>Marker750190 Chr5 SNP_site:16331157 Ref_Type:A
CACGACCAACACCTTGCCGCCCACTCCTTGCGCGCCAATCATGCCATTTTGGTATGTATGAGCTATCTTTGATATTTTGAXXXXXXXXXXA
AAATGAGTTTGAAGTTGATTTCTAAATCTGATTATTTATCAAATGTTCTAAAGAAAATTTGACGTTTTAGTGTCGAGTT
CACGACCAACGCCTTGCCGCCCACTCCTTGCGCGCCAATCATGCCATTTTGGTATGTATGAGCTATCTTTGATATTTTGAXXXXXXXXXXA
AAATGAGTTTGAAGTTGATTTCTAAATCTGATTATTTATCAAATGTTTTAAAGAAAATTTGACGTTTTAGTGTTGAGTT
>Marker750190 Chr5 SNP_site:16331408 Ref_Type:C
CACGACCAACACCTTGCCGCCCACTCCTTGCGCGCCAATCATGCCATTTTGGTATGTATGAGCTATCTTTGATATTTTGAXXXXXXXXXXA
AAATGAGTTTGAAGTTGATTTCTAAATCTGATTATTTATCAAATGTTCTAAAGAAAATTTGACGTTTTAGTGTCGAGTT
CACGACCAACGCCTTGCCGCCCACTCCTTGCGCGCCAATCATGCCATTTTGGTATGTATGAGCTATCTTTGATATTTTGAXXXXXXXXXXA
AAATGAGTTTGAAGTTGATTTCTAAATCTGATTATTTATCAAATGTTTTAAAGAAAATTTGACGTTTTAGTGTTGAGTT
>Marker750190 Chr5 SNP_site:16331434 Ref_Type:C
CACGACCAACACCTTGCCGCCCACTCCTTGCGCGCCAATCATGCCATTTTGGTATGTATGAGCTATCTTTGATATTTTGAXXXXXXXXXXA
AAATGAGTTTGAAGTTGATTTCTAAATCTGATTATTTATCAAATGTTCTAAAGAAAATTTGACGTTTTAGTGTCGAGTT
CACGACCAACGCCTTGCCGCCCACTCCTTGCGCGCCAATCATGCCATTTTGGTATGTATGAGCTATCTTTGATATTTTGAXXXXXXXXXXA
AAATGAGTTTGAAGTTGATTTCTAAATCTGATTATTTATCAAATGTTTTAAAGAAAATTTGACGTTTTAGTGTTGAGTT
>Marker750359 Chr5 SNP_site:2099553 Ref_Type:C
CACACGAGATTTCAAATAAATGTATTTCGTGAAATTGAACTTTGTTATATCGATTAATATTGTGAATACAATCTCTAGTAXXXXXXXXXXA
GAACATGATGATCTCGAAGTTGATAAGAACTTGCACGTCTTGAGAGCTTTATAAAAGTTTGAATCTTCAGTTCTTCAGT
CACACGAGATTTCAAATAAATGTATTTCGTGAAATTGAACTTTGTTATATTGATTAATATTGTGAATACAATCTCTAGTAXXXXXXXXXXA
GAAGATGATGATCTCGAAGTTGATAAGAACTTGCACGTCTTGAGAGCTTTATAAAAGTTTGAATCTTCAATTCTTCAGT
>Marker750359 Chr5 SNP_site:2099737 Ref_Type:C
CACACGAGATTTCAAATAAATGTATTTCGTGAAATTGAACTTTGTTATATCGATTAATATTGTGAATACAATCTCTAGTAXXXXXXXXXXA
GAACATGATGATCTCGAAGTTGATAAGAACTTGCACGTCTTGAGAGCTTTATAAAAGTTTGAATCTTCAGTTCTTCAGT
CACACGAGATTTCAAATAAATGTATTTCGTGAAATTGAACTTTGTTATATTGATTAATATTGTGAATACAATCTCTAGTAXXXXXXXXXXA
GAAGATGATGATCTCGAAGTTGATAAGAACTTGCACGTCTTGAGAGCTTTATAAAAGTTTGAATCTTCAATTCTTCAGT
>Marker750359 Chr5 SNP_site:2099803 Ref_Type:G
CACACGAGATTTCAAATAAATGTATTTCGTGAAATTGAACTTTGTTATATCGATTAATATTGTGAATACAATCTCTAGTAXXXXXXXXXXA
GAACATGATGATCTCGAAGTTGATAAGAACTTGCACGTCTTGAGAGCTTTATAAAAGTTTGAATCTTCAGTTCTTCAGT
CACACGAGATTTCAAATAAATGTATTTCGTGAAATTGAACTTTGTTATATTGATTAATATTGTGAATACAATCTCTAGTAXXXXXXXXXXA
GAAGATGATGATCTCGAAGTTGATAAGAACTTGCACGTCTTGAGAGCTTTATAAAAGTTTGAATCTTCAATTCTTCAGT
>Marker750587 Chr5 SNP_site:16236597 Ref_Type:G
ACTGCAAATATTGACCTCTGAAAATATGCCTTAGAGCAGAATTCTTATCCTTCCTCTATAGTCTCTTTTCGCACTTTACTXXXXXXXXXXC
AAATAAGGCTTCTAAGGTGCTTTTGATCATTACCTGTTGGAACTTTGTGGTCGGGTTACCACATTCAGAATACGGAGTG
ACTGCAAATATTGACCTCTGAAAATATGCCTTAGAGCAGAATTCTTATCCTTCCTCTATGGTCTCTTTTCTCACTTTACTXXXXXXXXXXC
AAATAAGGCTTCTAAGGTGCTTTTGATCATTACCTGTTGGAACTTTGTGGTCGGGTTACCACATTCAGAATACGGAGTG
>Marker750587 Chr5 SNP_site:16236608 Ref_Type:T
ACTGCAAATATTGACCTCTGAAAATATGCCTTAGAGCAGAATTCTTATCCTTCCTCTATAGTCTCTTTTCGCACTTTACTXXXXXXXXXXC
AAATAAGGCTTCTAAGGTGCTTTTGATCATTACCTGTTGGAACTTTGTGGTCGGGTTACCACATTCAGAATACGGAGTG
ACTGCAAATATTGACCTCTGAAAATATGCCTTAGAGCAGAATTCTTATCCTTCCTCTATGGTCTCTTTTCTCACTTTACTXXXXXXXXXXC
AAATAAGGCTTCTAAGGTGCTTTTGATCATTACCTGTTGGAACTTTGTGGTCGGGTTACCACATTCAGAATACGGAGTG
>Marker750633 Chr5 SNP_site:15068331 Ref_Type:A
CACTACTTAAAGTTTGAATAGTCTAAGATTTTTTAGTCTTTTTTTTCAATCACCCCTAAAGCTCTCAATCAATCATTGCAXXXXXXXXXXA
ATAGCCCAAAATCTCTGTATGTATTATTGATTAAATAATCAAATATTATCTAAATAAATTAAATATTTCCATGACGGTT

Create PDF with GO2PDF for free, if you wish to

CACTACTTAAAGTTTGAATAGTCTAAGATTTTTTAGTCTTTTTTTTCAATCACCCCTAAAGCTCTCAATCAATCATTGCAXXXXXXXXXXA
ATAGCCCAAAATCTCTGTATGTATTATTGATTAAATAATCAAATATTATCTAAATAAATTAAATATTTCCCTGACGGTT
>Marker751265 Chr5 SNP_site:2157054 Ref_Type:A
TACATTCTTGTGAGACCATGAGGTGAATGTGGGTTTATCACTTTCTTAGGTTTCTAATGAAAAAGAAGATACTTGTGAGTXXXXXXXXXXA
TTCTCTGTGAAGATATTATTGATTTCTGTAACATGCTAAAAGTCAATACTTTCACATTGGTGGCATACATGGCGTAAGT
TACATTCTTGTGAGACCATGAGGTGAATGTGGGTTTATCACTTTCTTAGGTTTCTTATGAAAAAGAAGATACTTGTGAGTXXXXXXXXXXA
TTCTCTGTGAAGATATTATTGATTTCTGTAACATGCTAAAAGTCAATACTTTCACATTGGTGGCATACATGGCGTAAGT
>Marker751620 Chr5 SNP_site:17451719 Ref_Type:C
CACAGACGCATGATTGAGGAAGCTAGTAAAGCTCATCAAAATAGATAAGAAAAAAAGAAAGAAACTAATTCAAGATTCCAXXXXXXXXXXA
TCCGAGTATCCACATGTATTTATCGTAACTTCTAAGAACAAAAATAAAAGAAAAAAAAGGGATACAGACAAAAAGAGTC
CACAGACGCATGATTGAGGAAGCTAGTAAAGCTCATCAAAATCGATAAGAAAAAAAGAAAGAAACCAATTCAAGATTCCAXXXXXXXXXXA
TCCGAGTATCCACATGTATTTATCGTAACTTCTAAGAACAAAAATAAAAGAAAAAAAAGGGATACAGACAAAAAGAGTC
>Marker751620 Chr5 SNP_site:17451742 Ref_Type:C
CACAGACGCATGATTGAGGAAGCTAGTAAAGCTCATCAAAATAGATAAGAAAAAAAGAAAGAAACTAATTCAAGATTCCAXXXXXXXXXXA
TCCGAGTATCCACATGTATTTATCGTAACTTCTAAGAACAAAAATAAAAGAAAAAAAAGGGATACAGACAAAAAGAGTC
CACAGACGCATGATTGAGGAAGCTAGTAAAGCTCATCAAAATCGATAAGAAAAAAAGAAAGAAACCAATTCAAGATTCCAXXXXXXXXXXA
TCCGAGTATCCACATGTATTTATCGTAACTTCTAAGAACAAAAATAAAAGAAAAAAAAGGGATACAGACAAAAAGAGTC
>Marker752023 Chr5 SNP_site:11722738 Ref_Type:C
CACCTTATTCAGATTGTTCCAAAAACTGACATTTCTCCTTTTTCTCGACTCAAAATCAAACCTTCTTTGCTCCAAGTTGTXXXXXXXXXXA
GACAGGTTGCATGGCTCGAGATTGACATGTATCACTTACATTTTGTTGGCATGCCTTAGTCTTACACCCCTTCCTAGTC
CACCTTATTCAGATTGTTCCAAAAACTGACATTTCTCCTTTTTCTCGACTCAAAATCAAACCTTCTTTGCTCCAAGTTGTXXXXXXXXXXA
GACAGGTTGCATGGCTTGAGATTGACATGTATCACTTACATTTTGTTGGCATGCCTTAGTCTTACACCCCTTCCTAGTC
>Marker752179 Chr5 SNP_site:16435483 Ref_Type:A
GACCAACTTTCTCTGACCATTTTTCAGTGATTGTTGCCAGCCTACTTTCATTGACAATTTTCTTGTGGTAGTCACTAATCXXXXXXXXXXG
GATAATATAACAAATTATGAATGACACTATTTAGCATTTGATAAGCTATAATGTGATGAGATTTAAAGTATGATGTGTA
GACCAACTTTCTCTGACCATTTTTCGGTGATTGTTGCCGGCCTACTTTCATTGACAATTTTCTTGTGGTAGTCACTAATCXXXXXXXXXXG
GATAATATAACAGATTATGAATGACACTATTTAGCATTTGATAAGCTATAATGTGATGAGATTTAAAGTATGATGTGTA
>Marker752179 Chr5 SNP_site:16435496 Ref_Type:A
GACCAACTTTCTCTGACCATTTTTCAGTGATTGTTGCCAGCCTACTTTCATTGACAATTTTCTTGTGGTAGTCACTAATCXXXXXXXXXXG
GATAATATAACAAATTATGAATGACACTATTTAGCATTTGATAAGCTATAATGTGATGAGATTTAAAGTATGATGTGTA
GACCAACTTTCTCTGACCATTTTTCGGTGATTGTTGCCGGCCTACTTTCATTGACAATTTTCTTGTGGTAGTCACTAATCXXXXXXXXXXG
GATAATATAACAGATTATGAATGACACTATTTAGCATTTGATAAGCTATAATGTGATGAGATTTAAAGTATGATGTGTA
>Marker752179 Chr5 SNP_site:16435644 Ref_Type:A
GACCAACTTTCTCTGACCATTTTTCAGTGATTGTTGCCAGCCTACTTTCATTGACAATTTTCTTGTGGTAGTCACTAATCXXXXXXXXXXG
GATAATATAACAAATTATGAATGACACTATTTAGCATTTGATAAGCTATAATGTGATGAGATTTAAAGTATGATGTGTA
GACCAACTTTCTCTGACCATTTTTCGGTGATTGTTGCCGGCCTACTTTCATTGACAATTTTCTTGTGGTAGTCACTAATCXXXXXXXXXXG
GATAATATAACAGATTATGAATGACACTATTTAGCATTTGATAAGCTATAATGTGATGAGATTTAAAGTATGATGTGTA
>Marker752222 Chr5 SNP_site:9649084 Ref_Type:T
CACTAAAACTAGCCAATGGGAAAAACCTAACAAGTATTTTAGCAAATCATAGGCCAACTTGAATCCATTTTCATAAAAATXXXXXXXXXXT
GCACGTTAAGCTCACTATTTTTGTAGTCCCAATTGCAATTTGATTTTTATCCATGACATATATAATAGGGTAGGATAGT
CACTAAAACTAGCCAATGGGAAAAACCTAACAAGTATTTTAGCAAATCATAGGCCAACTTGAATCCATTTTCATAAAAATXXXXXXXXXXT
GCACGTTAAGCTCACTATTTTTGTAGTCCCAATTGCAATTTGATTTTTATCCATGACTTATATAATAGGGTAGGATAGT
>Marker752402 Chr5 SNP_site:25366042 Ref_Type:G
ACTATATTTGCTAGCTGCTAAAATTTTCCTTCTGTTATAAGTTTTTGGTTGGCTTTTTTATTATATATGCTATGGTATATXXXXXXXXXXG

Create PDF with GO2PDF for free, if you wish to

AAGCCAAATTTTGAAAGGTAGAAAAAAGTAATTTTCAAAAACTTGTTATTGTTTTTAGAAATTGACTAGGATTTCAAGT
ACTATATTTGCTAGCTGCTAAAATTTTCCTTCTGTTGTAAGTTTTTGGTTGGCTTTTTTATTATATATGCTCTGGTATATXXXXXXXXXXG
AAGCCAAATTTTGAAAGGTAGAAAAAAGTAATTTTCAAAAACTTGTTATTGTTTTTAGAAATTGACTAGGATTTCAAGT
>Marker752402 Chr5 SNP_site:25366077 Ref_Type:C
ACTATATTTGCTAGCTGCTAAAATTTTCCTTCTGTTATAAGTTTTTGGTTGGCTTTTTTATTATATATGCTATGGTATATXXXXXXXXXXG
AAGCCAAATTTTGAAAGGTAGAAAAAAGTAATTTTCAAAAACTTGTTATTGTTTTTAGAAATTGACTAGGATTTCAAGT
ACTATATTTGCTAGCTGCTAAAATTTTCCTTCTGTTGTAAGTTTTTGGTTGGCTTTTTTATTATATATGCTCTGGTATATXXXXXXXXXXG
AAGCCAAATTTTGAAAGGTAGAAAAAAGTAATTTTCAAAAACTTGTTATTGTTTTTAGAAATTGACTAGGATTTCAAGT
>Marker752924 Chr5 SNP_site:23854723 Ref_Type:G
TACCCACATATATCAACGAGTTATGAGGCAAGAGATACATCAGATTGCACAAAGAATCTTTTAGTTGCTTAAAGTTACATXXXXXXXXXXA
GAAAGCTAGAGATTTTCTCAGAAGCTACTTTAATGGCAGGGCCCTCTTGTGGGGGGTAAAGACAAGCTCCAAGCTCGTC
TACCCACATATATCAACGAGTTATGAGGGAAGAGATACATCAGATTGCACAAAGAATCTTTTAGTTGCTTAAAGTTACATXXXXXXXXXXA
GAAAGCTAGAGATTTTCTCAGAAGCTACTTTAATGGCAGGGCCCTCTTGTGGGGGGTAAAGACAAGCTCCAAGCTCGTC
>Marker75301 Chr4 SNP_site:11493552 Ref_Type:C
GACCATAAACAAAACAACCAAAGTGAAGGATCGAAGATGGTGAGTCAGAGAGATAAAAATCAAAACCAAACTTCATTATGXXXXXXXXXXT
ATGAATGAATAGATAAGATGTTGAAAAGTGGAATGAGAGTGATGTCCAATCAAATCGATGGCAGAAAGAAACCAAAGGT
GACCATAAACAAAACAACCAAAGTGAAGGATCGAAGATGGTGAGTCAGAGAGATAAAAATCAAAACCAAATTTCATTATGXXXXXXXXXXT
ATGAATGAATAGATAAGATGTTGAAAAGTGGAATGAGAGTGATGTCCAATCAAATCGATGGCAGAAAGAAACCAAAGGT
>Marker753095 Chr5 SNP_site:21010167 Ref_Type:G
AACCATGTTCTTCAACAAATCTCTGGAATGTGTTGTAAATGTGCTGAAGAAACAGCTGCAGACTTGGTTGCTTACTGGAAXXXXXXXXXXT
TTGGTTACTAGTTTGTGGTTTTGTTTATACTAGTTGGTGCATTACAATATAATCGGTTCAGCATGCAAGATAATTTTGT
AACCATGTTCTTCAACAAATCTCTGGAATGTGTTGTGAATGTGCTGAAGAAACAGCTGCAGACTTGGTTGCTTACTGGAAXXXXXXXXXXT
TTGGTTACTAGTTCGTGGTTTTGTTTATACTAGTTGGTGCATTACAATATAATCGGTTCAGCATGCAAGATAATTTTGT
>Marker753095 Chr5 SNP_site:21010317 Ref_Type:C
AACCATGTTCTTCAACAAATCTCTGGAATGTGTTGTAAATGTGCTGAAGAAACAGCTGCAGACTTGGTTGCTTACTGGAAXXXXXXXXXXT
TTGGTTACTAGTTTGTGGTTTTGTTTATACTAGTTGGTGCATTACAATATAATCGGTTCAGCATGCAAGATAATTTTGT
AACCATGTTCTTCAACAAATCTCTGGAATGTGTTGTGAATGTGCTGAAGAAACAGCTGCAGACTTGGTTGCTTACTGGAAXXXXXXXXXXT
TTGGTTACTAGTTCGTGGTTTTGTTTATACTAGTTGGTGCATTACAATATAATCGGTTCAGCATGCAAGATAATTTTGT
>Marker754959 Chr5 SNP_site:17389170 Ref_Type:G
AACAAATAAAACAATGCAAATGAAATTCTTGATTGAAATCAAGTAAATTCACAAAAATGTTGAGCAAAAAGCTAGAAAATXXXXXXXXXXA
GTTTTAGCAAATATAATGAAAGAAAATTGCTGCCCTCTAGGATTGGATGTCAGAATTTACTATAGTATTTCTGGTTGTT
AACAAATAAAACAATGCAAATGAAATTCTTGATTGAAATCAAGTAAATTCACAAAAATGTTGAGCAAAAAGCTAGAAAATXXXXXXXXXXA
GTTTTAGCAAATATAATGAAAGGAAATTGCTGCCCTCTAGGATTGGATGTCAGAATTTACTATAGTATTTCTGGTTGTT
>Marker755656 Chr5 SNP_site:9170063 Ref_Type:A
ACCATTTTATCAGCTCAAGCGTTTATCACACACTCTTTTACAAAGTTGTAGCTATGCGGAGTAGTCTCAACACATTTAGAXXXXXXXXXXT
ATTCAAGATCACATAGCATGAATAGGGCATCTTATCCCATATCATATAGCAAGTATGAATTATTCTCATCGTATAGAGT
ACCATTTTATCAGCTCAAGCGTTTATCACACACTCTTTTACAGAGTTGTAGCTATGCGGAGTAGTCTCAACACATTTAGAXXXXXXXXXXT
ATTCAAGATCACATAGCATGAATAGGGCATCTTATCCCATATCATATAGCAAGTATGAATTATTCTCATCGTATAGAGT
>Marker755708 Chr5 SNP_site:11982196 Ref_Type:G
AACATTTCTTTCATACTTGGTGCTTGCATTATGGATAGCCCTTGAGCCTTCTCGGGTTTGTGATTTGTGGAATTTTATTAXXXXXXXXXXA
GGACTCACAATTTTACGACGGGTTCAAAAGAAAATCTCCTCTTTCTCAAAGTTGAAGAGTATTTGGTATAATCTTAGTA
AACATTTCTTTCATACTTGGTGCTTGCATTATGGATAGCCCTTGAGCCTTCTCGGGTTTGTGATTTGTGGAATTTTATTAXXXXXXXXXXA
GGACTCACGATTTTACGACCGGTTCAAAAGAAAATCTCCTCTTTCTCAAAGTTGAAGAGTATTTGGTATAATCTTAGTA
>Marker755708 Chr5 SNP_site:11982207 Ref_Type:C

Create PDF with GO2PDF for free, if you wish to

AACATTTCTTTCATACTTGGTGCTTGCATTATGGATAGCCCTTGAGCCTTCTCGGGTTTGTGATTTGTGGAATTTTATTAXXXXXXXXXXA
GGACTCACAATTTTACGACGGGTTCAAAAGAAAATCTCCTCTTTCTCAAAGTTGAAGAGTATTTGGTATAATCTTAGTA
AACATTTCTTTCATACTTGGTGCTTGCATTATGGATAGCCCTTGAGCCTTCTCGGGTTTGTGATTTGTGGAATTTTATTAXXXXXXXXXXA
GGACTCACGATTTTACGACCGGTTCAAAAGAAAATCTCCTCTTTCTCAAAGTTGAAGAGTATTTGGTATAATCTTAGTA
>Marker755888 Chr5 SNP_site:16512171 Ref_Type:A
AACATATTTTTTCAACTTCTTTTAATAATTTGCATACTTCTTGGGTAGGAAAGTTAAATTTTAAACCAAATTAAAAAGAGXXXXXXXXXXC
TAGGGAACATGAGAGAACATGGAAGAATTGGAGTTTGTGGAATGATATCACAATACAATCTTGAGAAGCCAGAAGGAGT
AACATATTTTTTCAACTTCTTTTAATAATTTGCATTCTTCTTGGGTAGGAAAGTTAAATTTTAAACCAAATTAAAAAGAGXXXXXXXXXXC
TAGGGAACATGAGAGAACATGGAAGAATTGGAGTTTGTGGAATGATATCACAATACAATCTTGAGAAGCCAGAAGGAGT
>Marker756024 Chr5 SNP_site:9281233 Ref_Type:C
CACAAAGTCATTGGCTGAGATTGTAAATTCTACATCTTTTTAAATAAGGGATTGGAAACAAAGAAAGATTCTGCATACCAXXXXXXXXXXC
ACCAGGAAAAATGTCATAAAACAATTAAATGCTTAGAGGTGCAACAGCTAGTTCTGTGTCTGACCTCATTTAGAAAGTA
CACAAAGTCATTGGCTGAGATTGTAAATTCTACATCTTTTTAAATAAGGGATTGGAAACAAAGAAAGCTTCTGCATACCAXXXXXXXXXXC
ACCAGGAAAAATGTCATAAAACAATTAAATGCTTAGAGGTGCAACAGCTAGTTCTGTGTCTGACCTCATTTAGAAAGTA
>Marker756102 Chr5 SNP_site:1438192 Ref_Type:G
AACAACCACTAAAACAATGATGTTCGAAAGAAGGAAATTTAATAGATGACACCCAAAAATTTAGTCTATAGTTGCATTGTXXXXXXXXXXC
ATGAATTCTACGTTTTCTTCAATGTGTCATAAAATGCGTTGATGTTGCCTTTGGATTTCACTGACTTACTCCAATCGGT
AACAACCACTAGAACAATGATGTTCGAAAGAAGGAAATTTAATAGATGACACCCAAAAATTTAGTTTATAGTTGCATTGTXXXXXXXXXXC
ATGAATTCTACGTTTTCTTCAATGTGTCATAAAATGCGTTGATGTTGCCTTTGGATTTCACTGACTTACTCCAATCGGT
>Marker756102 Chr5 SNP_site:1438246 Ref_Type:T
AACAACCACTAAAACAATGATGTTCGAAAGAAGGAAATTTAATAGATGACACCCAAAAATTTAGTCTATAGTTGCATTGTXXXXXXXXXXC
ATGAATTCTACGTTTTCTTCAATGTGTCATAAAATGCGTTGATGTTGCCTTTGGATTTCACTGACTTACTCCAATCGGT
AACAACCACTAGAACAATGATGTTCGAAAGAAGGAAATTTAATAGATGACACCCAAAAATTTAGTTTATAGTTGCATTGTXXXXXXXXXXC
ATGAATTCTACGTTTTCTTCAATGTGTCATAAAATGCGTTGATGTTGCCTTTGGATTTCACTGACTTACTCCAATCGGT
>Marker756318 Chr5 SNP_site:26852486 Ref_Type:T
ACAGGAGTTGATAGACTTCTTGCTTGCCATCAGTTTCAAATCCAGGAGCTCTAAGACCAATTTGGTGTAACCAGATGCTCXXXXXXXXXXA
CTTAATTTGCACAGTGACAACACTCAACTCGTGGCAAAGCTAAACATAGAAGGTCTCATCAAATTTAATCTAATCAAGT
ACAGGAGTTGATAGACTTCTTGCTTGCCATCAGTTTCAAATCTAGGAGCTCTAAGACCAATTTGGTGTAACCAGATGCTCXXXXXXXXXXA
CTTAATTTGCACAGTGACAACACTCAACTCGTGGCAAAGCTAAACATAGAAGGTCTCATCAAATTTAATCTAATCAAGT
>Marker756335 Chr5 SNP_site:23142524 Ref_Type:G
AACTGTTGTGTTGTTAATAATGATTGTTCTTTTTTTCTTTCTATATGAACGTAGATAAAGTGAGGGTTTGTGAATCAATTXXXXXXXXXXC
TCATTCTCACTTTCACTCTTTCACATTAATATTTAATTAATCTTTTCATAATTTCAATTATTAGTTATGGATATAAGTG
AACTGTTGTGTTGTTAATAATGATTGTTCTTTTTTTCTTTCTATATGAACGTAGATAAAGTGAGGGTTTGTGAATCAATTXXXXXXXXXXC
TCATTCTCACTTTCACTCTTTCACATTAATATTTAATTAATCTTTTCATAATTTCAATTATTAGTTATGGATATGAGTG
>Marker756701 Chr5 SNP_site:25525432 Ref_Type:G
GACCGATTCTGAGCTCCAAGCACGGCGAGAAAAGGGACTTTGCTATCGGTGTGACGAACCGTTCAGCAAGGGGCATCGCTXXXXXXXXXXT
AATGATGGGGAAAAACCTTCATCATTCAAGACTTCCCTTATACGCAACCTGTCAATTGAATGGCTGAAATTTCCTTGTA
GACCGGTTCTGAGCTCCAAGCACGGCGAGAAAAGGGACTTTGCTATCGGTGTGACGAACCGTTCAGCAAGGGGCATCGCTXXXXXXXXXXT
AATGATGGGGAAAAACCTTCATCATTCAAGACTTCCCTTATACGCAACCTGTCAATTGAATGGCTGAAATTTCCTTGTA
>Marker757606 Chr5 SNP_site:15687786 Ref_Type:A
ACTCTATTTATAATAGAAAGGCAAACTAGTCCTAATCCTAACTAATAAAAGAAACTAATTCTAATCCTAATTAATAAAAGXXXXXXXXXXT
AATACCCTATTCCTACTACATCATTCTATCCCTAAAAGAAAAAAAACTCATCCTCGAGTTTTAAAACGAAAATGAAGGT
ACTCTATTTATAATAGAAAGGCAAACTAGTCCTAATCCTAACTAATAAAAGAAACTAATTCTAATCCTAATTAATAAAAGXXXXXXXXXXT
AATACCCTATTCCTACTACATCATTCTATCCCTAAAAGAAAAAAAACTCGTCCTCAAGTTTTAAAACGAAAATGAAGGT

Create PDF with GO2PDF for free, if you wish to

>Marker757606 Chr5 SNP_site:15687792 Ref_Type:G

ACTCTATTTATAATAGAAAGGCAAACTAGTCCTAATCCTAACTAATAAAAGAAACTAATTCTAATCCTAATTAATAAAAGXXXXXXXXXXT
AATACCCTATTCCTACTACATCATTCTATCCCTAAAAGAAAAAAAACTCATCCTCGAGTTTTAAAACGAAAATGAAGGT
ACTCTATTTATAATAGAAAGGCAAACTAGTCCTAATCCTAACTAATAAAAGAAACTAATTCTAATCCTAATTAATAAAAGXXXXXXXXXXT
AATACCCTATTCCTACTACATCATTCTATCCCTAAAAGAAAAAAAACTCGTCCTCAAGTTTTAAAACGAAAATGAAGGT

>Marker757710 Chr5 SNP_site:13993866 Ref_Type:C

CACTGCTATCTCTTTTACGTTCATTCCTGGCCACTGCAGAATAAAATTCACAAGAGTTCCCATCTGAGCATACAACACCAXXXXXXXXXXC
GATCTATTAGTTCCTCCACCAATTTATAATTGTCTGTTGCAGCCATAGGTATGACCTGACAATAAAATAATGATATGTT
CACTGCTATCTCTTTTACGTTCATTCCTGGCCACTGCAGAATAAAATTCACAAGAGTTCCCATCTGAGCATACAACACCAXXXXXXXXXXC
GATCTATTAGTTCCTCCACCAATTTATAATTGTCTGTTGTAGCCATAGGTATGACCTGACAATAAAATAATGATATGTT

>Marker758125 Chr5 SNP_site:2477733 Ref_Type:C

ACCATCAACAAAATAACGGTAAAGTATCGAGATCTTATTCCTAGATTAGATGACACGTTGGATGAATTACATGGTGCAAAXXXXXXXXXXC
TTTTACGAGGGTAATGAATTATGTTTTGAGAGAATACATTGGAAAGTTTGTTATTGTATATTTTGATGATATTCTTGTT
ACCATCAACAAAATAACGGTAAAGTATCGAGATCTTATTCTTAGATTAGATGACACGTTGGATGAATTACATGGTGCAAAXXXXXXXXXXC
TTTTACGAGGGTAATGAATTATGTTTTGAGAGAATACATTGGAAAGTTTGTTATTGTATATTTTGATGATATTCTTGTT

>Marker758636 Chr5 SNP_site:9618987 Ref_Type:C

AACTTACAAGCGCAAATAATTACAGCAATTAATGAACAAATAATCAAATCCTCATATGAAGCGAATTCGCTGAGTTTCTAXXXXXXXXXXT
TCCATTGCGTAAAATGAAGATAGTGGAGAGGAATTGACGGTGACTTCTATATTGGGTTGCTGCCATTGTTGTTCTGGTG
AACTTACAAGCGCAAATAATTAGAGCAATTAATGAACAAATAATCAAATCCTCATATGAAGCGAATTCGATGAGTTTCTAXXXXXXXXXXT
TCCATTGCGTAAAATGAAGATAGTGGAGAGGAATTGACGGTGACTTCTATATTGGGTTGCTGCCATTGTTGTTCTGGTG

>Marker758636 Chr5 SNP_site:9619034 Ref_Type:C

AACTTACAAGCGCAAATAATTACAGCAATTAATGAACAAATAATCAAATCCTCATATGAAGCGAATTCGCTGAGTTTCTAXXXXXXXXXXT
TCCATTGCGTAAAATGAAGATAGTGGAGAGGAATTGACGGTGACTTCTATATTGGGTTGCTGCCATTGTTGTTCTGGTG
AACTTACAAGCGCAAATAATTAGAGCAATTAATGAACAAATAATCAAATCCTCATATGAAGCGAATTCGATGAGTTTCTAXXXXXXXXXXT
TCCATTGCGTAAAATGAAGATAGTGGAGAGGAATTGACGGTGACTTCTATATTGGGTTGCTGCCATTGTTGTTCTGGTG

>Marker758701 Chr5 SNP_site:26115970 Ref_Type:C

TACATAGATTATCACTTTAATGATTCTCCAATACCCCAATATGTTCTCCTTTTATTCGACCTAATCTCTACTTTTGTTTTXXXXXXXXXXT
AAACACAAAATCAAAACTATATACTAAATCATATTATGTGATGGAAACTTTATATTAAAGACATGAATCATATTATGTT
TACATAGATTATCACTTTAATGATTCTCCAATACCTCAATATGTTCTCCTTTTATTCGACCTAATCTCTACTTTTGTTTTXXXXXXXXXXT
AAACACAAAATCAAAACTATATACTAAATCATATTATGTGATGGAAACTTTATATTAAAGACATGAATCATATTATGTT

>Marker758825 Chr5 SNP_site:13849045 Ref_Type:C

AACATGAACACTTCAAATATAATAGATTAATCTAAGATCACTCATCGTAAATTTACAAGTTTTCCTCTCTTTTGTCTTTTXXXXXXXXXXG
ACACGACGATGAAGTATAACGTAATATGAACTCAGCAATTAGGTGTGTTTATATAGGAATATTTAACCAATTAAATGTT
AACATGAACACTTCAAATATAATAGATTAATCTAAGATCACTCATCGTAAATTTATAAGTTTTCCTCTCTTTTGTCTTTTXXXXXXXXXXG
ACACGACGATGAAGTATAACGTAATATGAACTCAGCAATTAGGTGTGTTTATATAGGAATATTTAACCAATTAAATGTT

>Marker758848 Chr5 SNP_site:9114310 Ref_Type:T

CACTTAGTTATTAGTTTTGGTTTAATCTTGATTCTCCACTTCAACTCTGATGTTTTTTCTTCACAACACATATCCCCTATXXXXXXXXXXT
AAAGCCTTCATTTTCGTTCATTTCAGATATCAAAAAACATTCTTGGTGTTGTTGTAATGCCCATGCCCAGGAGTCGAGT
CACTTAGTTATTAGTTTTGGTTTAATCTTGATTCTCCACTTCAACTCTGTTGTTTTTTCTTCACAACATATATCCCCTATXXXXXXXXXXT
AAAGCCTTCATTTTCGTTCATTTCAGATATCAAAAAACATTCTTGGTGTTGTTGTAATGCCCATGCCCAGGAGTCGAGT

>Marker758848 Chr5 SNP_site:9114329 Ref_Type:T

CACTTAGTTATTAGTTTTGGTTTAATCTTGATTCTCCACTTCAACTCTGATGTTTTTTCTTCACAACACATATCCCCTATXXXXXXXXXXT
AAAGCCTTCATTTTCGTTCATTTCAGATATCAAAAAACATTCTTGGTGTTGTTGTAATGCCCATGCCCAGGAGTCGAGT
CACTTAGTTATTAGTTTTGGTTTAATCTTGATTCTCCACTTCAACTCTGTTGTTTTTTCTTCACAACATATATCCCCTATXXXXXXXXXXT

Create PDF with GO2PDF for free, if you wish to

AAAGCCTTCATTTTCGTTCATTTCAGATATCAAAAAACATTCTTGGTGTTGTTGTAATGCCCATGCCCAGGAGTCGAGT

>Marker75987 Chr4 SNP_site:7697719 Ref_Type:C

CACGTTCAACCAAAGCCTTCTCATTTTCCTTTACCTAAAATTTTCAAATCATAAGAACATATTACATTCTTAGAGGCTATXXXXXXXXXXA

CACATAATGGAAGAAAGAAATCATAGTCCTATATGCATAAATAATGATACCAAAACATTAATATTAAAACTAAGTTGTT

CACGTTCAACCAAAGCCTTCTCATTTTCCTTTACCTAAAATTTTCAAATCATAAGAACATATTACATTCTTAGAGGCTATXXXXXXXXXXA

CACATAATGGAAGAAAGAAATTATAGTCCTATATGCATAAATAATGATACCAAAACATTTATATTAAAACTAAGTTGTT

>Marker75987 Chr4 SNP_site:7697757 Ref_Type:A

CACGTTCAACCAAAGCCTTCTCATTTTCCTTTACCTAAAATTTTCAAATCATAAGAACATATTACATTCTTAGAGGCTATXXXXXXXXXXA

CACATAATGGAAGAAAGAAATCATAGTCCTATATGCATAAATAATGATACCAAAACATTAATATTAAAACTAAGTTGTT

CACGTTCAACCAAAGCCTTCTCATTTTCCTTTACCTAAAATTTTCAAATCATAAGAACATATTACATTCTTAGAGGCTATXXXXXXXXXXA

CACATAATGGAAGAAAGAAATTATAGTCCTATATGCATAAATAATGATACCAAAACATTTATATTAAAACTAAGTTGTT

>Marker760218 Chr5 SNP_site:23402124 Ref_Type:C

TACATTTTTACGAGGCATTAAGAGAGCAAATTAGAGTGACATGACATGTCACCCTCTCTATTTCCTACCATCATATTTAGXXXXXXXXXXT

TTTTCCTAATACTTATAAAATAAACAAACTTGAGTCGTTTTTATAACCCTAATCAAATAGGATCTATGAGAGGGCCGTG

TACATTTTTATGAGGCATTAAGAGAGCAAATTAGAGTGACATGACATGTCACCCTCTCTATTTCCTACCATCATATTTAGXXXXXXXXXXT

TTTTCCTAATACTTATAAAATAAACAAACTTGAGTCGTTTTTATAACCCTAATCAAATAGGATCTATGAGAGGGCCGTG

>Marker760363 Chr5 SNP_site:14687856 Ref_Type:G

AACGTGTTGTGGGAGTCTCAACCATAGTTATTGAAAGTTAAAAAGGTCACTTACTTTCTTTAGTTAGATTATTGCCTCACXXXXXXXXXXA

TGATAGTCATTCCCAATAATCGCCCACGAATGACTACCATTGCATTCATGTGGAATTGTTAGATAATGATTATCATTGT

AACGTGTTGTGGGAGTCTCAACCATAGTTATTGAAAGTTAAAAAGGTCACTTACTTTCTTTAGTTAGATTATTGCCTCACXXXXXXXXXXA

TGATAGTCATTCCCAATAATCGCCCACTAATGACTACCATTGCATTAATGTGGAATTGTTAGATAATGATTATCATTGT

>Marker760363 Chr5 SNP_site:14687875 Ref_Type:C

AACGTGTTGTGGGAGTCTCAACCATAGTTATTGAAAGTTAAAAAGGTCACTTACTTTCTTTAGTTAGATTATTGCCTCACXXXXXXXXXXA

TGATAGTCATTCCCAATAATCGCCCACGAATGACTACCATTGCATTCATGTGGAATTGTTAGATAATGATTATCATTGT

AACGTGTTGTGGGAGTCTCAACCATAGTTATTGAAAGTTAAAAAGGTCACTTACTTTCTTTAGTTAGATTATTGCCTCACXXXXXXXXXXA

TGATAGTCATTCCCAATAATCGCCCACTAATGACTACCATTGCATTAATGTGGAATTGTTAGATAATGATTATCATTGT

>Marker760538 Chr5 SNP_site:23363970 Ref_Type:T

ACTTCTGCTTTTATACTCTACTTCTCTATTACTTTTCTTGTATCAGCAACAAGATGACTCATGTTTTTATATATTTTCTTXXXXXXXXXXA

TGAGCAACAAGTGTTTGCATGGATTGTCATTGTGTTTAAGAATGTAAAGTCTCTTTACTAACCATACCAAACATTTGTT

ACTTCTGCTTTTATACTTTACTTCTCTATTACTTTTCTTGTATCAGCAACAAGATGACTCATGTTTTTATATATTTTCTTXXXXXXXXXXA

TGAGCAACAAGTGTTTGCATGGATTGTCATTGTGTTTAAGAATCTAAAGTCTCTTTACTAACCATACCAAACATTTGTT

>Marker760538 Chr5 SNP_site:23364157 Ref_Type:C

ACTTCTGCTTTTATACTCTACTTCTCTATTACTTTTCTTGTATCAGCAACAAGATGACTCATGTTTTTATATATTTTCTTXXXXXXXXXXA

TGAGCAACAAGTGTTTGCATGGATTGTCATTGTGTTTAAGAATGTAAAGTCTCTTTACTAACCATACCAAACATTTGTT

ACTTCTGCTTTTATACTTTACTTCTCTATTACTTTTCTTGTATCAGCAACAAGATGACTCATGTTTTTATATATTTTCTTXXXXXXXXXXA

TGAGCAACAAGTGTTTGCATGGATTGTCATTGTGTTTAAGAATCTAAAGTCTCTTTACTAACCATACCAAACATTTGTT

>Marker76099 Chr4 SNP_site:4631969 Ref_Type:A

ACAAATTGGTGTGTGGATACTAATAATAGGGTTACGAATTTGATGTAATTGGAGTTGGTATGAAGCAGATTAAAGAAGAGXXXXXXXXXXT

TGAATGGATTGAATTACCCTATCTCTCCTTTGCCTTCTTTTGGAAGCTTTTGAATTTTGTTTCTTACTCAATTGGCGTA

ACAAATTGGTGTGTGGATACTAATAATAGGGTTACGAATTTGATGTAATTGGAGTTGGTATGGAGCAGATTAAAGAAGAGXXXXXXXXXXT

TGAATGGATTGAATTACCCTATCTCTCCTTTGCCTTCTTTTGGAAGCTTTTGAATTTTGTTTTTTACTCAATTGGCGTA

>Marker76099 Chr4 SNP_site:4632180 Ref_Type:C

ACAAATTGGTGTGTGGATACTAATAATAGGGTTACGAATTTGATGTAATTGGAGTTGGTATGAAGCAGATTAAAGAAGAGXXXXXXXXXXT

TGAATGGATTGAATTACCCTATCTCTCCTTTGCCTTCTTTTGGAAGCTTTTGAATTTTGTTTCTTACTCAATTGGCGTA

Create PDF with GO2PDF for free, if you wish to

ACAAATTGGTGTGTGGATACTAATAATAGGGTTACGAATTTGATGTAATTGGAGTTGGTATGGAGCAGATTAAAGAAGAGXXXXXXXXXXT
TGAATGGATTGAATTACCCTATCTCTCCTTTGCCTTCTTTTGGAAGCTTTTGAATTTTGTTTTTTACTCAATTGGCGTA
>Marker761041 Chr5 SNP_site:2545256 Ref_Type:G
CACATCATATGTGCTAGTTTCTAGTGGAGAACAAATATTTATGGGTAACTGCTCTACTTCAAAGGTTGAGGGACAATGCAXXXXXXXXXXA
TTTGATAAGTTTGTATTTTCCAAGAATGAGATTACATTGGAAATGGTTATTTGAGTGATGATCTCTTTAAATTAAATGT
CACATCATATGTGCTAGTTTCTAGTGGAGAACAAATATTTATGGGTAACTGCTCTACTTCAAAGGTTGAGGGACAATGCAXXXXXXXXXXA
TTTGATAAGTTTGTATTTTCCAAGAATGAGATTACATTGGAAATTGTTATTTGAGTGATGATCTCTTTAAATTAAATGT
>Marker761075 Chr5 SNP_site:9466774 Ref_Type:G
CACAAGCTTGATCTATTTTGTTCAATAAGGATGCCTCCTGGTAGTTAATTAAAGGCAAGAAAAATCCTATTTTCAATATCXXXXXXXXXXG
AAACCAAAGAGATGAAATGTGTTGCTGAGTAAAATTTAGATCATAACGTTCAGTGAGTGACTTAGTTATATCATGGAGT
CACAAGCTTGATCTGTTTTGTTCAATAAGGATGCCTCCTGGTAGTTAATTAAAGGCAAGAAAAATCCTATTTTCAATATCXXXXXXXXXXG
AAACCAAAGAGATGAAATGTGTTGCTGAGTAAAATTTAGATCATAACGTTCAGTGAGTGACTTAGTTATATCATGGAGT
>Marker761377 Chr5 SNP_site:11596102 Ref_Type:C
AACACATTGCAACTTTGATGTAGGGATCAATAGAAGAAAATCATTTACCTTAACTTTACTTAAACTTCTTTACCAAATCGXXXXXXXXXXC
TTACCCACAAAGAAGGAGAATCGAACCCAAACCAAACCTATTGAAAAATTCAAGGATTTGATCGTTATCCTTAAAAAGT
AACACATTGCAACTTTGATGTAGGGATCAATAGAAGAAAATCCTTTACCTTAACTTTACTTAAACTTCTTTACCAAACCGXXXXXXXXXXC
TTACCCACAAAGAAGGAGAATCGAACCCAAACCAAACCTATTGAAAAATTCAAGGATTTGATCGTTATCCTTCAAAAGT
>Marker761377 Chr5 SNP_site:11596137 Ref_Type:C
AACACATTGCAACTTTGATGTAGGGATCAATAGAAGAAAATCATTTACCTTAACTTTACTTAAACTTCTTTACCAAATCGXXXXXXXXXXC
TTACCCACAAAGAAGGAGAATCGAACCCAAACCAAACCTATTGAAAAATTCAAGGATTTGATCGTTATCCTTAAAAAGT
AACACATTGCAACTTTGATGTAGGGATCAATAGAAGAAAATCCTTTACCTTAACTTTACTTAAACTTCTTTACCAAACCGXXXXXXXXXXC
TTACCCACAAAGAAGGAGAATCGAACCCAAACCAAACCTATTGAAAAATTCAAGGATTTGATCGTTATCCTTCAAAAGT
>Marker761377 Chr5 SNP_site:11596308 Ref_Type:C
AACACATTGCAACTTTGATGTAGGGATCAATAGAAGAAAATCATTTACCTTAACTTTACTTAAACTTCTTTACCAAATCGXXXXXXXXXXC
TTACCCACAAAGAAGGAGAATCGAACCCAAACCAAACCTATTGAAAAATTCAAGGATTTGATCGTTATCCTTAAAAAGT
AACACATTGCAACTTTGATGTAGGGATCAATAGAAGAAAATCCTTTACCTTAACTTTACTTAAACTTCTTTACCAAACCGXXXXXXXXXXC
TTACCCACAAAGAAGGAGAATCGAACCCAAACCAAACCTATTGAAAAATTCAAGGATTTGATCGTTATCCTTCAAAAGT
>Marker76149 Chr4 SNP_site:11702204 Ref_Type:C
ACTTTTGATGATCTAATCTCTAGAACTGAGGTGGTGATGATGACTTGCTCGTGTATTTGCCTCAATGGAATCTGATTAATXXXXXXXXXXA
AGTTAGCGTGTTGCAAAGTGTAGCCAATAAGCAAGTCGATAGAATCTTCTCCTTTGACAAGGTAAGTTTCTGAAACGTT
ACTTTTGATGATCTAATGTCTAGAACTGAGGTGGTGATGATGACTTGCTCGTGTATTTGCCTCAATGGAATCTGATTAATXXXXXXXXXXA
AGTTAGCGTGTTGCAAAGTGTAGCCAATAAGCAAGTCGATAGAATCTTCTCCTTTGACAAGGTAAGTTTCTGAAACGTT
>Marker76165 Chr4 SNP_site:19291178 Ref_Type:G
ACGTATATTGTGCTCACAAATTGATTTCACAAATAAATTATAAATTTTGTGTTCGAAAGATGGCAAAAGAGCAGGAAAAGXXXXXXXXXXT
TCATCGAGATCAAACAACTAAACTTCGTGATGTTGTAAATGATTCACATGTCTTTAATATATGCACTGATAATGAAGGT
ACGTATATTGTGCTCACAAATTGATTTCACAAATAAGTTATAAATTTTGTGTTCAAAAGATGGCAAAAGAGCAGGAAAAGXXXXXXXXXXT
TCATCGAGATCAAACAACTAAACTTCGTGATGTTGTAAATGATTCACATGTCTTTAATATATGCACTGATAATGAAGGT
>Marker76165 Chr4 SNP_site:19291196 Ref_Type:A
ACGTATATTGTGCTCACAAATTGATTTCACAAATAAATTATAAATTTTGTGTTCGAAAGATGGCAAAAGAGCAGGAAAAGXXXXXXXXXXT
TCATCGAGATCAAACAACTAAACTTCGTGATGTTGTAAATGATTCACATGTCTTTAATATATGCACTGATAATGAAGGT
ACGTATATTGTGCTCACAAATTGATTTCACAAATAAGTTATAAATTTTGTGTTCAAAAGATGGCAAAAGAGCAGGAAAAGXXXXXXXXXXT
TCATCGAGATCAAACAACTAAACTTCGTGATGTTGTAAATGATTCACATGTCTTTAATATATGCACTGATAATGAAGGT
>Marker762427 Chr5 SNP_site:17435723 Ref_Type:T
ACTGTTATAATATATGAGTGCAACGTAAGATGTTATCAAGTATTACAATAATTAAAACACTGTCATAATTAATATACGAGXXXXXXXXXXT

Create PDF with GO2PDF for free, if you wish to

ATGTAAAAAAGTAATACTACGAATAATTATATAGGAATTGCCAGAGAGTCAGAGAGCGGACGCTGCAAGTAGTTTGGTA
ACTGTTATAATATATGAGTGCAACGTAAGATGTTATCAAGTATTACAATAATTAAAACACTGTCATAATTAATATATGATXXXXXXXXXXT
ATGTAAAAAAGTAATACTACGAATAATTATATAGGAATTGCCAGAGGGTCAGAGAGCGGACGCTGCAAGTAGTTTGGTA
>Marker762427 Chr5 SNP_site:17435726 Ref_Type:T
ACTGTTATAATATATGAGTGCAACGTAAGATGTTATCAAGTATTACAATAATTAAAACACTGTCATAATTAATATACGAGXXXXXXXXXXT
ATGTAAAAAAGTAATACTACGAATAATTATATAGGAATTGCCAGAGAGTCAGAGAGCGGACGCTGCAAGTAGTTTGGTA
ACTGTTATAATATATGAGTGCAACGTAAGATGTTATCAAGTATTACAATAATTAAAACACTGTCATAATTAATATATGATXXXXXXXXXXT
ATGTAAAAAAGTAATACTACGAATAATTATATAGGAATTGCCAGAGGGTCAGAGAGCGGACGCTGCAAGTAGTTTGGTA
>Marker762427 Chr5 SNP_site:17435929 Ref_Type:G
ACTGTTATAATATATGAGTGCAACGTAAGATGTTATCAAGTATTACAATAATTAAAACACTGTCATAATTAATATACGAGXXXXXXXXXXT
ATGTAAAAAAGTAATACTACGAATAATTATATAGGAATTGCCAGAGAGTCAGAGAGCGGACGCTGCAAGTAGTTTGGTA
ACTGTTATAATATATGAGTGCAACGTAAGATGTTATCAAGTATTACAATAATTAAAACACTGTCATAATTAATATATGATXXXXXXXXXXT
ATGTAAAAAAGTAATACTACGAATAATTATATAGGAATTGCCAGAGGGTCAGAGAGCGGACGCTGCAAGTAGTTTGGTA
>Marker76253 Chr4 SNP_site:6441036 Ref_Type:T
ACAAAACTCTTTGGAAGAGATTGTAAAATAAAGCTCACTTGATGTGCCTCATCAATGGGATCGCCATTTACTTCAACGATXXXXXXXXXXA
ATGAATCCATAATCTCTTTAGTCGTGGCTAAGGGTTCATGTTTCTTTGTGAAAACAACAGACAGGCTGGCAAGAATGTA
ACAAAACTCTTTGGAAGAGATTGTAAAATAAAGCTCACTTGATGTGCCTCATCAATGGGATCGCCATTTACTTCAACGATXXXXXXXXXXA
ATGAATCCATAATCTCTTTAGTCGTGGCTAAGGGTTCATGTTTCTTTGTGAAAACAACAGACATGCTGGCAAGAATGTA
>Marker762692 Chr5 SNP_site:23401761 Ref_Type:C
ACAAATGTTATGATGCAATGCCTTGTTATCACATGAGGTTTGATTTAGCCAAGATTTAAAGTCTGTCCATAAGTCTAAATXXXXXXXXXXT
AATTTTATTCAATAACCCTAAGTGATCGAGAGAAGAAAACTCAAAAGAGAACTCTAACTTAAATCTCTATGGTTTGGTT
ACAAATGTTATGATGCAATGCCTTGTTATCACATGAGGTTTGATTTAGCCAAGATTTAAAGTCTGTCCATAAGTCTAAATXXXXXXXXXXT
AATTTTATTCAATAACTCTAAGTGATCGAGAGAAGAAAACTCAAAAGAGAACTCTAACTTAAATCTCTATGGTTTGGTA
>Marker762692 Chr5 SNP_site:23401823 Ref_Type:T
ACAAATGTTATGATGCAATGCCTTGTTATCACATGAGGTTTGATTTAGCCAAGATTTAAAGTCTGTCCATAAGTCTAAATXXXXXXXXXXT
AATTTTATTCAATAACCCTAAGTGATCGAGAGAAGAAAACTCAAAAGAGAACTCTAACTTAAATCTCTATGGTTTGGTT
ACAAATGTTATGATGCAATGCCTTGTTATCACATGAGGTTTGATTTAGCCAAGATTTAAAGTCTGTCCATAAGTCTAAATXXXXXXXXXXT
AATTTTATTCAATAACTCTAAGTGATCGAGAGAAGAAAACTCAAAAGAGAACTCTAACTTAAATCTCTATGGTTTGGTA
>Marker76385 Chr4 SNP_site:13833297 Ref_Type:T
AACACAAACCAAGAGGCCGGTTGCCTATTTCAGCATAACACTGTGTATGAGGGATAGGGCACGACCTGTCTATGAGAGAGXXXXXXXXXXT
TTTGGGCTATTCTTTTGAGGTGGTGACCAGTTTGGAAAACAAGGCAGCCGATGCTTTGTCTAGAATGCCTCCTACAGTG
AACACAAACCAAGAGGCCGGTTGCCTATTTCAGCATAACACTGTGTATGAGGGATAGGGCACGACCTGTCTATGAGAGAGXXXXXXXXXXT
TTTGGGCTATTCTTTTGAGGTGGTGACCAGTTTGGAAAACAAGGCAGCTGATGCTTTGTCTAGAATGCCTCCTACAGTG
>Marker764144 Chr5 SNP_site:27742036 Ref_Type:A
AACAAAATGATAGAACAAGGGCTTTTTTTTCAACTAATAAACCAGCATGTAGAAAATAACATTCACTACTTGGCTAATACXXXXXXXXXXT
GTATCAACTCTTTGCTCCAATATGTTTTGAATTCCATATTGCGAAAACAACATTCTTTTGAACAAGTGGTTTTGAAGTC
AACAAAATGATAGAACAAGGGCTTTTTTTTCAACTAATAAACCAGCATGTAGAAAATAACATTCACTACTTGGCTAATACXXXXXXXXXXT
GTATCAACTCTTTGCTCCAATATGTTTTGAATTCCGTATTGCGAAAACAACATTCTTTTGAACAAGTGGTTTTGAAGTC
>Marker76443 Chr4 SNP_site:4013313 Ref_Type:C
AACTGTTGCTTCTTTCTAGATCCTCCTTCCCCATATAACTCATTCCATTGCCTCAATTCACTCATCATCGCTCCTTCTGCXXXXXXXXXXG
GGCAACTTGTGCAACTAGAATCATGTCGTTGGTTTGTTTTCTTTTTGCTGTCTTTTGTTTGAACAAATATTGGAACAGT
AACTGTTGCTTCTTTCTAGATCCTCCTTCCCCATATAACTCATTCCATTGCCTCAATTCACTCATCATTGCTCCTTCTGCXXXXXXXXXXG
GGCAACTTGTGCAACTAGAATCATGTCGTTGGTTTGTTTTCTTTTTGCTGTCTTTTGTTTGAACAAATATTGGAACAGT
>Marker764496 Chr5 SNP_site:23721692 Ref_Type:C

Create PDF with GO2PDF for free, if you wish to

AACAAGCTGCTCGCCTAGTCCAAGTAGATATTCAAAGAAGAGAAAGAATCAAGGAGTTAATAATAAATGGAAGTGATCTAXXXXXXXXXXA
GCTGGTGGAGAAGATGGAACCAGCATGAACAGAGATGCCCTCACTGGGAGCGAGATCTACTTGACATACAACACAAGTG
AACAAGCTGCTCGCCTCGTCCAAGTAGATATTCAAAGAAGAGAAAGAATCAAGGAGTTAATAATAAATGGAAGTGATCTAXXXXXXXXXXA
GCTGGTGGAGAAGATGGAACCAGCATGAACAGAGATGCCCTCACTGGGAGCAAGATCTACTTGACATACAACACAAGTG
>Marker764496 Chr5 SNP_site:23721900 Ref_Type:A
AACAAGCTGCTCGCCTAGTCCAAGTAGATATTCAAAGAAGAGAAAGAATCAAGGAGTTAATAATAAATGGAAGTGATCTAXXXXXXXXXXA
GCTGGTGGAGAAGATGGAACCAGCATGAACAGAGATGCCCTCACTGGGAGCGAGATCTACTTGACATACAACACAAGTG
AACAAGCTGCTCGCCTCGTCCAAGTAGATATTCAAAGAAGAGAAAGAATCAAGGAGTTAATAATAAATGGAAGTGATCTAXXXXXXXXXXA
GCTGGTGGAGAAGATGGAACCAGCATGAACAGAGATGCCCTCACTGGGAGCAAGATCTACTTGACATACAACACAAGTG
>Marker765199 Chr5 SNP_site:15695559 Ref_Type:A
GACTTTTTCACAATTTCACGACCATTTATAATGTTTGGTGTAGAATGAATTTGTTGAGCAAATGGTCATTTTCATATTTGXXXXXXXXXXG
TAACTAATAGCCTTTTCTTAAATATCTTCTTCTCGCATAATACTCTTTCGATGGATAAATTTTCTTGAATAACAATGTA
GACTTTTTCACAATTTCACGACCATTTCTAATGTTTGGTGTAGAATGAATTTGTTGAGCAAATGGTCATTTTCATATTTGXXXXXXXXXXG
TAACTAATAGCCTTTTCTTAAATATCTTCTTCTCGCATAATACTCTTTCGATGGATAAATTTTCTTGAATAACAATGTA
>Marker765311 Chr5 SNP_site:14527021 Ref_Type:A
TACTTCCAACACAGACAAAGCTCAGACACGTGAAAACTGAAGCCAATCTTATAAAATTTTTGGAAAACAAATGGCACAAGXXXXXXXXXXA
CGTACGTAAGGTCTTTAGTTAAAAGTTAAGCTGCAATTCAATCAGAAAATAAATATCCACAAACCCTTTAATTGATGTG
TACTTCCGACACAGACAAAGCTCAGACACGTGAAAACTGAAGCCAATCTTATAAAATTTTTGGAAAACAAATGGCACAAGXXXXXXXXXXA
CGTACGTAAGGTCTTTAGTTAAAAGTTAAGCTGCAATTCAATCAGAAAATAAATATCCACAAACCCTTTAGTTGATGTG
>Marker765311 Chr5 SNP_site:14527312 Ref_Type:A
TACTTCCAACACAGACAAAGCTCAGACACGTGAAAACTGAAGCCAATCTTATAAAATTTTTGGAAAACAAATGGCACAAGXXXXXXXXXXA
CGTACGTAAGGTCTTTAGTTAAAAGTTAAGCTGCAATTCAATCAGAAAATAAATATCCACAAACCCTTTAATTGATGTG
TACTTCCGACACAGACAAAGCTCAGACACGTGAAAACTGAAGCCAATCTTATAAAATTTTTGGAAAACAAATGGCACAAGXXXXXXXXXXA
CGTACGTAAGGTCTTTAGTTAAAAGTTAAGCTGCAATTCAATCAGAAAATAAATATCCACAAACCCTTTAGTTGATGTG
>Marker765463 Chr5 SNP_site:24516364 Ref_Type:T
CACAAATAACACCGTCACAGCGCCTACGTGTCAGTAACAGATTTTTTTCATTATAATTAGATCAAACCATGCCACCAAAAXXXXXXXXXXA
TTTCATTTTTAATTAAAAATGATATGCAATACACAAGACAAATTAATATATCATTTGGTGATATGGCCAAAATTTTAGT
CACAAATAACACCGTCACAGCGCCTACGTGTCAGTAACAGATTTTTTTCATTATAATTAGATCAAACCATGCCACCAAAAXXXXXXXXXXA
TTTCATTTTTAATTAAAAATGATATGCAATACACAAGATAAATTAATATATCATTTGATGATATGGCCAAAATTTTAGT
>Marker765463 Chr5 SNP_site:24516383 Ref_Type:A
CACAAATAACACCGTCACAGCGCCTACGTGTCAGTAACAGATTTTTTTCATTATAATTAGATCAAACCATGCCACCAAAAXXXXXXXXXXA
TTTCATTTTTAATTAAAAATGATATGCAATACACAAGACAAATTAATATATCATTTGGTGATATGGCCAAAATTTTAGT
CACAAATAACACCGTCACAGCGCCTACGTGTCAGTAACAGATTTTTTTCATTATAATTAGATCAAACCATGCCACCAAAAXXXXXXXXXXA
TTTCATTTTTAATTAAAAATGATATGCAATACACAAGATAAATTAATATATCATTTGATGATATGGCCAAAATTTTAGT
>Marker766095 Chr5 SNP_site:25461233 Ref_Type:A
ACAAAGAAATGAACACAAAATCCCAGAAAGGAACCTGAAAATCGTAGAACTTAGCAAACCGAGGCTTGCCCTCAGTATTCXXXXXXXXXXT
GCATAAAACATACCAAATACAAACTATAACAACTGCACGGTTCAGTGGAAAAAAATTCTTACAAATGATTTTTAAGGTA
ACAAAGAAATGAACACAAAATCCCAGAAAGGAACCTGAAAATCGTAGAACTTAGCAAACCGAGGCTTGCCCTCAGTATTCXXXXXXXXXXT
GCATAAAACATACCAAATACAAACTATAACAACTGCACGGTTCAGTGGAAAAAAATTCTTACAGATGATTTTTAAGGTA
>Marker766665 Chr5 SNP_site:15779622 Ref_Type:G
GACCTTTAAGCCTGGATTTTTATTGGACGTTTGTTTTTTTTCTTCAGTTTCCTTCAAAGAATATTGGTTTTGCAGAAAGAXXXXXXXXXXG
CTTTTGACGTCTTTGCTTGTTTTGGTTCTATTATATGTGTGTCACTCTATTATGGGTTCTAGCAAATTTGTTTCTTTGT
GACCTTTAAGCCTGGATTTTTATTGGACGTTTGTTTTTTTTCTTCAGTTTCCTTCAAAGAATATTGGTTTTGCAGAAAGAXXXXXXXXXXG
CTTTTGAGGTCTTTGCTTGTTTTGGTTCTATTATATGTGTGTCACTCTATTATGGGTTCTAGCAAATTTGTTTCTTTGT

Create PDF with GO2PDF for free, if you wish to

>Marker766681 Chr5 SNP_site:2477289 Ref_Type:G
TACTTTGCTTCCATTGTCTCTATTTGAAGTCCAATGTGATCAAATGAAATTAGAAAAGAAAAGAATTTGAAGAACAAGAGXXXXXXXXXXG
AAAAAAGAAAGAATGTGAGTTTGGTTATAAGAAAGGGGAAATTGAAAGACTTGTTTTCAAATGAAGCACCAAACTTTGT
TACTTTGCTTCCATTGTCTCTATTTGAAGTCCAATGTGATCAAATGAAATTAGAAAAGAAAGGAATTTGAAGAACAAGAGXXXXXXXXXXG
AAAAAAGAAAGAATGTGAGTTTGGTTATAAGAAAGGGGAAATTGAAAGACTTATTTTCAAATGAAGCACCAAACTTTGT
>Marker766681 Chr5 SNP_site:2477467 Ref_Type:A
TACTTTGCTTCCATTGTCTCTATTTGAAGTCCAATGTGATCAAATGAAATTAGAAAAGAAAAGAATTTGAAGAACAAGAGXXXXXXXXXXG
AAAAAAGAAAGAATGTGAGTTTGGTTATAAGAAAGGGGAAATTGAAAGACTTGTTTTCAAATGAAGCACCAAACTTTGT
TACTTTGCTTCCATTGTCTCTATTTGAAGTCCAATGTGATCAAATGAAATTAGAAAAGAAAGGAATTTGAAGAACAAGAGXXXXXXXXXXG
AAAAAAGAAAGAATGTGAGTTTGGTTATAAGAAAGGGGAAATTGAAAGACTTATTTTCAAATGAAGCACCAAACTTTGT
>Marker766791 Chr5 SNP_site:23153005 Ref_Type:G
CACAGTGAAAAATAGTCCTTTTATTGATTTGACATTTTGATTTCAAACGACAAAAATTGAATAGGCTGGAACATAGGAGCXXXXXXXXXXC
ATCTAGTAGGTTCTCTCTATGGATGTCAAGGGGTGAAACCTGTTGAATAGCACCAGGTAATTAAGTAATTTAGTAATGT
CACAGTGAAAAATAGTCCTTTTATTGATTTGACATTTTGATTTCAAACGACAAAAATTGAATAGGCTGGAACATAGGAGCXXXXXXXXXXC
ATCTAGTAGGTTCTCTCTGTGGATGTCAAGGGGTGAAACCTGTTGAATAGCACCAGGTAATTAAGTAATTTAGTAATGT
>Marker766822 Chr5 SNP_site:2163237 Ref_Type:A
ACTCGTTATGTGAAGCATGGTGTTCCATATTTTAAATATTAGGTCAGGAAATATTTTTCTTTTTTCTTTATTGAGGTTGTXXXXXXXXXXT
TATTTATGTGTTCTACCTCAGTGATTCGATAGGAATGTAAATTACCACTACTTAACTAACATGCAACATACATGTATGT
ACTCGTTATGTGAAGCATGGTGTTCCATCTTTTAAATATTAGGTCAGGAAATATTTTTCTTTTTTCTTTATTGAGGTTGTXXXXXXXXXXT
TATTTATGTGTTCTACCTCAGTGATTCGATAGGAATGTAAATTACCACTACTTAACTAACATGCAACATACATGTATGT
>Marker766863 Chr5 SNP_site:23345224 Ref_Type:T
CACTCAATACCTTCAACTTCTCATTTTTCCCCTTTCTAACAGCATTAAGATCATGGGAAGCCAAAGATATTGGAATGGCTXXXXXXXXXXT
ACAATTTGTTTGGTATCTCTATTAAATGATTGTTCTTGACAACCGTTGAGTGTATTGTCAAGATATATTTGTTTTTGTT
CACTCAATACCTTCAACTTCTCATTTTTCCCCTTTCTAACAGCATTAAGATCATGGGAAGCCAAAGATATTGGAATGGCTXXXXXXXXXXT
ACAATTTGTTTGGTATCTCTATTAAATGATTGTTCTTGACAACTGTTGAGTGTATTGTCAAGATATATTTGTTTTTGTT
>Marker767366 Chr5 SNP_site:14900754 Ref_Type:C
CACGCAAAGCTTTTTCCATTGTCATTCATTATAATTCACAAACATATCATCGTTACCGTTTTTTGTCCTATTTTCCAGTTXXXXXXXXXXG
ACGAGTGAAACCCGACTCTTCATCTTCTGATGAAGCTATGTCCCCTACATCTGTTCCCAACATTGTTGGGACAATTGGT
CACGCAAAGCTTTTTCCATTGTCATTCATTATAATTCACAAACATATCATCGTTACCGTTTTTTGTCCTATTTTCCAGTTXXXXXXXXXXG
ACGAGTGAAACCCGAGTCTTCATCTTCTGATGAAGCTATGTCCCCTACATCTGTTCCCAACATTGTTGGGACAATTGGT
>Marker767975 Chr5 SNP_site:363597 Ref_Type:C
ACTATGTCATGGAAGTCAGAAACTGCAGCATTTTTCTCATAGGAGCTGGAAACTCACGTTTAGGAATCGATGAGTAGAATXXXXXXXXXXA
TTCAAATTGTTGAAAACAAATGTGTTCTGCTTCAAACCAAAATTCGACACGACGTGGGTGCTAAATTGCTTCCAAGGTT
ACTATGTCATGGAAGTCAGAAACTGCAGCATTTTTCTCATAGGAGCTGGAAACTCACGTTTAGGAATCGATGAGTAGAATXXXXXXXXXXA
TTCAAATTGTTGAAAACAAATGTGTTCTGCTTCAAAGCAAAATTCGACAGGACGTGGGTGCTAAATTGCTTCCAAGGTT
>Marker767975 Chr5 SNP_site:363610 Ref_Type:C
ACTATGTCATGGAAGTCAGAAACTGCAGCATTTTTCTCATAGGAGCTGGAAACTCACGTTTAGGAATCGATGAGTAGAATXXXXXXXXXXA
TTCAAATTGTTGAAAACAAATGTGTTCTGCTTCAAACCAAAATTCGACACGACGTGGGTGCTAAATTGCTTCCAAGGTT
ACTATGTCATGGAAGTCAGAAACTGCAGCATTTTTCTCATAGGAGCTGGAAACTCACGTTTAGGAATCGATGAGTAGAATXXXXXXXXXXA
TTCAAATTGTTGAAAACAAATGTGTTCTGCTTCAAAGCAAAATTCGACAGGACGTGGGTGCTAAATTGCTTCCAAGGTT
>Marker768027 Chr5 SNP_site:14900295 Ref_Type:C
TACCGAGTAATGAGTTTTCTTACTTCGCATCTCTATTTCTCCACCACATTTATTTTCTCATGGTTGATTCAAGCATTAGGXXXXXXXXXXT
ATCTCTAGACATAACACAGCTAACTTGCATGCCTAATTGTGTTTCAACGTGCCAATGATGGTTCCCTTTAGAAACAGTC
TACCGAGTAATGAGTTTTCTTACTTCGCATCTCTATTTCTCCACCACATTTATTTTCTCATGGTTGATTCAAGTATTAGGXXXXXXXXXXT

Create PDF with GO2PDF for free, if you wish to

ATCTCTAGACATAACACAGCTAACTTGCATGCCTAATTGTGTTTCAACGTGCCAATGATGGTTCCCTTTAGAAACAGTC
>Marker770162 Chr5 SNP_site:182182 Ref_Type:T
AACGAATATTGATTAATCAGTTAATCTCGGATCGTTGAAGTCCAAGGTCTATAGATCTAGTAGGTTCCCTTGTTAGCTCAXXXXXXXXXXT
ATTTAGGATCTCAAAATTGATGGAAATAATGGTCAAAGTTATAAAAAGCCAAAATAGTGATTTCCGACTTTGAAAAGTC
AACGAATATTGATTAATCAGTTAATCTCGGATCGTTGAAGTCCATGGTTTATAGATCTAGTAGGTTCCCTTGTTAGCTCAXXXXXXXXXXT
ATTTAGGATCTCGAAATTGATGGAAATAATGGTCAAAGTTATAAAAAGCCAAAATAGTGATTTCCGACTTTGAAAAGTC
>Marker770162 Chr5 SNP_site:182186 Ref_Type:T
AACGAATATTGATTAATCAGTTAATCTCGGATCGTTGAAGTCCAAGGTCTATAGATCTAGTAGGTTCCCTTGTTAGCTCAXXXXXXXXXXT
ATTTAGGATCTCAAAATTGATGGAAATAATGGTCAAAGTTATAAAAAGCCAAAATAGTGATTTCCGACTTTGAAAAGTC
AACGAATATTGATTAATCAGTTAATCTCGGATCGTTGAAGTCCATGGTTTATAGATCTAGTAGGTTCCCTTGTTAGCTCAXXXXXXXXXXT
ATTTAGGATCTCGAAATTGATGGAAATAATGGTCAAAGTTATAAAAAGCCAAAATAGTGATTTCCGACTTTGAAAAGTC
>Marker770162 Chr5 SNP_site:182386 Ref_Type:G
AACGAATATTGATTAATCAGTTAATCTCGGATCGTTGAAGTCCAAGGTCTATAGATCTAGTAGGTTCCCTTGTTAGCTCAXXXXXXXXXXT
ATTTAGGATCTCAAAATTGATGGAAATAATGGTCAAAGTTATAAAAAGCCAAAATAGTGATTTCCGACTTTGAAAAGTC
AACGAATATTGATTAATCAGTTAATCTCGGATCGTTGAAGTCCATGGTTTATAGATCTAGTAGGTTCCCTTGTTAGCTCAXXXXXXXXXXT
ATTTAGGATCTCGAAATTGATGGAAATAATGGTCAAAGTTATAAAAAGCCAAAATAGTGATTTCCGACTTTGAAAAGTC
>Marker770437 Chr5 SNP_site:20982055 Ref_Type:G
TACTTTTTGAGAAAAAAATTGAAAAACAAGCCAATTTCCAGGGTGTTATATTTATGAAAATTTCGTGAAAATAATAAGATTXXXXXXXXXXA
ACACAAAAGCATAAGCTTTACCTACAACTTTCATGTCTCTCATCCCATTTCAACCTCATCCTTGGTTTCAAAATTAGTC
TACTTTTTGAGAAAAAAATTGAAAAACAAGCCAATTTCCAGGGTGTTATATTTATGAAAATTTCGTGAAAATAATAAGATTXXXXXXXXXXA
ACACAAAAGCATAAGCTTTACCTACAACTTTCATGTCTCTCATCCCATTTCAACCTCATCGTTGGTTTCAAAATTAGTC
>Marker77051 Chr4 SNP_site:5371212 Ref_Type:G
GACGTTCCTGACAATAGAAAATATGGGAAAAGTTATATATGGAATAAAATAGAAAGTATGCAAAAACTTATATATCAAACXXXXXXXXXXT
TAAGGATCCTTTCCAAATCTGCCATTTCTTTCAGTTCTCCTAAATATTACTTGCTGAAAATTGAGTTTATCATTAGGTT
GACGTTCCTGACAATAGAAAATATGGGAAAAGTTATATATGGAATAAAATAGAAAGTATGCAAAAAGTTATATATCAAACXXXXXXXXXXT
TAAGGATCCTTTCCAAATCTGCCATTTCTTTCAGTTCTCCTAAATATTACTTGCTGGAAATTGAGTATATCATTAGGTT
>Marker77051 Chr4 SNP_site:5371371 Ref_Type:G
GACGTTCCTGACAATAGAAAATATGGGAAAAGTTATATATGGAATAAAATAGAAAGTATGCAAAAACTTATATATCAAACXXXXXXXXXXT
TAAGGATCCTTTCCAAATCTGCCATTTCTTTCAGTTCTCCTAAATATTACTTGCTGAAAATTGAGTTTATCATTAGGTT
GACGTTCCTGACAATAGAAAATATGGGAAAAGTTATATATGGAATAAAATAGAAAGTATGCAAAAAGTTATATATCAAACXXXXXXXXXXT
TAAGGATCCTTTCCAAATCTGCCATTTCTTTCAGTTCTCCTAAATATTACTTGCTGGAAATTGAGTATATCATTAGGTT
>Marker77051 Chr4 SNP_site:5371381 Ref_Type:A
GACGTTCCTGACAATAGAAAATATGGGAAAAGTTATATATGGAATAAAATAGAAAGTATGCAAAAACTTATATATCAAACXXXXXXXXXXT
TAAGGATCCTTTCCAAATCTGCCATTTCTTTCAGTTCTCCTAAATATTACTTGCTGAAAATTGAGTTTATCATTAGGTT
GACGTTCCTGACAATAGAAAATATGGGAAAAGTTATATATGGAATAAAATAGAAAGTATGCAAAAAGTTATATATCAAACXXXXXXXXXXT
TAAGGATCCTTTCCAAATCTGCCATTTCTTTCAGTTCTCCTAAATATTACTTGCTGGAAATTGAGTATATCATTAGGTT
>Marker770577 Chr5 SNP_site:27770142 Ref_Type:C
AACAAAACACGAGAAGGAATTTATAATTGGACAACGGAATAGCAGAGAAAAGACAAAACATTAAATGAAAATGAAAAGAAXXXXXXXXXXT
CTCGGTTCCTCTTATTTTCTTCCATTGTAAATTATGAAGGGAAAATCTCTCTTGTCATGCAAGACAAAAGTTCTTAGTG
AACAAAACACGAGAAGGAATTTATAATTGGACAACGGAATAGCAGAGAAAAGACAAAACATTAAATGAAAATGAAAAGAAXXXXXXXXXXT
CTCGGTTCCTCTTATTTTTTTCCATTGTAAATTATGAAGGGAAAATCTCTCTTGTCATGCAAGACAAAAGTTCTTAGTG
>Marker77061 Chr4 SNP_site:5500089 Ref_Type:C
ACAGGAAGTGCGATGAGATCGAAAACAGTATCTGCACAATCAGAAATGAGAGAATCCGCAACAATCTTGATCTGATGAGCXXXXXXXXXXC
GTTCGCAACCGGAACCAAAACCTAATCTCAACCAACGACGAGGTTGCAAACACAAACAAAATGTCATAAATCAAACAGT

Create PDF with GO2PDF for free, if you wish to

ACAGGAAGTGCGATGAGATCGAAAACAGTATCTGCACAATCAGAAATGAGAGAATCCGCAACAATCTTGATCTGATGAGCXXXXXXXXXXC
GTTCGCAACCGGAACCAAAACCTAATCTCAACCAACGATGAGGTTGCAAACACAAACAAAATGTCAAAAATCTAACAGT
>Marker77061 Chr4 SNP_site:5500117 Ref_Type:T
ACAGGAAGTGCGATGAGATCGAAAACAGTATCTGCACAATCAGAAATGAGAGAATCCGCAACAATCTTGATCTGATGAGCXXXXXXXXXXC
GTTCGCAACCGGAACCAAAACCTAATCTCAACCAACGACGAGGTTGCAAACACAAACAAAATGTCATAAATCAAACAGT
ACAGGAAGTGCGATGAGATCGAAAACAGTATCTGCACAATCAGAAATGAGAGAATCCGCAACAATCTTGATCTGATGAGCXXXXXXXXXXC
GTTCGCAACCGGAACCAAAACCTAATCTCAACCAACGATGAGGTTGCAAACACAAACAAAATGTCAAAAATCTAACAGT
>Marker77061 Chr4 SNP_site:5500123 Ref_Type:A
ACAGGAAGTGCGATGAGATCGAAAACAGTATCTGCACAATCAGAAATGAGAGAATCCGCAACAATCTTGATCTGATGAGCXXXXXXXXXXC
GTTCGCAACCGGAACCAAAACCTAATCTCAACCAACGACGAGGTTGCAAACACAAACAAAATGTCATAAATCAAACAGT
ACAGGAAGTGCGATGAGATCGAAAACAGTATCTGCACAATCAGAAATGAGAGAATCCGCAACAATCTTGATCTGATGAGCXXXXXXXXXXC
GTTCGCAACCGGAACCAAAACCTAATCTCAACCAACGATGAGGTTGCAAACACAAACAAAATGTCAAAAATCTAACAGT
>Marker77152 Chr4 SNP_site:12616560 Ref_Type:A
TACCTAAGTCCTTCCCTTTAGGATTTGGAGTTGCAGAGGAAAAGAGAAACTATTCCCACCAAGGAAAATATATAGATAGAXXXXXXXXXXT
GCATTAAAGAGAAGTTCTAGGTGTAGGAGCGAAGCAATACGACTGGAGAACTCCACCAGAGGATAGTCTTCATGAGAGT
TACCTAAGTCCTTCCCTTTAGGATTTGGAGTTGCAGCGGAAAAGAGAAACTATTCCCACCAAGGAAAATATATAGATAGAXXXXXXXXXXT
GCATTAAAGAGAAGTTCCAGGTGTAGGAGCGAAGCAATACGACTGGAGAACTCCACCAGAAGATAGTCTTCATGAGAGT
>Marker77152 Chr4 SNP_site:12616773 Ref_Type:T
TACCTAAGTCCTTCCCTTTAGGATTTGGAGTTGCAGAGGAAAAGAGAAACTATTCCCACCAAGGAAAATATATAGATAGAXXXXXXXXXXT
GCATTAAAGAGAAGTTCTAGGTGTAGGAGCGAAGCAATACGACTGGAGAACTCCACCAGAGGATAGTCTTCATGAGAGT
TACCTAAGTCCTTCCCTTTAGGATTTGGAGTTGCAGCGGAAAAGAGAAACTATTCCCACCAAGGAAAATATATAGATAGAXXXXXXXXXXT
GCATTAAAGAGAAGTTCCAGGTGTAGGAGCGAAGCAATACGACTGGAGAACTCCACCAGAAGATAGTCTTCATGAGAGT
>Marker77152 Chr4 SNP_site:12616816 Ref_Type:G
TACCTAAGTCCTTCCCTTTAGGATTTGGAGTTGCAGAGGAAAAGAGAAACTATTCCCACCAAGGAAAATATATAGATAGAXXXXXXXXXXT
GCATTAAAGAGAAGTTCTAGGTGTAGGAGCGAAGCAATACGACTGGAGAACTCCACCAGAGGATAGTCTTCATGAGAGT
TACCTAAGTCCTTCCCTTTAGGATTTGGAGTTGCAGCGGAAAAGAGAAACTATTCCCACCAAGGAAAATATATAGATAGAXXXXXXXXXXT
GCATTAAAGAGAAGTTCCAGGTGTAGGAGCGAAGCAATACGACTGGAGAACTCCACCAGAAGATAGTCTTCATGAGAGT
>Marker772374 Chr5 SNP_site:14731287 Ref_Type:C
CACCTTTATGTATGTGTCGTCCAATGTCCCAAGATAATTCTGTATAATTGGAAAAACTGCAGCTAAGTGCTATGACCTCGXXXXXXXXXXA
GCACGAGGTTGAAATGACAAGAAACAGTCTCCTCAGACTGCACAAAATCTCTTTGGATGACTCTACTCTTCACATCGTG
CACCTTTATGTATGTGTCGTCCAATGTCCCAAGATAATTCTGTATAATTGGAAAAACTGCAGCTAAGTGCTATGACCTTGXXXXXXXXXXA
GCACGAGGTTGAAATGACAAGAAACAGTCTCCTCAAACTGCACAAAGTCTCTTTGGATGACTCTACTCTTCACATCGTG
>Marker772374 Chr5 SNP_site:14731454 Ref_Type:G
CACCTTTATGTATGTGTCGTCCAATGTCCCAAGATAATTCTGTATAATTGGAAAAACTGCAGCTAAGTGCTATGACCTCGXXXXXXXXXXA
GCACGAGGTTGAAATGACAAGAAACAGTCTCCTCAGACTGCACAAAATCTCTTTGGATGACTCTACTCTTCACATCGTG
CACCTTTATGTATGTGTCGTCCAATGTCCCAAGATAATTCTGTATAATTGGAAAAACTGCAGCTAAGTGCTATGACCTTGXXXXXXXXXXA
GCACGAGGTTGAAATGACAAGAAACAGTCTCCTCAAACTGCACAAAGTCTCTTTGGATGACTCTACTCTTCACATCGTG
>Marker772374 Chr5 SNP_site:14731465 Ref_Type:A
CACCTTTATGTATGTGTCGTCCAATGTCCCAAGATAATTCTGTATAATTGGAAAAACTGCAGCTAAGTGCTATGACCTCGXXXXXXXXXXA
GCACGAGGTTGAAATGACAAGAAACAGTCTCCTCAGACTGCACAAAATCTCTTTGGATGACTCTACTCTTCACATCGTG
CACCTTTATGTATGTGTCGTCCAATGTCCCAAGATAATTCTGTATAATTGGAAAAACTGCAGCTAAGTGCTATGACCTTGXXXXXXXXXXA
GCACGAGGTTGAAATGACAAGAAACAGTCTCCTCAAACTGCACAAAGTCTCTTTGGATGACTCTACTCTTCACATCGTG
>Marker772527 Chr5 SNP_site:2958002 Ref_Type:G
AACAGATTAATTTTTATATTTAAGTGATGATTGTGTGTGCCACTGCCAATGATAGGCTCAGCTTGCTAGTTAATTTACATXXXXXXXXXXT

Create PDF with GO2PDF for free, if you wish to

AAAATAAAGGGAATGTTAAATTTTAAAAAACAATCCTGCTTTCTAAAAGAATTGAAAGCTCAAAAGACATTCTAAGGTT
AACAGATTGATTTTTATATTTAAGTGATGATTGTGTGTGCCACTGCCAATGATAGGCTCAGCTTGCTAGTTAATTTACATXXXXXXXXXXT
AAAATAAAGGGAATGTTAAATTTTAAAAAACAATCCTGCTTTCTAAAAGAATTGAAAGCTCAAAAGACATTCTAAGGTT
>Marker772580 Chr5 SNP_site:16052371 Ref_Type:T
CACGTCTCTTCAGACTTCTACAATCATTGTGTCAAAAACAAAAATTGCCTTTCTAATTCAACTAAAATCTATAAATAATCXXXXXXXXXXT
AAACACCAAGGAAGTCACGTTTCTTGGTTGTTTTCATATATGGTCGAATCACCCATTCATTCTGAGCTTCCGTTTGGTC
CACGTCTTTTCAGACTTCTACAATCATTGTGTCAAAAACAAAAATTGCCTTTCTAATTCAACTAAAATCTATAAATAATCXXXXXXXXXXT
AAACACCAAGGAAGTCACGTTTCTTGGTTGTTTTCATATATGGTCGAATCACCCATTCATTCTGAGCTTCCGTTTGGTC
>Marker772618 Chr5 SNP_site:24753722 Ref_Type:A
ACTTTCACTTATAAGTCTGAATTTATGGCATCTCTGTATGGTTGCATGATGCTTTCTTATTTGCTTATGCTACTTTTCATXXXXXXXXXXA
TATTTAAGGACTTCACATCTTATAAGCTTCGAGTTCCTGTCACTGGGGGAATTATTTTGGATGAAACCTTTGAGAGGGT
ACTTTCACTTATAAGTCTGAATTTATGGCATCTCTGTATGGTTGCATGATGCTTTCTTATTTGCTTATGCTACTTTTCATXXXXXXXXXXA
TATTTAAGGACTTCACGTCTTATAAGCTTCGAGTTCCTGTCACTGGGGGAATTATTTTGGATGAAACCTTTGAGAGGGT
>Marker773329 Chr5 SNP_site:15575675 Ref_Type:A
ACATTTATATCATTTAGAAATTCAAATTTCAAGTTATCATTGATAAATTCATGAAAAACATATTGACATATTAGACACAAXXXXXXXXXXA
AAAATTAGCATATGTCTAGTAAAAAATTGTCATTCTTGCAAATATTGTTCGTTGTGCTAAATTATATTTAATCAATGTT
ACATTTATATCATTTAGAAATTCAAATTTCAAGTTATCATTGATAAATTCATGAAAAACATATTGACATATTAGACACAAXXXXXXXXXXA
AAATTTAGCATATGTCTAGTAAAAAATTGTCATTCTTGCAAATATTGTTCGTTGTGCTAAATTATATTTAATCAATGTT
>Marker773566 Chr5 SNP_site:14047905 Ref_Type:T
CACAAAGCTACCATTAGCTCAATTAACAACCTCTAACAATTAAATTTGTTGGAACAACTTCTTCTAGTATAGGAAATGAAXXXXXXXXXXG
TGTCTTAATTTTAACAAGGGAGGAAATGTTTTTTTTTATAAAGCTTGAACCACCTCACTACGTCACATTTGAATGGGTG
CACAAAGCTACCATTAGCTCAATTAACAACCTCTAACAATTAAATTTGTTGGAACAACTTCTTCTAGTATAGGAAATGAAXXXXXXXXXXG
TGTCTTAATTTTAACAAGGGAGGAAATGTTTTTTTTTATAAAGCTTGAACCACCTCACTATGTCACATTTGAATGGGTG
>Marker774268 Chr5 SNP_site:23185891 Ref_Type:A
AACAATAAACAAATCACTCTCCCAGGCTTAAAATTACTTAATTAGGATTTTTTCATCATTTAATTTCTAATCCTATCGTAXXXXXXXXXXA
ATTAAATGCTTTGAACTTAACTTCAAAGGTGCTAATTGACATGTAGCTCATATCTTCTTACTTTATTTTACAAAAAGTT
AACAATAAACAAATCACTCTCCCAGGCTTAAAATTACTTAATTAGGATTTTTTCATCATTTAGTTTCTAATCCTATCGTAXXXXXXXXXXA
ATTAAATGCTTTGAACTTAACTTCAAAGGTGCTAATTGACATGTAGCTCATATCTTCTTACTTTATTTTACAAAAAGTT
>Marker774340 Chr5 SNP_site:13023887 Ref_Type:G
AACCTTCATCCTCATCTCGTAGTGGTTCTAATAGCGTCGAATACTGATTACGAGCATCCGCGAGACTTTGTTTCAAAATCXXXXXXXXXXG
CTGGATTGCAGTTTTGGAAAAATTCAATTCCACTACTTGTTTGTATAGTTTTAAGTTCGATATGTTTTTACATTGTGTG
AACCTTCGTCCTCATCTCGTAGTGGTTCTAATAGCGTCGAATACTGATTACGAGCATCCGCGAGACTTTGTTTCAAAATCXXXXXXXXXXG
CTGGATTGCAGTTTTGGAAAAATTCAATTCCACTACTTGTTTGTATAGTTTTAAGTTCGATATGTTTTTACATTGTGTG
>Marker776452 Chr5 SNP_site:18129661 Ref_Type:A
TACTAAAATTGTGCGTATAGTTATAGATTGAAACAGTGACATAGAAAGTAGGAATGTTTAACTTCATTTGTTGCTTATTGXXXXXXXXXXT
CTTAACTACAATGGAGAAATTCAAGTAGGAGTGACCTTCACTCAGAAGGTAATTGACCATCTTTTAGTTATTGAATGTG
TACTAAAATTGTGCGTATAGTTATAGATTGAAACAGTGACATAGAAAGTAGGAATGTTTAACTTCATTTGTTGCTTATTGXXXXXXXXXXT
CTTAACTACAATGGAGAAATTCAAGTAGGAGTGACCTTCACTCAGAAGGTAATTGACCATCTTTTAGTTCTTGAATGTG
>Marker77657 Chr4 SNP_site:11408223 Ref_Type:A
ACTTTGAGTTTCTATTATTAATAAAGAAGTTTGTCTCTGTTTAAAAAAAAAACCAAAAAGCATACTTTTAAGTTCTTGAGXXXXXXXXXXA
GTTCCACTGATTGGGCTGACGATCGTTCTGTCCTCTCCGACCAAAGCATTCAAAATACTACTTTTACCAACATTAGGTC
ACTTTGAGTTTCTATTATTAATAAAGAAGTTTGTCTCTGTTTAAAAAAAAGACCAAAAAGCATACTTTAAAGTTCTTGAGXXXXXXXXXXA
GTTCCACTGATTGGGCTGACGATCGTTCTGTCCTCTCCGACCAAAGCATTCAAAATACTACTTTTACCAACATTAGGTC
>Marker77657 Chr4 SNP_site:11408241 Ref_Type:T

Create PDF with GO2PDF for free, if you wish to

ACTTTGAGTTTCTATTATTAATAAAGAAGTTTGTCTCTGTTTAAAAAAAAAACCAAAAAGCATACTTTTAAGTTCTTGAGXXXXXXXXXXA
GTTCCACTGATTGGGCTGACGATCGTTCTGTCCTCTCCGACCAAAGCATTCAAAATACTACTTTTACCAACATTAGGTC
ACTTTGAGTTTCTATTATTAATAAAGAAGTTTGTCTCTGTTTAAAAAAAAGACCAAAAAGCATACTTTAAAGTTCTTGAGXXXXXXXXXXA
GTTCCACTGATTGGGCTGACGATCGTTCTGTCCTCTCCGACCAAAGCATTCAAAATACTACTTTTACCAACATTAGGTC
>Marker776626 Chr5 SNP_site:25298410 Ref_Type:A
CACTTGGCTTGAATGCAACAATTGCCTCTTCAACAACAAAGAAACACGTAGGTATCTTCCTTGTTCATGTTCGTAACACTXXXXXXXXXXC
CCTCTCTTTGTAATTTCATCCTTTAGTGAAATTGTCTCTTAACAAAAAAGAAAAGTCTCTTAACAATAGTAATACCGTG
CACTTGGCTTGAATGCAACAATTGCCTCTTCCACAACAAAGAAACACGTAGATATCTTCCTTGTTCATGTTCGTAACACTXXXXXXXXXXC
CCTCTCTTTGTAATTTCATCCTTTAGTGAAATTGTCTCTTAACAAAAAAGAAAAGTCTCTTAACAATAGTAATACTGTG
>Marker776626 Chr5 SNP_site:25298430 Ref_Type:G
CACTTGGCTTGAATGCAACAATTGCCTCTTCAACAACAAAGAAACACGTAGGTATCTTCCTTGTTCATGTTCGTAACACTXXXXXXXXXXC
CCTCTCTTTGTAATTTCATCCTTTAGTGAAATTGTCTCTTAACAAAAAAGAAAAGTCTCTTAACAATAGTAATACCGTG
CACTTGGCTTGAATGCAACAATTGCCTCTTCCACAACAAAGAAACACGTAGATATCTTCCTTGTTCATGTTCGTAACACTXXXXXXXXXXC
CCTCTCTTTGTAATTTCATCCTTTAGTGAAATTGTCTCTTAACAAAAAAGAAAAGTCTCTTAACAATAGTAATACTGTG
>Marker776626 Chr5 SNP_site:25298639 Ref_Type:C
CACTTGGCTTGAATGCAACAATTGCCTCTTCAACAACAAAGAAACACGTAGGTATCTTCCTTGTTCATGTTCGTAACACTXXXXXXXXXXC
CCTCTCTTTGTAATTTCATCCTTTAGTGAAATTGTCTCTTAACAAAAAAGAAAAGTCTCTTAACAATAGTAATACCGTG
CACTTGGCTTGAATGCAACAATTGCCTCTTCCACAACAAAGAAACACGTAGATATCTTCCTTGTTCATGTTCGTAACACTXXXXXXXXXXC
CCTCTCTTTGTAATTTCATCCTTTAGTGAAATTGTCTCTTAACAAAAAAGAAAAGTCTCTTAACAATAGTAATACTGTG
>Marker777859 Chr5 SNP_site:1050409 Ref_Type:G
GACCAATGCACCTTATTGTTTATTGACTTCAATACGCTATGGAAACCTTTTATTTTCATTTACAATGCAGAGAAGAAAGTXXXXXXXXXXG
GGGAAGATGCAAGCATCTGTAGCACCTTGATTTCTTGGAAGTTGAAGGGTGCAAAGAATTTATAGGGATAAGGTTGGGT
GACCGATGCACCTTATTGTTTATTGACTTCAATACGCTATGGAAACCTTTTATTTTCATTTACAATGCAGAGAAGAAAGTXXXXXXXXXXG
GGGAAGATGCAAGCATCTGTAGCACCTTGATTTCTTGGAAGTTGAAGGGTGCAAAGAATTTATAAGGATAAGGTTGGGT
>Marker777859 Chr5 SNP_site:1050651 Ref_Type:A
GACCAATGCACCTTATTGTTTATTGACTTCAATACGCTATGGAAACCTTTTATTTTCATTTACAATGCAGAGAAGAAAGTXXXXXXXXXXG
GGGAAGATGCAAGCATCTGTAGCACCTTGATTTCTTGGAAGTTGAAGGGTGCAAAGAATTTATAGGGATAAGGTTGGGT
GACCGATGCACCTTATTGTTTATTGACTTCAATACGCTATGGAAACCTTTTATTTTCATTTACAATGCAGAGAAGAAAGTXXXXXXXXXXG
GGGAAGATGCAAGCATCTGTAGCACCTTGATTTCTTGGAAGTTGAAGGGTGCAAAGAATTTATAAGGATAAGGTTGGGT
>Marker7782 Chr4 SNP_site:6619583 Ref_Type:G
GACAAGCTCATTATCGAACATATTTCTTCTAAGGCCCAAGCTCCACGAATGAGTAACAATACACCAACATGCAGCCACATXXXXXXXXXXA
AAGCTGAGAATTTAAAGGTCTCCTTGTGCTTCAAAATACCAGCCCATATCTATAAGATTTTCCCCTATTCGGTTTAGTA
GACAAGCTCATTATCGAACATATTTCTTCTAAGGCCCAAGCTCCACGAATGAGTAACAATACACCAACATGCAGCCACATXXXXXXXXXXA
AAGCTGAGAATTTAAAGGTCTCCTTGTGCTTCAAAATACCAGCCCATATCTATAAGATTTTCCCCTGTTCGGTTTAGTA
>Marker778231 Chr5 SNP_site:21023952 Ref_Type:T
AACAAATTAAAGGAATTATTACATTTCTCTTCCATCCAAAACTAATTCAAACGTTCTTTTAAAAAAGTGAAACAATAAACXXXXXXXXXXC
CATGCCAAGAATTAAGTTTTGCTGACTGAGAAACTCGAGCTCGGCAGAAACTTCAGAACCATTTGCTTGTAAGGCTTGT
AACAAATTAAAGGAATTATTACATTTCTCTTCCATCCAAAACTAATTCAAATGTTCTTTTAAAAAAGTGAAACAATAAACXXXXXXXXXXC
CATGCCAAGAATTAAGTTTTGCTGACTGAGAAACTCGAGCTCGGCAGAAACTTCAGAACCATTTGCTTGTAAGGCTTGT
>Marker778263 Chr5 SNP_site:26146185 Ref_Type:G
TACATCATTTGATGTTTATATATAATTACTACTACTACTACTATTATTGAATCATATAGTTTAAAATGAAGAAATGAAAAXXXXXXXXXXT
TTCTTAGTGGCAATCTCACGAGTCTCAATGCTTTGCCAAAAATTCAATTTTCTTTTTTGTTTTGTTTCTTTAAATTAGT
TACATCATTTGATGTTTATATATAATTACTACTACTACTACTATTATTGAATCATATAGTTTAAAATGAAGAAATGAAAAXXXXXXXXXXT
TTCTTAGTGGCAATCTCACGAGTCTCGATGCTTTGCCAAAAATTCAATTTTCTTTTTTGTTTTGTTTCTTTAAATTAGT

Create PDF with GO2PDF for free, if you wish to

>Marker778270 Chr5 SNP_site:5154349 Ref_Type:G
ACCAAAAAGAAGCTTTAACCGAGCAATTTTACAACGTTCAGATCAATTGGTTTAGGTTTGATTCCTTTCAAAACAAATTTXXXXXXXXXXA
CAACTTGCAACAAGATGCCATGGAACAACAACAATCACAGAAAAGATGTTGAGAATCTTCATTGGCCGAGAAAAAAGTG
ACCAAAAAGAAGCTTTAACCGAGCAATTTTACAAGGTTCAGATCAATTGGTTTAGGTTTGATTCCTTTCAAAACAAATTTXXXXXXXXXXA
CAACTTGCAACAAGATGCCATGGAACAACAACAATCACAGAAAAGATGTTGAGAATCTTCATTGGCCGAGAAAAAAGTG
>Marker778795 Chr5 SNP_site:27305843 Ref_Type:C
ACCAAGATTGCATTCAATCATTGTTGTATTTCCCTGAAGCATTTGAAAATTTTGCAGCATAGTCTAATCAAATATCATTCXXXXXXXXXXC
TGCTAACTGCTACAATAATGATCAGGTATGAACAAAAACCAACTAAATATCTTATATTCATAATTCAATAATTTTGGTT
ACCAAGATTGCATTCAATCATTGTTGTATTTCCCTGAAGCATTTGAAACTTTTGCAGCATAGTCTAATCAAATATCATTCXXXXXXXXXXC
TGCTAACTGCTACAATAATGATCAGGTATGAACAAAAACCAACTAAATATCTTATATTCATAATTCAATAATTTTGGTT
>Marker778816 Chr5 SNP_site:21124607 Ref_Type:A
AACTTAAAATTTTACACAAGGGAGTTATGCATACAGAAATATGCCAGGCATGAAGAAAAACATTCACTCTTAAGAAAATAXXXXXXXXXXC
CAATCATTGTCACGAGTAGTGTCTGGGTGAGCACAAGTAAGAAATTAGATGATAAAAAATTTCAATCACAATTAAGGTT
AACTTAAAATTTTACACAAGGGAGTTATGCATACAGAAATATGCCAGGCATGAAGAAAAACATTCGCTCTTAAGAAAATAXXXXXXXXXXC
CAATCATTGTCACGAGTAGTGTCTGGGTGAGCATAAGTAAGAAATTAGATGATAAAAAATTTCAATCACAATTAAGGTT
>Marker778816 Chr5 SNP_site:21124754 Ref_Type:C
AACTTAAAATTTTACACAAGGGAGTTATGCATACAGAAATATGCCAGGCATGAAGAAAAACATTCACTCTTAAGAAAATAXXXXXXXXXXC
CAATCATTGTCACGAGTAGTGTCTGGGTGAGCACAAGTAAGAAATTAGATGATAAAAAATTTCAATCACAATTAAGGTT
AACTTAAAATTTTACACAAGGGAGTTATGCATACAGAAATATGCCAGGCATGAAGAAAAACATTCGCTCTTAAGAAAATAXXXXXXXXXXC
CAATCATTGTCACGAGTAGTGTCTGGGTGAGCATAAGTAAGAAATTAGATGATAAAAAATTTCAATCACAATTAAGGTT
>Marker778935 Chr5 SNP_site:16549979 Ref_Type:A
ACATAAAGAAGTAAAAGCATAGTTGAAGGGGAAATATGAGAAAAAGACCCCTAAACTCAAGGCATTTTGCTCTTAGACTTXXXXXXXXXXC
AAGGCCCAAGGTTTTGAGCCTTGGAGATTCACAAAAAAAATGCGCCTTATTTTGTTAGCCTCACTTTTTCAAGCTCGTG
ACATAAAGAAGTAAAAGCATAGTTGAAGGGGAAATATGAGAAAAAGACCCCTAAACTCAAGGCATTTTGCTCTTAGACTTXXXXXXXXXXC
AAGGCCCAAGGTTTTGAGCCTTGGAGCTTCACAAAAAAAATGCGCCTTATTTTGTTAGCCTCACTTTTTCAAGCTCGTG
>Marker77899 Chr4 SNP_site:10768206 Ref_Type:C
AACTGATTTCATTTTGATGAACCAGTTAGTGATGAACTACCCCCAATTCAATATCTCTGGTCCAATTTACTGAATTTGATXXXXXXXXXXT
CTTCCTAGTTCAATCATTAGTGGTCATCTACTGAGAATTAATTTTCTATACAGTAGAGTAAGGTTGATTTGTCCAGGTG
AACTGATTTCATTTTGATGAATCAGTTAGTGATGAACTACCCCCAATTCAATATCTCTGGTCCAATTTACTGAATTTGATXXXXXXXXXXT
CTTCCTAGTTCAATCATTAGTGGTCATCTACTGAGAATTAATTTTCTATACAGTAGAGTAAGGTTGATTTGTCCAGGTG
>Marker779015 Chr5 SNP_site:12453946 Ref_Type:G
ACTCGGTAACTGACATAGTTGATTAAGAGTTTCATTTAACTGAGGAATCTACCTTCTCAGCTAAGTGCCCGGAGCATGAAXXXXXXXXXXG
CTTTTTGTTGAAAGGGCAGGCACATTTAAGTTGCAGAAGACCTATCTTATTCTTTTAGATGACTACATTGATAACTGTA
ACTCGGTAACTGACATAGTTGATTAAGAGTTTCATTTAACTGGGGAATCTACCTTCTCAGCTAAGTGCCCGGAGCATGAAXXXXXXXXXXG
CTTTTTGTTGAAAGGGCAGGCACATTTAAGTTGCAGAAGACCTATCTTATTCTTTTAGATGACTACATTGATAACTGTA
>Marker779481 Chr5 SNP_site:9656668 Ref_Type:T
TACACAGTTCATTTTTTCGCAGCACCTCCAAAGTCTTCCCCAAGTGTAACAAATGTTCCTCAAAATGCTTGTTGTATATTXXXXXXXXXXG
CTTTCTAAATGTTGTTTTCTCAATATCGTCCCCATACATGCAAATATGATGGTAGTTTTCCTTTAAATCGATCTTAGTG
TACACAGTTCATTTTTTCGCAGCACCTCCAAAGTCTTCCCCAAGTGTAACAAATGTTCCTCAAAATGCTTGTTGTATATTXXXXXXXXXXG
CTTTCTAAATGTTGTTTTCTCAATATCTTCCCCATACATGCAAATATGATGGTAGTTTTCCTTTAAATCGATCTTAGTG
>Marker779760 Chr5 SNP_site:25336542 Ref_Type:T
TACACATCATCCAAGAGGTTTAGAAATAACTTTGCAATCCATCTTTGATTTAAATAATATTTGAAAACAAAACCAATTTTXXXXXXXXXXA
ACTTTTTTTTTTATATTATTATTATTATTTATTTACTTTTTAGAGAACTAAGCCAAAAATAACATAGCCAATCTTTTAAGTT
TACACATCATCCAAGAGGTTTAGAAATAACTTTGCAATCCATCTTTGTTTTAAATAATATTTGAAAACAAAACCAATTTTXXXXXXXXXXA

Create PDF with GO2PDF for free, if you wish to

ACTTTTTTTTTTATATTATTATTATTTATTTACTTTTTAGAGAACTAAGCCAAAAATAACATAGCCAATCTTTTATGTT
>Marker779760 Chr5 SNP_site:25336737 Ref_Type:T
TACACATCATCCAAGAGGTTTAGAAATAACTTTGCAATCCATCTTTGATTTAAATAATATTTGAAAACAAAACCAATTTTXXXXXXXXXXXA
ACTTTTTTTTTTATATTATTATTATTTATTTACTTTTTAGAGAACTAAGCCAAAAATAACATAGCCAATCTTTTAAGTT
TACACATCATCCAAGAGGTTTAGAAATAACTTTGCAATCCATCTTTGTTTTAAATAATATTTGAAAACAAAACCAATTTTXXXXXXXXXXXA
ACTTTTTTTTTTATATTATTATTATTTATTTACTTTTTAGAGAACTAAGCCAAAAATAACATAGCCAATCTTTTATGTT
>Marker780622 Chr5 SNP_site:15821100 Ref_Type:A
GACCTACCCTTTGGATGTAAAGCAAAAGACTGTAAATGGGTTTTGAGAAAGAAACTCAAACTTGATGGATCAGTAGATAAXXXXXXXXXXXA
GATGAATGTTAAGACTGTTTTCCTAAATGGTGATTTAGAAGAAGAGATATATATGGAACAACCTGAAGGTTTCATAGTT
GACCTACCCTTTGGATGTAAAGCAAAAGACTGTAAATGGGTTTTGAGAAAGAAACTCAAACTTGATGGATCCGTAGATAAXXXXXXXXXXXA
TATGAATGTTAAGACTGTTTTCCTAAATGGTGATTTAGAAGAAGAGATATATATGGAACAACCTGAAGGTTTCATAGTT
>Marker780622 Chr5 SNP_site:15821257 Ref_Type:G
GACCTACCCTTTGGATGTAAAGCAAAAGACTGTAAATGGGTTTTGAGAAAGAAACTCAAACTTGATGGATCAGTAGATAAXXXXXXXXXXXA
GATGAATGTTAAGACTGTTTTCCTAAATGGTGATTTAGAAGAAGAGATATATATGGAACAACCTGAAGGTTTCATAGTT
GACCTACCCTTTGGATGTAAAGCAAAAGACTGTAAATGGGTTTTGAGAAAGAAACTCAAACTTGATGGATCCGTAGATAAXXXXXXXXXXXA
TATGAATGTTAAGACTGTTTTCCTAAATGGTGATTTAGAAGAAGAGATATATATGGAACAACCTGAAGGTTTCATAGTT
>Marker780693 Chr5 SNP_site:13770093 Ref_Type:G
CACCATTCGAAACTCGTAATCACCTTCTCTGTTCGAAAACACGTAACACATTCACCTCTGTTCGAAAACGCGTGAAGACAXXXXXXXXXXXT
TCTTCTTTTCAATTTTACTTACTTGTAATCGAACTGTGTTACAGATAATCGAAGAAGGGAGTCTTCAATTCAGAAAGGT
CACCATTCGAAACTCGTAATCACCTTCTCTGTTCGAAAACGCGTAACACATTCACCTCTGTTCGAAAACGCGTGAAGACAXXXXXXXXXXXT
TCTTCTTTTCAATTTTACTTACTTGTAATCGAACTGTGTTACAGATAATCGAAGAAGGGAGTCTTCAATTCAGAAAGGT
>Marker781096 Chr5 SNP_site:17193239 Ref_Type:T
ACAAGAGCCTAAGTTTTTACATTCTATAAACTGACTTACGATGTGAGTCTTCAGTTTTTGTCCGTACGGCTATATGGTCCXXXXXXXXXXXT
GTTGCCACGTAGAGGGCACGAAGGGGTGGTCAGGGAGGCAGAGGAGTAGGCGTAATCAGCTAGAAGGTCAGCCTGTTGT
ACAAGAGCCTAAGTTTTTACATTCTATAAACTGACTTACGATGTGAGTCTTCAGTTTTTGTTCATACGGCTATATGGTCCXXXXXXXXXXXT
GTTGCCACGTAGAGGGCACGAAGGGGTGGTCAGGGAGGCAGAGGAGTAGGCGTAATCAGCTAGAAGGTCAGCCTGTTGT
>Marker781096 Chr5 SNP_site:17193241 Ref_Type:A
ACAAGAGCCTAAGTTTTTACATTCTATAAACTGACTTACGATGTGAGTCTTCAGTTTTTGTCCGTACGGCTATATGGTCCXXXXXXXXXXXT
GTTGCCACGTAGAGGGCACGAAGGGGTGGTCAGGGAGGCAGAGGAGTAGGCGTAATCAGCTAGAAGGTCAGCCTGTTGT
ACAAGAGCCTAAGTTTTTACATTCTATAAACTGACTTACGATGTGAGTCTTCAGTTTTTGTTCATACGGCTATATGGTCCXXXXXXXXXXXT
GTTGCCACGTAGAGGGCACGAAGGGGTGGTCAGGGAGGCAGAGGAGTAGGCGTAATCAGCTAGAAGGTCAGCCTGTTGT
>Marker782533 Chr5 SNP_site:16074323 Ref_Type:G
ACTTTTTCTTTGTGTTACAGTGGAAAAAGGAGATGGCATCCCACGACGAACCTCCTGGCTATGATCCTCCGACAACAGATXXXXXXXXXXXA
GTTTATATATTGTGGGGATTTCTTTTGAAGAGCTTAATGGGAGTTAATTTTTTTGTTTCTTTTCCCTGGGTGATATGGTC
ACTTTTTCTTTGTGTTACAGTGGAAAAAGGAGATGGCATCCCACGACGAACCTCCTGGCTATGATCCTCCGACAACATATXXXXXXXXXXXA
GTTTATATATTGTGGGGATTTCTTTTGAAGAGCTTAATGGGAGTTAATTTTTTTGTTTCTTTTCCCTGGGTGATATGGTC
>Marker78315 Chr4 SNP_site:9984389 Ref_Type:G
ACATCCCTTAACCAGAAAGATAAAGCAATCGTCCAAGGAAGCTATGGCGGTAAAGAGAAGGGCGTTGACGAGCCTTCAACXXXXXXXXXXXC
CACGGTCCAATTGGGTTTGGTGATCACGAACATGGAACGCTTGGGCCAAATCAGGAGCAAAATGTGACCTGTGAACAGT
ACATCCCTTAACCAGAAAGATGAAGCAATCGTCCAAGGAAGCTATGGCGGTAAAGAGAAGGGCGTTGACGAGCCTTCAACXXXXXXXXXXXC
CACGGTCCAATTGGGTTTGGTGATCACGAACATGGAACGCTTGGGCCAAATCAGGAGCAAAATGTGACCTGTGAACAGT
>Marker783940 Chr5 SNP_site:11992601 Ref_Type:T
TACTCCATCTAGAGAAAACTTGTTGCAGATGAAAAGATAAAAGCATGTCGTATAGAGATAGCAAACCATATATTAGATGTXXXXXXXXXXXT
CAAGTATAAAGGGGTCAGGATTATGGTCTTGCCCAAAGTAATTTTGAGAATGAAATCCAACAAGTTGCTTAATTTGGGT

Create PDF with GO2PDF for free, if you wish to

TACTCCATCTAGAGAAAACTTTTTGCAGATGAAAAGATAAAAGCATGTCGTATAGAGATAGCAAACCATATATTAGATGTXXXXXXXXXXT
CAAGTATAAAGGGGTCAGGATTATGGTCTTGCCCAAAGTAATTTTGAGAATGAAATCCAACAAGTTGCTTAATTTGGGT
>Marker784324 Chr5 SNP_site:18468453 Ref_Type:C
AACGGTAAATACATATCTGGTCAAATATTACATTTTTTACAATTTTCATAATTTTTTTTAAATGCTTAATTCTATTACAAXXXXXXXXXXXT
CCTAAGAAGAAAATGCTTAGCTGATTGCATATTTGCACCCTATTTCCCCCCTGAAGAGCCAACCAAATTTGCCAATGTT
AACGGTAAATATATATCTTGTCAAATATTACATTTTTTACAATTTTCATAATTTTTTTTAAATGCTTAATTCTATTACAAXXXXXXXXXXXT
CCTAAGAAGAAAATGCTTAGCTGATTGCATATTTGCACCCTATTTCCCCCCTGAAGAGCCAACCAAATTTGCCAATGTT
>Marker784324 Chr5 SNP_site:18468460 Ref_Type:G
AACGGTAAATACATATCTGGTCAAATATTACATTTTTTACAATTTTCATAATTTTTTTTAAATGCTTAATTCTATTACAAXXXXXXXXXXXT
CCTAAGAAGAAAATGCTTAGCTGATTGCATATTTGCACCCTATTTCCCCCCTGAAGAGCCAACCAAATTTGCCAATGTT
AACGGTAAATATATATCTTGTCAAATATTACATTTTTTACAATTTTCATAATTTTTTTTAAATGCTTAATTCTATTACAAXXXXXXXXXXXT
CCTAAGAAGAAAATGCTTAGCTGATTGCATATTTGCACCCTATTTCCCCCCTGAAGAGCCAACCAAATTTGCCAATGTT
>Marker784344 Chr5 SNP_site:1524776 Ref_Type:A
ACTGAACTGAAAGAATGATATGATGGTATCTTGAATTGTAAATTGATCAATTGACTATTTAAAATGTTAGAATGGACAAAXXXXXXXXXXXC
ATCCTTCGGGAGCACAAGATTGATATATGCATCCTTTGGGAGCGTCCTACGAGATCACGAGACTGTTATGATGCAGGGT
ACTGAACTGAAAGAATGATATGATGGTATCTTGAATTGTAAATTGATCAATTGACTATTTAAAATGTTAGAATGGACAAAXXXXXXXXXXXC
ATCCTTCGGGAGCACAAGATTGATATGTGCATCCTTTGGGAGCGTCCTACGAGATCACGAGACTGTTATGATGCAGGGT
>Marker784352 Chr5 SNP_site:26831201 Ref_Type:T
ACTTCTTGTTCTTCAATTTATTTCCTGGTCATTATTAGTTGTCCAGTTGTGATTTGTGATCTATTTTGTTATTTTACTTTXXXXXXXXXXC
AAATAAGTTTGTTGCATTTATTATTATGATGAAAGTCAGGTCAATGTTGGGAAGATGTGCATTTATTTTTCCCCAGAGT
ACTTCTTGTTCTTCAATTTATTTCTTGGTCATTATTAGTTGTCCAGTTGTGATTTGTGATCTATTTTGTTATTTTACTTTXXXXXXXXXXC
AAATAAGTTTGTTGCATTTATTATTATGATGAAAGTCAGGTCAATGTTGGGAAGATGTGCATTTATTTTTCCCCAGAGT
>Marker784859 Chr5 SNP_site:14569015 Ref_Type:G
AACCTAGGAAGAGTTCTTTTTAACTAAGTGATTGAATTTTAAAATAATTTATTTTAAAAATATTCATGTGTTTGACAACTXXXXXXXXXXT
CAACCGACCTCCAACCACTATTTTTTTAAGCGGTCATCGTTGGCCAACCTCTGAGCATTGTTTTACGATGAGCTCTGTT
AACCTAGGAAGAGTTCTTTTTAACTAAGTGATTGAATTTTAAAATAATTTATTTTAAAAATATTCATGTGTTTTACAACTXXXXXXXXXXT
CAACCGACCTCCAACCACTATTTTTTTAAGCGGTCATCGTTGGCCAACCTCTGAGCATTGTTTTACGATGAGCTCTGTT
>Marker785644 Chr5 SNP_site:24552538 Ref_Type:A
AACCCTAATTTGTAGATTTCATTCACAATTTTATAAATTTGTATTTTAAATTCTATAGCATTTGAAATTTCCAATACTAAXXXXXXXXXXXA
TTAACTTTATAAATTGGAATATAAGAAAATTTACATTTCCTTCCTTCTTTAATTGTTTTTCAATCTAAAATGTCACGTC
AACCCTAATTTGTAGATTTCATTCACAATTTTATAAATTTGTATTTTAAATTCTATAGCATTTGAAATTTCCAATACTAAXXXXXXXXXXXA
TTAACTTTATAAATTGGAATATTAGAAAATTTACCTTTCCTTCCTTCTTTAATTGTTTTTCAATCTAAAATGTCACGTC
>Marker785644 Chr5 SNP_site:24552550 Ref_Type:A
AACCCTAATTTGTAGATTTCATTCACAATTTTATAAATTTGTATTTTAAATTCTATAGCATTTGAAATTTCCAATACTAAXXXXXXXXXXXA
TTAACTTTATAAATTGGAATATAAGAAAATTTACATTTCCTTCCTTCTTTAATTGTTTTTCAATCTAAAATGTCACGTC
AACCCTAATTTGTAGATTTCATTCACAATTTTATAAATTTGTATTTTAAATTCTATAGCATTTGAAATTTCCAATACTAAXXXXXXXXXXXA
TTAACTTTATAAATTGGAATATTAGAAAATTTACCTTTCCTTCCTTCTTTAATTGTTTTTCAATCTAAAATGTCACGTC
>Marker785685 Chr5 SNP_site:12004482 Ref_Type:A
TACCTTGAATGCACTATATGAACTCTTGAAAGGAAGATGAGCATAGAACAAATATATCAACCACATGATAGTCAATATGTXXXXXXXXXXG
TCGTGAAGGAATTCCAAAAAAGTCATACGATAAATGCGAAATTATTCAGCAAATTGAAAATAAATTCGAATATTGTTGT
TACCTTGAATGCACTATATGAACTCTTGAAAGGAAGATGAGCATAGAACAAATATATCAACCACATGATAGTCAATATGTXXXXXXXXXXG
TCGTGAAGGAATTCCAAAACAGTCATACGATAAATGCAAAATTATTCAGCAAATTGAAAATAAATTCGAATATTGTTGT
>Marker785685 Chr5 SNP_site:12004500 Ref_Type:G
TACCTTGAATGCACTATATGAACTCTTGAAAGGAAGATGAGCATAGAACAAATATATCAACCACATGATAGTCAATATGTXXXXXXXXXXG

Create PDF with GO2PDF for free, if you wish to

TCGTGAAGGAATTCCAAAAAAGTCATACGATAAATGCGAAATTATTCAGCAAATTGAAAATAAATTCGAATATTGTTGT
TACCTTGAATGCACTATATGAACTCTTGAAAGGAAGATGAGCATAGAACAAATATATCAACCACATGATAGTCAATATGTXXXXXXXXXXG
TCGTGAAGGAATTCCAAAACAGTCATACGATAAATGCAAAATTATTCAGCAAATTGAAAATAAATTCGAATATTGTTGT
>Marker785707 Chr5 SNP_site:23640132 Ref_Type:G
ACTCTGTATGGGAGCTTCAGAAATCACACTTCATTAGACACTCAACCCAATATGAGAGCTTTTCTGAATTCTTTTGACTAXXXXXXXXXXXT
ACAGCTTCCTAGTCACTCTAGCCCAAACACCACAATACAAAGCATCGATTACGTTTCAAGCAACCGGGAGATGGATGTG
ACTCTGTATGGGAGCTTGAGAAATCACACTTCATTAGACACTCAACCCAATATGAGAGCTTTTCTGAATTCTTTTGACTAXXXXXXXXXXXA
ACAGCTTCCTAGTCACTCTAGCCCAAACACCACAATACAAAGCATCGATTACGTTTCAAGCAACCGGGAGATGGATGTG
>Marker785707 Chr5 SNP_site:23640362 Ref_Type:A
ACTCTGTATGGGAGCTTCAGAAATCACACTTCATTAGACACTCAACCCAATATGAGAGCTTTTCTGAATTCTTTTGACTAXXXXXXXXXXXT
ACAGCTTCCTAGTCACTCTAGCCCAAACACCACAATACAAAGCATCGATTACGTTTCAAGCAACCGGGAGATGGATGTG
ACTCTGTATGGGAGCTTGAGAAATCACACTTCATTAGACACTCAACCCAATATGAGAGCTTTTCTGAATTCTTTTGACTAXXXXXXXXXXXA
ACAGCTTCCTAGTCACTCTAGCCCAAACACCACAATACAAAGCATCGATTACGTTTCAAGCAACCGGGAGATGGATGTG
>Marker78609 Chr4 SNP_site:18157537 Ref_Type:C
CACCCTTAAATGGGAAACCTTTAACAATATGGTAGTCATCAACACCTCTAATTGGAGCATTTGCACCAAATATTCTCAAAXXXXXXXXXXXT
TTGATTACGAAATCAAATCTTTGAAGGAAGAAAATCCAGCGTGCGTGCATTCTGCTGATATTTTTTTTAGATGGGAGGT
CACCCTTAAATGGGAAACCTTTAACAATATGGTAGTCATCAACACCTGTAATTGGAGCATTTGCATCAAATATTCTCAAAXXXXXXXXXXXT
TTGATTACGAAATCAAATCTTTGAAGGAAGAAAATCCAGCGTGCGTGCATTCTGCTGATATTTTTTTTAGATGGGAGGT
>Marker78609 Chr4 SNP_site:18157555 Ref_Type:C
CACCCTTAAATGGGAAACCTTTAACAATATGGTAGTCATCAACACCTCTAATTGGAGCATTTGCACCAAATATTCTCAAAXXXXXXXXXXXT
TTGATTACGAAATCAAATCTTTGAAGGAAGAAAATCCAGCGTGCGTGCATTCTGCTGATATTTTTTTTAGATGGGAGGT
CACCCTTAAATGGGAAACCTTTAACAATATGGTAGTCATCAACACCTGTAATTGGAGCATTTGCATCAAATATTCTCAAAXXXXXXXXXXXT
TTGATTACGAAATCAAATCTTTGAAGGAAGAAAATCCAGCGTGCGTGCATTCTGCTGATATTTTTTTTAGATGGGAGGT
>Marker786357 Chr5 SNP_site:16413793 Ref_Type:C
ACATTCGGTAATAGTTCTTCACTGCTAACACTGAACACTGAATCTCCATTATCAGACCCTTCATCTACTGCCCTTGAAATXXXXXXXXXXC
GAGAGTAAGAAGCTTCACACAACTCTCTTCCAATAAATCTGAGGTTTCCAAGGCTGTCCATGCATTGCGGAAGGACGTA
ACATTCGGTAATAGTTCTTCACTGCTAACACTGAACACTGAATCTCCATTATCAGACCCTTCATCTACTGCCCTTGAAATXXXXXXXXXXC
GAGAGTAAGAAGCTTCACACAACTCTCTTCCAATAAATCTGAGGTTTCCAAGGCTGTCCATGCATTGCGGAAGGATGTA
>Marker78656 Chr4 SNP_site:11856360 Ref_Type:A
CACCTCTTCATCTAGCTGCTAGAAGTGGTTCTTTAGAATGTGTTCGAGAATTACTTGCGTGGGGAGCAGAAAGATTACAAXXXXXXXXXXXA
CTGGTTATGCTTATACCATTTCAATCGTTGGCACTGCGAATTACGTGAGCTACATAAGCACATAAGTTCTGAATACGTA
CACCTCTTCATCTAGCTGCTAGAAGTGGTTCTTTAGAATGTGTTCGAGAATTACTTGCGTGGGGAGCAGAAAGATTACAAXXXXXXXXXXXA
CTGGTTATGCTTATACCATTTCAATCGTTGGCGCTGCGAATTACGTGAGCTACATAAGCACATAAGTTCTGAATACGTA
>Marker786725 Chr5 SNP_site:21004979 Ref_Type:G
AACAAAGTGTAAAGGTTGAAACATGGAATACTAAATTGAAACAAAATTTAAATTCAATGATAAAGTTGTAGCGTTTTGGAXXXXXXXXXXXA
GCATTATTGATGATGTTGAAGGTAGTAGGCCTATAAAACCTTACAACACAAACATAAAGTCTAAATTTGAATGGTTGTT
AACAAAGTGTAAAGGTTGAAACATGGAATACTAAATTGAAACAAAATTTAAATTGAATGATAAAGTTGTAGCGTTTTGGAXXXXXXXXXXXA
GCATTATTGATGATGTTGAAGGTAGTAGGCCTATATAACCTTACAACACAAACATAAAGTCTAAATTTGAATGGTTGTT
>Marker786725 Chr5 SNP_site:21005162 Ref_Type:T
AACAAAGTGTAAAGGTTGAAACATGGAATACTAAATTGAAACAAAATTTAAATTCAATGATAAAGTTGTAGCGTTTTGGAXXXXXXXXXXXA
GCATTATTGATGATGTTGAAGGTAGTAGGCCTATAAAACCTTACAACACAAACATAAAGTCTAAATTTGAATGGTTGTT
AACAAAGTGTAAAGGTTGAAACATGGAATACTAAATTGAAACAAAATTTAAATTGAATGATAAAGTTGTAGCGTTTTGGAXXXXXXXXXXXA
GCATTATTGATGATGTTGAAGGTAGTAGGCCTATATAACCTTACAACACAAACATAAAGTCTAAATTTGAATGGTTGTT
>Marker786998 Chr5 SNP_site:25395521 Ref_Type:A

Create PDF with GO2PDF for free, if you wish to

TACTCCACCTGACATGTTCTCTACATGTTTCACAAAGTTCTATGCTCCTCTAAGATATTCATTGGTTGCTCTATTAAAAAXXXXXXXXXXT
ATCTTCAAATTATTGCTACAAGAATGTGAAATATAATTGAGAACCAACTCTATCCAAAGTGAAATCGAGAATACTAGTA
TACTCCACCTGACATGTTCTCTACGTGTTTCACAAAGTTCTATGCTCCTCTAAGATATTCATTGGTTGCTCTATTAAAAAXXXXXXXXXXT
ATCTTCAAATTATTGATACAAGATTGTGAAATATAATTGAGAACCAACTCTATCCAAAGTGAAATCGAGAATACTAGTA
>Marker786998 Chr5 SNP_site:25395702 Ref_Type:C
TACTCCACCTGACATGTTCTCTACATGTTTCACAAAGTTCTATGCTCCTCTAAGATATTCATTGGTTGCTCTATTAAAAAXXXXXXXXXXT
ATCTTCAAATTATTGCTACAAGAATGTGAAATATAATTGAGAACCAACTCTATCCAAAGTGAAATCGAGAATACTAGTA
TACTCCACCTGACATGTTCTCTACGTGTTTCACAAAGTTCTATGCTCCTCTAAGATATTCATTGGTTGCTCTATTAAAAAXXXXXXXXXXT
ATCTTCAAATTATTGATACAAGATTGTGAAATATAATTGAGAACCAACTCTATCCAAAGTGAAATCGAGAATACTAGTA
>Marker786998 Chr5 SNP_site:25395710 Ref_Type:A
TACTCCACCTGACATGTTCTCTACATGTTTCACAAAGTTCTATGCTCCTCTAAGATATTCATTGGTTGCTCTATTAAAAAXXXXXXXXXXT
ATCTTCAAATTATTGCTACAAGAATGTGAAATATAATTGAGAACCAACTCTATCCAAAGTGAAATCGAGAATACTAGTA
TACTCCACCTGACATGTTCTCTACGTGTTTCACAAAGTTCTATGCTCCTCTAAGATATTCATTGGTTGCTCTATTAAAAAXXXXXXXXXXT
ATCTTCAAATTATTGATACAAGATTGTGAAATATAATTGAGAACCAACTCTATCCAAAGTGAAATCGAGAATACTAGTA
>Marker78702 Chr4 SNP_site:11583255 Ref_Type:T
ACTGCAGCAACCTGATATATTTACCATTAGAGCAAGTTGTAAGCTTAAAATGATTGAAATTGCTAAAAGAAATGAAATTGXXXXXXXXXXA
TAAACCAAACCCTCTGGCTTTGTGACAGTTGTTCATCCTTTCTAATTCCTAGAATTTAAGCCCTTTATTTATTATTGTG
ACTGCAGCAACCTGATATATTTACCATTAGAGCAAGTTGTAAGCTTAAAATGATTGAAATTGCTATAAGAAATGAAATTGXXXXXXXXXXA
TAAACCAAACCCTCTGGCTTTGTGACAGTTGTTCATCCTTTCTAATTCCTAGAATTTAAGCCCTTTATTTATTATTGTG
>Marker78758 Chr4 SNP_site:11631810 Ref_Type:C
CACTAGCCTTATCCTTTTCTTGATCTTTAGTGGATTGTGCAGCAGAAGGCTTAGAGCTAGTGTTGATATTCCACGGTCCCXXXXXXXXXXT
AAATTGATTCTTGCACCCAACCGCTGGAATTCTTCTATATAATCAGCAACTATTCTTGTTCCTTGCCGGCAATTATGGT
CACTAGCCTTATCCTTTTCTTGATCTTTAGTGGATTGTGCAGCAGAAGGCTTAGAGCTAGTGTTGATATTCCACGGTCCCXXXXXXXXXXT
AAATTGATTCTTGCACCCAACCGCTGGAATTCTTCTATATAATCAGCAATTATTCTTGTTCCTTGCCGGCAATTATGGT
>Marker78771 Chr4 SNP_site:10886056 Ref_Type:G
ACTTATATATGATAAATAAACTAAATAGTATTCACAAATATAATTCATGCATTCACGAAACGGGACGAATAACTTAGTTTXXXXXXXXXXA
GAGTTGAGTTTTATAAAGGGGGTATCAATATGGATTAAGATTCTAAAATAAAGACCCAAAAACAAGACAAATTTATTGT
ACTTATATATGATAAATAAACTAAATAGTATTCGCAAATATAATTCATGCATTCACGAAACGGGACGAATAACTTAGTTTXXXXXXXXXXA
GAGTTGAGTTTTATAAAGGGGGTATCAATATGGATTAAGATTCTAAAATAAAGACCCAAAAACAAGACAAATTTATTGT
>Marker787744 Chr5 SNP_site:27078113 Ref_Type:A
CACACTCTGTAAGACTCGTGTGAAAACTGCAACTACAACATTGTTAGGTTTTCAAGGAGATTTAACAATGATTGATTTTCXXXXXXXXXXA
AGTTGAGAGCCGACAAAGATAAGAATGTTGTTCTAGTTTTAGGTTGTGAATTATGTGTCCAATTATGAACCCGTTGGTT
CACACTCTGTAAGACTCGTGTGAAAACTGCAACTACAACATTGTTAGGTTTTCAAGGAGATTTAACAATGATTGATTTTCXXXXXXXXXXA
AGTTGAGAGCCGACAGAGATAAGAATGTTGTTCTAGTTTTAGGTTGTGAATTATGTGTCCAATTATGAACCCGTTGGTT
>Marker787810 Chr5 SNP_site:3914997 Ref_Type:T
CACCAGTTTGTATCTGTATTGATTCACTGCTTATTTATCTCTCCTGCTAGTTGTGGATTTACTTGCCATGATATGTAGCTXXXXXXXXXXG
GTGCAATTTGCGACAAACCACATAGGTAAGTTTATGTGATAGATCATTTATTGCATAATGAAAACTTCTAATCTCTGTG
CACCAGTTTGTATTTGTATTGATTCACTGCTTATTTATCTCTCCTGCTAGTTGTGGATTTACTTGCCATGATATGTAGCTXXXXXXXXXXG
GTGCAATTTGCGACAAACCACATAGGTAAGTTTATGTGATAGATCATTTATTGCATAATGAAAACTTCTAATCTCTGTG
>Marker788070 Chr5 SNP_site:12491096 Ref_Type:C
AACAATTTTAAAATTTGCAATAATATTTCTATCAATTTCCACCTAATTTGATTATTCCAAAATTACAAGACTAATCCAACXXXXXXXXXXT
TGGATGATCTTGAATTCAAATACAAACTTAGATCCCACAACCTAAACCAAAATACAATAATGTCAAGTTAAAATGGGTT
AACAATTTTAAAATTTGCAATAATATTTCTATCAATTTCCACCTAATTTGATTATTCTAAAATTACAAGACTAATCCAACXXXXXXXXXXT
TGGATGATCTTGAATTCAAATACAAACTTAGATCCCACAACCTAAACCAAAATACAATAATGTCAAGTTAAAATGGGTT

Create PDF with GO2PDF for free, if you wish to

>Marker788114 Chr5 SNP_site:7883741 Ref_Type:C

AACAACACTAAACCAAATTCATTTTAGTAAAGAAAAAATCGTTATCAACTTCTAACATCCATTTATGTATAGCTAATTGTXXXXXXXXXXT
AAAAAATAGTTGGATTAAATTAGTGAATAGAAATATAATTAAATTTACCTTAATCTATTCAGTTTAAAAACACAGTGTT
AACACCACTAAACCAAATTCATTTTAGTAAAGAAAAAATCGTTATCAACTTCTAACATCCATTTATGTATAGCTAATTGTXXXXXXXXXXT
AAAAAATAGTTGGATTAAATTAGTGAATAGAAATATAATTAAATTTACCTTAATCTATTCAGTTTAAAAACACAGTGTT

>Marker788148 Chr5 SNP_site:26778879 Ref_Type:T

AACATTAAAAATATGAATTGGTAGCATGTTTTGGAGTGGGAAGTCTATGCATCATGAAATTTGCTATAGCTTGTCAGTATXXXXXXXXXXT
CTAGAACTCAGTGGTGATAATTCATTTTTGGGGATAATGCAGAGACTAGGAATGCGACAGGTGGCTTCAGACGGTGGTG
AACATTAAAAATATGAATTGGTAGCATGTTTTGGAGTGGGAAGTCTATGCATCATGAAATTTGCTATAGCTTGTCAGTATXXXXXXXXXXT
CTAGAACTCAGTGGTGATAATTCATTTTTGGGGATAATGCAGAGACTAGGAATGCGTCAGGTGGCTTCAGACGGTGGTG

>Marker788511 Chr5 SNP_site:8388660 Ref_Type:C

AACGTTTGAGGTTAATTTCAGTTGTATTCGTATCATTTTATAAAAGGTCGCTTCTAGCTACCTACCTACCTACCTAGCTAXXXXXXXXXXG
ATATGACTGGTAAAGAAAAATAAATATCAAACATGAGAATTATACTAATAACGCCCTTCACTACCAACAGTTTTAAGTA
AACGTTTGAGGTTAATTTCAGTTGTATTCGTATCATTTTATAAAAGGTCGCTTCTAGCTAGCTACCTACCTACCTAGCTAXXXXXXXXXXG
ATATGACTGGTAAAGAAAAATAAATATCAAACATGAGAATTATACTAATAACGCCCTTCACTACCAACAGTTTTAAGTA

>Marker788933 Chr5 SNP_site:26217272 Ref_Type:T

TACATGTTAGATGTTTCATGAATTGAATCCATATTTTTAACTTATAATAGTTAGGAAATAGGCGATGTTTTAATTTGTTTXXXXXXXXXXA
ATTTGCTTATAATAAATGTTGCAATTTTTTTCAATCCAATCATGACAACTAAGATAGATTAGACCCTCTCACTTTAAGT
TACATGTTAGATGTTTCATGAATTGAATCCATATTTTTAACTTATAATAGTTAGGAAATAGTCGATGTTCTAATTTGTTTXXXXXXXXXXA
ATTTGCTTATAATAAATGTTGCAATTTTTTTCAATCCAATCATGACAACTAAGATAGATTAGACCCTCCCACTTTAAGT

>Marker788933 Chr5 SNP_site:26217280 Ref_Type:C

TACATGTTAGATGTTTCATGAATTGAATCCATATTTTTAACTTATAATAGTTAGGAAATAGGCGATGTTTTAATTTGTTTXXXXXXXXXXA
ATTTGCTTATAATAAATGTTGCAATTTTTTTCAATCCAATCATGACAACTAAGATAGATTAGACCCTCTCACTTTAAGT
TACATGTTAGATGTTTCATGAATTGAATCCATATTTTTAACTTATAATAGTTAGGAAATAGTCGATGTTCTAATTTGTTTXXXXXXXXXXA
ATTTGCTTATAATAAATGTTGCAATTTTTTTCAATCCAATCATGACAACTAAGATAGATTAGACCCTCCCACTTTAAGT

>Marker788933 Chr5 SNP_site:26217466 Ref_Type:C

TACATGTTAGATGTTTCATGAATTGAATCCATATTTTTAACTTATAATAGTTAGGAAATAGGCGATGTTTTAATTTGTTTXXXXXXXXXXA
ATTTGCTTATAATAAATGTTGCAATTTTTTTCAATCCAATCATGACAACTAAGATAGATTAGACCCTCTCACTTTAAGT
TACATGTTAGATGTTTCATGAATTGAATCCATATTTTTAACTTATAATAGTTAGGAAATAGTCGATGTTCTAATTTGTTTXXXXXXXXXXA
ATTTGCTTATAATAAATGTTGCAATTTTTTTCAATCCAATCATGACAACTAAGATAGATTAGACCCTCCCACTTTAAGT

>Marker79028 Chr4 SNP_site:19383499 Ref_Type:G

ACAAGAGGATCTAAATATTAAAGACGAGTTAATTTGACTATAACGACAAATTTAAACTATTTATTAGTTGAAATGATTGTXXXXXXXXXXA
AGCACCGTTTGATTAAATCTATGGCATTGCCTAGTATTTAAAAATCAACCTTAAATTTTTGACTATTGATTAATTGGTC
ACAGGAGGATCTAAATATTAAAGACGAGTTAATTTGACTATAACGACAAATTTAAACTATTTATTAGTTGAAATGATTGTXXXXXXXXXXA
AGCACCGTTTGACTAAATCTATGGCATTGCCTAGTATTTAAAAATCAACCTTAAATTTTTGACTATTGATTAATTGGTC

>Marker79028 Chr4 SNP_site:19383681 Ref_Type:C

ACAAGAGGATCTAAATATTAAAGACGAGTTAATTTGACTATAACGACAAATTTAAACTATTTATTAGTTGAAATGATTGTXXXXXXXXXXA
AGCACCGTTTGATTAAATCTATGGCATTGCCTAGTATTTAAAAATCAACCTTAAATTTTTGACTATTGATTAATTGGTC
ACAGGAGGATCTAAATATTAAAGACGAGTTAATTTGACTATAACGACAAATTTAAACTATTTATTAGTTGAAATGATTGTXXXXXXXXXXA
AGCACCGTTTGACTAAATCTATGGCATTGCCTAGTATTTAAAAATCAACCTTAAATTTTTGACTATTGATTAATTGGTC

>Marker790327 Chr5 SNP_site:12887692 Ref_Type:T

AACAAGGATTGTTGATGAAGCTGGTCCCTCAACAAGAGTTGTTGAATCCAACACATCAGGTTAGTCTCATCTTTCTTAATXXXXXXXXXXA
TTGTCCTATAAACAGACAATGAATGGTGTCGATAAAGATTAATGGGTCAAAGCCATGAACCTTGAAATGGAGTTTATGT
AACAAGGATTGTTGATGAAGCTGGTCCCTCATCAAGAGTTGTTGAATCCAACACATCAGGTTAGTCTCATCTTTCTTAATXXXXXXXXXXA

Create PDF with GO2PDF for free, if you wish to

TTGTCCTATAAACAGGCAATGAATGGTGTCGATAAAGATCAATGGGTCAAAGCCATGAACCTTGAAATGGAGTTTATGT
>Marker790327 Chr5 SNP_site:12887863 Ref_Type:G
AACAAGGATTGTTGATGAAGCTGGTCCCTCAACAAGAGTTGTTGAATCCAACACATCAGGTTAGTCTCATCTTTCTTAATXXXXXXXXXXA
TTGTCCTATAAACAGACAATGAATGGTGTCGATAAAGATTAATGGGTCAAAGCCATGAACCTTGAAATGGAGTTTATGT
AACAAGGATTGTTGATGAAGCTGGTCCCTCATCAAGAGTTGTTGAATCCAACACATCAGGTTAGTCTCATCTTTCTTAATXXXXXXXXXXA
TTGTCCTATAAACAGGCAATGAATGGTGTCGATAAAGATCAATGGGTCAAAGCCATGAACCTTGAAATGGAGTTTATGT
>Marker790327 Chr5 SNP_site:12887887 Ref_Type:C
AACAAGGATTGTTGATGAAGCTGGTCCCTCAACAAGAGTTGTTGAATCCAACACATCAGGTTAGTCTCATCTTTCTTAATXXXXXXXXXXA
TTGTCCTATAAACAGACAATGAATGGTGTCGATAAAGATTAATGGGTCAAAGCCATGAACCTTGAAATGGAGTTTATGT
AACAAGGATTGTTGATGAAGCTGGTCCCTCATCAAGAGTTGTTGAATCCAACACATCAGGTTAGTCTCATCTTTCTTAATXXXXXXXXXXA
TTGTCCTATAAACAGGCAATGAATGGTGTCGATAAAGATCAATGGGTCAAAGCCATGAACCTTGAAATGGAGTTTATGT
>Marker7905 Chr4 SNP_site:11628438 Ref_Type:G
ACAGGAATTGACAGATGATATTGGAGAACTTGAGAACCAAAAGGATCGACTGGAGGCTGAATTGAAAAAGGTTGGGTTTCXXXXXXXXXXG
ATTGGTTGTGCTTTTATTTATTTTCCATATTTTTCCAATGGGAAGTTCGTATACGCTTCATGGGTTACTTTAGTGTGTT
ACAGGAATTGACAGATGATATTGGAGAACTTGAGAACCAAAAGGATCGGCTGGAGGCTGAATTGAAAAAGGTTGGATTTCXXXXXXXXXXG
ATTGGTTGTGCTTTTATTTATTTTCCATATTTTTCCAATGGGAAGTTCGTATATGCTTCATGGGTTACTTTAGTGTGTT
>Marker7905 Chr4 SNP_site:11628465 Ref_Type:A
ACAGGAATTGACAGATGATATTGGAGAACTTGAGAACCAAAAGGATCGACTGGAGGCTGAATTGAAAAAGGTTGGGTTTCXXXXXXXXXXG
ATTGGTTGTGCTTTTATTTATTTTCCATATTTTTCCAATGGGAAGTTCGTATACGCTTCATGGGTTACTTTAGTGTGTT
ACAGGAATTGACAGATGATATTGGAGAACTTGAGAACCAAAAGGATCGGCTGGAGGCTGAATTGAAAAAGGTTGGATTTCXXXXXXXXXXG
ATTGGTTGTGCTTTTATTTATTTTCCATATTTTTCCAATGGGAAGTTCGTATATGCTTCATGGGTTACTTTAGTGTGTT
>Marker7905 Chr4 SNP_site:11628625 Ref_Type:T
ACAGGAATTGACAGATGATATTGGAGAACTTGAGAACCAAAAGGATCGACTGGAGGCTGAATTGAAAAAGGTTGGGTTTCXXXXXXXXXXG
ATTGGTTGTGCTTTTATTTATTTTCCATATTTTTCCAATGGGAAGTTCGTATACGCTTCATGGGTTACTTTAGTGTGTT
ACAGGAATTGACAGATGATATTGGAGAACTTGAGAACCAAAAGGATCGGCTGGAGGCTGAATTGAAAAAGGTTGGATTTCXXXXXXXXXXG
ATTGGTTGTGCTTTTATTTATTTTCCATATTTTTCCAATGGGAAGTTCGTATATGCTTCATGGGTTACTTTAGTGTGTT
>Marker791000 Chr5 SNP_site:23345616 Ref_Type:A
ACATGCCTACCAAAGATTTCTACCACTTTCTGGAATTTAATTCTAAAAAATGATTCCTCTTAAGAATTTCTTAAAACTTTXXXXXXXXXXT
TTCTGATTGAAGCCAACTGCAGAAGACAGATTCCCTAAAATTGTTATTTTCATGTTCTGGTGTTTGTTTTTGCAATGTT
ACATGCCTACCAAAGATTTCTACCACTTTCTGGAATTTAATTCTAAAAAATGATTCCTCTTAAGAATTTCTTTAAACTTTXXXXXXXXXXT
TTCTGATTGAAGCCAACTGCAGAAGACAGATTCCCTAAAATTGTTATTTTCATGTTCTGGTGTTTGTTTTTGCAATGTT
>Marker79121 Chr4 SNP_site:5843108 Ref_Type:T
ACTTTATATCGAACTCTTAAATCTGGGACCCTCTATAACTTACTTGAAGAAATGAAAGTGAGATTCCCACTGGATTTTTCXXXXXXXXXXG
TAACGAGTGCTTCATGAAAGTTATAGGACATTGAATAAGATTTCCAACCATATCCAGTAGGGCTTTTAGTTCCATCTGT
ACTTTATATCGAACTCTTAAATCTGGGACCCTCTATAACTTACTTGAAGAAATGAAAGTGAGATTCCCACTGGATTTTTCXXXXXXXXXXG
TAACGAGTGCTTCATGAAAGTTATAGGACATTGAATAAGATTTCCAACCATATTCAATAGGGCTTTTAGTTCCATCTGT
>Marker79121 Chr4 SNP_site:5843111 Ref_Type:A
ACTTTATATCGAACTCTTAAATCTGGGACCCTCTATAACTTACTTGAAGAAATGAAAGTGAGATTCCCACTGGATTTTTCXXXXXXXXXXG
TAACGAGTGCTTCATGAAAGTTATAGGACATTGAATAAGATTTCCAACCATATCCAGTAGGGCTTTTAGTTCCATCTGT
ACTTTATATCGAACTCTTAAATCTGGGACCCTCTATAACTTACTTGAAGAAATGAAAGTGAGATTCCCACTGGATTTTTCXXXXXXXXXXG
TAACGAGTGCTTCATGAAAGTTATAGGACATTGAATAAGATTTCCAACCATATTCAATAGGGCTTTTAGTTCCATCTGT
>Marker792101 Chr5 SNP_site:1222989 Ref_Type:A
GACAAGAACTTTAGCTATCTCCAGGTAAAAGATTCTACTATTAGACCCTTGATATTATGTTTCCCCTTATTTTGCATTAAXXXXXXXXXXA
TGAAATATACCATGGTTGAGATATATTTTGATTGAAATATGATATTTTTGCGATGTTATATGTATATGCCTGTGTGGTC

Create PDF with GO2PDF for free, if you wish to

GACAAGAACTTTAGCTATCTCCAGGTAAAAGATTCTACTATTAGACCCTTGATATTATGTTTCCCCTTATTTTGCATTAAXXXXXXXXXXXA
TGAAATATACCATGGTTGAGATATATTTTGATTGAGATATGATATTTTTGCGATGTTATATGTATATGCCTGTGTGGTC
>Marker792146 Chr5 SNP_site:14418468 Ref_Type:G
AACCACACATAAGTATGTAATGACCAACTATATGTGTGTTCAAGGATATATATGCAATATGTGTTAGTTATTTGACAGAAXXXXXXXXXXXT
ATTTCAAATTGTATAATTTTTCTTTTGAAAAAAAAAAGCTCTTCACACGCAAAATGTGTCAAGTATTACCTAAGTTGTC
AACCACACATAAGTATGTAATGACCAACTGTATGTGTGTTCAAGGATATATATGCAATATGTGTTAGTTATTTGACAGAAXXXXXXXXXXXT
ATTTCAAATTGTATAATTTTTCTTTTGAAAAAAAAAAGCTCTTGACACGCAAAATGTGTCAAGTATTACCTAAGTTGTC
>Marker792146 Chr5 SNP_site:14418722 Ref_Type:G
AACCACACATAAGTATGTAATGACCAACTATATGTGTGTTCAAGGATATATATGCAATATGTGTTAGTTATTTGACAGAAXXXXXXXXXXXT
ATTTCAAATTGTATAATTTTTCTTTTGAAAAAAAAAAGCTCTTCACACGCAAAATGTGTCAAGTATTACCTAAGTTGTC
AACCACACATAAGTATGTAATGACCAACTGTATGTGTGTTCAAGGATATATATGCAATATGTGTTAGTTATTTGACAGAAXXXXXXXXXXXT
ATTTCAAATTGTATAATTTTTCTTTTGAAAAAAAAAAGCTCTTGACACGCAAAATGTGTCAAGTATTACCTAAGTTGTC
>Marker792751 Chr5 SNP_site:23383969 Ref_Type:A
CACACTCCTGCAAGAGAAGCAATGATTCTCTCAGTTCTTGTCACTGCATCATGGATGTTCGCTGTGATACTTCTAACATGXXXXXXXXXXXA
CCGATATGCTGCAATTCGAGCAGCATTTGGAACAGCTTTTGTTACAAGTGAATTCCAAGTAGGAACCTAGTATTTTGTG
CACACTCCTGCGAGAGAAGCAATGATTCTCTCAGTTCTTGTCACTGCATCATGGATGTTCGCTGTGATACTTCTAACATGXXXXXXXXXXXA
CCGATATGCTGCAATTCGAGCAGCATTTGGAACAGCTTTTGTTACAAGTGAATTCCAAGTAGGAACCTAGTATTTTGTG
>Marker79285 Chr4 SNP_site:3166941 Ref_Type:T
TACACCAGACCTCTGTCCCAACAGGAAATGCTGCTTTCCACAAATCTTCCTGCATTCAGTAATTGCTATTCGTAAATCAAXXXXXXXXXXXA
AAAAAAAACAAACAAACTTCATTTCCAAGTTCAAGTAAATCATTAACAAAGCAAAATACAAAAGTTTATTGAAGCTCGT
TACACCAGACCTCTGTCCCAACAGGAAATGCTGCTTTCCACAAATCTTCCTGCATTCAGTAATTGCTATTCGTAAATCAAXXXXXXXXXXXT
TAAAAAAACAAACAAACTTCATTTCCAAGTTCAAGTAAATCATTAACAAAGCAAAATACAAAAGTTTATTGAAGCTCGT
>Marker79285 Chr4 SNP_site:3166942 Ref_Type:T
TACACCAGACCTCTGTCCCAACAGGAAATGCTGCTTTCCACAAATCTTCCTGCATTCAGTAATTGCTATTCGTAAATCAAXXXXXXXXXXXA
AAAAAAAACAAACAAACTTCATTTCCAAGTTCAAGTAAATCATTAACAAAGCAAAATACAAAAGTTTATTGAAGCTCGT
TACACCAGACCTCTGTCCCAACAGGAAATGCTGCTTTCCACAAATCTTCCTGCATTCAGTAATTGCTATTCGTAAATCAAXXXXXXXXXXXT
TAAAAAAACAAACAAACTTCATTTCCAAGTTCAAGTAAATCATTAACAAAGCAAAATACAAAAGTTTATTGAAGCTCGT
>Marker79371 Chr4 SNP_site:18665952 Ref_Type:C
ACTTGTCTTTAAAGATTTGTAGATGCAGAATGTGAGATATATTGCAAATACTTTGTGTCCATACATGTGCTGTTTTGACCXXXXXXXXXXXC
GATCATCGACAATAGCAAATACGTAATGATGTTTTGTCCATTGTCATCCTCCCACTGAATGTAAAACGTTCAGTTGGTT
ACTTGTCTTTAAAGATTTGTAGATGTAGAATGTGAGATATATTGCAAATACTTTGTGTCCATACATGTGCTGTTTTGACCXXXXXXXXXXXC
GATAATCGACAATAGCAAATACGTAATGATGTTTTGTCCATTGTCATCCTCCCACTGAATGTAAAACGTTCAGTTGGTT
>Marker79371 Chr4 SNP_site:18666101 Ref_Type:C
ACTTGTCTTTAAAGATTTGTAGATGCAGAATGTGAGATATATTGCAAATACTTTGTGTCCATACATGTGCTGTTTTGACCXXXXXXXXXXXC
GATCATCGACAATAGCAAATACGTAATGATGTTTTGTCCATTGTCATCCTCCCACTGAATGTAAAACGTTCAGTTGGTT
ACTTGTCTTTAAAGATTTGTAGATGTAGAATGTGAGATATATTGCAAATACTTTGTGTCCATACATGTGCTGTTTTGACCXXXXXXXXXXXC
GATAATCGACAATAGCAAATACGTAATGATGTTTTGTCCATTGTCATCCTCCCACTGAATGTAAAACGTTCAGTTGGTT
>Marker79388 Chr4 SNP_site:16580524 Ref_Type:C
ACTATAGAGGAAGTTGGCAGTCCTGATGTTCAAGGAATTCAAGACTCAAAAATTGAATCAGTTGTAAGTGCAAGTAGTGAXXXXXXXXXXXA
AATCAGAGACGAAATCCCCAACACTAGCACGACATCATTGAAGCTTGTGGCTTATCTTTCTTTTGAGTTCTATAATGTT
ACTATAGAGGAAGTTGGCAGTCCTGATGTTCAAGGAATTCAAGACTCAAAAATTGAATTAGTTGTAAGTGCAAGTAGTGAXXXXXXXXXXXA
AATCAGAGACGAAATCCCCAACACTAGCACGACATCATTGAAGCTTGTGGCTTATCTTTCTTTTGAGTTCTATAATGTT
>Marker793882 Chr5 SNP_site:535605 Ref_Type:G
AACCACAACCAAAAAAAGAAAACAACAACAACAAGTTAGCTTGCTTTTAAATATAAACAGTAAAAAATAGGAATTAGACCXXXXXXXXXXXT

Create PDF with GO2PDF for free, if you wish to

TCGAGAGACGACCAGCAGAATAACTCATAGAACCAAAGCTAAACAATCTTAAACAAATATAGTCTTAGGAAAGAATGTA
AACCACAACCAAAAAAAGAAAACAACAACAACAAGTTAGCTTGCTTTTAAATATAAACAGTGAAAAATAGGAATTAGACCXXXXXXXXXXT
TCGAGAGACGACCAGCAGAATAACTCATAGAACCAAAGCTAAACAATCTTAAACAAATATAGTCTTAGGAAAGAATGTA
>Marker794617 Chr5 SNP_site:27080447 Ref_Type:A
ACAATACTGCAAAATGCCCTCTAAGTTGAGAGTAGGTTCCATTTGTCAAAGACTCTCCATCATGTAAGAGGAGATGCCATXXXXXXXXXXG
AGGTTGAGGCATAATGCTACTTGATAAGGTGGTAGCCTAAGTAGCCTTAAGGAATAATCCTCCACGAATCAATATCGTG
ACAATACTGCAAAATGCCCTCTAAGTTGAGAGTAGGTTCCATTTGTCAAAGACTCTCCATCATGTAAGAGGAGATGCCATXXXXXXXXXXG
AGGTTGAGGCATAATGCTACTTGATAAGGTGGTAGCCTAAGTAGCCTTAAGGAATAATCCTCCACGAATCATTATCGTG
>Marker79463 Chr4 SNP_site:16270598 Ref_Type:A
ACTAGTAATAGATAAGCTTAAGATCATTGCACAACGCATGAAAACAAGCATTAGCACGAAAGCAAGATGTAATGTGAGCCXXXXXXXXXXA
TAAGACAAACTATAATCTGGTATGTGCAAGTCATTAATAGTCTGTAGTGAAAGACTGAAAGCAATAACAAATTTTAGTT
ACTAGTAATAGATAAGCTTAAGATCATTGCACAACGCATGAAAACAAGCATTAGCACGAAAGCAAGATGTAATGTGAGCCXXXXXXXXXXA
TAAGACAAACTATAATCTGGTATGTGCAAGTCATTAATAGTCTGTAGTGAAAGACTGAAAGCAATAACTAATTTTAGTT
>Marker795409 Chr5 SNP_site:1379107 Ref_Type:C
ACAGTAGTAAAAGAACACAGACGAACGAATGCTTCGAACTCAAACTTTTGTCTAAAAGGTCAGAGGATATAACAAGTCAAXXXXXXXXXXT
AAATATGACACCAAAATCAAATCGAAAAATAATATAAATAGGAAAAGAAATGTCTTGCAAATTTTACAACTTTCACGTA
ACAGTAGTAAAAGAACACAGACGAACGACTGCTTCGAACTCAAACTTTTGTCTAAAAGGTCAGAGGATATAACAAGTCAAXXXXXXXXXXT
AAATATGACACCAAAATCAAATCGAAAAATAATATAAATAGGAAAAGAAATGTCTTGCAAATTTTACAACTTTCACGTA
>Marker79552 Chr4 SNP_site:21191874 Ref_Type:T
TACTTTGTTCCCTCTTGAAAGCTCCACCCCTTTCTCATACTTCAATTGTCTTTGTTCCAGAGCTGCTATTGGAATGCTTGXXXXXXXXXXT
GTTCCACAACCAGCAGCAGCAGAAGGCGCTAAAATAATATTGAAAGACCTAAGCGTTCTTAGCAAGGATAGCCAGGAGT
TACTTTGTTCCCTCTTGAAAGCTCCACCCCTTTCTCATACTTCAATTGTCTTTGTTCCAGTGCTGCTATTGGAACGCTTGXXXXXXXXXXT
GTTCCACAACCAGCAGCAGCAGAAGGCGGTAAAATAATATTGAAAGACCTAAGCGTTCTTAGCAAGGATAGCCAGGAGT
>Marker79552 Chr4 SNP_site:21191888 Ref_Type:C
TACTTTGTTCCCTCTTGAAAGCTCCACCCCTTTCTCATACTTCAATTGTCTTTGTTCCAGAGCTGCTATTGGAATGCTTGXXXXXXXXXXT
GTTCCACAACCAGCAGCAGCAGAAGGCGCTAAAATAATATTGAAAGACCTAAGCGTTCTTAGCAAGGATAGCCAGGAGT
TACTTTGTTCCCTCTTGAAAGCTCCACCCCTTTCTCATACTTCAATTGTCTTTGTTCCAGTGCTGCTATTGGAACGCTTGXXXXXXXXXXT
GTTCCACAACCAGCAGCAGCAGAAGGCGGTAAAATAATATTGAAAGACCTAAGCGTTCTTAGCAAGGATAGCCAGGAGT
>Marker79552 Chr4 SNP_site:21192089 Ref_Type:G
TACTTTGTTCCCTCTTGAAAGCTCCACCCCTTTCTCATACTTCAATTGTCTTTGTTCCAGAGCTGCTATTGGAATGCTTGXXXXXXXXXXT
GTTCCACAACCAGCAGCAGCAGAAGGCGCTAAAATAATATTGAAAGACCTAAGCGTTCTTAGCAAGGATAGCCAGGAGT
TACTTTGTTCCCTCTTGAAAGCTCCACCCCTTTCTCATACTTCAATTGTCTTTGTTCCAGTGCTGCTATTGGAACGCTTGXXXXXXXXXXT
GTTCCACAACCAGCAGCAGCAGAAGGCGGTAAAATAATATTGAAAGACCTAAGCGTTCTTAGCAAGGATAGCCAGGAGT
>Marker795562 Chr5 SNP_site:11831733 Ref_Type:A
TACTTGATAACAACCTAGGCAGCCAGTGACCCAGAAAATTTAAACTTAAACAAGTCACATAGCAAAACTATCAGTCAAACXXXXXXXXXXC
CGAACACGAATGCAATCAGGATCCACAAATACAGTGTAATTGACCCCAAACAGATACAACATATAAAATGTTATAGGTA
TACTTGATAACAACCTAGGCAGCCAGTGACCCAGAAAATTTAAACTTAAACAAGTCACGTAGCAAAACTATCAGTCAAACXXXXXXXXXXG
CGAACACGAATGCAACCAGGATCCACAAATACAGTGTAATTGACCCCAAACAGATACAACATATAAAATGTTATAGGTA
>Marker795562 Chr5 SNP_site:11831842 Ref_Type:C
TACTTGATAACAACCTAGGCAGCCAGTGACCCAGAAAATTTAAACTTAAACAAGTCACATAGCAAAACTATCAGTCAAACXXXXXXXXXXC
CGAACACGAATGCAATCAGGATCCACAAATACAGTGTAATTGACCCCAAACAGATACAACATATAAAATGTTATAGGTA
TACTTGATAACAACCTAGGCAGCCAGTGACCCAGAAAATTTAAACTTAAACAAGTCACGTAGCAAAACTATCAGTCAAACXXXXXXXXXXG
CGAACACGAATGCAACCAGGATCCACAAATACAGTGTAATTGACCCCAAACAGATACAACATATAAAATGTTATAGGTA
>Marker795562 Chr5 SNP_site:11831858 Ref_Type:T

Create PDF with GO2PDF for free, if you wish to

TACTTGATAACAACCTAGGCAGCCAGTGACCCAGAAAATTTAAACTTAAACAAGTCACATAGCAAAACTATCAGTCAAACXXXXXXXXXXXC
CGAACACGAATGCAATCAGGATCCACAAATACAGTGTAATTGACCCCAAACAGATACAACATATAAAATGTTATAGGTA
TACTTGATAACAACCTAGGCAGCCAGTGACCCAGAAAATTTAAACTTAAACAAGTCACGTAGCAAAACTATCAGTCAAACXXXXXXXXXXXG
CGAACACGAATGCAACCAGGATCCACAAATACAGTGTAATTGACCCCAAACAGATACAACATATAAAATGTTATAGGTA
>Marker79564 Chr4 SNP_site:7509658 Ref_Type:T
GACAACTAGGCTACTCAAAAGGTATTGGTTGGGGTCCATGGAAAAAGTCTCGAAACATTGCAGACAATTCTTCATCTACAXXXXXXXXXXXG
AAAATGCCGACAGATGAAAGAAATGACGATGATTGAAGATTTTACTCGGGCACAGAGAGGACCTTGATTCTATTGTGGT
GACAACTAGGCTACTCAAAAGGTATTGGTTGGGGTCCATGGAAAAAGTTTCGAAACATTGCAGACAATTCTTCATCTACAXXXXXXXXXXXG
AAAATGCCGACAGATGAAAGAAATGACGATGATTGAAGATTTTACTCGGGCACAGAGAGGACCTTGATTCTATTGTGGT
>Marker796043 Chr5 SNP_site:13097718 Ref_Type:T
TACGGTTACTATGCAACAATTTAATTAGTCTCTACTATATACACTAGAATACTTTTTTTAAAAAAAAAAAATTTGATTAAXXXXXXXXXXXG
ATTAGAATATGTGACTAAATGATCAAATGGGTCGTGGGTTAGGTCAGGTCAAGGGTGTCCAGTTCTACCAAAAGGATGT
TACGGTTACTATGCAACAATTTAATTAGTCTCTACTATATACACTAGAATACTTTTTTTTTTAAAAAAAAATTTGATTAAXXXXXXXXXXXG
ATTAGAATATGTGACTAAATGATCAAATGGGTCGTGGGTTAGGTCAGGTCAAGGGTGTCCAGTTCTACCAAAAGGATGT
>Marker796043 Chr5 SNP_site:13097719 Ref_Type:T
TACGGTTACTATGCAACAATTTAATTAGTCTCTACTATATACACTAGAATACTTTTTTTAAAAAAAAAAAATTTGATTAAXXXXXXXXXXXG
ATTAGAATATGTGACTAAATGATCAAATGGGTCGTGGGTTAGGTCAGGTCAAGGGTGTCCAGTTCTACCAAAAGGATGT
TACGGTTACTATGCAACAATTTAATTAGTCTCTACTATATACACTAGAATACTTTTTTTTTTAAAAAAAAATTTGATTAAXXXXXXXXXXXG
ATTAGAATATGTGACTAAATGATCAAATGGGTCGTGGGTTAGGTCAGGTCAAGGGTGTCCAGTTCTACCAAAAGGATGT
>Marker796043 Chr5 SNP_site:13097720 Ref_Type:T
TACGGTTACTATGCAACAATTTAATTAGTCTCTACTATATACACTAGAATACTTTTTTTAAAAAAAAAAAATTTGATTAAXXXXXXXXXXXG
ATTAGAATATGTGACTAAATGATCAAATGGGTCGTGGGTTAGGTCAGGTCAAGGGTGTCCAGTTCTACCAAAAGGATGT
TACGGTTACTATGCAACAATTTAATTAGTCTCTACTATATACACTAGAATACTTTTTTTTTTAAAAAAAAATTTGATTAAXXXXXXXXXXXG
ATTAGAATATGTGACTAAATGATCAAATGGGTCGTGGGTTAGGTCAGGTCAAGGGTGTCCAGTTCTACCAAAAGGATGT
>Marker796513 Chr5 SNP_site:25646013 Ref_Type:G
CACTAACTAATCATGAGTATAATACTCTATACGAAGTTTGGCATTCATACACCGATATCAACTCATGAGTTTTGATTGCAXXXXXXXXXXXG
GTTTTCCAAGTCGGTAAGAGAGAAAAGGACGACGACGCTCCAAAATTTCAACGTTTCATCTCCTTTGTCCAAGCTTGTG
CACTAACTAATCATGAGTATAATACTCTATACGAAGTTTGGCATTCATACACCGATATCAACTCATGAGTTTTGGTTGCAXXXXXXXXXXXG
GTTTTCCAAGTCGGTAAGAGAGAAAAGGACGACGACGCTCCAAAATTTCAACGTTTCATCTCCTTTGTCCAAGCTTGTG
>Marker79703 Chr4 SNP_site:1568112 Ref_Type:G
ACTGAATTTTCCCTGGAGTATGTGATTGATCTTTTTCCCCCCTTCCCTTTCAAGATCCCAAACTAGAAGGCCTGAACAGAXXXXXXXXXXXC
TTCAATCAGCCAAAATGTCAGTCTAAGCAATACAGATAGGACTCGACACTGTCATTTGCTAATTTTAGAAAAACCAGTA
ACTGAATTTTCCCTGGAGTATGTGATTGATCTTTTTCCCCCCTTCCCTTTCAAGATCCCAAACTAGAAGGCCTGAACAGAXXXXXXXXXXXC
TTCAATCAGCCAAAATGTCAGTCTAAGCAATACAGATAGGACTCGGCACTGTCATTTGCTAATTTTAAAAAAACCAGTA
>Marker79703 Chr4 SNP_site:1568134 Ref_Type:A
ACTGAATTTTCCCTGGAGTATGTGATTGATCTTTTTCCCCCCTTCCCTTTCAAGATCCCAAACTAGAAGGCCTGAACAGAXXXXXXXXXXXC
TTCAATCAGCCAAAATGTCAGTCTAAGCAATACAGATAGGACTCGACACTGTCATTTGCTAATTTTAGAAAAACCAGTA
ACTGAATTTTCCCTGGAGTATGTGATTGATCTTTTTCCCCCCTTCCCTTTCAAGATCCCAAACTAGAAGGCCTGAACAGAXXXXXXXXXXXC
TTCAATCAGCCAAAATGTCAGTCTAAGCAATACAGATAGGACTCGGCACTGTCATTTGCTAATTTTAAAAAAACCAGTA
>Marker797107 Chr5 SNP_site:6218040 Ref_Type:G
GACAAAAGCAAGAAGAGAATTTGCTTGGTTTGGTGACTAATATTTAGATGAATTTAGCTACTCTTAGGAAGTATCCAATAXXXXXXXXXXXT
GAAGATGATGGTGTGTATCAGTGTATGCGTTTCCAGCTTGTTCTAATGATGATAGGCGATAGTGTGGTATAGTTCATGT
GACAAAAGCAGGAAGAGAATTTGCTTGGTTTGGTGACTAATATTTAGATGAATTTAGCTACTCTTAGGAAGTATCCAATAXXXXXXXXXXXT
GAAGATGATGGTGTGTATCAGTGTATGTGTTTCCAGCTTGTTCTAATGATGATAGGCGATAGTGTGGTATAGTTCATGT

Create PDF with GO2PDF for free, if you wish to

>Marker797107 Chr5 SNP_site:6218228 Ref_Type:T
GACAAAAGCAAGAAGAGAATTTGCTTGGTTTGGTGACTAATATTTAGATGAATTTAGCTACTCTTAGGAAGTATCCAATAXXXXXXXXXXT
GAAGATGATGGTGTGTATCAGTGTATGCGTTTCCAGCTTGTTCTAATGATGATAGGCGATAGTGTGGTATAGTTCATGT
GACAAAAGCAGGAAGAGAATTTGCTTGGTTTGGTGACTAATATTTAGATGAATTTAGCTACTCTTAGGAAGTATCCAATAXXXXXXXXXXT
GAAGATGATGGTGTGTATCAGTGTATGTGTTTCCAGCTTGTTCTAATGATGATAGGCGATAGTGTGGTATAGTTCATGT
>Marker797510 Chr5 SNP_site:3116150 Ref_Type:C
ACAAAGGATTTAGGCTCCCAAAAACAACTGAAGTGCTAATATAAAAAATATAGTTATGTTAGATTCTGTGAAGTTGGCAAXXXXXXXXXXC
AATAATTTTATCGGTCTTATTCCTATTTCAGTGAGTGACCGTTTTGGAAAGTAGGGCAGCTTCTTCATGATTCCATTGT
ACAAAGGATTTAGGCTCCCAAAAACAACTGAAGTGCTAATATAAAAAATATAGTTATGTTAGATTCTGTGAAGTTGGCAAXXXXXXXXXXC
AATAATTTTATCGGTCTTCTTCCTATTTCAGTGAGTGACCGTTTTGGAAAGTAGGGCAGCTTCTTCATGATTCCATTGT
>Marker798351 Chr5 SNP_site:2556596 Ref_Type:C
ACCAAAAAGGAAAATACTAATGGAAAATACTAATAATCCATAGAACTCTAATTTTCCTTTAACACCATTCAATATTAAATXXXXXXXXXXA
ATGAGAAAATGGTGAAAAAAGTTCGAAGAAAATGTTGTTAGGTAATCGATGTAGGGAGAATTATATTATTCTTTGGGTA
ACCAAAAAGGAAAATACTAATGGAAAATACTAATAATCCATAGAACTGTAATTTTCCTTTAACACCATTCAATATTAAATXXXXXXXXXXA
ATGAGAAAATGGTGAAAAAAGTTCGAAGAAAATGTTGTTAGGTAATCGATGTAGGGAGGATTATATTATTCTTTGGGTA
>Marker798351 Chr5 SNP_site:2556792 Ref_Type:G
ACCAAAAAGGAAAATACTAATGGAAAATACTAATAATCCATAGAACTCTAATTTTCCTTTAACACCATTCAATATTAAATXXXXXXXXXXA
ATGAGAAAATGGTGAAAAAAGTTCGAAGAAAATGTTGTTAGGTAATCGATGTAGGGAGAATTATATTATTCTTTGGGTA
ACCAAAAAGGAAAATACTAATGGAAAATACTAATAATCCATAGAACTGTAATTTTCCTTTAACACCATTCAATATTAAATXXXXXXXXXXA
ATGAGAAAATGGTGAAAAAAGTTCGAAGAAAATGTTGTTAGGTAATCGATGTAGGGAGGATTATATTATTCTTTGGGTA
>Marker79842 Chr4 SNP_site:6056903 Ref_Type:A
ACGATAGACGTGTGTCCGGTAATTGAATTGTCTCTAAACTCGGTGGTAGGACTCACTACACCAGGAGCTTTCAAGGTCAAXXXXXXXXXXC
AATGATGACCATCGTGGAAGCTTCCTACCCTTGGAGCTCCACAACTTGGATATGGTTTTGGGTATGCAGTGGCTAGGTA
ACGATAGACGTGTGTCCGGTAGTTGAATTGTCTCTAAACTCGGTGGTAGGACTCACTACACCAGGAGCTTTCAAGGTCAAXXXXXXXXXXC
AATGATGACCATCGTGGAAGCTTCCTACCATTGGAGCTCCACAACTTGGATATGGTTTTGGGTATGCAGTGGCTAGGTA
>Marker79842 Chr4 SNP_site:6057148 Ref_Type:C
ACGATAGACGTGTGTCCGGTAATTGAATTGTCTCTAAACTCGGTGGTAGGACTCACTACACCAGGAGCTTTCAAGGTCAAXXXXXXXXXXC
AATGATGACCATCGTGGAAGCTTCCTACCCTTGGAGCTCCACAACTTGGATATGGTTTTGGGTATGCAGTGGCTAGGTA
ACGATAGACGTGTGTCCGGTAGTTGAATTGTCTCTAAACTCGGTGGTAGGACTCACTACACCAGGAGCTTTCAAGGTCAAXXXXXXXXXXC
AATGATGACCATCGTGGAAGCTTCCTACCATTGGAGCTCCACAACTTGGATATGGTTTTGGGTATGCAGTGGCTAGGTA
>Marker799497 Chr5 SNP_site:23350459 Ref_Type:G
CACGTTTAATTCTTCTTTGATAATTTTGTCACAGACCTTTTTCCACCTTCTATCATTATCAGTTTGATGCAGAAAAATTTXXXXXXXXXXC
TTTTTTTGCTTAGAAAATGGTGAAGAGAAGGAGGAGGTTAAACATTCTAACAATGACTTCAAAGATGATTATGTAAGTA
CACGTTTAATTCTTCTTTGATAATTTTTTCATAGACCTTTTTCCACCTTCTATCATTATCAGTTTGATGCAGAAAAATTTXXXXXXXXXXC
TTTTTTTGCTTAGAAAATGGTGAAGAGAAGGAGGAGGTTAAACATTCTAACAATGACTTCAAAGATGATTATGTAAGTA
>Marker799497 Chr5 SNP_site:23350463 Ref_Type:C
CACGTTTAATTCTTCTTTGATAATTTTGTCACAGACCTTTTTCCACCTTCTATCATTATCAGTTTGATGCAGAAAAATTTXXXXXXXXXXC
TTTTTTTGCTTAGAAAATGGTGAAGAGAAGGAGGAGGTTAAACATTCTAACAATGACTTCAAAGATGATTATGTAAGTA
CACGTTTAATTCTTCTTTGATAATTTTTTCATAGACCTTTTTCCACCTTCTATCATTATCAGTTTGATGCAGAAAAATTTXXXXXXXXXXC
TTTTTTTGCTTAGAAAATGGTGAAGAGAAGGAGGAGGTTAAACATTCTAACAATGACTTCAAAGATGATTATGTAAGTA
>Marker799604 Chr5 SNP_site:14661397 Ref_Type:T
TACCAAAGATAGAGGTCTATCACTGATATGGACTATGTTGAAAATATTGGTCTATCTGATAGACCATCAACGATAGAAATXXXXXXXXXXT
CATATACCTAAGAATCCAAAAGCACTCTTAACTCCTATGTTTTTGGACTATTTTTCGCCATTTTCGATATTGATAAAGT
TACCAAAGATAGAGGTCTATCACTGATATGGACTATGTTGAAAATATTGGTCTATCTGATAGACCATCAACGATAGAAATXXXXXXXXXXT

Create PDF with GO2PDF for free, if you wish to

CATATACCTAAGAATCCTAAAGCACTCTTAACTCCTATGTTTTTGGACTATTTTTCGCCATTTTCGATATTGATAAAGT
>Marker79982 Chr4 SNP_site:10847068 Ref_Type:A
ACTTCACCCCAGTAACTTGAAATAAATAATAAGAGAAACCTTTTTTTAACCCAAAATAGAAAAATGCACACTGTGCTGTCXXXXXXXXXXC
CACCTACAATTTTAGCCGATTAAAAAGCTTTCTTACCCAAGTTCATTAGAGGAAATGGGCTTTGAGAAAAAAATGGGTT
ACTTCACCCCAGTAACTTGAAATAAATAATAAGAGAAACCTTTTTTTAACCCAAAATAGAAAAATGCACACTGTGCTGTCXXXXXXXXXXC
CACCTACAATTTTAGCCGGTTAAAAAGCTTTCTTACCCAAGTTCATTAGAGGAAATGGGCTTTGAGAAAAAAATGGGTT
>Marker799865 Chr5 SNP_site:14377661 Ref_Type:G
TACCTTTTAGGAGGGTTCTCTCTACATTGACCCTTAGGTTGTTCCTCATTTTTTGTGGAATATGCTTCTCTGAAGTTTCTXXXXXXXXXXC
TCATGGCACACTAAGGTGCAGTGGCCCTCTGGAGCCAGGCGCACAAAAGGCGCAAGGCTTTTCTTCTCTTTTTGAGGTG
TACCTTTTAGGAGGGTTCTCTCTACATTGACCCTTAGGTTGTTCCTCATTTTTTGTGGAATATGCTTCTCTGAAGTTTCTXXXXXXXXXXC
TCATGGCGCACTAAGGTGCAGTGGCCCTCTGGAGCCAGGCGCACAAAAGGCGCAAGGCTTTTCTTCTCTTTTTGAGGTG
>Marker800054 Chr5 SNP_site:3266922 Ref_Type:A
ACCCGCCTCAAGAAGCAATATTACGCAGTAGCCAAATATTAATGCCAAAAGAAGTTTAAAGAATTGTAATTGTAATTTTCXXXXXXXXXXT
TTGATATTTAAGTTATGCGAAAAGAAATAAGTTGTTTCGCTAATGGCTAGACGTTTAATGTGTTACCTCGCAAGAAGTG
ACCCGCCTCAAGAAGCAATATTACGCAGTAGCCAAATATTAGTGCCAAAAGAAGTTTAAAGAATTGTAATTGTAATTTTCXXXXXXXXXXT
TTGATATTTAAGTTATGCGAAAAGAAATAAGTTGTTTCGCTAATGGCTAGACGTTTAATGTGTTACCTCGCAAGAAGTG
>Marker800342 Chr5 SNP_site:6883896 Ref_Type:G
TACTTGTATTTACTCTGCTTTTATTTATTTTCAGGGTTAAGTTGGATATATAACAAACTATATCATACATAAATGATTTCXXXXXXXXXXG
GCACCCAAAAGTGACATTAAAAAATGCTTTCGTAACAATTTGAAAATATCAATATCAATGTAAAAGGGAAATTTTGGTT
TACTTGTATTTACTCTGCTTTTATTTATTTTCAGGGTTAAGTTGGATATATAACAAACTATATCATACATAAATGATTTCXXXXXXXXXXG
GCACCCAAAAGTTACATTATAAAATGCTTTCGTAACAATTTGAAAATATCAATATCAATGTAAAAGGGAAATTTTGGTT
>Marker800342 Chr5 SNP_site:6883903 Ref_Type:A
TACTTGTATTTACTCTGCTTTTATTTATTTTCAGGGTTAAGTTGGATATATAACAAACTATATCATACATAAATGATTTCXXXXXXXXXXG
GCACCCAAAAGTGACATTAAAAAATGCTTTCGTAACAATTTGAAAATATCAATATCAATGTAAAAGGGAAATTTTGGTT
TACTTGTATTTACTCTGCTTTTATTTATTTTCAGGGTTAAGTTGGATATATAACAAACTATATCATACATAAATGATTTCXXXXXXXXXXG
GCACCCAAAAGTTACATTATAAAATGCTTTCGTAACAATTTGAAAATATCAATATCAATGTAAAAGGGAAATTTTGGTT
>Marker800481 Chr5 SNP_site:16734620 Ref_Type:A
CACGCGAAAAAAAACTGGAGACTCAAGAACAACTAGTGGTTTCTTCTTTATTGATTATCAAGAAAAATTAAAAGGGTAGAXXXXXXXXXXG
TAAAGGGAATATTCAAGAAGTTAGGGTGAAAATTCCTTCATCTATAATTTCTTATCAAGTTGTTGTTCTTGTAGTTGTG
CACGCGAAAAAAAACTGGAGACTCAAGAACATCTAGTGGTTTCTTCTTTATTGATTATCAAGAAAAATTAAAAGGGTAGAXXXXXXXXXXG
TAAAGGGAATATTCAAGAAGTTAGGGTGAAAATTCCTTCATCTATAATTTCTTATCAAGTTGTTGTTCTTGTAGTTGTG
>Marker801055 Chr5 SNP_site:2417594 Ref_Type:A
GACTCTAAATAGCAGATCATCTCTAAAACAAAAAACAAATAGAAGGTTTGGAAGGGGAACAATCATCTCATACCAAATGGXXXXXXXXXXA
TTTTAAAATTTATGAACTACTGATTTTGAGAGAACTTTTTATTTGATAATCAATTTTGACTAAGATATGGTAGATTGGT
GACTCTTACTAGCAGATCATCTCTAAAACAAAAAACAAATAGAAGGTTTGGAAGGGGAACAATCATCTCATACCAAATGAXXXXXXXXXXA
TTTTAAAATTTATGAACTACTGATTTTGAGAGAACTTTTTATTTGATAATCAATTTTGACTAAGATATGGTAGATTGGT
>Marker801055 Chr5 SNP_site:2417596 Ref_Type:A
GACTCTAAATAGCAGATCATCTCTAAAACAAAAAACAAATAGAAGGTTTGGAAGGGGAACAATCATCTCATACCAAATGGXXXXXXXXXXA
TTTTAAAATTTATGAACTACTGATTTTGAGAGAACTTTTTATTTGATAATCAATTTTGACTAAGATATGGTAGATTGGT
GACTCTTACTAGCAGATCATCTCTAAAACAAAAAACAAATAGAAGGTTTGGAAGGGGAACAATCATCTCATACCAAATGAXXXXXXXXXXA
TTTTAAAATTTATGAACTACTGATTTTGAGAGAACTTTTTATTTGATAATCAATTTTGACTAAGATATGGTAGATTGGT
>Marker801055 Chr5 SNP_site:2417667 Ref_Type:G
GACTCTAAATAGCAGATCATCTCTAAAACAAAAAACAAATAGAAGGTTTGGAAGGGGAACAATCATCTCATACCAAATGGXXXXXXXXXXA
TTTTAAAATTTATGAACTACTGATTTTGAGAGAACTTTTTATTTGATAATCAATTTTGACTAAGATATGGTAGATTGGT

Create PDF with GO2PDF for free, if you wish to

GACTCTTACTAGCAGATCATCTCTAAAACAAAAAACAAATAGAAGGTTTGGAAGGGGAACAATCATCTCATACCAAATGAXXXXXXXXXXA
TTTTAAAATTTATGAACTACTGATTTTGAGAGAACTTTTTATTTGATAATCAATTTTGACTAAGATATGGTAGATTGGT
>Marker801403 Chr5 SNP_site:26455205 Ref_Type:A
CACTTCCACCAACTACACGATCCATTTGGATCATATTTGTATTGATATATGTTTATATTCTTCATATAAAAGAAGAAGCTXXXXXXXXXXG
CTCTACATGATATAATATAATAACAAATACATAGTCATACCGTTCATCTATACAATACTAAAACATCACTCAAAACAGT
CACTTCCACCAACTACACGATCCATTTGGATCATATTTGTATTGATATATGTTTATATTCTTCATATAGAAGAAGAAGCTXXXXXXXXXXG
CTCTACATGATATAATATAATAACAAATACATAGTCATACCGTTCATCTATACAATACTAAAACATCACTCAAAACAGT
>Marker801650 Chr5 SNP_site:8737461 Ref_Type:A
AACTCTTAGGGACGCTAGAGCTAAAAGTATGCTACTTTTCTCTAGCTAAATTGTTTTATAACGATTTTAAGGGTATTTTAXXXXXXXXXXC
GAATTTTAATATACGATGTCTAGGTGATAAACCAGTTTTATTACCTCAGATCTCTCACCCATGCTCATAAAATTAGGTT
CACTCTTAGGGATGCTAGAGCTAAAAGTATGCTACTTTTCTCTAGCTAAATTGTTTTATAACGATTTTAAGGGTATTTTAXXXXXXXXXXC
GAATTTTAATATACGATGTCTAGGTGATAAACCAGATTTATTACCTCAGATCTCTCACCCATGCTCATAAAATTAGGTT
>Marker801650 Chr5 SNP_site:8737473 Ref_Type:C
AACTCTTAGGGACGCTAGAGCTAAAAGTATGCTACTTTTCTCTAGCTAAATTGTTTTATAACGATTTTAAGGGTATTTTAXXXXXXXXXXC
GAATTTTAATATACGATGTCTAGGTGATAAACCAGTTTTATTACCTCAGATCTCTCACCCATGCTCATAAAATTAGGTT
CACTCTTAGGGATGCTAGAGCTAAAAGTATGCTACTTTTCTCTAGCTAAATTGTTTTATAACGATTTTAAGGGTATTTTAXXXXXXXXXXC
GAATTTTAATATACGATGTCTAGGTGATAAACCAGATTTATTACCTCAGATCTCTCACCCATGCTCATAAAATTAGGTT
>Marker801650 Chr5 SNP_site:8737653 Ref_Type:T
AACTCTTAGGGACGCTAGAGCTAAAAGTATGCTACTTTTCTCTAGCTAAATTGTTTTATAACGATTTTAAGGGTATTTTAXXXXXXXXXXC
GAATTTTAATATACGATGTCTAGGTGATAAACCAGTTTTATTACCTCAGATCTCTCACCCATGCTCATAAAATTAGGTT
CACTCTTAGGGATGCTAGAGCTAAAAGTATGCTACTTTTCTCTAGCTAAATTGTTTTATAACGATTTTAAGGGTATTTTAXXXXXXXXXXC
GAATTTTAATATACGATGTCTAGGTGATAAACCAGATTTATTACCTCAGATCTCTCACCCATGCTCATAAAATTAGGTT
>Marker802044 Chr5 SNP_site:13504927 Ref_Type:A
AACCTATGCCTAATTTAACCTTTTCACTTACTTGCACATCTTCATTGTGTAACATTGTTGGTTGGACAATACTTGGGGCGXXXXXXXXXXA
ATTGGAGATATAGATTGACAAATCTCGATGATGCAAATGTTTTCATCATGTTTGTTGGATTGAATAAAGTTAATTTGGT
AACCTCTGCCTAATTTAACCTTTTCACTTACTTGCACATCTTCATTGTGTAACATTGTTGGTTGGACAATACTTGGGGCCXXXXXXXXXXA
ATTGGAGATATAGATTGACAAATCTCGATGATGCAAATGTTTTCATCATGTTTGTTGGATTGAATAAAGTTAATTTGGT
>Marker802044 Chr5 SNP_site:13505001 Ref_Type:G
AACCTATGCCTAATTTAACCTTTTCACTTACTTGCACATCTTCATTGTGTAACATTGTTGGTTGGACAATACTTGGGGCGXXXXXXXXXXA
ATTGGAGATATAGATTGACAAATCTCGATGATGCAAATGTTTTCATCATGTTTGTTGGATTGAATAAAGTTAATTTGGT
AACCTCTGCCTAATTTAACCTTTTCACTTACTTGCACATCTTCATTGTGTAACATTGTTGGTTGGACAATACTTGGGGCCXXXXXXXXXXA
ATTGGAGATATAGATTGACAAATCTCGATGATGCAAATGTTTTCATCATGTTTGTTGGATTGAATAAAGTTAATTTGGT
>Marker8022 Chr4 SNP_site:203912 Ref_Type:C
TACAACTCAACCATATTTCCAAAACTCCATCCCCTAAACACAACTAACCTTTCAGCTTATGACAAATTGCTTGGCGGAGTXXXXXXXXXXA
AAACTTGGACATTGAACACTAAAAATTTTAGCATAAGATAATACCTCAGTAATTGGGGCAGGTGTATCAAAATCAGGTT
TACAACTCAACCATATTTCCAAAACTCCATCCCCTAAACACAACTAACCTTTCAGCTTATGACAAATTGCTTGGCGGAGTXXXXXXXXXXA
AAACTTGGACATTGAACACTAAAAATTTTAGTATAAGATAATACCTCAGTAATTGGGGCAGGTGTATCAAAATCAGGTT
>Marker802377 Chr5 SNP_site:16957613 Ref_Type:A
TACAGTTGCCGAAGATTGGGTAGGGTGCCCAGTTCAGGAGGAATTTTCCCTGAAAGGCGATTTTCATTTAAATGGAGATAXXXXXXXXXXT
AGATTAAAATGCAAGAAATTGGTAAAACAATTTATACCCGAAATTAAAATTACTTACAAATGAGTTAGGCCCTTTAGTG
TACAGTTGCCGAAGATTGGGTAGGGTGCCCAGTTCAGGAGGAATTTTCCCTGAAAGGCGATTTTCATTTAAATGGAGATAXXXXXXXXXXT
AGATTAAAATGCAAGAAATTGGTAAAGCAATTTATACCCGAAATTAAAATTACTTACAAATGAGTTAGGCCCTTTAGTG
>Marker802693 Chr5 SNP_site:13182470 Ref_Type:G
GACTTTTCACTTTCGGGAGCATAATGATTCTAATATCTAAGATTCAAAAATGGAGGGTTACACTTACAAGAATTACTAAAXXXXXXXXXXA

Create PDF with GO2PDF for free, if you wish to

GGAGGAATAATCGACTACCCTTCGGCGGGGGTTATTCTAAACCTTTGAAATATCATTGCAAAACTTTATAATGATGGGT
GACTTTTCACTTTCGGGAGCATAATGATTCTAATATCTGAGATTCAAAAATGGAGGGTTACACTTACAAGAATTACTAAAXXXXXXXXXXA
GGAGGAATAATCGACTACCCTTCGGCGGGGGTTATTCTAAACCTTTGAAATATCATTGCAAAACTTTATAATGATGGGT
>Marker803009 Chr5 SNP_site:21126099 Ref_Type:T
CACCCGAACCCATGTAAGGTGCAGAATAGGAGTAACCCTGAGGAGCTTGAACGGGTTTGGCAGCAGATTTCTGGAGAGAGXXXXXXXXXXC
AACATTTATTTTATGAAAGAAATGTATAAGAAGTGTAGAGAGACTGGTCAAGGAATGTTATAAGTAACTGGCGAAAGTG
CACCCGAACCCATGTAAGGTGCAGAATAGGAGTAACCCTGAGGAGCTTGAACGGGTTTGGCAGCAGATTTCTGGAGAGAGXXXXXXXXXXT
AACTTTTATTTTATGAAAGAAATGTATAAGAAGTGTAGAGAGACTGGTCAAGGAATGTTATAAGTAACTGGCGAAAGTG
>Marker803009 Chr5 SNP_site:21126103 Ref_Type:T
CACCCGAACCCATGTAAGGTGCAGAATAGGAGTAACCCTGAGGAGCTTGAACGGGTTTGGCAGCAGATTTCTGGAGAGAGXXXXXXXXXXC
AACATTTATTTTATGAAAGAAATGTATAAGAAGTGTAGAGAGACTGGTCAAGGAATGTTATAAGTAACTGGCGAAAGTG
CACCCGAACCCATGTAAGGTGCAGAATAGGAGTAACCCTGAGGAGCTTGAACGGGTTTGGCAGCAGATTTCTGGAGAGAGXXXXXXXXXXT
AACTTTTATTTTATGAAAGAAATGTATAAGAAGTGTAGAGAGACTGGTCAAGGAATGTTATAAGTAACTGGCGAAAGTG
>Marker803131 Chr5 SNP_site:25770245 Ref_Type:T
TACTCCTTTATAGTTTCTTTACTTATAAAAAGTTTGTATTATCAAAACCGATATCATTCTATCATATATTACAAATTGGTXXXXXXXXXXG
AAACACTAGTTGTTAGGAGTATTAAGATATAGTAAAGCGTTGAGTAAATGTTGGTAAAGCATAATTCAAAATTTGGTGT
TACTCCTTTATAGTTTCTTTACTTATAAAAAGTTTGTATTATCAAAATCGATATTATTCTATCATATATTACAAATTGATXXXXXXXXXXG
AAACACTAGTTGTTAGGAGTATTAAGATATAGTAAAGCGTTGAGTAAATGTTGGTAAAGCATAATTCAAAATTTGGTGT
>Marker803131 Chr5 SNP_site:25770252 Ref_Type:T
TACTCCTTTATAGTTTCTTTACTTATAAAAAGTTTGTATTATCAAAACCGATATCATTCTATCATATATTACAAATTGGTXXXXXXXXXXG
AAACACTAGTTGTTAGGAGTATTAAGATATAGTAAAGCGTTGAGTAAATGTTGGTAAAGCATAATTCAAAATTTGGTGT
TACTCCTTTATAGTTTCTTTACTTATAAAAAGTTTGTATTATCAAAATCGATATTATTCTATCATATATTACAAATTGATXXXXXXXXXXG
AAACACTAGTTGTTAGGAGTATTAAGATATAGTAAAGCGTTGAGTAAATGTTGGTAAAGCATAATTCAAAATTTGGTGT
>Marker803131 Chr5 SNP_site:25770276 Ref_Type:A
TACTCCTTTATAGTTTCTTTACTTATAAAAAGTTTGTATTATCAAAACCGATATCATTCTATCATATATTACAAATTGGTXXXXXXXXXXG
AAACACTAGTTGTTAGGAGTATTAAGATATAGTAAAGCGTTGAGTAAATGTTGGTAAAGCATAATTCAAAATTTGGTGT
TACTCCTTTATAGTTTCTTTACTTATAAAAAGTTTGTATTATCAAAATCGATATTATTCTATCATATATTACAAATTGATXXXXXXXXXXG
AAACACTAGTTGTTAGGAGTATTAAGATATAGTAAAGCGTTGAGTAAATGTTGGTAAAGCATAATTCAAAATTTGGTGT
>Marker803269 Chr5 SNP_site:3493399 Ref_Type:A
CACTTAAGCAGTTTTGAATATTTTAAAAATTAGAATTAATCCTTGCTTTGATAATTTTTTTTCACAAGTAGTCCTCGTAGXXXXXXXXXXA
TCTTCCTCTCTAATGATGACAAGAAGAGGTTTAGGCCATTCTAACAAGTTTTTGATGAGAATTTTAAATTTATTTTAGT
CACTTAAGCAGTTTTGAATATTTTAAAAATTAGAATTAATCCTTGCTTTGATAATTTTTTTTCACAAGTAGTCCTCGTAGXXXXXXXXXXA
TCTTCCTCTCTAATGATGACAAGAAGAGGTTTAGGCCATTCTCACAAGTTTTTGATGAGAATTTTAAATTTATTTTAGT
>Marker803274 Chr5 SNP_site:4586391 Ref_Type:G
AACTTTAAAAAAATGTGCAAATTATTGTAAAAATTAAGAAAAATAGAATTTTATCTTAAAAATGAAAAAAAAAAAGAAAAXXXXXXXXXXA
TAGACGTGAAGGCACTGAAAACCCTAGCCGAGGGTGTGGATTCCAAAGAATAATCCCTTGGAGGAACGTTATCGGATGT
AACTTTAAAAAAATGTGCAAATTATTGTAAAAATTAAGAAAAATAGAATTTTATCTTAAAAATGAAAAAAAAAAGAAAAAXXXXXXXXXXA
TAGACGTGAAGGCACTGAAAACCCTAGCCGAGGGTGTGGATTCCAAAGAATAATCCCTTGGAGGAACGTTATCGGATGT
>Marker803274 Chr5 SNP_site:4586392 Ref_Type:A
AACTTTAAAAAAATGTGCAAATTATTGTAAAAATTAAGAAAAATAGAATTTTATCTTAAAAATGAAAAAAAAAAAGAAAAXXXXXXXXXXA
TAGACGTGAAGGCACTGAAAACCCTAGCCGAGGGTGTGGATTCCAAAGAATAATCCCTTGGAGGAACGTTATCGGATGT
AACTTTAAAAAAATGTGCAAATTATTGTAAAAATTAAGAAAAATAGAATTTTATCTTAAAAATGAAAAAAAAAAGAAAAAXXXXXXXXXXA
TAGACGTGAAGGCACTGAAAACCCTAGCCGAGGGTGTGGATTCCAAAGAATAATCCCTTGGAGGAACGTTATCGGATGT
>Marker803500 Chr5 SNP_site:16762431 Ref_Type:C

Create PDF with GO2PDF for free, if you wish to

TACTACCGGTTCCTGTTTTAATTTAGGCGATCCTCTCATTTTCTAGTATAGTAATAAACAAACTATTGTCTCTCGTTTCAXXXXXXXXXXT
TGCTCACAATGATATTTTTCATGAGAGGACAAAACACATAAAAAATGACTGTCACCATCTCCAAGGCTCTACCCTAGTC
TACTACCGGTTCCTGTTTTAATTTAGGCGATTATCTCATTTTCTAGTATAGTAAGAAACAAACTATTGTCTCTCGTTTCAXXXXXXXXXXT
TGCTCACAATGATATTTTTCATGAGAGGACAAAACACATAAAAAATGACTGTCACCATCTCCAAGGCTCTACCCTAGTC
>Marker803500 Chr5 SNP_site:16762432 Ref_Type:C
TACTACCGGTTCCTGTTTTAATTTAGGCGATCCTCTCATTTTCTAGTATAGTAATAAACAAACTATTGTCTCTCGTTTCAXXXXXXXXXXT
TGCTCACAATGATATTTTTCATGAGAGGACAAAACACATAAAAAATGACTGTCACCATCTCCAAGGCTCTACCCTAGTC
TACTACCGGTTCCTGTTTTAATTTAGGCGATTATCTCATTTTCTAGTATAGTAAGAAACAAACTATTGTCTCTCGTTTCAXXXXXXXXXXT
TGCTCACAATGATATTTTTCATGAGAGGACAAAACACATAAAAAATGACTGTCACCATCTCCAAGGCTCTACCCTAGTC
>Marker803500 Chr5 SNP_site:16762454 Ref_Type:T
TACTACCGGTTCCTGTTTTAATTTAGGCGATCCTCTCATTTTCTAGTATAGTAATAAACAAACTATTGTCTCTCGTTTCAXXXXXXXXXXT
TGCTCACAATGATATTTTTCATGAGAGGACAAAACACATAAAAAATGACTGTCACCATCTCCAAGGCTCTACCCTAGTC
TACTACCGGTTCCTGTTTTAATTTAGGCGATTATCTCATTTTCTAGTATAGTAAGAAACAAACTATTGTCTCTCGTTTCAXXXXXXXXXXT
TGCTCACAATGATATTTTTCATGAGAGGACAAAACACATAAAAAATGACTGTCACCATCTCCAAGGCTCTACCCTAGTC
>Marker804126 Chr5 SNP_site:25837531 Ref_Type:T
TACTTACTTTTCATCTCTGCATGCTTTGAGAACAAGGAAGTTTTAGCAAACTCTACTTCTTTGGAATTAGGCTCCACCTGXXXXXXXXXXC
AACTTTACCTTCCCGATTAATGTAATTTTTGCGCACGTCGGGTTTTCTGGATCAATCTTGCCACAAGTTCCAATAGGGT
TACTTACTTTTCATCTCTGCATGCTTTGAGAACAAGGAAGTTTTAGCAAACTCTACTTCTTTGGAATTAGGCTCCATCTGXXXXXXXXXXC
AACTTTACCTTCCCGATTAACGTAATTTTTGCGCACGTCGGGTTTTCTGGATCAATCTTGCCACAAGTTCCAATAGGGT
>Marker804126 Chr5 SNP_site:25837643 Ref_Type:C
TACTTACTTTTCATCTCTGCATGCTTTGAGAACAAGGAAGTTTTAGCAAACTCTACTTCTTTGGAATTAGGCTCCACCTGXXXXXXXXXXC
AACTTTACCTTCCCGATTAATGTAATTTTTGCGCACGTCGGGTTTTCTGGATCAATCTTGCCACAAGTTCCAATAGGGT
TACTTACTTTTCATCTCTGCATGCTTTGAGAACAAGGAAGTTTTAGCAAACTCTACTTCTTTGGAATTAGGCTCCATCTGXXXXXXXXXXC
AACTTTACCTTCCCGATTAACGTAATTTTTGCGCACGTCGGGTTTTCTGGATCAATCTTGCCACAAGTTCCAATAGGGT
>Marker804473 Chr5 SNP_site:188408 Ref_Type:A
AACTTATTCCACGTATGTCTCGTCGTGATGATAGATTTTCTTACTGCAAGCACTAACTCACCAACTTGAAATGATCGGAGXXXXXXXXXXA
TAGTGTAGCATTGTCTTCAGTAGTTAGCCCTTCTTGGATTGCCATTCTCAAGGATGGAATTTCTCTCTCTAGCGGGAGT
AACTTATTCCACGTATGTCTCGTCGTGATGATAGATTTTCTTACTGCACGCACTAACTCACCAACTTGAAATTATCGGAGXXXXXXXXXXA
TAGTGTAGCATTGTCTTCCGTAGTTAGCCCTTCTTGGATTGCCATTCTCAAGGATGGAATTTCTCTCTCTAGCGGGAGT
>Marker804473 Chr5 SNP_site:188432 Ref_Type:G
AACTTATTCCACGTATGTCTCGTCGTGATGATAGATTTTCTTACTGCAAGCACTAACTCACCAACTTGAAATGATCGGAGXXXXXXXXXXA
TAGTGTAGCATTGTCTTCAGTAGTTAGCCCTTCTTGGATTGCCATTCTCAAGGATGGAATTTCTCTCTCTAGCGGGAGT
AACTTATTCCACGTATGTCTCGTCGTGATGATAGATTTTCTTACTGCACGCACTAACTCACCAACTTGAAATTATCGGAGXXXXXXXXXXA
TAGTGTAGCATTGTCTTCCGTAGTTAGCCCTTCTTGGATTGCCATTCTCAAGGATGGAATTTCTCTCTCTAGCGGGAGT
>Marker804473 Chr5 SNP_site:188563 Ref_Type:A
AACTTATTCCACGTATGTCTCGTCGTGATGATAGATTTTCTTACTGCAAGCACTAACTCACCAACTTGAAATGATCGGAGXXXXXXXXXXA
TAGTGTAGCATTGTCTTCAGTAGTTAGCCCTTCTTGGATTGCCATTCTCAAGGATGGAATTTCTCTCTCTAGCGGGAGT
AACTTATTCCACGTATGTCTCGTCGTGATGATAGATTTTCTTACTGCACGCACTAACTCACCAACTTGAAATTATCGGAGXXXXXXXXXXA
TAGTGTAGCATTGTCTTCCGTAGTTAGCCCTTCTTGGATTGCCATTCTCAAGGATGGAATTTCTCTCTCTAGCGGGAGT
>Marker804513 Chr5 SNP_site:7402420 Ref_Type:G
ACCAAGCAAGAATCCATATGTTTAATTATTATGATAAGAGTTGAGATTAATTATGAGTGGAAAATGAAAATAATCTATGAXXXXXXXXXXA
TTTTGTTGGAAAACAATTCCATTTTTTTAAAAGAATAAATAATTTTGAAAAAATTTGTTGTAGATCACAAATAAATGTT
ACCAAGCAAGAATCCATATGTTTAATTATTATGATAAGAGTTGAGATTAATTATGAGTGGAAAATGAAAATAATCTATGGXXXXXXXXXXA
TTTTGTTGGAAAACAATTCCATTTTTTTAAAAGAATAAATAATTTTGAAAAAATTTGTTGTAGATCACAAATAAATGTT

Create PDF with GO2PDF for free, if you wish to

>Marker804654 Chr5 SNP_site:1493061 Ref_Type:T
TACTCAGACATTTGGAGTCGATTACTCTTAAATTTTTCCACTAGTTGCAAAACTAAATACCGTTAGAGTTCTGCTAACTTXXXXXXXXXXA
GTAGAGTTTGATCATCAGGCCCACAAACAACAATCCTTATATGCGCGAAAATGGTCACCGAAGCACGGTTTGATAGGTT
TACTCAGACATTTGGAGTCGATTACTCTTAAATTTTTTCACTAGTTGCAAAACTAAATACCGTTAGAGTTCTGCTAACTTXXXXXXXXXXA
GTAGAGTTTGATCATCAGGCCTACAAACAACAATCCTTATATGCACGAAAATGGTCACCGAAGCACGGTTTGATAGGTT
>Marker804654 Chr5 SNP_site:1493228 Ref_Type:T
TACTCAGACATTTGGAGTCGATTACTCTTAAATTTTTCCACTAGTTGCAAAACTAAATACCGTTAGAGTTCTGCTAACTTXXXXXXXXXXA
GTAGAGTTTGATCATCAGGCCCACAAACAACAATCCTTATATGCGCGAAAATGGTCACCGAAGCACGGTTTGATAGGTT
TACTCAGACATTTGGAGTCGATTACTCTTAAATTTTTTCACTAGTTGCAAAACTAAATACCGTTAGAGTTCTGCTAACTTXXXXXXXXXXA
GTAGAGTTTGATCATCAGGCCTACAAACAACAATCCTTATATGCACGAAAATGGTCACCGAAGCACGGTTTGATAGGTT
>Marker804654 Chr5 SNP_site:1493251 Ref_Type:A
TACTCAGACATTTGGAGTCGATTACTCTTAAATTTTTCCACTAGTTGCAAAACTAAATACCGTTAGAGTTCTGCTAACTTXXXXXXXXXXA
GTAGAGTTTGATCATCAGGCCCACAAACAACAATCCTTATATGCGCGAAAATGGTCACCGAAGCACGGTTTGATAGGTT
TACTCAGACATTTGGAGTCGATTACTCTTAAATTTTTTCACTAGTTGCAAAACTAAATACCGTTAGAGTTCTGCTAACTTXXXXXXXXXXA
GTAGAGTTTGATCATCAGGCCTACAAACAACAATCCTTATATGCACGAAAATGGTCACCGAAGCACGGTTTGATAGGTT
>Marker80529 Chr4 SNP_site:16555293 Ref_Type:A
AACAAACCCAACACCATTTTCATATATATGTTTTTCAATCCTCCATTACTGATATTAATTTAGTGCGTCCCAGGAATTCTXXXXXXXXXXA
GTGTCACCAAGTCAGAATCTCTATATCCAATCTCCAACAGTTTCTCTCCTCTCGAGCACTCAACAACGTTCCTTGGGTG
AACAAACCCAGCACCATTTTCATATATATGTTTTTCAATCCTCCATTACTGATATTAATTTAGTGCGTCCCAGGAATTCTXXXXXXXXXXA
GTGTCACCAAGTCAGAATCTCTATATCCAATCTCCAACAGTTTCTCTCCTCTCGAGCACACAACAACGTTCCTTGGGTG
>Marker80529 Chr4 SNP_site:16555546 Ref_Type:T
AACAAACCCAACACCATTTTCATATATATGTTTTTCAATCCTCCATTACTGATATTAATTTAGTGCGTCCCAGGAATTCTXXXXXXXXXXA
GTGTCACCAAGTCAGAATCTCTATATCCAATCTCCAACAGTTTCTCTCCTCTCGAGCACTCAACAACGTTCCTTGGGTG
AACAAACCCAGCACCATTTTCATATATATGTTTTTCAATCCTCCATTACTGATATTAATTTAGTGCGTCCCAGGAATTCTXXXXXXXXXXA
GTGTCACCAAGTCAGAATCTCTATATCCAATCTCCAACAGTTTCTCTCCTCTCGAGCACACAACAACGTTCCTTGGGTG
>Marker805334 Chr5 SNP_site:25190783 Ref_Type:T
AACATCCCGATGTTAAGAATCCTCAAATTCACTCAACATTTGAGGTTGGTGAATATTCGGCATAGTCCAACCCTAATGTGXXXXXXXXXXC
TCGTTCCCTACTTTATCATTTTCCTATGTGAATGAGAATGCTAGTCCAACCACAATTGGAGCTATTGTCCAGTCAGGTA
AACATCCCGATGTTAAGAATCCTCAAATTCACTCAACATTTGAGGTTGGTGAATATTCGGCATAGTCCAACCCTAATGTGXXXXXXXXXXC
TCGTTCCCTACTTTATCATTTTCCTATGTGAATGAGAATGTTAGTCCAACCACAATTGGAGCTATTGTCCAGTCAGGTA
>Marker805469 Chr5 SNP_site:15779126 Ref_Type:A
CACCGCACAAACCTGGAGTGTTTGGAGGTTTTGTGATAACCTAAATTTTATCTTTGTTGCCTTATACCCTTCTGTTTGACXXXXXXXXXXT
CCTGAATAATTTTTGCAGTCTCTTTCCAGAAATAATAATTTTGTCATGCTATGGTTTTATTTTTATTATTTTCATTGTT
CACCGCACAACCCTGGAGTTTTTGGAGGTTTTGTGATAACCTAAATTTTATCTTTGTTGCCTTATACCCTTCTGTTTGACXXXXXXXXXXT
CCTGAATAATTTTTGCAGTCTCTTTCCAGAAATAATAATTTTGTCATGCTATGGTTTTATTTTTATTATTTTCATTGTT
>Marker805469 Chr5 SNP_site:15779135 Ref_Type:G
CACCGCACAAACCTGGAGTGTTTGGAGGTTTTGTGATAACCTAAATTTTATCTTTGTTGCCTTATACCCTTCTGTTTGACXXXXXXXXXXT
CCTGAATAATTTTTGCAGTCTCTTTCCAGAAATAATAATTTTGTCATGCTATGGTTTTATTTTTATTATTTTCATTGTT
CACCGCACAACCCTGGAGTTTTTGGAGGTTTTGTGATAACCTAAATTTTATCTTTGTTGCCTTATACCCTTCTGTTTGACXXXXXXXXXXT
CCTGAATAATTTTTGCAGTCTCTTTCCAGAAATAATAATTTTGTCATGCTATGGTTTTATTTTTATTATTTTCATTGTT
>Marker805933 Chr5 SNP_site:4051158 Ref_Type:A
ACTTAGACTTGAGATTCTATGGATTACTTTGGCTACCAGATGTAGTAGGGTGATGGTGACTGTCTAGTTTCAATTCAAGTXXXXXXXXXXC
AAATCAGTCCAAACACCCACATTGGACTAAATCAATTAGCAATCATCAAATGTAGTTTTCATTTTGTTTCCTCTTATGT
ACTTAGACTTGAGATTCTATGGATTACTTTGGCTACCAGATGTAGTAGGGTGATGGTGACTGTCTAGTTTCAATTCAAGTXXXXXXXXXXC

Create PDF with GO2PDF for free, if you wish to

AAATCAGTCCAAACTCCCACATTGGACTAAATCAATTAGCAATCATCAAATGTAGTTTTCATTTTGTTTCCTCTTATGT
>Marker806006 Chr5 SNP_site:14559172 Ref_Type:C
ACGTGATGACAGCCATACTAAAAATTGAAATGAAGGCACACAAATCTATAAGTTAAATATGTAAAAAAAACAAAATTAACXXXXXXXXXXA
ACCCTATCTAACTTAATTATAATACAACGATACTAGATGGACACATAGAATCCACTGACTACGCAACTACCACTGAGTG
ACGTGATGACAGCCATACTAAAAATTGAAATGAAGGCACACAAATCTATAAGTTAAATATGTAAAAAAAACAAAATTAACXXXXXXXXXXA
ACCCTATCTAACTTAATTATAATACAACGATACTAGATGGACACATAGAATCCACTGACTACGCAACTACCATTGAGTG
>Marker806228 Chr5 SNP_site:6937380 Ref_Type:A
TACCTATACTCATGGAAGCTCAGCCCTAATTGAAGACAATTAAACTGATGTCTTGTAGTTCATAAGTTAGCAACTCTAGCXXXXXXXXXXG
GTCCAAATACATCACAGATTAATTTTCAGTTTTTGATGTCTTATGGCTAGCATCCCGTCACTATTAATATAAGCAGAGT
TACCTATACTCATGGAAGCTCAGCCCTAATTGAAGACAATTAAACTGATGTCTTGTAGTTCATAAGTTAGCAACTCTAGCXXXXXXXXXXG
GTCCAAATACATCACAGATTAATTTTCAGTTTTTGATGTCTTATGGCTAGCATCCCGTCACTGTTAATATAAGCAGAGT
>Marker806257 Chr5 SNP_site:3800975 Ref_Type:G
GACAAAAGATAGACAAACCATACAAATTGAATGAAACTAAATCGGCAGAGTAGTCGCTACCTCTGATTTCTACAATATAGXXXXXXXXXXA
AAAGCATGAATCGTTTGAGGTTGGGTTAAGTCTGTAAAGAAGATTTTGAAACATTTCAAATTTTTTGTTAAAGAGAGTA
GACAAAAGATAGACAAACCATACAAATTGAATGAAACTAAATCGGCAGAGTAGTCGCTACCTCTGATTTCTACAATATAGXXXXXXXXXXA
AAAGCATGAATCGTTTGAGGTTGGGTTAAGTCTGTAAATAAGATTTTGAAACATTTCAAATTTTTTGTTAAAGAGAGTA
>Marker806793 Chr5 SNP_site:26789994 Ref_Type:A
ACCCTCCAACTATATTATTTGAATGCTTTACTCCAATCATCCGCTCTTCTCTATCAACCCTTTTCGTCAGTAAATGATCTXXXXXXXXXXG
ACCCCAGTCCAATACGTAAAGAAAAAGCAGAATAGAGACAAGCTAGTCATGAAATGAAATGAAAAGCTTGCCCTTTGTA
ACCCTCCAACTATATTATTTGAATGCTTTACTCCAATCATCCGCTCTTCTCTATCAACCCTTTTCGTCGGTAAATGATCTXXXXXXXXXXG
ACCCCAGTCCAATACGTAAAGAAAAAGCAGAATAGAGACAAGCTAGTCATGAAATGAAATGAAAAGCTTGCCCTTTGTA
>Marker806982 Chr5 SNP_site:14849680 Ref_Type:G
CACTGGCTGATCTCGTTGTTCAATAAGCTCATGTTGTAGTTCTAGGAATGACGACTTGGACCTGCACCTATGGTTATTAGXXXXXXXXXXC
CTGAAAATTGGCAGCTCAAGTAGTGAGCGCAAAGGAAGTGCCAATCATCCAAACGATTGACCAATTTGTTCGATGGGTT
CACTGGCTGATCTCGTTGTTCAATAAGCTCATGTTGTAGTTCTAGGAATGACGACTTGGACCTGCACCTATGGTTATTATXXXXXXXXXXC
CTGAAAATTGGCAGCTCAAGTAGTGAGCGCAAAGGAAGTGCCAATCATCCAAACGATTGACCAATTTGTTTGATGGGTT
>Marker806982 Chr5 SNP_site:14849871 Ref_Type:C
CACTGGCTGATCTCGTTGTTCAATAAGCTCATGTTGTAGTTCTAGGAATGACGACTTGGACCTGCACCTATGGTTATTAGXXXXXXXXXXC
CTGAAAATTGGCAGCTCAAGTAGTGAGCGCAAAGGAAGTGCCAATCATCCAAACGATTGACCAATTTGTTCGATGGGTT
CACTGGCTGATCTCGTTGTTCAATAAGCTCATGTTGTAGTTCTAGGAATGACGACTTGGACCTGCACCTATGGTTATTATXXXXXXXXXXC
CTGAAAATTGGCAGCTCAAGTAGTGAGCGCAAAGGAAGTGCCAATCATCCAAACGATTGACCAATTTGTTTGATGGGTT
>Marker807213 Chr5 SNP_site:8117934 Ref_Type:G
CACAACCCTAAACACTTTATCGTTTCTCTTTCCATAGATCCCACAACAAAGCACATATCCTCACAAACCAAAGAAAGCACXXXXXXXXXXT
TGCAAACTAGCCCTCTAAGAGAAAGTGATCTAGGTTTTCCTTTGCCTTCCGATAAAGAATGCAACAAAAAGGTTTTGTT
CACAACCCTAAACACTTTATCGTTTCTCTTTCCATAGATCCCACAACAAAGCACATATCCTCACAAACCAAAGAAAGCGCXXXXXXXXXXT
TGCAAACTAGCCCTCTAAGAGAAAGTGATCTAGGTTTTCCTTTTCCTTCCGATAAAGAATGCAACAAAAAGGTTTTGTT
>Marker807213 Chr5 SNP_site:8118097 Ref_Type:T
CACAACCCTAAACACTTTATCGTTTCTCTTTCCATAGATCCCACAACAAAGCACATATCCTCACAAACCAAAGAAAGCACXXXXXXXXXXT
TGCAAACTAGCCCTCTAAGAGAAAGTGATCTAGGTTTTCCTTTGCCTTCCGATAAAGAATGCAACAAAAAGGTTTTGTT
CACAACCCTAAACACTTTATCGTTTCTCTTTCCATAGATCCCACAACAAAGCACATATCCTCACAAACCAAAGAAAGCGCXXXXXXXXXXT
TGCAAACTAGCCCTCTAAGAGAAAGTGATCTAGGTTTTCCTTTTCCTTCCGATAAAGAATGCAACAAAAAGGTTTTGTT
>Marker807594 Chr5 SNP_site:8461836 Ref_Type:T
AACGTTATATCCTGTGAAAGTAAAATTTGGAAATTGTTGGTTCGATTTGAAATATCTTGTTTTTCTCATAATATATATTTXXXXXXXXXXC
TGGCTCGATGTCTAGGTATCCAAAAAAGGGACCAAAAAATTCATGATTCTAATTTACAAATATCATTATGGGAATGAGT

Create PDF with GO2PDF for free, if you wish to

AACGTTATATCCTGTGAAAGTAAAATTTGTAAATTGTTGGTTCGATTTGAAATATCTTGTTTTTCTCATAATATATATTTXXXXXXXXXXC
TGGCTCGATGTCTAGGTATCCAAAAAAGGGACCAAAAAATTCATGATTCTAATTTACAAATATCATTATGGGAATGAGT
>Marker807641 Chr5 SNP_site:15906279 Ref_Type:A
AACTGTGCGTTAATAACTAGTTGTGTCTTTTGTGTATTTTGTTATATTTATCAATTTAGTATAAGCTCTAAGAGACTAAAXXXXXXXXXXA
TAAAATGATATCTATAAATTTGTTTTAGATGGATTCTTTTCAAATTCAAGAAAACTTCAAACGAATAATTGATCACAGT
AACTGTGCGTTAATAACTAGTTGTGTCTTTTGTGTATTTTGTTATATTTATCAATTTAGTATAAGCTCTAAGAGACTAAAXXXXXXXXXXA
TAAAATGATATCTATAACTTTGTTTTAGATGGATTCTTTTCAAATTCAAGAAAACTTCAAACGAATAATTGATCACAGT
>Marker807643 Chr5 SNP_site:14589496 Ref_Type:G
TACTTATACCTTCTTCAATCCATCTATGTCTTTCTTGCTCATATGTCCTTATGTTATTTGTAGTTTATGTTTTTTATATGXXXXXXXXXXA
GATAACTTTTATCATTTGTATCCTGACAGGAGAACACATGTGGGTATCAAACGCTGAGAAGTTGCTTCCGTTAAACGTG
TACTTATACCTTCTTCAATCCATCTATGTCTTTCTTGCTCATATGTCCTTATGTTATTTGTAGTTTATGTTTTTTATATGXXXXXXXXXXA
GATAACTTTTATCATTTGTATCCTGACAGGAGAACACATGTGGGTATCAAACGCTGAGAGGTTGCTTCCGTTAAACGTG
>Marker808090 Chr5 SNP_site:25168964 Ref_Type:A
ACTTATTTATGAGTTATTTACATTTTTTACCCAGAAGTTCTTTCACTGAAGAAGAAAATTTGGAATAAAAATGTGACTGAXXXXXXXXXXG
AGGGAAAAAAAATTGATCCACAGTCTGCCCAAAATGGCCAAACATGTTTGATGTTAATAAGAAAATCGAGCTTGATGGGT
ACTTATTTATGAGTTATTTACATTTTTTACCCAGAAGTTCTTTCACTGAAGAAGAAAATTTGGAATAAAAGTGTGACTGAXXXXXXXXXXG
AGGGAAAAAAAATTGATCCACAGTCTGCCCAAAATGGCCAAACATGTTTGATGTTAATAAGAAAATTGAGCTTGATGGGT
>Marker808090 Chr5 SNP_site:25169171 Ref_Type:C
ACTTATTTATGAGTTATTTACATTTTTTACCCAGAAGTTCTTTCACTGAAGAAGAAAATTTGGAATAAAAATGTGACTGAXXXXXXXXXXG
AGGGAAAAAAAATTGATCCACAGTCTGCCCAAAATGGCCAAACATGTTTGATGTTAATAAGAAAATCGAGCTTGATGGGT
ACTTATTTATGAGTTATTTACATTTTTTACCCAGAAGTTCTTTCACTGAAGAAGAAAATTTGGAATAAAAGTGTGACTGAXXXXXXXXXXG
AGGGAAAAAAAATTGATCCACAGTCTGCCCAAAATGGCCAAACATGTTTGATGTTAATAAGAAAATTGAGCTTGATGGGT
>Marker8083 Chr4 SNP_site:18232445 Ref_Type:A
AACCTGGAGACACCTTAACCAAAACATAACCAACAAAAATTTATACAACCACGGTTCCAAAAGCTTACACAAACTGTAGAXXXXXXXXXXG
TGGGTAAAACATTTTGGAAGGGTAAGCTATAAAGCTCAGTGAGTGACTCGGTTTCAAATAGAATTTTAAAGGTAAGAGT
AACCTGGAGACACCTTAACCAAAACATAACCAACAAAAATTTATACAACCACGGTTCCAAAAGCTTACACAAACTGTAGTXXXXXXXXXXG
TGGGTAAAACATTTTGGAAGGGTAAGATATAAAGCTCAGTGAGTGACTCGGTTTCAAATAGAATTTTAAAGGTAAGAGT
>Marker8083 Chr4 SNP_site:18232598 Ref_Type:C
AACCTGGAGACACCTTAACCAAAACATAACCAACAAAAATTTATACAACCACGGTTCCAAAAGCTTACACAAACTGTAGAXXXXXXXXXXG
TGGGTAAAACATTTTGGAAGGGTAAGCTATAAAGCTCAGTGAGTGACTCGGTTTCAAATAGAATTTTAAAGGTAAGAGT
AACCTGGAGACACCTTAACCAAAACATAACCAACAAAAATTTATACAACCACGGTTCCAAAAGCTTACACAAACTGTAGTXXXXXXXXXXG
TGGGTAAAACATTTTGGAAGGGTAAGATATAAAGCTCAGTGAGTGACTCGGTTTCAAATAGAATTTTAAAGGTAAGAGT
>Marker80836 Chr4 SNP_site:10727555 Ref_Type:C
TACAAATCAAAAACTCCAATTGACATATTTGACATCAATCAAGTTTAACATAAGGATCAACAAATTGACTAGAGAGCTTTXXXXXXXXXXC
AGATCATTGGACATTTTTCTGTATTATATTTCAGGAAAGTAGAACATGAAATGGATGATACCTTAAGATCTCTGTGGTA
TACAAATCAAAAACTCCAATTGACATATTTGACATCAATCAAGTTTAACATCAGGATCAACAAATTGACTAGAGAGCTTTXXXXXXXXXXC
AGATCATTGGACATTTTTCTGTATTATATTTCAGGAAAGTAGAACATGAAATGGATGATACCTTAAGATCTCTGTGGTA
>Marker808570 Chr5 SNP_site:6227479 Ref_Type:A
AACTCATGTTCATAATTCGTTTTGTTGTTTATTAATTTTGAAAGACCAGCACACTCGAGACCAGTGTATAATCTAGGAGCXXXXXXXXXXA
GAACATAACTAAAAAATGATTCCCGCAGACCTATTCGAATCACAAAGTGTCCTCCATCATCAATAAACCCCCCTTTGTT
AACTCGTGTTCATAATTCGTTTTGTTGTTTATTAATTTTGAAAGACCAGCACACTCGAGACCAGTGTATAATCTAGGAGCXXXXXXXXXXA
GAACATAACTAAAAAATGATTCCCGCAGACCTATTCGAATCACAAAGTGTCCTCCATCATCAATAAACCCCCCTTTGTT
>Marker808666 Chr5 SNP_site:9024828 Ref_Type:T
ACCCTACCTAATAATGACTGTTTTTTTCTCTATTCTCATTTTATATCTTTTTCAAACATAATCAAAATCCTCTAACTTTAXXXXXXXXXXT

Create PDF with GO2PDF for free, if you wish to

TATATTATTATAATCTCCTCACGTAATCTTCACAATCTCCTCACGAAATCAAATTAAAGTCAGGACTACCACTAGTGTT
ACCCTACCTAATAATGACTGTTTTTTTCTCTATTCTCATTTTATATCTTTTTCAAACATAATCAAAATCCTCTAACTTTAXXXXXXXXXXT
TATATTATTATAATCTCCTCATGTAATCTTCACAATCTCCTCACGAAATCAAATTAAAATCAGGACTACCACTAGTGTT
>Marker808666 Chr5 SNP_site:9024865 Ref_Type:A
ACCCTACCTAATAATGACTGTTTTTTTCTCTATTCTCATTTTATATCTTTTTCAAACATAATCAAAATCCTCTAACTTTAXXXXXXXXXXT
TATATTATTATAATCTCCTCACGTAATCTTCACAATCTCCTCACGAAATCAAATTAAAGTCAGGACTACCACTAGTGTT
ACCCTACCTAATAATGACTGTTTTTTTCTCTATTCTCATTTTATATCTTTTTCAAACATAATCAAAATCCTCTAACTTTAXXXXXXXXXXT
TATATTATTATAATCTCCTCATGTAATCTTCACAATCTCCTCACGAAATCAAATTAAAATCAGGACTACCACTAGTGTT
>Marker808823 Chr5 SNP_site:6291902 Ref_Type:C
AACGAATCCCAACGGATGATTCAAGAAACACATCAGTCTGTTGTGGCTGCTCTTTTGCTTGGAATAAAACAGTTCGTCTTXXXXXXXXXXG
AGGAGATGCTTGAAAAGTCCAAAGCAGCAGAAACTGTGGAAATTACTGAAGAAATATGTTCATCTAGAGGCTCCTACGT
AACGAATCCCAACGGATGATTCAAGAAACACATCAGTCTGTTGTGGCTGTTCTTTTGCTTGGAATAAAACAGTTCGTCTTXXXXXXXXXXG
AGGAGATGCTTGAAAAGTCCAAAGCAGCAGAAACTGTGGAAATTACTGAAGAAATATGTTCATCTAGAGGCTCCTACGT
>Marker809288 Chr5 SNP_site:2056959 Ref_Type:A
AACTGAACTTATATTATAGAAAGGTTCATTTTAGATAAATAAATTGCGTTTTGGGTAAATTTTAGGTTAAATTAAATAAAXXXXXXXXXXT
TACAATTCTCTAAGAAAATTTGAAACTTGAAAGGGATGAGATAAATGGTATAGATCAATGGTGTAGTGATCATCATGTC
AACTGAACTTATATTATAGAAAGGTTCATTTTAGATAAATAAATTGCGTTTTGGGTAAATTTTAGGTTAAATTAAATAAAXXXXXXXXXXT
TACAATTCTCTAAGAAAATTTGAATCTTGAAAGGGATGAGATAAATGGTATAGATCAATGGTGTAGTGATCATCATGTC
>Marker809442 Chr5 SNP_site:14446633 Ref_Type:C
ACCTCCTACTCTAATTCTTAATGTCTTGAAAGCTAATATCATACAAACCATGCACAACAGAATTGAGTTAGAATTTTAATXXXXXXXXXXT
TTCTTTCCATGTCCAAGATCAATAATTCTTACCATATTGAGCATTGATGCATGACCATCCCAAACACAAAGGTTGGGTT
ACCTCCTACTCTAATTCTTAATGTCTTGAAAGCTAATATCATACAAACCATGCACAACAGCATTGAGTTAGAATTTTAATXXXXXXXXXXT
TTCTTTCCATGTCCAAGATCAATAATTCTTACCATATTGAGCATTGATGCATGACCATCCCAAACACAAAGGTTGGGTT
>Marker809755 Chr5 SNP_site:24055919 Ref_Type:C
CACTATTTTTACATCCAACTGACATAGTATTAACAACGAGTTAAGAAACCTCAAGGGGACGAGGCAGAGGTTCTTAAGAGXXXXXXXXXXG
TAATCCGGGAGAGTTGATACGAATAACGCAAGTGAGAAATTTGAGTAATTTAGTGAGAAAACATCCTTAATTATTGGTG
CACTATTTTTACATCCAACTGACATAGTATTAACAACGAGTTAAGAAACCTCAAGGGGACGAGGCAGAGGTTCTTAAGAGXXXXXXXXXXG
TAATCCGGGAGAGTTGATACGAATAACGCAAGTGAGAAATTTGAGTAATTTAGTGAGAAAACATCTTTAATTATTGGTG
>Marker810384 Chr5 SNP_site:131386 Ref_Type:G
TACTATTTCCTTCTCAAATTAAAATAGTTTGATATCTCAAAACATATACTATTATAACCCTAACTATAACAATCCAAAACXXXXXXXXXXA
TCGGTTTCTATCACTCAGAGTATCATTGATAGACTTTTATTAATTTCTATCACTGATAGACGCTGAGGCTTTTGAGGTA
TACTATTTCCTTCTCAAATTAAAATAGTTTGATATCTCAAAACATATACTATTATAACCCTAACTATAACAATCCAAAACXXXXXXXXXXA
TCGGTTTCTATCACTCAGAGTATCATTGATAGACTTTTATTAATTTCTATCACTGATAGACGCTGGGGCTTTTGAGGTA
>Marker810504 Chr5 SNP_site:17381831 Ref_Type:T
TACTATTATTGTTACCATTTGACTCTAAGTAGGATCAAAATTGTGACAGTAACAGTTAAATGTAAAGATAAAATAACAAAXXXXXXXXXXC
CTTAATCAGATTATTTGATTTGATTACAAATTTGGAGGTAGGACCTACGATTGTTCATTAAAATTACAAAACACAAGTA
TACTATTATTGTTACCATTTGACTCTAAGTAGGATCAAAATTGTGACTGTAACAATTAAATGTAAAGATAAAATAACAAAXXXXXXXXXXC
CTTAATCAGATTATTTGATTTGATTACAAATTTGGAGGTAGGACCTACGATTGTTCATTAAAATTACAAAACACAAGTA
>Marker810504 Chr5 SNP_site:17381838 Ref_Type:A
TACTATTATTGTTACCATTTGACTCTAAGTAGGATCAAAATTGTGACAGTAACAGTTAAATGTAAAGATAAAATAACAAAXXXXXXXXXXC
CTTAATCAGATTATTTGATTTGATTACAAATTTGGAGGTAGGACCTACGATTGTTCATTAAAATTACAAAACACAAGTA
TACTATTATTGTTACCATTTGACTCTAAGTAGGATCAAAATTGTGACTGTAACAATTAAATGTAAAGATAAAATAACAAAXXXXXXXXXXC
CTTAATCAGATTATTTGATTTGATTACAAATTTGGAGGTAGGACCTACGATTGTTCATTAAAATTACAAAACACAAGTA
>Marker810508 Chr5 SNP_site:7554163 Ref_Type:A

Create PDF with GO2PDF for free, if you wish to

ACTTTTGCTATGTAATGAAAAATGGTAAATGTAGATTCATAAAACCATAACATATTTGGAGTTGTAACACTTCAAGCTGAXXXXXXXXXXA
AAATGGTCGTATAAATTAATTTCTTCTTAAAAGTAAAACAAAGAAACTAAATTAATGTTTGATATATTACCCACTTGTA
ACTTTTGCTATGTAATGAAGAATGGTAAATGTGGATTCATAAAACCATAACATATTTGGAGTTGTAACACTTCAAGCTGAXXXXXXXXXXA
AAATGGTCGTATAAATTAATTTTTTCTTAAAAGTAAAACAAAGAAACTAAATTAATGTTTGATATATTACCCACTTGTA
>Marker810508 Chr5 SNP_site:7554176 Ref_Type:A
ACTTTTGCTATGTAATGAAAAATGGTAAATGTAGATTCATAAAACCATAACATATTTGGAGTTGTAACACTTCAAGCTGAXXXXXXXXXXA
AAATGGTCGTATAAATTAATTTCTTCTTAAAAGTAAAACAAAGAAACTAAATTAATGTTTGATATATTACCCACTTGTA
ACTTTTGCTATGTAATGAAGAATGGTAAATGTGGATTCATAAAACCATAACATATTTGGAGTTGTAACACTTCAAGCTGAXXXXXXXXXXA
AAATGGTCGTATAAATTAATTTTTTCTTAAAAGTAAAACAAAGAAACTAAATTAATGTTTGATATATTACCCACTTGTA
>Marker810508 Chr5 SNP_site:7554372 Ref_Type:C
ACTTTTGCTATGTAATGAAAAATGGTAAATGTAGATTCATAAAACCATAACATATTTGGAGTTGTAACACTTCAAGCTGAXXXXXXXXXXA
AAATGGTCGTATAAATTAATTTCTTCTTAAAAGTAAAACAAAGAAACTAAATTAATGTTTGATATATTACCCACTTGTA
ACTTTTGCTATGTAATGAAGAATGGTAAATGTGGATTCATAAAACCATAACATATTTGGAGTTGTAACACTTCAAGCTGAXXXXXXXXXXA
AAATGGTCGTATAAATTAATTTTTTCTTAAAAGTAAAACAAAGAAACTAAATTAATGTTTGATATATTACCCACTTGTA
>Marker8123 Chr4 SNP_site:14005228 Ref_Type:T
CACTGTCGCATTATTCTTATTGTTGAAAGAATTTTGGGAAGGATACTTACCTCTAGCATACCCCGTAAAGTTAGGCTCATXXXXXXXXXXG
ATCGGATAAAGGTGCCATCAATTTATCGAATTGGTTACGATATTCCTCCACCATATCCTTGAGATTCGACCAATCGGTA
CACTGTCGCATTATTCTTATTGTTGAAAGAATTTTGGGAAGGATACTTACCTCTAGCATACCCCGTAAAGTTAGGCTCATXXXXXXXXXXG
ATCGGATAAAGGTGCCATCAATTTATCGAATTGGTTACGATATTCCTCCACCATATCCTTGAGATTCGACCAATTGGTA
>Marker812445 Chr5 SNP_site:1425251 Ref_Type:A
ACTTAAACTAAGAACCACTATAATTGTCTCTAGGGTTGTTTATCTTCTTCAATCAGCCTCTCCTTATTTTGATTACAAGTXXXXXXXXXXT
GTTTTTTGTTTTTTTTAAAAGTAGGGTGATACAACTTTGGATGAGAGATTATTTAACTCGGTATCACTTATTTTCAAGT
ACTTAATCTAAGAACCACTATAATTGTCTCTAGGGTTGTTTATCTTCTTCAATCAGCCTCTCCTTATTTTGATTACAAGTXXXXXXXXXXT
GTTTTTTGTTTTTTTTAAAAGTAGGGTGATACAACTTTGGATGAGAGATTATTTAACTCGGTATCACTTATTTTCAAGT
>Marker812478 Chr5 SNP_site:25757725 Ref_Type:C
AACACACACCTATAGAAATTTCTAAATGGCTAAATTATATATGTATATATATATAAAACAGCCTTGAACTTTTATAATCCXXXXXXXXXXG
TCTTTGTTTGAAATTCTAAAAATTGATATTACAAGTGAGACGGAGGATAGCTTTGAAACACTGATGAAAGTTGAGGGTT
AACACACACCTATAGAAATTTCTAAATGGCTAAATTATATATGTATATATATATAAAATAGCCTTGAACTTTTATAATCCXXXXXXXXXXG
TCTTTGTTTGAAATTCTAAAAATTGATATTACAAGTGAGACGGAGGATAGCTTTGAAACACTGATGAAACTTGAGGGTT
>Marker812478 Chr5 SNP_site:25757928 Ref_Type:G
AACACACACCTATAGAAATTTCTAAATGGCTAAATTATATATGTATATATATATAAAACAGCCTTGAACTTTTATAATCCXXXXXXXXXXG
TCTTTGTTTGAAATTCTAAAAATTGATATTACAAGTGAGACGGAGGATAGCTTTGAAACACTGATGAAAGTTGAGGGTT
AACACACACCTATAGAAATTTCTAAATGGCTAAATTATATATGTATATATATATAAAATAGCCTTGAACTTTTATAATCCXXXXXXXXXXG
TCTTTGTTTGAAATTCTAAAAATTGATATTACAAGTGAGACGGAGGATAGCTTTGAAACACTGATGAAACTTGAGGGTT
>Marker813231 Chr5 SNP_site:3506253 Ref_Type:T
ACTTGACCCTCCTTAAACCAATTGCCTGCCTTCCTCTCATCCCACTCCTATATTAATTCCTAGGTTAATAATTAACACGCXXXXXXXXXXG
AGAACTTCTAGAAGAGTGTTTGTTGATCAATGAAAGGTTGAAAGGAAAAGAGCATCCCAGTTCAGTGAAGCACCTTGTA
ACTTGACCCTCCTTAAACCAATTGCCTGCCTTCCTCTTATCCCACTCATATATTAATTCCTAGGTTAATAATTAACACGCXXXXXXXXXXG
AGAACTTCTAGAAGAGTGTTTGTTGATCAATGAAAGGTTGAAAGGAAAAGAGCATCCCAGTTCAGTGAAGCACCTTGTA
>Marker813231 Chr5 SNP_site:3506263 Ref_Type:A
ACTTGACCCTCCTTAAACCAATTGCCTGCCTTCCTCTCATCCCACTCCTATATTAATTCCTAGGTTAATAATTAACACGCXXXXXXXXXXG
AGAACTTCTAGAAGAGTGTTTGTTGATCAATGAAAGGTTGAAAGGAAAAGAGCATCCCAGTTCAGTGAAGCACCTTGTA
ACTTGACCCTCCTTAAACCAATTGCCTGCCTTCCTCTTATCCCACTCATATATTAATTCCTAGGTTAATAATTAACACGCXXXXXXXXXXG
AGAACTTCTAGAAGAGTGTTTGTTGATCAATGAAAGGTTGAAAGGAAAAGAGCATCCCAGTTCAGTGAAGCACCTTGTA

Create PDF with GO2PDF for free, if you wish to

>Marker813438 Chr5 SNP_site:16693666 Ref_Type:A
CACACCCCAGCCAACCACAAAAAAAACCCTTTTTCTCTGAAGGGCGGATAGAGGAGGAACTCCTCAAGGATAGCTCAAACXXXXXXXXXXT
CTTCCTTCACCTTCCGAAAAAGCAAACAAAAAGGTCCAACAAGAAGAGTCCTCCTCTTAGCAAGCCTATCAACAGTGTT
CACACCCCAGCCAACCACAAAAAAAACCCTTTTTCTCTGAAGGGCGGATAGAGGAGGAACTCCTCAAGGATAGCTCGAACXXXXXXXXXXT
CTTCCTTCACCTTCCGAAAAAGCAAACAAAAAGGTCCAACAAGAAGAGTCCTCCTCTTAGCAAGCCTATCAACAGTGTT
>Marker813514 Chr5 SNP_site:6827227 Ref_Type:A
AACAACAACGTTGAGAGTTAGACATAAATTTTGCTATATCTAATTAATGTATTTGTAATTATTTTTTATTAAAATGATAAXXXXXXXXXXT
GGAGATTATAATTTGGTGATATTTTCAATAGATTATCTGGTATGAAAGGAGTGAGATTTTTGTTAAAGTAATAAAAGTC
AACAACAACGTTGAGAGTTAGACATAAATTTTGCTATATCTAATTAATGTATTTGTAATTATTTTTTATTAAAATGATAAXXXXXXXXXXT
GGAGGTTATAATTTGGTGATATTTTCAATAGATTATCTGGTATGAAAGGAGTGAGATTTTTGTTAAAGTAATAAAAGTC
>Marker814305 Chr5 SNP_site:16554752 Ref_Type:T
AACAATTTAGCATCAATGTTTGACAAAAATGAGACCTCAATTATTCATCTACAATAATATTAAATTGCCGAGACCCTAAGXXXXXXXXXXG
GGAATTCCAATATCGAACATCATATATCGTTATATATGGAAAGAAAACACTTAACGATAACTCAGAGATGCAACAAGTC
AACAATTTAGCATCAATGTTTGACAAAAATGAGACCTCAATTATTCATCTACAATAATATTAAATTGCCGAGACCCTAAGXXXXXXXXXXG
GGAATTCCAATATCGAACATCATATATCGTTATATATGGAAAGAAAACACTTATCGATAACTCAGAGATGCAACAAGTC
>Marker814426 Chr5 SNP_site:23830730 Ref_Type:A
ACCTTCATTATTTTTGTGTTCTTTTAGTTTTCTCTAACAGTAATCACATGCAGCAAAATGAGAAATCATTCTCTCTCAAAXXXXXXXXXXT
TTCTGTATAGAACTCGGTGAATGCTCGAGAGTCGAGGATTCCATATCCATGGATAGTTTTCAATGAGAAGATTAAAGTG
ACCTTCATTATTTTTGTGTTCTTTTAGTTTTCTCTAACAGTAATCACATGCAGCAAAATGAGAAATCGTTTTCTCTCAAAXXXXXXXXXXT
TTCTGTATAGAACTCGGTGAATGCTCGAGAGTCGAGGATTCCATATCCATGGATAGTTTTCAATGAGAAGATTAAAGTG
>Marker814426 Chr5 SNP_site:23830733 Ref_Type:C
ACCTTCATTATTTTTGTGTTCTTTTAGTTTTCTCTAACAGTAATCACATGCAGCAAAATGAGAAATCATTCTCTCTCAAAXXXXXXXXXXT
TTCTGTATAGAACTCGGTGAATGCTCGAGAGTCGAGGATTCCATATCCATGGATAGTTTTCAATGAGAAGATTAAAGTG
ACCTTCATTATTTTTGTGTTCTTTTAGTTTTCTCTAACAGTAATCACATGCAGCAAAATGAGAAATCGTTTTCTCTCAAAXXXXXXXXXXT
TTCTGTATAGAACTCGGTGAATGCTCGAGAGTCGAGGATTCCATATCCATGGATAGTTTTCAATGAGAAGATTAAAGTG
>Marker814890 Chr5 SNP_site:21412 Ref_Type:A
AACTACCACTATATAGTGAGAGTAACAACATGAACTACTATTTTTTTTCTTTTTTCTTTGTTTTTTTTGTGTGGTCACTTXXXXXXXXXXT
CGAAAGATCTAGAATTTTTTGTAGTAGTTGAGTAAAGAATGGTATCTGTTGGCTTCACACCGTATCTTGGACTAAGAGT
AACTACCACTATATAGTGAGAGTAACAACATGAACTACTATTTTTTTTCTTTTTTCTTTGTTTTTTTTGTGTGGTCACTTXXXXXXXXXXT
CGAAAGATCTAGAATTTTTTGTAGTAGTTGTGTAAAGAATGGTATCTGTTGGCTTCACACCGTATCTTGGACTAAGAGT
>Marker814903 Chr5 SNP_site:23816224 Ref_Type:C
CACTTTATTGATGCAAGTAGTTGGAAAAATAAAATATTTCAACAAAAAAACGAGTGATTTGAATCATACCTACATAATTCXXXXXXXXXXA
TTGGATAACGACTGTGGATTTTTGTAGGTCTGTTTTATTAATTGTTAAATTTAATTTTATCATATAGTATGGCATGAGT
CACTTTATTGATGCAAGTAGTTGGAAAAATAAAATATTTCAACAAAAAAACGAGTGATTTGAATCATACCTACATAATTCXXXXXXXXXXA
TTGGATAACGACTGTGGATTTTTGTAGGTCTGTTTTATTAATTGTTACATTTAATTTTATCATATAGTATGGCATGAGT
>Marker815084 Chr5 SNP_site:26399151 Ref_Type:T
CACACTCACGCTTTCAACCACAGCAGGATCTGTGTGCTTGTTTCTGTGGCCATAGCCAAATAGGAAAAGCTCCTAAAACAXXXXXXXXXXC
CCCACCATCATCCATCAACTTCCGCACTTTCTGGACTCCATCCCACTCACCTTGTGATGCATATATGTTAGAAAGGAGT
CACACTCACGCTTTCAACCACAGCTGGATCTGTGTGCTTGTTTCTGTGGCCATAGCCAAATAGGAAAAGATCCTAAAACAXXXXXXXXXXC
CCCACCATCATCCATCAACTTCCGCACTTTCTGGACTCCATCCCACTCACCTTGTGATGCATATATGTTAGAAAGGAGT
>Marker815084 Chr5 SNP_site:26399196 Ref_Type:A
CACACTCACGCTTTCAACCACAGCAGGATCTGTGTGCTTGTTTCTGTGGCCATAGCCAAATAGGAAAAGCTCCTAAAACAXXXXXXXXXXC
CCCACCATCATCCATCAACTTCCGCACTTTCTGGACTCCATCCCACTCACCTTGTGATGCATATATGTTAGAAAGGAGT
CACACTCACGCTTTCAACCACAGCTGGATCTGTGTGCTTGTTTCTGTGGCCATAGCCAAATAGGAAAAGATCCTAAAACAXXXXXXXXXXC

Create PDF with GO2PDF for free, if you wish to

CCCACCATCATCCATCAACTTCCGCACTTTCTGGACTCCATCCCACTCACCTTGTGATGCATATATGTTAGAAAGGAGT
>Marker816259 Chr5 SNP_site:9967404 Ref_Type:T
CACTAATATTTAAAATGATATTGATCGTAGTGATGTTATTTTATTTATATATCATTGAGATGTTCTAACTATATATATTAXXXXXXXXXXT
GGGGTGAAATGACGAAAATATCCTTATTTAATTTTACTTCACATTTGCTCTTCTCTACCAACCTCTCTCTCTTCAATGT
CACTAATATTTAAAATGATATTGATTGTAGTGATGTTATTTTATTTATATATCATTGAGATGTTCTAACTATATATATTAXXXXXXXXXXT
GGGGTGAAATGACGAAAATATCCTTATTTAATTTTACTTCACATTTGCTCTTCTCTACCAACCTCTCTCTCTTCAATGT
>Marker816300 Chr5 SNP_site:24645431 Ref_Type:T
ACTATTAATATTTTCACTTGTATCATAATTTTGATGCACAAAGAAGTTCGGACATAAATTAATATCAATTTATATGCTACXXXXXXXXXXT
TAGGAACATCATCTTTAACAAGATGGGGAAGCTCATTTCTATCCTCCTCCTTAACAAGAAGGCATACTCCAGACACGTT
ACTATTAATATTTTCACTTGTATCATAATTTTGATGCACAAAGAAGTTTGGACATAAATTAATATCAATTTATATGCTACXXXXXXXXXXT
TAGGAACATCATCTTTAACAAGATGGGGAAGCTCATTTCTATCCTCCTCCTTAACAAGAAGGCATACTCCAGACACGTT
>Marker816457 Chr5 SNP_site:3786552 Ref_Type:C
TACCAAAATGTATTGTAAAATGAAAATGGAACGCCCCAAAACATCAACTTGCAGAAAGGATGAGGTGAATAGTATTCCTCXXXXXXXXXXG
CAGAAATTCGAGACAAATTATTCAGCAAAAACAAACTACACGTCACAAATCAACAGGAAGAAAGTCACAGAAACAGAGT
TACCAAAATGTATTGTAAAATGAAAATGGAACGCCCCAAAATATCAACTTGCAGATAGGATGAGGTGAATAGTATTCCTCXXXXXXXXXXG
CAGAAATTCGAGACAAATTATTCAGCAAAAACAAACTACACGTCACAAATCAACAGGAAGAAAGTCACAGAAACAGAGT
>Marker816457 Chr5 SNP_site:3786566 Ref_Type:A
TACCAAAATGTATTGTAAAATGAAAATGGAACGCCCCAAAACATCAACTTGCAGAAAGGATGAGGTGAATAGTATTCCTCXXXXXXXXXXG
CAGAAATTCGAGACAAATTATTCAGCAAAAACAAACTACACGTCACAAATCAACAGGAAGAAAGTCACAGAAACAGAGT
TACCAAAATGTATTGTAAAATGAAAATGGAACGCCCCAAAATATCAACTTGCAGATAGGATGAGGTGAATAGTATTCCTCXXXXXXXXXXG
CAGAAATTCGAGACAAATTATTCAGCAAAAACAAACTACACGTCACAAATCAACAGGAAGAAAGTCACAGAAACAGAGT
>Marker8165 Chr4 SNP_site:21640002 Ref_Type:A
TACCTAATACAGTTCATCCGAGATGAGGGCTAAATTATGAGCAAATGGAATTTGAAAATTTTCATTTGCATGAGGAGTAGXXXXXXXXXXA
ATAGCAAAGATGAGCATATAAACCAATTGATGAAATCATTTACCTCAAAATTTATAGATGAAAATATATGTTTATAAGT
TACCTCATACAGTTCATCCGAGATGAGGGCTAAATTATGAGCAAATGGAATTTGAAAATTTTCATTTGCATGAGGAGTAGXXXXXXXXXXA
ATAGCAAAGATGAGCATATAAACCAATTGATGAAATCATTTACCTCAAAATTTATAGATGAAAATATATGTTTATAAGT
>Marker816618 Chr5 SNP_site:24827014 Ref_Type:T
TACTTTCTTCAAGGGATAAGGCTTAAAAAAAAATCAAAATTTCAGTCTTATTAAATAAGATGTGATAAGAAAGGAATCTGXXXXXXXXXXA
GCATTGTCTGTCCAATCAACCATTTTTCTAAGCTTTTTACAAAATTTAATATTAGTGGGAAACTTAACAGTAATTGGTA
TACTTTCTTCAAGGGATAAGGCTTAAAAAAAAATCAAAATTTCAGTCTTATTAAATAAGATGTGATAAGAAAGGAATCTGXXXXXXXXXXA
GCATTGTCTGTTCAATCAACCATTTTTCTAAGCTTTTTACAAAATTTAATATTAGTGGGAAACTTAACAGTAATTGGTA
>Marker816761 Chr5 SNP_site:25172045 Ref_Type:G
AACATTCTGATGTGGATCATGCTTTTCGGGTCCCTAAATTGTGCAGAAATTCTTCAAAAGAAATCTCCAAGCAGGTGCCTXXXXXXXXXXT
AGTTTCTTCTCCATCTTTGATATTGCTCGGGTATTCGACGCCTCCCTCAGCTCCTTGGTGCTTTAGCTATTGGAGGGTC
AACATTCTGATGTGGATCATGCTTTTCGGGTCCCTAAATTGTGCAGAAATTCTTCAAAAGAAATCTCCAAGCAGGTGCCTXXXXXXXXXXT
AGTTTCTTCTCCATCTTTGATATTGCTCGGGTATTCGACGCGTCCCTCAGCTCCTTGGTGCTTTAGCTATTGGAGGGTC
>Marker81733 Chr4 SNP_site:5064148 Ref_Type:C
CACTAACACACATAATGACAATAAGGTGGGTGAGAATGACGAGTCTGAGAATAACGAATCCAAGACAGTTTTTCTTGAAGXXXXXXXXXXA
CAAAACCAAGTAGGACCATTTAGAAAACATTAGTAAATATGATCTTTACCTTGATCTTCCTATTGCCCTGAGGAAAGGT
CACTAACACACATAATGACAATAAGGTGGGTGAGAATGATGAGTCTGAGAATAACGAATCCAAGACAGTTTTTCTTGAAGXXXXXXXXXXA
CAAAACCAAGTAGGACCATTTAGAAAACATTAGTAAATATGATCTTTACCTTGATCTTCCTATTGCCCTGAGGAAAGGT
>Marker818354 Chr5 SNP_site:27269654 Ref_Type:T
CACTCTCTCCTAACTTAGCTCACATTTTCGCTAATCCTATACTATATGACTGTCAAGAAAACTCGGAGAGCTTTTAGAGTXXXXXXXXXXT
TGGTTTAAGTGATCCAAATAGCAGAAATTTTGAAAACAACGTTTTCATATCTGGATTTTGAATTTGAAATCTTGATGTT

Create PDF with GO2PDF for free, if you wish to

CACTCTCTCCTAACTTAGCTCACATTTTCGCTAATCCTATACTATTTGACTGTCAAGAAAACTCGGAGAGCTTTTAGAGTXXXXXXXXXXT
TGGTTTAAGTGATCCAAATAGCAGAAATTTTGAAAACAACGTTTTCATATCTGGATTTTGAATTTGAAATCTTGATGTT
>Marker81869 Chr4 SNP_site:309876 Ref_Type:G
ACATTCAGCTTTTTGTCAAAGCACTTGGAGGAAACGTCAGTGGATCAGATAAAAGAATTCTATTCCTTTGTGGGGTAAGCXXXXXXXXXXC
ACAACTGTTCAAGAATTTTCTCACTTCAAAACAACAAACATTTTTTTGTAACGCCTGTGAGGGTATGAGTATCTAGGTC
ACATTCAGCTTTTTGTCAAGGCACTTGGAGGAAACGTCAGTGGATCAGATAAAAGAATTCTATTCCTTTGTGGGGTAAGCXXXXXXXXXXC
ACAACTGTTCAAGAATTTTCTCACTTCAAAACAACAAACATTTTTTTGTAACGCCTGTGAGGGTATGAGTATCTAGGTC
>Marker818891 Chr5 SNP_site:13052967 Ref_Type:A
ACCATAAATTAGAAAAAAATAATCAAAATTCGATATTATATTAATAAAGTAAATCTCAGATGGCTACAAGGTATATCCCCXXXXXXXXXXG
TTAGTTCTATAATATGAATTTGTTGTATATAAATGTTTTTATTTGTAAAGAAAATTATTACAAAACATATTACTTTTGT
ACCATAAATTAGAAAAAAATAATCAAAATTCGATATTATATTAATAAAGTAAATCTCAGATGGCTACAAGGTATATCCCCXXXXXXXXXXG
TTAGTTCTATTATATGAATTTGTTGTATATAAATGTTTTTATTTGTAAAGAAAATTATTACAAAACATATTACTTTTGT
>Marker818975 Chr5 SNP_site:12904394 Ref_Type:T
AACTGCCTAGGCTTAGTGTTGTAGCTGAATATGGGATACGATCTCTGTTCAAGATAATTTGCAGGAGCAGATGATGTTGAXXXXXXXXXXC
TTTAATCAAGATTTACGTGGGTAGAGCAATATTTACAACCAAATAACTAAAACAAGCTAATTAACAACTAACAAATAGT
AACTGCCTAGGCTTAGTGTTGTAGCTGAATATGGGATACGATCTCTGTTCAAGATAATTTGTTGGAGCAGATGATGTTGAXXXXXXXXXXC
TTTAATCAAGATTTACGTGGGTAGAGCAATATTTACTACCAAATAACTAAAACAAGCTAATTAACAACTAACAAATAGT
>Marker818975 Chr5 SNP_site:12904395 Ref_Type:T
AACTGCCTAGGCTTAGTGTTGTAGCTGAATATGGGATACGATCTCTGTTCAAGATAATTTGCAGGAGCAGATGATGTTGAXXXXXXXXXXC
TTTAATCAAGATTTACGTGGGTAGAGCAATATTTACAACCAAATAACTAAAACAAGCTAATTAACAACTAACAAATAGT
AACTGCCTAGGCTTAGTGTTGTAGCTGAATATGGGATACGATCTCTGTTCAAGATAATTTGTTGGAGCAGATGATGTTGAXXXXXXXXXXC
TTTAATCAAGATTTACGTGGGTAGAGCAATATTTACTACCAAATAACTAAAACAAGCTAATTAACAACTAACAAATAGT
>Marker818975 Chr5 SNP_site:12904542 Ref_Type:T
AACTGCCTAGGCTTAGTGTTGTAGCTGAATATGGGATACGATCTCTGTTCAAGATAATTTGCAGGAGCAGATGATGTTGAXXXXXXXXXXC
TTTAATCAAGATTTACGTGGGTAGAGCAATATTTACAACCAAATAACTAAAACAAGCTAATTAACAACTAACAAATAGT
AACTGCCTAGGCTTAGTGTTGTAGCTGAATATGGGATACGATCTCTGTTCAAGATAATTTGTTGGAGCAGATGATGTTGAXXXXXXXXXXC
TTTAATCAAGATTTACGTGGGTAGAGCAATATTTACTACCAAATAACTAAAACAAGCTAATTAACAACTAACAAATAGT
>Marker819170 Chr5 SNP_site:660684 Ref_Type:A
AACGTATGCCTAAAACGACCACCCAAATGAAGAGATGTGACCTAAAAAATGAAACTATAGATTTATATTTGCATGATGACXXXXXXXXXXG
TCCATTTTAGCTCCATTTTCTTCTTAACATTCGATTTTGCCTATTTACTACTTGAGACGTATAAAATAATAATGAAGTA
AACGTATGCCTAAAACGACCACCCAAATGAAGAGATGTGACCTAAAAACTGAAACTATAGATTTATATTTGCATGATGACXXXXXXXXXXG
TCCATTTTAGCTCCATTTTCTTCTTAACATTCGATTTTGCCTATTTACTACTTGAGACGTATAAAATAATAATGAAGTA
>Marker819280 Chr5 SNP_site:18214917 Ref_Type:A
GACTTGGTCTTATTTAAACTTAGGAATTAGAAAGATATGTGTGTTTGGTCTTTAAGTTCTAACAATCTATTTTTTTTTCCXXXXXXXXXXT
AATTTTGTTTTAAAAATCAAATAAAATAAATACTTGAGGATCCTTTTATACTTACTTTTAAAAGCTCACCAACTTGAGT
GACTTGGTCTTATTTAAACTTAGGAATTAGAAAGATATGTGTGTTTGGTCTTTAAGTTCTAACAATCTATTTTTTTTTCCXXXXXXXXXXT
AATTTTGTTTTAAAAATCAAATAAAATAAATACTTGAGGATCCTTTTATACTTACTTTTACAAGCTCACCAACTTGAGT
>Marker819412 Chr5 SNP_site:16718770 Ref_Type:T
AACAAAAATGCAGCTTTTTGTTTTCAAATTCAGCGTCGGATCCAATTAGCTAAAGGTAGGGTTTGCCAATTGTAATTATTXXXXXXXXXXA
ATTTATAATTTTAAAAGTTTAACAATTTAATTAAATTAACACTAGGACTAAATTGGTAAATATAATCAAGGTTTATGTT
AACAAAAATGCAGCTTTTTGTTTTCAAATTCAGCGTCGGATCCAATTAGCTAATGGTAGGGTTTGCCAATTGTAATTATTXXXXXXXXXXA
ATTTATAATTTTAAAACTTTAACAATTTAATTAAATTAACACTAGGACTAAATTGGTAAATATAATCAAGGTTTATGTT
>Marker819412 Chr5 SNP_site:16718929 Ref_Type:C
AACAAAAATGCAGCTTTTTGTTTTCAAATTCAGCGTCGGATCCAATTAGCTAAAGGTAGGGTTTGCCAATTGTAATTATTXXXXXXXXXXA

Create PDF with GO2PDF for free, if you wish to

ATTTATAATTTTAAAAGTTTAACAATTTAATTAAATTAACACTAGGACTAAATTGGTAAATATAATCAAGGTTTATGTT
AACAAAAATGCAGCTTTTTGTTTTCAAATTCAGCGTCGGATCCAATTAGCTAATGGTAGGGTTTGCCAATTGTAATTATTXXXXXXXXXXA
ATTTATAATTTTAAAACTTTAACAATTTAATTAAATTAACACTAGGACTAAATTGGTAAATATAATCAAGGTTTATGTT
>Marker819788 Chr5 SNP_site:27782076 Ref_Type:A
TACTTACTTAAGAAAAATGTTAAAATGTAAGTATATGGTAAGAAAATGATAGAAAATAGACTTAATTTTTAAAAACTAAAXXXXXXXXXXA
AAGGACAATACAACCATTTCTCATTTAAGAGATGGTTAAAAAAAGTGTATAGATAGACAACAAAATAGCATTCAACGTA
TACTTACTTAAGAAAAATGTTAAAATGTAAGTATATGGTAAGAAAATGATAGAAAATAGACTTAATTTTTAAAAACTAAAXXXXXXXXXXA
AAGGACAATACAACCATTTCTCATTTAAGAGATGGTTAAAAATAGTGTATAGATAGACAACAAAATAGCATTCAACGTA
>Marker820585 Chr5 SNP_site:4362073 Ref_Type:A
AACTATATTCAACAAAATGGTGAAGGCCATTGGAGAGCTTTGCCTAAGAAAGCTGGTGAGTTTCCTTCTATTATTCTAATXXXXXXXXXXC
AACATAACAGCTGACGAAGATGACTTGATCATAAAGCTACATTCACTTCTCGGCAATCGTTGGTCATTGATCGCTGGTA
AACTATATTCAACAAAATGGTGAAGGCCATTGGAGAGCTTTGCCTAAGAAAGCTGGTGAGTTTCCTTCTATTATTCTAATXXXXXXXXXXC
AGCATAACAGCTGACGAAGATGACTTGATCATAAAGCTACATTCACTTCTCGGCAATCGTTGGTCATTGATCGCTGGTA
>Marker820596 Chr5 SNP_site:18212903 Ref_Type:C
AACAGAACAGCATGTATTGAGAAAGCAACATCACTTGCAGCAACAGGTATCATCTGAGAGATCCAAGAAAATCAACAACAXXXXXXXXXXG
TAATCAGAAAACAAAAGGTGAAAGGATAGAAAACTCAATACATGAACATTTATCGACTAATGAAGTAATCAAGCTAGTG
AACAGAACAGCATGTATTGAGAAAGCAACATCATTTGCAGCAACAGGTATCATCTGAGAGATCCAAGAAAATCAACAACAXXXXXXXXXXG
TAATCAGAAAACAAAAGGTGAAAGGATAGAAAACTCAATACATGAACATTTATCGACTAATGAAGTAATCAAGCTAGTG
>Marker820876 Chr5 SNP_site:9278701 Ref_Type:G
CACCCCTGCCTGAGTTTGCAGCTCATCAGAAGAAACCACGCGTGAGCTTCATGCGCCACAAGAAGAGGTTGTCTCATCCCXXXXXXXXXXC
TTGGCCCATAGGGAGGTTTGCTCTAAATAGTTTTCCTTGTTTATCTCACTGTATTATTCTATTTATTCCCCCCTCTTGT
CACCCCTGCCTGAGTTTGCAGCTCATCAGAAGAAACCACGCGTGAGCTTCATGCGCCGTAAGAAGAGGTTGTCTCGTCCCXXXXXXXXXXC
TTGGCCCATAGGGAGGTTTGCTCTAAATAGTTTTCCTTGTTTATCTCACTGTATTATTCTATTTATTCCCCCCTCTTGT
>Marker820876 Chr5 SNP_site:9278702 Ref_Type:T
CACCCCTGCCTGAGTTTGCAGCTCATCAGAAGAAACCACGCGTGAGCTTCATGCGCCACAAGAAGAGGTTGTCTCATCCCXXXXXXXXXXC
TTGGCCCATAGGGAGGTTTGCTCTAAATAGTTTTCCTTGTTTATCTCACTGTATTATTCTATTTATTCCCCCCTCTTGT
CACCCCTGCCTGAGTTTGCAGCTCATCAGAAGAAACCACGCGTGAGCTTCATGCGCCGTAAGAAGAGGTTGTCTCGTCCCXXXXXXXXXXC
TTGGCCCATAGGGAGGTTTGCTCTAAATAGTTTTCCTTGTTTATCTCACTGTATTATTCTATTTATTCCCCCCTCTTGT
>Marker820876 Chr5 SNP_site:9278719 Ref_Type:G
CACCCCTGCCTGAGTTTGCAGCTCATCAGAAGAAACCACGCGTGAGCTTCATGCGCCACAAGAAGAGGTTGTCTCATCCCXXXXXXXXXXC
TTGGCCCATAGGGAGGTTTGCTCTAAATAGTTTTCCTTGTTTATCTCACTGTATTATTCTATTTATTCCCCCCTCTTGT
CACCCCTGCCTGAGTTTGCAGCTCATCAGAAGAAACCACGCGTGAGCTTCATGCGCCGTAAGAAGAGGTTGTCTCGTCCCXXXXXXXXXXC
TTGGCCCATAGGGAGGTTTGCTCTAAATAGTTTTCCTTGTTTATCTCACTGTATTATTCTATTTATTCCCCCCTCTTGT
>Marker821351 Chr5 SNP_site:20934602 Ref_Type:A
AACGCGGCACTGACTCATCATTGGACGAACTCAAATGTGTTCATACTTCTTTGGTTTGGAGCCCAAATAACATCCTTTTAXXXXXXXXXXT
AACAAATACACAATACACGAGATAGTATAAATATTCTTAATGAAAACATAACATTCTCCTTCTTTATCAGGCATGTGTA
AACGCGGCACTGACTCATCATTGGACGAGCTCAAATGTGTTCATACTTCTTTGGTTTGGAGCCCAAATAACATCCTTTTAXXXXXXXXXXT
AACAAATACACAATACACGAGATAGTATAAATATTCTTAATGAAAACAGAACATTCTCCTTCTTTATCAGGCATGTGTA
>Marker821351 Chr5 SNP_site:20934805 Ref_Type:T
AACGCGGCACTGACTCATCATTGGACGAACTCAAATGTGTTCATACTTCTTTGGTTTGGAGCCCAAATAACATCCTTTTAXXXXXXXXXXT
AACAAATACACAATACACGAGATAGTATAAATATTCTTAATGAAAACATAACATTCTCCTTCTTTATCAGGCATGTGTA
AACGCGGCACTGACTCATCATTGGACGAGCTCAAATGTGTTCATACTTCTTTGGTTTGGAGCCCAAATAACATCCTTTTAXXXXXXXXXXT
AACAAATACACAATACACGAGATAGTATAAATATTCTTAATGAAAACAGAACATTCTCCTTCTTTATCAGGCATGTGTA
>Marker822160 Chr5 SNP_site:12084935 Ref_Type:C

Create PDF with GO2PDF for free, if you wish to

CACATTCCACCTTCAAGAAGGTGAATTTCAAGTCAAGCGGTAACGTTAGGCCTCTGATTATCCACGCATATAAACACAGGXXXXXXXXXXT

ATATAAGGAATGTTTTAGGGATTGATGTAGGTATGTTTTATTTTGGTAGAAGCTCTTGTGGCTTTTAGATGGAGAAGTT

CACATTCCACCTTCAAGAAGGTGAATTTCAAGTCAAGCGGTAATGTTAGGCCTCTGATTATCCACGCATATAAACACAGGXXXXXXXXXXT

ATATAAGGAATGTTTTAGGGATTGATGTAGGTATGTTTTATTTTGGTAGAAGCTCTTGTGGCTTTTAGATGGAGAAGTT

>Marker82290 Chr4 SNP_site:12507516 Ref_Type:G

CACATATGGGATTCGAACAAAATTCATATTATGTAGGTTAAAAATTCAACTGTCTGAAGACACAATCAGTTCAAAAGTCAXXXXXXXXXXC

ACTCTATCTTCTTACACATTATCTTCACCTTCCTCTTCGTCTTCATCGTAATACTCGTCTTCGGCTGTGGCGTCTTGGT

CACATATGGGATTCGAACAAAATTCATATTATGTAGGTTAAAAATTCAACTGTCTGAAGACACAATCAGTTCAAAAGTCAXXXXXXXXXXC

ACTCTATCTTCTTACACATTATCTTCAGCTTCCTCTTCGTCTTCATCGTAATACTCGTCTTCGGCTGTGGCGTCTTGGT

>Marker82292 Chr4 SNP_site:21814531 Ref_Type:C

ACTAATTTAGGTATCAAAGTGTGATAGGTTTGACATCACATCGATTCGAGAATCCATTCTTATTTCTATTAATTTAGAGAXXXXXXXXXXT

TGATGAATTATTTTATCTATGACTGCTTCAATGCATGTTGAATTTTGACAAAACAAATTAGAACTATAGCTTAGAAGTT

ACTAATTTAGGTATCAAAGTGTGATAGGTTTGACATCACATCGATTCGAGAATCCATTCTTATTTCTATTAATTTAGAGAXXXXXXXXXXT

TGATGAATTATTTTATCTATGATTGCTTCAATGCATGTTGAATTTTGACAAAACAAATTAGAACTATAGCTTAGAAGTT

>Marker823459 Chr5 SNP_site:16319117 Ref_Type:C

ACCTAATGGACCCACATTCCCCCGCATTGCCTGCAACCTGGCTATGCTACCATAACAACTCTTGATAGTTGAAAAAGTTGXXXXXXXXXXC

TGATTTATAATCAGCCTTATTAATGCAAGTGCTTTATTCTCAAGTTCATCTCTTGTGCTAGAGATCATATTGAAAAGTT

ACTTAATGGACCCACATTCCCCCGCATTGCCTGCAACCTGGCTATGCTACCATAACAACTCTTGATAGTTGAAAAAGTTGXXXXXXXXXXC

TGATTTATAATCAGCCTTATTAATGCAAGTGCTTTATTCTCAAGTTCATCTCTTGTGCTAGAGATCATATTGAAAAGTT

>Marker823618 Chr5 SNP_site:3689568 Ref_Type:G

CACTGGTGGTCAAGAACTCATCCGGACTGGGATATCAAAACCTGTATCGAGCTTCCTGTCTTTGCAATCTATTCTGAAGCXXXXXXXXXXT

GATTTCTGGAGGGCAGTAGAAGAATGTGTAGCAATATCAGAGCCTTTCCTGAGAGTGTTGAGAGAAGTGTGTGGGGGTA

CACTGGTGGTCAAGAACTCATCCGGACTGGGATATCGAAACCTGTATCAAGCTTCCTGTCTTTGCAATCTATTCTGAAGCXXXXXXXXXXC

GATTTCTGGAGGGCAGTAGAAGAATGTGTAGCAATATCAGAGCCTTTCCTGAGAGTGTTGAGAGAAGTGTGTGGGGGTA

>Marker823618 Chr5 SNP_site:3689580 Ref_Type:A

CACTGGTGGTCAAGAACTCATCCGGACTGGGATATCAAAACCTGTATCGAGCTTCCTGTCTTTGCAATCTATTCTGAAGCXXXXXXXXXXT

GATTTCTGGAGGGCAGTAGAAGAATGTGTAGCAATATCAGAGCCTTTCCTGAGAGTGTTGAGAGAAGTGTGTGGGGGTA

CACTGGTGGTCAAGAACTCATCCGGACTGGGATATCGAAACCTGTATCAAGCTTCCTGTCTTTGCAATCTATTCTGAAGCXXXXXXXXXXC

GATTTCTGGAGGGCAGTAGAAGAATGTGTAGCAATATCAGAGCCTTTCCTGAGAGTGTTGAGAGAAGTGTGTGGGGGTA

>Marker823618 Chr5 SNP_site:3689715 Ref_Type:T

CACTGGTGGTCAAGAACTCATCCGGACTGGGATATCAAAACCTGTATCGAGCTTCCTGTCTTTGCAATCTATTCTGAAGCXXXXXXXXXXT

GATTTCTGGAGGGCAGTAGAAGAATGTGTAGCAATATCAGAGCCTTTCCTGAGAGTGTTGAGAGAAGTGTGTGGGGGTA

CACTGGTGGTCAAGAACTCATCCGGACTGGGATATCGAAACCTGTATCAAGCTTCCTGTCTTTGCAATCTATTCTGAAGCXXXXXXXXXXC

GATTTCTGGAGGGCAGTAGAAGAATGTGTAGCAATATCAGAGCCTTTCCTGAGAGTGTTGAGAGAAGTGTGTGGGGGTA

>Marker824091 Chr5 SNP_site:14713815 Ref_Type:A

ACTACATTCTCTTTTACTTTTATCCATTTACGTGCAAGACAAAGATAATAGAGCGAAATGAGTATAAAAAGTCTCTTTATXXXXXXXXXXC

GGTGCCTGACTTGCTTATCTCATCGTGTAGCAATGCACCTTATGCCTTTGCCATCCAATCTGTTTCTTTAAGACTGAGT

ACTACATTCTCTTTTACTTTTATCCATTTACGTGCAAGACAAAGATAATAGAGCGGAATGAGTATAAAAAGTCTCTTTATXXXXXXXXXXC

GGCGCCTGACTTGCTTATCTCATCGTGTAGCAATGCACCTTATGCCTTTGCCATCCAATCTGTTTCTTTAAGACTGAGT

>Marker824091 Chr5 SNP_site:14713942 Ref_Type:T

ACTACATTCTCTTTTACTTTTATCCATTTACGTGCAAGACAAAGATAATAGAGCGAAATGAGTATAAAAAGTCTCTTTATXXXXXXXXXXC

GGTGCCTGACTTGCTTATCTCATCGTGTAGCAATGCACCTTATGCCTTTGCCATCCAATCTGTTTCTTTAAGACTGAGT

ACTACATTCTCTTTTACTTTTATCCATTTACGTGCAAGACAAAGATAATAGAGCGGAATGAGTATAAAAAGTCTCTTTATXXXXXXXXXXC

GGCGCCTGACTTGCTTATCTCATCGTGTAGCAATGCACCTTATGCCTTTGCCATCCAATCTGTTTCTTTAAGACTGAGT

Create PDF with GO2PDF for free, if you wish to

>Marker824509 Chr5 SNP_site:9424053 Ref_Type:G
GACAGATGTTGCTTTACATCGAATGATCGCTCTTCAGCCCTCAAATGAGTGAATAGGAATAAGAAAATGAGAAAAAAAATXXXXXXXXXXT
TATTTGCCAATTTTGCCTTCCATCTTTTGATTGTGACTTTATTTGCCAATTTGCCCCCTTATGAGTTCATAGGGTTGTA
GACAGATGTTGCTTTACATCGAATGATCGCTCTTCAGCCCTCAAATGAGTGAATAGGAATAAGAAAATGAGAAGAAAAATXXXXXXXXXXT
TATTTGCCAATTTTGCCTTCCATCTTTTGATTGTGACTTTATTTGCCAATTTGCCCCCTTATGAGTTCATAGGGTTGTA
>Marker825640 Chr5 SNP_site:8675859 Ref_Type:C
GACACGGCTCATCAGATTAGCACTCGTTCGTCTTCTTTGTTTTTTTTATCTTACTTTTTTGCTCTTCTAATGTGGGTCTCXXXXXXXXXXC
ATCACTTTTATGTATTTTGTTATCTATCTTTTATGATTATTTTCAAAATCAAAGTCAAATACAAGAAAGCTACAAGGTG
GACACGGCTCATCAGATTAGCACTCGTTCGTCTTCTTTGTTTTTTTTATCTTACTTTTTTGCTTTTCTAATGTGGGTCTCXXXXXXXXXXC
ATCACTATTATGTATTTTGTTATCTATCTTTTATGATTATTTTCAAAATCAAAGTCAAATACAAGAAAGCTACAAGGTG
>Marker825640 Chr5 SNP_site:8675967 Ref_Type:T
GACACGGCTCATCAGATTAGCACTCGTTCGTCTTCTTTGTTTTTTTTATCTTACTTTTTTGCTCTTCTAATGTGGGTCTCXXXXXXXXXXC
ATCACTTTTATGTATTTTGTTATCTATCTTTTATGATTATTTTCAAAATCAAAGTCAAATACAAGAAAGCTACAAGGTG
GACACGGCTCATCAGATTAGCACTCGTTCGTCTTCTTTGTTTTTTTTATCTTACTTTTTTGCTTTTCTAATGTGGGTCTCXXXXXXXXXXC
ATCACTATTATGTATTTTGTTATCTATCTTTTATGATTATTTTCAAAATCAAAGTCAAATACAAGAAAGCTACAAGGTG
>Marker825812 Chr5 SNP_site:13784574 Ref_Type:T
CACCCTAAAGGGGGGAGCTGTGGTATGACGTAGAATCAAGTAAGGATGCATTACAGACTCTACTATGATGGCTGAGTGCGXXXXXXXXXXA
AAAGTGGGGCATATTGTTGATCCATTTACAAAGACTCACATGGCTAAACTGTTCGAGGGTCTTCTAGAAAGTCTAGGTC
CACCCTAAAGGGGGGAGCTGTGGTATGACGTAGAATCAAGTAAGGATGCATTACAGACTCTACTATGATGGTTGAGTGCGXXXXXXXXXXA
AAAGTGGGGCATATTGTTGATCCATTTACACAGACTCACATGGCTAAACTGTTCGAGGGTCTTCTAGAAAGTCTAGGTC
>Marker825812 Chr5 SNP_site:13784718 Ref_Type:C
CACCCTAAAGGGGGGAGCTGTGGTATGACGTAGAATCAAGTAAGGATGCATTACAGACTCTACTATGATGGCTGAGTGCGXXXXXXXXXXA
AAAGTGGGGCATATTGTTGATCCATTTACAAAGACTCACATGGCTAAACTGTTCGAGGGTCTTCTAGAAAGTCTAGGTC
CACCCTAAAGGGGGGAGCTGTGGTATGACGTAGAATCAAGTAAGGATGCATTACAGACTCTACTATGATGGTTGAGTGCGXXXXXXXXXXA
AAAGTGGGGCATATTGTTGATCCATTTACACAGACTCACATGGCTAAACTGTTCGAGGGTCTTCTAGAAAGTCTAGGTC
>Marker825841 Chr5 SNP_site:2652123 Ref_Type:G
TACTAGTGAAGCATAACAAGTTTATTAGAAGTGCATTAAGTGTATCAAGGCCTAAAGAATATCAAGTGTATAGAGAAGAAXXXXXXXXXXT
GTATATCGAGTGTATCAAATGTAACAAGGAGTATTAGATGTATCAAGGGATATTAGATTTATCGGATATATCAAATGTA
TACTAGTGAAGCATAACAAGTTTATTAGAAGTGCATTAAGTGTATCAAGGCCTAAAGAATATCAAGTGTATAGAGAAGAAXXXXXXXXXXT
GTATATCGAGTGTATCGAATGTAACAAGGAGTATTAGATGTATCAAGGGATGTTAGATTTATCGGATATATCAAATGTA
>Marker825841 Chr5 SNP_site:2652158 Ref_Type:G
TACTAGTGAAGCATAACAAGTTTATTAGAAGTGCATTAAGTGTATCAAGGCCTAAAGAATATCAAGTGTATAGAGAAGAAXXXXXXXXXXT
GTATATCGAGTGTATCAAATGTAACAAGGAGTATTAGATGTATCAAGGGATATTAGATTTATCGGATATATCAAATGTA
TACTAGTGAAGCATAACAAGTTTATTAGAAGTGCATTAAGTGTATCAAGGCCTAAAGAATATCAAGTGTATAGAGAAGAAXXXXXXXXXXT
GTATATCGAGTGTATCGAATGTAACAAGGAGTATTAGATGTATCAAGGGATGTTAGATTTATCGGATATATCAAATGTA
>Marker826474 Chr5 SNP_site:15886789 Ref_Type:C
CACCAGAGTTTAAAAGAATTACATGTATATAACTTATCTTTTCGACACAATATCCATAATTACAACAAAATCACGTCACTXXXXXXXXXXT
AGATCTAACTCCTAGATAATAAAAGCATATCACTTATAGATTTTGAAATACTTTAATTTTTTTTTAAAAAACGTTGGTC
CACTAGAGTTTAAAAGAATTACATGTATATAACTTATCTTTTCGACACAATATCCATAATTACAACAAAATCACGTCACTXXXXXXXXXXT
AGATCTAACTCCTAGATAATAAAAGCATATCACTTATAGATTTTGAAATACTTTAATTTTTTTTTAAAAAACGTTGGTC
>Marker827429 Chr5 SNP_site:16000897 Ref_Type:T
AACCTCTTGGTTCTCAGCATCAACTTATTCATTTTGCATTATATGATCTACCATGCCTTCAAACCCCGTGCAGACTCCTTXXXXXXXXXXT
CAAAAGAACATTAATAGTTTTTAAAAGTAAGACTTTGATTCGTAACATTTTTCTATGCTCAATGTGTTTGAAAATTGTG
AACCTCTTGGTTCTCAGCATCAACTTATTCATTTTGCATTATATGATCTACCATGCCTTCAAACCCCGTGCAGACTCCTTXXXXXXXXXXT

Create PDF with GO2PDF for free, if you wish to

CAAAAGAATATTAATAGTTTTTAAAAGTAAGACTTTGATTCGTAACATTTTTCTATGCTCAATGTGTTTGAAAATTGTG
>Marker827560 Chr5 SNP_site:11650717 Ref_Type:G
ACCATGAATCATCTAATGAACAACTGGTTTAAGGTCCAACCTATAAACTGAATCCCCTCTCAAGCTAATGAGAGGATGGGXXXXXXXXXXA
GGGAAGACATATTGGGTCAGTGTTGATGAGCTGACCTCACCTATGCAGATCTAAGAATAATTTCGTCTGAATATGAGTT
ACCATGAATCATCTGATGAACAACTGGTTTAAGGTCCAACCTATAAACTGAATCCCCTCTCAAGCTAATGAGAGGGTGGGXXXXXXXXXXA
GGGAAGACATATTGGGTCAGTGTTGATGAGCTGACCTCACCTATGCAGATCTAAGAATAATTTCGTCTGAATATGAGTT
>Marker827560 Chr5 SNP_site:11650778 Ref_Type:G
ACCATGAATCATCTAATGAACAACTGGTTTAAGGTCCAACCTATAAACTGAATCCCCTCTCAAGCTAATGAGAGGATGGGXXXXXXXXXXA
GGGAAGACATATTGGGTCAGTGTTGATGAGCTGACCTCACCTATGCAGATCTAAGAATAATTTCGTCTGAATATGAGTT
ACCATGAATCATCTGATGAACAACTGGTTTAAGGTCCAACCTATAAACTGAATCCCCTCTCAAGCTAATGAGAGGGTGGGXXXXXXXXXXA
GGGAAGACATATTGGGTCAGTGTTGATGAGCTGACCTCACCTATGCAGATCTAAGAATAATTTCGTCTGAATATGAGTT
>Marker827820 Chr5 SNP_site:13448063 Ref_Type:A
CACTTGCTGCTCTCTACTATGACTATTTATAACATATGACTTTGTGTAGTAAATACTATTTTACAACACTTTTTCTATAAXXXXXXXXXXT
ACAAACATAAACTATATCATAATCCATACTAAAATATATAACATGTTCTTGAATATCAATTATTATAACTTGCTTATGT
CACTTGCTGCTCTCTACTATGACTATTTATAACATATGACTTTGTGTAGTAAATACTATTTTACAACACTTTTTCTATAAXXXXXXXXXXT
ACGAACATAAACTATATCATAATCCATACTAAAATATATAACATGTTCTTGAATATCAATTATTATAACTTGCTTATGT
>Marker828775 Chr5 SNP_site:5977536 Ref_Type:C
CACTATGATGTCGAAAATTAATTTTTGATAGCTGATCAGTATTCATTACATTTTATGATTTCTTTTAGGTAACAACAAATXXXXXXXXXXA
TGGGAGTCCTCACCCTTCTCTTCGTTCTAACGAAGAAACTAACCTTCAATATATACTACATATGTATAGGTTCAAGGGT
CACTATGATGTCGAAAATTAATTTTTGATAGCTGATCAGTATTCATTACATTTTATGATTTCTTTTAGGTAACAACAAATXXXXXXXXXXA
TGGGAGTCCTCACCCTTCTCTTTGTTCTAACGAAGAAACTAACCTTCAATATATACTACATATGTATAGGTTCAAGGGT
>Marker832090 Chr7 SNP_site:15456787 Ref_Type:A
CACAATTTTGCTGTTTCTCATGGTCTTGAAACTTCATCGAGTAGAAATAACAAAGTTTTGTCTTTTGGTATAGAAATTGAXXXXXXXXXXG
CATTGTTTCAAATGGTGGCAAATTTGGTTTTAAGTGTAATACCAATGTTGTTGCTTCTCATGGTTTTGGAATTCAGGGT
CACAATTTTGCTGTTTCTCGTGGTCTTGAAACTTCATCGAGTAGAAATAACAAAGTTTTGTCTTTTGGTATAGAAATTGAXXXXXXXXXXG
CATTGTTTCAAATGGTGGCAAATTTGGTTTTAAGAGTAATACCAATGTTGTTGCTTCTCATGGTTTTGGAATTCAGGGT
>Marker832090 Chr7 SNP_site:15456981 Ref_Type:T
CACAATTTTGCTGTTTCTCATGGTCTTGAAACTTCATCGAGTAGAAATAACAAAGTTTTGTCTTTTGGTATAGAAATTGAXXXXXXXXXXG
CATTGTTTCAAATGGTGGCAAATTTGGTTTTAAGTGTAATACCAATGTTGTTGCTTCTCATGGTTTTGGAATTCAGGGT
CACAATTTTGCTGTTTCTCGTGGTCTTGAAACTTCATCGAGTAGAAATAACAAAGTTTTGTCTTTTGGTATAGAAATTGAXXXXXXXXXXG
CATTGTTTCAAATGGTGGCAAATTTGGTTTTAAGAGTAATACCAATGTTGTTGCTTCTCATGGTTTTGGAATTCAGGGT
>Marker832230 Chr7 SNP_site:3331797 Ref_Type:T
GACACATTTCTTAGTTTGATTCCGAAGGAAAAGGTCATTTAATTAAATACTTTTTAGAAAACTTTTATACATGGTTTTTGXXXXXXXXXXC
ATGATATTACTGATTAGCTAATTTGCATATGCCATTCATTATTTTATGGTATCTAAAACAAAAGGATGTTTATAAAAGT
GACACATTTCTTAGTTTGTTTCCGAAGGAAAAGGTCATTTAATTAAATACTTTTTAGAAAACTTTTATACATGGTTTTTGXXXXXXXXXXC
ATGATATTACTGATTAGCTCATTTGCATATGCCATTCATTATCTTATGGTATCTAAAACAAAAGGATGTTTATAAAAGT
>Marker832230 Chr7 SNP_site:3331999 Ref_Type:C
GACACATTTCTTAGTTTGATTCCGAAGGAAAAGGTCATTTAATTAAATACTTTTTAGAAAACTTTTATACATGGTTTTTGXXXXXXXXXXC
ATGATATTACTGATTAGCTAATTTGCATATGCCATTCATTATTTTATGGTATCTAAAACAAAAGGATGTTTATAAAAGT
GACACATTTCTTAGTTTGTTTCCGAAGGAAAAGGTCATTTAATTAAATACTTTTTAGAAAACTTTTATACATGGTTTTTGXXXXXXXXXXC
ATGATATTACTGATTAGCTCATTTGCATATGCCATTCATTATCTTATGGTATCTAAAACAAAAGGATGTTTATAAAAGT
>Marker832230 Chr7 SNP_site:3332022 Ref_Type:C
GACACATTTCTTAGTTTGATTCCGAAGGAAAAGGTCATTTAATTAAATACTTTTTAGAAAACTTTTATACATGGTTTTTGXXXXXXXXXXC
ATGATATTACTGATTAGCTAATTTGCATATGCCATTCATTATTTTATGGTATCTAAAACAAAAGGATGTTTATAAAAGT

Create PDF with GO2PDF for free, if you wish to

GACACATTTCTTAGTTTGTTTCCGAAGGAAAAGGTCATTTAATTAAATACTTTTTAGAAAACTTTTATACATGGTTTTTGXXXXXXXXXXC
ATGATATTACTGATTAGCTCATTTGCATATGCCATTCATTATCTTATGGTATCTAAAACAAAAGGATGTTTATAAAAGT
>Marker832385 Chr7 SNP_site:9967471 Ref_Type:C
CACTAGATTAAACATTTGAATCAAGCAAGATAAGATTGGTTTCATTTTGTTGGACTGACATCTATTCCATAGTTGGGGTTXXXXXXXXXXA
TAAATAAACAATGTGTTTTCGAAAACCATAAAAAAATGTCATATAATCCAACACCAAAAAAGATACTATTAACAATGTT
CACTAGATTAAACATTTGAATCAAGCAAGATAAGATTGGTTTCATTTTGTTGGACTGACATCTATTCCATAGTTGGGGTTXXXXXXXXXXA
TAAATAAACAATGTGTTTTCGAAAACCATAAAAAAATGTCATATAATCCAATACCAAAAAAGATACTATTAACAATGTT
>Marker832625 Chr7 SNP_site:8638615 Ref_Type:A
TACTAATCAAAAGGAATTAGTTAGACCAGTGAAGAATCGCTTTACTACAACTTGCATGACATTATGATGGAAGCCAACTAXXXXXXXXXXA
AGAAGCTCGACCATTTATGAGGTTATGGATAGAGCTAATGAGACCATCGCTAAATCCTTTGATGGTAAGGAAGAAAAGT
TACTAATCAAAAGGAATTAGTTAGACCGGTGAAGAATCGCTTTACTACAACTTGCATGACATTATGATGGAAGCCAACTAXXXXXXXXXXA
AGAAGCTCGACCATTTATGAGGTTATGGATAGAGCTAATGAGACCATCGCTAAATCCTTTGATGGTAAGGAAGAAAAGT
>Marker833154 Chr7 SNP_site:15463131 Ref_Type:G
AACTAAATTTTTGGACGGAGTTGGACCCAAGTGCTTTAAAGTCAATTTTTGGACGGAGTTGGATCAATTCAAACGGTCTGXXXXXXXXXXC
TATTTGTAACATTAACAACATCAGTTTGGCTTATATGAACACCATCAGTGTTTGGGCTATTACCAGGAGCAACTATGTG
AACTAAATTTTTGGACGGAGTTGGACCCAAGTGCTTTAAAGTCAATTTTTGGACGGAGTTGGATCAATTCAAACGGTCTTXXXXXXXXXXC
TATTTGTAACATTAACAACATCAGTTTGGCTTATATGAACACCATCAGTGTTTGGGCTATTACCAGGAGCAACTATGTG
>Marker833272 Chr7 SNP_site:2486602 Ref_Type:A
TACAAAAATTAATTTTAAAAAATAGAGATCCAGTAATAAAATTTGATTGAAAGAGAGTAAAAATAAGACATCTCTTACTTXXXXXXXXXXT
CTACCAAAATTATTGCTTATAATGTTTTCACAATTTAAAACGAACATCTTTCTTTATTGTTCTTTTTAGATTTTAAGTG
TACAAAAATTAATTTTAAAAAATAGAGATCCAGTAATAAAATTTGATTGAAAGAGAGTTAAAATAAGACATCTCTTACTTXXXXXXXXXXT
CTACCAAAATTATTGCTTATAATGTTTTCACAATTTAAAACGAACATCTTTCATTATTGTTCTTTTTAGATTTTAAGTG
>Marker833272 Chr7 SNP_site:2486768 Ref_Type:T
TACAAAAATTAATTTTAAAAAATAGAGATCCAGTAATAAAATTTGATTGAAAGAGAGTAAAAATAAGACATCTCTTACTTXXXXXXXXXXT
CTACCAAAATTATTGCTTATAATGTTTTCACAATTTAAAACGAACATCTTTCTTTATTGTTCTTTTTAGATTTTAAGTG
TACAAAAATTAATTTTAAAAAATAGAGATCCAGTAATAAAATTTGATTGAAAGAGAGTTAAAATAAGACATCTCTTACTTXXXXXXXXXXT
CTACCAAAATTATTGCTTATAATGTTTTCACAATTTAAAACGAACATCTTTCATTATTGTTCTTTTTAGATTTTAAGTG
>Marker833465 Chr7 SNP_site:15191680 Ref_Type:G
TACTCTATCAACTTTAATTCTTTAACAATTTATTTTATAATACTTATGCATACTTTTACTACACAACAATTTAGTTTTTTXXXXXXXXXXA
AATGGCTACATTTGTTTTGTGAATAAAATTTGTCACTATTGTGGTAGAGAACATGAGTTTAGTATGTATGGTAGTTTGT
TACTCTATCAACTTTAATTCTTTAACAATTTATTTTATAATACTTATGCATACTTTTACTACACAACAATTTAGTTTTTTXXXXXXXXXXA
AATGGCTACATTTGTTTTGTGAATAAAATTTGTCACTATTGTGGTAGAGAACATGAGTTTAGTATGTATGTTAGTTTGT
>Marker833705 Chr7 SNP_site:16627154 Ref_Type:T
ACATCGCCGTCGATTCCCATTTGTTCCTGATTCACTTCAATTTCCTCTACTTCAATCTCAAATCTCAATCCCAACTTCTCXXXXXXXXXXT
TTCTCACTGTATTCAAGGGTTTCCGCCCGGTTGCGAGCTGGCTATGGACTACGAGCCCTATGATAGTAGCGGTAAAGTT
ACATCGCCGTCGATTCCCATTTGTTCCTGATTCACTTCAATTTTCTCTACTTCAATCTCAAATCTCAATCCCAACTTCTCXXXXXXXXXXT
TTCTCACTGTATTCAAGGGTTTCCGCCCGGTTGCGAGCTGGCTATGGACTACGAGCCCTATGATAGTAGCGGTAAAGTT
>Marker833896 Chr7 SNP_site:4724040 Ref_Type:C
ACCTTTTATTTGTAATGTTGTATTTATTTGAAAAATTAATAGAACATTATATTTATTGTTTTCCCATTATTATTGCGTCAXXXXXXXXXXA
CATAAGATATAAGATTAATAATTGATGTGATTGTTCATTCTAAAGTGGAAGATTCGATCTTCATCCTCACAATTGTTGT
ACCTTTTATTTGTAATGTTGTATTTATTTGAAAAATTAATAGAACATTATATTTATTGTTTTCCCATTATTATTGCGTCAXXXXXXXXXXA
CATAAGATATAAGATTAATAATTGATGTGATTGTTCATTCTAAAGTGGAAGATTGGATCTTCATCCTCACAATTGTTGT
>Marker83400 Chr4 SNP_site:14051922 Ref_Type:C
GACAAGTTACTAAGAGCCTAAAGATGACTAAGCTATTTTGAAAAGTAATATAATTAGGAGAAGAGGTCAAGAAAGGAGGGXXXXXXXXXXT

Create PDF with GO2PDF for free, if you wish to

GAGTAATTTTTCAAAATTAAGATTGATTTAAACTTCCTTCCAACGTTTCTAGTATTTTGTGATTTGTGTGTTTCATGTT
GACAAGTTACTAAGAGCTTAAAGATGACTAAGCTATTTTGAAAAGTAATATAATTAGGAGAAGAGGTCAAGAAAGGAGGGXXXXXXXXXXT
GAGTAATTTTTCAAAATTAAGATTGATTTAAACTTCCTTCCAACGTTTCTAGTATTTTGTGATTTGTGTGTTTCATGTT
>Marker834143 Chr7 SNP_site:7645406 Ref_Type:T
TACACTAATTTTTCATTGTTATTAAAATTAATCAAACTTTTTCTGCATCATACTTATTTTAAGTTAGTCACTAGAAAGTAXXXXXXXXXXA
CCAATTCTAAACAAAATTTCAAAGTTAAGAGAAAACCAACACATTCTTTTGGCAAGCTATTGTTCATCATTTGATAGTT
TACACTAATTTTTCATTGTTATTAAAATTAATCAAACTTTTTCTGCATCATACTTATTTTAAGTTAGTCACTAGAAAGTAXXXXXXXXXXA
CCAATTCTAAACAAAATTTCAAAGTTAAGAGAAAACCAACACATTCTTTTTGCAAGCTATTGTTCATCATTTGATAGTT
>Marker834265 Chr7 SNP_site:13344054 Ref_Type:G
CACCCCATTGTTTATATACCTCTAAATTCCTGGCAGAGATGCTATCATCTATACTCGTTAATTGTCCATTTACACTAGAAXXXXXXXXXXC
CAATGTTGAAAACAAGATTGTAAATGACATAAGCGGCAGCCATACAAACCAAACACAAGAACCTATTTTAAAGAATGTA
CACCCCATTGTTTATATACCTCTAAATTCCTGGCAGAGATGCTATCATCTATACTCGTTAATTGTCCATTTACACTAGAAXXXXXXXXXXC
CAATGTTGAAAACAAGATTGTAAATGACATAAGCGGCAGCGATACAAACCAAACACAAGAACCTATTTTAAAGAATGTA
>Marker834274 Chr7 SNP_site:12695156 Ref_Type:A
ACTATTATGGTAAAACTATGGATACGACTCACTTTGTATTTGATACAAATGCCATGATTTAATACATTCATGTAGGTGACXXXXXXXXXXA
CATAAGTATTTAAAAATACATCTTACATAGATATCATGCCTGTTTTATGCGATTGCCATGTTTAAATGCAGGATAAGTG
ACTATTCTGGTAAAACTATGGATACGACTCACTTTGTATTTGATACAAATGTCATGATTTAATACATTCATGTAGGTGACXXXXXXXXXXA
CATAAGTATTTAAAAATACATCTTACATAGATATCATGCCTGTTTTATGCGATTGCCATGTTTAAATGCAGGATAAGTG
>Marker834274 Chr7 SNP_site:12695201 Ref_Type:C
ACTATTATGGTAAAACTATGGATACGACTCACTTTGTATTTGATACAAATGCCATGATTTAATACATTCATGTAGGTGACXXXXXXXXXXA
CATAAGTATTTAAAAATACATCTTACATAGATATCATGCCTGTTTTATGCGATTGCCATGTTTAAATGCAGGATAAGTG
ACTATTCTGGTAAAACTATGGATACGACTCACTTTGTATTTGATACAAATGTCATGATTTAATACATTCATGTAGGTGACXXXXXXXXXXA
CATAAGTATTTAAAAATACATCTTACATAGATATCATGCCTGTTTTATGCGATTGCCATGTTTAAATGCAGGATAAGTG
>Marker834359 Chr7 SNP_site:445293 Ref_Type:T
ACTTAGAATCTTGTCCTTTTGTATTTCTATGCTTCCATGTGAAATGTGCTGTATTCGACAGCTTTGGGTCTAGAGATCTTXXXXXXXXXXG
AGGGGACTCTTGTATGTGTTGGCATGTCGTGGTTTTGACTTCACACATCGACCAGGCACACGGGACTCTCGGATCTGTT
ACTTAGAATCTTGTCCTTTTGTATTTCTATGCTTCCATGTGAAATGTGCTGTATTCGACAGCTTTGGGTCTAGAGATCTTXXXXXXXXXXG
AGGGGACTCTTGTATGTGTTGGCATGTCGTGGTTTTGACTTCACACATCGACCAGGCACACGGGACTTTCGGATCTGTT
>Marker83525 Chr4 SNP_site:18847861 Ref_Type:T
ACCTCCCCGTGAGATGACACACGAACGCCTAGTAAGCGGAACAACTCTTTTTCTTACCTTTTTACAACGCATTTCTTAAGXXXXXXXXXXT
GATAAGCACAAAAGAAAAACACAGCAGAAATAACAAATTAAAATTCCTCTCTTTATTAAATGAACACTTTTACAAAGTT
ACCTCCCTGTGAGATGACACACGAACGCCTAGTAAGCGGAACAACTCTTTTTCTTACCTTTTTACAACGCATTTCTTAAGXXXXXXXXXXT
GATAAGCACAAAAGAAAAACACAGCAGAAATAACAAATTAAAATTCCTCTCTTTATTAAATGAACACTTTTACAAAGTT
>Marker835313 Chr7 SNP_site:15269577 Ref_Type:T
ACATCACTTGCTCTAGAGGAGAACCAATCATACTCATGGCATCATGGAAGCAGGGTTTCCTCTTTTATGATGCATGAACAXXXXXXXXXXT
TTCTATAATCATATTTTCACAGGAGTGTGTTAGGGGAATTCTAAGAGACAAGACTATCATACTTGTGACTCACCAAGTT
ACATCACTTGCTCTAGAGGAGAACCAATCATACTCATGGCATCATGGAAGCAGGGTTTCTTCTTTTATGATGCATGAACAXXXXXXXXXXT
TTCTATAATCATATTTTCACAGGAGTGTGTTAGGGGAATTCTAAGAGACAAGACTATCATACTTGTGACTCACCAAGTT
>Marker835808 Chr7 SNP_site:6233947 Ref_Type:T
CACCAAGATTCATTGATTGACTTATGGGTTATCTGCAGGTTGTATTGAAGGAAAGCAAAAATTCCCATGGAGAATTAGACXXXXXXXXXXA
ACGAACCAAAAATCAGCGAATATGGGATCACAAAGTTTCTAGACGCTAAGAGAGTTCATCTTCTTTCCTCCAAGGGGTA
CACCAAGATTCATTGATTGACTTATGGGTTATCTGCAGGTTGTATTGAAGGAAAGCAAAAATTCCCATGGAGAATTAGACXXXXXXXXXXA
ATGAACCAAAAATCAGCGAATATGGGATCACAAAGTTTCTAGACGCTAAGAGAGTTCATCTTCTTTCCTCCAAGGGGTA
>Marker835816 Chr7 SNP_site:3471207 Ref_Type:T

Create PDF with GO2PDF for free, if you wish to

AACGTATCTTACATATAAAGTTTGTATAAGACTTGACAACAAAATAATTATTCTCTATTTGAAAATTTGATAATGGAAGAXXXXXXXXXXC
AATGCCACAAGATTTCAATCAAACTCCATTCATTTATATATACTTCTGAAGTGAAGATGCAAAAAGGTAACAATCAGTA
AACGTATCTTACATATAAAGTTTGTATAAGACTTGACAACAAAATAATTATTCTCTATTTGAAAATTTGATAATTGAAGAXXXXXXXXXXC
AATGCCACAAGATTTCAATCAAACTCCATTCATTTATATATACTTCTGAAGTGAAGATGCAAAAAGGTAACAATCAGTA
>Marker836054 Chr7 SNP_site:12196240 Ref_Type:A
CACAATGCTATTAGTTGTAAATGTAATTCTTCCTGTCATTGTAGATTTCCAGTATGCCTTGGACACTTGAATGATCAATTXXXXXXXXXXG
TTAGAAGTGACTTTTTATTTTTTTAGTTTATATTGTGTCACAATTCATTTCTCTGCTAAGTTATGTAAATAACTCAGTT
CACAATGCTATTAGTTGTAAATGTAATTCTTCCTGTCATTGTAGATTTCCAGTATGCCTTGGACACTTGAATGATCAATTXXXXXXXXXXG
TTAGAAGTGACTTTTTCTTTTTTTAGTTTATATTGTGTCACAATTCATCTCTCTGCTAAGTTATGTAAATAACTCAGTT
>Marker836054 Chr7 SNP_site:12196272 Ref_Type:T
CACAATGCTATTAGTTGTAAATGTAATTCTTCCTGTCATTGTAGATTTCCAGTATGCCTTGGACACTTGAATGATCAATTXXXXXXXXXXG
TTAGAAGTGACTTTTTATTTTTTTAGTTTATATTGTGTCACAATTCATTTCTCTGCTAAGTTATGTAAATAACTCAGTT
CACAATGCTATTAGTTGTAAATGTAATTCTTCCTGTCATTGTAGATTTCCAGTATGCCTTGGACACTTGAATGATCAATTXXXXXXXXXXG
TTAGAAGTGACTTTTTCTTTTTTTAGTTTATATTGTGTCACAATTCATCTCTCTGCTAAGTTATGTAAATAACTCAGTT
>Marker836158 Chr7 SNP_site:16624834 Ref_Type:T
AACAACGAAACTTCATGTTTCAAATAACACTTTAAGTAATACCGCTTCCCATAAAACAAACTAGATGAGTGATGATTAGCXXXXXXXXXXA
GGCTTAAGAAACAGTTCTTTCAGAAGTCCAAAACTTGAAGATTGAACACGGTAAAATGAATTTTGATATACAAAATGTT
AACAACGAAACTTCATGTTTCAAATAACACTTTAAGTAATACTGCTTCCCATAAAACAAACTAGATGAGTGATGATTAGCXXXXXXXXXXA
GGCTTAAGAAACAGTTCTTTCAGAAATCCAAAACTTGAAGAATGAACACGGTAAAATGAATTTTGATATACAAAATGTT
>Marker836158 Chr7 SNP_site:16625021 Ref_Type:A
AACAACGAAACTTCATGTTTCAAATAACACTTTAAGTAATACCGCTTCCCATAAAACAAACTAGATGAGTGATGATTAGCXXXXXXXXXXA
GGCTTAAGAAACAGTTCTTTCAGAAGTCCAAAACTTGAAGATTGAACACGGTAAAATGAATTTTGATATACAAAATGTT
AACAACGAAACTTCATGTTTCAAATAACACTTTAAGTAATACTGCTTCCCATAAAACAAACTAGATGAGTGATGATTAGCXXXXXXXXXXA
GGCTTAAGAAACAGTTCTTTCAGAAATCCAAAACTTGAAGAATGAACACGGTAAAATGAATTTTGATATACAAAATGTT
>Marker836158 Chr7 SNP_site:16625037 Ref_Type:A
AACAACGAAACTTCATGTTTCAAATAACACTTTAAGTAATACCGCTTCCCATAAAACAAACTAGATGAGTGATGATTAGCXXXXXXXXXXA
GGCTTAAGAAACAGTTCTTTCAGAAGTCCAAAACTTGAAGATTGAACACGGTAAAATGAATTTTGATATACAAAATGTT
AACAACGAAACTTCATGTTTCAAATAACACTTTAAGTAATACTGCTTCCCATAAAACAAACTAGATGAGTGATGATTAGCXXXXXXXXXXA
GGCTTAAGAAACAGTTCTTTCAGAAATCCAAAACTTGAAGAATGAACACGGTAAAATGAATTTTGATATACAAAATGTT
>Marker836330 Chr7 SNP_site:13383441 Ref_Type:A
GACCAACCAAAAGGCTTCAAAAGGAGATCCCAAAATTGTCGAGCAGAAGGACAATGAATAAAAAAATGAACATGAGATTTXXXXXXXXXXC
CATCCAAATGATCGAATTAAAATCCCTTTCAGAAGGCTCGAGAGCACCTGTCAAGTCATCAATCATAGATTTCACAGTA
GACCAACCAAAAGGCTTCAAAAGGAGATCCCATAATTGTCGAGCAGAAGGACAATGAATAAAAAAATGAACATGAGATTTXXXXXXXXXXC
CATCCAAATGATCGAATTAAAATCCCTTTCAGAAGGCTCGAGAGCACCTGTCAAGTCATCAATCATAGATTTCACAGTA
>Marker836440 Chr7 SNP_site:8127034 Ref_Type:C
CACCTTTAACTAAAGGGAAGATGAAAAGCATTATCATAGAGACTAGGTAAGTTCTCTCCTTTCTCTCCTCTCTCTTTTCC-XXXXXXXXXX
CAAGATGAAGATTTTGAGGCAAATAAAATGCTGCCATTATCAAGCTCTGCTGATTTCAAAAAAAGATGGATAGCTTTGTG
CACCTTTAACTAAAGGGAAGATGAAAAGCATTATCATAGAGACTAGGTAAGTTCTCTCCTTTCTCTCCTCTCT-TTTCCCTXXXXXXXXXX
CAAGATGAAGATTTTGAGGCAAATAAAATGCTGCCATTATCAAGCTCTGCTGATTTCAAAAAAAGATGGATAGCTTTGTG
>Marker83654 Chr4 SNP_site:7535823 Ref_Type:T
TACCACGACTACAAGATGAAGATCGATCTTCCTACATATAGTGGCAAGAGTAGTATAGAGTCCTTTTTGTTGGGATTTATXXXXXXXXXXT
TGAACAGTATGTAGAGACATATATGGATCGATGTTCAAAATCAGCTTAAAGGGTCTATAGTATAGGAATAAGGTTGGGT
TACCACGACTACAAGATGAAGATCGATCTTCCTACATATAGTGGCAAGAGTAGTATAGAGTCCTTTTTGTTGGGATTTATXXXXXXXXXXT
TGAACAGTATGTAGAGACATATATGGATCGATGTTCAAAATCAGCTTAAATGGTCTATAGTATAGGAATAAGGTTGGGT

Create PDF with GO2PDF for free, if you wish to

>Marker83705 Chr4 SNP_site:10996622 Ref_Type:G
ACCACACGGCGCACAATGTTGGATACGTCGGGGCATGATTCGTCGTAAAACGTGGGGCAGAGCTGAGCTTGAGAAGCTCTXXXXXXXXXXT
TTTTTTTTGTTACTAAGAAAAAAAATAATTGGGTTATGAATATGACAGGGTGGTGGTGGCTAAGTCACCGGCGAGAGTT
ACCACACGGCGCACAATGTTGGATACGTCGGGGCATGATTCGTCGTAAAACGTGGGGCAGAGCTGAGCTTGAGAAGCTCTXXXXXXXXXXT
TTTTTTTTGTTACTAAGAAAAAGAATAATTGGGTTATGAATATGACAAGGTGGTGGTGGCTAAGTCACCGGCGAGAGTT
>Marker83705 Chr4 SNP_site:10996647 Ref_Type:A
ACCACACGGCGCACAATGTTGGATACGTCGGGGCATGATTCGTCGTAAAACGTGGGGCAGAGCTGAGCTTGAGAAGCTCTXXXXXXXXXXT
TTTTTTTTGTTACTAAGAAAAAAAATAATTGGGTTATGAATATGACAGGGTGGTGGTGGCTAAGTCACCGGCGAGAGTT
ACCACACGGCGCACAATGTTGGATACGTCGGGGCATGATTCGTCGTAAAACGTGGGGCAGAGCTGAGCTTGAGAAGCTCTXXXXXXXXXXT
TTTTTTTTGTTACTAAGAAAAAGAATAATTGGGTTATGAATATGACAAGGTGGTGGTGGCTAAGTCACCGGCGAGAGTT
>Marker837129 Chr7 SNP_site:643126 Ref_Type:C
CACTACATGTATTTACCTTTGTTTGTTTTGTATTAGAGTATGCCTTCTTGTCCTCCTTTAAGTGCAACCACGTGTAACTTXXXXXXXXXXA
TGAACTGCCCAACTGCTTTGATCAAACAAGTGTGATTGTGAAACTTGATACAATGAGATGTAGAACATGAATTGGGGTG
CACTACATGTATTTACCTTTGTTTGTTTTGTATTAGAGTATGCCTTCTTGTCCTCCTTTAAGTGCAAGCACGTGTAACTTXXXXXXXXXXA
TGAACTGCCCAACTGCTTTGATCAAACAAGTGTGATTGTGAAACTTGATACAATGAGATGTAGAACATGAATTGGGGTG
>Marker837272 Chr7 SNP_site:16420219 Ref_Type:C
CACATTCTTTAACTTTCTTTTCTGTTGTGGGGTGGGGGATGGTGCCAGCCAGCAAAGGGTGGATTTTAGTTCCCTTGTCTXXXXXXXXXXC
CTAGTTCGTCCCATTATTTCAGCTTACTTAAAGATTACATTTGTATATGTTTCTGCATCTTCATTAATTTCACCGAGTG
CACATTCTTTAACTTTCTTTTTTGTTGTGGGGTGGGGGTTGGTGCCAGCCAGCAAAGGGTGGATTTTAGTTCCCTTGTCTXXXXXXXXXXC
CTAGTTCGTCCCATTATTTCAGCTTACTTAAAGATTACATTTGTATATGTTTCTGCATCTTCATTAATTTCACCGAGTG
>Marker837272 Chr7 SNP_site:16420236 Ref_Type:A
CACATTCTTTAACTTTCTTTTCTGTTGTGGGGTGGGGGATGGTGCCAGCCAGCAAAGGGTGGATTTTAGTTCCCTTGTCTXXXXXXXXXXC
CTAGTTCGTCCCATTATTTCAGCTTACTTAAAGATTACATTTGTATATGTTTCTGCATCTTCATTAATTTCACCGAGTG
CACATTCTTTAACTTTCTTTTTTGTTGTGGGGTGGGGGTTGGTGCCAGCCAGCAAAGGGTGGATTTTAGTTCCCTTGTCTXXXXXXXXXXC
CTAGTTCGTCCCATTATTTCAGCTTACTTAAAGATTACATTTGTATATGTTTCTGCATCTTCATTAATTTCACCGAGTG
>Marker83770 Chr4 SNP_site:17935367 Ref_Type:T
AACTTAAGATAAGGAAGTGTTAGTTTTGTGTGTCATGGTAACCGGAAAAGGGAAGACTAAAATGTTAAGGTTTAAAAGTTXXXXXXXXXXT
AGGACGCATGCTAGGTAGCAAGATAGGAGAAGATAGAGGTTATTTTGGCGTTTGATCGGTGGTTGAGAAGAACAAAGTG
AACTTAAGATAAGGAAGTGTTAGTTTTGTGTGTCATGGTAACTGGAAAAGGGAAGACTAAAATGTTAAGGTTTAAAAGTTXXXXXXXXXXT
AGGACGCATGCTAGGTAGCAAGATAGGAGAAGATAGAGGTTATTTTGGCGTTTGATCGGTGGTTGAGAAGAACAAAGTG
>Marker838006 Chr7 SNP_site:13477853 Ref_Type:A
ACTGCATCACCAGCAATTTCATTGTCCAAGTAAAATCACAGTTTCATGGACTTTGCAAATGAAATAGAGTCGATCTCAGAXXXXXXXXXXC
AGGACAAAATTAACGATAACTTTTGTGTCAAAATCAGTTTCAAATGCAAAAATGGAAGTTGAATGAAACAGTGTCTGTG
ACTGCATCACCAGCAATTTCATTGTCCAAGTAAAATCACAGTTTCATGGACTTTGCAAATGAAATAGAGTCGATCTCAGAXXXXXXXXXXC
AGGACAAAATTAACGATGACTTTTGTGTCAAAATCAGTTTCAAATGCAAAAATGGAAGTTGAATGAAACAGTGTCTGTG
>Marker83816 Chr4 SNP_site:8399691 Ref_Type:A
ACCAAAGATCATCTCATTACCATATTTCTTCATTTTCAATTCAAACTCAAATACATTTTAAGAATTTAAGATCATAAGAGXXXXXXXXXXA
CTTTCCTGTAGTTCCTTGTCAAATTTGACCTTATGATGGATCTAAATCTTCGAAACTCTTTCACTTCTTTCCTTAAGTA
ACCAAAGCTCATCTCATTACCATATTTCTTCATTTTCAATTCAAACTCAAATACATTTTAAGAATTTAAGATCATAAGAGXXXXXXXXXXA
CTTTCCTGTAGTTCCTTGTCAAATTTGACCTTCTGATGGATCTAAATCTTCGAAACTCTTTCACTTCTTTCCTTAAGTA
>Marker83816 Chr4 SNP_site:8399936 Ref_Type:A
ACCAAAGATCATCTCATTACCATATTTCTTCATTTTCAATTCAAACTCAAATACATTTTAAGAATTTAAGATCATAAGAGXXXXXXXXXXA
CTTTCCTGTAGTTCCTTGTCAAATTTGACCTTATGATGGATCTAAATCTTCGAAACTCTTTCACTTCTTTCCTTAAGTA
ACCAAAGCTCATCTCATTACCATATTTCTTCATTTTCAATTCAAACTCAAATACATTTTAAGAATTTAAGATCATAAGAGXXXXXXXXXXA

Create PDF with GO2PDF for free, if you wish to

CTTTCCTGTAGTTCCTTGTCAAATTTGACCTTCTGATGGATCTAAATCTTCGAAACTCTTTCACTTCTTTCCTTAAGTA
>Marker838431 Chr7 SNP_site:15545035 Ref_Type:T
AACGATTGTATGATGATGATGATCATTGTCCAAACCCTCACTATTTTCTTCTCTTTTCTTTAAATCAAGATTAGGATCAGXXXXXXXXXXG
TTCTCTTCCGTATGTGGTTGGAGTATCTCGAACGACGTTGAACGATGGCACTATCCTTGAAGTTGATTCAATTTGAGGT
AACGATTGTATGATGATGATGATCATTGTCCAAACCCTCACTATTTTCTTCTCTTTTCTTTAAATCAAGATTAGGATCAGXXXXXXXXXXG
TTCTCTTCCGTATGTGGTTGGAGTATCTCGAACGACGTTGAACGATGGCTCTATCCTTGAAGTTGATTCAATTTGAGGT
>Marker838436 Chr7 SNP_site:15484032 Ref_Type:G
AACAAATCGTGGGAGTGAAGAGCAGGGGATTGCGACGGCGCCAGATCAAAGATCGAAGCTTTGAGCTCTGCAATGCTGCCXXXXXXXXXXA
ATTGGGGAAAAGAAGAGAGTTTAGAGAAACAGAGGTCTGGGAAGAGAGTGCCATTGCAAAGGGATCGAGCAGTTTGGTG
AACAAATCGTGGGAGTGAAGAGCAGGGGATTGCGACGGCGCCAGATCGAAGATCGAAGCTTTGAGCTCTGCAATGCTGCCXXXXXXXXXXA
ATTGGGGAAAAGAAGAGAGTTTAGAGAAACAGAGGTCTGGGAAGAGAGTGCCATTGCAAAGGGATCGAGCAGTTTGGTG
>Marker838494 Chr7 SNP_site:15150505 Ref_Type:C
GACAAAATTGTAGAAGCCAATGGAAGCTATGAAGATGAACCAAATGCATATAATCGGAGCAAATGTGTAGCCAACTTTGTXXXXXXXXXXT
CAAAAAACTTAATTAATTACTACTAATTGAAAAATCATCTGCCTATCAATCCTAAATGTAATTGAATTATTTGTTAGTA
GACAAAATTGTAGAAGCCAATGGAAGCTATGAAGATGAACCAAATGCATATAATCGGAGCAAATGTGTAGCCAACTTTGTXXXXXXXXXXT
CAAAAAACTTAATTAATTACTACTAATTGAAAAATCATTTGCGTATCAATCCTAAATGTAATTGAATTATTTGTTAGTA
>Marker838494 Chr7 SNP_site:15150509 Ref_Type:C
GACAAAATTGTAGAAGCCAATGGAAGCTATGAAGATGAACCAAATGCATATAATCGGAGCAAATGTGTAGCCAACTTTGTXXXXXXXXXXT
CAAAAAACTTAATTAATTACTACTAATTGAAAAATCATCTGCCTATCAATCCTAAATGTAATTGAATTATTTGTTAGTA
GACAAAATTGTAGAAGCCAATGGAAGCTATGAAGATGAACCAAATGCATATAATCGGAGCAAATGTGTAGCCAACTTTGTXXXXXXXXXXT
CAAAAAACTTAATTAATTACTACTAATTGAAAAATCATTTGCGTATCAATCCTAAATGTAATTGAATTATTTGTTAGTA
>Marker83863 Chr4 SNP_site:11200850 Ref_Type:T
ACTATATTTCTTAGCTATACTACTCAACCTTATCTTTTCCAAAACAAAAGCAATATATTAATACTAATTTTACTTTGTATXXXXXXXXXXA
ATTGAAAAAAAATATGGAGCATGTTTATTTTTTAATAGCCATATTGAGGAGATATTTCATTTAATTTACACATCCATGT
ACTATATTTCTTAGCTATACTACTCAACCTTATCTTTTCCAAAACAAAAGCAATATATTAATACTAATTTTACTTTGTATXXXXXXXXXXA
ATTGAAAAAATATATGGAGCATGTTTATTTTTTAATAGCCATATTGAGGAGATATTTCATTTAATTTACACATCCATGT
>Marker838801 Chr7 SNP_site:4810362 Ref_Type:T
AACATATCAAAATTTAAAAATTTATCTTCCATATGTCAATAAAAATTTAATACATATAGTATTCACAATGCTTTCTAGTCXXXXXXXXXXC
ATTTTTGTCAATCATGAAATCACTAACTATGCAATGCAGAGAAGGGCCAAAGAAAGAAAGAAGAACAAAAGATTTTGGT
AACATATCAAAATTTAAAAATTTATCTTCCATATGTCAATAAAAATTTAATACATATAGTATTCATAATGCTTTCTAGTCXXXXXXXXXXC
ATTTTTGTCAATCATGAAATCACTAACTATGCAATGCAGAGAAGGGCCAAAGAAAGAAAGAAGACCAAAAGATTTTGGT
>Marker838801 Chr7 SNP_site:4810535 Ref_Type:C
AACATATCAAAATTTAAAAATTTATCTTCCATATGTCAATAAAAATTTAATACATATAGTATTCACAATGCTTTCTAGTCXXXXXXXXXXC
ATTTTTGTCAATCATGAAATCACTAACTATGCAATGCAGAGAAGGGCCAAAGAAAGAAAGAAGAACAAAAGATTTTGGT
AACATATCAAAATTTAAAAATTTATCTTCCATATGTCAATAAAAATTTAATACATATAGTATTCATAATGCTTTCTAGTCXXXXXXXXXXC
ATTTTTGTCAATCATGAAATCACTAACTATGCAATGCAGAGAAGGGCCAAAGAAAGAAAGAAGACCAAAAGATTTTGGT
>Marker838934 Chr7 SNP_site:14285885 Ref_Type:A
AACTACATTCTCTTTGAAATGATTTCTTTTTCGTTTCGATGGGGCTCAGTCACCGCCACAATGTTAAACAAAAAAGAAAAXXXXXXXXXXT
AATGATTGAGTTTTGTTTTATTGGTTGAATTTAGGAATAATGATGATTTATGTGTATGAATATATACATATGATATGTT
AACTACGTTCTCTTTGAAATGATTTCTTTTTCGTTTCGATGGGGCTCAGTCACCGCCACAATGTTAAACAAAAAAAAAAAXXXXXXXXXXT
AATGATTGAGTTTTGTTTTATTGGTTGAATTTAGGAATAATGATGATTTATGTGTATGAATATATACATATGATATGTT
>Marker838934 Chr7 SNP_site:14285954 Ref_Type:G
AACTACATTCTCTTTGAAATGATTTCTTTTTCGTTTCGATGGGGCTCAGTCACCGCCACAATGTTAAACAAAAAAGAAAAXXXXXXXXXXT
AATGATTGAGTTTTGTTTTATTGGTTGAATTTAGGAATAATGATGATTTATGTGTATGAATATATACATATGATATGTT

Create PDF with GO2PDF for free, if you wish to

AACTACGTTCTCTTTGAAATGATTTCTTTTTCGTTTCGATGGGGCTCAGTCACCGCCACAATGTTAAACAAAAAAAAAAAXXXXXXXXXXT
AATGATTGAGTTTTGTTTTATTGGTTGAATTTAGGAATAATGATGATTTATGTGTATGAATATATACATATGATATGTT
>Marker839242 Chr7 SNP_site:16338016 Ref_Type:A
ACTTTTAAAAAAGAAAAAAAATATATGTAAAAATAGGGTATGGAATTTAGGTTTTTTCTAAGCGAATTAACAAAATTTTAXXXXXXXXXXC
TAAAACTTACATTCACACATACATAATATTGCAAGTTTTATAAAGTTCAAAGTTTATTTTATAATTTATATTTTTTAGT
ACTTTTAAAAAAGAAAAAAAATATATGTAAAAATAGGGTATGGAATTTAGGTTTTTTCTAAGCGAATTAACAAAATTTTAXXXXXXXXXXC
TAAAACTTACATTCACACATACATAATATTGCAAGTTTTATAAAGTTCAAAGTTTATTTTATAATTTTTATTTTTTAGT
>Marker839292 Chr7 SNP_site:6235458 Ref_Type:G
AACCTTAGCTCCAAGGGTTCAATTTATATAGCTCATAAACTCCTCATACCCAACCCAAGATCATATCTTACTTCCAAACTXXXXXXXXXXT
GGTTATCATCATTTCTTAGAACTTGCTCGTTTAAACTTTACATAAGATCTCGGTAACTTCTAATAAATCCAGAAAAGTT
AACCTTAGCTCCAAGGGTTCAATTTATATAGCTCATAAACTCCTCATACCCAACCCAATATCATATCTTACTTCCAAACTXXXXXXXXXXT
GGTTATCATTATTTCTTAGAACTTGCTCGTTTAAACTTTACATAAGATCTCGGTAACTTCTAATAAATCCAGAAAAGTT
>Marker839292 Chr7 SNP_site:6235618 Ref_Type:C
AACCTTAGCTCCAAGGGTTCAATTTATATAGCTCATAAACTCCTCATACCCAACCCAAGATCATATCTTACTTCCAAACTXXXXXXXXXXT
GGTTATCATCATTTCTTAGAACTTGCTCGTTTAAACTTTACATAAGATCTCGGTAACTTCTAATAAATCCAGAAAAGTT
AACCTTAGCTCCAAGGGTTCAATTTATATAGCTCATAAACTCCTCATACCCAACCCAATATCATATCTTACTTCCAAACTXXXXXXXXXXT
GGTTATCATTATTTCTTAGAACTTGCTCGTTTAAACTTTACATAAGATCTCGGTAACTTCTAATAAATCCAGAAAAGTT
>Marker839470 Chr7 SNP_site:15640445 Ref_Type:T
AACCAATGATAGATGGAGAAGCTGGAGATCTTAGAGTTAGTGTTTTTCCTATTATTCAACTTGTTCTTTTGTGATTGTTTXXXXXXXXXXC
AATTTAATGTTGTTAATAGTGTCCCCATGCGATACTTTATTTTTAACAATTAATCGATGTGAGATTATTTGCCTTTGTG
AACCAATGATAGATGGAGAAGCTGGAGATCTTAGAGTTAGTGTTTTTCCTATTATTCAACTTGTTCTTTTGTGATTGTTTXXXXXXXXXXC
AATTTAATGTTGTTAATAGTGTTCCCATGCAATACTTTATTTTTAACAACTAATCGATGTGAGATTATTTGCCTTTGTG
>Marker839470 Chr7 SNP_site:15640453 Ref_Type:A
AACCAATGATAGATGGAGAAGCTGGAGATCTTAGAGTTAGTGTTTTTCCTATTATTCAACTTGTTCTTTTGTGATTGTTTXXXXXXXXXXC
AATTTAATGTTGTTAATAGTGTCCCCATGCGATACTTTATTTTTAACAATTAATCGATGTGAGATTATTTGCCTTTGTG
AACCAATGATAGATGGAGAAGCTGGAGATCTTAGAGTTAGTGTTTTTCCTATTATTCAACTTGTTCTTTTGTGATTGTTTXXXXXXXXXXC
AATTTAATGTTGTTAATAGTGTTCCCATGCAATACTTTATTTTTAACAACTAATCGATGTGAGATTATTTGCCTTTGTG
>Marker839470 Chr7 SNP_site:15640472 Ref_Type:C
AACCAATGATAGATGGAGAAGCTGGAGATCTTAGAGTTAGTGTTTTTCCTATTATTCAACTTGTTCTTTTGTGATTGTTTXXXXXXXXXXC
AATTTAATGTTGTTAATAGTGTCCCCATGCGATACTTTATTTTTAACAATTAATCGATGTGAGATTATTTGCCTTTGTG
AACCAATGATAGATGGAGAAGCTGGAGATCTTAGAGTTAGTGTTTTTCCTATTATTCAACTTGTTCTTTTGTGATTGTTTXXXXXXXXXXC
AATTTAATGTTGTTAATAGTGTTCCCATGCAATACTTTATTTTTAACAACTAATCGATGTGAGATTATTTGCCTTTGTG
>Marker839540 Chr7 SNP_site:17241515 Ref_Type:C
TACTGGTGACATTTCTACAACAATGAATCTGATGGTTTTACATCTAAAAACTGATGAAGGCATGAACATGAACTACCAGAXXXXXXXXXXA
AGCAGTCTCAGATTATAACCACAAACACATATGCAACAAATCATAACATGTGGTATTATGCTTACTGCGGTAGGGGGTA
TACTGGTGACATTTCTACAACAATGAATCTGATGGTTTTACATCTAAAAACTGATGAAGGTATGAACATGAACTACCAGAXXXXXXXXXXA
AGCAGTCTCAGATTATAACCACAAACACATATGCAACAAATCATAACATGTGGTATTATTCTTACTGCGGTAGGGGGTA
>Marker839540 Chr7 SNP_site:17241723 Ref_Type:G
TACTGGTGACATTTCTACAACAATGAATCTGATGGTTTTACATCTAAAAACTGATGAAGGCATGAACATGAACTACCAGAXXXXXXXXXXA
AGCAGTCTCAGATTATAACCACAAACACATATGCAACAAATCATAACATGTGGTATTATGCTTACTGCGGTAGGGGGTA
TACTGGTGACATTTCTACAACAATGAATCTGATGGTTTTACATCTAAAAACTGATGAAGGTATGAACATGAACTACCAGAXXXXXXXXXXA
AGCAGTCTCAGATTATAACCACAAACACATATGCAACAAATCATAACATGTGGTATTATTCTTACTGCGGTAGGGGGTA
>Marker839897 Chr7 SNP_site:16938905 Ref_Type:C
ACTTGGCCTCTGAAACATTCTTAAGCATAACCATCGATGTTCCTCGCTCTTTTACGATTCGCTCGTCATTTGGCAAATTGXXXXXXXXXXA

Create PDF with GO2PDF for free, if you wish to

AACTACTGGTCTCCACAAGCATTTTTAATAATCAACATTCAATTTCTTGGGGGATGATAGTTCTCAATTCAGCGTAGTT
ACTTGGCCTCTGAAACATTCTTAAGCATAACCATTGATGTTCCTCGCTCTTTTACTATTCGCTCGTCATTTGGCAAATTGXXXXXXXXXXA
AACTACTGGTCTCCACAAGCATTTTTAATAATCAACATTCAATTTCTTGGGGGATGATAGTTCTCAATTCAGCGTAGTT
>Marker839897 Chr7 SNP_site:16938926 Ref_Type:G
ACTTGGCCTCTGAAACATTCTTAAGCATAACCATCGATGTTCCTCGCTCTTTTACGATTCGCTCGTCATTTGGCAAATTGXXXXXXXXXXA
AACTACTGGTCTCCACAAGCATTTTTAATAATCAACATTCAATTTCTTGGGGGATGATAGTTCTCAATTCAGCGTAGTT
ACTTGGCCTCTGAAACATTCTTAAGCATAACCATTGATGTTCCTCGCTCTTTTACTATTCGCTCGTCATTTGGCAAATTGXXXXXXXXXXA
AACTACTGGTCTCCACAAGCATTTTTAATAATCAACATTCAATTTCTTGGGGGATGATAGTTCTCAATTCAGCGTAGTT
>Marker840166 Chr7 SNP_site:13984719 Ref_Type:A
CACTTGAATCCTTGTTGAGATATAAGATGCACTCTTGAATGTTAATACTTTATTGGCATTTTGGTTCTGGTTGCAGAAGAXXXXXXXXXXG
CCAACTTCGTAACTAGTTTTGATTTTCCATCTTACTAAACTGACTTTGGATTGCTTTAAATTGTTTCCAAGAGGGTGTA
CACTTGAATCCTTGTTGAGATATAAGATGCACTCTTGAATGTTAATACTTTATTGGCATTTTGGTTCTGGTTGCAGAAGAXXXXXXXXXXG
CCAACTTCGTAACTAGTTTTGATTTTCCGTCTTACTAAACTGACTTTGGATTGCTTTAAGTTGTTTCCAAGAGGGTGTA
>Marker840166 Chr7 SNP_site:13984750 Ref_Type:A
CACTTGAATCCTTGTTGAGATATAAGATGCACTCTTGAATGTTAATACTTTATTGGCATTTTGGTTCTGGTTGCAGAAGAXXXXXXXXXXG
CCAACTTCGTAACTAGTTTTGATTTTCCATCTTACTAAACTGACTTTGGATTGCTTTAAATTGTTTCCAAGAGGGTGTA
CACTTGAATCCTTGTTGAGATATAAGATGCACTCTTGAATGTTAATACTTTATTGGCATTTTGGTTCTGGTTGCAGAAGAXXXXXXXXXXG
CCAACTTCGTAACTAGTTTTGATTTTCCGTCTTACTAAACTGACTTTGGATTGCTTTAAGTTGTTTCCAAGAGGGTGTA
>Marker840548 Chr7 SNP_site:5673431 Ref_Type:T
ACAACATAGGTAGCGACCTTAGAGAACAATAGAGCAGACATGAAAATAGTATGCAAAGAGAAAATCAATGTAGTTCTAGAXXXXXXXXXXA
TGTTAAAATTATACTTCCTGCTAAACAAATATCCCAACACAAATATTCAAGAAATTCACAAGACAAACACCCTTGTGTA
ACAACATAGGTAGCGACCTTAGAGAACAATAGTGCAGACATGAAAATAGTATGCAAAGAGAAAATCAATGTAGTTCTAGAXXXXXXXXXXA
TGTTAAAATTATACTTCCTGCTAAACAAATATCCCAACACAAATATTCAAGAAATTCACAAGACAAACACCCTTGTGTA
>Marker840881 Chr7 SNP_site:13959685 Ref_Type:G
CACATGGAGGCCACATTCACAAATTTTCTATCAGTGATAATTATAATAATAATTAGGGCAATTTAAAAAATGTCATACTTXXXXXXXXXXA
CTTAAAATTCAATTAAAATACAAAAGAGCGATGGACTAGAAGAAAGGCTTCCAATTGAGTGAAATACAAGATGCATTGT
CACATGGAGGCCACATTCACAAATTTTCTATCAGTGATAATTATAATAATAATTAGGGCAATTTAAAAAATGTCATACTTXXXXXXXXXXA
CTTAAAATTCGATTAAAATACAAAAGAGAGATGGACTAGAAGAAAGGCTTCCAATTGAGTGAAATACAAGATGCATTGT
>Marker840881 Chr7 SNP_site:13959703 Ref_Type:A
CACATGGAGGCCACATTCACAAATTTTCTATCAGTGATAATTATAATAATAATTAGGGCAATTTAAAAAATGTCATACTTXXXXXXXXXXA
CTTAAAATTCAATTAAAATACAAAAGAGCGATGGACTAGAAGAAAGGCTTCCAATTGAGTGAAATACAAGATGCATTGT
CACATGGAGGCCACATTCACAAATTTTCTATCAGTGATAATTATAATAATAATTAGGGCAATTTAAAAAATGTCATACTTXXXXXXXXXXA
CTTAAAATTCGATTAAAATACAAAAGAGAGATGGACTAGAAGAAAGGCTTCCAATTGAGTGAAATACAAGATGCATTGT
>Marker841157 Chr7 SNP_site:1128440 Ref_Type:T
CACTTCTTCTATCACTATCAAACCCATAAAATATGAAAATATCGATAGAAATTTAAAACTATGAATAATTTAAAATCTTAXXXXXXXXXXT
GTTAATTATGTAATCATGTTTTACAAGATGATTGGAAGCCTTGTAATTACGCATATTACTGATTGTTAATAATTAGTGT
CACTTCTTCTATCACTATCAAACCCATAAAATATGAAAATATCGATAGAAATTTAAAACTATGAATAATTTAAAATCTTAXXXXXXXXXXT
GTTAATTATGTAATCATGTTTTACAAGATGATTGGAAGCCTTGTAATTACGCATATTATTGATTGTTAATAATTAGTGT
>Marker841294 Chr7 SNP_site:15203051 Ref_Type:T
AACCATGAAATGGTAACAAAAACTACCTTTAACATCAAAAGTAATAAAGGTGGAGCATACAGAAGAATGTTCATCTTTATXXXXXXXXXXA
AAAGAAATAAGGCCAATGCAGCATGGAGAAGAGTCATGGCAAAGCAGTCATTGAATAGACGGAGCATAAAGATGGAGTG
AACCATGAAATGGTAATAAAAACTACCTTTAACATCAAAAGTAATAAAGGTGGAGCATACAGAAGAATGTTCATCTTTATXXXXXXXXXXA
AAAGAAATAAGGCCAATGCAGCATGGAGAAGAGTCATGGCAAAGCAGTCATTGAATAGACGGAGCATAAAGATGGAGTG
>Marker841630 Chr7 SNP_site:8122663 Ref_Type:T

Create PDF with GO2PDF for free, if you wish to

ACCATTCCAGCACAAATGTAAAGTCAAGGAACAGGGGGAGTTGCGAATATTCGTAGTGCAAAAATATAACGAAGTGGAGAXXXXXXXXXXT
AAAGGGGAGGATCAAAGGAAAGGAAGTGATAGTATTAATCGATTGTGGTGCTACACAAAATTTTATATCTGAGAAGGTT
ACCATTCCAGCACAAATGTAAAGTCAAGGAACAGGGGGAGTTGCGAATATTCGTAGTGCAAAAATATAACGAAGTGGAGAXXXXXXXXXXT
AAAGGGGAGGATCAAAGGAAAGGAAGTGATAGTATTAATCGATTGTGGTGCTACACAAAATTTTATATTTGAGAAGGTT
>Marker841829 Chr7 SNP_site:13288601 Ref_Type:A
ACAGACCATTGCAACAAATGGCATCACAGGCTGAAGAATGGCTACGATTGGCTTTGCATAAGAATTAAGAACGAGTCCAAXXXXXXXXXXA
TACACCATTTCTTGATCGTAGCAAGAGGAGATAAAATTTAGTTATAGCTCAGCAAGAGGAAAGTATGCGATATAACAGT
ACAGACCATTGCAACAAATGGCATCACAGGCTGAAGAATGGCTACGATTGGCTTTGCATAAGAATTAAGAACGAGTCCAAXXXXXXXXXXA
TACACCATTTCTTGATCGTAGCGAGAGGAGATAAAATTTAGTTATAGCTCAGCAAGAGGAAAGTATGCGATATAACAGT
>Marker841962 Chr7 SNP_site:15859179 Ref_Type:A
AACTACCTTCAGTTATTTATGGAAATGAACATGTTTGTTCATGATGTATAACTTTTCATTGTGGCATTTTTATCTAAAGAXXXXXXXXXXA
ATTGTCTCTTTGCCTATTCTGATTTCTAATGTTGTGATTTTTCTTGTTTCAGATTCCTCAATTTGTTTTAGAAGCTGTT
AACTACCTTCAGTTATTTATGGAAATGAACATGTTTGTTCATGATGTATATCTTTTCATTGTGACATTTTTATCTAAAGAXXXXXXXXXXA
ATTGTCTCTTTGCCTATTCTGATTTCTAATGTTGTGATTTTTCTTGTTTCAGATTCCTCAATTTGTTTTAGAAGCTGTT
>Marker841962 Chr7 SNP_site:15859192 Ref_Type:G
AACTACCTTCAGTTATTTATGGAAATGAACATGTTTGTTCATGATGTATAACTTTTCATTGTGGCATTTTTATCTAAAGAXXXXXXXXXXA
ATTGTCTCTTTGCCTATTCTGATTTCTAATGTTGTGATTTTTCTTGTTTCAGATTCCTCAATTTGTTTTAGAAGCTGTT
AACTACCTTCAGTTATTTATGGAAATGAACATGTTTGTTCATGATGTATATCTTTTCATTGTGACATTTTTATCTAAAGAXXXXXXXXXXA
ATTGTCTCTTTGCCTATTCTGATTTCTAATGTTGTGATTTTTCTTGTTTCAGATTCCTCAATTTGTTTTAGAAGCTGTT
>Marker842295 Chr7 SNP_site:15695010 Ref_Type:G
AACGTGTATAACGAGAGAGAGCTTTTGACATTTAGCATGAGTGACTCGAATGGCCTTGCTAGACAATGTAGAAGCAGAATXXXXXXXXXXA
TAGGTTCGTCCCGGGTATGATGAAGTTGATCTCATTGCCATGTTTGTCATTGGTAGGTTGGAGAGGTAAGATTGTGGGT
AACGTGTATAACGAGAGAGAGCTTTTGACATTTAGCATGAGTGACTCGAATGGCCTTGCTAGACAATGTAGAAGCAGAATXXXXXXXXXXA
TAGGTTCGTCCGGGGTATGATGAAGTTGATCTCATTGCCATGTTTGTCATTGGTAGGTTGGAGAGGTAAGATTGTGGGT
>Marker843159 Chr7 SNP_site:54983 Ref_Type:G
AACGACAAGAAGGATTCGTGACATGCCATATAGGAACACAATTTTACTTCTGCTTTTCATTGATCATTTTTAGTGTTTTGXXXXXXXXXXA
TCTTTAAAGTAATGAATTTAGCTTAGTATAAAAAGTTGAGTTGTTACATAAAAGGGCTAAGTAGTTTGTTAAAAGTGTT
AACGACAAGAAGGATTCGTGACATGCCATATAGGAACACAATTTTACTTCTGCTTTTCATTGATCATTTTTAGTGTTTTGXXXXXXXXXXA
TCTTTAAAGTAATGAATTTAGCTTAGTATAAAGAGTTGAGTTGTTACATAAAAGGGCTAAGTAGTTTGTTAAAAGTGTT
>Marker84320 Chr4 SNP_site:21190154 Ref_Type:T
ACATTAACTGTGGATTTTTTTTTCTTTCTAAAAGACGTGTTTCCTGTGATATAAACGAAGAATGCATAAGAGCGAGAAGGXXXXXXXXXXC
TTATGGGCAGTTCCATTTTCGTTTTTTGAACTATTTTCTTTGGAATTATACTGGGCATTGGATATACGCTGTATTTGTC
ACATTAACTGTGGATTTTTTTTTCTTTCTAAAAGACGTGTTTCCTGTGATATAAACGAAGAATGCATAATAGCGAGAAGGXXXXXXXXXXC
TTATGGGCAGTTCCATTTTCGTTTTTTGAACTATTTTCTTTGGAATTATACTGGGCATTGGATATACGCCGTATTTGTC
>Marker84320 Chr4 SNP_site:21190323 Ref_Type:C
ACATTAACTGTGGATTTTTTTTTCTTTCTAAAAGACGTGTTTCCTGTGATATAAACGAAGAATGCATAAGAGCGAGAAGGXXXXXXXXXXC
TTATGGGCAGTTCCATTTTCGTTTTTTGAACTATTTTCTTTGGAATTATACTGGGCATTGGATATACGCTGTATTTGTC
ACATTAACTGTGGATTTTTTTTTCTTTCTAAAAGACGTGTTTCCTGTGATATAAACGAAGAATGCATAATAGCGAGAAGGXXXXXXXXXXC
TTATGGGCAGTTCCATTTTCGTTTTTTGAACTATTTTCTTTGGAATTATACTGGGCATTGGATATACGCCGTATTTGTC
>Marker843667 Chr7 SNP_site:18717701 Ref_Type:C
AACTCAGTATTGGGTATTTCAGATATATATTTATAACTAAAATTCTGATTTTGATTGGTCTTTTTTAGAGAGAGAGAAAAXXXXXXXXXXT
ACCACGTTTGTGACGAGCTAAAAACTTAAAACAATGGGTTTGTATTGATATGTCATATTGATGCTTTTGTATAATTGTT
AACTCAGTATTGGGTATTTCAGATATATATTTATAACTAAAATTCTGATTTTGATTGGTCTTTTTTAGAGAGAGAGAAAAXXXXXXXXXXT
ACCACGTTTGTGACGAGCTAACAACTTAAAACAATGGGTTTGTATTGATATGTCATATTGATGCTTTTGTATAATTGTT

Create PDF with GO2PDF for free, if you wish to

>Marker843736 Chr7 SNP_site:16302147 Ref_Type:A

TACCCTTTATAGTAAATCCATTAATAATGAATACATAAATTTTTAATCTTGACGTCATAATATATAACAAATTAGGAAATXXXXXXXXXXA

GATGTGATAAGTGATCCCATAACGAAATCGTGGCAATGAAATGTTACAGGAAGGTGACGCGTGGCACTTGCCTTGCTGT

TACCCTTTATAGTAAATCCATTAATAATGAATACATAAATTTTTAATCTTGACGTCATAATCTATAACAAATTAGGAAATXXXXXXXXXXA

GATGTGATAAGTGATCCCATAACGAAATCGTGGCAATGAAATGTTACAGGAAGGTGACGCGTGGCACTTGCCTTGCTGT

>Marker844453 Chr7 SNP_site:8386766 Ref_Type:A

TACTTTTTTCCCTTTTCCTTTCTGGCCACTCCGGCATGGATGTATTAAAATCGGTTCATCTTTTTCCAGCCTAATCATTTXXXXXXXXXXT

CCCTTGCCAAATATACAACAAGTCCAAGCTAGTTGAAGGAAATTCAGATAGATCATCAATCCTGACAAGATTGTTCGTA

TACTTTTTTCCCTTTTCCTTTCTGGCCACTCCGGCATGGATGTATTAAAATCGGTTCATCTTTTTCCAGCCTAATCATTTXXXXXXXXXXT

CCCTTGCCAAATATACAACAAGTCCAAGCTAGTTGAAGGAAATTCAGATAGATCATCGATCCTGACAAGATTGTTCGTA

>Marker844720 Chr7 SNP_site:15784986 Ref_Type:T

TACCTCAGTAAAGTTACGCCCCCACCTGGAACGTGTCCCTCCCTTGCGTTCCCAGGCGCGAACGCGACTTGGGCAGCTCCXXXXXXXXXXC

TTGTCATATGATATAAGAACGCAACACTTAAGAACTCGAGGGAACTTAGCAATTTTATCAACTCCTTAACAAACTTAGT

TACCTCAGTAAAGTTACGCCCCCACCTGGAACGTGTCCCTCCCTTGCGTTCCCAGGCGCGAACGCGACTTGGGCAGCTCCXXXXXXXXXXC

TTGTCATATGATATAAGAACGCAACACTTAAGAACTCGAGGGAACTTAGCAATTTTATTAACTCCTTAACAAACTTAGT

>Marker845139 Chr7 SNP_site:13855171 Ref_Type:C

CACTAAAACTCGTGCGCCTACACTTCTCAACAAAATAGGGTGGCTGAGAACCGTCATCTTCTGGAAGTAGCCCACTTCTTXXXXXXXXXXA

GACTCCATTAGATTGTCTTAAAGAGTCATATCCTACTACTCGTCTAATCTATGAGGTTCCCTTTCGTGTGTTTGGGTGT

CACTAAAACTCGTGCGCCTACACTTCTCAACAAAATAGGGTGGCTGAGAACCGTCATCTTCTGGAAGTAGCCCACTTCTTXXXXXXXXXXA

GACTCCCTTAGATTGTCTTAAAGAGTCATATCCTACTACTCGTCTAATCTATGAGGTTCCCTTTCGTGTGTTTGGGTGT

>Marker845219 Chr7 SNP_site:99818 Ref_Type:T

TACCTCCTAAAGGCACTCCCAAATATTGGATGGGAAGAAAGTTTATGGAGAAACCCCATTTTGATGTCACGCAATTTGTTXXXXXXXXXXC

AGGAGGTGAGTGAGGTTGTGTTTCTTGTTGAAACTTACACCTTTAATCAAGTTATCTTTTTCCAATTGATTGAGGAGTA

TACCTCCTAAAGGCACTCCCAAATATTGGATGGGAAGAAAGTTTATGGAGAAACCCTATTTTGATGTCACGCAATTTGTTXXXXXXXXXXC

AGGAGGTGAGTGAGGTTGTGTTTCTTATTGAAACTTACACCTTTAATCAAGTTATCTTTTTCCAATTGATTGAGGAGTA

>Marker845219 Chr7 SNP_site:100017 Ref_Type:A

TACCTCCTAAAGGCACTCCCAAATATTGGATGGGAAGAAAGTTTATGGAGAAACCCCATTTTGATGTCACGCAATTTGTTXXXXXXXXXXC

AGGAGGTGAGTGAGGTTGTGTTTCTTGTTGAAACTTACACCTTTAATCAAGTTATCTTTTTCCAATTGATTGAGGAGTA

TACCTCCTAAAGGCACTCCCAAATATTGGATGGGAAGAAAGTTTATGGAGAAACCCTATTTTGATGTCACGCAATTTGTTXXXXXXXXXXC

AGGAGGTGAGTGAGGTTGTGTTTCTTATTGAAACTTACACCTTTAATCAAGTTATCTTTTTCCAATTGATTGAGGAGTA

>Marker845596 Chr7 SNP_site:248160 Ref_Type:G

TACAAGAAAATAACAATTTGGAAATGATTGATCACTAAAGCTCTAAAACTTCTCAGAATGGAATTACTTACTTCATTGTAXXXXXXXXXXA

ATTTGATAGCAGCCAGAAGCAAGGACGAAAAATAAGCTAATAAGGAAAAAGATAACTGTAAAGCAGAAGTCATGCCGTG

TACAAGAAAATAACAATTTGGAAATGATTGATCACTGAAGCTCTAAAACTTCTCAGAATGGAATTACTTACTTCATTGTAXXXXXXXXXXA

ATTTGATAGCAGCCAGAAGCAAGGACGAAAAATAAGCTAATAAGGAAAAAGATAACTGTAAAGCAGAAGTCATGCCGTG

>Marker84567 Chr4 SNP_site:16488402 Ref_Type:A

ACCTTTCCAACTCCAAGTTTCAAGACTGTTGACGTCTCGTGGAGTCGGAGTGGGTTGAGACACTTATGATGTAGAAATCAXXXXXXXXXXC

CTGCGTTGTTTAACTCGTTGCCGAAATTGAGAAAGAGAGCATATGTGTCATCGAAAGTATTTGGGAATAAATACATAGT

ACCTTTCCAACTCCAAGTTTCAAGACTGTTGACGTCTCGTGGAGTCGGAGTGGGTTGAGACACTTATGATGTAGAAATCAXXXXXXXXXXC

CTGCGTTGTTTAACTCGTTGCCGAAATTGAGAAAGAGAGCGTATGTGTCATCGAAAGTATTTGGGAATAAATACATAGT

>Marker845883 Chr7 SNP_site:10162011 Ref_Type:A

GACTATTTTGAAACTTCTAGTCTAGTCGTGAAGAAATCAATAGAAAGAATTGTCCTTACCCTAGTTGCTACTTGTAATTGXXXXXXXXXXT

TGTTAAATAAATAAGTCCTTGTATGAATTTAAACAAGCACCTTGAGCATATAGTTTAATTATTTTACAACTCACTTGTT

GACTATTTTGAAACTTCTAGTCTAGTCGTGAAGAAATCAATAGTAAGAATTGTCCTTACCCTAGTTGCTACTTGTAATTGXXXXXXXXXXT

Create PDF with GO2PDF for free, if you wish to

TGTTAAATAAATAAGTCCTTGTATGAATTTAAACAAGCACCTTGAGCATATAGTTTAATTATTTTACAACTCACTTGTT
>Marker846192 Chr7 SNP_site:8279476 Ref_Type:T
AACGATTTTCCTAACAAAATAGGAAGATGTGAATTCGGAAATGAAATATCTATTGCTACGTTGATATATCCGTATAATTAXXXXXXXXXXT
TAGTTTGATTATAAAAATAAATTAACTATTAATTTAAATAGTTATTATGAACTTATCAACACAAAAATATTTGTTGGGT
AACGATTTTCCTAACAAAATAGGAAGATGTGAATTCGGAAATGAAATATCTATTGCTACGTTGATATATCCGTATAATTAXXXXXXXXXXT
TAGTTTGATTTTAAAAATAAATTAACTATTAATTTAAATAGTTATTATGAACTTATCAACACAAAAATATTTGTTGGGT
>Marker846202 Chr7 SNP_site:3095174 Ref_Type:C
CACATTTAATTGTTCAAATATGTTGTTTGAAATGTTAATTAAGACATTAATTGGTTGAAGAAATAACATATATTATATATXXXXXXXXXXT
TCATGTTAGCGGCTTCTTAAAGATGATAAAGGATCCACTCCCAATATATACATATATATGAATATAAAATCATAGTGTG
CACATTTAATTGTTTAAATATGTTGTTTGAAATGTTAATTAAGACATTAATTGGTTGAAGAAATAACATATATTTTATATXXXXXXXXXXT
TCATGTTAGCGGCTTCTTAAAGATGATAAAGGATCCACTCCCAATATATACATATATATGAATATAAAATCATAGTGTG
>Marker846202 Chr7 SNP_site:3095234 Ref_Type:A
CACATTTAATTGTTCAAATATGTTGTTTGAAATGTTAATTAAGACATTAATTGGTTGAAGAAATAACATATATTATATATXXXXXXXXXXT
TCATGTTAGCGGCTTCTTAAAGATGATAAAGGATCCACTCCCAATATATACATATATATGAATATAAAATCATAGTGTG
CACATTTAATTGTTTAAATATGTTGTTTGAAATGTTAATTAAGACATTAATTGGTTGAAGAAATAACATATATTTTATATXXXXXXXXXXT
TCATGTTAGCGGCTTCTTAAAGATGATAAAGGATCCACTCCCAATATATACATATATATGAATATAAAATCATAGTGTG
>Marker846328 Chr7 SNP_site:14806620 Ref_Type:A
CACTCTTCAAACTTGAACCTCAACTTCATCTAGAAAGAGAGAAAAAACCCCTAAGAACATGGGATCCAAATAAATAAGTGXXXXXXXXXXA
CATTTAGGCCTAGCTTGTGAGATGAGTTTGGTAGTCAAATCTCAAACACTTATGCCTGGCTCGTGAGATGAGTCTGGTA
CACTCTTCAAACTTGAACCTCAACTTCATCTAGAAAGAGAGAAAAAACCCCTAAGAACATGGGATCCAAATAAATAAGTGXXXXXXXXXXA
CATTTAGGCCTAGCTTGTGAGATGAGTTTGGTAGTCGAATCTCAAACACTTATGCTTGGCTCGTGAGATGAGTCTGGTA
>Marker846328 Chr7 SNP_site:14806639 Ref_Type:C
CACTCTTCAAACTTGAACCTCAACTTCATCTAGAAAGAGAGAAAAAACCCCTAAGAACATGGGATCCAAATAAATAAGTGXXXXXXXXXXA
CATTTAGGCCTAGCTTGTGAGATGAGTTTGGTAGTCAAATCTCAAACACTTATGCCTGGCTCGTGAGATGAGTCTGGTA
CACTCTTCAAACTTGAACCTCAACTTCATCTAGAAAGAGAGAAAAAACCCCTAAGAACATGGGATCCAAATAAATAAGTGXXXXXXXXXXA
CATTTAGGCCTAGCTTGTGAGATGAGTTTGGTAGTCGAATCTCAAACACTTATGCTTGGCTCGTGAGATGAGTCTGGTA
>Marker848777 Chr7 SNP_site:4856076 Ref_Type:A
CACTATTTAAAAATTGACTAGAAAACGAAATTAGAATCTAGATATCATTACAACGTATCAATCAGTCTAAACTTTCATCAXXXXXXXXXXA
CTTATTTTCCTTTTCATTCTTTTAAATCTTGTGTTTGATAGATGAATGATGTTGGATGTATTCTATATTTTTGAACGTT
CACTATTTGAAAATTGACTAGAAAACGAAATTAGAATCTAGATATCATTACAACCTATCAATCAGTCTAAACTTTCATCAXXXXXXXXXXA
CTTATTTTCCTTTTCATTCTTTTAAATCTTGTGTTTGATAGATGAATGATGTTGGATGTATTCTATATTTTTTAACGTT
>Marker848777 Chr7 SNP_site:4856122 Ref_Type:G
CACTATTTAAAAATTGACTAGAAAACGAAATTAGAATCTAGATATCATTACAACGTATCAATCAGTCTAAACTTTCATCAXXXXXXXXXXA
CTTATTTTCCTTTTCATTCTTTTAAATCTTGTGTTTGATAGATGAATGATGTTGGATGTATTCTATATTTTTGAACGTT
CACTATTTGAAAATTGACTAGAAAACGAAATTAGAATCTAGATATCATTACAACCTATCAATCAGTCTAAACTTTCATCAXXXXXXXXXXA
CTTATTTTCCTTTTCATTCTTTTAAATCTTGTGTTTGATAGATGAATGATGTTGGATGTATTCTATATTTTTTAACGTT
>Marker848777 Chr7 SNP_site:4856346 Ref_Type:G
CACTATTTAAAAATTGACTAGAAAACGAAATTAGAATCTAGATATCATTACAACGTATCAATCAGTCTAAACTTTCATCAXXXXXXXXXXA
CTTATTTTCCTTTTCATTCTTTTAAATCTTGTGTTTGATAGATGAATGATGTTGGATGTATTCTATATTTTTGAACGTT
CACTATTTGAAAATTGACTAGAAAACGAAATTAGAATCTAGATATCATTACAACCTATCAATCAGTCTAAACTTTCATCAXXXXXXXXXXA
CTTATTTTCCTTTTCATTCTTTTAAATCTTGTGTTTGATAGATGAATGATGTTGGATGTATTCTATATTTTTTAACGTT
>Marker84888 Chr4 SNP_site:6082938 Ref_Type:G
ACATGATTTTTATGAATGGTTTCAAGCTTAGATTTTATAATGAAATTTATATATGAGAACGTTATTATCGACTTTTTAAAXXXXXXXXXXC
AAACATGTTTTAACGTTATTATCGCCTAGTATTACTACATGACTAAGAAGTTGAGTCCGAGGTTTTATGATACCATGTG

Create PDF with GO2PDF for free, if you wish to

ACATGATTTTTATGAATGGTTTCAAGCTTAGATTTTATAATGAAATTTATATATGAGAACGTTATTATCGACTTTTTAAAXXXXXXXXXXXC
AAACATGTTTTAACGTTATTATCGCCTAGTATTACTACATGACTAGGAAGTTGAGTCCGAGGCTTTATGATACCATGTG
>Marker84888 Chr4 SNP_site:6082955 Ref_Type:C
ACATGATTTTTATGAATGGTTTCAAGCTTAGATTTTATAATGAAATTTATATATGAGAACGTTATTATCGACTTTTTAAAXXXXXXXXXXXC
AAACATGTTTTAACGTTATTATCGCCTAGTATTACTACATGACTAAGAAGTTGAGTCCGAGGTTTTATGATACCATGTG
ACATGATTTTTATGAATGGTTTCAAGCTTAGATTTTATAATGAAATTTATATATGAGAACGTTATTATCGACTTTTTAAAXXXXXXXXXXXC
AAACATGTTTTAACGTTATTATCGCCTAGTATTACTACATGACTAGGAAGTTGAGTCCGAGGCTTTATGATACCATGTG
>Marker849250 Chr7 SNP_site:6906 Ref_Type:G
AACTCAAAAGATGATATTCTTTTCTTCTTTTCTGTTTTGATTATTGGATGGTGATTCAAAATATATATGTCGTTTAACAAXXXXXXXXXXXA
ATTTGATTTTTTGGGCACTAGTTCCTTTCTATCATGTCGAATATTCTTTGGTTGGTTTTTGAGATTTTTGTATTTTGGT
AACTCAAAAGATGATATTCTTTTCTTCTTTTCTGTTTTGATTATTGGATGGTGATTCAAAATATATATGTCGTTTAACAAXXXXXXXXXXXA
ATTTGGTTTTTTGGGCACTAGTTCCTTTCTATCATGTCGAATATTCTTTGGTTGGTTTTTGAGATTTTTGTATTTTGGT
>Marker849560 Chr7 SNP_site:12206245 Ref_Type:C
GACTTCAATGCCATAAATTAAAAGGTTTCAATATAACTTATTTTCTTATGTTTATAGTATTTTGTGAGAGTTGTATCAACXXXXXXXXXXXA
TAGCATCCGCAGAGACTCAGTTCAACTTAAACTAGCCAACAGTATGAAGAATGACAATCCAACTTCCTGTATGTTTGTT
GACTTCAATGCCATAAATTAAAAGGTTTCAATATAACTTATTTTCTTATGTTTATAGTATTTTGTGAGAGTTGTATCAACXXXXXXXXXXXA
TAGCATCCGCAGAGACTCAGTTCAACTTAAACTAGCCAACAGTATGAAGCATGACAATCCAACTTCCTGTATGTTTGTT
>Marker849628 Chr7 SNP_site:16835809 Ref_Type:T
GACTTAGGTATATATATATATGGAGTGACTTTTTTTTATTTTGTTGAACAGCAAACATTGGAGGCATTGCTTAAAGCAGGCTAXXXXXXXXXXXC
TTTTAAATTTATTTATGACATTTGTTCATATCAGATTTTTCATAGTTTTCAAAAAGCCAATTCTTGTATTTTTTATTGTT
GACTTAGGTATATATTTATGGAGTGACTTTTTTTTATTTTGTTGAACAGCAAACATTGGAGGCATTGCTTAAAGCAGGCTAXXXXXXXXXXXC
TTTTAAATTTATTTATGACATTTGTTCATATCAGATTTTTCATAGTTTTTAAAAAGCCAATTCTTGTATTTTTTATTGTT
>Marker849628 Chr7 SNP_site:16836020 Ref_Type:T
GACTTAGGTATATATATATATGGAGTGACTTTTTTTTATTTTGTTGAACAGCAAACATTGGAGGCATTGCTTAAAGCAGGCTAXXXXXXXXXXXC
TTTTAAATTTATTTATGACATTTGTTCATATCAGATTTTTCATAGTTTTCAAAAAGCCAATTCTTGTATTTTTTATTGTT
GACTTAGGTATATATTTATGGAGTGACTTTTTTTTATTTTGTTGAACAGCAAACATTGGAGGCATTGCTTAAAGCAGGCTAXXXXXXXXXXXC
TTTTAAATTTATTTATGACATTTGTTCATATCAGATTTTTCATAGTTTTTAAAAAGCCAATTCTTGTATTTTTTATTGTT
>Marker849953 Chr7 SNP_site:6465216 Ref_Type:G
AACATATATTCTATTAAATTTCACTTTTCAAACTATTTTAATTTTAAAAATAAGTTATCTTGAAAATATTTGGGTGTTCTXXXXXXXXXXXT
CAAAATCATTAAAGATGAAACTCAATTATTCTCAAATATTAAGTAGTGACTAAAATATTGAAATTATTTACAAACAGGT
AACATATATTCTATTAAATTTCACTTTTCAAGCTATTTTAATTTTAAAAATAAGTTATCTTGAAAATATTTGGGTGTTCTXXXXXXXXXXXT
CAAAATCATTAAACATGAAACTCAATTATTCTCAAATATTAAGTAGTGACTAAAATATTGAAATTATTTACAAACAGGT
>Marker849953 Chr7 SNP_site:6465375 Ref_Type:G
AACATATATTCTATTAAATTTCACTTTTCAAACTATTTTAATTTTAAAAATAAGTTATCTTGAAAATATTTGGGTGTTCTXXXXXXXXXXXT
CAAAATCATTAAAGATGAAACTCAATTATTCTCAAATATTAAGTAGTGACTAAAATATTGAAATTATTTACAAACAGGT
AACATATATTCTATTAAATTTCACTTTTCAAGCTATTTTAATTTTAAAAATAAGTTATCTTGAAAATATTTGGGTGTTCTXXXXXXXXXXXT
CAAAATCATTAAACATGAAACTCAATTATTCTCAAATATTAAGTAGTGACTAAAATATTGAAATTATTTACAAACAGGT
>Marker850146 Chr7 SNP_site:18133852 Ref_Type:C
ACAGACGATGTCCAATGCTGCATTTCCATTTTTGATGCCTGTGTTCGTGATTCTTTCGAATATCATTTCTGACTCTAGATXXXXXXXXXXXT
CTCGATTCAAGAACTGGCCGATAGTCATGTTTTAAAGCGTTAGCTAAATATCTGAAATCACATCCTGAAAGATGAAGGT
ACAGACGATGTCCAATGCTGCATTTCCATTTTTGATGCCTGTGTTCGTGATTCTTTCGAATATCATTTCTGATTCTAGATXXXXXXXXXXXT
CTCGATTCAAGAACTGGCCGATAGTCATGTTTTAAAGCGTTAGCTAAATATCTGAAATCACATCCTGAAAGATGAAGGT
>Marker85020 Chr4 SNP_site:19479564 Ref_Type:C
TACAAATACACTATATGTGATATTTGACATCATTATTTGCATGCAAATACACTGCAAAAAAACACGTATTATCCTACATAXXXXXXXXXXXT

Create PDF with GO2PDF for free, if you wish to

TTTACCGACCCAGCAGGTGCATAATCAACAACCACAACAAAATCATTGTCAGAAAATTTCGACATAATCAACATAAGTC
TACAAATACACTATATGTGATATTTGACATCATTATTTGCATGCAAATACACTGCAAAAAAACACGTATTATCCTACATAXXXXXXXXXXT
TTTACTGACCCAGCAGGTGCATAATCAACAACCACAACAAAATCATTGTCAGAAAATTTCGACATAATCAACATAAGTC
>Marker850466 Chr7 SNP_site:16358644 Ref_Type:T
CACTAACTATTCTTGAGATTTCCTGGTATTTTTTCTCACTAATGTTATTCCTTGCATTCGTTCAGTAAAATTCATCAATGXXXXXXXXXXT
CTTCCCTACTCTTTTTAAGTAGCTTTTCGTTGGTCCGACCATGCAAAGATAATGGATTGTAGTTTAATACAAGAAGGTT
CACTAACTATTCTTGAGATTTCCTGGTATTTTTTCTCACTAATGTTATTCCTTGCATTCGTTCAGTAAAATTCATCAATGXXXXXXXXXXT
TTTCCCTACTCTTTTTAAGTAGCTTTTCGTTGGTCCGACCATGCAAAGATAATGGATTGTAGTTTAATACAAGAAGGTT
>Marker851723 Chr7 SNP_site:4708715 Ref_Type:C
ACTGGCATGAAATAGAGAATAAAGCAGTTGGTGCCCCTTCATATTTACCTTCAGAGGTAATTTTAACATCTCTCTCTATCXXXXXXXXXXT
TGGATAAATGATCGGCTAAAGGGCATTTGTATTTGGTAGTAACCAAGGGTCCCTTCTCGTTTATTTCCGCCAAAACAGT
ACTGGCATGAAATAGAGAATAAAGCAGTTGGTGCCCCTTCATATTTACCTTCAGAGGTAATTTTAACATCTCTCTCTATTXXXXXXXXXXT
TGGATAAATGATCGCCTAAAGGGCATTTGTATTTGGTAGTAACCAAGGGTCCCTTCTCGTTTATTTCCGCCAAAACAGT
>Marker851723 Chr7 SNP_site:4708877 Ref_Type:G
ACTGGCATGAAATAGAGAATAAAGCAGTTGGTGCCCCTTCATATTTACCTTCAGAGGTAATTTTAACATCTCTCTCTATCXXXXXXXXXXT
TGGATAAATGATCGGCTAAAGGGCATTTGTATTTGGTAGTAACCAAGGGTCCCTTCTCGTTTATTTCCGCCAAAACAGT
ACTGGCATGAAATAGAGAATAAAGCAGTTGGTGCCCCTTCATATTTACCTTCAGAGGTAATTTTAACATCTCTCTCTATTXXXXXXXXXXT
TGGATAAATGATCGCCTAAAGGGCATTTGTATTTGGTAGTAACCAAGGGTCCCTTCTCGTTTATTTCCGCCAAAACAGT
>Marker85194 Chr4 SNP_site:8051604 Ref_Type:C
AACATCAAGATTGACCCGTATGCTGGTGCAGAAAGTGGAAGGCTGCAACACAAGAACTGAAGTTAAGTCCTTACTTCTGTXXXXXXXXXXT
ATAAATTATGTAAATCATGGAGTTTGAATAAAATTAATGTAACTTGTAAAGCCTGCAGTTGGATAAACGATTTAACAGT
AACATCAAGATTGACCCGTATGCTGGTGCAGAAAGTGGAAGGCTGCAACACAAGAACTGAAGTTAAGTGCTTACTTCTGTXXXXXXXXXXT
ATAAATTATGTAAATCATGGAGTTTGAATAAAATTAATGTAACTTGTAAAGCCTGCAGTTGGATAAACGATTTAACAGT
>Marker85206 Chr4 SNP_site:23318588 Ref_Type:T
GACTTAGCTCTAATCCCTGTGCTTGTGATAATTTCTTGTCAAGATGAATACTCCATGATCTATTTTGTTCTCTTACATGCXXXXXXXXXXA
CCTCAAGTGGTCTTACCCAAGCAGTTGAAAGGGTTGTTCCGCGACCATTACAATTTTTAGTGAGGTTTCTTGCGTGGTT
GACTTAGCTCTAATCCTTGTGCTTGTGATAATTTCTTGTCAAGATGAATACTCCATGATCTATTTTGTTCTCTTATATGCXXXXXXXXXXA
CCTCAAGTGGTCTTACCCAAGCAGTTGAAAGGGTTGTTCCGCGACCATTACAATTTTTAGTGAGGTTTCTTGCGTGGTT
>Marker85206 Chr4 SNP_site:23318647 Ref_Type:T
GACTTAGCTCTAATCCCTGTGCTTGTGATAATTTCTTGTCAAGATGAATACTCCATGATCTATTTTGTTCTCTTACATGCXXXXXXXXXXA
CCTCAAGTGGTCTTACCCAAGCAGTTGAAAGGGTTGTTCCGCGACCATTACAATTTTTAGTGAGGTTTCTTGCGTGGTT
GACTTAGCTCTAATCCTTGTGCTTGTGATAATTTCTTGTCAAGATGAATACTCCATGATCTATTTTGTTCTCTTATATGCXXXXXXXXXXA
CCTCAAGTGGTCTTACCCAAGCAGTTGAAAGGGTTGTTCCGCGACCATTACAATTTTTAGTGAGGTTTCTTGCGTGGTT
>Marker85295 Chr4 SNP_site:18658371 Ref_Type:A
CACCCTTTTATTTCTCTTTTATTCCTATGGTTCTGTTTATTTCTTCTTTAGAATCCCGGAAACTAAACATTTGAAAAACCXXXXXXXXXXC
CATTGAGAAAATCACAAATCCATCTATGATCTATCTAATATACAGAGTTTCTGTTTCTTACCAAAAGACCATAAATGTA
CACCCTTTTATTTCTCTTTTATTCCTGTGGTTCTGTTTATTTCTTCTTTAGAATCCCGGAAACTAAACATTTGAAAAACCXXXXXXXXXXC
CATTGAGAAAATCACAAATCCATCTATGATCTATCTAATATACAGAGTTTCTGTTTCTTACCAAAAGACCATAAATGTA
>Marker852986 Chr7 SNP_site:7523533 Ref_Type:C
ACCAACCATATACTTGACCTGCTGATAGTTGTAAGATTCCTCAAATCATTAGTTCTGATAGTTCTGCCTCTATTCATGCTXXXXXXXXXXG
GTTCTAATCCCTCCGTTTTTGATGTTCATGGAAAAACTCTGAAAGAAAAGGCATTGTTACTACGGAGTCTTCCAAGAGT
ACCAACCATATACTTGACCTGCTGATAGTTGTAAGATTCCTCAAATCATTAGTTCTGATAGTTCTGCCTTTATTCATGCTXXXXXXXXXXG
GTTCTAATCCCTCCGTTTTTTATGTTCATGGAAAAACTCTGAAAGAAAAGGCATTGTTACTACGGAGTCTTCCAAGAGT
>Marker852986 Chr7 SNP_site:7523661 Ref_Type:G

Create PDF with GO2PDF for free, if you wish to

ACCAACCATATACTTGACCTGCTGATAGTTGTAAGATTCCTCAAATCATTAGTTCTGATAGTTCTGCCTCTATTCATGCTXXXXXXXXXXG
GTTCTAATCCCTCCGTTTTTGATGTTCATGGAAAAACTCTGAAAGAAAAGGCATTGTTACTACGGAGTCTTCCAAGAGT
ACCAACCATATACTTGACCTGCTGATAGTTGTAAGATTCCTCAAATCATTAGTTCTGATAGTTCTGCCTTTATTCATGCTXXXXXXXXXXG
GTTCTAATCCCTCCGTTTTTTATGTTCATGGAAAAACTCTGAAAGAAAAGGCATTGTTACTACGGAGTCTTCCAAGAGT
>Marker853155 Chr7 SNP_site:2643306 Ref_Type:G
AACCTTAACATTAATTTCACGTCTTCATAACCTCTCTTATTGAAATATGATATAGTTTCATTAATGTCTCATTTCTAAACXXXXXXXXXXA
TGTATGAGAGTCTAAAGTTTAAATTAATACAACTTCGAAATCAAGAATTAAAATAGATACAATTTCAAATTCAAAAGTT
AACCTTAACATTAATTTCACGTCTTCGTAACCTCTCTTATTGAAATATGATATAGTTTCATTAATGTCTCATTCCTAAACXXXXXXXXXXA
TGTATGAGAGTCTAAAGTTTAAATTAATACAACTTCGAAATCAAGAATTTAAATAGATACAATTTCAAATTCAAAAGTT
>Marker853155 Chr7 SNP_site:2643353 Ref_Type:C
AACCTTAACATTAATTTCACGTCTTCATAACCTCTCTTATTGAAATATGATATAGTTTCATTAATGTCTCATTTCTAAACXXXXXXXXXXA
TGTATGAGAGTCTAAAGTTTAAATTAATACAACTTCGAAATCAAGAATTAAAATAGATACAATTTCAAATTCAAAAGTT
AACCTTAACATTAATTTCACGTCTTCGTAACCTCTCTTATTGAAATATGATATAGTTTCATTAATGTCTCATTCCTAAACXXXXXXXXXXA
TGTATGAGAGTCTAAAGTTTAAATTAATACAACTTCGAAATCAAGAATTTAAATAGATACAATTTCAAATTCAAAAGTT
>Marker853155 Chr7 SNP_site:2643524 Ref_Type:T
AACCTTAACATTAATTTCACGTCTTCATAACCTCTCTTATTGAAATATGATATAGTTTCATTAATGTCTCATTTCTAAACXXXXXXXXXXA
TGTATGAGAGTCTAAAGTTTAAATTAATACAACTTCGAAATCAAGAATTAAAATAGATACAATTTCAAATTCAAAAGTT
AACCTTAACATTAATTTCACGTCTTCGTAACCTCTCTTATTGAAATATGATATAGTTTCATTAATGTCTCATTCCTAAACXXXXXXXXXXA
TGTATGAGAGTCTAAAGTTTAAATTAATACAACTTCGAAATCAAGAATTTAAATAGATACAATTTCAAATTCAAAAGTT
>Marker853236 Chr7 SNP_site:12786460 Ref_Type:G
AACCATTACTTCTTAACTTGGGTATGGGTCTTACAATTGGTCTCTGTAGAGGTTTCTGGTGTATTATTGAGGATTTTAATXXXXXXXXXXG
ATCCTCTTTTGTTGCTTCTAAGTTTGATAGATTCCTTCTCTTCAAGCATTGGGTAGATTTTTTTCAAAATGTTGTTGTG
AACCATTACTTCTTAACTTGGGTATGGGTCTTACAATTGGTCTCTGTAGAGGTTTCTGGTGTATTATTGGGGATTTTAATXXXXXXXXXXG
ATCCTCTTTTGTTGCTTCTAAGTTTGATAGATTCCTTCTCTTCAAGCATTGGGTAGATTTTTTTCAAAATGTTGTTGTG
>Marker853346 Chr7 SNP_site:9772747 Ref_Type:T
TACGTTGATGCAACGTCAAGAATTTTCAAACAATTAAAATGAACAGCAAGCACAACTATACGGCCATTAGGCATTATATCXXXXXXXXXXA
TAATGATAAATTGGCTATAAAGGCACCAACAAATTTGCACAGTTGGCTGTTGTATGCAAGAGAAATACATTTACCTGTG
TACGTTGATGCAACGTCAAGAATTTTCAAACAATTAAAATGAACAGCAAGCACAACTATATGGCCATTAGGCATTATATCXXXXXXXXXXA
TAATGATAAATTGGCTATAAAGGCACCAACAAATTTGCACAGTTGGCTGTTGTATGCAAGAGAAATACATTTACCTGTG
>Marker853570 Chr7 SNP_site:16965811 Ref_Type:T
ACTAGAAATCATATATACATCTTCCATCAGCATCATCTCATGTTTCTTGTTCCTGCCTCCGATCACCAAACTTGAATCTTXXXXXXXXXXG
TGTCTGCACAGAAGCTGGAATGTATGCCATCCGAGCTCGAAGGAAGACTGTGACAGAGAAGGACTTCCTTGACGCAGTG
ACTAGAAATCATATATACATCTTCCATTAGCATCATCTCATGTTTCTTGTTCCTGCCTCCGATCACCAAACTTGAATCTTXXXXXXXXXXG
TGTCTGCACAGAAGCTGGAATGTATGCCATCCGAGCTCGAAGGAAGACTGTGACAGAGAAGGACTTCCTTGACGCAGTG
>Marker854190 Chr7 SNP_site:13562337 Ref_Type:T
GACAAGAGGAGACGGTGTGGCCTGACATATGCCTCTAGACTGCATTGTGATAAGTGAAGTAATAGAGATGATATCTAATTXXXXXXXXXXG
GACAAGAAATATGGAATGTCCGGGGAAGTGGATAGAGATCAAACTTTTGGCTCCTGGTTTGTTTTTATGTTTCTTTGTG
GACAAGAGGAGACGGTGTGGCCTGACATATGCCTCTAGACTGCATTGTGATAAGTGAAGTAATAGTGATGATATCTAATGXXXXXXXXXXG
GACAAGAAATATGGAATGTCCGGGGAAGTGGATAGAGATCAAACTTTTGGCTCCTGGTTTGTTTTTATGTTTCTTTGTG
>Marker854190 Chr7 SNP_site:13562351 Ref_Type:G
GACAAGAGGAGACGGTGTGGCCTGACATATGCCTCTAGACTGCATTGTGATAAGTGAAGTAATAGAGATGATATCTAATTXXXXXXXXXXG
GACAAGAAATATGGAATGTCCGGGGAAGTGGATAGAGATCAAACTTTTGGCTCCTGGTTTGTTTTTATGTTTCTTTGTG
GACAAGAGGAGACGGTGTGGCCTGACATATGCCTCTAGACTGCATTGTGATAAGTGAAGTAATAGTGATGATATCTAATGXXXXXXXXXXG
GACAAGAAATATGGAATGTCCGGGGAAGTGGATAGAGATCAAACTTTTGGCTCCTGGTTTGTTTTTATGTTTCTTTGTG

Create PDF with GO2PDF for free, if you wish to

>Marker854440 Chr7 SNP_site:8117616 Ref_Type:A
ACTTCTAGATCTGAGGGAGGACAACCAGGCGGCGAGGGGAGCGAGGTCCCACACGAGCTAGCATCTCCAGAGAAGTAAAGXXXXXXXXXXG
AGAAGAGGTTGGCCTATCGATACTATTCTTCCAACTACTTTGTAACCCAACCTTACAACAGTAGAGACATGTCGAGGTG
ACTTCTAGATCTGAGGGAGGACAACCAGGCGGCGAGGGGAGCGAGGTCCCACACGAGCTAGCATCTCCGGAGAAGTAAAGXXXXXXXXXXG
AGAAGAGGTTGGCCTATCGATACTATTCTTCCAACTACTTTGTAACCCAACCTTACAACAGTAGAGACATGTCGAGGTG
>Marker854623 Chr7 SNP_site:6245613 Ref_Type:C
ACATTTGTTCGTCAATCCCAAGATTTTCTTTGATTATAATTTATCCTTTTGGTGGCTTCAACAGTTGCTCCCAAATGTTCXXXXXXXXXXT
GTCATGATTTCTTTAGATGTTCTTTAAAAGAGAAAGAGATGAAAGGTAGAGATTGTCCCACTAGGCGTGCCAAACTGTT
ACATTTGTTCGTGAATCCCAAGATTTTCTTTGATTATAATTTATCCTTTTGGTGGCTTCAACAGTTGCTCCCAAATGTTCXXXXXXXXXXT
GTCATGATTTCTTTAGATGTTCTTTAAAAGAGAAAGATATGAAAGGTAGAGATTGTCCCACTAGGCGTGCCAAACTGTT
>Marker854623 Chr7 SNP_site:6245832 Ref_Type:G
ACATTTGTTCGTCAATCCCAAGATTTTCTTTGATTATAATTTATCCTTTTGGTGGCTTCAACAGTTGCTCCCAAATGTTCXXXXXXXXXXT
GTCATGATTTCTTTAGATGTTCTTTAAAAGAGAAAGAGATGAAAGGTAGAGATTGTCCCACTAGGCGTGCCAAACTGTT
ACATTTGTTCGTGAATCCCAAGATTTTCTTTGATTATAATTTATCCTTTTGGTGGCTTCAACAGTTGCTCCCAAATGTTCXXXXXXXXXXT
GTCATGATTTCTTTAGATGTTCTTTAAAAGAGAAAGATATGAAAGGTAGAGATTGTCCCACTAGGCGTGCCAAACTGTT
>Marker854977 Chr7 SNP_site:14409457 Ref_Type:T
ACTTCTCAAGTCTTGCAATTCTAAAGATGAGTATAAAACAACTTGTCGTATTAAACACTATATATTCTCATTCAAACGTGXXXXXXXXXXG
CTTTTACCTACAACAATGACACAAATTAATTTTTTAATTTAAATAATGGTAAAAATATACTCACTTTCGTGAGATTGTA
ACTTCTCAAGTCTTGCAATTCTAAAGATGAGTATAAAACAACTTGTCGTATTAAACACTATATATTCTCATTCAAACGTGXXXXXXXXXXG
CTTTTACCTACAACAATGACACAAATTAATTTTTTAATTTAAATAATGGTAAAAATATACTCACTTTTGTGAGATTGTA
>Marker85526 Chr4 SNP_site:20043757 Ref_Type:T
GACCAAAAGTGCTCCAGCACCCAGGGATTGTGCAGCACCAGCAAACTACAAAAAGCTTGAAAATTAGTAAAGAAAAGAACXXXXXXXXXXT
ATTGAATTTTTGTAAGATAATTTAAAGGAGATAGTATTCTATGGTCAGAACGTCATATACAGACATACATAAATAGGTA
GACCAAAAGTGCTCCAGCACCCAGGGATTGTGCAGCACCAGCAAACTACAAAAAGCTTGAAAATTAGTAAAGAAAAGAACXXXXXXXXXXT
ATTGAATTTTTGTAAGATAATTTAAAGGAGATAGTATTCTATGGTCAGAACGTCATATACATACATACATAAATAGGTA
>Marker855505 Chr7 SNP_site:15655211 Ref_Type:T
TACATCTATTTTTAACCTCATTCGACACACGAGTTATTTTTCCCTTTACAATCCAAAACATTCATAATTTTCAAACTATTXXXXXXXXXXA
ATCAACATTTATTCACAATATATTCTAATCATACCACAATGAAAACTTTATTTGCTAATTATAACGATCTCAATCAAGT
TACATCTATTTTTAACCTCATTCGACACACGAGTTATTTTTCCCTTTACAATCCAAAACATTCATAATTTTCAAACTATTXXXXXXXXXXA
ATCAACATTTATTCACAATATATTCTAATCATACCACAATGAAAATTTTATTTGCTAATTATAACGATCTCAATCAAGT
>Marker855936 Chr7 SNP_site:15972170 Ref_Type:C
GACCCATGTGGTAGAAAGTGATCGAATTTTTGTTGCTGGTGTTGGTTTGCTTCAATGAAAAGTTTCTTTTATTTGTTGTTXXXXXXXXXXA
CCAATCTTAATGTGGTTTATGATCTTTTTTTTCTAAATTTACTTTCCAAATTGGATATATATAATTTTTTCATGATGTT
GACCCATGTGGTAGAAAGTGATCGAATTTTTGTTGCTGGTGTTGGTTTGCTTCAATGAAAAGTTTCTTTTATTTGTTGTTXXXXXXXXXXA
CCAATCTTAATGTGGTTTATGATTTTTTTTTTTCTAAATTTACTTTCCAAATTGGATATATATAACTTTTTCATGATGTT
>Marker855936 Chr7 SNP_site:15972211 Ref_Type:T
GACCCATGTGGTAGAAAGTGATCGAATTTTTGTTGCTGGTGTTGGTTTGCTTCAATGAAAAGTTTCTTTTATTTGTTGTTXXXXXXXXXXA
CCAATCTTAATGTGGTTTATGATCTTTTTTTTCTAAATTTACTTTCCAAATTGGATATATATAATTTTTTCATGATGTT
GACCCATGTGGTAGAAAGTGATCGAATTTTTGTTGCTGGTGTTGGTTTGCTTCAATGAAAAGTTTCTTTTATTTGTTGTTXXXXXXXXXXA
CCAATCTTAATGTGGTTTATGATTTTTTTTTTTCTAAATTTACTTTCCAAATTGGATATATATAACTTTTTCATGATGTT
>Marker85695 Chr4 SNP_site:16555224 Ref_Type:T
AACCCAACCCAACCCAAACTAAAAAATCCGGGTTGGACAGGGTTAGGTTGGCGGGTTGGTCTTGTTTTTTTTCTTCATTAXXXXXXXXXXA
GACATCCATATTGAAAATTTAAAAAAAAAAATTGCTATAAGAGCCAACATGCAATAGATTCATATTGGATTTTTTGGTG
AACCCAACCCAACCCAAACTAAAAAATCCGGGTTGGACAGGGTTAGGTTGGCGGGTTGGTCTTGTTTTTTTTCTTCATTAXXXXXXXXXXA

Create PDF with GO2PDF for free, if you wish to

GACATCCATATTGAAAATTTTAAAAAAAAGATTGCTATAAGAGCCAACATGCAATAGATTCATAATGGATTTTTTGGTG
>Marker85695 Chr4 SNP_site:16555233 Ref_Type:G
AACCCAACCCAACCCAAACTAAAAAATCCGGGTTGGACAGGGTTAGGTTGGCGGGTTGGTCTTGTTTTTTTTCTTCATTAXXXXXXXXXXA
GACATCCATATTGAAAATTTAAAAAAAAAAATTGCTATAAGAGCCAACATGCAATAGATTCATATTGGATTTTTTGGTG
AACCCAACCCAACCCAAACTAAAAAATCCGGGTTGGACAGGGTTAGGTTGGCGGGTTGGTCTTGTTTTTTTTCTTCATTAXXXXXXXXXXA
GACATCCATATTGAAAATTTTAAAAAAAAGATTGCTATAAGAGCCAACATGCAATAGATTCATAATGGATTTTTTGGTG
>Marker85695 Chr4 SNP_site:16555268 Ref_Type:A
AACCCAACCCAACCCAAACTAAAAAATCCGGGTTGGACAGGGTTAGGTTGGCGGGTTGGTCTTGTTTTTTTTCTTCATTAXXXXXXXXXXA
GACATCCATATTGAAAATTTAAAAAAAAAAATTGCTATAAGAGCCAACATGCAATAGATTCATATTGGATTTTTTGGTG
AACCCAACCCAACCCAAACTAAAAAATCCGGGTTGGACAGGGTTAGGTTGGCGGGTTGGTCTTGTTTTTTTTCTTCATTAXXXXXXXXXXA
GACATCCATATTGAAAATTTTAAAAAAAAGATTGCTATAAGAGCCAACATGCAATAGATTCATAATGGATTTTTTGGTG
>Marker857256 Chr7 SNP_site:24757 Ref_Type:T
CACAACATTACTTCGTATTTACTTTTTTGAATGAATGCATTCCTTTTATATTATATACTTAAGATGATGGTGCTAATTAGXXXXXXXXXXT
AACTAGCAAGTTTAAAATGTATTTAACGAAAATGACTTTGAAAGCAAAAGTCAAAGCTAAGGTTTCTTAATTTCCTAGT
CACAACATTACTTCGTATTTACTTTTTTGAATGAATGCATTCCTTTTATATTATATATTTAAGATGATGGTGCTAATTAGXXXXXXXXXXT
AACTAGCAAGTTTAAAATGTATTTAACGAAAATGACTTTGAAAGCAAAAGTCAAAGCTAAGGTTTCTTAATTTCCTAGT
>Marker857279 Chr7 SNP_site:15386417 Ref_Type:C
TACATGGTTTGATAAAACCTTTATAAAAGTTAGAATATAAATGCTCCAGGAAGTAAATAAATTGAACAAGAATAAGGGGCXXXXXXXXXXT
ATCAATAGGCTCTCCAAGCACGTTGAAATTCAGCCAAGAGTCGCTCCCCAATTGAAACACTTAGAGGAGCTCTCGTGTC
TACATGGTTTGATAAAACCTTTATAAAAGTTCGAATATAAATGCTCCGGGAAGTAAATAAATTGAACAAGAATAAGGGGCXXXXXXXXXXT
ATCAATAGGCTCTCCAAGCACGTTGAAATTCAGCCAAGAGTCGCTCCCCAATTGAAACACTTAGAGGAGTTCTCGTGTC
>Marker857279 Chr7 SNP_site:15386433 Ref_Type:G
TACATGGTTTGATAAAACCTTTATAAAAGTTAGAATATAAATGCTCCAGGAAGTAAATAAATTGAACAAGAATAAGGGGCXXXXXXXXXXT
ATCAATAGGCTCTCCAAGCACGTTGAAATTCAGCCAAGAGTCGCTCCCCAATTGAAACACTTAGAGGAGCTCTCGTGTC
TACATGGTTTGATAAAACCTTTATAAAAGTTCGAATATAAATGCTCCGGGAAGTAAATAAATTGAACAAGAATAAGGGGCXXXXXXXXXXT
ATCAATAGGCTCTCCAAGCACGTTGAAATTCAGCCAAGAGTCGCTCCCCAATTGAAACACTTAGAGGAGTTCTCGTGTC
>Marker857279 Chr7 SNP_site:15386639 Ref_Type:T
TACATGGTTTGATAAAACCTTTATAAAAGTTAGAATATAAATGCTCCAGGAAGTAAATAAATTGAACAAGAATAAGGGGCXXXXXXXXXXT
ATCAATAGGCTCTCCAAGCACGTTGAAATTCAGCCAAGAGTCGCTCCCCAATTGAAACACTTAGAGGAGCTCTCGTGTC
TACATGGTTTGATAAAACCTTTATAAAAGTTCGAATATAAATGCTCCGGGAAGTAAATAAATTGAACAAGAATAAGGGGCXXXXXXXXXXT
ATCAATAGGCTCTCCAAGCACGTTGAAATTCAGCCAAGAGTCGCTCCCCAATTGAAACACTTAGAGGAGTTCTCGTGTC
>Marker857444 Chr7 SNP_site:7254053 Ref_Type:T
TACCTGTGGTTGTGAGCGACAACATCGAGCTTCCATTTGAAGACATGGTGGATTACTCAGAATTCTCTGTTTTTGTAGCTXXXXXXXXXXT
TGAATATGAAAATGGCCATCCGGGTGGTATTGGACCAGTTCCTCCAGATGGTGCTGTAAATCACATATGGAGAAAAGTG
TACCTGTGGTTGTGAGCGACAACATTGAGCTTCCATTTGAAGACATGGTGGATTACTCAGAATTCTCTGTTTTTGTAGCTXXXXXXXXXXT
TGAATATGAAAATGGCCATCCGGGTGGTATTGGACCAGTTCCTCCAGATGGTGCTGTAAATCACATATGGAGAAAAGTG
>Marker85749 Chr4 SNP_site:6607976 Ref_Type:G
AACGACAGAGGAGATGAGATGAGGAATGTGAATGGCATAGAAGATGGGGAATCTAGGGTTGTAGAAGATGAAGAAGTTGGXXXXXXXXXXC
TCCTTACATTTCTCGTGTTATTTCATCTCCATTTTATTTAGACGGGGAAACGAGAAAAGAAAAAAGAAGAAAGAAGGTA
AACGACAGAGGAGATGAGATGAGGAATGTGAATGGCATAGAAGATGGGGAATCTAGGGTTGTAGAAGATGAAGAAGTTGGXXXXXXXXXXC
TCCTTAGATTTCTCGTGTTATTTCGTCTCCATTTTATTTAGACGGGGAAACGAGAAAAGAAAAAAGAAGAAAGAAGGTA
>Marker85749 Chr4 SNP_site:6607994 Ref_Type:G
AACGACAGAGGAGATGAGATGAGGAATGTGAATGGCATAGAAGATGGGGAATCTAGGGTTGTAGAAGATGAAGAAGTTGGXXXXXXXXXXC
TCCTTACATTTCTCGTGTTATTTCATCTCCATTTTATTTAGACGGGGAAACGAGAAAAGAAAAAAGAAGAAAGAAGGTA

Create PDF with GO2PDF for free, if you wish to

AACGACAGAGGAGATGAGATGAGGAATGTGAATGGCATAGAAGATGGGGAATCTAGGGTTGTAGAAGATGAAGAAGTTGGXXXXXXXXXXC
TCCTTAGATTTCTCGTGTTATTTCGTCTCCATTTTATTTAGACGGGGAAACGAGAAAAGAAAAAAGAAGAAAGAAGGTA
>Marker857599 Chr7 SNP_site:15215501 Ref_Type:T
GACAACATATTGATTCATTTAAATTATTGAAAGAAGGGAAAGGAGAAAATGAAAGACAACTATGGAAAATCTCAATTAATXXXXXXXXXXT
GAAGAAAAGCAAAAATTACCTTTCAATTTTGTTTTGCATTGAAAAACCAAACAAAATGAAATCGATCCAAGCGTATGTG
GACAACATATTGATTCATTTAAATTATTGAAAGAATGGAAAGGAGAAAATGAAAGACAACTATGGAAAATCTCAATTAATXXXXXXXXXXT
GAAGAAAAGCAAAAATTACCTTTCAATTTTGTTTTGCATTGAAAAACCAAACAAAATGAAATCGATCCAAGCGTATGTG
>Marker857893 Chr7 SNP_site:14016445 Ref_Type:G
ACAAGGAAAATGCGAAGGGAACCCACATGAGACTCGAGTTACATCAACAAGTTCAAGTTGAGCACACGATGAACTTAGATXXXXXXXXXXA
TGTTCTCAATCAACGGAAATATGAACAGCTGGATAATTCAGAGGATTAACAATTTGACACAAGCTAGAGCAACAAAGTC
ACAAGGAAAATGCGAAGGGAACCGACATGAGACTCGAGTTACATCAACAAGTTCAAGTTGAGCACACGATGAACTTAGATXXXXXXXXXXA
TGTTCTCAATCAACGGAAATATGAACAGCTGGATAATTCAGAGGATTAACAATTTGACACAAGCTAGAGCAACAAAGTC
>Marker858098 Chr7 SNP_site:17288206 Ref_Type:T
TACAGTAGCAGCGTATGAACAACATTCCATAACTATTGATATTTGCACAACCAAGACAACGTCAATCACAGGTCCAATAGXXXXXXXXXXA
TTTTCAATATAATCTCAAAACTCTGCCTAACACGTGAGCTCTAATTTCCATCTCGACTAATCTCATGACACAAATAGTT
TACAGTAGCAGCGTATGAACAACATTCCATAACTATTGATATTTGCACAACCAAGACAACGTCAATCACAGGTCCAATATXXXXXXXXXXA
TTTTCAATATAATCTCAAAACTCTGCCTAACACGTGAGCTCTAATTTCCATCTCGACTAATCTCATGACACAAATAGTT
>Marker858600 Chr7 SNP_site:14721744 Ref_Type:T
ACATCTACACTGGGTCTCACAATTCTTGTGTTTATATCTATGATTTGGTAAGTCACTTGACATTTGTTTTTGTGCATCTTXXXXXXXXXXA
TTTATAGTTCCGACCGTCCTTAAGAAGATAAAATAAGAATATTAGTCCAATTGCACACTTGGAAAAATTAATTTTTGGT
ACATCTACACTGGGTCTCACAATTCTTGTGTTTATATCTATGATTTGGTAAGTCACTTGACATTTGTTTTTGTGCATCTTXXXXXXXXXXA
TTTATAGTTCTGACCGTCCTTAAGAAGATAAAATAAGAATATTAGTCCAATTGCACACTTGGAAAAATTAATTTTTGGT
>Marker858629 Chr7 SNP_site:2449427 Ref_Type:C
CACCTATATTCATGAACCACCCTACTTCAAGAATATGACCATGGACCCTCCAGGAGCACATGGCGTTAAGGATGCTTACTXXXXXXXXXXT
TATTGACCGTGGAGTGGATCGCAAGGATTTTAATTCTTATGGAAGTAGGCGAGGCAATGATGAGGTGATGGCAAGGGGT
CACCTATATTCATGAACCACCCTACTTCAAGAATATGACCATGGATCCTCCAGGAGCACATGGCGTTAAGGATGCTTACTXXXXXXXXXXT
TCTTGACCGTGGAGTGGATCGCAAGGATTTTAATTCTTATGGAAGTAGGCGAGGCAATGATGAGGTGATGGCAAGGGGT
>Marker858629 Chr7 SNP_site:2449551 Ref_Type:A
CACCTATATTCATGAACCACCCTACTTCAAGAATATGACCATGGACCCTCCAGGAGCACATGGCGTTAAGGATGCTTACTXXXXXXXXXXT
TATTGACCGTGGAGTGGATCGCAAGGATTTTAATTCTTATGGAAGTAGGCGAGGCAATGATGAGGTGATGGCAAGGGGT
CACCTATATTCATGAACCACCCTACTTCAAGAATATGACCATGGATCCTCCAGGAGCACATGGCGTTAAGGATGCTTACTXXXXXXXXXXT
TCTTGACCGTGGAGTGGATCGCAAGGATTTTAATTCTTATGGAAGTAGGCGAGGCAATGATGAGGTGATGGCAAGGGGT
>Marker858828 Chr7 SNP_site:5263063 Ref_Type:A
ACAAGAGATCAAATAAGATCAAACCTCCAACTATAGAGTGGAAGAAAATGCCACTTCTCTTTGTAAGCTAAGTTTTTTTAXXXXXXXXXXC
TTATTGTGTAGCAACTTGTTCTTTGTATTTTATAAACATATTTAGACCCTAATATATACATATGAACAAAAAGAAAGTG
ACGAGAGATCAAATAAGATCAAACCTCCAACTATAGAGTGGAAGAAAATGCCACTTGTCTTTGTAAGCTAAGTTTTTTTAXXXXXXXXXXC
TTATTGTGTAGCAACTTGTTCTTTGTATTTTATAAACATATTTAGACCCTAATATATACATATGAACAAAAAGAAAGTG
>Marker858828 Chr7 SNP_site:5263117 Ref_Type:C
ACAAGAGATCAAATAAGATCAAACCTCCAACTATAGAGTGGAAGAAAATGCCACTTCTCTTTGTAAGCTAAGTTTTTTTAXXXXXXXXXXC
TTATTGTGTAGCAACTTGTTCTTTGTATTTTATAAACATATTTAGACCCTAATATATACATATGAACAAAAAGAAAGTG
ACGAGAGATCAAATAAGATCAAACCTCCAACTATAGAGTGGAAGAAAATGCCACTTGTCTTTGTAAGCTAAGTTTTTTTAXXXXXXXXXXC
TTATTGTGTAGCAACTTGTTCTTTGTATTTTATAAACATATTTAGACCCTAATATATACATATGAACAAAAAGAAAGTG
>Marker85899 Chr4 SNP_site:19245437 Ref_Type:A
AACCAGTTGTCAAGGCCATAATAAATGCAATGGTTGAATGTAATTTGCCGCCTGAAATGGTTACCAATCAGCAGATATGCXXXXXXXXXXG

Create PDF with GO2PDF for free, if you wish to

AATGCATTTTGTTGTTGGAAGACCAGATACACATTGTTCAAGATGGTCTTAAATCAAATTGTTTTCCTGGATTGAGGGT
AACCAGTTGTCAAGGCCATAATAAATGCAATGGTTGAATGTAATTTGCCGCCTGAAATGGTTACCAATCAGCAGATGTGCXXXXXXXXXXG
AATGCATTTTGTTGTTGGAAGACCAGATACACATTGTTCAAGATGGTCTTAAATCAAATTGTTTTCCTGGATTGAGGGT
>Marker859268 Chr7 SNP_site:16721455 Ref_Type:G
AACATTTACTGGGAGAACATATCTGGCGATGAAAATGGGATTTGTTAATGGACTATAAGGACATTATGGACTTACAATATXXXXXXXXXXT
TCAAGACTTTCGCTCAGCTTCATGATTCTTGCAAGTTACTTAAAGTTTAAAGATAGGCCAGAACCATCTTACGATAGTG
AACATTTACTGGGAGAACATATCTGGCGATGAAAATGGGATTTGTTAATGGACTATAGGGACATTATGGACTTACAATATXXXXXXXXXXT
TCAAGACTTTCGCTCAGCTTCATGATTCTTGCAGGTTACTTAAAGTTTAAAGATAGGCCAGAACCATCTTACGATAGTG
>Marker859268 Chr7 SNP_site:16721638 Ref_Type:G
AACATTTACTGGGAGAACATATCTGGCGATGAAAATGGGATTTGTTAATGGACTATAAGGACATTATGGACTTACAATATXXXXXXXXXXT
TCAAGACTTTCGCTCAGCTTCATGATTCTTGCAAGTTACTTAAAGTTTAAAGATAGGCCAGAACCATCTTACGATAGTG
AACATTTACTGGGAGAACATATCTGGCGATGAAAATGGGATTTGTTAATGGACTATAGGGACATTATGGACTTACAATATXXXXXXXXXXT
TCAAGACTTTCGCTCAGCTTCATGATTCTTGCAGGTTACTTAAAGTTTAAAGATAGGCCAGAACCATCTTACGATAGTG
>Marker859618 Chr7 SNP_site:12720681 Ref_Type:T
ACTCCCACTGAATTGAACTGTAAACTGAGAAATTTTGATGATAAAGTTCTTGTTGATAAAGAACAATATCAACGCCTTGTXXXXXXXXXXC
GACTCCTAGAAAAGGATTGATGTTCAGAAAGAAGGACAAAAACTATTGAGGCTTATACTAGCTCAAACTGGACAGGGTT
ACTCCCATTGAATTGAACTGTAAACTGAGAAATTTTGATGATAAAGTTCTTGTTGATAAAGAACAATATCAACGCCTTGTXXXXXXXXXXC
GACTCCTAGAAAAGGATTGATGTTCAGAAAGAAGGACAAAAACTATTGAGGCTTATACTAGCTCAAACTGGACAGGGTT
>Marker859677 Chr7 SNP_site:11921263 Ref_Type:A
ACAGTGACCAAAGACCTATATTCTTTATATATAGACTACATATTCATACCTCTTCAATCCACCATTCTTAACGTTTCATCXXXXXXXXXXA
AGGTTAATGCACAAGAAGGAGTTTGGTGACAAATTTCACCTTAGCGAGGTAGCTTCCAACATTTGTATCCCGAAAGGTG
ACAGTGACCAAAGACCTATATTCTTTATATATAGACTGCATATTCATACCTCTTCAATCCACCATTCTTAACGTTTCATCXXXXXXXXXXA
AGGTTAATGCACAAGAAGGAGTTTGGTGACAAATTTCACCTTAGCGAGGTAGCTTCCAACATTTGTATCCCGAAAGGTA
>Marker859677 Chr7 SNP_site:11921529 Ref_Type:T
ACAGTGACCAAAGACCTATATTCTTTATATATAGACTACATATTCATACCTCTTCAATCCACCATTCTTAACGTTTCATCXXXXXXXXXXA
AGGTTAATGCACAAGAAGGAGTTTGGTGACAAATTTCACCTTAGCGAGGTAGCTTCCAACATTTGTATCCCGAAAGGTG
ACAGTGACCAAAGACCTATATTCTTTATATATAGACTGCATATTCATACCTCTTCAATCCACCATTCTTAACGTTTCATCXXXXXXXXXXA
AGGTTAATGCACAAGAAGGAGTTTGGTGACAAATTTCACCTTAGCGAGGTAGCTTCCAACATTTGTATCCCGAAAGGTA
>Marker859818 Chr7 SNP_site:5256193 Ref_Type:T
ACCAATACGCAAAGGCGAAACAAGACATTGTGATAGAGAGCCATGATTCTGCTTTCGAGATCAAGAAAGGGGAAATGATTXXXXXXXXXXA
AGCAGTGTCCGGCGAGGGATCTGGTGGTGCTGATGTGCAGAGTAGTGTTGGTGGAACTTTTCCTCCGTTATGAGACGTT
ACCAATACGCAAAGGCGAAACAAGACATTGTGATAGAGAGCCATGATTCTGCTTTCGAGATCAAGAAAGGGGAAATGATTXXXXXXXXXXA
AGCAGTGTCCGGCGAGGGATCTGGTGGTGCTGATGTGCAGAGTAGTGTTGGTGGAACTTTTCCTCCGTTATGATACGTT
>Marker85989 Chr4 SNP_site:12760917 Ref_Type:G
TACAGGAATTTTGTAGTGACGGATCATAAATATCTCATTTCTTTTCAGAAAGGTTGGGAAGCGAAAGGCTGTCGTTAAAGXXXXXXXXXXT
ATTTGTTTGATTTTCTTAAGGAGAAAAATTCTCTTTTTTGTTTAAATTTTATATAACAGCGAGGAGTTCAGAAAGGAGT
TACAGGAATTTTGTAGTGACGGATCATAAATATCTCATTTCTTTTCAGAAAGGTTGGGAAGCGAAAGGCTGTCGTTAAAGXXXXXXXXXXT
ATTTGTTTGATTTTCTTAAGGGGAAAAATTCTCTTTTTTGTTTAAATTTTATATAACAGCGAGGAGTTCAGAAAGGAGT
>Marker861374 Chr7 SNP_site:12924156 Ref_Type:T
CACAATAATCCCTCGAAGCTCTTCCATTTTTAGAATCTCCAATCCAGTTCGTGTCTTCTCTACTAACCCTCCAACACTATXXXXXXXXXXA
ACCAACCCCATCCATTAATGATCCCTTCAACGTCTCCACTAGTCCTCCTATGACTGAACCAGGTTCGTTAAACATCGTA
CACAATAATCCCTCGAAGCTTTTCCATTTTTAGAATCTCCAATCCAGTTCGTGTCTTCTCTACTAACCCTCCAACACTATXXXXXXXXXXA
ACCAACCCCATCCATTAATGATCCCTTCAACGTCTCCACTAGTCCTCCTATGACTGAACCAGGTTCGTTAAACATCGTA
>Marker861474 Chr7 SNP_site:6451899 Ref_Type:C

Create PDF with GO2PDF for free, if you wish to

TACCAAGTTGCAGCTATGCGAAGTAGTCTCAACGCATTTACCAAAATTTCACCAATACCATCACGCATAATGCATCTTCCXXXXXXXXXXXA
TATCAGATCACATAGCGAGTATGAGATATCTCACACTAGAACACGACAAGTATAAATGAGGCATCTCATCGCATAGGGT
TACCAAGTTGCAGCTATGCGAAGTAGTCTCAACGCATTTACCAAAATTTCACCAATACCATCACGCATAATGCATCTTCCXXXXXXXXXXXA
TATCAGATCACATAGTGAGTATGAGATATCTCACACTAGAACACGACAAGTATAAATGAGGCATCTCATCGCATAGGGT
>Marker861565 Chr7 SNP_site:17190717 Ref_Type:T
ACTAGGATTGAACGGTTCGGCTGCAGCAGAAAGCTTGCTAGCATTCGTTTCTGCAGGCTTTTCCGAAGGAGATGAACAGGXXXXXXXXXXXT
GATTACAGCTCTGGGGTTCAGCTACTTTCTCTTCTAACTCAATAACATTCTCCGTATCAACCAGTTGCCTTAGAACAGT
ACTAGGATTGAACGGTTCGGCTGCAGCAGAAAGCTTGCTAGCATTCGTTTCTGCAGGCTTTTCCGAAGGAGATGAACAGGXXXXXXXXXXXT
TATTACAGCTCTGGGGTTCAGCTACTTTCTCTTCTAACTCAATAACATTCTCCGTATCAACCAGTTGCCTTAGAACAGT
>Marker86166 Chr4 SNP_site:3613120 Ref_Type:C
ACATCATTAATTTATGTTCATCAAAATACGTGACAAAGTTGTATTTTATGGAAAAAATTATGCTGAACTTGCTTGGGACTXXXXXXXXXXXG
GTTAAATTGCAAATTTAGTTTTATAGTTTGAAAAAAAAGCTAGAATTTAGTTTCTATGATTTATAAGTTGAATTTAGTC
ACATCATTAATTTATGTTCATCAAAATACGTGACAAAGTTGTATTTTATGGAAAAAATTATGCTGAACTTGCTTGGGACTXXXXXXXXXXXG
GTTAAATTGTAAATTTGGTTTTATAGTTTGAAAAAAAAGCTAGAATTTAGTTTTTATGATTTATAAGTTGAATTTAGTC
>Marker86166 Chr4 SNP_site:3613127 Ref_Type:A
ACATCATTAATTTATGTTCATCAAAATACGTGACAAAGTTGTATTTTATGGAAAAAATTATGCTGAACTTGCTTGGGACTXXXXXXXXXXXG
GTTAAATTGCAAATTTAGTTTTATAGTTTGAAAAAAAAGCTAGAATTTAGTTTCTATGATTTATAAGTTGAATTTAGTC
ACATCATTAATTTATGTTCATCAAAATACGTGACAAAGTTGTATTTTATGGAAAAAATTATGCTGAACTTGCTTGGGACTXXXXXXXXXXXG
GTTAAATTGTAAATTTGGTTTTATAGTTTGAAAAAAAAGCTAGAATTTAGTTTTTATGATTTATAAGTTGAATTTAGTC
>Marker86166 Chr4 SNP_site:3613164 Ref_Type:C
ACATCATTAATTTATGTTCATCAAAATACGTGACAAAGTTGTATTTTATGGAAAAAATTATGCTGAACTTGCTTGGGACTXXXXXXXXXXXG
GTTAAATTGCAAATTTAGTTTTATAGTTTGAAAAAAAAGCTAGAATTTAGTTTCTATGATTTATAAGTTGAATTTAGTC
ACATCATTAATTTATGTTCATCAAAATACGTGACAAAGTTGTATTTTATGGAAAAAATTATGCTGAACTTGCTTGGGACTXXXXXXXXXXXG
GTTAAATTGTAAATTTGGTTTTATAGTTTGAAAAAAAAGCTAGAATTTAGTTTTTATGATTTATAAGTTGAATTTAGTC
>Marker862044 Chr7 SNP_site:13010784 Ref_Type:G
ACTCTATTTGTTGATGTAACTTTGAAGGCATTGTTTCTTCTCATCAATTGGTTAGCATAAGTATAGACACATTGGCCCATXXXXXXXXXXXT
TAATTGTTAATCGATATGCGAACCTATTGAGAGGAGTTATTGCTGTTGGAAGGGCTACACAAGTGCAACAAAGACATGT
ACTCTATTTGTTGATGTAACTTTGAAGGCATTGTTTCTTCTCATCAATTGGTTAGCATAAGTATGGACACATTGGCCCATXXXXXXXXXXXT
TAATTGTTAATCGATATGCGAACCTATTGAGAGGAGTTATTGCTGTTGGAAGGGCTACACAAGTGCAACAAAGACATGT
>Marker862349 Chr7 SNP_site:13222799 Ref_Type:A
GACAAAATGAAGGAGATAAATAGGGTTTAGACTTCCACATGCCCATGAAATTTACTCATTTGACTCTGAAGCAGCCAAGAXXXXXXXXXXXA
TTGAAGTGAGAGCTACTAAACGGTAGATGGCAAGGATGCTTTCTCCTCTCTCTTTATTGACCATTGCTCTGTCGGTTGT
GACAAAATGAAGGAGATAAATAGGGTTTAGACTTCCACATGCCCATGAAATTTACTCATTTGTCTCTGAAGCAGCCAAGAXXXXXXXXXXXA
TTGAAGTGAGAGCTACTAAACGGTAGATGGCAAGGATGCTTTCTCCTCTCTCTTTATTGACCATTGCTCTGTCGGTTGT
>Marker862569 Chr7 SNP_site:865561 Ref_Type:T
GACTCAGCGCTCAGCTATGTTTTGACTTTAATTCTCAGAAGAACCTCATAATAAATGATGCTACTCTTCTCCTATTGGTCXXXXXXXXXXXA
AATAGGTAATGCATAACTCTTTGAAATCGTGCAACTAGGAAAGTAGAGAATGTCATTTTCCACGTTTCTCACAAACGTA
GACTCAGCGCTCAGCTATGTTTTGACTTTAATTCTCAGAAGAACCTTATAATAAATGATGCTACTCTTCTCCTATTGGTCXXXXXXXXXXXA
AATAGGTAATGCATAACTCTTTGAAATCGTGCAACTAGGAAAGTAGAGAATGTCATTTTCCACGTTTCTCACATACGTA
>Marker862569 Chr7 SNP_site:865766 Ref_Type:T
GACTCAGCGCTCAGCTATGTTTTGACTTTAATTCTCAGAAGAACCTCATAATAAATGATGCTACTCTTCTCCTATTGGTCXXXXXXXXXXXA
AATAGGTAATGCATAACTCTTTGAAATCGTGCAACTAGGAAAGTAGAGAATGTCATTTTCCACGTTTCTCACAAACGTA
GACTCAGCGCTCAGCTATGTTTTGACTTTAATTCTCAGAAGAACCTTATAATAAATGATGCTACTCTTCTCCTATTGGTCXXXXXXXXXXXA
AATAGGTAATGCATAACTCTTTGAAATCGTGCAACTAGGAAAGTAGAGAATGTCATTTTCCACGTTTCTCACATACGTA

Create PDF with GO2PDF for free, if you wish to

>Marker862658 Chr7 SNP_site:3254718 Ref_Type:T
TACTTCAGATGATCATTTTCTTGAAAACAAGCATAGCCACAAGGAAAGCATAATATTGTCTAGTAATTGTTCTAATCATGXXXXXXXXXXA
GTTAATAAATGCATCTCAGGTAGTAGAAAAGTATTGTTGGGCATGCAAGAAAAAATATATAATAAATAAACATTGATGT
TACTTCAGATGATCATTTTCTTGAAAACAAGCATAGCCACAAGGAAAGCATAATATTGTCTAGTAATTGTTCTAATCATGXXXXXXXXXXA
GTTAATAAATGCATCTTAGGTAATAGAAAAGTATTGTTGGGCATGCAAGAAAAAATATATAATAAATAAACATTGATGT
>Marker862658 Chr7 SNP_site:3254724 Ref_Type:A
TACTTCAGATGATCATTTTCTTGAAAACAAGCATAGCCACAAGGAAAGCATAATATTGTCTAGTAATTGTTCTAATCATGXXXXXXXXXXA
GTTAATAAATGCATCTCAGGTAGTAGAAAAGTATTGTTGGGCATGCAAGAAAAAATATATAATAAATAAACATTGATGT
TACTTCAGATGATCATTTTCTTGAAAACAAGCATAGCCACAAGGAAAGCATAATATTGTCTAGTAATTGTTCTAATCATGXXXXXXXXXXA
GTTAATAAATGCATCTTAGGTAATAGAAAAGTATTGTTGGGCATGCAAGAAAAAATATATAATAAATAAACATTGATGT
>Marker862930 Chr7 SNP_site:18671390 Ref_Type:T
AACATTAAAACAACAATGACAAATTTATATGCACAAAATGGGCGCATTAGCTGTCACCTTTTGGACTTAAATCAAATCCCXXXXXXXXXXA
TCTTCGCTTTAAAGTATTTTATTGGGGTTGTTTGATGCTCAAGCATGGATTATAGACCTATTAGATTTTATGATGTGTG
AACATTAAAACAATAATGACAAATTTATATGCACAAAATGGGCGCATTAGCTGTCACCTTTTGGACTTAAATCAAATCCCXXXXXXXXXXA
TATTCGCTTTAAAGTATTTTATTGGGGTTGTTTGATGCTCAAGCATGGATTATAGACCTATTAGATTTTATGATGTGTG
>Marker862930 Chr7 SNP_site:18671546 Ref_Type:A
AACATTAAAACAACAATGACAAATTTATATGCACAAAATGGGCGCATTAGCTGTCACCTTTTGGACTTAAATCAAATCCCXXXXXXXXXXA
TCTTCGCTTTAAAGTATTTTATTGGGGTTGTTTGATGCTCAAGCATGGATTATAGACCTATTAGATTTTATGATGTGTG
AACATTAAAACAATAATGACAAATTTATATGCACAAAATGGGCGCATTAGCTGTCACCTTTTGGACTTAAATCAAATCCCXXXXXXXXXXA
TATTCGCTTTAAAGTATTTTATTGGGGTTGTTTGATGCTCAAGCATGGATTATAGACCTATTAGATTTTATGATGTGTG
>Marker863195 Chr7 SNP_site:13354261 Ref_Type:G
AACCTAACGAAGTAACCAAAATGATTCTGGTTGTTTTAAACTTGAGATGGAGAAGACACCCTACAACTGCAGATAAGGGTXXXXXXXXXXC
TGCACATCTGTTCGTTGCCATCGAGGAATCTGTGAGGGAAAAGTTCGATCTCCATGCATAGGTGCAAATGCTGAAAGTT
AACCTAACGAAGTAACCAAAATGATTCTGGTTGTTTTAAACTTGAGATGGAGAAGACACCCTACAACTGCAGATAATGGTXXXXXXXXXXC
TGCACATCTGTTCGTTGCCATCGAGGAATCTGTGAGGGAAAAGTTCGATCTCCATGCATAGGTGCAAATGCTGAAAGTT
>Marker863215 Chr7 SNP_site:3609004 Ref_Type:T
ACTAGAACTCCACGGGATCTACTTTTAGTGAAGGAAGAATACCATGCTCGTTTCAAGCGGTCAATTGAAGAAGATGTCGCXXXXXXXXXXG
TTTGCTCGACACTATTTGATTCTTATTTCATTTTCAACAGTTATCTAAACCATTAGAAATTATTAACTATCCTGGTGTA
ACTAGAACTCCACGGGATCTACTTTTAGTGAAGGAAGAATACCATGCTCGTTTCAAGCGGTCAATTGAAGAAGATGTCGCXXXXXXXXXXG
TTTGCTCGACACTATTTGATTCTTATTTCATTTTCAACAGTTATCTAAACCATTAGAAATTATTAACTTTCCTGGTGTA
>Marker863532 Chr7 SNP_site:14250201 Ref_Type:G
GACCTCTAGAAATTGCAAATCTCAAAAGAAAGAGTTCTTGTGGTGTCAATGCCACACCTGATATCCAACTTGGAGATTTTXXXXXXXXXXT
TAGACATAAAATCATTTATAGATTTTCTTTTTTAATAATTCTTCCCTCGGATTGAATTCAAATCTTCGAACAATGAGTA
GACCTCTAGAAATTGCAAATCTCAAAAGAAAGAGTTCTTGTGGTGTCAATGGCACACCTGATATCCAACTTGGAGATTTTXXXXXXXXXXT
TAGACATAAAATCATTTATAGATTTTCTTTTTTAATAATTCTTCCCTCGGATTGAATTCAAATCTTCGAACAATGAGTA
>Marker863548 Chr7 SNP_site:6299380 Ref_Type:A
ACCTTATCCTAGTAACACTATTGATATGTCTCACTTTGTATTTGATACAAACGCAATGATCAATCGCGTTCGTGTAATTGXXXXXXXXXXC
TAGGATGACCTACGTAACTTAGTCTTAATCCTAAGTGTGTTATGAACTTTTGTTCGCGAGGGATTGTCCTTTGATTTGT
ACCTTATCCTAGTAACACTATTGATATGTCTCACTTTGTATTTGATACAAACGCAATGATCAATCGCGTTCGTGTAATTGXXXXXXXXXXC
TAGGATGACCTACGTAACTTAGTCTTAATCCTGAGTGTGTTATGAACTTTTGTTCGCGAGGGATTGTCCTTTGATTTGT
>Marker86388 Chr4 SNP_site:6637718 Ref_Type:C
TACAAGTTAGAATTCCAATCTACTACGTGGCGTCAAACTCATTTATGTAGCCATCTATTCAATAGGTAATAAGAAGTTTCXXXXXXXXXXA
ACCATGAAATAAGAAAATCCCAACAAAAATAGAGAAACAGAAACACTATTCTGTTATAATGATAAATTCCAAATGAGTT
TACAAGTTAGAATTCCAATCTACTACGTGGCGTCAAACTCATTTATGTAGCCATCTATTCAATAGGTAATAAGAAGTTTCXXXXXXXXXXA

Create PDF with GO2PDF for free, if you wish to

ACCATGAAATAAGAAAATCCCAATAAAAATAGAGAAACAGAAACACTACTCTGTTATAATGATAAATTCCAAATGAGTT

>Marker86388 Chr4 SNP_site:6637743 Ref_Type:T

TACAAGTTAGAATTCCAATCTACTACGTGGCGTCAAACTCATTTATGTAGCCATCTATTCAATAGGTAATAAGAAGTTTCXXXXXXXXXXA

ACCATGAAATAAGAAAATCCCAACAAAAATAGAGAAACAGAAACACTATTCTGTTATAATGATAAATTCCAAATGAGTT

TACAAGTTAGAATTCCAATCTACTACGTGGCGTCAAACTCATTTATGTAGCCATCTATTCAATAGGTAATAAGAAGTTTCXXXXXXXXXXA

ACCATGAAATAAGAAAATCCCAATAAAAATAGAGAAACAGAAACACTACTCTGTTATAATGATAAATTCCAAATGAGTT

>Marker864137 Chr7 SNP_site:3870548 Ref_Type:G

TACCATCTGAACCTCGGCCTCCGGCTTGCTCAACGAGAAGGCTAAGAGGATTTGCCTCATATACTAGACGAAGATGATCTXXXXXXXXXXG

GATTTTTGCCTTTCCCCTGTCTGATTGTGTCAATATATCTCCGCAATCCTTCTGGCCAGTCGAAATATCTTGCATCGTT

TACCATCTGAACCTCGGCCTCCGGCTTGCTCAACGAGAAGGCTAAGAGGGTTTGCCTCATATACTAGACGAAGATGATCTXXXXXXXXXXG

GATTTTTGCCTTTCCCCTGTCTGATTGTGTCAATATATCGCCGCAATCCTTCTGGCCAGTCGAAATATCTTGCATCGTT

>Marker864137 Chr7 SNP_site:3870706 Ref_Type:G

TACCATCTGAACCTCGGCCTCCGGCTTGCTCAACGAGAAGGCTAAGAGGATTTGCCTCATATACTAGACGAAGATGATCTXXXXXXXXXXG

GATTTTTGCCTTTCCCCTGTCTGATTGTGTCAATATATCTCCGCAATCCTTCTGGCCAGTCGAAATATCTTGCATCGTT

TACCATCTGAACCTCGGCCTCCGGCTTGCTCAACGAGAAGGCTAAGAGGGTTTGCCTCATATACTAGACGAAGATGATCTXXXXXXXXXXG

GATTTTTGCCTTTCCCCTGTCTGATTGTGTCAATATATCGCCGCAATCCTTCTGGCCAGTCGAAATATCTTGCATCGTT

>Marker864245 Chr7 SNP_site:6923144 Ref_Type:A

AACCAGGCATATATCTTATGTAAAGTTTATTTCTAAATATTTATGCAATTAAAATATAAGCTACACATTTTTACACTCTTXXXXXXXXXXC

TCTTCCTTAAAAACTTACCTTCCCTCAGCATTTTTAAATTTCTCAAAATGCGATCAAGTTTTATTCATTATTTAGGGTT

AACCAGGCATATATCTTATGTAAAGTTTATTTCTAAATATTTATGCAATTAAAATATAAGCTACGCATTTTTACACTCTTXXXXXXXXXXC

TCTTCCTTAAAAACTTACCTTCCCTCAGCATTTTTAAATTTCTCAAAATGCGATCAAGTTTTATTCATTATTTAGGGTT

>Marker864248 Chr7 SNP_site:14806013 Ref_Type:G

ACTATCTTTTCTAATTGCCTTATTTAAAACCCTTTTGTTTGAGATTCTTCATATGATCATCACTCTCATGTGTATAAGATXXXXXXXXXXA

TCATTTTACTGATGCAAATTTTGCTTCCTCTATACACTATTTTGTTTAGTGGGAAACAGGAAGCAGAAAAACCCATGTA

ACTATCTTTTCTAATTGCCTTATTTAAAACCCTTTTGTTTGAGGTTCTTCATATGATCATCACTCTCATGTGTATAAGATXXXXXXXXXXA

TCATTTTACTGATGCAAATTTTGCTTCCTCTATACACTATTTTGTTTAGTGGGAAACAGGAAGCAGAAAAACCCATGTA

>Marker864810 Chr7 SNP_site:15573953 Ref_Type:C

AACAACACTCCTACTCTTTCATAAATCATGATGACATCTTGTTTCTTATATATAAACTTAATTTCAATTTCCTAAATAGCXXXXXXXXXXC

TTCTAAATTAAAAACACACAAATGTAATACTATTTGATAGCGATATATAATTATTAATACGTGTTTTAATAAGATGGTA

AACAACACTCCTACTCTTTTATAAATCATGATGACATCTTGTTTCTTATATATAAACTTAATTTCAATTTCCTAAATAGCXXXXXXXXXXC

TTCTAAATTAAAAACACACAAATGTAATACTATTTGATAGCGATATATAATTATTAATACGTGTTTTAATAAGATGGTA

>Marker865008 Chr7 SNP_site:5691559 Ref_Type:G

ACATTATCTTTGATAAATACACTTATTGTTGGACCATTATTGATGATCTTTATTGTATATTATGTTAATAAAAGAACTATXXXXXXXXXXT

GAGCTCATTTATTTATTATTTCTGATTTTATCTTACGGTTTTGCTCTTATGATTGGTATATACATAAATGAATTATGTA

ACATTATCTTTGATAAATACACTTATTGTTGGACCATTATTGATTATCTTTATTGTATATTATGTTAATAAAAGAACTATXXXXXXXXXXT

GAGCTCATTTATTTATTGTTTCTGATTTTATCTTACGGTTTTGCTCTTATGATTGGTATATACATAAATGAATTATGTA

>Marker865008 Chr7 SNP_site:5691693 Ref_Type:A

ACATTATCTTTGATAAATACACTTATTGTTGGACCATTATTGATGATCTTTATTGTATATTATGTTAATAAAAGAACTATXXXXXXXXXXT

GAGCTCATTTATTTATTATTTCTGATTTTATCTTACGGTTTTGCTCTTATGATTGGTATATACATAAATGAATTATGTA

ACATTATCTTTGATAAATACACTTATTGTTGGACCATTATTGATTATCTTTATTGTATATTATGTTAATAAAAGAACTATXXXXXXXXXXT

GAGCTCATTTATTTATTGTTTCTGATTTTATCTTACGGTTTTGCTCTTATGATTGGTATATACATAAATGAATTATGTA

>Marker865263 Chr7 SNP_site:13006456 Ref_Type:A

TACTAATGGGATTCTGCATCGAATGCTTTGGTGGATTTGTAGGTAGATTTTCTCGTCGTCATCTACTGATGGGTTTCGGTXXXXXXXXXXG

GCAGACTTCCATTTGCGGTCGGGGTTGAGCATGTTGAAGCCTTTGAAGACGTTGATGATGCCAAGAATGAGAATTGAGT

Create PDF with GO2PDF for free, if you wish to

TACTAATGGGATTCTGCATCGAATGCTTTGGTGGATTTGTTGGTAGATTTTCTCGTCGTCATCTACTGATGGGTTTCGGTXXXXXXXXXXG
GCAGACTTCCATTTGCGGTCGGGGTTGAGCATGTTGAAGCCTTTGAAGACGTTGATGATGCCAAGAATGAGAATTGAGT
>Marker865592 Chr7 SNP_site:12207115 Ref_Type:C
CACCATTCTTATCATGCTGTCAATGTTGTTAAAAGGCTATTTAGTGTAGCCTAGTGATAAAAGGAGATCAATAAAACCAAXXXXXXXXXXXA
TCCGTATCCTAATCAATCAAGGGAACTTATCCTAATTCTAATTAATCAAGGATTGTAATCTGATAGACCTCCTATCGTT
CACCATTCTTATCATGCTGTCAATGTTGTTAAAAGGCTATTTAGTGTAGCTTAGTGATAAAAGGAGATCAATAAAACCAAXXXXXXXXXXXA
TCCGTATCCTAATCAATCAAGGGAACTTATCCTAATTCTAATTAATCAAGGATTGTAATCTGATAGACCTCCTATCGTT
>Marker865924 Chr7 SNP_site:12689122 Ref_Type:T
TACCATTAAAACCTTTCTCTTTCTCATGAATAAATAGTCTTAAGAGAGTAATTTAATTAAGTTGCTCAAGCATTACTCCAXXXXXXXXXXXT
ATATATATTGCATAGCAAGAATTAAAAAAGATTGTTACAAATATATCTAAACGCCGTCTAACCTATTTGTAAATATAGT
TACCATTAAAACCTTTCTCTTTCTCATGAATAAATAGTCTTAAGAGAGTAATTTAATTAAGTTGCTCAAGCATTACTCCAXXXXXXXXXXXT
ATATATATTGCATAGCAATAATTAAAAAAGATCGTTACAAATATATCTAAATGCCGTCTAACCTATTTGTAAATATAGT
>Marker865924 Chr7 SNP_site:12689136 Ref_Type:C
TACCATTAAAACCTTTCTCTTTCTCATGAATAAATAGTCTTAAGAGAGTAATTTAATTAAGTTGCTCAAGCATTACTCCAXXXXXXXXXXXT
ATATATATTGCATAGCAAGAATTAAAAAAGATTGTTACAAATATATCTAAACGCCGTCTAACCTATTTGTAAATATAGT
TACCATTAAAACCTTTCTCTTTCTCATGAATAAATAGTCTTAAGAGAGTAATTTAATTAAGTTGCTCAAGCATTACTCCAXXXXXXXXXXXT
ATATATATTGCATAGCAATAATTAAAAAAGATCGTTACAAATATATCTAAATGCCGTCTAACCTATTTGTAAATATAGT
>Marker865924 Chr7 SNP_site:12689155 Ref_Type:T
TACCATTAAAACCTTTCTCTTTCTCATGAATAAATAGTCTTAAGAGAGTAATTTAATTAAGTTGCTCAAGCATTACTCCAXXXXXXXXXXXT
ATATATATTGCATAGCAAGAATTAAAAAAGATTGTTACAAATATATCTAAACGCCGTCTAACCTATTTGTAAATATAGT
TACCATTAAAACCTTTCTCTTTCTCATGAATAAATAGTCTTAAGAGAGTAATTTAATTAAGTTGCTCAAGCATTACTCCAXXXXXXXXXXXT
ATATATATTGCATAGCAATAATTAAAAAAGATCGTTACAAATATATCTAAATGCCGTCTAACCTATTTGTAAATATAGT
>Marker866023 Chr7 SNP_site:15890910 Ref_Type:C
ACAACCTATAAAACAAGTATGAGTAACAAAAAGGACTACTTGACCTAAATTTAAGATATAGAGAATTCTAACCTTTCTATXXXXXXXXXXXT
ATTTTCTAGAAAAATTATTAGTCCAAGCTAAGTATGCTTAACTTTGATGTTCCTATGTCATTGAAAATTTTAAAAAGTG
ACAACCTATAAAACAAGTATGAGTAACAAAAAGGACTATTTGACCTAAATTTAAGATATAGAGAATTCTAACCTTTCTATXXXXXXXXXXXT
ATTTTCTAGAAAAATTATTAGTCCAAGCTAAGTATGCTTAACTTTGATGTTCTTATGTCATTGAAAATTTTAAAAAGTG
>Marker866023 Chr7 SNP_site:15891144 Ref_Type:C
ACAACCTATAAAACAAGTATGAGTAACAAAAAGGACTACTTGACCTAAATTTAAGATATAGAGAATTCTAACCTTTCTATXXXXXXXXXXXT
ATTTTCTAGAAAAATTATTAGTCCAAGCTAAGTATGCTTAACTTTGATGTTCCTATGTCATTGAAAATTTTAAAAAGTG
ACAACCTATAAAACAAGTATGAGTAACAAAAAGGACTATTTGACCTAAATTTAAGATATAGAGAATTCTAACCTTTCTATXXXXXXXXXXXT
ATTTTCTAGAAAAATTATTAGTCCAAGCTAAGTATGCTTAACTTTGATGTTCTTATGTCATTGAAAATTTTAAAAAGTG
>Marker866250 Chr7 SNP_site:16285416 Ref_Type:A
ACTTTATTAAAAGCAACATTTTCTTATTGTTGCAATAGCATGTCTTGTTTTCCATTTTATAAGTTGATTATGAAAAGCAAXXXXXXXXXXXA
ATCCTGCTATAACATGTTATTCTCCCTATTTTCCCTAATTCAAATTAACATGTTTTGTGCTTCTTATCAGCAAATAAGT
ACTTTATTAAAAGCAACATTTTCTTATTGTTGCAATAGCATGTCTTGTTTTCCATTTTATAAGTTGATTATGAAAAGCAAXXXXXXXXXXXA
ATCCTGCTATTACATGTTATTCTCCCTATTTTCCCTAATTCAAATTAACATGTTTTGTGCTTCTTATCAGCAAATAAGT
>Marker866252 Chr7 SNP_site:3741089 Ref_Type:T
ACTTACAATAGTGAAAAATTATCAGCTACATTGTGGCGGCCCAACAGAATAATTTCTCAATAAACACCAACTACCTTTGTXXXXXXXXXXG
AAAATAGAGGCAGAAATGAAAAAGAATGATTATCCAAAACAGCCATTCTTGCTATGGAGTTTGAGTCTCGGTCCCTTGT
ACTTACAATAGTGAAAAATTATCAGCTACATTGTGGCGGCTCAACAGAATAATTTCTCAATAAACACCAACTACCTTTGTXXXXXXXXXXG
AAAATAGAGGCAGAAATGAAAAAGAATGATTATCCAAAACAGCCATTCTTGCTATGGAGTTTGAGTCTCGGTCCCTTGT
>Marker866603 Chr7 SNP_site:17171729 Ref_Type:A
CACCTGCGAAATGATTTTTTGATATTTGGACATATTGAAGGCCTTTTAAACTACCAATCCAACTAGGAATTTTGCCTGATXXXXXXXXXXXT

Create PDF with GO2PDF for free, if you wish to

TCTAAAAAAGGAATGTAGTTGAACATAGAAATTTGTAAATCTCCAGAAAAGTTGTTATTGGATAGATCCAACCAGTGTA
CACCTGCGAAATGATTTTTTTGATATTTGGACATATTGAAGGCCTTTTAAACTACCAATCCAACTAGGAATTTTGCCTGATXXXXXXXXXXT
TCTAAAAAAGGGATGTAGTTGAACATAGAAATTTGTAAATCTCCAGAAAAGTTGTTATTGGATAGATCCAACCAGTGTA
>Marker866618 Chr7 SNP_site:14385389 Ref_Type:C
AACCTTTAGGCCATTTAAGACTTCAGAGCCAAGATCTCTAGCAACAAAAGTGATGCTTTTGGAATACAGTTTCAATTTTCXXXXXXXXXXC
AACTCACAAGCTCTTATATAAGCTTCCCTACTTATTTATCTTCACATAAATGCATTACTCAGTTTCTTATCATAAAAGT
AACCTTTAGGCCATTTAAGACTTCAGAGCCAAGATCTCTAGCAACAAAAGTGATGCTTTTGGAATACAGTTTCAATTTTCXXXXXXXXXXC
AACTCACAAGCTCTTATATAAGCTTCCTTACTTATTTATCTTCACATAAATGCATTACTCAGTTTCTTATCATAAAAGT
>Marker866664 Chr7 SNP_site:15677884 Ref_Type:C
CACAAAAAAGATCAGAAAAGATTCATCATATTCATCGAGAAACTGTAAAACACAAACACAAAAGTGAAAAAGAAATACAAXXXXXXXXXXT
AAAAGGAGAAAAAAAGATAATGAATAATGAGGTTTAAAATTTGCTGAGCATTTACTGAAAAACCAACAGAAACCATGTC
CACAAAAAAGATCAGAAAAGATTCATCATATTCATTGAGAAACTGTAAAACACAAACACAAAAGTGAAAAAGAAATACAAXXXXXXXXXXT
AAAAGGAGAAAAAAAGATAATGAATAATGAGGTTTAAAATTTGCTGAGCATTTACTGAAAAACCAACAGAAACCATGTC
>Marker86669 Chr4 SNP_site:10028356 Ref_Type:G
GACAAACAATTGATTATCCCTCAAAACCGCAAAACCATTCCCAAATGTTTCATATGCTCATCTATATCAGCACTATAGACXXXXXXXXXXT
GACTAAGAACTCATAGTGGCCTTCGTGTGTGCAAAACACTGTCTTCTCCACATCATCCTCCTTCATCCTTATTTGTGGT
GACAAACAATTGATTATCCCTCAAAACCGCAAAACCATTCCCAAATGTTTCATATGCTCATCTATATCAGCACTATAGACXXXXXXXXXXT
GACTAAGAACTCATAGTGGCCTTCGTGTGTGCGAAACACTGTCTTCTCCACATCATCCTCCTTCATCCTTATTTGTGGT
>Marker866753 Chr7 SNP_site:16773214 Ref_Type:T
AACTTTGAAGAAGGTAGAGAAGGGTGGAAGAGGTTGAAAGCCAATAGGCAAGATCGCTGATATTTTCTTGACTCTGAAAAXXXXXXXXXXG
ATCTGATTGTGTGAATAATTCTGTCAAATATATTTGTCCTCTCTGCTTCAAAAGATCTCCATTGAAGAAGTGTCTTGTA
AACTTTGAAGAAGGTAGAGAAGGGTGGAAGAGGTTGAAAGCCAATAGGCAAGATCGCTGATATTTTCTTGACTCTGAAAAXXXXXXXXXXG
ATCTGATTGTGTGAATAATTCTGTCAAATATATTTGTCTTCTCTGCTTCAAAAGATCTCCATTGAAGAAGTGTCTTGTA
>Marker867233 Chr7 SNP_site:14078830 Ref_Type:T
TACACGTTCTTGATTAGGATGAAAGGAACCCGATGGAGTTATCAGATGAAAACTGGATGAATTGTTCTCCACAGAATCTGXXXXXXXXXXT
TGAGACAAAATAACTGTGTGCAGTTTCGTCCTGTTCAAGGCATTATCACAAACTCCAGCTGTCACAACACGCATTAGGT
TACACGTTCTTTATTAGGATGAAAGGAACCCGATGGAGTTATCAGATGAAAACTGGATGAATTGTTCTCCACAGAATCTGXXXXXXXXXXT
TGAGACAAAATAACTGTGTGCAGTTTCGTCCTGTTCAAGGCATTATCACAAACTCCAGCTGTCACAACACGCATTAGGT
>Marker867418 Chr7 SNP_site:3485013 Ref_Type:T
ACTTTCAGTAAATGCCAAATTGAGTTCTACACAAAAAATTTATTTCAAATTTAATTTCCAACAACATTTGGTCGGTAACTXXXXXXXXXXA
ACCTGAAACTATAATAAGCAACATTACAGAGGCTATACTGCTGGAAAACCTGCTTTAATACGAATACTATGTCCATGTT
ACTTTCAGTAAATGCCAAATTGAGTTCTACACAAAAAATTTATTTCAAATTTAATTTCCAACAACATTTTGTCGGTAACTXXXXXXXXXXA
ACCTGAAACTATAATAAGCAACATTACAGAGGCTATACTGCTGGAAAACCTGCTTTAATACGAATACTATGTCCATGTT
>Marker867459 Chr7 SNP_site:7400963 Ref_Type:A
AACAATATTCAGTTGTTCATGCAACCATTACAAAACTTAACACTAACTCTATGATAAGATCCTGAGGTTGAGAATGTGCAXXXXXXXXXXA
AAGTTTTCACTTGTTGGAGAAATTGAGAGTCCCACGTTGGAAAAACTAAGGAGAATCATACTCCATATAAAATAGATGT
AACAATATTCAGTTGTTCATGCAACCATTACAAAACTTAACACTAACTCTATGATAAGATCCTGAGGTTGAGAATGTGCAXXXXXXXXXXA
AAGTTTTCACTTGTTGGAGAAATTGAGAGTCCCACGTTGGAAAAACTAAGGAGACTCATACTCCATATAAAATAGATGT
>Marker867565 Chr7 SNP_site:3166007 Ref_Type:C
CACCTGCAAGCAAAGAGGAACATGCACACTGATGTGATGTAAGGTATAATCACTCCTATGCTTAAGTTAGAAGGTTAAAGXXXXXXXXXXT
CCCTTTTTAACCCAGCCTATATTGGACTTTCGATCACTTAGGCCTCCTCTTGGGCTCGACCTTGATAGAGGTCAAGGTC
CACGTGCAAGCAAAGAGGAACATGCACACTGATGTGATGTAAGGTATAATCACTCCTATGCTTAAGTTAGAAGGTTAAAGXXXXXXXXXXT
CCCTTTTTAACCTAGCCTATATTGGACTTTCGATCACTTAGGCCTCCCCTTGGGCTCGACCTTGATAGAGGTCAAGGTC
>Marker867565 Chr7 SNP_site:3166215 Ref_Type:C

Create PDF with GO2PDF for free, if you wish to

CACCTGCAAGCAAAGAGGAACATGCACACTGATGTGATGTAAGGTATAATCACTCCTATGCTTAAGTTAGAAGGTTAAAGXXXXXXXXXXT
CCCTTTTTAACCCAGCCTATATTGGACTTTCGATCACTTAGGCCTCCTCTTGGGCTCGACCTTGATAGAGGTCAAGGTC
CACGTGCAAGCAAAGAGGAACATGCACACTGATGTGATGTAAGGTATAATCACTCCTATGCTTAAGTTAGAAGGTTAAAGXXXXXXXXXXT
CCCTTTTTAACCTAGCCTATATTGGACTTTCGATCACTTAGGCCTCCCCTTGGGCTCGACCTTGATAGAGGTCAAGGTC
>Marker867565 Chr7 SNP_site:3166250 Ref_Type:T
CACCTGCAAGCAAAGAGGAACATGCACACTGATGTGATGTAAGGTATAATCACTCCTATGCTTAAGTTAGAAGGTTAAAGXXXXXXXXXXT
CCCTTTTTAACCCAGCCTATATTGGACTTTCGATCACTTAGGCCTCCTCTTGGGCTCGACCTTGATAGAGGTCAAGGTC
CACGTGCAAGCAAAGAGGAACATGCACACTGATGTGATGTAAGGTATAATCACTCCTATGCTTAAGTTAGAAGGTTAAAGXXXXXXXXXXT
CCCTTTTTAACCTAGCCTATATTGGACTTTCGATCACTTAGGCCTCCCCTTGGGCTCGACCTTGATAGAGGTCAAGGTC
>Marker867783 Chr7 SNP_site:16361024 Ref_Type:C
TACTACCTTCATATGTGTTTGTCTAAACTGCATATTTTTCAATGAAAGGAAGCTTTCATAGATCTGAAACTTCATTTTTGXXXXXXXXXXA
TTTTACTGTGCTTCATTTTAAGATAATTTTTCCACTCTATCATCCAATATACAGAAGTAGTATAGATTCAAAATCAGTC
TACTACCTTTATATGTGTTTGTCTAAACTGCATATTTTTCAATGAAAGGAAGCTTTCATAGATCTGAAACTTCATTTTTGXXXXXXXXXXA
TTTTACTGTGCTTCATTTTAAGATAATTTTTCCGCTCTATCATCCAATATACAGAAGTAGTATAGATTCAAAATCAGTC
>Marker867783 Chr7 SNP_site:16361260 Ref_Type:A
TACTACCTTCATATGTGTTTGTCTAAACTGCATATTTTTCAATGAAAGGAAGCTTTCATAGATCTGAAACTTCATTTTTGXXXXXXXXXXA
TTTTACTGTGCTTCATTTTAAGATAATTTTTCCACTCTATCATCCAATATACAGAAGTAGTATAGATTCAAAATCAGTC
TACTACCTTTATATGTGTTTGTCTAAACTGCATATTTTTCAATGAAAGGAAGCTTTCATAGATCTGAAACTTCATTTTTGXXXXXXXXXXA
TTTTACTGTGCTTCATTTTAAGATAATTTTTCCGCTCTATCATCCAATATACAGAAGTAGTATAGATTCAAAATCAGTC
>Marker867870 Chr7 SNP_site:3916492 Ref_Type:C
CACATTACTGTGTTTAGAGTGCAGAGTTGTTTTGAGTTCGTGTTTGCGTTTGTGGTGCAGAGTTGTTTTGAGTTCATGTTXXXXXXXXXXT
ATTCAATAATTTTACCATTCTTTTTTTTGAATGTTTGAATAAGATATGAATTGCATTTTTTCTTTCTTATGGTGATGTA
CACATTACTGTGTTTAGAGTGCAGAGTTGTTTTGAGTTTGTGTTTGCGTTTGTGGTGCAGAGTTGTTTTGAGTTCATGTTXXXXXXXXXXT
ATTCAATAATTTTACCATTCTTTTTTTTGAATGTTTGAATAAAATATGAATTGCATTTTTTCTTTCTTATGGTGATGTA
>Marker867870 Chr7 SNP_site:3916686 Ref_Type:G
CACATTACTGTGTTTAGAGTGCAGAGTTGTTTTGAGTTCGTGTTTGCGTTTGTGGTGCAGAGTTGTTTTGAGTTCATGTTXXXXXXXXXXT
ATTCAATAATTTTACCATTCTTTTTTTTGAATGTTTGAATAAGATATGAATTGCATTTTTTCTTTCTTATGGTGATGTA
CACATTACTGTGTTTAGAGTGCAGAGTTGTTTTGAGTTTGTGTTTGCGTTTGTGGTGCAGAGTTGTTTTGAGTTCATGTTXXXXXXXXXXT
ATTCAATAATTTTACCATTCTTTTTTTTGAATGTTTGAATAAAATATGAATTGCATTTTTTCTTTCTTATGGTGATGTA
>Marker868191 Chr7 SNP_site:18079820 Ref_Type:G
ACATAATTGCAAAACATTGTTTTCATTCTACTGTTTCTTTTCAACTTTTCTCGAGTTGTCTATTTTTTACATCAATTTACXXXXXXXXXXT
TTTTAGGAGTTAATAATAAAAGAAACCAAAATCTAGAGGAATAGACAGGACTTGGATTGAATGCAATATTATATATGTT
ACATAATTGCAAAACATTGTTTTCGTTCTACTGTTTCTTTTCAACTTTTCTCGAGTTGTCTATTTTTTACATCAATTTACXXXXXXXXXXT
TTTTAGGAGTTAATAATAAAAGAAACCAAAAACTAGAGGAATAGACAGGACTTGGATTGAATGCAATATTATATATGTT
>Marker868191 Chr7 SNP_site:18080012 Ref_Type:A
ACATAATTGCAAAACATTGTTTTCATTCTACTGTTTCTTTTCAACTTTTCTCGAGTTGTCTATTTTTTACATCAATTTACXXXXXXXXXXT
TTTTAGGAGTTAATAATAAAAGAAACCAAAATCTAGAGGAATAGACAGGACTTGGATTGAATGCAATATTATATATGTT
ACATAATTGCAAAACATTGTTTTCGTTCTACTGTTTCTTTTCAACTTTTCTCGAGTTGTCTATTTTTTACATCAATTTACXXXXXXXXXXT
TTTTAGGAGTTAATAATAAAAGAAACCAAAAACTAGAGGAATAGACAGGACTTGGATTGAATGCAATATTATATATGTT
>Marker868964 Chr7 SNP_site:5517141 Ref_Type:C
CACGTTTTTCTATACCTGGGAGTTTTAAACGTTGTCTTCCATTATATCTTATTTTCTAGATTTTTAATTTGTGTATCTTGXXXXXXXXXXC
AGAATGGATGTAGAGAAATGAAATATATATATATATATTTGATGTTTTTCCATAACATTTCTTATCCCTATTATTGGGT
CACGTTTTTCTATACTTGGGAGTTTTAAATGTTGTCTTCCATTATATCTTATTTTCTAGATTTTTAATTTGTGTATCTTGXXXXXXXXXXC
AGAATGGATGTAGAGAAATGAAATATATATATATATAGTTGATGTTTTTCCATAACATTTCTTATCCCTATTATTGGGT

Create PDF with GO2PDF for free, if you wish to

>Marker868964 Chr7 SNP_site:5517155 Ref_Type:T
CACGTTTTTCTATACCTGGGAGTTTTAAACGTTGTCTTCCATTATATCTTATTTTCTAGATTTTTAATTTGTGTATCTTGXXXXXXXXXXC
AGAATGGATGTAGAGAAATGAAATATATATATATATATTTGATGTTTTTCCATAACATTTCTTATCCCTATTATTGGGT
CACGTTTTTCTATACTTGGGAGTTTTAAATGTTGTCTTCCATTATATCTTATTTTCTAGATTTTTAATTTGTGTATCTTGXXXXXXXXXXC
AGAATGGATGTAGAGAAATGAAATATATATATATATAGTTGATGTTTTTCCATAACATTTCTTATCCCTATTATTGGGT
>Marker868964 Chr7 SNP_site:5517353 Ref_Type:T
CACGTTTTTCTATACCTGGGAGTTTTAAACGTTGTCTTCCATTATATCTTATTTTCTAGATTTTTAATTTGTGTATCTTGXXXXXXXXXXC
AGAATGGATGTAGAGAAATGAAATATATATATATATATTTGATGTTTTTCCATAACATTTCTTATCCCTATTATTGGGT
CACGTTTTTCTATACTTGGGAGTTTTAAATGTTGTCTTCCATTATATCTTATTTTCTAGATTTTTAATTTGTGTATCTTGXXXXXXXXXXC
AGAATGGATGTAGAGAAATGAAATATATATATATATAGTTGATGTTTTTCCATAACATTTCTTATCCCTATTATTGGGT
>Marker869549 Chr7 SNP_site:17110623 Ref_Type:G
AACTTTTGAAGTGGTAATAATATTTTTGCTAATTTTGAGAATAATGATATAAACTTTACATCATTTAAGTTAAACATAGAXXXXXXXXXXA
ATATATCGTGTCACACTTCCTATAAGACAAAAGCACGAGCTAAACAATGTTAGAGTTTGAATTAGAATAAAAAGTAGTA
AACTTTTTAAGTGGTAATAATATTTTTGCTAATTTTGAGAATAATGATATAAACTTTACATCATTTAAGTTAAACATAGAXXXXXXXXXXA
ATATATCGTGTCACACTTCCTATAAGACAAAAGCACGAGCTAAACAATGTTAGAGTTTGAATTAGAATAAAAAGTAGTA
>Marker869818 Chr7 SNP_site:17083244 Ref_Type:T
AACATGTCCCGAACCGCCTTCACCCAAAACATTGCTTTTGCTGAAGTTATCAGTTGCTGCTTCCAACAATTTATAGTCGAXXXXXXXXXXA
AGATTAAGTTGGTTTAAATTCTTGCTGATTATAAATAAAGCAAGAAAAGAGCATAATCAAACTAATGGGAAAGGAAAGT
AACATGTCCCGAACCGCCTTCTCCCAAAACATTGCTTTTGCTGAAGTTATCAGTTGCTGCTTCCAACAATTTATAGTCGAXXXXXXXXXXA
AGATTAAGTTGGTTTAAATTCTTGCTGATTATAAATAAAGCAAGAAAAGAGCATAATCAAACTAATGGGAAAGGAAAGT
>Marker870650 Chr7 SNP_site:4514242 Ref_Type:A
CACTATCTGCTTTGCCATGAATGAAGATATAATTTAGTCACAACACTGTTGCTGAGCAAACAAAGGAACAAATTGATACGXXXXXXXXXXA
ACCACTTGGAATGTCTCTAACAATTTTCCGAGTTGGTGGGCATGTTGCTCCTCATCATTCACTCCGTCAAGCATTATGT
TACTATCTGCTTTGCCATGAATGAAGATATAATTTAGTCACAACACTGTTGCTGAGCAAACAAAGGAACAAATTGATACGXXXXXXXXXXA
ACCACTTGGAATGTCTCTAACAATTTTCCGAGTTGGTGGGCATGTTGCTCCTCATCATTCACTCCGTCAAGCATTATGT
>Marker870754 Chr7 SNP_site:14387176 Ref_Type:T
ACCTCAACATATCAACGGCATACACATGATTTTATCGTTTAGTTATAGGACCGAAAGAAAAAAAAAACTAACTTAAACAGXXXXXXXXXXG
ATGGGCAACTATCACAACAAATCAAGTCTCCACCATCTCCACAAATTCCACAAGTATCATCATTAGGATCTTCGACGTC
ACCTCAACATATCAACGGCTTACACATGATTTTATCGTTTAGTTATAGGACCGAAAGAAAAAAAAAACTAACTTAAACAGXXXXXXXXXXG
ATGGGCAACTATCACAACAAATCAAGTCTCCACCATCTCCACAAATTCCACAAGTATCATCATTAGGATCTTCGACGTC
>Marker870921 Chr7 SNP_site:13083377 Ref_Type:G
TACAAAATTGTTAATGGAAATCTTTGTGTCATCGATATCTCAAAAGACTGCTATAATTAATTGATGTGTGTGCGTTACCCXXXXXXXXXXG
GTTTAGTGTTTAACGATGAGAATGGAGGGAGAGCAAGGAGGCATATTGAGGAATGAAGATTAGGAACTGCAAATGGAGT
TACAAAATTGTTAATGGAAATCTTTGTGTCATCGATATCTCAAAAGACTGCTATAATTAATTGATGTGTGTGCGTTACCCXXXXXXXXXXG
GTTTAGTGTTTAACGATGAGAATGGAGGGAGAGCAAGGATGCATATTGAGGAATGAAGATTAGGAACTGCAAATGGAGT
>Marker871417 Chr7 SNP_site:3927626 Ref_Type:G
ACCGAGAACAATAACGTCGTATTCTTCATCCATGGGAGCAGGAAGGAAGAGGAAAGGAGAGATCGAGAGAGAAATGAAAGXXXXXXXXXXC
GCTACTATTATCAAATGACAAACCTCTTTTTCTTTTTCTTTTTTTAATATCAATATTTTTGTAGAAATTTTGCTGTGTA
ACCGAGAACAATAACGTCGTATTCTTCATCCATGGGAGCAGGAAGGAAGAGGAAAGGAGAGATCGAGAGAGAAATGAAAGXXXXXXXXXXC
GCTACTATTATCAAATGACAAACCTCTTTTTCTTTTTCTTTTTTTAATATCAATATTTTTGTAGAAATTTTTCTGTGTA
>Marker871478 Chr7 SNP_site:3086291 Ref_Type:G
AACAAATAAGACTATATATATATAAAAAAGAATAGGTTAAATGAATCAGACACATGTGCTTGAAAGTGTATGGATTCCTAATXXXXXXXXXXC
AAGTGGAAGCTGCTTGCAATGCAACAGTTTCATGTGCTGATATTCTTGCTCTAGCCACGCGTGATGGAGTCGTTTTGGT
AACAAATAAGACTATATATATATAAAAAAGAATAGGTTAAATGAATCAGACACATGTGCTTGAAAGTGTATGGATTCCTAATXXXXXXXXXXC

Create PDF with GO2PDF for free, if you wish to

AAGTGGAAGCTGCTTGCAATGCAACAGTTTCATGTGCTGATATTCTTGCTCTAGCCACGCGTGATGGGGTCGTTTTGGT
>Marker872336 Chr7 SNP_site:13031221 Ref_Type:G
ACCAACAGGACTAACACTTCCGTCAAACACCAACACGCCTCTTGATTTCAAATTATCCGGTTTAAACTCATGCACTGCTGXXXXXXXXXXG
ATTCGGATGCAAGGTCCCAGAAGGGAAAGGAGAGCGTGGGGGAAGGACGAACGGAGTTATAAGAAGTAATGTTGAAAGT
ACCAACAGGACTAACACTTCCGTCAAACACCAACACGCCTCTTGATTTCAAATTATCCGGTTTAAACTCATGCACTGCTGXXXXXXXXXXG
ATTGGGATGCAAGGTCCCAGAAGGGAAAGGAGAGCGTGGGGGAAGGACGAACGGAGTTATAAGAAGTAATGTTGAAAGT
>Marker872350 Chr7 SNP_site:1110190 Ref_Type:C
AACACAAGAGTATTAAGTTATGGCCTTCTCTTTTGTGTGTATATATATTGACTTCTTTTTTCTAAAATAATTTTAAAAAGXXXXXXXXXXT
AGATTTATTTGGTTCAAATACCATAATTCTAATAAGGATAATTTTTTGTTTGAAAAAAAAGAATCATGCTAGGGTTGTG
AACACAAGAGTATTAAGTTCTGGCCTTCTCTTTTGTGTATATATATATTGACTTCTTTTTTCTAAAATAATTTTAAAAAGXXXXXXXXXXT
AGATTTATTTGGTTCAAATACCATAATTCTAATAAGGATAATTTTTTCTTTGAAAAAAAAGAATCATGCTAGGGTTGTG
>Marker872350 Chr7 SNP_site:1110209 Ref_Type:A
AACACAAGAGTATTAAGTTATGGCCTTCTCTTTTGTGTGTATATATATTGACTTCTTTTTTCTAAAATAATTTTAAAAAGXXXXXXXXXXT
AGATTTATTTGGTTCAAATACCATAATTCTAATAAGGATAATTTTTTGTTTGAAAAAAAAGAATCATGCTAGGGTTGTG
AACACAAGAGTATTAAGTTCTGGCCTTCTCTTTTGTGTATATATATATTGACTTCTTTTTTCTAAAATAATTTTAAAAAGXXXXXXXXXXT
AGATTTATTTGGTTCAAATACCATAATTCTAATAAGGATAATTTTTTCTTTGAAAAAAAAGAATCATGCTAGGGTTGTG
>Marker872350 Chr7 SNP_site:1110398 Ref_Type:C
AACACAAGAGTATTAAGTTATGGCCTTCTCTTTTGTGTGTATATATATTGACTTCTTTTTTCTAAAATAATTTTAAAAAGXXXXXXXXXXT
AGATTTATTTGGTTCAAATACCATAATTCTAATAAGGATAATTTTTTGTTTGAAAAAAAAGAATCATGCTAGGGTTGTG
AACACAAGAGTATTAAGTTCTGGCCTTCTCTTTTGTGTATATATATATTGACTTCTTTTTTCTAAAATAATTTTAAAAAGXXXXXXXXXXT
AGATTTATTTGGTTCAAATACCATAATTCTAATAAGGATAATTTTTTCTTTGAAAAAAAAGAATCATGCTAGGGTTGTG
>Marker872560 Chr7 SNP_site:243053 Ref_Type:T
AACTGATTCCAATTTCAGTTTCTCATTCTCAATCTCCTGCATGAAAAAACGGGCTTCAGACAATCAGACAAATAGTCATTXXXXXXXXXXT
ATTATGGTCTCGTCCAATTATGATAGTGACGCGATAGATGAAGCATCAAGAACCAGTAATCTACAAATCTACTATAGTC
AACTGATTCCAATTTCAGTTTCTCATTCTCAATCTCCTGCATGAAAAAACGGGCTTCAGACTATCAGACAAATAGTCATTXXXXXXXXXXT
ATTATGGTCTCGTCCAATTATGATAGTGACGCGATAGATGAAGCATCAAGAACCAGTAATCTACAAATCTACTATAGTC
>Marker872909 Chr7 SNP_site:1723592 Ref_Type:A
CACCAAGTTTGAGCAATCAGTTTGACGAGGTTTGGTTCCCTACCTCTTTTATCCTTATGTTCTTGAACTTCATTAAGCAAXXXXXXXXXXG
CTGTGAAACTAGCATATCAAAATATAACAAATTTTTACAGGTAACGGATAAAGAATGTGCTGATTCTACTATTTTAGTG
CACCAAGTTTGAGCAATCAGTTTGACGAGGTTTGGTTCCCTGCCTCTTTTATCCTTATGTTCTTGAACTTCATTAAGCAAXXXXXXXXXXG
CTGTGAAACTAGCATATCAAAATATAACAAATTTTTACAGGTAACGGATAAAGAATGTGCTGATTCTACTATTTTAGTG
>Marker872924 Chr7 SNP_site:13036519 Ref_Type:C
CACTTCAACGAATCAAATTTTTCGAGGATCTCGTCCAATAGGTATTTAAGACCAAAAGATTGGGAAATTGTTTATTTTATXXXXXXXXXXT
TTAAAAACAATGTTCTTATTTTTCTTAGGCAAAGAAAAAGAGTAAAAATTAATTTTATTTTTTACAAAATGAGAGAGTT
CACTTCAACGAATCAAATTTTTCGAGGATCTCGTCCAATAGGTATTTAAGACCAAAAGATTGGGAAATTGTTTATTTTATXXXXXXXXXXT
TTAAAAAGAATGTTCTTATTTTTCTTAGGCAAAGAAAAAGAGTAAAAATTAATTTTATTTTTTACAAAATGAGAGAGTT
>Marker873124 Chr7 SNP_site:13364086 Ref_Type:T
CACCTTTTAAAGACGGACAAAAAACCCTTCTTCGCTCATTAATGACGGTTCATCCCTCCTTCCCTTCCCATTTTCTCTATXXXXXXXXXXC
AAACTTGTTTTAAATCATTAGAAATTAATTTTGGTTCGTTTTAAAATGTATAAATAAAAAATTGGGTTATTATAAATGT
CACCTTTTAAAGACGGACAAAAAACTCTTCTTCGCTCATTAATGACGGTTCATCCCTCCTTACCTTCCCATTTTCTCTATXXXXXXXXXXC
AAACTTGTTTTAAATCATTAGAAATTAATTTTGGTTCGTTTTAAAATGTATAAATAAAAAATTGGGTTATTATAAATGT
>Marker873124 Chr7 SNP_site:13364122 Ref_Type:A
CACCTTTTAAAGACGGACAAAAAACCCTTCTTCGCTCATTAATGACGGTTCATCCCTCCTTCCCTTCCCATTTTCTCTATXXXXXXXXXXC
AAACTTGTTTTAAATCATTAGAAATTAATTTTGGTTCGTTTTAAAATGTATAAATAAAAAATTGGGTTATTATAAATGT

Create PDF with GO2PDF for free, if you wish to

CACCTTTTAAAGACGGACAAAAAACTCTTCTTCGCTCATTAATGACGGTTCATCCCTCCTTACCTTCCCATTTTCTCTATXXXXXXXXXXC
AAACTTGTTTTAAATCATTAGAAATTAATTTTGGTTCGTTTTAAAATGTATAAATAAAAAATTGGGTTATTATAAATGT
>Marker873382 Chr7 SNP_site:17675529 Ref_Type:T
ACTTGTGCAAAGCTTCGAAACACTAGATCATAATCCAGCGCACCGATCCCGGTTATTTTCAATATTTACGGTCAATCATCXXXXXXXXXXA
AATCCCATATGAAGAGGTGGTTCTTGCAATTTGAATTTCGAGCTCAGACATCTGCACCAAAATATGTTTTCATTATGGT
ACTTGTGTAAAGCTTCGAAACACTAGATCATAATCCAGCGCACCGATCCCGGTTATTTTCAATATTTACGGTCAATCATCXXXXXXXXXXA
AATCCCATATGAAGAGGTGGTTCTTGCAATTTGAATTTCGAGCTCAGACATCTGCACCAAAATATGTTTTCATTATGGT
>Marker874099 Chr7 SNP_site:748431 Ref_Type:G
CACTTGCTTCACTCCCTTCATTCCTTCCAACGCTCTCTTCACTTTCCTCTCACATCCTTCGCAGTCTATTCGAATTTTTAXXXXXXXXXXC
CCATTTTCGTTACTAACACATAGATAAGAATTCAAATTACTGACATTTATATATCATGAAATAACTCCTTGTTTTTGGT
CACTTGCTTCACTCCCTTCATTCCTTCCAACGCTCTCTTCACTTTCCTCTCACATCCTTCGCAGTCTATTCGAATTTTTAXXXXXXXXXXC
CCATTTTCGTTGCTAACACATGGATAAGAATTCAAATTACTGACATTTATATATCATGAAATAACTCCTTGTTTTTGGT
>Marker874099 Chr7 SNP_site:748441 Ref_Type:A
CACTTGCTTCACTCCCTTCATTCCTTCCAACGCTCTCTTCACTTTCCTCTCACATCCTTCGCAGTCTATTCGAATTTTTAXXXXXXXXXXC
CCATTTTCGTTACTAACACATAGATAAGAATTCAAATTACTGACATTTATATATCATGAAATAACTCCTTGTTTTTGGT
CACTTGCTTCACTCCCTTCATTCCTTCCAACGCTCTCTTCACTTTCCTCTCACATCCTTCGCAGTCTATTCGAATTTTTAXXXXXXXXXXC
CCATTTTCGTTGCTAACACATGGATAAGAATTCAAATTACTGACATTTATATATCATGAAATAACTCCTTGTTTTTGGT
>Marker874606 Chr7 SNP_site:13702896 Ref_Type:G
ACTCCATCTCCAAACGCATGTTCTCACTGATCTCGTGCTTGGATCCATTTAACTCGGACTTGTCTGCCTGAGAATCTTGTXXXXXXXXXXG
CTATATCAAAGGGTAAGTCAATAAGTTGGCTAAGAAAATTATTCATAAAAACCACAATGCAATACCTCGGCCAAAGGTG
ACTCCATCTCCAAACGCATGTTCTCACTGATCTCGTGCTTGGATCCATTTAACTCGGACTTGTCTGCCTGAGAATCTTGTXXXXXXXXXXG
CTATATCAAAGGGTAAGTCAATAAGTTGGCTGAGAAAATTATTCATAAAAACCACAATGCAATACCTCGGCCAAAGGTG
>Marker874996 Chr7 SNP_site:3706440 Ref_Type:C
TACCTGGTCTTTCTTTTAATCTAATTTAAACGAGAGTAACATATGGAGATTATTGCTTTTAATATGCTATTATTGCCTTTXXXXXXXXXXG
TGATATTGCAATTAGCTTTCGTTAGGTATGCGAGACAGCTTCCTCTCATTTAAAGATATACCTTTCCTTTTCTATTGTG
TACCTGGTCTTTCTTTTAATCTAATTTAAACGAGAGTAACATATGGAGATTATTGCTTTTAATATGCTATTATTGCCTTTXXXXXXXXXXG
TGATATTGCAATTAGCTTTCGTTAGGTATGCGAGACAGCTTCCTCTCATTTAAAGATATACCTTTCCTTTTTTATTGTG
>Marker875014 Chr7 SNP_site:13622983 Ref_Type:T
ACCGTTTCGAACAGACAAGGCCTGGAACGATTAGTGTCCGACCAAAATTTCGTAGGTAAATGGCTGAAGCATCCATCATTXXXXXXXXXXT
ATTCTTAACCTTAAATGTCAAAATGATGGAGTTTAACCCTCGAATAAGTATCGCCAAAAAGTTCTATCCTATGTATGTA
ACCGTTTCGAACAGACAAGGCCTGGAACGATTAGTGTCCGACCAAAATTTCGTAGGTAAATGGCTGAAGCATCCATCATTXXXXXXXXXXT
ATTCTTAACCTTAAATGTCAAAATGATGGAGTTTAACCCTCTAATAAGTATCGCCAAAAAGTTCTATCCTATGTATGTA
>Marker875148 Chr7 SNP_site:15714385 Ref_Type:A
AACCCCATGTGGTGGATTAACAAAGACCTCTCCACTTAGCGAGGAGGGAATAATAGCTAAAATAGTGGAAAAGGGGAGAAXXXXXXXXXXC
GCTTCATAAAAGGAAAATATATTGGACAACAAATAGAAGGAAGATCAACAGATACAAAAAGCAATAGAGGCATCTTAGT
AACCCCATGTGGTGGATTAACAAAGACCTCTCCACTTAGCGAGGAGGGAGTAATAGCTAAAATAGTGGAAAAGGGGAGAAXXXXXXXXXXC
GCTTCATAAAAGGAAAATATATTGGACAACAAATAGAAGGAAAATCAACAGATACAAAAAGCAATAGAGGCATCTTAGT
>Marker875148 Chr7 SNP_site:15714591 Ref_Type:G
AACCCCATGTGGTGGATTAACAAAGACCTCTCCACTTAGCGAGGAGGGAATAATAGCTAAAATAGTGGAAAAGGGGAGAAXXXXXXXXXXC
GCTTCATAAAAGGAAAATATATTGGACAACAAATAGAAGGAAGATCAACAGATACAAAAAGCAATAGAGGCATCTTAGT
AACCCCATGTGGTGGATTAACAAAGACCTCTCCACTTAGCGAGGAGGGAGTAATAGCTAAAATAGTGGAAAAGGGGAGAAXXXXXXXXXXC
GCTTCATAAAAGGAAAATATATTGGACAACAAATAGAAGGAAAATCAACAGATACAAAAAGCAATAGAGGCATCTTAGT
>Marker875296 Chr7 SNP_site:8207902 Ref_Type:G
AACACTGACCTATCTGACTCTCCAGGAAGCAATGCGTAAAGCTGAAGCGGCTGGAAATGATGATTGTCCTGTTAAAATTAXXXXXXXXXXT

Create PDF with GO2PDF for free, if you wish to

GGATTTTCTCTAAAATTTAGTGACTTAAAAGCTTCTCAGGAATTACCGAAGGTGGGTCATGAGCACAGTGCCTGGAGTG
AACACTGACCTATCTGACTCTCCAGGAAGCAATGCGTAAAGCTGAAGCGGCTGGAAATGATGATTGTCCTGTTAAAATTAXXXXXXXXXXT
GGATTTTCTCTAAAATTTAGTGACTTAAAAGCTTCTCAGGAATTACCGAAGGTGGGTCATGAGCACAGTGCGTGGAGTG
>Marker875298 Chr7 SNP_site:3357077 Ref_Type:A
TACTAACTCTCATTCATCGACATACTTAACTGCCAAGGACACATTTACTGCATAAGAAACCATGTTTGAGTTGTAGTTTAXXXXXXXXXXT
GAGATATTATATTAGAAATATCATCATAATTATTTTAACATGGTTTAAGTTTAAAATAAATGTGATCTCATATTGAGGT
TACTAACTCTCATTCATCGACATACTTAACTGCCAAGGACACATTTACTGCATAAGAAACCATGTTTGAGTTGTAGTTTAXXXXXXXXXXT
GAGATCTTATATTAGAAATATCATCATAATTATTTTAACATGGTTTAAGTTTAAAATAAATGTGATCTCATATTGAGGT
>Marker875641 Chr7 SNP_site:8721188 Ref_Type:T
AACAGTTATTTATGATAATCTTGGCTGTCAATGGTGAAGATTTTATACAAATTATAGCTTAAATTTCAAATTCCAAATTTXXXXXXXXXXT
TTTGAAAACAGTGTATTTTCCTATATATATGAGAGACCTCCAGTTGTCTATGTGCCATTGCATGTGTGCTCCCAGAGTA
AACAGTTATTTATGATAATCTTGGCTGTCAATGGTGAAGATTTTATACAAATTATAGCTTAAATTTCAAATTCCAAATTTXXXXXXXXXXT
TTTGAAAACAGTGTATTTTCCTATATATATGTGAGACCTCCAGTTGTCTATGTGCCATTGCGTGTGTGCTCCCAGAGTA
>Marker875641 Chr7 SNP_site:8721218 Ref_Type:G
AACAGTTATTTATGATAATCTTGGCTGTCAATGGTGAAGATTTTATACAAATTATAGCTTAAATTTCAAATTCCAAATTTXXXXXXXXXXT
TTTGAAAACAGTGTATTTTCCTATATATATGAGAGACCTCCAGTTGTCTATGTGCCATTGCATGTGTGCTCCCAGAGTA
AACAGTTATTTATGATAATCTTGGCTGTCAATGGTGAAGATTTTATACAAATTATAGCTTAAATTTCAAATTCCAAATTTXXXXXXXXXXT
TTTGAAAACAGTGTATTTTCCTATATATATGTGAGACCTCCAGTTGTCTATGTGCCATTGCGTGTGTGCTCCCAGAGTA
>Marker875676 Chr7 SNP_site:12958205 Ref_Type:C
ACATCATTCGATCTCTGGTAGCCATAACTATTTTTTCAACTAAACGAGCAATTGTCTCTAATATGTTTGTATCAAATTCTXXXXXXXXXXT
TTTAGTTACTATAATAAGAGCTCATTTTGATACCAATTAAACTATCGGTCTCAACATTTCATATATAGGTTGTTCAAGT
ACATCATTTGATCTCTGGTAGCCATAACTATTTTTTCAACTAAACGAGCAATTGTCTCTAATATGTTTGTATCAAATTCTXXXXXXXXXXT
TTTAGTTACTATAACAAGAGCTCATTTTGATACCAATTAAACTATCGGTCTCAACATTTCATATATAGGTTGTTCAAGT
>Marker875676 Chr7 SNP_site:12958376 Ref_Type:T
ACATCATTCGATCTCTGGTAGCCATAACTATTTTTTCAACTAAACGAGCAATTGTCTCTAATATGTTTGTATCAAATTCTXXXXXXXXXXT
TTTAGTTACTATAATAAGAGCTCATTTTGATACCAATTAAACTATCGGTCTCAACATTTCATATATAGGTTGTTCAAGT
ACATCATTTGATCTCTGGTAGCCATAACTATTTTTTCAACTAAACGAGCAATTGTCTCTAATATGTTTGTATCAAATTCTXXXXXXXXXXT
TTTAGTTACTATAACAAGAGCTCATTTTGATACCAATTAAACTATCGGTCTCAACATTTCATATATAGGTTGTTCAAGT
>Marker875813 Chr7 SNP_site:4180855 Ref_Type:C
AACATAACCATGACAGCTTTTGGTGAAGAAATTCCTGCAGATTGCAAAACTGCTGGTCGTGATCCATCTCCATAGAGAACXXXXXXXXXXA
CGCTTCATGAAAATTACTGTCAAAATTAGAAAAAGGAACTACTTTACGAGAACATCATGCCATTGCAAATAAAAGAAGT
AACATAACCATGACAGCTTTTGGTGAAGAAATTCCTGCAGATTGCAAAACTGCTGGTCGTGATCCATCTCCATAGAGAACXXXXXXXXXXA
CGCTTCATGAAAATTACTGTCAAACTTAGAAAAAGGAACTACTTTACGAGAACATCATGCCATTGCAAATAAAAGAAGT
>Marker875819 Chr7 SNP_site:11358759 Ref_Type:A
AACACAGAGGGGGGAGGGGGGTAGAGTTTAGACAACGGGACCTTTTGCTTGGGTTATTTAGCATAATTGGTTAGAATGTTXXXXXXXXXXG
TGCATCATCGCTAAGAGGGACGTGTTCGACAATTGAGTGTGGGTAAGATTCTAAAATTAATTATATGATTAACAGGGTA
AACACAGAGGGGGGAGGGGGGTAGAGTTTAGACAACGGGACCTTTTGCTTGGGTTATTTAGCATAATTGGTTAGAATGTTXXXXXXXXXXG
TGCATCATCGCTAAGAGGGACGTGTTCGACAATTGAGTGTGGGTAAGATTCTAAAATTTATTATATGATTAACAGGGTA
>Marker876105 Chr7 SNP_site:9641391 Ref_Type:A
ACATATCTCACAAGTGCAATCATCAGCAGGAAGCTGGTTTTGGCAAGCAATATTTCTACACCTTTTATACACAAAAACCTXXXXXXXXXXT
CTTATCAATGTTTTTCCAGTTAAATCAGTTCTAGACTGAACAAGCTTTTGCAACATAAAAATCTCGTCTCTCTGTTGTG
ACATATCTCACAAGTGCAATCATCAGCAGGAAGCTGGTTTTGGCAAGCAATATTTCTACACCTTTTATACACAAAAACCTXXXXXXXXXXT
CTTATCAATGTTTTTCCAGTTAAATCAGTTCTAGGCTGAACAAGCTTTTGCAACATAAAAATCTCGTCTCTCTGTTGTG
>Marker876119 Chr7 SNP_site:10152130 Ref_Type:C

Create PDF with GO2PDF for free, if you wish to

ACTTTATTATTCTAATTCGTTGTTTTTATATAGTGTCCTCTACAAGTCAGAAGCATCGAATGTGGATACTATGCCATGAGXXXXXXXXXXA
TCTAATTTGTTGAACCTTATTTTGCATGTTTATAGATTGATACACGAAAGTCATATTCACATGCCAAGTTAGATGAGGT
ACTTTATTATTCTAATTCGTTGTTTTTATATAGTGTCCTCTACAAGTCAGAAGCATTGAATGTGGATACTATGCCATGAGXXXXXXXXXXA
TCTAATTTGTTGAACCTTATTTTGCATGTTTATAGATTGATACACGAAAGTCATATTCACATGCCAAGTTAGATGAGGT
>Marker876702 Chr7 SNP_site:5709565 Ref_Type:A
TACTCAACCAAAATGATCTCACAAGATGGAGAAAATGGGAGAGAAGAATTTTCAAAGATTGAGATTTTTTTTTTCCTTGXXXXXXXXXXT
CATGCAACCATGTGGAGGCATGATCACCTAGACACTCAACGCATATTTCCTTACGAGATAGCACGTTGAACACTTAGTA
TACTCAACCAAAATGATCTCACGAGATGGAGAAAATGGGAGAGAAGAATTTTCAAAGATTGAGATTTTTTTTTTCCTTGXXXXXXXXXXT
CATGCAACCATGTGGAGGCATGATCACCTAGACACTCAACGCATATTTCCTTACGAGATAGCACGTTGAACACTTAGTA
>Marker876790 Chr7 SNP_site:4491939 Ref_Type:T
AACAAAAGAACAAGTATCATAGAACCACTGCAGGAACTTAAACAACAGTGAAGGAATACTGCAAAGTATATTAGCAATTGXXXXXXXXXXC
TTTTGTCACCTTCTTCATGTTAAATCCTACCTGCAGATGAAAGTATGATTTGAGTCGAACTGTAGGAACAAGCTATGGT
AACAAAAGAACAAGTATCATAGAACCACTGCAGGAACTTAAACAACAGTGAAGGAATACTGCAAAGTATATTAGCAATTGXXXXXXXXXXC
TTTTGTCACCTTCTTCATGTTAAATCCTACCTGCAGATGAAAGTATGATTTGAGTTGAACTGTAGGAACAAGCTATGGT
>Marker87684 Chr4 SNP_site:1507899 Ref_Type:A
CACCTTCACCGGAAGAGGTGGCATCTGCTTCATTACTTCCGGCATTTCATTTAAGCTACAAGCATGAAGATTGAAGATAAXXXXXXXXXXT
CGGGTTTTAATGCTAAAAATCCTGACTGCTACTACGGGGAAGAACTCCTGCAACTAGGTCGATTTTACCAAGAGCTAGT
CACCTTCACCGGAAGTGGTGGCATCTGCTTCATTACTTCCGGCATTTCATTTAAGCTACAAGCATGTAGATTGAAGATAAXXXXXXXXXXT
CGGGTTTTAATGCTAAAAATCCTGACTGCTACTACGGGGAAGAATTCCTGCAACTAGGTCGATTTTACCAAGAGCTAGT
>Marker87684 Chr4 SNP_site:1507950 Ref_Type:A
CACCTTCACCGGAAGAGGTGGCATCTGCTTCATTACTTCCGGCATTTCATTTAAGCTACAAGCATGAAGATTGAAGATAAXXXXXXXXXXT
CGGGTTTTAATGCTAAAAATCCTGACTGCTACTACGGGGAAGAACTCCTGCAACTAGGTCGATTTTACCAAGAGCTAGT
CACCTTCACCGGAAGTGGTGGCATCTGCTTCATTACTTCCGGCATTTCATTTAAGCTACAAGCATGTAGATTGAAGATAAXXXXXXXXXXT
CGGGTTTTAATGCTAAAAATCCTGACTGCTACTACGGGGAAGAATTCCTGCAACTAGGTCGATTTTACCAAGAGCTAGT
>Marker87684 Chr4 SNP_site:1508127 Ref_Type:C
CACCTTCACCGGAAGAGGTGGCATCTGCTTCATTACTTCCGGCATTTCATTTAAGCTACAAGCATGAAGATTGAAGATAAXXXXXXXXXXT
CGGGTTTTAATGCTAAAAATCCTGACTGCTACTACGGGGAAGAACTCCTGCAACTAGGTCGATTTTACCAAGAGCTAGT
CACCTTCACCGGAAGTGGTGGCATCTGCTTCATTACTTCCGGCATTTCATTTAAGCTACAAGCATGTAGATTGAAGATAAXXXXXXXXXXT
CGGGTTTTAATGCTAAAAATCCTGACTGCTACTACGGGGAAGAATTCCTGCAACTAGGTCGATTTTACCAAGAGCTAGT
>Marker877150 Chr7 SNP_site:5119190 Ref_Type:G
AACGTATGAACAAAAATTATTGACATATTGTCCAAAAAATACCTCTAGACTTCTAAAAAAATTGCTTACATTATACTAAAXXXXXXXXXXC
AACATCGAAAAGTTGAATAAAAAACCAACACCATAAGTATTTTCATACATAGATAAGAGACAATTTGTATAATTTAGTA
AACTTATGAACAAAAATTATTGACACATTGTCCAAAAAATACCTCTAGACTTCTAAAAAAATTGCTTACGTTATACTAAAXXXXXXXXXXC
AACATCGAAAAGTTGAATAAAAAACCAACACCATAAGTATTTTCATACATAGATAAGAGACAATTTGTATAATTTAGTA
>Marker877150 Chr7 SNP_site:5119212 Ref_Type:T
AACGTATGAACAAAAATTATTGACATATTGTCCAAAAAATACCTCTAGACTTCTAAAAAAATTGCTTACATTATACTAAAXXXXXXXXXXC
AACATCGAAAAGTTGAATAAAAAACCAACACCATAAGTATTTTCATACATAGATAAGAGACAATTTGTATAATTTAGTA
AACTTATGAACAAAAATTATTGACACATTGTCCAAAAAATACCTCTAGACTTCTAAAAAAATTGCTTACGTTATACTAAAXXXXXXXXXXC
AACATCGAAAAGTTGAATAAAAAACCAACACCATAAGTATTTTCATACATAGATAAGAGACAATTTGTATAATTTAGTA
>Marker877150 Chr7 SNP_site:5119256 Ref_Type:A
AACGTATGAACAAAAATTATTGACATATTGTCCAAAAAATACCTCTAGACTTCTAAAAAAATTGCTTACATTATACTAAAXXXXXXXXXXC
AACATCGAAAAGTTGAATAAAAAACCAACACCATAAGTATTTTCATACATAGATAAGAGACAATTTGTATAATTTAGTA
AACTTATGAACAAAAATTATTGACACATTGTCCAAAAAATACCTCTAGACTTCTAAAAAAATTGCTTACGTTATACTAAAXXXXXXXXXXC
AACATCGAAAAGTTGAATAAAAAACCAACACCATAAGTATTTTCATACATAGATAAGAGACAATTTGTATAATTTAGTA

Create PDF with GO2PDF for free, if you wish to

>Marker87739 Chr4 SNP_site:21856663 Ref_Type:G
TACCCATCAAGCTTTTTGATGTTATACATTTAGGAATGTTATGATGAATATCTGTTGATTCTATTTTGGCAGGTTGCAGAXXXXXXXXXXA
ATATAGCTTTGTTGGTTCATTCGACGACCTTGGACCTTACGGGAGAGGACGATATAGGGATGCCATGGCACCAGAGGTA
TACCCATCAAGCTTTTTGATGTTATACATTTGGGAATGTTATGATGAATATCTGTTGATTCTATTTTGGCAGGTTGCAGAXXXXXXXXXXA
ATATAGCTTTGTTGGTTCATTCGACGACCTTGGACCTTACGGGAGAGGACGATATAGGGATGCCATGGCACCAGAGGTA
>Marker877618 Chr7 SNP_site:13038225 Ref_Type:G
ACCTTCCATCTATATGCCATGATTATCGTGAGGTGGTCACCTATGACGACTTTATTGAATATTTGTTTTCAACATGGAAGXXXXXXXXXXG
ATTACATCAAGGAAACCTCGCAACCGATCCTCCACTACCAATAACTAACAGATGGATCTATCATAAAATCAATGTTGTA
ACCTTCCATCTATATGCCATGATTATCGTGAGGTGGTCGCCTATGACGACTTTATTGAATATTTGTTTTCAACATGGAAGXXXXXXXXXXG
ATTACATCAAGGAAACCTCGCAACCGATCCTCCACTACCAATAACTAACACATGGATCTATCATAAAATCAATGTTGTA
>Marker877618 Chr7 SNP_site:13038414 Ref_Type:C
ACCTTCCATCTATATGCCATGATTATCGTGAGGTGGTCACCTATGACGACTTTATTGAATATTTGTTTTCAACATGGAAGXXXXXXXXXXG
ATTACATCAAGGAAACCTCGCAACCGATCCTCCACTACCAATAACTAACAGATGGATCTATCATAAAATCAATGTTGTA
ACCTTCCATCTATATGCCATGATTATCGTGAGGTGGTCGCCTATGACGACTTTATTGAATATTTGTTTTCAACATGGAAGXXXXXXXXXXG
ATTACATCAAGGAAACCTCGCAACCGATCCTCCACTACCAATAACTAACACATGGATCTATCATAAAATCAATGTTGTA
>Marker877773 Chr7 SNP_site:13820081 Ref_Type:C
AACATGGTAAATTGATGTTTTATCATATAACTAAAAAAATTCGGTGTCTTTTAAATTTTGATCGTTGCCATCTTGACACAXXXXXXXXXXT
ATTTTTTTGGGTTTTATGATTCGTTGTGGAAGTATTTGCTTACAAATTTGTAGAGTTTATTTTAAAAAATTGTTGTTGT
AACATGGTAAATTGATGTTTTATCATATAACTAAAAAAATTCGGTGTCTTTTAAATTTTGATCGTTGCCATCTTGACACAXXXXXXXXXXT
ATTTTTTTGGGTTTTATGATTTGTTGTGGAAGTATTTGCTTACAAATTTGTAGAGTTTATTTTAAAAAATTGTTGTTGT
>Marker87798 Chr4 SNP_site:7773355 Ref_Type:T
ACTAACTTTATATTTGATTGAAAACAAATAGCGATGTGACAAAAAGTAGTAGATGTGTTCAAATAACATGACATCCCAAAXXXXXXXXXXG
ATAGTTGAGGTATTTCTTTCTATTACCTAAATATGGTATTAGAACTCATATGGTTATTGGGTCCAATCATGTTTGAGTA
ACTAACTTTATATTTGATTGAAAACAAATAGCGATGTGACAAAAAGTAGTAGATGTGTTCAAATAACATGACATCCCAAAXXXXXXXXXXG
ATAGTTGAGGTATTTCTTTCTATTACCTAAATATGGTATTAGAACTCATATGGTTATTGGGTCCAATCATGTTTTAGTA
>Marker878205 Chr7 SNP_site:13987396 Ref_Type:T
AACCATTTGGTGATAATTAAATCACACGAGTTCGATCAATTTAAACCTTTCAACTAATAAGCCACCACTGGGAGATGATTXXXXXXXXXXA
AATTAATTGGTTTAATTGAAACCACAGTATAAATGGATATTTGGCTTCACATAAATTAATAAATTTAGTAGAATTTGTA
AACCATTTGGTGATAATTAAATCACACGAGTTCGATCAATTTAAACCTTTCAACTAATAATCCACCACTGGGAGATGATTXXXXXXXXXXA
AATTAATTGGTTTAATTGAAACCACAGTATAAATGGATATTTGGCTTCACATAAATTAATAAATTTAGTAGAATTTGTA
>Marker87842 Chr4 SNP_site:18204547 Ref_Type:T
ACTATCCTGTGCAAATTCAATGTCGAACAATTCTAGCATGTTGCCTACTACACAATCTCATCATTCGCGAGATGACAAATXXXXXXXXXXG
GGATGATTTGGCCGGAGAGATGTTCAATGAATGGCAGTTGCGTAACGAATAGGCTTTCGTGTCGATTTTGTATTCATGT
ACTATCTTGTGCAAATTCAATGTCGAACAATTCTAGCATGTTTCCTACTACACAATCTCATCATTCGCGAGATGACAAATXXXXXXXXXXG
GGATGATTTGGCCGGAGAGATGTTCAATGAATGGCAGTTGCGTAACGAATAGGCTTTCGTGTCGATTTTGTATTCCTGT
>Marker87842 Chr4 SNP_site:18204583 Ref_Type:T
ACTATCCTGTGCAAATTCAATGTCGAACAATTCTAGCATGTTGCCTACTACACAATCTCATCATTCGCGAGATGACAAATXXXXXXXXXXG
GGATGATTTGGCCGGAGAGATGTTCAATGAATGGCAGTTGCGTAACGAATAGGCTTTCGTGTCGATTTTGTATTCATGT
ACTATCTTGTGCAAATTCAATGTCGAACAATTCTAGCATGTTTCCTACTACACAATCTCATCATTCGCGAGATGACAAATXXXXXXXXXXG
GGATGATTTGGCCGGAGAGATGTTCAATGAATGGCAGTTGCGTAACGAATAGGCTTTCGTGTCGATTTTGTATTCCTGT
>Marker87842 Chr4 SNP_site:18204800 Ref_Type:A
ACTATCCTGTGCAAATTCAATGTCGAACAATTCTAGCATGTTGCCTACTACACAATCTCATCATTCGCGAGATGACAAATXXXXXXXXXXG
GGATGATTTGGCCGGAGAGATGTTCAATGAATGGCAGTTGCGTAACGAATAGGCTTTCGTGTCGATTTTGTATTCATGT
ACTATCTTGTGCAAATTCAATGTCGAACAATTCTAGCATGTTTCCTACTACACAATCTCATCATTCGCGAGATGACAAATXXXXXXXXXXG

Create PDF with GO2PDF for free, if you wish to

GGATGATTTGGCCGGAGAGATGTTCAATGAATGGCAGTTGCGTAACGAATAGGCTTTCGTGTCGATTTTGTATTCCTGT

>Marker878733 Chr7 SNP_site:13752973 Ref_Type:A

ACTCAACCTAAGAGATTATCATAGTTGTTCAATCAAATCACATTCATTTGATGTCACAAAGAGATGGAGAGATTCTTTCCXXXXXXXXXXA

TGTGTAAAAATGTCACTCTTTTCATTTATGGAAATTTGAATATGTCAATACATGCACTTGATGAAGGGATAATAGGGTA

ACTCAACCTAAGAGATTATCATAGTTGTTCAATCAAATCACATTCATTTGATGTCACAAAGAGATGGAGAGATTCTTTCCXXXXXXXXXXA

TGTGTAAAAATGTCACTCTTTTCATTTATGGCAATTTGAATATGTCAATACATGCACTTGATGAAGGGATAATAGGGTA

>Marker879082 Chr7 SNP_site:13119984 Ref_Type:T

ACCTTTAGCTCATTAACGTGTATGATTGAGTGAATATTGGTTCTTTTGAGTTGCTTAGGACATTTCAAGAGGGTCAATTGXXXXXXXXXXG

TTGTTTGGAATCATCGACAACTTTTTCTTTGAATTAGCTAATTGGAAGTTGAATGGATCCTTTTTTGGTCCAAAATGTT

ACCTTTAGCTCATTAACGTGTATGATTGAGTGAATATTGGTTCTTTTGAGTTGCTTAGGACATTTCAAGAGGGTCAATTGXXXXXXXXXXG

TTGTTTGGAATCATCGACAACTTTTTCTTTGAATTAGCTAATTGGAAGTTGAATGGATCCTTTTTTGTTCCAAAATGTT

>Marker879268 Chr7 SNP_site:1563501 Ref_Type:T

TACTCTGCTCGGAGGCAGTGGGTTCCAGTGTATTTGCGGGACACGTTTTTTGCTGAAATGTCTATTACACAGCGAAGTGAXXXXXXXXXXT

CTCCAATGGAAAAACAAGTTTCTGAGCTTTACACCAGGAAGCTATTCTCGAGGTTCCAAGAGGAGTTAGTTGGGACGTT

TACTCTGCTCGGAGGCAGTGGGTTCCAGTGTATTTGCGGGATACGTTTTTTGCTGAAATGTCTATTACACAGCGAAGTGAXXXXXXXXXXT

CTCCAATGGAAAAACAAGTTTCTGAGCTTTACACCAGGAAGCTATTCTCGAGGTTCCAAGAGGAGTTAGTTGGGACGTT

>Marker87930 Chr4 SNP_site:10520646 Ref_Type:C

AACCCAATCTAATTATTGAATGAAACATATCGTTTGGACCGGGATGTTGGCACTCTTCTAAAAAAAGGAGAGGTGGAGACXXXXXXXXXXT

CAAGATAAGAGCGGTGATTTTAAAAGAAGAATATTGAAAAAGAGAGAAGGGAGAGGTAGAAGATGGCTAGGTTGATGTT

AACCCAATCTAATTATTGAATGAAACATATCGTTTGGACCGGGATGTTGGCATTCTTCTAAAAAAAGGAGAGGTGGAGACXXXXXXXXXXT

CAAGATAAGAGCGGTGATTTTAAAAGAAGAATATTGAAAAAGAGAGAAGGGAGAGGTAGAAGATGGCTAGGTTGATGTT

>Marker879372 Chr7 SNP_site:559245 Ref_Type:G

AACCTCTTTTATAAACGTGTCGATGAAGTGGGTAGAAATCGCTGTCATTATCGTAATGTTGTTATGTAGGAAACATTTTTXXXXXXXXXXT

TACGAAGATTTTAGTGTTAAATCATTTTCTTCGAATTGCTCTTCCAAGATTAACAAAACATCATTTTTAGATCATAAGT

AACCTCTTTTATAAACGTGTCGATGAAGTGGGTAGAGATCGCAGTCATTATCGTAATGTTGTTATGTAGGAAACATTTTTXXXXXXXXXXT

TACGAAGACTTTAGTGTTAAATCATTTTCTTCGAATTGCTCTTCCAAGATTAACAAAACATCATTTTTAGATCATAAGT

>Marker879372 Chr7 SNP_site:559251 Ref_Type:A

AACCTCTTTTATAAACGTGTCGATGAAGTGGGTAGAAATCGCTGTCATTATCGTAATGTTGTTATGTAGGAAACATTTTTXXXXXXXXXXT

TACGAAGATTTTAGTGTTAAATCATTTTCTTCGAATTGCTCTTCCAAGATTAACAAAACATCATTTTTAGATCATAAGT

AACCTCTTTTATAAACGTGTCGATGAAGTGGGTAGAGATCGCAGTCATTATCGTAATGTTGTTATGTAGGAAACATTTTTXXXXXXXXXXT

TACGAAGACTTTAGTGTTAAATCATTTTCTTCGAATTGCTCTTCCAAGATTAACAAAACATCATTTTTAGATCATAAGT

>Marker879372 Chr7 SNP_site:559404 Ref_Type:C

AACCTCTTTTATAAACGTGTCGATGAAGTGGGTAGAAATCGCTGTCATTATCGTAATGTTGTTATGTAGGAAACATTTTTXXXXXXXXXXT

TACGAAGATTTTAGTGTTAAATCATTTTCTTCGAATTGCTCTTCCAAGATTAACAAAACATCATTTTTAGATCATAAGT

AACCTCTTTTATAAACGTGTCGATGAAGTGGGTAGAGATCGCAGTCATTATCGTAATGTTGTTATGTAGGAAACATTTTTXXXXXXXXXXT

TACGAAGACTTTAGTGTTAAATCATTTTCTTCGAATTGCTCTTCCAAGATTAACAAAACATCATTTTTAGATCATAAGT

>Marker879670 Chr7 SNP_site:1247223 Ref_Type:T

AACCAAGAATAATCCAATCTCCATCTTATATGTTGTTAAATAAAAAAAAATTATACTCTTCTCAAATAGTATTAAATTTTXXXXXXXXXXA

TATGAATTATAAGAAAATTTACAGAATATCGCTGATGTAAAGAGTAATATAAATAGTATAAGAGAAACTCTATAAAAGT

AACCAAGAATAATCCAATCTCCATCTTATATGTTGTTAAATAAAAAAAAATTATACTCTTCTCATATAGTATTAAATTTTXXXXXXXXXXA

TATGAATTATAAGAAAATTTACAGAATATCGCTGATGTAAAGAGTAATATAAATAGTATAAGAGAAACTCTATAAAAGT

>Marker879690 Chr7 SNP_site:5386500 Ref_Type:C

ACTTGTTCTGCTATTCCACTGAATAAAGATCACTTCCAGTTGGTGTTCTATCAGCCCATTAACCCAATCTTCTTCTTCCAXXXXXXXXXXT

CTTTTCACCATTGTTATATAAGAACCCAACTTGCCCAACACTTTTCTTCTTATTTCGGACTATTTTTCCTCTTTTCGTT

Create PDF with GO2PDF for free, if you wish to

ACTTGTTCTGCTATTCCATTGAATAAAGATCACTTCCAGTTGGTGTTCTATCAGCCCATTAACCCAATCTTCTTCTTCCAXXXXXXXXXXT
CTTTTCACCATTGTTATATAAGAACCCAACTTGCCCAACACTTTTCTTCTTATTTCGGACTATTTTTCCTCTTTTCGTT
>Marker879992 Chr7 SNP_site:8233986 Ref_Type:C
AACAACTATATCTTTAATTATTTTTGTTACGTAGTCAATTCAAACAAGTCATTCTAATATCTTTCAATCAGTTATGGATTXXXXXXXXXXC
CATTTAGTCATTCATTATCTTATTTCAGTGTCTAATCATCTAAATATACAAATCACTTTCATCATTTCATTAGAGAGTT
AACAACTATATCTTTAATTATTTTTGTTATGTAGTCAATTCAAACAAGTCATTCTAATATCTTTCAATCAGTTATGGATTXXXXXXXXXXC
CATTTAGTCATTCATTATTTTATTTCAGTGTCTAATCATCTAAATATACAAATCACTTTCATCATTTCATTAGAGAGTT
>Marker879992 Chr7 SNP_site:8234175 Ref_Type:C
AACAACTATATCTTTAATTATTTTTGTTACGTAGTCAATTCAAACAAGTCATTCTAATATCTTTCAATCAGTTATGGATTXXXXXXXXXXC
CATTTAGTCATTCATTATCTTATTTCAGTGTCTAATCATCTAAATATACAAATCACTTTCATCATTTCATTAGAGAGTT
AACAACTATATCTTTAATTATTTTTGTTATGTAGTCAATTCAAACAAGTCATTCTAATATCTTTCAATCAGTTATGGATTXXXXXXXXXXC
CATTTAGTCATTCATTATTTTATTTCAGTGTCTAATCATCTAAATATACAAATCACTTTCATCATTTCATTAGAGAGTT
>Marker880215 Chr7 SNP_site:13224749 Ref_Type:C
ACTTTATATGTAAATGCAATGCTTTTCCTGGCTGTCATGCAGTATTTGAAAGAACAAAGGCTTCCTCCTCAGACATTCATXXXXXXXXXXA
ACACCTTTTACTTTCTTATTTTATCTTCCATGTTCTCTCTGGTTTATGTTTGAGAATCTTTTTCACAAACGTGAAGGGT
ACTTTATATGTAAATGCAATGCTTTTCCTGGCTGTCATGCAGTATTTGAAAGAACAAAGGCTTCCTCCTCAGACATTCATXXXXXXXXXXA
ACACTTTTTACTTTCTTATTTTATCTTTCATGTTCTCTCTGGTTTATGTTTGAGAATCTTTTTCACAAACGTGAAGGGT
>Marker880215 Chr7 SNP_site:13224772 Ref_Type:C
ACTTTATATGTAAATGCAATGCTTTTCCTGGCTGTCATGCAGTATTTGAAAGAACAAAGGCTTCCTCCTCAGACATTCATXXXXXXXXXXA
ACACCTTTTACTTTCTTATTTTATCTTCCATGTTCTCTCTGGTTTATGTTTGAGAATCTTTTTCACAAACGTGAAGGGT
ACTTTATATGTAAATGCAATGCTTTTCCTGGCTGTCATGCAGTATTTGAAAGAACAAAGGCTTCCTCCTCAGACATTCATXXXXXXXXXXA
ACACTTTTTACTTTCTTATTTTATCTTTCATGTTCTCTCTGGTTTATGTTTGAGAATCTTTTTCACAAACGTGAAGGGT
>Marker880282 Chr7 SNP_site:7686806 Ref_Type:C
ACCACCTGCTTTGTATTTGAGAGAGAACACCCATTTGCATCCCACATTTTTGTGTCCCTTGGGTAGAGCACAAATTCCCCXXXXXXXXXXG
AGTCAAGGCTTGCGGTAAAAGCTCTGAACTGTGGTGAGAGATTATCATAGGAAACATAGTTACAAATGGGATGTTTAGT
ACCACCTGCTTTGTATTTGAGAGAGAACACCCATTTGCATCCCACATTTTTGTGTCCCTTGGGTAGAGCACAAATTCCCTXXXXXXXXXXG
AGTCAAGGCTTGCGGTAAAAGCTCTGAACTGTGGTGAGAGATTATCATAGGAAACATAGTTACAAATGGGATGTTTAGT
>Marker880307 Chr7 SNP_site:2071762 Ref_Type:G
GACATACTTAGTCTTCATGTTGCATTGTTGTCATTTTAATTGCAGAGCCGTTTGGCCCGTTTCTTTATATATTGCCTCTTXXXXXXXXXXC
CCCTGCTCCCAAACAAACTTGTGTATGTTTGTATGTAATTAATGTTTATTGTGTAATATTATCTAAACGAATGGTTGTA
GACATACTTAGTCTTCATGTTGCATTGTTGTCATTTTAATTGCATAGCCGTTTGGCCCGTTTCTTTATATATTGCCTCTTXXXXXXXXXXC
CCCTGCTCCCAAACAAACTTGTGTATGTTTGTATGTAATTAATGTTTATTGTGTAATATTATCTAAACAAATGGTTGTA
>Marker880307 Chr7 SNP_site:2071994 Ref_Type:G
GACATACTTAGTCTTCATGTTGCATTGTTGTCATTTTAATTGCAGAGCCGTTTGGCCCGTTTCTTTATATATTGCCTCTTXXXXXXXXXXC
CCCTGCTCCCAAACAAACTTGTGTATGTTTGTATGTAATTAATGTTTATTGTGTAATATTATCTAAACGAATGGTTGTA
GACATACTTAGTCTTCATGTTGCATTGTTGTCATTTTAATTGCATAGCCGTTTGGCCCGTTTCTTTATATATTGCCTCTTXXXXXXXXXXC
CCCTGCTCCCAAACAAACTTGTGTATGTTTGTATGTAATTAATGTTTATTGTGTAATATTATCTAAACAAATGGTTGTA
>Marker880325 Chr7 SNP_site:6278667 Ref_Type:C
TACCATTGTGGATTTGAAAACATATTGGGCATAAATCAGACCCATTTATTTTAGCCTCCTAAGCAAAACAAGTATTCTATXXXXXXXXXXT
AGAGTTTAAAACCACATGATTGATGATACAACTTCATCATATATTAGACATGATTGTGAGGGTAGATGAGTTGATAAGT
TACCATTGTGGATTTGAAAACATATTGGGCATAAATTAGACCCATTTATTTTAGCCTCCTAAGCAAAACAAGTATTCTATXXXXXXXXXXT
AGAGTTTAAAACCACATGATTGATGATACAACTTCATCATATATTAGACATGATTGTGAGGGTAGATGGGTTGATAAGT
>Marker880325 Chr7 SNP_site:6278880 Ref_Type:G
TACCATTGTGGATTTGAAAACATATTGGGCATAAATCAGACCCATTTATTTTAGCCTCCTAAGCAAAACAAGTATTCTATXXXXXXXXXXT

Create PDF with GO2PDF for free, if you wish to

AGAGTTTAAAACCACATGATTGATGATACAACTTCATCATATATTAGACATGATTGTGAGGGTAGATGAGTTGATAAGT
TACCATTGTGGATTTGAAAACATATTGGGCATAAATTAGACCCATTTATTTTAGCCTCCTAAGCAAAACAAGTATTCTATXXXXXXXXXXT
AGAGTTTAAAACCACATGATTGATGATACAACTTCATCATATATTAGACATGATTGTGAGGGTAGATGGGTTGATAAGT
>Marker880503 Chr7 SNP_site:3870807 Ref_Type:T
TACTGAATATATTTGCCCTGAAGAATAAGAAAGCAGTTGAAATAGGGTCCAAAAGATCCCCCTTATTGTCTCTCGAAGTGXXXXXXXXXXA
TAATCATTCTATTCACATTAGAAAAGATTCTTTTGGCTAATTCTCCAAATATTCATAACATTTGAGTGCAGTGTAGGTC
TACTGAATATATTTGCCCTGAAGAATAAGAAAGCAGTTGAAATAGGGTCCAAAAGATCCCCTTTATTGTCTCTCGAAGTGXXXXXXXXXXA
TAATCATTCTATTCACATTAGAAAAGATTCTTTTGGCTAATTCTCCAAATATTCATAACATTTGAGTGCAGTGTAGGTC
>Marker880935 Chr7 SNP_site:15839301 Ref_Type:C
ACTAAATCCTTGCTATTGGGTTTCTGGGTTATTCGATTTGGCTCATTTTAGGTTATTTTCCATTTTATGTTTGTGTTTAAXXXXXXXXXXT
GTTCTTGTTTTTATCATTTTTTTGGAGTGTTTTTTTAAGTTTGTTTTGGCATTACCAAAGTTGTTGTTTGGTTATTGGT
ACTAAATCTTTGCTATTGGGTTTCTGGGTTATTCGATTTGGCTCATTTTAGGTTATTTTCCATTTTATGTTTGTGTTTAAXXXXXXXXXXT
GTTCTTGTTTTTATCATTTTTTTGGAGTGTTTTTTTAAGTTTGTTTTGGCATTACCAAAGTTGTTGTTTGGTTATTGGT
>Marker881010 Chr7 SNP_site:13854284 Ref_Type:C
ACATGCCACCGTCTGACTAGAAATTGCCTCACACGCTGCCTCTGTTTGACGCATGGGCCCACGTGCCAACGTCGTTTCATXXXXXXXXXXC
ACTCTTTACAGCAACCGTTTTTCTATGGTAACGGGAATGGCCAACCCCATAACAAATCAGACATTGAAGCAGGCAAGTC
ACATGTCACCGTCTGACTAGAAATTGCCTCACACGCTGCCTCTGTTTGACGCATGGGCCCACGTGCCAACGTCGTTTCATXXXXXXXXXXC
ACTCTTTACAGCAACCGTTTTTCTATGGTAACGGGACTGGCCAACCCCATAACAAATCAGACATTGAAGCAGGCAAGTC
>Marker881010 Chr7 SNP_site:13854523 Ref_Type:A
ACATGCCACCGTCTGACTAGAAATTGCCTCACACGCTGCCTCTGTTTGACGCATGGGCCCACGTGCCAACGTCGTTTCATXXXXXXXXXXC
ACTCTTTACAGCAACCGTTTTTCTATGGTAACGGGAATGGCCAACCCCATAACAAATCAGACATTGAAGCAGGCAAGTC
ACATGTCACCGTCTGACTAGAAATTGCCTCACACGCTGCCTCTGTTTGACGCATGGGCCCACGTGCCAACGTCGTTTCATXXXXXXXXXXC
ACTCTTTACAGCAACCGTTTTTCTATGGTAACGGGACTGGCCAACCCCATAACAAATCAGACATTGAAGCAGGCAAGTC
>Marker88103 Chr4 SNP_site:5327591 Ref_Type:T
AACTTTTGACTTTGAAAAGTCAAGCTTTAACTGACTTTATATTCAAATGTGATTTGAATTTCAGAAAAATAAATGTAAATXXXXXXXXXXC
TTAATTGGTCAAATGACCATGGACTAAAGTTAGTGGGTTTTGTTGGATAATTTCACTAACACTTAGTGGGACAAGTGTT
AACTTTTGACTTTGAAAAGTCAAGCTTTAATTGACTTTATATTCAAATGTGATTTGAATTTCAGAAAAATAAATGTAAATXXXXXXXXXXC
TTAATTGGTCAAATGACCATGGACTAAAGTTAGTGGGTTTTGTTGGATAATTTCACTAACACTTAGTGGGACAAGTGTT
>Marker881047 Chr7 SNP_site:8856270 Ref_Type:A
AACCCAACCTCTTACTTCTAGCTACCCGACTCAACTTTTTTTCTCCAATTTTCTAACTCAATCCAATCCAACAAAAAACTXXXXXXXXXXA
TTCTCTTTTCACTTCTCATTAATTATTTGTATTGAAAATGATAAACTAACAAAACACCGACTCATGTAGAAACCCAAGT
AACCCAACCTCTTACTTCTGGCTACCCGACTCAACTTTTTTTCTCCAATTTTTTAACTCAATCCAATCCAACAAAAAACTXXXXXXXXXXA
TTCTCTTTTCACTTCTCATTAATTATTTGTATTGAAAATGATAAACTAACAAAACACCGACTCATGTAGAAACCCAAGT
>Marker881047 Chr7 SNP_site:8856303 Ref_Type:C
AACCCAACCTCTTACTTCTAGCTACCCGACTCAACTTTTTTTCTCCAATTTTCTAACTCAATCCAATCCAACAAAAAACTXXXXXXXXXXA
TTCTCTTTTCACTTCTCATTAATTATTTGTATTGAAAATGATAAACTAACAAAACACCGACTCATGTAGAAACCCAAGT
AACCCAACCTCTTACTTCTGGCTACCCGACTCAACTTTTTTTCTCCAATTTTTTAACTCAATCCAATCCAACAAAAAACTXXXXXXXXXXA
TTCTCTTTTCACTTCTCATTAATTATTTGTATTGAAAATGATAAACTAACAAAACACCGACTCATGTAGAAACCCAAGT
>Marker881102 Chr7 SNP_site:5273416 Ref_Type:C
CACAAAACAATATATCACCTATGATATCAATAATAAATCATGGGTTTTTGAGAAATACGACATCACATCTACTACTCAGCXXXXXXXXXXA
AAAGATGAAAATGGCTATTTATCAAATTGAAACTTATCATACATGATACACTCAAAATTTATGTTACTTTTCCTAAGTG
CACAAAACAATATATCACCTATGATATCAATAATAAATCATGGGTTTTTGAGAAATACGACATCACATCTACTATTCAGCXXXXXXXXXXA
AAAGATGAAAATGGCTATTTATCAAATTGAAACTTATCATACATGATACACTCAAAATTTATGTTACTTTTCCTAAGTG
>Marker881385 Chr7 SNP_site:3707329 Ref_Type:T

Create PDF with GO2PDF for free, if you wish to

GACTAATGCTCTTGAACTGTTACCCAATTTGTTATACACATGGAAGGTCGTCGTTGTAAAGTGAATATAATTACATGTAAXXXXXXXXXXG
TATCTAACCCTTTAGATAATTACTTTCCAAAATTTTAAAAGTCTTATTAAAAACCGTTTAGACCCGACCATGAAGGAGT
GACTAATGTTCTTGAACTGTTACCCAATTTGTTATACACATGGAAGGTCGTCGTTGTAAAGTGAATATAATTACATGTAAXXXXXXXXXXG
TATCTAACCCTTTAGATAATTACTTTCCAAAATTTTAAAAGTCTTATTAAAAACCGTTTAGACCCGACCATGAAGGAGT
>Marker882050 Chr7 SNP_site:9954956 Ref_Type:G
CACGAATATTGTAATATGGGACTGTATTTTATGGATGTGGTTTCCTAGAAAATGGATATTTGAGCATAACTGAGTTTTTTXXXXXXXXXXT
TGGTCAAGACGTGTTTCACTGGCAAGCAACAATTATGGGTCCAGCAGATAGTCCTTATGCGGGGGGTTTATTTTCAGTG
CACGAATATTGTAATATGGGACTGTATTTTATGGATGTGGTTTCCTAGAAAATGGATATTTGAGCATAACTGAGTTTTTTXXXXXXXXXXT
TGGTCAAGACGTGTTTCACTGGCAAGCAACAATTATGGGTCCAGCAGATAGTCCTTATGCTGGGGGTTTATTTTCAGTG
>Marker882098 Chr7 SNP_site:14705497 Ref_Type:T
ACTTATGAGATCGATGATGATATAATGGATGAATCGACTTCTTCATTAACAAAACTTAGTTGAAAAATAAGAAAAGACAAXXXXXXXXXXA
TAAGTTTTAATCTAACACGTATATTTTATCAATATATCCATTAAATGTTTCATAAAATTGAGACCTCAAGATTTTGGTT
ACTTATGAGATCGATGATGATATAATGGATGAATCGACTTCTTCATTAACAAAACTTAGTTGAAAAATAAGAAAAGACAAXXXXXXXXXXA
TAAGTTTTAATCTAACACGTATATTTTATCATTATATCCATTAAATGTTTCATAAAATTGAGACCTCAAGATTTTGGTT
>Marker882746 Chr7 SNP_site:3512761 Ref_Type:C
AACAGTTTGTGGAAAGAAAAGTATAGGTTGAAAGAAAAAGACACATATTTTTCTATTCTCAGGATTTAAATTCAAAAATGXXXXXXXXXXA
TGATTCATGATACTCTCTTTCAATATTATCATTAGTTATATCACACAAACTTACAAAATCATTATTTGTTTTGTTAGTT
AACAGTTTGTGGAAAGAAAAGTATAGGTTGAAAGAAAAAGACACATATTTTTCTATTCTCAGGATTTAAATTCAAAAATGXXXXXXXXXXA
TGATTCATGATACTCTCTTTTAATATTATCATTAGTTATATCACACAAACTTACAAAATCATTATTTGTTTTGTTAGTT
>Marker883223 Chr7 SNP_site:7749271 Ref_Type:A
TACAATGCAACTAATTGAAGCAAGTAGAGAAGAGTGCCTTGGTGTGATCAATGTTTGGTTGTTCGAAAGTAAATATAGATXXXXXXXXXXC
TATCACAACGAAGAATAGTAGTAGAGTCTTGGGAAAACTCATGTTTTGCATACAACCTCATAAGTAGCATCAATAGGGT
TACAATGCAACTAATTGAAGCAAGTAGAGAAGAGTGCCTTGGTGTGATCAATGTTTGGTTGTTCGAAAGTAAATATAGATXXXXXXXXXXC
TATCACAACGAAGAATAGTAGTAGAGTCTTGGGGAGCCTCATGTTTTGCATACAACCTCATAAGTAGCATCAATAGGGT
>Marker883223 Chr7 SNP_site:7749273 Ref_Type:A
TACAATGCAACTAATTGAAGCAAGTAGAGAAGAGTGCCTTGGTGTGATCAATGTTTGGTTGTTCGAAAGTAAATATAGATXXXXXXXXXXC
TATCACAACGAAGAATAGTAGTAGAGTCTTGGGAAAACTCATGTTTTGCATACAACCTCATAAGTAGCATCAATAGGGT
TACAATGCAACTAATTGAAGCAAGTAGAGAAGAGTGCCTTGGTGTGATCAATGTTTGGTTGTTCGAAAGTAAATATAGATXXXXXXXXXXC
TATCACAACGAAGAATAGTAGTAGAGTCTTGGGGAGCCTCATGTTTTGCATACAACCTCATAAGTAGCATCAATAGGGT
>Marker883223 Chr7 SNP_site:7749274 Ref_Type:A
TACAATGCAACTAATTGAAGCAAGTAGAGAAGAGTGCCTTGGTGTGATCAATGTTTGGTTGTTCGAAAGTAAATATAGATXXXXXXXXXXC
TATCACAACGAAGAATAGTAGTAGAGTCTTGGGAAAACTCATGTTTTGCATACAACCTCATAAGTAGCATCAATAGGGT
TACAATGCAACTAATTGAAGCAAGTAGAGAAGAGTGCCTTGGTGTGATCAATGTTTGGTTGTTCGAAAGTAAATATAGATXXXXXXXXXXC
TATCACAACGAAGAATAGTAGTAGAGTCTTGGGGAGCCTCATGTTTTGCATACAACCTCATAAGTAGCATCAATAGGGT
>Marker883543 Chr7 SNP_site:14193255 Ref_Type:T
TACCAGTAGGTAGAAGCTAGAGTAGTATATAATTGATCTCTTTTAGAATGAGTATTTTTAAATCCAAATCTATTTGGAGTXXXXXXXXXXA
GAAGGATGGTTTGCATAAGGATGGTAATTATTGAAAGGAGAGACAAAGAGCACACACTAATTTACGTGGAAACCTGAGT
TACCAGTAGGTAGAAGCTAGAGTTGTATATAATTGATCTCTTTTAGAATGAGTATTTTTAAATCCAAATCTATTTGGAGTXXXXXXXXXXG
GAAGGATGGTTTGCATAAGGATGGTAATTATTGAAAGGAGAGACAAAGAGCACACACTAATTTACGTGGAAACCTGAGT
>Marker883543 Chr7 SNP_site:14193458 Ref_Type:G
TACCAGTAGGTAGAAGCTAGAGTAGTATATAATTGATCTCTTTTAGAATGAGTATTTTTAAATCCAAATCTATTTGGAGTXXXXXXXXXXA
GAAGGATGGTTTGCATAAGGATGGTAATTATTGAAAGGAGAGACAAAGAGCACACACTAATTTACGTGGAAACCTGAGT
TACCAGTAGGTAGAAGCTAGAGTTGTATATAATTGATCTCTTTTAGAATGAGTATTTTTAAATCCAAATCTATTTGGAGTXXXXXXXXXXG
GAAGGATGGTTTGCATAAGGATGGTAATTATTGAAAGGAGAGACAAAGAGCACACACTAATTTACGTGGAAACCTGAGT

Create PDF with GO2PDF for free, if you wish to

>Marker883557 Chr7 SNP_site:14074621 Ref_Type:T
GACTTCAGATCTGTTGATGGATCAAACTCTGCAGCTGATGCAAAGGATTCCACAGATGGTGGAGCAGCTGAAGAAGATTTXXXXXXXXXXXA
GAGAAGCAGAGCTATGAGAATCTATTGGTTGCAAGGGTTCCGGAGCGTCTTTTCTTGTTCTGATGCTAAACTCATTTGT
GACTTCAGATCTGTTGTTGGATCAAACTCTGCAGCTGATGCAAAGGATTCCACAGATGGTGGAGCAGCTGAAGAAGATTTXXXXXXXXXXXA
GAGAAGCAGAGCTATGAGAATCTATTGGTTGCAAGGGTTCCGGAGCGTCTTTTCTTGTTCTGATGCTAAACTCATTTGT
>Marker883804 Chr7 SNP_site:5689669 Ref_Type:C
ACCTAAAAACAAGGTTTTAAATCGACATTATTGGCCTCTTTAATGTGTTTAAACCGATATCAAAGCCAACTGACATTAAAXXXXXXXXXXXA
AAGATGTTGGTTGCCAACCAACATTAAAGGCTAAATTTCTTCTAGTGCTTCTTTTCAAGTTGTTGTTCCTGTAGTTGTT
ACCTAAAAACAAGGTTTTAAATTGACATTATTGGCCTCTTTAATGTGTTTAAACCGATATCAAAGCCAACTAACATTAAAXXXXXXXXXXXA
AAGATGTTGGTTGCCAACCAACATTAAAGGCTAAATTTCTTCTAGTGCTTCTTTTCAAGTTGTTGTTCCTGTAGTTGTT
>Marker883804 Chr7 SNP_site:5689718 Ref_Type:G
ACCTAAAAACAAGGTTTTAAATCGACATTATTGGCCTCTTTAATGTGTTTAAACCGATATCAAAGCCAACTGACATTAAAXXXXXXXXXXXA
AAGATGTTGGTTGCCAACCAACATTAAAGGCTAAATTTCTTCTAGTGCTTCTTTTCAAGTTGTTGTTCCTGTAGTTGTT
ACCTAAAAACAAGGTTTTAAATTGACATTATTGGCCTCTTTAATGTGTTTAAACCGATATCAAAGCCAACTAACATTAAAXXXXXXXXXXXA
AAGATGTTGGTTGCCAACCAACATTAAAGGCTAAATTTCTTCTAGTGCTTCTTTTCAAGTTGTTGTTCCTGTAGTTGTT
>Marker884498 Chr7 SNP_site:16719985 Ref_Type:C
AACAAGGTTCATCTCTTATTATGTTGAGACATGGTTACCGAAGATGAAGAAGGAGAAGAATAAGAAGAAGATAGCAGAGTXXXXXXXXXXXT
TACCTGCATTAACAAAATAAATGACAGTAAAAGTAAGTTAATATTAACTAGATGAATCCTTGCTTTAAGGAAACATGTA
AACAAGGTTCATCTCTTATTATGTTGAGACATGGTTACCGAAGATGAAGAAGGAGAAGAATAAGAAGAAGATAGCAGAGTXXXXXXXXXXXT
TACCTGCATTAACAAAATAAATGACAGTAAAAGTAAGTTAATATTAACTAGATGAATCCTTGTTTTAAGGAAACATGTA
>Marker885005 Chr7 SNP_site:15401629 Ref_Type:A
ACTCTCTTGGATCCTTCTTCTATTCCTCACAACCAAATATATAATGCCTTAAGTTTTTATGATCATCTATATATACAATTXXXXXXXXXXXA
TTTAAGTTGTGTATTTCTCATTTTCTCATCAAGAAATTTATGTGTAATATTCTTTTTATTTCTCACAATTGTATTAAGT
ACTCTCTTGGATCCTTCTTCTATTCCTCACAACCAAATATATAATGCCTTAAGTTTTTATGATCATCTATATATACAATTXXXXXXXXXXXC
TTTAAGTTGTGTATTTCTCATTTTCTCATCAAGAAATTTATGTGTAATATTCTTTTTATTTCTCACAATTGTATTAAGT
>Marker885447 Chr7 SNP_site:261929 Ref_Type:A
AACCTAAACTCTGATTTGTTGGTTGAGTTTTCTTTTTAATTATTCTCTTTGCTTATTCTGATTGATTGAAGTCCTTATTAXXXXXXXXXXXA
AAACAGTGACATTTCTGGTTGGTTGGTTTTGTTTATAGAGAGATATAGCATCTGGAACTTGATCGAATTTCCATCTGTA
AACCTAAACTCTGATTTGTTGGTTGAGTTTTCTTTTTAATTATTCTCTTTGCTTATTCTGATTGATTGGAGTCCTTATTAXXXXXXXXXXXA
AAACAATGACATTTCTGGTTGGTTGGTTTTGTTTATAGAGAGATATAGCATCTGGAACTTGATCGAATTTCCATCTGTA
>Marker885447 Chr7 SNP_site:262046 Ref_Type:G
AACCTAAACTCTGATTTGTTGGTTGAGTTTTCTTTTTAATTATTCTCTTTGCTTATTCTGATTGATTGAAGTCCTTATTAXXXXXXXXXXXA
AAACAGTGACATTTCTGGTTGGTTGGTTTTGTTTATAGAGAGATATAGCATCTGGAACTTGATCGAATTTCCATCTGTA
AACCTAAACTCTGATTTGTTGGTTGAGTTTTCTTTTTAATTATTCTCTTTGCTTATTCTGATTGATTGGAGTCCTTATTAXXXXXXXXXXXA
AAACAATGACATTTCTGGTTGGTTGGTTTTGTTTATAGAGAGATATAGCATCTGGAACTTGATCGAATTTCCATCTGTA
>Marker885600 Chr7 SNP_site:704474 Ref_Type:C
GACGTTCAAGTTAATTAGATATTTGCTTTACCATCTATTGTTTTAACTTGATTGAATGAAAGGTGTTACAATTTTTTTCTXXXXXXXXXXXT
TTAGGAAAGGGATGTGACACAATTACCTACATTATATTATATGAATGAAACATATTTAAATGTAATGAAACATATAGTA
GACGTTCAAGTTAATTAGATATTTGCTTTACCATCTATTGTTTTAACTTGATTGAATGAAAGGTGTTACAATTTTTTTTCXXXXXXXXXXXT
TTAGGAAAGGGATGTGACACAATTACCTACATTATATTATATGAATGAAACATATTTAAATGTAATGAAACATATAGTA
>Marker885600 Chr7 SNP_site:704475 Ref_Type:T
GACGTTCAAGTTAATTAGATATTTGCTTTACCATCTATTGTTTTAACTTGATTGAATGAAAGGTGTTACAATTTTTTTCTXXXXXXXXXXXT
TTAGGAAAGGGATGTGACACAATTACCTACATTATATTATATGAATGAAACATATTTAAATGTAATGAAACATATAGTA
GACGTTCAAGTTAATTAGATATTTGCTTTACCATCTATTGTTTTAACTTGATTGAATGAAAGGTGTTACAATTTTTTTTCXXXXXXXXXXXT

Create PDF with GO2PDF for free, if you wish to

TTAGGAAAGGGATGTGACACAATTACCTACATTATATTATATGAATGAAACATATTTAAATGTAATGAAACATATAGTA
>Marker885657 Chr7 SNP_site:5079899 Ref_Type:T
TACATTCATGATCACTATGTATAAACTCAATAATCGAATTGTGTTGTTTATTAATTACAAATTTGAGCGTCTTTATGTATXXXXXXXXXXT
GTAGTTAAACATTGACTTAGAAAAGGTTAAATTTTATTCCAACTACAAAATGATGGTGATGAGTTTGCAACCCTAAGTG
TACATTCATGATCACTATGTATAAACTCAATAATCGAATTGTGTTGTTTATTAATTACAAATTTGAGTGTCTTTATGTATXXXXXXXXXXT
GTAGTTAAACATTGACTTAGAAAAGGTTAAATTTTATTCCAACTACAAAATGATGGTGATGAGTTTGCAACCCTAAGTG
>Marker886137 Chr7 SNP_site:15607568 Ref_Type:A
AACCCTCTCATAGAGTTGGCCTTTTATCTTTTCTCAATTAATTCATTACAACTTTTCTAAATCAGAACCTAAATGTGAAGXXXXXXXXXXT
TCTTCTTTAATTACTTGAGAGTTTGTATATATACTTAAAAACATTATGAAGGGGATGACTTATGTTTAATACATATGGT
AACCCTCTCATAGAGTTGGCCTTTTCTCTTTTCTCAATTAATTCATTACAACTTTTCTAAATCAGAACCTAAATGTGAAGXXXXXXXXXXT
TCTTCTTTAATTACTTGAGAGTTTGTATATATACTTAAAAACATTATGAAGGGGATGATTTATGTTTAATACATATGGT
>Marker886137 Chr7 SNP_site:15607780 Ref_Type:C
AACCCTCTCATAGAGTTGGCCTTTTATCTTTTCTCAATTAATTCATTACAACTTTTCTAAATCAGAACCTAAATGTGAAGXXXXXXXXXXT
TCTTCTTTAATTACTTGAGAGTTTGTATATATACTTAAAAACATTATGAAGGGGATGACTTATGTTTAATACATATGGT
AACCCTCTCATAGAGTTGGCCTTTTCTCTTTTCTCAATTAATTCATTACAACTTTTCTAAATCAGAACCTAAATGTGAAGXXXXXXXXXXT
TCTTCTTTAATTACTTGAGAGTTTGTATATATACTTAAAAACATTATGAAGGGGATGATTTATGTTTAATACATATGGT
>Marker887014 Chr7 SNP_site:8037115 Ref_Type:G
TACAATCTTGAACCAAATAACAGATTGAAAAGAAACTAAAAGAAAATTTACAGATGAGGAGAAAAGACGATGGAAAGAAAXXXXXXXXXXT
TACCGGTTTGTTATATTTATGAAAATTAACCAATAAATAAGTATATAGCCAACATGAGCTTAACTCAACTGGCACCGGT
TACAATCTTGAACCAAATAACAGATTGAAAAGAAACTAAAAGAAAATTTGCAGATGAGGAGAAAAGACGATGGAAAGAAAXXXXXXXXXXT
TACCGGTTTGTTATATTTATGAAAATTAACCAATAAATAAGTATATAGCCAACATGAGCTTAACTCAACTGGCACCGGT
>Marker887040 Chr7 SNP_site:15422229 Ref_Type:T
GACATCCGTTTGGAATAATATAACGGTAGAATTTAATAGCAAAAAATATATACAATTTCCAATAGTCATCAATTTAAAGAXXXXXXXXXXT
CAAAAACTGACCCACCATTCTCATTTAGATCAACATTTGCCTTAATGTCATTCACCCTGCCGCCAATGTTGGGAAGAGT
GACATCCGTTTGGAATAATATAACGGTAGAATTTAATAGCAAAAAATATATACAATTTCCTATAGTCATCAATTTAAAGAXXXXXXXXXXT
CAAAAACTGACCCACCATTCTCATTTAGATCAACATTTGCCTTAATGTCATTCACCGTGCCGCCAATGTTGGGAAGAGT
>Marker887040 Chr7 SNP_site:15422407 Ref_Type:C
GACATCCGTTTGGAATAATATAACGGTAGAATTTAATAGCAAAAAATATATACAATTTCCAATAGTCATCAATTTAAAGAXXXXXXXXXXT
CAAAAACTGACCCACCATTCTCATTTAGATCAACATTTGCCTTAATGTCATTCACCCTGCCGCCAATGTTGGGAAGAGT
GACATCCGTTTGGAATAATATAACGGTAGAATTTAATAGCAAAAAATATATACAATTTCCTATAGTCATCAATTTAAAGAXXXXXXXXXXT
CAAAAACTGACCCACCATTCTCATTTAGATCAACATTTGCCTTAATGTCATTCACCGTGCCGCCAATGTTGGGAAGAGT
>Marker887225 Chr7 SNP_site:15630733 Ref_Type:C
GACTTTGACAGTATCTTTCGGTTCTATATATTTATTCTTCTTGTTTTAAGTCCTCCACAATAATGTTGTGTGCTCCAATAXXXXXXXXXXT
TTTATAGAAAGCATCAATAAAATATTGGATGCTGATGAATGTAATGTTCCTTGAGGTAAAATATAAGTTTGTTGATGTC
GACTTTGACAGTATCTTTCGGTTCTATATATTTATTCTTCTTGTTTTAAGTCCTCCACAATAATGTTGTGTGCTCCAATAXXXXXXXXXXT
TTTATAGAAAGCATCAATAAAATATTGGATGCTGATGAATGTAATGTTCGTTGAGGTAAAATATAAGTTTGTTGATGTC
>Marker887577 Chr7 SNP_site:15859623 Ref_Type:T
GACCTGGATAGTCCAGATAAACGGAAGAAGATGATGGTTGCATGCACTCAGCCTCGCAGGGTGGCAGCAATGTCAGTTTCXXXXXXXXXXT
CCTTGTGCCTGCTCATTGCCACTATCTTTTCTCTATATAATTTGAAATTTTATTCTCTACTTGATTGTAAATTTGTTGT
GACCTGGATAGTCCAGATAAACGGAAGAAGATGATGGTTGCATGCACTCAGCCTCGCAGGGTGGCAGCAATGTCAGTTTCXXXXXXXXXXT
CCTTGTGCCTGCTCATTGCCACTATCTTTTCTCTATATAATTTGAAATTTTATTCTTTACTTGATTGTAAATTTGTTGT
>Marker888279 Chr7 SNP_site:4496430 Ref_Type:G
TACCACATCACCAGTTGGTAAGTTTCTGAAATTGTTGTCTGTCAAACACATTCGATAACATCCCCATGGTGCCCATGCCGXXXXXXXXXXC
ACTACGTGAAGCATAGAAAATTCTTGGATCAATTGATTATGAGATCCATTCACAAACAATTGGGTTCCTCAAGGGCGTA

Create PDF with GO2PDF for free, if you wish to

TACCACATCACCAGTTGGTAAGTTTCTGAAATTGTTGTCTGTCAAACACATTCGATAACATCCCCATGGTGCCCATGCCGXXXXXXXXXXC
ACTACGTGAAGCATAGAAAATTCTTGGATCAATTGATTATGGGATCCATTCACAAACAATTGGGTTCCTCAAGGGCGTA
>Marker888317 Chr7 SNP_site:8096492 Ref_Type:G
CACTAGTTTTGTTTTGGAAACTTTAGTCAAGTGATTGATTACTTTAGAACGCAAAAGAGCACCCAAATATCTCGACCTTTXXXXXXXXXXT
GGGCATGTGGATAAATCCTCCTTTAGAGTTAAGTTAATAAGAAGCAAAAAAGGAATACTGCTTTCACAAAGAAAATGTG
CACTAGTTTTGTTTTGGAAACTTTAGTCAAGTGATTGATTACTTTAGAACGCAAAAGAGCACCCAAATATCTCGACCTTTXXXXXXXXXXT
GGGCATGTGGATAAATCCTCCTTTAGAGTTAAGTTAATAAGAAGCAAAAAAGGAATACTGCTTTCACAAATAAAATGTG
>Marker88855 Chr4 SNP_site:1644731 Ref_Type:T
TACATATAACCATTAACTTTAGAGTCTGGTGACCCTTTTCTAGAAAAAATGGATACTTTTATCAGCAAAATTAAACAGTGXXXXXXXXXXT
ATAGGTAATGTAATCTGCATTAAAAGCATAAAACGGTGATTGGCATACATATCATTGTAGCTATGGACAACCTTCTGTT
TACATATAACCATTAACTTTAGAGTCTGGTGACCCTTTTCTAGAAAAAATGGATATTTTTATCAGCAAAATTAAACAGTGXXXXXXXXXXT
ATAGGTAATGTAATCTGCATTAAAAGCATAAAACGGTGATTGGCATACATATCATTGTAGCTATGGACAACCTTCTGTT
>Marker888844 Chr7 SNP_site:194874 Ref_Type:A
ACTTGTTCCAACAACAATGGAACTTCTATTGAATCTCCACCAGTTTGTCGTGATTCAAACTCCCTTTGCTGCTCTACAGCXXXXXXXXXXC
CAAGAAACGTTCAATCTTACCGTTATTCAATCATGGATCCAAAAACAGAAAAACAACTAAATAATGCTTAGACAAAGTA
ACTTGTTCCAACAACAATGGAACTTCTATTGAATCTCCACCAGTTTGTCGTGATTCAAACTCCCTTTGCTGCTCTACAGCXXXXXXXXXXC
CAAGAAACGTTCAATCTTACCGTTATTCAATCATGGATCCAAAAACAGAAAAACAACTAGATAATGCTTAGACAAAGTA
>Marker888899 Chr7 SNP_site:4515093 Ref_Type:T
AACAGTTAAACAGAACTCAAAGGGAGAGAAAAAGGGTAAAAGACGATAGTAACCGACTTTCTGAACTCCCATTTCTTTAGXXXXXXXXXXC
TCCCTATTCAATAATTGTTATAATTATGGTAAATCTGAATGCCTTTCTATCTGACACCACTGCATCGATGGGAAGATGT
AACAGTTAAACAGAACTCAAAGGGAGAGAAAAAGGGTAAAAGACGATAGTAACCGACTTTCTGAACTCCCATTTCTTTAGXXXXXXXXXXC
TCCCTATTCAATAATTGTTATAATTATTGCAAATCTGAATGCCTTTCTATCTGACACCACTGCATCGATGGGAAGATGT
>Marker888899 Chr7 SNP_site:4515095 Ref_Type:C
AACAGTTAAACAGAACTCAAAGGGAGAGAAAAAGGGTAAAAGACGATAGTAACCGACTTTCTGAACTCCCATTTCTTTAGXXXXXXXXXXC
TCCCTATTCAATAATTGTTATAATTATGGTAAATCTGAATGCCTTTCTATCTGACACCACTGCATCGATGGGAAGATGT
AACAGTTAAACAGAACTCAAAGGGAGAGAAAAAGGGTAAAAGACGATAGTAACCGACTTTCTGAACTCCCATTTCTTTAGXXXXXXXXXXC
TCCCTATTCAATAATTGTTATAATTATTGCAAATCTGAATGCCTTTCTATCTGACACCACTGCATCGATGGGAAGATGT
>Marker889235 Chr7 SNP_site:35979 Ref_Type:C
ACCATACCCACTTGTAAGAGTTTGATAAGGACCTCAACTTTAAAATTTAATCAACAATCATTTATATAATCGACCGCACGXXXXXXXXXXT
TCTATTATATATTTTTACAATAGGATTAAGATCAAACACACGGATGAGATCAACTTATATGATGTAAAAGTTTAAAAGT
ACCATACCCACTTGTAAGAGTTTGATAAGGACCTCAACTTTAAAATTTAATTAACAATCATTTATATAATCGACCGCACGXXXXXXXXXXT
TCTATTATATATTTTTACAATAGGATTAAGATCAAACACACGGATGAGATCAACTTATATGATGTAAAAGTTTAAAAGT
>Marker889265 Chr7 SNP_site:3784102 Ref_Type:T
GACTCCTTTGGAAGTTCCTAATAGAGTTTGGGAAGATATATCCATGGATTTCGTGGAAGGGTTGTCGAAAGCCAATGGAAXXXXXXXXXXA
ATAGTGTCGGATATCTCTGTCCTTAAGTCACTTTTGGAGGGAGTTGTTCAGACAGGCTGATACACGGCTGAATTATAGT
GACTCCTTTGGAAGTTCCTAATAGAGTTTGGGAAGATATATCCATGGATTTCGTGGAAGGGTTGTCGAAAGCCAATGGAAXXXXXXXXXXA
ATAGTGTCGGATATCTCTGTCCTTAAGTCACTTTTGGAGGGAGTTGTTCAGACAGGCTGATACATGGCTGAATTATAGT
>Marker889605 Chr7 SNP_site:4591267 Ref_Type:C
ACGTTTATTTAAACCGTGTTTCAAAACTAACCACATAAGTAATTCTTGTATATATAAATTTAAAATAACCCATTTTAAATXXXXXXXXXXA
TGAAGAAGGAGCGATCATGTTTCTCAATGACTTAAGTTGGACGTAAAATCAATGTCTCTCAAAGTAAGTTGATATCCGT
ACGTTTATTTAAACCGTGTTTCAAAACTAACCACATAAGTAATTCTTGTATATATAAATTTAAAATAACCGATTTTGAATXXXXXXXXXXA
TGAAGAAGGAGCGATCATGTTTCTCAATGACTTAAGTTAGACGTAAAATCAATGTCTCTCAAAGTAAGTTGATATCCGT
>Marker889605 Chr7 SNP_site:4591273 Ref_Type:A
ACGTTTATTTAAACCGTGTTTCAAAACTAACCACATAAGTAATTCTTGTATATATAAATTTAAAATAACCCATTTTAAATXXXXXXXXXXA

Create PDF with GO2PDF for free, if you wish to

TGAAGAAGGAGCGATCATGTTTCTCAATGACTTAAGTTGGACGTAAAATCAATGTCTCTCAAAGTAAGTTGATATCCGT
ACGTTTATTTAAACCGTGTTTCAAAACTAACCACATAAGTAATTCTTGTATATATAAATTTAAAATAACCGATTTTGAATXXXXXXXXXXA
TGAAGAAGGAGCGATCATGTTTCTCAATGACTTAAGTTAGACGTAAAATCAATGTCTCTCAAAGTAAGTTGATATCCGT
>Marker889605 Chr7 SNP_site:4591446 Ref_Type:G
ACGTTTATTTAAACCGTGTTTCAAAACTAACCACATAAGTAATTCTTGTATATATAAATTTAAAATAACCCATTTTAAATXXXXXXXXXXA
TGAAGAAGGAGCGATCATGTTTCTCAATGACTTAAGTTGGACGTAAAATCAATGTCTCTCAAAGTAAGTTGATATCCGT
ACGTTTATTTAAACCGTGTTTCAAAACTAACCACATAAGTAATTCTTGTATATATAAATTTAAAATAACCGATTTTGAATXXXXXXXXXXA
TGAAGAAGGAGCGATCATGTTTCTCAATGACTTAAGTTAGACGTAAAATCAATGTCTCTCAAAGTAAGTTGATATCCGT
>Marker889685 Chr7 SNP_site:7250793 Ref_Type:T
AACCTCCCTACTGATCTCAATGATCATGGAACGTGTGGCTTTCATAAAGTTCATGTTCGTGGAAACGGTTTTAAAGGGTCXXXXXXXXXXA
TTGAGAATTAAATATGCTATCCTTCACAAGATTGTGTGTCACTACTATATTTATTTATTGTTTTCATTGTTGTTTTGTT
AACCTCCCTACTGATCTCAATGATCATGGAACGTGTGGCTTTCATAAAGTTCATGTTCGTGGAAACTGTTTTAAAGGGTCXXXXXXXXXXA
TTGAGAATTAAATATGCTATCCTTCACAAGATTGTGTGTCACTGCTATATTTACTTATTGTTTTCATTGTTGTTTTGTT
>Marker889685 Chr7 SNP_site:7251004 Ref_Type:G
AACCTCCCTACTGATCTCAATGATCATGGAACGTGTGGCTTTCATAAAGTTCATGTTCGTGGAAACGGTTTTAAAGGGTCXXXXXXXXXXA
TTGAGAATTAAATATGCTATCCTTCACAAGATTGTGTGTCACTACTATATTTATTTATTGTTTTCATTGTTGTTTTGTT
AACCTCCCTACTGATCTCAATGATCATGGAACGTGTGGCTTTCATAAAGTTCATGTTCGTGGAAACTGTTTTAAAGGGTCXXXXXXXXXXA
TTGAGAATTAAATATGCTATCCTTCACAAGATTGTGTGTCACTGCTATATTTACTTATTGTTTTCATTGTTGTTTTGTT
>Marker889685 Chr7 SNP_site:7251014 Ref_Type:C
AACCTCCCTACTGATCTCAATGATCATGGAACGTGTGGCTTTCATAAAGTTCATGTTCGTGGAAACGGTTTTAAAGGGTCXXXXXXXXXXA
TTGAGAATTAAATATGCTATCCTTCACAAGATTGTGTGTCACTACTATATTTATTTATTGTTTTCATTGTTGTTTTGTT
AACCTCCCTACTGATCTCAATGATCATGGAACGTGTGGCTTTCATAAAGTTCATGTTCGTGGAAACTGTTTTAAAGGGTCXXXXXXXXXXA
TTGAGAATTAAATATGCTATCCTTCACAAGATTGTGTGTCACTGCTATATTTACTTATTGTTTTCATTGTTGTTTTGTT
>Marker88985 Chr4 SNP_site:14138188 Ref_Type:A
GACATTAAACAAGAGATTTAGAGGTTGAAAAATGTTTATAGGCTTAATTTTAAGAAACCAAAACAAACAACGAAATGGTTXXXXXXXXXXA
TAATTAAGTGTATCAGCGATAGAAATCTGATAGGGCTCTTATGGCCTATCAATGATAGATTAATATTTACAATATGGTA
GACATTAAACAAGAGATTTAGAGGTTGAAAAATGTTTATAGGCTTAATTTTAAGAAACCAAAACAAACAACGAGATGGTTXXXXXXXXXXA
TAATTAAGTGTATCAACGATAGAAATCTGATAGGGCTCTTATGGCCTATCAATGATAGATTAATATTTACAATATGGTA
>Marker88985 Chr4 SNP_site:14138339 Ref_Type:G
GACATTAAACAAGAGATTTAGAGGTTGAAAAATGTTTATAGGCTTAATTTTAAGAAACCAAAACAAACAACGAAATGGTTXXXXXXXXXXA
TAATTAAGTGTATCAGCGATAGAAATCTGATAGGGCTCTTATGGCCTATCAATGATAGATTAATATTTACAATATGGTA
GACATTAAACAAGAGATTTAGAGGTTGAAAAATGTTTATAGGCTTAATTTTAAGAAACCAAAACAAACAACGAGATGGTTXXXXXXXXXXA
TAATTAAGTGTATCAACGATAGAAATCTGATAGGGCTCTTATGGCCTATCAATGATAGATTAATATTTACAATATGGTA
>Marker889931 Chr7 SNP_site:4295616 Ref_Type:C
ACCTGATTTGTCTTCTTTAGTGGGATTGAGATTCCTGAGTTTGAATAACAGTGGGTTTTCTGGAGATTTTCCATGGAAATXXXXXXXXXXG
GATTGGGAACTTGTCTTTGCTTGAGAATCTTGAACTCTCACAAAATAAACTCACTGGTGAAATTCCTTATGAGATTGTG
ACCTGATTTGTCTTCTTTAGTGGGATTGAGATTCTTGAGTTTGAATAACAGTGGGTTTTCTGGAGATTTTCCATGGAAATXXXXXXXXXXG
GATTGGGAACTTGTCTTTGCTTGAGAATCTTGAACTCTCACAAAATAAACTCACTGGTGAAATTCCTTATGAGATTGTG
>Marker890314 Chr7 SNP_site:8706533 Ref_Type:A
TACAATAATTCCTTCTCTCCTAACGATGTTAATTAGGTGGGATTAACTCTTATCTAATAAAATACAAAAGCACTAAACAAXXXXXXXXXXT
TTCTCATGGCCAGAACAATAATAATACCTTATCAAAGCAGAAGCAGCATATGCTCATAATACTCTCTGCCACTATTGTA
TACAATAATTCCTTCTCTCCTAACGATGTTAATTAGGTGGGATTAACTCTTATCTAATAAAATACAAAAGCACTAAACAAXXXXXXXXXXT
TTCTCATGGCCAGAACAATAATAATACCTTATCAAAGCAGAAGCAGCATATGCTCATAATACTCTCTGCCGCTATTGTA
>Marker890937 Chr7 SNP_site:7728909 Ref_Type:A

Create PDF with GO2PDF for free, if you wish to

CACTTAAACCCTCCTATCTTCAATAACTGCGTCAAAGGAGCCGCAATTGTCCCATAATTCTGGACAAACTTGCGATAGTAXXXXXXXXXXXT
AATATGTCCCAAGTAATCTACTCGAGACTGAGCAAAGCTACATTTCTTCTTATTTGCATATAGTTCATTATTCCTTAGT
CACTTAAACCCTCCTATCTTCAATAACTGCGTCAAAGGAGCCGCAATTGTCCCATAATTCTGGACAAACTTGCGATAGTAXXXXXXXXXXXT
AATATGTCCCAAGTAATCTACTCGAGACTGAGCAAAGCTACATTTCTTCTTATTTGCATATAGTTCATTCTTCCTCAGT
>Marker890937 Chr7 SNP_site:7728915 Ref_Type:T
CACTTAAACCCTCCTATCTTCAATAACTGCGTCAAAGGAGCCGCAATTGTCCCATAATTCTGGACAAACTTGCGATAGTAXXXXXXXXXXXT
AATATGTCCCAAGTAATCTACTCGAGACTGAGCAAAGCTACATTTCTTCTTATTTGCATATAGTTCATTATTCCTTAGT
CACTTAAACCCTCCTATCTTCAATAACTGCGTCAAAGGAGCCGCAATTGTCCCATAATTCTGGACAAACTTGCGATAGTAXXXXXXXXXXXT
AATATGTCCCAAGTAATCTACTCGAGACTGAGCAAAGCTACATTTCTTCTTATTTGCATATAGTTCATTCTTCCTCAGT
>Marker891982 Chr7 SNP_site:491366 Ref_Type:G
ACCTGGATATTTAGTGCAAATGATCTCTTAAATAATAACAGCTTGAGTTAAAATTCTGGAAGAGCTTAAATTAGTATAAAXXXXXXXXXXXT
ATTGTCCCCAAAATGTTTGCCATCGGAGATACTTCAGAAACCATTTAATATGAATAGGAAAAGCTTGGATGCCTTTGTT
ACCTGGATATTTAGTGCAAATGATCTCTTAAATAATAACAGCTTGAGTTAAAATTCTGGAAGAGCTTAAATTAGTATAAAXXXXXXXXXXXT
ATTGTCCCCAAAATGTTTGCCATCGGATATACTTCAGAAATCATTTAATATGAATAGGAAAAGCTTGGATGCCTTTGTT
>Marker891982 Chr7 SNP_site:491379 Ref_Type:C
ACCTGGATATTTAGTGCAAATGATCTCTTAAATAATAACAGCTTGAGTTAAAATTCTGGAAGAGCTTAAATTAGTATAAAXXXXXXXXXXXT
ATTGTCCCCAAAATGTTTGCCATCGGAGATACTTCAGAAACCATTTAATATGAATAGGAAAAGCTTGGATGCCTTTGTT
ACCTGGATATTTAGTGCAAATGATCTCTTAAATAATAACAGCTTGAGTTAAAATTCTGGAAGAGCTTAAATTAGTATAAAXXXXXXXXXXXT
ATTGTCCCCAAAATGTTTGCCATCGGATATACTTCAGAAATCATTTAATATGAATAGGAAAAGCTTGGATGCCTTTGTT
>Marker891997 Chr7 SNP_site:12696168 Ref_Type:G
GACCAAGACGCCGAAAAGTTGTTATCCTTAAATGAGAAGCACTCTCAGCAAAATATATAAGTATTACTAGATATAAACCAXXXXXXXXXXXA
TTGCAGTGAAGTGGTCATAGAACTTGCTCGCATGATAACATGGAACCTTCCTTCACCACCTTCTTAACGCAAGTTAGTT
GACCAAGACGCCGAAAAGTTGTTGTCCTTAAATGAGAAGCACTCTCAGCAAAATATATAAGTATTACTAGATATAAACCAXXXXXXXXXXXA
TTGCAGTGAAGTGGTCATAGAACTTGCTCGCATGATAACATGGAACCTTCCTTCACCACCTTCTTAACGCAAGTTAGTT
>Marker892052 Chr7 SNP_site:9467042 Ref_Type:C
CACCTTCCCGAGAAAGATATATATTGATATCCAAGAGATAATATGCCGAAGAAGTGATATGACAAGCAGGTGACTCTTCTXXXXXXXXXXXT
TTAGATTAAAGTGTGTTTTCTACACATTTACCACAAATTGGGCATACTATAGATCATGTGTCATAACGTGATTGATGTA
CACCTTCCTGAGAAAGATATATATTGATATCCAAGAGATAATATGCGGAAGAAGTGATATGACAAGCAAGTGACTCTTCTXXXXXXXXXXXT
TTAGATTAAAGTGTGTTTTCTACACATTTACCACAAATTGGGCATACTATAGATCATGTGTCATAACGTGATTGATGTA
>Marker892052 Chr7 SNP_site:9467080 Ref_Type:C
CACCTTCCCGAGAAAGATATATATTGATATCCAAGAGATAATATGCCGAAGAAGTGATATGACAAGCAGGTGACTCTTCTXXXXXXXXXXXT
TTAGATTAAAGTGTGTTTTCTACACATTTACCACAAATTGGGCATACTATAGATCATGTGTCATAACGTGATTGATGTA
CACCTTCCTGAGAAAGATATATATTGATATCCAAGAGATAATATGCGGAAGAAGTGATATGACAAGCAAGTGACTCTTCTXXXXXXXXXXXT
TTAGATTAAAGTGTGTTTTCTACACATTTACCACAAATTGGGCATACTATAGATCATGTGTCATAACGTGATTGATGTA
>Marker892052 Chr7 SNP_site:9467102 Ref_Type:G
CACCTTCCCGAGAAAGATATATATTGATATCCAAGAGATAATATGCCGAAGAAGTGATATGACAAGCAGGTGACTCTTCTXXXXXXXXXXXT
TTAGATTAAAGTGTGTTTTCTACACATTTACCACAAATTGGGCATACTATAGATCATGTGTCATAACGTGATTGATGTA
CACCTTCCTGAGAAAGATATATATTGATATCCAAGAGATAATATGCGGAAGAAGTGATATGACAAGCAAGTGACTCTTCTXXXXXXXXXXXT
TTAGATTAAAGTGTGTTTTCTACACATTTACCACAAATTGGGCATACTATAGATCATGTGTCATAACGTGATTGATGTA
>Marker892324 Chr7 SNP_site:55614 Ref_Type:C
AACCAAAAATCTTAAAGAAGAAAATACTTCATTAACACGTAATAGAATTCTAAACGATCATATCTTATAGTCTAAATGAAXXXXXXXXXXXA
TAATGTTGTTGATTATGGTAAGAGATCATATAGTCTAATGTAAATGATCATTAAAAACATAAAATCAAACGATTGTGTT
AACCAAAAATCTTAAAGAAGAAAATACTTCATTAACACGTAATAGAATTCTAAACGATCATATCTTATAGTCTAAATGAAXXXXXXXXXXXA
TAATGTTGTTGATTATGGTAAGAGATCATATAGTTTAATGTAAATGATCATTAAAAACATAAAATCAAACGATTGTGTT

Create PDF with GO2PDF for free, if you wish to

>Marker893216 Chr7 SNP_site:3324540 Ref_Type:T
ACCTTCCAACAGGTAACTTTCATTATCACTACTTTCATCATCACCCCATTGTTTTCTAGCATTTTTAAGAGAGTAAATTAXXXXXXXXXXC
ACATGCTTACAATTATAACTGAATGATGCAGATGTGGATAACGAAGGCTGAATACGACGAGTCGGGTCCAGCTATAGTT
ACCTTCCAACAGGTAACTTTCATTTTCACTACTTTCATCATCACCCCATTGTTTTCTAGCATTTTTAAGAGAGTAGATTAXXXXXXXXXXC
ACATGCTTACAATTATAACTGAATGATGCAGATGTGGATAACGAAGGCTGAATACGACGAGTCGGGTCCAGCTATAGTT
>Marker893216 Chr7 SNP_site:3324591 Ref_Type:G
ACCTTCCAACAGGTAACTTTCATTATCACTACTTTCATCATCACCCCATTGTTTTCTAGCATTTTTAAGAGAGTAAATTAXXXXXXXXXXC
ACATGCTTACAATTATAACTGAATGATGCAGATGTGGATAACGAAGGCTGAATACGACGAGTCGGGTCCAGCTATAGTT
ACCTTCCAACAGGTAACTTTCATTTTCACTACTTTCATCATCACCCCATTGTTTTCTAGCATTTTTAAGAGAGTAGATTAXXXXXXXXXXC
ACATGCTTACAATTATAACTGAATGATGCAGATGTGGATAACGAAGGCTGAATACGACGAGTCGGGTCCAGCTATAGTT
>Marker893295 Chr7 SNP_site:7264711 Ref_Type:C
AACAAGTATGGATACTAGGCACCAAGAGGTTGTTGTCCGTAATTGTGAAGCTCTATACGTCTTATAAGTGAAATCTTCGAXXXXXXXXXXA
TTGCATCAAGGTTGTTGTGTTACTTAATTGAATTACAATGCATATACATTCATTCGCTTTTTTCGCATACAAGTTAGTT
AACAAGTATGGATACTAGGCACCAAGAGGTTGTTGTCCGTAATTGTGAAGCTCTATACGTCTTATAAGTGAAATCTTTCAXXXXXXXXXXA
TTGCATCAAGGTTGTTGTGTTACTTAATTGAATTACAATGCATATACATTCATTCGCTTTTTTCGCATACAAGTTAGTT
>Marker893295 Chr7 SNP_site:7264712 Ref_Type:G
AACAAGTATGGATACTAGGCACCAAGAGGTTGTTGTCCGTAATTGTGAAGCTCTATACGTCTTATAAGTGAAATCTTCGAXXXXXXXXXXA
TTGCATCAAGGTTGTTGTGTTACTTAATTGAATTACAATGCATATACATTCATTCGCTTTTTTCGCATACAAGTTAGTT
AACAAGTATGGATACTAGGCACCAAGAGGTTGTTGTCCGTAATTGTGAAGCTCTATACGTCTTATAAGTGAAATCTTTCAXXXXXXXXXXA
TTGCATCAAGGTTGTTGTGTTACTTAATTGAATTACAATGCATATACATTCATTCGCTTTTTTCGCATACAAGTTAGTT
>Marker893313 Chr7 SNP_site:9155605 Ref_Type:C
CACTTCTCTTCCTTCCTATCTTTGGGGTGATGTCATTCTTACTGTCGCTCATCTCATAAATCACATGCCTTATCGTGTCCXXXXXXXXXXT
AAAGCTCAAGCTTGTGTGTTTGTTGGATATCCTCTGCACCAATGAGACTATAAATGCTTTCATCCATCTTCACGTTAGT
CACTTCTCTTCCTTCCTATCTTTGGGGTGATGTCATTCTTACTGTCGCTCATCTCATAAATCACATGTCTTATCGTGTCCXXXXXXXXXXT
AAAGCTCAAGCTTGTGTGTTTGTTGGATATCCTCTGCACCAATGAGACTATAAATGCTTTCATCCATCTTCACGTTAGT
>Marker893628 Chr7 SNP_site:4916741 Ref_Type:T
TACTTTTTACTAATGGTTTAAAAAAACCAAGCAAAAATTTGAAAACTAAAAGTAGCTTTTAAAAGAATGCTTTTGATTTTXXXXXXXXXXC
AGCAAATAGGCTAAGAGATTCCATAAACAGACCACACCCACTGAAGCAACTAGCTGTAAATTAAGGCCAGAGATATGTT
TACTTTTTACTAATGGTTTAAAAAAACCAAGCAAAAATTTGAAAACTAAAAGTAGCTTTTAAAAGAATGCTTTTGATTTTXXXXXXXXXXC
AGCAAATAGGCTAAGAGATTCCATAAACAGACCACACCTACTGAAGCAACTAGCTGTAAATTAAGGCCAGAGATATGTT
>Marker893695 Chr7 SNP_site:5509110 Ref_Type:A
CACATAAAAGTTAGCATCATCTCAATGAAACAACTAATTATTTGAAAACCATTTAATAGTATGGAAATCATAATCATCAAXXXXXXXXXXA
AAAGCACTCACCACACTTAAACAAAAATCCTCCTCTTCCTTCTCTTCTCGTGGAACCTTCTTGCGGAACAAATCTTTGT
CACATAAAAGTTAGCATCATCTCAATGAAACAGCTAATTATTTGAAAACCATTTAATAGTATGGAAATCATAATCATCAAXXXXXXXXXXA
AAAGCACTCACCACACTTAAACAAAAATCCTCCTCTTCCTTCTCTTCTCGTGGAACCTTCTTGCGGAACAAATCTTTGT
>Marker894288 Chr7 SNP_site:9431633 Ref_Type:T
AACCTAAACGTCAAGTTTAATAAATACCTACCAAAAAAGTTTATTAAAAAACAAAAAAATGAAACCCTTCAAAGACAAGTXXXXXXXXXXA
GATGTGAAATTTTGAAATTTTTAACATTGTTCTAAATTAGCTCATTTCTTTGTAAGTTATAGCATTGGTCATTTTTTTGT
AACCTAAACGTCAAGTTTAATAAATACCTACCAAAAAAGTTTATTAAAAAACAAAAAAATGAAACCCTTCAAAGACATGTXXXXXXXXXXA
GATGTGAAATTTTGAAATTTTTAACATTGTTCTAAATTAGCTCATTTCTTTGTAGGCTATAGCATTGGTCATTTTTTTGT
>Marker894288 Chr7 SNP_site:9431788 Ref_Type:G
AACCTAAACGTCAAGTTTAATAAATACCTACCAAAAAAGTTTATTAAAAAACAAAAAAATGAAACCCTTCAAAGACAAGTXXXXXXXXXXA
GATGTGAAATTTTGAAATTTTTAACATTGTTCTAAATTAGCTCATTTCTTTGTAAGTTATAGCATTGGTCATTTTTTTGT
AACCTAAACGTCAAGTTTAATAAATACCTACCAAAAAAGTTTATTAAAAAACAAAAAAATGAAACCCTTCAAAGACATGTXXXXXXXXXXA

Create PDF with GO2PDF for free, if you wish to

GATGTGAAATTTTGAAATTTTTAACATTGTTCTAAATTAGCTCATTTCTTTGTAGGCTATAGCATTGGTCATTTTTTGT
>Marker894288 Chr7 SNP_site:9431790 Ref_Type:C
AACCTAAACGTCAAGTTTAATAAATACCTACCAAAAAAGTTTATTAAAAAACAAAAAAATGAAACCCTTCAAAGACAAGTXXXXXXXXXXA
GATGTGAAATTTTGAAATTTTTAACATTGTTCTAAATTAGCTCATTTCTTTGTAAGTTATAGCATTGGTCATTTTTTGT
AACCTAAACGTCAAGTTTAATAAATACCTACCAAAAAAGTTTATTAAAAAACAAAAAAATGAAACCCTTCAAAGACATGTXXXXXXXXXXA
GATGTGAAATTTTGAAATTTTTAACATTGTTCTAAATTAGCTCATTTCTTTGTAGGCTATAGCATTGGTCATTTTTTGT
>Marker89444 Chr4 SNP_site:3719407 Ref_Type:T
TACATCCGGATAGACACCCTCTCCAAAAACGTTCTTACAAAGATCCAGATGATATCTGTCATTAGGCAAGCTATTATGAAXXXXXXXXXXC
CGGAAGAATACTTTGCATCCACTTTAGATGAACGCATGCTATCATTTGCTGGTGCAACCTGGAAATATGCAACTTCAGT
TACATCCGGATAGACACCCTCTCCAAAAACGTTCTTACAAAGATCCAGATGATATCTGTCATTAGGCAAGCTATTATGAAXXXXXXXXXXC
TGGAAGAATACTTTGCATCCACTTTAGATGAACGCATGCTATCATTTGCTGGTGCAACCTGGAAATATGCAACTTCAGT
>Marker89445 Chr4 SNP_site:9698338 Ref_Type:G
TACCCCCACCGTTGTGCAGACAGACCTTCGACAACCCAAAGGACTGAGAATGGTCTCGGCCATGCAACTGAGAGAGGGCCXXXXXXXXXXC
AGGATTCACTAGACCTGTAAGAGATCCGTATCAAGCACAAGACTTCTTACGGAAAGAAGAGAATGGACGGTTGTGTGTC
TACCCCCGCCGTTGTGCAGACAGACCTTCGACAACCCAAAGGACTGAGAATGGTCTCGGCCATGCAACTGAGAGAGGGCCXXXXXXXXXXC
AGGATTCACTAGACCTGTAAGAGATCCGTATCAAGCACAAGACTTCTTACGGAAAGAAGAGAATGGACGATTGTGTGTC
>Marker89445 Chr4 SNP_site:9698634 Ref_Type:A
TACCCCCACCGTTGTGCAGACAGACCTTCGACAACCCAAAGGACTGAGAATGGTCTCGGCCATGCAACTGAGAGAGGGCCXXXXXXXXXXC
AGGATTCACTAGACCTGTAAGAGATCCGTATCAAGCACAAGACTTCTTACGGAAAGAAGAGAATGGACGGTTGTGTGTC
TACCCCCGCCGTTGTGCAGACAGACCTTCGACAACCCAAAGGACTGAGAATGGTCTCGGCCATGCAACTGAGAGAGGGCCXXXXXXXXXXC
AGGATTCACTAGACCTGTAAGAGATCCGTATCAAGCACAAGACTTCTTACGGAAAGAAGAGAATGGACGATTGTGTGTC
>Marker894760 Chr7 SNP_site:14221045 Ref_Type:A
GACTTGGAAGAGACCAAGGAGAGGGTAAAGATGTTAGAGGAATCTTGTCAATCTCTGTTGGGAGAGAAATCAACTCTTTCXXXXXXXXXXA
TTTTCTGATGCAATTGCTGAACTTGAAGCATTGAAGTTGAAATCAAAGGATCTAGAAGATTCCTGCCAATTGCTGGGTC
GACTTGGAAGAGACCAAGGAGAGGGTAAAGATGTTAGAGGAATCTTGTCAATCTCTGTTGGGAGAGAAATCAACTCTTTCXXXXXXXXXXA
TTTTCTGATGCAATTGCTGAACTTGAAGCATTGAAGTTGAAATCAAAGGATCTGGAAGATTCCTGCCAATTGCTGGGTC
>Marker894969 Chr7 SNP_site:15334710 Ref_Type:G
AACAAATTATTATTTCCAAATGCATCTGCAGCGAGGACACAACACTTCCAGAAATTTGTTCAACTCATGAAAACTTGAGAXXXXXXXXXXG
TGGGTGGAGTGACTATTATAGCCCATCCCATGTTTGGGGCTCCAATTATAATAGTTAATATTTTTAACCATTATAATGT
AACAAATTATTGTTTCCAAATGCATCTGCAGCGAGGACACAACACTTCCAGAAATCTGTTCAACTCATGAAAACTTGAGAXXXXXXXXXXG
TGGGTGGAGTGACTATTATAGCCCATCCCATGTTTGGGGCTCCAATTATAATAGTTAATATTTTTAACCATTATAATGT
>Marker894969 Chr7 SNP_site:15334754 Ref_Type:C
AACAAATTATTATTTCCAAATGCATCTGCAGCGAGGACACAACACTTCCAGAAATTTGTTCAACTCATGAAAACTTGAGAXXXXXXXXXXG
TGGGTGGAGTGACTATTATAGCCCATCCCATGTTTGGGGCTCCAATTATAATAGTTAATATTTTTAACCATTATAATGT
AACAAATTATTGTTTCCAAATGCATCTGCAGCGAGGACACAACACTTCCAGAAATCTGTTCAACTCATGAAAACTTGAGAXXXXXXXXXXG
TGGGTGGAGTGACTATTATAGCCCATCCCATGTTTGGGGCTCCAATTATAATAGTTAATATTTTTAACCATTATAATGT
>Marker895118 Chr7 SNP_site:12224316 Ref_Type:C
CACGCTTCTCATCTGTCGGCTCAATCACTTGTATATGAACCTGAATTTGGAACATCAACCAACAAGTATGAGTTATTAAAXXXXXXXXXXA
TCCCTATTTGATATGCAAATAATTGACATTGATTATCTAAAGCATAGAACAAGTAATTTTCAAGCTAAGCTTTCCAGTA
CACGCTTCTCATCTGTCGGCTCAATCACTTGTATATGAACTTGAATTTGGAACATCAACCAACAAGTATGAGTTATTAAAXXXXXXXXXXA
TCCCTATTTGATATGCAAATAATTGACATTGATTATCTAAAGCATAGAACAAGTAATTTTCAAGCTAAGCTTTCCAGTA
>Marker895380 Chr7 SNP_site:4263065 Ref_Type:A
GACTGACCATTGACCTTTCGGAGATTAGAGATTCAAGGATTCTCTCACCACTTATGAAATTATTTCCTGGTTGTCTTCAAXXXXXXXXXXA
TCCATGTATAATTGAATTATTAGAATTACTACTATAAGTCTATAAGAAAGGAAAACTGACGTGGGTCTGTATCTCTGGT

Create PDF with GO2PDF for free, if you wish to

GACTGACCATTGGCCTTTCGGAGATTAGAGATTCAAGGATTCTCTCACCACTTATGAAATTATTTCCTGGTTGTCTTCAAXXXXXXXXXXA

TCCATGTATAATTGAATTATTAGAATTACTACTATAAGTCTATAAGAAAGGAAAACTGACGTGGGTCTGTATCTCTGGT

>Marker89573 Chr4 SNP_site:1184522 Ref_Type:T

TACAAATTCTAGATAAGTAGTGTAAATTAAAATCCTTACCAAGTAATCTAAACCCAAGGAAAAGAGGAACTCACAGCTCGXXXXXXXXXXA

TCCATTTCAATGCAAATGATGAACACAGAGAAGCCACCATTTGAGCTCCAATATACAATGGCACCTGCAAACATATGTA

TACAAATTCTAGATAAGTAGTGTAAATTAAAATCCTTACTAAGTAATCTAAACCCAAGGAAAAGAGGAACTCACAGCTCGXXXXXXXXXXG

TCCATTTCAATGCAAATGATGAACACAGAGAAGCCACCATTTGAGCTCCAATATACAATGGCACCTGCAAACATATGTA

>Marker89573 Chr4 SNP_site:1184687 Ref_Type:G

TACAAATTCTAGATAAGTAGTGTAAATTAAAATCCTTACCAAGTAATCTAAACCCAAGGAAAAGAGGAACTCACAGCTCGXXXXXXXXXXA

TCCATTTCAATGCAAATGATGAACACAGAGAAGCCACCATTTGAGCTCCAATATACAATGGCACCTGCAAACATATGTA

TACAAATTCTAGATAAGTAGTGTAAATTAAAATCCTTACTAAGTAATCTAAACCCAAGGAAAAGAGGAACTCACAGCTCGXXXXXXXXXXG

TCCATTTCAATGCAAATGATGAACACAGAGAAGCCACCATTTGAGCTCCAATATACAATGGCACCTGCAAACATATGTA

>Marker895773 Chr7 SNP_site:17068439 Ref_Type:C

TACTCTTTCATCCTTTGGATCCATTAACTGGGGAAGATTTTAGTGTTCATCACTTTTTTTCAATTTATATTATTACCAAAXXXXXXXXXXT

GATTTTGCTATTTACTTACAATTTTGAAATCGAAGTAGCCCTTTCTTTTAGGTTATCCATAGAATAGGTGGCTGTTGTA

TACTCTTTCATCCTTTGGATCCATTAATTGGGGAAGATTTTGGTGTTCATCACTTTTTTTCAATTTATATTATTACCAAAXXXXXXXXXXT

GATTTTGCTATTTACTTACAATTTTGAAATCGAAGTAGCCCTTTCTTTTAGGTTATCCATAGAATAGGTGGCTGTTGTA

>Marker895773 Chr7 SNP_site:17068453 Ref_Type:A

TACTCTTTCATCCTTTGGATCCATTAACTGGGGAAGATTTTAGTGTTCATCACTTTTTTTCAATTTATATTATTACCAAAXXXXXXXXXXT

GATTTTGCTATTTACTTACAATTTTGAAATCGAAGTAGCCCTTTCTTTTAGGTTATCCATAGAATAGGTGGCTGTTGTA

TACTCTTTCATCCTTTGGATCCATTAATTGGGGAAGATTTTGGTGTTCATCACTTTTTTTCAATTTATATTATTACCAAAXXXXXXXXXXT

GATTTTGCTATTTACTTACAATTTTGAAATCGAAGTAGCCCTTTCTTTTAGGTTATCCATAGAATAGGTGGCTGTTGTA

>Marker895918 Chr7 SNP_site:13882629 Ref_Type:C

CACCTGCACAAATTGGCTCTAAATTTTGATTGCAGAAGCATTCGTATAAGGAAACTGGACGACATGATAGTAATCTTCATXXXXXXXXXXC

TGTAGATTATAAGGTAAAGGAAATCAAAGATCAACTGATAAGAGCAAAAGCCTACCTAAGTTTTGCCCCACCAGGTAGT

CACCTGCACAAATTGGCTCTAAATTTTGATTGCAGAAGCATTTGTATAAGGAAACTGGACGACATGATAGTAATCTTCATXXXXXXXXXXC

TGTAGATTATAAGGTAAAGGAAATCAAAGATCAACTGATAAGAGCAAAAGCCTACCTAAGTTTTGCCCCACCAGGTAGT

>Marker895974 Chr7 SNP_site:3123567 Ref_Type:G

CACTAGGCTACTAGGCGGCGCAATTGATACACTGTTGTTGAAATTGGAAGAAGCTGATCCTCTTCGCTCTGTTCGTTCGAXXXXXXXXXXT

TTGGCTGGAAATCTTCAAGGCAAACGGCGCATCTCTTGCCTTCTTCATCACCATTGCTGTTGTGCCTCTCATTGGAGTT

CACTAGGCTACTAGGCGGCGCAATTGATACACTGTTGTTGAAATTGTAAGAAGCTGATCCTCTTCGCTCTGTTCGTTCGAXXXXXXXXXXT

TTGGCTGGAAATCTTCAAGGCAAACGGCGCATCTCTTGCCTTCTTCATCACCATTGCTGTTGTGCCTCTCATTGGAGTT

>Marker895980 Chr7 SNP_site:2341786 Ref_Type:G

ACCCTCTCATCAATGATAAAATTGATAATATCTATGTAGGAAGGAGAGCAGAATTAGTCTCTCCCATAAATTTGTGGGATXXXXXXXXXXT

AAATGAAAGTAACTATCTATACCAACAAAGACTCTTTCCGTTTTTGGTGGTTCAATTATAAGAATTTCAAAATTAAGTG

ACCCTCTCATCAATGATAAAATTGATAATATCTATGTAGGAAGGAGAGCAGAATTAGTCTCTCCCATAAATTTGTGGGATXXXXXXXXXXT

AAATGAAAGTAACTATCTATACCAACAAAGACTCTTTCCTTTTTTGGTGGTTCAATTATAAGAATTTCAAAATTAAGTG

>Marker896082 Chr7 SNP_site:581598 Ref_Type:T

CACCAATTTTGTTTCCATCTATTTCATTGCTTTTCCAAAGAAACCTAAGAATCAACTGATCCTTTGCAATTTGAAAAGACXXXXXXXXXXA

TTCGTGTAGATTGTGCGTATCAGTAAGGTAAAGATTTGGTTAAAGTGTTTATTTAACGCATTTGCAATCCATTTGAGTT

CACCAATTTTGTTTCCATCTATTTCATTGCTTTTCCAAAGAAACCTAAGAATCAACTGATTCTTTGAAATTTGAAAAGACXXXXXXXXXXA

TTCGTGTAGATTGTGTGTATCAGTAAGGTAAAGATTTGGTTAAAGTGTTTATTTAACGCATTTGCAATCCATTTGAGTT

>Marker896082 Chr7 SNP_site:581604 Ref_Type:A

CACCAATTTTGTTTCCATCTATTTCATTGCTTTTCCAAAGAAACCTAAGAATCAACTGATCCTTTGCAATTTGAAAAGACXXXXXXXXXXA

Create PDF with GO2PDF for free, if you wish to

TTCGTGTAGATTGTGCGTATCAGTAAGGTAAAGATTTGGTTAAAGTGTTTATTTAACGCATTTGCAATCCATTTGAGTT
CACCAATTTTGTTTCCATCTATTTCATTGCTTTTCCAAAGAAACCTAAGAATCAACTGATTCTTTGAAATTTGAAAAGACXXXXXXXXXXA
TTCGTGTAGATTGTGTGTATCAGTAAGGTAAAGATTTGGTTAAAGTGTTTATTTAACGCATTTGCAATCCATTTGAGTT
>Marker896082 Chr7 SNP_site:581757 Ref_Type:T
CACCAATTTTGTTTCCATCTATTTCATTGCTTTTCCAAAGAAACCTAAGAATCAACTGATCCTTTGCAATTTGAAAAGACXXXXXXXXXXA
TTCGTGTAGATTGTGCGTATCAGTAAGGTAAAGATTTGGTTAAAGTGTTTATTTAACGCATTTGCAATCCATTTGAGTT
CACCAATTTTGTTTCCATCTATTTCATTGCTTTTCCAAAGAAACCTAAGAATCAACTGATTCTTTGAAATTTGAAAAGACXXXXXXXXXXA
TTCGTGTAGATTGTGTGTATCAGTAAGGTAAAGATTTGGTTAAAGTGTTTATTTAACGCATTTGCAATCCATTTGAGTT
>Marker896565 Chr7 SNP_site:14809364 Ref_Type:T
AACTTCATCTGTCCTGGACGTGCTTCACACTCTTCATGGTTAGAACAAATTTCAACACTTGCAGAATTGGCCTGCACTTCXXXXXXXXXXA
ATGAGTTGTGCAATTTAAAATAATAGTCAGAATTGCAGCCAGATGGTGTTTTTAATTCTCATGCGCCTCTTTTTTAGTC
AACTTCATCTGTCCTGGACGTGCTTCACACTCTTCATGGTTAGAACAAATTTCAACACTTGCAGAATTGGCCTGCACTTCXXXXXXXXXXA
ATGAGTTGTTCAATTTAAAATAATAGTCAGAATTGCAGCCAGATGGTGTTTTTAATTCTCATGCGCCTCTTTTTTAGTC
>Marker896955 Chr7 SNP_site:13412975 Ref_Type:T
ACAACAATTTAAACATTAGGGTCTCTTTCTCTTATATTTTTTCTCAATTCAATTCAATGTCAAATGTTTGTTTTCTTCTTXXXXXXXXXXT
ATGTTCCTCAAACAAAATTTTAGCCTCATACATCCAATGCTTGATGAAGCAAACAGAAGCACACTATATTCAGTTAGTT
ACAACAATTTAAACATTAGGGTCTCTTTCTCTTATATTTTTTCTCAATTCAATTCAATGTCAAATGTTTGTTTTCTTCTTXXXXXXXXXXT
ATGTTCCTCAAACAAAATTTTAGCCTCATACATTCAATGCTTGATGAAGCAAAGAGAAGCACACTATATTCAGTTAGTT
>Marker896955 Chr7 SNP_site:13412995 Ref_Type:G
ACAACAATTTAAACATTAGGGTCTCTTTCTCTTATATTTTTTCTCAATTCAATTCAATGTCAAATGTTTGTTTTCTTCTTXXXXXXXXXXT
ATGTTCCTCAAACAAAATTTTAGCCTCATACATCCAATGCTTGATGAAGCAAACAGAAGCACACTATATTCAGTTAGTT
ACAACAATTTAAACATTAGGGTCTCTTTCTCTTATATTTTTTCTCAATTCAATTCAATGTCAAATGTTTGTTTTCTTCTTXXXXXXXXXXT
ATGTTCCTCAAACAAAATTTTAGCCTCATACATTCAATGCTTGATGAAGCAAAGAGAAGCACACTATATTCAGTTAGTT
>Marker896999 Chr7 SNP_site:6296834 Ref_Type:G
ACCCGCCACAATAGTAACCTCCATCCAATCCAACCATGATAGCATACTAAATACACGGTCCGGAACAGCTCACATGTGATXXXXXXXXXXA
TCCATAAGGAAACACGCGGCCAAATGAAGCTCACGCATACTTAGCACCTACCACAGTAACATCCATCCATTCCTATGTG
ACCCGCCACGATAGTAACCTCCATCCAATCCAACCATGATAGCATACTAAATACACGGTCCGGAACAGCTCACATGTGATXXXXXXXXXXA
TCCATAAGGAAACACGCGGCCAAATGAAGCTCACGCATACTTAGCACCTACCACAGTAACATCCATCCATTCCTATGTG
>Marker897104 Chr7 SNP_site:9098850 Ref_Type:A
ACTCTTCTTTGTGCATAATCCTATTTGTAACGACAACATATATTTCAATAATATACACAAAATTAACAGATTCATTAGTTXXXXXXXXXXT
TGAACCCCCATTTTATTAAACCAATAACCATGTATGTAAAAAGTTTCTTTAGTTTTTGAAGTTTGTAAGGAACTTTAGT
ACTCTTCTTTGTGCATAATCCTATTTGTAACGACAACATATATTTCAATAATATACACAAAATTAACAGATTCATTAGTTXXXXXXXXXXT
TGAACCCCCATTTTATTAAACCAATAACCATGTATGTAAAAAGTTTCTTTAGTTTTTGAAGTTTGTAAGGAACTTTGGT
>Marker897156 Chr7 SNP_site:7650943 Ref_Type:C
TACTAAAGCATAGTTACATTTCTATGTGGAGATAGCTTTTTAGCATGATCAGAAATAAAAATTTCACTAGGATATATTAGXXXXXXXXXXG
CACGGGAGCCTGTATAAAGAAGTTAATACATCAATTCATCTGATACATGATATTCATTTGAAGAATGGAAACCACTGTT
TACTAAAGCATAGTTACATTTCTATGTGGAGATAGCTTTTTAGCATGATCAGAAATAAAAATTTCACTCGGATATATTAGXXXXXXXXXXG
CACGGGAGCCTGTATAAAGAAGTTAATACATCAATTCATCTGATACATGATATTCATTTGAAGAATGGAAACCACTGTT
>Marker897217 Chr7 SNP_site:13897756 Ref_Type:C
CACTTCATTTTGGGTGTGATGCAAAATTGACGAGAATTCCATTTGTTTTAAGAAGTTTATTATTTTCACTTGTTAAGTTGXXXXXXXXXXA
TATATGCTTCATACTTTAATATAGTGAAGATTACTTCTTGTGGGTGGAAATGTTGCCCAACTTTTATATGAACACTGTT
TACTTCATTTTGGGTGTGATGCAAAATTGACGAGAATTCCATTTGTTTTAAGAAGTTTATTATTTTCACTTGTTAAGTTGXXXXXXXXXXA
TATATGCTTCATACTTTAATATAGTGAAGATTACTTCTTGTGGGTGGAAATGTTGCCCAACTTTTATATGAACACTGTT
>Marker897719 Chr7 SNP_site:9829104 Ref_Type:T

Create PDF with GO2PDF for free, if you wish to

ACCATTTTAACCTCTCTAGTGGTTCCTTCTTAAGCCTTTTTACGTTGAGAAATTGACAAAACTAGCACTTTTTTTTTTATXXXXXXXXXXC
CCAACTGGGTATTTCGAACGCTACATATTCTTCAAAAACATTAAACTCCAAACACTTTGCTCATTTAGGACGAGAAGTG
ACCATTTTAACCTCTCTAGTGGTTCCTTCTTAAGCCTTTTTACGTTGAGAAATTGACAAAACTAGCACTTTTTTTTTTATXXXXXXXXXXT
CCAACTTGGTATTTCGAACGCTACATATTCTTCAAAAACATTAAACTCCAAACACTTTGCTCATTTAGGACGAGAAGTG
>Marker897719 Chr7 SNP_site:9829111 Ref_Type:T
ACCATTTTAACCTCTCTAGTGGTTCCTTCTTAAGCCTTTTTACGTTGAGAAATTGACAAAACTAGCACTTTTTTTTTTATXXXXXXXXXXC
CCAACTGGGTATTTCGAACGCTACATATTCTTCAAAAACATTAAACTCCAAACACTTTGCTCATTTAGGACGAGAAGTG
ACCATTTTAACCTCTCTAGTGGTTCCTTCTTAAGCCTTTTTACGTTGAGAAATTGACAAAACTAGCACTTTTTTTTTTATXXXXXXXXXXT
CCAACTTGGTATTTCGAACGCTACATATTCTTCAAAAACATTAAACTCCAAACACTTTGCTCATTTAGGACGAGAAGTG
>Marker898042 Chr7 SNP_site:12647569 Ref_Type:T
CACCTTCCACATCACGTCCATTCCTTTACTCTCATATACAAATCGAATCCTACACCTATCAATATCCCTCAACCCAAGAAXXXXXXXXXXT
CACTCTCCAACTCGAACCAAAACTTCTGGTTCCTCTCATATACTAACTGAATCAGGACTTCATTAGAACATCTAAAAGT
CACCTTCCACATCACGTCCATTTCTTTACTCTCATATACAAATCGAATCCTACACCTATCAATATCCCTCAACCCAAGAAXXXXXXXXXXT
CACTCTCCAACTCGAACCAAAACTTCTGGTTCCTCTCATATACTAACTGAATCAGGACTTCATTAGAACATCTAAAAGT
>Marker898220 Chr7 SNP_site:5485982 Ref_Type:G
CACGAACCTTGCTGCAATCGTCATCGAACACTCGCTGCTGTTGCTGCATGCTCGCTTCTTCAATTGCCTCCCTCTCTAGTXXXXXXXXXXC
TCATTGTGGTTAATGGGTCCAATCAGAGGGTTGATCGAGAAAGAAAAAGAAATTTGAGAGAAAGCTCGATTCGAATGGT
CACGAACCTTGCTGCAATCGTCATCGAACACTCGCTGCTGTTGCTGCATGCTCGCTTCTTCAATTGCCTCCCTCTCTAGTXXXXXXXXXXC
TCATTGTGGTTAATGGGTCCAATCAGAGGGTTGATCGAGAAAGAAGAAGAAATTTGAGAGAAAGCTCGATTCGAATGGT
>Marker898503 Chr7 SNP_site:553782 Ref_Type:A
GACGTAAGTATATGTAACCACTAGAGCCTTGCAATTAAGAAAATAAATGCAACCACAAAGGTAAGAAACTTAACCTTCACXXXXXXXXXXT
CTTGCCAGCTTACCTATCTCAATTTATCTTTTACCAAGGATCAAATAGACATAAACATCGTAAATTAAACTAGTAGGTT
GACGTAAGTATATGTAACCACTAGAGCCTTGCAATTAAGAAAATAAATGCAACCACAAAGGTAAGAAACTTAACCTTCACXXXXXXXXXXT
CTTGCCAGCTTACCTATCTCAATTTATCTTTTACCAAGGATCAAATAGACATAAACATCGTAAATTAAACTGGTAGGTT
>Marker898509 Chr7 SNP_site:3881774 Ref_Type:A
CACCATCAACCCATCGTGGATTGCGAAAACCTGGCTCAGCTAGCCTACCTTGTCCTTTATATCTAGCCACCTAGGATAAAXXXXXXXXXXA
GGAAGCTGGTATCTTTTTCCGACCTTTGCCCGAAATGCCACCTGTAAAACCATGACAATCATCAATACTACTCCACAGT
CACCATCAACCCATCGTGGATTGCGAAAACCTGGCTCAGCTAGCCTACCTTGTCCTTTATATCTAGCCACCTGGGATAAAXXXXXXXXXXA
GGAAGCTGGTATCTTTTTCCGACCTTTGCCCGAAATGCCACCTGTAAAACCATGACAATCATCAATACTACTCCACAGT
>Marker898576 Chr7 SNP_site:3853141 Ref_Type:T
CACAATTTGAACAAATTACGAAAAAATCTAAATTGTAAAATAAAACGAACTTCAAGATTGCATATCATAAAGAAGGATTGXXXXXXXXXXA
AGTAATGCACAATTTACGAAGATCATTGACACCCAATTGAAAGCCAAACCTTGTCCGGCGATACAGAATCAAAAGCGTA
CACAATTTGAACAAATTACGAAAAAATCTAAATTGTAAAATAAAACGAACTTCAAGATTGCATATCATAAAGAAGGATTGXXXXXXXXXXA
AGTAATGCACAATTTACGAAGATCATTGACACTCAATTGAAAGCCAAACCTTGTCCGGCGATACAGAATCAAAAGCGTA
>Marker89858 Chr4 SNP_site:18622913 Ref_Type:G
CACAACGGCAAGCTCCTCCCCAAAAATCCAAAAAGACCAAACCACAAAGTCTAAATTGCCTGCTTCCTCCTACCAAATCCXXXXXXXXXXT
CTAACTCTTGTCTCCAAATCAACCAACCAAACCAAACCATTGAGCCATGAGGGATCCAATAAGAAGATTTTCCAAGAGT
CACGACGGCAAGCTCCTCCCCAAAAATCCAAAAAGACCAAACCACAAAGTCTAAATTGCCTGCTTCCTCCTACCAAATCCXXXXXXXXXXT
CTAACTCTTGTCTCCAAATCAACCAACCAAACCAAACCATTGAGCCATGAGGGATCCAATAAGAAGATTTTCCAAGAGT
>Marker898582 Chr7 SNP_site:8210742 Ref_Type:C
CACAACCTTTGCTTCCCCTCCTACTATACCTTTTACCATTTAATTTCTTCCATTCCTAATCAAAAGGGATCTCCTTTCCTXXXXXXXXXXT
CTGGAACAATAATGAATATAGCACATTTAGTCACCTGACACCTATTAATCCGTAAATACAATGGAGTGTAACAGAAGTC
CACAACCTTTGCTTCCCCTCCTACTATACCTTTTACCATTTAATTTCTTCCATTCCTAATCAAAAGGGATCTCCTTTCCTXXXXXXXXXXT
CTGGAACAATAATGAATATAGCACATTTAGTCACCTGACACTTATTAATCCGTAAATACAATGGAGTGTAACAGAAGTC

Create PDF with GO2PDF for free, if you wish to

>Marker89860 Chr4 SNP_site:7520011 Ref_Type:T

ACTTTACTACTACTAAGATTGTAAAAATAAAGTATATTGTTTGAGTTTGAGTTTCATAGATTCTATCTACATGGTCATGAXXXXXXXXXXXG

AATCAGCTTCAATTGTTAGAAGTTTAGCACTCTGTGTTATGAAAAACAATCAAAGAAGTTGGGTGTTCAAAGTAAGAGT

ACTTTACTACTACTAAGATTGTAAAAATAAAGTATATTGTTTGAGTTTGAGTTTCATAGATTCTATCTACATGGTCATGAXXXXXXXXXXXT

AATCAGCTTCAATTGTTAGAAGTTTAGCACTCTGTGTTATGAAAAACAATCAAAGAAGTTGGGTGTTCAAAGTAAGAGT

>Marker89886 Chr4 SNP_site:16993306 Ref_Type:T

AACACTTTGACTATTTCCATCAATTTCGAGCTTTTGAATATGAATCCATATTCATATTTTTAATATTTAAACCACATTTAXXXXXXXXXXXA

TGGAGCTATAGATCATGAGCTTCAATGATTCAAAATTAACTGACTAAACTTTTTTAGACTAAGCAAATCAACATTCGTT

AACATTTTGACTATTTCCATCAATTTCGAGCTTTTGAATATGAATCCATATTCATATTTTTAATATTTAAACCACATTTAXXXXXXXXXXXA

TGGAGCTATAGATCATGAGCTTCAATAATTCAAAATTAACTGACTAAACTTTTTTAAACTAAGCAAATCAACATTCGTT

>Marker89886 Chr4 SNP_site:16993525 Ref_Type:A

AACACTTTGACTATTTCCATCAATTTCGAGCTTTTGAATATGAATCCATATTCATATTTTTAATATTTAAACCACATTTAXXXXXXXXXXXA

TGGAGCTATAGATCATGAGCTTCAATGATTCAAAATTAACTGACTAAACTTTTTTAGACTAAGCAAATCAACATTCGTT

AACATTTTGACTATTTCCATCAATTTCGAGCTTTTGAATATGAATCCATATTCATATTTTTAATATTTAAACCACATTTAXXXXXXXXXXXA

TGGAGCTATAGATCATGAGCTTCAATAATTCAAAATTAACTGACTAAACTTTTTTAAACTAAGCAAATCAACATTCGTT

>Marker89886 Chr4 SNP_site:16993555 Ref_Type:G

AACACTTTGACTATTTCCATCAATTTCGAGCTTTTGAATATGAATCCATATTCATATTTTTAATATTTAAACCACATTTAXXXXXXXXXXXA

TGGAGCTATAGATCATGAGCTTCAATGATTCAAAATTAACTGACTAAACTTTTTTAGACTAAGCAAATCAACATTCGTT

AACATTTTGACTATTTCCATCAATTTCGAGCTTTTGAATATGAATCCATATTCATATTTTTAATATTTAAACCACATTTAXXXXXXXXXXXA

TGGAGCTATAGATCATGAGCTTCAATAATTCAAAATTAACTGACTAAACTTTTTTAAACTAAGCAAATCAACATTCGTT

>Marker899077 Chr7 SNP_site:7256763 Ref_Type:C

AACGACGTATTGCGAGATTGTAGTTTTAGTTGAAGAAGTTAGTTTTTCGACTTGATTCCTCTAGTGTTAGATGAATTGGAXXXXXXXXXXXT

ATCCAGTGGTTAAAGGAAGATTATCACTACATGCTTAATTTCAATGTTAAAGGATGAGAAAATGTTGAAAAAGGATTGT

AACGACGTATTGCGAGATTGTAGTTTTAGTTGAAGAAGTTAGTTTTTCGACTTGATTCCTCTAGTGTTAGATGAATTGGAXXXXXXXXXXXT

ATCTAGTGGTTAAAGGAAGATTATCACTAGATGCTTAATTTCAATGTTAAAGGATGAGAAAATGTTGAAAAAGGATTGT

>Marker899077 Chr7 SNP_site:7256789 Ref_Type:C

AACGACGTATTGCGAGATTGTAGTTTTAGTTGAAGAAGTTAGTTTTTCGACTTGATTCCTCTAGTGTTAGATGAATTGGAXXXXXXXXXXXT

ATCCAGTGGTTAAAGGAAGATTATCACTACATGCTTAATTTCAATGTTAAAGGATGAGAAAATGTTGAAAAAGGATTGT

AACGACGTATTGCGAGATTGTAGTTTTAGTTGAAGAAGTTAGTTTTTCGACTTGATTCCTCTAGTGTTAGATGAATTGGAXXXXXXXXXXXT

ATCTAGTGGTTAAAGGAAGATTATCACTAGATGCTTAATTTCAATGTTAAAGGATGAGAAAATGTTGAAAAAGGATTGT

>Marker899128 Chr7 SNP_site:5254100 Ref_Type:A

ACTTTAAAATGCTACTGATAAAATTATACTTCAGGACAAAAACAAGTGGAAAAATGTGGCAAGACTAATTTTGATGCAAAXXXXXXXXXXXA

TTCAAAAAACTTTTCATATTTATTTTGCATAAAATAGTATAGATTTAAAAAACAAAACTTAGAAATAATAACTGATAGT

ACTTTAAAATGCTACTGATAAAATTATACTTCAGGACAAAAACAAGTGGAAAAATGTGGCAAGACTAATTTTGATGCAAAXXXXXXXXXXXA

TTCAAAAAACTTTTCATATTTATTTTGCATAAAATAGTATAGATTTAAAAAACAACACTTAGAAATAATAACTGATAGT

>Marker900174 Chr7 SNP_site:16246536 Ref_Type:C

AACAAAATGTGCGTGCATTAGTGCATTTTTAGTGATAAAGACTTGGTTGATTTCGTTGTTATTTAAATTAATATATTTGTXXXXXXXXXXXA

GTTCAAGTTTAGATGTTTGATTCTCCACATCATGCTTTAATACAACATTTTTGTTAAAAAGATTAAGCCTTTGATTGTG

AACAAAATGTGCGTGCATTAGTGCATTTTTAGTGATAAAGACTTGGTTGATTTTGTTGTTATTTAAATTAATATATTTGTXXXXXXXXXXXA

GTTCAAGTTTAGATGTTTGATTCTCCACATCATGCTTTAATACAATATTTTTGTTAAAAAGATTAAGCCTTTGATTGTG

>Marker900174 Chr7 SNP_site:16246723 Ref_Type:C

AACAAAATGTGCGTGCATTAGTGCATTTTTAGTGATAAAGACTTGGTTGATTTCGTTGTTATTTAAATTAATATATTTGTXXXXXXXXXXXA

GTTCAAGTTTAGATGTTTGATTCTCCACATCATGCTTTAATACAACATTTTTGTTAAAAAGATTAAGCCTTTGATTGTG

AACAAAATGTGCGTGCATTAGTGCATTTTTAGTGATAAAGACTTGGTTGATTTTGTTGTTATTTAAATTAATATATTTGTXXXXXXXXXXXA

Create PDF with GO2PDF for free, if you wish to

GTTCAAGTTTAGATGTTTGATTCTCCACATCATGCTTTAATACAATATTTTTGTTAAAAAGATTAAGCCTTTGATTGTG
>Marker900427 Chr7 SNP_site:18054150 Ref_Type:C
TACCTAGGGCCTTATGAGACAATGGAGCAGTCTAAGCATACGAGGAGTTTCTGAATGTAGGATATGAATTAAACAATGGAXXXXXXXXXXXA
TGGAATATGATAGTTTCATTTTCAGATGCAAAATACCAACTTTTGTGTGAGTTAAAAGAATTGACTTGTTAAGAAAAGT
TACCTAGGGCCTTATGAGACAATGGAGCAGTCTAAGCATACGAGGAGTTTCTGAATGTAGGATATGAATTAAACAATGGAXXXXXXXXXXXA
TGGCATATGATAGTTTCATTTTCAGATGCAAAATACCAACTTTTGTGTGAGTTAAAAGAATTGACTTGTTAAGAAAAGT
>Marker900613 Chr7 SNP_site:16232267 Ref_Type:T
AACAACTCCAAGCAATGAAAGTCAGTGACTATTCCTAAGACACATTGAGTATAATGCCATTTAATTAAACCTAAACCCTAXXXXXXXXXXXT
CCTTTATTTTCTACTCTTCAGGAATCAAGATTGTATGCATTTCTTTTCTATTATTTTAGCTCTTAACGCTTTTGATAGT
AACAACTCTAAGCAATGAAAGTCAGTGACTATTCCTAAGACACATTGAGTATAATGCCATTTAATTAAACCTAAACCCTAXXXXXXXXXXXT
CCTTTATTTTCTACTCTTCAGGAATCAAGATTGTATGCATTTCTTTTCTATTATTTTAGCTCTTAACGCTTTTGATAGT
>Marker900998 Chr7 SNP_site:7547669 Ref_Type:T
TACATTTAAAGTAATTATATTTCTTTAGAATCTTTTCTACATAGTGAGATTGATACAAAGAAATTTCCTTCTCATACCCTXXXXXXXXXXXA
AACACAATATTGTTTAAAGATTGTATTTTCGAACTTATGGTAGATGCATTTGTCACTTCCATTAACTTTGAAACATGTA
TACATTTAAAGTAATTATATTTTTTTAGAATCTTTTCTACATAGTGAGATTGATACAAAGAAATTTCCTTCTCATACCCTXXXXXXXXXXXA
AACACAATATTGTGTAAAGATTGTATTTTCGAACTTATGGTAGATGCATTTGTCACTTCCATTAACTTTGAAACATGTA
>Marker900998 Chr7 SNP_site:7547831 Ref_Type:G
TACATTTAAAGTAATTATATTTCTTTAGAATCTTTTCTACATAGTGAGATTGATACAAAGAAATTTCCTTCTCATACCCTXXXXXXXXXXXA
AACACAATATTGTTTAAAGATTGTATTTTCGAACTTATGGTAGATGCATTTGTCACTTCCATTAACTTTGAAACATGTA
TACATTTAAAGTAATTATATTTTTTTAGAATCTTTTCTACATAGTGAGATTGATACAAAGAAATTTCCTTCTCATACCCTXXXXXXXXXXXA
AACACAATATTGTGTAAAGATTGTATTTTCGAACTTATGGTAGATGCATTTGTCACTTCCATTAACTTTGAAACATGTA
>Marker901233 Chr7 SNP_site:15336187 Ref_Type:T
ACTTAACCTCAACATTCTCCTCCTTCATCGCACTCTCAATCCTCTCCTCTGTCTCCATTTCGTCGTGTGAAACCTCGTAGXXXXXXXXXXXA
TCACAAATGCGGCTCGGAACGGTCCAGTTTTATCAAACCCTGACGAGGATTTACCATAATCCCTCGGATCGAAACAGTA
ACTTAACCTCAACATTCTCCTCCTTCATCGCACTCTCAATCCTCTCCTCTGTTTCCATTTCGTCGTGTGAAACCTCGTAGXXXXXXXXXXXA
TCACAAATGCGGCTCGGAACGGTCCAGTTTTATCAAACCCTGACGAGGATTTACCATAATCCCTCGGATCGAAACAGTA
>Marker901270 Chr7 SNP_site:13982648 Ref_Type:T
CACACCGTTCCATGGTTTTCTATTCATCAAGAAGTAGATTCCAACACTTTCATATCTTTGGCAGGTAGTGGTTCAGCAACXXXXXXXXXXXC
AAAATCTTATTATTTGGTGTCCTATCACATTATTCACAGGGAGAAAGCCCGGCAGTGGTGCTGTTGATAACACAGGGTC
CACACCGTTCCATGGTTTTCTATTCATCAAGAAGTAGATTCCAACACTTTCATATCTTTTGCAGGTAGTGGTTCAGCAACXXXXXXXXXXXC
AAAATCTTATTATTTGGTGTCCTATCACATTATTCACAGGGAGAAAGCCCGGCAGTGGTGCTGTTGATAACACAGGGTC
>Marker901522 Chr7 SNP_site:7634982 Ref_Type:T
GACTAATTACATAGATTAGGTTATTTAAGAGATGTTTTTCAAATGTTCAAAGACTTTTTTTGCTAATTGTTAGTTTTTTCXXXXXXXXXXXG
CTCATTCGTTTAATATGGCATTCTTAAATATAAGTTTAGGGTTTATAATGGAACACTTTTCTTTTATAGTAGTGTAGTC
GACTAATTACATAGATTAGGTTATTTAAGAGATGTTTTTCAAATGTTCAAAGACTTTTTTTGCTAATTGTTAGTTTTTTCXXXXXXXXXXXG
CTCATTCGTTTAATATGGCATTCTTAAATATAAGTTTAGGGTTTATAATTGAACACTTTTCTTTTATAGTAGTGTAGTC
>Marker901947 Chr7 SNP_site:15307108 Ref_Type:C
CACAATTTAATACTTTGATTCCAACTTCACTCCAACGATGTCTCTGCCTCCATCTTCATCTGTTTAAACAAACTCAATGTXXXXXXXXXXXC
TCTTGCTCTTGCTTCGGCTCCTGCTCCTGCTCCTGCTAGGGCTTTTGCTAGCATCACGCCTTTTGGGACTTTTACTAGT
CACAATTTAATACTTTGATTCCAACTTCACTCCAACGCTGTCTCTGCCTCCATCTTCATCTGTTTAAACAAACTCAATGTXXXXXXXXXXXC
TTTTGCTCTTGCTTCGGCTCCTGCTCCTGCTCCTGCTAGGGCTTTTGCTAGCATCACGCCTTTTGGGACTTTTACTAGT
>Marker901947 Chr7 SNP_site:15307274 Ref_Type:T
CACAATTTAATACTTTGATTCCAACTTCACTCCAACGATGTCTCTGCCTCCATCTTCATCTGTTTAAACAAACTCAATGTXXXXXXXXXXXC
TCTTGCTCTTGCTTCGGCTCCTGCTCCTGCTCCTGCTAGGGCTTTTGCTAGCATCACGCCTTTTGGGACTTTTACTAGT

Create PDF with GO2PDF for free, if you wish to

CACAATTTAATACTTTGATTCCAACTTCACTCCAACGCTGTCTCTGCCTCCATCTTCATCTGTTTAAACAAACTCAATGTXXXXXXXXXXC
TTTTGCTCTTGCTTCGGCTCCTGCTCCTGCTCCTGCTAGGGCTTTTGCTAGCATCACGCCTTTTGGGACTTTTACTAGT
>Marker902017 Chr7 SNP_site:9614458 Ref_Type:T
TACAATGAAACAGAACAACATGAACTATTCACAATATCAAACTGGCCCACATTAAGGTCGTAATAAAACAAAACAAAAGGXXXXXXXXXXT
TAGCAAGTCTAGCAAATCTAATATGATAAGAAGCTAAACATAACCTCCATCCTAGGCATTTTATCCTCAAATATTATGT
TACAATGAAACAGAACAACATGAACTATTCACAATATCAAACTTGCCCACATTAAGTTCGTAATAAAACAAAACAAAAGGXXXXXXXXXXT
TAGCAAGTCTAGCAAATCTAATATGATAAGAAGCTAAACATAACCTCCATCCTAGGCATTTTATCCTCAAATATTATGT
>Marker902017 Chr7 SNP_site:9614471 Ref_Type:T
TACAATGAAACAGAACAACATGAACTATTCACAATATCAAACTGGCCCACATTAAGGTCGTAATAAAACAAAACAAAAGGXXXXXXXXXXT
TAGCAAGTCTAGCAAATCTAATATGATAAGAAGCTAAACATAACCTCCATCCTAGGCATTTTATCCTCAAATATTATGT
TACAATGAAACAGAACAACATGAACTATTCACAATATCAAACTTGCCCACATTAAGTTCGTAATAAAACAAAACAAAAGGXXXXXXXXXXT
TAGCAAGTCTAGCAAATCTAATATGATAAGAAGCTAAACATAACCTCCATCCTAGGCATTTTATCCTCAAATATTATGT
>Marker902247 Chr7 SNP_site:102408 Ref_Type:T
TACCGTGAGGTTTATCCCTTCCATATCGAAAATTTCTTTTTTTGTTTGACTCTGCTTTCCCCTTTCTTCTTATGTATTTGXXXXXXXXXXG
GGGCACACTTCTAGGGTCAAAGAAATATATTTTATTCTTTGGAGAAGTGAAGGAAACCTTTCTCTTTGAATTTTGAGTA
TACCGTGAGGTTTATCCCTTCCATATCGAAAATTTCTTTTTTTGTTTGACTCTGTTTTCCCCTTTCTTCTTATGTATTTGXXXXXXXXXXG
GGGCACACTTCTAGGGTCAAAGAAATATATTTTATTCTTTGGAGAAGTGAAGGAAACCTTTCTCTTTGAATTTTGAGTA
>Marker902875 Chr7 SNP_site:6225831 Ref_Type:A
CACAACATACCTCACTATCGTCTATCCTTATTGGTTAATCCTGGTTAGATTTCATATGTTTCCTCCTTGAACTCTTACTTXXXXXXXXXXA
CATAAAAAGGTAATTTCATTTGTAGAGTATTCGTTTTTATAACCTAATGAAAATTCGTTTTTATGTCACGGCCAGAAGT
CACAACATACCTCACTATCGTCTATCCTTATTGGTTCATCCTGGTTAGATTTCATATGTTTCCTCCTTGAACTCTTACTTXXXXXXXXXXA
CATAAAAAGGTAATTTCATTTGTAAAGTATTCGTTTTTATAACCTAATGAAAATTCGTTTTTATGTCACGGCCAGAAGT
>Marker902875 Chr7 SNP_site:6226016 Ref_Type:G
CACAACATACCTCACTATCGTCTATCCTTATTGGTTAATCCTGGTTAGATTTCATATGTTTCCTCCTTGAACTCTTACTTXXXXXXXXXXA
CATAAAAAGGTAATTTCATTTGTAGAGTATTCGTTTTTATAACCTAATGAAAATTCGTTTTTATGTCACGGCCAGAAGT
CACAACATACCTCACTATCGTCTATCCTTATTGGTTCATCCTGGTTAGATTTCATATGTTTCCTCCTTGAACTCTTACTTXXXXXXXXXXA
CATAAAAAGGTAATTTCATTTGTAAAGTATTCGTTTTTATAACCTAATGAAAATTCGTTTTTATGTCACGGCCAGAAGT
>Marker903041 Chr7 SNP_site:6945247 Ref_Type:T
ACTTGAATAGTTCAAACATAAAAACAAACATGATAGCAAGTTGTTAGGCTATGATTGCCATGTAGGTGAGTGCAGGTAAAXXXXXXXXXXA
GCTATTGTTGGAAACGACCTGAATAATCTGCAACAGATTTTAAGAGAACGCCATCGTCAAAGATCTGTATTACAGCGTC
ACTTGAATAGTTCAAACATAAAAACAAACATGATAGTAAGTTGATAGGCTATGATTGCCATGTAGGTGAGTGCAGGTAAAXXXXXXXXXXA
GCTATTGTTGGAAACGACCTGAATAATCTGCAACAGATTTTAAGAGAACGTCATCGTCAAAGATCTGTATTACAGCGTC
>Marker903041 Chr7 SNP_site:6945254 Ref_Type:A
ACTTGAATAGTTCAAACATAAAAACAAACATGATAGCAAGTTGTTAGGCTATGATTGCCATGTAGGTGAGTGCAGGTAAAXXXXXXXXXXA
GCTATTGTTGGAAACGACCTGAATAATCTGCAACAGATTTTAAGAGAACGCCATCGTCAAAGATCTGTATTACAGCGTC
ACTTGAATAGTTCAAACATAAAAACAAACATGATAGTAAGTTGATAGGCTATGATTGCCATGTAGGTGAGTGCAGGTAAAXXXXXXXXXXA
GCTATTGTTGGAAACGACCTGAATAATCTGCAACAGATTTTAAGAGAACGTCATCGTCAAAGATCTGTATTACAGCGTC
>Marker903041 Chr7 SNP_site:6945463 Ref_Type:T
ACTTGAATAGTTCAAACATAAAAACAAACATGATAGCAAGTTGTTAGGCTATGATTGCCATGTAGGTGAGTGCAGGTAAAXXXXXXXXXXA
GCTATTGTTGGAAACGACCTGAATAATCTGCAACAGATTTTAAGAGAACGCCATCGTCAAAGATCTGTATTACAGCGTC
ACTTGAATAGTTCAAACATAAAAACAAACATGATAGTAAGTTGATAGGCTATGATTGCCATGTAGGTGAGTGCAGGTAAAXXXXXXXXXXA
GCTATTGTTGGAAACGACCTGAATAATCTGCAACAGATTTTAAGAGAACGTCATCGTCAAAGATCTGTATTACAGCGTC
>Marker903562 Chr7 SNP_site:3831377 Ref_Type:C
TACTAGTTCTCCATTTGAATTGACACATGAGACCTACATCCAAATTTGTAATGTAATAACGTAACCCGATTGCACCTGAGXXXXXXXXXXT

Create PDF with GO2PDF for free, if you wish to

ATTGTTACTGTTACTGCTTGATCCTCAGAGGTAGAGGTAGCAGTAACAGTAAATTTGACAAAGTTCATAATTTTAAGTT
TACTAGTTCTGCATTTGAATTGACACATGAAACCTACATCCAAATTTGTAATGTAATAACGTAATCCGATTGCACCTGAGXXXXXXXXXXT
ATTGTTACTGTTACTGCTTGATCCTCAGAGGTAGAGGTAGCAGTAACAGTAAATTTGACAAAGTTCATAATTTTAAGTT
>Marker903562 Chr7 SNP_site:3831397 Ref_Type:G
TACTAGTTCTCCATTTGAATTGACACATGAGACCTACATCCAAATTTGTAATGTAATAACGTAACCCGATTGCACCTGAGXXXXXXXXXXT
ATTGTTACTGTTACTGCTTGATCCTCAGAGGTAGAGGTAGCAGTAACAGTAAATTTGACAAAGTTCATAATTTTAAGTT
TACTAGTTCTGCATTTGAATTGACACATGAAACCTACATCCAAATTTGTAATGTAATAACGTAATCCGATTGCACCTGAGXXXXXXXXXXT
ATTGTTACTGTTACTGCTTGATCCTCAGAGGTAGAGGTAGCAGTAACAGTAAATTTGACAAAGTTCATAATTTTAAGTT
>Marker903562 Chr7 SNP_site:3831431 Ref_Type:C
TACTAGTTCTCCATTTGAATTGACACATGAGACCTACATCCAAATTTGTAATGTAATAACGTAACCCGATTGCACCTGAGXXXXXXXXXXT
ATTGTTACTGTTACTGCTTGATCCTCAGAGGTAGAGGTAGCAGTAACAGTAAATTTGACAAAGTTCATAATTTTAAGTT
TACTAGTTCTGCATTTGAATTGACACATGAAACCTACATCCAAATTTGTAATGTAATAACGTAATCCGATTGCACCTGAGXXXXXXXXXXT
ATTGTTACTGTTACTGCTTGATCCTCAGAGGTAGAGGTAGCAGTAACAGTAAATTTGACAAAGTTCATAATTTTAAGTT
>Marker903830 Chr7 SNP_site:4260218 Ref_Type:A
AACTTCTTAACTTATTACTGACTATCCAAACGAAAAAAATAGACAAATTATAGCAGAAATGAAAACAGCAAAGGGAAAGGXXXXXXXXXXC
ACATAAAAAACTCCACTATTCTTCCATTACATGAAAATTTTCATGTCGGAAGAAAGAAATAGATGGATATAGGGAGGGT
AACTTCTTAACTTATTACTGACTATCCAAACGAAAAAAATAGACAAATTATAGCAGAAATGAAAACAGCAAAGGGAAAGGXXXXXXXXXXC
ACATACAAAACTCCACTATTCTTCCATTACATGAAAATTTTCATGTCGGAAGAAAGAAATAGATGGATATAGGGAGGGT
>Marker904159 Chr7 SNP_site:7704845 Ref_Type:G
CACACACTTCTGAGGGAGGGTGCATATGTTTATATACAGAAAGTGGTGCTGACATTCTGGCGTGCATCATTAACATTCTGXXXXXXXXXXG
CTTGCTCTTTTTCACCTAATATCCTGCGATAAAACATGTTGAAACCTCGTAAAAACAGGGATGAAGTTTTTTTGCGGTA
CACACACTTCTGAGGGAGGGTGCATATGTTTATATACAGAAAGTGGTGCTGACATTCTGGCGTGCATCATTAACATTCTGXXXXXXXXXXG
CTTGCTCTTTTTCGCCTAATATCCTGCGATAAAACATGTTGAAACCTCGTAAAAACAGGGATGAAGTTTTTTTGTGGTA
>Marker904159 Chr7 SNP_site:7704906 Ref_Type:T
CACACACTTCTGAGGGAGGGTGCATATGTTTATATACAGAAAGTGGTGCTGACATTCTGGCGTGCATCATTAACATTCTGXXXXXXXXXXG
CTTGCTCTTTTTCACCTAATATCCTGCGATAAAACATGTTGAAACCTCGTAAAAACAGGGATGAAGTTTTTTTGCGGTA
CACACACTTCTGAGGGAGGGTGCATATGTTTATATACAGAAAGTGGTGCTGACATTCTGGCGTGCATCATTAACATTCTGXXXXXXXXXXG
CTTGCTCTTTTTCGCCTAATATCCTGCGATAAAACATGTTGAAACCTCGTAAAAACAGGGATGAAGTTTTTTTGTGGTA
>Marker904187 Chr7 SNP_site:15405901 Ref_Type:C
TACGCGTAACTAAAGATGTGAAAGATTATCCCTTTGGAACGCCTGTTCAAATCTCTGTTGATGTCACAAGGAAAAATAAAXXXXXXXXXXC
TAAAATGCTAATCATTTTTGGTTCTCACAGGATTTTAATGAAGAAGGAAGTTTCTTCTATTCTATGTTTTGCATGTGTT
TACGTGTAACTAAAGATGTGAAAGATTATCCCTTTGGAACGCCTGTTCAAATCTCTGTTGATGTCACAAGGAAAAATAAAXXXXXXXXXXC
TAAAATGCTAATCATTTTTGGTTCTCACAGGATTTTAATGAAGAAGGAAGTTTCTTCTATTCTATGTTTTGCATGTGTT
>Marker904654 Chr7 SNP_site:6479274 Ref_Type:A
ACCAACTTCCTTTATTTGAAGAGACAACTAACAAACAACCTATCGGCCATCCATACCAGAATCATCCATAAATAGAGAAGXXXXXXXXXXG
CGCGCAACAAATTCATATATCCATAATCCATAAGCATTTAGATCATATTATAGGCCAAATCACATCAAAATATAAAGTC
ACCAACTTCCTTTATTTGAAGAGACAACTAACAAACAACCTATCGGCCATCCATACCAGAATCATCCCTAAATAGAGAAGXXXXXXXXXXG
CGCGCAACAAATTCATATATCCATAATCCATAAGCATTTAGATCATATTATAGGCCAAATCACATCAAAATATAAAGTC
>Marker904669 Chr7 SNP_site:14807188 Ref_Type:C
ACCTGCAACCGTTGGTTGCCATAGAGTCATAGCATTGGACATCTGAGGGGTTGGTGATTGGCATTGATCATACTCTGGCCXXXXXXXXXXT
GTCATTATATCATCAATACAAGAACTGAAACAAGGCTACAACACTGGAAACACGTCTGTTTTCAATCACCAACTCAAGT
ACCTGCAACCGTTGGTTGCCATAGAGTCATAGCATTGGACATCTGAGGGGTTGGTGATTGGCATTGATCATACTCTGGCCXXXXXXXXXXT
GTCATTATATCATCAATACAAGAACTGAAACAAGGCTACAACACTGGAAACACGTCTGTTTTCAATCACCAATTCAAGT
>Marker90479 Chr4 SNP_site:7168426 Ref_Type:T

Create PDF with GO2PDF for free, if you wish to

ACCCGCCACGATAGTAACCTCCATCCAATCCGACCATGATAGCACACTAAATACACGACTCGATATATCTCACACATGATXXXXXXXXXXC
ACAAGGAAACACATGGCCAAACGAAGCTCACGCATATTTAGCACCTACCATAGTAACAACATCCACCCACTCCTATGTG
ACCCGCCACGATAGTAACCTCCATCCAATCCGACCATGATAGCACACTAAATACACGACTCGATATATCTCACACATGATXXXXXXXXXXC
ACAAGGAAACACATGGCCAAACGAAGCTCACGCATATTTAGCACCTACCATAGTAACAACATCCACCCACTCTTATGTG
>Marker905074 Chr7 SNP_site:13837847 Ref_Type:T
ACAACATACTAGTTTTTTATTTTGAAGATTTAGGGTTTTCCATCGATAAAAATCATTGGTAGGATGACAGACTCTCATCAXXXXXXXXXXA
AGGGTAATCCAAATGTAATTCATTCCATCGGGTTGTTGTCAAATTGAATTGAGAGTTTTAGAATTTGATATTAGGGGTT
ACAACATACTAGTTTTTTATTTTTAAGATTTAGGGTTTTCCATCAATAAAAATCATTGGTAGGATGACAGACTCTCGTCAXXXXXXXXXXA
AGGGTAATCCAAATGTAATTCATTCCATCGGGTTGTTGTCAAATTGAATTGAGAGTTTTAGAATTTGATATTAGGGGTT
>Marker905074 Chr7 SNP_site:13837868 Ref_Type:A
ACAACATACTAGTTTTTTATTTTGAAGATTTAGGGTTTTCCATCGATAAAAATCATTGGTAGGATGACAGACTCTCATCAXXXXXXXXXXA
AGGGTAATCCAAATGTAATTCATTCCATCGGGTTGTTGTCAAATTGAATTGAGAGTTTTAGAATTTGATATTAGGGGTT
ACAACATACTAGTTTTTTATTTTTAAGATTTAGGGTTTTCCATCAATAAAAATCATTGGTAGGATGACAGACTCTCGTCAXXXXXXXXXXA
AGGGTAATCCAAATGTAATTCATTCCATCGGGTTGTTGTCAAATTGAATTGAGAGTTTTAGAATTTGATATTAGGGGTT
>Marker905074 Chr7 SNP_site:13837900 Ref_Type:G
ACAACATACTAGTTTTTTATTTTGAAGATTTAGGGTTTTCCATCGATAAAAATCATTGGTAGGATGACAGACTCTCATCAXXXXXXXXXXA
AGGGTAATCCAAATGTAATTCATTCCATCGGGTTGTTGTCAAATTGAATTGAGAGTTTTAGAATTTGATATTAGGGGTT
ACAACATACTAGTTTTTTATTTTTAAGATTTAGGGTTTTCCATCAATAAAAATCATTGGTAGGATGACAGACTCTCGTCAXXXXXXXXXXA
AGGGTAATCCAAATGTAATTCATTCCATCGGGTTGTTGTCAAATTGAATTGAGAGTTTTAGAATTTGATATTAGGGGTT
>Marker90588 Chr4 SNP_site:18319528 Ref_Type:T
AACCCCACAGCTCTAGTTGAAGGATCAATAAATAATATCCGAGCATTCATCTATACAAGAAAAATATATATGTATATACAXXXXXXXXXXA
AATGTGCTTCAAACAGCAGCACATAAGAAAAACCATTAATTAAGTGGCAGAGGCAACAATTTAATCATCAAAGAAAGTA
AACCCCACAGCTCTAGTTGAAGGATCAATAAATAATATCCGAGCATTCATCTATACATGAAAAATATATATGTATATACAXXXXXXXXXXA
AATGTGCTTCAAACAGCAGCACATAAGAAAAACCATTAATTAAGTGGCAGAGGCAACAATTTAATCATCAAAGAAAGTA
>Marker905983 Chr7 SNP_site:6137961 Ref_Type:G
CACCTACAAGAGTGAGGTCATCAACTATTTACTTCTTTTATGCGAGATAATCTTCCATTCTTATAGTGCCTTTCCTTGTGXXXXXXXXXXT
TGTGGATCGTGCCATCGATCCAAACAACCAACCAAGTAAGGCTTTGATATGTGGTTATCCAATCGTCATGTTCATTGTT
CACCTACAAGAGTGAGGTCATCAACTATTTACTTCTTTTATGCGAGATAATCTTCCATTCTTGTAGTGCCTTTCCTTGTGXXXXXXXXXXT
TGTGGATCGTGCCATCAATCCAAACAACCAACCAAGTAAGGCTTTGATATGTGGTTATCCAATCGTCATGTTCATTGTT
>Marker905983 Chr7 SNP_site:6138105 Ref_Type:A
CACCTACAAGAGTGAGGTCATCAACTATTTACTTCTTTTATGCGAGATAATCTTCCATTCTTATAGTGCCTTTCCTTGTGXXXXXXXXXXT
TGTGGATCGTGCCATCGATCCAAACAACCAACCAAGTAAGGCTTTGATATGTGGTTATCCAATCGTCATGTTCATTGTT
CACCTACAAGAGTGAGGTCATCAACTATTTACTTCTTTTATGCGAGATAATCTTCCATTCTTGTAGTGCCTTTCCTTGTGXXXXXXXXXXT
TGTGGATCGTGCCATCAATCCAAACAACCAACCAAGTAAGGCTTTGATATGTGGTTATCCAATCGTCATGTTCATTGTT
>Marker906409 Chr7 SNP_site:15634136 Ref_Type:A
ACAGGGAGAAACATCTGGGAGCAAACTTATTGTGCTAATGCTTTAAATGCCTCTAAATAATATTATGGCATGCACAAGAGXXXXXXXXXXT
TGACATGGAGAATATAGTCTAATTCAAACCAAGTAAGTTGTATTTAAAAAAATTTAGCACGTTGAAAATAAAATGAGTT
ACAGGGAGTAACATCTGGGAGCAAACTTATTGTGCTAATGCTTTAAATGCCTCTAAATAATATTATGGCATGCACAAGAGXXXXXXXXXXT
TGACATGGAGAATATCGTCTAATTCAAACCAAGTAAGTTGTATTTAAAAAAATTTAGCACGTTGAAAATAAAATGAGTT
>Marker906409 Chr7 SNP_site:15634352 Ref_Type:A
ACAGGGAGAAACATCTGGGAGCAAACTTATTGTGCTAATGCTTTAAATGCCTCTAAATAATATTATGGCATGCACAAGAGXXXXXXXXXXT
TGACATGGAGAATATAGTCTAATTCAAACCAAGTAAGTTGTATTTAAAAAAATTTAGCACGTTGAAAATAAAATGAGTT
ACAGGGAGTAACATCTGGGAGCAAACTTATTGTGCTAATGCTTTAAATGCCTCTAAATAATATTATGGCATGCACAAGAGXXXXXXXXXXT
TGACATGGAGAATATCGTCTAATTCAAACCAAGTAAGTTGTATTTAAAAAAATTTAGCACGTTGAAAATAAAATGAGTT

Create PDF with GO2PDF for free, if you wish to

>Marker906683 Chr7 SNP_site:3730945 Ref_Type:T
ACCATACATTTTCTTGATTTTGTCATTTCTAATAAGGTTAATGTCAATTATACACTTTTGTCTAAAACTAACAGTAGTTTXXXXXXXXXXT
TCTACTATTGGTGATTCCAAATTAATAAAGTCTTGCTTTCAAACCTCTCCAATTAAGGCTAATATTATTAATATAGAGT
ACCATACATTTTCTTGATTTTGTCATTTCTAATAATGTTAATGTCAATTATACACTTTTGTCTAAAACTAACAGTAGTTTXXXXXXXXXXT
TCTACTATTGGTGATTCCAAATTAATAAAGTCTTGCTTTCAAACCTCTCCAATTAAGGCTAATATTATTAATATAGAGT
>Marker90681 Chr4 SNP_site:15131149 Ref_Type:T
ACCCAAGGGACCAAATATAAAAATGACTAAAACCAATGTCTTCAACTTCACTTCTTCATAATTTGATTAGACAGGTCGAAXXXXXXXXXXC
AACATTCCATTTGACCTCAACCTTATCTCATACATGAACATTACAACAAAATCCTCTCCAACAAAATTATAAATTCTGT
ACCCAAGGGACCAAATATAAAAATGACTAAAACCAATGTCTTCAACTTCACTTCTTCATAATTTGATTAGACAGGTCGAAXXXXXXXXXXC
AACATTCCATTTGACCTCAACCTTATCTCATACATGAACATTACAATAAAATCCTCTCCAACAAAATTATAAATTCTGT
>Marker907288 Chr7 SNP_site:13336584 Ref_Type:A
ACAAAGACAAACCTCAGATAACAATTTCTCCTCTTTATCATAGAGATTTGAAAGCTCCCCAACCAGTTCAGAATGCTTTCXXXXXXXXXXT
CCTTCACCACAAAAAGACTAAAAATACAAATTCAATCCAAAATCTTCCCAATTCTCTAATTAGTCTTCAAAAATAAGTT
ACAAAGACAAACCTCAGATAACAATTTCTCCTCTTTATCATAGAGATTTGAAAGCTCCCCTACCAGTTCAGAATGCTTTCXXXXXXXXXXT
TCTTCATCACAAAAAGACTAAAAATACAAATTCAATCCAAAATCTTCCCAATTCTCTAATTAGTCTTCAAAAATAAGTT
>Marker907288 Chr7 SNP_site:13336737 Ref_Type:T
ACAAAGACAAACCTCAGATAACAATTTCTCCTCTTTATCATAGAGATTTGAAAGCTCCCCAACCAGTTCAGAATGCTTTCXXXXXXXXXXT
CCTTCACCACAAAAAGACTAAAAATACAAATTCAATCCAAAATCTTCCCAATTCTCTAATTAGTCTTCAAAAATAAGTT
ACAAAGACAAACCTCAGATAACAATTTCTCCTCTTTATCATAGAGATTTGAAAGCTCCCCTACCAGTTCAGAATGCTTTCXXXXXXXXXXT
TCTTCATCACAAAAAGACTAAAAATACAAATTCAATCCAAAATCTTCCCAATTCTCTAATTAGTCTTCAAAAATAAGTT
>Marker907288 Chr7 SNP_site:13336743 Ref_Type:C
ACAAAGACAAACCTCAGATAACAATTTCTCCTCTTTATCATAGAGATTTGAAAGCTCCCCAACCAGTTCAGAATGCTTTCXXXXXXXXXXT
CCTTCACCACAAAAAGACTAAAAATACAAATTCAATCCAAAATCTTCCCAATTCTCTAATTAGTCTTCAAAAATAAGTT
ACAAAGACAAACCTCAGATAACAATTTCTCCTCTTTATCATAGAGATTTGAAAGCTCCCCTACCAGTTCAGAATGCTTTCXXXXXXXXXXT
TCTTCATCACAAAAAGACTAAAAATACAAATTCAATCCAAAATCTTCCCAATTCTCTAATTAGTCTTCAAAAATAAGTT
>Marker908435 Chr7 SNP_site:15692993 Ref_Type:T
ACTTTTGGATTGGCATCCATTCCCATTCCCACTTCCCTTTTCCTCCTCTTCATCAAAAATCTCCATTTTTTCCCTCTCTCXXXXXXXXXXT
TTTCCATTGTTCATAACTACTGTTTGTGGATCACAACTCTATTCTTCAACAACTGCTTTTCATCCTCATATCACATGTA
ACTTTTGGATTGGCATCCATTCCCATTCCCACTTCCCTTTTCCTCCTCTTCATCAAAAATCTCCATTTTTTCCCTCTCTCXXXXXXXXXXT
TTTCCATTGTTCATAACTACTGTTTGTGGATCATAACTCTATTCTTCAACAACTGCTTTTCATCCTCATATCACATGTA
>Marker908483 Chr7 SNP_site:15516836 Ref_Type:C
AACTATAGATAGGAACCCATATTCAAATAATGCTAAAGAAGATATCATTGGTTTTCCTTCCGGTATAAAAAATAGTAACTXXXXXXXXXXC
AAATAGTGAAAATAAGGTTTTATAAAAGAAATGTCATGGCCAAAGTTATATGAGCATACCAGCCTTATAAAAGGTAGTA
AACTATAGATAGGAACCCATATTCAAATAATGCTAAAGAAGATATCATTGGTTTTCCTTCCGGTATAAAAAATAGTAACTXXXXXXXXXXC
AAATAGTGAAAATAAGGTTTTATAAAAGAAATGTCATGGCCAAAGTTATATGAGCATATCAGCCTTATAAAAGGTAGTA
>Marker909009 Chr7 SNP_site:6942539 Ref_Type:G
GACAGAGATCATACTAATCACAAGACTCGGAATGACAAACATCTCAGGCTCTCACCCAAGATATAGCATTTCTAAATTGTXXXXXXXXXXA
TAAAACTGTGCCCAAGTTCAGGCTCTCAGCCAAGGAACTAGATCATATTCCCAAAAAACTCACTCTCCCCAATTCAAGT
GACAGAGATCATACTAATCACAATACTCGGAATGACAAACATCTCAGGCTCTCACCCAAGATATAGCATTTCTAAATTGTXXXXXXXXXXA
TAAAACTGTGCCCAAGTTCAGGCTCTCAGCCAAGGAACTAGATCATATTCCCAAAAAACTCACTCTCCCCAATTCAAGT
>Marker909057 Chr7 SNP_site:15297701 Ref_Type:A
TACTATCAATGCTTAATTTTTTTAAAAAAAGATCAATCCACCTAAAAGTCTTTTAAAGAATTGCTCAACTTTATGAACCTAXXXXXXXXXXG
AAGTAGATGGATGATCATATGTGAATGCAGGGCTTTTTTGGGGAGTGTGAGTGACTATTGCGCTCATCAAGCTTCATGT
TACTATCAATGCTTAATTTTTTTAAAAAAAGATCAATCCACTTAAAAGCCTTTTAAAGAATTGCTCAACTTTATGAACCTAXXXXXXXXXXG

Create PDF with GO2PDF for free, if you wish to

AAGTAGATGGATGATCATATGTGAATGCAGGGCTTTTTTGGGGAGTGTGAGTGACTATTGCGCTCATCAAGCTTCATGT
>Marker909057 Chr7 SNP_site:15297719 Ref_Type:C
TACTATCAATGCTTAATTTTTTAAAAAAAGATCAATCCACCTAAAAGTCTTTTAAAGAATTGCTCAACTTTATGAACCTAXXXXXXXXXXG
AAGTAGATGGATGATCATATGTGAATGCAGGGCTTTTTTGGGGAGTGTGAGTGACTATTGCGCTCATCAAGCTTCATGT
TACTATCAATGCTTAATTTTTTTAAAAAAGATCAATCCACTTAAAAGCCTTTTAAAGAATTGCTCAACTTTATGAACCTAXXXXXXXXXXG
AAGTAGATGGATGATCATATGTGAATGCAGGGCTTTTTTGGGGAGTGTGAGTGACTATTGCGCTCATCAAGCTTCATGT
>Marker909057 Chr7 SNP_site:15297726 Ref_Type:T
TACTATCAATGCTTAATTTTTTAAAAAAAGATCAATCCACCTAAAAGTCTTTTAAAGAATTGCTCAACTTTATGAACCTAXXXXXXXXXXG
AAGTAGATGGATGATCATATGTGAATGCAGGGCTTTTTTGGGGAGTGTGAGTGACTATTGCGCTCATCAAGCTTCATGT
TACTATCAATGCTTAATTTTTTTAAAAAAGATCAATCCACTTAAAAGCCTTTTAAAGAATTGCTCAACTTTATGAACCTAXXXXXXXXXXG
AAGTAGATGGATGATCATATGTGAATGCAGGGCTTTTTTGGGGAGTGTGAGTGACTATTGCGCTCATCAAGCTTCATGT
>Marker909152 Chr7 SNP_site:9936772 Ref_Type:T
AACTCAGTTGGAAGATTTATTAATTATTTGGGCTGTATATTTGTAATCTTGCTATAGAGAAGGTTTATACCATCAGGTCAXXXXXXXXXXA
CAGTTTGCGTGGCGGCCGAGGCCACCATCCTTCTTGAGTCCAGAAAAAGAGGAAGAGATAGCGAAGAATTTGAAGAAGT
AACTCAGTTGGAAGATTTATTAATTATTTGGGCTGTATATTTGTAATCTTGCTATAGAGAAGGTTTATACCATCAGGTCAXXXXXXXXXXA
CAGTTTGCGTGGCGGCCGAGGCCACCATCCTTTTTGAGTCCAGAAAAAGAGGAAGAGATAGCGAAGAATTTGAAGAAGT
>Marker909539 Chr7 SNP_site:620389 Ref_Type:G
AACAAATTAAATACAAACCTTTTTTATATACCACAAGGGAAAAAAAGGAAAAAGGTGACCAAAATATATGAAAGGGATGAXXXXXXXXXXA
TTTAAAAAGATATATCAGATAGATGAACTGTTTAAATCGACAAAACTGCTAAAAATATTTTTAAATATAGCAAAAGGTA
AACAAATTAAATACAAACCTTTTTTATATACCACAAGGGAAAAAAAGGAAAAAGGTGACCAAAATATATGAAAGGGATGAXXXXXXXXXXA
TTTAAAAGGATATATCAGATAGATGAACTGTTTAAATCAACAAAACTGCTAAAAATATTTTTAAATATAGCAAAAGGTA
>Marker909539 Chr7 SNP_site:620420 Ref_Type:A
AACAAATTAAATACAAACCTTTTTTATATACCACAAGGGAAAAAAAGGAAAAAGGTGACCAAAATATATGAAAGGGATGAXXXXXXXXXXA
TTTAAAAAGATATATCAGATAGATGAACTGTTTAAATCGACAAAACTGCTAAAAATATTTTTAAATATAGCAAAAGGTA
AACAAATTAAATACAAACCTTTTTTATATACCACAAGGGAAAAAAAGGAAAAAGGTGACCAAAATATATGAAAGGGATGAXXXXXXXXXXA
TTTAAAAGGATATATCAGATAGATGAACTGTTTAAATCAACAAAACTGCTAAAAATATTTTTAAATATAGCAAAAGGTA
>Marker909553 Chr7 SNP_site:15411195 Ref_Type:A
CACTAGAAACCTTGCTTTGTAAATGGGCATGATAACTTTACAAGCAACGAACTTGATACACTTTTGAGAACTTCTCAAAGXXXXXXXXXXC
GCCTGATCACAAAACTAAATCAAGCTTTCACCTCGAGAGACCAGACAAACCAGCCAACAAGAAGAACGTCGAATTGGGT
CACTAGAAACCTTGCTTTGTAAATGGGCATGATCACTTTACAAGCAACGAACTTGATACACTTTTGAGAACTTCTCAAAGXXXXXXXXXXC
GCCCGATCACAAAACTAAATCAAGCTTTCACCTCGAGAGACTAGACAAACCAGCCAACAAGAAGAACGTCGAATTGGGT
>Marker909553 Chr7 SNP_site:15411335 Ref_Type:T
CACTAGAAACCTTGCTTTGTAAATGGGCATGATAACTTTACAAGCAACGAACTTGATACACTTTTGAGAACTTCTCAAAGXXXXXXXXXXC
GCCTGATCACAAAACTAAATCAAGCTTTCACCTCGAGAGACCAGACAAACCAGCCAACAAGAAGAACGTCGAATTGGGT
CACTAGAAACCTTGCTTTGTAAATGGGCATGATCACTTTACAAGCAACGAACTTGATACACTTTTGAGAACTTCTCAAAGXXXXXXXXXXC
GCCCGATCACAAAACTAAATCAAGCTTTCACCTCGAGAGACTAGACAAACCAGCCAACAAGAAGAACGTCGAATTGGGT
>Marker909553 Chr7 SNP_site:15411373 Ref_Type:C
CACTAGAAACCTTGCTTTGTAAATGGGCATGATAACTTTACAAGCAACGAACTTGATACACTTTTGAGAACTTCTCAAAGXXXXXXXXXXC
GCCTGATCACAAAACTAAATCAAGCTTTCACCTCGAGAGACCAGACAAACCAGCCAACAAGAAGAACGTCGAATTGGGT
CACTAGAAACCTTGCTTTGTAAATGGGCATGATCACTTTACAAGCAACGAACTTGATACACTTTTGAGAACTTCTCAAAGXXXXXXXXXXC
GCCCGATCACAAAACTAAATCAAGCTTTCACCTCGAGAGACTAGACAAACCAGCCAACAAGAAGAACGTCGAATTGGGT
>Marker909646 Chr7 SNP_site:7520745 Ref_Type:G
ACATTAGGCAAAGCTCTTAAGCATGATGCAATTTTAGGGCTACCAACACTGAATGTAAAGAGTCATTTATTCTGTGAAGAXXXXXXXXXXC
TTCACTTGGATAAGATTCTTAAGGAGTAAATCTGAGACAACTAAAGTATGTATAAATATATGCCGAAGATTACAACGTG

Create PDF with GO2PDF for free, if you wish to

ACATTAGGCAAAGCTCTTAAGCATGATGCAATTTTAGGGCTACCAACACTGAATGTAAAGAGTCATTTATTCTGTGAAGAXXXXXXXXXXC
TTCACTTGGATAAGATTCTTAAGGAGTAAATCTGAGACAGCTAAAGTATGTATAAATATATGCCGAAGATTACAACGTG
>Marker910372 Chr7 SNP_site:4897172 Ref_Type:G
ACTCGTAGTTTAGTTTAATTTTAGGATACATGTGTATTTGGTCCCTATGACTTCAGTTTCTATCGTAGATACCAAACGTAXXXXXXXXXXT
TAAATTAAGTTATTAACAACCCAACTACCATTTTCATAGTTGAACCAATGATGACCAAATATTTCGAATCAATTATGGT
ACTCGTGGTTTAGTTTAATTTTAGGATACATGTGTATTTGGTCCCTATGACTTCAGTTTCTATCGTAGATACCAAACGTAXXXXXXXXXXT
TAAATTAAGTTATTAACAACCCAACTACCATTTTCATAGTTGAACCAATGATGACCAAATATTTCGAATCAATTATGGT
>Marker911000 Chr7 SNP_site:4139918 Ref_Type:A
AACAAGGATATTTCAACGGTGCTGGTTGGCATGAACTCTGTCGGACAGGTATCTCTCAATAATCTCATTGTTTGGTATTCXXXXXXXXXXC
ACTATTGTCCTTAAGATTTCAATAATGTTTAATTTTAGTCCTTAAAGTAACAGAATCGTTTGTGACTAAACGGAAGGTT
AACAAGGATATTTCAGCGGTGCTGGTTGGCATGAACTCTGTCGGACAGGTATCTCTCAATAATCTCATTGTTTGGTATTCXXXXXXXXXXC
ACTATTGTCCTTAAGATTTCAATAATGTTTAATTTTAGTCCTTAAAGTAACAGAATCGTTTGTGACTAAACGGAAGGTT
>Marker911069 Chr7 SNP_site:16317832 Ref_Type:T
AACTCCAAAGTTAATGGTAAATTGATATATGTGGAATTATGCAATTCAATAATTATTCTTTAAGGAAGGAGTTTAGAGAGXXXXXXXXXXT
AACCTTACTCCTTCCTTCTCAACCAAAATCACCTATATACATATTTTCAAATAACATTCATAGTTACATATCCTTAAGT
AACTCCAAAGTTAATGGTAAATTGATATATGTGGAATTATGTAATTCAATAATTATTCTTTAAGGAAGGAGTTTAGAGAGXXXXXXXXXXT
AACCTTACTCCTTCCTTCTTAACCAAAATCACCTATATACATATTTTCAAATAACATTCATAGTTACATATCCTTAAGT
>Marker911069 Chr7 SNP_site:16318006 Ref_Type:T
AACTCCAAAGTTAATGGTAAATTGATATATGTGGAATTATGCAATTCAATAATTATTCTTTAAGGAAGGAGTTTAGAGAGXXXXXXXXXXT
AACCTTACTCCTTCCTTCTCAACCAAAATCACCTATATACATATTTTCAAATAACATTCATAGTTACATATCCTTAAGT
AACTCCAAAGTTAATGGTAAATTGATATATGTGGAATTATGTAATTCAATAATTATTCTTTAAGGAAGGAGTTTAGAGAGXXXXXXXXXXT
AACCTTACTCCTTCCTTCTTAACCAAAATCACCTATATACATATTTTCAAATAACATTCATAGTTACATATCCTTAAGT
>Marker911826 Chr7 SNP_site:13103166 Ref_Type:A
AACGTGCTAGTTGTGTCCTACAAAAAGAGAGAGCAATCTTACTATTTGAGGGGAATACTAGAAAATATCAATACCAATATXXXXXXXXXXT
ATGATAGAAGAATCGCCACCATCCAATTTGACTGAGCTTGAAATTTACATGAGCTCTACTACACTTTATCATAGTTGTT
AACGTGCTAGTTGTGTCCTACAAAAAGAGAGAGCAATCTTACTATTTGAGGGGAATACTAGAAAATATCAATACCAATATXXXXXXXXXXT
ATGATTGAAGAATCGCCACCATCCAATTTGACTGAGCTTGAAATTTACATGAGCTCTACTACACTTTATCATAGTTGTT
>Marker912306 Chr7 SNP_site:8190749 Ref_Type:G
CACTGAGGAATGATTTGTTATTCTATAAACAGAGATTGATGTTACATCCGAATTTGGATTCTCTTGTAATTATAATCTGAXXXXXXXXXXA
CAGAAAAGTCGTCCGGTTCAAAATAGGGGAATTGTTGGCCACATTGAAGGTCGCTGCTGTTCATAGATGTAGGAAGGTC
CACTGAGGAATGATTTGTTATTCTATAAACAGAGATTGATGTTACATCCGAATTTGGATTCTCTTGTAATTATAATCTGAXXXXXXXXXXA
CAGAAAAGTCGTCCGGTTCAAAATAGGGGAATTGTTGGCGACATTGAAGGTCGCTGCTGTTCATAGATCTAGGAAGGTC
>Marker912306 Chr7 SNP_site:8190778 Ref_Type:C
CACTGAGGAATGATTTGTTATTCTATAAACAGAGATTGATGTTACATCCGAATTTGGATTCTCTTGTAATTATAATCTGAXXXXXXXXXXA
CAGAAAAGTCGTCCGGTTCAAAATAGGGGAATTGTTGGCCACATTGAAGGTCGCTGCTGTTCATAGATGTAGGAAGGTC
CACTGAGGAATGATTTGTTATTCTATAAACAGAGATTGATGTTACATCCGAATTTGGATTCTCTTGTAATTATAATCTGAXXXXXXXXXXA
CAGAAAAGTCGTCCGGTTCAAAATAGGGGAATTGTTGGCGACATTGAAGGTCGCTGCTGTTCATAGATCTAGGAAGGTC
>Marker912670 Chr7 SNP_site:14010652 Ref_Type:C
AACCAGAAGAAATGTGTTATAGGACACTCAACAATACAGTATTTGGGACATTGGATTTCAAGCCGATGGAGAGAAATCCAXXXXXXXXXXT
TATTGCAAAATAACGCTTTCAAATGGAGTGAAGAGGCCACCTCGACATTCGAAATACGGAAGAGAGCTATGATAGTAGT
AACCAGAAGAAATGTGTTATAGGACACTCAAGAATACAGTATTTGGGACATTGGATTTCAAGCCGATGGAGAGAAATCCAXXXXXXXXXXT
TATTGCAAAATAAGGCTTTCAAATGGAGTGAAGAGGCCACCTCGACATTCGAAATACGGAAGAGAGCTATGATAGTAGT
>Marker912670 Chr7 SNP_site:14010828 Ref_Type:C
AACCAGAAGAAATGTGTTATAGGACACTCAACAATACAGTATTTGGGACATTGGATTTCAAGCCGATGGAGAGAAATCCAXXXXXXXXXXT

Create PDF with GO2PDF for free, if you wish to

TATTGCAAAATAACGCTTTCAAATGGAGTGAAGAGGCCACCTCGACATTCGAAATACGGAAGAGAGCTATGATAGTAGT
AACCAGAAGAAATGTGTTATAGGACACTCAAGAATACAGTATTTGGGACATTGGATTTCAAGCCGATGGAGAGAAATCCAXXXXXXXXXXXT
TATTGCAAAATAAGGCTTTCAAATGGAGTGAAGAGGCCACCTCGACATTCGAAATACGGAAGAGAGCTATGATAGTAGT
>Marker912973 Chr7 SNP_site:7493067 Ref_Type:A
TACTGGATTTGAGTTATTAAGACAAAACTAATAATATAATCAATAACAATTATTACAATAATAACACTTTATTAATAACAXXXXXXXXXXXT
ACTTCGATAATGATTTCGTTTTTACATCTATATGTTTTCAAATTTATGTTTGTTTTCTTCTAATTCTTCAATTATAGTT
TACTGGATTTGGGTTATTAAGACAAAACTAATAATATAATCAATAACAATTATTACAATAATAACACTTTATTAATAACAXXXXXXXXXXXT
ACTTCGATAATGATTTCGTTTTTACATCTATGTGTTTTCAAATTTATGTTTGTTTTCTTCTAATTCTTCAATTATAGTT
>Marker912973 Chr7 SNP_site:7493251 Ref_Type:A
TACTGGATTTGAGTTATTAAGACAAAACTAATAATATAATCAATAACAATTATTACAATAATAACACTTTATTAATAACAXXXXXXXXXXXT
ACTTCGATAATGATTTCGTTTTTACATCTATATGTTTTCAAATTTATGTTTGTTTTCTTCTAATTCTTCAATTATAGTT
TACTGGATTTGGGTTATTAAGACAAAACTAATAATATAATCAATAACAATTATTACAATAATAACACTTTATTAATAACAXXXXXXXXXXXT
ACTTCGATAATGATTTCGTTTTTACATCTATGTGTTTTCAAATTTATGTTTGTTTTCTTCTAATTCTTCAATTATAGTT
>Marker913394 Chr7 SNP_site:13772237 Ref_Type:G
ACAATTATACTTGACATGTAAAAAACGTCAAGTATATGAAACTACTTGACGTTTTTTAAATGTCAAGTAATCTAGTGTCAXXXXXXXXXXXT
TCCCTTTCAAATTGAAAATTGTATGATTTGAAATACCCATATAATTATATTGCATTTGTTTTGAAGTCTAACAATGGTA
ACAATTATACTTGACATGTAAAAAGCGTCAAGTATATGAAACTACTTGACGTTTTTTAAATGTCAAGTAATCTAGTGTCAXXXXXXXXXXXT
TCCCTTTCAAATTGAAAATTGTATGATTTGAAATACCCATATAATTATATTACATCTGTTTTGAAGTCTAACAATGGTA
>Marker913394 Chr7 SNP_site:13772465 Ref_Type:A
ACAATTATACTTGACATGTAAAAAACGTCAAGTATATGAAACTACTTGACGTTTTTTAAATGTCAAGTAATCTAGTGTCAXXXXXXXXXXXT
TCCCTTTCAAATTGAAAATTGTATGATTTGAAATACCCATATAATTATATTGCATTTGTTTTGAAGTCTAACAATGGTA
ACAATTATACTTGACATGTAAAAAGCGTCAAGTATATGAAACTACTTGACGTTTTTTAAATGTCAAGTAATCTAGTGTCAXXXXXXXXXXXT
TCCCTTTCAAATTGAAAATTGTATGATTTGAAATACCCATATAATTATATTACATCTGTTTTGAAGTCTAACAATGGTA
>Marker913394 Chr7 SNP_site:13772469 Ref_Type:C
ACAATTATACTTGACATGTAAAAAACGTCAAGTATATGAAACTACTTGACGTTTTTTAAATGTCAAGTAATCTAGTGTCAXXXXXXXXXXXT
TCCCTTTCAAATTGAAAATTGTATGATTTGAAATACCCATATAATTATATTGCATTTGTTTTGAAGTCTAACAATGGTA
ACAATTATACTTGACATGTAAAAAGCGTCAAGTATATGAAACTACTTGACGTTTTTTAAATGTCAAGTAATCTAGTGTCAXXXXXXXXXXXT
TCCCTTTCAAATTGAAAATTGTATGATTTGAAATACCCATATAATTATATTACATCTGTTTTGAAGTCTAACAATGGTA
>Marker913627 Chr7 SNP_site:3881372 Ref_Type:A
TACATATGTGTGTAACCAATTGAAATCATCAATTGGTTAGGAAAGGAACCCAAACTGTGCAAAGATGTAGATGTAATAAAXXXXXXXXXXXC
CTCAACCGATTAAAAAACACCAGGAAATGAAATTCAGGAGCCCAATAAACAAATCCAACGACAGGTCCCCCCTTTGATGT
TACATATGTGTGTAACCAATTGAAATCATCAATTGGTTAGGAAAGGAACCCAAACTGTGCAAAGATGTAGATGTTATAAAXXXXXXXXXXXC
CTCAACCGATTAAAAAACACCAGGAAATGAAATTCAGGAGCCCAATAAACAAATCCAACGACAGGTCCCCCCTTTGATGT
>Marker913937 Chr7 SNP_site:4040426 Ref_Type:A
AACCAAACAAGAAAAGTTTATTCTGTGTGCAGATTTTCTGCTTGATTATTTAGTTGTATTTCTTTGCTATTTCTTCATGAXXXXXXXXXXXC
TGATCTTGACATCCGAACTGCTATCCAGAATGCAACTGGTCCTAAATCTGCTTTATTTGTTCCCGAAGTAGGTTATGGT
AACCAAACAAGAAAAGTTTATTCTGTGTGCAGATTTTCTGCTTGATTATTTAGTTGTATTTCTTTGCTATTTCTTCATGCXXXXXXXXXXXC
TGATCTTGACATCCGAACTGCTATCCAGAATGCAACTGGTCCTAAATCTGCTTTATTTGTTCCCGAAGTAGGTTATGGT
>Marker914897 Chr7 SNP_site:13471145 Ref_Type:G
TACAAAAGGTTTATGGCTTTAGGTTCTCGACTTATTCTTCTTCTTCCAATAAACCCCTTTTACGAGAAACACAAATTGATXXXXXXXXXXXT
AAAAAATTTCAACTAGGGCTCAAAATATTCCCTTGTATAGAAAAAAAGAAACTTAACTGGTTATTTTCTTGCTGCAGTG
TACAAAAGGTTTATGGCTTTAGGTTCTTGACTTATTCTTCTTCTTCCAATAAACCCCTTTTACGAGAAACACAAATTGATXXXXXXXXXXXT
AAAAAATTTCAACTAGGGCTCAAAATATTCCCTTGTATAGAAAAAAAGAAACTTAACTGGTTATTTTCTTGCTGCAGTG
>Marker914912 Chr7 SNP_site:10073462 Ref_Type:A

Create PDF with GO2PDF for free, if you wish to

AACCTTAACTACCCATGGGCTTTCATCAAGGCCCAAGCCCACTATTTCTAAGAATTAATAATCAGAAAAAAGCCAAAACAXXXXXXXXXXC
TTAAGGAACAAGAAGAAGTCTTATAATGCTATTTTAATGAAAATTTAAATAAATTCCCTTAATATACAATTAGAGCGTG
AACCTTAACTACCCATGGGCTTTCATCAAGGCCCAAGCCCACTATTTCTAAGAATTAATAATCAGAACAAAGCCAAAACAXXXXXXXXXXC
TTAAGGAACAAGAAGAAGTCTTATAATGCTATTTTAATGAAAATTTAAATAAATTCCCTTAATATACAATTAGAGCGTG
>Marker914933 Chr7 SNP_site:5443629 Ref_Type:G
TACACATACATCACATTAAATGATTCTAATAGCGACCTAATCAATTCAACACACATTAAGTCTAGAAAACAAACCAAGAGXXXXXXXXXXA
TTGAGATTTATGCACAAAGATGCTATTATCTCAAGTAAAACATAGAAGAATTAGTAAAGTCCAACATGAATTCAAAGTA
TACACATACATCACATTAAATGATTCTAATAGCGACCTAATCAATTCAACACAGATTAAATCTAGAAAACAAACCAAGACXXXXXXXXXXA
TTGAGATTTATGCACAAAGATGCTATTATCTCAAGTAAAACATAGAAGAATTAGTAAAGTCCAACATGAATTCAAAGTA
>Marker914933 Chr7 SNP_site:5443635 Ref_Type:A
TACACATACATCACATTAAATGATTCTAATAGCGACCTAATCAATTCAACACACATTAAGTCTAGAAAACAAACCAAGAGXXXXXXXXXXA
TTGAGATTTATGCACAAAGATGCTATTATCTCAAGTAAAACATAGAAGAATTAGTAAAGTCCAACATGAATTCAAAGTA
TACACATACATCACATTAAATGATTCTAATAGCGACCTAATCAATTCAACACAGATTAAATCTAGAAAACAAACCAAGACXXXXXXXXXXA
TTGAGATTTATGCACAAAGATGCTATTATCTCAAGTAAAACATAGAAGAATTAGTAAAGTCCAACATGAATTCAAAGTA
>Marker914933 Chr7 SNP_site:5443655 Ref_Type:C
TACACATACATCACATTAAATGATTCTAATAGCGACCTAATCAATTCAACACACATTAAGTCTAGAAAACAAACCAAGAGXXXXXXXXXXA
TTGAGATTTATGCACAAAGATGCTATTATCTCAAGTAAAACATAGAAGAATTAGTAAAGTCCAACATGAATTCAAAGTA
TACACATACATCACATTAAATGATTCTAATAGCGACCTAATCAATTCAACACAGATTAAATCTAGAAAACAAACCAAGACXXXXXXXXXXA
TTGAGATTTATGCACAAAGATGCTATTATCTCAAGTAAAACATAGAAGAATTAGTAAAGTCCAACATGAATTCAAAGTA
>Marker915166 Chr7 SNP_site:14313571 Ref_Type:C
AACCTCTGCCTGTTTCCTCTTAACTCATTTAGGTGGGAGATAAATTGATTAAGTTCCTCGATCTTCCCTTTTCTTTTCTTXXXXXXXXXXA
ACAAACAAGAAGAAAACAAGAAACTAATGCATGAGCACTGGCAGATTGAAGAGATATAAGTTCTATAATCTTTATCGTT
AACCTCTGCCTGTTTCCTCTTAACTCATTTAGGTGGGAGATAAATTGATTAAGTTCCTCGATCTTCCCTTTTCTTTTTTTXXXXXXXXXXA
ACAAACAAGAAGAAAACAAGAAACTAATGCATGAGCACTGGTAGATTGAAGAGATATAAGTTCTATCATCTTTATCGTT
>Marker915166 Chr7 SNP_site:14313734 Ref_Type:C
AACCTCTGCCTGTTTCCTCTTAACTCATTTAGGTGGGAGATAAATTGATTAAGTTCCTCGATCTTCCCTTTTCTTTTCTTXXXXXXXXXXA
ACAAACAAGAAGAAAACAAGAAACTAATGCATGAGCACTGGCAGATTGAAGAGATATAAGTTCTATAATCTTTATCGTT
AACCTCTGCCTGTTTCCTCTTAACTCATTTAGGTGGGAGATAAATTGATTAAGTTCCTCGATCTTCCCTTTTCTTTTTTTXXXXXXXXXXA
ACAAACAAGAAGAAAACAAGAAACTAATGCATGAGCACTGGTAGATTGAAGAGATATAAGTTCTATCATCTTTATCGTT
>Marker915166 Chr7 SNP_site:14313759 Ref_Type:A
AACCTCTGCCTGTTTCCTCTTAACTCATTTAGGTGGGAGATAAATTGATTAAGTTCCTCGATCTTCCCTTTTCTTTTCTTXXXXXXXXXXA
ACAAACAAGAAGAAAACAAGAAACTAATGCATGAGCACTGGCAGATTGAAGAGATATAAGTTCTATAATCTTTATCGTT
AACCTCTGCCTGTTTCCTCTTAACTCATTTAGGTGGGAGATAAATTGATTAAGTTCCTCGATCTTCCCTTTTCTTTTTTTXXXXXXXXXXA
ACAAACAAGAAGAAAACAAGAAACTAATGCATGAGCACTGGTAGATTGAAGAGATATAAGTTCTATCATCTTTATCGTT
>Marker915267 Chr7 SNP_site:15565107 Ref_Type:C
GACTTTACCATTCTTCACAACATCACCACCATCTACGTTCCACATAATAACATTTTCTCCCCTTCCCTCACCAGATATCAXXXXXXXXXXT
AAAATTATGTTTCCTGAAATGGTGAATCAAGGGAGGTTTGATCAGAAAAACAATCTAATATCTGAAAAGCAGGGAGAGT
GACTTTACCATTTTTCACAACATCACCACCATCTACGTTCCACATAATAACATTTTCTCCCCTTCCCTCACCAGATATCAXXXXXXXXXXT
AAAATTATGTTTCCTGAAATGGTGAATCAAGGGAGGTTTCATCAGAAAAACAATCTAATATCTGAAAAGCAGGGAGAGT
>Marker915267 Chr7 SNP_site:15565371 Ref_Type:G
GACTTTACCATTCTTCACAACATCACCACCATCTACGTTCCACATAATAACATTTTCTCCCCTTCCCTCACCAGATATCAXXXXXXXXXXT
AAAATTATGTTTCCTGAAATGGTGAATCAAGGGAGGTTTGATCAGAAAAACAATCTAATATCTGAAAAGCAGGGAGAGT
GACTTTACCATTTTTCACAACATCACCACCATCTACGTTCCACATAATAACATTTTCTCCCCTTCCCTCACCAGATATCAXXXXXXXXXXT
AAAATTATGTTTCCTGAAATGGTGAATCAAGGGAGGTTTCATCAGAAAAACAATCTAATATCTGAAAAGCAGGGAGAGT

Create PDF with GO2PDF for free, if you wish to

>Marker915708 Chr7 SNP_site:6563650 Ref_Type:T
CACAACAACAATGATGGAATCAATTCAATAAACTTGATCTACATGATATTGTGACTGGAGCAGGGTATTTCAAAAGGTAAXXXXXXXXXXXT
CTCTGAAGAAATCTTCCCAGACATTTACCTTTTCTCCGAGAAACAAGACGCCACAGATGCAGAATGTTCTAGACAGGGT
CACAACAACAATGATGGAATCAATTCAATAAACTTGATCTACTTGATATTGTGACTGGAGCAGGGTATTTCAAAAGGTAAXXXXXXXXXXXT
CTCTGAAGAAATCTTCCCAGACATTTACCTTTTCTCCGAGAAACAAGACGCCACAGATGCAGAATGTTCTAGACAGGGT
>Marker915709 Chr7 SNP_site:6752638 Ref_Type:G
ACCCTAGCCTTGTAATCCTTTATACCCAATGCTTCAGTCAAAATGTCTTCACTGTCATTTATTGTAGCTAATTCATCCTAXXXXXXXXXXXC
ATCATCAAAATAAGTATTATTTTTGCTTTCCATGCTTCGTTCCATAGAGTTGAACGATAGAAAAAATCACTCGGTAGTT
ACCCTAGCCTTGTGTTCCTTTATACCCAATGCTTCAGTCAAAATATCTTCACTGTCATTTATTGTAGCTAATTCATCCTAXXXXXXXXXXXC
ATCATCAAAATAAGTATTATTTTTGCTTTCCATGCTTCGTTCCATAGAGTTGAACGATAGAAAAAATCACTCGGTAGTT
>Marker915709 Chr7 SNP_site:6752639 Ref_Type:T
ACCCTAGCCTTGTAATCCTTTATACCCAATGCTTCAGTCAAAATGTCTTCACTGTCATTTATTGTAGCTAATTCATCCTAXXXXXXXXXXXC
ATCATCAAAATAAGTATTATTTTTGCTTTCCATGCTTCGTTCCATAGAGTTGAACGATAGAAAAAATCACTCGGTAGTT
ACCCTAGCCTTGTGTTCCTTTATACCCAATGCTTCAGTCAAAATATCTTCACTGTCATTTATTGTAGCTAATTCATCCTAXXXXXXXXXXXC
ATCATCAAAATAAGTATTATTTTTGCTTTCCATGCTTCGTTCCATAGAGTTGAACGATAGAAAAAATCACTCGGTAGTT
>Marker915709 Chr7 SNP_site:6752669 Ref_Type:A
ACCCTAGCCTTGTAATCCTTTATACCCAATGCTTCAGTCAAAATGTCTTCACTGTCATTTATTGTAGCTAATTCATCCTAXXXXXXXXXXXC
ATCATCAAAATAAGTATTATTTTTGCTTTCCATGCTTCGTTCCATAGAGTTGAACGATAGAAAAAATCACTCGGTAGTT
ACCCTAGCCTTGTGTTCCTTTATACCCAATGCTTCAGTCAAAATATCTTCACTGTCATTTATTGTAGCTAATTCATCCTAXXXXXXXXXXXC
ATCATCAAAATAAGTATTATTTTTGCTTTCCATGCTTCGTTCCATAGAGTTGAACGATAGAAAAAATCACTCGGTAGTT
>Marker91594 Chr4 SNP_site:16440585 Ref_Type:C
GACTCTGTGAGACCCATTTCTAATTTGGAGGAAGTAAATTTAGCAAAGTATTAAGAAGTTGGCGTTGAATAAATCCGCAAXXXXXXXXXXXG
GCATTTTGGGTAGTCTTCACAAGAAGAACCAAGTGTTTCATGGAGAAGAAGTCTGTGAGGAAGCATTGGAGTTTAAGTT
GACTCTGTGAGACCCATTTCTAATTTGGAGGAAGTAAATTTAGCAAAGTATTAAGAAGTTGGGGTTGAATAAATCCGCAGXXXXXXXXXXXG
GCATTTTGGGTAGTCTTCACAAGAAGAACCAAGTGTTTCATGGAGAAGAAGTCTGTGAGGAAGCATTGGAGTTTAAGTT
>Marker91594 Chr4 SNP_site:16440602 Ref_Type:A
GACTCTGTGAGACCCATTTCTAATTTGGAGGAAGTAAATTTAGCAAAGTATTAAGAAGTTGGCGTTGAATAAATCCGCAAXXXXXXXXXXXG
GCATTTTGGGTAGTCTTCACAAGAAGAACCAAGTGTTTCATGGAGAAGAAGTCTGTGAGGAAGCATTGGAGTTTAAGTT
GACTCTGTGAGACCCATTTCTAATTTGGAGGAAGTAAATTTAGCAAAGTATTAAGAAGTTGGGGTTGAATAAATCCGCAGXXXXXXXXXXXG
GCATTTTGGGTAGTCTTCACAAGAAGAACCAAGTGTTTCATGGAGAAGAAGTCTGTGAGGAAGCATTGGAGTTTAAGTT
>Marker915976 Chr7 SNP_site:178252 Ref_Type:A
TACAGGTATGAACAAAAGTTAATTTTACCTCTTTACCCCACTACTCTATATTTATGATTGGCCTTTCTCCCTTGCACTAAXXXXXXXXXXXA
GTTAATTGTATGACTAGCAATATTTATTAAGAATTACAAATATAATAGAACTTATTAACTTTAGATTCAATGGTTGGTA
TACAGGTATGAACAAAAGTTAATTTTACCTCTTTACCCCACTACTCTATGTTTATGATTGGCCTTTCTCCCTTGCACTAAXXXXXXXXXXXA
GTTAATTGTATGACTAGCAATATTTATTAAGAATTACAAATATAATAGAACTTATTAACTTTAGATTCAATGGTTGGTA
>Marker916215 Chr7 SNP_site:14306406 Ref_Type:C
AACATAGCTTAAAGTTTTCAAAAACATAGGCCAGAATAGCTTCAGCTTAAGCACAATACTCTACAAGAAACCCTTTCTAGXXXXXXXXXXXC
TGAAGCCCACAAATATATGAATGGGCTGCACACATGACTGAAGTGATGCAGTCTTCAAAATGTTAATTAATGCAATAGT
AACATAGCTTAAAGTTTTCAAAAACATAGGCCAGAATAGCTTCAGCTTAAGCACAATACTCTACAAGAAACCCTTTCTAGXXXXXXXXXXXC
TGAAGCCCACAAATATATGAATGGGCTGCACATATGACTGAAGTGATGCAGTCTTCAAAATGTTAATTAATGCAATAGT
>Marker916567 Chr7 SNP_site:4462217 Ref_Type:C
TACCTCCAATGCAGATTCACATGGATGATATGGTCAAGAGCAAGATTTCAGGGTTTTGTCAAAACTATTCTCAATATCGAXXXXXXXXXXXA
TGGCTGGGGAGTTCAAAAATTGAAGGATTCTGAGAAAGTGCTGAAAGAAGAAAATGATTCCCAGGAGGGGCATTTGAGT
TACCTCCAATGCAGATTCACATGGATGATATGGTCAAGAGCAAGATTTCAGGGTTTTGTCAAAACTATTCTCAATATCGAXXXXXXXXXXXA

Create PDF with GO2PDF for free, if you wish to

TGGCTGGGGAGTTCAACAATTGAAGGATTCTGAGAAAGTGCTGAAAGAAGAAAATGATTCCCAGGAGGGGCATTTGAGT
>Marker917078 Chr7 SNP_site:6299478 Ref_Type:C
ACGGGTGAGAGTGGCCAGATCGCTGACTCAATAAGCTTATCATTTTGCAGACAAGACCGAGTGGAGAGTTGGGAACATAAXXXXXXXXXXXA
CGAGAGGGTTTCTGATTATTGGTAGAACTATAAATAGGTTGTTCATTGGAGGAGCACTGATATTTAAGTATTAAAGGTA
ACGGGTGAGAGTGGCCAGATCGCTGACTCAATAAGCTTATCATTTTGCAGATAAGACCGAGTGGAGAGTTGGGAACATAAXXXXXXXXXXXA
CGAGAGGGTTTCTGATTATTGGTAGAACTATAAATAGGTTGTTCATTGGAGGAGCACTGATATTTAAGTATTAAAGGTA
>Marker917113 Chr7 SNP_site:15328565 Ref_Type:A
TACTTCAATGGAGGACATCAAATAAACTTAAATTCTTGAGAAAGCTCTAAACTTTTTAGTCATTTTCAAAAAAAAAAAAAXXXXXXXXXXXA
TAGGCATGGTTTTAAATGCTGAGAGAATGGCACCTTTGAAAGAAAATAGGCTAATTTCTTATTGTTTATTATTTTCGTC
TACTTCAATGGAGGACATCAAATAAACTTAAATTCTTGAGAAAGCTCTAAACTTTTTAGTCATTTTCAAAAAAAAAAAAAXXXXXXXXXXXA
TAGGCATGGTTTTAAATGCTGAGAGAATGGCACCTTTGAAAGAAAATTGGCTAATTTCTTATTGTTTATTATTTTCGTC
>Marker918253 Chr7 SNP_site:15901456 Ref_Type:A
GACATGCAATGGAATGAACATAATATTGCTTTTGAATTTACACTGACTCTACTACAAATCAGTCTTCCTTAGACTAATACXXXXXXXXXXXA
TTAATACTCTCTTTTGGTTTCTCCTCCATTGGAAATTTTGAATTACTCTCTTTTGGCTGCTCCACTAAAATTGCAGGGT
GACATGCAATGGAATGAACATAATATTGCTTTTGAATTTACACTGACTCTACTACAAATCAGTCTTCCTTAGACTAATGCXXXXXXXXXXXA
TTAATACTCTCTTTTGGTTTCTCCTCCATTGGAAATTTTGAATTACTCTCTTTTGGCTGCTCCACTAAAATTGCAGGGT
>Marker91867 Chr4 SNP_site:22819939 Ref_Type:C
ACCAGTCCTTCTGTTCCATATTTGAATTCAATGGCTAAATCACAAGCCAAAGAACATGTTCATAACACAATCTAATAGTCXXXXXXXXXXXA
GAGAACTTTGGCATATAATACATTCATGGAATTATGCAAAAGAAGTATAAGTGGGTCCTTGGATATTGTAAGCTATGTT
ACCAGTCCTTCTGTTCCATATTTGAATTCAATGGCTAAATCACAAGCCAAAGAACATGTTCATAACACAATCTAATAGTCXXXXXXXXXXXA
GAGAACTTTGGCATATCATACATTCATGGAATTATGCAAAAGAAGTATAAGTGGGTCCTTGGATATTGTAAGCTATGTT
>Marker919117 Chr7 SNP_site:14284164 Ref_Type:A
TACATATGGCAGGACAAAGAAGATGAAAAAAAGATCAGTGATATCAACAGGGACACATGATTAAACAAAACATCCTTTCCXXXXXXXXXXXA
TCTGGTTGGGACTCAGTTCTGTGTTATTCCTGTTAGTGAGGGCCTCAAGGTTTTGGTTGGTTTCTGTTTTTTCTAATGT
TACATATGGCAGGACAAAGAAGATGATAAAAAGATCAGTGATATCAACAGGGACACATGATTAAACAAAACATCCTTTCCXXXXXXXXXXXA
TCTGGTTGGGACTCAGTTCTGTGTTATTCCTGTTAGTGAGGGCCTCAAGGTTTTGGTTGGTTTCTGTTTTTTCTAATGT
>Marker919165 Chr7 SNP_site:16451847 Ref_Type:G
AACGGATTGGTATCAAAGGCACAGAAAGTTCCCGAAGAAGGATGGGTCATGCAAGATGGAACTCCATGGCCCGGAAATAAXXXXXXXXXXXG
TAAAATGCGTGCAGTTTTCTTATTGAATACAAACTCATCGTCTCATTTACACGTCAGGTTTTCCTAGGGCAAAACGGTG
AACGGATTGGTATCAAAGGCACAGAAAGTTCCCGAAGAAGGATGGGTCATGCAAGATGGAACTCCATGGCCCGGAAATAAXXXXXXXXXXXG
TAAGATGCGTGCAGTTTTCTTATTGAATACAAACTCATCGTCTCATTTACACGTCAGGTTTTCCTAGGGCAAAACGGTG
>Marker919808 Chr7 SNP_site:15191721 Ref_Type:C
ACTTTTAATTATGATATCCATTCTTTGAAACACATATAATCACATGAATAGTCTTTCCATCTTTTTTTTCTTTTAGAATTAXXXXXXXXXXXT
AGCATTGATAAATAAATAATTAGTTTCTCTTCATGTCAATATAATTATTCCAAATTAAAACTTTAGTTTAATTTTAGTC
ACTTTTAATTATGATATCCATTCTTTGAAACATATATAATCACATGAATAGTCTTTCCATCTTTTTTTTCTTTTAGAATTAXXXXXXXXXXXT
AGCATTGATAAATAAATAATTAGTTTCTCTTGATGTCAATATAATTATTCCAAATTAAAACTTTAGTTTAATTTTAGTC
>Marker919808 Chr7 SNP_site:15191894 Ref_Type:C
ACTTTTAATTATGATATCCATTCTTTGAAACACATATAATCACATGAATAGTCTTTCCATCTTTTTTTTCTTTTAGAATTAXXXXXXXXXXXT
AGCATTGATAAATAAATAATTAGTTTCTCTTCATGTCAATATAATTATTCCAAATTAAAACTTTAGTTTAATTTTAGTC
ACTTTTAATTATGATATCCATTCTTTGAAACATATATAATCACATGAATAGTCTTTCCATCTTTTTTTTCTTTTAGAATTAXXXXXXXXXXXT
AGCATTGATAAATAAATAATTAGTTTCTCTTGATGTCAATATAATTATTCCAAATTAAAACTTTAGTTTAATTTTAGTC
>Marker919892 Chr7 SNP_site:4609889 Ref_Type:A
ACAACAATTATAATTATAAAAATAGAGATCCAACAATTCAATTTCTAAAAAAAAAAAAGAGAGAGAGAGAGAGAATTACAXXXXXXXXXXXC
AAATCAATAAAAGACAAAAGCTATAAAGATAAGAACTAATTCATCCATTGAGTTCTTCATTTCCAATCCAATCCACGTC

Create PDF with GO2PDF for free, if you wish to

ACAACAATTATAATTATAAAAATAGAGATCCAACAATTCAATTTCTAAAAAAAAAAGAGAGAGAGAGAGAGAGAATTACAXXXXXXXXXXC
AAATCAATAAAAGACAAAAGCTATAAAGATAAGAACTAATTCATCCATTGAGTTCTTCATTTCCAATCCAATCCACGTC
>Marker920343 Chr7 SNP_site:13612560 Ref_Type:C
ACAACCAACTAGAACCATCCAGCAAACAGCAATCCAATCCACTTCTTAGAAACATAACACATATTCGTAAAAATAGAACAXXXXXXXXXXC
TTCAACTACTGATGGTAGGGCAAGGAGATTTGTGCAACCAGCAATGAGTAGTTGAAAGATGCCCAATGGAGAGAGTGTG
ACAACCAACTAGAATCATCCAGCAAACAGCAATCCAATCCACTTCTTAGAAACATAACACATATTCGTAAAAATAGAACAXXXXXXXXXXC
TTCAACTACTGATGGTAGGGCAAGGAGATTTGTGCAACCAGCAATGAGTAGTTGAAAGATGCCCAATGGAGAGAGTGTG
>Marker920648 Chr7 SNP_site:6229692 Ref_Type:C
TACACCAATATGATTAATGGACGACGATGATGTTAAATTTCTACTTTTTTGAGTCAGATCATACTAGACTTCAAATATTCXXXXXXXXXXG
GGTGATAGAAATGAGCCAAATATTTTTTTCATCAATGGTCTCAATGAAAAACAATAACATCAACTCATGAAATTTATGT
TACACCAATCTGATTAATGGACGACGATGATGTTAAATTTCTACTTTTTTGAGTCAGATCATACTAGACTTCAAATATTCXXXXXXXXXXG
GGTGATAGAAATGAGCCAAATATTTTTTTCATCAGTGGTCTCAATGAAAAACAATAACATCAACTCATGAAATTTATGT
>Marker920648 Chr7 SNP_site:6229890 Ref_Type:G
TACACCAATATGATTAATGGACGACGATGATGTTAAATTTCTACTTTTTTGAGTCAGATCATACTAGACTTCAAATATTCXXXXXXXXXXG
GGTGATAGAAATGAGCCAAATATTTTTTTCATCAATGGTCTCAATGAAAAACAATAACATCAACTCATGAAATTTATGT
TACACCAATCTGATTAATGGACGACGATGATGTTAAATTTCTACTTTTTTGAGTCAGATCATACTAGACTTCAAATATTCXXXXXXXXXXG
GGTGATAGAAATGAGCCAAATATTTTTTTCATCAGTGGTCTCAATGAAAAACAATAACATCAACTCATGAAATTTATGT
>Marker920862 Chr7 SNP_site:6324714 Ref_Type:G
ACTGGAGTAACAGATGGATGACGCGACTGAGACAAAAGAAGGCAAGGACCGTGTGTGGTTGGGGATTATCGACGAGTCAAXXXXXXXXXXG
CTCGGAAAGGAAAACGATTGAACGGTGGTGGTTCCAACCACTAAAACGAACATTGAAAGCTTCAAAGGATCTGTAGGTC
ACTGGAGTAACGGATGGATGACGCGACTGAGACAAAAGAAGGCAAGGACCGTGTGTGGTTGGGGATTATCGACGAGTCAAXXXXXXXXXXG
CTCGGAAAGGAAAACGATTGAACGGTGGTGGTTCCAACCACTAAAACGAACATTGAAAGCTTCAAAGGATCTGTAGGTC
>Marker920983 Chr7 SNP_site:9068002 Ref_Type:G
AACTCAAAATTTTCATTTAACAATCCCCTACTTGATTTTTTTTTTTACTATTAATCCCCCACCTGAAATTTTTTAAATGTGXXXXXXXXXXA
TTTCACACACAACATTATTCGAGTGAAGTTCTAAATTTGCTAGTGCTTTAAGGCCTTGCACGTATATCCTAGTTTAGTG
AACTCAAAATTTTCATTTAACAATCCCCTACTTGATTTTTTTTTTTACTATTAATCCCCCACGTGAAATTTTTTAAATGTGXXXXXXXXXXA
TTTCACACACAACATTATTCGAGTGAAGTTCTAAATTTGCTAGTGCTTTAAGGCCTTGCACGTATATCCTAGTTTAGTG
>Marker921113 Chr7 SNP_site:13539044 Ref_Type:T
TACCATTCCTGAATTAAAAATTAACAACAAAATCACTACTTCGAGAACTTGCTACAACTTTTGTTCCTTAACTTTCAACTXXXXXXXXXXC
TCTCTCTTTCTGAAGCAAGATATCAATTCTATTTTATAGCTCATACCAAACTATTTCATCCAATTGTCTGCTTCTTTGT
TACCATTCCTGAATTAAAAATTAACAACAAAATCACTACTTCGAGAACTTGCTACAACTTTTGTTTCTTAACTTTCAACTXXXXXXXXXXC
TCTCTCTTTCTGAAGCAAGATATCAATTCTATTTTATAGCTCATACCAAACTATTTCATCCAATTGTCTGCTTCTTTGT
>Marker921439 Chr7 SNP_site:8697660 Ref_Type:A
ACTCCATTCCTTAGTTTTAGACAGAGTCTTATACAGAAAATTAAGCACAATCATGATACATGGTTCTAGAAAAGTGAATTXXXXXXXXXXA
TCCATATTTTTTTGTAGTTTAGCTTCTACCGAACTTCCTGTTTCTATAAATTTCTTTCCTGCCTCATCCGATTGTTGTG
ACTCCATTCCTTAGTTTTAGACAGAGTCTTATACAGAAAATTAAGCACAATCATGATACATGGTTCTAGAAAAGTGAATTXXXXXXXXXXA
TCCATTTTTTTTTGTAGTTTAGCTTCTACCGAACTTCCTGTTTCTATAAATTTCTTTCCTGCCTCATCTGATTGTTGTG
>Marker921439 Chr7 SNP_site:8697723 Ref_Type:C
ACTCCATTCCTTAGTTTTAGACAGAGTCTTATACAGAAAATTAAGCACAATCATGATACATGGTTCTAGAAAAGTGAATTXXXXXXXXXXA
TCCATATTTTTTTGTAGTTTAGCTTCTACCGAACTTCCTGTTTCTATAAATTTCTTTCCTGCCTCATCCGATTGTTGTG
ACTCCATTCCTTAGTTTTAGACAGAGTCTTATACAGAAAATTAAGCACAATCATGATACATGGTTCTAGAAAAGTGAATTXXXXXXXXXXA
TCCATTTTTTTTTGTAGTTTAGCTTCTACCGAACTTCCTGTTTCTATAAATTTCTTTCCTGCCTCATCTGATTGTTGTG
>Marker921663 Chr7 SNP_site:11072028 Ref_Type:C
AACAATAGTGTTATAAAATTCATCTTTCTAACCACTCACTTATTTCAACTATAAAGCATGAACTCTTGATGCTACTACTAXXXXXXXXXXT

Create PDF with GO2PDF for free, if you wish to

GGTTGCTAGCATTTAACACTTGATAAGGAAAATGTATGACAGGATTCACTTTATATAACCCTTATGGATAAGTATCAGT
AACAATAGTGTTATAAAATTCATCTTTCTAACCACTCACTTATTTCAACTATAAAGCATGAACTCTTGATGCTACTACTAXXXXXXXXXXT
GGTTGCTAGCATTTAACACTTGATAAGGAAAATGTATGACAGGATTCACTTTATATAATCCTTATGGATAAGTATCAGT
>Marker923095 Chr7 SNP_site:17235307 Ref_Type:C
CACACACAAGAACTGCTAGATTTGCTGGTCAAATGCGAAAATAAAATTCAAACTCGTATTAAGATTGGGCTGAATTCAAAXXXXXXXXXXT
TTCTATACTCCCAAGGAAATTGGAGGTCTTGGCATGCTATCCATGGGTCACATATTGATCCCACAAAGTGATCTTCGGT
CACACACAAGAACTGCTAGATTTGCTGGTCAAATGCGAAAATAAAATTCAAACTCGTATTAAGATTGGGCTGAATTCAAAXXXXXXXXXXT
TTCTATACTCCCAAGGAAATTGGAGGTCTTGGCATGTTATCCATGGGTCACATATTGATCCCACAAAGTGATCTTCGGT
>Marker923283 Chr7 SNP_site:7538343 Ref_Type:G
ACCGTAATTTATCATTAATTTATCTTTCATTAGCATGCCAAGTTAAGTTTTAGCACATTCAACTCTTTTAAACATCCATTXXXXXXXXXXG
AATTGCATTGGCATATTTCTTTCGATACAAGCAACAATCATTAAGGCATGCATGAATCTTTTAGTAATCCATTCCTAGT
ACCGTAATTTATCATTAATTTATCTTTCATTAGCATGCCAAGTTAAGTTTTAGCACATTCAACTCTTTTAAACATCCGTTXXXXXXXXXXC
AATTGCATTGGCATATTTCTTTCGATACAAGCAACAATCATTAAGGCATGCATGAATCTTTTAGTAATCTATTCCTAGT
>Marker923283 Chr7 SNP_site:7538453 Ref_Type:C
ACCGTAATTTATCATTAATTTATCTTTCATTAGCATGCCAAGTTAAGTTTTAGCACATTCAACTCTTTTAAACATCCATTXXXXXXXXXXG
AATTGCATTGGCATATTTCTTTCGATACAAGCAACAATCATTAAGGCATGCATGAATCTTTTAGTAATCCATTCCTAGT
ACCGTAATTTATCATTAATTTATCTTTCATTAGCATGCCAAGTTAAGTTTTAGCACATTCAACTCTTTTAAACATCCGTTXXXXXXXXXXC
AATTGCATTGGCATATTTCTTTCGATACAAGCAACAATCATTAAGGCATGCATGAATCTTTTAGTAATCTATTCCTAGT
>Marker923283 Chr7 SNP_site:7538523 Ref_Type:T
ACCGTAATTTATCATTAATTTATCTTTCATTAGCATGCCAAGTTAAGTTTTAGCACATTCAACTCTTTTAAACATCCATTXXXXXXXXXXG
AATTGCATTGGCATATTTCTTTCGATACAAGCAACAATCATTAAGGCATGCATGAATCTTTTAGTAATCCATTCCTAGT
ACCGTAATTTATCATTAATTTATCTTTCATTAGCATGCCAAGTTAAGTTTTAGCACATTCAACTCTTTTAAACATCCGTTXXXXXXXXXXC
AATTGCATTGGCATATTTCTTTCGATACAAGCAACAATCATTAAGGCATGCATGAATCTTTTAGTAATCTATTCCTAGT
>Marker923374 Chr7 SNP_site:13247432 Ref_Type:A
ACTATTTCAAGGTAACCAATAAATTTTCTTTTGATTTAAGTAAGAAGATGAATATTGATGGGAAATGCTACATACCTACCXXXXXXXXXXG
CTGACTAGAAGACTTCAAAAAGATGTAAAACTAATTTTAACATACCTGTGAGGCAACATCAACGCCAATTTCATCGAGT
ACTATTTCAAGGTAACCAATAAATTTTCTTTTGATTTATGTAAGAAGATGAATATTGATGGGAAATGCTACATACCTACCXXXXXXXXXXG
CTGACTAGAAGACTTCAAAAAGATGTAAAACTAATTTTAACATACCTGTGAGGCAACATCAACGCCAATTTCATCGAGT
>Marker923421 Chr7 SNP_site:15716776 Ref_Type:A
ACTTAATTAATTAAATTTTACGCAAATTTTACTAATATCCATCAATAACATTTCAATACTTTACAGTTTCAGTGTGGTGTXXXXXXXXXXA
TTCGACCCTTTGAACTACCAACCCAAGCATAGCTCAAATGCTACCGATATAGCTAGACTAAGAAGACTAAGAAGTAGTG
ACTTAATTAGTTAAATTTTACGCAAATTTTACTAATATCCATCAATAACATTTCAATACTTTACCGTTTCAGTGTGGTGTXXXXXXXXXXA
TTCGACCCTTTGAACTACCAACCCAAGCATAGCTCAAATGCTACCGATATAGCTAGACTAAGAAGACTAAGAAGTAGTG
>Marker923421 Chr7 SNP_site:15716831 Ref_Type:A
ACTTAATTAATTAAATTTTACGCAAATTTTACTAATATCCATCAATAACATTTCAATACTTTACAGTTTCAGTGTGGTGTXXXXXXXXXXA
TTCGACCCTTTGAACTACCAACCCAAGCATAGCTCAAATGCTACCGATATAGCTAGACTAAGAAGACTAAGAAGTAGTG
ACTTAATTAGTTAAATTTTACGCAAATTTTACTAATATCCATCAATAACATTTCAATACTTTACCGTTTCAGTGTGGTGTXXXXXXXXXXA
TTCGACCCTTTGAACTACCAACCCAAGCATAGCTCAAATGCTACCGATATAGCTAGACTAAGAAGACTAAGAAGTAGTG
>Marker924022 Chr7 SNP_site:1762977 Ref_Type:T
AACTAACAGAATTGTCGTCTTCTGATAGAAAAATTAATAAATAAATAATCAGATCCATAACAAATTAAATGAACTTTTTGXXXXXXXXXXT
AACTAAACTTTAACAAAATATTAAAATTATGAAACTCAAACATAGTTAGATCTGTATATTAATGATTATAATGTAAGTT
AACTAACAGAATTGTCGTTTTCTGATAGAAAAACTAATAAATAAATAATCAGATCCATAACAAATTAAATGAACTTTTTGXXXXXXXXXXT
AACTAAACTTTAACAAAATATTAAAATTATGAAACTCAAACATAGTTAGATCTGTATATTAATGATTATAATGTAAGTT
>Marker924022 Chr7 SNP_site:1762992 Ref_Type:C

Create PDF with GO2PDF for free, if you wish to

AACTAACAGAATTGTCGTCTTCTGATAGAAAAATTAATAAATAAATAATCAGATCCATAACAAATTAAATGAACTTTTTGXXXXXXXXXXXT
AACTAAACTTTAACAAAATATTAAAATTATGAAACTCAAACATAGTTAGATCTGTATATTAATGATTATAATGTAAGTT
AACTAACAGAATTGTCGTTTTCTGATAGAAAAACTAATAAATAAATAATCAGATCCATAACAAATTAAATGAACTTTTTGXXXXXXXXXXXT
AACTAAACTTTAACAAAATATTAAAATTATGAAACTCAAACATAGTTAGATCTGTATATTAATGATTATAATGTAAGTT
>Marker924187 Chr7 SNP_site:17160702 Ref_Type:G
ACTCAATTGATGTGAGTTTGGAAATGGTTGCATTTGGAATTTTTCCACTGAATTGATTAGATGAGATATCAAGAAGTTTAXXXXXXXXXXXA
GCTTCTTAAACTCTAAAAGAAATCAAAAGTTAAAACTAAGCAATCTAAATTCATATTTCTTAATAATATTTATATAGTA
ACTCAATTGATGTGAGTTTGGAAATGGTTGCGTTTGGAATTTTTCCACTGAATTGATTAGATGAGATATCAAGAAGTTTAXXXXXXXXXXXA
GTTTCTTAAACTCTAAAAGAAATCAAAAGTTAAAACTAAGCAATCTAAATTCATATTTCTTAATAATATTTATGTAGTA
>Marker924187 Chr7 SNP_site:17160880 Ref_Type:T
ACTCAATTGATGTGAGTTTGGAAATGGTTGCATTTGGAATTTTTCCACTGAATTGATTAGATGAGATATCAAGAAGTTTAXXXXXXXXXXXA
GCTTCTTAAACTCTAAAAGAAATCAAAAGTTAAAACTAAGCAATCTAAATTCATATTTCTTAATAATATTTATATAGTA
ACTCAATTGATGTGAGTTTGGAAATGGTTGCGTTTGGAATTTTTCCACTGAATTGATTAGATGAGATATCAAGAAGTTTAXXXXXXXXXXXA
GTTTCTTAAACTCTAAAAGAAATCAAAAGTTAAAACTAAGCAATCTAAATTCATATTTCTTAATAATATTTATGTAGTA
>Marker924187 Chr7 SNP_site:17160952 Ref_Type:G
ACTCAATTGATGTGAGTTTGGAAATGGTTGCATTTGGAATTTTTCCACTGAATTGATTAGATGAGATATCAAGAAGTTTAXXXXXXXXXXXA
GCTTCTTAAACTCTAAAAGAAATCAAAAGTTAAAACTAAGCAATCTAAATTCATATTTCTTAATAATATTTATATAGTA
ACTCAATTGATGTGAGTTTGGAAATGGTTGCGTTTGGAATTTTTCCACTGAATTGATTAGATGAGATATCAAGAAGTTTAXXXXXXXXXXXA
GTTTCTTAAACTCTAAAAGAAATCAAAAGTTAAAACTAAGCAATCTAAATTCATATTTCTTAATAATATTTATGTAGTA
>Marker924642 Chr7 SNP_site:5113065 Ref_Type:A
CACACTGAAAACATTTAAATGCCGCAAATCCTGTCGAATAGGGTTTGCAATATCAACAGGAGACACTGACCATGGTAACGXXXXXXXXXXXG
TCCAAAAATTTCAGTTAATGAGAAAAGGCACTGAAAGATTTTCCACCACCTCACCCCCACGTATTTCGGATTAACTAGT
CACACTGAAAACATTTAAATGCCGCAAATCCTGTCGAATAGGGTTTGCAATATCAACAGGAGACACTGACCATGGTAACGXXXXXXXXXXXG
TCCAAAAATTTCAGTTAATGAGAAAAGGCACTGAAAGATTTTCCACCTCCTCACCCCCACGTATTTCGGATTAACTAGT
>Marker925384 Chr7 SNP_site:436651 Ref_Type:C
ACCTTTAAGTTCGTTGATTTCAACAGATTGTTCCGTTGTTAGTGTCTCTAAAATGTCATTTCTCATTGCTCATTCCTGCAXXXXXXXXXXXC
ATTGAGCTCGTTGAAATCCATAAGCCGCATGGTTCGACTCTGGATCTCTGCCGTCCATCAATTTTTTTCCTATTAACGTC
ACCTTTAAGTTCGTTGATTTCAACAGATTGTTCCGTTGTTAGTGTCTCTAAAATGTCATTTCTCATTGCTCATTTCTGCAXXXXXXXXXXXC
ATTGAGCTCGTTGAAATCCATAAGCCGCATGGTTCGACTCTGGATCTCTGCCGTCCATCAATTTTTTTCCTATTAACGTC
>Marker925607 Chr7 SNP_site:15760211 Ref_Type:C
AACCATTTGGGAATTCGATTAAACTACCACCTCGAGCAGATGATGCTTCTTTGCTAGACTGAAGAGCTCTTTGACGTGTGXXXXXXXXXXXT
GATGCACCATAATTCTCCATACTAGAAATGCTGGAAAGTTTCCGCTGCTTCTTGCTTGGTTCTTCACCATCATCACGGT
AACCATTTGGGAATTCGATTAAACTACCACCTCGAGCAGATGATGCTTCTTTGCTAGACTGAAGAGCTCTTTGACGTGTGXXXXXXXXXXXT
GATGCACCATAATTCTCCATATTAGAAATGCTGGAAAGTTTCCGCTGCTTCTTGCTTGGTTCTTCACCATCATCACGGT
>Marker925750 Chr7 SNP_site:16319305 Ref_Type:G
ACTAACCCAGTGTTCTTGCTATTGACTTATGAATTGATTGAAAAATTTCTGTCCCAAGAACTTAGGCGAGAATTTGTGGGXXXXXXXXXXXA
ATACAGTAAAACACAACTTTGCATTCAAAGCTCCAAATTACATTCATGAATGGCACTTTGAACAAGGAAAGAAAAGTT
ACTAACCCAGTGTTCTTGCTATTGACTTATGAATTGATTGAAAAATTTCTGTCCCAAGAACTTAGGCGAGAATTTGTGGGXXXXXXXXXXXA
ATACAGTAAAACACGACTTTGCATTCAAAGCTCCAAATTACATTCATGAATGGCACTTTGAACAAGGAAAGAAAAGTT
>Marker925940 Chr7 SNP_site:7542766 Ref_Type:C
AACACTCAAAGAAGCTACCTTATCTACTTACTCAATAAGGAAAAGAAGGAATTACATGCATCTTGTGAAATTATATTCTCXXXXXXXXXXXT
CGTGTGAAATATTCTCTTTAATAAAAGATATTTAGAAAAAATAACGACTCGAACAAAAATATGTATAGTTGAAGATGGT
AACACTTAAAGAAGCTACCTTATCTACTTACTCAATAAGGAAAAGAAGGAATTACATGCATCTTGTGAAATTATATTCTCXXXXXXXXXXXT
CGTGTGAAATATTCTCTTTAATAAAATATATTTAGAAAAAATAACGACTCGAACAAAAATATGTATAGTTGAAGATGGT

Create PDF with GO2PDF for free, if you wish to

>Marker925940 Chr7 SNP_site:7542983 Ref_Type:G
AACACTCAAAGAAGCTACCTTATCTACTTACTCAATAAGGAAAAGAAGGAATTACATGCATCTTGTGAAATTATATTCTCXXXXXXXXXXXT
CGTGTGAAATATTCTCTTTAATAAAAGATATTTAGAAAAAATAACGACTCGAACAAAAATATGTATAGTTGAAGATGGT
AACACTTAAAGAAGCTACCTTATCTACTTACTCAATAAGGAAAAGAAGGAATTACATGCATCTTGTGAAATTATATTCTCXXXXXXXXXXXT
CGTGTGAAATATTCTCTTTAATAAAATATATTTAGAAAAAATAACGACTCGAACAAAAATATGTATAGTTGAAGATGGT
>Marker92600 Chr4 SNP_site:20385723 Ref_Type:A
AACCTTTCATCAATTACATCCATGATCCAACAATCTATGTTGTTTGTTTTGATAATTGATGAGAAACGAAAACATAAAAGXXXXXXXXXXXA
GGTAAGCTCAAAGGGAAGGGGCGACTTCTCCTCTCTTCGTCCATAAATCTCACAAGCTTGTTAAGAAATGGGCGTTTGT
AACCTTTCATCAATTACATCCATGATCCGACAATCTATGTTGTTTGTTTTGATAATTGATGAGAAACGAAAACATAAAAGXXXXXXXXXXXA
GGTAAGCTCAAAGGGAAGGGGCGACTTCTCCTCTCTTCGTCCATAAATCTCACAAGCTTGTTAAGAAATGGGCGTTTGT
>Marker926230 Chr7 SNP_site:5240258 Ref_Type:C
ACCAAGCACCAGACAAGACCTTGTAGCCTATTTTAGAGGGCTAGCCTCTCCTCTAAAAAATGCAGAATAGTCCATTTACTXXXXXXXXXXXT
TGAGACATTTGAGGATTGGAGATTTCTTCTGGACAGATGCATTGTTGAGAAAATATGAATTAGCTACTACCTTTCAAGT
ACCAAGCACTAGACAAGACCTTGTAGCCTATTTTAGAGGGCTAGCCTCTCCTCTAAAAAATGCAGAATAGTCCATTTACTXXXXXXXXXXXT
TGAGACATTTGAGGATTGGAGATTTCTTCTGGACAGATGCATTGTTGAGAAAATATGAATTAGCTACTACCTTTCAAGT
>Marker926750 Chr7 SNP_site:13392326 Ref_Type:C
TACCTGTCATGGATATTTATATATGCAAGATGCGTGTAATGTCATATAGATTATGAAAGAACTGCAACATGATCATAGTTXXXXXXXXXXXT
ATTTTCAAAGATGAAAGGGTTAAATTCCCATTCTCCATATTTTCTACATTTAACATTAAAGGAACTAATGCTAATTGTT
TACCTGTCATGGATATTTATATATGCAAGATGGGTGTAATGTCATATAGATTATGAAAGAACAGCAACATGATCATAGTTXXXXXXXXXXXT
ATTTTCAAAGATGAAAGGGTTAAATTCCCATTCTCCATATTTTCTACATTTAACATTAAAGGAACTAATGCTAATTGTT
>Marker926750 Chr7 SNP_site:13392356 Ref_Type:A
TACCTGTCATGGATATTTATATATGCAAGATGCGTGTAATGTCATATAGATTATGAAAGAACTGCAACATGATCATAGTTXXXXXXXXXXXT
ATTTTCAAAGATGAAAGGGTTAAATTCCCATTCTCCATATTTTCTACATTTAACATTAAAGGAACTAATGCTAATTGTT
TACCTGTCATGGATATTTATATATGCAAGATGGGTGTAATGTCATATAGATTATGAAAGAACAGCAACATGATCATAGTTXXXXXXXXXXXT
ATTTTCAAAGATGAAAGGGTTAAATTCCCATTCTCCATATTTTCTACATTTAACATTAAAGGAACTAATGCTAATTGTT
>Marker927469 Chr7 SNP_site:7435766 Ref_Type:A
TACATAAAAATCCAAGGAATCAGAAGTATAACCTACCACAAATTACCAAGAAGTGACAAATCAGAGAGCAATAACTCTTCXXXXXXXXXXXA
ATAGAGAGTGAAAAAGGGGACCTTCTTGTTTTGCATCTACTCCATGAACGATGGATGGCTGGAACACGAACAACGAGTA
TACATAAAAATCCAAGGAATCAGAAGTATAACCTACCACAAATTACCAAGAAGTGACAAATCAGAGAGCAATAACTCTTCXXXXXXXXXXXA
ATAGAGAGTGAAAAAGGGGACCTTCTTGTTTTGCATCTACTCCATGAACGATGGATGGCTGGAACACGAGCAACGAGTA
>Marker927594 Chr7 SNP_site:15522221 Ref_Type:G
AACGTGGAAGCAAATATCTTAGAGACAGATGAGGCAACCGCAACATCACGTGCAGTGAGTTTGTCCGTTGTCGTATTGTCXXXXXXXXXXXA
TTTTTTCATATGTTGGAAACTGAATCGCAACGTGACTTACACCGGCCAAAGCAGGCACAAGACCACTGCAGAACACGGT
AACGTGGAAGCAAATATCTTAGAGACAGATGAGGCAACCGCAACATCACGTGCAGTGAGTTTGTCCGTTGTCGTATTGTCXXXXXXXXXXXA
TTTTTTCATATGTTGGAAACTGAATCGCGACGTGACTTACACCGGCCAAAGCAGGCACAAGACCACTGCAGAACACGGT
>Marker92779 Chr4 SNP_site:4495937 Ref_Type:A
CACCATATAACAATCTAAAACTAGTGACAAATATGTATTCTAAAAACATCCTCTTTCATGACGTTGTTTTTCCTATGTTCXXXXXXXXXXXA
AGGAAGGAAGGGAAAAATTGACCATAGTAAGGAATAAATTGAAATAAAATAACCCACTGTGCCTATATATAAATTTGTG
CACCATATAACAATCTAAATCTAGTGACAAATATGTATTCTAAAAACATCCTCTTTCATGACGTTGTTTTTCCTATGTTCXXXXXXXXXXXA
AGGAAGGAAGGGAAAAATTGACCATAGTAAGGAATAAATTGAAATAAAATAACCCACTGTGCCTATATATAAATTTGTG
>Marker928144 Chr7 SNP_site:15788670 Ref_Type:T
ACCTTTCTTAAGCAGTTACTTCATAAGAGTTAATTAAGTTGGAAGTTATTAATTGTAGTTTATTGAGTATTTATTTGGTTXXXXXXXXXXXT
CGAAGGTGTCAAAATTATAGTGGATGGTCATGAAAGAGCAGTCACTGACAAGGAAGGATTCTACAAGCTCGATCAGGTA
ACCTTTCTTAAGCAGTTACTTCATAAGAGTTAATTAAGTTGGTAGTTATTAATTGTAGTTTATTGAGTATTTATTTGGTTXXXXXXXXXXXT

Create PDF with GO2PDF for free, if you wish to

CGAAGGTGTCAAAATTATAGTGGATGGTCATGAAAGAGCAGTCACTGACAAGGAAGGATTCTACAAGCTCGATCAGGTA
>Marker928448 Chr7 SNP_site:1683713 Ref_Type:G
TACTTTTCAGAAATCTATTCTTGAATGGTAAGTGGATCATGATTCATGTGATAATTGTCATTTCATATGCTTTAATGGAAXXXXXXXXXXG
TTCATGAACTTCAGTTATTTATTACATGGTTCATCCAAAATAAAAAAGAGAAAAAGCAAGAGGTTCTTAGTTGCTTGTT
TACTTTTCAGAAATCTATTCTTGAATGGTAAGTGGATCATGATTCATGTGATAATTGTCATTTCATATGCTTTAATGGAAXXXXXXXXXXG
TTCATGAACTTCAGTTATTTATTACATGGTTCATCCAAAATAAAAAGGAGAAAAAGCAAGAGGTTCTTAGTTGTTTGTT
>Marker928448 Chr7 SNP_site:1683740 Ref_Type:T
TACTTTTCAGAAATCTATTCTTGAATGGTAAGTGGATCATGATTCATGTGATAATTGTCATTTCATATGCTTTAATGGAAXXXXXXXXXXG
TTCATGAACTTCAGTTATTTATTACATGGTTCATCCAAAATAAAAAAGAGAAAAAGCAAGAGGTTCTTAGTTGCTTGTT
TACTTTTCAGAAATCTATTCTTGAATGGTAAGTGGATCATGATTCATGTGATAATTGTCATTTCATATGCTTTAATGGAAXXXXXXXXXXG
TTCATGAACTTCAGTTATTTATTACATGGTTCATCCAAAATAAAAAGGAGAAAAAGCAAGAGGTTCTTAGTTGTTTGTT
>Marker928838 Chr7 SNP_site:13279175 Ref_Type:G
TACAAATTTTCTAATTCTATTGTCTTTTTCTTTAATGATGACCAACATAGAGGAGGAGAAAAAGATGAATGCAACCTACAXXXXXXXXXXT
TTGCATTTATAAATTGTTAAAGAATGAATTGATAGGATGAAATTCAATAACAGCAAATCAAAAGTTATAAGAATATGTT
TACAAATTTTCTAATTCTATTGTCTTTTTCTTTAATGATGACCAACATAGAGGAGGAGAAAAAGATGAATGCAACCTACAXXXXXXXXXXT
TTGCATTTATAAATTGTTAAAGAATGAATTGATAGGATGAAATTCAATAACAGCAAATCAAAAGTTATAAGAATGTGTT
>Marker929060 Chr7 SNP_site:2580050 Ref_Type:A
ACAACACCAAAAAGATCTTGAAACTCAGATTAAAGAAAAGACCTTCACTTTAATAATTGACCACTCACATAAGCTCCACCXXXXXXXXXXC
TTTAAATTCACCCATTATATATCAATTGAGTTATGGTATGAGTTAGTTATTAAATGTAAAAGAATGTTCTTGGTTGGTA
ACAACACCAAAAAGATCTTGAAACTCAGATTAAAGAAAAGGCCTTCACTTTAATAATTGACCACTCACATAAGCTCCACCXXXXXXXXXXC
TTTAAATTCACCCATTATATATCAATTGAGTTATGGTATGAGTTAGTTATTAAATGTAAAAGAATGTTCTTGGTTGGTA
>Marker929082 Chr7 SNP_site:13466603 Ref_Type:G
ACTGAGCTTGTGAAGAAAATAAAGAGGCCTGTTTCTCAGTAATTAGATCCTTCTCTGTCTGAAACAAGGTATTGAGATCAXXXXXXXXXXG
AATGGAGTTTGGGTAGGAGAACCTCTAGAAAGTTTTACATTTGAATTAAGCAAGGAGAAAACTGATTATTTAAAATGTG
ACTGAGCTTGTGAAGAAAATAAAGAGGCCTGTTTCTCAGTAATTAGATCCTTCTCTGTCTGAAACAAGGTATTGAGATCAXXXXXXXXXXG
AATGGAGTTTGGGTAGGAGAACCTCTAGAAAGTTTTACATTTGAATTAGGCAAGGAGAAAACTGATTATTTAAAATGTG
>Marker929720 Chr7 SNP_site:1659933 Ref_Type:C
CACACTTCCATGCAGAATAGAGCAAAATCATTGAGCTCTTATATTGAGAATTTGCAAATGTTGAAGTTGAATTATGATCGXXXXXXXXXXT
ATTGATTGAACCACTGTGATAGGAGTTTCTTGGTCTCACCATTGGGCGATACCTTCTTGAATTTGTGTTATGTTGTGTA
CACACTTCCATGCAGAATAGAGCAAAATCATTGAGCTCTTATATTGAGAATTTGCAAATGTTGAAGTTGAATTATGATCGXXXXXXXXXXT
ATTGATTGAACCACTGTGATAGGAGTTTCTTGGTCTCACCATTGGGCGATACCTTCTTGACTTTGTGTTATGTTGTGTA
>Marker929739 Chr7 SNP_site:13156131 Ref_Type:T
TACTTAGGTCCAAGCTTGAATCACAAAGGGCATAGATGTTTGAGTTTTACTGTTTGGCTGTATATATCTCGAGATGTGCAXXXXXXXXXXT
AGCAAAATTTAGTATGGTTAAACTGGCGATTATAGAATTTTTTGGATTTTTTTTTACCCGTTCCTTTAAGTAGGATAGT
TACTTAGGTCCAAGCTTGAATCACAAAGGGCATAGATGTTTGAGTTTTACTGTTTGGCTGTATATATCTTGAGATGTGCAXXXXXXXXXXT
AGCAAAATTTAGTATGGTTAAACTGGCGGTTATAGAATTTTTTGGATTTTTTTTTACCCGTTCCTTTAAGTAGGATAGT
>Marker929739 Chr7 SNP_site:13156264 Ref_Type:G
TACTTAGGTCCAAGCTTGAATCACAAAGGGCATAGATGTTTGAGTTTTACTGTTTGGCTGTATATATCTCGAGATGTGCAXXXXXXXXXXT
AGCAAAATTTAGTATGGTTAAACTGGCGATTATAGAATTTTTTGGATTTTTTTTTACCCGTTCCTTTAAGTAGGATAGT
TACTTAGGTCCAAGCTTGAATCACAAAGGGCATAGATGTTTGAGTTTTACTGTTTGGCTGTATATATCTTGAGATGTGCAXXXXXXXXXXT
AGCAAAATTTAGTATGGTTAAACTGGCGGTTATAGAATTTTTTGGATTTTTTTTTACCCGTTCCTTTAAGTAGGATAGT
>Marker930068 Chr7 SNP_site:17972920 Ref_Type:G
CACCTATCTTGGACCGAATATCCGTTTGGACTTAACGGGCTTTGATACCATGTTTGATAATATAGGGTTCCATCTCAAAAXXXXXXXXXXA
ACAATTTCACCACCAAGATAACAGACACTGTCCCAAAAAAACAAATAACAAAATGTTGCAGAGATAGATAGAAACAAGT

Create PDF with GO2PDF for free, if you wish to

CACCTATCTTGGACCGAATATCCGTTTGGACTTAACGGGCTTTGATACCATGTTTGATAGTATAGGGTTCCATCTCAAAAXXXXXXXXXXXA
ACAATTTCACCACCAAGATAACAGACACTGTCCCAAAAAAACAAATAACAAAATGTTGCAGAGATAGATAGAAACAAGT
>Marker930379 Chr7 SNP_site:584667 Ref_Type:A
TACAAATGCTGAAAGAAGAACATAGAACTGGGAAAAATGGACAAAACACAAGCCACAAGGATGTAGCAACAGAGGACTTTXXXXXXXXXXC
TTTTCATTATTTCTGTCAAACTTTTTCCAAATTATATCCAAAATGACAGAATCATTCTCGAAAAACTTAATGCTCTAGT
TACAAATGCTGAAAGAAGAACATAGAACTGGGAAAAATGGACAAAACACAAGCCACAAGGATGTAGCAACAGAGGACTTTXXXXXXXXXXC
TTTTCATTATTTCTGTCAAACTTTTTCCAAATTATATCCAAAATGGCAGAATCATTCTCGAAAAACTTAATGCTCTAGT
>Marker930388 Chr7 SNP_site:14086226 Ref_Type:T
CACCACGACCATTGAGATCTTCACCTAAGACTGATTTTTCTTCATAACTAGCTGTCTTTTCCCTCTGACTGGCTTCATCAXXXXXXXXXXXA
AACAGCCATCAGATTTAACACCTCTTCATTTCATGAAAGTAATAGTTTCATGATAAGTAAATATGATTGAATCATCGTC
CACCATGACCATTGAGATCTTCACCTAAGACTGATTTTTCTTCATAACTAGCTGTCTTTTCCCTCTGACTGGCTTCATCAXXXXXXXXXXXA
AACAGCCATCAAATTTAACACCTCTTCATTTCATGAAAGTAATAGTTTCATGATAAGTAAATATGATTGAATCATCGTC
>Marker930388 Chr7 SNP_site:14086453 Ref_Type:A
CACCACGACCATTGAGATCTTCACCTAAGACTGATTTTTCTTCATAACTAGCTGTCTTTTCCCTCTGACTGGCTTCATCAXXXXXXXXXXXA
AACAGCCATCAGATTTAACACCTCTTCATTTCATGAAAGTAATAGTTTCATGATAAGTAAATATGATTGAATCATCGTC
CACCATGACCATTGAGATCTTCACCTAAGACTGATTTTTCTTCATAACTAGCTGTCTTTTCCCTCTGACTGGCTTCATCAXXXXXXXXXXXA
AACAGCCATCAAATTTAACACCTCTTCATTTCATGAAAGTAATAGTTTCATGATAAGTAAATATGATTGAATCATCGTC
>Marker931016 Chr7 SNP_site:4883653 Ref_Type:A
ACTAAAAATGAATTAAAAGAGAGAAAATGAAATAAAGGGAAGATTAGGTGATGTATGTTAGAAAAATAACCCAAATGAAAXXXXXXXXXXXT
TGTGCCCTGGGCTGGACTCTAGGCCAACTGGGCTTTGGAGCCCAAAACTCCATTTTGATGGGCTTAGCCCAGTTTAGTT
ACTAAAAATGAATTAAAAGAGAGAAAATGAAATAAAGGGAAGATTAGGTGGTGTATGTTAGAAAAATAACCCAAATGAAAXXXXXXXXXXXT
TGTGCCCTGGGCTGGACTCTAGGCCAACTGGGCTTTGGAGCCCAAAACTCCATTTTGATGGGCTTAGCCCAGTTTAGTT
>Marker931469 Chr7 SNP_site:12927475 Ref_Type:G
TACATTGGTTTAGAACTTGGGAGATTGGTGTCTTATGGTGGTGAAAATGTTATGAACTACACAATGCATATGGAACAGGAXXXXXXXXXXXA
GAGGTTCAAATTCCCAACAAGGCATGTTTCTCAACTAGAAAAAGGACATATATTTCGTTTAGCTTCTCTTTTTTTGGGT
TACATTGGTTTAGAACTTGGGAGATTGGTGTCTTATGGTGGTGAAAATGTTATGAACTACAGAATGCATATGGAACAGGAXXXXXXXXXXXA
GAGGTTCAAATTCCCAACAAGGCATGTTTCTCAACTAGAAAAAGGACATATATTTCGTTTAGCTTCTCTTTTTTTGGGT
>Marker932232 Chr7 SNP_site:407474 Ref_Type:T
AACCACTACCACTCTAATGCTTAAGTCAGAAAATTGGAACGCTGAAGAGAGAATTAGTGTTGATTTTTAAAACCTACTTCXXXXXXXXXXT
TAGAATTTTATCGCTTTCTAACCAAGCCTATGTGCGACCTGTTTGCCCGTGGGTTTTGCTAATGAGCTTGGTCAAGGTC
AACCACTACCACTCTAATGCTTAAGTCAGAAAATTGGAACGCTGAAGAGAGAATTAGTGTTGATTTTTAAAACCTACTTCXXXXXXXXXXT
TAGAATTTTATCGCTTTCTAACCAAGCCTATGTTCGACCTGTTTGCCCGTGGGTTTTGCTAATGAGCTTGGTCAAGGTC
>Marker932281 Chr7 SNP_site:15616872 Ref_Type:C
AACTATACAGATAACAGAGCAAAAATATTCTTTTCCACTACTCTGAACCCAATTATCGTCAAGTTCATTGGCCAATCAGTXXXXXXXXXXG
ATAGTGATGATCCTTTTTTACATGGCAATGGGTAATGGGTAATGGAAAAAAAACTGAACTGGATAAGCTGAAGAGAAGT
AACTATACAGATAACAGAGCAAAAATATTGTTTTCCACTACTCTGAACCCAATTATCGTCAAGTTCATTGGCCAATCAGTXXXXXXXXXXG
ATAGTGATGATCCTTTTTTACATGGCAATGGGTAATGGGTAATGGGAAAAAAACTGAACTGGATAAGCTGAAGAGAAGT
>Marker932281 Chr7 SNP_site:15617058 Ref_Type:A
AACTATACAGATAACAGAGCAAAAATATTCTTTTCCACTACTCTGAACCCAATTATCGTCAAGTTCATTGGCCAATCAGTXXXXXXXXXXG
ATAGTGATGATCCTTTTTTACATGGCAATGGGTAATGGGTAATGGAAAAAAAACTGAACTGGATAAGCTGAAGAGAAGT
AACTATACAGATAACAGAGCAAAAATATTGTTTTCCACTACTCTGAACCCAATTATCGTCAAGTTCATTGGCCAATCAGTXXXXXXXXXXG
ATAGTGATGATCCTTTTTTACATGGCAATGGGTAATGGGTAATGGGAAAAAAACTGAACTGGATAAGCTGAAGAGAAGT
>Marker933498 Chr7 SNP_site:13974500 Ref_Type:A
ACCCTCCACTTGAACTTTCTATCTCGCTCTTCTTTACAGCCAAACAATTCTTTCGGTATGATCGAACAGGTGGCCATCCAXXXXXXXXXXXA

Create PDF with GO2PDF for free, if you wish to

GCTTGTTGATGATCTCTTATTAAAAAGATGAGTAGAACCATCATATAATTTTTGTAAGATAATAAATCATTATAAAGTT
ACCCTCCACTTGAACTTTCTATCTCGCTCTTCTTTACAGCCAAACAATTCTTTCGGTATGATCGAACAGGTGGCCATCCAXXXXXXXXXXXA
GCTTGTTGATGATCTCTTATTAAAAAGATGAGTAGCACCATCATATAATTTTTGTAAGATAATAAATCATTATAAAGTT
>Marker933587 Chr7 SNP_site:13282151 Ref_Type:G
CACTTTTCTGCTTTTTTTTGTGAGGAAGTTATTGCATCAAAAGAAAGGAAAACAGACTGGGTATGGGCAAGGAAAAGAAGXXXXXXXXXXC
GCATTGAGGCAATCTCTTCACGAAGTTGTCCCAGGATCTAAATGAACGAGTTCGATTACAATCAGACAGATAAAAATGT
CACTTTTCTGCTTTTTTTTGTGAGGAAGTTATTGCATCAAAAGAAAGGAAAACAGACTGGGTATGGGCAAGGAAAAGAAGXXXXXXXXXXC
GCATTGAGGCAGTCTCTTCACGAAGTTGTCCCAGGATCTAAATGAACGAGTTCGATTACAATCAGACAGATAAAAATGT
>Marker933986 Chr7 SNP_site:440988 Ref_Type:G
ACCTAACTTTCTTAATTCATGGTTATGTGTAGGCTCATATAACAATTTTAAACTAAACAATGAAAAGTGAGCATACTTGAXXXXXXXXXXXT
CATGTCACAGTCATTAAACATAATGTGATTGAGTAATTGGTAGCTCTTTAGCTGTTTAGTGGAGCATTTGAGATGGGTC
ACCTAACTTTCTTAATTCATGGTTATGTGTAGGCTCATATAACAATTTTAAGCTAAACAATGAAAAGTGAGCATACTTGAXXXXXXXXXXXT
CATGTCACAGTCATTAAACATAATATGATTGAGTAATTGGTAGCTCTTTAGCTCTTTAGTGGAGCATTTGAGATGGGTC
>Marker933986 Chr7 SNP_site:441193 Ref_Type:A
ACCTAACTTTCTTAATTCATGGTTATGTGTAGGCTCATATAACAATTTTAAACTAAACAATGAAAAGTGAGCATACTTGAXXXXXXXXXXXT
CATGTCACAGTCATTAAACATAATGTGATTGAGTAATTGGTAGCTCTTTAGCTGTTTAGTGGAGCATTTGAGATGGGTC
ACCTAACTTTCTTAATTCATGGTTATGTGTAGGCTCATATAACAATTTTAAGCTAAACAATGAAAAGTGAGCATACTTGAXXXXXXXXXXXT
CATGTCACAGTCATTAAACATAATATGATTGAGTAATTGGTAGCTCTTTAGCTCTTTAGTGGAGCATTTGAGATGGGTC
>Marker933986 Chr7 SNP_site:441222 Ref_Type:C
ACCTAACTTTCTTAATTCATGGTTATGTGTAGGCTCATATAACAATTTTAAACTAAACAATGAAAAGTGAGCATACTTGAXXXXXXXXXXXT
CATGTCACAGTCATTAAACATAATGTGATTGAGTAATTGGTAGCTCTTTAGCTGTTTAGTGGAGCATTTGAGATGGGTC
ACCTAACTTTCTTAATTCATGGTTATGTGTAGGCTCATATAACAATTTTAAGCTAAACAATGAAAAGTGAGCATACTTGAXXXXXXXXXXXT
CATGTCACAGTCATTAAACATAATATGATTGAGTAATTGGTAGCTCTTTAGCTCTTTAGTGGAGCATTTGAGATGGGTC
>Marker934157 Chr7 SNP_site:15599108 Ref_Type:A
CACACATACTCCAAATTGCGAAACCCTAAATAAAAAATCCACCAAAGCAAAATGAAATGGAAAACCCAAAGGTGGCCGACXXXXXXXXXXXA
AAATCTTCACTCTACCCTTAGCTACCAAACATTATTAATCTTATCATATATAATATAATAATGTAGCTCTATAATATGT
CACACATACTCCAAATTGCGAAACCCTAAATGAAAAATCCACCAAAGCAAAATTAAATCGAAAACCCAAAGGTGGCCGACXXXXXXXXXXXA
AAATCTTCACTCTACCCTTAGCTACCAAACATTATTAATCTTATCATATATAATATAATAATGTAGCTCTATAATATGT
>Marker934157 Chr7 SNP_site:15599130 Ref_Type:G
CACACATACTCCAAATTGCGAAACCCTAAATAAAAAATCCACCAAAGCAAAATGAAATGGAAAACCCAAAGGTGGCCGACXXXXXXXXXXXA
AAATCTTCACTCTACCCTTAGCTACCAAACATTATTAATCTTATCATATATAATATAATAATGTAGCTCTATAATATGT
CACACATACTCCAAATTGCGAAACCCTAAATGAAAAATCCACCAAAGCAAAATTAAATCGAAAACCCAAAGGTGGCCGACXXXXXXXXXXXA
AAATCTTCACTCTACCCTTAGCTACCAAACATTATTAATCTTATCATATATAATATAATAATGTAGCTCTATAATATGT
>Marker934157 Chr7 SNP_site:15599135 Ref_Type:G
CACACATACTCCAAATTGCGAAACCCTAAATAAAAAATCCACCAAAGCAAAATGAAATGGAAAACCCAAAGGTGGCCGACXXXXXXXXXXXA
AAATCTTCACTCTACCCTTAGCTACCAAACATTATTAATCTTATCATATATAATATAATAATGTAGCTCTATAATATGT
CACACATACTCCAAATTGCGAAACCCTAAATGAAAAATCCACCAAAGCAAAATTAAATCGAAAACCCAAAGGTGGCCGACXXXXXXXXXXXA
AAATCTTCACTCTACCCTTAGCTACCAAACATTATTAATCTTATCATATATAATATAATAATGTAGCTCTATAATATGT
>Marker934570 Chr7 SNP_site:15524037 Ref_Type:T
GACAATGTTCATCTGCACAAATGCCACGAGTGTCCGTGAAAAAAATGCATAAAGTCGTAAATTGGAAAAAAAAAAACAG-AXXXXXXXXXX
ACTACTACTATTCTTTAACAAGTGAAAACAAACCAAATTATACACAATCCAAACACCGAGGTAAAGCTTGCTTCAAGGTT
GACAATGTTCATCTGCACAAATGCCACGAGTGTCCGTGAAAAAAATGCATAAAGTCGTAAATTGG-AAAAAAAAACAGAAXXXXXXXXXXX
ACTACTACTATTCTTTAACAAGTGAAAACAAACCAAATTATACACAATCCAAACACCGAGGTAAAGCTTGTTTCAAGGTT
>Marker934821 Chr7 SNP_site:2441131 Ref_Type:C

Create PDF with GO2PDF for free, if you wish to

TACTGGAAGCTTCACTGTTGTAGAGAAACGAAGCCAACTTTAGGCCCACGAGGCGCGAGAAGAGAAAACCAAGTTGAACAXXXXXXXXXXXA
TTGGAGAACCGCAACTATTCTTTTGCCTTTAGGACACCGTGGTCTCCACATGATTATTAATATAGAATGCAATGTTTGT
TACTGGAAGCTTCACTGTTGTAGAGAAACGAAGCCAACTTTAGGCCCACGAGGCGCGAGAAGAGAAAACCAAGTTGAATAXXXXXXXXXXXA
TTGGAGAACCGCAACTATTCTTTTGCCTTTAGGACACCGTGGTCTCCACATGATTATTAATATAGAATGCAATGTTTGT
>Marker93533 Chr4 SNP_site:21146608 Ref_Type:C
TACTTGTGTTAGTTATTATTACTTGTTTTTGCTATTCTTGTAGTTGTCATTTTACATCATGTCATATTTTTTTAATTATTXXXXXXXXXXXA
TTTGTAGGATTTATTGTCCATAGATAGGGTCTTCTCTTTTCAAACTTTCTTTTCATCCGTAACAATTAAATGCTGAGTA
TACTTGTGTTAGTTATTATTACTTGTTTTTGCTATTCTTGTAGTTGTCATTTTACATTATGTTATATTTTTTTAATTATTXXXXXXXXXXXA
TTTGTAGGATTTATTGTCCATAGATAGGGTCTTCTCTTTTCAAACTTTCTTTTCATCCGTAACAATTAAATGCTAAGTA
>Marker93533 Chr4 SNP_site:21146613 Ref_Type:C
TACTTGTGTTAGTTATTATTACTTGTTTTTGCTATTCTTGTAGTTGTCATTTTACATCATGTCATATTTTTTTAATTATTXXXXXXXXXXXA
TTTGTAGGATTTATTGTCCATAGATAGGGTCTTCTCTTTTCAAACTTTCTTTTCATCCGTAACAATTAAATGCTGAGTA
TACTTGTGTTAGTTATTATTACTTGTTTTTGCTATTCTTGTAGTTGTCATTTTACATTATGTTATATTTTTTTAATTATTXXXXXXXXXXXA
TTTGTAGGATTTATTGTCCATAGATAGGGTCTTCTCTTTTCAAACTTTCTTTTCATCCGTAACAATTAAATGCTAAGTA
>Marker93533 Chr4 SNP_site:21146793 Ref_Type:G
TACTTGTGTTAGTTATTATTACTTGTTTTTGCTATTCTTGTAGTTGTCATTTTACATCATGTCATATTTTTTTAATTATTXXXXXXXXXXXA
TTTGTAGGATTTATTGTCCATAGATAGGGTCTTCTCTTTTCAAACTTTCTTTTCATCCGTAACAATTAAATGCTGAGTA
TACTTGTGTTAGTTATTATTACTTGTTTTTGCTATTCTTGTAGTTGTCATTTTACATTATGTTATATTTTTTTAATTATTXXXXXXXXXXXA
TTTGTAGGATTTATTGTCCATAGATAGGGTCTTCTCTTTTCAAACTTTCTTTTCATCCGTAACAATTAAATGCTAAGTA
>Marker93674 Chr4 SNP_site:18989037 Ref_Type:C
AACTCTTTTCTTTCTTTTATTTATTTCTCGATATTAAATTTTCAATTTTACAAATGTATCAATAGAATATCAATATTGATXXXXXXXXXXXA
TTAAGACTTTATTTAAAGTATCCTAATTGAATTGCATTTTGAGAAACTTATTATTGGTGTGTATCTATCTTGCTATGTT
AACTCTTTTCTTTCTTTTATTTATTTCTCGATATTAAATTTTCAATTTTACAAATGTATCAATAGAATATCAATATTGATXXXXXXXXXXXA
TTAAGACTTTATTTAAAGTATCCTAATTGAATTGCATTTTGAGAAACTTATTATTGGTGTGTATCTATCTTGGTATGTT
>Marker93740 Chr4 SNP_site:16679145 Ref_Type:A
AACAGATTTACAAATAACTAAATATCCAAATCTCACTGTTTTTCGATTATACTATTTAATTATATCAATACTCCAAACTCXXXXXXXXXXXC
TTTTGCTTGATACTATGTTAAATCCACCCATCAACCCAAAAGCTTAAGTTTATAAGTTATAGTTAAATTAATTATCAGT
AACAGATTTACAAATAACTAAATATCCAAATCTCACTGTTTTTCGATTATACTATTTAATTATATCAATACTCCAAACTCXXXXXXXXXXXC
TTTTGCTTGATACTATGTTAAATCCACCCATCAACCCAAAAGCTTAAGTTTATAAGTTATAGTTAAGTTAATTATCAGT
>Marker93858 Chr4 SNP_site:8926767 Ref_Type:T
ACAATTACAGCATTGATACGTCGTATTTTGTCAATATTATCTAGCAGAAAGGAATCAGAAGGGAAAAACCCCTTATTTACXXXXXXXXXXXA
ACAAAAACAATACTGTCATTTCTTAGACCCATATACTATCTTATCTTTCTGGACGTGGGTCAGAAGTTGCAAGTTTGTT
ACAATTACAGCATTGATATGTCGTATTTTGTCAATATTATCTAGCAGAAAGGAATCAGAAGGGAAAAACCCCTTATTTACXXXXXXXXXXXA
ACAAAAACAATACTGTCATTTCTTAGACCCATATACTATCTTATCTTTCTGGACGTGGGTCAGAAGTTGCAAGTTTGTT
>Marker938653 Chr2 SNP_site:17315691 Ref_Type:T
CACAAATGTAGAATGAAAAAGGAAAATACAAGATAACAGAGACATACCTCCGAGACAGTAAGTCCAAGATCCCGAGTTATXXXXXXXXXXXC
TTCTCTATCTGATCTTTTAGTTCATTCTAGTTTTTTCCATTGAAAAACTCAAACCCAGTAACTGAAAAACGAACTAGTA
CACAAATGTAGAATGAAAAAGGAAAATACAAGATAACAGAGACATACCTCTGAGACAGTAAGTCCAAGATCCCGAGTTATXXXXXXXXXXXC
TTCTCTATCTGATCTTTTAGTTCATTCTAGTTTTTTCCATTGAAAAACTCAAACCCAGTAACTGAAAAACGAACTAGTA
>Marker938678 Chr2 SNP_site:14091032 Ref_Type:C
GACAAATTTCTTACATAAATTAAATGTTTATCATTTAGACGTCTTTAGAAACATTAATACCATGTTATGTGTGAGGGGTTXXXXXXXXXXXT
TTTTTTTCTTTTAGTTTTAGACTTGGGAACATTCTACTTCAAACATGGTTTTTTTTTTCAAGTTCAAACTTTGCGAGTA
GACAAATTTCTTACATAAATTAAATGTTTATCATTTAGACGTCTTTAGAAACATTAATCCCATGTTATGTGTGAGGGGTTXXXXXXXXXXXT
TTTTTTTCTTTTAGTTTTAGACTTGGGAACATTCTACTTCAAACATGGTTTTTTTTTTCAAGTTCAAACTTTGCGAGTA

Create PDF with GO2PDF for free, if you wish to

>Marker938811 Chr2 SNP_site:4461764 Ref_Type:C
AACAGTCCCCATGCAGGTATCACCGCTTATTGTAGATTGATGATATGGAGTAGTCTAGAGAGCTCTACATAGAAAATTAAXXXXXXXXXXXA
CTTATTTTCATTTTCACCTCACTCATTCCAAAAACTTAATCATATGAAATTATTCATTTTCTTATTGTGACCTTTTGTG
AACAGTCCCCATGCAGGTATCACTGCTTATTGTAGATTGATGATATGGAGTAGTCTAGAGAGCTCTACGTAGAAAATTAAXXXXXXXXXXXA
CTTATTTTCATTTTCACCTCACTCATTCCGAAAACTTAATCATATGAAATTATTCATTTTCTTATTGTGACCTTTTGTG
>Marker938811 Chr2 SNP_site:4461809 Ref_Type:A
AACAGTCCCCATGCAGGTATCACCGCTTATTGTAGATTGATGATATGGAGTAGTCTAGAGAGCTCTACATAGAAAATTAAXXXXXXXXXXXA
CTTATTTTCATTTTCACCTCACTCATTCCAAAAACTTAATCATATGAAATTATTCATTTTCTTATTGTGACCTTTTGTG
AACAGTCCCCATGCAGGTATCACTGCTTATTGTAGATTGATGATATGGAGTAGTCTAGAGAGCTCTACGTAGAAAATTAAXXXXXXXXXXXA
CTTATTTTCATTTTCACCTCACTCATTCCGAAAACTTAATCATATGAAATTATTCATTTTCTTATTGTGACCTTTTGTG
>Marker938811 Chr2 SNP_site:4461944 Ref_Type:A
AACAGTCCCCATGCAGGTATCACCGCTTATTGTAGATTGATGATATGGAGTAGTCTAGAGAGCTCTACATAGAAAATTAAXXXXXXXXXXXA
CTTATTTTCATTTTCACCTCACTCATTCCAAAAACTTAATCATATGAAATTATTCATTTTCTTATTGTGACCTTTTGTG
AACAGTCCCCATGCAGGTATCACTGCTTATTGTAGATTGATGATATGGAGTAGTCTAGAGAGCTCTACGTAGAAAATTAAXXXXXXXXXXXA
CTTATTTTCATTTTCACCTCACTCATTCCGAAAACTTAATCATATGAAATTATTCATTTTCTTATTGTGACCTTTTGTG
>Marker93885 Chr4 SNP_site:10043029 Ref_Type:A
ACATTCAGATCTATGTAGTCTTATGAAGGTTAAAGTAAGAGGAGAGTTTGAATATTTCATCACTTTTACTAATGCTTACTXXXXXXXXXXXA
GGTGGAGAGTATATGGATTTGAAATTTCAAGACTATTTGATGGAATGTGAAATTGTATCTCAACTCTCAGCACCTGGTA
ACATTCAGATCTATGTAGTCTTATGAAGGTTAAAGTAAGAGGAGGGTTTGAATATTTCATCACTTTTACTAATGCTTACTXXXXXXXXXXXA
GGTGGAGAGTATATGAATTTGAAATTTCAAGACTATTTGATGGAATGTGAAATTGTATCTCAACTCTCAGCACCTGGTA
>Marker93885 Chr4 SNP_site:10043202 Ref_Type:G
ACATTCAGATCTATGTAGTCTTATGAAGGTTAAAGTAAGAGGAGAGTTTGAATATTTCATCACTTTTACTAATGCTTACTXXXXXXXXXXXA
GGTGGAGAGTATATGGATTTGAAATTTCAAGACTATTTGATGGAATGTGAAATTGTATCTCAACTCTCAGCACCTGGTA
ACATTCAGATCTATGTAGTCTTATGAAGGTTAAAGTAAGAGGAGGGTTTGAATATTTCATCACTTTTACTAATGCTTACTXXXXXXXXXXXA
GGTGGAGAGTATATGAATTTGAAATTTCAAGACTATTTGATGGAATGTGAAATTGTATCTCAACTCTCAGCACCTGGTA
>Marker93909 Chr4 SNP_site:19315716 Ref_Type:C
CACCATCAAATAGAATTTTTCCTCCAAAAACATCCTTATGGTTCAGTAGGTTTGATGTGTATTAGATTTAATAGGTAATCXXXXXXXXXXXA
GTTTCTAACATGTATATTGGATTTGAAGAACTTTTTCTTGCATAATATATTGATGCATCTGTTATAATGCTTACAGGTA
CACCATCAAATAGAATTTTTCCTCCAAAAACATCTTTATGGTTCAGTAGGTTTGATGTGTATTAGATTTAATAGGTAATCXXXXXXXXXXXA
GTTTCTAACATGTATATTGGATTTGAAGAACTTTTTCTTGCATAATATATTGATGCATCTGTTATAATGCTTACAGGTA
>Marker939332 Chr2 SNP_site:17609873 Ref_Type:G
CACACCAAGATTTTTTATGTCTCATATTTCTAGCTTCTAATCTTGCTTCGTCACCATACCCATGAATACTTTGAAGAATTXXXXXXXXXXXT
TCTCCACAGGATCCGGCTTCATTCTCACTTGCTCGGGCAGTCCATGCCCATATGATTGCTTCCGGTTTCAAGCCTCGTG
CACACCAAGATTTTTTATGTCTCATATTTCTAGCTTCTAATCTTGCTTCGTCACCATACCCATGAATACTTTGAAGAATTXXXXXXXXXXXT
TCTCCACAGGATCCGGCTTCATTCTCACTTGCTCGGGCGGTCCATGCCCATATGATTGCCTCCGGTTTCAAGCCTCGTG
>Marker939332 Chr2 SNP_site:17609894 Ref_Type:C
CACACCAAGATTTTTTATGTCTCATATTTCTAGCTTCTAATCTTGCTTCGTCACCATACCCATGAATACTTTGAAGAATTXXXXXXXXXXXT
TCTCCACAGGATCCGGCTTCATTCTCACTTGCTCGGGCAGTCCATGCCCATATGATTGCTTCCGGTTTCAAGCCTCGTG
CACACCAAGATTTTTTATGTCTCATATTTCTAGCTTCTAATCTTGCTTCGTCACCATACCCATGAATACTTTGAAGAATTXXXXXXXXXXXT
TCTCCACAGGATCCGGCTTCATTCTCACTTGCTCGGGCGGTCCATGCCCATATGATTGCCTCCGGTTTCAAGCCTCGTG
>Marker939580 Chr2 SNP_site:13778347 Ref_Type:G
ACTCTCTTGATAACATTCATTTTTACTCTCTTGATATTCTTTCCTTTGAGTTATTTGGAGAATATGGTGTATATTTTTAAXXXXXXXXXXXA
ACCGTCTAAACTACTATTTGTTTAGCTTTTTGCGAAAGCAAGAACAACTTTGAGCTCGGTTTTGAGCATTGAAACTGGT
ACTCTCTTGATAACATTCATTTTTACTCTCTTGATATTCTTTCCTTTGAGTTGTTTGGAGAATATGGTGTATATTTTTAAXXXXXXXXXXXA

Create PDF with GO2PDF for free, if you wish to

ACCGTCTAAACTACTATTTGTTTAGCTTTTTGCGAAAGCAAGAACAACTTTGAGCTCGGTTTTGAGCATTGAAACTGGT
>Marker940705 Chr2 SNP_site:21926330 Ref_Type:C
ACCCTTTCCAAAAGCTCCATTCTGCTTCACTCGAAGACCACGACACTGCCTTGTCCATAAAAGCTGATCAAAATATTCCTXXXXXXXXXXA
GCAACTTTGAAATTGAGAGAGATTACCTGAACTTGGTGAGGTGAATGAGGAGAAAGGAGAGCAGAGATTTCATTGGGGT
ACCCTTTCCAAAAGCTCCATTCTGCTTCACTCGAAGACCACGACACTGCCTTGTCCATACAAGCTGATCAAAATATTCCTXXXXXXXXXXA
GCAACTTTGAAATTGAGAGAGATTACCTGAACTTGGTGAGGTGAATGAGGAGAAAGGAGAGCAGAGATTTCATTGGGGT
>Marker941505 Chr2 SNP_site:13580855 Ref_Type:T
CACAACTTCCATGCTCCTTGTCTGCAATATGGTCTAAACTTTTGTTTTCTGGTAACTTTTTATTATGTGTGGGGCAGGTGXXXXXXXXXXT
ATCTTAACTGACTCTTCCGGCAAGAGAAGAAAGGTTATGCTGAACAGCTCAACTATTTCAGAAGAGCGATTGTGTGGTT
CACAACTTCCATGCTCCTTGTCTGCAATATGGTCTAAACTTTTGTTTTCTGGTAACTTTTTATTATGTGTGGGGCAGGTGXXXXXXXXXXT
ATCTTAACTGACTCTTCTGGCAAGAGAAGAAAGGTTATGCTGAACAGCTCAACTATTTCAGAAGAGCGATTGTCTGGTT
>Marker941505 Chr2 SNP_site:13580911 Ref_Type:C
CACAACTTCCATGCTCCTTGTCTGCAATATGGTCTAAACTTTTGTTTTCTGGTAACTTTTTATTATGTGTGGGGCAGGTGXXXXXXXXXXT
ATCTTAACTGACTCTTCCGGCAAGAGAAGAAAGGTTATGCTGAACAGCTCAACTATTTCAGAAGAGCGATTGTGTGGTT
CACAACTTCCATGCTCCTTGTCTGCAATATGGTCTAAACTTTTGTTTTCTGGTAACTTTTTATTATGTGTGGGGCAGGTGXXXXXXXXXXT
ATCTTAACTGACTCTTCTGGCAAGAGAAGAAAGGTTATGCTGAACAGCTCAACTATTTCAGAAGAGCGATTGTCTGGTT
>Marker94212 Chr4 SNP_site:21064215 Ref_Type:G
TACTAGCCTTACCCATCCTCATCCACACTCTTTTTAGCGGGTAGTAGATATTGCCCTTCCACATTTTTGTATTTCGAGCGXXXXXXXXXXT
CAAGTTAAACTATGTTCGATTTAGTTCTAATATAATATTTCAATCTACAAATTTCACATAATTTAACAAAAAAAGAAGT
TACTAGCCTTACCCATCCTCATCCACGCTCTTTTTAGCGGGTAGTAGATATTGCCCTTCCACATTTTTGTATTTCGAGCGXXXXXXXXXXT
CAAGTTAAACTATGTTCGATTTAGTTCTAATATAATATTTCAATCTACAAATTTCACATAATTTAACAAAAAAAGAAGT
>Marker942567 Chr2 SNP_site:10896092 Ref_Type:T
AACCCAATCGCGAACGCAAAAGATATGGTAAAAATACCCAAAAGAAGAAGATTGACGGGATGCCTCTGATAGTAACAAGAXXXXXXXXXXA
AAGGCGTAAGAATGAGAAGGATGTATAAAGCCAAGCCCGCCCCAGTTGAGCTAAAGAAAGTAGAAATTGGTCGGACGTA
AACCCAATCGCGAACGCAAAAGATATGGTAAAAATACCCAAAAGAAGAAGATTGACGGGATGCCTCTGATAGTAACAAGAXXXXXXXXXXA
AAGGTGTAAGAATGAGAAGGATGTATAAAGCCAAGCCCGCCCCAGTTGAGCTAAAGAAAGTAGAAATTGGTCGGACGTA
>Marker94312 Chr4 SNP_site:20611417 Ref_Type:A
GACATCACCAAAAGATCCTCTTCCAATAAGCTCCAAAGAACTAAATCTTGCTGCCACTGCCTCTGCTATACTCGCCACATXXXXXXXXXXC
TCATCTCCAGATTTCCCTCATTTTTTGTAAGAAACAATCCAACCAGCAACCTCAAATCCACGACCAAAGTATTTATGTT
GACATCACCAAAAGATCCTCTTCCAATAAGCTCCAAAGAACTAAATCTTGCTGCCACTGCCTCTGCTATACTCGCCACGTXXXXXXXXXXC
TCATCTCCAGATTTCCCTCATTTTTTGTAAGAAACAATCCAACCAGCAACCTCAAATCCACGACCAAAGTATTTATGTT
>Marker943459 Chr2 SNP_site:17290535 Ref_Type:C
ACTCTTCTGCCTTTCTTTTTTATGTGGAATCAAACCCAAAATCACTTCTAAGATTCTTCTTCCTTTGCTTTCCTTTGATCXXXXXXXXXXC
AATTTTCCACTATCATGTTTGTTTTCTTGTAGTAATGTCATTTCTATCAATTACCGAACACAAACTTAGAGATAAGAGT
ACTCTTCTGCCTTTCTTTTTTATGTGGAATCAAACCCAAAATCACTTCTAAGATTCTTCTTCCTTTGCTTTCCTTTGATCXXXXXXXXXXC
AATTTTTCACTATCATGTTTGTTTTCTTGTGTTAATGTCATTTCTATCAATTACCGAACACAAACTTAGAGATAAGAGT
>Marker943459 Chr2 SNP_site:17290559 Ref_Type:A
ACTCTTCTGCCTTTCTTTTTTATGTGGAATCAAACCCAAAATCACTTCTAAGATTCTTCTTCCTTTGCTTTCCTTTGATCXXXXXXXXXXC
AATTTTCCACTATCATGTTTGTTTTCTTGTAGTAATGTCATTTCTATCAATTACCGAACACAAACTTAGAGATAAGAGT
ACTCTTCTGCCTTTCTTTTTTATGTGGAATCAAACCCAAAATCACTTCTAAGATTCTTCTTCCTTTGCTTTCCTTTGATCXXXXXXXXXXC
AATTTTTCACTATCATGTTTGTTTTCTTGTGTTAATGTCATTTCTATCAATTACCGAACACAAACTTAGAGATAAGAGT
>Marker943459 Chr2 SNP_site:17290560 Ref_Type:G
ACTCTTCTGCCTTTCTTTTTTATGTGGAATCAAACCCAAAATCACTTCTAAGATTCTTCTTCCTTTGCTTTCCTTTGATCXXXXXXXXXXC
AATTTTCCACTATCATGTTTGTTTTCTTGTAGTAATGTCATTTCTATCAATTACCGAACACAAACTTAGAGATAAGAGT

Create PDF with GO2PDF for free, if you wish to

ACTCTTCTGCCTTTCTTTTTTATGTGGAATCAAACCCAAAATCACTTCTAAGATTCTTCTTCCTTTGCTTTCCTTTGATCXXXXXXXXXXC
AATTTTTCACTATCATGTTTGTTTTCTTGTGTTAATGTCATTTCTATCAATTACCGAACACAAACTTAGAGATAAGAGT
>Marker943571 Chr2 SNP_site:6810873 Ref_Type:C
CACACTTTTCTGACCAAACAAACTTACCATTCTTATCCTCAATAAAATCTCTCCAATTCAATATCCAATTAGATATTGTTXXXXXXXXXXT
GGCTCACAAAATTGACTTTCTATATTTATTTTAATGTTCCAATAATATGATATTTTAGTGTGACAGTGTTAAACATAGT
CACACTTTTCTGACCAAACAAACTTACCATTCTTATCCTCAATAAAATCTCTCCAATTCAATATCCAATTAGATATTGTTXXXXXXXXXXT
GGCTCACAAAATTGACTTTCTATATTTATTTTAATGTTTCAATAATATGATATTTTAGTGTGACAGTGTTAAACATAGT
>Marker943594 Chr2 SNP_site:22640940 Ref_Type:A
CACCAAGTTCTTTAACCAAAAAAAAAAAGAAAAGAAAAGAAAACATCTGGTAGTCAAATTAGGGAAGCCATAGAAATAGTXXXXXXXXXXA
AAATAGTGAGTATAAAAATACCCTACAAAGATGCACTACTAGTCTCGCTATGTGAATCGTTGGCTTTATATTAGGAGGT
CACCAAGTTCTTTAACCAAAAAAAAAAAGAAAAGAAAAGAAAACATCTGGTAGTCAAATTAGGGAAGCCATAGAAATAGTXXXXXXXXXXA
AAATGGTGAGTATAAAAATACCCTACAAAGATGCACTACTAGTCTCGCTATGTGAATCGTTGGCTTTATATTAGGAGGT
>Marker943655 Chr2 SNP_site:10989871 Ref_Type:A
AACTCTCAAACGAAATGATGATGTGACTTTTACATGTGAATGACTAGTTAGACAGTCAAATAAGATGATAACTTTTAAAAXXXXXXXXXXT
TGAAGTATTGACATTTTACTTAAATAAATTGTGCTTGTATTTACTTTTTTAATTATTGTTTTACATTGTCGTGGATGTT
AACTCTCAAACGGAATGATGATGTGACTTTTACATGTGAATGACTAGTTAGACAGTCAAATAAGATGATAACTTTTAAAAXXXXXXXXXXT
TGAAGTATTGGCATTTTACTTAAATAAATTGTGCTTGTATTTACTTTTTTAATTATTGTTTTACATTGTCGTGGATGTT
>Marker943655 Chr2 SNP_site:10990052 Ref_Type:A
AACTCTCAAACGAAATGATGATGTGACTTTTACATGTGAATGACTAGTTAGACAGTCAAATAAGATGATAACTTTTAAAAXXXXXXXXXXT
TGAAGTATTGACATTTTACTTAAATAAATTGTGCTTGTATTTACTTTTTTAATTATTGTTTTACATTGTCGTGGATGTT
AACTCTCAAACGGAATGATGATGTGACTTTTACATGTGAATGACTAGTTAGACAGTCAAATAAGATGATAACTTTTAAAAXXXXXXXXXXT
TGAAGTATTGGCATTTTACTTAAATAAATTGTGCTTGTATTTACTTTTTTAATTATTGTTTTACATTGTCGTGGATGTT
>Marker944450 Chr2 SNP_site:3307312 Ref_Type:T
TACAGAAGCATGCAGGAAGATGTAAGAGCCTCTTTGGAACACGTTACATCTAAGATCTCGGTCACTCTTGATTTCTGGAAXXXXXXXXXXC
CTGTGGAGGTCTAGAGATCTTTCACTCTATTGTGAAAGTTCTTAAGATGTATAACATAGAAAGTAGAATTCTTTCATGT
TACAGAAGCATGCAGGAAGATGTAAGAGCCTCTTTGGAACACGTTTCATCTAAGATCTCGGTCACTCTTGATTTCTGGAAXXXXXXXXXXC
CTGTGGAGGTCTAGAGATCTTTCACTCTATTGTGAAAGTTCTTAAGATGTATAACATAGAAAGTAGAATTCTTTCATGT
>Marker944695 Chr2 SNP_site:20140355 Ref_Type:A
ACCTTGTGCATGCTTCTGAAACTAAATGTTCATTGCATGACAGCATATTGGGACGATGTGAAAGGAGAAGTTGTTTTCTGXXXXXXXXXXT
TTAAACTTCTATAGGGCTTAATGACTCCAATTGTGAGGAATGCCGATTTGAAGACCATATCGGCAATTTCTTCAGAGGT
ACCTTGTGCATGCTTCTGAAACTAAATGTTCATTGCATGACAGCATATTGGGACGATGTGAAAGGAGAAGTTGTTTTCTGXXXXXXXXXXT
TTAAACTTCTGTAGGGCTTAATGACTCCAATTGTGAGGAATGCCGATTTGAAGACCATATCGGCAATTTCTTCAGAGGT
>Marker945474 Chr2 SNP_site:13784320 Ref_Type:A
GACCGGTCAATTCCAAAGGTTAATTCTACACTATACTATTCAACACACTCCATAATACTAAACCTGATAGTGAGAAAGAAXXXXXXXXXXA
ACAATAAGAAAAGGAGTAAGGGAAAAGGTCCAAGCAATACCGACCTCAGGTTTGGTAATATTGTTAGTGATGTTTTGTC
GACCGGTCAATTCCCAAGGTTAATTCTACACTATACTATTCAACACACTCCATAATACTAAACTTGATAGTGAGAAAGAAXXXXXXXXXXA
ACAATAAGAAAAGGAGTAAGGGAAAAGGTCCAAGCAATACCGACCTCAGGTTTGGTAATATTGTTAGTGATGTTTTGTC
>Marker945474 Chr2 SNP_site:13784369 Ref_Type:C
GACCGGTCAATTCCAAAGGTTAATTCTACACTATACTATTCAACACACTCCATAATACTAAACCTGATAGTGAGAAAGAAXXXXXXXXXXA
ACAATAAGAAAAGGAGTAAGGGAAAAGGTCCAAGCAATACCGACCTCAGGTTTGGTAATATTGTTAGTGATGTTTTGTC
GACCGGTCAATTCCCAAGGTTAATTCTACACTATACTATTCAACACACTCCATAATACTAAACTTGATAGTGAGAAAGAAXXXXXXXXXXA
ACAATAAGAAAAGGAGTAAGGGAAAAGGTCCAAGCAATACCGACCTCAGGTTTGGTAATATTGTTAGTGATGTTTTGTC
>Marker945545 Chr2 SNP_site:15606595 Ref_Type:G
GACATTATTTTAGTATTCTAATTATAATGATTAATCTTATTCTTCGTTTGTTGGCTTGAAATATATACGAAAAAGAACCAXXXXXXXXXXC

Create PDF with GO2PDF for free, if you wish to

TTAAACTAATAGATTAGGATAAATTTAATTATATTAGGTTAAGAATTTCTTAAACAAATACTTAATTCCCGATATTGTT

GACATTATTTTAGTATTCTAATTATAATGGTTAATCTTATTCTTCGTTTGTTGGCTTGAAATATATACGAAAAAGAACCAXXXXXXXXXXC

TTAAACTAATAGGTTAGGATAAATTTAATTATATTAGGTTAAGAATTTCTTAAACAAATACTTAATTCCCGATATTGTT

>Marker945545 Chr2 SNP_site:15606780 Ref_Type:G

GACATTATTTTAGTATTCTAATTATAATGATTAATCTTATTCTTCGTTTGTTGGCTTGAAATATATACGAAAAAGAACCAXXXXXXXXXXC

TTAAACTAATAGATTAGGATAAATTTAATTATATTAGGTTAAGAATTTCTTAAACAAATACTTAATTCCCGATATTGTT

GACATTATTTTAGTATTCTAATTATAATGGTTAATCTTATTCTTCGTTTGTTGGCTTGAAATATATACGAAAAAGAACCAXXXXXXXXXXC

TTAAACTAATAGGTTAGGATAAATTTAATTATATTAGGTTAAGAATTTCTTAAACAAATACTTAATTCCCGATATTGTT

>Marker945800 Chr2 SNP_site:138530 Ref_Type:T

GACTTTCTTAGTCGCCTCACCACCTAGCTGCATCTTTATCACAGCAGCTCCTACATACAACTTTTTCTTCCTACTTCAAAXXXXXXXXXXA

TCTCGTCAATAAATAGCAATTTCTCCTTACTAAAGAAATCATAATAATGAGATAGCTCATTAAAAAATAGCCTATGGTC

GACTTTCTTAGTCGCCTCACCACCTAGCTGCATCTTTATCACAGCAGCTCCTACATACAACTTTTTCTTCCTACTTCAAAXXXXXXXXXXA

TCTCGTCAATAAATAGCAATTTCTCCTTACTAAAGAAATTATAATAATGAGATAGCTCATTAAAAAATAGCCTATGGTC

>Marker945922 Chr2 SNP_site:13430826 Ref_Type:A

TACATAAAAACATAATGGTTGGAGACAGTGTAGTAGAGAAAGAGGACCAGAATTGAAAAGAATGTTAGGGATGGAAGAGAXXXXXXXXXXC

ACTTCCCATTATGGCATGAACCGAGTTCTTTCTCTCTACACGCATCAAAGGGCCCTTGGATATTCTCAAAATCTCAAGT

TACATAAAAACATAATGGTTGGAGACAGTGTAGTAGAGAAAGAGGACCAGAATTGAAAAGAATGTTAGGGATGGAAGAGAXXXXXXXXXXC

ACTTCCCATTATGGCATGAGCCGAGTTCTTTCTCTCTACACGCATCAAAGGGCCCTTGGATATTCTCAAAATCTCAAGT

>Marker946492 Chr2 SNP_site:11444063 Ref_Type:A

CACCGTGAATTAAATCTCATTTGGAAGGTGGAATTTCTTTGTTCGTTGGTTATATAGTCTTCAAAATCGAACCTCTGATTXXXXXXXXXXT

TTTTGTTGGGCCTTATTAAAGACTTTTTGTTGTTTCCTGTAGCTAACTCAATAGATCGAGGCCGGAGACTACATCTTGT

CACCGTGAATTAAATCTCATTTGGAAGGTGGAATTTCTTTGTTCGTTGGTTATATTGTCTTCAAAATCGAACCTCTGATTXXXXXXXXXXT

TTTTGTTGGGCCTTATTAAAGACTTTTTGTTGTTTCCTGTAGCTAACTCAATAGATCGAGGCCGGAGACTACATCTTGT

>Marker946564 Chr2 SNP_site:10943092 Ref_Type:T

AACCTTGTCATTGCACTCTTCAGTTTGCGCACTCGGTCCAAGTTACGACTGCTAATCTGCATACAAATGCCAAATTTATTXXXXXXXXXXG

AGAACAATTATTTCCATTGATGTGAAGTCTTTTGGAGTGAAACCAAAAAGAAAATCCAGGAGGGTATATGTCCAAAGTG

AACCTTGTCATTGCACTCTTCAGTTTGCGCACTCGGTCCAAGTTACGACTGCTAATCTGCATACAAATGCCAAATTTATTXXXXXXXXXXG

AGAACAATTATTTCCATTGATGTGAAGTCTTTTGGAGTGAAACCAAAAAGAAAATCTAGGAGGGTATATGTCCAAAGTG

>Marker947710 Chr2 SNP_site:285356 Ref_Type:G

ACATTCCGCAGTCTGTAAGGTTTGAAGATGAGAGTGAGACCCAACATTAACATAGACGGAAATATAACAATAATAA-AATGXXXXXXXXXX

AAACATCACCTGTATATTTGTCATCTATTGTGATATTTATTAATTGATTAATATTTTACTTCATGCCGCTACATCAAGTA

ACATTCCGCAGTCTGTAAGTTTTGAAGATGAGAGTGAGACCCAACATTAACATAGACGGAAATACAACAATAACAATAAT-XXXXXXXXX

AAACATCACCTGTATATTTGTCATCTATTGTGATATTTATTAATTGATTAATATTTTACTTCATGCCGCTACATCAAGTA

>Marker947710 Chr2 SNP_site:285401 Ref_Type:T

ACATTCCGCAGTCTGTAAGGTTTGAAGATGAGAGTGAGACCCAACATTAACATAGACGGAAATATAACAATAATAA-AATGXXXXXXXXXX

AAACATCACCTGTATATTTGTCATCTATTGTGATATTTATTAATTGATTAATATTTTACTTCATGCCGCTACATCAAGTA

ACATTCCGCAGTCTGTAAGTTTTGAAGATGAGAGTGAGACCCAACATTAACATAGACGGAAATACAACAATAACAATAAT-XXXXXXXXX

AAACATCACCTGTATATTTGTCATCTATTGTGATATTTATTAATTGATTAATATTTTACTTCATGCCGCTACATCAAGTA

>Marker947710 Chr2 SNP_site:285410 Ref_Type:T

ACATTCCGCAGTCTGTAAGGTTTGAAGATGAGAGTGAGACCCAACATTAACATAGACGGAAATATAACAATAATAA-AATGXXXXXXXXXX

AAACATCACCTGTATATTTGTCATCTATTGTGATATTTATTAATTGATTAATATTTTACTTCATGCCGCTACATCAAGTA

ACATTCCGCAGTCTGTAAGTTTTGAAGATGAGAGTGAGACCCAACATTAACATAGACGGAAATACAACAATAACAATAAT-XXXXXXXXX

AAACATCACCTGTATATTTGTCATCTATTGTGATATTTATTAATTGATTAATATTTTACTTCATGCCGCTACATCAAGTA

>Marker948275 Chr2 SNP_site:21009859 Ref_Type:T

Create PDF with GO2PDF for free, if you wish to

ACATTCATGAAACAATACTACTCATGGAATTGAGAGGATACAACCCTACTCATATTGTCACAACCATTTGTTCCAACAGTXXXXXXXXXXC
TTAATGATCTTCAAATGTATGTCAGGTTACCGTTAGTGGTTATGCCGTGGCAGGAGGTGGGCGGGGAATAGAGAGAGTA
ACATTCATGAAACAATACTACTCATGGAATTGAGAGGATACAACCCTACTCATATTGTCACAACCATTTGTTCCAACAGTXXXXXXXXXXC
TTAATGATCTTCAAATGTATGTCAGGTTACCGTTAGTGGTTATGCCGTGGCAGGAGGTGGGCGGGGAATAGAGAGAGTG
>Marker948282 Chr2 SNP_site:22824582 Ref_Type:G
ACAAGTCATTACTTGATAGGCAATTCAAGATGGTTCCAGCGACTTGCAATGTGTTGTTAGAGGTTTTGCTTAAGCATGAAXXXXXXXXXXG
AAGGTGGGAACTCAACCAAAGTCAAGGCCTTTTGCTATGGACGTTGCAGGGTATAATAATATCATTGCAAGGTTTTGTG
ACAAGTCGTTACTTGATAGGCAATTCAAGATGGTTCCAGCGACTTGCAATGTGTTGTTAGAGGTTTTGCTTAAGCATGAAXXXXXXXXXXG
AAGGTGGGAACTCAACCAAAGTCAAGGCCTTTTGCTATGGACGTTGCAGGGTATAATAATATCATTGCAAGGTTTTGTG
>Marker94889 Chr4 SNP_site:1518432 Ref_Type:C
TACCTAACATTTCCTTGCCAACCCTAGATCTAGATATAAACAGAGTTCTCAAGTAGGAAATCATTTCCAACAAGAGGCTCXXXXXXXXXXT
GTAGGAGTTGAAATTAATTGTTAGTCATTAGTTATTTGACAGTTAGTTAGTTTTACCTACTGGTTGGTTTCTTTTTGTG
TACCTAATATTTCCTTGCCAACCCTAGATCTAGATATAAACAGAGTTCTCAAGTAGGAAGTCATTTCCAACAAGAGGCTCXXXXXXXXXXT
GTAGGAGTTGAAATTAATTGTTAGTCATTAGTTATTTGACAGTTAGTTAGTTTTACCTACTGGTTGGTTTCTTTTTGTG
>Marker94889 Chr4 SNP_site:1518484 Ref_Type:G
TACCTAACATTTCCTTGCCAACCCTAGATCTAGATATAAACAGAGTTCTCAAGTAGGAAATCATTTCCAACAAGAGGCTCXXXXXXXXXXT
GTAGGAGTTGAAATTAATTGTTAGTCATTAGTTATTTGACAGTTAGTTAGTTTTACCTACTGGTTGGTTTCTTTTTGTG
TACCTAATATTTCCTTGCCAACCCTAGATCTAGATATAAACAGAGTTCTCAAGTAGGAAGTCATTTCCAACAAGAGGCTCXXXXXXXXXXT
GTAGGAGTTGAAATTAATTGTTAGTCATTAGTTATTTGACAGTTAGTTAGTTTTACCTACTGGTTGGTTTCTTTTTGTG
>Marker949256 Chr2 SNP_site:8003860 Ref_Type:C
GACGAATAAGTGCTCTATTGATAAAGGCAATGACAAGTATTATTATATGTTGATTCCTTTCAATCATGGAAAAAAAGAATXXXXXXXXXXT
CTTGAGTCTATTTTAATTTTAGTCGGGACTTATTGTTACATGGAGCAATTAGGAGAAACTATCTTTTGCAGATCTAGTT
GACGAATAAGTGCTCTATTGATAAAGGCAATGACAAGTATTATTATATGTTGATTCCTTTCAATCATGGAAAAAAAGAATXXXXXXXXXXT
GTTGAGTCTATTTTAATTTTAGTCGGGACTTATTGTTACATGGAGCAATTAGGAGAAACTATCTTTTGCAGATCTAGTT
>Marker949328 Chr2 SNP_site:19099141 Ref_Type:G
AACAAATTTAATGTGGAAAACTATAATAGAAAAAATGGGATGAATTTAAAGAAAACAAATAAAATAAAGATAGTTACAACXXXXXXXXXXT
AGATACGAAAATAAAGAAAAATCTTTATAGTTAAATTGTCTATGTTTGTATTTAATGAAAACCTTGTGACATTATGGTG
AACAAATTTAATGTGGAAAACTATAATAGAAAAAATGGGATGAATTTAAAGAAAACAAATAAAATAAAGATAGTTACAACXXXXXXXXXXT
AGATACGAAAATAAAGAAAAATCTTTATAGTTAAATTGTCTATGTTTGTATTTAATGAAAACCTTGTTACATTATGGTG
>Marker94949 Chr4 SNP_site:6297184 Ref_Type:T
CACCTTTTAGGATACAAATGATGAAAATGCGCTACGAGAACATCAAATTTCCTCTCTAATTAAGGAGAACAACCTTTTGGXXXXXXXXXXT
CTCTCGAAGAGAATCAAGTTTTGCAATTAAGCTCAATCTAAACACCATGAGTCTTATAACTAAAGAACCCTTGTCAAGT
CACCTTTTATAATACAAATGATGAAAATGCGCTACGAGAACATCAAATTTCCTCTCTAATTAAGGAGAACAACCTTTTGGXXXXXXXXXXT
CTCTCGAAGAGAATCAAGTTTTGCAATTAAGCTCAATCTAAACACCATGAGTCTTATAACTAAAGAACCCTTATCAAGT
>Marker94949 Chr4 SNP_site:6297185 Ref_Type:A
CACCTTTTAGGATACAAATGATGAAAATGCGCTACGAGAACATCAAATTTCCTCTCTAATTAAGGAGAACAACCTTTTGGXXXXXXXXXXT
CTCTCGAAGAGAATCAAGTTTTGCAATTAAGCTCAATCTAAACACCATGAGTCTTATAACTAAAGAACCCTTGTCAAGT
CACCTTTTATAATACAAATGATGAAAATGCGCTACGAGAACATCAAATTTCCTCTCTAATTAAGGAGAACAACCTTTTGGXXXXXXXXXXT
CTCTCGAAGAGAATCAAGTTTTGCAATTAAGCTCAATCTAAACACCATGAGTCTTATAACTAAAGAACCCTTATCAAGT
>Marker94949 Chr4 SNP_site:6297466 Ref_Type:A
CACCTTTTAGGATACAAATGATGAAAATGCGCTACGAGAACATCAAATTTCCTCTCTAATTAAGGAGAACAACCTTTTGGXXXXXXXXXXT
CTCTCGAAGAGAATCAAGTTTTGCAATTAAGCTCAATCTAAACACCATGAGTCTTATAACTAAAGAACCCTTGTCAAGT
CACCTTTTATAATACAAATGATGAAAATGCGCTACGAGAACATCAAATTTCCTCTCTAATTAAGGAGAACAACCTTTTGGXXXXXXXXXXT
CTCTCGAAGAGAATCAAGTTTTGCAATTAAGCTCAATCTAAACACCATGAGTCTTATAACTAAAGAACCCTTATCAAGT

Create PDF with GO2PDF for free, if you wish to

>Marker949757 Chr2 SNP_site:16032006 Ref_Type:G
AACATGCTTCAGGGATGCAGAAGTGTTGATGAGTTTGAGAGACTAAACAAGATCGACGAAGGCACCTATGGCATTGTGTTXXXXXXXXXXXA
TTCCATCACCCATCCATTGTTGATGTGAAGGAAGTTGTGGTGGGGAATAGTCTTGATAGCATTTTTATGGCCATGGAGT
AACATGCTTCAGGGATGCAGAAGTGTTGATGAGTTTGAGAGACTAAACAAGATCGACGAAGGCACCTATGGCATTGTGTTXXXXXXXXXXXA
TTCCATCACCCATCCATTGTTGATGTGAAGGAAGTTGTGGTGGGTAATAGTCTTGATAGCATTTTTATGGCCATGGAGT
>Marker950067 Chr2 SNP_site:8007081 Ref_Type:C
TACTAGTAAAAGGAAATAGCCCAACCTAAATCAAACTACAAAATAAAAGAGAAATAAGTTCCTAATTTGGCAATTTCAAAXXXXXXXXXXXA
TCTCATAACTCATTCAGAGTAAAACTGGAGGCCCTATATTTGCTGGACCCTGCATATCTACTTCCATTCCTTCGCAGGT
TACTAGTAAAAGGAAATAGCCCAACCTAAATCAAACTACAAAATAAAAGAGAAATAAGTTCCTAATTTGGCAATTTCAAAXXXXXXXXXXXA
TCTCATAACTCATTCAGAGTAAAACTGGAGGCCCTATATTTGCTGGACCCTGCATATCTACTTCGATTCCTTCGCAGGT
>Marker950165 Chr2 SNP_site:15609922 Ref_Type:G
AACATTTTCAATTTCCATCATAATATATATGAAGATACCTTCATAATAAATTTACAGAGACGAAGCAACATGATTGAGATXXXXXXXXXXXC
AGTTCCGGAATAGTATCGAATACTTCTAAGTTAGTGAAAACACTTTTCAAATTTATCATAATCTTTAGGCTAAAAAGTA
AACATTTTCAATTTCCATCATAATATATATGAAGATACCTTCATAATAAATTTACAGAGACGAAGCAACATGATTGAGATXXXXXXXXXXXC
AGTTCCGGAATAGTATCGAATACTTCTAAGTTAGTGAAAACACTTTTCAAGTTTATCATAATCTTTAGGCTAAAAAGTA
>Marker950420 Chr2 SNP_site:21342984 Ref_Type:C
AACTCCATGTTTCAGCGGGTGAATAATGTATTTACTGGCCTCTCTTATTCACAAATTCTCTGCATGGAATCAATCTAATCXXXXXXXXXXXT
ACTTGAGACCCAATCCTGTTGTGTTTTCTATTTTATGCCATTTGCTGAAAACTGGCTGGTTTCTCTTGGGACCCATGTC
AACTCCATGTTTCAGCGGGTGAATAATGTATTTACTGGCCTCTCTTATTCACAAATTCTCTGCATGGAATCAATCTAATCXXXXXXXXXXXT
ACTTGAGACCTAATCCTGTTGTGTTTTCTATTTTATGCCATTTGCTGAAAACTGGCTGGTTTCTCTTGGGACCCATGTC
>Marker950565 Chr2 SNP_site:15684790 Ref_Type:G
CACATCCTTGAGCGTTTAAATTTCTTCTAATTAATTAATGAAAACTTTGTATCTTGCTTAAAAAAAAGACTATACATTCATXXXXXXXXXXXT
AATGCTAATATCTAGGAGACCTGCAAAACTGGTTCAATTAAATAAAGATAGCAAGACAATTTACAATTTACTGTTTGTT
CACGTCCTTGAGCGTTTAAATTTCTTCTAATTAATTAATGAAAACTTTGTATCTTGCTTAAAAAAAAGACTATACATTCATXXXXXXXXXXXT
AATGCTAATATCTAGGAGACCTGCAAAACTGGTTCAATTAAATAAAGATAGCAAGACAATTTACAATTTACTGTTTGTT
>Marker951177 Chr2 SNP_site:19191710 Ref_Type:A
AACGATTTCAATGACCTCCAAAGAATATGAATCAATCCTTATTTTCCACTTGTTGAGTGTCGTCGTATAACGTAATTCATXXXXXXXXXXXT
AAGAAAGGTGGTGAAACAGATATATTAAATTAATGAACGAGAATATAAGCAGACAAATAAAAAACCCAAATGCATAGTT
AACGATTTCAATGACCTCCAAAGAATTTGAATCAATCCTTATTTTCCACTTGTTGAGTGTCGTCGTATAACGTAATTCATXXXXXXXXXXXT
AAGAAAGGTGGTGAAACAGATATATTAAATTAATGAACGAGAATATAAGCAGACAAATAAATAAGCCAAATGCATAGTT
>Marker951177 Chr2 SNP_site:19191921 Ref_Type:A
AACGATTTCAATGACCTCCAAAGAATATGAATCAATCCTTATTTTCCACTTGTTGAGTGTCGTCGTATAACGTAATTCATXXXXXXXXXXXT
AAGAAAGGTGGTGAAACAGATATATTAAATTAATGAACGAGAATATAAGCAGACAAATAAAAAACCCAAATGCATAGTT
AACGATTTCAATGACCTCCAAAGAATTTGAATCAATCCTTATTTTCCACTTGTTGAGTGTCGTCGTATAACGTAATTCATXXXXXXXXXXXT
AAGAAAGGTGGTGAAACAGATATATTAAATTAATGAACGAGAATATAAGCAGACAAATAAATAAGCCAAATGCATAGTT
>Marker951177 Chr2 SNP_site:19191924 Ref_Type:C
AACGATTTCAATGACCTCCAAAGAATATGAATCAATCCTTATTTTCCACTTGTTGAGTGTCGTCGTATAACGTAATTCATXXXXXXXXXXXT
AAGAAAGGTGGTGAAACAGATATATTAAATTAATGAACGAGAATATAAGCAGACAAATAAAAAACCCAAATGCATAGTT
AACGATTTCAATGACCTCCAAAGAATTTGAATCAATCCTTATTTTCCACTTGTTGAGTGTCGTCGTATAACGTAATTCATXXXXXXXXXXXT
AAGAAAGGTGGTGAAACAGATATATTAAATTAATGAACGAGAATATAAGCAGACAAATAAATAAGCCAAATGCATAGTT
>Marker951516 Chr2 SNP_site:22660875 Ref_Type:A
ACTTGAAGTTGAACGACCCGTTTAATAACGCAGGAGGATTGCCTTCGATGGGTGACACTTTAGGCTATTCTCATTACATAXXXXXXXXXXXC
TATATCAATCGCGTGCCCCAAGAGGGGACATTCCTATCTTACTATATGTCGTTCGGTTCAGCTGCATCATCAGTGTGTT
ACTTGATGTTGAACGACCCGTTTAATAACGCAGGAGGATTGCCTTCGATGGGTGACACTTTAGGCTATTCTCATTACATAXXXXXXXXXXXC

Create PDF with GO2PDF for free, if you wish to

TATATCAATCGCGTGCCCCAAGAGGGGACATTCCTATCTTACTATATGTCGTTCGATTCAGCTGCATCATCAGTGTGTT

>Marker951516 Chr2 SNP_site:22661148 Ref_Type:G

ACTTGAAGTTGAACGACCCGTTTAATAACGCAGGAGGATTGCCTTCGATGGGTGACACTTTAGGCTATTCTCATTACATAXXXXXXXXXXC

TATATCAATCGCGTGCCCCAAGAGGGGACATTCCTATCTTACTATATGTCGTTCGGTTCAGCTGCATCATCAGTGTGTT

ACTTGATGTTGAACGACCCGTTTAATAACGCAGGAGGATTGCCTTCGATGGGTGACACTTTAGGCTATTCTCATTACATAXXXXXXXXXXC

TATATCAATCGCGTGCCCCAAGAGGGGACATTCCTATCTTACTATATGTCGTTCGATTCAGCTGCATCATCAGTGTGTT

>Marker953875 Chr2 SNP_site:8006686 Ref_Type:A

CACACTTCTAATGGATAGTCCTCCAGTCTTCATACTCCTAACTACGGGATAAAGACAGGTCTATTCTTGCCTTCTAATGGXXXXXXXXXXG

ACAAAGAGGTATCCCCCAGAAGGAAGTGTGGTATTCAATTCCCTTTTATTCAAGAAAAGCAAGAAAGCCTATGGTTGGT

CACACTTCTAATGGATAGTCCTCCAGTCTTCATACTCCTAACTACGGGATAAAGACAGGTCTATTCTTGCCTTCTAATGGXXXXXXXXXXG

ACGAAGAGGTATCCCCCAGAAGGAAGTGTGGTATTCAATTCCCTTTTATTCAAGAAAAGCAAGAAAGCCTATGGTTGGT

>Marker954192 Chr2 SNP_site:7795737 Ref_Type:G

ACTTAAAATTTTCAAAACAGTTCGCAATGACCCAAAATCATGTCATGACATTGCCTGACGCATATGGGTGGGCGGCAGGGXXXXXXXXXXT

CTTGGTCGTGTGTCTGGCGTCTTACGCAACCTTCCTCTCTTGGTCGTGCATCTCGCGCATAGGTCATGTGTCCAATGTC

ACTTAAAATTTTCAAAACAGTTCGCAATGACCCAAAGTCATGTCATGACATTGCCTGACGCATATGGGTGGGCGGCAGGGXXXXXXXXXXT

CTTGGTCGTGTGTCCGGCGTCTTACGCAACCTTCCTCTCTTGGTCGTGCATCTCGCGCATAGGTCATGTGTCCAATGTC

>Marker954192 Chr2 SNP_site:7795903 Ref_Type:C

ACTTAAAATTTTCAAAACAGTTCGCAATGACCCAAAATCATGTCATGACATTGCCTGACGCATATGGGTGGGCGGCAGGGXXXXXXXXXXT

CTTGGTCGTGTGTCTGGCGTCTTACGCAACCTTCCTCTCTTGGTCGTGCATCTCGCGCATAGGTCATGTGTCCAATGTC

ACTTAAAATTTTCAAAACAGTTCGCAATGACCCAAAGTCATGTCATGACATTGCCTGACGCATATGGGTGGGCGGCAGGGXXXXXXXXXXT

CTTGGTCGTGTGTCCGGCGTCTTACGCAACCTTCCTCTCTTGGTCGTGCATCTCGCGCATAGGTCATGTGTCCAATGTC

>Marker954519 Chr2 SNP_site:10836324 Ref_Type:A

AACTCCTGTATTTTCAAAATTCTCATCCCAAGTAGTATTGATGAAAAAGCAGTCGATTAGCGATCTTGCTAAGGAAGAACXXXXXXXXXXT

CACTTCCTACACACCATGCCCCAATACAATAATTTGACAAGGAAAGGAGCTCGGGGCAAACAAATTTTCTTTTCTTGTA

AACTCCTGTATTTTCAAAATTCTCATCCCGAGTAGTATTGATGAAAAAGCAGTCGATTAGCGATCTTGCTAAGAAAGAACXXXXXXXXXXT

CACTTCCTACACACCATGCCCCAATACAATAATTTGACAAGGAAAGGAGCTCGGGGCAAACAAATTTTCTTTTCTTGTA

>Marker954519 Chr2 SNP_site:10836368 Ref_Type:G

AACTCCTGTATTTTCAAAATTCTCATCCCAAGTAGTATTGATGAAAAAGCAGTCGATTAGCGATCTTGCTAAGGAAGAACXXXXXXXXXXT

CACTTCCTACACACCATGCCCCAATACAATAATTTGACAAGGAAAGGAGCTCGGGGCAAACAAATTTTCTTTTCTTGTA

AACTCCTGTATTTTCAAAATTCTCATCCCGAGTAGTATTGATGAAAAAGCAGTCGATTAGCGATCTTGCTAAGAAAGAACXXXXXXXXXXT

CACTTCCTACACACCATGCCCCAATACAATAATTTGACAAGGAAAGGAGCTCGGGGCAAACAAATTTTCTTTTCTTGTA

>Marker95464 Chr4 SNP_site:18970837 Ref_Type:C

GACACCATGGATTGAACCATGACCTCTTAATTAGTTATTGAGACTTTCTCCTATTTTTACTACTAGGCCAATCCATGATGXXXXXXXXXXT

GATTGTGATGGTAATTTAGCCAGACGTCGTTTAGGAACAAATTGTCTATGGCATGGAAAATTGGAAATGAAGTGAAGTG

GACACCATGGATTGAACCATGACCTCTTAATTAGTTATTGAGACTTTCTCCTATTTTTACTACTAGGTCAATCAATGATGXXXXXXXXXXT

GATTGTGATGGTAATTTAGCCAGACGTCGTTTAGGAACAAATTGTCTATGGCATGGAAAATTGGAAATGAAGTGAAGTG

>Marker95464 Chr4 SNP_site:18970843 Ref_Type:C

GACACCATGGATTGAACCATGACCTCTTAATTAGTTATTGAGACTTTCTCCTATTTTTACTACTAGGCCAATCCATGATGXXXXXXXXXXT

GATTGTGATGGTAATTTAGCCAGACGTCGTTTAGGAACAAATTGTCTATGGCATGGAAAATTGGAAATGAAGTGAAGTG

GACACCATGGATTGAACCATGACCTCTTAATTAGTTATTGAGACTTTCTCCTATTTTTACTACTAGGTCAATCAATGATGXXXXXXXXXXT

GATTGTGATGGTAATTTAGCCAGACGTCGTTTAGGAACAAATTGTCTATGGCATGGAAAATTGGAAATGAAGTGAAGTG

>Marker954724 Chr2 SNP_site:14417486 Ref_Type:T

AACATGTTTAAATTATGAACATAATCACACATTTACATATTTAGATAATGGTAAAGTTCCAATATAATTTTGGCATGTTTXXXXXXXXXXT

TGGTCAAATAATCTTTGTAGGATGATATTAAAAAAATACCAATTATGGATAAATGCAAATGTGTTTTCCACCAAGGAGT

Create PDF with GO2PDF for free, if you wish to

AACATGTTTAAATTATGAACATAATCACACATTTACATATTTAGATAATGGTAAAGTTCCAATATAATTTTGGCATTTTTXXXXXXXXXXT
TGGTCAAATAATCTTTGTAGGATGATATTAAAAAAATACCAATTATGGATAAATGCAAATGTGTTTTCCACCAAGGAGT
>Marker954810 Chr2 SNP_site:2538993 Ref_Type:C
AACAAATTATCCAATTTCATAACCTTATCTAAAACCTACTCCCTTTCAAACATTAGAGTTTAAATTTTGAATTTTTCTTTXXXXXXXXXXC
AAATTTTCTCTCATTGGAGGTAAGCTTGACTATTTGCTATTTGTCTCTATCGATTATATGATTTTTAGGAGCATTTGTT
AACAAATTATCCAATTTTATAACCTTATCTAAAACCTACTCCCTTTCAAACATTAGAGTTTAAATTTTGAATCTTTCTTTXXXXXXXXXXC
AAATTTTCTCTCATTGGAGGTAAGCTTGACTATTTGCTATTTGTCTCTATCAATTATATGATTTTTAGGAGCATTTGTT
>Marker954810 Chr2 SNP_site:2539048 Ref_Type:T
AACAAATTATCCAATTTCATAACCTTATCTAAAACCTACTCCCTTTCAAACATTAGAGTTTAAATTTTGAATTTTTCTTTXXXXXXXXXXC
AAATTTTCTCTCATTGGAGGTAAGCTTGACTATTTGCTATTTGTCTCTATCGATTATATGATTTTTAGGAGCATTTGTT
AACAAATTATCCAATTTTATAACCTTATCTAAAACCTACTCCCTTTCAAACATTAGAGTTTAAATTTTGAATCTTTCTTTXXXXXXXXXXC
AAATTTTCTCTCATTGGAGGTAAGCTTGACTATTTGCTATTTGTCTCTATCAATTATATGATTTTTAGGAGCATTTGTT
>Marker954810 Chr2 SNP_site:2539198 Ref_Type:G
AACAAATTATCCAATTTCATAACCTTATCTAAAACCTACTCCCTTTCAAACATTAGAGTTTAAATTTTGAATTTTTCTTTXXXXXXXXXXC
AAATTTTCTCTCATTGGAGGTAAGCTTGACTATTTGCTATTTGTCTCTATCGATTATATGATTTTTAGGAGCATTTGTT
AACAAATTATCCAATTTTATAACCTTATCTAAAACCTACTCCCTTTCAAACATTAGAGTTTAAATTTTGAATCTTTCTTTXXXXXXXXXXC
AAATTTTCTCTCATTGGAGGTAAGCTTGACTATTTGCTATTTGTCTCTATCAATTATATGATTTTTAGGAGCATTTGTT
>Marker955112 Chr2 SNP_site:17723320 Ref_Type:G
CACCTCCATCCATCCACAATGGTCCAAAATCCTTTGAAAAGGAAAGGCTTCCTAAGCTTATTTTGGGCAGATGTAGATGGXXXXXXXXXXT
GTAATAGATATCTCCTTGAACTGAATTAAAATATGGTAAAATCAATCTATAAGCCACATAAGTTTTAACCAACAAAGTT
CACCTCCATCCATCCACAATTGTCCAAAATCCTTTGAAAAGGAAAGGCTTCCTAAGCTTATTTTGGGCAGATGTAGATGGXXXXXXXXXXT
GTAATAGATATCTCCTTGAACTGAATTAAAATATGGTAAAATCAATCTATAAGCCACATAAGTTTTAACCAACAAAGTT
>Marker95535 Chr4 SNP_site:7293456 Ref_Type:T
AACTTAAGAATGTCGAAAGCAAGAATATAAAGCATAAATCAACCCTATTGCTGATAGTAGTGATAGTAATGAGAATGAAGXXXXXXXXXXT
GACGGGTTCTGATTACACAATGTGTTGCATGTGCCACGAATTTCATATAATCTTTTGTCGATTAGCAAGATTACTCGTG
AACTTAAGAATGTCGAAAGCAAGAATATAAAGCATAAATCAATCCTATTGCTGATAGTAGTGATAGTAATGAGAATGAAGXXXXXXXXXXT
GACGGGTTCTCATTACACAATGTGTTGCATGTGCCACGAATTTCATATAATCTTTTGTCGATTAGCAAGATTACTCGTG
>Marker95535 Chr4 SNP_site:7293619 Ref_Type:C
AACTTAAGAATGTCGAAAGCAAGAATATAAAGCATAAATCAACCCTATTGCTGATAGTAGTGATAGTAATGAGAATGAAGXXXXXXXXXXT
GACGGGTTCTGATTACACAATGTGTTGCATGTGCCACGAATTTCATATAATCTTTTGTCGATTAGCAAGATTACTCGTG
AACTTAAGAATGTCGAAAGCAAGAATATAAAGCATAAATCAATCCTATTGCTGATAGTAGTGATAGTAATGAGAATGAAGXXXXXXXXXXT
GACGGGTTCTCATTACACAATGTGTTGCATGTGCCACGAATTTCATATAATCTTTTGTCGATTAGCAAGATTACTCGTG
>Marker955654 Chr2 SNP_site:6753359 Ref_Type:A
AACAATCTACTTCAAAAGATATTAATAAAACATTGAAATTGAGAGTTTCATAAATGTCATTGAATTTAGAAACAAGGGCAXXXXXXXXXXT
TATGAACTTGAGTTTGCATTTTGGTCACATTTAGGGAGCTAGCTATTTTTATCTATGTTTATTTAGACCATATGTTGTG
AACAATCTACTTCAAAAGCTACTAATAAAACATTGAAATTGAGAGTTTCATAAATGTCATTGAATTTAGAAACAAGGGCAXXXXXXXXXXT
TATGAACTTGAGTTTGCATTTTGGTCACATTTAGGGAGCTAGCTATTTTTATCTATGTTTATTTAGACCATATGTTGTG
>Marker955654 Chr2 SNP_site:6753362 Ref_Type:T
AACAATCTACTTCAAAAGATATTAATAAAACATTGAAATTGAGAGTTTCATAAATGTCATTGAATTTAGAAACAAGGGCAXXXXXXXXXXT
TATGAACTTGAGTTTGCATTTTGGTCACATTTAGGGAGCTAGCTATTTTTATCTATGTTTATTTAGACCATATGTTGTG
AACAATCTACTTCAAAAGCTACTAATAAAACATTGAAATTGAGAGTTTCATAAATGTCATTGAATTTAGAAACAAGGGCAXXXXXXXXXXT
TATGAACTTGAGTTTGCATTTTGGTCACATTTAGGGAGCTAGCTATTTTTATCTATGTTTATTTAGACCATATGTTGTG
>Marker956175 Chr2 SNP_site:22100791 Ref_Type:G
CACACACACTAATTGATTATTTAATTTCTCCGGAAAAGAGTGTCCTTTTAGCTTCCCATTTATATAAACATCACCAAAGAXXXXXXXXXXT

Create PDF with GO2PDF for free, if you wish to

CTCATCAAACATCCTCTCCATAGAATCCTTCACAACCTCTCCAAAAGCTACGGACATGTCTTCTCTCTTCGATTTGGTT
CACACACAGTAATTGATTATTTAATTTCTCCGGAAAAGAGTGTCCTTTTAGCTTCCCATTTATATAAACATCACCAAAGAXXXXXXXXXXT
CTCATCAAACATCCTCTCCATAGAATCCTTCACAACCTCTCCAAAAGCTACGGACATGTCTTCTCTCTTCGATTTGGTT
>Marker9562 Chr4 SNP_site:3182431 Ref_Type:C
TACGGTTTCATGAATACTTATTTTTATGGTAGGTATTTTTGAGCATCCATAATTACTTTAATTGAAGTGTGGAGATAGAAXXXXXXXXXXT
ATTGGAAGTAGCAGCAATTATATTCTTTTATTTGGGAAATGAAGAAAATAAACCAACTTGGTGACTCTCCACCATTGTT
TACGGTTTCATGAATACTTATTTTTATGGTAGGTATTTTTGAGCATCCATAATTACTTTAATTGAAGTGTGGAGATAGAAXXXXXXXXXXT
ATTGGAAGTAGCAGCAATTATATTCTTTTATTTGGGAAATGAAGAAAATACACCAACTTGGTGACTCTCCACCATTGTT
>Marker95620 Chr4 SNP_site:529446 Ref_Type:C
GACCAAGTTTAAATTTGTTAGGATAAAAATTATAAATCTAATCTTACTTGCATACTTTTTAAAGTTAATAACATTCATTAXXXXXXXXXXA
CTAACATAGTAATAAGTAGTGAATTGTGTTTAGATTTGAGGATTGATTAAACATATAATAGGGGTTTAATTAATAAAGT
GACCAAGTTTAAATTTGTTAGGATAAAAATTATAAATCTAATCTTACTTGCATACTTTTTAAAGTTAATAACATTCATTCXXXXXXXXXXA
CTAACATAGTAATAAGTAGTGAATTGTGTTTAGATTTGAGGATTGATTAAACATATAATAGGGGTTTAATTAATAAAGT
>Marker956864 Chr2 SNP_site:4558142 Ref_Type:A
ACTTAGAAATATTTAGACCGATTCTACTGTATTTTTTGCAATTTGAACTGTGAGTTATGTTATAGAAGATGTGTTGGGATXXXXXXXXXXT
TTCAATTTTATTACAATTTAGTCCCTAAACCTTACTGCATAAAAATTTAAACAATTTAGTTCCTACGGCAGAAATTAGT
ACTTAGAAATATTTAGACCGATTCTACTGTATTTTTTGCAATTTGAACTGTGAGTTATGTTATAGAAGATGTGTTGGGATXXXXXXXXXXT
TTCAATTTTATTACAATTTAGTCCCTAAACCTTACTGCATAACAATTTAAACAATTTAGTTCCTACGGCAGAAATTAGT
>Marker957015 Chr2 SNP_site:22098405 Ref_Type:T
AACATATCATATCCATTATGATCAATATATATCTTCTCAAGTTCAAACAATTCATCATTTAAAGCATCTCTCAACCAATGXXXXXXXXXXA
GTTGTAGAAAGTGCAACACACTTCTGCAACCTTGACTACCAAGGCCCTAACCACCTGTAAATGTCATCAACCAAATGTA
AACATATCATATCCATTATGATCAATATATATCTTCTCAAGTTCAAACAATTCATCATTTAAAGCATCTCTCAACCAATGXXXXXXXXXXA
GTTGTAGAAAGTGCAACACACTTCTGCAACCTTGACTACCAAGGCCCTAACCACTTGTAAATGTCATCAACCAAATGTA
>Marker957075 Chr2 SNP_site:20373448 Ref_Type:T
GACTAGGGAGGTATTAATCAACGATTATTATGTATAAACTATTCAAACAAAGATAGCACATAAATCATCTAACATAAGATXXXXXXXXXXA
ATACGTCTGGAATATATAAGATAAAATCTGCCTGTAGTCGCCAGTCTTAATACCAGTATTGGGATCAATAAGGTAAGGT
GACTAGGGAGGTATTAATCAACGATTATTATGTATAAACTATTCAAACAAAGATAGCACATAAATCATCTAACATAAGATXXXXXXXXXXA
ATATGTCTGGAATATATAAGATAAAATCTGCCTGTAGTCGCCAGTCTTAATACCAGTATTGGGATCAATAAGGTAAGGT
>Marker95765 Chr4 SNP_site:15120154 Ref_Type:G
ACTTCAAAAGTTATCATCTTTTGTATTATATTTTCTTTGAATCTCTTCAATTTTTTACGCTCATCTTTCTATATTTCCATXXXXXXXXXXT
AAAAATTGGTGTATTCCCAAAAAAAAATGACTTCTAATAGGACTAATTATAAACACTATACAATTGTTTTCTATCAGGTT
ACTTCAAAAGTTATCATCTTTTGTATTATATTTTCTTTGAATGTCTTCAATTTTTTACGCTCATCTTTCTATATTTCCATXXXXXXXXXXT
AAAAATTGGTGTATTCCAAAAAAAAAATGACTTCTAATAGGACTAATTATAAACACTACACAATTGTTTTCTATCAGGTT
>Marker95765 Chr4 SNP_site:15120324 Ref_Type:A
ACTTCAAAAGTTATCATCTTTTGTATTATATTTTCTTTGAATCTCTTCAATTTTTTACGCTCATCTTTCTATATTTCCATXXXXXXXXXXT
AAAAATTGGTGTATTCCCAAAAAAAAATGACTTCTAATAGGACTAATTATAAACACTATACAATTGTTTTCTATCAGGTT
ACTTCAAAAGTTATCATCTTTTGTATTATATTTTCTTTGAATGTCTTCAATTTTTTACGCTCATCTTTCTATATTTCCATXXXXXXXXXXT
AAAAATTGGTGTATTCCAAAAAAAAAATGACTTCTAATAGGACTAATTATAAACACTACACAATTGTTTTCTATCAGGTT
>Marker95765 Chr4 SNP_site:15120364 Ref_Type:C
ACTTCAAAAGTTATCATCTTTTGTATTATATTTTCTTTGAATCTCTTCAATTTTTTACGCTCATCTTTCTATATTTCCATXXXXXXXXXXT
AAAAATTGGTGTATTCCCAAAAAAAAATGACTTCTAATAGGACTAATTATAAACACTATACAATTGTTTTCTATCAGGTT
ACTTCAAAAGTTATCATCTTTTGTATTATATTTTCTTTGAATGTCTTCAATTTTTTACGCTCATCTTTCTATATTTCCATXXXXXXXXXXT
AAAAATTGGTGTATTCCAAAAAAAAAATGACTTCTAATAGGACTAATTATAAACACTACACAATTGTTTTCTATCAGGTT
>Marker957867 Chr2 SNP_site:20399163 Ref_Type:A

Create PDF with GO2PDF for free, if you wish to

AACTTCAATGTGCGTTGCCCTATCATGTAGAACTGGATTATGAGTTAGCAATAGCAGCCTTATTATCACAATAAACTCGTXXXXXXXXXXXT
TTTGTTTTTTACTACATCGTGTAACTAAATTTCCTCCAACAAATGAACAATAGCTAGAAGTAAATCTTTTATCAGTAGT
AACTTCAATGTGCGTTGCCCTATCATGTAGAACTGGATTATGAGTTAGCAATAGCAGCCTTATTATCACAATAAACTCGTXXXXXXXXXXXT
TTTGTTTTTTACTACGTCGTGTAACTAAATTTCCTCCAACAAATGAACAATAGCTAGAAGTAAATCTTTTATCAGTAGT
>Marker957978 Chr2 SNP_site:3740114 Ref_Type:C
AACTCATAGATACGATAATTAGCAAGGAGCACATATAATTTTACTCTAATTCTAAATGTGCTTCCAGTTCATTGCGAGGTXXXXXXXXXXXC
TATCAATAAATTATTTGACGTCAGTATTTATACAGAATATCCAGTTAAGAAAATGAGAAGTTGAACAACAAACAAGGTG
AACTCATAGATACGATAATTAGCAAGGAGCACATATAATTTTACTCTAATTCTAAATGTGCTTCCAGTTCATTGCGAGGTXXXXXXXXXXXC
TATCAATAAATTATTTGACGTCAGTATTTATATAGAATATCCAGTTAAGAAAATGAGAAGTTGAACAACAAACAAGGTG
>Marker958864 Chr2 SNP_site:22082957 Ref_Type:G
ACTCTGGCAAGCCACCAAGAAATGCTTTTCATCCCATATTTTGAACTCGTATTTTCTTTGAATAGCATGTCTTTAATCCTXXXXXXXXXXXA
TATTGAATTTATGTCAATTCAGAAGTTGTTTGGTCCTACCTGAATATTAGTGAATTTGCCAAATAAATCGGACCCGGTA
ACTCTGGCAAGCCACCAAGAAATGCTTTTCATCCCATATTTTGAACTCGTATTTTCTTTGAATAGCATGTCTTTAATCCTXXXXXXXXXXXA
TATTGAATTTATGTCAATTCATAAGTTGTTTGGTCCTACCTGAATATTAGTGAATTTGCCAAATAAATCGGACCCGGTA
>Marker959409 Chr2 SNP_site:17017752 Ref_Type:G
ACCGTCAATACCCGCCGCCGCATCAGCTTAATGGCCAGCAGCACCAGCAGCCACACCAGGGATCCACCAGCGAGAACAAGXXXXXXXXXXXT
AGAATATTAATGCATACCAATTCATTTATTGACATTTTATTGATATCTACTGTTCTGGATTTGTTTGGCTAATTTTGTT
ACCGTCAATACCCGCCGCCGCATCAGCTTAATGGCCAGCAGCACCAGCAGCCACACCAGGGATCCACCAGCGAGAACAAGXXXXXXXXXXXT
AGAATATTAATGCATACCAATTCATTTATTGGCATTTTATTGATATCTACTGTTCTGGATTTGTTTGGCTAATTTTGTT
>Marker959943 Chr2 SNP_site:17308299 Ref_Type:T
TACCTTTTGCTTTGTAAGTCTTTCGCCGTCTCTTGTTTCTCCTTTATCTCATCTTTCGGTATTTTCTTACATTGCATCCGXXXXXXXXXXXC
GAGCACTGGAGTCGAGGTTCAATCTCACAGTGTTGCTTGTGTTTCTCTTTGTATTCGTTCTAAATTCCTCGTGTTTGTA
TACCTTTTGCTTTGTAAGTCTTTCGCCGTCTCTTGTTTCTCCTTTATCTCATCTTTCGGTATTTTCTTACATTGCATCCGXXXXXXXXXXXC
GAGCACTGGAGTCGAGGTTCAATCTCACAGTGTTGCTTGTGTTTCTCTTTGTATTCGTTCTAAATTCCTTTTGTTTGTA
>Marker959943 Chr2 SNP_site:17308300 Ref_Type:T
TACCTTTTGCTTTGTAAGTCTTTCGCCGTCTCTTGTTTCTCCTTTATCTCATCTTTCGGTATTTTCTTACATTGCATCCGXXXXXXXXXXXC
GAGCACTGGAGTCGAGGTTCAATCTCACAGTGTTGCTTGTGTTTCTCTTTGTATTCGTTCTAAATTCCTCGTGTTTGTA
TACCTTTTGCTTTGTAAGTCTTTCGCCGTCTCTTGTTTCTCCTTTATCTCATCTTTCGGTATTTTCTTACATTGCATCCGXXXXXXXXXXXC
GAGCACTGGAGTCGAGGTTCAATCTCACAGTGTTGCTTGTGTTTCTCTTTGTATTCGTTCTAAATTCCTTTTGTTTGTA
>Marker960129 Chr2 SNP_site:21590720 Ref_Type:G
TACAATCTTAGTAACCAAACCTAAAAGCCCATGAGAAAATTATTCTTTGTGATTGTAATAATTTTTTTTTATCCATGGTTXXXXXXXXXXXA
ATGTTTATAGCCTTTTGATGTATATACTGTTCTCAATTATGATGATAATTGGATTATTGTATTTATGTTTTATGTAGTT
TACAATCTTAGTAACCAAACGTAAAAGCCCATGAGAAAATTATTCTTTGTGATTGTAATAATTTTTTTTTATCCATGGTTXXXXXXXXXXXA
ATGTTTATAGCCTTTTGATGTATATACTGTTCTCAATTATGATGATAATTGGATTATTGTATTTATGTTTTATGTAGTT
>Marker960518 Chr2 SNP_site:3732504 Ref_Type:A
CACCCATGTGTCTAATTTTATTTTAATTTAGATAAGAATCATATCTTTGATAAAAATGAATTAAGGGGAAACCCCAACACXXXXXXXXXXXC
TCTGAAAAAACGTCCGTTGCGCTCGCCTCAAAAGTCCAAAAGAAAGAACACATAATTGCCAACCAACCCATTTTTCAGT
CACCCATGTGTCTAATTTTATTTTAATTTAGATAAGAATCATATCTTTGATAAAAATGAATTAAGGGGAAACCCCAACACXXXXXXXXXXXC
TCTGAAAAAACGTCCGTTGCGCTCGCCTCAAAAGTCCAAAAGAAAGCACACATAATTGCCAACCAACCCATTTTTCAGT
>Marker96054 Chr4 SNP_site:15549935 Ref_Type:C
GACTCCATTGTCAATGTCGAATCATACATTTTCCATAGTGTTTCAGCTTTCGTTACCACTCTCCATTACTTGGAGCGTAAXXXXXXXXXXXT
TCATGGGAAAGATGGTTTTCGGTGGAGTTTTGGGATTTAATTAAGTCACAGTTTGGTCAGCTACCAAAAAAGACATGTG
GACTCCATTGTCAATGTCGAATCATACCTTTTCCATAGTGTTTCAGCTTTCGTTACCACTCTCCATAACTTGGAGCGTAAXXXXXXXXXXXT
TCATGGGAAAGCTGGTTTTCGGTGGAGTTTTGGGATTTAATTAAGTCACAGTTTGGTCAGCTACCAAAAAAGACATGTG

Create PDF with GO2PDF for free, if you wish to

>Marker96054 Chr4 SNP_site:15549974 Ref_Type:A
GACTCCATTGTCAATGTCGAATCATACATTTTCCATAGTGTTTCAGCTTTCGTTACCACTCTCCATTACTTGGAGCGTAAXXXXXXXXXXXT
TCATGGGAAAGATGGTTTTCGGTGGAGTTTTGGGATTTAATTAAGTCACAGTTTGGTCAGCTACCAAAAAAGACATGTG
GACTCCATTGTCAATGTCGAATCATACCTTTTCCATAGTGTTTCAGCTTTCGTTACCACTCTCCATAACTTGGAGCGTAAXXXXXXXXXXXT
TCATGGGAAAGCTGGTTTTCGGTGGAGTTTTGGGATTTAATTAAGTCACAGTTTGGTCAGCTACCAAAAAAGACATGTG
>Marker96054 Chr4 SNP_site:15550096 Ref_Type:C
GACTCCATTGTCAATGTCGAATCATACATTTTCCATAGTGTTTCAGCTTTCGTTACCACTCTCCATTACTTGGAGCGTAAXXXXXXXXXXXT
TCATGGGAAAGATGGTTTTCGGTGGAGTTTTGGGATTTAATTAAGTCACAGTTTGGTCAGCTACCAAAAAAGACATGTG
GACTCCATTGTCAATGTCGAATCATACCTTTTCCATAGTGTTTCAGCTTTCGTTACCACTCTCCATAACTTGGAGCGTAAXXXXXXXXXXXT
TCATGGGAAAGCTGGTTTTCGGTGGAGTTTTGGGATTTAATTAAGTCACAGTTTGGTCAGCTACCAAAAAAGACATGTG
>Marker961440 Chr2 SNP_site:17037657 Ref_Type:T
AACACGTGAGATTCAAACACAAAAGTTCTCATACTTTAAAAACATTTCAACCAGAGTTTCTAAACTTTATTTCGTAGATCXXXXXXXXXXXA
CAGAACTTTTAAGAATCTATTTGATACTTTAAAAAATTCATAAATTATAAAATAAAAAACAAAAGTTAAAAGAAACGTT
AACACGTGAGATTCAAACACAAAAGTTCTCATACTTTAAAAACATTTCAACCAGAGTTTCTAAACTTTATTTTGTAGATCXXXXXXXXXXXA
CAGAACTTTTAAGAATCTATTTGATACTTTAAAAAATTCATAAATTATAAAATAAAAAACAAAAGTTAAAAGAAACGTT
>Marker962390 Chr2 SNP_site:3028905 Ref_Type:C
ACAGTGACACAATCTTTGCGGTTAGGATTGAACATCTACTTCCCTTCAAGAATATTCATAGCATGGGAAGGTGATTTGGAXXXXXXXXXXXA
AATAAAGCAATTCGTATTAAAAAATAAAGTGATTCATGTTTAATAAAAAACTTCACATATTTAGTGATTGGAAGAAGTG
ACAGTGACACAATCTTTGCGGTTAGGATTGAACATCTACTTCCCTTCAAGAATATTCATAGTATGGGAAGGTGATTTGGAXXXXXXXXXXXA
AATAAAGCAATTCGTATTAAAAAATAAAGTGATTCATGTTTAATAAAAAACTTCACATATTTAGTGATTGGAAGAAGTG
>Marker962710 Chr2 SNP_site:14541741 Ref_Type:A
ACGTATATTCAACGACCACTTTTAAATTATAAAAGAAAAGAATGATTAAATTGTTGGGTTTTGCAATATAATTATTATTAXXXXXXXXXXXG
AAGCTCAAAATGGACGGCTATGATTCTTCTCATCTTTGTCATACCTCTTGGGTCACTTCCATGGGCTGCCCAGCAGGTG
ACGTATATTCAACGACCACTTTTTAATTATAAAAGAAAAGAATGATTAAATTGTTGGGTTTTGCAATATAATTATTATTAXXXXXXXXXXXG
AAGCTCAAAATGGACGGCTATGATTCTTCTCATCTTTGTCATACCTCTTGGGTCACTTCCATGGGCTGCCCAGCAGGTG
>Marker96287 Chr4 SNP_site:21086133 Ref_Type:C
AACTCCTGTGCGAGAATCGAGGCCAGGGGGAGTGACACGTGTCCCGAGAAGCCTCTCAACGACGCCGTTTTTGTAGAGTCXXXXXXXXXXXT
GAAGAGGTTTGATTTGAAATTATCATATAATTGGTGAAGAGATATATGTCAAACTCTATCCTTTATAATTTCAAATGTT
AACTCCTGTGCGAGAATCGAGGCCAGGGGGAGTGACACGTGTCCCGAGAAGCCTCTCAACGACGCCGTTTTTGTAGAGTCXXXXXXXXXXXT
GAAGAGGTTTGATTTGAAATTATCATATAATTGGTGAAGAGATATATGTGAAACTCTATCCTTTATAATTTCAAATGTT
>Marker96295 Chr4 SNP_site:5036519 Ref_Type:C
CACAACAGTTCATGCGCTGAAGTCAAAAGGATAGGAATATGAAGCAGTTAAAATAATGGAAGTTAATAGCTGCAGGAATCXXXXXXXXXXXC
CATGAGGTAGATAATATTTTACCAAATCTTATGATACAACCAATTCCATGCAAAAACTAGGGCTGGTTTAGGCATTGTT
CACAACAGTTTATGCGCTGAAGTCAAAAGGATAGGAATGTGAAGCAGTTAAAATAATGGAAGTTAATAGCTGCAGGAATCXXXXXXXXXXXC
CATGAGGTAGATAATATTTTACCAAATCTTATGATACAACCAATTCCATGAAAAAACTAGGGCTGGTTTAGGCATTGTT
>Marker96295 Chr4 SNP_site:5036547 Ref_Type:G
CACAACAGTTCATGCGCTGAAGTCAAAAGGATAGGAATATGAAGCAGTTAAAATAATGGAAGTTAATAGCTGCAGGAATCXXXXXXXXXXXC
CATGAGGTAGATAATATTTTACCAAATCTTATGATACAACCAATTCCATGCAAAAACTAGGGCTGGTTTAGGCATTGTT
CACAACAGTTTATGCGCTGAAGTCAAAAGGATAGGAATGTGAAGCAGTTAAAATAATGGAAGTTAATAGCTGCAGGAATCXXXXXXXXXXXC
CATGAGGTAGATAATATTTTACCAAATCTTATGATACAACCAATTCCATGAAAAAACTAGGGCTGGTTTAGGCATTGTT
>Marker96295 Chr4 SNP_site:5036729 Ref_Type:C
CACAACAGTTCATGCGCTGAAGTCAAAAGGATAGGAATATGAAGCAGTTAAAATAATGGAAGTTAATAGCTGCAGGAATCXXXXXXXXXXXC
CATGAGGTAGATAATATTTTACCAAATCTTATGATACAACCAATTCCATGCAAAAACTAGGGCTGGTTTAGGCATTGTT
CACAACAGTTTATGCGCTGAAGTCAAAAGGATAGGAATGTGAAGCAGTTAAAATAATGGAAGTTAATAGCTGCAGGAATCXXXXXXXXXXXC

Create PDF with GO2PDF for free, if you wish to

CATGAGGTAGATAATATTTTACCAAATCTTATGATACAACCAATTCCATGAAAAAACTAGGGCTGGTTTAGGCATTGTT
>Marker963376 Chr2 SNP_site:13210827 Ref_Type:C
AACTATCTGGATCCTGTTTTTAACATTTTTGTTTTAGAATGGACAATCGATTAATTAATTATTATTTTCTTAAAAAAAATXXXXXXXXXXG
TAATTTACAACAATGAGTAATTAATTCGTTACTTTATGTTAGTGATGCTGCTGAATTATCAAACAAATTTCCGACAGTT
AACTATCTGGATCCTGTTTTTAACATTTTTGTTTTAGAATGGACAATTGATTAATTAATTATTATTTTCTTAAAAAAAATXXXXXXXXXXG
TAATTTACAACAATGAGTAATTAATTCGTTACTTTATGTTAGTGATGCTGCTGAATTATCAAACAAATTTCCGACAGTT
>Marker963381 Chr2 SNP_site:12597721 Ref_Type:A
AACATGTTATCCTATTGTGGAAGATAAATATTTTATTCTTCATAAACCCGAGAAATACTAGAAATGATTTGTCTTGAAGCXXXXXXXXXXA
AAAAAAAAGCGGCATAGGTCAAGAAAGCCAGTTCATAAATAAGTTCTGATGCTCGTGTTGAGAGAGATGGAAGAAATGT
AACATGTTATCCTATTGTGGAAGATAAATATTTTATTCTTCATAAACCCGAGAAATACTAGCAATGATTTGTCTTGAAGCXXXXXXXXXXA
AAAAAAAAGCGGCATAGGTCAAGAAAGCCAGTTCATAAATAAGTTCTGATGCTCGTGTTGAGAGAGATGGAAGAAATGT
>Marker964090 Chr2 SNP_site:4412554 Ref_Type:T
GACTCCTATTAGTGATGTGCTCTACCACTAATAGACTCCAAGAGTTAGATATAAATTTTGTTATATTTGCAAATTCTTTTXXXXXXXXXXG
CTATTTCTTTGAAATTTGACTAAAATGAAATCTTGTAATATAATATAGGCCTTTCAATTTATAGGATTTAGACTTTGTT
GACTCCTATTAGTGATGTGCTCTACCACTAATAGACTCCAAGAGTTAGATATAAATTTTGTTATATTTGTAAATTCTTTTXXXXXXXXXXG
CTATTTCTTTGAAATTTGACTAAAATGAAATCTTGTAATATAATATAGGCCTTTTAATTTATAGGATTTAGACTTTGTT
>Marker964090 Chr2 SNP_site:4412756 Ref_Type:T
GACTCCTATTAGTGATGTGCTCTACCACTAATAGACTCCAAGAGTTAGATATAAATTTTGTTATATTTGCAAATTCTTTTXXXXXXXXXXG
CTATTTCTTTGAAATTTGACTAAAATGAAATCTTGTAATATAATATAGGCCTTTCAATTTATAGGATTTAGACTTTGTT
GACTCCTATTAGTGATGTGCTCTACCACTAATAGACTCCAAGAGTTAGATATAAATTTTGTTATATTTGTAAATTCTTTTXXXXXXXXXXG
CTATTTCTTTGAAATTTGACTAAAATGAAATCTTGTAATATAATATAGGCCTTTTAATTTATAGGATTTAGACTTTGTT
>Marker964869 Chr2 SNP_site:20352951 Ref_Type:G
AACTTTCAAGAGATCTGGGTTATTGACAAAAACTGAATGCTTGATGGTGTTATTTCGAAAAGAGTCAAAGGGGGAATTTTXXXXXXXXXXA
TATTCTTTCTTTCACCCGAGGAGGGCCATATTACACATTCTACAAACCAATCTTTGGATGAATGGATCTTGGCAAGGTG
AACTTTCAAGAGATCTGGGTTATTGACAAAAACTGAATGCTTGATGGTGTTATTTCGAAAAGAGTCAAAGGGGGAATTTTXXXXXXXXXXA
TATTCTTTCTTTCACCCGAGGAGGGCCATGTTACACATTCTACAAACCAATCTTTGGATGAATGGATCTTGGCAAGGTG
>Marker965203 Chr2 SNP_site:22651830 Ref_Type:C
AACTTGCTATTTGTGTCCAGGTATGTTATTCTAAATATATAAAGCCTTCTGTCCCAGTTCTCAAACAAGTTATATAATTAXXXXXXXXXXA
AAAATCATTAAGTTGATAAACATCAAATGTTGGGAAGGTAAAATCATGACATATGTGTAAAGAAGCCAAATAAATGGTG
AACTTGCTATTTGTGTCCAGGTATGTTATTCTAAATATATAAAGCCTTCTGTCCCAGTTCTCAAACAAGTTATATAATTAXXXXXXXXXXA
AAAATGATTAAGTTGATAAACATCAAATGTTGGGAAGGTAAAATCATGACATATGTGTAAAGAAGCCAAATAAATGGTG
>Marker965278 Chr2 SNP_site:22068221 Ref_Type:T
AACCAAGTCGTAGTTCTTCTCTGTTTTATTTACATTTCCATCCATTGTTCATATCACTTGTATTAGATATATTAGATTATXXXXXXXXXXC
TAAAGATGTAATTATTTTGTTTGACTTGCGGTGAAGCTACGCTTAACACATCAAAAGATTAAGTTAGGTGATGGTAGTT
AACCAAGTCGTAGTTCTTCTCTGTTTTATTTACATTTCCATCCATTGTTCATATCACTTGTATTAGATATATTAGATTATXXXXXXXXXXC
TAAAGATGTAATTATTTTGTTTGACTTGCGGTGAAGCTACGCTTAACACATCAAAATATTAAGTTAGGTGATGGTAGTT
>Marker965557 Chr2 SNP_site:3032522 Ref_Type:G
CACATGGATTTATGAGATGGAATAAATAGTAACAACTATCTTTACTTTCTGTATAGTTGTAAATAATAAAATAAAACATAXXXXXXXXXXA
AAATGTAATCTGAATTACTAGAAATTAGATATGTGTTGAAAAATTGAGTCTAAACATCTCTCCCACATCTCTTATTGTC
CACATGGATTTATGAGATGGAATAAATAGTAACAACTATCTTTACTTTCTGTATAGTTGTAAATAATAAAATAAAACATAXXXXXXXXXXA
AAATGTAATCTGAATTACTAGAAATTAGATATGTGTTGAAAAATTGAGTCTAAACATCTCTCCCACATCTCTTGTTGTC
>Marker965827 Chr2 SNP_site:4341823 Ref_Type:C
AACCTTGAATAGCAATAAACAAAGAACGATTCAACATAAAAAAGTTGGTTATCATCATTCAACAACACTACGATGTTAATXXXXXXXXXXA
TGAAAGGGTCAAATTAGAGACAAAACCCCCCATGGTCAAAAGCTTAATTGAGAATTTGTCAAAAGCAAAACTTGTAGGT

Create PDF with GO2PDF for free, if you wish to

AACCTTGAATAGCAATAAACAAAGAAGGATTCAACATAAAAAAGTTGGTTATCATCATTCAACAACACTACGATGTTAATXXXXXXXXXXA

TGAAAGGGTCAAATTAGAGACAAAACCCCCCATGGTCAAAAGCTTAATTGAGAATTTGTCAAAAGCAAAACTTGTAGGT

>Marker96625 Chr4 SNP_site:7398434 Ref_Type:G

TACAAAATGGTTAGAGCATGTGTATAGATTATAGAAAGTTAAACGAGGTAACTGCTTGTTTTGGGTTTGTATGCACATGCXXXXXXXXXXC

CAAATGCAAGCAAAGAGAGGTTTCCTGATAAATTCAGGGTTGAACACAAGGAATTAATTTTCCTAATGATCTTATCGTG

TACAAAATGGTTAGAGCATGTGTATAGATTATAGAAAGTTAAACGAGGTAACTGCTTGTTTTGGGTTTGTATGCACATGCXXXXXXXXXXC

CAAATGCAAGCGAAGAGAGGTTTCCTGATAAATTCAGGGTTGAACACAAGAAATTAATTTTCCTAATGATCTTATTGTG

>Marker96625 Chr4 SNP_site:7398473 Ref_Type:A

TACAAAATGGTTAGAGCATGTGTATAGATTATAGAAAGTTAAACGAGGTAACTGCTTGTTTTGGGTTTGTATGCACATGCXXXXXXXXXXC

CAAATGCAAGCAAAGAGAGGTTTCCTGATAAATTCAGGGTTGAACACAAGGAATTAATTTTCCTAATGATCTTATCGTG

TACAAAATGGTTAGAGCATGTGTATAGATTATAGAAAGTTAAACGAGGTAACTGCTTGTTTTGGGTTTGTATGCACATGCXXXXXXXXXXC

CAAATGCAAGCGAAGAGAGGTTTCCTGATAAATTCAGGGTTGAACACAAGAAATTAATTTTCCTAATGATCTTATTGTG

>Marker96625 Chr4 SNP_site:7398498 Ref_Type:T

TACAAAATGGTTAGAGCATGTGTATAGATTATAGAAAGTTAAACGAGGTAACTGCTTGTTTTGGGTTTGTATGCACATGCXXXXXXXXXXC

CAAATGCAAGCAAAGAGAGGTTTCCTGATAAATTCAGGGTTGAACACAAGGAATTAATTTTCCTAATGATCTTATCGTG

TACAAAATGGTTAGAGCATGTGTATAGATTATAGAAAGTTAAACGAGGTAACTGCTTGTTTTGGGTTTGTATGCACATGCXXXXXXXXXXC

CAAATGCAAGCGAAGAGAGGTTTCCTGATAAATTCAGGGTTGAACACAAGAAATTAATTTTCCTAATGATCTTATTGTG

>Marker966879 Chr2 SNP_site:3926424 Ref_Type:G

TACATTGATGCTGTTTAACTTATTTTCTCTCCAACATAAGTCGATCTTCCATTCTTCATGTTTAACTTCCTTAGTTTTAGXXXXXXXXXXA

TTGGTAAAGTAATATTAATTATTTCCATTTAAGATAAGATACAACGGGACTATCTTTCCATAATTTATAAAGGAAGGTT

TACATTGATGCTGTTTAACTTATTTTCTCTCCAACATAAGTGGATCTTCCATTCTTCATGTTTAACTTCCTTAGTTTTAGXXXXXXXXXXA

TTGGTAAAGTAATATTAATTATTTCCATTTAAGATAAGATACAACGGGACTATCTTTCCATAATTTATAAAGGAAGGTT

>Marker967273 Chr2 SNP_site:15723047 Ref_Type:T

GACCAACATGCGGAAGGAGAAGAGGACGAAAGGGGACGGCGAAGGCAACGCGGTGAGTGGGCCTCAAAATAGAAAACCTCXXXXXXXXXXA

CCAACAATTCTAAAACTGCTTCCTTAACCTCCAATTCCATACTAACAATCTAAACCTTTATCCCCCATCCTCATTTGTA

GACCAACATGCGGAAGGAGAAGAGGACGAAAGGGGACGGCGAAGGCAACGCGGTGAGTGGGCCTCAAAATAGATAACCTCXXXXXXXXXXA

CCAACAATTCTAAAACTGCTTCCTTAACCTCCAATTCCATACTAACTATCTAAACCTTTATCCCCCATCCTCATTTGTA

>Marker967273 Chr2 SNP_site:15723230 Ref_Type:T

GACCAACATGCGGAAGGAGAAGAGGACGAAAGGGGACGGCGAAGGCAACGCGGTGAGTGGGCCTCAAAATAGAAAACCTCXXXXXXXXXXA

CCAACAATTCTAAAACTGCTTCCTTAACCTCCAATTCCATACTAACAATCTAAACCTTTATCCCCCATCCTCATTTGTA

GACCAACATGCGGAAGGAGAAGAGGACGAAAGGGGACGGCGAAGGCAACGCGGTGAGTGGGCCTCAAAATAGATAACCTCXXXXXXXXXXA

CCAACAATTCTAAAACTGCTTCCTTAACCTCCAATTCCATACTAACTATCTAAACCTTTATCCCCCATCCTCATTTGTA

>Marker967856 Chr2 SNP_site:3641426 Ref_Type:T

ACCATTTATTTCTGCATTATATTTTTACTAGGAAAGCATGGAACACTTTGTTTAGAATCTTTAATTTGGAGGTTTGCCTTXXXXXXXXXXA

TAGTAGAATTTTTTAAGACAATTATAATTCTTTTGATTCTTTTTGGACTGTGTTGCAACGCACAACTTCTTGGTGGAGT

ACCATTTATTTCTGCATTATATTTTTACTAGGAAAGCATGGAACACTTTGTTTAGAATCTTTAATTTGGAGGTTTGCCTTXXXXXXXXXXA

TAGTAGAATTTTTTAAGACAATTATAATTCTTTTGATTCTTTTTGGACTGTGTTGCAATGCACAACTTCTTGGTGGAGT

>Marker967911 Chr2 SNP_site:382909 Ref_Type:G

AACGCTTCTCTCAGAAGAATGAATCAAAGGACCGAGCCCCGAATCTTGCTCGTCCTTTCAAGATCTTTTGCTTTCCAACAXXXXXXXXXX-

TTTAAAGAAAATCATGATTCATTGTGTTTCGCAACGATATAATAGAAAGTATAAATTTTCATCACATAAGTATAAGTGTG

AACGCTTCTCTCAGAAGAATGAATCAAAGGACCGAGCCCCGAATCTTGCTCGTCCTTTCAAGATCTTTTGCTTTCCAACAXXXXXXXXXXT

TTTAAAG-AAATCATGATTCATTGTGTTTCGCAACGATATAATAGAAAGTATAAATTTTCATCACATATGTATAAGTGTG

>Marker968847 Chr2 SNP_site:13948998 Ref_Type:C

TACTTAAACTAACAAAACCCAAAATTATTTATGACTATTGTTTTTCATAAATTTCATATTTATTCTTTGTAATTTTTTAAXXXXXXXXXXC

Create PDF with GO2PDF for free, if you wish to

TTATATTACTAACTTTTCATATTATTTTTTCTTCTTATTCCCATATTTCATTGTTTGTAATTTCTTCATTTCCTTTTGT
TACTTAAACTAACAAAACCCAAAATTATTTATGACTATTGTTTTTCATAAATTTCATATTTATTCTTTGTAATTTTTTAAXXXXXXXXXXXC
TTATATTATTAACTTTTCATATTATTTTTTCTTCTTATTCCCATATTTCATTGTTTGTAATTTCTTCATTTCTTTTTGT
>Marker968847 Chr2 SNP_site:13949062 Ref_Type:C
TACTTAAACTAACAAAACCCAAAATTATTTATGACTATTGTTTTTCATAAATTTCATATTTATTCTTTGTAATTTTTTAAXXXXXXXXXXXC
TTATATTACTAACTTTTCATATTATTTTTTCTTCTTATTCCCATATTTCATTGTTTGTAATTTCTTCATTTCCTTTTGT
TACTTAAACTAACAAAACCCAAAATTATTTATGACTATTGTTTTTCATAAATTTCATATTTATTCTTTGTAATTTTTTAAXXXXXXXXXXXC
TTATATTATTAACTTTTCATATTATTTTTTCTTCTTATTCCCATATTTCATTGTTTGTAATTTCTTCATTTCTTTTTGT
>Marker968990 Chr2 SNP_site:16924800 Ref_Type:G
ACCATTTGACAGCAAAAATCAAGCCTCAACATCATCAATATCAGCTAGCCCGATACATAATTTGCTATTACACCCAATTCXXXXXXXXXXXA
GAATAATAGGATCACTTTTTTCCTAAAAAAAATACATTTCAAGTTCATTGTTGATTGGTATAAGATTTAGATCTCTTGT
ACCATTTGACAGCAAAAATCAAGCCTCAACATCATCAATATCAGCTATCCCGATACATAATTTGCTATTACACCCAATTCXXXXXXXXXXXA
GAATAATAGGATCACTTTTTTCCTAAAAAAAATACATTTCAAGTTCATTGTTGATTGGTATAAGATTTAGATCTCTTGT
>Marker969044 Chr2 SNP_site:21325547 Ref_Type:G
ACTATACGATGAGATACATGAGAGATATAATCACTAATGGGAGCATAGTAGTTACAAATTCGGTATGTTGTGTTTAAATGXXXXXXXXXXXA
ATGCATCTAGCTAATTTCTTAGGTGGCCACATGTGATTATGGTGATTAGCTAAGTTTTTTTTCTTTTTTGGGTTAGGTA
ACTATACGATGAGATACATGAGAGATATAATCACTAATGGGAGCATAGTAGTTACAGATTCGGTATGTTGTGTTTAAATGXXXXXXXXXXXA
ATGCAGCTAGCTAATTTCTTAGGTGGCCACATGTGATTATGGTGATTAGCTAAGTTTTTTTTCTTTTTTGGGTTAGGTA
>Marker969044 Chr2 SNP_site:21325694 Ref_Type:G
ACTATACGATGAGATACATGAGAGATATAATCACTAATGGGAGCATAGTAGTTACAAATTCGGTATGTTGTGTTTAAATGXXXXXXXXXXXA
ATGCATCTAGCTAATTTCTTAGGTGGCCACATGTGATTATGGTGATTAGCTAAGTTTTTTTTCTTTTTTGGGTTAGGTA
ACTATACGATGAGATACATGAGAGATATAATCACTAATGGGAGCATAGTAGTTACAGATTCGGTATGTTGTGTTTAAATGXXXXXXXXXXXA
ATGCAGCTAGCTAATTTCTTAGGTGGCCACATGTGATTATGGTGATTAGCTAAGTTTTTTTTCTTTTTTGGGTTAGGTA
>Marker969459 Chr2 SNP_site:19195633 Ref_Type:A
ACCAACATCGCCTGGGGGTGGATACAGAAGTGCAGTGGGTCGCATTTTTTTCAAGACAAAGGAAATGATTCAGGCTGTGGXXXXXXXXXXXT
GAGCCCTCCATTGGTAAATATATCAGAAAATCAAATCAATGGCTGGAACATAGCTGACATAGCCTCTGGATTTTTGGTT
ACCAACATCGCCTGGGGGTGGATACAGAAGTGCAGTGGGTCGCATTTTTTTCCAGACAAAGGAAATGATTCAGGCTGTGGXXXXXXXXXXXT
GAGCCCTCCATTGGTAAATATATCAGAAAATCAAATCAATGGCTGGAACATAGCTGACATAGCCTCTGGATTTTTGGTT
>Marker969891 Chr2 SNP_site:1692641 Ref_Type:A
AACCTAACTGTCTCACCGAGATTAGTGTCAAAATTAGCGGGAAAATAAAATTAAAAAATTTAATTTAAATAAAGTTTTTAXXXXXXXXXXXT
TATTATATAAATGTTACTTCACAAAAAAGTCCAATATGTAATTCCCAAAATTTGACTCCATACATTACCAATATTTGTC
AACCTAACTGTCTCACCGAGATTAGTGTCAAAATTAGCGGGAAAATAAAATTAAAAATTTTAATTTAAATAAAGTTTTTAXXXXXXXXXXXT
TATTATATAAATGTTACTTCACAAAAAAGTCCAATATGTAATTCCCAAAATTTGACTCCATACATTACCAATATTTGTC
>Marker970327 Chr2 SNP_site:13452550 Ref_Type:G
GACCAGAAACTAAATCAAGATCAATTCTTCATTCAAAGGACAAGAACAGAGATATGAAGAAAGGAATCTTATGATATTGCXXXXXXXXXXXA
GTTTGAAAGCAAATAAAAACAGAGTAAGAACTAAAACTAAGAGGAAATTTCAAAAATTTTCACCAGATTAAAATTAGTC
GACCAGAAACTAAATCAAGATCAATTCTTCATTCAAAGGACAAGAACAGAGATATGAAGAAAGGAATCTTATGATATTGCXXXXXXXXXXXA
GTTTGAAAGCAAATAAAAACAGGGTAAGAACTAAAACTAAGAGGAAATTTCAAAAATTTTCACCAGATTAAAATTAGTC
>Marker97106 Chr4 SNP_site:15502576 Ref_Type:T
ACTTGAGTTGATCTTTAGTCGAGAATATCCTTCAGAGTCTCATTCATCTGAAAAATCCAAATTAAATTCTTAATTAATCTXXXXXXXXXXXG
ATGTAGTGCTGACTAGACATAAATGATACGTGTGAAAGAATGTATTGGTAAGGTCGTCAAAATCAAGCAATAATATGTT
ACTTGAGTTGATCTTTAGTCGAGAATATCCTTCAGAGTCTCATTCATCTGAAAAATCCAAATTAAATTCTTAATTAATCTXXXXXXXXXXXG
ATGTAGTGCTGACTAGACATAAATGATATGTGTGAAAGAATGTATTGGTAAGGTCGTCAAAATCAAGCAATAATATGTT
>Marker971129 Chr2 SNP_site:22190892 Ref_Type:G

Create PDF with GO2PDF for free, if you wish to

ACAACTAATACTTTTTTACTTATTTTGAAAGAAAACTACTATGGAAATGAAAATAATTTGATATTTAATAGTAATTTATCXXXXXXXXXXC
ATGTGTAGAATTTGACGAGTGATAGAAGAGTCAATTGAAAACATTAATGCATAGGACAATATTGTTAATTTTTAAAGTT
ACAACTAATACTTTTTTACTTATTTTGAAAGAAAACTACTATGGAAATGAAAATAATTTGATATTTAATAGTAATTTATCXXXXXXXXXXC
ATGTTTAGAATTTGTCGAGTGATAGAAGAGTCAATTGAAAACATTAATGTATAGGACAATATTGTTAATTTTTAAAGTT
>Marker971129 Chr2 SNP_site:22190902 Ref_Type:A
ACAACTAATACTTTTTTACTTATTTTGAAAGAAAACTACTATGGAAATGAAAATAATTTGATATTTAATAGTAATTTATCXXXXXXXXXXC
ATGTGTAGAATTTGACGAGTGATAGAAGAGTCAATTGAAAACATTAATGCATAGGACAATATTGTTAATTTTTAAAGTT
ACAACTAATACTTTTTTACTTATTTTGAAAGAAAACTACTATGGAAATGAAAATAATTTGATATTTAATAGTAATTTATCXXXXXXXXXXC
ATGTTTAGAATTTGTCGAGTGATAGAAGAGTCAATTGAAAACATTAATGTATAGGACAATATTGTTAATTTTTAAAGTT
>Marker971129 Chr2 SNP_site:22190937 Ref_Type:C
ACAACTAATACTTTTTTACTTATTTTGAAAGAAAACTACTATGGAAATGAAAATAATTTGATATTTAATAGTAATTTATCXXXXXXXXXXC
ATGTGTAGAATTTGACGAGTGATAGAAGAGTCAATTGAAAACATTAATGCATAGGACAATATTGTTAATTTTTAAAGTT
ACAACTAATACTTTTTTACTTATTTTGAAAGAAAACTACTATGGAAATGAAAATAATTTGATATTTAATAGTAATTTATCXXXXXXXXXXC
ATGTTTAGAATTTGTCGAGTGATAGAAGAGTCAATTGAAAACATTAATGTATAGGACAATATTGTTAATTTTTAAAGTT
>Marker971696 Chr2 SNP_site:2989630 Ref_Type:A
AACTAGGAAATATCAAATATTTTATCTTTTTCTTGGTTCTGATGGATTTAAATTTCAACAGCTCCTTTGAGCGGCCCCCTXXXXXXXXXXC
CAAGTCACTTGAAAGTTTTTGCAGCTAATCAAGAGCAGGAAACTTCTACTGTTCCCTTATCTCTTGTTGCTGCGGAGTC
AACTAGGAAATGTCAAATATTTTATCTTTTTCTTGGTTCTGATGGATTTAAATTTCAACAGCTCCTTTGAGCGGCCCCCTXXXXXXXXXXC
CAAGTCACTTGAAAGTTTTTGCAGCTAATCAAGAGCAGGAAACTTCTACTGTTCCCTTATCTCTTGTTGCTGCGGAGTC
>Marker971868 Chr2 SNP_site:20645876 Ref_Type:G
ACGACGAAGGAACAAGACCAATAAGTGTAGCCAAAAGGAAAACATGAAAAGGTATATCGACAATGGGGGATGCCAAGTTTXXXXXXXXXXT
ATGAAATGAGGTTACTTTTCAATTACCTCAGATTGGAAAGTTTTCAATTTTTCAGGCCACATCCAAGAAACAAAAGGTC
ACGACGAAGGAACAAGACCAATGAGTGTAGCCAAAAGGAAAACATGAAAAGGTATATCGACAATGGGGGATGCCAAGTTTXXXXXXXXXXT
ATGAAATGAGGTTACTTTTCAATTACCTCAGATTGGAAAGTTTTCAATTTTTCAGGCCACATCCAAGAAACAAAAGGTC
>Marker972088 Chr2 SNP_site:14218332 Ref_Type:C
AACAAGAACAAATATATATACAAGTGACATTTAATACTTAACTATGTTCATTCCGTCTTTACTTACGGGATGCGTAGAGTXXXXXXXXXXT
ATCATCTACAGTTGTATCGTCTTAAATACTGATATAATTAGTTTGACATTTTGACAACTTGTGTATATATATGCTTTGT
AACAAGAACAAATATATATACAAGTGACATTTAATACTTAACTATGTTCATTCCGTCTTTACTTACGGGATGCGTAGAGTXXXXXXXXXXT
ATCATCTACAGTTGTATCGTCTTAAATACTGATATAATTAGTTTGACATTTTGACAAGTTGTGTATATATATGCTTTGT
>Marker97211 Chr4 SNP_site:8151511 Ref_Type:C
ACTTTATTCTATTTTTGCCTTTTTATTATTTTATATTTTTCCTCAAATGTGTTTGTCAATCTTTGAATTCAATATTATTGXXXXXXXXXXG
ATTTCTTTCCCTCAATGTTTGAATTCAAATGTGCCTTGTTTTGGGTATAATAACCTTGCAAGGGAAGGTGAAAACTAGT
ACTTTCTTCTATTTTTGCCCTTTTATTATTTTATATTTTTCCTCAAATGTGTTTGTCAATCTTTGAATTCAATATTATTGXXXXXXXXXXG
ATTTCTTTCCCTCAATGTTTGAATTCAAATGTGCCTTGTGTTGGGTATAATAACCTTGCAAGGGAAGGTGAAAACTAGT
>Marker97211 Chr4 SNP_site:8151525 Ref_Type:C
ACTTTATTCTATTTTTGCCTTTTTATTATTTTATATTTTTCCTCAAATGTGTTTGTCAATCTTTGAATTCAATATTATTGXXXXXXXXXXG
ATTTCTTTCCCTCAATGTTTGAATTCAAATGTGCCTTGTTTTGGGTATAATAACCTTGCAAGGGAAGGTGAAAACTAGT
ACTTTCTTCTATTTTTGCCCTTTTATTATTTTATATTTTTCCTCAAATGTGTTTGTCAATCTTTGAATTCAATATTATTGXXXXXXXXXXG
ATTTCTTTCCCTCAATGTTTGAATTCAAATGTGCCTTGTGTTGGGTATAATAACCTTGCAAGGGAAGGTGAAAACTAGT
>Marker97211 Chr4 SNP_site:8151709 Ref_Type:G
ACTTTATTCTATTTTTGCCTTTTTATTATTTTATATTTTTCCTCAAATGTGTTTGTCAATCTTTGAATTCAATATTATTGXXXXXXXXXXG
ATTTCTTTCCCTCAATGTTTGAATTCAAATGTGCCTTGTTTTGGGTATAATAACCTTGCAAGGGAAGGTGAAAACTAGT
ACTTTCTTCTATTTTTGCCCTTTTATTATTTTATATTTTTCCTCAAATGTGTTTGTCAATCTTTGAATTCAATATTATTGXXXXXXXXXXG
ATTTCTTTCCCTCAATGTTTGAATTCAAATGTGCCTTGTGTTGGGTATAATAACCTTGCAAGGGAAGGTGAAAACTAGT

Create PDF with GO2PDF for free, if you wish to

>Marker972846 Chr2 SNP_site:3337939 Ref_Type:A
GACAGTTTTTAACCGTCATTACTCCTGTAAGAATTTATAACTACCCTAATGAAAACTGCAACATTAGATCATTAACACAGXXXXXXXXXXA
CAGTTTTTACCCCCATTAGTCTTTGTCAAATTAAGATGTCCCGTATTAATCTAAGGTTAAGCCGTTGATGGTTTCTTGT
GACAGTTTTTAACCGTCATTACTCCTGTAAGAATTTATAACTACCCTAATGAAAACTGCAACATTAGATCATTAACACAGXXXXXXXXXXA
CAGTTTTTACCCCCATTAGTCTTTGTCAAATTAAGATGTCCCGTATTAATCTAAGGTTGAGCCGTTGATGGTTTCTTGT
>Marker97342 Chr4 SNP_site:11033739 Ref_Type:T
ACTTGTTGGAGAGCTATGATTGACGTCGATCACTAATATAATAGAGTAATTGATAAATATCTGGAAGTCTATACAAAACAXXXXXXXXXXG
GTATGAAACTTTTTTTCTTTTGTTTTGGAGAAATAGAGAAAAGAAAAGACAGAAAGAAGAGAAAGGGCCAGCGTTGGTT
ACTTGTTGGAGAGCTATGATTGACGTCGATCACTAATATAATAGAGTAATTGATAAATATCTGGAAGTCTATACAAAACAXXXXXXXXXXG
GTATGAAACTTTTTTTCTTTTGTTTTGGAGAAATAGAGAAAAGAAAAGATAGAAAGAAGAGAAAGGGCCAGCGTTGGTT
>Marker973662 Chr2 SNP_site:440827 Ref_Type:A
ACTGTGTTATACTTTTCTTCTGTGAGTTGTGACTCCAGGATAGATATGATCGGCATGCAAACAAATTATTCTGAAAAATGXXXXXXXXXXC
TAAAAGTTTGTAAATTCATCGGAATATGATGGCACCAATGCACTGTTCTATTGGTGGAGATAGAAATATCTTTGATGTT
ACTGTGTTATACTTTTCTTCTGTGAGTTGTGACTCCAGGATAGATATGATCGGCATGCAAACAATTTATTCTGAAAAATGXXXXXXXXXXC
TAAAAGTTCGTAAATTCATCGGAATATGATGGCACCAATGCACTGTTCTATTGGTGGAGATAGAAATATCTTTGATGTT
>Marker973662 Chr2 SNP_site:440942 Ref_Type:T
ACTGTGTTATACTTTTCTTCTGTGAGTTGTGACTCCAGGATAGATATGATCGGCATGCAAACAAATTATTCTGAAAAATGXXXXXXXXXXC
TAAAAGTTTGTAAATTCATCGGAATATGATGGCACCAATGCACTGTTCTATTGGTGGAGATAGAAATATCTTTGATGTT
ACTGTGTTATACTTTTCTTCTGTGAGTTGTGACTCCAGGATAGATATGATCGGCATGCAAACAATTTATTCTGAAAAATGXXXXXXXXXXC
TAAAAGTTCGTAAATTCATCGGAATATGATGGCACCAATGCACTGTTCTATTGGTGGAGATAGAAATATCTTTGATGTT
>Marker973722 Chr2 SNP_site:2364225 Ref_Type:A
ACCAAGACTGCTCCCGAATTATAGCAGATGAACGAAATTCAGAGGTATTCCTCTAATTTTGGTTGAGCTTTGACTCAAAGXXXXXXXXXXA
TAATTTTCTTTCGTAAGTCGGTTACTTGGAAGGAAACTAAATTGTTGTTTCCCATTTGCAAAAAAACAATTTTTTTTGT
ACCAAGACTGCTCCCGAATTATAGCAGATGGACGAAATTCAGAGGTCTTCCTCTAATTTTCGTTGAGCTTTGACTCAAAGXXXXXXXXXXA
TAATTTTCTTTCGTAAGTCGGTTACTTGGAAGGAAACTAAATTGTTGTTTCCCATTTGCAAAAAAACAATTTTTTTTGT
>Marker973722 Chr2 SNP_site:2364241 Ref_Type:A
ACCAAGACTGCTCCCGAATTATAGCAGATGAACGAAATTCAGAGGTATTCCTCTAATTTTGGTTGAGCTTTGACTCAAAGXXXXXXXXXXA
TAATTTTCTTTCGTAAGTCGGTTACTTGGAAGGAAACTAAATTGTTGTTTCCCATTTGCAAAAAAACAATTTTTTTTGT
ACCAAGACTGCTCCCGAATTATAGCAGATGGACGAAATTCAGAGGTCTTCCTCTAATTTTCGTTGAGCTTTGACTCAAAGXXXXXXXXXXA
TAATTTTCTTTCGTAAGTCGGTTACTTGGAAGGAAACTAAATTGTTGTTTCCCATTTGCAAAAAAACAATTTTTTTTGT
>Marker973722 Chr2 SNP_site:2364255 Ref_Type:G
ACCAAGACTGCTCCCGAATTATAGCAGATGAACGAAATTCAGAGGTATTCCTCTAATTTTGGTTGAGCTTTGACTCAAAGXXXXXXXXXXA
TAATTTTCTTTCGTAAGTCGGTTACTTGGAAGGAAACTAAATTGTTGTTTCCCATTTGCAAAAAAACAATTTTTTTTGT
ACCAAGACTGCTCCCGAATTATAGCAGATGGACGAAATTCAGAGGTCTTCCTCTAATTTTCGTTGAGCTTTGACTCAAAGXXXXXXXXXXA
TAATTTTCTTTCGTAAGTCGGTTACTTGGAAGGAAACTAAATTGTTGTTTCCCATTTGCAAAAAAACAATTTTTTTTGT
>Marker974690 Chr2 SNP_site:3153909 Ref_Type:T
GACTGGATTCATATACTTTTGGCTTTTGATCTTCTTGGCAAGCAAGGACATTGATGCTTTGTTGTTCTGAGAAAACCAATXXXXXXXXXXG
TTGATTTCAAACCAAACCTACCTCGTGAGTCTCTCTCTTCTCTCTATCTGGAAAATAAATGAAGTAGTTCAACTTTGTT
GACTGGATTCATATACTTTTGGCTTTTGATCTTCTTGGCAAGTAAGGAGATTGATGCTTTGTTGTTCTGAGAAAACCAATXXXXXXXXXXG
TTGATTTCAAACCAAACCTACCTCGTGAGTCTCTCTCTTCTCTCTATCTGGAAAATAAATGAAGTAGTTCAACTTTGTT
>Marker974690 Chr2 SNP_site:3153915 Ref_Type:C
GACTGGATTCATATACTTTTGGCTTTTGATCTTCTTGGCAAGCAAGGACATTGATGCTTTGTTGTTCTGAGAAAACCAATXXXXXXXXXXG
TTGATTTCAAACCAAACCTACCTCGTGAGTCTCTCTCTTCTCTCTATCTGGAAAATAAATGAAGTAGTTCAACTTTGTT
GACTGGATTCATATACTTTTGGCTTTTGATCTTCTTGGCAAGTAAGGAGATTGATGCTTTGTTGTTCTGAGAAAACCAATXXXXXXXXXXG

Create PDF with GO2PDF for free, if you wish to

TTGATTTCAAACCAAACCTACCTCGTGAGTCTCTCTCTTCTCTCTATCTGGAAAATAAATGAAGTAGTTCAACTTTGTT

>Marker974881 Chr2 SNP_site:20472979 Ref_Type:C

CACTCAAGAATGCGGGTTGAGTTGAACTCTAGTGTGAAAATTGGGAAAGGAGGATCTTTGAGTTCTGGGACTGAGGATACXXXXXXXXXXG

CTTGCTAGGGAATGCCCTAGTAATGAGAGTCTAAGAAAGGAATACAAACTTGTCTGCGAATTGTCTTTGGATGAGAAGT

CACTCAAGAATGCGGGTTGAGTTGAATTCTAGTGTGAAAATTGGGAAAGGAGGATCTTTGAGTTCTGGGACTGAGGATACXXXXXXXXXXG

CTTGCTAGGGAATGCCCTAGTAATGAGAGTCTAAAAAAGGAATACAAACTTGTCTGCGAATTGTCTTTGGATGAGAAGT

>Marker974881 Chr2 SNP_site:20473194 Ref_Type:G

CACTCAAGAATGCGGGTTGAGTTGAACTCTAGTGTGAAAATTGGGAAAGGAGGATCTTTGAGTTCTGGGACTGAGGATACXXXXXXXXXXG

CTTGCTAGGGAATGCCCTAGTAATGAGAGTCTAAGAAAGGAATACAAACTTGTCTGCGAATTGTCTTTGGATGAGAAGT

CACTCAAGAATGCGGGTTGAGTTGAATTCTAGTGTGAAAATTGGGAAAGGAGGATCTTTGAGTTCTGGGACTGAGGATACXXXXXXXXXXG

CTTGCTAGGGAATGCCCTAGTAATGAGAGTCTAAAAAAGGAATACAAACTTGTCTGCGAATTGTCTTTGGATGAGAAGT

>Marker974887 Chr2 SNP_site:13928734 Ref_Type:G

ACATGACTTTCAAGTTAAACATTGTATCAAAAAAAATATTTTTGCTGCATCAAGAAAGACCAAATTTGTAGTAGTGATTGXXXXXXXXXXC

TCTCACAAACCTTGCTTCAACAACAATGAAGAGGGCAACTTAAAGATCAACTATAATTTTGAATCACTTTTCACACGTT

ACATGACTTTCAAGTTAAACATTGTATCAAGAAAAATATTTTTGTTGCATCAAGAAAGACCAAATTTGTAGTAGTGATTGXXXXXXXXXXC

TCTCACAAACCTTGCTTCAACAACAATGAAGAGGGCAACTTAAAGATCAACTATAATTTTGAATCACTTTTCACACGTT

>Marker974887 Chr2 SNP_site:13928748 Ref_Type:T

ACATGACTTTCAAGTTAAACATTGTATCAAAAAAAATATTTTTGCTGCATCAAGAAAGACCAAATTTGTAGTAGTGATTGXXXXXXXXXXC

TCTCACAAACCTTGCTTCAACAACAATGAAGAGGGCAACTTAAAGATCAACTATAATTTTGAATCACTTTTCACACGTT

ACATGACTTTCAAGTTAAACATTGTATCAAGAAAAATATTTTTGTTGCATCAAGAAAGACCAAATTTGTAGTAGTGATTGXXXXXXXXXXC

TCTCACAAACCTTGCTTCAACAACAATGAAGAGGGCAACTTAAAGATCAACTATAATTTTGAATCACTTTTCACACGTT

>Marker9754 Chr4 SNP_site:15756555 Ref_Type:G

TACCTATATATTTATCTTTTTATTTCTTTTATATAGTTGCATTGATGTTTGTGCCTCAAGACGTCTCAGCTGCCCAAAGAXXXXXXXXXXA

TGCTTGTTATTGTTTCTTTGATTGCAGTGGTCAAAAGAATCGATCTGCATACTCGCATCTTCCTGATTTAATGTAATGT

TACCTATATATTTATCTTTTTATTTCTTTTATATAGTTGCATTGATGTTTGTGCCTCAAGACGTCTCAGCTGCCCAAAGAXXXXXXXXXXA

TGCTTGTTATTGTTTCTTTGATTGCGGTGGTCAAAAGAATCGATCTGCATACTCGCATCTTCCTTATTTAATGTAATGT

>Marker9754 Chr4 SNP_site:15756594 Ref_Type:T

TACCTATATATTTATCTTTTTATTTCTTTTATATAGTTGCATTGATGTTTGTGCCTCAAGACGTCTCAGCTGCCCAAAGAXXXXXXXXXXA

TGCTTGTTATTGTTTCTTTGATTGCAGTGGTCAAAAGAATCGATCTGCATACTCGCATCTTCCTGATTTAATGTAATGT

TACCTATATATTTATCTTTTTATTTCTTTTATATAGTTGCATTGATGTTTGTGCCTCAAGACGTCTCAGCTGCCCAAAGAXXXXXXXXXXA

TGCTTGTTATTGTTTCTTTGATTGCGGTGGTCAAAAGAATCGATCTGCATACTCGCATCTTCCTTATTTAATGTAATGT

>Marker97590 Chr4 SNP_site:16321330 Ref_Type:A

AACTTGTGAAAAAAATTTCAAGTTATCAAATTTATGAACACATCTATATCATAATAAAAAGATGTTTTCATAAATTAAAAXXXXXXXXXXA

AGATATGTTCATTAATTATTTATAACATGTCATACATTTTACTTTAAGAAAAGAACAATAACATGGTGCATCTAGAGTG

AACTTGTGAAGAAAATATCAAGTTATCAAATTTATGAACACATCTATATCATAATAAAAAGATGTTTTCATAAATTAAAAXXXXXXXXXXA

AGATATGTTCATTAATTATTTATAACATGTCATACATTTTACTTTAAGAAAAGAACAATAACATGGTGCATCTAGAGTG

>Marker97590 Chr4 SNP_site:16321336 Ref_Type:T

AACTTGTGAAAAAAATTTCAAGTTATCAAATTTATGAACACATCTATATCATAATAAAAAGATGTTTTCATAAATTAAAAXXXXXXXXXXA

AGATATGTTCATTAATTATTTATAACATGTCATACATTTTACTTTAAGAAAAGAACAATAACATGGTGCATCTAGAGTG

AACTTGTGAAGAAAATATCAAGTTATCAAATTTATGAACACATCTATATCATAATAAAAAGATGTTTTCATAAATTAAAAXXXXXXXXXXA

AGATATGTTCATTAATTATTTATAACATGTCATACATTTTACTTTAAGAAAAGAACAATAACATGGTGCATCTAGAGTG

>Marker97615 Chr4 SNP_site:8155595 Ref_Type:T

AACAAACTGTTAATCCAACTAGATCCGTGAATAATGATATAGAAATCATGAAAGCATGCATCTCTGAGATAGAAAATAAGXXXXXXXXXXT

GATATTACTTTAGGAAGTGGAATAATTTTCAATGATAATGATGATCCAAAGTCTTCTAGTGATCATGTTTCCTTGTGTG

Create PDF with GO2PDF for free, if you wish to

AACAAACTGTTAATCCAATTAGATCCGTGAATAATGATATAGAAATCATGAAAGCATCCATCTCTGAGATAGAAAATAAGXXXXXXXXXXT
GATATTACTTTAGGAAGTGGAATAATTTTCAATGATAATGATGATCCAAAGTCTTATAGTGATCATGTTTCCTTGTGTG
>Marker97615 Chr4 SNP_site:8155634 Ref_Type:C
AACAAACTGTTAATCCAACTAGATCCGTGAATAATGATATAGAAATCATGAAAGCATGCATCTCTGAGATAGAAAATAAGXXXXXXXXXXT
GATATTACTTTAGGAAGTGGAATAATTTTCAATGATAATGATGATCCAAAGTCTTCTAGTGATCATGTTTCCTTGTGTG
AACAAACTGTTAATCCAATTAGATCCGTGAATAATGATATAGAAATCATGAAAGCATCCATCTCTGAGATAGAAAATAAGXXXXXXXXXXT
GATATTACTTTAGGAAGTGGAATAATTTTCAATGATAATGATGATCCAAAGTCTTATAGTGATCATGTTTCCTTGTGTG
>Marker97615 Chr4 SNP_site:8155798 Ref_Type:A
AACAAACTGTTAATCCAACTAGATCCGTGAATAATGATATAGAAATCATGAAAGCATGCATCTCTGAGATAGAAAATAAGXXXXXXXXXXT
GATATTACTTTAGGAAGTGGAATAATTTTCAATGATAATGATGATCCAAAGTCTTCTAGTGATCATGTTTCCTTGTGTG
AACAAACTGTTAATCCAATTAGATCCGTGAATAATGATATAGAAATCATGAAAGCATCCATCTCTGAGATAGAAAATAAGXXXXXXXXXXT
GATATTACTTTAGGAAGTGGAATAATTTTCAATGATAATGATGATCCAAAGTCTTATAGTGATCATGTTTCCTTGTGTG
>Marker976464 Chr2 SNP_site:13820176 Ref_Type:T
ACTTTATTCTATTTTAGTTCAACTTTTCGAGTGAAACAAACCTAAATTAAAAAAGCTAATTGTTCTCTTTTCTCCAAAATXXXXXXXXXXA
AATATCCGAGACATTAAAATTAAATTTATATGTAGATGAATTGTATCTAGTAGATAATGACAATGAGCTAAAACACAGT
ACTTTATTCTATTTTAGTTCAACTTTTCGAGTGAAACAAACCTAAATTAAAAAAGCTAATTGTTCTCTTTTCTCCAAAATXXXXXXXXXXA
AATATCCGAGACATTAAAATTAAATTTATATGTAGATGAATTGTATCTAGTATATAATGACAATGAGCTAAAACACAGT
>Marker97662 Chr4 SNP_site:14261005 Ref_Type:G
AACCAAATCATTTCAACTGTCAGTTGAGTTACAATTTATTAAAGAGTGGAAAAAAAATAAAAATAAAAAAGTGGTCTAATXXXXXXXXXXG
ACCATATCCCCTTTTTTACTTTTCCTACTAATTTCAAAGAGGCCAAAAAGAGACTATGAATTGACTCACCACACCTGTA
AACCAAATCATTTCAACTGTCAGTTGAGTTACAATTTATTAAAGAGTGGAAAGAAAATAAAAATAAAAAAGTGGTCTAATXXXXXXXXXXG
ACCATATCCCCTTTTTTACTTTTCCTACTAATTTCAAAGAGGCCAAAAAGAGACTATGAATTGACTCACCACACCTGTA
>Marker976681 Chr2 SNP_site:18525900 Ref_Type:A
ACTAAAAAGATAGCTATACCTATATAAGTTTCCTAAAACAAAAGTTAATAAACATACTTTTTTAGTTTTTTATTGACAATXXXXXXXXXXA
TAAATTAATAGTTGTCTATTTGTGTTTTAAAAATATTCATTTTGTTTCCATATTTTAAAAAAGTGATAACTTAAAAGTG
ACTAGAAAGATAGCTATACCTATATAAGTTTCCTAAAACAAAAGTTAATAAACATACTTTTTTAGTTTTTTATTGACAATXXXXXXXXXXA
TAAATTAATAGTTGTCTATTTGTGTTTTAAAAATATTCATTTTGTTTCCATATTTTAAAAAAGTGATAACTTAAAAGTG
>Marker976971 Chr2 SNP_site:2023112 Ref_Type:T
ACTTATTTTTTCAATCTAATATCAAAGTTGTCCAAGATTCCATTACCTCTTCCACTTTTCCTTCAACAATTCTATCTTTTXXXXXXXXXXG
CTGATATTGGAGTAAAAACCATTTTTTTGATAAAGCAAGCTCAGGAAAATCCTCTACTTTCGTAAATAAATCTCTAAGTT
ACTTATTTTTTCAATCTAATATCAAAGTTGTCCAAGATTCCATTACCTCTTCCTCTTTTCCTTTAACAATTCTATCTTTTXXXXXXXXXXG
CTGATATTGGAGTAAAAACCATTTTTTTGATAAAGCAAGCTCAGGAAAATCCTCTACGTTCGTAAATAAATCTCTAAGTT
>Marker976971 Chr2 SNP_site:2023122 Ref_Type:T
ACTTATTTTTTCAATCTAATATCAAAGTTGTCCAAGATTCCATTACCTCTTCCACTTTTCCTTCAACAATTCTATCTTTTXXXXXXXXXXG
CTGATATTGGAGTAAAAACCATTTTTTTGATAAAGCAAGCTCAGGAAAATCCTCTACTTTCGTAAATAAATCTCTAAGTT
ACTTATTTTTTCAATCTAATATCAAAGTTGTCCAAGATTCCATTACCTCTTCCTCTTTTCCTTTAACAATTCTATCTTTTXXXXXXXXXXG
CTGATATTGGAGTAAAAACCATTTTTTTGATAAAGCAAGCTCAGGAAAATCCTCTACGTTCGTAAATAAATCTCTAAGTT
>Marker976971 Chr2 SNP_site:2023333 Ref_Type:G
ACTTATTTTTTCAATCTAATATCAAAGTTGTCCAAGATTCCATTACCTCTTCCACTTTTCCTTCAACAATTCTATCTTTTXXXXXXXXXXG
CTGATATTGGAGTAAAAACCATTTTTTTGATAAAGCAAGCTCAGGAAAATCCTCTACTTTCGTAAATAAATCTCTAAGTT
ACTTATTTTTTCAATCTAATATCAAAGTTGTCCAAGATTCCATTACCTCTTCCTCTTTTCCTTTAACAATTCTATCTTTTXXXXXXXXXXG
CTGATATTGGAGTAAAAACCATTTTTTTGATAAAGCAAGCTCAGGAAAATCCTCTACGTTCGTAAATAAATCTCTAAGTT
>Marker97712 Chr4 SNP_site:18325384 Ref_Type:G
ACTACGCCATGCAGTGCATCTCAGAATCCACCCACTTTTGGTTCTGAATTTGGAACTTTCAACCCTTCCACACTCAACTTXXXXXXXXXXA

Create PDF with GO2PDF for free, if you wish to

GAAAAAAAAATTGTCGATTTTGCAGCTTTTTACTTCCATCTTTGTGAGTTAAGAATGGTTGCTAGGGAGATGCCAAAGT
ACTACGCCATGCAGTGCATCTCAGAATCCACCCACTTTTGGTTCTGAATTTGGAACTTTCAACCCTTCCACACTCAACTTXXXXXXXXXXA
GAAAAAAAAATTGTCGATTTTGCAGCTTTTTACTTCCATCTTTGTGAGTTAAGAATGGTTGCTAGGTAGATGTCAAAGT
>Marker97712 Chr4 SNP_site:18325390 Ref_Type:C
ACTACGCCATGCAGTGCATCTCAGAATCCACCCACTTTTGGTTCTGAATTTGGAACTTTCAACCCTTCCACACTCAACTTXXXXXXXXXXA
GAAAAAAAAATTGTCGATTTTGCAGCTTTTTACTTCCATCTTTGTGAGTTAAGAATGGTTGCTAGGGAGATGCCAAAGT
ACTACGCCATGCAGTGCATCTCAGAATCCACCCACTTTTGGTTCTGAATTTGGAACTTTCAACCCTTCCACACTCAACTTXXXXXXXXXXA
GAAAAAAAAATTGTCGATTTTGCAGCTTTTTACTTCCATCTTTGTGAGTTAAGAATGGTTGCTAGGTAGATGTCAAAGT
>Marker97794 Chr4 SNP_site:11947662 Ref_Type:A
AACTTTTGATCACATTTATAATACATTTAGTCTGTTAATTGGATGTGTTTAAACCTATCATTTCTTGGGGTCGGATTATGXXXXXXXXXXT
GATCACAGGTTGCTGGTATATGAGTTCATGCAAAAGGGCAGTGCAGAAAATCATCTTTTCAGAAGTGAGTTTTCGAGTA
AACTTTTGATCACATTTATAATACATTTAGTCTGTTAATTGGATGTGTTTGAACCTATCATTTCTTGGGGTCGGATTATGXXXXXXXXXXT
GATCACAGGTTGCTGGTATATGAGTTCATGCAAAAGGGCAGTGCAGAAAATCATCTTTTCAGAAGTGAGTTTTCGAGTA
>Marker97819 Chr4 SNP_site:6207643 Ref_Type:C
ACAACCTATAGACCAGATCTTATTCAAGTAAGGTTGTGAGAAACAGAAACTGAAATTTAAACTAAAACAGTGTAATAAAAXXXXXXXXXXC
AATACATCTTCATCTTGAAGCTGAAGTTTGCAAATCATGTGAAGGACTCTACATCTTCTAGAATCTCCTTTGTCGAGTT
ACAACCTATAGACCAGATCTTATTCAAGTAAGGTTGTGAGAAACAGAAACTGAAATTTAAACTAAAACAGTGTAATAAAAXXXXXXXXXXC
AATACATCTTCATCTTGAAGCTGAAGTTTGCAAATCATGTGAAGGACTTTACATCTTCTAGAATCTCCTTTGTCGAGTT
>Marker97834 Chr4 SNP_site:1904865 Ref_Type:T
ACTGTTGATGCTGATACATTGTTGTTATCAGTAATCTTTAAAGACTGCACGTGATACTTTCAAAGCTTTTCAGAAATGACXXXXXXXXXXT
CAGCAGAACTGTCCTGTTAATTATGATGTTCATCATATAGCTAAAGCTCAGTTAGGAGATGAGAGTAGTTGAAAAGGTT
ACTGTTGATGCTGATACATTGTTGTTATCAGTAATCTTTAAAGACTGCACGTGATACTTTCAAAGCTTTTCAGAAATGACXXXXXXXXXXT
CAGCAGAACTTTCCTGTTAATTACGATGTTCATCATATAGCTAAAGCTCAGTTAGGAGATGAGAGTAGTTGAAAAGGTT
>Marker97834 Chr4 SNP_site:1904878 Ref_Type:C
ACTGTTGATGCTGATACATTGTTGTTATCAGTAATCTTTAAAGACTGCACGTGATACTTTCAAAGCTTTTCAGAAATGACXXXXXXXXXXT
CAGCAGAACTGTCCTGTTAATTATGATGTTCATCATATAGCTAAAGCTCAGTTAGGAGATGAGAGTAGTTGAAAAGGTT
ACTGTTGATGCTGATACATTGTTGTTATCAGTAATCTTTAAAGACTGCACGTGATACTTTCAAAGCTTTTCAGAAATGACXXXXXXXXXXT
CAGCAGAACTTTCCTGTTAATTACGATGTTCATCATATAGCTAAAGCTCAGTTAGGAGATGAGAGTAGTTGAAAAGGTT
>Marker978408 Chr2 SNP_site:17340034 Ref_Type:C
ACTTGCTGAGGAGATGGTTGGTGTTGGTTAATTCTTTTAATGGATTTTCTTTTCAAGAAAGATAAAAAAAGACGGTCAATXXXXXXXXXXC
TTTAAATAAAATACTTGAAAACATACAATAAAATAAACACAACCAGTTATTTGATGGTATAAATTGTTGTATTAAGGTG
ACTTGTTGAGGAGATGGTTGGTGTTGGTTAATTCTTTTAATGGATTTTCTTTTCAAGAAAGATAAAAAAAGACAGTCAATXXXXXXXXXXC
TTTAAATAAAATACTTGAAAACATACAATAAAATAAACACAACCAGTTATTTGATGGTATAAATTGTTGTATTAAGGTG
>Marker978408 Chr2 SNP_site:17340102 Ref_Type:G
ACTTGCTGAGGAGATGGTTGGTGTTGGTTAATTCTTTTAATGGATTTTCTTTTCAAGAAAGATAAAAAAAGACGGTCAATXXXXXXXXXXC
TTTAAATAAAATACTTGAAAACATACAATAAAATAAACACAACCAGTTATTTGATGGTATAAATTGTTGTATTAAGGTG
ACTTGTTGAGGAGATGGTTGGTGTTGGTTAATTCTTTTAATGGATTTTCTTTTCAAGAAAGATAAAAAAAGACAGTCAATXXXXXXXXXXC
TTTAAATAAAATACTTGAAAACATACAATAAAATAAACACAACCAGTTATTTGATGGTATAAATTGTTGTATTAAGGTG
>Marker978433 Chr2 SNP_site:13581391 Ref_Type:T
ACATGAGGCATCGAGAAAGTTTTGTTTATTCATGTCGGATAGGTGTTCCAGGCCAAATAAAATAATTTTTTTCCTAATTAXXXXXXXXXXT
AGGAATTAAGGTAAAAGGAACAAGAAAAAGCTGGTATTTAGGTTGAATATCAGTTGGTTGGAGTCACTTATTAAAGAGT
ACATGAGGCATCGAGAAAGTTTTGTTTATTCATGTCGGATAGGTGTTCCATGCCAAATAAAATTATTTTTTTCCTAATTAXXXXXXXXXXT
AGGAATTAAGGTAAAAGGAACAAGAAAAAGCTCGTATTTAGGTTGAATATCAGTTGGTTGGAGTCACTTATTAAAGAGT
>Marker978433 Chr2 SNP_site:13581404 Ref_Type:T

Create PDF with GO2PDF for free, if you wish to

ACATGAGGCATCGAGAAAGTTTTGTTTATTCATGTCGGATAGGTGTTCCAGGCCAAATAAAATAATTTTTTTCCTAATTAXXXXXXXXXXT
AGGAATTAAGGTAAAAGGAACAAGAAAAAGCTGGTATTTAGGTTGAATATCAGTTGGTTGGAGTCACTTATTAAAGAGT
ACATGAGGCATCGAGAAAGTTTTGTTTATTCATGTCGGATAGGTGTTCCATGCCAAATAAAATTATTTTTTTCCTAATTAXXXXXXXXXXT
AGGAATTAAGGTAAAAGGAACAAGAAAAAGCTCGTATTTAGGTTGAATATCAGTTGGTTGGAGTCACTTATTAAAGAGT
>Marker978433 Chr2 SNP_site:13581567 Ref_Type:C
ACATGAGGCATCGAGAAAGTTTTGTTTATTCATGTCGGATAGGTGTTCCAGGCCAAATAAAATAATTTTTTTCCTAATTAXXXXXXXXXXT
AGGAATTAAGGTAAAAGGAACAAGAAAAAGCTGGTATTTAGGTTGAATATCAGTTGGTTGGAGTCACTTATTAAAGAGT
ACATGAGGCATCGAGAAAGTTTTGTTTATTCATGTCGGATAGGTGTTCCATGCCAAATAAAATTATTTTTTTCCTAATTAXXXXXXXXXXT
AGGAATTAAGGTAAAAGGAACAAGAAAAAGCTCGTATTTAGGTTGAATATCAGTTGGTTGGAGTCACTTATTAAAGAGT
>Marker978636 Chr2 SNP_site:13794772 Ref_Type:A
AACTATGACAATTATGGTAACTATACTTTAACTATACTATACTACTTTCATCTCTCCTATTGTTTCTACTTTTCTTGATTXXXXXXXXXXT
ATACATTTAGGAATCGCAATGTATAATTAAGCATGAGAAGCTAAATTAGTTACACTATAAATTTAGGTTTTATGTAGTA
AACTATGACAATTATGGTAACTATACTTTAACTATACTATACTACTTTCATCTCTCCTATTGTTTCTACTTTTCTTGATTXXXXXXXXXXT
ATACATTTAGGAATCGCTATGTATAATTAAGCACGAGAAGCTAAATTAGTTACACTATAAATTTAGGTTTTATGCAGTA
>Marker978636 Chr2 SNP_site:13794788 Ref_Type:T
AACTATGACAATTATGGTAACTATACTTTAACTATACTATACTACTTTCATCTCTCCTATTGTTTCTACTTTTCTTGATTXXXXXXXXXXT
ATACATTTAGGAATCGCAATGTATAATTAAGCATGAGAAGCTAAATTAGTTACACTATAAATTTAGGTTTTATGTAGTA
AACTATGACAATTATGGTAACTATACTTTAACTATACTATACTACTTTCATCTCTCCTATTGTTTCTACTTTTCTTGATTXXXXXXXXXXT
ATACATTTAGGAATCGCTATGTATAATTAAGCACGAGAAGCTAAATTAGTTACACTATAAATTTAGGTTTTATGCAGTA
>Marker978636 Chr2 SNP_site:13794829 Ref_Type:T
AACTATGACAATTATGGTAACTATACTTTAACTATACTATACTACTTTCATCTCTCCTATTGTTTCTACTTTTCTTGATTXXXXXXXXXXT
ATACATTTAGGAATCGCAATGTATAATTAAGCATGAGAAGCTAAATTAGTTACACTATAAATTTAGGTTTTATGTAGTA
AACTATGACAATTATGGTAACTATACTTTAACTATACTATACTACTTTCATCTCTCCTATTGTTTCTACTTTTCTTGATTXXXXXXXXXXT
ATACATTTAGGAATCGCTATGTATAATTAAGCACGAGAAGCTAAATTAGTTACACTATAAATTTAGGTTTTATGCAGTA
>Marker978721 Chr2 SNP_site:10339746 Ref_Type:T
AACATTACTCTAAAGGAAAATTAAAGTTTATGTTGTTTTGAAAATTTCTACATTTGCATCTACTTGGGCAAGATTCGAATXXXXXXXXXXT
TATAAAACTATCCCAAAATAAAAAATGTTTTGACTTACCATTTTAATAGACTTAATAAGGAATATAATAATTAATTGTA
AACATTACTCTAAAGGAAAATTAAAGTTTATGTTGTTTTGAAAATTTCTACATTTGCATCTACTTTGGCAAGATTCGAATXXXXXXXXXXT
TATAAAACTATCCCAAAATAAAAAATGTTTTGACTTACCATTTTAAAAGACTTAATAAGGAATATAATAATTAATTGTA
>Marker978721 Chr2 SNP_site:10339919 Ref_Type:A
AACATTACTCTAAAGGAAAATTAAAGTTTATGTTGTTTTGAAAATTTCTACATTTGCATCTACTTGGGCAAGATTCGAATXXXXXXXXXXT
TATAAAACTATCCCAAAATAAAAAATGTTTTGACTTACCATTTTAATAGACTTAATAAGGAATATAATAATTAATTGTA
AACATTACTCTAAAGGAAAATTAAAGTTTATGTTGTTTTGAAAATTTCTACATTTGCATCTACTTTGGCAAGATTCGAATXXXXXXXXXXT
TATAAAACTATCCCAAAATAAAAAATGTTTTGACTTACCATTTTAAAAGACTTAATAAGGAATATAATAATTAATTGTA
>Marker979042 Chr2 SNP_site:6527855 Ref_Type:C
TACACATAACACGCGCAGACACACCCAACTCGTAATTCAGTATTACCTGTCGAACGGAAATGCTCACATCAGTTCTAACCXXXXXXXXXXT
CATCATCACTACCACCCTCAAGCAATGCTTTTCCAGAAGTTCCAGCAACCTGCAAAACAGAAACTAAGACACAGACGTA
TACACATAACACGCGCAGACACACCCAACTTGTAATTCAGTATTACCTGTCGAACGGAAATGCTCACATCAGTTCTAACCXXXXXXXXXXT
CATCATCACTACCACCCTCAAGCAATGCTTTTCCAGAAGTTCCAGCAACCTGCAAAACAGAAACTAAGACACAGACGTA
>Marker979154 Chr2 SNP_site:3831165 Ref_Type:T
GACATAGTCGTAGAATTTTTCCTTCTTTCCATGGTATCTAACTCAAAAAATTTGGCAGTCATATAGTTTTCGCAGAGGATXXXXXXXXXXG
TTTTTCCTCTTGGATTTGCTATAAACCAAAGGTGACTATTATGCATCTGTTCTCTCTTCAGAAATTCAGGAATGAGGTT
GACATAGTCGTAGAATTTTTCCTTCTTTCCATGGTATCTAACTCAAAAATTTTGGCAGTCATATAGTTTTCGCAGAGGATXXXXXXXXXXG
TTTTTCCTCTTGGATTTGCTATAAACCAAAGGTGACTATTATGCATCTGTTCTCTCTTCAGAAATTCAGGAATGAGGTT

Create PDF with GO2PDF for free, if you wish to

>Marker979256 Chr2 SNP_site:17759200 Ref_Type:C
AACCCACGCCGTTGGATTCCACCTACAAGTAACTTCAATGGAGAAATTAATTAGTAAAGTAATTAAATTTTTAGAAAAGAXXXXXXXXXXXA
TGGGATAATATTTTGGAGAGATCAGTGATAGAAAGGGTTGGTTTTATAGCAAAAAAATCTGAAGCTGTGTGGTTAAGTG
AACCCACGCCGTTGGATTCCACCTACAAGTAATTTCAATGGAGAAATTAATTAGTAAAGTAATTAAATTTTTAGAAAAGAXXXXXXXXXXXA
TGGGATAATATTTTGGAGAGATCAGTGATAGAAAGGGTTGGTTTTATAGCAAAAAAATCTGAAGCTGTGTGGTTAAGTG
>Marker979469 Chr2 SNP_site:20525838 Ref_Type:C
AACCAAAGTAAAAAAATTAGACCAATTATATGTTTTCTTTATTTTATTTTATTTAAACAAACATATACTATTTTAAACTAXXXXXXXXXXXA
AAATATTTGGTAGAGAGGAACAAAATACTTTATAATTATACTCTTTTAAATAAAACATTGACTATCAACATAATACGTA
AACCAAAGTAAAAAAATTAGACCAATTATATGTTTTCTTTATTTTATTTTATTTAAACAAACATATACTATTTTAAACTAXXXXXXXXXXXA
AAATATTTGGTAGAGAGGAACAAAATACTTTATAATTATACTCTTTTAAATAAAACATTGACTATCAACATAATATGTA
>Marker979715 Chr2 SNP_site:13841884 Ref_Type:C
ACCAAACAAGACAATGGAAAATAAAAGCAACAGAAATTACAGCATAAATTAAAATCAGTCTTACAATAATCTTCTCTAAGXXXXXXXXXXXG
TCATCGTCCAACGATCAATTCAAGTATAGAGACTAAAATTATACTATATTATAACTATAATAGGGTATAAATTATAGTT
ACCAAACAAGACAATGGAAAATAAAAGCAACAGAAATTACAGCATAAATTAAAATCAGTGTTACAATAATCTTCTCTAAGXXXXXXXXXXXG
TCATCGTCCAACGATCAATTCAAGTATAGAGACTAAAATTATACAATATTATAACTATAATAGGGTATAAATTATAGTT
>Marker979715 Chr2 SNP_site:13842081 Ref_Type:T
ACCAAACAAGACAATGGAAAATAAAAGCAACAGAAATTACAGCATAAATTAAAATCAGTCTTACAATAATCTTCTCTAAGXXXXXXXXXXXG
TCATCGTCCAACGATCAATTCAAGTATAGAGACTAAAATTATACTATATTATAACTATAATAGGGTATAAATTATAGTT
ACCAAACAAGACAATGGAAAATAAAAGCAACAGAAATTACAGCATAAATTAAAATCAGTGTTACAATAATCTTCTCTAAGXXXXXXXXXXXG
TCATCGTCCAACGATCAATTCAAGTATAGAGACTAAAATTATACAATATTATAACTATAATAGGGTATAAATTATAGTT
>Marker979814 Chr2 SNP_site:18993713 Ref_Type:G
ACCAACAGTTAATTAAAAATTAAAAGAGGAAGTTAAGAAGAGGAAAGAAGGGGATACAGTTACTTGGAAGACAGTTACCGXXXXXXXXXXXA
AGGCTGGTGTTCTGTGTTTTGGTTTGGTTTTTAGTATTTTGTTTTGTGTGTTGTTTTTGATATTTTGGATTAGGAGGTT
ACCAACAGTTGATTAAAAATTAAAAGAGGAAGTTAAGAAGAGGAAAGAAGGGGATACAGTTACTTGGAAGACAGTTACTGXXXXXXXXXXXA
AGGCTGGTGTTCTGTGTTTTGGTTTGGTTTTTAGTATTTTGTTTTGTGTGTTGTTTTTGATATTTTGGATTAGGAGGTT
>Marker979814 Chr2 SNP_site:18993781 Ref_Type:T
ACCAACAGTTAATTAAAAATTAAAAGAGGAAGTTAAGAAGAGGAAAGAAGGGGATACAGTTACTTGGAAGACAGTTACCGXXXXXXXXXXXA
AGGCTGGTGTTCTGTGTTTTGGTTTGGTTTTTAGTATTTTGTTTTGTGTGTTGTTTTTGATATTTTGGATTAGGAGGTT
ACCAACAGTTGATTAAAAATTAAAAGAGGAAGTTAAGAAGAGGAAAGAAGGGGATACAGTTACTTGGAAGACAGTTACTGXXXXXXXXXXXA
AGGCTGGTGTTCTGTGTTTTGGTTTGGTTTTTAGTATTTTGTTTTGTGTGTTGTTTTTGATATTTTGGATTAGGAGGTT
>Marker981747 Chr2 SNP_site:7342433 Ref_Type:T
ACTTTATTCAAGGTTCAACGTCATCTTTTTATAATTTGATGTGATTATCTTGGTGACTTTTATGTAAAATAATGTTGCCTXXXXXXXXXXXA
GGGGAGAAATTAAATACCCAATTAAGGTTTGCGACTTTCTGGTTTATTTTTCCAACCTGGCTTTTGGAATTCCAACGTT
ACTTTATTCAAGGTTCAACGTCATCTTTTTATAATTTTATGTGATTATCTTGGTGACTTTTATGTAAAATAATGTTGCCTXXXXXXXXXXXA
GGGGAGAAATTAAATACCCAATTAAGGTTTGCGACTTTCTGGTTTATTTTTCCAACCTGGCTTTTGGAATTCCAACGTT
>Marker982058 Chr2 SNP_site:22842236 Ref_Type:T
ACTTATCGCTTAGTAGTTGAACTTAAGTTAAGTGTTGAAAGCTAAAGGGTTAAGTATTTCCCTTACAATGAGTTGTTAAAXXXXXXXXXXXT
TGTAATATAGTTTATTAGTGATAGACTTTAACAAATTTTGTTATATTTGCAATTTTGTTTTTGGAAATTTTGCTATGTA
ACTTATCGTTTAGTAGTTGAACTTAAGTTAAGTGTTGAAAGCTAAAGGGTTAAGTATTTCCCTTACAATGAGTTGTTAAAXXXXXXXXXXXT
TGTAATATAGTTTATTAGTGATAAACTTTAACAAATTTTGTTATATTTGCAATTTTGTTTTTGGAAATTTTGCTATGTA
>Marker982058 Chr2 SNP_site:22842486 Ref_Type:A
ACTTATCGCTTAGTAGTTGAACTTAAGTTAAGTGTTGAAAGCTAAAGGGTTAAGTATTTCCCTTACAATGAGTTGTTAAAXXXXXXXXXXXT
TGTAATATAGTTTATTAGTGATAGACTTTAACAAATTTTGTTATATTTGCAATTTTGTTTTTGGAAATTTTGCTATGTA
ACTTATCGTTTAGTAGTTGAACTTAAGTTAAGTGTTGAAAGCTAAAGGGTTAAGTATTTCCCTTACAATGAGTTGTTAAAXXXXXXXXXXXT

Create PDF with GO2PDF for free, if you wish to

TGTAATATAGTTTATTAGTGATAAACTTTAACAAATTTTGTTATATTTGCAATTTTGTTTTTGGAAATTTTGCTATGTA
>Marker982219 Chr2 SNP_site:3006182 Ref_Type:T
CACTTGGGATTTAACTGACGATTGAGATGGCAGTATTGAACTCGATGTTGAATATATAGGAACTGTGATGGCGGGATGGTXXXXXXXXXXT
CCCGGGCATGGGAAAGTTCGTGGGTTTGATATAAACCCACCCGGATAGCCAAATTTTCAAGATTAATGAAAATACTGTG
CACTTGGGATTTAACTGACGATTGAGATGGCTGAATTGAACTCGATGTTGAATATATAGGAACTGTGATGGCGGGATGGTXXXXXXXXXXT
CCCGGGCATGGGAAAGTTCGTGGGTTTGATATAAACCCACCCTGATAGCCAAATTTTCAAGATTAATGAAAATACTGTG
>Marker982219 Chr2 SNP_site:3006184 Ref_Type:A
CACTTGGGATTTAACTGACGATTGAGATGGCAGTATTGAACTCGATGTTGAATATATAGGAACTGTGATGGCGGGATGGTXXXXXXXXXXT
CCCGGGCATGGGAAAGTTCGTGGGTTTGATATAAACCCACCCGGATAGCCAAATTTTCAAGATTAATGAAAATACTGTG
CACTTGGGATTTAACTGACGATTGAGATGGCTGAATTGAACTCGATGTTGAATATATAGGAACTGTGATGGCGGGATGGTXXXXXXXXXXT
CCCGGGCATGGGAAAGTTCGTGGGTTTGATATAAACCCACCCTGATAGCCAAATTTTCAAGATTAATGAAAATACTGTG
>Marker982219 Chr2 SNP_site:3006439 Ref_Type:T
CACTTGGGATTTAACTGACGATTGAGATGGCAGTATTGAACTCGATGTTGAATATATAGGAACTGTGATGGCGGGATGGTXXXXXXXXXXT
CCCGGGCATGGGAAAGTTCGTGGGTTTGATATAAACCCACCCGGATAGCCAAATTTTCAAGATTAATGAAAATACTGTG
CACTTGGGATTTAACTGACGATTGAGATGGCTGAATTGAACTCGATGTTGAATATATAGGAACTGTGATGGCGGGATGGTXXXXXXXXXXT
CCCGGGCATGGGAAAGTTCGTGGGTTTGATATAAACCCACCCTGATAGCCAAATTTTCAAGATTAATGAAAATACTGTG
>Marker98294 Chr4 SNP_site:6779999 Ref_Type:T
ACTTAAAAAAACCTATGTATGATATTATGGATAGCAGGGAATGTAAATGTCCTTGTAGAACTTGCCTATGAGAATCTCTTXXXXXXXXXXT
TTTAAATTCTATCAAGATAAACGTTGATAGAAGCCTGTCAATATCTATCAACTATAAACTTTTGCAAGTTTTTAGGAGT
ACTTAAAAAAACCTATGTATGATATTATGGATAGCAGGGAATGTAAATGTCCTTGTAGAACTTGCCTATGAGAATCTCTTXXXXXXXXXXT
TTTAAATTCTATCAAGATAAACGTTGATAGAAGCCTGTCAATATCTATCAACTATAAACTTTTGCAATTTTTTAGGAGT
>Marker982998 Chr2 SNP_site:22585699 Ref_Type:G
ACTTTAAACAACCTAAAAGCAAGACTTAAATAGCCACCCAATAAAACTCTAATGCATTTTAACAAGTTTAAATTAATAGTXXXXXXXXXXA
AATTGATCCAACCGAAATTACAAACAACTTACGAAAGAGATTGACTTACTAATTAAGAGAATAGCTACATATTATAGTG
ACTTTAAACAACCTAAAAGCAAGACTTAAATAGCCACCCAATAAAACTCTAATGCATTTTAACAAGTTTAAATTAATAGTXXXXXXXXXXA
AATTGATCCAACCGAAATTACGAACAACTTACGAAAGAGATTGACTTACTAATTAAGAGAATAGCTACATATTATAGTG
>Marker983042 Chr2 SNP_site:21909485 Ref_Type:C
ACAGCTTCATTTTTGTTGTTGAGTAATTATTCTTGGTTTAGCAGATTGCGTCCATTACCAATTCTGGGGGATTTATAGTTXXXXXXXXXXT
TCTGAAGCAACGTTCTTTCTTACCCCTTTCTATACTTATTTTTTCAAATTTTCTTCTCATTTGGTTAGTTGGTTTCGTG
ACAGTTTCATTTTTGTTGTTGAGTAATTATTCTTGGTTTAGCAGATTGCGTCCATTACCAATTCTGGGGGATTTATAGTTXXXXXXXXXXT
TCTGAAGCAACGTTCTTTCTTACCCCTTTCTATACTTATTTTTTCAATTTTTCTTCTCATTTGGTTAGTTGGTTTCGTG
>Marker983042 Chr2 SNP_site:21909710 Ref_Type:A
ACAGCTTCATTTTTGTTGTTGAGTAATTATTCTTGGTTTAGCAGATTGCGTCCATTACCAATTCTGGGGGATTTATAGTTXXXXXXXXXXT
TCTGAAGCAACGTTCTTTCTTACCCCTTTCTATACTTATTTTTTCAAATTTTCTTCTCATTTGGTTAGTTGGTTTCGTG
ACAGTTTCATTTTTGTTGTTGAGTAATTATTCTTGGTTTAGCAGATTGCGTCCATTACCAATTCTGGGGGATTTATAGTTXXXXXXXXXXT
TCTGAAGCAACGTTCTTTCTTACCCCTTTCTATACTTATTTTTTCAATTTTTCTTCTCATTTGGTTAGTTGGTTTCGTG
>Marker983456 Chr2 SNP_site:4534965 Ref_Type:T
TACACCTTTTCAATCTATCATTTTGTGACTTCTCACCTTTTAATGTGTTATCAATAGTTAGAGTTGTGATAGGTTCTTGAXXXXXXXXXXG
GCTAATGCACGCAAGGAGGAGTTTGGTAAAAAACTTCACCTTAGCGAGGCAACTTTCAACATTTGTATCCCGAAAGGTG
TACACCTTTTCAATCTATCATTTTGTGACTTCTCACCTTTTAATGTGTTATCAATAGTTAGAGTTGTGATAGGTTCTTGAXXXXXXXXXXG
GCTAATGCACGCAAGGAGGAGTTTGGTAAAAAATTTCACCTTAGCGAGGCAACTTTCAACATTTGTATCCCGAAAGGTG
>Marker983474 Chr2 SNP_site:17042785 Ref_Type:A
GACTAAAAAACATATAGAATTAATCTGAATTAGTCATTATATATAATATATTGCAAAAAAATAAATAAATGAGAAGTATAXXXXXXXXXXT
CTATTCTTTTGTTCTCTAAAATAAGAAAACAAGAAAAGAGAAGAAAGGAAACAAGAATTTCTCAACCCGTTCTGATTGT

Create PDF with GO2PDF for free, if you wish to

GACTAAAAAACATATAGAATTAATCTGAATTAGTCATTATATATAATATATTGCAAGAAAATAAATAAATGAGAAGTATAXXXXXXXXXXT
CTATTCTTTTGTTCTCTAAAATAAGAAAACAAGAAAAGAGAAGAAAGGAAACAAGAATTTCTCAACCCGTTCTGATTGT
>Marker983658 Chr2 SNP_site:5807706 Ref_Type:C
ACACAGAATTGGAAATTTTAATGTCTTTTTGTTATTGGGTTTTTCAAATAGATTGAAGAGTCAATTGGATGTTGAGAAAAXXXXXXXXXXC
TGGAAGTCGGTCTTAGACCCATGATAAAAGATTATATTGCTCGTGTAGTAATTGATCGTTACAATGTTATGATTGAAGT
ACACAGAATTGGAAATTTTAATGTCTTTTTGTTATTGGGTTTTTCAAATAGATTGAAGAGTCAATTGGATGTTGAGAAAAXXXXXXXXXXT
TGGAGGTCGGTCTTAGACCCATGATAAAAGATTATATTGCTCGTGTAGTAATTGATCGTTACAATGTTATGATTGAAGT
>Marker983658 Chr2 SNP_site:5807711 Ref_Type:A
ACACAGAATTGGAAATTTTAATGTCTTTTTGTTATTGGGTTTTTCAAATAGATTGAAGAGTCAATTGGATGTTGAGAAAAXXXXXXXXXXC
TGGAAGTCGGTCTTAGACCCATGATAAAAGATTATATTGCTCGTGTAGTAATTGATCGTTACAATGTTATGATTGAAGT
ACACAGAATTGGAAATTTTAATGTCTTTTTGTTATTGGGTTTTTCAAATAGATTGAAGAGTCAATTGGATGTTGAGAAAAXXXXXXXXXXT
TGGAGGTCGGTCTTAGACCCATGATAAAAGATTATATTGCTCGTGTAGTAATTGATCGTTACAATGTTATGATTGAAGT
>Marker984050 Chr2 SNP_site:22306053 Ref_Type:C
TACTTTACCAGACCTATACCTGAGCTTTGCTTGAAACTCAACGTTTCTGTTTTTACACTGAAACTAGATTCACTCGAGCAXXXXXXXXXXC
CACTTTTCTTAAACTTTGTGCTGTTCTTTAAAATGTTCCCGTCTATATTCCTTCTTTCTATCATGTATATTAATCTGTG
TACTTTACCAGACCTATACCTGAGCTTTGCTTGAAACTCAACGTTTCTGTTTTTACACTGAAACTAGATTCACTCGAGCAXXXXXXXXXXC
CACTTTTCTTAAACTTTGTGCTGTTCTTTAAAATGTTCTCGTCTATATTCCTTCTTTCTATCATGTATATTAATCTGTG
>Marker984240 Chr2 SNP_site:9365497 Ref_Type:A
TACTACTTTTAAAAATGGAAATCAAAATTATACTATTTTTTTAAAAATCAAATTTCCGGTAATGTTCCTATCTTTCAATGXXXXXXXXXXT
TTTTTAAAAGAAAATGGTTAAAAATAGCACATTTTCGTTCTTTTTATTATTTCTAGAAAAACCCTTAGTTGAATTCGTG
TACTACTTTTAAAAATGGAAATCAAAATTATACTATTTTTTTTATAATCAAATTTCCGGTAATGTTCCTATCTTTCAATGXXXXXXXXXXT
TTTCTAAAAGAAAATGGTTAAAAATAGCACATTTTCGTTCTTTTTATTATTTCTAGAAAAACCCTTAGTTGAATTCGTG
>Marker984240 Chr2 SNP_site:9365499 Ref_Type:A
TACTACTTTTAAAAATGGAAATCAAAATTATACTATTTTTTTAAAAATCAAATTTCCGGTAATGTTCCTATCTTTCAATGXXXXXXXXXXT
TTTTTAAAAGAAAATGGTTAAAAATAGCACATTTTCGTTCTTTTTATTATTTCTAGAAAAACCCTTAGTTGAATTCGTG
TACTACTTTTAAAAATGGAAATCAAAATTATACTATTTTTTTTATAATCAAATTTCCGGTAATGTTCCTATCTTTCAATGXXXXXXXXXXT
TTTCTAAAAGAAAATGGTTAAAAATAGCACATTTTCGTTCTTTTTATTATTTCTAGAAAAACCCTTAGTTGAATTCGTG
>Marker984240 Chr2 SNP_site:9365679 Ref_Type:T
TACTACTTTTAAAAATGGAAATCAAAATTATACTATTTTTTTAAAAATCAAATTTCCGGTAATGTTCCTATCTTTCAATGXXXXXXXXXXT
TTTTTAAAAGAAAATGGTTAAAAATAGCACATTTTCGTTCTTTTTATTATTTCTAGAAAAACCCTTAGTTGAATTCGTG
TACTACTTTTAAAAATGGAAATCAAAATTATACTATTTTTTTTATAATCAAATTTCCGGTAATGTTCCTATCTTTCAATGXXXXXXXXXXT
TTTCTAAAAGAAAATGGTTAAAAATAGCACATTTTCGTTCTTTTTATTATTTCTAGAAAAACCCTTAGTTGAATTCGTG
>Marker98438 Chr4 SNP_site:22490843 Ref_Type:C
ACAAATAGCGAAAAGATTTATGAAAATAAAATTTGTAAAATGCAATTTTTAAAGTGATGATCATTTAATAACTTACGATGXXXXXXXXXXA
AAATCGAATAACCTGTGACAGCTATTTAATAACCCTCTAATTGTTGTTTAATATATCTAATTGTCAAATTTATTTAGTG
ACAAATAGCGAAAAGATTTATGAAAATAAAATTTGTAAAATGCAATTTTTAAAGTGATGATCATTTAATAATTTACGATGXXXXXXXXXXA
AAATCGAATAACCTGTGACAGCTATTTAATAACCCTCTAATTGTTGTTTAATATATCTAATTGTCAAATTTATTTAGTG
>Marker984728 Chr2 SNP_site:17333824 Ref_Type:A
AACCAAATAACTCGAGGAATTTATATAAATACAAAAAAAAGAAAAGTATTTACATCAAATAACAAAAAAGAATTGATATTXXXXXXXXXXA
ACAATGGTAATGGATTAACTTGTCATCAGCTTGGGGATGTAGCTCAGATGGTAGAGCGCTCGCTTAGCATGCGAGAGGT
AACCAAATAACTCGAGGAATTTATATAAATACAAAAAAATGGAAAGTATTTACATCAAATAACAAAAAAGAATTGATATTXXXXXXXXXXA
ACAATGGTAATGGATTAACTTGTCATCAGCTTGGGGATGTAGCTCAGATGGTAGAGCGCTCGCTTAGCATGCGAGAGGT
>Marker984728 Chr2 SNP_site:17333826 Ref_Type:A
AACCAAATAACTCGAGGAATTTATATAAATACAAAAAAAAGAAAAGTATTTACATCAAATAACAAAAAAGAATTGATATTXXXXXXXXXXA

Create PDF with GO2PDF for free, if you wish to

ACAATGGTAATGGATTAACTTGTCATCAGCTTGGGGATGTAGCTCAGATGGTAGAGCGCTCGCTTAGCATGCGAGAGGT

AACCAAATAACTCGAGGAATTTATATAAATACAAAAAAATGGAAAGTATTTACATCAAATAACAAAAAAGAATTGATATTXXXXXXXXXXA

ACAATGGTAATGGATTAACTTGTCATCAGCTTGGGGATGTAGCTCAGATGGTAGAGCGCTCGCTTAGCATGCGAGAGGT

>Marker985193 Chr2 SNP_site:3725101 Ref_Type:A

AACCATATTCCTATTAAAATCATCAAGCACCTACAGCAGCTAATCATGACCAAGTAGCTCAGTGGGAAGAAAAGGTAGGCXXXXXXXXXXC

AAAACATGCCTAGACAAAATCGATGAACGAAGAGTAATGACAAAGCGCACTGCATAAAGCTCTACAACTAGATGTTGTG

AACCATATTCCTATTAAAATCATCAAGCACCTACAGCAGCTAATCATGACCAAGTAGCTCAGTGGGAAGAAAAGGTAGGCXXXXXXXXXXC

AAAGCATGCCTAGACAAAATCGATGAAAGAAGAGTAATGACAAAGCGCACTGCATAAAGCTCTACAACTAGATGTTGTG

>Marker985193 Chr2 SNP_site:3725125 Ref_Type:C

AACCATATTCCTATTAAAATCATCAAGCACCTACAGCAGCTAATCATGACCAAGTAGCTCAGTGGGAAGAAAAGGTAGGCXXXXXXXXXXC

AAAACATGCCTAGACAAAATCGATGAACGAAGAGTAATGACAAAGCGCACTGCATAAAGCTCTACAACTAGATGTTGTG

AACCATATTCCTATTAAAATCATCAAGCACCTACAGCAGCTAATCATGACCAAGTAGCTCAGTGGGAAGAAAAGGTAGGCXXXXXXXXXXC

AAAGCATGCCTAGACAAAATCGATGAAAGAAGAGTAATGACAAAGCGCACTGCATAAAGCTCTACAACTAGATGTTGTG

>Marker985288 Chr2 SNP_site:13768134 Ref_Type:T

TACTGTTTTTTTAATAGGAAATCCCAACAAAGTTTGTGAGAGAATATGGTGTAAAAGCTTTTGCAACTAACCAAATATGTXXXXXXXXXXA

CTCTGTTGGATTGGAGTATTTTCTCGTCTTTAAATACGAACGTCAATCTTCGAGCTTTTACGTGGTTATCTTTGATCGT

TACTGTTTTTTTAATAGGAAATCCCAACAAAGTTTGTGAGAGAATATGGTGTAAAAGCTTTTGCAACTAACCAAATATGTXXXXXXXXXXA

CTCTGTTGGATTGGAGTATTTTCTCGTCTTTAAATACGAATGTCAATCTTCGAGCTTTTACGTGGTTATCTTTGATCGT

>Marker985590 Chr2 SNP_site:14405233 Ref_Type:A

ACCCAAAGCCCTATAATACCTCCTATTTGAAAGAGTTTTCAATGTCGATCACAAACCGATATTTAACATCAGCTTTTGCAXXXXXXXXXXT

CTCTTTCAGCCCTCCAAAGTTGCTTCCAGCTACCACCCTCCTACCTATAAGCAAAGTTTTTAAAAGGTCGTCATTTGTT

ACCCAAAGCCCTATAATACCTCCTATTTGAAAGAGTTTTCGATGTCGATCACAAACCGATATTTAACATCAGCTTTTGCAXXXXXXXXXXT

CTCTTTCAGCCCTCCAAAGTTGCTTCCAGCTACCACCCTCCTACCTATAAGCAAAGTTTTTAAAAGGTCGTCATTTGTT

>Marker985686 Chr2 SNP_site:427518 Ref_Type:A

AACCATGATCATCCAACCCTTTCTTTCTCTCCCTGGCTTCTCTCTCTCACCTGAGAAAGTTCTCATCCATTTCCCTTTGGXXXXXXXXXXT

ATTCAAAAAAATTTATCATTGATGTATATAATAATTATTTTGAAAATGTAATACCAAACTGTTCATATGTCTTAAAAGT

AACCATGATCATCCAACCCTTTCTTTCTCTCCCTGGCTTCTCTCTCTCACCTGAGAAAGTTCTCATCCATTTCCCTTTGGXXXXXXXXXXT

ATTCACAAAAATTTATCATTGATGTATATAATAATTATTTTGAAAATGTAATACCAAACTGTTCATATGTCTTAAAAGT

>Marker986034 Chr2 SNP_site:22577276 Ref_Type:A

TACCGTCAGGGGAACTTGAGTTTATATATTTAAGTTATGGTGATAACTTGTTAATATAACTTTAGGATTAAATTACAAACXXXXXXXXXXG

CTGAAGGATAATATTTATTGTCTCACCCATTTTTCATATTGCTTGTCATATTCATAACCGTATCTCTCTTCGATCAAGT

TACCGTCAGGGGAGCTTGAGTTTATATATTTAAGTTATGGTGATAACTTGTTAATATAACTTTAGGATTAAATTACAAACXXXXXXXXXXG

CTGAAGGATAATATTTATTGTCTCACCCATTTTTCATATTGCTTGTCATATTCATAACCGTATCTCTCTTCGATCAAGT

>Marker986273 Chr2 SNP_site:19047261 Ref_Type:C

ACTGTATAACGTTGGACAAAACTACTTGTTGTTTAGCATACCCGTTTATTTATCTCAACTTGCATTTTCATTTCTAGATAXXXXXXXXXXG

TGGATGTGGAAAAAGGTATTGGGTTGAACTTTAGGTTGTAAGAATCATTATTTGATTGAGTTGAATTGGGGAAATTAGT

ACTGTATAATGTTGGACAAAACTACTTGTTGTTTAGCATACCCGTTTATTTATCTCAACTTGCATTTTCATTTCTAGATAXXXXXXXXXXG

TGGATGTGGAAAAAGGTATTGGGTTGAACTTTAGGTTGTAAGAATCATTATTTGATTGAGTTGAATTGGGGAAATTAGT

>Marker986452 Chr2 SNP_site:6796270 Ref_Type:T

CACCTAAAGCCTGTTGAAAGTTGTTGGCAACCCCAGGATTATCTCCCAGACCCAGAATCTGACGGTTTCTACGACCAAGTXXXXXXXXXXT

GCAAGCCTCACCTCGTGGGCAATTTGGACAAGAGCATGGACTGCGGAAGAGAATAGACATGGTGACCTTTTGAATAAGT

CACCTAAAGCCTGTTGAAAGTTGTTGGCAACCTCAGGATTATCTCCCAGACCCAGAATCTGACGGTTTCTACGACCAAGTXXXXXXXXXXT

GCAAGCCTCACCTCGTGGGCAATTTGGACAAGAGCATGGACTGCGGAAGAGAATAGACATGGTGACCTTTTGAATAAGT

>Marker986467 Chr2 SNP_site:3260582 Ref_Type:C

Create PDF with GO2PDF for free, if you wish to

ACTGTAGCCACAGATATCTGGGCCAAACATGATGCTGCAGGAAACAAAAAGCACATAATCATAAAGAAAGTCATGAGATTXXXXXXXXXXA
CCTCCACAATCAAGCTTCTGTTCGTTCTTGACGGAAAATTGGAAAACAAGAATGTTGTCTTTGTTGCTGAATTCAGGGT
ACTGTAGCCACAGATATCTGGGCCAAACATGATGCTGCAGGCAACAAAAAACACATAATCATAAAGAAAGTCATGAGATTXXXXXXXXXXA
CCTCCACAATCAAGCTTCTGTTCGTTCTTGACGGAAAATTGGAAAACAAGAATGTTGTCTTTGTTGCTGAATTCAGGGT
>Marker986467 Chr2 SNP_site:3260591 Ref_Type:A
ACTGTAGCCACAGATATCTGGGCCAAACATGATGCTGCAGGAAACAAAAAGCACATAATCATAAAGAAAGTCATGAGATTXXXXXXXXXXA
CCTCCACAATCAAGCTTCTGTTCGTTCTTGACGGAAAATTGGAAAACAAGAATGTTGTCTTTGTTGCTGAATTCAGGGT
ACTGTAGCCACAGATATCTGGGCCAAACATGATGCTGCAGGCAACAAAAAACACATAATCATAAAGAAAGTCATGAGATTXXXXXXXXXXA
CCTCCACAATCAAGCTTCTGTTCGTTCTTGACGGAAAATTGGAAAACAAGAATGTTGTCTTTGTTGCTGAATTCAGGGT
>Marker986903 Chr2 SNP_site:7999395 Ref_Type:T
ACAGTTGATATCAACTCATATTTTTTGTTACATTCTTATTTAATGGATGACCAAATATAATCTCTTTCTGTGTAGGCGTAXXXXXXXXXXG
TGAAACTCTGCCAATACTGCTATGGGATGCACTTGTCAAGCTATTACCAGAAACCACTATTTTGAATTTGTATGGTAGT
ACAGTTGATATCAACTCATATTTTTTGTTACATTCTTATTTAATGGATGACCAAATATAATCTCTTTCTTTGTAGGCGTAXXXXXXXXXXG
TGAAACTCTGCCAATACTGCTATGGGATGCACTTGTCAAGCTATTACCAGAAACCACTATTTTGAATTTGTATGGTAGT
>Marker987118 Chr2 SNP_site:16489178 Ref_Type:G
ACCTTTCAACCTCGCCATACACATAAAGATAATAATAAACTATGAAATAAAAAAAATAAAGATATGTTGTCATATGACCTXXXXXXXXXXA
TTGTATAGATAAGTCTATCGAATAACTTAAGATTTTTTCAAGTCATAATGATTATAGAGTCTCAAACACAAGTTTAGTT
ACCTTTCAACCTCGCCATACACATAAAGATAATAATAAACTATGAAATAAAAAAAATAAAGATATTTTATCATATGACCTXXXXXXXXXXA
TTGTATAGATAAGTCTATCGAATAACTTAAGATTTTTTCAAGTCATAATGATTATAGAGTCTCAAACACAAGTTTAGTT
>Marker987118 Chr2 SNP_site:16489181 Ref_Type:G
ACCTTTCAACCTCGCCATACACATAAAGATAATAATAAACTATGAAATAAAAAAAATAAAGATATGTTGTCATATGACCTXXXXXXXXXXA
TTGTATAGATAAGTCTATCGAATAACTTAAGATTTTTTCAAGTCATAATGATTATAGAGTCTCAAACACAAGTTTAGTT
ACCTTTCAACCTCGCCATACACATAAAGATAATAATAAACTATGAAATAAAAAAAATAAAGATATTTTATCATATGACCTXXXXXXXXXXA
TTGTATAGATAAGTCTATCGAATAACTTAAGATTTTTTCAAGTCATAATGATTATAGAGTCTCAAACACAAGTTTAGTT
>Marker987138 Chr2 SNP_site:2891491 Ref_Type:T
ACTCTACTAATGACACCATATTTGTTATGTTGAGGGTGTGGCAAACTTAGTTCAATCACCAAAGGCTTCAATGTCCCATCXXXXXXXXXXT
TGCTTCATTGCCTATTATCACATAATTAAGAAAGAAAATTGATGATCCAAATCCAAATTAGTATAGTTTAGCAGTAGTC
ACTCTACTAATGACACCATATTTGTTATGTTGAGGGTGTGGCAAACTTAGTTCAATCACCAAAGGCTTCAATGTCCCATCXXXXXXXXXXT
TGCTTCATTGCCTATTATCACATAATTAAGAAAGAAAATTGATGATCCAAATCCAAATTAGTATAGTTTAGCAGTAGTT
>Marker987408 Chr2 SNP_site:19190190 Ref_Type:T
ACTATGGTTTGAGACCCTTTTTCTATTCATTTTCAAGTCGCATCAAATATCCAACAAGGCTGCTTTACTACAATAACTGCXXXXXXXXXXA
TGCATGCACGTATATAGAAAATCTGTCATCGTGGCCCTGAAAGACAAGAACTGGGTTAAGCACCTAACTCAATAAAGTC
ACTATGGTTTGAGACCCTTTTTCTATTCATTTTCAAGTCGCATCAAATATCTAACAAGGCTGCTTTACTACAATAACTGCXXXXXXXXXXA
TGCATGCACGTATATAGAAAATCTGTCATCGTGGCCCTGAAAGACAAGAACTGGGTTAAGCACCTAACTCAATAAAGTC
>Marker987443 Chr2 SNP_site:6581246 Ref_Type:C
AACAGCATGAAGGTAGGGGGAGAGACCGAGAGATACAGGAAAATTAAACTTACATTCGTCTTCCATGTCAATGAAATCTAXXXXXXXXXXG
ATAATTTTCAAGCAAGCAACCGCAACAGCTTCAAAAAAGTAGTTATGCTCAAATATCAACACAAAAAACGCAGAGGGTA
AACAGCATGAAGGTAGGGGGAGAGACCGAGAGATACAGGAAAATTAAACTTACATTCGTCTTCCATGTCAATGAAATCTAXXXXXXXXXXG
ATAATTTTCAAGCAAGCAACCGCAACAGCTTCAAAAAAGTAGTTATGCTCAAATATCAACACAAAAAAGGCAGAGGGTA
>Marker988287 Chr2 SNP_site:10844916 Ref_Type:G
ACATTCATGTTCATGCTTACTCACGTGAGATCACGTGCCCAAACGGAATTCTCATCTGCTTCAAAGTTTGGACTAAAATTXXXXXXXXXXA
TTTGTTTGGTAACAATTTTAATTAGTTAGATTCTAATTTAAACAATTGTTTAAACTTTGTTTGATGCTATGTATATGTA
ACATTCATGTTCATGCTTACTCACGTGAGATCACGTGCCCAAAGGTATTTCTCATCTGCTTCAAAGTTTGGACTAAAATTXXXXXXXXXXA
TTTGTTTGGTAACAATTTTAATTAGTTAGATTCTAATTTAAACAATTGTTTAAACTTTGTTTGATGCTATGTATATGTA

Create PDF with GO2PDF for free, if you wish to

>Marker988287 Chr2 SNP_site:10844918 Ref_Type:T
ACATTCATGTTCATGCTTACTCACGTGAGATCACGTGCCCAAACGGAATTCTCATCTGCTTCAAAGTTTGGACTAAAATTXXXXXXXXXXA
TTTGTTTGGTAACAATTTTAATTAGTTAGATTCTAATTTAAACAATTGTTTAAACTTTGTTTGATGCTATGTATATGTA
ACATTCATGTTCATGCTTACTCACGTGAGATCACGTGCCCAAAGGTATTTCTCATCTGCTTCAAAGTTTGGACTAAAATTXXXXXXXXXXA
TTTGTTTGGTAACAATTTTAATTAGTTAGATTCTAATTTAAACAATTGTTTAAACTTTGTTTGATGCTATGTATATGTA
>Marker988287 Chr2 SNP_site:10844920 Ref_Type:T
ACATTCATGTTCATGCTTACTCACGTGAGATCACGTGCCCAAACGGAATTCTCATCTGCTTCAAAGTTTGGACTAAAATTXXXXXXXXXXA
TTTGTTTGGTAACAATTTTAATTAGTTAGATTCTAATTTAAACAATTGTTTAAACTTTGTTTGATGCTATGTATATGTA
ACATTCATGTTCATGCTTACTCACGTGAGATCACGTGCCCAAAGGTATTTCTCATCTGCTTCAAAGTTTGGACTAAAATTXXXXXXXXXXA
TTTGTTTGGTAACAATTTTAATTAGTTAGATTCTAATTTAAACAATTGTTTAAACTTTGTTTGATGCTATGTATATGTA
>Marker989047 Chr2 SNP_site:4481986 Ref_Type:C
ACATCAAATTGCAGGTGAGATACCTTTTGAGTTATTTTTTAGTTCTAGGTTCAGGTTCGATTCTGTTGGAGGAGTTTATTXXXXXXXXXXG
TATATTTTAGTCATTTATCAATGTCCTACAGATAATGGACCATTCCAGTCATTTAGTGGCTTTTGTAATTTATGATGTG
ACATCAAATTGCAGGTGAGATACCTTTTGAGTTATTTTTTAGTTCTAGGTTCAGGTTCGATTCTGTTGGAGGAGTTTATTXXXXXXXXXXG
TATATTTTAGTCATTTATCAATGTCCTACAGATAATGGATCATTCCAGTCATTTAGTGGCTTTTGTAATTTATGATGTG
>Marker989051 Chr2 SNP_site:12718473 Ref_Type:C
CACCGTGAAAAGGTCGTATGTTGCACAAGAGCCCAAAAAGAATCAAAAGAGCTAAAATTATCATCGACACAAAACTGAAAXXXXXXXXXXXA
CATGCCATAAGAGCTCATGAAGTCTAAATAGGCATTTGGTTCGAAAAATTAAAAATTAGGATCCAATGAAAGAATTGTG
CACCGTGAAAAGGTCGTATGTTGCACAAGAGCCCAAAAAGAATCAAAAGAGCTAAAATTATCATCGACACAAAACTGAAAXXXXXXXXXXXA
CATGCTATAAGAGCTCATGAAGTCTAAATAGGCATTTGGTTCGAAAAATTAAAAATTAGGATCCAATGAAAGAATTGTG
>Marker989345 Chr2 SNP_site:2874647 Ref_Type:C
AACATCATAGTCGTAAATTCTATCCCATTCTTGACGCTCTCCTTTTCCATCTCCTCTTAGATTCAATAGCTCTTCTTCTCXXXXXXXXXXT
TAAACAAACAAAAAATCAACTAATTAATTACCTTGTTGGCAAAGAAAATTCGATCTTTTCTGTATCTTCTTTGAGGGTA
AACATCATAGTCGTAAATTCTATCCCATTCTTGACGCTCTCCTTTTCCATCTCCTCTTAGATTCAATAGCTCTTCTTCTCXXXXXXXXXXT
TAAACAAACAAAAAATCAACTAATTAATTACCTTGTTGGCAAAGAAAATTCGATCTTTTTTGTATCTTCTTTGAGGGTA
>Marker989401 Chr2 SNP_site:20306091 Ref_Type:T
CACAACGATGTCATCCTCATGGCCATTGATCATACCCTCACACTCTTAACTACTGACACTAATTGGTGATTCTGTTACAGXXXXXXXXXXC
TAAAGGATCAGAAACTGACCTGGTTTTTCCAGATGGTGCTTGAATGAAAGAGCTGCAGTTTTCTAAAAGATCTGGGGGT
CACAACGATGTCATCCTCATGGCCATTGATCATACCCTCACACTCTTAACTACTGACACTAATTGGTGATTCTGTTACATXXXXXXXXXXC
TAAAGGATCAGAAACTGACCTGGTTTTTCCAGATGGTGCTTGAATGAAAGAGTTGCAGTTTTCTAAAAGATCTGGGGGT
>Marker989401 Chr2 SNP_site:20306249 Ref_Type:T
CACAACGATGTCATCCTCATGGCCATTGATCATACCCTCACACTCTTAACTACTGACACTAATTGGTGATTCTGTTACAGXXXXXXXXXXC
TAAAGGATCAGAAACTGACCTGGTTTTTCCAGATGGTGCTTGAATGAAAGAGCTGCAGTTTTCTAAAAGATCTGGGGGT
CACAACGATGTCATCCTCATGGCCATTGATCATACCCTCACACTCTTAACTACTGACACTAATTGGTGATTCTGTTACATXXXXXXXXXXC
TAAAGGATCAGAAACTGACCTGGTTTTTCCAGATGGTGCTTGAATGAAAGAGTTGCAGTTTTCTAAAAGATCTGGGGGT
>Marker991665 Chr2 SNP_site:12853563 Ref_Type:T
GACAACAACCATACAGGTTATTGAAAGTGAGATTGGAGAATTAGTGAGAGTATTATAGTAATTTTACGCACATACAAATTXXXXXXXXXXC
AAACATTTTTAACTTTTTCTTTTTCAAAATTAAACGAAAACTAAATCAAATTAAACACACTAGATCTTTTTAAATTGGT
GACAACAACCATACAGGTTATTGAAAGTGAGATTGGATAATTAGTGAGAGTATTATAGTAATTTTACGCACATACAAATTXXXXXXXXXXC
AAACATTTTTAACTTTTTCTTTTTCAAAATTAAATGAAAACTAAATCAAATTAAACACACTAGATCTTTTTAAATTGGT
>Marker991665 Chr2 SNP_site:12853754 Ref_Type:T
GACAACAACCATACAGGTTATTGAAAGTGAGATTGGAGAATTAGTGAGAGTATTATAGTAATTTTACGCACATACAAATTXXXXXXXXXXC
AAACATTTTTAACTTTTTCTTTTTCAAAATTAAACGAAAACTAAATCAAATTAAACACACTAGATCTTTTTAAATTGGT
GACAACAACCATACAGGTTATTGAAAGTGAGATTGGATAATTAGTGAGAGTATTATAGTAATTTTACGCACATACAAATTXXXXXXXXXXC

Create PDF with GO2PDF for free, if you wish to

AAACATTTTTAACTTTTTCTTTTTCAAAATTAAATGAAAACTAAATCAAATTAAACACACTAGATCTTTTTAAATTGGT
>Marker992080 Chr2 SNP_site:6513912 Ref_Type:A
ACATTGGAAATGTGATACAAATCATAATCCTAACTCACAAATTTACATGAGTGCCAATAGCAAGAAAAGTGCCAAAAATGXXXXXXXXXXT
AACCAAACAACCACCCAATAATATGAAGTAACCATAAACTGAGAGAAACATTCTAAACAAACTGAGAATGATGGAGAGT
ACATTGGAAATGTGATACAAGTCATAATCCTAACTCACAAATTTACATGAGTGCCAATAGCAAGAAAAGTGCCAAAAATGXXXXXXXXXXT
AACCAAACAACCACCCAATAATATGAAGTAACCATAAACTGAGAGAAACATTCTAAACAAACTGAGAATGATGGAGAGT
>Marker99245 Chr4 SNP_site:22919386 Ref_Type:A
CACAGGATAAAAGAACAAACGCTTACATCAGATTTATTTTGCAGGGGAACGAGTGCTGCAACAAGTTCTTTAATGCTTGGXXXXXXXXXXG
TCATAAAATGTTAATAAAATATTTGCTAGTGAAAGCATTTGGCTAGTTGCTAGTGACACTCATAAAATATTTGTAAAGT
CACAGGATAAAAGAACAAACGCTTACGTCAGATTTATTTTGCAGGGGAACGAGTGCTGCAACAAGTTCTTTAATGCTTGGXXXXXXXXXXG
TCATAAAATGTTAATAAAATATTTGCTAGTGAAAGCATTTGGCTAGTTGCTAGTGACACTCATAAAATATTTGTAAAGT
>Marker992828 Chr2 SNP_site:1830353 Ref_Type:T
ACTGGGACGACAAATATGGCACCCATAGTGTTTCACCCGATGTAAGATTTTAAACATTGTATATCATCTCTTGTAGTTCGXXXXXXXXXXG
AACCATGCAATTAGCAATTCATTTCTATTGGACGACGATTCAAGGTTAGTATCTTTTTACCAGGATTTGAAGGACAGTA
ACTGGGACGACAAATATGGCACCCATAGTGTTTCACCCGATGTAAGATTTTAAACATTGTATTTCATCTCTTGTAGTTCGXXXXXXXXXXG
AACCATGCAATTAGCAATTCATTTCTATTAGACGACGATTCAAGGTTAGTATCTTTTTACCAGGATTTGAAGGACAGTA
>Marker992828 Chr2 SNP_site:1830524 Ref_Type:A
ACTGGGACGACAAATATGGCACCCATAGTGTTTCACCCGATGTAAGATTTTAAACATTGTATATCATCTCTTGTAGTTCGXXXXXXXXXXG
AACCATGCAATTAGCAATTCATTTCTATTGGACGACGATTCAAGGTTAGTATCTTTTTACCAGGATTTGAAGGACAGTA
ACTGGGACGACAAATATGGCACCCATAGTGTTTCACCCGATGTAAGATTTTAAACATTGTATTTCATCTCTTGTAGTTCGXXXXXXXXXXG
AACCATGCAATTAGCAATTCATTTCTATTAGACGACGATTCAAGGTTAGTATCTTTTTACCAGGATTTGAAGGACAGTA
>Marker993231 Chr2 SNP_site:11138921 Ref_Type:T
AACACTTTTGATAGACTTGCGAGGAGGAAAACTTTGCTTGTGGGGCCTTTTTGCTGCATTCTTTGTTGAAGGCAGTGGAAXXXXXXXXXXG
TTTGTGCGACGATCGAGGAGCTCCTCTTCCATCCGCTTTTTCAGAGAGAAATGGTGTTTTCTATAGCTTGTTGGATGTG
AACACTTTTGATAGACTTGCGAGGAGGAAAACTTTGCTTGTGGGGCCTTTTTGCTGCATTCTTTGTTGAAGGCAGTGGAAXXXXXXXXXXG
TTTGTGTGACGATCAAGGAGCTCCTCTTCCATCCGCTTTTTCAGAGAGAAATGGTGTTTTCTATAGCTTGTTGGATGTG
>Marker993231 Chr2 SNP_site:11138929 Ref_Type:A
AACACTTTTGATAGACTTGCGAGGAGGAAAACTTTGCTTGTGGGGCCTTTTTGCTGCATTCTTTGTTGAAGGCAGTGGAAXXXXXXXXXXG
TTTGTGCGACGATCGAGGAGCTCCTCTTCCATCCGCTTTTTCAGAGAGAAATGGTGTTTTCTATAGCTTGTTGGATGTG
AACACTTTTGATAGACTTGCGAGGAGGAAAACTTTGCTTGTGGGGCCTTTTTGCTGCATTCTTTGTTGAAGGCAGTGGAAXXXXXXXXXXG
TTTGTGTGACGATCAAGGAGCTCCTCTTCCATCCGCTTTTTCAGAGAGAAATGGTGTTTTCTATAGCTTGTTGGATGTG
>Marker99342 Chr4 SNP_site:5789765 Ref_Type:C
AACTTTTATTCAGTCCTAGCATTCAACCAATGGCCATTGTAAAAGGCATCTTTATTTGCTTTCTTCTCAAACTAATGCCGXXXXXXXXXXC
ACCAAGAAATCCGACAAACAACATCTTCCCATGAGAAATTTAATGATGTTGGAAAAGAAGTGCCGAGCTAAAAGTAGTG
AACTTTTATTCAGTCCTAGCATTCAACCAATGGCCATTGTAAAAGGTATCTTTATTTGCTTTCTTCTCAAACTAATGCCGXXXXXXXXXXC
ACCAAGAAATCCGACAAACAACATCTTCCCATGAGAAATTTAATGATGTTGGAAAAGAAGTGCCGAGCTAAAAGTAGTG
>Marker993527 Chr2 SNP_site:15594036 Ref_Type:G
AACATCCATCTTTCCTTCTAGATTTGTTTTAAGTTATTAGGTATATTTTGTCAATATTTTAAAAATTAAAGTGAAAATTTXXXXXXXXXXT
AAGGAAATAACATTGTTTATTTATACTCCCAACCTAAGTTATTACATATGTTATTAGTATTTTTACACATATTGGAGTA
AACATCCATCTTTCCTTCTAGATTTGTTTTAAGTTATTAGGTATATTTTGTCAATATTTTAAAAATTAAAGTTAAAATTTXXXXXXXXXXT
AAGGAAATAACATTGTTTATTTATACTCCCAACCTAAGTTATTACATATGTTATTAGTATTTTTACACATATTGGAGTG
>Marker993527 Chr2 SNP_site:15594258 Ref_Type:T
AACATCCATCTTTCCTTCTAGATTTGTTTTAAGTTATTAGGTATATTTTGTCAATATTTTAAAAATTAAAGTGAAAATTTXXXXXXXXXXT
AAGGAAATAACATTGTTTATTTATACTCCCAACCTAAGTTATTACATATGTTATTAGTATTTTTACACATATTGGAGTA

Create PDF with GO2PDF for free, if you wish to

AACATCCATCTTTCCTTCTAGATTTGTTTTAAGTTATTAGGTATATTTTGTCAATATTTTAAAAATTAAAGTTAAAATTTXXXXXXXXXXT
AAGGAAATAACATTGTTTATTTATACTCCCAACCTAAGTTATTACATATGTTATTAGTATTTTTACACATATTGGAGTG
>Marker994133 Chr2 SNP_site:11354137 Ref_Type:T
CACAAAAGAATGAATCCCATCCTAAGACCACACCCGGCGACCTTCTCTGTTATTACCACACTCAATGCGTCCAGACAAGGXXXXXXXXXXA
TATGAGATTACAGAAGGGATTCGGAACTACGAAAACAATTTCATTGGCTATAAGAGATGCAAGACTAAACTTTGATAGT
CACAAAAGAATGAATCCCATCCTAAGACCACACCCGGCGACCTTCTCTGTTATTACCACACTCAATGCGTCCAGACAAGGXXXXXXXXXXA
TATGAGATTACAGAATGGAGTCGGAACTACGAAAACAATTTCATTGGCTATAAGAGATGCAAGACTAAACTTTGATAGT
>Marker994133 Chr2 SNP_site:11354141 Ref_Type:G
CACAAAAGAATGAATCCCATCCTAAGACCACACCCGGCGACCTTCTCTGTTATTACCACACTCAATGCGTCCAGACAAGGXXXXXXXXXXA
TATGAGATTACAGAAGGGATTCGGAACTACGAAAACAATTTCATTGGCTATAAGAGATGCAAGACTAAACTTTGATAGT
CACAAAAGAATGAATCCCATCCTAAGACCACACCCGGCGACCTTCTCTGTTATTACCACACTCAATGCGTCCAGACAAGGXXXXXXXXXXA
TATGAGATTACAGAATGGAGTCGGAACTACGAAAACAATTTCATTGGCTATAAGAGATGCAAGACTAAACTTTGATAGT
>Marker995135 Chr2 SNP_site:10946200 Ref_Type:A
TACTTAATCAAACTTGTAATTATTGGTTAATATTTTGGTTTTAAATAGTATGAAGATTTCAAATTGCGGTAAGAGTAGGTXXXXXXXXXXA
ATATTAAACACAAGAAACCAAATCTAGAGATCAACCAAAATGTATCTTTAGTGCCATATCATCCATTTATGTTTGGGTG
TACTTAATCAAACTTGTAATTATTGGTTAATATTTTGGTTTTAGATAGTATGAAGATTTCAAATTGTGGTAAGAGTAGGTXXXXXXXXXXA
ATATTAAACACAAGAAACCAAATCTAGAGATCAACCAAAATGTATCTTTAGTGCAATATCATCCATTTATGTTTGGGTG
>Marker995135 Chr2 SNP_site:10946223 Ref_Type:C
TACTTAATCAAACTTGTAATTATTGGTTAATATTTTGGTTTTAAATAGTATGAAGATTTCAAATTGCGGTAAGAGTAGGTXXXXXXXXXXA
ATATTAAACACAAGAAACCAAATCTAGAGATCAACCAAAATGTATCTTTAGTGCCATATCATCCATTTATGTTTGGGTG
TACTTAATCAAACTTGTAATTATTGGTTAATATTTTGGTTTTAGATAGTATGAAGATTTCAAATTGTGGTAAGAGTAGGTXXXXXXXXXXA
ATATTAAACACAAGAAACCAAATCTAGAGATCAACCAAAATGTATCTTTAGTGCAATATCATCCATTTATGTTTGGGTG
>Marker995135 Chr2 SNP_site:10946407 Ref_Type:C
TACTTAATCAAACTTGTAATTATTGGTTAATATTTTGGTTTTAAATAGTATGAAGATTTCAAATTGCGGTAAGAGTAGGTXXXXXXXXXXA
ATATTAAACACAAGAAACCAAATCTAGAGATCAACCAAAATGTATCTTTAGTGCCATATCATCCATTTATGTTTGGGTG
TACTTAATCAAACTTGTAATTATTGGTTAATATTTTGGTTTTAGATAGTATGAAGATTTCAAATTGTGGTAAGAGTAGGTXXXXXXXXXXA
ATATTAAACACAAGAAACCAAATCTAGAGATCAACCAAAATGTATCTTTAGTGCAATATCATCCATTTATGTTTGGGTG
>Marker995289 Chr2 SNP_site:3027852 Ref_Type:C
GACATCCACCTGTATTTTGAGAAAGCTGGGGAGTAAGAACTCCGACTCTTTTCTTTGTTATTTATTCTTTTTGCTTCCTCXXXXXXXXXXG
CTTGACTTAGCTTCATAATACTTTGTTCAATTTTCTGGTTAGAACTTAGTAACTATGGTTCTGAACGAAATACAAAGTT
GACATCCATCTGTATTTTGAGAAAGCTGGGGAGTAAGAACTCCGACTCTTTTCTTTGTTATTTATTCTTTTTGCTTCCTCXXXXXXXXXXG
CTTGACTTAGCTTCATAATACTTTGTTCAATTTTCTGGTTAGAACTTAGTAACTATGGTTCTGAACGAAATACAAAGTT
>Marker995453 Chr2 SNP_site:13416023 Ref_Type:A
AACGCTTCTCTACTCCTTTTTCGATTTCAATTTCGTTTTCACCAAACCTCTCCATTTCTTCTCCACTACTCCTCTCCGTTXXXXXXXXXXT
TAGATATACCATTAAATGTGTATGACCCATCTCTTTGATGCCATTGAGATTTTTCAGCAAACAAATTGATGCATATGTC
AACGCTTCTCTACTCCTTTTTCGATTTCAATTTCGTTTTCACCAAACCTCTCCATTTCTTCTCCACTACTCCTCTCCGTTXXXXXXXXXXT
TAGATATACCATTAAATGTGTCTGACCCATCTCTTTGATGCCATTGAGATTTTTCAGCAAACAAATTGATGCATATGTC
>Marker995782 Chr2 SNP_site:1918188 Ref_Type:T
ACTGATCTTCGGATTAATTTTTGCTTTAGATTTTGAATTTAAATCATCATCAAACCCTTTGTCACATATTCAGTGAAATAXXXXXXXXXXT
AATTAAAGTTTAATCAGAAGTTCCAAAAAATTAGCCACGTGGTGCCGACGTGGGTTCGAAATCGCGTGCAACGCACGTG
ACTGTTCTTCGGATTAATTTTTGCTTTAGATTTTGAATTTAAATCATCATCAAACCCTTTGTCACATATTCAATGAAATAXXXXXXXXXXT
AATTAAAGTTTAATCAGAAGTTCCAAAAAATTAGCCACGTGGTGCCGACGTGGGTTCGAAATCGCGTGCAACGCACGTG
>Marker995782 Chr2 SNP_site:1918256 Ref_Type:A
ACTGATCTTCGGATTAATTTTTGCTTTAGATTTTGAATTTAAATCATCATCAAACCCTTTGTCACATATTCAGTGAAATAXXXXXXXXXXT

Create PDF with GO2PDF for free, if you wish to

AATTAAAGTTTAATCAGAAGTTCCAAAAAATTAGCCACGTGGTGCCGACGTGGGTTCGAAATCGCGTGCAACGCACGTG
ACTGTTCTTCGGATTAATTTTTGCTTTAGATTTTGAATTTAAATCATCATCAAACCCTTTGTCACATATTCAATGAAATAXXXXXXXXXXT
AATTAAAGTTTAATCAGAAGTTCCAAAAAATTAGCCACGTGGTGCCGACGTGGGTTCGAAATCGCGTGCAACGCACGTG
>Marker996141 Chr2 SNP_site:15945846 Ref_Type:C
TACAATCCCATGGTCTATTTCTTCCAACTAAACGGAGATTCAAACTTCACTGAAATCGAAATTAAAACCCACCTCAAAACXXXXXXXXXXC
CTTTCTTAGAAACTCGCATCAAATCCACACTCCACGACGTCTTTTCCATGTCCTTTCCGGAGGATCTCAACTGCCTTGT
TACAATCCCATGGTTTATTTCTTCCAACTAAACGGAGATTCAAACTTCACTGAAATCGAAATTAAAACCCACCTCAAAACXXXXXXXXXXC
CTTTCTTAGAAACTCGCGTCAAATCCACACTCCACGACGTCTTTTCCATGTCCTTTCCGGAGGATCTCAACTGCCTTGT
>Marker996141 Chr2 SNP_site:15946021 Ref_Type:A
TACAATCCCATGGTCTATTTCTTCCAACTAAACGGAGATTCAAACTTCACTGAAATCGAAATTAAAACCCACCTCAAAACXXXXXXXXXXC
CTTTCTTAGAAACTCGCATCAAATCCACACTCCACGACGTCTTTTCCATGTCCTTTCCGGAGGATCTCAACTGCCTTGT
TACAATCCCATGGTTTATTTCTTCCAACTAAACGGAGATTCAAACTTCACTGAAATCGAAATTAAAACCCACCTCAAAACXXXXXXXXXXC
CTTTCTTAGAAACTCGCGTCAAATCCACACTCCACGACGTCTTTTCCATGTCCTTTCCGGAGGATCTCAACTGCCTTGT
>Marker996745 Chr2 SNP_site:17313284 Ref_Type:A
TACCTTCAGAAAGAATGCCACAGCAACAATCAAAAGGCTAAGAGTCATTCCAACAGAGGAGATCTGAAATAAAATTATAAXXXXXXXXXXT
AAAAAACATACAAGTCGTTGCTGAAAAGCAGAGTTCAGATAGAGCAAATCAATCACTAAGCACATCGATTTTAGATTGT
TACCTTCAGAAAGAATGCCACAGCAACGATCAAAAGGCTAAGAGTCATTCCAACAGAGGAGATCTGAAATAAAATTATAAXXXXXXXXXXT
AAAAAACATACAAGTCGTTGCTGAAAAGCAGAGTTCAGATAGAGCAAATCAATCACTAAGCACATCGATTTTAGATTGT
>Marker99802 Chr4 SNP_site:12795550 Ref_Type:T
CACCTGCAGCCTCTAAAAGCACAGTAAGAACCATCAAAAGGTTGTTTTAAAGAAAATGTTCTTACCTAACTATGTATGATXXXXXXXXXXG
ATACTGTTTCACATCTGAAAAGAGAAATGAAAGGGCTCCTTATCGTTATCAAGTTAAGAGATCCTTTTTTCGTTTTAGT
CACCTGCAGCCTCTAAAAGCACAGTAAGAACCATCAAAAGGTTTTTTTAAAGAAAATGTTCTTACCTAACTATGTATGATXXXXXXXXXXG
ATACTGTTTCACATCTGAAAAGAGAAATGAAAGGGCTCCTTATCGTTATCAAGTTAAGAGATCCTTTTTTCGTTTTAGT
>Marker998407 Chr2 SNP_site:3829061 Ref_Type:T
AACTAAAACCACCAAACATATTTGATATACTAGAAGGAAGTTTGTTGGTGGTGCGCTAGACCCTTCCATTTTCAGAGGGGXXXXXXXXXXT
TGAAAACGAAATGCAAGAATTTCAAGGATGTTTCTCATCTTTCGGTAGTTTTTTGCATTTGATTTATTTCATACATTGT
AACTAAAACCACCAAACATATTTGATTTACTAGAAGGAAGTTTGTTGGTGGTGCGCTAGACCCTTCCATTTTCAGAGGAGXXXXXXXXXXT
TGAAAACGAAATGCAAGAATTTCAAGGATGTTTCTCATCTTTCGGTAGTTTTTTGCATTTGATTTATTTCATACATTGT
>Marker998407 Chr2 SNP_site:3829113 Ref_Type:A
AACTAAAACCACCAAACATATTTGATATACTAGAAGGAAGTTTGTTGGTGGTGCGCTAGACCCTTCCATTTTCAGAGGGGXXXXXXXXXXT
TGAAAACGAAATGCAAGAATTTCAAGGATGTTTCTCATCTTTCGGTAGTTTTTTGCATTTGATTTATTTCATACATTGT
AACTAAAACCACCAAACATATTTGATTTACTAGAAGGAAGTTTGTTGGTGGTGCGCTAGACCCTTCCATTTTCAGAGGAGXXXXXXXXXXT
TGAAAACGAAATGCAAGAATTTCAAGGATGTTTCTCATCTTTCGGTAGTTTTTTGCATTTGATTTATTTCATACATTGT
>Marker998575 Chr2 SNP_site:7728874 Ref_Type:G
TACTTGGTCATGGTAGTGAAGTTAGTCATTGGATTCCACAAAAGGTAAGTGGTCAGATGGAGGGGATTCGTGTATCTTATXXXXXXXXXXT
TAGAACAACAAAAGTTGCCTGTGGTGTTTGGCACACTGCTGCTGCTGTTGAAGCAATTAATGAACTTTCTGATTCTGGT
TACTTGGTCATGGTAGTGAAGTTAGTCATTGGATTCCACAAAAGGTAGGTGGTCAGATGGAGGGGATTCGTGTATCTTATXXXXXXXXXXT
TAGAACAACAAAAGTTGCCTGTGGTGTTTGGCACACTGCTGCTGCTGTTGAAGCAATTAATGAACTTTCTGATTCTGGT
>Marker999994 Chr2 SNP_site:1148018 Ref_Type:A
ACATTTTGTCTGTCTTAAAAAATTAATTGCTCCTTGATTTGACCTAATTCTTGCAGCCTTTGGATGTCGTCCTCTCTCAAXXXXXXXXXXT
CTTCTTTTTCTCAATTGTCGTAGTATGGCAAATATGAAAATATTAGAAGAATATTGAATTTTTATGTTTATAATATGTT
ACATTTTGTCTGTCTTAAAAAATTAATTGCTCCTTGATTTGACCTAATTCTTGCAGCCTTTGGATGTCGTCCTCTCTCAAXXXXXXXXXXT
CTTCTTTTTCTCAATTGTCGTTGTATGGCAAATATGAAAATATTAGAAGAATATTGAATTTTTATGTTTATAATATGTT

Create PDF with GO2PDF for free, if you wish to
