# Supplementary material for: A High-Density Genetic Linkage Map for Cucumber (Cucumis sativus L.): Based on Specific Length Amplified Fragment (SLAF) Sequencing and QTL Analysis of Fruit Traits in Cucumber
Source: Front Plant Sci. 2016 Apr 19;7:437. doi: 10.3389/fpls.2016.00437 (PMC4835494; doi:10.3389/fpls.2016.00437)
Supplement: TABLE S3 — Physical position of mapped markers. [file Table_3.PDF]

Marker_ID	ChrID	start_posi	end_posi	Genetic_distance
Marker322919	Chr1	28618593	28618850	0
Marker324932	Chr1	28822680	28822920	0
Marker329746	Chr1	28657843	28658112	0
Marker339297	Chr1	28788500	28788769	0
Marker340443	Chr1	28788171	28788426	0
Marker387747	Chr1	28666854	28667100	0
Marker390747	Chr1	28825698	28825955	0
Marker411361	Chr1	28770850	28771137	0
Marker423145	Chr1	28933049	28933341	0
Marker427323	Chr1	28603615	28603868	0
Marker433546	Chr1	28859154	28859448	0
Marker438701	Chr1	28593613	28593856	0
Marker310501	Chr1	28579610	28579877	0.329
Marker321528	Chr1	28383844	28384106	0.656
Marker434603	Chr1	28387937	28388173	0.656
Marker341596	Chr1	28362413	28362726	0.983
Marker422733	Chr1	28352212	28352476	1.309
Marker426713	Chr1	28253272	28253534	1.309
Marker360132	Chr1	28178700	28178963	1.636
Marker393565	Chr1	28177471	28177728	1.636
Marker338748	Chr1	28147094	28147355	2.617
Marker399658	Chr1	27924094	27924403	2.617
Marker359941	Chr1	27863311	27863632	3.597
Marker415659	Chr1	27850965	27851228	3.924
Marker390130	Chr1	27846053	27846320	4.251
Marker304912	Chr1	27688267	27688524	5.231
Marker312708	Chr1	27401926	27402203	5.885
Marker323131	Chr1	27394132	27394378	5.885
Marker328495	Chr1	27406693	27406964	5.885
Marker392382	Chr1	27393114	27393365	5.885
Marker431956	Chr1	27420523	27420818	5.885
Marker306063	Chr1	27392432	27392691	6.539
Marker378872	Chr1	27311443	27311758	6.865
Marker399909	Chr1	27151438	27151697	6.865
Marker415440	Chr1	27129716	27129987	7.519
Marker322788	Chr1	27080518	27080818	7.846
Marker443322	Chr1	27127399	27127667	7.846
Marker345789	Chr1	26186418	26186691	8.175
Marker384086	Chr1	26585460	26585707	8.175
Marker389209	Chr1	26248918	26249182	8.175
Marker398034	Chr1	26585726	26586009	8.175
Marker454949	Chr1	26141652	26141958	8.828
Marker378110	Chr1	26087229	26087492	9.155
Marker425488	Chr1	26122148	26122439	9.155
Marker343318	Chr1	26086429	26086693	9.482
Marker313855	Chr1	26072446	26072768	9.809
Marker331025	Chr1	26069324	26069608	10.136
Marker359007	Chr1	26043982	26044260	10.136
Marker382136	Chr1	26024780	26025035	10.136
Marker447888	Chr1	26040648	26040936	10.136
Marker333213	Chr1	25998272	25998591	10.462
Marker389841	Chr1	26018448	26018738	10.462
Marker403330	Chr1	25988537	25988808	10.462

Create PDF with GO2PDF for free, if you wish to

Marker432214	Chr1	25987312	25987584	10.791
Marker412858	Chr1	25964278	25964591	11.118
Marker421111	Chr1	25962718	25963011	11.118
Marker304726	Chr1	25853797	25854089	11.445
Marker309837	Chr1	25907835	25908093	11.445
Marker343885	Chr1	25909117	25909391	11.445
Marker352948	Chr1	25837681	25837964	11.445
Marker372328	Chr1	25894828	25895072	11.445
Marker398718	Chr1	25884170	25884449	11.445
Marker402275	Chr1	25953918	25954213	11.445
Marker436867	Chr1	25912149	25912469	11.445
Marker458117	Chr1	25923602	25923878	11.445
Marker337990	Chr1	25832603	25832867	11.772
Marker417839	Chr1	25827869	25828160	11.772
Marker322333	Chr1	25756846	25757112	12.752
Marker323973	Chr1	25612149	25612414	12.752
Marker361920	Chr1	25824775	25825048	12.752
Marker365520	Chr1	25585297	25585589	12.752
Marker399152	Chr1	25747114	25747375	12.752
Marker454349	Chr1	25712962	25713277	12.752
Marker459435	Chr1	25574167	25574466	12.752
Marker400277	Chr1	25523969	25524241	13.406
Marker410571	Chr1	25526145	25526426	13.406
Marker433210	Chr1	25568758	25569007	13.406
Marker309762	Chr1	25519774	25520034	13.733
Marker339217	Chr1	25503189	25503462	14.713
Marker375837	Chr1	25491420	25491723	14.713
Marker346856	Chr1	25488404	25488683	15.04
Marker309769	Chr1	25357349	25357626	15.367
Marker405828	Chr1	25227094	25227359	15.367
Marker326838	Chr1	25223258	25223565	15.694
Marker424356	Chr1	25187300	25187577	15.694
Marker386167	Chr1	25118265	25118523	16.02
Marker418227	Chr1	25180975	25181291	16.02
Marker321924	Chr1	25090361	25090613	16.347
Marker400734	Chr1	25080396	25080657	17.001
Marker307653	Chr1	25067161	25067413	17.655
Marker386072	Chr1	24822967	24823238	18.308
Marker320187	Chr1	24674221	24674472	18.962
Marker418615	Chr1	24667228	24667500	18.962
Marker426825	Chr1	24631124	24631415	19.615
Marker453414	Chr1	24657920	24658205	19.615
Marker337374	Chr1	24592155	24592439	20.269
Marker431451	Chr1	24231473	24231761	21.056
Marker457517	Chr1	24209086	24209330	21.056
Marker388496	Chr1	24193406	24193659	21.382
Marker350326	Chr1	24173704	24173970	21.709
Marker359924	Chr1	24170631	24170935	22.036
Marker347603	Chr1	24115170	24115450	22.363
Marker397147	Chr1	24151610	24151887	22.363
Marker306066	Chr1	23958091	23958394	22.69
Marker395727	Chr1	23991045	23991344	22.69
Marker416188	Chr1	23922354	23922639	23.67
Marker312290	Chr1	23911880	23912148	24.324

Create PDF with GO2PDF for free, if you wish to

Marker332030	Chr1	23845113	23845417	24.651
Marker344372	Chr1	23875215	23875495	24.651
Marker382913	Chr1	23513312	23513589	25.631
Marker333003	Chr1	23449889	23450162	26.612
Marker367544	Chr1	23281869	23282123	27.265
Marker326141	Chr1	23267311	23267581	28.052
Marker451366	Chr1	23247033	23247331	28.705
Marker405968	Chr1	23113974	23114251	29.686
Marker354216	Chr1	23013889	23014161	30.013
Marker357159	Chr1	22987419	22987664	30.013
Marker434832	Chr1	22777300	22777549	30.339
Marker441336	Chr1	22766389	22766640	31.32
Marker375691	Chr1	22658557	22658821	31.647
Marker420651	Chr1	22642969	22643246	31.974
Marker453787	Chr1	22621894	22622214	31.974
Marker445616	Chr1	22472427	22472706	32.3
Marker381527	Chr1	22417106	22417414	32.954
Marker415046	Chr1	22224663	22224913	33.608
Marker438324	Chr1	22073597	22073876	33.934
Marker391279	Chr1	21891363	21891609	34.588
Marker313525	Chr1	21860951	21861215	35.048
Marker315017	Chr1	21844511	21844798	35.375
Marker418013	Chr1	21591993	21592249	36.355
Marker420414	Chr1	21583082	21583387	36.682
Marker316802	Chr1	21362512	21362768	37.336
Marker351292	Chr1	21325701	21325961	37.336
Marker456658	Chr1	21314035	21314355	37.989
Marker337853	Chr1	21027456	21027701	39.297
Marker363661	Chr1	21215194	21215447	39.297
Marker367764	Chr1	21183652	21183902	39.297
Marker368497	Chr1	21242546	21242842	39.297
Marker398547	Chr1	21024822	21025122	39.297
Marker408458	Chr1	21028882	21029142	39.297
Marker420443	Chr1	21191839	21192082	39.297
Marker457608	Chr1	21261331	21261585	39.297
Marker395557	Chr1	21015722	21016034	39.623
Marker314609	Chr1	20952578	20952843	40.277
Marker346373	Chr1	20940017	20940336	40.931
Marker357085	Chr1	20693352	20693626	41.258
Marker380691	Chr1	20691585	20691862	41.258
Marker397523	Chr1	20612322	20612611	41.584
Marker382626	Chr1	20469920	20470176	42.892
Marker413445	Chr1	20524141	20524429	42.892
Marker457128	Chr1	20469563	20469877	43.219
Marker443736	Chr1	20468819	20469079	44.526
Marker337939	Chr1	20460316	20460564	44.986
Marker392028	Chr1	20451132	20451434	45.313
Marker415086	Chr1	19817844	19818115	45.639
Marker345442	Chr1	19737527	19737848	45.968
Marker437639	Chr1	19721121	19721409	45.968
Marker417138	Chr1	19558861	19559140	46.295
Marker367053	Chr1	19549622	19549872	46.949
Marker437180	Chr1	19556823	19557080	46.949
Marker318357	Chr1	19520509	19520787	47.276

Create PDF with GO2PDF for free, if you wish to

Marker401062	Chr1	19398791	19399086	47.602
Marker369853	Chr1	19398323	19398613	48.256
Marker383305	Chr1	19018224	19018523	48.583
Marker404396	Chr1	19180313	19180583	48.583
Marker446762	Chr1	19368369	19368618	48.583
Marker459111	Chr1	18955772	18956042	49.89
Marker324977	Chr1	18938630	18938909	50.217
Marker439505	Chr1	18936002	18936260	50.546
Marker417104	Chr1	18748035	18748310	51.2
Marker322318	Chr1	18664142	18664428	51.921
Marker417014	Chr1	17968966	17969212	51.921
Marker439885	Chr1	17914850	17915127	51.921
Marker455594	Chr1	17483746	17484000	52.248
Marker345832	Chr1	17477874	17478157	52.574
Marker426791	Chr1	17483425	17483746	52.574
Marker428201	Chr1	17419822	17420094	52.901
Marker388601	Chr1	17318858	17319124	53.882
Marker439186	Chr1	17173267	17173558	53.882
Marker322442	Chr1	17155076	17155383	54.341
Marker363991	Chr1	17094758	17095047	54.341
Marker334549	Chr1	17083748	17084000	55.649
Marker409297	Chr1	17048546	17048834	56.109
Marker440466	Chr1	17020920	17021177	56.435
Marker388130	Chr1	16930034	16930296	57.089
Marker375483	Chr1	16916369	16916656	57.416
Marker385878	Chr1	16904062	16904342	57.743
Marker401428	Chr1	16851685	16851945	58.396
Marker414230	Chr1	16883096	16883343	58.396
Marker359692	Chr1	16842407	16842658	59.704
Marker395787	Chr1	16841110	16841427	60.031
Marker382262	Chr1	16833778	16834052	60.357
Marker321070	Chr1	16816172	16816443	60.684
Marker360930	Chr1	16247169	16247468	61.144
Marker382154	Chr1	16476982	16477237	61.144
Marker418696	Chr1	16463618	16463880	61.144
Marker397643	Chr1	16175886	16176142	61.797
Marker434739	Chr1	16103139	16103425	62.126
Marker432892	Chr1	16100656	16100933	62.78
Marker398763	Chr1	16094520	16094822	63.107
Marker422747	Chr1	16010516	16010782	63.107
Marker365870	Chr1	15826781	15827069	64.087
Marker413009	Chr1	16002528	16002832	64.087
Marker438950	Chr1	15466949	15467219	65.068
Marker329937	Chr1	15299735	15299982	65.395
Marker412990	Chr1	15312167	15312457	65.395
Marker342411	Chr1	15244749	15245051	65.722
Marker408295	Chr1	15243149	15243431	65.722
Marker379621	Chr1	15228296	15228567	66.375
Marker383257	Chr1	15142842	15143092	67.029
Marker308380	Chr1	14744108	14744376	67.356
Marker330324	Chr1	14850992	14851278	67.356
Marker452204	Chr1	14708169	14708426	67.356
Marker407772	Chr1	14692275	14692554	68.009
Marker417930	Chr1	14609670	14609930	68.336

Create PDF with GO2PDF for free, if you wish to

Marker379207	Chr1	14581621	14581892	68.663
Marker320278	Chr1	14323207	14323501	69.316
Marker461184	Chr1	14581028	14581284	69.316
Marker348286	Chr1	14315452	14315750	69.643
Marker431768	Chr1	14228353	14228649	69.643
Marker319000	Chr1	13799448	13799710	69.972
Marker443082	Chr1	13455225	13455487	70.299
Marker406537	Chr1	13441705	13442006	70.626
Marker439130	Chr1	13146636	13146882	70.953
Marker366572	Chr1	12927348	12927645	71.606
Marker320100	Chr1	12861427	12861699	72.26
Marker332785	Chr1	12737201	12737498	73.567
Marker319654	Chr1	12597840	12598121	74.221
Marker445371	Chr1	12354629	12354905	74.548
Marker338400	Chr1	12354343	12354629	75.201
Marker387434	Chr1	12320975	12321283	75.528
Marker314131	Chr1	12294633	12294943	76.182
Marker460711	Chr1	12178653	12178927	76.509
Marker401174	Chr1	11923480	11923725	76.835
Marker452900	Chr1	11948077	11948357	76.835
Marker454089	Chr1	11976795	11977053	76.835
Marker414421	Chr1	11766849	11767149	77.162
Marker354508	Chr1	11709194	11709461	78.47
Marker339038	Chr1	11103784	11104058	78.797
Marker447104	Chr1	11132031	11132311	78.797
Marker384917	Chr1	11100423	11100690	79.45
Marker425579	Chr1	10900463	10900766	79.45
Marker346744	Chr1	10845847	10846133	79.777
Marker343315	Chr1	10727389	10727686	80.104
Marker358029	Chr1	10677303	10677562	80.104
Marker326633	Chr1	10630100	10630416	81.084
Marker311295	Chr1	10350048	10350337	81.738
Marker460809	Chr1	10459858	10460099	81.738
Marker322036	Chr1	10069809	10070120	82.065
Marker425076	Chr1	10113230	10113527	82.065
Marker360845	Chr1	10053776	10054034	82.392
Marker369382	Chr1	10047304	10047558	82.718
Marker347961	Chr1	9857985	9858255	83.045
Marker454484	Chr1	9766494	9766758	83.372
Marker313097	Chr1	9590736	9591032	84.026
Marker315368	Chr1	9680656	9680911	84.026
Marker407091	Chr1	9570884	9571192	84.352
Marker365332	Chr1	9452598	9452883	84.679
Marker309411	Chr1	9364306	9364591	85.008
Marker349000	Chr1	9292342	9292585	85.335
Marker422656	Chr1	9305600	9305885	85.335
Marker410782	Chr1	9172496	9172814	85.662
Marker320680	Chr1	9090805	9091059	85.991
Marker414452	Chr1	9000383	9000662	86.644
Marker436936	Chr1	9008543	9008829	86.644
Marker332198	Chr1	8795272	8795546	87.298
Marker382229	Chr1	8978748	8979005	87.298
Marker425363	Chr1	8707866	8708126	87.625
Marker453692	Chr1	8693445	8693717	87.625

Create PDF with GO2PDF for free, if you wish to

Marker340733	Chr1	8689191	8689468	88.278
Marker448346	Chr1	8681128	8681441	88.605
Marker454627	Chr1	8512330	8512585	88.932
Marker461092	Chr1	8459182	8459449	88.932
Marker349127	Chr1	8435815	8436103	89.259
Marker367708	Chr1	8397324	8397581	89.586
Marker377265	Chr1	8258377	8258643	89.912
Marker386227	Chr1	8258752	8259013	89.912
Marker370245	Chr1	8252006	8252283	90.893
Marker439021	Chr1	8226283	8226558	91.22
Marker320417	Chr1	8132837	8133099	91.547
Marker456289	Chr1	7822266	7822549	92.2
Marker323148	Chr1	7750371	7750634	92.527
Marker350824	Chr1	7716038	7716346	93.181
Marker414867	Chr1	7541788	7542050	93.507
Marker359713	Chr1	7529000	7529280	94.488
Marker428067	Chr1	7533237	7533531	94.488
Marker318361	Chr1	7520420	7520699	95.795
Marker438395	Chr1	7469582	7469893	96.122
Marker447064	Chr1	7433422	7433689	96.122
Marker322040	Chr1	7408397	7408648	96.776
Marker452938	Chr1	7311572	7311866	96.776
Marker311335	Chr1	7242080	7242368	97.429
Marker352813	Chr1	7279644	7279937	97.429
Marker452637	Chr1	7295196	7295490	97.429
Marker411001	Chr1	7069051	7069317	98.737
Marker322422	Chr1	7063741	7063989	99.064
Marker309055	Chr1	6956590	6956842	99.391
Marker423446	Chr1	6971387	6971703	99.391
Marker390710	Chr1	6945394	6945644	99.717
Marker403491	Chr1	6921678	6921931	99.717
Marker451353	Chr1	6953340	6953619	99.717
Marker380169	Chr1	6878164	6878446	100.371
Marker323950	Chr1	6831068	6831351	100.698
Marker374325	Chr1	6614148	6614418	100.698
Marker376344	Chr1	6852140	6852404	100.698
Marker395618	Chr1	6656638	6656931	100.698
Marker406971	Chr1	6592877	6593186	100.698
Marker322944	Chr1	6578797	6579096	101.025
Marker358824	Chr1	6590014	6590323	101.025
Marker459336	Chr1	6577333	6577599	101.025
Marker332882	Chr1	6530561	6530821	101.678
Marker428573	Chr1	6506780	6507042	101.678
Marker428001	Chr1	6501147	6501423	102.005
Marker458436	Chr1	6469658	6469904	102.659
Marker337742	Chr1	6450692	6450990	103.312
Marker360566	Chr1	6463254	6463504	103.312
Marker347385	Chr1	6436305	6436579	104.293
Marker336478	Chr1	6251179	6251481	104.62
Marker370370	Chr1	6424657	6424927	104.62
Marker398880	Chr1	6200093	6200398	104.62
Marker335524	Chr1	6147406	6147696	104.946
Marker457847	Chr1	6146013	6146273	104.946
Marker330843	Chr1	6145199	6145455	105.927

Create PDF with GO2PDF for free, if you wish to

Marker351762	Chr1	5957977	5958270	106.581
Marker422019	Chr1	6053247	6053498	106.581
Marker356416	Chr1	5931614	5931895	106.907
Marker360007	Chr1	5902011	5902284	106.907
Marker397653	Chr1	5892380	5892640	108.215
Marker429225	Chr1	5506375	5506630	109.073
Marker430872	Chr1	5354144	5354400	109.727
Marker309520	Chr1	5016619	5016884	110.381
Marker376067	Chr1	4924602	4924845	110.707
Marker349789	Chr1	4905840	4906088	111.494
Marker419492	Chr1	4800645	4800925	111.821
Marker361157	Chr1	4706262	4706562	112.801
Marker328363	Chr1	4696945	4697201	113.455
Marker312526	Chr1	4667395	4667673	113.782
Marker313528	Chr1	4643269	4643542	114.762
Marker338605	Chr1	4603888	4604162	115.222
Marker340770	Chr1	4603061	4603324	115.222
Marker399422	Chr1	4556763	4557056	116.202
Marker452248	Chr1	4549851	4550147	117.51
Marker404292	Chr1	4447104	4447357	118.163
Marker401300	Chr1	4200060	4200352	118.49
Marker373811	Chr1	4196870	4197141	118.817
Marker394231	Chr1	4170689	4170958	119.471
Marker405296	Chr1	4189425	4189685	119.471
Marker411882	Chr1	4104624	4104929	119.797
Marker439635	Chr1	4120447	4120741	119.797
Marker388243	Chr1	3867984	3868257	120.451
Marker307032	Chr1	3695170	3695464	121.432
Marker355094	Chr1	3715120	3715390	121.432
Marker395182	Chr1	3692489	3692747	121.432
Marker448560	Chr1	3714852	3715120	121.432
Marker452468	Chr1	3584094	3584352	121.432
Marker336156	Chr1	3578058	3578317	122.085
Marker436670	Chr1	3536608	3536888	122.085
Marker449218	Chr1	3416232	3416501	123.393
Marker345840	Chr1	3371088	3371349	123.852
Marker306703	Chr1	2783645	2783918	124.179
Marker338812	Chr1	2746101	2746376	124.179
Marker328659	Chr1	2744944	2745199	124.506
Marker447791	Chr1	2667817	2668089	125.487
Marker437657	Chr1	2611193	2611465	125.813
Marker305536	Chr1	2574300	2574593	126.14
Marker435658	Chr1	2567537	2567809	127.121
Marker321979	Chr1	2461397	2461709	127.774
Marker355607	Chr1	2459511	2459756	127.774
Marker369850	Chr1	2045432	2045698	128.103
Marker343313	Chr1	2037840	2038118	128.43
Marker362183	Chr1	1968744	1968998	128.757
Marker404055	Chr1	1980882	1981141	128.757
Marker384336	Chr1	1763139	1763431	129.737
Marker324914	Chr1	1520943	1521208	130.391
Marker351845	Chr1	1745725	1745983	130.391
Marker353204	Chr1	1628134	1628392	130.391
Marker364209	Chr1	1305712	1306008	131.045

Create PDF with GO2PDF for free, if you wish to

Marker340833	Chr1	1258533	1258799	131.371
Marker369636	Chr1	1234515	1234787	132.352
Marker311736	Chr1	1153601	1153886	132.679
Marker317240	Chr1	1208596	1208880	132.679
Marker366028	Chr1	1172701	1172970	132.679
Marker332201	Chr1	1131763	1132064	133.332
Marker355366	Chr1	1131459	1131763	133.659
Marker449445	Chr1	1110536	1110796	133.986
Marker380306	Chr1	1089434	1089698	134.313
Marker388606	Chr1	961382	961655	134.313
Marker411449	Chr1	1089016	1089265	134.313
Marker339234	Chr1	924261	924511	135.293
Marker416016	Chr1	952827	953093	135.293
Marker418577	Chr1	959686	959974	135.293
Marker436008	Chr1	904296	904583	135.293
Marker450697	Chr1	868856	869093	135.62
Marker404849	Chr1	858511	858765	135.947
Marker313585	Chr1	262858	263163	136.274
Marker319607	Chr1	795613	795899	136.274
Marker357839	Chr1	846993	847262	136.274
Marker359019	Chr1	257321	257624	136.274
Marker419531	Chr1	757442	757709	136.274
Marker427665	Chr1	775200	775457	136.274
Marker316167	Chr1	96627	96887	136.927
Marker336282	Chr1	28435	28707	136.927
Marker397435	Chr1	69214	69479	136.927
Marker458277	Chr1	227504	227814	136.927
Marker945800	Chr2	138278	138571	0
Marker1017507	Chr2	251361	251639	0
Marker947710	Chr2	285338	285602	0.654
Marker1038315	Chr2	261466	261766	0.654
Marker1050122	Chr2	375185	375443	0.98
Marker1053604	Chr2	295969	296265	0.98
Marker1054392	Chr2	300569	300855	0.98
Marker967911	Chr2	382666	382921	1.307
Marker985686	Chr2	427298	427593	1.307
Marker1057334	Chr2	375443	375708	1.307
Marker973662	Chr2	440764	441014	1.961
Marker1000474	Chr2	953749	954022	1.961
Marker1025782	Chr2	445086	445349	1.961
Marker1051190	Chr2	441660	441925	1.961
Marker1036355	Chr2	1051339	1051598	2.615
Marker1039291	Chr2	1065541	1065802	3.268
Marker1036520	Chr2	1109571	1109807	3.922
Marker1054132	Chr2	1103027	1103333	3.922
Marker999994	Chr2	1147813	1148077	4.575
Marker1004028	Chr2	1274376	1274645	5.229
Marker1001042	Chr2	1608835	1609104	6.537
Marker969891	Chr2	1692585	1692887	6.863
Marker1051888	Chr2	1796119	1796387	7.844
Marker992828	Chr2	1830292	1830575	8.171
Marker1038311	Chr2	1916154	1916396	9.151
Marker995782	Chr2	1918185	1918466	9.938
Marker973722	Chr2	2364196	2364450	10.591

Create PDF with GO2PDF for free, if you wish to

Marker976971	Chr2	2023060	2023357	10.591
Marker1022979	Chr2	2320959	2321251	10.591
Marker1050856	Chr2	2391367	2391637	10.918
Marker1010387	Chr2	2415652	2415946	11.572
Marker1023740	Chr2	2506863	2507146	11.572
Marker954810	Chr2	2538977	2539227	12.225
Marker1021668	Chr2	2591357	2591599	12.552
Marker1015621	Chr2	2729991	2730256	12.879
Marker1011198	Chr2	2735764	2736008	13.206
Marker989345	Chr2	2874376	2874668	13.533
Marker987138	Chr2	2891219	2891493	13.859
Marker1013904	Chr2	2931048	2931274	14.186
Marker971696	Chr2	2989620	2989883	14.513
Marker1002381	Chr2	2980353	2980628	14.513
Marker1023327	Chr2	2949348	2949603	14.513
Marker1045542	Chr2	2947298	2947581	14.513
Marker982219	Chr2	3006152	3006477	14.84
Marker995289	Chr2	3027845	3028106	15.167
Marker1055921	Chr2	3015538	3015831	15.167
Marker962390	Chr2	3028845	3029133	15.493
Marker1047116	Chr2	3030301	3030549	15.493
Marker965557	Chr2	3032228	3032529	15.82
Marker1011758	Chr2	3128618	3128883	15.82
Marker974690	Chr2	3153868	3154140	16.147
Marker1014237	Chr2	3154796	3155057	17.454
Marker1007754	Chr2	3213296	3213596	18.108
Marker1021357	Chr2	3257500	3257748	18.762
Marker1048205	Chr2	3250391	3250679	18.762
Marker986467	Chr2	3260542	3260831	19.221
Marker944450	Chr2	3307268	3307529	19.548
Marker972846	Chr2	3337668	3337961	20.202
Marker1050540	Chr2	3373788	3374039	20.202
Marker1009348	Chr2	3391120	3391399	20.529
Marker1031035	Chr2	3395590	3395896	20.529
Marker1007361	Chr2	3469420	3469670	20.855
Marker1036917	Chr2	3532119	3532371	21.509
Marker967856	Chr2	3641170	3641448	21.836
Marker1003070	Chr2	3635300	3635596	21.836
Marker1003574	Chr2	3680269	3680528	21.836
Marker1023594	Chr2	3681949	3682191	21.836
Marker985193	Chr2	3724882	3725178	22.163
Marker960518	Chr2	3732243	3732538	22.49
Marker1042938	Chr2	3739147	3739445	22.816
Marker957978	Chr2	3739868	3740162	23.143
Marker998407	Chr2	3829036	3829321	23.143
Marker1025679	Chr2	3804961	3805218	23.143
Marker1061565	Chr2	3780896	3781203	23.143
Marker979154	Chr2	3831117	3831369	24.124
Marker966879	Chr2	3926384	3926698	24.45
Marker1026032	Chr2	3948153	3948441	24.777
Marker1016996	Chr2	4090948	4091205	25.431
Marker1021435	Chr2	4332797	4333057	25.431
Marker1039967	Chr2	4338870	4339124	25.891
Marker965827	Chr2	4341798	4342086	26.217

Create PDF with GO2PDF for free, if you wish to

Marker938811	Chr2	4461742	4461995	26.544
Marker964090	Chr2	4412486	4412782	26.544
Marker1052937	Chr2	4377288	4377565	26.544
Marker989047	Chr2	4481760	4482027	26.871
Marker1040282	Chr2	4520301	4520573	26.871
Marker1046649	Chr2	4495404	4495669	26.871
Marker983456	Chr2	4534750	4535012	27.525
Marker956864	Chr2	4557880	4558180	28.178
Marker1033329	Chr2	4592288	4592592	28.832
Marker1054527	Chr2	4627390	4627655	29.159
Marker1058749	Chr2	4919536	4919810	29.485
Marker1036026	Chr2	4920658	4920916	30.466
Marker1019718	Chr2	4927650	4927943	30.926
Marker1023421	Chr2	4939701	4939957	31.579
Marker1023924	Chr2	5019013	5019279	32.56
Marker1021698	Chr2	5551536	5551817	33.281
Marker983658	Chr2	5807492	5807787	33.935
Marker992080	Chr2	6513893	6514155	34.261
Marker979042	Chr2	6527826	6528073	35.569
Marker1038655	Chr2	6533551	6533853	36.355
Marker1004685	Chr2	6551462	6551722	37.142
Marker1036145	Chr2	6553578	6553850	37.469
Marker987443	Chr2	6580935	6581258	38.122
Marker955654	Chr2	6753342	6753637	38.776
Marker943571	Chr2	6810592	6810915	39.103
Marker986452	Chr2	6796239	6796537	39.103
Marker1061616	Chr2	6922917	6923156	39.43
Marker981747	Chr2	7342397	7342698	39.756
Marker998575	Chr2	7728828	7729130	40.41
Marker1048808	Chr2	7762928	7763176	41.064
Marker1013153	Chr2	7774504	7774787	42.698
Marker954192	Chr2	7795702	7795969	43.352
Marker1040715	Chr2	7811464	7811715	44.332
Marker949256	Chr2	8003643	8003940	44.659
Marker986903	Chr2	7999327	7999594	44.659
Marker953875	Chr2	8006472	8006764	44.986
Marker950067	Chr2	8006824	8007097	45.64
Marker1021272	Chr2	8036250	8036563	47.407
Marker1030162	Chr2	8043836	8044095	47.407
Marker1043928	Chr2	8058429	8058697	48.388
Marker984240	Chr2	9365456	9365756	49.695
Marker1003363	Chr2	9373594	9373847	51.003
Marker1015029	Chr2	9391529	9391775	51.462
Marker978721	Chr2	10339682	10339953	51.789
Marker1003649	Chr2	10834041	10834309	52.116
Marker954519	Chr2	10836296	10836601	53.423
Marker988287	Chr2	10844874	10845153	53.423
Marker1008492	Chr2	10873073	10873320	53.75
Marker942567	Chr2	10895885	10896168	54.404
Marker1050291	Chr2	10919517	10919810	54.731
Marker946564	Chr2	10942830	10943116	55.711
Marker995135	Chr2	10946158	10946433	55.711
Marker1025196	Chr2	10941407	10941681	55.711
Marker1055698	Chr2	10950011	10950320	55.711

Create PDF with GO2PDF for free, if you wish to

Marker1040957	Chr2	10959350	10959610	56.038
Marker1005320	Chr2	10983701	10983991	56.365
Marker943655	Chr2	10989860	10990122	56.692
Marker1046677	Chr2	10997676	10997979	57.018
Marker1011348	Chr2	10997979	10998274	57.345
Marker1011427	Chr2	11040289	11040549	57.999
Marker1004780	Chr2	11127120	11127398	58.326
Marker993231	Chr2	11138739	11138995	58.652
Marker1017378	Chr2	11149814	11150093	59.306
Marker1057109	Chr2	11183132	11183389	59.633
Marker994133	Chr2	11353949	11354202	59.96
Marker1001741	Chr2	11210635	11210888	59.96
Marker1020777	Chr2	11354914	11355175	60.287
Marker946492	Chr2	11444009	11444266	60.613
Marker1003285	Chr2	11448647	11448895	61.594
Marker1033829	Chr2	11570817	11571082	61.923
Marker997547	Chr2	11973954	11974234	62.25
Marker1002719	Chr2	12508732	12509033	62.903
Marker1018030	Chr2	12489316	12489595	62.903
Marker1031143	Chr2	12517580	12517829	63.557
Marker1040965	Chr2	12551706	12551991	65.191
Marker957716	Chr2	12565649	12565903	66.959
Marker1026691	Chr2	12578665	12578929	67.286
Marker963381	Chr2	12597661	12597957	67.939
Marker1004061	Chr2	12643823	12644078	68.266
Marker1042052	Chr2	12647004	12647311	68.593
Marker1047871	Chr2	12686871	12687132	68.593
Marker989051	Chr2	12718271	12718548	69.247
Marker991665	Chr2	12853527	12853800	69.573
Marker1042795	Chr2	12863375	12863640	69.573
Marker1035013	Chr2	12916834	12917084	70.227
Marker963376	Chr2	13210781	13211063	70.554
Marker995453	Chr2	13415799	13416082	70.554
Marker1036790	Chr2	13159667	13159920	70.554
Marker1053917	Chr2	13278661	13278913	70.554
Marker1035095	Chr2	13419543	13419802	71.861
Marker945922	Chr2	13430600	13430887	72.188
Marker970327	Chr2	13452330	13452608	72.188
Marker1015528	Chr2	13511139	13511399	72.842
Marker1046808	Chr2	13482846	13483119	72.842
Marker1054704	Chr2	13579243	13579529	73.169
Marker941505	Chr2	13580626	13580918	73.495
Marker978433	Chr2	13581342	13581615	73.822
Marker1032720	Chr2	13624841	13625124	74.149
Marker1020178	Chr2	13751378	13751675	74.476
Marker985288	Chr2	13767905	13768174	75.262
Marker939580	Chr2	13778296	13778572	75.589
Marker945474	Chr2	13784307	13784593	75.916
Marker978636	Chr2	13794524	13794835	75.916
Marker1024444	Chr2	13792010	13792288	75.916
Marker976464	Chr2	13819967	13820204	76.243
Marker979715	Chr2	13841826	13842117	76.57
Marker974887	Chr2	13928705	13928962	76.896
Marker1026563	Chr2	13911222	13911504	76.896

Create PDF with GO2PDF for free, if you wish to

Marker1031170	Chr2	13930084	13930393	76.896
Marker968847	Chr2	13948812	13949070	77.223
Marker1008490	Chr2	14024545	14024797	78.204
Marker938678	Chr2	14090975	14091259	79.184
Marker1014874	Chr2	14153541	14153792	79.184
Marker972088	Chr2	14218057	14218355	79.511
Marker1000814	Chr2	14270739	14271018	80.165
Marker1037290	Chr2	14344653	14344936	80.165
Marker985590	Chr2	14405194	14405448	81.145
Marker1000252	Chr2	14406918	14407186	81.472
Marker954724	Chr2	14417411	14417715	82.453
Marker962710	Chr2	14541719	14542013	82.779
Marker1044100	Chr2	14422401	14422693	82.779
Marker1012064	Chr2	14549390	14549692	83.106
Marker1060160	Chr2	15107161	15107406	84.087
Marker1061839	Chr2	15455200	15455498	84.413
Marker1057406	Chr2	15495461	15495739	85.2
Marker1032722	Chr2	15509203	15509454	85.527
Marker993527	Chr2	15593965	15594260	86.507
Marker945545	Chr2	15606567	15606848	88.142
Marker1029536	Chr2	15604579	15604848	88.142
Marker950165	Chr2	15609679	15609952	88.795
Marker950565	Chr2	15684788	15685048	88.795
Marker1040196	Chr2	15685318	15685621	88.795
Marker967273	Chr2	15722975	15723264	89.122
Marker1028391	Chr2	15926206	15926517	89.449
Marker996141	Chr2	15945833	15946084	90.103
Marker1033991	Chr2	15996563	15996848	90.43
Marker1056430	Chr2	16028337	16028634	90.889
Marker949757	Chr2	16031768	16032042	91.216
Marker1036940	Chr2	16284141	16284395	91.87
Marker1048738	Chr2	16469286	16469546	92.85
Marker987118	Chr2	16489114	16489360	93.177
Marker968990	Chr2	16924754	16925037	93.506
Marker1011283	Chr2	16994144	16994458	93.966
Marker1058833	Chr2	17008286	17008575	94.292
Marker959409	Chr2	17017511	17017801	95.6
Marker961440	Chr2	17037586	17037875	95.6
Marker983474	Chr2	17042730	17043001	95.6
Marker1044273	Chr2	17062710	17063002	95.927
Marker943459	Chr2	17290347	17290609	96.907
Marker1034815	Chr2	17079415	17079672	96.907
Marker1048282	Chr2	17063499	17063783	96.907
Marker959943	Chr2	17308032	17308310	98.215
Marker996745	Chr2	17313258	17313541	98.868
Marker938653	Chr2	17315642	17315952	99.195
Marker984728	Chr2	17333786	17334040	99.522
Marker978408	Chr2	17340030	17340333	100.829
Marker1028124	Chr2	17345311	17345566	101.156
Marker1056942	Chr2	17593582	17593824	101.81
Marker1003554	Chr2	17600195	17600482	102.463
Marker939332	Chr2	17609651	17609915	103.771
Marker1058717	Chr2	17709214	17709475	104.751
Marker955112	Chr2	17723301	17723567	105.405

Create PDF with GO2PDF for free, if you wish to

Marker979256	Chr2	17759169	17759450	105.405
Marker1057882	Chr2	17761786	17762037	106.453
Marker976681	Chr2	18525897	18526202	107.894
Marker1001047	Chr2	18416558	18416830	107.894
Marker1046248	Chr2	18553120	18553408	108.874
Marker1053846	Chr2	18957729	18958028	109.528
Marker1056657	Chr2	18962411	18962661	109.855
Marker979814	Chr2	18993704	18993962	111.162
Marker986273	Chr2	19047253	19047551	111.489
Marker1034245	Chr2	19081086	19081334	112.142
Marker949328	Chr2	19098874	19099154	112.929
Marker987408	Chr2	19190140	19190402	113.256
Marker951177	Chr2	19191685	19191940	113.909
Marker1047132	Chr2	19194755	19195040	113.909
Marker969459	Chr2	19195582	19195853	115.217
Marker1019327	Chr2	19206564	19206850	115.217
Marker1027585	Chr2	19335907	19336168	115.544
Marker1045106	Chr2	19503899	19504161	116.524
Marker1053673	Chr2	19801794	19802075	116.853
Marker1015430	Chr2	20136379	20136645	117.507
Marker1018557	Chr2	20018466	20018718	117.507
Marker1050643	Chr2	20139855	20140149	118.16
Marker944695	Chr2	20140162	20140425	118.814
Marker1035180	Chr2	20144434	20144752	118.814
Marker1013728	Chr2	20200896	20201167	119.141
Marker1032207	Chr2	20291017	20291294	119.468
Marker1032341	Chr2	20279056	20279303	119.468
Marker964869	Chr2	20352717	20353002	119.795
Marker989401	Chr2	20306013	20306277	119.795
Marker957075	Chr2	20373238	20373525	120.448
Marker957867	Chr2	20398952	20399228	120.448
Marker974881	Chr2	20472954	20473240	120.448
Marker979469	Chr2	20525565	20525843	121.102
Marker1007299	Chr2	20503177	20503472	121.102
Marker1055984	Chr2	20492353	20492601	121.102
Marker1045858	Chr2	20593879	20594163	121.429
Marker1057468	Chr2	20597845	20598101	121.755
Marker1005178	Chr2	20611583	20611854	122.082
Marker1015945	Chr2	20615436	20615707	122.736
Marker971868	Chr2	20645855	20646146	123.063
Marker1025046	Chr2	20642992	20643243	123.063
Marker1002940	Chr2	20908137	20908412	123.389
Marker1037620	Chr2	20990006	20990310	124.043
Marker1002169	Chr2	20997596	20997863	125.351
Marker948275	Chr2	21009578	21009861	126.331
Marker1012536	Chr2	21054065	21054331	126.658
Marker1033292	Chr2	21190592	21190879	126.987
Marker1048816	Chr2	21119173	21119447	126.987
Marker1036551	Chr2	21300397	21300664	128.294
Marker969044	Chr2	21325492	21325769	129.275
Marker1015754	Chr2	21337768	21338061	130.255
Marker950420	Chr2	21342802	21343054	130.582
Marker1035842	Chr2	21395569	21395826	130.582
Marker960129	Chr2	21590701	21591003	131.042

Create PDF with GO2PDF for free, if you wish to

Marker1008595	Chr2	21810950	21811231	131.695
Marker1020292	Chr2	21852640	21852901	132.349
Marker1046943	Chr2	21879746	21880024	132.676
Marker1021718	Chr2	21891130	21891414	133.33
Marker1050448	Chr2	21907773	21908021	133.983
Marker983042	Chr2	21909482	21909743	134.31
Marker940705	Chr2	21926272	21926562	134.637
Marker1010011	Chr2	22033862	22034115	134.637
Marker1017050	Chr2	22029329	22029616	134.637
Marker958864	Chr2	22082702	22083016	135.29
Marker965278	Chr2	22067958	22068245	135.29
Marker957015	Chr2	22098167	22098431	135.944
Marker1050568	Chr2	22089982	22090280	135.944
Marker956175	Chr2	22100784	22101102	136.598
Marker971129	Chr2	22190702	22190968	136.924
Marker1017240	Chr2	22215112	22215409	137.905
Marker984050	Chr2	22305834	22306095	138.559
Marker1011028	Chr2	22327419	22327676	138.559
Marker943594	Chr2	22640754	22641016	139.539
Marker948282	Chr2	22824576	22824885	139.539
Marker951516	Chr2	22660870	22661173	139.539
Marker965203	Chr2	22651629	22651905	139.539
Marker982058	Chr2	22842229	22842543	139.539
Marker982998	Chr2	22585436	22585758	139.539
Marker986034	Chr2	22577264	22577569	139.539
Marker1004021	Chr2	22665313	22665634	139.539
Marker1032851	Chr2	22663070	22663359	139.539
Marker1045696	Chr2	22640497	22640754	139.539
Marker1059258	Chr2	22355765	22356027	139.539
Marker603540	Chr3	221252	221525	0
Marker620650	Chr3	217402	217687	0
Marker496362	Chr3	265365	265664	0.981
Marker648037	Chr3	277314	277612	0.981
Marker648548	Chr3	323626	323878	0.981
Marker562951	Chr3	346312	346611	1.307
Marker495966	Chr3	354949	355194	1.634
Marker514917	Chr3	443940	444191	1.634
Marker549828	Chr3	499767	500045	1.961
Marker573381	Chr3	451135	451384	1.961
Marker577769	Chr3	470185	470438	1.961
Marker595939	Chr3	496460	496726	1.961
Marker554229	Chr3	518702	518954	2.288
Marker627082	Chr3	650104	650387	2.941
Marker554170	Chr3	663552	663824	3.595
Marker519662	Chr3	767737	768000	3.922
Marker561011	Chr3	778133	778453	4.902
Marker584877	Chr3	784433	784673	4.902
Marker478667	Chr3	811447	811710	5.556
Marker562045	Chr3	792213	792491	5.556
Marker642193	Chr3	818338	818616	5.883
Marker518212	Chr3	861513	861768	6.536
Marker616617	Chr3	926218	926527	6.863
Marker503065	Chr3	1017131	1017426	7.517
Marker557775	Chr3	968764	969082	7.517

Create PDF with GO2PDF for free, if you wish to

Marker463807	Chr3	1161975	1162219	8.17
Marker481511	Chr3	1047072	1047336	8.17
Marker518134	Chr3	1045702	1045958	8.17
Marker571673	Chr3	1037945	1038233	8.17
Marker483785	Chr3	1165903	1166214	9.151
Marker563668	Chr3	1546055	1546323	9.805
Marker617589	Chr3	1550967	1551268	10.131
Marker481912	Chr3	1662983	1663301	10.458
Marker576482	Chr3	1742612	1742896	10.785
Marker496610	Chr3	1780588	1780839	11.439
Marker463106	Chr3	1800354	1800635	12.092
Marker553007	Chr3	2109009	2109269	12.419
Marker466763	Chr3	2169542	2169823	12.879
Marker499767	Chr3	2353352	2353616	13.532
Marker522251	Chr3	2328651	2328930	13.532
Marker478618	Chr3	2462163	2462443	13.861
Marker631603	Chr3	2463927	2464201	13.861
Marker518171	Chr3	2522682	2522925	14.188
Marker574220	Chr3	2578869	2579101	14.188
Marker611822	Chr3	2500805	2501098	14.188
Marker644887	Chr3	2585719	2585960	14.188
Marker628577	Chr3	2625821	2626083	15.169
Marker570971	Chr3	2687105	2687397	15.495
Marker671749	Chr3	2683545	2683812	15.495
Marker649324	Chr3	2708631	2708914	16.476
Marker505510	Chr3	2710573	2710892	16.803
Marker593866	Chr3	2743923	2744198	18.11
Marker612162	Chr3	2719854	2720122	18.11
Marker592492	Chr3	2817874	2818127	18.764
Marker650951	Chr3	2969301	2969579	19.744
Marker590648	Chr3	3111645	3111895	20.398
Marker481176	Chr3	3114116	3114408	20.725
Marker494320	Chr3	3113066	3113323	20.725
Marker559707	Chr3	3179564	3179846	21.052
Marker489996	Chr3	3228762	3229037	21.378
Marker622285	Chr3	3244676	3244979	22.032
Marker489772	Chr3	3328297	3328563	22.359
Marker539333	Chr3	3397233	3397485	22.359
Marker596602	Chr3	3294170	3294427	22.359
Marker667506	Chr3	3685282	3685584	23.666
Marker558518	Chr3	3794910	3795229	24.647
Marker588078	Chr3	3794509	3794793	24.647
Marker591557	Chr3	3846461	3846705	24.647
Marker555227	Chr3	3861261	3861528	25.301
Marker509450	Chr3	3906771	3907034	25.954
Marker667073	Chr3	4028808	4029075	26.281
Marker644852	Chr3	4059424	4059698	27.261
Marker643752	Chr3	4126448	4126724	27.588
Marker465079	Chr3	4128169	4128485	27.915
Marker668985	Chr3	4132305	4132607	27.915
Marker625516	Chr3	4192798	4193076	28.242
Marker485276	Chr3	4299783	4300067	28.569
Marker524868	Chr3	4221765	4222032	28.569
Marker562355	Chr3	4702024	4702285	29.222

Create PDF with GO2PDF for free, if you wish to

Marker599751	Chr3	4705340	4705590	29.222
Marker507325	Chr3	4872053	4872313	29.549
Marker537256	Chr3	4734567	4734847	29.549
Marker504376	Chr3	5259365	5259636	29.876
Marker494048	Chr3	5341554	5341825	30.53
Marker540786	Chr3	5445949	5446209	30.856
Marker554667	Chr3	5369179	5369470	30.856
Marker582904	Chr3	5449944	5450235	31.183
Marker627676	Chr3	5489851	5490117	31.837
Marker496898	Chr3	5582919	5583177	32.164
Marker542894	Chr3	5549176	5549460	32.164
Marker579205	Chr3	5551486	5551799	32.164
Marker618462	Chr3	5614282	5614558	32.49
Marker575863	Chr3	5615897	5616156	33.471
Marker648596	Chr3	5645588	5645859	33.798
Marker652562	Chr3	5686762	5687052	33.798
Marker567527	Chr3	5748240	5748549	34.451
Marker638811	Chr3	5767489	5767753	34.778
Marker484510	Chr3	5815914	5816172	35.105
Marker533275	Chr3	5862722	5862983	35.432
Marker553914	Chr3	5878662	5878946	36.085
Marker645135	Chr3	5881338	5881611	36.412
Marker503967	Chr3	6235449	6235702	36.739
Marker618323	Chr3	6102866	6103146	36.739
Marker478460	Chr3	6568977	6569292	38.046
Marker565646	Chr3	6622438	6622714	38.046
Marker579512	Chr3	6620425	6620675	38.046
Marker601090	Chr3	6654920	6655207	38.7
Marker616200	Chr3	6688490	6688739	39.354
Marker516546	Chr3	6693467	6693785	40.007
Marker545827	Chr3	6715135	6715422	40.661
Marker640996	Chr3	6740013	6740281	41.315
Marker559641	Chr3	6785303	6785585	42.295
Marker625673	Chr3	6867834	6868105	42.622
Marker626989	Chr3	6859426	6859676	42.622
Marker549515	Chr3	6917243	6917488	42.949
Marker605371	Chr3	6917788	6918107	43.276
Marker611413	Chr3	6922417	6922680	43.276
Marker490029	Chr3	7000696	7000943	43.602
Marker484859	Chr3	7017481	7017760	44.256
Marker642234	Chr3	7099907	7100193	44.91
Marker587385	Chr3	7140310	7140618	45.563
Marker592359	Chr3	7122317	7122577	45.563
Marker516527	Chr3	7244267	7244568	45.89
Marker573624	Chr3	7252086	7252349	46.544
Marker618984	Chr3	7266078	7266394	46.544
Marker495686	Chr3	7277668	7277929	46.87
Marker576880	Chr3	7391667	7391946	46.87
Marker648233	Chr3	7277362	7277668	46.87
Marker625238	Chr3	8010678	8010958	47.524
Marker536941	Chr3	8134968	8135234	47.851
Marker553857	Chr3	8127176	8127460	47.851
Marker515791	Chr3	8135308	8135583	48.831
Marker534270	Chr3	8156699	8157010	50.139

Create PDF with GO2PDF for free, if you wish to

Marker487508	Chr3	8162593	8162854	50.793
Marker600097	Chr3	8169888	8170150	51.773
Marker591591	Chr3	8250160	8250427	52.1
Marker631189	Chr3	8221003	8221266	52.1
Marker467875	Chr3	8262511	8262773	52.754
Marker489458	Chr3	8419230	8419514	52.754
Marker583938	Chr3	8407350	8407642	52.754
Marker667496	Chr3	8356225	8356504	52.754
Marker580275	Chr3	8609469	8609733	53.08
Marker495863	Chr3	8776372	8776627	53.407
Marker533186	Chr3	8813502	8813814	53.407
Marker612827	Chr3	8807047	8807324	53.407
Marker670015	Chr3	8814070	8814382	53.734
Marker468478	Chr3	9075734	9076020	54.061
Marker603112	Chr3	9119142	9119396	55.041
Marker532201	Chr3	9148337	9148601	55.368
Marker490272	Chr3	9386529	9386813	55.695
Marker497563	Chr3	9433127	9433393	55.695
Marker503234	Chr3	9178051	9178305	55.695
Marker611176	Chr3	9328248	9328506	55.695
Marker648667	Chr3	9440355	9440622	57.002
Marker587087	Chr3	9495755	9496043	57.329
Marker619259	Chr3	9502648	9502942	57.983
Marker543731	Chr3	9532329	9532599	58.31
Marker643861	Chr3	9546039	9546344	58.963
Marker527481	Chr3	9547792	9548052	59.29
Marker484392	Chr3	9590097	9590339	59.944
Marker625603	Chr3	9576288	9576559	59.944
Marker566653	Chr3	9619164	9619445	60.27
Marker586437	Chr3	9624996	9625250	60.27
Marker625365	Chr3	9638081	9638369	60.924
Marker663980	Chr3	9648508	9648801	61.905
Marker557049	Chr3	9732353	9732592	62.231
Marker629012	Chr3	9840635	9840901	62.231
Marker510939	Chr3	9873780	9874049	62.558
Marker613716	Chr3	9890555	9890831	63.539
Marker501347	Chr3	9939125	9939405	63.866
Marker648578	Chr3	9995888	9996141	63.866
Marker470406	Chr3	9998256	9998524	64.192
Marker601688	Chr3	10075685	10075994	64.192
Marker479285	Chr3	10265397	10265665	64.846
Marker558401	Chr3	10739361	10739626	65.173
Marker650408	Chr3	10733010	10733286	65.173
Marker567739	Chr3	10740566	10740828	65.5
Marker605191	Chr3	11123761	11124031	66.153
Marker562727	Chr3	11177127	11177395	66.807
Marker629770	Chr3	11143546	11143795	66.807
Marker603662	Chr3	11185878	11186136	67.787
Marker491693	Chr3	11191700	11191972	68.114
Marker501939	Chr3	11187051	11187370	68.114
Marker524425	Chr3	11227703	11227990	68.441
Marker465315	Chr3	11275224	11275543	69.095
Marker586926	Chr3	11265574	11265836	69.095
Marker639661	Chr3	11258010	11258306	69.095

Create PDF with GO2PDF for free, if you wish to

Marker466288	Chr3	11332552	11332815	69.421
Marker478488	Chr3	11467225	11467489	69.421
Marker547027	Chr3	11356316	11356597	69.421
Marker557614	Chr3	11420715	11420969	69.421
Marker482547	Chr3	11470236	11470490	69.748
Marker577879	Chr3	11468448	11468693	69.748
Marker471809	Chr3	11501614	11501880	70.075
Marker526476	Chr3	11558293	11558561	70.402
Marker513707	Chr3	11574517	11574766	71.055
Marker543451	Chr3	11821580	11821857	71.382
Marker608501	Chr3	11810677	11810933	71.382
Marker628294	Chr3	11575755	11576026	71.382
Marker462725	Chr3	11857867	11858144	72.036
Marker657322	Chr3	11829892	11830191	72.036
Marker517322	Chr3	11865253	11865522	72.363
Marker652039	Chr3	11899848	11900145	72.363
Marker536779	Chr3	11919991	11920259	72.689
Marker625755	Chr3	12020193	12020475	72.689
Marker664198	Chr3	12024165	12024436	73.149
Marker501742	Chr3	12035523	12035805	73.803
Marker640656	Chr3	12035241	12035523	73.803
Marker524214	Chr3	12057014	12057281	74.13
Marker597456	Chr3	12353554	12353807	74.13
Marker541750	Chr3	12491117	12491379	74.456
Marker653766	Chr3	12388302	12388568	74.456
Marker651738	Chr3	12522583	12522837	75.11
Marker486679	Chr3	12533681	12533949	75.764
Marker572606	Chr3	12687694	12687980	76.417
Marker583236	Chr3	12603878	12604170	76.417
Marker669000	Chr3	12576293	12576547	76.417
Marker505000	Chr3	12789960	12790243	76.744
Marker669551	Chr3	12840363	12840644	76.744
Marker501793	Chr3	12891079	12891343	77.398
Marker539519	Chr3	12865489	12865743	77.398
Marker473709	Chr3	12905247	12905538	77.724
Marker612644	Chr3	12992962	12993259	78.051
Marker482665	Chr3	12993259	12993512	78.705
Marker468272	Chr3	13016300	13016567	79.032
Marker566171	Chr3	13060492	13060753	79.359
Marker506791	Chr3	13145829	13146141	80.012
Marker553301	Chr3	13202253	13202532	80.339
Marker539563	Chr3	13409979	13410252	81.646
Marker592553	Chr3	13273811	13274096	81.646
Marker487192	Chr3	13906942	13907234	82.3
Marker632698	Chr3	13996378	13996639	82.3
Marker512622	Chr3	14049707	14049992	83.281
Marker530745	Chr3	14040168	14040452	83.281
Marker605112	Chr3	14086255	14086545	83.281
Marker661591	Chr3	14023345	14023607	83.281
Marker577667	Chr3	14118978	14119231	83.607
Marker482838	Chr3	14131316	14131603	83.934
Marker624471	Chr3	14281371	14281643	84.261
Marker623146	Chr3	14288790	14289101	84.588
Marker476305	Chr3	15413726	15413984	84.915

Create PDF with GO2PDF for free, if you wish to

Marker584428	Chr3	15245760	15246017	84.915
Marker608768	Chr3	15219620	15219884	84.915
Marker647283	Chr3	15567468	15567759	85.244
Marker504251	Chr3	15967127	15967408	85.897
Marker653752	Chr3	15934160	15934472	85.897
Marker558233	Chr3	16327219	16327534	86.551
Marker635888	Chr3	16372286	16372549	87.204
Marker546960	Chr3	16372747	16373034	87.858
Marker559418	Chr3	16392928	16393198	88.185
Marker487999	Chr3	16427581	16427868	88.512
Marker504217	Chr3	16450384	16450653	88.512
Marker509818	Chr3	17065419	17065708	88.838
Marker572638	Chr3	17288969	17289311	89.165
Marker540256	Chr3	17350867	17351139	89.492
Marker557893	Chr3	17390990	17391251	90.146
Marker587075	Chr3	17508970	17509221	90.146
Marker501399	Chr3	17992282	17992568	90.473
Marker583828	Chr3	18099601	18099868	91.126
Marker542722	Chr3	18419075	18419363	92.434
Marker515764	Chr3	18485117	18485390	92.76
Marker566778	Chr3	18481718	18482003	92.76
Marker461861	Chr3	18495051	18495317	93.087
Marker586874	Chr3	18500924	18501194	94.656
Marker525127	Chr3	18502081	18502339	95.637
Marker615555	Chr3	18538610	18538875	95.637
Marker616638	Chr3	18759571	18759829	95.964
Marker560910	Chr3	18767109	18767357	96.685
Marker663910	Chr3	18776065	18776348	96.685
Marker527959	Chr3	19111795	19112067	97.012
Marker490165	Chr3	19280669	19280944	97.338
Marker629392	Chr3	19302129	19302400	97.338
Marker607357	Chr3	19445803	19446092	97.665
Marker547729	Chr3	20472832	20473095	97.992
Marker611143	Chr3	19987794	19988092	97.992
Marker646428	Chr3	20530778	20531054	99.367
Marker462897	Chr3	20565647	20565908	100.021
Marker463148	Chr3	21051804	21052056	100.348
Marker503189	Chr3	21077683	21077965	100.807
Marker467252	Chr3	21674489	21674779	101.134
Marker594469	Chr3	21130427	21130710	101.134
Marker653778	Chr3	21694034	21694305	101.461
Marker482814	Chr3	21694479	21694779	102.114
Marker656252	Chr3	21703405	21703695	102.114
Marker496895	Chr3	21711731	21712036	102.768
Marker654184	Chr3	21720031	21720362	103.095
Marker508390	Chr3	21738885	21739136	103.422
Marker525237	Chr3	21724920	21725213	103.422
Marker570797	Chr3	21725623	21725920	103.422
Marker546457	Chr3	22018620	22018904	104.402
Marker480039	Chr3	22125740	22126025	104.729
Marker607262	Chr3	22126795	22127070	105.056
Marker666034	Chr3	22295974	22296276	105.383
Marker505432	Chr3	22320138	22320432	106.036
Marker647434	Chr3	22772782	22773028	107.017

Create PDF with GO2PDF for free, if you wish to

Marker601502	Chr3	22857825	22858130	107.344
Marker608952	Chr3	22890507	22890758	108.324
Marker598057	Chr3	23243225	23243469	108.978
Marker525428	Chr3	23537654	23537911	109.958
Marker596591	Chr3	23542391	23542679	110.285
Marker474374	Chr3	23542708	23542997	110.939
Marker622452	Chr3	23772516	23772770	111.265
Marker572333	Chr3	23801676	23801970	112.573
Marker615712	Chr3	24230222	24230463	112.9
Marker651103	Chr3	24212299	24212564	112.9
Marker616317	Chr3	24283147	24283411	113.227
Marker641536	Chr3	24376706	24376993	113.553
Marker644563	Chr3	24635919	24636187	113.553
Marker582056	Chr3	24798485	24798758	113.88
Marker509256	Chr3	24969328	24969631	114.207
Marker532147	Chr3	24991969	24992252	114.536
Marker660916	Chr3	25057556	25057835	114.863
Marker661197	Chr3	24994739	24995010	114.863
Marker568490	Chr3	25139159	25139444	115.19
Marker640351	Chr3	25156942	25157205	115.19
Marker541042	Chr3	25200041	25200299	115.516
Marker539528	Chr3	25243625	25243911	115.843
Marker503126	Chr3	25303516	25303796	116.17
Marker582197	Chr3	25369432	25369716	117.15
Marker648932	Chr3	25487399	25487688	117.804
Marker481853	Chr3	25577984	25578236	118.785
Marker469939	Chr3	25605559	25605864	119.438
Marker563008	Chr3	25821204	25821518	119.765
Marker502910	Chr3	26014045	26014313	120.092
Marker646395	Chr3	26099923	26100197	120.419
Marker468306	Chr3	26167074	26167384	120.745
Marker486720	Chr3	26105728	26106018	120.745
Marker533223	Chr3	26235434	26235744	121.399
Marker588496	Chr3	26323394	26323677	122.053
Marker472756	Chr3	26378366	26378644	122.38
Marker568273	Chr3	26384389	26384651	122.706
Marker488542	Chr3	26742692	26742990	123.36
Marker608040	Chr3	26764174	26764435	123.82
Marker624332	Chr3	26757113	26757424	123.82
Marker519948	Chr3	26769324	26769640	124.149
Marker657659	Chr3	27079908	27080215	124.802
Marker501246	Chr3	27114707	27115010	125.129
Marker465562	Chr3	27118753	27119071	125.456
Marker572516	Chr3	27132787	27133043	125.783
Marker600518	Chr3	27162836	27163132	126.109
Marker607363	Chr3	27156873	27157134	126.109
Marker637432	Chr3	27145397	27145690	126.109
Marker641490	Chr3	27248285	27248562	126.436
Marker489943	Chr3	27270676	27270950	126.763
Marker547611	Chr3	27264465	27264781	126.763
Marker616226	Chr3	27263447	27263740	126.763
Marker499631	Chr3	27372557	27372853	127.09
Marker576684	Chr3	27429282	27429586	127.417
Marker583852	Chr3	27462254	27462531	127.417

Create PDF with GO2PDF for free, if you wish to

Marker587816	Chr3	27472264	27472574	128.07
Marker649099	Chr3	27531204	27531454	128.397
Marker560488	Chr3	27601972	27602215	128.724
Marker637283	Chr3	27614954	27615217	128.724
Marker501782	Chr3	27628596	27628873	129.051
Marker619176	Chr3	27645582	27645853	129.704
Marker646096	Chr3	27716955	27717240	130.031
Marker488638	Chr3	27741879	27742147	130.358
Marker494292	Chr3	27718805	27719053	130.358
Marker612325	Chr3	27751510	27751802	130.358
Marker665023	Chr3	27948929	27949222	131.012
Marker549995	Chr3	27951438	27951684	131.665
Marker604311	Chr3	27974422	27974698	131.992
Marker514226	Chr3	27996007	27996275	132.646
Marker562720	Chr3	27996275	27996541	132.646
Marker495856	Chr3	27997581	27997880	133.299
Marker568991	Chr3	28104762	28105051	133.953
Marker597071	Chr3	28024538	28024811	133.953
Marker510802	Chr3	28197059	28197354	134.607
Marker521131	Chr3	28346919	28347173	134.607
Marker559541	Chr3	28404235	28404485	134.933
Marker463935	Chr3	28501028	28501311	135.914
Marker594644	Chr3	28547688	28547942	136.567
Marker473697	Chr3	28780889	28781179	137.548
Marker647268	Chr3	28943230	28943499	138.528
Marker655093	Chr3	28953855	28954131	139.182
Marker469720	Chr3	29091943	29092239	140.49
Marker471936	Chr3	29254002	29254262	141.143
Marker603611	Chr3	29265703	29265986	141.47
Marker467835	Chr3	29277164	29277479	141.797
Marker586878	Chr3	29338555	29338826	142.777
Marker471326	Chr3	29343746	29344013	143.104
Marker585795	Chr3	29345799	29346078	145.066
Marker599312	Chr3	29366933	29367190	145.066
Marker567482	Chr3	29384089	29384347	145.393
Marker617610	Chr3	29494763	29495020	145.72
Marker496412	Chr3	29584034	29584291	146.046
Marker462748	Chr3	29652610	29652888	146.7
Marker591057	Chr3	29681990	29682268	148.989
Marker559494	Chr3	29733786	29734101	149.643
Marker541267	Chr3	29743609	29743879	150.95
Marker644836	Chr3	29777888	29778195	150.95
Marker500175	Chr3	29783096	29783346	151.931
Marker548207	Chr3	29781676	29781935	151.931
Marker467397	Chr3	29795374	29795661	152.584
Marker541694	Chr3	29807489	29807729	152.911
Marker574117	Chr3	30094869	30095128	153.238
Marker608260	Chr3	30303351	30303610	153.565
Marker524093	Chr3	30417445	30417713	153.892
Marker500044	Chr3	30427758	30428024	154.218
Marker530667	Chr3	30445763	30446034	154.545
Marker539358	Chr3	30513990	30514261	154.872
Marker561793	Chr3	30633826	30634109	154.872
Marker606557	Chr3	30583547	30583844	154.872

Create PDF with GO2PDF for free, if you wish to

Marker621930	Chr3	30569637	30569921	154.872
Marker648999	Chr3	30483911	30484212	154.872
Marker487258	Chr3	30643098	30643362	155.199
Marker567944	Chr3	30646246	30646514	155.199
Marker498763	Chr3	30652722	30653005	155.528
Marker515978	Chr3	30657414	30657669	155.528
Marker538060	Chr3	30688844	30689122	155.855
Marker569378	Chr3	30751014	30751311	156.508
Marker575607	Chr3	30796542	30796844	156.508
Marker611432	Chr3	30939324	30939585	156.837
Marker515722	Chr3	30993888	30994146	157.491
Marker556320	Chr3	30999013	30999248	157.491
Marker561048	Chr3	30983755	30984030	157.491
Marker641519	Chr3	31164850	31165136	157.491
Marker570172	Chr3	31232182	31232453	157.818
Marker591491	Chr3	31213537	31213817	157.818
Marker536810	Chr3	31242229	31242474	158.277
Marker608258	Chr3	31243422	31243681	159.585
Marker628994	Chr3	31337295	31337559	159.585
Marker492820	Chr3	31348659	31348954	159.912
Marker517095	Chr3	31350161	31350448	159.912
Marker554070	Chr3	31349191	31349489	159.912
Marker563087	Chr3	31349894	31350161	159.912
Marker578350	Chr3	31368022	31368269	160.238
Marker496735	Chr3	31413224	31413512	160.565
Marker555296	Chr3	31430265	31430522	160.565
Marker608481	Chr3	31369894	31370191	160.565
Marker639761	Chr3	31430695	31430967	160.565
Marker529413	Chr3	31460995	31461234	160.892
Marker649430	Chr3	31461279	31461543	160.892
Marker528224	Chr3	31462019	31462281	161.219
Marker580667	Chr3	31481887	31482194	161.219
Marker599688	Chr3	31484898	31485182	161.219
Marker629903	Chr3	31501945	31502240	161.219
Marker500976	Chr3	31510524	31510797	161.546
Marker475476	Chr3	31525302	31525562	162.199
Marker547319	Chr3	31637570	31637886	162.199
Marker668750	Chr3	31680423	31680709	162.199
Marker561935	Chr3	31716733	31717001	162.853
Marker616129	Chr3	31718723	31719022	163.507
Marker469102	Chr3	31732663	31732947	164.487
Marker462042	Chr3	31904122	31904406	165.141
Marker540057	Chr3	31873947	31874205	165.141
Marker559740	Chr3	32167088	32167353	165.467
Marker523145	Chr3	32321144	32321404	166.121
Marker539995	Chr3	32182988	32183228	166.121
Marker607722	Chr3	32188825	32189086	166.121
Marker637509	Chr3	32349933	32350210	166.121
Marker653522	Chr3	32351237	32351497	166.121
Marker467394	Chr3	32530472	32530763	166.775
Marker469831	Chr3	32455327	32455650	166.775
Marker547393	Chr3	32631448	32631732	166.775
Marker530311	Chr3	32652300	32652612	167.428
Marker545801	Chr3	32651988	32652252	167.428

Create PDF with GO2PDF for free, if you wish to

Marker627366	Chr3	32665430	32665739	167.888
Marker508338	Chr3	32752087	32752363	168.215
Marker474803	Chr3	32795218	32795488	168.542
Marker544133	Chr3	32780339	32780621	168.542
Marker621798	Chr3	32791057	32791329	168.542
Marker484437	Chr3	33184864	33185158	168.868
Marker643781	Chr3	33183498	33183770	168.868
Marker610420	Chr3	33192575	33192872	169.195
Marker524307	Chr3	33229333	33229627	169.849
Marker573723	Chr3	33389745	33390034	169.849
Marker635205	Chr3	33389229	33389534	169.849
Marker639704	Chr3	33392722	33392986	169.849
Marker598064	Chr3	33421411	33421659	170.176
Marker653248	Chr3	33421134	33421411	170.176
Marker575257	Chr3	33428934	33429201	170.829
Marker614301	Chr3	33464246	33464519	170.829
Marker500988	Chr3	33683384	33683641	171.156
Marker529842	Chr3	33555260	33555572	171.156
Marker467328	Chr3	33692855	33693115	171.81
Marker563120	Chr3	33700066	33700353	171.81
Marker622723	Chr3	33713827	33714075	171.81
Marker483092	Chr3	33727074	33727368	172.463
Marker493115	Chr3	33810122	33810419	172.79
Marker516646	Chr3	33857052	33857330	172.79
Marker523560	Chr3	33805724	33806007	172.79
Marker584592	Chr3	33901469	33901759	172.79
Marker545182	Chr3	33908911	33909163	173.771
Marker474904	Chr3	33941649	33941903	174.097
Marker567290	Chr3	33920236	33920538	174.097
Marker613118	Chr3	33945550	33945798	174.097
Marker570073	Chr3	33972064	33972351	174.424
Marker469128	Chr3	34021549	34021857	174.751
Marker637791	Chr3	34001719	34001990	174.751
Marker622564	Chr3	34039366	34039630	175.078
Marker510951	Chr3	34112947	34113239	175.732
Marker523763	Chr3	34101048	34101314	175.732
Marker586703	Chr3	34080946	34081272	175.732
Marker562210	Chr3	34120219	34120509	176.058
Marker475834	Chr3	34121627	34121905	176.385
Marker553910	Chr3	34126024	34126272	176.385
Marker487940	Chr3	34215946	34216238	177.693
Marker526334	Chr3	34248464	34248728	177.693
Marker532061	Chr3	34127967	34128226	177.693
Marker568090	Chr3	34256393	34256693	177.693
Marker600016	Chr3	34198160	34198419	177.693
Marker628988	Chr3	34325132	34325429	177.693
Marker488718	Chr3	34354968	34355237	178.019
Marker602924	Chr3	34371845	34372085	178.019
Marker597166	Chr3	34389477	34389783	178.346
Marker620644	Chr3	34372085	34372396	178.346
Marker475627	Chr3	34397078	34397357	178.673
Marker618214	Chr3	34399215	34399504	179.327
Marker554972	Chr3	34406163	34406431	179.653
Marker630326	Chr3	34404885	34405138	179.653

Create PDF with GO2PDF for free, if you wish to

Marker635075	Chr3	34399764	34400020	179.653
Marker538133	Chr3	34537672	34537950	179.98
Marker548945	Chr3	34499841	34500115	179.98
Marker577846	Chr3	34454832	34455143	179.98
Marker657231	Chr3	34407216	34407517	179.98
Marker659904	Chr3	34561439	34561753	179.98
Marker666166	Chr3	34518082	34518338	179.98
Marker481579	Chr3	34580800	34581047	180.634
Marker480875	Chr3	34653885	34654176	180.961
Marker667459	Chr3	34675423	34675669	180.961
Marker538988	Chr3	34680364	34680639	181.288
Marker669849	Chr3	34677900	34678193	181.288
Marker484086	Chr3	34756117	34756433	181.614
Marker469899	Chr3	34759715	34759980	182.595
Marker496483	Chr3	34812529	34812827	182.922
Marker629937	Chr3	34818514	34818778	182.922
Marker564514	Chr3	34868579	34868853	183.248
Marker666780	Chr3	34834809	34835077	183.248
Marker507292	Chr3	34922937	34923188	183.575
Marker535635	Chr3	35018825	35019116	183.575
Marker609529	Chr3	34884100	34884361	183.575
Marker609817	Chr3	34972571	34972834	183.575
Marker469731	Chr3	35048902	35049209	183.902
Marker527100	Chr3	35051121	35051408	184.556
Marker662447	Chr3	35075033	35075291	185.536
Marker507486	Chr3	35092964	35093255	186.19
Marker605521	Chr3	35110444	35110728	186.517
Marker531029	Chr3	35111353	35111658	187.17
Marker665331	Chr3	35127654	35127913	187.497
Marker591355	Chr3	35172937	35173193	187.824
Marker518645	Chr3	35528525	35528812	188.151
Marker529918	Chr3	35533388	35533654	188.151
Marker537365	Chr3	35374006	35374297	188.151
Marker576004	Chr3	35323408	35323680	188.151
Marker645938	Chr3	35196707	35196996	188.151
Marker585484	Chr3	35630386	35630657	188.477
Marker595412	Chr3	35620393	35620655	188.477
Marker647047	Chr3	35627663	35627926	188.477
Marker477622	Chr3	35675072	35675343	189.131
Marker621666	Chr3	35664518	35664811	189.131
Marker563110	Chr3	35676892	35677168	189.591
Marker646840	Chr3	35706490	35706799	189.591
Marker472427	Chr3	35803563	35803862	189.918
Marker511940	Chr3	35786119	35786389	189.918
Marker566930	Chr3	35807798	35808064	189.918
Marker645205	Chr3	35808734	35809006	189.918
Marker646410	Chr3	35812586	35812880	190.571
Marker489878	Chr3	35845380	35845629	190.898
Marker596193	Chr3	35862610	35862904	190.898
Marker618738	Chr3	36032504	36032747	190.898
Marker482420	Chr3	36065580	36065892	191.225
Marker583204	Chr3	36058116	36058426	191.225
Marker510562	Chr3	36092320	36092570	191.878
Marker560135	Chr3	36096060	36096320	192.205

Create PDF with GO2PDF for free, if you wish to

Marker632582	Chr3	36104481	36104773	192.205
Marker642491	Chr3	36432198	36432455	192.205
Marker644003	Chr3	36438550	36438843	192.534
Marker652066	Chr3	36438267	36438550	192.534
Marker471870	Chr3	36467196	36467490	193.515
Marker520039	Chr3	36446123	36446411	193.515
Marker592420	Chr3	36515730	36515971	194.168
Marker579342	Chr3	36525005	36525299	194.495
Marker508178	Chr3	36542891	36543157	195.149
Marker570325	Chr3	36538212	36538505	195.149
Marker558477	Chr3	36551068	36551336	195.802
Marker575770	Chr3	36602326	36602577	195.802
Marker593205	Chr3	36668008	36668271	196.456
Marker563164	Chr3	36702809	36703091	197.11
Marker646407	Chr3	36711354	36711629	197.11
Marker669588	Chr3	36710088	36710341	197.11
Marker475426	Chr3	36742841	36743082	197.436
Marker581311	Chr3	36740973	36741245	197.436
Marker475640	Chr3	36743082	36743359	197.763
Marker633918	Chr3	36812800	36813091	198.744
Marker486515	Chr3	36949802	36950045	199.397
Marker524719	Chr3	36982925	36983179	199.724
Marker481167	Chr3	37086986	37087309	200.051
Marker594509	Chr3	37105837	37106112	200.378
Marker579929	Chr3	37202109	37202353	200.705
Marker527832	Chr3	37243488	37243749	201.031
Marker509971	Chr3	37246863	37247109	202.012
Marker533698	Chr3	37333499	37333750	202.012
Marker594418	Chr3	37344628	37344883	202.339
Marker487609	Chr3	37362891	37363162	202.666
Marker584642	Chr3	37500116	37500385	202.666
Marker584206	Chr3	37502231	37502537	203.319
Marker550134	Chr3	37550779	37551057	203.646
Marker560554	Chr3	37837905	37838198	203.973
Marker587880	Chr3	37866668	37866962	204.3
Marker518023	Chr3	37866962	37867249	204.626
Marker627448	Chr3	37871661	37871975	204.626
Marker495734	Chr3	37878823	37879112	204.953
Marker580760	Chr3	37874459	37874758	204.953
Marker534880	Chr3	37882660	37882916	205.934
Marker643000	Chr3	38198452	38198719	206.261
Marker467020	Chr3	38366652	38366932	206.914
Marker475386	Chr3	38350805	38351063	206.914
Marker492316	Chr3	38572141	38572426	207.241
Marker657555	Chr3	38586291	38586538	207.241
Marker462243	Chr3	38732190	38732436	207.568
Marker621508	Chr3	38625773	38626051	207.568
Marker639956	Chr3	38733863	38734110	207.568
Marker661381	Chr3	38620323	38620598	207.568
Marker541489	Chr3	38741671	38741955	208.221
Marker469434	Chr3	38755363	38755647	208.548
Marker553874	Chr3	38769784	38770062	208.548
Marker635432	Chr3	38768388	38768652	208.548
Marker463038	Chr3	38772806	38773087	209.202

Create PDF with GO2PDF for free, if you wish to

Marker477183	Chr3	38883787	38884043	209.202
Marker520519	Chr3	38931539	38931815	209.202
Marker540931	Chr3	39009128	39009417	209.202
Marker591571	Chr3	38930359	38930609	209.202
Marker598416	Chr3	38844811	38845096	209.202
Marker633407	Chr3	38772325	38772586	209.202
Marker645957	Chr3	38989649	38989923	209.202
Marker566944	Chr3	39012450	39012718	209.855
Marker484381	Chr3	39113812	39114093	210.182
Marker524981	Chr3	39018277	39018538	210.182
Marker536247	Chr3	39014414	39014719	210.182
Marker610758	Chr3	39074966	39075283	210.182
Marker511002	Chr3	39120303	39120550	210.836
Marker562962	Chr3	39118866	39119126	210.836
Marker606636	Chr3	39119314	39119569	210.836
Marker468180	Chr3	39244389	39244688	211.163
Marker512118	Chr3	39295256	39295542	211.489
Marker659576	Chr3	39287004	39287286	211.489
Marker579731	Chr3	39310544	39310816	211.816
Marker488627	Chr3	39607232	39607518	212.143
Marker510748	Chr3	39496479	39496726	212.143
Marker511188	Chr3	39323113	39323411	212.143
Marker518033	Chr3	39586612	39586891	212.143
Marker519569	Chr3	39753004	39753299	212.143
Marker531800	Chr3	39389007	39389304	212.143
Marker542398	Chr3	39350336	39350624	212.143
Marker581709	Chr3	39679024	39679274	212.143
Marker604284	Chr3	39740366	39740656	212.143
Marker639000	Chr3	39386803	39387101	212.143
Marker649971	Chr3	39614354	39614598	212.143
Marker20552	Chr4	23091633	23091894	0
Marker31803	Chr4	22892563	22892877	0
Marker38619	Chr4	23400650	23400925	0
Marker51406	Chr4	22913776	22914022	0
Marker60959	Chr4	23332824	23333122	0
Marker72525	Chr4	23398610	23398859	0
Marker85206	Chr4	23318573	23318857	0
Marker99245	Chr4	22919361	22919667	0
Marker120766	Chr4	23120934	23121220	0
Marker126685	Chr4	23056845	23057115	0
Marker126837	Chr4	23097044	23097344	0
Marker124553	Chr4	22891049	22891330	0.654
Marker91867	Chr4	22819726	22820003	1.307
Marker39046	Chr4	22700304	22700564	1.961
Marker61597	Chr4	22689171	22689434	2.615
Marker72295	Chr4	22687663	22687937	2.941
Marker13242	Chr4	22648999	22649266	3.268
Marker26389	Chr4	22657562	22657832	3.268
Marker55242	Chr4	22330029	22330354	4.382
Marker98438	Chr4	22490773	22491063	4.382
Marker106789	Chr4	22223933	22224213	4.382
Marker74771	Chr4	21893999	21894303	5.035
Marker87739	Chr4	21856633	21856942	5.035
Marker56931	Chr4	21826459	21826774	5.689

Create PDF with GO2PDF for free, if you wish to

Marker82292	Chr4	21814304	21814589	6.016
Marker13326	Chr4	21805238	21805522	6.342
Marker8165	Chr4	21639998	21640253	6.669
Marker19633	Chr4	21623635	21623933	6.669
Marker7294	Chr4	21621667	21621931	6.996
Marker114255	Chr4	21598793	21599058	7.323
Marker54856	Chr4	21341557	21341870	7.65
Marker20562	Chr4	21249642	21249928	7.976
Marker79552	Chr4	21191815	21192141	7.976
Marker84320	Chr4	21190086	21190334	7.976
Marker110834	Chr4	21180321	21180580	8.303
Marker93533	Chr4	21146552	21146799	8.63
Marker57308	Chr4	21067419	21067691	8.957
Marker96287	Chr4	21085883	21086164	8.957
Marker94212	Chr4	21064190	21064476	9.284
Marker109212	Chr4	21035954	21036255	9.61
Marker22683	Chr4	20988529	20988772	9.937
Marker113401	Chr4	20987075	20987336	9.937
Marker14084	Chr4	20870732	20871015	10.264
Marker49019	Chr4	20870005	20870252	10.264
Marker20640	Chr4	20868639	20868878	10.591
Marker48194	Chr4	20851689	20851976	10.918
Marker16158	Chr4	20803593	20803866	11.244
Marker24415	Chr4	20805070	20805331	11.244
Marker24745	Chr4	20814837	20815125	11.244
Marker15171	Chr4	20779520	20779813	11.704
Marker18424	Chr4	20794649	20794936	11.704
Marker45074	Chr4	20758934	20759205	11.704
Marker27982	Chr4	20637512	20637820	12.031
Marker48930	Chr4	20456727	20456975	12.031
Marker94312	Chr4	20611340	20611595	12.031
Marker38717	Chr4	20454830	20455110	12.358
Marker92600	Chr4	20385696	20385960	12.358
Marker111975	Chr4	20362621	20362861	12.685
Marker45484	Chr4	20311294	20311551	13.471
Marker70097	Chr4	20124152	20124404	13.471
Marker116274	Chr4	20049031	20049317	13.798
Marker72110	Chr4	20047621	20047871	14.125
Marker85526	Chr4	20043502	20043776	14.451
Marker43843	Chr4	19904507	19904807	15.432
Marker26265	Chr4	19885895	19886154	16.219
Marker50847	Chr4	19883046	19883311	17.199
Marker67607	Chr4	19683066	19683362	17.199
Marker85020	Chr4	19479393	19479639	17.199
Marker111659	Chr4	19459256	19459513	17.526
Marker56917	Chr4	19446959	19447243	17.853
Marker79028	Chr4	19383497	19383749	18.506
Marker10491	Chr4	19301888	19302177	19.16
Marker93909	Chr4	19315683	19315985	19.16
Marker24691	Chr4	19292379	19292691	19.487
Marker118052	Chr4	19291789	19292086	19.487
Marker10815	Chr4	19271719	19271998	20.14
Marker76165	Chr4	19291143	19291426	20.14
Marker85899	Chr4	19245362	19245657	20.14

Create PDF with GO2PDF for free, if you wish to

Marker107254	Chr4	19254315	19254604	20.14
Marker124955	Chr4	19210365	19210648	20.14
Marker74354	Chr4	19147408	19147714	20.467
Marker44411	Chr4	18999926	19000238	20.794
Marker71461	Chr4	19138179	19138448	20.794
Marker93674	Chr4	18988743	18989045	20.794
Marker95464	Chr4	18970771	18971047	21.121
Marker42259	Chr4	18851619	18851869	21.448
Marker79371	Chr4	18665928	18666178	22.101
Marker83525	Chr4	18847855	18848113	22.101
Marker85295	Chr4	18658346	18658603	22.428
Marker89858	Chr4	18622911	18623174	22.428
Marker120354	Chr4	18622149	18622424	22.428
Marker6611	Chr4	18579121	18579389	22.755
Marker22068	Chr4	18584158	18584477	22.755
Marker49202	Chr4	18603339	18603592	22.755
Marker49820	Chr4	18580699	18580945	22.755
Marker70216	Chr4	18447478	18447748	23.082
Marker112948	Chr4	18506865	18507119	23.082
Marker120713	Chr4	18518177	18518435	23.082
Marker61010	Chr4	18325421	18325712	23.735
Marker64118	Chr4	18447202	18447478	23.735
Marker90588	Chr4	18319472	18319774	24.062
Marker97712	Chr4	18325088	18325398	24.062
Marker119551	Chr4	18305204	18305494	24.062
Marker129475	Chr4	18317939	18318193	24.062
Marker30833	Chr4	18256583	18256829	24.389
Marker112853	Chr4	18298293	18298546	24.389
Marker8083	Chr4	18232367	18232652	24.716
Marker10414	Chr4	18117656	18117911	24.716
Marker72798	Chr4	18156793	18157067	24.716
Marker78609	Chr4	18157491	18157777	24.716
Marker87842	Chr4	18204542	18204805	24.716
Marker118751	Chr4	18096765	18097033	25.042
Marker13979	Chr4	18080608	18080875	25.696
Marker117183	Chr4	18061199	18061520	26.35
Marker45298	Chr4	18028173	18028473	26.677
Marker66303	Chr4	18060935	18061199	26.677
Marker5704	Chr4	17528152	17528439	27.33
Marker56294	Chr4	17612752	17613049	27.33
Marker70626	Chr4	18020053	18020355	27.33
Marker83770	Chr4	17935326	17935623	27.33
Marker116003	Chr4	17987968	17988244	27.33
Marker116100	Chr4	17405092	17405383	27.33
Marker123378	Chr4	17582345	17582612	27.33
Marker18645	Chr4	17336143	17336406	27.984
Marker73671	Chr4	17223038	17223305	28.77
Marker119618	Chr4	17249464	17249769	28.77
Marker125459	Chr4	17203270	17203523	28.77
Marker11510	Chr4	17198720	17198993	29.097
Marker30762	Chr4	17149838	17150085	29.097
Marker105850	Chr4	17045616	17045883	29.097
Marker115048	Chr4	17092664	17092931	29.097
Marker56001	Chr4	17035890	17036145	29.424

Create PDF with GO2PDF for free, if you wish to

Marker73145	Chr4	16762070	16762326	30.078
Marker74018	Chr4	16708885	16709175	30.078
Marker89886	Chr4	16993303	16993579	30.078
Marker93740	Chr4	16678907	16679159	30.078
Marker48929	Chr4	16648637	16648908	30.404
Marker66302	Chr4	16632771	16633067	30.404
Marker37014	Chr4	16629458	16629771	31.712
Marker60989	Chr4	16623950	16624226	31.712
Marker109592	Chr4	16622132	16622409	31.712
Marker79388	Chr4	16580467	16580743	32.039
Marker80529	Chr4	16555284	16555567	32.692
Marker21074	Chr4	16529753	16530035	33.019
Marker30896	Chr4	16551036	16551320	33.019
Marker56699	Chr4	16506448	16506740	33.019
Marker85695	Chr4	16555005	16555284	33.019
Marker119820	Chr4	16506068	16506354	33.346
Marker52338	Chr4	16493119	16493402	33.673
Marker68666	Chr4	16456526	16456812	33.673
Marker84567	Chr4	16488184	16488442	33.673
Marker91594	Chr4	16440524	16440796	33.673
Marker106971	Chr4	16429539	16429793	33.673
Marker54098	Chr4	16333592	16333850	34.326
Marker68063	Chr4	16269882	16270176	35.307
Marker79463	Chr4	16270359	16270610	35.307
Marker97590	Chr4	16321321	16321581	35.307
Marker48653	Chr4	16264106	16264352	36.093
Marker3285	Chr4	16234951	16235239	36.42
Marker31740	Chr4	16207663	16207932	36.747
Marker63740	Chr4	16040972	16041231	37.074
Marker108160	Chr4	16193393	16193682	37.074
Marker115526	Chr4	15788371	15788632	37.727
Marker9754	Chr4	15756332	15756610	38.056
Marker55043	Chr4	15752532	15752810	38.056
Marker60722	Chr4	15671082	15671359	38.383
Marker96054	Chr4	15549909	15550165	38.71
Marker97106	Chr4	15502314	15502628	39.037
Marker27562	Chr4	15429049	15429305	39.364
Marker13650	Chr4	15413516	15413857	39.69
Marker90681	Chr4	15130891	15131183	40.017
Marker95765	Chr4	15120113	15120387	41.652
Marker13764	Chr4	15110019	15110324	42.305
Marker115981	Chr4	15079979	15080249	42.959
Marker116562	Chr4	14978818	14979113	43.286
Marker56919	Chr4	14971413	14971673	44.266
Marker72078	Chr4	14862653	14862912	44.266
Marker104999	Chr4	14827625	14827939	44.266
Marker49131	Chr4	14703828	14704146	44.726
Marker70639	Chr4	14682641	14682955	45.38
Marker111776	Chr4	14679004	14679288	45.38
Marker97662	Chr4	14260954	14261227	46.033
Marker106706	Chr4	14404243	14404513	46.033
Marker63448	Chr4	14229828	14230075	46.687
Marker101999	Chr4	14151838	14152105	47.014
Marker88985	Chr4	14138116	14138404	47.667

Create PDF with GO2PDF for free, if you wish to

Marker39931	Chr4	14137176	14137423	47.994
Marker11011	Chr4	14090481	14090748	48.321
Marker62496	Chr4	14066762	14067051	48.648
Marker83400	Chr4	14051906	14052173	48.648
Marker71378	Chr4	14046302	14046576	48.975
Marker8123	Chr4	14004933	14005234	49.301
Marker112112	Chr4	13991091	13991422	49.301
Marker73273	Chr4	13852113	13852392	49.628
Marker76385	Chr4	13833037	13833329	49.955
Marker58638	Chr4	13828630	13828933	50.282
Marker114460	Chr4	13814143	13814432	50.609
Marker55523	Chr4	13706854	13707114	50.935
Marker4282	Chr4	13606472	13606724	51.722
Marker125099	Chr4	13625246	13625490	51.722
Marker53990	Chr4	13545076	13545355	52.049
Marker12313	Chr4	13445890	13446162	52.375
Marker17423	Chr4	13506131	13506403	52.375
Marker105793	Chr4	13425836	13426130	52.375
Marker38062	Chr4	13333829	13334098	53.029
Marker33791	Chr4	13258140	13258448	53.356
Marker46113	Chr4	13261598	13261886	53.356
Marker73855	Chr4	13249376	13249650	53.683
Marker24279	Chr4	13135537	13135803	54.01
Marker103074	Chr4	13127325	13127575	54.01
Marker32525	Chr4	13097172	13097461	54.99
Marker23361	Chr4	13093946	13094201	55.317
Marker73728	Chr4	13080255	13080542	55.97
Marker13417	Chr4	12884571	12884865	56.624
Marker101271	Chr4	13072316	13072600	56.624
Marker13842	Chr4	12835440	12835735	58.586
Marker17503	Chr4	12841959	12842222	58.586
Marker73031	Chr4	12829038	12829303	59.893
Marker34185	Chr4	12822605	12822884	60.547
Marker49608	Chr4	12798356	12798600	60.874
Marker58122	Chr4	12815456	12815754	60.874
Marker59598	Chr4	12817376	12817663	60.874
Marker61945	Chr4	12795775	12796037	61.201
Marker99802	Chr4	12795508	12795775	61.527
Marker85989	Chr4	12760677	12760976	61.854
Marker65404	Chr4	12716499	12716755	62.508
Marker57386	Chr4	12704385	12704659	62.835
Marker113531	Chr4	12697070	12697364	63.161
Marker26363	Chr4	12683538	12683836	63.815
Marker38800	Chr4	12671899	12672168	63.815
Marker39728	Chr4	12631774	12632056	64.142
Marker61746	Chr4	12557654	12557912	64.796
Marker77152	Chr4	12616525	12616836	64.796
Marker110677	Chr4	12552049	12552350	65.449
Marker82290	Chr4	12507292	12507569	65.776
Marker120883	Chr4	12491014	12491280	66.103
Marker4017	Chr4	12468498	12468806	66.432
Marker101419	Chr4	12393417	12393676	66.432
Marker121640	Chr4	12316864	12317122	66.432
Marker33207	Chr4	12222610	12222925	67.085

Create PDF with GO2PDF for free, if you wish to

Marker52515	Chr4	12191731	12191988	67.739
Marker128890	Chr4	12160115	12160364	67.739
Marker5942	Chr4	12015499	12015790	68.066
Marker39553	Chr4	11946833	11947109	68.393
Marker97794	Chr4	11947613	11947895	68.393
Marker113622	Chr4	11929149	11929432	69.046
Marker118567	Chr4	11907634	11907931	69.7
Marker124221	Chr4	11875655	11875929	70.027
Marker64666	Chr4	11873025	11873266	70.353
Marker25820	Chr4	11811303	11811583	70.68
Marker78656	Chr4	11856146	11856408	70.68
Marker74393	Chr4	11776535	11776836	71.988
Marker7439	Chr4	11752992	11753242	72.315
Marker128991	Chr4	11741242	11741543	72.315
Marker43570	Chr4	11704703	11705017	72.641
Marker64188	Chr4	11653402	11653686	72.641
Marker76149	Chr4	11702188	11702438	72.641
Marker40288	Chr4	11649704	11649999	72.968
Marker78758	Chr4	11631525	11631841	73.295
Marker100685	Chr4	11628834	11629092	73.622
Marker7905	Chr4	11628391	11628652	73.949
Marker58493	Chr4	11601588	11601848	73.949
Marker68731	Chr4	11623003	11623301	73.949
Marker78702	Chr4	11583191	11583486	74.602
Marker116475	Chr4	11539124	11539414	74.929
Marker26415	Chr4	11537049	11537346	75.256
Marker17099	Chr4	11525909	11526157	75.583
Marker75301	Chr4	11493483	11493764	76.563
Marker103351	Chr4	11418873	11419176	76.563
Marker77657	Chr4	11408174	11408433	76.89
Marker54630	Chr4	11394745	11395030	77.87
Marker13330	Chr4	11392335	11392598	78.197
Marker118103	Chr4	11312518	11312808	78.197
Marker55063	Chr4	11199717	11200009	78.524
Marker67953	Chr4	11294914	11295172	78.524
Marker83863	Chr4	11200661	11200920	78.524
Marker110882	Chr4	11203001	11203279	78.524
Marker97342	Chr4	11033471	11033770	78.851
Marker83705	Chr4	10996410	10996680	79.504
Marker127087	Chr4	11007837	11008110	79.504
Marker5195	Chr4	10887045	10887369	80.158
Marker78771	Chr4	10886024	10886303	80.485
Marker32628	Chr4	10878284	10878533	81.139
Marker19658	Chr4	10851050	10851317	81.465
Marker79982	Chr4	10846815	10847130	81.465
Marker45093	Chr4	10845948	10846209	81.792
Marker53941	Chr4	10783468	10783776	82.119
Marker77899	Chr4	10768186	10768477	82.773
Marker80836	Chr4	10727505	10727809	83.099
Marker45070	Chr4	10696173	10696449	83.426
Marker87930	Chr4	10520595	10520895	83.426
Marker40343	Chr4	10507154	10507435	83.753
Marker93885	Chr4	10042986	10043267	84.407
Marker105396	Chr4	10067380	10067657	84.407

Create PDF with GO2PDF for free, if you wish to

Marker86669	Chr4	10028136	10028404	84.733
Marker60423	Chr4	9978029	9978336	85.06
Marker78315	Chr4	9984369	9984629	85.06
Marker29116	Chr4	9760099	9760383	85.714
Marker102009	Chr4	9828071	9828364	85.714
Marker48805	Chr4	9755393	9755696	86.041
Marker108079	Chr4	9706145	9706418	86.694
Marker24695	Chr4	9680016	9680277	87.348
Marker89445	Chr4	9698332	9698645	87.348
Marker22790	Chr4	9647564	9647867	87.675
Marker126948	Chr4	9669547	9669785	87.675
Marker28424	Chr4	9629850	9630086	88.328
Marker100836	Chr4	8927029	8927276	88.982
Marker93858	Chr4	8926750	8927029	89.962
Marker16151	Chr4	8919176	8919492	90.289
Marker127422	Chr4	8809120	8809423	90.943
Marker55977	Chr4	8771502	8771812	91.923
Marker28924	Chr4	8705424	8705699	92.25
Marker46197	Chr4	8676658	8676920	92.577
Marker34548	Chr4	8590402	8590678	93.231
Marker56865	Chr4	8412083	8412374	93.557
Marker83816	Chr4	8399685	8399984	93.884
Marker52675	Chr4	8390930	8391179	94.211
Marker116923	Chr4	8379694	8379979	94.211
Marker71094	Chr4	8294943	8295208	94.538
Marker71244	Chr4	8158347	8158607	94.867
Marker97615	Chr4	8155578	8155823	95.52
Marker97211	Chr4	8151507	8151750	95.847
Marker19684	Chr4	8112205	8112464	96.174
Marker15147	Chr4	7981481	7981743	97.482
Marker33376	Chr4	8016789	8017090	97.482
Marker85194	Chr4	8051537	8051801	97.482
Marker41805	Chr4	7968165	7968441	97.808
Marker123749	Chr4	7958657	7958936	97.808
Marker10958	Chr4	7949875	7950121	98.135
Marker20723	Chr4	7935978	7936243	98.462
Marker18683	Chr4	7909848	7910143	99.442
Marker46302	Chr4	7926761	7927025	99.442
Marker56960	Chr4	7922256	7922539	99.442
Marker102614	Chr4	7807726	7808034	99.442
Marker75987	Chr4	7697487	7697778	99.769
Marker87798	Chr4	7773099	7773361	99.769
Marker108661	Chr4	7604713	7605019	100.098
Marker120304	Chr4	7609330	7609591	100.098
Marker24214	Chr4	7556132	7556398	100.752
Marker123715	Chr4	7563761	7564013	100.752
Marker83654	Chr4	7535547	7535853	101.732
Marker89860	Chr4	7519776	7520092	101.732
Marker105743	Chr4	7542500	7542769	101.732
Marker6518	Chr4	7403247	7403489	102.386
Marker79564	Chr4	7509611	7509882	102.386
Marker96625	Chr4	7398196	7398503	102.713
Marker16548	Chr4	7324242	7324510	103.693
Marker54247	Chr4	7319771	7320063	103.693

Create PDF with GO2PDF for free, if you wish to

Marker65056	Chr4	7359225	7359498	103.693
Marker95535	Chr4	7293415	7293689	104.02
Marker43411	Chr4	7281254	7281504	104.349
Marker45220	Chr4	7176201	7176468	105.003
Marker122022	Chr4	7259630	7259897	105.003
Marker57282	Chr4	7169675	7169931	105.33
Marker90479	Chr4	7168137	7168434	105.983
Marker53505	Chr4	7167885	7168137	106.31
Marker101500	Chr4	7007073	7007315	106.964
Marker60345	Chr4	7006649	7007073	107.617
Marker69461	Chr4	6887416	6887684	107.944
Marker27820	Chr4	6820386	6820664	108.598
Marker121748	Chr4	6820136	6820386	109.251
Marker98294	Chr4	6779703	6780012	109.58
Marker126428	Chr4	6674753	6675003	110.234
Marker86388	Chr4	6637528	6637775	110.561
Marker128013	Chr4	6626885	6627142	110.887
Marker7782	Chr4	6619324	6619597	111.214
Marker85749	Chr4	6607787	6608050	111.868
Marker18681	Chr4	6508408	6508702	112.522
Marker76253	Chr4	6440771	6441053	113.175
Marker122945	Chr4	6371501	6371769	114.156
Marker94949	Chr4	6297176	6297474	114.809
Marker104898	Chr4	6321788	6322051	114.809
Marker54662	Chr4	6285683	6285940	115.136
Marker11362	Chr4	6254109	6254374	115.463
Marker22455	Chr4	6252110	6252421	115.463
Marker97819	Chr4	6207421	6207675	116.117
Marker118476	Chr4	6236882	6237192	116.117
Marker109282	Chr4	6192418	6192681	116.443
Marker27416	Chr4	6188877	6189180	117.097
Marker106267	Chr4	6165310	6165581	117.097
Marker70541	Chr4	6116928	6117217	117.424
Marker71093	Chr4	6109484	6109770	117.424
Marker18306	Chr4	6071458	6071775	117.751
Marker84888	Chr4	6082717	6082973	117.751
Marker119299	Chr4	6069896	6070160	118.077
Marker51550	Chr4	6067430	6067718	118.404
Marker15874	Chr4	6046289	6046544	119.058
Marker35761	Chr4	6059115	6059409	119.058
Marker79842	Chr4	6056883	6057199	119.058
Marker123090	Chr4	6004487	6004741	119.058
Marker123923	Chr4	5982553	5982877	119.058
Marker31602	Chr4	5955952	5956198	119.385
Marker79121	Chr4	5842881	5843135	120.365
Marker129505	Chr4	5790901	5791214	120.692
Marker61303	Chr4	5671423	5671688	121.999
Marker99342	Chr4	5789720	5790001	121.999
Marker77061	Chr4	5499870	5500131	122.328
Marker47870	Chr4	5433056	5433313	122.655
Marker124132	Chr4	5434781	5435028	122.655
Marker127316	Chr4	5429275	5429553	122.982
Marker57114	Chr4	5425893	5426202	123.309
Marker127404	Chr4	5427224	5427473	123.309

Create PDF with GO2PDF for free, if you wish to

Marker68878	Chr4	5424968	5425231	123.636
Marker19259	Chr4	5412301	5412620	123.962
Marker49009	Chr4	5382950	5383274	125.27
Marker33896	Chr4	5350388	5350657	125.597
Marker72609	Chr4	5356865	5357184	125.597
Marker77051	Chr4	5371147	5371395	125.597
Marker88103	Chr4	5327562	5327821	125.923
Marker27377	Chr4	5316840	5317146	126.25
Marker31765	Chr4	5261149	5261415	126.904
Marker20644	Chr4	5251838	5252152	127.231
Marker81733	Chr4	5064110	5064360	127.558
Marker35963	Chr4	4972997	4973315	127.884
Marker44211	Chr4	4899076	4899350	127.884
Marker49726	Chr4	4979197	4979496	127.884
Marker96295	Chr4	5036510	5036759	127.884
Marker6481	Chr4	4840954	4841214	128.211
Marker48024	Chr4	4874005	4874331	128.211
Marker28855	Chr4	4794764	4795019	128.865
Marker105524	Chr4	4716777	4717072	128.865
Marker63965	Chr4	4712038	4712282	129.192
Marker15346	Chr4	4645145	4645395	130.172
Marker61156	Chr4	4656523	4656791	130.172
Marker36295	Chr4	4636089	4636385	130.499
Marker76099	Chr4	4631908	4632198	131.153
Marker100438	Chr4	4627183	4627458	131.479
Marker27176	Chr4	4590879	4591169	131.806
Marker92779	Chr4	4495919	4496239	132.133
Marker125079	Chr4	4489824	4490100	132.787
Marker50323	Chr4	4447977	4448240	133.113
Marker127794	Chr4	4443517	4443814	134.748
Marker126855	Chr4	4410740	4411053	135.208
Marker7005	Chr4	4271212	4271455	135.861
Marker64707	Chr4	4270733	4270989	136.188
Marker20138	Chr4	4186883	4187128	136.515
Marker44177	Chr4	4112428	4112728	136.515
Marker103169	Chr4	4057226	4057489	136.842
Marker24310	Chr4	4054176	4054421	137.168
Marker124645	Chr4	4049578	4049836	137.495
Marker3624	Chr4	4034849	4035119	138.803
Marker76443	Chr4	4013246	4013490	139.13
Marker48054	Chr4	3930008	3930322	139.456
Marker89444	Chr4	3719239	3719487	140.11
Marker123929	Chr4	3769202	3769485	140.11
Marker86166	Chr4	3612924	3613191	140.437
Marker25864	Chr4	3605520	3605791	141.09
Marker17601	Chr4	3189817	3190125	141.55
Marker9562	Chr4	3182206	3182461	141.877
Marker79285	Chr4	3166743	3167022	142.531
Marker57561	Chr4	3158820	3159138	143.511
Marker107444	Chr4	3136416	3136691	145.279
Marker60817	Chr4	3112212	3112492	146.913
Marker127300	Chr4	2667117	2667386	147.894
Marker103747	Chr4	2514012	2514271	148.547
Marker72286	Chr4	2503756	2504041	149.201

Create PDF with GO2PDF for free, if you wish to

Marker7446	Chr4	2502715	2502971	150.508
Marker97834	Chr4	1904652	1904935	150.835
Marker22480	Chr4	1901632	1901923	151.816
Marker60513	Chr4	1814600	1814876	151.816
Marker67015	Chr4	1808107	1808349	152.469
Marker45133	Chr4	1797454	1797730	153.123
Marker113318	Chr4	1692319	1692600	153.45
Marker68142	Chr4	1647911	1648194	154.103
Marker88855	Chr4	1644677	1644960	154.103
Marker69017	Chr4	1632791	1633036	154.43
Marker34851	Chr4	1572874	1573175	155.084
Marker120517	Chr4	1572015	1572266	155.084
Marker79703	Chr4	1567879	1568147	156.391
Marker121801	Chr4	1533321	1533640	156.391
Marker12900	Chr4	1530125	1530381	156.718
Marker11576	Chr4	1518873	1519119	157.372
Marker94889	Chr4	1518426	1518725	158.025
Marker14377	Chr4	1512172	1512445	158.485
Marker87684	Chr4	1507885	1508163	158.485
Marker101320	Chr4	1507375	1507668	158.812
Marker108916	Chr4	1429920	1430201	158.812
Marker60883	Chr4	1428132	1428396	159.792
Marker37640	Chr4	1224095	1224355	160.119
Marker117010	Chr4	1196862	1197161	160.446
Marker6029	Chr4	1178405	1178719	160.773
Marker14837	Chr4	1187407	1187697	160.773
Marker89573	Chr4	1184484	1184768	160.773
Marker6235	Chr4	1161441	1161692	161.1
Marker54753	Chr4	1156581	1156852	161.1
Marker126857	Chr4	1141474	1141787	161.426
Marker61467	Chr4	1077105	1077365	162.08
Marker24535	Chr4	853905	854164	163.061
Marker6085	Chr4	596183	596442	163.714
Marker11303	Chr4	841282	841555	163.714
Marker41649	Chr4	446618	446912	163.714
Marker95620	Chr4	529368	529610	163.714
Marker40151	Chr4	383814	384086	164.368
Marker26880	Chr4	215326	215600	165.348
Marker81869	Chr4	309858	310146	165.348
Marker8022	Chr4	203661	203961	165.675
Marker17092	Chr4	211077	211374	165.675
Marker24410	Chr4	168575	168827	165.675
Marker27245	Chr4	209793	210077	165.675
Marker29108	Chr4	77990	78294	165.675
Marker29547	Chr4	209476	209793	165.675
Marker35867	Chr4	115372	115613	165.675
Marker121698	Chr4	47764	48046	165.675
Marker718888	Chr5	27644708	27645019	0
Marker719020	Chr5	27067487	27067750	0
Marker725043	Chr5	27822732	27823019	0
Marker725906	Chr5	27782267	27782563	0
Marker764144	Chr5	27741809	27742081	0
Marker770577	Chr5	27769890	27770204	0
Marker778795	Chr5	27305796	27306050	0

Create PDF with GO2PDF for free, if you wish to

Marker787744	Chr5	27077902	27078178	0
Marker794617	Chr5	27080166	27080456	0
Marker818354	Chr5	27269610	27269868	0
Marker819788	Chr5	27781850	27782114	0
Marker756318	Chr5	26852445	26852703	0.327
Marker784352	Chr5	26831178	26831441	0.327
Marker727206	Chr5	26782142	26782402	0.98
Marker806793	Chr5	26789927	26790190	0.98
Marker788148	Chr5	26778629	26778903	1.961
Marker700742	Chr5	26763063	26763336	2.421
Marker716860	Chr5	26456069	26456324	2.421
Marker801403	Chr5	26455138	26455443	2.747
Marker815084	Chr5	26399128	26399431	3.074
Marker788933	Chr5	26217212	26217478	3.728
Marker778263	Chr5	26145947	26146239	4.055
Marker725579	Chr5	26085155	26085447	4.381
Marker758701	Chr5	26115936	26116223	4.381
Marker804126	Chr5	25837456	25837703	5.035
Marker742930	Chr5	25809046	25809322	5.362
Marker803131	Chr5	25770199	25770460	5.689
Marker812478	Chr5	25757668	25757939	6.016
Marker709994	Chr5	25729837	25730093	6.342
Marker742682	Chr5	25729537	25729837	6.342
Marker725820	Chr5	25641056	25641359	6.996
Marker796513	Chr5	25645940	25646218	6.996
Marker678453	Chr5	25568686	25569000	7.65
Marker717560	Chr5	25573200	25573465	7.65
Marker691798	Chr5	25460378	25460630	7.976
Marker704856	Chr5	25499097	25499416	7.976
Marker749101	Chr5	25479756	25480037	7.976
Marker756701	Chr5	25525428	25525688	7.976
Marker766095	Chr5	25460966	25461250	7.976
Marker698721	Chr5	25438040	25438327	8.303
Marker786998	Chr5	25395498	25395767	8.303
Marker698853	Chr5	25318230	25318535	8.957
Marker735101	Chr5	25383516	25383802	8.957
Marker736587	Chr5	25370898	25371165	8.957
Marker752402	Chr5	25366007	25366266	8.957
Marker779760	Chr5	25336496	25336742	8.957
Marker776626	Chr5	25298380	25298644	9.284
Marker684046	Chr5	25276889	25277131	9.937
Marker745914	Chr5	25195044	25195298	9.937
Marker805334	Chr5	25190529	25190823	10.591
Marker816761	Chr5	25171834	25172084	10.591
Marker808090	Chr5	25168895	25169186	11.245
Marker742447	Chr5	25089387	25089630	11.704
Marker725009	Chr5	25020379	25020646	12.358
Marker730936	Chr5	24847899	24848173	12.358
Marker816618	Chr5	24826830	24827083	12.685
Marker692010	Chr5	24802562	24802852	13.011
Marker698195	Chr5	24719522	24719811	13.011
Marker706001	Chr5	24822962	24823269	13.011
Marker728444	Chr5	24804262	24804546	13.011
Marker735986	Chr5	24726760	24727034	13.011

Create PDF with GO2PDF for free, if you wish to

Marker772618	Chr5	24753545	24753786	13.011
Marker701302	Chr5	24561175	24561467	13.338
Marker734308	Chr5	24591819	24592098	13.338
Marker816300	Chr5	24645384	24645638	13.338
Marker735397	Chr5	24541021	24541276	13.665
Marker785644	Chr5	24552337	24552596	13.665
Marker695531	Chr5	24329756	24330010	13.994
Marker765463	Chr5	24516171	24516406	13.994
Marker687846	Chr5	24178737	24178989	14.321
Marker809755	Chr5	24055682	24055934	14.648
Marker712437	Chr5	24012749	24013006	14.974
Marker752924	Chr5	23854696	23855002	15.628
Marker725050	Chr5	23838687	23838973	15.955
Marker814426	Chr5	23830664	23830971	16.608
Marker814903	Chr5	23815943	23816257	16.935
Marker689933	Chr5	23793176	23793428	17.262
Marker718697	Chr5	23768431	23768673	17.589
Marker710770	Chr5	23734827	23735142	17.916
Marker742081	Chr5	23748909	23749206	17.916
Marker678115	Chr5	23709556	23709822	18.242
Marker764496	Chr5	23721677	23721929	18.242
Marker690094	Chr5	23411937	23412234	18.569
Marker785707	Chr5	23640116	23640443	18.569
Marker760218	Chr5	23402115	23402386	18.896
Marker762692	Chr5	23401520	23401825	19.223
Marker792751	Chr5	23383959	23384232	19.223
Marker760538	Chr5	23363954	23364194	19.55
Marker791000	Chr5	23345545	23345800	19.876
Marker799497	Chr5	23350433	23350676	19.876
Marker766863	Chr5	23345003	23345261	20.857
Marker696234	Chr5	23286178	23286487	21.511
Marker687102	Chr5	23191707	23192016	21.837
Marker774268	Chr5	23185830	23186114	22.164
Marker766791	Chr5	23152785	23153067	22.491
Marker756335	Chr5	23142221	23142530	23.145
Marker803009	Chr5	21125936	21126180	23.471
Marker778231	Chr5	21023902	21024161	23.798
Marker778816	Chr5	21124543	21124801	23.798
Marker753095	Chr5	21010132	21010384	24.258
Marker740314	Chr5	21008835	21009125	24.912
Marker770437	Chr5	20981813	20982075	26.873
Marker786725	Chr5	21004926	21005207	26.873
Marker821351	Chr5	20934575	20934837	28.641
Marker745959	Chr5	20906197	20906466	29.295
Marker717837	Chr5	20144324	20144581	30.735
Marker711270	Chr5	18500204	18500457	31.062
Marker784324	Chr5	18468443	18468745	31.389
Marker819280	Chr5	18214630	18214937	31.715
Marker820596	Chr5	18212871	18213128	33.35
Marker684533	Chr5	18202920	18203190	34.004
Marker741148	Chr5	18145393	18145692	34.657
Marker739933	Chr5	18135527	18135785	34.984
Marker776452	Chr5	18129399	18129672	35.313
Marker743513	Chr5	18125148	18125444	35.642

Create PDF with GO2PDF for free, if you wish to

Marker751620	Chr5	17451678	17451937	35.969
Marker762427	Chr5	17435648	17435963	36.428
Marker754959	Chr5	17389003	17389228	37.082
Marker810504	Chr5	17381785	17382057	37.409
Marker728101	Chr5	17227991	17228272	38.063
Marker705235	Chr5	17109481	17109731	38.716
Marker781096	Chr5	17193179	17193464	38.716
Marker802377	Chr5	16957408	16957667	39.043
Marker715122	Chr5	16916578	16916867	39.37
Marker720409	Chr5	16759608	16759855	39.37
Marker803500	Chr5	16762401	16762681	39.37
Marker800481	Chr5	16734590	16734843	39.829
Marker819412	Chr5	16718718	16718993	40.156
Marker813438	Chr5	16693591	16693845	40.483
Marker685403	Chr5	16576518	16576789	41.791
Marker814305	Chr5	16554509	16554779	42.444
Marker755888	Chr5	16512137	16512430	43.098
Marker778935	Chr5	16549793	16550033	43.098
Marker752179	Chr5	16435459	16435712	43.751
Marker786357	Chr5	16413526	16413798	43.751
Marker750190	Chr5	16331148	16331441	44.405
Marker823459	Chr5	16319116	16319395	45.059
Marker715172	Chr5	16076719	16076995	45.712
Marker750587	Chr5	16236539	16236807	45.712
Marker782533	Chr5	16074247	16074514	46.366
Marker735652	Chr5	16060876	16061132	46.693
Marker772580	Chr5	16052365	16052651	47.02
Marker742828	Chr5	16034589	16034850	47.346
Marker684358	Chr5	15915211	15915479	48
Marker714810	Chr5	15986648	15986953	48
Marker827429	Chr5	16000694	16000969	48
Marker728332	Chr5	15896490	15896774	48.654
Marker807641	Chr5	15906046	15906342	48.654
Marker826474	Chr5	15886787	15887088	48.654
Marker693655	Chr5	15878774	15879046	48.98
Marker735039	Chr5	15886088	15886348	48.98
Marker780622	Chr5	15821030	15821337	49.307
Marker738061	Chr5	15805289	15805575	49.634
Marker766665	Chr5	15779403	15779695	50.288
Marker805469	Chr5	15779117	15779403	50.941
Marker757606	Chr5	15687530	15687817	51.595
Marker765199	Chr5	15695533	15695783	51.595
Marker773329	Chr5	15575488	15575752	51.595
Marker712279	Chr5	15574513	15574763	51.922
Marker737981	Chr5	15563538	15563807	52.249
Marker711008	Chr5	15532629	15532887	52.902
Marker677844	Chr5	15495701	15495950	53.229
Marker721331	Chr5	15446810	15447100	53.883
Marker734324	Chr5	15120834	15121140	54.209
Marker750633	Chr5	15068046	15068341	54.536
Marker739889	Chr5	14909613	14909870	54.863
Marker767366	Chr5	14900541	14900819	54.863
Marker768027	Chr5	14900223	14900541	55.517
Marker806982	Chr5	14849602	14849881	55.517

Create PDF with GO2PDF for free, if you wish to

Marker733958	Chr5	14770868	14771151	55.843
Marker772374	Chr5	14731210	14731499	55.843
Marker687426	Chr5	14703316	14703591	56.17
Marker824091	Chr5	14713761	14714020	56.17
Marker688861	Chr5	14700101	14700367	56.497
Marker741497	Chr5	14690282	14690597	56.497
Marker760363	Chr5	14687637	14687909	57.151
Marker677182	Chr5	14591206	14591499	57.804
Marker678914	Chr5	14645187	14645440	57.804
Marker784859	Chr5	14568943	14569209	57.804
Marker799604	Chr5	14661168	14661460	57.804
Marker806006	Chr5	14558933	14559180	57.804
Marker807643	Chr5	14589201	14589517	57.804
Marker721921	Chr5	14489956	14490240	58.131
Marker738900	Chr5	14519064	14519313	58.131
Marker765311	Chr5	14527015	14527322	58.131
Marker792146	Chr5	14418440	14418759	58.785
Marker809442	Chr5	14446574	14446864	58.785
Marker799865	Chr5	14377477	14377734	59.765
Marker699409	Chr5	14087642	14087955	60.419
Marker773566	Chr5	14047649	14047925	60.746
Marker677903	Chr5	14037516	14037792	61.073
Marker712609	Chr5	14036602	14036892	61.073
Marker748292	Chr5	14003242	14003495	61.073
Marker757710	Chr5	13993618	13993907	61.073
Marker700806	Chr5	13873387	13873657	61.399
Marker702308	Chr5	13878421	13878693	61.399
Marker704358	Chr5	13968033	13968330	61.399
Marker710347	Chr5	13966292	13966558	61.399
Marker713920	Chr5	13981728	13981999	61.399
Marker721680	Chr5	13858927	13859223	62.053
Marker687344	Chr5	13784214	13784504	62.38
Marker710266	Chr5	13813484	13813784	62.38
Marker758825	Chr5	13848991	13849269	62.38
Marker825812	Chr5	13784504	13784768	62.38
Marker728330	Chr5	13592128	13592405	62.707
Marker780693	Chr5	13770054	13770379	62.707
Marker802044	Chr5	13504923	13505213	63.033
Marker710926	Chr5	13477635	13477902	63.36
Marker734081	Chr5	13485445	13485752	63.36
Marker744016	Chr5	13497042	13497324	63.36
Marker827820	Chr5	13447892	13448141	63.36
Marker718491	Chr5	13327374	13327626	63.687
Marker718339	Chr5	13238601	13238877	64.014
Marker722265	Chr5	13203401	13203676	64.667
Marker741797	Chr5	13156626	13156895	64.667
Marker796043	Chr5	13097660	13097954	64.667
Marker802693	Chr5	13182433	13182690	64.667
Marker774340	Chr5	13023881	13024124	64.994
Marker790327	Chr5	12887662	12887928	64.994
Marker818891	Chr5	13052792	13053037	64.994
Marker818975	Chr5	12904334	12904586	64.994
Marker714792	Chr5	12884504	12884782	66.302
Marker721588	Chr5	12838562	12838847	66.628

Create PDF with GO2PDF for free, if you wish to

Marker748718	Chr5	12744846	12745109	67.282
Marker743804	Chr5	12584116	12584406	68.794
Marker788070	Chr5	12491040	12491282	69.908
Marker779015	Chr5	12453905	12454180	70.888
Marker731989	Chr5	12146863	12147140	72.002
Marker704897	Chr5	12104535	12104818	73.309
Marker822160	Chr5	12084893	12085141	73.963
Marker748554	Chr5	12056516	12056792	74.29
Marker785685	Chr5	12004261	12004543	74.617
Marker783940	Chr5	11992581	11992869	75.924
Marker755708	Chr5	11981962	11982268	76.251
Marker795562	Chr5	11831676	11831923	77.231
Marker741454	Chr5	11821246	11821509	77.558
Marker727462	Chr5	11810117	11810362	78.018
Marker752023	Chr5	11722529	11722802	78.018
Marker690717	Chr5	11639597	11639906	78.998
Marker698374	Chr5	11652133	11652378	78.998
Marker827560	Chr5	11650704	11650989	78.998
Marker761377	Chr5	11596061	11596316	79.652
Marker717647	Chr5	11532452	11532719	80.306
Marker730915	Chr5	11483737	11484011	80.959
Marker705418	Chr5	11440581	11440827	81.613
Marker693409	Chr5	11390859	11391125	82.267
Marker736094	Chr5	10585452	10585735	82.593
Marker706661	Chr5	10488268	10488524	82.92
Marker744951	Chr5	10101960	10102220	83.574
Marker690064	Chr5	10019195	10019458	83.901
Marker733718	Chr5	10017692	10017975	83.901
Marker747621	Chr5	10011793	10012065	84.227
Marker733093	Chr5	9979478	9979753	84.881
Marker816259	Chr5	9967380	9967682	84.881
Marker716345	Chr5	9887221	9887517	85.21
Marker718978	Chr5	9802035	9802339	85.537
Marker729669	Chr5	9803877	9804133	85.537
Marker680014	Chr5	9713147	9713414	85.864
Marker752222	Chr5	9648824	9649107	86.19
Marker779481	Chr5	9656428	9656721	86.19
Marker758636	Chr5	9618966	9619262	86.844
Marker728952	Chr5	9584320	9584581	87.498
Marker761075	Chr5	9466761	9467003	87.824
Marker824509	Chr5	9423981	9424226	87.824
Marker678056	Chr5	9340139	9340402	88.805
Marker756024	Chr5	9281167	9281444	89.459
Marker820876	Chr5	9278645	9278907	89.785
Marker755656	Chr5	9170022	9170288	90.439
Marker758848	Chr5	9114262	9114539	90.439
Marker698681	Chr5	8918344	8918595	91.42
Marker808666	Chr5	9024638	9024887	91.42
Marker729432	Chr5	8862441	8862694	91.746
Marker700069	Chr5	8862182	8862441	92.073
Marker750084	Chr5	8794170	8794469	92.4
Marker715740	Chr5	8726288	8726566	92.727
Marker801650	Chr5	8737462	8737698	92.727
Marker825640	Chr5	8675797	8676041	93.054

Create PDF with GO2PDF for free, if you wish to

Marker700797	Chr5	8673283	8673552	94.034
Marker807594	Chr5	8461808	8462075	94.688
Marker711829	Chr5	8278655	8278942	95.341
Marker788511	Chr5	8388601	8388872	95.341
Marker689843	Chr5	8224064	8224324	95.668
Marker788114	Chr5	7883738	7883986	95.668
Marker807213	Chr5	8117857	8118134	95.668
Marker733522	Chr5	7793166	7793447	95.995
Marker677698	Chr5	7562185	7562460	96.649
Marker810508	Chr5	7554145	7554430	97.956
Marker724768	Chr5	7436880	7437171	98.283
Marker694909	Chr5	6964853	6965092	98.61
Marker804513	Chr5	7402342	7402615	98.61
Marker806228	Chr5	6937133	6937398	98.61
Marker800342	Chr5	6883681	6883964	99.263
Marker689014	Chr5	6830139	6830436	99.917
Marker813514	Chr5	6827039	6827303	100.571
Marker681975	Chr5	6605173	6605444	101.878
Marker690249	Chr5	6440627	6440884	101.878
Marker712691	Chr5	6551074	6551380	101.878
Marker737383	Chr5	6416470	6416747	101.878
Marker736933	Chr5	6362817	6363092	102.532
Marker735127	Chr5	6227727	6228010	102.991
Marker808823	Chr5	6291854	6292128	102.991
Marker808570	Chr5	6227475	6227727	103.645
Marker705808	Chr5	6226121	6226399	103.972
Marker797107	Chr5	6218031	6218281	104.952
Marker684690	Chr5	6181438	6181745	105.279
Marker730753	Chr5	6170134	6170461	105.933
Marker746815	Chr5	6073654	6073928	107.24
Marker729637	Chr5	5992869	5993145	107.894
Marker828723	Chr5	5805320	5805618	108.942
Marker828775	Chr5	5977287	5977594	108.942
Marker739850	Chr5	5235063	5235303	109.269
Marker778270	Chr5	5154316	5154608	109.596
Marker705844	Chr5	5015581	5015844	110.249
Marker721308	Chr5	5012960	5013228	110.249
Marker803274	Chr5	4586318	4586566	110.903
Marker820585	Chr5	4361857	4362152	112.21
Marker805933	Chr5	4050919	4051224	112.864
Marker702073	Chr5	3921239	3921512	113.191
Marker743423	Chr5	3920051	3920324	113.518
Marker787810	Chr5	3914985	3915270	114.171
Marker738568	Chr5	3839435	3839705	114.825
Marker733437	Chr5	3830630	3830910	115.805
Marker725567	Chr5	3827548	3827799	116.132
Marker806257	Chr5	3800720	3801017	116.786
Marker816457	Chr5	3786512	3786779	117.439
Marker823618	Chr5	3689533	3689796	117.766
Marker744336	Chr5	3535531	3535798	118.42
Marker740883	Chr5	3526064	3526337	119.4
Marker813231	Chr5	3506217	3506491	121.168
Marker803269	Chr5	3493157	3493437	123.525
Marker800054	Chr5	3266882	3267203	124.187

Create PDF with GO2PDF for free, if you wish to

Marker700067	Chr5	3236334	3236609	125.372
Marker712860	Chr5	3182644	3182912	126.026
Marker797510	Chr5	3115928	3116212	126.353
Marker772527	Chr5	2957995	2958247	128.382
Marker722284	Chr5	2936479	2936739	129.363
Marker730364	Chr5	2816956	2817256	130.997
Marker740874	Chr5	2807097	2807373	133.287
Marker725429	Chr5	2788285	2788544	134.073
Marker705185	Chr5	2714528	2714826	136.035
Marker708861	Chr5	2706421	2706685	137.342
Marker825841	Chr5	2651916	2652187	138.323
Marker692722	Chr5	2604739	2605014	138.976
Marker722787	Chr5	2601832	2602123	139.63
Marker720050	Chr5	2553596	2553864	139.957
Marker798351	Chr5	2556550	2556814	139.957
Marker761041	Chr5	2545012	2545292	140.284
Marker682242	Chr5	2543361	2543643	140.611
Marker758125	Chr5	2477694	2477984	140.611
Marker766681	Chr5	2477229	2477495	140.611
Marker682555	Chr5	2447229	2447509	140.937
Marker704781	Chr5	2431431	2431715	141.591
Marker705259	Chr5	2345981	2346236	141.591
Marker721705	Chr5	2377061	2377376	141.591
Marker725592	Chr5	2437962	2438224	141.591
Marker732418	Chr5	2288077	2288363	141.591
Marker733414	Chr5	2389471	2389724	141.591
Marker801055	Chr5	2417589	2417855	141.591
Marker743899	Chr5	2256957	2257272	141.918
Marker722017	Chr5	2192176	2192476	142.571
Marker735777	Chr5	2118384	2118653	142.571
Marker751265	Chr5	2157000	2157285	142.571
Marker766822	Chr5	2163210	2163466	142.571
Marker750359	Chr5	2099504	2099814	142.898
Marker716783	Chr5	2099254	2099504	143.225
Marker739775	Chr5	2083024	2083304	143.225
Marker729262	Chr5	1594728	1594995	143.552
Marker809288	Chr5	2056750	2057015	143.552
Marker784344	Chr5	1524541	1524830	144.205
Marker719378	Chr5	1499000	1499291	144.859
Marker804654	Chr5	1493025	1493287	144.859
Marker728297	Chr5	1460789	1461059	145.186
Marker756102	Chr5	1438182	1438473	145.186
Marker706072	Chr5	1410942	1411217	145.513
Marker812445	Chr5	1425246	1425557	145.513
Marker679435	Chr5	1386385	1386631	146.166
Marker709393	Chr5	1355877	1356123	146.82
Marker795409	Chr5	1379080	1379344	146.82
Marker684273	Chr5	1354208	1354471	147.474
Marker691632	Chr5	1106066	1106329	147.8
Marker792101	Chr5	1222768	1223034	147.8
Marker706405	Chr5	751854	752156	148.127
Marker732574	Chr5	1096866	1097155	148.127
Marker777859	Chr5	1050406	1050667	148.127
Marker677609	Chr5	702290	702568	148.781

Create PDF with GO2PDF for free, if you wish to

Marker678436	Chr5	690352	690634	148.781
Marker681840	Chr5	695305	695585	148.781
Marker793882	Chr5	535545	535827	149.761
Marker819170	Chr5	660637	660910	149.761
Marker687707	Chr5	202253	202513	150.088
Marker702976	Chr5	201289	201544	150.088
Marker767975	Chr5	363352	363641	150.088
Marker703429	Chr5	191190	191470	150.415
Marker804473	Chr5	188361	188625	150.415
Marker716247	Chr5	32893	33175	150.742
Marker770162	Chr5	182139	182454	150.742
Marker810384	Chr5	131139	131401	150.742
Marker715406	Chr5	30463	30733	151.069
Marker814890	Chr5	21177	21462	151.069
Marker153181	Chr6	176908	177184	0
Marker231826	Chr6	53559	53818	0
Marker181937	Chr6	378032	378315	0.327
Marker191269	Chr6	461305	461569	0.327
Marker227459	Chr6	749027	749309	0.327
Marker239805	Chr6	416756	417025	0.327
Marker240752	Chr6	720867	721133	0.327
Marker250724	Chr6	442637	442897	0.327
Marker145136	Chr6	775132	775448	0.654
Marker150693	Chr6	977645	977958	0.654
Marker167750	Chr6	1147737	1148010	0.98
Marker263814	Chr6	1157974	1158247	0.98
Marker239176	Chr6	1169863	1170104	1.307
Marker193623	Chr6	1173042	1173329	1.634
Marker160945	Chr6	1212401	1212674	1.961
Marker179368	Chr6	1376857	1377153	1.961
Marker239287	Chr6	1414584	1414866	1.961
Marker159347	Chr6	1421679	1421935	2.288
Marker190287	Chr6	1430473	1430741	3.595
Marker208350	Chr6	1468246	1468489	3.922
Marker240420	Chr6	1492142	1492399	4.249
Marker285969	Chr6	1496022	1496279	4.576
Marker186080	Chr6	1890432	1890712	5.229
Marker219688	Chr6	1892271	1892537	5.229
Marker195832	Chr6	2049129	2049412	6.21
Marker273620	Chr6	1991555	1991835	6.21
Marker146512	Chr6	2125526	2125800	6.536
Marker146957	Chr6	2128193	2128467	6.536
Marker176045	Chr6	2084282	2084597	6.536
Marker276715	Chr6	2127918	2128170	6.536
Marker250506	Chr6	2132142	2132438	7.19
Marker264223	Chr6	2142052	2142316	7.517
Marker233002	Chr6	2218233	2218502	8.497
Marker295115	Chr6	2171334	2171617	8.497
Marker205962	Chr6	2218502	2218756	8.824
Marker214874	Chr6	2334376	2334639	8.824
Marker215305	Chr6	2334798	2335077	8.824
Marker145011	Chr6	2347774	2348054	9.151
Marker164857	Chr6	2449772	2450050	9.805
Marker263600	Chr6	2446086	2446389	9.805

Create PDF with GO2PDF for free, if you wish to

Marker273170	Chr6	2466376	2466666	10.131
Marker279757	Chr6	2457437	2457697	10.131
Marker189848	Chr6	2512583	2512842	10.458
Marker226221	Chr6	2525597	2525861	10.458
Marker249080	Chr6	2502247	2502520	10.458
Marker199401	Chr6	2677920	2678190	11.112
Marker251722	Chr6	2684626	2684895	11.112
Marker162062	Chr6	2686364	2686661	11.439
Marker267025	Chr6	2799126	2799368	11.439
Marker292404	Chr6	2712688	2712959	11.439
Marker154098	Chr6	3085345	3085627	11.765
Marker192942	Chr6	3051065	3051351	11.765
Marker216706	Chr6	2931860	2932138	11.765
Marker273390	Chr6	2801494	2801779	11.765
Marker275699	Chr6	2863388	2863632	11.765
Marker167669	Chr6	3183707	3183970	12.092
Marker247350	Chr6	3186647	3186908	12.746
Marker250468	Chr6	3222037	3222315	13.073
Marker152887	Chr6	3303243	3303503	13.726
Marker226152	Chr6	3325114	3325376	13.726
Marker254817	Chr6	3339224	3339500	13.726
Marker180451	Chr6	3357324	3357585	14.053
Marker190733	Chr6	3362866	3363148	14.38
Marker207411	Chr6	3414752	3415034	14.707
Marker223472	Chr6	3427259	3427544	14.707
Marker196480	Chr6	3452473	3452779	15.166
Marker214163	Chr6	3454671	3454924	15.166
Marker235217	Chr6	3430006	3430326	15.166
Marker248786	Chr6	3510000	3510325	15.493
Marker234992	Chr6	3530393	3530676	15.82
Marker202612	Chr6	3534471	3534720	16.147
Marker224789	Chr6	3533490	3533778	16.147
Marker188663	Chr6	3542242	3542526	16.474
Marker261597	Chr6	3584546	3584786	17.127
Marker276124	Chr6	3584834	3585096	17.781
Marker192044	Chr6	3598587	3598849	18.435
Marker283567	Chr6	3643568	3643846	18.435
Marker200273	Chr6	3716298	3716586	19.415
Marker258651	Chr6	3866165	3866418	19.415
Marker247911	Chr6	3868681	3868925	19.742
Marker194505	Chr6	3897427	3897675	20.395
Marker194920	Chr6	3934153	3934406	21.049
Marker226758	Chr6	3977774	3978075	21.376
Marker218952	Chr6	4009917	4010190	22.03
Marker185556	Chr6	4146171	4146418	22.356
Marker282266	Chr6	4901714	4902005	23.664
Marker287115	Chr6	4897219	4897506	23.664
Marker294940	Chr6	4465555	4465850	23.664
Marker214382	Chr6	4921676	4921974	23.991
Marker250529	Chr6	4913240	4913546	23.991
Marker209644	Chr6	4922309	4922585	24.317
Marker180347	Chr6	5004487	5004771	24.646
Marker256042	Chr6	4936899	4937170	24.646
Marker272619	Chr6	5007378	5007670	24.973

Create PDF with GO2PDF for free, if you wish to

Marker187904	Chr6	5012157	5012437	25.954
Marker229963	Chr6	5095766	5096008	25.954
Marker255321	Chr6	5270580	5270834	26.281
Marker194300	Chr6	5281972	5282291	26.607
Marker218807	Chr6	5291513	5291776	26.607
Marker215173	Chr6	5296440	5296726	27.261
Marker201469	Chr6	5336476	5336784	27.588
Marker146275	Chr6	5343467	5343751	29.222
Marker252770	Chr6	5345233	5345516	30.203
Marker270206	Chr6	5353228	5353512	30.203
Marker171317	Chr6	5360177	5360467	30.856
Marker191350	Chr6	5407425	5407705	30.856
Marker240739	Chr6	5869704	5870004	31.837
Marker225421	Chr6	5874845	5875105	32.164
Marker285317	Chr6	5875386	5875649	32.491
Marker259162	Chr6	5875649	5875920	33.798
Marker291339	Chr6	6153510	6153771	34.452
Marker289051	Chr6	7042466	7042741	35.565
Marker183327	Chr6	7058327	7058612	37.006
Marker205268	Chr6	7064512	7064830	37.659
Marker153569	Chr6	7072438	7072735	38.313
Marker228352	Chr6	7137666	7137968	38.967
Marker209614	Chr6	7190449	7190705	39.293
Marker214524	Chr6	7188125	7188379	39.293
Marker151432	Chr6	7258505	7258769	39.947
Marker183718	Chr6	7298711	7298961	39.947
Marker200653	Chr6	7244700	7244970	39.947
Marker227774	Chr6	7191147	7191415	39.947
Marker187297	Chr6	7302514	7302769	40.927
Marker210840	Chr6	7315863	7316130	41.254
Marker266233	Chr6	7322870	7323147	41.254
Marker198455	Chr6	7335205	7335469	41.908
Marker285351	Chr6	7336051	7336316	41.908
Marker188348	Chr6	7461301	7461572	42.562
Marker164070	Chr6	7479855	7480136	42.888
Marker172611	Chr6	7723310	7723560	43.215
Marker180350	Chr6	7916986	7917237	43.215
Marker214693	Chr6	7898237	7898484	43.215
Marker293969	Chr6	7737304	7737571	43.215
Marker194932	Chr6	7927148	7927452	44.196
Marker249221	Chr6	7983885	7984141	44.522
Marker208544	Chr6	7997483	7997753	44.849
Marker297846	Chr6	8002598	8002878	45.176
Marker218451	Chr6	8031465	8031757	45.503
Marker185477	Chr6	8041585	8041860	45.83
Marker161457	Chr6	8260357	8260656	46.81
Marker165285	Chr6	8403189	8403449	46.81
Marker165671	Chr6	8735027	8735293	46.81
Marker181337	Chr6	8736590	8736851	46.81
Marker222398	Chr6	8676424	8676673	46.81
Marker254911	Chr6	8450314	8450569	46.81
Marker288011	Chr6	8159303	8159544	46.81
Marker148176	Chr6	8756327	8756582	47.137
Marker273652	Chr6	8765922	8766172	47.791

Create PDF with GO2PDF for free, if you wish to

Marker280893	Chr6	8770194	8770444	47.791
Marker264617	Chr6	8841468	8841716	48.117
Marker257197	Chr6	8842052	8842338	48.444
Marker248082	Chr6	8843136	8843394	48.771
Marker281759	Chr6	8846786	8847059	49.231
Marker200248	Chr6	8857482	8857785	49.884
Marker251021	Chr6	8849588	8849880	49.884
Marker156199	Chr6	8872020	8872279	50.538
Marker179503	Chr6	8896817	8897121	50.538
Marker192382	Chr6	8863523	8863792	50.538
Marker218302	Chr6	8876835	8877104	50.538
Marker256934	Chr6	8860047	8860335	50.538
Marker165778	Chr6	9033559	9033830	50.865
Marker284491	Chr6	8897161	8897443	50.865
Marker201941	Chr6	9045780	9046067	51.518
Marker280361	Chr6	9039769	9040085	51.518
Marker189938	Chr6	9066355	9066624	51.845
Marker193617	Chr6	9081275	9081551	52.499
Marker206975	Chr6	9111912	9112219	52.826
Marker179536	Chr6	9225575	9225849	53.152
Marker184907	Chr6	9245266	9245531	53.152
Marker147191	Chr6	9256091	9256365	53.806
Marker245730	Chr6	9263581	9263844	53.806
Marker242800	Chr6	9266537	9266826	54.787
Marker193059	Chr6	9286333	9286580	55.44
Marker177307	Chr6	9379791	9380059	55.767
Marker207309	Chr6	9483340	9483628	56.094
Marker179482	Chr6	9491339	9491591	56.421
Marker216324	Chr6	9508839	9509131	56.747
Marker144284	Chr6	9703252	9703511	57.074
Marker285890	Chr6	9701018	9701272	57.074
Marker148257	Chr6	9781044	9781328	57.401
Marker170100	Chr6	9773344	9773600	57.401
Marker243412	Chr6	9792571	9792837	57.401
Marker164858	Chr6	9819189	9819454	58.055
Marker256207	Chr6	9882874	9883180	58.055
Marker167996	Chr6	9945329	9945637	58.381
Marker270228	Chr6	10193306	10193608	58.381
Marker272585	Chr6	10208203	10208481	58.381
Marker168925	Chr6	10280642	10280906	59.035
Marker240285	Chr6	10283827	10284075	59.035
Marker158579	Chr6	10284075	10284360	59.362
Marker295485	Chr6	10299650	10299920	60.016
Marker153948	Chr6	10321106	10321400	61.323
Marker277404	Chr6	10321400	10321681	61.323
Marker201297	Chr6	10466952	10467244	61.65
Marker222357	Chr6	10323337	10323597	61.65
Marker229660	Chr6	10425931	10426168	61.65
Marker250947	Chr6	10370952	10371201	61.65
Marker232934	Chr6	10475165	10475457	62.303
Marker282299	Chr6	10599013	10599280	62.957
Marker298632	Chr6	10519573	10519893	62.957
Marker142747	Chr6	10940781	10941111	63.611
Marker172483	Chr6	11254633	11254891	63.611

Create PDF with GO2PDF for free, if you wish to

Marker273529	Chr6	11262537	11262856	63.938
Marker277271	Chr6	11497066	11497337	64.918
Marker144100	Chr6	11507932	11508244	65.245
Marker169379	Chr6	11579267	11579550	65.245
Marker212876	Chr6	11589405	11589686	65.245
Marker147821	Chr6	11661937	11662243	66.225
Marker265447	Chr6	11676025	11676306	66.552
Marker214294	Chr6	11828256	11828520	67.533
Marker189293	Chr6	11839587	11839873	67.859
Marker273003	Chr6	12180190	12180454	68.186
Marker219360	Chr6	12415759	12416042	68.513
Marker284407	Chr6	12417524	12417803	68.84
Marker266525	Chr6	12706836	12707122	69.494
Marker181955	Chr6	12727779	12728024	69.82
Marker196811	Chr6	12818623	12818938	70.147
Marker205093	Chr6	12902562	12902860	70.147
Marker297311	Chr6	12838844	12839196	70.147
Marker196495	Chr6	12992360	12992615	70.607
Marker262939	Chr6	12912751	12913003	70.607
Marker292153	Chr6	13155999	13156306	70.607
Marker145082	Chr6	13244675	13244906	70.934
Marker151881	Chr6	13246101	13246376	70.934
Marker289826	Chr6	13266071	13266364	70.934
Marker249705	Chr6	13270051	13270345	71.26
Marker291778	Chr6	13269780	13270051	71.26
Marker155031	Chr6	13345902	13346170	71.587
Marker159685	Chr6	13352141	13352412	71.587
Marker190056	Chr6	13298276	13298547	71.587
Marker244111	Chr6	13393905	13394190	71.587
Marker296234	Chr6	13383379	13383640	71.587
Marker173283	Chr6	13410958	13411258	72.241
Marker186131	Chr6	13397426	13397700	72.241
Marker199389	Chr6	13449429	13449700	72.894
Marker200270	Chr6	13461239	13461507	73.221
Marker178517	Chr6	13462576	13462888	73.548
Marker149392	Chr6	13516978	13517242	73.875
Marker239624	Chr6	13559994	13560276	73.875
Marker143500	Chr6	13653540	13653809	74.202
Marker193495	Chr6	13651382	13651639	74.202
Marker214942	Chr6	13609611	13609864	74.202
Marker191036	Chr6	13813705	13813978	74.528
Marker188659	Chr6	13834281	13834539	74.855
Marker151896	Chr6	13964590	13964843	75.509
Marker176244	Chr6	13975426	13975702	75.836
Marker238349	Chr6	13980360	13980645	75.836
Marker162130	Chr6	13998802	13999065	76.163
Marker152468	Chr6	14003737	14004020	76.489
Marker189506	Chr6	14027364	14027628	76.816
Marker199175	Chr6	14043924	14044180	78.778
Marker148861	Chr6	14101087	14101346	79.105
Marker256255	Chr6	14365739	14366048	79.105
Marker247042	Chr6	15092739	15093029	79.891
Marker267262	Chr6	15145234	15145540	79.891
Marker293539	Chr6	15127581	15127876	79.891

Create PDF with GO2PDF for free, if you wish to

Marker197111	Chr6	15644283	15644549	80.218
Marker209058	Chr6	15171317	15171606	80.218
Marker193566	Chr6	15649521	15649802	80.545
Marker272239	Chr6	15669213	15669531	81.525
Marker178479	Chr6	15766303	15766550	81.852
Marker238174	Chr6	15803515	15803795	81.852
Marker295987	Chr6	15835129	15835388	82.833
Marker146689	Chr6	15845752	15846013	83.159
Marker153818	Chr6	15844306	15844565	83.159
Marker164395	Chr6	15980079	15980357	83.159
Marker174765	Chr6	15845179	15845429	83.159
Marker270264	Chr6	15841794	15842028	83.159
Marker164287	Chr6	16161789	16162050	83.486
Marker233972	Chr6	16327600	16327896	83.815
Marker239971	Chr6	16366223	16366467	84.796
Marker189074	Chr6	16511710	16512010	85.123
Marker278549	Chr6	16630067	16630334	85.123
Marker289818	Chr6	16623149	16623435	85.123
Marker178228	Chr6	16635104	16635379	85.449
Marker191847	Chr6	16712419	16712722	86.103
Marker233549	Chr6	16722428	16722687	86.757
Marker142079	Chr6	16737256	16737521	87.083
Marker231632	Chr6	16744953	16745247	87.41
Marker281931	Chr6	16753369	16753629	88.064
Marker179098	Chr6	16755216	16755502	88.391
Marker297473	Chr6	16878377	16878617	89.044
Marker153579	Chr6	16886356	16886644	90.025
Marker156835	Chr6	16892523	16892789	90.354
Marker176511	Chr6	16970625	16970906	90.354
Marker232049	Chr6	16891864	16892117	90.354
Marker255727	Chr6	16937322	16937577	90.354
Marker195883	Chr6	17127177	17127463	90.681
Marker144669	Chr6	17155406	17155666	91.334
Marker186537	Chr6	17211438	17211692	91.334
Marker267530	Chr6	17257557	17257849	91.661
Marker168372	Chr6	17340876	17341178	91.988
Marker181101	Chr6	17352300	17352538	91.988
Marker282253	Chr6	17383857	17384131	92.315
Marker207863	Chr6	17930759	17931057	92.641
Marker160664	Chr6	17931293	17931552	93.295
Marker249274	Chr6	18040585	18040836	93.622
Marker274882	Chr6	17968965	17969231	93.622
Marker175174	Chr6	18047215	18047499	94.275
Marker206194	Chr6	18083314	18083614	94.275
Marker218367	Chr6	18217722	18217991	94.929
Marker185660	Chr6	18333629	18333881	95.256
Marker232068	Chr6	18277252	18277502	95.256
Marker146807	Chr6	18406969	18407239	95.91
Marker184629	Chr6	18383506	18383777	95.91
Marker299383	Chr6	18436974	18437268	96.563
Marker162526	Chr6	18440838	18441117	97.217
Marker276855	Chr6	18449637	18449941	97.217
Marker196412	Chr6	18469987	18470287	98.524
Marker185278	Chr6	18484189	18484442	99.178

Create PDF with GO2PDF for free, if you wish to

Marker221483	Chr6	18775257	18775515	100.158
Marker262609	Chr6	18585437	18585703	100.158
Marker257104	Chr6	18780121	18780445	100.812
Marker185507	Chr6	18816916	18817184	101.466
Marker225895	Chr6	18988140	18988417	101.466
Marker298968	Chr6	18814782	18815047	101.466
Marker154034	Chr6	18988783	18989082	102.773
Marker148455	Chr6	19021289	19021537	104.081
Marker198038	Chr6	19037096	19037397	105.388
Marker259639	Chr6	19057651	19057945	105.715
Marker179581	Chr6	19810560	19810859	107.35
Marker195333	Chr6	19065805	19066081	107.35
Marker151238	Chr6	20935769	20936042	108.33
Marker287666	Chr6	20979243	20979498	110.098
Marker146779	Chr6	21015072	21015349	111.078
Marker219574	Chr6	21460102	21460353	111.405
Marker249671	Chr6	21529625	21529879	112.192
Marker265707	Chr6	21545284	21545539	112.192
Marker170613	Chr6	21615508	21615759	113.172
Marker237194	Chr6	21625326	21625630	113.499
Marker237659	Chr6	21796199	21796503	114.152
Marker258632	Chr6	21804167	21804401	114.806
Marker231683	Chr6	21864895	21865176	115.787
Marker225276	Chr6	21945743	21946054	116.113
Marker145156	Chr6	21948595	21948850	116.767
Marker245488	Chr6	21986028	21986291	117.094
Marker276974	Chr6	21978776	21979073	117.094
Marker201458	Chr6	21995471	21995745	117.747
Marker187399	Chr6	22090428	22090723	118.534
Marker182935	Chr6	22133661	22133938	119.515
Marker257943	Chr6	22156024	22156339	120.168
Marker298441	Chr6	22354027	22354327	121.149
Marker163591	Chr6	22527997	22528256	122.262
Marker291532	Chr6	22530315	22530574	123.376
Marker274933	Chr6	22565940	22566268	124.029
Marker156079	Chr6	22571779	22572044	124.683
Marker255854	Chr6	22584171	22584428	125.143
Marker194651	Chr6	22589722	22590021	126.123
Marker168182	Chr6	22640220	22640486	126.777
Marker260236	Chr6	24206972	24207243	127.757
Marker296284	Chr6	24231152	24231433	128.411
Marker258945	Chr6	24504392	24504653	128.738
Marker146416	Chr6	24809881	24810150	129.718
Marker192686	Chr6	24852786	24853079	130.178
Marker280799	Chr6	25354768	25355045	130.505
Marker184837	Chr6	25371040	25371331	130.831
Marker224949	Chr6	25438853	25439098	131.485
Marker146191	Chr6	25460500	25460786	132.466
Marker165302	Chr6	26078214	26078490	132.792
Marker275952	Chr6	26226582	26226845	133.446
Marker253564	Chr6	26240445	26240696	133.773
Marker211004	Chr6	26256581	26256848	134.426
Marker245068	Chr6	26268571	26268819	134.886
Marker243579	Chr6	26275751	26275994	135.54

Create PDF with GO2PDF for free, if you wish to

Marker162159	Chr6	26369754	26370013	135.867
Marker279846	Chr6	26359018	26359284	135.867
Marker290224	Chr6	26302754	26303003	135.867
Marker299092	Chr6	26358741	26358995	135.867
Marker250030	Chr6	26385014	26385314	136.52
Marker190420	Chr6	26390382	26390627	136.847
Marker196896	Chr6	26467504	26467760	137.176
Marker228219	Chr6	26491188	26491480	137.176
Marker237800	Chr6	26396823	26397088	137.176
Marker247319	Chr6	26495364	26495656	137.503
Marker152976	Chr6	26541493	26541792	137.83
Marker235121	Chr6	26554923	26555216	138.81
Marker201963	Chr6	26623540	26623813	139.137
Marker217283	Chr6	26634046	26634318	139.137
Marker168110	Chr6	26690300	26690572	139.79
Marker168390	Chr6	26707641	26707900	140.117
Marker159460	Chr6	26754128	26754432	140.444
Marker211978	Chr6	26726077	26726329	140.444
Marker246176	Chr6	26764398	26764660	140.444
Marker285192	Chr6	26728522	26728767	140.444
Marker163969	Chr6	26777365	26777618	141.098
Marker149091	Chr6	26791051	26791339	141.425
Marker293319	Chr6	26840049	26840324	141.751
Marker241415	Chr6	26854640	26854895	142.078
Marker232072	Chr6	26861507	26861776	144.04
Marker160266	Chr6	26946237	26946501	144.367
Marker239111	Chr6	26939656	26939942	144.367
Marker270085	Chr6	26873250	26873553	144.367
Marker245400	Chr6	26948491	26948754	144.694
Marker277158	Chr6	27319130	27319412	145.347
Marker270861	Chr6	27323366	27323640	146.001
Marker232308	Chr6	27355391	27355657	146.654
Marker235643	Chr6	27355818	27356098	147.308
Marker147869	Chr6	27417013	27417300	147.635
Marker189162	Chr6	27428755	27429045	147.635
Marker239498	Chr6	27425110	27425408	147.635
Marker184419	Chr6	27436357	27436642	148.288
Marker248234	Chr6	27452600	27452876	148.615
Marker224105	Chr6	27464008	27464275	148.942
Marker174885	Chr6	27488878	27489153	149.269
Marker152487	Chr6	27550856	27551129	149.923
Marker198515	Chr6	27552297	27552560	149.923
Marker232023	Chr6	27670439	27670754	150.249
Marker216436	Chr6	27789348	27789618	150.576
Marker256666	Chr6	27804195	27804483	150.576
Marker294808	Chr6	27716631	27716917	150.576
Marker146044	Chr6	27882498	27882774	150.903
Marker205080	Chr6	27901678	27901958	151.23
Marker295589	Chr6	27901958	27902272	151.557
Marker181480	Chr6	27988416	27988650	151.883
Marker169181	Chr6	27997742	27998002	152.537
Marker250064	Chr6	28010757	28011023	152.864
Marker253786	Chr6	28009502	28009827	152.864
Marker169482	Chr6	28146849	28147170	153.191

Create PDF with GO2PDF for free, if you wish to

Marker170554	Chr6	28084366	28084626	153.191
Marker181798	Chr6	28145933	28146204	153.191
Marker189493	Chr6	28158083	28158351	153.517
Marker269979	Chr6	28159651	28159931	153.844
Marker276235	Chr6	28159292	28159562	153.844
Marker154708	Chr6	28162754	28163066	154.171
Marker179016	Chr6	28196741	28197023	154.171
Marker193227	Chr6	28249920	28250221	154.498
Marker251666	Chr6	28295467	28295755	154.498
Marker148272	Chr6	28345529	28345818	154.825
Marker228838	Chr6	28357290	28357550	154.825
Marker142319	Chr6	28533263	28533547	155.151
Marker169083	Chr6	28543625	28543910	155.151
Marker190212	Chr6	28563719	28564001	155.151
Marker190477	Chr6	29015682	29015925	155.151
Marker200695	Chr6	28357648	28357894	155.151
Marker239314	Chr6	28371099	28371402	155.151
Marker271836	Chr6	28523606	28523905	155.151
Marker287892	Chr6	28590632	28590880	155.151
Marker296240	Chr6	28553919	28554207	155.151
Marker297511	Chr6	28687490	28687769	155.151
Marker297909	Chr6	28597035	28597289	155.151
Marker843159	Chr7	54741	55031	0
Marker849250	Chr7	6735	6981	0
Marker857256	Chr7	24701	24991	0
Marker889235	Chr7	35929	36211	0
Marker892324	Chr7	55402	55660	0.327
Marker845219	Chr7	99763	100071	0.654
Marker902247	Chr7	102355	102650	0.654
Marker915976	Chr7	178204	178511	0.98
Marker845596	Chr7	248125	248414	1.307
Marker872560	Chr7	242993	243297	1.307
Marker885447	Chr7	261862	262121	1.307
Marker888844	Chr7	194635	194895	1.307
Marker932232	Chr7	407221	407521	1.634
Marker925384	Chr7	436578	436837	1.961
Marker933986	Chr7	440938	441249	1.961
Marker834359	Chr7	445044	445306	2.288
Marker891982	Chr7	491150	491419	2.614
Marker898503	Chr7	553523	553791	3.595
Marker879372	Chr7	559210	559476	4.902
Marker896082	Chr7	581539	581822	5.883
Marker909539	Chr7	620212	620462	6.863
Marker930379	Chr7	584408	584702	6.863
Marker837129	Chr7	643060	643361	7.517
Marker885600	Chr7	704397	704692	8.498
Marker874099	Chr7	748256	748500	9.151
Marker862569	Chr7	865516	865773	10.459
Marker872350	Chr7	1110172	1110431	11.112
Marker841157	Chr7	1128204	1128462	11.766
Marker879670	Chr7	1247160	1247418	11.766
Marker879268	Chr7	1563461	1563775	12.093
Marker929720	Chr7	1659670	1659953	13.4
Marker872909	Chr7	1723552	1723839	14.054

Create PDF with GO2PDF for free, if you wish to

Marker928448	Chr7	1683481	1683747	14.054
Marker924022	Chr7	1762960	1763219	15.102
Marker880307	Chr7	2071719	2072006	16.082
Marker895980	Chr7	2341520	2341827	16.409
Marker934821	Chr7	2441054	2441317	17.39
Marker858629	Chr7	2449383	2449630	17.717
Marker833272	Chr7	2486545	2486796	18.043
Marker929060	Chr7	2580011	2580265	19.091
Marker853155	Chr7	2643281	2643555	20.072
Marker871478	Chr7	3086013	3086304	20.399
Marker846202	Chr7	3095161	3095408	21.379
Marker867565	Chr7	3166005	3166283	21.379
Marker895974	Chr7	3123522	3123786	21.379
Marker862658	Chr7	3254503	3254782	21.706
Marker893216	Chr7	3324517	3324779	22.687
Marker832230	Chr7	3331780	3332060	23.34
Marker875298	Chr7	3356889	3357152	23.34
Marker835816	Chr7	3471134	3471417	23.994
Marker863215	Chr7	3608739	3609016	24.321
Marker867418	Chr7	3484945	3485242	24.321
Marker882746	Chr7	3512578	3512821	24.321
Marker874996	Chr7	3706152	3706449	24.974
Marker881385	Chr7	3707322	3707580	25.628
Marker866252	Chr7	3741050	3741313	26.282
Marker889265	Chr7	3783822	3784118	26.282
Marker906683	Chr7	3730911	3731177	26.282
Marker898576	Chr7	3852897	3853189	26.608
Marker903562	Chr7	3831368	3831631	26.608
Marker864137	Chr7	3870500	3870747	26.935
Marker880503	Chr7	3870747	3870995	26.935
Marker913627	Chr7	3881299	3881560	27.262
Marker898509	Chr7	3881703	3881980	27.589
Marker867870	Chr7	3916455	3916724	27.916
Marker871417	Chr7	3927333	3927635	27.916
Marker913937	Chr7	4040348	4040599	27.916
Marker911000	Chr7	4139904	4140156	28.242
Marker875813	Chr7	4180614	4180911	29.683
Marker903830	Chr7	4259970	4260293	29.683
Marker889931	Chr7	4295583	4295893	30.336
Marker895380	Chr7	4263054	4263329	30.336
Marker916567	Chr7	4462039	4462281	31.644
Marker876790	Chr7	4491679	4491964	31.971
Marker888279	Chr7	4496198	4496469	31.971
Marker870650	Chr7	4514243	4514524	32.43
Marker888899	Chr7	4514851	4515146	32.757
Marker889605	Chr7	4591198	4591488	33.084
Marker919892	Chr7	4609834	4610104	33.411
Marker851723	Chr7	4708637	4708943	33.738
Marker833896	Chr7	4723798	4724066	34.064
Marker838801	Chr7	4810298	4810551	34.718
Marker848777	Chr7	4856069	4856354	35.372
Marker910372	Chr7	4897167	4897436	36.025
Marker931016	Chr7	4883604	4883856	36.025
Marker893628	Chr7	4916481	4916783	36.679

Create PDF with GO2PDF for free, if you wish to

Marker885657	Chr7	5079833	5080089	37.006
Marker877150	Chr7	5119188	5119438	37.659
Marker899128	Chr7	5253876	5254125	37.659
Marker924642	Chr7	5112854	5113098	37.659
Marker926230	Chr7	5240250	5240515	37.659
Marker858828	Chr7	5263062	5263312	37.986
Marker859818	Chr7	5255884	5256200	37.986
Marker881102	Chr7	5273343	5273609	38.313
Marker879690	Chr7	5386483	5386747	38.967
Marker898220	Chr7	5485746	5486017	38.967
Marker914933	Chr7	5443577	5443862	38.967
Marker840548	Chr7	5673400	5673675	39.293
Marker868964	Chr7	5517127	5517396	39.293
Marker893695	Chr7	5509079	5509367	39.293
Marker883804	Chr7	5689648	5689926	39.62
Marker865008	Chr7	5691516	5691756	40.601
Marker876702	Chr7	5709544	5709837	40.601
Marker905983	Chr7	6137900	6138169	40.601
Marker902875	Chr7	6225796	6226072	41.908
Marker920648	Chr7	6229684	6229936	42.235
Marker835808	Chr7	6233750	6234026	42.562
Marker839292	Chr7	6235401	6235689	42.562
Marker854623	Chr7	6245602	6245875	42.889
Marker863548	Chr7	6299165	6299428	43.542
Marker880325	Chr7	6278632	6278892	43.542
Marker896999	Chr7	6296826	6297510	43.542
Marker917078	Chr7	6299428	6299695	43.542
Marker920862	Chr7	6324704	6324994	44.196
Marker849953	Chr7	6465186	6465442	44.523
Marker861474	Chr7	6451704	6451964	44.523
Marker904654	Chr7	6479208	6479466	44.85
Marker915708	Chr7	6563609	6563877	45.83
Marker864245	Chr7	6923081	6923376	46.157
Marker915709	Chr7	6752626	6752883	46.157
Marker909009	Chr7	6942517	6942777	46.484
Marker903041	Chr7	6945212	6945493	46.81
Marker889685	Chr7	7250728	7251041	48.118
Marker857444	Chr7	7254029	7254295	48.445
Marker893295	Chr7	7264635	7264909	49.098
Marker899077	Chr7	7256582	7256840	49.098
Marker867459	Chr7	7400705	7400989	49.425
Marker912973	Chr7	7493057	7493300	49.752
Marker927469	Chr7	7435533	7435777	49.752
Marker852986	Chr7	7523465	7523721	50.079
Marker909646	Chr7	7520464	7520786	50.079
Marker923283	Chr7	7538267	7538534	50.406
Marker900998	Chr7	7547648	7547898	51.059
Marker925940	Chr7	7542761	7543037	51.059
Marker834143	Chr7	7645164	7645436	51.386
Marker901522	Chr7	7634737	7635013	51.386
Marker897156	Chr7	7650876	7651150	51.713
Marker880282	Chr7	7686728	7686986	52.366
Marker904159	Chr7	7704611	7704912	52.366
Marker883223	Chr7	7749065	7749318	52.693

Create PDF with GO2PDF for free, if you wish to

Marker887014	Chr7	8037067	8037331	52.693
Marker890937	Chr7	7728653	7728920	52.693
Marker888317	Chr7	8096236	8096502	53.02
Marker854440	Chr7	8117549	8117845	53.347
Marker836440	Chr7	8126958	8127250	53.674
Marker841630	Chr7	8122394	8122675	53.674
Marker912306	Chr7	8190507	8190790	53.674
Marker875296	Chr7	8207603	8207911	54
Marker898582	Chr7	8210495	8210781	54
Marker879992	Chr7	8233958	8234237	54.327
Marker846192	Chr7	8279277	8279546	54.654
Marker832625	Chr7	8638589	8638880	54.981
Marker844453	Chr7	8386520	8386789	54.981
Marker921439	Chr7	8697472	8697735	55.961
Marker875641	Chr7	8721004	8721237	56.288
Marker890314	Chr7	8706249	8706543	56.288
Marker881047	Chr7	8856252	8856553	56.615
Marker897104	Chr7	9098621	9098854	56.615
Marker920983	Chr7	9067942	9068206	56.615
Marker893313	Chr7	9155539	9155842	56.942
Marker894288	Chr7	9431557	9431814	57.269
Marker834593	Chr7	9464634	9464928	57.595
Marker892052	Chr7	9467035	9467275	57.595
Marker876105	Chr7	9641180	9641437	58.576
Marker902017	Chr7	9614416	9614707	58.576
Marker853346	Chr7	9772688	9772980	58.903
Marker897719	Chr7	9828880	9829185	59.23
Marker909152	Chr7	9936559	9936820	59.883
Marker882050	Chr7	9954705	9954976	61.191
Marker832385	Chr7	9967232	9967500	61.517
Marker914912	Chr7	10073396	10073655	61.844
Marker876119	Chr7	10152075	10152333	63.152
Marker845883	Chr7	10161969	10162237	63.805
Marker921663	Chr7	11071773	11072050	64.132
Marker875819	Chr7	11358483	11358781	64.786
Marker859677	Chr7	11921227	11921531	65.766
Marker836054	Chr7	12196043	12196304	66.747
Marker849560	Chr7	12206004	12206276	67.727
Marker865592	Chr7	12207066	12207360	68.054
Marker834274	Chr7	12695151	12695428	68.381
Marker865924	Chr7	12688901	12689184	68.381
Marker895118	Chr7	12224277	12224579	68.381
Marker898042	Chr7	12647548	12647819	68.381
Marker891997	Chr7	12696146	12696409	69.035
Marker853236	Chr7	12786392	12786688	69.361
Marker859618	Chr7	12720675	12720966	69.361
Marker861374	Chr7	12924137	12924438	69.688
Marker931469	Chr7	12927415	12927686	70.015
Marker862044	Chr7	13010721	13011005	70.342
Marker865263	Chr7	13006417	13006688	70.342
Marker872336	Chr7	13031028	13031298	70.342
Marker872924	Chr7	13036344	13036592	70.342
Marker875676	Chr7	12958198	12958442	70.342
Marker877618	Chr7	13038188	13038444	70.342

Create PDF with GO2PDF for free, if you wish to

Marker870921	Chr7	13083149	13083418	70.669
Marker879082	Chr7	13119738	13119997	70.995
Marker911826	Chr7	13102966	13103241	70.995
Marker862349	Chr7	13222738	13222990	71.322
Marker880215	Chr7	13224535	13224825	71.322
Marker929739	Chr7	13156063	13156316	71.322
Marker923374	Chr7	13247395	13247657	71.649
Marker928838	Chr7	13278863	13279181	71.649
Marker834265	Chr7	13343796	13344094	71.976
Marker841829	Chr7	13288398	13288659	71.976
Marker863195	Chr7	13354186	13354466	71.976
Marker873124	Chr7	13364062	13364377	71.976
Marker907288	Chr7	13336525	13336817	71.976
Marker933587	Chr7	13281959	13282220	71.976
Marker836330	Chr7	13383410	13383685	72.303
Marker896955	Chr7	13412750	13413022	72.303
Marker926750	Chr7	13392295	13392617	72.303
Marker838006	Chr7	13477667	13477916	72.629
Marker854190	Chr7	13562273	13562551	72.629
Marker914897	Chr7	13471119	13471400	72.629
Marker921113	Chr7	13538980	13539283	72.629
Marker929082	Chr7	13466354	13466635	72.629
Marker875014	Chr7	13622742	13623022	72.956
Marker920343	Chr7	13612547	13612792	72.956
Marker874606	Chr7	13702649	13702945	73.61
Marker877773	Chr7	13819878	13820140	73.937
Marker878733	Chr7	13752703	13753022	73.937
Marker913394	Chr7	13772214	13772494	73.937
Marker881010	Chr7	13854280	13854567	74.59
Marker905074	Chr7	13837825	13838114	74.59
Marker845139	Chr7	13854987	13855245	74.917
Marker895918	Chr7	13882588	13882854	75.244
Marker840881	Chr7	13959449	13959755	75.571
Marker897217	Chr7	13897757	13898036	75.571
Marker840166	Chr7	13984468	13984771	75.897
Marker901270	Chr7	13982590	13982871	75.897
Marker933498	Chr7	13974262	13974545	75.897
Marker857893	Chr7	14016423	14016685	76.551
Marker878205	Chr7	13987337	13987593	76.551
Marker912670	Chr7	14010622	14010895	76.551
Marker883557	Chr7	14074606	14074887	77.205
Marker867233	Chr7	14078820	14079113	78.185
Marker930388	Chr7	14086222	14086522	78.185
Marker863532	Chr7	14250151	14250403	78.512
Marker883543	Chr7	14193233	14193539	78.512
Marker894760	Chr7	14220822	14221072	78.512
Marker919117	Chr7	14284139	14284434	79.166
Marker838934	Chr7	14285880	14286131	79.495
Marker916215	Chr7	14306177	14306454	80.148
Marker915166	Chr7	14313495	14313773	80.475
Marker866618	Chr7	14385153	14385442	80.802
Marker870754	Chr7	14387158	14387445	80.802
Marker854977	Chr7	14409224	14409470	81.129
Marker882098	Chr7	14705280	14705546	81.782

Create PDF with GO2PDF for free, if you wish to

Marker858600	Chr7	14721533	14721814	82.436
Marker864248	Chr7	14805971	14806271	83.09
Marker846328	Chr7	14806368	14806664	83.743
Marker904669	Chr7	14806920	14807196	84.203
Marker896565	Chr7	14809144	14809435	84.53
Marker838494	Chr7	15150283	15150547	84.989
Marker833465	Chr7	15191395	15191690	85.316
Marker841294	Chr7	15203036	15203316	85.643
Marker919808	Chr7	15191690	15191943	85.643
Marker857599	Chr7	15215467	15215738	85.972
Marker835313	Chr7	15269519	15269775	86.626
Marker894969	Chr7	15334700	15334993	86.626
Marker901233	Chr7	15336136	15336425	86.626
Marker901947	Chr7	15307072	15307353	86.626
Marker909057	Chr7	15297680	15297950	86.626
Marker917113	Chr7	15328327	15328598	86.626
Marker857279	Chr7	15386387	15386650	86.952
Marker885005	Chr7	15401418	15401710	86.952
Marker904187	Chr7	15405898	15406194	86.952
Marker909553	Chr7	15411163	15411412	87.279
Marker832090	Chr7	15456769	15457027	87.606
Marker833154	Chr7	15463053	15463332	87.606
Marker887040	Chr7	15422170	15422431	87.606
Marker838436	Chr7	15483986	15484309	88.26
Marker908483	Chr7	15516590	15516858	88.26
Marker927594	Chr7	15522003	15522273	88.26
Marker838431	Chr7	15544819	15545066	88.586
Marker915267	Chr7	15565096	15565412	88.586
Marker934570	Chr7	15523773	15524047	88.586
Marker864810	Chr7	15573935	15574188	88.913
Marker886137	Chr7	15607544	15607802	88.913
Marker934157	Chr7	15599078	15599340	88.913
Marker932281	Chr7	15616844	15617093	89.567
Marker887225	Chr7	15630487	15630764	89.894
Marker839470	Chr7	15640219	15640503	90.22
Marker855505	Chr7	15655010	15655246	90.22
Marker906409	Chr7	15634129	15634417	90.22
Marker866664	Chr7	15677850	15678160	90.547
Marker842295	Chr7	15694795	15695079	90.874
Marker908435	Chr7	15692790	15693040	90.874
Marker875148	Chr7	15714337	15714629	91.201
Marker923421	Chr7	15716768	15717089	91.528
Marker925607	Chr7	15759955	15760270	91.854
Marker844720	Chr7	15784721	15785008	92.508
Marker928144	Chr7	15788629	15788891	92.835
Marker880935	Chr7	15839294	15839560	93.162
Marker841962	Chr7	15859130	15859378	93.815
Marker866023	Chr7	15890873	15891172	94.478
Marker887577	Chr7	15859378	15859647	94.478
Marker855936	Chr7	15971978	15972227	94.804
Marker918253	Chr7	15901379	15901634	94.804
Marker900174	Chr7	16246484	16246758	95.131
Marker900613	Chr7	16232260	16232558	95.131
Marker866250	Chr7	16285204	16285486	96.439

Create PDF with GO2PDF for free, if you wish to

Marker843736	Chr7	16302087	16302343	96.766
Marker911069	Chr7	16317792	16318067	97.092
Marker925750	Chr7	16319094	16319371	97.092
Marker839242	Chr7	16337760	16338029	97.746
Marker837272	Chr7	16420199	16420458	98.073
Marker850466	Chr7	16358452	16358724	98.073
Marker867783	Chr7	16361016	16361307	98.073
Marker919165	Chr7	16451603	16451924	98.073
Marker836158	Chr7	16624793	16625076	98.4
Marker833705	Chr7	16627112	16627394	98.726
Marker884498	Chr7	16719752	16720003	98.726
Marker859268	Chr7	16721399	16721685	99.053
Marker849628	Chr7	16835795	16836051	99.38
Marker866753	Chr7	16772971	16773256	99.38
Marker905862	Chr7	16777921	16778175	99.38
Marker839897	Chr7	16938872	16939156	99.707
Marker853570	Chr7	16965785	16966077	100.034
Marker866603	Chr7	17171534	17171798	100.36
Marker869549	Chr7	17110617	17110859	100.36
Marker869818	Chr7	17083224	17083497	100.36
Marker895773	Chr7	17068413	17068710	100.36
Marker924187	Chr7	17160672	17160959	100.36
Marker839540	Chr7	17241456	17241744	100.687
Marker843667	Chr7	18717470	18717760	100.687
Marker850146	Chr7	18133781	18134055	100.687
Marker858098	Chr7	17288128	17288387	100.687
Marker861565	Chr7	17190506	17190797	100.687
Marker862930	Chr7	18671378	18671625	100.687
Marker868191	Chr7	18079797	18080061	100.687
Marker873382	Chr7	17675523	17675782	100.687
Marker886149	Chr7	17676144	17676428	100.687
Marker900427	Chr7	18053938	18054227	100.687
Marker923095	Chr7	17235084	17235351	100.687
Marker930068	Chr7	17972862	17973118	100.687

Create PDF with GO2PDF for free, if you wish to
